# Supplementary material for: Revision and Extension of a Generally Applicable Group-Additivity Method for the Calculation of the Standard Heat of Combustion and Formation of Organic Molecules
Source: Molecules. 2021 Oct 10;26(20):6101. doi: 10.3390/molecules26206101 (PMC8539152; doi:10.3390/molecules26206101)
Supplement: Supplementary file 1 [file molecules-26-06101-s001.zip › Table S2. Experimental vs. calculated deltaH_c) Data Table.pdf]

**Table S2. Experimental vs. calculated deltaH_c) Data Table.**		
**Molecule Name**	**DeltaH°(c) exp**	**DeltaH°(c) calc**
(-)-Dimethyl D-tartrate	-2593	-2576.3
(1,1-Dimethylpentyloxymethyl)oxirane	-6355.8	-6365.5
(1,1-Dimethylpropoxymethyl)oxirane	-5045.8	-5050.7
(1,2-Propadienylsulphonyl)-benzene	-5183.6	-5204.6
(1-Chloroethyl)benzene	-4399.8	-4401.4
(1-Methylvinyl)cyclopropane	-3849	-3853.2
(2,2-Dimethylpropoxymethyl)oxirane	-5048.5	-5062.9
(2-Fluoro-2,2-dinitroethoxy)(2,2,2-trinitroethoxy)methane	-2419.1	-2383.6
(2-Fluoro-2,2-dinitroethyl) acrylate	-2278.3	-2286.5
(2-Methylphenoxy)trimethylsilane	-6684	-6689.1
(2-Methylpropoxymethyl)oxirane	-4400.9	-4413.7
(3-(N-(1,2,4-Triazolo[4,3-b]-1,2,4,5-tetrazin-3-yl)amino-4-aminofurazan	-3197.4	-3186.3
(3-(N-(1,2,4-Triazolo[4,3-b]-1,2,4,5-tetrazin-3-yl)amino-4-azidofurazan	-3250.9	-3268.2
(3-(N-(1,2,4-Triazolo[4,3-b]-1,2,4,5-tetrazin-3-yl)amino-4-nitrofurazan	-2945.9	-2972.7
(3-Aminopropyl)-dibutylborane	-8319.5	-8362.4
(3-Aminopropyl)-dipropylborane	-7039	-7055.5
(3-Methylbutoxymethyl)oxirane	-5054.4	-5069.8
(3-Methylphenoxy)trimethylsilane	-6683	-6689.1
(4-Methylphenoxy)trimethylsilane	-6686	-6689.1
(9Z)-hexadecenoic acid	-9877.3	-9849.7
(9Z)-Octadecenoic acid	-11160.7	-11156.7
(Benzyloxy-NNO-azoxy)-phenylmethane	-7645.4	-7636.6
(Chloromethyl)benzene	-3708.7	-3749.2
(Diethylamino)acetonitrile	-4092.2	-4091.5
(Diethylamino)trimethylsilane	-6082.7	-6071.7
(Dimethylamino)acetonitrile	-2786.3	-2784.2
(Dinitromethyl)benzene	-3571	-3573.1
(E)-(2-Phenylethenyl)-sulphonylbenzene	-7686.4	-7667.6
(E)-1-Methyl-1,2-cyclopentanediol	-3539.5	-3544.2
(E)-1-Methyl-4-(1-propenyl-sulphonyl)-benzene	-5959.7	-5950.1
(E)-1-Methyl-4-(2-phenylethenyl)sulphonylbenzene	-8328	-8310.5
(E)-2,3-Dimethylbicyclo[2.2.1]heptane	-5681.9	-5682.2
(Ethoxymethyl)oxirane	-3102.4	-3110.1
(Ethylthio)benzene	-5204.6	-5187.5
(Fluoromethyl)benzene	-3762.8	-3759.3
(Iodomethyl)benzene	-3809.47	-3818.2
(L)-Alanine	-1621	-1623.8
(L)-Cysteine	-2263	-2251.3

(L)-Cystine	-4248	-4241.2
(L)-Histidine	-3180.6	-3165.9
(L)-Hydroxyproline	-2594.1	-2567.6
(L)-Methionine	-3564.1	-3564.2
(Pentyloxymethyl)oxirane	-5071	-5070.5
(trans-1-Butenyl)benzene	-5639.6	-5693.9
(Z)-1,2,3,4-Tetrahydronaphthalene-1,2-diol	-5238.4	-5227.7
(Z)-1,2,3,4-Tetrahydronaphthalene-2,3-diol	-5228.7	-5227.7
(Z)-1,2-Bis(benzylthio)ethylene	-9937	-9920.4
(Z)-1-Methyl-1,2-cyclopentanediol	-3549.1	-3544.2
(Z)-1-Methyl-4-(2-phenylethenyl)sulphonylbenzene	-8329.5	-8307.9
(Z,Z,Z)-1,4,7-Cyclononatriene	-5400.7	-5358.7
[1,8]Paracyclophane	-11898	-11888
[2,2]Metacyclophane	-8661.3	-8615.4
[3,3]Paracyclophane	-9967.8	-9922.4
1-(1-Butenyl)piperidine	-5887.4	-5850.8
1-(1-Nitro-1H-pyrazol-3-yl)-1H-tetrazole	-2573.9	-2520.7
1-(2-Aminophenyl)pyrrole	-5489.5	-5500.1
1-(2-Hydroxycyclopentyl)-2-propanone	-4000.54	-3968.6
1-(2-Methylphenyl)ethanone	-4796.1	-4794.4
1-(2-Propenyl)piperidine	-5255.4	-5269.1
1-(2-Pyridinyl)ethanone	-3653.2	-3649.3
1-(3,4-Dimethoxyphenyl)naphthalene	-9197.4	-9191.7
1-(3-Formyl-4-methoxyphenyl)naphthalene	-8935.2	-8937.2
1(3H)-Isobenzofuranone	-3693.2	-3705.5
1-(3-Methoxyphenyl)naphthalene	-8675.7	-8678.5
1-(4-Chlorophenyl)pyrrole	-5209.7	-5226.7
1-(4-Ethylphenyl)-2-phenylethane	-8886.58	-8873.3
1-(4-Fluorophenyl)pyrrole	-5204.8	-5235.6
1-(4-Formylphenyl)naphthalene	-8421.4	-8423.9
1-(4-Hydroxyphenyl)butan-1-one	-5233.1	-5247.3
1-(4-Hydroxyphenyl)heptan-1-one	-7184.8	-7207.7
1-(4-Hydroxyphenyl)hexan-1-one	-6534	-6554.2
1-(4-Hydroxyphenyl)pentan-1-one	-5882	-5900.7
1-(4-Iodophenyl)pyrrole	-5289.2	-5301.6
1-(4'-Methoxybenzylidene)-2-phenazin-1-oylhydrazine	-10578	-10585.2
1-(4-Methoxyphenyl)naphthalene	-8671.9	-8678.5
1-(4-Methylphenyl)pyrrole	-6004.7	-6022.3
1-(Biphenyl-4-yl)naphthalene	-11156	-11171.2
1,1,1,2,2,3,3-Heptafluoro-7,7-dimethyloctan-4,5-dione	-4738	-4753.8
1,1,1,2,2-Pentafluoropropane	-1367.51	-1344.9
1,1,1,2,3,3,3-Heptafluoropropane	-987.76	-989.6

1,1,1,2,3,3-Hexafluoropropane	-1176.14	-1185.6
1,1,1,2-Tetrachloroethane	-973.9	-988.7
1,1,1,2-Tetraphenylethane	-13606	-13598.3
1,1,1,3,5,5,5-Heptanitropentane	-2528	-2514.2
1,1,1,3-Tetrachloropropane	-1638	-1642.2
1,1,1,4-Tetranitrobutane	-2242	-2270.4
1,1,1-Trichloro-3,3,3-trifluoropropane	-1234.3	-1277
1,1,1-Trichlorofluoroethane	-977.83	-998.8
1,1,1-Trifluoro-4-(2-thienyl)-4-hydroxy-3-buten-2-one	-4039.9	-4067.4
1,1,1-Trifluoro-4-(2-thienyl)-4-mercapto-3-buten-2-one	-4865.5	-4891.1
1,1,1-Trifluoro-5,5-dimethylhexan-2,4-dione	-4126.2	-4116.4
1,1,1-Trifluoro-5-methylheptan-2,4-dione	-4138.6	-4125.9
1,1,1-Trifluoro-5-methylhexan-2,4-dione	-3469	-3471.1
1,1,1-Trifluoroacetylacetone	-2170.32	-2163.6
1,1,1-Trifluoroethane	-992.88	-1026.2
1,1,1-Trifluorohexan-2,4-dione	-2835.2	-2817.1
1,1,1-Trimethoxy-2-chloroethane	-2903.5	-2905.1
1,1,1-Trimethoxyethane	-3072.99	-3062.8
1,1,1-Trinitroethane	-1119.68	-1149.8
1,1,1-Triphenylethane	-10599	-10583.3
1,1,2,2-Tetrachloroethane	-972.8	-989.9
1,1,2,2-Tetrafluoro-1,2-diphenylethane	-6870.7	-6902.3
1,1,2,2-Tetrafluoroethane	-875.7	-895.4
1,1,2,2-Tetramethylcyclopropane	-4635.6	-4670
1,1,2,2-Tetraphenylethane	-13600.32	-13594.3
1,1,2,2-Tetra-p-tolylethane	-16166	-16163.3
1,1,2-Trichloroethane	-1098.1	-1111.8
1,1,2-Trifluoro-1,2-diphenylethane	-7069.1	-7069.8
1,1,2-Trimethylcyclopropane	-3979.9	-4018.2
1,1,2-Triphenylethane	-10572	-10587.6
1,1,2-Tris(2-methylphenyl)ethane	-12513.4	-12532.4
1,1,2-Tris(4-methylphenyl)ethane	-12505.43	-12513.7
1,1,3,3,5,5-Hexaphenyl-7,7-dimethylcyclotetrasiloxane	-22112	-22090.2
1,1,3,3-Tetramethylguanidinium nitrate	-3656.51	-3656.5
1,1,3,3-Tetranitrobutane	-2217.3	-2189.2
1,1,4,4-Tetramethylcyclodecane	-9172.7	-9159.8
1,1,4,4-Tetraphenyl-1,3-butadiene	-14501.77	-14526.3
1,1,4,4-Tetraphenylbutane	-14897	-14899.9
1,1,4,6,7-Pentamethylindan	-8197.93	-8217.4
1,1,4,6-Tetramethylindane	-7561.4	-7560.3
1,1,4,7-Tetramethylindane	-7564.2	-7574.5
1,1,5,5-Tetramethylcyclodecane	-9182.2	-9150.9

1,10-Decanediol	-6400.3	-6408.9
1,12-Dimethylbenz[a]anthracene	-10293.62	-10266.5
1,12-Dimethylbenzo[c]phenanthrene	-10311	-10254.9
1,1'-Bicycloheptyl	-8929.1	-8905.1
1,1'-Binaphthyl	-10040	-10060.4
1,1-Bis(4-methylphenyl)ethane	-8841.4	-8856.9
1,1-Bis(4-tolyl)ethylene	-8674.43	-8690.1
1,1-Bis(difluoroamino)heptane	-5192.9	-5161.1
1,1-Di(methoxy-NNOazoxy)ethane	-3027.5	-3048.4
1,1-Dibutoxypropane	-7155.2	-7142.9
1,1-Dichloro-1-fluoroethane	-1068.96	-1091.7
1,1-Dichlorocyclopropane	-1772.2	-1770.1
1,1-Dichloroethane	-1246.8	-1269.5
1,1-Dichlorotetrafluoroethane	-567.03	-568.8
1,1-Dicyclohexylbutane	-10220	-10212.6
1,1-Dicyclohexylhexane	-11560	-11519.6
1,1-Diethoxyethane	-3870.5	-3875.6
1,1-Diethoxynon-2-yne	-8143.7	-8148.7
1,1-Diethoxyoct-2-yne	-7492.3	-7495.2
1,1-Diethylcyclohexane	-6520.83	-6537.5
1,1-Diethynylcyclopropane	-4113	-4132
1,1-Difluoroethane	-1209.85	-1222.2
1,1-Difluoroethylene	-1091.87	-1117
1,1-Diisopropoxyethane	-5155.17	-5163.5
1,1-Dimethoxy-2-methylpropane	-3888.6	-3898.7
1,1-Dimethoxy-2-phenylcyclopropane	-6103.95	-6096.6
1,1-Dimethoxybutane	-3896.68	-3899.4
1,1-Dimethoxycyclobutane	-3733.71	-3734.3
1,1-Dimethoxycyclopentane	-4312.86	-4315.6
1,1-Dimethoxypentane	-4549.98	-4552.9
1,1-Dimethoxypropane	-3241.69	-3245.9
1,1-Dimethyl-2-ethylcyclopropane	-4668.36	-4671.7
1,1-Dimethyl-2-hexylcyclopropane	-7284.7	-7285.6
1,1-Dimethyl-2-propylcyclopropane	-5322.35	-5325.2
1,1-Dimethyl-3-phenylurea	-5054.3	-5048.8
1,1-Dimethyl-4-penten-2-yn-1-ylhydroperoxide	-4247.4	-4210.5
1,1-Dimethylazoethane	-5645.1	-5668.3
1,1-Dimethylcyclohexane	-5219.65	-5224
1,1-Dimethylcyclopentane	-4586.51	-4589.9
1,1-Dimethylcyclopropane	-3363.4	-3365.4
1,1-Dimethylindane	-6275.6	-6274.4
1,1-Dinitroethane	-1211.39	-1215.2

1,1-Dinitropropane	-1867.4	-1868.7
1,1-Di-o-tolylethane	-8848.45	-8856.9
1,1-Di-o-tolylethylene	-8677.53	-8707.5
1,1'-Diphenyl-1,1'-bicyclohexyl	-13624	-13617.4
1,1'-Diphenyl-1,1'-bicyclopentyl	-12343	-12331
1,1-Diphenyl-3,3,5,5,7,7-hexamethylcyclotetrasiloxane	-12593	-12593
1,1-Diphenylbutane	-8901.5	-8878
1,1-Diphenylcyclopropane	-8089	-8081.2
1,1-Diphenylethane	-7558.6	-7571
1,1-Diphenylethylene	-7396.5	-7404.3
1,1-Diphenylhexane	-10230	-10184.9
1,1-Diphenylpropane	-8245	-8224.5
1,1-Ethanediol diacetate	-2918.7	-2906.1
1,1'-Oxybis(2,4,4-trinitro-4-fluoro-2-azabutane)	-3199.1	-3201.5
1,2,2,3-Tetrachloropropane	-1594.9	-1591
1,2,2-Trichloropropane	-1778.3	-1748.7
1,2,3,4,5,6,7,8-Octahydroanthracene	-7962.1	-7965.3
1,2,3,4-Tetrachlorobenzene	-2642.2	-2653.4
1',2',3',4'-Tetrahydro-1',2-binaphthylmethane	-11173.5	-11180.5
1',2',3',4'-Tetrahydro-1,2-dinaphthylmethane	-11166	-11177.9
1,2,3,4-Tetrahydro-5-hydroperoxynaphthalene	-5468.2	-5478.7
1,2,3,4-Tetrahydronaphthalene	-5617.5	-5614.7
1,2,3,4-Tetrahydro-N-methylcarbazole	-7258.3	-7273.7
1,2,3,4-Tetrahydroquinoline	-5130.3	-5103.2
1,2,3,4-Tetraphenyl-1,3-butadiene	-14521	-14523.7
1,2,3,4-Tetraphenylbenzene	-15280	-15267.1
1,2,3,4-Tetraphenylnaphthalene	-17219	-17170.2
1,2,3,5-Tetrachlorobenzene	-2634.2	-2653.4
1,2,3,5-Tetraethylbenzene	-8462.69	-8458.7
1,2,3,5-Tetramethylbenzene	-5843.52	-5835.7
1,2,3,6,7,8-Hexahydropyrene	-8538.8	-8556.2
1,2,3-Benzenetriol	-2667.4	-2638.3
1,2,3-Propanetricarboxylic acid	-1961.9	-1955.1
1,2,3-Propanetriol	-1654.3	-1641.1
1,2,3-Trichlorobenzene	-2793.8	-2806.1
1,2,3-Trichloropropane	-1735	-1725.5
1,2,3-Trichloropropene	-1578.4	-1585.3
1,2,3-Triethylbenzene	-7169.14	-7158.4
1,2,3-Trimethoxybenzene	-4817.7	-4803.8
1,2,3-Trimethylcyclopentene	-5034.33	-5035.5
1,2,3-Triphenylbenzene	-12259	-12266.3
1,2,4,5-Tetrachlorobenzene	-2631.2	-2653.4

1,2,4,5-Tetrafluorobenzene	-2679	-2688.8
1,2,4,5-Tetramethylbenzene	-5837.3	-5835.7
1,2,4,5-Tetraphenylbenzene	-15261	-15267.1
1,2,4-Benzenetricarboxylic acid	-3219.9	-3189
1,2,4-Butanetriol trinitrate	-2177	-2177.2
1,2,4-Triazole	-1328.9	-1364.6
1,2,4-Triazolo[1,5-a]pyrimidine	-2797.3	-2809.6
1,2,4-Trichlorobenzene	-2810.5	-2806.1
1,2,4-Triethylbenzene	-7165.41	-7159.7
1,2,4-Trihydroxybenzene	-2654.8	-2638.3
1,2,4-Trimethoxybenzene	-4820.1	-4803.8
1,2,4-Trimethylbenzene	-5198.28	-5192.8
1,2,4-Triphenylbenzene	-12260	-12271.6
1,2,4-Tri-t-butylbenzene	-11126.6	-11093.1
1,2,5,6-Diacetone-mannitol	-6594.51	-6606.8
1,2,5,6-Tetrahydropyridine	-3287.3	-3266.5
1,2,5-Pentanetriol trinitrate	-2814	-2828
1,2,5-Trimethylpyrrole	-4313.4	-4305.7
1,2-Anhydro-3,4,5,6-alloinositol	-2884	-2867.4
1,2-Benzenedicarboxylic acid, di-2-propenyl ester	-6964.37	-6925.2
1,2-Bis(2-cyano-2-propyl)-hydrazine	-5268.3	-5287.7
1,2-Bis(2-tolyl)ethane	-8834.55	-8857.6
1,2-Bis(4-tolyl)ethane	-8826.5	-8857.6
1,2-Bis(difluoroamino)-2-methylpropane	-3153.4	-3190.8
1,2-Bis(difluoroamino)-4-methylpentane	-4522.3	-4518.5
1,2-Bis(methylnitramino)ethane	-2911.1	-2941
1,2-Butadiene	-2572.14	-2573.5
1,2-Butanediamine	-3170.9	-3172.3
1,2-Butanediol	-2481.6	-2478.6
1,2-Cyclohexanedione dioxime	-3657.6	-3632.3
1,2-Di-(5-tetrazolyl)ethane	-2877.6	-2900
1,2-Diaminobenzene	-3504	-3479.8
1,2-Dibenzoylethane	-8041.4	-8055.7
1,2-Dibenzoylhydrazine	-7022.4	-7013.5
1,2-Dibromobutane	-2571.8	-2548
1,2-Dibromododecane	-7746.54	-7775.7
1,2-Dibromoethane	-1237.2	-1241.6
1,2-Dibromotetrafluoroethane	-631.79	-639.2
1,2-Dichloro-1,1,2-trifluoroethane	-761.06	-776.3
1,2-Dichlorobenzene	-2957.8	-2958.7
1,2-Dichlorobutane	-2533.3	-2536.7
1,2-Dicyclohexylbutane	-10260	-10219.2

1,2-Dicyclohexylethane	-8849.09	-8905.1
1,2-Diethylbenzene	-5871.39	-5859.4
1,2-Difluorobenzene	-2960.7	-2976.4
1,2-Difluoroethane	-1259.15	-1253.8
1,2-Dihydro-3H-1,2,4-triazol-3-one	-1073.3	-1076.4
1,2-Dihydronaphthalene	-5439.33	-5434.7
1,2-Dihydroxy-3-isopropyl-6-methylbenzene	-5464.2	-5439
1,2-Dihydroxy-9,10-anthracenedione	-6066.6	-6068.1
1,2-Dihydroxybenzene	-2856.3	-2846.9
1,2-Dihydroxynaphthalene	-4768.6	-4744.7
1,2-Diiodobenzene	-3120	-3108.5
1,2-Diiodoethane	-1368	-1371.7
1,2-Dimethoxypropane	-3262.69	-3270.3
1,2-Dimethyl-1,2-diphenlethylene glycol dimethyl ether	-9929.9	-9920.2
1,2-Dimethylbenzene	-4552.6	-4552.5
1,2-Dimethylhydrazine	-1983	-1971.3
1,2'-Dinaphthylmethane	-10712	-10716.5
1,2-Dinitrobenzene	-2944.2	-2927.8
1,2-Dinitroethane	-1180	-1176.5
1,2-Diphenylbenzimidazole	-9774	-9768.4
1,2-Diphenylethane	-7562.6	-7571.7
1,2-Diphenylhydrazine	-6643.8	-6622.8
1,2-Epoxy-4-oxa-5-phenylpentane	-5838.4	-5836.7
1,2-Ethanediamine	-1868.6	-1863
1,2-Ethanediol	-1179.5	-1181.1
1,2-Ethanediol dinitrate	-1123.3	-1101.1
1,2-Ethanedithiol	-2796.7	-2804.1
1,2-Hexanediol	-3784.8	-3785.5
1,2-Hydrazinedicarboxamide	-1151	-1160
1,2-Naphthoquinone	-4629.2	-4611.7
1,2-Pentadiene	-3263.78	-3226.9
1,2-Pentanediol	-3135.8	-3132
1,2-Propanediol	-1824.6	-1825.1
1,2-Propanediol 1-methacrylate	-3843.2	-3838.8
1,2-Propanediol dinitrate	-1742.5	-1745.1
1,3,3-Trimethyltricyclo[2.2.1.02,6]heptane	-6155.1	-6182.7
1,3,3-Triphenyl-2-propen-1-one	-10640	-10636.2
1,3,5,5-Tetramethylbarbituric acid	-4170.3	-4161.4
1,3,5,6-Tetramethyluracil	-4379.7	-4395.1
1,3,5,7-Tetramethyladamantane	-8582.97	-8633.7
1,3,5-Benzenetricarboxylic acid	-3209	-3191.6
1,3,5-Cycloheptatriene	-4042.66	-4034.4

1,3,5-Trichlorobenzene	-2785.4	-2806.1
1,3,5-Trihydroxybenzene	-2634	-2638.3
1,3,5-Trimethyl-1H-pyrazole	-3812.64	-3837.7
1,3,5-Trimethyl-2-nitrobenzene	-5043.8	-5024.7
1,3,5-Trimethyl-4-nitrosopyrazole	-3759.2	-3746
1,3,5-Trimethyladamantane	-7939	-7984.6
1,3,5-Trimethylbarbituric acid	-3517	-3512.3
1,3,5-Trimethylbenzene	-5193.1	-5192.8
1,3,5-Trimethylhexahydro-s-triazine	-4529.2	-4528.2
1,3,5-Trimethyluracil	-3733	-3752.2
1,3,5-Trinitro-1,3,5-triazacycloheptane	-2749.1	-2728.9
1,3,5-Trinitrobenzene	-2754.47	-2759.7
1,3,5-Trioxane	-1518	-1511.5
1,3,5-Triphenylbenzene	-12279.34	-12278.1
1,3,5-Triphenyltriazine-2,4,6-trione	-10063	-10053.8
1,3,5-Tri-t-butylbenzene	-11058.23	-11062.8
1,3,5-Trithiane	-3926.53	-3921.4
1,3,6-Trioxocane	-2880.7	-2866.8
1,3-Benzenedicarbonitrile	-3992.2	-3991.3
1,3-Benzothiazole-2-thione	-4767.6	-4759
1,3-Benzoxazole-2-thione	-4005.6	-4033.7
1,3-Butanediol dinitrate	-2377	-2398.5
1,3-Butyleneglycol	-2502.2	-2478.6
1,3-Cyclohexadiene	-3575.8	-3560.9
1,3-Cyclohexanedione	-3079	-3097.8
1,3-Diacetin	-3349.4	-3399.9
1,3-Diamino-2,4,6-trinitrobenzene	-2978	-2971.8
1,3-Diaminobenzene	-3497	-3477.2
1,3-Dibromo-2-methylpropane	-2538.39	-2545.2
1,3-Dibromobutane	-2527.99	-2548
1,3-Dichloro-2-propanol	-1700	-1693.6
1,3-Dichlorobenzene	-2959.13	-2958.7
1,3-Dichlorobutane	-2527.3	-2536.7
1,3-Dichloropropane	-1885.3	-1887.1
1,3-Dichloro-trans-2-butene	-2356.06	-2373
1,3-Dicyclohexylbutane	-10220	-10215.2
1,3-Diethoxypropane	-4559.1	-4562.9
1,3-Diethyl-1,3-diphenylurea	-9427	-9392.7
1,3-Diethyl-2-thiobarbituric acid	-5022.9	-5014.3
1,3-Diethylbarbituric acid	-4151.7	-4164.2
1,3-Diethylbenzene	-5866.37	-5862
1,3-Difluorobenzene	-2946.7	-2976.4

1,3-Dihydroisobenzofuran	-4207.6	-4190.3
1,3-Dihydroxy-2,2-di(ethoxy-NNO-azoxy)propane	-4622.9	-4617.4
1,3-Dihydroxy-2,2-di(methoxy-NNO-azoxy)propane	-3353.1	-3334.2
1,3-Dihydroxyacetone	-1436	-1423.1
1,3-Dihydroxybenzene	-2847.9	-2846.9
1,3-Dihydroxynaphthalene	-4751.2	-4744.7
1,3-Diiodobenzene	-3119.6	-3108.5
1,3-Diiodocyclobutane	-2566.1	-2554.4
1,3-Diiodopropane	-2029.1	-2025.2
1,3-Diisopropylbenzene	-7167.06	-7166.2
1,3-Dimethoxybenzene	-4297	-4290.5
1,3-Dimethyl-5-fluorouracil	-2965.3	-2999.5
1,3-Dimethyladamantane	-7299.1	-7335.5
1,3-Dimethylbenzene	-4551.6	-4549.8
1,3-Dimethyluracil	-3096.21	-3110.1
1,3-Dinitro-1,3-diazacyclopentane	-2037.7	-2051.9
1,3-Dinitro-2-imidazolidinone	-1615	-1653.5
1,3-Dinitrobenzene	-2927.78	-2927.8
1,3-Dinitropropane	-1815.4	-1829.9
1,3-Dioxane	-2342.4	-2337.5
1,3-Dioxepane	-3009	-2990.9
1,3-Dioxolan-2-one	-1171.32	-1175.4
1,3-Dioxolane	-1700.8	-1698
1,3-Diphenyl-1-triazene	-6625.8	-6595.2
1,3-Diphenyl-2,3-epoxy-1-propanone	-7522	-7534.2
1,3-Diphenyl-3-ethoxy-2-propen-1-one	-8791.9	-8759
1,3-Diphenylacetone	-7819.5	-7813.7
1,3-Diphenylbutane	-8912	-8884.5
1,3-Diphenylpropane	-8245	-8227.8
1,3-Di-t-butyl urea	-5900.1	-5875.9
1,3-Di-t-butylbenzene	-8471.7	-8464.5
1,3-Dithiane	-3913.94	-3897.2
1,3-Dithiane sulfoxide	-3725.6	-3738.2
1,3-Dithiolane	-3254.15	-3253
1,3-Oxazolidine-2-thione	-2317.7	-2339
1,3-Propanediol	-1843	-1834.6
1,3-Propanediol dinitrate	-1743	-1754.6
1,3-Thiazolidine-2-thione	-3084.91	-3083.8
1,4,4a,8a-Tetrahydro-1,4-methanonaphthalene-5,8-dione	-5593.6	-5569.4
1,4,5,6-Tetrahydropyrimidine	-2696.8	-2694.9
1,4,5,8-Naphthalenetetracarboxylic dianhydride	-5154	-5104.9
1,4,5,8-Tetrahydro-1,6-methanonaphthalene	-6384.4	-6434.4

1,4,5,8-Tetramethylnaphthalene	-7777.6	-7768.2
1,4,7,10,13,16-Hexaoxacyclooctadecane	-7071	-7081.1
1,4-Anthraquinone	-6473.1	-6509.5
1,4-Benzenedicarbonitrile	-3988.2	-3991.3
1,4-Benzoquinone	-2748.98	-2737.9
1,4-Benzothiazin-3-one	-4602.8	-4615
1,4-Bis-(2-hydroxyethyl)piperazine	-5256.5	-5270.2
1,4-Bis(hydroxymethyl)benzene	-4183	-4181.9
1,4-Bis(hydroxymethyl)-cyclohexane	-4853.75	-4853.2
1,4-Bis(methoxycarbonyl)-cyclohexane	-5277	-5328.9
1,4-Butanediol dinitrate	-2387	-2408
1,4-Butanedithiol	-4104.8	-4111
1,4-Cyclohexadiene	-3570.5	-3560.9
1,4-Cyclohexanedione	-3096.8	-3097.8
1,4-Diaminobenzene	-3507.4	-3477.2
1,4-Dibromobenzene	-2942.1	-2962.2
1,4-Dibromobutane	-2535.69	-2548.5
1,4-Dichlorobenzene	-2939.9	-2958.7
1,4-Dichlorobutane	-2534	-2540.5
1,4-Dicyano-2-butyne	-3301.18	-3303.3
1,4-Dicyanobenzene di-N-oxide	-4057.2	-4044
1,4-Dicyclohexylbutane	-10220	-10212
1,4-Dicyclopropylbuta-1,3-diyne	-5867.9	-5895.1
1,4-Diethylbenzene	-5863	-5862
1,4-Difluorobenzene	-2948.4	-2976.4
1,4-Dihydronaphthalene	-5452.03	-5434.7
1,4-Dihydroxy-2,2,6,6-Tetramethylpiperidine	-5811.5	-5799.2
1,4-Dihydroxybenzene	-2849.6	-2846.9
1,4-Dihydroxynaphthalene	-4742.41	-4744.7
1,4-Diiodobenzene	-3093.2	-3108.5
1,4-Diiodobutane	-2687.4	-2678.6
1,4-Diisocyanatobutane	-3220.4	-3217.4
1,4-Dimethoxybenzene	-4283.8	-4290.5
1,4-Dimethyl-2,5-piperazinedione	-3352.1	-3363.7
1,4-Dimethyl-2,6,7-trioxabicyclo[2.2.2]octane	-3838.44	-3842.1
1,4-Dimethylbenzene	-4552.6	-4549.8
1,4-Dimethylbicyclo[2.2.1]heptane	-5664.7	-5670.3
1,4-Dimethylpiperazine	-4328.1	-4330.9
1,4-Dinitrobenzene	-2902	-2927.8
1,4-Dinitroimidazole	-1600.9	-1587.3
1,4-Dinitropiperazine	-2666.2	-2694
1,4-Dinitropyrazole	-1644.2	-1652.8

1,4-Dioxane	-2362.23	-2360.4
1,4-Dioxane-2,5-dione	-1441.8	-1390.9
1,4-Dioxane-2,6-dione	-1445.3	-1414.9
1,4-Dioxaspiro[4.4]nonane	-4054.98	-4061
1,4-Dioxatetralin	-4036.7	-4024.6
1,4-Diphenylbutane	-8858.7	-8883.9
1,4-Diphenylnaphthalene	-11167	-11168.6
1,4-Di-t-butylbenzene	-8469.95	-8463.2
1,4-Di-t-butylperoxy pyromellitate	-8566.1	-8590.3
1,4-Dithiane	-3851.4	-3873.2
1,4-D-Mannonolactone	-2550.8	-2549.1
1,4-Naphthoquinone	-4612.39	-4611.7
1,4-Oxycyclohexane	-3566.3	-3584.1
1,4-Pentadiene	-3193.28	-3187
1,5,5-Trimethylbarbituric acid	-3478.1	-3473.5
1,5-Cyclooctadiene	-4887	-4879.4
1,5-Dihydroxyisoquinoline	-4194.1	-4202.4
1,5-Dimethyltetrazole	-2228.2	-2250.3
1,5-Dinitronaphthalene	-4834.2	-4825.6
1,5-Diphenylformazan	-7288.2	-7268.9
1,5-Hexadiene	-3844.3	-3840.5
1,5-Hexadiyne	-3604.87	-3622.5
1,5-Naphthalenediamine	-5406.4	-5380.2
1,5-Pentanediol	-3153.49	-3141.5
1,5-Pentanediol dinitrate	-3035	-3061.5
1,5-Pentanedithiol	-4757.1	-4764.5
1,6-Anhydro-beta-D-glucopyranose	-2832	-2820.1
1,6-Bis(methylnitramino)hexane	-5524.5	-5554.9
1,6-Dicyclohexylhexane	-11560	-11518.9
1,6-Dimethyl-4-isopropylnaphthaIene	-8346	-8399.4
1,6-Diphenylhexane	-10230	-10185.6
1,6-Hexanediamine	-4445.81	-4476.9
1,6-Hexanedioic acid	-2802.4	-2827.1
1,7-Difluoro-1,1,3,5,7,7-hexanitro-3,5-diazaheptane	-2688.6	-2686.5
1,7-Difluoro-1,1,5,7,7-pentanitro-5-aza-3-oxaheptane	-2520	-2515.2
1,7-Heptanediol	-4467	-4448.5
1,7-Octadiyne	-4914.69	-4929.5
1,8-Bis(biphenyl-4-yl)naphthalene	-17186	-17175.4
1,8-Cyclotetradecadiyne	-8520.83	-8546.3
1,8-Di(3,4-dimethoxyphenyl)naphthalene	-13270	-13221.7
1,8-Di(3-formyl-4-methoxyphenyl)naphthalene	-12731	-12709.9
1,8-Di(4-methoxyphenyl)naphthalene	-12218	-12189.9

1,8-Diaminonaphthalene	-5429.6	-5377.6
1,8-Difluoro-1,1,3,6,8,8-hexanitro-3,6-diazaoctane	-3335.2	-3342.6
1,8-Dimethylnaphthalene	-6463.2	-6470.8
1,8-Dinitronaphthalene	-4838.8	-4825.6
1,8-Diphenylnaphthalene	-11187	-11163.4
1,8-Octanediol	-5097.57	-5101.9
1,9-Nonanediol	-5746.47	-5755.4
1,I-Dimethylazoxyethane	-5559.6	-5539.1
10-Undecenoic acid	-6615.1	-6601.2
11-Butyldocosane	-17233	-17239.5
11-Cyclohexylheneicosane	-17652	-17647.5
11-Cyclopentylheneicosane	-17026	-17015.3
11-Decylheneicosane	-20492	-20506.9
11-Phenylheneicosane	-16991	-16973
12-Crown-4	-4738.1	-4726
13,14-Dihydroxybehenic acid	-13544	-13566.2
13-Cyclohexylpentacosane	-20280.9	-20264
15-Crown-5	-5903	-5900.9
1a,2a,4b-1,2,4-Trimethylcyclopentane	-5215.4	-5241
1-Acetyl-2-naphthol	-5853.9	-5855.5
1-Acetyladamantane	-6916.96	-6928.3
1-Adamantanol	-5828.4	-5834.1
1-Adamantyl isocyanide	-6464.1	-6477.8
1-Allyl-5-allylaminotetrazole	-4680	-4664.5
1-Allyl-5-aminotetrazole	-2841.6	-2840.4
1-Amino-1-cyclopropylethane	-3478	-3523.8
1-Amino-2-(2-hydroxyethoxy)ethane	-2710.2	-2702.2
1-Aminoadamantane	-6169.2	-6175.7
1-Azabicyclo[2.2.2]octane	-4557.4	-4531.4
1-Azidohexane	-4371	-4367.1
1-Azidooctane	-5674	-5674
1-Azidopentane	-3691	-3713.6
1-Benzopyran-4-one	-4169.3	-4220
1-Benzosuberone	-5878	-5856.6
1-Benzoyl-2-phenylacetylene	-7486.8	-7512.5
1-Benzoylglycerol	-4876	-4878.4
1-Benzyl-2-piperidone	-6649	-6651.1
1-Benzyl-4-piperidinol	-6922	-6941.2
1-Benzyl-4-piperidone	-6729.7	-6723.2
1-Benzylimidazole	-5506.1	-5489
1-Benzylpyrazole	-5567	-5572.9
1-Bromo-2-chloro-1,1,2-trifluoroethane	-806.3	-789.8

1-Bromo-4-chlorobenzene	-2963.7	-2960.5
1-Bromobutane	-2716.5	-2702.3
1-Bromodecane	-6574.66	-6623.1
1-Bromododecane	-7950.3	-7930
1-Bromohexadecane	-10568	-10543.9
1-Bromonaphthalene	-5047.1	-5010.9
1-Bromooctane	-5332.6	-5316.2
1-Bromopentane	-3350.19	-3355.7
1-Bromopropane	-2056.8	-2048.8
1-Butanethiol	-3483.3	-3483.5
1-Butanol	-2677.6	-2672
1-Butene	-2698.38	-2694.8
1-Butoxy-2,3-epoxypropane	-4413.36	-4417
1-Butoxy-3-t-butylperoxy-2-propanol	-7016.8	-7018.3
1-Butyl-1-methylpyrrolidinium dicyanamide	-7244.8	-7250.1
1-Butyl-3-methylimidazolium chloride	-5232.3	-5206.6
1-Butyl-3-methylimidazolium dicyanoamide	-6273.9	-6271.6
1-Butyl-3-methylimidazolium nitrate	-5013.2	-5017.8
1-Butylamine	-3018	-3013
1-Butyltetralin	-8222.4	-8230.5
1-Butyne	-2596.6	-2585.8
1-Carboxyadamantane	-5976.57	-6018.4
1-Carboxyphenazine	-6096.8	-6062.8
1-Chloro-1,1-difluoroethane	-1056.72	-1080.6
1-Chloro-1-fluoroethane	-1242.4	-1244.7
1-Chloro-1-methylcyclohexane	-4392.1	-4401.1
1-Chloro-1-methylcyclopentane	-3760.3	-3766.9
1-Chloro-3-isocyanatobenzene	-3277	-3276.4
1-Chloro-4-(trifluoromethyl)benzene	-3208.3	-3231.4
1-Chlorobutane	-2704.1	-2698.3
1-Chlorododecane	-7926.4	-7926
1-Chloronaphthalene	-5009.2	-5009.2
1-Chlorooctadecane	-11850	-11846.8
1-Chlorooctane	-5310	-5312.2
1-Chloropentane	-3349.9	-3351.7
1-Chloropropane	-2025	-2044.8
1-Cyanoacetylpiperidine	-4646	-4667.5
1-Cyanoadamantane	-6388.8	-6407.1
1-Cyanonaphthalene	-5517.5	-5525.5
1-Cyclohexenecarbonitrile	-4091.6	-4103.6
1-Cyclohexyl-1-propanone	-5440	-5466.4
1-Cyclopropyl-1,3-pentadiyne	-4724.2	-4727.9

1-Cyclopropyl-2-methylbenzene	-5725.4	-5717.1
1-Decanethiol	-7405.1	-7404.3
1-Decene	-6619.6	-6615.6
1-Decyl-3-methylimidazolium bromide	-9105.2	-9127.4
1-Decylazide	-6965.3	-6981
1-Decylnaphthalene	-11723.49	-11686
1-Decyne	-6502.42	-6506.6
1-Dimethylamino-2-propyne	-3451.4	-3446.8
1-Docosanoic acid	-13971	-13950.5
1-Dodecanol	-7909.4	-7899.8
1-Dodecene	-7925.9	-7922.5
1-Dodecyl-3-methylimidazolium bromide	-10406	-10434.4
1-Eicosene	-13136.67	-13150.3
1-Ethanol-3-methyl-imidazolium dicyanoamide	-4793	-4780.7
1-Ethoxy-2-nitrobenzene	-4275.6	-4250.7
1-Ethyl-1-methylcyclohexane	-5877.95	-5882.7
1-Ethyl-1-methylcyclopentane	-5244.55	-5246
1-Ethyl-2-phenylindole	-8543.2	-8583.5
1-Ethyl-3-methylimidazolium chloride	-3886.2	-3899.7
1-Ethyl-3-methylimidazolium dicyanamide	-4955.4	-4964.7
1-Ethyl-3-methylimidazolium nitrate	-3697.5	-3710.9
1-Ethyl-4-methyl-1,3-cyclohexadiene	-5498.4	-5501
1-Ethyl-4-nitro-1,2,3-triazole	-2559.1	-2541
1-Ethyl-4-piperidone	-4367.1	-4368
1-Ethyl-8-methylnaphthalene	-7142.1	-7124.3
1-Ethyladamantane	-7322.3	-7345
1-Ethylcyclohexene	-5042.1	-5039
1-Ethylcyclopentene	-4411.2	-4396.8
1-Ethylimidazole	-3155.7	-3131.1
1-Ethylpiperidine	-4776.8	-4779.5
1-Ethylpyrazole	-3190.2	-3215
1-Ethyltetralin	-6920.8	-6923.5
1-Ethylthiooctane	-7413.04	-7406.5
1-Fluoro-1,1,3,5,5-pentanitro-3-azaheptane	-3423	-3422.4
1-Fluoro-1,1-dinitroethane	-1087.2	-1098.8
1-Fluorododecane	-7921.6	-7936.2
1-Fluoroheptane	-4692.4	-4668.8
1-Fluorononane	-5962.5	-5975.7
1-Fluorooctane	-5318	-5322.3
1-Fluoropropane	-2070.13	-2054.9
1-Fluorotetradecane	-9227.1	-9243.1
1-Glyceryl laurate	-9035.2	-9055.2

1-Glyceryl stearate	-12928	-12976
1H-Benzotriazole	-3325.6	-3331.2
1-Heptadecanol	-11119.67	-11167.1
1-Heptadecene	-11206.67	-11189.9
1-Heptanethiol	-5446.46	-5443.9
1-Heptanol	-4637.6	-4632.4
1-Heptene	-4660.66	-4655.2
1-Heptylhydroperoxide	-4701.6	-4695.4
1-Heptyne	-4539.74	-4546.2
1-Hexadecanol	-10510	-10513.7
1-Hexanethiol	-4792	-4790.4
1-Hexanol	-3984.37	-3979
1-Hexen-5-one	-3595.34	-3590.2
1-Hexene	-4004.56	-4001.7
1-Hexyl-1,2,3,4-tetrahydronaphthalene	-9553.29	-9537.4
1-Hexylhydroperoxide	-4065.2	-4041.9
1-Hexyne	-3916.34	-3892.7
1H-Indazole	-3778.6	-3788.8
1H-Indazole-3-carboxylic acid	-3751.3	-3751.7
1H-Indazole-5-carboxylic acid	-3746.8	-3766.4
1H-Naphtho[2,3-d][1,2,3]triazole	-5209.7	-5229
1H-Purine	-2708.6	-2715.1
1H-Pyrazole	-1871.85	-1884.8
1-Hydroxy-2,2,6,6-tetramethyl-4-piperidinone	-5593	-5581.2
1-Hydroxycumene	-5006.2	-5007.5
1-Hydroxyisoquinoline	-4395.1	-4411
1-Hydroxytetralin	-5408.7	-5421.2
1-Indanol	-4775.7	-4781.7
1-Iodohexane	-4074.36	-4074.3
1-Iodonaphthalene	-5096.9	-5084.1
1-Isopropoxy-2-propanol	-3835.18	-3833.2
1-Isopropyl-6-methylindane	-7574.3	-7574.4
1-Isopropyl-8-methylnaphthalene	-7798.6	-7777.1
1-Methoxy-2-propanone	-2335.78	-2329.7
1-Methoxy-a-D-glucopyranoside	-3529.2	-3524.7
1-Methoxycarbonyladamantane	-6721.7	-6740.2
1-Methoxydecane	-7315.1	-7315.5
1-Methyl-1,2,3,4,-tetrahydro-1-naphthol	-6085.2	-6067
1-Methyl-1H-pyrazole	-2553.3	-2564
1-Methyl-1-phenylhydrazine	-4331.3	-4307.9
1-Methyl-2-phenylindole	-7891.6	-7928.6
1-Methyl-2-piperidinemethanol	-4598.3	-4595

1-Methyl-2-piperidone	-3635.6	-3642.3
1-Methyl-3,4-dinitropyrazole	-2252.9	-2249.1
1-Methyl-3-pentylimidazolium chloride	-5904.3	-5860.1
1-Methyl-3-phenoxybenzene	-6776.2	-6778
1-Methyl-4-(1-methylethenylsulphonyl)benzene	-5966.8	-5946.6
1-Methyl-4-(2-methyl-1-propenylsulphonyl)-benzene	-6589.8	-6592.1
1-Methyl-4-(2-propynyl-sulphonyl)benzene	-5859.7	-5854.7
1-Methyl-4-benzylbenzene	-7571.5	-7561.2
1-Methyl-4-isopropylcyclohexene	-6352.12	-6339.3
1-Methyl-4-nitroimidazole	-2288.1	-2293.9
1-Methyl-4-piperidone	-3713.9	-3714.3
1-Methyl-5-phenyltetrazole	-4583.7	-4610.7
1-Methyladamantane	-6668.39	-6686.3
1-Methylcyclohexanol	-4366.66	-4371.8
1-Methylcyclohexene	-4388.3	-4382.9
1-Methylcyclohexylhydroperoxide	-4428.2	-4434.7
1-Methylcyclopentanol	-3724.8	-3737.7
1-Methylcyclopentene	-3753.8	-3743.3
1-Methyldiamantane	-8805.42	-8806.6
1-Methylimidazole	-2498.3	-2480.1
1-Methylindazole	-4457.2	-4468
1-Methylindole	-4924.6	-4923.9
1-Methylindole-3-carboxylic acid	-4861.8	-4898
1-Methylnaphthalene	-5805.88	-5804.7
1-Methylnorbornane	-5018	-5025.8
1-Methylnorcamphor	-4611.1	-4629.5
1-Methylpropyl 2-pentenoate	-5321.7	-5325
1-Methylpropyl 3-pentenoate	-5329.3	-5325
1-Methylpropyl 4-pentenoate	-5335.1	-5346.4
1-Methylpyrrole	-3030.3	-3019.8
1-Methyltetralin	-6270.1	-6267.4
1-Methyltetralin-1-hydroperoxide	-6172.2	-6127.3
1-Methyltetrazole	-1593.4	-1620.9
1-Monocaprin	-7727.6	-7748.3
1-Naphthaleneacetic acid	-5792	-5790.3
1-Naphthalenecarboxylic acid	-5142.08	-5136.8
1-Naphthol	-4951.1	-4953.3
1-Naphthyl acetate	-5862.7	-5856
1-Naphthylamine	-5285.9	-5271
1-Naphthylisocyanate	-5355.5	-5326.9
1-Nitro-2,6-diisopropylbenzene	-6990.2	-6994.2
1-Nitro-2-isopropylbenzene	-5041.6	-5042.4

1-Nitro-2-nitrosobenzene	-3038.7	-3034.3
1-Nitro-2-propanone	-1600	-1604.7
1-Nitroadamantane	-5819.9	-5835.5
1-Nitrobutane	-2669.5	-2669.7
1-Nitronaphthalene	-4985.4	-4993.7
1-Nitropentane	-3324	-3323.2
1-Nitropropane	-2014	-2016.2
1-Nitropyrazole	-1792	-1836.5
1-Nitroso-2-naphthol	-4891.7	-4891.4
1-Nonadecanoic acid	-11999.68	-11990.1
1-Nonadecene	-12516.49	-12496.8
1-Nonanol	-5946.3	-5939.4
1-Nonene	-5969.12	-5962.1
1-Nonylnaphthalene	-11067.39	-11035.1
1-Nonyne	-5848.4	-5853.1
1-Norbornyl cyanide	-4733.4	-4746.6
1-Norbornylisocyanide	-4825.4	-4817.3
1-Octadecanol	-11820	-11820.6
1-Octadecene	-11861.52	-11843.4
1-Octanethiol	-6102.29	-6097.4
1-Octanol	-5293.6	-5285.9
1-Octen-3-yne	-5007.02	-5007.5
1-Octyl-3-methylimidazolium bromide	-7837.8	-7820.5
1-Octyne	-5191.53	-5199.7
1-Palmitoylglycerol	-11626	-11669.1
1-Pentadecanoic acid	-9335.21	-9376.2
1-Pentadecanol	-9817.7	-9860.2
1-Pentadecene	-9895.79	-9883
1-Pentanethiol	-4137.8	-4137
1-Pentanol	-3330.91	-3325.5
1-Pentene	-3351.94	-3348.2
1-Pentyne	-3257.35	-3239.3
1-Phenyl-1,3-butanedione	-5032.7	-5044.3
1-Phenyl-1,3-butanedione (enol form)	-5032.7	-5034.9
1-Phenyl-1-propanol	-5040.3	-5023
1-Phenyl-1-propanone	-4803.7	-4802.4
1-Phenyl-2-butene	-5672.2	-5687.4
1-Phenyl-2-nitropropene	-4816.9	-4852.8
1-Phenyl-2-propanol	-5039.91	-5020.4
1-Phenyl-3-(methylamino)-2-butene-1-one	-6033.6	-6035.5
1-Phenyl-3-butene	-5683.95	-5708.7
1-Phenyl-4,7-dioxaspiro[2.4]heptane	-5865.03	-5839.9

1-Phenylcyclohexane-cis-1,2-diol	-6540	-6536.2
1-Phenylcyclohexane-cis-1,2-diol diacetate	-8302.3	-8295
1-Phenylcyclohexene	-6710.62	-6743.3
1-Phenylcyclopentane-cis-1,2-diol	-5910.3	-5888.1
1-Phenyldodecane	-11113.44	-11097.7
1-Phenylethylformate	-4626.96	-4626.2
1-Phenylheneicosane	-16978	-16976.3
1-Phenylimidazole	-4865	-4839.7
1-Phenylindene	-7771.33	-7797.3
1-Phenylnaphthalene	-8177.2	-8165.2
1-Phenyloct-2-yn-1-ol	-7964.2	-7986.6
1-Phenylpenta-1-yn-3-one	-5804.9	-5808.1
1-Phenylpyrazole	-4909.24	-4920.1
1-Phenylpyrrole	-5384.2	-5379.4
1-Phenyltetrazole	-3976.6	-3977
1-Phenyl-trans-1,2-cyclohexanediol	-6543.8	-6532.3
1-Phthalazinone	-3977.3	-3984.1
1-Piperidinecarboxamide	-3715.1	-3729.6
1-Piperidineethanol	-4595.2	-4595.5
1-Piperidinoacetonitrile	-4478.9	-4497.6
1-Piperidinocyclohexanecarbonitrile	-7496.1	-7506.3
1-Propanethiol	-2828.2	-2830
1-Propanol	-2021.2	-2018.6
1-Propylamine	-2366.8	-2359.5
1-Propylnaphthalene	-7114.6	-7114.3
1-Pyrenecarboxaldehyde	-8112.5	-8130.8
1-t-Butoxy-2-[2-(t-butoxy)propoxy]propane	-8992.84	-8951.8
1-t-Butyl-4-ethylbenzene	-7168	-7163.9
1-t-Butylperoxy-3-ethoxy-2-propanol	-5697.5	-5708.7
1-t-Butylperoxy-3-methoxy-2-propanol	-5058.1	-5067.2
1-t-Butylperoxy-3-pentoxy-2-propanol	-7672.3	-7669.1
1-t-Butylperoxy-3-propoxy-2-propanol	-6359	-6364.8
1-t-Butylperoxycyclohexanol	-6208.8	-6201.5
1-Tetradecene	-9236.81	-9229.5
1-Tetradecyl-3-methylimidazolium bromide	-11718	-11741.3
1-Tetradecylamine	-9521.45	-9547.7
1-Tetralone	-5154.7	-5203.2
1-Thiaindan	-4955.91	-4934.8
1-trans-5-trans-9-cis-Cyclododecatriene	-7324.5	-7315.9
1-Triacontene	-19632.22	-19685
1-Tridecanol	-8517.8	-8553.2
1-Tridecene	-8583.82	-8576

1-Undecanol	-7253.7	-7246.3
1-Undecene	-7276.93	-7269.1
1-Xylylazo-2-naphthol	-9395	-9432
2-(1-Cyclohexenyl)cyclohexanone	-7005.9	-6995.9
2-(1H-Indol-3-yl)ethanol	-5338.9	-5348.3
2-(1H-Indol-3-yl)ethanol	-5338.9	-5347.4
2-(1-Hydroxy-1-methylethyl)-2-methyl-1,3-dioxane	-4726.1	-4715.6
2-(2-(2-Methoxyethoxy)ethoxy)ethanol	-4258.18	-4264.1
2-(2-Adamantylidene)adamantane	-11632	-11652.5
2-(2-Hydroxyphenyl)benzothiazole	-6977.2	-6973.4
2-(2-Hydroxyphenyl)benzoxazole	-6198.3	-6216.3
2-(2-Methoxyethoxy)ethanol	-3095	-3083.9
2-(5H)-Furanone	-1832.9	-1827.1
2-(5H)-Thiophenone	-2625.4	-2644.5
2-(Biphenyl-4-yl)naphthalene	-11142	-11173.9
2-(Butylnitroamino)-ethanol nitrate	-4026.6	-3979
2-(Diacetoxymethyl)-5-nitrofuran	-3930	-3911.5
2-(Dimethylamino)acetophenone	-5677	-5663.3
2-(Dimethylamino)propiophenone	-6337.7	-6319.1
2-(Phenoxymethyl)oxirane	-4801.2	-4799.7
2,10-Dimethyl-2,10-undecanediol	-8287.54	-8329.7
2,11-Dimethyl-2,11-dodecanediol	-8956.9	-8983.1
2,2,2-Trifluoroethanol	-821.53	-842.2
2,2,2-Trinitro-1-phenylethane	-4130	-4161.1
2,2',2''-Tripyridine	-7772.3	-7760.9
2,2,3,3,3-Pentafluoro-1-propanol	-1149.7	-1160.9
2,2,3,3,4,4,4-Heptafluoro-1-butanol	-1474	-1479.7
2,2,3,3-Tetrafluoro-1-propanol	-1353.4	-1357
2,2,3,3-Tetrafluoropropyl-1',1',2',3',3'-hexafluoropropyl ether	-2320.4	-2319.4
2,2,3,3-Tetramethylbutane	-5451.5	-5459.8
2,2,3,3-Tetramethylhexane	-6780.49	-6769.4
2,2,3,3-Tetramethylpentane	-6125.89	-6110.7
2,2,3,4-Tetramethylpentane	-6122.2	-6116.9
2,2,3-Trimethylhexane	-6117.2	-6116.3
2,2,3-Trimethylpentane	-5467.47	-5461.5
2,2,4,4,5,-Pentamethylhexane	-7434.8	-7430
2,2',4,4',6,6'-Hexamethylazobenzene N,N'-dioxide	-10267.64	-10260.1
2,2,4,4,6,8,8-Heptamethylnonane	-10685.85	-10688.4
2,2,4,4,6-Pentamethyl-1,3-dioxane	-5547	-5544.9
2,2',4,4',6-Pentanitrobenzophenone	-5716	-5666.3
2,2,4,4-Tetramethyl-1,3-cyclobutanedione	-4483.2	-4487.5
2,2,4,4-Tetramethylpentane	-6117.8	-6119.6

2,2,4-Trimethyl-1,3-dioxane	-4256.06	-4256.4
2,2,4-Trimethyl-3-pentanone	-5053.2	-5047.4
2,2,4-Trimethylhexane	-6117	-6115
2,2,5,5-Tetramethylheptane	-7412.6	-7429.4
2,2,5,5-Tetramethylhexane	-6760.45	-6766.8
2,2,5-Trimethylhexane	-6110.87	-6117.6
2,2',6,6'-Tetraethylazobenzene N,N'-dioxide	-11593.23	-11599.9
2,2,6,6-Tetramethyl-3,5-heptanedione	-6599.2	-6597.2
2,2,6,6-Tetramethyl-4-piperidone	-5636.9	-5628.5
2,2',6,6'-Tetramethylazobenzene N,N'-dioxide	-8974.85	-8974.2
2,2,6-Trimethylheptane-3,5-dione	-5943.82	-5949.4
2,2,6-Trimethylheptane-3,5-dione (enol form)	-5943.5	-5933.5
2,2,7,7-Tetramethylocta-3,5-diyne	-7455.6	-7466.1
2,2’-Diaminodiethylamine	-3358.3	-3349.1
2,2'-Bi-4-picoline	-6546.7	-6551.4
2,2'-Bipyridine	-5264.5	-5265.6
2,2'-Biquinoline	-9049.4	-9066.6
2,2-Bis(tertbutylperoxy)butane	-7826.8	-7840.1
2,2'-Diaminodiphenyldisulfide	-7743.9	-7733.4
2,2-Dichloro-1,1,1-trifluoroethane	-726.31	-746.6
2,2-Dichloro-1,1,2-trifluoroethane	-768.23	-764.9
2,2'-Dichlorobiphenyl	-5945.5	-5959.5
2,2-Dichloropropane	-1879.5	-1906.4
2,2-Dicyclohexylbutane	-10240	-10218.8
2,2-Diethoxypropane	-4506.08	-4508
2,2'-Difluorobiphenyl	-5927.9	-5977.2
2,2-Difluoroethanol	-1028.2	-1038.2
2,2-Difluoroethyl acetate	-1906	-1917.7
2,2-Dimethoxy-3,3-dimethylbutane	-5200	-5185.6
2,2-Dimethoxy-3-methylbutane	-4532.4	-4533.8
2,2-Dimethoxybutane	-3879.68	-3878.4
2,2-Dimethoxypentane	-4535.38	-4531.9
2,2-Dimethoxypropane	-3225.99	-3224.9
2,2-Dimethyl-1,3-dioxane	-3607.2	-3612.4
2,2-Dimethyl-1,3-dioxolane	-2968.48	-2972.9
2,2-Dimethyl-1,3-propanediol	-3131.3	-3133.9
2,2-Dimethyl-3,5-heptanedione	-5301.1	-5292.7
2,2-Dimethyl-3-ethylpentane	-6127.2	-6117.6
2,2-Dimethyladamantane	-7329.18	-7344.3
2,2-Dimethylheptane	-6115.97	-6115.7
2,2-Dimethylhexane	-5462.49	-5462.2
2,2-Dimethyloctane	-6770.84	-6771.8

2,2-Dimethylpentane	-4806.3	-4811.4
2,2-Dimethylpropane	-3494.79	-3501.8
2,2-Dimethylsuccinic acid	-2802.4	-2819.4
2,2-Dimethylthiirane	-3297.4	-3328.1
2,2-Dinitroadamantane	-5685.2	-5704.2
2,2'-Dinitroazobenzene N,N'-dioxide	-6086.46	-6066.4
2,2-Dinitropropane	-1850.3	-1869.5
2,2-diphenyl-1,3-dioxolane	-7700.74	-7674.7
2,2-Diphenyl-dimethoxymethane	-7924.16	-7940.7
2,2'-Dipyridyl N,N'-oxide	-5197	-5197.8
2,2'-Dipyridyl N-oxide	-5249.1	-5234.3
2,2'-Dipyrrolylmethane	-5100.26	-5054
2,2'-Oxybisethanol dinitrate	-2261	-2281.3
2,3,3,4-Tetramethylpentane	-6126.27	-6109.1
2,3,3-Trimethyl-1-butene	-4640.86	-4636.1
2,3,3-Trimethylhexane	-6118.8	-6115
2,3,3-Trimethylpentane	-5470.86	-5461.5
2,3,4,5,6-Pentafluorotoluene	-3194.4	-3188
2,3,4,5-Tetrafluoroaniline	-2801.2	-2795.4
2,3,4,5-Tetramethylbenzoic acid	-5819	-5810.7
2,3,4,6-Tetrafluoroaniline	-2811.1	-2795.4
2,3,4,6-Tetramethylbenzoic acid	-5825.8	-5810.7
2,3,4,6-Tetranitroaniline	-2742.5	-2697.7
2,3,4-Trichloroaniline	-2939.3	-2912.7
2,3,4-Trifluoroaniline	-2955	-2939.2
2,3,4-Trimethylbenzoic acid	-5167.25	-5167.8
2,3,4-Trimethylpentane	-5469.36	-5465.2
2,3,5,6-Tetrabromo-p-xylene	-3920.7	-3946.3
2,3,5,6-Tetrachloronitrobenzene	-2531.5	-2485.4
2,3,5,6-tetrachloro-p-xylene	-3926.2	-3939.3
2,3,5,6-Tetrafluoroaniline	-2797.7	-2795.4
2,3,5,6-Tetramethylbenzoic acid	-5823.5	-5810.7
2,3,5,6-Tetramethylpyrazine N,N'-dioxide	-4756.6	-4778.4
2,3,5-Trichlorohydroquinone	-2418.7	-2389
2,3,5-Trichloropyridine	-2315.6	-2311.2
2,3,5-Trimethylbenzoic acid	-5165.15	-5167.8
2,3,5-Trimethylhexane	-6115.8	-6116
2,3,5-Trimethylpyrazine	-4204.1	-4185.1
2,3,5-Trimethylpyrazine N,N'-dioxide	-4113.2	-4130.6
2,3,5-Trinitrotoluene	-3447	-3402.6
2,3,6,7-Tetrachloroquinoxaline	-3564.1	-3566.9
2,3,6-Trifluoroaniline	-2940.2	-2939.2

2,3,6-Trimethylbenzoic acid	-5174.5	-5167.8
2,3,6-Tris(phenylimino)-1,3,5-triphenyl-1,3,5-triazacyclohexane	-20256	-20244.6
2,3-5,6-Dibenzoxalene	-7795.6	-7824.3
2,3-Bis(difluoroamino)-2-methylbutane	-3848.7	-3846.6
2,3-Butandione	-2066.1	-2033
2,3-Butyleneglycol	-2463.7	-2469.1
2,3-Dibromobutane	-2536.39	-2547.4
2,3-Dichloro-1-propanol	-1704	-1699.3
2,3-Dichloroaniline	-3074.7	-3065.3
2,3-Dichlorobutane	-2523.9	-2532.9
2,3-Dichlorohydroquinone	-2563.8	-2541.6
2,3-Dichlorophenol	-2756.8	-2750.2
2,3-Dichloropropene	-1726.2	-1738.3
2,3-Dichloropyridine	-2459.5	-2463.8
2,3-Dichloroquinoxaline	-3878.1	-3872.1
2,3-Difluoroaniline	-3092.8	-3083
2,3-Difluorophenol	-2761.6	-2767.9
2,3-Dihydro-1,1,4,6-tetramethyl-1H-indene	-7556.3	-7560.3
2,3-Dihydro-1H-indole	-4492.4	-4459.1
2,3-Dihydrobenzofuran	-4191.6	-4175.4
2,3-Dihydrofuran	-2328.5	-2284.3
2,3-Dihydrothiophene	-3086.8	-3044.8
2,3-Dihydrothiophene 1,1-dioxide	-2710.4	-2690.4
2,3-Dihydroxyanisole	-3387.7	-3360.2
2,3-Dihydroxybenzoic acid	-2828.9	-2821.6
2,3-Dihydroxypyridine	-2309.1	-2304.6
2,3-Dihydroxytoluene	-3505.4	-3489.8
2,3-Dimethoxy-2,3-diphenylsuccinnonitrile	-9393.2	-9352.6
2,3-Dimethoxybenzaldehyde	-4557.4	-4549.2
2,3-Dimethoxybenzoic acid	-4283.8	-4265.2
2,3-Dimethoxyphenol	-4115.09	-4082
2,3-Dimethyl-1,3-butadiene	-3807.14	-3822.9
2,3-Dimethyl-1-butene	-3985.56	-3989.6
2,3-Dimethyl-1-hexene	-5302.35	-5299.1
2,3-Dimethyl-1-pentene	-4639	-4648.3
2,3-Dimethyl-2,3-bis(4-t-butylphenyl)butane	-15360	-15387.9
2,3-Dimethyl-2,3-bis(phenylazo)butane	-10589	-10623.7
2,3-Dimethyl-2,3-dinitrobutane	-3765	-3760.8
2,3-Dimethyl-2-butanethiol	-4777	-4773.8
2,3-Dimethylbutane	-4157.38	-4161.5
2,3-Dimethylhexane	-5471.78	-5465.9
2,3-Dimethylindole	-5515.04	-5520.9

2,3-Dimethyloctane	-6779.65	-6772.8
2,3-Dimethylpentane	-4811.49	-4809.8
2,3-Dimethylpyrazine	-3577.8	-3550.6
2,3-Dimethylpyrazine N,N'-dioxide	-3481.7	-3482.8
2,3-Dimethylquinoxaline	-5451.3	-5453.8
2,3-Dimethylquinoxaline 1,4-dioxide	-5389.3	-5404.6
2,3-Dinitroaniline	-3064	-3034.1
2,3-Diphenylbutane	-8933	-8878.6
2,3-Diphenylsuccinic acid	-7556.8	-7536.2
2,3-Lutidine	-4062.9	-4050.2
2,3-Naphthalenediol	-4761.9	-4744.7
2,3-Pentadiene	-3246	-3208.2
2,3-Pyridinedicarboxylic acid	-2736	-2714.4
2,3-Xylenol	-4339.19	-4341.3
2,4,4,6,6-Pentamethyl-1,3-dioxane	-5554.5	-5551.6
2,4,4-Trimethyl-2-nitropentane	-5273.6	-5261.1
2,4,4-Trimethyl-2-pentene	-5296	-5285
2,4,4-Trimethylhexane	-6121.4	-6121.5
2,4,5,6-Tetrachloropyrimidine	-1672.2	-1644.9
2,4,5,7-Tetramethyl-4,5-bis(4-t-butylphenyl)octane	-19301	-19304.7
2,4,5,7-Tetramethylphenanthrene	-9672.8	-9652.6
2,4,5-Trichloroaniline	-2942.2	-2912.7
2,4,5-Trifluoroaniline	-2924.2	-2939.2
2,4,5-Trimethylacetophenone	-6077.3	-6080.3
2,4,5-Trimethylaniline	-5318.7	-5299.3
2,4,5-Trinitrotoluene	-3454	-3402.6
2,4,6,-Trinitro-1,3,5,-tris(methylnitramino)benzene	-4945.9	-4953.7
2,4,6-Collidine	-4689.1	-4684.7
2,4,6-Cycloheptatrien-1-one	-3602	-3622.8
2,4,6-Triaminopyrimidine	-2522.3	-2510.7
2,4,6-Triazido-s-triazine	-2234.3	-2221
2,4,6-Tribromoaniline	-2917.7	-2917.9
2,4,6-Tribromo-m-cresol	-3275.7	-3245.7
2,4,6-Tribromophenol	-2626.9	-2602.8
2,4,6-Trichloroaniline	-2923.3	-2912.7
2,4,6-Trichloronitrobenzene	-2659.2	-2638
2,4,6-Trichloropyrimidine	-1818	-1797.6
2,4,6-Triethoxy-1,3,5-triazine	-5100.3	-5122.7
2,4,6-Trifluoroaniline	-2919.1	-2939.2
2,4,6-Triisopropylphenol	-8942.5	-8904.2
2,4,6-Trimethoxybenzonitrile	-5150	-5167.4
2,4,6-Trimethoxybenzonitrile N-oxide	-5187.1	-5193.8

2,4,6-Trimethoxy-s-triazine	-3168.7	-3198
2,4,6-Trimethyl-1,3-Dioxane	-4267.1	-4267.9
2,4,6-Trimethylbenzonitrile	-5530.7	-5556.4
2,4,6-Trimethylbenzonitrile N-oxide	-5563.1	-5582.8
2,4,6-Trinitroanisol	-3284.1	-3273
2,4,6-Trinitrobenzaldehyde	-3052.6	-3018.4
2,4,6-Trinitrobenzoic acid	-2773.7	-2734.7
2,4,6-Trinitro-N-methylaniline	-3562.8	-3547.2
2,4,6-Trinitrophenetol	-3956.5	-3914.5
2,4,6-Trinitrophenol	-2570	-2550.9
2,4,6-Trinitrophenylhydrazine	-3112.1	-3108.6
2,4,6-Trinitroresorcinol	-2356.9	-2342.1
2,4,6-Triphenoxy-s-triazine	-10289	-10263.6
2,4,6-Triphenylpyridine	-11757	-11766.2
2,4,6-Triphenyltriazine	-10717.5	-10713.3
2,4,6-Tri-t-butylnitrobenzene	-10926.4	-10888.2
2,4,6-Tri-t-butylnitrosobenzene	-11019	-10997.3
2,4,6-Tri-t-butylphenol	-10870	-10851.7
2,4'-Bipyridine	-5274.7	-5276.6
2,4-Diaminoazobenzene	-6717.4	-6709.4
2,4-Diaminopyrimidine	-2433.7	-2427
2,4-Diazidopyrimidine	-2564	-2590.7
2,4-Dibromo-6-methylphenol	-3412.3	-3396.6
2,4-Dibromoaniline	-3058	-3068.8
2,4-Dibromophenol	-2747.8	-2753.7
2,4-dichloro-1-trifluoromethylbenzene	-3048.74	-3078.8
2,4-Dichloro-5-methylpyrimidine	-2602.1	-2596.8
2,4-Dichloro-6-(ethylamino)-s-triazine	-2833.5	-2841.2
2,4-Dichloro-6-methylpyrimidine	-2575.8	-2588.4
2',4'-Dichloroacetophenone	-3864.7	-3843.6
2,4-Dichloroaniline	-3070.4	-3065.3
2,4-Dichloroanisole	-3474.9	-3472
2,4-Dichloronitrobenzene	-2789.7	-2790.6
2,4-Dichlorophenol	-2753.7	-2750.2
2,4-Dichloropyrimidine	-1956.2	-1953.9
2,4-Difluorophenol	-2761.9	-2767.9
2,4-Dihydroxyacetophenone	-3720.77	-3734.1
2,4-Dihydroxybenzophenone	-6052	-6092
2,4-Diisopropylphenol	-6979	-6951.1
2,4-Dimethoxy-6-(2,2-dinitro-2-fluoroethoxy)-1,3,5-triazine	-3380.2	-3389.3
2',4'-Dimethoxyacetophenone	-5163.5	-5175.4
2,4-Dimethoxybenzaldehyde	-4530.2	-4549.2

2,4-Dimethoxybenzoic acid	-4258.6	-4265.2
2,4-Dimethoxyphenol	-4084.7	-4082
2,4-Dimethyl-1-pentene	-4641.6	-4640.4
2,4-Dimethyl-2-pentene	-4635.5	-4626.9
2,4-Dimethyl-3-ethylpentane	-6130.1	-6125.2
2,4-Dimethyl-3-pentanone	-4405.64	-4398.3
2,4-Dimethyl-5-oxo-1H-pyrrol-3-carboxylic acid ethyl ester	-4714.9	-4719.1
2,4-Dimethylacetanilide	-5505.76	-5526.4
2,4-Dimethylbenzoic acid	-4512.3	-4524.8
2,4-Dimethylcyclohexene	-5002.5	-5033
2,4-Dimethylhexane	-5467.4	-5471.1
2,4-Dimethyloctane	-6776	-6778
2,4-Dimethylpentane	-4809.98	-4809.8
2,4-Dimethylphenylacetic acid	-5158.05	-5178.3
2,4-Dimethylpyrrole	-3632.2	-3616.8
2,4-Dinitro-1,3-dimethylbenzene	-4205.8	-4213.7
2,4-Dinitro-1-naphthol	-4611.2	-4616.8
2,4-Dinitro-2,4-diazapentane	-2273.9	-2285
2,4-Dinitroaniline	-3010	-3034.1
2,4-Dinitroanisol	-3466.6	-3441.1
2,4-Dinitro-N-methylaniline	-3705	-3715
2,4-Dinitrophenetole	-4065.9	-4082.6
2,4-Dinitrophenol	-2720	-2719.3
2,4-Dinitrophenoxyethanol	-3895.3	-3898.7
2,4-Dinitrophenylhydrazine	-3272.6	-3279
2,4-Dinitrophenylmethylnitramine	-3628.2	-3659.1
2,4-Dinitroresorcinol	-2511.9	-2510.2
2,4-Dinitrosobenzene-1,3-diol	-2699.7	-2723.5
2,4-Dinitrotoluene	-3586	-3570.7
2,4-Di-t-butylphenol	-8255	-8253.4
2,4-Hexadiyne	-3553.7	-3560.6
2,4-Hexanedione	-3281	-3327.9
2,4-Imidazolidinedione	-1303.6	-1299.2
2,4-Lutidine	-4059.6	-4050.2
2,4-Pentanediol	-3128.64	-3122.3
2,4-Toluene diisocyanate	-4235	-4237
2,4-Xylenol	-4348.4	-4341.3
2,4-Xylidine	-4681.2	-4656.4
2,5,8-Trimethyltetralin	-7552.5	-7553.3
2,5-Aldehydine	-4708.7	-4706.3
2,5-Bis(azidomethyl)-3-nitrooxazolidine	-3616.1	-3602
2,5-Diamino-1,3,4-thiadiazole	-1956.5	-1980

2,5-Dibromoaniline	-3064	-3068.8
2,5-Dibromonitrobenzene	-2791.78	-2794.1
2,5-Dibromopyridine	-2460.4	-2462.3
2,5-Dichloro-1,4-benzoquinone	-2443	-2447.2
2,5-Dichloro-4-nitroaniline	-2894.4	-2897.2
2',5'-Dichloroacetophenone	-3860.5	-3843.6
2,5-Dichloroaniline	-3061.2	-3065.3
2,5-Dichlorohydroquinone	-2547	-2541.6
2,5-Dichlorophenol	-2748.1	-2750.2
2,5-Dichloropyridine	-2447.8	-2463.8
2,5-Dichlorostyrene	-4088.3	-4096.5
2,5-Difluoroaniline	-3084.3	-3083
2,5-Difluoronitrobenzene	-2801.1	-2808.3
2,5-Difluorophenol	-2745.8	-2767.9
2,5-Dihydro-3-methylthiophene 1,1-dioxide	-3357.4	-3331.9
2,5-Dihydrofuran	-2366.3	-2316.4
2,5-Dihydroperoxy-2,5-dimethylhex-3-yne	-4856.8	-4881.8
2,5-Dihydrothiophene	-3081.1	-3072.9
2,5-Dihydroxybenzoic acid	-2825.7	-2821.6
2,5-Dihydroxytoluene	-3492	-3489.8
2,5-Diisopropylphenol	-6966	-6953.8
2',5'-Dimethoxyacetophenone	-5194.5	-5178
2,5-Dimethoxybenzaldehyde	-4539.56	-4549.2
2,5-Dimethoxybenzoic acid	-4276	-4265.2
2,5-Dimethyl-1-phenylpyrrole	-6667.91	-6673.1
2,5-Dimethyl-2,4-hexadiene	-5089.13	-5110.2
2,5-Dimethyl-2,5-hexanediol	-5039	-5062.3
2,5-Dimethyl-2H-tetrazole	-2240.5	-2264.1
2,5-Dimethyl-2-hydroperoxide-5-tert-butylperoxyhexane	-7807.5	-7828.7
2,5-Dimethyl-2-t-butylperoxy-5-trimethylsilylperoxyhexyne	-10576	-10537.7
2,5-Dimethyl-3-furancarboxylic acid	-3297.5	-3316.8
2,5-Dimethyl-3-hexyne-2,5-diol	-4763.2	-4755.9
2,5-Dimethyl-5-t-butylperoxy-2-hexanol	-7746.6	-7765.8
2,5-Dimethylbenzothiazole	-5465.3	-5467.3
2,5-Dimethyldiphenylmethane	-8213.9	-8204.1
2,5-Dimethylhexane	-5464	-5468.5
2,5-Dimethylhexane-2,5-dihydroperoxide	-5157.2	-5188.2
2,5-Dimethyloctane	-6775.54	-6770.2
2,5-Dimethylphenylacetonitrile	-5563	-5567
2,5-Dimethylpyrazine	-3568.9	-3550.6
2,5-Dimethylpyrazine N,N'-dioxide	-3481.1	-3482.8
2,5-Dimethylpyrrole	-3630.5	-3617.7

2,5-Dimethylpyrrole-3-carboxylic ethylester	-4924.39	-4955.2
2,5-Dimethylthiophene	-4116.8	-4096
2,5-Dinitroaniline	-3031	-3034.1
2,5-Dinitrotoluene	-3578	-3570.7
2,5-Diphenyl-1,3,4-oxadiazole	-7103.6	-7082.5
2,5-Diphenylpyridine	-8735.7	-8773.8
2,5-Diphenyltetrazole	-6939.8	-6908.6
2,5-Di-t-butylphenol	-8276	-8254.7
2,5-Furandione	-1389.5	-1366.4
2,5-Lutidine	-4062.2	-4050.2
2,5-Pyrrolidinedione	-1829.6	-1845.2
2,5-Xylenol	-4330.6	-4341.3
2,5-Xylidine	-4684.4	-4656.4
2,6-Diaminopyridine	-2963.8	-2940.2
2,6-Dibromoaniline	-3061	-3068.8
2,6-Dibromophenol	-2756.2	-2753.7
2,6-Dibromopyridine	-2449.6	-2453.6
2,6-Dichloro-4-nitroaniline	-2872.7	-2897.2
2,6-Dichloroaniline	-3068.9	-3065.3
2,6-Dichloroanisole	-3493.9	-3472
2,6-Dichlorobenzoquinone	-2449.7	-2447.2
2,6-Dichlorohydroquinone	-2551	-2541.6
2,6-Dichlorophenol	-2757.73	-2750.2
2,6-Dichloropyrazine	-1981.6	-1968.9
2,6-Dichloropyridine	-2442.9	-2460.1
2,6-Diethylaniline	-5998.66	-5968.6
2,6-Diethylnaphthalene	-7733.9	-7759.8
2,6-Difluoroaniline	-3086.4	-3083
2,6-Difluorophenol	-2742.5	-2767.9
2,6-Dihydroxybenzoic acid	-2827.2	-2821.4
2,6-Diisopropylnaphthalene	-9032.4	-9060.1
2,6-Diisopropylphenol	-6962	-6953.8
2',6'-Dimethoxyacetophenone	-5185.5	-5175.4
2,6-Dimethoxybenzaldehyde	-4542.8	-4549.2
2,6-Dimethoxybenzoic acid	-4276.9	-4265.2
2,6-Dimethoxyphenol	-4061.89	-4082
2,6-Dimethyl-4-heptanol	-5934.63	-5927.2
2,6-Dimethyl-4-pyrone	-3549.6	-3580
2,6-Dimethylbenzoic acid	-4530.1	-4524.8
2,6-Dimethylbenzonitrile	-4890.1	-4913.5
2,6-Dimethylcyclohexanol	-5044.2	-5027.6
2,6-Dimethylcyclohexanone	-4765.6	-4812.2

2,6-Dimethylheptan-4-one	-5709.65	-5705.2
2,6-Dimethylheptane	-6118.05	-6116.7
2,6-dimethylheptane-3,5-dione	-5301.4	-5300.3
2,6-Dimethylnaphthalene	-6431.4	-6447.7
2,6-Dimethyloctane	-6776.01	-6772.8
2,6-Dimethylpiperidine	-4744.7	-4747.6
2,6-Dimethylquinoline	-5937.1	-5950.7
2,6-Dinitro-4-methylphenol	-3372.8	-3361.9
2,6-Dinitroaniline	-3025	-3033.9
2,6-Dinitroanisol	-3425.37	-3441.1
2,6-Dinitrophenol	-2723.1	-2719
2,6-Dinitrotoluene	-3574	-3570.7
2,6-Diphenylpyridine	-8746.4	-8762.8
2,6-Di-t-butyl-4-phenylphenol	-11280	-11272.8
2,6-Di-t-butylnaphthalene	-10351	-10363.7
2,6-Di-t-butyl-p-cresol	-8916	-8921.3
2,6-Di-t-butylphenol	-8280	-8266.8
2,6-Lutidine	-4053.5	-4041.8
2,6-Naphthalenedicarboxylic acid	-5095.1	-5111.8
2,6-Pyridinedicarboxylic acid	-2724	-2708.6
2,6-Xylenol	-4339.9	-4341.3
2,7-Dibromofluorene	-6329.7	-6357.9
2,7-Dihydroxynaphthalene	-4752.3	-4744.7
2,7-Dimethyl-2,7-octanediol	-6353.96	-6369.3
2,7-Dimethylbenzo[1,2-d,5,4-d]bisoxazole	-4870.9	-4870.6
2,7-Dimethylnaphthalene	-6431.6	-6447.7
2,7-Dimethyloctane	-6747.1	-6770.2
2,7-Dimethylphenanthrene	-8333.4	-8348.1
2,7-dimethylquinoline	-5935	-5950.7
2,7-Di-t-butyl-9,9-dimethylxanthene	-12997.19	-13026.4
2,7-Di-t-butylfluorene	-11838	-11861.5
2,9-Dimethyl-2,9-decanediol	-7653.9	-7676.2
2.5-Dichloronitrobenzene	-2789.9	-2790.6
2:3,6:7-Dibenzobicyclo[3.2.2]nona-2,6-dien-4-one	-8616.6	-8608.6
2-Acetoxyanisole	-4522.1	-4471.4
2-Acetoxycinnamic acid	-5062.2	-5062.7
2-Acetoxynaphthalene	-5847	-5856
2-Acetyl-1-naphthol	-5828.4	-5840.5
2-Acetyl-3-methylquinoxaline N,N'-dioxide	-5673.9	-5663.9
2-Acetyl-3-methylthiophene	-4351.9	-4348.8
2-Acetyl-4-methylthiophene	-4344.5	-4348.8
2-Acetyl-5-methylthiophene	-4341.9	-4338

2-Acetylbenzofuran	-4838.8	-4850.2
2-Acetylfuran	-2958.1	-2961.7
2-Acetylpyrrole	-3197.9	-3216.7
2-Acetylthiophene	-3702.5	-3706.8
2-Adamantanol	-5838	-5847.6
2-Adamantanone	-5625	-5625.7
2-Allyl-5-aminotetrazole	-2859.2	-2854.1
2-Amino-1,3,4-triazole	-1435.5	-1445.4
2-Amino-4,6-bis(2,2-dinitro-2-fluoroethoxy)-1,3,5-triazine	-3197.8	-3173.1
2-Amino-4,6-dimethylpyrimidine	-3613.9	-3612.3
2-Amino-4,6-dinitrophenol	-2834	-2825.6
2-Amino-4-methylbenzothiazole	-4896.9	-4886.8
2-Amino-4-methylpyrimidine	-2977.6	-2977.8
2-Amino-4-nitroanisole	-3666	-3715.7
2-Amino-5-nitroanisole	-3701	-3715.7
2-Amino-5-nitropyridine	-2673.1	-2688.4
2-Amino-6-chlorobenzothiazole	-4090.5	-4091.2
2-Amino-6-methylbenzothiazole	-4906.9	-4886.8
2-Aminoacridine	-6715.55	-6680.4
2-Aminobenzamide	-3678	-3674.8
2-Aminobenzenesulfonamide	-3749.4	-3746.5
2-Aminobenzimidazole	-3780.6	-3807.2
2-Aminobenzothiazole	-4273.6	-4243.8
2-Aminobenzoxazole	-3516.9	-3536.1
2-Aminobutyric acid	-2254	-2279.9
2-aminodiphenyl ether	-6238.7	-6241.6
2-Aminofluorene	-6768.9	-6766.3
2-Aminophenol	-3162.41	-3162
2-Aminopyridine	-2864.4	-2856.5
2-Anisaldehyde	-4025	-4035.9
2-Azidoethanol	-1597.2	-1569.2
2-Azidomethyl-3-nitrooxazolidine	-2756.2	-2753.9
2-Benzimidazolethiol	-4262.6	-4271.9
2-Benzoyloxynaphthalene	-8219.9	-8213.8
2-Benzylbenzimidazole	-7327.1	-7371.7
2-Biphenyl methanol	-6727.3	-6743.7
2-Bromoacetophenone	-4014.6	-3998
2-Bromoaniline	-3224	-3219.7
2-Bromoanisole	-3631.5	-3626.4
2-Bromobenzoic acid	-3092.37	-3088.1
2-Bromobenzonitrile	-3469.5	-3476.8
2-Bromobutane	-2705.2	-2701.7

2-Bromofluorene	-6490.7	-6508.8
2-Bromonaphthalene	-5029.9	-5010.9
2-Bromonitrobenzene	-2939.99	-2945
2-Bromophenol	-2920.1	-2904.6
2-Bromopropane	-2052	-2048.2
2-Bromopyridine	-2623.2	-2613.2
2-Butanethiol	-3476.58	-3470.9
2-Butanol	-2662.4	-2665.1
2-Butanone	-2445.8	-2444.5
2-Butene-1-imine	-2681.36	-2663.7
2-Butenyl p-tolyl sulphone	-6583.4	-6598.4
2-Butyl-1-decanol	-9235.54	-9211.3
2-Butyl-1-nonanol	-8583.24	-8557.8
2-Butyl-1-octanol	-7909.7	-7904.3
2-Butylamine	-3010.6	-3010.1
2-Butyldiphenylmethane	-9519	-9524.2
2-Butyloctanoic acid	-7395.8	-7412.5
2-Butylthiophene	-5424.5	-5425.2
2-Butyne	-2576.8	-2554.8
2-Carbamoyl-3-methylquinoxaline N-oxide	-5106.8	-5088.2
2-Carboxyadamantane	-5992.47	-6019.4
2-Carboxypyridine	-2735.2	-2739.4
2-Carboxypyridine N-oxide	-2713.4	-2705.2
2-Carene	-6199.1	-6195.2
2-Chloro-1,1,1,2-tetrafluoroethane	-722.96	-721.9
2-Chloro-1,1,1-trifluoroethane	-875.24	-868.4
2-Chloro-1,1-difluoroethylene	-971.4	-964
2-Chloro-1,3-propanediol	-1687.47	-1673
2-Chloro-1-ethylbenzene	-4403.2	-4410.4
2-Chloro-2,3-dimethylbutane	-3982.4	-3987.7
2-Chloro-2,4-dimethylpentane	-4647.1	-4639.9
2-Chloro-2,5-cyclohexadiene-1,4-dione	-2592.8	-2592.5
2-Chloro-2-methylbutane	-3327.5	-3334.9
2-Chloro-2-methylpentane	-3980.4	-3988.4
2-Chloro-2-methylpropane	-2692.8	-2678.8
2-Chloro-3-hydroxypyridine	-2396.8	-2407.9
2-Chloro-4,6-dinitroaniline	-2855.3	-2881.5
2-Chloro-4-nitroaniline	-3024.4	-3049.8
2-Chloro-5-nitroaniline	-3028.2	-3049.8
2-Chloro-6-(trichloromethyl)pyridine	-2830.8	-2848.3
2-Chloroacetophenone	-4012.6	-3996.2
2-Chloroaniline	-3237.5	-3217.9

2-Chloroanisole	-3624.5	-3624.6
2-Chlorobenzaldehyde	-3374.4	-3370
2-Chlorobenzenesulfonamide	-3526.7	-3487.3
2-Chlorobenzoic acid	-3087.7	-3086.4
2-Chlorobenzonitrile	-3472.1	-3475
2-Chlorobenzylidene-2-methylphenylacetonitrile	-8229	-8247.4
2-Chlorobiphenyl	-6072	-6112.1
2-Chlorobutane	-2685.4	-2694.4
2-Chlorobutanoic acid	-2022.5	-2026.5
2-Chlorodibenzo-p-dioxin	-5574.8	-5584.3
2-Chloroethanol	-1191.4	-1207.4
2-Chloroethyl ethyl ether	-2549.16	-2571.5
2-Chloroethyl vinyl ether	-2390	-2378.1
2-Chloronaphthalene	-5014.5	-5009.2
2-Chloronitrobenzene	-2937.6	-2943.3
2-Chlorophenol	-2927.92	-2902.8
2-Chlorophenyl isocyanate	-3272	-3276.4
2-Chlorophenylacetic acid	-3723.9	-3739.8
2-Chloropropane	-2028.4	-2041
2-Chloropropanoic acid	-1393	-1373
2-Chloropropene	-1897.95	-1896
2-Chloropyrazine	-2149.4	-2125.3
2-Chloropyridine	-2616.3	-2616.5
2-Chloropyrimidine	-2111.4	-2110.2
2-Chloroquinoline	-4492.5	-4517
2-Chlorotoluene	-3750	-3754.3
2-Cyano-2-nitroadamantane	-6238.4	-6206.4
2-Cyanoadamantane	-6389.3	-6408.1
2-Cyanobenzoic acid	-3597.4	-3602.7
2-Cyanobiphenyl	-6634.6	-6631.1
2-Cyanobutane	-3213.8	-3226.9
2-Cyclohexene-1-one	-3335.82	-3329.3
2-Cyclohexylcyclohexanone	-7189.49	-7184
2-Cyclohexylidenecyclohexanone	-6987.7	-6996.7
2-Cyclopenten-1-one	-2674.5	-2685.2
2-Cyclopropyl-1-butene	-4555.8	-4509.3
2-Cyclopropyl-1-pentene	-5123	-5162.8
2-Cyclopropyl-2-butene	-4534.8	-4489.2
2-Cyclopropyl-2-hexene	-5816.2	-5794.9
2-Cyclopropyl-2-pentene	-5143.9	-5138.8
2-Cyclopropyl-3-methyl-1-butene	-5164.9	-5159.4
2-Decyne	-6484.1	-6475.7

2-Diethylaminoethanol	-4198.3	-4192
2-Dodecanone	-7676	-7672.3
2-Ethoxy-4,4,5,5-tetramethyl-1,3-dioxolane	-5390.6	-5403.9
2-Ethoxycarbonyl-3-methylquinoxaline 1,4-dioxide	-6125.7	-6100
2-Ethoxycarbonylaniline	-4724.5	-4708.7
2-Ethoxycinnamic acid	-5514.1	-5523.4
2-Ethoxyethanol	-2556.98	-2545.3
2-Ethoxyethylacetate	-3415.76	-3424.7
2-Ethoxynaphthalene	-6303.1	-6316.6
2-Ethyl-1-butanol	-3982.37	-3983.5
2-Ethyl-1-butene	-3991.65	-3995.5
2-Ethyl-1-hexanoic acid	-4803.25	-4803.9
2-Ethyl-1-hexanol	-5287.7	-5287.8
2-Ethyl-1-pentene	-4646.76	-4647.7
2-Ethyl-2-hexenal	-4907.73	-4894.2
2-Ethyl-2-methyl-1,3-dioxolane	-3626.2	-3629
2-Ethyl-2-nitro-1,3-propanediol	-2938	-2936.5
2-Ethyl-3-hydroxy-4-pyrone	-3385.6	-3364.6
2-Ethyl-3-methyl-1-butene	-4644.5	-4645.7
2-Ethylacrolein	-2946.6	-2955.2
2-Ethylanthracene	-8357.3	-8356
2-Ethylbenzoic acid	-4529.4	-4538
2-Ethylbiphenyl	-7585.2	-7566.4
2-Ethyldiphenylmethane	-8217	-8217.3
2-Ethylhexanal	-5089.85	-5084.9
2-Ethylhexyl acetate	-6169.95	-6169.9
2-Ethylimidazole	-3089.6	-3079.6
2-Ethyl-m-xylene	-5859.76	-5848.8
2-Ethylnaphthalene	-6500	-6460.8
2-Ethylnitrobenzene	-4388	-4394.9
2-Ethyloxazoline	-3060.6	-3027.5
2-Ethylpiperidine	-4740.3	-4753.2
2-Ethyl-p-xylene	-5855.07	-5848.8
2-Ethylpyridine	-4042.3	-4060.8
2-Ethylthioethanol	-3317.12	-3301.7
2-Ethylthiophene	-4125.71	-4118.3
2-Ethyltoluene	-5213.68	-5205.9
2-Fluorenylaldehyde	-6892.7	-6918.4
2-Fluoroaniline	-3238.5	-3226.8
2-Fluoroanisole	-3632.2	-3633.5
2-Fluorobenzoic acid	-3097	-3095.2
2-Fluorobenzonitrile	-3473.2	-3483.9

2-Fluoroethanol	-1210.53	-1217.5
2-Fluoronitrobenzene	-2949.9	-2952.1
2-Fluorophenol	-2908	-2911.7
2-Fluoropropane	-2062.53	-2035.3
2-Fluorotoluene	-3779	-3763.1
2-Formylbenzoic acid	-3476.71	-3497.7
2-Formylimidazole	-2044.9	-2041.9
2-Formylpyrrole	-2575.7	-2590.5
2-Furanacrolein	-3430	-3459.8
2-Furanacrylic acid	-3192.5	-3178.7
2-Furanacrylonitrile	-3562.5	-3564.8
2-Furancarbonitrile	-2458.3	-2437.8
2-Furancarboxylic acid hydrazide	-2619.5	-2618.3
2-Furoic acid	-2042.3	-2049.2
2-Heptanone	-4410.36	-4404.9
2-Heptylhydroperoxide	-4698.2	-4685.9
2-Heptyne	-4520.7	-4515.2
2-Hexanol	-3972.78	-3969.5
2-Hexyldecanoic acid	-10065	-10026.4
2-Hexylhydroperoxide	-4054.7	-4032.4
2-Hexylthiophene	-6735.6	-6732.2
2-Hydroxy-4-methylquinoline	-5032.2	-5056.6
2-Hydroxy-6-methylpyridine	-3149.2	-3147.7
2-Hydroxyacetophenone	-3941.77	-3942.7
2-Hydroxybenzalaniline N-oxide	-6629.56	-6634.2
2-Hydroxybenzaldehyde	-3331.27	-3313.9
2-Hydroxybenzamide	-3354.78	-3359.7
2-Hydroxybenzonitrile	-3415	-3419.1
2-Hydroxybiphenyl	-6063	-6058.9
2-Hydroxydiphenylether	-5930.2	-5926.5
2-Hydroxydiphenylmethane	-6761	-6709.7
2-Hydroxyethylacrylate	-2553.08	-2552.8
2-Hydroxyethylmethacrylate	-3165.14	-3194.8
2-Hydroxymethyl-1,3-dioxane	-2773.6	-2796
2-Hydroxymethyl-1,3-propanediol	-2260.6	-2300.5
2-Hydroxynicotinic acid	-2494.6	-2487.9
2-Hydroxyphenazine di-N-oxide	-5792.66	-5822.2
2-Hydroxypropylacrylate	-3210.24	-3196.7
2-Hydroxyquinoline	-4397.1	-4413.7
2-Imidazolidinone	-1764.7	-1765.7
2-Indanol	-4772.6	-4781.7
2-Iodoaniline	-3306.4	-3292.8

2-Iodoanisole	-3704.8	-3699.5
2-Iodobenzoic acid	-3167	-3161.3
2-Iodobenzonitrile	-3554.4	-3549.9
2-Iodonaphthalene	-5082.09	-5084.1
2-Iodonitrobenzene	-3020.6	-3018.2
2-Iodophenol	-2979.8	-2977.7
2-Iodopropane	-2106.58	-2123.8
2-Iodotoluene	-3833.4	-3829.2
2-Isopropyl-5-methylphenol	-5657	-5644.9
2-Isopropyl-6-tert-butylphenol	-7644	-7602.9
2-Isopropylbenzimidazole	-5618.5	-5664
2-Isopropylbenzoic acid	-5189.4	-5185.5
2-Isopropyl-p-cresol	-5661	-5647.5
2-Isopropylthiophene	-4773.7	-4768.5
2-Isopropyltoluene	-5862.6	-5858.7
2-Mercapto-5-methoxybenzimidazole	-4768.3	-4785.1
2-Mercaptoethanol	-1999.05	-1992.6
2-Methoxy-1,3-dioxolane	-2187.34	-2185.5
2-Methoxy-1-naphthaldehyde	-5942.4	-5936.4
2-Methoxy-1-propanol	-2555.73	-2547.7
2-Methoxy-4,6-bis(2,2-dinitro-2-fluoroethoxy)-1,3,5-triazine	-3599.7	-3580.6
2-Methoxy-5-nitrophenol	-3380.9	-3400.6
2-Methoxyacetophenone	-4672	-4662.1
2-Methoxybenzamide	-4068.7	-4081.8
2-Methoxycarbonyl-3-methylquinoxaline N,N'-dioxide	-5490.8	-5458.5
2-Methoxynaphthalene	-5659.9	-5675.1
2-Methoxy-p-cresol	-4214.4	-4211.6
2-Methoxyphenol	-3574.71	-3568.7
2-Methoxypyridine	-3275.91	-3235
2-Methoxytetrahydropyran	-3633.8	-3645.4
2-Methyl-1,2-propanediamine	-3157.2	-3150.8
2-Methyl-1,2-propanediol	-2464	-2465.6
2-Methyl-1,3-dioxane	-2960.3	-2980
2-Methyl-1,3-dioxolane	-2332.3	-2340.5
2-Methyl-1,3-pentanediol	-3787.28	-3787.1
2-Methyl-1,3-propanediol	-2499.3	-2484.7
2-Methyl-1-butanethiol	-4130.4	-4136.3
2-Methyl-1-butanol	-3328.39	-3324.8
2-Methyl-1-dodecanol	-8570.74	-8552.6
2-Methyl-1-heptene	-5298.5	-5297.2
2-Methyl-1-hexanol	-4640.08	-4631.7
2-Methyl-1-hexene	-4645.96	-4643.7

2-Methyl-1H-indene	-5428.61	-5427.9
2-Methyl-1-naphthoxazole	-5958	-5965.1
2-Methyl-1-nonene	-6609.24	-6604.2
2-Methyl-1-octene	-5953.05	-5950.7
2-Methyl-1-pentene	-3988.8	-3990.3
2-Methyl-1-propanethiol	-3473.5	-3480.2
2-Methyl-1-propanol	-2670.9	-2671.3
2-Methyl-1-tridecanol	-9236.24	-9206
2-Methyl-2-((nitrooxy)methyl)-1,3-propanediol dinitrate	-2811	-2829.8
2-Methyl-2,3,3-trinitrobutane	-2932.7	-2976.7
2-Methyl-2,3,3-trinitropentane	-3642.8	-3627.6
2-Methyl-2-butanamine	-3656.6	-3647.3
2-Methyl-2-butanol	-3303.1	-3305.7
2-Methyl-2-nitro-1,3-propanediol	-2281.8	-2282.5
2-Methyl-2-nitro-1,3-propanediol dinitrate	-2203.2	-2203
2-Methyl-2-nitro-1-phenyl-1-propanol	-5479.3	-5468.6
2-Methyl-2-nitro-1-propanol	-2451.7	-2466.7
2-Methyl-2-nitro-3-phenyl-1-propanol	-5449.1	-5480.7
2-Methyl-2-pentanethiol	-4775.6	-4771.9
2-Methyl-2-pentenal	-3590.94	-3587.3
2-Methyl-2-pentene	-3980.23	-3971.5
2-Methyl-2-propanol	-2644	-2649.6
2-Methyl-2-propene-1-imine	-2677.26	-2673.7
2-Methyl-2-propenyl p-tolyl sulphone	-6583.5	-6605.7
2-Methyl-3-(phenylmethyl)quinoxaline-1,4-dioxide	-8399.5	-8415.9
2-Methyl-3,5-diisopropylphenol	-7607	-7603.2
2-Methyl-3-hydroxypyridine	-3187.9	-3198.8
2-Methyl-4-(2,6,6-trimethyl-2-cyclohexen-1-yl)-2-butenal	-8420.7	-8379.7
2-Methyl-4-quinolinol	-5059	-5099.3
2-Methyl-5-hexen-3-yn-2-ol	-4169.2	-4147.6
2-Methyl-6-nitrobenzoxazole	-3865.8	-3899.2
2-Methyl-6-t-butylaniline	-6649.6	-6626.5
2-Methyl-8-hydroxyquinoline	-5091.5	-5099.3
2-Methyl-8-quinolinol	-5091.5	-5107.7
2-Methylacetanilide	-4874.77	-4883.5
2-Methyladamantane	-6688.99	-6691.3
2-Methylalanine	-2265.9	-2269
2-Methylaminoethanol	-2213.82	-2200.2
2-Methylbenzaldehyde	-4181.09	-4165.6
2-Methylbenzenesulfonamide	-4286.3	-4282.8
2-Methylbenzoic acid	-3874.9	-3881.9
2-Methylbenzoquinone	-3361	-3379.9

2-Methylbenzothiazole	-4829.3	-4824.4
2-Methylbenzyl cyanide	-4921	-4924
2-Methylbenzylalcohol	-4373.59	-4365.9
2-Methylbicyclo[2.2.2]-2-octene	-5484.2	-5441
2-Methylbutanedioic acid	-2175	-2170.3
2-Methylbutyl-2-nitrite	-3291	-3316
2-Methylbutyraldehyde	-3127.47	-3124.5
2-Methylbutyric acid	-2842.2	-2840.8
2-Methylenebicyclo[2.2.1]heptane	-4859	-4852.8
2-Methylene-bicyclo[2.2.2]octane	-5491.7	-5459.8
2-Methylenecyclohexanol	-4194.94	-4208.2
2-Methylfumaric acid	-2000.6	-1984.8
2-Methylheptane	-5465.2	-5469.2
2-Methylhexanal	-4441.09	-4431.5
2-Methylhexane	-4811.4	-4813.1
2-Methylhexanoic acid	-4144.66	-4147.8
2-Methylimidazole	-2432.9	-2426.2
2-Methylindole	-4891.9	-4883.5
2-Methyllactic acid	-1976.2	-1981.7
2-Methylnaphth[2,3-d]oxazole	-5955.9	-5965.1
2-Methylnaphthalene	-5806.46	-5804.7
2-Methylnaphtho[1,2-d]oxazole	-5965.6	-5965.1
2-Methylnaphtho[1,2-d]thiazole	-6720.8	-6722.2
2-Methylnonane	-6769.5	-6776.1
2-Methylnorbornene	-4867.5	-4849.3
2-Methyloctanal	-5747.95	-5738.4
2-Methyloctane	-6123.57	-6120
2-Methyloxazoline	-2404.9	-2374
2-Methylpentane	-4157	-4159.6
2-Methylpiperidine	-4094	-4097.1
2-Methylpropanal	-2468.3	-2468.4
2-Methylpropane	-2868.5	-2852.7
2-Methylpropanoic acid	-2185	-2184.8
2-Methylpropyl 2-pentenoate	-5325.5	-5328.5
2-Methylpropyl 3-Pentenoate	-5326.82	-5328.5
2-Methylpropyl 4-pentenoate	-5343.92	-5347.3
2-Methylpropylamine	-3015.5	-3009.7
2-Methylpyrrole	-2993.24	-2974.8
2-Methylquinoline	-5316.49	-5307.8
2-Methylquinoxaline N,N'-dioxide	-4747.8	-4756.8
2-Methylresorcinol	-3504.3	-3489.8
2-Methyltetrazole	-1640.2	-1625.4

2-Methylthiolane	-3893.6	-3903
2-Methylthiophene	-3474.3	-3464.8
2-Monopalmitin	-11685	-11669.1
2-Naphthaleneacetic acid	-5779.3	-5790.3
2-Naphthalenecarbonitrile	-5513.6	-5525.5
2-Naphthalenecarboxylic acid	-5138	-5139.4
2-Naphthol	-4953.1	-4953.3
2-Naphthylamine	-5279	-5268.4
2-n-Hexoxyethanol	-5164.07	-5159.1
2-Nitro-2-isopropyl-1,3-propanetriol	-3598	-3594.2
2-Nitro-2-propyl-1,3-propanetriol	-3596.1	-3592.3
2-Nitroacetanilide	-4077.5	-4072.2
2-Nitroadamantane	-5841	-5840.4
2-Nitroaniline	-3206.3	-3202.2
2-Nitrobenzaldehyde	-3358.7	-3354.6
2-Nitrobenzamide	-3388	-3397.8
2-Nitrobenzenesulfonamide	-3479.2	-3471.8
2-Nitrobenzoic acid	-3077.7	-3070.6
2-Nitrobutane	-2652.7	-2661.9
2-Nitrocinnamic acid	-4162	-4200.5
2-Nitrocinnamide	-4539.8	-4530
2-nitrodiphenyl ether	-5981.3	-5967
2-Nitrodiphenylamine	-6228	-6237.4
2-Nitroethanol	-1210	-1178.8
2-Nitrofluorene	-6450	-6491.6
2-Nitrofuran	-1899	-1928.9
2-Nitroisobutane	-2630	-2651
2-Nitro-m-xylene	-4383.5	-4381.7
2-Nitrophenol	-2885.1	-2887.1
2-Nitropropane	-1998.8	-2005.8
2-Nitroso-1-naphthol	-4887.8	-4891.4
2-Nitro-t-butylbenzene	-5714	-5691.6
2-Nitrotoluene	-3733.2	-3738.8
2-Nonanone	-5721.35	-5715.8
2-Nonyn-1-ol	-5614.1	-5638.2
2-Nonyne	-5829.7	-5822.2
2-Nonynoic acid	-5159.3	-5154.3
2-Norbornanone	-3967.1	-3980.4
2-Norbornanonedimethylketal	-5400.92	-5399.1
2-Octanone	-5050.1	-5058.4
2-Octylthiophene	-8046	-8039.1
2-Octyn-1-ol	-4993.2	-4984.7

2-Octyne	-5171.63	-5168.7
2-Octynoic acid	-4535.9	-4500.8
2-Oxetanone	-1423.2	-1446.9
2-Oxiranylmethanol	-1740	-1750.6
2-Oxoglutaric acid	-1796.25	-1762.1
2-Pentanol	-3315.4	-3316
2-Pentylthiophene	-6079.5	-6078.7
2-Pentyne	-3241.85	-3208.3
2-Phenyl-1,3-dioxolane	-4703.3	-4684.4
2-Phenyl-1-propanol	-5037.08	-5031.8
2-Phenyl-2,5,5-trimethyl-1,3-dioxane	-7278.74	-7269.6
2-Phenyl-3-methyl-2-butene	-6329.1	-6320.6
2-Phenyl-5,5-dimethyl-1,3-dioxane	-6616.44	-6637.1
2-Phenylaniline	-6388	-6374
2-Phenylbenzimidazole	-6679.8	-6718.3
2-Phenylbenzoic acid	-6200.05	-6239.8
2-Phenylbenzothiazole	-7183.9	-7182.2
2-Phenylbenzoxazole	-6408.7	-6425.2
2-Phenylcarbamoyl-3-methylquinoxaline N,N'-dioxide	-8096.4	-8095.8
2-Phenylcarbamoyl-3-methylquinoxaline N-oxide	-8127.1	-8120.7
2-Phenylglycine	-4005.1	-3984.3
2-Phenylhex-2-enenitrile	-6759.8	-6706.2
2-Phenylindole	-7221.3	-7236.7
2-Phenylnaphthalene	-8151.3	-8167.8
2-Phenylpent-2-enenitrile	-6103.7	-6052.7
2-Phenylpyrrole	-5360.5	-5332.6
2-Phenylsuccinic acid	-4523.3	-4528.2
2-Phenyltoluene	-6917.1	-6907.7
2-Picoline	-3418.2	-3407.3
2-Picoline N-oxide	-3361.9	-3373.4
2-Piperidineethanol	-4566.2	-4573.1
2-Piperidinemethanol	-3890.7	-3913.1
2-Piperidone	-2947.2	-2951.2
2-Propanethiol	-2821.89	-2817.4
2-Propanol	-2007.3	-2009.1
2-Propylpentanoic acid	-4792.4	-4803.9
2-Propylthiophene	-4779.94	-4771.8
2-Propyltoluene	-5867.41	-5856.8
2-Pyrazinecarboxylic acid	-2268	-2248.2
2-Pyridinealdoxime	-3297.1	-3285.6
2-Pyridinecarbonitrile	-3142.7	-3128.1
2-Pyridone	-2517.62	-2557.4

2-Pyrrolidone	-2309.7	-2311.7
2-Quinolinecarbonitrile	-5042.29	-5028.6
2-Stearoylglycerol	-12944	-12976
2-t-Butoxycarbonyl-3-methylquinoxaline N-oxide	-7408.3	-7409.2
2-t-Butyl-4-methylphenol	-6312.8	-6294
2-t-Butyl-5-methylphenol	-6314.8	-6296.7
2-t-Butyl-6-phenylphenol	-8704.4	-8671.9
2-t-Butylbenzoic acid	-5857.4	-5839.9
2-t-Butylnaphthalene	-7769.5	-7760.1
2-t-Butylperoxyethanol	-3853.1	-3884.6
2-t-Butylphenol	-5660.1	-5651.1
2-Tetralone	-5216.1	-5203.2
2-Thiaadamantane	-6000.8	-5994.8
2-Thiocresol	-4547.08	-4515.4
2-Thiopheneacetamide	-3766.2	-3777.3
2-Thiopheneacetic acid	-3457.3	-3450.4
2-Thiopheneacetonitrile	-3845.2	-3839
2-Thiophenecarbaldehyde	-3079.2	-3080.6
2-Thiophenecarbonitrile	-3196.7	-3185.6
2-Thiophenecarboxamide	-3113	-3123.8
2-Thiophenecarboxylic acid	-2790.8	-2799.5
2-Thiophenecarboxylic acid hydrazide	-3363.1	-3366.1
2-Thiouracil	-2563.6	-2584.3
2-Thioxo-4-imidazolidinone	-2149.6	-2149.4
2-t-Pentylperoxyethanol	-4510.4	-4540.7
2-Trichloroacetylpyrrole	-2810.7	-2814.1
2-Trifluoroacetylpyrrole	-2692.6	-2693.9
2-Vinyl-5-norbornene	-5373	-5349.7
2-Vinylaziridine	-2775.16	-2734.7
2-Vinylfuran	-3210.2	-3209.3
2-Vinylpyridine	-3913.4	-3899.6
3-(1-Methyl-2-pyrrolidinyl)pyridine	-6003.6	-5989.6
3-(2-Furyl)-2-propenal	-3432.47	-3476.3
3-(5-(2-Nitrophenyl)-2-furyl)acrylic acid	-6003	-5991.5
3-(5-(3-Nitrophenyl)-2-furyl)acrylic acid	-5989	-5991.5
3-(5-(4-Nitrophenyl)-2-furyl)acrylic acid	-5970.6	-5991.5
3-(Dimethylamino)acrylaldehyde	-3079	-3108.7
3-(Dimethylamino)propylamine	-3876	-3876.6
3-(Ethylsulphonyl)-1-propene	-3595.35	-3594.9
3-(Methylamino)propionitrile	-2776.6	-2758.4
3-(Methylamino)propylamine	-3208.9	-3194.6
3-(Methylmercapto)-propanal	-3110.46	-3102.6

3-(Triethoxysilyl)-1-propanamine	-6659.03	-6651.4
3-(Trifluoromethyl)acetanilide	-4364.7	-4360.6
3,3,3-Trifluoropropene	-1517.05	-1518.4
3,3,4-Trimethylhexane	-6122.3	-6117.6
3,3,5,5-Tetramethylheptane	-7438.1	-7432
3,3,5-Trimethylheptane	-6779.2	-6773.7
3,3,6,6-Tetramethyl-1,7-octadiyne	-7510.54	-7533.3
3,3,6,6-Tetramethyloctane	-8070.7	-8086.8
3,3-Bis(chloromethyl)oxetane	-2898.3	-2915.5
3,3'-Bitolyl	-7529.9	-7555.9
3,3'-Dihydroxy-4,4'-diaminodiphenylmethane	-6738.1	-6714.3
3,3-Dimethyl-1,4-pentadiyne	-4249.18	-4268.3
3,3-Dimethyl-1-butene	-3991.36	-3994.1
3,3-Dimethyl-1-pentene	-4647.3	-4650.2
3,3-Dimethylbutanoic acid	-3514.76	-3487.4
3,3-Dimethylhexane	-5466.84	-5464.9
3,3-Dimethyloxetane	-3212.2	-3231
3,3-Dimethylpentane	-4810.4	-4811.4
3,3'-Dinitrobenzophenone	-6154.6	-6173.2
3,3'-Oxydi-1,2-propanediol tetranitrate	-3161.9	-3121.2
3,4,4'-Trichlorocarbanilide	-6239.3	-6248.9
3,4,5,6-Tetramethylphenanthrene	-9682.4	-9645
3,4,5-Trichloroaniline	-2935.8	-2912.7
3,4,5-Trifluoroaniline	-2935.7	-2939.2
3,4,5-Trimethoxybenzoic acid	-4798.9	-4778.8
3,4,5-Trimethoxytoluene	-5475	-5446.7
3,4,5-Trimethylbenzoic acid	-5152.95	-5167.8
3',4'-Dichloroacetophenone	-3825.8	-3843.6
3,4-Dichloroaniline	-3073.7	-3065.3
3,4-Dichloronitrobenzene	-2771	-2790.6
3,4-Dichlorophenol	-2748.6	-2750.2
3,4-Dichlorphenylisocyanate	-3105	-3123.7
3,4-Diethyl-3,4-bis(4-t-butylphenyl)-hexane	-18041	-18029.6
3,4-Diethyl-3,4-dimethoxyhexane	-7866	-7826.3
3,4-Difluoroaniline	-3094.4	-3083
3,4-Difluoronitrobenzene	-2790.1	-2808.3
3,4-Difluorophenol	-2755.7	-2767.9
3,4-Dihydro-1H-2-benzopyran	-4850.5	-4834.4
3,4-Dihydro-2H-1,5-benzodioxepin	-4729.5	-4678
3,4-Dihydro-2H-1-benzopyran	-4831.6	-4819.6
3,4-Dihydro-2H-1-benzopyran-2-one	-4367.1	-4358.9
3,4-Dihydro-2H-pyran-2-carbaldehyde	-3229.9	-3188.2

3,4-Dihydro-4H-pyran	-2963.1	-2928.4
3,4-Dihydroxytoluene	-3504.6	-3489.8
3',4'-Dimethoxyacetophenone	-5165.5	-5175.4
3,4-Dimethoxybenzaldehyde	-4529.46	-4549.2
3,4-Dimethoxybenzoic acid	-4256.3	-4265.5
3,4-Dimethoxyphenylacetonitrile	-5301	-5307.7
3,4-Dimethyl-1,2,5-oxadiazole	-2488	-2505.2
3,4-Dimethyl-1-pentene	-4647.3	-4651.2
3,4-Dimethylbenzoic acid	-4505.26	-4524.8
3,4-Dimethylhexane	-5472.54	-5467.2
3,4-Dinitro-1-trinitromethyl-1H-pyrazole	-1980.6	-1966.1
3,4-Dinitropyrazole	-1587.44	-1569.9
3,4-Dinitrotoluene	-3598	-3570.7
3,4-Diphenyl-1,2,5-oxadiazole	-7267.2	-7220.9
3,4-Lutidine	-4061.8	-4058.6
3,4-Methylenedioxycinnamic acid	-4472.3	-4460.1
3,4-Xylenol	-4334.9	-4341.3
3,5,3',5'-Tetramethyl-4,4'-diethyldipyrrylmethene	-10092	-10054.3
3,5,5-Trimethyl-1-hexanol	-5943	-5934.9
3,5,5-Trimethyl-2-cyclohexenone	-5265.1	-5270.7
3,5,7-Trioxa-1,1,1,9,9,9-hexanitrononane	-2907.5	-2938.3
3,5,7-Trioxanonane	-3736.1	-3736.9
3,5-Dibromo-4-hydroxybenzonitrile	-3131	-3117.3
3,5-Dibromophenol	-2754.2	-2753.7
3,5-Dichloroaniline	-3065.2	-3065.3
3,5-Dichloroanisole	-3458	-3472
3,5-Dichloronitrobenzene	-2766.5	-2790.6
3,5-Dichlorophenol	-2749.1	-2750.2
3,5-Dichloropyridine	-2458.4	-2467.5
3,5-Diethylbenzoic acid	-5821.7	-5835.7
3,5-Difluoroaniline	-3057.7	-3083
3,5-Difluorophenol	-2735.2	-2767.9
3,5-Dihydroxybenzoic acid	-2825.3	-2821.9
3',5'-Diisopropyl-4,4-dimethyl-3-phenyl-1,2-benzocyclobuten-3-ol	-12440	-12407
3,5-Diisopropylphenol	-6958	-6957.7
3,5-Dimethoxybenzaldehyde	-4532.96	-4549.2
3,5-Dimethoxybenzoic acid	-4246.7	-4265.5
3,5-Dimethyl-1-phenyl-4-nitrosopyrazole	-6110.4	-6106.1
3,5-Dimethyl-2-cyclohexen-1-one	-4648.8	-4621.5
3,5-Dimethyl-4-nitrosopyrazole	-3079.2	-3066.8
3,5-Dimethyl-cyclohexanol	-5010	-5027.6
3,5-Dimethylheptan-4-one	-5705.5	-5710.4

3,5-Dimethylisoxazole	-2905	-2898.4
3,5-Dimethylisoxazole-4-carboxylic acid	-2874.9	-2872.5
3,5-Dimethylpyrazole	-3129.8	-3158.5
3,5-Dimethylpyridine N-oxide	-4009.7	-4011.5
3,5-Dimethylpyrrole-2-carboxylic ethylester	-4928.59	-4955.2
3,5-Dinitro-1-trinitromethyl-1H-pyrazole	-1960.9	-1963.6
3,5-Dinitroaniline	-3037	-3034.4
3,5-Dinitrobenzoic acid	-2894	-2902.8
3,5-Dinitropyrazole	-1561.12	-1567.4
3,5-Dinitrotoluene	-3571.3	-3570.7
3,5-Diphenylisoxazole	-7619.5	-7614.1
3,5-Diphenylpyridine	-8751.3	-8783.5
3,5-Di-t-butyl-2-hydroxybenzaldehyde	-8502.6	-8511.8
3,5-Di-t-butylbenzoic acid	-8428.22	-8439.5
3,5-Di-t-butylcatechol	-8082.7	-8043.5
3,5-Di-t-butyl-o-quinone	-7959.4	-7927.4
3,5-Di-t-butylphenol	-8242	-8256
3,5-Dithiaheptane	-4770.5	-4792.8
3,5-Lupetidine	-4743.3	-4746.8
3,5-Lutidine	-4066	-4058.6
3,5-Xylenol	-4332.8	-4341.3
3,6-Dibutanal-1,2,4,5-tetroxane	-5588.5	-5554.6
3,6-Diethyloct-4-yne-3,6-diol	-7390	-7369.8
3,6-Diphenyl-1,2-dithiin	-9455	-9460.3
3,6-Diphenyl-4-cyclohexene-1,2-dicarboxylic anhydride	-9759.6	-9752.2
3,6-Di-t-butyl-o-quinone	-7957.4	-7932.7
3,6-Dithiaoctane	-5423.3	-5422.3
3,7,11,15-Tetramethyl-1-hexadecyn-3-ol	-12860	-12840.7
3,7,11-Trimethyl-1-dodecyn-3-ol	-9567.3	-9571.4
3,7-Dimethyl-1-thiaindene sulfone	-5724	-5685.9
3,7-Dinitroso-1,3,5,7-Tetraazabicyclo[3.3.1]nonane	-3624.9	-3608.7
3,9-Dimethylbenzo[a]anthracene	-10239.82	-10245.9
3,9-Dodecadiyne	-7497.28	-7481.4
3,9-Perylenedicarboxylic acid	-9741.6	-9722.5
3,beta-Dinitrostyrene	-4008.4	-4014.3
3-Acetoxycinnamic acid	-5062.5	-5062.7
3-Acetyl-2,5-dimethylfuran	-4225.1	-4226.7
3-Acetyl-2,5-dimethylthiophene	-4994.7	-4980
3-Acetyl-2-methyl-5-phenylthiophene	-7328.3	-7340.5
3-Acetyl-2-oxazolidinone	-2350.2	-2341.8
3-Acetylpyridine	-3653.7	-3657.7
3-Acetylthiophene	-3691.5	-3717.6

3-Amino-1-nitroguanidine	-1130.3	-1134.8
3-Amino-4-phenyl-1,2,5-oxadiazole	-4369.4	-4326.1
3-Amino-5-methylisoxazole	-2369.7	-2349.9
3-Aminoacetophenone	-4261	-4258.1
3-Aminobenzamide	-3670.4	-3675.1
3-aminodiphenyl ether	-6247.9	-6241.6
3-Aminophenol	-3161.2	-3162
3-Aminopyridine	-2885.2	-2879.4
3-Aminoquinoline	-4790.2	-4779.9
3-Anisaldehyde	-4037.77	-4035.9
3-Azabicyclo[3.2.2]nonane	-5190.2	-5158.2
3-Azido-1,2,4-triazole	-1515.2	-1527.3
3-Azido-5-ethyl-1,2,4-triazole	-2798.9	-2814.8
3-Azido-5-methyl-1,2,4-triazole	-2146.2	-2161.4
3-Azido-5-phenyl-1,2,4-triazole	-4540.6	-4519.2
3-Benzylphenol	-6761	-6709.7
3-Bromoacetophenone	-4002.1	-3998
3-Bromoaniline	-3241.1	-3219.7
3-Bromoanisole	-3626.5	-3626.4
3-Bromobenzoic acid	-3077.67	-3088.1
3-Bromobenzonitrile	-3467.2	-3476.8
3-Bromonitrobenzene	-2920.19	-2945
3-Bromopyridine	-2632.2	-2621.9
3-Bromoquinoline	-4546.9	-4522.4
3-Buten-2-one	-2295.1	-2285.9
3-Butene-2-imine	-2676.76	-2659.8
3-Butenyl 4-tolyl sulphone	-6592.3	-6617.1
3-Butylthiophene	-5431.1	-5436.1
3-Butyne-1-amine	-2780.06	-2742.8
3-Butyne-2-amine	-2779.06	-2739.9
3-Butynoic acid	-1905.33	-1917.9
3-Butynylbenzene	-5612.8	-5599.7
3-Carboxyphenol	-3021.6	-3030.4
3-Carene	-6192.2	-6196.5
3-Chloro-1,1,1-trifluoropropane	-1504.2	-1521.9
3-Chloro-1-propanol	-1861.27	-1860.8
3-Chloro-3-methylpentane	-3984.4	-3993.6
3-Chloro-4-methylphenylisocyanate	-3900	-3919.3
3-Chloro-5-hydroxypyridine	-2402	-2411.6
3-Chloroacetophenone	-4000.7	-3998.9
3-Chloroaniline	-3221.8	-3217.9
3-Chloroanisole	-3618.4	-3624.6

3-Chlorobenzaldehyde	-3366.9	-3370
3-Chlorobenzoic acid	-3070.34	-3086.4
3-Chlorobenzonitrile	-3466.2	-3475
3-Chlorobenzoylchloride	-3183.61	-3181.8
3-Chlorobutanoic acid	-2037	-2026.5
3-Chloronitrobenzene	-2913.9	-2943.3
3-Chlorophenol	-2910	-2902.8
3-Chlorophenylacetic acid	-3726.4	-3739.8
3-Chloropropionic acid	-1370	-1376.9
3-Chloropyridine	-2622.5	-2620.2
3-Chlorotoluene	-3751	-3754.3
3-Cyanobenzoic acid	-3592.5	-3602.7
3-Cyanophenol	-3415	-3419.1
3-Cyanopyridine N-oxide	-3104.8	-3089.3
3-Cyclohexene-1-carboxaldehyde	-4024.41	-4006.7
3-Cyclohexyleicosane	-16997	-16996.7
3-Decyne	-6482.4	-6475.7
3-Dimethylaminopropanenitrile	-3440.3	-3440.3
3-Ethyl-1-heptanol	-5963.47	-5946.5
3-Ethyl-1-hexene	-5313.75	-5313.2
3-Ethyl-1-pentene	-4654.36	-4654.5
3-Ethyl-2-methyl-1-pentene	-5300.45	-5296.5
3-Ethyl-2-methylpentane	-5474.79	-5469.8
3-Ethyl-2-pentene	-4640.6	-4628.9
3-Ethyl-3-methylpentane	-5467.7	-5467.5
3-Ethylbenzoic acid	-4524.9	-4538
3-Ethyldiphenylmethane	-8196	-8214.6
3-Ethylheptanal	-5748.65	-5743.7
3-Ethylheptane	-6128.69	-6122.6
3-Ethylhexane	-5473.96	-5469.2
3-Ethyl-o-xylene	-5859.38	-5848.8
3-Ethylpentane	-4814	-4813.1
3-Ethylphenol	-4365.9	-4351.8
3-Ethyltoluene	-5211.38	-5205.9
3-Fluoroacetanilide	-4092	-4096.8
3-Fluoroaniline	-3227.4	-3226.8
3-Fluoroanisole	-3619.6	-3633.5
3-Fluorobenzoic acid	-3085.9	-3095.2
3-Fluorobenzonitrile	-3474.1	-3483.9
3-Fluorobenzotrifluoride	-3211.7	-3240.2
3-Fluoronitrobenzene	-2929	-2952.1
3-Fluorophenetole	-4269.4	-4275

3-Fluorophenol	-2911.32	-2911.7
3-Formyl-6-isopropylchromone	-6415.1	-6429.6
3-Formyl-6-methylchromone	-5116.8	-5120.7
3-Formylchromone	-4452.4	-4477.8
3-Furanacrylic acid	-3151.8	-3192.6
3-Furancarboxaldehyde	-2339.2	-2349.3
3H-1,2-Benzodithiole-3-thione	-5276	-5290.9
3-Heptanone	-4409.96	-4404.9
3-Heptylhydroperoxide	-4697.8	-4685.9
3-Heptyne	-4518.7	-4515.2
3-Hexanone	-3758.56	-3751.4
3-Hexylhydroperoxide	-4059.7	-4035
3-Hexylthiophene	-6736.4	-6743
3-Hydroxy-1,3,3-triphenyl-1-propanone	-10600	-10623.6
3-Hydroxy-2-methylpropionaldehyde	-2291.31	-2284.5
3-Hydroxy-2-naphthalenecarboxylic acid	-4930.4	-4928
3-Hydroxy-6-methylpyridine	-3195.5	-3198.8
3-Hydroxyacetophenone	-3923.67	-3943
3-Hydroxybenzaldehyde	-3298.3	-3314.1
3-Hydroxybiphenyl	-6063	-6061.5
3-Hydroxybutyric acid	-2039.8	-1994.3
3-Hydroxycinnamic acid	-4152.1	-4160
3-Hydroxycoumarin	-3939.5	-3935.7
3-Hydroxydiphenylether	-5936.8	-5926.5
3-Hydroxypyridine	-2550.1	-2564.2
3-Hydroxypyridine N-oxide	-2511.4	-2517.1
3-Iodoaniline	-3301.5	-3292.8
3-Iodoanisole	-3704.3	-3699.5
3-Iodobenzoic acid	-3153.8	-3161.3
3-Iodobenzonitrile	-3556.9	-3549.9
3-Iodonitrobenzene	-2999.3	-3018.2
3-Iodophenol	-2980	-2977.7
3-Iodopropionic acid	-1435.1	-1445.9
3-Iodotoluene	-3834.2	-3829.2
3-Isochromanone	-4351.2	-4349.7
3-Isopropylbenzoic acid	-5174.25	-5190.8
3-Isopropyltoluene	-5857.3	-5856.1
3-lsopropyl-1,2-benzenediol	-4812.3	-4796.1
3-Menthene	-6350.8	-6339.3
3-Mercaptopropyl-triethoxysilane	-7086.4	-7121.9
3-Mercaptopropyl-trimethoxysilane	-5167.92	-5197.3
3-Methoxy-1,3,3-triphenylpropyne	-11450	-11451.6

3-Methoxy-2-nitrobenzoic acid	-3597	-3584.2
3-Methoxy-4-hydroxybenzaldehyde	-3828	-3827.4
3-Methoxy-4-nitrobenzoic acid	-3565.7	-3584.2
3-Methoxyacetophenone	-4665.2	-4662.1
3-Methoxybenzamide	-4061.8	-4081.8
3-Methoxydiphenylether	-6643.7	-6648.3
3-Methoxyphenol	-3584.5	-3568.7
3-Methoxypropionitrile	-2457.5	-2461.9
3-Methoxysalicylaldehyde	-3800.5	-3827.1
3-Methoxytoluene	-4421.9	-4420.2
3-Methyl-1,2-butadiene	-3214.12	-3215.5
3-Methyl-1-butanethiol	-4133.1	-4136.3
3-Methyl-1-butene	-3345.1	-3344.9
3-Methyl-1-butyne	-3249.35	-3235.9
3-Methyl-1-hexene	-4657.46	-4654.5
3-Methyl-1H-pyrazole	-2507.35	-2515.6
3-Methyl-1-pentanol	-3983.88	-3980.9
3-Methyl-1-pentene	-4000.6	-4001
3-Methyl-2,5-furandione	-2036.36	-2008.4
3-Methyl-2-butanethiol	-4126	-4123.7
3-Methyl-2-butanol	-3315.1	-3317.9
3-Methyl-2-butanone	-3097	-3094.6
3-Methyl-2-cyclopenten-1-one	-3308.6	-3327.2
3-Methyl-2-phenylbutane-2-ol	-6336.5	-6313.7
3-Methyl-2-thiophenecarbonitrile	-3835.2	-3827.6
3-Methyl-2-thiophenecarboxyaldehyde	-3722.3	-3722.6
3-Methyl-2-thiophenecarboxylic acid	-3426.9	-3438.9
3-Methyl-3-pyrazoline-5-one	-2280.7	-2287.8
3-Methyl-3-t-amylperoxy-1-triphenylsilyl-1-butyne	-16603	-16597.1
3-Methyl-3-t-butylperoxy-1-triphenylsilyl-1-butyne	-15933	-15938.3
3-Methyl-3-t-hexylperoxy-1-triphenylsilyl-1-butyne	-17250	-17250.5
3-Methyl-4-isopropylphenol	-5644.38	-5650.1
3-Methyl-4-nitropyridine-1-oxide	-3201.6	-3200.5
3-Methyl-4-phenyl-1,2,5-oxadiazole	-4872.7	-4874.6
3-Methyl-5-phenylisoxazole	-5239.6	-5256.3
3-Methylbenzaldehyde	-4170.87	-4165.6
3-Methylbenzoic acid	-3866.5	-3881.9
3-Methylbenzylalcohol	-4382.99	-4365.9
3-Methylbutanoic acid	-2837.37	-2838.2
3-Methylbutyl 2-chlorobutanoate	-5310.8	-5349.6
3-Methylbutyl 3-chlorobutyrate	-5350.5	-5349.6
3-Methylbutyl 3-chloropropionate	-4722.1	-4699.9

3-Methylbutyl 4-chlorobutyrate	-5338.4	-5353.4
3-Methylbutyl chloroacetate	-4057	-4046.5
3-Methylbutyl trichloroacetate	-3805	-3801.6
3-Methyl-cis-2-pentene	-3984.29	-3975.4
3-Methylcyclohexanone	-4196.1	-4159.5
3-Methylcyclohexylamine	-4718.3	-4725
3-Methylcyclopentanone	-3516	-3515.3
3-Methylcyclopentene	-3766.5	-3751.5
3-Methyldiamantane	-8792.42	-8807.6
3-Methyldihydro-2,5-furandione	-2205.1	-2201.2
3-Methylenecyclobutanenitrile	-3569.4	-3563.6
3-Methyleneheptane	-5301.93	-5302.5
3-Methylheptane	-5469.37	-5471.8
3-Methylhexanal	-4443.17	-4431.5
3-Methylhexane	-4818.14	-4815.7
3-Methylisoxazole	-2284.5	-2272.9
3-Methyl-N-(2-methylphenyl)-2-quinoxalinecarboxamide N,N'-dioxide	-8761.9	-8738.7
3-Methylnonane	-6780.37	-6778.7
3-Methyloctane	-6125.64	-6120
3-Methylpentane	-4162.4	-4159.6
3-Methylpicric acid	-3212.7	-3193.8
3-Methylpiperidine	-4095.3	-4096.7
3-Methylpyridine	-3423.4	-3415.7
3-Methylpyrrole	-2990.44	-2973.9
3-Methylquinoxaline-2-carboxamide 1,4-dioxide	-5074.4	-5063.3
3-Methylsulfolane	-3513.35	-3529.3
3-Methylthiolane	-3896.4	-3912.3
3-Methylthiophene	-3472.8	-3475.7
3-Methyl-trans-2-pentene	-3984.2	-3975.4
3-Methylundecane	-8088.35	-8085.7
3-Nitraza-1,5-pentanediisocyanate	-3242.3	-3257.5
3-Nitro-2-butanol	-2471.7	-2468.1
3-Nitro-3-(4-nitrophenyl)pentane	-6127.5	-6151.6
3-Nitroacetanilide	-4059.1	-4072.5
3-Nitroacetophenone	-3950	-3980.8
3-Nitroaniline	-3190.3	-3202.5
3-Nitrobenzaldehyde	-3350	-3354.6
3-Nitrobenzamide	-3366.6	-3400.4
3-Nitrobenzenesulfonamide	-3454.2	-3471.8
3-Nitrobenzoic acid	-3055	-3070.9
3-Nitrobenzophenone	-6324.5	-6341.3
3-Nitrobenzotrifluoride	-3228	-3216

3-Nitrobiphenyl	-6073.5	-6101.9
3-Nitrocinnamic acid	-4171	-4200.5
3-nitrodiphenyl ether	-5961.3	-5967
3-Nitrophenetol	-4225.3	-4250.7
3-Nitrophenol	-2880	-2887.4
3-Nitrophthalic acid	-3070	-3045.9
3-Nitrophthalic anhydride	-3095.3	-3072.1
3-Nitrophthalimide	-3394.1	-3361.7
3-Nitro-p-toluidine	-3828.9	-3845.4
3-Nitrotoluene	-3724.8	-3738.8
3-Nonanone	-5720.75	-5711.9
3-Nonyne	-5828.1	-5822.2
3-Octanone	-5052	-5058.4
3-Octylthiophene	-8030.2	-8049.9
3-Octyne	-5170.93	-5168.7
3-Oxabicyclo[3.2.2]nonane	-4873.2	-4852.2
3-Pentanol	-3314.66	-3316
3-Pentynoic acid	-2534.6	-2540.4
3-Phenyl-1-butene	-5710	-5710.6
3-Phenyl-2-propyn-1-ol	-4764.7	-4728.7
3-Phenyl-5-phenoxymethyl-2-N-phenyliminooxazolidine	-11441	-11430
3-Phenyl-5-phenoxymethyl-2-oxazolidinone	-8022.4	-8020.7
3-Phenylamino-2-naphthoic acid	-8291.5	-8278.6
3-Phenylcyclobutanone	-5307.8	-5308.4
3-Phenylcyclobutenone	-5106.3	-5120.3
3-Phenylisoxazole	-4617.5	-4630.7
3-Phenylpropene	-5062.09	-5052.6
3-Phenylpropiolic acid	-4277.3	-4244.8
3-Phenyltoluene	-6916.2	-6912.9
3-Picoline N-oxide	-3366.4	-3368.5
3-Picrylamino-1,2,4-triazole	-4014	-3969.1
3-Piperidinecarboxamide	-3741.5	-3758.3
3-Piperidinemethanol	-3895.3	-3912.7
3-Propyltoluene	-5863.65	-5856.8
3-Pyridinecarbonitrile	-3128.3	-3136.5
3-Quinolinecarbonitrile	-5038.09	-5037
3-Sulfolene	-2716.64	-2689.9
3-t-Butylbenzoic acid	-5829.3	-5839.9
3-t-Butylperoxy-1,2-propanediol	-4325.4	-4344.6
3-t-Butylphenol	-5661	-5656.4
3-Thiacyclohexanone	-3531	-3485.5
3-Thiopheneacetic acid	-3444.1	-3461.2

3-Thiopheneacetonitrile	-3844.4	-3849.9
3-Thiophenecarbonitrile	-3195.2	-3196.4
3-Thiophenecarboxyaldehyde	-3081.2	-3091.4
3-Thiophenecarboxylic acid	-2787.5	-2810.4
3-Thiopropionic acid	-2173.41	-2162.1
3-Trifluoromethylaniline	-3476.7	-3490.6
3-Trifluoromethylbenzoic acid	-3377	-3359
3-Trifluoromethylphenol	-3193.9	-3175.5
4-(1H-Tetrazol-5-yl)-3-tetrazene-2-carboximidamide	-2120	-2112.5
4,4'-(Dimethoxy)azobenzene	-7554.2	-7523
4,4,4-Trifluoro-1-(2-furanyl)-butane-1,3-dione	-3335.3	-3331.6
4,4,4-Trifluoro-1-(2-naphthyl)-butane-1,3-dione	-6419.8	-6421.9
4,4,4-Trinitrobutyronitrile	-2150.5	-2177.5
4,4,6,6-Tetramethyl-1,3-dioxane	-4916.7	-4909.1
4,4,6,6-Tetramethylnonane	-8752.3	-8737.6
4,4,6-Trimethyl-1,3-dioxane	-4258.1	-4268.6
4,4'-Azobis(4-cyano-1-pentanol)	-7357.1	-7361
4,4'-Bipyridine	-5265.2	-5287.6
4,4'-Bis-(N-carbazolyl) -1,1'-biphenyl	-18114	-18158
4,4'-Diaminodiphenyldisulfide	-7738.7	-7733.4
4,4'-Dichlorobiphenyl	-5927.1	-5964.7
4,4'-Diethoxyazoxybenzene	-8705	-8707.7
4,4'-Difluorobiphenyl	-5927.1	-5982.4
4,4'-Diisocyanatobiphenyl	-6602.4	-6600.1
4,4'-Diisocyanatodiphenylmethane	-7279	-7248.3
4,4-Dimethyl-1,3-cyclohexanedione	-4363.5	-4399.7
4,4-Dimethyl-1-pentene	-4647.96	-4650.2
4,4'-Dimethylbiphenyl	-7526.1	-7555.9
4,4'-Dinitrodiphenyl ether	-5753.8	-5798.9
4,4'-Dinitrohydrazobenzene	-6265.1	-6286.7
4,4'-Di-t-butylbiphenyl	-11471.63	-11471.9
4,4'-Oxybis(benzoylhydrazide)	-7207.8	-7228.7
4,5,9,10-Tetrahydropyrene	-8322.2	-8352.2
4,5-Benzo-1,3-dithiole-2-thione	-5256	-5246.8
4,5-Dichloro-2-nitroaniline	-2880.1	-2896.9
4,5-Diethyl-4,5-bis-(4-tert-butylphenyl)-octane	-19331	-19307.4
4,5-Dimethyl-1,3-dioxane	-3624.4	-3634.2
4,5-Dimethyl-2-furaldehyde	-3603.4	-3600.4
4,5-Dimethylphenanthrene	-8385.9	-8368.6
4,5-Dimethylpyrrole-3-carboxylic ethylester	-4932.79	-4954.3
4,5-Tetramethylene-1,2-dithiole-3-thione	-5754.3	-5738.9
4,5-Tetramethylene-1,3-dithiolan-2-thione	-5911.5	-5929.4

4,6-Dichloro-2-methylpyrimidine	-2581.2	-2585.3
4,6-Dichloro-5-methylpyrimidine	-2590.4	-2605.8
4,6-Dichloropyrimidine	-1956.6	-1962.9
4,6-Diethyl-4,6-dimethylnonane	-10068.2	-10055.1
4,6-Dimethylindane	-6265.7	-6261
4,6-Dinitro-m-xylene	-4189.4	-4213.7
4,6-Dinitro-o-cresol	-3332.9	-3361.9
4,6-Dinitroresorcinol	-2495.18	-2510.2
4,7,7-Trimethylbicyclo[4.1.0]heptan-3-ol	-6195.5	-6191.2
4,7-Dichloroquinoline	-4353.3	-4368
4,7-Dimethylindane	-6263.7	-6261
4,beta-Dinitrostyrene	-4027.5	-4014.3
4-Acetoxycinnamic acid	-5076.3	-5065.3
4-Acetylanisole	-4669.8	-4662.1
4-Acetylphenol	-3927.1	-3943
4-Acetylpyridine	-3652.3	-3660.3
4-Amino-3-azido-1,2,4-triazole	-1788.2	-1776.9
4-Amino-3-azido-5-ethyl-1,2,4-triazole	-3072.3	-3064.4
4-Amino-3-azido-5-methyl-1,2,4-triazole	-2380.6	-2410.9
4-Amino-3-azido-5-phenyl-1,2,4-triazole	-4819	-4771.4
4-Amino-3-furazanecarboxamidoxime	-1932	-1917.5
4-Aminoacetanilide	-4341.3	-4364.5
4-Aminoacetophenone	-4252.2	-4255.5
4-Aminoazobenzene	-6617.4	-6603.1
4-Aminobenzamide	-3666.6	-3675.1
4-Aminobenzenesulfonamide	-3729.3	-3746.5
4-Aminobutyric acid	-2283.9	-2287.8
4-aminodiphenyl ether	-6239.4	-6241.6
4-Aminohexanoic acid	-3572.7	-3589.5
4-Aminoindane	-5107.3	-5081.7
4-Aminophenol	-3167.4	-3162
4-Aminopyridine	-2866.8	-2879.4
4-Benzyloxyphenol	-6580.1	-6568.1
4-Benzylpiperidine	-7100.2	-7110.6
4-Biphenylamine	-6379.59	-6376.6
4-Biphenylmethanol	-6713.7	-6729
4-Bromoacetophenone	-3982.7	-3998
4-Bromoaniline	-3227	-3219.7
4-Bromoanisole	-3626.3	-3626.4
4-Bromobenzoic acid	-3091.8	-3088.1
4-Bromobenzonitrile	-3463.3	-3476.8
4-Bromobiphenyl	-6123.4	-6119.1

4-Bromonitrobenzene	-2913.59	-2945
4-Bromophenol	-2901.2	-2904.6
4-Bromotoluene	-3747.89	-3756
4-Butoxybenzoic acid	-5682.3	-5700.7
4-Carboxyphenol	-3019.97	-3033.1
4-Carboxypyridine N-oxide	-2696.3	-2700.6
4-Chloro-2,6-dinitroaniline	-2879.4	-2881.2
4-Chloro-3-nitroaniline	-3052.7	-3049.8
4-Chloroacetophenone	-3991.4	-3996.2
4-Chloroaniline	-3221.8	-3217.9
4-Chloroanisole	-3621.7	-3624.6
4-Chlorobenzaldehyde	-3346.4	-3370
4-Chlorobenzoic acid	-3066	-3086.4
4-Chlorobenzonitrile	-3463.6	-3475
4-Chlorobutanoic acid	-2028	-2030.4
4-Chloroethylbenzene	-4405.6	-4410.4
4-Chloronitrobenzene	-2919.8	-2943.3
4-Chlorophenol	-2901.6	-2902.8
4-Chlorophenylacetic acid	-3729	-3739.8
4-Chlorophenylisocyanate	-3259	-3276.4
4-Chloroquinoline	-4508.4	-4520.7
4-Chlorotoluene	-3750	-3754.3
4-Chromanone	-4395.8	-4408.1
4-Cumylphenol	-4983	-5002
4-Cyanobenzoic acid	-3592.8	-3602.7
4-Cyanopyridine	-3143	-3136.5
4-Cyanopyridine N-oxide	-3096.7	-3089.3
4-Cyanothiazole	-2684.1	-2674
4-Decyne	-6480.4	-6475.7
4-Dichloromethyl-4-methyl-2,5-cyclohexadienone	-4130.2	-4169.1
4-Dimethylaminoazobenzene	-7989.8	-7977.6
4-Dimethylaminobenzaldehyde	-4977	-5001.1
4-Dimethylaminonitrosobenzene	-4680.2	-4680.9
4-Dimethylaminopyridine	-4235.9	-4262.8
4-Ethoxyaniline	-4550.7	-4525.4
4-Ethoxybenzoic acid	-4365.2	-4393.8
4-Ethyl-3,5-dimethyl-1H-pyrrole-2-carboxaldehyde	-5144.6	-5174.9
4-Ethylbenzoic acid	-4519.6	-4535.4
4-Ethyldiphenylmethane	-8196	-8217.3
4-Ethyl-m-xylene	-5855.78	-5846.2
4-Ethylnitrobenzene	-4379	-4392.3
4-Ethyl-o-xylene	-5853.86	-5846.2

4-Ethylphenol	-4352.82	-4351.8
4-Ethyltoluene	-5210.29	-5203.3
4-Fluoro-2-nitroacetanilide	-3917	-3928.4
4-Fluoro-2-nitrophenol	-2731.1	-2743.3
4-Fluoro-3-nitroaniline	-3083	-3058.7
4-Fluoroacetanilide	-4094	-4096.8
4-Fluoroaniline	-3233.3	-3226.8
4-Fluoroanisole	-3624.9	-3633.5
4-Fluorobenzoic acid	-3094.7	-3095.2
4-Fluorobenzonitrile	-3451.5	-3483.9
4-Fluoronitrobenzene	-2921.9	-2952.1
4-Fluorophenetole	-4283	-4275
4-Fluorophenol	-2914.8	-2911.7
4-Fluorotoluene	-3747.2	-3763.1
4-Formylbenzoic acid	-3463.34	-3497.7
4-Formylmorpholine	-2886.06	-2881.7
4-Heptanone	-4412.36	-4404.9
4-Heptylhydroperoxide	-4710.8	-4688.5
4-Hydroxy-3-methoxybenzaldehyde	-3838	-3827.4
4-Hydroxy-4-methyl-2-pentanone	-3548.26	-3544.8
4-Hydroxyazobenzene	-6318.4	-6288
4-Hydroxybenzaldehyde	-3317.37	-3314.1
4-Hydroxybenzonitrile	-3412.36	-3419.1
4-Hydroxybiphenyl	-6067	-6061.5
4-Hydroxycoumarin	-3919.2	-3935.7
4-Hydroxydiphenylether	-5911.7	-5926.5
4-Hydroxynicotinic acid	-2496.6	-2539
4'-Hydroxypropiophenone	-4576.8	-4593.8
4-Hydroxypyridine	-2537.5	-2564.2
4-Hydroxystyrene	-4184.6	-4193.2
4-Hydroxytetrahydropyran	-2905.6	-2947.1
4-Iodoaniline	-3302.3	-3292.8
4-Iodoanisole	-3685	-3699.5
4-Iodobenzoic acid	-3154.2	-3161.3
4-Iodobenzonitrile	-3551.3	-3549.9
4-Iodonitrobenzene	-2989.9	-3018.2
4-Iodophenol	-2980.3	-2977.7
4-Iodotoluene	-3822.5	-3829.2
4-Isopropyl-2-methylphenol	-5653	-5647.5
4-Isopropylbenzoic acid	-5170.05	-5188.2
4-Isopropylbenzophenone	-8464	-8458.5
4-Isopropylbenzylidine-t-butylamine N-oxide	-8357.7	-8344.3

4-Isopropyldicyclohexylmethane	-10200	-10210.6
4-Isopropyltropolone	-5313.05	-5330.5
4-Methoxy-2-nitrobenzoic acid	-3571	-3584.2
4-Methoxy-2-nitrophenol	-3390.7	-3400.4
4-Methoxyazobenzene	-7014.5	-7009.8
4-Methoxybenzaldehyde	-4032.47	-4035.9
4-Methoxybenzamide	-4061	-4081.8
4-Methoxybenzoic acid	-3729.7	-3752.3
4-Methoxybenzylideneacetone	-5763.5	-5791.7
4-Methoxycarbonylhomocubane	-5989.8	-6018.6
4-Methoxyphenol	-3564.98	-3568.7
4-Methoxyphenylacetic acid	-4395.66	-4405.7
4-Methoxypyridine	-3288.11	-3286.1
4-Methyl-1,3-dioxane	-2980.6	-2984.1
4-Methyl-1-heptene	-5310.65	-5308
4-Methyl-1-hexene	-4657.06	-4654.5
4-Methyl-1H-pyrazole	-2517.5	-2526.8
4-Methyl-1-octanol	-5941.57	-5941.3
4-Methyl-1-pentene	-3998.72	-3998.4
4-Methyl-1-phenyl-2,6,7-trioxabicyclo[2.2.2]octane	-6234.02	-6200
4-Methyl-2,5-diisopropylphenol	-7607	-7596.7
4-Methyl-2,6,7-trioxabicyclo[2.2.2]octane	-3218.54	-3216.8
4-Methyl-2-phenylpent-2-enenitrile	-6754.5	-6702.9
4-Methyl-2-pyrazoline-5-one	-2286.2	-2291.6
4-Methyl-3,5-diisopropylphenol	-7607	-7599.3
4-Methyl-4-t-butylperoxy-1-triphenylsiloxy-2-butyne	-16305	-16294.9
4-Methylacetophenone	-4787.4	-4791.8
4-Methylbenzaldehyde	-4173.71	-4165.6
4-Methylbenzoic acid	-3868	-3881.9
4-Methylbenzoic anhydride	-7776	-7785.4
4-Methylbenzophenone	-7146	-7152.3
4-Methylbenzoyl chloride	-3972.5	-3977.3
4-Methyl-cis-2-pentene	-3991.73	-3984.9
4-Methylcyclohexene	-4395	-4393.6
4-Methylcyclopentene	-3772.6	-3751.5
4-Methyldiamantane	-8791.32	-8802.7
4-Methyldibenzothiophene	-7217.7	-7256.7
4-Methylene-2-oxetanone	-1913.4	-1903.2
4-Methylheptane	-5468.9	-5466.6
4-Methylnonane	-6779.86	-6778.7
4-Methyl-N-phenylaniline	-7026.6	-7046
4-Methyloctane	-6124.57	-6120

4-Methylpent-3-en-2-one	-3569.2	-3560
4-Methylphenanthrene	-7720	-7719.9
4-Methylphthalic acid	-3857.5	-3856.9
4-Methylpiperidine	-4095.4	-4096.7
4-Methylpyridine	-3418.3	-3415.7
4-Methylpyridine N-oxide	-3367.1	-3368.5
4-Methylquinoline	-5315.9	-5316.2
4-Methylresorcinol	-3496.3	-3489.8
4-Methylthiazole	-2958.8	-2953.3
4-Methyl-trans-2-pentene	-3987.21	-3979.6
4-Morpholineethanamine	-4153.3	-4156.2
4-Morpholinepropanamine	-4809.3	-4809.7
4-N,N-diethylamino-7-nitrobenzofurazan	-5784.1	-5781.8
4-N,N-Dimethylamino-7-nitrobenzofurazan	-4464.1	-4477.2
4-Nitroacetanilide	-4053.7	-4072.5
4-Nitroacetophenone	-3952.4	-3983.4
4-Nitroaniline	-3186.2	-3202.5
4-Nitrobenzaldehyde	-3339.8	-3354.6
4-Nitrobenzamide	-3370.2	-3400.4
4-Nitrobenzenesulfonamide	-3450.1	-3471.8
4-Nitrobenzofuroxan	-2992.4	-2979.5
4-Nitrobenzoic acid	-3049.75	-3073.5
4-Nitrobenzophenone	-6327.8	-6341.3
4-Nitrobenzylidene-2-methylphenylacetonitrile	-8186	-8232
4-Nitrobenzylideneaniline	-6682.3	-6701.9
4-Nitrobenzylidene-t-butylamine	-6287.7	-6285.2
4-Nitrobenzylidine-t-butylamine N-oxide	-6224	-6227.1
4-Nitrobiphenyl	-6048.8	-6101.9
4-Nitrocatechol	-2666.59	-2678.8
4-Nitrocinnamic acid	-4147	-4200.5
4-nitrodiphenyl ether	-5931.1	-5967
4-Nitroguaiacol	-3378	-3400.6
4-Nitroimidazole	-1603.3	-1605.9
4-Nitrophenetole	-4211.9	-4250.7
4-Nitrophenol	-2879.6	-2887.4
4-Nitrophenyl azide	-3243.38	-3284.4
4-Nitrophthalic acid	-3074.9	-3045.9
4-Nitrophthalic anhydride	-3077.2	-3072.1
4-Nitrophthalimide	-3380.8	-3361.7
4-Nitropyrazole	-1678.5	-1701.1
4-Nitropyridine N-oxide	-2556.3	-2557.5
4-Nitroso-1-naphthol	-4871.4	-4891.7

4-Nitrosodiphenylamine	-6364.4	-6341.6
4-Nitrosophenol	-2987.1	-2993.9
4-Nitrostilbene	-7183.9	-7228.9
4-Nitrotoluene	-3708	-3738.8
4-Nonanone	-5719.85	-5711.9
4-Nonylphenol	-8951.51	-8928.7
4-Nonyne	-5828.1	-5822.2
4-Octanone	-5065.25	-5058.4
4-Octyloxybenzoic acid	-8278.9	-8314.6
4-Octyne	-5169.63	-5168.7
4-Oxopentanoic acid	-2427.1	-2430.1
4-Pentenoic acid	-2680.3	-2680.3
4-Phenyl-3-butyn-2-one	-5173.1	-5154.7
4-Phenylazophenol (hydrazono form)	-6314.1	-6275.2
4-Phenylbut-3-en-2-one	-5282.3	-5278.5
4-Phenylcyclohexene	-6761.7	-6754.1
4-Phenyltoluene	-6888.6	-6912.9
4-Piperidinecarboxamide	-3736.6	-3758.3
4-Piperidinemethanol	-3891.3	-3912.7
4-piperidine-piperidine	-6648.3	-6676.6
4-Propyltoluene	-5864.82	-5856.8
4-Quinolinol	-4427.7	-4464.8
4-t-Butyl-2,6-xylenol	-6945	-6942.2
4-t-Butyl-2-methylphenol	-6314	-6299.3
4-t-Butylbenzoic acid	-5830.7	-5837.3
4-t-Butyl-benzophenone	-9127	-9107.7
4-t-Butylbiphenyl	-8842.5	-8870.9
4-t-Butylcatechol	-5461.9	-5447.8
4-t-Butyldiphenyl ether	-8705.2	-8733.4
4-t-Butylphenol	-5625.8	-5653.7
4-Thianone	-3485	-3485.5
4-t-Octylphenol	-8266.6	-8290.2
4-t-Pentylphenol	-6318.82	-6308.5
4-Vinylcyclohexene	-4889.8	-4885.9
5-(2-Nitrophenyl)-2-furaldehyde oxime	-5458.8	-5411
5-(2-Nitrophenyl)-furan-2-carbaldehyde	-5137	-5148.2
5-(3-Nitrophenyl)-2-furaldehyde oxime	-5442	-5411
5-(3-Nitrophenyl)-furan-2-carbaldehyde	-5127.4	-5148.2
5-(4-Nitrophenyl)-2-furaldehyde oxime	-5422.2	-5411
5-(4-Nitrophenyl)-furan-2-carbaldehyde	-5106.4	-5148.2
5-(Dimethylamino)-2,4-pentadienal	-4199	-4224.1
5,12-Dihydronaphthacene	-9190	-9206.3

5,12-Naphthacenedione	-8375.21	-8383.3
5,5,7,7-Tetramethylundecane	-10049.4	-10044.6
5,5'-Bitetrazole	-1604.6	-1593.1
5,5-Dimethyl-1,3-cyclohexanedione	-4379.7	-4385.4
5,5-Dimethyl-1,3-dioxan-2-one	-3119.3	-3118.9
5,5-Dimethyl-1,3-dioxane	-3614.7	-3636.8
5,5-Dimethylhydantoin	-2583.7	-2584.4
5,5'-Hydrazotetrazole	-1887.4	-1912.2
5,5'-Methylenebis(2-keto-1,3-dimethyltetrahydro-1,3,5-triazine	-7078.3	-7080.6
5,5'-Spirobi-1,3-dioxane	-3767	-3771.7
5,6,6a,7,8,12b,12c-Octahydrodibenzo[a,i]biphenylene	-10808	-10832.5
5,6,7,8-Tetrahydroquinoline	-5127	-5115
5,6-Dihydro-6-methyluracil	-2592.4	-2598.6
5,6-Dimethoxy-1-indanone	-5570.7	-5588.1
5,6-Dimethylchrysene	-10296.1	-10274.9
5,6-Dimethyluracil	-2995.8	-3019.1
5,7-Dibromo-8-hydroxyquinoline	-4171.9	-4163
5,7-Dichloro-8-hydroxyquinoline	-4145.3	-4159.5
5,7-Diiodo-8-hydroxyquinoline	-4349.7	-4309.3
5,7-Dodecadiyne	-7480.96	-7481.4
5,8-Dimethylbenzo[c]phenanthrene	-10265	-10260.1
5-Acetamidotetrazole	-1891.2	-1887.9
5-Acetyl-2-thiophenecarboxylic acid	-3666.2	-3672.7
5-Amino-1H-1,2,4-triazole	-1436.5	-1445.4
5-Amino-1-methyltetrazole	-1696.2	-1697.1
5-Amino-1-phenyltetrazole	-4068.7	-4069.5
5-Amino-2-mercaptobenzimidazole	-4379	-4378.4
5-Amino-2-methyltetrazole	-1708.4	-1706.2
5-Amino-3,4-dimethyl-isoxazole	-3028.2	-3050.8
5-Aminohexanoic acid	-3578.47	-3586.8
5-Aminoindane	-5100.9	-5081.7
5-Aminoindazole	-3901.8	-3895.4
5-Aminoquinoline	-4790.5	-4782.5
5-Aminotetrazole	-1030.1	-1017.9
5-Aminouracil	-1847.2	-1810.5
5-Aminovaleric acid	-2937	-2941.2
5-Azabicyclo[2.1.0]pentane	-2801.76	-2758.2
5-Azidomethyl-3-nitrooxazolidine	-2771.2	-2728.6
5-Bromoindole	-4079.9	-4085
5-Bromoindoline	-4321.1	-4308.2
5-Butyldocosane	-17247.16	-17239.5
5-Carboxy-1,2,3-triazole	-1364	-1372

5-Chloro-2-methylbenzoxazole	-3883.6	-3914.7
5-Chloro-2-nitroaniline	-3031.1	-3049.6
5-Chloro-6-hydroxynicotinic acid	-2340.5	-2335.5
5-Chloro-8-hydroxyquinoline	-4298.5	-4312.1
5-Chlorobenzofurazan N-oxide	-3010.6	-3017.8
5-Chlorouracil	-1585.2	-1588.8
5-Cyano-5H-dibenzo[a,d]cycloheptene	-8188	-8152.9
5-Cyanotetrazole	-1332	-1291.8
5-Decyne	-6479.2	-6475.7
5-Diallylaminotetrazole	-4680.8	-4680.2
5-Dimethylaminotetrazole	-2365.1	-2389.8
5-Ethyl-2-thiophenecarboxaldehyde	-4367.6	-4365.3
5-Ethyl-5-methylhydantoin	-3227.6	-3237.9
5-Ethylidene-2-norbornene	-5362.28	-5318.1
5-Ethyl-m-xylene	-5852.1	-5848.8
5-Ethylnorbornane	-5688	-5680.9
5-Fluoro-2-methylbenzothiazole	-4673.5	-4680.6
5-Fluoro-2-methylbenzoxazole	-3924.1	-3923.5
5-Guanylaminotetrazole	-1672.2	-1686
5-Hexen-3-one	-3591	-3590.2
5-Hexyl-2-methyl-m-dioxane	-6903	-6900.1
5-Hydroxy-1,3-dioxane	-2149.9	-2144
5-Hydroxyisoquinoline	-4455.2	-4462.1
5-Hydroxymethylene-5H-6,7-dihydrodibenzo[a.c]cyclohepten-6-one	-7812.1	-7796.9
5-Hydroxynicotinic acid	-2540.1	-2539.2
5-Hydroxypentanal	-2918.6	-2941.2
5-Indanol	-4764	-4766.6
5-Iodosalicylic acid	-2958	-2952.4
5-Isopropyl-2-methylphenol	-5665	-5652.8
5-Isopropylbarbituric acid	-3467	-3439.9
5-Isopropyl-m-cresol	-5639.88	-5647.5
5-Methoxy-1-tetralone	-5700.9	-5716.4
5-Methoxybenzofurazan	-3670	-3688.6
5-Methoxybenzofurazan N-oxide	-3664.4	-3641.7
5-Methoxyindane	-5492.8	-5488.4
5-Methoxytetrazole	-1427.8	-1419.5
5-Methyl-1-heptene	-5310.7	-5310.6
5-Methyl-1-hexene	-4655.37	-4654.5
5-Methyl-2,3-dihydrofuran	-2945.9	-2909.8
5-Methyl-2-phenylhex-2-enenitrile	-7406.4	-7357.7
5-Methyl-2-phenyltetrazole	-4566.04	-4550.8
5-Methyl-2-thiophenecarboxyaldehyde	-3727	-3711.8

5-Methyl-2-thiophenecarboxylic acid	-3424.8	-3430.7
5-Methyl-2-thiouracil	-3206.2	-3226.3
5-Methyl-3-phenylisoxazole	-5235	-5256.3
5-Methyl-5-phenylhydantoin	-4985.9	-4942.3
5-Methylaminotetrazole	-1704.9	-1698.8
5-Methylbenzofuroxan	-3788.5	-3805.2
5-Methylcaprolactam	-4251.7	-4254.9
5-Methylhydantoin	-1946	-1949.8
5-Methylisoxazole	-2261.7	-2267.6
5-Methylisoxazole-3-carboxylic acid	-2255.7	-2230.5
5-Methyl-N-nitro-1H-1,2,4-triazol-3-amine	-1948.4	-1982.1
5-Methylnonane	-6773.96	-6781.4
5-Methylresorcinol	-3496.4	-3489.8
5-Methyltetrazole	-1543.1	-1571.1
5-Methyluracil	-2371.2	-2376.2
5-Nitro-8-hydroxyquinoline	-4283.4	-4296.7
5-Nitroaminotetrazole	-931.4	-920.5
5-Nitrofurancarboxylic acid	-1879	-1886.5
5-Nitrofurfural	-2169	-2170.2
5-Nitroimidazole	-1603.3	-1605.9
5-Nitroindole	-4032	-4067.8
5-Nitroindoline	-4274.5	-4291
5-Nitro-o-toluidine	-3809.3	-3845.4
5-Nitrouracil	-1579.8	-1550.5
5-Nonanone	-5719.91	-5711.9
5-Phenyl-1,2-dithiole-3-thione	-6392	-6392.5
5-Phenylaminotetrazole	-4062.9	-4050.5
5-Phenylisoxazole	-4622.5	-4625.5
5-Phenyltetrazole	-3910	-3928.9
5-Tetrazolylurethane	-2355.9	-2352.5
6,10,14-Trimethylpentadeca-3,5-diene-2-one	-11209	-11228.1
6,10-Dimethyl-2-undecanone	-8274.6	-8328.3
6,10-Dimethyl-4,5,9-undecatrien-2-one	-7781.6	-7821.6
6,13-Pentacenedione	-10299	-10281.1
6,19-Methanobenzocyclooctadecen-21-one	-13075	-13093.3
6,6-(Tetramethylene-3´-oxa)-7a-(nitroxymethyl)-3-oxoperhydroimidazo[1,5-c]oxazol-6-ium nitrate	-5384.8	-5376.5
6,6-(Tetramethylene-3´-oxa)-7a-methyl-3-oxoperhydroimidazo[1,5-c]oxazol-6-ium nitrate	-5587.5	-5604.8
6,6-Dimethyl-1,5,7-trioxaspirononane	-4624	-4640.9
6,6-Dimethyl-1-phenyl-4,8-dioxaspiro[2.5]octane	-7784.61	-7783.4
6,6-Dimethylfulvene	-4667.3	-4664.9

6,6'-Diphenylfulvene	-9387.46	-9388.5
6,6-Paracyclophane	-13825	-13848.4
6,6-Pentamethylene-7a-(nitroxymethyl)-3-oxoperhydroimidazo[1,5-c]oxazol-6-ium nitrate	-6166.4	-6159
6,7-Dihydro-4(5H)-benzofuranone	-3996	-4012.4
6-Amino-1,3-dimethyluracil	-3186.6	-3198.7
6-Amino-1-hexanol	-4107.8	-4135.9
6-Amino-1-methyluracil	-2496	-2513.3
6-Aminohexaneamide	-3964.08	-3978.9
6-Aminoindazole	-3896	-3895.4
6-Aminopurine	-2781.1	-2798.8
6-Aminoquinoline	-4785.3	-4779.9
6-Aminouracil	-1810.3	-1822.7
6-Aza-2-thiothymine	-2800.9	-2768.1
6-Azathymine	-1963.7	-1917.9
6-Azauracil	-1322.5	-1287.1
6-Chloro-2-benzoxazolone	-3002.8	-3006.8
6-Chloro-2-hydroxypyridine	-2366.1	-2356.8
6-Chloroquinoline	-4508.2	-4520.7
6H-Dibenzo[b,d]pyran	-6481	-6516.1
6-Hydroxyhexanoic acid	-3292.76	-3311
6-Hydroxynicotinic acid	-2485.6	-2488.2
6-Methoxy-1-indanone	-5048.3	-5074.9
6-Methoxy-alpha-tetralone	-5695.9	-5716.4
6-Methoxyquinoline	-5192.4	-5186.6
6-Methoxyquinoline N-oxide	-5133.8	-5148.7
6-Methyl-1-heptene	-5308.85	-5308
6-Methyl-1-indanone	-5184.1	-5201.9
6-Methyl-2-pyridone	-3149.2	-3200.3
6-Methyl-2-thiouracil	-3191.4	-3227.3
6-Methylquinoline	-5314.6	-5316.2
6-Methyluracil	-2351.7	-2377.1
6-Oxabicyclo[3.1.0]hexane	-2980.1	-2999.2
6-Phenyluracil	-4748.4	-4740.2
6-t-Butyl-1,1-dimethylindane	-8878.5	-8875.3
6-Undecanone	-7024.6	-7018.8
7,12-Dimethylbenz[a]anthracene	-10307.4	-10277.5
7,7,8,8-Tetracyanoquinodimethane	-5958.9	-5958.9
7,7-Dimethoxybicyclo[2.2.1]heptane	-5408.32	-5399.1
7-Bromo-5-chloro-8-hydroxyquinoline	-4130.2	-4161.2
7-Hexadecylspiro[4.5]decane	-16787	-16765.6
7-Methoxy-1-tetralone	-5698.2	-5716.4

7-Methyl-1-octene	-5963.65	-5961.4
7-Methylcaprolactam	-4250.1	-4266.9
7-Norbornanone	-4002.4	-3951.2
7-Oxabicyclo[4.1.0]heptane	-3624.9	-3638.7
7-Phenylhept-4-yn-3-one	-7064.7	-7115.1
8-Aminocaprylic acid	-4884	-4901.6
8-Aminoquinoline	-4779.2	-4779.9
8-Methyl-1-nonanol	-6600.83	-6592.1
8-Methyl-1-nonene	-6617.74	-6614.9
8-Methylquinoline	-5324.6	-5316.2
8-Oxabicyclo[5.1.0]octane	-4936.4	-4968.8
8-Oxatricyclo[3,2,1,0(1,5)]octane	-4174.3	-4207.6
8-Oxypurine	-2476.1	-2431.6
8-Quinolinol	-4461.9	-4464.8
9(10H)-Anthracenone	-6863	-6897
9,10(3',4')-Furanoanthracene-12,14-dione	-8380	-8371.3
9,10-Anthraquinone	-6468.9	-6485.5
9,10-Benzophenanthrene	-8949.9	-8965.3
9,10-Dihydroanthracene	-7290.5	-7308.5
9,10-Dihydrophenanthrene	-7307.65	-7311.1
9,10-Dimethylanthracene	-8367.2	-8368.6
9,10-Dimethylphenanthrene	-8344.6	-8359.7
9,10-Diphenylanthracene	-13112.4	-13061.2
9,10-Octalin	-6076.8	-6089.7
9,10-Phenanthrenediol	-6694.8	-6647.8
9,9'-Dimethyl-9,9'-bifluorenyl	-14366	-14374.1
9,9-Dimethylxanthene	-7805.24	-7827.2
9-Acridinamine	-6704	-6685.7
9-Anthroic acid	-7064.5	-7034.6
9-Cyclohexyleicosane	-16989	-16996.7
9-Ethyl-9H-carbazole	-7437.6	-7482.8
9-Fluorenecarboxylic acid	-6632	-6641.9
9H-Fluoren-9-one	-6222.8	-6243.5
9-Hydroxy-1,4-anthraquinone	-6273.2	-6300.7
9-Methoxy-1,4-anthraquinone	-7038.8	-7040.1
9-Methyl-9H-fluorene	-7289.3	-7309.8
9-Methylanthracene	-7714.7	-7707.8
9-Nitroanthracene	-6906.9	-6891.5
9-Oxatetracyclo[5.4.0.0(3,10).0(4,8)]undeca-5-en-2-one	-5121.7	-5127.6
9-Phenyl-9-phosphafluorene	-9982.2	-9991.6
9-Phenyleicosane	-16329	-16330
Abametapir	-6546.7	-6551.4

Acenaphthene	-6222	-6214.8
Acenaphthylene	-6052.2	-6025.6
Acetadote	-3118	-3150
Acetaldehyde	-1167.2	-1164.8
Acetaldehyde thiosemicarbazone	-2847.4	-2816.3
Acetaldol	-2285.56	-2278.3
Acetaldoxime	-1425	-1427.6
Acetamide	-1191.5	-1208
Acetanilide	-4228.08	-4240.6
Acetic acid	-875.12	-881.1
Acetic anhydride	-1794.2	-1783.8
Acetoacetanilide	-5107.51	-5135.7
Acetoacetyldiphenylamine	-8211.9	-8183.5
Acetol	-1625.23	-1607
Acetone di-n-propylhydrazone	-6336.2	-6327.4
Acetone ethylisopropylhydrazone	-5667.2	-5672.3
Acetone glycerol	-3390	-3432.9
Acetone isopropylhydrazone	-4340.5	-4321.1
Acetone methylhydrazone	-3043.9	-3019.5
Acetone oxime	-2052	-2058.4
Acetone propylhydrazone	-4355.5	-4324
Acetone thiosemicarbazide	-3483.8	-3447.1
Acetonitrile	-1256.33	-1269.8
Acetophenone	-4151.6	-4151.5
Acetophenone ethylene glycol ketal	-5306.3	-5316.8
Acetophenone oxime	-4414	-4418.9
Acetovanillone	-4444.7	-4456.2
Acetoxyacetic acid	-1553.45	-1576.6
Acetoxyl	-6568.7	-6551.2
Acetylacetone	-2687	-2686.5
Acetylacetone (enol form)	-2685.4	-2674.5
Acetylchloride	-967.15	-976.6
Acetylfluoride	-947.5	-968.2
Acetylmesitylene	-6062.2	-6082.9
Aconitic acid	-1987.1	-1967.8
Acrylamide	-1683	-1702.9
Acrylic acid	-1374	-1373.4
Actiprofen	-7145.62	-7145.2
Adamantane	-6033.35	-6037.2
Adiponitrile	-3591.62	-3604.4
Adipoyl-bis(N,N-diethylthiourea)	-11143	-11144.3
Adrucil	-1599.7	-1623.5

AIBN	-5109.1	-5109.8
Alanine anhydride	-3292.8	-3282.8
Alanylalanine	-3267.5	-3265.2
Alanylglycine	-2618.9	-2614.7
Alanylphenylalanine	-6301.1	-6281.8
Allantoin	-1714	-1711.7
all-trans-1,5,9-Cyclododecatriene	-7321.2	-7304.3
Allyl acetate	-2726.65	-2736.7
Allyl alcohol	-1868.28	-1857.3
Allyl bromide	-1886.98	-1887.6
Allyl chloride	-1888.15	-1883.6
Allyl chloroacetate	-2606	-2579
Allyl cyanide	-2406.4	-2415.5
Allyl dichloroacetate	-2479	-2457.2
Allyl ethyl sulfide	-3969	-3977.9
Allyl ethyl sulfoxide	-3826.2	-3818.9
Allyl iodide	-1950.56	-1952.6
Allyl phenyl ether	-4932.9	-4911.1
Allyl sulfide	-4468.9	-4470.2
Allylamine	-2204	-2198.3
Allylcyclopentane	-5087.83	-5086.5
Allylmethylsulfone	-2936.46	-2942.7
Allyl-t-butylsulfide	-5269.94	-5268.9
Allyltrichloroacetate	-2356	-2334.1
alpha,alpha'-Dihydroxy-4-diisopropylbenzene	-6751.6	-6750.9
alpha,alpha-Dimethyl-p-isopropylbenzyl alcohol	-6950.9	-6959.2
alpha,beta-Glucooctanoic-1,4-lactone	-3461.7	-3475.4
Alpha-caran-3-ol	-6178.1	-6201.5
alpha-Chlorohydrin	-1679	-1667.4
alpha-D-Glucose	-2805	-2802.4
alpha-Fluorocinnamic acid	-4240.6	-4255.4
alpha-Ionone	-7697.3	-7703.9
alpha-Methylstyrene	-5044.52	-5043.8
alpha-Phellandrene	-6165.9	-6161.9
alpha-Phenyl-1-piperidinoacetonitrile	-7499.5	-7506
alpha-Pinene	-6205.3	-6208.2
alpha-Santonin	-7890.2	-7904.8
Amfepramone	-7635.3	-7626.4
AMFOX-7	-1819.9	-1833.5
Aminomethylcumulene	-2774.47	-2730.5
Ammelide	-1259	-1275.6
Amyl acetate	-4205.56	-4204.9

Amyl formate	-3585.48	-3584.8
Amylamine	-3675.38	-3666.5
Amylbenzene	-6530.17	-6523.4
Androstane	-11740	-11698.6
Androsterone	-11095	-11085.1
Anethole	-5544.5	-5549.8
Angelic acid	-2656.9	-2650.1
Aniline	-3384.51	-3370.6
Aniline-4-sulfonic acid	-3351.2	-3357.5
Anthracene	-7054.5	-7059.6
Anthracene photodimer	-14169	-14119.5
Anthracene-9,10-endoperoxide	-7025.8	-6984.7
anti-Tricyclo[4.2.0.02.5]octane	-5032	-5024.4
Arabinose	-2340.4	-2342.4
Arabinosylhypoxanthine	-4802.2	-4790.5
Arabitol	-2559.8	-2561.1
Arachic alcohol	-13130	-13127.5
Arachidic acid	-12655.9	-12643.6
Asparagine	-1928.5	-1936
Aspartic acid	-1602.9	-1609.1
Aspirin	-3926.71	-3932.9
Atrazine	-5049.51	-5056.6
Azepane	-4129.7	-4100
Azidobenzene	-3421.99	-3452.5
Aziridine	-1591.36	-1591.9
Azocane	-4799.3	-4773.4
Azocyclohexane N,N'-dioxide	-7625	-7658.8
Azodicarbonamide	-1066.9	-1038.9
Azomethane	-1794.41	-1796
Azomethane N,N'-dioxide	-1646.41	-1630.7
Azo-t-butane N,N'-dioxide	-5514.77	-5512.7
Barbital	-4110	-4092.4
Barbituric acid	-1501.6	-1486.2
Behenyl alcohol	-14450	-14434.5
Benzal 3-hydrazino-5-methyl-1,2,4-triazole	-5765	-5761.7
Benzal diacetate	-5270.5	-5264
Benzalacetophenone	-7626.2	-7637.6
Benzalaniline	-6872.7	-6870
Benzaldehyde	-3527.47	-3522.7
Benzaldehyde diethylacetal	-6246.87	-6233.4
Benzaldehyde dimethyl acetal	-4951.68	-4950.3
Benzamide	-3554.8	-3568.5

Benzanilide	-6598.8	-6601.1
Benzanthrone	-8114	-8144
Benzene	-3267.62	-3264
Benzenecarbothioamide	-4401.4	-4398.2
Benzenesulfonamide	-3643.3	-3639.9
Benzenethiol	-3887.2	-3872.5
Benzhydryl chloride	-6766.8	-6756.7
Benzil	-6784	-6748.7
Benzil dioxime	-7277.2	-7283.5
Benzimidazole	-3691	-3726.4
Benzimidazole-2-thione	-4262.6	-4293
Benzo[a]anthracene	-8969	-8960.1
Benzo[a]phenanthrene	-8943.5	-8962.7
Benzo[a]pyrene	-9759.1	-9769.9
Benzo[b]furan	-3970.7	-3982.7
Benzo[b]triphenylene	-10842	-10863.1
Benzo[c]cinnoline	-6144.8	-6164
Benzo[c]phenanthrene	-8983	-8969
Benzo[e]pyrene	-9745.7	-9772.5
Benzo[f]quinoline	-6552.5	-6573.8
Benzo[g,h,i]perylene	-10529	-10579.7
Benzo[g]quinoline	-6550.8	-6571.1
Benzo[h]quinoline	-6562	-6571.1
Benzo[k]fluoranthene	-9757.6	-9797.2
Benzo-15-crown-5	-7585.1	-7565.1
Benzocyclobutane	-4447	-4405.8
Benzofuran-2-carboxylic acid	-3943.9	-3940.3
Benzofurazan	-3168.5	-3210
Benzofurazan N-oxide	-3151.6	-3163.2
Benzoic acid	-3229.2	-3239
Benzoic anhydride	-6507.4	-6499.5
Benzoin	-6976	-6966.7
Benzonitrile	-3632.3	-3627.7
Benzophenone	-6515.5	-6518.3
Benzophenone anilide	-9872.9	-9856.1
Benzophenoneimine	-6860.4	-6888.5
Benzothiazole	-4215.1	-4230.6
Benzotrifluoride	-3355.66	-3384
Benzoyl chloride	-3332.6	-3334.4
Benzoyl nitrile	-3901.5	-3869.6
Benzoyl(cyclohexyloxy)-carbonylperoxide	-7054.2	-7026.3
Benzoylacetonitrile	-4549.7	-4523.1

Benzoylformic acid	-3518.35	-3483.6
Benzoylglycylglycine	-5216.4	-5223.3
Benzyl acetate	-4605.26	-4602.4
Benzyl alcohol	-3743	-3722.9
Benzyl benzoate	-6955.85	-6960.2
Benzyl chloroformate	-3797.6	-3779.2
Benzyl ethyl ether	-5094.3	-5087.1
Benzyl formate	-3989.14	-3982.3
Benzyl isonitrile	-4373.7	-4363
Benzyl mercaptan	-4546.9	-4534.4
Benzyl methyl sulfone	-4808.3	-4808.3
Benzylamine	-4075	-4066.5
Benzylaniline	-7079.58	-7060.3
Benzylazide	-4123	-4111.1
Benzylbromide	-3757.89	-3753.2
Benzyldiphenylamine	-10091	-10102.6
Benzylethylamine	-5391.5	-5393
Benzylethylsulfide	-5853.2	-5843.5
Benzylidenemalonic acid	-4363.1	-4340.1
Benzylidene-p-isopropylphenylacetonitrile	-9746	-9706.3
Benzylmethylsulfide	-5205.1	-5191.2
beta-Alanine	-1622.9	-1634.3
beta-Butylaminoisobutyronitrile	-5356.3	-5366.5
beta-Cyanopropiophenone	-5149.9	-5176.6
beta-D-Fructose	-2809.8	-2793.5
beta-HCH	-2959.5	-2951.4
beta-Hydroxynaphthaldehyde	-5209.2	-5214.3
beta-Isopropyl-beta-propiolactone	-3368	-3400.7
beta-Nitrostyrene	-4179	-4182.4
beta-Resorcylic acid	-2814.1	-2821.6
Bicyclo[1.1.0]butane	-2626.85	-2576.6
Bicyclo[2.2.2]oct-2-ene	-4839.7	-4799
Bicyclo[2.2.2]octan-2-one	-4589	-4567.5
Bicyclo[2.2.2]octane	-5001.93	-4979
Bicyclo[3.1.0]hexane	-3831.34	-3791.8
Bicyclo[3.2.2]non-6-ene	-5490.9	-5456.4
Bicyclo[3.3.1]non-2-ene	-5445.1	-5452.5
Bicyclo[3.3.1]nonane	-5650.1	-5644
Bicyclo[3.3.2]decane	-6343.4	-6302.8
Bicyclo[4.2.1]non-3-ene	-5496.3	-5466.4
Bicyclo[4.4.1]undeca-1,3,5,7,9-pentaene	-6015.81	-6027.9
Bicyclo[4.4.1]undeca-1,3,5,8-tetralene	-6230	-6224.1

Bicyclo[5.1.0]octane	-5092.13	-5087.4
Bicyclohexane	-7597.81	-7598.1
Bicyclopentyl	-6333.02	-6335.1
Bicyclopropyl	-3886.1	-3883.6
Biphenyl	-6276	-6270
Bis(1-ethylhexyl) o-phthalate	-13791	-13768.6
Bis(2,2,2-Trinitroethyl)urea	-2522.5	-2506.4
Bis(2,4,5-trimethylphenyl)-methane	-10759.5	-10775.8
Bis(2-chloroethyl) ether	-2408.13	-2413.8
Bis(2-ethylhexyl) adipate	-13404.14	-13404.5
Bis(2-hydroxyethyl)methylamine	-3354.23	-3351.8
Bis-(2-hydroxyethyl)terephalate	-5527.42	-5572.8
Bis(2-methylbenzoyl) peroxide	-7802	-7837.1
Bis(2-methylphenoxy)dimethylsilane	-9425	-9420.4
Bis(2-methylphenyl)acetylene	-8537.3	-8556.4
Bis(3-methylphenoxy)dimethylsilane	-9424	-9425.6
Bis(4-aminophenyl) ether	-6376.1	-6348.2
Bis(4-methylbenzoyl) peroxide	-7851	-7837.1
Bis(4-methylphenoxy)dimethylsilane	-9424	-9420.4
Bis(4-methylphenyl)acetylene	-8528.3	-8556.4
Bis(chloromethyl)ether	-1124.16	-1128.5
Bis(cyclohexyl)amine	-7782.4	-7784.4
Bis(hydroxymethyl)urea	-1606.8	-1626
Bis(isopropyl) peroxide	-4092.8	-4072
Bis(piperidino)methane	-7326.5	-7348.6
Bis(trifluoroacetyl)methane	-1673.7	-1640.7
Bisphenol A	-7820	-7808.3
Bisphenol AP	-10194	-10166.2
Bisphenol E	-7163.2	-7155.2
Bisphenol F	-6502.4	-6501.1
Brassidic acid	-13775	-13770.6
Bromobenzene	-3111.9	-3113.1
Bromoethane	-1424.7	-1395.3
Bromopentafluoroethane	-569.72	-571.3
Bromoxynil octanoate	-7946.7	-7940.8
Brucine	-12279	-12235.1
Bullvalene	-5626.5	-5603
But-1-enyl-4-tolyl sulphone	-6595.7	-6603.5
Butadiyne	-2333.91	-2315.6
Butanal	-2479.4	-2471.8
Butanamide	-2496.3	-2515
Butane	-2857.9	-2856

Butane-1,2,3-triyl trinitrate	-2175.7	-2167.7
Butane-1,2,4-triol	-2260.1	-2294.3
Butanedioic acid	-1495	-1520.2
Butanoic acid	-2185	-2188.1
Butanoic anhydride	-4433.21	-4397.7
Butyl 1,1-dimethylpropyl ether	-5971.5	-5976.8
Butyl 2-methylbutyrate	-5504.5	-5511.1
Butyl 2-pentenoate	-5333.3	-5331.9
Butyl 3-pentenoate	-5345.6	-5331.9
Butyl 4-oxopentanoate	-5091.7	-5100.4
Butyl 4-pentenoate	-5352.3	-5350.6
Butyl anthranilate	-6029.7	-6015.6
Butyl butanoate	-4844.2	-4858.4
Butyl crotonate	-4684.53	-4678.4
Butyl ethyl sulfide	-4794.9	-4792.6
Butyl isopropylamine	-4980.9	-4995.3
Butyl lactate	-4026	-4011.4
Butyl oleate	-13843	-13827
Butyl pentanoate	-5504.85	-5511.9
Butyl pivalate	-5497.7	-5504.2
Butyl propanoate	-4200.3	-4204.9
Butyl t-butyl ether	-5317.3	-5320.7
Butyl vinyl ether	-3857	-3842.8
Butylbenzene	-5876.68	-5869.9
Butylcellosolve	-3867.38	-3852.2
Butylcyclohexane	-6530.3	-6534
Butylcyclopentane	-5903.95	-5903.8
Butylene carbonate	-2470.8	-2484.5
Butylisobutyl amine	-5647.7	-5645.7
Butylisopropylether	-4678.94	-4680.2
Butylmethylamine	-3684.2	-3691.1
Butylmethylketone	-3753.8	-3751.4
Butylsilane	-4027.7	-4033.4
Butyraldehyde n-butylhydrazone	-5676.4	-5653.6
Butyric acid methylester	-2900	-2909.9
Butyronitrile	-2568.2	-2576.7
Butyrophenone	-5464.95	-5455.8
Caffeic acid	-3934.9	-3951.5
Caffeine	-4255.08	-4216.9
Camphene	-6148.4	-6157.4
Camphor	-5910.7	-5914.2
Camphor oxime	-6196.9	-6181.6

Capraldehyde	-6404.1	-6392.6
Caproic aldehyde	-3786.93	-3778.7
Caprolactone	-3315.38	-3311.9
Caprylamide	-5104.48	-5128.9
Caprylene	-5313.85	-5308.7
Caprylic aldehyde	-5103.3	-5085.6
Caprylonitrile	-5188.04	-5190.6
Carane	-6393.4	-6404.6
Carbanilide	-6713.7	-6706.9
Carbazole	-6133.5	-6139.9
Carbitol	-3738.5	-3725.4
Carbon suboxide	-1059.1	-1094.7
Carbonothioic dihydrazide	-1961	-1979.1
Carvenone	-5895.7	-5930.4
Cellobiose	-5642.1	-5619.8
Cetane	-10699	-10697.6
Cetene	-10539	-10536.4
Cetylmethacrylate	-12567.05	-12527.4
Chloral	-780.19	-762.2
Chloranil	-2156	-2156.6
Chlorfenidim	-4861.8	-4893.6
Chloroacetaldehyde	-986.46	-1007.1
Chloroacetamide	-1044	-1050.3
Chloroacetic acid	-726.8	-723.4
Chloroacetic acid ethylester	-2089.44	-2086.8
Chloroacetyl chloride	-833.72	-818.8
Chlorobenzene	-3112.7	-3111.4
Chlorocyclohexane	-3747.6	-3759.2
Chloroethane	-1413.1	-1391.3
Chloroethylene	-1240.05	-1253.6
Chlorohydroquinone	-2702	-2694.3
Chloromethyl methyl ether	-1289.18	-1287.3
Chloromethylacetate	-1451	-1444.1
Chloropentafluorobenzene	-2428.7	-2392.4
Chloroxynil	-3059	-3113.8
Cholest-5-en-3-ol	-16524	-16553.6
Cholesteryl methyl ether	-17226	-17253.6
Cinnamamide	-4720.6	-4698.1
cis- Cyclooctene	-5078.33	-5059.4
cis,cis-1,4-Diphenylbutadiene	-8501.31	-8518.7
cis-1-(Cyclohexylmethyl)-2-ethylcyclohexane	-9548	-9564.4
cis-1-(Cyclohexylmethyl)-4-ethylcyclohexane	-9548	-9561.8

cis-1,2-Bis(2-tolyl)ethylene	-8663.4	-8680.2
cis-1,2-Cyclohexanedicarboxylic acid	-3869	-3885.3
cis-1,2-Cyclohexanediol	-3523.56	-3533.9
cis-1,2-Cyclohexanol diacetate	-5280.6	-5292.7
cis-1,2-Cyclopentanediol	-2909	-2903.7
cis-1,2-Dichloroethylene	-1123.32	-1100.6
cis-1,2-Diethylcyclopropane	-4678.66	-4678.6
cis-1,2-Dimethyl-3,3-dichlorocyclopropane	-3071.3	-3073
cis-1,2-Dimethylcyclohexane	-5226.55	-5227.7
cis-1,2-Dimethylcyclopentane	-4593.29	-4593.5
cis-1,2-Dimethylcyclopropane	-3370.4	-3369.1
cis-1,2-Diphenylcyclopropane	-8082.2	-8084.8
cis-1,2-Divinylcyclobutane	-4987.3	-4996.7
cis-1,2-Indandiol diacetate	-6341.7	-6347
cis-1,2-Indanediol	-4592	-4588.2
cis-1,3-Dimethylcyclohexane	-5219.1	-5221.1
cis-1,3-Dimethylcyclopentane	-4587.3	-4590.9
cis-1,4-Cyclohexanedicarboxylic acid	-3866.9	-3885.3
cis-1,4-Dimethylcyclohexane	-5222.8	-5225
cis-1,4-Di-t-butyl-cyclohexane	-9145.4	-9142.4
cis-1-Ethyl-2-methylcyclohexane	-5877.8	-5879.8
cis-1-Ethyl-2-methylcyclopentane	-5247.55	-5249.6
cis-1-Ethyl-3-methylcyclohexane	-5871.05	-5877.2
cis-1-Ethyl-3-methylcyclopentane	-5243.95	-5245
cis-1-Ethyl-4-Methylcyclohexane	-5879.25	-5881.1
cis-1-Methyl-1,2-cyclohexanediol	-4147	-4178.4
cis-1-Methyl-1,2-dicyclopropylcyclopropane	-6355.9	-6350.1
cis-1-Phenyl-3,3-dimethyl-1-butene	-7023.4	-6998.2
cis-1-Phenylcyclopropane-2-carboxylic acid	-5047.13	-5056.4
cis-2,2,4,6-Tetramethyl-1,3-dioxane	-4895.4	-4900.4
cis-2,2,5,5-Tetramethyl-3-hexene	-6629.8	-6609.9
cis-2,2-Dimethyl-3-hexene	-5298.4	-5296.4
cis-2,3-Dimethylthiirane	-3297.4	-3324.1
cis-2,4,6-Trimethyl-1,3,5-trioxane	-3394.2	-3439
cis-2,4-Dimethyl-1,3-dioxane	-3610.8	-3624
cis-2,5-Dimethyl-3-hexene	-5287.31	-5297.5
cis-2-Butene	-2709.4	-2676
cis-2-Butene-1,4-diol	-2346.28	-2308.1
cis-2-Decalone	-5867.2	-5874.4
cis-2-Heptene	-4650.2	-4636.4
cis-2-Hexahydroindanone	-5235.3	-5239.6
cis-2-Hexene	-3994.87	-3983

cis-2-Methoxycinnamic acid	-4889.4	-4879.2
cis-2-Methyl-5-ethyl-1,3-dioxane	-4236.3	-4286.2
cis-2-Methylcyclohexanol	-4380.2	-4381.4
cis-2-Methylcyclopentanol	-3744	-3747.3
cis-2-Octene	-5302.66	-5289.9
cis-2-Pentene	-3343.2	-3329.5
cis-2-pentenenitrile	-3039.8	-3050.2
cis-3,4-Dihydro-3,4-dimethyl-2,5-furandione	-2846.4	-2853.9
cis-3,4-Dimethyl-2-pentene	-4633.8	-4624.3
cis-3,5-Dimethylpyrrolizidine	-5856.8	-5872.7
cis-3-Decen-1-yne	-6325.1	-6326.6
cis-3-Heptene	-4654.25	-4636.4
cis-3-Hexene	-3999.86	-3983
cis-3-Methyl-3-hexene	-4639.4	-4625
cis-3-Methylcyclohexanol	-4334.7	-4377.5
cis-3-Octene	-5306.05	-5289.9
cis-3-Penten-1-yne	-3053.41	-3059.3
cis-4,4-Dimethyl-2-pentene	-4650.1	-4643
cis-4,4'-Dimethylstilbene	-8686.43	-8677.6
cis-4,4'-Dinitrostilbene	-7012.4	-7055.6
cis-4,6-Dimethyl-1,3-dioxane	-3601.3	-3625.4
cis-4-Coumaric acid	-4174	-4157.4
cis-4-Hydroxycyclohexanecarboxylic lactone	-3717.5	-3707.9
cis-4-Methoxycinnamic acid	-4910.3	-4879.2
cis-4-Methylcyclohexanol	-4338	-4381.4
cis-4-Octene	-5306.45	-5289.9
cis-8-Methyl-2-hydrindanone	-5873.8	-5888.7
cis-9-Methyldecalin	-6943.1	-6937.7
cis-Azobenzene	-6514.1	-6496.5
cis-Benzaldehyde oxime	-3782.48	-3785.4
cis-Bicyclo[3.3.0]octan-2-one	-4578.6	-4581.4
cis-Bicyclo[3.3.0]octane	-5016	-5016.2
cis-Bicyclo[4,2,0]octane	-5084.2	-5077
cis-Bicyclo[4.3.0]nona-3,7-diene	-5320.9	-5286.5
cis-Bicyclo[6.1.0]nonane	-5751.12	-5751.1
cis-Butenedinitrile	-2129.31	-2117.5
cis-Cyclodecene	-6391.7	-6376
cis-Cyclohexane-1,2-dicarboxylic anhydride	-3914.8	-3916.1
cis-Cyclohexane-1,3-dicarboxylic acid	-3869	-3887.9
cis-Cyclohexane-1,3-dicarboxylic acid dimethyl ester	-5375.4	-5328.9
cis-Cyclononene	-5749.8	-5721.2
cis-Decalin	-6292.52	-6285.9

cis-Dihydro-3,4-diethyl-2,5-furandione	-4169.7	-4162.9
cis-Heptacyclene	-12008	-12030.2
cis-Hydrindan	-5659.02	-5655.7
cis-Octahydroinden-1-one	-5214.9	-5230.3
cis-Oxolane-3,4-diol diacetate	-3885.1	-3859
cis-Oxolane-3,4-diol dinitrate	-2025.5	-2020.2
cis-Perfluorobicyclo[4.3.0]nonane	-3151.4	-3148.2
cis-Stilbene	-7404	-7391.7
cis-Tetralin-1,2-diol	-5228.3	-5227.7
Citraconic acid	-2002	-1982.2
Citric acid monohydrate	-1953.1	-1969
Colamine	-1514.15	-1522.1
Coproporphyrin I tetramethylester	-20871	-20846
Cortisone	-11204	-11186.1
Coumarin	-4139.2	-4179
Creatine	-2325.6	-2318.4
Creatinine	-2359.8	-2354.6
Crotonaldehyde	-2294.45	-2291.8
Crotonic acid	-2002.1	-2008.1
Cryofluorane	-589.9	-612.2
Cubane	-4833.27	-4821
Cumene	-5218.95	-5210.5
Cumulene	-1944.4	-1938.8
Cumyl chloride	-5063.6	-5039.3
Cumyl perbenzoate	-8351	-8312.6
Cumylhydroperoxide	-5098.4	-5073
Cyanamide	-738.14	-728.8
Cyanoacetamide	-1577	-1582.2
Cyanoacetic acid	-1254	-1255.4
Cyanoethylene	-1756.4	-1762.1
Cyanoguanidine	-1377.6	-1396.9
Cyanuric acid	-905.8	-911.2
Cyclam	-7249	-7265.6
Cyclobutane	-2722.3	-2711.9
Cyclobutane-1,3-dione	-1886	-1888.9
Cyclobutanecarbonitrile	-3071	-3082.8
Cyclobutanecarboxylic acid	-2685.6	-2694.1
Cyclobutanol	-2518.2	-2518.5
Cyclobutanone	-2292.1	-2300.4
Cyclobutylamine	-2867.79	-2866
Cyclodeca-1,2,6,7-tetraene	-5932.9	-5932.1
Cyclodecane	-6586.2	-6556

Cyclodecanone	-6144.3	-6153.5
Cyclododecane	-7845.4	-7855.3
Cyclododecanone	-7436.1	-7445.1
Cyclogeraniolane	-5882.3	-5874.2
Cycloheptadecane	-11126.1	-11120.8
Cycloheptadecanone	-10727	-10722.7
Cycloheptane	-4597	-4579.5
Cycloheptanol	-4362	-4380.8
Cycloheptene	-4428.3	-4394.3
Cyclohexadecane	-10466	-10466
Cyclohexane	-3922.5	-3920.8
Cyclohexane-1,1-dicarboxylic acid	-3893.6	-3886.9
Cyclohexanecarbonitrile	-4279.5	-4291.7
Cyclohexanecarboxylic acid	-3883.7	-3905.7
Cyclohexanemethanol	-4377.3	-4390.9
Cyclohexanethiol	-4540.45	-4535.7
Cyclohexanol	-3737	-3727.4
Cyclohexanone	-3517.6	-3509.3
Cyclohexanone oxime	-3779.9	-3776.7
Cyclohexene	-3751.9	-3740.8
Cyclohexyl acetate	-4598.85	-4606.8
Cyclohexyl butanoate	-5912.4	-5913.7
Cyclohexyl formate	-3977.02	-3986.7
Cyclohexyl iodide	-3835.9	-3842.1
Cyclohexyl methyl carbonate	-4440	-4435.9
Cyclohexylamine	-4074.07	-4074.9
Cyclohexylhydroperoxide	-3805.6	-3790.3
Cyclohexylisocyanate	-4128.89	-4098.6
Cyclononane	-5931.4	-5902.7
Cyclononanone	-5493.4	-5494.2
Cyclooctane	-5265.7	-5227.8
Cyclooctanone	-4822.9	-4818.9
Cyclooctatetraene	-4545.7	-4507.8
Cyclopentadecane	-9814	-9820.4
Cyclopentadecanone	-9412.3	-9416.5
Cyclopentadiene	-2932.83	-2912
Cyclopentane	-3293.1	-3290.6
Cyclopentanethiol	-3911.85	-3905.5
Cyclopentanol	-3096.7	-3097.1
Cyclopentanone	-2873.5	-2865.1
Cyclopentene	-3115.2	-3101.3
Cyclopentyl methyl sulfide	-4568.2	-4567.6

Cyclopentylacetic acid	-3920.81	-3926.3
Cyclopentylamine	-3444.5	-3444.7
Cyclopentylcycloheptane	-7640	-7621.4
Cyclopentylcyclohexane	-6956.7	-6963.2
Cyclopropane	-2074.48	-2066.2
Cyclopropanecarbonitrile	-2429.4	-2437.1
Cyclopropanecarboxamide	-2385.66	-2375.3
Cyclopropanecarboxylic acid	-2035	-2048.4
Cyclopropylacethylene	-3086.1	-3099.6
Cyclopropylaldimine	-2726.66	-2704
Cyclopropylamine	-2226.7	-2220.2
Cyclopropylbenzene	-5076.4	-5074.2
Cyclopropylethan-1-one oxime	-3226.3	-3225.6
Cyclotetradecane	-9138.7	-9168
Cyclotridecane	-8521.8	-8517.2
Cycloundecane	-7237.3	-7194.7
Cycloundecanone	-6800.7	-6789
Cytosine	-2067.3	-2085.4
DAAF	-2575.6	-2571.7
DABCO	-4104.3	-4083.8
DADP	-3644.5	-3667.2
DCP	-10062	-10074
Decahydroazulene	-6324	-6309.2
Decalin-9-hydroperoxide	-6158.4	-6145.8
Decane	-6777.9	-6776.8
Decanedioic acid	-5425	-5441
Decanenitrile	-6492.1	-6497.6
Decyl 2-cyanoacrylate	-8380.6	-8327.3
Decylbenzene	-9793	-9790.8
Decylcyclohexane	-10451	-10457.4
Decylcyclopentane	-9829.61	-9827.2
delta-HCH	-2959.5	-2951.4
Desmotroposantonin methyl ether	-8486.3	-8518.4
Desoxyribose	-2527.3	-2556.8
D-Fucose	-2978.9	-2986.3
D-Galactose	-2805.6	-2802.4
D-Glucaric acid 1,4-lactone	-2068.5	-2065.2
D-Gluconolactone	-2533.8	-2535.4
D-Glucose 2,3,4,5,6-pentaacetate	-7190.9	-7229.5
d-Glycero-gulo-heptono-1,4-lactone	-2981.9	-3014.6
D-Gulose	-2817.3	-2802.4
Di-(2-chloroethoxy)methane	-2907.92	-2917.6

Di(2-ethylhexyl)amine	-10889.83	-10885.9
Di(2-pyridyl)ketone	-4668.3	-4654
Di(ethoxy-NNO-azoxy)methane	-3681.1	-3686.6
Di(methoxy-NNO-azoxy)methane	-2401.2	-2403.5
Diacetin	-3348.9	-3399.9
Diacetoxymethane	-2273.8	-2263.6
Diacyldiperoxide I	-13872	-13884.4
Diacylperoxide II	-14536	-14537.9
Diacylperoxide III	-4761.3	-4735.9
Diacylperoxide IV	-9966.8	-9963.6
Diacylperoxide V	-15164	-15191.4
Diacylperoxide VI	-7364.8	-7349.7
Diacylperoxide VII	-8668	-8656.7
Diacylperoxide VIII	-17774	-17805.3
Dialuric acid	-1316.3	-1292.7
Diamantan-1-ol	-7938.6	-7954.4
Diamantan-3-ol	-7953.7	-7964
Diamantan-4-ol	-7932.5	-7950.5
Diamantane	-8131.2	-8153.5
Diamylamine	-6964.76	-6956
Dibenz[a,h]anthracene	-10836	-10860.5
Dibenzamide	-6836.3	-6797.7
Dibenzo[a,e]cyclooctene-5,11-dicarbonitrile	-8983	-8981
Dibenzo[b,d]thiophene	-6592.1	-6613.8
Dibenzo[b,e]pyridine	-6593.8	-6573.8
Dibenzofuran	-5836.3	-5873.9
Dibenzo-p-dioxin	-5716.7	-5736.9
Dibenzosuberenone	-7326	-7370.5
Dibenzosuberone	-7568.8	-7550.5
Dibenzoylmethane	-7386	-7394.1
Dibenzoylmethane (keto form)	-7398.5	-7404.8
Dibenzyl carbonate	-7436.8	-7430.9
Dibenzylamine	-7786	-7750.9
Dibenzylideneazine	-7563	-7576.1
Dibenzylsulfide	-8216.35	-8201.4
Dibenzylsulfone	-7829.6	-7818.4
Dibutoxymethane	-5850.5	-5846.9
Dibutyl decanedioate	-10793.57	-10781.6
Dibutyl phthalate	-8598	-8557.2
Dibutylamine	-5651.2	-5649
Dibutyldiazene	-5684.27	-5711.9
Dibutyldisulfide	-6702.3	-6705.5

Dibutylperoxide	-5343.67	-5397.9
Dibutylsulfate	-5422.16	-5413.2
Dibutylsulfide	-6102	-6099.5
Dibutyrylperoxide	-4479.1	-4449.4
Dichlorfenidim	-4690.31	-4740.9
Dichloroacetaldehyde	-900.65	-885.3
Dichloroacetic acid	-624	-601.6
Dichloroacetyl chloride	-717.29	-697
Dichlorodiethylsilane	-3367.48	-3379.9
Dichlorodimethylsilane	-2058.19	-2053.7
Dichlorodiphenylsilane	-6810.36	-6802.3
Dicumyl	-10188	-10186
Dicyanomethane	-1654.2	-1644
Di-cyclohexadiene	-7035	-6995.7
Dicyclohexanone diperoxide	-7114.3	-7103.8
Dicyclohexyl carbonate	-7429.5	-7439.7
Dicyclohexyl phthalate	-10655	-10679.5
Dicyclohexylsulfide	-8216.8	-8209.2
Dicyclopentylmethane	-6986.6	-6991.2
Dicyclopropyldinitromethane	-4218.1	-4204
DIDP	-16407.22	-16397.5
Diethanolamine	-2652.3	-2667.2
Diethoxydimethylsilane	-4769	-4774.2
Diethyl cellosolve	-3913.35	-3909.4
Diethyl cis-cyclohexane-1,3-dicarboxylate	-6647	-6612
Diethyl disulfide	-4089.9	-4091.6
Diethyl maleate	-4068.13	-4066.9
Diethyl oxalate	-2981.3	-2939.9
Diethyl phthalate	-5946	-5944.6
Diethyl succinate	-4239.26	-4246.9
Diethyl sulfate	-2792.3	-2799.3
Diethyl sulfite	-3004.8	-3008.6
Diethyl sulphide	-3486.1	-3485.7
Diethyl tartrate	-3877.3	-3859.4
Diethylacetamide	-3883.6	-3887.1
Diethylamine	-3035	-3035.2
Diethylaminoacetone	-4617.3	-4612.7
Diethylammonium diethyldithiocarbamate	-7639.6	-7650
Diethylcarbonate	-2715.2	-2715.2
Diethylcyanamide	-3412.5	-3402.7
Diethyldimethylsilane	-5285	-5291.9
Diethyldipropylsilane	-7922	-7925.1

Diethylene glycol dimethyl ether	-3805.5	-3806.5
Diethyleneglycol	-2374.7	-2361.3
Diethyleneglycol-di-n-butylether	-7719.97	-7703.5
Diethylketone	-3102.3	-3098
Diethylnitramine	-2897	-2896
Diethylperoxide	-2781.9	-2784
Diethylsulfone	-3090	-3102.7
Diethylsulfoxide	-3337.5	-3326.7
Difluoroacetal	-876.9	-838
Difluoroacetic acid	-562.96	-554.3
Difluoroacetyl fluoride	-655.7	-641.3
Diformylhydrazine	-1027.7	-1029.9
Dihexyl phthalate	-11177.97	-11171.1
Dihexyladipate	-10788.67	-10781.6
Dihydro-2(3H)-thiophenone	-2800	-2819.8
Dihydrodicyclopentadiene	-5912	-5933.9
Diisoamyl phthalate	-9857.9	-9868
Diisobutyl azelate	-10092	-10121.5
Diisobutyl disulfide	-6697	-6698.9
Diisobutyl phthalate	-8556.6	-8550.6
Diisobutyl sulfone	-5701.4	-5715.2
Diisobutylamine	-5638.1	-5642.4
Diisobutylammonium diisobutyldithiocarbamate	-12891	-12868.4
Diisobutylene	-5292.45	-5312.7
Diisobutylsulfide	-6093.2	-6092.9
Di-isooctyl phthalate	-13797.65	-13783.6
Diisopentyl sulfide	-7404.3	-7405.1
Diisopentylamine	-6974.3	-6954.6
Diisopentylsulfide	-7399.1	-7391.2
Diisopropanolamine	-3934.98	-3955.2
Diisopropenyldiacetylene	-5811.83	-5829.2
Diisopropyl ether	-4010.4	-4028.8
Diisopropyl sebacate	-9406	-9455.7
Diisopropyl suberate	-8151.3	-8148.7
Diisopropyl sulfide	-4782.7	-4772.6
Diisopropylamine	-4333.8	-4341.5
Diisopropylammonium diisopropyldithiocarbamate	-10260	-10252.6
Diisopropylcyanamide	-4704	-4703.8
Diisopropyldiazene	-4400.58	-4399.1
Dimedone	-4379.7	-4397.1
Dimethoxane	-4278.63	-4285.6
Dimethoxymethane	-1947.2	-1950

Dimethyl 1,1-cyclopropanedicarboxylate	-3463	-3475.9
Dimethyl 1,2-cyclopentanedicarboxylate	-4676.5	-4675.4
Dimethyl carbonate	-1429.3	-1432.1
Dimethyl carbonotrithioate	-3836	-3827.3
Dimethyl cubane-1,4-dicarboxylate	-6218.1	-6227
Dimethyl cyclobutane-1,2-dicarboxylate	-4119	-4120
Dimethyl disulfide	-2788.3	-2787.1
Dimethyl fumarate	-2777.1	-2783.8
Dimethyl glutarate	-3612	-3617.2
Dimethyl hexanedioate	-4265.1	-4270.7
Dimethyl isophthalate	-4651.5	-4657.6
Dimethyl maleate	-2802.6	-2783.8
Dimethyl malonate	-2311.2	-2310.3
Dimethyl methylmalonate	-2963.9	-2960.5
Dimethyl oxalate	-1675.6	-1656.8
Dimethyl phthalate	-4680.4	-4657.6
Dimethyl pimelate	-4920.4	-4924.2
Dimethyl pyridine-2,6-dicarboxylate	-4151.9	-4149.6
Dimethyl sebacate	-6844.2	-6884.6
Dimethyl suberate	-5574.3	-5577.7
Dimethyl succinate	-2959	-2963.8
Dimethyl sulfate	-1512.49	-1516.3
Dimethyl sulfide	-2181.5	-2181.1
Dimethyl sulfite	-1723.2	-1725.5
Dimethyl sulfone	-1796.7	-1798.1
Dimethyl-2,6-naphthalinedicarboxylate	-6527.6	-6555.4
Dimethylacetal	-2585	-2592.5
Dimethylacetamide	-2583.89	-2579.9
Dimethylamine	-1744.6	-1733.2
Dimethylaminomethanol	-2214.81	-2218.9
Dimethylene urethane	-1464.8	-1464.7
Dimethylether	-1461.4	-1446.1
Dimethylglyoxime	-2555.6	-2579.3
Dimethylmaleic anhydride	-2637.2	-2650.4
Dimethylmalononitrile	-2960.2	-2943.3
Dimethylnitramine	-1574	-1594
Dimethylparabanic acid	-2253.3	-2208.7
Dimethylperoxide	-1519.91	-1500.9
Dimethylpropiolactone	-2716.7	-2746.2
Dimethylterephthalate	-4631.66	-4675
Dimethylvinylcarbinol	-3145.1	-3141.8
Dimethylvinylethynylmethanol butyl ether	-6836	-6818.7

Di-n-butylether	-5346.47	-5343.1
Di-n-butylsulfite	-5633.66	-5622.4
Di-n-butylsulfone	-5712.7	-5716.6
Di-n-Decylphthalate	-16407.12	-16398.9
Di-n-hexylether	-7961.59	-7957
Dinitrofluorophenylethane	-3410.8	-3456.7
Dinitromethane	-574.89	-570.3
Di-n-nonylphthalate	-15109.74	-15091.9
Di-n-octyladipate	-13403.44	-13395.5
Di-n-octylether	-10579.97	-10570.9
Di-n-propyl ketone n-propylhydrazone	-6972.2	-6954.7
Di-n-propyl phthalate	-7275.12	-7250.3
Di-n-propyldiazene N-oxide	-4274.2	-4272.6
Di-n-propylsulfate	-4108.17	-4106.3
Di-n-propylsulfite	-4317.3	-4315.5
Di-n-propylsulfone	-4416	-4409.6
Di-n-undecyl phthalate	-17715.71	-17705.8
Dioctyl sulfide	-11329	-11327.3
Dioctylamine	-10891	-10876.8
Dipentaerythritol	-5507	-5530.9
Dipentyl phthalate	-9875	-9865.5
Dipentyl sulfide	-7419.7	-7406.5
Diphencyprone	-7529.8	-7474.5
Diphenyl ether	-6136.4	-6135.1
Diphenyl phthalate	-9388.4	-9368
Diphenyl sulfide	-6916.9	-6888.6
Diphenyl(phenylethynyl)carbinol	-10720	-10729
Diphenylacetamide	-7324.1	-7290.7
Diphenylamine	-6409.5	-6414.7
Diphenylbutadiyne	-8243	-8276.3
Diphenylcarbonate	-6143.6	-6142.5
Diphenyldisulfide	-7509.9	-7520.3
Diphenylmethane	-6921.4	-6918.2
Diphenylmethanol	-6725.45	-6724.8
Diphenylmethylsilane	-8104	-8116.1
Diphenylnitrone	-6840.16	-6842.7
Diphenylsulfone	-6528.5	-6554.3
Diphenylsulfoxide	-6763.4	-6789.9
Dipropionamide	-3382.2	-3385
Dipropionyl peroxide	-3172.7	-3142.5
Dipropyl disulfide	-5395	-5398.6
Dipropyl ether	-4035.96	-4036.2

Dipropyl sulfide	-4794.2	-4792.6
Dipropylamine	-4351.69	-4342.1
Dipropylammonium dipropyldithiocarbamate	-10252	-10271.7
Dipropyldiazene	-4376.28	-4404.9
Dipropyldibutylsilane	-10555	-10546.8
Dipropyldiethoxysilane	-7406	-7407.4
Dipropylmethylsilane	-5990	-6003.3
Dipropylnitramine	-4201.5	-4203
Dipropylsulfoxide	-4634.6	-4633.6
Di-p-tolyl sulfone	-7805.9	-7840.1
Di-s-butylamine	-5635.8	-5653.7
Di-s-butylether	-5322.87	-5326.8
Di-s-butylsulfide	-6106.06	-6082.2
Dispiro{cyclopropane-6,1'-pentacyclo[6.3.1.0(2,7).0(3,5).0(9,11)]-dodecane-12,1"-cyclopropane}	-9471	-9439.7
Di-t-amylperoxypyromellitate	-9945.4	-9907.7
Di-t-butyl ether	-5324.76	-5306.1
Di-t-butyl peroxymuconate	-7881.4	-7893.5
Di-t-butyl sulfone	-5682.5	-5679.4
Di-t-butyldisulfide	-6673.94	-6663.1
Di-t-butylsulfide	-6075.5	-6065
Dithiodilactic acid	-4030.6	-4037.5
Dithiooxamide	-2541.9	-2526.5
Divinyl ether	-2393.35	-2366.5
Divinyl sulfone	-2826.5	-2790.6
d-Leucylglycylglycine	-5592.5	-5567.8
D-Mannose	-2812.67	-2802.4
DMSO	-2037.3	-2022.1
Dodecane	-8086.5	-8083.8
Dodecanedioic acid	-6741.51	-6747.9
Dodecylcyclohexane	-11760.6	-11761.8
Dodecylcyclopentane	-11135	-11128.9
Dopa	-4177.8	-4218
D-Psicose	-2807.7	-2793.2
D-Ribonolactone	-2100.1	-2089.4
D-Ribose	-2347.4	-2361
Drometrizole	-6744	-6790.3
DTBP	-5343.47	-5353.1
Dulcitol	-3017.3	-3025.8
Dulcose	-3015.3	-3022.9
Durylic acid	-5165.15	-5167.8
EDNA	-1552.9	-1565

Eicosane	-13316	-13311.5
Elaidic acid	-11153.2	-11156.7
Ellagic acid	-4988.8	-5006.6
Enanthic acid	-4148.3	-4148.5
Enantholactam	-4267.2	-4258.2
endo-1,4a,8,8a-Tetrahydro-1,4-ethanonaphthalene-5,8-dione	-6215.3	-6161.2
endo-2-Cyanonorbornane	-4740.4	-4747.6
endo-2-Methyl-7-oxabicyclo[2.2.1]heptane	-4219.3	-4236.9
endo-2-Methylnorbornane	-5026.8	-5029.4
endo-Dicyclopentadienone	-5348.3	-5336.7
Epiandrosterone	-11137	-11086.4
Epichlorohydrin	-1761.6	-1772.2
Epoxycycloheptane	-4272.1	-4294.8
epsilon-Aminocaproic acid	-3582.2	-3594.7
epsilon-Caprolactam	-3605.7	-3604.7
epsilon-Caprothiolactam	-4431.3	-4439.7
Erucic acid	-13804	-13770.6
Erythritol	-2118	-2103.7
Ethane	-1552.01	-1549.1
Ethanediol diacetate	-2927.31	-2939.9
Ethanethiol	-2174.6	-2176.6
Ethanol	-1367.7	-1365.1
Ethene	-1411.9	-1406.6
Ethoxyacetic acid	-2069	-2061.3
Ethoxyacetonitrile	-2458.5	-2450
Ethoxybenzene	-4427.69	-4418.8
Ethoxytrimethylsilane	-4371	-4362.1
Ethoxytriphenylsilane	-11446	-11485.1
Ethriol	-3611	-3608.6
Ethyl (Z)-3-pentenoate	-4035.54	-4024.9
Ethyl 1H-indole-2-carboxylate	-5554.2	-5578.2
Ethyl 2,4,5-trimethyl-1H-pyrrole-3-carboxylate	-5589	-5597.2
Ethyl 2,4-pentadienoate	-3848.31	-3863.7
Ethyl 2-chlorobutanoate	-3338	-3389.9
Ethyl 2-chloropropionate	-2737	-2736.4
Ethyl 2-cyanoacetoacetate	-3504	-3510.8
Ethyl 2-cyanopropanoate	-3276.8	-3268.9
Ethyl 2-ethylacetoacetate	-4432.3	-4447.5
Ethyl 2-methylbutanoate	-4195	-4215.8
Ethyl 2-methylene-3-butenoate	-3848.31	-3873.6
Ethyl 2-nonynoate	-6494.8	-6517.6
Ethyl 2-octynoate	-5837.9	-5864.2

Ethyl 2-pentynoate	-3884.71	-3903.7
Ethyl 2-thiopheneacetate	-4810	-4813.7
Ethyl 2-thiophenecarboxylate	-4161	-4160.3
Ethyl 3,5-dimethyl-4-propyl-1H-pyrrole-2-carboxylate	-6868.5	-6904.2
Ethyl 3-aminobenzoate	-4724.8	-4708.9
Ethyl 3-chlorobutanoate	-3384	-3389.9
Ethyl 3-chloropropionate	-2750	-2740.2
Ethyl 3-ethoxycrotonate	-4501.6	-4491.6
Ethyl 3-hydroxybenzoate	-4379.5	-4393.8
Ethyl 3-pentynoate	-3898.8	-3903.7
Ethyl 3-phenylpropiolate	-5606.1	-5608.1
Ethyl 3-thiopheneacetate	-4820.3	-4824.6
Ethyl 4,4,4-trinitrobutyrate	-3133	-3152.2
Ethyl 4,5-dimethyl-1H-pyrrole-2-carboxylate	-4950.1	-4955.2
Ethyl 4-aminobenzoate	-4698.5	-4708.9
Ethyl 4-chlorobutyrate	-3390.3	-3393.7
Ethyl 4-fluorobenzoate	-4436.9	-4458.6
Ethyl 4-hydroxybenzoate	-4372.3	-4393.8
Ethyl 4-pentenoate	-4038	-4043.7
Ethyl acetate	-2237.9	-2244.5
Ethyl acetoacetate	-3180.5	-3139.9
Ethyl acrylate	-2715	-2736.7
Ethyl azide	-1769.47	-1753.2
Ethyl azidoacetate	-2436.9	-2448.6
Ethyl benzoate	-4594.15	-4602.4
Ethyl but-3-yne- 1-carboxylate	-3904.8	-3934.7
Ethyl butanoate	-3545	-3551.4
Ethyl caprylate	-6130	-6165.3
Ethyl chloroformate	-1413.75	-1421.3
Ethyl cis-2-pentenoate	-4029	-4024.9
Ethyl crotonate	-3372.64	-3371.5
Ethyl cyanoacetate	-2638	-2618.7
Ethyl cyclobutanecarboxylate	-4042	-4057.5
Ethyl decanoate	-7448	-7472.3
Ethyl dichloroacetate	-1976	-1964.9
Ethyl difluoroacetate	-1939.1	-1917.7
Ethyl dimethyldithiocarbamate	-4694.4	-4672.8
Ethyl dodecanoate	-8778	-8779.2
Ethyl elaidate	-12528	-12520
Ethyl ether	-2732.1	-2729.2
Ethyl ethoxyacetate	-3436.5	-3424.7
Ethyl ethoxymethyl ketone	-3636.1	-3624.7

Ethyl formate	-1618.88	-1624.4
Ethyl glycylglycinate	-3370.6	-3392.3
Ethyl hexadecanoate	-11368	-11393.1
Ethyl hydrocinnamate	-5906.3	-5911.9
Ethyl hydroperoxide	-1402.4	-1428
Ethyl iodide	-1462.5	-1460.4
Ethyl isobutanoate	-3540.02	-3548.1
Ethyl isonitrile	-2005	-2005.1
Ethyl isopropyl sulfide	-4131.3	-4131.8
Ethyl isovalerate	-4184.4	-4204.2
Ethyl lactate	-2703.89	-2704.5
Ethyl levulinate	-3783.7	-3793.4
Ethyl methacrylate	-3371.4	-3378.8
Ethyl methyl ketone oxime	-2727	-2711.9
Ethyl methyl sulfide	-2833.6	-2833.4
Ethyl methyl sulfite	-2360.06	-2367
Ethyl methyl sulphone	-2441.4	-2450.4
Ethyl myristate	-10067	-10086.1
Ethyl N,N-dimethylglycinate	-3764.2	-3758.9
Ethyl N,N-diphenylcarbamate	-7765.5	-7752.7
Ethyl nitroacetate	-2087	-2060.8
Ethyl oleate	-12525	-12520
Ethyl orthoformate	-4365.5	-4362.2
Ethyl pentanoate	-4201.6	-4204.9
Ethyl phenylacetate	-5243.9	-5255.8
Ethyl pivalate	-4188.5	-4197.3
Ethyl propanoate	-2894	-2898
Ethyl propiolate	-2659	-2627.8
Ethyl propyl sulfide	-4142.7	-4139.1
Ethyl salicylate	-4396.8	-4393.5
Ethyl succinic acid	-2801	-2829
Ethyl t-amyl ether	-4675.51	-4669.8
Ethyl t-butyl ether	-4013.98	-4013.8
Ethyl t-butyl ketone	-4399.3	-4397.3
Ethyl t-butyl sulfoxide	-4618.4	-4615.1
Ethyl trans-2-pentenoate	-4020	-4024.9
Ethyl trichloroacetate	-1872	-1841.9
Ethyl vinyl ether	-2540	-2535.8
Ethyl(1,1-dimethylpropyl)malononitrile	-6206.8	-6213.5
Ethylamine	-1714.5	-1706
Ethylbenzene	-4563.9	-4563
Ethylcarbamate	-1663.8	-1672.6

Ethylcarbamide	-1966.1	-1973.6
Ethylcyclobutane	-4019.81	-4015.5
Ethylcyclohexane	-5226.22	-5227.1
Ethylcyclopentane	-4591.9	-4578.2
Ethylcyclopropane	-3371.9	-3369.8
Ethyldibutylsilane	-7963	-7978.6
Ethyldidecylsilane	-15853	-15820.2
Ethyldiethylcarbamate	-4306	-4350.4
Ethyldihexylsilane	-10596	-10592.5
Ethyldiisobutylsilane	-7949	-7969.3
Ethyldiisopentylsilane	-9262	-9284.1
Ethyldioctylsilane	-13224	-13206.3
Ethyldipentylsilane	-9282	-9285.5
Ethyldiphenylsilane	-8763	-8783.2
Ethyldipropylsilane	-6649	-6671.6
Ethylene glycol monobutyl ether acetate	-4728.05	-4731.6
Ethylenedichloride	-1246.4	-1233.6
Ethyleneglycoldiacrylate	-3917.48	-3924.4
Ethylenethiourea	-2603.6	-2615.9
Ethylepoxide	-2548.5	-2583.4
Ethylidene diurethan	-4453.9	-4447.8
Ethylidenecyclohexane	-5045.4	-5039
Ethylidenecyclopentane	-4412.8	-4392.2
Ethyl-isoamylketone	-5063.95	-5057.7
Ethylisobutylether	-4031.28	-4032.9
Ethylisopropylether	-3369.19	-3373.2
Ethylisopropylketone	-3752.86	-3748.1
Ethylmalonic acid	-2172.2	-2172.7
Ethylmethylether	-2108.8	-2087.7
Ethyl-n-hexylether	-5343.36	-5343.1
Ethylnitrate	-1311.2	-1325.1
Ethylnitrite	-1394.23	-1375.4
Ethyl-n-propyldisulfide	-4743.56	-4745.1
Ethyl-n-propylether	-3381.4	-3382.7
Ethylsilane	-2696.3	-2726.5
Ethyl-t-butylsulfide	-4779.87	-4776.7
Ethyltripropylsilane	-8583	-8581.1
Ethyne	-1300.5	-1309.8
Ethynylbenzene	-4291.72	-4290.2
Etioporphin I	-18025	-18015.2
Etioporphyrin II	-18053	-18007.4
exo-2-Methyl-7-oxabicyclo[2.2.1]heptane	-4215.7	-4234.3

exo-2-Methylnorbornane	-5026.4	-5029.4
exo-4-Hydroxy-endo-endo-tetracyclo[6.2.1.1.3,6.02,7]dodecane	-7009.7	-6999.8
exo-Bicyclo[2.2.1]heptane-2-carbonitrile	-4739.2	-4747.6
exo-Dicyclopentadiene	-5766.8	-5752.9
exo-Norbornene oxide	-4085.7	-4094.6
Ferulic acid	-4666	-4673.3
Flavanone	-7405.1	-7409.9
Flavone	-7176.9	-7206.1
Fluoranthene	-7906.2	-7899.4
Fluorene	-6635	-6659.7
Fluorene-9-methanol	-7114.5	-7125.8
Fluoroacetal	-1054.7	-1017.2
Fluoroacetic acid	-714.84	-733.5
Fluorobenzene	-3103.9	-3120.2
Fluorocyclohexane	-3738.5	-3753.6
Fluoroethane	-1411.83	-1401.4
Formaldehyde diethylacetal	-3234.59	-3233.1
Formamide	-568.57	-576.7
Formanilide	-3605.7	-3609.3
Formic acid	-254.81	-261
Formylurea	-866.67	-883.1
FOX-7	-1152	-1150
Freon 113	-641.6	-623.3
Fumaric acid	-1334.7	-1340.2
Furacilin	-2995	-2971.5
Furan	-2088	-2091.5
Furfural	-2340.8	-2332.8
Furfuranol	-2550.6	-2533.1
Furfurylideneacetone	-4054.07	-4086
Galactaric acid	-2022.2	-2055.8
Gallic acid	-2601.47	-2613.3
gamma-Butyrolactone	-2012.46	-2016.3
gamma-Valerolactone	-2649.6	-2660.3
Gluconic acid	-2489.1	-2539.5
Glucose pentaacetate	-7190.9	-7199.5
Glutamic acid	-2277	-2265.5
Glutamine	-2572.8	-2592.3
Glutaraldehyde	-2747.08	-2741
Glutaric anhydride	-2206.5	-2195.2
Glutarimide	-2480.3	-2489.4
Glyceraldehyde	-1448	-1440.8
Glycerol 1,3-dinitrate	-1562.4	-1561.1

Glycerol formal	-2162.08	-2158
Glycerol trinitrate	-1525.47	-1521.1
Glycerol trioleate	-35100	-35112.2
Glyceryl-1-nitrate	-1605.4	-1601.1
Glyceryl-2-benzoate	-4880.5	-4878.4
Glyceryl-2-caprate	-7740.8	-7748.3
Glyceryl-2-laurate	-9043.6	-9057.8
Glyceryltribenzoate	-11413	-11361.3
Glycidyl butyrate	-3909.2	-3932.3
Glycidyl isopropyl ether	-3735.66	-3754.1
Glycidyl methacrylate	-3728.3	-3759.6
Glycidyl propyl ether	-3758.9	-3763.6
Glycine	-978.6	-980.8
Glycine anhydride	-1976.5	-1981.7
Glycolaldehyde	-955.39	-980.8
Glycoluril	-1938.7	-1958.8
Glycylalanylphenylalanine	-7300.5	-7277.5
Glycylglycine	-1971.8	-1971.7
Glycylglycine-N-carboxylic acid	-1969.9	-1964
Glycylglycylglycylglycine	-3965	-3953.3
Glycyl-L-phenylalanine	-5645.1	-5637.5
Glyme	-2623.6	-2626.3
Glyoxime	-1272	-1306.1
Guaiazulene	-8469.5	-8418.3
Guajen	-6434.78	-6447.7
Guanazole	-1548	-1526.3
Guanidine	-1052.1	-1065.9
Guanine	-2498.2	-2512.7
Hemellitic acid	-4523.66	-4524.8
Hemimellitene	-5201.55	-5192.8
Hemimellitic acid	-3238.7	-3189
Heneicosane	-13908	-13965
Heneicosylcyclopentane	-17014	-17015.4
Heptadecane	-11351	-11351.1
Heptadecylbenzene	-14334.7	-14362.4
Heptane	-4816	-4816.4
Heptanedioic acid	-3460.2	-3480.6
Heptyl bromide	-4679.9	-4662.7
Heptylbenzene	-7838.66	-7827.7
Heptylcyclohexane	-8478.5	-8494.4
Heptylcyclopentane	-7861.3	-7866.8
Hexa-2-ene-1,6-dioic acid	-2633.5	-2647.1

Hexa-3-ene-1,6-dioic acid	-2633.5	-2647.1
Hexachlorobenzene	-2361	-2348.2
Hexachlorobutadiene	-1691.7	-1676.5
Hexachloroethane	-727	-743.8
Hexachloropropylene	-1248.6	-1210.2
Hexacyclododecane	-6773.4	-6798.2
Hexadecylbenzene	-13682.73	-13711.6
Hexadecylcyclohexane	-14386	-14375.6
Hexadecylcyclopentane	-13755	-13742.8
Hexaethylbenzene	-11027	-11058.1
Hexafluorobenzene	-2445.6	-2401.3
Hexaglycine	-5930	-5935
Hexahydrofarnesyl acetone	-11602	-11598.8
Hexamethylbenzene	-7137.92	-7137.2
Hexamethylcyclotrisiloxane	-5858.31	-5887.2
Hexamethyl-dewar benzene	-7385	-7374.6
Hexamethyleneglycol	-3794.88	-3795
Hexamethylenetetramine	-4202.82	-4231.4
Hexanamide	-3796	-3821.9
Hexane	-4166.08	-4162.9
Hexanenitrile	-3878.46	-3883.7
Hexaphenylethane	-19765.5	-19721.1
Hexyl bromide	-4024.6	-4009.2
Hexyl hexanoate	-7472.8	-7472.3
Hexylbenzene	-7184.46	-7174.3
Hexylcyclohexane	-7847.2	-7840.9
Hexylcyclopentane	-7207.5	-7208.1
Hippuric acid	-4221.3	-4232.5
HMX	-2774	-2763.8
HNS	-6424.7	-6383.2
Homocubane-4-carboxylic acid	-5285	-5296.8
Hydantoic acid	-1289.5	-1317.3
Hydrazine	-622.7	-608.1
Hydrazinecarbothioamide	-1712.4	-1734.2
Hydrazinecarboxamide	-882.4	-884.1
Hydrocinnamyl alcohol	-5042.18	-5032.5
Hydrogenperoxide	-98.02	-72
Hydroxyacetic acid	-695.43	-697.2
Hydroxyacetonitrile	-1074.81	-1085.8
Hydroxyisobutyric acid	-1974.5	-2003.1
Hydroxytyrosol	-3955.2	-3959.3
Hydurilic acid	-2755.8	-2723.6

Hypoxanthine	-2431.1	-2431.8
Imidazole	-1801.9	-1792.1
Imidodicarbonic diamide	-938	-945.2
Indane	-4982.47	-4975.1
Indene	-4795.5	-4785.9
Indole	-4235.13	-4235.9
Indole-2-carboxylic acid	-4217.9	-4210.9
Indole-3-carboxylic acid	-4195.9	-4210
Inositol	-2771.1	-2765
Iodobenzene	-3192.8	-3186.3
Ioxynil	-3219	-3263.6
Isatin	-3594.5	-3576.4
Isoamyl acetate	-4199.87	-4204.2
Isobutyl 2-chlorobutyrate	-4653.9	-4693.5
Isobutyl 2-chloropropionate	-4033	-4040
Isobutyl 3-chlorobutyrate	-4691.9	-4696.1
Isobutyl 3-chloropropionate	-4056	-4043.9
Isobutyl 4-chlorobutyrate	-4684.8	-4697.3
Isobutyl acetate	-3534	-3548.1
Isobutyl butanoate	-4837.1	-4855.1
Isobutyl chloride	-2693.66	-2694.9
Isobutyl chloroacetate	-3399	-3390.4
Isobutyl dichloroacetate	-3274	-3268.6
Isobutyl formate	-2923.1	-2928
Isobutyl isobutyrate	-4844.28	-4851.7
Isobutyl t-butylether	-5317.37	-5317.4
Isobutyl trichloroacetate	-3154	-3145.5
Isobutyl valerate	-5498.15	-5508.5
Isobutyl vinyl ether	-3818	-3839.5
Isobutylbenzene	-5870.09	-5866.6
Isobutylcyclohexane	-6534.24	-6533.3
Isobutylene	-2681.68	-2683.3
Isobutylmethylether	-3388.86	-3391.3
Isobutylnitrite	-2675	-2679.1
iso-Butylsilane	-4036.1	-4030.1
Isobutyraldehyde isobutylhydrazone	-5662.2	-5647
Isobutyramide	-2491.7	-2514.3
Isobutyronitrile	-2560.6	-2573.4
Isocinnamic acid	-4366	-4366
Isocumene	-5218	-5216.5
Isoleucine	-3583.7	-3588.8
Isonicotinamide	-3070.5	-3077.3

Isonicotinamide N-oxide	-3019.7	-3030.1
Isonicotinic acid	-2727.5	-2747.8
Isooctane	-5465.21	-5462.8
Isoorotic acid	-1705.9	-1708.2
Isopentane	-3506.59	-3508.8
Isopentene	-3337.84	-3336.8
Isopentyl 2-chloropropanoate	-4688.2	-4696.1
Isopentyl alcohol	-3328.59	-3324.8
Isopentyl butanoate	-5491.1	-5511.1
Isopentyl chloride	-3355.4	-3351
Isopentyl crotonate	-5327.5	-5331.2
Isopentyl dichloroacetate	-3932	-3924.6
Isopentyl isovalerate	-6153.2	-6165.4
Isopentylamine	-3639	-3665.8
Isophorone diisocyanate	-6870.46	-6881.8
Isophthalamide	-3854.6	-3873
Isophthalic acid	-3217.85	-3219.2
Isophthaloylchloride	-3397.7	-3404.9
Isoprene	-3161.15	-3178.2
Isopropanolamine	-2174.31	-2166
Isopropenylacetate	-2726.55	-2700.7
Isopropenylacetylene	-3056	-3066.6
Isopropoxytrimethylsilane	-5031	-5008.7
Isopropyl 2-chloropropanoate	-3396	-3384.2
Isopropyl 2-pentenoate	-4673.6	-4668.9
Isopropyl 4-pentenoate	-4687.8	-4687.7
Isopropyl benzoate	-5231.7	-5246.3
Isopropyl butyrate	-4184.4	-4195.4
Isopropyl chloroacetate	-2733	-2730.7
Isopropyl crotonate	-4012.5	-4015.4
Isopropyl cyclohexanoate	-5875.3	-5921.4
Isopropyl decanoate	-8125.2	-8116.2
Isopropyl dichloroacetate	-2618	-2608.9
Isopropyl hexanoate	-5460.9	-5502.4
Isopropyl laurate	-9373.6	-9425.8
Isopropyl methyl ether	-2753.15	-2731.7
Isopropyl methyl sulfide	-3480.8	-3476.9
Isopropyl myristate	-10736.17	-10735.4
iso-Propyl nitrite	-2017	-2019.4
Isopropyl octanoate	-6792.5	-6809.3
Isopropyl palmitate	-12082.33	-12039.7
Isopropyl pentanoate	-4846.15	-4848.9

Isopropyl propionate	-3531	-3541.9
Isopropyl t-butyl ether	-4648.3	-4663
Isopropyl trichloroacetate	-2492	-2485.8
Isopropyl vinyl ether	-3181	-3179.8
Isopropylacetate	-2869.8	-2893.7
Isopropylacrylate	-3373.34	-3384.7
Isopropylamine	-2356.1	-2356.6
Isopropylcarbamide	-2613.7	-2624.2
Isopropylcyclohexane	-5878.7	-5882.4
Isopropylcyclopentane	-5248.26	-5244.4
Isopropylnitrate	-1951	-1969
Isopropylthioacetate	-3703.15	-3702.8
Isoquinoline	-4686.46	-4670.6
Isoserine	-1438.2	-1440.5
Isovaleraldehyde	-3122.47	-3121.9
Isovaleramide	-3149.7	-3165.1
Isovaleronitrile	-3246	-3226.9
Isoxazole	-1654	-1642
Isoxylic acid	-4514.6	-4524.8
Ketene	-1026.07	-984.5
Kyanmethin	-3607.8	-3616.2
Lactic acid	-1344.9	-1341.1
Lactide	-2712.5	-2678.8
Lactonitrile	-1757.47	-1729.8
Lactose	-5629.45	-5603.5
Lauric acid	-7377	-7415.8
Lauric peroxyacid	-7476.8	-7488.4
Leucine	-3581.17	-3583.5
Leucine anhydride	-7201.7	-7204.8
Lindane	-2961.8	-2951.4
Linoleic acid	-10990.21	-10976.7
Lophine	-10831	-10785.6
Maleic acid	-1355.2	-1340.2
Malic acid	-1328.8	-1326.4
Malonamide	-1495.21	-1520.5
Malonic acid	-861.15	-866.4
Malonyl dihydrazide	-1991.2	-2004.8
Maltose	-5649.2	-5608.2
Mandelic acid	-3711	-3699
m-Anisic acid	-3737.9	-3752.3
Mannitol hexanitrate	-2807	-2783.7
m-Anthranilic acid	-3346.5	-3345.6

m-Chlorobenzyliden-5,6,7,8-tetrahydronaphthyl-2-acetonitrile	-9986.8	-9955.1
m-Cresol	-3706.4	-3698.4
m-Cresylacetate	-4600	-4601.1
m-Cumenol	-4993.6	-5004.6
Melamine	-1966.4	-1975.3
Menthol	-6318.51	-6339.1
Mercaptoacetic acid	-1507.41	-1508.6
Mesitoic acid	-5172.3	-5167.8
Mesitol	-4983	-4986.8
Mesitylenic acid	-4508.06	-4524.8
Meso-2,3-diethyl-2,3-dimethylsuccinonitrile	-6188.1	-6216.1
meso-2,3-Diethylbutanedioic acid	-4130	-4132.6
Meso-2,3-dimethyl-butanedioic acid	-2812.7	-2823.1
meso-Tartaric acid	-1152.6	-1135.6
Metanilic acid	-3357.3	-3357.5
Methacrolein	-2293	-2299.1
Methacrylamide	-2327.3	-2344.9
Methacrylic acid	-2016.7	-2015.4
Methanethiol	-1521.8	-1524.3
Methanol	-726	-723.5
Methoxyacetic acid	-1412.34	-1419.8
Methoxybenzene	-3782.9	-3777.3
Methyl (dimethylamino) acetate	-3125.3	-3117.4
Methyl (Z) 11-eicosenoate	-13190	-13185.4
Methyl (Z) 9-octadecenoate	-11832.4	-11878.5
Methyl 1H-indazole-6-carboxylate	-4488	-4485.6
Methyl 1H-indole-3-carboxylate	-4896.9	-4931.8
Methyl 1-methyl-2-pyrrolecarboxylate	-3711.3	-3716.7
Methyl 2,4-dimethyl-1H-pyrrole-3-carboxylate	-4297.8	-4312.7
Methyl 2,4-dimethyl-1H-pyrrole-5-carboxylate	-4307	-4313.6
Methyl 2-aminobenzoate	-4079.5	-4067.1
Methyl 2-chloropropanoate	-2105	-2094.8
Methyl 2-furoate	-2769	-2771
Methyl 2-hexyldecanoate	-10743	-10753.5
Methyl 2-hexynoate	-3943.8	-3915.7
Methyl 2-iodobenzoate	-3905.3	-3883.1
Methyl 2-methoxybenzoate	-4487.5	-4474.1
Methyl 2-methylbutyrate	-3541.8	-3562.7
Methyl 2-methylene-3,3-dimethylbutanoate	-4730.9	-4693.9
Methyl 2-methylstearate	-12686	-12711.2
Methyl 2-thiopheneacetate	-4169.4	-4172.2
Methyl 2-thiophenecarboxylate	-3519.3	-3518.7

Methyl 3-chlorobutyrate	-2735	-2748.3
Methyl 3-chloropropionate	-2098	-2098.7
Methyl 3-hydroxybenzoate	-3741.12	-3752.3
Methyl 3-iodobenzoate	-3870.2	-3883.1
Methyl 3-methylbutyrate	-3537.2	-3560
Methyl 3-t-butylperoxycarbonylpropanoate	-4953.9	-4955.1
Methyl 3-thiopheneacetate	-4171.4	-4183
Methyl 4-chlorobenzoate	-3788.5	-3808.2
Methyl 4-chlorobutyrate	-2733	-2752.2
Methyl 4-formylbenzoate	-4211.94	-4219.5
Methyl 4-hydroxybenzoate	-3731.52	-3752.3
Methyl 4-iodobenzoate	-3861.8	-3883.1
Methyl 4-methoxybenzoate	-4453.2	-4474.1
Methyl 4-methylbenzoate	-4580.76	-4603.7
Methyl 5-cis-dodecenoate	-7949.6	-7957.7
Methyl 5-nitro-2-furoate	-2605	-2608.3
Methyl acetate	-1593.9	-1602.9
Methyl acrylate	-2069.4	-2095.2
Methyl benzoate	-3960.3	-3960.8
Methyl bicyclobutane-1-carboxylate	-3303.6	-3279.6
Methyl carbamate	-1029.9	-1031.1
Methyl cellosolve	-1909.08	-1903.7
Methyl centralite	-8116.1	-8093.3
Methyl chlorocarbonate	-778.41	-779.8
Methyl crotonate	-2728	-2729.9
Methyl cyclobutanecarboxylate	-3397.7	-3416
Methyl cyclohexanecarboxylate	-4595.9	-4624.9
Methyl cyclopropanecarboxylate	-2764.9	-2770.2
Methyl decanoate	-6836.9	-6830.7
Methyl dichloroacetate	-1316	-1323.4
Methyl dimethyldithiocarbamate	-3991	-4020.6
Methyl dimethylmalonate	-3611.1	-3609.6
Methyl ferulate	-5366.3	-5395.1
Methyl formate	-980.13	-982.9
Methyl hexadecanoate	-10718.33	-10751.5
Methyl hexanoate	-4218	-4216.8
Methyl hydroxyacetate	-1428.6	-1419
Methyl isoamyl ketone	-4407.63	-4404.2
Methyl isobutanoate	-2897.21	-2906.6
Methyl isobutyl ketone	-3694.6	-3748.1
Methyl isopropyl sulfone	-3103.3	-3091.3
Methyl laurate	-8143.7	-8137.7

Methyl levulinate	-3138.9	-3151.9
Methyl linolenate	-11506	-11518.5
Methyl methacrylate	-2724.6	-2737.2
Methyl methylthiomethyl sulfoxide	-3330.77	-3329.2
Methyl N,N-diethyldithiocarbamate	-5320.8	-5322.6
Methyl nonanoate	-6181.3	-6177.2
Methyl octanoate	-5527.5	-5523.8
Methyl palmitoleate	-10548	-10571.6
Methyl pentadecanoate	-10105	-10098.1
Methyl pentafluoropropanoate	-1434.6	-1398.8
Methyl pentyl sulfide	-4800.1	-4793.8
Methyl phenylpropiolate	-5009.9	-4966.6
Methyl pivalate	-3546	-3555.7
Methyl propanoate	-2245.6	-2256.4
Methyl propyl sulfide	-3489.08	-3486.9
Methyl p-tolyl sulfone	-4796	-4819.1
Methyl pyruvate	-1876.6	-1844.9
Methyl t-butyl sulfide	-4127.2	-4124.4
Methyl t-butylacetate	-4199.2	-4209.2
Methyl tetradecanoate	-9452.5	-9444.6
Methyl tetryl	-4160	-4134
Methyl t-pentyl ether	-4020.1	-4028.3
Methyl trichloroacetate	-1211	-1200.3
Methyl tridecanoate	-8798.8	-8791.1
Methyl undecanoate	-7486.5	-7484.2
Methyl valerate	-3564.3	-3563.4
Methyl vanillate	-4255.4	-4265.5
Methyl Z 13-docoseneoate	-14452	-14492.4
Methyl Z,Z-9,12-octadecadieneoate	-11690	-11698.5
Methyl(1,1,2-trimethylpropyl)malononitrile	-6239	-6210.2
Methylacetamide	-1867.7	-1888.9
Methylacetoacetate	-2487.7	-2498.4
Methylacetylacetone (enolform)	-3353.2	-3316.5
Methylamine	-1060.8	-1055.1
Methylbenzene	-3909.8	-3906.9
Methylcarbamide	-1316.8	-1325.3
Methylchloroacetate	-1451	-1445.2
Methylchloroform	-1108	-1146.4
Methylcyanoacetate	-1979.1	-1977.2
Methylcyclobutane	-3354.49	-3362.1
Methylcyclohexane	-4568.48	-4571
Methylcyclopentane	-3938.3	-3917.5

Methylcyclopropane	-2719.1	-2716.3
Methylcyclopropylketone	-2956.2	-2958.3
Methyldibutylsilane	-7306	-7310.2
Methyldidecylsilane	-15189	-15151.8
Methyldiethylsilane	-4673	-4693.7
Methyldihexylsilane	-9934	-9924.1
Methyldipentylsilane	-8619	-8617.1
Methylelaidate	-11894	-11878.5
Methylenanthate	-4867.4	-4870.3
Methylene-bis(N,N-dimethylurea)	-4493.5	-4488.6
Methylenecyclobutane	-3204.7	-3192.7
Methylenecyclohexane	-4412.4	-4401.6
Methylenecyclopentane	-3772.66	-3757.5
Methylenesuccinic acid	-1983.9	-2001
Methylglyoxal 2,4-dinitrophenylosazone	-7795	-7810.4
Methylglyoxime	-1915	-1936.9
Methylhydrazine	-1305.2	-1289.7
Methylhydroperoxide	-769.8	-786.5
Methylisocyanate	-1124.58	-1078.8
Methylisonitrile	-1333	-1352.3
Methylketene	-1648.8	-1619.2
Methyllactate	-2072.68	-2062.9
Methylnitrate	-666.5	-683.5
Methylnitrite	-756.7	-733.9
Methyl-n-pentylether	-4049.68	-4048.1
Methyl-n-propylketone	-3101.5	-3098
Methyloxiran	-1917.4	-1929.9
Methylphenylsulfone	-4154.8	-4176.2
Methylprednisolone	-11899	-11875.3
Methylprednisolone aceponate	-14304	-14303.7
Methylpropylether	-2739.31	-2741.2
Methylsalicylate	-3749.24	-3752
Methylthiirane	-2651.6	-2683.2
Methyl-t-pentylsulfide	-4787.18	-4780.5
Methyltriethylsilane	-5946	-5955
Methyltriphenoxysilane	-10220	-10218.4
Methyltripropylsilane	-7918	-7923.3
Methyltris(2-methylphenoxy)silane	-12159	-12147.1
Methyltris(3-methylphenoxy)silane	-12150	-12147.1
Methyltris(4-methylphenoxy)silane	-12166	-12147.1
Methyltrivinylsilane	-5269.76	-5259.3
m-Nitrobenzylidene-p-isopropylphenylacetonitrile	-9506.5	-9540.8

Monoacetin	-2488.2	-2520.5
Monophenyl succinic anhydride	-4576.9	-4561.6
Monothiodibenzoylmethane	-8213.6	-8217.9
Morpholine	-2673	-2666.3
MTBE	-3369	-3372.2
m-Terphenyl	-9282.86	-9274.7
m-Toluamide	-4194.9	-4211.4
m-Toluidine	-4047	-4013.5
Mustard gas	-3163.5	-3170.2
Myrcene	-6240.4	-6255.4
Myristic acid t-butylperoxyester	-11440	-11435.9
Myristonitrile	-9107.7	-9111.4
Myristyl alcohol	-9168.4	-9206.7
N-(1-Cyclohexenyl)piperidine	-6944.2	-6916.7
N-(2-Aminoethyl)ethanolamine	-3011.03	-3008.2
N-(2-Methylphenyl)-N'-phenylurea	-7367	-7349.8
N-(2-Methylpropylidene)-butylamine	-5417	-5420.8
N-(3-Methylphenyl)-acetamide	-4922.4	-4883.5
N-(3-Phenoxy-2-hydroxypropyl)aniline	-8034	-8031.9
N-(4-Isopropylphenylmethylene)benzenamine N-oxide	-8780	-8794.5
N-(4-Methoxyphenylmethylene)benzenamine N-oxide	-7346.1	-7356
N-(Diethylaminothiocarbonyl)benzamidine	-7752.3	-7750.2
N-(Diethylaminothiocarbonyl)benzimido ethyl ester	-8799.2	-8768.7
N-(Diethylaminothiocarbonyl)-N',N'-diethylbenzamidine	-10435	-10426.7
N-(Diethylaminothiocarbonyl)-N'-ethylbenzamidine	-9072.7	-9079.5
N-(Diethylaminothiocarbonyl)-N'-phenylbenzamidine	-10801	-10780.1
N-(Hydroxyethyl)piperazine	-4134.3	-4121.2
N,N'-(1,3-Phenylene)bis(phthalimide)	-9910.9	-9890.6
N,N-(Dimethyl)thiobenzamide	-5757	-5770.1
N,N,2,2-Tetramethylpropionamide	-4557.1	-4542.4
N,N,N',N'-Tetrafluoro-1,2-cyclohexanediamine	-4288	-4271.6
N,N’-Bis(salicylaldehydo)ethylenediimine	-8371.4	-8369.6
N,N'-Bis(2-fluoro-2,2-dinitroethyl)ethanediamide	-2599.5	-2629.7
N,N-Bis(2-hydroxyethyl)ethylenediamine	-4118.9	-4153.3
N,N-Bis(3-phenoxy-2-hydroxypropyl)aniline	-12720	-12709.2
N,N'-Bis(acetylacetone)cyclohexanediimine	-9449.9	-9475.1
N,N'-Bis(benzoylacetone)cyclohexanediimine	-14176	-14194.7
N,N'-Bis-(m-methoxyphenyl)terephtalamide	-10966	-10964.6
N,N'-Bis(o-methoxyphenyl)terephthalamide	-11007	-10964.6
N,N'-Bis(salicylaldehydo)cyclohexanediimine	-10733	-10743.4
N,N'-Bis(salicylaldehydo)propylenediimine	-9036.2	-9023.1
N,N'-Bis(salicylaldehydo)tetramethylenediimine	-9693.8	-9676.5

N,N-Dibutylaminotriethylsilane	-10684	-10684.1
N,N-Diethylaminotriethylsilane	-8064.4	-8070.2
N,N-Diethylaniline	-6080.6	-6049.7
N,N-Diethylhydroxylamine	-2971.3	-2995.5
N,N-Diethyl-N'-isobutanoylthiourea	-6354.8	-6342.1
N,N-Diethyl-N'-isovaleroylthiourea	-6977	-7000.8
N,N-Diethyl-N'-pivaloylthiourea	-6968.4	-6991.2
N,N-Diethylthiobenzamide	-7064	-7072.1
N,N'-Diethylurea	-3302.8	-3305.5
N,N-Diethylurea	-3310.3	-3318.3
N,N-Difluorobenzylamine	-4078.4	-4085.2
N,N-Diisobutylaminotriethylsilane	-10667	-10680.1
N,N-Dimethyl-1-adamantylcarboxamide	-7733.2	-7731.4
N,N-Dimethyl-3-nitroaniline	-4557.2	-4574.4
N,N-Dimethyl-3-toluidine	-5413.9	-5397
N,N-Dimethyl-4-nitroaniline	-4541.8	-4585.9
N,N-Dimethylaminoacetone	-3315.9	-3305.5
N,N-Dimethylaminotrimethylsilane	-4771.1	-4763.2
N,N-Dimethylaniline	-4757.5	-4745.1
N,N-Dimethylbenzamide	-4938	-4940.4
N,N-Dimethylbutyramide	-3893	-3886.9
N,N-Dimethylcyclohexylamine	-5442.8	-5437.6
N,N-Dimethylethanolamine	-2891.24	-2882.1
N,N-Dimethylformamide	-1943.1	-1948.6
N,N-Dimethylglycine bisulfate	-2610.6	-2604.7
N,N-Dimethylglycine methyl ester bisulfate	-3323.2	-3329.1
N,N-Dimethylglycine methyl ester sulfate	-6765.2	-6790.2
N,N-Dimethylglycine sulfate	-5371.6	-5346.6
N,N'-Dimethyl-N,N'-dinitro-ethanediamide	-2126.2	-2116.5
N,N'-Dimethyl-N,N'-dinitrourea	-1928.6	-1891.3
N,N-Dimethyl-N',N'-diphenylurea	-8102.6	-8093.7
N,N'-Dimethyl-N-phenylurea	-5038.8	-5045.8
N,N-Dimethylpropionamide	-3236.6	-3233.4
N,N-Dimethyl-p-toluidine	-5411.2	-5397
N,N'-Dimethylurea	-2010.2	-2003.5
N,N-Dimethylurea	-2004.8	-2013.7
N,N'-Dinitrosopiperazine	-2810	-2805.1
N,N-Diphenyl-N'-ethylurea	-8037	-8053.7
N,N-Diphenyl-N'-methylurea	-7403	-7402.7
N,N'-Diphenyl-p-phenylenediamine	-9545.16	-9547.5
N,N-Diphenylurea	-6707.9	-6721.8
N,N-Diphenyluretidione	-6806.9	-6800.6

N,N'-Di-t-butylethylenediamine	-7105.08	-7102.1
N,N-Ethylene bis(N',N,-diphenyl urea)	-14539	-14558.4
N,N-Methylacetylcarbamide	-2154.3	-2199.5
N,N'-Trimethyleneurea	-2404.7	-2409.9
N-Acetamido-N'-nitroguanidine	-1988.7	-1966.5
N-Acetylacetamide	-2085	-2078
N-Acetylbenzamide	-4454.5	-4438.5
N-Acetylbenzenesulfonamide	-4304.1	-4301.7
N-Acetylurea	-1493.3	-1511.8
N-Amino-N'-nitroguanidine	-1131	-1131
n-Amyl ether	-6644	-6650.1
Naphthalene	-5157.3	-5161.8
Naphthazarin	-4196.8	-4194
N-Benzoyl-D-phenylalanine	-7936.6	-7897
N-Benzoyl-L-phenylalanine	-7941.4	-7895.6
N-Benzoyl-N',N'-diethylurea	-6529.6	-6546.1
N-Benzoyl-N',N'-diisobutylurea	-9143.4	-9159.9
N-Benzoyl-N-methylbenzamide	-7520.9	-7485.7
N-Benzoyl-o-aminodiphenylamine	-9753	-9741.5
N-Benzoylthiocarbamic O-hexyl ester	-8414	-8391.3
N-Benzoylthiocarbamic O-butyl ester	-7103.8	-7084.3
N-Benzoylthiocarbamic O-ethylester	-5784.7	-5777.4
N-Benzylbenzaldehyde imine	-7550.8	-7526
N-Benzyl-pivalophenone imine	-10131	-10129
n-Butyl acetate	-3549.56	-3551.4
n-Butyl acrylate	-4047	-4043.7
n-Butyl methyl ether	-3394.39	-3394.6
n-Butyl-2-chloropropanoate	-4066.12	-4043.3
n-Butyl-3-chlorobutanoate	-4707.02	-4696.8
n-Butyl-3-chloropropanoate	-4079.92	-4047.2
n-Butyl-4-chlorobutanoate	-4699.92	-4700.7
n-Butylbenzoate	-5904.59	-5909.3
n-Butylchloroacetate	-3418.3	-3393.7
n-Butyldichloroacetate	-3283.3	-3271.9
n-Butylethylether	-4036.08	-4036.2
n-Butylformate	-2929.77	-2931.3
n-Butylmalonic acid	-3475.7	-3477
n-Butylmethacrylate	-4680.94	-4685.7
n-Butylmethylsulfide	-4142.1	-4140.3
n-Butylmethylsulfone	-3751.57	-3757.3
n-Butylnitrite	-2678	-2682.4
n-Butylnonanoate	-8139.54	-8125.7

n-Butylpentylamide	-5791.9	-5807.2
N-Butylpiperidine	-6089.36	-6086.4
n-Butylstearate	-13972	-14007
n-Butyltrichloroacetate	-3168.2	-3148.8
n-Butylurea	-3263.1	-3280.6
N-Carboxymethylglycine	-1657.1	-1673.6
n-Crotylacetate	-3382.64	-3371.5
N-Cyclohexyl-2,4,6-trimethylbenzaldehyde imine	-9455.2	-9465.7
N-Cyclohexylpiperidine	-7134.1	-7150.9
N-Cyclopentylpiperidine	-6507.2	-6520.7
n-Decanoic acid	-6084.24	-6108.9
n-Decyl alcohol	-6600.69	-6592.8
n-Decylacetate	-7478.28	-7472.3
n-Decylamine	-6951.78	-6933.8
n-Decylformate	-6864	-6852.2
n-Dodecanal	-7712.28	-7699.5
n-Dodecylamine	-8218.38	-8240.7
n-Dodecylmercaptan	-8718.75	-8711.3
Neoheptanoic acid	-4824.82	-4800.9
Neohexane	-4150.98	-4157.9
Neohexanoic acid	-4163.56	-4143.5
Neopentyl alcohol	-3302.98	-3317.8
Neopentyl mercaptan	-4119.4	-4129.3
Neopentyl t-butyl ketone	-6350.7	-6350
N-Ethylaniline	-4724.2	-4702.4
N-Ethylmorpholine	-3998.2	-3999.2
N-Ethyl-m-toluidine	-5370	-5345.4
N-Ethyl-N'-nitroguanidine	-2219	-2224.3
N-Formamido-N'-nitroguanidine	-1355.7	-1335.2
N-Formylleucine	-3841.6	-3850.9
N-Glycyl-dl-valine	-3920	-3925.8
n-Heptaldehyde	-4447.2	-4432.2
n-Heptylacetate	-5515.48	-5511.9
n-Heptylamine	-4949.7	-4973.4
n-Heptylformate	-4895.37	-4891.8
n-Heptyl-n-undecylphthalate	-15107.54	-15091.9
n-Hexanoic acid	-3490.4	-3495
n-Hexylacetate	-4860.45	-4858.4
n-Hexylamine	-4292.8	-4319.9
n-Hexylformate	-4240.07	-4238.3
Nicotinamide N-oxide	-3019.4	-3030.1
Nicotinic acid	-2730.67	-2747.8

Nicotinic acid N-oxide	-2698.2	-2700.6
N-Isopropylaminotriethylsilane	-7385.8	-7397.3
N-Isopropyl-N'-phenylurea	-5650.6	-5656.7
Nitroacetic acid methylester	-1431.1	-1416.7
Nitrobenzene	-3094.9	-3095.9
Nitrocyclohexane	-3719	-3724.1
Nitroethane	-1361.6	-1362.8
Nitroethylene	-1183.28	-1187.2
Nitroguanidine	-896.2	-892.5
Nitroguanylazide	-977.8	-974.4
Nitromethane	-709	-710
Nitropenta	-2572.4	-2605.9
Nitrophenylmethane	-3732.1	-3720.6
N-Leucylglycine	-4574.8	-4577
N-Methyl-1,8-Naphthalimide	-6100.04	-6115.6
N-Methyl-2,3-5,6-dibenzazalene	-8752.9	-8787.5
N-Methyl-2-pyrrolidone	-2993.7	-3002.7
N-Methyl-4-nitroaniline	-3867	-3883.4
N-Methylaniline	-4073	-4054.1
N-Methylbenzaldehydeimine	-4540.2	-4517.1
N-Methylcaprolactam	-4308.6	-4295.7
N-Methylcarbazole	-6791.4	-6827.9
N-Methylcyclohexylamine	-4752.05	-4755.6
N-Methyl-di(2-propynyl)-amine	-4507	-4481
N-Methyldiphenylamine	-7094	-7093.7
N-Methylformamide	-1261.12	-1257.6
N-Methylindoline	-5130.8	-5150.1
N-Methylmaleimide	-2352.8	-2343.9
N-Methylmorpholine	-3344	-3345.6
N-Methyl-N'-nitroguanidine	-1576	-1573.4
N-Methyl-N'-phenylurea	-4354.8	-4355.2
N-Methyl-N-phenylurethane	-5409.1	-5404.7
N-Methyl-octahydroindole	-5814.1	-5856.5
N-Methylphthalimide	-4217	-4217.7
N-Methylpiperidine	-4123	-4125.9
N-Methylpropargylamine	-2810.26	-2767.4
N-Methylpropionamide	-2539.7	-2542.4
N-Methylsuccinimide	-2498.1	-2533.2
N-Methyl-trimethylsilane	-4096	-4097.2
N-Nitro-1-butanamine	-2887	-2864
N-Nitro-1H-1,2,4-triazol-3-amine	-1328.2	-1348.1
N-Nitrobis(2-hydroxyethyl)-amine dinitrate	-2459.9	-2448.1

N-Nitrodibutylamine	-5510.7	-5513.8
N-Nitroethylamine	-1552.9	-1557
N-Nitrophthalamide	-3495	-3511.1
N-Nitropiperidine	-3305.97	-3307.4
N-Nitrosodiisobutylamine	-5515	-5558.8
N-Nitrosodimethylamine	-1650	-1649.6
N-Nitrosomorpholine	-2579	-2582.7
N-Nitrosopiperidine	-3367.87	-3362.9
N-Nitrosuccinimide	-1829.2	-1826.6
n-Nonanal	-5750.22	-5739.1
n-Nonylamine	-6296.74	-6280.3
n-Nonylformate	-6209.01	-6198.7
n-Nonylmercaptan	-6757.89	-6750.9
n-Octanoic acid	-4802.3	-4802
n-Octylacetate	-6169.69	-6165.3
n-Octylamine	-5635.4	-5626.9
N-Octylaminotriethylsilane	-10669	-10661
n-Octylformate	-5551.7	-5545.2
Nonadecane	-12662	-12658.1
Nonane	-6124.6	-6123.4
Nonanedioic acid	-4775.6	-4787.5
Nonylbenzene	-9148.44	-9134.7
Nonylcyclohexane	-9806	-9801.3
Nonylcyclopentane	-9168.9	-9168.5
Nopinene	-6214.1	-6224.9
Norbornane	-4377.44	-4376.7
Norbornene	-4213	-4207.3
Norcarane	-4433	-4433.9
Norleucine	-3582.2	-3586.8
Norpempidine	-6050.1	-6029.7
Nortricyclene	-4227.1	-4224.4
n-Pentyl-2-mercaptan	-4135.77	-4124.4
n-Pentylmalononitrile	-4931.8	-4908
N-Phenethylazetidine	-6594	-6581.9
N-Phenyl-2-naphthylamine	-8313.9	-8301
N-Phenylanthranilic acid	-6380.7	-6380.5
N-Phenylglycine	-4037.6	-4034.5
N-Phenylpiperidine	-6478.4	-6461
N-Propenylpiperidine	-5234.4	-5197.3
n-Propyl acetate	-2894.65	-2898
n-Propyl benzoate	-5256.15	-5255.8
n-Propyl iodide	-2115.38	-2113.8

n-Propylacrylate	-3385.57	-3390.2
n-Propylcarbamate	-2308.6	-2326.1
n-Propylchloroacetate	-2753	-2740.2
n-Propylformate	-2273.98	-2277.9
n-Propylmethacrylate	-4025.84	-4032.2
n-Propylnitrate	-1966	-1978.5
N-Propylpiperidine	-5426.9	-5432.9
N-sec-Pentylaminotriethylsilane	-8694.6	-8710.8
N-Stearoyldextramine	-16133	-16111.8
N-t-Butylaminotriethylsilane	-8054.1	-8033.2
N-t-Butyl-N'-phenylurea	-6292	-6291.4
n-Undecanal	-7058.67	-7046
n-Undecanoic acid	-6741.83	-6762.4
n-Undecylamine	-7607.24	-7587.3
n-Undecylmercaptan	-8063.27	-8057.8
n-Valeraldehyde	-3166	-3125.2
o-Anisic acid	-3752.8	-3752
o-Anthranilic acid	-3353.83	-3345.3
Ocotoic acid	-1717.5	-1709.2
o-Cresol	-3695.8	-3698.4
Oct-2-ynamide	-4817.9	-4827.7
Octadecane	-12009	-12004.6
Octadecyl isocyanate	-12146	-12185.3
Octadecylbenzene	-14987.09	-15018.5
Octahydro-1,2,4-metheno-3H-cyclobuta[cd]pentalen-3-one	-5417	-5398.6
Octahydro-1,3,6-ethanylylidenecyclobuta[cd]indene-2,8(1H)-dione	-6197.7	-6177.4
Octahydroindole	-5174.3	-5181.8
Octamethylenimine	-5461.3	-5422.4
Octane	-5470.2	-5469.9
Octanedioic acid	-4110.9	-4134
Octanone-3-oxime	-5349.3	-5325.7
Octanone-4-oxime	-5339.4	-5325.7
Octylbenzene	-8492.95	-8483.8
Octylcyclohexane	-9151.7	-9147.9
Octylcyclopentane	-8515	-8520.3
o-Cumenol	-5022.9	-5002
omega,4'-Dichloroacetophenone	-3829.6	-3838.5
omega-Bromoacetophenone	-3994.9	-3995.2
omega-Chloroacetophenone	-3992.5	-3991.2
Orthanilic acid	-3363.9	-3357.3
Orthoformic acid trimethyl ester	-2436.3	-2437.5
o-Terphenyl	-9266.5	-9265.6

o-Toluamide	-4200	-4211.4
o-Toluidine	-4036	-4013.5
Oxacyclohexadecan-2-one	-9196	-9208.7
Oxalamide	-851.7	-867
Oxalic acid	-242.9	-213.2
Oxalic acid dihydrate	-217.71	-240.9
Oxalylchloride	-417	-404.1
Oxalyldihydrazide	-1369.7	-1351.6
Oxamic acid	-545.12	-540.1
Oxetane	-1927.9	-1931.7
Oxindole	-3982	-3992.6
Oxirane	-1262.9	-1285.9
p-Acetotoluide	-4920.7	-4883.5
Palmitic acid	-10028.6	-10029.7
p-Anisylchloride	-3837.9	-3847.7
p-Anthranilic acid	-3344	-3345.6
Papaverine	-10375	-10369.5
Paracetamol	-4041.24	-4049.4
p-Azoxyanisole	-7408.6	-7424.6
p-Benzidine	-6508	-6483.2
PBN	-6416.4	-6395.2
p-Chlorobenzyliden-5,6,7,8-tetrahydronaphthyl-2-acetonitrile	-9982.2	-9955.1
PCNB	-2380.6	-2332.7
p-Cresol	-3701.1	-3698.4
p-Cymene	-5861.85	-5853.5
p-Diacetylbenzene diethyl ketal	-10465	-10467.8
Pelargonic acid	-5458	-5455.4
Pentachlorobenzene	-2502.1	-2500.8
Pentachloroethane	-862.3	-866.9
Pentachlorofluoroethane	-692.06	-689.1
Pentachlorophenol	-2329.7	-2292.2
Pentacyclo[5.4.02,603,1005,9]undecane-8,11-dione	-5559.5	-5547.2
Pentacyclo[6.3.1.0(2,7).0(3,5).0(9,11)]dodecane	-7136.3	-7114.5
Pentacyclo[6.3.1.13,6.02,7.09,11]tridecane	-7713.9	-7722.3
Pentadecane	-10046	-10044.2
Pentadecylbenzene	-13032.37	-13055.5
Pentadecylcyclohexane	-13732	-13722.2
Pentadecylcyclopentane	-13100	-13089.3
Pentaerythritol	-2763.7	-2765.6
Pentaethylbenzene	-9760.54	-9763
Pentafluoroaniline	-2669	-2651.6
Pentafluorobenzene	-2557	-2545.1

Pentafluorobenzoic acid	-2552.8	-2520.1
Pentafluoroethane	-698.32	-699.3
Pentafluorophenol	-2349.7	-2336.5
Pentamethoxycarbonylbenzene	-6794.7	-6748
Pentamethylbenzene	-6486.03	-6478.6
Pentamethylbenzoic acid	-6477.34	-6453.6
Pentane	-3511.49	-3509.5
Pentanedioic acid	-2152.4	-2173.6
Pentanenitrile	-3220.7	-3230.2
Pentanoic acid	-2838.8	-2841.6
Pentaphenylbenzene	-18309	-18267.9
Pentyl Elaidate	-14498.6	-14480.4
Pentylcyclohexane	-7189.8	-7187.5
Pentylcyclopentane	-6558	-6554.6
Perchlorocyclopentadiene	-2030	-2034.7
Perfluoro-2,7-dimethyloctane	-3184.6	-3184.7
Perfluoro-2-methyl-2-pentene	-1993.7	-1957.5
Perfluoroacetone	-763.12	-745.3
Perfluorobutane	-1161.72	-1140.8
Perfluorobutanoic acid	-1009.53	-995.7
Perfluoro-cis-decalin	-3466.7	-3453
Perfluorocyclohexene	-2186.1	-2180.7
Perfluoroethane	-485.44	-503.3
Perfluoroheptanoic acid	-1941.65	-1952
Perfluorohexadecane	-4952.88	-4965.7
Perfluoromethylcyclohexane	-2256.2	-2296.9
Perfluoro-n-decane	-3047.65	-3053.2
Perfluoro-n-octane	-2415.57	-2415.7
Perfluoropropene	-1144.12	-1118.2
Perfluoro-trans-decalin	-3466.7	-3453
Perflutren	-787.18	-822
Perhydroanthracene	-8645.7	-8651.1
Perhydrotriquinacene	-6066.9	-6093
Peroxyhexadecanoic acid	-10074	-10102.3
Peroxyoctadecanoic acid	-11378	-11409.3
Peroxytetradecanoic acid	-8766.7	-8795.4
Perseitol	-3496.8	-3481.1
Perylene	-9772.8	-9772.5
Perylene-1,12-quinone	-9258	-9273.7
Phenaceturic acid	-4878.5	-4883.3
Phenanthrene	-7055.92	-7062.3
Phenanthrene-9,10-dione	-6497.8	-6488.1

Phenanthridine	-6542.8	-6573.8
Phenazine	-6101.25	-6088
Phenazine oxide	-6062.7	-6059.4
Phenethyl alcohol	-4398.68	-4379
Phenol	-3055.5	-3055.4
Phenothiazine	-6777	-6752.1
Phenoxathiin	-6498.7	-6481.4
Phenoxazine	-6006.3	-6007.6
Phenoxyacetic acid	-3780.3	-3750.9
Phenoxytrimethylsilane	-6041	-6046.2
Phenyl benzoate	-6309	-6316
Phenyl benzyl ketone	-7153	-7162.8
Phenyl vinyl ether	-4265.2	-4252.1
Phenyl vinyl sulfone	-4682.6	-4672.4
Phenylacetamide	-4224.3	-4219.4
Phenylacetate	-3959.37	-3958.1
Phenylacetic acid	-3890.1	-3892.5
Phenylacetone	-4818.8	-4802.4
Phenylalanine	-4646.3	-4635.1
Phenylalanine-N-carboxylic acid dimethyl ester	-6099.4	-6081.3
Phenylalanine-N-carboxylic anhydride	-4670.8	-4665.9
Phenylcyclobutenedione	-4708.4	-4708.8
Phenylcyclohexane	-6936.84	-6934.1
Phenylformate	-3343.4	-3340.7
Phenylfurazan	-4243.4	-4232.2
Phenylglyoxime	-4276.9	-4294.8
Phenylhydrazine	-3645.4	-3615.5
Phenylisocyanate	-3408	-3429
Phenylpropargyl aldehyde	-4527.9	-4528.5
Phenylpropiolamide	-4589.4	-4571.7
Phenylurea	-3679.3	-3674.3
Phlorol	-4368.39	-4351.8
Phthalamide	-3857.3	-3867.8
Phthalazine	-4267	-4258.1
Phthalhydrazide	-3758.4	-3744.2
Phthalic acid	-3222.1	-3214
Phthalic anhydride	-3259.4	-3240.2
Phthalic heptylnonylester	-13799.95	-13785
Phthalimide	-3544.9	-3529.7
Phthalonitrile	-4000.3	-3991.3
Phyllopyrrole	-5596.1	-5555.3
Picolinamide	-3064.3	-3066.3

Picramide	-2860.1	-2865.8
Pinacolone	-3750.16	-3747.7
Piperazine	-2962.2	-2972.2
Piperidine	-3453.9	-3446.5
Piperonal	-3645	-3611.5
Pivalamide	-3139.9	-3160.8
Pivalic anhydride	-5732.02	-5689.3
Pivalone	-5722.9	-5704.4
Pivalonitrile	-3213.9	-3222.6
Pivalophenone	-6120.7	-6119
PMC	-7859.3	-7832.2
p-Menthadiene	-6175.1	-6169.9
p-Nitrobenzylidene-p-isopropylphenylacetonitrile	-9527.2	-9540.8
p-Quaterphenyl	-12244	-12282.1
Prehnitene	-5849.67	-5835.7
Progesterone	-12008	-11987.2
Proline	-2746.2	-2742.4
Propanal	-1817.5	-1818.3
Propanal dipropylhydrazone	-6350.3	-6343.5
Propanal propylhydrazone	-4369.4	-4346.7
Propanamide	-1842.75	-1861.5
Propane	-2204.5	-2202.5
Propane-1,2-dithiol	-3450.77	-3444.9
Propane-1,3-dithiol	-3451.3	-3457.5
Propanenitrile	-1918	-1923.3
Propanoic acid	-1528.3	-1534.6
Propanoic anhydride	-3113.64	-3090.8
Propanolamine	-2177.07	-2175.5
Propanone	-1816.5	-1791
Propargylamine	-2100.8	-2089.3
Propazine	-5688.3	-5707.2
Propenal	-1667.56	-1657.1
Propham	-5351	-5349.1
Propiolic acid	-1274.14	-1264.4
Propoxytrimethylsilane	-5032	-5015.6
Propyl 2-chlorobutanoate	-4005.3	-4043.3
Propyl 2-pentenoate	-4687.4	-4678.4
Propyl 3-chlorobutanoate	-4045.9	-4043.3
Propyl 3-chloropropanoate	-3419.2	-3393.7
Propyl 3-pentenoate	-4697.9	-4678.4
Propyl 4-chlorobutanoate	-4044.7	-4047.2
Propyl 4-pentenoate	-4705	-4697.2

Propyl butyrate	-4187.8	-4204.9
Propyl chloroformate	-2065.3	-2074.8
Propyl crotonate	-4026.3	-4024.9
Propyl cyclohexanoate	-5871.9	-5919.9
Propyl decanoate	-8093.9	-8125.7
Propyl dichloroacetate	-2636	-2618.4
Propyl dodecanoate	-9422.1	-9432.7
Propyl hexanoate	-5471.9	-5511.9
Propyl isobutanoate	-4194.06	-4201.6
Propyl nitrite	-2030	-2028.9
Propyl non-2-ynoate	-7192.3	-7171.1
Propyl octanoate	-6801.8	-6818.8
Propyl oleate	-13197.4	-13173.5
Propyl palmitate	-12003	-12046.6
Propyl propanoate	-3551.26	-3551.4
Propyl trichloroacetate	-2516	-2495.3
Propyl valerate	-4851.8	-4858.4
Propylcyclohexane	-5880.75	-5880.5
Propylcyclopentane	-5245.9	-5231.7
Propylene	-2058.1	-2041.3
Propylenecarbonate	-1817.4	-1819.4
Propylenediamine	-2513.5	-2513.6
Propyleneglycol-1-monomethylether-2-acetate	-3415.53	-3432.3
Propyleneglycol-2-monomethylether-1-acetate	-3415.53	-3429.7
Propylisonitrile	-2673	-2658.6
Propylmalonic acid	-2827.2	-2823.8
Propylnitramine	-2216	-2210.5
Propyltributylsilane	-11211	-11197.6
Propyltriethylsilane	-7266	-7271.6
Propyne	-1938.7	-1932.3
Protoadamantane	-6070.9	-6060.4
Protocatechuic acid	-2823.7	-2824.5
Protoporphyrin dimethylester	-19099	-19130.9
Pseudoionone	-7760.5	-7774.5
p-Terphenyl	-9277.76	-9276
p-Toluamide	-4190.1	-4211.4
p-Toluidine	-4020	-4013.5
p-Tolyl propadienyl sulphone	-5821	-5847.5
p-Tolyl vinyl sulfone	-5328.4	-5315.4
Pulegone	-5933.3	-5922.3
Purine	-2708.6	-2715.1
Purpurin	-5866.4	-5859.3

Pyracene	-7308.8	-7267.9
Pyrazinamide	-2588.6	-2575.1
Pyrazine	-2285.5	-2281.6
Pyrazine N,N'-dioxide	-2215.3	-2187.3
Pyrene	-7850.7	-7869.5
Pyridazine	-2370.6	-2360.3
Pyridine	-2784.2	-2772.8
Pyridine N-oxide	-2692.5	-2725.6
Pyridine-2,5-dicarboxylic acid	-2724.9	-2714.4
Pyridine-2,6-dicarbonyl-bis(N,N-diethylthiourea)	-11041	-11028.4
Pyrimidine	-2293.8	-2275.6
Pyromellitic dianhydride	-3267	-3216.4
Pyrrole	-2350	-2331.8
Pyrrole-2-aldoxime	-2842.4	-2853.3
Pyrrolidine	-2816.7	-2816.3
Pyrrolizidine	-4564.2	-4569
Pyruvaldehyde	-1443	-1406.8
Pyruvic acid	-1163.4	-1123.1
Quadricyclane	-4200	-4160.8
Quinazoline	-4172	-4176.1
Quinizarin	-6057.4	-6067.9
Quinoline	-4683.2	-4673.3
Quinoxaline	-4179.3	-4184.8
Quinoxaline N,N'-dioxide	-4120.7	-4109
R134a	-872.31	-878.6
RDX	-2092	-2072.8
Refrigerant 115	-555.29	-557.7
Refrigerant 216	-946.47	-930.9
Rhamnose	-3004.9	-2986.3
Ribitol	-2564	-2566.3
Rufigallol	-5230.6	-5233.7
Sabinene	-6181.9	-6225.3
Saccharose	-5637.4	-5615.6
Salicylaldoxime	-3573.8	-3576.6
Salicylanilide	-6383.96	-6392.2
Salicylic acid	-3029.6	-3030.2
Salol	-6109.1	-6107.2
Sarcosine	-1675.1	-1688.8
Sarcosine-N-carboxylic anhydride	-1686.9	-1695.1
s-Butenylacetate	-3383.74	-3335.4
s-Butyl butyrate	-4845.75	-4856.7
s-Butyl crotonate	-4674.4	-4668.9

s-Butyl pentanoate	-5493.95	-5505
s-Butyl propionate	-4185.3	-4195.4
s-Butylacetate	-3534.72	-3549.8
s-Butylacrylate	-4035.74	-4038.1
s-Butylbenzene	-5873.48	-5869.2
s-Butylformate	-2916.21	-2921.9
s-Butylmethylether	-3379.75	-3385.1
s-Butylnitrite	-2672	-2672.9
s-Butyl-t-butylether	-5306.87	-5316.4
sec-Butylurea	-3269.3	-3280.3
Serine	-1438.89	-1439.6
Serylserine	-2898.9	-2897.2
S-Ethyl thioacetate	-3090.46	-3061.9
S-Ethyl trifluorothioacetate	-2561.5	-2539.1
Simazine	-4403.7	-4406.1
Simvastatin	-14030.11	-14036.5
Skatole	-4878.45	-4877.9
Sorbic acid	-3125.8	-3135.1
Sorbitol	-3037	-3021.1
s-Phenethyl alcohol	-4371.2	-4369.5
Spiro[2.2]pentane	-3270.6	-3229.1
Spiro[4.4]nonane	-5688.3	-5678
Spiro[4.5]decane	-6307.5	-6310.8
Spiro[5,5]undecane	-6942.4	-6946.3
Spiro[5.6]dodecane	-7613.2	-7598.4
S-Propylthioacetate	-3707.25	-3715.4
Squaric acid	-1261.6	-1222.4
Stearic acid	-11336.8	-11336.7
s-trans-1,3-Butadiene	-2521.65	-2533.6
s-Triazine	-1781	-1772.4
Strychnine	-11244	-11205.4
Styrene	-4395	-4401.8
Suberone	-4170.2	-4165.4
Succindiamide	-2137.8	-2173.9
Succinonitrile	-2285.4	-2297.5
Succinyl anhydride	-1544.9	-1551
Succinyl dihydrazide	-2638.3	-2658.5
Sulcatone	-4837.8	-4867
Sulfacetamide	-4405.8	-4408.2
Sulfolane	-2879.92	-2879.2
Sylvestrene	-6136.3	-6181.5
syn-Tricyclo[4.2.0.02.5]octane	-5056	-5029.6

t-Amyl acetate	-4173.6	-4185.1
t-Amyl butyrate	-5489.2	-5492
t-Amyl mercaptan	-4124.57	-4118.4
Tartaric acid	-1150.1	-1135.3
TATB	-3079	-3077.9
TBCP	-7710.5	-7710.9
t-Butyl crotonoate	-4678.2	-4656
t-Butyl Decanoate	-8764.3	-8775.5
t-Butyl dodecanoate	-10058.9	-10063.7
t-Butyl ethyl sulfone	-4390.1	-4388.4
t-Butyl glycidyl ether	-4388.46	-4394.6
t-Butyl octanoate	-7453	-7449.8
t-Butyl peroxydecanoate	-8828.3	-8822.1
t-Butyl peroxydodecanoate	-10138	-10129
t-Butyl phenyl carbonate	-5731.3	-5713.4
t-Butyl p-isopropylcumylperoxide	-9666.54	-9665.3
t-Butyl tetradecanoate	-11373.6	-11370.7
t-Butyl-(E)-peroxy 2-butenoate	-4735.3	-4721.3
t-Butyl(phenylacetylenyl)ketone	-7115.4	-7107.4
t-Butyl-1,1,3,3-tetra(methylbutyl)diazene	-8265	-8278.5
t-Butylacetate	-3521.5	-3529
t-Butylacetylene	-3868.1	-3885.1
t-Butylamine	-2997.6	-2991.2
t-Butylbenzene	-5869.18	-5864.9
t-Butylcarbamide	-3267.8	-3258.8
t-Butyldifluoroamine	-2986	-3012.5
t-Butyldimethylvinylethynylperoxide	-6872.2	-6853.7
t-Butylformamide	-3201	-3193.8
t-Butylformate	-2899.32	-2908.9
t-Butylhydroperoxide	-2710	-2712.5
t-Butylmalononitrile	-4250.6	-4246.9
t-Butylmercaptan	-3467.08	-3462.3
t-Butylmethacrylate	-4654.13	-4663.3
t-Butylmethylsulfone	-3731.37	-3736.2
t-Butylnitrite	-2654	-2659.9
t-Butylperoxymethanol	-3204.4	-3208.2
t-Butylperoxymethyloxirane	-4436	-4449.4
t-Butylperoxytrimethylsilane	-5688	-5725.2
t-Dodecylmercaptan	-8707.1	-8692.7
TEGDN	-3440	-3461.4
Terebic acid	-3257.3	-3283.1
Terephthalaldehyde	-3764.82	-3781.4

Terephthalamide	-3858.3	-3873
Terephthalic acid	-3224.5	-3214
Terephthaloyl chloride	-3382	-3404.9
Tetrabis(2,2,2-trinitroethyl)orthocarbonate	-3918	-3898
Tetrabutoxysilane	-10847	-10817.7
Tetracene	-8956.9	-8957.5
Tetrachloroethylene	-830.9	-840.2
Tetrachlorohydroquinone	-2277	-2236.3
Tetraconic acid	-3332.7	-3280.3
Tetracyclo[6,2,1,02,7,03,5]undecane	-6619.1	-6598.8
Tetra-cyclopentadiene	-11426	-11418
Tetradecahydroacridine	-8150.1	-8182.8
Tetradecane	-9399.84	-9390.7
Tetradecanoic acid	-8683	-8722.8
Tetradecylbenzene	-12426.32	-12404.6
Tetradecylcyclohexane	-13077	-13068.7
Tetradecylcyclopentane	-12446	-12435.9
Tetraethoxymethane	-5486.3	-5495.1
Tetraethylammonium nitrate	-5573.4	-5590.6
Tetraethylmethane	-6128.78	-6123.6
Tetraethylpyromellitate	-8644.4	-8620.1
Tetraethylurea	-6019	-5994.8
Tetrafluoroethene	-823.52	-827.4
Tetraglycol	-4742.67	-4721.6
Tetrahydro-2,6-diphenyl-4H-thiopyran-4-one	-9518.6	-9488.2
Tetrahydro-2H-pyran-2-one	-2651.65	-2655.9
Tetrahydro-alpha--tri-cyclopentadiene	-8935.4	-8946.8
Tetrahydrodicyclopentadiene	-6109	-6111.6
Tetrahydrofuran	-2505.4	-2487.1
Tetrahydrofurfuryl alcohol	-2963	-2965.7
Tetrahydropyran	-3143	-3140.6
Tetraisobutylsucccinonitrile	-12750	-12740.1
Tetrakis(2-methylphenoxy)silane	-14894	-14882.3
Tetrakis(3-methylphenoxy)silane	-14883	-14882.3
Tetrakis(4-methylphenoxy)silane	-14886	-14882.3
Tetrakis(o-aminophenyl)porphyrin	-22648	-22683.1
Tetrakis(p-tolyl)porphyrin	-24798	-24849.5
Tetralin-1-hydroperoxide	-5468.2	-5484.1
Tetramethyl orthocarbonate	-2917.89	-2929
Tetramethylammonium nitrate	-2960.5	-2958.5
Tetramethylbutanedinitrile	-4878	-4903.9
Tetramethylene glycol	-2495.5	-2488

Tetramethylethene	-3975.86	-3960.1
Tetramethylglycollide	-4010	-3959.9
Tetramethylguanidine	-3835.7	-3814.9
Tetramethyl-p-phenylenediamine	-6261.3	-6226.2
Tetramethylpyrazine	-4823.2	-4819.6
Tetramethylpyromellitate	-6069.1	-6051.2
Tetramethylsilane	-3973	-3965.6
Tetramethylthiacyclopropane	-4598.15	-4619.1
Tetramethylthiourea	-4246.7	-4240.9
Tetramethylurea	-3420.4	-3390.8
Tetramethyoxysilane	-3007	-3023.7
Tetra-n-butylammonium nitrate	-10841	-10818.4
Tetranitromethane	-430.82	-442.6
Tetraphenoxysilane	-12270	-12310.6
Tetraphenylethylene	-13401	-13391.5
Tetraphenylmethane	-12943	-12933.3
Tetraphenylporphyrin	-22257	-22277.8
Tetrapropoxysilane	-8216	-8203.8
Tetra-p-tolylethene	-15978	-15968.4
Tetra-t-amylperoxypyromellitate	-16629.8	-16644.9
Tetravinylsilane	-5678	-5690.6
Tetrazole	-916.91	-941.7
Tetryl	-3502.3	-3491
Theobromine	-3538.7	-3524.3
Thiamazole	-3038.8	-3025.6
Thianaphthene	-4708.2	-4723.7
Thiane	-3892.5	-3897
Thianthrene	-7250	-7226
Thiepane	-4568.3	-4550.5
Thietane	-2666.6	-2663.6
Thiirane	-2012.6	-2042.4
Thioacetamide	-2033.4	-2037.8
Thioacetic acid	-1742.6	-1746.9
Thioanisole	-4534	-4539.2
Thiobarbituric acid	-2350.9	-2336.3
Thiolactic acid	-2174.6	-2149.5
Thiolan-3-one	-2848	-2832
Thiolane	-3248.7	-3262.1
Thionaphthene-2-carboxylic acid	-4675.6	-4689.6
Thiophene	-2830.1	-2833.6
Thiophenedicarboxylic acid	-2762.3	-2765.5
Thiourea	-1478.03	-1491.9

Thioxanthene	-7264.2	-7267.2
Thioxanthone	-6840.5	-6855.7
Threonine	-2087.1	-2089.1
Thujone	-5991.1	-5981.1
Thymoquinone	-5319.1	-5328.2
Tiglic acid	-2621.8	-2650.1
Tiglic aldehyde	-2879	-2933.8
TME	-2941.8	-2949.6
TNC	-5459	-5467.5
t-Nonylmercaptan	-6740.33	-6732.3
TNT	-3402	-3402.6
TNX	-4068.1	-4045.6
Tolane	-7250	-7270.6
t-Pentylbenzene	-6522.4	-6517.1
trans-(+)-Bicyclo-[6.1.0]nonane	-5749.72	-5743.5
trans,trans-1,4-Diphenylbutadiene	-8475.7	-8523.9
Trans-1,1'-dimethyl-5,5'-azotetrazole	-3186.2	-3158.9
trans-1,2-Bis(2-tolyl)ethylene	-8668.7	-8682.8
trans-1,2-Cyclohexanedicarboxylic acid	-3861.6	-3885.3
trans-1,2-Cyclohexanediol	-3527.76	-3533.9
trans-1,2-Cyclohexanediol diacetate	-5287.3	-5292.7
trans-1,2-Cyclopentanediol diacetate	-4661	-4639.2
trans-1,2-Dibenzoylethylene	-7896.5	-7880.9
trans-1,2-Dibromocyclohexane	-3632.2	-3612.2
trans-1,2-Dichloroethylene	-1095.8	-1100.6
trans-1,2-Diethylcyclopropane	-4672.1	-4673.4
trans-1,2-Dimethyl-3,3-dichlorocyclopropane	-3067.9	-3070.4
trans-1,2-Dimethylcyclohexane	-5220.15	-5223.7
trans-1,2-Dimethylcyclopentane	-4587.35	-4590.9
trans-1,2-Dimethylcyclopropane	-3366	-3366.5
trans-1,2-Diphenylcyclopropane	-8070	-8082.2
trans-1,2-Divinylcyclobutane	-4964.3	-4996.7
trans-1,2-Indandiol diacetate	-6356.8	-6347
trans-1,2-Indanediol	-4585	-4588.2
trans-1,3,5-Hexatriene	-3630	-3660.5
trans-1,3-Dimethylcyclohexane	-5222.65	-5225
trans-1,3-Dimethylcyclopentane	-4590.49	-4590.9
trans-1,3-Pentadiene	-3189.05	-3168.3
Trans-1,4-bis(1,1-dimethylethyl)cyclohexane	-9132.21	-9147.6
trans-1,4-Dichloro-2-butene	-2365.78	-2360.6
trans-1,4-Diethylcyclohexane	-6531.84	-6533.3
trans-1,4-Dimethylcyclohexane	-5215.95	-5221.1

trans-1-Ethyl-2-methylcyclohexane	-5877.95	-5883.8
trans-1-Ethyl-2-methylcyclopentane	-5243.25	-5247
trans-1-Ethyl-3-methylcyclopentane	-5242.35	-5247
trans-1-Ethyl-4-methylcyclohexane	-5871.75	-5877.2
trans-1-Methyl-1,2-Cyclohexanediol	-4157	-4177
trans-1-Methyl-1,2-dicyclopropylcyclopropane	-6353.7	-6352.7
trans-1-Phenyl-3,3-dimethyl-1-butene	-6990.8	-6989.3
trans-1-Propenylbenzene	-4985.2	-5036.5
trans-2,2,4,6-Tetramethyl-1,3-dioxane	-4908.4	-4904.3
trans-2,2,5,5-Tetramethyl-3-hexene	-6585.9	-6581.5
Trans-2,2'-diethyl-5,5'-azotetrazole	-4448.5	-4469.8
trans-2,2-Dimethyl-3-hexene	-5276.9	-5282.2
Trans-2,2'-dimethyl-5,5'-azotetrazole	-3186.2	-3167.8
trans-2,3-Dimethoxycinnamic acid	-5383.2	-5395.1
trans-2,3-Dimethylthiirane	-3292.2	-3324.1
trans-2,4-Dimethoxycinnamic acid	-5360.6	-5395.1
trans-2,5-Diisopropyl-1,3-dioxane	-6192	-6242.6
trans-2,5-Dimethoxycinnamic acid	-5378.4	-5395.1
trans-2,5-Dimethyl-3-hexene	-5275.5	-5283.3
trans-2-Butene	-2685.88	-2676
trans-2-Butene-1,4-diol	-2317.58	-2308.1
trans-2-Butenedinitrile	-2128	-2117.5
trans-2-Decalone	-5858	-5874.4
trans-2-Decene	-6610.84	-6596.8
trans-2-Dodecene	-7918.53	-7903.8
trans-2-Eicosene	-13149.28	-13131.5
trans-2-Ethyl-5-methyl-1,3-dioxane	-4247	-4283.6
trans-2-Heptene	-4645.8	-4636.4
trans-2-Hexahydroindanone	-5237.1	-5239.6
trans-2-Hexene	-3993.26	-3983
trans-2-Hexyl-5-methyl-1,3-dioxane	-6893	-6897.5
trans-2-Methoxycinnamic acid	-4863.5	-4881.8
trans-2-Methyl-5-butyl-1,3-dioxane	-5566	-5593.2
trans-2-Methyl-5-ethyl-1,3-dioxane	-4265	-4286.2
trans-2-Methylcyclohexanol	-4380.2	-4377.5
trans-2-Methyldecalin	-6922	-6940
trans-2-Octene	-5302.66	-5289.9
trans-2-Pentadecene	-9877.41	-9864.2
trans-2-Pentene	-3335.9	-3329.5
trans-2-Pentenenitrile	-3044.84	-3050.2
trans-2-Pentenoic acid	-2666.55	-2661.6
trans-2-Phenyl-1-cyclopropanecarboxylic acid	-5035.63	-5056.4

trans-3,4-Dihydro-3,4-dimethyl-2,5-furandione	-2839	-2856
trans-3,4-Dimethoxycinnamic acid	-5379.6	-5395.1
trans-3,4-Dimethyl-2-pentene	-4633.5	-4624.3
trans-3,5-Dimethoxycinnamic acid	-5377.3	-5395.1
trans-3,5-Diphenyl-1,2,4-trioxolane	-7105.1	-7072.5
trans-3-Decen-1-yne	-6326.36	-6326.6
trans-3-Heptene	-4649.26	-4636.4
trans-3-Hexene	-3992.66	-3983
trans-3-Hexenoic acid	-3338	-3315
trans-3-Methyl-3-hexene	-4642.6	-4625
trans-3-Methylcyclohexanol	-4356.5	-4381.4
trans-3-Octene	-5303.97	-5289.9
trans-3-Penten-1-yine	-3055.08	-3059.3
trans-3-Pentenenitrile	-3048.8	-3050.2
trans-3-Pentenoic acid	-2676.1	-2661.6
trans-4,4-Dimethyl-2-pentene	-4636.85	-4628.8
trans-4,4'-Dimethylstilbene	-8646.43	-8682.8
trans-4,6-Dimethyl-1,3-dioxane	-3613.5	-3628.1
trans-4-Coumaric acid	-4152	-4160
trans-4-Methoxycinnamic acid	-4878.1	-4881.8
trans-4-Methylcyclohexanol	-4325.3	-4377.5
trans-4-Octene	-5303.25	-5289.9
trans-8-Methyl-2-hydrindanone	-5888.2	-5892.6
trans-9-Decalol	-6063	-6086.8
trans-9-Methyldecalin	-6937.2	-6940.3
trans-Aconitic acid	-1985.8	-1967.8
trans-allo-Ocimene	-6201.9	-6214
trans-Azobenzene	-6471.8	-6496.5
trans-Azobenzene N,N'-dioxide	-6396.78	-6402.5
trans-Bicyclo[3.3.0]octan-2-one	-4602.8	-4609.4
trans-Bicyclo[3.3.0]octane	-5040	-5025.5
trans-Cinnamic acid	-4359.3	-4368.6
trans-Crotononitrile	-2385.24	-2396.8
trans-Cyclodecene	-6405.7	-6362.6
trans-Cyclohexane-1,2-dicarboxylic anhydride	-3919.6	-3930.1
trans-Cyclohexane-1,3-dicarboxylic acid diethyl ester	-6639	-6612
trans-Cyclohexane-1,3-dicarboxylic acid dimethyl ester	-5367.5	-5328.9
trans-Cyclohexane-1,4-dicarboxylic acid	-3867.81	-3885.3
trans-Cyclononene	-5761.8	-5706.5
trans-Cyclopentane-1,2-diol	-2909.07	-2903.7
trans-Decahydroquinoline	-5780.3	-5812
trans-Decalin	-6281.32	-6285.9

trans-Dihydro-3,4-diethyl-2,5-furandione	-4163	-4168.1
trans-Diphenyldiazene N-oxide	-6394.7	-6398.1
trans-Geraniol	-6160.9	-6210
trans-Heptacyclene	-12056.4	-12030.2
trans-Hydrindan	-5652	-5655.7
trans-Octahydroinden-1-one	-5193.6	-5239.6
trans-Oxolane-3,4-diol dinitrate	-2045.5	-2020.2
trans-Perfluorobicyclo[4,3.0]nonane	-3147.5	-3157.6
trans-Stilbene	-7364.6	-7397
trans-Tetralin-1,2-diol	-5222.9	-5230.3
trans-Tetralin-2,3-diol	-5223.3	-5230.3
Tratramide	-1783.6	-1786.5
Trehalose	-5630.61	-5606.1
Tribenzylamine	-11406	-11461.7
Tributylamine	-8299.2	-8294.2
Tributylsilane	-9279	-9285.5
Trichloroacetamide	-779.1	-805.4
Trichloroacetic acid	-490.8	-478.5
Trichloroacetylchloride	-597.05	-573.9
Trichloroethylene	-947.7	-970.4
Trichlorotoluene	-3494.9	-3504.3
Tricyclene	-6146.7	-6187.3
Tricyclo[6.2.2.0(2,7)]dodecane	-7367.6	-7349.4
Tricyclopropylmethane	-6380.8	-6363.6
Tridecanal	-8366.7	-8353
Tridecane	-8739	-8737.2
Tridecanoic acid	-8030.72	-8069.3
Tridecanolactone	-7895.3	-7901.7
Tridecylbenzene	-11769.29	-11748.6
Tridecylcyclohexane	-12422	-12415.2
Tridecylcyclopentane	-11791	-11782.4
Triethanolamine	-3840.6	-3821.4
Triethoxymethylsilane	-5176	-5177.9
Triethoxyphenylsilane	-7558	-7552.2
Triethyl phosphate	-4115.6	-4104
Triethylamine	-4377.1	-4373.4
Triethylborane	-4975.6	-4938.1
Triethyleneglycol	-3557.6	-3541.5
Triethylsilane	-5325.6	-5356.8
Triethylsuccinic anhydride	-5476.1	-5468.7
Trifluoroacetal	-671.1	-641.9
Trifluoroacetamide	-659.17	-685.1

Trifluoroacetic acid	-397.2	-358.3
Trifluoroacetyl fluoride	-451.6	-445.3
Triheptylsilane	-15191	-15166.7
Trihexylamine	-12232.32	-12213.7
Trihexylsilane	-13222	-13206.3
Trihydroxyglutaric acid	-1624.5	-1595.6
Triisoamylamine	-10277	-10251.2
Triisobutyl phosphate	-7988.4	-8014.8
Triisobutylamine	-8249.2	-8284.2
Triisobutylsilane	-9268	-9275.5
Triisopentylborane	-10802	-10817.2
Triisopentylsilane	-11244	-11243.8
Trilactic acid	-3999.1	-4020
Trilinolein	-34556	-34584.2
Trimellitic anhydride	-3221.19	-3217.8
Trimethoprim	-7657.1	-7623.6
Trimethoxymethylsilane	-3250	-3253.2
Trimethyl 1,3,5-benzenetricarboxylate	-5340.3	-5354.4
Trimethyl isocyanurate	-2968.5	-2975.2
Trimethyl orthobenzoate	-5444	-5420.7
Trimethyl trimellitate	-5386.4	-5354.4
Trimethylacetic acid	-2832.1	-2833.9
Trimethylamine	-2422.5	-2412.5
Trimethylene urethane	-2116.5	-2108.9
Trimethylethylene	-3330.39	-3318
Trimethylthiirane	-3940.9	-3971.6
Trimethylurea	-2672.7	-2694.5
Trimethylvinylsilane	-4398	-4396.9
Trimyristin	-27842	-27804.3
Tri-n-butylborane	-8893.5	-8858.9
Tri-n-decylamine	-20071	-20056.6
Tri-n-nonylamine	-18109	-18096.2
Tri-n-octylamine	-16145	-16135.8
Trinonylsilane	-19140	-19087.6
Tri-n-pentylamine	-10259.09	-10254.6
Trioctylsilane	-17164	-17127.2
Tripalmitolein	-31179	-31185.2
Tripentylsilane	-11251	-11245.9
Triphenylamine	-9467.77	-9450.7
Triphenylethylene	-10397	-10394.2
Triphenylmethanol	-9760.9	-9725.8
Triphenylphosphane	-10299	-10289.6

Tripropyl phosphate	-6079.2	-6064.4
Tripropylamine	-6335.66	-6336.4
Tripropylborane	-6901.1	-6898.5
Tripropylsilane	-7306	-7325.1
Triptane	-4808.06	-4808.1
Triptycene	-10088	-10088.4
Tris(2,2,2-trinitroethyl)orthoformate	-3172	-3164.3
Tris(2-aminoethyl)amine	-4859.9	-4844.3
Tris(2-methylphenyl)ethylene	-12328.2	-12337.8
Tris(4-methylphenyl)ethylene	-12324.2	-12323
Tris-(carboethoxy)methane	-4969.4	-4939
Tris(hydroxymethyl)nitromethane	-2118	-2098.8
Tritane	-9934.1	-9926.3
Tri-t-Butylperoxymethylsilane	-9243.3	-9267.2
Tri-t-Butylperoxyvinylsilane	-9722	-9698.5
Tritetralin	-10319	-10321.2
Tritylazide	-10113.04	-10112
Trivinylsilane	-4642	-4661.2
Trolox	-7194.5	-7161.7
Trometamol	-2428.27	-2439.3
Tropolone	-3382	-3379.6
Tryptophane	-5629.4	-5606.1
Tyrosine	-4428.1	-4429.2
Tyrosol	-4156.6	-4167.9
Undecane	-7430.9	-7430.3
Undecanedioic acid	-6087.5	-6094.4
Undecanonitrile	-7145.3	-7151
Undecylbenzene	-10457.25	-10441.6
Undecylcyclohexane	-11114	-11108.3
Uracil	-1721.3	-1734.1
Urea	-645	-641.8
Uridine	-4096.8	-4092.8
Valeramide	-3159.2	-3168.4
Valine	-2933.9	-2932.7
Valylphenylalanine	-7601.7	-7590.6
Vanillic acid	-3542.9	-3543.7
Vanillyl alcohol	-4033.1	-4027.6
Vat Blue 1	-7594.9	-7565.6
Veratrole	-4286.9	-4290.5
Vidarabine	-5139.4	-5171.5
Vinyl acetate	-2086	-2075.2
Vinylacetylene	-2380	-2424.6

Vinylcyclohexane	-5063.63	-5063.2
Vinylcyclopentane	-4434.7	-4433
Vinylcyclopropane	-3215.45	-3208.6
Vinylene carbonate	-1006.5	-979.2
Vinylethylenecarbonate	-2271.53	-2323.2
Vinylformate	-1460	-1455.1
Vinylidenechloride	-1096	-1123.4
Vinylisopentyl ether	-4490	-4495.5
Vinylpivalate	-4043.44	-4027.9
Vinylpropionate	-2727.49	-2728.6
Vinylsilane	-2524.6	-2494.6
Vinyltriethoxysilane	-5605	-5609.1
Vinyltrimethoxysilane	-3696.92	-3684.4
Vinyltripropoxysilane	-7583	-7569.5
Vitamin B3	-3083.78	-3077.3
Vitamin C	-2339.8	-2326.2
Vitamin E acetate	-18536	-18537.5
Vitamin K3	-5238	-5253.7
Xanthene	-6494.6	-6522.7
Xanthine	-2152.5	-2148.3
Xanthone	-6071.63	-6111.2
Xylitol	-2564	-2574.5
Xylose	-2340.4	-2342.4
