# Supplementary material for: Revision and Extension of a Generally Applicable Group-Additivity Method for the Calculation of the Standard Heat of Combustion and Formation of Organic Molecules
Source: Molecules. 2021 Oct 10;26(20):6101. doi: 10.3390/molecules26206101 (PMC8539152; doi:10.3390/molecules26206101)
Supplement: Supplementary file 1 [file molecules-26-06101-s001.zip › Table S3. Experimental vs. calculated deltaH_f) Data Table.pdf]

Table S3. Experimental vs. calculated deltaH_f) Data Table.		
**Molecule Name**	**DeltaH°(f) exp**	**DeltaH°(f) calc**
(-)-Dimethyl D-tartrate	-1199.70	-1216.40
(1,1-Dimethylpentyloxymethyl)oxirane	-442.10	-432.40
(1,1-Dimethylpropoxymethyl)oxirane	-392.60	-387.70
(1,2-Propadienylsulphonyl)-benzene	-106.80	-85.80
(1-Chloroethyl)benzene	-60.10	-58.50
(1-Methylvinyl)cyclopropane	56.30	60.50
(2,2-Dimethylpropoxymethyl)oxirane	-389.90	-375.50
(2-Fluoro-2,2-dinitroethoxy)(2,2,2-trinitroethoxy)methane	-581.50	-617.00
(2-Fluoro-2,2-dinitroethyl) acrylate	-579.25	-571.00
(2-Methylphenoxy)trimethylsilane	-389.72	-384.60
(2-Methylpropoxymethyl)oxirane	-357.70	-344.90
(3-(N-(1,2,4-Triazolo[4,3-b]-1,2,4,5-tetrazin-3-yl)amino-4-aminofurazan	656.50	645.40
(3-(N-(1,2,4-Triazolo[4,3-b]-1,2,4,5-tetrazin-3-yl)amino-4-azidofurazan	996.00	1013.30
(3-(N-(1,2,4-Triazolo[4,3-b]-1,2,4,5-tetrazin-3-yl)amino-4-nitrofurazan	691.00	717.80
(3-Aminopropyl)-dibutylborane	-395.97	-353.10
(3-Aminopropyl)-dipropylborane	-316.88	-300.40
(3-Methylbutoxymethyl)oxirane	-384.00	-368.60
(3-Methylphenoxy)trimethylsilane	-390.72	-384.60
(4-Methylphenoxy)trimethylsilane	-387.72	-384.60
(9Z)-hexadecenoic acid	-713.40	-741.00
(9Z)-Octadecenoic acid	-789.57	-793.60
(Benzyloxy-NNO-azoxy)-phenylmethane	130.43	121.60
(Chloromethyl)benzene	-33.00	-30.90
(Diethylamino)acetonitrile	13.40	12.70
(Diethylamino)trimethylsilane	-238.74	-249.70
(Dimethylamino)acetonitrile	67.10	65.00
(Dinitromethyl)benzene	-43.50	-41.40
(E)-(2-Phenylethenyl)-sulphonylbenzene	-144.90	-163.70
(E)-1-Methyl-1,2-cyclopentanediol	-539.30	-534.60
(E)-1-Methyl-4-(1-propenyl-sulphonyl)-benzene	-296.50	-306.10
(E)-1-Methyl-4-(2-phenylethenyl)sulphonylbenzene	-183.10	-200.60
(E)-2,3-Dimethylbicyclo[2.2.1]heptane	-150.20	-149.90
(Ethoxymethyl)oxirane	-296.60	-288.90
(Ethylthio)benzene	21.93	4.80
(Fluoromethyl)benzene	-168.30	-171.80
(Iodomethyl)benzene	53.00	61.70
(L)-Alanine	-561.39	-558.60
(L)-Cysteine	-534.10	-533.50

(L)-Cystine	-1035.53	-1042.30
(L)-Histidine	-469.13	-483.80
(L)-Hydroxyproline	-661.10	-688.40
(L)-Methionine	-580.26	-580.20
(Pentyloxymethyl)oxirane	-367.40	-367.90
(trans-1-Butenyl)benzene	-14.30	40.00
(Z)-1,2,3,4-Tetrahydronaphthalene-1,2-diol	-415.50	-426.20
(Z)-1,2,3,4-Tetrahydronaphthalene-2,3-diol	-425.20	-426.20
(Z)-1,2-Bis(benzylthio)ethylene	143.70	127.10
(Z)-1-Methyl-1,2-cyclopentanediol	-529.70	-534.60
(Z)-1-Methyl-4-(2-phenylethenyl)sulphonylbenzene	-181.60	-203.20
(Z,Z,Z)-1,4,7-Cyclononatriene	140.60	98.60
[1,8]Paracyclophane	-89.50	-99.50
[2,2]Metacyclophane	72.80	26.90
[3,3]Paracyclophane	19.70	-25.70
1-(1-Butenyl)piperidine	-87.70	-124.30
1-(1-Nitro-1H-pyrazol-3-yl)-1H-tetrazole	569.80	516.60
1-(2-Aminophenyl)pyrrole	121.70	132.30
1-(2-Hydroxycyclopentyl)-2-propanone	-472.00	-503.90
1-(2-Methylphenyl)ethanone	-178.00	-179.70
1-(2-Propenyl)piperidine	-39.90	-26.20
1-(2-Pyridinyl)ethanone	-104.30	-108.20
1-(3,4-Dimethoxyphenyl)naphthalene	-178.70	-184.40
1-(3-Formyl-4-methoxyphenyl)naphthalene	-154.90	-152.90
1(3H)-Isobenzofuranone	-315.00	-302.70
1-(3-Methoxyphenyl)naphthalene	-20.60	-17.80
1-(4-Chlorophenyl)pyrrole	105.31	122.30
1-(4-Ethylphenyl)-2-phenylethane	12.03	-1.30
1-(4-Fluorophenyl)pyrrole	-50.64	-19.80
1-(4-Formylphenyl)naphthalene	11.10	13.60
1-(4-Hydroxyphenyl)butan-1-one	-420.80	-406.60
1-(4-Hydroxyphenyl)heptan-1-one	-508.40	-485.50
1-(4-Hydroxyphenyl)hexan-1-one	-479.40	-459.20
1-(4-Hydroxyphenyl)pentan-1-one	-451.60	-432.90
1-(4-Iodophenyl)pyrrole	208.40	220.80
1-(4'-Methoxybenzylidene)-2-phenazin-1-oylhydrazine	20.60	27.80
1-(4-Methoxyphenyl)naphthalene	-24.40	-17.80
1-(4-Methylphenyl)pyrrole	100.10	117.70
1-(Biphenyl-4-yl)naphthalene	204.83	220.00
1,1,1,2,2,3,3-Heptafluoro-7,7-dimethyloctan-4,5-dione	-1988.30	-1972.50
1,1,1,2,2-Pentafluoropropane	-1111.00	-1133.60
1,1,1,2,3,3,3-Heptafluoropropane	-1552.00	-1550.20

1,1,1,2,3,3-Hexafluoropropane	-1333.00	-1323.50
1,1,1,2-Tetrachloroethane	-193.40	-175.20
1,1,1,2-Tetraphenylethane	221.70	214.00
1,1,1,3,5,5,5-Heptanitropentane	-155.90	-169.70
1,1,1,3-Tetrachloropropane	-208.70	-201.50
1,1,1,4-Tetranitrobutane	-191.20	-162.80
1,1,1-Trichloro-3,3,3-trifluoropropane	-821.70	-779.00
1,1,1-Trichlorofluoroethane	-337.10	-316.10
1,1,1-Trifluoro-4-(2-thienyl)-4-hydroxy-3-buten-2-one	-948.60	-921.10
1,1,1-Trifluoro-4-(2-thienyl)-4-mercapto-3-buten-2-one	-725.40	-699.80
1,1,1-Trifluoro-5,5-dimethylhexan-2,4-dione	-1118.00	-1127.80
1,1,1-Trifluoro-5-methylheptan-2,4-dione	-1105.60	-1118.30
1,1,1-Trifluoro-5-methylhexan-2,4-dione	-1095.40	-1093.30
1,1,1-Trifluoroacetylacetone	-1034.50	-1041.20
1,1,1-Trifluoroethane	-744.60	-711.30
1,1,1-Trifluorohexan-2,4-dione	-1049.40	-1067.50
1,1,1-Trimethoxy-2-chloroethane	-661.10	-659.50
1,1,1-Trimethoxyethane	-612.00	-622.20
1,1,1-Trinitroethane	-96.90	-66.80
1,1,1-Triphenylethane	149.40	133.70
1,1,2,2-Tetrachloroethane	-202.40	-174.00
1,1,2,2-Tetrafluoro-1,2-diphenylethane	-766.80	-735.20
1,1,2,2-Tetrafluoroethane	-892.40	-872.70
1,1,2,2-Tetramethylcyclopropane	-119.80	-88.60
1,1,2,2-Tetraphenylethane	216.00	210.00
1,1,2,2-Tetra-p-tolylethane	62.50	59.80
1,1,2-Trichloroethane	-186.20	-172.50
1,1,2-Trifluoro-1,2-diphenylethane	-537.70	-537.00
1,1,2-Trimethylcyclopropane	-96.20	-60.60
1,1,2-Triphenylethane	130.20	138.00
1,1,2-Tris(2-methylphenyl)ethane	24.40	43.40
1,1,2-Tris(4-methylphenyl)ethane	16.40	24.70
1,1,3,3,5,5-Hexaphenyl-7,7-dimethylcyclotetrasiloxane	-1391.05	-1412.90
1,1,3,3-Tetramethylguanidinium nitrate	-314.50	-314.50
1,1,3,3-Tetranitrobutane	-215.90	-244.00
1,1,4,4-Tetramethylcyclodecane	-344.41	-357.30
1,1,4,4-Tetraphenyl-1,3-butadiene	329.90	354.40
1,1,4,4-Tetraphenylbutane	153.10	156.00
1,1,4,6,7-Pentamethylindan	-175.10	-155.60
1,1,4,6-Tetramethylindane	-131.80	-132.90
1,1,4,7-Tetramethylindane	-129.00	-118.70
1,1,5,5-Tetramethylcyclodecane	-334.91	-366.20

1,10-Decanediol	-683.66	-675.10
1,12-Dimethylbenz[a]anthracene	130.00	102.90
1,12-Dimethylbenzo[c]phenanthrene	147.40	91.30
1,1'-Bicycloheptyl	-302.00	-326.00
1,1'-Binaphthyl	162.40	182.80
1,1-Bis(4-methylphenyl)ethane	-33.20	-17.70
1,1-Bis(4-tolyl)ethylene	85.90	101.60
1,1-Bis(difluoroamino)heptane	-260.19	-292.00
1,1-Di(methoxy-NNOazoxy)ethane	22.30	43.20
1,1-Dibutoxypropane	-608.60	-620.90
1,1-Dichloro-1-fluoroethane	-366.40	-343.70
1,1-Dichlorocyclopropane	-26.30	-28.40
1,1-Dichloroethane	-163.30	-135.20
1,1-Dichlorotetrafluoroethane	-960.20	-958.40
1,1-Dicyclohexylbutane	-370.70	-378.10
1,1-Dicyclohexylhexane	-390.30	-430.70
1,1-Diethoxyethane	-491.41	-489.20
1,1-Diethoxynon-2-yne	-407.60	-402.60
1,1-Diethoxyoct-2-yne	-379.20	-376.30
1,1-Diethylcyclohexane	-277.11	-260.40
1,1-Diethynylcyclopropane	498.50	517.50
1,1-Difluoroethane	-497.00	-484.70
1,1-Difluoroethylene	-328.96	-303.80
1,1-Diisopropoxyethane	-569.20	-560.90
1,1-Dimethoxy-2-methylpropane	-476.20	-466.10
1,1-Dimethoxy-2-phenylcyclopropane	-229.70	-237.00
1,1-Dimethoxybutane	-468.10	-465.40
1,1-Dimethoxycyclobutane	-345.05	-344.50
1,1-Dimethoxycyclopentane	-445.70	-443.00
1,1-Dimethoxypentane	-494.60	-491.70
1,1-Dimethoxypropane	-443.30	-439.10
1,1-Dimethyl-2-ethylcyclopropane	-90.20	-86.90
1,1-Dimethyl-2-hexylcyclopropane	-193.00	-192.10
1,1-Dimethyl-2-propylcyclopropane	-116.00	-113.20
1,1-Dimethyl-3-phenylurea	-205.80	-211.30
1,1-Dimethyl-4-penten-2-yn-1-ylhydroperoxide	60.89	24.00
1,1-Dimethylazoethane	-79.30	-56.10
1,1-Dimethylcyclohexane	-218.70	-214.40
1,1-Dimethylcyclopentane	-172.05	-168.70
1,1-Dimethylcyclopropane	-33.30	-33.60
1,1-Dimethylindane	-53.60	-59.20
1,1-Dinitroethane	-148.20	-144.40

1,1-Dinitropropane	-171.98	-170.70
1,1-Di-o-tolylethane	-26.10	-17.70
1,1-Di-o-tolylethylene	89.00	119.00
1,1'-Diphenyl-1,1'-bicyclohexyl	-116.90	-123.50
1,1'-Diphenyl-1,1'-bicyclopentyl	-38.30	-50.30
1,1-Diphenyl-3,3,5,5,7,7-hexamethylcyclotetrasiloxane	-1890.51	-1890.50
1,1-Diphenylbutane	26.90	3.40
1,1-Diphenylcyclopropane	180.30	172.50
1,1-Diphenylethane	48.66	56.00
1,1-Diphenylethylene	172.42	175.40
1,1-Diphenylhexane	-4.10	-49.20
1,1-Diphenylpropane	50.20	29.70
1,1-Ethanediol diacetate	-874.00	-886.60
1,1'-Oxybis(2,4,4-trinitro-4-fluoro-2-azabutane)	-654.90	-652.50
1,2,2,3-Tetrachloropropane	-251.80	-252.70
1,2,2-Trichloropropane	-185.80	-215.40
1,2,3,4,5,6,7,8-Octahydroanthracene	-124.90	-121.70
1,2,3,4-Tetrachlorobenzene	-96.80	-85.60
1',2',3',4'-Tetrahydro-1',2-binaphthylmethane	44.10	51.10
1',2',3',4'-Tetrahydro-1,2-dinaphthylmethane	36.60	48.50
1,2,3,4-Tetrahydro-5-hydroperoxynaphthalene	-185.70	-175.20
1,2,3,4-Tetrahydronaphthalene	-36.40	-39.20
1,2,3,4-Tetrahydro-N-methylcarbazole	-5.90	9.50
1,2,3,4-Tetrahydroquinoline	13.20	-13.90
1,2,3,4-Tetraphenyl-1,3-butadiene	349.10	351.80
1,2,3,4-Tetraphenylbenzene	320.60	307.70
1,2,3,4-Tetraphenylnaphthalene	398.47	349.70
1,2,3,5-Tetrachlorobenzene	-104.80	-85.60
1,2,3,5-Tetraethylbenzene	-196.36	-200.40
1,2,3,5-Tetramethylbenzene	-96.36	-104.20
1,2,3,6,7,8-Hexahydropyrene	-49.70	-32.30
1,2,3-Benzenetriol	-553.30	-582.40
1,2,3-Propanetricarboxylic acid	-1544.90	-1551.60
1,2,3-Propanetriol	-669.60	-684.30
1,2,3-Trichlorobenzene	-65.60	-53.30
1,2,3-Trichloropropane	-230.60	-238.60
1,2,3-Trichloropropene	-101.80	-92.80
1,2,3-Triethylbenzene	-130.32	-141.10
1,2,3-Trimethoxybenzene	-442.40	-456.30
1,2,3-Trimethylcyclopentene	-118.00	-116.80
1,2,3-Triphenylbenzene	234.30	241.60
1,2,4,5-Tetrachlorobenzene	-107.80	-85.60

1,2,4,5-Tetrafluorobenzene	-664.20	-654.40
1,2,4,5-Tetramethylbenzene	-98.99	-104.20
1,2,4,5-Tetraphenylbenzene	301.60	307.70
1,2,4-Benzenetricarboxylic acid	-1179.20	-1213.00
1,2,4-Butanetriol trinitrate	-399.17	-399.00
1,2,4-Triazole	112.30	148.00
1,2,4-Triazolo[1,5-a]pyrimidine	256.39	268.70
1,2,4-Trichlorobenzene	-48.90	-53.30
1,2,4-Triethylbenzene	-134.05	-139.80
1,2,4-Trihydroxybenzene	-563.80	-582.40
1,2,4-Trimethoxybenzene	-440.00	-456.30
1,2,4-Trimethylbenzene	-61.80	-67.30
1,2,4-Triphenylbenzene	235.30	246.90
1,2,4-Tri-t-butylbenzene	-251.60	-285.10
1,2,5,6-Diacetone-mannitol	-1277.00	-1264.70
1,2,5,6-Tetrahydropyridine	33.00	10.50
1,2,5-Pentanetriol trinitrate	-442.00	-428.00
1,2,5-Trimethylpyrrole	-16.10	-23.80
1,2-Anhydro-3,4,5,6-alloinositol	-908.70	-925.30
1,2-Benzenedicarboxylic acid, di-2-propenyl ester	-550.60	-589.80
1,2-Bis(2-cyano-2-propyl)-hydrazine	116.00	135.40
1,2-Bis(2-tolyl)ethane	-40.00	-17.00
1,2-Bis(4-tolyl)ethane	-48.10	-17.00
1,2-Bis(difluoroamino)-2-methylpropane	-260.30	-222.90
1,2-Bis(difluoroamino)-4-methylpentane	-250.99	-254.80
1,2-Bis(methylnitramino)ethane	-94.10	-64.20
1,2-Butadiene	138.99	140.30
1,2-Butanediamine	-120.20	-118.90
1,2-Butanediol	-523.60	-526.60
1,2-Cyclohexanedione dioxime	-135.10	-160.40
1,2-Di-(5-tetrazolyl)ethane	444.64	466.80
1,2-Diaminobenzene	-2.72	-26.90
1,2-Dibenzoylethane	-255.60	-246.80
1,2-Dibenzoylhydrazine	-206.54	-215.40
1,2-Dibromobutane	-142.10	-128.00
1,2-Dibromododecane	-367.80	-338.60
1,2-Dibromoethane	-79.20	-74.80
1,2-Dibromotetrafluoroethane	-807.10	-799.70
1,2-Dichloro-1,1,2-trifluoroethane	-735.55	-720.30
1,2-Dichlorobenzene	-22.00	-21.10
1,2-Dichlorobutane	-231.00	-227.60
1,2-Dicyclohexylbutane	-330.70	-371.50

1,2-Dicyclohexylethane	-382.00	-326.00
1,2-Diethylbenzene	-68.49	-80.50
1,2-Difluorobenzene	-319.20	-305.50
1,2-Difluoroethane	-447.70	-453.10
1,2-Dihydro-3H-1,2,4-triazol-3-one	-143.30	-140.20
1,2-Dihydronaphthalene	71.50	66.90
1,2-Dihydroxy-3-isopropyl-6-methylbenzene	-475.70	-500.90
1,2-Dihydroxy-9,10-anthracenedione	-590.30	-588.80
1,2-Dihydroxybenzene	-364.40	-373.80
1,2-Dihydroxynaphthalene	-313.20	-337.10
1,2-Diiodobenzene	187.00	175.80
1,2-Diiodoethane	9.30	14.10
1,2-Dimethoxypropane	-422.30	-414.70
1,2-Dimethyl-1,2-diphenlethylene glycol dimethyl ether	-304.20	-313.90
1,2-Dimethylbenzene	-24.35	-27.80
1,2-Dimethylhydrazine	52.70	39.70
1,2'-Dinaphthylmethane	154.60	159.10
1,2-Dinitrobenzene	9.52	-6.90
1,2-Dinitroethane	-179.00	-183.10
1,2-Diphenylbenzimidazole	290.20	284.60
1,2-Diphenylethane	51.55	56.70
1,2-Diphenylhydrazine	202.40	181.40
1,2-Epoxy-4-oxa-5-phenylpentane	-209.20	-210.90
1,2-Ethanediamine	-63.00	-68.60
1,2-Ethanediol	-455.11	-464.50
1,2-Ethanediol dinitrate	-236.30	-258.50
1,2-Ethanedithiol	-53.72	-46.30
1,2-Hexanediol	-580.00	-579.30
1,2-Hydrazinedicarboxamide	-494.61	-485.60
1,2-Naphthoquinone	-166.60	-184.10
1,2-Pentadiene	150.83	114.00
1,2-Pentanediol	-549.20	-553.00
1,2-Propanediol	-500.74	-500.30
1,2-Propanediol 1-methacrylate	-629.34	-633.70
1,2-Propanediol dinitrate	-296.90	-294.30
1,3,3-Trimethyltricyclo[2.2.1.02,6]heptane	-70.80	-43.20
1,3,3-Triphenyl-2-propen-1-one	82.60	78.80
1,3,5,5-Tetramethylbarbituric acid	-696.00	-704.90
1,3,5,6-Tetramethyluracil	-486.60	-471.20
1,3,5,7-Tetramethyladamantane	-362.10	-311.40
1,3,5-Benzenetricarboxylic acid	-1190.00	-1210.40
1,3,5-Cycloheptatriene	142.17	133.90

1,3,5-Trichlorobenzene	-74.00	-53.30
1,3,5-Trihydroxybenzene	-584.60	-582.40
1,3,5-Trimethyl-1H-pyrazole	19.90	45.00
1,3,5-Trimethyl-2-nitrobenzene	-73.27	-92.40
1,3,5-Trimethyl-4-nitrosopyrazole	109.50	96.30
1,3,5-Trimethyladamantane	-326.28	-280.70
1,3,5-Trimethylbarbituric acid	-669.50	-674.20
1,3,5-Trimethylbenzene	-63.43	-67.30
1,3,5-Trimethylhexahydro-s-triazine	21.40	20.40
1,3,5-Trimethyluracil	-453.50	-434.30
1,3,5-Trinitro-1,3,5-triazacycloheptane	29.92	9.70
1,3,5-Trinitrobenzene	-37.20	-32.00
1,3,5-Trioxane	-521.38	-527.90
1,3,5-Triphenylbenzene	254.60	253.40
1,3,5-Triphenyltriazine-2,4,6-trione	-351.40	-360.60
1,3,5-Tri-t-butylbenzene	-320.00	-315.40
1,3,5-Trithiane	80.00	74.90
1,3,6-Trioxocane	-515.90	-532.20
1,3-Benzenedicarbonitrile	270.00	269.10
1,3-Benzothiazole-2-thione	91.40	82.80
1,3-Benzoxazole-2-thione	-68.20	-40.10
1,3-Butanediol dinitrate	-342.20	-320.70
1,3-Butyleneglycol	-501.00	-526.60
1,3-Cyclohexadiene	69.10	54.20
1,3-Cyclohexanedione	-427.70	-408.90
1,3-Diacetin	-1120.70	-1072.60
1,3-Diamino-2,4,6-trinitrobenzene	-99.69	-105.90
1,3-Diaminobenzene	-9.72	-29.50
1,3-Dibromo-2-methylpropane	-137.60	-130.80
1,3-Dibromobutane	-148.00	-128.00
1,3-Dichloro-2-propanol	-385.30	-390.90
1,3-Dichlorobenzene	-20.70	-21.10
1,3-Dichlorobutane	-237.30	-227.60
1,3-Dichloropropane	-199.90	-197.40
1,3-Dichloro-trans-2-butene	-122.25	-105.30
1,3-Dicyclohexylbutane	-370.70	-375.50
1,3-Diethoxypropane	-485.50	-481.70
1,3-Diethyl-1,3-diphenylurea	-127.30	-161.60
1,3-Diethyl-2-thiobarbituric acid	-445.79	-454.40
1,3-Diethylbarbituric acid	-714.61	-702.10
1,3-Diethylbenzene	-73.51	-77.90
1,3-Difluorobenzene	-343.90	-305.50

1,3-Dihydroisobenzofuran	-86.70	-104.00
1,3-Dihydroxy-2,2-di(ethoxy-NNO-azoxy)propane	-421.68	-427.20
1,3-Dihydroxy-2,2-di(methoxy-NNO-azoxy)propane	-331.89	-350.80
1,3-Dihydroxyacetone	-603.40	-616.30
1,3-Dihydroxybenzene	-372.80	-373.80
1,3-Dihydroxynaphthalene	-330.60	-337.10
1,3-Diiodobenzene	187.00	175.80
1,3-Diiodocyclobutane	135.00	123.30
1,3-Diiodopropane	-9.00	-12.20
1,3-Diisopropylbenzene	-132.40	-133.30
1,3-Dimethoxybenzene	-283.30	-289.80
1,3-Dimethyl-5-fluorouracil	-572.00	-537.80
1,3-Dimethyladamantane	-286.40	-250.00
1,3-Dimethylbenzene	-25.36	-30.50
1,3-Dimethyluracil	-410.51	-396.60
1,3-Dinitro-1,3-diazacyclopentane	-1.68	12.50
1,3-Dinitro-2-imidazolidinone	-138.40	-99.90
1,3-Dinitrobenzene	-6.90	-6.90
1,3-Dinitropropane	-207.10	-209.50
1,3-Dioxane	-376.77	-381.70
1,3-Dioxepane	-390.00	-408.10
1,3-Dioxolan-2-one	-582.04	-578.00
1,3-Dioxolane	-337.20	-341.40
1,3-Diphenyl-1-triazene	327.40	296.80
1,3-Diphenyl-2,3-epoxy-1-propanone	-100.70	-88.50
1,3-Diphenyl-3-ethoxy-2-propen-1-one	-190.40	-223.30
1,3-Diphenylacetone	-89.20	-95.00
1,3-Diphenylbutane	37.40	9.90
1,3-Diphenylpropane	50.20	33.00
1,3-Di-t-butyl urea	-504.10	-528.30
1,3-Di-t-butylbenzene	-187.40	-194.60
1,3-Dithiane	-10.00	-26.70
1,3-Dithiane sulfoxide	-198.34	-185.70
1,3-Dithiolane	10.00	8.90
1,3-Oxazolidine-2-thione	-181.10	-159.80
1,3-Propanediol	-480.80	-490.80
1,3-Propanediol dinitrate	-296.40	-284.80
1,3-Thiazolidine-2-thione	-16.23	-17.30
1,4,4a,8a-Tetrahydro-1,4-methanonaphthalene-5,8-dione	-168.00	-192.20
1,4,5,6-Tetrahydropyrimidine	-22.40	-24.30
1,4,5,8-Naphthalenetetracarboxylic dianhydride	-930.90	-980.00
1,4,5,8-Tetrahydro-1,6-methanonaphthalene	50.80	100.80

1,4,5,8-Tetramethylnaphthalene	-23.40	-32.80
1,4,7,10,13,16-Hexaoxacyclooctadecane	-1086.50	-1076.40
1,4-Anthraquinone	-183.80	-147.40
1,4-Benzenedicarbonitrile	265.98	269.10
1,4-Benzoquinone	-185.70	-196.80
1,4-Benzothiazin-3-one	-150.80	-138.60
1,4-Bis-(2-hydroxyethyl)piperazine	-467.87	-454.20
1,4-Bis(hydroxymethyl)benzene	-397.30	-398.40
1,4-Bis(hydroxymethyl)-cyclohexane	-584.60	-585.20
1,4-Bis(methoxycarbonyl)-cyclohexane	-948.90	-897.00
1,4-Butanediol dinitrate	-332.20	-311.20
1,4-Butanedithiol	-105.70	-99.00
1,4-Cyclohexadiene	63.80	54.20
1,4-Cyclohexanedione	-409.90	-408.90
1,4-Diaminobenzene	0.68	-29.50
1,4-Dibromobenzene	50.61	70.70
1,4-Dibromobutane	-140.30	-127.50
1,4-Dichlorobenzene	-39.90	-21.10
1,4-Dichlorobutane	-229.80	-223.80
1,4-Dicyano-2-butyne	366.50	368.60
1,4-Dicyanobenzene di-N-oxide	334.98	321.80
1,4-Dicyclohexylbutane	-370.70	-378.70
1,4-Dicyclopropylbuta-1,3-diyne	500.10	527.30
1,4-Diethylbenzene	-72.84	-77.90
1,4-Difluorobenzene	-342.30	-305.50
1,4-Dihydronaphthalene	84.20	66.90
1,4-Dihydroxy-2,2,6,6-Tetramethylpiperidine	-449.70	-462.00
1,4-Dihydroxybenzene	-371.10	-373.80
1,4-Dihydroxynaphthalene	-339.40	-337.10
1,4-Diiodobenzene	160.70	175.80
1,4-Diiodobutane	-29.80	-38.60
1,4-Diisocyanatobutane	-286.30	-289.30
1,4-Dimethoxybenzene	-296.50	-289.80
1,4-Dimethyl-2,5-piperazinedione	-440.60	-429.00
1,4-Dimethyl-2,6,7-trioxabicyclo[2.2.2]octane	-634.10	-630.40
1,4-Dimethylbenzene	-24.35	-30.50
1,4-Dimethylbicyclo[2.2.1]heptane	-167.40	-161.80
1,4-Dimethylpiperazine	-36.68	-33.90
1,4-Dinitrobenzene	-32.68	-6.90
1,4-Dinitroimidazole	133.60	120.00
1,4-Dinitropiperazine	-52.98	-25.20
1,4-Dinitropyrazole	176.90	185.50

1,4-Dioxane	-355.13	-358.80
1,4-Dioxane-2,5-dione	-705.30	-756.20
1,4-Dioxane-2,6-dione	-701.83	-732.20
1,4-Dioxaspiro[4.4]nonane	-417.56	-411.50
1,4-Dioxatetralin	-257.60	-269.70
1,4-Diphenylbutane	-9.90	9.30
1,4-Diphenylnaphthalene	215.83	217.40
1,4-Di-t-butylbenzene	-189.10	-195.90
1,4-Di-t-butylperoxy pyromellitate	-1668.00	-1643.80
1,4-Dithiane	-72.50	-50.70
1,4-D-Mannonolactone	-1241.90	-1243.60
1,4-Naphthoquinone	-183.40	-184.10
1,4-Oxycyclohexane	-226.40	-208.60
1,4-Pentadiene	80.33	74.10
1,5,5-Trimethylbarbituric acid	-708.40	-713.00
1,5-Cyclooctadiene	20.69	13.10
1,5-Dihydroxyisoquinoline	-350.90	-342.60
1,5-Dimethyltetrazole	188.82	210.90
1,5-Dinitronaphthalene	30.50	29.80
1,5-Diphenylformazan	453.00	433.70
1,5-Hexadiene	51.56	47.80
1,5-Hexadiyne	384.17	401.80
1,5-Naphthalenediamine	38.60	12.40
1,5-Pentanediol	-531.50	-543.50
1,5-Pentanediol dinitrate	-364.00	-337.50
1,5-Pentanedithiol	-132.70	-125.30
1,6-Anhydro-beta-D-glucopyranose	-960.74	-972.60
1,6-Bis(methylnitramino)hexane	-199.87	-169.50
1,6-Dicyclohexylhexane	-390.30	-431.40
1,6-Dimethyl-4-isopropylnaphthaIene	-134.80	-81.40
1,6-Diphenylhexane	-4.10	-48.50
1,6-Hexanediamine	-205.00	-173.90
1,6-Hexanedioic acid	-990.34	-965.60
1,7-Difluoro-1,1,3,5,7,7-hexanitro-3,5-diazaheptane	-485.60	-487.70
1,7-Difluoro-1,1,5,7,7-pentanitro-5-aza-3-oxaheptane	-654.20	-659.00
1,7-Heptanediol	-577.60	-596.10
1,7-Octadiyne	334.40	349.20
1,8-Bis(biphenyl-4-yl)naphthalene	365.47	354.90
1,8-Cyclotetradecadiyne	147.80	173.30
1,8-Di(3,4-dimethoxyphenyl)naphthalene	-400.30	-448.60
1,8-Di(3-formyl-4-methoxyphenyl)naphthalene	-367.30	-388.40
1,8-Di(4-methoxyphenyl)naphthalene	-92.80	-120.90

1,8-Diaminonaphthalene	61.80	9.80
1,8-Difluoro-1,1,3,6,8,8-hexanitro-3,6-diazaoctane	-518.80	-511.40
1,8-Dimethylnaphthalene	26.10	29.40
1,8-Dinitronaphthalene	46.20	29.80
1,8-Diphenylnaphthalene	235.83	212.20
1,8-Octanediol	-626.80	-622.50
1,9-Nonanediol	-657.70	-648.80
1,I-Dimethylazoxyethane	-164.77	-185.30
10-Undecenoic acid	-577.78	-590.50
11-Butyldocosane	-717.60	-721.20
11-Cyclohexylheneicosane	-690.70	-706.90
11-Cyclopentylheneicosane	-648.60	-659.30
11-Decylheneicosane	-867.63	-852.70
11-Phenylheneicosane	-505.40	-523.40
12-Crown-4	-700.30	-712.40
13,14-Dihydroxybehenic acid	-1411.00	-1389.30
13-Cyclohexylpentacosane	-792.70	-809.60
15-Crown-5	-894.90	-897.00
1a,2a,4b-1,2,4-Trimethylcyclopentane	-223.00	-197.40
1-Acetyl-2-naphthol	-301.50	-299.90
1-Acetyladamantane	-382.50	-371.20
1-Adamantanol	-397.50	-391.80
1-Adamantyl isocyanide	-12.60	1.10
1-Allyl-5-allylaminotetrazole	350.44	335.00
1-Allyl-5-aminotetrazole	265.44	264.20
1-Amino-1-cyclopropylethane	-64.00	-18.20
1-Amino-2-(2-hydroxyethoxy)ethane	-438.01	-446.00
1-Aminoadamantane	-199.70	-193.20
1-Azabicyclo[2.2.2]octane	-58.10	-84.10
1-Azidohexane	149.20	145.30
1-Azidooctane	92.60	92.60
1-Azidopentane	149.00	171.60
1-Benzopyran-4-one	-232.70	-182.00
1-Benzosuberone	-169.60	-191.00
1-Benzoyl-2-phenylacetylene	150.10	175.80
1-Benzoylglycerol	-777.30	-775.50
1-Benzyl-2-piperidone	-221.43	-219.30
1-Benzyl-4-piperidinol	-234.45	-215.30
1-Benzyl-4-piperidone	-140.73	-147.20
1-Benzylimidazole	138.27	121.20
1-Benzylpyrazole	199.17	205.10
1-Bromo-2-chloro-1,1,2-trifluoroethane	-675.30	-662.60

1-Bromo-4-chlorobenzene	28.00	24.80
1-Bromobutane	-143.80	-138.30
1-Bromodecane	-344.70	-296.30
1-Bromododecane	-344.70	-348.90
1-Bromohexadecane	-444.50	-454.20
1-Bromonaphthalene	116.39	93.70
1-Bromooctane	-245.10	-243.60
1-Bromopentane	-170.20	-164.70
1-Bromopropane	-121.90	-112.00
1-Butanethiol	-124.70	-124.10
1-Butanol	-327.62	-333.20
1-Butene	-20.80	-24.40
1-Butoxy-2,3-epoxypropane	-345.20	-341.60
1-Butoxy-3-t-butylperoxy-2-propanol	-747.00	-745.50
1-Butyl-1-methylpyrrolidinium dicyanamide	53.09	58.40
1-Butyl-3-methylimidazolium chloride	-85.62	-111.30
1-Butyl-3-methylimidazolium dicyanoamide	191.01	188.70
1-Butyl-3-methylimidazolium nitrate	-282.14	-277.50
1-Butylamine	-130.21	-135.20
1-Butyltetralin	-150.60	-142.50
1-Butyne	141.88	152.60
1-Carboxyadamantane	-643.10	-601.30
1-Carboxyphenazine	-166.33	-200.30
1-Chloro-1,1-difluoroethane	-529.70	-505.80
1-Chloro-1-fluoroethane	-313.40	-311.10
1-Chloro-1-methylcyclohexane	-246.00	-237.00
1-Chloro-1-methylcyclopentane	-198.00	-191.40
1-Chloro-3-isocyanatobenzene	-74.00	-74.60
1-Chloro-4-(trifluoromethyl)benzene	-663.63	-640.50
1-Chlorobutane	-188.20	-186.50
1-Chlorododecane	-392.30	-397.10
1-Chloronaphthalene	49.80	47.80
1-Chlorooctadecane	-544.10	-555.10
1-Chlorooctane	-293.90	-291.70
1-Chloropentane	-214.70	-212.90
1-Chloropropane	-160.60	-160.20
1-Cyanoacetylpiperidine	-220.30	-198.80
1-Cyanoadamantane	-87.90	-69.60
1-Cyanonaphthalene	184.93	192.90
1-Cyclohexenecarbonitrile	48.10	60.10
1-Cyclohexyl-1-propanone	-392.10	-365.70
1-Cyclopropyl-1,3-pentadiyne	429.90	433.60

1-Cyclopropyl-2-methylbenzene	71.50	63.20
1-Decanethiol	-276.50	-282.00
1-Decene	-173.80	-182.30
1-Decyl-3-methylimidazolium bromide	-247.31	-225.10
1-Decylazide	24.40	40.10
1-Decylnaphthalene	-156.26	-193.80
1-Decyne	-9.50	-5.30
1-Dimethylamino-2-propyne	195.40	190.80
1-Docosanoic acid	-983.90	-1005.00
1-Dodecanol	-534.15	-543.70
1-Dodecene	-226.20	-235.00
1-Dodecyl-3-methylimidazolium bromide	-306.10	-277.70
1-Eicosene	-459.21	-445.60
1-Ethanol-3-methyl-imidazolium dicyanoamide	69.70	57.40
1-Ethoxy-2-nitrobenzene	-161.68	-186.60
1-Ethyl-1-methylcyclohexane	-240.20	-235.40
1-Ethyl-1-methylcyclopentane	-193.80	-192.40
1-Ethyl-2-phenylindole	97.70	138.00
1-Ethyl-3-methylimidazolium chloride	-72.13	-58.60
1-Ethyl-3-methylimidazolium dicyanamide	232.10	241.40
1-Ethyl-3-methylimidazolium nitrate	-238.30	-224.90
1-Ethyl-4-methyl-1,3-cyclohexadiene	-47.70	-45.10
1-Ethyl-4-nitro-1,2,3-triazole	125.90	107.80
1-Ethyl-4-piperidone	-248.45	-247.50
1-Ethyl-8-methylnaphthalene	20.90	3.10
1-Ethyladamantane	-263.20	-240.50
1-Ethylcyclohexene	-106.69	-113.30
1-Ethylcyclopentene	-58.28	-75.70
1-Ethylimidazole	42.80	18.20
1-Ethylpiperidine	-124.77	-122.10
1-Ethylpyrazole	77.30	102.10
1-Ethyltetralin	-92.60	-89.90
1-Ethylthiooctane	-273.30	-279.80
1-Fluoro-1,1,3,5,5-pentanitro-3-azaheptane	-400.40	-401.00
1-Fluoro-1,1-dinitroethane	-303.00	-291.40
1-Fluorododecane	-552.60	-538.00
1-Fluoroheptane	-382.80	-406.40
1-Fluorononane	-472.30	-459.10
1-Fluorooctane	-437.00	-432.70
1-Fluoropropane	-285.90	-301.10
1-Fluorotetradecane	-606.70	-590.70
1-Glyceryl laurate	-1161.70	-1141.70

1-Glyceryl stearate	-1347.70	-1299.70
1H-Benzotriazole	247.91	253.50
1-Heptadecanol	-722.85	-675.40
1-Heptadecene	-349.83	-366.60
1-Heptanethiol	-200.50	-203.10
1-Heptanol	-403.30	-412.20
1-Heptene	-97.90	-103.40
1-Heptylhydroperoxide	-343.00	-349.20
1-Heptyne	67.20	73.70
1-Hexadecanol	-686.50	-649.00
1-Hexanethiol	-175.70	-176.80
1-Hexanol	-380.41	-385.80
1-Hexen-5-one	-197.40	-202.50
1-Hexene	-74.20	-77.10
1-Hexyl-1,2,3,4-tetrahydronaphthalene	-179.33	-195.20
1-Hexylhydroperoxide	-299.60	-322.90
1-Hexyne	123.60	100.00
1H-Indazole	164.13	174.30
1H-Indazole-3-carboxylic acid	-256.90	-256.50
1H-Indazole-5-carboxylic acid	-261.40	-241.80
1H-Naphtho[2,3-d][1,2,3]triazole	270.90	290.20
1H-Purine	167.70	174.20
1H-Pyrazole	118.49	131.40
1-Hydroxy-2,2,6,6-tetramethyl-4-piperidinone	-382.10	-393.90
1-Hydroxycumene	-250.40	-252.60
1-Hydroxyisoquinoline	-149.90	-134.00
1-Hydroxytetralin	-245.20	-232.70
1-Indanol	-198.40	-192.40
1-Iodohexane	-146.40	-146.50
1-Iodonaphthalene	162.00	146.30
1-Isopropoxy-2-propanol	-529.60	-531.60
1-Isopropyl-6-methylindane	-118.90	-118.80
1-Isopropyl-8-methylnaphthalene	-2.40	-23.90
1-Methoxy-2-propanone	-383.40	-389.50
1-Methoxy-a-D-glucopyranoside	-1229.40	-1233.90
1-Methoxycarbonyladamantane	-577.80	-559.30
1-Methoxydecane	-448.70	-448.30
1-Methyl-1,2,3,4,-tetrahydro-1-naphthol	-248.40	-266.60
1-Methyl-1H-pyrazole	120.15	130.80
1-Methyl-1-phenylhydrazine	144.80	121.40
1-Methyl-2-phenylindole	125.90	162.90
1-Methyl-2-piperidinemethanol	-303.27	-306.60

1-Methyl-2-piperidone	-300.15	-293.50
1-Methyl-3,4-dinitropyrazole	105.80	102.00
1-Methyl-3-pentylimidazolium chloride	-93.41	-137.60
1-Methyl-3-phenoxybenzene	-59.00	-57.20
1-Methyl-4-(1-methylethenylsulphonyl)benzene	-289.40	-309.60
1-Methyl-4-(2-methyl-1-propenylsulphonyl)-benzene	-346.20	-343.90
1-Methyl-4-(2-propynyl-sulphonyl)benzene	-110.50	-115.50
1-Methyl-4-benzylbenzene	56.50	46.20
1-Methyl-4-isopropylcyclohexene	-159.80	-172.60
1-Methyl-4-nitroimidazole	-2.00	3.80
1-Methyl-4-piperidone	-221.85	-221.50
1-Methyl-5-phenyltetrazole	292.20	316.40
1-Methyladamantane	-237.30	-219.40
1-Methylcyclohexanol	-391.90	-386.80
1-Methylcyclohexene	-81.17	-89.60
1-Methylcyclohexylhydroperoxide	-330.40	-323.90
1-Methylcyclopentanol	-354.00	-341.10
1-Methylcyclopentene	-36.44	-49.40
1-Methyldiamantane	-247.40	-246.20
1-Methylimidazole	65.15	46.90
1-Methylindazole	162.90	173.70
1-Methylindole	93.60	92.90
1-Methylindole-3-carboxylic acid	-363.00	-326.80
1-Methylnaphthalene	44.27	43.10
1-Methylnorbornane	-134.30	-126.50
1-Methylnorcamphor	-255.20	-236.80
1-Methylpropyl 2-pentenoate	-510.40	-507.10
1-Methylpropyl 3-pentenoate	-502.80	-507.10
1-Methylpropyl 4-pentenoate	-497.00	-485.70
1-Methylpyrrole	62.38	49.90
1-Methyltetralin	-63.50	-66.20
1-Methyltetralin-1-hydroperoxide	-161.45	-206.30
1-Methyltetrazole	233.80	261.30
1-Monocaprin	-1109.70	-1089.00
1-Naphthaleneacetic acid	-359.20	-365.10
1-Naphthalenecarboxylic acid	-333.50	-338.80
1-Naphthol	-130.71	-128.50
1-Naphthyl acetate	-292.70	-299.40
1-Naphthylamine	61.08	46.20
1-Naphthylisocyanate	22.90	-5.70
1-Nitro-2,6-diisopropylbenzene	-166.30	-162.30
1-Nitro-2-isopropylbenzene	-75.50	-74.70

1-Nitro-2-nitrosobenzene	104.00	99.60
1-Nitro-2-propanone	-295.00	-291.70
1-Nitroadamantane	-263.00	-247.40
1-Nitrobutane	-192.50	-192.50
1-Nitronaphthalene	46.60	54.90
1-Nitropentane	-215.40	-218.80
1-Nitropropane	-167.20	-166.20
1-Nitropyrazole	181.70	226.20
1-Nitroso-2-naphthol	-47.10	-47.40
1-Nonadecanoic acid	-916.40	-926.00
1-Nonadecene	-399.59	-419.30
1-Nonanol	-453.60	-464.80
1-Nonene	-149.03	-156.00
1-Nonylnaphthalene	-132.57	-164.90
1-Nonyne	16.30	21.00
1-Norbornyl cyanide	10.10	23.30
1-Norbornylisocyanide	102.10	94.00
1-Octadecanol	-702.30	-701.70
1-Octadecene	-374.77	-392.90
1-Octanethiol	-224.47	-229.40
1-Octanol	-426.60	-438.50
1-Octen-3-yne	140.71	141.20
1-Octyl-3-methylimidazolium bromide	-155.12	-172.40
1-Octyne	39.20	47.40
1-Palmitoylglycerol	-1290.10	-1247.00
1-Pentadecanoic acid	-861.70	-820.70
1-Pentadecanol	-658.20	-622.70
1-Pentadecene	-301.12	-313.90
1-Pentanethiol	-149.69	-150.40
1-Pentanol	-354.08	-359.50
1-Pentene	-47.03	-50.80
1-Pentyne	144.40	126.40
1-Phenyl-1,3-butanedione	-335.10	-323.50
1-Phenyl-1,3-butanedione (enol form)	-335.10	-332.90
1-Phenyl-1-propanol	-219.78	-237.10
1-Phenyl-1-propanone	-170.40	-171.70
1-Phenyl-2-butene	18.30	33.50
1-Phenyl-2-nitropropene	-14.15	21.80
1-Phenyl-2-propanol	-220.17	-239.70
1-Phenyl-3-(methylamino)-2-butene-1-one	-157.00	-155.10
1-Phenyl-3-butene	30.10	54.80
1-Phenyl-4,7-dioxaspiro[2.4]heptane	-182.60	-207.70

1-Phenylcyclohexane-cis-1,2-diol	-473.40	-477.20
1-Phenylcyclohexane-cis-1,2-diol diacetate	-858.30	-865.60
1-Phenylcyclohexene	-16.80	15.90
1-Phenylcyclopentane-cis-1,2-diol	-423.30	-445.50
1-Phenyldodecane	-264.79	-280.50
1-Phenylethylformate	-347.10	-347.90
1-Phenylheneicosane	-518.40	-520.10
1-Phenylimidazole	177.00	151.70
1-Phenylindene	148.61	174.60
1-Phenylnaphthalene	160.70	148.70
1-Phenyloct-2-yn-1-ol	-122.80	-100.40
1-Phenylpenta-1-yn-3-one	43.30	46.50
1-Phenylpyrazole	221.20	232.10
1-Phenylpyrrole	159.52	154.60
1-Phenyltetrazole	362.16	362.50
1-Phenyl-trans-1,2-cyclohexanediol	-469.60	-481.10
1-Phthalazinone	-30.90	-24.10
1-Piperidinecarboxamide	-363.70	-349.20
1-Piperidineethanol	-306.37	-306.10
1-Piperidinoacetonitrile	6.40	25.10
1-Piperidinocyclohexanecarbonitrile	-89.40	-79.20
1-Propanethiol	-99.90	-97.80
1-Propanol	-304.21	-306.80
1-Propylamine	-101.47	-108.90
1-Propylnaphthalene	-6.60	-6.90
1-Pyrenecarboxaldehyde	-11.70	6.60
1-t-Butoxy-2-[2-(t-butoxy)propoxy]propane	-810.30	-851.30
1-t-Butyl-4-ethylbenzene	-131.46	-135.60
1-t-Butylperoxy-3-ethoxy-2-propanol	-706.70	-695.50
1-t-Butylperoxy-3-methoxy-2-propanol	-666.30	-657.20
1-t-Butylperoxy-3-pentoxy-2-propanol	-771.20	-774.40
1-t-Butylperoxy-3-propoxy-2-propanol	-725.00	-719.20
1-t-Butylperoxycyclohexanol	-589.10	-596.40
1-Tetradecene	-280.30	-287.60
1-Tetradecyl-3-methylimidazolium bromide	-353.69	-330.40
1-Tetradecylamine	-424.70	-398.40
1-Tetralone	-213.10	-164.60
1-Thiaindan	59.26	38.10
1-trans-5-trans-9-cis-Cyclododecatriene	25.00	16.40
1-Triacontene	-761.60	-708.80
1-Tridecanol	-599.40	-570.10
1-Tridecene	-253.50	-261.30

1-Undecanol	-504.80	-517.50
1-Undecene	-200.80	-208.60
1-Xylylazo-2-naphthol	18.90	55.90
2-(1-Cyclohexenyl)cyclohexanone	-293.56	-303.60
2-(1H-Indol-3-yl)ethanol	-171.94	-162.50
2-(1H-Indol-3-yl)ethanol	-171.90	-163.40
2-(1-Hydroxy-1-methylethyl)-2-methyl-1,3-dioxane	-712.30	-722.80
2-(2-(2-Methoxyethoxy)ethoxy)ethanol	-786.40	-780.50
2-(2-Adamantylidene)adamantane	-247.80	-227.30
2-(2-Hydroxyphenyl)benzothiazole	-31.30	-35.10
2-(2-Hydroxyphenyl)benzoxazole	-207.80	-189.80
2-(2-Methoxyethoxy)ethanol	-589.99	-601.10
2-(5H)-Furanone	-314.20	-320.00
2-(5H)-Thiophenone	-124.10	-105.00
2-(Biphenyl-4-yl)naphthalene	190.83	222.70
2-(Butylnitroamino)-ethanol nitrate	-195.20	-242.80
2-(Diacetoxymethyl)-5-nitrofuran	-901.00	-919.50
2-(Dimethylamino)acetophenone	-119.90	-133.60
2-(Dimethylamino)propiophenone	-139.00	-157.60
2-(Phenoxymethyl)oxirane	-172.90	-174.40
2,10-Dimethyl-2,10-undecanediol	-835.80	-793.60
2,11-Dimethyl-2,11-dodecanediol	-846.20	-820.00
2,2,2-Trifluoroethanol	-932.40	-895.30
2,2,2-Trinitro-1-phenylethane	-21.30	9.80
2,2',2''-Tripyridine	292.60	281.20
2,2,3,3,3-Pentafluoro-1-propanol	-1328.80	-1317.60
2,2,3,3,4,4,4-Heptafluoro-1-butanol	-1745.60	-1739.90
2,2,3,3-Tetrafluoro-1-propanol	-1114.90	-1090.90
2,2,3,3-Tetrafluoropropyl-1',1',2',3',3'-hexafluoropropyl ether	-2350.60	-2351.60
2,2,3,3-Tetramethylbutane	-272.87	-264.60
2,2,3,3-Tetramethylhexane	-303.47	-314.60
2,2,3,3-Tetramethylpentane	-278.28	-293.50
2,2,3,4-Tetramethylpentane	-277.70	-287.30
2,2,3-Trimethylhexane	-287.00	-287.90
2,2,3-Trimethylpentane	-256.90	-262.90
2,2,4,4,5,-Pentamethylhexane	-329.00	-333.80
2,2',4,4',6,6'-Hexamethylazobenzene N,N'-dioxide	33.50	26.00
2,2,4,4,6,8,8-Heptamethylnonane	-476.87	-474.30
2,2,4,4,6-Pentamethyl-1,3-dioxane	-571.10	-573.20
2,2',4,4',6-Pentanitrobenzophenone	-118.10	-167.80
2,2,4,4-Tetramethyl-1,3-cyclobutanedione	-379.90	-378.80
2,2,4,4-Tetramethylpentane	-279.99	-284.60

2,2,4-Trimethyl-1,3-dioxane	-502.50	-502.20
2,2,4-Trimethyl-3-pentanone	-385.20	-391.00
2,2,4-Trimethylhexane	-287.20	-289.20
2,2,5,5-Tetramethylheptane	-351.20	-334.40
2,2,5,5-Tetramethylhexane	-323.51	-317.20
2,2,5-Trimethylhexane	-293.30	-286.60
2,2',6,6'-Tetraethylazobenzene N,N'-dioxide	-0.50	6.20
2,2,6,6-Tetramethyl-3,5-heptanedione	-592.50	-594.50
2,2,6,6-Tetramethyl-4-piperidone	-338.20	-346.60
2,2',6,6'-Tetramethylazobenzene N,N'-dioxide	100.30	99.60
2,2,6-Trimethylheptane-3,5-dione	-568.10	-562.50
2,2,6-Trimethylheptane-3,5-dione (enol form)	-568.40	-578.40
2,2,7,7-Tetramethylocta-3,5-diyne	156.10	166.60
2,2’-Diaminodiethylamine	-75.93	-85.10
2,2'-Bi-4-picoline	105.30	110.00
2,2'-Bipyridine	186.10	183.80
2,2'-Biquinoline	245.40	262.60
2,2-Bis(tertbutylperoxy)butane	-616.70	-603.40
2,2'-Diaminodiphenyldisulfide	97.73	87.20
2,2-Dichloro-1,1,1-trifluoroethane	-770.30	-750.00
2,2-Dichloro-1,1,2-trifluoroethane	-728.38	-731.70
2,2'-Dichlorobiphenyl	30.94	45.00
2,2-Dichloropropane	-205.80	-178.10
2,2-Dicyclohexylbutane	-350.70	-371.90
2,2-Diethoxypropane	-538.50	-536.60
2,2'-Difluorobiphenyl	-288.70	-239.40
2,2-Difluoroethanol	-678.70	-668.70
2,2-Difluoroethyl acetate	-874.40	-862.70
2,2-Dimethoxy-3,3-dimethylbutane	-524.40	-538.80
2,2-Dimethoxy-3-methylbutane	-512.20	-510.80
2,2-Dimethoxybutane	-485.10	-486.40
2,2-Dimethoxypentane	-509.20	-512.70
2,2-Dimethoxypropane	-459.00	-460.10
2,2-Dimethyl-1,3-dioxane	-468.90	-466.40
2,2-Dimethyl-1,3-dioxolane	-430.49	-426.10
2,2-Dimethyl-1,3-propanediol	-553.70	-551.10
2,2-Dimethyl-3,5-heptanedione	-531.00	-539.40
2,2-Dimethyl-3-ethylpentane	-272.70	-286.60
2,2-Dimethyladamantane	-256.30	-241.20
2,2-Dimethylheptane	-288.20	-288.50
2,2-Dimethylhexane	-261.88	-262.20
2,2-Dimethyloctane	-313.12	-312.20

2,2-Dimethylpentane	-238.28	-233.20
2,2-Dimethylpropane	-190.20	-183.20
2,2-Dimethylsuccinic acid	-990.30	-973.30
2,2-Dimethylthiirane	-24.16	6.50
2,2-Dinitroadamantane	-254.70	-235.70
2,2'-Dinitroazobenzene N,N'-dioxide	217.10	197.00
2,2-Dinitropropane	-189.08	-169.90
2,2-diphenyl-1,3-dioxolane	-208.00	-234.00
2,2-Diphenyl-dimethoxymethane	-270.60	-254.10
2,2'-Dipyridyl N,N'-oxide	115.19	116.00
2,2'-Dipyridyl N-oxide	167.29	152.50
2,2'-Dipyrrolylmethane	126.20	79.90
2,2'-Oxybisethanol dinitrate	-458.20	-437.90
2,3,3,4-Tetramethylpentane	-277.90	-295.10
2,3,3-Trimethyl-1-butene	-117.70	-122.50
2,3,3-Trimethylhexane	-285.40	-289.20
2,3,3-Trimethylpentane	-253.51	-262.90
2,3,4,5,6-Pentafluorotoluene	-883.80	-865.60
2,3,4,5-Tetrafluoroaniline	-685.00	-690.80
2,3,4,5-Tetramethylbenzoic acid	-514.60	-522.90
2,3,4,6-Tetrafluoroaniline	-675.10	-690.80
2,3,4,6-Tetramethylbenzoic acid	-507.80	-522.90
2,3,4,6-Tetranitroaniline	-49.17	-94.00
2,3,4-Trichloroaniline	-63.11	-89.70
2,3,4-Trifluoroaniline	-500.58	-516.40
2,3,4-Trimethylbenzoic acid	-486.60	-486.10
2,3,4-Trimethylpentane	-255.01	-259.20
2,3,5,6-Tetrabromo-p-xylene	-1.20	24.40
2,3,5,6-Tetrachloronitrobenzene	-64.50	-110.60
2,3,5,6-tetrachloro-p-xylene	-172.40	-159.30
2,3,5,6-Tetrafluoroaniline	-688.50	-690.80
2,3,5,6-Tetramethylbenzoic acid	-510.10	-522.90
2,3,5,6-Tetramethylpyrazine N,N'-dioxide	-109.71	-87.90
2,3,5-Trichlorohydroquinone	-440.70	-470.40
2,3,5-Trichloropyridine	-7.02	-11.40
2,3,5-Trimethylbenzoic acid	-488.70	-486.10
2,3,5-Trimethylhexane	-288.40	-288.20
2,3,5-Trimethylpyrazine	17.60	-1.40
2,3,5-Trimethylpyrazine N,N'-dioxide	-73.31	-55.90
2,3,5-Trinitrotoluene	-24.50	-68.90
2,3,6,7-Tetrachloroquinoxaline	37.59	40.40
2,3,6-Trifluoroaniline	-515.38	-516.40

2,3,6-Trimethylbenzoic acid	-479.40	-486.10
2,3,6-Tris(phenylimino)-1,3,5-triphenyl-1,3,5-triazacyclohexane	608.55	597.10
2,3-5,6-Dibenzoxalene	65.10	93.80
2,3-Bis(difluoroamino)-2-methylbutane	-244.80	-246.90
2,3-Butandione	-367.05	-400.20
2,3-Butyleneglycol	-541.50	-536.10
2,3-Dibromobutane	-139.60	-128.60
2,3-Dichloro-1-propanol	-381.50	-385.20
2,3-Dichloroaniline	-48.14	-57.50
2,3-Dichlorobutane	-240.40	-231.40
2,3-Dichlorohydroquinone	-416.00	-438.20
2,3-Dichlorophenol	-223.03	-229.60
2,3-Dichloropropene	-73.47	-60.20
2,3-Dichloropyridine	16.45	20.80
2,3-Dichloroquinoxaline	110.72	104.70
2,3-Difluoroaniline	-332.15	-342.00
2,3-Difluorophenol	-520.34	-514.00
2,3-Dihydro-1,1,4,6-tetramethyl-1H-indene	-136.90	-132.90
2,3-Dihydro-1H-indole	55.10	21.80
2,3-Dihydrobenzofuran	-102.70	-118.90
2,3-Dihydrofuran	-104.70	-148.90
2,3-Dihydrothiophene	52.90	9.30
2,3-Dihydrothiophene 1,1-dioxide	-325.10	-345.10
2,3-Dihydroxyanisole	-512.80	-540.30
2,3-Dihydroxybenzoic acid	-785.60	-792.90
2,3-Dihydroxypyridine	-374.80	-379.30
2,3-Dihydroxytoluene	-395.10	-410.70
2,3-Dimethoxy-2,3-diphenylsuccinnonitrile	17.10	-23.50
2,3-Dimethoxybenzaldehyde	-416.70	-424.90
2,3-Dimethoxybenzoic acid	-690.30	-708.90
2,3-Dimethoxyphenol	-465.20	-498.30
2,3-Dimethyl-1,3-butadiene	14.40	30.20
2,3-Dimethyl-1-butene	-93.20	-89.20
2,3-Dimethyl-1-hexene	-136.00	-139.30
2,3-Dimethyl-1-pentene	-119.60	-110.30
2,3-Dimethyl-2,3-bis(4-t-butylphenyl)butane	-312.50	-284.60
2,3-Dimethyl-2,3-bis(phenylazo)butane	354.90	389.60
2,3-Dimethyl-2,3-dinitrobutane	-313.76	-318.00
2,3-Dimethyl-2-butanethiol	-190.20	-193.40
2,3-Dimethylbutane	-207.40	-203.30
2,3-Dimethylhexane	-252.59	-258.50
2,3-Dimethylindole	4.20	10.10

2,3-Dimethyloctane	-304.31	-311.20
2,3-Dimethylpentane	-233.09	-234.80
2,3-Dimethylpyrazine	71.10	43.90
2,3-Dimethylpyrazine N,N'-dioxide	-25.02	-23.90
2,3-Dimethylquinoxaline	83.50	86.00
2,3-Dimethylquinoxaline 1,4-dioxide	21.50	36.80
2,3-Dinitroaniline	-12.00	-43.60
2,3-Diphenylbutane	58.40	4.00
2,3-Diphenylsuccinic acid	-745.71	-766.30
2,3-Lutidine	19.34	6.70
2,3-Naphthalenediol	-319.90	-337.10
2,3-Pentadiene	133.10	95.30
2,3-Pyridinedicarboxylic acid	-735.50	-757.10
2,3-Xylenol	-241.10	-239.00
2,4,4,6,6-Pentamethyl-1,3-dioxane	-563.60	-566.50
2,4,4-Trimethyl-2-nitropentane	-307.80	-320.30
2,4,4-Trimethyl-2-pentene	-142.40	-153.40
2,4,4-Trimethylhexane	-282.80	-282.70
2,4,5,6-Tetrachloropyrimidine	6.80	-20.50
2,4,5,7-Tetramethyl-4,5-bis(4-t-butylphenyl)octane	-450.30	-446.60
2,4,5,7-Tetramethylphenanthrene	10.70	-9.50
2,4,5-Trichloroaniline	-60.21	-89.70
2,4,5-Trifluoroaniline	-531.38	-516.40
2,4,5-Trimethylacetophenone	-256.30	-253.30
2,4,5-Trimethylaniline	-84.40	-103.80
2,4,5-Trinitrotoluene	-17.50	-68.90
2,4,6,-Trinitro-1,3,5,-tris(methylnitramino)benzene	114.90	122.70
2,4,6-Collidine	-34.20	-38.60
2,4,6-Cycloheptatrien-1-one	-12.50	8.30
2,4,6-Triaminopyrimidine	-53.87	-65.50
2,4,6-Triazido-s-triazine	1053.00	1039.70
2,4,6-Tribromoaniline	47.80	48.00
2,4,6-Tribromo-m-cresol	-131.00	-161.00
2,4,6-Tribromophenol	-100.00	-124.10
2,4,6-Trichloroaniline	-79.11	-89.70
2,4,6-Trichloronitrobenzene	-57.20	-78.40
2,4,6-Trichloropyrimidine	32.17	11.80
2,4,6-Triethoxy-1,3,5-triazine	-588.81	-566.40
2,4,6-Trifluoroaniline	-536.48	-516.40
2,4,6-Triisopropylphenol	-396.30	-434.60
2,4,6-Trimethoxybenzonitrile	-360.84	-343.40
2,4,6-Trimethoxybenzonitrile N-oxide	-323.74	-317.00

2,4,6-Trimethoxy-s-triazine	-481.03	-451.70
2,4,6-Trimethyl-1,3-Dioxane	-491.50	-490.70
2,4,6-Trimethylbenzonitrile	19.86	45.60
2,4,6-Trimethylbenzonitrile N-oxide	52.26	72.00
2,4,6-Trinitroanisol	-157.30	-198.50
2,4,6-Trinitrobenzaldehyde	-132.84	-167.00
2,4,6-Trinitrobenzoic acid	-411.70	-450.70
2,4,6-Trinitro-N-methylaniline	-49.30	-67.30
2,4,6-Trinitrophenetol	-204.60	-236.80
2,4,6-Trinitrophenol	-217.90	-240.80
2,4,6-Trinitrophenylhydrazine	36.80	30.90
2,4,6-Trinitroresorcinol	-434.80	-449.60
2,4,6-Triphenoxy-s-triazine	-125.40	-150.80
2,4,6-Triphenylpyridine	269.00	278.20
2,4,6-Triphenyltriazine	303.11	298.90
2,4,6-Tri-t-butylnitrobenzene	-308.82	-347.00
2,4,6-Tri-t-butylnitrosobenzene	-216.20	-237.90
2,4,6-Tri-t-butylphenol	-508.20	-526.50
2,4'-Bipyridine	192.90	194.80
2,4-Diaminoazobenzene	276.00	268.00
2,4-Diaminopyrimidine	0.55	-6.20
2,4-Diazidopyrimidine	702.90	729.60
2,4-Dibromo-6-methylphenol	-159.00	-174.70
2,4-Dibromoaniline	23.50	34.30
2,4-Dibromophenol	-143.70	-137.80
2,4-dichloro-1-trifluoromethylbenzene	-702.75	-672.70
2,4-Dichloro-5-methylpyrimidine	16.00	10.70
2,4-Dichloro-6-(ethylamino)-s-triazine	-38.60	-30.90
2,4-Dichloro-6-methylpyrimidine	-10.30	2.30
2',4'-Dichloroacetophenone	-188.70	-209.80
2,4-Dichloroaniline	-52.44	-57.50
2,4-Dichloroanisole	-184.70	-187.60
2,4-Dichloronitrobenzene	-47.10	-46.20
2,4-Dichlorophenol	-226.13	-229.60
2,4-Dichloropyrimidine	49.93	47.60
2,4-Difluorophenol	-520.04	-514.00
2,4-Dihydroxyacetophenone	-573.50	-560.20
2,4-Dihydroxybenzophenone	-497.20	-457.20
2,4-Diisopropylphenol	-320.50	-348.40
2,4-Dimethoxy-6-(2,2-dinitro-2-fluoroethoxy)-1,3,5-triazine	-693.93	-684.80
2',4'-Dimethoxyacetophenone	-490.40	-478.50
2,4-Dimethoxybenzaldehyde	-443.90	-424.90

2,4-Dimethoxybenzoic acid	-715.50	-708.90
2,4-Dimethoxyphenol	-495.60	-498.30
2,4-Dimethyl-1-pentene	-117.00	-118.20
2,4-Dimethyl-2-pentene	-123.10	-131.70
2,4-Dimethyl-3-ethylpentane	-274.10	-279.00
2,4-Dimethyl-3-pentanone	-352.92	-360.30
2,4-Dimethyl-5-oxo-1H-pyrrol-3-carboxylic acid ethyl ester	-688.20	-684.00
2,4-Dimethylacetanilide	-291.10	-270.50
2,4-Dimethylbenzoic acid	-458.50	-449.30
2,4-Dimethylcyclohexene	-149.80	-119.30
2,4-Dimethylhexane	-257.00	-253.30
2,4-Dimethyloctane	-308.00	-306.00
2,4-Dimethylpentane	-234.60	-234.80
2,4-Dimethylphenylacetic acid	-495.80	-475.60
2,4-Dimethylpyrrole	-17.50	-32.90
2,4-Dinitro-1,3-dimethylbenzene	-88.47	-80.60
2,4-Dinitro-1-naphthol	-172.00	-179.00
2,4-Dinitro-2,4-diazapentane	-51.50	-40.40
2,4-Dinitroaniline	-65.70	-43.60
2,4-Dinitroanisol	-186.60	-173.40
2,4-Dinitro-N-methylaniline	-52.48	-42.50
2,4-Dinitrophenetole	-228.37	-211.70
2,4-Dinitrophenol	-214.68	-215.40
2,4-Dinitrophenoxyethanol	-398.97	-395.60
2,4-Dinitrophenylhydrazine	51.90	58.30
2,4-Dinitrophenylmethylnitramine	13.70	44.60
2,4-Dinitroresorcinol	-422.80	-424.50
2,4-Dinitrosobenzene-1,3-diol	-235.00	-211.20
2,4-Dinitrotoluene	-28.47	-43.80
2,4-Di-t-butylphenol	-404.10	-405.70
2,4-Hexadiyne	333.00	339.90
2,4-Hexanedione	-511.70	-464.80
2,4-Imidazolidinedione	-449.76	-454.20
2,4-Lutidine	16.12	6.70
2,4-Pentanediol	-556.35	-562.70
2,4-Toluene diisocyanate	-167.00	-165.00
2,4-Xylenol	-228.78	-239.00
2,4-Xylidine	-42.10	-66.90
2,5,8-Trimethyltetralin	-140.70	-139.90
2,5-Aldehydine	-14.60	-17.00
2,5-Bis(azidomethyl)-3-nitrooxazolidine	503.20	489.10
2,5-Diamino-1,3,4-thiadiazole	-5.50	18.00

2,5-Dibromoaniline	29.50	34.30
2,5-Dibromonitrobenzene	43.30	45.60
2,5-Dibromopyridine	105.69	107.60
2,5-Dichloro-1,4-benzoquinone	-250.80	-246.60
2,5-Dichloro-4-nitroaniline	-85.40	-82.60
2',5'-Dichloroacetophenone	-192.90	-209.80
2,5-Dichloroaniline	-61.64	-57.50
2,5-Dichlorohydroquinone	-432.80	-438.20
2,5-Dichlorophenol	-231.73	-229.60
2,5-Dichloropyridine	4.75	20.80
2,5-Dichlorostyrene	34.90	43.10
2,5-Difluoroaniline	-340.65	-342.00
2,5-Difluoronitrobenzene	-337.83	-330.60
2,5-Difluorophenol	-536.14	-514.00
2,5-Dihydro-3-methylthiophene 1,1-dioxide	-357.90	-383.40
2,5-Dihydrofuran	-66.90	-116.80
2,5-Dihydroperoxy-2,5-dimethylhex-3-yne	-295.50	-270.50
2,5-Dihydrothiophene	47.00	37.40
2,5-Dihydroxybenzoic acid	-788.80	-792.90
2,5-Dihydroxytoluene	-408.50	-410.70
2,5-Diisopropylphenol	-333.50	-345.70
2',5'-Dimethoxyacetophenone	-459.35	-475.90
2,5-Dimethoxybenzaldehyde	-434.50	-424.90
2,5-Dimethoxybenzoic acid	-698.10	-708.90
2,5-Dimethyl-1-phenylpyrrole	83.50	88.70
2,5-Dimethyl-2,4-hexadiene	-63.20	-42.10
2,5-Dimethyl-2,5-hexanediol	-685.40	-662.10
2,5-Dimethyl-2H-tetrazole	201.10	224.70
2,5-Dimethyl-2-hydroperoxide-5-tert-butylperoxyhexane	-636.00	-614.80
2,5-Dimethyl-2-t-butylperoxy-5-trimethylsilylperoxyhexyne	-468.74	-507.00
2,5-Dimethyl-3-furancarboxylic acid	-603.00	-583.70
2,5-Dimethyl-3-hexyne-2,5-diol	-389.10	-396.40
2,5-Dimethyl-5-t-butylperoxy-2-hexanol	-696.90	-677.70
2,5-Dimethylbenzothiazole	31.90	33.90
2,5-Dimethyldiphenylmethane	19.10	9.30
2,5-Dimethylhexane	-260.37	-255.90
2,5-Dimethylhexane-2,5-dihydroperoxide	-567.20	-536.20
2,5-Dimethyloctane	-308.42	-313.80
2,5-Dimethylphenylacetonitrile	52.20	56.20
2,5-Dimethylpyrazine	62.18	43.90
2,5-Dimethylpyrazine N,N'-dioxide	-25.62	-23.90
2,5-Dimethylpyrrole	-19.20	-32.00

2,5-Dimethylpyrrole-3-carboxylic ethylester	-478.70	-447.90
2,5-Dimethylthiophene	7.70	-13.10
2,5-Dinitroaniline	-44.30	-43.60
2,5-Dinitrotoluene	-36.50	-43.80
2,5-Diphenyl-1,3,4-oxadiazole	160.70	139.60
2,5-Diphenylpyridine	182.40	220.50
2,5-Diphenyltetrazole	390.60	359.40
2,5-Di-t-butylphenol	-383.10	-404.40
2,5-Furandione	-471.61	-494.70
2,5-Lutidine	18.63	6.70
2,5-Pyrrolidinedione	-460.54	-444.90
2,5-Xylenol	-249.70	-239.00
2,5-Xylidine	-38.90	-66.90
2,6-Diaminopyridine	-6.50	-29.70
2,6-Dibromoaniline	26.50	34.30
2,6-Dibromophenol	-135.30	-137.80
2,6-Dibromopyridine	94.89	98.90
2,6-Dichloro-4-nitroaniline	-107.10	-82.60
2,6-Dichloroaniline	-53.94	-57.50
2,6-Dichloroanisole	-165.70	-187.60
2,6-Dichlorobenzoquinone	-244.11	-246.60
2,6-Dichlorohydroquinone	-428.80	-438.20
2,6-Dichlorophenol	-222.10	-229.60
2,6-Dichloropyrazine	75.33	62.60
2,6-Dichloropyridine	-0.15	17.10
2,6-Diethylaniline	-84.23	-114.30
2,6-Diethylnaphthalene	-67.09	-41.20
2,6-Difluoroaniline	-338.55	-342.00
2,6-Difluorophenol	-539.44	-514.00
2,6-Dihydroxybenzoic acid	-787.30	-793.10
2,6-Diisopropylnaphthalene	-128.18	-100.50
2,6-Diisopropylphenol	-337.50	-345.70
2',6'-Dimethoxyacetophenone	-468.40	-478.50
2,6-Dimethoxybenzaldehyde	-431.30	-424.90
2,6-Dimethoxybenzoic acid	-697.20	-708.90
2,6-Dimethoxyphenol	-518.40	-498.30
2,6-Dimethyl-4-heptanol	-469.54	-477.00
2,6-Dimethyl-4-pyrone	-350.90	-320.50
2,6-Dimethylbenzoic acid	-440.70	-449.30
2,6-Dimethylbenzonitrile	59.05	82.50
2,6-Dimethylcyclohexanol	-394.20	-410.80
2,6-Dimethylcyclohexanone	-386.70	-340.10

2,6-Dimethylheptan-4-one	-408.50	-412.90
2,6-Dimethylheptane	-286.12	-287.50
2,6-dimethylheptane-3,5-dione	-530.70	-531.80
2,6-Dimethylnaphthalene	-5.69	6.30
2,6-Dimethyloctane	-307.95	-311.20
2,6-Dimethylpiperidine	-156.87	-154.00
2,6-Dimethylquinoline	32.50	46.10
2,6-Dinitro-4-methylphenol	-241.70	-252.60
2,6-Dinitroaniline	-50.60	-43.80
2,6-Dinitroanisol	-189.10	-173.40
2,6-Dinitrophenol	-210.00	-215.70
2,6-Dinitrotoluene	-40.47	-43.80
2,6-Diphenylpyridine	193.10	209.50
2,6-Di-t-butyl-4-phenylphenol	-313.70	-320.90
2,6-Di-t-butylnaphthalene	-170.16	-156.50
2,6-Di-t-butyl-p-cresol	-422.80	-417.50
2,6-Di-t-butylphenol	-379.10	-392.30
2,6-Lutidine	12.60	-1.70
2,6-Naphthalenedicarboxylic acid	-774.30	-757.60
2,6-Pyridinedicarboxylic acid	-747.46	-762.90
2,6-Xylenol	-240.39	-239.00
2,7-Dibromofluorene	109.76	138.00
2,7-Dihydroxynaphthalene	-329.50	-337.10
2,7-Dimethyl-2,7-octanediol	-730.00	-714.70
2,7-Dimethylbenzo[1,2-d,5,4-d]bisoxazole	-210.90	-211.20
2,7-Dimethylnaphthalene	-5.40	6.30
2,7-Dimethyloctane	-336.90	-313.80
2,7-Dimethylphenanthrene	30.90	45.60
2,7-dimethylquinoline	30.40	46.10
2,7-Di-t-butyl-9,9-dimethylxanthene	-349.90	-320.70
2,7-Di-t-butylfluorene	-149.50	-126.00
2,9-Dimethyl-2,9-decanediol	-789.60	-767.30
2.5-Dichloronitrobenzene	-46.92	-46.20
2:3,6:7-Dibenzobicyclo[3.2.2]nona-2,6-dien-4-one	-79.70	-87.70
2-Acetoxyanisole	-452.00	-502.70
2-Acetoxycinnamic acid	-699.40	-698.90
2-Acetoxynaphthalene	-308.40	-299.40
2-Acetyl-1-naphthol	-327.00	-314.90
2-Acetyl-3-methylquinoxaline N,N'-dioxide	-87.71	-97.70
2-Acetyl-3-methylthiophene	-151.00	-154.10
2-Acetyl-4-methylthiophene	-158.40	-154.10
2-Acetyl-5-methylthiophene	-161.00	-164.90

2-Acetylbenzofuran	-243.00	-231.60
2-Acetylfuran	-262.60	-259.00
2-Acetylpyrrole	-165.80	-147.00
2-Acetylthiophene	-120.58	-116.30
2-Adamantanol	-387.90	-378.30
2-Adamantanone	-314.90	-314.20
2-Allyl-5-aminotetrazole	283.03	277.90
2-Amino-1,3,4-triazole	75.90	85.80
2-Amino-4,6-bis(2,2-dinitro-2-fluoroethoxy)-1,3,5-triazine	-763.94	-788.60
2-Amino-4,6-dimethylpyrimidine	-35.83	-37.40
2-Amino-4,6-dinitrophenol	-243.69	-252.10
2-Amino-4-methylbenzothiazole	0.20	-9.90
2-Amino-4-methylpyrimidine	7.66	7.90
2-Amino-4-nitroanisole	-234.50	-184.80
2-Amino-5-nitroanisole	-199.50	-184.80
2-Amino-5-nitropyridine	-10.80	4.50
2-Amino-6-chlorobenzothiazole	-5.90	-5.20
2-Amino-6-methylbenzothiazole	10.20	-9.90
2-Aminoacridine	166.40	131.20
2-Aminobenzamide	-222.50	-225.70
2-Aminobenzenesulfonamide	-359.70	-362.60
2-Aminobenzimidazole	23.10	49.70
2-Aminobenzothiazole	56.70	26.90
2-Aminobenzoxazole	-97.60	-78.40
2-Aminobutyric acid	-608.20	-582.30
2-aminodiphenyl ether	-59.70	-56.80
2-Aminofluorene	76.70	74.10
2-Aminophenol	-201.30	-201.70
2-Aminopyridine	37.47	29.60
2-Anisaldehyde	-266.50	-258.40
2-Azidoethanol	94.60	66.60
2-Azidomethyl-3-nitrooxazolidine	180.00	177.74
2-Benzimidazolethiol	45.70	55.00
2-Benzoyloxynaphthalene	-190.40	-196.50
2-Benzylbenzimidazole	98.20	142.80
2-Biphenyl methanol	-107.90	-91.50
2-Bromoacetophenone	-115.06	-131.70
2-Bromoaniline	24.89	20.60
2-Bromoanisole	-104.39	-109.50
2-Bromobenzoic acid	-357.50	-361.80
2-Bromobenzonitrile	162.64	169.90
2-Bromobutane	-155.10	-138.90

2-Bromofluorene	106.15	124.30
2-Bromonaphthalene	112.69	93.70
2-Bromonitrobenzene	26.90	31.90
2-Bromophenol	-136.00	-151.50
2-Bromopropane	-130.50	-112.60
2-Bromopyridine	103.89	93.90
2-Butanethiol	-131.00	-136.70
2-Butanol	-342.82	-340.10
2-Butanone	-273.30	-274.70
2-Butene-1-imine	105.20	87.50
2-Butenyl p-tolyl sulphone	-352.60	-337.60
2-Butyl-1-decanol	-567.60	-591.80
2-Butyl-1-nonanol	-540.10	-565.50
2-Butyl-1-octanol	-533.85	-539.20
2-Butylamine	-137.49	-138.10
2-Butyldiphenylmethane	-35.30	-30.10
2-Butyloctanoic acid	-761.70	-745.00
2-Butylthiophene	-44.19	-43.50
2-Butyne	119.08	121.60
2-Carbamoyl-3-methylquinoxaline N-oxide	-118.02	-136.60
2-Carboxyadamantane	-627.20	-600.30
2-Carboxypyridine	-342.49	-338.30
2-Carboxypyridine N-oxide	-364.29	-372.50
2-Carene	-26.80	-30.70
2-Chloro-1,1,1,2-tetrafluoroethane	-924.70	-925.80
2-Chloro-1,1,1-trifluoroethane	-741.80	-748.60
2-Chloro-1,1-difluoroethylene	-329.00	-336.40
2-Chloro-1,3-propanediol	-517.50	-532.00
2-Chloro-1-ethylbenzene	-56.70	-49.50
2-Chloro-2,3-dimethylbutane	-262.00	-256.70
2-Chloro-2,4-dimethylpentane	-277.00	-284.20
2-Chloro-2,5-cyclohexadiene-1,4-dione	-221.40	-221.70
2-Chloro-2-methylbutane	-235.00	-229.70
2-Chloro-2-methylpentane	-264.00	-256.00
2-Chloro-2-methylpropane	-211.30	-206.00
2-Chloro-3-hydroxypyridine	-166.70	-155.60
2-Chloro-4,6-dinitroaniline	-102.00	-75.80
2-Chloro-4-nitroaniline	-75.90	-50.50
2-Chloro-5-nitroaniline	-72.10	-50.50
2-Chloro-6-(trichloromethyl)pyridine	-51.20	-33.70
2-Chloroacetophenone	-161.23	-177.60
2-Chloroaniline	-4.60	-25.40

2-Chloroanisole	-155.56	-155.50
2-Chlorobenzaldehyde	-119.60	-124.00
2-Chlorobenzenesulfonamide	-319.00	-358.40
2-Chlorobenzoic acid	-404.83	-407.60
2-Chlorobenzonitrile	121.07	124.00
2-Chlorobenzylidene-2-methylphenylacetonitrile	189.90	208.30
2-Chlorobiphenyl	37.10	77.20
2-Chlorobutane	-192.80	-190.40
2-Chlorobutanoic acid	-576.20	-572.20
2-Chlorodibenzo-p-dioxin	-174.10	-164.60
2-Chloroethanol	-295.40	-317.80
2-Chloroethyl ethyl ether	-335.60	-313.30
2-Chloroethyl vinyl ether	-208.70	-220.60
2-Chloronaphthalene	53.12	47.80
2-Chloronitrobenzene	-19.66	-14.00
2-Chlorophenol	-172.35	-197.50
2-Chlorophenyl isocyanate	-79.00	-74.60
2-Chlorophenylacetic acid	-449.93	-434.00
2-Chloropropane	-172.10	-164.00
2-Chloropropanoic acid	-525.95	-545.90
2-Chloropropene	-21.00	-22.90
2-Chloropyrazine	122.70	98.60
2-Chloropyridine	52.82	53.00
2-Chloropyrimidine	84.70	83.50
2-Chloroquinoline	67.90	92.40
2-Chlorotoluene	-30.10	-25.80
2-Cyano-2-nitroadamantane	-95.20	-127.20
2-Cyanoadamantane	-87.40	-68.60
2-Cyanobenzoic acid	-267.80	-262.50
2-Cyanobiphenyl	228.50	225.00
2-Cyanobutane	-42.20	-29.10
2-Cyclohexene-1-one	-170.90	-177.40
2-Cyclohexylcyclohexanone	-395.99	-401.50
2-Cyclohexylidenecyclohexanone	-311.80	-302.80
2-Cyclopenten-1-one	-152.40	-141.70
2-Cyclopropyl-1-butene	83.30	36.80
2-Cyclopropyl-1-pentene	-29.30	10.50
2-Cyclopropyl-2-butene	62.30	16.70
2-Cyclopropyl-2-hexene	-15.90	-37.20
2-Cyclopropyl-2-pentene	-8.40	-13.50
2-Cyclopropyl-3-methyl-1-butene	12.60	7.10
2-Decyne	-27.80	-36.20

2-Diethylaminoethanol	-309.50	-315.80
2-Dodecanone	-481.50	-485.20
2-Ethoxy-4,4,5,5-tetramethyl-1,3-dioxolane	-727.50	-714.20
2-Ethoxycarbonyl-3-methylquinoxaline 1,4-dioxide	-315.70	-341.40
2-Ethoxycarbonylaniline	-392.60	-408.40
2-Ethoxycinnamic acid	-533.53	-524.20
2-Ethoxyethanol	-448.22	-459.90
2-Ethoxyethylacetate	-663.00	-654.10
2-Ethoxynaphthalene	-138.30	-124.80
2-Ethyl-1-butanol	-382.41	-381.30
2-Ethyl-1-butene	-87.11	-83.30
2-Ethyl-1-hexanoic acid	-635.10	-634.50
2-Ethyl-1-hexanol	-436.70	-436.60
2-Ethyl-1-pentene	-111.80	-110.90
2-Ethyl-2-hexenal	-244.60	-258.10
2-Ethyl-2-methyl-1,3-dioxolane	-452.56	-449.80
2-Ethyl-2-nitro-1,3-propanediol	-603.98	-605.50
2-Ethyl-3-hydroxy-4-pyrone	-514.89	-535.90
2-Ethyl-3-methyl-1-butene	-114.06	-112.90
2-Ethylacrolein	-166.30	-157.70
2-Ethylanthracene	54.80	53.50
2-Ethylbenzoic acid	-444.70	-436.10
2-Ethylbiphenyl	70.20	51.40
2-Ethyldiphenylmethane	22.20	22.50
2-Ethylhexanal	-348.50	-353.50
2-Ethylhexyl acetate	-627.99	-628.00
2-Ethylimidazole	-23.30	-33.30
2-Ethyl-m-xylene	-80.12	-91.10
2-Ethylnaphthalene	58.60	19.40
2-Ethylnitrobenzene	-49.28	-42.40
2-Ethyloxazoline	-195.40	-228.50
2-Ethylpiperidine	-161.27	-148.40
2-Ethyl-p-xylene	-84.81	-91.10
2-Ethylpyridine	-1.20	17.30
2-Ethylthioethanol	-290.46	-305.90
2-Ethylthiophene	16.61	9.20
2-Ethyltoluene	-46.40	-54.20
2-Fluorenylaldehyde	-50.20	-24.50
2-Fluoroaniline	-155.83	-167.50
2-Fluoroanisole	-298.92	-297.60
2-Fluorobenzoic acid	-548.09	-549.90
2-Fluorobenzonitrile	-28.88	-18.20

2-Fluoroethanol	-465.70	-458.70
2-Fluoronitrobenzene	-158.41	-156.20
2-Fluorophenol	-343.32	-339.60
2-Fluoropropane	-293.50	-320.70
2-Fluorotoluene	-152.10	-168.00
2-Formylbenzoic acid	-531.54	-510.50
2-Formylimidazole	-102.20	-105.20
2-Formylpyrrole	-106.40	-93.40
2-Furanacrolein	-182.00	-154.70
2-Furanacrylic acid	-421.97	-435.80
2-Furanacrylonitrile	91.00	93.30
2-Furancarbonitrile	60.40	39.90
2-Furancarboxylic acid hydrazide	-207.40	-208.60
2-Furoic acid	-498.40	-491.70
2-Heptanone	-348.20	-353.70
2-Heptylhydroperoxide	-346.40	-358.70
2-Heptyne	48.20	42.70
2-Hexanol	-392.00	-395.30
2-Hexyldecanoic acid	-811.70	-850.30
2-Hexylhydroperoxide	-310.10	-332.40
2-Hexylthiophene	-92.68	-96.10
2-Hydroxy-4-methylquinoline	-192.60	-168.20
2-Hydroxy-6-methylpyridine	-214.51	-216.00
2-Hydroxyacetophenone	-352.50	-351.60
2-Hydroxybenzalaniline N-oxide	-62.60	-58.00
2-Hydroxybenzaldehyde	-283.20	-300.60
2-Hydroxybenzamide	-402.70	-397.80
2-Hydroxybenzonitrile	-56.50	-52.40
2-Hydroxybiphenyl	-92.40	-96.50
2-Hydroxydiphenylether	-225.20	-228.90
2-Hydroxydiphenylmethane	-74.20	-125.50
2-Hydroxyethylacrylate	-559.87	-560.10
2-Hydroxyethylmethacrylate	-627.60	-597.90
2-Hydroxymethyl-1,3-dioxane	-625.40	-603.00
2-Hydroxymethyl-1,3-propanediol	-744.60	-704.70
2-Hydroxynicotinic acid	-583.10	-589.80
2-Hydroxyphenazine di-N-oxide	-76.70	-47.20
2-Hydroxypropylacrylate	-582.50	-596.00
2-Hydroxyquinoline	-147.90	-131.30
2-Imidazolidinone	-274.68	-273.70
2-Indanol	-201.50	-192.40
2-Iodoaniline	86.71	73.10

2-Iodoanisole	-51.67	-57.00
2-Iodobenzoic acid	-303.45	-309.20
2-Iodobenzonitrile	226.96	222.50
2-Iodonaphthalene	144.30	146.30
2-Iodonitrobenzene	86.93	84.50
2-Iodophenol	-95.80	-99.00
2-Iodopropane	-74.80	-57.60
2-Iodotoluene	76.90	72.70
2-Isopropyl-5-methylphenol	-280.00	-295.00
2-Isopropyl-6-tert-butylphenol	-335.30	-376.40
2-Isopropylbenzimidazole	-35.40	10.10
2-Isopropylbenzoic acid	-464.50	-468.40
2-Isopropyl-p-cresol	-278.90	-292.40
2-Isopropylthiophene	-15.20	-20.40
2-Isopropyltoluene	-73.30	-81.20
2-Mercapto-5-methoxybenzimidazole	-128.40	-111.60
2-Mercaptoethanol	-248.94	-255.40
2-Methoxy-1,3-dioxolane	-531.84	-533.70
2-Methoxy-1-naphthaldehyde	-213.00	-219.00
2-Methoxy-1-propanol	-449.47	-457.50
2-Methoxy-4,6-bis(2,2-dinitro-2-fluoroethoxy)-1,3,5-triazine	-898.82	-917.90
2-Methoxy-5-nitrophenol	-376.60	-356.90
2-Methoxyacetophenone	-302.10	-312.00
2-Methoxybenzamide	-368.58	-355.50
2-Methoxycarbonyl-3-methylquinoxaline N,N'-dioxide	-270.81	-303.10
2-Methoxynaphthalene	-101.70	-86.50
2-Methoxy-p-cresol	-365.90	-368.70
2-Methoxyphenol	-325.78	-331.80
2-Methoxypyridine	-87.80	-128.70
2-Methoxytetrahydropyran	-442.30	-433.40
2-Methyl-1,2-propanediamine	-133.90	-140.40
2-Methyl-1,2-propanediol	-539.70	-539.60
2-Methyl-1,3-dioxane	-436.40	-419.00
2-Methyl-1,3-dioxolane	-385.10	-378.70
2-Methyl-1,3-pentanediol	-577.50	-577.70
2-Methyl-1,3-propanediol	-505.90	-520.50
2-Methyl-1-butanethiol	-157.00	-151.10
2-Methyl-1-butanol	-356.60	-360.20
2-Methyl-1-dodecanol	-552.60	-570.70
2-Methyl-1-heptene	-139.90	-141.20
2-Methyl-1-hexanol	-404.50	-412.90
2-Methyl-1-hexene	-112.60	-114.90

2-Methyl-1H-indene	60.78	60.10
2-Methyl-1-naphthoxazole	-54.40	-47.30
2-Methyl-1-nonene	-188.70	-193.70
2-Methyl-1-octene	-165.10	-167.40
2-Methyl-1-pentene	-89.96	-88.50
2-Methyl-1-propanethiol	-132.00	-127.40
2-Methyl-1-propanol	-334.32	-333.90
2-Methyl-1-tridecanol	-566.90	-597.10
2-Methyl-2-((nitrooxy)methyl)-1,3-propanediol dinitrate	-445.00	-426.20
2-Methyl-2,3,3-trinitrobutane	-323.26	-279.30
2-Methyl-2,3,3-trinitropentane	-292.95	-308.20
2-Methyl-2-butanamine	-171.40	-180.70
2-Methyl-2-butanol	-381.90	-379.30
2-Methyl-2-nitro-1,3-propanediol	-575.30	-579.70
2-Methyl-2-nitro-1,3-propanediol dinitrate	-373.00	-373.20
2-Methyl-2-nitro-1-phenyl-1-propanol	-317.56	-328.30
2-Methyl-2-nitro-1-propanol	-410.00	-395.50
2-Methyl-2-nitro-3-phenyl-1-propanol	-347.76	-316.20
2-Methyl-2-pentanethiol	-191.60	-195.30
2-Methyl-2-pentenal	-201.80	-205.40
2-Methyl-2-pentene	-98.53	-107.30
2-Methyl-2-propanol	-359.48	-355.60
2-Methyl-2-propene-1-imine	101.10	97.50
2-Methyl-2-propenyl p-tolyl sulphone	-352.50	-330.30
2-Methyl-3-(phenylmethyl)quinoxaline-1,4-dioxide	97.00	113.40
2-Methyl-3,5-diisopropylphenol	-372.30	-376.10
2-Methyl-3-hydroxypyridine	-175.80	-164.90
2-Methyl-4-(2,6,6-trimethyl-2-cyclohexen-1-yl)-2-butenal	-238.40	-279.40
2-Methyl-4-quinolinol	-165.80	-125.50
2-Methyl-5-hexen-3-yn-2-ol	-17.30	-38.90
2-Methyl-6-nitrobenzoxazole	-142.40	-109.00
2-Methyl-6-t-butylaniline	-113.10	-136.20
2-Methyl-8-hydroxyquinoline	-133.30	-125.50
2-Methyl-8-quinolinol	-133.30	-117.10
2-Methylacetanilide	-242.30	-233.60
2-Methyladamantane	-216.70	-214.40
2-Methylalanine	-596.30	-593.20
2-Methylaminoethanol	-254.59	-268.20
2-Methylbenzaldehyde	-113.18	-128.70
2-Methylbenzenesulfonamide	-359.59	-363.10
2-Methylbenzoic acid	-416.50	-412.40
2-Methylbenzoquinone	-253.50	-234.60

2-Methylbenzothiazole	75.70	70.80
2-Methylbenzyl cyanide	90.00	93.00
2-Methylbenzylalcohol	-206.70	-214.40
2-Methylbicyclo[2.2.2]-2-octene	-61.90	-105.10
2-Methylbutanedioic acid	-937.95	-942.60
2-Methylbutyl-2-nitrite	-251.00	-226.00
2-Methylbutyraldehyde	-271.50	-274.50
2-Methylbutyric acid	-556.80	-558.20
2-Methylenebicyclo[2.2.1]heptane	-7.30	-13.50
2-Methylene-bicyclo[2.2.2]octane	-54.40	-86.30
2-Methylenecyclohexanol	-277.60	-264.30
2-Methylfumaric acid	-826.30	-842.10
2-Methylheptane	-255.01	-255.20
2-Methylhexanal	-317.47	-327.10
2-Methylhexane	-229.49	-231.50
2-Methylhexanoic acid	-613.90	-610.80
2-Methylimidazole	-0.30	-7.00
2-Methylindole	60.70	52.50
2-Methyllactic acid	-742.98	-737.50
2-Methylnaphth[2,3-d]oxazole	-56.50	-47.30
2-Methylnaphthalene	44.85	43.10
2-Methylnaphtho[1,2-d]oxazole	-46.80	-47.30
2-Methylnaphtho[1,2-d]thiazole	106.00	107.40
2-Methylnonane	-314.46	-307.90
2-Methylnorbornene	1.20	-17.00
2-Methyloctanal	-370.20	-379.70
2-Methyloctane	-280.60	-284.20
2-Methyloxazoline	-171.30	-202.20
2-Methylpentane	-204.64	-205.20
2-Methylpiperidine	-127.80	-124.70
2-Methylpropanal	-248.90	-250.80
2-Methylpropane	-153.47	-152.50
2-Methylpropanoic acid	-534.18	-534.40
2-Methylpropyl 2-pentenoate	-506.60	-503.60
2-Methylpropyl 3-Pentenoate	-505.30	-503.60
2-Methylpropyl 4-pentenoate	-488.20	-484.80
2-Methylpropylamine	-132.60	-138.50
2-Methylpyrrole	23.30	4.90
2-Methylquinoline	95.15	83.00
2-Methylquinoxaline N,N'-dioxide	59.76	68.80
2-Methylresorcinol	-396.20	-410.70
2-Methyltetrazole	280.60	265.80

2-Methylthiolane	-105.00	-98.40
2-Methylthiophene	44.60	35.50
2-Monopalmitin	-1231.10	-1247.00
2-Naphthaleneacetic acid	-371.90	-365.10
2-Naphthalenecarbonitrile	181.03	192.90
2-Naphthalenecarboxylic acid	-346.10	-336.20
2-Naphthol	-128.71	-128.50
2-Naphthylamine	54.18	43.60
2-n-Hexoxyethanol	-560.30	-565.30
2-Nitro-2-isopropyl-1,3-propanetriol	-623.77	-627.60
2-Nitro-2-propyl-1,3-propanetriol	-625.67	-629.50
2-Nitroacetanilide	-216.77	-222.10
2-Nitroadamantane	-241.90	-242.50
2-Nitroaniline	-26.10	-18.50
2-Nitrobenzaldehyde	-112.76	-116.90
2-Nitrobenzamide	-226.50	-216.70
2-Nitrobenzenesulfonamide	-343.90	-351.30
2-Nitrobenzoic acid	-393.76	-400.90
2-Nitrobutane	-207.60	-200.30
2-Nitrocinnamic acid	-379.90	-344.50
2-Nitrocinnamide	-148.20	-158.00
2-nitrodiphenyl ether	-31.10	-45.40
2-Nitrodiphenylamine	72.60	82.00
2-Nitroethanol	-351.00	-323.80
2-Nitrofluorene	43.90	85.50
2-Nitrofuran	-104.10	-75.20
2-Nitroisobutane	-229.80	-211.20
2-Nitro-m-xylene	-53.80	-55.60
2-Nitrophenol	-192.59	-190.60
2-Nitropropane	-180.30	-176.60
2-Nitroso-1-naphthol	-51.00	-47.40
2-Nitro-t-butylbenzene	-82.90	-105.30
2-Nitrotoluene	-24.28	-18.70
2-Nonanone	-396.80	-402.30
2-Nonyn-1-ol	-218.00	-193.90
2-Nonyne	-2.40	-9.90
2-Nonynoic acid	-386.80	-391.80
2-Norbornanone	-219.40	-206.10
2-Norbornanonedimethylketal	-431.20	-433.00
2-Octanone	-372.70	-380.00
2-Octylthiophene	-141.87	-148.80
2-Octyn-1-ol	-159.10	-167.60

2-Octyne	19.30	16.40
2-Octynoic acid	-330.40	-365.50
2-Oxetanone	-329.90	-306.50
2-Oxiranylmethanol	-299.40	-288.80
2-Oxoglutaric acid	-1030.68	-1064.80
2-Pentanol	-365.20	-369.00
2-Pentylthiophene	-68.99	-69.80
2-Pentyne	128.90	95.40
2-Phenyl-1,3-dioxolane	-267.40	-289.70
2-Phenyl-1-propanol	-223.00	-228.30
2-Phenyl-2,5,5-trimethyl-1,3-dioxane	-414.50	-423.60
2-Phenyl-3-methyl-2-butene	-4.50	-13.00
2-Phenyl-5,5-dimethyl-1,3-dioxane	-397.00	-376.30
2-Phenylaniline	93.80	75.60
2-Phenylbenzimidazole	130.60	169.10
2-Phenylbenzoic acid	-349.10	-309.40
2-Phenylbenzothiazole	175.40	173.70
2-Phenylbenzoxazole	2.60	19.10
2-Phenylcarbamoyl-3-methylquinoxaline N,N'-dioxide	-63.10	-63.70
2-Phenylcarbamoyl-3-methylquinoxaline N-oxide	-32.40	-38.80
2-Phenylglycine	-432.08	-453.00
2-Phenylhex-2-enenitrile	175.40	121.80
2-Phenylindole	135.40	150.80
2-Phenylnaphthalene	134.81	151.30
2-Phenylpent-2-enenitrile	199.10	148.10
2-Phenylpyrrole	144.44	107.80
2-Phenylsuccinic acid	-844.50	-839.60
2-Phenyltoluene	86.50	72.50
2-Picoline	54.49	43.60
2-Picoline N-oxide	-1.81	9.70
2-Piperidineethanol	-335.37	-328.50
2-Piperidinemethanol	-331.07	-308.70
2-Piperidone	-308.76	-304.80
2-Propanethiol	-105.90	-110.40
2-Propanol	-318.07	-316.30
2-Propylpentanoic acid	-646.00	-634.50
2-Propylthiophene	-8.96	-17.10
2-Propyltoluene	-72.47	-83.10
2-Pyrazinecarboxylic acid	-272.91	-292.70
2-Pyridinealdoxime	76.40	64.90
2-Pyridinecarbonitrile	208.00	193.40
2-Pyridone	-166.30	-126.50

2-Pyrrolidone	-266.46	-264.50
2-Quinolinecarbonitrile	246.50	232.80
2-Stearoylglycerol	-1331.70	-1299.70
2-t-Butoxycarbonyl-3-methylquinoxaline N-oxide	-392.69	-391.80
2-t-Butyl-4-methylphenol	-306.90	-325.70
2-t-Butyl-5-methylphenol	-304.90	-323.00
2-t-Butyl-6-phenylphenol	-170.20	-202.70
2-t-Butylbenzoic acid	-476.20	-493.70
2-t-Butylnaphthalene	-31.50	-40.90
2-t-Butylperoxyethanol	-511.70	-480.20
2-t-Butylphenol	-279.80	-288.80
2-Tetralone	-151.70	-164.60
2-Thiaadamantane	-147.70	-153.70
2-Thiocresol	44.20	12.50
2-Thiopheneacetamide	-199.89	-188.80
2-Thiopheneacetic acid	-365.78	-372.70
2-Thiopheneacetonitrile	165.13	158.90
2-Thiophenecarbaldehyde	-64.10	-62.70
2-Thiophenecarbonitrile	196.42	185.30
2-Thiophenecarboxamide	-173.30	-162.50
2-Thiophenecarboxylic acid	-352.49	-343.80
2-Thiophenecarboxylic acid hydrazide	-66.20	-63.20
2-Thiouracil	-185.90	-165.20
2-Thioxo-4-imidazolidinone	-206.14	-206.30
2-t-Pentylperoxyethanol	-534.20	-503.90
2-Trichloroacetylpyrrole	-191.70	-188.30
2-Trifluoroacetylpyrrole	-763.00	-761.70
2-Vinyl-5-norbornene	112.90	89.60
2-Vinylaziridine	199.00	158.50
2-Vinylfuran	-10.50	-11.40
2-Vinylpyridine	157.10	142.10
3-(1-Methyl-2-pyrrolidinyl)pyridine	63.72	49.70
3-(2-Furyl)-2-propenal	-182.00	-138.20
3-(5-(2-Nitrophenyl)-2-furyl)acrylic acid	-403.10	-414.60
3-(5-(3-Nitrophenyl)-2-furyl)acrylic acid	-417.10	-414.60
3-(5-(4-Nitrophenyl)-2-furyl)acrylic acid	-435.50	-414.60
3-(Dimethylamino)acrylaldehyde	-177.00	-147.30
3-(Dimethylamino)propylamine	-95.01	-94.40
3-(Ethylsulphonyl)-1-propene	-406.00	-406.50
3-(Methylamino)propionitrile	57.40	39.20
3-(Methylamino)propylamine	-82.30	-96.60
3-(Methylmercapto)-propanal	-211.10	-219.00

3-(Triethoxysilyl)-1-propanamine	-1022.00	-1029.60
3-(Trifluoromethyl)acetanilide	-844.20	-848.30
3,3,3-Trifluoropropene	-614.20	-612.80
3,3,4-Trimethylhexane	-281.90	-286.60
3,3,5,5-Tetramethylheptane	-325.70	-331.80
3,3,5-Trimethylheptane	-304.76	-310.30
3,3,6,6-Tetramethyl-1,7-octadiyne	211.08	233.80
3,3,6,6-Tetramethyloctane	-372.80	-356.70
3,3-Bis(chloromethyl)oxetane	-259.80	-242.60
3,3'-Bitolyl	20.00	40.90
3,3'-Dihydroxy-4,4'-diaminodiphenylmethane	-383.10	-406.90
3,3-Dimethyl-1,4-pentadiyne	348.69	367.80
3,3-Dimethyl-1-butene	-87.40	-84.70
3,3-Dimethyl-1-pentene	-111.30	-108.40
3,3-Dimethylbutanoic acid	-564.00	-591.40
3,3-Dimethylhexane	-257.53	-259.50
3,3-Dimethyloxetane	-186.77	-168.00
3,3-Dimethylpentane	-234.18	-233.20
3,3'-Dinitrobenzophenone	-108.50	-89.90
3,3'-Oxydi-1,2-propanediol tetranitrate	-630.80	-671.50
3,4,4'-Trichlorocarbanilide	-234.60	-225.00
3,4,5,6-Tetramethylphenanthrene	20.30	-17.10
3,4,5-Trichloroaniline	-66.61	-89.70
3,4,5-Trifluoroaniline	-519.88	-516.40
3,4,5-Trimethoxybenzoic acid	-855.00	-875.10
3,4,5-Trimethoxytoluene	-464.90	-493.20
3,4,5-Trimethylbenzoic acid	-500.90	-486.10
3',4'-Dichloroacetophenone	-227.60	-209.80
3,4-Dichloroaniline	-49.14	-57.50
3,4-Dichloronitrobenzene	-65.82	-46.20
3,4-Dichlorophenol	-231.23	-229.60
3,4-Dichlorphenylisocyanate	-125.60	-106.90
3,4-Diethyl-3,4-bis(4-t-butylphenyl)-hexane	-350.70	-362.10
3,4-Diethyl-3,4-dimethoxyhexane	-577.50	-617.20
3,4-Difluoroaniline	-330.55	-342.00
3,4-Difluoronitrobenzene	-348.83	-330.60
3,4-Difluorophenol	-526.24	-514.00
3,4-Dihydro-1H-2-benzopyran	-123.60	-139.70
3,4-Dihydro-2H-1,5-benzodioxepin	-244.60	-296.10
3,4-Dihydro-2H-1-benzopyran	-142.50	-154.50
3,4-Dihydro-2H-1-benzopyran-2-one	-320.90	-329.10
3,4-Dihydro-2H-pyran-2-carbaldehyde	-276.82	-318.50

3,4-Dihydro-4H-pyran	-149.80	-184.50
3,4-Dihydroxytoluene	-395.90	-410.70
3',4'-Dimethoxyacetophenone	-488.35	-478.50
3,4-Dimethoxybenzaldehyde	-444.60	-424.90
3,4-Dimethoxybenzoic acid	-717.80	-708.60
3,4-Dimethoxyphenylacetonitrile	-209.84	-203.10
3,4-Dimethyl-1,2,5-oxadiazole	54.80	72.00
3,4-Dimethyl-1-pentene	-111.30	-107.40
3,4-Dimethylbenzoic acid	-468.80	-449.30
3,4-Dimethylhexane	-251.83	-257.20
3,4-Dinitro-1-trinitromethyl-1H-pyrazole	262.50	248.00
3,4-Dinitropyrazole	120.10	102.60
3,4-Dinitrotoluene	-16.50	-43.80
3,4-Diphenyl-1,2,5-oxadiazole	324.30	278.00
3,4-Lutidine	18.30	15.10
3,4-Methylenedioxycinnamic acid	-609.50	-621.70
3,4-Xylenol	-245.40	-239.00
3,5,3',5'-Tetramethyl-4,4'-diethyldipyrrylmethene	-34.40	-72.10
3,5,5-Trimethyl-1-hexanol	-461.20	-469.30
3,5,5-Trimethyl-2-cyclohexenone	-281.00	-275.40
3,5,7-Trioxa-1,1,1,9,9,9-hexanitrononane	-599.20	-568.40
3,5,7-Trioxanonane	-628.70	-627.90
3,5-Dibromo-4-hydroxybenzonitrile	-11.30	-25.00
3,5-Dibromophenol	-137.30	-137.80
3,5-Dichloroaniline	-57.64	-57.50
3,5-Dichloroanisole	-201.60	-187.60
3,5-Dichloronitrobenzene	-70.32	-46.20
3,5-Dichlorophenol	-230.73	-229.60
3,5-Dichloropyridine	15.35	24.50
3,5-Diethylbenzoic acid	-511.90	-497.90
3,5-Difluoroaniline	-367.25	-342.00
3,5-Difluorophenol	-546.74	-514.00
3,5-Dihydroxybenzoic acid	-789.20	-792.60
3',5'-Diisopropyl-4,4-dimethyl-3-phenyl-1,2-benzocyclobuten-3-ol	-227.30	-260.30
3,5-Diisopropylphenol	-341.50	-341.80
3,5-Dimethoxybenzaldehyde	-441.10	-424.90
3,5-Dimethoxybenzoic acid	-727.40	-708.60
3,5-Dimethyl-1-phenyl-4-nitrosopyrazole	205.80	201.50
3,5-Dimethyl-2-cyclohexen-1-one	-217.50	-244.80
3,5-Dimethyl-4-nitrosopyrazole	109.30	96.90
3,5-Dimethyl-cyclohexanol	-428.40	-410.80
3,5-Dimethylheptan-4-one	-412.60	-407.70

3,5-Dimethylisoxazole	-63.20	-71.50
3,5-Dimethylisoxazole-4-carboxylic acid	-488.80	-491.20
3,5-Dimethylpyrazole	16.90	45.60
3,5-Dimethylpyridine N-oxide	-33.80	-32.00
3,5-Dimethylpyrrole-2-carboxylic ethylester	-474.50	-447.90
3,5-Dinitro-1-trinitromethyl-1H-pyrazole	242.80	245.50
3,5-Dinitroaniline	-38.90	-43.30
3,5-Dinitrobenzoic acid	-434.50	-425.70
3,5-Dinitropyrazole	93.78	100.10
3,5-Dinitrotoluene	-43.17	-43.80
3,5-Diphenylisoxazole	139.80	134.40
3,5-Diphenylpyridine	198.00	230.20
3,5-Di-t-butyl-2-hydroxybenzaldehyde	-550.20	-541.00
3,5-Di-t-butylbenzoic acid	-624.60	-613.30
3,5-Di-t-butylcatechol	-576.40	-615.60
3,5-Di-t-butyl-o-quinone	-413.60	-445.60
3,5-Di-t-butylphenol	-417.10	-403.10
3,5-Dithiaheptane	-119.30	-97.00
3,5-Lupetidine	-158.27	-154.80
3,5-Lutidine	22.50	15.10
3,5-Xylenol	-247.50	-239.00
3,6-Dibutanal-1,2,4,5-tetroxane	-637.40	-671.30
3,6-Diethyloct-4-yne-3,6-diol	-481.50	-501.70
3,6-Diphenyl-1,2-dithiin	233.70	239.00
3,6-Diphenyl-4-cyclohexene-1,2-dicarboxylic anhydride	-404.00	-411.40
3,6-Di-t-butyl-o-quinone	-415.60	-440.30
3,6-Dithiaoctane	-146.30	-147.30
3,7,11,15-Tetramethyl-1-hexadecyn-3-ol	-449.90	-469.20
3,7,11-Trimethyl-1-dodecyn-3-ol	-343.60	-339.50
3,7-Dimethyl-1-thiaindene sulfone	-246.20	-284.30
3,7-Dinitroso-1,3,5,7-Tetraazabicyclo[3.3.1]nonane	225.93	209.70
3,9-Dimethylbenzo[a]anthracene	76.20	82.30
3,9-Dodecadiyne	197.82	181.90
3,9-Perylenedicarboxylic acid	-637.50	-656.60
3,beta-Dinitrostyrene	0.15	6.10
3-Acetoxycinnamic acid	-699.10	-698.90
3-Acetyl-2,5-dimethylfuran	-355.20	-353.60
3-Acetyl-2,5-dimethylthiophene	-188.00	-202.70
3-Acetyl-2-methyl-5-phenylthiophene	-109.26	-97.10
3-Acetyl-2-oxazolidinone	-619.70	-628.10
3-Acetylpyridine	-103.80	-99.80
3-Acetylthiophene	-131.58	-105.50

3-Amino-1-nitroguanidine	21.50	26.00
3-Amino-4-phenyl-1,2,5-oxadiazole	218.10	174.80
3-Amino-5-methylisoxazole	-63.50	-83.30
3-Aminoacetophenone	-173.30	-179.20
3-Aminobenzamide	-230.10	-225.40
3-aminodiphenyl ether	-50.50	-56.80
3-Aminophenol	-202.50	-201.70
3-Aminopyridine	58.30	52.50
3-Aminoquinoline	102.20	91.90
3-Anisaldehyde	-256.50	-258.40
3-Azabicyclo[3.2.2]nonane	-105.10	-137.10
3-Azido-1,2,4-triazole	441.71	453.70
3-Azido-5-ethyl-1,2,4-triazole	365.93	381.60
3-Azido-5-methyl-1,2,4-triazole	392.72	408.00
3-Azido-5-phenyl-1,2,4-triazole	532.14	511.00
3-Benzylphenol	-74.20	-125.50
3-Bromoacetophenone	-127.56	-131.70
3-Bromoaniline	41.99	20.60
3-Bromoanisole	-109.39	-109.50
3-Bromobenzoic acid	-372.20	-361.80
3-Bromobenzonitrile	160.34	169.90
3-Bromonitrobenzene	7.10	31.90
3-Bromopyridine	112.89	102.60
3-Bromoquinoline	166.50	142.00
3-Buten-2-one	-138.10	-147.30
3-Butene-2-imine	100.60	83.60
3-Butenyl 4-tolyl sulphone	-343.70	-318.90
3-Butylthiophene	-37.59	-32.60
3-Butyne-1-amine	203.90	166.60
3-Butyne-2-amine	202.90	163.70
3-Butynoic acid	-241.80	-229.20
3-Butynylbenzene	245.00	231.90
3-Carboxyphenol	-592.90	-584.10
3-Carene	-33.70	-29.40
3-Chloro-1,1,1-trifluoropropane	-792.60	-774.90
3-Chloro-1-propanol	-343.70	-344.20
3-Chloro-3-methylpentane	-260.00	-250.80
3-Chloro-4-methylphenylisocyanate	-130.80	-111.50
3-Chloro-5-hydroxypyridine	-161.50	-151.90
3-Chloroacetophenone	-173.13	-174.90
3-Chloroaniline	-20.30	-25.40
3-Chloroanisole	-161.66	-155.50

3-Chlorobenzaldehyde	-127.10	-124.00
3-Chlorobenzoic acid	-423.70	-407.60
3-Chlorobenzonitrile	115.17	124.00
3-Chlorobenzoylchloride	-190.00	-191.80
3-Chlorobutanoic acid	-561.70	-572.20
3-Chloronitrobenzene	-43.36	-14.00
3-Chlorophenol	-190.27	-197.50
3-Chlorophenylacetic acid	-447.43	-434.00
3-Chloropropionic acid	-549.30	-542.00
3-Chloropyridine	59.02	56.70
3-Chlorotoluene	-29.10	-25.80
3-Cyanobenzoic acid	-272.70	-262.50
3-Cyanophenol	-56.50	-52.40
3-Cyanopyridine N-oxide	170.12	154.60
3-Cyclohexene-1-carboxaldehyde	-162.10	-179.80
3-Cyclohexyleicosane	-677.60	-677.90
3-Decyne	-29.50	-36.20
3-Dimethylaminopropanenitrile	41.33	41.30
3-Ethyl-1-heptanol	-440.70	-457.70
3-Ethyl-1-hexene	-124.60	-125.20
3-Ethyl-1-pentene	-104.20	-104.10
3-Ethyl-2-methyl-1-pentene	-137.90	-141.90
3-Ethyl-2-methylpentane	-249.58	-254.60
3-Ethyl-2-pentene	-118.00	-129.70
3-Ethyl-3-methylpentane	-256.70	-256.90
3-Ethylbenzoic acid	-449.20	-436.10
3-Ethyldiphenylmethane	1.20	19.80
3-Ethylheptanal	-369.50	-374.40
3-Ethylheptane	-275.48	-281.60
3-Ethylhexane	-250.41	-255.20
3-Ethyl-o-xylene	-80.50	-91.10
3-Ethylpentane	-230.58	-231.50
3-Ethylphenol	-214.41	-228.50
3-Ethyltoluene	-48.70	-54.20
3-Fluoroacetanilide	-375.90	-371.10
3-Fluoroaniline	-166.93	-167.50
3-Fluoroanisole	-311.52	-297.60
3-Fluorobenzoic acid	-559.19	-549.90
3-Fluorobenzonitrile	-27.98	-18.20
3-Fluorobenzotrifluoride	-811.30	-782.80
3-Fluoronitrobenzene	-179.31	-156.20
3-Fluorophenetole	-341.50	-335.90

3-Fluorophenol	-340.00	-339.60
3-Formyl-6-isopropylchromone	-420.10	-405.60
3-Formyl-6-methylchromone	-358.80	-354.90
3-Formylchromone	-343.40	-318.00
3-Furanacrylic acid	-462.67	-421.90
3-Furancarboxaldehyde	-201.71	-191.60
3H-1,2-Benzodithiole-3-thione	140.40	155.30
3-Heptanone	-348.60	-353.70
3-Heptylhydroperoxide	-346.80	-358.70
3-Heptyne	46.20	42.70
3-Hexanone	-320.20	-327.40
3-Hexylhydroperoxide	-305.10	-329.80
3-Hexylthiophene	-91.88	-85.30
3-Hydroxy-1,3,3-triphenyl-1-propanone	-243.40	-219.80
3-Hydroxy-2-methylpropionaldehyde	-427.87	-434.70
3-Hydroxy-2-naphthalenecarboxylic acid	-547.70	-547.60
3-Hydroxy-6-methylpyridine	-168.20	-164.90
3-Hydroxyacetophenone	-370.60	-351.30
3-Hydroxybenzaldehyde	-316.20	-300.40
3-Hydroxybiphenyl	-92.40	-93.90
3-Hydroxybutyric acid	-679.52	-724.90
3-Hydroxycinnamic acid	-535.90	-528.00
3-Hydroxycoumarin	-462.50	-466.30
3-Hydroxydiphenylether	-218.60	-228.90
3-Hydroxypyridine	-132.00	-119.70
3-Hydroxypyridine N-oxide	-172.52	-166.80
3-Iodoaniline	81.81	73.10
3-Iodoanisole	-52.17	-57.00
3-Iodobenzoic acid	-315.40	-309.20
3-Iodobenzonitrile	229.46	222.50
3-Iodonitrobenzene	65.63	84.50
3-Iodophenol	-96.68	-99.00
3-Iodopropionic acid	-460.00	-449.50
3-Iodotoluene	79.10	72.70
3-Isochromanone	-336.80	-338.30
3-Isopropylbenzoic acid	-479.60	-463.10
3-Isopropyltoluene	-82.58	-83.80
3-lsopropyl-1,2-benzenediol	-447.80	-464.00
3-Menthene	-161.10	-172.60
3-Mercaptopropyl-triethoxysilane	-1054.00	-1018.50
3-Mercaptopropyl-trimethoxysilane	-933.10	-903.70
3-Methoxy-1,3,3-triphenylpropyne	212.80	214.40

3-Methoxy-2-nitrobenzoic acid	-554.30	-567.10
3-Methoxy-4-hydroxybenzaldehyde	-453.40	-466.90
3-Methoxy-4-nitrobenzoic acid	-585.60	-567.10
3-Methoxyacetophenone	-308.90	-312.00
3-Methoxybenzamide	-375.50	-355.50
3-Methoxydiphenylether	-191.50	-186.90
3-Methoxyphenol	-316.00	-331.80
3-Methoxypropionitrile	-118.70	-114.30
3-Methoxysalicylaldehyde	-493.77	-467.20
3-Methoxytoluene	-158.40	-160.10
3-Methyl-1,2-butadiene	101.17	102.60
3-Methyl-1-butanethiol	-154.27	-151.10
3-Methyl-1-butene	-53.90	-54.10
3-Methyl-1-butyne	136.40	123.00
3-Methyl-1-hexene	-101.10	-104.10
3-Methyl-1H-pyrazole	74.20	82.40
3-Methyl-1-pentanol	-380.90	-383.90
3-Methyl-1-pentene	-78.16	-77.80
3-Methyl-2,5-furandione	-504.55	-532.50
3-Methyl-2-butanethiol	-158.80	-163.70
3-Methyl-2-butanol	-369.90	-367.10
3-Methyl-2-butanone	-299.50	-304.40
3-Methyl-2-cyclopenten-1-one	-198.10	-179.50
3-Methyl-2-phenylbutane-2-ol	-283.20	-306.00
3-Methyl-2-thiophenecarbonitrile	155.13	147.50
3-Methyl-2-thiophenecarboxyaldehyde	-100.80	-100.50
3-Methyl-2-thiophenecarboxylic acid	-396.18	-384.20
3-Methyl-3-pyrazoline-5-one	-152.50	-145.40
3-Methyl-3-t-amylperoxy-1-triphenylsilyl-1-butyne	153.20	147.30
3-Methyl-3-t-butylperoxy-1-triphenylsilyl-1-butyne	162.99	168.30
3-Methyl-3-t-hexylperoxy-1-triphenylsilyl-1-butyne	120.40	120.90
3-Methyl-4-isopropylphenol	-295.50	-289.80
3-Methyl-4-nitropyridine-1-oxide	-19.10	-20.20
3-Methyl-4-phenyl-1,2,5-oxadiazole	184.70	186.60
3-Methyl-5-phenylisoxazole	14.80	31.50
3-Methylbenzaldehyde	-123.40	-128.70
3-Methylbenzoic acid	-424.90	-412.40
3-Methylbenzylalcohol	-197.30	-214.40
3-Methylbutanoic acid	-561.60	-560.80
3-Methylbutyl 2-chlorobutanoate	-686.90	-648.10
3-Methylbutyl 3-chlorobutyrate	-647.20	-648.10
3-Methylbutyl 3-chloropropionate	-595.80	-618.00

3-Methylbutyl 4-chlorobutyrate	-659.30	-644.30
3-Methylbutyl chloroacetate	-581.10	-591.60
3-Methylbutyl trichloroacetate	-592.30	-595.70
3-Methyl-cis-2-pentene	-94.47	-103.40
3-Methylcyclohexanone	-276.40	-313.00
3-Methylcyclohexylamine	-183.30	-176.60
3-Methylcyclopentanone	-276.70	-277.40
3-Methylcyclopentene	-24.90	-41.20
3-Methyldiamantane	-260.40	-245.20
3-Methyldihydro-2,5-furandione	-621.80	-625.70
3-Methylenecyclobutanenitrile	205.70	199.90
3-Methyleneheptane	-136.42	-135.90
3-Methylheptane	-255.00	-252.60
3-Methylhexanal	-315.39	-327.10
3-Methylhexane	-226.44	-228.90
3-Methylisoxazole	-5.60	-17.20
3-Methyl-N-(2-methylphenyl)-2-quinoxalinecarboxamide N,N'-dioxide	-77.40	-100.60
3-Methylnonane	-303.59	-305.30
3-Methyloctane	-278.53	-284.20
3-Methylpentane	-202.38	-205.20
3-Methylpicric acid	-258.76	-277.70
3-Methylpiperidine	-126.47	-125.10
3-Methylpyridine	59.69	52.00
3-Methylpyrrole	20.50	4.00
3-Methylquinoxaline-2-carboxamide 1,4-dioxide	-150.40	-161.50
3-Methylsulfolane	-488.00	-472.10
3-Methylthiolane	-105.00	-89.10
3-Methylthiophene	43.10	46.40
3-Methyl-trans-2-pentene	-94.56	-103.40
3-Methylundecane	-355.20	-357.80
3-Nitraza-1,5-pentanediisocyanate	-264.40	-249.20
3-Nitro-2-butanol	-390.00	-394.10
3-Nitro-3-(4-nitrophenyl)pentane	-206.10	-182.00
3-Nitroacetanilide	-235.17	-221.80
3-Nitroacetophenone	-201.30	-170.50
3-Nitroaniline	-30.40	-18.20
3-Nitrobenzaldehyde	-120.00	-116.90
3-Nitrobenzamide	-247.90	-214.10
3-Nitrobenzenesulfonamide	-368.90	-351.30
3-Nitrobenzoic acid	-414.00	-400.60
3-Nitrobenzophenone	-81.60	-64.80
3-Nitrobenzotrifluoride	-621.30	-633.30

3-Nitrobiphenyl	65.10	89.50
3-Nitrocinnamic acid	-370.30	-344.50
3-nitrodiphenyl ether	-51.10	-45.40
3-Nitrophenetol	-211.98	-186.60
3-Nitrophenol	-197.69	-190.30
3-Nitrophthalic acid	-795.20	-819.30
3-Nitrophthalic anhydride	-483.90	-507.10
3-Nitrophthalimide	-328.10	-360.50
3-Nitro-p-toluidine	-71.59	-55.10
3-Nitrotoluene	-32.68	-18.70
3-Nonanone	-397.40	-406.20
3-Nonyne	-4.00	-9.90
3-Octanone	-371.80	-380.00
3-Octylthiophene	-157.67	-138.00
3-Octyne	18.60	16.40
3-Oxabicyclo[3.2.2]nonane	-279.10	-300.10
3-Pentanol	-370.33	-369.00
3-Pentynoic acid	-292.30	-286.50
3-Phenyl-1-butene	56.10	56.70
3-Phenyl-2-propyn-1-ol	76.70	40.70
3-Phenyl-5-phenoxymethyl-2-N-phenyliminooxazolidine	-82.20	-93.20
3-Phenyl-5-phenoxymethyl-2-oxazolidinone	-423.10	-424.80
3-Phenylamino-2-naphthoic acid	-261.77	-274.70
3-Phenylcyclobutanone	-60.00	-59.40
3-Phenylcyclobutenone	24.50	38.50
3-Phenylisoxazole	72.50	85.70
3-Phenylpropene	88.03	78.50
3-Phenylpropiolic acid	-124.70	-157.20
3-Phenyltoluene	85.56	77.70
3-Picoline N-oxide	2.69	4.80
3-Picrylamino-1,2,4-triazole	148.80	103.90
3-Piperidinecarboxamide	-337.30	-320.50
3-Piperidinemethanol	-326.50	-309.10
3-Propyltoluene	-76.23	-83.10
3-Pyridinecarbonitrile	193.40	201.80
3-Quinolinecarbonitrile	242.30	241.20
3-Sulfolene	-318.90	-345.60
3-t-Butylbenzoic acid	-504.30	-493.70
3-t-Butylperoxy-1,2-propanediol	-719.20	-700.00
3-t-Butylphenol	-278.90	-283.50
3-Thiacyclohexanone	-184.30	-229.80
3-Thiopheneacetic acid	-378.98	-361.90

3-Thiopheneacetonitrile	164.33	169.80
3-Thiophenecarbonitrile	194.92	196.10
3-Thiophenecarboxyaldehyde	-62.10	-51.90
3-Thiophenecarboxylic acid	-355.79	-332.90
3-Thiopropionic acid	-468.36	-479.70
3-Trifluoromethylaniline	-658.70	-644.80
3-Trifluoromethylbenzoic acid	-1009.10	-1027.10
3-Trifluoromethylphenol	-798.50	-816.90
4-(1H-Tetrazol-5-yl)-3-tetrazene-2-carboximidamide	474.40	466.90
4,4'-(Dimethoxy)azobenzene	39.20	8.00
4,4,4-Trifluoro-1-(2-furanyl)-butane-1,3-dione	-1050.80	-1054.50
4,4,4-Trifluoro-1-(2-naphthyl)-butane-1,3-dione	-901.00	-898.90
4,4,4-Trinitrobutyronitrile	3.40	30.40
4,4,6,6-Tetramethyl-1,3-dioxane	-521.65	-529.30
4,4,6,6-Tetramethylnonane	-371.00	-385.70
4,4,6-Trimethyl-1,3-dioxane	-500.50	-490.00
4,4'-Azobis(4-cyano-1-pentanol)	-228.40	-224.50
4,4'-Bipyridine	183.40	205.80
4,4'-Bis-(N-carbazolyl) -1,1'-biphenyl	505.90	549.90
4,4'-Diaminodiphenyldisulfide	92.53	87.20
4,4'-Dichlorobiphenyl	16.54	50.20
4,4'-Diethoxyazoxybenzene	-169.55	-166.90
4,4'-Difluorobiphenyl	-289.50	-234.20
4,4'-Diisocyanatobiphenyl	-54.50	-56.80
4,4'-Diisocyanatodiphenylmethane	-57.70	-88.40
4,4-Dimethyl-1,3-cyclohexanedione	-502.80	-466.60
4,4-Dimethyl-1-pentene	-110.60	-108.40
4,4'-Dimethylbiphenyl	16.20	40.90
4,4'-Dinitrodiphenyl ether	-115.60	-70.50
4,4'-Dinitrohydrazobenzene	109.72	131.30
4,4'-Di-t-butylbiphenyl	-122.10	-121.80
4,4'-Oxybis(benzoylhydrazide)	-307.20	-286.30
4,5,9,10-Tetrahydropyrene	19.70	49.70
4,5-Benzo-1,3-dithiole-2-thione	120.40	111.20
4,5-Dichloro-2-nitroaniline	-99.70	-82.90
4,5-Diethyl-4,5-bis-(4-tert-butylphenyl)-octane	-420.26	-443.90
4,5-Dimethyl-1,3-dioxane	-454.40	-444.60
4,5-Dimethyl-2-furaldehyde	-297.10	-300.10
4,5-Dimethylphenanthrene	83.40	66.10
4,5-Dimethylpyrrole-3-carboxylic ethylester	-470.30	-448.80
4,5-Tetramethylene-1,2-dithiole-3-thione	46.70	31.30
4,5-Tetramethylene-1,3-dithiolan-2-thione	-82.20	-64.30

4,6-Dichloro-2-methylpyrimidine	-4.90	-0.80
4,6-Dichloro-5-methylpyrimidine	4.30	19.70
4,6-Dichloropyrimidine	50.33	56.60
4,6-Diethyl-4,6-dimethylnonane	-414.70	-427.80
4,6-Dimethylindane	-67.90	-72.60
4,6-Dinitro-m-xylene	-104.90	-80.60
4,6-Dinitro-o-cresol	-281.60	-252.60
4,6-Dinitroresorcinol	-439.50	-424.50
4,7,7-Trimethylbicyclo[4.1.0]heptan-3-ol	-316.40	-320.70
4,7-Dichloroquinoline	49.14	63.80
4,7-Dimethylindane	-69.90	-72.60
4,beta-Dinitrostyrene	19.30	6.10
4-Acetoxycinnamic acid	-685.30	-696.30
4-Acetylanisole	-304.30	-312.00
4-Acetylphenol	-364.30	-351.30
4-Acetylpyridine	-105.20	-97.20
4-Amino-3-azido-1,2,4-triazole	571.50	560.30
4-Amino-3-azido-5-ethyl-1,2,4-triazole	496.14	488.20
4-Amino-3-azido-5-methyl-1,2,4-triazole	483.99	514.50
4-Amino-3-azido-5-phenyl-1,2,4-triazole	667.70	620.10
4-Amino-3-furazanecarboxamidoxime	35.60	21.10
4-Aminoacetanilide	-239.00	-215.80
4-Aminoacetophenone	-182.10	-181.80
4-Aminoazobenzene	319.00	304.70
4-Aminobenzamide	-233.89	-225.40
4-Aminobenzenesulfonamide	-379.80	-362.60
4-Aminobutyric acid	-581.00	-574.40
4-aminodiphenyl ether	-59.00	-56.80
4-Aminohexanoic acid	-646.20	-632.30
4-Aminoindane	-9.80	-35.40
4-Aminophenol	-196.30	-201.70
4-Aminopyridine	39.90	52.50
4-Benzyloxyphenol	-255.10	-267.10
4-Benzylpiperidine	-56.25	-45.90
4-Biphenylamine	81.20	78.20
4-Biphenylmethanol	-121.50	-106.20
4-Bromoacetophenone	-146.96	-131.70
4-Bromoaniline	27.89	20.60
4-Bromoanisole	-109.59	-109.50
4-Bromobenzoic acid	-358.07	-361.80
4-Bromobenzonitrile	156.44	169.90
4-Bromobiphenyl	132.60	128.30

4-Bromonitrobenzene	0.50	31.90
4-Bromophenol	-154.90	-151.50
4-Bromotoluene	12.00	20.10
4-Butoxybenzoic acid	-651.30	-632.90
4-Carboxyphenol	-594.50	-581.40
4-Carboxypyridine N-oxide	-381.39	-377.10
4-Chloro-2,6-dinitroaniline	-77.90	-76.10
4-Chloro-3-nitroaniline	-47.60	-50.50
4-Chloroacetophenone	-182.43	-177.60
4-Chloroaniline	-21.48	-25.40
4-Chloroanisole	-158.36	-155.50
4-Chlorobenzaldehyde	-147.60	-124.00
4-Chlorobenzoic acid	-428.04	-407.60
4-Chlorobenzonitrile	112.57	124.00
4-Chlorobutanoic acid	-570.70	-568.30
4-Chloroethylbenzene	-54.30	-49.50
4-Chloronitrobenzene	-37.46	-14.00
4-Chlorophenol	-198.67	-197.50
4-Chlorophenylacetic acid	-444.83	-434.00
4-Chlorophenylisocyanate	-92.00	-74.60
4-Chloroquinoline	83.80	96.10
4-Chlorotoluene	-30.10	-25.80
4-Chromanone	-292.20	-279.90
4-Cumylphenol	-277.10	-258.10
4-Cyanobenzoic acid	-272.40	-262.50
4-Cyanopyridine	208.30	201.80
4-Cyanopyridine N-oxide	162.80	154.60
4-Cyanothiazole	220.35	210.50
4-Decyne	-31.50	-36.20
4-Dichloromethyl-4-methyl-2,5-cyclohexadienone	-209.20	-170.30
4-Dimethylaminoazobenzene	331.80	319.60
4-Dimethylaminobenzaldehyde	-137.00	-116.00
4-Dimethylaminonitrosobenzene	99.90	100.60
4-Dimethylaminopyridine	49.40	76.30
4-Ethoxyaniline	-172.60	-197.90
4-Ethoxybenzoic acid	-608.90	-580.30
4-Ethyl-3,5-dimethyl-1H-pyrrole-2-carboxaldehyde	-258.50	-228.20
4-Ethylbenzoic acid	-454.50	-438.70
4-Ethyldiphenylmethane	1.20	22.50
4-Ethyl-m-xylene	-84.10	-93.70
4-Ethylnitrobenzene	-58.30	-45.00
4-Ethyl-o-xylene	-86.02	-93.70

4-Ethylphenol	-227.47	-228.50
4-Ethyltoluene	-49.79	-56.80
4-Fluoro-2-nitroacetanilide	-407.90	-396.50
4-Fluoro-2-nitrophenol	-377.20	-365.00
4-Fluoro-3-nitroaniline	-168.30	-192.60
4-Fluoroacetanilide	-373.90	-371.10
4-Fluoroaniline	-161.03	-167.50
4-Fluoroanisole	-306.22	-297.60
4-Fluorobenzoic acid	-550.39	-549.90
4-Fluorobenzonitrile	-50.58	-18.20
4-Fluoronitrobenzene	-186.41	-156.20
4-Fluorophenetole	-327.90	-335.90
4-Fluorophenol	-336.52	-339.60
4-Fluorotoluene	-186.90	-168.00
4-Formylbenzoic acid	-544.91	-510.50
4-Formylmorpholine	-369.90	-374.30
4-Heptanone	-346.20	-353.70
4-Heptylhydroperoxide	-333.80	-356.10
4-Hydroxy-3-methoxybenzaldehyde	-456.30	-466.90
4-Hydroxy-4-methyl-2-pentanone	-530.50	-534.00
4-Hydroxyazobenzene	163.00	132.60
4-Hydroxybenzaldehyde	-297.10	-300.40
4-Hydroxybenzonitrile	-59.10	-52.40
4-Hydroxybiphenyl	-88.40	-93.90
4-Hydroxycoumarin	-482.80	-466.30
4-Hydroxydiphenylether	-243.70	-228.90
4-Hydroxynicotinic acid	-581.10	-538.70
4'-Hydroxypropiophenone	-397.30	-380.30
4-Hydroxypyridine	-146.40	-119.70
4-Hydroxystyrene	-109.67	-101.10
4-Hydroxytetrahydropyran	-493.37	-451.90
4-Iodoaniline	82.61	73.10
4-Iodoanisole	-71.47	-57.00
4-Iodobenzoic acid	-316.10	-309.20
4-Iodobenzonitrile	223.86	222.50
4-Iodonitrobenzene	56.23	84.50
4-Iodophenol	-96.38	-99.00
4-Iodotoluene	67.40	72.70
4-Isopropyl-2-methylphenol	-286.90	-292.40
4-Isopropylbenzoic acid	-483.80	-465.70
4-Isopropylbenzophenone	-124.50	-130.00
4-Isopropylbenzylidine-t-butylamine N-oxide	-158.34	-171.70

4-Isopropyldicyclohexylmethane	-390.70	-380.10
4-Isopropyltropolone	-340.80	-323.40
4-Methoxy-2-nitrobenzoic acid	-580.30	-567.10
4-Methoxy-2-nitrophenol	-366.80	-357.10
4-Methoxyazobenzene	179.30	174.60
4-Methoxybenzaldehyde	-261.80	-258.40
4-Methoxybenzamide	-376.30	-355.50
4-Methoxybenzoic acid	-564.60	-542.00
4-Methoxybenzylideneacetone	-284.10	-255.90
4-Methoxycarbonylhomocubane	-57.80	-29.00
4-Methoxyphenol	-335.51	-331.80
4-Methoxyphenylacetic acid	-578.40	-568.40
4-Methoxypyridine	-75.60	-77.60
4-Methyl-1,3-dioxane	-418.40	-414.90
4-Methyl-1-heptene	-127.70	-130.40
4-Methyl-1-hexene	-101.50	-104.10
4-Methyl-1H-pyrazole	84.30	93.60
4-Methyl-1-octanol	-462.60	-462.90
4-Methyl-1-pentene	-80.04	-80.40
4-Methyl-1-phenyl-2,6,7-trioxabicyclo[2.2.2]octane	-493.40	-527.40
4-Methyl-2,5-diisopropylphenol	-372.30	-382.60
4-Methyl-2,6,7-trioxabicyclo[2.2.2]octane	-574.20	-575.90
4-Methyl-2-phenylpent-2-enenitrile	170.10	118.50
4-Methyl-2-pyrazoline-5-one	-147.00	-141.60
4-Methyl-3,5-diisopropylphenol	-372.30	-380.00
4-Methyl-4-t-butylperoxy-1-triphenylsiloxy-2-butyne	-144.80	-154.90
4-Methylacetophenone	-186.66	-182.30
4-Methylbenzaldehyde	-120.56	-128.70
4-Methylbenzoic acid	-426.27	-412.40
4-Methylbenzoic anhydride	-526.50	-517.10
4-Methylbenzophenone	-82.90	-76.60
4-Methylbenzoyl chloride	-201.30	-196.50
4-Methyl-cis-2-pentene	-87.03	-93.90
4-Methylcyclohexene	-77.50	-78.90
4-Methylcyclopentene	-20.10	-41.20
4-Methyldiamantane	-261.50	-250.10
4-Methyldibenzothiophene	66.20	105.20
4-Methylene-2-oxetanone	-233.73	-243.90
4-Methylheptane	-251.63	-257.80
4-Methylnonane	-304.10	-305.30
4-Methyl-N-phenylaniline	48.40	67.80
4-Methyloctane	-279.60	-284.20

4-Methylpent-3-en-2-one	-220.98	-232.70
4-Methylphenanthrene	97.30	97.20
4-Methylphthalic acid	-830.50	-831.10
4-Methylpiperidine	-126.37	-125.10
4-Methylpyridine	54.59	52.00
4-Methylpyridine N-oxide	5.60	4.80
4-Methylquinoline	91.10	91.40
4-Methylresorcinol	-404.20	-410.70
4-Methylthiazole	66.27	60.80
4-Methyl-trans-2-pentene	-91.55	-99.20
4-Morpholineethanamine	-211.50	-208.60
4-Morpholinepropanamine	-235.30	-234.90
4-N,N-diethylamino-7-nitrobenzofurazan	130.25	127.90
4-N,N-Dimethylamino-7-nitrobenzofurazan	169.83	182.90
4-Nitroacetanilide	-240.57	-221.80
4-Nitroacetophenone	-198.86	-167.90
4-Nitroaniline	-42.00	-18.20
4-Nitrobenzaldehyde	-131.66	-116.90
4-Nitrobenzamide	-244.30	-214.10
4-Nitrobenzenesulfonamide	-373.00	-351.30
4-Nitrobenzofuroxan	200.73	187.80
4-Nitrobenzoic acid	-421.71	-398.00
4-Nitrobenzophenone	-78.30	-64.80
4-Nitrobenzylidene-2-methylphenylacetonitrile	169.50	215.50
4-Nitrobenzylideneaniline	133.15	152.70
4-Nitrobenzylidene-t-butylamine	-45.95	-48.40
4-Nitrobenzylidine-t-butylamine N-oxide	-109.65	-106.50
4-Nitrobiphenyl	40.50	89.50
4-Nitrocatechol	-411.10	-398.90
4-Nitrocinnamic acid	-395.00	-344.50
4-nitrodiphenyl ether	-81.30	-45.40
4-Nitroguaiacol	-379.50	-356.90
4-Nitroimidazole	-7.00	-4.40
4-Nitrophenetole	-225.38	-186.60
4-Nitrophenol	-198.09	-190.30
4-Nitrophenyl azide	308.70	349.70
4-Nitrophthalic acid	-790.30	-819.30
4-Nitrophthalic anhydride	-502.01	-507.10
4-Nitrophthalimide	-341.40	-360.50
4-Nitropyrazole	68.20	90.80
4-Nitropyridine N-oxide	15.39	16.60
4-Nitroso-1-naphthol	-67.40	-47.10

4-Nitrosodiphenylamine	213.00	186.20
4-Nitrosophenol	-90.60	-83.80
4-Nitrostilbene	98.00	143.00
4-Nitrotoluene	-49.48	-18.70
4-Nonanone	-398.30	-406.20
4-Nonylphenol	-387.33	-410.10
4-Nonyne	-4.00	-9.90
4-Octanone	-373.10	-380.00
4-Octyloxybenzoic acid	-773.90	-738.20
4-Octyne	17.30	16.40
4-Oxopentanoic acid	-685.85	-682.80
4-Pentenoic acid	-432.60	-432.60
4-Phenyl-3-butyn-2-one	91.30	72.90
4-Phenylazophenol (hydrazono form)	158.70	119.80
4-Phenylbut-3-en-2-one	-85.50	-89.30
4-Phenylcyclohexene	34.30	26.70
4-Phenyltoluene	58.00	77.70
4-Piperidinecarboxamide	-342.20	-320.50
4-Piperidinemethanol	-330.50	-309.10
4-piperidine-piperidine	-149.64	-121.30
4-Propyltoluene	-75.06	-83.10
4-Quinolinol	-117.30	-80.20
4-t-Butyl-2,6-xylenol	-354.50	-357.30
4-t-Butyl-2-methylphenol	-305.70	-320.40
4-t-Butylbenzoic acid	-502.90	-496.30
4-t-Butyl-benzophenone	-141.30	-160.60
4-t-Butylbiphenyl	-32.10	-3.70
4-t-Butylcatechol	-474.00	-492.10
4-t-Butyldiphenyl ether	-169.40	-141.20
4-t-Butylphenol	-310.50	-286.20
4-Thianone	-230.30	-229.80
4-t-Octylphenol	-392.45	-368.90
4-t-Pentylphenol	-300.85	-311.20
4-Vinylcyclohexene	23.50	19.60
5-(2-Nitrophenyl)-2-furaldehyde oxime	-16.80	-64.60
5-(2-Nitrophenyl)-furan-2-carbaldehyde	-195.57	-184.40
5-(3-Nitrophenyl)-2-furaldehyde oxime	-33.60	-64.60
5-(3-Nitrophenyl)-furan-2-carbaldehyde	-205.17	-184.40
5-(4-Nitrophenyl)-2-furaldehyde oxime	-53.38	-64.60
5-(4-Nitrophenyl)-furan-2-carbaldehyde	-226.17	-184.40
5-(Dimethylamino)-2,4-pentadienal	-130.50	-105.40
5,12-Dihydronaphthacene	106.00	116.20

5,12-Naphthacenedione	-142.80	-134.70
5,5,7,7-Tetramethylundecane	-433.50	-438.30
5,5'-Bitetrazole	531.00	519.50
5,5-Dimethyl-1,3-cyclohexanedione	-486.60	-480.90
5,5-Dimethyl-1,3-dioxan-2-one	-673.40	-673.80
5,5-Dimethyl-1,3-dioxane	-461.30	-442.00
5,5-Dimethylhydantoin	-533.30	-528.50
5,5'-Hydrazotetrazole	565.64	552.60
5,5'-Methylenebis(2-keto-1,3-dimethyltetrahydro-1,3,5-triazine	-399.40	-397.10
5,5'-Spirobi-1,3-dioxane	-705.50	-700.80
5,6,6a,7,8,12b,12c-Octahydrodibenzo[a,i]biphenylene	72.33	96.80
5,6,7,8-Tetrahydroquinoline	9.90	-2.10
5,6-Dihydro-6-methyluracil	-520.50	-514.30
5,6-Dimethoxy-1-indanone	-476.90	-459.50
5,6-Dimethylchrysene	132.50	111.30
5,6-Dimethyluracil	-510.90	-487.60
5,7-Dibromo-8-hydroxyquinoline	-43.90	-52.80
5,7-Dichloro-8-hydroxyquinoline	-158.90	-144.70
5,7-Diiodo-8-hydroxyquinoline	92.70	52.30
5,7-Dodecadiyne	181.50	181.90
5,8-Dimethylbenzo[c]phenanthrene	101.40	96.50
5-Acetamidotetrazole	-5.00	-8.50
5-Acetyl-2-thiophenecarboxylic acid	-550.66	-544.20
5-Amino-1H-1,2,4-triazole	76.80	85.80
5-Amino-1-methyltetrazole	193.64	194.50
5-Amino-1-phenyltetrazole	311.08	312.00
5-Amino-2-mercaptobenzimidazole	19.10	18.50
5-Amino-2-methyltetrazole	206.80	203.60
5-Amino-3,4-dimethyl-isoxazole	-84.70	-62.10
5-Aminohexanoic acid	-643.30	-635.00
5-Aminoindane	-16.20	-35.40
5-Aminoindazole	144.30	137.90
5-Aminoquinoline	102.50	94.50
5-Aminotetrazole	207.30	195.10
5-Aminouracil	-442.90	-479.60
5-Aminovaleric acid	-604.10	-600.80
5-Azabicyclo[2.1.0]pentane	225.60	182.00
5-Azidomethyl-3-nitrooxazolidine	195.00	152.40
5-Bromoindole	93.20	98.30
5-Bromoindoline	48.40	35.50
5-Butyldocosane	-713.50	-721.20
5-Carboxy-1,2,3-triazole	-246.30	-238.30

5-Chloro-2-methylbenzoxazole	-147.20	-116.10
5-Chloro-2-nitroaniline	-69.20	-50.70
5-Chloro-6-hydroxynicotinic acid	-616.80	-621.80
5-Chloro-8-hydroxyquinoline	-126.10	-112.50
5-Chlorobenzofurazan N-oxide	196.35	203.60
5-Chlorouracil	-441.50	-437.90
5-Cyano-5H-dibenzo[a,d]cycloheptene	314.50	279.40
5-Cyanotetrazole	402.00	361.20
5-Decyne	-32.70	-36.20
5-Diallylaminotetrazole	351.27	350.70
5-Dimethylaminotetrazole	182.54	207.40
5-Ethyl-2-thiophenecarboxaldehyde	-135.30	-137.60
5-Ethyl-5-methylhydantoin	-565.22	-554.80
5-Ethylidene-2-norbornene	102.20	58.00
5-Ethyl-m-xylene	-87.78	-91.10
5-Ethylnorbornane	-144.10	-151.20
5-Fluoro-2-methylbenzothiazole	-110.80	-103.70
5-Fluoro-2-methylbenzoxazole	-257.80	-258.40
5-Guanylaminotetrazole	169.57	183.40
5-Hexen-3-one	-201.70	-202.50
5-Hexyl-2-methyl-m-dioxane	-574.70	-577.60
5-Hydroxy-1,3-dioxane	-569.30	-575.20
5-Hydroxyisoquinoline	-89.80	-82.90
5-Hydroxymethylene-5H-6,7-dihydrodibenzo[a.c]cyclohepten-6-one	-204.40	-219.60
5-Hydroxynicotinic acid	-537.60	-538.50
5-Hydroxypentanal	-480.23	-457.80
5-Indanol	-210.10	-207.50
5-Iodosalicylic acid	-512.50	-518.10
5-Isopropyl-2-methylphenol	-274.90	-287.10
5-Isopropylbarbituric acid	-719.51	-746.60
5-Isopropyl-m-cresol	-300.00	-292.40
5-Methoxy-1-tetralone	-346.70	-331.20
5-Methoxybenzofurazan	55.53	74.10
5-Methoxybenzofurazan N-oxide	49.93	27.20
5-Methoxyindane	-161.10	-165.50
5-Methoxytetrazole	69.10	59.90
5-Methyl-1-heptene	-127.70	-127.80
5-Methyl-1-hexene	-103.19	-104.10
5-Methyl-2,3-dihydrofuran	-167.00	-203.10
5-Methyl-2-phenylhex-2-enenitrile	142.20	93.50
5-Methyl-2-phenyltetrazole	271.77	256.50
5-Methyl-2-thiophenecarboxyaldehyde	-96.10	-111.30

5-Methyl-2-thiophenecarboxylic acid	-398.28	-392.40
5-Methyl-2-thiouracil	-223.10	-203.00
5-Methyl-3-phenylisoxazole	10.20	31.50
5-Methyl-5-phenylhydantoin	-381.90	-425.50
5-Methylaminotetrazole	202.64	196.20
5-Methylbenzofuroxan	174.03	190.70
5-Methylcaprolactam	-363.83	-360.60
5-Methylhydantoin	-486.92	-483.40
5-Methylisoxazole	-28.44	-22.50
5-Methylisoxazole-3-carboxylic acid	-428.20	-453.40
5-Methyl-N-nitro-1H-1,2,4-triazol-3-amine	52.00	85.70
5-Methylnonane	-310.00	-302.60
5-Methylresorcinol	-404.10	-410.70
5-Methyltetrazole	183.50	211.50
5-Methyluracil	-455.73	-450.70
5-Nitro-8-hydroxyquinoline	-118.60	-105.30
5-Nitroaminotetrazole	251.60	240.70
5-Nitrofurancarboxylic acid	-516.80	-511.40
5-Nitrofurfural	-228.90	-227.70
5-Nitroimidazole	-7.00	-4.40
5-Nitroindole	23.80	59.60
5-Nitroindoline	-19.80	-3.30
5-Nitro-o-toluidine	-91.19	-55.10
5-Nitrouracil	-424.30	-453.60
5-Nonanone	-398.24	-406.20
5-Phenyl-1,2-dithiole-3-thione	182.80	183.30
5-Phenylaminotetrazole	305.22	293.00
5-Phenylisoxazole	77.50	80.50
5-Phenyltetrazole	295.53	314.40
5-Tetrazolylurethane	-220.23	-223.70
6,10,14-Trimethylpentadeca-3,5-diene-2-one	-455.20	-436.10
6,10-Dimethyl-2-undecanone	-562.70	-509.00
6,10-Dimethyl-4,5,9-undecatrien-2-one	-197.70	-157.70
6,13-Pentacenedione	-80.10	-98.00
6,19-Methanobenzocyclooctadecen-21-one	-272.10	-253.80
6,6-(Tetramethylene-3´-oxa)-7a-(nitroxymethyl)-3-oxoperhydroimidazo[1,5-c]oxazol-6-ium nitrate	-841.10	-849.40
6,6-(Tetramethylene-3´-oxa)-7a-methyl-3-oxoperhydroimidazo[1,5-c]oxazol-6-ium nitrate	-781.41	-764.10
6,6-Dimethyl-1,5,7-trioxaspirononane	-528.33	-511.40
6,6-Dimethyl-1-phenyl-4,8-dioxaspiro[2.5]octane	-302.40	-303.60
6,6-Dimethylfulvene	87.00	84.60

6,6'-Diphenylfulvene	297.40	298.40
6,6-Paracyclophane	-201.90	-178.50
6,6-Pentamethylene-7a-(nitroxymethyl)-3-oxoperhydroimidazo[1,5-c]oxazol-6-ium nitrate	-739.29	-746.70
6,7-Dihydro-4(5H)-benzofuranone	-298.30	-281.90
6-Amino-1,3-dimethyluracil	-463.10	-451.00
6-Amino-1-hexanol	-400.00	-371.90
6-Amino-1-methyluracil	-473.90	-456.60
6-Aminohexaneamide	-400.70	-385.90
6-Aminoindazole	138.50	137.90
6-Aminopurine	97.18	114.90
6-Aminoquinoline	97.30	91.90
6-Aminouracil	-479.80	-467.40
6-Aza-2-thiothymine	-91.63	-124.40
6-Azathymine	-326.44	-372.20
6-Azauracil	-287.85	-323.20
6-Chloro-2-benzoxazolone	-348.20	-344.20
6-Chloro-2-hydroxypyridine	-197.40	-206.70
6-Chloroquinoline	83.60	96.10
6H-Dibenzo[b,d]pyran	-68.20	-33.10
6-Hydroxyhexanoic acid	-786.00	-767.80
6-Hydroxynicotinic acid	-592.10	-589.50
6-Methoxy-1-indanone	-319.50	-292.90
6-Methoxy-alpha-tetralone	-351.70	-331.20
6-Methoxyquinoline	-32.42	-38.20
6-Methoxyquinoline N-oxide	-91.02	-76.10
6-Methyl-1-heptene	-129.50	-130.40
6-Methyl-1-indanone	-183.70	-165.90
6-Methyl-2-pyridone	-214.50	-163.40
6-Methyl-2-thiouracil	-237.90	-202.00
6-Methylquinoline	89.80	91.40
6-Methyluracil	-475.23	-449.80
6-Oxabicyclo[3.1.0]hexane	-132.80	-113.70
6-Phenyluracil	-333.41	-341.60
6-t-Butyl-1,1-dimethylindane	-174.30	-177.50
6-Undecanone	-448.10	-458.90
7,12-Dimethylbenz[a]anthracene	143.80	113.90
7,7,8,8-Tetracyanoquinodimethane	661.60	661.60
7,7-Dimethoxybicyclo[2.2.1]heptane	-423.80	-433.00
7-Bromo-5-chloro-8-hydroxyquinoline	-129.80	-98.80
7-Hexadecylspiro[4.5]decane	-601.60	-623.00
7-Methoxy-1-tetralone	-349.40	-331.20

7-Methyl-1-octene	-154.50	-156.70
7-Methylcaprolactam	-365.40	-348.60
7-Norbornanone	-184.10	-235.30
7-Oxabicyclo[4.1.0]heptane	-167.80	-154.00
7-Phenylhept-4-yn-3-one	-56.50	-6.10
8-Aminocaprylic acid	-697.40	-679.80
8-Aminoquinoline	91.20	91.90
8-Methyl-1-nonanol	-483.13	-491.90
8-Methyl-1-nonene	-180.20	-183.00
8-Methylquinoline	103.23	91.40
8-Oxabicyclo[5.1.0]octane	-215.90	-183.50
8-Oxatricyclo[3,2,1,0(1,5)]octane	-298.20	-264.90
8-Oxypurine	-64.40	-109.30
8-Quinolinol	-82.10	-80.20
9(10H)-Anthracenone	-79.92	-45.90
9,10(3',4')-Furanoanthracene-12,14-dione	-424.00	-432.70
9,10-Anthraquinone	-188.00	-171.40
9,10-Benzophenanthrene	151.80	161.30
9,10-Dihydroanthracene	66.40	79.60
9,10-Dihydrophenanthrene	78.70	82.20
9,10-Dimethylanthracene	64.70	66.10
9,10-Dimethylphenanthrene	47.70	57.20
9,10-Diphenylanthracene	308.70	248.90
9,10-Octalin	-149.10	-136.20
9,10-Phenanthrenediol	-248.10	-295.10
9,9'-Dimethyl-9,9'-bifluorenyl	194.10	202.20
9,9-Dimethylxanthene	-103.50	-81.50
9-Acridinamine	159.20	136.50
9-Anthroic acid	-272.20	-302.10
9-Cyclohexyleicosane	-685.60	-677.90
9-Ethyl-9H-carbazole	65.60	110.80
9-Fluorenecarboxylic acid	-310.90	-301.00
9H-Fluoren-9-one	-40.30	-19.60
9-Hydroxy-1,4-anthraquinone	-383.70	-356.20
9-Methoxy-1,4-anthraquinone	-297.90	-296.60
9-Methyl-9H-fluorene	60.40	80.90
9-Methylanthracene	92.00	85.10
9-Nitroanthracene	107.00	91.60
9-Oxatetracyclo[5.4.0.0(3,10).0(4,8)]undeca-5-en-2-one	-246.10	-240.20
9-Phenyl-9-phosphafluorene	184.28	193.70
9-Phenyleicosane	-487.60	-486.60
Abametapir	109.60	110.00

Acenaphthene	66.62	59.40
Acenaphthylene	182.84	156.20
Acetadote	-740.30	-708.30
Acetaldehyde	-192.43	-194.80
Acetaldehyde thiosemicarbazone	62.62	31.50
Acetaldol	-433.62	-440.90
Acetaldoxime	-77.60	-75.00
Acetamide	-311.10	-294.60
Acetanilide	-209.20	-196.70
Acetic acid	-484.45	-478.50
Acetic anhydride	-625.72	-649.40
Acetoacetanilide	-403.33	-375.10
Acetoacetyldiphenylamine	-233.60	-262.00
Acetol	-414.15	-432.40
Acetone di-n-propylhydrazone	-68.00	-76.80
Acetone ethylisopropylhydrazone	-57.20	-52.10
Acetone glycerol	-682.45	-645.90
Acetone isopropylhydrazone	-24.30	-43.70
Acetone methylhydrazone	38.70	14.30
Acetone oxime	-130.40	-124.00
Acetone propylhydrazone	-9.30	-40.80
Acetone thiosemicarbazide	19.23	-17.50
Acetonitrile	40.60	53.20
Acetophenone	-142.64	-142.80
Acetophenone ethylene glycol ketal	-343.80	-337.10
Acetophenone oxime	-23.30	-18.40
Acetovanillone	-529.40	-517.90
Acetoxyacetic acid	-879.70	-856.60
Acetoxyl	-374.22	-391.70
Acetylacetone	-425.90	-426.40
Acetylacetone (enol form)	-427.60	-438.40
Acetylchloride	-272.00	-262.60
Acetylfluoride	-442.70	-422.00
Acetylmesitylene	-271.40	-250.70
Aconitic acid	-1233.80	-1252.90
Acrylamide	-212.08	-193.50
Acrylic acid	-379.36	-380.00
Actiprofen	-547.62	-548.00
Adamantane	-192.55	-188.70
Adiponitrile	84.90	97.70
Adipoyl-bis(N,N-diethylthiourea)	-652.40	-651.10
Adrucil	-578.10	-554.30

AIBN	242.79	243.50
Alanine anhydride	-499.94	-509.90
Alanylalanine	-811.30	-813.60
Alanylglycine	-780.07	-784.30
Alanylphenylalanine	-712.34	-731.60
Allantoin	-719.15	-721.50
all-trans-1,5,9-Cyclododecatriene	21.70	4.80
Allyl acetate	-386.30	-376.20
Allyl alcohol	-171.10	-182.10
Allyl bromide	12.20	12.80
Allyl chloride	-30.80	-35.30
Allyl chloroacetate	-386.50	-413.50
Allyl cyanide	117.70	125.40
Allyl dichloroacetate	-393.10	-414.90
Allyl ethyl sulfide	-32.35	-23.50
Allyl ethyl sulfoxide	-175.15	-182.50
Allyl iodide	55.20	57.20
Allyl phenyl ether	-41.20	-63.00
Allyl sulfide	73.80	75.10
Allylamine	-10.00	15.90
Allylcyclopentane	-64.50	-65.80
Allylmethylsulfone	-385.10	-378.90
Allyl-t-butylsulfide	-91.00	-92.00
Allyltrichloroacetate	-395.30	-417.50
alpha,alpha'-Dihydroxy-4-diisopropylbenzene	-547.90	-548.60
alpha,alpha-Dimethyl-p-isopropylbenzyl alcohol	-348.60	-340.30
alpha,beta-Glucooctanoic-1,4-lactone	-1690.60	-1676.90
Alpha-caran-3-ol	-333.80	-310.40
alpha-Chlorohydrin	-525.30	-537.60
alpha-D-Glucose	-1273.76	-1276.40
alpha-Fluorocinnamic acid	-478.10	-463.30
alpha-Ionone	-282.00	-275.40
alpha-Methylstyrene	70.46	69.70
alpha-Phellandrene	-60.00	-64.00
alpha-Phenyl-1-piperidinoacetonitrile	92.30	98.80
alpha-Pinene	-16.40	-17.70
alpha-Santonin	-590.60	-576.00
Amfepramone	-200.90	-209.80
AMFOX-7	-219.50	-205.90
Aminomethylcumulene	198.30	154.30
Ammelide	-494.40	-477.80
Amyl acetate	-553.00	-553.70

Amyl formate	-493.28	-494.00
Amylamine	-152.62	-161.50
Amylbenzene	-89.50	-96.30
Androstane	-314.01	-359.40
Androsterone	-678.26	-686.90
Anethole	-109.40	-104.10
Angelic acid	-455.94	-462.80
Aniline	20.80	6.90
Aniline-4-sulfonic acid	-614.89	-608.60
Anthracene	111.58	116.70
Anthracene photodimer	283.20	233.70
Anthracene-9,10-endoperoxide	82.90	41.80
anti-Tricyclo[4.2.0.02.5]octane	165.70	158.10
Arabinose	-1058.60	-1056.60
Arabinosylhypoxanthine	-851.65	-863.40
Arabitol	-1125.00	-1123.90
Arachic alcohol	-751.90	-754.40
Arachidic acid	-940.00	-952.30
Asparagine	-789.21	-783.20
Aspartic acid	-973.30	-967.10
Aspirin	-761.33	-755.10
Atrazine	-125.40	-118.30
Azepane	-92.10	-121.80
Azidobenzene	344.30	374.80
Aziridine	91.90	89.30
Azocane	-102.30	-128.20
Azocyclohexane N,N'-dioxide	-246.50	-212.70
Azodicarbonamide	-292.66	-320.70
Azomethane	148.80	150.40
Azomethane N,N'-dioxide	0.80	-14.90
Azo-t-butane N,N'-dioxide	-209.60	-211.70
Barbital	-756.31	-773.90
Barbituric acid	-645.53	-660.90
Behenyl alcohol	-791.50	-807.00
Benzal 3-hydrazino-5-methyl-1,2,4-triazole	254.20	250.90
Benzal diacetate	-777.10	-783.60
Benzalacetophenone	3.50	14.90
Benzalaniline	180.54	177.80
Benzaldehyde	-87.00	-91.80
Benzaldehyde diethylacetal	-372.80	-386.30
Benzaldehyde dimethyl acetal	-308.40	-309.80
Benzamide	-202.60	-189.00

Benzanilide	-93.40	-91.10
Benzanthrone	-10.20	19.80
Benzene	49.00	43.30
Benzenecarbothioamide	41.50	38.30
Benzenesulfonamide	-322.79	-326.20
Benzenethiol	63.70	49.40
Benzhydryl chloride	52.10	42.00
Benzil	-154.00	-194.20
Benzil dioxime	48.30	54.60
Benzimidazole	79.50	111.90
Benzimidazole-2-thione	45.70	76.10
Benzo[a]anthracene	170.80	156.10
Benzo[a]phenanthrene	145.30	158.70
Benzo[a]pyrene	167.50	178.30
Benzo[b]furan	-37.50	-25.50
Benzo[b]triphenylene	184.80	198.00
Benzo[c]cinnoline	275.40	294.60
Benzo[c]phenanthrene	184.90	165.00
Benzo[e]pyrene	154.10	180.90
Benzo[f]quinoline	150.60	167.70
Benzo[g,h,i]perylene	149.90	200.60
Benzo[g]quinoline	144.70	165.00
Benzo[h]quinoline	160.20	165.00
Benzo[k]fluoranthene	172.40	205.60
Benzo-15-crown-5	-787.90	-807.90
Benzocyclobutane	152.70	111.50
Benzofuran-2-carboxylic acid	-458.10	-461.70
Benzofurazan	233.80	275.30
Benzofurazan N-oxide	216.90	228.50
Benzoic acid	-385.31	-375.50
Benzoic anhydride	-435.52	-443.40
Benzoin	-252.90	-262.20
Benzonitrile	163.18	156.20
Benzophenone	-34.50	-30.90
Benzophenone anilide	246.06	229.30
Benzophenoneimine	168.24	196.30
Benzothiazole	141.30	156.80
Benzotrifluoride	-636.70	-608.40
Benzoyl chloride	-158.00	-159.60
Benzoyl nitrile	36.30	4.40
Benzoyl(cyclohexyloxy)-carbonylperoxide	-746.79	-774.70
Benzoylacetonitrile	4.70	-21.90

Benzoylformic acid	-487.22	-524.60
Benzoylglycylglycine	-831.23	-824.30
Benzyl acetate	-368.80	-371.70
Benzyl alcohol	-154.90	-177.60
Benzyl benzoate	-273.10	-268.70
Benzyl chloroformate	-376.20	-394.60
Benzyl ethyl ether	-165.78	-173.00
Benzyl formate	-305.13	-312.00
Benzyl isonitrile	222.40	211.70
Benzyl mercaptan	43.54	31.50
Benzyl methyl sulfone	-374.37	-374.40
Benzylamine	31.50	23.00
Benzylaniline	101.40	82.10
Benzylazide	365.50	353.60
Benzylbromide	22.00	17.30
Benzyldiphenylamine	178.10	189.70
Benzylethylamine	-11.60	-10.10
Benzylethylsulfide	-5.20	-19.00
Benzylidenemalonic acid	-718.70	-741.70
Benzylidene-p-isopropylphenylacetonitrile	226.90	187.20
Benzylmethylsulfide	26.20	8.50
beta-Alanine	-559.50	-548.10
beta-Butylaminoisobutyronitrile	-82.10	-71.90
beta-Cyanopropiophenone	-74.90	-48.20
beta-D-Fructose	-1268.96	-1285.30
beta-HCH	-396.66	-404.80
beta-Hydroxynaphthaldehyde	-266.40	-261.30
beta-Isopropyl-beta-propiolactone	-424.70	-392.00
beta-Nitrostyrene	27.74	31.10
beta-Resorcylic acid	-800.37	-792.90
Bicyclo[1.1.0]butane	193.70	143.40
Bicyclo[2.2.2]oct-2-ene	-26.60	-67.30
Bicyclo[2.2.2]octan-2-one	-277.30	-298.80
Bicyclo[2.2.2]octane	-150.40	-173.30
Bicyclo[3.1.0]hexane	38.60	-0.90
Bicyclo[3.2.2]non-6-ene	-55.20	-89.70
Bicyclo[3.3.1]non-2-ene	-101.00	-93.60
Bicyclo[3.3.1]nonane	-182.00	-188.10
Bicyclo[3.3.2]decane	-168.50	-209.10
Bicyclo[4.2.1]non-3-ene	-49.80	-79.70
Bicyclo[4.4.1]undeca-1,3,5,7,9-pentaene	254.20	266.30
Bicyclo[4.4.1]undeca-1,3,5,8-tetralene	182.40	176.50

Bicyclo[5.1.0]octane	-60.20	-64.90
Bicyclohexane	-273.70	-273.40
Bicyclopentyl	-178.90	-176.80
Bicyclopropyl	93.40	90.90
Biphenyl	120.62	114.60
Bis(1-ethylhexyl) o-phthalate	-1093.90	-1116.30
Bis(2,2,2-Trinitroethyl)urea	-304.43	-320.50
Bis(2,4,5-trimethylphenyl)-methane	-154.40	-138.10
Bis(2-chloroethyl) ether	-356.20	-350.50
Bis(2-ethylhexyl) adipate	-1265.30	-1264.90
Bis(2-hydroxyethyl)methylamine	-473.77	-476.20
Bis-(2-hydroxyethyl)terephalate	-1200.00	-1154.60
Bis(2-methylbenzoyl) peroxide	-500.50	-465.40
Bis(2-methylphenoxy)dimethylsilane	-583.40	-588.00
Bis(2-methylphenyl)acetylene	234.80	253.90
Bis(3-methylphenoxy)dimethylsilane	-584.40	-582.80
Bis(4-aminophenyl) ether	-65.30	-93.20
Bis(4-methylbenzoyl) peroxide	-451.50	-465.40
Bis(4-methylphenoxy)dimethylsilane	-584.40	-588.00
Bis(4-methylphenyl)acetylene	225.80	253.90
Bis(chloromethyl)ether	-280.58	-276.20
Bis(cyclohexyl)amine	-226.80	-230.10
Bis(hydroxymethyl)urea	-718.60	-699.40
Bis(isopropyl) peroxide	-272.00	-292.80
Bis(piperidino)methane	-151.23	-129.10
Bis(trifluoroacetyl)methane	-1623.00	-1656.00
Bisphenol A	-374.76	-386.50
Bisphenol AP	-255.60	-283.40
Bisphenol E	-351.80	-359.80
Bisphenol F	-332.80	-334.10
Brassidic acid	-895.98	-898.80
Bromobenzene	60.90	57.00
Bromoethane	-90.50	-85.70
Bromopentafluoroethane	-1064.40	-1062.80
Bromoxynil octanoate	-347.90	-353.80
Brucine	-496.55	-539.90
Bullvalene	258.67	235.20
But-1-enyl-4-tolyl sulphone	-340.30	-332.50
Butadiyne	472.80	454.50
Butanal	-239.90	-247.40
Butanamide	-365.89	-347.20
Butane	-147.30	-149.20

Butane-1,2,3-triyl trinitrate	-400.50	-408.50
Butane-1,2,4-triol	-745.10	-710.90
Butanedioic acid	-938.15	-913.00
Butanoic acid	-534.19	-531.10
Butanoic anhydride	-719.12	-754.60
Butyl 1,1-dimethylpropyl ether	-432.70	-427.40
Butyl 2-methylbutyrate	-613.60	-607.00
Butyl 2-pentenoate	-498.80	-500.20
Butyl 3-pentenoate	-486.50	-500.20
Butyl 4-oxopentanoate	-740.40	-731.70
Butyl 4-pentenoate	-479.80	-481.50
Butyl anthranilate	-447.00	-461.10
Butyl butanoate	-575.39	-580.00
Butyl crotonate	-467.80	-473.90
Butyl ethyl sulfide	-172.27	-174.60
Butyl isopropylamine	-206.70	-192.30
Butyl lactate	-732.60	-747.20
Butyl oleate	-816.90	-842.40
Butyl pentanoate	-613.30	-606.20
Butyl pivalate	-620.40	-613.90
Butyl propanoate	-549.90	-553.70
Butyl t-butyl ether	-407.10	-403.70
Butyl vinyl ether	-221.80	-236.00
Butylbenzene	-63.20	-70.00
Butylcellosolve	-497.40	-512.60
Butylcyclohexane	-263.09	-263.90
Butylcyclopentane	-214.20	-214.30
Butylene carbonate	-642.15	-628.40
Butylisobutyl amine	-219.70	-221.70
Butylisopropylether	-365.64	-364.40
Butylmethylamine	-143.80	-136.90
Butylmethylketone	-322.01	-327.40
Butylsilane	-108.86	-105.60
Butyraldehyde n-butylhydrazone	-48.00	-70.80
Butyric acid methylester	-499.00	-489.10
Butyronitrile	-5.80	0.50
Butyrophenone	-188.90	-198.10
Caffeic acid	-753.10	-736.50
Caffeine	-325.21	-363.40
Camphene	-77.50	-68.50
Camphor	-315.20	-311.70
Camphor oxime	-172.00	-187.30

Capraldehyde	-393.84	-405.30
Caproic aldehyde	-291.83	-300.10
Caprolactone	-477.36	-480.80
Caprylamide	-476.88	-452.50
Caprylene	-124.50	-129.70
Caprylic aldehyde	-334.94	-352.80
Caprylonitrile	-107.30	-104.70
Carane	-118.50	-107.30
Carbanilide	-121.47	-128.30
Carbazole	121.13	127.50
Carbitol	-626.28	-639.40
Carbon suboxide	-122.20	-86.60
Carbonothioic dihydrazide	106.80	124.90
Carvenone	-330.20	-295.50
Cellobiose	-2229.50	-2251.70
Cetane	-456.30	-465.10
Cetene	-330.50	-340.30
Cetylmethacrylate	-742.81	-782.50
Chloral	-218.10	-236.10
Chloranil	-296.90	-296.30
Chlorfenidim	-277.80	-246.00
Chloroacetaldehyde	-252.69	-232.10
Chloroacetamide	-338.50	-331.90
Chloroacetic acid	-512.35	-515.80
Chloroacetic acid ethylester	-509.30	-511.90
Chloroacetyl chloride	-285.00	-299.90
Chlorobenzene	11.50	11.10
Chlorocyclohexane	-210.70	-199.10
Chloroethane	-136.80	-133.90
Chloroethylene	0.90	14.40
Chlorohydroquinone	-398.30	-406.00
Chloromethyl methyl ether	-236.00	-237.90
Chloromethylacetate	-467.90	-474.80
Chloropentafluorobenzene	-850.40	-861.00
Chloroxynil	-171.60	-116.80
Cholest-5-en-3-ol	-674.80	-656.80
Cholesteryl methyl ether	-664.10	-636.50
Cinnamamide	-110.40	-132.90
cis- Cyclooctene	-74.00	-92.90
cis,cis-1,4-Diphenylbutadiene	198.80	216.20
cis-1-(Cyclohexylmethyl)-2-ethylcyclohexane	-362.90	-346.50
cis-1-(Cyclohexylmethyl)-4-ethylcyclohexane	-362.90	-349.10

cis-1,2-Bis(2-tolyl)ethylene	74.90	91.70
cis-1,2-Cyclohexanedicarboxylic acid	-997.30	-981.00
cis-1,2-Cyclohexanediol	-555.20	-544.90
cis-1,2-Cyclohexanol diacetate	-945.30	-933.20
cis-1,2-Cyclopentanediol	-490.00	-495.30
cis-1,2-Dichloroethylene	4.60	-18.10
cis-1,2-Diethylcyclopropane	-79.90	-80.00
cis-1,2-Dimethyl-3,3-dichlorocyclopropane	-86.80	-85.10
cis-1,2-Dimethylcyclohexane	-211.80	-210.70
cis-1,2-Dimethylcyclopentane	-165.27	-165.10
cis-1,2-Dimethylcyclopropane	-26.30	-29.90
cis-1,2-Diphenylcyclopropane	173.50	176.10
cis-1,2-Divinylcyclobutane	124.30	130.40
cis-1,2-Indandiol diacetate	-779.50	-774.20
cis-1,2-Indanediol	-382.10	-385.90
cis-1,3-Dimethylcyclohexane	-219.25	-217.30
cis-1,3-Dimethylcyclopentane	-171.30	-167.70
cis-1,4-Cyclohexanedicarboxylic acid	-999.40	-981.00
cis-1,4-Dimethylcyclohexane	-215.60	-213.40
cis-1,4-Di-t-butyl-cyclohexane	-371.70	-374.70
cis-1-Ethyl-2-methylcyclohexane	-240.30	-238.30
cis-1-Ethyl-2-methylcyclopentane	-190.80	-188.80
cis-1-Ethyl-3-methylcyclohexane	-247.10	-240.90
cis-1-Ethyl-3-methylcyclopentane	-194.40	-193.40
cis-1-Ethyl-4-Methylcyclohexane	-238.90	-237.00
cis-1-Methyl-1,2-cyclohexanediol	-611.60	-580.20
cis-1-Methyl-1,2-dicyclopropylcyclopropane	130.00	124.20
cis-1-Phenyl-3,3-dimethyl-1-butene	10.00	-15.20
cis-1-Phenylcyclopropane-2-carboxylic acid	-320.70	-311.40
cis-2,2,4,6-Tetramethyl-1,3-dioxane	-543.00	-538.00
cis-2,2,5,5-Tetramethyl-3-hexene	-168.10	-188.00
cis-2,2-Dimethyl-3-hexene	-140.00	-142.00
cis-2,3-Dimethylthiirane	-24.16	2.50
cis-2,4,6-Trimethyl-1,3,5-trioxane	-681.80	-639.80
cis-2,4-Dimethyl-1,3-dioxane	-468.00	-454.80
cis-2,5-Dimethyl-3-hexene	-151.04	-140.90
cis-2-Butene	-29.66	-43.20
cis-2-Butene-1,4-diol	-372.90	-411.10
cis-2-Decalone	-358.70	-351.50
cis-2-Heptene	-105.14	-122.20
cis-2-Hexahydroindanone	-310.80	-306.50
cis-2-Hexene	-83.89	-95.80

cis-2-Methoxycinnamic acid	-478.40	-488.60
cis-2-Methyl-5-ethyl-1,3-dioxane	-522.30	-472.40
cis-2-Methylcyclohexanol	-378.36	-377.20
cis-2-Methylcyclopentanol	-334.80	-331.50
cis-2-Octene	-135.69	-148.50
cis-2-Pentene	-53.35	-69.50
cis-2-pentenenitrile	69.90	80.30
cis-3,4-Dihydro-3,4-dimethyl-2,5-furandione	-660.30	-652.80
cis-3,4-Dimethyl-2-pentene	-124.80	-134.30
cis-3,5-Dimethylpyrrolizidine	-118.30	-102.40
cis-3-Decen-1-yne	99.20	100.70
cis-3-Heptene	-104.31	-122.20
cis-3-Hexene	-78.90	-95.80
cis-3-Methyl-3-hexene	-119.20	-133.60
cis-3-Methylcyclohexanol	-399.90	-381.10
cis-3-Octene	-132.30	-148.50
cis-3-Penten-1-yne	226.48	232.40
cis-4,4-Dimethyl-2-pentene	-105.31	-115.60
cis-4,4'-Dimethylstilbene	97.90	89.10
cis-4,4'-Dinitrostilbene	69.50	112.70
cis-4,6-Dimethyl-1,3-dioxane	-477.50	-453.40
cis-4-Coumaric acid	-514.00	-530.60
cis-4-Hydroxycyclohexanecarboxylic lactone	-469.00	-478.60
cis-4-Methoxycinnamic acid	-457.50	-488.60
cis-4-Methylcyclohexanol	-418.44	-377.20
cis-4-Octene	-131.90	-148.50
cis-8-Methyl-2-hydrindanone	-352.10	-337.20
cis-9-Methyldecalin	-248.60	-254.00
cis-Azobenzene	358.72	341.10
cis-Benzaldehyde oxime	25.00	27.90
cis-Bicyclo[3.3.0]octan-2-one	-287.70	-284.90
cis-Bicyclo[3.3.0]octane	-136.30	-136.10
cis-Bicyclo[4,2,0]octane	-68.10	-75.30
cis-Bicyclo[4.3.0]nona-3,7-diene	60.80	26.40
cis-Bicyclo[6.1.0]nonane	-81.00	-81.00
cis-Butenedinitrile	268.20	256.40
cis-Cyclodecene	-120.20	-135.90
cis-Cyclohexane-1,2-dicarboxylic anhydride	-665.50	-664.20
cis-Cyclohexane-1,3-dicarboxylic acid	-997.30	-978.40
cis-Cyclohexane-1,3-dicarboxylic acid dimethyl ester	-850.50	-897.00
cis-Cyclononene	-82.30	-110.90
cis-Decalin	-219.40	-226.00

cis-Dihydro-3,4-diethyl-2,5-furandione	-696.60	-703.40
cis-Heptacyclene	269.30	291.50
cis-Hydrindan	-173.10	-176.40
cis-Octahydroinden-1-one	-331.20	-315.80
cis-Oxolane-3,4-diol diacetate	-981.20	-1007.30
cis-Oxolane-3,4-diol dinitrate	-407.70	-413.00
cis-Perfluorobicyclo[4.3.0]nonane	-3170.70	-3173.90
cis-Stilbene	183.30	162.80
cis-Tetralin-1,2-diol	-425.60	-426.20
Citraconic acid	-824.80	-844.70
Citric acid monohydrate	-1839.70	-1823.70
Colamine	-274.47	-266.50
Coproporphyrin I tetramethylester	-1458.30	-1483.40
Cortisone	-1067.60	-1087.40
Coumarin	-262.80	-223.00
Creatine	-536.00	-543.80
Creatinine	-216.37	-221.60
Crotonaldehyde	-138.70	-141.40
Crotonic acid	-430.82	-425.10
Cryofluorane	-960.20	-915.00
Cubane	539.00	526.70
Cumene	-41.13	-49.60
Cumulene	191.04	185.40
Cumyl chloride	-76.00	-100.30
Cumyl perbenzoate	-237.53	-275.90
Cumylhydroperoxide	-161.68	-187.10
Cyanamide	58.80	49.00
Cyanoacetamide	-176.40	-171.20
Cyanoacetic acid	-356.30	-354.90
Cyanoethylene	147.10	151.80
Cyanoguanidine	21.30	37.30
Cyanuric acid	-704.55	-699.10
Cyclam	-121.00	-104.40
Cyclobutane	3.18	-7.30
Cyclobutane-1,3-dione	-261.10	-258.20
Cyclobutanecarbonitrile	103.00	112.90
Cyclobutanecarboxylic acid	-427.30	-418.80
Cyclobutanol	-201.00	-200.70
Cyclobutanone	-141.10	-132.80
Cyclobutylamine	5.60	3.80
Cyclodeca-1,2,6,7-tetraene	279.00	278.20
Cyclodecane	-211.70	-241.90

Cyclodecanone	-367.60	-358.40
Cyclododecane	-312.10	-302.20
Cyclododecanone	-435.40	-426.40
Cyclogeraniolane	-235.80	-243.90
Cycloheptadecane	-430.40	-435.70
Cycloheptadecanone	-543.50	-547.80
Cycloheptane	-156.88	-179.10
Cycloheptanol	-396.60	-377.80
Cycloheptene	-44.20	-78.20
Cyclohexadecane	-410.70	-410.70
Cyclohexane	-156.29	-158.00
Cyclohexane-1,1-dicarboxylic acid	-972.70	-979.40
Cyclohexanecarbonitrile	-47.20	-37.80
Cyclohexanecarboxylic acid	-588.80	-566.80
Cyclohexanemethanol	-381.30	-367.70
Cyclohexanethiol	-140.70	-145.40
Cyclohexanol	-348.60	-351.40
Cyclohexanone	-276.10	-283.40
Cyclohexanone oxime	-155.90	-159.10
Cyclohexene	-38.20	-51.90
Cyclohexyl acetate	-553.48	-545.50
Cyclohexyl butanoate	-599.50	-598.20
Cyclohexyl formate	-495.52	-485.80
Cyclohexyl iodide	-98.80	-92.60
Cyclohexyl methyl carbonate	-712.33	-716.40
Cyclohexylamine	-147.70	-146.90
Cyclohexylhydroperoxide	-273.20	-288.50
Cyclohexylisocyanate	-200.64	-230.90
Cyclononane	-181.20	-215.40
Cyclononanone	-338.70	-337.90
Cyclooctane	-169.90	-210.60
Cyclooctanone	-329.43	-333.40
Cyclooctatetraene	254.51	213.50
Cyclopentadecane	-382.90	-376.50
Cyclopentadecanone	-498.60	-494.40
Cyclopentadiene	105.90	85.10
Cyclopentane	-105.84	-108.40
Cyclopentanethiol	-89.50	-95.90
Cyclopentanol	-302.30	-301.90
Cyclopentanone	-240.20	-247.80
Cyclopentene	4.27	-11.60
Cyclopentyl methyl sulfide	-112.90	-113.50

Cyclopentylacetic acid	-551.73	-546.20
Cyclopentylamine	-95.14	-97.30
Cyclopentylcycloheptane	-231.50	-250.10
Cyclopentylcyclohexane	-235.00	-228.50
Cyclopropane	35.10	26.80
Cyclopropanecarbonitrile	140.80	147.00
Cyclopropanecarboxamide	-190.50	-200.90
Cyclopropanecarboxylic acid	-396.50	-384.80
Cyclopropylacethylene	259.20	272.70
Cyclopropylaldimine	150.50	127.80
Cyclopropylamine	45.80	37.80
Cyclopropylbenzene	100.30	100.10
Cyclopropylethan-1-one oxime	-29.70	-30.40
Cyclotetradecane	-378.40	-349.10
Cyclotridecane	-315.50	-320.10
Cycloundecane	-240.40	-283.00
Cycloundecanone	-391.00	-402.70
Cytosine	-221.30	-204.70
DAAF	428.50	424.60
DABCO	28.20	5.00
DADP	-434.26	-411.60
DCP	-172.14	-160.10
Decahydroazulene	-187.90	-202.70
Decalin-9-hydroperoxide	-353.52	-366.10
Decane	-329.57	-307.20
Decanedioic acid	-1082.60	-1070.90
Decanenitrile	-158.40	-157.30
Decyl 2-cyanoacrylate	-421.50	-474.80
Decylbenzene	-225.64	-227.80
Decylcyclohexane	-418.40	-419.30
Decylcyclopentane	-367.30	-369.70
delta-HCH	-396.66	-404.80
Desmotroposantonin methyl ether	-674.30	-642.20
Desoxyribose	-871.70	-842.20
D-Fucose	-1099.90	-1092.50
D-Galactose	-1273.20	-1276.40
D-Glucaric acid 1,4-lactone	-1438.20	-1441.50
D-Gluconolactone	-1258.90	-1257.30
D-Glucose 2,3,4,5,6-pentaacetate	-2255.70	-2217.10
d-Glycero-gulo-heptono-1,4-lactone	-1490.60	-1457.90
D-Gulose	-1261.50	-1276.40
Di-(2-chloroethoxy)methane	-536.20	-526.50

Di(2-ethylhexyl)amine	-415.90	-419.80
Di(2-pyridyl)ketone	-19.68	-34.00
Di(ethoxy-NNO-azoxy)methane	-3.89	1.60
Di(methoxy-NNO-azoxy)methane	75.80	78.10
Diacetin	-1123.60	-1072.60
Diacetoxymethane	-839.10	-849.30
Diacyldiperoxide I	-1584.99	-1572.60
Diacylperoxide II	-1600.78	-1598.90
Diacylperoxide III	-1178.58	-1204.00
Diacylperoxide IV	-1411.43	-1414.60
Diacylperoxide V	-1652.58	-1625.20
Diacylperoxide VI	-1294.25	-1309.40
Diacylperoxide VII	-1350.64	-1361.90
Diacylperoxide VIII	-1761.75	-1730.50
Dialuric acid	-830.66	-854.40
Diamantan-1-ol	-434.40	-418.60
Diamantan-3-ol	-419.30	-409.00
Diamantan-4-ol	-440.50	-422.50
Diamantane	-241.80	-219.50
Diamylamine	-262.21	-271.00
Dibenz[a,h]anthracene	178.80	195.40
Dibenzamide	-249.60	-288.20
Dibenzo[a,e]cyclooctene-5,11-dicarbonitrile	465.00	463.00
Dibenzo[b,d]thiophene	104.20	142.10
Dibenzo[b,e]pyridine	179.40	167.70
Dibenzofuran	-29.20	4.50
Dibenzo-p-dioxin	-152.70	-132.50
Dibenzosuberenone	-10.70	33.80
Dibenzosuberone	-53.92	-72.20
Dibenzoylmethane	-236.70	-228.60
Dibenzoylmethane (keto form)	-224.20	-217.90
Dibenzyl carbonate	-471.94	-477.80
Dibenzylamine	128.00	92.90
Dibenzylideneazine	334.10	347.20
Dibenzylsulfide	99.00	84.00
Dibenzylsulfone	-282.60	-298.90
Dibutoxymethane	-553.70	-557.30
Dibutyl decanedioate	-1156.70	-1168.70
Dibutyl phthalate	-843.00	-889.40
Dibutylamine	-212.30	-218.40
Dibutyldiazene	-40.10	-12.50
Dibutyldisulfide	-222.90	-223.60

Dibutylperoxide	-380.70	-326.50
Dibutylsulfate	-904.60	-913.60
Dibutylsulfide	-220.50	-227.30
Dibutyrylperoxide	-673.20	-702.90
Dichlorfenidim	-328.90	-278.30
Dichloroacetaldehyde	-218.07	-233.40
Dichloroacetic acid	-496.30	-517.10
Dichloroacetyl chloride	-281.00	-301.30
Dichlorodiethylsilane	-530.70	-518.30
Dichlorodimethylsilane	-480.40	-484.90
Dichlorodiphenylsilane	-238.00	-246.10
Dicumyl	-39.30	-48.10
Dicyanomethane	187.90	176.70
Di-cyclohexadiene	21.60	-17.70
Dicyclohexanone diperoxide	-471.18	-481.70
Dicyclohexyl carbonate	-835.78	-825.60
Dicyclohexyl phthalate	-938.70	-914.20
Dicyclohexylsulfide	-257.09	-264.70
Dicyclopentylmethane	-205.10	-200.50
Dicyclopropyldinitromethane	31.60	17.50
DIDP	-1196.90	-1206.60
Diethanolamine	-493.80	-481.00
Diethoxydimethylsilane	-729.63	-724.40
Diethyl cellosolve	-451.43	-455.40
Diethyl cis-cyclohexane-1,3-dicarboxylate	-938.50	-973.50
Diethyl disulfide	-120.06	-118.40
Diethyl maleate	-798.18	-799.40
Diethyl oxalate	-809.73	-852.80
Diethyl phthalate	-781.42	-782.80
Diethyl succinate	-913.07	-905.40
Diethyl sulfate	-813.20	-808.30
Diethyl sulfite	-600.70	-599.00
Diethyl sulphide	-120.10	-121.90
Diethyl tartrate	-1275.00	-1292.90
Diethylacetamide	-338.20	-334.70
Diethylamine	-131.00	-113.00
Diethylaminoacetone	-284.30	-288.90
Diethylammonium diethyldithiocarbamate	-255.36	-245.00
Diethylcarbonate	-682.65	-683.80
Diethylcyanamide	13.50	3.70
Diethyldimethylsilane	-213.63	-206.70
Diethyldipropylsilane	-295.81	-292.70

Diethylene glycol dimethyl ether	-559.30	-558.30
Diethyleneglycol	-628.50	-643.90
Diethyleneglycol-di-n-butylether	-723.58	-740.00
Diethylketone	-296.72	-301.00
Diethylnitramine	-106.20	-109.20
Diethylperoxide	-223.30	-221.20
Diethylsulfone	-515.50	-504.90
Diethylsulfoxide	-268.00	-280.90
Difluoroacetal	-543.90	-582.80
Difluoroacetic acid	-857.90	-866.50
Difluoroacetyl fluoride	-795.80	-810.20
Diformylhydrazine	-331.90	-329.70
Dihexyl phthalate	-987.80	-994.70
Dihexyladipate	-1161.60	-1168.70
Dihydro-2(3H)-thiophenone	-235.50	-215.70
Dihydrodicyclopentadiene	-27.90	-6.00
Diisoamyl phthalate	-948.30	-938.20
Diisobutyl azelate	-1178.48	-1149.00
Diisobutyl disulfide	-232.14	-230.20
Diisobutyl phthalate	-890.00	-896.00
Diisobutyl sulfone	-625.36	-611.60
Diisobutylamine	-225.40	-225.00
Diisobutylammonium diisobutyldithiocarbamate	-442.31	-464.90
Diisobutylene	-145.90	-125.70
Diisobutylsulfide	-229.20	-233.90
Di-isooctyl phthalate	-1087.30	-1101.30
Diisopentyl sulfide	-282.04	-281.20
Diisopentylamine	-252.70	-272.40
Diisopentylsulfide	-282.00	-295.10
Diisopropanolamine	-572.82	-552.60
Diisopropenyldiacetylene	444.00	461.40
Diisopropyl ether	-351.50	-336.00
Diisopropyl sebacate	-1184.70	-1135.00
Diisopropyl suberate	-1079.80	-1082.40
Diisopropyl sulfide	-181.60	-194.60
Diisopropylamine	-171.00	-166.30
Diisopropylammonium diisopropyldithiocarbamate	-354.13	-361.50
Diisopropylcyanamide	-54.60	-54.80
Diisopropyldiazene	35.80	34.30
Dimedone	-486.60	-469.20
Dimethoxane	-873.70	-866.70
Dimethoxymethane	-378.20	-375.40

Dimethyl 1,1-cyclopropanedicarboxylate	-723.50	-710.60
Dimethyl 1,2-cyclopentanedicarboxylate	-869.60	-870.70
Dimethyl carbonate	-610.08	-607.30
Dimethyl carbonotrithioate	-10.50	-19.20
Dimethyl cubane-1,4-dicarboxylate	-223.30	-214.40
Dimethyl cyclobutane-1,2-dicarboxylate	-747.30	-746.30
Dimethyl disulfide	-62.05	-63.30
Dimethyl fumarate	-729.30	-722.90
Dimethyl glutarate	-860.54	-855.30
Dimethyl hexanedioate	-887.18	-881.60
Dimethyl isophthalate	-730.90	-710.20
Dimethyl maleate	-680.85	-722.90
Dimethyl malonate	-801.75	-802.60
Dimethyl methylmalonate	-828.80	-832.20
Dimethyl oxalate	-756.30	-776.40
Dimethyl phthalate	-683.80	-710.20
Dimethyl pimelate	-911.70	-907.90
Dimethyl pyridine-2,6-dicarboxylate	-679.10	-681.40
Dimethyl sebacate	-1027.30	-986.90
Dimethyl suberate	-937.60	-934.20
Dimethyl succinate	-833.74	-828.90
Dimethyl sulfate	-735.50	-731.70
Dimethyl sulfide	-65.40	-66.90
Dimethyl sulfite	-523.60	-522.50
Dimethyl sulfone	-451.29	-449.90
Dimethyl-2,6-naphthalinedicarboxylate	-701.35	-673.50
Dimethylacetal	-420.20	-412.70
Dimethylacetamide	-278.30	-282.30
Dimethylamine	-43.90	-55.40
Dimethylaminomethanol	-253.60	-249.50
Dimethylene urethane	-431.60	-431.70
Dimethylether	-184.10	-199.50
Dimethylglyoxime	-163.60	-139.90
Dimethylmaleic anhydride	-574.50	-570.30
Dimethylmalononitrile	133.30	116.40
Dimethylnitramine	-71.61	-51.60
Dimethylparabanic acid	-573.59	-618.20
Dimethylperoxide	-125.70	-144.70
Dimethylpropiolactone	-396.20	-366.70
Dimethylterephthalate	-710.00	-692.80
Dimethylvinylcarbinol	-253.87	-257.20
Dimethylvinylethynylmethanol butyl ether	-69.70	-87.00

Di-n-butylether	-377.90	-381.30
Di-n-butylsulfite	-693.10	-704.40
Di-n-butylsulfone	-610.20	-610.20
Di-n-Decylphthalate	-1197.00	-1205.20
Di-n-hexylether	-481.96	-486.50
Dinitrofluorophenylethane	-234.30	-188.40
Dinitromethane	-104.90	-109.50
Di-n-nonylphthalate	-1134.80	-1152.60
Di-n-octyladipate	-1266.00	-1273.90
Di-n-octylether	-582.75	-591.80
Di-n-propyl ketone n-propylhydrazone	-111.80	-129.30
Di-n-propyl phthalate	-811.89	-836.70
Di-n-propyldiazene N-oxide	-90.58	-92.20
Di-n-propylsulfate	-859.00	-860.90
Di-n-propylsulfite	-646.80	-651.70
Di-n-propylsulfone	-548.19	-557.60
Di-n-undecyl phthalate	-1248.00	-1257.90
Dioctyl sulfide	-436.11	-437.80
Dioctylamine	-407.30	-428.90
Dipentaerythritol	-1572.30	-1553.10
Dipentyl phthalate	-931.20	-940.70
Dipentyl sulfide	-266.64	-279.80
Diphencyprone	193.10	137.80
Diphenyl ether	-32.11	-20.30
Diphenyl phthalate	-489.20	-509.60
Diphenyl sulfide	163.40	130.80
Diphenyl(phenylethynyl)carbinol	162.60	171.60
Diphenylacetamide	-47.90	-81.30
Diphenylamine	111.10	116.30
Diphenylbutadiyne	518.40	545.80
Diphenylcarbonate	-401.20	-406.70
Diphenyldisulfide	149.75	160.20
Diphenylmethane	86.23	83.00
Diphenylmethanol	-109.72	-110.40
Diphenylmethylsilane	134.98	147.10
Diphenylnitrone	148.00	150.50
Diphenylsulfone	-225.00	-203.50
Diphenylsulfoxide	9.70	32.10
Dipropionamide	-553.60	-550.80
Dipropionyl peroxide	-620.00	-650.20
Dipropyl disulfide	-171.50	-171.00
Dipropyl ether	-328.82	-328.60

Dipropyl sulfide	-169.80	-174.60
Dipropylamine	-156.11	-165.70
Dipropylammonium dipropyldithiocarbamate	-362.13	-342.40
Dipropyldiazene	11.50	40.10
Dipropyldibutylsilane	-381.98	-390.20
Dipropyldiethoxysilane	-811.81	-810.40
Dipropylmethylsilane	-188.43	-175.10
Dipropylnitramine	-163.28	-161.80
Dipropylsulfoxide	-329.40	-333.60
Di-p-tolyl sulfone	-311.45	-277.20
Di-s-butylamine	-231.60	-213.70
Di-s-butylether	-401.50	-397.60
Di-s-butylsulfide	-220.70	-244.60
Dispiro{cyclopropane-6,1'-pentacyclo[6.3.1.0(2,7).0(3,5).0(9,11)]-dodecane-12,1"-cyclopropane}	310.40	279.10
Di-t-amylperoxypyromellitate	-1648.30	-1686.00
Di-t-butyl ether	-399.61	-418.30
Di-t-butyl peroxymuconate	-777.70	-765.60
Di-t-butyl sulfone	-644.26	-647.40
Di-t-butyldisulfide	-255.20	-266.00
Di-t-butylsulfide	-251.26	-261.80
Dithiodilactic acid	-966.73	-960.00
Dithiooxamide	-20.80	-37.90
Divinyl ether	-39.80	-66.70
Divinyl sulfone	-209.00	-244.90
d-Leucylglycylglycine	-1062.40	-1087.10
D-Mannose	-1266.09	-1276.40
DMSO	-203.40	-225.90
Dodecane	-387.73	-359.70
Dodecanedioic acid	-1130.00	-1123.60
Dodecylcyclohexane	-475.69	-474.50
Dodecylcyclopentane	-421.50	-427.60
Dopa	-939.30	-899.10
D-Psicose	-1271.10	-1285.60
D-Ribonolactone	-1012.80	-1023.50
D-Ribose	-1051.57	-1038.00
Drometrizole	51.80	98.10
DTBP	-380.90	-371.30
Dulcitol	-1347.40	-1339.00
Dulcose	-1349.50	-1341.90
Durylic acid	-488.70	-486.10
EDNA	-92.71	-80.60

Eicosane	-565.90	-570.40
Elaidic acid	-797.07	-793.60
Ellagic acid	-1382.08	-1364.30
Enanthic acid	-610.23	-610.10
Enantholactam	-348.34	-357.30
endo-1,4a,8,8a-Tetrahydro-1,4-ethanonaphthalene-5,8-dione	-226.10	-280.20
endo-2-Cyanonorbornane	17.10	24.30
endo-2-Methyl-7-oxabicyclo[2.2.1]heptane	-253.20	-235.60
endo-2-Methylnorbornane	-125.50	-122.90
endo-Dicyclopentadienone	-19.50	-31.10
Epiandrosterone	-636.39	-685.60
Epichlorohydrin	-149.00	-146.70
Epoxycycloheptane	-200.40	-177.70
epsilon-Aminocaproic acid	-639.10	-627.10
epsilon-Caprolactam	-330.05	-331.10
epsilon-Caprothiolactam	-106.80	-98.40
Erucic acid	-866.67	-898.80
Erythritol	-887.20	-901.50
Ethane	-93.60	-96.50
Ethanediol diacetate	-865.43	-852.80
Ethanethiol	-73.60	-71.40
Ethanol	-277.88	-280.50
Ethene	52.34	47.00
Ethoxyacetic acid	-650.20	-657.90
Ethoxyacetonitrile	-117.70	-126.20
Ethoxybenzene	-152.60	-161.50
Ethoxytrimethylsilane	-447.84	-456.70
Ethoxytriphenylsilane	-137.49	-98.40
Ethriol	-753.80	-756.20
Ethyl (Z)-3-pentenoate	-437.00	-447.60
Ethyl 1H-indole-2-carboxylate	-350.40	-326.40
Ethyl 2,4,5-trimethyl-1H-pyrrole-3-carboxylate	-493.90	-485.70
Ethyl 2,4-pentadienoate	-338.20	-322.80
Ethyl 2-chlorobutanoate	-620.30	-568.40
Ethyl 2-chloropropionate	-541.50	-542.10
Ethyl 2-cyanoacetoacetate	-539.50	-532.70
Ethyl 2-cyanopropanoate	-372.90	-380.80
Ethyl 2-ethylacetoacetate	-720.00	-704.80
Ethyl 2-methylbutanoate	-563.56	-542.80
Ethyl 2-methylene-3-butenoate	-338.20	-312.90
Ethyl 2-nonynoate	-410.90	-388.10
Ethyl 2-octynoate	-388.00	-361.70

Ethyl 2-pentynoate	-301.80	-282.80
Ethyl 2-thiopheneacetate	-372.67	-369.00
Ethyl 2-thiophenecarboxylate	-341.88	-342.60
Ethyl 3,5-dimethyl-4-propyl-1H-pyrrole-2-carboxylate	-574.00	-538.30
Ethyl 3-aminobenzoate	-392.30	-408.20
Ethyl 3-chlorobutanoate	-574.30	-568.40
Ethyl 3-chloropropionate	-528.50	-538.30
Ethyl 3-ethoxycrotonate	-650.70	-660.70
Ethyl 3-hydroxybenzoate	-594.60	-580.30
Ethyl 3-pentynoate	-287.70	-282.80
Ethyl 3-phenylpropiolate	-155.50	-153.50
Ethyl 3-thiopheneacetate	-362.37	-358.10
Ethyl 4,4,4-trinitrobutyrate	-516.70	-497.50
Ethyl 4,5-dimethyl-1H-pyrrole-2-carboxylate	-453.00	-447.90
Ethyl 4-aminobenzoate	-418.57	-408.20
Ethyl 4-chlorobutyrate	-568.00	-564.60
Ethyl 4-fluorobenzoate	-567.80	-546.10
Ethyl 4-hydroxybenzoate	-601.80	-580.30
Ethyl 4-pentenoate	-434.50	-428.80
Ethyl acetate	-478.80	-474.70
Ethyl acetoacetate	-612.24	-652.80
Ethyl acrylate	-370.60	-376.20
Ethyl azide	266.87	250.60
Ethyl azidoacetate	-139.30	-127.60
Ethyl benzoate	-379.91	-371.70
Ethyl but-3-yne- 1-carboxylate	-281.70	-251.80
Ethyl butanoate	-528.40	-527.40
Ethyl caprylate	-667.90	-632.60
Ethyl chloroformate	-505.20	-497.60
Ethyl cis-2-pentenoate	-443.50	-447.60
Ethyl crotonate	-420.10	-421.20
Ethyl cyanoacetate	-330.30	-351.20
Ethyl cyclobutanecarboxylate	-430.50	-415.00
Ethyl decanoate	-709.50	-685.20
Ethyl dichloroacetate	-502.30	-513.40
Ethyl difluoroacetate	-841.30	-862.70
Ethyl dimethyldithiocarbamate	-52.30	-73.90
Ethyl dodecanoate	-739.10	-737.90
Ethyl elaidate	-781.90	-789.90
Ethyl ether	-271.20	-276.00
Ethyl ethoxyacetate	-642.30	-654.10
Ethyl ethoxymethyl ketone	-442.70	-454.10

Ethyl formate	-420.50	-415.00
Ethyl glycylglycinate	-708.20	-686.50
Ethyl hexadecanoate	-868.30	-843.20
Ethyl hydrocinnamate	-427.30	-421.70
Ethyl hydroperoxide	-198.90	-217.60
Ethyl iodide	-39.09	-41.20
Ethyl isobutanoate	-538.74	-530.70
Ethyl isonitrile	108.40	108.70
Ethyl isopropyl sulfide	-156.07	-155.60
Ethyl isovalerate	-571.30	-554.40
Ethyl lactate	-695.08	-694.50
Ethyl levulinate	-688.80	-679.10
Ethyl methacrylate	-421.34	-413.90
Ethyl methyl ketone oxime	-135.20	-150.30
Ethyl methyl sulfide	-91.60	-94.40
Ethyl methyl sulfite	-567.73	-560.80
Ethyl methyl sulphone	-486.39	-477.40
Ethyl myristate	-809.70	-790.60
Ethyl N,N-dimethylglycinate	-457.60	-462.90
Ethyl N,N-diphenylcarbamate	-286.20	-299.00
Ethyl nitroacetate	-489.20	-515.40
Ethyl oleate	-775.80	-789.90
Ethyl orthoformate	-675.80	-682.40
Ethyl pentanoate	-557.00	-553.70
Ethyl phenylacetate	-410.00	-398.10
Ethyl pivalate	-570.10	-561.30
Ethyl propanoate	-504.97	-501.00
Ethyl propiolate	-167.90	-199.10
Ethyl propyl sulfide	-144.67	-148.30
Ethyl salicylate	-577.30	-580.60
Ethyl succinic acid	-991.70	-963.70
Ethyl t-amyl ether	-369.07	-374.80
Ethyl t-butyl ether	-350.80	-351.00
Ethyl t-butyl ketone	-359.30	-361.30
Ethyl t-butyl sulfoxide	-348.77	-352.10
Ethyl trans-2-pentenoate	-452.50	-447.60
Ethyl trichloroacetate	-485.90	-516.00
Ethyl vinyl ether	-166.60	-183.40
Ethyl(1,1-dimethylpropyl)malononitrile	-19.10	-12.40
Ethylamine	-74.11	-82.60
Ethylbenzene	-21.51	-17.30
Ethylcarbamate	-518.59	-509.80

Ethylcarbamide	-357.80	-351.80
Ethylcyclobutane	-58.95	-63.30
Ethylcyclohexane	-212.13	-211.30
Ethylcyclopentane	-163.43	-180.40
Ethylcyclopropane	-24.80	-29.20
Ethyldibutylsilane	-254.81	-239.20
Ethyldidecylsilane	-522.34	-555.10
Ethyldiethylcarbamate	-592.30	-551.20
Ethyldihexylsilane	-340.98	-344.50
Ethyldiisobutylsilane	-268.81	-248.50
Ethyldiisopentylsilane	-315.40	-293.30
Ethyldioctylsilane	-432.16	-449.90
Ethyldipentylsilane	-295.40	-291.90
Ethyldiphenylsilane	114.19	134.40
Ethyldipropylsilane	-209.22	-186.60
Ethylene glycol monobutyl ether acetate	-710.30	-706.80
Ethylenedichloride	-167.40	-171.10
Ethyleneglycoldiacrylate	-662.81	-655.90
Ethylenethiourea	-38.20	-25.90
Ethylepoxide	-168.90	-135.80
Ethylidene diurethan	-984.50	-990.60
Ethylidenecyclohexane	-106.90	-113.30
Ethylidenecyclopentane	-59.70	-80.30
Ethyl-isoamylketone	-374.40	-380.70
Ethylisobutylether	-333.50	-331.90
Ethylisopropylether	-315.80	-311.80
Ethylisopropylketone	-325.90	-330.70
Ethylmalonic acid	-940.70	-940.20
Ethylmethylether	-216.40	-237.70
Ethyl-n-hexylether	-381.01	-381.30
Ethylnitrate	-190.40	-177.50
Ethylnitrite	-108.37	-127.20
Ethyl-n-propyldisulfide	-146.20	-144.70
Ethyl-n-propylether	-303.59	-302.30
Ethylsilane	-83.74	-53.00
Ethyl-t-butylsulfide	-187.30	-190.50
Ethyltripropylsilane	-314.60	-316.50
Ethyne	226.88	236.20
Ethynylbenzene	283.47	282.00
Etioporphin I	-10.13	-19.90
Etioporphyrin II	18.42	-27.70
exo-2-Methyl-7-oxabicyclo[2.2.1]heptane	-256.80	-238.20

exo-2-Methylnorbornane	-125.90	-122.90
exo-4-Hydroxy-endo-endo-tetracyclo[6.2.1.1.3,6.02,7]dodecane	-289.80	-299.70
exo-Bicyclo[2.2.1]heptane-2-carbonitrile	15.90	24.30
exo-Dicyclopentadiene	112.90	99.00
exo-Norbornene oxide	-100.80	-91.90
Ferulic acid	-701.83	-694.50
Flavanone	-217.60	-212.80
Flavone	-159.80	-130.60
Fluoranthene	175.73	168.90
Fluorene	90.20	110.50
Fluorene-9-methanol	-114.40	-103.10
Fluoroacetal	-335.50	-373.00
Fluoroacetic acid	-675.40	-656.70
Fluorobenzene	-150.60	-131.10
Fluorocyclohexane	-370.90	-355.80
Fluoroethane	-264.40	-274.80
Formaldehyde diethylacetal	-450.40	-451.90
Formamide	-254.00	-246.10
Formanilide	-151.46	-148.20
Formic acid	-424.70	-418.80
Formylurea	-494.04	-476.50
FOX-7	-207.60	-209.60
Freon 113	-755.00	-752.90
Fumaric acid	-811.57	-806.90
Furacilin	-225.70	-249.20
Furan	-59.13	-55.60
Furfural	-201.60	-208.10
Furfuranol	-276.20	-293.80
Furfurylideneacetone	-240.20	-208.30
Galactaric acid	-1771.00	-1736.90
Gallic acid	-1013.00	-1001.20
gamma-Butyrolactone	-420.69	-416.90
gamma-Valerolactone	-463.30	-452.60
Gluconic acid	-1588.10	-1539.30
Glucose pentaacetate	-2249.40	-2247.10
Glutamic acid	-978.96	-990.50
Glutamine	-826.06	-806.70
Glutaraldehyde	-365.87	-371.90
Glutaric anhydride	-620.43	-631.70
Glutarimide	-489.60	-480.50
Glyceraldehyde	-591.38	-598.60
Glycerol 1,3-dinitrate	-477.00	-478.30

Glycerol formal	-557.10	-561.20
Glycerol trinitrate	-370.90	-375.30
Glycerol trioleate	-2218.10	-2205.90
Glyceryl-1-nitrate	-577.00	-581.30
Glyceryl-2-benzoate	-772.80	-775.50
Glyceryl-2-caprate	-1096.50	-1089.00
Glyceryl-2-laurate	-1153.40	-1139.10
Glyceryltribenzoate	-895.98	-949.50
Glycidyl butyrate	-563.30	-540.20
Glycidyl isopropyl ether	-343.10	-324.70
Glycidyl methacrylate	-458.20	-426.90
Glycidyl propyl ether	-319.90	-315.20
Glycine	-524.00	-521.80
Glycine anhydride	-456.65	-451.50
Glycolaldehyde	-404.20	-378.80
Glycoluril	-494.50	-474.40
Glycylalanylphenylalanine	-929.52	-952.50
Glycylglycine	-747.39	-747.50
Glycylglycine-N-carboxylic acid	-1143.05	-1148.90
Glycylglycylglycylglycine	-1187.30	-1199.00
Glycyl-L-phenylalanine	-688.50	-696.10
Glyme	-379.56	-378.90
Glyoxime	-90.50	-53.50
Guaiazulene	-11.30	-62.50
Guajen	-6.62	6.30
Guanazole	45.40	23.70
Guanidine	-56.00	-42.90
Guanine	-183.90	-171.20
Hemellitic acid	-450.40	-449.30
Hemimellitene	-58.53	-67.30
Hemimellitic acid	-1160.00	-1213.00
Heneicosane	-653.70	-596.70
Heneicosylcyclopentane	-660.60	-659.20
Heptadecane	-479.50	-491.40
Heptadecylbenzene	-442.50	-414.80
Heptane	-238.24	-228.20
Heptanedioic acid	-1009.80	-991.90
Heptyl bromide	-218.40	-217.30
Heptylbenzene	-140.60	-151.60
Heptylcyclohexane	-358.80	-342.90
Heptylcyclopentane	-296.23	-290.70
Hexa-2-ene-1,6-dioic acid	-875.04	-859.60

Hexa-3-ene-1,6-dioic acid	-875.04	-859.60
Hexachlorobenzene	-141.77	-149.90
Hexachlorobutadiene	-18.90	-34.10
Hexachloroethane	-196.01	-179.20
Hexachloropropylene	-68.18	-106.60
Hexacyclododecane	46.00	70.80
Hexadecylbenzene	-414.67	-385.80
Hexadecylcyclohexane	-569.50	-579.90
Hexadecylcyclopentane	-520.70	-532.90
Hexaethylbenzene	-329.60	-320.10
Hexafluorobenzene	-991.30	-1003.10
Hexaglycine	-1655.50	-1650.50
Hexahydrofarnesyl acetone	-634.30	-637.50
Hexamethylbenzene	-161.54	-162.30
Hexamethylcyclotrisiloxane	-1622.00	-1593.10
Hexamethyl-dewar benzene	85.50	75.10
Hexamethyleneglycol	-569.90	-569.80
Hexamethylenetetramine	124.06	152.60
Hexanamide	-425.77	-399.90
Hexane	-198.70	-201.90
Hexanenitrile	-57.29	-52.10
Hexaphenylethane	511.80	467.40
Hexyl bromide	-194.20	-191.00
Hexyl hexanoate	-684.70	-685.20
Hexylbenzene	-115.00	-125.20
Hexylcyclohexane	-310.33	-316.60
Hexylcyclopentane	-270.23	-269.60
Hippuric acid	-609.72	-598.50
HMX	54.82	44.60
HNS	53.80	12.30
Homocubane-4-carboxylic acid	-82.83	-71.00
Hydantoic acid	-749.44	-722.10
Hydrazine	50.70	36.10
Hydrazinecarbothioamide	-23.85	23.00
Hydrazinecarboxamide	-226.40	-224.70
Hydrocinnamyl alcohol	-217.90	-227.60
Hydrogenperoxide	-188.00	-214.00
Hydroxyacetic acid	-664.03	-662.40
Hydroxyacetonitrile	-141.77	-130.80
Hydroxyisobutyric acid	-744.83	-716.10
Hydroxytyrosol	-625.10	-621.00
Hydurilic acid	-1252.70	-1284.60

Hypoxanthine	-109.86	-109.10
Imidazole	48.54	38.70
Imidodicarbonic diamide	-563.70	-557.40
Indane	11.70	1.00
Indene	110.60	97.90
Indole	86.60	84.60
Indole-2-carboxylic acid	-327.10	-334.10
Indole-3-carboxylic acid	-349.10	-335.00
Inositol	-1307.66	-1313.80
Iodobenzene	117.10	109.60
Ioxynil	35.60	80.20
Isatin	-270.73	-288.80
Isoamyl acetate	-558.69	-554.40
Isobutyl 2-chlorobutyrate	-664.00	-624.40
Isobutyl 2-chloropropionate	-605.10	-598.10
Isobutyl 3-chlorobutyrate	-626.00	-621.80
Isobutyl 3-chloropropionate	-582.10	-594.20
Isobutyl 4-chlorobutyrate	-633.10	-620.60
Isobutyl acetate	-536.06	-530.70
Isobutyl butanoate	-601.30	-583.30
Isobutyl chloride	-191.10	-189.90
Isobutyl chloroacetate	-559.30	-567.90
Isobutyl dichloroacetate	-563.90	-569.30
Isobutyl formate	-475.87	-471.00
Isobutyl isobutyrate	-594.07	-586.70
Isobutyl t-butylether	-407.00	-407.00
Isobutyl trichloroacetate	-563.50	-572.00
Isobutyl valerate	-620.00	-609.60
Isobutyl vinyl ether	-260.80	-239.30
Isobutylbenzene	-69.79	-73.30
Isobutylcyclohexane	-263.70	-264.60
Isobutylene	-37.50	-35.90
Isobutylmethylether	-296.13	-293.70
Isobutylnitrite	-187.20	-183.10
iso-Butylsilane	-104.67	-108.90
Isobutyraldehyde isobutylhydrazone	-62.20	-77.40
Isobutyramide	-370.50	-347.90
Isobutyronitrile	-13.80	-2.80
Isocinnamic acid	-322.00	-322.00
Isocumene	-38.33	-43.60
Isoleucine	-638.07	-633.00
Isonicotinamide	-150.20	-143.40

Isonicotinamide N-oxide	-201.00	-190.60
Isonicotinic acid	-350.19	-329.90
Isooctane	-259.16	-261.60
Isoorotic acid	-835.00	-832.70
Isopentane	-178.40	-176.20
Isopentene	-61.13	-62.20
Isopentyl 2-chloropropanoate	-629.70	-621.80
Isopentyl alcohol	-356.40	-360.20
Isopentyl butanoate	-627.00	-607.00
Isopentyl chloride	-209.20	-213.60
Isopentyl crotonate	-504.60	-500.90
Isopentyl dichloroacetate	-585.70	-593.10
Isopentyl isovalerate	-644.74	-632.50
Isopentylamine	-189.00	-162.20
Isophorone diisocyanate	-429.00	-417.70
Isophthalamide	-439.70	-421.30
Isophthalic acid	-790.40	-789.00
Isophthaloylchloride	-322.00	-362.50
Isoprene	48.20	65.30
Isopropanolamine	-294.10	-302.40
Isopropenylacetate	-386.40	-412.20
Isopropenylacetylene	229.10	239.70
Isopropoxytrimethylsilane	-467.63	-489.90
Isopropyl 2-chloropropanoate	-562.30	-574.10
Isopropyl 2-pentenoate	-478.70	-483.40
Isopropyl 4-pentenoate	-464.50	-464.60
Isopropyl benzoate	-422.15	-407.60
Isopropyl butyrate	-574.20	-563.20
Isopropyl chloroacetate	-545.50	-547.80
Isopropyl crotonate	-460.00	-457.10
Isopropyl cyclohexanoate	-636.60	-590.50
Isopropyl decanoate	-712.10	-721.10
Isopropyl dichloroacetate	-540.10	-549.20
Isopropyl hexanoate	-657.20	-615.70
Isopropyl laurate	-823.30	-771.10
Isopropyl methyl ether	-252.05	-273.50
Isopropyl methyl sulfide	-124.70	-130.70
Isopropyl myristate	-820.33	-821.10
iso-Propyl nitrite	-165.40	-163.00
Isopropyl octanoate	-685.20	-668.40
Isopropyl palmitate	-833.75	-876.40
Isopropyl pentanoate	-592.20	-589.50

Isopropyl propionate	-547.80	-536.90
Isopropyl t-butyl ether	-396.28	-381.60
Isopropyl trichloroacetate	-545.70	-551.90
Isopropyl vinyl ether	-218.00	-219.20
Isopropylacetate	-526.90	-505.30
Isopropylacrylate	-419.40	-408.00
Isopropylamine	-112.30	-111.80
Isopropylcarbamide	-391.50	-381.00
Isopropylcyclohexane	-239.45	-235.70
Isopropylcyclopentane	-190.09	-194.00
Isopropylnitrate	-229.70	-213.40
Isopropylthioacetate	-298.20	-298.60
Isoquinoline	145.10	125.60
Isoserine	-744.41	-741.90
Isovaleraldehyde	-276.50	-277.10
Isovaleramide	-392.30	-376.90
Isovaleronitrile	-10.00	-29.10
Isoxazole	44.78	31.70
Isoxylic acid	-456.10	-449.30
Ketene	-47.50	-89.10
Kyanmethin	-41.93	-33.50
Lactic acid	-694.00	-698.30
Lactide	-792.10	-827.90
Lactonitrile	-138.90	-166.60
Lactose	-2242.06	-2268.00
Lauric acid	-738.55	-741.70
Lauric peroxyacid	-680.73	-669.10
Leucine	-640.60	-638.30
Leucine anhydride	-669.81	-666.70
Lindane	-394.36	-404.80
Linoleic acid	-674.04	-687.50
Lophine	272.00	228.20
Maleic acid	-790.57	-806.90
Malic acid	-1104.40	-1106.80
Malonamide	-544.17	-518.90
Malonic acid	-891.62	-887.00
Malonyl dihydrazide	-334.20	-320.60
Maltose	-2222.40	-2263.30
Mandelic acid	-583.27	-595.30
m-Anisic acid	-556.40	-542.00
Mannitol hexanitrate	-699.70	-723.00
m-Anthranilic acid	-411.14	-411.90

m-Chlorobenzyliden-5,6,7,8-tetrahydronaphthyl-2-acetonitrile	194.40	162.70
m-Cresol	-194.14	-202.10
m-Cresylacetate	-374.10	-373.00
m-Cumenol	-266.50	-255.50
Melamine	-72.98	-64.10
Menthol	-479.43	-458.80
Mercaptoacetic acid	-454.56	-453.40
Mesitoic acid	-481.60	-486.10
Mesitol	-277.10	-273.30
Mesitylenic acid	-466.00	-449.30
Meso-2,3-diethyl-2,3-dimethylsuccinonitrile	-37.80	-9.80
meso-2,3-Diethylbutanedioic acid	-1022.30	-1019.70
Meso-2,3-dimethyl-butanedioic acid	-980.00	-969.60
meso-Tartaric acid	-1280.70	-1297.60
Metanilic acid	-608.79	-608.60
Methacrolein	-138.60	-134.10
Methacrylamide	-248.90	-231.30
Methacrylic acid	-416.45	-417.80
Methanethiol	-46.70	-43.90
Methanol	-238.82	-242.30
Methoxyacetic acid	-627.04	-619.60
Methoxybenzene	-114.80	-123.20
Methyl (dimethylamino) acetate	-416.70	-424.60
Methyl (Z) 11-eicosenoate	-799.70	-804.30
Methyl (Z) 9-octadecenoate	-734.50	-751.60
Methyl 1H-indazole-6-carboxylate	-200.00	-202.40
Methyl 1H-indole-3-carboxylate	-327.90	-293.00
Methyl 1-methyl-2-pyrrolecarboxylate	-332.20	-326.80
Methyl 2,4-dimethyl-1H-pyrrole-3-carboxylate	-425.50	-410.60
Methyl 2,4-dimethyl-1H-pyrrole-5-carboxylate	-416.30	-409.70
Methyl 2-aminobenzoate	-357.80	-370.20
Methyl 2-chloropropanoate	-493.70	-503.90
Methyl 2-furoate	-450.00	-449.70
Methyl 2-hexyldecanoate	-813.50	-803.00
Methyl 2-hexynoate	-242.70	-270.80
Methyl 2-iodobenzoate	-244.90	-267.10
Methyl 2-methoxybenzoate	-486.60	-500.00
Methyl 2-methylbutyrate	-536.96	-516.10
Methyl 2-methylene-3,3-dimethylbutanoate	-421.40	-458.40
Methyl 2-methylstearate	-909.90	-884.70
Methyl 2-thiopheneacetate	-333.48	-330.70
Methyl 2-thiophenecarboxylate	-303.78	-304.40

Methyl 3-chlorobutyrate	-543.50	-530.20
Methyl 3-chloropropionate	-500.70	-500.00
Methyl 3-hydroxybenzoate	-553.15	-542.00
Methyl 3-iodobenzoate	-280.00	-267.10
Methyl 3-methylbutyrate	-541.60	-518.80
Methyl 3-t-butylperoxycarbonylpropanoate	-878.20	-877.00
Methyl 3-thiopheneacetate	-331.48	-319.90
Methyl 4-chlorobenzoate	-385.30	-365.60
Methyl 4-chlorobutyrate	-545.50	-526.30
Methyl 4-formylbenzoate	-476.10	-468.50
Methyl 4-hydroxybenzoate	-562.75	-542.00
Methyl 4-iodobenzoate	-288.40	-267.10
Methyl 4-methoxybenzoate	-520.90	-500.00
Methyl 4-methylbenzoate	-393.30	-370.40
Methyl 5-cis-dodecenoate	-601.70	-593.60
Methyl 5-nitro-2-furoate	-472.70	-469.40
Methyl acetate	-445.80	-436.50
Methyl acrylate	-362.20	-338.00
Methyl benzoate	-343.50	-333.50
Methyl bicyclobutane-1-carboxylate	-203.10	-227.10
Methyl carbamate	-472.70	-471.50
Methyl cellosolve	-416.32	-421.70
Methyl centralite	-78.70	-101.50
Methyl chlorocarbonate	-460.74	-459.40
Methyl crotonate	-384.90	-383.00
Methyl cyclobutanecarboxylate	-395.00	-376.70
Methyl cyclohexanecarboxylate	-556.40	-527.40
Methyl cyclopropanecarboxylate	-348.00	-342.70
Methyl decanoate	-640.87	-647.00
Methyl dichloroacetate	-482.50	-475.10
Methyl dimethyldithiocarbamate	-76.00	-46.40
Methyl dimethylmalonate	-861.40	-862.90
Methyl ferulate	-681.33	-652.50
Methyl formate	-379.32	-376.70
Methyl hexadecanoate	-838.17	-805.00
Methyl hexanoate	-540.56	-541.80
Methyl hydroxyacetate	-610.80	-620.40
Methyl isoamyl ketone	-350.93	-354.40
Methyl isobutanoate	-501.76	-492.40
Methyl isobutyl ketone	-384.20	-330.70
Methyl isopropyl sulfone	-504.28	-516.30
Methyl laurate	-693.00	-699.60

Methyl levulinate	-653.80	-640.80
Methyl linolenate	-552.00	-539.50
Methyl methacrylate	-382.40	-375.70
Methyl methylthiomethyl sulfoxide	-199.40	-201.00
Methyl N,N-diethyldithiocarbamate	-105.70	-103.90
Methyl nonanoate	-616.67	-620.70
Methyl octanoate	-590.63	-594.30
Methyl palmitoleate	-722.50	-698.90
Methyl pentadecanoate	-771.84	-778.60
Methyl pentafluoropropanoate	-1437.70	-1473.50
Methyl pentyl sulfide	-167.07	-173.40
Methyl phenylpropiolate	-71.90	-115.20
Methyl pivalate	-532.80	-523.10
Methyl propanoate	-471.60	-462.80
Methyl propyl sulfide	-118.50	-120.70
Methyl p-tolyl sulfone	-386.67	-363.60
Methyl pyruvate	-556.60	-588.30
Methyl t-butyl sulfide	-157.10	-163.00
Methyl t-butylacetate	-559.40	-549.40
Methyl tetradecanoate	-744.41	-752.30
Methyl tetryl	8.74	-17.30
Methyl t-pentyl ether	-344.70	-336.50
Methyl trichloroacetate	-467.10	-477.80
Methyl tridecanoate	-718.29	-726.00
Methyl undecanoate	-671.00	-673.30
Methyl valerate	-514.52	-515.40
Methyl vanillate	-718.70	-708.60
Methyl Z 13-docoseneoate	-897.20	-856.80
Methyl Z,Z-9,12-octadecadieneoate	-654.00	-645.50
Methyl(1,1,2-trimethylpropyl)malononitrile	13.10	-15.70
Methylacetamide	-314.70	-293.50
Methylacetoacetate	-623.20	-614.50
Methylacetylacetone (enolform)	-439.50	-476.20
Methylamine	-47.31	-53.70
Methylbenzene	9.31	6.40
Methylcarbamide	-328.81	-320.30
Methylchloroacetate	-487.00	-473.70
Methylchloroform	-177.30	-137.90
Methylcyanoacetate	-311.04	-312.90
Methylcyclobutane	-44.48	-36.90
Methylcyclohexane	-190.08	-187.60
Methylcyclopentane	-137.90	-161.30

Methylcyclopropane	1.70	-2.90
Methylcyclopropylketone	-156.70	-154.60
Methyldibutylsilane	-232.01	-227.80
Methyldidecylsilane	-506.54	-543.70
Methyldiethylsilane	-145.84	-125.10
Methyldihexylsilane	-323.19	-333.10
Methyldipentylsilane	-278.60	-280.50
Methylelaidate	-736.04	-751.60
Methylenanthate	-567.44	-568.10
Methylene-bis(N,N-dimethylurea)	-551.10	-556.00
Methylenecyclobutane	93.85	79.80
Methylenecyclohexane	-61.30	-70.90
Methylenecyclopentane	-20.08	-35.20
Methylenesuccinic acid	-841.80	-825.90
Methylglyoxal 2,4-dinitrophenylosazone	172.30	187.70
Methylglyoxime	-126.80	-102.50
Methylhydrazine	54.20	37.90
Methylhydroperoxide	-196.00	-179.30
Methylisocyanate	-92.00	-137.80
Methylisonitrile	132.70	135.70
Methylketene	-104.60	-134.20
Methyllactate	-646.50	-656.30
Methylnitrate	-156.30	-139.30
Methylnitrite	-66.10	-88.90
Methyl-n-pentylether	-315.10	-316.70
Methyl-n-propylketone	-297.47	-301.00
Methyloxiran	-123.00	-109.50
Methylphenylsulfone	-345.40	-326.70
Methylprednisolone	-1054.30	-1078.00
Methylprednisolone aceponate	-1476.20	-1476.50
Methylpropylether	-265.89	-264.00
Methylsalicylate	-545.03	-542.30
Methylthiirane	11.30	41.40
Methyl-t-pentylsulfide	-179.99	-186.70
Methyltriethylsilane	-232.43	-223.40
Methyltriphenoxysilane	-683.70	-685.30
Methyltripropylsilane	-299.81	-294.50
Methyltris(2-methylphenoxy)silane	-784.08	-796.00
Methyltris(3-methylphenoxy)silane	-793.08	-796.00
Methyltris(4-methylphenoxy)silane	-777.08	-796.00
Methyltrivinylsilane	-50.60	-61.10
m-Nitrobenzylidene-p-isopropylphenylacetonitrile	130.40	164.70

Monoacetin	-909.10	-878.50
Monophenyl succinic anhydride	-504.90	-520.20
Monothiodibenzoylmethane	-11.50	-7.20
Morpholine	-189.19	-195.90
MTBE	-315.40	-312.80
m-Terphenyl	192.80	184.60
m-Toluamide	-242.38	-225.90
m-Toluidine	3.50	-30.00
Mustard gas	-203.20	-196.50
Myrcene	14.50	29.50
Myristic acid t-butylperoxyester	-796.29	-800.40
Myristonitrile	-260.20	-262.70
Myristyl alcohol	-628.18	-596.40
N-(1-Cyclohexenyl)piperidine	-104.50	-132.00
N-(2-Aminoethyl)ethanolamine	-280.19	-283.00
N-(2-Methylphenyl)-N'-phenylurea	-148.00	-165.20
N-(2-Methylpropylidene)-butylamine	-164.40	-160.60
N-(3-Methylphenyl)-acetamide	-194.70	-233.60
N-(3-Phenoxy-2-hydroxypropyl)aniline	-303.80	-305.90
N-(4-Isopropylphenylmethylene)benzenamine N-oxide	48.46	63.00
N-(4-Methoxyphenylmethylene)benzenamine N-oxide	-25.86	-16.00
N-(Diethylaminothiocarbonyl)benzamidine	-6.50	-8.60
N-(Diethylaminothiocarbonyl)benzimido ethyl ester	-176.20	-206.70
N-(Diethylaminothiocarbonyl)-N',N'-diethylbenzamidine	-43.00	-51.30
N-(Diethylaminothiocarbonyl)-N'-ethylbenzamidine	-45.70	-38.90
N-(Diethylaminothiocarbonyl)-N'-phenylbenzamidine	107.50	86.60
N-(Hydroxyethyl)piperazine	-230.48	-243.60
N,N'-(1,3-Phenylene)bis(phthalimide)	-468.20	-488.50
N,N-(Dimethyl)thiobenzamide	37.50	50.60
N,N,2,2-Tetramethylpropionamide	-344.50	-359.20
N,N,N',N'-Tetrafluoro-1,2-cyclohexanediamine	-199.27	-215.70
N,N’-Bis(salicylaldehydo)ethylenediimine	-217.10	-218.90
N,N'-Bis(2-fluoro-2,2-dinitroethyl)ethanediamide	-968.50	-938.30
N,N-Bis(2-hydroxyethyl)ethylenediamine	-531.91	-497.50
N,N-Bis(3-phenoxy-2-hydroxypropyl)aniline	-591.80	-602.60
N,N'-Bis(acetylacetone)cyclohexanediimine	-568.70	-543.50
N,N'-Bis(benzoylacetone)cyclohexanediimine	-352.41	-333.70
N,N'-Bis-(m-methoxyphenyl)terephtalamide	-557.20	-558.60
N,N'-Bis(o-methoxyphenyl)terephthalamide	-516.20	-558.60
N,N'-Bis(salicylaldehydo)cyclohexanediimine	-288.70	-278.30
N,N'-Bis(salicylaldehydo)propylenediimine	-232.10	-245.20
N,N'-Bis(salicylaldehydo)tetramethylenediimine	-254.30	-271.60

N,N-Dibutylaminotriethylsilane	-395.99	-395.90
N,N-Diethylaminotriethylsilane	-296.42	-290.60
N,N-Diethylaniline	-2.30	-33.20
N,N-Diethylhydroxylamine	-176.91	-152.70
N,N-Diethyl-N'-isobutanoylthiourea	-365.73	-378.40
N,N-Diethyl-N'-isovaleroylthiourea	-423.32	-399.50
N,N-Diethyl-N'-pivaloylthiourea	-431.92	-409.10
N,N-Diethylthiobenzamide	-15.00	-6.90
N,N'-Diethylurea	-382.20	-379.50
N,N-Diethylurea	-372.20	-366.70
N,N-Difluorobenzylamine	-26.35	-19.50
N,N-Diisobutylaminotriethylsilane	-412.99	-399.90
N,N-Dimethyl-1-adamantylcarboxamide	-389.10	-390.90
N,N-Dimethyl-3-nitroaniline	-23.10	-5.90
N,N-Dimethyl-3-toluidine	10.80	-6.10
N,N-Dimethyl-4-nitroaniline	-38.50	5.60
N,N-Dimethylaminoacetone	-226.10	-236.50
N,N-Dimethylaminotrimethylsilane	-190.70	-198.60
N,N-Dimethylaniline	46.00	21.80
N,N-Dimethylbenzamide	-179.10	-176.70
N,N-Dimethylbutyramide	-328.80	-334.90
N,N-Dimethylcyclohexylamine	-138.56	-143.80
N,N-Dimethylethanolamine	-256.97	-266.10
N,N-Dimethylformamide	-239.20	-233.80
N,N-Dimethylglycine bisulfate	-1139.99	-1145.90
N,N-Dimethylglycine methyl ester bisulfate	-1107.19	-1101.30
N,N-Dimethylglycine methyl ester sulfate	-1207.17	-1182.20
N,N-Dimethylglycine sulfate	-1241.18	-1266.20
N,N'-Dimethyl-N,N'-dinitro-ethanediamide	-307.00	-316.70
N,N'-Dimethyl-N,N'-dinitrourea	-110.80	-148.10
N,N-Dimethyl-N',N'-diphenylurea	-92.20	-101.10
N,N'-Dimethyl-N-phenylurea	-221.30	-214.30
N,N-Dimethylpropionamide	-305.40	-308.60
N,N-Dimethyl-p-toluidine	8.10	-6.10
N,N'-Dimethylurea	-315.20	-321.90
N,N-Dimethylurea	-319.10	-311.70
N,N'-Dinitrosopiperazine	90.80	85.90
N,N-Diphenyl-N'-ethylurea	-157.80	-141.10
N,N-Diphenyl-N'-methylurea	-112.00	-112.30
N,N'-Diphenyl-p-phenylenediamine	169.08	171.40
N,N-Diphenylurea	-127.30	-113.40
N,N-Diphenyluretidione	-136.02	-142.30

N,N'-Di-t-butylethylenediamine	-264.90	-267.90
N,N-Ethylene bis(N',N,-diphenyl urea)	-204.90	-185.50
N,N-Methylacetylcarbamide	-564.90	-519.70
N,N'-Trimethyleneurea	-314.50	-309.30
N-Acetamido-N'-nitroguanidine	-193.60	-215.90
N-Acetylacetamide	-489.86	-498.20
N-Acetylbenzamide	-376.50	-392.50
N-Acetylbenzenesulfonamide	-735.60	-738.00
N-Acetylurea	-544.74	-527.60
N-Amino-N'-nitroguanidine	22.10	22.20
n-Amyl ether	-425.12	-433.90
Naphthalene	78.00	80.00
Naphthazarin	-599.00	-601.80
N-Benzoyl-D-phenylalanine	-508.90	-548.50
N-Benzoyl-L-phenylalanine	-504.10	-549.90
N-Benzoyl-N',N'-diethylurea	-483.80	-467.30
N-Benzoyl-N',N'-diisobutylurea	-589.20	-572.70
N-Benzoyl-N-methylbenzamide	-244.80	-280.00
N-Benzoyl-o-aminodiphenylamine	-16.90	-28.40
N-Benzoylthiocarbamic O-hexyl ester	-418.40	-441.10
N-Benzoylthiocarbamic O-butyl ester	-369.00	-388.50
N-Benzoylthiocarbamic O-ethylester	-328.50	-335.80
N-Benzylbenzaldehyde imine	178.84	154.00
N-Benzyl-pivalophenone imine	39.87	37.90
n-Butyl acetate	-529.20	-527.40
n-Butyl acrylate	-425.50	-428.80
n-Butyl methyl ether	-290.60	-290.40
n-Butyl-2-chloropropanoate	-572.00	-594.80
n-Butyl-3-chlorobutanoate	-610.90	-621.10
n-Butyl-3-chloropropanoate	-558.20	-590.90
n-Butyl-4-chlorobutanoate	-618.00	-617.20
n-Butylbenzoate	-429.06	-424.30
n-Butylchloroacetate	-538.40	-564.60
n-Butyldichloroacetate	-550.20	-566.00
n-Butylethylether	-328.70	-328.60
n-Butylformate	-469.20	-467.70
n-Butylmalonic acid	-996.80	-995.50
n-Butylmethacrylate	-471.39	-466.60
n-Butylmethylsulfide	-142.90	-147.10
n-Butylmethylsulfone	-535.80	-530.10
n-Butylnitrite	-184.20	-179.80
n-Butylnonanoate	-697.78	-711.60

n-Butylpentylamide	-469.30	-454.00
N-Butylpiperidine	-171.80	-174.80
n-Butylstearate	-978.16	-948.50
n-Butyltrichloroacetate	-546.40	-568.70
n-Butylurea	-419.50	-404.40
N-Carboxymethylglycine	-919.00	-902.60
n-Crotylacetate	-410.10	-421.20
N-Cyclohexyl-2,4,6-trimethylbenzaldehyde imine	-134.41	-123.90
N-Cyclohexylpiperidine	-200.62	-183.80
N-Cyclopentylpiperidine	-147.73	-134.20
n-Decanoic acid	-713.70	-689.00
n-Decyl alcohol	-478.10	-491.20
n-Decylacetate	-679.25	-685.20
n-Decylamine	-275.19	-293.20
n-Decylformate	-613.73	-625.50
n-Dodecanal	-445.25	-458.00
n-Dodecylamine	-368.18	-345.90
n-Dodecylmercaptan	-327.18	-334.60
Neoheptanoic acid	-613.53	-637.50
Neohexane	-213.80	-206.90
Neohexanoic acid	-595.00	-615.10
Neopentyl alcohol	-382.01	-367.20
Neopentyl mercaptan	-168.00	-158.10
Neopentyl t-butyl ketone	-447.20	-447.90
N-Ethylaniline	4.00	-20.90
N-Ethylmorpholine	-223.57	-222.60
N-Ethyl-m-toluidine	-33.10	-57.70
N-Ethyl-N'-nitroguanidine	-106.40	-101.10
N-Formamido-N'-nitroguanidine	-146.96	-167.40
N-Formylleucine	-773.95	-764.60
N-Glycyl-dl-valine	-838.60	-832.80
n-Heptaldehyde	-311.50	-326.40
n-Heptylacetate	-602.67	-606.20
n-Heptylamine	-266.00	-214.20
n-Heptylformate	-542.98	-546.60
n-Heptyl-n-undecylphthalate	-1137.00	-1152.60
n-Hexanoic acid	-588.36	-583.80
n-Hexylacetate	-577.90	-580.00
n-Hexylamine	-239.00	-187.90
n-Hexylformate	-518.49	-520.30
Nicotinamide N-oxide	-201.30	-190.60
Nicotinic acid	-347.02	-329.90

Nicotinic acid N-oxide	-379.49	-377.10
N-Isopropylaminotriethylsilane	-295.23	-283.70
N-Isopropyl-N'-phenylurea	-289.30	-283.20
Nitroacetic acid methylester	-464.00	-479.70
Nitrobenzene	12.50	18.20
Nitrocyclohexane	-216.80	-211.70
Nitroethane	-141.00	-139.80
Nitroethylene	-33.30	-29.40
Nitroguanidine	-69.62	-73.30
Nitroguanylazide	298.00	294.60
Nitromethane	-113.00	-112.80
Nitropenta	-538.60	-507.00
Nitrophenylmethane	-22.80	-36.90
N-Leucylglycine	-863.60	-861.40
N-Methyl-1,8-Naphthalimide	-306.10	-290.50
N-Methyl-2,3-5,6-dibenzazalene	199.60	234.20
N-Methyl-2-pyrrolidone	-262.26	-253.30
N-Methyl-4-nitroaniline	-33.49	-17.10
N-Methylaniline	33.40	10.60
N-Methylbenzaldehydeimine	102.92	79.80
N-Methylcaprolactam	-306.89	-319.80
N-Methylcarbazole	103.70	135.70
N-Methylcyclohexylamine	-149.52	-146.00
N-Methyl-di(2-propynyl)-amine	463.50	437.50
N-Methyldiphenylamine	120.50	115.50
N-Methylformamide	-241.48	-245.00
N-Methylindoline	13.70	33.00
N-Methylmaleimide	-331.10	-340.00
N-Methylmorpholine	-197.98	-196.40
N-Methyl-N'-nitroguanidine	-69.60	-72.20
N-Methyl-N'-phenylurea	-225.50	-225.10
N-Methyl-N-phenylurethane	-387.80	-392.20
N-Methyl-octahydroindole	-161.00	-118.60
N-Methylphthalimide	-328.00	-327.30
N-Methylpiperidine	-98.77	-95.90
N-Methylpropargylamine	234.10	191.20
N-Methylpropionamide	-322.50	-319.80
N-Methylsuccinimide	-471.80	-436.70
N-Methyl-trimethylsilane	-186.06	-184.90
N-Nitro-1-butanamine	-118.20	-141.20
N-Nitro-1H-1,2,4-triazol-3-amine	111.60	131.50
N-Nitrobis(2-hydroxyethyl)-amine dinitrate	-259.28	-271.10

N-Nitrodibutylamine	-213.70	-210.60
N-Nitroethylamine	-92.71	-88.60
N-Nitrophthalamide	-227.20	-211.10
N-Nitropiperidine	-93.00	-91.60
N-Nitrosodiisobutylamine	-209.40	-165.60
N-Nitrosodimethylamine	4.40	4.00
N-Nitrosomorpholine	-140.20	-136.50
N-Nitrosopiperidine	-31.10	-36.10
N-Nitrosuccinimide	-317.93	-320.50
n-Nonanal	-367.93	-379.00
n-Nonylamine	-250.44	-266.90
n-Nonylformate	-588.93	-599.20
n-Nonylmercaptan	-248.66	-255.70
n-Octanoic acid	-636.10	-636.40
n-Octylacetate	-628.25	-632.60
n-Octylamine	-228.10	-240.50
N-Octylaminotriethylsilane	-410.99	-419.00
n-Octylformate	-566.45	-572.90
Nonadecane	-530.90	-544.00
Nonane	-296.44	-280.80
Nonanedioic acid	-1056.52	-1044.60
Nonylbenzene	-190.40	-204.10
Nonylcyclohexane	-390.91	-395.60
Nonylcyclopentane	-348.21	-348.60
Nopinene	-7.70	-1.00
Norbornane	-95.10	-95.80
Norbornene	29.70	20.80
Norcarane	-36.00	-38.60
Norleucine	-639.32	-635.00
Norpempidine	-206.90	-231.50
Nortricyclene	40.60	37.90
n-Pentyl-2-mercaptan	-151.60	-163.00
n-Pentylmalononitrile	65.50	41.70
N-Phenethylazetidine	117.30	105.20
N-Phenyl-2-naphthylamine	154.40	141.50
N-Phenylanthranilic acid	-311.50	-311.70
N-Phenylglycine	-396.71	-402.80
N-Phenylpiperidine	1.74	-15.70
N-Propenylpiperidine	-60.90	-98.00
n-Propyl acetate	-504.32	-501.00
n-Propyl benzoate	-397.70	-398.10
n-Propyl iodide	-66.00	-67.60

n-Propylacrylate	-407.17	-402.50
n-Propylcarbamate	-552.60	-536.10
n-Propylchloroacetate	-525.50	-538.30
n-Propylformate	-445.20	-441.30
n-Propylmethacrylate	-446.70	-440.30
n-Propylnitrate	-214.50	-203.90
N-Propylpiperidine	-154.46	-148.50
N-sec-Pentylaminotriethylsilane	-346.01	-329.80
N-Stearoyldextramine	-1077.40	-1098.60
N-t-Butylaminotriethylsilane	-306.72	-327.60
N-t-Butyl-N'-phenylurea	-327.70	-328.30
n-Undecanal	-419.06	-431.70
n-Undecanoic acid	-735.90	-715.30
n-Undecylamine	-299.52	-319.50
n-Undecylmercaptan	-302.87	-308.30
n-Valeraldehyde	-293.08	-273.80
o-Anisic acid	-541.50	-542.30
o-Anthranilic acid	-401.10	-412.20
Ocotoic acid	-823.40	-831.70
o-Cresol	-204.73	-202.10
Oct-2-ynamide	-191.40	-181.60
Octadecane	-567.43	-517.70
Octadecyl isocyanate	-627.10	-587.80
Octadecylbenzene	-469.90	-438.50
Octahydro-1,2,4-metheno-3H-cyclobuta[cd]pentalen-3-one	49.20	30.80
Octahydro-1,3,6-ethanylylidenecyclobuta[cd]indene-2,8(1H)-dione	-243.70	-264.00
Octahydroindole	-121.00	-113.50
Octamethylenimine	-120.10	-159.00
Octane	-254.20	-254.50
Octanedioic acid	-1038.00	-1018.30
Octanone-3-oxime	-232.10	-255.70
Octanone-4-oxime	-242.00	-255.70
Octylbenzene	-166.10	-175.30
Octylcyclohexane	-365.41	-369.20
Octylcyclopentane	-322.32	-317.00
o-Cumenol	-237.20	-258.10
omega,4'-Dichloroacetophenone	-223.80	-214.90
omega-Bromoacetophenone	-134.76	-134.50
omega-Chloroacetophenone	-181.30	-182.60
Orthanilic acid	-602.19	-608.80
Orthoformic acid trimethyl ester	-568.90	-567.70
o-Terphenyl	179.80	175.50

o-Toluamide	-237.28	-225.90
o-Toluidine	-4.72	-30.00
Oxacyclohexadecan-2-one	-714.90	-702.20
Oxalamide	-507.89	-492.60
Oxalic acid	-828.93	-860.40
Oxalic acid dihydrate	-1427.70	-1404.70
Oxalylchloride	-415.70	-428.60
Oxalyldihydrazide	-295.20	-294.00
Oxamic acid	-671.56	-676.50
Oxetane	-111.48	-107.70
Oxindole	-169.26	-158.70
Oxirane	-78.00	-73.70
p-Acetotoluide	-196.40	-233.60
Palmitic acid	-848.10	-847.00
p-Anisylchloride	-335.90	-326.10
p-Anthranilic acid	-413.66	-411.90
Papaverine	-502.30	-509.20
Paracetamol	-396.04	-387.90
p-Azoxyanisole	-106.40	-90.40
p-Benzidine	66.60	41.80
PBN	-60.26	-81.50
p-Chlorobenzyliden-5,6,7,8-tetrahydronaphthyl-2-acetonitrile	189.80	162.70
PCNB	-94.90	-142.80
p-Cresol	-199.42	-202.10
p-Cymene	-78.03	-86.40
p-Diacetylbenzene diethyl ketal	-913.20	-910.40
Pelargonic acid	-660.13	-662.70
Pentachlorobenzene	-116.40	-117.70
Pentachloroethane	-203.00	-176.50
Pentachlorofluoroethane	-382.00	-385.00
Pentachlorophenol	-292.40	-326.30
Pentacyclo[5.4.02,603,1005,9]undecane-8,11-dione	-202.10	-214.40
Pentacyclo[6.3.1.0(2,7).0(3,5).0(9,11)]dodecane	122.90	101.10
Pentacyclo[6.3.1.13,6.02,7.09,11]tridecane	20.70	29.10
Pentadecane	-473.50	-438.70
Pentadecylbenzene	-385.24	-362.10
Pentadecylcyclohexane	-543.70	-553.50
Pentadecylcyclopentane	-495.90	-506.60
Pentaerythritol	-921.10	-919.40
Pentaethylbenzene	-258.10	-255.60
Pentafluoroaniline	-847.82	-865.20
Pentafluorobenzene	-816.80	-828.70

Pentafluorobenzoic acid	-1239.60	-1247.50
Pentafluoroethane	-1100.40	-1099.40
Pentafluorophenol	-1024.10	-1037.30
Pentamethoxycarbonylbenzene	-1793.83	-1840.50
Pentamethylbenzene	-133.64	-141.10
Pentamethylbenzoic acid	-536.10	-559.80
Pentane	-173.50	-175.50
Pentanedioic acid	-960.62	-939.30
Pentanenitrile	-33.10	-25.80
Pentanoic acid	-560.17	-557.40
Pentaphenylbenzene	414.90	373.80
Pentyl Elaidate	-850.60	-868.80
Pentylcyclohexane	-287.93	-290.20
Pentylcyclopentane	-239.94	-243.30
Perchlorocyclopentadiene	-74.33	-69.60
Perfluoro-2,7-dimethyloctane	-4573.05	-4573.00
Perfluoro-2-methyl-2-pentene	-2452.50	-2488.70
Perfluoroacetone	-1460.00	-1477.80
Perfluorobutane	-2149.70	-2170.60
Perfluorobutanoic acid	-1924.00	-1937.80
Perfluoro-cis-decalin	-3596.42	-3610.10
Perfluorocyclohexene	-1913.10	-1918.30
Perfluoroethane	-1343.90	-1326.00
Perfluoroheptanoic acid	-3215.00	-3204.60
Perfluorohexadecane	-7251.00	-7238.20
Perfluoromethylcyclohexane	-2931.10	-2890.40
Perfluoro-n-decane	-4710.00	-4704.50
Perfluoro-n-octane	-3860.00	-3859.90
Perfluoropropene	-1079.00	-1104.90
Perfluoro-trans-decalin	-3596.40	-3610.10
Perflutren	-1783.20	-1748.40
Perhydroanthracene	-299.40	-294.00
Perhydrotriquinacene	-159.00	-132.90
Peroxyhexadecanoic acid	-802.70	-774.40
Peroxyoctadecanoic acid	-858.29	-827.00
Peroxytetradecanoic acid	-750.41	-721.70
Perseitol	-1547.90	-1563.50
Perylene	181.22	180.90
Perylene-1,12-quinone	-47.60	-31.90
Phenaceturic acid	-632.21	-627.50
Phenanthrene	113.00	119.40
Phenanthrene-9,10-dione	-159.10	-168.80

Phenanthridine	141.90	167.70
Phenazine	231.89	218.60
Phenazine oxide	193.30	190.00
Phenethyl alcohol	-181.61	-201.30
Phenol	-165.17	-165.30
Phenothiazine	162.20	137.30
Phenoxathiin	27.00	9.70
Phenoxazine	-6.10	-4.80
Phenoxyacetic acid	-514.14	-543.40
Phenoxytrimethylsilane	-352.93	-347.70
Phenyl benzoate	-240.20	-233.20
Phenyl benzyl ketone	-75.90	-66.10
Phenyl vinyl ether	-29.10	-42.20
Phenyl vinyl sulfone	-214.10	-224.30
Phenylacetamide	-213.00	-217.90
Phenylacetate	-334.90	-336.20
Phenylacetic acid	-404.17	-401.80
Phenylacetone	-151.90	-171.70
Phenylalanine	-466.90	-482.00
Phenylalanine-N-carboxylic acid dimethyl ester	-771.00	-789.10
Phenylalanine-N-carboxylic anhydride	-554.02	-558.90
Phenylcyclobutenedione	-87.40	-87.00
Phenylcyclohexane	-76.60	-79.30
Phenylformate	-271.10	-273.80
Phenylfurazan	235.20	224.00
Phenylglyoxime	-17.40	0.50
Phenylhydrazine	141.00	108.80
Phenylisocyanate	-61.08	-42.50
Phenylpropargyl aldehyde	125.90	126.50
Phenylpropiolamide	44.40	26.70
Phenylurea	-221.19	-226.20
Phlorol	-208.96	-228.50
Phthalamide	-437.10	-426.50
Phthalazine	258.75	249.90
Phthalhydrazide	-249.80	-264.00
Phthalic acid	-786.15	-794.20
Phthalic anhydride	-460.10	-482.00
Phthalic heptylnonylester	-1085.00	-1099.90
Phthalimide	-320.33	-335.50
Phthalonitrile	281.40	269.10
Phyllopyrrole	-93.00	-133.80
Picolinamide	-156.40	-154.40

Picramide	-72.80	-68.90
Pinacolone	-328.60	-331.10
Piperazine	-43.00	-33.00
Piperidine	-86.40	-95.50
Piperonal	-363.20	-396.70
Pivalamide	-402.10	-381.20
Pivalic anhydride	-779.90	-822.60
Pivalone	-395.20	-413.70
Pivalonitrile	-42.10	-33.40
Pivalophenone	-212.90	-214.60
PMC	-513.70	-540.80
p-Menthadiene	-50.80	-56.00
p-Nitrobenzylidene-p-isopropylphenylacetonitrile	151.10	164.70
p-Quaterphenyl	227.00	257.40
Prehnitene	-90.21	-104.20
Progesterone	-552.66	-572.30
Proline	-512.20	-513.60
Propanal	-221.90	-221.10
Propanal dipropylhydrazone	-53.90	-60.70
Propanal propylhydrazone	4.60	-18.10
Propanamide	-339.64	-320.90
Propane	-120.90	-122.90
Propane-1,2-dithiol	-79.40	-85.30
Propane-1,3-dithiol	-78.84	-72.70
Propanenitrile	21.63	26.90
Propanoic acid	-511.08	-504.80
Propanoic anhydride	-679.10	-701.90
Propanolamine	-291.34	-292.90
Propanone	-248.10	-248.40
Propargylamine	204.40	192.90
Propazine	-166.40	-147.50
Propenal	-85.80	-96.30
Propham	-445.90	-447.80
Propiolic acid	-193.20	-202.90
Propoxytrimethylsilane	-466.63	-483.00
Propyl 2-chlorobutanoate	-632.80	-594.80
Propyl 2-pentenoate	-464.90	-473.90
Propyl 3-chlorobutanoate	-592.20	-594.80
Propyl 3-chloropropanoate	-539.10	-564.60
Propyl 3-pentenoate	-454.40	-473.90
Propyl 4-chlorobutanoate	-593.40	-590.90
Propyl 4-pentenoate	-447.30	-455.10

Propyl butyrate	-570.80	-553.70
Propyl chloroformate	-533.40	-523.90
Propyl crotonate	-446.20	-447.60
Propyl cyclohexanoate	-640.00	-592.00
Propyl decanoate	-743.40	-711.60
Propyl dichloroacetate	-522.10	-539.70
Propyl dodecanoate	-774.80	-764.20
Propyl hexanoate	-646.20	-606.20
Propyl isobutanoate	-564.50	-557.00
Propyl nitrite	-152.40	-153.50
Propyl non-2-ynoate	-393.20	-414.40
Propyl octanoate	-675.90	-658.90
Propyl oleate	-792.30	-816.20
Propyl palmitate	-913.10	-869.50
Propyl propanoate	-527.50	-527.40
Propyl trichloroacetate	-521.70	-542.40
Propyl valerate	-586.60	-580.00
Propylcyclohexane	-237.40	-237.60
Propylcyclopentane	-188.82	-206.70
Propylene	4.00	1.90
Propylenecarbonate	-615.75	-613.80
Propylenediamine	-97.80	-97.80
Propyleneglycol-1-monomethylether-2-acetate	-663.23	-646.50
Propyleneglycol-2-monomethylether-1-acetate	-663.23	-649.10
Propylisonitrile	96.80	82.40
Propylmalonic acid	-965.50	-968.90
Propylnitramine	-109.40	-114.90
Propyltributylsilane	-405.78	-419.20
Propyltriethylsilane	-272.01	-266.40
Propyne	187.00	178.90
Protoadamantane	-155.00	-165.50
Protocatechuic acid	-790.80	-790.00
Protoporphyrin dimethylester	-511.20	-479.30
Pseudoionone	-218.80	-204.80
p-Terphenyl	187.70	185.90
p-Toluamide	-247.18	-225.90
p-Toluidine	-23.50	-30.00
p-Tolyl propadienyl sulphone	-149.20	-122.70
p-Tolyl vinyl sulfone	-248.00	-261.00
Pulegone	-292.60	-303.60
Purine	167.69	174.20
Purpurin	-790.50	-797.60

Pyracene	84.70	39.00
Pyrazinamide	-95.30	-108.80
Pyrazine	138.37	134.50
Pyrazine N,N'-dioxide	68.17	40.20
Pyrene	125.20	139.00
Pyridazine	223.47	213.20
Pyridine	100.20	88.90
Pyridine N-oxide	8.60	41.70
Pyridine-2,5-dicarboxylic acid	-746.60	-757.10
Pyridine-2,6-dicarbonyl-bis(N,N-diethylthiourea)	-433.20	-445.80
Pyrimidine	145.90	128.50
Pyromellitic dianhydride	-956.75	-1007.30
Pyrrole	59.86	41.70
Pyrrole-2-aldoxime	15.49	26.40
Pyrrolidine	-41.32	-45.90
Pyrrolizidine	-51.30	-46.50
Pyruvaldehyde	-309.10	-346.60
Pyruvic acid	-589.96	-630.30
Quadricyclane	299.50	260.30
Quinazoline	163.80	167.90
Quinizarin	-595.80	-589.00
Quinoline	138.17	128.30
Quinoxaline	171.10	176.60
Quinoxaline N,N'-dioxide	112.45	100.80
R134a	-895.79	-889.50
RDX	52.62	33.40
Refrigerant 115	-1123.00	-1120.60
Refrigerant 216	-1321.80	-1337.40
Rhamnose	-1073.90	-1092.50
Ribitol	-1121.00	-1118.70
Rufigallol	-1426.30	-1423.20
Sabinene	-44.00	-0.60
Saccharose	-2234.11	-2255.90
Salicylaldoxime	-183.70	-180.90
Salicylanilide	-308.20	-300.00
Salicylic acid	-584.87	-584.30
Salol	-440.10	-442.00
Sarcosine	-513.30	-493.60
Sarcosine-N-carboxylic anhydride	-603.24	-595.00
s-Butenylacetate	-409.00	-457.30
s-Butyl butyrate	-592.60	-581.70
s-Butyl crotonate	-477.90	-483.40

s-Butyl pentanoate	-624.20	-613.10
s-Butyl propionate	-573.30	-563.20
s-Butylacetate	-544.04	-529.00
s-Butylacrylate	-436.80	-434.40
s-Butylbenzene	-66.40	-70.70
s-Butylformate	-482.76	-477.10
s-Butylmethylether	-305.24	-299.90
s-Butylnitrite	-190.20	-189.30
s-Butyl-t-butylether	-417.50	-408.00
sec-Butylurea	-415.70	-404.70
Serine	-743.50	-742.80
Serylserine	-1179.80	-1181.60
S-Ethyl thioacetate	-231.10	-259.70
S-Ethyl trifluorothioacetate	-851.90	-874.30
Simazine	-91.40	-89.00
Simvastatin	-1248.61	-1242.20
Skatole	47.40	46.90
Sorbic acid	-380.92	-371.60
Sorbitol	-1327.78	-1343.70
s-Phenethyl alcohol	-209.09	-210.80
Spiro[2.2]pentane	157.65	116.20
Spiro[4.4]nonane	-143.80	-154.10
Spiro[4.5]decane	-204.40	-201.10
Spiro[5,5]undecane	-244.50	-245.40
Spiro[5.6]dodecane	-258.30	-273.10
S-Propylthioacetate	-294.10	-286.00
Squaric acid	-599.51	-638.70
Stearic acid	-899.49	-899.60
s-trans-1,3-Butadiene	88.50	100.40
s-Triazine	170.65	162.10
Strychnine	-171.46	-210.10
Styrene	103.85	107.50
Suberone	-302.30	-307.10
Succindiamide	-581.55	-545.30
Succinonitrile	139.70	150.40
Succinyl anhydride	-602.06	-596.10
Succinyl dihydrazide	-366.90	-346.70
Sulcatone	-314.50	-285.30
Sulfacetamide	-776.90	-774.50
Sulfolane	-441.64	-442.40
Sylvestrene	-89.60	-44.40
syn-Tricyclo[4.2.0.02.5]octane	189.70	163.30

t-Amyl acetate	-585.00	-573.50
t-Amyl butyrate	-628.90	-626.10
t-Amyl mercaptan	-162.80	-169.00
Tartaric acid	-1283.30	-1297.90
TATB	-141.70	-142.80
TBCP	-268.76	-268.40
t-Butyl crotonoate	-474.10	-496.30
t-Butyl Decanoate	-752.80	-741.60
t-Butyl dodecanoate	-817.80	-813.00
t-Butyl ethyl sulfone	-577.07	-578.80
t-Butyl glycidyl ether	-370.10	-364.00
t-Butyl octanoate	-704.50	-707.70
t-Butyl peroxydecanoate	-688.81	-695.00
t-Butyl peroxydodecanoate	-738.70	-747.70
t-Butyl phenyl carbonate	-602.35	-620.20
t-Butyl p-isopropylcumylperoxide	-352.10	-353.30
t-Butyl tetradecanoate	-862.70	-865.60
t-Butyl-(E)-peroxy 2-butenoate	-417.00	-431.00
t-Butyl(phenylacetylenyl)ketone	-5.80	-13.80
t-Butyl-1,1,3,3-tetra(methylbutyl)diazene	-178.50	-165.00
t-Butylacetate	-554.50	-549.80
t-Butylacetylene	77.90	92.40
t-Butylamine	-150.60	-157.00
t-Butylbenzene	-70.70	-75.00
t-Butylcarbamide	-417.20	-426.20
t-Butyldifluoroamine	-223.45	-197.00
t-Butyldimethylvinylethynylperoxide	-33.50	-52.00
t-Butylformamide	-340.98	-348.20
t-Butylformate	-499.65	-490.10
t-Butylhydroperoxide	-293.60	-292.70
t-Butylmalononitrile	64.10	60.40
t-Butylmercaptan	-140.50	-145.30
t-Butylmethacrylate	-498.20	-489.00
t-Butylmethylsulfone	-556.00	-551.20
t-Butylnitrite	-208.20	-202.30
t-Butylperoxymethanol	-480.60	-476.80
t-Butylperoxymethyloxirane	-322.60	-309.20
t-Butylperoxytrimethylsilane	-490.43	-453.20
t-Dodecylmercaptan	-338.83	-353.20
TEGDN	-638.76	-617.40
Terebic acid	-929.47	-903.40
Terephthalaldehyde	-243.43	-226.80

Terephthalamide	-436.00	-421.30
Terephthalic acid	-783.75	-794.20
Terephthaloyl chloride	-385.40	-362.50
Tetrabis(2,2,2-trinitroethyl)orthocarbonate	-770.00	-790.00
Tetrabutoxysilane	-1449.57	-1478.90
Tetracene	158.78	153.50
Tetrachloroethylene	-48.60	-37.70
Tetrachlorohydroquinone	-462.00	-502.70
Tetraconic acid	-854.11	-906.20
Tetracyclo[6,2,1,02,7,03,5]undecane	-0.60	-20.90
Tetra-cyclopentadiene	118.30	110.30
Tetradecahydroacridine	-258.20	-225.50
Tetradecane	-403.30	-412.40
Tetradecanoic acid	-789.21	-794.30
Tetradecylbenzene	-311.49	-333.20
Tetradecylcyclohexane	-518.90	-527.20
Tetradecylcyclopentane	-470.10	-480.20
Tetraethoxymethane	-914.60	-909.10
Tetraethylammonium nitrate	-436.99	-419.80
Tetraethylmethane	-275.39	-280.60
Tetraethylpyromellitate	-1589.70	-1614.00
Tetraethylurea	-380.00	-409.40
Tetrafluoroethene	-658.56	-654.70
Tetraglycol	-981.70	-1002.80
Tetrahydro-2,6-diphenyl-4H-thiopyran-4-one	-66.10	-96.50
Tetrahydro-2H-pyran-2-one	-461.30	-457.00
Tetrahydro-alpha--tri-cyclopentadiene	-117.40	-106.00
Tetrahydrodicyclopentadiene	-116.90	-114.30
Tetrahydrofuran	-213.95	-232.10
Tetrahydrofurfuryl alcohol	-435.85	-433.30
Tetrahydropyran	-255.81	-258.40
Tetraisobutylsucccinonitrile	-273.80	-283.70
Tetrakis(2-methylphenoxy)silane	-983.76	-995.50
Tetrakis(3-methylphenoxy)silane	-994.76	-995.50
Tetrakis(4-methylphenoxy)silane	-991.76	-995.50
Tetrakis(o-aminophenyl)porphyrin	459.64	494.70
Tetrakis(p-tolyl)porphyrin	462.51	514.00
Tetralin-1-hydroperoxide	-185.70	-169.80
Tetramethyl orthocarbonate	-767.10	-756.00
Tetramethylammonium nitrate	-330.70	-332.70
Tetramethylbutanedinitrile	11.70	37.60
Tetramethylene glycol	-509.70	-517.20

Tetramethylethene	-102.90	-118.70
Tetramethylglycollide	-856.31	-906.40
Tetramethylguanidine	7.70	-13.10
Tetramethyl-p-phenylenediamine	35.40	0.30
Tetramethylpyrazine	-43.10	-46.70
Tetramethylpyromellitate	-1445.87	-1463.80
Tetramethylsilane	-166.00	-173.40
Tetramethylthiacyclopropane	-83.00	-62.00
Tetramethylthiourea	-38.10	-46.50
Tetramethylurea	-262.20	-294.20
Tetramethyoxysilane	-1132.05	-1115.30
Tetra-n-butylammonium nitrate	-607.74	-630.30
Tetranitromethane	82.44	48.80
Tetraphenoxysilane	-888.58	-848.00
Tetraphenylethylene	311.50	293.20
Tetraphenylmethane	247.20	228.80
Tetraphenylporphyrin	640.68	661.50
Tetrapropoxysilane	-1361.40	-1373.60
Tetra-p-tolylethene	160.50	150.90
Tetra-t-amylperoxypyromellitate	-1761.90	-1746.80
Tetravinylsilane	-36.14	-23.50
Tetrazole	237.00	261.90
Tetryl	41.00	19.50
Theobromine	-361.50	-376.20
Thiamazole	3.30	-9.90
Thianaphthene	100.60	113.10
Thiane	-106.30	-104.40
Thianthrene	186.60	151.90
Thiepane	-112.85	-130.60
Thietane	24.83	21.80
Thiirane	52.00	80.40
Thioacetamide	-70.60	-67.20
Thioacetic acid	-219.35	-215.10
Thioanisole	31.12	36.30
Thiobarbituric acid	-398.62	-413.20
Thiolactic acid	-468.40	-492.30
Thiolan-3-one	-187.50	-203.50
Thiolane	-72.90	-59.50
Thionaphthene-2-carboxylic acid	-328.80	-314.80
Thiophene	80.20	84.10
Thiophenedicarboxylic acid	-774.76	-771.60
Thiourea	-89.10	-76.30

Thioxanthene	112.70	115.70
Thioxanthone	-25.00	-9.80
Threonine	-775.09	-773.10
Thujone	-234.80	-244.80
Thymoquinone	-334.80	-325.70
Tiglic acid	-491.11	-462.80
Tiglic aldehyde	-233.90	-179.10
TME	-743.20	-735.40
TNC	18.70	27.20
t-Nonylmercaptan	-266.22	-274.30
TNT	-69.46	-68.90
TNX	-83.16	-105.70
Tolane	312.40	327.70
t-Pentylbenzene	-97.30	-102.60
trans-(+)-Bicyclo-[6.1.0]nonane	-82.40	-88.60
trans,trans-1,4-Diphenylbutadiene	178.80	221.40
Trans-1,1'-dimethyl-5,5'-azotetrazole	753.05	725.70
trans-1,2-Bis(2-tolyl)ethylene	80.20	94.30
trans-1,2-Cyclohexanedicarboxylic acid	-1004.70	-981.00
trans-1,2-Cyclohexanediol	-551.00	-544.90
trans-1,2-Cyclohexanediol diacetate	-938.60	-933.20
trans-1,2-Cyclopentanediol diacetate	-885.10	-906.90
trans-1,2-Dibenzoylethylene	-119.99	-135.60
trans-1,2-Dibromocyclohexane	-162.80	-137.40
trans-1,2-Dichloroethylene	-22.90	-18.10
trans-1,2-Diethylcyclopropane	-86.46	-85.20
trans-1,2-Dimethyl-3,3-dichlorocyclopropane	-90.20	-87.70
trans-1,2-Dimethylcyclohexane	-218.20	-214.70
trans-1,2-Dimethylcyclopentane	-171.21	-167.70
trans-1,2-Dimethylcyclopropane	-30.70	-32.50
trans-1,2-Diphenylcyclopropane	161.30	173.50
trans-1,2-Divinylcyclobutane	101.30	130.40
trans-1,2-Indandiol diacetate	-764.40	-774.20
trans-1,2-Indanediol	-389.10	-385.90
trans-1,3,5-Hexatriene	123.30	153.80
trans-1,3-Dimethylcyclohexane	-215.70	-213.40
trans-1,3-Dimethylcyclopentane	-168.07	-167.70
trans-1,3-Pentadiene	76.10	55.40
Trans-1,4-bis(1,1-dimethylethyl)cyclohexane	-384.90	-369.50
trans-1,4-Dichloro-2-butene	-112.53	-117.70
trans-1,4-Diethylcyclohexane	-266.10	-264.60
trans-1,4-Dimethylcyclohexane	-222.40	-217.30

trans-1-Ethyl-2-methylcyclohexane	-240.20	-234.30
trans-1-Ethyl-2-methylcyclopentane	-195.10	-191.40
trans-1-Ethyl-3-methylcyclopentane	-196.00	-191.40
trans-1-Ethyl-4-methylcyclohexane	-246.40	-240.90
trans-1-Methyl-1,2-Cyclohexanediol	-601.60	-581.60
trans-1-Methyl-1,2-dicyclopropylcyclopropane	127.80	126.80
trans-1-Phenyl-3,3-dimethyl-1-butene	-22.60	-24.10
trans-1-Propenylbenzene	11.10	62.40
trans-2,2,4,6-Tetramethyl-1,3-dioxane	-530.00	-534.10
trans-2,2,5,5-Tetramethyl-3-hexene	-212.00	-216.40
Trans-2,2'-diethyl-5,5'-azotetrazole	655.65	677.10
trans-2,2-Dimethyl-3-hexene	-161.50	-156.20
Trans-2,2'-dimethyl-5,5'-azotetrazole	753.05	734.60
trans-2,3-Dimethoxycinnamic acid	-664.40	-652.50
trans-2,3-Dimethylthiirane	-29.36	2.50
trans-2,4-Dimethoxycinnamic acid	-687.00	-652.50
trans-2,5-Diisopropyl-1,3-dioxane	-605.90	-555.30
trans-2,5-Dimethoxycinnamic acid	-669.20	-652.50
trans-2,5-Dimethyl-3-hexene	-159.24	-155.10
trans-2-Butene	-33.30	-43.20
trans-2-Butene-1,4-diol	-401.60	-411.10
trans-2-Butenedinitrile	266.90	256.40
trans-2-Decalone	-367.90	-351.50
trans-2-Decene	-187.10	-201.10
trans-2-Dodecene	-239.00	-253.70
trans-2-Eicosene	-446.60	-464.40
trans-2-Ethyl-5-methyl-1,3-dioxane	-511.60	-475.00
trans-2-Heptene	-109.54	-122.20
trans-2-Hexahydroindanone	-309.00	-306.50
trans-2-Hexene	-85.50	-95.80
trans-2-Hexyl-5-methyl-1,3-dioxane	-584.70	-580.20
trans-2-Methoxycinnamic acid	-504.30	-486.00
trans-2-Methyl-5-butyl-1,3-dioxane	-552.10	-524.90
trans-2-Methyl-5-ethyl-1,3-dioxane	-493.60	-472.40
trans-2-Methylcyclohexanol	-415.80	-381.10
trans-2-Methyldecalin	-269.70	-251.70
trans-2-Octene	-135.69	-148.50
trans-2-Pentadecene	-319.50	-332.70
trans-2-Pentene	-63.07	-69.50
trans-2-Pentenenitrile	74.90	80.30
trans-2-Pentenoic acid	-446.40	-451.30
trans-2-Phenyl-1-cyclopropanecarboxylic acid	-332.20	-311.40

trans-3,4-Dihydro-3,4-dimethyl-2,5-furandione	-667.70	-650.70
trans-3,4-Dimethoxycinnamic acid	-668.00	-652.50
trans-3,4-Dimethyl-2-pentene	-125.10	-134.30
trans-3,5-Dimethoxycinnamic acid	-670.30	-652.50
trans-3,5-Diphenyl-1,2,4-trioxolane	-123.80	-156.40
trans-3-Decen-1-yne	100.46	100.70
trans-3-Heptene	-109.30	-122.20
trans-3-Hexene	-86.10	-95.80
trans-3-Hexenoic acid	-461.39	-477.70
trans-3-Methyl-3-hexene	-116.00	-133.60
trans-3-Methylcyclohexanol	-402.06	-377.20
trans-3-Octene	-134.38	-148.50
trans-3-Penten-1-yine	228.15	232.40
trans-3-Pentenenitrile	78.90	80.30
trans-3-Pentenoic acid	-436.80	-451.30
trans-4,4-Dimethyl-2-pentene	-121.71	-129.80
trans-4,4'-Dimethylstilbene	57.90	94.30
trans-4,6-Dimethyl-1,3-dioxane	-465.30	-450.70
trans-4-Coumaric acid	-536.00	-528.00
trans-4-Methoxycinnamic acid	-489.70	-486.00
trans-4-Methylcyclohexanol	-433.30	-381.10
trans-4-Octene	-135.10	-148.50
trans-8-Methyl-2-hydrindanone	-337.70	-333.30
trans-9-Decalol	-448.90	-425.10
trans-9-Methyldecalin	-254.50	-251.40
trans-Aconitic acid	-1234.90	-1252.90
trans-allo-Ocimene	-24.00	-11.90
trans-Azobenzene	316.42	341.10
trans-Azobenzene N,N'-dioxide	241.40	247.10
trans-Bicyclo[3.3.0]octan-2-one	-263.50	-256.90
trans-Bicyclo[3.3.0]octane	-112.30	-126.80
trans-Cinnamic acid	-325.30	-319.40
trans-Crotononitrile	95.10	106.70
trans-Cyclodecene	-106.20	-149.30
trans-Cyclohexane-1,2-dicarboxylic anhydride	-660.70	-650.20
trans-Cyclohexane-1,3-dicarboxylic acid diethyl ester	-946.50	-973.50
trans-Cyclohexane-1,3-dicarboxylic acid dimethyl ester	-858.40	-897.00
trans-Cyclohexane-1,4-dicarboxylic acid	-998.50	-981.00
trans-Cyclononene	-70.30	-125.60
trans-Cyclopentane-1,2-diol	-489.90	-495.30
trans-Decahydroquinoline	-194.80	-163.10
trans-Decalin	-230.60	-226.00

trans-Dihydro-3,4-diethyl-2,5-furandione	-703.30	-698.20
trans-Diphenyldiazene N-oxide	243.40	242.70
trans-Geraniol	-351.00	-301.90
trans-Heptacyclene	317.70	291.50
trans-Hydrindan	-176.40	-176.40
trans-Octahydroinden-1-one	-352.50	-306.50
trans-Oxolane-3,4-diol dinitrate	-387.70	-413.00
trans-Perfluorobicyclo[4,3.0]nonane	-3174.60	-3164.50
trans-Stilbene	135.65	168.10
trans-Tetralin-1,2-diol	-431.00	-423.60
trans-Tetralin-2,3-diol	-430.60	-423.60
Tratramide	-935.75	-932.70
Trehalose	-2240.90	-2265.40
Tribenzylamine	133.55	189.30
Tributylamine	-287.36	-292.40
Tributylsilane	-298.40	-291.90
Trichloroacetamide	-362.20	-335.90
Trichloroacetic acid	-507.49	-519.80
Trichloroacetylchloride	-280.80	-304.00
Trichloroethylene	-52.00	-27.90
Trichlorotoluene	-44.30	-34.90
Tricyclene	-79.20	-38.60
Tricyclo[6.2.2.0(2,7)]dodecane	-217.90	-236.10
Tricyclopropylmethane	154.90	137.70
Tridecanal	-470.62	-484.30
Tridecane	-414.43	-386.10
Tridecanoic acid	-806.60	-768.00
Tridecanolactone	-656.00	-649.60
Tridecylbenzene	-288.73	-309.40
Tridecylcyclohexane	-494.10	-500.90
Tridecylcyclopentane	-445.30	-453.90
Triethanolamine	-667.20	-686.40
Triethoxymethylsilane	-1002.43	-1000.50
Triethoxyphenylsilane	-875.31	-881.10
Triethyl phosphate	-1243.50	-1254.70
Triethylamine	-169.00	-134.40
Triethylborane	-194.60	-235.40
Triethyleneglycol	-807.20	-823.30
Triethylsilane	-171.66	-141.80
Triethylsuccinic anhydride	-749.80	-757.20
Trifluoroacetal	-780.40	-809.60
Trifluoroacetamide	-935.30	-909.40

Trifluoroacetic acid	-1069.90	-1093.20
Trifluoroacetyl fluoride	-1030.50	-1036.80
Triheptylsilane	-504.54	-528.80
Trihexylamine	-433.00	-451.60
Trihexylsilane	-434.16	-449.90
Trihydroxyglutaric acid	-1490.50	-1517.30
Triisoamylamine	-348.94	-374.70
Triisobutyl phosphate	-1448.60	-1422.60
Triisobutylamine	-337.36	-302.40
Triisobutylsilane	-309.40	-301.90
Triisopentylborane	-489.63	-474.40
Triisopentylsilane	-372.78	-373.00
Trilactic acid	-1547.00	-1526.10
Trilinolein	-1904.10	-1875.90
Trimellitic anhydride	-894.81	-898.20
Trimethoprim	-429.91	-463.40
Trimethoxymethylsilane	-889.04	-885.80
Trimethyl 1,3,5-benzenetricarboxylate	-1101.10	-1087.00
Trimethyl isocyanurate	-681.20	-674.50
Trimethyl orthobenzoate	-495.88	-519.20
Trimethyl trimellitate	-1055.00	-1087.00
Trimethylacetic acid	-564.40	-565.10
Trimethylamine	-46.06	-55.90
Trimethylene urethane	-459.70	-467.30
Trimethylethylene	-68.58	-81.00
Trimethylthiirane	-60.45	-29.80
Trimethylurea	-330.50	-310.70
Trimethylvinylsilane	-134.82	-135.90
Trimyristin	-2176.70	-2214.40
Tri-n-butylborane	-348.34	-393.30
Tri-n-decylamine	-751.85	-766.20
Tri-n-nonylamine	-674.47	-687.30
Tri-n-octylamine	-587.50	-608.30
Trinonylsilane	-634.30	-686.70
Tri-n-pentylamine	-366.85	-371.30
Trioctylsilane	-570.92	-607.70
Tripalmitolein	-2060.40	-2054.20
Tripentylsilane	-365.78	-370.90
Triphenylamine	234.70	217.60
Triphenylethylene	233.38	230.60
Triphenylmethanol	-2.50	-44.10
Triphenylphosphane	215.06	205.70

Tripropyl phosphate	-1318.80	-1333.60
Tripropylamine	-206.90	-210.80
Tripropylborane	-311.77	-314.40
Tripropylsilane	-232.01	-212.90
Triptane	-236.52	-236.50
Triptycene	210.40	210.80
Tris(2,2,2-trinitroethyl)orthoformate	-585.50	-593.20
Tris(2-aminoethyl)amine	-76.90	-92.50
Tris(2-methylphenyl)ethylene	125.20	134.80
Tris(4-methylphenyl)ethylene	121.20	120.00
Tris-(carboethoxy)methane	-1256.50	-1286.90
Tris(hydroxymethyl)nitromethane	-742.20	-763.40
Tritane	167.70	156.40
Tri-t-Butylperoxymethylsilane	-1013.89	-990.00
Tri-t-Butylperoxyvinylsilane	-928.96	-952.50
Tritetralin	-201.20	-199.00
Tritylazide	486.20	485.20
Trivinylsilane	1.43	20.60
Trolox	-892.50	-925.30
Trometamol	-719.94	-708.90
Tropolone	-239.20	-234.90
Tryptophane	-418.23	-441.50
Tyrosine	-685.10	-687.90
Tyrosol	-423.70	-412.40
Undecane	-356.27	-333.50
Undecanedioic acid	-1104.20	-1097.30
Undecanonitrile	-189.40	-183.70
Undecylbenzene	-241.18	-256.80
Undecylcyclohexane	-442.50	-448.20
Uracil	-424.40	-413.00
Urea	-320.82	-324.00
Uridine	-1163.30	-1167.30
Valeramide	-382.78	-373.60
Valine	-608.08	-609.30
Valylphenylalanine	-771.33	-782.40
Vanillic acid	-751.40	-750.60
Vanillyl alcohol	-547.20	-552.70
Vat Blue 1	-133.98	-164.90
Veratrole	-290.40	-289.80
Vidarabine	-657.50	-625.40
Vinyl acetate	-350.80	-358.00
Vinylacetylene	304.60	277.50

Vinylcyclohexane	-88.70	-89.10
Vinylcyclopentane	-37.80	-39.50
Vinylcyclopropane	102.50	95.70
Vinylene carbonate	-460.80	-488.10
Vinylethylenecarbonate	-555.40	-503.70
Vinylformate	-293.36	-298.30
Vinylidenechloride	-23.90	4.70
Vinylisopentyl ether	-268.60	-263.10
Vinylpivalate	-429.10	-444.60
Vinylpropionate	-385.46	-384.30
Vinylsilane	29.31	1.20
Vinyltriethoxysilane	-967.20	-963.10
Vinyltrimethoxysilane	-835.90	-848.40
Vinyltripropoxysilane	-1028.58	-1042.10
Vitamin B3	-136.92	-143.40
Vitamin C	-1166.92	-1180.50
Vitamin E acetate	-1107.50	-1106.00
Vitamin K3	-237.60	-221.90
Xanthene	-54.60	-26.50
Xanthine	-388.41	-392.60
Xanthone	-191.50	-151.90
Xylitol	-1118.60	-1110.50
Xylose	-1058.40	-1056.60
