# Supplementary material for: Revision and Extension of a Generally Applicable Group-Additivity Method for the Calculation of the Standard Heat of Combustion and Formation of Organic Molecules
Source: Molecules. 2021 Oct 10;26(20):6101. doi: 10.3390/molecules26206101 (PMC8539152; doi:10.3390/molecules26206101)
Supplement: Supplementary file 1 [file Table S1. Compounds List for deltaH_c) Calculations.pdf › Table S1. Compounds List for deltaH_c) Calculations.pdf]

```
Triolein
  ChemBrain IXL

167166  0  0  0  0  0  0  0  0  1 V2000
   39.5295   24.9513   -6.1677 C   0  0  0  0  0  0  0  0  0  0  0  0  0
   38.4889   24.1489   -5.3628 C   0  0  0  0  0  0  0  0  0  0  0  0  0
   37.0732   24.6461   -5.7139 C   0  0  0  0  0  0  0  0  0  0  0  0  0
   36.0327   23.8437   -4.9089 C   0  0  0  0  0  0  0  0  0  0  0  0  0
   34.6171   24.3425   -5.2581 C   0  0  0  0  0  0  0  0  0  0  0  0  0
   33.5767   23.5402   -4.4528 C   0  0  0  0  0  0  0  0  0  0  0  0  0
   32.1612   24.0410   -4.7993 C   0  0  0  0  0  0  0  0  0  0  0  0  0
   31.1211   23.2391   -3.9932 C   0  0  0  0  0  0  0  0  0  0  0  0  0
   29.7618   23.7228   -4.3228 C   0  0  0  0  0  0  0  0  0  0  0  0  0
   28.6964   23.1662   -3.7421 C   0  0  0  0  0  0  0  0  0  0  0  0  0
   28.8749   22.0679   -2.7664 C   0  0  0  0  0  0  0  0  0  0  0  0  0
   27.4981   21.6225   -2.2366 C   0  0  0  0  0  0  0  0  0  0  0  0  0
   27.6834   20.4708   -1.2297 C   0  0  0  0  0  0  0  0  0  0  0  0  0
   26.3088   20.0407   -0.6820 C   0  0  0  0  0  0  0  0  0  0  0  0  0
   26.4927   18.8750    0.3090 C   0  0  0  0  0  0  0  0  0  0  0  0  0
   25.1214   18.4641    0.8794 C   0  0  0  0  0  0  0  0  0  0  0  0  0
   25.2953   17.2465    1.8078 C   0  0  0  0  0  0  0  0  0  0  0  0  0
   24.0127   16.9565    2.4869 C   0  0  0  0  0  0  0  0  0  0  0  0  0
   23.9925   16.2337    3.4696 O   0  0  0  0  0  0  0  0  0  0  0  0  0
   20.3332   17.5199    0.5354 C   0  0  0  0  0  0  0  0  0  0  0  0  0
   20.3189   17.7978    2.0511 C   0  0  0  0  0  0  0  0  0  0  0  0  0
   21.5496   17.1408    2.7055 C   0  0  0  0  0  0  0  0  0  0  0  0  0
   22.7630   17.5393    1.9775 O   0  0  0  0  0  0  0  0  0  0  0  0  0
   20.3564   19.2488    2.2835 O   0  0  0  0  0  0  0  0  0  0  0  0  0
   21.6223   17.9452   -0.0286 O   0  0  0  0  0  0  0  0  0  0  0  0  0
   22.8635   38.8837    5.4905 C   0  0  0  0  0  0  0  0  0  0  0  0  0
   22.7276   37.3551    5.6303 C   0  0  0  0  0  0  0  0  0  0  0  0  0
   22.6563   36.7156    4.2300 C   0  0  0  0  0  0  0  0  0  0  0  0  0
   22.5175   35.1872    4.3699 C   0  0  0  0  0  0  0  0  0  0  0  0  0
   22.4592   34.5464    2.9696 C   0  0  0  0  0  0  0  0  0  0  0  0  0
   22.3174   33.0184    3.1096 C   0  0  0  0  0  0  0  0  0  0  0  0  0
   22.2769   32.3758    1.7096 C   0  0  0  0  0  0  0  0  0  0  0  0  0
   22.1340   30.8479    1.8499 C   0  0  0  0  0  0  0  0  0  0  0  0  0
   22.1061   30.2296    0.5056 C   0  0  0  0  0  0  0  0  0  0  0  0  0
   21.8973   28.9171    0.3788 C   0  0  0  0  0  0  0  0  0  0  0  0  0
   21.6912   28.0811    1.5825 C   0  0  0  0  0  0  0  0  0  0  0  0  0
   21.5183   26.6088    1.1619 C   0  0  0  0  0  0  0  0  0  0  0  0  0
   21.2897   25.7404    2.4143 C   0  0  0  0  0  0  0  0  0  0  0  0  0
   21.1165   24.2680    1.9940 C   0  0  0  0  0  0  0  0  0  0  0  0  0
   20.8604   23.4039    3.2439 C   0  0  0  0  0  0  0  0  0  0  0  0  0
   20.6823   21.9322    2.8234 C   0  0  0  0  0  0  0  0  0  0  0  0  0
   20.3034   21.0881    4.0557 C   0  0  0  0  0  0  0  0  0  0  0  0  0
   20.3266   19.6526    3.6966 C   0  0  0  0  0  0  0  0  0  0  0  0  0
   20.3211   18.8043    4.5734 O   0  0  0  0  0  0  0  0  0  0  0  0  0
    1.6832   23.3969    6.6127 C   0  0  0  0  0  0  0  0  0  0  0  0  0
    3.1349   22.9458    6.3602 C   0  0  0  0  0  0  0  0  0  0  0  0  0
    3.5327   23.2830    4.9101 C   0  0  0  0  0  0  0  0  0  0  0  0  0
    4.9834   22.8293    4.6567 C   0  0  0  0  0  0  0  0  0  0  0  0  0
    5.3859   23.1792    3.2109 C   0  0  0  0  0  0  0  0  0  0  0  0  0
    6.8364   22.7249    2.9573 C   0  0  0  0  0  0  0  0  0  0  0  0  0
    7.2476   23.0977    1.5197 C   0  0  0  0  0  0  0  0  0  0  0  0  0
    8.7043   22.6604    1.2722 C   0  0  0  0  0  0  0  0  0  0  0  0  0
    9.0922   22.9990   -0.1154 C   0  0  0  0  0  0  0  0  0  0  0  0  0
   10.2768   22.6088   -0.5915 C   0  0  0  0  0  0  0  0  0  0  0  0  0
   11.1941   21.8207    0.2617 C   0  0  0  0  0  0  0  0  0  0  0  0  0
   12.5011   21.5453   -0.5068 C   0  0  0  0  0  0  0  0  0  0  0  0  0
   13.4598   20.7324    0.3848 C   0  0  0  0  0  0  0  0  0  0  0  0  0
   14.7612   20.4450   -0.3888 C   0  0  0  0  0  0  0  0  0  0  0  0  0
   15.7147   19.6210    0.4981 C   0  0  0  0  0  0  0  0  0  0  0  0  0
```

17.0159	19.3333	-0.2757	C	0	0	0	0	0	0	0	0	0	0	0	0	0
17.9702	18.5109	0.6117	C	0	0	0	0	0	0	0	0	0	0	0	0	0
19.2376	18.2732	-0.1147	C	0	0	0	0	0	0	0	0	0	0	0	0	0
19.3792	18.6984	-1.2494	O	0	0	0	0	0	0	0	0	0	0	0	0	0
22.2485	18.3404	0.6179	H	0	0	0	0	0	0	0	0	0	0	0	0	0
39.4442	26.0024	-5.9213	H	0	0	0	0	0	0	0	0	0	0	0	0	0
39.3512	24.8132	-7.2270	H	0	0	0	0	0	0	0	0	0	0	0	0	0
40.5245	24.6021	-5.9206	H	0	0	0	0	0	0	0	0	0	0	0	0	0
38.5739	23.0978	-5.6095	H	0	0	0	0	0	0	0	0	0	0	0	0	0
38.6675	24.2866	-4.3035	H	0	0	0	0	0	0	0	0	0	0	0	0	0
36.9882	25.6972	-5.4672	H	0	0	0	0	0	0	0	0	0	0	0	0	0
36.8946	24.5083	-6.7731	H	0	0	0	0	0	0	0	0	0	0	0	0	0
36.1166	22.7929	-5.1569	H	0	0	0	0	0	0	0	0	0	0	0	0	0
36.2124	23.9801	-3.8496	H	0	0	0	0	0	0	0	0	0	0	0	0	0
34.5332	25.3934	-5.0102	H	0	0	0	0	0	0	0	0	0	0	0	0	0
34.4372	24.2059	-6.3173	H	0	0	0	0	0	0	0	0	0	0	0	0	0
33.6591	22.4896	-4.7023	H	0	0	0	0	0	0	0	0	0	0	0	0	0
33.7581	23.6749	-3.3936	H	0	0	0	0	0	0	0	0	0	0	0	0	0
32.0790	25.0918	-4.5502	H	0	0	0	0	0	0	0	0	0	0	0	0	0
31.9796	23.9058	-5.8584	H	0	0	0	0	0	0	0	0	0	0	0	0	0
31.2013	22.1888	-4.2446	H	0	0	0	0	0	0	0	0	0	0	0	0	0
31.3047	23.3719	-2.9342	H	0	0	0	0	0	0	0	0	0	0	0	0	0
29.6315	24.5283	-5.0349	H	0	0	0	0	0	0	0	0	0	0	0	0	0
27.7015	23.5183	-3.9852	H	0	0	0	0	0	0	0	0	0	0	0	0	0
29.4845	22.4140	-1.9409	H	0	0	0	0	0	0	0	0	0	0	0	0	0
29.3635	21.2312	-3.2500	H	0	0	0	0	0	0	0	0	0	0	0	0	0
26.8854	21.2843	-3.0631	H	0	0	0	0	0	0	0	0	0	0	0	0	0
27.0130	22.4569	-1.7454	H	0	0	0	0	0	0	0	0	0	0	0	0	0
28.3098	20.8040	-0.4114	H	0	0	0	0	0	0	0	0	0	0	0	0	0
28.1536	19.6303	-1.7251	H	0	0	0	0	0	0	0	0	0	0	0	0	0
25.6773	19.7214	-1.5018	H	0	0	0	0	0	0	0	0	0	0	0	0	0
25.8450	20.8770	-0.1735	H	0	0	0	0	0	0	0	0	0	0	0	0	0
27.1411	19.1878	1.1180	H	0	0	0	0	0	0	0	0	0	0	0	0	0
26.9375	18.0315	-0.2045	H	0	0	0	0	0	0	0	0	0	0	0	0	0
24.4548	18.2055	0.0659	H	0	0	0	0	0	0	0	0	0	0	0	0	0
24.7020	19.2895	1.4412	H	0	0	0	0	0	0	0	0	0	0	0	0	0
26.0551	17.4630	2.5486	H	0	0	0	0	0	0	0	0	0	0	0	0	0
25.5965	16.3864	1.2226	H	0	0	0	0	0	0	0	0	0	0	0	0	0
20.1951	16.4599	0.3615	H	0	0	0	0	0	0	0	0	0	0	0	0	0
19.4162	17.3859	2.4851	H	0	0	0	0	0	0	0	0	0	0	0	0	0
21.4451	16.0634	2.6704	H	0	0	0	0	0	0	0	0	0	0	0	0	0
21.6252	17.4628	3.7367	H	0	0	0	0	0	0	0	0	0	0	0	0	0
22.0043	39.2755	4.9603	H	0	0	0	0	0	0	0	0	0	0	0	0	0
23.7656	39.1169	4.9385	H	0	0	0	0	0	0	0	0	0	0	0	0	0
22.9161	39.3329	6.4746	H	0	0	0	0	0	0	0	0	0	0	0	0	0
21.8242	37.1217	6.1801	H	0	0	0	0	0	0	0	0	0	0	0	0	0
23.5855	36.9635	6.1627	H	0	0	0	0	0	0	0	0	0	0	0	0	0
23.5605	36.9472	3.6809	H	0	0	0	0	0	0	0	0	0	0	0	0	0
21.7995	37.1086	3.6969	H	0	0	0	0	0	0	0	0	0	0	0	0	0
21.6086	34.9553	4.9112	H	0	0	0	0	0	0	0	0	0	0	0	0	0
23.3699	34.7954	4.9110	H	0	0	0	0	0	0	0	0	0	0	0	0	0
23.3690	34.7766	2.4291	H	0	0	0	0	0	0	0	0	0	0	0	0	0
21.6080	34.9398	2.4278	H	0	0	0	0	0	0	0	0	0	0	0	0	0
21.4013	32.7879	3.6393	H	0	0	0	0	0	0	0	0	0	0	0	0	0
23.1623	32.6266	3.6624	H	0	0	0	0	0	0	0	0	0	0	0	0	0
23.1934	32.6056	1.1803	H	0	0	0	0	0	0	0	0	0	0	0	0	0
21.4325	32.7681	1.1566	H	0	0	0	0	0	0	0	0	0	0	0	0	0
21.2137	30.6178	2.3722	H	0	0	0	0	0	0	0	0	0	0	0	0	0
22.9744	30.4566	2.4098	H	0	0	0	0	0	0	0	0	0	0	0	0	0
22.2550	30.8418	-0.3753	H	0	0	0	0	0	0	0	0	0	0	0	0	0
21.8788	28.4642	-0.6047	H	0	0	0	0	0	0	0	0	0	0	0	0	0
20.8031	28.4139	2.1054	H	0	0	0	0	0	0	0	0	0	0	0	0	0
22.5500	28.1714	2.2361	H	0	0	0	0	0	0	0	0	0	0	0	0	0

22.4104 26.2730 0.6478 H 0 0 0 0 0 0 0 0 0 0 0 0
20.6653 26.5200 0.5005 H 0 0 0 0 0 0 0 0 0 0 0 0
20.3977 26.0763 2.9285 H 0 0 0 0 0 0 0 0 0 0 0 0
22.1426 25.8291 3.0756 H 0 0 0 0 0 0 0 0 0 0 0 0
22.0160 23.9263 1.4969 H 0 0 0 0 0 0 0 0 0 0 0 0
20.2753 24.1823 1.3173 H 0 0 0 0 0 0 0 0 0 0 0 0
19.9629 23.7479 3.7429 H 0 0 0 0 0 0 0 0 0 0 0 0
21.7032 23.4870 3.9190 H 0 0 0 0 0 0 0 0 0 0 0 0
21.6094 21.5620 2.4034 H 0 0 0 0 0 0 0 0 0 0 0 0
19.8963 21.8613 2.0817 H 0 0 0 0 0 0 0 0 0 0 0 0
19.3095 21.3610 4.3884 H 0 0 0 0 0 0 0 0 0 0 0 0
21.0139 21.2727 4.8520 H 0 0 0 0 0 0 0 0 0 0 0 0
1.0206 22.8805 5.9293 H 0 0 0 0 0 0 0 0 0 0 0 0
1.4045 23.1620 7.6325 H 0 0 0 0 0 0 0 0 0 0 0 0
1.6044 24.4653 6.4539 H 0 0 0 0 0 0 0 0 0 0 0 0
3.7973 23.4605 7.0452 H 0 0 0 0 0 0 0 0 0 0 0 0
3.2133 21.8770 6.5168 H 0 0 0 0 0 0 0 0 0 0 0 0
2.8691 22.7698 4.2251 H 0 0 0 0 0 0 0 0 0 0 0 0
3.4563 24.3520 4.7541 H 0 0 0 0 0 0 0 0 0 0 0 0
5.6459 23.3351 5.3483 H 0 0 0 0 0 0 0 0 0 0 0 0
5.0577 21.7589 4.8031 H 0 0 0 0 0 0 0 0 0 0 0 0
4.7232 22.6738 2.5193 H 0 0 0 0 0 0 0 0 0 0 0 0
5.3121 24.2497 3.0646 H 0 0 0 0 0 0 0 0 0 0 0 0
7.4970 23.2166 3.6607 H 0 0 0 0 0 0 0 0 0 0 0 0
6.9064 21.6518 3.0859 H 0 0 0 0 0 0 0 0 0 0 0 0
6.5954 22.5954 0.8159 H 0 0 0 0 0 0 0 0 0 0 0 0
7.1649 24.1694 1.3870 H 0 0 0 0 0 0 0 0 0 0 0 0
9.3584 23.1746 1.9656 H 0 0 0 0 0 0 0 0 0 0 0 0
8.7903 21.5910 1.4204 H 0 0 0 0 0 0 0 0 0 0 0 0
8.4150 23.5633 -0.7445 H 0 0 0 0 0 0 0 0 0 0 0 0
10.5666 22.8689 -1.6020 H 0 0 0 0 0 0 0 0 0 0 0 0
10.7247 20.8806 0.5243 H 0 0 0 0 0 0 0 0 0 0 0 0
11.4161 22.3776 1.1637 H 0 0 0 0 0 0 0 0 0 0 0 0
12.9672 22.4854 -0.7748 H 0 0 0 0 0 0 0 0 0 0 0 0
12.2798 20.9829 -1.4055 H 0 0 0 0 0 0 0 0 0 0 0 0
12.9898 19.7966 0.6610 H 0 0 0 0 0 0 0 0 0 0 0 0
13.6889 21.2990 1.2789 H 0 0 0 0 0 0 0 0 0 0 0 0
15.2359 21.3808 -0.6569 H 0 0 0 0 0 0 0 0 0 0 0 0
14.5310 19.8865 -1.2876 H 0 0 0 0 0 0 0 0 0 0 0 0
15.2399 18.6853 0.7664 H 0 0 0 0 0 0 0 0 0 0 0 0
15.9451 20.1797 1.3968 H 0 0 0 0 0 0 0 0 0 0 0 0
17.4900 20.2689 -0.5453 H 0 0 0 0 0 0 0 0 0 0 0 0
16.7856 18.7734 -1.1737 H 0 0 0 0 0 0 0 0 0 0 0 0
17.5094 17.5612 0.8540 H 0 0 0 0 0 0 0 0 0 0 0 0
18.1742 19.0565 1.5248 H 0 0 0 0 0 0 0 0 0 0 0 0
1 2 1 0 0 0 0
2 3 1 0 0 0 0
3 4 1 0 0 0 0
4 5 1 0 0 0 0
5 6 1 0 0 0 0
6 7 1 0 0 0 0
7 8 1 0 0 0 0
8 9 1 0 0 0 0
9 10 2 0 0 0 0
10 11 1 0 0 0 0
11 12 1 0 0 0 0
12 13 1 0 0 0 0
13 14 1 0 0 0 0
14 15 1 0 0 0 0
15 16 1 0 0 0 0
16 17 1 0 0 0 0
17 18 1 0 0 0 0
18 19 2 0 0 0 0

20	21	1	0	0	0	0
21	22	1	0	0	0	0
22	23	1	0	0	0	0
21	24	1	0	0	0	0
20	25	1	0	0	0	0
23	18	1	0	0	0	0
26	27	1	0	0	0	0
27	28	1	0	0	0	0
28	29	1	0	0	0	0
29	30	1	0	0	0	0
30	31	1	0	0	0	0
31	32	1	0	0	0	0
32	33	1	0	0	0	0
33	34	1	0	0	0	0
34	35	2	0	0	0	0
35	36	1	0	0	0	0
36	37	1	0	0	0	0
37	38	1	0	0	0	0
38	39	1	0	0	0	0
39	40	1	0	0	0	0
40	41	1	0	0	0	0
41	42	1	0	0	0	0
42	43	1	0	0	0	0
43	44	2	0	0	0	0
43	24	1	0	0	0	0
45	46	1	0	0	0	0
46	47	1	0	0	0	0
47	48	1	0	0	0	0
48	49	1	0	0	0	0
49	50	1	0	0	0	0
50	51	1	0	0	0	0
51	52	1	0	0	0	0
52	53	1	0	0	0	0
53	54	2	0	0	0	0
54	55	1	0	0	0	0
55	56	1	0	0	0	0
56	57	1	0	0	0	0
57	58	1	0	0	0	0
58	59	1	0	0	0	0
59	60	1	0	0	0	0
60	61	1	0	0	0	0
61	62	1	0	0	0	0
62	63	2	0	0	0	0
62	20	1	0	0	0	0
25	64	1	0	0	0	0
1	65	1	0	0	0	0
1	66	1	0	0	0	0
1	67	1	0	0	0	0
2	68	1	0	0	0	0
2	69	1	0	0	0	0
3	70	1	0	0	0	0
3	71	1	0	0	0	0
4	72	1	0	0	0	0
4	73	1	0	0	0	0
5	74	1	0	0	0	0
5	75	1	0	0	0	0
6	76	1	0	0	0	0
6	77	1	0	0	0	0
7	78	1	0	0	0	0
7	79	1	0	0	0	0
8	80	1	0	0	0	0
8	81	1	0	0	0	0
9	82	1	0	0	0	0

10 83	1	0	0	0	0
11 84	1	0	0	0	0
11 85	1	0	0	0	0
12 86	1	0	0	0	0
12 87	1	0	0	0	0
13 88	1	0	0	0	0
13 89	1	0	0	0	0
14 90	1	0	0	0	0
14 91	1	0	0	0	0
15 92	1	0	0	0	0
15 93	1	0	0	0	0
16 94	1	0	0	0	0
16 95	1	0	0	0	0
17 96	1	0	0	0	0
17 97	1	0	0	0	0
20 98	1	0	0	0	0
21 99	1	0	0	0	0
22100	1	0	0	0	0
22101	1	0	0	0	0
26102	1	0	0	0	0
26103	1	0	0	0	0
26104	1	0	0	0	0
27105	1	0	0	0	0
27106	1	0	0	0	0
28107	1	0	0	0	0
28108	1	0	0	0	0
29109	1	0	0	0	0
29110	1	0	0	0	0
30111	1	0	0	0	0
30112	1	0	0	0	0
31113	1	0	0	0	0
31114	1	0	0	0	0
32115	1	0	0	0	0
32116	1	0	0	0	0
33117	1	0	0	0	0
33118	1	0	0	0	0
34119	1	0	0	0	0
35120	1	0	0	0	0
36121	1	0	0	0	0
36122	1	0	0	0	0
37123	1	0	0	0	0
37124	1	0	0	0	0
38125	1	0	0	0	0
38126	1	0	0	0	0
39127	1	0	0	0	0
39128	1	0	0	0	0
40129	1	0	0	0	0
40130	1	0	0	0	0
41131	1	0	0	0	0
41132	1	0	0	0	0
42133	1	0	0	0	0
42134	1	0	0	0	0
45135	1	0	0	0	0
45136	1	0	0	0	0
45137	1	0	0	0	0
46138	1	0	0	0	0
46139	1	0	0	0	0
47140	1	0	0	0	0
47141	1	0	0	0	0
48142	1	0	0	0	0
48143	1	0	0	0	0
49144	1	0	0	0	0
49145	1	0	0	0	0

50146 1 0 0 0 0
50147 1 0 0 0 0
51148 1 0 0 0 0
51149 1 0 0 0 0
52150 1 0 0 0 0
52151 1 0 0 0 0
53152 1 0 0 0 0
54153 1 0 0 0 0
55154 1 0 0 0 0
55155 1 0 0 0 0
56156 1 0 0 0 0
56157 1 0 0 0 0
57158 1 0 0 0 0
57159 1 0 0 0 0
58160 1 0 0 0 0
58161 1 0 0 0 0
59162 1 0 0 0 0
59163 1 0 0 0 0
60164 1 0 0 0 0
60165 1 0 0 0 0
61166 1 0 0 0 0
61167 1 0 0 0 0
M END
> <Alias name>
Glycerol trioleate

> <deltaH°(c)> kJ/mol
-35100

$$$$
Trilinolein
ChemBrain IXL

161160 0 0 0 0 0 0 0 0 1 V2000
14.2368 58.1676 5.0846 C 0 0 0 0 0 0 0 0 0 0 0 0
15.4544 58.8456 4.4218 C 0 0 0 0 0 0 0 0 0 0 0 0
14.7228 57.1748 6.1587 C 0 0 0 0 0 0 0 0 0 0 0 0
17.7332 58.3432 3.5734 C 0 0 0 0 0 0 0 0 0 0 0 0
16.4298 57.8205 4.0160 O 0 0 0 0 0 0 0 0 0 0 0 0
15.2005 55.9452 5.5099 O 0 0 0 0 0 0 0 0 0 0 0 0
18.7841 57.4128 3.0963 C 0 0 0 0 0 0 0 0 0 0 0 0
12.1117 57.0301 4.4914 C 0 0 0 0 0 0 0 0 0 0 0 0
13.4580 57.4455 4.0648 O 0 0 0 0 0 0 0 0 0 0 0 0
18.2193 55.9793 3.0522 C 0 0 0 0 0 0 0 0 0 0 0 0
19.3289 54.9960 2.6188 C 0 0 0 0 0 0 0 0 0 0 0 0
18.7504 53.5661 2.5659 C 0 0 0 0 0 0 0 0 0 0 0 0
19.8753 52.5554 2.2479 C 0 0 0 0 0 0 0 0 0 0 0 0
19.2794 51.1299 2.2008 C 0 0 0 0 0 0 0 0 0 0 0 0
20.4065 50.0939 1.9877 C 0 0 0 0 0 0 0 0 0 0 0 0
19.8601 48.7259 2.1899 C 0 0 0 0 0 0 0 0 0 0 0 0
11.1871 56.3920 3.5235 C 0 0 0 0 0 0 0 0 0 0 0 0
11.8720 56.2763 2.1437 C 0 0 0 0 0 0 0 0 0 0 0 0
10.9330 55.5308 1.1705 C 0 0 0 0 0 0 0 0 0 0 0 0
11.6023 55.4113 -0.2157 C 0 0 0 0 0 0 0 0 0 0 0 0
10.6627 54.6417 -1.1699 C 0 0 0 0 0 0 0 0 0 0 0 0
11.3190 54.5168 -2.5608 C 0 0 0 0 0 0 0 0 0 0 0 0
10.3765 53.7359 -3.5030 C 0 0 0 0 0 0 0 0 0 0 0 0
10.3652 52.9597 -5.8179 C 0 0 0 0 0 0 0 0 0 0 0 0
20.5172 47.6564 1.7191 C 0 0 0 0 0 0 0 0 0 0 0 0
9.0374 52.3539 -5.5642 C 0 0 0 0 0 0 0 0 0 0 0 0
8.5621 51.6743 -6.7915 C 0 0 0 0 0 0 0 0 0 0 0 0
7.3627 51.0864 -6.8089 C 0 0 0 0 0 0 0 0 0 0 0 0
6.5071 51.1118 -5.6005 C 0 0 0 0 0 0 0 0 0 0 0 0

5.2668	50.2247	-5.8298	C	0	0	0	0	0	0	0	0	0	0	0	0	0
4.3749	50.2527	-4.5726	C	0	0	0	0	0	0	0	0	0	0	0	0	0
3.1384	49.3608	-4.7995	C	0	0	0	0	0	0	0	0	0	0	0	0	0
2.2483	49.3866	-3.5415	C	0	0	0	0	0	0	0	0	0	0	0	0	0
21.8313	47.8184	1.0428	C	0	0	0	0	0	0	0	0	0	0	0	0	0
22.3908	46.4779	0.7265	C	0	0	0	0	0	0	0	0	0	0	0	0	0
23.5289	46.3664	0.0279	C	0	0	0	0	0	0	0	0	0	0	0	0	0
24.2443	47.5816	-0.4398	C	0	0	0	0	0	0	0	0	0	0	0	0	0
25.4771	47.1632	-1.2739	C	0	0	0	0	0	0	0	0	0	0	0	0	0
26.2380	48.4217	-1.7454	C	0	0	0	0	0	0	0	0	0	0	0	0	0
27.4642	47.9973	-2.5823	C	0	0	0	0	0	0	0	0	0	0	0	0	0
28.2266	49.2520	-3.0546	C	0	0	0	0	0	0	0	0	0	0	0	0	0
10.9994	53.6152	-4.8416	C	0	0	0	0	0	0	0	0	0	0	0	0	0
15.2296	54.7570	6.3754	C	0	0	0	0	0	0	0	0	0	0	0	0	0
15.7645	53.4770	5.8583	C	0	0	0	0	0	0	0	0	0	0	0	0	0
15.5044	53.3866	4.3414	C	0	0	0	0	0	0	0	0	0	0	0	0	0
15.9696	52.0114	3.8200	C	0	0	0	0	0	0	0	0	0	0	0	0	0
15.8143	51.9640	2.2861	C	0	0	0	0	0	0	0	0	0	0	0	0	0
16.3084	50.6005	1.7583	C	0	0	0	0	0	0	0	0	0	0	0	0	0
16.3291	50.6241	0.2153	C	0	0	0	0	0	0	0	0	0	0	0	0	0
16.9199	49.3004	-0.3159	C	0	0	0	0	0	0	0	0	0	0	0	0	0
16.8986	49.3109	-1.7992	C	0	0	0	0	0	0	0	0	0	0	0	0	0
17.5767	48.3861	-2.4880	C	0	0	0	0	0	0	0	0	0	0	0	0	0
18.3183	47.3184	-1.7738	C	0	0	0	0	0	0	0	0	0	0	0	0	0
18.9963	46.4438	-2.7615	C	0	0	0	0	0	0	0	0	0	0	0	0	0
19.6353	45.3408	-2.3565	C	0	0	0	0	0	0	0	0	0	0	0	0	0
19.7022	45.0060	-0.9133	C	0	0	0	0	0	0	0	0	0	0	0	0	0
20.7981	43.9438	-0.6826	C	0	0	0	0	0	0	0	0	0	0	0	0	0
20.8087	43.5257	0.8024	C	0	0	0	0	0	0	0	0	0	0	0	0	0
21.9597	42.5285	1.0471	C	0	0	0	0	0	0	0	0	0	0	0	0	0
22.0170	42.1651	2.5442	C	0	0	0	0	0	0	0	0	0	0	0	0	0
14.8128	54.8260	7.5200	O	0	0	0	0	0	0	0	0	0	0	0	0	0
17.9485	59.5439	3.5993	O	0	0	0	0	0	0	0	0	0	0	0	0	0
11.7557	57.2113	5.6442	O	0	0	0	0	0	0	0	0	0	0	0	0	0
13.6123	58.9218	5.5475	H	0	0	0	0	0	0	0	0	0	0	0	0	0
15.9173	59.5245	5.1277	H	0	0	0	0	0	0	0	0	0	0	0	0	0
15.1287	59.3992	3.5499	H	0	0	0	0	0	0	0	0	0	0	0	0	0
13.9040	56.9360	6.8263	H	0	0	0	0	0	0	0	0	0	0	0	0	0
15.5309	57.6220	6.7244	H	0	0	0	0	0	0	0	0	0	0	0	0	0
19.6302	57.4471	3.7714	H	0	0	0	0	0	0	0	0	0	0	0	0	0
19.1023	57.7063	2.1035	H	0	0	0	0	0	0	0	0	0	0	0	0	0
17.4022	55.9359	2.3428	H	0	0	0	0	0	0	0	0	0	0	0	0	0
17.8579	55.7042	4.0352	H	0	0	0	0	0	0	0	0	0	0	0	0	0
20.1425	55.0312	3.3329	H	0	0	0	0	0	0	0	0	0	0	0	0	0
19.6960	55.2744	1.6385	H	0	0	0	0	0	0	0	0	0	0	0	0	0
17.9915	53.5137	1.7952	H	0	0	0	0	0	0	0	0	0	0	0	0	0
18.3078	53.3219	3.5236	H	0	0	0	0	0	0	0	0	0	0	0	0	0
20.6351	52.6051	3.0183	H	0	0	0	0	0	0	0	0	0	0	0	0	0
20.3167	52.7953	1.2883	H	0	0	0	0	0	0	0	0	0	0	0	0	0
18.5711	51.0635	1.3845	H	0	0	0	0	0	0	0	0	0	0	0	0	0
18.7721	50.9218	3.1347	H	0	0	0	0	0	0	0	0	0	0	0	0	0
21.2029	50.2743	2.6996	H	0	0	0	0	0	0	0	0	0	0	0	0	0
20.7947	50.1836	0.9806	H	0	0	0	0	0	0	0	0	0	0	0	0	0
18.9257	48.5929	2.7203	H	0	0	0	0	0	0	0	0	0	0	0	0	0
10.2914	56.9936	3.4312	H	0	0	0	0	0	0	0	0	0	0	0	0	0
10.9219	55.4035	3.8774	H	0	0	0	0	0	0	0	0	0	0	0	0	0
12.7992	55.7259	2.2449	H	0	0	0	0	0	0	0	0	0	0	0	0	0
12.0796	57.2671	1.7586	H	0	0	0	0	0	0	0	0	0	0	0	0	0
10.0044	56.0798	1.0754	H	0	0	0	0	0	0	0	0	0	0	0	0	0
10.7274	54.5404	1.5571	H	0	0	0	0	0	0	0	0	0	0	0	0	0
12.5389	54.8759	-0.1204	H	0	0	0	0	0	0	0	0	0	0	0	0	0
11.7902	56.4011	-0.6133	H	0	0	0	0	0	0	0	0	0	0	0	0	0
9.7255	55.1766	-1.2610	H	0	0	0	0	0	0	0	0	0	0	0	0	0

10.4753	53.6528	-0.7703	H	0	0	0	0	0	0	0	0	0	0	0	0	0
12.2596	53.9876	-2.4706	H	0	0	0	0	0	0	0	0	0	0	0	0	0
11.4978	55.5051	-2.9663	H	0	0	0	0	0	0	0	0	0	0	0	0	0
9.4350	54.2639	-3.5899	H	0	0	0	0	0	0	0	0	0	0	0	0	0
10.1994	52.7474	-3.0975	H	0	0	0	0	0	0	0	0	0	0	0	0	0
10.8223	52.8711	-6.7958	H	0	0	0	0	0	0	0	0	0	0	0	0	0
20.0885	46.6687	1.8322	H	0	0	0	0	0	0	0	0	0	0	0	0	0
8.3343	53.1290	-5.2856	H	0	0	0	0	0	0	0	0	0	0	0	0	0
9.1173	51.6329	-4.7602	H	0	0	0	0	0	0	0	0	0	0	0	0	0
9.1875	51.6538	-7.6754	H	0	0	0	0	0	0	0	0	0	0	0	0	0
7.0158	50.5910	-7.7073	H	0	0	0	0	0	0	0	0	0	0	0	0	0
7.0676	50.7364	-4.7533	H	0	0	0	0	0	0	0	0	0	0	0	0	0
6.1918	52.1288	-5.4027	H	0	0	0	0	0	0	0	0	0	0	0	0	0
4.7069	50.5992	-6.6779	H	0	0	0	0	0	0	0	0	0	0	0	0	0
5.5819	49.2073	-6.0261	H	0	0	0	0	0	0	0	0	0	0	0	0	0
4.9361	49.8825	-3.7236	H	0	0	0	0	0	0	0	0	0	0	0	0	0
4.0560	51.2694	-4.3789	H	0	0	0	0	0	0	0	0	0	0	0	0	0
2.5760	49.7321	-5.6472	H	0	0	0	0	0	0	0	0	0	0	0	0	0
3.4573	48.3444	-4.9951	H	0	0	0	0	0	0	0	0	0	0	0	0	0
1.3799	48.7592	-3.7006	H	0	0	0	0	0	0	0	0	0	0	0	0	0
2.8108	49.0161	-2.6935	H	0	0	0	0	0	0	0	0	0	0	0	0	0
1.9284	50.4028	-3.3463	H	0	0	0	0	0	0	0	0	0	0	0	0	0
22.5120	48.3490	1.6974	H	0	0	0	0	0	0	0	0	0	0	0	0	0
21.6994	48.3806	0.1263	H	0	0	0	0	0	0	0	0	0	0	0	0	0
21.8759	45.5868	1.0624	H	0	0	0	0	0	0	0	0	0	0	0	0	0
23.9294	45.3857	-0.1970	H	0	0	0	0	0	0	0	0	0	0	0	0	0
23.5801	48.1790	-1.0521	H	0	0	0	0	0	0	0	0	0	0	0	0	0
24.5673	48.1624	0.4154	H	0	0	0	0	0	0	0	0	0	0	0	0	0
26.1332	46.5526	-0.6659	H	0	0	0	0	0	0	0	0	0	0	0	0	0
25.1525	46.5945	-2.1364	H	0	0	0	0	0	0	0	0	0	0	0	0	0
25.5822	49.0346	-2.3513	H	0	0	0	0	0	0	0	0	0	0	0	0	0
26.5680	48.9885	-0.8836	H	0	0	0	0	0	0	0	0	0	0	0	0	0
28.1197	47.3839	-1.9766	H	0	0	0	0	0	0	0	0	0	0	0	0	0
27.1342	47.4304	-3.4441	H	0	0	0	0	0	0	0	0	0	0	0	0	0
27.5716	49.8664	-3.6598	H	0	0	0	0	0	0	0	0	0	0	0	0	0
29.0857	48.9523	-3.6428	H	0	0	0	0	0	0	0	0	0	0	0	0	0
28.5590	49.8183	-2.1934	H	0	0	0	0	0	0	0	0	0	0	0	0	0
11.9695	54.0587	-5.0290	H	0	0	0	0	0	0	0	0	0	0	0	0	0
15.2728	52.6520	6.3587	H	0	0	0	0	0	0	0	0	0	0	0	0	0
16.8301	53.4294	6.0454	H	0	0	0	0	0	0	0	0	0	0	0	0	0
16.0539	54.1697	3.8338	H	0	0	0	0	0	0	0	0	0	0	0	0	0
14.4453	53.5061	4.1492	H	0	0	0	0	0	0	0	0	0	0	0	0	0
15.3656	51.2323	4.2684	H	0	0	0	0	0	0	0	0	0	0	0	0	0
17.0091	51.8586	4.0827	H	0	0	0	0	0	0	0	0	0	0	0	0	0
16.4007	52.7575	1.8397	H	0	0	0	0	0	0	0	0	0	0	0	0	0
14.7715	52.0955	2.0250	H	0	0	0	0	0	0	0	0	0	0	0	0	0
15.6410	49.8183	2.0986	H	0	0	0	0	0	0	0	0	0	0	0	0	0
17.3075	50.4098	2.1301	H	0	0	0	0	0	0	0	0	0	0	0	0	0
16.9382	51.4526	-0.1243	H	0	0	0	0	0	0	0	0	0	0	0	0	0
15.3190	50.7431	-0.1568	H	0	0	0	0	0	0	0	0	0	0	0	0	0
16.3283	48.4692	0.0479	H	0	0	0	0	0	0	0	0	0	0	0	0	0
17.9408	49.1958	0.0301	H	0	0	0	0	0	0	0	0	0	0	0	0	0
16.3327	50.0693	-2.3255	H	0	0	0	0	0	0	0	0	0	0	0	0	0
17.5842	48.4172	-3.5705	H	0	0	0	0	0	0	0	0	0	0	0	0	0
17.6259	46.7293	-1.1850	H	0	0	0	0	0	0	0	0	0	0	0	0	0
19.0573	47.7665	-1.1209	H	0	0	0	0	0	0	0	0	0	0	0	0	0
18.9739	46.6990	-3.8137	H	0	0	0	0	0	0	0	0	0	0	0	0	0
20.1050	44.6905	-3.0839	H	0	0	0	0	0	0	0	0	0	0	0	0	0
19.9386	45.8978	-0.3461	H	0	0	0	0	0	0	0	0	0	0	0	0	0
18.7459	44.6145	-0.5887	H	0	0	0	0	0	0	0	0	0	0	0	0	0
20.5958	43.0770	-1.2996	H	0	0	0	0	0	0	0	0	0	0	0	0	0
21.7631	44.3577	-0.9475	H	0	0	0	0	0	0	0	0	0	0	0	0	0
20.9517	44.4015	1.4231	H	0	0	0	0	0	0	0	0	0	0	0	0	0

```
   19.8652   43.0563    1.0522 H   0  0  0  0  0  0  0  0  0  0  0  0
   21.7902   41.6315    0.4644 H   0  0  0  0  0  0  0  0  0  0  0  0
   22.8980   42.9801    0.7494 H   0  0  0  0  0  0  0  0  0  0  0  0
   22.1840   43.0622    3.1275 H   0  0  0  0  0  0  0  0  0  0  0  0
   22.8275   41.4672    2.7155 H   0  0  0  0  0  0  0  0  0  0  0  0
   21.0803   41.7103    2.8416 H   0  0  0  0  0  0  0  0  0  0  0  0
  1  9  1  0  0  0  0
  1  2  1  0  0  0  0
  1  3  1  0  0  0  0
  2  5  1  0  0  0  0
  3  6  1  0  0  0  0
  4  5  1  0  0  0  0
  4  7  1  0  0  0  0
  4 62  2  0  0  0  0
  6 43  1  0  0  0  0
  7 10  1  0  0  0  0
  8  9  1  0  0  0  0
  8 17  1  0  0  0  0
  8 63  2  0  0  0  0
 10 11  1  0  0  0  0
 11 12  1  0  0  0  0
 12 13  1  0  0  0  0
 13 14  1  0  0  0  0
 14 15  1  0  0  0  0
 15 16  1  0  0  0  0
 16 25  2  0  0  0  0
 17 18  1  0  0  0  0
 18 19  1  0  0  0  0
 19 20  1  0  0  0  0
 20 21  1  0  0  0  0
 21 22  1  0  0  0  0
 22 23  1  0  0  0  0
 23 42  1  0  0  0  0
 24 26  1  0  0  0  0
 24 42  2  0  0  0  0
 25 34  1  0  0  0  0
 26 27  1  0  0  0  0
 27 28  2  0  0  0  0
 28 29  1  0  0  0  0
 29 30  1  0  0  0  0
 30 31  1  0  0  0  0
 31 32  1  0  0  0  0
 32 33  1  0  0  0  0
 34 35  1  0  0  0  0
 35 36  2  0  0  0  0
 36 37  1  0  0  0  0
 37 38  1  0  0  0  0
 38 39  1  0  0  0  0
 39 40  1  0  0  0  0
 40 41  1  0  0  0  0
 43 44  1  0  0  0  0
 43 61  2  0  0  0  0
 44 45  1  0  0  0  0
 45 46  1  0  0  0  0
 46 47  1  0  0  0  0
 47 48  1  0  0  0  0
 48 49  1  0  0  0  0
 49 50  1  0  0  0  0
 50 51  1  0  0  0  0
 51 52  2  0  0  0  0
 52 53  1  0  0  0  0
 53 54  1  0  0  0  0
 54 55  2  0  0  0  0
```

55 56 1 0 0 0 0
56 57 1 0 0 0 0
57 58 1 0 0 0 0
58 59 1 0 0 0 0
59 60 1 0 0 0 0
1 64 1 0 0 0 0
2 65 1 0 0 0 0
2 66 1 0 0 0 0
3 67 1 0 0 0 0
3 68 1 0 0 0 0
7 69 1 0 0 0 0
7 70 1 0 0 0 0
10 71 1 0 0 0 0
10 72 1 0 0 0 0
11 73 1 0 0 0 0
11 74 1 0 0 0 0
12 75 1 0 0 0 0
12 76 1 0 0 0 0
13 77 1 0 0 0 0
13 78 1 0 0 0 0
14 79 1 0 0 0 0
14 80 1 0 0 0 0
15 81 1 0 0 0 0
15 82 1 0 0 0 0
16 83 1 0 0 0 0
17 84 1 0 0 0 0
17 85 1 0 0 0 0
18 86 1 0 0 0 0
18 87 1 0 0 0 0
19 88 1 0 0 0 0
19 89 1 0 0 0 0
20 90 1 0 0 0 0
20 91 1 0 0 0 0
21 92 1 0 0 0 0
21 93 1 0 0 0 0
22 94 1 0 0 0 0
22 95 1 0 0 0 0
23 96 1 0 0 0 0
23 97 1 0 0 0 0
24 98 1 0 0 0 0
25 99 1 0 0 0 0
26100 1 0 0 0 0
26101 1 0 0 0 0
27102 1 0 0 0 0
28103 1 0 0 0 0
29104 1 0 0 0 0
29105 1 0 0 0 0
30106 1 0 0 0 0
30107 1 0 0 0 0
31108 1 0 0 0 0
31109 1 0 0 0 0
32110 1 0 0 0 0
32111 1 0 0 0 0
33112 1 0 0 0 0
33113 1 0 0 0 0
33114 1 0 0 0 0
34115 1 0 0 0 0
34116 1 0 0 0 0
35117 1 0 0 0 0
36118 1 0 0 0 0
37119 1 0 0 0 0
37120 1 0 0 0 0
38121 1 0 0 0 0

```
 38122  1  0  0  0  0
 39123  1  0  0  0  0
 39124  1  0  0  0  0
 40125  1  0  0  0  0
 40126  1  0  0  0  0
 41127  1  0  0  0  0
 41128  1  0  0  0  0
 41129  1  0  0  0  0
 42130  1  0  0  0  0
 44131  1  0  0  0  0
 44132  1  0  0  0  0
 45133  1  0  0  0  0
 45134  1  0  0  0  0
 46135  1  0  0  0  0
 46136  1  0  0  0  0
 47137  1  0  0  0  0
 47138  1  0  0  0  0
 48139  1  0  0  0  0
 48140  1  0  0  0  0
 49141  1  0  0  0  0
 49142  1  0  0  0  0
 50143  1  0  0  0  0
 50144  1  0  0  0  0
 51145  1  0  0  0  0
 52146  1  0  0  0  0
 53147  1  0  0  0  0
 53148  1  0  0  0  0
 54149  1  0  0  0  0
 55150  1  0  0  0  0
 56151  1  0  0  0  0
 56152  1  0  0  0  0
 57153  1  0  0  0  0
 57154  1  0  0  0  0
 58155  1  0  0  0  0
 58156  1  0  0  0  0
 59157  1  0  0  0  0
 59158  1  0  0  0  0
 60159  1  0  0  0  0
 60160  1  0  0  0  0
 60161  1  0  0  0  0
M  END
>  <Alias name>
Trilinolein

>  <deltaH°(c)>   kJ/mol
-34556

$$$$
Tripalmitolein
  ChemBrain IXL

149148  0  0  0  0  0  0  0  0  1 V2000
   19.5820   89.4822    4.7096 C   0  0  0  0  0  0  0  0  0  0  0  0
   18.3455   90.0166    3.9566 C   0  0  0  0  0  0  0  0  0  0  0  0
   17.0668   89.5970    4.7121 C   0  0  0  0  0  0  0  0  0  0  0  0
   22.0622   89.4127    4.6100 C   0  0  0  0  0  0  0  0  0  0  0  0
   23.3648   89.7438    3.9843 C   0  0  0  0  0  0  0  0  0  0  0  0
   23.1314   90.5672    2.7006 C   0  0  0  0  0  0  0  0  0  0  0  0
   24.4912   90.9089    2.0578 C   0  0  0  0  0  0  0  0  0  0  0  0
   24.2644   91.7323    0.7741 C   0  0  0  0  0  0  0  0  0  0  0  0
   25.6266   92.0732    0.1365 C   0  0  0  0  0  0  0  0  0  0  0  0
   25.4039   92.8961   -1.1481 C   0  0  0  0  0  0  0  0  0  0  0  0
   22.0315   88.7570    5.6386 O   0  0  0  0  0  0  0  0  0  0  0  0
```

20.8057	89.8735	3.9925	O	0	0	0	0	0	0	0	0	0	0	0	0	0
26.7677	93.2362	-1.7824	C	0	0	0	0	0	0	0	0	0	0	0	0	0
31.4291	94.4102	-3.8083	C	0	0	0	0	0	0	0	0	0	0	0	0	0
34.8889	95.2245	-5.3612	C	0	0	0	0	0	0	0	0	0	0	0	0	0
33.8750	94.6596	-4.3475	C	0	0	0	0	0	0	0	0	0	0	0	0	0
32.4429	94.9748	-4.8226	C	0	0	0	0	0	0	0	0	0	0	0	0	0
27.6057	94.4642	-3.7217	C	0	0	0	0	0	0	0	0	0	0	0	0	0
28.9836	94.1609	-3.2675	C	0	0	0	0	0	0	0	0	0	0	0	0	0
26.5566	94.0264	-3.0186	C	0	0	0	0	0	0	0	0	0	0	0	0	0
29.9969	94.7258	-4.2826	C	0	0	0	0	0	0	0	0	0	0	0	0	0
18.4128	91.4875	3.8762	O	0	0	0	0	0	0	0	0	0	0	0	0	0
15.8826	90.0968	3.9963	O	0	0	0	0	0	0	0	0	0	0	0	0	0
18.4483	92.1903	5.1725	C	0	0	0	0	0	0	0	0	0	0	0	0	0
18.5180	93.6699	5.2149	C	0	0	0	0	0	0	0	0	0	0	0	0	0
18.5312	94.2314	3.7784	C	0	0	0	0	0	0	0	0	0	0	0	0	0
18.6028	95.7700	3.8318	C	0	0	0	0	0	0	0	0	0	0	0	0	0
18.6139	96.3368	2.3987	C	0	0	0	0	0	0	0	0	0	0	0	0	0
18.6835	97.8754	2.4580	C	0	0	0	0	0	0	0	0	0	0	0	0	0
18.6913	98.4476	1.0271	C	0	0	0	0	0	0	0	0	0	0	0	0	0
18.4204	91.5542	6.2135	O	0	0	0	0	0	0	0	0	0	0	0	0	0
18.7592	99.9863	1.0916	C	0	0	0	0	0	0	0	0	0	0	0	0	0
19.0612	105.1809	1.4143	C	0	0	0	0	0	0	0	0	0	0	0	0	0
19.2073	109.0494	1.6487	C	0	0	0	0	0	0	0	0	0	0	0	0	0
19.1823	107.5883	2.1376	C	0	0	0	0	0	0	0	0	0	0	0	0	0
19.0854	106.6420	0.9249	C	0	0	0	0	0	0	0	0	0	0	0	0	0
18.8506	101.8610	-0.4713	C	0	0	0	0	0	0	0	0	0	0	0	0	0
18.9419	102.7733	0.6928	C	0	0	0	0	0	0	0	0	0	0	0	0	0
18.7641	100.5410	-0.2824	C	0	0	0	0	0	0	0	0	0	0	0	0	0
18.9653	104.2340	0.2021	C	0	0	0	0	0	0	0	0	0	0	0	0	0
14.5895	89.7508	4.6147	C	0	0	0	0	0	0	0	0	0	0	0	0	0
13.3206	90.1977	3.9900	C	0	0	0	0	0	0	0	0	0	0	0	0	0
13.6252	91.0069	2.7118	C	0	0	0	0	0	0	0	0	0	0	0	0	0
12.3010	91.4708	2.0721	C	0	0	0	0	0	0	0	0	0	0	0	0	0
12.5998	92.2845	0.7970	C	0	0	0	0	0	0	0	0	0	0	0	0	0
11.2732	92.7514	0.1646	C	0	0	0	0	0	0	0	0	0	0	0	0	0
11.5680	93.5716	-1.1070	C	0	0	0	0	0	0	0	0	0	0	0	0	0
14.5620	89.0950	5.6433	O	0	0	0	0	0	0	0	0	0	0	0	0	0
10.2397	94.0410	-1.7340	C	0	0	0	0	0	0	0	0	0	0	0	0	0
5.6968	95.5901	-3.7758	C	0	0	0	0	0	0	0	0	0	0	0	0	0
2.3263	96.8070	-5.2600	C	0	0	0	0	0	0	0	0	0	0	0	0	0
3.2831	96.0749	-4.2992	C	0	0	0	0	0	0	0	0	0	0	0	0	0
4.7402	96.3232	-4.7364	C	0	0	0	0	0	0	0	0	0	0	0	0	0
9.5117	95.3367	-3.6733	C	0	0	0	0	0	0	0	0	0	0	0	0	0
8.1099	95.1023	-3.2528	C	0	0	0	0	0	0	0	0	0	0	0	0	0
10.5201	94.8342	-2.9543	C	0	0	0	0	0	0	0	0	0	0	0	0	0
7.1540	95.8366	-4.2135	C	0	0	0	0	0	0	0	0	0	0	0	0	0
19.6021	89.8970	5.7098	H	0	0	0	0	0	0	0	0	0	0	0	0	0
19.5288	88.4022	4.7684	H	0	0	0	0	0	0	0	0	0	0	0	0	0
18.3256	89.6016	2.9566	H	0	0	0	0	0	0	0	0	0	0	0	0	0
17.0220	88.5167	4.7719	H	0	0	0	0	0	0	0	0	0	0	0	0	0
17.0858	90.0128	5.7119	H	0	0	0	0	0	0	0	0	0	0	0	0	0
23.9614	90.3216	4.6792	H	0	0	0	0	0	0	0	0	0	0	0	0	0
23.8876	88.8287	3.7352	H	0	0	0	0	0	0	0	0	0	0	0	0	0
22.5373	89.9892	2.0035	H	0	0	0	0	0	0	0	0	0	0	0	0	0
22.6087	91.4828	2.9485	H	0	0	0	0	0	0	0	0	0	0	0	0	0
25.0851	91.4859	2.7559	H	0	0	0	0	0	0	0	0	0	0	0	0	0
25.0138	89.9930	1.8110	H	0	0	0	0	0	0	0	0	0	0	0	0	0
23.6725	91.1550	0.0745	H	0	0	0	0	0	0	0	0	0	0	0	0	0
23.7414	92.6482	1.0200	H	0	0	0	0	0	0	0	0	0	0	0	0	0
26.2181	92.6503	0.8365	H	0	0	0	0	0	0	0	0	0	0	0	0	0
26.1497	91.1570	-0.1082	H	0	0	0	0	0	0	0	0	0	0	0	0	0
24.8136	92.3189	-1.8489	H	0	0	0	0	0	0	0	0	0	0	0	0	0
24.8807	93.8123	-0.9039	H	0	0	0	0	0	0	0	0	0	0	0	0	0

27.3576	93.8137	-1.0813	H	0	0	0	0	0	0	0	0	0	0	0	0
27.2912	92.3197	-2.0252	H	0	0	0	0	0	0	0	0	0	0	0	0
31.5968	94.8643	-2.8395	H	0	0	0	0	0	0	0	0	0	0	0	0
31.5555	93.3374	-3.7308	H	0	0	0	0	0	0	0	0	0	0	0	0
34.7624	96.2972	-5.4387	H	0	0	0	0	0	0	0	0	0	0	0	0
34.7221	94.7703	-6.3300	H	0	0	0	0	0	0	0	0	0	0	0	0
35.8949	95.0029	-5.0271	H	0	0	0	0	0	0	0	0	0	0	0	0
34.0421	95.1137	-3.3787	H	0	0	0	0	0	0	0	0	0	0	0	0
34.0016	93.5868	-4.2700	H	0	0	0	0	0	0	0	0	0	0	0	0
32.2757	94.5204	-5.7913	H	0	0	0	0	0	0	0	0	0	0	0	0
32.3164	96.0476	-4.9003	H	0	0	0	0	0	0	0	0	0	0	0	0
27.4485	95.0414	-4.6245	H	0	0	0	0	0	0	0	0	0	0	0	0
29.1524	94.6148	-2.2988	H	0	0	0	0	0	0	0	0	0	0	0	0
29.1103	93.0881	-3.1904	H	0	0	0	0	0	0	0	0	0	0	0	0
25.5509	94.2495	-3.3528	H	0	0	0	0	0	0	0	0	0	0	0	0
29.8290	94.2715	-5.2513	H	0	0	0	0	0	0	0	0	0	0	0	0
29.8704	95.7986	-4.3601	H	0	0	0	0	0	0	0	0	0	0	0	0
19.4218	93.9733	5.7285	H	0	0	0	0	0	0	0	0	0	0	0	0
17.6559	94.0559	5.7445	H	0	0	0	0	0	0	0	0	0	0	0	0
17.6272	93.9294	3.2640	H	0	0	0	0	0	0	0	0	0	0	0	0
19.3941	93.8477	3.2482	H	0	0	0	0	0	0	0	0	0	0	0	0
17.7408	96.1527	4.3641	H	0	0	0	0	0	0	0	0	0	0	0	0
19.5071	96.0713	4.3458	H	0	0	0	0	0	0	0	0	0	0	0	0
17.7099	96.0356	1.8840	H	0	0	0	0	0	0	0	0	0	0	0	0
19.4768	95.9562	1.8661	H	0	0	0	0	0	0	0	0	0	0	0	0
17.8220	98.2549	2.9934	H	0	0	0	0	0	0	0	0	0	0	0	0
19.5884	98.1760	2.9716	H	0	0	0	0	0	0	0	0	0	0	0	0
17.7866	98.1472	0.5131	H	0	0	0	0	0	0	0	0	0	0	0	0
19.5535	98.0700	0.4916	H	0	0	0	0	0	0	0	0	0	0	0	0
17.8980	100.3630	1.6295	H	0	0	0	0	0	0	0	0	0	0	0	0
19.6645	100.2861	1.6049	H	0	0	0	0	0	0	0	0	0	0	0	0
18.2040	105.0303	2.0587	H	0	0	0	0	0	0	0	0	0	0	0	0
19.9690	104.9701	1.9659	H	0	0	0	0	0	0	0	0	0	0	0	0
18.2995	109.2612	1.0975	H	0	0	0	0	0	0	0	0	0	0	0	0
19.2755	109.7140	2.5009	H	0	0	0	0	0	0	0	0	0	0	0	0
20.0645	109.1998	1.0041	H	0	0	0	0	0	0	0	0	0	0	0	0
18.3252	107.4380	2.7822	H	0	0	0	0	0	0	0	0	0	0	0	0
20.0901	107.3767	2.6889	H	0	0	0	0	0	0	0	0	0	0	0	0
19.9423	106.7926	0.2801	H	0	0	0	0	0	0	0	0	0	0	0	0
18.1773	106.8532	0.3738	H	0	0	0	0	0	0	0	0	0	0	0	0
18.8540	102.2643	-1.4764	H	0	0	0	0	0	0	0	0	0	0	0	0
18.0845	102.6230	1.3372	H	0	0	0	0	0	0	0	0	0	0	0	0
19.8496	102.5638	1.2451	H	0	0	0	0	0	0	0	0	0	0	0	0
18.6975	99.8760	-1.1347	H	0	0	0	0	0	0	0	0	0	0	0	0
19.8224	104.3847	-0.4425	H	0	0	0	0	0	0	0	0	0	0	0	0
18.0572	104.4443	-0.3494	H	0	0	0	0	0	0	0	0	0	0	0	0
12.7210	89.3329	3.7343	H	0	0	0	0	0	0	0	0	0	0	0	0
12.7753	90.8202	4.6886	H	0	0	0	0	0	0	0	0	0	0	0	0
14.2259	91.8715	2.9662	H	0	0	0	0	0	0	0	0	0	0	0	0
14.1669	90.3843	2.0104	H	0	0	0	0	0	0	0	0	0	0	0	0
11.7017	90.6059	1.8159	H	0	0	0	0	0	0	0	0	0	0	0	0
11.7583	92.0899	2.7757	H	0	0	0	0	0	0	0	0	0	0	0	0
13.2016	93.1478	1.0524	H	0	0	0	0	0	0	0	0	0	0	0	0
13.1384	91.6648	0.0908	H	0	0	0	0	0	0	0	0	0	0	0	0
10.6731	91.8878	-0.0941	H	0	0	0	0	0	0	0	0	0	0	0	0
10.7329	93.3668	0.8733	H	0	0	0	0	0	0	0	0	0	0	0	0
12.1699	94.4341	-0.8488	H	0	0	0	0	0	0	0	0	0	0	0	0
12.1052	92.9558	-1.8176	H	0	0	0	0	0	0	0	0	0	0	0	0
9.6390	93.1782	-1.9944	H	0	0	0	0	0	0	0	0	0	0	0	0
9.7013	94.6536	-1.0214	H	0	0	0	0	0	0	0	0	0	0	0	0
5.4877	94.5277	-3.7994	H	0	0	0	0	0	0	0	0	0	0	0	0
5.5541	95.9637	-2.7694	H	0	0	0	0	0	0	0	0	0	0	0	0
1.3027	96.6322	-4.9526	H	0	0	0	0	0	0	0	0	0	0	0	0

```
    2.5339   97.8697   -5.2360 H   0  0  0  0  0  0  0  0  0  0  0  0
    2.4698   96.4337   -6.2664 H   0  0  0  0  0  0  0  0  0  0  0  0
    3.0752   95.0123   -4.3230 H   0  0  0  0  0  0  0  0  0  0  0  0
    3.1395   96.4483   -3.2928 H   0  0  0  0  0  0  0  0  0  0  0  0
    4.9483   97.3857   -4.7120 H   0  0  0  0  0  0  0  0  0  0  0  0
    4.8835   95.9502   -5.7429 H   0  0  0  0  0  0  0  0  0  0  0  0
    9.7194   95.9160   -4.5646 H   0  0  0  0  0  0  0  0  0  0  0  0
    7.8990   94.0403   -3.2760 H   0  0  0  0  0  0  0  0  0  0  0  0
    7.9675   95.4766   -2.2465 H   0  0  0  0  0  0  0  0  0  0  0  0
   11.5434   95.0070   -3.2638 H   0  0  0  0  0  0  0  0  0  0  0  0
    7.3634   96.8989   -4.1893 H   0  0  0  0  0  0  0  0  0  0  0  0
    7.2966   95.4633   -5.2201 H   0  0  0  0  0  0  0  0  0  0  0  0
  1 12  1  0  0  0  0
  1  2  1  0  0  0  0
  2  3  1  0  0  0  0
  2 22  1  0  0  0  0
  3 23  1  0  0  0  0
  4  5  1  0  0  0  0
  4 12  1  0  0  0  0
  4 11  2  0  0  0  0
  5  6  1  0  0  0  0
  6  7  1  0  0  0  0
  7  8  1  0  0  0  0
  8  9  1  0  0  0  0
  9 10  1  0  0  0  0
 10 13  1  0  0  0  0
 13 20  1  0  0  0  0
 14 17  1  0  0  0  0
 14 21  1  0  0  0  0
 15 16  1  0  0  0  0
 16 17  1  0  0  0  0
 18 19  1  0  0  0  0
 18 20  2  0  0  0  0
 19 21  1  0  0  0  0
 22 24  1  0  0  0  0
 23 41  1  0  0  0  0
 24 25  1  0  0  0  0
 24 31  2  0  0  0  0
 25 26  1  0  0  0  0
 26 27  1  0  0  0  0
 27 28  1  0  0  0  0
 28 29  1  0  0  0  0
 29 30  1  0  0  0  0
 30 32  1  0  0  0  0
 32 39  1  0  0  0  0
 33 36  1  0  0  0  0
 33 40  1  0  0  0  0
 34 35  1  0  0  0  0
 35 36  1  0  0  0  0
 37 38  1  0  0  0  0
 37 39  2  0  0  0  0
 38 40  1  0  0  0  0
 41 42  1  0  0  0  0
 41 48  2  0  0  0  0
 42 43  1  0  0  0  0
 43 44  1  0  0  0  0
 44 45  1  0  0  0  0
 45 46  1  0  0  0  0
 46 47  1  0  0  0  0
 47 49  1  0  0  0  0
 49 56  1  0  0  0  0
 50 53  1  0  0  0  0
 50 57  1  0  0  0  0
```

51	52	1	0	0	0	0
52	53	1	0	0	0	0
54	55	1	0	0	0	0
54	56	2	0	0	0	0
55	57	1	0	0	0	0
1	58	1	0	0	0	0
1	59	1	0	0	0	0
2	60	1	0	0	0	0
3	61	1	0	0	0	0
3	62	1	0	0	0	0
5	63	1	0	0	0	0
5	64	1	0	0	0	0
6	65	1	0	0	0	0
6	66	1	0	0	0	0
7	67	1	0	0	0	0
7	68	1	0	0	0	0
8	69	1	0	0	0	0
8	70	1	0	0	0	0
9	71	1	0	0	0	0
9	72	1	0	0	0	0
10	73	1	0	0	0	0
10	74	1	0	0	0	0
13	75	1	0	0	0	0
13	76	1	0	0	0	0
14	77	1	0	0	0	0
14	78	1	0	0	0	0
15	79	1	0	0	0	0
15	80	1	0	0	0	0
15	81	1	0	0	0	0
16	82	1	0	0	0	0
16	83	1	0	0	0	0
17	84	1	0	0	0	0
17	85	1	0	0	0	0
18	86	1	0	0	0	0
19	87	1	0	0	0	0
19	88	1	0	0	0	0
20	89	1	0	0	0	0
21	90	1	0	0	0	0
21	91	1	0	0	0	0
25	92	1	0	0	0	0
25	93	1	0	0	0	0
26	94	1	0	0	0	0
26	95	1	0	0	0	0
27	96	1	0	0	0	0
27	97	1	0	0	0	0
28	98	1	0	0	0	0
28	99	1	0	0	0	0
29	100	1	0	0	0	0
29	101	1	0	0	0	0
30	102	1	0	0	0	0
30	103	1	0	0	0	0
32	104	1	0	0	0	0
32	105	1	0	0	0	0
33	106	1	0	0	0	0
33	107	1	0	0	0	0
34	108	1	0	0	0	0
34	109	1	0	0	0	0
34	110	1	0	0	0	0
35	111	1	0	0	0	0
35	112	1	0	0	0	0
36	113	1	0	0	0	0
36	114	1	0	0	0	0
37	115	1	0	0	0	0

```
 38116  1  0  0  0  0
 38117  1  0  0  0  0
 39118  1  0  0  0  0
 40119  1  0  0  0  0
 40120  1  0  0  0  0
 42121  1  0  0  0  0
 42122  1  0  0  0  0
 43123  1  0  0  0  0
 43124  1  0  0  0  0
 44125  1  0  0  0  0
 44126  1  0  0  0  0
 45127  1  0  0  0  0
 45128  1  0  0  0  0
 46129  1  0  0  0  0
 46130  1  0  0  0  0
 47131  1  0  0  0  0
 47132  1  0  0  0  0
 49133  1  0  0  0  0
 49134  1  0  0  0  0
 50135  1  0  0  0  0
 50136  1  0  0  0  0
 51137  1  0  0  0  0
 51138  1  0  0  0  0
 51139  1  0  0  0  0
 52140  1  0  0  0  0
 52141  1  0  0  0  0
 53142  1  0  0  0  0
 53143  1  0  0  0  0
 54144  1  0  0  0  0
 55145  1  0  0  0  0
 55146  1  0  0  0  0
 56147  1  0  0  0  0
 57148  1  0  0  0  0
 57149  1  0  0  0  0
M  END
>  <Alias name>
Tripalmitolein

>  <deltaH°(c)>   kJ/mol
-31179

$$$$
Trimyristin
  ChemBrain IXL

137136  0  0  0  0  0  0  0  0  1 V2000
   16.7289   11.1526    4.0969 C   0  0  0  0  0  0  0  0  0  0  0  0  0
   18.0030   10.8324    3.2914 C   0  0  0  0  0  0  0  0  0  0  0  0  0
   16.6154   12.6767    4.2946 C   0  0  0  0  0  0  0  0  0  0  0  0  0
   15.5511   10.6644    3.3652 O   0  0  0  0  0  0  0  0  0  0  0  0  0
   18.1129    9.3782    3.1060 O   0  0  0  0  0  0  0  0  0  0  0  0  0
   15.3996   12.9820    5.0625 O   0  0  0  0  0  0  0  0  0  0  0  0  0
   15.3583   11.2552    2.0331 C   0  0  0  0  0  0  0  0  0  0  0  0  0
   19.3087    8.9334    2.3757 C   0  0  0  0  0  0  0  0  0  0  0  0  0
   15.1832   14.4088    5.3423 C   0  0  0  0  0  0  0  0  0  0  0  0  0
   14.2061   10.8433    1.2005 C   0  0  0  0  0  0  0  0  0  0  0  0  0
   16.1482   12.0851    1.6137 O   0  0  0  0  0  0  0  0  0  0  0  0  0
   20.2873    9.9282    1.8825 C   0  0  0  0  0  0  0  0  0  0  0  0  0
   19.4945    7.7443    2.1762 O   0  0  0  0  0  0  0  0  0  0  0  0  0
   16.1516   15.4145    4.8512 C   0  0  0  0  0  0  0  0  0  0  0  0  0
   14.2052   14.7640    5.9793 O   0  0  0  0  0  0  0  0  0  0  0  0  0
   13.3557    9.8160    1.9726 C   0  0  0  0  0  0  0  0  0  0  0  0  0
   21.4031    9.2074    1.1013 C   0  0  0  0  0  0  0  0  0  0  0  0  0
```

15.6884	16.8225	5.2729	C	0	0	0	0	0	0	0	0	0	0	0	0	0
12.1556	9.3877	1.1061	C	0	0	0	0	0	0	0	0	0	0	0	0	0
22.4261	10.2423	0.5941	C	0	0	0	0	0	0	0	0	0	0	0	0	0
16.6939	17.8701	4.7571	C	0	0	0	0	0	0	0	0	0	0	0	0	0
11.3055	8.3600	1.8780	C	0	0	0	0	0	0	0	0	0	0	0	0	0
23.5412	9.5216	-0.1881	C	0	0	0	0	0	0	0	0	0	0	0	0	0
16.2291	19.2783	5.1766	C	0	0	0	0	0	0	0	0	0	0	0	0	0
10.1059	7.9310	1.0111	C	0	0	0	0	0	0	0	0	0	0	0	0	0
24.5697	10.5553	-0.6866	C	0	0	0	0	0	0	0	0	0	0	0	0	0
17.2331	20.3263	4.6584	C	0	0	0	0	0	0	0	0	0	0	0	0	0
9.2556	6.9035	1.7831	C	0	0	0	0	0	0	0	0	0	0	0	0	0
25.6820	9.8351	-1.4732	C	0	0	0	0	0	0	0	0	0	0	0	0	0
16.7668	21.7345	5.0756	C	0	0	0	0	0	0	0	0	0	0	0	0	0
8.0563	6.4741	0.9160	C	0	0	0	0	0	0	0	0	0	0	0	0	0
26.7142	10.8679	-1.9656	C	0	0	0	0	0	0	0	0	0	0	0	0	0
17.7691	22.7829	4.5551	C	0	0	0	0	0	0	0	0	0	0	0	0	0
7.2055	5.4473	1.6884	C	0	0	0	0	0	0	0	0	0	0	0	0	0
27.8217	10.1487	-2.7600	C	0	0	0	0	0	0	0	0	0	0	0	0	0
17.3014	24.1913	4.9702	C	0	0	0	0	0	0	0	0	0	0	0	0	0
6.0062	5.0178	0.8212	C	0	0	0	0	0	0	0	0	0	0	0	0	0
28.8559	11.1810	-3.2494	C	0	0	0	0	0	0	0	0	0	0	0	0	0
18.3025	25.2399	4.4478	C	0	0	0	0	0	0	0	0	0	0	0	0	0
5.1547	3.9920	1.5941	C	0	0	0	0	0	0	0	0	0	0	0	0	0
29.9586	10.4630	-4.0514	C	0	0	0	0	0	0	0	0	0	0	0	0	0
17.8339	26.6484	4.8616	C	0	0	0	0	0	0	0	0	0	0	0	0	0
3.9554	3.5626	0.7269	C	0	0	0	0	0	0	0	0	0	0	0	0	0
30.9937	11.4950	-4.5393	C	0	0	0	0	0	0	0	0	0	0	0	0	0
18.8343	27.6971	4.3379	C	0	0	0	0	0	0	0	0	0	0	0	0	0
3.1034	2.5375	1.5001	C	0	0	0	0	0	0	0	0	0	0	0	0	0
32.0934	10.7777	-5.3461	C	0	0	0	0	0	0	0	0	0	0	0	0	0
18.3652	29.1056	4.7512	C	0	0	0	0	0	0	0	0	0	0	0	0	0
1.9041	2.1081	0.6330	C	0	0	0	0	0	0	0	0	0	0	0	0	0
33.1292	11.8096	-5.8328	C	0	0	0	0	0	0	0	0	0	0	0	0	0
19.3654	30.1544	4.2273	C	0	0	0	0	0	0	0	0	0	0	0	0	0
16.7808	10.6661	5.0631	H	0	0	0	0	0	0	0	0	0	0	0	0	0
18.8706	11.1943	3.8291	H	0	0	0	0	0	0	0	0	0	0	0	0	0
17.9499	11.3166	2.3241	H	0	0	0	0	0	0	0	0	0	0	0	0	0
17.4829	13.0363	4.8340	H	0	0	0	0	0	0	0	0	0	0	0	0	0
16.5638	13.1632	3.3284	H	0	0	0	0	0	0	0	0	0	0	0	0	0
13.6009	11.7106	0.9674	H	0	0	0	0	0	0	0	0	0	0	0	0	0
14.5659	10.3966	0.2819	H	0	0	0	0	0	0	0	0	0	0	0	0	0
20.7190	10.4574	2.7230	H	0	0	0	0	0	0	0	0	0	0	0	0	0
19.7875	10.6335	1.2300	H	0	0	0	0	0	0	0	0	0	0	0	0	0
17.1275	15.2133	5.2756	H	0	0	0	0	0	0	0	0	0	0	0	0	0
16.2100	15.3621	3.7710	H	0	0	0	0	0	0	0	0	0	0	0	0	0
13.9608	8.9485	2.2054	H	0	0	0	0	0	0	0	0	0	0	0	0	0
12.9964	10.2626	2.8915	H	0	0	0	0	0	0	0	0	0	0	0	0	0
20.9721	8.6826	0.2577	H	0	0	0	0	0	0	0	0	0	0	0	0	0
21.8994	8.4984	1.7523	H	0	0	0	0	0	0	0	0	0	0	0	0	0
14.7109	17.0222	4.8515	H	0	0	0	0	0	0	0	0	0	0	0	0	0
15.6336	16.8760	6.3532	H	0	0	0	0	0	0	0	0	0	0	0	0	0
11.5503	10.2551	0.8736	H	0	0	0	0	0	0	0	0	0	0	0	0	0
12.5150	8.9415	0.1870	H	0	0	0	0	0	0	0	0	0	0	0	0	0
22.8578	10.7666	1.4377	H	0	0	0	0	0	0	0	0	0	0	0	0	0
21.9297	10.9519	-0.0562	H	0	0	0	0	0	0	0	0	0	0	0	0	0
17.6710	17.6716	5.1800	H	0	0	0	0	0	0	0	0	0	0	0	0	0
16.7502	17.8154	3.6770	H	0	0	0	0	0	0	0	0	0	0	0	0	0
11.9110	7.4928	2.1109	H	0	0	0	0	0	0	0	0	0	0	0	0	0
10.9457	8.8063	2.7967	H	0	0	0	0	0	0	0	0	0	0	0	0	0
23.1104	9.0033	-1.0359	H	0	0	0	0	0	0	0	0	0	0	0	0	0
24.0329	8.8069	0.4602	H	0	0	0	0	0	0	0	0	0	0	0	0	0
15.2512	19.4760	4.7553	H	0	0	0	0	0	0	0	0	0	0	0	0	0
16.1748	19.3336	6.2568	H	0	0	0	0	0	0	0	0	0	0	0	0	0

9.5005	8.7982	0.7780	H	0	0	0	0	0	0	0	0	0	0	0	0	0
10.4657	7.4844	0.0924	H	0	0	0	0	0	0	0	0	0	0	0	0	0
25.0031	11.0711	0.1613	H	0	0	0	0	0	0	0	0	0	0	0	0	0
24.0772	11.2721	-1.3321	H	0	0	0	0	0	0	0	0	0	0	0	0	0
18.2106	20.1297	5.0812	H	0	0	0	0	0	0	0	0	0	0	0	0	0
17.2889	20.2697	3.5783	H	0	0	0	0	0	0	0	0	0	0	0	0	0
9.8611	6.0364	2.0165	H	0	0	0	0	0	0	0	0	0	0	0	0	0
8.8955	7.3501	2.7016	H	0	0	0	0	0	0	0	0	0	0	0	0	0
25.2492	9.3234	-2.3239	H	0	0	0	0	0	0	0	0	0	0	0	0	0
26.1711	9.1147	-0.8292	H	0	0	0	0	0	0	0	0	0	0	0	0	0
15.7884	21.9303	4.6546	H	0	0	0	0	0	0	0	0	0	0	0	0	0
16.7130	21.7917	6.1558	H	0	0	0	0	0	0	0	0	0	0	0	0	0
7.4512	7.3412	0.6820	H	0	0	0	0	0	0	0	0	0	0	0	0	0
8.4164	6.0267	-0.0022	H	0	0	0	0	0	0	0	0	0	0	0	0	0
27.1517	11.3751	-1.1146	H	0	0	0	0	0	0	0	0	0	0	0	0	0
26.2239	11.5920	-2.6045	H	0	0	0	0	0	0	0	0	0	0	0	0	0
18.7472	22.5881	4.9774	H	0	0	0	0	0	0	0	0	0	0	0	0	0
17.8242	22.7246	3.4750	H	0	0	0	0	0	0	0	0	0	0	0	0	0
7.8106	4.5802	1.9225	H	0	0	0	0	0	0	0	0	0	0	0	0	0
6.8453	5.8947	2.6065	H	0	0	0	0	0	0	0	0	0	0	0	0	0
27.3846	9.6436	-3.6124	H	0	0	0	0	0	0	0	0	0	0	0	0	0
28.3104	9.4228	-2.1218	H	0	0	0	0	0	0	0	0	0	0	0	0	0
16.3227	24.3854	4.5492	H	0	0	0	0	0	0	0	0	0	0	0	0	0
17.2479	24.2501	6.0503	H	0	0	0	0	0	0	0	0	0	0	0	0	0
5.4016	5.8851	0.5864	H	0	0	0	0	0	0	0	0	0	0	0	0	0
6.3664	4.5697	-0.0966	H	0	0	0	0	0	0	0	0	0	0	0	0	0
29.2977	11.6816	-2.3966	H	0	0	0	0	0	0	0	0	0	0	0	0	0
28.3661	11.9106	-3.8823	H	0	0	0	0	0	0	0	0	0	0	0	0	0
19.2810	25.0465	4.8697	H	0	0	0	0	0	0	0	0	0	0	0	0	0
18.3570	25.1803	3.3678	H	0	0	0	0	0	0	0	0	0	0	0	0	0
5.7593	3.1247	1.8288	H	0	0	0	0	0	0	0	0	0	0	0	0	0
4.7945	4.4402	2.5118	H	0	0	0	0	0	0	0	0	0	0	0	0	0
29.5170	9.9634	-4.9049	H	0	0	0	0	0	0	0	0	0	0	0	0	0
30.4476	9.7325	-3.4188	H	0	0	0	0	0	0	0	0	0	0	0	0	0
16.8550	26.8414	4.4404	H	0	0	0	0	0	0	0	0	0	0	0	0	0
17.7804	26.7082	5.9416	H	0	0	0	0	0	0	0	0	0	0	0	0	0
3.3511	4.4300	0.4916	H	0	0	0	0	0	0	0	0	0	0	0	0	0
4.3156	3.1139	-0.1906	H	0	0	0	0	0	0	0	0	0	0	0	0	0
31.4381	11.9918	-3.6857	H	0	0	0	0	0	0	0	0	0	0	0	0	0
30.5040	12.2277	-5.1687	H	0	0	0	0	0	0	0	0	0	0	0	0	0
19.8131	27.5044	4.7595	H	0	0	0	0	0	0	0	0	0	0	0	0	0
18.8881	27.6369	3.2580	H	0	0	0	0	0	0	0	0	0	0	0	0	0
3.7077	1.6701	1.7354	H	0	0	0	0	0	0	0	0	0	0	0	0	0
2.7432	2.9862	2.4176	H	0	0	0	0	0	0	0	0	0	0	0	0	0
31.6491	10.2817	-6.2002	H	0	0	0	0	0	0	0	0	0	0	0	0	0
32.5825	10.0444	-4.7169	H	0	0	0	0	0	0	0	0	0	0	0	0	0
17.3863	29.2982	4.3299	H	0	0	0	0	0	0	0	0	0	0	0	0	0
18.3116	29.1658	5.8312	H	0	0	0	0	0	0	0	0	0	0	0	0	0
2.2642	1.6594	-0.2846	H	0	0	0	0	0	0	0	0	0	0	0	0	0
1.3053	1.3876	1.1763	H	0	0	0	0	0	0	0	0	0	0	0	0	0
1.2998	2.9755	0.3977	H	0	0	0	0	0	0	0	0	0	0	0	0	0
32.6399	12.5434	-6.4612	H	0	0	0	0	0	0	0	0	0	0	0	0	0
33.9015	11.3056	-6.4006	H	0	0	0	0	0	0	0	0	0	0	0	0	0
33.5742	12.3049	-4.9787	H	0	0	0	0	0	0	0	0	0	0	0	0	0
19.0357	31.1443	4.5176	H	0	0	0	0	0	0	0	0	0	0	0	0	0
19.4191	30.0940	3.1473	H	0	0	0	0	0	0	0	0	0	0	0	0	0
20.3443	29.9618	4.6487	H	0	0	0	0	0	0	0	0	0	0	0	0	0

1	2	1	0	0	0	0
1	3	1	0	0	0	0
1	4	1	0	0	0	0
2	5	1	0	0	0	0
3	6	1	0	0	0	0
4	7	1	0	0	0	0

5	8	1	0	0	0	0
6	9	1	0	0	0	0
7	10	1	0	0	0	0
7	11	2	0	0	0	0
8	12	1	0	0	0	0
8	13	2	0	0	0	0
9	14	1	0	0	0	0
9	15	2	0	0	0	0
10	16	1	0	0	0	0
12	17	1	0	0	0	0
14	18	1	0	0	0	0
16	19	1	0	0	0	0
17	20	1	0	0	0	0
18	21	1	0	0	0	0
19	22	1	0	0	0	0
20	23	1	0	0	0	0
21	24	1	0	0	0	0
22	25	1	0	0	0	0
23	26	1	0	0	0	0
24	27	1	0	0	0	0
25	28	1	0	0	0	0
26	29	1	0	0	0	0
27	30	1	0	0	0	0
28	31	1	0	0	0	0
29	32	1	0	0	0	0
30	33	1	0	0	0	0
31	34	1	0	0	0	0
32	35	1	0	0	0	0
33	36	1	0	0	0	0
34	37	1	0	0	0	0
35	38	1	0	0	0	0
36	39	1	0	0	0	0
37	40	1	0	0	0	0
38	41	1	0	0	0	0
39	42	1	0	0	0	0
40	43	1	0	0	0	0
41	44	1	0	0	0	0
42	45	1	0	0	0	0
43	46	1	0	0	0	0
44	47	1	0	0	0	0
45	48	1	0	0	0	0
46	49	1	0	0	0	0
47	50	1	0	0	0	0
48	51	1	0	0	0	0
1	52	1	0	0	0	0
2	53	1	0	0	0	0
2	54	1	0	0	0	0
3	55	1	0	0	0	0
3	56	1	0	0	0	0
10	57	1	0	0	0	0
10	58	1	0	0	0	0
12	59	1	0	0	0	0
12	60	1	0	0	0	0
14	61	1	0	0	0	0
14	62	1	0	0	0	0
16	63	1	0	0	0	0
16	64	1	0	0	0	0
17	65	1	0	0	0	0
17	66	1	0	0	0	0
18	67	1	0	0	0	0
18	68	1	0	0	0	0
19	69	1	0	0	0	0
19	70	1	0	0	0	0

20	71	1	0	0	0	0
20	72	1	0	0	0	0
21	73	1	0	0	0	0
21	74	1	0	0	0	0
22	75	1	0	0	0	0
22	76	1	0	0	0	0
23	77	1	0	0	0	0
23	78	1	0	0	0	0
24	79	1	0	0	0	0
24	80	1	0	0	0	0
25	81	1	0	0	0	0
25	82	1	0	0	0	0
26	83	1	0	0	0	0
26	84	1	0	0	0	0
27	85	1	0	0	0	0
27	86	1	0	0	0	0
28	87	1	0	0	0	0
28	88	1	0	0	0	0
29	89	1	0	0	0	0
29	90	1	0	0	0	0
30	91	1	0	0	0	0
30	92	1	0	0	0	0
31	93	1	0	0	0	0
31	94	1	0	0	0	0
32	95	1	0	0	0	0
32	96	1	0	0	0	0
33	97	1	0	0	0	0
33	98	1	0	0	0	0
34	99	1	0	0	0	0
34	100	1	0	0	0	0
35	101	1	0	0	0	0
35	102	1	0	0	0	0
36	103	1	0	0	0	0
36	104	1	0	0	0	0
37	105	1	0	0	0	0
37	106	1	0	0	0	0
38	107	1	0	0	0	0
38	108	1	0	0	0	0
39	109	1	0	0	0	0
39	110	1	0	0	0	0
40	111	1	0	0	0	0
40	112	1	0	0	0	0
41	113	1	0	0	0	0
41	114	1	0	0	0	0
42	115	1	0	0	0	0
42	116	1	0	0	0	0
43	117	1	0	0	0	0
43	118	1	0	0	0	0
44	119	1	0	0	0	0
44	120	1	0	0	0	0
45	121	1	0	0	0	0
45	122	1	0	0	0	0
46	123	1	0	0	0	0
46	124	1	0	0	0	0
47	125	1	0	0	0	0
47	126	1	0	0	0	0
48	127	1	0	0	0	0
48	128	1	0	0	0	0
49	129	1	0	0	0	0
49	130	1	0	0	0	0
49	131	1	0	0	0	0
50	132	1	0	0	0	0
50	133	1	0	0	0	0

```
 50134  1  0  0  0  0
 51135  1  0  0  0  0
 51136  1  0  0  0  0
 51137  1  0  0  0  0
M  END
>  <Alias name>
Trimyristin

>  <deltaH°(c)>   kJ/mol
-27842

$$$$
Tetrakis(p-tolyl)porphyrin
  ChemBrain IXL

 90 98  0  0  0  0  0  0  0  0  0  1 V2000
    8.3171    8.5460    0.9156 C   0  0  0  0  0  0  0  0  0  0  0  0
    9.2435    7.6267    0.9834 C   0  0  0  0  0  0  0  0  0  0  0  0
   10.5469    8.2669    0.7316 C   0  0  0  0  0  0  0  0  0  0  0  0
   10.3134    9.5923    0.4949 N   0  0  0  0  0  0  0  0  0  0  0  0
    8.9643    9.8243    0.5881 C   0  0  0  0  0  0  0  0  0  0  0  0
   11.7638    7.5808    0.7526 C   0  0  0  0  0  0  0  0  0  0  0  0
   14.6630    9.6481    0.0178 C   0  0  0  0  0  0  0  0  0  0  0  0
   15.2511    8.4161    0.3066 C   0  0  0  0  0  0  0  0  0  0  0  0
   14.2283    7.5128    0.6332 C   0  0  0  0  0  0  0  0  0  0  0  0
   13.0058    8.1869    0.5387 C   0  0  0  0  0  0  0  0  0  0  0  0
   13.2850    9.5037    0.1881 N   0  0  0  0  0  0  0  0  0  0  0  0
   15.3535   10.7913   -0.3791 C   0  0  0  0  0  0  0  0  0  0  0  0
   14.6844   11.9977   -0.5700 C   0  0  0  0  0  0  0  0  0  0  0  0
   15.3301   13.2735   -0.9099 C   0  0  0  0  0  0  0  0  0  0  0  0
   14.4029   14.1913   -0.9850 C   0  0  0  0  0  0  0  0  0  0  0  0
   13.1005   13.5526   -0.7248 C   0  0  0  0  0  0  0  0  0  0  0  0
   13.3353   12.2296   -0.4764 N   0  0  0  0  0  0  0  0  0  0  0  0
   11.8835   14.2380   -0.7496 C   0  0  0  0  0  0  0  0  0  0  0  0
   10.6420   13.6336   -0.5278 C   0  0  0  0  0  0  0  0  0  0  0  0
    9.4199   14.3095   -0.6142 C   0  0  0  0  0  0  0  0  0  0  0  0
    8.3981   13.4080   -0.2799 C   0  0  0  0  0  0  0  0  0  0  0  0
    8.9861   12.1751    0.0046 C   0  0  0  0  0  0  0  0  0  0  0  0
   10.3631   12.3172   -0.1757 N   0  0  0  0  0  0  0  0  0  0  0  0
    8.2965   11.0331    0.4065 C   0  0  0  0  0  0  0  0  0  0  0  0
   11.9847   18.4113   -1.5603 C   0  0  0  0  0  0  0  0  0  0  0  0
   11.1400   17.9178   -0.5659 C   0  0  0  0  0  0  0  0  0  0  0  0
   11.1051   16.5511   -0.3002 C   0  0  0  0  0  0  0  0  0  0  0  0
   11.9129   15.6826   -1.0305 C   0  0  0  0  0  0  0  0  0  0  0  0
   12.7520   16.1734   -2.0271 C   0  0  0  0  0  0  0  0  0  0  0  0
   12.7905   17.5392   -2.2911 C   0  0  0  0  0  0  0  0  0  0  0  0
    4.0713   11.2127    1.1077 C   0  0  0  0  0  0  0  0  0  0  0  0
    4.8879   12.2778    1.4902 C   0  0  0  0  0  0  0  0  0  0  0  0
    6.2653   12.2241    1.2564 C   0  0  0  0  0  0  0  0  0  0  0  0
    6.8289   11.1022    0.6419 C   0  0  0  0  0  0  0  0  0  0  0  0
    6.0072   10.0430    0.2487 C   0  0  0  0  0  0  0  0  0  0  0  0
    4.6312   10.0966    0.4858 C   0  0  0  0  0  0  0  0  0  0  0  0
   19.5863   10.6231   -1.0342 C   0  0  0  0  0  0  0  0  0  0  0  0
   19.0152   11.7429   -0.4295 C   0  0  0  0  0  0  0  0  0  0  0  0
   17.6365   11.7931   -0.2080 C   0  0  0  0  0  0  0  0  0  0  0  0
   16.8235   10.7265   -0.5992 C   0  0  0  0  0  0  0  0  0  0  0  0
   17.3981    9.6015   -1.1976 C   0  0  0  0  0  0  0  0  0  0  0  0
   18.7782    9.5511   -1.4156 C   0  0  0  0  0  0  0  0  0  0  0  0
   11.6618    3.4000    1.5246 C   0  0  0  0  0  0  0  0  0  0  0  0
   10.8506    4.2645    2.2585 C   0  0  0  0  0  0  0  0  0  0  0  0
   10.8893    5.6329    2.0075 C   0  0  0  0  0  0  0  0  0  0  0  0
   11.7340    6.1337    1.0205 C   0  0  0  0  0  0  0  0  0  0  0  0
   12.5472    5.2727    0.2873 C   0  0  0  0  0  0  0  0  0  0  0  0
```

```
  12.5122    3.9037    0.5402 C   0  0  0  0  0  0  0  0  0  0  0  0
   2.5482   11.2685    1.3723 C   0  0  0  0  0  0  0  0  0  0  0  0
  11.6181    1.8914    1.8014 C   0  0  0  0  0  0  0  0  0  0  0  0
  21.1125   10.5708   -1.2810 C   0  0  0  0  0  0  0  0  0  0  0  0
  12.0281   19.9172   -1.8517 C   0  0  0  0  0  0  0  0  0  0  0  0
  12.6052   10.2452    0.1091 H   0  0  0  0  0  0  0  0  0  0  0  0
  11.0421   11.5743   -0.1026 H   0  0  0  0  0  0  0  0  0  0  0  0
   7.2567    8.3928    1.0772 H   0  0  0  0  0  0  0  0  0  0  0  0
   9.0812    6.5751    1.1854 H   0  0  0  0  0  0  0  0  0  0  0  0
  16.3115    8.1963    0.2851 H   0  0  0  0  0  0  0  0  0  0  0  0
  14.3621    6.4763    0.9158 H   0  0  0  0  0  0  0  0  0  0  0  0
  16.3902   13.4260   -1.0740 H   0  0  0  0  0  0  0  0  0  0  0  0
  14.5641   15.2410   -1.1976 H   0  0  0  0  0  0  0  0  0  0  0  0
   9.2858   15.3460   -0.8968 H   0  0  0  0  0  0  0  0  0  0  0  0
   7.3382   13.6297   -0.2503 H   0  0  0  0  0  0  0  0  0  0  0  0
  10.5123   18.5954   -0.0014 H   0  0  0  0  0  0  0  0  0  0  0  0
  10.4515   16.1639    0.4726 H   0  0  0  0  0  0  0  0  0  0  0  0
  13.3738   15.4923   -2.5958 H   0  0  0  0  0  0  0  0  0  0  0  0
  13.4453   17.9231   -3.0627 H   0  0  0  0  0  0  0  0  0  0  0  0
   4.4530   13.1474    1.9689 H   0  0  0  0  0  0  0  0  0  0  0  0
   6.8955   13.0531    1.5521 H   0  0  0  0  0  0  0  0  0  0  0  0
   6.4369    9.1786   -0.2407 H   0  0  0  0  0  0  0  0  0  0  0  0
   3.9976    9.2703    0.1864 H   0  0  0  0  0  0  0  0  0  0  0  0
  19.6421   12.5747   -0.1313 H   0  0  0  0  0  0  0  0  0  0  0  0
  17.1979   12.6606    0.2679 H   0  0  0  0  0  0  0  0  0  0  0  0
  16.7743    8.7676   -1.4930 H   0  0  0  0  0  0  0  0  0  0  0  0
  19.2217    8.6788   -1.8811 H   0  0  0  0  0  0  0  0  0  0  0  0
  10.1914    3.8728    3.0224 H   0  0  0  0  0  0  0  0  0  0  0  0
  10.2636    6.3080    2.5788 H   0  0  0  0  0  0  0  0  0  0  0  0
  13.2051    5.6677   -0.4778 H   0  0  0  0  0  0  0  0  0  0  0  0
  13.1442    3.2319   -0.0265 H   0  0  0  0  0  0  0  0  0  0  0  0
   2.1902   10.2799    1.6345 H   0  0  0  0  0  0  0  0  0  0  0  0
   2.3473   11.9527    2.1880 H   0  0  0  0  0  0  0  0  0  0  0  0
   2.0383   11.6115    0.4800 H   0  0  0  0  0  0  0  0  0  0  0  0
  12.6164    1.4812    1.7203 H   0  0  0  0  0  0  0  0  0  0  0  0
  11.2366    1.7195    2.7998 H   0  0  0  0  0  0  0  0  0  0  0  0
  10.9702    1.4114    1.0793 H   0  0  0  0  0  0  0  0  0  0  0  0
  21.4711   11.5601   -1.5395 H   0  0  0  0  0  0  0  0  0  0  0  0
  21.3244    9.8866   -2.0939 H   0  0  0  0  0  0  0  0  0  0  0  0
  21.6127   10.2294   -0.3826 H   0  0  0  0  0  0  0  0  0  0  0  0
  11.0300   20.3283   -1.7730 H   0  0  0  0  0  0  0  0  0  0  0  0
  12.4081   20.0794   -2.8523 H   0  0  0  0  0  0  0  0  0  0  0  0
  12.6772   20.4040   -1.1352 H   0  0  0  0  0  0  0  0  0  0  0  0
  1  2  2  0  0  0  0
  2  3  1  0  0  0  0
  3  4  4  0  0  0  0
  4  5  4  0  0  0  0
  5  1  1  0  0  0  0
  7  8  4  0  0  0  0
  8  9  4  0  0  0  0
  9 10  4  0  0  0  0
 10 11  1  0  0  0  0
 11  7  1  0  0  0  0
 12 13  4  0  0  0  0
 13 14  1  0  0  0  0
 14 15  2  0  0  0  0
 15 16  1  0  0  0  0
 16 17  4  0  0  0  0
 17 13  4  0  0  0  0
 16 18  4  0  0  0  0
 18 19  4  0  0  0  0
 19 20  4  0  0  0  0
 20 21  4  0  0  0  0
```

21	22	4	0	0	0	0
22	23	1	0	0	0	0
23	19	1	0	0	0	0
24	5	4	0	0	0	0
6	3	4	0	0	0	0
22	24	4	0	0	0	0
10	6	4	0	0	0	0
7	12	4	0	0	0	0
25	26	4	0	0	0	0
26	27	4	0	0	0	0
27	28	4	0	0	0	0
28	29	4	0	0	0	0
29	30	4	0	0	0	0
30	25	4	0	0	0	0
31	32	4	0	0	0	0
32	33	4	0	0	0	0
33	34	4	0	0	0	0
34	35	4	0	0	0	0
35	36	4	0	0	0	0
36	31	4	0	0	0	0
37	38	4	0	0	0	0
38	39	4	0	0	0	0
39	40	4	0	0	0	0
40	41	4	0	0	0	0
41	42	4	0	0	0	0
42	37	4	0	0	0	0
43	44	4	0	0	0	0
44	45	4	0	0	0	0
45	46	4	0	0	0	0
46	47	4	0	0	0	0
47	48	4	0	0	0	0
48	43	4	0	0	0	0
40	12	1	0	0	0	0
46	6	1	0	0	0	0
28	18	1	0	0	0	0
34	24	1	0	0	0	0
31	49	1	0	0	0	0
43	50	1	0	0	0	0
37	51	1	0	0	0	0
25	52	1	0	0	0	0
11	53	1	0	0	0	0
23	54	1	0	0	0	0
1	55	1	0	0	0	0
2	56	1	0	0	0	0
8	57	1	0	0	0	0
9	58	1	0	0	0	0
14	59	1	0	0	0	0
15	60	1	0	0	0	0
20	61	1	0	0	0	0
21	62	1	0	0	0	0
26	63	1	0	0	0	0
27	64	1	0	0	0	0
29	65	1	0	0	0	0
30	66	1	0	0	0	0
32	67	1	0	0	0	0
33	68	1	0	0	0	0
35	69	1	0	0	0	0
36	70	1	0	0	0	0
38	71	1	0	0	0	0
39	72	1	0	0	0	0
41	73	1	0	0	0	0
42	74	1	0	0	0	0
44	75	1	0	0	0	0

```
 45 76  1  0  0  0  0
 47 77  1  0  0  0  0
 48 78  1  0  0  0  0
 49 79  1  0  0  0  0
 49 80  1  0  0  0  0
 49 81  1  0  0  0  0
 50 82  1  0  0  0  0
 50 83  1  0  0  0  0
 50 84  1  0  0  0  0
 51 85  1  0  0  0  0
 51 86  1  0  0  0  0
 51 87  1  0  0  0  0
 52 88  1  0  0  0  0
 52 89  1  0  0  0  0
 52 90  1  0  0  0  0
M  END
>  <Alias name>
Tetrakis(p-tolyl)porphyrin

>  <deltaH°(c)>   kJ/mol
-24798

$$$$
Tetrakis(o-aminophenyl)porphyrin
  ChemBrain IXL

 86 94  0  0  0  0  0  0  0  0  1 V2000
   14.2072    7.6814   -0.8157 C   0  0  0  0  0  0  0  0  0  0  0  0
   13.3579    6.7381   -1.1167 C   0  0  0  0  0  0  0  0  0  0  0  0
   11.9981    7.2618   -0.9116 C   0  0  0  0  0  0  0  0  0  0  0  0
   12.1158    8.5526   -0.4776 N   0  0  0  0  0  0  0  0  0  0  0  0
   13.4456    8.8675   -0.3983 C   0  0  0  0  0  0  0  0  0  0  0  0
   10.8421    6.5203   -1.1377 C   0  0  0  0  0  0  0  0  0  0  0  0
    7.7957    8.3933   -0.4884 C   0  0  0  0  0  0  0  0  0  0  0  0
    7.2911    7.1699   -0.9357 C   0  0  0  0  0  0  0  0  0  0  0  0
    8.3785    6.3325   -1.2305 C   0  0  0  0  0  0  0  0  0  0  0  0
    9.5556    7.0392   -0.9648 C   0  0  0  0  0  0  0  0  0  0  0  0
    9.1874    8.3041   -0.5121 N   0  0  0  0  0  0  0  0  0  0  0  0
    7.0151    9.4815   -0.0968 C   0  0  0  0  0  0  0  0  0  0  0  0
    7.5885   10.6799    0.3207 C   0  0  0  0  0  0  0  0  0  0  0  0
    6.8267   11.8636    0.7455 C   0  0  0  0  0  0  0  0  0  0  0  0
    7.6774   12.7995    1.0714 C   0  0  0  0  0  0  0  0  0  0  0  0
    9.0384   12.2738    0.8726 C   0  0  0  0  0  0  0  0  0  0  0  0
    8.9201   10.9893    0.4185 N   0  0  0  0  0  0  0  0  0  0  0  0
   10.1972   13.0090    1.1218 C   0  0  0  0  0  0  0  0  0  0  0  0
   11.4869   12.4892    0.9489 C   0  0  0  0  0  0  0  0  0  0  0  0
   12.6653   13.2003    1.2009 C   0  0  0  0  0  0  0  0  0  0  0  0
   13.7533   12.3704    0.8805 C   0  0  0  0  0  0  0  0  0  0  0  0
   13.2478   11.1468    0.4336 C   0  0  0  0  0  0  0  0  0  0  0  0
   11.8550   11.2223    0.4995 N   0  0  0  0  0  0  0  0  0  0  0  0
   14.0237   10.0684   -0.0036 C   0  0  0  0  0  0  0  0  0  0  0  0
    9.7839   17.0127    2.5333 C   0  0  0  0  0  0  0  0  0  0  0  0
   10.6167   16.7415    1.4468 C   0  0  0  0  0  0  0  0  0  0  0  0
   10.7352   15.4367    0.9674 C   0  0  0  0  0  0  0  0  0  0  0  0
   10.0446   14.4007    1.5946 C   0  0  0  0  0  0  0  0  0  0  0  0
    9.2003   14.6741    2.6699 C   0  0  0  0  0  0  0  0  0  0  0  0
    9.0711   15.9794    3.1411 C   0  0  0  0  0  0  0  0  0  0  0  0
   18.2882   10.3939   -0.0800 C   0  0  0  0  0  0  0  0  0  0  0  0
   17.6784    9.2914    0.5166 C   0  0  0  0  0  0  0  0  0  0  0  0
   16.2876    9.1865    0.5310 C   0  0  0  0  0  0  0  0  0  0  0  0
   15.5025   10.1848   -0.0486 C   0  0  0  0  0  0  0  0  0  0  0  0
   16.1147   11.2760   -0.6705 C   0  0  0  0  0  0  0  0  0  0  0  0
   17.5071   11.3865   -0.6723 C   0  0  0  0  0  0  0  0  0  0  0  0
```

```
   2.7490    9.1802   -0.1135 C   0  0  0  0  0  0  0  0  0  0  0  0
   3.3735   10.2678   -0.7233 C   0  0  0  0  0  0  0  0  0  0  0  0
   4.7665   10.3522   -0.7432 C   0  0  0  0  0  0  0  0  0  0  0  0
   5.5362    9.3641   -0.1243 C   0  0  0  0  0  0  0  0  0  0  0  0
   4.9102    8.2671    0.4698 C   0  0  0  0  0  0  0  0  0  0  0  0
   3.5170    8.1771    0.4777 C   0  0  0  0  0  0  0  0  0  0  0  0
  11.2455    2.4911   -2.4673 C   0  0  0  0  0  0  0  0  0  0  0  0
  11.9584    3.5098   -3.0997 C   0  0  0  0  0  0  0  0  0  0  0  0
  11.8329    4.8250   -2.6566 C   0  0  0  0  0  0  0  0  0  0  0  0
  10.9932    5.1215   -1.5839 C   0  0  0  0  0  0  0  0  0  0  0  0
  10.3032    4.1019   -0.9325 C   0  0  0  0  0  0  0  0  0  0  0  0
  10.4171    2.7872   -1.3837 C   0  0  0  0  0  0  0  0  0  0  0  0
  11.5463   15.1678   -0.1417 N   0  0  0  0  0  0  0  0  0  0  0  0
  15.3380   12.2601   -1.2923 N   0  0  0  0  0  0  0  0  0  0  0  0
   5.3897   11.4291   -1.3847 N   0  0  0  0  0  0  0  0  0  0  0  0
   9.4975    4.3993    0.1731 N   0  0  0  0  0  0  0  0  0  0  0  0
   9.8253    9.0398   -0.2471 H   0  0  0  0  0  0  0  0  0  0  0  0
  11.2181   10.4692    0.2867 H   0  0  0  0  0  0  0  0  0  0  0  0
  12.0651   15.9138   -0.5800 H   0  0  0  0  0  0  0  0  0  0  0  0
  11.6119   14.2277   -0.5027 H   0  0  0  0  0  0  0  0  0  0  0  0
  15.7838   13.0592   -1.7190 H   0  0  0  0  0  0  0  0  0  0  0  0
  14.3342   12.1713   -1.3144 H   0  0  0  0  0  0  0  0  0  0  0  0
   4.8313   12.1566   -1.8066 H   0  0  0  0  0  0  0  0  0  0  0  0
   6.3961   11.4783   -1.4254 H   0  0  0  0  0  0  0  0  0  0  0  0
   8.9799    3.6655    0.6322 H   0  0  0  0  0  0  0  0  0  0  0  0
   9.4346    5.3486    0.5108 H   0  0  0  0  0  0  0  0  0  0  0  0
  15.2865    7.6041   -0.8650 H   0  0  0  0  0  0  0  0  0  0  0  0
  13.6098    5.7420   -1.4583 H   0  0  0  0  0  0  0  0  0  0  0  0
   6.2440    6.9126   -1.0376 H   0  0  0  0  0  0  0  0  0  0  0  0
   8.3167    5.3165   -1.5998 H   0  0  0  0  0  0  0  0  0  0  0  0
   5.7472   11.9457    0.7816 H   0  0  0  0  0  0  0  0  0  0  0  0
   7.4254   13.7910    1.4270 H   0  0  0  0  0  0  0  0  0  0  0  0
  12.7279   14.2122    1.5812 H   0  0  0  0  0  0  0  0  0  0  0  0
  14.8006   12.6326    0.9676 H   0  0  0  0  0  0  0  0  0  0  0  0
   9.6908   18.0253    2.9049 H   0  0  0  0  0  0  0  0  0  0  0  0
  11.1707   17.5437    0.9760 H   0  0  0  0  0  0  0  0  0  0  0  0
   8.6446   13.8712    3.1393 H   0  0  0  0  0  0  0  0  0  0  0  0
   8.4177   16.1904    3.9785 H   0  0  0  0  0  0  0  0  0  0  0  0
  19.3683   10.4796   -0.0834 H   0  0  0  0  0  0  0  0  0  0  0  0
  18.2848    8.5160    0.9691 H   0  0  0  0  0  0  0  0  0  0  0  0
  15.8157    8.3284    0.9930 H   0  0  0  0  0  0  0  0  0  0  0  0
  17.9817   12.2437   -1.1341 H   0  0  0  0  0  0  0  0  0  0  0  0
   1.6675    9.1146   -0.0991 H   0  0  0  0  0  0  0  0  0  0  0  0
   2.7768   11.0473   -1.1818 H   0  0  0  0  0  0  0  0  0  0  0  0
   5.5050    7.4855    0.9248 H   0  0  0  0  0  0  0  0  0  0  0  0
   3.0318    7.3280    0.9431 H   0  0  0  0  0  0  0  0  0  0  0  0
  11.3350    1.4709   -2.8174 H   0  0  0  0  0  0  0  0  0  0  0  0
  12.6089    3.2795   -3.9344 H   0  0  0  0  0  0  0  0  0  0  0  0
  12.3875    5.6173   -3.1452 H   0  0  0  0  0  0  0  0  0  0  0  0
   9.8632    1.9969   -0.8936 H   0  0  0  0  0  0  0  0  0  0  0  0
  1  2  2  0  0  0  0
  2  3  1  0  0  0  0
  3  4  4  0  0  0  0
  4  5  4  0  0  0  0
  5  1  1  0  0  0  0
  7  8  4  0  0  0  0
  8  9  4  0  0  0  0
  9 10  4  0  0  0  0
 10 11  1  0  0  0  0
 11  7  1  0  0  0  0
 12 13  4  0  0  0  0
 13 14  1  0  0  0  0
 14 15  2  0  0  0  0
```

15	16	1	0	0	0	0
16	17	4	0	0	0	0
17	13	4	0	0	0	0
16	18	4	0	0	0	0
18	19	4	0	0	0	0
19	20	4	0	0	0	0
20	21	4	0	0	0	0
21	22	4	0	0	0	0
22	23	1	0	0	0	0
23	19	1	0	0	0	0
24	5	4	0	0	0	0
6	3	4	0	0	0	0
22	24	4	0	0	0	0
10	6	4	0	0	0	0
7	12	4	0	0	0	0
25	26	4	0	0	0	0
26	27	4	0	0	0	0
27	28	4	0	0	0	0
28	29	4	0	0	0	0
29	30	4	0	0	0	0
30	25	4	0	0	0	0
31	32	4	0	0	0	0
32	33	4	0	0	0	0
33	34	4	0	0	0	0
34	35	4	0	0	0	0
35	36	4	0	0	0	0
36	31	4	0	0	0	0
37	38	4	0	0	0	0
38	39	4	0	0	0	0
39	40	4	0	0	0	0
40	41	4	0	0	0	0
41	42	4	0	0	0	0
42	37	4	0	0	0	0
43	44	4	0	0	0	0
44	45	4	0	0	0	0
45	46	4	0	0	0	0
46	47	4	0	0	0	0
47	48	4	0	0	0	0
48	43	4	0	0	0	0
40	12	1	0	0	0	0
46	6	1	0	0	0	0
28	18	1	0	0	0	0
34	24	1	0	0	0	0
11	53	1	0	0	0	0
23	54	1	0	0	0	0
27	49	1	0	0	0	0
35	50	1	0	0	0	0
39	51	1	0	0	0	0
47	52	1	0	0	0	0
49	55	1	0	0	0	0
49	56	1	0	0	0	0
50	57	1	0	0	0	0
50	58	1	0	0	0	0
51	59	1	0	0	0	0
51	60	1	0	0	0	0
52	61	1	0	0	0	0
52	62	1	0	0	0	0
1	63	1	0	0	0	0
2	64	1	0	0	0	0
8	65	1	0	0	0	0
9	66	1	0	0	0	0
14	67	1	0	0	0	0
15	68	1	0	0	0	0

```
 20 69  1  0  0  0  0
 21 70  1  0  0  0  0
 25 71  1  0  0  0  0
 26 72  1  0  0  0  0
 29 73  1  0  0  0  0
 30 74  1  0  0  0  0
 31 75  1  0  0  0  0
 32 76  1  0  0  0  0
 33 77  1  0  0  0  0
 36 78  1  0  0  0  0
 37 79  1  0  0  0  0
 38 80  1  0  0  0  0
 41 81  1  0  0  0  0
 42 82  1  0  0  0  0
 43 83  1  0  0  0  0
 44 84  1  0  0  0  0
 45 85  1  0  0  0  0
 48 86  1  0  0  0  0
M  END
>  <Alias name>
Tetrakis(o-aminophenyl)porphyrin

>  <deltaH°(c)>   kJ/mol
-22648

$$$$
Tetraphenylporphyrin
  ChemBrain IXL

 78 86  0  0  0  0  0  0  0  0  1 V2000
    7.8262    7.5416   -1.2095 C   0  0  0  0  0  0  0  0  0  0  0  0
    8.7880    6.6781   -1.0150 C   0  0  0  0  0  0  0  0  0  0  0  0
    9.9794    7.4119   -0.5534 C   0  0  0  0  0  0  0  0  0  0  0  0
    9.6544    8.7393   -0.5168 N   0  0  0  0  0  0  0  0  0  0  0  0
    8.3490    8.8832   -0.9135 C   0  0  0  0  0  0  0  0  0  0  0  0
   11.1903    6.7974   -0.2239 C   0  0  0  0  0  0  0  0  0  0  0  0
   13.7963    9.1063    0.8181 C   0  0  0  0  0  0  0  0  0  0  0  0
   14.4191    7.8691    0.9899 C   0  0  0  0  0  0  0  0  0  0  0  0
   13.5012    6.8686    0.6375 C   0  0  0  0  0  0  0  0  0  0  0  0
   12.3128    7.4896    0.2388 C   0  0  0  0  0  0  0  0  0  0  0  0
   12.4947    8.8615    0.3792 N   0  0  0  0  0  0  0  0  0  0  0  0
   14.3987   10.3422    1.0413 C   0  0  0  0  0  0  0  0  0  0  0  0
   13.6748   11.5255    0.9130 C   0  0  0  0  0  0  0  0  0  0  0  0
   14.2008   12.8681    1.1983 C   0  0  0  0  0  0  0  0  0  0  0  0
   13.2417   13.7325    0.9950 C   0  0  0  0  0  0  0  0  0  0  0  0
   12.0486   12.9978    0.5392 C   0  0  0  0  0  0  0  0  0  0  0  0
   12.3700   11.6694    0.5142 N   0  0  0  0  0  0  0  0  0  0  0  0
   10.8397   13.6131    0.2041 C   0  0  0  0  0  0  0  0  0  0  0  0
    9.7138   12.9202   -0.2494 C   0  0  0  0  0  0  0  0  0  0  0  0
    8.5222   13.5404   -0.6396 C   0  0  0  0  0  0  0  0  0  0  0  0
    7.6013   12.5392   -0.9825 C   0  0  0  0  0  0  0  0  0  0  0  0
    8.2259   11.3023   -0.8148 C   0  0  0  0  0  0  0  0  0  0  0  0
    9.5312   11.5480   -0.3872 N   0  0  0  0  0  0  0  0  0  0  0  0
    7.6221   10.0659   -1.0317 C   0  0  0  0  0  0  0  0  0  0  0  0
   10.5872   17.8456    0.5829 C   0  0  0  0  0  0  0  0  0  0  0  0
    9.5206   17.0450    0.9936 C   0  0  0  0  0  0  0  0  0  0  0  0
    9.6029   15.6596    0.8700 C   0  0  0  0  0  0  0  0  0  0  0  0
   10.7494   15.0778    0.3338 C   0  0  0  0  0  0  0  0  0  0  0  0
   11.8132   15.8751   -0.0808 C   0  0  0  0  0  0  0  0  0  0  0  0
   11.7341   17.2599    0.0460 C   0  0  0  0  0  0  0  0  0  0  0  0
    3.4717    9.8797   -2.0719 C   0  0  0  0  0  0  0  0  0  0  0  0
    3.9128   10.8055   -1.1266 C   0  0  0  0  0  0  0  0  0  0  0  0
    5.2671   10.8708   -0.7902 C   0  0  0  0  0  0  0  0  0  0  0  0
```

```
6.1822 10.0068 -1.3972 C 0 0 0 0 0 0 0 0 0 0 0 0
5.7389 9.0861 -2.3498 C 0 0 0 0 0 0 0 0 0 0 0 0
4.3842 9.0206 -2.6836 C 0 0 0 0 0 0 0 0 0 0 0 0
18.5356 10.5230 2.1342 C 0 0 0 0 0 0 0 0 0 0 0 0
18.1036 9.5909 1.1908 C 0 0 0 0 0 0 0 0 0 0 0 0
16.7538 9.5281 0.8365 C 0 0 0 0 0 0 0 0 0 0 0 0
15.8339 10.4001 1.4245 C 0 0 0 0 0 0 0 0 0 0 0 0
16.2681 11.3277 2.3746 C 0 0 0 0 0 0 0 0 0 0 0 0
17.6183 11.3909 2.7261 C 0 0 0 0 0 0 0 0 0 0 0 0
11.4625 2.5707 -0.6505 C 0 0 0 0 0 0 0 0 0 0 0 0
10.3108 3.1451 -0.1117 C 0 0 0 0 0 0 0 0 0 0 0 0
10.2252 4.5280 0.0313 C 0 0 0 0 0 0 0 0 0 0 0 0
11.2871 5.3346 -0.3697 C 0 0 0 0 0 0 0 0 0 0 0 0
12.4385 4.7640 -0.9075 C 0 0 0 0 0 0 0 0 0 0 0 0
12.5275 3.3806 -1.0470 C 0 0 0 0 0 0 0 0 0 0 0 0
11.7875 9.5647 0.2258 H 0 0 0 0 0 0 0 0 0 0 0 0
10.2402 10.8453 -0.2398 H 0 0 0 0 0 0 0 0 0 0 0 0
6.8177 7.3082 -1.5291 H 0 0 0 0 0 0 0 0 0 0 0 0
8.7222 5.6085 -1.1704 H 0 0 0 0 0 0 0 0 0 0 0 0
15.4328 7.7092 1.3364 H 0 0 0 0 0 0 0 0 0 0 0 0
13.6788 5.8013 0.6743 H 0 0 0 0 0 0 0 0 0 0 0 0
15.2094 13.1014 1.5174 H 0 0 0 0 0 0 0 0 0 0 0 0
13.3105 14.8033 1.1404 H 0 0 0 0 0 0 0 0 0 0 0 0
8.3444 14.6076 -0.6775 H 0 0 0 0 0 0 0 0 0 0 0 0
6.5844 12.6984 -1.3197 H 0 0 0 0 0 0 0 0 0 0 0 0
10.5248 18.9210 0.6806 H 0 0 0 0 0 0 0 0 0 0 0 0
8.6297 17.4992 1.4079 H 0 0 0 0 0 0 0 0 0 0 0 0
8.7771 15.0353 1.1903 H 0 0 0 0 0 0 0 0 0 0 0 0
12.7009 15.4177 -0.5014 H 0 0 0 0 0 0 0 0 0 0 0 0
12.5621 17.8801 -0.2718 H 0 0 0 0 0 0 0 0 0 0 0 0
2.4201 9.8280 -2.3311 H 0 0 0 0 0 0 0 0 0 0 0 0
3.2037 11.4744 -0.6532 H 0 0 0 0 0 0 0 0 0 0 0 0
5.6071 11.5921 -0.0582 H 0 0 0 0 0 0 0 0 0 0 0 0
6.4458 8.4221 -2.8303 H 0 0 0 0 0 0 0 0 0 0 0 0
4.0412 8.3019 -3.4185 H 0 0 0 0 0 0 0 0 0 0 0 0
19.5838 10.5728 2.4072 H 0 0 0 0 0 0 0 0 0 0 0 0
18.8165 8.9153 0.7327 H 0 0 0 0 0 0 0 0 0 0 0 0
16.4210 8.8026 0.1054 H 0 0 0 0 0 0 0 0 0 0 0 0
15.5574 11.9988 2.8395 H 0 0 0 0 0 0 0 0 0 0 0 0
17.9543 12.1147 3.4592 H 0 0 0 0 0 0 0 0 0 0 0 0
11.5299 1.4968 -0.7608 H 0 0 0 0 0 0 0 0 0 0 0 0
9.4841 2.5176 0.1952 H 0 0 0 0 0 0 0 0 0 0 0 0
9.3339 4.9765 0.4538 H 0 0 0 0 0 0 0 0 0 0 0 0
13.2631 5.3956 -1.2165 H 0 0 0 0 0 0 0 0 0 0 0 0
13.4222 2.9352 -1.4626 H 0 0 0 0 0 0 0 0 0 0 0 0
1 2 2 0 0 0 0
2 3 1 0 0 0 0
3 4 4 0 0 0 0
4 5 4 0 0 0 0
5 1 1 0 0 0 0
7 8 4 0 0 0 0
8 9 4 0 0 0 0
9 10 4 0 0 0 0
10 11 1 0 0 0 0
11 7 1 0 0 0 0
12 13 4 0 0 0 0
13 14 1 0 0 0 0
14 15 2 0 0 0 0
15 16 1 0 0 0 0
16 17 4 0 0 0 0
17 13 4 0 0 0 0
16 18 4 0 0 0 0
18 19 4 0 0 0 0
```

19	20	4	0	0	0	0
20	21	4	0	0	0	0
21	22	4	0	0	0	0
22	23	1	0	0	0	0
23	19	1	0	0	0	0
24	5	4	0	0	0	0
6	3	4	0	0	0	0
22	24	4	0	0	0	0
10	6	4	0	0	0	0
7	12	4	0	0	0	0
25	26	4	0	0	0	0
26	27	4	0	0	0	0
27	28	4	0	0	0	0
28	29	4	0	0	0	0
29	30	4	0	0	0	0
30	25	4	0	0	0	0
31	32	4	0	0	0	0
32	33	4	0	0	0	0
33	34	4	0	0	0	0
34	35	4	0	0	0	0
35	36	4	0	0	0	0
36	31	4	0	0	0	0
37	38	4	0	0	0	0
38	39	4	0	0	0	0
39	40	4	0	0	0	0
40	41	4	0	0	0	0
41	42	4	0	0	0	0
42	37	4	0	0	0	0
43	44	4	0	0	0	0
44	45	4	0	0	0	0
45	46	4	0	0	0	0
46	47	4	0	0	0	0
47	48	4	0	0	0	0
48	43	4	0	0	0	0
40	12	1	0	0	0	0
46	6	1	0	0	0	0
28	18	1	0	0	0	0
34	24	1	0	0	0	0
11	49	1	0	0	0	0
23	50	1	0	0	0	0
1	51	1	0	0	0	0
2	52	1	0	0	0	0
8	53	1	0	0	0	0
9	54	1	0	0	0	0
14	55	1	0	0	0	0
15	56	1	0	0	0	0
20	57	1	0	0	0	0
21	58	1	0	0	0	0
25	59	1	0	0	0	0
26	60	1	0	0	0	0
27	61	1	0	0	0	0
29	62	1	0	0	0	0
30	63	1	0	0	0	0
31	64	1	0	0	0	0
32	65	1	0	0	0	0
33	66	1	0	0	0	0
35	67	1	0	0	0	0
36	68	1	0	0	0	0
37	69	1	0	0	0	0
38	70	1	0	0	0	0
39	71	1	0	0	0	0
41	72	1	0	0	0	0
42	73	1	0	0	0	0

43 74 1 0 0 0 0
44 75 1 0 0 0 0
45 76 1 0 0 0 0
47 77 1 0 0 0 0
48 78 1 0 0 0 0
M END
> <Alias name>
Tetraphenylporphyrin

> <deltaH°(c)>   kJ/mol
-22257

$$$$
1,1,3,3,5,5-Hexaphenyl-7,7-dimethylcyclotetrasiloxane
  ChemBrain IXL

82 88 0 0 0 0 0 0 0 0 1 V2000
    6.6813   9.3418   0.2275 Si 0 0 0 0 0 0 0 0 0 0 0 0
    7.1372   7.9325   0.9064 O   0 0 0 0 0 0 0 0 0 0 0 0
    7.5000   6.7468   -0.1505 Si 0 0 0 0 0 0 0 0 0 0 0 0
    8.8638   7.1360   -0.9528 O   0 0 0 0 0 0 0 0 0 0 0 0
   10.1119   7.5866   -0.0071 Si 0 0 0 0 0 0 0 0 0 0 0 0
    9.7616   9.0062   0.7140 O   0 0 0 0 0 0 0 0 0 0 0 0
    9.2484   10.1824   -0.2914 Si 0 0 0 0 0 0 0 0 0 0 0 0
    7.7831   9.7886   -0.8880 O   0 0 0 0 0 0 0 0 0 0 0 0
    9.1263   11.7890   0.6438 C   0 0 0 0 0 0 0 0 0 0 0 0
   10.4591   10.3715   -1.6947 C   0 0 0 0 0 0 0 0 0 0 0 0
   10.7970   4.3094   3.2236 C   0 0 0 0 0 0 0 0 0 0 0 0
   10.0106   5.4238   3.5163 C   0 0 0 0 0 0 0 0 0 0 0 0
    9.8204   6.4155   2.5539 C   0 0 0 0 0 0 0 0 0 0 0 0
   10.4041   6.2854   1.2936 C   0 0 0 0 0 0 0 0 0 0 0 0
   11.2065   5.1807   1.0074 C   0 0 0 0 0 0 0 0 0 0 0 0
   11.3965   4.1888   1.9697 C   0 0 0 0 0 0 0 0 0 0 0 0
   13.9814   7.9888   -2.5576 C   0 0 0 0 0 0 0 0 0 0 0 0
   12.9381   7.1737   -2.9973 C   0 0 0 0 0 0 0 0 0 0 0 0
   11.7636   7.0806   -2.2504 C   0 0 0 0 0 0 0 0 0 0 0 0
   11.6444   7.7790   -1.0488 C   0 0 0 0 0 0 0 0 0 0 0 0
   12.6732   8.6216   -0.6273 C   0 0 0 0 0 0 0 0 0 0 0 0
   13.8475   8.7156   -1.3744 C   0 0 0 0 0 0 0 0 0 0 0 0
    6.3828   12.6255   3.5106 C   0 0 0 0 0 0 0 0 0 0 0 0
    5.6398   12.7441   2.3360 C   0 0 0 0 0 0 0 0 0 0 0 0
    5.7270   11.7582   1.3529 C   0 0 0 0 0 0 0 0 0 0 0 0
    6.5597   10.6552   1.5432 C   0 0 0 0 0 0 0 0 0 0 0 0
    7.3012   10.5356   2.7187 C   0 0 0 0 0 0 0 0 0 0 0 0
    7.2133   11.5210   3.7022 C   0 0 0 0 0 0 0 0 0 0 0 0
    3.5277   9.5633   -2.4393 C   0 0 0 0 0 0 0 0 0 0 0 0
    2.5304   8.8190   -1.8089 C   0 0 0 0 0 0 0 0 0 0 0 0
    2.7800   8.2252   -0.5715 C   0 0 0 0 0 0 0 0 0 0 0 0
    4.0266   8.3766   0.0360 C   0 0 0 0 0 0 0 0 0 0 0 0
    5.0218   9.1263   -0.5914 C   0 0 0 0 0 0 0 0 0 0 0 0
    4.7745   9.7143   -1.8320 C   0 0 0 0 0 0 0 0 0 0 0 0
    8.1265   2.7478   2.1440 C   0 0 0 0 0 0 0 0 0 0 0 0
    8.6811   2.9184   0.8754 C   0 0 0 0 0 0 0 0 0 0 0 0
    8.4990   4.1199   0.1904 C   0 0 0 0 0 0 0 0 0 0 0 0
    7.7578   5.1488   0.7718 C   0 0 0 0 0 0 0 0 0 0 0 0
    7.2099   4.9811   2.0437 C   0 0 0 0 0 0 0 0 0 0 0 0
    7.3915   3.7794   2.7284 C   0 0 0 0 0 0 0 0 0 0 0 0
    3.9815   6.2893   -3.1604 C   0 0 0 0 0 0 0 0 0 0 0 0
    5.0940   7.0595   -3.4998 C   0 0 0 0 0 0 0 0 0 0 0 0
    6.1561   7.1834   -2.6038 C   0 0 0 0 0 0 0 0 0 0 0 0
    6.0997   6.5481   -1.3632 C   0 0 0 0 0 0 0 0 0 0 0 0
    4.9948   5.7642   -1.0307 C   0 0 0 0 0 0 0 0 0 0 0 0
    3.9324   5.6406   -1.9264 C   0 0 0 0 0 0 0 0 0 0 0 0

```
  8.4793   11.6587    1.5023 H   0  0  0  0  0  0  0  0  0  0  0  0
 10.1123   12.0886    0.9770 H   0  0  0  0  0  0  0  0  0  0  0  0
  8.7165   12.5538   -0.0044 H   0  0  0  0  0  0  0  0  0  0  0  0
 11.4186   10.6868   -1.3036 H   0  0  0  0  0  0  0  0  0  0  0  0
 10.0913   11.1148   -2.3912 H   0  0  0  0  0  0  0  0  0  0  0  0
 10.5713    9.4229   -2.2050 H   0  0  0  0  0  0  0  0  0  0  0  0
 10.9422    3.5380    3.9697 H   0  0  0  0  0  0  0  0  0  0  0  0
  9.5477    5.5190    4.4908 H   0  0  0  0  0  0  0  0  0  0  0  0
  9.2195    7.2864    2.7851 H   0  0  0  0  0  0  0  0  0  0  0  0
 11.6819    5.0933    0.0383 H   0  0  0  0  0  0  0  0  0  0  0  0
 12.0093    3.3250    1.7433 H   0  0  0  0  0  0  0  0  0  0  0  0
 14.8953    8.0572   -3.1347 H   0  0  0  0  0  0  0  0  0  0  0  0
 13.0400    6.6134   -3.9185 H   0  0  0  0  0  0  0  0  0  0  0  0
 10.9446    6.4661   -2.6033 H   0  0  0  0  0  0  0  0  0  0  0  0
 12.5603    9.2026    0.2796 H   0  0  0  0  0  0  0  0  0  0  0  0
 14.6552    9.3529   -1.0360 H   0  0  0  0  0  0  0  0  0  0  0  0
  6.3150   13.3908    4.2739 H   0  0  0  0  0  0  0  0  0  0  0  0
  4.9952   13.6015    2.1872 H   0  0  0  0  0  0  0  0  0  0  0  0
  5.1484   11.8491    0.4420 H   0  0  0  0  0  0  0  0  0  0  0  0
  7.9453    9.6778    2.8677 H   0  0  0  0  0  0  0  0  0  0  0  0
  7.7897   11.4287    4.6144 H   0  0  0  0  0  0  0  0  0  0  0  0
  3.3341   10.0239   -3.4002 H   0  0  0  0  0  0  0  0  0  0  0  0
  1.5623    8.7022   -2.2802 H   0  0  0  0  0  0  0  0  0  0  0  0
  2.0060    7.6466   -0.0826 H   0  0  0  0  0  0  0  0  0  0  0  0
  4.2217    7.9122    0.9947 H   0  0  0  0  0  0  0  0  0  0  0  0
  5.5503   10.2881   -2.3236 H   0  0  0  0  0  0  0  0  0  0  0  0
  8.2666    1.8144    2.6752 H   0  0  0  0  0  0  0  0  0  0  0  0
  9.2530    2.1181    0.4223 H   0  0  0  0  0  0  0  0  0  0  0  0
  8.9331    4.2540   -0.7927 H   0  0  0  0  0  0  0  0  0  0  0  0
  6.6435    5.7839    2.4994 H   0  0  0  0  0  0  0  0  0  0  0  0
  6.9619    3.6473    3.7137 H   0  0  0  0  0  0  0  0  0  0  0  0
  3.1560    6.1949   -3.8550 H   0  0  0  0  0  0  0  0  0  0  0  0
  5.1331    7.5613   -4.4587 H   0  0  0  0  0  0  0  0  0  0  0  0
  7.0243    7.7729   -2.8715 H   0  0  0  0  0  0  0  0  0  0  0  0
  4.9616    5.2517   -0.0772 H   0  0  0  0  0  0  0  0  0  0  0  0
  3.0697    5.0411   -1.6637 H   0  0  0  0  0  0  0  0  0  0  0  0
  1  2  1  0  0  0  0
  2  3  1  0  0  0  0
  3  4  1  0  0  0  0
  4  5  1  0  0  0  0
  5  6  1  0  0  0  0
  6  7  1  0  0  0  0
  7  8  1  0  0  0  0
  8  1  1  0  0  0  0
  7  9  1  0  0  0  0
  7 10  1  0  0  0  0
 11 12  4  0  0  0  0
 12 13  4  0  0  0  0
 13 14  4  0  0  0  0
 14 15  4  0  0  0  0
 15 16  4  0  0  0  0
 16 11  4  0  0  0  0
 17 18  4  0  0  0  0
 18 19  4  0  0  0  0
 19 20  4  0  0  0  0
 20 21  4  0  0  0  0
 21 22  4  0  0  0  0
 22 17  4  0  0  0  0
 14  5  1  0  0  0  0
 20  5  1  0  0  0  0
 23 24  4  0  0  0  0
 24 25  4  0  0  0  0
 25 26  4  0  0  0  0
```

```
 26 27  4  0  0  0  0
 27 28  4  0  0  0  0
 28 23  4  0  0  0  0
 29 30  4  0  0  0  0
 30 31  4  0  0  0  0
 31 32  4  0  0  0  0
 32 33  4  0  0  0  0
 33 34  4  0  0  0  0
 34 29  4  0  0  0  0
 33  1  1  0  0  0  0
 26  1  1  0  0  0  0
 35 36  4  0  0  0  0
 36 37  4  0  0  0  0
 37 38  4  0  0  0  0
 38 39  4  0  0  0  0
 39 40  4  0  0  0  0
 40 35  4  0  0  0  0
 41 42  4  0  0  0  0
 42 43  4  0  0  0  0
 43 44  4  0  0  0  0
 44 45  4  0  0  0  0
 45 46  4  0  0  0  0
 46 41  4  0  0  0  0
 44  3  1  0  0  0  0
 38  3  1  0  0  0  0
  9 47  1  0  0  0  0
  9 48  1  0  0  0  0
  9 49  1  0  0  0  0
 10 50  1  0  0  0  0
 10 51  1  0  0  0  0
 10 52  1  0  0  0  0
 11 53  1  0  0  0  0
 12 54  1  0  0  0  0
 13 55  1  0  0  0  0
 15 56  1  0  0  0  0
 16 57  1  0  0  0  0
 17 58  1  0  0  0  0
 18 59  1  0  0  0  0
 19 60  1  0  0  0  0
 21 61  1  0  0  0  0
 22 62  1  0  0  0  0
 23 63  1  0  0  0  0
 24 64  1  0  0  0  0
 25 65  1  0  0  0  0
 27 66  1  0  0  0  0
 28 67  1  0  0  0  0
 29 68  1  0  0  0  0
 30 69  1  0  0  0  0
 31 70  1  0  0  0  0
 32 71  1  0  0  0  0
 34 72  1  0  0  0  0
 35 73  1  0  0  0  0
 36 74  1  0  0  0  0
 37 75  1  0  0  0  0
 39 76  1  0  0  0  0
 40 77  1  0  0  0  0
 41 78  1  0  0  0  0
 42 79  1  0  0  0  0
 43 80  1  0  0  0  0
 45 81  1  0  0  0  0
 46 82  1  0  0  0  0
M  END
>  <Alias name>
```

1,1,3,3,5,5-Hexaphenyl-7,7-dimethylcyclotetrasiloxane

> <deltaH°(c)> kJ/mol
-22112

$$$$
Bis(N-(N',N'-diethylaminothiocarbonyl)-N'-phenylbenzamidinato)nickel(II)
ChemBrain IXL

85 90 0 0 0 0 0 0 0 0 1 V2000
14.1693 13.3039 -1.2128 C 0 0 0 0 0 0 0 0 0 0 0 0
12.8768 13.2971 -0.6880 C 0 0 0 0 0 0 0 0 0 0 0 0
12.1972 12.0900 -0.5244 C 0 0 0 0 0 0 0 0 0 0 0 0
12.8040 10.8918 -0.9010 C 0 0 0 0 0 0 0 0 0 0 0 0
14.0913 10.8998 -1.4372 C 0 0 0 0 0 0 0 0 0 0 0 0
14.7714 12.1066 -1.5997 C 0 0 0 0 0 0 0 0 0 0 0 0
12.0833 9.6118 -0.7324 C 0 0 0 0 0 0 0 0 0 0 0 0
10.8084 9.5798 0.0323 N 0 5 0 0 0 0 0 0 0 0 0 0
12.5687 8.5451 -1.2457 N 0 0 0 0 0 0 0 0 0 0 0 0
12.3362 7.2945 -0.6464 C 0 0 0 0 0 0 0 0 0 0 0 0
13.3981 6.5397 -0.1336 N 0 0 0 0 0 0 0 0 0 0 0 0
10.7570 6.6932 -0.5434 S 0 0 0 0 0 0 0 0 0 0 0 0
13.1731 5.6354 1.0057 C 0 0 0 0 0 0 0 0 0 0 0 0
14.4986 4.9484 1.3878 C 0 0 0 0 0 0 0 0 0 0 0 0
9.3257 8.4818 -0.5776 Ni 0 0 0 0 0 0 0 0 0 0 0 0
4.8086 4.4017 -4.0000 C 0 0 0 0 0 0 0 0 0 0 0 0
4.4124 5.7189 -4.2304 C 0 0 0 0 0 0 0 0 0 0 0 0
5.1300 6.7714 -3.6630 C 0 0 0 0 0 0 0 0 0 0 0 0
6.2536 6.5063 -2.8804 C 0 0 0 0 0 0 0 0 0 0 0 0
6.6973 5.1942 -2.7200 C 0 0 0 0 0 0 0 0 0 0 0 0
5.9668 4.1403 -3.2681 C 0 0 0 0 0 0 0 0 0 0 0 0
6.9741 7.6181 -2.2228 C 0 0 0 0 0 0 0 0 0 0 0 0
7.7991 7.3499 -1.0071 N 0 5 0 0 0 0 0 0 0 0 0 0
6.8767 8.7984 -2.7078 N 0 0 0 0 0 0 0 0 0 0 0 0
6.7317 9.9078 -1.8589 C 0 0 0 0 0 0 0 0 0 0 0 0
5.5622 10.6775 -1.8698 N 0 0 0 0 0 0 0 0 0 0 0 0
7.9825 10.3187 -0.8122 S 0 0 0 0 0 0 0 0 0 0 0 0
5.1163 11.3455 -0.6370 C 0 0 0 0 0 0 0 0 0 0 0 0
3.8073 12.1111 -0.9088 C 0 0 0 0 0 0 0 0 0 0 0 0
4.7869 10.8273 -3.1094 C 0 0 0 0 0 0 0 0 0 0 0 0
5.4952 10.0766 -4.2526 C 0 0 0 0 0 0 0 0 0 0 0 0
14.7328 6.6444 -0.7360 C 0 0 0 0 0 0 0 0 0 0 0 0
14.6748 7.5949 -1.9462 C 0 0 0 0 0 0 0 0 0 0 0 0
9.4939 10.8482 3.3250 C 0 0 0 0 0 0 0 0 0 0 0 0
9.5511 10.2280 2.0764 C 0 0 0 0 0 0 0 0 0 0 0 0
10.7427 10.2303 1.3506 C 0 0 0 0 0 0 0 0 0 0 0 0
11.8765 10.8508 1.8756 C 0 0 0 0 0 0 0 0 0 0 0 0
11.8351 11.4172 3.1496 C 0 0 0 0 0 0 0 0 0 0 0 0
10.6407 11.4260 3.8699 C 0 0 0 0 0 0 0 0 0 0 0 0
6.6460 4.2044 1.6116 C 0 0 0 0 0 0 0 0 0 0 0 0
7.7680 4.9763 1.9111 C 0 0 0 0 0 0 0 0 0 0 0 0
8.1332 6.0281 1.0715 C 0 0 0 0 0 0 0 0 0 0 0 0
7.3539 6.3287 -0.0447 C 0 0 0 0 0 0 0 0 0 0 0 0
6.1487 5.6585 -0.2488 C 0 0 0 0 0 0 0 0 0 0 0 0
5.8207 4.5617 0.5463 C 0 0 0 0 0 0 0 0 0 0 0 0
14.7052 14.2387 -1.3199 H 0 0 0 0 0 0 0 0 0 0 0 0
12.4011 14.2288 -0.4077 H 0 0 0 0 0 0 0 0 0 0 0 0
11.1985 12.0832 -0.1050 H 0 0 0 0 0 0 0 0 0 0 0 0
14.5627 9.9690 -1.7274 H 0 0 0 0 0 0 0 0 0 0 0 0
15.7667 12.1139 -2.0262 H 0 0 0 0 0 0 0 0 0 0 0 0
12.4432 4.8837 0.7312 H 0 0 0 0 0 0 0 0 0 0 0 0
12.8048 6.2042 1.8508 H 0 0 0 0 0 0 0 0 0 0 0 0
15.2383 5.7009 1.6311 H 0 0 0 0 0 0 0 0 0 0 0 0

14.8500	4.3529	0.5543	H	0	0	0	0	0	0	0	0	0	0	0	0
14.3389	4.3082	2.2469	H	0	0	0	0	0	0	0	0	0	0	0	0
4.2172	3.5823	-4.3891	H	0	0	0	0	0	0	0	0	0	0	0	0
3.5480	5.9243	-4.8497	H	0	0	0	0	0	0	0	0	0	0	0	0
4.8150	7.7941	-3.8299	H	0	0	0	0	0	0	0	0	0	0	0	0
7.6085	4.9939	-2.1701	H	0	0	0	0	0	0	0	0	0	0	0	0
6.2987	3.1195	-3.1255	H	0	0	0	0	0	0	0	0	0	0	0	0
4.9469	10.6039	0.1339	H	0	0	0	0	0	0	0	0	0	0	0	0
5.8788	12.0405	-0.3074	H	0	0	0	0	0	0	0	0	0	0	0	0
3.0181	11.4064	-1.1390	H	0	0	0	0	0	0	0	0	0	0	0	0
3.5352	12.6835	-0.0303	H	0	0	0	0	0	0	0	0	0	0	0	0
3.9486	12.7817	-1.7470	H	0	0	0	0	0	0	0	0	0	0	0	0
4.7086	11.8777	-3.3614	H	0	0	0	0	0	0	0	0	0	0	0	0
3.7954	10.4154	-2.9669	H	0	0	0	0	0	0	0	0	0	0	0	0
6.5400	10.3603	-4.2779	H	0	0	0	0	0	0	0	0	0	0	0	0
5.4147	9.0093	-4.0879	H	0	0	0	0	0	0	0	0	0	0	0	0
5.0287	10.3326	-5.1959	H	0	0	0	0	0	0	0	0	0	0	0	0
15.4298	7.0334	-0.0040	H	0	0	0	0	0	0	0	0	0	0	0	0
15.0599	5.6647	-1.0614	H	0	0	0	0	0	0	0	0	0	0	0	0
13.7391	7.4521	-2.4726	H	0	0	0	0	0	0	0	0	0	0	0	0
15.5005	7.3810	-2.6130	H	0	0	0	0	0	0	0	0	0	0	0	0
14.7433	8.6199	-1.6036	H	0	0	0	0	0	0	0	0	0	0	0	0
8.5592	10.8812	3.8712	H	0	0	0	0	0	0	0	0	0	0	0	0
8.6707	9.7450	1.6709	H	0	0	0	0	0	0	0	0	0	0	0	0
12.7890	10.8927	1.2940	H	0	0	0	0	0	0	0	0	0	0	0	0
12.7300	11.8500	3.5794	H	0	0	0	0	0	0	0	0	0	0	0	0
10.6038	11.8813	4.8520	H	0	0	0	0	0	0	0	0	0	0	0	0
6.4159	3.3285	2.2056	H	0	0	0	0	0	0	0	0	0	0	0	0
8.3549	4.7591	2.7949	H	0	0	0	0	0	0	0	0	0	0	0	0
9.0205	6.6105	1.2863	H	0	0	0	0	0	0	0	0	0	0	0	0
5.4683	5.9896	-1.0237	H	0	0	0	0	0	0	0	0	0	0	0	0
4.9263	3.9879	0.3368	H	0	0	0	0	0	0	0	0	0	0	0	0

1	2	4	0	0	0	0
2	3	4	0	0	0	0
3	4	4	0	0	0	0
4	5	4	0	0	0	0
5	6	4	0	0	0	0
6	1	4	0	0	0	0
4	7	1	0	0	0	0
7	8	1	0	0	0	0
7	9	2	0	0	0	0
9	10	1	0	0	0	0
10	11	1	0	0	0	0
10	12	2	0	0	0	0
11	13	1	0	0	0	0
13	14	1	0	0	0	0
15	12	1	0	0	0	0
15	8	1	0	0	0	0
16	17	4	0	0	0	0
17	18	4	0	0	0	0
18	19	4	0	0	0	0
19	20	4	0	0	0	0
20	21	4	0	0	0	0
21	16	4	0	0	0	0
19	22	1	0	0	0	0
22	23	1	0	0	0	0
22	24	2	0	0	0	0
24	25	1	0	0	0	0
25	26	1	0	0	0	0
25	27	2	0	0	0	0
26	28	1	0	0	0	0
28	29	1	0	0	0	0
27	15	1	0	0	0	0

 23 15 1 0 0 0 0
 26 30 1 0 0 0 0
 30 31 1 0 0 0 0
 11 32 1 0 0 0 0
 32 33 1 0 0 0 0
 34 35 4 0 0 0 0
 35 36 4 0 0 0 0
 36 37 4 0 0 0 0
 37 38 4 0 0 0 0
 38 39 4 0 0 0 0
 39 34 4 0 0 0 0
 40 41 4 0 0 0 0
 41 42 4 0 0 0 0
 42 43 4 0 0 0 0
 43 44 4 0 0 0 0
 44 45 4 0 0 0 0
 45 40 4 0 0 0 0
 36 8 1 0 0 0 0
 43 23 1 0 0 0 0
  1 46 1 0 0 0 0
  2 47 1 0 0 0 0
  3 48 1 0 0 0 0
  5 49 1 0 0 0 0
  6 50 1 0 0 0 0
 13 51 1 0 0 0 0
 13 52 1 0 0 0 0
 14 53 1 0 0 0 0
 14 54 1 0 0 0 0
 14 55 1 0 0 0 0
 16 56 1 0 0 0 0
 17 57 1 0 0 0 0
 18 58 1 0 0 0 0
 20 59 1 0 0 0 0
 21 60 1 0 0 0 0
 28 61 1 0 0 0 0
 28 62 1 0 0 0 0
 29 63 1 0 0 0 0
 29 64 1 0 0 0 0
 29 65 1 0 0 0 0
 30 66 1 0 0 0 0
 30 67 1 0 0 0 0
 31 68 1 0 0 0 0
 31 69 1 0 0 0 0
 31 70 1 0 0 0 0
 32 71 1 0 0 0 0
 32 72 1 0 0 0 0
 33 73 1 0 0 0 0
 33 74 1 0 0 0 0
 33 75 1 0 0 0 0
 34 76 1 0 0 0 0
 35 77 1 0 0 0 0
 37 78 1 0 0 0 0
 38 79 1 0 0 0 0
 39 80 1 0 0 0 0
 40 81 1 0 0 0 0
 41 82 1 0 0 0 0
 42 83 1 0 0 0 0
 44 84 1 0 0 0 0
 45 85 1 0 0 0 0
M END
> <Alias name>

Bis(N-(N',N'-diethylaminothiocarbonyl)-N'-phenylbenzamidinato)nickel(II)

```
>  <deltaH°(c)>   kJ/mol
-21386.1

$$$$
Coproporphyrin (I) tetramethylester
  ChemBrain IXL

 98102  0  0  0  0  0  0  0  0  1 V2000
    8.7887   11.9913    0.2234 C   0  0  0  0  0  0  0  0  0  0  0  0
    7.6254   11.0578    0.3000 C   0  0  0  0  0  0  0  0  0  0  0  0
    8.6577   13.3848    0.0946 C   0  0  0  0  0  0  0  0  0  0  0  0
    9.9291   11.2217    0.2961 N   0  0  0  0  0  0  0  0  0  0  0  0
    8.0852    9.8431    0.3982 C   0  0  0  0  0  0  0  0  0  0  0  0
    6.0885   11.3871    0.2768 C   0  0  0  0  0  0  0  0  0  0  0  0
    9.7131   14.3122    0.0500 C   0  0  0  0  0  0  0  0  0  0  0  0
    9.5492    9.8966    0.3981 C   0  0  0  0  0  0  0  0  0  0  0  0
    7.2303    8.5642    0.4986 C   0  0  0  0  0  0  0  0  0  0  0  0
    5.8316   12.8924    0.1953 C   0  0  0  0  0  0  0  0  0  0  0  0
    9.5294   15.7095   -0.1136 C   0  0  0  0  0  0  0  0  0  0  0  0
   11.0953   14.0971    0.1552 N   0  0  0  0  0  0  0  0  0  0  0  0
   10.3611    8.7887    0.4836 C   0  0  0  0  0  0  0  0  0  0  0  0
    4.3132   13.1557    0.2371 C   0  0  0  0  0  0  0  0  0  0  0  0
   10.7890   16.3109   -0.0914 C   0  0  0  0  0  0  0  0  0  0  0  0
    8.2074   16.5427   -0.2997 C   0  0  0  0  0  0  0  0  0  0  0  0
   11.7457   15.3162    0.0757 C   0  0  0  0  0  0  0  0  0  0  0  0
   11.7080    8.9745    0.5047 C   0  0  0  0  0  0  0  0  0  0  0  0
    3.4367   12.1635   -0.1192 O   0  0  0  0  0  0  0  0  0  0  0  0
    3.8954   14.2480    0.5879 O   0  0  0  0  0  0  0  0  0  0  0  0
   11.0716   17.8196   -0.2295 C   0  0  0  0  0  0  0  0  0  0  0  0
    6.9696   15.6465   -0.3589 C   0  0  0  0  0  0  0  0  0  0  0  0
   13.0936   15.5006    0.1523 C   0  0  0  0  0  0  0  0  0  0  0  0
   12.6925    7.9900    0.5617 C   0  0  0  0  0  0  0  0  0  0  0  0
   12.3331   10.2025    0.4775 N   0  0  0  0  0  0  0  0  0  0  0  0
    5.7252   16.5143   -0.6278 C   0  0  0  0  0  0  0  0  0  0  0  0
   13.8952   14.3971    0.2904 C   0  0  0  0  0  0  0  0  0  0  0  0
   13.9354    8.6255    0.5813 C   0  0  0  0  0  0  0  0  0  0  0  0
   12.4550    6.4670    0.5934 C   0  0  0  0  0  0  0  0  0  0  0  0
   13.7170   10.0055    0.5314 C   0  0  0  0  0  0  0  0  0  0  0  0
    5.5336   17.6719    0.0811 O   0  0  0  0  0  0  0  0  0  0  0  0
    4.9134   16.1685   -1.4716 O   0  0  0  0  0  0  0  0  0  0  0  0
   13.5000   13.0800    0.3341 N   0  0  0  0  0  0  0  0  0  0  0  0
   15.3559   14.4329    0.4142 C   0  0  0  0  0  0  0  0  0  0  0  0
   15.3076    7.9261    0.6453 C   0  0  0  0  0  0  0  0  0  0  0  0
   13.0518    5.8290   -0.6779 C   0  0  0  0  0  0  0  0  0  0  0  0
   14.7670   10.9188    0.5431 C   0  0  0  0  0  0  0  0  0  0  0  0
   14.6250   12.3013    0.4667 C   0  0  0  0  0  0  0  0  0  0  0  0
   15.7867   13.2081    0.5180 C   0  0  0  0  0  0  0  0  0  0  0  0
   16.2298   15.7022    0.4213 C   0  0  0  0  0  0  0  0  0  0  0  0
   12.8226    4.3051   -0.6474 C   0  0  0  0  0  0  0  0  0  0  0  0
   17.2619   12.7827    0.6705 C   0  0  0  0  0  0  0  0  0  0  0  0
   11.6414    3.8006   -0.1693 O   0  0  0  0  0  0  0  0  0  0  0  0
   13.6947    3.5514   -1.0498 O   0  0  0  0  0  0  0  0  0  0  0  0
   18.0535   13.2118   -0.5810 C   0  0  0  0  0  0  0  0  0  0  0  0
   19.5299   12.7991   -0.4221 C   0  0  0  0  0  0  0  0  0  0  0  0
   19.8470   11.6231    0.2066 O   0  0  0  0  0  0  0  0  0  0  0  0
   20.4130   13.5213   -0.8569 O   0  0  0  0  0  0  0  0  0  0  0  0
    2.0102   12.5223   -0.0394 C   0  0  0  0  0  0  0  0  0  0  0  0
    4.3147   18.4261   -0.2582 C   0  0  0  0  0  0  0  0  0  0  0  0
   11.5300    2.3317   -0.1857 C   0  0  0  0  0  0  0  0  0  0  0  0
   21.2868   11.3275    0.3039 C   0  0  0  0  0  0  0  0  0  0  0  0
   11.5914   13.1826    0.2753 H   0  0  0  0  0  0  0  0  0  0  0  0
   11.8203   11.1144    0.4256 H   0  0  0  0  0  0  0  0  0  0  0  0
    7.6945   13.7630    0.0265 H   0  0  0  0  0  0  0  0  0  0  0  0
```

```
  5.6364   10.9047   -0.5811 H   0  0  0  0  0  0  0  0  0  0  0  0
  5.6329   11.0005    1.1799 H   0  0  0  0  0  0  0  0  0  0  0  0
  7.8710    7.6945    0.4265 H   0  0  0  0  0  0  0  0  0  0  0  0
  6.7100    8.5515    1.4481 H   0  0  0  0  0  0  0  0  0  0  0  0
  6.5085    8.5485   -0.3088 H   0  0  0  0  0  0  0  0  0  0  0  0
  6.3032   13.3847    1.0355 H   0  0  0  0  0  0  0  0  0  0  0  0
  6.2348   13.2766   -0.7308 H   0  0  0  0  0  0  0  0  0  0  0  0
  9.9379    7.7935    0.5339 H   0  0  0  0  0  0  0  0  0  0  0  0
  8.2765   17.1073   -1.2212 H   0  0  0  0  0  0  0  0  0  0  0  0
  8.1045   17.2321    0.5291 H   0  0  0  0  0  0  0  0  0  0  0  0
 10.1358   18.3645   -0.2224 H   0  0  0  0  0  0  0  0  0  0  0  0
 11.5906   18.0057   -1.1615 H   0  0  0  0  0  0  0  0  0  0  0  0
 11.6872   18.1487    0.5984 H   0  0  0  0  0  0  0  0  0  0  0  0
  7.0830   14.9257   -1.1581 H   0  0  0  0  0  0  0  0  0  0  0  0
  6.8502   15.1349    0.5850 H   0  0  0  0  0  0  0  0  0  0  0  0
 13.5211   16.4946    0.1053 H   0  0  0  0  0  0  0  0  0  0  0  0
 11.3915    6.2675    0.6323 H   0  0  0  0  0  0  0  0  0  0  0  0
 12.9339    6.0450    1.4682 H   0  0  0  0  0  0  0  0  0  0  0  0
 15.9888    8.5208    1.2409 H   0  0  0  0  0  0  0  0  0  0  0  0
 15.7043    7.8175   -0.3565 H   0  0  0  0  0  0  0  0  0  0  0  0
 15.1935    6.9482    1.0963 H   0  0  0  0  0  0  0  0  0  0  0  0
 14.1146    6.0326   -0.7187 H   0  0  0  0  0  0  0  0  0  0  0  0
 12.5704    6.2489   -1.5524 H   0  0  0  0  0  0  0  0  0  0  0  0
 15.7714   10.5233    0.6172 H   0  0  0  0  0  0  0  0  0  0  0  0
 15.6639   16.5278    0.8344 H   0  0  0  0  0  0  0  0  0  0  0  0
 16.5285   15.9404   -0.5919 H   0  0  0  0  0  0  0  0  0  0  0  0
 17.1113   15.5303    1.0266 H   0  0  0  0  0  0  0  0  0  0  0  0
 17.6856   13.2591    1.5458 H   0  0  0  0  0  0  0  0  0  0  0  0
 17.3190   11.7079    0.7821 H   0  0  0  0  0  0  0  0  0  0  0  0
 17.6356   12.7291   -1.4557 H   0  0  0  0  0  0  0  0  0  0  0  0
 17.9905   14.2862   -0.6972 H   0  0  0  0  0  0  0  0  0  0  0  0
  1.4074   11.6737   -0.3384 H   0  0  0  0  0  0  0  0  0  0  0  0
  1.7639   12.7993    0.9783 H   0  0  0  0  0  0  0  0  0  0  0  0
  1.8103   13.3574   -0.6994 H   0  0  0  0  0  0  0  0  0  0  0  0
  3.4446   17.8077   -0.0751 H   0  0  0  0  0  0  0  0  0  0  0  0
  4.2596   19.3174    0.3545 H   0  0  0  0  0  0  0  0  0  0  0  0
  4.3438   18.7080   -1.3036 H   0  0  0  0  0  0  0  0  0  0  0  0
 12.3168    1.9030    0.4229 H   0  0  0  0  0  0  0  0  0  0  0  0
 10.5662    2.0384    0.2114 H   0  0  0  0  0  0  0  0  0  0  0  0
 11.6252    1.9736   -1.2035 H   0  0  0  0  0  0  0  0  0  0  0  0
 21.7807   12.1160    0.8582 H   0  0  0  0  0  0  0  0  0  0  0  0
 21.7107   11.2654   -0.6909 H   0  0  0  0  0  0  0  0  0  0  0  0
 21.4276   10.3837    0.8157 H   0  0  0  0  0  0  0  0  0  0  0  0
  1  2  1  0  0  0  0
  1  3  4  0  0  0  0
  1  4  4  0  0  0  0
  2  5  2  0  0  0  0
  2  6  1  0  0  0  0
  3  7  4  0  0  0  0
  4  8  4  0  0  0  0
  5  9  1  0  0  0  0
  5  8  1  0  0  0  0
  6 10  1  0  0  0  0
  7 11  4  0  0  0  0
  7 12  1  0  0  0  0
  8 13  4  0  0  0  0
 10 14  1  0  0  0  0
 11 15  4  0  0  0  0
 11 16  1  0  0  0  0
 12 17  1  0  0  0  0
 13 18  4  0  0  0  0
 14 19  1  0  0  0  0
 14 20  2  0  0  0  0
```

15	21	1	0	0	0	0
15	17	4	0	0	0	0
16	22	1	0	0	0	0
17	23	4	0	0	0	0
18	24	4	0	0	0	0
18	25	1	0	0	0	0
22	26	1	0	0	0	0
23	27	4	0	0	0	0
24	28	4	0	0	0	0
24	29	1	0	0	0	0
25	30	1	0	0	0	0
26	31	1	0	0	0	0
26	32	2	0	0	0	0
27	33	4	0	0	0	0
27	34	1	0	0	0	0
28	35	1	0	0	0	0
28	30	4	0	0	0	0
29	36	1	0	0	0	0
30	37	4	0	0	0	0
33	38	4	0	0	0	0
34	39	2	0	0	0	0
34	40	1	0	0	0	0
36	41	1	0	0	0	0
37	38	4	0	0	0	0
38	39	1	0	0	0	0
39	42	1	0	0	0	0
41	43	1	0	0	0	0
41	44	2	0	0	0	0
42	45	1	0	0	0	0
45	46	1	0	0	0	0
46	47	1	0	0	0	0
46	48	2	0	0	0	0
12	53	1	0	0	0	0
19	49	1	0	0	0	0
25	54	1	0	0	0	0
31	50	1	0	0	0	0
43	51	1	0	0	0	0
47	52	1	0	0	0	0
3	55	1	0	0	0	0
6	56	1	0	0	0	0
6	57	1	0	0	0	0
9	58	1	0	0	0	0
9	59	1	0	0	0	0
9	60	1	0	0	0	0
10	61	1	0	0	0	0
10	62	1	0	0	0	0
13	63	1	0	0	0	0
16	64	1	0	0	0	0
16	65	1	0	0	0	0
21	66	1	0	0	0	0
21	67	1	0	0	0	0
21	68	1	0	0	0	0
22	69	1	0	0	0	0
22	70	1	0	0	0	0
23	71	1	0	0	0	0
29	72	1	0	0	0	0
29	73	1	0	0	0	0
35	74	1	0	0	0	0
35	75	1	0	0	0	0
35	76	1	0	0	0	0
36	77	1	0	0	0	0
36	78	1	0	0	0	0
37	79	1	0	0	0	0

40 80 1 0 0 0 0
40 81 1 0 0 0 0
40 82 1 0 0 0 0
42 83 1 0 0 0 0
42 84 1 0 0 0 0
45 85 1 0 0 0 0
45 86 1 0 0 0 0
49 87 1 0 0 0 0
49 88 1 0 0 0 0
49 89 1 0 0 0 0
50 90 1 0 0 0 0
50 91 1 0 0 0 0
50 92 1 0 0 0 0
51 93 1 0 0 0 0
51 94 1 0 0 0 0
51 95 1 0 0 0 0
52 96 1 0 0 0 0
52 97 1 0 0 0 0
52 98 1 0 0 0 0
M END
> <Alias name>
Coproporphyrin I tetramethylester

> <deltaH°(c)> kJ/mol
-20871

$$$$
11-Decylheneicosane
ChemBrain IXL

95 94 0 0 0 0 0 0 0 0 1 V2000
27.5361 20.0263 -0.0963 C 0 0 0 0 0 0 0 0 0 0 0 0
26.2728 20.1063 0.7826 C 0 0 0 0 0 0 0 0 0 0 0 0
25.0193 20.0146 -0.1095 C 0 0 0 0 0 0 0 0 0 0 0 0
23.7560 20.0942 0.7697 C 0 0 0 0 0 0 0 0 0 0 0 0
22.5026 20.0018 -0.1223 C 0 0 0 0 0 0 0 0 0 0 0 0
21.2393 20.0802 0.7570 C 0 0 0 0 0 0 0 0 0 0 0 0
19.9859 19.9862 -0.1348 C 0 0 0 0 0 0 0 0 0 0 0 0
18.7226 20.0622 0.7446 C 0 0 0 0 0 0 0 0 0 0 0 0
17.4695 19.9656 -0.1468 C 0 0 0 0 0 0 0 0 0 0 0 0
16.2055 20.0385 0.7344 C 0 0 0 0 0 0 0 0 0 0 0 0
14.9443 19.9627 -0.1564 C 0 0 0 0 0 0 0 0 0 0 0 0
13.6838 20.0422 0.7352 C 0 0 0 0 0 0 0 0 0 0 0 0
12.4191 19.9717 -0.1451 C 0 0 0 0 0 0 0 0 0 0 0 0
11.1668 20.0710 0.7472 C 0 0 0 0 0 0 0 0 0 0 0 0
9.9028 19.9966 -0.1313 C 0 0 0 0 0 0 0 0 0 0 0 0
8.6502 20.0927 0.7614 C 0 0 0 0 0 0 0 0 0 0 0 0
7.3862 20.0155 -0.1168 C 0 0 0 0 0 0 0 0 0 0 0 0
6.1335 20.1096 0.7760 C 0 0 0 0 0 0 0 0 0 0 0 0
4.8694 20.0308 -0.1020 C 0 0 0 0 0 0 0 0 0 0 0 0
3.6167 20.1241 0.7910 C 0 0 0 0 0 0 0 0 0 0 0 0
2.3527 20.0447 -0.0869 C 0 0 0 0 0 0 0 0 0 0 0 0
14.9421 18.6316 -0.9513 C 0 0 0 0 0 0 0 0 0 0 0 0
14.9396 17.4364 0.0259 C 0 0 0 0 0 0 0 0 0 0 0 0
14.9366 16.1199 -0.7746 C 0 0 0 0 0 0 0 0 0 0 0 0
14.9333 14.9261 0.1998 C 0 0 0 0 0 0 0 0 0 0 0 0
14.9297 13.6093 -0.6008 C 0 0 0 0 0 0 0 0 0 0 0 0
14.9257 12.4155 0.3737 C 0 0 0 0 0 0 0 0 0 0 0 0
14.9217 11.0987 -0.4269 C 0 0 0 0 0 0 0 0 0 0 0 0
14.9174 9.9049 0.5477 C 0 0 0 0 0 0 0 0 0 0 0 0
14.9132 8.5879 -0.2529 C 0 0 0 0 0 0 0 0 0 0 0 0
14.9089 7.3941 0.7215 C 0 0 0 0 0 0 0 0 0 0 0 0
27.5361 20.8460 -0.8040 H 0 0 0 0 0 0 0 0 0 0 0 0

27.5433	19.0858	-0.6332	H	0	0	0	0	0	0	0	0	0	0	0	0	0
28.4167	20.0907	0.5307	H	0	0	0	0	0	0	0	0	0	0	0	0	0
26.2657	21.0468	1.3195	H	0	0	0	0	0	0	0	0	0	0	0	0	0
26.2730	19.2866	1.4903	H	0	0	0	0	0	0	0	0	0	0	0	0	0
25.0266	19.0742	-0.6466	H	0	0	0	0	0	0	0	0	0	0	0	0	0
25.0190	20.8345	-0.8170	H	0	0	0	0	0	0	0	0	0	0	0	0	0
23.7485	21.0348	1.3065	H	0	0	0	0	0	0	0	0	0	0	0	0	0
23.7565	19.2745	1.4775	H	0	0	0	0	0	0	0	0	0	0	0	0	0
22.5105	19.0616	-0.6597	H	0	0	0	0	0	0	0	0	0	0	0	0	0
22.5015	20.8220	-0.8296	H	0	0	0	0	0	0	0	0	0	0	0	0	0
21.2307	21.0208	1.2937	H	0	0	0	0	0	0	0	0	0	0	0	0	0
21.2408	19.2606	1.4648	H	0	0	0	0	0	0	0	0	0	0	0	0	0
19.9952	19.0462	-0.6726	H	0	0	0	0	0	0	0	0	0	0	0	0	0
19.9835	20.8065	-0.8419	H	0	0	0	0	0	0	0	0	0	0	0	0	0
18.7124	21.0028	1.2813	H	0	0	0	0	0	0	0	0	0	0	0	0	0
18.7259	19.2427	1.4526	H	0	0	0	0	0	0	0	0	0	0	0	0	0
17.4819	19.0265	-0.6844	H	0	0	0	0	0	0	0	0	0	0	0	0	0
17.4650	20.7863	-0.8533	H	0	0	0	0	0	0	0	0	0	0	0	0	0
16.2027	20.9728	1.2820	H	0	0	0	0	0	0	0	0	0	0	0	0	0
16.2049	19.2123	1.4334	H	0	0	0	0	0	0	0	0	0	0	0	0	0
14.9453	20.7941	-0.8503	H	0	0	0	0	0	0	0	0	0	0	0	0	0
13.6830	19.2164	1.4347	H	0	0	0	0	0	0	0	0	0	0	0	0	0
13.6893	20.9768	1.2822	H	0	0	0	0	0	0	0	0	0	0	0	0	0
12.4248	20.7922	-0.8518	H	0	0	0	0	0	0	0	0	0	0	0	0	0
12.4044	19.0325	-0.6825	H	0	0	0	0	0	0	0	0	0	0	0	0	0
11.1626	19.2519	1.4556	H	0	0	0	0	0	0	0	0	0	0	0	0	0
11.1791	21.0120	1.2833	H	0	0	0	0	0	0	0	0	0	0	0	0	0
9.9059	20.8168	-0.8386	H	0	0	0	0	0	0	0	0	0	0	0	0	0
9.8917	19.0565	-0.6687	H	0	0	0	0	0	0	0	0	0	0	0	0	0
8.6482	19.2735	1.4697	H	0	0	0	0	0	0	0	0	0	0	0	0	0
8.6604	21.0336	1.2976	H	0	0	0	0	0	0	0	0	0	0	0	0	0
7.3875	20.8353	-0.8245	H	0	0	0	0	0	0	0	0	0	0	0	0	0
7.3767	19.0751	-0.6539	H	0	0	0	0	0	0	0	0	0	0	0	0	0
6.1327	19.2903	1.4843	H	0	0	0	0	0	0	0	0	0	0	0	0	0
6.1424	21.0504	1.3124	H	0	0	0	0	0	0	0	0	0	0	0	0	0
4.8700	20.8504	-0.8100	H	0	0	0	0	0	0	0	0	0	0	0	0	0
4.8609	19.0902	-0.6388	H	0	0	0	0	0	0	0	0	0	0	0	0	0
3.6164	19.3047	1.4991	H	0	0	0	0	0	0	0	0	0	0	0	0	0
3.6251	21.0648	1.3275	H	0	0	0	0	0	0	0	0	0	0	0	0	0
2.3441	19.1040	-0.6233	H	0	0	0	0	0	0	0	0	0	0	0	0	0
1.4726	20.1103	0.5408	H	0	0	0	0	0	0	0	0	0	0	0	0	0
2.3528	20.8641	-0.7950	H	0	0	0	0	0	0	0	0	0	0	0	0	0
15.8247	18.5836	-1.5754	H	0	0	0	0	0	0	0	0	0	0	0	0	0
14.0595	18.5869	-1.5758	H	0	0	0	0	0	0	0	0	0	0	0	0	0
14.0560	17.4805	0.6491	H	0	0	0	0	0	0	0	0	0	0	0	0	0
15.8235	17.4766	0.6490	H	0	0	0	0	0	0	0	0	0	0	0	0	0
15.8210	16.0746	-1.3981	H	0	0	0	0	0	0	0	0	0	0	0	0	0
14.0525	16.0790	-1.3987	H	0	0	0	0	0	0	0	0	0	0	0	0	0
14.0491	14.9718	0.8235	H	0	0	0	0	0	0	0	0	0	0	0	0	0
15.8177	14.9669	0.8236	H	0	0	0	0	0	0	0	0	0	0	0	0	0
15.8140	13.5635	-1.2243	H	0	0	0	0	0	0	0	0	0	0	0	0	0
14.0455	13.5688	-1.2249	H	0	0	0	0	0	0	0	0	0	0	0	0	0
14.0415	12.4615	0.9973	H	0	0	0	0	0	0	0	0	0	0	0	0	0
15.8100	12.4559	0.9976	H	0	0	0	0	0	0	0	0	0	0	0	0	0
15.8060	11.0526	-1.0504	H	0	0	0	0	0	0	0	0	0	0	0	0	0
14.0375	11.0584	-1.0509	H	0	0	0	0	0	0	0	0	0	0	0	0	0
14.0331	9.9511	1.1712	H	0	0	0	0	0	0	0	0	0	0	0	0	0
15.8017	9.9451	1.1717	H	0	0	0	0	0	0	0	0	0	0	0	0	0
15.7975	8.5416	-0.8764	H	0	0	0	0	0	0	0	0	0	0	0	0	0
14.0290	8.5476	-0.8769	H	0	0	0	0	0	0	0	0	0	0	0	0	0
15.7931	7.4342	1.3455	H	0	0	0	0	0	0	0	0	0	0	0	0	0
14.9059	6.4689	0.1587	H	0	0	0	0	0	0	0	0	0	0	0	0	0
14.0245	7.4402	1.3450	H	0	0	0	0	0	0	0	0	0	0	0	0	0

1	2	1	0	0	0	0
2	3	1	0	0	0	0
3	4	1	0	0	0	0
4	5	1	0	0	0	0
5	6	1	0	0	0	0
6	7	1	0	0	0	0
7	8	1	0	0	0	0
8	9	1	0	0	0	0
9	10	1	0	0	0	0
10	11	1	0	0	0	0
11	12	1	0	0	0	0
12	13	1	0	0	0	0
13	14	1	0	0	0	0
14	15	1	0	0	0	0
15	16	1	0	0	0	0
16	17	1	0	0	0	0
17	18	1	0	0	0	0
18	19	1	0	0	0	0
19	20	1	0	0	0	0
20	21	1	0	0	0	0
11	22	1	0	0	0	0
22	23	1	0	0	0	0
23	24	1	0	0	0	0
24	25	1	0	0	0	0
25	26	1	0	0	0	0
26	27	1	0	0	0	0
27	28	1	0	0	0	0
28	29	1	0	0	0	0
29	30	1	0	0	0	0
30	31	1	0	0	0	0
1	32	1	0	0	0	0
1	33	1	0	0	0	0
1	34	1	0	0	0	0
2	35	1	0	0	0	0
2	36	1	0	0	0	0
3	37	1	0	0	0	0
3	38	1	0	0	0	0
4	39	1	0	0	0	0
4	40	1	0	0	0	0
5	41	1	0	0	0	0
5	42	1	0	0	0	0
6	43	1	0	0	0	0
6	44	1	0	0	0	0
7	45	1	0	0	0	0
7	46	1	0	0	0	0
8	47	1	0	0	0	0
8	48	1	0	0	0	0
9	49	1	0	0	0	0
9	50	1	0	0	0	0
10	51	1	0	0	0	0
10	52	1	0	0	0	0
11	53	1	0	0	0	0
12	54	1	0	0	0	0
12	55	1	0	0	0	0
13	56	1	0	0	0	0
13	57	1	0	0	0	0
14	58	1	0	0	0	0
14	59	1	0	0	0	0
15	60	1	0	0	0	0
15	61	1	0	0	0	0
16	62	1	0	0	0	0
16	63	1	0	0	0	0
17	64	1	0	0	0	0

```
 17 65  1  0  0  0  0
 18 66  1  0  0  0  0
 18 67  1  0  0  0  0
 19 68  1  0  0  0  0
 19 69  1  0  0  0  0
 20 70  1  0  0  0  0
 20 71  1  0  0  0  0
 21 72  1  0  0  0  0
 21 73  1  0  0  0  0
 21 74  1  0  0  0  0
 22 75  1  0  0  0  0
 22 76  1  0  0  0  0
 23 77  1  0  0  0  0
 23 78  1  0  0  0  0
 24 79  1  0  0  0  0
 24 80  1  0  0  0  0
 25 81  1  0  0  0  0
 25 82  1  0  0  0  0
 26 83  1  0  0  0  0
 26 84  1  0  0  0  0
 27 85  1  0  0  0  0
 27 86  1  0  0  0  0
 28 87  1  0  0  0  0
 28 88  1  0  0  0  0
 29 89  1  0  0  0  0
 29 90  1  0  0  0  0
 30 91  1  0  0  0  0
 30 92  1  0  0  0  0
 31 93  1  0  0  0  0
 31 94  1  0  0  0  0
 31 95  1  0  0  0  0
M  END
>  <Alias name>
11-Decylheneicosane

>  <deltaH°(c)>   kJ/mol
-20492

$$$$
13-Cyclohexylpentacosane
  ChemBrain IXL

 93 93  0  0  0  0  0  0  0  0  1 V2000
   11.8926    9.4150   -1.5692 C   0  0  0  0  0  0  0  0  0  0  0  0
   12.7957    8.4164   -0.8005 C   0  0  0  0  0  0  0  0  0  0  0  0
   13.6993    9.1873    0.1917 C   0  0  0  0  0  0  0  0  0  0  0  0
   11.9144    7.4078   -0.0210 C   0  0  0  0  0  0  0  0  0  0  0  0
   10.9992   10.1872   -0.5748 C   0  0  0  0  0  0  0  0  0  0  0  0
   14.5693   10.2018   -0.5786 C   0  0  0  0  0  0  0  0  0  0  0  0
   12.8184    6.3975    0.7196 C   0  0  0  0  0  0  0  0  0  0  0  0
   10.9992    6.6497   -1.0080 C   0  0  0  0  0  0  0  0  0  0  0  0
   10.1076   11.1769   -1.3489 C   0  0  0  0  0  0  0  0  0  0  0  0
   15.4848   10.9483    0.4109 C   0  0  0  0  0  0  0  0  0  0  0  0
   11.9424    5.3905    1.4911 C   0  0  0  0  0  0  0  0  0  0  0  0
   10.1272    5.6420   -0.2327 C   0  0  0  0  0  0  0  0  0  0  0  0
    9.2144   11.9477   -0.3574 C   0  0  0  0  0  0  0  0  0  0  0  0
   16.3544   11.9617   -0.3582 C   0  0  0  0  0  0  0  0  0  0  0  0
   11.0334    4.6356    0.5021 C   0  0  0  0  0  0  0  0  0  0  0  0
    8.3235   12.9386   -1.1316 C   0  0  0  0  0  0  0  0  0  0  0  0
   17.2701   12.7084    0.6312 C   0  0  0  0  0  0  0  0  0  0  0  0
    7.4297   13.7088   -0.1403 C   0  0  0  0  0  0  0  0  0  0  0  0
   18.1398   13.7217   -0.1382 C   0  0  0  0  0  0  0  0  0  0  0  0
    6.5396   14.7005   -0.9145 C   0  0  0  0  0  0  0  0  0  0  0  0
```

19.0557	14.4683	0.8511 C	0	0	0	0	0	0	0	0	0	0	0	0	0
5.6452	15.4702	0.0767 C	0	0	0	0	0	0	0	0	0	0	0	0	0
19.9255	15.4814	0.0816 C	0	0	0	0	0	0	0	0	0	0	0	0	0
4.7557	16.4624	-0.6975 C	0	0	0	0	0	0	0	0	0	0	0	0	0
20.8414	16.2281	1.0708 C	0	0	0	0	0	0	0	0	0	0	0	0	0
3.8608	17.2318	0.2936 C	0	0	0	0	0	0	0	0	0	0	0	0	0
21.7112	17.2412	0.3012 C	0	0	0	0	0	0	0	0	0	0	0	0	0
2.9716	18.2243	-0.4807 C	0	0	0	0	0	0	0	0	0	0	0	0	0
22.6273	17.9880	1.2905 C	0	0	0	0	0	0	0	0	0	0	0	0	0
2.0765	18.9934	0.5102 C	0	0	0	0	0	0	0	0	0	0	0	0	0
23.4970	19.0010	0.5210 C	0	0	0	0	0	0	0	0	0	0	0	0	0
11.2683	8.8724	-2.2666 H	0	0	0	0	0	0	0	0	0	0	0	0	0
12.5124	10.1150	-2.1138 H	0	0	0	0	0	0	0	0	0	0	0	0	0
13.4161	7.8789	-1.5068 H	0	0	0	0	0	0	0	0	0	0	0	0	0
13.0828	9.7126	0.9095 H	0	0	0	0	0	0	0	0	0	0	0	0	0
14.3405	8.4882	0.7121 H	0	0	0	0	0	0	0	0	0	0	0	0	0
11.3059	7.9403	0.6983 H	0	0	0	0	0	0	0	0	0	0	0	0	0
10.3752	9.4893	-0.0319 H	0	0	0	0	0	0	0	0	0	0	0	0	0
11.6217	10.7329	0.1223 H	0	0	0	0	0	0	0	0	0	0	0	0	0
15.1761	9.6776	-1.3065 H	0	0	0	0	0	0	0	0	0	0	0	0	0
13.9315	10.9137	-1.0859 H	0	0	0	0	0	0	0	0	0	0	0	0	0
13.4560	6.9260	1.4159 H	0	0	0	0	0	0	0	0	0	0	0	0	0
13.4307	5.8668	0.0010 H	0	0	0	0	0	0	0	0	0	0	0	0	0
11.6078	6.1196	-1.7301 H	0	0	0	0	0	0	0	0	0	0	0	0	0
10.3606	7.3550	-1.5231 H	0	0	0	0	0	0	0	0	0	0	0	0	0
10.7314	11.8763	-1.8917 H	0	0	0	0	0	0	0	0	0	0	0	0	0
9.4847	10.6316	-2.0471 H	0	0	0	0	0	0	0	0	0	0	0	0	0
14.8781	11.4724	1.1389 H	0	0	0	0	0	0	0	0	0	0	0	0	0
16.1238	10.2365	0.9188 H	0	0	0	0	0	0	0	0	0	0	0	0	0
11.3309	5.9209	2.2106 H	0	0	0	0	0	0	0	0	0	0	0	0	0
12.5775	4.6829	2.0094 H	0	0	0	0	0	0	0	0	0	0	0	0	0
9.4866	5.1112	-0.9259 H	0	0	0	0	0	0	0	0	0	0	0	0	0
9.5175	6.1722	0.4884 H	0	0	0	0	0	0	0	0	0	0	0	0	0
8.5900	11.2484	0.1847 H	0	0	0	0	0	0	0	0	0	0	0	0	0
9.8373	12.4923	0.3414 H	0	0	0	0	0	0	0	0	0	0	0	0	0
16.9610	11.4375	-1.0863 H	0	0	0	0	0	0	0	0	0	0	0	0	0
15.7154	12.6735	-0.8661 H	0	0	0	0	0	0	0	0	0	0	0	0	0
10.4201	3.9270	1.0446 H	0	0	0	0	0	0	0	0	0	0	0	0	0
11.6446	4.1067	-0.2186 H	0	0	0	0	0	0	0	0	0	0	0	0	0
8.9480	13.6382	-1.6733 H	0	0	0	0	0	0	0	0	0	0	0	0	0
7.7011	12.3940	-1.8309 H	0	0	0	0	0	0	0	0	0	0	0	0	0
16.6635	13.2328	1.3592 H	0	0	0	0	0	0	0	0	0	0	0	0	0
17.9091	11.9966	1.1392 H	0	0	0	0	0	0	0	0	0	0	0	0	0
6.8047	13.0093	0.4009 H	0	0	0	0	0	0	0	0	0	0	0	0	0
8.0521	14.2528	0.5594 H	0	0	0	0	0	0	0	0	0	0	0	0	0
18.7464	13.1973	-0.8662 H	0	0	0	0	0	0	0	0	0	0	0	0	0
17.5009	14.4334	-0.6461 H	0	0	0	0	0	0	0	0	0	0	0	0	0
7.1647	15.4003	-1.4552 H	0	0	0	0	0	0	0	0	0	0	0	0	0
5.9176	14.1565	-1.6146 H	0	0	0	0	0	0	0	0	0	0	0	0	0
18.4492	14.9928	1.5791 H	0	0	0	0	0	0	0	0	0	0	0	0	0
19.6946	13.7566	1.3591 H	0	0	0	0	0	0	0	0	0	0	0	0	0
5.0197	14.7704	0.6170 H	0	0	0	0	0	0	0	0	0	0	0	0	0
6.2672	16.0138	0.7771 H	0	0	0	0	0	0	0	0	0	0	0	0	0
20.5320	14.9569	-0.6464 H	0	0	0	0	0	0	0	0	0	0	0	0	0
19.2866	16.1932	-0.4264 H	0	0	0	0	0	0	0	0	0	0	0	0	0
5.3812	17.1624	-1.2375 H	0	0	0	0	0	0	0	0	0	0	0	0	0
4.1340	15.9189	-1.3982 H	0	0	0	0	0	0	0	0	0	0	0	0	0
20.2350	16.7527	1.7988 H	0	0	0	0	0	0	0	0	0	0	0	0	0
21.4803	15.5164	1.5789 H	0	0	0	0	0	0	0	0	0	0	0	0	0
3.2352	16.5317	0.8334 H	0	0	0	0	0	0	0	0	0	0	0	0	0
4.4825	17.7750	0.9945 H	0	0	0	0	0	0	0	0	0	0	0	0	0
22.3177	16.7167	-0.4267 H	0	0	0	0	0	0	0	0	0	0	0	0	0
21.0723	17.9529	-0.2068 H	0	0	0	0	0	0	0	0	0	0	0	0	0

3.5972 18.9245 -1.0203 H 0 0 0 0 0 0 0 0 0 0 0 0
2.3500 17.6811 -1.1817 H 0 0 0 0 0 0 0 0 0 0 0 0
22.0208 18.5126 2.0184 H 0 0 0 0 0 0 0 0 0 0 0 0
23.2662 17.2764 1.7985 H 0 0 0 0 0 0 0 0 0 0 0 0
1.4507 18.2934 1.0498 H 0 0 0 0 0 0 0 0 0 0 0 0
2.6980 19.5367 1.2113 H 0 0 0 0 0 0 0 0 0 0 0 0
1.4518 19.6908 -0.0340 H 0 0 0 0 0 0 0 0 0 0 0 0
22.8582 19.7128 0.0130 H 0 0 0 0 0 0 0 0 0 0 0 0
24.1035 18.4766 -0.2069 H 0 0 0 0 0 0 0 0 0 0 0 0
24.1405 19.5256 1.2163 H 0 0 0 0 0 0 0 0 0 0 0 0
1 2 1 0 0 0 0
1 5 1 0 0 0 0
2 3 1 0 0 0 0
2 4 1 0 0 0 0
3 6 1 0 0 0 0
7 4 1 0 0 0 0
4 8 1 0 0 0 0
5 9 1 0 0 0 0
6 10 1 0 0 0 0
11 7 1 0 0 0 0
8 12 1 0 0 0 0
9 13 1 0 0 0 0
10 14 1 0 0 0 0
15 11 1 0 0 0 0
12 15 1 0 0 0 0
13 16 1 0 0 0 0
14 17 1 0 0 0 0
16 18 1 0 0 0 0
17 19 1 0 0 0 0
18 20 1 0 0 0 0
19 21 1 0 0 0 0
20 22 1 0 0 0 0
21 23 1 0 0 0 0
22 24 1 0 0 0 0
23 25 1 0 0 0 0
24 26 1 0 0 0 0
25 27 1 0 0 0 0
26 28 1 0 0 0 0
27 29 1 0 0 0 0
28 30 1 0 0 0 0
29 31 1 0 0 0 0
1 32 1 0 0 0 0
1 33 1 0 0 0 0
2 34 1 0 0 0 0
3 35 1 0 0 0 0
3 36 1 0 0 0 0
4 37 1 0 0 0 0
5 38 1 0 0 0 0
5 39 1 0 0 0 0
6 40 1 0 0 0 0
6 41 1 0 0 0 0
7 42 1 0 0 0 0
7 43 1 0 0 0 0
8 44 1 0 0 0 0
8 45 1 0 0 0 0
9 46 1 0 0 0 0
9 47 1 0 0 0 0
10 48 1 0 0 0 0
10 49 1 0 0 0 0
11 50 1 0 0 0 0
11 51 1 0 0 0 0
12 52 1 0 0 0 0
12 53 1 0 0 0 0

```
 13 54  1  0  0  0  0
 13 55  1  0  0  0  0
 14 56  1  0  0  0  0
 14 57  1  0  0  0  0
 15 58  1  0  0  0  0
 15 59  1  0  0  0  0
 16 60  1  0  0  0  0
 16 61  1  0  0  0  0
 17 62  1  0  0  0  0
 17 63  1  0  0  0  0
 18 64  1  0  0  0  0
 18 65  1  0  0  0  0
 19 66  1  0  0  0  0
 19 67  1  0  0  0  0
 20 68  1  0  0  0  0
 20 69  1  0  0  0  0
 21 70  1  0  0  0  0
 21 71  1  0  0  0  0
 22 72  1  0  0  0  0
 22 73  1  0  0  0  0
 23 74  1  0  0  0  0
 23 75  1  0  0  0  0
 24 76  1  0  0  0  0
 24 77  1  0  0  0  0
 25 78  1  0  0  0  0
 25 79  1  0  0  0  0
 26 80  1  0  0  0  0
 26 81  1  0  0  0  0
 27 82  1  0  0  0  0
 27 83  1  0  0  0  0
 28 84  1  0  0  0  0
 28 85  1  0  0  0  0
 29 86  1  0  0  0  0
 29 87  1  0  0  0  0
 30 88  1  0  0  0  0
 30 89  1  0  0  0  0
 30 90  1  0  0  0  0
 31 91  1  0  0  0  0
 31 92  1  0  0  0  0
 31 93  1  0  0  0  0
M  END
>  <Alias name>
13-Cyclohexylpentacosane

>  <deltaH°(c)>   kJ/mol
-20280.9

$$$$
2,3,6-Tris(phenylimino)-1,3,5-triphenyl-1,3,5-triazacyclohexane
  ChemBrain IXL

 75 81  0  0  0  0  0  0  0  0  1 V2000
    7.9486    9.5818    0.0544 C   0  0  0  0  0  0  0  0  0  0  0  0
    9.3038    9.5905    0.2776 N   0  0  0  0  0  0  0  0  0  0  0  0
   10.0591    8.3995    0.0457 C   0  0  0  0  0  0  0  0  0  0  0  0
    9.3926    7.2139   -0.0334 N   0  0  0  0  0  0  0  0  0  0  0  0
    8.0435    7.2108   -0.4978 C   0  0  0  0  0  0  0  0  0  0  0  0
    7.3352    8.3886   -0.4846 N   0  0  0  0  0  0  0  0  0  0  0  0
   11.3426    8.3862   -0.0953 N   0  0  0  0  0  0  0  0  0  0  0  0
    7.4570    6.1501   -0.9359 N   0  0  0  0  0  0  0  0  0  0  0  0
    7.2866   10.6545    0.3449 N   0  0  0  0  0  0  0  0  0  0  0  0
   11.4458    3.7415    1.1331 C   0  0  0  0  0  0  0  0  0  0  0  0
   12.0601    4.6697    0.2925 C   0  0  0  0  0  0  0  0  0  0  0  0
```

```
11.3732    5.8206   -0.0957 C   0  0  0  0  0  0  0  0  0  0  0  0
10.0686    6.0436    0.3527 C   0  0  0  0  0  0  0  0  0  0  0  0
 9.4570    5.1163    1.1976 C   0  0  0  0  0  0  0  0  0  0  0  0
10.1449    3.9648    1.5857 C   0  0  0  0  0  0  0  0  0  0  0  0
 9.4018    2.4341   -1.1176 C   0  0  0  0  0  0  0  0  0  0  0  0
 8.1886    2.5561   -0.4397 C   0  0  0  0  0  0  0  0  0  0  0  0
 7.5451    3.7929   -0.3778 C   0  0  0  0  0  0  0  0  0  0  0  0
 8.1157    4.9105   -0.9913 C   0  0  0  0  0  0  0  0  0  0  0  0
 9.3277    4.7862   -1.6725 C   0  0  0  0  0  0  0  0  0  0  0  0
 9.9709    3.5487   -1.7342 C   0  0  0  0  0  0  0  0  0  0  0  0
13.9442   10.8106    0.9301 C   0  0  0  0  0  0  0  0  0  0  0  0
13.6655   11.8777    0.0762 C   0  0  0  0  0  0  0  0  0  0  0  0
12.6116   11.7858   -0.8333 C   0  0  0  0  0  0  0  0  0  0  0  0
11.8351   10.6271   -0.8892 C   0  0  0  0  0  0  0  0  0  0  0  0
12.1103    9.5604   -0.0332 C   0  0  0  0  0  0  0  0  0  0  0  0
13.1682    9.6518    0.8745 C   0  0  0  0  0  0  0  0  0  0  0  0
 4.3497    9.2881   -2.4839 C   0  0  0  0  0  0  0  0  0  0  0  0
 3.4525    8.3007   -2.0747 C   0  0  0  0  0  0  0  0  0  0  0  0
 3.8456    7.3481   -1.1348 C   0  0  0  0  0  0  0  0  0  0  0  0
 5.1354    7.3836   -0.6040 C   0  0  0  0  0  0  0  0  0  0  0  0
 6.0332    8.3750   -1.0098 C   0  0  0  0  0  0  0  0  0  0  0  0
 5.6404    9.3240   -1.9530 C   0  0  0  0  0  0  0  0  0  0  0  0
11.3103   11.9430    2.3135 C   0  0  0  0  0  0  0  0  0  0  0  0
11.0120   13.1537    1.6873 C   0  0  0  0  0  0  0  0  0  0  0  0
10.1500   13.1776    0.5912 C   0  0  0  0  0  0  0  0  0  0  0  0
 9.5871   11.9906    0.1212 C   0  0  0  0  0  0  0  0  0  0  0  0
 9.8888   10.7770    0.7456 C   0  0  0  0  0  0  0  0  0  0  0  0
10.7475   10.7551    1.8436 C   0  0  0  0  0  0  0  0  0  0  0  0
 3.1337   10.9718   -0.1696 C   0  0  0  0  0  0  0  0  0  0  0  0
 3.9858   11.8984   -0.7708 C   0  0  0  0  0  0  0  0  0  0  0  0
 5.3665   11.7883   -0.6015 C   0  0  0  0  0  0  0  0  0  0  0  0
 5.8972   10.7490    0.1661 C   0  0  0  0  0  0  0  0  0  0  0  0
 5.0436    9.8256    0.7709 C   0  0  0  0  0  0  0  0  0  0  0  0
 3.6624    9.9361    0.6014 C   0  0  0  0  0  0  0  0  0  0  0  0
11.9788    2.8473    1.4342 H   0  0  0  0  0  0  0  0  0  0  0  0
13.0706    4.4966   -0.0590 H   0  0  0  0  0  0  0  0  0  0  0  0
11.8529    6.5420   -0.7460 H   0  0  0  0  0  0  0  0  0  0  0  0
 8.4492    5.2896    1.5520 H   0  0  0  0  0  0  0  0  0  0  0  0
 9.6686    3.2439    2.2385 H   0  0  0  0  0  0  0  0  0  0  0  0
 9.9017    1.4739   -1.1650 H   0  0  0  0  0  0  0  0  0  0  0  0
 7.7463    1.6905    0.0386 H   0  0  0  0  0  0  0  0  0  0  0  0
 6.6019    3.8856    0.1467 H   0  0  0  0  0  0  0  0  0  0  0  0
 9.7690    5.6498   -2.1533 H   0  0  0  0  0  0  0  0  0  0  0  0
10.9127    3.4534   -2.2609 H   0  0  0  0  0  0  0  0  0  0  0  0
14.7629   10.8818    1.6362 H   0  0  0  0  0  0  0  0  0  0  0  0
14.2674   12.7776    0.1196 H   0  0  0  0  0  0  0  0  0  0  0  0
12.3960   12.6143   -1.4964 H   0  0  0  0  0  0  0  0  0  0  0  0
11.0195   10.5558   -1.5967 H   0  0  0  0  0  0  0  0  0  0  0  0
13.3873    8.8224    1.5363 H   0  0  0  0  0  0  0  0  0  0  0  0
 4.0442   10.0271   -3.2139 H   0  0  0  0  0  0  0  0  0  0  0  0
 2.4509    8.2739   -2.4867 H   0  0  0  0  0  0  0  0  0  0  0  0
 3.1492    6.5809   -0.8171 H   0  0  0  0  0  0  0  0  0  0  0  0
 5.4404    6.6410    0.1237 H   0  0  0  0  0  0  0  0  0  0  0  0
 6.3365   10.0882   -2.2732 H   0  0  0  0  0  0  0  0  0  0  0  0
11.9791   11.9251    3.1647 H   0  0  0  0  0  0  0  0  0  0  0  0
11.4498   14.0751    2.0521 H   0  0  0  0  0  0  0  0  0  0  0  0
 9.9178   14.1178    0.1049 H   0  0  0  0  0  0  0  0  0  0  0  0
 8.9153   12.0109   -0.7288 H   0  0  0  0  0  0  0  0  0  0  0  0
10.9771    9.8168    2.3312 H   0  0  0  0  0  0  0  0  0  0  0  0
 2.0616   11.0565   -0.3017 H   0  0  0  0  0  0  0  0  0  0  0  0
 3.5754   12.7031   -1.3689 H   0  0  0  0  0  0  0  0  0  0  0  0
 6.0272   12.5104   -1.0661 H   0  0  0  0  0  0  0  0  0  0  0  0
 5.4527    9.0236    1.3718 H   0  0  0  0  0  0  0  0  0  0  0  0
```

```
    3.0004    9.2174    1.0685 H   0  0  0  0  0  0  0  0  0  0  0  0
  1  2  1  0  0  0  0
  2  3  1  0  0  0  0
  3  4  1  0  0  0  0
  4  5  1  0  0  0  0
  5  6  1  0  0  0  0
  6  1  1  0  0  0  0
  3  7  2  0  0  0  0
  5  8  2  0  0  0  0
  1  9  2  0  0  0  0
 10 11  4  0  0  0  0
 11 12  4  0  0  0  0
 12 13  4  0  0  0  0
 13 14  4  0  0  0  0
 14 15  4  0  0  0  0
 15 10  4  0  0  0  0
 16 17  4  0  0  0  0
 17 18  4  0  0  0  0
 18 19  4  0  0  0  0
 19 20  4  0  0  0  0
 20 21  4  0  0  0  0
 21 16  4  0  0  0  0
 22 23  4  0  0  0  0
 23 24  4  0  0  0  0
 24 25  4  0  0  0  0
 25 26  4  0  0  0  0
 26 27  4  0  0  0  0
 27 22  4  0  0  0  0
 28 29  4  0  0  0  0
 29 30  4  0  0  0  0
 30 31  4  0  0  0  0
 31 32  4  0  0  0  0
 32 33  4  0  0  0  0
 33 28  4  0  0  0  0
 13  4  1  0  0  0  0
 26  7  1  0  0  0  0
 19  8  1  0  0  0  0
 34 35  4  0  0  0  0
 35 36  4  0  0  0  0
 36 37  4  0  0  0  0
 37 38  4  0  0  0  0
 38 39  4  0  0  0  0
 39 34  4  0  0  0  0
 32  6  1  0  0  0  0
 40 41  4  0  0  0  0
 41 42  4  0  0  0  0
 42 43  4  0  0  0  0
 43 44  4  0  0  0  0
 44 45  4  0  0  0  0
 45 40  4  0  0  0  0
 38  2  1  0  0  0  0
 43  9  1  0  0  0  0
 10 46  1  0  0  0  0
 11 47  1  0  0  0  0
 12 48  1  0  0  0  0
 14 49  1  0  0  0  0
 15 50  1  0  0  0  0
 16 51  1  0  0  0  0
 17 52  1  0  0  0  0
 18 53  1  0  0  0  0
 20 54  1  0  0  0  0
 21 55  1  0  0  0  0
 22 56  1  0  0  0  0
```

```
 23 57  1  0  0  0  0
 24 58  1  0  0  0  0
 25 59  1  0  0  0  0
 27 60  1  0  0  0  0
 28 61  1  0  0  0  0
 29 62  1  0  0  0  0
 30 63  1  0  0  0  0
 31 64  1  0  0  0  0
 33 65  1  0  0  0  0
 34 66  1  0  0  0  0
 35 67  1  0  0  0  0
 36 68  1  0  0  0  0
 37 69  1  0  0  0  0
 39 70  1  0  0  0  0
 40 71  1  0  0  0  0
 41 72  1  0  0  0  0
 42 73  1  0  0  0  0
 44 74  1  0  0  0  0
 45 75  1  0  0  0  0
M  END
>  <Alias name>
2,3,6-Tris(phenylimino)-1,3,5-triphenyl-1,3,5-triazacyclohexane

>  <deltaH°(c)>   kJ/mol
-20256

$$$$
Tri-n-decylamine
  ChemBrain IXL

 94 93  0  0  0  0  0  0  0  0  1 V2000
   19.7618   18.8368   -0.3364 C   0  0  0  0  0  0  0  0  0  0  0  0
   18.3074   18.8533    0.1729 C   0  0  0  0  0  0  0  0  0  0  0  0
   17.5907   17.5653   -0.2770 C   0  0  0  0  0  0  0  0  0  0  0  0
   16.1369   17.5811    0.2340 C   0  0  0  0  0  0  0  0  0  0  0  0
   15.4206   16.2922   -0.2139 C   0  0  0  0  0  0  0  0  0  0  0  0
   13.9649   16.3087    0.3004 C   0  0  0  0  0  0  0  0  0  0  0  0
   13.2566   15.0946   -0.1590 N   0  0  0  0  0  0  0  0  0  0  0  0
   20.4788   20.1242    0.1148 C   0  0  0  0  0  0  0  0  0  0  0  0
   21.9336   20.1073   -0.3933 C   0  0  0  0  0  0  0  0  0  0  0  0
   22.6508   21.3945    0.0585 C   0  0  0  0  0  0  0  0  0  0  0  0
   13.2479    7.5888   -0.0420 C   0  0  0  0  0  0  0  0  0  0  0  0
   13.9778    8.8597    0.4345 C   0  0  0  0  0  0  0  0  0  0  0  0
   13.2312   10.1052   -0.0817 C   0  0  0  0  0  0  0  0  0  0  0  0
   13.9601   11.3760    0.3965 C   0  0  0  0  0  0  0  0  0  0  0  0
   13.2126   12.6215   -0.1177 C   0  0  0  0  0  0  0  0  0  0  0  0
   13.9430   13.8937    0.3637 C   0  0  0  0  0  0  0  0  0  0  0  0
   13.9938    6.3434    0.4755 C   0  0  0  0  0  0  0  0  0  0  0  0
   13.2633    5.0724    0.0001 C   0  0  0  0  0  0  0  0  0  0  0  0
   14.0089    3.8270    0.5182 C   0  0  0  0  0  0  0  0  0  0  0  0
    6.7605   18.8523   -0.3369 C   0  0  0  0  0  0  0  0  0  0  0  0
    7.4849   17.6049    0.2055 C   0  0  0  0  0  0  0  0  0  0  0  0
    8.9481   17.6094   -0.2782 C   0  0  0  0  0  0  0  0  0  0  0  0
    9.6729   16.3630    0.2659 C   0  0  0  0  0  0  0  0  0  0  0  0
   11.1366   16.3684   -0.2159 C   0  0  0  0  0  0  0  0  0  0  0  0
   11.8619   15.1201    0.3315 C   0  0  0  0  0  0  0  0  0  0  0  0
    5.2977   18.8484    0.1482 C   0  0  0  0  0  0  0  0  0  0  0  0
    4.5735   20.0965   -0.3931 C   0  0  0  0  0  0  0  0  0  0  0  0
    3.1108   20.0929    0.0925 C   0  0  0  0  0  0  0  0  0  0  0  0
   24.1057   21.3775   -0.4492 C   0  0  0  0  0  0  0  0  0  0  0  0
   13.2784    2.5560    0.0433 C   0  0  0  0  0  0  0  0  0  0  0  0
    2.3867   21.3411   -0.4483 C   0  0  0  0  0  0  0  0  0  0  0  0
   20.2769   17.9753    0.0704 H   0  0  0  0  0  0  0  0  0  0  0  0
```

```
19.7650 18.7819 -1.4180 H 0 0 0 0 0 0 0 0 0 0 0 0
17.7920 19.7143 -0.2345 H 0 0 0 0 0 0 0 0 0 0 0 0
18.3043 18.9091 1.2544 H 0 0 0 0 0 0 0 0 0 0 0 0
18.1068 16.7043 0.1294 H 0 0 0 0 0 0 0 0 0 0 0 0
17.5926 17.5100 -1.3586 H 0 0 0 0 0 0 0 0 0 0 0 0
15.6203 18.4414 -0.1733 H 0 0 0 0 0 0 0 0 0 0 0 0
16.1351 17.6377 1.3156 H 0 0 0 0 0 0 0 0 0 0 0 0
15.9388 15.4340 0.1919 H 0 0 0 0 0 0 0 0 0 0 0 0
15.4209 16.2366 -1.2953 H 0 0 0 0 0 0 0 0 0 0 0 0
13.4620 17.1854 -0.0834 H 0 0 0 0 0 0 0 0 0 0 0 0
13.9653 16.3388 1.3832 H 0 0 0 0 0 0 0 0 0 0 0 0
19.9642 20.9857 -0.2924 H 0 0 0 0 0 0 0 0 0 0 0 0
20.4749 20.1794 1.1964 H 0 0 0 0 0 0 0 0 0 0 0 0
22.4481 19.2456 0.0137 H 0 0 0 0 0 0 0 0 0 0 0 0
21.9376 20.0525 -1.4750 H 0 0 0 0 0 0 0 0 0 0 0 0
22.1365 22.2562 -0.3487 H 0 0 0 0 0 0 0 0 0 0 0 0
22.6466 21.4494 1.1400 H 0 0 0 0 0 0 0 0 0 0 0 0
13.2233 7.5710 -1.1246 H 0 0 0 0 0 0 0 0 0 0 0 0
12.2353 7.5886 0.3422 H 0 0 0 0 0 0 0 0 0 0 0 0
14.0031 8.8770 1.5170 H 0 0 0 0 0 0 0 0 0 0 0 0
14.9901 8.8603 0.0497 H 0 0 0 0 0 0 0 0 0 0 0 0
13.2069 10.0887 -1.1643 H 0 0 0 0 0 0 0 0 0 0 0 0
12.2185 10.1039 0.3022 H 0 0 0 0 0 0 0 0 0 0 0 0
13.9855 11.3917 1.4791 H 0 0 0 0 0 0 0 0 0 0 0 0
14.9725 11.3780 0.0117 H 0 0 0 0 0 0 0 0 0 0 0 0
13.1889 12.6066 -1.2003 H 0 0 0 0 0 0 0 0 0 0 0 0
12.2014 12.6170 0.2657 H 0 0 0 0 0 0 0 0 0 0 0 0
13.9444 13.9208 1.4465 H 0 0 0 0 0 0 0 0 0 0 0 0
14.9622 13.8766 0.0033 H 0 0 0 0 0 0 0 0 0 0 0 0
14.0190 6.3617 1.5580 H 0 0 0 0 0 0 0 0 0 0 0 0
15.0062 6.3432 0.0908 H 0 0 0 0 0 0 0 0 0 0 0 0
13.2384 5.0539 -1.0825 H 0 0 0 0 0 0 0 0 0 0 0 0
12.2508 5.0729 0.3845 H 0 0 0 0 0 0 0 0 0 0 0 0
14.0340 3.8457 1.6007 H 0 0 0 0 0 0 0 0 0 0 0 0
15.0213 3.8264 0.1336 H 0 0 0 0 0 0 0 0 0 0 0 0
7.2580 19.7440 0.0239 H 0 0 0 0 0 0 0 0 0 0 0 0
6.7819 18.8400 -1.4196 H 0 0 0 0 0 0 0 0 0 0 0 0
6.9879 16.7131 -0.1559 H 0 0 0 0 0 0 0 0 0 0 0 0
7.4627 17.6169 1.2882 H 0 0 0 0 0 0 0 0 0 0 0 0
9.4447 18.5018 0.0823 H 0 0 0 0 0 0 0 0 0 0 0 0
8.9705 17.5962 -1.3609 H 0 0 0 0 0 0 0 0 0 0 0 0
9.1771 15.4705 -0.0956 H 0 0 0 0 0 0 0 0 0 0 0 0
9.6493 16.3757 1.3485 H 0 0 0 0 0 0 0 0 0 0 0 0
11.6297 17.2611 0.1440 H 0 0 0 0 0 0 0 0 0 0 0 0
11.1601 16.3538 -1.2985 H 0 0 0 0 0 0 0 0 0 0 0 0
11.3456 14.2322 -0.0061 H 0 0 0 0 0 0 0 0 0 0 0 0
11.8601 15.1480 1.4143 H 0 0 0 0 0 0 0 0 0 0 0 0
4.7999 17.9571 -0.2132 H 0 0 0 0 0 0 0 0 0 0 0 0
5.2764 18.8600 1.2309 H 0 0 0 0 0 0 0 0 0 0 0 0
5.0716 20.9878 -0.0320 H 0 0 0 0 0 0 0 0 0 0 0 0
4.5944 20.0848 -1.4759 H 0 0 0 0 0 0 0 0 0 0 0 0
2.6126 19.2018 -0.2687 H 0 0 0 0 0 0 0 0 0 0 0 0
3.0899 20.1043 1.1752 H 0 0 0 0 0 0 0 0 0 0 0 0
24.6094 22.2820 -0.1316 H 0 0 0 0 0 0 0 0 0 0 0 0
24.1100 21.3227 -1.5308 H 0 0 0 0 0 0 0 0 0 0 0 0
24.6201 20.5159 -0.0420 H 0 0 0 0 0 0 0 0 0 0 0 0
13.8024 1.6811 0.4075 H 0 0 0 0 0 0 0 0 0 0 0 0
12.2660 2.5565 0.4279 H 0 0 0 0 0 0 0 0 0 0 0 0
13.2533 2.5372 -1.0393 H 0 0 0 0 0 0 0 0 0 0 0 0
1.3590 21.3383 -0.1069 H 0 0 0 0 0 0 0 0 0 0 0 0
2.8847 22.2323 -0.0869 H 0 0 0 0 0 0 0 0 0 0 0 0
2.4074 21.3297 -1.5310 H 0 0 0 0 0 0 0 0 0 0 0 0
1 2 1 0 0 0 0
```

2	3	1	0	0	0	0
3	4	1	0	0	0	0
4	5	1	0	0	0	0
5	6	1	0	0	0	0
6	7	1	0	0	0	0
1	8	1	0	0	0	0
8	9	1	0	0	0	0
9	10	1	0	0	0	0
11	12	1	0	0	0	0
12	13	1	0	0	0	0
13	14	1	0	0	0	0
14	15	1	0	0	0	0
15	16	1	0	0	0	0
11	17	1	0	0	0	0
17	18	1	0	0	0	0
18	19	1	0	0	0	0
20	21	1	0	0	0	0
21	22	1	0	0	0	0
22	23	1	0	0	0	0
23	24	1	0	0	0	0
24	25	1	0	0	0	0
20	26	1	0	0	0	0
26	27	1	0	0	0	0
27	28	1	0	0	0	0
16	7	1	0	0	0	0
25	7	1	0	0	0	0
10	29	1	0	0	0	0
19	30	1	0	0	0	0
28	31	1	0	0	0	0
1	32	1	0	0	0	0
1	33	1	0	0	0	0
2	34	1	0	0	0	0
2	35	1	0	0	0	0
3	36	1	0	0	0	0
3	37	1	0	0	0	0
4	38	1	0	0	0	0
4	39	1	0	0	0	0
5	40	1	0	0	0	0
5	41	1	0	0	0	0
6	42	1	0	0	0	0
6	43	1	0	0	0	0
8	44	1	0	0	0	0
8	45	1	0	0	0	0
9	46	1	0	0	0	0
9	47	1	0	0	0	0
10	48	1	0	0	0	0
10	49	1	0	0	0	0
11	50	1	0	0	0	0
11	51	1	0	0	0	0
12	52	1	0	0	0	0
12	53	1	0	0	0	0
13	54	1	0	0	0	0
13	55	1	0	0	0	0
14	56	1	0	0	0	0
14	57	1	0	0	0	0
15	58	1	0	0	0	0
15	59	1	0	0	0	0
16	60	1	0	0	0	0
16	61	1	0	0	0	0
17	62	1	0	0	0	0
17	63	1	0	0	0	0
18	64	1	0	0	0	0
18	65	1	0	0	0	0

```
 19 66  1  0  0  0  0
 19 67  1  0  0  0  0
 20 68  1  0  0  0  0
 20 69  1  0  0  0  0
 21 70  1  0  0  0  0
 21 71  1  0  0  0  0
 22 72  1  0  0  0  0
 22 73  1  0  0  0  0
 23 74  1  0  0  0  0
 23 75  1  0  0  0  0
 24 76  1  0  0  0  0
 24 77  1  0  0  0  0
 25 78  1  0  0  0  0
 25 79  1  0  0  0  0
 26 80  1  0  0  0  0
 26 81  1  0  0  0  0
 27 82  1  0  0  0  0
 27 83  1  0  0  0  0
 28 84  1  0  0  0  0
 28 85  1  0  0  0  0
 29 86  1  0  0  0  0
 29 87  1  0  0  0  0
 29 88  1  0  0  0  0
 30 89  1  0  0  0  0
 30 90  1  0  0  0  0
 30 91  1  0  0  0  0
 31 92  1  0  0  0  0
 31 93  1  0  0  0  0
 31 94  1  0  0  0  0
M  END
>  <Alias name>
Tri-n-decylamine

>  <deltaH°(c)>   kJ/mol
-20071

$$$$
Hexaphenylethane
  ChemBrain IXL

 68 73  0  0  0  0  0  0  0  0  1 V2000
    9.9359    6.6948    0.9564 C   0  0  0  0  0  0  0  0  0  0  0  0
    9.3901    7.9909    1.5276 C   0  0  0  0  0  0  0  0  0  0  0  0
    7.9002    7.9401    1.7976 C   0  0  0  0  0  0  0  0  0  0  0  0
    7.0947    7.0299    1.1959 C   0  0  0  0  0  0  0  0  0  0  0  0
    7.6636    6.0227    0.2518 C   0  0  0  0  0  0  0  0  0  0  0  0
    9.1320    5.7829    0.3546 C   0  0  0  0  0  0  0  0  0  0  0  0
    6.8537    5.3312   -0.6897 C   0  0  0  0  0  0  0  0  0  0  0  0
    5.3914    5.5619   -0.7949 C   0  0  0  0  0  0  0  0  0  0  0  0
    7.4135    4.3307   -1.6334 C   0  0  0  0  0  0  0  0  0  0  0  0
    9.6494    9.0943    0.5450 C   0  0  0  0  0  0  0  0  0  0  0  0
    9.1968   10.3809    1.1298 C   0  0  0  0  0  0  0  0  0  0  0  0
    8.8901    8.8021   -0.6953 C   0  0  0  0  0  0  0  0  0  0  0  0
   11.1016    9.1647    0.2505 C   0  0  0  0  0  0  0  0  0  0  0  0
    9.4896    8.0800   -1.7286 C   0  0  0  0  0  0  0  0  0  0  0  0
    8.7715    7.7979   -2.8917 C   0  0  0  0  0  0  0  0  0  0  0  0
    7.4531    8.2350   -3.0212 C   0  0  0  0  0  0  0  0  0  0  0  0
    6.8522    8.9540   -1.9877 C   0  0  0  0  0  0  0  0  0  0  0  0
    7.5699    9.2363   -0.8245 C   0  0  0  0  0  0  0  0  0  0  0  0
   11.5515    9.8429   -0.8819 C   0  0  0  0  0  0  0  0  0  0  0  0
   12.9173    9.9088   -1.1601 C   0  0  0  0  0  0  0  0  0  0  0  0
   13.8346    9.2974   -0.3050 C   0  0  0  0  0  0  0  0  0  0  0  0
   13.3863    8.6204    0.8292 C   0  0  0  0  0  0  0  0  0  0  0  0
```

12.0202 8.5546 1.1079 C 0 0 0 0 0 0 0 0 0 0 0 0
8.9529 11.4768 0.3029 C 0 0 0 0 0 0 0 0 0 0 0 0
8.5265 12.6862 0.8534 C 0 0 0 0 0 0 0 0 0 0 0 0
8.3444 12.8008 2.2319 C 0 0 0 0 0 0 0 0 0 0 0 0
8.5888 11.7059 3.0608 C 0 0 0 0 0 0 0 0 0 0 0 0
9.0154 10.4960 2.5105 C 0 0 0 0 0 0 0 0 0 0 0 0
4.8083 5.6518 -2.0604 C 0 0 0 0 0 0 0 0 0 0 0 0
3.4330 5.8470 -2.1858 C 0 0 0 0 0 0 0 0 0 0 0 0
2.6365 5.9464 -1.0455 C 0 0 0 0 0 0 0 0 0 0 0 0
3.2158 5.8488 0.2204 C 0 0 0 0 0 0 0 0 0 0 0 0
4.5930 5.6528 0.3464 C 0 0 0 0 0 0 0 0 0 0 0 0
8.7014 4.4689 -2.1534 C 0 0 0 0 0 0 0 0 0 0 0 0
9.1956 3.5217 -3.0536 C 0 0 0 0 0 0 0 0 0 0 0 0
8.4009 2.4406 -3.4377 C 0 0 0 0 0 0 0 0 0 0 0 0
7.1120 2.3058 -2.9233 C 0 0 0 0 0 0 0 0 0 0 0 0
6.6183 3.2510 -2.0242 C 0 0 0 0 0 0 0 0 0 0 0 0
10.9979 6.4944 1.0260 H 0 0 0 0 0 0 0 0 0 0 0 0
9.9084 8.2129 2.4530 H 0 0 0 0 0 0 0 0 0 0 0 0
7.4656 8.6554 2.4844 H 0 0 0 0 0 0 0 0 0 0 0 0
6.0324 7.0295 1.4064 H 0 0 0 0 0 0 0 0 0 0 0 0
9.5575 4.8713 -0.0485 H 0 0 0 0 0 0 0 0 0 0 0 0
10.5123 7.7383 -1.6279 H 0 0 0 0 0 0 0 0 0 0 0 0
9.2378 7.2394 -3.6942 H 0 0 0 0 0 0 0 0 0 0 0 0
6.8959 8.0162 -3.9243 H 0 0 0 0 0 0 0 0 0 0 0 0
5.8284 9.2931 -2.0883 H 0 0 0 0 0 0 0 0 0 0 0 0
7.1021 9.7927 -0.0218 H 0 0 0 0 0 0 0 0 0 0 0 0
10.8405 10.3182 -1.5452 H 0 0 0 0 0 0 0 0 0 0 0 0
13.2655 10.4347 -2.0400 H 0 0 0 0 0 0 0 0 0 0 0 0
14.8947 9.3484 -0.5218 H 0 0 0 0 0 0 0 0 0 0 0 0
14.0990 8.1459 1.4935 H 0 0 0 0 0 0 0 0 0 0 0 0
11.6729 8.0299 1.9899 H 0 0 0 0 0 0 0 0 0 0 0 0
9.0946 11.3890 -0.7663 H 0 0 0 0 0 0 0 0 0 0 0 0
8.3368 13.5362 0.2105 H 0 0 0 0 0 0 0 0 0 0 0 0
8.0132 13.7400 2.6585 H 0 0 0 0 0 0 0 0 0 0 0 0
8.4473 11.7952 4.1316 H 0 0 0 0 0 0 0 0 0 0 0 0
9.2058 9.6462 3.1552 H 0 0 0 0 0 0 0 0 0 0 0 0
5.4252 5.5697 -2.9467 H 0 0 0 0 0 0 0 0 0 0 0 0
2.9838 5.9213 -3.1689 H 0 0 0 0 0 0 0 0 0 0 0 0
1.5683 6.0993 -1.1429 H 0 0 0 0 0 0 0 0 0 0 0 0
2.5962 5.9252 1.1054 H 0 0 0 0 0 0 0 0 0 0 0 0
5.0405 5.5716 1.3288 H 0 0 0 0 0 0 0 0 0 0 0 0
9.3172 5.3092 -1.8595 H 0 0 0 0 0 0 0 0 0 0 0 0
10.1964 3.6256 -3.4542 H 0 0 0 0 0 0 0 0 0 0 0 0
8.7849 1.7059 -4.1355 H 0 0 0 0 0 0 0 0 0 0 0 0
6.4941 1.4673 -3.2218 H 0 0 0 0 0 0 0 0 0 0 0 0
5.6156 3.1472 -1.6281 H 0 0 0 0 0 0 0 0 0 0 0 0
2 1 1 0 0 0 0
3 2 1 0 0 0 0
4 3 2 0 0 0 0
5 4 1 0 0 0 0
6 5 1 0 0 0 0
1 6 2 0 0 0 0
5 7 2 0 0 0 0
7 8 1 0 0 0 0
7 9 1 0 0 0 0
2 10 1 0 0 0 0
10 11 1 0 0 0 0
10 12 1 0 0 0 0
10 13 1 0 0 0 0
14 12 4 0 0 0 0
15 14 4 0 0 0 0
16 15 4 0 0 0 0
17 16 4 0 0 0 0

```
 18 17  4  0  0  0  0
 12 18  4  0  0  0  0
 19 13  4  0  0  0  0
 20 19  4  0  0  0  0
 21 20  4  0  0  0  0
 22 21  4  0  0  0  0
 23 22  4  0  0  0  0
 13 23  4  0  0  0  0
 24 11  4  0  0  0  0
 25 24  4  0  0  0  0
 26 25  4  0  0  0  0
 27 26  4  0  0  0  0
 28 27  4  0  0  0  0
 11 28  4  0  0  0  0
 29  8  4  0  0  0  0
 30 29  4  0  0  0  0
 31 30  4  0  0  0  0
 32 31  4  0  0  0  0
 33 32  4  0  0  0  0
  8 33  4  0  0  0  0
 34  9  4  0  0  0  0
 35 34  4  0  0  0  0
 36 35  4  0  0  0  0
 37 36  4  0  0  0  0
 38 37  4  0  0  0  0
  9 38  4  0  0  0  0
  1 39  1  0  0  0  0
  2 40  1  0  0  0  0
  3 41  1  0  0  0  0
  4 42  1  0  0  0  0
  6 43  1  0  0  0  0
 14 44  1  0  0  0  0
 15 45  1  0  0  0  0
 16 46  1  0  0  0  0
 17 47  1  0  0  0  0
 18 48  1  0  0  0  0
 19 49  1  0  0  0  0
 20 50  1  0  0  0  0
 21 51  1  0  0  0  0
 22 52  1  0  0  0  0
 23 53  1  0  0  0  0
 24 54  1  0  0  0  0
 25 55  1  0  0  0  0
 26 56  1  0  0  0  0
 27 57  1  0  0  0  0
 28 58  1  0  0  0  0
 29 59  1  0  0  0  0
 30 60  1  0  0  0  0
 31 61  1  0  0  0  0
 32 62  1  0  0  0  0
 33 63  1  0  0  0  0
 34 64  1  0  0  0  0
 35 65  1  0  0  0  0
 36 66  1  0  0  0  0
 37 67  1  0  0  0  0
 38 68  1  0  0  0  0
M  END
>  <Alias name>
Hexaphenylethane

>  <deltaH°(c)>   kJ/mol
-19765.5
```

$$$$
1-Triacontene
  ChemBrain IXL

 90 89 0 0 0 0 0 0 0 0 1 V2000
   11.0166   20.3283   -0.1312 C   0 0 0 0 0 0 0 0 0 0 0 0
   12.1347   19.2679   -0.1209 C   0 0 0 0 0 0 0 0 0 0 0 0
   13.5070   19.9688   -0.1366 C   0 0 0 0 0 0 0 0 0 0 0 0
   14.6257   18.9091   -0.1231 C   0 0 0 0 0 0 0 0 0 0 0 0
   15.9976   19.6108   -0.1400 C   0 0 0 0 0 0 0 0 0 0 0 0
   17.1170   18.5518   -0.1225 C   0 0 0 0 0 0 0 0 0 0 0 0
   18.4885   19.2542   -0.1409 C   0 0 0 0 0 0 0 0 0 0 0 0
   19.6084   18.1959   -0.1187 C   0 0 0 0 0 0 0 0 0 0 0 0
   20.9796   18.8989   -0.1387 C   0 0 0 0 0 0 0 0 0 0 0 0
   22.1000   17.8412   -0.1109 C   0 0 0 0 0 0 0 0 0 0 0 0
   23.4709   18.5447   -0.1321 C   0 0 0 0 0 0 0 0 0 0 0 0
   24.5917   17.4876   -0.0978 C   0 0 0 0 0 0 0 0 0 0 0 0
   25.9625   18.1915   -0.1198 C   0 0 0 0 0 0 0 0 0 0 0 0
   27.0835   17.1350   -0.0784 C   0 0 0 0 0 0 0 0 0 0 0 0
   28.4541   17.8392   -0.1004 C   0 0 0 0 0 0 0 0 0 0 0 0
   29.5753   16.7831   -0.0519 C   0 0 0 0 0 0 0 0 0 0 0 0
   30.9458   17.4875   -0.0735 C   0 0 0 0 0 0 0 0 0 0 0 0
   32.0672   16.4319   -0.0185 C   0 0 0 0 0 0 0 0 0 0 0 0
   33.4376   17.1364   -0.0397 C   0 0 0 0 0 0 0 0 0 0 0 0
   34.5590   16.0811   0.0201 C   0 0 0 0 0 0 0 0 0 0 0 0
   35.9294   16.7857   -0.0012 C   0 0 0 0 0 0 0 0 0 0 0 0
   37.0509   15.7304   0.0613 C   0 0 0 0 0 0 0 0 0 0 0 0
   38.4213   16.4350   0.0396 C   0 0 0 0 0 0 0 0 0 0 0 0
    9.6439   19.6283   -0.1163 C   0 0 0 0 0 0 0 0 0 0 0 0
    8.5265   20.6893   -0.1241 C   0 0 0 0 0 0 0 0 0 0 0 0
    7.1534   19.9900   -0.1097 C   0 0 0 0 0 0 0 0 0 0 0 0
    6.0365   21.0517   -0.1158 C   0 0 0 0 0 0 0 0 0 0 0 0
    4.6630   20.3529   -0.1016 C   0 0 0 0 0 0 0 0 0 0 0 0
    3.5899   21.3729   -0.1067 C   0 0 0 0 0 0 0 0 0 0 0 0
    2.3094   20.9950   -0.1081 C   0 0 0 0 0 0 0 0 0 0 0 0
   11.1095   20.9592   0.7442 H   0 0 0 0 0 0 0 0 0 0 0 0
   11.1027   20.9350   -1.0241 H   0 0 0 0 0 0 0 0 0 0 0 0
   12.0417   18.6366   -0.9959 H   0 0 0 0 0 0 0 0 0 0 0 0
   12.0491   18.6618   0.7725 H   0 0 0 0 0 0 0 0 0 0 0 0
   13.5990   20.6018   0.7373 H   0 0 0 0 0 0 0 0 0 0 0 0
   13.5933   20.5734   -1.0310 H   0 0 0 0 0 0 0 0 0 0 0 0
   14.5335   18.2755   -0.9965 H   0 0 0 0 0 0 0 0 0 0 0 0
   14.5401   18.3052   0.7718 H   0 0 0 0 0 0 0 0 0 0 0 0
   16.0887   20.2465   0.7321 H   0 0 0 0 0 0 0 0 0 0 0 0
   16.0841   20.2127   -1.0361 H   0 0 0 0 0 0 0 0 0 0 0 0
   17.0256   17.9153   -0.9940 H   0 0 0 0 0 0 0 0 0 0 0 0
   17.0312   17.9507   0.7743 H   0 0 0 0 0 0 0 0 0 0 0 0
   18.5785   19.8931   0.7289 H   0 0 0 0 0 0 0 0 0 0 0 0
   18.5754   19.8529   -1.0392 H   0 0 0 0 0 0 0 0 0 0 0 0
   19.5180   17.5561   -0.9879 H   0 0 0 0 0 0 0 0 0 0 0 0
   19.5223   17.5981   0.7802 H   0 0 0 0 0 0 0 0 0 0 0 0
   21.0684   19.5415   0.7286 H   0 0 0 0 0 0 0 0 0 0 0 0
   21.0671   19.4939   -1.0393 H   0 0 0 0 0 0 0 0 0 0 0 0
   22.0109   17.1979   -0.9775 H   0 0 0 0 0 0 0 0 0 0 0 0
   22.0131   17.2470   0.7904 H   0 0 0 0 0 0 0 0 0 0 0 0
   23.5582   19.1912   0.7324 H   0 0 0 0 0 0 0 0 0 0 0 0
   23.5595   19.1357   -1.0353 H   0 0 0 0 0 0 0 0 0 0 0 0
   24.5043   16.8405   -0.9618 H   0 0 0 0 0 0 0 0 0 0 0 0
   24.5035   16.8973   0.8058 H   0 0 0 0 0 0 0 0 0 0 0 0
   26.0479   18.8421   0.7418 H   0 0 0 0 0 0 0 0 0 0 0 0
   26.0525   18.7783   -1.0255 H   0 0 0 0 0 0 0 0 0 0 0 0
   26.9982   16.4840   -0.9397 H   0 0 0 0 0 0 0 0 0 0 0 0
   26.9935   16.5486   0.8276 H   0 0 0 0 0 0 0 0 0 0 0 0

```
  28.5374   18.4936    0.7585 H   0  0  0  0  0  0  0  0  0  0  0  0
  28.5460   18.4220   -1.0086 H   0  0  0  0  0  0  0  0  0  0  0  0
  29.4923   16.1285   -0.9106 H   0  0  0  0  0  0  0  0  0  0  0  0
  29.4833   16.2005    0.8564 H   0  0  0  0  0  0  0  0  0  0  0  0
  31.0271   18.1452    0.7831 H   0  0  0  0  0  0  0  0  0  0  0  0
  31.0396   18.0669   -0.9837 H   0  0  0  0  0  0  0  0  0  0  0  0
  31.9861   15.7740   -0.8749 H   0  0  0  0  0  0  0  0  0  0  0  0
  31.9732   15.8527    0.8918 H   0  0  0  0  0  0  0  0  0  0  0  0
  33.5173   17.7966    0.8151 H   0  0  0  0  0  0  0  0  0  0  0  0
  33.5329   17.7132   -0.9514 H   0  0  0  0  0  0  0  0  0  0  0  0
  34.4793   15.4207   -0.8346 H   0  0  0  0  0  0  0  0  0  0  0  0
  34.4637   15.5045    0.9319 H   0  0  0  0  0  0  0  0  0  0  0  0
  36.0084   17.4473    0.8526 H   0  0  0  0  0  0  0  0  0  0  0  0
  36.0254   17.3610   -0.9138 H   0  0  0  0  0  0  0  0  0  0  0  0
  36.9718   15.0687   -0.7923 H   0  0  0  0  0  0  0  0  0  0  0  0
  36.9551   15.1555    0.9740 H   0  0  0  0  0  0  0  0  0  0  0  0
  38.5004   17.0970    0.8931 H   0  0  0  0  0  0  0  0  0  0  0  0
  38.5174   17.0097   -0.8732 H   0  0  0  0  0  0  0  0  0  0  0  0
  39.2091   15.6933    0.0839 H   0  0  0  0  0  0  0  0  0  0  0  0
   9.5502   18.9987   -0.9925 H   0  0  0  0  0  0  0  0  0  0  0  0
   9.5583   19.0204    0.7759 H   0  0  0  0  0  0  0  0  0  0  0  0
   8.6202   21.3186    0.7524 H   0  0  0  0  0  0  0  0  0  0  0  0
   8.6125   21.2975   -1.0160 H   0  0  0  0  0  0  0  0  0  0  0  0
   7.0591   19.3617   -0.9867 H   0  0  0  0  0  0  0  0  0  0  0  0
   7.0678   19.3810    0.7818 H   0  0  0  0  0  0  0  0  0  0  0  0
   6.1310   21.6799    0.7613 H   0  0  0  0  0  0  0  0  0  0  0  0
   6.1224   21.6609   -1.0071 H   0  0  0  0  0  0  0  0  0  0  0  0
   4.5684   19.7250   -0.9790 H   0  0  0  0  0  0  0  0  0  0  0  0
   4.5773   19.7432    0.7894 H   0  0  0  0  0  0  0  0  0  0  0  0
   3.8435   22.4258   -0.1092 H   0  0  0  0  0  0  0  0  0  0  0  0
   2.0553   19.9422   -0.1056 H   0  0  0  0  0  0  0  0  0  0  0  0
   1.5247   21.7413   -0.1118 H   0  0  0  0  0  0  0  0  0  0  0  0
  1  2  1  0  0  0  0
  2  3  1  0  0  0  0
  3  4  1  0  0  0  0
  4  5  1  0  0  0  0
  5  6  1  0  0  0  0
  6  7  1  0  0  0  0
  7  8  1  0  0  0  0
  8  9  1  0  0  0  0
  9 10  1  0  0  0  0
 10 11  1  0  0  0  0
 11 12  1  0  0  0  0
 12 13  1  0  0  0  0
 13 14  1  0  0  0  0
 14 15  1  0  0  0  0
 15 16  1  0  0  0  0
 16 17  1  0  0  0  0
 17 18  1  0  0  0  0
 18 19  1  0  0  0  0
 19 20  1  0  0  0  0
 20 21  1  0  0  0  0
 21 22  1  0  0  0  0
 22 23  1  0  0  0  0
  1 24  1  0  0  0  0
 24 25  1  0  0  0  0
 25 26  1  0  0  0  0
 26 27  1  0  0  0  0
 27 28  1  0  0  0  0
 28 29  1  0  0  0  0
 29 30  2  0  0  0  0
  1 31  1  0  0  0  0
  1 32  1  0  0  0  0
```

```
  2 33  1  0  0  0  0
  2 34  1  0  0  0  0
  3 35  1  0  0  0  0
  3 36  1  0  0  0  0
  4 37  1  0  0  0  0
  4 38  1  0  0  0  0
  5 39  1  0  0  0  0
  5 40  1  0  0  0  0
  6 41  1  0  0  0  0
  6 42  1  0  0  0  0
  7 43  1  0  0  0  0
  7 44  1  0  0  0  0
  8 45  1  0  0  0  0
  8 46  1  0  0  0  0
  9 47  1  0  0  0  0
  9 48  1  0  0  0  0
 10 49  1  0  0  0  0
 10 50  1  0  0  0  0
 11 51  1  0  0  0  0
 11 52  1  0  0  0  0
 12 53  1  0  0  0  0
 12 54  1  0  0  0  0
 13 55  1  0  0  0  0
 13 56  1  0  0  0  0
 14 57  1  0  0  0  0
 14 58  1  0  0  0  0
 15 59  1  0  0  0  0
 15 60  1  0  0  0  0
 16 61  1  0  0  0  0
 16 62  1  0  0  0  0
 17 63  1  0  0  0  0
 17 64  1  0  0  0  0
 18 65  1  0  0  0  0
 18 66  1  0  0  0  0
 19 67  1  0  0  0  0
 19 68  1  0  0  0  0
 20 69  1  0  0  0  0
 20 70  1  0  0  0  0
 21 71  1  0  0  0  0
 21 72  1  0  0  0  0
 22 73  1  0  0  0  0
 22 74  1  0  0  0  0
 23 75  1  0  0  0  0
 23 76  1  0  0  0  0
 23 77  1  0  0  0  0
 24 78  1  0  0  0  0
 24 79  1  0  0  0  0
 25 80  1  0  0  0  0
 25 81  1  0  0  0  0
 26 82  1  0  0  0  0
 26 83  1  0  0  0  0
 27 84  1  0  0  0  0
 27 85  1  0  0  0  0
 28 86  1  0  0  0  0
 28 87  1  0  0  0  0
 29 88  1  0  0  0  0
 30 89  1  0  0  0  0
 30 90  1  0  0  0  0
M  END
>  <Alias name>
1-Triacontene

>  <deltaH°(c)>   kJ/mol
```

-19632.22

$$$$
4,5-Diethyl-4,5-bis-(4-tert-butylphenyl)-octane
  ChemBrain IXL

```
 82 83  0  0  0  0  0  0  0  0  1 V2000
    5.2225    7.6099    0.8455 C   0  0  0  0  0  0  0  0  0  0  0  0
    5.5784    8.7540    1.5623 C   0  0  0  0  0  0  0  0  0  0  0  0
    5.9970    7.1920   -0.2416 C   0  0  0  0  0  0  0  0  0  0  0  0
    6.7106    9.4869    1.1971 C   0  0  0  0  0  0  0  0  0  0  0  0
    7.4853    9.0688    0.1097 C   0  0  0  0  0  0  0  0  0  0  0  0
    7.0882   10.7094    1.9655 C   0  0  0  0  0  0  0  0  0  0  0  0
    7.1295    7.9257   -0.6067 C   0  0  0  0  0  0  0  0  0  0  0  0
    5.6169    5.9677   -1.0083 C   0  0  0  0  0  0  0  0  0  0  0  0
    6.9326   10.4382    3.4767 C   0  0  0  0  0  0  0  0  0  0  0  0
    6.1680   11.8765    1.5569 C   0  0  0  0  0  0  0  0  0  0  0  0
    8.5560   11.0860    1.6649 C   0  0  0  0  0  0  0  0  0  0  0  0
    5.8726    6.2242   -2.5186 C   0  0  0  0  0  0  0  0  0  0  0  0
    6.5474    4.7595   -0.5143 C   0  0  0  0  0  0  0  0  0  0  0  0
    4.0859    5.7027   -0.7915 C   0  0  0  0  0  0  0  0  0  0  0  0
    5.0223    7.4213   -2.9943 C   0  0  0  0  0  0  0  0  0  0  0  0
    7.9655    5.1372   -0.7887 C   0  0  0  0  0  0  0  0  0  0  0  0
    6.3905    4.5204    1.0286 C   0  0  0  0  0  0  0  0  0  0  0  0
    6.2272    3.4427   -1.2711 C   0  0  0  0  0  0  0  0  0  0  0  0
    2.0213    4.5174   -1.6237 C   0  0  0  0  0  0  0  0  0  0  0  0
    3.5351    4.6996   -1.8410 C   0  0  0  0  0  0  0  0  0  0  0  0
    8.7338    5.7511    0.2052 C   0  0  0  0  0  0  0  0  0  0  0  0
    8.5312    4.8800   -2.0408 C   0  0  0  0  0  0  0  0  0  0  0  0
   10.0599    6.1048   -0.0515 C   0  0  0  0  0  0  0  0  0  0  0  0
   10.6264    5.8469   -1.3035 C   0  0  0  0  0  0  0  0  0  0  0  0
    9.8566    5.2332   -2.2975 C   0  0  0  0  0  0  0  0  0  0  0  0
   12.0460    6.2259   -1.5767 C   0  0  0  0  0  0  0  0  0  0  0  0
    5.0693    3.3801    2.8524 C   0  0  0  0  0  0  0  0  0  0  0  0
    5.2090    3.5542    1.3283 C   0  0  0  0  0  0  0  0  0  0  0  0
    7.2994    2.3797   -0.9386 C   0  0  0  0  0  0  0  0  0  0  0  0
   12.6049    5.3659   -2.7321 C   0  0  0  0  0  0  0  0  0  0  0  0
   12.8988    5.9905   -0.3113 C   0  0  0  0  0  0  0  0  0  0  0  0
   12.1133    7.7148   -1.9696 C   0  0  0  0  0  0  0  0  0  0  0  0
    4.3449    7.0465    1.1336 H   0  0  0  0  0  0  0  0  0  0  0  0
    4.9753    9.0727    2.4022 H   0  0  0  0  0  0  0  0  0  0  0  0
    8.3627    9.6306   -0.1790 H   0  0  0  0  0  0  0  0  0  0  0  0
    7.7334    7.6082   -1.4461 H   0  0  0  0  0  0  0  0  0  0  0  0
    5.9019   10.1922    3.6960 H   0  0  0  0  0  0  0  0  0  0  0  0
    7.5701    9.6107    3.7625 H   0  0  0  0  0  0  0  0  0  0  0  0
    7.2182   11.3224    4.0330 H   0  0  0  0  0  0  0  0  0  0  0  0
    5.1387   11.6169    1.7710 H   0  0  0  0  0  0  0  0  0  0  0  0
    6.4402   12.7636    2.1155 H   0  0  0  0  0  0  0  0  0  0  0  0
    6.2787   12.0694    0.4968 H   0  0  0  0  0  0  0  0  0  0  0  0
    8.8313   11.9565    2.2475 H   0  0  0  0  0  0  0  0  0  0  0  0
    8.6640   11.3097    0.6113 H   0  0  0  0  0  0  0  0  0  0  0  0
    9.2027   10.2574    1.9269 H   0  0  0  0  0  0  0  0  0  0  0  0
    5.6026    5.3435   -3.0854 H   0  0  0  0  0  0  0  0  0  0  0  0
    6.9187    6.4438   -2.6757 H   0  0  0  0  0  0  0  0  0  0  0  0
    3.5548    6.6397   -0.9038 H   0  0  0  0  0  0  0  0  0  0  0  0
    3.9195    5.3305    0.1926 H   0  0  0  0  0  0  0  0  0  0  0  0
    3.9731    7.2019   -2.8445 H   0  0  0  0  0  0  0  0  0  0  0  0
    5.2912    8.3036   -2.4271 H   0  0  0  0  0  0  0  0  0  0  0  0
    5.2069    7.5989   -4.0464 H   0  0  0  0  0  0  0  0  0  0  0  0
    7.3016    4.0739    1.4074 H   0  0  0  0  0  0  0  0  0  0  0  0
    6.2265    5.4671    1.5261 H   0  0  0  0  0  0  0  0  0  0  0  0
    6.2216    3.6271   -2.3369 H   0  0  0  0  0  0  0  0  0  0  0  0
    5.2681    3.0765   -0.9634 H   0  0  0  0  0  0  0  0  0  0  0  0
```

1.6324	3.8224	-2.3575	H	0	0	0	0	0	0	0	0	0	0	0	0	0
1.8438	4.1286	-0.6286	H	0	0	0	0	0	0	0	0	0	0	0	0	0
1.5234	5.4730	-1.7321	H	0	0	0	0	0	0	0	0	0	0	0	0	0
4.0190	3.7591	-1.7385	H	0	0	0	0	0	0	0	0	0	0	0	0	0
3.7089	5.0871	-2.8360	H	0	0	0	0	0	0	0	0	0	0	0	0	0
8.3010	5.9535	1.1764	H	0	0	0	0	0	0	0	0	0	0	0	0	0
7.9414	4.4058	-2.8142	H	0	0	0	0	0	0	0	0	0	0	0	0	0
10.6489	6.5796	0.7216	H	0	0	0	0	0	0	0	0	0	0	0	0	0
10.2867	5.0307	-3.2684	H	0	0	0	0	0	0	0	0	0	0	0	0	0
4.2516	2.7029	3.0649	H	0	0	0	0	0	0	0	0	0	0	0	0	0
5.9888	2.9734	3.2550	H	0	0	0	0	0	0	0	0	0	0	0	0	0
4.8696	4.3418	3.3087	H	0	0	0	0	0	0	0	0	0	0	0	0	0
4.3011	3.9481	0.9341	H	0	0	0	0	0	0	0	0	0	0	0	0	0
5.4080	2.5917	0.8760	H	0	0	0	0	0	0	0	0	0	0	0	0	0
8.2713	2.7334	-1.2541	H	0	0	0	0	0	0	0	0	0	0	0	0	0
7.0659	1.4583	-1.4572	H	0	0	0	0	0	0	0	0	0	0	0	0	0
7.3105	2.1995	0.1289	H	0	0	0	0	0	0	0	0	0	0	0	0	0
13.6429	5.6234	-2.9033	H	0	0	0	0	0	0	0	0	0	0	0	0	0
12.0349	5.5542	-3.6324	H	0	0	0	0	0	0	0	0	0	0	0	0	0
12.5319	4.3175	-2.4703	H	0	0	0	0	0	0	0	0	0	0	0	0	0
12.5296	6.6104	0.4948	H	0	0	0	0	0	0	0	0	0	0	0	0	0
13.9304	6.2470	-0.5191	H	0	0	0	0	0	0	0	0	0	0	0	0	0
12.8373	4.9484	-0.0231	H	0	0	0	0	0	0	0	0	0	0	0	0	0
11.5187	7.8805	-2.8596	H	0	0	0	0	0	0	0	0	0	0	0	0	0
13.1422	7.9900	-2.1666	H	0	0	0	0	0	0	0	0	0	0	0	0	0
11.7266	8.3200	-1.1592	H	0	0	0	0	0	0	0	0	0	0	0	0	0

1	2	4	0	0	0	0
3	1	4	0	0	0	0
2	4	4	0	0	0	0
7	3	4	0	0	0	0
3	8	1	0	0	0	0
4	5	4	0	0	0	0
4	6	1	0	0	0	0
5	7	4	0	0	0	0
6	9	1	0	0	0	0
6	10	1	0	0	0	0
6	11	1	0	0	0	0
8	12	1	0	0	0	0
8	13	1	0	0	0	0
8	14	1	0	0	0	0
12	15	1	0	0	0	0
13	16	1	0	0	0	0
13	17	1	0	0	0	0
13	18	1	0	0	0	0
14	20	1	0	0	0	0
16	21	4	0	0	0	0
22	16	4	0	0	0	0
17	28	1	0	0	0	0
18	29	1	0	0	0	0
19	20	1	0	0	0	0
21	23	4	0	0	0	0
25	22	4	0	0	0	0
23	24	4	0	0	0	0
24	25	4	0	0	0	0
24	26	1	0	0	0	0
26	30	1	0	0	0	0
26	31	1	0	0	0	0
26	32	1	0	0	0	0
27	28	1	0	0	0	0
1	33	1	0	0	0	0
2	34	1	0	0	0	0
5	35	1	0	0	0	0
7	36	1	0	0	0	0

```
  9 37  1  0  0  0  0
  9 38  1  0  0  0  0
  9 39  1  0  0  0  0
 10 40  1  0  0  0  0
 10 41  1  0  0  0  0
 10 42  1  0  0  0  0
 11 43  1  0  0  0  0
 11 44  1  0  0  0  0
 11 45  1  0  0  0  0
 12 46  1  0  0  0  0
 12 47  1  0  0  0  0
 14 48  1  0  0  0  0
 14 49  1  0  0  0  0
 15 50  1  0  0  0  0
 15 51  1  0  0  0  0
 15 52  1  0  0  0  0
 17 53  1  0  0  0  0
 17 54  1  0  0  0  0
 18 55  1  0  0  0  0
 18 56  1  0  0  0  0
 19 57  1  0  0  0  0
 19 58  1  0  0  0  0
 19 59  1  0  0  0  0
 20 60  1  0  0  0  0
 20 61  1  0  0  0  0
 21 62  1  0  0  0  0
 22 63  1  0  0  0  0
 23 64  1  0  0  0  0
 25 65  1  0  0  0  0
 27 66  1  0  0  0  0
 27 67  1  0  0  0  0
 27 68  1  0  0  0  0
 28 69  1  0  0  0  0
 28 70  1  0  0  0  0
 29 71  1  0  0  0  0
 29 72  1  0  0  0  0
 29 73  1  0  0  0  0
 30 74  1  0  0  0  0
 30 75  1  0  0  0  0
 30 76  1  0  0  0  0
 31 77  1  0  0  0  0
 31 78  1  0  0  0  0
 31 79  1  0  0  0  0
 32 80  1  0  0  0  0
 32 81  1  0  0  0  0
 32 82  1  0  0  0  0
M  END
>  <Alias name>
4,5-Diethyl-4,5-bis-(4-tert-butylphenyl)-octane

>  <deltaH°(c)>   kJ/mol
-19331

$$$$
2,4,5,7-Tetramethyl-4,5-bis(4-t-butylphenyl)octane
  ChemBrain IXL

 82 83  0  0  0  0  0  0  0  0  1 V2000
    7.3373    7.9114   -1.0699 C   0  0  0  0  0  0  0  0  0  0  0  0
    6.7795    8.8981   -0.2549 C   0  0  0  0  0  0  0  0  0  0  0  0
    6.8036    6.6187   -1.0748 C   0  0  0  0  0  0  0  0  0  0  0  0
    5.6836    8.6000    0.5620 C   0  0  0  0  0  0  0  0  0  0  0  0
    5.1496    7.3087    0.5573 C   0  0  0  0  0  0  0  0  0  0  0  0
```

5.0856	9.6549	1.4346	C	0	0	0	0	0	0	0	0	0	0	0	0	0
5.7065	6.3225	-0.2568	C	0	0	0	0	0	0	0	0	0	0	0	0	0
7.4029	5.5599	-1.9500	C	0	0	0	0	0	0	0	0	0	0	0	0	0
4.5257	9.0101	2.7217	C	0	0	0	0	0	0	0	0	0	0	0	0	0
6.1649	10.6918	1.8153	C	0	0	0	0	0	0	0	0	0	0	0	0	0
3.9433	10.3601	0.6774	C	0	0	0	0	0	0	0	0	0	0	0	0	0
8.4909	4.7612	-1.1341	C	0	0	0	0	0	0	0	0	0	0	0	0	0
6.2711	4.5951	-2.4454	C	0	0	0	0	0	0	0	0	0	0	0	0	0
8.0874	6.2352	-3.1569	C	0	0	0	0	0	0	0	0	0	0	0	0	0
9.5711	5.7107	-0.7288	C	0	0	0	0	0	0	0	0	0	0	0	0	0
7.8646	4.1308	0.1670	C	0	0	0	0	0	0	0	0	0	0	0	0	0
9.0976	3.6567	-2.0273	C	0	0	0	0	0	0	0	0	0	0	0	0	0
5.2984	5.3284	-3.4147	C	0	0	0	0	0	0	0	0	0	0	0	0	0
9.3905	6.5461	0.3772	C	0	0	0	0	0	0	0	0	0	0	0	0	0
10.7672	5.7694	-1.4523	C	0	0	0	0	0	0	0	0	0	0	0	0	0
10.3992	7.4346	0.7574	C	0	0	0	0	0	0	0	0	0	0	0	0	0
11.5940	7.4922	0.0339	C	0	0	0	0	0	0	0	0	0	0	0	0	0
11.7741	6.6563	-1.0725	C	0	0	0	0	0	0	0	0	0	0	0	0	0
12.6707	8.4423	0.4418	C	0	0	0	0	0	0	0	0	0	0	0	0	0
7.0942	2.8052	-0.1389	C	0	0	0	0	0	0	0	0	0	0	0	0	0
8.0859	1.6161	-0.2243	C	0	0	0	0	0	0	0	0	0	0	0	0	0
6.0953	2.5340	1.0028	C	0	0	0	0	0	0	0	0	0	0	0	0	0
14.0065	8.0378	-0.2214	C	0	0	0	0	0	0	0	0	0	0	0	0	0
12.8349	8.4094	1.9756	C	0	0	0	0	0	0	0	0	0	0	0	0	0
12.2918	9.8686	-0.0029	C	0	0	0	0	0	0	0	0	0	0	0	0	0
3.9549	4.5743	-3.4400	C	0	0	0	0	0	0	0	0	0	0	0	0	0
5.8818	5.3506	-4.8541	C	0	0	0	0	0	0	0	0	0	0	0	0	0
8.1847	8.1499	-1.6983	H	0	0	0	0	0	0	0	0	0	0	0	0	0
7.1981	9.8948	-0.2571	H	0	0	0	0	0	0	0	0	0	0	0	0	0
4.3025	7.0706	1.1855	H	0	0	0	0	0	0	0	0	0	0	0	0	0
5.2868	5.3277	-0.2525	H	0	0	0	0	0	0	0	0	0	0	0	0	0
5.3219	8.4922	3.2421	H	0	0	0	0	0	0	0	0	0	0	0	0	0
4.1178	9.7816	3.3630	H	0	0	0	0	0	0	0	0	0	0	0	0	0
3.7450	8.3068	2.4645	H	0	0	0	0	0	0	0	0	0	0	0	0	0
5.7330	11.4366	2.4723	H	0	0	0	0	0	0	0	0	0	0	0	0	0
6.5359	11.1737	0.9207	H	0	0	0	0	0	0	0	0	0	0	0	0	0
6.9816	10.1934	2.3229	H	0	0	0	0	0	0	0	0	0	0	0	0	0
3.1835	9.6349	0.4133	H	0	0	0	0	0	0	0	0	0	0	0	0	0
3.5086	11.1243	1.3099	H	0	0	0	0	0	0	0	0	0	0	0	0	0
4.3350	10.8168	-0.2232	H	0	0	0	0	0	0	0	0	0	0	0	0	0
6.7083	3.7461	-2.9538	H	0	0	0	0	0	0	0	0	0	0	0	0	0
5.7131	4.2498	-1.5933	H	0	0	0	0	0	0	0	0	0	0	0	0	0
7.3889	6.9046	-3.6386	H	0	0	0	0	0	0	0	0	0	0	0	0	0
8.4126	5.4788	-3.8599	H	0	0	0	0	0	0	0	0	0	0	0	0	0
8.9444	6.8003	-2.8154	H	0	0	0	0	0	0	0	0	0	0	0	0	0
7.1842	4.8449	0.6062	H	0	0	0	0	0	0	0	0	0	0	0	0	0
8.6550	3.9166	0.8758	H	0	0	0	0	0	0	0	0	0	0	0	0	0
9.8787	3.1407	-1.4834	H	0	0	0	0	0	0	0	0	0	0	0	0	0
8.3255	2.9540	-2.3082	H	0	0	0	0	0	0	0	0	0	0	0	0	0
9.5155	4.1029	-2.9193	H	0	0	0	0	0	0	0	0	0	0	0	0	0
5.1330	6.3407	-3.0674	H	0	0	0	0	0	0	0	0	0	0	0	0	0
8.4671	6.5052	0.9409	H	0	0	0	0	0	0	0	0	0	0	0	0	0
10.9150	5.1272	-2.3087	H	0	0	0	0	0	0	0	0	0	0	0	0	0
10.2542	8.0794	1.6143	H	0	0	0	0	0	0	0	0	0	0	0	0	0
12.6952	6.6949	-1.6374	H	0	0	0	0	0	0	0	0	0	0	0	0	0
6.5600	2.8942	-1.0623	H	0	0	0	0	0	0	0	0	0	0	0	0	0
8.6361	1.5393	0.7053	H	0	0	0	0	0	0	0	0	0	0	0	0	0
7.5334	0.6999	-0.3913	H	0	0	0	0	0	0	0	0	0	0	0	0	0
8.7730	1.7682	-1.0337	H	0	0	0	0	0	0	0	0	0	0	0	0	0
5.5568	1.6164	0.8011	H	0	0	0	0	0	0	0	0	0	0	0	0	0
6.6331	2.4389	1.9381	H	0	0	0	0	0	0	0	0	0	0	0	0	0
5.3939	3.3564	1.0717	H	0	0	0	0	0	0	0	0	0	0	0	0	0
14.7884	8.7140	0.1014	H	0	0	0	0	0	0	0	0	0	0	0	0	0

13.9069	8.0911	-1.2980	H	0	0	0	0	0	0	0	0	0	0	0	0
14.2623	7.0260	0.0686	H	0	0	0	0	0	0	0	0	0	0	0	0
11.9075	8.7098	2.4454	H	0	0	0	0	0	0	0	0	0	0	0	0
13.6244	9.0906	2.2684	H	0	0	0	0	0	0	0	0	0	0	0	0
13.0903	7.4049	2.2899	H	0	0	0	0	0	0	0	0	0	0	0	0
12.1788	9.8928	-1.0800	H	0	0	0	0	0	0	0	0	0	0	0	0
11.3586	10.1567	0.4645	H	0	0	0	0	0	0	0	0	0	0	0	0
13.0723	10.5585	0.2936	H	0	0	0	0	0	0	0	0	0	0	0	0
4.1164	3.5606	-3.7855	H	0	0	0	0	0	0	0	0	0	0	0	0
3.5342	4.5530	-2.4424	H	0	0	0	0	0	0	0	0	0	0	0	0
3.2701	5.0793	-4.1099	H	0	0	0	0	0	0	0	0	0	0	0	0
5.1821	5.8444	-5.5169	H	0	0	0	0	0	0	0	0	0	0	0	0
6.0403	4.3342	-5.1927	H	0	0	0	0	0	0	0	0	0	0	0	0
6.8131	5.8776	-4.8665	H	0	0	0	0	0	0	0	0	0	0	0	0

1	2	4	0	0	0	0
3	1	4	0	0	0	0
2	4	4	0	0	0	0
7	3	4	0	0	0	0
3	8	1	0	0	0	0
4	5	4	0	0	0	0
4	6	1	0	0	0	0
5	7	4	0	0	0	0
6	9	1	0	0	0	0
6	10	1	0	0	0	0
6	11	1	0	0	0	0
8	12	1	0	0	0	0
8	13	1	0	0	0	0
8	14	1	0	0	0	0
12	15	1	0	0	0	0
12	16	1	0	0	0	0
12	17	1	0	0	0	0
13	18	1	0	0	0	0
15	19	4	0	0	0	0
20	15	4	0	0	0	0
16	25	1	0	0	0	0
18	31	1	0	0	0	0
18	32	1	0	0	0	0
19	21	4	0	0	0	0
23	20	4	0	0	0	0
21	22	4	0	0	0	0
22	23	4	0	0	0	0
22	24	1	0	0	0	0
24	28	1	0	0	0	0
24	29	1	0	0	0	0
24	30	1	0	0	0	0
25	26	1	0	0	0	0
25	27	1	0	0	0	0
1	33	1	0	0	0	0
2	34	1	0	0	0	0
5	35	1	0	0	0	0
7	36	1	0	0	0	0
9	37	1	0	0	0	0
9	38	1	0	0	0	0
9	39	1	0	0	0	0
10	40	1	0	0	0	0
10	41	1	0	0	0	0
10	42	1	0	0	0	0
11	43	1	0	0	0	0
11	44	1	0	0	0	0
11	45	1	0	0	0	0
13	46	1	0	0	0	0
13	47	1	0	0	0	0
14	48	1	0	0	0	0

```
 14 49  1  0  0  0  0
 14 50  1  0  0  0  0
 16 51  1  0  0  0  0
 16 52  1  0  0  0  0
 17 53  1  0  0  0  0
 17 54  1  0  0  0  0
 17 55  1  0  0  0  0
 18 56  1  0  0  0  0
 19 57  1  0  0  0  0
 20 58  1  0  0  0  0
 21 59  1  0  0  0  0
 23 60  1  0  0  0  0
 25 61  1  0  0  0  0
 26 62  1  0  0  0  0
 26 63  1  0  0  0  0
 26 64  1  0  0  0  0
 27 65  1  0  0  0  0
 27 66  1  0  0  0  0
 27 67  1  0  0  0  0
 28 68  1  0  0  0  0
 28 69  1  0  0  0  0
 28 70  1  0  0  0  0
 29 71  1  0  0  0  0
 29 72  1  0  0  0  0
 29 73  1  0  0  0  0
 30 74  1  0  0  0  0
 30 75  1  0  0  0  0
 30 76  1  0  0  0  0
 31 77  1  0  0  0  0
 31 78  1  0  0  0  0
 31 79  1  0  0  0  0
 32 80  1  0  0  0  0
 32 81  1  0  0  0  0
 32 82  1  0  0  0  0
M  END
>  <Alias name>
2,4,5,7-Tetramethyl-4,5-bis(4-t-butylphenyl)octane

>  <deltaH°(c)>   kJ/mol
-19301

$$$$
Trinonylsilane
  ChemBrain IXL

 86 85  0  0  0  0  0  0  0  0  1 V2000
    9.3009    3.0198    3.2614 Si  0  0  0  0  0  0  0  0  0  0  0  0
    9.3209    4.5345    4.3858 C   0  0  0  0  0  0  0  0  0  0  0  0
    9.1838    5.8345    3.5660 C   0  0  0  0  0  0  0  0  0  0  0  0
    9.3288    7.0474    4.5048 C   0  0  0  0  0  0  0  0  0  0  0  0
    9.1901    8.3470    3.6883 C   0  0  0  0  0  0  0  0  0  0  0  0
    9.3382    9.5614    4.6252 C   0  0  0  0  0  0  0  0  0  0  0  0
    9.2007   10.8604    3.8074 C   0  0  0  0  0  0  0  0  0  0  0  0
    9.3508   12.0755    4.7433 C   0  0  0  0  0  0  0  0  0  0  0  0
    9.2139   13.3743    3.9249 C   0  0  0  0  0  0  0  0  0  0  0  0
    3.5991    7.4430   -4.1951 C   0  0  0  0  0  0  0  0  0  0  0  0
    3.6212    6.3083   -3.1523 C   0  0  0  0  0  0  0  0  0  0  0  0
    4.9701    6.3216   -2.4071 C   0  0  0  0  0  0  0  0  0  0  0  0
    4.9924    5.1875   -1.3639 C   0  0  0  0  0  0  0  0  0  0  0  0
    6.3407    5.2026   -0.6179 C   0  0  0  0  0  0  0  0  0  0  0  0
    6.3634    4.0696    0.4264 C   0  0  0  0  0  0  0  0  0  0  0  0
    7.7107    4.0877    1.1733 C   0  0  0  0  0  0  0  0  0  0  0  0
    7.7316    2.9511    2.2166 C   0  0  0  0  0  0  0  0  0  0  0  0
```

10.8522	2.9285	2.1916 C	0	0	0	0	0	0	0	0	0	0	0	0
11.0146	4.2059	1.3415 C	0	0	0	0	0	0	0	0	0	0	0	0
12.2218	4.0410	0.3987 C	0	0	0	0	0	0	0	0	0	0	0	0
12.3893	5.3179	-0.4478 C	0	0	0	0	0	0	0	0	0	0	0	0
13.5963	5.1546	-1.3917 C	0	0	0	0	0	0	0	0	0	0	0	0
13.7633	6.4323	-2.2372 C	0	0	0	0	0	0	0	0	0	0	0	0
14.9700	6.2697	-3.1818 C	0	0	0	0	0	0	0	0	0	0	0	0
15.1368	7.5478	-4.0268 C	0	0	0	0	0	0	0	0	0	0	0	0
16.3433	7.3855	-4.9717 C	0	0	0	0	0	0	0	0	0	0	0	0
9.3648	14.5896	4.8602 C	0	0	0	0	0	0	0	0	0	0	0	0
2.2501	7.4305	-4.9398 C	0	0	0	0	0	0	0	0	0	0	0	0
9.2993	1.8250	4.1346 H	0	0	0	0	0	0	0	0	0	0	0	0
8.4945	4.4703	5.0827 H	0	0	0	0	0	0	0	0	0	0	0	0
10.2519	4.5543	4.9386 H	0	0	0	0	0	0	0	0	0	0	0	0
9.9480	5.8734	2.8151 H	0	0	0	0	0	0	0	0	0	0	0	0
8.2154	5.8632	3.0976 H	0	0	0	0	0	0	0	0	0	0	0	0
8.5555	7.0124	5.2622 H	0	0	0	0	0	0	0	0	0	0	0	0
10.3015	7.0217	4.9802 H	0	0	0	0	0	0	0	0	0	0	0	0
9.9621	8.3805	2.9295 H	0	0	0	0	0	0	0	0	0	0	0	0
8.2167	8.3734	3.2143 H	0	0	0	0	0	0	0	0	0	0	0	0
8.5658	9.5287	5.3836 H	0	0	0	0	0	0	0	0	0	0	0	0
10.3114	9.5348	5.0997 H	0	0	0	0	0	0	0	0	0	0	0	0
9.9724	10.8923	3.0482 H	0	0	0	0	0	0	0	0	0	0	0	0
8.2271	10.8876	3.3339 H	0	0	0	0	0	0	0	0	0	0	0	0
8.5790	12.0440	5.5024 H	0	0	0	0	0	0	0	0	0	0	0	0
10.3244	12.0483	5.2169 H	0	0	0	0	0	0	0	0	0	0	0	0
9.9853	13.4055	3.1654 H	0	0	0	0	0	0	0	0	0	0	0	0
8.2401	13.4018	3.4516 H	0	0	0	0	0	0	0	0	0	0	0	0
3.7265	8.3952	-3.6952 H	0	0	0	0	0	0	0	0	0	0	0	0
4.4043	7.2972	-4.9044 H	0	0	0	0	0	0	0	0	0	0	0	0
3.4944	5.3560	-3.6522 H	0	0	0	0	0	0	0	0	0	0	0	0
2.8157	6.4540	-2.4432 H	0	0	0	0	0	0	0	0	0	0	0	0
5.0970	7.2741	-1.9075 H	0	0	0	0	0	0	0	0	0	0	0	0
5.7756	6.1758	-3.1162 H	0	0	0	0	0	0	0	0	0	0	0	0
4.8668	4.2349	-1.8635 H	0	0	0	0	0	0	0	0	0	0	0	0
4.1862	5.3328	-0.6555 H	0	0	0	0	0	0	0	0	0	0	0	0
6.4664	6.1556	-0.1191 H	0	0	0	0	0	0	0	0	0	0	0	0
7.1468	5.0568	-1.3263 H	0	0	0	0	0	0	0	0	0	0	0	0
6.2397	3.1162	-0.0723 H	0	0	0	0	0	0	0	0	0	0	0	0
5.5564	4.2144	1.1339 H	0	0	0	0	0	0	0	0	0	0	0	0
7.8324	5.0335	1.6633 H	0	0	0	0	0	0	0	0	0	0	0	0
8.5092	3.9415	0.4669 H	0	0	0	0	0	0	0	0	0	0	0	0
7.6901	2.0001	1.7004 H	0	0	0	0	0	0	0	0	0	0	0	0
6.8690	3.0421	2.8649 H	0	0	0	0	0	0	0	0	0	0	0	0
11.7189	2.8204	2.8318 H	0	0	0	0	0	0	0	0	0	0	0	0
10.7830	2.0678	1.5380 H	0	0	0	0	0	0	0	0	0	0	0	0
10.1293	4.3708	0.7597 H	0	0	0	0	0	0	0	0	0	0	0	0
11.1837	5.0487	1.9888 H	0	0	0	0	0	0	0	0	0	0	0	0
13.1176	3.8760	0.9844 H	0	0	0	0	0	0	0	0	0	0	0	0
12.0564	3.1935	-0.2550 H	0	0	0	0	0	0	0	0	0	0	0	0
11.4932	5.4830	-1.0331 H	0	0	0	0	0	0	0	0	0	0	0	0
12.5545	6.1652	0.2061 H	0	0	0	0	0	0	0	0	0	0	0	0
14.4925	4.9892	-0.8066 H	0	0	0	0	0	0	0	0	0	0	0	0
13.4310	4.3077	-2.0461 H	0	0	0	0	0	0	0	0	0	0	0	0
12.8669	6.5978	-2.8220 H	0	0	0	0	0	0	0	0	0	0	0	0
13.9288	7.2791	-1.5827 H	0	0	0	0	0	0	0	0	0	0	0	0
15.8664	6.1041	-2.5970 H	0	0	0	0	0	0	0	0	0	0	0	0
14.8045	5.4231	-3.8365 H	0	0	0	0	0	0	0	0	0	0	0	0
14.2403	7.7135	-4.6114 H	0	0	0	0	0	0	0	0	0	0	0	0
15.3024	8.3944	-3.3721 H	0	0	0	0	0	0	0	0	0	0	0	0
16.4602	8.2837	-5.5652 H	0	0	0	0	0	0	0	0	0	0	0	0
16.1776	6.5390	-5.6265 H	0	0	0	0	0	0	0	0	0	0	0	0
17.2398	7.2198	-4.3872 H	0	0	0	0	0	0	0	0	0	0	0	0

```
    8.5933   14.5586    5.6197 H   0  0  0  0  0  0  0  0  0  0  0  0
    9.2684   15.5020    4.2849 H   0  0  0  0  0  0  0  0  0  0  0  0
   10.3385   14.5623    5.3334 H   0  0  0  0  0  0  0  0  0  0  0  0
    2.1226    6.4783   -5.4398 H   0  0  0  0  0  0  0  0  0  0  0  0
    1.4448    7.5765   -4.2306 H   0  0  0  0  0  0  0  0  0  0  0  0
    2.2349    8.2278   -5.6724 H   0  0  0  0  0  0  0  0  0  0  0  0
  1  2  1  0  0  0  0
  2  3  1  0  0  0  0
  3  4  1  0  0  0  0
  4  5  1  0  0  0  0
  5  6  1  0  0  0  0
  6  7  1  0  0  0  0
  7  8  1  0  0  0  0
  8  9  1  0  0  0  0
 10 11  1  0  0  0  0
 11 12  1  0  0  0  0
 12 13  1  0  0  0  0
 13 14  1  0  0  0  0
 14 15  1  0  0  0  0
 15 16  1  0  0  0  0
 16 17  1  0  0  0  0
 17  1  1  0  0  0  0
 18 19  1  0  0  0  0
 19 20  1  0  0  0  0
 20 21  1  0  0  0  0
 21 22  1  0  0  0  0
 22 23  1  0  0  0  0
 23 24  1  0  0  0  0
 24 25  1  0  0  0  0
 18  1  1  0  0  0  0
 25 26  1  0  0  0  0
  9 27  1  0  0  0  0
 10 28  1  0  0  0  0
  1 29  1  0  0  0  0
  2 30  1  0  0  0  0
  2 31  1  0  0  0  0
  3 32  1  0  0  0  0
  3 33  1  0  0  0  0
  4 34  1  0  0  0  0
  4 35  1  0  0  0  0
  5 36  1  0  0  0  0
  5 37  1  0  0  0  0
  6 38  1  0  0  0  0
  6 39  1  0  0  0  0
  7 40  1  0  0  0  0
  7 41  1  0  0  0  0
  8 42  1  0  0  0  0
  8 43  1  0  0  0  0
  9 44  1  0  0  0  0
  9 45  1  0  0  0  0
 10 46  1  0  0  0  0
 10 47  1  0  0  0  0
 11 48  1  0  0  0  0
 11 49  1  0  0  0  0
 12 50  1  0  0  0  0
 12 51  1  0  0  0  0
 13 52  1  0  0  0  0
 13 53  1  0  0  0  0
 14 54  1  0  0  0  0
 14 55  1  0  0  0  0
 15 56  1  0  0  0  0
 15 57  1  0  0  0  0
 16 58  1  0  0  0  0
```

```
 16 59  1  0  0  0  0
 17 60  1  0  0  0  0
 17 61  1  0  0  0  0
 18 62  1  0  0  0  0
 18 63  1  0  0  0  0
 19 64  1  0  0  0  0
 19 65  1  0  0  0  0
 20 66  1  0  0  0  0
 20 67  1  0  0  0  0
 21 68  1  0  0  0  0
 21 69  1  0  0  0  0
 22 70  1  0  0  0  0
 22 71  1  0  0  0  0
 23 72  1  0  0  0  0
 23 73  1  0  0  0  0
 24 74  1  0  0  0  0
 24 75  1  0  0  0  0
 25 76  1  0  0  0  0
 25 77  1  0  0  0  0
 26 78  1  0  0  0  0
 26 79  1  0  0  0  0
 26 80  1  0  0  0  0
 27 81  1  0  0  0  0
 27 82  1  0  0  0  0
 27 83  1  0  0  0  0
 28 84  1  0  0  0  0
 28 85  1  0  0  0  0
 28 86  1  0  0  0  0
M  END
>  <Alias name>
Trinonylsilane

>  <deltaH°(c)>   kJ/mol
-19140

$$$$
Protoporphyrin dimethylester
  ChemBrain IXL

 82 86  0  0  0  0  0  0  0  0  1 V2000
   12.3163   11.1902   -0.1408 C   0  0  0  0  0  0  0  0  0  0  0  0  0
   13.3619   12.1200   -0.1229 C   0  0  0  0  0  0  0  0  0  0  0  0  0
   12.4814    9.8050   -0.1966 C   0  0  0  0  0  0  0  0  0  0  0  0  0
   11.1216   11.9077   -0.0964 N   0  0  0  0  0  0  0  0  0  0  0  0  0
   12.8002   13.4038   -0.0738 C   0  0  0  0  0  0  0  0  0  0  0  0  0
   14.8724   11.8055   -0.1495 C   0  0  0  0  0  0  0  0  0  0  0  0  0
   11.3939    8.9290   -0.1962 C   0  0  0  0  0  0  0  0  0  0  0  0  0
   11.4099   13.2700   -0.0607 C   0  0  0  0  0  0  0  0  0  0  0  0  0
   13.5798   14.7322   -0.0392 C   0  0  0  0  0  0  0  0  0  0  0  0  0
   15.6549   13.0403    0.3482 C   0  0  0  0  0  0  0  0  0  0  0  0  0
   11.4969    7.4615   -0.2739 C   0  0  0  0  0  0  0  0  0  0  0  0  0
   10.0622    9.2431   -0.1313 N   0  0  0  0  0  0  0  0  0  0  0  0  0
   10.5228   14.3454   -0.0218 C   0  0  0  0  0  0  0  0  0  0  0  0  0
   17.1628   12.7215    0.3931 C   0  0  0  0  0  0  0  0  0  0  0  0  0
   10.2880    6.9680   -0.2733 C   0  0  0  0  0  0  0  0  0  0  0  0  0
   12.7949    6.6379   -0.3393 C   0  0  0  0  0  0  0  0  0  0  0  0  0
    9.3360    8.0829   -0.1699 C   0  0  0  0  0  0  0  0  0  0  0  0  0
    9.1371   14.1843    0.0061 C   0  0  0  0  0  0  0  0  0  0  0  0  0
   17.6390   11.5748   -0.1704 O   0  0  0  0  0  0  0  0  0  0  0  0  0
   17.9325   13.4982    0.9345 O   0  0  0  0  0  0  0  0  0  0  0  0  0
    9.9209    5.4749   -0.3690 C   0  0  0  0  0  0  0  0  0  0  0  0  0
   12.5406    5.2471    0.2776 C   0  0  0  0  0  0  0  0  0  0  0  0  0
    7.9529    7.9203   -0.1191 C   0  0  0  0  0  0  0  0  0  0  0  0  0
```

```
8.1836   15.3036    0.0292 C   0  0  0  0  0  0  0  0  0  0  0  0
8.4109   13.0224    0.0150 N   0  0  0  0  0  0  0  0  0  0  0  0
13.8618    4.4604    0.3451 C   0  0  0  0  0  0  0  0  0  0  0  0
7.0684    8.9932   -0.0233 C   0  0  0  0  0  0  0  0  0  0  0  0
6.9720   14.8088    0.0394 C   0  0  0  0  0  0  0  0  0  0  0  0
8.5447   16.7402    0.0412 C   0  0  0  0  0  0  0  0  0  0  0  0
7.0773   13.3377    0.0382 C   0  0  0  0  0  0  0  0  0  0  0  0
13.8438    3.0953    0.3146 O   0  0  0  0  0  0  0  0  0  0  0  0
14.9234    5.0557    0.4262 O   0  0  0  0  0  0  0  0  0  0  0  0
7.3542   10.3567    0.0056 N   0  0  0  0  0  0  0  0  0  0  0  0
5.6824    8.8542    0.0627 C   0  0  0  0  0  0  0  0  0  0  0  0
5.6639   15.6278    0.0483 C   0  0  0  0  0  0  0  0  0  0  0  0
7.5909   17.6734    0.0646 C   0  0  0  0  0  0  0  0  0  0  0  0
5.9897   12.4580    0.0601 C   0  0  0  0  0  0  0  0  0  0  0  0
6.1597   11.0704    0.0879 C   0  0  0  0  0  0  0  0  0  0  0  0
5.1213   10.1345    0.1754 C   0  0  0  0  0  0  0  0  0  0  0  0
4.9073    7.5233    0.0216 C   0  0  0  0  0  0  0  0  0  0  0  0
3.6818   10.4239    0.3800 C   0  0  0  0  0  0  0  0  0  0  0  0
3.2720   11.6686    0.6352 C   0  0  0  0  0  0  0  0  0  0  0  0
19.0935   11.3884   -0.0564 C   0  0  0  0  0  0  0  0  0  0  0  0
12.5063    2.4935    0.1968 C   0  0  0  0  0  0  0  0  0  0  0  0
10.1968   11.5046   -0.0837 H   0  0  0  0  0  0  0  0  0  0  0  0
8.2791   10.7613    0.0061 H   0  0  0  0  0  0  0  0  0  0  0  0
13.4837    9.3975   -0.2414 H   0  0  0  0  0  0  0  0  0  0  0  0
15.0787   10.9610    0.4961 H   0  0  0  0  0  0  0  0  0  0  0  0
15.1754   11.5695   -1.1627 H   0  0  0  0  0  0  0  0  0  0  0  0
12.8824   15.5583    0.0272 H   0  0  0  0  0  0  0  0  0  0  0  0
14.1689   14.8288   -0.9428 H   0  0  0  0  0  0  0  0  0  0  0  0
14.2358   14.7434    0.8223 H   0  0  0  0  0  0  0  0  0  0  0  0
15.4828   13.8709   -0.3252 H   0  0  0  0  0  0  0  0  0  0  0  0
15.3138   13.3058    1.3411 H   0  0  0  0  0  0  0  0  0  0  0  0
10.9273   15.3501   -0.0128 H   0  0  0  0  0  0  0  0  0  0  0  0
13.5746    7.1440    0.2150 H   0  0  0  0  0  0  0  0  0  0  0  0
13.0998    6.5247   -1.3731 H   0  0  0  0  0  0  0  0  0  0  0  0
10.2738    4.9591    0.5154 H   0  0  0  0  0  0  0  0  0  0  0  0
8.8450    5.3718   -0.4419 H   0  0  0  0  0  0  0  0  0  0  0  0
10.3852    5.0445   -1.2475 H   0  0  0  0  0  0  0  0  0  0  0  0
11.8297    4.7056   -0.3351 H   0  0  0  0  0  0  0  0  0  0  0  0
12.1393    5.3641    1.2765 H   0  0  0  0  0  0  0  0  0  0  0  0
7.5479    6.9165   -0.1550 H   0  0  0  0  0  0  0  0  0  0  0  0
9.5880   17.0344    0.0309 H   0  0  0  0  0  0  0  0  0  0  0  0
4.8157   14.9552    0.0374 H   0  0  0  0  0  0  0  0  0  0  0  0
5.6277   16.2404    0.9414 H   0  0  0  0  0  0  0  0  0  0  0  0
5.6309   16.2651   -0.8274 H   0  0  0  0  0  0  0  0  0  0  0  0
6.5485   17.3827    0.0710 H   0  0  0  0  0  0  0  0  0  0  0  0
7.8611   18.7230    0.0770 H   0  0  0  0  0  0  0  0  0  0  0  0
4.9854   12.8639    0.0574 H   0  0  0  0  0  0  0  0  0  0  0  0
5.6074    6.6970    0.0612 H   0  0  0  0  0  0  0  0  0  0  0  0
4.2375    7.4695    0.8704 H   0  0  0  0  0  0  0  0  0  0  0  0
4.3341    7.4670   -0.8953 H   0  0  0  0  0  0  0  0  0  0  0  0
2.9571    9.6187    0.3220 H   0  0  0  0  0  0  0  0  0  0  0  0
2.2155   11.8723    0.7758 H   0  0  0  0  0  0  0  0  0  0  0  0
3.9919   12.4740    0.7020 H   0  0  0  0  0  0  0  0  0  0  0  0
19.6022   12.2094   -0.5469 H   0  0  0  0  0  0  0  0  0  0  0  0
19.3761   10.4555   -0.5276 H   0  0  0  0  0  0  0  0  0  0  0  0
19.3734   11.3639    0.9898 H   0  0  0  0  0  0  0  0  0  0  0  0
12.5942    1.4140    0.2027 H   0  0  0  0  0  0  0  0  0  0  0  0
11.8930    2.8102    1.0315 H   0  0  0  0  0  0  0  0  0  0  0  0
12.0468    2.8131   -0.7304 H   0  0  0  0  0  0  0  0  0  0  0  0
1  2  4  0  0  0  0
1  3  4  0  0  0  0
1  4  1  0  0  0  0
2  5  4  0  0  0  0
```

2	6	1	0	0	0	0
3	7	4	0	0	0	0
4	8	1	0	0	0	0
5	9	1	0	0	0	0
5	8	4	0	0	0	0
6	10	1	0	0	0	0
7	11	1	0	0	0	0
7	12	4	0	0	0	0
8	13	4	0	0	0	0
10	14	1	0	0	0	0
11	15	2	0	0	0	0
11	16	1	0	0	0	0
12	17	4	0	0	0	0
13	18	4	0	0	0	0
14	19	1	0	0	0	0
14	20	2	0	0	0	0
15	21	1	0	0	0	0
15	17	1	0	0	0	0
16	22	1	0	0	0	0
17	23	4	0	0	0	0
18	24	1	0	0	0	0
18	25	4	0	0	0	0
22	26	1	0	0	0	0
23	27	4	0	0	0	0
24	28	2	0	0	0	0
24	29	1	0	0	0	0
25	30	4	0	0	0	0
26	31	1	0	0	0	0
26	32	2	0	0	0	0
27	33	1	0	0	0	0
27	34	4	0	0	0	0
28	35	1	0	0	0	0
28	30	1	0	0	0	0
29	36	2	0	0	0	0
30	37	4	0	0	0	0
33	38	1	0	0	0	0
34	39	4	0	0	0	0
34	40	1	0	0	0	0
37	38	4	0	0	0	0
38	39	4	0	0	0	0
39	41	1	0	0	0	0
41	42	2	0	0	0	0
4	45	1	0	0	0	0
19	43	1	0	0	0	0
31	44	1	0	0	0	0
33	46	1	0	0	0	0
3	47	1	0	0	0	0
6	48	1	0	0	0	0
6	49	1	0	0	0	0
9	50	1	0	0	0	0
9	51	1	0	0	0	0
9	52	1	0	0	0	0
10	53	1	0	0	0	0
10	54	1	0	0	0	0
13	55	1	0	0	0	0
16	56	1	0	0	0	0
16	57	1	0	0	0	0
21	58	1	0	0	0	0
21	59	1	0	0	0	0
21	60	1	0	0	0	0
22	61	1	0	0	0	0
22	62	1	0	0	0	0
23	63	1	0	0	0	0

```
 29 64  1  0  0  0  0
 35 65  1  0  0  0  0
 35 66  1  0  0  0  0
 35 67  1  0  0  0  0
 36 68  1  0  0  0  0
 36 69  1  0  0  0  0
 37 70  1  0  0  0  0
 40 71  1  0  0  0  0
 40 72  1  0  0  0  0
 40 73  1  0  0  0  0
 41 74  1  0  0  0  0
 42 75  1  0  0  0  0
 42 76  1  0  0  0  0
 43 77  1  0  0  0  0
 43 78  1  0  0  0  0
 43 79  1  0  0  0  0
 44 80  1  0  0  0  0
 44 81  1  0  0  0  0
 44 82  1  0  0  0  0
M  END
>  <Alias name>
Protoporphyrin dimethylester

>  <deltaH°(c)>   kJ/mol
-19099

$$$$
Vitamin E acetate
  ChemBrain IXL

 86 87  0  0  0  0  0  0  0  0  1 V2000
    9.5989    9.9397    0.9088 C   0  0  0  0  0  0  0  0  0  0  0  0
    9.9543   11.2465    1.3037 C   0  0  0  0  0  0  0  0  0  0  0  0
    8.2327    9.6299    0.4163 O   0  0  0  0  0  0  0  0  0  0  0  0
   10.5422    8.9163    0.9806 C   0  0  0  0  0  0  0  0  0  0  0  0
   11.2432   11.5061    1.7668 C   0  0  0  0  0  0  0  0  0  0  0  0
    8.9714   12.3633    1.2333 C   0  0  0  0  0  0  0  0  0  0  0  0
    7.3645   10.7865    0.1623 C   0  0  0  0  0  0  0  0  0  0  0  0
   11.8319    9.1835    1.4416 C   0  0  0  0  0  0  0  0  0  0  0  0
   10.1723    7.5451    0.5666 C   0  0  0  0  0  0  0  0  0  0  0  0
   12.1909   10.4825    1.8078 C   0  0  0  0  0  0  0  0  0  0  0  0
   11.6048   12.8705    2.2149 C   0  0  0  0  0  0  0  0  0  0  0  0
    7.5436   11.8054    1.2862 C   0  0  0  0  0  0  0  0  0  0  0  0
    5.8897   10.3276    0.1091 C   0  0  0  0  0  0  0  0  0  0  0  0
    7.7479   11.4340   -1.1832 C   0  0  0  0  0  0  0  0  0  0  0  0
   12.8198    8.0882    1.5419 C   0  0  0  0  0  0  0  0  0  0  0  0
   13.5682   10.7732    2.2367 O   0  0  0  0  0  0  0  0  0  0  0  0
    5.4871    9.7202    1.4682 C   0  0  0  0  0  0  0  0  0  0  0  0
   14.1644    9.7755    3.1384 C   0  0  0  0  0  0  0  0  0  0  0  0
    3.9930    9.3328    1.4376 C   0  0  0  0  0  0  0  0  0  0  0  0
   15.6173    9.8077    3.4222 C   0  0  0  0  0  0  0  0  0  0  0  0
   13.4616    8.9210    3.6526 O   0  0  0  0  0  0  0  0  0  0  0  0
    3.5685    8.8128    2.8261 C   0  0  0  0  0  0  0  0  0  0  0  0
    4.3652    7.5358    3.1640 C   0  0  0  0  0  0  0  0  0  0  0  0
    2.0605    8.4924    2.8185 C   0  0  0  0  0  0  0  0  0  0  0  0
    3.9623    6.4008    2.1987 C   0  0  0  0  0  0  0  0  0  0  0  0
    4.8684    5.1760    2.4444 C   0  0  0  0  0  0  0  0  0  0  0  0
    4.3855    3.9901    1.5830 C   0  0  0  0  0  0  0  0  0  0  0  0
    4.2628    4.4344    0.1123 C   0  0  0  0  0  0  0  0  0  0  0  0
    3.0109    3.5116    2.0900 C   0  0  0  0  0  0  0  0  0  0  0  0
    5.6180    4.9817   -0.3778 C   0  0  0  0  0  0  0  0  0  0  0  0
    5.5039    5.3697   -1.8656 C   0  0  0  0  0  0  0  0  0  0  0  0
    6.8426    5.9599   -2.3502 C   0  0  0  0  0  0  0  0  0  0  0  0
```

6.7452	6.2833	-3.8540	C	0	0	0	0	0	0	0	0	0	0	0	0	0	0
7.1498	7.2506	-1.5664	C	0	0	0	0	0	0	0	0	0	0	0	0	0	0
9.1269	13.0325	2.0707	H	0	0	0	0	0	0	0	0	0	0	0	0	0	0
9.1108	12.9076	0.3075	H	0	0	0	0	0	0	0	0	0	0	0	0	0	0
10.6161	6.8302	1.2478	H	0	0	0	0	0	0	0	0	0	0	0	0	0	0
9.0942	7.4409	0.5857	H	0	0	0	0	0	0	0	0	0	0	0	0	0	0
10.5349	7.3609	-0.4370	H	0	0	0	0	0	0	0	0	0	0	0	0	0	0
11.0446	13.1173	3.1086	H	0	0	0	0	0	0	0	0	0	0	0	0	0	0
11.3682	13.5818	1.4329	H	0	0	0	0	0	0	0	0	0	0	0	0	0	0
12.6650	12.9108	2.4308	H	0	0	0	0	0	0	0	0	0	0	0	0	0	0
6.8336	12.6139	1.1589	H	0	0	0	0	0	0	0	0	0	0	0	0	0	0
7.3771	11.3242	2.2419	H	0	0	0	0	0	0	0	0	0	0	0	0	0	0
5.7716	9.5826	-0.6680	H	0	0	0	0	0	0	0	0	0	0	0	0	0	0
5.2549	11.1783	-0.1078	H	0	0	0	0	0	0	0	0	0	0	0	0	0	0
7.1255	12.3039	-1.3555	H	0	0	0	0	0	0	0	0	0	0	0	0	0	0
7.5989	10.7196	-1.9833	H	0	0	0	0	0	0	0	0	0	0	0	0	0	0
8.7879	11.7342	-1.1562	H	0	0	0	0	0	0	0	0	0	0	0	0	0	0
13.8066	8.4701	1.3129	H	0	0	0	0	0	0	0	0	0	0	0	0	0	0
12.5632	7.3056	0.8385	H	0	0	0	0	0	0	0	0	0	0	0	0	0	0
12.8123	7.6864	2.5475	H	0	0	0	0	0	0	0	0	0	0	0	0	0	0
5.6552	10.4476	2.2525	H	0	0	0	0	0	0	0	0	0	0	0	0	0	0
6.0845	8.8378	1.6601	H	0	0	0	0	0	0	0	0	0	0	0	0	0	0
3.8358	8.5575	0.6981	H	0	0	0	0	0	0	0	0	0	0	0	0	0	0
3.3998	10.2021	1.1810	H	0	0	0	0	0	0	0	0	0	0	0	0	0	0
15.9541	10.8361	3.4654	H	0	0	0	0	0	0	0	0	0	0	0	0	0	0
16.1502	9.2861	2.6368	H	0	0	0	0	0	0	0	0	0	0	0	0	0	0
15.8106	9.3243	4.3721	H	0	0	0	0	0	0	0	0	0	0	0	0	0	0
3.7714	9.5715	3.5719	H	0	0	0	0	0	0	0	0	0	0	0	0	0	0
4.1503	7.2361	4.1822	H	0	0	0	0	0	0	0	0	0	0	0	0	0	0
5.4248	7.7352	3.0631	H	0	0	0	0	0	0	0	0	0	0	0	0	0	0
1.8536	7.7457	2.0623	H	0	0	0	0	0	0	0	0	0	0	0	0	0	0
1.7672	8.1135	3.7900	H	0	0	0	0	0	0	0	0	0	0	0	0	0	0
1.5011	9.3935	2.5988	H	0	0	0	0	0	0	0	0	0	0	0	0	0	0
4.0789	6.7374	1.1760	H	0	0	0	0	0	0	0	0	0	0	0	0	0	0
2.9291	6.1271	2.3737	H	0	0	0	0	0	0	0	0	0	0	0	0	0	0
4.8263	4.9013	3.4912	H	0	0	0	0	0	0	0	0	0	0	0	0	0	0
5.8884	5.4232	2.1777	H	0	0	0	0	0	0	0	0	0	0	0	0	0	0
5.0987	3.1782	1.6559	H	0	0	0	0	0	0	0	0	0	0	0	0	0	0
3.9751	3.5865	-0.4972	H	0	0	0	0	0	0	0	0	0	0	0	0	0	0
3.5104	5.2086	0.0309	H	0	0	0	0	0	0	0	0	0	0	0	0	0	0
2.6820	2.6665	1.4975	H	0	0	0	0	0	0	0	0	0	0	0	0	0	0
3.0923	3.2146	3.1283	H	0	0	0	0	0	0	0	0	0	0	0	0	0	0
2.2926	4.3169	2.0000	H	0	0	0	0	0	0	0	0	0	0	0	0	0	0
5.8871	5.8546	0.2039	H	0	0	0	0	0	0	0	0	0	0	0	0	0	0
6.3794	4.2202	-0.2602	H	0	0	0	0	0	0	0	0	0	0	0	0	0	0
5.2651	4.4900	-2.4503	H	0	0	0	0	0	0	0	0	0	0	0	0	0	0
4.7200	6.1069	-1.9867	H	0	0	0	0	0	0	0	0	0	0	0	0	0	0
7.6352	5.2403	-2.1861	H	0	0	0	0	0	0	0	0	0	0	0	0	0	0
5.9495	6.9995	-4.0183	H	0	0	0	0	0	0	0	0	0	0	0	0	0	0
6.5334	5.3756	-4.4055	H	0	0	0	0	0	0	0	0	0	0	0	0	0	0
7.6839	6.7021	-4.1949	H	0	0	0	0	0	0	0	0	0	0	0	0	0	0
6.3491	7.9640	-1.7178	H	0	0	0	0	0	0	0	0	0	0	0	0	0	0
8.0820	7.6740	-1.9195	H	0	0	0	0	0	0	0	0	0	0	0	0	0	0
7.2342	7.0210	-0.5115	H	0	0	0	0	0	0	0	0	0	0	0	0	0	0

1	3	1	0	0	0	0
1	4	4	0	0	0	0
2	5	4	0	0	0	0
2	6	1	0	0	0	0
3	7	1	0	0	0	0
4	8	4	0	0	0	0
4	9	1	0	0	0	0
5	10	4	0	0	0	0
5	11	1	0	0	0	0

6	12	1	0	0	0	0
7	13	1	0	0	0	0
7	14	1	0	0	0	0
7	12	1	0	0	0	0
8	15	1	0	0	0	0
8	10	4	0	0	0	0
10	16	1	0	0	0	0
13	17	1	0	0	0	0
16	18	1	0	0	0	0
17	19	1	0	0	0	0
18	20	1	0	0	0	0
18	21	2	0	0	0	0
19	22	1	0	0	0	0
22	23	1	0	0	0	0
22	24	1	0	0	0	0
23	25	1	0	0	0	0
25	26	1	0	0	0	0
26	27	1	0	0	0	0
27	28	1	0	0	0	0
27	29	1	0	0	0	0
28	30	1	0	0	0	0
30	31	1	0	0	0	0
31	32	1	0	0	0	0
32	33	1	0	0	0	0
32	34	1	0	0	0	0
1	2	4	0	0	0	0
6	35	1	0	0	0	0
6	36	1	0	0	0	0
9	37	1	0	0	0	0
9	38	1	0	0	0	0
9	39	1	0	0	0	0
11	40	1	0	0	0	0
11	41	1	0	0	0	0
11	42	1	0	0	0	0
12	43	1	0	0	0	0
12	44	1	0	0	0	0
13	45	1	0	0	0	0
13	46	1	0	0	0	0
14	47	1	0	0	0	0
14	48	1	0	0	0	0
14	49	1	0	0	0	0
15	50	1	0	0	0	0
15	51	1	0	0	0	0
15	52	1	0	0	0	0
17	53	1	0	0	0	0
17	54	1	0	0	0	0
19	55	1	0	0	0	0
19	56	1	0	0	0	0
20	57	1	0	0	0	0
20	58	1	0	0	0	0
20	59	1	0	0	0	0
22	60	1	0	0	0	0
23	61	1	0	0	0	0
23	62	1	0	0	0	0
24	63	1	0	0	0	0
24	64	1	0	0	0	0
24	65	1	0	0	0	0
25	66	1	0	0	0	0
25	67	1	0	0	0	0
26	68	1	0	0	0	0
26	69	1	0	0	0	0
27	70	1	0	0	0	0
28	71	1	0	0	0	0

```
 28 72  1  0  0  0  0
 29 73  1  0  0  0  0
 29 74  1  0  0  0  0
 29 75  1  0  0  0  0
 30 76  1  0  0  0  0
 30 77  1  0  0  0  0
 31 78  1  0  0  0  0
 31 79  1  0  0  0  0
 32 80  1  0  0  0  0
 33 81  1  0  0  0  0
 33 82  1  0  0  0  0
 33 83  1  0  0  0  0
 34 84  1  0  0  0  0
 34 85  1  0  0  0  0
 34 86  1  0  0  0  0
M  END
>  <Alias name>
Vitamin E acetate

>  <deltaH°(c)>   kJ/mol
-18536

$$$$
Pentaphenylbenzene
  ChemBrain IXL

 62 67  0  0  0  0  0  0  0  0  1 V2000
    8.9125    8.8064    0.0000 C   0  0  0  0  0  0  0  0  0  0  0  0
    9.6072    7.6011    0.0037 C   0  0  0  0  0  0  0  0  0  0  0  0
    8.9059    6.3861   -0.0033 C   0  0  0  0  0  0  0  0  0  0  0  0
    7.5144    6.3858   -0.0023 C   0  0  0  0  0  0  0  0  0  0  0  0
    6.8193    7.5913   -0.0001 C   0  0  0  0  0  0  0  0  0  0  0  0
    7.5214    8.8060   -0.0050 C   0  0  0  0  0  0  0  0  0  0  0  0
    3.2541    6.5925   -0.7194 C   0  0  0  0  0  0  0  0  0  0  0  0
    2.5482    7.5391    0.0232 C   0  0  0  0  0  0  0  0  0  0  0  0
    3.2372    8.5048    0.7574 C   0  0  0  0  0  0  0  0  0  0  0  0
    4.6323    8.5238    0.7491 C   0  0  0  0  0  0  0  0  0  0  0  0
    5.3383    7.5769    0.0067 C   0  0  0  0  0  0  0  0  0  0  0  0
    4.6489    6.6116   -0.7280 C   0  0  0  0  0  0  0  0  0  0  0  0
    5.4314   12.5292   -0.0307 C   0  0  0  0  0  0  0  0  0  0  0  0
    5.0578   11.5144   -0.9121 C   0  0  0  0  0  0  0  0  0  0  0  0
    5.7405   10.2978   -0.9049 C   0  0  0  0  0  0  0  0  0  0  0  0
    6.7954   10.0955   -0.0148 C   0  0  0  0  0  0  0  0  0  0  0  0
    7.1704   11.1113    0.8648 C   0  0  0  0  0  0  0  0  0  0  0  0
    6.4879   12.3277    0.8575 C   0  0  0  0  0  0  0  0  0  0  0  0
   11.4858    3.7398   -0.7652 C   0  0  0  0  0  0  0  0  0  0  0  0
   10.9950    2.6604   -0.0301 C   0  0  0  0  0  0  0  0  0  0  0  0
    9.8236    2.7989    0.7146 C   0  0  0  0  0  0  0  0  0  0  0  0
    9.1431    4.0166    0.7243 C   0  0  0  0  0  0  0  0  0  0  0  0
    9.6334    5.0961   -0.0114 C   0  0  0  0  0  0  0  0  0  0  0  0
   10.8052    4.9577   -0.7558 C   0  0  0  0  0  0  0  0  0  0  0  0
   13.8767    7.6503    0.0320 C   0  0  0  0  0  0  0  0  0  0  0  0
   13.1836    6.8154    0.9089 C   0  0  0  0  0  0  0  0  0  0  0  0
   11.7887    6.7992    0.9009 C   0  0  0  0  0  0  0  0  0  0  0  0
   11.0870    7.6164    0.0144 C   0  0  0  0  0  0  0  0  0  0  0  0
   11.7801    8.4529   -0.8607 C   0  0  0  0  0  0  0  0  0  0  0  0
   13.1748    8.4692   -0.8526 C   0  0  0  0  0  0  0  0  0  0  0  0
   11.4478   11.4669    0.8466 C   0  0  0  0  0  0  0  0  0  0  0  0
   11.0479   12.5042    0.0040 C   0  0  0  0  0  0  0  0  0  0  0  0
    9.9503   12.3335   -0.8399 C   0  0  0  0  0  0  0  0  0  0  0  0
    9.2528   11.1254   -0.8413 C   0  0  0  0  0  0  0  0  0  0  0  0
    9.6526   10.0880    0.0014 C   0  0  0  0  0  0  0  0  0  0  0  0
   10.7501   10.2589    0.8454 C   0  0  0  0  0  0  0  0  0  0  0  0
```

```
    6.9727    5.4481   -0.0031 H   0  0  0  0  0  0  0  0  0  0  0  0
    2.7193    5.8427   -1.2894 H   0  0  0  0  0  0  0  0  0  0  0  0
    1.4653    7.5245    0.0297 H   0  0  0  0  0  0  0  0  0  0  0  0
    2.6892    9.2397    1.3339 H   0  0  0  0  0  0  0  0  0  0  0  0
    5.1671    9.2735    1.3190 H   0  0  0  0  0  0  0  0  0  0  0  0
    5.1969    5.8768   -1.3049 H   0  0  0  0  0  0  0  0  0  0  0  0
    4.9011   13.4735   -0.0361 H   0  0  0  0  0  0  0  0  0  0  0  0
    4.2377   11.6710   -1.6019 H   0  0  0  0  0  0  0  0  0  0  0  0
    5.4516    9.5105   -1.5901 H   0  0  0  0  0  0  0  0  0  0  0  0
    7.9917   10.9553    1.5534 H   0  0  0  0  0  0  0  0  0  0  0  0
    6.7781   13.1156    1.5415 H   0  0  0  0  0  0  0  0  0  0  0  0
   12.3952    3.6323   -1.3432 H   0  0  0  0  0  0  0  0  0  0  0  0
   11.5235    1.7151   -0.0374 H   0  0  0  0  0  0  0  0  0  0  0  0
    9.4425    1.9610    1.2853 H   0  0  0  0  0  0  0  0  0  0  0  0
    8.2338    4.1241    1.3028 H   0  0  0  0  0  0  0  0  0  0  0  0
   11.1863    5.7955   -1.3264 H   0  0  0  0  0  0  0  0  0  0  0  0
   14.9596    7.6625    0.0380 H   0  0  0  0  0  0  0  0  0  0  0  0
   13.7286    6.1799    1.5959 H   0  0  0  0  0  0  0  0  0  0  0  0
   11.2506    6.1524    1.5827 H   0  0  0  0  0  0  0  0  0  0  0  0
   11.2351    9.0898   -1.5465 H   0  0  0  0  0  0  0  0  0  0  0  0
   13.7128    9.1176   -1.5332 H   0  0  0  0  0  0  0  0  0  0  0  0
   12.2999   11.5994    1.5018 H   0  0  0  0  0  0  0  0  0  0  0  0
   11.5894   13.4421    0.0050 H   0  0  0  0  0  0  0  0  0  0  0  0
    9.6397   13.1388   -1.4941 H   0  0  0  0  0  0  0  0  0  0  0  0
    8.4008   10.9928   -1.4967 H   0  0  0  0  0  0  0  0  0  0  0  0
   11.0605    9.4537    1.4997 H   0  0  0  0  0  0  0  0  0  0  0  0
  1  2  4  0  0  0  0
  2  3  4  0  0  0  0
  3  4  4  0  0  0  0
  4  5  4  0  0  0  0
  5  6  4  0  0  0  0
  6  1  4  0  0  0  0
  7  8  4  0  0  0  0
  8  9  4  0  0  0  0
  9 10  4  0  0  0  0
 10 11  4  0  0  0  0
 11 12  4  0  0  0  0
 12  7  4  0  0  0  0
 13 14  4  0  0  0  0
 14 15  4  0  0  0  0
 15 16  4  0  0  0  0
 16 17  4  0  0  0  0
 17 18  4  0  0  0  0
 18 13  4  0  0  0  0
 19 20  4  0  0  0  0
 20 21  4  0  0  0  0
 21 22  4  0  0  0  0
 22 23  4  0  0  0  0
 23 24  4  0  0  0  0
 24 19  4  0  0  0  0
 25 26  4  0  0  0  0
 26 27  4  0  0  0  0
 27 28  4  0  0  0  0
 28 29  4  0  0  0  0
 29 30  4  0  0  0  0
 30 25  4  0  0  0  0
 16  6  1  0  0  0  0
 11  5  1  0  0  0  0
 23  3  1  0  0  0  0
 31 32  4  0  0  0  0
 32 33  4  0  0  0  0
 33 34  4  0  0  0  0
 34 35  4  0  0  0  0
```

35 36 4 0 0 0 0
36 31 4 0 0 0 0
35 1 1 0 0 0 0
28 2 1 0 0 0 0
4 37 1 0 0 0 0
7 38 1 0 0 0 0
8 39 1 0 0 0 0
9 40 1 0 0 0 0
10 41 1 0 0 0 0
12 42 1 0 0 0 0
13 43 1 0 0 0 0
14 44 1 0 0 0 0
15 45 1 0 0 0 0
17 46 1 0 0 0 0
18 47 1 0 0 0 0
19 48 1 0 0 0 0
20 49 1 0 0 0 0
21 50 1 0 0 0 0
22 51 1 0 0 0 0
24 52 1 0 0 0 0
25 53 1 0 0 0 0
26 54 1 0 0 0 0
27 55 1 0 0 0 0
29 56 1 0 0 0 0
30 57 1 0 0 0 0
31 58 1 0 0 0 0
32 59 1 0 0 0 0
33 60 1 0 0 0 0
34 61 1 0 0 0 0
36 62 1 0 0 0 0
M END
> <Alias name>
Pentaphenylbenzene

> <deltaH°(c)>   kJ/mol
-18309

$$$$
4,4'-Bis-(N-carbazolyl) -1,1'-biphenyl
ChemBrain IXL

62 69 0 0 0 0 0 0 0 0 1 V2000
12.8783   9.6587   -0.2146 C   0 0 0 0 0 0 0 0 0 0 0 0
11.7985   10.5304   -0.0107 C   0 0 0 0 0 0 0 0 0 0 0 0
12.0611   11.9044   0.1089 C   0 0 0 0 0 0 0 0 0 0 0 0
13.3831   12.3981   0.0299 C   0 0 0 0 0 0 0 0 0 0 0 0
14.4833   11.5321   -0.1783 C   0 0 0 0 0 0 0 0 0 0 0 0
14.1994   10.1511   -0.3008 C   0 0 0 0 0 0 0 0 0 0 0 0
19.3169   11.7050   -0.2468 C   0 0 0 0 0 0 0 0 0 0 0 0
19.5781   10.3563   -0.0584 C   0 0 0 0 0 0 0 0 0 0 0 0
18.5135   9.4761   0.0807 C   0 0 0 0 0 0 0 0 0 0 0 0
17.2138   9.9668   0.0274 C   0 0 0 0 0 0 0 0 0 0 0 0
17.0160   11.3328   -0.1695 C   0 0 0 0 0 0 0 0 0 0 0 0
18.0014   12.1208   -0.2904 C   0 0 0 0 0 0 0 0 0 0 0 0
8.7940   8.1881   0.6163 C   0 0 0 0 0 0 0 0 0 0 0 0
7.6807   8.9811   0.2513 C   0 0 0 0 0 0 0 0 0 0 0 0
7.9464   10.2952   -0.2020 C   0 0 0 0 0 0 0 0 0 0 0 0
9.2666   10.7930   -0.2828 C   0 0 0 0 0 0 0 0 0 0 0 0
10.3608   9.9912   0.0803 C   0 0 0 0 0 0 0 0 0 0 0 0
10.1133   8.6851   0.5284 C   0 0 0 0 0 0 0 0 0 0 0 0
17.5409   15.7342   -0.7913 C   0 0 0 0 0 0 0 0 0 0 0 0
18.2403   14.5481   -0.6282 C   0 0 0 0 0 0 0 0 0 0 0 0
17.5245   13.3790   -0.4636 C   0 0 0 0 0 0 0 0 0 0 0 0

```
16.2579   13.3316   -0.4506 C   0  0  0  0  0  0  0  0  0  0  0  0  0
15.4981   14.4894   -0.6149 C   0  0  0  0  0  0  0  0  0  0  0  0  0
16.1525   15.7050   -0.7851 C   0  0  0  0  0  0  0  0  0  0  0  0  0
5.9226    7.1658    0.4567 C   0  0  0  0  0  0  0  0  0  0  0  0  0
6.6917    6.0045    0.5173 C   0  0  0  0  0  0  0  0  0  0  0  0  0
6.0480    4.7787    0.6471 C   0  0  0  0  0  0  0  0  0  0  0  0  0
4.6611    4.7424    0.7083 C   0  0  0  0  0  0  0  0  0  0  0  0  0
3.9523    5.9321    0.6371 C   0  0  0  0  0  0  0  0  0  0  0  0  0
4.6580    7.1115    0.5094 C   0  0  0  0  0  0  0  0  0  0  0  0  0
3.6431   11.0354    0.2239 C   0  0  0  0  0  0  0  0  0  0  0  0  0
4.9464   10.5487    0.2234 C   0  0  0  0  0  0  0  0  0  0  0  0  0
5.1516    9.1729    0.3241 C   0  0  0  0  0  0  0  0  0  0  0  0  0
4.1723    8.3754    0.4222 C   0  0  0  0  0  0  0  0  0  0  0  0  0
2.8542    8.7864    0.4303 C   0  0  0  0  0  0  0  0  0  0  0  0  0
2.5842   10.1428    0.3284 C   0  0  0  0  0  0  0  0  0  0  0  0  0
6.2761    8.4493    0.3416 N   0  0  0  0  0  0  0  0  0  0  0  0  0
15.8953   12.0568   -0.2653 N   0  0  0  0  0  0  0  0  0  0  0  0  0
12.6959    8.5945   -0.3072 H   0  0  0  0  0  0  0  0  0  0  0  0  0
11.2395   12.5946    0.2639 H   0  0  0  0  0  0  0  0  0  0  0  0  0
13.5484   13.4494    0.1309 H   0  0  0  0  0  0  0  0  0  0  0  0  0
14.9969    9.4600   -0.4623 H   0  0  0  0  0  0  0  0  0  0  0  0  0
20.1267   12.4155   -0.3569 H   0  0  0  0  0  0  0  0  0  0  0  0  0
20.5983    9.9946   -0.0203 H   0  0  0  0  0  0  0  0  0  0  0  0  0
18.6930    8.4179    0.2294 H   0  0  0  0  0  0  0  0  0  0  0  0  0
16.3851    9.2972    0.1375 H   0  0  0  0  0  0  0  0  0  0  0  0  0
8.6405    7.1904    0.9674 H   0  0  0  0  0  0  0  0  0  0  0  0  0
7.1352   10.9302   -0.4914 H   0  0  0  0  0  0  0  0  0  0  0  0  0
9.4371   11.8062   -0.6293 H   0  0  0  0  0  0  0  0  0  0  0  0  0
10.9448    8.0504    0.8106 H   0  0  0  0  0  0  0  0  0  0  0  0  0
18.0701   16.6705   -0.9219 H   0  0  0  0  0  0  0  0  0  0  0  0  0
19.3232   14.5392   -0.6304 H   0  0  0  0  0  0  0  0  0  0  0  0  0
14.4269   14.4545   -0.6121 H   0  0  0  0  0  0  0  0  0  0  0  0  0
15.5848   16.6193   -0.9117 H   0  0  0  0  0  0  0  0  0  0  0  0  0
7.7615    6.0447    0.4652 H   0  0  0  0  0  0  0  0  0  0  0  0  0
6.6229    3.8618    0.6998 H   0  0  0  0  0  0  0  0  0  0  0  0  0
4.1402    3.7981    0.8099 H   0  0  0  0  0  0  0  0  0  0  0  0  0
2.8702    5.9353    0.6803 H   0  0  0  0  0  0  0  0  0  0  0  0  0
3.4562   12.0998    0.1437 H   0  0  0  0  0  0  0  0  0  0  0  0  0
5.7722   11.2284    0.1469 H   0  0  0  0  0  0  0  0  0  0  0  0  0
2.0494    8.0666    0.5146 H   0  0  0  0  0  0  0  0  0  0  0  0  0
1.5616   10.5010    0.3303 H   0  0  0  0  0  0  0  0  0  0  0  0  0
1  2  4  0  0  0  0
2  3  4  0  0  0  0
3  4  4  0  0  0  0
4  5  4  0  0  0  0
5  6  4  0  0  0  0
6  1  4  0  0  0  0
7  8  4  0  0  0  0
8  9  4  0  0  0  0
9 10  4  0  0  0  0
10 11  4  0  0  0  0
11 12  4  0  0  0  0
12  7  4  0  0  0  0
13 14  4  0  0  0  0
14 15  4  0  0  0  0
15 16  4  0  0  0  0
16 17  4  0  0  0  0
17 18  4  0  0  0  0
18 13  4  0  0  0  0
19 20  4  0  0  0  0
20 21  4  0  0  0  0
21 22  4  0  0  0  0
22 23  4  0  0  0  0
```

```
 23 24  4  0  0  0  0
 24 19  4  0  0  0  0
 25 26  4  0  0  0  0
 26 27  4  0  0  0  0
 27 28  4  0  0  0  0
 28 29  4  0  0  0  0
 29 30  4  0  0  0  0
 30 25  4  0  0  0  0
 31 32  4  0  0  0  0
 32 33  4  0  0  0  0
 33 34  4  0  0  0  0
 34 35  4  0  0  0  0
 35 36  4  0  0  0  0
 36 31  4  0  0  0  0
 17  2  1  0  0  0  0
 14 37  1  0  0  0  0
  5 38  1  0  0  0  0
 25 37  1  0  0  0  0
 33 37  1  0  0  0  0
 22 38  1  0  0  0  0
 11 38  1  0  0  0  0
 12 21  1  0  0  0  0
 30 34  1  0  0  0  0
  1 39  1  0  0  0  0
  3 40  1  0  0  0  0
  4 41  1  0  0  0  0
  6 42  1  0  0  0  0
  7 43  1  0  0  0  0
  8 44  1  0  0  0  0
  9 45  1  0  0  0  0
 10 46  1  0  0  0  0
 13 47  1  0  0  0  0
 15 48  1  0  0  0  0
 16 49  1  0  0  0  0
 18 50  1  0  0  0  0
 19 51  1  0  0  0  0
 20 52  1  0  0  0  0
 23 53  1  0  0  0  0
 24 54  1  0  0  0  0
 26 55  1  0  0  0  0
 27 56  1  0  0  0  0
 28 57  1  0  0  0  0
 29 58  1  0  0  0  0
 31 59  1  0  0  0  0
 32 60  1  0  0  0  0
 35 61  1  0  0  0  0
 36 62  1  0  0  0  0
M  END
>  <Alias name>
4,4'-Bis-(N-carbazolyl) -1,1'-biphenyl

>  <deltaH°(c)>   kJ/mol
-18114

$$$$
Tri-n-nonylamine
  ChemBrain IXL

 85 84  0  0  0  0  0  0  0  0  1 V2000
    5.5166    4.9727   -0.3108 C   0  0  0  0  0  0  0  0  0  0  0  0
    6.9664    4.8768    0.2031 C   0  0  0  0  0  0  0  0  0  0  0  0
    7.7642    6.1046   -0.2774 C   0  0  0  0  0  0  0  0  0  0  0  0
    9.2138    6.0088    0.2368 C   0  0  0  0  0  0  0  0  0  0  0  0
```

10.0115	7.2369	-0.2430	C	0	0	0	0	0	0	0	0	0	0	0	0
11.4635	7.1397	0.2728	C	0	0	0	0	0	0	0	0	0	0	0	0
12.2485	8.2927	-0.2181	N	0	0	0	0	0	0	0	0	0	0	0	0
4.7187	3.7450	0.1700	C	0	0	0	0	0	0	0	0	0	0	0	0
3.2688	3.8411	-0.3436	C	0	0	0	0	0	0	0	0	0	0	0	0
2.4708	2.6136	0.1372	C	0	0	0	0	0	0	0	0	0	0	0	0
12.7395	15.7825	-0.3103	C	0	0	0	0	0	0	0	0	0	0	0	0
11.9312	14.5750	0.2032	C	0	0	0	0	0	0	0	0	0	0	0	0
12.5958	13.2701	-0.2771	C	0	0	0	0	0	0	0	0	0	0	0	0
11.7878	12.0626	0.2368	C	0	0	0	0	0	0	0	0	0	0	0	0
12.4527	10.7579	-0.2429	C	0	0	0	0	0	0	0	0	0	0	0	0
11.6423	9.5490	0.2726	C	0	0	0	0	0	0	0	0	0	0	0	0
12.0751	17.0874	0.1704	C	0	0	0	0	0	0	0	0	0	0	0	0
12.8835	18.2951	-0.3429	C	0	0	0	0	0	0	0	0	0	0	0	0
12.2193	19.5999	0.1378	C	0	0	0	0	0	0	0	0	0	0	0	0
18.4899	4.1232	-0.3088	C	0	0	0	0	0	0	0	0	0	0	0	0
17.8479	5.4268	0.2047	C	0	0	0	0	0	0	0	0	0	0	0	0
16.3856	5.5035	-0.2759	C	0	0	0	0	0	0	0	0	0	0	0	0
15.7436	6.8068	0.2379	C	0	0	0	0	0	0	0	0	0	0	0	0
14.2813	6.8833	-0.2422	C	0	0	0	0	0	0	0	0	0	0	0	0
13.6394	8.1895	0.2731	C	0	0	0	0	0	0	0	0	0	0	0	0
19.9520	4.0464	0.1722	C	0	0	0	0	0	0	0	0	0	0	0	0
20.5941	2.7427	-0.3411	C	0	0	0	0	0	0	0	0	0	0	0	0
22.0561	2.6658	0.1399	C	0	0	0	0	0	0	0	0	0	0	0	0
5.5185	5.0007	-1.3934	H	0	0	0	0	0	0	0	0	0	0	0	0
5.0567	5.8750	0.0728	H	0	0	0	0	0	0	0	0	0	0	0	0
6.9645	4.8486	1.2857	H	0	0	0	0	0	0	0	0	0	0	0	0
7.4264	3.9746	-0.1807	H	0	0	0	0	0	0	0	0	0	0	0	0
7.7664	6.1327	-1.3601	H	0	0	0	0	0	0	0	0	0	0	0	0
7.3040	7.0068	0.1062	H	0	0	0	0	0	0	0	0	0	0	0	0
9.2116	5.9804	1.3195	H	0	0	0	0	0	0	0	0	0	0	0	0
9.6742	5.1068	-0.1470	H	0	0	0	0	0	0	0	0	0	0	0	0
10.0141	7.2648	-1.3256	H	0	0	0	0	0	0	0	0	0	0	0	0
9.5501	8.1369	0.1402	H	0	0	0	0	0	0	0	0	0	0	0	0
11.4621	7.1383	1.3559	H	0	0	0	0	0	0	0	0	0	0	0	0
11.9082	6.2224	-0.0874	H	0	0	0	0	0	0	0	0	0	0	0	0
4.7170	3.7171	1.2527	H	0	0	0	0	0	0	0	0	0	0	0	0
5.1786	2.8427	-0.2137	H	0	0	0	0	0	0	0	0	0	0	0	0
3.2704	3.8690	-1.4262	H	0	0	0	0	0	0	0	0	0	0	0	0
2.8089	4.7434	0.0400	H	0	0	0	0	0	0	0	0	0	0	0	0
2.9305	1.7112	-0.2464	H	0	0	0	0	0	0	0	0	0	0	0	0
2.4690	2.5857	1.2199	H	0	0	0	0	0	0	0	0	0	0	0	0
1.4521	2.6813	-0.2238	H	0	0	0	0	0	0	0	0	0	0	0	0
13.7507	15.7296	0.0737	H	0	0	0	0	0	0	0	0	0	0	0	0
12.7632	15.7670	-1.3929	H	0	0	0	0	0	0	0	0	0	0	0	0
10.9200	14.6278	-0.1810	H	0	0	0	0	0	0	0	0	0	0	0	0
11.9073	14.5907	1.2858	H	0	0	0	0	0	0	0	0	0	0	0	0
13.6071	13.2176	0.1069	H	0	0	0	0	0	0	0	0	0	0	0	0
12.6194	13.2543	-1.3597	H	0	0	0	0	0	0	0	0	0	0	0	0
10.7767	12.1150	-0.1475	H	0	0	0	0	0	0	0	0	0	0	0	0
11.7639	12.0787	1.3194	H	0	0	0	0	0	0	0	0	0	0	0	0
13.4626	10.7075	0.1407	H	0	0	0	0	0	0	0	0	0	0	0	0
12.4760	10.7417	-1.3254	H	0	0	0	0	0	0	0	0	0	0	0	0
10.6257	9.6226	-0.0880	H	0	0	0	0	0	0	0	0	0	0	0	0
11.6414	9.5509	1.3557	H	0	0	0	0	0	0	0	0	0	0	0	0
11.0639	17.1404	-0.2138	H	0	0	0	0	0	0	0	0	0	0	0	0
12.0513	17.1028	1.2530	H	0	0	0	0	0	0	0	0	0	0	0	0
13.8947	18.2421	0.0411	H	0	0	0	0	0	0	0	0	0	0	0	0
12.9072	18.2797	-1.4255	H	0	0	0	0	0	0	0	0	0	0	0	0
11.2081	19.6530	-0.2463	H	0	0	0	0	0	0	0	0	0	0	0	0
12.1956	19.6153	1.2204	H	0	0	0	0	0	0	0	0	0	0	0	0
12.7875	20.4482	-0.2230	H	0	0	0	0	0	0	0	0	0	0	0	0
18.4648	4.1106	-1.3914	H	0	0	0	0	0	0	0	0	0	0	0	0

```
  17.9385    3.2738    0.0750 H   0  0  0  0  0  0  0  0  0  0  0  0
  17.8731    5.4395    1.2873 H   0  0  0  0  0  0  0  0  0  0  0  0
  18.3991    6.2763   -0.1792 H   0  0  0  0  0  0  0  0  0  0  0  0
  16.3604    5.4910   -1.3586 H   0  0  0  0  0  0  0  0  0  0  0  0
  15.8345    4.6539    0.1078 H   0  0  0  0  0  0  0  0  0  0  0  0
  15.7692    6.8194    1.3205 H   0  0  0  0  0  0  0  0  0  0  0  0
  16.2946    7.6565   -0.1461 H   0  0  0  0  0  0  0  0  0  0  0  0
  14.2561    6.8713   -1.3248 H   0  0  0  0  0  0  0  0  0  0  0  0
  13.7327    6.0339    0.1412 H   0  0  0  0  0  0  0  0  0  0  0  0
  13.6411    8.1893    1.3562 H   0  0  0  0  0  0  0  0  0  0  0  0
  14.2115    9.0332   -0.0872 H   0  0  0  0  0  0  0  0  0  0  0  0
  19.9770    4.0592    1.2548 H   0  0  0  0  0  0  0  0  0  0  0  0
  20.5034    4.8959   -0.2117 H   0  0  0  0  0  0  0  0  0  0  0  0
  20.5692    2.7300   -1.4237 H   0  0  0  0  0  0  0  0  0  0  0  0
  20.0427    1.8933    0.0427 H   0  0  0  0  0  0  0  0  0  0  0  0
  22.6076    3.5151   -0.2440 H   0  0  0  0  0  0  0  0  0  0  0  0
  22.5070    1.7497   -0.2209 H   0  0  0  0  0  0  0  0  0  0  0  0
  22.0811    2.6784    1.2225 H   0  0  0  0  0  0  0  0  0  0  0  0
  1  2  1  0  0  0  0
  2  3  1  0  0  0  0
  3  4  1  0  0  0  0
  4  5  1  0  0  0  0
  5  6  1  0  0  0  0
  6  7  1  0  0  0  0
  1  8  1  0  0  0  0
  8  9  1  0  0  0  0
  9 10  1  0  0  0  0
 11 12  1  0  0  0  0
 12 13  1  0  0  0  0
 13 14  1  0  0  0  0
 14 15  1  0  0  0  0
 15 16  1  0  0  0  0
 11 17  1  0  0  0  0
 17 18  1  0  0  0  0
 18 19  1  0  0  0  0
 20 21  1  0  0  0  0
 21 22  1  0  0  0  0
 22 23  1  0  0  0  0
 23 24  1  0  0  0  0
 24 25  1  0  0  0  0
 20 26  1  0  0  0  0
 26 27  1  0  0  0  0
 27 28  1  0  0  0  0
 16  7  1  0  0  0  0
 25  7  1  0  0  0  0
  1 29  1  0  0  0  0
  1 30  1  0  0  0  0
  2 31  1  0  0  0  0
  2 32  1  0  0  0  0
  3 33  1  0  0  0  0
  3 34  1  0  0  0  0
  4 35  1  0  0  0  0
  4 36  1  0  0  0  0
  5 37  1  0  0  0  0
  5 38  1  0  0  0  0
  6 39  1  0  0  0  0
  6 40  1  0  0  0  0
  8 41  1  0  0  0  0
  8 42  1  0  0  0  0
  9 43  1  0  0  0  0
  9 44  1  0  0  0  0
 10 45  1  0  0  0  0
 10 46  1  0  0  0  0
```

```
 10 47  1  0  0  0  0
 11 48  1  0  0  0  0
 11 49  1  0  0  0  0
 12 50  1  0  0  0  0
 12 51  1  0  0  0  0
 13 52  1  0  0  0  0
 13 53  1  0  0  0  0
 14 54  1  0  0  0  0
 14 55  1  0  0  0  0
 15 56  1  0  0  0  0
 15 57  1  0  0  0  0
 16 58  1  0  0  0  0
 16 59  1  0  0  0  0
 17 60  1  0  0  0  0
 17 61  1  0  0  0  0
 18 62  1  0  0  0  0
 18 63  1  0  0  0  0
 19 64  1  0  0  0  0
 19 65  1  0  0  0  0
 19 66  1  0  0  0  0
 20 67  1  0  0  0  0
 20 68  1  0  0  0  0
 21 69  1  0  0  0  0
 21 70  1  0  0  0  0
 22 71  1  0  0  0  0
 22 72  1  0  0  0  0
 23 73  1  0  0  0  0
 23 74  1  0  0  0  0
 24 75  1  0  0  0  0
 24 76  1  0  0  0  0
 25 77  1  0  0  0  0
 25 78  1  0  0  0  0
 26 79  1  0  0  0  0
 26 80  1  0  0  0  0
 27 81  1  0  0  0  0
 27 82  1  0  0  0  0
 28 83  1  0  0  0  0
 28 84  1  0  0  0  0
 28 85  1  0  0  0  0
M  END
>  <Alias name>
Tri-n-nonylamine

>  <deltaH°(c)>   kJ/mol
-18109

$$$$
Etioporphyrin II
  ChemBrain IXL

 74 78  0  0  0  0  0  0  0  0  1 V2000
   18.9408    9.6253    0.2944 C   0  0  0  0  0  0  0  0  0  0  0  0
   20.1598    8.9511    0.2962 C   0  0  0  0  0  0  0  0  0  0  0  0
   18.7456   10.9751    0.2997 C   0  0  0  0  0  0  0  0  0  0  0  0
   17.9313    8.6823    0.2860 N   0  0  0  0  0  0  0  0  0  0  0  0
   19.8931    7.5789    0.2884 C   0  0  0  0  0  0  0  0  0  0  0  0
   21.5564    9.6023    0.3051 C   0  0  0  0  0  0  0  0  0  0  0  0
   17.4514   11.4405    0.2972 C   0  0  0  0  0  0  0  0  0  0  0  0
   18.5049    7.4064    0.2821 C   0  0  0  0  0  0  0  0  0  0  0  0
   20.9455    6.4529    0.2870 C   0  0  0  0  0  0  0  0  0  0  0  0
   22.1494    9.5643   -1.1162 C   0  0  0  0  0  0  0  0  0  0  0  0
   17.0633   12.8548    0.3022 C   0  0  0  0  0  0  0  0  0  0  0  0
   16.2978   10.6870    0.2899 N   0  0  0  0  0  0  0  0  0  0  0  0
```

```
17.9184    6.1407    0.2737 C   0  0  0  0  0  0  0  0  0  0  0  0
15.7608   12.9206    0.2978 C   0  0  0  0  0  0  0  0  0  0  0  0
18.0286   14.0564    0.3110 C   0  0  0  0  0  0  0  0  0  0  0  0
15.2254   11.5466    0.2900 C   0  0  0  0  0  0  0  0  0  0  0  0
16.5467    5.8873    0.2677 C   0  0  0  0  0  0  0  0  0  0  0  0
14.9230   14.2145    0.3003 C   0  0  0  0  0  0  0  0  0  0  0  0
18.0573   14.7022   -1.0875 C   0  0  0  0  0  0  0  0  0  0  0  0
13.8537   11.2931    0.2843 C   0  0  0  0  0  0  0  0  0  0  0  0
16.0111    4.5133    0.2588 C   0  0  0  0  0  0  0  0  0  0  0  0
15.4745    6.7471    0.2683 N   0  0  0  0  0  0  0  0  0  0  0  0
13.2671   10.0273    0.2767 C   0  0  0  0  0  0  0  0  0  0  0  0
14.7087    4.5793    0.2545 C   0  0  0  0  0  0  0  0  0  0  0  0
16.8483    3.2194    0.2551 C   0  0  0  0  0  0  0  0  0  0  0  0
14.3206    5.9938    0.2605 C   0  0  0  0  0  0  0  0  0  0  0  0
13.8405    8.7513    0.2723 N   0  0  0  0  0  0  0  0  0  0  0  0
11.8789    9.8551    0.2718 C   0  0  0  0  0  0  0  0  0  0  0  0
13.7441    3.3772    0.2449 C   0  0  0  0  0  0  0  0  0  0  0  0
17.3430    2.9324   -1.1753 C   0  0  0  0  0  0  0  0  0  0  0  0
13.0262    6.4590    0.2585 C   0  0  0  0  0  0  0  0  0  0  0  0
12.8309    7.8086    0.2647 C   0  0  0  0  0  0  0  0  0  0  0  0
11.6120    8.4830    0.2646 C   0  0  0  0  0  0  0  0  0  0  0  0
10.8267   10.9813    0.2739 C   0  0  0  0  0  0  0  0  0  0  0  0
10.2153    7.8320    0.2577 C   0  0  0  0  0  0  0  0  0  0  0  0
 9.6308    7.8865   -1.1667 C   0  0  0  0  0  0  0  0  0  0  0  0
16.9137    8.9204    0.2832 H   0  0  0  0  0  0  0  0  0  0  0  0
14.8580    8.5130    0.2742 H   0  0  0  0  0  0  0  0  0  0  0  0
19.5845   11.6598    0.3056 H   0  0  0  0  0  0  0  0  0  0  0  0
22.2041    9.0581    0.9811 H   0  0  0  0  0  0  0  0  0  0  0  0
21.4735   10.6305    0.6343 H   0  0  0  0  0  0  0  0  0  0  0  0
20.8275    5.8498   -0.6046 H   0  0  0  0  0  0  0  0  0  0  0  0
20.8102    5.8315    1.1636 H   0  0  0  0  0  0  0  0  0  0  0  0
21.9379    6.8860    0.3012 H   0  0  0  0  0  0  0  0  0  0  0  0
23.1309   10.0218   -1.1101 H   0  0  0  0  0  0  0  0  0  0  0  0
21.5017   10.1086   -1.7923 H   0  0  0  0  0  0  0  0  0  0  0  0
22.2324    8.5359   -1.4454 H   0  0  0  0  0  0  0  0  0  0  0  0
18.5822    5.2850    0.2718 H   0  0  0  0  0  0  0  0  0  0  0  0
17.6918   14.7848    1.0381 H   0  0  0  0  0  0  0  0  0  0  0  0
19.0234   13.7186    0.5730 H   0  0  0  0  0  0  0  0  0  0  0  0
14.1048   14.1133    1.0025 H   0  0  0  0  0  0  0  0  0  0  0  0
15.5480   15.0492    0.5923 H   0  0  0  0  0  0  0  0  0  0  0  0
14.5272   14.3899   -0.6923 H   0  0  0  0  0  0  0  0  0  0  0  0
17.0624   15.0404   -1.3495 H   0  0  0  0  0  0  0  0  0  0  0  0
18.7355   15.5464   -1.0814 H   0  0  0  0  0  0  0  0  0  0  0  0
18.3941   13.9738   -1.8147 H   0  0  0  0  0  0  0  0  0  0  0  0
13.1899   12.1488    0.2861 H   0  0  0  0  0  0  0  0  0  0  0  0
16.2381    2.3933    0.5986 H   0  0  0  0  0  0  0  0  0  0  0  0
17.6991    3.3380    0.9144 H   0  0  0  0  0  0  0  0  0  0  0  0
13.8467    2.8398   -0.6897 H   0  0  0  0  0  0  0  0  0  0  0  0
13.9825    2.7166    1.0692 H   0  0  0  0  0  0  0  0  0  0  0  0
12.7258    3.7302    0.3483 H   0  0  0  0  0  0  0  0  0  0  0  0
17.9314    2.0233   -1.1781 H   0  0  0  0  0  0  0  0  0  0  0  0
16.4920    2.8137   -1.8346 H   0  0  0  0  0  0  0  0  0  0  0  0
17.9533    3.7585   -1.5188 H   0  0  0  0  0  0  0  0  0  0  0  0
12.1875    5.7743    0.2523 H   0  0  0  0  0  0  0  0  0  0  0  0
10.9497   11.5916   -0.6122 H   0  0  0  0  0  0  0  0  0  0  0  0
 9.8342   10.5483    0.2790 H   0  0  0  0  0  0  0  0  0  0  0  0
10.9572   11.5954    1.1562 H   0  0  0  0  0  0  0  0  0  0  0  0
10.2961    6.8000    0.5755 H   0  0  0  0  0  0  0  0  0  0  0  0
 9.5636    8.3686    0.9360 H   0  0  0  0  0  0  0  0  0  0  0  0
 8.6492    7.4293   -1.1717 H   0  0  0  0  0  0  0  0  0  0  0  0
 9.5499    8.9187   -1.4844 H   0  0  0  0  0  0  0  0  0  0  0  0
10.2825    7.3500   -1.8451 H   0  0  0  0  0  0  0  0  0  0  0  0
1  2  4  0  0  0  0
```

1	3	4	0	0	0	0
1	4	1	0	0	0	0
2	5	4	0	0	0	0
2	6	1	0	0	0	0
3	7	4	0	0	0	0
4	8	1	0	0	0	0
5	9	1	0	0	0	0
5	8	4	0	0	0	0
6	10	1	0	0	0	0
7	11	1	0	0	0	0
7	12	4	0	0	0	0
8	13	4	0	0	0	0
11	14	2	0	0	0	0
11	15	1	0	0	0	0
12	16	4	0	0	0	0
13	17	4	0	0	0	0
14	18	1	0	0	0	0
14	16	1	0	0	0	0
15	19	1	0	0	0	0
16	20	4	0	0	0	0
17	21	1	0	0	0	0
17	22	4	0	0	0	0
20	23	4	0	0	0	0
21	24	2	0	0	0	0
21	25	1	0	0	0	0
22	26	4	0	0	0	0
23	27	1	0	0	0	0
23	28	4	0	0	0	0
24	29	1	0	0	0	0
24	26	1	0	0	0	0
25	30	1	0	0	0	0
26	31	4	0	0	0	0
27	32	1	0	0	0	0
28	33	4	0	0	0	0
28	34	1	0	0	0	0
31	32	4	0	0	0	0
32	33	4	0	0	0	0
33	35	1	0	0	0	0
35	36	1	0	0	0	0
4	37	1	0	0	0	0
27	38	1	0	0	0	0
3	39	1	0	0	0	0
6	40	1	0	0	0	0
6	41	1	0	0	0	0
9	42	1	0	0	0	0
9	43	1	0	0	0	0
9	44	1	0	0	0	0
10	45	1	0	0	0	0
10	46	1	0	0	0	0
10	47	1	0	0	0	0
13	48	1	0	0	0	0
15	49	1	0	0	0	0
15	50	1	0	0	0	0
18	51	1	0	0	0	0
18	52	1	0	0	0	0
18	53	1	0	0	0	0
19	54	1	0	0	0	0
19	55	1	0	0	0	0
19	56	1	0	0	0	0
20	57	1	0	0	0	0
25	58	1	0	0	0	0
25	59	1	0	0	0	0
29	60	1	0	0	0	0

```
 29 61  1  0  0  0  0
 29 62  1  0  0  0  0
 30 63  1  0  0  0  0
 30 64  1  0  0  0  0
 30 65  1  0  0  0  0
 31 66  1  0  0  0  0
 34 67  1  0  0  0  0
 34 68  1  0  0  0  0
 34 69  1  0  0  0  0
 35 70  1  0  0  0  0
 35 71  1  0  0  0  0
 36 72  1  0  0  0  0
 36 73  1  0  0  0  0
 36 74  1  0  0  0  0
M  END
>  <Alias name>
Etioporphyrin II

>  <deltaH°(c)>   kJ/mol
-18053

$$$$
3,4-Diethyl-3,4-bis(4-t-butylphenyl)-hexane
  ChemBrain IXL

 76 77  0  0  0  0  0  0  0  0  1 V2000
    5.5578    7.7412   -0.5955 C   0  0  0  0  0  0  0  0  0  0  0  0
    5.8871    9.0555   -0.2640 C   0  0  0  0  0  0  0  0  0  0  0  0
    5.6737    6.7312    0.3596 C   0  0  0  0  0  0  0  0  0  0  0  0
    6.3251    9.3609    1.0247 C   0  0  0  0  0  0  0  0  0  0  0  0
    6.4310    8.3525    1.9828 C   0  0  0  0  0  0  0  0  0  0  0  0
    6.6775   10.7545    1.3754 C   0  0  0  0  0  0  0  0  0  0  0  0
    6.1014    7.0383    1.6513 C   0  0  0  0  0  0  0  0  0  0  0  0
    5.3438    5.3338    0.0024 C   0  0  0  0  0  0  0  0  0  0  0  0
    6.0198   11.1282    2.7180 C   0  0  0  0  0  0  0  0  0  0  0  0
    8.2081   10.8814    1.5003 C   0  0  0  0  0  0  0  0  0  0  0  0
    6.1714   11.7052    0.2733 C   0  0  0  0  0  0  0  0  0  0  0  0
    6.5227    4.7108   -0.7673 C   0  0  0  0  0  0  0  0  0  0  0  0
    4.0829    5.3133   -0.8834 C   0  0  0  0  0  0  0  0  0  0  0  0
    7.7592    4.8459    0.0341 C   0  0  0  0  0  0  0  0  0  0  0  0
    6.2375    3.2185   -1.0254 C   0  0  0  0  0  0  0  0  0  0  0  0
    6.7003    5.4404   -2.1131 C   0  0  0  0  0  0  0  0  0  0  0  0
    3.5096    3.8835   -0.9278 C   0  0  0  0  0  0  0  0  0  0  0  0
    3.0279    6.2715   -0.2974 C   0  0  0  0  0  0  0  0  0  0  0  0
    7.7986    4.3626    1.3420 C   0  0  0  0  0  0  0  0  0  0  0  0
    8.8849    5.4572   -0.5179 C   0  0  0  0  0  0  0  0  0  0  0  0
    8.9552    4.5141    2.1068 C   0  0  0  0  0  0  0  0  0  0  0  0
   10.0763    5.1384    1.5596 C   0  0  0  0  0  0  0  0  0  0  0  0
   10.0417    5.6087    0.2468 C   0  0  0  0  0  0  0  0  0  0  0  0
   11.3023    5.3018    2.3719 C   0  0  0  0  0  0  0  0  0  0  0  0
    5.8504    2.5327    0.2991 C   0  0  0  0  0  0  0  0  0  0  0  0
    7.6238    4.6131   -3.0282 C   0  0  0  0  0  0  0  0  0  0  0  0
   12.5277    5.3840    1.4413 C   0  0  0  0  0  0  0  0  0  0  0  0
   11.4559    4.0954    3.3183 C   0  0  0  0  0  0  0  0  0  0  0  0
   11.1997    6.5960    3.2019 C   0  0  0  0  0  0  0  0  0  0  0  0
    2.6932    5.8467    1.1456 C   0  0  0  0  0  0  0  0  0  0  0  0
    5.2121    7.5049   -1.5943 H   0  0  0  0  0  0  0  0  0  0  0  0
    5.8028    9.8388   -1.0071 H   0  0  0  0  0  0  0  0  0  0  0  0
    6.7689    8.5901    2.9839 H   0  0  0  0  0  0  0  0  0  0  0  0
    6.1777    6.2564    2.3968 H   0  0  0  0  0  0  0  0  0  0  0  0
    5.1604    4.7640    0.9049 H   0  0  0  0  0  0  0  0  0  0  0  0
    4.9442   11.0360    2.6311 H   0  0  0  0  0  0  0  0  0  0  0  0
    6.3778   10.4620    3.4931 H   0  0  0  0  0  0  0  0  0  0  0  0
```

```
6.2751 12.1492 2.9732 H 0 0 0 0 0 0 0 0 0 0 0 0
8.4656 11.8997 1.7640 H 0 0 0 0 0 0 0 0 0 0 0 0
8.6699 10.6256 0.5546 H 0 0 0 0 0 0 0 0 0 0 0 0
8.5646 10.2080 2.2699 H 0 0 0 0 0 0 0 0 0 0 0 0
5.0988 11.5979 0.1686 H 0 0 0 0 0 0 0 0 0 0 0 0
6.4074 12.7274 0.5418 H 0 0 0 0 0 0 0 0 0 0 0 0
6.6522 11.4582 -0.6652 H 0 0 0 0 0 0 0 0 0 0 0 0
4.3410 5.6301 -1.8863 H 0 0 0 0 0 0 0 0 0 0 0 0
5.4226 3.1238 -1.7326 H 0 0 0 0 0 0 0 0 0 0 0 0
7.1239 2.7464 -1.4308 H 0 0 0 0 0 0 0 0 0 0 0 0
5.7347 5.5607 -2.5886 H 0 0 0 0 0 0 0 0 0 0 0 0
7.1414 6.4142 -1.9404 H 0 0 0 0 0 0 0 0 0 0 0 0
4.2017 3.2331 -1.4483 H 0 0 0 0 0 0 0 0 0 0 0 0
3.3642 3.5213 0.0825 H 0 0 0 0 0 0 0 0 0 0 0 0
2.5602 3.8912 -1.4487 H 0 0 0 0 0 0 0 0 0 0 0 0
2.1303 6.2337 -0.9022 H 0 0 0 0 0 0 0 0 0 0 0 0
3.4192 7.2814 -0.2949 H 0 0 0 0 0 0 0 0 0 0 0 0
6.9313 3.8697 1.7635 H 0 0 0 0 0 0 0 0 0 0 0 0
8.8609 5.8141 -1.5402 H 0 0 0 0 0 0 0 0 0 0 0 0
8.9829 4.1471 3.1253 H 0 0 0 0 0 0 0 0 0 0 0 0
10.9128 6.0914 -0.1787 H 0 0 0 0 0 0 0 0 0 0 0 0
6.6808 2.5906 0.9918 H 0 0 0 0 0 0 0 0 0 0 0 0
4.9890 3.0317 0.7257 H 0 0 0 0 0 0 0 0 0 0 0 0
5.6091 1.4939 0.1107 H 0 0 0 0 0 0 0 0 0 0 0 0
8.5725 4.4508 -2.5318 H 0 0 0 0 0 0 0 0 0 0 0 0
7.7882 5.1491 -3.9548 H 0 0 0 0 0 0 0 0 0 0 0 0
7.1597 3.6580 -3.2411 H 0 0 0 0 0 0 0 0 0 0 0 0
12.4350 6.2503 0.7979 H 0 0 0 0 0 0 0 0 0 0 0 0
12.5814 4.4882 0.8351 H 0 0 0 0 0 0 0 0 0 0 0 0
13.4281 5.4714 2.0368 H 0 0 0 0 0 0 0 0 0 0 0 0
12.3644 4.2044 3.8976 H 0 0 0 0 0 0 0 0 0 0 0 0
11.5063 3.1837 2.7358 H 0 0 0 0 0 0 0 0 0 0 0 0
10.6049 4.0501 3.9865 H 0 0 0 0 0 0 0 0 0 0 0 0
10.3367 6.5394 3.8537 H 0 0 0 0 0 0 0 0 0 0 0 0
12.0957 6.7142 3.7987 H 0 0 0 0 0 0 0 0 0 0 0 0
11.0946 7.4442 2.5367 H 0 0 0 0 0 0 0 0 0 0 0 0
3.5994 5.8378 1.7387 H 0 0 0 0 0 0 0 0 0 0 0 0
1.9882 6.5482 1.5742 H 0 0 0 0 0 0 0 0 0 0 0 0
2.2569 4.8555 1.1381 H 0 0 0 0 0 0 0 0 0 0 0 0
1 2 4 0 0 0 0
3 1 4 0 0 0 0
2 4 4 0 0 0 0
7 3 4 0 0 0 0
3 8 1 0 0 0 0
4 5 4 0 0 0 0
4 6 1 0 0 0 0
5 7 4 0 0 0 0
6 9 1 0 0 0 0
6 10 1 0 0 0 0
6 11 1 0 0 0 0
8 12 1 0 0 0 0
8 13 1 0 0 0 0
12 14 1 0 0 0 0
12 15 1 0 0 0 0
12 16 1 0 0 0 0
13 17 1 0 0 0 0
13 18 1 0 0 0 0
14 19 4 0 0 0 0
20 14 4 0 0 0 0
15 25 1 0 0 0 0
16 26 1 0 0 0 0
18 30 1 0 0 0 0
19 21 4 0 0 0 0
```

23 20 4 0 0 0 0
21 22 4 0 0 0 0
22 23 4 0 0 0 0
22 24 1 0 0 0 0
24 27 1 0 0 0 0
24 28 1 0 0 0 0
24 29 1 0 0 0 0
1 31 1 0 0 0 0
2 32 1 0 0 0 0
5 33 1 0 0 0 0
7 34 1 0 0 0 0
8 35 1 0 0 0 0
9 36 1 0 0 0 0
9 37 1 0 0 0 0
9 38 1 0 0 0 0
10 39 1 0 0 0 0
10 40 1 0 0 0 0
10 41 1 0 0 0 0
11 42 1 0 0 0 0
11 43 1 0 0 0 0
11 44 1 0 0 0 0
13 45 1 0 0 0 0
15 46 1 0 0 0 0
15 47 1 0 0 0 0
16 48 1 0 0 0 0
16 49 1 0 0 0 0
17 50 1 0 0 0 0
17 51 1 0 0 0 0
17 52 1 0 0 0 0
18 53 1 0 0 0 0
18 54 1 0 0 0 0
19 55 1 0 0 0 0
20 56 1 0 0 0 0
21 57 1 0 0 0 0
23 58 1 0 0 0 0
25 59 1 0 0 0 0
25 60 1 0 0 0 0
25 61 1 0 0 0 0
26 62 1 0 0 0 0
26 63 1 0 0 0 0
26 64 1 0 0 0 0
27 65 1 0 0 0 0
27 66 1 0 0 0 0
27 67 1 0 0 0 0
28 68 1 0 0 0 0
28 69 1 0 0 0 0
28 70 1 0 0 0 0
29 71 1 0 0 0 0
29 72 1 0 0 0 0
29 73 1 0 0 0 0
30 74 1 0 0 0 0
30 75 1 0 0 0 0
30 76 1 0 0 0 0
M END
> <Alias name>
3,4-Diethyl-3,4-bis(4-t-butylphenyl)-hexane

> <deltaH°(c)>   kJ/mol
-18041

$$$$
Etioporphin I
ChemBrain IXL

74 78 0 0 0 0 0 0 0 0 1 V2000
    9.6283   5.4146   -0.1942 C   0 0 0 0 0 0 0 0 0 0 0 0
    9.0079   4.0619   -0.1076 C   0 0 0 0 0 0 0 0 0 0 0 0
   10.9961   5.6356   -0.3904 C   0 0 0 0 0 0 0 0 0 0 0 0
    8.6166   6.3349   -0.0470 N   0 0 0 0 0 0 0 0 0 0 0 0
    7.7271   4.2151   0.0772 C   0 0 0 0 0 0 0 0 0 0 0 0
    9.6988   2.6531   -0.2099 C   0 0 0 0 0 0 0 0 0 0 0 0
   11.6120   6.8842   -0.4774 C   0 0 0 0 0 0 0 0 0 0 0 0
    7.4322   5.6502   0.1224 C   0 0 0 0 0 0 0 0 0 0 0 0
    6.6958   3.0791   0.2234 C   0 0 0 0 0 0 0 0 0 0 0 0
   11.2121   2.7756   -0.4299 C   0 0 0 0 0 0 0 0 0 0 0 0
   12.9917   7.0007   -0.6747 C   0 0 0 0 0 0 0 0 0 0 0 0
   11.0956   8.1835   -0.4025 N   0 0 0 0 0 0 0 0 0 0 0 0
    6.1784   6.1818   0.3041 C   0 0 0 0 0 0 0 0 0 0 0 0
   13.3108   8.3593   -0.7198 C   0 0 0 0 0 0 0 0 0 0 0 0
   13.9873   5.8328   -0.8175 C   0 0 0 0 0 0 0 0 0 0 0 0
   12.1334   9.0833   -0.5507 C   0 0 0 0 0 0 0 0 0 0 0 0
    6.0489   7.5373   0.3248 C   0 0 0 0 0 0 0 0 0 0 0 0
   14.7191   8.9522   -0.9212 C   0 0 0 0 0 0 0 0 0 0 0 0
   12.0039   10.4389   -0.5315 C   0 0 0 0 0 0 0 0 0 0 0 0
    4.8720   8.2614   0.4966 C   0 0 0 0 0 0 0 0 0 0 0 0
    7.0866   8.4367   0.1753 N   0 0 0 0 0 0 0 0 0 0 0 0
   15.5221   8.8233   0.3873 C   0 0 0 0 0 0 0 0 0 0 0 0
   10.7501   10.9707   -0.3510 C   0 0 0 0 0 0 0 0 0 0 0 0
    5.1914   9.6200   0.4515 C   0 0 0 0 0 0 0 0 0 0 0 0
    3.4641   7.6689   0.7008 C   0 0 0 0 0 0 0 0 0 0 0 0
    6.5707   9.7362   0.2514 C   0 0 0 0 0 0 0 0 0 0 0 0
   10.4548   12.4056   -0.3088 C   0 0 0 0 0 0 0 0 0 0 0 0
    9.5661   10.2858   -0.1803 N   0 0 0 0 0 0 0 0 0 0 0 0
    4.1964   10.7881   0.5970 C   0 0 0 0 0 0 0 0 0 0 0 0
    2.6507   7.8189   -0.5990 C   0 0 0 0 0 0 0 0 0 0 0 0
    7.1866   10.9849   0.1631 C   0 0 0 0 0 0 0 0 0 0 0 0
    9.1739   12.5588   -0.1240 C   0 0 0 0 0 0 0 0 0 0 0 0
   11.4863   13.5413   -0.4580 C   0 0 0 0 0 0 0 0 0 0 0 0
    8.5541   11.2060   -0.0344 C   0 0 0 0 0 0 0 0 0 0 0 0
    8.4822   13.9675   -0.0244 C   0 0 0 0 0 0 0 0 0 0 0 0
    6.9688   13.8447   0.1944 C   0 0 0 0 0 0 0 0 0 0 0 0
   10.0980   8.4660   -0.2593 H   0 0 0 0 0 0 0 0 0 0 0 0
    8.0839   8.1541   0.0304 H   0 0 0 0 0 0 0 0 0 0 0 0
   11.6242   4.7872   -0.4815 H   0 0 0 0 0 0 0 0 0 0 0 0
    9.2652   2.1074   -1.0386 H   0 0 0 0 0 0 0 0 0 0 0 0
    9.5190   2.1050   0.7065 H   0 0 0 0 0 0 0 0 0 0 0 0
    7.1797   2.2030   0.6371 H   0 0 0 0 0 0 0 0 0 0 0 0
    5.8994   3.3974   0.8846 H   0 0 0 0 0 0 0 0 0 0 0 0
    6.2835   2.8386   -0.7486 H   0 0 0 0 0 0 0 0 0 0 0 0
   11.4018   3.3112   -1.3516 H   0 0 0 0 0 0 0 0 0 0 0 0
   11.6452   1.7853   -0.4960 H   0 0 0 0 0 0 0 0 0 0 0 0
   11.6572   3.3058   0.4027 H   0 0 0 0 0 0 0 0 0 0 0 0
    5.3154   5.5394   0.4276 H   0 0 0 0 0 0 0 0 0 0 0 0
   14.0575   5.3014   0.1234 H   0 0 0 0 0 0 0 0 0 0 0 0
   14.9621   6.2200   -1.0869 H   0 0 0 0 0 0 0 0 0 0 0 0
   13.6417   5.1562   -1.5893 H   0 0 0 0 0 0 0 0 0 0 0 0
   14.6358   9.9972   -1.1921 H   0 0 0 0 0 0 0 0 0 0 0 0
   15.2276   8.4139   -1.7113 H   0 0 0 0 0 0 0 0 0 0 0 0
   12.8669   11.0813   -0.6552 H   0 0 0 0 0 0 0 0 0 0 0 0
   16.5117   9.2396   0.2459 H   0 0 0 0 0 0 0 0 0 0 0 0
   15.6055   7.7780   0.6582 H   0 0 0 0 0 0 0 0 0 0 0 0
   15.0135   9.3615   1.1775 H   0 0 0 0 0 0 0 0 0 0 0 0
    3.5470   6.6199   0.9556 H   0 0 0 0 0 0 0 0 0 0 0 0
    2.9632   8.1967   1.5028 H   0 0 0 0 0 0 0 0 0 0 0 0
    4.1026   11.3024   -0.3513 H   0 0 0 0 0 0 0 0 0 0 0 0
    3.2292   10.4035   0.8962 H   0 0 0 0 0 0 0 0 0 0 0 0

4.5583	11.4791	1.3482	H	0	0	0	0	0	0	0	0	0	0	0	0
1.6613	7.4028	-0.4557	H	0	0	0	0	0	0	0	0	0	0	0	0
2.5676	8.8683	-0.8538	H	0	0	0	0	0	0	0	0	0	0	0	0
3.1515	7.2912	-1.4012	H	0	0	0	0	0	0	0	0	0	0	0	0
6.5584	11.8332	0.2544	H	0	0	0	0	0	0	0	0	0	0	0	0
11.0397	14.4781	-0.1488	H	0	0	0	0	0	0	0	0	0	0	0	0
11.7962	13.6135	-1.4931	H	0	0	0	0	0	0	0	0	0	0	0	0
12.3480	13.3299	0.1628	H	0	0	0	0	0	0	0	0	0	0	0	0
8.9149	14.5147	0.8039	H	0	0	0	0	0	0	0	0	0	0	0	0
8.6627	14.5143	-0.9415	H	0	0	0	0	0	0	0	0	0	0	0	0
6.5249	13.3116	-0.6369	H	0	0	0	0	0	0	0	0	0	0	0	0
6.7785	13.3120	1.1176	H	0	0	0	0	0	0	0	0	0	0	0	0
6.5350	14.8349	0.2568	H	0	0	0	0	0	0	0	0	0	0	0	0

1	2	1	0	0	0	0
1	3	4	0	0	0	0
1	4	4	0	0	0	0
2	5	2	0	0	0	0
2	6	1	0	0	0	0
3	7	4	0	0	0	0
4	8	4	0	0	0	0
5	9	1	0	0	0	0
5	8	1	0	0	0	0
6	10	1	0	0	0	0
7	11	4	0	0	0	0
7	12	1	0	0	0	0
8	13	4	0	0	0	0
11	14	4	0	0	0	0
11	15	1	0	0	0	0
12	16	1	0	0	0	0
13	17	4	0	0	0	0
14	18	1	0	0	0	0
14	16	4	0	0	0	0
16	19	4	0	0	0	0
17	20	4	0	0	0	0
17	21	1	0	0	0	0
18	22	1	0	0	0	0
19	23	4	0	0	0	0
20	24	4	0	0	0	0
20	25	1	0	0	0	0
21	26	1	0	0	0	0
23	27	1	0	0	0	0
23	28	4	0	0	0	0
24	29	1	0	0	0	0
24	26	4	0	0	0	0
25	30	1	0	0	0	0
26	31	4	0	0	0	0
27	32	2	0	0	0	0
27	33	1	0	0	0	0
28	34	4	0	0	0	0
31	34	4	0	0	0	0
32	35	1	0	0	0	0
32	34	1	0	0	0	0
35	36	1	0	0	0	0
12	37	1	0	0	0	0
21	38	1	0	0	0	0
3	39	1	0	0	0	0
6	40	1	0	0	0	0
6	41	1	0	0	0	0
9	42	1	0	0	0	0
9	43	1	0	0	0	0
9	44	1	0	0	0	0
10	45	1	0	0	0	0
10	46	1	0	0	0	0

10 47 1 0 0 0 0
13 48 1 0 0 0 0
15 49 1 0 0 0 0
15 50 1 0 0 0 0
15 51 1 0 0 0 0
18 52 1 0 0 0 0
18 53 1 0 0 0 0
19 54 1 0 0 0 0
22 55 1 0 0 0 0
22 56 1 0 0 0 0
22 57 1 0 0 0 0
25 58 1 0 0 0 0
25 59 1 0 0 0 0
29 60 1 0 0 0 0
29 61 1 0 0 0 0
29 62 1 0 0 0 0
30 63 1 0 0 0 0
30 64 1 0 0 0 0
30 65 1 0 0 0 0
31 66 1 0 0 0 0
33 67 1 0 0 0 0
33 68 1 0 0 0 0
33 69 1 0 0 0 0
35 70 1 0 0 0 0
35 71 1 0 0 0 0
36 72 1 0 0 0 0
36 73 1 0 0 0 0
36 74 1 0 0 0 0
M END
> <Alias name>
Etioporphin I

> <deltaH°(c)>   kJ/mol
-18025

$$$$
Diacylperoxide VIII
ChemBrain IXL

92 91 0 0 0 0 0 0 0 0 1 V2000
24.4431   19.1294   -2.1255 C   0 0 0 0 0 0 0 0 0 0 0 0
25.8596   18.7764   -1.8689 C   0 0 0 0 0 0 0 0 0 0 0 0
26.6984   18.9554   -2.7367 O   0 0 0 0 0 0 0 0 0 0 0 0
26.2558   18.2104   -0.5687 O   0 0 0 0 0 0 0 0 0 0 0 0
16.6378   18.9368   2.0502 C   0 0 0 0 0 0 0 0 0 0 0 0
15.2234   19.3006   1.7975 C   0 0 0 0 0 0 0 0 0 0 0 0
14.3839   19.1188   2.6640 O   0 0 0 0 0 0 0 0 0 0 0 0
14.8301   19.8810   0.5027 O   0 0 0 0 0 0 0 0 0 0 0 0
11.6818   22.0607   -0.0463 C   0 0 0 0 0 0 0 0 0 0 0 0
13.0877   21.6174   0.1076 C   0 0 0 0 0 0 0 0 0 0 0 0
13.9950   22.4281   0.0169 O   0 0 0 0 0 0 0 0 0 0 0 0
13.3908   20.2006   0.3697 O   0 0 0 0 0 0 0 0 0 0 0 0
29.4175   16.0558   0.0019 C   0 0 0 0 0 0 0 0 0 0 0 0
28.0089   16.4887   -0.1569 C   0 0 0 0 0 0 0 0 0 0 0 0
27.1068   15.6731   -0.0593 O   0 0 0 0 0 0 0 0 0 0 0 0
27.6970   17.9011   -0.4320 O   0 0 0 0 0 0 0 0 0 0 0 0
17.4861   19.2620   0.8042 C   0 0 0 0 0 0 0 0 0 0 0 0
23.5939   18.7977   -0.8817 C   0 0 0 0 0 0 0 0 0 0 0 0
22.1226   19.1680   -1.1505 C   0 0 0 0 0 0 0 0 0 0 0 0
21.2746   18.8383   0.0932 C   0 0 0 0 0 0 0 0 0 0 0 0
19.8038   19.2120   -0.1742 C   0 0 0 0 0 0 0 0 0 0 0 0
18.9561   18.8849   1.0704 C   0 0 0 0 0 0 0 0 0 0 0 0
10.7379   20.8517   0.1129 C   0 0 0 0 0 0 0 0 0 0 0 0

30.3522	17.2736	-0.1438 C	0	0	0	0	0	0	0	0	0	0	0	0	0
39.0098	19.1119	0.0338 C	0	0	0	0	0	0	0	0	0	0	0	0	0
37.5450	19.5651	-0.1197 C	0	0	0	0	0	0	0	0	0	0	0	0	0
36.6117	18.3482	0.0333 C	0	0	0	0	0	0	0	0	0	0	0	0	0
35.1471	18.8015	-0.1218 C	0	0	0	0	0	0	0	0	0	0	0	0	0
34.2137	17.5846	0.0294 C	0	0	0	0	0	0	0	0	0	0	0	0	0
32.7495	18.0376	-0.1293 C	0	0	0	0	0	0	0	0	0	0	0	0	0
31.8159	16.8206	0.0190 C	0	0	0	0	0	0	0	0	0	0	0	0	0
2.0633	19.0885	-0.0004 C	0	0	0	0	0	0	0	0	0	0	0	0	0
3.5250	18.6230	0.1466 C	0	0	0	0	0	0	0	0	0	0	0	0	0
4.4681	19.8305	-0.0197 C	0	0	0	0	0	0	0	0	0	0	0	0	0
5.9297	19.3652	0.1291 C	0	0	0	0	0	0	0	0	0	0	0	0	0
6.8727	20.5728	-0.0355 C	0	0	0	0	0	0	0	0	0	0	0	0	0
8.3340	20.1080	0.1172 C	0	0	0	0	0	0	0	0	0	0	0	0	0
9.2771	21.3160	-0.0444 C	0	0	0	0	0	0	0	0	0	0	0	0	0
24.0781	18.5625	-2.9730 H	0	0	0	0	0	0	0	0	0	0	0	0	0
24.3688	20.1882	-2.3409 H	0	0	0	0	0	0	0	0	0	0	0	0	0
17.0095	19.5009	2.8967 H	0	0	0	0	0	0	0	0	0	0	0	0	0
16.7047	17.8775	2.2655 H	0	0	0	0	0	0	0	0	0	0	0	0	0
11.5467	22.4968	-1.0284 H	0	0	0	0	0	0	0	0	0	0	0	0	0
11.4519	22.7998	0.7112 H	0	0	0	0	0	0	0	0	0	0	0	0	0
29.5508	15.6141	0.9817 H	0	0	0	0	0	0	0	0	0	0	0	0	0
29.6577	15.3239	-0.7593 H	0	0	0	0	0	0	0	0	0	0	0	0	0
17.1160	18.6967	-0.0422 H	0	0	0	0	0	0	0	0	0	0	0	0	0
17.4188	20.3212	0.5881 H	0	0	0	0	0	0	0	0	0	0	0	0	0
23.9593	19.3637	-0.0337 H	0	0	0	0	0	0	0	0	0	0	0	0	0
23.6662	17.7386	-0.6671 H	0	0	0	0	0	0	0	0	0	0	0	0	0
21.7566	18.6013	-1.9977 H	0	0	0	0	0	0	0	0	0	0	0	0	0
22.0505	20.2268	-1.3662 H	0	0	0	0	0	0	0	0	0	0	0	0	0
21.6421	19.4034	0.9408 H	0	0	0	0	0	0	0	0	0	0	0	0	0
21.3448	17.7791	0.3077 H	0	0	0	0	0	0	0	0	0	0	0	0	0
19.4356	18.6458	-1.0208 H	0	0	0	0	0	0	0	0	0	0	0	0	0
19.7340	20.2709	-0.3902 H	0	0	0	0	0	0	0	0	0	0	0	0	0
19.3258	19.4494	1.9175 H	0	0	0	0	0	0	0	0	0	0	0	0	0
19.0239	17.8256	1.2853 H	0	0	0	0	0	0	0	0	0	0	0	0	0
10.8738	20.4144	1.0944 H	0	0	0	0	0	0	0	0	0	0	0	0	0
10.9657	20.1133	-0.6459 H	0	0	0	0	0	0	0	0	0	0	0	0	0
30.2183	17.7163	-1.1231 H	0	0	0	0	0	0	0	0	0	0	0	0	0
30.1138	18.0050	0.6186 H	0	0	0	0	0	0	0	0	0	0	0	0	0
39.6654	19.9671	-0.0738 H	0	0	0	0	0	0	0	0	0	0	0	0	0
39.2431	18.3799	-0.7295 H	0	0	0	0	0	0	0	0	0	0	0	0	0
39.1504	18.6702	1.0126 H	0	0	0	0	0	0	0	0	0	0	0	0	0
37.4046	20.0069	-1.0984 H	0	0	0	0	0	0	0	0	0	0	0	0	0
37.3118	20.2971	0.6436 H	0	0	0	0	0	0	0	0	0	0	0	0	0
36.7513	17.9070	1.0125 H	0	0	0	0	0	0	0	0	0	0	0	0	0
36.8456	17.6158	-0.7294 H	0	0	0	0	0	0	0	0	0	0	0	0	0
35.0079	19.2436	-1.1006 H	0	0	0	0	0	0	0	0	0	0	0	0	0
34.9128	19.5332	0.6414 H	0	0	0	0	0	0	0	0	0	0	0	0	0
34.3512	17.1436	1.0090 H	0	0	0	0	0	0	0	0	0	0	0	0	0
34.4494	16.8520	-0.7326 H	0	0	0	0	0	0	0	0	0	0	0	0	0
32.6127	18.4802	-1.1082 H	0	0	0	0	0	0	0	0	0	0	0	0	0
32.5131	18.7690	0.6337 H	0	0	0	0	0	0	0	0	0	0	0	0	0
31.9506	16.3794	0.9988 H	0	0	0	0	0	0	0	0	0	0	0	0	0
32.0539	16.0883	-0.7425 H	0	0	0	0	0	0	0	0	0	0	0	0	0
1.4008	18.2399	0.1165 H	0	0	0	0	0	0	0	0	0	0	0	0	0
1.8415	19.8271	0.7600 H	0	0	0	0	0	0	0	0	0	0	0	0	0
1.9205	19.5256	-0.9809 H	0	0	0	0	0	0	0	0	0	0	0	0	0
3.6676	18.1859	1.1271 H	0	0	0	0	0	0	0	0	0	0	0	0	0
3.7467	17.8844	-0.6137 H	0	0	0	0	0	0	0	0	0	0	0	0	0
4.3263	20.2671	-1.0006 H	0	0	0	0	0	0	0	0	0	0	0	0	0
4.2456	20.5697	0.7400 H	0	0	0	0	0	0	0	0	0	0	0	0	0
6.0711	18.9279	1.1097 H	0	0	0	0	0	0	0	0	0	0	0	0	0
6.1527	18.6268	-0.6311 H	0	0	0	0	0	0	0	0	0	0	0	0	0

```
    6.7332   21.0088   -1.0169 H   0  0  0  0  0  0  0  0  0  0  0  0
    6.6481   21.3122    0.7233 H   0  0  0  0  0  0  0  0  0  0  0  0
    8.4728   19.6705    1.0981 H   0  0  0  0  0  0  0  0  0  0  0  0
    8.5593   19.3698   -0.6425 H   0  0  0  0  0  0  0  0  0  0  0  0
    9.1404   21.7519   -1.0263 H   0  0  0  0  0  0  0  0  0  0  0  0
    9.0497   22.0553    0.7137 H   0  0  0  0  0  0  0  0  0  0  0  0
  1  2  1  0  0  0  0
  2  3  2  0  0  0  0
  2  4  1  0  0  0  0
  5  6  1  0  0  0  0
  6  7  2  0  0  0  0
  6  8  1  0  0  0  0
  9 10  1  0  0  0  0
 10 11  2  0  0  0  0
 10 12  1  0  0  0  0
 13 14  1  0  0  0  0
 14 15  2  0  0  0  0
 14 16  1  0  0  0  0
 16  4  1  0  0  0  0
 12  8  1  0  0  0  0
  5 17  1  0  0  0  0
  1 18  1  0  0  0  0
 19 20  1  0  0  0  0
 20 21  1  0  0  0  0
 21 22  1  0  0  0  0
 19 18  1  0  0  0  0
 22 17  1  0  0  0  0
  9 23  1  0  0  0  0
 13 24  1  0  0  0  0
 25 26  1  0  0  0  0
 26 27  1  0  0  0  0
 27 28  1  0  0  0  0
 28 29  1  0  0  0  0
 29 30  1  0  0  0  0
 30 31  1  0  0  0  0
 32 33  1  0  0  0  0
 33 34  1  0  0  0  0
 34 35  1  0  0  0  0
 35 36  1  0  0  0  0
 36 37  1  0  0  0  0
 37 38  1  0  0  0  0
 38 23  1  0  0  0  0
 31 24  1  0  0  0  0
  1 39  1  0  0  0  0
  1 40  1  0  0  0  0
  5 41  1  0  0  0  0
  5 42  1  0  0  0  0
  9 43  1  0  0  0  0
  9 44  1  0  0  0  0
 13 45  1  0  0  0  0
 13 46  1  0  0  0  0
 17 47  1  0  0  0  0
 17 48  1  0  0  0  0
 18 49  1  0  0  0  0
 18 50  1  0  0  0  0
 19 51  1  0  0  0  0
 19 52  1  0  0  0  0
 20 53  1  0  0  0  0
 20 54  1  0  0  0  0
 21 55  1  0  0  0  0
 21 56  1  0  0  0  0
 22 57  1  0  0  0  0
 22 58  1  0  0  0  0
```

```
 23 59  1  0  0  0  0
 23 60  1  0  0  0  0
 24 61  1  0  0  0  0
 24 62  1  0  0  0  0
 25 63  1  0  0  0  0
 25 64  1  0  0  0  0
 25 65  1  0  0  0  0
 26 66  1  0  0  0  0
 26 67  1  0  0  0  0
 27 68  1  0  0  0  0
 27 69  1  0  0  0  0
 28 70  1  0  0  0  0
 28 71  1  0  0  0  0
 29 72  1  0  0  0  0
 29 73  1  0  0  0  0
 30 74  1  0  0  0  0
 30 75  1  0  0  0  0
 31 76  1  0  0  0  0
 31 77  1  0  0  0  0
 32 78  1  0  0  0  0
 32 79  1  0  0  0  0
 32 80  1  0  0  0  0
 33 81  1  0  0  0  0
 33 82  1  0  0  0  0
 34 83  1  0  0  0  0
 34 84  1  0  0  0  0
 35 85  1  0  0  0  0
 35 86  1  0  0  0  0
 36 87  1  0  0  0  0
 36 88  1  0  0  0  0
 37 89  1  0  0  0  0
 37 90  1  0  0  0  0
 38 91  1  0  0  0  0
 38 92  1  0  0  0  0
M  END
>  <Alias name>
Diacylperoxide VIII

>  <deltaH°(c)>   kJ/mol
-17774

$$$$
Di-n-undecylphthalate
  ChemBrain IXL

 84 84  0  0  0  0  0  0  0  0  1 V2000
    2.7593    6.3618   -0.3832 C   0  0  0  0  0  0  0  0  0  0  0  0
    2.4720    7.5652   -1.0305 C   0  0  0  0  0  0  0  0  0  0  0  0
    3.4945    8.4729   -1.3109 C   0  0  0  0  0  0  0  0  0  0  0  0
    4.8112    8.1854   -0.9462 C   0  0  0  0  0  0  0  0  0  0  0  0
    5.1051    6.9557   -0.2891 C   0  0  0  0  0  0  0  0  0  0  0  0
    4.0710    6.0590   -0.0140 C   0  0  0  0  0  0  0  0  0  0  0  0
    5.8844    9.1938   -1.2688 C   0  0  0  0  0  0  0  0  0  0  0  0
    5.7421    9.9322   -2.2296 O   0  0  0  0  0  0  0  0  0  0  0  0
    7.1162    9.3098   -0.4330 O   0  0  0  0  0  0  0  0  0  0  0  0
    6.5075    6.5888    0.1280 C   0  0  0  0  0  0  0  0  0  0  0  0
    6.6725    5.8474    1.0828 O   0  0  0  0  0  0  0  0  0  0  0  0
    7.6987    7.1112   -0.6069 O   0  0  0  0  0  0  0  0  0  0  0  0
    8.1271   10.2899   -0.9402 C   0  0  0  0  0  0  0  0  0  0  0  0
    9.3147   10.4140    0.0637 C   0  0  0  0  0  0  0  0  0  0  0  0
   10.3841   11.3977   -0.5081 C   0  0  0  0  0  0  0  0  0  0  0  0
   11.6010   11.4805    0.4588 C   0  0  0  0  0  0  0  0  0  0  0  0
   12.6453   12.4924   -0.0953 C   0  0  0  0  0  0  0  0  0  0  0  0
```

13.8605	12.5718	0.8681	C	0	0	0	0	0	0	0	0	0	0	0	0
14.8921	13.5966	0.3276	C	0	0	0	0	0	0	0	0	0	0	0	0
16.0982	13.6802	1.2960	C	0	0	0	0	0	0	0	0	0	0	0	0
9.0184	6.7321	-0.0100	C	0	0	0	0	0	0	0	0	0	0	0	0
10.1890	7.2347	-0.9117	C	0	0	0	0	0	0	0	0	0	0	0	0
11.5555	6.8853	-0.2400	C	0	0	0	0	0	0	0	0	0	0	0	0
12.7292	7.4366	-1.1018	C	0	0	0	0	0	0	0	0	0	0	0	0
14.0885	7.0554	-0.4454	C	0	0	0	0	0	0	0	0	0	0	0	0
15.2591	7.6049	-1.3058	C	0	0	0	0	0	0	0	0	0	0	0	0
16.6145	7.2008	-0.6671	C	0	0	0	0	0	0	0	0	0	0	0	0
17.7793	7.7751	-1.5123	C	0	0	0	0	0	0	0	0	0	0	0	0
17.1230	14.7099	0.7640	C	0	0	0	0	0	0	0	0	0	0	0	0
19.1321	7.3523	-0.8912	C	0	0	0	0	0	0	0	0	0	0	0	0
18.3180	14.8011	1.7395	C	0	0	0	0	0	0	0	0	0	0	0	0
19.3384	15.8321	1.2125	C	0	0	0	0	0	0	0	0	0	0	0	0
20.2867	8.0572	-1.6389	C	0	0	0	0	0	0	0	0	0	0	0	0
21.6388	7.6015	-1.0503	C	0	0	0	0	0	0	0	0	0	0	0	0
1.9627	5.6616	-0.1672	H	0	0	0	0	0	0	0	0	0	0	0	0
1.4534	7.7949	-1.3156	H	0	0	0	0	0	0	0	0	0	0	0	0
3.2659	9.4046	-1.8138	H	0	0	0	0	0	0	0	0	0	0	0	0
4.2879	5.1243	0.4884	H	0	0	0	0	0	0	0	0	0	0	0	0
8.5029	9.9532	-1.8985	H	0	0	0	0	0	0	0	0	0	0	0	0
7.6579	11.2588	-1.0603	H	0	0	0	0	0	0	0	0	0	0	0	0
8.9488	10.7911	1.0106	H	0	0	0	0	0	0	0	0	0	0	0	0
9.7644	9.4404	0.2129	H	0	0	0	0	0	0	0	0	0	0	0	0
10.7179	11.0435	-1.4756	H	0	0	0	0	0	0	0	0	0	0	0	0
9.9454	12.3820	-0.6178	H	0	0	0	0	0	0	0	0	0	0	0	0
11.2646	11.8090	1.4342	H	0	0	0	0	0	0	0	0	0	0	0	0
12.0588	10.5031	0.5462	H	0	0	0	0	0	0	0	0	0	0	0	0
12.9812	12.1656	-1.0718	H	0	0	0	0	0	0	0	0	0	0	0	0
12.1893	13.4711	-0.1804	H	0	0	0	0	0	0	0	0	0	0	0	0
13.5233	12.8841	1.8486	H	0	0	0	0	0	0	0	0	0	0	0	0
14.3267	11.5970	0.9413	H	0	0	0	0	0	0	0	0	0	0	0	0
15.2361	13.2813	-0.6497	H	0	0	0	0	0	0	0	0	0	0	0	0
14.4256	14.5708	0.2485	H	0	0	0	0	0	0	0	0	0	0	0	0
15.7537	13.9883	2.2754	H	0	0	0	0	0	0	0	0	0	0	0	0
16.5699	12.7081	1.3694	H	0	0	0	0	0	0	0	0	0	0	0	0
9.1080	7.1779	0.9729	H	0	0	0	0	0	0	0	0	0	0	0	0
9.0743	5.6541	0.0800	H	0	0	0	0	0	0	0	0	0	0	0	0
10.1306	6.7535	-1.8802	H	0	0	0	0	0	0	0	0	0	0	0	0
10.1132	8.3075	-1.0370	H	0	0	0	0	0	0	0	0	0	0	0	0
11.5952	7.3300	0.7468	H	0	0	0	0	0	0	0	0	0	0	0	0
11.6488	5.8096	-0.1530	H	0	0	0	0	0	0	0	0	0	0	0	0
12.6778	7.0100	-2.0958	H	0	0	0	0	0	0	0	0	0	0	0	0
12.6520	8.5147	-1.1683	H	0	0	0	0	0	0	0	0	0	0	0	0
14.1413	7.4826	0.5485	H	0	0	0	0	0	0	0	0	0	0	0	0
14.1667	5.9772	-0.3783	H	0	0	0	0	0	0	0	0	0	0	0	0
15.1958	7.1932	-2.3054	H	0	0	0	0	0	0	0	0	0	0	0	0
15.1933	8.6846	-1.3563	H	0	0	0	0	0	0	0	0	0	0	0	0
16.6713	7.5958	0.3397	H	0	0	0	0	0	0	0	0	0	0	0	0
16.6910	6.1209	-0.6355	H	0	0	0	0	0	0	0	0	0	0	0	0
17.7130	7.3939	-2.5238	H	0	0	0	0	0	0	0	0	0	0	0	0
17.7135	8.8559	-1.5297	H	0	0	0	0	0	0	0	0	0	0	0	0
17.4766	14.3971	-0.2107	H	0	0	0	0	0	0	0	0	0	0	0	0
16.6501	15.6807	0.6817	H	0	0	0	0	0	0	0	0	0	0	0	0
19.1539	7.6351	0.1540	H	0	0	0	0	0	0	0	0	0	0	0	0
19.2493	6.2791	-0.9771	H	0	0	0	0	0	0	0	0	0	0	0	0
17.9646	15.1105	2.7154	H	0	0	0	0	0	0	0	0	0	0	0	0
18.7938	13.8315	1.8191	H	0	0	0	0	0	0	0	0	0	0	0	0
18.8622	16.8010	1.1274	H	0	0	0	0	0	0	0	0	0	0	0	0
19.6980	15.5199	0.2398	H	0	0	0	0	0	0	0	0	0	0	0	0
20.1725	15.8992	1.9010	H	0	0	0	0	0	0	0	0	0	0	0	0
20.2464	7.7997	-2.6901	H	0	0	0	0	0	0	0	0	0	0	0	0

```
  20.1880    9.1297   -1.5263 H   0  0  0  0  0  0  0  0  0  0  0  0
  22.4403    8.1761   -1.4994 H   0  0  0  0  0  0  0  0  0  0  0  0
  21.7881    6.5495   -1.2600 H   0  0  0  0  0  0  0  0  0  0  0  0
  21.6383    7.7595    0.0211 H   0  0  0  0  0  0  0  0  0  0  0  0
  1  2  4  0  0  0  0
  2  3  4  0  0  0  0
  3  4  4  0  0  0  0
  4  5  4  0  0  0  0
  5  6  4  0  0  0  0
  6  1  4  0  0  0  0
  7  8  2  0  0  0  0
  7  9  1  0  0  0  0
  7  4  1  0  0  0  0
 10 11  2  0  0  0  0
 10 12  1  0  0  0  0
 10  5  1  0  0  0  0
  9 13  1  0  0  0  0
 13 14  1  0  0  0  0
 14 15  1  0  0  0  0
 15 16  1  0  0  0  0
 16 17  1  0  0  0  0
 17 18  1  0  0  0  0
 18 19  1  0  0  0  0
 19 20  1  0  0  0  0
 21 22  1  0  0  0  0
 22 23  1  0  0  0  0
 23 24  1  0  0  0  0
 24 25  1  0  0  0  0
 25 26  1  0  0  0  0
 26 27  1  0  0  0  0
 27 28  1  0  0  0  0
 21 12  1  0  0  0  0
 20 29  1  0  0  0  0
 28 30  1  0  0  0  0
 29 31  1  0  0  0  0
 31 32  1  0  0  0  0
 30 33  1  0  0  0  0
 33 34  1  0  0  0  0
  1 35  1  0  0  0  0
  2 36  1  0  0  0  0
  3 37  1  0  0  0  0
  6 38  1  0  0  0  0
 13 39  1  0  0  0  0
 13 40  1  0  0  0  0
 14 41  1  0  0  0  0
 14 42  1  0  0  0  0
 15 43  1  0  0  0  0
 15 44  1  0  0  0  0
 16 45  1  0  0  0  0
 16 46  1  0  0  0  0
 17 47  1  0  0  0  0
 17 48  1  0  0  0  0
 18 49  1  0  0  0  0
 18 50  1  0  0  0  0
 19 51  1  0  0  0  0
 19 52  1  0  0  0  0
 20 53  1  0  0  0  0
 20 54  1  0  0  0  0
 21 55  1  0  0  0  0
 21 56  1  0  0  0  0
 22 57  1  0  0  0  0
 22 58  1  0  0  0  0
 23 59  1  0  0  0  0
```

```
 23 60  1  0  0  0  0
 24 61  1  0  0  0  0
 24 62  1  0  0  0  0
 25 63  1  0  0  0  0
 25 64  1  0  0  0  0
 26 65  1  0  0  0  0
 26 66  1  0  0  0  0
 27 67  1  0  0  0  0
 27 68  1  0  0  0  0
 28 69  1  0  0  0  0
 28 70  1  0  0  0  0
 29 71  1  0  0  0  0
 29 72  1  0  0  0  0
 30 73  1  0  0  0  0
 30 74  1  0  0  0  0
 31 75  1  0  0  0  0
 31 76  1  0  0  0  0
 32 77  1  0  0  0  0
 32 78  1  0  0  0  0
 32 79  1  0  0  0  0
 33 80  1  0  0  0  0
 33 81  1  0  0  0  0
 34 82  1  0  0  0  0
 34 83  1  0  0  0  0
 34 84  1  0  0  0  0
M  END
>  <Alias name>
Di-n-undecyl phthalate

>  <deltaH°(c)>   kJ/mol
-17715.71

$$$$
11-Cyclohexylheneicosane
  ChemBrain IXL

 81 81  0  0  0  0  0  0  0  0  1 V2000
   19.8327    9.2362   -8.6634 C   0  0  0  0  0  0  0  0  0  0  0  0
   19.4136   10.0705   -7.4374 C   0  0  0  0  0  0  0  0  0  0  0  0
   18.0821    9.5311   -6.8794 C   0  0  0  0  0  0  0  0  0  0  0  0
   17.6650   10.3629   -5.6509 C   0  0  0  0  0  0  0  0  0  0  0  0
   16.3365    9.8197   -5.0896 C   0  0  0  0  0  0  0  0  0  0  0  0
   15.9249   10.6440   -3.8542 C   0  0  0  0  0  0  0  0  0  0  0  0
   14.6034   10.0918   -3.2855 C   0  0  0  0  0  0  0  0  0  0  0  0
   14.2014   10.9031   -2.0383 C   0  0  0  0  0  0  0  0  0  0  0  0
   12.8911   10.3367   -1.4580 C   0  0  0  0  0  0  0  0  0  0  0  0
   12.5029   11.1309   -0.1938 C   0  0  0  0  0  0  0  0  0  0  0  0
   11.1908   10.5627    0.3954 C   0  0  0  0  0  0  0  0  0  0  0  0
   10.8303   11.3590    1.6827 C   0  0  0  0  0  0  0  0  0  0  0  0
    9.3090   11.2524    1.9753 C   0  0  0  0  0  0  0  0  0  0  0  0
    8.9847   12.0144    3.2747 C   0  0  0  0  0  0  0  0  0  0  0  0
    7.4787   11.8944    3.5788 C   0  0  0  0  0  0  0  0  0  0  0  0
    7.1587   12.6379    4.8903 C   0  0  0  0  0  0  0  0  0  0  0  0
    5.6563   12.5015    5.2052 C   0  0  0  0  0  0  0  0  0  0  0  0
    5.3394   13.2332    6.5241 C   0  0  0  0  0  0  0  0  0  0  0  0
    3.8392   13.0873    6.8451 C   0  0  0  0  0  0  0  0  0  0  0  0
    3.5236   13.8139    8.1672 C   0  0  0  0  0  0  0  0  0  0  0  0
    2.0243   13.6648    8.4905 C   0  0  0  0  0  0  0  0  0  0  0  0
   12.6375    8.8442    1.6045 C   0  0  0  0  0  0  0  0  0  0  0  0
   11.3930    9.0372    0.7112 C   0  0  0  0  0  0  0  0  0  0  0  0
   10.1665    8.4590    1.4351 C   0  0  0  0  0  0  0  0  0  0  0  0
   10.3746    6.9507    1.6548 C   0  0  0  0  0  0  0  0  0  0  0  0
   11.6183    6.7330    2.5426 C   0  0  0  0  0  0  0  0  0  0  0  0
```

12.8601	7.3353	1.8499	C	0	0	0	0	0	0	0	0	0	0	0	0
20.7684	9.6153	-9.0552	H	0	0	0	0	0	0	0	0	0	0	0	0
19.0672	9.3058	-9.4263	H	0	0	0	0	0	0	0	0	0	0	0	0
19.9571	8.2010	-8.3702	H	0	0	0	0	0	0	0	0	0	0	0	0
19.2893	11.1056	-7.7306	H	0	0	0	0	0	0	0	0	0	0	0	0
20.1793	10.0008	-6.6747	H	0	0	0	0	0	0	0	0	0	0	0	0
18.2060	8.4952	-6.5886	H	0	0	0	0	0	0	0	0	0	0	0	0
17.3157	9.6029	-7.6412	H	0	0	0	0	0	0	0	0	0	0	0	0
18.4331	10.2934	-4.8905	H	0	0	0	0	0	0	0	0	0	0	0	0
17.5377	11.3983	-5.9419	H	0	0	0	0	0	0	0	0	0	0	0	0
15.5664	9.8952	-5.8473	H	0	0	0	0	0	0	0	0	0	0	0	0
16.4626	8.7823	-4.8053	H	0	0	0	0	0	0	0	0	0	0	0	0
16.6991	10.5742	-3.1001	H	0	0	0	0	0	0	0	0	0	0	0	0
15.7909	11.6802	-4.1392	H	0	0	0	0	0	0	0	0	0	0	0	0
14.7353	9.0523	-3.0119	H	0	0	0	0	0	0	0	0	0	0	0	0
13.8257	10.1719	-4.0350	H	0	0	0	0	0	0	0	0	0	0	0	0
14.0568	11.9407	-2.3128	H	0	0	0	0	0	0	0	0	0	0	0	0
14.9852	10.8322	-1.2944	H	0	0	0	0	0	0	0	0	0	0	0	0
13.0333	9.2952	-1.2004	H	0	0	0	0	0	0	0	0	0	0	0	0
12.1025	10.4220	-2.1953	H	0	0	0	0	0	0	0	0	0	0	0	0
12.3595	12.1727	-0.4527	H	0	0	0	0	0	0	0	0	0	0	0	0
13.2937	11.0491	0.5401	H	0	0	0	0	0	0	0	0	0	0	0	0
10.3965	10.6811	-0.3312	H	0	0	0	0	0	0	0	0	0	0	0	0
11.0819	12.4019	1.5341	H	0	0	0	0	0	0	0	0	0	0	0	0
11.3954	10.9715	2.5201	H	0	0	0	0	0	0	0	0	0	0	0	0
9.0267	10.2266	2.0868	H	0	0	0	0	0	0	0	0	0	0	0	0
8.7540	11.6891	1.1543	H	0	0	0	0	0	0	0	0	0	0	0	0
9.5523	11.5888	4.0930	H	0	0	0	0	0	0	0	0	0	0	0	0
9.2480	13.0583	3.1572	H	0	0	0	0	0	0	0	0	0	0	0	0
6.9099	12.3314	2.7673	H	0	0	0	0	0	0	0	0	0	0	0	0
7.2143	10.8494	3.6833	H	0	0	0	0	0	0	0	0	0	0	0	0
7.7369	12.2078	5.6988	H	0	0	0	0	0	0	0	0	0	0	0	0
7.4119	13.6854	4.7830	H	0	0	0	0	0	0	0	0	0	0	0	0
5.0774	12.9387	4.4012	H	0	0	0	0	0	0	0	0	0	0	0	0
5.4024	11.4533	5.3044	H	0	0	0	0	0	0	0	0	0	0	0	0
5.5869	14.2827	6.4233	H	0	0	0	0	0	0	0	0	0	0	0	0
5.9237	12.7999	7.3264	H	0	0	0	0	0	0	0	0	0	0	0	0
3.5914	12.0375	6.9423	H	0	0	0	0	0	0	0	0	0	0	0	0
3.2545	13.5236	6.0447	H	0	0	0	0	0	0	0	0	0	0	0	0
4.1101	13.3791	8.9670	H	0	0	0	0	0	0	0	0	0	0	0	0
3.7690	14.8642	8.0694	H	0	0	0	0	0	0	0	0	0	0	0	0
1.7788	12.6145	8.5883	H	0	0	0	0	0	0	0	0	0	0	0	0
1.8029	14.1752	9.4195	H	0	0	0	0	0	0	0	0	0	0	0	0
1.4375	14.0997	7.6908	H	0	0	0	0	0	0	0	0	0	0	0	0
12.4860	9.3463	2.5512	H	0	0	0	0	0	0	0	0	0	0	0	0
13.5058	9.2586	1.1103	H	0	0	0	0	0	0	0	0	0	0	0	0
11.5400	8.5022	-0.2182	H	0	0	0	0	0	0	0	0	0	0	0	0
9.2793	8.6206	0.8355	H	0	0	0	0	0	0	0	0	0	0	0	0
10.0535	8.9420	2.3932	H	0	0	0	0	0	0	0	0	0	0	0	0
10.5209	6.4622	0.6994	H	0	0	0	0	0	0	0	0	0	0	0	0
9.5038	6.5333	2.1449	H	0	0	0	0	0	0	0	0	0	0	0	0
11.7703	5.6722	2.6975	H	0	0	0	0	0	0	0	0	0	0	0	0
11.4678	7.2192	3.4986	H	0	0	0	0	0	0	0	0	0	0	0	0
13.7285	7.1966	2.4817	H	0	0	0	0	0	0	0	0	0	0	0	0
13.0207	6.8366	0.9020	H	0	0	0	0	0	0	0	0	0	0	0	0

1	2	1	0	0	0	0
2	3	1	0	0	0	0
3	4	1	0	0	0	0
4	5	1	0	0	0	0
5	6	1	0	0	0	0
6	7	1	0	0	0	0
7	8	1	0	0	0	0
8	9	1	0	0	0	0

9	10	1	0	0	0	0
10	11	1	0	0	0	0
11	12	1	0	0	0	0
12	13	1	0	0	0	0
13	14	1	0	0	0	0
14	15	1	0	0	0	0
15	16	1	0	0	0	0
16	17	1	0	0	0	0
17	18	1	0	0	0	0
18	19	1	0	0	0	0
19	20	1	0	0	0	0
20	21	1	0	0	0	0
22	23	1	0	0	0	0
23	24	1	0	0	0	0
24	25	1	0	0	0	0
25	26	1	0	0	0	0
26	27	1	0	0	0	0
27	22	1	0	0	0	0
23	11	1	0	0	0	0
1	28	1	0	0	0	0
1	29	1	0	0	0	0
1	30	1	0	0	0	0
2	31	1	0	0	0	0
2	32	1	0	0	0	0
3	33	1	0	0	0	0
3	34	1	0	0	0	0
4	35	1	0	0	0	0
4	36	1	0	0	0	0
5	37	1	0	0	0	0
5	38	1	0	0	0	0
6	39	1	0	0	0	0
6	40	1	0	0	0	0
7	41	1	0	0	0	0
7	42	1	0	0	0	0
8	43	1	0	0	0	0
8	44	1	0	0	0	0
9	45	1	0	0	0	0
9	46	1	0	0	0	0
10	47	1	0	0	0	0
10	48	1	0	0	0	0
11	49	1	0	0	0	0
12	50	1	0	0	0	0
12	51	1	0	0	0	0
13	52	1	0	0	0	0
13	53	1	0	0	0	0
14	54	1	0	0	0	0
14	55	1	0	0	0	0
15	56	1	0	0	0	0
15	57	1	0	0	0	0
16	58	1	0	0	0	0
16	59	1	0	0	0	0
17	60	1	0	0	0	0
17	61	1	0	0	0	0
18	62	1	0	0	0	0
18	63	1	0	0	0	0
19	64	1	0	0	0	0
19	65	1	0	0	0	0
20	66	1	0	0	0	0
20	67	1	0	0	0	0
21	68	1	0	0	0	0
21	69	1	0	0	0	0
21	70	1	0	0	0	0
22	71	1	0	0	0	0

```
 22 72  1  0  0  0  0
 23 73  1  0  0  0  0
 24 74  1  0  0  0  0
 24 75  1  0  0  0  0
 25 76  1  0  0  0  0
 25 77  1  0  0  0  0
 26 78  1  0  0  0  0
 26 79  1  0  0  0  0
 27 80  1  0  0  0  0
 27 81  1  0  0  0  0
M  END
>  <Alias name>
11-Cyclohexylheneicosane

>  <deltaH°(c)>   kJ/mol
-17652

$$$$
3-Methyl-3-t-hexylperoxy-1-triphenylsilyl-1-butyne
  ChemBrain IXL

 66 68  0  0  0  0  0  0  0  0  1 V2000
    6.6405    2.7144   -1.7450 C   0  0  0  0  0  0  0  0  0  0  0  0
    6.5165    3.7412   -2.6818 C   0  0  0  0  0  0  0  0  0  0  0  0
    7.0054    5.0150   -2.3895 C   0  0  0  0  0  0  0  0  0  0  0  0
    7.6182    5.2623   -1.1602 C   0  0  0  0  0  0  0  0  0  0  0  0
    7.7422    4.2354   -0.2233 C   0  0  0  0  0  0  0  0  0  0  0  0
    7.2533    2.9615   -0.5157 C   0  0  0  0  0  0  0  0  0  0  0  0
    7.2203    6.9405    1.8615 C   0  0  0  0  0  0  0  0  0  0  0  0
    8.3466    7.1974    1.0793 C   0  0  0  0  0  0  0  0  0  0  0  0
    9.5300    7.6284    1.6808 C   0  0  0  0  0  0  0  0  0  0  0  0
    9.5865    7.8021    3.0642 C   0  0  0  0  0  0  0  0  0  0  0  0
    8.4597    7.5449    3.8463 C   0  0  0  0  0  0  0  0  0  0  0  0
    7.2766    7.1141    3.2448 C   0  0  0  0  0  0  0  0  0  0  0  0
    8.2707    6.9650   -0.7697 Si  0  0  0  0  0  0  0  0  0  0  0  0
   12.5446    7.4427   -2.5522 C   0  0  0  0  0  0  0  0  0  0  0  0
   11.9697    6.1753   -2.4484 C   0  0  0  0  0  0  0  0  0  0  0  0
   10.6886    6.0318   -1.9142 C   0  0  0  0  0  0  0  0  0  0  0  0
    9.9823    7.1560   -1.4838 C   0  0  0  0  0  0  0  0  0  0  0  0
   10.5570    8.4233   -1.5876 C   0  0  0  0  0  0  0  0  0  0  0  0
   11.8382    8.5667   -2.1218 C   0  0  0  0  0  0  0  0  0  0  0  0
    7.1388    8.2445   -1.5170 C   0  0  0  0  0  0  0  0  0  0  0  0
    6.4087    9.0703   -2.0009 C   0  0  0  0  0  0  0  0  0  0  0  0
    5.4765   10.1287   -2.6225 C   0  0  0  0  0  0  0  0  0  0  0  0
    6.0397   10.5670   -3.9887 C   0  0  0  0  0  0  0  0  0  0  0  0
    4.2528    6.8434   -1.6974 C   0  0  0  0  0  0  0  0  0  0  0  0
    3.4826    7.1611   -2.9996 C   0  0  0  0  0  0  0  0  0  0  0  0
    4.1319    9.5597   -2.8097 O   0  0  0  0  0  0  0  0  0  0  0  0
    3.6328    5.6044   -1.0142 C   0  0  0  0  0  0  0  0  0  0  0  0
    4.0763    8.3411   -3.6485 O   0  0  0  0  0  0  0  0  0  0  0  0
    5.3908   11.3504   -1.6874 C   0  0  0  0  0  0  0  0  0  0  0  0
    3.5659    5.9555   -3.9585 C   0  0  0  0  0  0  0  0  0  0  0  0
    2.0043    7.4527   -2.6672 C   0  0  0  0  0  0  0  0  0  0  0  0
    4.3912    5.3032    0.2907 C   0  0  0  0  0  0  0  0  0  0  0  0
    6.2611    1.7258   -1.9718 H   0  0  0  0  0  0  0  0  0  0  0  0
    6.0410    3.5495   -3.6356 H   0  0  0  0  0  0  0  0  0  0  0  0
    6.9091    5.8118   -3.1166 H   0  0  0  0  0  0  0  0  0  0  0  0
    8.2176    4.4271    0.7306 H   0  0  0  0  0  0  0  0  0  0  0  0
    7.3495    2.1647    0.2112 H   0  0  0  0  0  0  0  0  0  0  0  0
    6.3031    6.6064    1.3953 H   0  0  0  0  0  0  0  0  0  0  0  0
   10.4045    7.8280    1.0741 H   0  0  0  0  0  0  0  0  0  0  0  0
   10.5046    8.1365    3.5309 H   0  0  0  0  0  0  0  0  0  0  0  0
    8.5034    7.6797    4.9198 H   0  0  0  0  0  0  0  0  0  0  0  0
```

6.4021	6.9144	3.8516	H	0	0	0	0	0	0	0	0	0	0	0	0
13.5388	7.5540	-2.9667	H	0	0	0	0	0	0	0	0	0	0	0	0
12.5179	5.3032	-2.7824	H	0	0	0	0	0	0	0	0	0	0	0	0
10.2426	5.0483	-1.8336	H	0	0	0	0	0	0	0	0	0	0	0	0
10.0089	9.2955	-1.2535	H	0	0	0	0	0	0	0	0	0	0	0	0
12.2843	9.5501	-2.2023	H	0	0	0	0	0	0	0	0	0	0	0	0
5.3866	11.3121	-4.4261	H	0	0	0	0	0	0	0	0	0	0	0	0
6.0996	9.7087	-4.6466	H	0	0	0	0	0	0	0	0	0	0	0	0
7.0283	10.9880	-3.8537	H	0	0	0	0	0	0	0	0	0	0	0	0
4.1924	7.6920	-1.0274	H	0	0	0	0	0	0	0	0	0	0	0	0
5.2889	6.6437	-1.9321	H	0	0	0	0	0	0	0	0	0	0	0	0
3.7050	4.7521	-1.6771	H	0	0	0	0	0	0	0	0	0	0	0	0
2.5928	5.7988	-0.7882	H	0	0	0	0	0	0	0	0	0	0	0	0
4.7384	12.0954	-2.1257	H	0	0	0	0	0	0	0	0	0	0	0	0
4.9945	11.0432	-0.7276	H	0	0	0	0	0	0	0	0	0	0	0	0
6.3795	11.7712	-1.5518	H	0	0	0	0	0	0	0	0	0	0	0	0
4.6043	5.7428	-4.1817	H	0	0	0	0	0	0	0	0	0	0	0	0
3.1125	5.0903	-3.4923	H	0	0	0	0	0	0	0	0	0	0	0	0
3.0398	6.1874	-4.8760	H	0	0	0	0	0	0	0	0	0	0	0	0
1.5579	6.5807	-2.2073	H	0	0	0	0	0	0	0	0	0	0	0	0
1.9466	8.2910	-1.9839	H	0	0	0	0	0	0	0	0	0	0	0	0
1.4705	7.6925	-3.5783	H	0	0	0	0	0	0	0	0	0	0	0	0
4.3165	6.1541	0.9560	H	0	0	0	0	0	0	0	0	0	0	0	0
5.4315	5.1105	0.0668	H	0	0	0	0	0	0	0	0	0	0	0	0
3.9574	4.4331	0.7674	H	0	0	0	0	0	0	0	0	0	0	0	0

1	2	4	0	0	0	0
2	3	4	0	0	0	0
3	4	4	0	0	0	0
4	5	4	0	0	0	0
5	6	4	0	0	0	0
6	1	4	0	0	0	0
7	8	4	0	0	0	0
8	9	4	0	0	0	0
9	10	4	0	0	0	0
10	11	4	0	0	0	0
11	12	4	0	0	0	0
12	7	4	0	0	0	0
4	13	1	0	0	0	0
13	8	1	0	0	0	0
14	15	4	0	0	0	0
15	16	4	0	0	0	0
16	17	4	0	0	0	0
17	18	4	0	0	0	0
18	19	4	0	0	0	0
19	14	4	0	0	0	0
17	13	1	0	0	0	0
13	20	1	0	0	0	0
20	21	3	0	0	0	0
21	22	1	0	0	0	0
24	25	1	0	0	0	0
24	27	1	0	0	0	0
25	28	1	0	0	0	0
28	26	1	0	0	0	0
23	22	1	0	0	0	0
22	29	1	0	0	0	0
26	22	1	0	0	0	0
25	30	1	0	0	0	0
25	31	1	0	0	0	0
27	32	1	0	0	0	0
1	33	1	0	0	0	0
2	34	1	0	0	0	0
3	35	1	0	0	0	0
5	36	1	0	0	0	0

```
  6 37  1  0  0  0  0
  7 38  1  0  0  0  0
  9 39  1  0  0  0  0
 10 40  1  0  0  0  0
 11 41  1  0  0  0  0
 12 42  1  0  0  0  0
 14 43  1  0  0  0  0
 15 44  1  0  0  0  0
 16 45  1  0  0  0  0
 18 46  1  0  0  0  0
 19 47  1  0  0  0  0
 23 48  1  0  0  0  0
 23 49  1  0  0  0  0
 23 50  1  0  0  0  0
 24 51  1  0  0  0  0
 24 52  1  0  0  0  0
 27 53  1  0  0  0  0
 27 54  1  0  0  0  0
 29 55  1  0  0  0  0
 29 56  1  0  0  0  0
 29 57  1  0  0  0  0
 30 58  1  0  0  0  0
 30 59  1  0  0  0  0
 30 60  1  0  0  0  0
 31 61  1  0  0  0  0
 31 62  1  0  0  0  0
 31 63  1  0  0  0  0
 32 64  1  0  0  0  0
 32 65  1  0  0  0  0
 32 66  1  0  0  0  0
M  END
>  <Alias name>
3-Methyl-3-t-hexylperoxy-1-triphenylsilyl-1-butyne

>  <deltaH°(c)>   kJ/mol
-17250

$$$$
5-Butyldocosane
  ChemBrain IXL

 80 79  0  0  0  0  0  0  0  0  1 V2000
   28.8280   11.9531    0.0000 C   0  0  0  0  0  0  0  0  0  0  0  0
   27.5326   11.7773   -0.8158 C   0  0  0  0  0  0  0  0  0  0  0  0
   26.3140   12.0097    0.0985 C   0  0  0  0  0  0  0  0  0  0  0  0
   25.0177   11.8340   -0.7189 C   0  0  0  0  0  0  0  0  0  0  0  0
   23.7910   12.0433    0.1984 C   0  0  0  0  0  0  0  0  0  0  0  0
   22.4976   11.8573   -0.6277 C   0  0  0  0  0  0  0  0  0  0  0  0
   21.2672   12.0577    0.2807 C   0  0  0  0  0  0  0  0  0  0  0  0
   19.9818   11.8507   -0.5435 C   0  0  0  0  0  0  0  0  0  0  0  0
   18.7521   12.0538    0.3629 C   0  0  0  0  0  0  0  0  0  0  0  0
   17.4664   11.8503   -0.4621 C   0  0  0  0  0  0  0  0  0  0  0  0
   16.2367   12.0570    0.4434 C   0  0  0  0  0  0  0  0  0  0  0  0
   14.9510   11.8572   -0.3824 C   0  0  0  0  0  0  0  0  0  0  0  0
   13.7213   12.0674    0.5223 C   0  0  0  0  0  0  0  0  0  0  0  0
   12.4356   11.8711   -0.3044 C   0  0  0  0  0  0  0  0  0  0  0  0
   11.2058   12.0846    0.5996 C   0  0  0  0  0  0  0  0  0  0  0  0
    9.9201   11.8913   -0.2277 C   0  0  0  0  0  0  0  0  0  0  0  0
    8.6904   12.1072    0.6757 C   0  0  0  0  0  0  0  0  0  0  0  0
    7.4047   11.9160   -0.1522 C   0  0  0  0  0  0  0  0  0  0  0  0
    6.1750   12.1335    0.7509 C   0  0  0  0  0  0  0  0  0  0  0  0
    4.8892   11.9436   -0.0773 C   0  0  0  0  0  0  0  0  0  0  0  0
    3.6594   12.1618    0.8257 C   0  0  0  0  0  0  0  0  0  0  0  0
```

2.3737 11.9724 -0.0024 C 0 0 0 0 0 0 0 0 0 0 0 0
23.8261 13.4698 0.8050 C 0 0 0 0 0 0 0 0 0 0 0 0
23.7940 14.5218 -0.3246 C 0 0 0 0 0 0 0 0 0 0 0 0
23.8288 15.9345 0.2898 C 0 0 0 0 0 0 0 0 0 0 0 0
23.7965 16.9856 -0.8366 C 0 0 0 0 0 0 0 0 0 0 0 0
28.8665 12.9569 0.4046 H 0 0 0 0 0 0 0 0 0 0 0 0
29.6842 11.7899 -0.6427 H 0 0 0 0 0 0 0 0 0 0 0 0
28.8438 11.2360 0.8114 H 0 0 0 0 0 0 0 0 0 0 0 0
27.5170 12.4944 -1.6272 H 0 0 0 0 0 0 0 0 0 0 0 0
27.4942 10.7735 -1.2204 H 0 0 0 0 0 0 0 0 0 0 0 0
26.3294 11.2922 0.9096 H 0 0 0 0 0 0 0 0 0 0 0 0
26.3535 13.0127 0.5029 H 0 0 0 0 0 0 0 0 0 0 0 0
24.9965 12.5588 -1.5222 H 0 0 0 0 0 0 0 0 0 0 0 0
24.9882 10.8347 -1.1353 H 0 0 0 0 0 0 0 0 0 0 0 0
23.8132 11.3135 0.9982 H 0 0 0 0 0 0 0 0 0 0 0 0
22.4745 12.5816 -1.4315 H 0 0 0 0 0 0 0 0 0 0 0 0
22.4780 10.8577 -1.0439 H 0 0 0 0 0 0 0 0 0 0 0 0
21.2959 11.3398 1.0911 H 0 0 0 0 0 0 0 0 0 0 0 0
21.2768 13.0608 0.6865 H 0 0 0 0 0 0 0 0 0 0 0 0
19.9542 12.5672 -1.3551 H 0 0 0 0 0 0 0 0 0 0 0 0
19.9701 10.8462 -0.9481 H 0 0 0 0 0 0 0 0 0 0 0 0
18.7784 11.3360 1.1734 H 0 0 0 0 0 0 0 0 0 0 0 0
18.7651 13.0577 0.7691 H 0 0 0 0 0 0 0 0 0 0 0 0
17.4413 12.5668 -1.2738 H 0 0 0 0 0 0 0 0 0 0 0 0
17.4522 10.8458 -0.8667 H 0 0 0 0 0 0 0 0 0 0 0 0
16.2604 11.3391 1.2540 H 0 0 0 0 0 0 0 0 0 0 0 0
16.2523 13.0608 0.8496 H 0 0 0 0 0 0 0 0 0 0 0 0
14.9285 12.5737 -1.1942 H 0 0 0 0 0 0 0 0 0 0 0 0
14.9342 10.8527 -0.7870 H 0 0 0 0 0 0 0 0 0 0 0 0
13.7425 11.3496 1.3329 H 0 0 0 0 0 0 0 0 0 0 0 0
13.7394 13.0712 0.9284 H 0 0 0 0 0 0 0 0 0 0 0 0
12.4155 12.5878 -1.1161 H 0 0 0 0 0 0 0 0 0 0 0 0
12.4163 10.8668 -0.7090 H 0 0 0 0 0 0 0 0 0 0 0 0
11.2248 11.3668 1.4104 H 0 0 0 0 0 0 0 0 0 0 0 0
11.2262 13.0884 1.0056 H 0 0 0 0 0 0 0 0 0 0 0 0
9.9020 12.6081 -1.0393 H 0 0 0 0 0 0 0 0 0 0 0 0
9.8988 10.8870 -0.6326 H 0 0 0 0 0 0 0 0 0 0 0 0
8.7077 11.3895 1.4866 H 0 0 0 0 0 0 0 0 0 0 0 0
8.7125 13.1110 1.0815 H 0 0 0 0 0 0 0 0 0 0 0 0
7.3879 12.6331 -0.9636 H 0 0 0 0 0 0 0 0 0 0 0 0
7.3820 10.9119 -0.5572 H 0 0 0 0 0 0 0 0 0 0 0 0
6.1912 11.4160 1.5619 H 0 0 0 0 0 0 0 0 0 0 0 0
6.1980 13.1374 1.1565 H 0 0 0 0 0 0 0 0 0 0 0 0
4.8732 12.6609 -0.8885 H 0 0 0 0 0 0 0 0 0 0 0 0
4.8659 10.9396 -0.4826 H 0 0 0 0 0 0 0 0 0 0 0 0
3.6752 11.4444 1.6368 H 0 0 0 0 0 0 0 0 0 0 0 0
3.6828 13.1657 1.2312 H 0 0 0 0 0 0 0 0 0 0 0 0
2.3501 10.9684 -0.4079 H 0 0 0 0 0 0 0 0 0 0 0 0
1.5097 12.1258 0.6323 H 0 0 0 0 0 0 0 0 0 0 0 0
2.3577 12.6898 -0.8135 H 0 0 0 0 0 0 0 0 0 0 0 0
22.9678 13.6080 1.4492 H 0 0 0 0 0 0 0 0 0 0 0 0
24.7317 13.5922 1.3844 H 0 0 0 0 0 0 0 0 0 0 0 0
24.6536 14.3880 -0.9681 H 0 0 0 0 0 0 0 0 0 0 0 0
22.8873 14.4034 -0.9034 H 0 0 0 0 0 0 0 0 0 0 0 0
22.9689 16.0693 0.9340 H 0 0 0 0 0 0 0 0 0 0 0 0
24.7361 16.0540 0.8689 H 0 0 0 0 0 0 0 0 0 0 0 0
22.8892 16.8662 -1.4159 H 0 0 0 0 0 0 0 0 0 0 0 0
23.8209 17.9783 -0.4047 H 0 0 0 0 0 0 0 0 0 0 0 0
24.6565 16.8509 -1.4809 H 0 0 0 0 0 0 0 0 0 0 0 0
1 2 1 0 0 0 0
2 3 1 0 0 0 0
3 4 1 0 0 0 0
4 5 1 0 0 0 0

5	6	1	0	0	0	0
6	7	1	0	0	0	0
7	8	1	0	0	0	0
8	9	1	0	0	0	0
9	10	1	0	0	0	0
10	11	1	0	0	0	0
11	12	1	0	0	0	0
12	13	1	0	0	0	0
13	14	1	0	0	0	0
14	15	1	0	0	0	0
15	16	1	0	0	0	0
16	17	1	0	0	0	0
17	18	1	0	0	0	0
18	19	1	0	0	0	0
19	20	1	0	0	0	0
20	21	1	0	0	0	0
21	22	1	0	0	0	0
5	23	1	0	0	0	0
23	24	1	0	0	0	0
24	25	1	0	0	0	0
25	26	1	0	0	0	0
1	27	1	0	0	0	0
1	28	1	0	0	0	0
1	29	1	0	0	0	0
2	30	1	0	0	0	0
2	31	1	0	0	0	0
3	32	1	0	0	0	0
3	33	1	0	0	0	0
4	34	1	0	0	0	0
4	35	1	0	0	0	0
5	36	1	0	0	0	0
6	37	1	0	0	0	0
6	38	1	0	0	0	0
7	39	1	0	0	0	0
7	40	1	0	0	0	0
8	41	1	0	0	0	0
8	42	1	0	0	0	0
9	43	1	0	0	0	0
9	44	1	0	0	0	0
10	45	1	0	0	0	0
10	46	1	0	0	0	0
11	47	1	0	0	0	0
11	48	1	0	0	0	0
12	49	1	0	0	0	0
12	50	1	0	0	0	0
13	51	1	0	0	0	0
13	52	1	0	0	0	0
14	53	1	0	0	0	0
14	54	1	0	0	0	0
15	55	1	0	0	0	0
15	56	1	0	0	0	0
16	57	1	0	0	0	0
16	58	1	0	0	0	0
17	59	1	0	0	0	0
17	60	1	0	0	0	0
18	61	1	0	0	0	0
18	62	1	0	0	0	0
19	63	1	0	0	0	0
19	64	1	0	0	0	0
20	65	1	0	0	0	0
20	66	1	0	0	0	0
21	67	1	0	0	0	0
21	68	1	0	0	0	0

```
 22 69  1  0  0  0  0
 22 70  1  0  0  0  0
 22 71  1  0  0  0  0
 23 72  1  0  0  0  0
 23 73  1  0  0  0  0
 24 74  1  0  0  0  0
 24 75  1  0  0  0  0
 25 76  1  0  0  0  0
 25 77  1  0  0  0  0
 26 78  1  0  0  0  0
 26 79  1  0  0  0  0
 26 80  1  0  0  0  0
M  END
>  <Alias name>
5-Butyldocosane

>  <deltaH°(c)>   kJ/mol
-17247.16

$$$$
11-Butyldocosane
  ChemBrain IXL

 80 79  0  0  0  0  0  0  0  0  1 V2000
   28.7647   16.8379   -0.1002 C   0  0  0  0  0  0  0  0  0  0  0  0
   27.4704   17.0153   -0.9174 C   0  0  0  0  0  0  0  0  0  0  0  0
   26.2502   16.7972   -0.0014 C   0  0  0  0  0  0  0  0  0  0  0  0
   24.9558   16.9742   -0.8189 C   0  0  0  0  0  0  0  0  0  0  0  0
   23.7355   16.7560    0.0968 C   0  0  0  0  0  0  0  0  0  0  0  0
   22.4414   16.9319   -0.7213 C   0  0  0  0  0  0  0  0  0  0  0  0
   21.2209   16.7130    0.1938 C   0  0  0  0  0  0  0  0  0  0  0  0
   19.9271   16.8870   -0.6253 C   0  0  0  0  0  0  0  0  0  0  0  0
   18.7063   16.6670    0.2889 C   0  0  0  0  0  0  0  0  0  0  0  0
   17.4122   16.8386   -0.5329 C   0  0  0  0  0  0  0  0  0  0  0  0
   16.1826   16.6407    0.3830 C   0  0  0  0  0  0  0  0  0  0  0  0
   14.8922   16.8204   -0.4492 C   0  0  0  0  0  0  0  0  0  0  0  0
   13.6582   16.6294    0.4565 C   0  0  0  0  0  0  0  0  0  0  0  0
   12.3763   16.8312   -0.3746 C   0  0  0  0  0  0  0  0  0  0  0  0
   11.1424   16.6366    0.5279 C   0  0  0  0  0  0  0  0  0  0  0  0
    9.8608   16.8368   -0.3043 C   0  0  0  0  0  0  0  0  0  0  0  0
    8.6264   16.6393    0.5970 C   0  0  0  0  0  0  0  0  0  0  0  0
    7.3452   16.8387   -0.2360 C   0  0  0  0  0  0  0  0  0  0  0  0
    6.1105   16.6392    0.6644 C   0  0  0  0  0  0  0  0  0  0  0  0
    4.8294   16.8382   -0.1689 C   0  0  0  0  0  0  0  0  0  0  0  0
    3.5944   16.6379    0.7311 C   0  0  0  0  0  0  0  0  0  0  0  0
    2.3135   16.8369   -0.1022 C   0  0  0  0  0  0  0  0  0  0  0  0
   16.2135   15.2203    1.0040 C   0  0  0  0  0  0  0  0  0  0  0  0
   16.1834   14.1570   -0.1149 C   0  0  0  0  0  0  0  0  0  0  0  0
   16.2142   12.7505    0.5137 C   0  0  0  0  0  0  0  0  0  0  0  0
   16.1840   11.6882   -0.6020 C   0  0  0  0  0  0  0  0  0  0  0  0
   28.7848   17.5609    0.7058 H   0  0  0  0  0  0  0  0  0  0  0  0
   29.6219   16.9912   -0.7441 H   0  0  0  0  0  0  0  0  0  0  0  0
   28.7972   15.8369    0.3118 H   0  0  0  0  0  0  0  0  0  0  0  0
   27.4505   16.2923   -1.7234 H   0  0  0  0  0  0  0  0  0  0  0  0
   27.4381   18.0163   -1.3295 H   0  0  0  0  0  0  0  0  0  0  0  0
   26.2699   17.5205    0.8045 H   0  0  0  0  0  0  0  0  0  0  0  0
   26.2826   15.7963    0.4109 H   0  0  0  0  0  0  0  0  0  0  0  0
   24.9362   16.2510   -1.6249 H   0  0  0  0  0  0  0  0  0  0  0  0
   24.9233   17.9752   -1.2312 H   0  0  0  0  0  0  0  0  0  0  0  0
   23.7546   17.4796    0.9023 H   0  0  0  0  0  0  0  0  0  0  0  0
   23.7684   15.7553    0.5096 H   0  0  0  0  0  0  0  0  0  0  0  0
   22.4227   16.2084   -1.5271 H   0  0  0  0  0  0  0  0  0  0  0  0
   22.4084   17.9327   -1.1339 H   0  0  0  0  0  0  0  0  0  0  0  0
```

21.2387 17.4373 0.9988 H 0 0 0 0 0 0 0 0 0 0 0 0
21.2545 15.7127 0.6073 H 0 0 0 0 0 0 0 0 0 0 0 0
19.9099 16.1632 -1.4307 H 0 0 0 0 0 0 0 0 0 0 0 0
19.8932 17.8876 -1.0383 H 0 0 0 0 0 0 0 0 0 0 0 0
18.7222 17.3921 1.0931 H 0 0 0 0 0 0 0 0 0 0 0 0
18.7420 15.6678 0.7029 H 0 0 0 0 0 0 0 0 0 0 0 0
17.3912 16.1066 -1.3298 H 0 0 0 0 0 0 0 0 0 0 0 0
17.3863 17.8341 -0.9585 H 0 0 0 0 0 0 0 0 0 0 0 0
16.2036 17.3784 1.1754 H 0 0 0 0 0 0 0 0 0 0 0 0
14.8712 16.0889 -1.2465 H 0 0 0 0 0 0 0 0 0 0 0 0
14.8751 17.8162 -0.8744 H 0 0 0 0 0 0 0 0 0 0 0 0
13.6848 17.3542 1.2607 H 0 0 0 0 0 0 0 0 0 0 0 0
13.6649 15.6298 0.8708 H 0 0 0 0 0 0 0 0 0 0 0 0
12.3513 16.1082 -1.1806 H 0 0 0 0 0 0 0 0 0 0 0 0
12.3675 17.8324 -0.7873 H 0 0 0 0 0 0 0 0 0 0 0 0
11.1666 17.3601 1.3335 H 0 0 0 0 0 0 0 0 0 0 0 0
11.1517 15.6356 0.9412 H 0 0 0 0 0 0 0 0 0 0 0 0
9.8379 16.1147 -1.1110 H 0 0 0 0 0 0 0 0 0 0 0 0
9.8506 17.8384 -0.7160 H 0 0 0 0 0 0 0 0 0 0 0 0
8.6488 17.3618 1.4034 H 0 0 0 0 0 0 0 0 0 0 0 0
8.6369 15.6378 1.0091 H 0 0 0 0 0 0 0 0 0 0 0 0
7.3236 16.1170 -1.0432 H 0 0 0 0 0 0 0 0 0 0 0 0
7.3341 17.8406 -0.6471 H 0 0 0 0 0 0 0 0 0 0 0 0
6.1319 17.3610 1.4715 H 0 0 0 0 0 0 0 0 0 0 0 0
6.1216 15.6374 1.0756 H 0 0 0 0 0 0 0 0 0 0 0 0
4.8084 16.1169 -0.9765 H 0 0 0 0 0 0 0 0 0 0 0 0
4.8180 17.8403 -0.5796 H 0 0 0 0 0 0 0 0 0 0 0 0
3.6154 17.3592 1.5386 H 0 0 0 0 0 0 0 0 0 0 0 0
3.6058 15.6359 1.1417 H 0 0 0 0 0 0 0 0 0 0 0 0
2.2925 16.1156 -0.9098 H 0 0 0 0 0 0 0 0 0 0 0 0
1.4459 16.6960 0.5304 H 0 0 0 0 0 0 0 0 0 0 0 0
2.3020 17.8390 -0.5127 H 0 0 0 0 0 0 0 0 0 0 0 0
15.3529 15.0899 1.6468 H 0 0 0 0 0 0 0 0 0 0 0 0
17.1171 15.1023 1.5875 H 0 0 0 0 0 0 0 0 0 0 0 0
17.0453 14.2831 -0.7571 H 0 0 0 0 0 0 0 0 0 0 0 0
15.2788 14.2708 -0.6979 H 0 0 0 0 0 0 0 0 0 0 0 0
15.3519 12.6235 1.1565 H 0 0 0 0 0 0 0 0 0 0 0 0
17.1194 12.6356 1.0971 H 0 0 0 0 0 0 0 0 0 0 0 0
17.0462 11.8151 -1.2448 H 0 0 0 0 0 0 0 0 0 0 0 0
16.2056 10.6998 -0.1600 H 0 0 0 0 0 0 0 0 0 0 0 0
15.2787 11.8030 -1.1854 H 0 0 0 0 0 0 0 0 0 0 0 0
1 2 1 0 0 0 0
2 3 1 0 0 0 0
3 4 1 0 0 0 0
4 5 1 0 0 0 0
5 6 1 0 0 0 0
6 7 1 0 0 0 0
7 8 1 0 0 0 0
8 9 1 0 0 0 0
9 10 1 0 0 0 0
10 11 1 0 0 0 0
11 12 1 0 0 0 0
12 13 1 0 0 0 0
13 14 1 0 0 0 0
14 15 1 0 0 0 0
15 16 1 0 0 0 0
16 17 1 0 0 0 0
17 18 1 0 0 0 0
18 19 1 0 0 0 0
19 20 1 0 0 0 0
20 21 1 0 0 0 0
21 22 1 0 0 0 0
23 24 1 0 0 0 0

```
 24 25  1  0  0  0  0
 25 26  1  0  0  0  0
 23 11  1  0  0  0  0
  1 27  1  0  0  0  0
  1 28  1  0  0  0  0
  1 29  1  0  0  0  0
  2 30  1  0  0  0  0
  2 31  1  0  0  0  0
  3 32  1  0  0  0  0
  3 33  1  0  0  0  0
  4 34  1  0  0  0  0
  4 35  1  0  0  0  0
  5 36  1  0  0  0  0
  5 37  1  0  0  0  0
  6 38  1  0  0  0  0
  6 39  1  0  0  0  0
  7 40  1  0  0  0  0
  7 41  1  0  0  0  0
  8 42  1  0  0  0  0
  8 43  1  0  0  0  0
  9 44  1  0  0  0  0
  9 45  1  0  0  0  0
 10 46  1  0  0  0  0
 10 47  1  0  0  0  0
 11 48  1  0  0  0  0
 12 49  1  0  0  0  0
 12 50  1  0  0  0  0
 13 51  1  0  0  0  0
 13 52  1  0  0  0  0
 14 53  1  0  0  0  0
 14 54  1  0  0  0  0
 15 55  1  0  0  0  0
 15 56  1  0  0  0  0
 16 57  1  0  0  0  0
 16 58  1  0  0  0  0
 17 59  1  0  0  0  0
 17 60  1  0  0  0  0
 18 61  1  0  0  0  0
 18 62  1  0  0  0  0
 19 63  1  0  0  0  0
 19 64  1  0  0  0  0
 20 65  1  0  0  0  0
 20 66  1  0  0  0  0
 21 67  1  0  0  0  0
 21 68  1  0  0  0  0
 22 69  1  0  0  0  0
 22 70  1  0  0  0  0
 22 71  1  0  0  0  0
 23 72  1  0  0  0  0
 23 73  1  0  0  0  0
 24 74  1  0  0  0  0
 24 75  1  0  0  0  0
 25 76  1  0  0  0  0
 25 77  1  0  0  0  0
 26 78  1  0  0  0  0
 26 79  1  0  0  0  0
 26 80  1  0  0  0  0
M  END
>  <Alias name>
11-Butyldocosane

>  <deltaH°(c)>   kJ/mol
-17233
```

```
$$$$
Cholesteryl methyl ether
  ChemBrain IXL

 77 80  0  0  0  0  0  0  0  0  1 V2000
   10.1262   11.7790   -0.0794 C   0  0  0  0  0  0  0  0  0  0  0  0
   11.4423   11.0634   -0.5752 C   0  0  0  0  0  0  0  0  0  0  0  0
    9.0353   11.3261   -1.0568 C   0  0  0  0  0  0  0  0  0  0  0  0
   10.9773   10.0242   -1.6494 C   0  0  0  0  0  0  0  0  0  0  0  0
    9.4419    9.9658   -1.5019 C   0  0  0  0  0  0  0  0  0  0  0  0
   11.6279    8.6322   -1.3871 C   0  0  0  0  0  0  0  0  0  0  0  0
    7.6063   11.2715   -0.4931 C   0  0  0  0  0  0  0  0  0  0  0  0
    9.0797    8.9356   -0.4137 C   0  0  0  0  0  0  0  0  0  0  0  0
    8.6930    9.6414   -2.7612 C   0  0  0  0  0  0  0  0  0  0  0  0
    7.1582    9.5648   -2.3970 C   0  0  0  0  0  0  0  0  0  0  0  0
    6.6678   10.9054   -1.6639 C   0  0  0  0  0  0  0  0  0  0  0  0
    7.1942   12.6534    0.0415 C   0  0  0  0  0  0  0  0  0  0  0  0
    5.2160   10.7093   -1.1508 C   0  0  0  0  0  0  0  0  0  0  0  0
    5.7248   12.6666    0.4178 C   0  0  0  0  0  0  0  0  0  0  0  0
    4.8292   11.7711   -0.0989 C   0  0  0  0  0  0  0  0  0  0  0  0
    3.4388   11.8310    0.3821 C   0  0  0  0  0  0  0  0  0  0  0  0
    4.2608   10.7902   -2.3861 C   0  0  0  0  0  0  0  0  0  0  0  0
    5.0920    9.3119   -0.5181 C   0  0  0  0  0  0  0  0  0  0  0  0
    2.9187   11.4446   -2.0081 C   0  0  0  0  0  0  0  0  0  0  0  0
    2.5456   11.0655   -0.5770 C   0  0  0  0  0  0  0  0  0  0  0  0
    2.7403    9.6254   -0.3818 O   0  0  0  0  0  0  0  0  0  0  0  0
    1.9513    8.7690   -1.2721 C   0  0  0  0  0  0  0  0  0  0  0  0
   13.1793    8.7728   -1.3665 C   0  0  0  0  0  0  0  0  0  0  0  0
   11.2244    7.6575   -2.5136 C   0  0  0  0  0  0  0  0  0  0  0  0
   13.8134    7.4883   -0.7681 C   0  0  0  0  0  0  0  0  0  0  0  0
   15.3584    7.6256   -0.7438 C   0  0  0  0  0  0  0  0  0  0  0  0
   15.9620    6.5106    0.1447 C   0  0  0  0  0  0  0  0  0  0  0  0
   15.5410    5.1331   -0.4021 C   0  0  0  0  0  0  0  0  0  0  0  0
   17.5029    6.6145    0.1371 C   0  0  0  0  0  0  0  0  0  0  0  0
   10.2451   12.8545   -0.1187 H   0  0  0  0  0  0  0  0  0  0  0  0
    9.8840   11.4624    0.9279 H   0  0  0  0  0  0  0  0  0  0  0  0
   11.9283   10.5568    0.2499 H   0  0  0  0  0  0  0  0  0  0  0  0
   12.1162   11.7855   -1.0212 H   0  0  0  0  0  0  0  0  0  0  0  0
    9.0435   11.9878   -1.9142 H   0  0  0  0  0  0  0  0  0  0  0  0
   11.2405   10.3743   -2.6405 H   0  0  0  0  0  0  0  0  0  0  0  0
   11.2871    8.2460   -0.4353 H   0  0  0  0  0  0  0  0  0  0  0  0
    7.5384   10.5281    0.2911 H   0  0  0  0  0  0  0  0  0  0  0  0
    8.0105    8.9458   -0.2476 H   0  0  0  0  0  0  0  0  0  0  0  0
    9.5911    9.1881    0.5071 H   0  0  0  0  0  0  0  0  0  0  0  0
    9.3857    7.9471   -0.7367 H   0  0  0  0  0  0  0  0  0  0  0  0
    9.0263    8.6870   -3.1517 H   0  0  0  0  0  0  0  0  0  0  0  0
    8.8581   10.4177   -3.4978 H   0  0  0  0  0  0  0  0  0  0  0  0
    6.5845    9.4255   -3.3046 H   0  0  0  0  0  0  0  0  0  0  0  0
    6.9923    8.7202   -1.7390 H   0  0  0  0  0  0  0  0  0  0  0  0
    6.6764   11.7162   -2.3822 H   0  0  0  0  0  0  0  0  0  0  0  0
    7.7897   12.8921    0.9132 H   0  0  0  0  0  0  0  0  0  0  0  0
    7.3715   13.3985   -0.7268 H   0  0  0  0  0  0  0  0  0  0  0  0
    5.3725   13.4120    1.1191 H   0  0  0  0  0  0  0  0  0  0  0  0
    3.1175   12.8655    0.4270 H   0  0  0  0  0  0  0  0  0  0  0  0
    3.3765   11.3898    1.3674 H   0  0  0  0  0  0  0  0  0  0  0  0
    4.0767    9.7904   -2.7593 H   0  0  0  0  0  0  0  0  0  0  0  0
    4.7356   11.3776   -3.1638 H   0  0  0  0  0  0  0  0  0  0  0  0
    5.8139    9.2125    0.2823 H   0  0  0  0  0  0  0  0  0  0  0  0
    5.2809    8.5567   -1.2712 H   0  0  0  0  0  0  0  0  0  0  0  0
    4.0925    9.1837   -0.1212 H   0  0  0  0  0  0  0  0  0  0  0  0
    3.0083   12.5214   -2.0855 H   0  0  0  0  0  0  0  0  0  0  0  0
    2.1470   11.0980   -2.6852 H   0  0  0  0  0  0  0  0  0  0  0  0
```

1.5101	11.3215	-0.3921	H	0	0	0	0	0	0	0	0	0	0	0	0
2.1406	7.7303	-1.0339	H	0	0	0	0	0	0	0	0	0	0	0	0
0.8984	8.9841	-1.1395	H	0	0	0	0	0	0	0	0	0	0	0	0
2.2326	8.9611	-2.3000	H	0	0	0	0	0	0	0	0	0	0	0	0
13.5416	8.9143	-2.3785	H	0	0	0	0	0	0	0	0	0	0	0	0
13.4568	9.6270	-0.7624	H	0	0	0	0	0	0	0	0	0	0	0	0
11.7201	6.7058	-2.3629	H	0	0	0	0	0	0	0	0	0	0	0	0
10.1522	7.5122	-2.4994	H	0	0	0	0	0	0	0	0	0	0	0	0
11.5203	8.0702	-3.4712	H	0	0	0	0	0	0	0	0	0	0	0	0
13.4499	7.3441	0.2411	H	0	0	0	0	0	0	0	0	0	0	0	0
13.5385	6.6345	-1.3752	H	0	0	0	0	0	0	0	0	0	0	0	0
15.7457	7.5328	-1.7515	H	0	0	0	0	0	0	0	0	0	0	0	0
15.6292	8.5938	-0.3411	H	0	0	0	0	0	0	0	0	0	0	0	0
15.5990	6.6218	1.1588	H	0	0	0	0	0	0	0	0	0	0	0	0
15.9547	4.3534	0.2261	H	0	0	0	0	0	0	0	0	0	0	0	0
15.9123	5.0170	-1.4129	H	0	0	0	0	0	0	0	0	0	0	0	0
14.4609	5.0602	-0.4036	H	0	0	0	0	0	0	0	0	0	0	0	0
17.8017	7.5885	0.5036	H	0	0	0	0	0	0	0	0	0	0	0	0
17.8694	6.4806	-0.8732	H	0	0	0	0	0	0	0	0	0	0	0	0
17.9196	5.8449	0.7769	H	0	0	0	0	0	0	0	0	0	0	0	0

2	1	1	0	0	0	0
1	3	1	0	0	0	0
4	2	1	0	0	0	0
3	5	1	0	0	0	0
3	7	1	0	0	0	0
5	4	1	0	0	0	0
4	6	1	0	0	0	0
5	8	1	0	0	0	0
9	5	1	0	0	0	0
6	23	1	0	0	0	0
6	24	1	0	0	0	0
7	12	1	0	0	0	0
7	11	1	0	0	0	0
10	9	1	0	0	0	0
11	10	1	0	0	0	0
13	11	1	0	0	0	0
12	14	1	0	0	0	0
15	13	1	0	0	0	0
17	13	1	0	0	0	0
13	18	1	0	0	0	0
14	15	2	0	0	0	0
15	16	1	0	0	0	0
16	20	1	0	0	0	0
19	17	1	0	0	0	0
20	19	1	0	0	0	0
20	21	1	0	0	0	0
21	22	1	0	0	0	0
23	25	1	0	0	0	0
25	26	1	0	0	0	0
26	27	1	0	0	0	0
27	28	1	0	0	0	0
27	29	1	0	0	0	0
1	30	1	0	0	0	0
1	31	1	0	0	0	0
2	32	1	0	0	0	0
2	33	1	0	0	0	0
3	34	1	0	0	0	0
4	35	1	0	0	0	0
6	36	1	0	0	0	0
7	37	1	0	0	0	0
8	38	1	0	0	0	0
8	39	1	0	0	0	0
8	40	1	0	0	0	0

```
  9 41  1  0  0  0  0
  9 42  1  0  0  0  0
 10 43  1  0  0  0  0
 10 44  1  0  0  0  0
 11 45  1  0  0  0  0
 12 46  1  0  0  0  0
 12 47  1  0  0  0  0
 14 48  1  0  0  0  0
 16 49  1  0  0  0  0
 16 50  1  0  0  0  0
 17 51  1  0  0  0  0
 17 52  1  0  0  0  0
 18 53  1  0  0  0  0
 18 54  1  0  0  0  0
 18 55  1  0  0  0  0
 19 56  1  0  0  0  0
 19 57  1  0  0  0  0
 20 58  1  0  0  0  0
 22 59  1  0  0  0  0
 22 60  1  0  0  0  0
 22 61  1  0  0  0  0
 23 62  1  0  0  0  0
 23 63  1  0  0  0  0
 24 64  1  0  0  0  0
 24 65  1  0  0  0  0
 24 66  1  0  0  0  0
 25 67  1  0  0  0  0
 25 68  1  0  0  0  0
 26 69  1  0  0  0  0
 26 70  1  0  0  0  0
 27 71  1  0  0  0  0
 28 72  1  0  0  0  0
 28 73  1  0  0  0  0
 28 74  1  0  0  0  0
 29 75  1  0  0  0  0
 29 76  1  0  0  0  0
 29 77  1  0  0  0  0
M  END
>  <Alias name>
Cholesteryl methyl ether

>  <deltaH°(c)>   kJ/mol
-17226

$$$$
1,2,3,4-Tetraphenylnaphthalene
  ChemBrain IXL

 58 63  0  0  0  0  0  0  0  0  1 V2000
    3.0804    4.4944   -0.5050 C   0  0  0  0  0  0  0  0  0  0  0  0
    3.9638    5.4957   -0.1015 C   0  0  0  0  0  0  0  0  0  0  0  0
    5.1678    5.6790   -0.7815 C   0  0  0  0  0  0  0  0  0  0  0  0
    5.4866    4.8641   -1.8678 C   0  0  0  0  0  0  0  0  0  0  0  0
    4.6033    3.8627   -2.2713 C   0  0  0  0  0  0  0  0  0  0  0  0
    3.4003    3.6777   -1.5898 C   0  0  0  0  0  0  0  0  0  0  0  0
   10.6079   11.7735    1.7034 C   0  0  0  0  0  0  0  0  0  0  0  0
   10.6170   10.5297    2.3350 C   0  0  0  0  0  0  0  0  0  0  0  0
    9.7245    9.5363    1.9319 C   0  0  0  0  0  0  0  0  0  0  0  0
    8.8245    9.7861    0.8959 C   0  0  0  0  0  0  0  0  0  0  0  0
    8.8136   11.0306    0.2660 C   0  0  0  0  0  0  0  0  0  0  0  0
    9.7060   12.0240    0.6691 C   0  0  0  0  0  0  0  0  0  0  0  0
   11.5207    5.6737    1.2455 C   0  0  0  0  0  0  0  0  0  0  0  0
   10.1672    5.9660    1.0759 C   0  0  0  0  0  0  0  0  0  0  0  0
```

```
  9.7832    7.1284    0.4069 C   0  0  0  0  0  0  0  0  0  0  0  0
 10.7527    7.9996   -0.0903 C   0  0  0  0  0  0  0  0  0  0  0  0
 12.1062    7.7073    0.0794 C   0  0  0  0  0  0  0  0  0  0  0  0
 12.4902    6.5443    0.7472 C   0  0  0  0  0  0  0  0  0  0  0  0
  8.8726    2.4819   -0.9337 C   0  0  0  0  0  0  0  0  0  0  0  0
  7.7006    2.6779   -0.2029 C   0  0  0  0  0  0  0  0  0  0  0  0
  7.2388    3.9714    0.0410 C   0  0  0  0  0  0  0  0  0  0  0  0
  7.9494    5.0691   -0.4451 C   0  0  0  0  0  0  0  0  0  0  0  0
  9.1207    4.8731   -1.1771 C   0  0  0  0  0  0  0  0  0  0  0  0
  9.5826    3.5794   -1.4208 C   0  0  0  0  0  0  0  0  0  0  0  0
  3.8229    9.6191   -0.0351 C   0  0  0  0  0  0  0  0  0  0  0  0
  4.7101   10.6154    0.3728 C   0  0  0  0  0  0  0  0  0  0  0  0
  6.0630   10.3192    0.5395 C   0  0  0  0  0  0  0  0  0  0  0  0
  6.5288    9.0266    0.2984 C   0  0  0  0  0  0  0  0  0  0  0  0
  5.6416    8.0301   -0.1094 C   0  0  0  0  0  0  0  0  0  0  0  0
  4.2887    8.3264   -0.2762 C   0  0  0  0  0  0  0  0  0  0  0  0
  6.1074    6.7374   -0.3503 C   0  0  0  0  0  0  0  0  0  0  0  0
  7.4591    6.4415   -0.1850 C   0  0  0  0  0  0  0  0  0  0  0  0
  8.3472    7.4388    0.2254 C   0  0  0  0  0  0  0  0  0  0  0  0
  7.8819    8.7304    0.4649 C   0  0  0  0  0  0  0  0  0  0  0  0
  2.1463    4.3510    0.0239 H   0  0  0  0  0  0  0  0  0  0  0  0
  3.7148    6.1308    0.7397 H   0  0  0  0  0  0  0  0  0  0  0  0
  6.4198    5.0090   -2.3980 H   0  0  0  0  0  0  0  0  0  0  0  0
  4.8516    3.2289   -3.1136 H   0  0  0  0  0  0  0  0  0  0  0  0
  2.7147    2.9001   -1.9028 H   0  0  0  0  0  0  0  0  0  0  0  0
 11.3010   12.5446    2.0161 H   0  0  0  0  0  0  0  0  0  0  0  0
 11.3170   10.3353    3.1382 H   0  0  0  0  0  0  0  0  0  0  0  0
  9.7303    8.5712    2.4234 H   0  0  0  0  0  0  0  0  0  0  0  0
  8.1123   11.2254   -0.5360 H   0  0  0  0  0  0  0  0  0  0  0  0
  9.6986   12.9897    0.1790 H   0  0  0  0  0  0  0  0  0  0  0  0
 11.8189    4.7708    1.7640 H   0  0  0  0  0  0  0  0  0  0  0  0
  9.4147    5.2906    1.4636 H   0  0  0  0  0  0  0  0  0  0  0  0
 10.4547    8.9029   -0.6081 H   0  0  0  0  0  0  0  0  0  0  0  0
 12.8588    8.3833   -0.3072 H   0  0  0  0  0  0  0  0  0  0  0  0
 13.5410    6.3172    0.8787 H   0  0  0  0  0  0  0  0  0  0  0  0
  9.2313    1.4776   -1.1228 H   0  0  0  0  0  0  0  0  0  0  0  0
  7.1493    1.8257    0.1751 H   0  0  0  0  0  0  0  0  0  0  0  0
  6.3285    4.1235    0.6077 H   0  0  0  0  0  0  0  0  0  0  0  0
  9.6714    5.7251   -1.5561 H   0  0  0  0  0  0  0  0  0  0  0  0
 10.4925    3.4272   -1.9882 H   0  0  0  0  0  0  0  0  0  0  0  0
  2.7726    9.8491   -0.1647 H   0  0  0  0  0  0  0  0  0  0  0  0
  4.3484   11.6189    0.5601 H   0  0  0  0  0  0  0  0  0  0  0  0
  6.7517   11.0927    0.8561 H   0  0  0  0  0  0  0  0  0  0  0  0
  3.6000    7.5529   -0.5927 H   0  0  0  0  0  0  0  0  0  0  0  0
  1  2  4  0  0  0  0
  2  3  4  0  0  0  0
  3  4  4  0  0  0  0
  4  5  4  0  0  0  0
  5  6  4  0  0  0  0
  6  1  4  0  0  0  0
  7  8  4  0  0  0  0
  8  9  4  0  0  0  0
  9 10  4  0  0  0  0
 10 11  4  0  0  0  0
 11 12  4  0  0  0  0
 12  7  4  0  0  0  0
 13 14  4  0  0  0  0
 14 15  4  0  0  0  0
 15 16  4  0  0  0  0
 16 17  4  0  0  0  0
 17 18  4  0  0  0  0
 18 13  4  0  0  0  0
 19 20  4  0  0  0  0
```

20 21 4 0 0 0 0
21 22 4 0 0 0 0
22 23 4 0 0 0 0
23 24 4 0 0 0 0
24 19 4 0 0 0 0
25 26 4 0 0 0 0
26 27 4 0 0 0 0
27 28 4 0 0 0 0
28 29 4 0 0 0 0
29 30 4 0 0 0 0
30 25 4 0 0 0 0
29 31 4 0 0 0 0
31 32 4 0 0 0 0
32 33 4 0 0 0 0
33 34 4 0 0 0 0
34 28 4 0 0 0 0
3 31 1 0 0 0 0
22 32 1 0 0 0 0
15 33 1 0 0 0 0
10 34 1 0 0 0 0
1 35 1 0 0 0 0
2 36 1 0 0 0 0
4 37 1 0 0 0 0
5 38 1 0 0 0 0
6 39 1 0 0 0 0
7 40 1 0 0 0 0
8 41 1 0 0 0 0
9 42 1 0 0 0 0
11 43 1 0 0 0 0
12 44 1 0 0 0 0
13 45 1 0 0 0 0
14 46 1 0 0 0 0
16 47 1 0 0 0 0
17 48 1 0 0 0 0
18 49 1 0 0 0 0
19 50 1 0 0 0 0
20 51 1 0 0 0 0
21 52 1 0 0 0 0
23 53 1 0 0 0 0
24 54 1 0 0 0 0
25 55 1 0 0 0 0
26 56 1 0 0 0 0
27 57 1 0 0 0 0
30 58 1 0 0 0 0
M END
> <Alias name>
1,2,3,4-Tetraphenylnaphthalene

> <deltaH°(c)>   kJ/mol
-17219

$$$$
1,8-Bis(biphenyl-4-yl)naphthalene
ChemBrain IXL

58 63 0 0 0 0 0 0 0 0 1 V2000
9.6035   6.8962   1.1749 C   0 0 0 0 0 0 0 0 0 0 0 0
8.9281   7.8977   1.8835 C   0 0 0 0 0 0 0 0 0 0 0 0
8.9544   9.2207   1.4307 C   0 0 0 0 0 0 0 0 0 0 0 0
9.6685   9.5364   0.2683 C   0 0 0 0 0 0 0 0 0 0 0 0
10.3433   8.5344   -0.4399 C   0 0 0 0 0 0 0 0 0 0 0 0
10.3118   7.2127   0.0120 C   0 0 0 0 0 0 0 0 0 0 0 0
9.1938   2.7013   -2.7293 C   0 0 0 0 0 0 0 0 0 0 0 0

8.5816	2.4623	-1.4987	C	0 0 0 0 0 0 0 0 0 0 0 0 0
8.0627	3.5269	-0.7568	C	0 0 0 0 0 0 0 0 0 0 0 0 0
8.1551	4.8337	-1.2447	C	0 0 0 0 0 0 0 0 0 0 0 0 0
8.7692	5.0696	-2.4781	C	0 0 0 0 0 0 0 0 0 0 0 0 0
9.2878	4.0041	-3.2191	C	0 0 0 0 0 0 0 0 0 0 0 0 0
6.9362	5.7305	0.7534	C	0 0 0 0 0 0 0 0 0 0 0 0 0
6.4177	6.7975	1.4976	C	0 0 0 0 0 0 0 0 0 0 0 0 0
6.5650	8.1136	1.0424	C	0 0 0 0 0 0 0 0 0 0 0 0 0
7.2269	8.3528	-0.1664	C	0 0 0 0 0 0 0 0 0 0 0 0 0
7.7450	7.2851	-0.9103	C	0 0 0 0 0 0 0 0 0 0 0 0 0
7.6009	5.9724	-0.4515	C	0 0 0 0 0 0 0 0 0 0 0 0 0
11.7141	3.8306	-0.9730	C	0 0 0 0 0 0 0 0 0 0 0 0 0
11.0418	4.8305	-0.2650	C	0 0 0 0 0 0 0 0 0 0 0 0 0
11.0309	6.1435	-0.7442	C	0 0 0 0 0 0 0 0 0 0 0 0 0
11.6957	6.4509	-1.9349	C	0 0 0 0 0 0 0 0 0 0 0 0 0
12.3677	5.4501	-2.6420	C	0 0 0 0 0 0 0 0 0 0 0 0 0
12.3767	4.1407	-2.1608	C	0 0 0 0 0 0 0 0 0 0 0 0 0
4.6157	9.2866	2.0501	C	0 0 0 0 0 0 0 0 0 0 0 0 0
4.0408	10.3264	2.7805	C	0 0 0 0 0 0 0 0 0 0 0 0 0
4.8414	11.3435	3.3050	C	0 0 0 0 0 0 0 0 0 0 0 0 0
6.2242	11.3288	3.1055	C	0 0 0 0 0 0 0 0 0 0 0 0 0
6.8237	10.2898	2.3758	C	0 0 0 0 0 0 0 0 0 0 0 0 0
5.9956	9.2540	1.8375	C	0 0 0 0 0 0 0 0 0 0 0 0 0
8.2431	10.3005	2.1944	C	0 0 0 0 0 0 0 0 0 0 0 0 0
9.0050	11.3391	2.7340	C	0 0 0 0 0 0 0 0 0 0 0 0 0
8.3891	12.3633	3.4529	C	0 0 0 0 0 0 0 0 0 0 0 0 0
7.0049	12.3576	3.6387	C	0 0 0 0 0 0 0 0 0 0 0 0 0
9.5782	5.8729	1.5285	H	0 0 0 0 0 0 0 0 0 0 0 0 0
8.3857	7.6479	2.7852	H	0 0 0 0 0 0 0 0 0 0 0 0 0
9.7013	10.5603	-0.0843	H	0 0 0 0 0 0 0 0 0 0 0 0 0
10.8927	8.7841	-1.3409	H	0 0 0 0 0 0 0 0 0 0 0 0 0
9.5962	1.8750	-3.3047	H	0 0 0 0 0 0 0 0 0 0 0 0 0
8.5091	1.4499	-1.1189	H	0 0 0 0 0 0 0 0 0 0 0 0 0
7.5884	3.3385	0.1978	H	0 0 0 0 0 0 0 0 0 0 0 0 0
8.8438	6.0795	-2.8604	H	0 0 0 0 0 0 0 0 0 0 0 0 0
9.7637	4.1889	-4.1751	H	0 0 0 0 0 0 0 0 0 0 0 0 0
6.8213	4.7136	1.1120	H	0 0 0 0 0 0 0 0 0 0 0 0 0
5.8986	6.6028	2.4283	H	0 0 0 0 0 0 0 0 0 0 0 0 0
7.3371	9.3659	-0.5287	H	0 0 0 0 0 0 0 0 0 0 0 0 0
8.2585	7.4759	-1.8446	H	0 0 0 0 0 0 0 0 0 0 0 0 0
11.7214	2.8131	-0.5999	H	0 0 0 0 0 0 0 0 0 0 0 0 0
10.5284	4.5871	0.6563	H	0 0 0 0 0 0 0 0 0 0 0 0 0
11.6902	7.4660	-2.3108	H	0 0 0 0 0 0 0 0 0 0 0 0 0
12.8825	5.6901	-3.5649	H	0 0 0 0 0 0 0 0 0 0 0 0 0
12.8983	3.3645	-2.7096	H	0 0 0 0 0 0 0 0 0 0 0 0 0
3.9889	8.4990	1.6465	H	0 0 0 0 0 0 0 0 0 0 0 0 0
2.9705	10.3436	2.9417	H	0 0 0 0 0 0 0 0 0 0 0 0 0
4.3881	12.1467	3.8692	H	0 0 0 0 0 0 0 0 0 0 0 0 0
10.0802	11.3498	2.5919	H	0 0 0 0 0 0 0 0 0 0 0 0 0
8.9868	13.1653	3.8671	H	0 0 0 0 0 0 0 0 0 0 0 0 0
6.5349	13.1545	4.1982	H	0 0 0 0 0 0 0 0 0 0 0 0 0

1	2	4	0	0	0	0
2	3	4	0	0	0	0
3	4	4	0	0	0	0
4	5	4	0	0	0	0
5	6	4	0	0	0	0
6	1	4	0	0	0	0
7	8	4	0	0	0	0
8	9	4	0	0	0	0
9	10	4	0	0	0	0
10	11	4	0	0	0	0
11	12	4	0	0	0	0
12	7	4	0	0	0	0

13 14 4 0 0 0 0
14 15 4 0 0 0 0
15 16 4 0 0 0 0
16 17 4 0 0 0 0
17 18 4 0 0 0 0
18 13 4 0 0 0 0
19 20 4 0 0 0 0
20 21 4 0 0 0 0
21 22 4 0 0 0 0
22 23 4 0 0 0 0
23 24 4 0 0 0 0
24 19 4 0 0 0 0
25 26 4 0 0 0 0
26 27 4 0 0 0 0
27 28 4 0 0 0 0
28 29 4 0 0 0 0
29 30 4 0 0 0 0
30 25 4 0 0 0 0
29 31 4 0 0 0 0
31 32 4 0 0 0 0
32 33 4 0 0 0 0
33 34 4 0 0 0 0
34 28 4 0 0 0 0
3 31 1 0 0 0 0
21 6 1 0 0 0 0
15 30 1 0 0 0 0
10 18 1 0 0 0 0
1 35 1 0 0 0 0
2 36 1 0 0 0 0
4 37 1 0 0 0 0
5 38 1 0 0 0 0
7 39 1 0 0 0 0
8 40 1 0 0 0 0
9 41 1 0 0 0 0
11 42 1 0 0 0 0
12 43 1 0 0 0 0
13 44 1 0 0 0 0
14 45 1 0 0 0 0
16 46 1 0 0 0 0
17 47 1 0 0 0 0
19 48 1 0 0 0 0
20 49 1 0 0 0 0
22 50 1 0 0 0 0
23 51 1 0 0 0 0
24 52 1 0 0 0 0
25 53 1 0 0 0 0
26 54 1 0 0 0 0
27 55 1 0 0 0 0
32 56 1 0 0 0 0
33 57 1 0 0 0 0
34 58 1 0 0 0 0
M END
> <Alias name>
1,8-Bis(biphenyl-4-yl)naphthalene

> <deltaH°(c)>   kJ/mol
-17186

$$$$
Trioctylsilane
  ChemBrain IXL

77 76 0 0 0 0 0 0 0 0 1 V2000

3.9216	8.6956	2.6144	Si	0	0	0	0	0	0	0	0	0	0	0	0	0
3.9037	7.0728	1.6522	C	0	0	0	0	0	0	0	0	0	0	0	0	0
5.2380	6.8540	0.9088	C	0	0	0	0	0	0	0	0	0	0	0	0	0
5.1380	5.5812	0.0465	C	0	0	0	0	0	0	0	0	0	0	0	0	0
6.4705	5.3569	-0.6942	C	0	0	0	0	0	0	0	0	0	0	0	0	0
6.3711	4.0840	-1.5571	C	0	0	0	0	0	0	0	0	0	0	0	0	0
7.7039	3.8598	-2.2976	C	0	0	0	0	0	0	0	0	0	0	0	0	0
7.6046	2.5868	-3.1607	C	0	0	0	0	0	0	0	0	0	0	0	0	0
8.9374	2.3626	-3.9010	C	0	0	0	0	0	0	0	0	0	0	0	0	0
14.1965	8.8729	4.0658	C	0	0	0	0	0	0	0	0	0	0	0	0	0
12.8378	8.7899	4.7881	C	0	0	0	0	0	0	0	0	0	0	0	0	0
11.6995	8.8605	3.7514	C	0	0	0	0	0	0	0	0	0	0	0	0	0
10.3406	8.7777	4.4737	C	0	0	0	0	0	0	0	0	0	0	0	0	0
9.2027	8.8481	3.4370	C	0	0	0	0	0	0	0	0	0	0	0	0	0
7.8437	8.7656	4.1589	C	0	0	0	0	0	0	0	0	0	0	0	0	0
6.7066	8.8357	3.1217	C	0	0	0	0	0	0	0	0	0	0	0	0	0
5.3476	8.7573	3.8482	C	0	0	0	0	0	0	0	0	0	0	0	0	0
3.9349	10.1884	1.4607	C	0	0	0	0	0	0	0	0	0	0	0	0	0
5.1469	10.1416	0.5070	C	0	0	0	0	0	0	0	0	0	0	0	0	0
5.1845	11.4327	-0.3329	C	0	0	0	0	0	0	0	0	0	0	0	0	0
6.3925	11.3882	-1.2887	C	0	0	0	0	0	0	0	0	0	0	0	0	0
6.4316	12.6800	-2.1281	C	0	0	0	0	0	0	0	0	0	0	0	0	0
7.6402	12.6356	-3.0833	C	0	0	0	0	0	0	0	0	0	0	0	0	0
7.6800	13.9278	-3.9224	C	0	0	0	0	0	0	0	0	0	0	0	0	0
8.8885	13.8834	-4.8774	C	0	0	0	0	0	0	0	0	0	0	0	0	0
2.6652	8.7561	3.3941	H	0	0	0	0	0	0	0	0	0	0	0	0	0
3.0922	7.0938	0.9355	H	0	0	0	0	0	0	0	0	0	0	0	0	0
3.7479	6.2529	2.3422	H	0	0	0	0	0	0	0	0	0	0	0	0	0
6.0326	6.7352	1.6245	H	0	0	0	0	0	0	0	0	0	0	0	0	0
5.4451	7.6954	0.2775	H	0	0	0	0	0	0	0	0	0	0	0	0	0
4.3386	5.6959	-0.6751	H	0	0	0	0	0	0	0	0	0	0	0	0	0
4.9307	4.7295	0.6826	H	0	0	0	0	0	0	0	0	0	0	0	0	0
7.2698	5.2422	0.0275	H	0	0	0	0	0	0	0	0	0	0	0	0	0
6.6778	6.2087	-1.3301	H	0	0	0	0	0	0	0	0	0	0	0	0	0
5.5719	4.1987	-2.2790	H	0	0	0	0	0	0	0	0	0	0	0	0	0
6.1637	3.2322	-0.9213	H	0	0	0	0	0	0	0	0	0	0	0	0	0
8.5030	3.7450	-1.5757	H	0	0	0	0	0	0	0	0	0	0	0	0	0
7.9114	4.7117	-2.9333	H	0	0	0	0	0	0	0	0	0	0	0	0	0
6.8057	2.7017	-3.8827	H	0	0	0	0	0	0	0	0	0	0	0	0	0
7.3971	1.7350	-2.5251	H	0	0	0	0	0	0	0	0	0	0	0	0	0
9.1450	3.2145	-4.5368	H	0	0	0	0	0	0	0	0	0	0	0	0	0
8.8674	1.4682	-4.5075	H	0	0	0	0	0	0	0	0	0	0	0	0	0
9.7365	2.2477	-3.1791	H	0	0	0	0	0	0	0	0	0	0	0	0	0
14.2603	9.8079	3.5229	H	0	0	0	0	0	0	0	0	0	0	0	0	0
14.2879	8.0459	3.3725	H	0	0	0	0	0	0	0	0	0	0	0	0	0
14.9962	8.8232	4.7943	H	0	0	0	0	0	0	0	0	0	0	0	0	0
12.7465	9.6169	5.4813	H	0	0	0	0	0	0	0	0	0	0	0	0	0
12.7741	7.8550	5.3309	H	0	0	0	0	0	0	0	0	0	0	0	0	0
11.7908	8.0335	3.0582	H	0	0	0	0	0	0	0	0	0	0	0	0	0
11.7633	9.7954	3.2084	H	0	0	0	0	0	0	0	0	0	0	0	0	0
10.2492	9.6048	5.1668	H	0	0	0	0	0	0	0	0	0	0	0	0	0
10.2769	7.8429	5.0168	H	0	0	0	0	0	0	0	0	0	0	0	0	0
9.2940	8.0209	2.7440	H	0	0	0	0	0	0	0	0	0	0	0	0	0
9.2666	9.7828	2.8937	H	0	0	0	0	0	0	0	0	0	0	0	0	0
7.7520	9.5930	4.8516	H	0	0	0	0	0	0	0	0	0	0	0	0	0
7.7796	7.8310	4.7022	H	0	0	0	0	0	0	0	0	0	0	0	0	0
6.8004	8.0170	2.4359	H	0	0	0	0	0	0	0	0	0	0	0	0	0
6.7739	9.7659	2.5851	H	0	0	0	0	0	0	0	0	0	0	0	0	0
5.3212	7.8684	4.4663	H	0	0	0	0	0	0	0	0	0	0	0	0	0
5.2351	9.6315	4.4772	H	0	0	0	0	0	0	0	0	0	0	0	0	0
3.0255	10.1906	0.8726	H	0	0	0	0	0	0	0	0	0	0	0	0	0
3.9779	11.0943	2.0525	H	0	0	0	0	0	0	0	0	0	0	0	0	0
6.0531	10.0602	1.0740	H	0	0	0	0	0	0	0	0	0	0	0	0	0

```
 5.0546    9.2945   -0.1500 H   0  0  0  0  0  0  0  0  0  0  0  0
 4.2712   11.5155   -0.9089 H   0  0  0  0  0  0  0  0  0  0  0  0
 5.2757   12.2885    0.3245 H   0  0  0  0  0  0  0  0  0  0  0  0
 7.3057   11.3044   -0.7126 H   0  0  0  0  0  0  0  0  0  0  0  0
 6.3009   10.5329   -1.9466 H   0  0  0  0  0  0  0  0  0  0  0  0
 5.5187   12.7638   -2.7046 H   0  0  0  0  0  0  0  0  0  0  0  0
 6.5229   13.5354   -1.4701 H   0  0  0  0  0  0  0  0  0  0  0  0
 8.5531   12.5513   -2.5068 H   0  0  0  0  0  0  0  0  0  0  0  0
 7.5488   11.7804   -3.7416 H   0  0  0  0  0  0  0  0  0  0  0  0
 6.7672   14.0120   -4.4990 H   0  0  0  0  0  0  0  0  0  0  0  0
 7.7713   14.7829   -3.2642 H   0  0  0  0  0  0  0  0  0  0  0  0
 8.9163   14.7914   -5.4668 H   0  0  0  0  0  0  0  0  0  0  0  0
 8.7972   13.0283   -5.5358 H   0  0  0  0  0  0  0  0  0  0  0  0
 9.8014   13.7992   -4.3008 H   0  0  0  0  0  0  0  0  0  0  0  0
  1  2  1  0  0  0  0
  2  3  1  0  0  0  0
  3  4  1  0  0  0  0
  4  5  1  0  0  0  0
  5  6  1  0  0  0  0
  6  7  1  0  0  0  0
  7  8  1  0  0  0  0
  8  9  1  0  0  0  0
 10 11  1  0  0  0  0
 11 12  1  0  0  0  0
 12 13  1  0  0  0  0
 13 14  1  0  0  0  0
 14 15  1  0  0  0  0
 15 16  1  0  0  0  0
 16 17  1  0  0  0  0
 17  1  1  0  0  0  0
 18 19  1  0  0  0  0
 19 20  1  0  0  0  0
 20 21  1  0  0  0  0
 21 22  1  0  0  0  0
 22 23  1  0  0  0  0
 23 24  1  0  0  0  0
 24 25  1  0  0  0  0
 18  1  1  0  0  0  0
  1 26  1  0  0  0  0
  2 27  1  0  0  0  0
  2 28  1  0  0  0  0
  3 29  1  0  0  0  0
  3 30  1  0  0  0  0
  4 31  1  0  0  0  0
  4 32  1  0  0  0  0
  5 33  1  0  0  0  0
  5 34  1  0  0  0  0
  6 35  1  0  0  0  0
  6 36  1  0  0  0  0
  7 37  1  0  0  0  0
  7 38  1  0  0  0  0
  8 39  1  0  0  0  0
  8 40  1  0  0  0  0
  9 41  1  0  0  0  0
  9 42  1  0  0  0  0
  9 43  1  0  0  0  0
 10 44  1  0  0  0  0
 10 45  1  0  0  0  0
 10 46  1  0  0  0  0
 11 47  1  0  0  0  0
 11 48  1  0  0  0  0
 12 49  1  0  0  0  0
 12 50  1  0  0  0  0
```

```
 13 51  1  0  0  0  0
 13 52  1  0  0  0  0
 14 53  1  0  0  0  0
 14 54  1  0  0  0  0
 15 55  1  0  0  0  0
 15 56  1  0  0  0  0
 16 57  1  0  0  0  0
 16 58  1  0  0  0  0
 17 59  1  0  0  0  0
 17 60  1  0  0  0  0
 18 61  1  0  0  0  0
 18 62  1  0  0  0  0
 19 63  1  0  0  0  0
 19 64  1  0  0  0  0
 20 65  1  0  0  0  0
 20 66  1  0  0  0  0
 21 67  1  0  0  0  0
 21 68  1  0  0  0  0
 22 69  1  0  0  0  0
 22 70  1  0  0  0  0
 23 71  1  0  0  0  0
 23 72  1  0  0  0  0
 24 73  1  0  0  0  0
 24 74  1  0  0  0  0
 25 75  1  0  0  0  0
 25 76  1  0  0  0  0
 25 77  1  0  0  0  0
M  END
>  <Alias name>
Trioctylsilane

>  <deltaH°(c)>   kJ/mol
-17164

$$$$
11-Cyclopentylheneicosane
  ChemBrain IXL

 78 78  0  0  0  0  0  0  0  0  1 V2000
   24.4124    7.4003    0.0000 C   0  0  0  0  0  0  0  0  0  0  0  0
   22.9762    7.4865   -0.5517 C   0  0  0  0  0  0  0  0  0  0  0  0
   22.2341    8.6545    0.1269 C   0  0  0  0  0  0  0  0  0  0  0  0
   20.7981    8.7412   -0.4257 C   0  0  0  0  0  0  0  0  0  0  0  0
   20.0557    9.9088    0.2530 C   0  0  0  0  0  0  0  0  0  0  0  0
   18.6205    9.9962   -0.3012 C   0  0  0  0  0  0  0  0  0  0  0  0
   17.8773   11.1632    0.3777 C   0  0  0  0  0  0  0  0  0  0  0  0
   16.4429   11.2512   -0.1786 C   0  0  0  0  0  0  0  0  0  0  0  0
   15.6985   12.4169    0.5008 C   0  0  0  0  0  0  0  0  0  0  0  0
   14.2635   12.5044   -0.0586 C   0  0  0  0  0  0  0  0  0  0  0  0
   13.4982   13.6525    0.6425 C   0  0  0  0  0  0  0  0  0  0  0  0
   12.0584   13.7100    0.0921 C   0  0  0  0  0  0  0  0  0  0  0  0
   11.2959   12.4384    0.5157 C   0  0  0  0  0  0  0  0  0  0  0  0
    9.8559   12.4978   -0.0294 C   0  0  0  0  0  0  0  0  0  0  0  0
    9.0960   11.2237    0.3876 C   0  0  0  0  0  0  0  0  0  0  0  0
    7.6579   11.2792   -0.1636 C   0  0  0  0  0  0  0  0  0  0  0  0
    6.9002   10.0020    0.2483 C   0  0  0  0  0  0  0  0  0  0  0  0
    5.4638   10.0549   -0.3072 C   0  0  0  0  0  0  0  0  0  0  0  0
    4.7075    8.7758    0.1014 C   0  0  0  0  0  0  0  0  0  0  0  0
    3.2716    8.8274   -0.4562 C   0  0  0  0  0  0  0  0  0  0  0  0
    2.5158    7.5477   -0.0489 C   0  0  0  0  0  0  0  0  0  0  0  0
   14.2247   15.4102   -1.0795 C   0  0  0  0  0  0  0  0  0  0  0  0
   14.2504   15.0097    0.4022 C   0  0  0  0  0  0  0  0  0  0  0  0
   13.5338   16.1560    1.1252 C   0  0  0  0  0  0  0  0  0  0  0  0
```

12.5053	16.7364	0.0966	C	0	0	0	0	0	0	0	0	0	0	0	0
12.8666	16.0966	-1.2824	C	0	0	0	0	0	0	0	0	0	0	0	0
24.9342	8.3269	-0.2050	H	0	0	0	0	0	0	0	0	0	0	0	0
24.9335	6.5795	-0.4769	H	0	0	0	0	0	0	0	0	0	0	0	0
24.3789	7.2338	1.0696	H	0	0	0	0	0	0	0	0	0	0	0	0
23.0099	7.6530	-1.6213	H	0	0	0	0	0	0	0	0	0	0	0	0
22.4546	6.5598	-0.3469	H	0	0	0	0	0	0	0	0	0	0	0	0
22.1999	8.4877	1.1964	H	0	0	0	0	0	0	0	0	0	0	0	0
22.7560	9.5812	-0.0774	H	0	0	0	0	0	0	0	0	0	0	0	0
20.8324	8.9082	-1.4952	H	0	0	0	0	0	0	0	0	0	0	0	0
20.2763	7.8144	-0.2217	H	0	0	0	0	0	0	0	0	0	0	0	0
20.0203	9.7412	1.3224	H	0	0	0	0	0	0	0	0	0	0	0	0
20.5781	10.8355	0.0499	H	0	0	0	0	0	0	0	0	0	0	0	0
18.6561	10.1643	-1.3705	H	0	0	0	0	0	0	0	0	0	0	0	0
18.0983	9.0692	-0.0987	H	0	0	0	0	0	0	0	0	0	0	0	0
17.8403	10.9944	1.4468	H	0	0	0	0	0	0	0	0	0	0	0	0
18.4001	12.0900	0.1763	H	0	0	0	0	0	0	0	0	0	0	0	0
16.4802	11.4209	-1.2475	H	0	0	0	0	0	0	0	0	0	0	0	0
15.9206	10.3239	0.0217	H	0	0	0	0	0	0	0	0	0	0	0	0
15.6597	12.2463	1.5695	H	0	0	0	0	0	0	0	0	0	0	0	0
16.2217	13.3427	0.3016	H	0	0	0	0	0	0	0	0	0	0	0	0
14.3025	12.6930	-1.1235	H	0	0	0	0	0	0	0	0	0	0	0	0
13.7514	11.5684	0.1220	H	0	0	0	0	0	0	0	0	0	0	0	0
13.4620	13.4556	1.7068	H	0	0	0	0	0	0	0	0	0	0	0	0
12.0864	13.7640	-0.9835	H	0	0	0	0	0	0	0	0	0	0	0	0
11.5531	14.5783	0.4884	H	0	0	0	0	0	0	0	0	0	0	0	0
11.2705	12.3757	1.5966	H	0	0	0	0	0	0	0	0	0	0	0	0
11.7966	11.5665	0.1153	H	0	0	0	0	0	0	0	0	0	0	0	0
9.8817	12.5651	-1.1100	H	0	0	0	0	0	0	0	0	0	0	0	0
9.3523	13.3673	0.3746	H	0	0	0	0	0	0	0	0	0	0	0	0
9.0662	11.1583	1.4683	H	0	0	0	0	0	0	0	0	0	0	0	0
9.6024	10.3542	-0.0128	H	0	0	0	0	0	0	0	0	0	0	0	0
7.6881	11.3483	-1.2440	H	0	0	0	0	0	0	0	0	0	0	0	0
7.1498	12.1465	0.2396	H	0	0	0	0	0	0	0	0	0	0	0	0
6.8673	9.9343	1.3287	H	0	0	0	0	0	0	0	0	0	0	0	0
7.4104	9.1348	-0.1524	H	0	0	0	0	0	0	0	0	0	0	0	0
5.4969	10.1250	-1.3875	H	0	0	0	0	0	0	0	0	0	0	0	0
4.9526	10.9207	0.0952	H	0	0	0	0	0	0	0	0	0	0	0	0
4.6730	8.7063	1.1816	H	0	0	0	0	0	0	0	0	0	0	0	0
5.2196	7.9100	-0.2999	H	0	0	0	0	0	0	0	0	0	0	0	0
3.3062	8.8977	-1.5363	H	0	0	0	0	0	0	0	0	0	0	0	0
2.7591	9.6926	-0.0543	H	0	0	0	0	0	0	0	0	0	0	0	0
3.0283	6.6824	-0.4508	H	0	0	0	0	0	0	0	0	0	0	0	0
2.4810	7.4773	1.0313	H	0	0	0	0	0	0	0	0	0	0	0	0
1.5070	7.5842	-0.4409	H	0	0	0	0	0	0	0	0	0	0	0	0
15.0166	16.1245	-1.2675	H	0	0	0	0	0	0	0	0	0	0	0	0
14.3341	14.5502	-1.7269	H	0	0	0	0	0	0	0	0	0	0	0	0
15.2689	14.9218	0.7555	H	0	0	0	0	0	0	0	0	0	0	0	0
13.0479	15.8229	2.0338	H	0	0	0	0	0	0	0	0	0	0	0	0
14.2630	16.9231	1.3547	H	0	0	0	0	0	0	0	0	0	0	0	0
11.4922	16.4654	0.3640	H	0	0	0	0	0	0	0	0	0	0	0	0
12.6053	17.8140	0.0612	H	0	0	0	0	0	0	0	0	0	0	0	0
12.1336	15.3509	-1.5222	H	0	0	0	0	0	0	0	0	0	0	0	0
12.9044	16.8409	-2.0679	H	0	0	0	0	0	0	0	0	0	0	0	0

1	2	1	0	0	0	0
2	3	1	0	0	0	0
3	4	1	0	0	0	0
4	5	1	0	0	0	0
5	6	1	0	0	0	0
6	7	1	0	0	0	0
7	8	1	0	0	0	0
8	9	1	0	0	0	0
9	10	1	0	0	0	0

10	11	1	0	0	0	0
11	12	1	0	0	0	0
12	13	1	0	0	0	0
13	14	1	0	0	0	0
14	15	1	0	0	0	0
15	16	1	0	0	0	0
16	17	1	0	0	0	0
17	18	1	0	0	0	0
18	19	1	0	0	0	0
19	20	1	0	0	0	0
20	21	1	0	0	0	0
22	23	1	0	0	0	0
23	24	1	0	0	0	0
24	25	1	0	0	0	0
26	22	1	0	0	0	0
23	11	1	0	0	0	0
25	26	1	0	0	0	0
1	27	1	0	0	0	0
1	28	1	0	0	0	0
1	29	1	0	0	0	0
2	30	1	0	0	0	0
2	31	1	0	0	0	0
3	32	1	0	0	0	0
3	33	1	0	0	0	0
4	34	1	0	0	0	0
4	35	1	0	0	0	0
5	36	1	0	0	0	0
5	37	1	0	0	0	0
6	38	1	0	0	0	0
6	39	1	0	0	0	0
7	40	1	0	0	0	0
7	41	1	0	0	0	0
8	42	1	0	0	0	0
8	43	1	0	0	0	0
9	44	1	0	0	0	0
9	45	1	0	0	0	0
10	46	1	0	0	0	0
10	47	1	0	0	0	0
11	48	1	0	0	0	0
12	49	1	0	0	0	0
12	50	1	0	0	0	0
13	51	1	0	0	0	0
13	52	1	0	0	0	0
14	53	1	0	0	0	0
14	54	1	0	0	0	0
15	55	1	0	0	0	0
15	56	1	0	0	0	0
16	57	1	0	0	0	0
16	58	1	0	0	0	0
17	59	1	0	0	0	0
17	60	1	0	0	0	0
18	61	1	0	0	0	0
18	62	1	0	0	0	0
19	63	1	0	0	0	0
19	64	1	0	0	0	0
20	65	1	0	0	0	0
20	66	1	0	0	0	0
21	67	1	0	0	0	0
21	68	1	0	0	0	0
21	69	1	0	0	0	0
22	70	1	0	0	0	0
22	71	1	0	0	0	0
23	72	1	0	0	0	0

 24 73 1 0 0 0 0
 24 74 1 0 0 0 0
 25 75 1 0 0 0 0
 25 76 1 0 0 0 0
 26 77 1 0 0 0 0
 26 78 1 0 0 0 0
M END
> <Alias name>
11-Cyclopentylheneicosane

> <deltaH°(c)>   kJ/mol
-17026

$$$$
Heneicosylcyclopentane
  ChemBrain IXL

 78 78 0 0 0 0 0 0 0 0 1 V2000
    6.1297   14.0257   0.1039 C   0 0 0 0 0 0 0 0 0 0 0 0
    4.9808   12.9985   0.2406 C   0 0 0 0 0 0 0 0 0 0 0 0
    3.6069   13.7035   0.0799 C   0 0 0 0 0 0 0 0 0 0 0 0
    7.4819   13.3043   0.2640 C   0 0 0 0 0 0 0 0 0 0 0 0
    3.4017   14.7985   1.1269 C   0 0 0 0 0 0 0 0 0 0 0 0
    3.4921   14.4299   -1.2542 C   0 0 0 0 0 0 0 0 0 0 0 0
    8.6283   14.3251   0.1278 C   0 0 0 0 0 0 0 0 0 0 0 0
    2.4185   15.8210   0.4666 C   0 0 0 0 0 0 0 0 0 0 0 0
    2.3512   15.4231   -1.0464 C   0 0 0 0 0 0 0 0 0 0 0 0
    9.9806   13.6034   0.2876 C   0 0 0 0 0 0 0 0 0 0 0 0
   11.1272   14.6241   0.1510 C   0 0 0 0 0 0 0 0 0 0 0 0
   12.4793   13.9020   0.3103 C   0 0 0 0 0 0 0 0 0 0 0 0
   13.6262   14.9224   0.1735 C   0 0 0 0 0 0 0 0 0 0 0 0
   14.9781   14.1999   0.3322 C   0 0 0 0 0 0 0 0 0 0 0 0
   16.1253   15.2199   0.1949 C   0 0 0 0 0 0 0 0 0 0 0 0
   17.4771   14.4969   0.3531 C   0 0 0 0 0 0 0 0 0 0 0 0
   18.6245   15.5165   0.2154 C   0 0 0 0 0 0 0 0 0 0 0 0
   19.9761   14.7931   0.3731 C   0 0 0 0 0 0 0 0 0 0 0 0
   21.1238   15.8123   0.2349 C   0 0 0 0 0 0 0 0 0 0 0 0
   22.4753   15.0886   0.3922 C   0 0 0 0 0 0 0 0 0 0 0 0
   23.6231   16.1075   0.2537 C   0 0 0 0 0 0 0 0 0 0 0 0
   24.9745   15.3835   0.4107 C   0 0 0 0 0 0 0 0 0 0 0 0
   26.1225   16.4022   0.2720 C   0 0 0 0 0 0 0 0 0 0 0 0
   27.4738   15.6781   0.4289 C   0 0 0 0 0 0 0 0 0 0 0 0
   28.6220   16.6968   0.2901 C   0 0 0 0 0 0 0 0 0 0 0 0
   29.9732   15.9727   0.4470 C   0 0 0 0 0 0 0 0 0 0 0 0
    6.0853   14.4916   -0.8707 H   0 0 0 0 0 0 0 0 0 0 0 0
    6.0340   14.7816   0.8703 H   0 0 0 0 0 0 0 0 0 0 0 0
    5.0318   12.5312   1.2162 H   0 0 0 0 0 0 0 0 0 0 0 0
    5.0858   12.2405   -0.5257 H   0 0 0 0 0 0 0 0 0 0 0 0
    2.8058   12.9792   0.1596 H   0 0 0 0 0 0 0 0 0 0 0 0
    7.5285   12.8373   1.2401 H   0 0 0 0 0 0 0 0 0 0 0 0
    7.5800   12.5467   -0.5037 H   0 0 0 0 0 0 0 0 0 0 0 0
    3.0064   14.4051   2.0550 H   0 0 0 0 0 0 0 0 0 0 0 0
    4.3468   15.2896   1.3037 H   0 0 0 0 0 0 0 0 0 0 0 0
    4.4071   14.9695   -1.4520 H   0 0 0 0 0 0 0 0 0 0 0 0
    3.2754   13.7433   -2.0630 H   0 0 0 0 0 0 0 0 0 0 0 0
    8.5815   14.7923   -0.8482 H   0 0 0 0 0 0 0 0 0 0 0 0
    8.5304   15.0825   0.8956 H   0 0 0 0 0 0 0 0 0 0 0 0
    1.4299   15.7471   0.9022 H   0 0 0 0 0 0 0 0 0 0 0 0
    2.8076   16.8242   0.5884 H   0 0 0 0 0 0 0 0 0 0 0 0
    1.4137   14.9140   -1.2327 H   0 0 0 0 0 0 0 0 0 0 0 0
    2.4412   16.2875   -1.6925 H   0 0 0 0 0 0 0 0 0 0 0 0
   10.0273   13.1364   1.2636 H   0 0 0 0 0 0 0 0 0 0 0 0
   10.0783   12.8459   -0.4802 H   0 0 0 0 0 0 0 0 0 0 0 0

```
 11.0802   15.0913   -0.8249 H   0  0  0  0  0  0  0  0  0  0  0  0
 11.0297   15.3815    0.9190 H   0  0  0  0  0  0  0  0  0  0  0  0
 12.5263   13.4350    1.2863 H   0  0  0  0  0  0  0  0  0  0  0  0
 12.5766   13.1445   -0.4575 H   0  0  0  0  0  0  0  0  0  0  0  0
 13.5790   15.3897   -0.8024 H   0  0  0  0  0  0  0  0  0  0  0  0
 13.5291   15.6797    0.9415 H   0  0  0  0  0  0  0  0  0  0  0  0
 15.0254   13.7328    1.3082 H   0  0  0  0  0  0  0  0  0  0  0  0
 15.0749   13.4423   -0.4356 H   0  0  0  0  0  0  0  0  0  0  0  0
 16.0779   15.6872   -0.7809 H   0  0  0  0  0  0  0  0  0  0  0  0
 16.0288   15.9772    0.9630 H   0  0  0  0  0  0  0  0  0  0  0  0
 17.5246   14.0298    1.3291 H   0  0  0  0  0  0  0  0  0  0  0  0
 17.5733   13.7393   -0.4148 H   0  0  0  0  0  0  0  0  0  0  0  0
 18.5769   15.9839   -0.7605 H   0  0  0  0  0  0  0  0  0  0  0  0
 18.5286   16.2739    0.9835 H   0  0  0  0  0  0  0  0  0  0  0  0
 20.0238   14.3260    1.3490 H   0  0  0  0  0  0  0  0  0  0  0  0
 20.0718   14.0355   -0.3949 H   0  0  0  0  0  0  0  0  0  0  0  0
 21.0760   16.2797   -0.7409 H   0  0  0  0  0  0  0  0  0  0  0  0
 21.0284   16.5698    1.0030 H   0  0  0  0  0  0  0  0  0  0  0  0
 22.5231   14.6214    1.3681 H   0  0  0  0  0  0  0  0  0  0  0  0
 22.5705   14.3310   -0.3758 H   0  0  0  0  0  0  0  0  0  0  0  0
 23.5752   16.5748   -0.7222 H   0  0  0  0  0  0  0  0  0  0  0  0
 23.5280   16.8651    1.0218 H   0  0  0  0  0  0  0  0  0  0  0  0
 25.0224   14.9163    1.3866 H   0  0  0  0  0  0  0  0  0  0  0  0
 25.0695   14.6259   -0.3573 H   0  0  0  0  0  0  0  0  0  0  0  0
 26.0745   16.8695   -0.7038 H   0  0  0  0  0  0  0  0  0  0  0  0
 26.0276   17.1598    1.0401 H   0  0  0  0  0  0  0  0  0  0  0  0
 27.5218   15.2109    1.4048 H   0  0  0  0  0  0  0  0  0  0  0  0
 27.5686   14.9204   -0.3391 H   0  0  0  0  0  0  0  0  0  0  0  0
 28.5740   17.1640   -0.6857 H   0  0  0  0  0  0  0  0  0  0  0  0
 28.5272   17.4544    1.0581 H   0  0  0  0  0  0  0  0  0  0  0  0
 30.7798   16.6887    0.3494 H   0  0  0  0  0  0  0  0  0  0  0  0
 30.0681   15.2151   -0.3211 H   0  0  0  0  0  0  0  0  0  0  0  0
 30.0213   15.5055    1.4228 H   0  0  0  0  0  0  0  0  0  0  0  0
  1  2  1  0  0  0  0
  1  4  1  0  0  0  0
  2  3  1  0  0  0  0
  5  3  1  0  0  0  0
  3  6  1  0  0  0  0
  4  7  1  0  0  0  0
  8  5  1  0  0  0  0
  6  9  1  0  0  0  0
  7 10  1  0  0  0  0
  9  8  1  0  0  0  0
 10 11  1  0  0  0  0
 11 12  1  0  0  0  0
 12 13  1  0  0  0  0
 13 14  1  0  0  0  0
 14 15  1  0  0  0  0
 15 16  1  0  0  0  0
 16 17  1  0  0  0  0
 17 18  1  0  0  0  0
 18 19  1  0  0  0  0
 19 20  1  0  0  0  0
 20 21  1  0  0  0  0
 21 22  1  0  0  0  0
 22 23  1  0  0  0  0
 23 24  1  0  0  0  0
 24 25  1  0  0  0  0
 25 26  1  0  0  0  0
  1 27  1  0  0  0  0
  1 28  1  0  0  0  0
  2 29  1  0  0  0  0
  2 30  1  0  0  0  0
```

```
  3 31  1  0  0  0  0
  4 32  1  0  0  0  0
  4 33  1  0  0  0  0
  5 34  1  0  0  0  0
  5 35  1  0  0  0  0
  6 36  1  0  0  0  0
  6 37  1  0  0  0  0
  7 38  1  0  0  0  0
  7 39  1  0  0  0  0
  8 40  1  0  0  0  0
  8 41  1  0  0  0  0
  9 42  1  0  0  0  0
  9 43  1  0  0  0  0
 10 44  1  0  0  0  0
 10 45  1  0  0  0  0
 11 46  1  0  0  0  0
 11 47  1  0  0  0  0
 12 48  1  0  0  0  0
 12 49  1  0  0  0  0
 13 50  1  0  0  0  0
 13 51  1  0  0  0  0
 14 52  1  0  0  0  0
 14 53  1  0  0  0  0
 15 54  1  0  0  0  0
 15 55  1  0  0  0  0
 16 56  1  0  0  0  0
 16 57  1  0  0  0  0
 17 58  1  0  0  0  0
 17 59  1  0  0  0  0
 18 60  1  0  0  0  0
 18 61  1  0  0  0  0
 19 62  1  0  0  0  0
 19 63  1  0  0  0  0
 20 64  1  0  0  0  0
 20 65  1  0  0  0  0
 21 66  1  0  0  0  0
 21 67  1  0  0  0  0
 22 68  1  0  0  0  0
 22 69  1  0  0  0  0
 23 70  1  0  0  0  0
 23 71  1  0  0  0  0
 24 72  1  0  0  0  0
 24 73  1  0  0  0  0
 25 74  1  0  0  0  0
 25 75  1  0  0  0  0
 26 76  1  0  0  0  0
 26 77  1  0  0  0  0
 26 78  1  0  0  0  0
M  END
>  <Alias name>
Heneicosylcyclopentane

>  <deltaH°(c)>   kJ/mol
-17014

$$$$
3-Cyclohexyleicosane
  ChemBrain IXL

 78 78  0  0  0  0  0  0  0  0  1 V2000
    5.7049   14.1731   -0.0891 C   0  0  0  0  0  0  0  0  0  0  0  0
    4.3614   13.8501    0.6079 C   0  0  0  0  0  0  0  0  0  0  0  0
    3.2321   14.6941   -0.0306 C   0  0  0  0  0  0  0  0  0  0  0  0
```

4.0398	12.3398	0.4498	C	0	0	0	0	0	0	0	0	0	0	0	0
6.8318	13.3258	0.5366	C	0	0	0	0	0	0	0	0	0	0	0	0
3.5391	16.1945	0.1530	C	0	0	0	0	0	0	0	0	0	0	0	0
2.7046	12.0185	1.1542	C	0	0	0	0	0	0	0	0	0	0	0	0
3.9288	11.9805	-1.0485	C	0	0	0	0	0	0	0	0	0	0	0	0
8.1706	13.6695	-0.1445	C	0	0	0	0	0	0	0	0	0	0	0	0
2.3966	10.5152	1.0087	C	0	0	0	0	0	0	0	0	0	0	0	0
3.6183	10.4771	-1.1938	C	0	0	0	0	0	0	0	0	0	0	0	0
9.2973	12.8247	0.4814	C	0	0	0	0	0	0	0	0	0	0	0	0
2.2859	10.1555	-0.4868	C	0	0	0	0	0	0	0	0	0	0	0	0
10.6362	13.1681	-0.2001	C	0	0	0	0	0	0	0	0	0	0	0	0
11.7633	12.3247	0.4270	C	0	0	0	0	0	0	0	0	0	0	0	0
13.1021	12.6680	-0.2547	C	0	0	0	0	0	0	0	0	0	0	0	0
14.2295	11.8258	0.3735	C	0	0	0	0	0	0	0	0	0	0	0	0
15.5682	12.1691	-0.3084	C	0	0	0	0	0	0	0	0	0	0	0	0
16.6960	11.3281	0.3208	C	0	0	0	0	0	0	0	0	0	0	0	0
18.0346	11.6714	-0.3613	C	0	0	0	0	0	0	0	0	0	0	0	0
19.1626	10.8313	0.2687	C	0	0	0	0	0	0	0	0	0	0	0	0
20.5012	11.1744	-0.4136	C	0	0	0	0	0	0	0	0	0	0	0	0
21.6294	10.3350	0.2170	C	0	0	0	0	0	0	0	0	0	0	0	0
22.9680	10.6780	-0.4654	C	0	0	0	0	0	0	0	0	0	0	0	0
24.0963	9.8388	0.1654	C	0	0	0	0	0	0	0	0	0	0	0	0
25.4348	10.1816	-0.5170	C	0	0	0	0	0	0	0	0	0	0	0	0
5.9332	15.2231	0.0388	H	0	0	0	0	0	0	0	0	0	0	0	0
5.6281	13.9480	-1.1449	H	0	0	0	0	0	0	0	0	0	0	0	0
4.4372	14.0911	1.6610	H	0	0	0	0	0	0	0	0	0	0	0	0
3.1638	14.4673	-1.0866	H	0	0	0	0	0	0	0	0	0	0	0	0
2.2915	14.4598	0.4500	H	0	0	0	0	0	0	0	0	0	0	0	0
4.8309	11.7555	0.9010	H	0	0	0	0	0	0	0	0	0	0	0	0
6.6154	12.2750	0.3953	H	0	0	0	0	0	0	0	0	0	0	0	0
6.8999	13.5414	1.5957	H	0	0	0	0	0	0	0	0	0	0	0	0
2.7390	16.7817	-0.2802	H	0	0	0	0	0	0	0	0	0	0	0	0
4.4711	16.4362	-0.3409	H	0	0	0	0	0	0	0	0	0	0	0	0
3.6208	16.4197	1.2091	H	0	0	0	0	0	0	0	0	0	0	0	0
1.9084	12.5942	0.7007	H	0	0	0	0	0	0	0	0	0	0	0	0
2.7803	12.2714	2.2043	H	0	0	0	0	0	0	0	0	0	0	0	0
3.1332	12.5579	-1.5012	H	0	0	0	0	0	0	0	0	0	0	0	0
4.8643	12.2039	-1.5442	H	0	0	0	0	0	0	0	0	0	0	0	0
8.3875	14.7212	-0.0040	H	0	0	0	0	0	0	0	0	0	0	0	0
8.1028	13.4531	-1.2035	H	0	0	0	0	0	0	0	0	0	0	0	0
3.1935	9.9379	1.4609	H	0	0	0	0	0	0	0	0	0	0	0	0
1.4608	10.2889	1.5044	H	0	0	0	0	0	0	0	0	0	0	0	0
4.4150	9.8988	-0.7425	H	0	0	0	0	0	0	0	0	0	0	0	0
3.5396	10.2248	-2.2439	H	0	0	0	0	0	0	0	0	0	0	0	0
9.0803	11.7729	0.3413	H	0	0	0	0	0	0	0	0	0	0	0	0
9.3652	13.0414	1.5404	H	0	0	0	0	0	0	0	0	0	0	0	0
1.4889	10.7329	-0.9389	H	0	0	0	0	0	0	0	0	0	0	0	0
2.0691	9.0995	-0.5888	H	0	0	0	0	0	0	0	0	0	0	0	0
10.8526	14.2202	-0.0609	H	0	0	0	0	0	0	0	0	0	0	0	0
10.5686	12.9504	-1.2588	H	0	0	0	0	0	0	0	0	0	0	0	0
11.5468	11.2726	0.2882	H	0	0	0	0	0	0	0	0	0	0	0	0
11.8309	12.5426	1.4857	H	0	0	0	0	0	0	0	0	0	0	0	0
13.3179	13.7203	-0.1168	H	0	0	0	0	0	0	0	0	0	0	0	0
13.0348	12.4490	-1.3132	H	0	0	0	0	0	0	0	0	0	0	0	0
14.0136	10.7735	0.2358	H	0	0	0	0	0	0	0	0	0	0	0	0
14.2969	12.0451	1.4319	H	0	0	0	0	0	0	0	0	0	0	0	0
15.7835	13.2217	-0.1716	H	0	0	0	0	0	0	0	0	0	0	0	0
15.5012	11.9490	-1.3667	H	0	0	0	0	0	0	0	0	0	0	0	0
16.4806	10.2756	0.1841	H	0	0	0	0	0	0	0	0	0	0	0	0
16.7631	11.5484	1.3790	H	0	0	0	0	0	0	0	0	0	0	0	0
18.2496	12.7241	-0.2253	H	0	0	0	0	0	0	0	0	0	0	0	0
17.9677	11.4503	-1.4194	H	0	0	0	0	0	0	0	0	0	0	0	0
18.9477	9.7785	0.1328	H	0	0	0	0	0	0	0	0	0	0	0	0

19.2296 11.0525 1.3267 H 0 0 0 0 0 0 0 0 0 0 0 0
20.7159 12.2273 -0.2782 H 0 0 0 0 0 0 0 0 0 0 0 0
20.4344 10.9527 -1.4715 H 0 0 0 0 0 0 0 0 0 0 0 0
21.4147 9.2821 0.0818 H 0 0 0 0 0 0 0 0 0 0 0 0
21.6963 10.5568 1.2749 H 0 0 0 0 0 0 0 0 0 0 0 0
23.1825 11.7309 -0.3305 H 0 0 0 0 0 0 0 0 0 0 0 0
22.9012 10.4559 -1.5233 H 0 0 0 0 0 0 0 0 0 0 0 0
23.8817 8.7859 0.0306 H 0 0 0 0 0 0 0 0 0 0 0 0
24.1632 10.0610 1.2232 H 0 0 0 0 0 0 0 0 0 0 0 0
25.6495 11.2346 -0.3821 H 0 0 0 0 0 0 0 0 0 0 0 0
25.3681 9.9594 -1.5748 H 0 0 0 0 0 0 0 0 0 0 0 0
26.2275 9.5919 -0.0735 H 0 0 0 0 0 0 0 0 0 0 0 0
1 2 1 0 0 0 0
1 5 1 0 0 0 0
2 3 1 0 0 0 0
2 4 1 0 0 0 0
3 6 1 0 0 0 0
7 4 1 0 0 0 0
4 8 1 0 0 0 0
5 9 1 0 0 0 0
10 7 1 0 0 0 0
8 11 1 0 0 0 0
9 12 1 0 0 0 0
13 10 1 0 0 0 0
11 13 1 0 0 0 0
12 14 1 0 0 0 0
14 15 1 0 0 0 0
15 16 1 0 0 0 0
16 17 1 0 0 0 0
17 18 1 0 0 0 0
18 19 1 0 0 0 0
19 20 1 0 0 0 0
20 21 1 0 0 0 0
21 22 1 0 0 0 0
22 23 1 0 0 0 0
23 24 1 0 0 0 0
24 25 1 0 0 0 0
25 26 1 0 0 0 0
1 27 1 0 0 0 0
1 28 1 0 0 0 0
2 29 1 0 0 0 0
3 30 1 0 0 0 0
3 31 1 0 0 0 0
4 32 1 0 0 0 0
5 33 1 0 0 0 0
5 34 1 0 0 0 0
6 35 1 0 0 0 0
6 36 1 0 0 0 0
6 37 1 0 0 0 0
7 38 1 0 0 0 0
7 39 1 0 0 0 0
8 40 1 0 0 0 0
8 41 1 0 0 0 0
9 42 1 0 0 0 0
9 43 1 0 0 0 0
10 44 1 0 0 0 0
10 45 1 0 0 0 0
11 46 1 0 0 0 0
11 47 1 0 0 0 0
12 48 1 0 0 0 0
12 49 1 0 0 0 0
13 50 1 0 0 0 0
13 51 1 0 0 0 0

```
 14 52  1  0  0  0  0
 14 53  1  0  0  0  0
 15 54  1  0  0  0  0
 15 55  1  0  0  0  0
 16 56  1  0  0  0  0
 16 57  1  0  0  0  0
 17 58  1  0  0  0  0
 17 59  1  0  0  0  0
 18 60  1  0  0  0  0
 18 61  1  0  0  0  0
 19 62  1  0  0  0  0
 19 63  1  0  0  0  0
 20 64  1  0  0  0  0
 20 65  1  0  0  0  0
 21 66  1  0  0  0  0
 21 67  1  0  0  0  0
 22 68  1  0  0  0  0
 22 69  1  0  0  0  0
 23 70  1  0  0  0  0
 23 71  1  0  0  0  0
 24 72  1  0  0  0  0
 24 73  1  0  0  0  0
 25 74  1  0  0  0  0
 25 75  1  0  0  0  0
 26 76  1  0  0  0  0
 26 77  1  0  0  0  0
 26 78  1  0  0  0  0
M  END
>  <Alias name>
3-Cyclohexyleicosane

>  <deltaH°(c)>   kJ/mol
-16997

$$$$
11-Phenylheneicosane
  ChemBrain IXL

 75 75  0  0  0  0  0  0  0  0  1 V2000
   16.0880   11.3669    0.5646 C   0  0  0  0  0  0  0  0  0  0  0  0
   16.7135   11.9984    1.8234 C   0  0  0  0  0  0  0  0  0  0  0  0
   18.1148   11.4008    2.0557 C   0  0  0  0  0  0  0  0  0  0  0  0
   14.6902   11.9708    0.3274 C   0  0  0  0  0  0  0  0  0  0  0  0
   14.0692   11.3473   -0.9376 C   0  0  0  0  0  0  0  0  0  0  0  0
   12.0511   11.3290   -2.4408 C   0  0  0  0  0  0  0  0  0  0  0  0
   12.6742   11.9561   -1.1786 C   0  0  0  0  0  0  0  0  0  0  0  0
   10.6593   11.9433   -2.6860 C   0  0  0  0  0  0  0  0  0  0  0  0
   20.1461   11.4430    3.5404 C   0  0  0  0  0  0  0  0  0  0  0  0
   18.7429   12.0370    3.3108 C   0  0  0  0  0  0  0  0  0  0  0  0
   20.7747   12.0801    4.7949 C   0  0  0  0  0  0  0  0  0  0  0  0
    8.6440   11.9299   -4.1930 C   0  0  0  0  0  0  0  0  0  0  0  0
   10.0350   11.3142   -3.9467 C   0  0  0  0  0  0  0  0  0  0  0  0
    8.0178   11.2974   -5.4510 C   0  0  0  0  0  0  0  0  0  0  0  0
   22.8066   12.1234    6.2788 C   0  0  0  0  0  0  0  0  0  0  0  0
   22.1784   11.4869    5.0238 C   0  0  0  0  0  0  0  0  0  0  0  0
   24.2103   11.5302    6.5077 C   0  0  0  0  0  0  0  0  0  0  0  0
   14.8095   13.4351    0.1481 C   0  0  0  0  0  0  0  0  0  0  0  0
   24.8382   12.1661    7.7631 C   0  0  0  0  0  0  0  0  0  0  0  0
   15.5784   13.9496   -0.8960 C   0  0  0  0  0  0  0  0  0  0  0  0
   15.6904   15.3298   -1.0653 C   0  0  0  0  0  0  0  0  0  0  0  0
   15.0336   16.1954   -0.1905 C   0  0  0  0  0  0  0  0  0  0  0  0
   14.2649   15.6808    0.8538 C   0  0  0  0  0  0  0  0  0  0  0  0
   14.1530   14.3007    1.0231 C   0  0  0  0  0  0  0  0  0  0  0  0
```

5.9992	11.2784	-6.9535	C	0	0	0	0	0	0	0	0	0	0	0	0
6.6268	11.9132	-5.6974	C	0	0	0	0	0	0	0	0	0	0	0	0
4.6083	11.8942	-7.2000	C	0	0	0	0	0	0	0	0	0	0	0	0
16.7188	11.5691	-0.2922	H	0	0	0	0	0	0	0	0	0	0	0	0
15.9999	10.2964	0.7034	H	0	0	0	0	0	0	0	0	0	0	0	0
16.0858	11.7909	2.6813	H	0	0	0	0	0	0	0	0	0	0	0	0
16.7959	13.0696	1.6870	H	0	0	0	0	0	0	0	0	0	0	0	0
18.7411	11.6044	1.1959	H	0	0	0	0	0	0	0	0	0	0	0	0
18.0319	10.3302	2.1966	H	0	0	0	0	0	0	0	0	0	0	0	0
14.0572	11.7620	1.1809	H	0	0	0	0	0	0	0	0	0	0	0	0
14.7046	11.5521	-1.7903	H	0	0	0	0	0	0	0	0	0	0	0	0
13.9776	10.2764	-0.8046	H	0	0	0	0	0	0	0	0	0	0	0	0
11.9542	10.2593	-2.3022	H	0	0	0	0	0	0	0	0	0	0	0	0
12.6882	11.5262	-3.2940	H	0	0	0	0	0	0	0	0	0	0	0	0
12.7661	13.0265	-1.3150	H	0	0	0	0	0	0	0	0	0	0	0	0
12.0398	11.7543	-0.3244	H	0	0	0	0	0	0	0	0	0	0	0	0
10.0228	11.7478	-1.8319	H	0	0	0	0	0	0	0	0	0	0	0	0
10.7564	13.0128	-2.8266	H	0	0	0	0	0	0	0	0	0	0	0	0
20.0663	10.3721	3.6808	H	0	0	0	0	0	0	0	0	0	0	0	0
20.7704	11.6487	2.6797	H	0	0	0	0	0	0	0	0	0	0	0	0
18.8226	13.1080	3.1713	H	0	0	0	0	0	0	0	0	0	0	0	0
18.1183	11.8306	4.1712	H	0	0	0	0	0	0	0	0	0	0	0	0
20.8537	13.1511	4.6549	H	0	0	0	0	0	0	0	0	0	0	0	0
20.1508	11.8737	5.6557	H	0	0	0	0	0	0	0	0	0	0	0	0
8.0078	11.7384	-3.3378	H	0	0	0	0	0	0	0	0	0	0	0	0
8.7427	12.9987	-4.3373	H	0	0	0	0	0	0	0	0	0	0	0	0
10.6722	11.5086	-4.8005	H	0	0	0	0	0	0	0	0	0	0	0	0
9.9366	10.2449	-3.8056	H	0	0	0	0	0	0	0	0	0	0	0	0
8.6540	11.4889	-6.3062	H	0	0	0	0	0	0	0	0	0	0	0	0
7.9190	10.2286	-5.3067	H	0	0	0	0	0	0	0	0	0	0	0	0
22.8856	13.1945	6.1393	H	0	0	0	0	0	0	0	0	0	0	0	0
22.1825	11.9165	7.1394	H	0	0	0	0	0	0	0	0	0	0	0	0
22.0994	10.4158	5.1633	H	0	0	0	0	0	0	0	0	0	0	0	0
22.8024	11.6938	4.1632	H	0	0	0	0	0	0	0	0	0	0	0	0
24.1313	10.4591	6.6466	H	0	0	0	0	0	0	0	0	0	0	0	0
24.8345	11.7376	5.6473	H	0	0	0	0	0	0	0	0	0	0	0	0
24.9172	13.2373	7.6242	H	0	0	0	0	0	0	0	0	0	0	0	0
25.8247	11.7493	7.9240	H	0	0	0	0	0	0	0	0	0	0	0	0
24.2139	11.9587	8.6235	H	0	0	0	0	0	0	0	0	0	0	0	0
16.0885	13.2776	-1.5751	H	0	0	0	0	0	0	0	0	0	0	0	0
16.2871	15.7292	-1.8760	H	0	0	0	0	0	0	0	0	0	0	0	0
15.1205	17.2668	-0.3220	H	0	0	0	0	0	0	0	0	0	0	0	0
13.7551	16.3529	1.5330	H	0	0	0	0	0	0	0	0	0	0	0	0
13.5564	13.9012	1.8339	H	0	0	0	0	0	0	0	0	0	0	0	0
6.6348	11.4679	-7.8097	H	0	0	0	0	0	0	0	0	0	0	0	0
5.9002	10.2100	-6.8069	H	0	0	0	0	0	0	0	0	0	0	0	0
5.9913	11.7236	-4.8412	H	0	0	0	0	0	0	0	0	0	0	0	0
6.7258	12.9817	-5.8440	H	0	0	0	0	0	0	0	0	0	0	0	0
4.1670	11.4478	-8.0825	H	0	0	0	0	0	0	0	0	0	0	0	0
3.9729	11.7051	-6.3436	H	0	0	0	0	0	0	0	0	0	0	0	0
4.7074	12.9626	-7.3470	H	0	0	0	0	0	0	0	0	0	0	0	0

6	8	1	0	0	0	0
8	13	1	0	0	0	0
9	10	1	0	0	0	0
9	11	1	0	0	0	0
11	16	1	0	0	0	0
12	13	1	0	0	0	0
12	14	1	0	0	0	0
14	26	1	0	0	0	0
15	16	1	0	0	0	0
15	17	1	0	0	0	0
17	19	1	0	0	0	0
20	18	4	0	0	0	0

1	2	1	0	0	0	0
21	20	4	0	0	0	0
1	4	1	0	0	0	0
22	21	4	0	0	0	0
2	3	1	0	0	0	0
23	22	4	0	0	0	0
3	10	1	0	0	0	0
24	23	4	0	0	0	0
18	24	4	0	0	0	0
25	26	1	0	0	0	0
4	5	1	0	0	0	0
5	7	1	0	0	0	0
6	7	1	0	0	0	0
25	27	1	0	0	0	0
18	4	1	0	0	0	0
1	28	1	0	0	0	0
1	29	1	0	0	0	0
2	30	1	0	0	0	0
2	31	1	0	0	0	0
3	32	1	0	0	0	0
3	33	1	0	0	0	0
4	34	1	0	0	0	0
5	35	1	0	0	0	0
5	36	1	0	0	0	0
6	37	1	0	0	0	0
6	38	1	0	0	0	0
7	39	1	0	0	0	0
7	40	1	0	0	0	0
8	41	1	0	0	0	0
8	42	1	0	0	0	0
9	43	1	0	0	0	0
9	44	1	0	0	0	0
10	45	1	0	0	0	0
10	46	1	0	0	0	0
11	47	1	0	0	0	0
11	48	1	0	0	0	0
12	49	1	0	0	0	0
12	50	1	0	0	0	0
13	51	1	0	0	0	0
13	52	1	0	0	0	0
14	53	1	0	0	0	0
14	54	1	0	0	0	0
15	55	1	0	0	0	0
15	56	1	0	0	0	0
16	57	1	0	0	0	0
16	58	1	0	0	0	0
17	59	1	0	0	0	0
17	60	1	0	0	0	0
19	61	1	0	0	0	0
19	62	1	0	0	0	0
19	63	1	0	0	0	0
20	64	1	0	0	0	0
21	65	1	0	0	0	0
22	66	1	0	0	0	0
23	67	1	0	0	0	0
24	68	1	0	0	0	0
25	69	1	0	0	0	0
25	70	1	0	0	0	0
26	71	1	0	0	0	0
26	72	1	0	0	0	0
27	73	1	0	0	0	0
27	74	1	0	0	0	0
27	75	1	0	0	0	0

M END
> <Alias name>
11-Phenylheneicosane

> <deltaH°(c)>   kJ/mol
-16991

$$$$
9-Cyclohexyleicosane
  ChemBrain IXL

```
 78 78  0  0  0  0  0  0  0  0  1 V2000
   19.5502    8.4366   -0.8923 C   0  0  0  0  0  0  0  0  0  0  0  0
   21.0160    8.2520   -0.4537 C   0  0  0  0  0  0  0  0  0  0  0  0
   21.6564    7.1109   -1.2682 C   0  0  0  0  0  0  0  0  0  0  0  0
   15.4531   13.9393   -1.0045 C   0  0  0  0  0  0  0  0  0  0  0  0
   18.9100    9.5776   -0.0780 C   0  0  0  0  0  0  0  0  0  0  0  0
   23.1221    6.9264   -0.8298 C   0  0  0  0  0  0  0  0  0  0  0  0
   15.4692   13.5400    0.4877 C   0  0  0  0  0  0  0  0  0  0  0  0
   16.2450   15.2489   -1.1937 C   0  0  0  0  0  0  0  0  0  0  0  0
   17.4444    9.7626   -0.5169 C   0  0  0  0  0  0  0  0  0  0  0  0
   14.8191   14.6615    1.3253 C   0  0  0  0  0  0  0  0  0  0  0  0
   15.5974   16.3685   -0.3537 C   0  0  0  0  0  0  0  0  0  0  0  0
   16.8044   10.9037    0.2972 C   0  0  0  0  0  0  0  0  0  0  0  0
   15.6127   15.9692    1.1358 C   0  0  0  0  0  0  0  0  0  0  0  0
   15.3377   11.0887   -0.1431 C   0  0  0  0  0  0  0  0  0  0  0  0
   14.6823   12.2173    0.6886 C   0  0  0  0  0  0  0  0  0  0  0  0
   13.2154   12.4085    0.2341 C   0  0  0  0  0  0  0  0  0  0  0  0
   12.4279   11.1018    0.4610 C   0  0  0  0  0  0  0  0  0  0  0  0
   10.9630   11.3038    0.0279 C   0  0  0  0  0  0  0  0  0  0  0  0
   10.1754    9.9987    0.2547 C   0  0  0  0  0  0  0  0  0  0  0  0
    8.7104   10.2007   -0.1790 C   0  0  0  0  0  0  0  0  0  0  0  0
    7.9227    8.8956    0.0479 C   0  0  0  0  0  0  0  0  0  0  0  0
    6.4579    9.0975   -0.3862 C   0  0  0  0  0  0  0  0  0  0  0  0
    5.6702    7.7925   -0.1592 C   0  0  0  0  0  0  0  0  0  0  0  0
    4.2054    7.9943   -0.5937 C   0  0  0  0  0  0  0  0  0  0  0  0
    3.4175    6.6892   -0.3666 C   0  0  0  0  0  0  0  0  0  0  0  0
    1.9529    6.8909   -0.8012 C   0  0  0  0  0  0  0  0  0  0  0  0
   19.5172    8.6829   -1.9464 H   0  0  0  0  0  0  0  0  0  0  0  0
   19.0031    7.5182   -0.7188 H   0  0  0  0  0  0  0  0  0  0  0  0
   21.0491    8.0056    0.6004 H   0  0  0  0  0  0  0  0  0  0  0  0
   21.5631    9.1704   -0.6271 H   0  0  0  0  0  0  0  0  0  0  0  0
   21.6232    7.3572   -2.3223 H   0  0  0  0  0  0  0  0  0  0  0  0
   21.1094    6.1925   -1.0947 H   0  0  0  0  0  0  0  0  0  0  0  0
   15.9094   13.1549   -1.5938 H   0  0  0  0  0  0  0  0  0  0  0  0
   14.4311   14.0866   -1.3287 H   0  0  0  0  0  0  0  0  0  0  0  0
   18.9428    9.3312    0.9761 H   0  0  0  0  0  0  0  0  0  0  0  0
   19.4573   10.4959   -0.2512 H   0  0  0  0  0  0  0  0  0  0  0  0
   23.6692    7.8447   -1.0034 H   0  0  0  0  0  0  0  0  0  0  0  0
   23.5718    6.1246   -1.4023 H   0  0  0  0  0  0  0  0  0  0  0  0
   23.1554    6.6800    0.2243 H   0  0  0  0  0  0  0  0  0  0  0  0
   16.4926   13.4010    0.8105 H   0  0  0  0  0  0  0  0  0  0  0  0
   17.2683   15.1015   -0.8710 H   0  0  0  0  0  0  0  0  0  0  0  0
   16.2333   15.5297   -2.2394 H   0  0  0  0  0  0  0  0  0  0  0  0
   17.4116   10.0088   -1.5710 H   0  0  0  0  0  0  0  0  0  0  0  0
   16.8970    8.8444   -0.3434 H   0  0  0  0  0  0  0  0  0  0  0  0
   13.7978   14.8103    1.0003 H   0  0  0  0  0  0  0  0  0  0  0  0
   14.8282   14.3822    2.3715 H   0  0  0  0  0  0  0  0  0  0  0  0
   14.5745   16.5167   -0.6771 H   0  0  0  0  0  0  0  0  0  0  0  0
   16.1546   17.2874   -0.4866 H   0  0  0  0  0  0  0  0  0  0  0  0
   16.8366   10.6571    1.3512 H   0  0  0  0  0  0  0  0  0  0  0  0
   17.3526   11.8205    0.1240 H   0  0  0  0  0  0  0  0  0  0  0  0
   15.1580   16.7558    1.7249 H   0  0  0  0  0  0  0  0  0  0  0  0
```

16.6355	15.8210	1.4595	H	0	0	0	0	0	0	0	0	0	0	0	0	0
15.3064	11.3484	-1.1933	H	0	0	0	0	0	0	0	0	0	0	0	0	0
14.7970	10.1649	0.0150	H	0	0	0	0	0	0	0	0	0	0	0	0	0
14.7002	11.9464	1.7369	H	0	0	0	0	0	0	0	0	0	0	0	0	0
13.1928	12.6644	-0.8172	H	0	0	0	0	0	0	0	0	0	0	0	0	0
12.7610	13.2047	0.8087	H	0	0	0	0	0	0	0	0	0	0	0	0	0
12.4607	10.8382	1.5109	H	0	0	0	0	0	0	0	0	0	0	0	0	0
12.8701	10.3065	-0.1248	H	0	0	0	0	0	0	0	0	0	0	0	0	0
10.9301	11.5674	-1.0220	H	0	0	0	0	0	0	0	0	0	0	0	0	0
10.5197	12.0994	0.6139	H	0	0	0	0	0	0	0	0	0	0	0	0	0
10.2080	9.7353	1.3047	H	0	0	0	0	0	0	0	0	0	0	0	0	0
10.6189	9.2031	-0.3311	H	0	0	0	0	0	0	0	0	0	0	0	0	0
8.6778	10.4640	-1.2290	H	0	0	0	0	0	0	0	0	0	0	0	0	0
8.2670	10.9964	0.4067	H	0	0	0	0	0	0	0	0	0	0	0	0	0
7.9550	8.6324	1.0979	H	0	0	0	0	0	0	0	0	0	0	0	0	0
8.3664	8.0998	-0.5376	H	0	0	0	0	0	0	0	0	0	0	0	0	0
6.4256	9.3606	-1.4363	H	0	0	0	0	0	0	0	0	0	0	0	0	0
6.0143	9.8934	0.1992	H	0	0	0	0	0	0	0	0	0	0	0	0	0
5.7023	7.5295	0.8908	H	0	0	0	0	0	0	0	0	0	0	0	0	0
6.1139	6.9966	-0.7445	H	0	0	0	0	0	0	0	0	0	0	0	0	0
4.1733	8.2573	-1.6438	H	0	0	0	0	0	0	0	0	0	0	0	0	0
3.7617	8.7903	-0.0085	H	0	0	0	0	0	0	0	0	0	0	0	0	0
3.4495	6.4263	0.6834	H	0	0	0	0	0	0	0	0	0	0	0	0	0
3.8612	5.8933	-0.9517	H	0	0	0	0	0	0	0	0	0	0	0	0	0
1.3995	5.9738	-0.6415	H	0	0	0	0	0	0	0	0	0	0	0	0	0
1.5090	7.6868	-0.2160	H	0	0	0	0	0	0	0	0	0	0	0	0	0
1.9208	7.1537	-1.8513	H	0	0	0	0	0	0	0	0	0	0	0	0	0

1	2	1	0	0	0	0
1	5	1	0	0	0	0
2	3	1	0	0	0	0
3	6	1	0	0	0	0
7	4	1	0	0	0	0
4	8	1	0	0	0	0
5	9	1	0	0	0	0
10	7	1	0	0	0	0
8	11	1	0	0	0	0
9	12	1	0	0	0	0
13	10	1	0	0	0	0
11	13	1	0	0	0	0
12	14	1	0	0	0	0
14	15	1	0	0	0	0
15	16	1	0	0	0	0
16	17	1	0	0	0	0
17	18	1	0	0	0	0
18	19	1	0	0	0	0
19	20	1	0	0	0	0
20	21	1	0	0	0	0
21	22	1	0	0	0	0
22	23	1	0	0	0	0
23	24	1	0	0	0	0
24	25	1	0	0	0	0
25	26	1	0	0	0	0
7	15	1	0	0	0	0
1	27	1	0	0	0	0
1	28	1	0	0	0	0
2	29	1	0	0	0	0
2	30	1	0	0	0	0
3	31	1	0	0	0	0
3	32	1	0	0	0	0
4	33	1	0	0	0	0
4	34	1	0	0	0	0
5	35	1	0	0	0	0
5	36	1	0	0	0	0

```
  6 37  1  0  0  0  0
  6 38  1  0  0  0  0
  6 39  1  0  0  0  0
  7 40  1  0  0  0  0
  8 41  1  0  0  0  0
  8 42  1  0  0  0  0
  9 43  1  0  0  0  0
  9 44  1  0  0  0  0
 10 45  1  0  0  0  0
 10 46  1  0  0  0  0
 11 47  1  0  0  0  0
 11 48  1  0  0  0  0
 12 49  1  0  0  0  0
 12 50  1  0  0  0  0
 13 51  1  0  0  0  0
 13 52  1  0  0  0  0
 14 53  1  0  0  0  0
 14 54  1  0  0  0  0
 15 55  1  0  0  0  0
 16 56  1  0  0  0  0
 16 57  1  0  0  0  0
 17 58  1  0  0  0  0
 17 59  1  0  0  0  0
 18 60  1  0  0  0  0
 18 61  1  0  0  0  0
 19 62  1  0  0  0  0
 19 63  1  0  0  0  0
 20 64  1  0  0  0  0
 20 65  1  0  0  0  0
 21 66  1  0  0  0  0
 21 67  1  0  0  0  0
 22 68  1  0  0  0  0
 22 69  1  0  0  0  0
 23 70  1  0  0  0  0
 23 71  1  0  0  0  0
 24 72  1  0  0  0  0
 24 73  1  0  0  0  0
 25 74  1  0  0  0  0
 25 75  1  0  0  0  0
 26 76  1  0  0  0  0
 26 77  1  0  0  0  0
 26 78  1  0  0  0  0
M  END
>  <Alias name>
9-Cyclohexyleicosane

>  <deltaH°(c)>   kJ/mol
-16989

$$$$
1-Phenylheneicosane
  ChemBrain IXL

 75 75  0  0  0  0  0  0  0  0  1 V2000
   16.3155   15.3366    0.0000 C   0  0  0  0  0  0  0  0  0  0  0  0
   17.5149   16.3046    0.0002 C   0  0  0  0  0  0  0  0  0  0  0  0
   18.8288   15.4980   -0.0002 C   0  0  0  0  0  0  0  0  0  0  0  0
   15.0031   16.1456    0.0003 C   0  0  0  0  0  0  0  0  0  0  0  0
   13.8025   15.1790    0.0000 C   0  0  0  0  0  0  0  0  0  0  0  0
   11.2899   15.0226    0.0001 C   0  0  0  0  0  0  0  0  0  0  0  0
   12.4908   15.9889    0.0003 C   0  0  0  0  0  0  0  0  0  0  0  0
    9.9782   15.8323    0.0003 C   0  0  0  0  0  0  0  0  0  0  0  0
   21.3420   15.6661   -0.0006 C   0  0  0  0  0  0  0  0  0  0  0  0
```

20.0260	16.4690	0.0000	C	0	0	0	0	0	0	0	0	0	0	0	0	0
22.5369	16.6406	-0.0005	C	0	0	0	0	0	0	0	0	0	0	0	0	0
7.4657	15.6748	0.0003	C	0	0	0	0	0	0	0	0	0	0	0	0	0
8.7776	14.8656	0.0001	C	0	0	0	0	0	0	0	0	0	0	0	0	0
6.2654	14.7077	0.0002	C	0	0	0	0	0	0	0	0	0	0	0	0	0
25.0484	16.8178	-0.0014	C	0	0	0	0	0	0	0	0	0	0	0	0	0
23.8547	15.8410	-0.0014	C	0	0	0	0	0	0	0	0	0	0	0	0	0
26.3665	16.0189	-0.0025	C	0	0	0	0	0	0	0	0	0	0	0	0	0
28.8416	16.2324	-0.0039	C	0	0	0	0	0	0	0	0	0	0	0	0	0
27.5638	16.9965	-0.0027	C	0	0	0	0	0	0	0	0	0	0	0	0	0
30.0593	16.9151	-0.0044	C	0	0	0	0	0	0	0	0	0	0	0	0	0
31.2603	16.2030	-0.0055	C	0	0	0	0	0	0	0	0	0	0	0	0	0
31.2455	14.8073	-0.0062	C	0	0	0	0	0	0	0	0	0	0	0	0	0
30.0301	14.1226	-0.0057	C	0	0	0	0	0	0	0	0	0	0	0	0	0
28.8294	14.8329	-0.0046	C	0	0	0	0	0	0	0	0	0	0	0	0	0
3.7533	14.5493	0.0003	C	0	0	0	0	0	0	0	0	0	0	0	0	0
4.9534	15.5166	0.0005	C	0	0	0	0	0	0	0	0	0	0	0	0	0
2.4414	15.3578	0.0006	C	0	0	0	0	0	0	0	0	0	0	0	0	0
16.3551	14.7123	0.8841	H	0	0	0	0	0	0	0	0	0	0	0	0	0
16.3550	14.7129	-0.8845	H	0	0	0	0	0	0	0	0	0	0	0	0	0
17.4747	16.9290	-0.8837	H	0	0	0	0	0	0	0	0	0	0	0	0	0
17.4749	16.9283	0.8847	H	0	0	0	0	0	0	0	0	0	0	0	0	0
18.8697	14.8739	0.8840	H	0	0	0	0	0	0	0	0	0	0	0	0	0
18.8695	14.8745	-0.8847	H	0	0	0	0	0	0	0	0	0	0	0	0	0
14.9639	16.7700	-0.8837	H	0	0	0	0	0	0	0	0	0	0	0	0	0
14.9640	16.7694	0.8847	H	0	0	0	0	0	0	0	0	0	0	0	0	0
13.8415	14.5548	0.8841	H	0	0	0	0	0	0	0	0	0	0	0	0	0
13.8415	14.5553	-0.8844	H	0	0	0	0	0	0	0	0	0	0	0	0	0
11.3288	14.3983	0.8841	H	0	0	0	0	0	0	0	0	0	0	0	0	0
11.3288	14.3988	-0.8844	H	0	0	0	0	0	0	0	0	0	0	0	0	0
12.4519	16.6132	-0.8838	H	0	0	0	0	0	0	0	0	0	0	0	0	0
12.4519	16.6128	0.8847	H	0	0	0	0	0	0	0	0	0	0	0	0	0
9.9391	16.4565	-0.8838	H	0	0	0	0	0	0	0	0	0	0	0	0	0
9.9392	16.4561	0.8847	H	0	0	0	0	0	0	0	0	0	0	0	0	0
21.3847	15.0420	0.8835	H	0	0	0	0	0	0	0	0	0	0	0	0	0
21.3843	15.0426	-0.8852	H	0	0	0	0	0	0	0	0	0	0	0	0	0
19.9841	17.0933	-0.8839	H	0	0	0	0	0	0	0	0	0	0	0	0	0
19.9845	17.0926	0.8844	H	0	0	0	0	0	0	0	0	0	0	0	0	0
22.4932	17.2648	-0.8844	H	0	0	0	0	0	0	0	0	0	0	0	0	0
22.4938	17.2640	0.8839	H	0	0	0	0	0	0	0	0	0	0	0	0	0
7.4264	16.2990	-0.8838	H	0	0	0	0	0	0	0	0	0	0	0	0	0
7.4265	16.2986	0.8848	H	0	0	0	0	0	0	0	0	0	0	0	0	0
8.8167	14.2413	0.8842	H	0	0	0	0	0	0	0	0	0	0	0	0	0
8.8166	14.2417	-0.8843	H	0	0	0	0	0	0	0	0	0	0	0	0	0
6.3047	14.0835	0.8843	H	0	0	0	0	0	0	0	0	0	0	0	0	0
6.3046	14.0839	-0.8842	H	0	0	0	0	0	0	0	0	0	0	0	0	0
25.0040	17.4419	-0.8853	H	0	0	0	0	0	0	0	0	0	0	0	0	0
25.0049	17.4410	0.8831	H	0	0	0	0	0	0	0	0	0	0	0	0	0
23.8988	15.2168	0.8827	H	0	0	0	0	0	0	0	0	0	0	0	0	0
23.8982	15.2176	-0.8860	H	0	0	0	0	0	0	0	0	0	0	0	0	0
26.4100	15.3947	0.8816	H	0	0	0	0	0	0	0	0	0	0	0	0	0
26.4090	15.3955	-0.8873	H	0	0	0	0	0	0	0	0	0	0	0	0	0
27.5177	17.6211	-0.8860	H	0	0	0	0	0	0	0	0	0	0	0	0	0
27.5188	17.6201	0.8815	H	0	0	0	0	0	0	0	0	0	0	0	0	0
30.0725	17.9976	-0.0038	H	0	0	0	0	0	0	0	0	0	0	0	0	0
32.2039	16.7336	-0.0059	H	0	0	0	0	0	0	0	0	0	0	0	0	0
32.1774	14.2556	-0.0071	H	0	0	0	0	0	0	0	0	0	0	0	0	0
30.0184	13.0395	-0.0063	H	0	0	0	0	0	0	0	0	0	0	0	0	0
27.8890	14.2979	-0.0042	H	0	0	0	0	0	0	0	0	0	0	0	0	0
3.7927	13.9251	0.8844	H	0	0	0	0	0	0	0	0	0	0	0	0	0
3.7926	13.9255	-0.8840	H	0	0	0	0	0	0	0	0	0	0	0	0	0
4.9140	16.1408	-0.8837	H	0	0	0	0	0	0	0	0	0	0	0	0	0
4.9141	16.1404	0.8849	H	0	0	0	0	0	0	0	0	0	0	0	0	0

1.5984	14.6780	0.0005	H	0	0	0	0	0	0	0	0	0	0	0	0
2.4019	15.9816	0.8850	H	0	0	0	0	0	0	0	0	0	0	0	0
2.4018	15.9820	-0.8835	H	0	0	0	0	0	0	0	0	0	0	0	0

6	8	1	0	0	0	0
8	13	1	0	0	0	0
9	10	1	0	0	0	0
9	11	1	0	0	0	0
11	16	1	0	0	0	0
12	13	1	0	0	0	0
12	14	1	0	0	0	0
14	26	1	0	0	0	0
15	16	1	0	0	0	0
15	17	1	0	0	0	0
17	19	1	0	0	0	0
18	19	1	0	0	0	0
20	18	4	0	0	0	0
1	2	1	0	0	0	0
21	20	4	0	0	0	0
1	4	1	0	0	0	0
22	21	4	0	0	0	0
2	3	1	0	0	0	0
23	22	4	0	0	0	0
3	10	1	0	0	0	0
24	23	4	0	0	0	0
18	24	4	0	0	0	0
25	26	1	0	0	0	0
4	5	1	0	0	0	0
5	7	1	0	0	0	0
6	7	1	0	0	0	0
25	27	1	0	0	0	0
1	28	1	0	0	0	0
1	29	1	0	0	0	0
2	30	1	0	0	0	0
2	31	1	0	0	0	0
3	32	1	0	0	0	0
3	33	1	0	0	0	0
4	34	1	0	0	0	0
4	35	1	0	0	0	0
5	36	1	0	0	0	0
5	37	1	0	0	0	0
6	38	1	0	0	0	0
6	39	1	0	0	0	0
7	40	1	0	0	0	0
7	41	1	0	0	0	0
8	42	1	0	0	0	0
8	43	1	0	0	0	0
9	44	1	0	0	0	0
9	45	1	0	0	0	0
10	46	1	0	0	0	0
10	47	1	0	0	0	0
11	48	1	0	0	0	0
11	49	1	0	0	0	0
12	50	1	0	0	0	0
12	51	1	0	0	0	0
13	52	1	0	0	0	0
13	53	1	0	0	0	0
14	54	1	0	0	0	0
14	55	1	0	0	0	0
15	56	1	0	0	0	0
15	57	1	0	0	0	0
16	58	1	0	0	0	0
16	59	1	0	0	0	0
17	60	1	0	0	0	0

17 61 1 0 0 0 0
19 62 1 0 0 0 0
19 63 1 0 0 0 0
20 64 1 0 0 0 0
21 65 1 0 0 0 0
22 66 1 0 0 0 0
23 67 1 0 0 0 0
24 68 1 0 0 0 0
25 69 1 0 0 0 0
25 70 1 0 0 0 0
26 71 1 0 0 0 0
26 72 1 0 0 0 0
27 73 1 0 0 0 0
27 74 1 0 0 0 0
27 75 1 0 0 0 0
M END
> <Alias name>
1-Phenylheneicosane

> <deltaH°(c)>   kJ/mol
-16978

$$$$
7-Hexadecylspiro(4,5)decane
  ChemBrain IXL

 76 77 0 0 0 0 0 0 0 0 1 V2000
    6.2884   10.1855   -0.2657 C   0 0 0 0 0 0 0 0 0 0 0 0
    7.5193   11.0670   0.0301 C   0 0 0 0 0 0 0 0 0 0 0 0
    5.0015   10.9366   0.1393 C   0 0 0 0 0 0 0 0 0 0 0 0
    8.8015   10.3040   -0.3552 C   0 0 0 0 0 0 0 0 0 0 0 0
    4.8785   12.2365   -0.6848 C   0 0 0 0 0 0 0 0 0 0 0 0
    3.7771   10.0374   -0.1338 C   0 0 0 0 0 0 0 0 0 0 0 0
   10.0315   11.1841   -0.0596 C   0 0 0 0 0 0 0 0 0 0 0 0
    3.5880   12.9792   -0.2850 C   0 0 0 0 0 0 0 0 0 0 0 0
    2.4891   10.7764   0.2844 C   0 0 0 0 0 0 0 0 0 0 0 0
   11.3139   10.4211   -0.4449 C   0 0 0 0 0 0 0 0 0 0 0 0
    2.3540   12.0810   -0.5305 C   0 0 0 0 0 0 0 0 0 0 0 0
    3.4323   14.2816   -1.0665 C   0 0 0 0 0 0 0 0 0 0 0 0
    3.6231   13.4172   1.1750 C   0 0 0 0 0 0 0 0 0 0 0 0
   12.5440   11.3012   -0.1493 C   0 0 0 0 0 0 0 0 0 0 0 0
    2.5957   15.2236   -0.1381 C   0 0 0 0 0 0 0 0 0 0 0 0
    2.5777   14.5260   1.2628 C   0 0 0 0 0 0 0 0 0 0 0 0
   13.8263   10.5382   -0.5348 C   0 0 0 0 0 0 0 0 0 0 0 0
   15.0563   11.4184   -0.2394 C   0 0 0 0 0 0 0 0 0 0 0 0
   16.3386   10.6555   -0.6252 C   0 0 0 0 0 0 0 0 0 0 0 0
   17.5687   11.5359   -0.3303 C   0 0 0 0 0 0 0 0 0 0 0 0
   18.8509   10.7732   -0.7165 C   0 0 0 0 0 0 0 0 0 0 0 0
   20.0810   11.6537   -0.4220 C   0 0 0 0 0 0 0 0 0 0 0 0
   21.3632   10.8912   -0.8086 C   0 0 0 0 0 0 0 0 0 0 0 0
   22.5933   11.7718   -0.5145 C   0 0 0 0 0 0 0 0 0 0 0 0
   23.8756   11.0093   -0.9013 C   0 0 0 0 0 0 0 0 0 0 0 0
   25.1056   11.8899   -0.6073 C   0 0 0 0 0 0 0 0 0 0 0 0
    6.3609   9.2653   0.3008 H   0 0 0 0 0 0 0 0 0 0 0 0
    6.2547   9.9557   -1.3235 H   0 0 0 0 0 0 0 0 0 0 0 0
    7.4570   11.9806   -0.5466 H   0 0 0 0 0 0 0 0 0 0 0 0
    7.5458   11.3073   1.0857 H   0 0 0 0 0 0 0 0 0 0 0 0
    5.0425   11.1795   1.1938 H   0 0 0 0 0 0 0 0 0 0 0 0
    8.8647   9.3897   0.2218 H   0 0 0 0 0 0 0 0 0 0 0 0
    8.7750   10.0636   -1.4108 H   0 0 0 0 0 0 0 0 0 0 0 0
    5.7335   12.8707   -0.4905 H   0 0 0 0 0 0 0 0 0 0 0 0
    4.8421   11.9925   -1.7393 H   0 0 0 0 0 0 0 0 0 0 0 0
    3.8682   9.1216   0.4369 H   0 0 0 0 0 0 0 0 0 0 0 0

```
3.7311 9.8012 -1.1897 H 0 0 0 0 0 0 0 0 0 0 0 0
9.9683 12.0984 -0.6366 H 0 0 0 0 0 0 0 0 0 0 0 0
10.0580 11.4246 0.9961 H 0 0 0 0 0 0 0 0 0 0 0 0
1.6321 10.1427 0.0936 H 0 0 0 0 0 0 0 0 0 0 0 0
2.5345 11.0113 1.3391 H 0 0 0 0 0 0 0 0 0 0 0 0
11.3771 9.5068 0.1322 H 0 0 0 0 0 0 0 0 0 0 0 0
11.2874 10.1806 -1.5005 H 0 0 0 0 0 0 0 0 0 0 0 0
2.2890 11.8406 -1.5844 H 0 0 0 0 0 0 0 0 0 0 0 0
1.4579 12.6065 -0.2251 H 0 0 0 0 0 0 0 0 0 0 0 0
4.4140 14.7156 -1.2112 H 0 0 0 0 0 0 0 0 0 0 0 0
2.9478 14.1275 -2.0226 H 0 0 0 0 0 0 0 0 0 0 0 0
3.3883 12.5999 1.8425 H 0 0 0 0 0 0 0 0 0 0 0 0
4.6005 13.8231 1.4046 H 0 0 0 0 0 0 0 0 0 0 0 0
12.4807 12.2155 -0.7263 H 0 0 0 0 0 0 0 0 0 0 0 0
12.5705 11.5416 0.9064 H 0 0 0 0 0 0 0 0 0 0 0 0
3.0748 16.1932 -0.0840 H 0 0 0 0 0 0 0 0 0 0 0 0
1.5809 15.3270 -0.5015 H 0 0 0 0 0 0 0 0 0 0 0 0
2.7920 15.2252 2.0614 H 0 0 0 0 0 0 0 0 0 0 0 0
1.6064 14.0719 1.4149 H 0 0 0 0 0 0 0 0 0 0 0 0
13.8896 9.6239 0.0424 H 0 0 0 0 0 0 0 0 0 0 0 0
13.7996 10.2976 -1.5904 H 0 0 0 0 0 0 0 0 0 0 0 0
14.9929 12.3327 -0.8164 H 0 0 0 0 0 0 0 0 0 0 0 0
15.0831 11.6588 0.8162 H 0 0 0 0 0 0 0 0 0 0 0 0
16.4023 9.7413 -0.0481 H 0 0 0 0 0 0 0 0 0 0 0 0
16.3117 10.4149 -1.6808 H 0 0 0 0 0 0 0 0 0 0 0 0
17.5049 12.4502 -0.9073 H 0 0 0 0 0 0 0 0 0 0 0 0
17.5958 11.7763 0.7253 H 0 0 0 0 0 0 0 0 0 0 0 0
18.9149 9.8590 -0.1394 H 0 0 0 0 0 0 0 0 0 0 0 0
18.8237 10.5325 -1.7721 H 0 0 0 0 0 0 0 0 0 0 0 0
20.0169 12.5680 -0.9990 H 0 0 0 0 0 0 0 0 0 0 0 0
20.1084 11.8942 0.6336 H 0 0 0 0 0 0 0 0 0 0 0 0
21.4274 9.9770 -0.2315 H 0 0 0 0 0 0 0 0 0 0 0 0
21.3357 10.6505 -1.8642 H 0 0 0 0 0 0 0 0 0 0 0 0
22.5289 12.6861 -1.0915 H 0 0 0 0 0 0 0 0 0 0 0 0
22.6209 12.0124 0.5411 H 0 0 0 0 0 0 0 0 0 0 0 0
23.9400 10.0952 -0.3242 H 0 0 0 0 0 0 0 0 0 0 0 0
23.8480 10.7687 -1.9568 H 0 0 0 0 0 0 0 0 0 0 0 0
25.0413 12.8041 -1.1845 H 0 0 0 0 0 0 0 0 0 0 0 0
26.0064 11.3540 -0.8792 H 0 0 0 0 0 0 0 0 0 0 0 0
25.1334 12.1305 0.4482 H 0 0 0 0 0 0 0 0 0 0 0 0
1 2 1 0 0 0 0
1 3 1 0 0 0 0
2 4 1 0 0 0 0
5 3 1 0 0 0 0
3 6 1 0 0 0 0
4 7 1 0 0 0 0
8 5 1 0 0 0 0
6 9 1 0 0 0 0
7 10 1 0 0 0 0
11 8 1 0 0 0 0
12 8 1 0 0 0 0
8 13 1 0 0 0 0
9 11 1 0 0 0 0
10 14 1 0 0 0 0
15 12 1 0 0 0 0
13 16 1 0 0 0 0
14 17 1 0 0 0 0
16 15 1 0 0 0 0
17 18 1 0 0 0 0
18 19 1 0 0 0 0
19 20 1 0 0 0 0
20 21 1 0 0 0 0
21 22 1 0 0 0 0
```

```
 22 23  1  0  0  0  0
 23 24  1  0  0  0  0
 24 25  1  0  0  0  0
 25 26  1  0  0  0  0
  1 27  1  0  0  0  0
  1 28  1  0  0  0  0
  2 29  1  0  0  0  0
  2 30  1  0  0  0  0
  3 31  1  0  0  0  0
  4 32  1  0  0  0  0
  4 33  1  0  0  0  0
  5 34  1  0  0  0  0
  5 35  1  0  0  0  0
  6 36  1  0  0  0  0
  6 37  1  0  0  0  0
  7 38  1  0  0  0  0
  7 39  1  0  0  0  0
  9 40  1  0  0  0  0
  9 41  1  0  0  0  0
 10 42  1  0  0  0  0
 10 43  1  0  0  0  0
 11 44  1  0  0  0  0
 11 45  1  0  0  0  0
 12 46  1  0  0  0  0
 12 47  1  0  0  0  0
 13 48  1  0  0  0  0
 13 49  1  0  0  0  0
 14 50  1  0  0  0  0
 14 51  1  0  0  0  0
 15 52  1  0  0  0  0
 15 53  1  0  0  0  0
 16 54  1  0  0  0  0
 16 55  1  0  0  0  0
 17 56  1  0  0  0  0
 17 57  1  0  0  0  0
 18 58  1  0  0  0  0
 18 59  1  0  0  0  0
 19 60  1  0  0  0  0
 19 61  1  0  0  0  0
 20 62  1  0  0  0  0
 20 63  1  0  0  0  0
 21 64  1  0  0  0  0
 21 65  1  0  0  0  0
 22 66  1  0  0  0  0
 22 67  1  0  0  0  0
 23 68  1  0  0  0  0
 23 69  1  0  0  0  0
 24 70  1  0  0  0  0
 24 71  1  0  0  0  0
 25 72  1  0  0  0  0
 25 73  1  0  0  0  0
 26 74  1  0  0  0  0
 26 75  1  0  0  0  0
 26 76  1  0  0  0  0
M  END
>  <Alias name>
7-Hexadecylspiro[4.5]decane

>  <deltaH°(c)>   kJ/mol
-16787

$$$$
Tetra-t-amylperoxypyromellitate
```

ChemBrain IXL


```
88 88  0  0  0  0  0  0  0  0  1 V2000
    8.6631    9.4912   -0.5096 C   0  0  0  0  0  0  0  0  0  0  0  0  0
    9.4343    8.3660   -0.1967 C   0  0  0  0  0  0  0  0  0  0  0  0  0
    9.2823   10.7170   -0.7226 C   0  0  0  0  0  0  0  0  0  0  0  0  0
    7.1903    9.3886   -0.6166 C   0  0  0  0  0  0  0  0  0  0  0  0  0
   10.8197    8.4273   -0.2927 C   0  0  0  0  0  0  0  0  0  0  0  0  0
    8.7877    7.1084    0.2370 C   0  0  0  0  0  0  0  0  0  0  0  0  0
   10.6696   10.7995   -0.7213 C   0  0  0  0  0  0  0  0  0  0  0  0  0
    6.5598    8.0878   -0.8786 O   0  0  0  0  0  0  0  0  0  0  0  0  0
    6.4993   10.3854   -0.4880 O   0  0  0  0  0  0  0  0  0  0  0  0  0
   11.4369    9.6337   -0.6038 C   0  0  0  0  0  0  0  0  0  0  0  0  0
    9.4559    5.8201   -0.0012 O   0  0  0  0  0  0  0  0  0  0  0  0  0
    7.7018    7.1403    0.7924 O   0  0  0  0  0  0  0  0  0  0  0  0  0
   11.3218   12.1236   -0.8442 C   0  0  0  0  0  0  0  0  0  0  0  0  0
    5.0802    8.1076   -0.9098 O   0  0  0  0  0  0  0  0  0  0  0  0  0
   12.9025    9.6725   -0.8086 C   0  0  0  0  0  0  0  0  0  0  0  0  0
    8.7115    4.6304    0.4682 O   0  0  0  0  0  0  0  0  0  0  0  0  0
   12.6882   12.3224   -0.3403 O   0  0  0  0  0  0  0  0  0  0  0  0  0
   10.7235   13.0511   -1.3637 O   0  0  0  0  0  0  0  0  0  0  0  0  0
   13.7687    8.7158   -0.1015 O   0  0  0  0  0  0  0  0  0  0  0  0  0
   13.3916   10.4965   -1.5641 O   0  0  0  0  0  0  0  0  0  0  0  0  0
   13.3034   13.6199   -0.7010 O   0  0  0  0  0  0  0  0  0  0  0  0  0
   15.1989    8.7637   -0.4806 O   0  0  0  0  0  0  0  0  0  0  0  0  0
    4.4261    7.3028    0.1315 C   0  0  0  0  0  0  0  0  0  0  0  0  0
    4.9135    7.7627    1.5195 C   0  0  0  0  0  0  0  0  0  0  0  0  0
    4.7802    5.8175   -0.0714 C   0  0  0  0  0  0  0  0  0  0  0  0  0
    2.8983    7.4843    0.0380 C   0  0  0  0  0  0  0  0  0  0  0  0  0
   13.7076   14.4549    0.4396 C   0  0  0  0  0  0  0  0  0  0  0  0  0
   12.5105   14.6377    1.3929 C   0  0  0  0  0  0  0  0  0  0  0  0  0
   14.1722   15.8337   -0.0707 C   0  0  0  0  0  0  0  0  0  0  0  0  0
   14.8641   13.7687    1.1926 C   0  0  0  0  0  0  0  0  0  0  0  0  0
   16.1405    8.7876    0.6490 C   0  0  0  0  0  0  0  0  0  0  0  0  0
   15.8501    7.5989    1.5871 C   0  0  0  0  0  0  0  0  0  0  0  0  0
   17.5830    8.6811    0.1178 C   0  0  0  0  0  0  0  0  0  0  0  0  0
   15.9750   10.1073    1.4263 C   0  0  0  0  0  0  0  0  0  0  0  0  0
    8.4425    3.6191   -0.5646 C   0  0  0  0  0  0  0  0  0  0  0  0  0
    9.7763    3.1049   -1.1422 C   0  0  0  0  0  0  0  0  0  0  0  0  0
    7.6656    2.4437    0.0582 C   0  0  0  0  0  0  0  0  0  0  0  0  0
    7.6037    4.2494   -1.6928 C   0  0  0  0  0  0  0  0  0  0  0  0  0
    6.3980    2.9774    0.7528 C   0  0  0  0  0  0  0  0  0  0  0  0  0
    2.5388    8.9600    0.2998 C   0  0  0  0  0  0  0  0  0  0  0  0  0
   16.1620   11.2971    0.4642 C   0  0  0  0  0  0  0  0  0  0  0  0  0
   11.3523   15.3212    0.6395 C   0  0  0  0  0  0  0  0  0  0  0  0  0
    8.6861   11.6048   -0.8890 H   0  0  0  0  0  0  0  0  0  0  0  0  0
   11.4162    7.5382   -0.1257 H   0  0  0  0  0  0  0  0  0  0  0  0  0
    4.6268    8.7948    1.6790 H   0  0  0  0  0  0  0  0  0  0  0  0  0
    5.9917    7.6755    1.5709 H   0  0  0  0  0  0  0  0  0  0  0  0  0
    4.4641    7.1410    2.2841 H   0  0  0  0  0  0  0  0  0  0  0  0  0
    5.8504    5.6847    0.0292 H   0  0  0  0  0  0  0  0  0  0  0  0  0
    4.4690    5.5042   -1.0602 H   0  0  0  0  0  0  0  0  0  0  0  0  0
    4.2706    5.2198    0.6739 H   0  0  0  0  0  0  0  0  0  0  0  0  0
    2.5609    7.2006   -0.9511 H   0  0  0  0  0  0  0  0  0  0  0  0  0
    2.4146    6.8584    0.7777 H   0  0  0  0  0  0  0  0  0  0  0  0  0
   12.1851   13.6700    1.7535 H   0  0  0  0  0  0  0  0  0  0  0  0  0
   12.8085   15.2540    2.2319 H   0  0  0  0  0  0  0  0  0  0  0  0  0
   13.3444   16.3373   -0.5548 H   0  0  0  0  0  0  0  0  0  0  0  0  0
   14.9800   15.7031   -0.7801 H   0  0  0  0  0  0  0  0  0  0  0  0  0
   14.5183   16.4298    0.7647 H   0  0  0  0  0  0  0  0  0  0  0  0  0
   15.1406   14.3668    2.0519 H   0  0  0  0  0  0  0  0  0  0  0  0  0
   15.7166   13.6699    0.5320 H   0  0  0  0  0  0  0  0  0  0  0  0  0
   14.5475   12.7868    1.5228 H   0  0  0  0  0  0  0  0  0  0  0  0  0
```

16.5392 7.6216 2.4227 H 0 0 0 0 0 0 0 0 0 0 0 0
14.8341 7.6693 1.9553 H 0 0 0 0 0 0 0 0 0 0 0 0
15.9734 6.6707 1.0428 H 0 0 0 0 0 0 0 0 0 0 0 0
17.7702 9.4858 -0.5824 H 0 0 0 0 0 0 0 0 0 0 0 0
17.7140 7.7296 -0.3824 H 0 0 0 0 0 0 0 0 0 0 0 0
18.2785 8.7530 0.9448 H 0 0 0 0 0 0 0 0 0 0 0 0
14.9841 10.1476 1.8617 H 0 0 0 0 0 0 0 0 0 0 0 0
16.7175 10.1588 2.2131 H 0 0 0 0 0 0 0 0 0 0 0 0
10.3321 3.9345 -1.5612 H 0 0 0 0 0 0 0 0 0 0 0 0
9.5765 2.3757 -1.9179 H 0 0 0 0 0 0 0 0 0 0 0 0
10.3572 2.6428 -0.3535 H 0 0 0 0 0 0 0 0 0 0 0 0
8.2919 1.9421 0.7857 H 0 0 0 0 0 0 0 0 0 0 0 0
7.3842 1.7444 -0.7194 H 0 0 0 0 0 0 0 0 0 0 0 0
7.3396 3.4873 -2.4154 H 0 0 0 0 0 0 0 0 0 0 0 0
6.7016 4.6790 -1.2751 H 0 0 0 0 0 0 0 0 0 0 0 0
8.1805 5.0253 -2.1809 H 0 0 0 0 0 0 0 0 0 0 0 0
5.8568 2.1527 1.1996 H 0 0 0 0 0 0 0 0 0 0 0 0
6.6793 3.6844 1.5235 H 0 0 0 0 0 0 0 0 0 0 0 0
5.7672 3.4704 0.0234 H 0 0 0 0 0 0 0 0 0 0 0 0
1.4657 9.0881 0.2291 H 0 0 0 0 0 0 0 0 0 0 0 0
2.8715 9.2423 1.2910 H 0 0 0 0 0 0 0 0 0 0 0 0
3.0264 9.5865 -0.4367 H 0 0 0 0 0 0 0 0 0 0 0 0
15.3329 11.3326 -0.2319 H 0 0 0 0 0 0 0 0 0 0 0 0
17.0884 11.1763 -0.0838 H 0 0 0 0 0 0 0 0 0 0 0 0
16.1961 12.2183 1.0319 H 0 0 0 0 0 0 0 0 0 0 0 0
11.0076 14.6736 -0.1571 H 0 0 0 0 0 0 0 0 0 0 0 0
10.5377 15.5126 1.3270 H 0 0 0 0 0 0 0 0 0 0 0 0
11.6977 16.2578 0.2194 H 0 0 0 0 0 0 0 0 0 0 0 0
1 2 4 0 0 0 0
1 3 4 0 0 0 0
1 4 1 0 0 0 0
2 5 4 0 0 0 0
2 6 1 0 0 0 0
3 7 4 0 0 0 0
4 8 1 0 0 0 0
4 9 2 0 0 0 0
5 10 4 0 0 0 0
6 11 1 0 0 0 0
6 12 2 0 0 0 0
7 13 1 0 0 0 0
7 10 4 0 0 0 0
8 14 1 0 0 0 0
10 15 1 0 0 0 0
11 16 1 0 0 0 0
13 17 1 0 0 0 0
13 18 2 0 0 0 0
15 19 1 0 0 0 0
15 20 2 0 0 0 0
17 21 1 0 0 0 0
19 22 1 0 0 0 0
14 23 1 0 0 0 0
23 24 1 0 0 0 0
23 25 1 0 0 0 0
23 26 1 0 0 0 0
21 27 1 0 0 0 0
27 28 1 0 0 0 0
27 29 1 0 0 0 0
27 30 1 0 0 0 0
22 31 1 0 0 0 0
31 32 1 0 0 0 0
31 33 1 0 0 0 0
31 34 1 0 0 0 0
16 35 1 0 0 0 0

35 36 1 0 0 0 0
35 37 1 0 0 0 0
35 38 1 0 0 0 0
37 39 1 0 0 0 0
26 40 1 0 0 0 0
34 41 1 0 0 0 0
28 42 1 0 0 0 0
3 43 1 0 0 0 0
5 44 1 0 0 0 0
24 45 1 0 0 0 0
24 46 1 0 0 0 0
24 47 1 0 0 0 0
25 48 1 0 0 0 0
25 49 1 0 0 0 0
25 50 1 0 0 0 0
26 51 1 0 0 0 0
26 52 1 0 0 0 0
28 53 1 0 0 0 0
28 54 1 0 0 0 0
29 55 1 0 0 0 0
29 56 1 0 0 0 0
29 57 1 0 0 0 0
30 58 1 0 0 0 0
30 59 1 0 0 0 0
30 60 1 0 0 0 0
32 61 1 0 0 0 0
32 62 1 0 0 0 0
32 63 1 0 0 0 0
33 64 1 0 0 0 0
33 65 1 0 0 0 0
33 66 1 0 0 0 0
34 67 1 0 0 0 0
34 68 1 0 0 0 0
36 69 1 0 0 0 0
36 70 1 0 0 0 0
36 71 1 0 0 0 0
37 72 1 0 0 0 0
37 73 1 0 0 0 0
38 74 1 0 0 0 0
38 75 1 0 0 0 0
38 76 1 0 0 0 0
39 77 1 0 0 0 0
39 78 1 0 0 0 0
39 79 1 0 0 0 0
40 80 1 0 0 0 0
40 81 1 0 0 0 0
40 82 1 0 0 0 0
41 83 1 0 0 0 0
41 84 1 0 0 0 0
41 85 1 0 0 0 0
42 86 1 0 0 0 0
42 87 1 0 0 0 0
42 88 1 0 0 0 0
M END
> <Alias name>
Tetra-t-amylperoxypyromellitate

> <deltaH°(c)>   kJ/mol
-16629.8

$$$$
3-Methyl-3-t-amylperoxy-1-triphenylsilyl-1-butyne
ChemBrain IXL

```
63 65  0  0  0  0  0  0  0  0  1 V2000
    6.6308    2.6711   -0.9133 C   0  0  0  0  0  0  0  0  0  0  0  0
    6.6659    3.4860   -2.0450 C   0  0  0  0  0  0  0  0  0  0  0  0
    7.1699    4.7836   -1.9549 C   0  0  0  0  0  0  0  0  0  0  0  0
    7.6374    5.2670   -0.7327 C   0  0  0  0  0  0  0  0  0  0  0  0
    7.6041    4.4515    0.3986 C   0  0  0  0  0  0  0  0  0  0  0  0
    7.1001    3.1538    0.3085 C   0  0  0  0  0  0  0  0  0  0  0  0
    8.7657    8.2786    1.8769 C   0  0  0  0  0  0  0  0  0  0  0  0
    9.1467    7.2308    1.0385 C   0  0  0  0  0  0  0  0  0  0  0  0
   10.1561    6.3528    1.4340 C   0  0  0  0  0  0  0  0  0  0  0  0
   10.7831    6.5215    2.6687 C   0  0  0  0  0  0  0  0  0  0  0  0
   10.4013    7.5687    3.5075 C   0  0  0  0  0  0  0  0  0  0  0  0
    9.3927    8.4473    3.1115 C   0  0  0  0  0  0  0  0  0  0  0  0
    8.3068    7.0035   -0.6110 Si  0  0  0  0  0  0  0  0  0  0  0  0
   11.3811    7.7303   -4.0300 C   0  0  0  0  0  0  0  0  0  0  0  0
   11.8093    7.6953   -2.7027 C   0  0  0  0  0  0  0  0  0  0  0  0
   10.8881    7.4763   -1.6782 C   0  0  0  0  0  0  0  0  0  0  0  0
    9.5387    7.2933   -1.9809 C   0  0  0  0  0  0  0  0  0  0  0  0
    9.1107    7.3273   -3.3082 C   0  0  0  0  0  0  0  0  0  0  0  0
   10.0319    7.5462   -4.3327 C   0  0  0  0  0  0  0  0  0  0  0  0
    6.9044    8.2225   -0.7699 C   0  0  0  0  0  0  0  0  0  0  0  0
    5.9990    9.0095   -0.8728 C   0  0  0  0  0  0  0  0  0  0  0  0
    4.8403   10.0167   -1.0047 C   0  0  0  0  0  0  0  0  0  0  0  0
    5.0134   10.8318   -2.3010 C   0  0  0  0  0  0  0  0  0  0  0  0
    4.3773    6.4461   -0.9399 C   0  0  0  0  0  0  0  0  0  0  0  0
    3.1916    6.9443   -1.7888 C   0  0  0  0  0  0  0  0  0  0  0  0
    3.5628    9.2908   -1.0494 O   0  0  0  0  0  0  0  0  0  0  0  0
    4.1306    4.9837   -0.5212 C   0  0  0  0  0  0  0  0  0  0  0  0
    3.4209    8.3433   -2.1775 O   0  0  0  0  0  0  0  0  0  0  0  0
    4.8476   10.9697    0.2062 C   0  0  0  0  0  0  0  0  0  0  0  0
    3.0641    6.0739   -3.0540 C   0  0  0  0  0  0  0  0  0  0  0  0
    1.8930    6.8464   -0.9650 C   0  0  0  0  0  0  0  0  0  0  0  0
    6.2393    1.6638   -0.9832 H   0  0  0  0  0  0  0  0  0  0  0  0
    6.3019    3.1111   -2.9937 H   0  0  0  0  0  0  0  0  0  0  0  0
    7.1983    5.4158   -2.8338 H   0  0  0  0  0  0  0  0  0  0  0  0
    7.9695    4.8258    1.3469 H   0  0  0  0  0  0  0  0  0  0  0  0
    7.0731    2.5212    1.1871 H   0  0  0  0  0  0  0  0  0  0  0  0
    7.9831    8.9611    1.5692 H   0  0  0  0  0  0  0  0  0  0  0  0
   10.4529    5.5402    0.7825 H   0  0  0  0  0  0  0  0  0  0  0  0
   11.5663    5.8395    2.9760 H   0  0  0  0  0  0  0  0  0  0  0  0
   10.8880    7.6996    4.4661 H   0  0  0  0  0  0  0  0  0  0  0  0
    9.0965    9.2605    3.7627 H   0  0  0  0  0  0  0  0  0  0  0  0
   12.0962    7.9004   -4.8253 H   0  0  0  0  0  0  0  0  0  0  0  0
   12.8568    7.8381   -2.4677 H   0  0  0  0  0  0  0  0  0  0  0  0
   11.2206    7.4484   -0.6479 H   0  0  0  0  0  0  0  0  0  0  0  0
    8.0634    7.1837   -3.5433 H   0  0  0  0  0  0  0  0  0  0  0  0
    9.6995    7.5732   -5.3631 H   0  0  0  0  0  0  0  0  0  0  0  0
    4.1991   11.5398   -2.3937 H   0  0  0  0  0  0  0  0  0  0  0  0
    5.0081   10.1621   -3.1520 H   0  0  0  0  0  0  0  0  0  0  0  0
    5.9547   11.3665   -2.2683 H   0  0  0  0  0  0  0  0  0  0  0  0
    4.4726    7.0636   -0.0554 H   0  0  0  0  0  0  0  0  0  0  0  0
    5.2885    6.5061   -1.5221 H   0  0  0  0  0  0  0  0  0  0  0  0
    4.9614    4.6352    0.0797 H   0  0  0  0  0  0  0  0  0  0  0  0
    3.2166    4.9229    0.0566 H   0  0  0  0  0  0  0  0  0  0  0  0
    4.0403    4.3653   -1.4057 H   0  0  0  0  0  0  0  0  0  0  0  0
    4.0353   11.6798    0.1116 H   0  0  0  0  0  0  0  0  0  0  0  0
    4.7223   10.3972    1.1169 H   0  0  0  0  0  0  0  0  0  0  0  0
    5.7901   11.5019    0.2410 H   0  0  0  0  0  0  0  0  0  0  0  0
    3.9844    6.1264   -3.6226 H   0  0  0  0  0  0  0  0  0  0  0  0
    2.2437    6.4368   -3.6608 H   0  0  0  0  0  0  0  0  0  0  0  0
    2.8747    5.0467   -2.7680 H   0  0  0  0  0  0  0  0  0  0  0  0
    1.7166    5.8137   -0.6905 H   0  0  0  0  0  0  0  0  0  0  0  0
```

```
   1.9879     7.4470    -0.0688 H    0  0  0  0  0  0  0  0  0  0  0  0
   1.0618     7.2097    -1.5567 H    0  0  0  0  0  0  0  0  0  0  0  0
  1  2  4  0  0  0  0
  2  3  4  0  0  0  0
  3  4  4  0  0  0  0
  4  5  4  0  0  0  0
  5  6  4  0  0  0  0
  6  1  4  0  0  0  0
  7  8  4  0  0  0  0
  8  9  4  0  0  0  0
  9 10  4  0  0  0  0
 10 11  4  0  0  0  0
 11 12  4  0  0  0  0
 12  7  4  0  0  0  0
  4 13  1  0  0  0  0
 13  8  1  0  0  0  0
 14 15  4  0  0  0  0
 15 16  4  0  0  0  0
 16 17  4  0  0  0  0
 17 18  4  0  0  0  0
 18 19  4  0  0  0  0
 19 14  4  0  0  0  0
 17 13  1  0  0  0  0
 13 20  1  0  0  0  0
 20 21  3  0  0  0  0
 21 22  1  0  0  0  0
 24 25  1  0  0  0  0
 24 27  1  0  0  0  0
 25 28  1  0  0  0  0
 28 26  1  0  0  0  0
 23 22  1  0  0  0  0
 22 29  1  0  0  0  0
 26 22  1  0  0  0  0
 25 30  1  0  0  0  0
 25 31  1  0  0  0  0
  1 32  1  0  0  0  0
  2 33  1  0  0  0  0
  3 34  1  0  0  0  0
  5 35  1  0  0  0  0
  6 36  1  0  0  0  0
  7 37  1  0  0  0  0
  9 38  1  0  0  0  0
 10 39  1  0  0  0  0
 11 40  1  0  0  0  0
 12 41  1  0  0  0  0
 14 42  1  0  0  0  0
 15 43  1  0  0  0  0
 16 44  1  0  0  0  0
 18 45  1  0  0  0  0
 19 46  1  0  0  0  0
 23 47  1  0  0  0  0
 23 48  1  0  0  0  0
 23 49  1  0  0  0  0
 24 50  1  0  0  0  0
 24 51  1  0  0  0  0
 27 52  1  0  0  0  0
 27 53  1  0  0  0  0
 27 54  1  0  0  0  0
 29 55  1  0  0  0  0
 29 56  1  0  0  0  0
 29 57  1  0  0  0  0
 30 58  1  0  0  0  0
 30 59  1  0  0  0  0
```

 30 60 1 0 0 0 0
 31 61 1 0 0 0 0
 31 62 1 0 0 0 0
 31 63 1 0 0 0 0
M END
> <Alias name>
3-Methyl-3-t-amylperoxy-1-triphenylsilyl-1-butyne

> <deltaH°(c)>   kJ/mol
-16603

$$$$
Bis(O-hexyl-N-benzoylthiocarbamato)nickel(II)
  ChemBrain IXL

 73 76 0 0 0 0 0 0 0 0 1 V2000
   11.0989   17.1453   -2.4848 C   0 0 0 0 0 0 0 0 0 0 0 0
   10.3968   16.0377   -2.0094 C   0 0 0 0 0 0 0 0 0 0 0 0
   10.9518   15.2339   -1.0105 C   0 0 0 0 0 0 0 0 0 0 0 0
   12.1668   15.5889   -0.4175 C   0 0 0 0 0 0 0 0 0 0 0 0
   12.8681   16.7014   -0.8950 C   0 0 0 0 0 0 0 0 0 0 0 0
   12.3368   17.4743   -1.9318 C   0 0 0 0 0 0 0 0 0 0 0 0
   12.7086   14.7801   0.7208 C   0 0 0 0 0 0 0 0 0 0 0 0
   12.0681   13.8152   1.1299 O   0 0 0 0 0 0 0 0 0 0 0 0
   13.9492   15.1345   1.3351 N   0 0 0 0 0 0 0 0 0 0 0 0
   14.9253   14.2948   1.3897 C   0 0 0 0 0 0 0 0 0 0 0 0
   16.3205   14.7890   1.4647 O   0 0 0 0 0 0 0 0 0 0 0 0
   14.5989   12.4497   1.3811 S   0 5 0 0 0 0 0 0 0 0 0 0
   17.3269   13.7661   1.8203 C   0 0 0 0 0 0 0 0 0 0 0 0
   18.7184   14.4276   1.9823 C   0 0 0 0 0 0 0 0 0 0 0 0
   12.5381   12.0453   0.7093 Ni 0 0 0 0 0 0 0 0 0 0 0 0
   13.8243   6.1391   -2.0634 C   0 0 0 0 0 0 0 0 0 0 0 0
   14.4738   6.6968   -0.9717 C   0 0 0 0 0 0 0 0 0 0 0 0
   14.0637   7.9333   -0.5061 C   0 0 0 0 0 0 0 0 0 0 0 0
   12.7625   8.3329   -0.7361 C   0 0 0 0 0 0 0 0 0 0 0 0
   12.0839   7.7912   -1.8293 C   0 0 0 0 0 0 0 0 0 0 0 0
   12.6410   6.7130   -2.5199 C   0 0 0 0 0 0 0 0 0 0 0 0
   12.1101   9.3255   0.1814 C   0 0 0 0 0 0 0 0 0 0 0 0
   12.8198   10.1560   0.7766 O   0 0 0 0 0 0 0 0 0 0 0 0
   10.6843   9.2950   0.3748 N   0 0 0 0 0 0 0 0 0 0 0 0
    9.9589   10.2836   0.0032 C   0 0 0 0 0 0 0 0 0 0 0 0
    8.4927   10.1786   -0.0859 O   0 0 0 0 0 0 0 0 0 0 0 0
   10.7429   11.8512   -0.4208 S   0 5 0 0 0 0 0 0 0 0 0 0
    7.7755   11.4890   -0.0911 C   0 0 0 0 0 0 0 0 0 0 0 0
    6.3414   11.2842   0.4318 C   0 0 0 0 0 0 0 0 0 0 0 0
    5.6415   12.6480   0.6426 C   0 0 0 0 0 0 0 0 0 0 0 0
    4.2055   12.3913   1.1483 C   0 0 0 0 0 0 0 0 0 0 0 0
   19.7674   13.3393   2.3150 C   0 0 0 0 0 0 0 0 0 0 0 0
   21.1914   13.9264   2.1878 C   0 0 0 0 0 0 0 0 0 0 0 0
    3.4929   13.7256   1.4340 C   0 0 0 0 0 0 0 0 0 0 0 0
    2.3093   13.4698   2.3906 C   0 0 0 0 0 0 0 0 0 0 0 0
   22.2298   12.8223   2.4888 C   0 0 0 0 0 0 0 0 0 0 0 0
   23.6542   13.3979   2.3495 C   0 0 0 0 0 0 0 0 0 0 0 0
   10.6828   17.7500   -3.2836 H   0 0 0 0 0 0 0 0 0 0 0 0
    9.4203   15.8014   -2.4156 H   0 0 0 0 0 0 0 0 0 0 0 0
   10.4393   14.3345   -0.6957 H   0 0 0 0 0 0 0 0 0 0 0 0
   13.8242   16.9654   -0.4618 H   0 0 0 0 0 0 0 0 0 0 0 0
   12.8861   18.3302   -2.3072 H   0 0 0 0 0 0 0 0 0 0 0 0
   17.3733   13.0195   1.0373 H   0 0 0 0 0 0 0 0 0 0 0 0
   17.0439   13.2928   2.7519 H   0 0 0 0 0 0 0 0 0 0 0 0
   18.6828   15.1527   2.7864 H   0 0 0 0 0 0 0 0 0 0 0 0
   18.9930   14.9243   1.0597 H   0 0 0 0 0 0 0 0 0 0 0 0
   14.2363   5.2661   -2.5554 H   0 0 0 0 0 0 0 0 0 0 0 0

15.2870	6.1708	-0.4868	H	0	0	0	0	0	0	0	0	0	0	0	0	0
14.7515	8.5801	0.0239	H	0	0	0	0	0	0	0	0	0	0	0	0	0
11.1332	8.2042	-2.1431	H	0	0	0	0	0	0	0	0	0	0	0	0	0
12.1567	6.3235	-3.4074	H	0	0	0	0	0	0	0	0	0	0	0	0	0
8.2977	12.1896	0.5502	H	0	0	0	0	0	0	0	0	0	0	0	0	0
7.7395	11.8833	-1.1025	H	0	0	0	0	0	0	0	0	0	0	0	0	0
5.7791	10.7022	-0.2860	H	0	0	0	0	0	0	0	0	0	0	0	0	0
6.3783	10.7537	1.3729	H	0	0	0	0	0	0	0	0	0	0	0	0	0
6.1876	13.2293	1.3769	H	0	0	0	0	0	0	0	0	0	0	0	0	0
5.6055	13.1896	-0.2959	H	0	0	0	0	0	0	0	0	0	0	0	0	0
3.6533	11.8444	0.3952	H	0	0	0	0	0	0	0	0	0	0	0	0	0
4.2466	11.8062	2.0581	H	0	0	0	0	0	0	0	0	0	0	0	0	0
19.6574	12.5111	1.6262	H	0	0	0	0	0	0	0	0	0	0	0	0	0
19.6127	12.9880	3.3277	H	0	0	0	0	0	0	0	0	0	0	0	0	0
21.3125	14.7382	2.8944	H	0	0	0	0	0	0	0	0	0	0	0	0	0
21.3409	14.2980	1.1815	H	0	0	0	0	0	0	0	0	0	0	0	0	0
4.1870	14.4179	1.8934	H	0	0	0	0	0	0	0	0	0	0	0	0	0
3.1233	14.1449	0.5060	H	0	0	0	0	0	0	0	0	0	0	0	0	0
2.2691	12.4155	2.6397	H	0	0	0	0	0	0	0	0	0	0	0	0	0
2.4433	14.0490	3.2952	H	0	0	0	0	0	0	0	0	0	0	0	0	0
1.3846	13.7614	1.9083	H	0	0	0	0	0	0	0	0	0	0	0	0	0
22.1012	12.0059	1.7889	H	0	0	0	0	0	0	0	0	0	0	0	0	0
22.0859	12.4575	3.4984	H	0	0	0	0	0	0	0	0	0	0	0	0	0
23.6072	14.4794	2.3352	H	0	0	0	0	0	0	0	0	0	0	0	0	0
24.0983	13.0435	1.4272	H	0	0	0	0	0	0	0	0	0	0	0	0	0
24.2582	13.0738	3.1885	H	0	0	0	0	0	0	0	0	0	0	0	0	0

1	2	4	0	0	0	0
2	3	4	0	0	0	0
3	4	4	0	0	0	0
4	5	4	0	0	0	0
5	6	4	0	0	0	0
6	1	4	0	0	0	0
4	7	1	0	0	0	0
7	8	2	0	0	0	0
7	9	1	0	0	0	0
9	10	2	0	0	0	0
10	11	1	0	0	0	0
10	12	1	0	0	0	0
11	13	1	0	0	0	0
13	14	1	0	0	0	0
15	12	1	0	0	0	0
15	8	1	0	0	0	0
16	17	4	0	0	0	0
17	18	4	0	0	0	0
18	19	4	0	0	0	0
19	20	4	0	0	0	0
20	21	4	0	0	0	0
21	16	4	0	0	0	0
19	22	1	0	0	0	0
22	23	2	0	0	0	0
22	24	1	0	0	0	0
24	25	2	0	0	0	0
25	26	1	0	0	0	0
25	27	1	0	0	0	0
26	28	1	0	0	0	0
28	29	1	0	0	0	0
27	15	1	0	0	0	0
23	15	1	0	0	0	0
29	30	1	0	0	0	0
30	31	1	0	0	0	0
14	32	1	0	0	0	0
32	33	1	0	0	0	0
31	34	1	0	0	0	0

```
 34 35  1  0  0  0  0
 33 36  1  0  0  0  0
 36 37  1  0  0  0  0
  1 38  1  0  0  0  0
  2 39  1  0  0  0  0
  3 40  1  0  0  0  0
  5 41  1  0  0  0  0
  6 42  1  0  0  0  0
 13 43  1  0  0  0  0
 13 44  1  0  0  0  0
 14 45  1  0  0  0  0
 14 46  1  0  0  0  0
 16 47  1  0  0  0  0
 17 48  1  0  0  0  0
 18 49  1  0  0  0  0
 20 50  1  0  0  0  0
 21 51  1  0  0  0  0
 28 52  1  0  0  0  0
 28 53  1  0  0  0  0
 29 54  1  0  0  0  0
 29 55  1  0  0  0  0
 30 56  1  0  0  0  0
 30 57  1  0  0  0  0
 31 58  1  0  0  0  0
 31 59  1  0  0  0  0
 32 60  1  0  0  0  0
 32 61  1  0  0  0  0
 33 62  1  0  0  0  0
 33 63  1  0  0  0  0
 34 64  1  0  0  0  0
 34 65  1  0  0  0  0
 35 66  1  0  0  0  0
 35 67  1  0  0  0  0
 35 68  1  0  0  0  0
 36 69  1  0  0  0  0
 36 70  1  0  0  0  0
 37 71  1  0  0  0  0
 37 72  1  0  0  0  0
 37 73  1  0  0  0  0
M  END
>  <Alias name>
Bis(O-hexyl-N-benzoylthiocarbamato)nickel(II)

>  <deltaH°(c)>   kJ/mol
-16576.2

$$$$
Cholesterol
  ChemBrain IXL

 74 77  0  0  0  0  0  0  0  0  1 V2000
    6.0430    8.8419    0.7633 C   0  0  0  0  0  0  0  0  0  0  0  0
    5.1734    8.6968   -0.5065 C   0  0  0  0  0  0  0  0  0  0  0  0
    3.7180    8.3894   -0.1087 C   0  0  0  0  0  0  0  0  0  0  0  0
    3.1255    9.5981    0.6339 C   0  0  0  0  0  0  0  0  0  0  0  0
    3.8481    9.7719    1.9800 C   0  0  0  0  0  0  0  0  0  0  0  0
    5.3680    9.8640    1.7391 C   0  0  0  0  0  0  0  0  0  0  0  0
    6.1569    7.4715    1.4593 C   0  0  0  0  0  0  0  0  0  0  0  0
    7.4400    9.3245    0.3513 C   0  0  0  0  0  0  0  0  0  0  0  0
    6.0688   10.8161    2.3910 C   0  0  0  0  0  0  0  0  0  0  0  0
    8.2417    9.7212    1.6208 C   0  0  0  0  0  0  0  0  0  0  0  0
    7.6148   10.9779    2.2373 C   0  0  0  0  0  0  0  0  0  0  0  0
    8.1820    8.1619   -0.4661 C   0  0  0  0  0  0  0  0  0  0  0  0
```

9.6579	10.0303	1.1878	C	0	0	0	0	0	0	0	0	0	0	0	0	0
9.7087	8.4919	-0.6687	C	0	0	0	0	0	0	0	0	0	0	0	0	0
10.2890	8.7959	0.6786	C	0	0	0	0	0	0	0	0	0	0	0	0	0
10.5857	10.5431	2.2976	C	0	0	0	0	0	0	0	0	0	0	0	0	0
9.9868	7.6303	1.6427	C	0	0	0	0	0	0	0	0	0	0	0	0	0
11.7939	9.0784	0.6861	C	0	0	0	0	0	0	0	0	0	0	0	0	0
12.0151	10.0297	1.9029	C	0	0	0	0	0	0	0	0	0	0	0	0	0
12.6095	7.7768	0.8483	C	0	0	0	0	0	0	0	0	0	0	0	0	0
1.6921	9.3746	0.8710	O	0	0	0	0	0	0	0	0	0	0	0	0	0
14.1189	8.0974	0.7051	C	0	0	0	0	0	0	0	0	0	0	0	0	0
12.3436	7.1641	2.2362	C	0	0	0	0	0	0	0	0	0	0	0	0	0
14.3835	8.7539	-0.6716	C	0	0	0	0	0	0	0	0	0	0	0	0	0
15.8834	9.1118	-0.7958	C	0	0	0	0	0	0	0	0	0	0	0	0	0
16.1518	9.7490	-2.1782	C	0	0	0	0	0	0	0	0	0	0	0	0	0
17.6555	10.0659	-2.3172	C	0	0	0	0	0	0	0	0	0	0	0	0	0
15.3402	11.0522	-2.3115	C	0	0	0	0	0	0	0	0	0	0	0	0	0
5.2046	9.6179	-1.0699	H	0	0	0	0	0	0	0	0	0	0	0	0	0
5.5573	7.8910	-1.1145	H	0	0	0	0	0	0	0	0	0	0	0	0	0
3.1360	8.1920	-0.9971	H	0	0	0	0	0	0	0	0	0	0	0	0	0
3.6957	7.5238	0.5362	H	0	0	0	0	0	0	0	0	0	0	0	0	0
3.2574	10.4884	0.0364	H	0	0	0	0	0	0	0	0	0	0	0	0	0
3.6348	8.9250	2.6149	H	0	0	0	0	0	0	0	0	0	0	0	0	0
3.5041	10.6767	2.4589	H	0	0	0	0	0	0	0	0	0	0	0	0	0
6.7776	7.5639	2.3378	H	0	0	0	0	0	0	0	0	0	0	0	0	0
6.5986	6.7579	0.7795	H	0	0	0	0	0	0	0	0	0	0	0	0	0
5.1726	7.1319	1.7480	H	0	0	0	0	0	0	0	0	0	0	0	0	0
7.3389	10.1909	-0.2850	H	0	0	0	0	0	0	0	0	0	0	0	0	0
5.5366	11.5056	3.0292	H	0	0	0	0	0	0	0	0	0	0	0	0	0
8.2369	8.9110	2.3353	H	0	0	0	0	0	0	0	0	0	0	0	0	0
8.0516	11.1523	3.2093	H	0	0	0	0	0	0	0	0	0	0	0	0	0
7.8202	11.8254	1.5990	H	0	0	0	0	0	0	0	0	0	0	0	0	0
7.7122	8.0524	-1.4320	H	0	0	0	0	0	0	0	0	0	0	0	0	0
8.0908	7.2331	0.0791	H	0	0	0	0	0	0	0	0	0	0	0	0	0
9.6273	10.7607	0.3931	H	0	0	0	0	0	0	0	0	0	0	0	0	0
9.8157	9.3489	-1.3161	H	0	0	0	0	0	0	0	0	0	0	0	0	0
10.2133	7.6400	-1.1009	H	0	0	0	0	0	0	0	0	0	0	0	0	0
10.2907	10.1332	3.2526	H	0	0	0	0	0	0	0	0	0	0	0	0	0
10.5710	11.6222	2.3305	H	0	0	0	0	0	0	0	0	0	0	0	0	0
10.4144	6.7188	1.2509	H	0	0	0	0	0	0	0	0	0	0	0	0	0
10.4173	7.8425	2.6106	H	0	0	0	0	0	0	0	0	0	0	0	0	0
8.9181	7.5126	1.7421	H	0	0	0	0	0	0	0	0	0	0	0	0	0
12.0782	9.5788	-0.2277	H	0	0	0	0	0	0	0	0	0	0	0	0	0
12.6455	10.8591	1.6200	H	0	0	0	0	0	0	0	0	0	0	0	0	0
12.4571	9.4866	2.7260	H	0	0	0	0	0	0	0	0	0	0	0	0	0
12.3182	7.0726	0.0829	H	0	0	0	0	0	0	0	0	0	0	0	0	0
1.4869	8.6150	1.4032	H	0	0	0	0	0	0	0	0	0	0	0	0	0
14.4183	8.7764	1.4900	H	0	0	0	0	0	0	0	0	0	0	0	0	0
14.6899	7.1838	0.7832	H	0	0	0	0	0	0	0	0	0	0	0	0	0
12.9851	6.3061	2.3792	H	0	0	0	0	0	0	0	0	0	0	0	0	0
12.5497	7.8985	3.0000	H	0	0	0	0	0	0	0	0	0	0	0	0	0
11.3109	6.8558	2.3018	H	0	0	0	0	0	0	0	0	0	0	0	0	0
14.1124	8.0642	-1.4572	H	0	0	0	0	0	0	0	0	0	0	0	0	0
13.7911	9.6524	-0.7616	H	0	0	0	0	0	0	0	0	0	0	0	0	0
16.1518	9.8124	-0.0190	H	0	0	0	0	0	0	0	0	0	0	0	0	0
16.4769	8.2154	-0.6922	H	0	0	0	0	0	0	0	0	0	0	0	0	0
15.8564	9.0600	-2.9557	H	0	0	0	0	0	0	0	0	0	0	0	0	0
17.8414	10.5131	-3.2831	H	0	0	0	0	0	0	0	0	0	0	0	0	0
18.2259	9.1534	-2.2275	H	0	0	0	0	0	0	0	0	0	0	0	0	0
17.9520	10.7539	-1.5392	H	0	0	0	0	0	0	0	0	0	0	0	0	0
14.2869	10.8307	-2.2268	H	0	0	0	0	0	0	0	0	0	0	0	0	0
15.6284	11.7375	-1.5281	H	0	0	0	0	0	0	0	0	0	0	0	0	0
15.5373	11.5022	-3.2738	H	0	0	0	0	0	0	0	0	0	0	0	0	0

1 2 1 0 0 0 0

2	3	1	0	0	0	0
3	4	1	0	0	0	0
4	5	1	0	0	0	0
5	6	1	0	0	0	0
6	1	1	0	0	0	0
1	7	1	0	0	0	0
1	8	1	0	0	0	0
6	9	2	0	0	0	0
10	8	1	0	0	0	0
9	11	1	0	0	0	0
11	10	1	0	0	0	0
8	12	1	0	0	0	0
10	13	1	0	0	0	0
12	14	1	0	0	0	0
14	15	1	0	0	0	0
15	13	1	0	0	0	0
13	16	1	0	0	0	0
15	17	1	0	0	0	0
15	18	1	0	0	0	0
16	19	1	0	0	0	0
19	18	1	0	0	0	0
18	20	1	0	0	0	0
4	21	1	0	0	0	0
20	22	1	0	0	0	0
20	23	1	0	0	0	0
22	24	1	0	0	0	0
24	25	1	0	0	0	0
25	26	1	0	0	0	0
26	27	1	0	0	0	0
26	28	1	0	0	0	0
2	29	1	0	0	0	0
2	30	1	0	0	0	0
3	31	1	0	0	0	0
3	32	1	0	0	0	0
4	33	1	0	0	0	0
5	34	1	0	0	0	0
5	35	1	0	0	0	0
7	36	1	0	0	0	0
7	37	1	0	0	0	0
7	38	1	0	0	0	0
8	39	1	0	0	0	0
9	40	1	0	0	0	0
10	41	1	0	0	0	0
11	42	1	0	0	0	0
11	43	1	0	0	0	0
12	44	1	0	0	0	0
12	45	1	0	0	0	0
13	46	1	0	0	0	0
14	47	1	0	0	0	0
14	48	1	0	0	0	0
16	49	1	0	0	0	0
16	50	1	0	0	0	0
17	51	1	0	0	0	0
17	52	1	0	0	0	0
17	53	1	0	0	0	0
18	54	1	0	0	0	0
19	55	1	0	0	0	0
19	56	1	0	0	0	0
20	57	1	0	0	0	0
21	58	1	0	0	0	0
22	59	1	0	0	0	0
22	60	1	0	0	0	0
23	61	1	0	0	0	0

```
 23 62  1  0  0  0  0
 23 63  1  0  0  0  0
 24 64  1  0  0  0  0
 24 65  1  0  0  0  0
 25 66  1  0  0  0  0
 25 67  1  0  0  0  0
 26 68  1  0  0  0  0
 27 69  1  0  0  0  0
 27 70  1  0  0  0  0
 27 71  1  0  0  0  0
 28 72  1  0  0  0  0
 28 73  1  0  0  0  0
 28 74  1  0  0  0  0
M  END
>  <Alias name>
Cholest-5-en-3-ol

>  <deltaH°(c)>   kJ/mol
-16524

$$$$
Diisodecylphthalate
  ChemBrain IXL

 78 78  0  0  0  0  0  0  0  0  1 V2000
    2.9308    4.9755    0.5453 C   0  0  0  0  0  0  0  0  0  0  0  0
    2.5233    6.1406   -0.1061 C   0  0  0  0  0  0  0  0  0  0  0  0
    3.4558    7.1312   -0.4167 C   0  0  0  0  0  0  0  0  0  0  0  0
    4.7985    6.9608   -0.0768 C   0  0  0  0  0  0  0  0  0  0  0  0
    5.2112    5.7819    0.5761 C   0  0  0  0  0  0  0  0  0  0  0  0
    4.2722    4.7968    0.8858 C   0  0  0  0  0  0  0  0  0  0  0  0
    5.7794    8.0345   -0.4151 C   0  0  0  0  0  0  0  0  0  0  0  0
    5.6169    8.7065   -1.4203 O   0  0  0  0  0  0  0  0  0  0  0  0
    6.9465    8.2932    0.4593 O   0  0  0  0  0  0  0  0  0  0  0  0
    6.6425    5.5691    0.9463 C   0  0  0  0  0  0  0  0  0  0  0  0
    6.9201    4.8759    1.9111 O   0  0  0  0  0  0  0  0  0  0  0  0
    7.7289    6.1896    0.1524 O   0  0  0  0  0  0  0  0  0  0  0  0
    7.8992    9.3069   -0.0546 C   0  0  0  0  0  0  0  0  0  0  0  0
    8.9951    9.5858    1.0057 C   0  0  0  0  0  0  0  0  0  0  0  0
    9.9911   10.6412    0.4585 C   0  0  0  0  0  0  0  0  0  0  0  0
   11.0723   10.9410    1.5259 C   0  0  0  0  0  0  0  0  0  0  0  0
   12.0499   12.0144    0.9884 C   0  0  0  0  0  0  0  0  0  0  0  0
   13.1333   12.3088    2.0524 C   0  0  0  0  0  0  0  0  0  0  0  0
   14.1064   13.3835    1.5169 C   0  0  0  0  0  0  0  0  0  0  0  0
   15.2139   13.6451    2.5612 C   0  0  0  0  0  0  0  0  0  0  0  0
    9.0942    5.9770    0.6918 C   0  0  0  0  0  0  0  0  0  0  0  0
   10.1454    6.5805   -0.2754 C   0  0  0  0  0  0  0  0  0  0  0  0
   11.5678    6.3614    0.3035 C   0  0  0  0  0  0  0  0  0  0  0  0
   12.6238    6.9555   -0.6614 C   0  0  0  0  0  0  0  0  0  0  0  0
   14.0413    6.7303   -0.0804 C   0  0  0  0  0  0  0  0  0  0  0  0
   15.0999    7.3009   -1.0535 C   0  0  0  0  0  0  0  0  0  0  0  0
   16.5136    7.0694   -0.4725 C   0  0  0  0  0  0  0  0  0  0  0  0
   17.5742    7.5999   -1.4620 C   0  0  0  0  0  0  0  0  0  0  0  0
   16.1853   14.7166    2.0248 C   0  0  0  0  0  0  0  0  0  0  0  0
   15.9883   12.3400    2.8286 C   0  0  0  0  0  0  0  0  0  0  0  0
   18.9838    7.3646   -0.8812 C   0  0  0  0  0  0  0  0  0  0  0  0
   17.4434    6.8560   -2.8052 C   0  0  0  0  0  0  0  0  0  0  0  0
    2.2051    4.2092    0.7868 H   0  0  0  0  0  0  0  0  0  0  0  0
    1.4823    6.2761   -0.3709 H   0  0  0  0  0  0  0  0  0  0  0  0
    3.1369    8.0342   -0.9227 H   0  0  0  0  0  0  0  0  0  0  0  0
    4.5856    3.8918    1.3914 H   0  0  0  0  0  0  0  0  0  0  0  0
    7.3662   10.2249   -0.2709 H   0  0  0  0  0  0  0  0  0  0  0  0
    8.3624    8.9380   -0.9615 H   0  0  0  0  0  0  0  0  0  0  0  0
```

8.5341	9.9607	1.9111	H	0	0	0	0	0	0	0	0	0	0	0	0	0	
9.5267	8.6683	1.2248	H	0	0	0	0	0	0	0	0	0	0	0	0	0	
10.4656	10.2588	-0.4369	H	0	0	0	0	0	0	0	0	0	0	0	0	0	
9.4565	11.5528	0.2215	H	0	0	0	0	0	0	0	0	0	0	0	0	0	
10.5972	11.3059	2.4280	H	0	0	0	0	0	0	0	0	0	0	0	0	0	
11.6204	10.0340	1.7487	H	0	0	0	0	0	0	0	0	0	0	0	0	0	
12.5224	11.6516	0.0839	H	0	0	0	0	0	0	0	0	0	0	0	0	0	
11.5031	12.9234	0.7699	H	0	0	0	0	0	0	0	0	0	0	0	0	0	
12.6611	12.6694	2.9579	H	0	0	0	0	0	0	0	0	0	0	0	0	0	
13.6826	11.4010	2.2690	H	0	0	0	0	0	0	0	0	0	0	0	0	0	
14.5553	13.0365	0.5945	H	0	0	0	0	0	0	0	0	0	0	0	0	0	
13.5628	14.3016	1.3305	H	0	0	0	0	0	0	0	0	0	0	0	0	0	
14.7655	13.9941	3.4832	H	0	0	0	0	0	0	0	0	0	0	0	0	0	
9.2758	4.9147	0.8023	H	0	0	0	0	0	0	0	0	0	0	0	0	0	
9.1777	6.4581	1.6584	H	0	0	0	0	0	0	0	0	0	0	0	0	0	
9.9625	7.6416	-0.3908	H	0	0	0	0	0	0	0	0	0	0	0	0	0	
10.0698	6.0941	-1.2401	H	0	0	0	0	0	0	0	0	0	0	0	0	0	
11.7479	5.3003	0.4246	H	0	0	0	0	0	0	0	0	0	0	0	0	0	
11.6442	6.8515	1.2663	H	0	0	0	0	0	0	0	0	0	0	0	0	0	
12.4473	8.0174	-0.7800	H	0	0	0	0	0	0	0	0	0	0	0	0	0	
12.5474	6.4673	-1.6251	H	0	0	0	0	0	0	0	0	0	0	0	0	0	
14.2113	5.6692	0.0547	H	0	0	0	0	0	0	0	0	0	0	0	0	0	
14.1232	7.2329	0.8755	H	0	0	0	0	0	0	0	0	0	0	0	0	0	
14.9337	8.3629	-1.1854	H	0	0	0	0	0	0	0	0	0	0	0	0	0	
15.0171	6.8011	-2.0108	H	0	0	0	0	0	0	0	0	0	0	0	0	0	
16.6687	6.0097	-0.3119	H	0	0	0	0	0	0	0	0	0	0	0	0	0	
16.6063	7.5942	0.4704	H	0	0	0	0	0	0	0	0	0	0	0	0	0	
17.4218	8.6604	-1.6204	H	0	0	0	0	0	0	0	0	0	0	0	0	0	
15.6511	15.6469	1.8767	H	0	0	0	0	0	0	0	0	0	0	0	0	0	
16.6028	14.3859	1.0818	H	0	0	0	0	0	0	0	0	0	0	0	0	0	
16.9849	14.8694	2.7397	H	0	0	0	0	0	0	0	0	0	0	0	0	0	
16.7960	12.5354	3.5236	H	0	0	0	0	0	0	0	0	0	0	0	0	0	
16.3958	11.9649	1.8979	H	0	0	0	0	0	0	0	0	0	0	0	0	0	
15.3180	11.6028	3.2523	H	0	0	0	0	0	0	0	0	0	0	0	0	0	
19.0880	7.9120	0.0474	H	0	0	0	0	0	0	0	0	0	0	0	0	0	
19.7282	7.7092	-1.5889	H	0	0	0	0	0	0	0	0	0	0	0	0	0	
19.1256	6.3072	-0.6947	H	0	0	0	0	0	0	0	0	0	0	0	0	0	
18.2277	7.1834	-3.4770	H	0	0	0	0	0	0	0	0	0	0	0	0	0	
17.5336	5.7899	-2.6376	H	0	0	0	0	0	0	0	0	0	0	0	0	0	
16.4779	7.0720	-3.2450	H	0	0	0	0	0	0	0	0	0	0	0	0	0	

1	2	4	0	0	0	0
2	3	4	0	0	0	0
3	4	4	0	0	0	0
4	5	4	0	0	0	0
5	6	4	0	0	0	0
6	1	4	0	0	0	0
7	8	2	0	0	0	0
7	9	1	0	0	0	0
7	4	1	0	0	0	0
10	11	2	0	0	0	0
10	12	1	0	0	0	0
10	5	1	0	0	0	0
9	13	1	0	0	0	0
13	14	1	0	0	0	0
14	15	1	0	0	0	0
15	16	1	0	0	0	0
16	17	1	0	0	0	0
17	18	1	0	0	0	0
18	19	1	0	0	0	0
19	20	1	0	0	0	0
21	22	1	0	0	0	0
22	23	1	0	0	0	0
23	24	1	0	0	0	0

24 25 1 0 0 0 0
25 26 1 0 0 0 0
26 27 1 0 0 0 0
27 28 1 0 0 0 0
21 12 1 0 0 0 0
20 29 1 0 0 0 0
20 30 1 0 0 0 0
28 31 1 0 0 0 0
28 32 1 0 0 0 0
1 33 1 0 0 0 0
2 34 1 0 0 0 0
3 35 1 0 0 0 0
6 36 1 0 0 0 0
13 37 1 0 0 0 0
13 38 1 0 0 0 0
14 39 1 0 0 0 0
14 40 1 0 0 0 0
15 41 1 0 0 0 0
15 42 1 0 0 0 0
16 43 1 0 0 0 0
16 44 1 0 0 0 0
17 45 1 0 0 0 0
17 46 1 0 0 0 0
18 47 1 0 0 0 0
18 48 1 0 0 0 0
19 49 1 0 0 0 0
19 50 1 0 0 0 0
20 51 1 0 0 0 0
21 52 1 0 0 0 0
21 53 1 0 0 0 0
22 54 1 0 0 0 0
22 55 1 0 0 0 0
23 56 1 0 0 0 0
23 57 1 0 0 0 0
24 58 1 0 0 0 0
24 59 1 0 0 0 0
25 60 1 0 0 0 0
25 61 1 0 0 0 0
26 62 1 0 0 0 0
26 63 1 0 0 0 0
27 64 1 0 0 0 0
27 65 1 0 0 0 0
28 66 1 0 0 0 0
29 67 1 0 0 0 0
29 68 1 0 0 0 0
29 69 1 0 0 0 0
30 70 1 0 0 0 0
30 71 1 0 0 0 0
30 72 1 0 0 0 0
31 73 1 0 0 0 0
31 74 1 0 0 0 0
31 75 1 0 0 0 0
32 76 1 0 0 0 0
32 77 1 0 0 0 0
32 78 1 0 0 0 0
M END
> <Alias name>
DIDP

> <deltaH°(c)>   kJ/mol
-16407.22

$$$$

```
Di-n-Decylphthalate
  ChemBrain IXL

 78 78  0  0  0  0  0  0  0  0  1 V2000
    2.5255   13.4211    2.8141 C   0  0  0  0  0  0  0  0  0  0  0  0
    2.4021   12.1033    3.2590 C   0  0  0  0  0  0  0  0  0  0  0  0
    3.5071   11.2509    3.2437 C   0  0  0  0  0  0  0  0  0  0  0  0
    4.7431   11.7084    2.7832 C   0  0  0  0  0  0  0  0  0  0  0  0
    4.8702   13.0544    2.3328 C   0  0  0  0  0  0  0  0  0  0  0  0
    3.7549   13.8940    2.3526 C   0  0  0  0  0  0  0  0  0  0  0  0
    5.9100   10.7539    2.7846 C   0  0  0  0  0  0  0  0  0  0  0  0
    5.9444    9.8525    3.6060 O   0  0  0  0  0  0  0  0  0  0  0  0
    7.0223   10.8870    1.7976 O   0  0  0  0  0  0  0  0  0  0  0  0
    6.1789   13.6068    1.8256 C   0  0  0  0  0  0  0  0  0  0  0  0
    6.1688   14.5121    1.0078 O   0  0  0  0  0  0  0  0  0  0  0  0
    7.4874   13.0710    2.3074 O   0  0  0  0  0  0  0  0  0  0  0  0
    8.1517    9.9250    1.9924 C   0  0  0  0  0  0  0  0  0  0  0  0
    9.1992   10.0787    0.8471 C   0  0  0  0  0  0  0  0  0  0  0  0
   10.3945    9.1061    1.0958 C   0  0  0  0  0  0  0  0  0  0  0  0
   11.4748    9.3003   -0.0069 C   0  0  0  0  0  0  0  0  0  0  0  0
   12.6436    8.2996    0.2200 C   0  0  0  0  0  0  0  0  0  0  0  0
   13.7228    8.4982   -0.8775 C   0  0  0  0  0  0  0  0  0  0  0  0
   14.8760    7.4840   -0.6672 C   0  0  0  0  0  0  0  0  0  0  0  0
   15.9508    7.6853   -1.7631 C   0  0  0  0  0  0  0  0  0  0  0  0
    8.6898   13.6572    1.6355 C   0  0  0  0  0  0  0  0  0  0  0  0
    9.9982   13.1077    2.2842 C   0  0  0  0  0  0  0  0  0  0  0  0
   11.2383   13.6834    1.5296 C   0  0  0  0  0  0  0  0  0  0  0  0
   12.5477   13.0934    2.1294 C   0  0  0  0  0  0  0  0  0  0  0  0
   13.7798   13.6973    1.3959 C   0  0  0  0  0  0  0  0  0  0  0  0
   15.0868   13.1224    2.0050 C   0  0  0  0  0  0  0  0  0  0  0  0
   16.3135   13.7113    1.2614 C   0  0  0  0  0  0  0  0  0  0  0  0
   17.6167   13.1662    1.8950 C   0  0  0  0  0  0  0  0  0  0  0  0
   17.0862    6.6543   -1.5718 C   0  0  0  0  0  0  0  0  0  0  0  0
   18.8322   13.6264    1.0580 C   0  0  0  0  0  0  0  0  0  0  0  0
   20.1346   13.1255    1.7188 C   0  0  0  0  0  0  0  0  0  0  0  0
   18.1620    6.8628   -2.6593 C   0  0  0  0  0  0  0  0  0  0  0  0
    1.6654   14.0778    2.8272 H   0  0  0  0  0  0  0  0  0  0  0  0
    1.4467   11.7417    3.6167 H   0  0  0  0  0  0  0  0  0  0  0  0
    3.4058   10.2298    3.5906 H   0  0  0  0  0  0  0  0  0  0  0  0
    3.8446   14.9170    2.0081 H   0  0  0  0  0  0  0  0  0  0  0  0
    7.7651    8.9130    1.9897 H   0  0  0  0  0  0  0  0  0  0  0  0
    8.6305   10.1224    2.9435 H   0  0  0  0  0  0  0  0  0  0  0  0
    9.5631   11.0980    0.8252 H   0  0  0  0  0  0  0  0  0  0  0  0
    8.7339    9.8417   -0.1016 H   0  0  0  0  0  0  0  0  0  0  0  0
   10.0375    8.0837    1.0725 H   0  0  0  0  0  0  0  0  0  0  0  0
   10.8306    9.3123    2.0656 H   0  0  0  0  0  0  0  0  0  0  0  0
   11.8553   10.3132    0.0359 H   0  0  0  0  0  0  0  0  0  0  0  0
   11.0331    9.1224   -0.9794 H   0  0  0  0  0  0  0  0  0  0  0  0
   12.2649    7.2860    0.1730 H   0  0  0  0  0  0  0  0  0  0  0  0
   13.0844    8.4746    1.1938 H   0  0  0  0  0  0  0  0  0  0  0  0
   14.1150    9.5059   -0.8192 H   0  0  0  0  0  0  0  0  0  0  0  0
   13.2786    8.3405   -1.8525 H   0  0  0  0  0  0  0  0  0  0  0  0
   14.4854    6.4756   -0.7268 H   0  0  0  0  0  0  0  0  0  0  0  0
   15.3217    7.6410    0.3073 H   0  0  0  0  0  0  0  0  0  0  0  0
   16.3574    8.6864   -1.6901 H   0  0  0  0  0  0  0  0  0  0  0  0
   15.5012    7.5490   -2.7388 H   0  0  0  0  0  0  0  0  0  0  0  0
    8.6665   14.7356    1.7367 H   0  0  0  0  0  0  0  0  0  0  0  0
    8.6754   13.3964    0.5845 H   0  0  0  0  0  0  0  0  0  0  0  0
   10.0072   12.0271    2.2195 H   0  0  0  0  0  0  0  0  0  0  0  0
   10.0388   13.4070    3.3241 H   0  0  0  0  0  0  0  0  0  0  0  0
   11.2518   14.7618    1.6302 H   0  0  0  0  0  0  0  0  0  0  0  0
   11.1745   13.4216    0.4806 H   0  0  0  0  0  0  0  0  0  0  0  0
   12.5478   12.0175    2.0063 H   0  0  0  0  0  0  0  0  0  0  0  0
```

```
   12.6025   13.3354    3.1834 H   0  0  0  0  0  0  0  0  0  0  0  0
   13.7742   14.7745    1.5084 H   0  0  0  0  0  0  0  0  0  0  0  0
   13.7323   13.4451    0.3436 H   0  0  0  0  0  0  0  0  0  0  0  0
   15.0883   12.0442    1.9033 H   0  0  0  0  0  0  0  0  0  0  0  0
   15.1418   13.3853    3.0540 H   0  0  0  0  0  0  0  0  0  0  0  0
   16.2992   14.7913    1.3405 H   0  0  0  0  0  0  0  0  0  0  0  0
   16.2739   13.4258    0.2174 H   0  0  0  0  0  0  0  0  0  0  0  0
   17.5829   12.0839    1.9158 H   0  0  0  0  0  0  0  0  0  0  0  0
   17.7119   13.5434    2.9057 H   0  0  0  0  0  0  0  0  0  0  0  0
   16.6819    5.6528   -1.6523 H   0  0  0  0  0  0  0  0  0  0  0  0
   17.5324    6.7857   -0.5939 H   0  0  0  0  0  0  0  0  0  0  0  0
   18.8471   14.7080    1.0072 H   0  0  0  0  0  0  0  0  0  0  0  0
   18.7563   13.2193    0.0573 H   0  0  0  0  0  0  0  0  0  0  0  0
   20.2625   13.6105    2.6787 H   0  0  0  0  0  0  0  0  0  0  0  0
   20.9777   13.3615    1.0802 H   0  0  0  0  0  0  0  0  0  0  0  0
   20.0787   12.0533    1.8608 H   0  0  0  0  0  0  0  0  0  0  0  0
   18.5788    7.8584   -2.5693 H   0  0  0  0  0  0  0  0  0  0  0  0
   17.7136    6.7464   -3.6383 H   0  0  0  0  0  0  0  0  0  0  0  0
   18.9496    6.1292   -2.5338 H   0  0  0  0  0  0  0  0  0  0  0  0
  1  2  4  0  0  0  0
  2  3  4  0  0  0  0
  3  4  4  0  0  0  0
  4  5  4  0  0  0  0
  5  6  4  0  0  0  0
  6  1  4  0  0  0  0
  7  8  2  0  0  0  0
  7  9  1  0  0  0  0
  7  4  1  0  0  0  0
 10 11  2  0  0  0  0
 10 12  1  0  0  0  0
 10  5  1  0  0  0  0
  9 13  1  0  0  0  0
 13 14  1  0  0  0  0
 14 15  1  0  0  0  0
 15 16  1  0  0  0  0
 16 17  1  0  0  0  0
 17 18  1  0  0  0  0
 18 19  1  0  0  0  0
 19 20  1  0  0  0  0
 21 22  1  0  0  0  0
 22 23  1  0  0  0  0
 23 24  1  0  0  0  0
 24 25  1  0  0  0  0
 25 26  1  0  0  0  0
 26 27  1  0  0  0  0
 27 28  1  0  0  0  0
 21 12  1  0  0  0  0
 20 29  1  0  0  0  0
 28 30  1  0  0  0  0
 30 31  1  0  0  0  0
 29 32  1  0  0  0  0
  1 33  1  0  0  0  0
  2 34  1  0  0  0  0
  3 35  1  0  0  0  0
  6 36  1  0  0  0  0
 13 37  1  0  0  0  0
 13 38  1  0  0  0  0
 14 39  1  0  0  0  0
 14 40  1  0  0  0  0
 15 41  1  0  0  0  0
 15 42  1  0  0  0  0
 16 43  1  0  0  0  0
 16 44  1  0  0  0  0
```

```
 17 45  1  0  0  0  0
 17 46  1  0  0  0  0
 18 47  1  0  0  0  0
 18 48  1  0  0  0  0
 19 49  1  0  0  0  0
 19 50  1  0  0  0  0
 20 51  1  0  0  0  0
 20 52  1  0  0  0  0
 21 53  1  0  0  0  0
 21 54  1  0  0  0  0
 22 55  1  0  0  0  0
 22 56  1  0  0  0  0
 23 57  1  0  0  0  0
 23 58  1  0  0  0  0
 24 59  1  0  0  0  0
 24 60  1  0  0  0  0
 25 61  1  0  0  0  0
 25 62  1  0  0  0  0
 26 63  1  0  0  0  0
 26 64  1  0  0  0  0
 27 65  1  0  0  0  0
 27 66  1  0  0  0  0
 28 67  1  0  0  0  0
 28 68  1  0  0  0  0
 29 69  1  0  0  0  0
 29 70  1  0  0  0  0
 30 71  1  0  0  0  0
 30 72  1  0  0  0  0
 31 73  1  0  0  0  0
 31 74  1  0  0  0  0
 31 75  1  0  0  0  0
 32 76  1  0  0  0  0
 32 77  1  0  0  0  0
 32 78  1  0  0  0  0
M  END
>  <Alias name>
Di-n-Decylphthalate

>  <deltaH°(c)>   kJ/mol
-16407.12

$$$$
9-Phenyleicosane
  ChemBrain IXL

 72 72  0  0  0  0  0  0  0  0  1 V2000
    9.0723    8.5912    1.7182 C   0  0  0  0  0  0  0  0  0  0  0  0  0
    8.0148    7.6058    1.1696 C   0  0  0  0  0  0  0  0  0  0  0  0  0
    7.1905    8.2949    0.0510 C   0  0  0  0  0  0  0  0  0  0  0  0  0
    8.6994    6.3981    0.6241 C   0  0  0  0  0  0  0  0  0  0  0  0  0
   10.0171    9.0288    0.5798 C   0  0  0  0  0  0  0  0  0  0  0  0  0
    6.5167    9.5662    0.6101 C   0  0  0  0  0  0  0  0  0  0  0  0  0
    8.3584    5.9013   -0.6390 C   0  0  0  0  0  0  0  0  0  0  0  0  0
    9.6873    5.7578    1.3764 C   0  0  0  0  0  0  0  0  0  0  0  0  0
   11.0807    9.9897    1.1411 C   0  0  0  0  0  0  0  0  0  0  0  0  0
    5.6938   10.2461   -0.5030 C   0  0  0  0  0  0  0  0  0  0  0  0  0
    9.0023    4.7712   -1.1443 C   0  0  0  0  0  0  0  0  0  0  0  0  0
   10.3307    4.6273    0.8694 C   0  0  0  0  0  0  0  0  0  0  0  0  0
   12.0254   10.4325    0.0079 C   0  0  0  0  0  0  0  0  0  0  0  0  0
    5.0321   11.5222    0.0554 C   0  0  0  0  0  0  0  0  0  0  0  0  0
    9.9883    4.1343   -0.3905 C   0  0  0  0  0  0  0  0  0  0  0  0  0
   13.0916   11.3875    0.5769 C   0  0  0  0  0  0  0  0  0  0  0  0  0
    4.2186   12.2117   -1.0580 C   0  0  0  0  0  0  0  0  0  0  0  0  0
```

14.0390	11.8353	-0.5519	C	0	0	0	0	0	0	0	0	0	0	0	0
3.5629	13.4899	-0.4978	C	0	0	0	0	0	0	0	0	0	0	0	0
15.1063	12.7855	0.0242	C	0	0	0	0	0	0	0	0	0	0	0	0
2.7530	14.1838	-1.6107	C	0	0	0	0	0	0	0	0	0	0	0	0
16.0562	13.2371	-1.1013	C	0	0	0	0	0	0	0	0	0	0	0	0
2.0993	15.4620	-1.0497	C	0	0	0	0	0	0	0	0	0	0	0	0
17.1241	14.1842	-0.5205	C	0	0	0	0	0	0	0	0	0	0	0	0
18.0753	14.6382	-1.6443	C	0	0	0	0	0	0	0	0	0	0	0	0
19.1435	15.5839	-1.0619	C	0	0	0	0	0	0	0	0	0	0	0	0
9.6475	8.1053	2.4957	H	0	0	0	0	0	0	0	0	0	0	0	0
8.5768	9.4607	2.1293	H	0	0	0	0	0	0	0	0	0	0	0	0
7.3514	7.3095	1.9724	H	0	0	0	0	0	0	0	0	0	0	0	0
7.8460	8.5649	-0.7671	H	0	0	0	0	0	0	0	0	0	0	0	0
6.4291	7.6135	-0.3074	H	0	0	0	0	0	0	0	0	0	0	0	0
9.4470	9.5321	-0.1905	H	0	0	0	0	0	0	0	0	0	0	0	0
10.5037	8.1578	0.1586	H	0	0	0	0	0	0	0	0	0	0	0	0
5.8622	9.2973	1.4298	H	0	0	0	0	0	0	0	0	0	0	0	0
7.2757	10.2511	0.9643	H	0	0	0	0	0	0	0	0	0	0	0	0
7.5946	6.3917	-1.2272	H	0	0	0	0	0	0	0	0	0	0	0	0
9.9547	6.1378	2.3537	H	0	0	0	0	0	0	0	0	0	0	0	0
11.6520	9.4853	1.9104	H	0	0	0	0	0	0	0	0	0	0	0	0
10.5936	10.8591	1.5650	H	0	0	0	0	0	0	0	0	0	0	0	0
6.3475	10.5091	-1.3256	H	0	0	0	0	0	0	0	0	0	0	0	0
4.9279	9.5654	-0.8537	H	0	0	0	0	0	0	0	0	0	0	0	0
8.7365	4.3886	-2.1221	H	0	0	0	0	0	0	0	0	0	0	0	0
11.0960	4.1325	1.4539	H	0	0	0	0	0	0	0	0	0	0	0	0
11.4557	10.9423	-0.7593	H	0	0	0	0	0	0	0	0	0	0	0	0
12.5099	9.5632	-0.4192	H	0	0	0	0	0	0	0	0	0	0	0	0
4.3731	11.2585	0.8734	H	0	0	0	0	0	0	0	0	0	0	0	0
5.7984	12.1985	0.4133	H	0	0	0	0	0	0	0	0	0	0	0	0
10.4878	3.2574	-0.7832	H	0	0	0	0	0	0	0	0	0	0	0	0
13.6606	10.8766	1.3438	H	0	0	0	0	0	0	0	0	0	0	0	0
12.6062	12.2554	1.0059	H	0	0	0	0	0	0	0	0	0	0	0	0
4.8771	12.4731	-1.8771	H	0	0	0	0	0	0	0	0	0	0	0	0
3.4495	11.5374	-1.4141	H	0	0	0	0	0	0	0	0	0	0	0	0
13.4715	12.3505	-1.3171	H	0	0	0	0	0	0	0	0	0	0	0	0
14.5227	10.9676	-0.9833	H	0	0	0	0	0	0	0	0	0	0	0	0
2.9024	13.2282	0.3196	H	0	0	0	0	0	0	0	0	0	0	0	0
4.3323	14.1624	-0.1388	H	0	0	0	0	0	0	0	0	0	0	0	0
15.6732	12.2695	0.7892	H	0	0	0	0	0	0	0	0	0	0	0	0
14.6220	13.6521	0.4569	H	0	0	0	0	0	0	0	0	0	0	0	0
3.4134	14.4453	-2.4282	H	0	0	0	0	0	0	0	0	0	0	0	0
1.9830	13.5121	-1.9693	H	0	0	0	0	0	0	0	0	0	0	0	0
15.4902	13.7557	-1.8652	H	0	0	0	0	0	0	0	0	0	0	0	0
16.5395	12.3706	-1.5354	H	0	0	0	0	0	0	0	0	0	0	0	0
1.4386	15.2006	-0.2322	H	0	0	0	0	0	0	0	0	0	0	0	0
2.8693	16.1337	-0.6909	H	0	0	0	0	0	0	0	0	0	0	0	0
1.5306	15.9496	-1.8316	H	0	0	0	0	0	0	0	0	0	0	0	0
17.6897	13.6650	0.2433	H	0	0	0	0	0	0	0	0	0	0	0	0
16.6403	15.0500	-0.0855	H	0	0	0	0	0	0	0	0	0	0	0	0
17.5100	15.1585	-2.4076	H	0	0	0	0	0	0	0	0	0	0	0	0
18.5585	13.7725	-2.0800	H	0	0	0	0	0	0	0	0	0	0	0	0
18.6602	16.4495	-0.6258	H	0	0	0	0	0	0	0	0	0	0	0	0
19.8120	15.9033	-1.8516	H	0	0	0	0	0	0	0	0	0	0	0	0
19.7089	15.0634	-0.2987	H	0	0	0	0	0	0	0	0	0	0	0	0

1	2	1	0	0	0	0
1	5	1	0	0	0	0
2	3	1	0	0	0	0
2	4	1	0	0	0	0
3	6	1	0	0	0	0
7	4	4	0	0	0	0
4	8	4	0	0	0	0
5	9	1	0	0	0	0

6	10	1	0	0	0	0
11	7	4	0	0	0	0
8	12	4	0	0	0	0
9	13	1	0	0	0	0
10	14	1	0	0	0	0
15	11	4	0	0	0	0
12	15	4	0	0	0	0
13	16	1	0	0	0	0
14	17	1	0	0	0	0
16	18	1	0	0	0	0
17	19	1	0	0	0	0
18	20	1	0	0	0	0
19	21	1	0	0	0	0
20	22	1	0	0	0	0
21	23	1	0	0	0	0
22	24	1	0	0	0	0
24	25	1	0	0	0	0
25	26	1	0	0	0	0
1	27	1	0	0	0	0
1	28	1	0	0	0	0
2	29	1	0	0	0	0
3	30	1	0	0	0	0
3	31	1	0	0	0	0
5	32	1	0	0	0	0
5	33	1	0	0	0	0
6	34	1	0	0	0	0
6	35	1	0	0	0	0
7	36	1	0	0	0	0
8	37	1	0	0	0	0
9	38	1	0	0	0	0
9	39	1	0	0	0	0
10	40	1	0	0	0	0
10	41	1	0	0	0	0
11	42	1	0	0	0	0
12	43	1	0	0	0	0
13	44	1	0	0	0	0
13	45	1	0	0	0	0
14	46	1	0	0	0	0
14	47	1	0	0	0	0
15	48	1	0	0	0	0
16	49	1	0	0	0	0
16	50	1	0	0	0	0
17	51	1	0	0	0	0
17	52	1	0	0	0	0
18	53	1	0	0	0	0
18	54	1	0	0	0	0
19	55	1	0	0	0	0
19	56	1	0	0	0	0
20	57	1	0	0	0	0
20	58	1	0	0	0	0
21	59	1	0	0	0	0
21	60	1	0	0	0	0
22	61	1	0	0	0	0
22	62	1	0	0	0	0
23	63	1	0	0	0	0
23	64	1	0	0	0	0
23	65	1	0	0	0	0
24	66	1	0	0	0	0
24	67	1	0	0	0	0
25	68	1	0	0	0	0
25	69	1	0	0	0	0
26	70	1	0	0	0	0
26	71	1	0	0	0	0

 26 72 1 0 0 0 0
M END
> <Alias name>
9-Phenyleicosane

> <deltaH°(c)>   kJ/mol
-16329

$$$$
4-Methyl-4-t-butylperoxy-1-triphenylsiloxy-2-butyne
  ChemBrain IXL

```
 64 66  0  0  0  0  0  0  0  0  1 V2000
   10.5684   10.8537    3.3401 C   0  0  0  0  0  0  0  0  0  0  0  0  0
    9.4844   10.0418    3.6789 C   0  0  0  0  0  0  0  0  0  0  0  0  0
    9.1758    8.9273    2.8969 C   0  0  0  0  0  0  0  0  0  0  0  0  0
    9.9513    8.6249    1.7763 C   0  0  0  0  0  0  0  0  0  0  0  0  0
   11.0352    9.4366    1.4373 C   0  0  0  0  0  0  0  0  0  0  0  0  0
   11.3438   10.5511    2.2192 C   0  0  0  0  0  0  0  0  0  0  0  0  0
    9.6553    8.7825   -1.5682 C   0  0  0  0  0  0  0  0  0  0  0  0  0
    9.9152    7.5120   -1.0516 C   0  0  0  0  0  0  0  0  0  0  0  0  0
   10.4565    6.5214   -1.8729 C   0  0  0  0  0  0  0  0  0  0  0  0  0
   10.7377    6.8014   -3.2112 C   0  0  0  0  0  0  0  0  0  0  0  0  0
   10.4777    8.0720   -3.7281 C   0  0  0  0  0  0  0  0  0  0  0  0  0
    9.9365    9.0626   -2.9065 C   0  0  0  0  0  0  0  0  0  0  0  0  0
    9.5402    7.1389    0.7333 Si  0  0  0  0  0  0  0  0  0  0  0  0  0
   12.0958    3.5095    2.1275 C   0  0  0  0  0  0  0  0  0  0  0  0  0
   12.7227    4.6788    1.6929 C   0  0  0  0  0  0  0  0  0  0  0  0  0
   11.9560    5.7678    1.2746 C   0  0  0  0  0  0  0  0  0  0  0  0  0
   10.5625    5.6872    1.2910 C   0  0  0  0  0  0  0  0  0  0  0  0  0
    9.9354    4.5181    1.7255 C   0  0  0  0  0  0  0  0  0  0  0  0  0
   10.7021    3.4292    2.1438 C   0  0  0  0  0  0  0  0  0  0  0  0  0
    5.6187    7.3279    0.6717 C   0  0  0  0  0  0  0  0  0  0  0  0  0
    4.5053    6.9647    0.8476 C   0  0  0  0  0  0  0  0  0  0  0  0  0
    3.0682    6.4949    1.0753 C   0  0  0  0  0  0  0  0  0  0  0  0  0
    2.4028    6.2170   -0.2747 C   0  0  0  0  0  0  0  0  0  0  0  0  0
    4.3426    3.1814    3.4091 C   0  0  0  0  0  0  0  0  0  0  0  0  0
    4.8741    3.6428    2.0498 C   0  0  0  0  0  0  0  0  0  0  0  0  0
    3.0732    5.2968    1.8606 O   0  0  0  0  0  0  0  0  0  0  0  0  0
    3.7919    4.1605    1.2651 O   0  0  0  0  0  0  0  0  0  0  0  0  0
    2.2874    7.5842    1.8139 C   0  0  0  0  0  0  0  0  0  0  0  0  0
    5.9275    4.7335    2.2577 C   0  0  0  0  0  0  0  0  0  0  0  0  0
    5.5081    2.4557    1.3212 C   0  0  0  0  0  0  0  0  0  0  0  0  0
    7.9592    6.7806    0.8962 O   0  0  0  0  0  0  0  0  0  0  0  0  0
    7.0563    7.7944    0.4459 C   0  0  0  0  0  0  0  0  0  0  0  0  0
   10.8078   11.7182    3.9467 H   0  0  0  0  0  0  0  0  0  0  0  0  0
    8.8830   10.2765    4.5483 H   0  0  0  0  0  0  0  0  0  0  0  0  0
    8.3349    8.2975    3.1596 H   0  0  0  0  0  0  0  0  0  0  0  0  0
   11.6367    9.2018    0.5678 H   0  0  0  0  0  0  0  0  0  0  0  0  0
   12.1846   11.1809    1.9564 H   0  0  0  0  0  0  0  0  0  0  0  0  0
    9.2355    9.5508   -0.9307 H   0  0  0  0  0  0  0  0  0  0  0  0  0
   10.6582    5.5358   -1.4719 H   0  0  0  0  0  0  0  0  0  0  0  0  0
   11.1575    6.0331   -3.8485 H   0  0  0  0  0  0  0  0  0  0  0  0  0
   10.6959    8.2892   -4.7663 H   0  0  0  0  0  0  0  0  0  0  0  0  0
    9.7349   10.0481   -3.3074 H   0  0  0  0  0  0  0  0  0  0  0  0  0
   12.6906    2.6648    2.4521 H   0  0  0  0  0  0  0  0  0  0  0  0  0
   13.8037    4.7411    1.6803 H   0  0  0  0  0  0  0  0  0  0  0  0  0
   12.4421    6.6749    0.9374 H   0  0  0  0  0  0  0  0  0  0  0  0  0
    8.8542    4.4559    1.7380 H   0  0  0  0  0  0  0  0  0  0  0  0  0
   10.2159    2.5222    2.4809 H   0  0  0  0  0  0  0  0  0  0  0  0  0
    2.9552    5.4460   -0.7975 H   0  0  0  0  0  0  0  0  0  0  0  0  0
    1.3845    5.8852   -0.1138 H   0  0  0  0  0  0  0  0  0  0  0  0  0
    2.3978    7.1229   -0.8682 H   0  0  0  0  0  0  0  0  0  0  0  0  0
```

```
    5.1595    2.7885    4.0018 H   0  0  0  0  0  0  0  0  0  0  0  0
    3.8936    4.0212    3.9250 H   0  0  0  0  0  0  0  0  0  0  0  0
    3.5986    2.4083    3.2623 H   0  0  0  0  0  0  0  0  0  0  0  0
    1.2691    7.2523    1.9748 H   0  0  0  0  0  0  0  0  0  0  0  0
    2.7581    7.7809    2.7692 H   0  0  0  0  0  0  0  0  0  0  0  0
    2.2823    8.4903    1.2205 H   0  0  0  0  0  0  0  0  0  0  0  0
    5.4788    5.5764    2.7684 H   0  0  0  0  0  0  0  0  0  0  0  0
    6.3090    5.0544    1.2968 H   0  0  0  0  0  0  0  0  0  0  0  0
    6.7407    4.3406    2.8555 H   0  0  0  0  0  0  0  0  0  0  0  0
    6.3252    2.0628    1.9138 H   0  0  0  0  0  0  0  0  0  0  0  0
    5.8840    2.7816    0.3592 H   0  0  0  0  0  0  0  0  0  0  0  0
    4.7640    1.6826    1.1749 H   0  0  0  0  0  0  0  0  0  0  0  0
    7.2312    8.7074    1.0017 H   0  0  0  0  0  0  0  0  0  0  0  0
    7.2152    7.9777   -0.6096 H   0  0  0  0  0  0  0  0  0  0  0  0
  1  2  4  0  0  0  0
  2  3  4  0  0  0  0
  3  4  4  0  0  0  0
  4  5  4  0  0  0  0
  5  6  4  0  0  0  0
  6  1  4  0  0  0  0
  7  8  4  0  0  0  0
  8  9  4  0  0  0  0
  9 10  4  0  0  0  0
 10 11  4  0  0  0  0
 11 12  4  0  0  0  0
 12  7  4  0  0  0  0
  4 13  1  0  0  0  0
 13  8  1  0  0  0  0
 14 15  4  0  0  0  0
 15 16  4  0  0  0  0
 16 17  4  0  0  0  0
 17 18  4  0  0  0  0
 18 19  4  0  0  0  0
 19 14  4  0  0  0  0
 17 13  1  0  0  0  0
 20 21  3  0  0  0  0
 21 22  1  0  0  0  0
 24 25  1  0  0  0  0
 25 27  1  0  0  0  0
 27 26  1  0  0  0  0
 23 22  1  0  0  0  0
 22 28  1  0  0  0  0
 26 22  1  0  0  0  0
 25 29  1  0  0  0  0
 25 30  1  0  0  0  0
 13 31  1  0  0  0  0
 31 32  1  0  0  0  0
 32 20  1  0  0  0  0
  1 33  1  0  0  0  0
  2 34  1  0  0  0  0
  3 35  1  0  0  0  0
  5 36  1  0  0  0  0
  6 37  1  0  0  0  0
  7 38  1  0  0  0  0
  9 39  1  0  0  0  0
 10 40  1  0  0  0  0
 11 41  1  0  0  0  0
 12 42  1  0  0  0  0
 14 43  1  0  0  0  0
 15 44  1  0  0  0  0
 16 45  1  0  0  0  0
 18 46  1  0  0  0  0
 19 47  1  0  0  0  0
```

23 48 1 0 0 0 0
23 49 1 0 0 0 0
23 50 1 0 0 0 0
24 51 1 0 0 0 0
24 52 1 0 0 0 0
24 53 1 0 0 0 0
28 54 1 0 0 0 0
28 55 1 0 0 0 0
28 56 1 0 0 0 0
29 57 1 0 0 0 0
29 58 1 0 0 0 0
29 59 1 0 0 0 0
30 60 1 0 0 0 0
30 61 1 0 0 0 0
30 62 1 0 0 0 0
32 63 1 0 0 0 0
32 64 1 0 0 0 0
M END
> <Alias name>
4-Methyl-4-t-butylperoxy-1-triphenylsiloxy-2-butyne

> <deltaH°(c)>   kJ/mol
-16305

$$$$
Bis(monothiodibenzoylmethanate)copper(II)
  ChemBrain IXL

 57 62 0 0 0 0 0 0 0 0 1 V2000
    8.9779   13.6117   2.3169 C   0 0 0 0 0 0 0 0 0 0 0 0 0
   10.0506   12.9706   1.6971 C   0 0 0 0 0 0 0 0 0 0 0 0 0
    9.8150   12.0050   0.7164 C   0 0 0 0 0 0 0 0 0 0 0 0 0
    8.5054   11.6822   0.3504 C   0 0 0 0 0 0 0 0 0 0 0 0 0
    7.4331   12.3208   0.9765 C   0 0 0 0 0 0 0 0 0 0 0 0 0
    7.6696   13.2865   1.9573 C   0 0 0 0 0 0 0 0 0 0 0 0 0
    8.2610   10.6599   -0.7045 C   0 0 0 0 0 0 0 0 0 0 0 0 0
    9.2319   10.1286   -1.2438 O   0 0 0 0 0 0 0 0 0 0 0 0 0
    6.8310   10.2885   -1.1063 C   0 0 0 0 0 0 0 0 0 0 0 0 0
    6.4303   8.9841   -1.2696 C   0 0 0 0 0 0 0 0 0 0 0 0 0
    7.5791   7.5589   -0.9401 S   0 5 0 0 0 0 0 0 0 0 0 0 0
    9.5833   8.2971   -0.9290 Cu 0 0 0 0 0 0 0 0 0 0 0 0 0
    4.1744   9.7036   -2.0329 C   0 0 0 0 0 0 0 0 0 0 0 0 0
    5.0606   8.6757   -1.7167 C   0 0 0 0 0 0 0 0 0 0 0 0 0
    4.6569   7.3459   -1.8224 C   0 0 0 0 0 0 0 0 0 0 0 0 0
    3.3654   7.0421   -2.2486 C   0 0 0 0 0 0 0 0 0 0 0 0 0
    2.4776   8.0693   -2.5677 C   0 0 0 0 0 0 0 0 0 0 0 0 0
    2.8818   9.4002   -2.4591 C   0 0 0 0 0 0 0 0 0 0 0 0 0
   15.2893   9.0944   -3.5660 C   0 0 0 0 0 0 0 0 0 0 0 0 0
   13.9966   9.4324   -3.9669 C   0 0 0 0 0 0 0 0 0 0 0 0 0
   12.8987   8.8079   -3.3777 C   0 0 0 0 0 0 0 0 0 0 0 0 0
   13.0940   7.8434   -2.3917 C   0 0 0 0 0 0 0 0 0 0 0 0 0
   14.3850   7.5081   -1.9857 C   0 0 0 0 0 0 0 0 0 0 0 0 0
   15.4835   8.1324   -2.5747 C   0 0 0 0 0 0 0 0 0 0 0 0 0
   11.9331   7.1763   -1.7766 C   0 0 0 0 0 0 0 0 0 0 0 0 0
   10.7911   7.4727   -2.1513 O   0 0 0 0 0 0 0 0 0 0 0 0 0
   12.1613   6.1459   -0.7053 C   0 0 0 0 0 0 0 0 0 0 0 0 0
   11.5931   6.2753   0.5111 C   0 0 0 0 0 0 0 0 0 0 0 0 0
   10.5828   7.7672   0.8899 S   0 5 0 0 0 0 0 0 0 0 0 0 0
   11.1674   5.3697   2.8043 C   0 0 0 0 0 0 0 0 0 0 0 0 0
   11.7682   5.2215   1.5510 C   0 0 0 0 0 0 0 0 0 0 0 0 0
   12.5315   4.0837   1.2788 C   0 0 0 0 0 0 0 0 0 0 0 0 0
   12.6976   3.1010   2.2574 C   0 0 0 0 0 0 0 0 0 0 0 0 0
   12.0990   3.2525   3.5086 C   0 0 0 0 0 0 0 0 0 0 0 0 0

```
11.3339 4.3865 3.7821 C 0 0 0 0 0 0 0 0 0 0 0 0
9.1609 14.3621 3.0774 H 0 0 0 0 0 0 0 0 0 0 0 0
11.0669 13.2224 1.9773 H 0 0 0 0 0 0 0 0 0 0 0 0
10.6490 11.5059 0.2391 H 0 0 0 0 0 0 0 0 0 0 0 0
6.4175 12.0674 0.7016 H 0 0 0 0 0 0 0 0 0 0 0 0
6.8370 13.7839 2.4397 H 0 0 0 0 0 0 0 0 0 0 0 0
6.1107 11.0809 -1.2663 H 0 0 0 0 0 0 0 0 0 0 0 0
4.4894 10.7365 -1.9476 H 0 0 0 0 0 0 0 0 0 0 0 0
5.3473 6.5490 -1.5732 H 0 0 0 0 0 0 0 0 0 0 0 0
3.0519 6.0090 -2.3317 H 0 0 0 0 0 0 0 0 0 0 0 0
1.4748 7.8336 -2.8997 H 0 0 0 0 0 0 0 0 0 0 0 0
2.1922 10.1974 -2.7057 H 0 0 0 0 0 0 0 0 0 0 0 0
16.1419 9.5786 -4.0243 H 0 0 0 0 0 0 0 0 0 0 0 0
13.8458 10.1795 -4.7355 H 0 0 0 0 0 0 0 0 0 0 0 0
11.8943 9.0716 -3.6856 H 0 0 0 0 0 0 0 0 0 0 0 0
14.5342 6.7630 -1.2132 H 0 0 0 0 0 0 0 0 0 0 0 0
16.4868 7.8705 -2.2626 H 0 0 0 0 0 0 0 0 0 0 0 0
12.7936 5.2908 -0.9113 H 0 0 0 0 0 0 0 0 0 0 0 0
10.5717 6.2480 3.0183 H 0 0 0 0 0 0 0 0 0 0 0 0
12.9951 3.9630 0.3084 H 0 0 0 0 0 0 0 0 0 0 0 0
13.2915 2.2201 2.0454 H 0 0 0 0 0 0 0 0 0 0 0 0
12.2284 2.4895 4.2678 H 0 0 0 0 0 0 0 0 0 0 0 0
10.8689 4.5039 4.7540 H 0 0 0 0 0 0 0 0 0 0 0 0
1 2 4 0 0 0 0
2 3 4 0 0 0 0
3 4 4 0 0 0 0
4 5 4 0 0 0 0
5 6 4 0 0 0 0
6 1 4 0 0 0 0
7 8 2 0 0 0 0
7 9 1 0 0 0 0
7 4 1 0 0 0 0
9 10 2 0 0 0 0
10 11 1 0 0 0 0
11 12 1 0 0 0 0
8 12 1 0 0 0 0
13 14 4 0 0 0 0
14 15 4 0 0 0 0
15 16 4 0 0 0 0
16 17 4 0 0 0 0
17 18 4 0 0 0 0
18 13 4 0 0 0 0
14 10 1 0 0 0 0
19 20 4 0 0 0 0
20 21 4 0 0 0 0
21 22 4 0 0 0 0
22 23 4 0 0 0 0
23 24 4 0 0 0 0
24 19 4 0 0 0 0
25 26 2 0 0 0 0
25 27 1 0 0 0 0
25 22 1 0 0 0 0
27 28 2 0 0 0 0
28 29 1 0 0 0 0
30 31 4 0 0 0 0
31 32 4 0 0 0 0
32 33 4 0 0 0 0
33 34 4 0 0 0 0
34 35 4 0 0 0 0
35 30 4 0 0 0 0
31 28 1 0 0 0 0
29 12 1 0 0 0 0
26 12 1 0 0 0 0
```

```
  1 36  1  0  0  0  0
  2 37  1  0  0  0  0
  3 38  1  0  0  0  0
  5 39  1  0  0  0  0
  6 40  1  0  0  0  0
  9 41  1  0  0  0  0
 13 42  1  0  0  0  0
 15 43  1  0  0  0  0
 16 44  1  0  0  0  0
 17 45  1  0  0  0  0
 18 46  1  0  0  0  0
 19 47  1  0  0  0  0
 20 48  1  0  0  0  0
 21 49  1  0  0  0  0
 23 50  1  0  0  0  0
 24 51  1  0  0  0  0
 27 52  1  0  0  0  0
 30 53  1  0  0  0  0
 32 54  1  0  0  0  0
 33 55  1  0  0  0  0
 34 56  1  0  0  0  0
 35 57  1  0  0  0  0
M  END
>  <Alias name>
Bis(monothiodibenzoylmethanate)copper(II)

>  <deltaH°(c)>   kJ/mol
-16199.8

$$$$
1,1,2,2-Tetra-p-tolylethane
  ChemBrain IXL

 60 63  0  0  0  0  0  0  0  0  1 V2000
    7.9389    6.2703   -0.0770 C   0  0  0  0  0  0  0  0  0  0  0  0
    6.7818    5.7289   -0.6382 C   0  0  0  0  0  0  0  0  0  0  0  0
    8.5939    5.5971    0.9544 C   0  0  0  0  0  0  0  0  0  0  0  0
    8.4752    7.5552   -0.5818 C   0  0  0  0  0  0  0  0  0  0  0  0
    6.3007    4.4974   -0.1929 C   0  0  0  0  0  0  0  0  0  0  0  0
    6.9675    3.8147    0.8246 C   0  0  0  0  0  0  0  0  0  0  0  0
    8.1128    4.3656    1.3998 C   0  0  0  0  0  0  0  0  0  0  0  0
    6.4599    2.5059    1.2940 C   0  0  0  0  0  0  0  0  0  0  0  0
    8.9301    8.4488    0.6142 C   0  0  0  0  0  0  0  0  0  0  0  0
    9.6303    7.2776   -1.4648 C   0  0  0  0  0  0  0  0  0  0  0  0
    7.7633    8.8011    1.4558 C   0  0  0  0  0  0  0  0  0  0  0  0
    9.5403    9.6961    0.1008 C   0  0  0  0  0  0  0  0  0  0  0  0
    6.8386    7.8221    1.8205 C   0  0  0  0  0  0  0  0  0  0  0  0
    5.7375    8.1613    2.6080 C   0  0  0  0  0  0  0  0  0  0  0  0
    5.5630    9.4787    3.0336 C   0  0  0  0  0  0  0  0  0  0  0  0
    6.4903   10.4564    2.6727 C   0  0  0  0  0  0  0  0  0  0  0  0
    4.3940    9.8394    3.8680 C   0  0  0  0  0  0  0  0  0  0  0  0
    7.5909   10.1173    1.8854 C   0  0  0  0  0  0  0  0  0  0  0  0
   10.8428   10.0390    0.4650 C   0  0  0  0  0  0  0  0  0  0  0  0
   11.4276   11.1981   -0.0458 C   0  0  0  0  0  0  0  0  0  0  0  0
   10.7038   12.0231   -0.9070 C   0  0  0  0  0  0  0  0  0  0  0  0
    9.3939   11.6915   -1.2534 C   0  0  0  0  0  0  0  0  0  0  0  0
   11.3254   13.2503   -1.4533 C   0  0  0  0  0  0  0  0  0  0  0  0
    8.8094   10.5325   -0.7428 C   0  0  0  0  0  0  0  0  0  0  0  0
   10.8016    6.7394   -0.9318 C   0  0  0  0  0  0  0  0  0  0  0  0
   11.9037    6.5138   -1.7564 C   0  0  0  0  0  0  0  0  0  0  0  0
   11.8280    6.8081   -3.1178 C   0  0  0  0  0  0  0  0  0  0  0  0
   10.6482    7.3229   -3.6556 C   0  0  0  0  0  0  0  0  0  0  0  0
   12.9992    6.5742   -3.9916 C   0  0  0  0  0  0  0  0  0  0  0  0
```

```
    9.5460    7.5483   -2.8307 C   0  0  0  0  0  0  0  0  0  0  0  0
    6.2571    6.2646   -1.4197 H   0  0  0  0  0  0  0  0  0  0  0  0
    9.4758    6.0305    1.4095 H   0  0  0  0  0  0  0  0  0  0  0  0
    7.7067    8.0703   -1.1448 H   0  0  0  0  0  0  0  0  0  0  0  0
    5.4094    4.0713   -0.6371 H   0  0  0  0  0  0  0  0  0  0  0  0
    8.6285    3.8373    2.1920 H   0  0  0  0  0  0  0  0  0  0  0  0
    7.0076    1.7098    0.8049 H   0  0  0  0  0  0  0  0  0  0  0  0
    6.5935    2.4303    2.3660 H   0  0  0  0  0  0  0  0  0  0  0  0
    5.4072    2.4200    1.0544 H   0  0  0  0  0  0  0  0  0  0  0  0
    9.6562    7.9107    1.2110 H   0  0  0  0  0  0  0  0  0  0  0  0
    6.9753    6.7990    1.4927 H   0  0  0  0  0  0  0  0  0  0  0  0
    5.0182    7.4019    2.8889 H   0  0  0  0  0  0  0  0  0  0  0  0
    6.3559   11.4787    3.0038 H   0  0  0  0  0  0  0  0  0  0  0  0
    3.8728    8.9387    4.1680 H   0  0  0  0  0  0  0  0  0  0  0  0
    4.7298   10.3725    4.7489 H   0  0  0  0  0  0  0  0  0  0  0  0
    3.7241   10.4709    3.2974 H   0  0  0  0  0  0  0  0  0  0  0  0
    8.3119   10.8761    1.6075 H   0  0  0  0  0  0  0  0  0  0  0  0
   11.4000    9.4054    1.1443 H   0  0  0  0  0  0  0  0  0  0  0  0
   12.4435   11.4574    0.2261 H   0  0  0  0  0  0  0  0  0  0  0  0
    8.8307   12.3339   -1.9188 H   0  0  0  0  0  0  0  0  0  0  0  0
   10.8515   14.1204   -1.0160 H   0  0  0  0  0  0  0  0  0  0  0  0
   11.1976   13.2718   -2.5285 H   0  0  0  0  0  0  0  0  0  0  0  0
   12.3819   13.2567   -1.2151 H   0  0  0  0  0  0  0  0  0  0  0  0
    7.7881   10.2820   -1.0012 H   0  0  0  0  0  0  0  0  0  0  0  0
   10.8553    6.4969    0.1223 H   0  0  0  0  0  0  0  0  0  0  0  0
   12.8182    6.1101   -1.3398 H   0  0  0  0  0  0  0  0  0  0  0  0
   10.5880    7.5475   -4.7134 H   0  0  0  0  0  0  0  0  0  0  0  0
   13.4605    7.5225   -4.2384 H   0  0  0  0  0  0  0  0  0  0  0  0
   12.6785    6.0812   -4.9009 H   0  0  0  0  0  0  0  0  0  0  0  0
   13.7158    5.9479   -3.4747 H   0  0  0  0  0  0  0  0  0  0  0  0
    8.6251    7.9335   -3.2508 H   0  0  0  0  0  0  0  0  0  0  0  0
  1  2  4  0  0  0  0
  3  1  4  0  0  0  0
  1  4  1  0  0  0  0
  2  5  4  0  0  0  0
  7  3  4  0  0  0  0
  4  9  1  0  0  0  0
  4 10  1  0  0  0  0
  5  6  4  0  0  0  0
  6  7  4  0  0  0  0
  6  8  1  0  0  0  0
  9 11  1  0  0  0  0
  9 12  1  0  0  0  0
 10 25  4  0  0  0  0
 30 10  4  0  0  0  0
 11 13  4  0  0  0  0
 18 11  4  0  0  0  0
 12 19  4  0  0  0  0
 24 12  4  0  0  0  0
 13 14  4  0  0  0  0
 14 15  4  0  0  0  0
 15 16  4  0  0  0  0
 15 17  1  0  0  0  0
 16 18  4  0  0  0  0
 19 20  4  0  0  0  0
 20 21  4  0  0  0  0
 21 22  4  0  0  0  0
 21 23  1  0  0  0  0
 22 24  4  0  0  0  0
 25 26  4  0  0  0  0
 26 27  4  0  0  0  0
 27 28  4  0  0  0  0
 27 29  1  0  0  0  0
```

```
 28 30  4  0  0  0  0
  2 31  1  0  0  0  0
  3 32  1  0  0  0  0
  4 33  1  0  0  0  0
  5 34  1  0  0  0  0
  7 35  1  0  0  0  0
  8 36  1  0  0  0  0
  8 37  1  0  0  0  0
  8 38  1  0  0  0  0
  9 39  1  0  0  0  0
 13 40  1  0  0  0  0
 14 41  1  0  0  0  0
 16 42  1  0  0  0  0
 17 43  1  0  0  0  0
 17 44  1  0  0  0  0
 17 45  1  0  0  0  0
 18 46  1  0  0  0  0
 19 47  1  0  0  0  0
 20 48  1  0  0  0  0
 22 49  1  0  0  0  0
 23 50  1  0  0  0  0
 23 51  1  0  0  0  0
 23 52  1  0  0  0  0
 24 53  1  0  0  0  0
 25 54  1  0  0  0  0
 26 55  1  0  0  0  0
 28 56  1  0  0  0  0
 29 57  1  0  0  0  0
 29 58  1  0  0  0  0
 29 59  1  0  0  0  0
 30 60  1  0  0  0  0
M  END
>  <Alias name>
1,1,2,2-Tetra-p-tolylethane

>  <deltaH°(c)>   kJ/mol
-16166

$$$$
Tri-n-octylamine
  ChemBrain IXL

 76 75  0  0  0  0  0  0  0  0  1 V2000
    1.9657    2.7379    0.3510 C   0  0  0  0  0  0  0  0  0  0  0  0
    3.4496    2.7115    0.7656 C   0  0  0  0  0  0  0  0  0  0  0  0
    4.1571    3.9690    0.2238 C   0  0  0  0  0  0  0  0  0  0  0  0
    5.6411    3.9427    0.6389 C   0  0  0  0  0  0  0  0  0  0  0  0
    6.3485    5.2003    0.0976 C   0  0  0  0  0  0  0  0  0  0  0  0
    7.8322    5.1743    0.5134 C   0  0  0  0  0  0  0  0  0  0  0  0
    8.5394    6.4324   -0.0269 C   0  0  0  0  0  0  0  0  0  0  0  0
   10.0254    6.4055    0.3911 C   0  0  0  0  0  0  0  0  0  0  0  0
   10.7227    7.5880   -0.1584 N   0  0  0  0  0  0  0  0  0  0  0  0
   10.8946   17.6074   -0.3818 C   0  0  0  0  0  0  0  0  0  0  0  0
   10.1772   16.3693    0.1903 C   0  0  0  0  0  0  0  0  0  0  0  0
   10.8666   15.0914   -0.3263 C   0  0  0  0  0  0  0  0  0  0  0  0
   10.1493   13.8533    0.2461 C   0  0  0  0  0  0  0  0  0  0  0  0
   10.8389   12.5754   -0.2700 C   0  0  0  0  0  0  0  0  0  0  0  0
   10.1222   11.3374    0.3033 C   0  0  0  0  0  0  0  0  0  0  0  0
   10.8124   10.0596   -0.2119 C   0  0  0  0  0  0  0  0  0  0  0  0
   10.0938    8.8202    0.3637 C   0  0  0  0  0  0  0  0  0  0  0  0
   19.2850    2.4160   -0.7952 C   0  0  0  0  0  0  0  0  0  0  0  0
   18.6199    3.6948   -0.2503 C   0  0  0  0  0  0  0  0  0  0  0  0
   17.1272    3.7016   -0.6342 C   0  0  0  0  0  0  0  0  0  0  0  0
```

16.4622	4.9804	-0.0889 C	0	0	0	0	0	0	0	0	0	0	0	0
14.9695	4.9869	-0.4724 C	0	0	0	0	0	0	0	0	0	0	0	0
14.3044	6.2653	0.0738 C	0	0	0	0	0	0	0	0	0	0	0	0
12.8116	6.2714	-0.3087 C	0	0	0	0	0	0	0	0	0	0	0	0
12.1464	7.5523	0.2396 C	0	0	0	0	0	0	0	0	0	0	0	0
1.4688	1.8543	0.7318 H	0	0	0	0	0	0	0	0	0	0	0	0
1.4924	3.6224	0.7593 H	0	0	0	0	0	0	0	0	0	0	0	0
1.8929	2.7556	-0.7294 H	0	0	0	0	0	0	0	0	0	0	0	0
3.5222	2.6937	1.8460 H	0	0	0	0	0	0	0	0	0	0	0	0
3.9227	1.8270	0.3574 H	0	0	0	0	0	0	0	0	0	0	0	0
4.0847	3.9867	-0.8566 H	0	0	0	0	0	0	0	0	0	0	0	0
3.6839	4.8535	0.6319 H	0	0	0	0	0	0	0	0	0	0	0	0
5.7135	3.9247	1.7193 H	0	0	0	0	0	0	0	0	0	0	0	0
6.1145	3.0583	0.2306 H	0	0	0	0	0	0	0	0	0	0	0	0
6.2767	5.2180	-0.9829 H	0	0	0	0	0	0	0	0	0	0	0	0
5.8747	6.0846	0.5054 H	0	0	0	0	0	0	0	0	0	0	0	0
7.9039	5.1560	1.5939 H	0	0	0	0	0	0	0	0	0	0	0	0
8.3061	4.2903	0.1051 H	0	0	0	0	0	0	0	0	0	0	0	0
8.4685	6.4500	-1.1073 H	0	0	0	0	0	0	0	0	0	0	0	0
8.0641	7.3143	0.3805 H	0	0	0	0	0	0	0	0	0	0	0	0
10.0963	6.4140	1.4719 H	0	0	0	0	0	0	0	0	0	0	0	0
10.4865	5.5058	0.0077 H	0	0	0	0	0	0	0	0	0	0	0	0
10.4100	18.5052	-0.0186 H	0	0	0	0	0	0	0	0	0	0	0	0
10.8476	17.5842	-1.4635 H	0	0	0	0	0	0	0	0	0	0	0	0
11.9303	17.6029	-0.0652 H	0	0	0	0	0	0	0	0	0	0	0	0
9.1415	16.3740	-0.1263 H	0	0	0	0	0	0	0	0	0	0	0	0
10.2241	16.3927	1.2720 H	0	0	0	0	0	0	0	0	0	0	0	0
11.9023	15.0869	-0.0099 H	0	0	0	0	0	0	0	0	0	0	0	0
10.8195	15.0678	-1.4080 H	0	0	0	0	0	0	0	0	0	0	0	0
9.1136	13.8576	-0.0705 H	0	0	0	0	0	0	0	0	0	0	0	0
10.1962	13.8770	1.3279 H	0	0	0	0	0	0	0	0	0	0	0	0
11.8748	12.5714	0.0462 H	0	0	0	0	0	0	0	0	0	0	0	0
10.7916	12.5513	-1.3517 H	0	0	0	0	0	0	0	0	0	0	0	0
9.0865	11.3410	-0.0133 H	0	0	0	0	0	0	0	0	0	0	0	0
10.1691	11.3619	1.3850 H	0	0	0	0	0	0	0	0	0	0	0	0
11.8468	10.0581	0.1037 H	0	0	0	0	0	0	0	0	0	0	0	0
10.7644	10.0348	-1.2935 H	0	0	0	0	0	0	0	0	0	0	0	0
9.0532	8.8446	0.0712 H	0	0	0	0	0	0	0	0	0	0	0	0
10.1644	8.8320	1.4445 H	0	0	0	0	0	0	0	0	0	0	0	0
20.3337	2.4115	-0.5253 H	0	0	0	0	0	0	0	0	0	0	0	0
19.1897	2.3910	-1.8737 H	0	0	0	0	0	0	0	0	0	0	0	0
18.7989	1.5468	-0.3696 H	0	0	0	0	0	0	0	0	0	0	0	0
18.7153	3.7198	0.8281 H	0	0	0	0	0	0	0	0	0	0	0	0
19.1060	4.5639	-0.6759 H	0	0	0	0	0	0	0	0	0	0	0	0
17.0318	3.6768	-1.7127 H	0	0	0	0	0	0	0	0	0	0	0	0
16.6412	2.8323	-0.2088 H	0	0	0	0	0	0	0	0	0	0	0	0
16.5579	5.0052	0.9896 H	0	0	0	0	0	0	0	0	0	0	0	0
16.9480	5.8496	-0.5146 H	0	0	0	0	0	0	0	0	0	0	0	0
14.8737	4.9626	-1.5508 H	0	0	0	0	0	0	0	0	0	0	0	0
14.4838	4.1173	-0.0471 H	0	0	0	0	0	0	0	0	0	0	0	0
14.4007	6.2898	1.1522 H	0	0	0	0	0	0	0	0	0	0	0	0
14.7897	7.1348	-0.3519 H	0	0	0	0	0	0	0	0	0	0	0	0
12.7155	6.2480	-1.3871 H	0	0	0	0	0	0	0	0	0	0	0	0
12.3286	5.4019	0.1158 H	0	0	0	0	0	0	0	0	0	0	0	0
12.2200	7.5626	1.3202 H	0	0	0	0	0	0	0	0	0	0	0	0
12.6543	8.4174	-0.1636 H	0	0	0	0	0	0	0	0	0	0	0	0

1	2	1	0	0	0	0
2	3	1	0	0	0	0
3	4	1	0	0	0	0
4	5	1	0	0	0	0
5	6	1	0	0	0	0
6	7	1	0	0	0	0
7	8	1	0	0	0	0

8	9	1	0	0	0	0
10	11	1	0	0	0	0
11	12	1	0	0	0	0
12	13	1	0	0	0	0
13	14	1	0	0	0	0
14	15	1	0	0	0	0
15	16	1	0	0	0	0
16	17	1	0	0	0	0
17	9	1	0	0	0	0
18	19	1	0	0	0	0
19	20	1	0	0	0	0
20	21	1	0	0	0	0
21	22	1	0	0	0	0
22	23	1	0	0	0	0
23	24	1	0	0	0	0
24	25	1	0	0	0	0
25	9	1	0	0	0	0
1	26	1	0	0	0	0
1	27	1	0	0	0	0
1	28	1	0	0	0	0
2	29	1	0	0	0	0
2	30	1	0	0	0	0
3	31	1	0	0	0	0
3	32	1	0	0	0	0
4	33	1	0	0	0	0
4	34	1	0	0	0	0
5	35	1	0	0	0	0
5	36	1	0	0	0	0
6	37	1	0	0	0	0
6	38	1	0	0	0	0
7	39	1	0	0	0	0
7	40	1	0	0	0	0
8	41	1	0	0	0	0
8	42	1	0	0	0	0
10	43	1	0	0	0	0
10	44	1	0	0	0	0
10	45	1	0	0	0	0
11	46	1	0	0	0	0
11	47	1	0	0	0	0
12	48	1	0	0	0	0
12	49	1	0	0	0	0
13	50	1	0	0	0	0
13	51	1	0	0	0	0
14	52	1	0	0	0	0
14	53	1	0	0	0	0
15	54	1	0	0	0	0
15	55	1	0	0	0	0
16	56	1	0	0	0	0
16	57	1	0	0	0	0
17	58	1	0	0	0	0
17	59	1	0	0	0	0
18	60	1	0	0	0	0
18	61	1	0	0	0	0
18	62	1	0	0	0	0
19	63	1	0	0	0	0
19	64	1	0	0	0	0
20	65	1	0	0	0	0
20	66	1	0	0	0	0
21	67	1	0	0	0	0
21	68	1	0	0	0	0
22	69	1	0	0	0	0
22	70	1	0	0	0	0
23	71	1	0	0	0	0

23 72 1 0 0 0 0
24 73 1 0 0 0 0
24 74 1 0 0 0 0
25 75 1 0 0 0 0
25 76 1 0 0 0 0
M END
> <Alias name>
Tri-n-octylamine

> <deltaH°(c)>   kJ/mol
-16145

$$$$
N-Stearoyldextramine
  ChemBrain IXL

80 80 0 0 0 0 0 0 0 0 1 V2000
26.0099   14.5655   0.6621 C   0 0 0 0 0 0 0 0 0 0 0 0
26.5630   13.4512   0.0295 C   0 0 0 0 0 0 0 0 0 0 0 0
25.5918   15.6612   -0.0937 C   0 0 0 0 0 0 0 0 0 0 0 0
26.6984   13.4333   -1.3596 C   0 0 0 0 0 0 0 0 0 0 0 0
27.0044   12.2885   0.8335 C   0 0 0 0 0 0 0 0 0 0 0 0
26.2801   14.5291   -2.1155 C   0 0 0 0 0 0 0 0 0 0 0 0
25.7265   15.6433   -1.4827 C   0 0 0 0 0 0 0 0 0 0 0 0
25.2851   16.7983   -2.2794 N   0 3 0 0 0 0 0 0 0 0 0 0
25.4039   16.7821   -3.5138 O   0 0 0 0 0 0 0 0 0 0 0 0
24.7934   17.7882   -1.7170 O   0 5 0 0 0 0 0 0 0 0 0 0
26.9421   10.0672   -1.0493 O   0 0 0 0 0 0 0 0 0 0 0 0
25.6850   10.6613   -0.5724 C   0 0 0 0 0 0 0 0 0 0 0 0
25.8885   11.2227   0.8491 C   0 0 0 0 0 0 0 0 0 0 0 0
24.6355   11.8352   1.3152 N   0 0 0 0 0 0 0 0 0 0 0 0
27.2838   12.7237   2.2102 O   0 0 0 0 0 0 0 0 0 0 0 0
23.6359   11.0417   1.8840 C   0 0 0 0 0 0 0 0 0 0 0 0
22.3737   11.6626   2.3354 C   0 0 0 0 0 0 0 0 0 0 0 0
23.8053   9.8394   2.0077 O   0 0 0 0 0 0 0 0 0 0 0 0
21.1774   10.8770   1.7654 C   0 0 0 0 0 0 0 0 0 0 0 0
19.8706   11.5505   2.2155 C   0 0 0 0 0 0 0 0 0 0 0 0
14.8629   11.3702   2.0744 C   0 0 0 0 0 0 0 0 0 0 0 0
16.1503   10.6616   1.6165 C   0 0 0 0 0 0 0 0 0 0 0 0
13.6329   10.5714   1.6074 C   0 0 0 0 0 0 0 0 0 0 0 0
17.3678   11.4555   2.1220 C   0 0 0 0 0 0 0 0 0 0 0 0
18.6654   10.7618   1.6733 C   0 0 0 0 0 0 0 0 0 0 0 0
8.5992   10.4146   1.7264 C   0 0 0 0 0 0 0 0 0 0 0 0
9.8480   11.2176   2.1335 C   0 0 0 0 0 0 0 0 0 0 0 0
7.3388   11.1490   2.2224 C   0 0 0 0 0 0 0 0 0 0 0 0
11.1154   10.4893   1.6471 C   0 0 0 0 0 0 0 0 0 0 0 0
12.3561   11.2911   2.0798 C   0 0 0 0 0 0 0 0 0 0 0 0
2.3161   11.0210   2.4485 C   0 0 0 0 0 0 0 0 0 0 0 0
3.5713   10.2807   1.9473 C   0 0 0 0 0 0 0 0 0 0 0 0
4.8282   11.0841   2.3316 C   0 0 0 0 0 0 0 0 0 0 0 0
6.0848   10.3456   1.8311 C   0 0 0 0 0 0 0 0 0 0 0 0
26.9028   9.6811   -1.9522 H   0 0 0 0 0 0 0 0 0 0 0 0
24.4949   12.8288   1.2214 H   0 0 0 0 0 0 0 0 0 0 0 0
27.5844   12.0119   2.8180 H   0 0 0 0 0 0 0 0 0 0 0 0
25.9049   14.5795   1.7399 H   0 0 0 0 0 0 0 0 0 0 0 0
25.1625   16.5253   0.3977 H   0 0 0 0 0 0 0 0 0 0 0 0
27.1276   12.5688   -1.8510 H   0 0 0 0 0 0 0 0 0 0 0 0
27.9027   11.8660   0.3997 H   0 0 0 0 0 0 0 0 0 0 0 0
26.3849   14.5148   -3.1931 H   0 0 0 0 0 0 0 0 0 0 0 0
25.3879   11.4607   -1.2379 H   0 0 0 0 0 0 0 0 0 0 0 0
24.9123   9.9029   -0.5529 H   0 0 0 0 0 0 0 0 0 0 0 0
26.1690   10.4173   1.5166 H   0 0 0 0 0 0 0 0 0 0 0 0
22.3315   11.6492   3.4173 H   0 0 0 0 0 0 0 0 0 0 0 0

```
 22.3314   12.6867    1.9856 H   0  0  0  0  0  0  0  0  0  0  0  0
 21.2054    9.8584    2.1325 H   0  0  0  0  0  0  0  0  0  0  0  0
 21.2284   10.8735    0.6835 H   0  0  0  0  0  0  0  0  0  0  0  0
 19.8299   11.5704    3.2974 H   0  0  0  0  0  0  0  0  0  0  0  0
 19.8385   12.5639    1.8352 H   0  0  0  0  0  0  0  0  0  0  0  0
 14.8564   11.4402    3.1550 H   0  0  0  0  0  0  0  0  0  0  0  0
 14.8291   12.3656    1.6490 H   0  0  0  0  0  0  0  0  0  0  0  0
 16.1724   10.6125    0.5348 H   0  0  0  0  0  0  0  0  0  0  0  0
 16.1770    9.6592    2.0254 H   0  0  0  0  0  0  0  0  0  0  0  0
 13.6327   10.5040    0.5264 H   0  0  0  0  0  0  0  0  0  0  0  0
 13.6656    9.5758    2.0325 H   0  0  0  0  0  0  0  0  0  0  0  0
 17.3414   11.5035    3.2035 H   0  0  0  0  0  0  0  0  0  0  0  0
 17.3375   12.4592    1.7163 H   0  0  0  0  0  0  0  0  0  0  0  0
 18.7065   10.7344    0.5914 H   0  0  0  0  0  0  0  0  0  0  0  0
 18.6895    9.7514    2.0626 H   0  0  0  0  0  0  0  0  0  0  0  0
  8.5637   10.3223    0.6480 H   0  0  0  0  0  0  0  0  0  0  0  0
  8.6423    9.4287    2.1726 H   0  0  0  0  0  0  0  0  0  0  0  0
  9.8790   11.3123    3.2119 H   0  0  0  0  0  0  0  0  0  0  0  0
  9.8047   12.2031    1.6864 H   0  0  0  0  0  0  0  0  0  0  0  0
  7.3808   11.2491    3.3000 H   0  0  0  0  0  0  0  0  0  0  0  0
  7.2930   12.1322    1.7705 H   0  0  0  0  0  0  0  0  0  0  0  0
 11.0947   10.4076    0.5673 H   0  0  0  0  0  0  0  0  0  0  0  0
 11.1541    9.4993    2.0844 H   0  0  0  0  0  0  0  0  0  0  0  0
 12.3702   11.3763    3.1593 H   0  0  0  0  0  0  0  0  0  0  0  0
 12.3171   12.2805    1.6411 H   0  0  0  0  0  0  0  0  0  0  0  0
  1.4324   10.4564    2.1791 H   0  0  0  0  0  0  0  0  0  0  0  0
  2.3641   11.1246    3.5255 H   0  0  0  0  0  0  0  0  0  0  0  0
  2.2701   12.0027    1.9932 H   0  0  0  0  0  0  0  0  0  0  0  0
  3.5238   10.1777    0.8704 H   0  0  0  0  0  0  0  0  0  0  0  0
  3.6172    9.2991    2.4023 H   0  0  0  0  0  0  0  0  0  0  0  0
  4.8753   11.1869    3.4087 H   0  0  0  0  0  0  0  0  0  0  0  0
  4.7819   12.0661    1.8771 H   0  0  0  0  0  0  0  0  0  0  0  0
  6.0407   10.2464    0.7536 H   0  0  0  0  0  0  0  0  0  0  0  0
  6.1301    9.3625    2.2831 H   0  0  0  0  0  0  0  0  0  0  0  0
  2  1  4  0  0  0  0
  1  3  4  0  0  0  0
  4  2  4  0  0  0  0
  2  5  1  0  0  0  0
  3  7  4  0  0  0  0
  6  4  4  0  0  0  0
  5 13  1  0  0  0  0
  5 15  1  0  0  0  0
  7  6  4  0  0  0  0
  7  8  1  0  0  0  0
  8  9  2  0  0  0  0
  8 10  1  0  0  0  0
 11 12  1  0  0  0  0
 12 13  1  0  0  0  0
 13 14  1  0  0  0  0
 14 16  1  0  0  0  0
 16 17  1  0  0  0  0
 16 18  2  0  0  0  0
 17 19  1  0  0  0  0
 19 20  1  0  0  0  0
 20 25  1  0  0  0  0
 21 22  1  0  0  0  0
 21 23  1  0  0  0  0
 22 24  1  0  0  0  0
 23 30  1  0  0  0  0
 24 25  1  0  0  0  0
 26 27  1  0  0  0  0
 26 28  1  0  0  0  0
 27 29  1  0  0  0  0
```

```
 28 34  1  0  0  0  0
 29 30  1  0  0  0  0
 31 32  1  0  0  0  0
 32 33  1  0  0  0  0
 33 34  1  0  0  0  0
 11 35  1  0  0  0  0
 14 36  1  0  0  0  0
 15 37  1  0  0  0  0
  1 38  1  0  0  0  0
  3 39  1  0  0  0  0
  4 40  1  0  0  0  0
  5 41  1  0  0  0  0
  6 42  1  0  0  0  0
 12 43  1  0  0  0  0
 12 44  1  0  0  0  0
 13 45  1  0  0  0  0
 17 46  1  0  0  0  0
 17 47  1  0  0  0  0
 19 48  1  0  0  0  0
 19 49  1  0  0  0  0
 20 50  1  0  0  0  0
 20 51  1  0  0  0  0
 21 52  1  0  0  0  0
 21 53  1  0  0  0  0
 22 54  1  0  0  0  0
 22 55  1  0  0  0  0
 23 56  1  0  0  0  0
 23 57  1  0  0  0  0
 24 58  1  0  0  0  0
 24 59  1  0  0  0  0
 25 60  1  0  0  0  0
 25 61  1  0  0  0  0
 26 62  1  0  0  0  0
 26 63  1  0  0  0  0
 27 64  1  0  0  0  0
 27 65  1  0  0  0  0
 28 66  1  0  0  0  0
 28 67  1  0  0  0  0
 29 68  1  0  0  0  0
 29 69  1  0  0  0  0
 30 70  1  0  0  0  0
 30 71  1  0  0  0  0
 31 72  1  0  0  0  0
 31 73  1  0  0  0  0
 31 74  1  0  0  0  0
 32 75  1  0  0  0  0
 32 76  1  0  0  0  0
 33 77  1  0  0  0  0
 33 78  1  0  0  0  0
 34 79  1  0  0  0  0
 34 80  1  0  0  0  0
M  END
>  <Alias name>
N-Stearoyldextramine

>  <deltaH°(c)>   kJ/mol
-16133

$$$$
Bis(monothiodibenzoylmethanate)zinc(II)
  ChemBrain IXL

 57 62  0  0  0  0  0  0  0  0  1 V2000
```

8.9794 13.5560 2.2593 C 0 0 0 0 0 0 0 0 0 0 0 0
10.0555 12.9114 1.6487 C 0 0 0 0 0 0 0 0 0 0 0 0
9.8250 11.9410 0.6726 C 0 0 0 0 0 0 0 0 0 0 0 0
8.5180 11.6145 0.3058 C 0 0 0 0 0 0 0 0 0 0 0 0
7.4418 12.2595 0.9181 C 0 0 0 0 0 0 0 0 0 0 0 0
7.6728 13.2301 1.8943 C 0 0 0 0 0 0 0 0 0 0 0 0
8.2790 10.5828 -0.7349 C 0 0 0 0 0 0 0 0 0 0 0 0
9.2541 10.0385 -1.2615 O 0 0 0 0 0 0 0 0 0 0 0 0
6.8498 10.2215 -1.1398 C 0 0 0 0 0 0 0 0 0 0 0 0
6.4539 8.9244 -1.3593 C 0 0 0 0 0 0 0 0 0 0 0 0
7.6300 7.4982 -1.1360 S 0 5 0 0 0 0 0 0 0 0 0 0
9.5703 8.2511 -0.9968 Zn 0 0 0 0 0 0 0 0 0 0 0 0
4.1569 9.6571 -2.0146 C 0 0 0 0 0 0 0 0 0 0 0 0
5.0673 8.6240 -1.7839 C 0 0 0 0 0 0 0 0 0 0 0 0
4.6729 7.2971 -1.9539 C 0 0 0 0 0 0 0 0 0 0 0 0
3.3717 7.0025 -2.3598 C 0 0 0 0 0 0 0 0 0 0 0 0
2.4634 8.0351 -2.5940 C 0 0 0 0 0 0 0 0 0 0 0 0
2.8558 9.3622 -2.4203 C 0 0 0 0 0 0 0 0 0 0 0 0
15.3085 9.0267 -3.5908 C 0 0 0 0 0 0 0 0 0 0 0 0
14.0226 9.3938 -3.9883 C 0 0 0 0 0 0 0 0 0 0 0 0
12.9130 8.7892 -3.4013 C 0 0 0 0 0 0 0 0 0 0 0 0
13.0905 7.8191 -2.4184 C 0 0 0 0 0 0 0 0 0 0 0 0
14.3742 7.4504 -2.0193 C 0 0 0 0 0 0 0 0 0 0 0 0
15.4842 8.0546 -2.6060 C 0 0 0 0 0 0 0 0 0 0 0 0
11.9202 7.1800 -1.7992 C 0 0 0 0 0 0 0 0 0 0 0 0
10.7805 7.4960 -2.1762 O 0 0 0 0 0 0 0 0 0 0 0 0
12.1374 6.1505 -0.7255 C 0 0 0 0 0 0 0 0 0 0 0 0
11.5268 6.2562 0.4742 C 0 0 0 0 0 0 0 0 0 0 0 0
10.4461 7.7076 0.8207 S 0 5 0 0 0 0 0 0 0 0 0 0
11.1003 5.3551 2.7712 C 0 0 0 0 0 0 0 0 0 0 0 0
11.7122 5.2086 1.5232 C 0 0 0 0 0 0 0 0 0 0 0 0
12.4959 4.0792 1.2650 C 0 0 0 0 0 0 0 0 0 0 0 0
12.6648 3.1028 2.2490 C 0 0 0 0 0 0 0 0 0 0 0 0
12.0519 3.2518 3.4931 C 0 0 0 0 0 0 0 0 0 0 0 0
11.2700 4.3775 3.7545 C 0 0 0 0 0 0 0 0 0 0 0 0
9.1584 14.3096 3.0170 H 0 0 0 0 0 0 0 0 0 0 0 0
11.0700 13.1643 1.9324 H 0 0 0 0 0 0 0 0 0 0 0 0
10.6611 11.4411 0.1991 H 0 0 0 0 0 0 0 0 0 0 0 0
6.4273 12.0074 0.6358 H 0 0 0 0 0 0 0 0 0 0 0 0
6.8375 13.7306 2.3689 H 0 0 0 0 0 0 0 0 0 0 0 0
6.1254 11.0156 -1.2551 H 0 0 0 0 0 0 0 0 0 0 0 0
4.4593 10.6873 -1.8790 H 0 0 0 0 0 0 0 0 0 0 0 0
5.3779 6.4951 -1.7706 H 0 0 0 0 0 0 0 0 0 0 0 0
3.0666 5.9722 -2.4933 H 0 0 0 0 0 0 0 0 0 0 0 0
1.4539 7.8064 -2.9108 H 0 0 0 0 0 0 0 0 0 0 0 0
2.1502 10.1637 -2.6005 H 0 0 0 0 0 0 0 0 0 0 0 0
16.1702 9.4962 -4.0466 H 0 0 0 0 0 0 0 0 0 0 0 0
13.8862 10.1481 -4.7524 H 0 0 0 0 0 0 0 0 0 0 0 0
11.9134 9.0725 -3.7086 H 0 0 0 0 0 0 0 0 0 0 0 0
14.5087 6.6947 -1.2540 H 0 0 0 0 0 0 0 0 0 0 0 0
16.4821 7.7694 -2.2971 H 0 0 0 0 0 0 0 0 0 0 0 0
12.7977 5.3154 -0.9159 H 0 0 0 0 0 0 0 0 0 0 0 0
10.4934 6.2278 2.9770 H 0 0 0 0 0 0 0 0 0 0 0 0
12.9731 3.9598 0.3017 H 0 0 0 0 0 0 0 0 0 0 0 0
13.2722 2.2286 2.0464 H 0 0 0 0 0 0 0 0 0 0 0 0
12.1829 2.4933 4.2563 H 0 0 0 0 0 0 0 0 0 0 0 0
10.7944 4.4928 4.7212 H 0 0 0 0 0 0 0 0 0 0 0 0
1 2 4 0 0 0 0
2 3 4 0 0 0 0
3 4 4 0 0 0 0
4 5 4 0 0 0 0
5 6 4 0 0 0 0
6 1 4 0 0 0 0

```
  7  8  2  0  0  0  0
  7  9  1  0  0  0  0
  7  4  1  0  0  0  0
  9 10  2  0  0  0  0
 10 11  1  0  0  0  0
 11 12  1  0  0  0  0
  8 12  1  0  0  0  0
 13 14  4  0  0  0  0
 14 15  4  0  0  0  0
 15 16  4  0  0  0  0
 16 17  4  0  0  0  0
 17 18  4  0  0  0  0
 18 13  4  0  0  0  0
 14 10  1  0  0  0  0
 19 20  4  0  0  0  0
 20 21  4  0  0  0  0
 21 22  4  0  0  0  0
 22 23  4  0  0  0  0
 23 24  4  0  0  0  0
 24 19  4  0  0  0  0
 25 26  2  0  0  0  0
 25 27  1  0  0  0  0
 25 22  1  0  0  0  0
 27 28  2  0  0  0  0
 28 29  1  0  0  0  0
 30 31  4  0  0  0  0
 31 32  4  0  0  0  0
 32 33  4  0  0  0  0
 33 34  4  0  0  0  0
 34 35  4  0  0  0  0
 35 30  4  0  0  0  0
 31 28  1  0  0  0  0
 29 12  1  0  0  0  0
 26 12  1  0  0  0  0
  1 36  1  0  0  0  0
  2 37  1  0  0  0  0
  3 38  1  0  0  0  0
  5 39  1  0  0  0  0
  6 40  1  0  0  0  0
  9 41  1  0  0  0  0
 13 42  1  0  0  0  0
 15 43  1  0  0  0  0
 16 44  1  0  0  0  0
 17 45  1  0  0  0  0
 18 46  1  0  0  0  0
 19 47  1  0  0  0  0
 20 48  1  0  0  0  0
 21 49  1  0  0  0  0
 23 50  1  0  0  0  0
 24 51  1  0  0  0  0
 27 52  1  0  0  0  0
 30 53  1  0  0  0  0
 32 54  1  0  0  0  0
 33 55  1  0  0  0  0
 34 56  1  0  0  0  0
 35 57  1  0  0  0  0
M  END
```
> <Alias name>
Bis(monothiodibenzoylmethanate)zinc(II)

> <deltaH°(c)>   kJ/mol
-16057.2

```
$$$$
Tetra-p-tolylethene
  ChemBrain IXL

 58 61  0  0  0  0  0  0  0  0  1 V2000
    7.3143    7.4087    0.0944 C   0  0  0  0  0  0  0  0  0  0  0  0
    7.3150    6.1184   -0.0749 C   0  0  0  0  0  0  0  0  0  0  0  0
    8.6158    8.1213    0.1885 C   0  0  0  0  0  0  0  0  0  0  0  0
    6.0119    8.1209    0.1895 C   0  0  0  0  0  0  0  0  0  0  0  0
    6.0137    5.4058   -0.1690 C   0  0  0  0  0  0  0  0  0  0  0  0
    8.6174    5.4063   -0.1700 C   0  0  0  0  0  0  0  0  0  0  0  0
    3.8184    5.1862   -1.1633 C   0  0  0  0  0  0  0  0  0  0  0  0
    5.0464    5.8446   -1.0758 C   0  0  0  0  0  0  0  0  0  0  0  0
    3.5541    4.0892   -0.3427 C   0  0  0  0  0  0  0  0  0  0  0  0
    5.7474    4.3101    0.6545 C   0  0  0  0  0  0  0  0  0  0  0  0
    4.5189    3.6512    0.5663 C   0  0  0  0  0  0  0  0  0  0  0  0
    2.2493    3.3900   -0.4367 C   0  0  0  0  0  0  0  0  0  0  0  0
   10.0906    3.6146    0.5188 C   0  0  0  0  0  0  0  0  0  0  0  0
    8.8615    4.2725    0.6077 C   0  0  0  0  0  0  0  0  0  0  0  0
   11.0782    4.0912   -0.3453 C   0  0  0  0  0  0  0  0  0  0  0  0
    9.6075    5.8834   -1.0320 C   0  0  0  0  0  0  0  0  0  0  0  0
   10.8360    5.2259   -1.1202 C   0  0  0  0  0  0  0  0  0  0  0  0
   12.3838    3.3929   -0.4400 C   0  0  0  0  0  0  0  0  0  0  0  0
   10.8116    8.3398    1.1820 C   0  0  0  0  0  0  0  0  0  0  0  0
    9.5835    7.6816    1.0945 C   0  0  0  0  0  0  0  0  0  0  0  0
   11.0756    9.4375    0.3623 C   0  0  0  0  0  0  0  0  0  0  0  0
    8.8818    9.2177   -0.6341 C   0  0  0  0  0  0  0  0  0  0  0  0
   10.1104    9.8764   -0.5460 C   0  0  0  0  0  0  0  0  0  0  0  0
   12.3805   10.1366    0.4563 C   0  0  0  0  0  0  0  0  0  0  0  0
    4.5396    9.9142   -0.4975 C   0  0  0  0  0  0  0  0  0  0  0  0
    5.7687    9.2563   -0.5864 C   0  0  0  0  0  0  0  0  0  0  0  0
    3.5510    9.4361    0.3647 C   0  0  0  0  0  0  0  0  0  0  0  0
    5.0209    7.6424    1.0496 C   0  0  0  0  0  0  0  0  0  0  0  0
    3.7923    8.3000    1.1378 C   0  0  0  0  0  0  0  0  0  0  0  0
    2.2453   10.1345    0.4594 C   0  0  0  0  0  0  0  0  0  0  0  0
    3.0702    5.5270   -1.8686 H   0  0  0  0  0  0  0  0  0  0  0  0
    5.2489    6.6970   -1.7121 H   0  0  0  0  0  0  0  0  0  0  0  0
    6.4936    3.9707    1.3620 H   0  0  0  0  0  0  0  0  0  0  0  0
    4.3144    2.7996    1.2038 H   0  0  0  0  0  0  0  0  0  0  0  0
    2.4137    2.3196   -0.4604 H   0  0  0  0  0  0  0  0  0  0  0  0
    1.6410    3.6426    0.4232 H   0  0  0  0  0  0  0  0  0  0  0  0
    1.7394    3.6968   -1.3420 H   0  0  0  0  0  0  0  0  0  0  0  0
   10.2778    2.7337    1.1208 H   0  0  0  0  0  0  0  0  0  0  0  0
    8.0976    3.9027    1.2802 H   0  0  0  0  0  0  0  0  0  0  0  0
    9.4223    6.7649   -1.6329 H   0  0  0  0  0  0  0  0  0  0  0  0
   11.6017    5.5966   -1.7908 H   0  0  0  0  0  0  0  0  0  0  0  0
   13.1835    4.1234   -0.4637 H   0  0  0  0  0  0  0  0  0  0  0  0
   12.4111    2.7988   -1.3455 H   0  0  0  0  0  0  0  0  0  0  0  0
   12.5112    2.7462    0.4196 H   0  0  0  0  0  0  0  0  0  0  0  0
   11.5600    7.9984    1.8867 H   0  0  0  0  0  0  0  0  0  0  0  0
    9.3812    6.8287    1.7302 H   0  0  0  0  0  0  0  0  0  0  0  0
    8.1354    9.5578   -1.3411 H   0  0  0  0  0  0  0  0  0  0  0  0
   10.3147   10.7286   -1.1827 H   0  0  0  0  0  0  0  0  0  0  0  0
   12.2163   11.2070    0.4801 H   0  0  0  0  0  0  0  0  0  0  0  0
   12.9889    9.8838   -0.4036 H   0  0  0  0  0  0  0  0  0  0  0  0
   12.8904    9.8297    1.3616 H   0  0  0  0  0  0  0  0  0  0  0  0
    4.3530   10.7962   -1.0981 H   0  0  0  0  0  0  0  0  0  0  0  0
    6.5333    9.6272   -1.2575 H   0  0  0  0  0  0  0  0  0  0  0  0
    5.2053    6.7598    1.6491 H   0  0  0  0  0  0  0  0  0  0  0  0
    3.0258    7.9281    1.8069 H   0  0  0  0  0  0  0  0  0  0  0  0
    1.4456    9.4039    0.4830 H   0  0  0  0  0  0  0  0  0  0  0  0
    2.2180   10.7285    1.3650 H   0  0  0  0  0  0  0  0  0  0  0  0
    2.1179   10.7811   -0.4002 H   0  0  0  0  0  0  0  0  0  0  0  0
```

```
  1  2  2  0  0  0  0
  1  3  1  0  0  0  0
  1  4  1  0  0  0  0
  2  5  1  0  0  0  0
  2  6  1  0  0  0  0
 20  3  4  0  0  0  0
  3 22  4  0  0  0  0
 26  4  4  0  0  0  0
  4 28  4  0  0  0  0
  8  5  4  0  0  0  0
  5 10  4  0  0  0  0
 14  6  4  0  0  0  0
  6 16  4  0  0  0  0
  7  8  4  0  0  0  0
  9  7  4  0  0  0  0
 11  9  4  0  0  0  0
  9 12  1  0  0  0  0
 10 11  4  0  0  0  0
 13 14  4  0  0  0  0
 15 13  4  0  0  0  0
 17 15  4  0  0  0  0
 15 18  1  0  0  0  0
 16 17  4  0  0  0  0
 19 20  4  0  0  0  0
 21 19  4  0  0  0  0
 23 21  4  0  0  0  0
 21 24  1  0  0  0  0
 22 23  4  0  0  0  0
 25 26  4  0  0  0  0
 27 25  4  0  0  0  0
 29 27  4  0  0  0  0
 27 30  1  0  0  0  0
 28 29  4  0  0  0  0
  7 31  1  0  0  0  0
  8 32  1  0  0  0  0
 10 33  1  0  0  0  0
 11 34  1  0  0  0  0
 12 35  1  0  0  0  0
 12 36  1  0  0  0  0
 12 37  1  0  0  0  0
 13 38  1  0  0  0  0
 14 39  1  0  0  0  0
 16 40  1  0  0  0  0
 17 41  1  0  0  0  0
 18 42  1  0  0  0  0
 18 43  1  0  0  0  0
 18 44  1  0  0  0  0
 19 45  1  0  0  0  0
 20 46  1  0  0  0  0
 22 47  1  0  0  0  0
 23 48  1  0  0  0  0
 24 49  1  0  0  0  0
 24 50  1  0  0  0  0
 24 51  1  0  0  0  0
 25 52  1  0  0  0  0
 26 53  1  0  0  0  0
 28 54  1  0  0  0  0
 29 55  1  0  0  0  0
 30 56  1  0  0  0  0
 30 57  1  0  0  0  0
 30 58  1  0  0  0  0
M  END
>  &lt;Alias name&gt;
```

Tetra-p-tolylethene

> <deltaH°(c)>   kJ/mol
-15978

$$$$
3-Methyl-3-t-butylperoxy-1-triphenylsilyl-1-butyne
  ChemBrain IXL

 60 62 0 0 0 0 0 0 0 0 1 V2000
    5.6520   3.2814   -2.2270 C   0 0 0 0 0 0 0 0 0 0 0 0
    5.4895   4.4613   -2.9533 C   0 0 0 0 0 0 0 0 0 0 0 0
    6.1812   5.6128   -2.5769 C   0 0 0 0 0 0 0 0 0 0 0 0
    7.0350   5.5845   -1.4740 C   0 0 0 0 0 0 0 0 0 0 0 0
    7.1979   4.4044   -0.7482 C   0 0 0 0 0 0 0 0 0 0 0 0
    6.5062   3.2529   -1.1245 C   0 0 0 0 0 0 0 0 0 0 0 0
    7.9851   7.5027   1.8376 C   0 0 0 0 0 0 0 0 0 0 0 0
    8.6340   6.9089   0.7549 C   0 0 0 0 0 0 0 0 0 0 0 0
    9.7886   6.1540   0.9622 C   0 0 0 0 0 0 0 0 0 0 0 0
   10.2943   5.9928   2.2522 C   0 0 0 0 0 0 0 0 0 0 0 0
    9.6455   6.5866   3.3350 C   0 0 0 0 0 0 0 0 0 0 0 0
    8.4908   7.3416   3.1277 C   0 0 0 0 0 0 0 0 0 0 0 0
    7.9582   7.1246   -0.9699 Si 0 0 0 0 0 0 0 0 0 0 0 0
   11.4748   7.8667   -3.9283 C   0 0 0 0 0 0 0 0 0 0 0 0
   11.5471   8.3529   -2.6228 C   0 0 0 0 0 0 0 0 0 0 0 0
   10.4933   8.1303   -1.7362 C   0 0 0 0 0 0 0 0 0 0 0 0
    9.3671   7.4217   -2.1552 C   0 0 0 0 0 0 0 0 0 0 0 0
    9.2949   6.9353   -3.4607 C   0 0 0 0 0 0 0 0 0 0 0 0
   10.3487   7.1579   -4.3472 C   0 0 0 0 0 0 0 0 0 0 0 0
    6.7958   8.5825   -1.0059 C   0 0 0 0 0 0 0 0 0 0 0 0
    6.0451   9.5235   -1.0293 C   0 0 0 0 0 0 0 0 0 0 0 0
    5.0839   10.7275   -1.0594 C   0 0 0 0 0 0 0 0 0 0 0 0
    5.2216   11.4612   -2.4075 C   0 0 0 0 0 0 0 0 0 0 0 0
    2.8025   7.9928   -1.4146 C   0 0 0 0 0 0 0 0 0 0 0 0
    3.7013   10.2547   -0.8978 O   0 0 0 0 0 0 0 0 0 0 0 0
    1.6060   8.1928   -0.4644 C   0 0 0 0 0 0 0 0 0 0 0 0
    3.9609   7.3252   -0.6484 C   0 0 0 0 0 0 0 0 0 0 0 0
    2.3789   7.0926   -2.5915 C   0 0 0 0 0 0 0 0 0 0 0 0
    3.2430   9.2974   -1.9294 O   0 0 0 0 0 0 0 0 0 0 0 0
    5.4341   11.6933   0.0892 C   0 0 0 0 0 0 0 0 0 0 0 0
    5.1149   2.3874   -2.5191 H   0 0 0 0 0 0 0 0 0 0 0 0
    4.8264   4.4834   -3.8093 H   0 0 0 0 0 0 0 0 0 0 0 0
    6.0555   6.5287   -3.1410 H   0 0 0 0 0 0 0 0 0 0 0 0
    7.8614   4.3823   0.1075 H   0 0 0 0 0 0 0 0 0 0 0 0
    6.6324   2.3369   -0.5608 H   0 0 0 0 0 0 0 0 0 0 0 0
    7.0887   8.0887   1.6767 H   0 0 0 0 0 0 0 0 0 0 0 0
   10.2923   5.6929   0.1216 H   0 0 0 0 0 0 0 0 0 0 0 0
   11.1907   5.4067   2.4132 H   0 0 0 0 0 0 0 0 0 0 0 0
   10.0381   6.4615   4.3365 H   0 0 0 0 0 0 0 0 0 0 0 0
    7.9871   7.8026   3.9683 H   0 0 0 0 0 0 0 0 0 0 0 0
   12.2929   8.0395   -4.6166 H   0 0 0 0 0 0 0 0 0 0 0 0
   12.4214   8.9032   -2.2975 H   0 0 0 0 0 0 0 0 0 0 0 0
   10.5496   8.5077   -0.7226 H   0 0 0 0 0 0 0 0 0 0 0 0
    8.4207   6.3849   -3.7858 H   0 0 0 0 0 0 0 0 0 0 0 0
   10.2926   6.7804   -5.3608 H   0 0 0 0 0 0 0 0 0 0 0 0
    4.5460   12.3073   -2.4287 H   0 0 0 0 0 0 0 0 0 0 0 0
    4.9753   10.7824   -3.2147 H   0 0 0 0 0 0 0 0 0 0 0 0
    6.2401   11.8094   -2.5267 H   0 0 0 0 0 0 0 0 0 0 0 0
    1.2822   7.2317   -0.0843 H   0 0 0 0 0 0 0 0 0 0 0 0
    1.9033   8.8262   0.3622 H   0 0 0 0 0 0 0 0 0 0 0 0
    0.7915   8.6610   -1.0031 H   0 0 0 0 0 0 0 0 0 0 0 0
    4.2596   7.9585   0.1778 H   0 0 0 0 0 0 0 0 0 0 0 0
    4.8013   7.1832   -1.3166 H   0 0 0 0 0 0 0 0 0 0 0 0

```
   3.6360    6.3647   -0.2679 H   0  0  0  0  0  0  0  0  0  0  0  0
   3.2196    6.9526   -3.2597 H   0  0  0  0  0  0  0  0  0  0  0  0
   2.0552    6.1312   -2.2123 H   0  0  0  0  0  0  0  0  0  0  0  0
   1.5643    7.5613   -3.1295 H   0  0  0  0  0  0  0  0  0  0  0  0
   4.7592   12.5400    0.0673 H   0  0  0  0  0  0  0  0  0  0  0  0
   5.3362   11.1780    1.0367 H   0  0  0  0  0  0  0  0  0  0  0  0
   6.4530   12.0406   -0.0292 H   0  0  0  0  0  0  0  0  0  0  0  0
  1  2  4  0  0  0  0
  2  3  4  0  0  0  0
  3  4  4  0  0  0  0
  4  5  4  0  0  0  0
  5  6  4  0  0  0  0
  6  1  4  0  0  0  0
  7  8  4  0  0  0  0
  8  9  4  0  0  0  0
  9 10  4  0  0  0  0
 10 11  4  0  0  0  0
 11 12  4  0  0  0  0
 12  7  4  0  0  0  0
  4 13  1  0  0  0  0
 13  8  1  0  0  0  0
 14 15  4  0  0  0  0
 15 16  4  0  0  0  0
 16 17  4  0  0  0  0
 17 18  4  0  0  0  0
 18 19  4  0  0  0  0
 19 14  4  0  0  0  0
 17 13  1  0  0  0  0
 13 20  1  0  0  0  0
 20 21  3  0  0  0  0
 21 22  1  0  0  0  0
 24 26  1  0  0  0  0
 24 27  1  0  0  0  0
 24 28  1  0  0  0  0
 29 25  1  0  0  0  0
 23 22  1  0  0  0  0
 22 30  1  0  0  0  0
 25 22  1  0  0  0  0
 24 29  1  0  0  0  0
  1 31  1  0  0  0  0
  2 32  1  0  0  0  0
  3 33  1  0  0  0  0
  5 34  1  0  0  0  0
  6 35  1  0  0  0  0
  7 36  1  0  0  0  0
  9 37  1  0  0  0  0
 10 38  1  0  0  0  0
 11 39  1  0  0  0  0
 12 40  1  0  0  0  0
 14 41  1  0  0  0  0
 15 42  1  0  0  0  0
 16 43  1  0  0  0  0
 18 44  1  0  0  0  0
 19 45  1  0  0  0  0
 23 46  1  0  0  0  0
 23 47  1  0  0  0  0
 23 48  1  0  0  0  0
 26 49  1  0  0  0  0
 26 50  1  0  0  0  0
 26 51  1  0  0  0  0
 27 52  1  0  0  0  0
 27 53  1  0  0  0  0
 27 54  1  0  0  0  0
```

```
 28 55  1  0  0  0  0
 28 56  1  0  0  0  0
 28 57  1  0  0  0  0
 30 58  1  0  0  0  0
 30 59  1  0  0  0  0
 30 60  1  0  0  0  0
M  END
>  <Alias name>
3-Methyl-3-t-butylperoxy-1-triphenylsilyl-1-butyne

>  <deltaH°(c)>   kJ/mol
-15933

$$$$
Ethyldidecylsilane
  ChemBrain IXL

 71 70  0  0  0  0  0  0  0  0  1 V2000
    4.5563    4.9932    1.8724 C   0  0  0  0  0  0  0  0  0  0  0  0
    3.3838    4.2071    1.2499 C   0  0  0  0  0  0  0  0  0  0  0  0
    3.4848    4.2175   -0.6339 Si  0  0  0  0  0  0  0  0  0  0  0  0
    3.4977    5.9755   -1.3199 C   0  0  0  0  0  0  0  0  0  0  0  0
    4.8128    6.6990   -0.9627 C   0  0  0  0  0  0  0  0  0  0  0  0
    4.7376    8.1618   -1.4395 C   0  0  0  0  0  0  0  0  0  0  0  0
    6.0581    8.8794   -1.0983 C   0  0  0  0  0  0  0  0  0  0  0  0
    5.9913   10.3403   -1.5834 C   0  0  0  0  0  0  0  0  0  0  0  0
    7.3165   11.0524   -1.2487 C   0  0  0  0  0  0  0  0  0  0  0  0
    7.2546   12.5120   -1.7388 C   0  0  0  0  0  0  0  0  0  0  0  0
    8.5823   13.2213   -1.4077 C   0  0  0  0  0  0  0  0  0  0  0  0
    8.5225   14.6804   -1.8999 C   0  0  0  0  0  0  0  0  0  0  0  0
    9.8508   15.3887   -1.5700 C   0  0  0  0  0  0  0  0  0  0  0  0
   16.3158    3.0967   -0.9977 C   0  0  0  0  0  0  0  0  0  0  0  0
   14.9991    2.5290   -1.5623 C   0  0  0  0  0  0  0  0  0  0  0  0
   13.8054    3.2415   -0.8972 C   0  0  0  0  0  0  0  0  0  0  0  0
   12.4884    2.6760   -1.4638 C   0  0  0  0  0  0  0  0  0  0  0  0
   11.2954    3.3926   -0.8021 C   0  0  0  0  0  0  0  0  0  0  0  0
    9.9779    2.8339   -1.3741 C   0  0  0  0  0  0  0  0  0  0  0  0
    8.7865    3.5601   -0.7203 C   0  0  0  0  0  0  0  0  0  0  0  0
    7.4681    3.0139   -1.3019 C   0  0  0  0  0  0  0  0  0  0  0  0
    6.2800    3.7556   -0.6606 C   0  0  0  0  0  0  0  0  0  0  0  0
    4.9606    3.2205   -1.2560 C   0  0  0  0  0  0  0  0  0  0  0  0
    2.2620    3.5571   -1.1425 H   0  0  0  0  0  0  0  0  0  0  0  0
    4.4779    6.0313    1.5997 H   0  0  0  0  0  0  0  0  0  0  0  0
    5.4882    4.5974    1.5190 H   0  0  0  0  0  0  0  0  0  0  0  0
    4.5142    4.9038    2.9508 H   0  0  0  0  0  0  0  0  0  0  0  0
    2.4518    4.6646    1.5577 H   0  0  0  0  0  0  0  0  0  0  0  0
    3.4107    3.1832    1.6015 H   0  0  0  0  0  0  0  0  0  0  0  0
    3.4006    5.9364   -2.3977 H   0  0  0  0  0  0  0  0  0  0  0  0
    2.6608    6.5246   -0.9068 H   0  0  0  0  0  0  0  0  0  0  0  0
    4.9630    6.6783    0.0989 H   0  0  0  0  0  0  0  0  0  0  0  0
    5.6356    6.2097   -1.4535 H   0  0  0  0  0  0  0  0  0  0  0  0
    4.5808    8.1847   -2.5109 H   0  0  0  0  0  0  0  0  0  0  0  0
    3.9152    8.6622   -0.9432 H   0  0  0  0  0  0  0  0  0  0  0  0
    6.2120    8.8616   -0.0264 H   0  0  0  0  0  0  0  0  0  0  0  0
    6.8803    8.3737   -1.5893 H   0  0  0  0  0  0  0  0  0  0  0  0
    5.8326   10.3580   -2.6546 H   0  0  0  0  0  0  0  0  0  0  0  0
    5.1729   10.8484   -1.0886 H   0  0  0  0  0  0  0  0  0  0  0  0
    7.4736   11.0378   -0.1773 H   0  0  0  0  0  0  0  0  0  0  0  0
    8.1349   10.5413   -1.7405 H   0  0  0  0  0  0  0  0  0  0  0  0
    7.0949   12.5266   -2.8099 H   0  0  0  0  0  0  0  0  0  0  0  0
    6.4384   13.0245   -1.2450 H   0  0  0  0  0  0  0  0  0  0  0  0
    8.7414   13.2078   -0.3366 H   0  0  0  0  0  0  0  0  0  0  0  0
    9.3985   12.7074   -1.9004 H   0  0  0  0  0  0  0  0  0  0  0  0
```

```
    8.3626   14.6939   -2.9709 H   0  0  0  0  0  0  0  0  0  0  0  0
    7.7070   15.1947   -1.4066 H   0  0  0  0  0  0  0  0  0  0  0  0
    9.8087   16.4140   -1.9160 H   0  0  0  0  0  0  0  0  0  0  0  0
   10.6663   14.8745   -2.0634 H   0  0  0  0  0  0  0  0  0  0  0  0
   10.0108   15.3754   -0.4990 H   0  0  0  0  0  0  0  0  0  0  0  0
   16.3687    4.1587   -1.2036 H   0  0  0  0  0  0  0  0  0  0  0  0
   17.1546    2.5961   -1.4653 H   0  0  0  0  0  0  0  0  0  0  0  0
   16.3494    2.9344    0.0725 H   0  0  0  0  0  0  0  0  0  0  0  0
   14.9657    2.6913   -2.6325 H   0  0  0  0  0  0  0  0  0  0  0  0
   14.9462    1.4671   -1.3564 H   0  0  0  0  0  0  0  0  0  0  0  0
   13.8377    3.0779    0.1729 H   0  0  0  0  0  0  0  0  0  0  0  0
   13.8595    4.3036   -1.1018 H   0  0  0  0  0  0  0  0  0  0  0  0
   12.4575    2.8370   -2.5343 H   0  0  0  0  0  0  0  0  0  0  0  0
   12.4323    1.6145   -1.2566 H   0  0  0  0  0  0  0  0  0  0  0  0
   11.3233    3.2277    0.2679 H   0  0  0  0  0  0  0  0  0  0  0  0
   11.3551    4.4547   -1.0053 H   0  0  0  0  0  0  0  0  0  0  0  0
    9.9535    2.9929   -2.4451 H   0  0  0  0  0  0  0  0  0  0  0  0
    9.9138    1.7733   -1.1649 H   0  0  0  0  0  0  0  0  0  0  0  0
    8.8055    3.3945    0.3498 H   0  0  0  0  0  0  0  0  0  0  0  0
    8.8569    4.6218   -0.9224 H   0  0  0  0  0  0  0  0  0  0  0  0
    7.4546    3.1699   -2.3735 H   0  0  0  0  0  0  0  0  0  0  0  0
    7.3904    1.9547   -1.0899 H   0  0  0  0  0  0  0  0  0  0  0  0
    6.2890    3.5874    0.4025 H   0  0  0  0  0  0  0  0  0  0  0  0
    6.3687    4.8047   -0.8590 H   0  0  0  0  0  0  0  0  0  0  0  0
    4.8384    2.1847   -0.9645 H   0  0  0  0  0  0  0  0  0  0  0  0
    5.0018    3.2838   -2.3363 H   0  0  0  0  0  0  0  0  0  0  0  0
  1  2  1  0  0  0  0
  2  3  1  0  0  0  0
  3  4  1  0  0  0  0
  4  5  1  0  0  0  0
  5  6  1  0  0  0  0
  6  7  1  0  0  0  0
  7  8  1  0  0  0  0
  8  9  1  0  0  0  0
  9 10  1  0  0  0  0
 10 11  1  0  0  0  0
 11 12  1  0  0  0  0
 12 13  1  0  0  0  0
 14 15  1  0  0  0  0
 15 16  1  0  0  0  0
 16 17  1  0  0  0  0
 17 18  1  0  0  0  0
 18 19  1  0  0  0  0
 19 20  1  0  0  0  0
 20 21  1  0  0  0  0
 21 22  1  0  0  0  0
 22 23  1  0  0  0  0
 23  3  1  0  0  0  0
  3 24  1  0  0  0  0
  1 25  1  0  0  0  0
  1 26  1  0  0  0  0
  1 27  1  0  0  0  0
  2 28  1  0  0  0  0
  2 29  1  0  0  0  0
  4 30  1  0  0  0  0
  4 31  1  0  0  0  0
  5 32  1  0  0  0  0
  5 33  1  0  0  0  0
  6 34  1  0  0  0  0
  6 35  1  0  0  0  0
  7 36  1  0  0  0  0
  7 37  1  0  0  0  0
  8 38  1  0  0  0  0
```

```
  8 39  1  0  0  0  0
  9 40  1  0  0  0  0
  9 41  1  0  0  0  0
 10 42  1  0  0  0  0
 10 43  1  0  0  0  0
 11 44  1  0  0  0  0
 11 45  1  0  0  0  0
 12 46  1  0  0  0  0
 12 47  1  0  0  0  0
 13 48  1  0  0  0  0
 13 49  1  0  0  0  0
 13 50  1  0  0  0  0
 14 51  1  0  0  0  0
 14 52  1  0  0  0  0
 14 53  1  0  0  0  0
 15 54  1  0  0  0  0
 15 55  1  0  0  0  0
 16 56  1  0  0  0  0
 16 57  1  0  0  0  0
 17 58  1  0  0  0  0
 17 59  1  0  0  0  0
 18 60  1  0  0  0  0
 18 61  1  0  0  0  0
 19 62  1  0  0  0  0
 19 63  1  0  0  0  0
 20 64  1  0  0  0  0
 20 65  1  0  0  0  0
 21 66  1  0  0  0  0
 21 67  1  0  0  0  0
 22 68  1  0  0  0  0
 22 69  1  0  0  0  0
 23 70  1  0  0  0  0
 23 71  1  0  0  0  0
M  END
>  <Alias name>
Ethyldidecylsilane

>  <deltaH°(c)>   kJ/mol
-15853

$$$$
2,3-Dimethyl-2,3-bis(4-tert-butylphenyl)butane
  ChemBrain IXL

 64 65  0  0  0  0  0  0  0  0  1 V2000
    6.3816    5.5323   -1.1745 C   0  0  0  0  0  0  0  0  0  0  0  0
    5.1723    5.8084   -0.5360 C   0  0  0  0  0  0  0  0  0  0  0  0
    7.0131    4.3049   -0.9721 C   0  0  0  0  0  0  0  0  0  0  0  0
    4.5932    4.8563    0.3033 C   0  0  0  0  0  0  0  0  0  0  0  0
    5.2227    3.6274    0.5026 C   0  0  0  0  0  0  0  0  0  0  0  0
    3.3109    5.1500    0.9821 C   0  0  0  0  0  0  0  0  0  0  0  0
    6.4319    3.3512   -0.1359 C   0  0  0  0  0  0  0  0  0  0  0  0
    8.2994    4.0142   -1.6449 C   0  0  0  0  0  0  0  0  0  0  0  0
    3.5893    5.6385    2.4169 C   0  0  0  0  0  0  0  0  0  0  0  0
    2.5567    6.2455    0.2038 C   0  0  0  0  0  0  0  0  0  0  0  0
    2.4516    3.8717    1.0324 C   0  0  0  0  0  0  0  0  0  0  0  0
    8.3549    2.5212   -2.0226 C   0  0  0  0  0  0  0  0  0  0  0  0
    9.4661    4.3467   -0.6926 C   0  0  0  0  0  0  0  0  0  0  0  0
    8.4170    4.8711   -2.9202 C   0  0  0  0  0  0  0  0  0  0  0  0
    9.3743    3.4573    0.5625 C   0  0  0  0  0  0  0  0  0  0  0  0
    9.3899    5.7707   -0.2948 C   0  0  0  0  0  0  0  0  0  0  0  0
   10.8045    4.0854   -1.4102 C   0  0  0  0  0  0  0  0  0  0  0  0
    8.4675    6.1775    0.6696 C   0  0  0  0  0  0  0  0  0  0  0  0
```

```
 10.2410    6.7061   -0.8839 C   0  0  0  0  0  0  0  0  0  0  0  0
  8.3926    7.5202    1.0410 C   0  0  0  0  0  0  0  0  0  0  0  0
  9.2416    8.4560    0.4497 C   0  0  0  0  0  0  0  0  0  0  0  0
 10.1662    8.0488   -0.5124 C   0  0  0  0  0  0  0  0  0  0  0  0
  9.1614    9.8809    0.8430 C   0  0  0  0  0  0  0  0  0  0  0  0
  7.9036   10.5161    0.2191 C   0  0  0  0  0  0  0  0  0  0  0  0
  9.0824    9.9864    2.3783 C   0  0  0  0  0  0  0  0  0  0  0  0
 10.4148   10.6238    0.3410 C   0  0  0  0  0  0  0  0  0  0  0  0
  6.8301    6.2708   -1.8274 H   0  0  0  0  0  0  0  0  0  0  0  0
  4.6831    6.7620   -0.6918 H   0  0  0  0  0  0  0  0  0  0  0  0
  4.7726    2.8877    1.1532 H   0  0  0  0  0  0  0  0  0  0  0  0
  6.9195    2.3965    0.0175 H   0  0  0  0  0  0  0  0  0  0  0  0
  2.6511    5.8528    2.9137 H   0  0  0  0  0  0  0  0  0  0  0  0
  4.1926    6.5372    2.3816 H   0  0  0  0  0  0  0  0  0  0  0  0
  4.1198    4.8688    2.9638 H   0  0  0  0  0  0  0  0  0  0  0  0
  1.6042    6.4375    0.6823 H   0  0  0  0  0  0  0  0  0  0  0  0
  2.3893    5.9151   -0.8139 H   0  0  0  0  0  0  0  0  0  0  0  0
  3.1464    7.1538    0.1961 H   0  0  0  0  0  0  0  0  0  0  0  0
  2.9728    3.1084    1.5969 H   0  0  0  0  0  0  0  0  0  0  0  0
  1.5052    4.0916    1.5109 H   0  0  0  0  0  0  0  0  0  0  0  0
  2.2730    3.5169    0.0249 H   0  0  0  0  0  0  0  0  0  0  0  0
  9.2876    2.3121   -2.5318 H   0  0  0  0  0  0  0  0  0  0  0  0
  8.2911    1.9191   -1.1246 H   0  0  0  0  0  0  0  0  0  0  0  0
  7.5252    2.2832   -2.6766 H   0  0  0  0  0  0  0  0  0  0  0  0
  7.5706    4.6743   -3.5666 H   0  0  0  0  0  0  0  0  0  0  0  0
  9.3338    4.6212   -3.4397 H   0  0  0  0  0  0  0  0  0  0  0  0
  8.4291    5.9199   -2.6506 H   0  0  0  0  0  0  0  0  0  0  0  0
 10.1877    3.6980    1.2357 H   0  0  0  0  0  0  0  0  0  0  0  0
  8.4291    3.6330    1.0611 H   0  0  0  0  0  0  0  0  0  0  0  0
  9.4415    2.4160    0.2727 H   0  0  0  0  0  0  0  0  0  0  0  0
 10.8570    4.6894   -2.3075 H   0  0  0  0  0  0  0  0  0  0  0  0
 10.8732    3.0377   -1.6757 H   0  0  0  0  0  0  0  0  0  0  0  0
 11.6240    4.3453   -0.7516 H   0  0  0  0  0  0  0  0  0  0  0  0
  7.8095    5.4509    1.1300 H   0  0  0  0  0  0  0  0  0  0  0  0
 10.9598    6.3899   -1.6297 H   0  0  0  0  0  0  0  0  0  0  0  0
  7.6753    7.8361    1.7884 H   0  0  0  0  0  0  0  0  0  0  0  0
 10.8260    8.7750   -0.9708 H   0  0  0  0  0  0  0  0  0  0  0  0
  7.0231    9.9907    0.5676 H   0  0  0  0  0  0  0  0  0  0  0  0
  7.8421   11.5571    0.5113 H   0  0  0  0  0  0  0  0  0  0  0  0
  7.9617   10.4469   -0.8601 H   0  0  0  0  0  0  0  0  0  0  0  0
  8.1979    9.4703    2.7306 H   0  0  0  0  0  0  0  0  0  0  0  0
  9.9631    9.5341    2.8171 H   0  0  0  0  0  0  0  0  0  0  0  0
  9.0305   11.0292    2.6663 H   0  0  0  0  0  0  0  0  0  0  0  0
 10.3692   11.6600    0.6526 H   0  0  0  0  0  0  0  0  0  0  0  0
 10.4556   10.5734   -0.7401 H   0  0  0  0  0  0  0  0  0  0  0  0
 11.3004   10.1601    0.7576 H   0  0  0  0  0  0  0  0  0  0  0  0
  1  2  4  0  0  0  0
  3  1  4  0  0  0  0
  2  4  4  0  0  0  0
  7  3  4  0  0  0  0
  3  8  1  0  0  0  0
  4  5  4  0  0  0  0
  4  6  1  0  0  0  0
  5  7  4  0  0  0  0
  6  9  1  0  0  0  0
  6 10  1  0  0  0  0
  6 11  1  0  0  0  0
  8 12  1  0  0  0  0
  8 13  1  0  0  0  0
  8 14  1  0  0  0  0
 13 15  1  0  0  0  0
 13 16  1  0  0  0  0
 13 17  1  0  0  0  0
```

```
 16 18  4  0  0  0  0
 19 16  4  0  0  0  0
 18 20  4  0  0  0  0
 22 19  4  0  0  0  0
 20 21  4  0  0  0  0
 21 22  4  0  0  0  0
 21 23  1  0  0  0  0
 23 24  1  0  0  0  0
 23 25  1  0  0  0  0
 23 26  1  0  0  0  0
  1 27  1  0  0  0  0
  2 28  1  0  0  0  0
  5 29  1  0  0  0  0
  7 30  1  0  0  0  0
  9 31  1  0  0  0  0
  9 32  1  0  0  0  0
  9 33  1  0  0  0  0
 10 34  1  0  0  0  0
 10 35  1  0  0  0  0
 10 36  1  0  0  0  0
 11 37  1  0  0  0  0
 11 38  1  0  0  0  0
 11 39  1  0  0  0  0
 12 40  1  0  0  0  0
 12 41  1  0  0  0  0
 12 42  1  0  0  0  0
 14 43  1  0  0  0  0
 14 44  1  0  0  0  0
 14 45  1  0  0  0  0
 15 46  1  0  0  0  0
 15 47  1  0  0  0  0
 15 48  1  0  0  0  0
 17 49  1  0  0  0  0
 17 50  1  0  0  0  0
 17 51  1  0  0  0  0
 18 52  1  0  0  0  0
 19 53  1  0  0  0  0
 20 54  1  0  0  0  0
 22 55  1  0  0  0  0
 24 56  1  0  0  0  0
 24 57  1  0  0  0  0
 24 58  1  0  0  0  0
 25 59  1  0  0  0  0
 25 60  1  0  0  0  0
 25 61  1  0  0  0  0
 26 62  1  0  0  0  0
 26 63  1  0  0  0  0
 26 64  1  0  0  0  0
M  END
>  <Alias name>
2,3-Dimethyl-2,3-bis(4-t-butylphenyl)butane

>  <deltaH°(c)>   kJ/mol
-15360

$$$$
1,2,3,4-Tetraphenylbenzene
  ChemBrain IXL

 52 56  0  0  0  0  0  0  0  0  1 V2000
    8.6598   10.4967    1.2596 C   0  0  0  0  0  0  0  0  0  0  0  0
    9.5297    9.5136    0.7878 C   0  0  0  0  0  0  0  0  0  0  0  0
    9.0230    8.3855    0.1416 C   0  0  0  0  0  0  0  0  0  0  0  0
```

```
    7.6490    8.2543   -0.0572 C   0  0  0  0  0  0  0  0  0  0  0  0
    6.7782    9.2331    0.4227 C   0  0  0  0  0  0  0  0  0  0  0  0
    7.2840   10.3566    1.0769 C   0  0  0  0  0  0  0  0  0  0  0  0
    4.5318   10.1935   -0.0590 C   0  0  0  0  0  0  0  0  0  0  0  0
    5.3178    9.0799    0.2380 C   0  0  0  0  0  0  0  0  0  0  0  0
    4.7272    7.8221    0.3609 C   0  0  0  0  0  0  0  0  0  0  0  0
    3.3500    7.6787    0.1918 C   0  0  0  0  0  0  0  0  0  0  0  0
    2.5636    8.7927   -0.1024 C   0  0  0  0  0  0  0  0  0  0  0  0
    3.1545   10.0501   -0.2279 C   0  0  0  0  0  0  0  0  0  0  0  0
   13.7424    9.9656    1.3184 C   0  0  0  0  0  0  0  0  0  0  0  0
   13.2428    9.4225    0.1345 C   0  0  0  0  0  0  0  0  0  0  0  0
   11.8667    9.2724   -0.0378 C   0  0  0  0  0  0  0  0  0  0  0  0
   10.9900    9.6676    0.9728 C   0  0  0  0  0  0  0  0  0  0  0  0
   11.4897   10.2080    2.1578 C   0  0  0  0  0  0  0  0  0  0  0  0
   12.8658   10.3582    2.3301 C   0  0  0  0  0  0  0  0  0  0  0  0
   11.6783    5.3354   -1.2293 C   0  0  0  0  0  0  0  0  0  0  0  0
   10.6651    5.8055   -2.0650 C   0  0  0  0  0  0  0  0  0  0  0  0
    9.7972    6.8014   -1.6168 C   0  0  0  0  0  0  0  0  0  0  0  0
    9.9429    7.3278   -0.3332 C   0  0  0  0  0  0  0  0  0  0  0  0
   10.9554    6.8569    0.5030 C   0  0  0  0  0  0  0  0  0  0  0  0
   11.8235    5.8611    0.0547 C   0  0  0  0  0  0  0  0  0  0  0  0
    6.1089    4.8556   -2.1333 C   0  0  0  0  0  0  0  0  0  0  0  0
    5.6538    6.1345   -2.4548 C   0  0  0  0  0  0  0  0  0  0  0  0
    6.1574    7.2450   -1.7770 C   0  0  0  0  0  0  0  0  0  0  0  0
    7.1156    7.0764   -0.7773 C   0  0  0  0  0  0  0  0  0  0  0  0
    7.5712    5.7976   -0.4563 C   0  0  0  0  0  0  0  0  0  0  0  0
    7.0676    4.6872   -1.1341 C   0  0  0  0  0  0  0  0  0  0  0  0
    9.0525   11.3687    1.7678 H   0  0  0  0  0  0  0  0  0  0  0  0
    6.6086   11.1199    1.4429 H   0  0  0  0  0  0  0  0  0  0  0  0
    4.9908   11.1694   -0.1584 H   0  0  0  0  0  0  0  0  0  0  0  0
    5.3380    6.9570    0.5876 H   0  0  0  0  0  0  0  0  0  0  0  0
    2.8912    6.7024    0.2890 H   0  0  0  0  0  0  0  0  0  0  0  0
    1.4943    8.6814   -0.2333 H   0  0  0  0  0  0  0  0  0  0  0  0
    2.5440   10.9149   -0.4566 H   0  0  0  0  0  0  0  0  0  0  0  0
   14.8107   10.0824    1.4520 H   0  0  0  0  0  0  0  0  0  0  0  0
   13.9234    9.1173   -0.6507 H   0  0  0  0  0  0  0  0  0  0  0  0
   11.4789    8.8492   -0.9562 H   0  0  0  0  0  0  0  0  0  0  0  0
   10.8093   10.5110    2.9440 H   0  0  0  0  0  0  0  0  0  0  0  0
   13.2537   10.7796    3.2493 H   0  0  0  0  0  0  0  0  0  0  0  0
   12.3523    4.5624   -1.5773 H   0  0  0  0  0  0  0  0  0  0  0  0
   10.5523    5.3973   -3.0618 H   0  0  0  0  0  0  0  0  0  0  0  0
    9.0102    7.1660   -2.2654 H   0  0  0  0  0  0  0  0  0  0  0  0
   11.0676    7.2644    1.5001 H   0  0  0  0  0  0  0  0  0  0  0  0
   12.6100    5.4960    0.7035 H   0  0  0  0  0  0  0  0  0  0  0  0
    5.7178    3.9936   -2.6594 H   0  0  0  0  0  0  0  0  0  0  0  0
    4.9095    6.2653   -3.2306 H   0  0  0  0  0  0  0  0  0  0  0  0
    5.8045    8.2379   -2.0270 H   0  0  0  0  0  0  0  0  0  0  0  0
    8.3159    5.6669    0.3191 H   0  0  0  0  0  0  0  0  0  0  0  0
    7.4210    3.6943   -0.8845 H   0  0  0  0  0  0  0  0  0  0  0  0
  1  2  4  0  0  0  0
  2  3  4  0  0  0  0
  3  4  4  0  0  0  0
  4  5  4  0  0  0  0
  5  6  4  0  0  0  0
  6  1  4  0  0  0  0
  7  8  4  0  0  0  0
  8  9  4  0  0  0  0
  9 10  4  0  0  0  0
 10 11  4  0  0  0  0
 11 12  4  0  0  0  0
 12  7  4  0  0  0  0
 13 14  4  0  0  0  0
 14 15  4  0  0  0  0
```

15 16 4 0 0 0 0
16 17 4 0 0 0 0
17 18 4 0 0 0 0
18 13 4 0 0 0 0
19 20 4 0 0 0 0
20 21 4 0 0 0 0
21 22 4 0 0 0 0
22 23 4 0 0 0 0
23 24 4 0 0 0 0
24 19 4 0 0 0 0
22 3 1 0 0 0 0
8 5 1 0 0 0 0
16 2 1 0 0 0 0
25 26 4 0 0 0 0
26 27 4 0 0 0 0
27 28 4 0 0 0 0
28 29 4 0 0 0 0
29 30 4 0 0 0 0
30 25 4 0 0 0 0
28 4 1 0 0 0 0
1 31 1 0 0 0 0
6 32 1 0 0 0 0
7 33 1 0 0 0 0
9 34 1 0 0 0 0
10 35 1 0 0 0 0
11 36 1 0 0 0 0
12 37 1 0 0 0 0
13 38 1 0 0 0 0
14 39 1 0 0 0 0
15 40 1 0 0 0 0
17 41 1 0 0 0 0
18 42 1 0 0 0 0
19 43 1 0 0 0 0
20 44 1 0 0 0 0
21 45 1 0 0 0 0
23 46 1 0 0 0 0
24 47 1 0 0 0 0
25 48 1 0 0 0 0
26 49 1 0 0 0 0
27 50 1 0 0 0 0
29 51 1 0 0 0 0
30 52 1 0 0 0 0
M END
> <Alias name>
1,2,3,4-Tetraphenylbenzene

> <deltaH°(c)> kJ/mol
-15280

$$$$
1,2,4,5-Tetraphenylbenzene
ChemBrain IXL

52 56 0 0 0 0 0 0 0 0 1 V2000
7.4658 8.7895 0.0000 C 0 0 0 0 0 0 0 0 0 0 0 0
8.8526 8.8064 -0.0105 C 0 0 0 0 0 0 0 0 0 0 0 0
9.5734 7.5952 0.0055 C 0 0 0 0 0 0 0 0 0 0 0 0
8.8970 6.3844 -0.0045 C 0 0 0 0 0 0 0 0 0 0 0 0
7.5101 6.3674 -0.0124 C 0 0 0 0 0 0 0 0 0 0 0 0
6.7894 7.5787 0.0082 C 0 0 0 0 0 0 0 0 0 0 0 0
5.6577 4.9018 -0.8058 C 0 0 0 0 0 0 0 0 0 0 0 0
6.8126 5.0633 -0.0414 C 0 0 0 0 0 0 0 0 0 0 0 0
7.3188 3.9912 0.6935 C 0 0 0 0 0 0 0 0 0 0 0 0

```
    6.6690    2.7578    0.6655 C   0  0  0  0  0  0  0  0  0  0  0  0
    5.5131    2.5962   -0.0979 C   0  0  0  0  0  0  0  0  0  0  0  0
    5.0075    3.6681   -0.8337 C   0  0  0  0  0  0  0  0  0  0  0  0
   10.8495   12.5779   -0.0900 C   0  0  0  0  0  0  0  0  0  0  0  0
   11.3517   11.5089   -0.8323 C   0  0  0  0  0  0  0  0  0  0  0  0
   10.7016   10.2751   -0.8064 C   0  0  0  0  0  0  0  0  0  0  0  0
    9.5501   10.1106   -0.0374 C   0  0  0  0  0  0  0  0  0  0  0  0
    9.0473   11.1799    0.7040 C   0  0  0  0  0  0  0  0  0  0  0  0
    9.6971   12.4133    0.6780 C   0  0  0  0  0  0  0  0  0  0  0  0
   13.8407    7.5512    0.0835 C   0  0  0  0  0  0  0  0  0  0  0  0
   13.1459    6.6125   -0.6788 C   0  0  0  0  0  0  0  0  0  0  0  0
   11.7518    6.6298   -0.7044 C   0  0  0  0  0  0  0  0  0  0  0  0
   11.0523    7.5861    0.0319 C   0  0  0  0  0  0  0  0  0  0  0  0
   11.7466    8.5239    0.7953 C   0  0  0  0  0  0  0  0  0  0  0  0
   13.1411    8.5068    0.8207 C   0  0  0  0  0  0  0  0  0  0  0  0
    2.5223    7.6225    0.0948 C   0  0  0  0  0  0  0  0  0  0  0  0
    3.2155    8.5621   -0.6679 C   0  0  0  0  0  0  0  0  0  0  0  0
    4.6095    8.5448   -0.6962 C   0  0  0  0  0  0  0  0  0  0  0  0
    5.3105    7.5877    0.0375 C   0  0  0  0  0  0  0  0  0  0  0  0
    4.6177    6.6490    0.8012 C   0  0  0  0  0  0  0  0  0  0  0  0
    3.2233    6.6661    0.8294 C   0  0  0  0  0  0  0  0  0  0  0  0
    6.9120    9.7202    0.0018 H   0  0  0  0  0  0  0  0  0  0  0  0
    9.4508    5.4537   -0.0062 H   0  0  0  0  0  0  0  0  0  0  0  0
    5.2659    5.7339   -1.3773 H   0  0  0  0  0  0  0  0  0  0  0  0
    8.2168    4.1168    1.2860 H   0  0  0  0  0  0  0  0  0  0  0  0
    7.0618    1.9257    1.2365 H   0  0  0  0  0  0  0  0  0  0  0  0
    5.0081    1.6385   -0.1196 H   0  0  0  0  0  0  0  0  0  0  0  0
    4.1101    3.5424   -1.4265 H   0  0  0  0  0  0  0  0  0  0  0  0
   11.3545   13.5356   -0.1101 H   0  0  0  0  0  0  0  0  0  0  0  0
   12.2464   11.6368   -1.4286 H   0  0  0  0  0  0  0  0  0  0  0  0
   11.0908    9.4453   -1.3830 H   0  0  0  0  0  0  0  0  0  0  0  0
    8.1520   11.0519    1.3000 H   0  0  0  0  0  0  0  0  0  0  0  0
    9.3070   13.2432    1.2540 H   0  0  0  0  0  0  0  0  0  0  0  0
   14.9233    7.5382    0.1033 H   0  0  0  0  0  0  0  0  0  0  0  0
   13.6888    5.8705   -1.2509 H   0  0  0  0  0  0  0  0  0  0  0  0
   11.2120    5.9007   -1.2961 H   0  0  0  0  0  0  0  0  0  0  0  0
   11.2032    9.2651    1.3679 H   0  0  0  0  0  0  0  0  0  0  0  0
   13.6806    9.2354    1.4127 H   0  0  0  0  0  0  0  0  0  0  0  0
    1.4397    7.6355    0.1166 H   0  0  0  0  0  0  0  0  0  0  0  0
    2.6714    9.3048   -1.2380 H   0  0  0  0  0  0  0  0  0  0  0  0
    5.1482    9.2747   -1.2881 H   0  0  0  0  0  0  0  0  0  0  0  0
    5.1623    5.9072    1.3719 H   0  0  0  0  0  0  0  0  0  0  0  0
    2.6849    5.9367    1.4216 H   0  0  0  0  0  0  0  0  0  0  0  0
  1  2  4  0  0  0  0
  2  3  4  0  0  0  0
  3  4  4  0  0  0  0
  4  5  4  0  0  0  0
  5  6  4  0  0  0  0
  6  1  4  0  0  0  0
  7  8  4  0  0  0  0
  8  9  4  0  0  0  0
  9 10  4  0  0  0  0
 10 11  4  0  0  0  0
 11 12  4  0  0  0  0
 12  7  4  0  0  0  0
 13 14  4  0  0  0  0
 14 15  4  0  0  0  0
 15 16  4  0  0  0  0
 16 17  4  0  0  0  0
 17 18  4  0  0  0  0
 18 13  4  0  0  0  0
 19 20  4  0  0  0  0
 20 21  4  0  0  0  0
```

21 22 4 0 0 0 0
22 23 4 0 0 0 0
23 24 4 0 0 0 0
24 19 4 0 0 0 0
22 3 1 0 0 0 0
8 5 1 0 0 0 0
16 2 1 0 0 0 0
25 26 4 0 0 0 0
26 27 4 0 0 0 0
27 28 4 0 0 0 0
28 29 4 0 0 0 0
29 30 4 0 0 0 0
30 25 4 0 0 0 0
28 6 1 0 0 0 0
1 31 1 0 0 0 0
4 32 1 0 0 0 0
7 33 1 0 0 0 0
9 34 1 0 0 0 0
10 35 1 0 0 0 0
11 36 1 0 0 0 0
12 37 1 0 0 0 0
13 38 1 0 0 0 0
14 39 1 0 0 0 0
15 40 1 0 0 0 0
17 41 1 0 0 0 0
18 42 1 0 0 0 0
19 43 1 0 0 0 0
20 44 1 0 0 0 0
21 45 1 0 0 0 0
23 46 1 0 0 0 0
24 47 1 0 0 0 0
25 48 1 0 0 0 0
26 49 1 0 0 0 0
27 50 1 0 0 0 0
29 51 1 0 0 0 0
30 52 1 0 0 0 0
M END
> <Alias name>
1,2,4,5-Tetraphenylbenzene

> <deltaH°(c)>   kJ/mol
-15261

$$$$
Bis(N-(N',N'-diethylaminothiocarbonyl)benzamidinato)nickel(II)
ChemBrain IXL

65 68 0 0 0 0 0 0 0 0 1 V2000
15.2285   11.1429   0.1972 C   0 0 0 0 0 0 0 0 0 0 0 0
15.3706   9.8600   -0.3316 C   0 0 0 0 0 0 0 0 0 0 0 0
14.3522   8.9221   -0.1667 C   0 0 0 0 0 0 0 0 0 0 0 0
13.1938   9.2686   0.5270 C   0 0 0 0 0 0 0 0 0 0 0 0
13.0893   10.5213   1.1278 C   0 0 0 0 0 0 0 0 0 0 0 0
14.0853   11.4748   0.9239 C   0 0 0 0 0 0 0 0 0 0 0 0
12.0763   8.3088   0.6261 C   0 0 0 0 0 0 0 0 0 0 0 0
10.8696   8.6738   1.4025 N   0 5 0 0 0 0 0 0 0 0 0 0
12.1630   7.1724   0.0421 N   0 0 0 0 0 0 0 0 0 0 0 0
11.3897   6.0788   0.4831 C   0 0 0 0 0 0 0 0 0 0 0 0
12.0124   4.9051   0.9395 N   0 0 0 0 0 0 0 0 0 0 0 0
9.6864   6.1803   0.4631 S   0 0 0 0 0 0 0 0 0 0 0 0
11.3556   4.0753   1.9609 C   0 0 0 0 0 0 0 0 0 0 0 0
12.2496   2.8632   2.2911 C   0 0 0 0 0 0 0 0 0 0 0 0
9.1180   8.3989   0.6589 Ni 0 0 0 0 0 0 0 0 0 0 0 0

3.4013	6.8118	-2.9173	C	0	0	0	0	0	0	0	0	0	0	0	0
3.8192	8.0720	-3.3425	C	0	0	0	0	0	0	0	0	0	0	0	0
4.9274	8.6727	-2.7488	C	0	0	0	0	0	0	0	0	0	0	0	0
5.5586	8.0522	-1.6734	C	0	0	0	0	0	0	0	0	0	0	0	0
5.2112	6.7497	-1.3220	C	0	0	0	0	0	0	0	0	0	0	0	0
4.1309	6.1279	-1.9456	C	0	0	0	0	0	0	0	0	0	0	0	0
6.5949	8.7759	-0.9072	C	0	0	0	0	0	0	0	0	0	0	0	0
7.2422	8.1089	0.2667	N	0	5	0	0	0	0	0	0	0	0	0	0
6.9191	9.9685	-1.2648	N	0	0	0	0	0	0	0	0	0	0	0	0
7.3702	10.9131	-0.3158	C	0	0	0	0	0	0	0	0	0	0	0	0
6.6467	12.0786	-0.0500	N	0	0	0	0	0	0	0	0	0	0	0	0
8.8020	10.6427	0.5137	S	0	0	0	0	0	0	0	0	0	0	0	0
6.7513	12.7109	1.2741	C	0	0	0	0	0	0	0	0	0	0	0	0
5.8515	13.9581	1.3238	C	0	0	0	0	0	0	0	0	0	0	0	0
5.7990	12.6789	-1.0888	C	0	0	0	0	0	0	0	0	0	0	0	0
5.9148	11.8571	-2.3863	C	0	0	0	0	0	0	0	0	0	0	0	0
13.3188	4.4995	0.3964	C	0	0	0	0	0	0	0	0	0	0	0	0
13.7607	5.4985	-0.6892	C	0	0	0	0	0	0	0	0	0	0	0	0
10.9632	8.9484	2.3690	H	0	0	0	0	0	0	0	0	0	0	0	0
6.6709	7.6000	0.9249	H	0	0	0	0	0	0	0	0	0	0	0	0
16.0054	11.8808	0.0437	H	0	0	0	0	0	0	0	0	0	0	0	0
16.2711	9.5925	-0.8700	H	0	0	0	0	0	0	0	0	0	0	0	0
14.4608	7.9255	-0.5770	H	0	0	0	0	0	0	0	0	0	0	0	0
12.2352	10.7535	1.7521	H	0	0	0	0	0	0	0	0	0	0	0	0
13.9712	12.4721	1.3295	H	0	0	0	0	0	0	0	0	0	0	0	0
10.4012	3.7280	1.5850	H	0	0	0	0	0	0	0	0	0	0	0	0
11.1986	4.6627	2.8570	H	0	0	0	0	0	0	0	0	0	0	0	0
13.2311	3.2094	2.5907	H	0	0	0	0	0	0	0	0	0	0	0	0
12.3410	2.2326	1.4153	H	0	0	0	0	0	0	0	0	0	0	0	0
11.8047	2.2959	3.0994	H	0	0	0	0	0	0	0	0	0	0	0	0
2.5112	6.3646	-3.3412	H	0	0	0	0	0	0	0	0	0	0	0	0
3.2837	8.5835	-4.1325	H	0	0	0	0	0	0	0	0	0	0	0	0
5.2974	9.6198	-3.1225	H	0	0	0	0	0	0	0	0	0	0	0	0
5.7795	6.2217	-0.5660	H	0	0	0	0	0	0	0	0	0	0	0	0
3.8588	5.1153	-1.6757	H	0	0	0	0	0	0	0	0	0	0	0	0
6.4356	12.0077	2.0352	H	0	0	0	0	0	0	0	0	0	0	0	0
7.7794	13.0003	1.4552	H	0	0	0	0	0	0	0	0	0	0	0	0
4.8299	13.6748	1.1048	H	0	0	0	0	0	0	0	0	0	0	0	0
5.9002	14.3984	2.3123	H	0	0	0	0	0	0	0	0	0	0	0	0
6.1921	14.6782	0.5907	H	0	0	0	0	0	0	0	0	0	0	0	0
6.1224	13.6955	-1.2753	H	0	0	0	0	0	0	0	0	0	0	0	0
4.7687	12.6835	-0.7552	H	0	0	0	0	0	0	0	0	0	0	0	0
6.9086	11.4327	-2.4574	H	0	0	0	0	0	0	0	0	0	0	0	0
5.1817	11.0602	-2.3754	H	0	0	0	0	0	0	0	0	0	0	0	0
5.7355	12.5010	-3.2384	H	0	0	0	0	0	0	0	0	0	0	0	0
14.0520	4.4856	1.1936	H	0	0	0	0	0	0	0	0	0	0	0	0
13.2378	3.5096	-0.0359	H	0	0	0	0	0	0	0	0	0	0	0	0
12.8870	5.9117	-1.1769	H	0	0	0	0	0	0	0	0	0	0	0	0
14.3750	4.9877	-1.4208	H	0	0	0	0	0	0	0	0	0	0	0	0
14.3318	6.2976	-0.2333	H	0	0	0	0	0	0	0	0	0	0	0	0

1	2	4	0	0	0	0
2	3	4	0	0	0	0
3	4	4	0	0	0	0
4	5	4	0	0	0	0
5	6	4	0	0	0	0
6	1	4	0	0	0	0
4	7	1	0	0	0	0
7	8	1	0	0	0	0
7	9	2	0	0	0	0
9	10	1	0	0	0	0
10	11	1	0	0	0	0
10	12	2	0	0	0	0
11	13	1	0	0	0	0

```
 13 14  1  0  0  0  0
 15 12  1  0  0  0  0
 15  8  1  0  0  0  0
 16 17  4  0  0  0  0
 17 18  4  0  0  0  0
 18 19  4  0  0  0  0
 19 20  4  0  0  0  0
 20 21  4  0  0  0  0
 21 16  4  0  0  0  0
 19 22  1  0  0  0  0
 22 23  1  0  0  0  0
 22 24  2  0  0  0  0
 24 25  1  0  0  0  0
 25 26  1  0  0  0  0
 25 27  2  0  0  0  0
 26 28  1  0  0  0  0
 28 29  1  0  0  0  0
 27 15  1  0  0  0  0
 23 15  1  0  0  0  0
 26 30  1  0  0  0  0
 30 31  1  0  0  0  0
 11 32  1  0  0  0  0
 32 33  1  0  0  0  0
  8 34  1  0  0  0  0
 23 35  1  0  0  0  0
  1 36  1  0  0  0  0
  2 37  1  0  0  0  0
  3 38  1  0  0  0  0
  5 39  1  0  0  0  0
  6 40  1  0  0  0  0
 13 41  1  0  0  0  0
 13 42  1  0  0  0  0
 14 43  1  0  0  0  0
 14 44  1  0  0  0  0
 14 45  1  0  0  0  0
 16 46  1  0  0  0  0
 17 47  1  0  0  0  0
 18 48  1  0  0  0  0
 20 49  1  0  0  0  0
 21 50  1  0  0  0  0
 28 51  1  0  0  0  0
 28 52  1  0  0  0  0
 29 53  1  0  0  0  0
 29 54  1  0  0  0  0
 29 55  1  0  0  0  0
 30 56  1  0  0  0  0
 30 57  1  0  0  0  0
 31 58  1  0  0  0  0
 31 59  1  0  0  0  0
 31 60  1  0  0  0  0
 32 61  1  0  0  0  0
 32 62  1  0  0  0  0
 33 63  1  0  0  0  0
 33 64  1  0  0  0  0
 33 65  1  0  0  0  0
M  END
>  <Alias name>
Bis(N-(N',N'-diethylaminothiocarbonyl)benzamidinato)nickel(II)

>  <deltaH°(c)>   kJ/mol
-15238.2

$$$$
```

Triheptylsilane
ChemBrain IXL

68 67 0 0 0 0 0 0 0 0 1 V2000
7.9515 2.9947 2.4784 Si 0 0 0 0 0 0 0 0 0 0 0 0
6.3853 2.9156 1.4295 C 0 0 0 0 0 0 0 0 0 0 0 0
6.3612 4.0481 0.3817 C 0 0 0 0 0 0 0 0 0 0 0 0
5.0177 4.0165 -0.3717 C 0 0 0 0 0 0 0 0 0 0 0 0
4.9915 5.1437 -1.4221 C 0 0 0 0 0 0 0 0 0 0 0 0
3.6476 5.1128 -2.1758 C 0 0 0 0 0 0 0 0 0 0 0 0
3.6215 6.2401 -3.2264 C 0 0 0 0 0 0 0 0 0 0 0 0
2.2777 6.2094 -3.9800 C 0 0 0 0 0 0 0 0 0 0 0 0
9.5070 2.9004 1.4148 C 0 0 0 0 0 0 0 0 0 0 0 0
9.6687 4.1708 0.5542 C 0 0 0 0 0 0 0 0 0 0 0 0
10.8822 4.0022 -0.3800 C 0 0 0 0 0 0 0 0 0 0 0 0
11.0494 5.2708 -1.2387 C 0 0 0 0 0 0 0 0 0 0 0 0
12.2629 5.1023 -2.1737 C 0 0 0 0 0 0 0 0 0 0 0 0
12.4301 6.3713 -3.0323 C 0 0 0 0 0 0 0 0 0 0 0 0
13.6433 6.2028 -3.9675 C 0 0 0 0 0 0 0 0 0 0 0 0
7.9642 4.5142 3.5968 C 0 0 0 0 0 0 0 0 0 0 0 0
7.8318 5.8115 2.7720 C 0 0 0 0 0 0 0 0 0 0 0 0
7.9629 7.0269 3.7097 C 0 0 0 0 0 0 0 0 0 0 0 0
7.8270 8.3248 2.8901 C 0 0 0 0 0 0 0 0 0 0 0 0
7.9584 9.5408 3.8277 C 0 0 0 0 0 0 0 0 0 0 0 0
7.8223 10.8388 3.0080 C 0 0 0 0 0 0 0 0 0 0 0 0
7.9538 12.0548 3.9453 C 0 0 0 0 0 0 0 0 0 0 0 0
7.9499 1.8044 3.3576 H 0 0 0 0 0 0 0 0 0 0 0 0
6.3494 1.9624 0.9167 H 0 0 0 0 0 0 0 0 0 0 0 0
5.5205 3.0050 2.0752 H 0 0 0 0 0 0 0 0 0 0 0 0
6.4733 4.9966 0.8686 H 0 0 0 0 0 0 0 0 0 0 0 0
7.1641 3.9052 -0.3202 H 0 0 0 0 0 0 0 0 0 0 0 0
4.9033 3.0597 -0.8660 H 0 0 0 0 0 0 0 0 0 0 0 0
4.2062 4.1591 0.3312 H 0 0 0 0 0 0 0 0 0 0 0 0
5.1061 6.1005 -0.9278 H 0 0 0 0 0 0 0 0 0 0 0 0
5.8029 5.0010 -2.1250 H 0 0 0 0 0 0 0 0 0 0 0 0
3.5329 4.1560 -2.6700 H 0 0 0 0 0 0 0 0 0 0 0 0
2.8361 5.2556 -1.4729 H 0 0 0 0 0 0 0 0 0 0 0 0
3.7364 7.1968 -2.7322 H 0 0 0 0 0 0 0 0 0 0 0 0
4.4329 6.0972 -3.9294 H 0 0 0 0 0 0 0 0 0 0 0 0
2.1628 5.2526 -4.4743 H 0 0 0 0 0 0 0 0 0 0 0 0
1.4663 6.3524 -3.2771 H 0 0 0 0 0 0 0 0 0 0 0 0
2.2597 7.0015 -4.7182 H 0 0 0 0 0 0 0 0 0 0 0 0
10.3718 2.8005 2.0589 H 0 0 0 0 0 0 0 0 0 0 0 0
9.4431 2.0340 0.7682 H 0 0 0 0 0 0 0 0 0 0 0 0
8.7862 4.3264 -0.0344 H 0 0 0 0 0 0 0 0 0 0 0 0
9.8303 5.0203 1.1944 H 0 0 0 0 0 0 0 0 0 0 0 0
11.7753 3.8470 0.2126 H 0 0 0 0 0 0 0 0 0 0 0 0
10.7244 3.1473 -1.0260 H 0 0 0 0 0 0 0 0 0 0 0 0
10.1562 5.4261 -1.8310 H 0 0 0 0 0 0 0 0 0 0 0 0
11.2074 6.1256 -0.5927 H 0 0 0 0 0 0 0 0 0 0 0 0
13.1561 4.9470 -1.5814 H 0 0 0 0 0 0 0 0 0 0 0 0
12.1049 4.2476 -2.8197 H 0 0 0 0 0 0 0 0 0 0 0 0
11.5368 6.5267 -3.6245 H 0 0 0 0 0 0 0 0 0 0 0 0
12.5882 7.2260 -2.3864 H 0 0 0 0 0 0 0 0 0 0 0 0
13.7605 7.0946 -4.5706 H 0 0 0 0 0 0 0 0 0 0 0 0
13.4852 5.3482 -4.6136 H 0 0 0 0 0 0 0 0 0 0 0 0
14.5367 6.0474 -3.3754 H 0 0 0 0 0 0 0 0 0 0 0 0
7.1337 4.4522 4.2890 H 0 0 0 0 0 0 0 0 0 0 0 0
8.8918 4.5365 4.1552 H 0 0 0 0 0 0 0 0 0 0 0 0
8.6039 5.8509 2.0292 H 0 0 0 0 0 0 0 0 0 0 0 0
6.8685 5.8361 2.2933 H 0 0 0 0 0 0 0 0 0 0 0 0
7.1825 6.9901 4.4597 H 0 0 0 0 0 0 0 0 0 0 0 0
8.9310 7.0059 4.1947 H 0 0 0 0 0 0 0 0 0 0 0 0

```
    8.6073    8.3614    2.1400 H   0  0  0  0  0  0  0  0  0  0  0  0
    6.8588    8.3458    2.4053 H   0  0  0  0  0  0  0  0  0  0  0  0
    7.1782    9.5041    4.5779 H   0  0  0  0  0  0  0  0  0  0  0  0
    8.9266    9.5199    4.3125 H   0  0  0  0  0  0  0  0  0  0  0  0
    8.6024   10.8754    2.2577 H   0  0  0  0  0  0  0  0  0  0  0  0
    6.8540   10.8598    2.5233 H   0  0  0  0  0  0  0  0  0  0  0  0
    7.1737   12.0184    4.6957 H   0  0  0  0  0  0  0  0  0  0  0  0
    7.8581   12.9667    3.3691 H   0  0  0  0  0  0  0  0  0  0  0  0
    8.9221   12.0340    4.4300 H   0  0  0  0  0  0  0  0  0  0  0  0
  2  1  1  0  0  0  0
  2  3  1  0  0  0  0
  3  4  1  0  0  0  0
  4  5  1  0  0  0  0
  5  6  1  0  0  0  0
  6  7  1  0  0  0  0
  7  8  1  0  0  0  0
  9 10  1  0  0  0  0
 10 11  1  0  0  0  0
 11 12  1  0  0  0  0
 12 13  1  0  0  0  0
 13 14  1  0  0  0  0
 14 15  1  0  0  0  0
 16 17  1  0  0  0  0
 17 18  1  0  0  0  0
 18 19  1  0  0  0  0
 19 20  1  0  0  0  0
 20 21  1  0  0  0  0
 21 22  1  0  0  0  0
  9  1  1  0  0  0  0
 16  1  1  0  0  0  0
  1 23  1  0  0  0  0
  2 24  1  0  0  0  0
  2 25  1  0  0  0  0
  3 26  1  0  0  0  0
  3 27  1  0  0  0  0
  4 28  1  0  0  0  0
  4 29  1  0  0  0  0
  5 30  1  0  0  0  0
  5 31  1  0  0  0  0
  6 32  1  0  0  0  0
  6 33  1  0  0  0  0
  7 34  1  0  0  0  0
  7 35  1  0  0  0  0
  8 36  1  0  0  0  0
  8 37  1  0  0  0  0
  8 38  1  0  0  0  0
  9 39  1  0  0  0  0
  9 40  1  0  0  0  0
 10 41  1  0  0  0  0
 10 42  1  0  0  0  0
 11 43  1  0  0  0  0
 11 44  1  0  0  0  0
 12 45  1  0  0  0  0
 12 46  1  0  0  0  0
 13 47  1  0  0  0  0
 13 48  1  0  0  0  0
 14 49  1  0  0  0  0
 14 50  1  0  0  0  0
 15 51  1  0  0  0  0
 15 52  1  0  0  0  0
 15 53  1  0  0  0  0
 16 54  1  0  0  0  0
 16 55  1  0  0  0  0
```

```
 17 56  1  0  0  0  0
 17 57  1  0  0  0  0
 18 58  1  0  0  0  0
 18 59  1  0  0  0  0
 19 60  1  0  0  0  0
 19 61  1  0  0  0  0
 20 62  1  0  0  0  0
 20 63  1  0  0  0  0
 21 64  1  0  0  0  0
 21 65  1  0  0  0  0
 22 66  1  0  0  0  0
 22 67  1  0  0  0  0
 22 68  1  0  0  0  0
M  END
>  <Alias name>
Triheptylsilane

>  <deltaH°(c)>   kJ/mol
-15191

$$$$
Methyldidecylsilane
  ChemBrain IXL

 68 67  0  0  0  0  0  0  0  0  1 V2000
    3.7841    4.3613    0.0000 Si  0  0  0  0  0  0  0  0  0  0  0  0
    5.2345    3.3629   -0.6497 C   0  0  0  0  0  0  0  0  0  0  0  0
    6.5661    4.0202   -0.2350 C   0  0  0  0  0  0  0  0  0  0  0  0
    7.7389    3.1249   -0.6784 C   0  0  0  0  0  0  0  0  0  0  0  0
    9.0704    3.7843   -0.2696 C   0  0  0  0  0  0  0  0  0  0  0  0
   10.2449    2.8931   -0.7176 C   0  0  0  0  0  0  0  0  0  0  0  0
   11.5759    3.5558   -0.3122 C   0  0  0  0  0  0  0  0  0  0  0  0
   12.7513    2.6671   -0.7632 C   0  0  0  0  0  0  0  0  0  0  0  0
    3.9110    4.4950    1.8552 C   0  0  0  0  0  0  0  0  0  0  0  0
    3.7095    6.0839   -0.7415 C   0  0  0  0  0  0  0  0  0  0  0  0
    4.9535    6.8977   -0.3323 C   0  0  0  0  0  0  0  0  0  0  0  0
    4.8159    8.3406   -0.8546 C   0  0  0  0  0  0  0  0  0  0  0  0
    6.0620    9.1528   -0.4514 C   0  0  0  0  0  0  0  0  0  0  0  0
    5.9289   10.5948   -0.9782 C   0  0  0  0  0  0  0  0  0  0  0  0
    7.1777   11.4046   -0.5785 C   0  0  0  0  0  0  0  0  0  0  0  0
    7.0472   12.8459   -1.1081 C   0  0  0  0  0  0  0  0  0  0  0  0
   14.0820    3.3317   -0.3597 C   0  0  0  0  0  0  0  0  0  0  0  0
   15.2578    2.4440   -0.8120 C   0  0  0  0  0  0  0  0  0  0  0  0
   16.5883    3.1092   -0.4093 C   0  0  0  0  0  0  0  0  0  0  0  0
    8.2976   13.6543   -0.7104 C   0  0  0  0  0  0  0  0  0  0  0  0
    8.1683   15.0954   -1.2412 C   0  0  0  0  0  0  0  0  0  0  0  0
    9.4191   15.9034   -0.8443 C   0  0  0  0  0  0  0  0  0  0  0  0
    2.5345    3.6492   -0.3484 H   0  0  0  0  0  0  0  0  0  0  0  0
    5.1896    2.3632   -0.2356 H   0  0  0  0  0  0  0  0  0  0  0  0
    5.1797    3.3058   -1.7297 H   0  0  0  0  0  0  0  0  0  0  0  0
    6.5908    4.1397    0.8411 H   0  0  0  0  0  0  0  0  0  0  0  0
    6.6563    4.9799   -0.7053 H   0  0  0  0  0  0  0  0  0  0  0  0
    7.7124    3.0019   -1.7541 H   0  0  0  0  0  0  0  0  0  0  0  0
    7.6542    2.1561   -0.2019 H   0  0  0  0  0  0  0  0  0  0  0  0
    9.0984    3.9045    0.8064 H   0  0  0  0  0  0  0  0  0  0  0  0
    9.1528    4.7547   -0.7433 H   0  0  0  0  0  0  0  0  0  0  0  0
   10.2155    2.7710   -1.7933 H   0  0  0  0  0  0  0  0  0  0  0  0
   10.1646    1.9235   -0.2419 H   0  0  0  0  0  0  0  0  0  0  0  0
   11.6063    3.6761    0.7637 H   0  0  0  0  0  0  0  0  0  0  0  0
   11.6548    4.5264   -0.7863 H   0  0  0  0  0  0  0  0  0  0  0  0
   12.7200    2.5457   -1.8389 H   0  0  0  0  0  0  0  0  0  0  0  0
   12.6735    1.6970   -0.2880 H   0  0  0  0  0  0  0  0  0  0  0  0
    3.0594    5.0447    2.2365 H   0  0  0  0  0  0  0  0  0  0  0  0
```

```
   4.8237    5.0153    2.1179 H   0  0  0  0  0  0  0  0  0  0  0  0
   3.9236    3.5026    2.2885 H   0  0  0  0  0  0  0  0  0  0  0  0
   3.6631    6.0115   -1.8210 H   0  0  0  0  0  0  0  0  0  0  0  0
   2.8224    6.5875   -0.3779 H   0  0  0  0  0  0  0  0  0  0  0  0
   5.0376    6.9117    0.7473 H   0  0  0  0  0  0  0  0  0  0  0  0
   5.8319    6.4507   -0.7554 H   0  0  0  0  0  0  0  0  0  0  0  0
   4.7277    8.3269   -1.9339 H   0  0  0  0  0  0  0  0  0  0  0  0
   3.9326    8.7974   -0.4258 H   0  0  0  0  0  0  0  0  0  0  0  0
   6.1485    9.1693    0.6280 H   0  0  0  0  0  0  0  0  0  0  0  0
   6.9454    8.6934   -0.8773 H   0  0  0  0  0  0  0  0  0  0  0  0
   5.8397   10.5781   -2.0574 H   0  0  0  0  0  0  0  0  0  0  0  0
   5.0472   11.0558   -0.5502 H   0  0  0  0  0  0  0  0  0  0  0  0
   7.2657   11.4232    0.5008 H   0  0  0  0  0  0  0  0  0  0  0  0
   8.0593   10.9421   -1.0046 H   0  0  0  0  0  0  0  0  0  0  0  0
   6.9577   12.8272   -2.1872 H   0  0  0  0  0  0  0  0  0  0  0  0
   6.1666   13.3093   -0.6807 H   0  0  0  0  0  0  0  0  0  0  0  0
  14.1136    3.4523    0.7161 H   0  0  0  0  0  0  0  0  0  0  0  0
  14.1591    4.3022   -0.8342 H   0  0  0  0  0  0  0  0  0  0  0  0
  15.2259    2.3231   -1.8877 H   0  0  0  0  0  0  0  0  0  0  0  0
  15.1811    1.4738   -0.3372 H   0  0  0  0  0  0  0  0  0  0  0  0
  16.6651    4.0795   -0.8841 H   0  0  0  0  0  0  0  0  0  0  0  0
  17.4144    2.4853   -0.7272 H   0  0  0  0  0  0  0  0  0  0  0  0
  16.6204    3.2301    0.6665 H   0  0  0  0  0  0  0  0  0  0  0  0
   8.3867   13.6738    0.3688 H   0  0  0  0  0  0  0  0  0  0  0  0
   9.1782   13.1902   -1.1370 H   0  0  0  0  0  0  0  0  0  0  0  0
   8.0787   15.0759   -2.3203 H   0  0  0  0  0  0  0  0  0  0  0  0
   7.2881   15.5598   -0.8142 H   0  0  0  0  0  0  0  0  0  0  0  0
   9.3281   16.9159   -1.2175 H   0  0  0  0  0  0  0  0  0  0  0  0
  10.2994   15.4390   -1.2713 H   0  0  0  0  0  0  0  0  0  0  0  0
   9.5088   15.9230    0.2348 H   0  0  0  0  0  0  0  0  0  0  0  0
  2  1  1  0  0  0  0
  2  3  1  0  0  0  0
  3  4  1  0  0  0  0
  4  5  1  0  0  0  0
  5  6  1  0  0  0  0
  6  7  1  0  0  0  0
  7  8  1  0  0  0  0
 10 11  1  0  0  0  0
 11 12  1  0  0  0  0
 12 13  1  0  0  0  0
 13 14  1  0  0  0  0
 14 15  1  0  0  0  0
 15 16  1  0  0  0  0
  9  1  1  0  0  0  0
 10  1  1  0  0  0  0
  8 17  1  0  0  0  0
 17 18  1  0  0  0  0
 18 19  1  0  0  0  0
 16 20  1  0  0  0  0
 20 21  1  0  0  0  0
 21 22  1  0  0  0  0
  1 23  1  0  0  0  0
  2 24  1  0  0  0  0
  2 25  1  0  0  0  0
  3 26  1  0  0  0  0
  3 27  1  0  0  0  0
  4 28  1  0  0  0  0
  4 29  1  0  0  0  0
  5 30  1  0  0  0  0
  5 31  1  0  0  0  0
  6 32  1  0  0  0  0
  6 33  1  0  0  0  0
  7 34  1  0  0  0  0
```

```
  7 35  1  0  0  0  0
  8 36  1  0  0  0  0
  8 37  1  0  0  0  0
  9 38  1  0  0  0  0
  9 39  1  0  0  0  0
  9 40  1  0  0  0  0
 10 41  1  0  0  0  0
 10 42  1  0  0  0  0
 11 43  1  0  0  0  0
 11 44  1  0  0  0  0
 12 45  1  0  0  0  0
 12 46  1  0  0  0  0
 13 47  1  0  0  0  0
 13 48  1  0  0  0  0
 14 49  1  0  0  0  0
 14 50  1  0  0  0  0
 15 51  1  0  0  0  0
 15 52  1  0  0  0  0
 16 53  1  0  0  0  0
 16 54  1  0  0  0  0
 17 55  1  0  0  0  0
 17 56  1  0  0  0  0
 18 57  1  0  0  0  0
 18 58  1  0  0  0  0
 19 59  1  0  0  0  0
 19 60  1  0  0  0  0
 19 61  1  0  0  0  0
 20 62  1  0  0  0  0
 20 63  1  0  0  0  0
 21 64  1  0  0  0  0
 21 65  1  0  0  0  0
 22 66  1  0  0  0  0
 22 67  1  0  0  0  0
 22 68  1  0  0  0  0
M  END
>  <Alias name>
Methyldidecylsilane

>  <deltaH°(c)>   kJ/mol
-15189

$$$$
Diacylperoxide V
  ChemBrain IXL

 80 79  0  0  0  0  0  0  0  0  1 V2000
   18.8722   17.6151   -1.5981 C   0  0  0  0  0  0  0  0  0  0  0  0
   20.3505   17.6820   -1.5685 C   0  0  0  0  0  0  0  0  0  0  0  0
   20.9634   18.0695   -2.5496 O   0  0  0  0  0  0  0  0  0  0  0  0
   21.0802   17.2814   -0.3569 O   0  0  0  0  0  0  0  0  0  0  0  0
   16.2981   16.4899    1.0754 C   0  0  0  0  0  0  0  0  0  0  0  0
   14.8186   16.4566    1.0586 C   0  0  0  0  0  0  0  0  0  0  0  0
   14.2065   16.0495    2.0323 O   0  0  0  0  0  0  0  0  0  0  0  0
   14.0865   16.9141   -0.1312 O   0  0  0  0  0  0  0  0  0  0  0  0
   10.4183   18.1211   -0.2194 C   0  0  0  0  0  0  0  0  0  0  0  0
   11.8973   18.0887   -0.2652 C   0  0  0  0  0  0  0  0  0  0  0  0
   12.5229   19.1101   -0.4974 O   0  0  0  0  0  0  0  0  0  0  0  0
   12.6125   16.8260   -0.0310 O   0  0  0  0  0  0  0  0  0  0  0  0
   24.6559   18.3717    0.6156 C   0  0  0  0  0  0  0  0  0  0  0  0
   23.1843   18.2172    0.5853 C   0  0  0  0  0  0  0  0  0  0  0  0
   22.4946   18.7793    1.4201 O   0  0  0  0  0  0  0  0  0  0  0  0
   22.5535   17.3865   -0.4506 O   0  0  0  0  0  0  0  0  0  0  0  0
   16.8154   17.0225   -0.2750 C   0  0  0  0  0  0  0  0  0  0  0  0
```

18.3553	17.0747	-0.2507	C	0	0	0	0	0	0	0	0	0	0	0	0
25.2843	17.5287	-0.5111	C	0	0	0	0	0	0	0	0	0	0	0	0
26.8154	17.7014	-0.4889	C	0	0	0	0	0	0	0	0	0	0	0	0
27.4436	16.8616	-1.6180	C	0	0	0	0	0	0	0	0	0	0	0	0
9.8804	16.6965	0.0173	C	0	0	0	0	0	0	0	0	0	0	0	0
8.3413	16.7332	0.0845	C	0	0	0	0	0	0	0	0	0	0	0	0
7.8036	15.3098	0.3288	C	0	0	0	0	0	0	0	0	0	0	0	0
28.9748	17.0338	-1.5952	C	0	0	0	0	0	0	0	0	0	0	0	0
6.2648	15.3477	0.4027	C	0	0	0	0	0	0	0	0	0	0	0	0
33.2939	15.6972	-3.8062	C	0	0	0	0	0	0	0	0	0	0	0	0
31.7626	15.5265	-3.8304	C	0	0	0	0	0	0	0	0	0	0	0	0
31.1343	16.3656	-2.7008	C	0	0	0	0	0	0	0	0	0	0	0	0
29.6030	16.1944	-2.7246	C	0	0	0	0	0	0	0	0	0	0	0	0
2.1125	12.5794	1.0545	C	0	0	0	0	0	0	0	0	0	0	0	0
3.6510	12.5406	0.9752	C	0	0	0	0	0	0	0	0	0	0	0	0
4.1886	13.9633	0.7272	C	0	0	0	0	0	0	0	0	0	0	0	0
5.7272	13.9248	0.6496	C	0	0	0	0	0	0	0	0	0	0	0	0
18.5566	16.9552	-2.3968	H	0	0	0	0	0	0	0	0	0	0	0	0
18.4694	18.6060	-1.7681	H	0	0	0	0	0	0	0	0	0	0	0	0
16.6355	17.1402	1.8731	H	0	0	0	0	0	0	0	0	0	0	0	0
16.6799	15.4898	1.2392	H	0	0	0	0	0	0	0	0	0	0	0	0
10.0363	18.4996	-1.1594	H	0	0	0	0	0	0	0	0	0	0	0	0
10.0962	18.7673	0.5877	H	0	0	0	0	0	0	0	0	0	0	0	0
25.0346	18.0347	1.5726	H	0	0	0	0	0	0	0	0	0	0	0	0
24.9125	19.4141	0.4725	H	0	0	0	0	0	0	0	0	0	0	0	0
16.4889	16.3650	-1.0712	H	0	0	0	0	0	0	0	0	0	0	0	0
16.4231	18.0176	-0.4447	H	0	0	0	0	0	0	0	0	0	0	0	0
18.6819	17.7276	0.5493	H	0	0	0	0	0	0	0	0	0	0	0	0
18.7478	16.0787	-0.0868	H	0	0	0	0	0	0	0	0	0	0	0	0
24.8976	17.8586	-1.4674	H	0	0	0	0	0	0	0	0	0	0	0	0
25.0365	16.4850	-0.3621	H	0	0	0	0	0	0	0	0	0	0	0	0
27.2024	17.3689	0.4664	H	0	0	0	0	0	0	0	0	0	0	0	0
27.0631	18.7455	-0.6350	H	0	0	0	0	0	0	0	0	0	0	0	0
27.0570	17.1944	-2.5733	H	0	0	0	0	0	0	0	0	0	0	0	0
27.1955	15.8175	-1.4721	H	0	0	0	0	0	0	0	0	0	0	0	0
10.2735	16.3127	0.9507	H	0	0	0	0	0	0	0	0	0	0	0	0
10.1910	16.0534	-0.7968	H	0	0	0	0	0	0	0	0	0	0	0	0
7.9481	17.1121	-0.8507	H	0	0	0	0	0	0	0	0	0	0	0	0
8.0307	17.3805	0.8954	H	0	0	0	0	0	0	0	0	0	0	0	0
8.2006	14.9291	1.2617	H	0	0	0	0	0	0	0	0	0	0	0	0
8.1102	14.6637	-0.4845	H	0	0	0	0	0	0	0	0	0	0	0	0
29.3614	16.7007	-0.6400	H	0	0	0	0	0	0	0	0	0	0	0	0
29.2228	18.0779	-1.7407	H	0	0	0	0	0	0	0	0	0	0	0	0
5.8678	15.7267	-0.5310	H	0	0	0	0	0	0	0	0	0	0	0	0
5.9583	15.9954	1.2147	H	0	0	0	0	0	0	0	0	0	0	0	0
33.5431	16.7412	-3.9505	H	0	0	0	0	0	0	0	0	0	0	0	0
33.7355	15.1076	-4.6001	H	0	0	0	0	0	0	0	0	0	0	0	0
33.6794	15.3627	-2.8510	H	0	0	0	0	0	0	0	0	0	0	0	0
31.3771	15.8609	-4.7856	H	0	0	0	0	0	0	0	0	0	0	0	0
31.5134	14.4825	-3.6860	H	0	0	0	0	0	0	0	0	0	0	0	0
31.5202	16.0315	-1.7456	H	0	0	0	0	0	0	0	0	0	0	0	0
31.3832	17.4096	-2.8455	H	0	0	0	0	0	0	0	0	0	0	0	0
29.2172	16.5283	-3.6799	H	0	0	0	0	0	0	0	0	0	0	0	0
29.3542	15.1504	-2.5796	H	0	0	0	0	0	0	0	0	0	0	0	0
1.7347	11.5795	1.2287	H	0	0	0	0	0	0	0	0	0	0	0	0
1.8092	13.2267	1.8680	H	0	0	0	0	0	0	0	0	0	0	0	0
1.7125	12.9592	0.1225	H	0	0	0	0	0	0	0	0	0	0	0	0
4.0511	12.1608	1.9072	H	0	0	0	0	0	0	0	0	0	0	0	0
3.9543	11.8932	0.1617	H	0	0	0	0	0	0	0	0	0	0	0	0
3.7895	14.3426	-0.2054	H	0	0	0	0	0	0	0	0	0	0	0	0
3.8843	14.6110	1.5401	H	0	0	0	0	0	0	0	0	0	0	0	0
6.1263	13.5448	1.5819	H	0	0	0	0	0	0	0	0	0	0	0	0
6.0315	13.2777	-0.1638	H	0	0	0	0	0	0	0	0	0	0	0	0

1	2	1	0	0	0	0
2	3	2	0	0	0	0
2	4	1	0	0	0	0
5	6	1	0	0	0	0
6	7	2	0	0	0	0
6	8	1	0	0	0	0
9	10	1	0	0	0	0
10	11	2	0	0	0	0
10	12	1	0	0	0	0
13	14	1	0	0	0	0
14	15	2	0	0	0	0
14	16	1	0	0	0	0
16	4	1	0	0	0	0
12	8	1	0	0	0	0
5	17	1	0	0	0	0
1	18	1	0	0	0	0
17	18	1	0	0	0	0
13	19	1	0	0	0	0
19	20	1	0	0	0	0
20	21	1	0	0	0	0
9	22	1	0	0	0	0
22	23	1	0	0	0	0
23	24	1	0	0	0	0
21	25	1	0	0	0	0
24	26	1	0	0	0	0
27	28	1	0	0	0	0
28	29	1	0	0	0	0
29	30	1	0	0	0	0
31	32	1	0	0	0	0
32	33	1	0	0	0	0
33	34	1	0	0	0	0
34	26	1	0	0	0	0
30	25	1	0	0	0	0
1	35	1	0	0	0	0
1	36	1	0	0	0	0
5	37	1	0	0	0	0
5	38	1	0	0	0	0
9	39	1	0	0	0	0
9	40	1	0	0	0	0
13	41	1	0	0	0	0
13	42	1	0	0	0	0
17	43	1	0	0	0	0
17	44	1	0	0	0	0
18	45	1	0	0	0	0
18	46	1	0	0	0	0
19	47	1	0	0	0	0
19	48	1	0	0	0	0
20	49	1	0	0	0	0
20	50	1	0	0	0	0
21	51	1	0	0	0	0
21	52	1	0	0	0	0
22	53	1	0	0	0	0
22	54	1	0	0	0	0
23	55	1	0	0	0	0
23	56	1	0	0	0	0
24	57	1	0	0	0	0
24	58	1	0	0	0	0
25	59	1	0	0	0	0
25	60	1	0	0	0	0
26	61	1	0	0	0	0
26	62	1	0	0	0	0
27	63	1	0	0	0	0
27	64	1	0	0	0	0

```
 27 65  1  0  0  0  0
 28 66  1  0  0  0  0
 28 67  1  0  0  0  0
 29 68  1  0  0  0  0
 29 69  1  0  0  0  0
 30 70  1  0  0  0  0
 30 71  1  0  0  0  0
 31 72  1  0  0  0  0
 31 73  1  0  0  0  0
 31 74  1  0  0  0  0
 32 75  1  0  0  0  0
 32 76  1  0  0  0  0
 33 77  1  0  0  0  0
 33 78  1  0  0  0  0
 34 79  1  0  0  0  0
 34 80  1  0  0  0  0
M  END
>  <Alias name>
Diacylperoxide V

>  <deltaH°(c)>   kJ/mol
-15164

$$$$
Di-n-nonylphthalate
  ChemBrain IXL

 72 72  0  0  0  0  0  0  0  0  1 V2000
    2.7432    5.7608   -0.4115 C   0  0  0  0  0  0  0  0  0  0  0  0
    2.4485    6.9722   -1.0395 C   0  0  0  0  0  0  0  0  0  0  0  0
    3.4659    7.8895   -1.3057 C   0  0  0  0  0  0  0  0  0  0  0  0
    4.7831    7.6017   -0.9453 C   0  0  0  0  0  0  0  0  0  0  0  0
    5.0835    6.3689   -0.3113 C   0  0  0  0  0  0  0  0  0  0  0  0
    4.0569    5.4606   -0.0484 C   0  0  0  0  0  0  0  0  0  0  0  0
    5.8525    8.6110   -1.2453 C   0  0  0  0  0  0  0  0  0  0  0  0
    5.7362    9.3394   -2.2174 O   0  0  0  0  0  0  0  0  0  0  0  0
    7.0506    8.7394   -0.3732 O   0  0  0  0  0  0  0  0  0  0  0  0
    6.4858    6.0135    0.0915 C   0  0  0  0  0  0  0  0  0  0  0  0
    6.6677    5.2648    1.0379 O   0  0  0  0  0  0  0  0  0  0  0  0
    7.6576    6.5573   -0.6475 O   0  0  0  0  0  0  0  0  0  0  0  0
    8.0766    9.7058   -0.8566 C   0  0  0  0  0  0  0  0  0  0  0  0
    9.2225    9.8355    0.1861 C   0  0  0  0  0  0  0  0  0  0  0  0
   10.3061   10.8148   -0.3488 C   0  0  0  0  0  0  0  0  0  0  0  0
   11.4682   10.9226    0.6736 C   0  0  0  0  0  0  0  0  0  0  0  0
   12.5301   11.9260    0.1531 C   0  0  0  0  0  0  0  0  0  0  0  0
   13.6892   12.0332    1.1740 C   0  0  0  0  0  0  0  0  0  0  0  0
   14.7379   13.0500    0.6650 C   0  0  0  0  0  0  0  0  0  0  0  0
   15.8913   13.1583    1.6871 C   0  0  0  0  0  0  0  0  0  0  0  0
    8.9840    6.1960   -0.0707 C   0  0  0  0  0  0  0  0  0  0  0  0
   10.1280    6.7368   -0.9753 C   0  0  0  0  0  0  0  0  0  0  0  0
   11.5047    6.3862   -0.3400 C   0  0  0  0  0  0  0  0  0  0  0  0
   12.6510    6.9556   -1.2180 C   0  0  0  0  0  0  0  0  0  0  0  0
   14.0214    6.5666   -0.6031 C   0  0  0  0  0  0  0  0  0  0  0  0
   15.1654    7.1679   -1.4564 C   0  0  0  0  0  0  0  0  0  0  0  0
   16.5317    6.7464   -0.8650 C   0  0  0  0  0  0  0  0  0  0  0  0
   17.6685    7.4653   -1.6254 C   0  0  0  0  0  0  0  0  0  0  0  0
   16.9323   14.1811    1.1852 C   0  0  0  0  0  0  0  0  0  0  0  0
   19.0330    7.0127   -1.0637 C   0  0  0  0  0  0  0  0  0  0  0  0
    1.9511    5.0525   -0.2056 H   0  0  0  0  0  0  0  0  0  0  0  0
    1.4286    7.2008   -1.3206 H   0  0  0  0  0  0  0  0  0  0  0  0
    3.2331    8.8280   -1.7937 H   0  0  0  0  0  0  0  0  0  0  0  0
    4.2810    4.5198    0.4392 H   0  0  0  0  0  0  0  0  0  0  0  0
    8.4852    9.3555   -1.7965 H   0  0  0  0  0  0  0  0  0  0  0  0
```

7.6157 10.6748 -1.0049 H 0 0 0 0 0 0 0 0 0 0 0 0
8.8217 10.2159 1.1174 H 0 0 0 0 0 0 0 0 0 0 0 0
9.6679 8.8633 0.3556 H 0 0 0 0 0 0 0 0 0 0 0 0
10.6903 10.4466 -1.2922 H 0 0 0 0 0 0 0 0 0 0 0 0
9.8659 11.7933 -0.4970 H 0 0 0 0 0 0 0 0 0 0 0 0
11.0815 11.2687 1.6240 H 0 0 0 0 0 0 0 0 0 0 0 0
11.9256 9.9496 0.8035 H 0 0 0 0 0 0 0 0 0 0 0 0
12.9182 11.5801 -0.7972 H 0 0 0 0 0 0 0 0 0 0 0 0
12.0736 12.8994 0.0231 H 0 0 0 0 0 0 0 0 0 0 0 0
13.2996 12.3651 2.1284 H 0 0 0 0 0 0 0 0 0 0 0 0
14.1561 11.0634 1.2929 H 0 0 0 0 0 0 0 0 0 0 0 0
15.1308 12.7173 -0.2878 H 0 0 0 0 0 0 0 0 0 0 0 0
14.2714 14.0199 0.5440 H 0 0 0 0 0 0 0 0 0 0 0 0
15.4980 13.4837 2.6422 H 0 0 0 0 0 0 0 0 0 0 0 0
16.3636 12.1905 1.8024 H 0 0 0 0 0 0 0 0 0 0 0 0
9.0749 6.6280 0.9183 H 0 0 0 0 0 0 0 0 0 0 0 0
9.0629 5.1180 0.0004 H 0 0 0 0 0 0 0 0 0 0 0 0
10.0579 6.2829 -1.9560 H 0 0 0 0 0 0 0 0 0 0 0 0
10.0374 7.8118 -1.0681 H 0 0 0 0 0 0 0 0 0 0 0 0
11.5637 6.8174 0.6518 H 0 0 0 0 0 0 0 0 0 0 0 0
11.6057 5.3101 -0.2709 H 0 0 0 0 0 0 0 0 0 0 0 0
12.5764 6.5472 -2.2181 H 0 0 0 0 0 0 0 0 0 0 0 0
12.5697 8.0345 -1.2622 H 0 0 0 0 0 0 0 0 0 0 0 0
14.0857 6.9514 0.4073 H 0 0 0 0 0 0 0 0 0 0 0 0
14.1152 5.4877 -0.5843 H 0 0 0 0 0 0 0 0 0 0 0 0
15.0859 6.8049 -2.4737 H 0 0 0 0 0 0 0 0 0 0 0 0
15.0891 8.2481 -1.4522 H 0 0 0 0 0 0 0 0 0 0 0 0
16.5709 7.0182 0.1825 H 0 0 0 0 0 0 0 0 0 0 0 0
16.6531 5.6747 -0.9643 H 0 0 0 0 0 0 0 0 0 0 0 0
17.6120 7.2172 -2.6782 H 0 0 0 0 0 0 0 0 0 0 0 0
17.5650 8.5360 -1.5012 H 0 0 0 0 0 0 0 0 0 0 0 0
16.4613 15.1495 1.0706 H 0 0 0 0 0 0 0 0 0 0 0 0
17.3269 13.8563 0.2304 H 0 0 0 0 0 0 0 0 0 0 0 0
17.7406 14.2557 1.9031 H 0 0 0 0 0 0 0 0 0 0 0 0
19.8249 7.5805 -1.5376 H 0 0 0 0 0 0 0 0 0 0 0 0
19.1750 5.9581 -1.2653 H 0 0 0 0 0 0 0 0 0 0 0 0
19.0578 7.1823 0.0056 H 0 0 0 0 0 0 0 0 0 0 0 0
1 2 4 0 0 0 0
2 3 4 0 0 0 0
3 4 4 0 0 0 0
4 5 4 0 0 0 0
5 6 4 0 0 0 0
6 1 4 0 0 0 0
7 8 2 0 0 0 0
7 9 1 0 0 0 0
7 4 1 0 0 0 0
10 11 2 0 0 0 0
10 12 1 0 0 0 0
10 5 1 0 0 0 0
9 13 1 0 0 0 0
13 14 1 0 0 0 0
14 15 1 0 0 0 0
15 16 1 0 0 0 0
16 17 1 0 0 0 0
17 18 1 0 0 0 0
18 19 1 0 0 0 0
19 20 1 0 0 0 0
21 22 1 0 0 0 0
22 23 1 0 0 0 0
23 24 1 0 0 0 0
24 25 1 0 0 0 0
25 26 1 0 0 0 0
26 27 1 0 0 0 0

```
 27 28  1  0  0  0  0
 21 12  1  0  0  0  0
 20 29  1  0  0  0  0
 28 30  1  0  0  0  0
  1 31  1  0  0  0  0
  2 32  1  0  0  0  0
  3 33  1  0  0  0  0
  6 34  1  0  0  0  0
 13 35  1  0  0  0  0
 13 36  1  0  0  0  0
 14 37  1  0  0  0  0
 14 38  1  0  0  0  0
 15 39  1  0  0  0  0
 15 40  1  0  0  0  0
 16 41  1  0  0  0  0
 16 42  1  0  0  0  0
 17 43  1  0  0  0  0
 17 44  1  0  0  0  0
 18 45  1  0  0  0  0
 18 46  1  0  0  0  0
 19 47  1  0  0  0  0
 19 48  1  0  0  0  0
 20 49  1  0  0  0  0
 20 50  1  0  0  0  0
 21 51  1  0  0  0  0
 21 52  1  0  0  0  0
 22 53  1  0  0  0  0
 22 54  1  0  0  0  0
 23 55  1  0  0  0  0
 23 56  1  0  0  0  0
 24 57  1  0  0  0  0
 24 58  1  0  0  0  0
 25 59  1  0  0  0  0
 25 60  1  0  0  0  0
 26 61  1  0  0  0  0
 26 62  1  0  0  0  0
 27 63  1  0  0  0  0
 27 64  1  0  0  0  0
 28 65  1  0  0  0  0
 28 66  1  0  0  0  0
 29 67  1  0  0  0  0
 29 68  1  0  0  0  0
 29 69  1  0  0  0  0
 30 70  1  0  0  0  0
 30 71  1  0  0  0  0
 30 72  1  0  0  0  0
M  END
>  <Alias name>
Di-n-nonylphthalate

>  <deltaH°(c)>   kJ/mol
-15109.74

$$$$
n-Heptyl-n-undecylphthalate
  ChemBrain IXL

 72 72  0  0  0  0  0  0  0  0  1 V2000
    2.4637    8.7770   -0.9239 C   0  0  0  0  0  0  0  0  0  0  0  0
    2.7701    7.5095   -0.4247 C   0  0  0  0  0  0  0  0  0  0  0  0
    4.0854    7.1866   -0.0872 C   0  0  0  0  0  0  0  0  0  0  0  0
    5.1048    8.1270   -0.2469 C   0  0  0  0  0  0  0  0  0  0  0  0
    4.7907    9.4229   -0.7526 C   0  0  0  0  0  0  0  0  0  0  0  0
```

3.4706	9.7293	-1.0874	C	0	0	0	0	0	0	0	0	0	0	0	0	0
6.5122	7.7342	0.1308	C	0	0	0	0	0	0	0	0	0	0	0	0	0
6.6853	6.8897	0.9945	O	0	0	0	0	0	0	0	0	0	0	0	0	0
7.6991	8.3539	-0.5337	O	0	0	0	0	0	0	0	0	0	0	0	0	0
5.8449	10.4826	-0.9456	C	0	0	0	0	0	0	0	0	0	0	0	0	0
5.6870	11.3341	-1.8052	O	0	0	0	0	0	0	0	0	0	0	0	0	0
7.0764	10.5143	-0.1032	O	0	0	0	0	0	0	0	0	0	0	0	0	0
9.0218	7.9227	0.0194	C	0	0	0	0	0	0	0	0	0	0	0	0	0
10.1895	8.5407	-0.8121	C	0	0	0	0	0	0	0	0	0	0	0	0	0
11.5569	8.1126	-0.1898	C	0	0	0	0	0	0	0	0	0	0	0	0	0
12.7301	8.7722	-0.9721	C	0	0	0	0	0	0	0	0	0	0	0	0	0
14.0882	8.2995	-0.3764	C	0	0	0	0	0	0	0	0	0	0	0	0	0
15.2611	8.9556	-1.1542	C	0	0	0	0	0	0	0	0	0	0	0	0	0
8.0667	11.5666	-0.4849	C	0	0	0	0	0	0	0	0	0	0	0	0	0
9.2573	11.5831	0.5203	C	0	0	0	0	0	0	0	0	0	0	0	0	0
10.3035	12.6469	0.0688	C	0	0	0	0	0	0	0	0	0	0	0	0	0
11.5231	12.6335	1.0297	C	0	0	0	0	0	0	0	0	0	0	0	0	0
12.5425	13.7188	0.5920	C	0	0	0	0	0	0	0	0	0	0	0	0	0
13.7495	13.7212	1.5603	C	0	0	0	0	0	0	0	0	0	0	0	0	0
14.7555	14.8135	1.1322	C	0	0	0	0	0	0	0	0	0	0	0	0	0
16.6135	8.4515	-0.5843	C	0	0	0	0	0	0	0	0	0	0	0	0	0
17.7829	9.1217	-1.3478	C	0	0	0	0	0	0	0	0	0	0	0	0	0
19.1314	8.6002	-0.7963	C	0	0	0	0	0	0	0	0	0	0	0	0	0
20.2945	9.3706	-1.4608	C	0	0	0	0	0	0	0	0	0	0	0	0	0
21.6409	8.8265	-0.9377	C	0	0	0	0	0	0	0	0	0	0	0	0	0
1.4425	9.0217	-1.1854	H	0	0	0	0	0	0	0	0	0	0	0	0	0
1.9853	6.7753	-0.2986	H	0	0	0	0	0	0	0	0	0	0	0	0	0
4.3168	6.2023	0.3008	H	0	0	0	0	0	0	0	0	0	0	0	0	0
3.2275	10.7105	-1.4763	H	0	0	0	0	0	0	0	0	0	0	0	0	0
9.1035	8.2506	1.0484	H	0	0	0	0	0	0	0	0	0	0	0	0	0
9.0901	6.8422	-0.0178	H	0	0	0	0	0	0	0	0	0	0	0	0	0
10.1320	8.1869	-1.8336	H	0	0	0	0	0	0	0	0	0	0	0	0	0
10.1102	9.6196	-0.8000	H	0	0	0	0	0	0	0	0	0	0	0	0	0
11.5950	8.4279	0.8459	H	0	0	0	0	0	0	0	0	0	0	0	0	0
11.6530	7.0351	-0.2404	H	0	0	0	0	0	0	0	0	0	0	0	0	0
12.6751	8.4850	-2.0147	H	0	0	0	0	0	0	0	0	0	0	0	0	0
12.6569	9.8498	-0.8908	H	0	0	0	0	0	0	0	0	0	0	0	0	0
14.1436	8.5868	0.6666	H	0	0	0	0	0	0	0	0	0	0	0	0	0
14.1614	7.2221	-0.4572	H	0	0	0	0	0	0	0	0	0	0	0	0	0
15.1915	8.6914	-2.2020	H	0	0	0	0	0	0	0	0	0	0	0	0	0
15.2056	10.0321	-1.0497	H	0	0	0	0	0	0	0	0	0	0	0	0	0
8.4419	11.3613	-1.4799	H	0	0	0	0	0	0	0	0	0	0	0	0	0
7.5808	12.5349	-0.4786	H	0	0	0	0	0	0	0	0	0	0	0	0	0
8.8926	11.8331	1.5085	H	0	0	0	0	0	0	0	0	0	0	0	0	0
9.7236	10.6071	0.5448	H	0	0	0	0	0	0	0	0	0	0	0	0	0
10.6368	12.4199	-0.9366	H	0	0	0	0	0	0	0	0	0	0	0	0	0
9.8468	13.6289	0.0823	H	0	0	0	0	0	0	0	0	0	0	0	0	0
11.1897	12.8390	2.0393	H	0	0	0	0	0	0	0	0	0	0	0	0	0
11.9975	11.6603	0.9976	H	0	0	0	0	0	0	0	0	0	0	0	0	0
12.8875	13.5058	-0.4123	H	0	0	0	0	0	0	0	0	0	0	0	0	0
12.0649	14.6907	0.6102	H	0	0	0	0	0	0	0	0	0	0	0	0	0
13.4047	13.9239	2.5666	H	0	0	0	0	0	0	0	0	0	0	0	0	0
14.2357	12.7540	1.5336	H	0	0	0	0	0	0	0	0	0	0	0	0	0
14.2652	15.7792	1.1383	H	0	0	0	0	0	0	0	0	0	0	0	0	0
15.1190	14.5996	0.1346	H	0	0	0	0	0	0	0	0	0	0	0	0	0
15.5894	14.8280	1.8245	H	0	0	0	0	0	0	0	0	0	0	0	0	0
16.6788	8.7038	0.4670	H	0	0	0	0	0	0	0	0	0	0	0	0	0
16.6760	7.3767	-0.7014	H	0	0	0	0	0	0	0	0	0	0	0	0	0
17.7092	8.8836	-2.4018	H	0	0	0	0	0	0	0	0	0	0	0	0	0
17.7311	10.1953	-1.2164	H	0	0	0	0	0	0	0	0	0	0	0	0	0
19.1658	8.7504	0.2757	H	0	0	0	0	0	0	0	0	0	0	0	0	0
19.2267	7.5439	-1.0157	H	0	0	0	0	0	0	0	0	0	0	0	0	0
20.2469	9.2411	-2.5349	H	0	0	0	0	0	0	0	0	0	0	0	0	0

```
 20.2133   10.4236   -1.2206 H   0  0  0  0  0  0  0  0  0  0  0  0
 22.4501    9.4386   -1.3181 H   0  0  0  0  0  0  0  0  0  0  0  0
 21.7730    7.8054   -1.2734 H   0  0  0  0  0  0  0  0  0  0  0  0
 21.6458    8.8541    0.1449 H   0  0  0  0  0  0  0  0  0  0  0  0
 1  2  4  0  0  0  0
 2  3  4  0  0  0  0
 3  4  4  0  0  0  0
 4  5  4  0  0  0  0
 5  6  4  0  0  0  0
 6  1  4  0  0  0  0
 7  8  2  0  0  0  0
 7  9  1  0  0  0  0
 7  4  1  0  0  0  0
10 11  2  0  0  0  0
10 12  1  0  0  0  0
10  5  1  0  0  0  0
 9 13  1  0  0  0  0
13 14  1  0  0  0  0
14 15  1  0  0  0  0
15 16  1  0  0  0  0
16 17  1  0  0  0  0
17 18  1  0  0  0  0
19 20  1  0  0  0  0
20 21  1  0  0  0  0
21 22  1  0  0  0  0
22 23  1  0  0  0  0
23 24  1  0  0  0  0
19 12  1  0  0  0  0
24 25  1  0  0  0  0
18 26  1  0  0  0  0
26 27  1  0  0  0  0
27 28  1  0  0  0  0
28 29  1  0  0  0  0
29 30  1  0  0  0  0
 1 31  1  0  0  0  0
 2 32  1  0  0  0  0
 3 33  1  0  0  0  0
 6 34  1  0  0  0  0
13 35  1  0  0  0  0
13 36  1  0  0  0  0
14 37  1  0  0  0  0
14 38  1  0  0  0  0
15 39  1  0  0  0  0
15 40  1  0  0  0  0
16 41  1  0  0  0  0
16 42  1  0  0  0  0
17 43  1  0  0  0  0
17 44  1  0  0  0  0
18 45  1  0  0  0  0
18 46  1  0  0  0  0
19 47  1  0  0  0  0
19 48  1  0  0  0  0
20 49  1  0  0  0  0
20 50  1  0  0  0  0
21 51  1  0  0  0  0
21 52  1  0  0  0  0
22 53  1  0  0  0  0
22 54  1  0  0  0  0
23 55  1  0  0  0  0
23 56  1  0  0  0  0
24 57  1  0  0  0  0
24 58  1  0  0  0  0
25 59  1  0  0  0  0
```

```
 25 60  1  0  0  0  0
 25 61  1  0  0  0  0
 26 62  1  0  0  0  0
 26 63  1  0  0  0  0
 27 64  1  0  0  0  0
 27 65  1  0  0  0  0
 28 66  1  0  0  0  0
 28 67  1  0  0  0  0
 29 68  1  0  0  0  0
 29 69  1  0  0  0  0
 30 70  1  0  0  0  0
 30 71  1  0  0  0  0
 30 72  1  0  0  0  0
M  END
>  <Alias name>
n-Heptyl-n-undecylphthalate

>  <deltaH°(c)>   kJ/mol
-15107.54

$$$$
Octadecylbenzene
  ChemBrain IXL

 66 66  0  0  0  0  0  0  0  0  1 V2000
   17.6195   11.7178   -2.3094 C   0  0  0  0  0  0  0  0  0  0  0  0
   16.6308   10.8529   -1.5029 C   0  0  0  0  0  0  0  0  0  0  0  0
   15.4174   11.7082   -1.0863 C   0  0  0  0  0  0  0  0  0  0  0  0
   14.4263   10.8363   -0.2906 C   0  0  0  0  0  0  0  0  0  0  0  0
   13.2162   11.6896    0.1395 C   0  0  0  0  0  0  0  0  0  0  0  0
   12.2233   10.8093    0.9246 C   0  0  0  0  0  0  0  0  0  0  0  0
   11.0165   11.6600    1.3695 C   0  0  0  0  0  0  0  0  0  0  0  0
   10.0234   10.7717    2.1459 C   0  0  0  0  0  0  0  0  0  0  0  0
    8.8200   11.6193    2.6067 C   0  0  0  0  0  0  0  0  0  0  0  0
   18.8370   10.8627   -2.7144 C   0  0  0  0  0  0  0  0  0  0  0  0
    7.8293   10.7232    3.3776 C   0  0  0  0  0  0  0  0  0  0  0  0
   19.8225   11.7232   -3.5299 C   0  0  0  0  0  0  0  0  0  0  0  0
    6.6298   11.5666    3.8541 C   0  0  0  0  0  0  0  0  0  0  0  0
   21.0437   10.8695   -3.9261 C   0  0  0  0  0  0  0  0  0  0  0  0
    5.6432   10.6640    4.6226 C   0  0  0  0  0  0  0  0  0  0  0  0
   22.0265   11.7269   -4.7479 C   0  0  0  0  0  0  0  0  0  0  0  0
   23.2497   10.8739   -5.1382 C   0  0  0  0  0  0  0  0  0  0  0  0
   24.2311   11.7291   -5.9635 C   0  0  0  0  0  0  0  0  0  0  0  0
    2.3264   12.9917    5.9696 C   0  0  0  0  0  0  0  0  0  0  0  0
    3.3594   13.5981    5.2547 C   0  0  0  0  0  0  0  0  0  0  0  0
    4.4431   12.8377    4.8145 C   0  0  0  0  0  0  0  0  0  0  0  0
    4.4936   11.4711    5.0893 C   0  0  0  0  0  0  0  0  0  0  0  0
    3.4605   10.8646    5.8042 C   0  0  0  0  0  0  0  0  0  0  0  0
    2.3768   11.6250    6.2443 C   0  0  0  0  0  0  0  0  0  0  0  0
   17.9496   12.5510   -1.7013 H   0  0  0  0  0  0  0  0  0  0  0  0
   17.1290   12.0922   -3.1993 H   0  0  0  0  0  0  0  0  0  0  0  0
   16.2951   10.0241   -2.1138 H   0  0  0  0  0  0  0  0  0  0  0  0
   17.1243   10.4717   -0.6174 H   0  0  0  0  0  0  0  0  0  0  0  0
   14.9278   12.0960   -1.9712 H   0  0  0  0  0  0  0  0  0  0  0  0
   15.7513   12.5321   -0.4677 H   0  0  0  0  0  0  0  0  0  0  0  0
   14.9195   10.4410    0.5888 H   0  0  0  0  0  0  0  0  0  0  0  0
   14.0870   10.0172   -0.9125 H   0  0  0  0  0  0  0  0  0  0  0  0
   12.7267   12.0912   -0.7391 H   0  0  0  0  0  0  0  0  0  0  0  0
   13.5536   12.5036    0.7691 H   0  0  0  0  0  0  0  0  0  0  0  0
   12.7169   10.3996    1.7973 H   0  0  0  0  0  0  0  0  0  0  0  0
   11.8803   10.0003    0.2915 H   0  0  0  0  0  0  0  0  0  0  0  0
   10.5255   12.0745    0.4977 H   0  0  0  0  0  0  0  0  0  0  0  0
   11.3577   12.4648    2.0089 H   0  0  0  0  0  0  0  0  0  0  0  0
```

10.5186 10.3487 3.0112 H 0 0 0 0 0 0 0 0 0 0 0 0
9.6761 9.9724 1.5029 H 0 0 0 0 0 0 0 0 0 0 0 0
9.1657 12.4161 3.2538 H 0 0 0 0 0 0 0 0 0 0 0 0
8.3253 12.0447 1.7422 H 0 0 0 0 0 0 0 0 0 0 0 0
19.3312 10.4939 -1.8240 H 0 0 0 0 0 0 0 0 0 0 0 0
18.5057 10.0253 -3.3162 H 0 0 0 0 0 0 0 0 0 0 0 0
7.4777 9.9321 2.7270 H 0 0 0 0 0 0 0 0 0 0 0 0
8.3282 10.2894 4.2353 H 0 0 0 0 0 0 0 0 0 0 0 0
19.3306 12.0863 -4.4237 H 0 0 0 0 0 0 0 0 0 0 0 0
20.1490 12.5640 -2.9305 H 0 0 0 0 0 0 0 0 0 0 0 0
6.1299 12.0008 2.9970 H 0 0 0 0 0 0 0 0 0 0 0 0
6.9801 12.3568 4.5067 H 0 0 0 0 0 0 0 0 0 0 0 0
21.5385 10.5106 -3.0320 H 0 0 0 0 0 0 0 0 0 0 0 0
20.7165 10.0257 -4.5209 H 0 0 0 0 0 0 0 0 0 0 0 0
6.1455 10.2249 5.4757 H 0 0 0 0 0 0 0 0 0 0 0 0
5.2894 9.8772 3.9680 H 0 0 0 0 0 0 0 0 0 0 0 0
21.5333 12.0822 -5.6443 H 0 0 0 0 0 0 0 0 0 0 0 0
22.3508 12.5730 -4.1547 H 0 0 0 0 0 0 0 0 0 0 0 0
23.7444 10.5209 -4.2418 H 0 0 0 0 0 0 0 0 0 0 0 0
22.9250 10.0262 -5.7289 H 0 0 0 0 0 0 0 0 0 0 0 0
24.5551 12.5774 -5.3733 H 0 0 0 0 0 0 0 0 0 0 0 0
23.7370 12.0811 -6.8606 H 0 0 0 0 0 0 0 0 0 0 0 0
25.0909 11.1297 -6.2364 H 0 0 0 0 0 0 0 0 0 0 0 0
1.4851 13.5820 6.3113 H 0 0 0 0 0 0 0 0 0 0 0 0
3.3203 14.6591 5.0414 H 0 0 0 0 0 0 0 0 0 0 0 0
5.2450 13.3085 4.2595 H 0 0 0 0 0 0 0 0 0 0 0 0
3.4996 9.8035 6.0174 H 0 0 0 0 0 0 0 0 0 0 0 0
1.5748 11.1541 6.7993 H 0 0 0 0 0 0 0 0 0 0 0 0
1 2 1 0 0 0 0
2 3 1 0 0 0 0
3 4 1 0 0 0 0
4 5 1 0 0 0 0
5 6 1 0 0 0 0
6 7 1 0 0 0 0
7 8 1 0 0 0 0
8 9 1 0 0 0 0
1 10 1 0 0 0 0
9 11 1 0 0 0 0
10 12 1 0 0 0 0
11 13 1 0 0 0 0
12 14 1 0 0 0 0
13 15 1 0 0 0 0
14 16 1 0 0 0 0
16 17 1 0 0 0 0
17 18 1 0 0 0 0
19 20 4 0 0 0 0
20 21 4 0 0 0 0
21 22 4 0 0 0 0
22 23 4 0 0 0 0
23 24 4 0 0 0 0
24 19 4 0 0 0 0
22 15 1 0 0 0 0
1 25 1 0 0 0 0
1 26 1 0 0 0 0
2 27 1 0 0 0 0
2 28 1 0 0 0 0
3 29 1 0 0 0 0
3 30 1 0 0 0 0
4 31 1 0 0 0 0
4 32 1 0 0 0 0
5 33 1 0 0 0 0
5 34 1 0 0 0 0
6 35 1 0 0 0 0

```
  6 36  1  0  0  0  0
  7 37  1  0  0  0  0
  7 38  1  0  0  0  0
  8 39  1  0  0  0  0
  8 40  1  0  0  0  0
  9 41  1  0  0  0  0
  9 42  1  0  0  0  0
 10 43  1  0  0  0  0
 10 44  1  0  0  0  0
 11 45  1  0  0  0  0
 11 46  1  0  0  0  0
 12 47  1  0  0  0  0
 12 48  1  0  0  0  0
 13 49  1  0  0  0  0
 13 50  1  0  0  0  0
 14 51  1  0  0  0  0
 14 52  1  0  0  0  0
 15 53  1  0  0  0  0
 15 54  1  0  0  0  0
 16 55  1  0  0  0  0
 16 56  1  0  0  0  0
 17 57  1  0  0  0  0
 17 58  1  0  0  0  0
 18 59  1  0  0  0  0
 18 60  1  0  0  0  0
 18 61  1  0  0  0  0
 19 62  1  0  0  0  0
 20 63  1  0  0  0  0
 21 64  1  0  0  0  0
 23 65  1  0  0  0  0
 24 66  1  0  0  0  0
M  END
>  <Alias name>
Octadecylbenzene

>  <deltaH°(c)>   kJ/mol
-14987.09

$$$$
1,1,4,4-Tetraphenylbutane
  ChemBrain IXL

 54 57  0  0  0  0  0  0  0  0  1 V2000
    7.5194    7.8394   -1.7599 C   0  0  0  0  0  0  0  0  0  0  0  0
    6.6750    6.5505   -1.7791 C   0  0  0  0  0  0  0  0  0  0  0  0
    5.2375    6.9026   -1.7906 C   0  0  0  0  0  0  0  0  0  0  0  0
    6.9761    5.7454   -0.5743 C   0  0  0  0  0  0  0  0  0  0  0  0
    9.0162    7.4730   -1.7645 C   0  0  0  0  0  0  0  0  0  0  0  0
    4.8138    8.0850   -2.3976 C   0  0  0  0  0  0  0  0  0  0  0  0
    4.3063    6.0520   -1.1944 C   0  0  0  0  0  0  0  0  0  0  0  0
    7.4232    4.4308   -0.7077 C   0  0  0  0  0  0  0  0  0  0  0  0
    6.8129    6.3013    0.6947 C   0  0  0  0  0  0  0  0  0  0  0  0
    9.8606    8.7620   -1.7758 C   0  0  0  0  0  0  0  0  0  0  0  0
    3.4598    8.4206   -2.4016 C   0  0  0  0  0  0  0  0  0  0  0  0
    2.9523    6.3877   -1.1985 C   0  0  0  0  0  0  0  0  0  0  0  0
    7.7114    3.6734    0.4279 C   0  0  0  0  0  0  0  0  0  0  0  0
    7.1011    5.5440    1.8303 C   0  0  0  0  0  0  0  0  0  0  0  0
    9.5608    9.5588   -0.5651 C   0  0  0  0  0  0  0  0  0  0  0  0
   11.2981    8.4100   -1.7912 C   0  0  0  0  0  0  0  0  0  0  0  0
    2.5291    7.5722   -1.8018 C   0  0  0  0  0  0  0  0  0  0  0  0
    7.5506    4.2301    1.6968 C   0  0  0  0  0  0  0  0  0  0  0  0
    9.7459    9.0013    0.7002 C   0  0  0  0  0  0  0  0  0  0  0  0
    9.0933   10.8672   -0.6893 C   0  0  0  0  0  0  0  0  0  0  0  0
```

```
11.7189    7.2221   -2.3894 C   0  0  0  0  0  0  0  0  0  0  0  0
12.2321    9.2661   -1.2076 C   0  0  0  0  0  0  0  0  0  0  0  0
9.4584    9.7506    1.8413 C   0  0  0  0  0  0  0  0  0  0  0  0
8.8058   11.6164    0.4517 C   0  0  0  0  0  0  0  0  0  0  0  0
13.0730    6.8867   -2.3973 C   0  0  0  0  0  0  0  0  0  0  0  0
13.5862    8.9308   -1.2156 C   0  0  0  0  0  0  0  0  0  0  0  0
8.9882   11.0580    1.7170 C   0  0  0  0  0  0  0  0  0  0  0  0
14.0066    7.7409   -1.8101 C   0  0  0  0  0  0  0  0  0  0  0  0
7.2906    8.4340   -2.6356 H   0  0  0  0  0  0  0  0  0  0  0  0
7.2898    8.4082   -0.8673 H   0  0  0  0  0  0  0  0  0  0  0  0
6.9131    5.9761   -2.6658 H   0  0  0  0  0  0  0  0  0  0  0  0
9.2495    6.8964   -0.8780 H   0  0  0  0  0  0  0  0  0  0  0  0
9.2415    6.8861   -2.6464 H   0  0  0  0  0  0  0  0  0  0  0  0
5.5361    8.7424   -2.8656 H   0  0  0  0  0  0  0  0  0  0  0  0
4.6345    5.1312   -0.7284 H   0  0  0  0  0  0  0  0  0  0  0  0
7.5464    3.9980   -1.6928 H   0  0  0  0  0  0  0  0  0  0  0  0
6.4625    7.3208    0.7984 H   0  0  0  0  0  0  0  0  0  0  0  0
9.6216    9.3424   -2.6583 H   0  0  0  0  0  0  0  0  0  0  0  0
3.1312    9.3399   -2.8705 H   0  0  0  0  0  0  0  0  0  0  0  0
2.2296    5.7286   -0.7334 H   0  0  0  0  0  0  0  0  0  0  0  0
8.0600    2.6533    0.3243 H   0  0  0  0  0  0  0  0  0  0  0  0
6.9760    5.9760    2.8154 H   0  0  0  0  0  0  0  0  0  0  0  0
1.4780    7.8330   -1.8044 H   0  0  0  0  0  0  0  0  0  0  0  0
7.7747    3.6423    2.5784 H   0  0  0  0  0  0  0  0  0  0  0  0
10.1127    7.9869    0.7967 H   0  0  0  0  0  0  0  0  0  0  0  0
8.9534   11.3013   -1.6716 H   0  0  0  0  0  0  0  0  0  0  0  0
10.9944    6.5603   -2.8476 H   0  0  0  0  0  0  0  0  0  0  0  0
11.9061   10.1911   -0.7483 H   0  0  0  0  0  0  0  0  0  0  0  0
9.6004    9.3172    2.8236 H   0  0  0  0  0  0  0  0  0  0  0  0
8.4412   12.6316    0.3553 H   0  0  0  0  0  0  0  0  0  0  0  0
13.3994    5.9633   -2.8594 H   0  0  0  0  0  0  0  0  0  0  0  0
14.3111    9.5942   -0.7602 H   0  0  0  0  0  0  0  0  0  0  0  0
8.7646   11.6396    2.6029 H   0  0  0  0  0  0  0  0  0  0  0  0
15.0577    7.4802   -1.8157 H   0  0  0  0  0  0  0  0  0  0  0  0
1  2  1  0  0  0  0
1  5  1  0  0  0  0
2  3  1  0  0  0  0
2  4  1  0  0  0  0
6  3  4  0  0  0  0
3  7  4  0  0  0  0
8  4  4  0  0  0  0
4  9  4  0  0  0  0
5 10  1  0  0  0  0
11  6  4  0  0  0  0
7 12  4  0  0  0  0
13  8  4  0  0  0  0
9 14  4  0  0  0  0
10 15  1  0  0  0  0
10 16  1  0  0  0  0
17 11  4  0  0  0  0
12 17  4  0  0  0  0
18 13  4  0  0  0  0
14 18  4  0  0  0  0
15 19  4  0  0  0  0
20 15  4  0  0  0  0
21 16  4  0  0  0  0
16 22  4  0  0  0  0
19 23  4  0  0  0  0
24 20  4  0  0  0  0
25 21  4  0  0  0  0
22 26  4  0  0  0  0
23 27  4  0  0  0  0
27 24  4  0  0  0  0
```

```
 28 25  4  0  0  0  0
 26 28  4  0  0  0  0
  1 29  1  0  0  0  0
  1 30  1  0  0  0  0
  2 31  1  0  0  0  0
  5 32  1  0  0  0  0
  5 33  1  0  0  0  0
  6 34  1  0  0  0  0
  7 35  1  0  0  0  0
  8 36  1  0  0  0  0
  9 37  1  0  0  0  0
 10 38  1  0  0  0  0
 11 39  1  0  0  0  0
 12 40  1  0  0  0  0
 13 41  1  0  0  0  0
 14 42  1  0  0  0  0
 17 43  1  0  0  0  0
 18 44  1  0  0  0  0
 19 45  1  0  0  0  0
 20 46  1  0  0  0  0
 21 47  1  0  0  0  0
 22 48  1  0  0  0  0
 23 49  1  0  0  0  0
 24 50  1  0  0  0  0
 25 51  1  0  0  0  0
 26 52  1  0  0  0  0
 27 53  1  0  0  0  0
 28 54  1  0  0  0  0
M  END
>  <Alias name>
1,1,4,4-Tetraphenylbutane

>  <deltaH°(c)>   kJ/mol
-14897

$$$$
Tetrakis(2-methylphenoxy)silane
  ChemBrain IXL

 61 64  0  0  0  0  0  0  0  0  1 V2000
    7.7774    7.4304   -0.0065 Si  0  0  0  0  0  0  0  0  0  0  0  0
    7.1473    7.0675   -1.4735 O   0  0  0  0  0  0  0  0  0  0  0  0
    7.7249    9.0505    0.2230 O   0  0  0  0  0  0  0  0  0  0  0  0
    9.3316    6.9203    0.0615 O   0  0  0  0  0  0  0  0  0  0  0  0
   12.0193    8.6147   -2.6566 C   0  0  0  0  0  0  0  0  0  0  0  0
   12.4549    7.6637   -1.7337 C   0  0  0  0  0  0  0  0  0  0  0  0
   11.5520    7.1021   -0.8290 C   0  0  0  0  0  0  0  0  0  0  0  0
   10.2094    7.4896   -0.8441 C   0  0  0  0  0  0  0  0  0  0  0  0
    9.7751    8.4454   -1.7728 C   0  0  0  0  0  0  0  0  0  0  0  0
   10.6802    9.0052   -2.6761 C   0  0  0  0  0  0  0  0  0  0  0  0
    3.6490    8.0429   -0.7891 C   0  0  0  0  0  0  0  0  0  0  0  0
    3.0594    7.7539   -2.0199 C   0  0  0  0  0  0  0  0  0  0  0  0
    3.8333    7.2386   -3.0599 C   0  0  0  0  0  0  0  0  0  0  0  0
    5.1978    7.0118   -2.8703 C   0  0  0  0  0  0  0  0  0  0  0  0
    5.7936    7.2997   -1.6394 C   0  0  0  0  0  0  0  0  0  0  0  0
    5.0130    7.8172   -0.5966 C   0  0  0  0  0  0  0  0  0  0  0  0
    8.6816    8.6385    2.4140 C   0  0  0  0  0  0  0  0  0  0  0  0
    9.1576    9.1355    3.6285 C   0  0  0  0  0  0  0  0  0  0  0  0
    9.1567   10.5094    3.8701 C   0  0  0  0  0  0  0  0  0  0  0  0
    8.6797   11.3882    2.8974 C   0  0  0  0  0  0  0  0  0  0  0  0
    8.2030   10.8940    1.6817 C   0  0  0  0  0  0  0  0  0  0  0  0
    8.2016    9.5186    1.4344 C   0  0  0  0  0  0  0  0  0  0  0  0
    6.0209    6.4610   -3.9842 C   0  0  0  0  0  0  0  0  0  0  0  0
```

7.6944 11.8366 0.6451 C 0 0 0 0 0 0 0 0 0 0 0 0
12.0225 6.0865 0.1552 C 0 0 0 0 0 0 0 0 0 0 0 0
6.9074 6.6853 1.1637 O 0 0 0 0 0 0 0 0 0 0 0 0
7.2997 3.2106 -0.0325 C 0 0 0 0 0 0 0 0 0 0 0 0
6.5696 2.5241 0.9380 C 0 0 0 0 0 0 0 0 0 0 0 0
5.9572 3.2262 1.9762 C 0 0 0 0 0 0 0 0 0 0 0 0
6.0741 4.6157 2.0452 C 0 0 0 0 0 0 0 0 0 0 0 0
6.8044 5.3084 1.0758 C 0 0 0 0 0 0 0 0 0 0 0 0
7.4184 4.5997 0.0340 C 0 0 0 0 0 0 0 0 0 0 0 0
5.4179 5.3613 3.1565 C 0 0 0 0 0 0 0 0 0 0 0 0
12.7205 9.0497 -3.3579 H 0 0 0 0 0 0 0 0 0 0 0 0
13.4946 7.3609 -1.7190 H 0 0 0 0 0 0 0 0 0 0 0 0
8.7382 8.7526 -1.7932 H 0 0 0 0 0 0 0 0 0 0 0 0
10.3424 9.7434 -3.3927 H 0 0 0 0 0 0 0 0 0 0 0 0
3.0479 8.4429 0.0180 H 0 0 0 0 0 0 0 0 0 0 0 0
2.0012 7.9295 -2.1680 H 0 0 0 0 0 0 0 0 0 0 0 0
3.3751 7.0144 -4.0152 H 0 0 0 0 0 0 0 0 0 0 0 0
5.4639 8.0434 0.3602 H 0 0 0 0 0 0 0 0 0 0 0 0
8.6849 7.5721 2.2331 H 0 0 0 0 0 0 0 0 0 0 0 0
9.5279 8.4537 4.3839 H 0 0 0 0 0 0 0 0 0 0 0 0
9.5262 10.8937 4.8127 H 0 0 0 0 0 0 0 0 0 0 0 0
8.6793 12.4546 3.0855 H 0 0 0 0 0 0 0 0 0 0 0 0
6.7693 7.1883 -4.2737 H 0 0 0 0 0 0 0 0 0 0 0 0
5.3827 6.2429 -4.8317 H 0 0 0 0 0 0 0 0 0 0 0 0
6.5092 5.5515 -3.6566 H 0 0 0 0 0 0 0 0 0 0 0 0
6.6708 11.5833 0.3980 H 0 0 0 0 0 0 0 0 0 0 0 0
7.7335 12.8494 1.0270 H 0 0 0 0 0 0 0 0 0 0 0 0
8.3092 11.7619 -0.2434 H 0 0 0 0 0 0 0 0 0 0 0 0
13.0563 6.2833 0.4111 H 0 0 0 0 0 0 0 0 0 0 0 0
11.9401 5.0979 -0.2791 H 0 0 0 0 0 0 0 0 0 0 0 0
11.4129 6.1402 1.0489 H 0 0 0 0 0 0 0 0 0 0 0 0
7.7750 2.6651 -0.8382 H 0 0 0 0 0 0 0 0 0 0 0 0
6.4783 1.4463 0.8854 H 0 0 0 0 0 0 0 0 0 0 0 0
5.3905 2.6929 2.7294 H 0 0 0 0 0 0 0 0 0 0 0 0
7.9859 5.1260 -0.7216 H 0 0 0 0 0 0 0 0 0 0 0 0
4.4896 4.8706 3.4220 H 0 0 0 0 0 0 0 0 0 0 0 0
6.0755 5.3795 4.0167 H 0 0 0 0 0 0 0 0 0 0 0 0
5.2116 6.3759 2.8383 H 0 0 0 0 0 0 0 0 0 0 0 0
2 1 1 0 0 0 0
3 1 1 0 0 0 0
4 1 1 0 0 0 0
5 6 4 0 0 0 0
6 7 4 0 0 0 0
7 8 4 0 0 0 0
8 9 4 0 0 0 0
9 10 4 0 0 0 0
10 5 4 0 0 0 0
8 4 1 0 0 0 0
11 12 4 0 0 0 0
12 13 4 0 0 0 0
13 14 4 0 0 0 0
14 15 4 0 0 0 0
15 16 4 0 0 0 0
16 11 4 0 0 0 0
15 2 1 0 0 0 0
17 18 4 0 0 0 0
18 19 4 0 0 0 0
19 20 4 0 0 0 0
20 21 4 0 0 0 0
21 22 4 0 0 0 0
22 17 4 0 0 0 0
22 3 1 0 0 0 0
14 23 1 0 0 0 0

21 24 1 0 0 0 0
 7 25 1 0 0 0 0
27 28 4 0 0 0 0
28 29 4 0 0 0 0
29 30 4 0 0 0 0
30 31 4 0 0 0 0
31 32 4 0 0 0 0
32 27 4 0 0 0 0
31 26 1 0 0 0 0
30 33 1 0 0 0 0
26 1 1 0 0 0 0
 5 34 1 0 0 0 0
 6 35 1 0 0 0 0
 9 36 1 0 0 0 0
10 37 1 0 0 0 0
11 38 1 0 0 0 0
12 39 1 0 0 0 0
13 40 1 0 0 0 0
16 41 1 0 0 0 0
17 42 1 0 0 0 0
18 43 1 0 0 0 0
19 44 1 0 0 0 0
20 45 1 0 0 0 0
23 46 1 0 0 0 0
23 47 1 0 0 0 0
23 48 1 0 0 0 0
24 49 1 0 0 0 0
24 50 1 0 0 0 0
24 51 1 0 0 0 0
25 52 1 0 0 0 0
25 53 1 0 0 0 0
25 54 1 0 0 0 0
27 55 1 0 0 0 0
28 56 1 0 0 0 0
29 57 1 0 0 0 0
32 58 1 0 0 0 0
33 59 1 0 0 0 0
33 60 1 0 0 0 0
33 61 1 0 0 0 0
M END
> <Alias name>
Tetrakis(2-methylphenoxy)silane

> <deltaH°(c)>   kJ/mol
-14894

$$$$
Tetrakis(4-methylphenoxy)silane
  ChemBrain IXL

 61 64 0 0 0 0 0 0 0 0 1 V2000
    7.8580   7.3189   -0.0811 Si 0 0 0 0 0 0 0 0 0 0 0 0
    7.4431   6.3216   -1.3121 O   0 0 0 0 0 0 0 0 0 0 0 0
    7.8979   8.8642   -0.6216 O   0 0 0 0 0 0 0 0 0 0 0 0
    9.3361   6.9004   0.4870 O   0 0 0 0 0 0 0 0 0 0 0 0
   12.5438   7.1154   -2.1853 C   0 0 0 0 0 0 0 0 0 0 0 0
   11.2619   7.4568   -2.6176 C   0 0 0 0 0 0 0 0 0 0 0 0
   10.1841   7.3884   -1.7333 C   0 0 0 0 0 0 0 0 0 0 0 0
   10.3870   6.9766   -0.4090 C   0 0 0 0 0 0 0 0 0 0 0 0
   11.6723   6.6360   0.0185 C   0 0 0 0 0 0 0 0 0 0 0 0
   12.7487   6.7051   -0.8675 C   0 0 0 0 0 0 0 0 0 0 0 0
    4.0494   7.6467   -1.9297 C   0 0 0 0 0 0 0 0 0 0 0 0
    3.6444   6.8254   -2.9825 C   0 0 0 0 0 0 0 0 0 0 0 0

```
    4.5062    5.8430   -3.4713 C   0  0  0  0  0  0  0  0  0  0  0  0
    5.7730    5.6811   -2.9080 C   0  0  0  0  0  0  0  0  0  0  0  0
    6.1835    6.5005   -1.8540 C   0  0  0  0  0  0  0  0  0  0  0  0
    5.3158    7.4866   -1.3649 C   0  0  0  0  0  0  0  0  0  0  0  0
    8.2441   11.1752   -0.0873 C   0  0  0  0  0  0  0  0  0  0  0  0
    8.5377   12.1765    0.8399 C   0  0  0  0  0  0  0  0  0  0  0  0
    8.7791   11.8449    2.1734 C   0  0  0  0  0  0  0  0  0  0  0  0
    8.7270   10.5114    2.5806 C   0  0  0  0  0  0  0  0  0  0  0  0
    8.4337    9.5084    1.6551 C   0  0  0  0  0  0  0  0  0  0  0  0
    8.1907    9.8387    0.3148 C   0  0  0  0  0  0  0  0  0  0  0  0
    6.7546    7.1909    1.1228 O   0  0  0  0  0  0  0  0  0  0  0  0
    5.4485    4.4988    3.2591 C   0  0  0  0  0  0  0  0  0  0  0  0
    6.1740    3.4025    2.7918 C   0  0  0  0  0  0  0  0  0  0  0  0
    7.0999    3.5675    1.7612 C   0  0  0  0  0  0  0  0  0  0  0  0
    7.3014    4.8283    1.1970 C   0  0  0  0  0  0  0  0  0  0  0  0
    6.5739    5.9314    1.6645 C   0  0  0  0  0  0  0  0  0  0  0  0
    5.6483    5.7605    2.6963 C   0  0  0  0  0  0  0  0  0  0  0  0
    5.9602    2.0558    3.3930 C   0  0  0  0  0  0  0  0  0  0  0  0
    2.2923    6.9974   -3.5848 C   0  0  0  0  0  0  0  0  0  0  0  0
    9.0924   12.9149    3.1623 C   0  0  0  0  0  0  0  0  0  0  0  0
   13.6932    7.1890   -3.1309 C   0  0  0  0  0  0  0  0  0  0  0  0
   11.1029    7.7752   -3.6404 H   0  0  0  0  0  0  0  0  0  0  0  0
    9.1928    7.6544   -2.0748 H   0  0  0  0  0  0  0  0  0  0  0  0
   11.8343    6.3173    1.0406 H   0  0  0  0  0  0  0  0  0  0  0  0
   13.7435    6.4401   -0.5317 H   0  0  0  0  0  0  0  0  0  0  0  0
    3.3806    8.4092   -1.5503 H   0  0  0  0  0  0  0  0  0  0  0  0
    4.1920    5.2055   -4.2884 H   0  0  0  0  0  0  0  0  0  0  0  0
    6.4397    4.9179   -3.2894 H   0  0  0  0  0  0  0  0  0  0  0  0
    5.6236    8.1264   -0.5489 H   0  0  0  0  0  0  0  0  0  0  0  0
    8.0572   11.4354   -1.1216 H   0  0  0  0  0  0  0  0  0  0  0  0
    8.5781   13.2115    0.5237 H   0  0  0  0  0  0  0  0  0  0  0  0
    8.9144   10.2540    3.6156 H   0  0  0  0  0  0  0  0  0  0  0  0
    8.3948    8.4766    1.9771 H   0  0  0  0  0  0  0  0  0  0  0  0
    4.7297    4.3710    4.0590 H   0  0  0  0  0  0  0  0  0  0  0  0
    7.6631    2.7165    1.3986 H   0  0  0  0  0  0  0  0  0  0  0  0
    8.0206    4.9495    0.3983 H   0  0  0  0  0  0  0  0  0  0  0  0
    5.0838    6.6096    3.0610 H   0  0  0  0  0  0  0  0  0  0  0  0
    6.9096    1.5406    3.4716 H   0  0  0  0  0  0  0  0  0  0  0  0
    5.5262    2.1652    4.3793 H   0  0  0  0  0  0  0  0  0  0  0  0
    5.2895    1.4826    2.7651 H   0  0  0  0  0  0  0  0  0  0  0  0
    1.8685    6.0251   -3.8043 H   0  0  0  0  0  0  0  0  0  0  0  0
    1.6503    7.5218   -2.8878 H   0  0  0  0  0  0  0  0  0  0  0  0
    2.3751    7.5700   -4.5002 H   0  0  0  0  0  0  0  0  0  0  0  0
    8.1757   13.4044    3.4668 H   0  0  0  0  0  0  0  0  0  0  0  0
    9.7565   13.6412    2.7102 H   0  0  0  0  0  0  0  0  0  0  0  0
    9.5726   12.4768    4.0286 H   0  0  0  0  0  0  0  0  0  0  0  0
   13.9613    8.2256   -3.2930 H   0  0  0  0  0  0  0  0  0  0  0  0
   13.4141    6.7365   -4.0744 H   0  0  0  0  0  0  0  0  0  0  0  0
   14.5398    6.6584   -2.7127 H   0  0  0  0  0  0  0  0  0  0  0  0
  2  1  1  0  0  0  0
  3  1  1  0  0  0  0
  4  1  1  0  0  0  0
  5  6  4  0  0  0  0
  6  7  4  0  0  0  0
  7  8  4  0  0  0  0
  8  9  4  0  0  0  0
  9 10  4  0  0  0  0
 10  5  4  0  0  0  0
  8  4  1  0  0  0  0
 11 12  4  0  0  0  0
 12 13  4  0  0  0  0
 13 14  4  0  0  0  0
 14 15  4  0  0  0  0
```

```
 15 16  4  0  0  0  0
 16 11  4  0  0  0  0
 15  2  1  0  0  0  0
 17 18  4  0  0  0  0
 18 19  4  0  0  0  0
 19 20  4  0  0  0  0
 20 21  4  0  0  0  0
 21 22  4  0  0  0  0
 22 17  4  0  0  0  0
 22  3  1  0  0  0  0
 24 25  4  0  0  0  0
 25 26  4  0  0  0  0
 26 27  4  0  0  0  0
 27 28  4  0  0  0  0
 28 29  4  0  0  0  0
 29 24  4  0  0  0  0
 28 23  1  0  0  0  0
 23  1  1  0  0  0  0
 25 30  1  0  0  0  0
 12 31  1  0  0  0  0
 19 32  1  0  0  0  0
  5 33  1  0  0  0  0
  6 34  1  0  0  0  0
  7 35  1  0  0  0  0
  9 36  1  0  0  0  0
 10 37  1  0  0  0  0
 11 38  1  0  0  0  0
 13 39  1  0  0  0  0
 14 40  1  0  0  0  0
 16 41  1  0  0  0  0
 17 42  1  0  0  0  0
 18 43  1  0  0  0  0
 20 44  1  0  0  0  0
 21 45  1  0  0  0  0
 24 46  1  0  0  0  0
 26 47  1  0  0  0  0
 27 48  1  0  0  0  0
 29 49  1  0  0  0  0
 30 50  1  0  0  0  0
 30 51  1  0  0  0  0
 30 52  1  0  0  0  0
 31 53  1  0  0  0  0
 31 54  1  0  0  0  0
 31 55  1  0  0  0  0
 32 56  1  0  0  0  0
 32 57  1  0  0  0  0
 32 58  1  0  0  0  0
 33 59  1  0  0  0  0
 33 60  1  0  0  0  0
 33 61  1  0  0  0  0
M  END
>  <Alias name>
Tetrakis(4-methylphenoxy)silane

>  <deltaH°(c)>   kJ/mol
-14886

$$$$
Tetrakis(3-methylphenoxy)silane
  ChemBrain IXL

 61 64  0  0  0  0  0  0  0  0  1 V2000
    7.7843    7.0780   -0.1939 Si  0  0  0  0  0  0  0  0  0  0  0  0
```

8.3741	7.1460	-1.7202	O	0	0	0	0	0	0	0	0	0	0	0	0	0
7.3488	8.5807	0.2899	O	0	0	0	0	0	0	0	0	0	0	0	0	0
8.9376	6.4928	0.8112	O	0	0	0	0	0	0	0	0	0	0	0	0	0
12.4605	8.7129	1.1771	C	0	0	0	0	0	0	0	0	0	0	0	0	0
11.4438	9.1664	0.3359	C	0	0	0	0	0	0	0	0	0	0	0	0	0
10.2635	8.4324	0.2068	C	0	0	0	0	0	0	0	0	0	0	0	0	0
10.0970	7.2384	0.9217	C	0	0	0	0	0	0	0	0	0	0	0	0	0
11.1180	6.7892	1.7622	C	0	0	0	0	0	0	0	0	0	0	0	0	0
12.2975	7.5248	1.8899	C	0	0	0	0	0	0	0	0	0	0	0	0	0
5.2884	8.2659	-3.4167	C	0	0	0	0	0	0	0	0	0	0	0	0	0
5.7372	8.3396	-4.7358	C	0	0	0	0	0	0	0	0	0	0	0	0	0
7.0593	8.0158	-5.0415	C	0	0	0	0	0	0	0	0	0	0	0	0	0
7.9337	7.6180	-4.0287	C	0	0	0	0	0	0	0	0	0	0	0	0	0
7.4899	7.5424	-2.7065	C	0	0	0	0	0	0	0	0	0	0	0	0	0
6.1611	7.8683	-2.4023	C	0	0	0	0	0	0	0	0	0	0	0	0	0
6.4108	9.9433	2.0247	C	0	0	0	0	0	0	0	0	0	0	0	0	0
5.8489	10.0745	3.2957	C	0	0	0	0	0	0	0	0	0	0	0	0	0
5.6681	8.9486	4.0993	C	0	0	0	0	0	0	0	0	0	0	0	0	0
6.0492	7.6902	3.6324	C	0	0	0	0	0	0	0	0	0	0	0	0	0
6.6114	7.5567	2.3616	C	0	0	0	0	0	0	0	0	0	0	0	0	0
6.7942	8.6862	1.5521	C	0	0	0	0	0	0	0	0	0	0	0	0	0
6.4765	6.0925	-0.1557	O	0	0	0	0	0	0	0	0	0	0	0	0	0
5.7756	2.5725	-0.9525	C	0	0	0	0	0	0	0	0	0	0	0	0	0
7.0310	2.1320	-1.3719	C	0	0	0	0	0	0	0	0	0	0	0	0	0
8.1108	3.0155	-1.3870	C	0	0	0	0	0	0	0	0	0	0	0	0	0
7.9361	4.3402	-0.9829	C	0	0	0	0	0	0	0	0	0	0	0	0	0
6.6759	4.7855	-0.5610	C	0	0	0	0	0	0	0	0	0	0	0	0	0
5.5986	3.8967	-0.5480	C	0	0	0	0	0	0	0	0	0	0	0	0	0
9.4520	2.5452	-1.8350	C	0	0	0	0	0	0	0	0	0	0	0	0	0
3.8761	8.6119	-3.0906	C	0	0	0	0	0	0	0	0	0	0	0	0	0
5.8558	6.4878	4.4912	C	0	0	0	0	0	0	0	0	0	0	0	0	0
11.6177	10.4355	-0.4254	C	0	0	0	0	0	0	0	0	0	0	0	0	0
13.3758	9.2829	1.2768	H	0	0	0	0	0	0	0	0	0	0	0	0	0
9.4790	8.7892	-0.4468	H	0	0	0	0	0	0	0	0	0	0	0	0	0
10.9942	5.8673	2.3167	H	0	0	0	0	0	0	0	0	0	0	0	0	0
13.0866	7.1729	2.5427	H	0	0	0	0	0	0	0	0	0	0	0	0	0
5.0596	8.6481	-5.5221	H	0	0	0	0	0	0	0	0	0	0	0	0	0
7.4075	8.0730	-6.0652	H	0	0	0	0	0	0	0	0	0	0	0	0	0
8.9594	7.3673	-4.2688	H	0	0	0	0	0	0	0	0	0	0	0	0	0
5.8072	7.8131	-1.3816	H	0	0	0	0	0	0	0	0	0	0	0	0	0
6.5500	10.8190	1.4031	H	0	0	0	0	0	0	0	0	0	0	0	0	0
5.5530	11.0511	3.6582	H	0	0	0	0	0	0	0	0	0	0	0	0	0
5.2320	9.0513	5.0852	H	0	0	0	0	0	0	0	0	0	0	0	0	0
6.9048	6.5784	2.0052	H	0	0	0	0	0	0	0	0	0	0	0	0	0
4.9377	1.8867	-0.9409	H	0	0	0	0	0	0	0	0	0	0	0	0	0
7.1674	1.1045	-1.6856	H	0	0	0	0	0	0	0	0	0	0	0	0	0
8.7770	5.0204	-0.9969	H	0	0	0	0	0	0	0	0	0	0	0	0	0
4.6230	4.2359	-0.2229	H	0	0	0	0	0	0	0	0	0	0	0	0	0
10.2183	2.9999	-1.2193	H	0	0	0	0	0	0	0	0	0	0	0	0	0
9.6061	2.8263	-2.8693	H	0	0	0	0	0	0	0	0	0	0	0	0	0
9.5066	1.4675	-1.7418	H	0	0	0	0	0	0	0	0	0	0	0	0	0
3.2221	8.2246	-3.8621	H	0	0	0	0	0	0	0	0	0	0	0	0	0
3.6079	8.1742	-2.1369	H	0	0	0	0	0	0	0	0	0	0	0	0	0
3.7718	9.6884	-3.0358	H	0	0	0	0	0	0	0	0	0	0	0	0	0
6.2263	6.6928	5.4879	H	0	0	0	0	0	0	0	0	0	0	0	0	0
6.3989	5.6512	4.0691	H	0	0	0	0	0	0	0	0	0	0	0	0	0
4.8014	6.2456	4.5408	H	0	0	0	0	0	0	0	0	0	0	0	0	0
11.1621	10.3355	-1.4029	H	0	0	0	0	0	0	0	0	0	0	0	0	0
11.1435	11.2469	0.1125	H	0	0	0	0	0	0	0	0	0	0	0	0	0
12.6739	10.6466	-0.5395	H	0	0	0	0	0	0	0	0	0	0	0	0	0

2	1	1	0	0	0	0
3	1	1	0	0	0	0
4	1	1	0	0	0	0

```
  5  6  4  0  0  0  0
  6  7  4  0  0  0  0
  7  8  4  0  0  0  0
  8  9  4  0  0  0  0
  9 10  4  0  0  0  0
 10  5  4  0  0  0  0
  8  4  1  0  0  0  0
 11 12  4  0  0  0  0
 12 13  4  0  0  0  0
 13 14  4  0  0  0  0
 14 15  4  0  0  0  0
 15 16  4  0  0  0  0
 16 11  4  0  0  0  0
 15  2  1  0  0  0  0
 17 18  4  0  0  0  0
 18 19  4  0  0  0  0
 19 20  4  0  0  0  0
 20 21  4  0  0  0  0
 21 22  4  0  0  0  0
 22 17  4  0  0  0  0
 22  3  1  0  0  0  0
 24 25  4  0  0  0  0
 25 26  4  0  0  0  0
 26 27  4  0  0  0  0
 27 28  4  0  0  0  0
 28 29  4  0  0  0  0
 29 24  4  0  0  0  0
 28 23  1  0  0  0  0
 23  1  1  0  0  0  0
 26 30  1  0  0  0  0
 11 31  1  0  0  0  0
 20 32  1  0  0  0  0
  6 33  1  0  0  0  0
  5 34  1  0  0  0  0
  7 35  1  0  0  0  0
  9 36  1  0  0  0  0
 10 37  1  0  0  0  0
 12 38  1  0  0  0  0
 13 39  1  0  0  0  0
 14 40  1  0  0  0  0
 16 41  1  0  0  0  0
 17 42  1  0  0  0  0
 18 43  1  0  0  0  0
 19 44  1  0  0  0  0
 21 45  1  0  0  0  0
 24 46  1  0  0  0  0
 25 47  1  0  0  0  0
 27 48  1  0  0  0  0
 29 49  1  0  0  0  0
 30 50  1  0  0  0  0
 30 51  1  0  0  0  0
 30 52  1  0  0  0  0
 31 53  1  0  0  0  0
 31 54  1  0  0  0  0
 31 55  1  0  0  0  0
 32 56  1  0  0  0  0
 32 57  1  0  0  0  0
 32 58  1  0  0  0  0
 33 59  1  0  0  0  0
 33 60  1  0  0  0  0
 33 61  1  0  0  0  0
M  END
>  <Alias name>
```

Tetrakis(3-methylphenoxy)silane

> <deltaH°(c)>   kJ/mol
-14883

$$$$
Bis(dibenzoylmethanate)copper(II)
  ChemBrain IXL

```
 57 62  0  0  0  0  0  0  0  0  1 V2000
    8.7643   13.4574    3.1099 C   0  0  0  0  0  0  0  0  0  0  0  0
    9.8550   12.7896    2.5525 C   0  0  0  0  0  0  0  0  0  0  0  0
    9.6526   11.8316    1.5574 C   0  0  0  0  0  0  0  0  0  0  0  0
    8.3584   11.5402    1.1169 C   0  0  0  0  0  0  0  0  0  0  0  0
    7.2677   12.2081    1.6778 C   0  0  0  0  0  0  0  0  0  0  0  0
    7.4710   13.1664    2.6732 C   0  0  0  0  0  0  0  0  0  0  0  0
    8.1547   10.5186    0.0543 C   0  0  0  0  0  0  0  0  0  0  0  0
    9.1358    9.9548   -0.3938 O   0  0  0  0  0  0  0  0  0  0  0  0
    6.7540   10.1919   -0.4688 C   0  0  0  0  0  0  0  0  0  0  0  0
    6.3738    8.9239   -0.8775 C   0  0  0  0  0  0  0  0  0  0  0  0
    7.2763    7.7128   -0.8053 O   0  5  0  0  0  0  0  0  0  0  0  0
    9.1564    8.0981   -0.5312 Cu  0  0  0  0  0  0  0  0  0  0  0  0
    4.1253    9.8067   -1.4554 C   0  0  0  0  0  0  0  0  0  0  0  0
    5.0159    8.7344   -1.4074 C   0  0  0  0  0  0  0  0  0  0  0  0
    4.6251    7.4782   -1.8626 C   0  0  0  0  0  0  0  0  0  0  0  0
    3.3397    7.2933   -2.3658 C   0  0  0  0  0  0  0  0  0  0  0  0
    2.4487    8.3658   -2.4127 C   0  0  0  0  0  0  0  0  0  0  0  0
    2.8430    9.6225   -1.9573 C   0  0  0  0  0  0  0  0  0  0  0  0
   14.8267    9.3776   -3.3018 C   0  0  0  0  0  0  0  0  0  0  0  0
   13.5227    9.7265   -3.6556 C   0  0  0  0  0  0  0  0  0  0  0  0
   12.4380    9.0515   -3.0876 C   0  0  0  0  0  0  0  0  0  0  0  0
   12.6539    8.0234   -2.1636 C   0  0  0  0  0  0  0  0  0  0  0  0
   13.9640    7.6783   -1.8095 C   0  0  0  0  0  0  0  0  0  0  0  0
   15.0477    8.3546   -2.3786 C   0  0  0  0  0  0  0  0  0  0  0  0
   11.4872    7.3002   -1.5583 C   0  0  0  0  0  0  0  0  0  0  0  0
   10.3501    7.6114   -1.8786 O   0  0  0  0  0  0  0  0  0  0  0  0
   11.7040    6.1962   -0.5629 C   0  0  0  0  0  0  0  0  0  0  0  0
   10.9672    6.1000    0.5835 C   0  0  0  0  0  0  0  0  0  0  0  0
    9.9269    7.1237    0.9754 O   0  5  0  0  0  0  0  0  0  0  0  0
   10.4675    4.8196    2.6466 C   0  0  0  0  0  0  0  0  0  0  0  0
   11.2067    4.9505    1.4751 C   0  0  0  0  0  0  0  0  0  0  0  0
   12.1700    3.9941    1.1437 C   0  0  0  0  0  0  0  0  0  0  0  0
   12.3901    2.9080    1.9828 C   0  0  0  0  0  0  0  0  0  0  0  0
   11.6490    2.7750    3.1553 C   0  0  0  0  0  0  0  0  0  0  0  0
   10.6880    3.7308    3.4875 C   0  0  0  0  0  0  0  0  0  0  0  0
    8.9213   14.2018    3.8816 H   0  0  0  0  0  0  0  0  0  0  0  0
   10.8592   13.0148    2.8924 H   0  0  0  0  0  0  0  0  0  0  0  0
   10.5007   11.3134    1.1264 H   0  0  0  0  0  0  0  0  0  0  0  0
    6.2640   11.9832    1.3416 H   0  0  0  0  0  0  0  0  0  0  0  0
    6.6246   13.6843    3.1070 H   0  0  0  0  0  0  0  0  0  0  0  0
    6.0320   10.9886   -0.5209 H   0  0  0  0  0  0  0  0  0  0  0  0
    4.4285   10.7815   -1.1039 H   0  0  0  0  0  0  0  0  0  0  0  0
    5.3203    6.6466   -1.8257 H   0  0  0  0  0  0  0  0  0  0  0  0
    3.0333    6.3182   -2.7199 H   0  0  0  0  0  0  0  0  0  0  0  0
    1.4515    8.2228   -2.8024 H   0  0  0  0  0  0  0  0  0  0  0  0
    2.1539   10.4551   -1.9934 H   0  0  0  0  0  0  0  0  0  0  0  0
   15.6679    9.9011   -3.7440 H   0  0  0  0  0  0  0  0  0  0  0  0
   13.3517   10.5220   -4.3717 H   0  0  0  0  0  0  0  0  0  0  0  0
   11.4286    9.3262   -3.3640 H   0  0  0  0  0  0  0  0  0  0  0  0
   14.1403    6.8865   -1.0931 H   0  0  0  0  0  0  0  0  0  0  0  0
   16.0610    8.0849   -2.1032 H   0  0  0  0  0  0  0  0  0  0  0  0
   12.4635    5.4588   -0.7646 H   0  0  0  0  0  0  0  0  0  0  0  0
    9.7218    5.5629    2.9027 H   0  0  0  0  0  0  0  0  0  0  0  0
```

12.7463	4.0937	0.2355	H	0	0	0	0	0	0	0	0	0	0	0	0
13.1364	2.1680	1.7237	H	0	0	0	0	0	0	0	0	0	0	0	0
11.8194	1.9304	3.8074	H	0	0	0	0	0	0	0	0	0	0	0	0
10.1138	3.6270	4.3977	H	0	0	0	0	0	0	0	0	0	0	0	0

1	2	4	0	0	0	0
2	3	4	0	0	0	0
3	4	4	0	0	0	0
4	5	4	0	0	0	0
5	6	4	0	0	0	0
6	1	4	0	0	0	0
7	8	2	0	0	0	0
7	9	1	0	0	0	0
7	4	1	0	0	0	0
9	10	2	0	0	0	0
10	11	1	0	0	0	0
11	12	1	0	0	0	0
8	12	1	0	0	0	0
13	14	4	0	0	0	0
14	15	4	0	0	0	0
15	16	4	0	0	0	0
16	17	4	0	0	0	0
17	18	4	0	0	0	0
18	13	4	0	0	0	0
14	10	1	0	0	0	0
19	20	4	0	0	0	0
20	21	4	0	0	0	0
21	22	4	0	0	0	0
22	23	4	0	0	0	0
23	24	4	0	0	0	0
24	19	4	0	0	0	0
25	26	2	0	0	0	0
25	27	1	0	0	0	0
25	22	1	0	0	0	0
27	28	2	0	0	0	0
28	29	1	0	0	0	0
30	31	4	0	0	0	0
31	32	4	0	0	0	0
32	33	4	0	0	0	0
33	34	4	0	0	0	0
34	35	4	0	0	0	0
35	30	4	0	0	0	0
31	28	1	0	0	0	0
29	12	1	0	0	0	0
26	12	1	0	0	0	0
1	36	1	0	0	0	0
2	37	1	0	0	0	0
3	38	1	0	0	0	0
5	39	1	0	0	0	0
6	40	1	0	0	0	0
9	41	1	0	0	0	0
13	42	1	0	0	0	0
15	43	1	0	0	0	0
16	44	1	0	0	0	0
17	45	1	0	0	0	0
18	46	1	0	0	0	0
19	47	1	0	0	0	0
20	48	1	0	0	0	0
21	49	1	0	0	0	0
23	50	1	0	0	0	0
24	51	1	0	0	0	0
27	52	1	0	0	0	0
30	53	1	0	0	0	0
32	54	1	0	0	0	0

```
 33 55  1  0  0  0  0
 34 56  1  0  0  0  0
 35 57  1  0  0  0  0
M  END
>  <Alias name>
Bis(dibenzoylmethanate)copper(II)

>  <deltaH°(c)>   kJ/mol
-14595.4

$$$$
N,N-Ethylene bis(N',N,-diphenyl urea)
  ChemBrain IXL

 60 63  0  0  0  0  0  0  0  0  1 V2000
    6.2558   10.5608    0.2018 N   0  0  0  0  0  0  0  0  0  0  0  0
    7.3220    9.6884    0.3799 C   0  0  0  0  0  0  0  0  0  0  0  0
    6.4665   11.7884   -0.4463 C   0  0  0  0  0  0  0  0  0  0  0  0
    4.9810   10.2085    0.6727 C   0  0  0  0  0  0  0  0  0  0  0  0
    8.6222   10.1790    0.3435 N   0  0  0  0  0  0  0  0  0  0  0  0
    7.1188    8.5002    0.5665 O   0  0  0  0  0  0  0  0  0  0  0  0
    9.7477    9.2507    0.3440 C   0  0  0  0  0  0  0  0  0  0  0  0
   11.0540   10.0444    0.3355 C   0  0  0  0  0  0  0  0  0  0  0  0
   12.1796    9.1161    0.3335 N   0  0  0  0  0  0  0  0  0  0  0  0
   13.4801    9.6067    0.3628 C   0  0  0  0  0  0  0  0  0  0  0  0
   14.5462    8.7326    0.1908 N   0  0  0  0  0  0  0  0  0  0  0  0
   13.6838   10.7966    0.5381 O   0  0  0  0  0  0  0  0  0  0  0  0
   15.8197    9.0851    0.6646 C   0  0  0  0  0  0  0  0  0  0  0  0
   14.3365    7.5030   -0.4540 C   0  0  0  0  0  0  0  0  0  0  0  0
    5.7170   12.9103   -0.0878 C   0  0  0  0  0  0  0  0  0  0  0  0
    5.9317   14.1293   -0.7333 C   0  0  0  0  0  0  0  0  0  0  0  0
    6.8960   14.2275   -1.7368 C   0  0  0  0  0  0  0  0  0  0  0  0
    7.6460   13.1071   -2.0948 C   0  0  0  0  0  0  0  0  0  0  0  0
    7.4316   11.8882   -1.4499 C   0  0  0  0  0  0  0  0  0  0  0  0
    4.8517    9.2502    1.6793 C   0  0  0  0  0  0  0  0  0  0  0  0
    3.5862    8.8964    2.1492 C   0  0  0  0  0  0  0  0  0  0  0  0
    2.4487    9.5002    1.6129 C   0  0  0  0  0  0  0  0  0  0  0  0
    2.5765   10.4577    0.6063 C   0  0  0  0  0  0  0  0  0  0  0  0
    3.8422   10.8117    0.1359 C   0  0  0  0  0  0  0  0  0  0  0  0
   16.9596    8.4803    0.1318 C   0  0  0  0  0  0  0  0  0  0  0  0
   18.2242    8.8344    0.6050 C   0  0  0  0  0  0  0  0  0  0  0  0
   18.3499    9.7934    1.6104 C   0  0  0  0  0  0  0  0  0  0  0  0
   17.2113   10.3987    2.1427 C   0  0  0  0  0  0  0  0  0  0  0  0
   15.9469   10.0448    1.6700 C   0  0  0  0  0  0  0  0  0  0  0  0
   13.3724    7.3999   -1.4584 C   0  0  0  0  0  0  0  0  0  0  0  0
   13.1593    6.1791   -2.1002 C   0  0  0  0  0  0  0  0  0  0  0  0
   13.9095    5.0601   -1.7381 C   0  0  0  0  0  0  0  0  0  0  0  0
   14.8726    5.1616   -0.7339 C   0  0  0  0  0  0  0  0  0  0  0  0
   15.0860    6.3825   -0.0916 C   0  0  0  0  0  0  0  0  0  0  0  0
    8.7871   11.1741    0.3159 H   0  0  0  0  0  0  0  0  0  0  0  0
   12.0146    8.1208    0.3099 H   0  0  0  0  0  0  0  0  0  0  0  0
    9.6997    8.6231   -0.5372 H   0  0  0  0  0  0  0  0  0  0  0  0
    9.7060    8.6318    1.2318 H   0  0  0  0  0  0  0  0  0  0  0  0
   11.1029   10.6705    1.2179 H   0  0  0  0  0  0  0  0  0  0  0  0
   11.0949   10.6648   -0.5512 H   0  0  0  0  0  0  0  0  0  0  0  0
    4.9688   12.8354    0.6913 H   0  0  0  0  0  0  0  0  0  0  0  0
    5.3495   14.9995   -0.4554 H   0  0  0  0  0  0  0  0  0  0  0  0
    7.0625   15.1736   -2.2378 H   0  0  0  0  0  0  0  0  0  0  0  0
    8.3949   13.1834   -2.8738 H   0  0  0  0  0  0  0  0  0  0  0  0
    8.0146   11.0193   -1.7282 H   0  0  0  0  0  0  0  0  0  0  0  0
    5.7339    8.7809    2.0961 H   0  0  0  0  0  0  0  0  0  0  0  0
    3.4869    8.1528    2.9306 H   0  0  0  0  0  0  0  0  0  0  0  0
    1.4663    9.2255    1.9780 H   0  0  0  0  0  0  0  0  0  0  0  0
```

```
   1.6931   10.9264    0.1900 H   0  0  0  0  0  0  0  0  0  0  0  0
   3.9402   11.5547   -0.6457 H   0  0  0  0  0  0  0  0  0  0  0  0
  16.8632    7.7363   -0.6490 H   0  0  0  0  0  0  0  0  0  0  0  0
  19.1085    8.3646    0.1918 H   0  0  0  0  0  0  0  0  0  0  0  0
  19.3314   10.0681    1.9777 H   0  0  0  0  0  0  0  0  0  0  0  0
  17.3089   11.1435    2.9232 H   0  0  0  0  0  0  0  0  0  0  0  0
  15.0637   10.5153    2.0837 H   0  0  0  0  0  0  0  0  0  0  0  0
  12.7893    8.2678   -1.7397 H   0  0  0  0  0  0  0  0  0  0  0  0
  12.4113    6.1003   -2.8797 H   0  0  0  0  0  0  0  0  0  0  0  0
  13.7440    4.1125   -2.2367 H   0  0  0  0  0  0  0  0  0  0  0  0
  15.4549    4.2925   -0.4529 H   0  0  0  0  0  0  0  0  0  0  0  0
  15.8333    6.4599    0.6882 H   0  0  0  0  0  0  0  0  0  0  0  0
  1  2  1  0  0  0  0
  1  3  1  0  0  0  0
  1  4  1  0  0  0  0
  2  5  1  0  0  0  0
  2  6  2  0  0  0  0
 15  3  4  0  0  0  0
  3 19  4  0  0  0  0
 20  4  4  0  0  0  0
  4 24  4  0  0  0  0
  5  7  1  0  0  0  0
  7  8  1  0  0  0  0
  8  9  1  0  0  0  0
  9 10  1  0  0  0  0
 10 11  1  0  0  0  0
 10 12  2  0  0  0  0
 11 13  1  0  0  0  0
 11 14  1  0  0  0  0
 25 13  4  0  0  0  0
 13 29  4  0  0  0  0
 30 14  4  0  0  0  0
 14 34  4  0  0  0  0
 16 15  4  0  0  0  0
 17 16  4  0  0  0  0
 18 17  4  0  0  0  0
 19 18  4  0  0  0  0
 21 20  4  0  0  0  0
 22 21  4  0  0  0  0
 23 22  4  0  0  0  0
 24 23  4  0  0  0  0
 26 25  4  0  0  0  0
 27 26  4  0  0  0  0
 28 27  4  0  0  0  0
 29 28  4  0  0  0  0
 31 30  4  0  0  0  0
 32 31  4  0  0  0  0
 33 32  4  0  0  0  0
 34 33  4  0  0  0  0
  5 35  1  0  0  0  0
  9 36  1  0  0  0  0
  7 37  1  0  0  0  0
  7 38  1  0  0  0  0
  8 39  1  0  0  0  0
  8 40  1  0  0  0  0
 15 41  1  0  0  0  0
 16 42  1  0  0  0  0
 17 43  1  0  0  0  0
 18 44  1  0  0  0  0
 19 45  1  0  0  0  0
 20 46  1  0  0  0  0
 21 47  1  0  0  0  0
 22 48  1  0  0  0  0
```

```
 23 49  1  0  0  0  0
 24 50  1  0  0  0  0
 25 51  1  0  0  0  0
 26 52  1  0  0  0  0
 27 53  1  0  0  0  0
 28 54  1  0  0  0  0
 29 55  1  0  0  0  0
 30 56  1  0  0  0  0
 31 57  1  0  0  0  0
 32 58  1  0  0  0  0
 33 59  1  0  0  0  0
 34 60  1  0  0  0  0
M  END
>  <Alias name>
N,N-Ethylene bis(N',N,-diphenyl urea)

>  <deltaH°(c)>   kJ/mol
-14539

$$$$
Diacylperoxide II
  ChemBrain IXL

 77 76  0  0  0  0  0  0  0  0  1 V2000
   13.1044   11.7775    0.8010 C   0  0  0  0  0  0  0  0  0  0  0  0
   12.5429   13.0773    1.2389 C   0  0  0  0  0  0  0  0  0  0  0  0
   11.3913   13.1404    1.6365 O   0  0  0  0  0  0  0  0  0  0  0  0
   13.3724   14.2917    1.1948 O   0  0  0  0  0  0  0  0  0  0  0  0
   15.1425   10.6163   -0.1156 C   0  0  0  0  0  0  0  0  0  0  0  0
   16.5442   10.7974   -0.5613 C   0  0  0  0  0  0  0  0  0  0  0  0
   17.1840    9.8399   -0.9639 O   0  0  0  0  0  0  0  0  0  0  0  0
   17.1656   12.1305   -0.5189 O   0  0  0  0  0  0  0  0  0  0  0  0
   20.9778   12.7621   -0.3588 C   0  0  0  0  0  0  0  0  0  0  0  0
   19.5431   12.6623   -0.0018 C   0  0  0  0  0  0  0  0  0  0  0  0
   19.1700   12.9775    1.1161 O   0  0  0  0  0  0  0  0  0  0  0  0
   18.5676   12.1834   -0.9939 O   0  0  0  0  0  0  0  0  0  0  0  0
   21.1859   12.2719   -1.8059 C   0  0  0  0  0  0  0  0  0  0  0  0
   22.6800   12.3778   -2.1739 C   0  0  0  0  0  0  0  0  0  0  0  0
   22.8901   11.8893   -3.6211 C   0  0  0  0  0  0  0  0  0  0  0  0
   24.3837   11.9977   -3.9884 C   0  0  0  0  0  0  0  0  0  0  0  0
   24.5942   11.5105   -5.4356 C   0  0  0  0  0  0  0  0  0  0  0  0
   26.0872   11.6208   -5.8032 C   0  0  0  0  0  0  0  0  0  0  0  0
   26.2974   11.1342   -7.2505 C   0  0  0  0  0  0  0  0  0  0  0  0
   12.7532   17.4395    3.3545 C   0  0  0  0  0  0  0  0  0  0  0  0
   13.3347   16.1935    2.8022 C   0  0  0  0  0  0  0  0  0  0  0  0
   14.3426   15.7190    3.2995 O   0  0  0  0  0  0  0  0  0  0  0  0
   12.6982   15.5277    1.6549 O   0  0  0  0  0  0  0  0  0  0  0  0
   11.5227   17.8494    2.5206 C   0  0  0  0  0  0  0  0  0  0  0  0
   10.9147   19.1414    3.1037 C   0  0  0  0  0  0  0  0  0  0  0  0
    9.6802   19.5495    2.2752 C   0  0  0  0  0  0  0  0  0  0  0  0
    9.0663   20.8345    2.8663 C   0  0  0  0  0  0  0  0  0  0  0  0
    7.8281   21.2392    2.0423 C   0  0  0  0  0  0  0  0  0  0  0  0
    7.2091   22.5186    2.6390 C   0  0  0  0  0  0  0  0  0  0  0  0
    5.9690   22.9212    1.8171 C   0  0  0  0  0  0  0  0  0  0  0  0
   27.7899   11.2454   -7.6184 C   0  0  0  0  0  0  0  0  0  0  0  0
    5.3476   24.1979    2.4163 C   0  0  0  0  0  0  0  0  0  0  0  0
   14.5632   11.9726    0.3377 C   0  0  0  0  0  0  0  0  0  0  0  0
   12.5146   11.3882   -0.0197 H   0  0  0  0  0  0  0  0  0  0  0  0
   13.0792   11.0775    1.6269 H   0  0  0  0  0  0  0  0  0  0  0  0
   15.1147    9.9181    0.7118 H   0  0  0  0  0  0  0  0  0  0  0  0
   14.5509   10.2276   -0.9353 H   0  0  0  0  0  0  0  0  0  0  0  0
   21.5610   12.1488    0.3170 H   0  0  0  0  0  0  0  0  0  0  0  0
   21.2988   13.7933   -0.2777 H   0  0  0  0  0  0  0  0  0  0  0  0
```

```
 20.6030   12.8849   -2.4821 H   0  0  0  0  0  0  0  0  0  0  0  0
 20.8662   11.2403   -1.8871 H   0  0  0  0  0  0  0  0  0  0  0  0
 23.2625   11.7642   -1.4979 H   0  0  0  0  0  0  0  0  0  0  0  0
 22.9998   13.4092   -2.0913 H   0  0  0  0  0  0  0  0  0  0  0  0
 22.3067   12.5019   -4.2972 H   0  0  0  0  0  0  0  0  0  0  0  0
 22.5722   10.8573   -3.7035 H   0  0  0  0  0  0  0  0  0  0  0  0
 24.9670   11.3844   -3.3127 H   0  0  0  0  0  0  0  0  0  0  0  0
 24.7020   13.0296   -3.9050 H   0  0  0  0  0  0  0  0  0  0  0  0
 24.0100   12.1229   -6.1113 H   0  0  0  0  0  0  0  0  0  0  0  0
 24.2774   10.4783   -5.5187 H   0  0  0  0  0  0  0  0  0  0  0  0
 26.6715   11.0080   -5.1279 H   0  0  0  0  0  0  0  0  0  0  0  0
 26.4043   12.6530   -5.7198 H   0  0  0  0  0  0  0  0  0  0  0  0
 25.7127   11.7466   -7.9257 H   0  0  0  0  0  0  0  0  0  0  0  0
 25.9811   10.1018   -7.3338 H   0  0  0  0  0  0  0  0  0  0  0  0
 13.4929   18.2298    3.3180 H   0  0  0  0  0  0  0  0  0  0  0  0
 12.4535   17.2732    4.3818 H   0  0  0  0  0  0  0  0  0  0  0  0
 10.7850   17.0571    2.5518 H   0  0  0  0  0  0  0  0  0  0  0  0
 11.8232   18.0229    1.4947 H   0  0  0  0  0  0  0  0  0  0  0  0
 11.6511   19.9347    3.0682 H   0  0  0  0  0  0  0  0  0  0  0  0
 10.6189   18.9694    4.1313 H   0  0  0  0  0  0  0  0  0  0  0  0
  8.9469   18.7530    2.3035 H   0  0  0  0  0  0  0  0  0  0  0  0
  9.9774   19.7302    1.2497 H   0  0  0  0  0  0  0  0  0  0  0  0
  9.7981   21.6322    2.8335 H   0  0  0  0  0  0  0  0  0  0  0  0
  8.7735   20.6553    3.8935 H   0  0  0  0  0  0  0  0  0  0  0  0
  7.0989   20.4388    2.0694 H   0  0  0  0  0  0  0  0  0  0  0  0
  8.1220   21.4250    1.0167 H   0  0  0  0  0  0  0  0  0  0  0  0
  7.9373   23.3196    2.6095 H   0  0  0  0  0  0  0  0  0  0  0  0
  6.9173   22.3337    3.6655 H   0  0  0  0  0  0  0  0  0  0  0  0
  5.2420   22.1189    1.8440 H   0  0  0  0  0  0  0  0  0  0  0  0
  6.2613   23.1091    0.7913 H   0  0  0  0  0  0  0  0  0  0  0  0
 28.1064   12.2778   -7.5352 H   0  0  0  0  0  0  0  0  0  0  0  0
 27.9375   10.9034   -8.6353 H   0  0  0  0  0  0  0  0  0  0  0  0
 28.3748   10.6330   -6.9432 H   0  0  0  0  0  0  0  0  0  0  0  0
  5.0553   24.0100    3.4420 H   0  0  0  0  0  0  0  0  0  0  0  0
  6.0745   25.0002    2.3892 H   0  0  0  0  0  0  0  0  0  0  0  0
  4.4762   24.4807    1.8386 H   0  0  0  0  0  0  0  0  0  0  0  0
 15.1537   12.3618    1.1578 H   0  0  0  0  0  0  0  0  0  0  0  0
 14.5891   12.6712   -0.4895 H   0  0  0  0  0  0  0  0  0  0  0  0
  1  2  1  0  0  0  0
  2  3  2  0  0  0  0
  2  4  1  0  0  0  0
  5  6  1  0  0  0  0
  6  7  2  0  0  0  0
  6  8  1  0  0  0  0
  9 10  1  0  0  0  0
 10 11  2  0  0  0  0
 10 12  1  0  0  0  0
 13  9  1  0  0  0  0
 13 14  1  0  0  0  0
 14 15  1  0  0  0  0
 15 16  1  0  0  0  0
 16 17  1  0  0  0  0
 17 18  1  0  0  0  0
 18 19  1  0  0  0  0
 20 21  1  0  0  0  0
 21 22  2  0  0  0  0
 21 23  1  0  0  0  0
 24 20  1  0  0  0  0
 24 25  1  0  0  0  0
 25 26  1  0  0  0  0
 26 27  1  0  0  0  0
 27 28  1  0  0  0  0
 28 29  1  0  0  0  0
```

```
 29 30  1  0  0  0  0
 23  4  1  0  0  0  0
 12  8  1  0  0  0  0
 19 31  1  0  0  0  0
 30 32  1  0  0  0  0
  5 33  1  0  0  0  0
 33  1  1  0  0  0  0
  1 34  1  0  0  0  0
  1 35  1  0  0  0  0
  5 36  1  0  0  0  0
  5 37  1  0  0  0  0
  9 38  1  0  0  0  0
  9 39  1  0  0  0  0
 13 40  1  0  0  0  0
 13 41  1  0  0  0  0
 14 42  1  0  0  0  0
 14 43  1  0  0  0  0
 15 44  1  0  0  0  0
 15 45  1  0  0  0  0
 16 46  1  0  0  0  0
 16 47  1  0  0  0  0
 17 48  1  0  0  0  0
 17 49  1  0  0  0  0
 18 50  1  0  0  0  0
 18 51  1  0  0  0  0
 19 52  1  0  0  0  0
 19 53  1  0  0  0  0
 20 54  1  0  0  0  0
 20 55  1  0  0  0  0
 24 56  1  0  0  0  0
 24 57  1  0  0  0  0
 25 58  1  0  0  0  0
 25 59  1  0  0  0  0
 26 60  1  0  0  0  0
 26 61  1  0  0  0  0
 27 62  1  0  0  0  0
 27 63  1  0  0  0  0
 28 64  1  0  0  0  0
 28 65  1  0  0  0  0
 29 66  1  0  0  0  0
 29 67  1  0  0  0  0
 30 68  1  0  0  0  0
 30 69  1  0  0  0  0
 31 70  1  0  0  0  0
 31 71  1  0  0  0  0
 31 72  1  0  0  0  0
 32 73  1  0  0  0  0
 32 74  1  0  0  0  0
 32 75  1  0  0  0  0
 33 76  1  0  0  0  0
 33 77  1  0  0  0  0
M  END
>  <Alias name>
Diacylperoxide II

>  <deltaH°(c)>   kJ/mol
-14536

$$$$
1,2,3,4-Tetraphenyl-1,3-butadiene
  ChemBrain IXL

 50 53  0  0  0  0  0  0  0  0  1 V2000
```

```
 7.6507   7.7592   0.1394 C   0  0  0  0  0  0  0  0  0  0  0  0
 9.0532   7.5969  -0.2383 C   0  0  0  0  0  0  0  0  0  0  0  0
 9.5690   6.3623  -0.3577 C   0  0  0  0  0  0  0  0  0  0  0  0
 9.8826   8.8034  -0.4824 C   0  0  0  0  0  0  0  0  0  0  0  0
 7.1264   8.9911   0.2255 C   0  0  0  0  0  0  0  0  0  0  0  0
 6.8303   6.5560   0.4208 C   0  0  0  0  0  0  0  0  0  0  0  0
10.9946   6.1704  -0.7401 C   0  0  0  0  0  0  0  0  0  0  0  0
10.6743   8.8925  -1.6286 C   0  0  0  0  0  0  0  0  0  0  0  0
 9.8641   9.8564   0.4339 C   0  0  0  0  0  0  0  0  0  0  0  0
 5.7020   9.1795   0.6058 C   0  0  0  0  0  0  0  0  0  0  0  0
 6.8337   5.4868  -0.4763 C   0  0  0  0  0  0  0  0  0  0  0  0
 6.0612   6.4868   1.5834 C   0  0  0  0  0  0  0  0  0  0  0  0
11.9777   7.0291  -0.2426 C   0  0  0  0  0  0  0  0  0  0  0  0
11.3532   5.1270  -1.5966 C   0  0  0  0  0  0  0  0  0  0  0  0
11.4473  10.0323  -1.8568 C   0  0  0  0  0  0  0  0  0  0  0  0
10.6369  10.9956   0.2054 C   0  0  0  0  0  0  0  0  0  0  0  0
 4.7251   8.3071   0.1216 C   0  0  0  0  0  0  0  0  0  0  0  0
 5.3379  10.2326   1.4472 C   0  0  0  0  0  0  0  0  0  0  0  0
 6.0679   4.3507  -0.2123 C   0  0  0  0  0  0  0  0  0  0  0  0
 5.2952   5.3503   1.8471 C   0  0  0  0  0  0  0  0  0  0  0  0
13.3146   6.8452  -0.6020 C   0  0  0  0  0  0  0  0  0  0  0  0
12.6901   4.9436  -1.9553 C   0  0  0  0  0  0  0  0  0  0  0  0
11.4285  11.0836  -0.9398 C   0  0  0  0  0  0  0  0  0  0  0  0
 3.3876   8.4871   0.4794 C   0  0  0  0  0  0  0  0  0  0  0  0
 4.0005  10.4122   1.8045 C   0  0  0  0  0  0  0  0  0  0  0  0
 5.2986   4.2824   0.9493 C   0  0  0  0  0  0  0  0  0  0  0  0
13.6705   5.8027  -1.4582 C   0  0  0  0  0  0  0  0  0  0  0  0
 3.0256   9.5394   1.3208 C   0  0  0  0  0  0  0  0  0  0  0  0
 8.9422   5.4987  -0.1745 H   0  0  0  0  0  0  0  0  0  0  0  0
 7.7454   9.8546   0.0166 H   0  0  0  0  0  0  0  0  0  0  0  0
10.6891   8.0775  -2.3413 H   0  0  0  0  0  0  0  0  0  0  0  0
 9.2496   9.7892   1.3230 H   0  0  0  0  0  0  0  0  0  0  0  0
 7.4311   5.5389  -1.3781 H   0  0  0  0  0  0  0  0  0  0  0  0
 6.0587   7.3148   2.2811 H   0  0  0  0  0  0  0  0  0  0  0  0
11.7036   7.8382   0.4223 H   0  0  0  0  0  0  0  0  0  0  0  0
10.5939   4.4593  -1.9831 H   0  0  0  0  0  0  0  0  0  0  0  0
12.0621  10.1008  -2.7460 H   0  0  0  0  0  0  0  0  0  0  0  0
10.6222  11.8120   0.9173 H   0  0  0  0  0  0  0  0  0  0  0  0
 5.0045   7.4904  -0.5319 H   0  0  0  0  0  0  0  0  0  0  0  0
 6.0935  10.9109   1.8230 H   0  0  0  0  0  0  0  0  0  0  0  0
 6.0707   3.5215  -0.9094 H   0  0  0  0  0  0  0  0  0  0  0  0
 4.6978   5.2971   2.7490 H   0  0  0  0  0  0  0  0  0  0  0  0
14.0763   7.5120  -0.2160 H   0  0  0  0  0  0  0  0  0  0  0  0
12.9666   4.1340  -2.6199 H   0  0  0  0  0  0  0  0  0  0  0  0
12.0286  11.9681  -1.1170 H   0  0  0  0  0  0  0  0  0  0  0  0
 2.6303   7.8096   0.1037 H   0  0  0  0  0  0  0  0  0  0  0  0
 3.7193  11.2294   2.4576 H   0  0  0  0  0  0  0  0  0  0  0  0
 4.7039   3.4004   1.1542 H   0  0  0  0  0  0  0  0  0  0  0  0
14.7081   5.6602  -1.7371 H   0  0  0  0  0  0  0  0  0  0  0  0
 1.9875   9.6789   1.5985 H   0  0  0  0  0  0  0  0  0  0  0  0
  1  2  1  0  0  0  0
  1  5  2  0  0  0  0
  1  6  1  0  0  0  0
  2  3  2  0  0  0  0
  2  4  1  0  0  0  0
  3  7  1  0  0  0  0
  8  4  4  0  0  0  0
  4  9  4  0  0  0  0
  5 10  1  0  0  0  0
 11  6  4  0  0  0  0
  6 12  4  0  0  0  0
  7 13  4  0  0  0  0
 14  7  4  0  0  0  0
```

15 8 4 0 0 0 0
 9 16 4 0 0 0 0
10 17 4 0 0 0 0
18 10 4 0 0 0 0
19 11 4 0 0 0 0
12 20 4 0 0 0 0
13 21 4 0 0 0 0
22 14 4 0 0 0 0
23 15 4 0 0 0 0
16 23 4 0 0 0 0
17 24 4 0 0 0 0
25 18 4 0 0 0 0
26 19 4 0 0 0 0
20 26 4 0 0 0 0
21 27 4 0 0 0 0
27 22 4 0 0 0 0
24 28 4 0 0 0 0
28 25 4 0 0 0 0
 3 29 1 0 0 0 0
 5 30 1 0 0 0 0
 8 31 1 0 0 0 0
 9 32 1 0 0 0 0
11 33 1 0 0 0 0
12 34 1 0 0 0 0
13 35 1 0 0 0 0
14 36 1 0 0 0 0
15 37 1 0 0 0 0
16 38 1 0 0 0 0
17 39 1 0 0 0 0
18 40 1 0 0 0 0
19 41 1 0 0 0 0
20 42 1 0 0 0 0
21 43 1 0 0 0 0
22 44 1 0 0 0 0
23 45 1 0 0 0 0
24 46 1 0 0 0 0
25 47 1 0 0 0 0
26 48 1 0 0 0 0
27 49 1 0 0 0 0
28 50 1 0 0 0 0
M END
> <Alias name>
1,2,3,4-Tetraphenyl-1,3-butadiene

> <deltaH°(c)>   kJ/mol
-14521

$$$$
1,1,4,4-Tetraphenyl-1,3-butadiene
  ChemBrain IXL

 50 53 0 0 0 0 0 0 0 0 1 V2000
    9.1103   7.8704   0.0539 C   0 0 0 0 0 0 0 0 0 0 0 0
   10.0529   6.8934   0.0291 C   0 0 0 0 0 0 0 0 0 0 0 0
   11.4893   7.2364   0.0290 C   0 0 0 0 0 0 0 0 0 0 0 0
    9.6659   5.4667   0.0010 C   0 0 0 0 0 0 0 0 0 0 0 0
    7.6421   7.5323   0.0539 C   0 0 0 0 0 0 0 0 0 0 0 0
   11.8999   8.5422   -0.2353 C   0 0 0 0 0 0 0 0 0 0 0 0
   12.4353   6.2462   0.2917 C   0 0 0 0 0 0 0 0 0 0 0 0
   10.2938   4.5982   -0.8912 C   0 0 0 0 0 0 0 0 0 0 0 0
    8.6856   4.9868   0.8693 C   0 0 0 0 0 0 0 0 0 0 0 0
    6.6995   8.5093   0.0291 C   0 0 0 0 0 0 0 0 0 0 0 0
   13.2585   8.8588   -0.2334 C   0 0 0 0 0 0 0 0 0 0 0 0

```
   13.7932    6.5623    0.2935 C   0  0  0  0  0  0  0  0  0  0  0  0
    9.9362    3.2508   -0.9213 C   0  0  0  0  0  0  0  0  0  0  0  0
    8.3276    3.6387    0.8393 C   0  0  0  0  0  0  0  0  0  0  0  0
    7.0865    9.9360    0.0010 C   0  0  0  0  0  0  0  0  0  0  0  0
    5.2630    8.1663    0.0290 C   0  0  0  0  0  0  0  0  0  0  0  0
   14.2051    7.8687    0.0312 C   0  0  0  0  0  0  0  0  0  0  0  0
    8.9528    2.7709   -0.0565 C   0  0  0  0  0  0  0  0  0  0  0  0
    8.0669   10.4158    0.8693 C   0  0  0  0  0  0  0  0  0  0  0  0
    6.4587   10.8045   -0.8912 C   0  0  0  0  0  0  0  0  0  0  0  0
    4.8524    6.8605   -0.2354 C   0  0  0  0  0  0  0  0  0  0  0  0
    4.3171    9.1565    0.2917 C   0  0  0  0  0  0  0  0  0  0  0  0
    8.4249   11.7639    0.8393 C   0  0  0  0  0  0  0  0  0  0  0  0
    6.8163   12.1519   -0.9212 C   0  0  0  0  0  0  0  0  0  0  0  0
    3.4939    6.5439   -0.2334 C   0  0  0  0  0  0  0  0  0  0  0  0
    2.9592    8.8404    0.2936 C   0  0  0  0  0  0  0  0  0  0  0  0
    7.7997   12.6318   -0.0564 C   0  0  0  0  0  0  0  0  0  0  0  0
    2.5473    7.5340    0.0312 C   0  0  0  0  0  0  0  0  0  0  0  0
    9.4184    8.9095    0.0742 H   0  0  0  0  0  0  0  0  0  0  0  0
    7.3339    6.4932    0.0742 H   0  0  0  0  0  0  0  0  0  0  0  0
   11.1645    9.3099   -0.4419 H   0  0  0  0  0  0  0  0  0  0  0  0
   12.1148    5.2315    0.4943 H   0  0  0  0  0  0  0  0  0  0  0  0
   11.0592    4.9705   -1.5607 H   0  0  0  0  0  0  0  0  0  0  0  0
    8.2026    5.6604    1.5665 H   0  0  0  0  0  0  0  0  0  0  0  0
   13.5783    9.8730   -0.4373 H   0  0  0  0  0  0  0  0  0  0  0  0
   14.5275    5.7937    0.4986 H   0  0  0  0  0  0  0  0  0  0  0  0
   10.4221    2.5774   -1.6159 H   0  0  0  0  0  0  0  0  0  0  0  0
    7.5645    3.2658    1.5113 H   0  0  0  0  0  0  0  0  0  0  0  0
   15.2598    8.1141    0.0329 H   0  0  0  0  0  0  0  0  0  0  0  0
    8.6748    1.7245   -0.0804 H   0  0  0  0  0  0  0  0  0  0  0  0
    8.5499    9.7422    1.5664 H   0  0  0  0  0  0  0  0  0  0  0  0
    5.6932   10.4322   -1.5607 H   0  0  0  0  0  0  0  0  0  0  0  0
    5.5879    6.0928   -0.4420 H   0  0  0  0  0  0  0  0  0  0  0  0
    4.6376   10.1712    0.4944 H   0  0  0  0  0  0  0  0  0  0  0  0
    9.1880   12.1368    1.5113 H   0  0  0  0  0  0  0  0  0  0  0  0
    6.3304   12.8253   -1.6158 H   0  0  0  0  0  0  0  0  0  0  0  0
    3.1740    5.5297   -0.4374 H   0  0  0  0  0  0  0  0  0  0  0  0
    2.2249    9.6091    0.4986 H   0  0  0  0  0  0  0  0  0  0  0  0
    8.0777   13.6782   -0.0803 H   0  0  0  0  0  0  0  0  0  0  0  0
    1.4926    7.2886    0.0329 H   0  0  0  0  0  0  0  0  0  0  0  0
  1  2  2  0  0  0  0
  1  5  1  0  0  0  0
  2  3  1  0  0  0  0
  2  4  1  0  0  0  0
  6  3  4  0  0  0  0
  3  7  4  0  0  0  0
  8  4  4  0  0  0  0
  4  9  4  0  0  0  0
  5 10  2  0  0  0  0
 11  6  4  0  0  0  0
  7 12  4  0  0  0  0
 13  8  4  0  0  0  0
  9 14  4  0  0  0  0
 10 15  1  0  0  0  0
 10 16  1  0  0  0  0
 17 11  4  0  0  0  0
 12 17  4  0  0  0  0
 18 13  4  0  0  0  0
 14 18  4  0  0  0  0
 15 19  4  0  0  0  0
 20 15  4  0  0  0  0
 21 16  4  0  0  0  0
 16 22  4  0  0  0  0
 19 23  4  0  0  0  0
```

```
 24 20  4  0  0  0  0
 25 21  4  0  0  0  0
 22 26  4  0  0  0  0
 23 27  4  0  0  0  0
 27 24  4  0  0  0  0
 28 25  4  0  0  0  0
 26 28  4  0  0  0  0
  1 29  1  0  0  0  0
  5 30  1  0  0  0  0
  6 31  1  0  0  0  0
  7 32  1  0  0  0  0
  8 33  1  0  0  0  0
  9 34  1  0  0  0  0
 11 35  1  0  0  0  0
 12 36  1  0  0  0  0
 13 37  1  0  0  0  0
 14 38  1  0  0  0  0
 17 39  1  0  0  0  0
 18 40  1  0  0  0  0
 19 41  1  0  0  0  0
 20 42  1  0  0  0  0
 21 43  1  0  0  0  0
 22 44  1  0  0  0  0
 23 45  1  0  0  0  0
 24 46  1  0  0  0  0
 25 47  1  0  0  0  0
 26 48  1  0  0  0  0
 27 49  1  0  0  0  0
 28 50  1  0  0  0  0
M  END
>  <Alias name>
1,1,4,4-Tetraphenyl-1,3-butadiene

>  <deltaH°(c)>   kJ/mol
-14501.77

$$$$
Pentyl Elaidate
  ChemBrain IXL

 69 68  0  0  0  0  0  0  0  0  1 V2000
   27.4904   16.4179    0.0000 C   0  0  0  0  0  0  0  0  0  0  0  0
   26.0294   16.9076    0.0100 C   0  0  0  0  0  0  0  0  0  0  0  0
   25.0803   15.6935   -0.0059 C   0  0  0  0  0  0  0  0  0  0  0  0
   23.6192   16.1834    0.0040 C   0  0  0  0  0  0  0  0  0  0  0  0
   22.6703   14.9694   -0.0124 C   0  0  0  0  0  0  0  0  0  0  0  0
   21.2094   15.4593   -0.0028 C   0  0  0  0  0  0  0  0  0  0  0  0
   20.2605   14.2454   -0.0201 C   0  0  0  0  0  0  0  0  0  0  0  0
   18.7994   14.7354   -0.0110 C   0  0  0  0  0  0  0  0  0  0  0  0
   17.8875   13.5696   -0.0285 C   0  0  0  0  0  0  0  0  0  0  0  0
   16.5658   13.7564   -0.0484 C   0  0  0  0  0  0  0  0  0  0  0  0
   15.6540   12.5907   -0.0661 C   0  0  0  0  0  0  0  0  0  0  0  0
   14.1939   13.0789    0.0006 C   0  0  0  0  0  0  0  0  0  0  0  0
   13.2454   11.8651   -0.0203 C   0  0  0  0  0  0  0  0  0  0  0  0
   11.7855   12.3527    0.0456 C   0  0  0  0  0  0  0  0  0  0  0  0
   10.8375   11.1387    0.0211 C   0  0  0  0  0  0  0  0  0  0  0  0
    9.3774   11.6254    0.0867 C   0  0  0  0  0  0  0  0  0  0  0  0
    8.4299   10.4103    0.0587 C   0  0  0  0  0  0  0  0  0  0  0  0
    7.0256   10.8749    0.1233 C   0  0  0  0  0  0  0  0  0  0  0  0
    6.1245   10.0665    0.2787 O   0  0  0  0  0  0  0  0  0  0  0  0
    6.7168   12.3104    0.0001 O   0  0  0  0  0  0  0  0  0  0  0  0
    5.2877   12.6521    0.0790 C   0  0  0  0  0  0  0  0  0  0  0  0
    5.1154   14.1767   -0.0616 C   0  0  0  0  0  0  0  0  0  0  0  0
```

3.6184	14.5340	0.0205	C	0	0	0	0	0	0	0	0	0	0	0	0
3.4455	16.0591	-0.1195	C	0	0	0	0	0	0	0	0	0	0	0	0
1.9486	16.4162	-0.0379	C	0	0	0	0	0	0	0	0	0	0	0	0
28.1571	17.2711	0.0111	H	0	0	0	0	0	0	0	0	0	0	0	0
27.6687	15.8321	-0.8933	H	0	0	0	0	0	0	0	0	0	0	0	0
27.6721	15.8063	0.8751	H	0	0	0	0	0	0	0	0	0	0	0	0
25.8478	17.5193	-0.8650	H	0	0	0	0	0	0	0	0	0	0	0	0
25.8512	17.4934	0.9033	H	0	0	0	0	0	0	0	0	0	0	0	0
25.2618	15.0819	0.8692	H	0	0	0	0	0	0	0	0	0	0	0	0
25.2585	15.1077	-0.8991	H	0	0	0	0	0	0	0	0	0	0	0	0
23.4377	16.7953	-0.8709	H	0	0	0	0	0	0	0	0	0	0	0	0
23.4409	16.7690	0.8974	H	0	0	0	0	0	0	0	0	0	0	0	0
22.8516	14.3576	0.8627	H	0	0	0	0	0	0	0	0	0	0	0	0
22.8488	14.3838	-0.9057	H	0	0	0	0	0	0	0	0	0	0	0	0
21.0282	16.0715	-0.8776	H	0	0	0	0	0	0	0	0	0	0	0	0
21.0307	16.0445	0.8908	H	0	0	0	0	0	0	0	0	0	0	0	0
20.4414	13.6333	0.8548	H	0	0	0	0	0	0	0	0	0	0	0	0
20.4394	13.6601	-0.9136	H	0	0	0	0	0	0	0	0	0	0	0	0
18.6188	15.3481	-0.8855	H	0	0	0	0	0	0	0	0	0	0	0	0
18.6204	15.3203	0.8828	H	0	0	0	0	0	0	0	0	0	0	0	0
18.2925	12.5650	-0.0252	H	0	0	0	0	0	0	0	0	0	0	0	0
16.1608	14.7610	-0.0518	H	0	0	0	0	0	0	0	0	0	0	0	0
15.8619	11.9568	0.7871	H	0	0	0	0	0	0	0	0	0	0	0	0
15.8051	12.0281	-0.9790	H	0	0	0	0	0	0	0	0	0	0	0	0
13.9863	13.7139	-0.8518	H	0	0	0	0	0	0	0	0	0	0	0	0
14.0422	13.6401	0.9144	H	0	0	0	0	0	0	0	0	0	0	0	0
13.4528	11.2302	0.8323	H	0	0	0	0	0	0	0	0	0	0	0	0
13.3976	11.3038	-0.9339	H	0	0	0	0	0	0	0	0	0	0	0	0
11.5787	12.9894	-0.8058	H	0	0	0	0	0	0	0	0	0	0	0	0
11.6325	12.9120	0.9603	H	0	0	0	0	0	0	0	0	0	0	0	0
11.0444	10.5019	0.8723	H	0	0	0	0	0	0	0	0	0	0	0	0
10.9908	10.5795	-0.8937	H	0	0	0	0	0	0	0	0	0	0	0	0
9.1710	12.2639	-0.7634	H	0	0	0	0	0	0	0	0	0	0	0	0
9.2233	12.1826	1.0026	H	0	0	0	0	0	0	0	0	0	0	0	0
8.6377	9.7713	0.9081	H	0	0	0	0	0	0	0	0	0	0	0	0
8.5840	9.8538	-0.8575	H	0	0	0	0	0	0	0	0	0	0	0	0
4.7526	12.1532	-0.7196	H	0	0	0	0	0	0	0	0	0	0	0	0
4.8919	12.3308	1.0345	H	0	0	0	0	0	0	0	0	0	0	0	0
5.6500	14.6755	0.7372	H	0	0	0	0	0	0	0	0	0	0	0	0
5.5115	14.4981	-1.0169	H	0	0	0	0	0	0	0	0	0	0	0	0
3.0839	14.0353	-0.7786	H	0	0	0	0	0	0	0	0	0	0	0	0
3.2223	14.2120	0.9756	H	0	0	0	0	0	0	0	0	0	0	0	0
3.9796	16.5578	0.6798	H	0	0	0	0	0	0	0	0	0	0	0	0
3.8418	16.3812	-1.0744	H	0	0	0	0	0	0	0	0	0	0	0	0
1.4144	15.9176	-0.8372	H	0	0	0	0	0	0	0	0	0	0	0	0
1.5522	16.0941	0.9171	H	0	0	0	0	0	0	0	0	0	0	0	0
1.8273	17.4878	-0.1361	H	0	0	0	0	0	0	0	0	0	0	0	0

1	2	1	0	0	0	0
2	3	1	0	0	0	0
3	4	1	0	0	0	0
4	5	1	0	0	0	0
5	6	1	0	0	0	0
6	7	1	0	0	0	0
7	8	1	0	0	0	0
8	9	1	0	0	0	0
9	10	2	0	0	0	0
10	11	1	0	0	0	0
11	12	1	0	0	0	0
12	13	1	0	0	0	0
13	14	1	0	0	0	0
14	15	1	0	0	0	0
15	16	1	0	0	0	0
16	17	1	0	0	0	0

17 18 1 0 0 0 0
18 19 2 0 0 0 0
18 20 1 0 0 0 0
20 21 1 0 0 0 0
21 22 1 0 0 0 0
22 23 1 0 0 0 0
23 24 1 0 0 0 0
24 25 1 0 0 0 0
1 26 1 0 0 0 0
1 27 1 0 0 0 0
1 28 1 0 0 0 0
2 29 1 0 0 0 0
2 30 1 0 0 0 0
3 31 1 0 0 0 0
3 32 1 0 0 0 0
4 33 1 0 0 0 0
4 34 1 0 0 0 0
5 35 1 0 0 0 0
5 36 1 0 0 0 0
6 37 1 0 0 0 0
6 38 1 0 0 0 0
7 39 1 0 0 0 0
7 40 1 0 0 0 0
8 41 1 0 0 0 0
8 42 1 0 0 0 0
9 43 1 0 0 0 0
10 44 1 0 0 0 0
11 45 1 0 0 0 0
11 46 1 0 0 0 0
12 47 1 0 0 0 0
12 48 1 0 0 0 0
13 49 1 0 0 0 0
13 50 1 0 0 0 0
14 51 1 0 0 0 0
14 52 1 0 0 0 0
15 53 1 0 0 0 0
15 54 1 0 0 0 0
16 55 1 0 0 0 0
16 56 1 0 0 0 0
17 57 1 0 0 0 0
17 58 1 0 0 0 0
21 59 1 0 0 0 0
21 60 1 0 0 0 0
22 61 1 0 0 0 0
22 62 1 0 0 0 0
23 63 1 0 0 0 0
23 64 1 0 0 0 0
24 65 1 0 0 0 0
24 66 1 0 0 0 0
25 67 1 0 0 0 0
25 68 1 0 0 0 0
25 69 1 0 0 0 0
M END
> <Alias name>
Pentyl Elaidate

> <deltaH°(c)> kJ/mol
-14498.6

$$$$
Methyl erucate
ChemBrain IXL

```
69 68  0  0  0  0  0  0  0  0  1 V2000
  12.3070   13.5668    0.0000 C   0  0  0  0  0  0  0  0  0  0  0  0
  13.1207   14.8750    0.0112 C   0  0  0  0  0  0  0  0  0  0  0  0
  10.8037   13.8995    0.0478 C   0  0  0  0  0  0  0  0  0  0  0  0
  14.6249   14.5466   -0.0307 C   0  0  0  0  0  0  0  0  0  0  0  0
   9.9859   12.5940    0.0371 C   0  0  0  0  0  0  0  0  0  0  0  0
  15.4355   15.8568   -0.0186 C   0  0  0  0  0  0  0  0  0  0  0  0
   8.4839   12.9316    0.0920 C   0  0  0  0  0  0  0  0  0  0  0  0
  16.9401   15.5305   -0.0570 C   0  0  0  0  0  0  0  0  0  0  0  0
   7.6621   11.6290    0.0800 C   0  0  0  0  0  0  0  0  0  0  0  0
  17.7196   16.7904   -0.0447 C   0  0  0  0  0  0  0  0  0  0  0  0
   6.1614   11.9707    0.1422 C   0  0  0  0  0  0  0  0  0  0  0  0
  19.0536   16.7517    0.0182 C   0  0  0  0  0  0  0  0  0  0  0  0
   5.3367   10.6701    0.1270 C   0  0  0  0  0  0  0  0  0  0  0  0
  19.7565   15.4488    0.0758 C   0  0  0  0  0  0  0  0  0  0  0  0
   3.8957   10.9995    0.1941 C   0  0  0  0  0  0  0  0  0  0  0  0
  21.2780   15.6818    0.0141 C   0  0  0  0  0  0  0  0  0  0  0  0
   3.3938   12.2384   -0.4246 O   0  0  0  0  0  0  0  0  0  0  0  0
   3.1248   10.2432    0.7627 O   0  0  0  0  0  0  0  0  0  0  0  0
  22.0076   14.3259    0.0730 C   0  0  0  0  0  0  0  0  0  0  0  0
  23.5293   14.5583    0.0099 C   0  0  0  0  0  0  0  0  0  0  0  0
  24.2590   13.2023    0.0677 C   0  0  0  0  0  0  0  0  0  0  0  0
  25.7810   13.4349    0.0035 C   0  0  0  0  0  0  0  0  0  0  0  0
  26.5110   12.0787    0.0608 C   0  0  0  0  0  0  0  0  0  0  0  0
  28.0329   12.3111   -0.0038 C   0  0  0  0  0  0  0  0  0  0  0  0
   1.9454   12.4619   -0.2930 C   0  0  0  0  0  0  0  0  0  0  0  0
  12.5723   12.9689    0.8632 H   0  0  0  0  0  0  0  0  0  0  0  0
  12.5260   13.0127   -0.9044 H   0  0  0  0  0  0  0  0  0  0  0  0
  12.8572   15.4712   -0.8537 H   0  0  0  0  0  0  0  0  0  0  0  0
  12.8979   15.4306    0.9137 H   0  0  0  0  0  0  0  0  0  0  0  0
  10.5390   14.4980   -0.8151 H   0  0  0  0  0  0  0  0  0  0  0  0
  10.5857   14.4541    0.9521 H   0  0  0  0  0  0  0  0  0  0  0  0
  14.8887   13.9507    0.8343 H   0  0  0  0  0  0  0  0  0  0  0  0
  14.8485   13.9917   -0.9335 H   0  0  0  0  0  0  0  0  0  0  0  0
  10.2528   11.9933    0.8978 H   0  0  0  0  0  0  0  0  0  0  0  0
  10.1993   12.0413   -0.8695 H   0  0  0  0  0  0  0  0  0  0  0  0
  15.1729   16.4518   -0.8846 H   0  0  0  0  0  0  0  0  0  0  0  0
  15.2096   16.4126    0.8831 H   0  0  0  0  0  0  0  0  0  0  0  0
   8.2177   13.5339   -0.7678 H   0  0  0  0  0  0  0  0  0  0  0  0
   8.2713   13.4838    0.9990 H   0  0  0  0  0  0  0  0  0  0  0  0
  17.2023   14.9352    0.8090 H   0  0  0  0  0  0  0  0  0  0  0  0
  17.1660   14.9749   -0.9588 H   0  0  0  0  0  0  0  0  0  0  0  0
   7.9307   11.0244    0.9375 H   0  0  0  0  0  0  0  0  0  0  0  0
   7.8701   11.0789   -0.8294 H   0  0  0  0  0  0  0  0  0  0  0  0
  17.2076   17.7438   -0.0868 H   0  0  0  0  0  0  0  0  0  0  0  0
   5.8937   12.5778   -0.7137 H   0  0  0  0  0  0  0  0  0  0  0  0
   5.9538   12.5191    1.0528 H   0  0  0  0  0  0  0  0  0  0  0  0
  19.6214   17.6738    0.0270 H   0  0  0  0  0  0  0  0  0  0  0  0
   5.6077   10.0608    0.9804 H   0  0  0  0  0  0  0  0  0  0  0  0
   5.5403   10.1240   -0.7858 H   0  0  0  0  0  0  0  0  0  0  0  0
  19.4511   14.8364   -0.7637 H   0  0  0  0  0  0  0  0  0  0  0  0
  19.5059   14.9441    1.0007 H   0  0  0  0  0  0  0  0  0  0  0  0
  21.5841   16.2933    0.8540 H   0  0  0  0  0  0  0  0  0  0  0  0
  21.5288   16.1868   -0.9105 H   0  0  0  0  0  0  0  0  0  0  0  0
  21.7007   13.7142   -0.7664 H   0  0  0  0  0  0  0  0  0  0  0  0
  21.7574   13.8212    0.9980 H   0  0  0  0  0  0  0  0  0  0  0  0
  23.8363   15.1696    0.8496 H   0  0  0  0  0  0  0  0  0  0  0  0
  23.7792   15.0636   -0.9149 H   0  0  0  0  0  0  0  0  0  0  0  0
  23.9515   12.5909   -0.7716 H   0  0  0  0  0  0  0  0  0  0  0  0
  24.0096   12.6973    0.9928 H   0  0  0  0  0  0  0  0  0  0  0  0
  26.0885   14.0460    0.8431 H   0  0  0  0  0  0  0  0  0  0  0  0
  26.0303   13.9402   -0.9213 H   0  0  0  0  0  0  0  0  0  0  0  0
  26.2033   11.4674   -0.7786 H   0  0  0  0  0  0  0  0  0  0  0  0
```

26.2619	11.5735	0.9857	H	0	0	0	0	0	0	0	0	0	0	0	0
28.3408	12.9223	0.8356	H	0	0	0	0	0	0	0	0	0	0	0	0
28.2821	12.8163	-0.9288	H	0	0	0	0	0	0	0	0	0	0	0	0
28.5456	11.3581	0.0364	H	0	0	0	0	0	0	0	0	0	0	0	0
1.4100	11.6458	-0.7624	H	0	0	0	0	0	0	0	0	0	0	0	0
1.6815	12.5105	0.7563	H	0	0	0	0	0	0	0	0	0	0	0	0
1.6786	13.3937	-0.7758	H	0	0	0	0	0	0	0	0	0	0	0	0

1	2	1	0	0	0	0
1	3	1	0	0	0	0
2	4	1	0	0	0	0
3	5	1	0	0	0	0
4	6	1	0	0	0	0
5	7	1	0	0	0	0
6	8	1	0	0	0	0
7	9	1	0	0	0	0
8	10	1	0	0	0	0
9	11	1	0	0	0	0
10	12	2	0	0	0	0
11	13	1	0	0	0	0
12	14	1	0	0	0	0
13	15	1	0	0	0	0
14	16	1	0	0	0	0
15	17	1	0	0	0	0
15	18	2	0	0	0	0
16	19	1	0	0	0	0
19	20	1	0	0	0	0
20	21	1	0	0	0	0
21	22	1	0	0	0	0
22	23	1	0	0	0	0
23	24	1	0	0	0	0
17	25	1	0	0	0	0
1	26	1	0	0	0	0
1	27	1	0	0	0	0
2	28	1	0	0	0	0
2	29	1	0	0	0	0
3	30	1	0	0	0	0
3	31	1	0	0	0	0
4	32	1	0	0	0	0
4	33	1	0	0	0	0
5	34	1	0	0	0	0
5	35	1	0	0	0	0
6	36	1	0	0	0	0
6	37	1	0	0	0	0
7	38	1	0	0	0	0
7	39	1	0	0	0	0
8	40	1	0	0	0	0
8	41	1	0	0	0	0
9	42	1	0	0	0	0
9	43	1	0	0	0	0
10	44	1	0	0	0	0
11	45	1	0	0	0	0
11	46	1	0	0	0	0
12	47	1	0	0	0	0
13	48	1	0	0	0	0
13	49	1	0	0	0	0
14	50	1	0	0	0	0
14	51	1	0	0	0	0
16	52	1	0	0	0	0
16	53	1	0	0	0	0
19	54	1	0	0	0	0
19	55	1	0	0	0	0
20	56	1	0	0	0	0
20	57	1	0	0	0	0

21 58 1 0 0 0 0
21 59 1 0 0 0 0
22 60 1 0 0 0 0
22 61 1 0 0 0 0
23 62 1 0 0 0 0
23 63 1 0 0 0 0
24 64 1 0 0 0 0
24 65 1 0 0 0 0
24 66 1 0 0 0 0
25 67 1 0 0 0 0
25 68 1 0 0 0 0
25 69 1 0 0 0 0
M END
> <Alias name>
Methyl Z 13-docoseneoate

> <deltaH°(c)>   kJ/mol
-14452

$$$$
Docosanol
  ChemBrain IXL

69 68 0 0 0 0 0 0 0 0 1 V2000
2.1780   11.7811   3.7620 O   0 0 0 0 0 0 0 0 0 0 0 0
3.5032   11.1899   3.5285 C   0 0 0 0 0 0 0 0 0 0 0 0
4.4910   12.2944   3.1057 C   0 0 0 0 0 0 0 0 0 0 0 0
27.2873   16.0503   -3.1112 C   0 0 0 0 0 0 0 0 0 0 0 0
25.8964   16.6690   -2.8702 C   0 0 0 0 0 0 0 0 0 0 0 0
18.7591   15.2088   -0.8875 C   0 0 0 0 0 0 0 0 0 0 0 0
11.6240   13.7507   1.1046 C   0 0 0 0 0 0 0 0 0 0 0 0
20.1496   14.5900   -1.1295 C   0 0 0 0 0 0 0 0 0 0 0 0
10.6360   12.6457   1.5266 C   0 0 0 0 0 0 0 0 0 0 0 0
21.1380   15.6954   -1.5492 C   0 0 0 0 0 0 0 0 0 0 0 0
9.2462   13.2651   1.7710 C   0 0 0 0 0 0 0 0 0 0 0 0
22.5286   15.0766   -1.7906 C   0 0 0 0 0 0 0 0 0 0 0 0
8.2583   12.1603   2.1934 C   0 0 0 0 0 0 0 0 0 0 0 0
23.5171   16.1821   -2.2100 C   0 0 0 0 0 0 0 0 0 0 0 0
6.8686   12.7797   2.4381 C   0 0 0 0 0 0 0 0 0 0 0 0
24.9078   15.5635   -2.4511 C   0 0 0 0 0 0 0 0 0 0 0 0
5.8807   11.6749   2.8608 C   0 0 0 0 0 0 0 0 0 0 0 0
15.3922   13.6173   0.1961 C   0 0 0 0 0 0 0 0 0 0 0 0
16.3804   14.7225   -0.2247 C   0 0 0 0 0 0 0 0 0 0 0 0
14.0021   14.2365   0.4394 C   0 0 0 0 0 0 0 0 0 0 0 0
17.7708   14.1035   -0.4673 C   0 0 0 0 0 0 0 0 0 0 0 0
13.0140   13.1314   0.8608 C   0 0 0 0 0 0 0 0 0 0 0 0
28.2758   17.1557   -3.5302 C   0 0 0 0 0 0 0 0 0 0 0 0
1.4817   11.1442   4.0372 H   0 0 0 0 0 0 0 0 0 0 0 0
3.4340   10.4475   2.7430 H   0 0 0 0 0 0 0 0 0 0 0 0
3.8547   10.7204   4.4389 H   0 0 0 0 0 0 0 0 0 0 0 0
4.5600   13.0368   3.8912 H   0 0 0 0 0 0 0 0 0 0 0 0
4.1395   12.7638   2.1953 H   0 0 0 0 0 0 0 0 0 0 0 0
27.6362   15.5806   -2.1999 H   0 0 0 0 0 0 0 0 0 0 0 0
27.2211   15.3084   -3.8973 H   0 0 0 0 0 0 0 0 0 0 0 0
25.5474   17.1387   -3.7815 H   0 0 0 0 0 0 0 0 0 0 0 0
25.9626   17.4110   -2.0841 H   0 0 0 0 0 0 0 0 0 0 0 0
18.8260   15.9510   -0.1017 H   0 0 0 0 0 0 0 0 0 0 0 0
18.4094   15.6783   -1.7987 H   0 0 0 0 0 0 0 0 0 0 0 0
11.2731   14.2201   0.1939 H   0 0 0 0 0 0 0 0 0 0 0 0
11.6923   14.4930   1.8902 H   0 0 0 0 0 0 0 0 0 0 0 0
20.4991   14.1202   -0.2184 H   0 0 0 0 0 0 0 0 0 0 0 0
20.0827   13.8480   -1.9155 H   0 0 0 0 0 0 0 0 0 0 0 0
10.5674   11.9035   0.7409 H   0 0 0 0 0 0 0 0 0 0 0 0

```
 10.9871   12.1762    2.4372 H   0  0  0  0  0  0  0  0  0  0  0  0
 20.7887   16.1650   -2.4605 H   0  0  0  0  0  0  0  0  0  0  0  0
 21.2046   16.4375   -0.7633 H   0  0  0  0  0  0  0  0  0  0  0  0
  8.8950   13.7345    0.8604 H   0  0  0  0  0  0  0  0  0  0  0  0
  9.3149   14.0075    2.5565 H   0  0  0  0  0  0  0  0  0  0  0  0
 22.8778   14.6068   -0.8795 H   0  0  0  0  0  0  0  0  0  0  0  0
 22.4621   14.3347   -2.5768 H   0  0  0  0  0  0  0  0  0  0  0  0
  8.1894   11.4180    1.4078 H   0  0  0  0  0  0  0  0  0  0  0  0
  8.6096   11.6907    3.1039 H   0  0  0  0  0  0  0  0  0  0  0  0
 23.1680   16.6518   -3.1213 H   0  0  0  0  0  0  0  0  0  0  0  0
 23.5835   16.9242   -1.4240 H   0  0  0  0  0  0  0  0  0  0  0  0
  6.9375   13.5221    3.2236 H   0  0  0  0  0  0  0  0  0  0  0  0
  6.5172   13.2491    1.5276 H   0  0  0  0  0  0  0  0  0  0  0  0
 25.2568   15.0937   -1.5398 H   0  0  0  0  0  0  0  0  0  0  0  0
 24.8415   14.8215   -3.2372 H   0  0  0  0  0  0  0  0  0  0  0  0
  5.8117   10.9326    2.0752 H   0  0  0  0  0  0  0  0  0  0  0  0
  6.2322   11.2055    3.7712 H   0  0  0  0  0  0  0  0  0  0  0  0
 15.3244   12.8753   -0.5897 H   0  0  0  0  0  0  0  0  0  0  0  0
 15.7425   13.1476    1.1069 H   0  0  0  0  0  0  0  0  0  0  0  0
 16.4479   15.4648    0.5610 H   0  0  0  0  0  0  0  0  0  0  0  0
 16.0304   15.1920   -1.1357 H   0  0  0  0  0  0  0  0  0  0  0  0
 13.6516   14.7059   -0.4715 H   0  0  0  0  0  0  0  0  0  0  0  0
 14.0700   14.9788    1.2250 H   0  0  0  0  0  0  0  0  0  0  0  0
 18.1206   13.6338    0.4437 H   0  0  0  0  0  0  0  0  0  0  0  0
 17.7034   13.3615   -1.2533 H   0  0  0  0  0  0  0  0  0  0  0  0
 12.9458   12.3893    0.0750 H   0  0  0  0  0  0  0  0  0  0  0  0
 13.3647   12.6618    1.7715 H   0  0  0  0  0  0  0  0  0  0  0  0
 28.3421   17.8977   -2.7441 H   0  0  0  0  0  0  0  0  0  0  0  0
 27.9270   17.6255   -4.4415 H   0  0  0  0  0  0  0  0  0  0  0  0
 29.2530   16.7208   -3.6994 H   0  0  0  0  0  0  0  0  0  0  0  0
  2  1  1  0  0  0  0
  3  2  1  0  0  0  0
  4  5  1  0  0  0  0
  5 16  1  0  0  0  0
  6 21  1  0  0  0  0
  7  9  1  0  0  0  0
  8  6  1  0  0  0  0
  9 11  1  0  0  0  0
 10  8  1  0  0  0  0
 11 13  1  0  0  0  0
 12 10  1  0  0  0  0
 13 15  1  0  0  0  0
 14 12  1  0  0  0  0
 15 17  1  0  0  0  0
 16 14  1  0  0  0  0
 17  3  1  0  0  0  0
 18 20  1  0  0  0  0
 19 18  1  0  0  0  0
 20 22  1  0  0  0  0
 21 19  1  0  0  0  0
 22  7  1  0  0  0  0
 23  4  1  0  0  0  0
  1 24  1  0  0  0  0
  2 25  1  0  0  0  0
  2 26  1  0  0  0  0
  3 27  1  0  0  0  0
  3 28  1  0  0  0  0
  4 29  1  0  0  0  0
  4 30  1  0  0  0  0
  5 31  1  0  0  0  0
  5 32  1  0  0  0  0
  6 33  1  0  0  0  0
  6 34  1  0  0  0  0
```

```
  7 35  1  0  0  0  0
  7 36  1  0  0  0  0
  8 37  1  0  0  0  0
  8 38  1  0  0  0  0
  9 39  1  0  0  0  0
  9 40  1  0  0  0  0
 10 41  1  0  0  0  0
 10 42  1  0  0  0  0
 11 43  1  0  0  0  0
 11 44  1  0  0  0  0
 12 45  1  0  0  0  0
 12 46  1  0  0  0  0
 13 47  1  0  0  0  0
 13 48  1  0  0  0  0
 14 49  1  0  0  0  0
 14 50  1  0  0  0  0
 15 51  1  0  0  0  0
 15 52  1  0  0  0  0
 16 53  1  0  0  0  0
 16 54  1  0  0  0  0
 17 55  1  0  0  0  0
 17 56  1  0  0  0  0
 18 57  1  0  0  0  0
 18 58  1  0  0  0  0
 19 59  1  0  0  0  0
 19 60  1  0  0  0  0
 20 61  1  0  0  0  0
 20 62  1  0  0  0  0
 21 63  1  0  0  0  0
 21 64  1  0  0  0  0
 22 65  1  0  0  0  0
 22 66  1  0  0  0  0
 23 67  1  0  0  0  0
 23 68  1  0  0  0  0
 23 69  1  0  0  0  0
M  END
>  <Alias name>
Behenyl alcohol

>  <deltaH°(c)>   kJ/mol
-14450

$$$$
Bis(dibenzoylmethanate)zinc(II)
  ChemBrain IXL

 57 62  0  0  0  0  0  0  0  0  1 V2000
    8.7328   13.3791    3.0229 C   0  0  0  0  0  0  0  0  0  0  0  0  0
    9.8211   12.6848    2.4934 C   0  0  0  0  0  0  0  0  0  0  0  0  0
    9.6190   11.7167    1.5088 C   0  0  0  0  0  0  0  0  0  0  0  0  0
    8.3278   11.4405    1.0532 C   0  0  0  0  0  0  0  0  0  0  0  0  0
    7.2396   12.1378    1.5817 C   0  0  0  0  0  0  0  0  0  0  0  0  0
    7.4424   13.1063    2.5666 C   0  0  0  0  0  0  0  0  0  0  0  0  0
    8.1236   10.4034    0.0104 C   0  0  0  0  0  0  0  0  0  0  0  0  0
    9.0990    9.8008   -0.4007 O   0  0  0  0  0  0  0  0  0  0  0  0  0
    6.7312   10.1081   -0.5371 C   0  0  0  0  0  0  0  0  0  0  0  0  0
    6.3349    8.8455   -0.9260 C   0  0  0  0  0  0  0  0  0  0  0  0  0
    7.2310    7.6315   -0.8182 O   0  5  0  0  0  0  0  0  0  0  0  0  0
    9.0636    8.0048   -0.5558 Zn  0  0  0  0  0  0  0  0  0  0  0  0  0
    4.0710    9.7385   -1.5100 C   0  0  0  0  0  0  0  0  0  0  0  0  0
    4.9664    8.6639   -1.4700 C   0  0  0  0  0  0  0  0  0  0  0  0  0
    4.5725    7.4141   -1.9445 C   0  0  0  0  0  0  0  0  0  0  0  0  0
    3.2874    7.2365   -2.4572 C   0  0  0  0  0  0  0  0  0  0  0  0  0
```

```
2.3958 8.3093 -2.4963 C 0 0 0 0 0 0 0 0 0 0 0 0
2.7882 9.5604 -2.0228 C 0 0 0 0 0 0 0 0 0 0 0 0
14.6717 9.3292 -3.3676 C 0 0 0 0 0 0 0 0 0 0 0 0
13.3652 9.6816 -3.7093 C 0 0 0 0 0 0 0 0 0 0 0 0
12.2841 9.0063 -3.1340 C 0 0 0 0 0 0 0 0 0 0 0 0
12.5073 7.9749 -2.2142 C 0 0 0 0 0 0 0 0 0 0 0 0
13.8193 7.6256 -1.8745 C 0 0 0 0 0 0 0 0 0 0 0 0
14.8988 8.3019 -2.4507 C 0 0 0 0 0 0 0 0 0 0 0 0
11.3489 7.2507 -1.5955 C 0 0 0 0 0 0 0 0 0 0 0 0
10.2018 7.5619 -1.8882 O 0 0 0 0 0 0 0 0 0 0 0 0
11.5872 6.1465 -0.6199 C 0 0 0 0 0 0 0 0 0 0 0 0
10.8558 6.0314 0.5203 C 0 0 0 0 0 0 0 0 0 0 0 0
9.7920 7.0359 0.9054 O 0 5 0 0 0 0 0 0 0 0 0 0
12.0839 3.9252 1.0573 C 0 0 0 0 0 0 0 0 0 0 0 0
11.1245 4.8807 1.4116 C 0 0 0 0 0 0 0 0 0 0 0 0
10.4188 4.7484 2.6041 C 0 0 0 0 0 0 0 0 0 0 0 0
10.6665 3.6611 3.4412 C 0 0 0 0 0 0 0 0 0 0 0 0
11.6215 2.7074 3.0854 C 0 0 0 0 0 0 0 0 0 0 0 0
12.3307 2.8408 1.8935 C 0 0 0 0 0 0 0 0 0 0 0 0
8.8895 14.1297 3.7880 H 0 0 0 0 0 0 0 0 0 0 0 0
10.8228 12.8973 2.8468 H 0 0 0 0 0 0 0 0 0 0 0 0
10.4647 11.1787 1.0980 H 0 0 0 0 0 0 0 0 0 0 0 0
6.2381 11.9275 1.2285 H 0 0 0 0 0 0 0 0 0 0 0 0
6.5980 13.6461 2.9771 H 0 0 0 0 0 0 0 0 0 0 0 0
6.0295 10.9226 -0.6223 H 0 0 0 0 0 0 0 0 0 0 0 0
4.3704 10.7100 -1.1436 H 0 0 0 0 0 0 0 0 0 0 0 0
5.2645 6.5816 -1.9149 H 0 0 0 0 0 0 0 0 0 0 0 0
2.9821 6.2661 -2.8249 H 0 0 0 0 0 0 0 0 0 0 0 0
1.3994 8.1709 -2.8941 H 0 0 0 0 0 0 0 0 0 0 0 0
2.0969 10.3937 -2.0532 H 0 0 0 0 0 0 0 0 0 0 0 0
15.5099 9.8533 -3.8143 H 0 0 0 0 0 0 0 0 0 0 0 0
13.1895 10.4798 -4.4213 H 0 0 0 0 0 0 0 0 0 0 0 0
11.2726 9.2831 -3.4015 H 0 0 0 0 0 0 0 0 0 0 0 0
14.0002 6.8302 -1.1638 H 0 0 0 0 0 0 0 0 0 0 0 0
15.9137 8.0288 -2.1856 H 0 0 0 0 0 0 0 0 0 0 0 0
12.3585 5.4245 -0.8301 H 0 0 0 0 0 0 0 0 0 0 0 0
12.6363 4.0242 0.1342 H 0 0 0 0 0 0 0 0 0 0 0 0
9.6785 5.4893 2.8797 H 0 0 0 0 0 0 0 0 0 0 0 0
10.1176 3.5571 4.3666 H 0 0 0 0 0 0 0 0 0 0 0 0
11.8118 1.8642 3.7345 H 0 0 0 0 0 0 0 0 0 0 0 0
13.0732 2.1022 1.6168 H 0 0 0 0 0 0 0 0 0 0 0 0
1 2 4 0 0 0 0
2 3 4 0 0 0 0
3 4 4 0 0 0 0
4 5 4 0 0 0 0
5 6 4 0 0 0 0
6 1 4 0 0 0 0
7 8 2 0 0 0 0
7 9 1 0 0 0 0
7 4 1 0 0 0 0
9 10 2 0 0 0 0
10 11 1 0 0 0 0
11 12 1 0 0 0 0
8 12 1 0 0 0 0
13 14 4 0 0 0 0
14 15 4 0 0 0 0
15 16 4 0 0 0 0
16 17 4 0 0 0 0
17 18 4 0 0 0 0
18 13 4 0 0 0 0
14 10 1 0 0 0 0
19 20 4 0 0 0 0
20 21 4 0 0 0 0
```

```
 21 22  4  0  0  0  0
 22 23  4  0  0  0  0
 23 24  4  0  0  0  0
 24 19  4  0  0  0  0
 25 26  2  0  0  0  0
 25 27  1  0  0  0  0
 25 22  1  0  0  0  0
 27 28  2  0  0  0  0
 28 29  1  0  0  0  0
 30 31  4  0  0  0  0
 31 32  4  0  0  0  0
 32 33  4  0  0  0  0
 33 34  4  0  0  0  0
 34 35  4  0  0  0  0
 35 30  4  0  0  0  0
 31 28  1  0  0  0  0
 29 12  1  0  0  0  0
 26 12  1  0  0  0  0
  1 36  1  0  0  0  0
  2 37  1  0  0  0  0
  3 38  1  0  0  0  0
  5 39  1  0  0  0  0
  6 40  1  0  0  0  0
  9 41  1  0  0  0  0
 13 42  1  0  0  0  0
 15 43  1  0  0  0  0
 16 44  1  0  0  0  0
 17 45  1  0  0  0  0
 18 46  1  0  0  0  0
 19 47  1  0  0  0  0
 20 48  1  0  0  0  0
 21 49  1  0  0  0  0
 23 50  1  0  0  0  0
 24 51  1  0  0  0  0
 27 52  1  0  0  0  0
 30 53  1  0  0  0  0
 32 54  1  0  0  0  0
 33 55  1  0  0  0  0
 34 56  1  0  0  0  0
 35 57  1  0  0  0  0
M  END
>  <Alias name>
Bis(dibenzoylmethanate)zinc(II)

>  <deltaH°(c)>   kJ/mol
-14412.5

$$$$
Hexadecylcyclohexane
  ChemBrain IXL

 66 66  0  0  0  0  0  0  0  0  1 V2000
   20.0767   12.9128    0.0000 C   0  0  0  0  0  0  0  0  0  0  0  0
   18.8549   12.0422   -0.3526 C   0  0  0  0  0  0  0  0  0  0  0  0
   17.5661   12.7623    0.0893 C   0  0  0  0  0  0  0  0  0  0  0  0
   16.3442   11.8926   -0.2651 C   0  0  0  0  0  0  0  0  0  0  0  0
   15.0552   12.6140    0.1744 C   0  0  0  0  0  0  0  0  0  0  0  0
   13.8332   11.7455   -0.1825 C   0  0  0  0  0  0  0  0  0  0  0  0
   12.5440   12.4683    0.2541 C   0  0  0  0  0  0  0  0  0  0  0  0
   11.3218   11.6012   -0.1053 C   0  0  0  0  0  0  0  0  0  0  0  0
   10.0325   12.3255    0.3284 C   0  0  0  0  0  0  0  0  0  0  0  0
   21.3655   12.1935   -0.4436 C   0  0  0  0  0  0  0  0  0  0  0  0
    8.8101   11.4595   -0.0333 C   0  0  0  0  0  0  0  0  0  0  0  0
```

```
 22.5872   13.0647   -0.0921 C   0  0  0  0  0  0  0  0  0  0  0  0
  7.5208   12.1850    0.3979 C   0  0  0  0  0  0  0  0  0  0  0  0
 23.8760   12.3457   -0.5365 C   0  0  0  0  0  0  0  0  0  0  0  0
  6.2975   11.3184    0.0342 C   0  0  0  0  0  0  0  0  0  0  0  0
  3.5792   14.0914    0.1958 C   0  0  0  0  0  0  0  0  0  0  0  0
  4.8758   13.3854   -0.2495 C   0  0  0  0  0  0  0  0  0  0  0  0
  5.0032   12.0315    0.4824 C   0  0  0  0  0  0  0  0  0  0  0  0
  3.7870   11.1464    0.1402 C   0  0  0  0  0  0  0  0  0  0  0  0
  2.4925   11.8546    0.5864 C   0  0  0  0  0  0  0  0  0  0  0  0
  2.3660   13.2044   -0.1471 C   0  0  0  0  0  0  0  0  0  0  0  0
 25.0976   13.2169   -0.1854 C   0  0  0  0  0  0  0  0  0  0  0  0
 20.0008   13.8649   -0.5105 H   0  0  0  0  0  0  0  0  0  0  0  0
 20.1055   13.0781    1.0699 H   0  0  0  0  0  0  0  0  0  0  0  0
 18.9302   11.0904    0.1587 H   0  0  0  0  0  0  0  0  0  0  0  0
 18.8268   11.8760   -1.4224 H   0  0  0  0  0  0  0  0  0  0  0  0
 17.4916   13.7146   -0.4211 H   0  0  0  0  0  0  0  0  0  0  0  0
 17.5935   12.9274    1.1593 H   0  0  0  0  0  0  0  0  0  0  0  0
 16.4177   10.9409    0.2465 H   0  0  0  0  0  0  0  0  0  0  0  0
 16.3178   11.7263   -1.3350 H   0  0  0  0  0  0  0  0  0  0  0  0
 14.9826   13.5663   -0.3362 H   0  0  0  0  0  0  0  0  0  0  0  0
 15.0808   12.7790    1.2444 H   0  0  0  0  0  0  0  0  0  0  0  0
 13.9046   10.7939    0.3295 H   0  0  0  0  0  0  0  0  0  0  0  0
 13.8088   11.5790   -1.2523 H   0  0  0  0  0  0  0  0  0  0  0  0
 12.4736   13.4207   -0.2567 H   0  0  0  0  0  0  0  0  0  0  0  0
 12.5674   12.6335    1.3242 H   0  0  0  0  0  0  0  0  0  0  0  0
 11.3911   10.6495    0.4069 H   0  0  0  0  0  0  0  0  0  0  0  0
 11.2996   11.4345   -1.1752 H   0  0  0  0  0  0  0  0  0  0  0  0
  9.9641   13.2777   -0.1829 H   0  0  0  0  0  0  0  0  0  0  0  0
 10.0538   12.4909    1.3984 H   0  0  0  0  0  0  0  0  0  0  0  0
 21.4418   11.2416    0.0674 H   0  0  0  0  0  0  0  0  0  0  0  0
 21.3362   12.0276   -1.5134 H   0  0  0  0  0  0  0  0  0  0  0  0
  8.8775   10.5078    0.4792 H   0  0  0  0  0  0  0  0  0  0  0  0
  8.7898   11.2927   -1.1032 H   0  0  0  0  0  0  0  0  0  0  0  0
 22.5105   14.0167   -0.6027 H   0  0  0  0  0  0  0  0  0  0  0  0
 22.6167   13.2302    0.9778 H   0  0  0  0  0  0  0  0  0  0  0  0
  7.4550   13.1364   -0.1136 H   0  0  0  0  0  0  0  0  0  0  0  0
  7.5406   12.3507    1.4679 H   0  0  0  0  0  0  0  0  0  0  0  0
 23.9528   11.3938   -0.0257 H   0  0  0  0  0  0  0  0  0  0  0  0
 23.8463   12.1800   -1.6063 H   0  0  0  0  0  0  0  0  0  0  0  0
  6.3730   10.3616    0.5359 H   0  0  0  0  0  0  0  0  0  0  0  0
  6.2713   11.1623   -1.0372 H   0  0  0  0  0  0  0  0  0  0  0  0
  3.4897   15.0398   -0.3192 H   0  0  0  0  0  0  0  0  0  0  0  0
  3.6103   14.2627    1.2647 H   0  0  0  0  0  0  0  0  0  0  0  0
  5.7261   14.0090   -0.0066 H   0  0  0  0  0  0  0  0  0  0  0  0
  4.8458   13.2163   -1.3188 H   0  0  0  0  0  0  0  0  0  0  0  0
  5.0362   12.2008    1.5516 H   0  0  0  0  0  0  0  0  0  0  0  0
  3.8750   10.1978    0.6549 H   0  0  0  0  0  0  0  0  0  0  0  0
  3.7546   10.9752   -0.9288 H   0  0  0  0  0  0  0  0  0  0  0  0
  1.6396   11.2321    0.3462 H   0  0  0  0  0  0  0  0  0  0  0  0
  2.5247   12.0261    1.6552 H   0  0  0  0  0  0  0  0  0  0  0  0
  2.3346   13.0330   -1.2160 H   0  0  0  0  0  0  0  0  0  0  0  0
  1.4560   13.7011    0.1657 H   0  0  0  0  0  0  0  0  0  0  0  0
 25.0209   14.1688   -0.6961 H   0  0  0  0  0  0  0  0  0  0  0  0
 26.0030   12.7116   -0.4978 H   0  0  0  0  0  0  0  0  0  0  0  0
 25.1274   13.3826    0.8845 H   0  0  0  0  0  0  0  0  0  0  0  0
  1  2  1  0  0  0  0
  2  3  1  0  0  0  0
  3  4  1  0  0  0  0
  4  5  1  0  0  0  0
  5  6  1  0  0  0  0
  6  7  1  0  0  0  0
  7  8  1  0  0  0  0
  8  9  1  0  0  0  0
```

1 10 1 0 0 0 0
9 11 1 0 0 0 0
10 12 1 0 0 0 0
11 13 1 0 0 0 0
12 14 1 0 0 0 0
13 15 1 0 0 0 0
16 17 1 0 0 0 0
17 18 1 0 0 0 0
18 19 1 0 0 0 0
19 20 1 0 0 0 0
20 21 1 0 0 0 0
21 16 1 0 0 0 0
18 15 1 0 0 0 0
14 22 1 0 0 0 0
1 23 1 0 0 0 0
1 24 1 0 0 0 0
2 25 1 0 0 0 0
2 26 1 0 0 0 0
3 27 1 0 0 0 0
3 28 1 0 0 0 0
4 29 1 0 0 0 0
4 30 1 0 0 0 0
5 31 1 0 0 0 0
5 32 1 0 0 0 0
6 33 1 0 0 0 0
6 34 1 0 0 0 0
7 35 1 0 0 0 0
7 36 1 0 0 0 0
8 37 1 0 0 0 0
8 38 1 0 0 0 0
9 39 1 0 0 0 0
9 40 1 0 0 0 0
10 41 1 0 0 0 0
10 42 1 0 0 0 0
11 43 1 0 0 0 0
11 44 1 0 0 0 0
12 45 1 0 0 0 0
12 46 1 0 0 0 0
13 47 1 0 0 0 0
13 48 1 0 0 0 0
14 49 1 0 0 0 0
14 50 1 0 0 0 0
15 51 1 0 0 0 0
15 52 1 0 0 0 0
16 53 1 0 0 0 0
16 54 1 0 0 0 0
17 55 1 0 0 0 0
17 56 1 0 0 0 0
18 57 1 0 0 0 0
19 58 1 0 0 0 0
19 59 1 0 0 0 0
20 60 1 0 0 0 0
20 61 1 0 0 0 0
21 62 1 0 0 0 0
21 63 1 0 0 0 0
22 64 1 0 0 0 0
22 65 1 0 0 0 0
22 66 1 0 0 0 0
M END
> <Alias name>
Hexadecylcyclohexane

> <deltaH°(c)> kJ/mol

-14386

$$$$
9,9'-Dimethyl-9,9'-bifluorenyl
  ChemBrain IXL

```
 50 55  0  0  0  0  0  0  0  0  1 V2000
    5.0129    6.9810    2.5622 C   0  0  0  0  0  0  0  0  0  0  0  0
    5.4206    5.6945    2.2019 C   0  0  0  0  0  0  0  0  0  0  0  0
    6.3251    5.5438    1.1557 C   0  0  0  0  0  0  0  0  0  0  0  0
    6.7787    6.5636    0.5332 C   0  0  0  0  0  0  0  0  0  0  0  0
    6.4172    7.8650    0.8382 C   0  0  0  0  0  0  0  0  0  0  0  0
    5.5179    8.0776    1.8716 C   0  0  0  0  0  0  0  0  0  0  0  0
    7.6297    6.1594   -0.4250 C   0  0  0  0  0  0  0  0  0  0  0  0
    7.7166    4.8832   -0.4094 C   0  0  0  0  0  0  0  0  0  0  0  0
    8.5384    4.2141   -1.3047 C   0  0  0  0  0  0  0  0  0  0  0  0
    9.2695    4.9569   -2.2304 C   0  0  0  0  0  0  0  0  0  0  0  0
    9.1612    6.3432   -2.2349 C   0  0  0  0  0  0  0  0  0  0  0  0
    8.3255    6.9558   -1.3151 C   0  0  0  0  0  0  0  0  0  0  0  0
    6.8826    4.3398    0.6128 C   0  0  0  0  0  0  0  0  0  0  0  0
    3.8113    2.1940    2.9600 C   0  0  0  0  0  0  0  0  0  0  0  0
    4.9300    2.3911    2.1517 C   0  0  0  0  0  0  0  0  0  0  0  0
    4.7885    3.1198    0.9778 C   0  0  0  0  0  0  0  0  0  0  0  0
    3.6525    3.5994    0.6373 C   0  0  0  0  0  0  0  0  0  0  0  0
    2.5049    3.4417    1.3915 C   0  0  0  0  0  0  0  0  0  0  0  0
    2.5856    2.7256    2.5748 C   0  0  0  0  0  0  0  0  0  0  0  0
    3.7726    4.2549   -0.5294 C   0  0  0  0  0  0  0  0  0  0  0  0
    4.9857    4.1919   -0.9304 C   0  0  0  0  0  0  0  0  0  0  0  0
    5.3711    4.7958   -2.1225 C   0  0  0  0  0  0  0  0  0  0  0  0
    4.4116    5.4745   -2.8773 C   0  0  0  0  0  0  0  0  0  0  0  0
    3.0981    5.5314   -2.4235 C   0  0  0  0  0  0  0  0  0  0  0  0
    2.7728    4.9103   -1.2277 C   0  0  0  0  0  0  0  0  0  0  0  0
    5.7810    3.4493    0.0042 C   0  0  0  0  0  0  0  0  0  0  0  0
    6.3444    2.1851   -0.6544 C   0  0  0  0  0  0  0  0  0  0  0  0
    7.7221    3.6104    1.6675 C   0  0  0  0  0  0  0  0  0  0  0  0
    4.3082    7.1268    3.3736 H   0  0  0  0  0  0  0  0  0  0  0  0
    5.0374    4.8324    2.7286 H   0  0  0  0  0  0  0  0  0  0  0  0
    6.8285    8.7024    0.2809 H   0  0  0  0  0  0  0  0  0  0  0  0
    5.2131    9.0870    2.1371 H   0  0  0  0  0  0  0  0  0  0  0  0
    8.6116    3.1377   -1.2869 H   0  0  0  0  0  0  0  0  0  0  0  0
    9.9173    4.4593   -2.9412 H   0  0  0  0  0  0  0  0  0  0  0  0
    9.7224    6.9382   -2.9484 H   0  0  0  0  0  0  0  0  0  0  0  0
    8.2207    8.0372   -1.2941 H   0  0  0  0  0  0  0  0  0  0  0  0
    3.8949    1.6312    3.8813 H   0  0  0  0  0  0  0  0  0  0  0  0
    5.8876    1.9841    2.4359 H   0  0  0  0  0  0  0  0  0  0  0  0
    1.5608    3.8685    1.0643 H   0  0  0  0  0  0  0  0  0  0  0  0
    1.7039    2.5828    3.1914 H   0  0  0  0  0  0  0  0  0  0  0  0
    6.3956    4.7428   -2.4617 H   0  0  0  0  0  0  0  0  0  0  0  0
    4.6857    5.9545   -3.8105 H   0  0  0  0  0  0  0  0  0  0  0  0
    2.3368    6.0546   -2.9968 H   0  0  0  0  0  0  0  0  0  0  0  0
    1.7550    4.9381   -0.8481 H   0  0  0  0  0  0  0  0  0  0  0  0
    6.9993    2.4643   -1.4656 H   0  0  0  0  0  0  0  0  0  0  0  0
    5.5278    1.5860   -1.0403 H   0  0  0  0  0  0  0  0  0  0  0  0
    6.8967    1.6136    0.0769 H   0  0  0  0  0  0  0  0  0  0  0  0
    8.2419    2.7838    1.2062 H   0  0  0  0  0  0  0  0  0  0  0  0
    8.4438    4.2998    2.0918 H   0  0  0  0  0  0  0  0  0  0  0  0
    7.0767    3.2421    2.4479 H   0  0  0  0  0  0  0  0  0  0  0  0
  1  2  4  0  0  0  0
  2  3  4  0  0  0  0
  3  4  4  0  0  0  0
  4  5  4  0  0  0  0
  5  6  4  0  0  0  0
  6  1  4  0  0  0  0
```

```
  7  8  4  0  0  0  0
  8  9  4  0  0  0  0
  9 10  4  0  0  0  0
 10 11  4  0  0  0  0
 11 12  4  0  0  0  0
 12  7  4  0  0  0  0
  4  7  1  0  0  0  0
  3 13  1  0  0  0  0
 13  8  1  0  0  0  0
 14 15  4  0  0  0  0
 15 16  4  0  0  0  0
 16 17  4  0  0  0  0
 17 18  4  0  0  0  0
 18 19  4  0  0  0  0
 19 14  4  0  0  0  0
 20 21  4  0  0  0  0
 21 22  4  0  0  0  0
 22 23  4  0  0  0  0
 23 24  4  0  0  0  0
 24 25  4  0  0  0  0
 25 20  4  0  0  0  0
 17 20  1  0  0  0  0
 16 26  1  0  0  0  0
 26 21  1  0  0  0  0
 26 13  1  0  0  0  0
 26 27  1  0  0  0  0
 13 28  1  0  0  0  0
  1 29  1  0  0  0  0
  2 30  1  0  0  0  0
  5 31  1  0  0  0  0
  6 32  1  0  0  0  0
  9 33  1  0  0  0  0
 10 34  1  0  0  0  0
 11 35  1  0  0  0  0
 12 36  1  0  0  0  0
 14 37  1  0  0  0  0
 15 38  1  0  0  0  0
 18 39  1  0  0  0  0
 19 40  1  0  0  0  0
 22 41  1  0  0  0  0
 23 42  1  0  0  0  0
 24 43  1  0  0  0  0
 25 44  1  0  0  0  0
 27 45  1  0  0  0  0
 27 46  1  0  0  0  0
 27 47  1  0  0  0  0
 28 48  1  0  0  0  0
 28 49  1  0  0  0  0
 28 50  1  0  0  0  0
M  END
>  <Alias name>
9,9'-Dimethyl-9,9'-bifluorenyl

>  <deltaH°(c)>   kJ/mol
-14366

$$$$
Heptadecylbenzene
  ChemBrain IXL

 63 63  0  0  0  0  0  0  0  0  1 V2000
   19.9075   12.9030    0.0000 C   0  0  0  0  0  0  0  0  0  0  0  0
   18.6963   13.8518   -0.0912 C   0  0  0  0  0  0  0  0  0  0  0  0
```

```
 17.3918   13.0346   -0.0024 C   0  0  0  0  0  0  0  0  0  0  0  0
 16.1845   13.9897   -0.0780 C   0  0  0  0  0  0  0  0  0  0  0  0
 14.8759   13.1780    0.0002 C   0  0  0  0  0  0  0  0  0  0  0  0
 13.6737   14.1409   -0.0643 C   0  0  0  0  0  0  0  0  0  0  0  0
 12.3603   13.3361   -0.0014 C   0  0  0  0  0  0  0  0  0  0  0  0
 11.1637   14.3070   -0.0611 C   0  0  0  0  0  0  0  0  0  0  0  0
  9.8457   13.5088   -0.0160 C   0  0  0  0  0  0  0  0  0  0  0  0
 21.2093   13.7235   -0.0947 C   0  0  0  0  0  0  0  0  0  0  0  0
  8.6531   14.4858   -0.0757 C   0  0  0  0  0  0  0  0  0  0  0  0
 22.4226   12.7791    0.0121 C   0  0  0  0  0  0  0  0  0  0  0  0
  7.3329   13.6910   -0.0477 C   0  0  0  0  0  0  0  0  0  0  0  0
 23.7231   13.6010   -0.0862 C   0  0  0  0  0  0  0  0  0  0  0  0
  6.1390   14.6716   -0.1090 C   0  0  0  0  0  0  0  0  0  0  0  0
 24.9372   12.6588    0.0310 C   0  0  0  0  0  0  0  0  0  0  0  0
 26.2371   13.4807   -0.0697 C   0  0  0  0  0  0  0  0  0  0  0  0
  2.4479   12.4988   -0.0615 C   0  0  0  0  0  0  0  0  0  0  0  0
  3.6602   11.8098   -0.0198 C   0  0  0  0  0  0  0  0  0  0  0  0
  4.8640   12.5147   -0.0351 C   0  0  0  0  0  0  0  0  0  0  0  0
  4.8581   13.9130   -0.0922 C   0  0  0  0  0  0  0  0  0  0  0  0
  3.6434   14.6000   -0.1339 C   0  0  0  0  0  0  0  0  0  0  0  0
  2.4394   13.8933   -0.1185 C   0  0  0  0  0  0  0  0  0  0  0  0
 19.8700   12.1897   -0.8140 H   0  0  0  0  0  0  0  0  0  0  0  0
 19.8816   12.3745    0.9449 H   0  0  0  0  0  0  0  0  0  0  0  0
 18.7330   14.5623    0.7253 H   0  0  0  0  0  0  0  0  0  0  0  0
 18.7250   14.3840   -1.0339 H   0  0  0  0  0  0  0  0  0  0  0  0
 17.3491   12.3318   -0.8253 H   0  0  0  0  0  0  0  0  0  0  0  0
 17.3669   12.4945    0.9359 H   0  0  0  0  0  0  0  0  0  0  0  0
 16.2254   14.6882    0.7487 H   0  0  0  0  0  0  0  0  0  0  0  0
 16.2145   14.5356   -1.0129 H   0  0  0  0  0  0  0  0  0  0  0  0
 14.8299   12.4858   -0.8314 H   0  0  0  0  0  0  0  0  0  0  0  0
 14.8476   12.6264    0.9318 H   0  0  0  0  0  0  0  0  0  0  0  0
 13.7165   14.8272    0.7724 H   0  0  0  0  0  0  0  0  0  0  0  0
 13.7093   14.7000   -0.9911 H   0  0  0  0  0  0  0  0  0  0  0  0
 12.3143   12.6536   -0.8410 H   0  0  0  0  0  0  0  0  0  0  0  0
 12.3242   12.7741    0.9238 H   0  0  0  0  0  0  0  0  0  0  0  0
 11.2052   14.9827    0.7842 H   0  0  0  0  0  0  0  0  0  0  0  0
 11.2080   14.8771   -0.9807 H   0  0  0  0  0  0  0  0  0  0  0  0
  9.8030   12.8345   -0.8625 H   0  0  0  0  0  0  0  0  0  0  0  0
  9.7998   12.9380    0.9033 H   0  0  0  0  0  0  0  0  0  0  0  0
 21.2412   14.4441    0.7131 H   0  0  0  0  0  0  0  0  0  0  0  0
 21.2395   14.2440   -1.0440 H   0  0  0  0  0  0  0  0  0  0  0  0
  8.6907   15.1534    0.7762 H   0  0  0  0  0  0  0  0  0  0  0  0
  8.7063   15.0640   -0.9898 H   0  0  0  0  0  0  0  0  0  0  0  0
 22.3902   12.0567   -0.7941 H   0  0  0  0  0  0  0  0  0  0  0  0
 22.3940   12.2609    0.9626 H   0  0  0  0  0  0  0  0  0  0  0  0
  7.2964   13.0236   -0.9000 H   0  0  0  0  0  0  0  0  0  0  0  0
  7.2793   13.1128    0.8667 H   0  0  0  0  0  0  0  0  0  0  0  0
 23.7519   14.3281    0.7159 H   0  0  0  0  0  0  0  0  0  0  0  0
 23.7547   14.1139   -1.0395 H   0  0  0  0  0  0  0  0  0  0  0  0
  6.1749   15.3353    0.7459 H   0  0  0  0  0  0  0  0  0  0  0  0
  6.1993   15.2544   -1.0195 H   0  0  0  0  0  0  0  0  0  0  0  0
 24.9077   11.9305   -0.7699 H   0  0  0  0  0  0  0  0  0  0  0  0
 24.9065   12.1476    0.9852 H   0  0  0  0  0  0  0  0  0  0  0  0
 26.2659   14.2101    0.7303 H   0  0  0  0  0  0  0  0  0  0  0  0
 26.2686   13.9908   -1.0246 H   0  0  0  0  0  0  0  0  0  0  0  0
 27.0900   12.8188    0.0143 H   0  0  0  0  0  0  0  0  0  0  0  0
  1.5137   11.9513   -0.0497 H   0  0  0  0  0  0  0  0  0  0  0  0
  3.6670   10.7276    0.0244 H   0  0  0  0  0  0  0  0  0  0  0  0
  5.8020   11.9764   -0.0027 H   0  0  0  0  0  0  0  0  0  0  0  0
  3.6351   15.6817   -0.1781 H   0  0  0  0  0  0  0  0  0  0  0  0
  1.4982   14.4273   -0.1508 H   0  0  0  0  0  0  0  0  0  0  0  0
  1  2  1  0  0  0  0
  2  3  1  0  0  0  0
```

```
  3  4  1  0  0  0  0
  4  5  1  0  0  0  0
  5  6  1  0  0  0  0
  6  7  1  0  0  0  0
  7  8  1  0  0  0  0
  8  9  1  0  0  0  0
  1 10  1  0  0  0  0
  9 11  1  0  0  0  0
 10 12  1  0  0  0  0
 11 13  1  0  0  0  0
 12 14  1  0  0  0  0
 13 15  1  0  0  0  0
 14 16  1  0  0  0  0
 16 17  1  0  0  0  0
 18 19  4  0  0  0  0
 19 20  4  0  0  0  0
 20 21  4  0  0  0  0
 21 22  4  0  0  0  0
 22 23  4  0  0  0  0
 23 18  4  0  0  0  0
 21 15  1  0  0  0  0
  1 24  1  0  0  0  0
  1 25  1  0  0  0  0
  2 26  1  0  0  0  0
  2 27  1  0  0  0  0
  3 28  1  0  0  0  0
  3 29  1  0  0  0  0
  4 30  1  0  0  0  0
  4 31  1  0  0  0  0
  5 32  1  0  0  0  0
  5 33  1  0  0  0  0
  6 34  1  0  0  0  0
  6 35  1  0  0  0  0
  7 36  1  0  0  0  0
  7 37  1  0  0  0  0
  8 38  1  0  0  0  0
  8 39  1  0  0  0  0
  9 40  1  0  0  0  0
  9 41  1  0  0  0  0
 10 42  1  0  0  0  0
 10 43  1  0  0  0  0
 11 44  1  0  0  0  0
 11 45  1  0  0  0  0
 12 46  1  0  0  0  0
 12 47  1  0  0  0  0
 13 48  1  0  0  0  0
 13 49  1  0  0  0  0
 14 50  1  0  0  0  0
 14 51  1  0  0  0  0
 15 52  1  0  0  0  0
 15 53  1  0  0  0  0
 16 54  1  0  0  0  0
 16 55  1  0  0  0  0
 17 56  1  0  0  0  0
 17 57  1  0  0  0  0
 17 58  1  0  0  0  0
 18 59  1  0  0  0  0
 19 60  1  0  0  0  0
 20 61  1  0  0  0  0
 22 62  1  0  0  0  0
 23 63  1  0  0  0  0
M  END
>  <Alias name>
```

Heptadecylbenzene

> <deltaH°(c)>   kJ/mol
-14334.7

$$$$
Methylprednisolone aceponate
  ChemBrain IXL

 70 73 0 0 0 0 0 0 0 0 1 V2000
    5.1383   8.8012   0.9157 O   0 0 0 0 0 0 0 0 0 0 0 0
    9.2539   6.0062   1.7645 O   0 0 0 0 0 0 0 0 0 0 0 0
    4.8745   7.0430   3.0173 O   0 0 0 0 0 0 0 0 0 0 0 0
    3.4120   4.9088   2.9059 O   0 0 0 0 0 0 0 0 0 0 0 0
   14.2618   8.5986   -1.3370 O   0 0 0 0 0 0 0 0 0 0 0 0
    2.8888   8.5789   0.3534 O   0 0 0 0 0 0 0 0 0 0 0 0
    2.0878   3.2080   3.7767 O   0 0 0 0 0 0 0 0 0 0 0 0
    7.0915   7.2328   -0.7695 C   0 0 0 0 0 0 0 0 0 0 0 0
    8.3955   6.6447   -0.9937 C   0 0 0 0 0 0 0 0 0 0 0 0
    9.3486   7.4840   -0.2035 C   0 0 0 0 0 0 0 0 0 0 0 0
    6.7405   7.0210   0.6583 C   0 0 0 0 0 0 0 0 0 0 0 0
    5.3027   7.3399   0.7516 C   0 0 0 0 0 0 0 0 0 0 0 0
   10.6437   7.1028   -0.5387 C   0 0 0 0 0 0 0 0 0 0 0 0
    9.0812   7.3917   1.3083 C   0 0 0 0 0 0 0 0 0 0 0 0
    7.6282   7.8509   1.5573 C   0 0 0 0 0 0 0 0 0 0 0 0
    5.9482   6.6376   -1.5516 C   0 0 0 0 0 0 0 0 0 0 0 0
    8.7834   6.6685   -2.5314 C   0 0 0 0 0 0 0 0 0 0 0 0
   10.9040   7.7733   -1.8263 C   0 0 0 0 0 0 0 0 0 0 0 0
    4.7036   6.8585   -0.6318 C   0 0 0 0 0 0 0 0 0 0 0 0
    9.8469   7.7877   -2.8431 C   0 0 0 0 0 0 0 0 0 0 0 0
    4.7206   6.6233   1.8827 C   0 0 0 0 0 0 0 0 0 0 0 0
    6.9489   5.5313   0.9964 C   0 0 0 0 0 0 0 0 0 0 0 0
   11.5547   7.4944   0.5791 C   0 0 0 0 0 0 0 0 0 0 0 0
   10.7296   5.5758   -0.7188 C   0 0 0 0 0 0 0 0 0 0 0 0
   12.0622   8.3723   -2.0732 C   0 0 0 0 0 0 0 0 0 0 0 0
   12.7169   8.0912   0.3346 C   0 0 0 0 0 0 0 0 0 0 0 0
   13.0970   8.3711   -1.0460 C   0 0 0 0 0 0 0 0 0 0 0 0
    3.8122   9.3389   0.5916 C   0 0 0 0 0 0 0 0 0 0 0 0
    3.9589   5.3972   1.6429 C   0 0 0 0 0 0 0 0 0 0 0 0
    9.1701   9.1697   -2.8571 C   0 0 0 0 0 0 0 0 0 0 0 0
    3.5963   10.8046   0.5556 C   0 0 0 0 0 0 0 0 0 0 0 0
    2.6038   3.6975   2.7863 C   0 0 0 0 0 0 0 0 0 0 0 0
    4.9569   11.5338   0.6219 C   0 0 0 0 0 0 0 0 0 0 0 0
    2.4156   3.0709   1.4631 C   0 0 0 0 0 0 0 0 0 0 0 0
    9.1883   5.8786   2.7372 H   0 0 0 0 0 0 0 0 0 0 0 0
    7.1441   8.2957   -0.9712 H   0 0 0 0 0 0 0 0 0 0 0 0
    8.4112   5.6267   -0.6262 H   0 0 0 0 0 0 0 0 0 0 0 0
    9.2163   8.5164   -0.5038 H   0 0 0 0 0 0 0 0 0 0 0 0
    9.7671   8.0413   1.8417 H   0 0 0 0 0 0 0 0 0 0 0 0
    7.5262   8.9008   1.3105 H   0 0 0 0 0 0 0 0 0 0 0 0
    7.3618   7.6868   2.5928 H   0 0 0 0 0 0 0 0 0 0 0 0
    6.1170   5.5770   -1.7146 H   0 0 0 0 0 0 0 0 0 0 0 0
    5.8252   7.1517   -2.4967 H   0 0 0 0 0 0 0 0 0 0 0 0
    7.8969   6.8496   -3.1206 H   0 0 0 0 0 0 0 0 0 0 0 0
    9.2005   5.7048   -2.8049 H   0 0 0 0 0 0 0 0 0 0 0 0
    4.0606   7.6201   -1.0515 H   0 0 0 0 0 0 0 0 0 0 0 0
    4.1611   5.9312   -0.5068 H   0 0 0 0 0 0 0 0 0 0 0 0
   10.2894   7.5989   -3.8157 H   0 0 0 0 0 0 0 0 0 0 0 0
    8.0062   5.2940   0.9456 H   0 0 0 0 0 0 0 0 0 0 0 0
    6.5824   5.3326   1.9941 H   0 0 0 0 0 0 0 0 0 0 0 0
    6.4083   4.9204   0.2838 H   0 0 0 0 0 0 0 0 0 0 0 0
   11.2706   7.2843   1.6013 H   0 0 0 0 0 0 0 0 0 0 0 0
   11.7454   5.3034   -0.9948 H   0 0 0 0 0 0 0 0 0 0 0 0

10.4696	5.0887	0.2140	H	0	0	0	0	0	0	0	0	0	0	0	0
10.0494	5.2615	-1.4934	H	0	0	0	0	0	0	0	0	0	0	0	0
12.2378	8.8589	-3.0265	H	0	0	0	0	0	0	0	0	0	0	0	0
13.3764	8.3698	1.1505	H	0	0	0	0	0	0	0	0	0	0	0	0
4.6113	4.6455	1.2159	H	0	0	0	0	0	0	0	0	0	0	0	0
3.1493	5.6056	0.9544	H	0	0	0	0	0	0	0	0	0	0	0	0
8.6565	9.3300	-1.9195	H	0	0	0	0	0	0	0	0	0	0	0	0
9.9235	9.9390	-2.9930	H	0	0	0	0	0	0	0	0	0	0	0	0
8.4592	9.2144	-3.6720	H	0	0	0	0	0	0	0	0	0	0	0	0
3.0892	11.0717	-0.3629	H	0	0	0	0	0	0	0	0	0	0	0	0
2.9889	11.0989	1.4014	H	0	0	0	0	0	0	0	0	0	0	0	0
4.7923	12.6050	0.6202	H	0	0	0	0	0	0	0	0	0	0	0	0
5.5568	11.2599	-0.2375	H	0	0	0	0	0	0	0	0	0	0	0	0
5.4756	11.2503	1.5294	H	0	0	0	0	0	0	0	0	0	0	0	0
3.2369	3.3485	0.8125	H	0	0	0	0	0	0	0	0	0	0	0	0
1.4829	3.4119	1.0321	H	0	0	0	0	0	0	0	0	0	0	0	0
2.3909	1.9953	1.5741	H	0	0	0	0	0	0	0	0	0	0	0	0
12	1	1	0	0	0	0										
1	28	1	0	0	0	0										
14	2	1	0	0	0	0										
3	21	2	0	0	0	0										
4	29	1	0	0	0	0										
4	32	1	0	0	0	0										
5	27	2	0	0	0	0										
6	28	2	0	0	0	0										
7	32	2	0	0	0	0										
8	9	1	0	0	0	0										
8	11	1	0	0	0	0										
8	16	1	0	0	0	0										
9	10	1	0	0	0	0										
9	17	1	0	0	0	0										
10	13	1	0	0	0	0										
10	14	1	0	0	0	0										
11	12	1	0	0	0	0										
11	15	1	0	0	0	0										
11	22	1	0	0	0	0										
12	19	1	0	0	0	0										
12	21	1	0	0	0	0										
13	18	1	0	0	0	0										
13	23	1	0	0	0	0										
13	24	1	0	0	0	0										
14	15	1	0	0	0	0										
16	19	1	0	0	0	0										
17	20	1	0	0	0	0										
18	20	1	0	0	0	0										
18	25	2	0	0	0	0										
20	30	1	0	0	0	0										
21	29	1	0	0	0	0										
23	26	2	0	0	0	0										
25	27	1	0	0	0	0										
26	27	1	0	0	0	0										
28	31	1	0	0	0	0										
31	33	1	0	0	0	0										
32	34	1	0	0	0	0										
2	35	1	0	0	0	0										
8	36	1	0	0	0	0										
9	37	1	0	0	0	0										
10	38	1	0	0	0	0										
14	39	1	0	0	0	0										
15	40	1	0	0	0	0										
15	41	1	0	0	0	0										
16	42	1	0	0	0	0										
16	43	1	0	0	0	0										

```
 17 44  1  0  0  0  0
 17 45  1  0  0  0  0
 19 46  1  0  0  0  0
 19 47  1  0  0  0  0
 20 48  1  0  0  0  0
 22 49  1  0  0  0  0
 22 50  1  0  0  0  0
 22 51  1  0  0  0  0
 23 52  1  0  0  0  0
 24 53  1  0  0  0  0
 24 54  1  0  0  0  0
 24 55  1  0  0  0  0
 25 56  1  0  0  0  0
 26 57  1  0  0  0  0
 29 58  1  0  0  0  0
 29 59  1  0  0  0  0
 30 60  1  0  0  0  0
 30 61  1  0  0  0  0
 30 62  1  0  0  0  0
 31 63  1  0  0  0  0
 31 64  1  0  0  0  0
 33 65  1  0  0  0  0
 33 66  1  0  0  0  0
 33 67  1  0  0  0  0
 34 68  1  0  0  0  0
 34 69  1  0  0  0  0
 34 70  1  0  0  0  0
M  END
>  <Alias name>
Methylprednisolone aceponate

>  <deltaH°(c)>   kJ/mol
-14304

$$$$
N,N'-Bis(benzoylacetone)cyclohexanediimine
  ChemBrain IXL

 60 62  0  0  0  0  0  0  0  0  1 V2000
    5.5235    6.7939    1.7123 C   0  0  0  0  0  0  0  0  0  0  0  0
    5.4187    7.5965    0.6507 C   0  0  0  0  0  0  0  0  0  0  0  0
    5.5411    9.0622    0.8160 C   0  0  0  0  0  0  0  0  0  0  0  0
    5.7686    7.3654    3.0443 O   0  0  0  0  0  0  0  0  0  0  0  0
    5.7023    9.5593    1.9735 N   0  0  0  0  0  0  0  0  0  0  0  0
    9.8487    9.7374   -0.3988 C   0  0  0  0  0  0  0  0  0  0  0  0
    9.7937    9.1602    0.8037 C   0  0  0  0  0  0  0  0  0  0  0  0
    8.8808    9.6935    1.8394 C   0  0  0  0  0  0  0  0  0  0  0  0
    8.9962   10.8972   -0.6971 O   0  0  0  0  0  0  0  0  0  0  0  0
    8.2152   10.7516    1.6153 N   0  0  0  0  0  0  0  0  0  0  0  0
    7.3185   11.2315    2.5774 C   0  0  0  0  0  0  0  0  0  0  0  0
    7.5082   12.7518    2.7434 C   0  0  0  0  0  0  0  0  0  0  0  0
    6.5212   13.2800    3.8024 C   0  0  0  0  0  0  0  0  0  0  0  0
    5.0774   12.9889    3.3493 C   0  0  0  0  0  0  0  0  0  0  0  0
    4.8877   11.4687    3.1833 C   0  0  0  0  0  0  0  0  0  0  0  0
    5.8747   10.9405    2.1243 C   0  0  0  0  0  0  0  0  0  0  0  0
    5.4755    9.9485   -0.3674 C   0  0  0  0  0  0  0  0  0  0  0  0
    8.7437    8.9938    3.1363 C   0  0  0  0  0  0  0  0  0  0  0  0
    5.1438    2.5672    1.2394 C   0  0  0  0  0  0  0  0  0  0  0  0
    4.9522    3.4115    0.1455 C   0  0  0  0  0  0  0  0  0  0  0  0
    5.0771    4.7923    0.2998 C   0  0  0  0  0  0  0  0  0  0  0  0
    5.3930    5.3289    1.5481 C   0  0  0  0  0  0  0  0  0  0  0  0
    5.5859    4.4846    2.6417 C   0  0  0  0  0  0  0  0  0  0  0  0
    5.4606    3.1038    2.4875 C   0  0  0  0  0  0  0  0  0  0  0  0
```

12.5025	8.2159	-3.3780	C	0	0	0	0	0	0	0	0	0	0	0	0
12.5472	7.7085	-2.0793	C	0	0	0	0	0	0	0	0	0	0	0	0
11.6800	8.2054	-1.1061	C	0	0	0	0	0	0	0	0	0	0	0	0
10.7681	9.2096	-1.4315	C	0	0	0	0	0	0	0	0	0	0	0	0
10.7228	9.7164	-2.7304	C	0	0	0	0	0	0	0	0	0	0	0	0
11.5904	9.2200	-3.7035	C	0	0	0	0	0	0	0	0	0	0	0	0
5.8353	8.3458	3.0700	H	0	0	0	0	0	0	0	0	0	0	0	0
8.4208	11.1913	0.0436	H	0	0	0	0	0	0	0	0	0	0	0	0
5.2442	7.1752	-0.3316	H	0	0	0	0	0	0	0	0	0	0	0	0
10.4169	8.3017	1.0213	H	0	0	0	0	0	0	0	0	0	0	0	0
7.5063	10.7389	3.5234	H	0	0	0	0	0	0	0	0	0	0	0	0
8.5229	12.9563	3.0618	H	0	0	0	0	0	0	0	0	0	0	0	0
7.3205	13.2444	1.7974	H	0	0	0	0	0	0	0	0	0	0	0	0
6.6545	14.3484	3.9190	H	0	0	0	0	0	0	0	0	0	0	0	0
6.7090	12.7873	4.7484	H	0	0	0	0	0	0	0	0	0	0	0	0
4.8896	13.4816	2.4033	H	0	0	0	0	0	0	0	0	0	0	0	0
4.3837	13.3602	4.0936	H	0	0	0	0	0	0	0	0	0	0	0	0
3.8729	11.2641	2.8649	H	0	0	0	0	0	0	0	0	0	0	0	0
5.0754	10.9761	4.1294	H	0	0	0	0	0	0	0	0	0	0	0	0
5.6869	11.4331	1.1783	H	0	0	0	0	0	0	0	0	0	0	0	0
4.6702	10.6614	-0.2404	H	0	0	0	0	0	0	0	0	0	0	0	0
5.2945	9.3537	-1.2542	H	0	0	0	0	0	0	0	0	0	0	0	0
6.4138	10.4788	-0.4737	H	0	0	0	0	0	0	0	0	0	0	0	0
9.3259	8.0808	3.1172	H	0	0	0	0	0	0	0	0	0	0	0	0
9.1032	9.6346	3.9320	H	0	0	0	0	0	0	0	0	0	0	0	0
7.7018	8.7540	3.3088	H	0	0	0	0	0	0	0	0	0	0	0	0
5.0465	1.4953	1.1197	H	0	0	0	0	0	0	0	0	0	0	0	0
4.7065	2.9949	-0.8235	H	0	0	0	0	0	0	0	0	0	0	0	0
4.9287	5.4477	-0.5495	H	0	0	0	0	0	0	0	0	0	0	0	0
5.8329	4.9010	3.6105	H	0	0	0	0	0	0	0	0	0	0	0	0
5.6094	2.4484	3.3367	H	0	0	0	0	0	0	0	0	0	0	0	0
13.1759	7.8303	-4.1335	H	0	0	0	0	0	0	0	0	0	0	0	0
13.2551	6.9288	-1.8266	H	0	0	0	0	0	0	0	0	0	0	0	0
11.7148	7.8115	-0.0978	H	0	0	0	0	0	0	0	0	0	0	0	0
10.0141	10.4953	-2.9834	H	0	0	0	0	0	0	0	0	0	0	0	0
11.5558	9.6140	-4.7117	H	0	0	0	0	0	0	0	0	0	0	0	0

1	2	2	0	0	0	0
2	3	1	0	0	0	0
1	4	1	0	0	0	0
3	5	2	0	0	0	0
6	7	2	0	0	0	0
7	8	1	0	0	0	0
6	9	1	0	0	0	0
8	10	2	0	0	0	0
11	12	1	0	0	0	0
12	13	1	0	0	0	0
13	14	1	0	0	0	0
14	15	1	0	0	0	0
15	16	1	0	0	0	0
16	11	1	0	0	0	0
5	16	1	0	0	0	0
10	11	1	0	0	0	0
3	17	1	0	0	0	0
8	18	1	0	0	0	0
19	20	4	0	0	0	0
20	21	4	0	0	0	0
21	22	4	0	0	0	0
22	23	4	0	0	0	0
23	24	4	0	0	0	0
24	19	4	0	0	0	0
25	26	4	0	0	0	0
26	27	4	0	0	0	0
27	28	4	0	0	0	0

```
 28 29  4  0  0  0  0
 29 30  4  0  0  0  0
 30 25  4  0  0  0  0
 28  6  1  0  0  0  0
 22  1  1  0  0  0  0
  4 31  1  0  0  0  0
  9 32  1  0  0  0  0
  2 33  1  0  0  0  0
  7 34  1  0  0  0  0
 11 35  1  0  0  0  0
 12 36  1  0  0  0  0
 12 37  1  0  0  0  0
 13 38  1  0  0  0  0
 13 39  1  0  0  0  0
 14 40  1  0  0  0  0
 14 41  1  0  0  0  0
 15 42  1  0  0  0  0
 15 43  1  0  0  0  0
 16 44  1  0  0  0  0
 17 45  1  0  0  0  0
 17 46  1  0  0  0  0
 17 47  1  0  0  0  0
 18 48  1  0  0  0  0
 18 49  1  0  0  0  0
 18 50  1  0  0  0  0
 19 51  1  0  0  0  0
 20 52  1  0  0  0  0
 21 53  1  0  0  0  0
 23 54  1  0  0  0  0
 24 55  1  0  0  0  0
 25 56  1  0  0  0  0
 26 57  1  0  0  0  0
 27 58  1  0  0  0  0
 29 59  1  0  0  0  0
 30 60  1  0  0  0  0
M  END
>  <Alias name>
N,N'-Bis(benzoylacetone)cyclohexanediimine

>  <deltaH°(c)>   kJ/mol
-14176

$$$$
Anthracene photodimer
  ChemBrain IXL

 48 54  0  0  0  0  0  0  0  0  1 V2000
    3.0630    5.2565    2.4524 C   0  0  0  0  0  0  0  0  0  0  0  0
    2.4331    5.7692    1.3186 C   0  0  0  0  0  0  0  0  0  0  0  0
    4.4092    4.8956    2.3972 C   0  0  0  0  0  0  0  0  0  0  0  0
    3.1502    5.9204    0.1312 C   0  0  0  0  0  0  0  0  0  0  0  0
    4.4968    5.5597    0.0813 C   0  0  0  0  0  0  0  0  0  0  0  0
    5.1215    5.0507    1.2074 C   0  0  0  0  0  0  0  0  0  0  0  0
    5.2732    5.7079   -1.1631 C   0  0  0  0  0  0  0  0  0  0  0  0
    6.5394    4.6765    1.1257 C   0  0  0  0  0  0  0  0  0  0  0  0
    6.7705    4.0261   -0.1769 C   0  0  0  0  0  0  0  0  0  0  0  0
    7.4081    5.9556    1.2215 C   0  0  0  0  0  0  0  0  0  0  0  0
    6.1455    4.5346   -1.3031 C   0  0  0  0  0  0  0  0  0  0  0  0
    7.6020    2.9110   -0.2818 C   0  0  0  0  0  0  0  0  0  0  0  0
    6.3431    3.9358   -2.5479 C   0  0  0  0  0  0  0  0  0  0  0  0
    6.1486    6.9829   -1.0729 C   0  0  0  0  0  0  0  0  0  0  0  0
    7.1750    2.8217   -2.6580 C   0  0  0  0  0  0  0  0  0  0  0  0
    7.8048    2.3089   -1.5242 C   0  0  0  0  0  0  0  0  0  0  0  0
```

```
 10.2623    5.8820   -1.2342 C   0  0  0  0  0  0  0  0  0  0  0  0
  9.6393    6.3904   -2.3737 C   0  0  0  0  0  0  0  0  0  0  0  0
  9.5376    5.7366   -0.0513 C   0  0  0  0  0  0  0  0  0  0  0  0
  8.2926    6.7528   -2.3289 C   0  0  0  0  0  0  0  0  0  0  0  0
  7.5714    6.6039   -1.1441 C   0  0  0  0  0  0  0  0  0  0  0  0
  8.1907    6.0991   -0.0131 C   0  0  0  0  0  0  0  0  0  0  0  0
  6.5307    7.1331    1.3509 C   0  0  0  0  0  0  0  0  0  0  0  0
  5.9118    7.6385    0.2200 C   0  0  0  0  0  0  0  0  0  0  0  0
  6.3248    7.7387    2.5906 C   0  0  0  0  0  0  0  0  0  0  0  0
  5.0797    8.7548    0.3130 C   0  0  0  0  0  0  0  0  0  0  0  0
  4.8695    9.3626    1.5507 C   0  0  0  0  0  0  0  0  0  0  0  0
  5.4924    8.8542    2.6902 C   0  0  0  0  0  0  0  0  0  0  0  0
  2.5072    5.1385    3.3751 H   0  0  0  0  0  0  0  0  0  0  0  0
  1.3877    6.0498    1.3599 H   0  0  0  0  0  0  0  0  0  0  0  0
  4.9002    4.4958    3.2767 H   0  0  0  0  0  0  0  0  0  0  0  0
  2.6619    6.3170   -0.7499 H   0  0  0  0  0  0  0  0  0  0  0  0
  4.6017    5.7765   -2.0130 H   0  0  0  0  0  0  0  0  0  0  0  0
  6.7916    3.9938    1.9308 H   0  0  0  0  0  0  0  0  0  0  0  0
  8.0717    5.8920    2.0779 H   0  0  0  0  0  0  0  0  0  0  0  0
  8.0894    2.5130    0.5992 H   0  0  0  0  0  0  0  0  0  0  0  0
  5.8517    4.3351   -3.4275 H   0  0  0  0  0  0  0  0  0  0  0  0
  5.9044    7.6606   -1.8846 H   0  0  0  0  0  0  0  0  0  0  0  0
  7.3316    2.3550   -3.6233 H   0  0  0  0  0  0  0  0  0  0  0  0
  8.4511    1.4437   -1.6081 H   0  0  0  0  0  0  0  0  0  0  0  0
 11.3081    5.6003   -1.2677 H   0  0  0  0  0  0  0  0  0  0  0  0
 10.2009    6.5040   -3.2931 H   0  0  0  0  0  0  0  0  0  0  0  0
 10.0201    5.3429    0.8356 H   0  0  0  0  0  0  0  0  0  0  0  0
  7.8084    7.1488   -3.2125 H   0  0  0  0  0  0  0  0  0  0  0  0
  6.8099    7.3440    3.4743 H   0  0  0  0  0  0  0  0  0  0  0  0
  4.5976    9.1491   -0.5739 H   0  0  0  0  0  0  0  0  0  0  0  0
  4.2229   10.2289    1.6267 H   0  0  0  0  0  0  0  0  0  0  0  0
  5.3302    9.3252    3.6521 H   0  0  0  0  0  0  0  0  0  0  0  0
  1  2  4  0  0  0  0
  3  1  4  0  0  0  0
  2  4  4  0  0  0  0
  4  5  4  0  0  0  0
  6  5  4  0  0  0  0
  5  7  1  0  0  0  0
  6  3  4  0  0  0  0
  8  6  1  0  0  0  0
  9  8  1  0  0  0  0
  8 10  1  0  0  0  0
 11  9  4  0  0  0  0
 12  9  4  0  0  0  0
 11 13  4  0  0  0  0
 11  7  1  0  0  0  0
  7 14  1  0  0  0  0
 13 15  4  0  0  0  0
 15 16  4  0  0  0  0
 16 12  4  0  0  0  0
 17 18  4  0  0  0  0
 19 17  4  0  0  0  0
 18 20  4  0  0  0  0
 20 21  4  0  0  0  0
 22 21  4  0  0  0  0
 21 14  1  0  0  0  0
 22 19  4  0  0  0  0
 10 22  1  0  0  0  0
 23 10  1  0  0  0  0
 24 23  4  0  0  0  0
 25 23  4  0  0  0  0
 24 26  4  0  0  0  0
 14 24  1  0  0  0  0
```

```
 26 27  4  0  0  0  0
 27 28  4  0  0  0  0
 28 25  4  0  0  0  0
  1 29  1  0  0  0  0
  2 30  1  0  0  0  0
  3 31  1  0  0  0  0
  4 32  1  0  0  0  0
  7 33  1  0  0  0  0
  8 34  1  0  0  0  0
 10 35  1  0  0  0  0
 12 36  1  0  0  0  0
 13 37  1  0  0  0  0
 14 38  1  0  0  0  0
 15 39  1  0  0  0  0
 16 40  1  0  0  0  0
 17 41  1  0  0  0  0
 18 42  1  0  0  0  0
 19 43  1  0  0  0  0
 20 44  1  0  0  0  0
 25 45  1  0  0  0  0
 26 46  1  0  0  0  0
 27 47  1  0  0  0  0
 28 48  1  0  0  0  0
M  END
>  <Alias name>
Anthracene photodimer

>  <deltaH°(c)>   kJ/mol
-14169

$$$$
Simvastatin
  ChemBrain IXL

 68 70  0  0  0  0  0  0  0  0  1 V2000
    8.6231    6.0785    0.9746 C   0  0  0  0  0  0  0  0  0  0  0  0
    8.1848    7.1893    1.8046 C   0  0  0  0  0  0  0  0  0  0  0  0
    8.2585    8.4072    1.3123 C   0  0  0  0  0  0  0  0  0  0  0  0
    8.7837    8.5895   -0.0389 C   0  0  0  0  0  0  0  0  0  0  0  0
    8.6211    7.5503   -1.0561 C   0  0  0  0  0  0  0  0  0  0  0  0
    8.0046    6.2694   -0.3936 C   0  0  0  0  0  0  0  0  0  0  0  0
    9.4122    9.6878   -0.3483 C   0  0  0  0  0  0  0  0  0  0  0  0
   10.0221    7.2283   -1.6679 C   0  0  0  0  0  0  0  0  0  0  0  0
   10.8348    8.5653   -1.9510 C   0  0  0  0  0  0  0  0  0  0  0  0
    9.9507    9.8045   -1.6753 C   0  0  0  0  0  0  0  0  0  0  0  0
    6.4858    6.4599   -0.1832 C   0  0  0  0  0  0  0  0  0  0  0  0
    8.1418    4.7520    1.5930 C   0  0  0  0  0  0  0  0  0  0  0  0
    5.7512    6.6053   -1.5114 C   0  0  0  0  0  0  0  0  0  0  0  0
    4.2351    6.6580   -1.2196 C   0  0  0  0  0  0  0  0  0  0  0  0
    3.9774    7.6662   -0.1612 O   0  0  0  0  0  0  0  0  0  0  0  0
    3.7710    5.2850   -0.7318 C   0  0  0  0  0  0  0  0  0  0  0  0
    2.2663    5.3263   -0.4858 C   0  0  0  0  0  0  0  0  0  0  0  0
    2.6746    7.5670    0.5639 C   0  0  0  0  0  0  0  0  0  0  0  0
    1.9651    6.2513    0.7168 C   0  0  0  0  0  0  0  0  0  0  0  0
    2.1791    8.5724    1.0453 O   0  0  0  0  0  0  0  0  0  0  0  0
    1.5889    5.8357   -1.6862 O   0  0  0  0  0  0  0  0  0  0  0  0
   12.2394    6.5666   -0.7535 C   0  0  0  0  0  0  0  0  0  0  0  0
   12.7538    7.2205   -1.6458 O   0  0  0  0  0  0  0  0  0  0  0  0
   10.7791    6.3901   -0.7199 O   0  0  0  0  0  0  0  0  0  0  0  0
   13.0863    5.9494    0.2944 C   0  0  0  0  0  0  0  0  0  0  0  0
   14.4323    6.6972    0.3749 C   0  0  0  0  0  0  0  0  0  0  0  0
   12.3694    6.0317    1.6606 C   0  0  0  0  0  0  0  0  0  0  0  0
   13.3401    4.4737   -0.0616 C   0  0  0  0  0  0  0  0  0  0  0  0
```

```
 12.1098    7.5081    2.0228 C   0  0  0  0  0  0  0  0  0  0  0  0
 10.7813   11.0996   -1.7816 C   0  0  0  0  0  0  0  0  0  0  0  0
  1.7213    5.2952   -2.4979 H   0  0  0  0  0  0  0  0  0  0  0  0
  9.7030    6.0802    0.8968 H   0  0  0  0  0  0  0  0  0  0  0  0
  7.7978    7.0176    2.8030 H   0  0  0  0  0  0  0  0  0  0  0  0
  7.9363    9.2601    1.8995 H   0  0  0  0  0  0  0  0  0  0  0  0
  7.9599    7.9085   -1.8346 H   0  0  0  0  0  0  0  0  0  0  0  0
  8.1911    5.4015   -1.0120 H   0  0  0  0  0  0  0  0  0  0  0  0
  9.5282   10.4877    0.3749 H   0  0  0  0  0  0  0  0  0  0  0  0
  9.8906    6.6886   -2.5953 H   0  0  0  0  0  0  0  0  0  0  0  0
 11.7042    8.5984   -1.3041 H   0  0  0  0  0  0  0  0  0  0  0  0
 11.1566    8.5785   -2.9829 H   0  0  0  0  0  0  0  0  0  0  0  0
  9.1392    9.8361   -2.3912 H   0  0  0  0  0  0  0  0  0  0  0  0
  6.0919    5.6006    0.3463 H   0  0  0  0  0  0  0  0  0  0  0  0
  6.3192    7.3492    0.4126 H   0  0  0  0  0  0  0  0  0  0  0  0
  8.6104    4.6139    2.5605 H   0  0  0  0  0  0  0  0  0  0  0  0
  7.0656    4.7802    1.7147 H   0  0  0  0  0  0  0  0  0  0  0  0
  8.4102    3.9313    0.9416 H   0  0  0  0  0  0  0  0  0  0  0  0
  6.0609    7.5196   -2.0020 H   0  0  0  0  0  0  0  0  0  0  0  0
  5.9726    5.7563   -2.1464 H   0  0  0  0  0  0  0  0  0  0  0  0
  3.6979    6.9265   -2.1211 H   0  0  0  0  0  0  0  0  0  0  0  0
  4.2815    5.0374    0.1912 H   0  0  0  0  0  0  0  0  0  0  0  0
  3.9963    4.5372   -1.4822 H   0  0  0  0  0  0  0  0  0  0  0  0
  1.9100    4.3259   -0.2689 H   0  0  0  0  0  0  0  0  0  0  0  0
  0.8970    6.4263    0.7715 H   0  0  0  0  0  0  0  0  0  0  0  0
  2.2948    5.7704    1.6287 H   0  0  0  0  0  0  0  0  0  0  0  0
 14.2553    7.7322    0.6405 H   0  0  0  0  0  0  0  0  0  0  0  0
 14.9291    6.6510   -0.5862 H   0  0  0  0  0  0  0  0  0  0  0  0
 15.0584    6.2336    1.1275 H   0  0  0  0  0  0  0  0  0  0  0  0
 11.4264    5.5027    1.6058 H   0  0  0  0  0  0  0  0  0  0  0  0
 12.9929    5.5787    2.4222 H   0  0  0  0  0  0  0  0  0  0  0  0
 13.9834    4.0272    0.6859 H   0  0  0  0  0  0  0  0  0  0  0  0
 12.3966    3.9424   -0.0901 H   0  0  0  0  0  0  0  0  0  0  0  0
 13.8175    4.4137   -1.0317 H   0  0  0  0  0  0  0  0  0  0  0  0
 11.4629    7.9544    1.2783 H   0  0  0  0  0  0  0  0  0  0  0  0
 13.0505    8.0440    2.0518 H   0  0  0  0  0  0  0  0  0  0  0  0
 11.6333    7.5628    2.9945 H   0  0  0  0  0  0  0  0  0  0  0  0
 10.1348   11.9559   -1.6145 H   0  0  0  0  0  0  0  0  0  0  0  0
 11.2203   11.1677   -2.7675 H   0  0  0  0  0  0  0  0  0  0  0  0
 11.5650   11.0899   -1.0356 H   0  0  0  0  0  0  0  0  0  0  0  0
  1  2  1  0  0  0  0
  2  3  2  0  0  0  0
  3  4  1  0  0  0  0
  4  5  1  0  0  0  0
  5  6  1  0  0  0  0
  6  1  1  0  0  0  0
  4  7  2  0  0  0  0
  5  8  1  0  0  0  0
  8  9  1  0  0  0  0
  9 10  1  0  0  0  0
 10  7  1  0  0  0  0
  6 11  1  0  0  0  0
  1 12  1  0  0  0  0
 11 13  1  0  0  0  0
 13 14  1  0  0  0  0
 14 15  1  0  0  0  0
 14 16  1  0  0  0  0
 16 17  1  0  0  0  0
 15 18  1  0  0  0  0
 17 19  1  0  0  0  0
 19 18  1  0  0  0  0
 18 20  2  0  0  0  0
 17 21  1  0  0  0  0
```

```
 22 23  2  0  0  0  0
 22 24  1  0  0  0  0
 22 25  1  0  0  0  0
 25 26  1  0  0  0  0
 25 27  1  0  0  0  0
 25 28  1  0  0  0  0
 27 29  1  0  0  0  0
 10 30  1  0  0  0  0
 24  8  1  0  0  0  0
 21 31  1  0  0  0  0
  1 32  1  0  0  0  0
  2 33  1  0  0  0  0
  3 34  1  0  0  0  0
  5 35  1  0  0  0  0
  6 36  1  0  0  0  0
  7 37  1  0  0  0  0
  8 38  1  0  0  0  0
  9 39  1  0  0  0  0
  9 40  1  0  0  0  0
 10 41  1  0  0  0  0
 11 42  1  0  0  0  0
 11 43  1  0  0  0  0
 12 44  1  0  0  0  0
 12 45  1  0  0  0  0
 12 46  1  0  0  0  0
 13 47  1  0  0  0  0
 13 48  1  0  0  0  0
 14 49  1  0  0  0  0
 16 50  1  0  0  0  0
 16 51  1  0  0  0  0
 17 52  1  0  0  0  0
 19 53  1  0  0  0  0
 19 54  1  0  0  0  0
 26 55  1  0  0  0  0
 26 56  1  0  0  0  0
 26 57  1  0  0  0  0
 27 58  1  0  0  0  0
 27 59  1  0  0  0  0
 28 60  1  0  0  0  0
 28 61  1  0  0  0  0
 28 62  1  0  0  0  0
 29 63  1  0  0  0  0
 29 64  1  0  0  0  0
 29 65  1  0  0  0  0
 30 66  1  0  0  0  0
 30 67  1  0  0  0  0
 30 68  1  0  0  0  0
M  END
>  <Alias name>
Simvastatin

>  <deltaH°(c)>   kJ/mol
-14030.11

$$$$
n-Butylstearate
  ChemBrain IXL

 68 67  0  0  0  0  0  0  0  0  1 V2000
   12.2748   14.0048    0.3295 C   0  0  0  0  0  0  0  0  0  0  0  0
   11.1475   14.8664    0.9307 C   0  0  0  0  0  0  0  0  0  0  0  0
   10.0538   13.9498    1.5123 C   0  0  0  0  0  0  0  0  0  0  0  0
    8.9270   14.8127    2.1129 C   0  0  0  0  0  0  0  0  0  0  0  0
```

7.8324	13.8971	2.6944	C	0	0	0	0	0	0	0	0	0	0	0	0
6.7059	14.7609	3.2945	C	0	0	0	0	0	0	0	0	0	0	0	0
5.6107	13.8459	3.8760	C	0	0	0	0	0	0	0	0	0	0	0	0
4.4845	14.7101	4.4758	C	0	0	0	0	0	0	0	0	0	0	0	0
3.3889	13.7954	5.0574	C	0	0	0	0	0	0	0	0	0	0	0	0
13.3673	14.9226	-0.2521	C	0	0	0	0	0	0	0	0	0	0	0	0
2.2628	14.6597	5.6571	C	0	0	0	0	0	0	0	0	0	0	0	0
14.4953	14.0625	-0.8539	C	0	0	0	0	0	0	0	0	0	0	0	0
15.5865	14.9819	-1.4352	C	0	0	0	0	0	0	0	0	0	0	0	0
16.7153	14.1236	-2.0374	C	0	0	0	0	0	0	0	0	0	0	0	0
17.8054	15.0445	-2.6181	C	0	0	0	0	0	0	0	0	0	0	0	0
18.9352	14.1877	-3.2202	C	0	0	0	0	0	0	0	0	0	0	0	0
20.0247	15.1104	-3.8005	C	0	0	0	0	0	0	0	0	0	0	0	0
21.1123	14.2898	-4.3797	C	0	0	0	0	0	0	0	0	0	0	0	0
22.0755	14.8337	-4.8956	O	0	0	0	0	0	0	0	0	0	0	0	0
21.0318	12.8194	-4.3333	O	0	0	0	0	0	0	0	0	0	0	0	0
22.1698	12.1109	-4.9398	C	0	0	0	0	0	0	0	0	0	0	0	0
21.9516	10.5907	-4.8193	C	0	0	0	0	0	0	0	0	0	0	0	0
23.1439	9.8489	-5.4545	C	0	0	0	0	0	0	0	0	0	0	0	0
22.9263	8.3282	-5.3340	C	0	0	0	0	0	0	0	0	0	0	0	0
12.7028	13.3792	1.1031	H	0	0	0	0	0	0	0	0	0	0	0	0
11.8711	13.3802	-0.4578	H	0	0	0	0	0	0	0	0	0	0	0	0
10.7200	15.4923	0.1571	H	0	0	0	0	0	0	0	0	0	0	0	0
11.5513	15.4909	1.7180	H	0	0	0	0	0	0	0	0	0	0	0	0
10.4810	13.3242	2.2863	H	0	0	0	0	0	0	0	0	0	0	0	0
9.6498	13.3253	0.7251	H	0	0	0	0	0	0	0	0	0	0	0	0
8.5003	15.4387	1.3390	H	0	0	0	0	0	0	0	0	0	0	0	0
9.3311	15.4371	2.9002	H	0	0	0	0	0	0	0	0	0	0	0	0
8.2589	13.2713	3.4686	H	0	0	0	0	0	0	0	0	0	0	0	0
7.4282	13.2727	1.9073	H	0	0	0	0	0	0	0	0	0	0	0	0
6.2797	15.3869	2.5203	H	0	0	0	0	0	0	0	0	0	0	0	0
7.1102	15.3852	4.0817	H	0	0	0	0	0	0	0	0	0	0	0	0
6.0368	13.2200	4.6503	H	0	0	0	0	0	0	0	0	0	0	0	0
5.2064	13.2216	3.0888	H	0	0	0	0	0	0	0	0	0	0	0	0
4.0585	15.3361	3.7014	H	0	0	0	0	0	0	0	0	0	0	0	0
4.8888	15.3345	5.2629	H	0	0	0	0	0	0	0	0	0	0	0	0
3.8148	13.1695	5.8317	H	0	0	0	0	0	0	0	0	0	0	0	0
2.9845	13.1711	4.2703	H	0	0	0	0	0	0	0	0	0	0	0	0
12.9389	15.5483	-1.0253	H	0	0	0	0	0	0	0	0	0	0	0	0
13.7707	15.5472	0.5353	H	0	0	0	0	0	0	0	0	0	0	0	0
1.4931	14.0168	6.0656	H	0	0	0	0	0	0	0	0	0	0	0	0
2.6671	15.2840	6.4443	H	0	0	0	0	0	0	0	0	0	0	0	0
1.8368	15.2856	4.8827	H	0	0	0	0	0	0	0	0	0	0	0	0
14.9242	13.4371	-0.0807	H	0	0	0	0	0	0	0	0	0	0	0	0
14.0921	13.4379	-1.6414	H	0	0	0	0	0	0	0	0	0	0	0	0
15.1573	15.6074	-2.2082	H	0	0	0	0	0	0	0	0	0	0	0	0
15.9892	15.6068	-0.6476	H	0	0	0	0	0	0	0	0	0	0	0	0
17.1450	13.4983	-1.2645	H	0	0	0	0	0	0	0	0	0	0	0	0
16.3130	13.4988	-2.8252	H	0	0	0	0	0	0	0	0	0	0	0	0
17.3755	15.6697	-3.3910	H	0	0	0	0	0	0	0	0	0	0	0	0
18.2072	15.6695	-1.8302	H	0	0	0	0	0	0	0	0	0	0	0	0
19.3653	13.5626	-2.4475	H	0	0	0	0	0	0	0	0	0	0	0	0
18.5336	13.5628	-4.0084	H	0	0	0	0	0	0	0	0	0	0	0	0
20.4254	15.7356	-3.0122	H	0	0	0	0	0	0	0	0	0	0	0	0
19.5941	15.7355	-4.5730	H	0	0	0	0	0	0	0	0	0	0	0	0
23.0822	12.3866	-4.4255	H	0	0	0	0	0	0	0	0	0	0	0	0
22.2485	12.3826	-5.9853	H	0	0	0	0	0	0	0	0	0	0	0	0
21.0394	10.3151	-5.3337	H	0	0	0	0	0	0	0	0	0	0	0	0
21.8728	10.3191	-3.7739	H	0	0	0	0	0	0	0	0	0	0	0	0
24.0562	10.1248	-4.9402	H	0	0	0	0	0	0	0	0	0	0	0	0
23.2226	10.1205	-6.4999	H	0	0	0	0	0	0	0	0	0	0	0	0
23.7642	7.8073	-5.7804	H	0	0	0	0	0	0	0	0	0	0	0	0
22.8477	8.0565	-4.2886	H	0	0	0	0	0	0	0	0	0	0	0	0

22.0141 8.0522 -5.8484 H 0 0 0 0 0 0 0 0 0 0 0 0
1 2 1 0 0 0 0
2 3 1 0 0 0 0
3 4 1 0 0 0 0
4 5 1 0 0 0 0
5 6 1 0 0 0 0
6 7 1 0 0 0 0
7 8 1 0 0 0 0
8 9 1 0 0 0 0
1 10 1 0 0 0 0
9 11 1 0 0 0 0
12 13 1 0 0 0 0
13 14 1 0 0 0 0
14 15 1 0 0 0 0
15 16 1 0 0 0 0
16 17 1 0 0 0 0
17 18 1 0 0 0 0
10 12 1 0 0 0 0
18 19 2 0 0 0 0
18 20 1 0 0 0 0
20 21 1 0 0 0 0
21 22 1 0 0 0 0
22 23 1 0 0 0 0
23 24 1 0 0 0 0
1 25 1 0 0 0 0
1 26 1 0 0 0 0
2 27 1 0 0 0 0
2 28 1 0 0 0 0
3 29 1 0 0 0 0
3 30 1 0 0 0 0
4 31 1 0 0 0 0
4 32 1 0 0 0 0
5 33 1 0 0 0 0
5 34 1 0 0 0 0
6 35 1 0 0 0 0
6 36 1 0 0 0 0
7 37 1 0 0 0 0
7 38 1 0 0 0 0
8 39 1 0 0 0 0
8 40 1 0 0 0 0
9 41 1 0 0 0 0
9 42 1 0 0 0 0
10 43 1 0 0 0 0
10 44 1 0 0 0 0
11 45 1 0 0 0 0
11 46 1 0 0 0 0
11 47 1 0 0 0 0
12 48 1 0 0 0 0
12 49 1 0 0 0 0
13 50 1 0 0 0 0
13 51 1 0 0 0 0
14 52 1 0 0 0 0
14 53 1 0 0 0 0
15 54 1 0 0 0 0
15 55 1 0 0 0 0
16 56 1 0 0 0 0
16 57 1 0 0 0 0
17 58 1 0 0 0 0
17 59 1 0 0 0 0
21 60 1 0 0 0 0
21 61 1 0 0 0 0
22 62 1 0 0 0 0
22 63 1 0 0 0 0

```
 23 64  1  0  0  0  0
 23 65  1  0  0  0  0
 24 66  1  0  0  0  0
 24 67  1  0  0  0  0
 24 68  1  0  0  0  0
M  END
>  <Alias name>
n-Butylstearate

>  <deltaH°(c)>   kJ/mol
-13972

$$$$
Behenic acid
  ChemBrain IXL

 68 67  0  0  0  0  0  0  0  0  1 V2000
   10.1572   11.9550    1.9775 C   0  0  0  0  0  0  0  0  0  0  0  0
   11.2426   12.9716    1.5737 C   0  0  0  0  0  0  0  0  0  0  0  0
    9.0270   12.6823    2.7313 C   0  0  0  0  0  0  0  0  0  0  0  0
   12.3731   12.2440    0.8206 C   0  0  0  0  0  0  0  0  0  0  0  0
    7.9421   11.6653    3.1359 C   0  0  0  0  0  0  0  0  0  0  0  0
   13.4593   13.2603    0.4179 C   0  0  0  0  0  0  0  0  0  0  0  0
    6.8121   12.3924    3.8901 C   0  0  0  0  0  0  0  0  0  0  0  0
   14.5902   12.5323   -0.3342 C   0  0  0  0  0  0  0  0  0  0  0  0
    5.7277   11.3752    4.2953 C   0  0  0  0  0  0  0  0  0  0  0  0
   15.6772   13.5482   -0.7356 C   0  0  0  0  0  0  0  0  0  0  0  0
    4.5980   12.1020    5.0505 C   0  0  0  0  0  0  0  0  0  0  0  0
   16.8088   12.8198   -1.4864 C   0  0  0  0  0  0  0  0  0  0  0  0
    3.5145   11.0845    5.4570 C   0  0  0  0  0  0  0  0  0  0  0  0
   17.8967   13.8352   -1.8863 C   0  0  0  0  0  0  0  0  0  0  0  0
    2.4310   11.7820    6.1851 C   0  0  0  0  0  0  0  0  0  0  0  0
   19.0289   13.1064   -2.6358 C   0  0  0  0  0  0  0  0  0  0  0  0
    2.5125   13.2323    6.4111 O   0  0  0  0  0  0  0  0  0  0  0  0
    1.4704   11.1534    6.5978 O   0  0  0  0  0  0  0  0  0  0  0  0
   20.1177   14.1215   -3.0344 C   0  0  0  0  0  0  0  0  0  0  0  0
   21.2504   13.3923   -3.7828 C   0  0  0  0  0  0  0  0  0  0  0  0
   22.3399   14.4071   -4.1802 C   0  0  0  0  0  0  0  0  0  0  0  0
   23.4729   13.6778   -4.9279 C   0  0  0  0  0  0  0  0  0  0  0  0
   24.5629   14.6924   -5.3245 C   0  0  0  0  0  0  0  0  0  0  0  0
   25.6961   13.9630   -6.0718 C   0  0  0  0  0  0  0  0  0  0  0  0
    1.7530   13.6198    6.9002 H   0  0  0  0  0  0  0  0  0  0  0  0
   10.5917   11.1992    2.6202 H   0  0  0  0  0  0  0  0  0  0  0  0
    9.7543   11.4850    1.0889 H   0  0  0  0  0  0  0  0  0  0  0  0
   11.6452   13.4420    2.4623 H   0  0  0  0  0  0  0  0  0  0  0  0
   10.8083   13.7271    0.9306 H   0  0  0  0  0  0  0  0  0  0  0  0
    8.5920   13.4375    2.0884 H   0  0  0  0  0  0  0  0  0  0  0  0
    9.4300   13.1528    3.6196 H   0  0  0  0  0  0  0  0  0  0  0  0
   11.9707   11.7745   -0.0685 H   0  0  0  0  0  0  0  0  0  0  0  0
   12.8067   11.4879    1.4633 H   0  0  0  0  0  0  0  0  0  0  0  0
    8.3772   10.9098    3.7784 H   0  0  0  0  0  0  0  0  0  0  0  0
    7.5389   11.1951    2.2475 H   0  0  0  0  0  0  0  0  0  0  0  0
   13.8612   13.7304    1.3069 H   0  0  0  0  0  0  0  0  0  0  0  0
   13.0258   14.0160   -0.2255 H   0  0  0  0  0  0  0  0  0  0  0  0
    6.3766   13.1475    3.2474 H   0  0  0  0  0  0  0  0  0  0  0  0
    7.2154   12.8631    4.7782 H   0  0  0  0  0  0  0  0  0  0  0  0
   14.1885   12.0632   -1.2238 H   0  0  0  0  0  0  0  0  0  0  0  0
   15.0228   11.7759    0.3088 H   0  0  0  0  0  0  0  0  0  0  0  0
    6.1633   10.6197    4.9375 H   0  0  0  0  0  0  0  0  0  0  0  0
    5.3239   10.9050    3.4072 H   0  0  0  0  0  0  0  0  0  0  0  0
   16.0783   14.0181    0.1540 H   0  0  0  0  0  0  0  0  0  0  0  0
   15.2449   14.3041   -1.3795 H   0  0  0  0  0  0  0  0  0  0  0  0
    4.1616   12.8568    4.4079 H   0  0  0  0  0  0  0  0  0  0  0  0
```

5.0020 12.5732 5.9380 H 0 0 0 0 0 0 0 0 0 0 0 0
16.4079 12.3509 -2.3765 H 0 0 0 0 0 0 0 0 0 0 0 0
17.2403 12.0631 -0.8429 H 0 0 0 0 0 0 0 0 0 0 0 0
3.1088 10.6151 4.5694 H 0 0 0 0 0 0 0 0 0 0 0 0
3.9514 10.3285 6.0976 H 0 0 0 0 0 0 0 0 0 0 0 0
18.2969 14.3049 -0.9963 H 0 0 0 0 0 0 0 0 0 0 0 0
17.4655 14.5913 -2.5307 H 0 0 0 0 0 0 0 0 0 0 0 0
18.6290 12.6376 -3.5264 H 0 0 0 0 0 0 0 0 0 0 0 0
19.4594 12.3496 -1.9917 H 0 0 0 0 0 0 0 0 0 0 0 0
20.5171 14.5909 -2.1439 H 0 0 0 0 0 0 0 0 0 0 0 0
19.6874 14.8778 -3.6792 H 0 0 0 0 0 0 0 0 0 0 0 0
20.8512 12.9237 -4.6738 H 0 0 0 0 0 0 0 0 0 0 0 0
21.6800 12.6354 -3.1383 H 0 0 0 0 0 0 0 0 0 0 0 0
22.7387 14.8761 -3.2893 H 0 0 0 0 0 0 0 0 0 0 0 0
21.9104 15.1638 -4.8251 H 0 0 0 0 0 0 0 0 0 0 0 0
23.0742 13.2094 -5.8192 H 0 0 0 0 0 0 0 0 0 0 0 0
23.9019 12.9206 -4.2833 H 0 0 0 0 0 0 0 0 0 0 0 0
24.9614 15.1610 -4.4332 H 0 0 0 0 0 0 0 0 0 0 0 0
24.1340 15.4494 -5.9694 H 0 0 0 0 0 0 0 0 0 0 0 0
26.4621 14.6760 -6.3507 H 0 0 0 0 0 0 0 0 0 0 0 0
25.2976 13.4942 -6.9631 H 0 0 0 0 0 0 0 0 0 0 0 0
26.1252 13.2060 -5.4269 H 0 0 0 0 0 0 0 0 0 0 0 0
1 2 1 0 0 0 0
1 3 1 0 0 0 0
2 4 1 0 0 0 0
3 5 1 0 0 0 0
4 6 1 0 0 0 0
5 7 1 0 0 0 0
6 8 1 0 0 0 0
7 9 1 0 0 0 0
8 10 1 0 0 0 0
9 11 1 0 0 0 0
10 12 1 0 0 0 0
11 13 1 0 0 0 0
12 14 1 0 0 0 0
13 15 1 0 0 0 0
14 16 1 0 0 0 0
15 17 1 0 0 0 0
15 18 2 0 0 0 0
16 19 1 0 0 0 0
19 20 1 0 0 0 0
20 21 1 0 0 0 0
21 22 1 0 0 0 0
22 23 1 0 0 0 0
23 24 1 0 0 0 0
17 25 1 0 0 0 0
1 26 1 0 0 0 0
1 27 1 0 0 0 0
2 28 1 0 0 0 0
2 29 1 0 0 0 0
3 30 1 0 0 0 0
3 31 1 0 0 0 0
4 32 1 0 0 0 0
4 33 1 0 0 0 0
5 34 1 0 0 0 0
5 35 1 0 0 0 0
6 36 1 0 0 0 0
6 37 1 0 0 0 0
7 38 1 0 0 0 0
7 39 1 0 0 0 0
8 40 1 0 0 0 0
8 41 1 0 0 0 0
9 42 1 0 0 0 0

```
  9 43  1  0  0  0  0
 10 44  1  0  0  0  0
 10 45  1  0  0  0  0
 11 46  1  0  0  0  0
 11 47  1  0  0  0  0
 12 48  1  0  0  0  0
 12 49  1  0  0  0  0
 13 50  1  0  0  0  0
 13 51  1  0  0  0  0
 14 52  1  0  0  0  0
 14 53  1  0  0  0  0
 16 54  1  0  0  0  0
 16 55  1  0  0  0  0
 19 56  1  0  0  0  0
 19 57  1  0  0  0  0
 20 58  1  0  0  0  0
 20 59  1  0  0  0  0
 21 60  1  0  0  0  0
 21 61  1  0  0  0  0
 22 62  1  0  0  0  0
 22 63  1  0  0  0  0
 23 64  1  0  0  0  0
 23 65  1  0  0  0  0
 24 66  1  0  0  0  0
 24 67  1  0  0  0  0
 24 68  1  0  0  0  0
M  END
>  <Alias name>
1-Docosanoic acid

>  <deltaH°(c)>   kJ/mol
-13971

$$$$
Bis(O-Butyl-N-benzoylthiocarbamato)nickel(II)
  ChemBrain IXL

 61 64  0  0  0  0  0  0  0  0  1 V2000
    8.7297   15.5737   -1.9554 C   0  0  0  0  0  0  0  0  0  0  0  0
    8.2725   14.7867   -0.8992 C   0  0  0  0  0  0  0  0  0  0  0  0
    9.1799   14.0539   -0.1356 C   0  0  0  0  0  0  0  0  0  0  0  0
   10.5233   14.0163   -0.5034 C   0  0  0  0  0  0  0  0  0  0  0  0
   10.9973   14.9062   -1.4653 C   0  0  0  0  0  0  0  0  0  0  0  0
   10.0955   15.6489   -2.2265 C   0  0  0  0  0  0  0  0  0  0  0  0
   11.4429   13.0325    0.1271 C   0  0  0  0  0  0  0  0  0  0  0  0
   11.0132   12.2823    1.0059 O   0  0  0  0  0  0  0  0  0  0  0  0
   12.8067   12.9719   -0.2838 N   0  0  0  0  0  0  0  0  0  0  0  0
   13.6258   12.1718    0.2874 C   0  0  0  0  0  0  0  0  0  0  0  0
   15.0089   12.5992    0.5762 O   0  0  0  0  0  0  0  0  0  0  0  0
   13.0810   10.4693    0.7538 S   0  5  0  0  0  0  0  0  0  0  0  0
   15.8066   11.6232    1.3450 C   0  0  0  0  0  0  0  0  0  0  0  0
   17.3123   11.8689    1.1053 C   0  0  0  0  0  0  0  0  0  0  0  0
   10.9269   10.4231    0.7712 Ni  0  0  0  0  0  0  0  0  0  0  0  0
   10.2620    4.5834   -2.1369 C   0  0  0  0  0  0  0  0  0  0  0  0
    9.0577    4.8157   -1.4753 C   0  0  0  0  0  0  0  0  0  0  0  0
    8.9498    5.8837   -0.5862 C   0  0  0  0  0  0  0  0  0  0  0  0
   10.0147    6.7688   -0.4370 C   0  0  0  0  0  0  0  0  0  0  0  0
   11.1154    6.6615   -1.2787 C   0  0  0  0  0  0  0  0  0  0  0  0
   11.3074    5.4936   -2.0086 C   0  0  0  0  0  0  0  0  0  0  0  0
    9.9759    7.8173    0.6129 C   0  0  0  0  0  0  0  0  0  0  0  0
   10.9714    8.5247    0.8130 O   0  0  0  0  0  0  0  0  0  0  0  0
    8.8001    7.9876    1.3786 N   0  0  0  0  0  0  0  0  0  0  0  0
    8.0313    8.9681    1.1408 C   0  0  0  0  0  0  0  0  0  0  0  0
```

```
  6.5675    8.8670    1.3561 O   0  0  0  0  0  0  0  0  0  0  0  0  0
  8.7539   10.5201    0.5244 S   0  5  0  0  0  0  0  0  0  0  0  0  0
  5.8081   10.1068    1.0563 C   0  0  0  0  0  0  0  0  0  0  0  0  0
  4.3447    9.9651    1.5458 C   0  0  0  0  0  0  0  0  0  0  0  0  0
  3.5731   11.2746    1.2463 C   0  0  0  0  0  0  0  0  0  0  0  0  0
  2.1109   11.1402    1.7254 C   0  0  0  0  0  0  0  0  0  0  0  0  0
 18.1341   10.8945    1.9780 C   0  0  0  0  0  0  0  0  0  0  0  0  0
 19.6394   11.1579    1.7726 C   0  0  0  0  0  0  0  0  0  0  0  0  0
  8.0248   16.1260   -2.5646 H   0  0  0  0  0  0  0  0  0  0  0  0  0
  7.2142   14.7447   -0.6723 H   0  0  0  0  0  0  0  0  0  0  0  0  0
  8.8417   13.5159    0.7415 H   0  0  0  0  0  0  0  0  0  0  0  0  0
 12.0628   15.0213   -1.6206 H   0  0  0  0  0  0  0  0  0  0  0  0  0
 10.4553   16.2831   -3.0271 H   0  0  0  0  0  0  0  0  0  0  0  0  0
 15.5514   10.6206    1.0258 H   0  0  0  0  0  0  0  0  0  0  0  0  0
 15.5884   11.7310    2.4003 H   0  0  0  0  0  0  0  0  0  0  0  0  0
 17.5598   12.8890    1.3717 H   0  0  0  0  0  0  0  0  0  0  0  0  0
 17.5444   11.7015    0.0607 H   0  0  0  0  0  0  0  0  0  0  0  0  0
 10.3846    3.6985   -2.7486 H   0  0  0  0  0  0  0  0  0  0  0  0  0
  8.2082    4.1683   -1.6518 H   0  0  0  0  0  0  0  0  0  0  0  0  0
  8.0414    6.0249   -0.0140 H   0  0  0  0  0  0  0  0  0  0  0  0  0
 11.8173    7.4822   -1.3667 H   0  0  0  0  0  0  0  0  0  0  0  0  0
 12.2650    5.2933   -2.4733 H   0  0  0  0  0  0  0  0  0  0  0  0  0
  6.2747   10.9443    1.5598 H   0  0  0  0  0  0  0  0  0  0  0  0  0
  5.8131   10.2826   -0.0127 H   0  0  0  0  0  0  0  0  0  0  0  0  0
  3.8688    9.1400    1.0303 H   0  0  0  0  0  0  0  0  0  0  0  0  0
  4.3366    9.7776    2.6124 H   0  0  0  0  0  0  0  0  0  0  0  0  0
  4.0470   12.0989    1.7648 H   0  0  0  0  0  0  0  0  0  0  0  0  0
  3.5860   11.4649    0.1801 H   0  0  0  0  0  0  0  0  0  0  0  0  0
  1.8635   10.0931    1.8436 H   0  0  0  0  0  0  0  0  0  0  0  0  0
  1.9936   11.6486    2.6749 H   0  0  0  0  0  0  0  0  0  0  0  0  0
  1.4484   11.5873    0.9930 H   0  0  0  0  0  0  0  0  0  0  0  0  0
 17.9047    9.8748    1.6937 H   0  0  0  0  0  0  0  0  0  0  0  0  0
 17.8812   11.0434    3.0205 H   0  0  0  0  0  0  0  0  0  0  0  0  0
 19.8163   12.2249    1.7245 H   0  0  0  0  0  0  0  0  0  0  0  0  0
 19.9642   10.6959    0.8485 H   0  0  0  0  0  0  0  0  0  0  0  0  0
 20.1958   10.7367    2.6015 H   0  0  0  0  0  0  0  0  0  0  0  0  0
  1  2  4  0  0  0  0
  2  3  4  0  0  0  0
  3  4  4  0  0  0  0
  4  5  4  0  0  0  0
  5  6  4  0  0  0  0
  6  1  4  0  0  0  0
  4  7  1  0  0  0  0
  7  8  2  0  0  0  0
  7  9  1  0  0  0  0
  9 10  2  0  0  0  0
 10 11  1  0  0  0  0
 10 12  1  0  0  0  0
 11 13  1  0  0  0  0
 13 14  1  0  0  0  0
 15 12  1  0  0  0  0
 15  8  1  0  0  0  0
 16 17  4  0  0  0  0
 17 18  4  0  0  0  0
 18 19  4  0  0  0  0
 19 20  4  0  0  0  0
 20 21  4  0  0  0  0
 21 16  4  0  0  0  0
 19 22  1  0  0  0  0
 22 23  2  0  0  0  0
 22 24  1  0  0  0  0
 24 25  2  0  0  0  0
 25 26  1  0  0  0  0
```

```
 25 27  1  0  0  0  0
 26 28  1  0  0  0  0
 28 29  1  0  0  0  0
 27 15  1  0  0  0  0
 23 15  1  0  0  0  0
 29 30  1  0  0  0  0
 30 31  1  0  0  0  0
 14 32  1  0  0  0  0
 32 33  1  0  0  0  0
  1 34  1  0  0  0  0
  2 35  1  0  0  0  0
  3 36  1  0  0  0  0
  5 37  1  0  0  0  0
  6 38  1  0  0  0  0
 13 39  1  0  0  0  0
 13 40  1  0  0  0  0
 14 41  1  0  0  0  0
 14 42  1  0  0  0  0
 16 43  1  0  0  0  0
 17 44  1  0  0  0  0
 18 45  1  0  0  0  0
 20 46  1  0  0  0  0
 21 47  1  0  0  0  0
 28 48  1  0  0  0  0
 28 49  1  0  0  0  0
 29 50  1  0  0  0  0
 29 51  1  0  0  0  0
 30 52  1  0  0  0  0
 30 53  1  0  0  0  0
 31 54  1  0  0  0  0
 31 55  1  0  0  0  0
 31 56  1  0  0  0  0
 32 57  1  0  0  0  0
 32 58  1  0  0  0  0
 33 59  1  0  0  0  0
 33 60  1  0  0  0  0
 33 61  1  0  0  0  0
M  END
>  <Alias name>
Bis(O-Butyl-N-benzoylthiocarbamato)nickel(II)

>  <deltaH°(c)>   kJ/mol
-13952.2

$$$$
Heneicosane
  ChemBrain IXL

 65 64  0  0  0  0  0  0  0  0  1 V2000
    2.2738   14.7085    7.8872 C   0  0  0  0  0  0  0  0  0  0  0  0
    2.9086   13.4370    7.2915 C   0  0  0  0  0  0  0  0  0  0  0  0
    4.0408   13.8328    6.3236 C   0  0  0  0  0  0  0  0  0  0  0  0
    4.6755   12.5611    5.7277 C   0  0  0  0  0  0  0  0  0  0  0  0
    5.8074   12.9569    4.7596 C   0  0  0  0  0  0  0  0  0  0  0  0
    6.4417   11.6852    4.1633 C   0  0  0  0  0  0  0  0  0  0  0  0
    7.5732   12.0811    3.1948 C   0  0  0  0  0  0  0  0  0  0  0  0
    8.2069   10.8094    2.5977 C   0  0  0  0  0  0  0  0  0  0  0  0
    9.3377   11.2053    1.6285 C   0  0  0  0  0  0  0  0  0  0  0  0
    9.9705    9.9337    1.0305 C   0  0  0  0  0  0  0  0  0  0  0  0
   11.1006   10.3296    0.0603 C   0  0  0  0  0  0  0  0  0  0  0  0
   11.7325    9.0581   -0.5388 C   0  0  0  0  0  0  0  0  0  0  0  0
   12.8617    9.4541   -1.5099 C   0  0  0  0  0  0  0  0  0  0  0  0
   13.4928    8.1826   -2.1100 C   0  0  0  0  0  0  0  0  0  0  0  0
```

```
  14.6213    8.5786   -3.0819 C   0  0  0  0  0  0  0  0  0  0  0  0
  15.2517    7.3071   -3.6828 C   0  0  0  0  0  0  0  0  0  0  0  0
  16.3796    7.7032   -4.6554 C   0  0  0  0  0  0  0  0  0  0  0  0
  17.0096    6.4317   -5.2568 C   0  0  0  0  0  0  0  0  0  0  0  0
  18.1372    6.8278   -6.2297 C   0  0  0  0  0  0  0  0  0  0  0  0
  18.7670    5.5563   -6.8315 C   0  0  0  0  0  0  0  0  0  0  0  0
  19.8945    5.9523   -7.8045 C   0  0  0  0  0  0  0  0  0  0  0  0
   1.4783   14.4302    8.5672 H   0  0  0  0  0  0  0  0  0  0  0  0
   3.0276   15.2708    8.4243 H   0  0  0  0  0  0  0  0  0  0  0  0
   1.8693   15.3182    7.0887 H   0  0  0  0  0  0  0  0  0  0  0  0
   2.1547   12.8748    6.7545 H   0  0  0  0  0  0  0  0  0  0  0  0
   3.3130   12.8274    8.0900 H   0  0  0  0  0  0  0  0  0  0  0  0
   4.7948   14.3949    6.8606 H   0  0  0  0  0  0  0  0  0  0  0  0
   3.6364   14.4425    5.5251 H   0  0  0  0  0  0  0  0  0  0  0  0
   3.9214   11.9989    5.1908 H   0  0  0  0  0  0  0  0  0  0  0  0
   5.0800   11.9514    6.5261 H   0  0  0  0  0  0  0  0  0  0  0  0
   6.5616   13.5188    5.2966 H   0  0  0  0  0  0  0  0  0  0  0  0
   5.4029   13.5669    3.9614 H   0  0  0  0  0  0  0  0  0  0  0  0
   5.6874   11.1232    3.6267 H   0  0  0  0  0  0  0  0  0  0  0  0
   6.8466   11.0754    4.9615 H   0  0  0  0  0  0  0  0  0  0  0  0
   8.3278   12.6426    3.7315 H   0  0  0  0  0  0  0  0  0  0  0  0
   7.1684   12.6914    2.3969 H   0  0  0  0  0  0  0  0  0  0  0  0
   7.4520   10.2476    2.0615 H   0  0  0  0  0  0  0  0  0  0  0  0
   8.6122   10.1994    3.3955 H   0  0  0  0  0  0  0  0  0  0  0  0
  10.0929   11.7665    2.1648 H   0  0  0  0  0  0  0  0  0  0  0  0
   8.9324   11.8160    0.8312 H   0  0  0  0  0  0  0  0  0  0  0  0
   9.2151    9.3722    0.4948 H   0  0  0  0  0  0  0  0  0  0  0  0
  10.3765    9.3233    1.8276 H   0  0  0  0  0  0  0  0  0  0  0  0
  11.8564   10.8904    0.5961 H   0  0  0  0  0  0  0  0  0  0  0  0
  10.6947   10.9407   -0.7364 H   0  0  0  0  0  0  0  0  0  0  0  0
  10.9764    8.4970   -1.0740 H   0  0  0  0  0  0  0  0  0  0  0  0
  12.1391    8.4473    0.2578 H   0  0  0  0  0  0  0  0  0  0  0  0
  13.6182   10.0145   -0.9747 H   0  0  0  0  0  0  0  0  0  0  0  0
  12.4553   10.0655   -2.3060 H   0  0  0  0  0  0  0  0  0  0  0  0
  12.7361    7.6219   -2.6447 H   0  0  0  0  0  0  0  0  0  0  0  0
  13.8999    7.5714   -1.3140 H   0  0  0  0  0  0  0  0  0  0  0  0
  15.3783    9.1387   -2.5472 H   0  0  0  0  0  0  0  0  0  0  0  0
  14.2143    9.1903   -3.8776 H   0  0  0  0  0  0  0  0  0  0  0  0
  14.4945    6.7468   -4.2172 H   0  0  0  0  0  0  0  0  0  0  0  0
  15.6592    6.6956   -2.8873 H   0  0  0  0  0  0  0  0  0  0  0  0
  17.1370    8.2632   -4.1210 H   0  0  0  0  0  0  0  0  0  0  0  0
  15.9722    8.3150   -5.4507 H   0  0  0  0  0  0  0  0  0  0  0  0
  16.2521    5.8716   -5.7910 H   0  0  0  0  0  0  0  0  0  0  0  0
  17.4172    5.8200   -4.4615 H   0  0  0  0  0  0  0  0  0  0  0  0
  18.8948    7.3878   -5.6955 H   0  0  0  0  0  0  0  0  0  0  0  0
  17.7296    7.4397   -7.0249 H   0  0  0  0  0  0  0  0  0  0  0  0
  18.0095    4.9963   -7.3656 H   0  0  0  0  0  0  0  0  0  0  0  0
  19.1748    4.9445   -6.0364 H   0  0  0  0  0  0  0  0  0  0  0  0
  19.4868    6.5641   -8.5997 H   0  0  0  0  0  0  0  0  0  0  0  0
  20.3368    5.0587   -8.2271 H   0  0  0  0  0  0  0  0  0  0  0  0
  20.6522    6.5122   -7.2704 H   0  0  0  0  0  0  0  0  0  0  0  0
  1  2  1  0  0  0  0
  2  3  1  0  0  0  0
  3  4  1  0  0  0  0
  4  5  1  0  0  0  0
  5  6  1  0  0  0  0
  6  7  1  0  0  0  0
  7  8  1  0  0  0  0
  8  9  1  0  0  0  0
  9 10  1  0  0  0  0
 10 11  1  0  0  0  0
 11 12  1  0  0  0  0
 12 13  1  0  0  0  0
```

```
 13 14  1  0  0  0  0
 14 15  1  0  0  0  0
 15 16  1  0  0  0  0
 16 17  1  0  0  0  0
 17 18  1  0  0  0  0
 18 19  1  0  0  0  0
 19 20  1  0  0  0  0
 20 21  1  0  0  0  0
  1 22  1  0  0  0  0
  1 23  1  0  0  0  0
  1 24  1  0  0  0  0
  2 25  1  0  0  0  0
  2 26  1  0  0  0  0
  3 27  1  0  0  0  0
  3 28  1  0  0  0  0
  4 29  1  0  0  0  0
  4 30  1  0  0  0  0
  5 31  1  0  0  0  0
  5 32  1  0  0  0  0
  6 33  1  0  0  0  0
  6 34  1  0  0  0  0
  7 35  1  0  0  0  0
  7 36  1  0  0  0  0
  8 37  1  0  0  0  0
  8 38  1  0  0  0  0
  9 39  1  0  0  0  0
  9 40  1  0  0  0  0
 10 41  1  0  0  0  0
 10 42  1  0  0  0  0
 11 43  1  0  0  0  0
 11 44  1  0  0  0  0
 12 45  1  0  0  0  0
 12 46  1  0  0  0  0
 13 47  1  0  0  0  0
 13 48  1  0  0  0  0
 14 49  1  0  0  0  0
 14 50  1  0  0  0  0
 15 51  1  0  0  0  0
 15 52  1  0  0  0  0
 16 53  1  0  0  0  0
 16 54  1  0  0  0  0
 17 55  1  0  0  0  0
 17 56  1  0  0  0  0
 18 57  1  0  0  0  0
 18 58  1  0  0  0  0
 19 59  1  0  0  0  0
 19 60  1  0  0  0  0
 20 61  1  0  0  0  0
 20 62  1  0  0  0  0
 21 63  1  0  0  0  0
 21 64  1  0  0  0  0
 21 65  1  0  0  0  0
M  END
>  <Alias name>
Heneicosane

>  <deltaH°(c)>   kJ/mol
-13908

$$$$
Diacyldiperoxide I
  ChemBrain IXL
```

```
74 73  0  0  0  0  0  0  0  0  1 V2000
   16.8329   15.5040    0.5537 C   0  0  0  0  0  0  0  0  0  0  0  0
   15.4030   15.8852    0.5738 C   0  0  0  0  0  0  0  0  0  0  0  0
   14.6560   15.4073    1.4117 O   0  0  0  0  0  0  0  0  0  0  0  0
   14.8828   16.8396   -0.4158 O   0  0  0  0  0  0  0  0  0  0  0  0
   17.5396   16.2221   -0.6123 C   0  0  0  0  0  0  0  0  0  0  0  0
   18.9701   15.8429   -0.6308 C   0  0  0  0  0  0  0  0  0  0  0  0
   19.7191   16.3282   -1.4626 O   0  0  0  0  0  0  0  0  0  0  0  0
   19.4885   14.8815    0.3530 O   0  0  0  0  0  0  0  0  0  0  0  0
   22.6703   12.7352   -0.0817 C   0  0  0  0  0  0  0  0  0  0  0  0
   21.2577   13.1623    0.0298 C   0  0  0  0  0  0  0  0  0  0  0  0
   20.3628   12.3367   -0.0473 O   0  0  0  0  0  0  0  0  0  0  0  0
   20.9328   14.5814    0.2334 O   0  0  0  0  0  0  0  0  0  0  0  0
   23.5898   13.9664    0.0336 C   0  0  0  0  0  0  0  0  0  0  0  0
   25.0604   13.5226   -0.0896 C   0  0  0  0  0  0  0  0  0  0  0  0
   25.9799   14.7537    0.0265 C   0  0  0  0  0  0  0  0  0  0  0  0
   27.4507   14.3093   -0.0921 C   0  0  0  0  0  0  0  0  0  0  0  0
   28.3703   15.5401    0.0263 C   0  0  0  0  0  0  0  0  0  0  0  0
   29.8413   15.0952   -0.0884 C   0  0  0  0  0  0  0  0  0  0  0  0
   30.7610   16.3258    0.0311 C   0  0  0  0  0  0  0  0  0  0  0  0
   11.7006   18.9876    0.0080 C   0  0  0  0  0  0  0  0  0  0  0  0
   13.1133   18.5603   -0.1023 C   0  0  0  0  0  0  0  0  0  0  0  0
   14.0078   19.3869   -0.0324 O   0  0  0  0  0  0  0  0  0  0  0  0
   13.4385   17.1399   -0.2961 O   0  0  0  0  0  0  0  0  0  0  0  0
   10.7804   17.7587   -0.1248 C   0  0  0  0  0  0  0  0  0  0  0  0
    9.3098   18.2025   -0.0022 C   0  0  0  0  0  0  0  0  0  0  0  0
    8.3895   16.9735   -0.1342 C   0  0  0  0  0  0  0  0  0  0  0  0
    6.9188   17.4179   -0.0156 C   0  0  0  0  0  0  0  0  0  0  0  0
    5.9985   16.1892   -0.1494 C   0  0  0  0  0  0  0  0  0  0  0  0
    4.5276   16.6341   -0.0347 C   0  0  0  0  0  0  0  0  0  0  0  0
    3.6073   15.4055   -0.1694 C   0  0  0  0  0  0  0  0  0  0  0  0
   32.2320   15.8807   -0.0815 C   0  0  0  0  0  0  0  0  0  0  0  0
    2.1363   15.8507   -0.0566 C   0  0  0  0  0  0  0  0  0  0  0  0
   17.2965   15.7920    1.4892 H   0  0  0  0  0  0  0  0  0  0  0  0
   16.9191   14.4324    0.4230 H   0  0  0  0  0  0  0  0  0  0  0  0
   17.0773   15.9327   -1.5479 H   0  0  0  0  0  0  0  0  0  0  0  0
   17.4519   17.2937   -0.4826 H   0  0  0  0  0  0  0  0  0  0  0  0
   22.9002   12.0372    0.7138 H   0  0  0  0  0  0  0  0  0  0  0  0
   22.8278   12.2554   -1.0397 H   0  0  0  0  0  0  0  0  0  0  0  0
   23.3565   14.6667   -0.7589 H   0  0  0  0  0  0  0  0  0  0  0  0
   23.4359   14.4433    0.9936 H   0  0  0  0  0  0  0  0  0  0  0  0
   25.2935   12.8218    0.7026 H   0  0  0  0  0  0  0  0  0  0  0  0
   25.2145   13.0461   -1.0499 H   0  0  0  0  0  0  0  0  0  0  0  0
   25.7490   15.4530   -0.7675 H   0  0  0  0  0  0  0  0  0  0  0  0
   25.8236   15.2319    0.9856 H   0  0  0  0  0  0  0  0  0  0  0  0
   27.6813   13.6087    0.7010 H   0  0  0  0  0  0  0  0  0  0  0  0
   27.6075   13.8324   -1.0518 H   0  0  0  0  0  0  0  0  0  0  0  0
   28.1417   16.2393   -0.7685 H   0  0  0  0  0  0  0  0  0  0  0  0
   28.2116   16.0185    0.9848 H   0  0  0  0  0  0  0  0  0  0  0  0
   30.0697   14.3953    0.7059 H   0  0  0  0  0  0  0  0  0  0  0  0
   30.0002   14.6175   -1.0472 H   0  0  0  0  0  0  0  0  0  0  0  0
   30.5335   17.0251   -0.7640 H   0  0  0  0  0  0  0  0  0  0  0  0
   30.6010   16.8044    0.9894 H   0  0  0  0  0  0  0  0  0  0  0  0
   11.4747   19.6947   -0.7806 H   0  0  0  0  0  0  0  0  0  0  0  0
   11.5395   19.4569    0.9706 H   0  0  0  0  0  0  0  0  0  0  0  0
   11.0100   17.0491    0.6605 H   0  0  0  0  0  0  0  0  0  0  0  0
   10.9375   17.2926   -1.0897 H   0  0  0  0  0  0  0  0  0  0  0  0
    9.0800   18.9116   -0.7878 H   0  0  0  0  0  0  0  0  0  0  0  0
    9.1528   18.6690    0.9625 H   0  0  0  0  0  0  0  0  0  0  0  0
    8.6174   16.2656    0.6531 H   0  0  0  0  0  0  0  0  0  0  0  0
    8.5486   16.5053   -1.0978 H   0  0  0  0  0  0  0  0  0  0  0  0
    6.6913   18.1267   -0.8022 H   0  0  0  0  0  0  0  0  0  0  0  0
    6.7595   17.8850    0.9484 H   0  0  0  0  0  0  0  0  0  0  0  0
```

```
    6.2241   15.4816    0.6389 H   0  0  0  0  0  0  0  0  0  0  0  0
    6.1598   15.7204   -1.1123 H   0  0  0  0  0  0  0  0  0  0  0  0
    4.3022   17.3422   -0.8225 H   0  0  0  0  0  0  0  0  0  0  0  0
    4.3661   17.1023    0.9285 H   0  0  0  0  0  0  0  0  0  0  0  0
    3.8318   14.6981    0.6193 H   0  0  0  0  0  0  0  0  0  0  0  0
    3.7697   14.9366   -1.1320 H   0  0  0  0  0  0  0  0  0  0  0  0
   32.8783   16.7456    0.0023 H   0  0  0  0  0  0  0  0  0  0  0  0
   32.3919   15.4020   -1.0397 H   0  0  0  0  0  0  0  0  0  0  0  0
   32.4595   15.1816    0.7137 H   0  0  0  0  0  0  0  0  0  0  0  0
    1.4895   14.9873   -0.1514 H   0  0  0  0  0  0  0  0  0  0  0  0
    1.9738   16.3196    0.9060 H   0  0  0  0  0  0  0  0  0  0  0  0
    1.9118   16.5582   -0.8452 H   0  0  0  0  0  0  0  0  0  0  0  0
  1  2  1  0  0  0  0
  2  3  2  0  0  0  0
  2  4  1  0  0  0  0
  5  6  1  0  0  0  0
  6  7  2  0  0  0  0
  6  8  1  0  0  0  0
  5  1  1  0  0  0  0
  9 10  1  0  0  0  0
 10 11  2  0  0  0  0
 10 12  1  0  0  0  0
 13  9  1  0  0  0  0
 13 14  1  0  0  0  0
 14 15  1  0  0  0  0
 15 16  1  0  0  0  0
 16 17  1  0  0  0  0
 17 18  1  0  0  0  0
 18 19  1  0  0  0  0
 20 21  1  0  0  0  0
 21 22  2  0  0  0  0
 21 23  1  0  0  0  0
 24 20  1  0  0  0  0
 24 25  1  0  0  0  0
 25 26  1  0  0  0  0
 26 27  1  0  0  0  0
 27 28  1  0  0  0  0
 28 29  1  0  0  0  0
 29 30  1  0  0  0  0
 23  4  1  0  0  0  0
 12  8  1  0  0  0  0
 19 31  1  0  0  0  0
 30 32  1  0  0  0  0
  1 33  1  0  0  0  0
  1 34  1  0  0  0  0
  5 35  1  0  0  0  0
  5 36  1  0  0  0  0
  9 37  1  0  0  0  0
  9 38  1  0  0  0  0
 13 39  1  0  0  0  0
 13 40  1  0  0  0  0
 14 41  1  0  0  0  0
 14 42  1  0  0  0  0
 15 43  1  0  0  0  0
 15 44  1  0  0  0  0
 16 45  1  0  0  0  0
 16 46  1  0  0  0  0
 17 47  1  0  0  0  0
 17 48  1  0  0  0  0
 18 49  1  0  0  0  0
 18 50  1  0  0  0  0
 19 51  1  0  0  0  0
 19 52  1  0  0  0  0
```

```
 20 53  1  0  0  0  0
 20 54  1  0  0  0  0
 24 55  1  0  0  0  0
 24 56  1  0  0  0  0
 25 57  1  0  0  0  0
 25 58  1  0  0  0  0
 26 59  1  0  0  0  0
 26 60  1  0  0  0  0
 27 61  1  0  0  0  0
 27 62  1  0  0  0  0
 28 63  1  0  0  0  0
 28 64  1  0  0  0  0
 29 65  1  0  0  0  0
 29 66  1  0  0  0  0
 30 67  1  0  0  0  0
 30 68  1  0  0  0  0
 31 69  1  0  0  0  0
 31 70  1  0  0  0  0
 31 71  1  0  0  0  0
 32 72  1  0  0  0  0
 32 73  1  0  0  0  0
 32 74  1  0  0  0  0
M  END
>  <Alias name>
Diacyldiperoxide I

>  <deltaH°(c)>   kJ/mol
-13872

$$$$
Butyl oleate
  ChemBrain IXL

 66 65  0  0  0  0  0  0  0  0  1 V2000
    2.1290    9.7444    5.7320 C   0  0  0  0  0  0  0  0  0  0  0  0
    3.4514    9.4516    4.9971 C   0  0  0  0  0  0  0  0  0  0  0  0
    4.1400   10.7803    4.6289 C   0  0  0  0  0  0  0  0  0  0  0  0
    5.4624   10.4873    3.8941 C   0  0  0  0  0  0  0  0  0  0  0  0
    6.1514   11.8158    3.5265 C   0  0  0  0  0  0  0  0  0  0  0  0
    7.4738   11.5225    2.7922 C   0  0  0  0  0  0  0  0  0  0  0  0
    8.1638   12.8507    2.4253 C   0  0  0  0  0  0  0  0  0  0  0  0
    9.4862   12.5562    1.6913 C   0  0  0  0  0  0  0  0  0  0  0  0
   10.1517   13.8323    1.3387 C   0  0  0  0  0  0  0  0  0  0  0  0
   11.3276   13.8211    0.7042 C   0  0  0  0  0  0  0  0  0  0  0  0
   11.9687   12.5327    0.3517 C   0  0  0  0  0  0  0  0  0  0  0  0
   13.2706   12.8013   -0.4275 C   0  0  0  0  0  0  0  0  0  0  0  0
   13.9334   11.4600   -0.7958 C   0  0  0  0  0  0  0  0  0  0  0  0
   15.2321   11.7265   -1.5806 C   0  0  0  0  0  0  0  0  0  0  0  0
   15.8911   10.3846   -1.9524 C   0  0  0  0  0  0  0  0  0  0  0  0
   17.1850   10.6496   -2.7452 C   0  0  0  0  0  0  0  0  0  0  0  0
   17.8417    9.3065   -3.1191 C   0  0  0  0  0  0  0  0  0  0  0  0
   19.0816    9.5581   -3.8881 C   0  0  0  0  0  0  0  0  0  0  0  0
   19.6622    8.6291   -4.4262 O   0  0  0  0  0  0  0  0  0  0  0  0
   19.6133   10.9274   -4.0034 O   0  0  0  0  0  0  0  0  0  0  0  0
   20.8492   11.0546   -4.7918 C   0  0  0  0  0  0  0  0  0  0  0  0
   21.2843   12.5320   -4.8300 C   0  0  0  0  0  0  0  0  0  0  0  0
   22.5785   12.6652   -5.6565 C   0  0  0  0  0  0  0  0  0  0  0  0
   23.0134   14.1430   -5.6959 C   0  0  0  0  0  0  0  0  0  0  0  0
    1.6454    8.8107    5.9907 H   0  0  0  0  0  0  0  0  0  0  0  0
    2.3345   10.3068    6.6345 H   0  0  0  0  0  0  0  0  0  0  0  0
    1.4779   10.3217    5.0873 H   0  0  0  0  0  0  0  0  0  0  0  0
    4.1024    8.8742    5.6418 H   0  0  0  0  0  0  0  0  0  0  0  0
    3.2459    8.8893    4.0947 H   0  0  0  0  0  0  0  0  0  0  0  0
```

3.4890 11.3577 3.9841 H 0 0 0 0 0 0 0 0 0 0 0 0
4.3454 11.3426 5.5314 H 0 0 0 0 0 0 0 0 0 0 0 0
6.1132 9.9095 4.5388 H 0 0 0 0 0 0 0 0 0 0 0 0
5.2570 9.9253 2.9914 H 0 0 0 0 0 0 0 0 0 0 0 0
5.5008 12.3936 2.8816 H 0 0 0 0 0 0 0 0 0 0 0 0
6.3567 12.3779 4.4292 H 0 0 0 0 0 0 0 0 0 0 0 0
8.1240 10.9441 3.4368 H 0 0 0 0 0 0 0 0 0 0 0 0
7.2684 10.9610 1.8891 H 0 0 0 0 0 0 0 0 0 0 0 0
7.5139 13.4292 1.7804 H 0 0 0 0 0 0 0 0 0 0 0 0
8.3693 13.4123 3.3283 H 0 0 0 0 0 0 0 0 0 0 0 0
10.1354 11.9768 2.3362 H 0 0 0 0 0 0 0 0 0 0 0 0
9.2803 11.9950 0.7883 H 0 0 0 0 0 0 0 0 0 0 0 0
9.6848 14.7751 1.5954 H 0 0 0 0 0 0 0 0 0 0 0 0
11.8123 14.7549 0.4473 H 0 0 0 0 0 0 0 0 0 0 0 0
11.2940 11.9503 -0.2634 H 0 0 0 0 0 0 0 0 0 0 0 0
12.1968 11.9835 1.2569 H 0 0 0 0 0 0 0 0 0 0 0 0
13.9470 13.3815 0.1880 H 0 0 0 0 0 0 0 0 0 0 0 0
13.0430 13.3519 -1.3319 H 0 0 0 0 0 0 0 0 0 0 0 0
13.2551 10.8785 -1.4078 H 0 0 0 0 0 0 0 0 0 0 0 0
14.1643 10.9110 0.1087 H 0 0 0 0 0 0 0 0 0 0 0 0
15.9121 12.3055 -0.9680 H 0 0 0 0 0 0 0 0 0 0 0 0
15.0013 12.2778 -2.4838 H 0 0 0 0 0 0 0 0 0 0 0 0
15.2084 9.8035 -2.5600 H 0 0 0 0 0 0 0 0 0 0 0 0
16.1270 9.8356 -1.0491 H 0 0 0 0 0 0 0 0 0 0 0 0
17.8688 11.2292 -2.1373 H 0 0 0 0 0 0 0 0 0 0 0 0
16.9490 11.1998 -3.6477 H 0 0 0 0 0 0 0 0 0 0 0 0
17.1553 8.7250 -3.7221 H 0 0 0 0 0 0 0 0 0 0 0 0
18.0819 8.7586 -2.2163 H 0 0 0 0 0 0 0 0 0 0 0 0
21.6310 10.4596 -4.3360 H 0 0 0 0 0 0 0 0 0 0 0 0
20.6712 10.7046 -5.8011 H 0 0 0 0 0 0 0 0 0 0 0 0
20.5023 13.1269 -5.2854 H 0 0 0 0 0 0 0 0 0 0 0 0
21.4628 12.8820 -3.8208 H 0 0 0 0 0 0 0 0 0 0 0 0
23.3607 12.0708 -5.2006 H 0 0 0 0 0 0 0 0 0 0 0 0
22.4002 12.3144 -6.6654 H 0 0 0 0 0 0 0 0 0 0 0 0
22.2313 14.7374 -6.1518 H 0 0 0 0 0 0 0 0 0 0 0 0
23.9227 14.2362 -6.2767 H 0 0 0 0 0 0 0 0 0 0 0 0
23.1919 14.4938 -4.6870 H 0 0 0 0 0 0 0 0 0 0 0 0
1 2 1 0 0 0 0
2 3 1 0 0 0 0
3 4 1 0 0 0 0
4 5 1 0 0 0 0
5 6 1 0 0 0 0
6 7 1 0 0 0 0
7 8 1 0 0 0 0
8 9 1 0 0 0 0
9 10 2 0 0 0 0
10 11 1 0 0 0 0
11 12 1 0 0 0 0
12 13 1 0 0 0 0
13 14 1 0 0 0 0
14 15 1 0 0 0 0
15 16 1 0 0 0 0
16 17 1 0 0 0 0
17 18 1 0 0 0 0
18 19 2 0 0 0 0
18 20 1 0 0 0 0
20 21 1 0 0 0 0
21 22 1 0 0 0 0
22 23 1 0 0 0 0
23 24 1 0 0 0 0
1 25 1 0 0 0 0
1 26 1 0 0 0 0
1 27 1 0 0 0 0

```
  2 28  1  0  0  0  0
  2 29  1  0  0  0  0
  3 30  1  0  0  0  0
  3 31  1  0  0  0  0
  4 32  1  0  0  0  0
  4 33  1  0  0  0  0
  5 34  1  0  0  0  0
  5 35  1  0  0  0  0
  6 36  1  0  0  0  0
  6 37  1  0  0  0  0
  7 38  1  0  0  0  0
  7 39  1  0  0  0  0
  8 40  1  0  0  0  0
  8 41  1  0  0  0  0
  9 42  1  0  0  0  0
 10 43  1  0  0  0  0
 11 44  1  0  0  0  0
 11 45  1  0  0  0  0
 12 46  1  0  0  0  0
 12 47  1  0  0  0  0
 13 48  1  0  0  0  0
 13 49  1  0  0  0  0
 14 50  1  0  0  0  0
 14 51  1  0  0  0  0
 15 52  1  0  0  0  0
 15 53  1  0  0  0  0
 16 54  1  0  0  0  0
 16 55  1  0  0  0  0
 17 56  1  0  0  0  0
 17 57  1  0  0  0  0
 21 58  1  0  0  0  0
 21 59  1  0  0  0  0
 22 60  1  0  0  0  0
 22 61  1  0  0  0  0
 23 62  1  0  0  0  0
 23 63  1  0  0  0  0
 24 64  1  0  0  0  0
 24 65  1  0  0  0  0
 24 66  1  0  0  0  0
M  END
>  <Alias name>
Butyl oleate

>  <deltaH°(c)>   kJ/mol
-13843

$$$$
6,6-Paracyclophane
  ChemBrain IXL

 56 58  0  0  0  0  0  0  0  0  1 V2000
    8.0913    6.8043   -1.8973 C   0  0  0  0  0  0  0  0  0  0  0  0
    8.0559    5.4097   -1.8953 C   0  0  0  0  0  0  0  0  0  0  0  0
    6.9344    7.5309   -2.1797 C   0  0  0  0  0  0  0  0  0  0  0  0
    9.3647    7.5176   -1.5977 C   0  0  0  0  0  0  0  0  0  0  0  0
    6.8634    4.7418   -2.1741 C   0  0  0  0  0  0  0  0  0  0  0  0
    5.7061    5.4685   -2.4547 C   0  0  0  0  0  0  0  0  0  0  0  0
    5.7419    6.8630   -2.4584 C   0  0  0  0  0  0  0  0  0  0  0  0
    4.4319    4.7551   -2.7506 C   0  0  0  0  0  0  0  0  0  0  0  0
    8.2998    4.9458    1.6594 C   0  0  0  0  0  0  0  0  0  0  0  0
    8.2967    3.8673    0.7746 C   0  0  0  0  0  0  0  0  0  0  0  0
    7.1337    5.2874    2.3447 C   0  0  0  0  0  0  0  0  0  0  0  0
    9.5474    5.7322    1.8723 C   0  0  0  0  0  0  0  0  0  0  0  0
```

```
   7.1043    3.1992    0.4957 C   0  0  0  0  0  0  0  0  0  0  0  0
   5.9173    3.6109    1.1021 C   0  0  0  0  0  0  0  0  0  0  0  0
   5.9413    4.6193    2.0657 C   0  0  0  0  0  0  0  0  0  0  0  0
   4.6252    2.9744    0.7209 C   0  0  0  0  0  0  0  0  0  0  0  0
   9.0519    8.8795   -0.9745 C   0  0  0  0  0  0  0  0  0  0  0  0
   7.9499    8.7192    0.0747 C   0  0  0  0  0  0  0  0  0  0  0  0
   8.0256    7.3178    0.6839 C   0  0  0  0  0  0  0  0  0  0  0  0
   9.2847    7.2036    1.5457 C   0  0  0  0  0  0  0  0  0  0  0  0
   3.2485    5.6292   -2.3304 C   0  0  0  0  0  0  0  0  0  0  0  0
   3.5304    6.2437   -0.9579 C   0  0  0  0  0  0  0  0  0  0  0  0
   4.4349    5.3063   -0.1554 C   0  0  0  0  0  0  0  0  0  0  0  0
   3.6532    4.0485    0.2289 C   0  0  0  0  0  0  0  0  0  0  0  0
   8.9544    4.8456   -1.6774 H   0  0  0  0  0  0  0  0  0  0  0  0
   6.9622    8.6135   -2.1825 H   0  0  0  0  0  0  0  0  0  0  0  0
   9.9532    6.9298   -0.9042 H   0  0  0  0  0  0  0  0  0  0  0  0
   9.9230    7.6599   -2.5147 H   0  0  0  0  0  0  0  0  0  0  0  0
   6.8359    3.6592   -2.1726 H   0  0  0  0  0  0  0  0  0  0  0  0
   4.8438    7.4271   -2.6776 H   0  0  0  0  0  0  0  0  0  0  0  0
   4.3707    4.5507   -3.8124 H   0  0  0  0  0  0  0  0  0  0  0  0
   4.4045    3.8221   -2.2013 H   0  0  0  0  0  0  0  0  0  0  0  0
   9.2191    3.5490    0.3047 H   0  0  0  0  0  0  0  0  0  0  0  0
   7.1542    6.0705    3.0925 H   0  0  0  0  0  0  0  0  0  0  0  0
   9.8592    5.6418    2.9055 H   0  0  0  0  0  0  0  0  0  0  0  0
  10.3282    5.3526    1.2250 H   0  0  0  0  0  0  0  0  0  0  0  0
   7.1000    2.3617   -0.1910 H   0  0  0  0  0  0  0  0  0  0  0  0
   5.0351    4.8832    2.5968 H   0  0  0  0  0  0  0  0  0  0  0  0
   4.7970    2.2538   -0.0692 H   0  0  0  0  0  0  0  0  0  0  0  0
   4.2024    2.4719    1.5821 H   0  0  0  0  0  0  0  0  0  0  0  0
   9.9438    9.2745   -0.5039 H   0  0  0  0  0  0  0  0  0  0  0  0
   8.7187    9.5619   -1.7466 H   0  0  0  0  0  0  0  0  0  0  0  0
   6.9831    8.8578   -0.3931 H   0  0  0  0  0  0  0  0  0  0  0  0
   8.0832    9.4593    0.8541 H   0  0  0  0  0  0  0  0  0  0  0  0
   8.0629    6.5811   -0.1090 H   0  0  0  0  0  0  0  0  0  0  0  0
   7.1508    7.1434    1.2980 H   0  0  0  0  0  0  0  0  0  0  0  0
   9.1441    7.7580    2.4654 H   0  0  0  0  0  0  0  0  0  0  0  0
  10.1304    7.6100    1.0049 H   0  0  0  0  0  0  0  0  0  0  0  0
   3.1072    6.4192   -3.0576 H   0  0  0  0  0  0  0  0  0  0  0  0
   2.3527    5.0229   -2.2774 H   0  0  0  0  0  0  0  0  0  0  0  0
   4.0231    7.1998   -1.0847 H   0  0  0  0  0  0  0  0  0  0  0  0
   2.5969    6.3862   -0.4276 H   0  0  0  0  0  0  0  0  0  0  0  0
   4.7717    5.8103    0.7420 H   0  0  0  0  0  0  0  0  0  0  0  0
   5.2918    5.0291   -0.7569 H   0  0  0  0  0  0  0  0  0  0  0  0
   3.1150    3.6797   -0.6356 H   0  0  0  0  0  0  0  0  0  0  0  0
   2.9499    4.2873    1.0171 H   0  0  0  0  0  0  0  0  0  0  0  0
  2  1  4  0  0  0  0
  1  3  4  0  0  0  0
  1  4  1  0  0  0  0
  5  2  4  0  0  0  0
  3  7  4  0  0  0  0
  4 17  1  0  0  0  0
  6  5  4  0  0  0  0
  7  6  4  0  0  0  0
  8  6  1  0  0  0  0
 21  8  1  0  0  0  0
  9 10  4  0  0  0  0
 11  9  4  0  0  0  0
 12  9  1  0  0  0  0
 10 13  4  0  0  0  0
 15 11  4  0  0  0  0
 20 12  1  0  0  0  0
 13 14  4  0  0  0  0
 14 15  4  0  0  0  0
 14 16  1  0  0  0  0
```

16 24 1 0 0 0 0
17 18 1 0 0 0 0
18 19 1 0 0 0 0
19 20 1 0 0 0 0
22 21 1 0 0 0 0
23 22 1 0 0 0 0
24 23 1 0 0 0 0
2 25 1 0 0 0 0
3 26 1 0 0 0 0
4 27 1 0 0 0 0
4 28 1 0 0 0 0
5 29 1 0 0 0 0
7 30 1 0 0 0 0
8 31 1 0 0 0 0
8 32 1 0 0 0 0
10 33 1 0 0 0 0
11 34 1 0 0 0 0
12 35 1 0 0 0 0
12 36 1 0 0 0 0
13 37 1 0 0 0 0
15 38 1 0 0 0 0
16 39 1 0 0 0 0
16 40 1 0 0 0 0
17 41 1 0 0 0 0
17 42 1 0 0 0 0
18 43 1 0 0 0 0
18 44 1 0 0 0 0
19 45 1 0 0 0 0
19 46 1 0 0 0 0
20 47 1 0 0 0 0
20 48 1 0 0 0 0
21 49 1 0 0 0 0
21 50 1 0 0 0 0
22 51 1 0 0 0 0
22 52 1 0 0 0 0
23 53 1 0 0 0 0
23 54 1 0 0 0 0
24 55 1 0 0 0 0
24 56 1 0 0 0 0
M END
> <Alias name>
6,6-Paracyclophane

> <deltaH°(c)>   kJ/mol
-13825

$$$$
Erucic acid
ChemBrain IXL

66 65 0 0 0 0 0 0 0 0 1 V2000
9.4420   11.2669   2.1423 C   0 0 0 0 0 0 0 0 0 0 0 0
10.2132   10.0176   1.6750 C   0 0 0 0 0 0 0 0 0 0 0 0
8.2242   10.8360   2.9822 C   0 0 0 0 0 0 0 0 0 0 0 0
11.4306   10.4510   0.8360 C   0 0 0 0 0 0 0 0 0 0 0 0
7.4532   12.0868   3.4461 C   0 0 0 0 0 0 0 0 0 0 0 0
12.2012   9.2030   0.3645 C   0 0 0 0 0 0 0 0 0 0 0 0
6.2359   11.6587   4.2881 C   0 0 0 0 0 0 0 0 0 0 0 0
13.4177   9.6388   -0.4744 C   0 0 0 0 0 0 0 0 0 0 0 0
5.4662   12.9112   4.7501 C   0 0 0 0 0 0 0 0 0 0 0 0
14.1591   8.4407   -0.9314 C   0 0 0 0 0 0 0 0 0 0 0 0
4.2500   12.4855   5.5949 C   0 0 0 0 0 0 0 0 0 0 0 0
15.2592   8.5786   -1.6767 C   0 0 0 0 0 0 0 0 0 0 0 0

3.4808	13.7408	6.0569	C	0	0	0	0	0	0	0	0	0	0	0	0
15.7394	9.9301	-2.0474	C	0	0	0	0	0	0	0	0	0	0	0	0
2.3106	13.3360	6.8720	C	0	0	0	0	0	0	0	0	0	0	0	0
17.0159	9.8067	-2.9008	C	0	0	0	0	0	0	0	0	0	0	0	0
1.4200	14.3593	7.4375	O	0	0	0	0	0	0	0	0	0	0	0	0
2.0851	12.1537	7.0744	O	0	0	0	0	0	0	0	0	0	0	0	0
17.5140	11.2133	-3.2843	C	0	0	0	0	0	0	0	0	0	0	0	0
18.7913	11.0913	-4.1370	C	0	0	0	0	0	0	0	0	0	0	0	0
19.2894	12.4982	-4.5198	C	0	0	0	0	0	0	0	0	0	0	0	0
20.5676	12.3765	-5.3716	C	0	0	0	0	0	0	0	0	0	0	0	0
21.0659	13.7837	-5.7544	C	0	0	0	0	0	0	0	0	0	0	0	0
22.3445	13.6621	-6.6057	C	0	0	0	0	0	0	0	0	0	0	0	0
1.6685	15.2872	7.2297	H	0	0	0	0	0	0	0	0	0	0	0	0
10.0932	11.8890	2.7438	H	0	0	0	0	0	0	0	0	0	0	0	0
9.1048	11.8267	1.2787	H	0	0	0	0	0	0	0	0	0	0	0	0
9.5624	9.3958	1.0727	H	0	0	0	0	0	0	0	0	0	0	0	0
10.5505	9.4574	2.5383	H	0	0	0	0	0	0	0	0	0	0	0	0
7.5734	10.2128	2.3814	H	0	0	0	0	0	0	0	0	0	0	0	0
8.5612	10.2779	3.8470	H	0	0	0	0	0	0	0	0	0	0	0	0
12.0822	11.0711	1.4391	H	0	0	0	0	0	0	0	0	0	0	0	0
11.0931	11.0135	-0.0257	H	0	0	0	0	0	0	0	0	0	0	0	0
8.1049	12.7107	4.0453	H	0	0	0	0	0	0	0	0	0	0	0	0
7.1156	12.6440	2.5810	H	0	0	0	0	0	0	0	0	0	0	0	0
11.5498	8.5830	-0.2389	H	0	0	0	0	0	0	0	0	0	0	0	0
12.5393	8.6404	1.2259	H	0	0	0	0	0	0	0	0	0	0	0	0
5.5840	11.0346	3.6893	H	0	0	0	0	0	0	0	0	0	0	0	0
6.5734	11.1023	5.1538	H	0	0	0	0	0	0	0	0	0	0	0	0
14.0694	10.2579	0.1298	H	0	0	0	0	0	0	0	0	0	0	0	0
13.0791	10.2034	-1.3344	H	0	0	0	0	0	0	0	0	0	0	0	0
6.1190	13.5361	5.3470	H	0	0	0	0	0	0	0	0	0	0	0	0
5.1276	13.4666	3.8842	H	0	0	0	0	0	0	0	0	0	0	0	0
13.8093	7.4519	-0.6613	H	0	0	0	0	0	0	0	0	0	0	0	0
3.5968	11.8608	4.9981	H	0	0	0	0	0	0	0	0	0	0	0	0
4.5886	11.9302	6.4609	H	0	0	0	0	0	0	0	0	0	0	0	0
15.8001	7.7017	-2.0101	H	0	0	0	0	0	0	0	0	0	0	0	0
4.1352	14.3658	6.6517	H	0	0	0	0	0	0	0	0	0	0	0	0
3.1421	14.2952	5.1906	H	0	0	0	0	0	0	0	0	0	0	0	0
14.9731	10.4420	-2.6163	H	0	0	0	0	0	0	0	0	0	0	0	0
15.9592	10.4939	-1.1491	H	0	0	0	0	0	0	0	0	0	0	0	0
17.7824	9.2948	-2.3323	H	0	0	0	0	0	0	0	0	0	0	0	0
16.7964	9.2438	-3.7997	H	0	0	0	0	0	0	0	0	0	0	0	0
16.7477	11.7250	-3.8533	H	0	0	0	0	0	0	0	0	0	0	0	0
17.7326	11.7764	-2.3854	H	0	0	0	0	0	0	0	0	0	0	0	0
19.5576	10.5794	-3.5681	H	0	0	0	0	0	0	0	0	0	0	0	0
18.5727	10.5286	-5.0362	H	0	0	0	0	0	0	0	0	0	0	0	0
18.5234	13.0099	-5.0893	H	0	0	0	0	0	0	0	0	0	0	0	0
19.5072	13.0611	-3.6205	H	0	0	0	0	0	0	0	0	0	0	0	0
21.3336	11.8648	-4.8021	H	0	0	0	0	0	0	0	0	0	0	0	0
20.3499	11.8136	-6.2709	H	0	0	0	0	0	0	0	0	0	0	0	0
20.3001	14.2952	-6.3242	H	0	0	0	0	0	0	0	0	0	0	0	0
21.2832	14.3467	-4.8551	H	0	0	0	0	0	0	0	0	0	0	0	0
22.6944	14.6510	-6.8746	H	0	0	0	0	0	0	0	0	0	0	0	0
22.1273	13.0991	-7.5050	H	0	0	0	0	0	0	0	0	0	0	0	0
23.1104	13.1508	-6.0358	H	0	0	0	0	0	0	0	0	0	0	0	0

1	2	1	0	0	0	0
1	3	1	0	0	0	0
2	4	1	0	0	0	0
3	5	1	0	0	0	0
4	6	1	0	0	0	0
5	7	1	0	0	0	0
6	8	1	0	0	0	0
7	9	1	0	0	0	0
8	10	1	0	0	0	0

```
  9 11  1  0  0  0  0
 10 12  2  0  0  0  0
 11 13  1  0  0  0  0
 12 14  1  0  0  0  0
 13 15  1  0  0  0  0
 14 16  1  0  0  0  0
 15 17  1  0  0  0  0
 15 18  2  0  0  0  0
 16 19  1  0  0  0  0
 19 20  1  0  0  0  0
 20 21  1  0  0  0  0
 21 22  1  0  0  0  0
 22 23  1  0  0  0  0
 23 24  1  0  0  0  0
 17 25  1  0  0  0  0
  1 26  1  0  0  0  0
  1 27  1  0  0  0  0
  2 28  1  0  0  0  0
  2 29  1  0  0  0  0
  3 30  1  0  0  0  0
  3 31  1  0  0  0  0
  4 32  1  0  0  0  0
  4 33  1  0  0  0  0
  5 34  1  0  0  0  0
  5 35  1  0  0  0  0
  6 36  1  0  0  0  0
  6 37  1  0  0  0  0
  7 38  1  0  0  0  0
  7 39  1  0  0  0  0
  8 40  1  0  0  0  0
  8 41  1  0  0  0  0
  9 42  1  0  0  0  0
  9 43  1  0  0  0  0
 10 44  1  0  0  0  0
 11 45  1  0  0  0  0
 11 46  1  0  0  0  0
 12 47  1  0  0  0  0
 13 48  1  0  0  0  0
 13 49  1  0  0  0  0
 14 50  1  0  0  0  0
 14 51  1  0  0  0  0
 16 52  1  0  0  0  0
 16 53  1  0  0  0  0
 19 54  1  0  0  0  0
 19 55  1  0  0  0  0
 20 56  1  0  0  0  0
 20 57  1  0  0  0  0
 21 58  1  0  0  0  0
 21 59  1  0  0  0  0
 22 60  1  0  0  0  0
 22 61  1  0  0  0  0
 23 62  1  0  0  0  0
 23 63  1  0  0  0  0
 24 64  1  0  0  0  0
 24 65  1  0  0  0  0
 24 66  1  0  0  0  0
M  END
>  <Alias name>
Erucic acid

>  <deltaH°(c)>   kJ/mol
-13804
```

```
$$$$
Phthalic heptylnonylester
  ChemBrain IXL

 66 66  0  0  0  0  0  0  0  0  1 V2000
    2.4286   10.9289    1.6191 C   0  0  0  0  0  0  0  0  0  0  0  0
    2.4004    9.5602    1.8904 C   0  0  0  0  0  0  0  0  0  0  0  0
    3.5781    8.8137    1.8450 C   0  0  0  0  0  0  0  0  0  0  0  0
    4.7875    9.4327    1.5279 C   0  0  0  0  0  0  0  0  0  0  0  0
    4.8173   10.8154    1.2596 C   0  0  0  0  0  0  0  0  0  0  0  0
    3.6351   11.5551    1.3040 C   0  0  0  0  0  0  0  0  0  0  0  0
    6.0344    8.6161    1.4805 C   0  0  0  0  0  0  0  0  0  0  0  0
    6.1549    7.6518    2.2184 O   0  0  0  0  0  0  0  0  0  0  0  0
    7.1258    8.9641    0.5447 O   0  0  0  0  0  0  0  0  0  0  0  0
    6.0984   11.5049    0.9244 C   0  0  0  0  0  0  0  0  0  0  0  0
    6.0849   12.4877    0.2014 O   0  0  0  0  0  0  0  0  0  0  0  0
    7.3856   11.0085    1.4629 O   0  0  0  0  0  0  0  0  0  0  0  0
    8.3485    8.1397    0.6782 C   0  0  0  0  0  0  0  0  0  0  0  0
    9.3662    8.5285   -0.4213 C   0  0  0  0  0  0  0  0  0  0  0  0
   10.6500    7.6784   -0.2616 C   0  0  0  0  0  0  0  0  0  0  0  0
   11.6509    8.0325   -1.3850 C   0  0  0  0  0  0  0  0  0  0  0  0
   12.9393    7.1971   -1.2116 C   0  0  0  0  0  0  0  0  0  0  0  0
   13.8863    7.4553   -2.4030 C   0  0  0  0  0  0  0  0  0  0  0  0
   15.1927    6.6590   -2.2055 C   0  0  0  0  0  0  0  0  0  0  0  0
   11.1015   11.9396    1.1616 C   0  0  0  0  0  0  0  0  0  0  0  0
   12.3679   11.4061    1.8733 C   0  0  0  0  0  0  0  0  0  0  0  0
   13.6112   12.1725    1.3624 C   0  0  0  0  0  0  0  0  0  0  0  0
   14.8762   11.6408    2.0737 C   0  0  0  0  0  0  0  0  0  0  0  0
   16.1163   12.4118    1.5693 C   0  0  0  0  0  0  0  0  0  0  0  0
   17.3804   11.8702    2.2701 C   0  0  0  0  0  0  0  0  0  0  0  0
   18.6177   12.6465    1.7744 C   0  0  0  0  0  0  0  0  0  0  0  0
    9.8521   11.1842    1.6818 C   0  0  0  0  0  0  0  0  0  0  0  0
    8.5860   11.7272    0.9725 C   0  0  0  0  0  0  0  0  0  0  0  0
    1.5132   11.5057    1.6532 H   0  0  0  0  0  0  0  0  0  0  0  0
    1.4635    9.0769    2.1363 H   0  0  0  0  0  0  0  0  0  0  0  0
    3.5539    7.7517    2.0564 H   0  0  0  0  0  0  0  0  0  0  0  0
    3.6543   12.6173    1.0934 H   0  0  0  0  0  0  0  0  0  0  0  0
    8.0902    7.0928    0.5751 H   0  0  0  0  0  0  0  0  0  0  0  0
    8.7908    8.3068    1.6527 H   0  0  0  0  0  0  0  0  0  0  0  0
    8.9317    8.3457   -1.3963 H   0  0  0  0  0  0  0  0  0  0  0  0
    9.6149    9.5783   -0.3282 H   0  0  0  0  0  0  0  0  0  0  0  0
   11.1015    7.8844    0.7010 H   0  0  0  0  0  0  0  0  0  0  0  0
   10.3966    6.6272   -0.3233 H   0  0  0  0  0  0  0  0  0  0  0  0
   11.2058    7.8130   -2.3475 H   0  0  0  0  0  0  0  0  0  0  0  0
   11.8948    9.0864   -1.3322 H   0  0  0  0  0  0  0  0  0  0  0  0
   13.4329    7.4817   -0.2905 H   0  0  0  0  0  0  0  0  0  0  0  0
   12.6852    6.1450   -1.1745 H   0  0  0  0  0  0  0  0  0  0  0  0
   13.4064    7.1388   -3.3208 H   0  0  0  0  0  0  0  0  0  0  0  0
   14.1137    8.5126   -2.4608 H   0  0  0  0  0  0  0  0  0  0  0  0
   15.7063    7.0206   -1.3231 H   0  0  0  0  0  0  0  0  0  0  0  0
   15.8295    6.7890   -3.0723 H   0  0  0  0  0  0  0  0  0  0  0  0
   14.9604    5.6084   -2.0828 H   0  0  0  0  0  0  0  0  0  0  0  0
   10.9934   12.9979    1.3647 H   0  0  0  0  0  0  0  0  0  0  0  0
   11.1970   11.7857    0.0938 H   0  0  0  0  0  0  0  0  0  0  0  0
   12.4832   10.3503    1.6614 H   0  0  0  0  0  0  0  0  0  0  0  0
   12.2693   11.5508    2.9420 H   0  0  0  0  0  0  0  0  0  0  0  0
   13.4960   13.2285    1.5736 H   0  0  0  0  0  0  0  0  0  0  0  0
   13.7107   12.0273    0.2938 H   0  0  0  0  0  0  0  0  0  0  0  0
   14.9951   10.5863    1.8579 H   0  0  0  0  0  0  0  0  0  0  0  0
   14.7749   11.7810    3.1428 H   0  0  0  0  0  0  0  0  0  0  0  0
   16.0026   13.4651    1.7940 H   0  0  0  0  0  0  0  0  0  0  0  0
   16.2128   12.2796    0.4987 H   0  0  0  0  0  0  0  0  0  0  0  0
   17.4984   10.8186    2.0397 H   0  0  0  0  0  0  0  0  0  0  0  0
```

```
   17.2812   11.9959    3.3412 H   0  0  0  0  0  0  0  0  0  0  0  0
   18.5048   13.6968    2.0133 H   0  0  0  0  0  0  0  0  0  0  0  0
   18.7126   12.5284    0.7021 H   0  0  0  0  0  0  0  0  0  0  0  0
   19.5054   12.2594    2.2600 H   0  0  0  0  0  0  0  0  0  0  0  0
    9.9573   10.1268    1.4732 H   0  0  0  0  0  0  0  0  0  0  0  0
    9.7594   11.3337    2.7504 H   0  0  0  0  0  0  0  0  0  0  0  0
    8.4828   12.7849    1.1816 H   0  0  0  0  0  0  0  0  0  0  0  0
    8.6819   11.5798   -0.0962 H   0  0  0  0  0  0  0  0  0  0  0  0
  1  2  4  0  0  0  0
  2  3  4  0  0  0  0
  3  4  4  0  0  0  0
  4  5  4  0  0  0  0
  5  6  4  0  0  0  0
  6  1  4  0  0  0  0
  7  8  2  0  0  0  0
  7  9  1  0  0  0  0
  7  4  1  0  0  0  0
 10 11  2  0  0  0  0
 10 12  1  0  0  0  0
 10  5  1  0  0  0  0
  9 13  1  0  0  0  0
 13 14  1  0  0  0  0
 14 15  1  0  0  0  0
 15 16  1  0  0  0  0
 16 17  1  0  0  0  0
 17 18  1  0  0  0  0
 18 19  1  0  0  0  0
 20 21  1  0  0  0  0
 21 22  1  0  0  0  0
 22 23  1  0  0  0  0
 23 24  1  0  0  0  0
 24 25  1  0  0  0  0
 25 26  1  0  0  0  0
 20 27  1  0  0  0  0
 27 28  1  0  0  0  0
 28 12  1  0  0  0  0
  1 29  1  0  0  0  0
  2 30  1  0  0  0  0
  3 31  1  0  0  0  0
  6 32  1  0  0  0  0
 13 33  1  0  0  0  0
 13 34  1  0  0  0  0
 14 35  1  0  0  0  0
 14 36  1  0  0  0  0
 15 37  1  0  0  0  0
 15 38  1  0  0  0  0
 16 39  1  0  0  0  0
 16 40  1  0  0  0  0
 17 41  1  0  0  0  0
 17 42  1  0  0  0  0
 18 43  1  0  0  0  0
 18 44  1  0  0  0  0
 19 45  1  0  0  0  0
 19 46  1  0  0  0  0
 19 47  1  0  0  0  0
 20 48  1  0  0  0  0
 20 49  1  0  0  0  0
 21 50  1  0  0  0  0
 21 51  1  0  0  0  0
 22 52  1  0  0  0  0
 22 53  1  0  0  0  0
 23 54  1  0  0  0  0
 23 55  1  0  0  0  0
```

```
 24 56  1  0  0  0  0
 24 57  1  0  0  0  0
 25 58  1  0  0  0  0
 25 59  1  0  0  0  0
 26 60  1  0  0  0  0
 26 61  1  0  0  0  0
 26 62  1  0  0  0  0
 27 63  1  0  0  0  0
 27 64  1  0  0  0  0
 28 65  1  0  0  0  0
 28 66  1  0  0  0  0
M  END
>  <Alias name>
Phthalic heptylnonylester

>  <deltaH°(c)>   kJ/mol
-13799.95

$$$$
Diisooctylphthalate
  ChemBrain IXL

 66 66  0  0  0  0  0  0  0  0  1 V2000
    2.4775    5.8614   -0.4834 C   0  0  0  0  0  0  0  0  0  0  0  0  0
    2.8178    4.7326    0.2636 C   0  0  0  0  0  0  0  0  0  0  0  0  0
    4.1391    4.5303    0.6646 C   0  0  0  0  0  0  0  0  0  0  0  0  0
    5.1263    5.4548    0.3202 C   0  0  0  0  0  0  0  0  0  0  0  0  0
    4.7805    6.6008   -0.4326 C   0  0  0  0  0  0  0  0  0  0  0  0  0
    3.4568    6.7929   -0.8307 C   0  0  0  0  0  0  0  0  0  0  0  0  0
    6.5363    5.2118    0.7617 C   0  0  0  0  0  0  0  0  0  0  0  0  0
    6.7468    4.6068    1.8001 O   0  0  0  0  0  0  0  0  0  0  0  0  0
    7.6817    5.6949   -0.0497 O   0  0  0  0  0  0  0  0  0  0  0  0  0
    5.8103    7.6172   -0.8175 C   0  0  0  0  0  0  0  0  0  0  0  0  0
    5.6693    8.2580   -1.8461 O   0  0  0  0  0  0  0  0  0  0  0  0  0
    6.9993    7.8577    0.0379 O   0  0  0  0  0  0  0  0  0  0  0  0  0
    9.0191    5.4578    0.5548 C   0  0  0  0  0  0  0  0  0  0  0  0  0
   10.1345    5.8888   -0.4357 C   0  0  0  0  0  0  0  0  0  0  0  0  0
   11.5261    5.6582    0.2147 C   0  0  0  0  0  0  0  0  0  0  0  0  0
   12.6432    6.0669   -0.7792 C   0  0  0  0  0  0  0  0  0  0  0  0  0
   14.0287    5.8430   -0.1245 C   0  0  0  0  0  0  0  0  0  0  0  0  0
   15.1438    6.1167   -1.1607 C   0  0  0  0  0  0  0  0  0  0  0  0  0
   16.5211    5.8314   -0.5251 C   0  0  0  0  0  0  0  0  0  0  0  0  0
   15.0826    7.5906   -1.6063 C   0  0  0  0  0  0  0  0  0  0  0  0  0
    7.9777    8.8277   -0.5174 C   0  0  0  0  0  0  0  0  0  0  0  0  0
    9.1091    9.0944    0.5117 C   0  0  0  0  0  0  0  0  0  0  0  0  0
   10.1144   10.1189   -0.0805 C   0  0  0  0  0  0  0  0  0  0  0  0  0
   11.2504   10.3920    0.9370 C   0  0  0  0  0  0  0  0  0  0  0  0  0
   12.2398   11.4236    0.3411 C   0  0  0  0  0  0  0  0  0  0  0  0  0
   13.3768   11.7001    1.3511 C   0  0  0  0  0  0  0  0  0  0  0  0  0
   14.3543   12.7356    0.7558 C   0  0  0  0  0  0  0  0  0  0  0  0  0
   14.1354   10.3916    1.6450 C   0  0  0  0  0  0  0  0  0  0  0  0  0
    1.4519    6.0147   -0.7941 H   0  0  0  0  0  0  0  0  0  0  0  0  0
    2.0555    4.0125    0.5326 H   0  0  0  0  0  0  0  0  0  0  0  0  0
    4.3996    3.6535    1.2447 H   0  0  0  0  0  0  0  0  0  0  0  0  0
    3.1893    7.6675   -1.4107 H   0  0  0  0  0  0  0  0  0  0  0  0  0
    9.1079    6.0336    1.4678 H   0  0  0  0  0  0  0  0  0  0  0  0  0
    9.1288    4.4045    0.7825 H   0  0  0  0  0  0  0  0  0  0  0  0  0
   10.0589    5.3014   -1.3422 H   0  0  0  0  0  0  0  0  0  0  0  0  0
   10.0184    6.9383   -0.6753 H   0  0  0  0  0  0  0  0  0  0  0  0  0
   11.6076    6.2573    1.1134 H   0  0  0  0  0  0  0  0  0  0  0  0  0
   11.6362    4.6111    0.4691 H   0  0  0  0  0  0  0  0  0  0  0  0  0
   12.5668    5.4643   -1.6758 H   0  0  0  0  0  0  0  0  0  0  0  0  0
   12.5318    7.1127   -1.0370 H   0  0  0  0  0  0  0  0  0  0  0  0  0
```

14.1442 6.5172 0.7150 H 0 0 0 0 0 0 0 0 0 0 0 0
14.1028 4.8196 0.2223 H 0 0 0 0 0 0 0 0 0 0 0 0
15.0036 5.4736 -2.0208 H 0 0 0 0 0 0 0 0 0 0 0 0
16.6853 6.5116 0.3017 H 0 0 0 0 0 0 0 0 0 0 0 0
17.2977 5.9721 -1.2676 H 0 0 0 0 0 0 0 0 0 0 0 0
16.5482 4.8109 -0.1638 H 0 0 0 0 0 0 0 0 0 0 0 0
14.1097 7.7986 -2.0329 H 0 0 0 0 0 0 0 0 0 0 0 0
15.2467 8.2335 -0.7503 H 0 0 0 0 0 0 0 0 0 0 0 0
15.8496 7.7755 -2.3488 H 0 0 0 0 0 0 0 0 0 0 0 0
8.4074 8.4239 -1.4257 H 0 0 0 0 0 0 0 0 0 0 0 0
7.4717 9.7584 -0.7428 H 0 0 0 0 0 0 0 0 0 0 0 0
8.6820 9.4936 1.4233 H 0 0 0 0 0 0 0 0 0 0 0 0
9.6250 8.1677 0.7300 H 0 0 0 0 0 0 0 0 0 0 0 0
10.5385 9.7202 -0.9938 H 0 0 0 0 0 0 0 0 0 0 0 0
9.5975 11.0453 -0.2988 H 0 0 0 0 0 0 0 0 0 0 0 0
10.8280 10.7851 1.8535 H 0 0 0 0 0 0 0 0 0 0 0 0
11.7758 9.4689 1.1485 H 0 0 0 0 0 0 0 0 0 0 0 0
12.6608 11.0311 -0.5762 H 0 0 0 0 0 0 0 0 0 0 0 0
11.7140 12.3467 0.1299 H 0 0 0 0 0 0 0 0 0 0 0 0
12.9567 12.0881 2.2708 H 0 0 0 0 0 0 0 0 0 0 0 0
13.8260 13.6603 0.5593 H 0 0 0 0 0 0 0 0 0 0 0 0
14.7673 12.3523 -0.1693 H 0 0 0 0 0 0 0 0 0 0 0 0
15.1569 12.9210 1.4596 H 0 0 0 0 0 0 0 0 0 0 0 0
14.9737 10.5996 2.2991 H 0 0 0 0 0 0 0 0 0 0 0 0
14.4986 9.9686 0.7166 H 0 0 0 0 0 0 0 0 0 0 0 0
13.4683 9.6877 2.1260 H 0 0 0 0 0 0 0 0 0 0 0 0
1 2 4 0 0 0 0
2 3 4 0 0 0 0
3 4 4 0 0 0 0
4 5 4 0 0 0 0
5 6 4 0 0 0 0
6 1 4 0 0 0 0
7 8 2 0 0 0 0
7 9 1 0 0 0 0
7 4 1 0 0 0 0
10 11 2 0 0 0 0
10 12 1 0 0 0 0
10 5 1 0 0 0 0
9 13 1 0 0 0 0
13 14 1 0 0 0 0
14 15 1 0 0 0 0
15 16 1 0 0 0 0
16 17 1 0 0 0 0
17 18 1 0 0 0 0
18 19 1 0 0 0 0
18 20 1 0 0 0 0
21 22 1 0 0 0 0
22 23 1 0 0 0 0
23 24 1 0 0 0 0
24 25 1 0 0 0 0
25 26 1 0 0 0 0
26 27 1 0 0 0 0
26 28 1 0 0 0 0
21 12 1 0 0 0 0
1 29 1 0 0 0 0
2 30 1 0 0 0 0
3 31 1 0 0 0 0
6 32 1 0 0 0 0
13 33 1 0 0 0 0
13 34 1 0 0 0 0
14 35 1 0 0 0 0
14 36 1 0 0 0 0
15 37 1 0 0 0 0

15 38 1 0 0 0 0
16 39 1 0 0 0 0
16 40 1 0 0 0 0
17 41 1 0 0 0 0
17 42 1 0 0 0 0
18 43 1 0 0 0 0
19 44 1 0 0 0 0
19 45 1 0 0 0 0
19 46 1 0 0 0 0
20 47 1 0 0 0 0
20 48 1 0 0 0 0
20 49 1 0 0 0 0
21 50 1 0 0 0 0
21 51 1 0 0 0 0
22 52 1 0 0 0 0
22 53 1 0 0 0 0
23 54 1 0 0 0 0
23 55 1 0 0 0 0
24 56 1 0 0 0 0
24 57 1 0 0 0 0
25 58 1 0 0 0 0
25 59 1 0 0 0 0
26 60 1 0 0 0 0
27 61 1 0 0 0 0
27 62 1 0 0 0 0
27 63 1 0 0 0 0
28 64 1 0 0 0 0
28 65 1 0 0 0 0
28 66 1 0 0 0 0
M END
> <Alias name>
Di-isooctyl phthalate

> <deltaH°(c)>   kJ/mol
-13797.65

$$$$
Bis(1-ethylhexyl) o-phthalate
  ChemBrain IXL

 66 66 0 0 0 0 0 0 0 0 1 V2000
    5.9476   3.8788   -2.2756 C   0 0 0 0 0 0 0 0 0 0 0 0
    6.5779   2.8540   -2.9834 C   0 0 0 0 0 0 0 0 0 0 0 0
    7.8848   2.4803   -2.6628 C   0 0 0 0 0 0 0 0 0 0 0 0
    8.5681   3.1298   -1.6332 C   0 0 0 0 0 0 0 0 0 0 0 0
    7.9492   4.1565   -0.9178 C   0 0 0 0 0 0 0 0 0 0 0 0
    6.6202   4.5352   -1.2437 C   0 0 0 0 0 0 0 0 0 0 0 0
    8.7148   4.8347   0.1847 C   0 0 0 0 0 0 0 0 0 0 0 0
    8.3241   6.1893   0.6714 O   0 0 0 0 0 0 0 0 0 0 0 0
    9.6718   4.2678   0.6869 O   0 0 0 0 0 0 0 0 0 0 0 0
    5.9166   5.6296   -0.4986 C   0 0 0 0 0 0 0 0 0 0 0 0
    6.0670   5.7364   0.7077 O   0 0 0 0 0 0 0 0 0 0 0 0
    5.0307   6.5844   -1.2146 O   0 0 0 0 0 0 0 0 0 0 0 0
    4.4387   7.6539   -0.3654 C   0 0 0 0 0 0 0 0 0 0 0 0
    5.5382   8.6612   0.0770 C   0 0 0 0 0 0 0 0 0 0 0 0
    3.3458   8.4067   -1.1561 C   0 0 0 0 0 0 0 0 0 0 0 0
    2.2033   7.4373   -1.5068 C   0 0 0 0 0 0 0 0 0 0 0 0
    6.0259   9.4901   -1.1333 C   0 0 0 0 0 0 0 0 0 0 0 0
    7.0454   10.5481   -0.6495 C   0 0 0 0 0 0 0 0 0 0 0 0
    7.4956   11.4184   -1.8414 C   0 0 0 0 0 0 0 0 0 0 0 0
    8.4812   12.4984   -1.3474 C   0 0 0 0 0 0 0 0 0 0 0 0
    9.0617   6.6829   1.8718 C   0 0 0 0 0 0 0 0 0 0 0 0
   10.5720   6.8008   1.5569 C   0 0 0 0 0 0 0 0 0 0 0 0

8.5179	8.0725	2.2908	C	0	0	0	0	0	0	0	0	0	0	0	0	0
7.0960	7.9235	2.8717	C	0	0	0	0	0	0	0	0	0	0	0	0	0
10.8054	7.8572	0.4574	C	0	0	0	0	0	0	0	0	0	0	0	0	0
12.3217	7.9968	0.2042	C	0	0	0	0	0	0	0	0	0	0	0	0	0
12.5717	9.0577	-0.8848	C	0	0	0	0	0	0	0	0	0	0	0	0	0
14.0889	9.1975	-1.1245	C	0	0	0	0	0	0	0	0	0	0	0	0	0
4.9337	4.1651	-2.5267	H	0	0	0	0	0	0	0	0	0	0	0	0	0
6.0521	2.3478	-3.7821	H	0	0	0	0	0	0	0	0	0	0	0	0	0
8.3695	1.6852	-3.2138	H	0	0	0	0	0	0	0	0	0	0	0	0	0
9.5814	2.8369	-1.3882	H	0	0	0	0	0	0	0	0	0	0	0	0	0
3.9951	7.2032	0.5139	H	0	0	0	0	0	0	0	0	0	0	0	0	0
6.3707	8.1232	0.5016	H	0	0	0	0	0	0	0	0	0	0	0	0	0
5.1263	9.3322	0.8218	H	0	0	0	0	0	0	0	0	0	0	0	0	0
2.9565	9.2168	-0.5506	H	0	0	0	0	0	0	0	0	0	0	0	0	0
3.7699	8.8089	-2.0668	H	0	0	0	0	0	0	0	0	0	0	0	0	0
2.5903	6.6265	-2.1103	H	0	0	0	0	0	0	0	0	0	0	0	0	0
1.7768	7.0376	-0.5950	H	0	0	0	0	0	0	0	0	0	0	0	0	0
1.4383	7.9671	-2.0611	H	0	0	0	0	0	0	0	0	0	0	0	0	0
5.1826	9.9908	-1.5904	H	0	0	0	0	0	0	0	0	0	0	0	0	0
6.4948	8.8365	-1.8573	H	0	0	0	0	0	0	0	0	0	0	0	0	0
7.9058	10.0516	-0.2187	H	0	0	0	0	0	0	0	0	0	0	0	0	0
6.5808	11.1786	0.0994	H	0	0	0	0	0	0	0	0	0	0	0	0	0
6.6312	11.8966	-2.2857	H	0	0	0	0	0	0	0	0	0	0	0	0	0
7.9829	10.7953	-2.5803	H	0	0	0	0	0	0	0	0	0	0	0	0	0
7.9921	13.1237	-0.6100	H	0	0	0	0	0	0	0	0	0	0	0	0	0
8.7978	13.1085	-2.1845	H	0	0	0	0	0	0	0	0	0	0	0	0	0
9.3451	12.0223	-0.9007	H	0	0	0	0	0	0	0	0	0	0	0	0	0
8.9243	5.9856	2.6887	H	0	0	0	0	0	0	0	0	0	0	0	0	0
11.1006	7.0991	2.4547	H	0	0	0	0	0	0	0	0	0	0	0	0	0
10.9466	5.8423	1.2223	H	0	0	0	0	0	0	0	0	0	0	0	0	0
9.1670	8.5000	3.0460	H	0	0	0	0	0	0	0	0	0	0	0	0	0
8.4917	8.7258	1.4274	H	0	0	0	0	0	0	0	0	0	0	0	0	0
6.4556	7.4468	2.1459	H	0	0	0	0	0	0	0	0	0	0	0	0	0
7.1353	7.3187	3.7687	H	0	0	0	0	0	0	0	0	0	0	0	0	0
6.7013	8.9035	3.1148	H	0	0	0	0	0	0	0	0	0	0	0	0	0
10.3113	7.5476	-0.4546	H	0	0	0	0	0	0	0	0	0	0	0	0	0
10.4055	8.8106	0.7805	H	0	0	0	0	0	0	0	0	0	0	0	0	0
12.8130	8.3000	1.1211	H	0	0	0	0	0	0	0	0	0	0	0	0	0
12.7220	7.0449	-0.1210	H	0	0	0	0	0	0	0	0	0	0	0	0	0
12.0873	8.7531	-1.8038	H	0	0	0	0	0	0	0	0	0	0	0	0	0
12.1682	10.0095	-0.5617	H	0	0	0	0	0	0	0	0	0	0	0	0	0
14.5728	9.5060	-0.2057	H	0	0	0	0	0	0	0	0	0	0	0	0	0
14.2664	9.9397	-1.8927	H	0	0	0	0	0	0	0	0	0	0	0	0	0
14.4935	8.2445	-1.4429	H	0	0	0	0	0	0	0	0	0	0	0	0	0

13	14	1	0	0	0	0
5	7	1	0	0	0	0
13	15	1	0	0	0	0
4	3	4	0	0	0	0
15	16	1	0	0	0	0
7	8	1	0	0	0	0
14	17	1	0	0	0	0
17	18	1	0	0	0	0
7	9	2	0	0	0	0
18	19	1	0	0	0	0
5	4	4	0	0	0	0
19	20	1	0	0	0	0
6	10	1	0	0	0	0
8	21	1	0	0	0	0
3	2	4	0	0	0	0
21	22	1	0	0	0	0
10	11	2	0	0	0	0
21	23	1	0	0	0	0
6	5	4	0	0	0	0

```
 23 24  1  0  0  0  0
 10 12  1  0  0  0  0
 22 25  1  0  0  0  0
  1  6  4  0  0  0  0
 25 26  1  0  0  0  0
 12 13  1  0  0  0  0
 26 27  1  0  0  0  0
  2  1  4  0  0  0  0
 27 28  1  0  0  0  0
  1 29  1  0  0  0  0
  2 30  1  0  0  0  0
  3 31  1  0  0  0  0
  4 32  1  0  0  0  0
 13 33  1  0  0  0  0
 14 34  1  0  0  0  0
 14 35  1  0  0  0  0
 15 36  1  0  0  0  0
 15 37  1  0  0  0  0
 16 38  1  0  0  0  0
 16 39  1  0  0  0  0
 16 40  1  0  0  0  0
 17 41  1  0  0  0  0
 17 42  1  0  0  0  0
 18 43  1  0  0  0  0
 18 44  1  0  0  0  0
 19 45  1  0  0  0  0
 19 46  1  0  0  0  0
 20 47  1  0  0  0  0
 20 48  1  0  0  0  0
 20 49  1  0  0  0  0
 21 50  1  0  0  0  0
 22 51  1  0  0  0  0
 22 52  1  0  0  0  0
 23 53  1  0  0  0  0
 23 54  1  0  0  0  0
 24 55  1  0  0  0  0
 24 56  1  0  0  0  0
 24 57  1  0  0  0  0
 25 58  1  0  0  0  0
 25 59  1  0  0  0  0
 26 60  1  0  0  0  0
 26 61  1  0  0  0  0
 27 62  1  0  0  0  0
 27 63  1  0  0  0  0
 28 64  1  0  0  0  0
 28 65  1  0  0  0  0
 28 66  1  0  0  0  0
M  END
>  <Alias name>
Bis(1-ethylhexyl) o-phthalate

>  <deltaH°(c)>   kJ/mol
-13791

$$$$
Brassidic acid
  ChemBrain IXL

 66 65  0  0  0  0  0  0  0  0  1 V2000
    9.2851   12.0866    2.6414 C   0  0  0  0  0  0  0  0  0  0  0  0
   10.2728   11.2337    1.8215 C   0  0  0  0  0  0  0  0  0  0  0  0
    8.3312   11.1629    3.4239 C   0  0  0  0  0  0  0  0  0  0  0  0
   11.2242   12.1617    1.0408 C   0  0  0  0  0  0  0  0  0  0  0  0
```

```
7.3421   12.0202    4.2376 C   0  0  0  0  0  0  0  0  0  0  0  0
12.2078   11.3129    0.2119 C   0  0  0  0  0  0  0  0  0  0  0  0
6.3870   11.1005    5.0232 C   0  0  0  0  0  0  0  0  0  0  0  0
13.1556   12.2441   -0.5696 C   0  0  0  0  0  0  0  0  0  0  0  0
5.3985   11.9617    5.8338 C   0  0  0  0  0  0  0  0  0  0  0  0
14.0947   11.4314   -1.3745 C   0  0  0  0  0  0  0  0  0  0  0  0
4.4430   11.0451    6.6222 C   0  0  0  0  0  0  0  0  0  0  0  0
15.0206   12.0310   -2.1265 C   0  0  0  0  0  0  0  0  0  0  0  0
3.4555   11.9086    7.4316 C   0  0  0  0  0  0  0  0  0  0  0  0
15.9594   11.2187   -2.9323 C   0  0  0  0  0  0  0  0  0  0  0  0
2.5380   11.0297    8.1906 C   0  0  0  0  0  0  0  0  0  0  0  0
16.9514   12.1488   -3.6569 C   0  0  0  0  0  0  0  0  0  0  0  0
1.5010   11.6177    9.0509 O   0  0  0  0  0  0  0  0  0  0  0  0
2.6422    9.8174    8.1024 O   0  0  0  0  0  0  0  0  0  0  0  0
17.9128   11.3054   -4.5166 C   0  0  0  0  0  0  0  0  0  0  0  0
18.9003   12.2368   -5.2459 C   0  0  0  0  0  0  0  0  0  0  0  0
19.8442   11.3962   -6.1274 C   0  0  0  0  0  0  0  0  0  0  0  0
20.8281   12.3287   -6.8604 C   0  0  0  0  0  0  0  0  0  0  0  0
21.7603   11.4901   -7.7561 C   0  0  0  0  0  0  0  0  0  0  0  0
22.7440   12.4228   -8.4890 C   0  0  0  0  0  0  0  0  0  0  0  0
1.4831   12.6004    9.0675 H   0  0  0  0  0  0  0  0  0  0  0  0
8.7103   12.7155    1.9728 H   0  0  0  0  0  0  0  0  0  0  0  0
9.8360   12.7081    3.3365 H   0  0  0  0  0  0  0  0  0  0  0  0
10.8486   10.6053    2.4897 H   0  0  0  0  0  0  0  0  0  0  0  0
9.7225   10.6124    1.1258 H   0  0  0  0  0  0  0  0  0  0  0  0
8.9049   10.5370    4.0962 H   0  0  0  0  0  0  0  0  0  0  0  0
7.7821   10.5389    2.7295 H   0  0  0  0  0  0  0  0  0  0  0  0
10.6473   12.7949    0.3783 H   0  0  0  0  0  0  0  0  0  0  0  0
11.7785   12.7785    1.7373 H   0  0  0  0  0  0  0  0  0  0  0  0
6.7684   12.6455    3.5647 H   0  0  0  0  0  0  0  0  0  0  0  0
7.8918   12.6453    4.9304 H   0  0  0  0  0  0  0  0  0  0  0  0
12.7869   10.6813    0.8743 H   0  0  0  0  0  0  0  0  0  0  0  0
11.6539   10.6947   -0.4838 H   0  0  0  0  0  0  0  0  0  0  0  0
6.9600   10.4765    5.6979 H   0  0  0  0  0  0  0  0  0  0  0  0
5.8375   10.4746    4.3310 H   0  0  0  0  0  0  0  0  0  0  0  0
12.5755   12.8810   -1.2259 H   0  0  0  0  0  0  0  0  0  0  0  0
13.7143   12.8571    0.1268 H   0  0  0  0  0  0  0  0  0  0  0  0
4.8253   12.5849    5.1586 H   0  0  0  0  0  0  0  0  0  0  0  0
5.9485   12.5888    6.5244 H   0  0  0  0  0  0  0  0  0  0  0  0
14.0308   10.3505   -1.3534 H   0  0  0  0  0  0  0  0  0  0  0  0
5.0158   10.4220    7.2979 H   0  0  0  0  0  0  0  0  0  0  0  0
3.8924   10.4180    5.9318 H   0  0  0  0  0  0  0  0  0  0  0  0
15.0847   13.1119   -2.1470 H   0  0  0  0  0  0  0  0  0  0  0  0
2.8823   12.5310    6.7556 H   0  0  0  0  0  0  0  0  0  0  0  0
4.0063   12.5366    8.1209 H   0  0  0  0  0  0  0  0  0  0  0  0
16.5045   10.5458   -2.2819 H   0  0  0  0  0  0  0  0  0  0  0  0
15.4040   10.6436   -3.6629 H   0  0  0  0  0  0  0  0  0  0  0  0
16.4049   12.8339   -4.2933 H   0  0  0  0  0  0  0  0  0  0  0  0
17.5195   12.7105   -2.9258 H   0  0  0  0  0  0  0  0  0  0  0  0
18.4628   10.6235   -3.8798 H   0  0  0  0  0  0  0  0  0  0  0  0
17.3445   10.7402   -5.2449 H   0  0  0  0  0  0  0  0  0  0  0  0
18.3492   12.9315   -5.8676 H   0  0  0  0  0  0  0  0  0  0  0  0
19.4822   12.7876   -4.5173 H   0  0  0  0  0  0  0  0  0  0  0  0
20.3981   10.7040   -5.5054 H   0  0  0  0  0  0  0  0  0  0  0  0
19.2622   10.8427   -6.8539 H   0  0  0  0  0  0  0  0  0  0  0  0
20.2736   13.0293   -7.4724 H   0  0  0  0  0  0  0  0  0  0  0  0
21.4190   12.8728   -6.1339 H   0  0  0  0  0  0  0  0  0  0  0  0
22.3149   10.7895   -7.1442 H   0  0  0  0  0  0  0  0  0  0  0  0
21.1695   10.9459   -8.4826 H   0  0  0  0  0  0  0  0  0  0  0  0
22.1893   13.1250   -9.0990 H   0  0  0  0  0  0  0  0  0  0  0  0
23.3975   11.8338   -9.1206 H   0  0  0  0  0  0  0  0  0  0  0  0
23.3366   12.9651   -7.7626 H   0  0  0  0  0  0  0  0  0  0  0  0
1  2  1  0  0  0  0
```

1	3	1	0	0	0	0
2	4	1	0	0	0	0
3	5	1	0	0	0	0
4	6	1	0	0	0	0
5	7	1	0	0	0	0
6	8	1	0	0	0	0
7	9	1	0	0	0	0
8	10	1	0	0	0	0
9	11	1	0	0	0	0
10	12	2	0	0	0	0
11	13	1	0	0	0	0
12	14	1	0	0	0	0
13	15	1	0	0	0	0
14	16	1	0	0	0	0
15	17	1	0	0	0	0
15	18	2	0	0	0	0
16	19	1	0	0	0	0
19	20	1	0	0	0	0
20	21	1	0	0	0	0
21	22	1	0	0	0	0
22	23	1	0	0	0	0
23	24	1	0	0	0	0
17	25	1	0	0	0	0
1	26	1	0	0	0	0
1	27	1	0	0	0	0
2	28	1	0	0	0	0
2	29	1	0	0	0	0
3	30	1	0	0	0	0
3	31	1	0	0	0	0
4	32	1	0	0	0	0
4	33	1	0	0	0	0
5	34	1	0	0	0	0
5	35	1	0	0	0	0
6	36	1	0	0	0	0
6	37	1	0	0	0	0
7	38	1	0	0	0	0
7	39	1	0	0	0	0
8	40	1	0	0	0	0
8	41	1	0	0	0	0
9	42	1	0	0	0	0
9	43	1	0	0	0	0
10	44	1	0	0	0	0
11	45	1	0	0	0	0
11	46	1	0	0	0	0
12	47	1	0	0	0	0
13	48	1	0	0	0	0
13	49	1	0	0	0	0
14	50	1	0	0	0	0
14	51	1	0	0	0	0
16	52	1	0	0	0	0
16	53	1	0	0	0	0
19	54	1	0	0	0	0
19	55	1	0	0	0	0
20	56	1	0	0	0	0
20	57	1	0	0	0	0
21	58	1	0	0	0	0
21	59	1	0	0	0	0
22	60	1	0	0	0	0
22	61	1	0	0	0	0
23	62	1	0	0	0	0
23	63	1	0	0	0	0
24	64	1	0	0	0	0
24	65	1	0	0	0	0

```
 24 66  1  0  0  0  0
M  END
>  <Alias name>
Brassidic acid

>  <deltaH°(c)>   kJ/mol
-13775

$$$$
Hexadecylcyclopentane
  ChemBrain IXL

 63 63  0  0  0  0  0  0  0  0  1 V2000
   22.4991   12.8691    0.4377 C   0  0  0  0  0  0  0  0  0  0  0  0  0
   23.7667   13.5411   -0.0689 C   0  0  0  0  0  0  0  0  0  0  0  0  0
   24.8556   12.4883    0.1215 C   0  0  0  0  0  0  0  0  0  0  0  0  0
   24.1387   11.1117   -0.0751 C   0  0  0  0  0  0  0  0  0  0  0  0  0
   22.6075   11.4300   -0.0615 C   0  0  0  0  0  0  0  0  0  0  0  0  0
   21.2341   13.5619   -0.1078 C   0  0  0  0  0  0  0  0  0  0  0  0  0
   19.9814   12.8207    0.3999 C   0  0  0  0  0  0  0  0  0  0  0  0  0
   18.7178   13.5153   -0.1438 C   0  0  0  0  0  0  0  0  0  0  0  0  0
   17.4655   12.7740    0.3633 C   0  0  0  0  0  0  0  0  0  0  0  0  0
   16.2019   13.4682   -0.1808 C   0  0  0  0  0  0  0  0  0  0  0  0  0
   14.9496   12.7265    0.3258 C   0  0  0  0  0  0  0  0  0  0  0  0  0
   13.6859   13.4204   -0.2189 C   0  0  0  0  0  0  0  0  0  0  0  0  0
   12.4336   12.6782    0.2871 C   0  0  0  0  0  0  0  0  0  0  0  0  0
   11.1700   13.3716   -0.2581 C   0  0  0  0  0  0  0  0  0  0  0  0  0
    9.9177   12.6290    0.2473 C   0  0  0  0  0  0  0  0  0  0  0  0  0
    8.6541   13.3220   -0.2984 C   0  0  0  0  0  0  0  0  0  0  0  0  0
    7.4019   12.5790    0.2066 C   0  0  0  0  0  0  0  0  0  0  0  0  0
    6.1382   13.2718   -0.3394 C   0  0  0  0  0  0  0  0  0  0  0  0  0
    4.8860   12.5287    0.1653 C   0  0  0  0  0  0  0  0  0  0  0  0  0
    3.6222   13.2214   -0.3809 C   0  0  0  0  0  0  0  0  0  0  0  0  0
    2.3700   12.4783    0.1237 C   0  0  0  0  0  0  0  0  0  0  0  0  0
   22.4967   12.8770    1.5207 H   0  0  0  0  0  0  0  0  0  0  0  0  0
   23.9878   14.4433    0.4878 H   0  0  0  0  0  0  0  0  0  0  0  0  0
   23.6590   13.7641   -1.1233 H   0  0  0  0  0  0  0  0  0  0  0  0  0
   25.2201   12.5504    1.1394 H   0  0  0  0  0  0  0  0  0  0  0  0  0
   25.6734   12.6175   -0.5763 H   0  0  0  0  0  0  0  0  0  0  0  0  0
   24.4056   10.4478    0.7378 H   0  0  0  0  0  0  0  0  0  0  0  0  0
   24.4051   10.6663   -1.0255 H   0  0  0  0  0  0  0  0  0  0  0  0  0
   22.2327   11.3886   -1.0768 H   0  0  0  0  0  0  0  0  0  0  0  0  0
   22.0600   10.7387    0.5670 H   0  0  0  0  0  0  0  0  0  0  0  0  0
   21.2507   13.5431   -1.1906 H   0  0  0  0  0  0  0  0  0  0  0  0  0
   21.2092   14.5889    0.2348 H   0  0  0  0  0  0  0  0  0  0  0  0  0
   19.9656   12.8385    1.4826 H   0  0  0  0  0  0  0  0  0  0  0  0  0
   20.0051   11.7941    0.0560 H   0  0  0  0  0  0  0  0  0  0  0  0  0
   18.7339   13.4978   -1.2265 H   0  0  0  0  0  0  0  0  0  0  0  0  0
   18.6938   14.5419    0.2004 H   0  0  0  0  0  0  0  0  0  0  0  0  0
   17.4493   12.7917    1.4461 H   0  0  0  0  0  0  0  0  0  0  0  0  0
   17.4898   11.7473    0.0194 H   0  0  0  0  0  0  0  0  0  0  0  0  0
   16.2184   13.4508   -1.2636 H   0  0  0  0  0  0  0  0  0  0  0  0  0
   16.1774   14.4948    0.1634 H   0  0  0  0  0  0  0  0  0  0  0  0  0
   14.9329   12.7443    1.4085 H   0  0  0  0  0  0  0  0  0  0  0  0  0
   14.9743   11.6999   -0.0181 H   0  0  0  0  0  0  0  0  0  0  0  0  0
   13.7029   13.4030   -1.3016 H   0  0  0  0  0  0  0  0  0  0  0  0  0
   13.6609   14.4469    0.1253 H   0  0  0  0  0  0  0  0  0  0  0  0  0
   12.4164   12.6959    1.3698 H   0  0  0  0  0  0  0  0  0  0  0  0  0
   12.4589   11.6516   -0.0568 H   0  0  0  0  0  0  0  0  0  0  0  0  0
   11.1874   13.3543   -1.3408 H   0  0  0  0  0  0  0  0  0  0  0  0  0
   11.1444   14.3981    0.0861 H   0  0  0  0  0  0  0  0  0  0  0  0  0
    9.9001   12.6466    1.3300 H   0  0  0  0  0  0  0  0  0  0  0  0  0
    9.9435   11.6024   -0.0967 H   0  0  0  0  0  0  0  0  0  0  0  0  0
```

```
    8.6719   13.3047   -1.3811 H   0  0  0  0  0  0  0  0  0  0  0  0
    8.6281   14.3485    0.0459 H   0  0  0  0  0  0  0  0  0  0  0  0
    7.3839   12.5965    1.2893 H   0  0  0  0  0  0  0  0  0  0  0  0
    7.4280   11.5525   -0.1375 H   0  0  0  0  0  0  0  0  0  0  0  0
    6.1563   13.2545   -1.4221 H   0  0  0  0  0  0  0  0  0  0  0  0
    6.1120   14.2983    0.0048 H   0  0  0  0  0  0  0  0  0  0  0  0
    4.8678   12.5461    1.2481 H   0  0  0  0  0  0  0  0  0  0  0  0
    4.9123   11.5021   -0.1788 H   0  0  0  0  0  0  0  0  0  0  0  0
    3.6404   13.2040   -1.4635 H   0  0  0  0  0  0  0  0  0  0  0  0
    3.5958   14.2478   -0.0367 H   0  0  0  0  0  0  0  0  0  0  0  0
    2.3962   11.4517   -0.2205 H   0  0  0  0  0  0  0  0  0  0  0  0
    2.3517   12.4956    1.2064 H   0  0  0  0  0  0  0  0  0  0  0  0
    1.4822   12.9652   -0.2602 H   0  0  0  0  0  0  0  0  0  0  0  0
  1  2  1  0  0  0  0
  2  3  1  0  0  0  0
  3  4  1  0  0  0  0
  4  5  1  0  0  0  0
  1  5  1  0  0  0  0
  6  7  1  0  0  0  0
  7  8  1  0  0  0  0
  8  9  1  0  0  0  0
  9 10  1  0  0  0  0
 10 11  1  0  0  0  0
 11 12  1  0  0  0  0
 12 13  1  0  0  0  0
 13 14  1  0  0  0  0
 14 15  1  0  0  0  0
 15 16  1  0  0  0  0
  1  6  1  0  0  0  0
 16 17  1  0  0  0  0
 17 18  1  0  0  0  0
 18 19  1  0  0  0  0
 19 20  1  0  0  0  0
 20 21  1  0  0  0  0
  1 22  1  0  0  0  0
  2 23  1  0  0  0  0
  2 24  1  0  0  0  0
  3 25  1  0  0  0  0
  3 26  1  0  0  0  0
  4 27  1  0  0  0  0
  4 28  1  0  0  0  0
  5 29  1  0  0  0  0
  5 30  1  0  0  0  0
  6 31  1  0  0  0  0
  6 32  1  0  0  0  0
  7 33  1  0  0  0  0
  7 34  1  0  0  0  0
  8 35  1  0  0  0  0
  8 36  1  0  0  0  0
  9 37  1  0  0  0  0
  9 38  1  0  0  0  0
 10 39  1  0  0  0  0
 10 40  1  0  0  0  0
 11 41  1  0  0  0  0
 11 42  1  0  0  0  0
 12 43  1  0  0  0  0
 12 44  1  0  0  0  0
 13 45  1  0  0  0  0
 13 46  1  0  0  0  0
 14 47  1  0  0  0  0
 14 48  1  0  0  0  0
 15 49  1  0  0  0  0
 15 50  1  0  0  0  0
```

16 51 1 0 0 0 0
16 52 1 0 0 0 0
17 53 1 0 0 0 0
17 54 1 0 0 0 0
18 55 1 0 0 0 0
18 56 1 0 0 0 0
19 57 1 0 0 0 0
19 58 1 0 0 0 0
20 59 1 0 0 0 0
20 60 1 0 0 0 0
21 61 1 0 0 0 0
21 62 1 0 0 0 0
21 63 1 0 0 0 0
M END
> <Alias name>
Hexadecylcyclopentane

> <deltaH°(c)>   kJ/mol
-13755

$$$$
Pentadecylcyclohexane
  ChemBrain IXL

63 63 0 0 0 0 0 0 0 0 1 V2000
19.8243   12.8816   -1.1711 C   0 0 0 0 0 0 0 0 0 0 0 0
18.6376   13.5006   -0.4071 C   0 0 0 0 0 0 0 0 0 0 0 0
17.3300   12.8114   -0.8433 C   0 0 0 0 0 0 0 0 0 0 0 0
16.1434   13.4299   -0.0789 C   0 0 0 0 0 0 0 0 0 0 0 0
14.8359   12.7398   -0.5139 C   0 0 0 0 0 0 0 0 0 0 0 0
13.6495   13.3578   0.2511 C   0 0 0 0 0 0 0 0 0 0 0 0
12.3422   12.6664   -0.1826 C   0 0 0 0 0 0 0 0 0 0 0 0
11.1559   13.2837   0.5832 C   0 0 0 0 0 0 0 0 0 0 0 0
9.8488   12.5911   0.1508 C   0 0 0 0 0 0 0 0 0 0 0 0
21.1320   13.5702   -0.7342 C   0 0 0 0 0 0 0 0 0 0 0 0
8.6626   13.2079   0.9172 C   0 0 0 0 0 0 0 0 0 0 0 0
22.3188   12.9511   -1.4980 C   0 0 0 0 0 0 0 0 0 0 0 0
7.3559   12.5141   0.4861 C   0 0 0 0 0 0 0 0 0 0 0 0
23.6265   13.6393   -1.0610 C   0 0 0 0 0 0 0 0 0 0 0 0
6.1689   13.1317   1.2539 C   0 0 0 0 0 0 0 0 0 0 0 0
3.5939   10.2744   0.6970 C   0 0 0 0 0 0 0 0 0 0 0 0
4.9112   10.9458   1.1353 C   0 0 0 0 0 0 0 0 0 0 0 0
4.8538   12.4545   0.8105 C   0 0 0 0 0 0 0 0 0 0 0 0
3.6695   13.0968   1.5617 C   0 0 0 0 0 0 0 0 0 0 0 0
2.3543   12.4237   1.1217 C   0 0 0 0 0 0 0 0 0 0 0 0
2.4125   10.9185   1.4489 C   0 0 0 0 0 0 0 0 0 0 0 0
19.6808   13.0214   -2.2354 H   0 0 0 0 0 0 0 0 0 0 0 0
19.8828   11.8229   -0.9506 H   0 0 0 0 0 0 0 0 0 0 0 0
18.7814   13.3613   0.6572 H   0 0 0 0 0 0 0 0 0 0 0 0
18.5787   14.5592   -0.6279 H   0 0 0 0 0 0 0 0 0 0 0 0
17.1860   12.9509   -1.9075 H   0 0 0 0 0 0 0 0 0 0 0 0
17.3890   11.7527   -0.6227 H   0 0 0 0 0 0 0 0 0 0 0 0
16.2879   13.2912   0.9855 H   0 0 0 0 0 0 0 0 0 0 0 0
16.0838   14.4884   -0.3001 H   0 0 0 0 0 0 0 0 0 0 0 0
14.6911   12.8789   -1.5782 H   0 0 0 0 0 0 0 0 0 0 0 0
14.8958   11.6812   -0.2931 H   0 0 0 0 0 0 0 0 0 0 0 0
13.7949   13.2195   1.3154 H   0 0 0 0 0 0 0 0 0 0 0 0
13.5889   14.4161   0.0295 H   0 0 0 0 0 0 0 0 0 0 0 0
12.1964   12.8050   -1.2468 H   0 0 0 0 0 0 0 0 0 0 0 0
12.4031   11.6080   0.0385 H   0 0 0 0 0 0 0 0 0 0 0 0
11.3023   13.1461   1.6473 H   0 0 0 0 0 0 0 0 0 0 0 0
11.0942   14.3419   0.3611 H   0 0 0 0 0 0 0 0 0 0 0 0
9.7020   12.7291   -0.9133 H   0 0 0 0 0 0 0 0 0 0 0 0

```
  9.9107   11.5329    0.3724 H   0  0  0  0  0  0  0  0  0  0  0  0
  21.2754   13.4305    0.3301 H   0  0  0  0  0  0  0  0  0  0  0  0
  21.0736   14.6289   -0.9547 H   0  0  0  0  0  0  0  0  0  0  0  0
  8.8100   13.0708    1.9814 H   0  0  0  0  0  0  0  0  0  0  0  0
  8.6000   14.2659    0.6948 H   0  0  0  0  0  0  0  0  0  0  0  0
  22.1756   13.0909   -2.5623 H   0  0  0  0  0  0  0  0  0  0  0  0
  22.3771   11.8923   -1.2777 H   0  0  0  0  0  0  0  0  0  0  0  0
  7.2084   12.6516   -0.5779 H   0  0  0  0  0  0  0  0  0  0  0  0
  7.4195   11.4569    0.7081 H   0  0  0  0  0  0  0  0  0  0  0  0
  23.5684   14.6980   -1.2813 H   0  0  0  0  0  0  0  0  0  0  0  0
  24.4603   13.2041   -1.5978 H   0  0  0  0  0  0  0  0  0  0  0  0
  23.7699   13.4994    0.0033 H   0  0  0  0  0  0  0  0  0  0  0  0
  6.3105   12.9838    2.3174 H   0  0  0  0  0  0  0  0  0  0  0  0
  6.1164   14.1924    1.0415 H   0  0  0  0  0  0  0  0  0  0  0  0
  3.4586   10.4071   -0.3692 H   0  0  0  0  0  0  0  0  0  0  0  0
  3.6342    9.2169    0.9267 H   0  0  0  0  0  0  0  0  0  0  0  0
  5.0479   10.8109    2.2011 H   0  0  0  0  0  0  0  0  0  0  0  0
  5.7389   10.4932    0.6053 H   0  0  0  0  0  0  0  0  0  0  0  0
  4.7201   12.5894   -0.2557 H   0  0  0  0  0  0  0  0  0  0  0  0
  3.8037   12.9637    2.6282 H   0  0  0  0  0  0  0  0  0  0  0  0
  3.6278   14.1543    1.3326 H   0  0  0  0  0  0  0  0  0  0  0  0
  2.2200   12.5564    0.0553 H   0  0  0  0  0  0  0  0  0  0  0  0
  1.5236   12.8758    1.6491 H   0  0  0  0  0  0  0  0  0  0  0  0
  1.4881   10.4463    1.1407 H   0  0  0  0  0  0  0  0  0  0  0  0
  2.5476   10.7858    2.5152 H   0  0  0  0  0  0  0  0  0  0  0  0
  1  2  1  0  0  0  0
  2  3  1  0  0  0  0
  3  4  1  0  0  0  0
  4  5  1  0  0  0  0
  5  6  1  0  0  0  0
  6  7  1  0  0  0  0
  7  8  1  0  0  0  0
  8  9  1  0  0  0  0
  1 10  1  0  0  0  0
  9 11  1  0  0  0  0
 10 12  1  0  0  0  0
 11 13  1  0  0  0  0
 12 14  1  0  0  0  0
 13 15  1  0  0  0  0
 16 17  1  0  0  0  0
 17 18  1  0  0  0  0
 18 19  1  0  0  0  0
 19 20  1  0  0  0  0
 20 21  1  0  0  0  0
 21 16  1  0  0  0  0
 18 15  1  0  0  0  0
  1 22  1  0  0  0  0
  1 23  1  0  0  0  0
  2 24  1  0  0  0  0
  2 25  1  0  0  0  0
  3 26  1  0  0  0  0
  3 27  1  0  0  0  0
  4 28  1  0  0  0  0
  4 29  1  0  0  0  0
  5 30  1  0  0  0  0
  5 31  1  0  0  0  0
  6 32  1  0  0  0  0
  6 33  1  0  0  0  0
  7 34  1  0  0  0  0
  7 35  1  0  0  0  0
  8 36  1  0  0  0  0
  8 37  1  0  0  0  0
  9 38  1  0  0  0  0
```

```
  9 39  1  0  0  0  0
 10 40  1  0  0  0  0
 10 41  1  0  0  0  0
 11 42  1  0  0  0  0
 11 43  1  0  0  0  0
 12 44  1  0  0  0  0
 12 45  1  0  0  0  0
 13 46  1  0  0  0  0
 13 47  1  0  0  0  0
 14 48  1  0  0  0  0
 14 49  1  0  0  0  0
 14 50  1  0  0  0  0
 15 51  1  0  0  0  0
 15 52  1  0  0  0  0
 16 53  1  0  0  0  0
 16 54  1  0  0  0  0
 17 55  1  0  0  0  0
 17 56  1  0  0  0  0
 18 57  1  0  0  0  0
 19 58  1  0  0  0  0
 19 59  1  0  0  0  0
 20 60  1  0  0  0  0
 20 61  1  0  0  0  0
 21 62  1  0  0  0  0
 21 63  1  0  0  0  0
M  END
>  <Alias name>
Pentadecylcyclohexane

>  <deltaH°(c)>   kJ/mol
-13732

$$$$
Hexadecylbenzene
  ChemBrain IXL

 60 60  0  0  0  0  0  0  0  0  1 V2000
   19.9556   13.1566    0.0000 C   0  0  0  0  0  0  0  0  0  0  0  0
   18.7130   14.0680    0.0083 C   0  0  0  0  0  0  0  0  0  0  0  0
   17.4395   13.2003    0.0013 C   0  0  0  0  0  0  0  0  0  0  0  0
   16.1968   14.1116    0.0106 C   0  0  0  0  0  0  0  0  0  0  0  0
   14.9234   13.2438    0.0031 C   0  0  0  0  0  0  0  0  0  0  0  0
   13.6807   14.1550    0.0124 C   0  0  0  0  0  0  0  0  0  0  0  0
   12.4074   13.2871    0.0048 C   0  0  0  0  0  0  0  0  0  0  0  0
   11.1646   14.1981    0.0135 C   0  0  0  0  0  0  0  0  0  0  0  0
    9.8914   13.3301    0.0068 C   0  0  0  0  0  0  0  0  0  0  0  0
   21.2292   14.0242    0.0067 C   0  0  0  0  0  0  0  0  0  0  0  0
    8.6486   14.2411    0.0146 C   0  0  0  0  0  0  0  0  0  0  0  0
   22.4718   13.1128   -0.0005 C   0  0  0  0  0  0  0  0  0  0  0  0
    7.3754   13.3730    0.0103 C   0  0  0  0  0  0  0  0  0  0  0  0
   23.7453   13.9804    0.0060 C   0  0  0  0  0  0  0  0  0  0  0  0
    6.1326   14.2839    0.0193 C   0  0  0  0  0  0  0  0  0  0  0  0
   24.9879   13.0690   -0.0003 C   0  0  0  0  0  0  0  0  0  0  0  0
    2.5005   13.2693    0.0075 C   0  0  0  0  0  0  0  0  0  0  0  0
    3.6530   14.0553    0.0115 C   0  0  0  0  0  0  0  0  0  0  0  0
    4.9099   13.4501    0.0169 C   0  0  0  0  0  0  0  0  0  0  0  0
    5.0143   12.0591    0.0200 C   0  0  0  0  0  0  0  0  0  0  0  0
    3.8618   11.2731    0.0160 C   0  0  0  0  0  0  0  0  0  0  0  0
    2.6049   11.8782    0.0096 C   0  0  0  0  0  0  0  0  0  0  0  0
   19.9443   12.5389   -0.8895 H   0  0  0  0  0  0  0  0  0  0  0  0
   19.9452   12.5240    0.8790 H   0  0  0  0  0  0  0  0  0  0  0  0
   18.7242   14.6855    0.8980 H   0  0  0  0  0  0  0  0  0  0  0  0
   18.7235   14.7008   -0.8705 H   0  0  0  0  0  0  0  0  0  0  0  0
```

17.4280 12.5832 -0.8886 H 0 0 0 0 0 0 0 0 0 0 0 0
17.4293 12.5671 0.8798 H 0 0 0 0 0 0 0 0 0 0 0 0
16.2081 14.7285 0.9007 H 0 0 0 0 0 0 0 0 0 0 0 0
16.2072 14.7451 -0.8678 H 0 0 0 0 0 0 0 0 0 0 0 0
14.9121 12.6270 -0.8871 H 0 0 0 0 0 0 0 0 0 0 0 0
14.9132 12.6103 0.8814 H 0 0 0 0 0 0 0 0 0 0 0 0
13.6919 14.7717 0.9026 H 0 0 0 0 0 0 0 0 0 0 0 0
13.6910 14.7886 -0.8658 H 0 0 0 0 0 0 0 0 0 0 0 0
12.3964 12.6700 -0.8851 H 0 0 0 0 0 0 0 0 0 0 0 0
12.3969 12.6538 0.8834 H 0 0 0 0 0 0 0 0 0 0 0 0
11.1759 14.8157 0.9031 H 0 0 0 0 0 0 0 0 0 0 0 0
11.1748 14.8309 -0.8654 H 0 0 0 0 0 0 0 0 0 0 0 0
9.8804 12.7121 -0.8825 H 0 0 0 0 0 0 0 0 0 0 0 0
9.8810 12.6977 0.8859 H 0 0 0 0 0 0 0 0 0 0 0 0
21.2401 14.6425 0.8958 H 0 0 0 0 0 0 0 0 0 0 0 0
21.2400 14.6563 -0.8727 H 0 0 0 0 0 0 0 0 0 0 0 0
8.6604 14.8603 0.9030 H 0 0 0 0 0 0 0 0 0 0 0 0
8.6581 14.8722 -0.8655 H 0 0 0 0 0 0 0 0 0 0 0 0
22.4608 12.4944 -0.8896 H 0 0 0 0 0 0 0 0 0 0 0 0
22.4610 12.4808 0.8789 H 0 0 0 0 0 0 0 0 0 0 0 0
7.3632 12.7544 -0.8786 H 0 0 0 0 0 0 0 0 0 0 0 0
7.3663 12.7412 0.8899 H 0 0 0 0 0 0 0 0 0 0 0 0
23.7560 14.5992 0.8948 H 0 0 0 0 0 0 0 0 0 0 0 0
23.7563 14.6120 -0.8737 H 0 0 0 0 0 0 0 0 0 0 0 0
6.1410 14.9149 -0.8609 H 0 0 0 0 0 0 0 0 0 0 0 0
6.1454 14.9033 0.9076 H 0 0 0 0 0 0 0 0 0 0 0 0
25.8829 13.6788 0.0039 H 0 0 0 0 0 0 0 0 0 0 0 0
24.9770 12.4499 -0.8889 H 0 0 0 0 0 0 0 0 0 0 0 0
24.9770 12.4377 0.8796 H 0 0 0 0 0 0 0 0 0 0 0 0
1.5247 13.7391 0.0027 H 0 0 0 0 0 0 0 0 0 0 0 0
3.5719 15.1352 0.0104 H 0 0 0 0 0 0 0 0 0 0 0 0
5.9900 11.5893 0.0256 H 0 0 0 0 0 0 0 0 0 0 0 0
3.9428 10.1931 0.0178 H 0 0 0 0 0 0 0 0 0 0 0 0
1.7101 11.2681 0.0064 H 0 0 0 0 0 0 0 0 0 0 0 0
1 2 1 0 0 0 0
2 3 1 0 0 0 0
3 4 1 0 0 0 0
4 5 1 0 0 0 0
5 6 1 0 0 0 0
6 7 1 0 0 0 0
7 8 1 0 0 0 0
8 9 1 0 0 0 0
1 10 1 0 0 0 0
9 11 1 0 0 0 0
10 12 1 0 0 0 0
11 13 1 0 0 0 0
12 14 1 0 0 0 0
13 15 1 0 0 0 0
14 16 1 0 0 0 0
17 18 4 0 0 0 0
18 19 4 0 0 0 0
19 20 4 0 0 0 0
20 21 4 0 0 0 0
21 22 4 0 0 0 0
22 17 4 0 0 0 0
19 15 1 0 0 0 0
1 23 1 0 0 0 0
1 24 1 0 0 0 0
2 25 1 0 0 0 0
2 26 1 0 0 0 0
3 27 1 0 0 0 0
3 28 1 0 0 0 0
4 29 1 0 0 0 0

```
  4 30  1  0  0  0  0
  5 31  1  0  0  0  0
  5 32  1  0  0  0  0
  6 33  1  0  0  0  0
  6 34  1  0  0  0  0
  7 35  1  0  0  0  0
  7 36  1  0  0  0  0
  8 37  1  0  0  0  0
  8 38  1  0  0  0  0
  9 39  1  0  0  0  0
  9 40  1  0  0  0  0
 10 41  1  0  0  0  0
 10 42  1  0  0  0  0
 11 43  1  0  0  0  0
 11 44  1  0  0  0  0
 12 45  1  0  0  0  0
 12 46  1  0  0  0  0
 13 47  1  0  0  0  0
 13 48  1  0  0  0  0
 14 49  1  0  0  0  0
 14 50  1  0  0  0  0
 15 51  1  0  0  0  0
 15 52  1  0  0  0  0
 16 53  1  0  0  0  0
 16 54  1  0  0  0  0
 16 55  1  0  0  0  0
 17 56  1  0  0  0  0
 18 57  1  0  0  0  0
 20 58  1  0  0  0  0
 21 59  1  0  0  0  0
 22 60  1  0  0  0  0
M  END
>  <Alias name>
Hexadecylbenzene

>  <deltaH°(c)>   kJ/mol
-13682.73

$$$$
1,1'-Diphenyl-1,1'-bicyclohexyl
  ChemBrain IXL

 54 57  0  0  0  0  0  0  0  0  1 V2000
   11.1017    6.6493    1.0140 C   0  0  0  0  0  0  0  0  0  0  0  0
   11.6973    6.6596   -0.2474 C   0  0  0  0  0  0  0  0  0  0  0  0
    9.7256    6.8418    1.1372 C   0  0  0  0  0  0  0  0  0  0  0  0
   10.9168    6.8629   -1.3856 C   0  0  0  0  0  0  0  0  0  0  0  0
    9.5406    7.0554   -1.2624 C   0  0  0  0  0  0  0  0  0  0  0  0
    8.9446    7.0418   -0.0012 C   0  0  0  0  0  0  0  0  0  0  0  0
    7.4837    7.2396    0.1290 C   0  0  0  0  0  0  0  0  0  0  0  0
    4.6654    6.0557    1.2539 C   0  0  0  0  0  0  0  0  0  0  0  0
    5.2996    6.0988    0.0121 C   0  0  0  0  0  0  0  0  0  0  0  0
    3.2872    6.2536    1.3405 C   0  0  0  0  0  0  0  0  0  0  0  0
    4.5549    6.3351   -1.1436 C   0  0  0  0  0  0  0  0  0  0  0  0
    6.7625    5.8938   -0.0792 C   0  0  0  0  0  0  0  0  0  0  0  0
    3.1768    6.5333   -1.0570 C   0  0  0  0  0  0  0  0  0  0  0  0
    2.5429    6.4928    0.1851 C   0  0  0  0  0  0  0  0  0  0  0  0
    5.9169    8.6877    1.4635 C   0  0  0  0  0  0  0  0  0  0  0  0
    6.2722    9.9930    0.7257 C   0  0  0  0  0  0  0  0  0  0  0  0
    7.1621    7.7826    1.5348 C   0  0  0  0  0  0  0  0  0  0  0  0
    7.2486    9.6829   -0.4255 C   0  0  0  0  0  0  0  0  0  0  0  0
    7.0052    8.2484   -0.9331 C   0  0  0  0  0  0  0  0  0  0  0  0
    7.1165    5.3309   -1.4693 C   0  0  0  0  0  0  0  0  0  0  0  0
```

```
    7.2104    4.8975    1.0078 C   0  0  0  0  0  0  0  0  0  0  0  0
    8.3528    4.4182   -1.3542 C   0  0  0  0  0  0  0  0  0  0  0  0
    7.9690    3.1250   -0.6093 C   0  0  0  0  0  0  0  0  0  0  0  0
    6.9693    3.4573    0.5153 C   0  0  0  0  0  0  0  0  0  0  0  0
   11.7077    6.4919    1.8977 H   0  0  0  0  0  0  0  0  0  0  0  0
   12.7656    6.5097   -0.3431 H   0  0  0  0  0  0  0  0  0  0  0  0
    9.2636    6.8360    2.1167 H   0  0  0  0  0  0  0  0  0  0  0  0
   11.3792    6.8714   -2.3649 H   0  0  0  0  0  0  0  0  0  0  0  0
    8.9350    7.2153   -2.1459 H   0  0  0  0  0  0  0  0  0  0  0  0
    5.2429    5.8685    2.1507 H   0  0  0  0  0  0  0  0  0  0  0  0
    2.7950    6.2217    2.3047 H   0  0  0  0  0  0  0  0  0  0  0  0
    5.0467    6.3648   -2.1080 H   0  0  0  0  0  0  0  0  0  0  0  0
    2.5989    6.7186   -1.9540 H   0  0  0  0  0  0  0  0  0  0  0  0
    1.4731    6.6469    0.2523 H   0  0  0  0  0  0  0  0  0  0  0  0
    5.1275    8.1741    0.9288 H   0  0  0  0  0  0  0  0  0  0  0  0
    5.5815    8.9199    2.4668 H   0  0  0  0  0  0  0  0  0  0  0  0
    5.3701   10.4377    0.3240 H   0  0  0  0  0  0  0  0  0  0  0  0
    6.7379   10.6837    1.4178 H   0  0  0  0  0  0  0  0  0  0  0  0
    6.9689    6.9550    2.2060 H   0  0  0  0  0  0  0  0  0  0  0  0
    8.0045    8.3559    1.9018 H   0  0  0  0  0  0  0  0  0  0  0  0
    8.2673    9.7716   -0.0687 H   0  0  0  0  0  0  0  0  0  0  0  0
    7.0871   10.3844   -1.2347 H   0  0  0  0  0  0  0  0  0  0  0  0
    7.5556    8.0927   -1.8528 H   0  0  0  0  0  0  0  0  0  0  0  0
    5.9476    8.1050   -1.1167 H   0  0  0  0  0  0  0  0  0  0  0  0
    7.3332    6.1487   -2.1454 H   0  0  0  0  0  0  0  0  0  0  0  0
    6.2798    4.7589   -1.8510 H   0  0  0  0  0  0  0  0  0  0  0  0
    6.6404    5.0698    1.9124 H   0  0  0  0  0  0  0  0  0  0  0  0
    8.2645    5.0371    1.2132 H   0  0  0  0  0  0  0  0  0  0  0  0
    9.1319    4.9331   -0.8058 H   0  0  0  0  0  0  0  0  0  0  0  0
    8.7123    4.1707   -2.3454 H   0  0  0  0  0  0  0  0  0  0  0  0
    8.8577    2.6774   -0.1817 H   0  0  0  0  0  0  0  0  0  0  0  0
    7.5133    2.4300   -1.3038 H   0  0  0  0  0  0  0  0  0  0  0  0
    5.9583    3.3696    0.1371 H   0  0  0  0  0  0  0  0  0  0  0  0
    7.1083    2.7665    1.3377 H   0  0  0  0  0  0  0  0  0  0  0  0
  2  1  4  0  0  0  0
  1  3  4  0  0  0  0
  4  2  4  0  0  0  0
  3  6  4  0  0  0  0
  5  4  4  0  0  0  0
  6  5  4  0  0  0  0
  6  7  1  0  0  0  0
 19  7  1  0  0  0  0
  7 17  1  0  0  0  0
  7 12  1  0  0  0  0
  9  8  4  0  0  0  0
  8 10  4  0  0  0  0
 11  9  4  0  0  0  0
  9 12  1  0  0  0  0
 10 14  4  0  0  0  0
 13 11  4  0  0  0  0
 12 20  1  0  0  0  0
 21 12  1  0  0  0  0
 14 13  4  0  0  0  0
 15 16  1  0  0  0  0
 17 15  1  0  0  0  0
 16 18  1  0  0  0  0
 18 19  1  0  0  0  0
 20 22  1  0  0  0  0
 24 21  1  0  0  0  0
 22 23  1  0  0  0  0
 23 24  1  0  0  0  0
  1 25  1  0  0  0  0
  2 26  1  0  0  0  0
```

```
  3 27  1  0  0  0  0
  4 28  1  0  0  0  0
  5 29  1  0  0  0  0
  8 30  1  0  0  0  0
 10 31  1  0  0  0  0
 11 32  1  0  0  0  0
 13 33  1  0  0  0  0
 14 34  1  0  0  0  0
 15 35  1  0  0  0  0
 15 36  1  0  0  0  0
 16 37  1  0  0  0  0
 16 38  1  0  0  0  0
 17 39  1  0  0  0  0
 17 40  1  0  0  0  0
 18 41  1  0  0  0  0
 18 42  1  0  0  0  0
 19 43  1  0  0  0  0
 19 44  1  0  0  0  0
 20 45  1  0  0  0  0
 20 46  1  0  0  0  0
 21 47  1  0  0  0  0
 21 48  1  0  0  0  0
 22 49  1  0  0  0  0
 22 50  1  0  0  0  0
 23 51  1  0  0  0  0
 23 52  1  0  0  0  0
 24 53  1  0  0  0  0
 24 54  1  0  0  0  0
M  END
>  <Alias name>
1,1'-Diphenyl-1,1'-bicyclohexyl

>  <deltaH°(c)>   kJ/mol
-13624

$$$$
1,1,1,2-Tetraphenylethane
  ChemBrain IXL

 48 51  0  0  0  0  0  0  0  0  1 V2000
    8.5706    3.7124   -0.2750 C   0  0  0  0  0  0  0  0  0  0  0  0  0
    8.1616    3.2154    0.9627 C   0  0  0  0  0  0  0  0  0  0  0  0  0
    7.3242    3.9770    1.7779 C   0  0  0  0  0  0  0  0  0  0  0  0  0
    6.8939    5.2345    1.3543 C   0  0  0  0  0  0  0  0  0  0  0  0  0
    7.2911    5.7254    0.1104 C   0  0  0  0  0  0  0  0  0  0  0  0  0
    8.1402    4.9700   -0.6986 C   0  0  0  0  0  0  0  0  0  0  0  0  0
    2.5477    7.0317   -0.5728 C   0  0  0  0  0  0  0  0  0  0  0  0  0
    3.2273    8.2196   -0.3028 C   0  0  0  0  0  0  0  0  0  0  0  0  0
    4.6199    8.2234   -0.2210 C   0  0  0  0  0  0  0  0  0  0  0  0  0
    5.3333    7.0406   -0.4162 C   0  0  0  0  0  0  0  0  0  0  0  0  0
    4.6532    5.8510   -0.6775 C   0  0  0  0  0  0  0  0  0  0  0  0  0
    3.2606    5.8472   -0.7593 C   0  0  0  0  0  0  0  0  0  0  0  0  0
    6.8119    7.0484   -0.3485 C   0  0  0  0  0  0  0  0  0  0  0  0  0
    8.0589   10.0672    2.4018 C   0  0  0  0  0  0  0  0  0  0  0  0  0
    7.1539    9.0856    2.8059 C   0  0  0  0  0  0  0  0  0  0  0  0  0
    6.7578    8.0914    1.9112 C   0  0  0  0  0  0  0  0  0  0  0  0  0
    7.2562    8.0866    0.6083 C   0  0  0  0  0  0  0  0  0  0  0  0  0
    8.1737    9.0585    0.2090 C   0  0  0  0  0  0  0  0  0  0  0  0  0
    8.5698   10.0529    1.1037 C   0  0  0  0  0  0  0  0  0  0  0  0  0
   11.6497    7.2598   -1.6435 C   0  0  0  0  0  0  0  0  0  0  0  0  0
   10.9178    7.0415   -0.4762 C   0  0  0  0  0  0  0  0  0  0  0  0  0
    9.5234    7.0690   -0.5115 C   0  0  0  0  0  0  0  0  0  0  0  0  0
    8.8612    7.3174   -1.7139 C   0  0  0  0  0  0  0  0  0  0  0  0  0
```

```
  9.5931    7.5325   -2.8818 C   0  0  0  0  0  0  0  0  0  0  0  0
 10.9873    7.5050   -2.8465 C   0  0  0  0  0  0  0  0  0  0  0  0
  7.3823    7.3534   -1.7528 C   0  0  0  0  0  0  0  0  0  0  0  0
  9.2223    3.1219   -0.9070 H   0  0  0  0  0  0  0  0  0  0  0  0
  8.4942    2.2383    1.2907 H   0  0  0  0  0  0  0  0  0  0  0  0
  7.0080    3.5921    2.7396 H   0  0  0  0  0  0  0  0  0  0  0  0
  6.2514    5.8299    1.9913 H   0  0  0  0  0  0  0  0  0  0  0  0
  8.4652    5.3598   -1.6553 H   0  0  0  0  0  0  0  0  0  0  0  0
  1.4666    7.0289   -0.6376 H   0  0  0  0  0  0  0  0  0  0  0  0
  2.6737    9.1390   -0.1568 H   0  0  0  0  0  0  0  0  0  0  0  0
  5.1472    9.1446   -0.0059 H   0  0  0  0  0  0  0  0  0  0  0  0
  5.2063    4.9304   -0.8168 H   0  0  0  0  0  0  0  0  0  0  0  0
  2.7329    4.9246   -0.9676 H   0  0  0  0  0  0  0  0  0  0  0  0
  8.3649   10.8404    3.0957 H   0  0  0  0  0  0  0  0  0  0  0  0
  6.7589    9.0954    3.8143 H   0  0  0  0  0  0  0  0  0  0  0  0
  6.0632    7.3233    2.2281 H   0  0  0  0  0  0  0  0  0  0  0  0
  8.5785    9.0411   -0.7953 H   0  0  0  0  0  0  0  0  0  0  0  0
  9.2740   10.8138    0.7906 H   0  0  0  0  0  0  0  0  0  0  0  0
 12.7321    7.2389   -1.6160 H   0  0  0  0  0  0  0  0  0  0  0  0
 11.4321    6.8506    0.4576 H   0  0  0  0  0  0  0  0  0  0  0  0
  8.9552    6.8975    0.3943 H   0  0  0  0  0  0  0  0  0  0  0  0
  9.0788    7.7209   -3.8161 H   0  0  0  0  0  0  0  0  0  0  0  0
 11.5554    7.6741   -3.7529 H   0  0  0  0  0  0  0  0  0  0  0  0
  7.0229    6.6119   -2.4556 H   0  0  0  0  0  0  0  0  0  0  0  0
  7.0547    8.3363   -2.0682 H   0  0  0  0  0  0  0  0  0  0  0  0
  1  2  4  0  0  0  0
  2  3  4  0  0  0  0
  3  4  4  0  0  0  0
  4  5  4  0  0  0  0
  5  6  4  0  0  0  0
  6  1  4  0  0  0  0
  7  8  4  0  0  0  0
  8  9  4  0  0  0  0
  9 10  4  0  0  0  0
 10 11  4  0  0  0  0
 11 12  4  0  0  0  0
 12  7  4  0  0  0  0
 10 13  1  0  0  0  0
 13  5  1  0  0  0  0
 14 15  4  0  0  0  0
 15 16  4  0  0  0  0
 16 17  4  0  0  0  0
 17 18  4  0  0  0  0
 18 19  4  0  0  0  0
 19 14  4  0  0  0  0
 20 21  4  0  0  0  0
 21 22  4  0  0  0  0
 22 23  4  0  0  0  0
 23 24  4  0  0  0  0
 24 25  4  0  0  0  0
 25 20  4  0  0  0  0
 23 26  1  0  0  0  0
 26 13  1  0  0  0  0
 17 13  1  0  0  0  0
  1 27  1  0  0  0  0
  2 28  1  0  0  0  0
  3 29  1  0  0  0  0
  4 30  1  0  0  0  0
  6 31  1  0  0  0  0
  7 32  1  0  0  0  0
  8 33  1  0  0  0  0
  9 34  1  0  0  0  0
 11 35  1  0  0  0  0
```

```
 12 36  1  0  0  0  0
 14 37  1  0  0  0  0
 15 38  1  0  0  0  0
 16 39  1  0  0  0  0
 18 40  1  0  0  0  0
 19 41  1  0  0  0  0
 20 42  1  0  0  0  0
 21 43  1  0  0  0  0
 22 44  1  0  0  0  0
 24 45  1  0  0  0  0
 25 46  1  0  0  0  0
 26 47  1  0  0  0  0
 26 48  1  0  0  0  0
M  END
>  <Alias name>
1,1,1,2-Tetraphenylethane

>  <deltaH°(c)>   kJ/mol
-13606

$$$$
1,1,2,2-Tetraphenylethane
  ChemBrain IXL

 48 51  0  0  0  0  0  0  0  0  1 V2000
    7.5484    9.4906    1.6333 C   0  0  0  0  0  0  0  0  0  0  0  0
    6.4214    9.4049    2.4510 C   0  0  0  0  0  0  0  0  0  0  0  0
    5.3162    8.6597    2.0396 C   0  0  0  0  0  0  0  0  0  0  0  0
    5.3380    8.0003    0.8104 C   0  0  0  0  0  0  0  0  0  0  0  0
    6.4644    8.0873   -0.0082 C   0  0  0  0  0  0  0  0  0  0  0  0
    7.5701    8.8315    0.4039 C   0  0  0  0  0  0  0  0  0  0  0  0
    2.4892    7.1030   -2.8011 C   0  0  0  0  0  0  0  0  0  0  0  0
    3.3588    6.0190   -2.9226 C   0  0  0  0  0  0  0  0  0  0  0  0
    4.6614    6.1111   -2.4317 C   0  0  0  0  0  0  0  0  0  0  0  0
    5.0961    7.2896   -1.8251 C   0  0  0  0  0  0  0  0  0  0  0  0
    4.2238    8.3707   -1.6962 C   0  0  0  0  0  0  0  0  0  0  0  0
    2.9215    8.2787   -2.1872 C   0  0  0  0  0  0  0  0  0  0  0  0
    6.4821    7.3958   -1.3170 C   0  0  0  0  0  0  0  0  0  0  0  0
    4.9214    3.3673    0.4835 C   0  0  0  0  0  0  0  0  0  0  0  0
    4.9838    3.7635    1.8196 C   0  0  0  0  0  0  0  0  0  0  0  0
    5.7376    4.8802    2.1815 C   0  0  0  0  0  0  0  0  0  0  0  0
    6.4286    5.6010    1.2072 C   0  0  0  0  0  0  0  0  0  0  0  0
    6.3638    5.2065   -0.1294 C   0  0  0  0  0  0  0  0  0  0  0  0
    5.6122    4.0882   -0.4908 C   0  0  0  0  0  0  0  0  0  0  0  0
   11.2003    6.2724   -0.0187 C   0  0  0  0  0  0  0  0  0  0  0  0
   10.6878    7.1169   -1.0038 C   0  0  0  0  0  0  0  0  0  0  0  0
    9.3472    7.0199   -1.3775 C   0  0  0  0  0  0  0  0  0  0  0  0
    8.5196    6.0777   -0.7666 C   0  0  0  0  0  0  0  0  0  0  0  0
    9.0320    5.2339    0.2191 C   0  0  0  0  0  0  0  0  0  0  0  0
   10.3724    5.3310    0.5928 C   0  0  0  0  0  0  0  0  0  0  0  0
    7.0981    5.9696   -1.1637 C   0  0  0  0  0  0  0  0  0  0  0  0
    8.4064   10.0689    1.9530 H   0  0  0  0  0  0  0  0  0  0  0  0
    6.4043    9.9168    3.4053 H   0  0  0  0  0  0  0  0  0  0  0  0
    4.4412    8.5929    2.6744 H   0  0  0  0  0  0  0  0  0  0  0  0
    4.4804    7.4209    0.4915 H   0  0  0  0  0  0  0  0  0  0  0  0
    8.4452    8.8976   -0.2305 H   0  0  0  0  0  0  0  0  0  0  0  0
    1.4783    7.0319   -3.1833 H   0  0  0  0  0  0  0  0  0  0  0  0
    3.0227    5.1060   -3.3982 H   0  0  0  0  0  0  0  0  0  0  0  0
    5.3349    5.2678   -2.5214 H   0  0  0  0  0  0  0  0  0  0  0  0
    4.5579    9.2815   -1.2147 H   0  0  0  0  0  0  0  0  0  0  0  0
    2.2461    9.1199   -2.0919 H   0  0  0  0  0  0  0  0  0  0  0  0
    7.0770    7.9695   -2.0169 H   0  0  0  0  0  0  0  0  0  0  0  0
    4.3367    2.5000    0.2026 H   0  0  0  0  0  0  0  0  0  0  0  0
```

```
    4.4470    3.2042    2.5759 H   0  0  0  0  0  0  0  0  0  0  0  0
    5.7863    5.1875    3.2188 H   0  0  0  0  0  0  0  0  0  0  0  0
    7.0154    6.4666    1.4884 H   0  0  0  0  0  0  0  0  0  0  0  0
    5.5650    3.7795   -1.5278 H   0  0  0  0  0  0  0  0  0  0  0  0
   12.2410    6.3478    0.2713 H   0  0  0  0  0  0  0  0  0  0  0  0
   11.3306    7.8477   -1.4785 H   0  0  0  0  0  0  0  0  0  0  0  0
    8.9491    7.6758   -2.1417 H   0  0  0  0  0  0  0  0  0  0  0  0
    8.3890    4.5033    0.6943 H   0  0  0  0  0  0  0  0  0  0  0  0
   10.7702    4.6754    1.3576 H   0  0  0  0  0  0  0  0  0  0  0  0
    7.0299    5.4469   -2.1097 H   0  0  0  0  0  0  0  0  0  0  0  0
  1  2  4  0  0  0  0
  2  3  4  0  0  0  0
  3  4  4  0  0  0  0
  4  5  4  0  0  0  0
  5  6  4  0  0  0  0
  6  1  4  0  0  0  0
  7  8  4  0  0  0  0
  8  9  4  0  0  0  0
  9 10  4  0  0  0  0
 10 11  4  0  0  0  0
 11 12  4  0  0  0  0
 12  7  4  0  0  0  0
 10 13  1  0  0  0  0
 13  5  1  0  0  0  0
 14 15  4  0  0  0  0
 15 16  4  0  0  0  0
 16 17  4  0  0  0  0
 17 18  4  0  0  0  0
 18 19  4  0  0  0  0
 19 14  4  0  0  0  0
 20 21  4  0  0  0  0
 21 22  4  0  0  0  0
 22 23  4  0  0  0  0
 23 24  4  0  0  0  0
 24 25  4  0  0  0  0
 25 20  4  0  0  0  0
 23 26  1  0  0  0  0
 26 18  1  0  0  0  0
 26 13  1  0  0  0  0
  1 27  1  0  0  0  0
  2 28  1  0  0  0  0
  3 29  1  0  0  0  0
  4 30  1  0  0  0  0
  6 31  1  0  0  0  0
  7 32  1  0  0  0  0
  8 33  1  0  0  0  0
  9 34  1  0  0  0  0
 11 35  1  0  0  0  0
 12 36  1  0  0  0  0
 13 37  1  0  0  0  0
 14 38  1  0  0  0  0
 15 39  1  0  0  0  0
 16 40  1  0  0  0  0
 17 41  1  0  0  0  0
 19 42  1  0  0  0  0
 20 43  1  0  0  0  0
 21 44  1  0  0  0  0
 22 45  1  0  0  0  0
 24 46  1  0  0  0  0
 25 47  1  0  0  0  0
 26 48  1  0  0  0  0
M  END
>  <Alias name>
```

```
1,1,2,2-Tetraphenylethane

>  <deltaH°(c)>   kJ/mol
-13600.32

$$$$
13,14-Dihydroxybehenic acid
  ChemBrain IXL

 70 69  0  0  0  0  0  0  0  0  1 V2000
   18.0624   14.2181    0.1466 C   0  0  0  0  0  0  0  0  0  0  0  0
   16.6594   14.1570   -0.4876 C   0  0  0  0  0  0  0  0  0  0  0  0
   18.4655   12.8158    0.6424 C   0  0  0  0  0  0  0  0  0  0  0  0
   16.2571   15.5616   -0.9775 C   0  0  0  0  0  0  0  0  0  0  0  0
   19.8677   12.8812    1.2783 C   0  0  0  0  0  0  0  0  0  0  0  0
   14.8531   15.5041   -1.6092 C   0  0  0  0  0  0  0  0  0  0  0  0
   20.2730   11.4812    1.7790 C   0  0  0  0  0  0  0  0  0  0  0  0
   14.4504   16.9134   -2.0931 C   0  0  0  0  0  0  0  0  0  0  0  0
   21.6749   11.5514    2.4155 C   0  0  0  0  0  0  0  0  0  0  0  0
   13.0442   16.8660   -2.7331 C   0  0  0  0  0  0  0  0  0  0  0  0
   22.0831   10.1535    2.9194 C   0  0  0  0  0  0  0  0  0  0  0  0
   12.0155   16.3775   -1.6916 C   0  0  0  0  0  0  0  0  0  0  0  0
   23.4859   10.2275    3.5563 C   0  0  0  0  0  0  0  0  0  0  0  0
   10.6100   16.3663   -2.3241 C   0  0  0  0  0  0  0  0  0  0  0  0
   23.8822    8.8857    4.0429 C   0  0  0  0  0  0  0  0  0  0  0  0
    9.5841   15.8680   -1.2878 C   0  0  0  0  0  0  0  0  0  0  0  0
   22.9666    7.7451    3.8869 O   0  0  0  0  0  0  0  0  0  0  0  0
   24.9691    8.7264    4.5736 O   0  0  0  0  0  0  0  0  0  0  0  0
    8.1777   15.8656   -1.9172 C   0  0  0  0  0  0  0  0  0  0  0  0
    7.1534   15.3591   -0.8834 C   0  0  0  0  0  0  0  0  0  0  0  0
    5.7460   15.3620   -1.5108 C   0  0  0  0  0  0  0  0  0  0  0  0
    4.7227   14.8487   -0.4793 C   0  0  0  0  0  0  0  0  0  0  0  0
    3.3147   14.8545   -1.1056 C   0  0  0  0  0  0  0  0  0  0  0  0
    2.2920   14.3374   -0.0754 C   0  0  0  0  0  0  0  0  0  0  0  0
   13.0554   15.9457   -3.8810 O   0  0  0  0  0  0  0  0  0  0  0  0
   12.0237   17.2824   -0.5324 O   0  0  0  0  0  0  0  0  0  0  0  0
   23.3044    6.8904    4.2356 H   0  0  0  0  0  0  0  0  0  0  0  0
   13.2829   15.0143   -3.6656 H   0  0  0  0  0  0  0  0  0  0  0  0
   11.7983   18.2178   -0.7338 H   0  0  0  0  0  0  0  0  0  0  0  0
   18.7776   14.5593   -0.5916 H   0  0  0  0  0  0  0  0  0  0  0  0
   18.0505   14.9065    0.9826 H   0  0  0  0  0  0  0  0  0  0  0  0
   15.9445   13.8132    0.2497 H   0  0  0  0  0  0  0  0  0  0  0  0
   16.6715   13.4716   -1.3261 H   0  0  0  0  0  0  0  0  0  0  0  0
   17.7500   12.4740    1.3801 H   0  0  0  0  0  0  0  0  0  0  0  0
   18.4796   12.1272   -0.1934 H   0  0  0  0  0  0  0  0  0  0  0  0
   16.9713   15.9047   -1.7158 H   0  0  0  0  0  0  0  0  0  0  0  0
   16.2475   16.2470   -0.1390 H   0  0  0  0  0  0  0  0  0  0  0  0
   20.5831   13.2214    0.5399 H   0  0  0  0  0  0  0  0  0  0  0  0
   19.8536   13.5722    2.1121 H   0  0  0  0  0  0  0  0  0  0  0  0
   14.1407   15.1575   -0.8719 H   0  0  0  0  0  0  0  0  0  0  0  0
   14.8636   14.8222   -2.4499 H   0  0  0  0  0  0  0  0  0  0  0  0
   19.5576   11.1406    2.5173 H   0  0  0  0  0  0  0  0  0  0  0  0
   20.2887   10.7900    0.9454 H   0  0  0  0  0  0  0  0  0  0  0  0
   15.1677   17.2604   -2.8265 H   0  0  0  0  0  0  0  0  0  0  0  0
   14.4396   17.5937   -1.2505 H   0  0  0  0  0  0  0  0  0  0  0  0
   22.3899   11.8915    1.6766 H   0  0  0  0  0  0  0  0  0  0  0  0
   21.6588   12.2442    3.2477 H   0  0  0  0  0  0  0  0  0  0  0  0
   12.7704   17.8579   -3.0704 H   0  0  0  0  0  0  0  0  0  0  0  0
   21.3683    9.8131    3.6585 H   0  0  0  0  0  0  0  0  0  0  0  0
   22.1001    9.4603    2.0874 H   0  0  0  0  0  0  0  0  0  0  0  0
   12.2737   15.3769   -1.3702 H   0  0  0  0  0  0  0  0  0  0  0  0
   24.2000   10.5682    2.8169 H   0  0  0  0  0  0  0  0  0  0  0  0
   23.4684   10.9216    4.3874 H   0  0  0  0  0  0  0  0  0  0  0  0
```

```
 10.3478   17.3689   -2.6387 H   0  0  0  0  0  0  0  0  0  0  0  0
 10.6056   15.7059   -3.1825 H   0  0  0  0  0  0  0  0  0  0  0  0
  9.8429   14.8630   -0.9782 H   0  0  0  0  0  0  0  0  0  0  0  0
  9.5929   16.5246   -0.4266 H   0  0  0  0  0  0  0  0  0  0  0  0
  7.9166   16.8717   -2.2213 H   0  0  0  0  0  0  0  0  0  0  0  0
  8.1703   15.2136   -2.7819 H   0  0  0  0  0  0  0  0  0  0  0  0
  7.4124   14.3515   -0.5825 H   0  0  0  0  0  0  0  0  0  0  0  0
  7.1636   16.0086   -0.0169 H   0  0  0  0  0  0  0  0  0  0  0  0
  5.4850   16.3705   -1.8072 H   0  0  0  0  0  0  0  0  0  0  0  0
  5.7369   14.7164   -2.3802 H   0  0  0  0  0  0  0  0  0  0  0  0
  4.9826   13.8394   -0.1847 H   0  0  0  0  0  0  0  0  0  0  0  0
  4.7333   15.4929    0.3912 H   0  0  0  0  0  0  0  0  0  0  0  0
  3.0538   15.8642   -1.3977 H   0  0  0  0  0  0  0  0  0  0  0  0
  3.3047   14.2124   -1.9775 H   0  0  0  0  0  0  0  0  0  0  0  0
  1.3025   14.3424   -0.5154 H   0  0  0  0  0  0  0  0  0  0  0  0
  2.5524   13.3275    0.2161 H   0  0  0  0  0  0  0  0  0  0  0  0
  2.3023   14.9791    0.7970 H   0  0  0  0  0  0  0  0  0  0  0  0
  1  2  1  0  0  0  0
  1  3  1  0  0  0  0
  2  4  1  0  0  0  0
  3  5  1  0  0  0  0
  4  6  1  0  0  0  0
  5  7  1  0  0  0  0
  6  8  1  0  0  0  0
  7  9  1  0  0  0  0
  8 10  1  0  0  0  0
  9 11  1  0  0  0  0
 10 12  1  0  0  0  0
 11 13  1  0  0  0  0
 12 14  1  0  0  0  0
 13 15  1  0  0  0  0
 14 16  1  0  0  0  0
 15 17  1  0  0  0  0
 15 18  2  0  0  0  0
 16 19  1  0  0  0  0
 19 20  1  0  0  0  0
 20 21  1  0  0  0  0
 21 22  1  0  0  0  0
 22 23  1  0  0  0  0
 23 24  1  0  0  0  0
 17 27  1  0  0  0  0
 10 25  1  0  0  0  0
 12 26  1  0  0  0  0
 25 28  1  0  0  0  0
 26 29  1  0  0  0  0
  1 30  1  0  0  0  0
  1 31  1  0  0  0  0
  2 32  1  0  0  0  0
  2 33  1  0  0  0  0
  3 34  1  0  0  0  0
  3 35  1  0  0  0  0
  4 36  1  0  0  0  0
  4 37  1  0  0  0  0
  5 38  1  0  0  0  0
  5 39  1  0  0  0  0
  6 40  1  0  0  0  0
  6 41  1  0  0  0  0
  7 42  1  0  0  0  0
  7 43  1  0  0  0  0
  8 44  1  0  0  0  0
  8 45  1  0  0  0  0
  9 46  1  0  0  0  0
  9 47  1  0  0  0  0
```

```
 10 48  1  0  0  0  0
 11 49  1  0  0  0  0
 11 50  1  0  0  0  0
 12 51  1  0  0  0  0
 13 52  1  0  0  0  0
 13 53  1  0  0  0  0
 14 54  1  0  0  0  0
 14 55  1  0  0  0  0
 16 56  1  0  0  0  0
 16 57  1  0  0  0  0
 19 58  1  0  0  0  0
 19 59  1  0  0  0  0
 20 60  1  0  0  0  0
 20 61  1  0  0  0  0
 21 62  1  0  0  0  0
 21 63  1  0  0  0  0
 22 64  1  0  0  0  0
 22 65  1  0  0  0  0
 23 66  1  0  0  0  0
 23 67  1  0  0  0  0
 24 68  1  0  0  0  0
 24 69  1  0  0  0  0
 24 70  1  0  0  0  0
M  END
>  <Alias name>
13,14-Dihydroxybehenic acid

>  <deltaH°(c)>   kJ/mol
-13544

$$$$
Di(2-ethylhexyl)adipate
  ChemBrain IXL

 68 67  0  0  0  0  0  0  0  0  1 V2000
    5.6694    5.3771    0.7014 C   0  0  0  0  0  0  0  0  0  0  0  0
    6.8673    6.1645    0.1358 C   0  0  0  0  0  0  0  0  0  0  0  0
    8.1076    5.2510    0.0918 C   0  0  0  0  0  0  0  0  0  0  0  0
    9.3052    6.0378   -0.4753 C   0  0  0  0  0  0  0  0  0  0  0  0
    4.4774    6.2536    0.7412 C   0  0  0  0  0  0  0  0  0  0  0  0
    3.4102    5.8143    1.1368 O   0  0  0  0  0  0  0  0  0  0  0  0
    4.5795    7.6534    0.3041 O   0  0  0  0  0  0  0  0  0  0  0  0
   10.4940    5.1577   -0.5266 C   0  0  0  0  0  0  0  0  0  0  0  0
   10.4095    3.9966   -0.1618 O   0  0  0  0  0  0  0  0  0  0  0  0
   11.7765    5.6826   -1.0169 O   0  0  0  0  0  0  0  0  0  0  0  0
    3.3286    8.4216    0.3830 C   0  0  0  0  0  0  0  0  0  0  0  0
    3.5717    9.8533   -0.1326 C   0  0  0  0  0  0  0  0  0  0  0  0
    4.6550   10.5321    0.7278 C   0  0  0  0  0  0  0  0  0  0  0  0
    4.1742   10.6128    2.1897 C   0  0  0  0  0  0  0  0  0  0  0  0
    5.2586   11.2892    3.0506 C   0  0  0  0  0  0  0  0  0  0  0  0
    4.7793   11.3664    4.5131 C   0  0  0  0  0  0  0  0  0  0  0  0
    2.2626   10.6612   -0.0416 C   0  0  0  0  0  0  0  0  0  0  0  0
    2.5073   12.0948   -0.5510 C   0  0  0  0  0  0  0  0  0  0  0  0
   11.7568    7.0969   -1.4171 C   0  0  0  0  0  0  0  0  0  0  0  0
   13.1604    7.5146   -1.8971 C   0  0  0  0  0  0  0  0  0  0  0  0
   13.1333    8.9897   -2.3420 C   0  0  0  0  0  0  0  0  0  0  0  0
   14.5356    9.4057   -2.8270 C   0  0  0  0  0  0  0  0  0  0  0  0
   14.5159   10.8893   -3.2434 C   0  0  0  0  0  0  0  0  0  0  0  0
   15.9172   11.3042   -3.7324 C   0  0  0  0  0  0  0  0  0  0  0  0
   13.5822    6.6276   -3.0845 C   0  0  0  0  0  0  0  0  0  0  0  0
   12.5858    6.8149   -4.2450 C   0  0  0  0  0  0  0  0  0  0  0  0
    5.4678    4.5222    0.0678 H   0  0  0  0  0  0  0  0  0  0  0  0
    5.9013    5.0374    1.7032 H   0  0  0  0  0  0  0  0  0  0  0  0
```

```
   7.0701    7.0183    0.7704 H   0  0  0  0  0  0  0  0  0  0  0  0
   6.6347    6.5059   -0.8653 H   0  0  0  0  0  0  0  0  0  0  0  0
   7.9044    4.3966   -0.5419 H   0  0  0  0  0  0  0  0  0  0  0  0
   8.3409    4.9105    1.0930 H   0  0  0  0  0  0  0  0  0  0  0  0
   9.5129    6.8883    0.1623 H   0  0  0  0  0  0  0  0  0  0  0  0
   9.0692    6.3844   -1.4739 H   0  0  0  0  0  0  0  0  0  0  0  0
   2.5717    7.9424   -0.2255 H   0  0  0  0  0  0  0  0  0  0  0  0
   2.9932    8.4605    1.4120 H   0  0  0  0  0  0  0  0  0  0  0  0
   3.9012    9.8147   -1.1635 H   0  0  0  0  0  0  0  0  0  0  0  0
   4.8402   11.5312    0.3531 H   0  0  0  0  0  0  0  0  0  0  0  0
   5.5692    9.9534    0.6799 H   0  0  0  0  0  0  0  0  0  0  0  0
   3.9872    9.6138    2.5639 H   0  0  0  0  0  0  0  0  0  0  0  0
   3.2610   11.1930    2.2378 H   0  0  0  0  0  0  0  0  0  0  0  0
   5.4435   12.2892    2.6781 H   0  0  0  0  0  0  0  0  0  0  0  0
   6.1726   10.7105    3.0002 H   0  0  0  0  0  0  0  0  0  0  0  0
   4.5926   10.3665    4.8850 H   0  0  0  0  0  0  0  0  0  0  0  0
   5.5422   11.8400    5.1185 H   0  0  0  0  0  0  0  0  0  0  0  0
   3.8664   11.9468    4.5637 H   0  0  0  0  0  0  0  0  0  0  0  0
   1.5026   10.1867   -0.6500 H   0  0  0  0  0  0  0  0  0  0  0  0
   1.9306   10.6959    0.9887 H   0  0  0  0  0  0  0  0  0  0  0  0
   1.5818   12.6557   -0.5090 H   0  0  0  0  0  0  0  0  0  0  0  0
   2.8616   12.0590   -1.5737 H   0  0  0  0  0  0  0  0  0  0  0  0
   3.2504   12.5773    0.0719 H   0  0  0  0  0  0  0  0  0  0  0  0
  11.0445    7.2348   -2.2211 H   0  0  0  0  0  0  0  0  0  0  0  0
  11.4683    7.7076   -0.5705 H   0  0  0  0  0  0  0  0  0  0  0  0
  13.8694    7.3947   -1.0872 H   0  0  0  0  0  0  0  0  0  0  0  0
  12.4216    9.1103   -3.1493 H   0  0  0  0  0  0  0  0  0  0  0  0
  12.8406    9.6134   -1.5064 H   0  0  0  0  0  0  0  0  0  0  0  0
  15.2511    9.2650   -2.0262 H   0  0  0  0  0  0  0  0  0  0  0  0
  14.8195    8.7962   -3.6760 H   0  0  0  0  0  0  0  0  0  0  0  0
  13.7983   11.0306   -4.0421 H   0  0  0  0  0  0  0  0  0  0  0  0
  14.2350   11.4991   -2.3936 H   0  0  0  0  0  0  0  0  0  0  0  0
  15.9029   12.3463   -4.0269 H   0  0  0  0  0  0  0  0  0  0  0  0
  16.1990   10.6931   -4.5810 H   0  0  0  0  0  0  0  0  0  0  0  0
  16.6344   11.1647   -2.9331 H   0  0  0  0  0  0  0  0  0  0  0  0
  13.5862    5.5894   -2.7761 H   0  0  0  0  0  0  0  0  0  0  0  0
  14.5750    6.9107   -3.4118 H   0  0  0  0  0  0  0  0  0  0  0  0
  11.5917    6.5395   -3.9152 H   0  0  0  0  0  0  0  0  0  0  0  0
  12.5878    7.8514   -4.5588 H   0  0  0  0  0  0  0  0  0  0  0  0
  12.8776    6.1854   -5.0765 H   0  0  0  0  0  0  0  0  0  0  0  0
  1  2  1  0  0  0  0
  2  3  1  0  0  0  0
  3  4  1  0  0  0  0
  5  6  2  0  0  0  0
  5  7  1  0  0  0  0
  8  9  2  0  0  0  0
  8 10  1  0  0  0  0
  5  1  1  0  0  0  0
  8  4  1  0  0  0  0
  7 11  1  0  0  0  0
 11 12  1  0  0  0  0
 12 13  1  0  0  0  0
 13 14  1  0  0  0  0
 14 15  1  0  0  0  0
 15 16  1  0  0  0  0
 12 17  1  0  0  0  0
 17 18  1  0  0  0  0
 19 20  1  0  0  0  0
 20 21  1  0  0  0  0
 21 22  1  0  0  0  0
 22 23  1  0  0  0  0
 23 24  1  0  0  0  0
 20 25  1  0  0  0  0
```

```
 25 26  1  0  0  0  0
 19 10  1  0  0  0  0
  1 27  1  0  0  0  0
  1 28  1  0  0  0  0
  2 29  1  0  0  0  0
  2 30  1  0  0  0  0
  3 31  1  0  0  0  0
  3 32  1  0  0  0  0
  4 33  1  0  0  0  0
  4 34  1  0  0  0  0
 11 35  1  0  0  0  0
 11 36  1  0  0  0  0
 12 37  1  0  0  0  0
 13 38  1  0  0  0  0
 13 39  1  0  0  0  0
 14 40  1  0  0  0  0
 14 41  1  0  0  0  0
 15 42  1  0  0  0  0
 15 43  1  0  0  0  0
 16 44  1  0  0  0  0
 16 45  1  0  0  0  0
 16 46  1  0  0  0  0
 17 47  1  0  0  0  0
 17 48  1  0  0  0  0
 18 49  1  0  0  0  0
 18 50  1  0  0  0  0
 18 51  1  0  0  0  0
 19 52  1  0  0  0  0
 19 53  1  0  0  0  0
 20 54  1  0  0  0  0
 21 55  1  0  0  0  0
 21 56  1  0  0  0  0
 22 57  1  0  0  0  0
 22 58  1  0  0  0  0
 23 59  1  0  0  0  0
 23 60  1  0  0  0  0
 24 61  1  0  0  0  0
 24 62  1  0  0  0  0
 24 63  1  0  0  0  0
 25 64  1  0  0  0  0
 25 65  1  0  0  0  0
 26 66  1  0  0  0  0
 26 67  1  0  0  0  0
 26 68  1  0  0  0  0
M  END
>  <Alias name>
Bis(2-ethylhexyl) adipate

>  <deltaH°(c)>   kJ/mol
-13404.14

$$$$
Di-n-octyladipate
  ChemBrain IXL

 68 67  0  0  0  0  0  0  0  0  1 V2000
   12.8528   15.6376    0.1428 C   0  0  0  0  0  0  0  0  0  0  0  0
   11.7430   14.5687    0.1866 C   0  0  0  0  0  0  0  0  0  0  0  0
   10.3706   15.2594    0.3042 C   0  0  0  0  0  0  0  0  0  0  0  0
    9.2585   14.1917    0.3480 C   0  0  0  0  0  0  0  0  0  0  0  0
   14.1739   14.9779    0.0293 C   0  0  0  0  0  0  0  0  0  0  0  0
   15.1886   15.6542   -0.0202 O   0  0  0  0  0  0  0  0  0  0  0  0
   14.2631   13.5077   -0.0238 O   0  0  0  0  0  0  0  0  0  0  0  0
```

```
7.9357 14.8551 0.4608 C 0 0 0 0 0 0 0 0 0 0 0 0
7.8644 16.0727 0.5037 O 0 0 0 0 0 0 0 0 0 0 0 0
6.7078 14.0469 0.5211 O 0 0 0 0 0 0 0 0 0 0 0 0
15.6274 12.9675 -0.1367 C 0 0 0 0 0 0 0 0 0 0 0 0
15.5692 11.4285 -0.1822 C 0 0 0 0 0 0 0 0 0 0 0 0
16.9986 10.8641 -0.3010 C 0 0 0 0 0 0 0 0 0 0 0 0
16.9418 9.3247 -0.3470 C 0 0 0 0 0 0 0 0 0 0 0 0
18.3715 8.7614 -0.4662 C 0 0 0 0 0 0 0 0 0 0 0 0
18.3154 7.2220 -0.5126 C 0 0 0 0 0 0 0 0 0 0 0 0
19.7453 6.6591 -0.6321 C 0 0 0 0 0 0 0 0 0 0 0 0
19.6895 5.1199 -0.6787 C 0 0 0 0 0 0 0 0 0 0 0 0
6.9126 12.5923 0.4617 C 0 0 0 0 0 0 0 0 0 0 0 0
5.5492 11.8798 0.5335 C 0 0 0 0 0 0 0 0 0 0 0 0
5.7621 10.3553 0.4709 C 0 0 0 0 0 0 0 0 0 0 0 0
4.3976 9.6434 0.5420 C 0 0 0 0 0 0 0 0 0 0 0 0
4.6097 8.1187 0.4789 C 0 0 0 0 0 0 0 0 0 0 0 0
3.2448 7.4072 0.5494 C 0 0 0 0 0 0 0 0 0 0 0 0
3.4564 5.8821 0.4859 C 0 0 0 0 0 0 0 0 0 0 0 0
2.0913 5.1707 0.5562 C 0 0 0 0 0 0 0 0 0 0 0 0
12.6978 16.2835 -0.7126 H 0 0 0 0 0 0 0 0 0 0 0 0
12.8213 16.2275 1.0505 H 0 0 0 0 0 0 0 0 0 0 0 0
11.8969 13.9231 1.0425 H 0 0 0 0 0 0 0 0 0 0 0 0
11.7738 13.9786 -0.7210 H 0 0 0 0 0 0 0 0 0 0 0 0
10.2173 15.9053 -0.5515 H 0 0 0 0 0 0 0 0 0 0 0 0
10.3403 15.8496 1.2119 H 0 0 0 0 0 0 0 0 0 0 0 0
9.4122 13.5460 1.2038 H 0 0 0 0 0 0 0 0 0 0 0 0
9.2896 13.6013 -0.5593 H 0 0 0 0 0 0 0 0 0 0 0 0
16.2119 13.2814 0.7193 H 0 0 0 0 0 0 0 0 0 0 0 0
16.0884 13.3381 -1.0440 H 0 0 0 0 0 0 0 0 0 0 0 0
14.9847 11.1144 -1.0381 H 0 0 0 0 0 0 0 0 0 0 0 0
15.1088 11.0577 0.7252 H 0 0 0 0 0 0 0 0 0 0 0 0
17.5829 11.1782 0.5551 H 0 0 0 0 0 0 0 0 0 0 0 0
17.4590 11.2355 -1.2082 H 0 0 0 0 0 0 0 0 0 0 0 0
16.3574 9.0104 -1.2029 H 0 0 0 0 0 0 0 0 0 0 0 0
16.4819 8.9531 0.5604 H 0 0 0 0 0 0 0 0 0 0 0 0
18.9558 9.0757 0.3898 H 0 0 0 0 0 0 0 0 0 0 0 0
18.8314 9.1333 -1.3734 H 0 0 0 0 0 0 0 0 0 0 0 0
17.7310 6.9077 -1.3685 H 0 0 0 0 0 0 0 0 0 0 0 0
17.8557 6.8500 0.3947 H 0 0 0 0 0 0 0 0 0 0 0 0
20.3296 6.9734 0.2238 H 0 0 0 0 0 0 0 0 0 0 0 0
20.2050 7.0312 -1.5393 H 0 0 0 0 0 0 0 0 0 0 0 0
20.6943 4.7247 -0.7627 H 0 0 0 0 0 0 0 0 0 0 0 0
19.2299 4.7476 0.2285 H 0 0 0 0 0 0 0 0 0 0 0 0
19.1053 4.8055 -1.5347 H 0 0 0 0 0 0 0 0 0 0 0 0
7.5278 12.2796 1.2964 H 0 0 0 0 0 0 0 0 0 0 0 0
7.4061 12.3346 -0.4673 H 0 0 0 0 0 0 0 0 0 0 0 0
4.9340 12.1929 -0.3009 H 0 0 0 0 0 0 0 0 0 0 0 0
5.0558 12.1375 1.4625 H 0 0 0 0 0 0 0 0 0 0 0 0
6.3768 10.0420 1.3057 H 0 0 0 0 0 0 0 0 0 0 0 0
6.2558 10.0976 -0.4579 H 0 0 0 0 0 0 0 0 0 0 0 0
3.7829 9.9572 -0.2925 H 0 0 0 0 0 0 0 0 0 0 0 0
3.9041 9.9011 1.4709 H 0 0 0 0 0 0 0 0 0 0 0 0
5.2241 7.8048 1.3136 H 0 0 0 0 0 0 0 0 0 0 0 0
5.1036 7.8611 -0.4499 H 0 0 0 0 0 0 0 0 0 0 0 0
2.6304 7.7215 -0.2853 H 0 0 0 0 0 0 0 0 0 0 0 0
2.7510 7.6648 1.4782 H 0 0 0 0 0 0 0 0 0 0 0 0
4.0706 5.5678 1.3206 H 0 0 0 0 0 0 0 0 0 0 0 0
3.9502 5.6245 -0.4428 H 0 0 0 0 0 0 0 0 0 0 0 0
2.2401 4.0990 0.5115 H 0 0 0 0 0 0 0 0 0 0 0 0
1.5974 5.4283 1.4849 H 0 0 0 0 0 0 0 0 0 0 0 0
1.4771 5.4850 -0.2786 H 0 0 0 0 0 0 0 0 0 0 0 0
1 2 1 0 0 0 0
2 3 1 0 0 0 0
```

3	4	1	0	0	0	0
5	6	2	0	0	0	0
5	7	1	0	0	0	0
8	9	2	0	0	0	0
8	10	1	0	0	0	0
5	1	1	0	0	0	0
8	4	1	0	0	0	0
7	11	1	0	0	0	0
11	12	1	0	0	0	0
12	13	1	0	0	0	0
13	14	1	0	0	0	0
14	15	1	0	0	0	0
15	16	1	0	0	0	0
16	17	1	0	0	0	0
17	18	1	0	0	0	0
19	20	1	0	0	0	0
20	21	1	0	0	0	0
21	22	1	0	0	0	0
22	23	1	0	0	0	0
23	24	1	0	0	0	0
24	25	1	0	0	0	0
25	26	1	0	0	0	0
19	10	1	0	0	0	0
1	27	1	0	0	0	0
1	28	1	0	0	0	0
2	29	1	0	0	0	0
2	30	1	0	0	0	0
3	31	1	0	0	0	0
3	32	1	0	0	0	0
4	33	1	0	0	0	0
4	34	1	0	0	0	0
11	35	1	0	0	0	0
11	36	1	0	0	0	0
12	37	1	0	0	0	0
12	38	1	0	0	0	0
13	39	1	0	0	0	0
13	40	1	0	0	0	0
14	41	1	0	0	0	0
14	42	1	0	0	0	0
15	43	1	0	0	0	0
15	44	1	0	0	0	0
16	45	1	0	0	0	0
16	46	1	0	0	0	0
17	47	1	0	0	0	0
17	48	1	0	0	0	0
18	49	1	0	0	0	0
18	50	1	0	0	0	0
18	51	1	0	0	0	0
19	52	1	0	0	0	0
19	53	1	0	0	0	0
20	54	1	0	0	0	0
20	55	1	0	0	0	0
21	56	1	0	0	0	0
21	57	1	0	0	0	0
22	58	1	0	0	0	0
22	59	1	0	0	0	0
23	60	1	0	0	0	0
23	61	1	0	0	0	0
24	62	1	0	0	0	0
24	63	1	0	0	0	0
25	64	1	0	0	0	0
25	65	1	0	0	0	0
26	66	1	0	0	0	0

```
 26 67  1  0  0  0  0
 26 68  1  0  0  0  0
M  END
>  <Alias name>
Di-n-octyladipate

>  <deltaH°(c)>   kJ/mol
-13403.44

$$$$
Tetraphenylethylene
  ChemBrain IXL

 46 49  0  0  0  0  0  0  0  0  1 V2000
   11.2481   11.1659   -0.9336 C   0  0  0  0  0  0  0  0  0  0  0  0
   12.2107   11.5360    0.0057 C   0  0  0  0  0  0  0  0  0  0  0  0
   12.6674   10.6060    0.9399 C   0  0  0  0  0  0  0  0  0  0  0  0
   12.1621    9.3053    0.9345 C   0  0  0  0  0  0  0  0  0  0  0  0
   11.2002    8.9344   -0.0060 C   0  0  0  0  0  0  0  0  0  0  0  0
   10.7426    9.8656   -0.9391 C   0  0  0  0  0  0  0  0  0  0  0  0
   10.6571    7.5561   -0.0119 C   0  0  0  0  0  0  0  0  0  0  0  0
   11.4890    6.4873   -0.0119 C   0  0  0  0  0  0  0  0  0  0  0  0
    6.4213    7.0164   -0.0294 C   0  0  0  0  0  0  0  0  0  0  0  0
    7.0154    7.8489    0.9192 C   0  0  0  0  0  0  0  0  0  0  0  0
    8.3994    8.0235    0.9246 C   0  0  0  0  0  0  0  0  0  0  0  0
    9.1895    7.3648   -0.0179 C   0  0  0  0  0  0  0  0  0  0  0  0
    8.5953    6.5335   -0.9676 C   0  0  0  0  0  0  0  0  0  0  0  0
    7.2112    6.3590   -0.9730 C   0  0  0  0  0  0  0  0  0  0  0  0
   15.7286    7.0165   -0.0293 C   0  0  0  0  0  0  0  0  0  0  0  0
   14.9405    7.6767   -0.9725 C   0  0  0  0  0  0  0  0  0  0  0  0
   13.5555    7.5061   -0.9671 C   0  0  0  0  0  0  0  0  0  0  0  0
   12.9585    6.6756   -0.0179 C   0  0  0  0  0  0  0  0  0  0  0  0
   13.7475    6.0142    0.9241 C   0  0  0  0  0  0  0  0  0  0  0  0
   15.1321    6.1850    0.9187 C   0  0  0  0  0  0  0  0  0  0  0  0
    9.9261    2.5137    0.0057 C   0  0  0  0  0  0  0  0  0  0  0  0
    9.4775    3.4429    0.9447 C   0  0  0  0  0  0  0  0  0  0  0  0
    9.9864    4.7419    0.9392 C   0  0  0  0  0  0  0  0  0  0  0  0
   10.9434    5.1117   -0.0060 C   0  0  0  0  0  0  0  0  0  0  0  0
   11.3932    4.1819   -0.9438 C   0  0  0  0  0  0  0  0  0  0  0  0
   10.8843    2.8832   -0.9384 C   0  0  0  0  0  0  0  0  0  0  0  0
   10.8931   11.8880   -1.6586 H   0  0  0  0  0  0  0  0  0  0  0  0
   12.6035   12.5454    0.0096 H   0  0  0  0  0  0  0  0  0  0  0  0
   13.4144   10.8936    1.6694 H   0  0  0  0  0  0  0  0  0  0  0  0
   12.5161    8.5839    1.6603 H   0  0  0  0  0  0  0  0  0  0  0  0
    9.9946    9.5788   -1.6679 H   0  0  0  0  0  0  0  0  0  0  0  0
    5.3469    6.8804   -0.0332 H   0  0  0  0  0  0  0  0  0  0  0  0
    6.4023    8.3596    1.6515 H   0  0  0  0  0  0  0  0  0  0  0  0
    8.8606    8.6706    1.6604 H   0  0  0  0  0  0  0  0  0  0  0  0
    9.2083    6.0240   -1.7006 H   0  0  0  0  0  0  0  0  0  0  0  0
    6.7498    5.7132   -1.7097 H   0  0  0  0  0  0  0  0  0  0  0  0
   16.8035    7.1495   -0.0332 H   0  0  0  0  0  0  0  0  0  0  0  0
   15.4038    8.3218   -1.7087 H   0  0  0  0  0  0  0  0  0  0  0  0
   12.9442    8.0179   -1.6998 H   0  0  0  0  0  0  0  0  0  0  0  0
   13.2849    5.3679    1.6596 H   0  0  0  0  0  0  0  0  0  0  0  0
   15.7440    5.6721    1.6505 H   0  0  0  0  0  0  0  0  0  0  0  0
    9.5306    1.5056    0.0097 H   0  0  0  0  0  0  0  0  0  0  0  0
    8.7340    3.1558    1.6779 H   0  0  0  0  0  0  0  0  0  0  0  0
    9.6388    5.4629    1.6685 H   0  0  0  0  0  0  0  0  0  0  0  0
   12.1379    4.4685   -1.6762 H   0  0  0  0  0  0  0  0  0  0  0  0
   11.2330    2.1618   -1.6671 H   0  0  0  0  0  0  0  0  0  0  0  0
  1  2  4  0  0  0  0
  2  3  4  0  0  0  0
  3  4  4  0  0  0  0
```

```
  4  5  4  0  0  0  0
  5  6  4  0  0  0  0
  6  1  4  0  0  0  0
  5  7  1  0  0  0  0
  7  8  2  0  0  0  0
  9 10  4  0  0  0  0
 10 11  4  0  0  0  0
 11 12  4  0  0  0  0
 12 13  4  0  0  0  0
 13 14  4  0  0  0  0
 14  9  4  0  0  0  0
 12  7  1  0  0  0  0
 15 16  4  0  0  0  0
 16 17  4  0  0  0  0
 17 18  4  0  0  0  0
 18 19  4  0  0  0  0
 19 20  4  0  0  0  0
 20 15  4  0  0  0  0
 18  8  1  0  0  0  0
 21 22  4  0  0  0  0
 22 23  4  0  0  0  0
 23 24  4  0  0  0  0
 24 25  4  0  0  0  0
 25 26  4  0  0  0  0
 26 21  4  0  0  0  0
 24  8  1  0  0  0  0
  1 27  1  0  0  0  0
  2 28  1  0  0  0  0
  3 29  1  0  0  0  0
  4 30  1  0  0  0  0
  6 31  1  0  0  0  0
  9 32  1  0  0  0  0
 10 33  1  0  0  0  0
 11 34  1  0  0  0  0
 13 35  1  0  0  0  0
 14 36  1  0  0  0  0
 15 37  1  0  0  0  0
 16 38  1  0  0  0  0
 17 39  1  0  0  0  0
 19 40  1  0  0  0  0
 20 41  1  0  0  0  0
 21 42  1  0  0  0  0
 22 43  1  0  0  0  0
 23 44  1  0  0  0  0
 25 45  1  0  0  0  0
 26 46  1  0  0  0  0
M  END
>  <Alias name>
Tetraphenylethylene

>  <deltaH°(c)>   kJ/mol
-13401

$$$$
Eicosane
  ChemBrain IXL

 62 61  0  0  0  0  0  0  0  0  1 V2000
    2.1374   13.5936    4.5312 C   0  0  0  0  0  0  0  0  0  0  0  0
    3.4137   14.2904    4.0197 C   0  0  0  0  0  0  0  0  0  0  0  0
    4.4518   13.2227    3.6216 C   0  0  0  0  0  0  0  0  0  0  0  0
    5.7313   13.9146    3.1124 C   0  0  0  0  0  0  0  0  0  0  0  0
    6.7691   12.8471    2.7143 C   0  0  0  0  0  0  0  0  0  0  0  0
```

```
  8.0484   13.5391    2.2048 C   0  0  0  0  0  0  0  0  0  0  0  0
  9.0862   12.4716    1.8067 C   0  0  0  0  0  0  0  0  0  0  0  0
 10.3653   13.1635    1.2967 C   0  0  0  0  0  0  0  0  0  0  0  0
 11.4031   12.0961    0.8986 C   0  0  0  0  0  0  0  0  0  0  0  0
 12.6820   12.7880    0.3879 C   0  0  0  0  0  0  0  0  0  0  0  0
 13.7198   11.7206   -0.0102 C   0  0  0  0  0  0  0  0  0  0  0  0
 14.9983   12.4125   -0.5216 C   0  0  0  0  0  0  0  0  0  0  0  0
 16.0361   11.3451   -0.9198 C   0  0  0  0  0  0  0  0  0  0  0  0
 17.3144   12.0371   -1.4318 C   0  0  0  0  0  0  0  0  0  0  0  0
 18.3522   10.9697   -1.8301 C   0  0  0  0  0  0  0  0  0  0  0  0
 19.6303   11.6616   -2.3426 C   0  0  0  0  0  0  0  0  0  0  0  0
 20.6681   10.5942   -2.7409 C   0  0  0  0  0  0  0  0  0  0  0  0
 21.9461   11.2862   -3.2537 C   0  0  0  0  0  0  0  0  0  0  0  0
 22.9842   10.2186   -3.6521 C   0  0  0  0  0  0  0  0  0  0  0  0
 24.2614   10.9155   -4.1612 C   0  0  0  0  0  0  0  0  0  0  0  0
  2.4141   12.6796    5.0422 H   0  0  0  0  0  0  0  0  0  0  0  0
  1.6288   14.2425    5.2334 H   0  0  0  0  0  0  0  0  0  0  0  0
  1.4872   13.3578    3.6979 H   0  0  0  0  0  0  0  0  0  0  0  0
  3.8272   14.9091    4.8065 H   0  0  0  0  0  0  0  0  0  0  0  0
  3.1754   14.9008    3.1575 H   0  0  0  0  0  0  0  0  0  0  0  0
  4.0434   12.5969    2.8376 H   0  0  0  0  0  0  0  0  0  0  0  0
  4.6903   12.6119    4.4835 H   0  0  0  0  0  0  0  0  0  0  0  0
  6.1396   14.5404    3.8964 H   0  0  0  0  0  0  0  0  0  0  0  0
  5.4928   14.5253    2.2504 H   0  0  0  0  0  0  0  0  0  0  0  0
  6.3606   12.2212    1.9306 H   0  0  0  0  0  0  0  0  0  0  0  0
  7.0078   12.2366    3.5764 H   0  0  0  0  0  0  0  0  0  0  0  0
  8.4568   14.1650    2.9886 H   0  0  0  0  0  0  0  0  0  0  0  0
  7.8097   14.1496    1.3428 H   0  0  0  0  0  0  0  0  0  0  0  0
  8.6776   11.8454    1.0232 H   0  0  0  0  0  0  0  0  0  0  0  0
  9.3252   11.8613    2.6689 H   0  0  0  0  0  0  0  0  0  0  0  0
 10.7739   13.7897    2.0802 H   0  0  0  0  0  0  0  0  0  0  0  0
 10.1263   13.7738    0.4346 H   0  0  0  0  0  0  0  0  0  0  0  0
 10.9944   11.4696    0.1154 H   0  0  0  0  0  0  0  0  0  0  0  0
 11.6425   11.4860    1.7608 H   0  0  0  0  0  0  0  0  0  0  0  0
 13.0908   13.4145    1.1711 H   0  0  0  0  0  0  0  0  0  0  0  0
 12.4426   13.3981   -0.4743 H   0  0  0  0  0  0  0  0  0  0  0  0
 13.3108   11.0939   -0.7931 H   0  0  0  0  0  0  0  0  0  0  0  0
 13.9595   11.1108    0.8521 H   0  0  0  0  0  0  0  0  0  0  0  0
 15.4073   13.0393    0.2612 H   0  0  0  0  0  0  0  0  0  0  0  0
 14.7586   13.0223   -1.3839 H   0  0  0  0  0  0  0  0  0  0  0  0
 15.6270   10.7181   -1.7023 H   0  0  0  0  0  0  0  0  0  0  0  0
 16.2763   10.7356   -0.0574 H   0  0  0  0  0  0  0  0  0  0  0  0
 17.7236   12.6641   -0.6493 H   0  0  0  0  0  0  0  0  0  0  0  0
 17.0743   12.6465   -2.2942 H   0  0  0  0  0  0  0  0  0  0  0  0
 17.9429   10.3424   -2.6123 H   0  0  0  0  0  0  0  0  0  0  0  0
 18.5927   10.3604   -0.9676 H   0  0  0  0  0  0  0  0  0  0  0  0
 20.0397   12.2889   -1.5604 H   0  0  0  0  0  0  0  0  0  0  0  0
 19.3899   12.2709   -3.2051 H   0  0  0  0  0  0  0  0  0  0  0  0
 20.2587    9.9668   -3.5229 H   0  0  0  0  0  0  0  0  0  0  0  0
 20.9088    9.9852   -1.8783 H   0  0  0  0  0  0  0  0  0  0  0  0
 22.3555   11.9137   -2.4717 H   0  0  0  0  0  0  0  0  0  0  0  0
 21.7054   11.8953   -4.1163 H   0  0  0  0  0  0  0  0  0  0  0  0
 22.5745    9.5915   -4.4342 H   0  0  0  0  0  0  0  0  0  0  0  0
 23.2229    9.6167   -2.7840 H   0  0  0  0  0  0  0  0  0  0  0  0
 24.4013   11.8402   -3.6150 H   0  0  0  0  0  0  0  0  0  0  0  0
 24.1666   11.1321   -5.2180 H   0  0  0  0  0  0  0  0  0  0  0  0
 25.1168   10.2756   -3.9835 H   0  0  0  0  0  0  0  0  0  0  0  0
  1  2  1  0  0  0  0
  2  3  1  0  0  0  0
  3  4  1  0  0  0  0
  4  5  1  0  0  0  0
  5  6  1  0  0  0  0
  6  7  1  0  0  0  0
```

7 8 1 0 0 0 0
8 9 1 0 0 0 0
9 10 1 0 0 0 0
10 11 1 0 0 0 0
11 12 1 0 0 0 0
12 13 1 0 0 0 0
13 14 1 0 0 0 0
14 15 1 0 0 0 0
15 16 1 0 0 0 0
16 17 1 0 0 0 0
17 18 1 0 0 0 0
18 19 1 0 0 0 0
19 20 1 0 0 0 0
1 21 1 0 0 0 0
1 22 1 0 0 0 0
1 23 1 0 0 0 0
2 24 1 0 0 0 0
2 25 1 0 0 0 0
3 26 1 0 0 0 0
3 27 1 0 0 0 0
4 28 1 0 0 0 0
4 29 1 0 0 0 0
5 30 1 0 0 0 0
5 31 1 0 0 0 0
6 32 1 0 0 0 0
6 33 1 0 0 0 0
7 34 1 0 0 0 0
7 35 1 0 0 0 0
8 36 1 0 0 0 0
8 37 1 0 0 0 0
9 38 1 0 0 0 0
9 39 1 0 0 0 0
10 40 1 0 0 0 0
10 41 1 0 0 0 0
11 42 1 0 0 0 0
11 43 1 0 0 0 0
12 44 1 0 0 0 0
12 45 1 0 0 0 0
13 46 1 0 0 0 0
13 47 1 0 0 0 0
14 48 1 0 0 0 0
14 49 1 0 0 0 0
15 50 1 0 0 0 0
15 51 1 0 0 0 0
16 52 1 0 0 0 0
16 53 1 0 0 0 0
17 54 1 0 0 0 0
17 55 1 0 0 0 0
18 56 1 0 0 0 0
18 57 1 0 0 0 0
19 58 1 0 0 0 0
19 59 1 0 0 0 0
20 60 1 0 0 0 0
20 61 1 0 0 0 0
20 62 1 0 0 0 0
M END
> <Alias name>
Eicosane

> <deltaH°(c)> kJ/mol
-13316

$$$$

1,8-Di(3,4-dimethoxyphenyl)naphthalene
ChemBrain IXL

```
 54 57  0  0  0  0  0  0  0  0  1 V2000
    5.6482   10.4348    0.8616 C   0  0  0  0  0  0  0  0  0  0  0  0
    4.3353   10.9046    0.9071 C   0  0  0  0  0  0  0  0  0  0  0  0
    3.2949   10.1393    0.3756 C   0  0  0  0  0  0  0  0  0  0  0  0
    3.5574    8.8980   -0.2106 C   0  0  0  0  0  0  0  0  0  0  0  0
    4.8734    8.4080   -0.2766 C   0  0  0  0  0  0  0  0  0  0  0  0
    5.9355    9.1964    0.2836 C   0  0  0  0  0  0  0  0  0  0  0  0
    5.1069    7.1372   -0.9015 C   0  0  0  0  0  0  0  0  0  0  0  0
    4.0327    6.4054   -1.4108 C   0  0  0  0  0  0  0  0  0  0  0  0
    2.7344    6.9077   -1.3258 C   0  0  0  0  0  0  0  0  0  0  0  0
    2.4980    8.1489   -0.7308 C   0  0  0  0  0  0  0  0  0  0  0  0
   10.0526    7.9031    0.2533 C   0  0  0  0  0  0  0  0  0  0  0  0
    9.0961    7.1564    0.9382 C   0  0  0  0  0  0  0  0  0  0  0  0
    7.7559    7.5703    0.9479 C   0  0  0  0  0  0  0  0  0  0  0  0
    7.3712    8.7364    0.2706 C   0  0  0  0  0  0  0  0  0  0  0  0
    8.3395    9.4865   -0.4068 C   0  0  0  0  0  0  0  0  0  0  0  0
    9.6755    9.0695   -0.4173 C   0  0  0  0  0  0  0  0  0  0  0  0
    9.5045    5.9317    1.6496 O   0  0  0  0  0  0  0  0  0  0  0  0
    8.4603    5.2999    2.4663 C   0  0  0  0  0  0  0  0  0  0  0  0
   11.4595    7.4575    0.2397 O   0  0  0  0  0  0  0  0  0  0  0  0
   12.3847    8.3115   -0.5228 C   0  0  0  0  0  0  0  0  0  0  0  0
    9.0467    5.4355   -1.2853 C   0  0  0  0  0  0  0  0  0  0  0  0
    8.0562    4.7543   -0.5799 C   0  0  0  0  0  0  0  0  0  0  0  0
    6.7768    5.3092   -0.4530 C   0  0  0  0  0  0  0  0  0  0  0  0
    6.4850    6.5513   -1.0309 C   0  0  0  0  0  0  0  0  0  0  0  0
    7.4807    7.2291   -1.7426 C   0  0  0  0  0  0  0  0  0  0  0  0
    8.7592    6.6732   -1.8673 C   0  0  0  0  0  0  0  0  0  0  0  0
    8.3631    3.4504    0.0297 O   0  0  0  0  0  0  0  0  0  0  0  0
    7.2337    2.7907    0.6966 C   0  0  0  0  0  0  0  0  0  0  0  0
   10.3943    4.8457   -1.4131 O   0  0  0  0  0  0  0  0  0  0  0  0
   11.3228    5.5741   -2.2941 C   0  0  0  0  0  0  0  0  0  0  0  0
    6.4501   11.0337    1.2769 H   0  0  0  0  0  0  0  0  0  0  0  0
    4.1229   11.8661    1.3564 H   0  0  0  0  0  0  0  0  0  0  0  0
    2.2794   10.5095    0.4180 H   0  0  0  0  0  0  0  0  0  0  0  0
    4.2095    5.4420   -1.8747 H   0  0  0  0  0  0  0  0  0  0  0  0
    1.9077    6.3327   -1.7227 H   0  0  0  0  0  0  0  0  0  0  0  0
    1.4886    8.5329   -0.6722 H   0  0  0  0  0  0  0  0  0  0  0  0
    7.0159    6.9868    1.4805 H   0  0  0  0  0  0  0  0  0  0  0  0
    8.0546   10.3931   -0.9251 H   0  0  0  0  0  0  0  0  0  0  0  0
   10.4201    9.6522   -0.9466 H   0  0  0  0  0  0  0  0  0  0  0  0
    8.8487    4.3887    2.9062 H   0  0  0  0  0  0  0  0  0  0  0  0
    8.1566    5.9790    3.2527 H   0  0  0  0  0  0  0  0  0  0  0  0
    7.6078    5.0645    1.8428 H   0  0  0  0  0  0  0  0  0  0  0  0
   12.3857    9.3090   -0.1020 H   0  0  0  0  0  0  0  0  0  0  0  0
   12.0665    8.3559   -1.5567 H   0  0  0  0  0  0  0  0  0  0  0  0
   13.3849    7.8968   -0.4706 H   0  0  0  0  0  0  0  0  0  0  0  0
    6.0104    4.7755    0.0943 H   0  0  0  0  0  0  0  0  0  0  0  0
    7.2623    8.1864   -2.1984 H   0  0  0  0  0  0  0  0  0  0  0  0
    9.5284    7.2033   -2.4163 H   0  0  0  0  0  0  0  0  0  0  0  0
    7.5572    1.8365    1.0947 H   0  0  0  0  0  0  0  0  0  0  0  0
    6.8764    3.4164    1.5045 H   0  0  0  0  0  0  0  0  0  0  0  0
    6.4357    2.6320   -0.0176 H   0  0  0  0  0  0  0  0  0  0  0  0
   11.4410    6.5885   -1.9350 H   0  0  0  0  0  0  0  0  0  0  0  0
   10.9268    5.5914   -3.3017 H   0  0  0  0  0  0  0  0  0  0  0  0
   12.2862    5.0766   -2.2936 H   0  0  0  0  0  0  0  0  0  0  0  0
  1  2  4  0  0  0  0
  2  3  4  0  0  0  0
  3  4  4  0  0  0  0
  4  5  4  0  0  0  0
  5  6  4  0  0  0  0
```

```
  6  1  4  0  0  0  0
  5  7  4  0  0  0  0
  7  8  4  0  0  0  0
  8  9  4  0  0  0  0
  4 10  4  0  0  0  0
 10  9  4  0  0  0  0
 11 12  4  0  0  0  0
 12 13  4  0  0  0  0
 13 14  4  0  0  0  0
 14 15  4  0  0  0  0
 15 16  4  0  0  0  0
 16 11  4  0  0  0  0
 14  6  1  0  0  0  0
 12 17  1  0  0  0  0
 17 18  1  0  0  0  0
 19 20  1  0  0  0  0
 19 11  1  0  0  0  0
 21 22  4  0  0  0  0
 22 23  4  0  0  0  0
 23 24  4  0  0  0  0
 24 25  4  0  0  0  0
 25 26  4  0  0  0  0
 26 21  4  0  0  0  0
 22 27  1  0  0  0  0
 27 28  1  0  0  0  0
 29 30  1  0  0  0  0
 29 21  1  0  0  0  0
 24  7  1  0  0  0  0
  1 31  1  0  0  0  0
  2 32  1  0  0  0  0
  3 33  1  0  0  0  0
  8 34  1  0  0  0  0
  9 35  1  0  0  0  0
 10 36  1  0  0  0  0
 13 37  1  0  0  0  0
 15 38  1  0  0  0  0
 16 39  1  0  0  0  0
 18 40  1  0  0  0  0
 18 41  1  0  0  0  0
 18 42  1  0  0  0  0
 20 43  1  0  0  0  0
 20 44  1  0  0  0  0
 20 45  1  0  0  0  0
 23 46  1  0  0  0  0
 25 47  1  0  0  0  0
 26 48  1  0  0  0  0
 28 49  1  0  0  0  0
 28 50  1  0  0  0  0
 28 51  1  0  0  0  0
 30 52  1  0  0  0  0
 30 53  1  0  0  0  0
 30 54  1  0  0  0  0
M  END
>  <Alias name>
1,8-Di(3,4-dimethoxyphenyl)naphthalene

>  <deltaH°(c)>   kJ/mol
-13270

$$$$
Ethyldioctylsilane
  ChemBrain IXL
```

59 58 0 0 0 0 0 0 0 0 1 V2000
13.5161 11.5301 0.6068 Si 0 0 0 0 0 0 0 0 0 0 0 0
13.5205 9.7836 1.3212 C 0 0 0 0 0 0 0 0 0 0 0 0
13.5821 11.5117 -1.2791 C 0 0 0 0 0 0 0 0 0 0 0 0
12.2057 9.0461 0.9924 C 0 0 0 0 0 0 0 0 0 0 0 0
12.0539 12.5386 1.2431 C 0 0 0 0 0 0 0 0 0 0 0 0
10.7207 11.9934 0.6896 C 0 0 0 0 0 0 0 0 0 0 0 0
9.5504 12.7682 1.3251 C 0 0 0 0 0 0 0 0 0 0 0 0
8.2170 12.2271 0.7737 C 0 0 0 0 0 0 0 0 0 0 0 0
7.0459 12.9999 1.4109 C 0 0 0 0 0 0 0 0 0 0 0 0
12.3989 10.7159 -1.8687 C 0 0 0 0 0 0 0 0 0 0 0 0
12.2982 7.5921 1.4934 C 0 0 0 0 0 0 0 0 0 0 0 0
10.9840 6.8540 1.1725 C 0 0 0 0 0 0 0 0 0 0 0 0
11.0752 5.4000 1.6748 C 0 0 0 0 0 0 0 0 0 0 0 0
5.7125 12.4576 0.8604 C 0 0 0 0 0 0 0 0 0 0 0 0
9.7602 4.6626 1.3550 C 0 0 0 0 0 0 0 0 0 0 0 0
9.8509 3.2085 1.8578 C 0 0 0 0 0 0 0 0 0 0 0 0
8.5359 2.4714 1.5385 C 0 0 0 0 0 0 0 0 0 0 0 0
4.5410 13.2296 1.4984 C 0 0 0 0 0 0 0 0 0 0 0 0
3.2077 12.6870 0.9483 C 0 0 0 0 0 0 0 0 0 0 0 0
14.7481 12.1992 1.0807 H 0 0 0 0 0 0 0 0 0 0 0 0
13.6313 9.8406 2.3969 H 0 0 0 0 0 0 0 0 0 0 0 0
14.3552 9.2323 0.9064 H 0 0 0 0 0 0 0 0 0 0 0 0
14.5086 11.0500 -1.5971 H 0 0 0 0 0 0 0 0 0 0 0 0
13.5481 12.5301 -1.6456 H 0 0 0 0 0 0 0 0 0 0 0 0
12.0431 9.0471 -0.0674 H 0 0 0 0 0 0 0 0 0 0 0 0
11.3852 9.5383 1.4842 H 0 0 0 0 0 0 0 0 0 0 0 0
12.1694 13.5685 0.9287 H 0 0 0 0 0 0 0 0 0 0 0 0
12.0376 12.4968 2.3251 H 0 0 0 0 0 0 0 0 0 0 0 0
10.6943 12.1249 -0.3779 H 0 0 0 0 0 0 0 0 0 0 0 0
10.6273 10.9521 0.9263 H 0 0 0 0 0 0 0 0 0 0 0 0
9.5747 12.6407 2.4003 H 0 0 0 0 0 0 0 0 0 0 0 0
9.6406 13.8201 1.0840 H 0 0 0 0 0 0 0 0 0 0 0 0
8.1921 12.3558 -0.3013 H 0 0 0 0 0 0 0 0 0 0 0 0
8.1278 11.1747 1.0135 H 0 0 0 0 0 0 0 0 0 0 0 0
7.0713 12.8718 2.4860 H 0 0 0 0 0 0 0 0 0 0 0 0
7.1344 14.0522 1.1705 H 0 0 0 0 0 0 0 0 0 0 0 0
12.4832 9.6821 -1.5829 H 0 0 0 0 0 0 0 0 0 0 0 0
11.4734 11.1160 -1.5040 H 0 0 0 0 0 0 0 0 0 0 0 0
12.4211 10.7900 -2.9488 H 0 0 0 0 0 0 0 0 0 0 0 0
12.4619 7.5889 2.5640 H 0 0 0 0 0 0 0 0 0 0 0 0
13.1228 7.0921 1.0006 H 0 0 0 0 0 0 0 0 0 0 0 0
10.8207 6.8566 0.1019 H 0 0 0 0 0 0 0 0 0 0 0 0
10.1594 7.3548 1.6646 H 0 0 0 0 0 0 0 0 0 0 0 0
11.2393 5.3975 2.7453 H 0 0 0 0 0 0 0 0 0 0 0 0
11.8992 4.8988 1.1821 H 0 0 0 0 0 0 0 0 0 0 0 0
5.6867 12.5862 -0.2146 H 0 0 0 0 0 0 0 0 0 0 0 0
5.6243 11.4051 1.1003 H 0 0 0 0 0 0 0 0 0 0 0 0
9.5963 4.6648 0.2845 H 0 0 0 0 0 0 0 0 0 0 0 0
8.9362 5.1640 1.8474 H 0 0 0 0 0 0 0 0 0 0 0 0
10.0152 3.2063 2.9282 H 0 0 0 0 0 0 0 0 0 0 0 0
10.6747 2.7069 1.3651 H 0 0 0 0 0 0 0 0 0 0 0 0
8.5998 1.4497 1.8919 H 0 0 0 0 0 0 0 0 0 0 0 0
7.7121 2.9729 2.0312 H 0 0 0 0 0 0 0 0 0 0 0 0
8.3716 2.4735 0.4681 H 0 0 0 0 0 0 0 0 0 0 0 0
4.5669 13.1012 2.5734 H 0 0 0 0 0 0 0 0 0 0 0 0
4.6289 14.2820 1.2584 H 0 0 0 0 0 0 0 0 0 0 0 0
3.1197 11.6346 1.1884 H 0 0 0 0 0 0 0 0 0 0 0 0
2.3848 13.2296 1.3968 H 0 0 0 0 0 0 0 0 0 0 0 0
3.1817 12.8155 -0.1267 H 0 0 0 0 0 0 0 0 0 0 0 0
1 2 1 0 0 0 0
3 1 1 0 0 0 0
4 2 1 0 0 0 0

```
  6  5  1  0  0  0  0
  5  1  1  0  0  0  0
  6  7  1  0  0  0  0
  7  8  1  0  0  0  0
  8  9  1  0  0  0  0
  3 10  1  0  0  0  0
  4 11  1  0  0  0  0
 11 12  1  0  0  0  0
 12 13  1  0  0  0  0
  9 14  1  0  0  0  0
 13 15  1  0  0  0  0
 15 16  1  0  0  0  0
 16 17  1  0  0  0  0
 14 18  1  0  0  0  0
 18 19  1  0  0  0  0
  1 20  1  0  0  0  0
  2 21  1  0  0  0  0
  2 22  1  0  0  0  0
  3 23  1  0  0  0  0
  3 24  1  0  0  0  0
  4 25  1  0  0  0  0
  4 26  1  0  0  0  0
  5 27  1  0  0  0  0
  5 28  1  0  0  0  0
  6 29  1  0  0  0  0
  6 30  1  0  0  0  0
  7 31  1  0  0  0  0
  7 32  1  0  0  0  0
  8 33  1  0  0  0  0
  8 34  1  0  0  0  0
  9 35  1  0  0  0  0
  9 36  1  0  0  0  0
 10 37  1  0  0  0  0
 10 38  1  0  0  0  0
 10 39  1  0  0  0  0
 11 40  1  0  0  0  0
 11 41  1  0  0  0  0
 12 42  1  0  0  0  0
 12 43  1  0  0  0  0
 13 44  1  0  0  0  0
 13 45  1  0  0  0  0
 14 46  1  0  0  0  0
 14 47  1  0  0  0  0
 15 48  1  0  0  0  0
 15 49  1  0  0  0  0
 16 50  1  0  0  0  0
 16 51  1  0  0  0  0
 17 52  1  0  0  0  0
 17 53  1  0  0  0  0
 17 54  1  0  0  0  0
 18 55  1  0  0  0  0
 18 56  1  0  0  0  0
 19 57  1  0  0  0  0
 19 58  1  0  0  0  0
 19 59  1  0  0  0  0
M  END
>  <Alias name>
Ethyldioctylsilane

>  <deltaH°(c)>   kJ/mol
-13224

$$$$
```

Trihexylsilane
 ChemBrain IXL

```
 59 58  0  0  0  0  0  0  0  0  1 V2000
    3.6956    7.2965   -1.8478 Si  0  0  0  0  0  0  0  0  0  0  0  0
    3.7257    8.7703   -0.6702 C   0  0  0  0  0  0  0  0  0  0  0  0
    5.1017    7.3795   -3.1032 C   0  0  0  0  0  0  0  0  0  0  0  0
    4.9532    8.7104    0.2628 C   0  0  0  0  0  0  0  0  0  0  0  0
    3.6931    5.6578   -0.9125 C   0  0  0  0  0  0  0  0  0  0  0  0
    5.0381    5.4277   -0.1922 C   0  0  0  0  0  0  0  0  0  0  0  0
    4.9514    4.1399    0.6491 C   0  0  0  0  0  0  0  0  0  0  0  0
    6.2944    3.9042    1.3672 C   0  0  0  0  0  0  0  0  0  0  0  0
    6.2080    2.6160    2.2089 C   0  0  0  0  0  0  0  0  0  0  0  0
    6.4725    7.4446   -2.3980 C   0  0  0  0  0  0  0  0  0  0  0  0
    7.5920    7.3948   -3.4553 C   0  0  0  0  0  0  0  0  0  0  0  0
    8.9631    7.4637   -2.7551 C   0  0  0  0  0  0  0  0  0  0  0  0
   10.0832    7.4138   -3.8125 C   0  0  0  0  0  0  0  0  0  0  0  0
    5.0004    9.9866    1.1248 C   0  0  0  0  0  0  0  0  0  0  0  0
    6.2230    9.9290    2.0612 C   0  0  0  0  0  0  0  0  0  0  0  0
    6.2706   11.2055    2.9234 C   0  0  0  0  0  0  0  0  0  0  0  0
   11.4543    7.4829   -3.1124 C   0  0  0  0  0  0  0  0  0  0  0  0
    7.5509    2.3802    2.9270 C   0  0  0  0  0  0  0  0  0  0  0  0
    7.4931   11.1479    3.8598 C   0  0  0  0  0  0  0  0  0  0  0  0
    2.4271    7.3688   -2.6065 H   0  0  0  0  0  0  0  0  0  0  0  0
    3.7578    9.6858   -1.2478 H   0  0  0  0  0  0  0  0  0  0  0  0
    2.8261    8.7613   -0.0675 H   0  0  0  0  0  0  0  0  0  0  0  0
    4.9794    8.2647   -3.7149 H   0  0  0  0  0  0  0  0  0  0  0  0
    5.0647    6.5017   -3.7364 H   0  0  0  0  0  0  0  0  0  0  0  0
    4.8739    7.8516    0.9060 H   0  0  0  0  0  0  0  0  0  0  0  0
    5.8503    8.6421   -0.3201 H   0  0  0  0  0  0  0  0  0  0  0  0
    3.5278    4.8493   -1.6137 H   0  0  0  0  0  0  0  0  0  0  0  0
    2.8921    5.6661   -0.1838 H   0  0  0  0  0  0  0  0  0  0  0  0
    5.2533    6.2582    0.4508 H   0  0  0  0  0  0  0  0  0  0  0  0
    5.8225    5.3223   -0.9210 H   0  0  0  0  0  0  0  0  0  0  0  0
    4.7361    3.2992    0.0012 H   0  0  0  0  0  0  0  0  0  0  0  0
    4.1621    4.2410    1.3837 H   0  0  0  0  0  0  0  0  0  0  0  0
    6.5097    4.7449    2.0151 H   0  0  0  0  0  0  0  0  0  0  0  0
    7.0837    3.8031    0.6326 H   0  0  0  0  0  0  0  0  0  0  0  0
    5.9926    1.7753    1.5611 H   0  0  0  0  0  0  0  0  0  0  0  0
    5.4187    2.7171    2.9436 H   0  0  0  0  0  0  0  0  0  0  0  0
    6.5486    8.3644   -1.8449 H   0  0  0  0  0  0  0  0  0  0  0  0
    6.5777    6.6130   -1.7296 H   0  0  0  0  0  0  0  0  0  0  0  0
    7.5187    6.4707   -4.0153 H   0  0  0  0  0  0  0  0  0  0  0  0
    7.4887    8.2353   -4.1304 H   0  0  0  0  0  0  0  0  0  0  0  0
    9.0363    8.3878   -2.1950 H   0  0  0  0  0  0  0  0  0  0  0  0
    9.0663    6.6232   -2.0800 H   0  0  0  0  0  0  0  0  0  0  0  0
   10.0101    6.4897   -4.3725 H   0  0  0  0  0  0  0  0  0  0  0  0
    9.9800    8.2542   -4.4877 H   0  0  0  0  0  0  0  0  0  0  0  0
    5.0791   10.8541    0.4813 H   0  0  0  0  0  0  0  0  0  0  0  0
    4.0958   10.0565    1.7161 H   0  0  0  0  0  0  0  0  0  0  0  0
    6.1442    9.0614    2.7046 H   0  0  0  0  0  0  0  0  0  0  0  0
    7.1276    9.8589    1.4698 H   0  0  0  0  0  0  0  0  0  0  0  0
    6.3493   12.0730    2.2800 H   0  0  0  0  0  0  0  0  0  0  0  0
    5.3660   11.2756    3.5148 H   0  0  0  0  0  0  0  0  0  0  0  0
   11.5276    8.4070   -2.5525 H   0  0  0  0  0  0  0  0  0  0  0  0
   12.2412    7.4478   -3.8556 H   0  0  0  0  0  0  0  0  0  0  0  0
   11.5577    6.6424   -2.4373 H   0  0  0  0  0  0  0  0  0  0  0  0
    7.7663    3.2209    3.5749 H   0  0  0  0  0  0  0  0  0  0  0  0
    8.3402    2.2790    2.1924 H   0  0  0  0  0  0  0  0  0  0  0  0
    7.4899    1.4750    3.5184 H   0  0  0  0  0  0  0  0  0  0  0  0
    7.5263   12.0449    4.4656 H   0  0  0  0  0  0  0  0  0  0  0  0
    8.3977   11.0778    3.2686 H   0  0  0  0  0  0  0  0  0  0  0  0
    7.4143   10.2804    4.5034 H   0  0  0  0  0  0  0  0  0  0  0  0
```

```
  1  2  1  0  0  0  0
  3  1  1  0  0  0  0
  4  2  1  0  0  0  0
  6  5  1  0  0  0  0
  5  1  1  0  0  0  0
  6  7  1  0  0  0  0
  7  8  1  0  0  0  0
  8  9  1  0  0  0  0
  3 10  1  0  0  0  0
 10 11  1  0  0  0  0
 11 12  1  0  0  0  0
 12 13  1  0  0  0  0
  4 14  1  0  0  0  0
 14 15  1  0  0  0  0
 15 16  1  0  0  0  0
 13 17  1  0  0  0  0
  9 18  1  0  0  0  0
 16 19  1  0  0  0  0
  1 20  1  0  0  0  0
  2 21  1  0  0  0  0
  2 22  1  0  0  0  0
  3 23  1  0  0  0  0
  3 24  1  0  0  0  0
  4 25  1  0  0  0  0
  4 26  1  0  0  0  0
  5 27  1  0  0  0  0
  5 28  1  0  0  0  0
  6 29  1  0  0  0  0
  6 30  1  0  0  0  0
  7 31  1  0  0  0  0
  7 32  1  0  0  0  0
  8 33  1  0  0  0  0
  8 34  1  0  0  0  0
  9 35  1  0  0  0  0
  9 36  1  0  0  0  0
 10 37  1  0  0  0  0
 10 38  1  0  0  0  0
 11 39  1  0  0  0  0
 11 40  1  0  0  0  0
 12 41  1  0  0  0  0
 12 42  1  0  0  0  0
 13 43  1  0  0  0  0
 13 44  1  0  0  0  0
 14 45  1  0  0  0  0
 14 46  1  0  0  0  0
 15 47  1  0  0  0  0
 15 48  1  0  0  0  0
 16 49  1  0  0  0  0
 16 50  1  0  0  0  0
 17 51  1  0  0  0  0
 17 52  1  0  0  0  0
 17 53  1  0  0  0  0
 18 54  1  0  0  0  0
 18 55  1  0  0  0  0
 18 56  1  0  0  0  0
 19 57  1  0  0  0  0
 19 58  1  0  0  0  0
 19 59  1  0  0  0  0
M  END
>  <Alias name>
Trihexylsilane

>  <deltaH°(c)>   kJ/mol
```

```
-13222

$$$$
Propyl oleate
  ChemBrain IXL

 63 62  0  0  0  0  0  0  0  0  1 V2000
   12.9429   10.1061    0.0000 C   0  0  0  0  0  0  0  0  0  0  0  0
   11.6198    9.9306    0.0618 C   0  0  0  0  0  0  0  0  0  0  0  0
   10.7175   11.1040    0.1260 C   0  0  0  0  0  0  0  0  0  0  0  0
    9.2524   10.6313    0.0605 C   0  0  0  0  0  0  0  0  0  0  0  0
    8.3158   11.8531    0.1276 C   0  0  0  0  0  0  0  0  0  0  0  0
    6.8504   11.3817    0.0618 C   0  0  0  0  0  0  0  0  0  0  0  0
    5.9143   12.6040    0.1294 C   0  0  0  0  0  0  0  0  0  0  0  0
    4.4486   12.1330    0.0636 C   0  0  0  0  0  0  0  0  0  0  0  0
    3.5126   13.3554    0.1317 C   0  0  0  0  0  0  0  0  0  0  0  0
   13.5107   11.4746   -0.0044 C   0  0  0  0  0  0  0  0  0  0  0  0
    2.0468   12.8846    0.0660 C   0  0  0  0  0  0  0  0  0  0  0  0
   15.0481   11.3947    0.0578 C   0  0  0  0  0  0  0  0  0  0  0  0
   15.6358   12.8190    0.0529 C   0  0  0  0  0  0  0  0  0  0  0  0
   17.1730   12.7414    0.1176 C   0  0  0  0  0  0  0  0  0  0  0  0
   17.7588   14.1663    0.1115 C   0  0  0  0  0  0  0  0  0  0  0  0
   19.2959   14.0904    0.1797 C   0  0  0  0  0  0  0  0  0  0  0  0
   19.8806   15.5163    0.1717 C   0  0  0  0  0  0  0  0  0  0  0  0
   21.3577   15.4467    0.2417 C   0  0  0  0  0  0  0  0  0  0  0  0
   22.0042   16.4582    0.4616 O   0  0  0  0  0  0  0  0  0  0  0  0
   22.0456   14.1585    0.0461 O   0  0  0  0  0  0  0  0  0  0  0  0
   23.5131   14.2125    0.1381 C   0  0  0  0  0  0  0  0  0  0  0  0
   24.0938   12.8025   -0.0823 C   0  0  0  0  0  0  0  0  0  0  0  0
   25.6308   12.8591    0.0139 C   0  0  0  0  0  0  0  0  0  0  0  0
   13.6008    9.2471   -0.0469 H   0  0  0  0  0  0  0  0  0  0  0  0
   11.2068    8.9295    0.0649 H   0  0  0  0  0  0  0  0  0  0  0  0
   10.8836   11.6365    1.0543 H   0  0  0  0  0  0  0  0  0  0  0  0
   10.9216   11.7625   -0.7093 H   0  0  0  0  0  0  0  0  0  0  0  0
    9.0858   10.0992   -0.8679 H   0  0  0  0  0  0  0  0  0  0  0  0
    9.0477    9.9731    0.8958 H   0  0  0  0  0  0  0  0  0  0  0  0
    8.4825   12.3851    1.0561 H   0  0  0  0  0  0  0  0  0  0  0  0
    8.5211   12.5115   -0.7075 H   0  0  0  0  0  0  0  0  0  0  0  0
    6.6835   10.8502   -0.8669 H   0  0  0  0  0  0  0  0  0  0  0  0
    6.6451   10.7232    0.8966 H   0  0  0  0  0  0  0  0  0  0  0  0
    6.0813   13.1355    1.0581 H   0  0  0  0  0  0  0  0  0  0  0  0
    6.1197   13.2626   -0.7054 H   0  0  0  0  0  0  0  0  0  0  0  0
    4.2816   11.6017   -0.8652 H   0  0  0  0  0  0  0  0  0  0  0  0
    4.2434   11.4742    0.8983 H   0  0  0  0  0  0  0  0  0  0  0  0
    3.6797   13.8867    1.0605 H   0  0  0  0  0  0  0  0  0  0  0  0
    3.7178   14.0142   -0.7029 H   0  0  0  0  0  0  0  0  0  0  0  0
   13.2125   11.9860   -0.9113 H   0  0  0  0  0  0  0  0  0  0  0  0
   13.1429   12.0207    0.8555 H   0  0  0  0  0  0  0  0  0  0  0  0
    1.8415   12.2257    0.9006 H   0  0  0  0  0  0  0  0  0  0  0  0
    1.3893   13.7436    0.1140 H   0  0  0  0  0  0  0  0  0  0  0  0
    1.8796   12.3534   -0.8629 H   0  0  0  0  0  0  0  0  0  0  0  0
   15.3469   10.8840    0.9649 H   0  0  0  0  0  0  0  0  0  0  0  0
   15.4167   10.8490   -0.8020 H   0  0  0  0  0  0  0  0  0  0  0  0
   15.3380   13.3291   -0.8549 H   0  0  0  0  0  0  0  0  0  0  0  0
   15.2656   13.3651    0.9118 H   0  0  0  0  0  0  0  0  0  0  0  0
   17.4710   12.2324    1.0258 H   0  0  0  0  0  0  0  0  0  0  0  0
   17.5435   12.1950   -0.7409 H   0  0  0  0  0  0  0  0  0  0  0  0
   17.4625   14.6745   -0.7978 H   0  0  0  0  0  0  0  0  0  0  0  0
   17.3862   14.7134    0.9686 H   0  0  0  0  0  0  0  0  0  0  0  0
   19.5923   13.5836    1.0898 H   0  0  0  0  0  0  0  0  0  0  0  0
   19.6689   13.5423   -0.6767 H   0  0  0  0  0  0  0  0  0  0  0  0
   19.5860   16.0219   -0.7396 H   0  0  0  0  0  0  0  0  0  0  0  0
   19.5046   16.0650    1.0264 H   0  0  0  0  0  0  0  0  0  0  0  0
```

```
  23.8004   14.5719    1.1186 H   0  0  0  0  0  0  0  0  0  0  0  0
  23.8980   14.8839   -0.6196 H   0  0  0  0  0  0  0  0  0  0  0  0
  23.8065   12.4430   -1.0625 H   0  0  0  0  0  0  0  0  0  0  0  0
  23.7092   12.1313    0.6755 H   0  0  0  0  0  0  0  0  0  0  0  0
  26.0155   13.5303   -0.7441 H   0  0  0  0  0  0  0  0  0  0  0  0
  26.0389   11.8680   -0.1408 H   0  0  0  0  0  0  0  0  0  0  0  0
  25.9182   13.2189    0.9941 H   0  0  0  0  0  0  0  0  0  0  0  0
  1  2  2  0  0  0  0
  2  3  1  0  0  0  0
  3  4  1  0  0  0  0
  4  5  1  0  0  0  0
  5  6  1  0  0  0  0
  6  7  1  0  0  0  0
  7  8  1  0  0  0  0
  8  9  1  0  0  0  0
  1 10  1  0  0  0  0
  9 11  1  0  0  0  0
 12 13  1  0  0  0  0
 13 14  1  0  0  0  0
 14 15  1  0  0  0  0
 15 16  1  0  0  0  0
 16 17  1  0  0  0  0
 17 18  1  0  0  0  0
 10 12  1  0  0  0  0
 18 19  2  0  0  0  0
 18 20  1  0  0  0  0
 20 21  1  0  0  0  0
 21 22  1  0  0  0  0
 22 23  1  0  0  0  0
  1 24  1  0  0  0  0
  2 25  1  0  0  0  0
  3 26  1  0  0  0  0
  3 27  1  0  0  0  0
  4 28  1  0  0  0  0
  4 29  1  0  0  0  0
  5 30  1  0  0  0  0
  5 31  1  0  0  0  0
  6 32  1  0  0  0  0
  6 33  1  0  0  0  0
  7 34  1  0  0  0  0
  7 35  1  0  0  0  0
  8 36  1  0  0  0  0
  8 37  1  0  0  0  0
  9 38  1  0  0  0  0
  9 39  1  0  0  0  0
 10 40  1  0  0  0  0
 10 41  1  0  0  0  0
 11 42  1  0  0  0  0
 11 43  1  0  0  0  0
 11 44  1  0  0  0  0
 12 45  1  0  0  0  0
 12 46  1  0  0  0  0
 13 47  1  0  0  0  0
 13 48  1  0  0  0  0
 14 49  1  0  0  0  0
 14 50  1  0  0  0  0
 15 51  1  0  0  0  0
 15 52  1  0  0  0  0
 16 53  1  0  0  0  0
 16 54  1  0  0  0  0
 17 55  1  0  0  0  0
 17 56  1  0  0  0  0
 21 57  1  0  0  0  0
```

```
 21 58  1  0  0  0  0
 22 59  1  0  0  0  0
 22 60  1  0  0  0  0
 23 61  1  0  0  0  0
 23 62  1  0  0  0  0
 23 63  1  0  0  0  0
M  END
>  <Alias name>
Propyl oleate

>  <deltaH°(c)>   kJ/mol
-13197.4

$$$$
Methyl (Z) 11-eicosenoate
  ChemBrain IXL

 63 62  0  0  0  0  0  0  0  0  1 V2000
   15.4680   12.5174   -0.0894 C   0  0  0  0  0  0  0  0  0  0  0  0
   14.7028   11.1808   -0.0341 C   0  0  0  0  0  0  0  0  0  0  0  0
   16.9820   12.2456    0.0012 C   0  0  0  0  0  0  0  0  0  0  0  0
   13.1883   11.4509   -0.1237 C   0  0  0  0  0  0  0  0  0  0  0  0
   17.7493   13.5807   -0.0587 C   0  0  0  0  0  0  0  0  0  0  0  0
   12.4545   10.1663   -0.0724 C   0  0  0  0  0  0  0  0  0  0  0  0
   19.2628   13.3062    0.0321 C   0  0  0  0  0  0  0  0  0  0  0  0
   11.1193   10.1588   -0.0847 C   0  0  0  0  0  0  0  0  0  0  0  0
   20.0327   14.6392   -0.0345 C   0  0  0  0  0  0  0  0  0  0  0  0
   10.3722   11.4350   -0.1496 C   0  0  0  0  0  0  0  0  0  0  0  0
   21.5457   14.3619    0.0568 C   0  0  0  0  0  0  0  0  0  0  0  0
    8.8595   11.1493   -0.0736 C   0  0  0  0  0  0  0  0  0  0  0  0
   22.3181   15.6927   -0.0172 C   0  0  0  0  0  0  0  0  0  0  0  0
    8.0814   12.4781   -0.1396 C   0  0  0  0  0  0  0  0  0  0  0  0
   23.7707   15.4246    0.0733 C   0  0  0  0  0  0  0  0  0  0  0  0
    6.5688   12.1924   -0.0613 C   0  0  0  0  0  0  0  0  0  0  0  0
   24.4977   16.1988    0.6738 O   0  0  0  0  0  0  0  0  0  0  0  0
   24.3374   14.2255   -0.5603 O   0  0  0  0  0  0  0  0  0  0  0  0
    5.7908   13.5213   -0.1261 C   0  0  0  0  0  0  0  0  0  0  0  0
    4.2783   13.2359   -0.0464 C   0  0  0  0  0  0  0  0  0  0  0  0
    3.5007   14.5650   -0.1106 C   0  0  0  0  0  0  0  0  0  0  0  0
    1.9883   14.2798   -0.0303 C   0  0  0  0  0  0  0  0  0  0  0  0
   25.7900   14.0662   -0.4010 C   0  0  0  0  0  0  0  0  0  0  0  0
   15.2468   13.0218   -1.0219 H   0  0  0  0  0  0  0  0  0  0  0  0
   15.1630   13.1428    0.7406 H   0  0  0  0  0  0  0  0  0  0  0  0
   14.9249   10.6761    0.8981 H   0  0  0  0  0  0  0  0  0  0  0  0
   15.0077   10.5558   -0.8643 H   0  0  0  0  0  0  0  0  0  0  0  0
   17.2033   11.7439    0.9351 H   0  0  0  0  0  0  0  0  0  0  0  0
   17.2860   11.6173   -0.8268 H   0  0  0  0  0  0  0  0  0  0  0  0
   12.9662   11.9566   -1.0553 H   0  0  0  0  0  0  0  0  0  0  0  0
   12.8830   12.0745    0.7074 H   0  0  0  0  0  0  0  0  0  0  0  0
   17.5286   14.0826   -0.9927 H   0  0  0  0  0  0  0  0  0  0  0  0
   17.4458   14.2091    0.7695 H   0  0  0  0  0  0  0  0  0  0  0  0
   13.0007    9.2323   -0.0248 H   0  0  0  0  0  0  0  0  0  0  0  0
   19.4837   12.8084    0.9681 H   0  0  0  0  0  0  0  0  0  0  0  0
   19.5649   12.6734   -0.7932 H   0  0  0  0  0  0  0  0  0  0  0  0
   10.5829    9.2187   -0.0472 H   0  0  0  0  0  0  0  0  0  0  0  0
   19.8127   15.1371   -0.9708 H   0  0  0  0  0  0  0  0  0  0  0  0
   19.7311   15.2723    0.7908 H   0  0  0  0  0  0  0  0  0  0  0  0
   10.6626   12.0660    0.6813 H   0  0  0  0  0  0  0  0  0  0  0  0
   10.5981   11.9386   -1.0815 H   0  0  0  0  0  0  0  0  0  0  0  0
   21.7661   13.8684    0.9953 H   0  0  0  0  0  0  0  0  0  0  0  0
   21.8460   13.7242   -0.7654 H   0  0  0  0  0  0  0  0  0  0  0  0
    8.5689   10.5191   -0.9051 H   0  0  0  0  0  0  0  0  0  0  0  0
    8.6338   10.6447    0.8578 H   0  0  0  0  0  0  0  0  0  0  0  0
```

```
  22.0997   16.1854   -0.9566 H   0  0  0  0  0  0  0  0  0  0  0  0
  22.0169   16.3313    0.8040 H   0  0  0  0  0  0  0  0  0  0  0  0
   8.3730   13.1088    0.6911 H   0  0  0  0  0  0  0  0  0  0  0  0
   8.3058   12.9819   -1.0716 H   0  0  0  0  0  0  0  0  0  0  0  0
   6.2769   11.5624   -0.8925 H   0  0  0  0  0  0  0  0  0  0  0  0
   6.3444   11.6878    0.8703 H   0  0  0  0  0  0  0  0  0  0  0  0
   6.0834   14.1517    0.7045 H   0  0  0  0  0  0  0  0  0  0  0  0
   6.0144   14.0254   -1.0582 H   0  0  0  0  0  0  0  0  0  0  0  0
   3.9856   12.6059   -0.8772 H   0  0  0  0  0  0  0  0  0  0  0  0
   4.0548   12.7315    0.8855 H   0  0  0  0  0  0  0  0  0  0  0  0
   3.7938   15.1952    0.7200 H   0  0  0  0  0  0  0  0  0  0  0  0
   3.7240   15.0692   -1.0427 H   0  0  0  0  0  0  0  0  0  0  0  0
   1.6952   13.6497   -0.8608 H   0  0  0  0  0  0  0  0  0  0  0  0
   1.7650   13.7756    0.9018 H   0  0  0  0  0  0  0  0  0  0  0  0
   1.4420   15.2139   -0.0754 H   0  0  0  0  0  0  0  0  0  0  0  0
  26.2967   14.9164   -0.8407 H   0  0  0  0  0  0  0  0  0  0  0  0
  26.1102   13.1586   -0.8975 H   0  0  0  0  0  0  0  0  0  0  0  0
  26.0331   14.0064    0.6527 H   0  0  0  0  0  0  0  0  0  0  0  0
  1  2  1  0  0  0  0
  1  3  1  0  0  0  0
  2  4  1  0  0  0  0
  3  5  1  0  0  0  0
  4  6  1  0  0  0  0
  5  7  1  0  0  0  0
  6  8  2  0  0  0  0
  7  9  1  0  0  0  0
  8 10  1  0  0  0  0
  9 11  1  0  0  0  0
 10 12  1  0  0  0  0
 11 13  1  0  0  0  0
 12 14  1  0  0  0  0
 13 15  1  0  0  0  0
 14 16  1  0  0  0  0
 15 17  2  0  0  0  0
 15 18  1  0  0  0  0
 16 19  1  0  0  0  0
 19 20  1  0  0  0  0
 20 21  1  0  0  0  0
 21 22  1  0  0  0  0
 18 23  1  0  0  0  0
  1 24  1  0  0  0  0
  1 25  1  0  0  0  0
  2 26  1  0  0  0  0
  2 27  1  0  0  0  0
  3 28  1  0  0  0  0
  3 29  1  0  0  0  0
  4 30  1  0  0  0  0
  4 31  1  0  0  0  0
  5 32  1  0  0  0  0
  5 33  1  0  0  0  0
  6 34  1  0  0  0  0
  7 35  1  0  0  0  0
  7 36  1  0  0  0  0
  8 37  1  0  0  0  0
  9 38  1  0  0  0  0
  9 39  1  0  0  0  0
 10 40  1  0  0  0  0
 10 41  1  0  0  0  0
 11 42  1  0  0  0  0
 11 43  1  0  0  0  0
 12 44  1  0  0  0  0
 12 45  1  0  0  0  0
 13 46  1  0  0  0  0
```

```
 13 47  1  0  0  0  0
 14 48  1  0  0  0  0
 14 49  1  0  0  0  0
 16 50  1  0  0  0  0
 16 51  1  0  0  0  0
 19 52  1  0  0  0  0
 19 53  1  0  0  0  0
 20 54  1  0  0  0  0
 20 55  1  0  0  0  0
 21 56  1  0  0  0  0
 21 57  1  0  0  0  0
 22 58  1  0  0  0  0
 22 59  1  0  0  0  0
 22 60  1  0  0  0  0
 23 61  1  0  0  0  0
 23 62  1  0  0  0  0
 23 63  1  0  0  0  0
M  END
>  <Alias name>
Methyl (Z) 11-eicosenoate

>  <deltaH°(c)>   kJ/mol
-13190

$$$$
trans-2-Eicosene
  ChemBrain IXL

 60 59  0  0  0  0  0  0  0  0  1 V2000
    2.0705   13.8631    0.0000 C   0  0  0  0  0  0  0  0  0  0  0  0  0
    3.3968   14.5190    0.0584 C   0  0  0  0  0  0  0  0  0  0  0  0  0
    4.5096   13.7809    0.0287 C   0  0  0  0  0  0  0  0  0  0  0  0  0
    5.8357   14.4372    0.0871 C   0  0  0  0  0  0  0  0  0  0  0  0  0
    6.9434   13.3665    0.0251 C   0  0  0  0  0  0  0  0  0  0  0  0  0
    8.3232   14.0509    0.0840 C   0  0  0  0  0  0  0  0  0  0  0  0  0
    9.4316   12.9813    0.0218 C   0  0  0  0  0  0  0  0  0  0  0  0  0
   10.8107   13.6673    0.0784 C   0  0  0  0  0  0  0  0  0  0  0  0  0
   11.9203   12.5992    0.0166 C   0  0  0  0  0  0  0  0  0  0  0  0  0
   13.2985   13.2873    0.0710 C   0  0  0  0  0  0  0  0  0  0  0  0  0
   14.4095   12.2207    0.0105 C   0  0  0  0  0  0  0  0  0  0  0  0  0
   15.7868   12.9108    0.0632 C   0  0  0  0  0  0  0  0  0  0  0  0  0
   16.8991   11.8455    0.0047 C   0  0  0  0  0  0  0  0  0  0  0  0  0
   18.2755   12.5372    0.0564 C   0  0  0  0  0  0  0  0  0  0  0  0  0
   19.3888   11.4729    0.0000 C   0  0  0  0  0  0  0  0  0  0  0  0  0
   20.7646   12.1657    0.0510 C   0  0  0  0  0  0  0  0  0  0  0  0  0
   21.8785   11.1020   -0.0038 C   0  0  0  0  0  0  0  0  0  0  0  0  0
   23.2540   11.7955    0.0466 C   0  0  0  0  0  0  0  0  0  0  0  0  0
   24.3682   10.7321   -0.0072 C   0  0  0  0  0  0  0  0  0  0  0  0  0
   25.7435   11.4256    0.0428 C   0  0  0  0  0  0  0  0  0  0  0  0  0
    2.1978   12.7875    0.0154 H   0  0  0  0  0  0  0  0  0  0  0  0  0
    1.4789   14.1686    0.8541 H   0  0  0  0  0  0  0  0  0  0  0  0  0
    1.5642   14.1535   -0.9122 H   0  0  0  0  0  0  0  0  0  0  0  0  0
    3.4644   15.5978    0.1253 H   0  0  0  0  0  0  0  0  0  0  0  0  0
    4.4421   12.7018   -0.0382 H   0  0  0  0  0  0  0  0  0  0  0  0  0
    5.9227   14.9933    1.0124 H   0  0  0  0  0  0  0  0  0  0  0  0  0
    5.9406   15.1140   -0.7518 H   0  0  0  0  0  0  0  0  0  0  0  0  0
    6.8559   12.8096   -0.8997 H   0  0  0  0  0  0  0  0  0  0  0  0  0
    6.8396   12.6904    0.8648 H   0  0  0  0  0  0  0  0  0  0  0  0  0
    8.4107   14.6077    1.0088 H   0  0  0  0  0  0  0  0  0  0  0  0  0
    8.4268   14.7270   -0.7556 H   0  0  0  0  0  0  0  0  0  0  0  0  0
    9.3435   12.4234   -0.9023 H   0  0  0  0  0  0  0  0  0  0  0  0  0
    9.3294   12.3061    0.8624 H   0  0  0  0  0  0  0  0  0  0  0  0  0
   10.8984   14.2257    1.0022 H   0  0  0  0  0  0  0  0  0  0  0  0  0
```

```
 10.9126   14.3424   -0.7623 H   0  0  0  0  0  0  0  0  0  0  0  0
 11.8322   12.0399   -0.9066 H   0  0  0  0  0  0  0  0  0  0  0  0
 11.8198   11.9250    0.8582 H   0  0  0  0  0  0  0  0  0  0  0  0
 13.3860   13.8475    0.9937 H   0  0  0  0  0  0  0  0  0  0  0  0
 13.3989   13.9610   -0.7710 H   0  0  0  0  0  0  0  0  0  0  0  0
 14.3218   11.6598   -0.9118 H   0  0  0  0  0  0  0  0  0  0  0  0
 14.3103   11.5477    0.8532 H   0  0  0  0  0  0  0  0  0  0  0  0
 15.8737   13.4728    0.9849 H   0  0  0  0  0  0  0  0  0  0  0  0
 15.8861   13.5829   -0.7801 H   0  0  0  0  0  0  0  0  0  0  0  0
 16.8121   11.2829   -0.9166 H   0  0  0  0  0  0  0  0  0  0  0  0
 16.8005   11.1739    0.8486 H   0  0  0  0  0  0  0  0  0  0  0  0
 18.3617   13.1010    0.9771 H   0  0  0  0  0  0  0  0  0  0  0  0
 18.3743   13.2079   -0.7881 H   0  0  0  0  0  0  0  0  0  0  0  0
 19.3026   10.9087   -0.9204 H   0  0  0  0  0  0  0  0  0  0  0  0
 19.2906   10.8026    0.8449 H   0  0  0  0  0  0  0  0  0  0  0  0
 20.8502   12.7309    0.9708 H   0  0  0  0  0  0  0  0  0  0  0  0
 20.8631   12.8353   -0.7945 H   0  0  0  0  0  0  0  0  0  0  0  0
 21.7928   10.5366   -0.9235 H   0  0  0  0  0  0  0  0  0  0  0  0
 21.7804   10.4328    0.8420 H   0  0  0  0  0  0  0  0  0  0  0  0
 23.3393   12.3615    0.9660 H   0  0  0  0  0  0  0  0  0  0  0  0
 23.3522   12.4644   -0.7995 H   0  0  0  0  0  0  0  0  0  0  0  0
 24.2828   10.1659   -0.9264 H   0  0  0  0  0  0  0  0  0  0  0  0
 24.2702   10.0634    0.8390 H   0  0  0  0  0  0  0  0  0  0  0  0
 25.8417   12.0941   -0.8035 H   0  0  0  0  0  0  0  0  0  0  0  0
 26.5262   10.6782    0.0052 H   0  0  0  0  0  0  0  0  0  0  0  0
 25.8290   11.9919    0.9620 H   0  0  0  0  0  0  0  0  0  0  0  0
  1  2  1  0  0  0  0
  2  3  2  0  0  0  0
  3  4  1  0  0  0  0
  4  5  1  0  0  0  0
  5  6  1  0  0  0  0
  6  7  1  0  0  0  0
  7  8  1  0  0  0  0
  8  9  1  0  0  0  0
  9 10  1  0  0  0  0
 10 11  1  0  0  0  0
 11 12  1  0  0  0  0
 12 13  1  0  0  0  0
 13 14  1  0  0  0  0
 14 15  1  0  0  0  0
 15 16  1  0  0  0  0
 16 17  1  0  0  0  0
 17 18  1  0  0  0  0
 18 19  1  0  0  0  0
 19 20  1  0  0  0  0
  1 21  1  0  0  0  0
  1 22  1  0  0  0  0
  1 23  1  0  0  0  0
  2 24  1  0  0  0  0
  3 25  1  0  0  0  0
  4 26  1  0  0  0  0
  4 27  1  0  0  0  0
  5 28  1  0  0  0  0
  5 29  1  0  0  0  0
  6 30  1  0  0  0  0
  6 31  1  0  0  0  0
  7 32  1  0  0  0  0
  7 33  1  0  0  0  0
  8 34  1  0  0  0  0
  8 35  1  0  0  0  0
  9 36  1  0  0  0  0
  9 37  1  0  0  0  0
 10 38  1  0  0  0  0
```

10 39 1 0 0 0 0
11 40 1 0 0 0 0
11 41 1 0 0 0 0
12 42 1 0 0 0 0
12 43 1 0 0 0 0
13 44 1 0 0 0 0
13 45 1 0 0 0 0
14 46 1 0 0 0 0
14 47 1 0 0 0 0
15 48 1 0 0 0 0
15 49 1 0 0 0 0
16 50 1 0 0 0 0
16 51 1 0 0 0 0
17 52 1 0 0 0 0
17 53 1 0 0 0 0
18 54 1 0 0 0 0
18 55 1 0 0 0 0
19 56 1 0 0 0 0
19 57 1 0 0 0 0
20 58 1 0 0 0 0
20 59 1 0 0 0 0
20 60 1 0 0 0 0
M END
> <Alias name>
trans-2-Eicosene

> <deltaH°(c)>   kJ/mol
-13149.28

$$$$
1-Eicosene
  ChemBrain IXL

 60 59 0 0 0 0 0 0 0 0 1 V2000
    2.1832   13.7107   3.7551 C   0 0 0 0 0 0 0 0 0 0 0 0
    3.4027   14.1076   3.3839 C   0 0 0 0 0 0 0 0 0 0 0 0
    4.4352   13.1041   3.0396 C   0 0 0 0 0 0 0 0 0 0 0 0
    5.7498   13.8222   2.6766 C   0 0 0 0 0 0 0 0 0 0 0 0
    6.8234   12.7771   2.3162 C   0 0 0 0 0 0 0 0 0 0 0 0
    8.1384   13.4945   1.9543 C   0 0 0 0 0 0 0 0 0 0 0 0
    9.2102   12.4488   1.5906 C   0 0 0 0 0 0 0 0 0 0 0 0
   10.5265   13.1649   1.2306 C   0 0 0 0 0 0 0 0 0 0 0 0
   11.5959   12.1183   0.8623 C   0 0 0 0 0 0 0 0 0 0 0 0
   12.9137   12.8329   0.5049 C   0 0 0 0 0 0 0 0 0 0 0 0
   13.9802   11.7853   0.1308 C   0 0 0 0 0 0 0 0 0 0 0 0
   15.2994   12.4982   -0.2244 C   0 0 0 0 0 0 0 0 0 0 0 0
   16.3628   11.4497   -0.6052 C   0 0 0 0 0 0 0 0 0 0 0 0
   17.6833   12.1610   -0.9588 C   0 0 0 0 0 0 0 0 0 0 0 0
   18.7435   11.1119   -1.3465 C   0 0 0 0 0 0 0 0 0 0 0 0
   20.0650   11.8221   -1.6989 C   0 0 0 0 0 0 0 0 0 0 0 0
   21.1229   10.7726   -2.0921 C   0 0 0 0 0 0 0 0 0 0 0 0
   22.4450   11.4821   -2.4436 C   0 0 0 0 0 0 0 0 0 0 0 0
   23.5017   10.4325   -2.8401 C   0 0 0 0 0 0 0 0 0 0 0 0
   24.8242   11.1415   -3.1907 C   0 0 0 0 0 0 0 0 0 0 0 0
    1.9491   12.6543   3.8045 H   0 0 0 0 0 0 0 0 0 0 0 0
    1.4281   14.4449   4.0070 H   0 0 0 0 0 0 0 0 0 0 0 0
    3.6364   15.1640   3.3347 H   0 0 0 0 0 0 0 0 0 0 0 0
    4.0996   12.5165   2.1940 H   0 0 0 0 0 0 0 0 0 0 0 0
    4.6025   12.4524   3.8883 H   0 0 0 0 0 0 0 0 0 0 0 0
    6.0864   14.4086   3.5226 H   0 0 0 0 0 0 0 0 0 0 0 0
    5.5825   14.4749   1.8286 H   0 0 0 0 0 0 0 0 0 0 0 0
    6.4869   12.1913   1.4698 H   0 0 0 0 0 0 0 0 0 0 0 0
    6.9901   12.1239   3.1638 H   0 0 0 0 0 0 0 0 0 0 0 0

```
   8.4763   14.0783    2.8016 H   0  0  0  0  0  0  0  0  0  0  0  0
   7.9713   14.1495    1.1081 H   0  0  0  0  0  0  0  0  0  0  0  0
   8.8727   11.8662    0.7423 H   0  0  0  0  0  0  0  0  0  0  0  0
   9.3759   11.7926    2.4361 H   0  0  0  0  0  0  0  0  0  0  0  0
  10.8660   13.7448    2.0800 H   0  0  0  0  0  0  0  0  0  0  0  0
  10.3602   13.8234    0.3870 H   0  0  0  0  0  0  0  0  0  0  0  0
  11.2568   11.5399    0.0118 H   0  0  0  0  0  0  0  0  0  0  0  0
  11.7604   11.4584    1.7051 H   0  0  0  0  0  0  0  0  0  0  0  0
  13.2553   13.4078    1.3567 H   0  0  0  0  0  0  0  0  0  0  0  0
  12.7485   13.4959   -0.3354 H   0  0  0  0  0  0  0  0  0  0  0  0
  13.6389   11.2118   -0.7221 H   0  0  0  0  0  0  0  0  0  0  0  0
  14.1437   11.1210    0.9703 H   0  0  0  0  0  0  0  0  0  0  0  0
  15.6436   13.0677    0.6300 H   0  0  0  0  0  0  0  0  0  0  0  0
  15.1351   13.1660   -1.0611 H   0  0  0  0  0  0  0  0  0  0  0  0
  16.0188   10.8813   -1.4605 H   0  0  0  0  0  0  0  0  0  0  0  0
  16.5258   10.7808    0.2307 H   0  0  0  0  0  0  0  0  0  0  0  0
  18.0300   12.7255   -0.1021 H   0  0  0  0  0  0  0  0  0  0  0  0
  17.5193   12.8334   -1.7919 H   0  0  0  0  0  0  0  0  0  0  0  0
  18.3969   10.5484   -2.2039 H   0  0  0  0  0  0  0  0  0  0  0  0
  18.9065   10.4387   -0.5140 H   0  0  0  0  0  0  0  0  0  0  0  0
  20.4139   12.3824   -0.8403 H   0  0  0  0  0  0  0  0  0  0  0  0
  19.9013   12.4981   -2.5290 H   0  0  0  0  0  0  0  0  0  0  0  0
  20.7741   10.2130   -2.9513 H   0  0  0  0  0  0  0  0  0  0  0  0
  21.2858   10.0959   -1.2625 H   0  0  0  0  0  0  0  0  0  0  0  0
  22.7950   12.0398   -1.5837 H   0  0  0  0  0  0  0  0  0  0  0  0
  22.2816   12.1605   -3.2718 H   0  0  0  0  0  0  0  0  0  0  0  0
  23.1519    9.8753   -3.7003 H   0  0  0  0  0  0  0  0  0  0  0  0
  23.6646    9.7536   -2.0122 H   0  0  0  0  0  0  0  0  0  0  0  0
  24.6613   11.8207   -4.0183 H   0  0  0  0  0  0  0  0  0  0  0  0
  25.5662   10.4038   -3.4697 H   0  0  0  0  0  0  0  0  0  0  0  0
  25.1744   11.6982   -2.3303 H   0  0  0  0  0  0  0  0  0  0  0  0
  1  2  2  0  0  0  0
  2  3  1  0  0  0  0
  3  4  1  0  0  0  0
  4  5  1  0  0  0  0
  5  6  1  0  0  0  0
  6  7  1  0  0  0  0
  7  8  1  0  0  0  0
  8  9  1  0  0  0  0
  9 10  1  0  0  0  0
 10 11  1  0  0  0  0
 11 12  1  0  0  0  0
 12 13  1  0  0  0  0
 13 14  1  0  0  0  0
 14 15  1  0  0  0  0
 15 16  1  0  0  0  0
 16 17  1  0  0  0  0
 17 18  1  0  0  0  0
 18 19  1  0  0  0  0
 19 20  1  0  0  0  0
  1 21  1  0  0  0  0
  1 22  1  0  0  0  0
  2 23  1  0  0  0  0
  3 24  1  0  0  0  0
  3 25  1  0  0  0  0
  4 26  1  0  0  0  0
  4 27  1  0  0  0  0
  5 28  1  0  0  0  0
  5 29  1  0  0  0  0
  6 30  1  0  0  0  0
  6 31  1  0  0  0  0
  7 32  1  0  0  0  0
  7 33  1  0  0  0  0
```

```
  8 34  1  0  0  0  0
  8 35  1  0  0  0  0
  9 36  1  0  0  0  0
  9 37  1  0  0  0  0
 10 38  1  0  0  0  0
 10 39  1  0  0  0  0
 11 40  1  0  0  0  0
 11 41  1  0  0  0  0
 12 42  1  0  0  0  0
 12 43  1  0  0  0  0
 13 44  1  0  0  0  0
 13 45  1  0  0  0  0
 14 46  1  0  0  0  0
 14 47  1  0  0  0  0
 15 48  1  0  0  0  0
 15 49  1  0  0  0  0
 16 50  1  0  0  0  0
 16 51  1  0  0  0  0
 17 52  1  0  0  0  0
 17 53  1  0  0  0  0
 18 54  1  0  0  0  0
 18 55  1  0  0  0  0
 19 56  1  0  0  0  0
 19 57  1  0  0  0  0
 20 58  1  0  0  0  0
 20 59  1  0  0  0  0
 20 60  1  0  0  0  0
M  END
>  <Alias name>
1-Eicosene

>  <deltaH°(c)>   kJ/mol
-13136.67

$$$$
1-Eicosanol
  ChemBrain IXL

 63 62  0  0  0  0  0  0  0  0  1 V2000
    2.2840   13.0378    0.0000 C   0  0  0  0  0  0  0  0  0  0  0  0
    3.5290   13.9454    0.0266 C   0  0  0  0  0  0  0  0  0  0  0  0
    4.8005   13.0750   -0.0066 C   0  0  0  0  0  0  0  0  0  0  0  0
    6.0456   13.9828    0.0201 C   0  0  0  0  0  0  0  0  0  0  0  0
    7.3169   13.1124   -0.0128 C   0  0  0  0  0  0  0  0  0  0  0  0
    8.5620   14.0202    0.0142 C   0  0  0  0  0  0  0  0  0  0  0  0
    9.8334   13.1497   -0.0181 C   0  0  0  0  0  0  0  0  0  0  0  0
   11.0784   14.0575    0.0095 C   0  0  0  0  0  0  0  0  0  0  0  0
   12.3498   13.1871   -0.0219 C   0  0  0  0  0  0  0  0  0  0  0  0
   13.5949   14.0948    0.0062 C   0  0  0  0  0  0  0  0  0  0  0  0
   14.8662   13.2243   -0.0242 C   0  0  0  0  0  0  0  0  0  0  0  0
   16.1113   14.1320    0.0046 C   0  0  0  0  0  0  0  0  0  0  0  0
   17.3827   13.2615   -0.0247 C   0  0  0  0  0  0  0  0  0  0  0  0
   18.6278   14.1692    0.0047 C   0  0  0  0  0  0  0  0  0  0  0  0
   19.8991   13.2987   -0.0238 C   0  0  0  0  0  0  0  0  0  0  0  0
   21.1442   14.2063    0.0061 C   0  0  0  0  0  0  0  0  0  0  0  0
   22.4155   13.3358   -0.0217 C   0  0  0  0  0  0  0  0  0  0  0  0
   23.6606   14.2435    0.0084 C   0  0  0  0  0  0  0  0  0  0  0  0
   24.9321   13.3728   -0.0191 C   0  0  0  0  0  0  0  0  0  0  0  0
   26.1771   14.2803    0.0112 C   0  0  0  0  0  0  0  0  0  0  0  0
   27.3895   13.4497   -0.0149 O   0  0  0  0  0  0  0  0  0  0  0  0
   28.2358   13.9494    0.0019 H   0  0  0  0  0  0  0  0  0  0  0  0
    2.2959   12.3832    0.8627 H   0  0  0  0  0  0  0  0  0  0  0  0
    1.3908   13.6496    0.0234 H   0  0  0  0  0  0  0  0  0  0  0  0
```

2.2903	12.4427	-0.9048	H	0	0	0	0	0	0	0	0	0	0	0	0
3.5170	14.6000	-0.8360	H	0	0	0	0	0	0	0	0	0	0	0	0
3.5225	14.5405	0.9314	H	0	0	0	0	0	0	0	0	0	0	0	0
4.8125	12.4204	0.8561	H	0	0	0	0	0	0	0	0	0	0	0	0
4.8070	12.4799	-0.9114	H	0	0	0	0	0	0	0	0	0	0	0	0
6.0338	14.6373	-0.8426	H	0	0	0	0	0	0	0	0	0	0	0	0
6.0390	14.5780	0.9249	H	0	0	0	0	0	0	0	0	0	0	0	0
7.3287	12.4578	0.8499	H	0	0	0	0	0	0	0	0	0	0	0	0
7.3237	12.5173	-0.9176	H	0	0	0	0	0	0	0	0	0	0	0	0
8.5505	14.6745	-0.8487	H	0	0	0	0	0	0	0	0	0	0	0	0
8.5551	14.6155	0.9189	H	0	0	0	0	0	0	0	0	0	0	0	0
9.8447	12.4952	0.8447	H	0	0	0	0	0	0	0	0	0	0	0	0
9.8405	12.5545	-0.9228	H	0	0	0	0	0	0	0	0	0	0	0	0
11.0674	14.7117	-0.8536	H	0	0	0	0	0	0	0	0	0	0	0	0
11.0710	14.6531	0.9140	H	0	0	0	0	0	0	0	0	0	0	0	0
12.3606	12.5327	0.8409	H	0	0	0	0	0	0	0	0	0	0	0	0
12.3575	12.5917	-0.9266	H	0	0	0	0	0	0	0	0	0	0	0	0
13.5844	14.7488	-0.8570	H	0	0	0	0	0	0	0	0	0	0	0	0
13.5869	14.6905	0.9106	H	0	0	0	0	0	0	0	0	0	0	0	0
14.8764	12.5701	0.8388	H	0	0	0	0	0	0	0	0	0	0	0	0
14.8745	12.6288	-0.9287	H	0	0	0	0	0	0	0	0	0	0	0	0
16.1015	14.7858	-0.8587	H	0	0	0	0	0	0	0	0	0	0	0	0
16.1027	14.7280	0.9089	H	0	0	0	0	0	0	0	0	0	0	0	0
17.3923	12.6075	0.8385	H	0	0	0	0	0	0	0	0	0	0	0	0
17.3915	12.6658	-0.9291	H	0	0	0	0	0	0	0	0	0	0	0	0
18.6184	14.8229	-0.8587	H	0	0	0	0	0	0	0	0	0	0	0	0
18.6186	14.7653	0.9089	H	0	0	0	0	0	0	0	0	0	0	0	0
19.9083	12.6448	0.8395	H	0	0	0	0	0	0	0	0	0	0	0	0
19.9084	12.7028	-0.9280	H	0	0	0	0	0	0	0	0	0	0	0	0
21.1352	14.8599	-0.8574	H	0	0	0	0	0	0	0	0	0	0	0	0
21.1347	14.8025	0.9102	H	0	0	0	0	0	0	0	0	0	0	0	0
22.4244	12.6821	0.8417	H	0	0	0	0	0	0	0	0	0	0	0	0
22.4252	12.7397	-0.9259	H	0	0	0	0	0	0	0	0	0	0	0	0
23.6519	14.8970	-0.8551	H	0	0	0	0	0	0	0	0	0	0	0	0
23.6509	14.8397	0.9125	H	0	0	0	0	0	0	0	0	0	0	0	0
24.9408	12.7193	0.8444	H	0	0	0	0	0	0	0	0	0	0	0	0
24.9419	12.7767	-0.9231	H	0	0	0	0	0	0	0	0	0	0	0	0
26.1674	14.8765	0.9153	H	0	0	0	0	0	0	0	0	0	0	0	0
26.1686	14.9339	-0.8523	H	0	0	0	0	0	0	0	0	0	0	0	0

1	2	1	0	0	0	0
2	3	1	0	0	0	0
3	4	1	0	0	0	0
4	5	1	0	0	0	0
5	6	1	0	0	0	0
6	7	1	0	0	0	0
7	8	1	0	0	0	0
8	9	1	0	0	0	0
9	10	1	0	0	0	0
10	11	1	0	0	0	0
11	12	1	0	0	0	0
12	13	1	0	0	0	0
13	14	1	0	0	0	0
14	15	1	0	0	0	0
15	16	1	0	0	0	0
16	17	1	0	0	0	0
17	18	1	0	0	0	0
18	19	1	0	0	0	0
19	20	1	0	0	0	0
20	21	1	0	0	0	0
21	22	1	0	0	0	0
1	23	1	0	0	0	0
1	24	1	0	0	0	0
1	25	1	0	0	0	0

```
  2 26  1  0  0  0  0
  2 27  1  0  0  0  0
  3 28  1  0  0  0  0
  3 29  1  0  0  0  0
  4 30  1  0  0  0  0
  4 31  1  0  0  0  0
  5 32  1  0  0  0  0
  5 33  1  0  0  0  0
  6 34  1  0  0  0  0
  6 35  1  0  0  0  0
  7 36  1  0  0  0  0
  7 37  1  0  0  0  0
  8 38  1  0  0  0  0
  8 39  1  0  0  0  0
  9 40  1  0  0  0  0
  9 41  1  0  0  0  0
 10 42  1  0  0  0  0
 10 43  1  0  0  0  0
 11 44  1  0  0  0  0
 11 45  1  0  0  0  0
 12 46  1  0  0  0  0
 12 47  1  0  0  0  0
 13 48  1  0  0  0  0
 13 49  1  0  0  0  0
 14 50  1  0  0  0  0
 14 51  1  0  0  0  0
 15 52  1  0  0  0  0
 15 53  1  0  0  0  0
 16 54  1  0  0  0  0
 16 55  1  0  0  0  0
 17 56  1  0  0  0  0
 17 57  1  0  0  0  0
 18 58  1  0  0  0  0
 18 59  1  0  0  0  0
 19 60  1  0  0  0  0
 19 61  1  0  0  0  0
 20 62  1  0  0  0  0
 20 63  1  0  0  0  0
M  END
>  <Alias name>
Arachic alcohol

>  <deltaH°(c)>   kJ/mol
-13130

$$$$
9,10-Diphenylanthracene
  ChemBrain IXL

 44 48  0  0  0  0  0  0  0  0  1 V2000
    9.2623    3.9547    0.4683 C   0  0  0  0  0  0  0  0  0  0  0  0
    7.8975    3.7465    0.2683 C   0  0  0  0  0  0  0  0  0  0  0  0
    7.0502    4.8336    0.0532 C   0  0  0  0  0  0  0  0  0  0  0  0
    7.5679    6.1289    0.0374 C   0  0  0  0  0  0  0  0  0  0  0  0
    8.9327    6.3371    0.2374 C   0  0  0  0  0  0  0  0  0  0  0  0
    9.7798    5.2500    0.4532 C   0  0  0  0  0  0  0  0  0  0  0  0
    8.6032    8.7196    0.0059 C   0  0  0  0  0  0  0  0  0  0  0  0
    7.2384    8.5114   -0.1941 C   0  0  0  0  0  0  0  0  0  0  0  0
    6.3912    9.5985   -0.4098 C   0  0  0  0  0  0  0  0  0  0  0  0
    6.9088   10.8938   -0.4249 C   0  0  0  0  0  0  0  0  0  0  0  0
    8.2736   11.1020   -0.2249 C   0  0  0  0  0  0  0  0  0  0  0  0
    9.1208   10.0149   -0.0099 C   0  0  0  0  0  0  0  0  0  0  0  0
    9.4503    7.6324    0.2216 C   0  0  0  0  0  0  0  0  0  0  0  0
```

```
    6.7207    7.2161   -0.1783 C   0  0  0  0  0  0  0  0  0  0  0  0
   13.6279    8.2697    0.8336 C   0  0  0  0  0  0  0  0  0  0  0  0
   12.6956    8.9950    1.5758 C   0  0  0  0  0  0  0  0  0  0  0  0
   11.3308    8.7868    1.3759 C   0  0  0  0  0  0  0  0  0  0  0  0
   10.8983    7.8533    0.4337 C   0  0  0  0  0  0  0  0  0  0  0  0
   11.8306    7.1281   -0.3085 C   0  0  0  0  0  0  0  0  0  0  0  0
   13.1954    7.3362   -0.1085 C   0  0  0  0  0  0  0  0  0  0  0  0
    2.9756    7.5115    0.1524 C   0  0  0  0  0  0  0  0  0  0  0  0
    4.3404    7.7197    0.3524 C   0  0  0  0  0  0  0  0  0  0  0  0
    5.2727    6.9952   -0.3905 C   0  0  0  0  0  0  0  0  0  0  0  0
    4.8403    6.0625   -1.3334 C   0  0  0  0  0  0  0  0  0  0  0  0
    3.4755    5.8543   -1.5334 C   0  0  0  0  0  0  0  0  0  0  0  0
    2.5431    6.5788   -0.7905 C   0  0  0  0  0  0  0  0  0  0  0  0
    9.9200    3.1107    0.6353 H   0  0  0  0  0  0  0  0  0  0  0  0
    7.4957    2.7408    0.2801 H   0  0  0  0  0  0  0  0  0  0  0  0
    5.9906    4.6721   -0.1015 H   0  0  0  0  0  0  0  0  0  0  0  0
   10.8393    5.4117    0.6089 H   0  0  0  0  0  0  0  0  0  0  0  0
    5.3317    9.4368   -0.5655 H   0  0  0  0  0  0  0  0  0  0  0  0
    6.2510   11.7379   -0.5919 H   0  0  0  0  0  0  0  0  0  0  0  0
    8.6754   12.1077   -0.2367 H   0  0  0  0  0  0  0  0  0  0  0  0
   10.1805   10.1764    0.1449 H   0  0  0  0  0  0  0  0  0  0  0  0
   14.6875    8.4313    0.9889 H   0  0  0  0  0  0  0  0  0  0  0  0
   13.0314    9.7197    2.3072 H   0  0  0  0  0  0  0  0  0  0  0  0
   10.6070    9.3498    1.9521 H   0  0  0  0  0  0  0  0  0  0  0  0
   11.4948    6.4034   -1.0399 H   0  0  0  0  0  0  0  0  0  0  0  0
   13.9192    6.7731   -0.6846 H   0  0  0  0  0  0  0  0  0  0  0  0
    2.2518    8.0741    0.7291 H   0  0  0  0  0  0  0  0  0  0  0  0
    4.6762    8.4438    1.0844 H   0  0  0  0  0  0  0  0  0  0  0  0
    5.5640    5.5001   -1.9102 H   0  0  0  0  0  0  0  0  0  0  0  0
    3.1397    5.1302   -2.2653 H   0  0  0  0  0  0  0  0  0  0  0  0
    1.4836    6.4172   -0.9457 H   0  0  0  0  0  0  0  0  0  0  0  0
  1  2  4  0  0  0  0
  2  3  4  0  0  0  0
  3  4  4  0  0  0  0
  4  5  4  0  0  0  0
  5  6  4  0  0  0  0
  6  1  4  0  0  0  0
  7  8  4  0  0  0  0
  8  9  4  0  0  0  0
  9 10  4  0  0  0  0
 10 11  4  0  0  0  0
 11 12  4  0  0  0  0
 12  7  4  0  0  0  0
  5 13  4  0  0  0  0
 13  7  4  0  0  0  0
  4 14  4  0  0  0  0
 14  8  4  0  0  0  0
 15 16  4  0  0  0  0
 16 17  4  0  0  0  0
 17 18  4  0  0  0  0
 18 19  4  0  0  0  0
 19 20  4  0  0  0  0
 20 15  4  0  0  0  0
 18 13  1  0  0  0  0
 21 22  4  0  0  0  0
 22 23  4  0  0  0  0
 23 24  4  0  0  0  0
 24 25  4  0  0  0  0
 25 26  4  0  0  0  0
 26 21  4  0  0  0  0
 23 14  1  0  0  0  0
  1 27  1  0  0  0  0
  2 28  1  0  0  0  0
```

```
  3 29  1  0  0  0  0
  6 30  1  0  0  0  0
  9 31  1  0  0  0  0
 10 32  1  0  0  0  0
 11 33  1  0  0  0  0
 12 34  1  0  0  0  0
 15 35  1  0  0  0  0
 16 36  1  0  0  0  0
 17 37  1  0  0  0  0
 19 38  1  0  0  0  0
 20 39  1  0  0  0  0
 21 40  1  0  0  0  0
 22 41  1  0  0  0  0
 24 42  1  0  0  0  0
 25 43  1  0  0  0  0
 26 44  1  0  0  0  0
M  END
>  <Alias name>
9,10-Diphenylanthracene

>  <deltaH°(c)>   kJ/mol
-13112.4

$$$$
Pentadecylcyclopentane
  ChemBrain IXL

 60 60  0  0  0  0  0  0  0  0  1 V2000
   21.2408   11.5783   -0.1666 C   0  0  0  0  0  0  0  0  0  0  0  0
   22.4720   10.8719    0.3814 C   0  0  0  0  0  0  0  0  0  0  0  0
   23.6017   11.8819    0.1967 C   0  0  0  0  0  0  0  0  0  0  0  0
   22.9303   13.2864    0.3512 C   0  0  0  0  0  0  0  0  0  0  0  0
   21.3893   13.0221    0.3082 C   0  0  0  0  0  0  0  0  0  0  0  0
   19.9401   10.9412    0.3625 C   0  0  0  0  0  0  0  0  0  0  0  0
   18.7263   11.7157   -0.1881 C   0  0  0  0  0  0  0  0  0  0  0  0
   17.4269   11.0768    0.3392 C   0  0  0  0  0  0  0  0  0  0  0  0
   16.2133   11.8507   -0.2114 C   0  0  0  0  0  0  0  0  0  0  0  0
   14.9140   11.2119    0.3164 C   0  0  0  0  0  0  0  0  0  0  0  0
   13.7003   11.9854   -0.2347 C   0  0  0  0  0  0  0  0  0  0  0  0
   12.4010   11.3467    0.2935 C   0  0  0  0  0  0  0  0  0  0  0  0
   11.1873   12.1198   -0.2580 C   0  0  0  0  0  0  0  0  0  0  0  0
    9.8881   11.4812    0.2704 C   0  0  0  0  0  0  0  0  0  0  0  0
    8.6742   12.2538   -0.2814 C   0  0  0  0  0  0  0  0  0  0  0  0
    7.3751   11.6152    0.2472 C   0  0  0  0  0  0  0  0  0  0  0  0
    6.1611   12.3876   -0.3050 C   0  0  0  0  0  0  0  0  0  0  0  0
    4.8620   11.7490    0.2238 C   0  0  0  0  0  0  0  0  0  0  0  0
    3.6480   12.5212   -0.3286 C   0  0  0  0  0  0  0  0  0  0  0  0
    2.3489   11.8828    0.2001 C   0  0  0  0  0  0  0  0  0  0  0  0
   21.2624   11.5496   -1.2490 H   0  0  0  0  0  0  0  0  0  0  0  0
   22.3329   10.6734    1.4370 H   0  0  0  0  0  0  0  0  0  0  0  0
   22.6732    9.9520   -0.1532 H   0  0  0  0  0  0  0  0  0  0  0  0
   24.3987   11.7374    0.9155 H   0  0  0  0  0  0  0  0  0  0  0  0
   23.9865   11.7873   -0.8112 H   0  0  0  0  0  0  0  0  0  0  0  0
   23.1910   13.7405    1.2991 H   0  0  0  0  0  0  0  0  0  0  0  0
   23.2387   13.9247   -0.4674 H   0  0  0  0  0  0  0  0  0  0  0  0
   20.8810   13.7200   -0.3454 H   0  0  0  0  0  0  0  0  0  0  0  0
   20.9937   13.0963    1.3137 H   0  0  0  0  0  0  0  0  0  0  0  0
   19.8864    9.9092    0.0387 H   0  0  0  0  0  0  0  0  0  0  0  0
   19.9329   10.9807    1.4447 H   0  0  0  0  0  0  0  0  0  0  0  0
   18.7786   12.7475    0.1367 H   0  0  0  0  0  0  0  0  0  0  0  0
   18.7341   11.6771   -1.2704 H   0  0  0  0  0  0  0  0  0  0  0  0
   17.3745   10.0449    0.0147 H   0  0  0  0  0  0  0  0  0  0  0  0
   17.4190   11.1156    1.4215 H   0  0  0  0  0  0  0  0  0  0  0  0
```

```
 16.2659   12.8827    0.1129 H   0  0  0  0  0  0  0  0  0  0  0  0
 16.2211   11.8116   -1.2937 H   0  0  0  0  0  0  0  0  0  0  0  0
 14.8616   10.1798   -0.0076 H   0  0  0  0  0  0  0  0  0  0  0  0
 14.9061   11.2513    1.3986 H   0  0  0  0  0  0  0  0  0  0  0  0
 13.7527   13.0176    0.0892 H   0  0  0  0  0  0  0  0  0  0  0  0
 13.7081   11.9458   -1.3169 H   0  0  0  0  0  0  0  0  0  0  0  0
 12.3488   10.3145   -0.0300 H   0  0  0  0  0  0  0  0  0  0  0  0
 12.3931   11.3868    1.3757 H   0  0  0  0  0  0  0  0  0  0  0  0
 11.2395   13.1521    0.0653 H   0  0  0  0  0  0  0  0  0  0  0  0
 11.1951   12.0796   -1.3402 H   0  0  0  0  0  0  0  0  0  0  0  0
  9.8360   10.4488   -0.0526 H   0  0  0  0  0  0  0  0  0  0  0  0
  9.8801   11.5217    1.3526 H   0  0  0  0  0  0  0  0  0  0  0  0
  8.7263   13.2863    0.0414 H   0  0  0  0  0  0  0  0  0  0  0  0
  8.6821   12.2132   -1.3636 H   0  0  0  0  0  0  0  0  0  0  0  0
  7.3231   10.5827   -0.0754 H   0  0  0  0  0  0  0  0  0  0  0  0
  7.3671   11.6561    1.3294 H   0  0  0  0  0  0  0  0  0  0  0  0
  6.2131   13.4201    0.0175 H   0  0  0  0  0  0  0  0  0  0  0  0
  6.1691   12.3466   -1.3872 H   0  0  0  0  0  0  0  0  0  0  0  0
  4.8102   10.7164   -0.0986 H   0  0  0  0  0  0  0  0  0  0  0  0
  4.8541   11.7901    1.3060 H   0  0  0  0  0  0  0  0  0  0  0  0
  3.6998   13.5538   -0.0063 H   0  0  0  0  0  0  0  0  0  0  0  0
  3.6559   12.4800   -1.4108 H   0  0  0  0  0  0  0  0  0  0  0  0
  2.2969   10.8502   -0.1222 H   0  0  0  0  0  0  0  0  0  0  0  0
  2.3408   11.9241    1.2823 H   0  0  0  0  0  0  0  0  0  0  0  0
  1.4960   12.4255   -0.1882 H   0  0  0  0  0  0  0  0  0  0  0  0
  1  2  1  0  0  0  0
  2  3  1  0  0  0  0
  3  4  1  0  0  0  0
  4  5  1  0  0  0  0
  1  5  1  0  0  0  0
  6  7  1  0  0  0  0
  7  8  1  0  0  0  0
  8  9  1  0  0  0  0
  9 10  1  0  0  0  0
 10 11  1  0  0  0  0
 11 12  1  0  0  0  0
 12 13  1  0  0  0  0
 13 14  1  0  0  0  0
 14 15  1  0  0  0  0
 15 16  1  0  0  0  0
  1  6  1  0  0  0  0
 16 17  1  0  0  0  0
 17 18  1  0  0  0  0
 18 19  1  0  0  0  0
 19 20  1  0  0  0  0
  1 21  1  0  0  0  0
  2 22  1  0  0  0  0
  2 23  1  0  0  0  0
  3 24  1  0  0  0  0
  3 25  1  0  0  0  0
  4 26  1  0  0  0  0
  4 27  1  0  0  0  0
  5 28  1  0  0  0  0
  5 29  1  0  0  0  0
  6 30  1  0  0  0  0
  6 31  1  0  0  0  0
  7 32  1  0  0  0  0
  7 33  1  0  0  0  0
  8 34  1  0  0  0  0
  8 35  1  0  0  0  0
  9 36  1  0  0  0  0
  9 37  1  0  0  0  0
 10 38  1  0  0  0  0
```

```
 10 39  1  0  0  0  0
 11 40  1  0  0  0  0
 11 41  1  0  0  0  0
 12 42  1  0  0  0  0
 12 43  1  0  0  0  0
 13 44  1  0  0  0  0
 13 45  1  0  0  0  0
 14 46  1  0  0  0  0
 14 47  1  0  0  0  0
 15 48  1  0  0  0  0
 15 49  1  0  0  0  0
 16 50  1  0  0  0  0
 16 51  1  0  0  0  0
 17 52  1  0  0  0  0
 17 53  1  0  0  0  0
 18 54  1  0  0  0  0
 18 55  1  0  0  0  0
 19 56  1  0  0  0  0
 19 57  1  0  0  0  0
 20 58  1  0  0  0  0
 20 59  1  0  0  0  0
 20 60  1  0  0  0  0
M  END
>  <Alias name>
Pentadecylcyclopentane

>  <deltaH°(c)>   kJ/mol
-13100

$$$$
Tetradecylcyclohexane
  ChemBrain IXL

 60 60  0  0  0  0  0  0  0  0  1 V2000
    3.3260   10.4955    3.5478 C   0  0  0  0  0  0  0  0  0  0  0  0
    2.2025    9.4427    3.4733 C   0  0  0  0  0  0  0  0  0  0  0  0
    2.7820    8.0494    3.7877 C   0  0  0  0  0  0  0  0  0  0  0  0
    3.8767    7.7038    2.7588 C   0  0  0  0  0  0  0  0  0  0  0  0
    5.0029    8.7554    2.8313 C   0  0  0  0  0  0  0  0  0  0  0  0
    4.4227   10.1522    2.5195 C   0  0  0  0  0  0  0  0  0  0  0  0
    6.0974    8.4053    1.7997 C   0  0  0  0  0  0  0  0  0  0  0  0
    7.2411    9.4369    1.8853 C   0  0  0  0  0  0  0  0  0  0  0  0
    8.3400    9.0702    0.8690 C   0  0  0  0  0  0  0  0  0  0  0  0
    9.4828   10.1006    0.9539 C   0  0  0  0  0  0  0  0  0  0  0  0
   10.5815    9.7343   -0.0628 C   0  0  0  0  0  0  0  0  0  0  0  0
   11.7240   10.7651    0.0217 C   0  0  0  0  0  0  0  0  0  0  0  0
   12.8224   10.3991   -0.9955 C   0  0  0  0  0  0  0  0  0  0  0  0
   13.9647   11.4302   -0.9114 C   0  0  0  0  0  0  0  0  0  0  0  0
   15.0629   11.0646   -1.9290 C   0  0  0  0  0  0  0  0  0  0  0  0
   16.2049   12.0960   -1.8453 C   0  0  0  0  0  0  0  0  0  0  0  0
   17.3029   11.7307   -2.8631 C   0  0  0  0  0  0  0  0  0  0  0  0
   18.4449   12.7622   -2.7796 C   0  0  0  0  0  0  0  0  0  0  0  0
   19.5428   12.3970   -3.7977 C   0  0  0  0  0  0  0  0  0  0  0  0
   20.6847   13.4284   -3.7143 C   0  0  0  0  0  0  0  0  0  0  0  0
    2.9185   11.4742    3.3274 H   0  0  0  0  0  0  0  0  0  0  0  0
    3.7527   10.4981    4.5432 H   0  0  0  0  0  0  0  0  0  0  0  0
    1.7756    9.4400    2.4780 H   0  0  0  0  0  0  0  0  0  0  0  0
    1.4328    9.6847    4.1955 H   0  0  0  0  0  0  0  0  0  0  0  0
    1.9924    7.3102    3.7364 H   0  0  0  0  0  0  0  0  0  0  0  0
    3.2097    8.0523    4.7827 H   0  0  0  0  0  0  0  0  0  0  0  0
    4.2829    6.7245    2.9795 H   0  0  0  0  0  0  0  0  0  0  0  0
    3.4489    7.7011    1.7639 H   0  0  0  0  0  0  0  0  0  0  0  0
    5.4316    8.7564    3.8259 H   0  0  0  0  0  0  0  0  0  0  0  0
```

```
    5.2109   10.8915    2.5745 H   0  0  0  0  0  0  0  0  0  0  0  0
    3.9967   10.1521    1.5238 H   0  0  0  0  0  0  0  0  0  0  0  0
    6.4878    7.4175    2.0112 H   0  0  0  0  0  0  0  0  0  0  0  0
    5.6724    8.4191    0.8037 H   0  0  0  0  0  0  0  0  0  0  0  0
    6.8565   10.4229    1.6595 H   0  0  0  0  0  0  0  0  0  0  0  0
    7.6578    9.4318    2.8848 H   0  0  0  0  0  0  0  0  0  0  0  0
    8.7258    8.0838    1.0948 H   0  0  0  0  0  0  0  0  0  0  0  0
    7.9233    9.0748   -0.1306 H   0  0  0  0  0  0  0  0  0  0  0  0
    9.0968   11.0871    0.7285 H   0  0  0  0  0  0  0  0  0  0  0  0
    9.8997   10.0958    1.9534 H   0  0  0  0  0  0  0  0  0  0  0  0
   10.9677    8.7480    0.1627 H   0  0  0  0  0  0  0  0  0  0  0  0
   10.1644    9.7389   -1.0623 H   0  0  0  0  0  0  0  0  0  0  0  0
   11.3377   11.7514   -0.2035 H   0  0  0  0  0  0  0  0  0  0  0  0
   12.1414   10.7603    1.0210 H   0  0  0  0  0  0  0  0  0  0  0  0
   13.2091    9.4129   -0.7702 H   0  0  0  0  0  0  0  0  0  0  0  0
   12.4050   10.4036   -1.9948 H   0  0  0  0  0  0  0  0  0  0  0  0
   13.5780   12.4165   -1.1364 H   0  0  0  0  0  0  0  0  0  0  0  0
   14.3825   11.4255    0.0878 H   0  0  0  0  0  0  0  0  0  0  0  0
   15.4498   10.0785   -1.7038 H   0  0  0  0  0  0  0  0  0  0  0  0
   14.6451   11.0690   -2.9281 H   0  0  0  0  0  0  0  0  0  0  0  0
   15.8179   13.0822   -2.0702 H   0  0  0  0  0  0  0  0  0  0  0  0
   16.6229   12.0914   -0.8462 H   0  0  0  0  0  0  0  0  0  0  0  0
   17.6901   10.7446   -2.6381 H   0  0  0  0  0  0  0  0  0  0  0  0
   16.8849   11.7351   -3.8622 H   0  0  0  0  0  0  0  0  0  0  0  0
   18.0577   13.7483   -3.0045 H   0  0  0  0  0  0  0  0  0  0  0  0
   18.8630   12.7577   -1.7806 H   0  0  0  0  0  0  0  0  0  0  0  0
   19.9301   11.4109   -3.5728 H   0  0  0  0  0  0  0  0  0  0  0  0
   19.1247   12.4014   -4.7966 H   0  0  0  0  0  0  0  0  0  0  0  0
   20.2975   14.4145   -3.9392 H   0  0  0  0  0  0  0  0  0  0  0  0
   21.4560   13.1716   -4.4297 H   0  0  0  0  0  0  0  0  0  0  0  0
   21.1029   13.4240   -2.7153 H   0  0  0  0  0  0  0  0  0  0  0  0
  1  2  1  0  0  0  0
  2  3  1  0  0  0  0
  3  4  1  0  0  0  0
  4  5  1  0  0  0  0
  5  6  1  0  0  0  0
  6  1  1  0  0  0  0
  5  7  1  0  0  0  0
  7  8  1  0  0  0  0
  8  9  1  0  0  0  0
  9 10  1  0  0  0  0
 10 11  1  0  0  0  0
 11 12  1  0  0  0  0
 12 13  1  0  0  0  0
 13 14  1  0  0  0  0
 14 15  1  0  0  0  0
 15 16  1  0  0  0  0
 16 17  1  0  0  0  0
 17 18  1  0  0  0  0
 18 19  1  0  0  0  0
 19 20  1  0  0  0  0
  1 21  1  0  0  0  0
  1 22  1  0  0  0  0
  2 23  1  0  0  0  0
  2 24  1  0  0  0  0
  3 25  1  0  0  0  0
  3 26  1  0  0  0  0
  4 27  1  0  0  0  0
  4 28  1  0  0  0  0
  5 29  1  0  0  0  0
  6 30  1  0  0  0  0
  6 31  1  0  0  0  0
  7 32  1  0  0  0  0
```

```
  7 33  1  0  0  0  0
  8 34  1  0  0  0  0
  8 35  1  0  0  0  0
  9 36  1  0  0  0  0
  9 37  1  0  0  0  0
 10 38  1  0  0  0  0
 10 39  1  0  0  0  0
 11 40  1  0  0  0  0
 11 41  1  0  0  0  0
 12 42  1  0  0  0  0
 12 43  1  0  0  0  0
 13 44  1  0  0  0  0
 13 45  1  0  0  0  0
 14 46  1  0  0  0  0
 14 47  1  0  0  0  0
 15 48  1  0  0  0  0
 15 49  1  0  0  0  0
 16 50  1  0  0  0  0
 16 51  1  0  0  0  0
 17 52  1  0  0  0  0
 17 53  1  0  0  0  0
 18 54  1  0  0  0  0
 18 55  1  0  0  0  0
 19 56  1  0  0  0  0
 19 57  1  0  0  0  0
 20 58  1  0  0  0  0
 20 59  1  0  0  0  0
 20 60  1  0  0  0  0
M  END
>  <Alias name>
Tetradecylcyclohexane

>  <deltaH°(c)>   kJ/mol
-13077

$$$$
6,19-Methanobenzocyclooctadecen-21-one
  ChemBrain IXL

 54 56  0  0  0  0  0  0  0  0  1 V2000
    4.2492    6.3739   -0.2470 C   0  0  0  0  0  0  0  0  0  0  0  0
    4.2583    7.8773   -0.1198 C   0  0  0  0  0  0  0  0  0  0  0  0
    3.2000    5.7810   -0.9670 C   0  0  0  0  0  0  0  0  0  0  0  0
    5.3080    5.3517    0.3748 C   0  0  0  0  0  0  0  0  0  0  0  0
    3.2178    8.5964   -0.7278 C   0  0  0  0  0  0  0  0  0  0  0  0
    5.3269    8.7659    0.6621 C   0  0  0  0  0  0  0  0  0  0  0  0
    2.2004    7.9468   -1.4334 C   0  0  0  0  0  0  0  0  0  0  0  0
    2.1916    6.5538   -1.5518 C   0  0  0  0  0  0  0  0  0  0  0  0
    6.6728    8.4088    0.9149 C   0  0  0  0  0  0  0  0  0  0  0  0
    7.3245    7.0502    0.4769 C   0  0  0  0  0  0  0  0  0  0  0  0
    7.5380    9.3908    1.6354 C   0  0  0  0  0  0  0  0  0  0  0  0
    6.6597    5.6443    0.6831 C   0  0  0  0  0  0  0  0  0  0  0  0
    8.4315    7.0876   -0.0356 O   0  0  0  0  0  0  0  0  0  0  0  0
    9.0256    8.9350    1.5708 C   0  0  0  0  0  0  0  0  0  0  0  0
    9.6640    9.3927    0.2354 C   0  0  0  0  0  0  0  0  0  0  0  0
   10.8414    8.4493   -0.1216 C   0  0  0  0  0  0  0  0  0  0  0  0
   11.7759    9.1398   -1.1384 C   0  0  0  0  0  0  0  0  0  0  0  0
   12.5959    8.0695   -1.8997 C   0  0  0  0  0  0  0  0  0  0  0  0
   13.0013    6.9301   -0.9305 C   0  0  0  0  0  0  0  0  0  0  0  0
   11.9371    5.8011   -0.9732 C   0  0  0  0  0  0  0  0  0  0  0  0
    7.5185    4.5422    1.2299 C   0  0  0  0  0  0  0  0  0  0  0  0
    8.9724    5.0658    1.4836 C   0  0  0  0  0  0  0  0  0  0  0  0
    9.7788    5.0660    0.1534 C   0  0  0  0  0  0  0  0  0  0  0  0
```

```
  11.1574    5.7592    0.3696 C   0  0  0  0  0  0  0  0  0  0  0  0
   3.1713    4.7034   -1.0706 H   0  0  0  0  0  0  0  0  0  0  0  0
   4.9661    4.3418    0.5674 H   0  0  0  0  0  0  0  0  0  0  0  0
   3.2031    9.6765   -0.6476 H   0  0  0  0  0  0  0  0  0  0  0  0
   4.9979    9.7331    1.0216 H   0  0  0  0  0  0  0  0  0  0  0  0
   1.4122    8.5286   -1.8922 H   0  0  0  0  0  0  0  0  0  0  0  0
   1.3964    6.0683   -2.1016 H   0  0  0  0  0  0  0  0  0  0  0  0
   7.4395   10.3651    1.1751 H   0  0  0  0  0  0  0  0  0  0  0  0
   7.2274    9.4498    2.6723 H   0  0  0  0  0  0  0  0  0  0  0  0
   9.5722    9.3747    2.3978 H   0  0  0  0  0  0  0  0  0  0  0  0
   9.0757    7.8560    1.6414 H   0  0  0  0  0  0  0  0  0  0  0  0
   8.9212    9.3576   -0.5512 H   0  0  0  0  0  0  0  0  0  0  0  0
  10.0327   10.4070    0.3394 H   0  0  0  0  0  0  0  0  0  0  0  0
  10.4529    7.5364   -0.5534 H   0  0  0  0  0  0  0  0  0  0  0  0
  11.3992    8.2142    0.7761 H   0  0  0  0  0  0  0  0  0  0  0  0
  12.4510    9.8062   -0.6145 H   0  0  0  0  0  0  0  0  0  0  0  0
  11.1835    9.7094   -1.8444 H   0  0  0  0  0  0  0  0  0  0  0  0
  13.4892    8.5266   -2.3111 H   0  0  0  0  0  0  0  0  0  0  0  0
  11.9976    7.6624   -2.7048 H   0  0  0  0  0  0  0  0  0  0  0  0
  13.0750    7.3208    0.0759 H   0  0  0  0  0  0  0  0  0  0  0  0
  13.9632    6.5286   -1.2320 H   0  0  0  0  0  0  0  0  0  0  0  0
  11.2467    5.9877   -1.7853 H   0  0  0  0  0  0  0  0  0  0  0  0
  12.4307    4.8490   -1.1323 H   0  0  0  0  0  0  0  0  0  0  0  0
   7.5499    3.7245    0.5214 H   0  0  0  0  0  0  0  0  0  0  0  0
   7.0975    4.1903    2.1651 H   0  0  0  0  0  0  0  0  0  0  0  0
   9.4665    4.4185    2.2012 H   0  0  0  0  0  0  0  0  0  0  0  0
   8.9300    6.0723    1.8795 H   0  0  0  0  0  0  0  0  0  0  0  0
   9.2226    5.6011   -0.6040 H   0  0  0  0  0  0  0  0  0  0  0  0
   9.9379    4.0444   -0.1709 H   0  0  0  0  0  0  0  0  0  0  0  0
  11.7311    5.2017    1.1015 H   0  0  0  0  0  0  0  0  0  0  0  0
  11.0021    6.7691    0.7275 H   0  0  0  0  0  0  0  0  0  0  0  0
  1  2  4  0  0  0  0
  1  3  4  0  0  0  0
  4  1  1  0  0  0  0
  5  2  4  0  0  0  0
  2  6  1  0  0  0  0
  3  8  4  0  0  0  0
 12  4  2  0  0  0  0
  7  5  4  0  0  0  0
  6  9  2  0  0  0  0
  8  7  4  0  0  0  0
  9 10  1  0  0  0  0
  9 11  1  0  0  0  0
 10 12  1  0  0  0  0
 10 13  2  0  0  0  0
 11 14  1  0  0  0  0
 14 15  1  0  0  0  0
 15 16  1  0  0  0  0
 16 17  1  0  0  0  0
 17 18  1  0  0  0  0
 18 19  1  0  0  0  0
 19 20  1  0  0  0  0
 20 24  1  0  0  0  0
 22 21  1  0  0  0  0
 23 22  1  0  0  0  0
 24 23  1  0  0  0  0
 21 12  1  0  0  0  0
  3 25  1  0  0  0  0
  4 26  1  0  0  0  0
  5 27  1  0  0  0  0
  6 28  1  0  0  0  0
  7 29  1  0  0  0  0
  8 30  1  0  0  0  0
```

```
 11 31  1  0  0  0  0
 11 32  1  0  0  0  0
 14 33  1  0  0  0  0
 14 34  1  0  0  0  0
 15 35  1  0  0  0  0
 15 36  1  0  0  0  0
 16 37  1  0  0  0  0
 16 38  1  0  0  0  0
 17 39  1  0  0  0  0
 17 40  1  0  0  0  0
 18 41  1  0  0  0  0
 18 42  1  0  0  0  0
 19 43  1  0  0  0  0
 19 44  1  0  0  0  0
 20 45  1  0  0  0  0
 20 46  1  0  0  0  0
 21 47  1  0  0  0  0
 21 48  1  0  0  0  0
 22 49  1  0  0  0  0
 22 50  1  0  0  0  0
 23 51  1  0  0  0  0
 23 52  1  0  0  0  0
 24 53  1  0  0  0  0
 24 54  1  0  0  0  0
M  END
>  <Alias name>
6,19-Methanobenzocyclooctadecen-21-one

>  <deltaH°(c)>   kJ/mol
-13075

$$$$
Pentadecylbenzene
  ChemBrain IXL

 57 57  0  0  0  0  0  0  0  0  1 V2000
   19.5593   13.5347   -0.7428 C   0  0  0  0  0  0  0  0  0  0  0  0
   18.2029   14.1558   -0.3558 C   0  0  0  0  0  0  0  0  0  0  0  0
   17.0920   13.0955   -0.4927 C   0  0  0  0  0  0  0  0  0  0  0  0
   15.7360   13.7233   -0.1152 C   0  0  0  0  0  0  0  0  0  0  0  0
   14.6225   12.6645   -0.2415 C   0  0  0  0  0  0  0  0  0  0  0  0
   13.2682   13.3015    0.1273 C   0  0  0  0  0  0  0  0  0  0  0  0
   12.1513   12.2447    0.0180 C   0  0  0  0  0  0  0  0  0  0  0  0
   10.7999   12.8914    0.3821 C   0  0  0  0  0  0  0  0  0  0  0  0
    9.6805   11.8358    0.2955 C   0  0  0  0  0  0  0  0  0  0  0  0
   20.6688   14.5955   -0.6000 C   0  0  0  0  0  0  0  0  0  0  0  0
    8.3316   12.4892    0.6596 C   0  0  0  0  0  0  0  0  0  0  0  0
   22.0250   13.9779   -0.9935 C   0  0  0  0  0  0  0  0  0  0  0  0
    7.2132   11.4314    0.5967 C   0  0  0  0  0  0  0  0  0  0  0  0
   23.1341   15.0381   -0.8478 C   0  0  0  0  0  0  0  0  0  0  0  0
    5.8621   12.0865    0.9634 C   0  0  0  0  0  0  0  0  0  0  0  0
    4.0449    8.7907    0.5480 C   0  0  0  0  0  0  0  0  0  0  0  0
    5.0660    9.7404    0.5866 C   0  0  0  0  0  0  0  0  0  0  0  0
    4.7773   11.0684    0.9203 C   0  0  0  0  0  0  0  0  0  0  0  0
    3.4641   11.4397    1.2144 C   0  0  0  0  0  0  0  0  0  0  0  0
    2.4430   10.4883    1.1754 C   0  0  0  0  0  0  0  0  0  0  0  0
    2.7335    9.1644    0.8423 C   0  0  0  0  0  0  0  0  0  0  0  0
   19.5190   13.1901   -1.7687 H   0  0  0  0  0  0  0  0  0  0  0  0
   19.7732   12.6982   -0.0890 H   0  0  0  0  0  0  0  0  0  0  0  0
   18.2447   14.5042    0.6688 H   0  0  0  0  0  0  0  0  0  0  0  0
   17.9881   14.9902   -1.0120 H   0  0  0  0  0  0  0  0  0  0  0  0
   17.0540   12.7420   -1.5157 H   0  0  0  0  0  0  0  0  0  0  0  0
   17.3024   12.2645    0.1691 H   0  0  0  0  0  0  0  0  0  0  0  0
```

15.7770	14.0832	0.9054	H	0	0	0	0	0	0	0	0	0	0	0	0
15.5241	14.5507	-0.7811	H	0	0	0	0	0	0	0	0	0	0	0	0
14.5846	12.2992	-1.2604	H	0	0	0	0	0	0	0	0	0	0	0	0
14.8295	11.8406	0.4303	H	0	0	0	0	0	0	0	0	0	0	0	0
13.3110	13.6765	1.1423	H	0	0	0	0	0	0	0	0	0	0	0	0
13.0583	14.1195	-0.5506	H	0	0	0	0	0	0	0	0	0	0	0	0
12.1094	11.8658	-0.9958	H	0	0	0	0	0	0	0	0	0	0	0	0
12.3575	11.4294	0.7005	H	0	0	0	0	0	0	0	0	0	0	0	0
10.8481	13.2830	1.3906	H	0	0	0	0	0	0	0	0	0	0	0	0
10.5888	13.6986	-0.3083	H	0	0	0	0	0	0	0	0	0	0	0	0
9.6314	11.4424	-0.7124	H	0	0	0	0	0	0	0	0	0	0	0	0
9.8901	11.0299	0.9880	H	0	0	0	0	0	0	0	0	0	0	0	0
20.7119	14.9366	0.4270	H	0	0	0	0	0	0	0	0	0	0	0	0
20.4521	15.4342	-1.2501	H	0	0	0	0	0	0	0	0	0	0	0	0
8.3870	12.8958	1.6618	H	0	0	0	0	0	0	0	0	0	0	0	0
8.1154	13.2856	-0.0416	H	0	0	0	0	0	0	0	0	0	0	0	0
21.9827	13.6388	-2.0211	H	0	0	0	0	0	0	0	0	0	0	0	0
22.2412	13.1381	-0.3448	H	0	0	0	0	0	0	0	0	0	0	0	0
7.1575	11.0244	-0.4055	H	0	0	0	0	0	0	0	0	0	0	0	0
7.4309	10.6355	1.2984	H	0	0	0	0	0	0	0	0	0	0	0	0
22.9175	15.8786	-1.4956	H	0	0	0	0	0	0	0	0	0	0	0	0
24.0868	14.6044	-1.1255	H	0	0	0	0	0	0	0	0	0	0	0	0
23.1774	15.3764	0.1801	H	0	0	0	0	0	0	0	0	0	0	0	0
5.9239	12.5033	1.9609	H	0	0	0	0	0	0	0	0	0	0	0	0
5.6421	12.8767	0.2565	H	0	0	0	0	0	0	0	0	0	0	0	0
4.2705	7.7634	0.2896	H	0	0	0	0	0	0	0	0	0	0	0	0
6.0818	9.4463	0.3577	H	0	0	0	0	0	0	0	0	0	0	0	0
3.2373	12.4663	1.4729	H	0	0	0	0	0	0	0	0	0	0	0	0
1.4251	10.7775	1.4036	H	0	0	0	0	0	0	0	0	0	0	0	0
1.9411	8.4270	0.8123	H	0	0	0	0	0	0	0	0	0	0	0	0

1	2	1	0	0	0	0
2	3	1	0	0	0	0
3	4	1	0	0	0	0
4	5	1	0	0	0	0
5	6	1	0	0	0	0
6	7	1	0	0	0	0
7	8	1	0	0	0	0
8	9	1	0	0	0	0
1	10	1	0	0	0	0
9	11	1	0	0	0	0
10	12	1	0	0	0	0
11	13	1	0	0	0	0
12	14	1	0	0	0	0
13	15	1	0	0	0	0
16	17	4	0	0	0	0
17	18	4	0	0	0	0
18	19	4	0	0	0	0
19	20	4	0	0	0	0
20	21	4	0	0	0	0
21	16	4	0	0	0	0
18	15	1	0	0	0	0
1	22	1	0	0	0	0
1	23	1	0	0	0	0
2	24	1	0	0	0	0
2	25	1	0	0	0	0
3	26	1	0	0	0	0
3	27	1	0	0	0	0
4	28	1	0	0	0	0
4	29	1	0	0	0	0
5	30	1	0	0	0	0
5	31	1	0	0	0	0
6	32	1	0	0	0	0
6	33	1	0	0	0	0

```
  7 34  1  0  0  0  0
  7 35  1  0  0  0  0
  8 36  1  0  0  0  0
  8 37  1  0  0  0  0
  9 38  1  0  0  0  0
  9 39  1  0  0  0  0
 10 40  1  0  0  0  0
 10 41  1  0  0  0  0
 11 42  1  0  0  0  0
 11 43  1  0  0  0  0
 12 44  1  0  0  0  0
 12 45  1  0  0  0  0
 13 46  1  0  0  0  0
 13 47  1  0  0  0  0
 14 48  1  0  0  0  0
 14 49  1  0  0  0  0
 14 50  1  0  0  0  0
 15 51  1  0  0  0  0
 15 52  1  0  0  0  0
 16 53  1  0  0  0  0
 17 54  1  0  0  0  0
 19 55  1  0  0  0  0
 20 56  1  0  0  0  0
 21 57  1  0  0  0  0
M  END
>  <Alias name>
Pentadecylbenzene

>  <deltaH°(c)>   kJ/mol
-13032.37

$$$$
2,7-Di-t-butyl-9,9-dimethylxanthene
  ChemBrain IXL

 54 56  0  0  0  0  0  0  0  0  1 V2000
    7.1981    7.5253    0.1636 C   0  0  0  0  0  0  0  0  0  0  0  0
    8.4616    6.7213    0.4088 C   0  0  0  0  0  0  0  0  0  0  0  0
    7.2239    8.9265    0.1177 C   0  0  0  0  0  0  0  0  0  0  0  0
    5.9762    6.8639   -0.0128 C   0  0  0  0  0  0  0  0  0  0  0  0
    9.7200    7.5327    0.1616 C   0  0  0  0  0  0  0  0  0  0  0  0
    8.4644    5.5062   -0.5341 C   0  0  0  0  0  0  0  0  0  0  0  0
    8.4641    6.2385    1.8619 C   0  0  0  0  0  0  0  0  0  0  0  0
    8.4531    9.6474    0.4380 O   0  0  0  0  0  0  0  0  0  0  0  0
    6.0761    9.6433   -0.2319 C   0  0  0  0  0  0  0  0  0  0  0  0
    4.8056    7.5928   -0.2631 C   0  0  0  0  0  0  0  0  0  0  0  0
    9.6860    8.9337    0.1159 C   0  0  0  0  0  0  0  0  0  0  0  0
   10.9456    6.8785   -0.0167 C   0  0  0  0  0  0  0  0  0  0  0  0
    4.8594    8.9808   -0.3825 C   0  0  0  0  0  0  0  0  0  0  0  0
   10.8290    9.6573   -0.2353 C   0  0  0  0  0  0  0  0  0  0  0  0
   12.1114    7.6143   -0.2687 C   0  0  0  0  0  0  0  0  0  0  0  0
   12.0494    9.0019   -0.3879 C   0  0  0  0  0  0  0  0  0  0  0  0
   13.4162    6.9220   -0.4088 C   0  0  0  0  0  0  0  0  0  0  0  0
   13.2859    5.4787    0.1069 C   0  0  0  0  0  0  0  0  0  0  0  0
   14.4873    7.6677    0.4140 C   0  0  0  0  0  0  0  0  0  0  0  0
   13.8358    6.9022   -1.8936 C   0  0  0  0  0  0  0  0  0  0  0  0
    3.5048    6.8928   -0.4010 C   0  0  0  0  0  0  0  0  0  0  0  0
    3.6013    5.5038    0.2526 C   0  0  0  0  0  0  0  0  0  0  0  0
    2.3997    7.7137    0.2962 C   0  0  0  0  0  0  0  0  0  0  0  0
    3.1571    6.7356   -1.8958 C   0  0  0  0  0  0  0  0  0  0  0  0
    5.9358    5.7848    0.0448 H   0  0  0  0  0  0  0  0  0  0  0  0
    9.3499    4.9117   -0.3539 H   0  0  0  0  0  0  0  0  0  0  0  0
    7.5825    4.9067   -0.3527 H   0  0  0  0  0  0  0  0  0  0  0  0
```

8.4628 5.8491 -1.5634 H 0 0 0 0 0 0 0 0 0 0 0 0
9.3494 5.6444 2.0424 H 0 0 0 0 0 0 0 0 0 0 0 0
7.5822 5.6400 2.0440 H 0 0 0 0 0 0 0 0 0 0 0 0
8.4626 7.0961 2.5250 H 0 0 0 0 0 0 0 0 0 0 0 0
6.1304 10.7160 -0.3856 H 0 0 0 0 0 0 0 0 0 0 0 0
10.9924 5.7996 0.0407 H 0 0 0 0 0 0 0 0 0 0 0 0
3.9561 9.5436 -0.5920 H 0 0 0 0 0 0 0 0 0 0 0 0
10.7682 10.7297 -0.3887 H 0 0 0 0 0 0 0 0 0 0 0 0
12.9491 9.5700 -0.5987 H 0 0 0 0 0 0 0 0 0 0 0 0
14.2447 4.9812 0.0337 H 0 0 0 0 0 0 0 0 0 0 0 0
12.9644 5.4932 1.1408 H 0 0 0 0 0 0 0 0 0 0 0 0
12.5562 4.9469 -0.4907 H 0 0 0 0 0 0 0 0 0 0 0 0
14.5948 8.6775 0.0365 H 0 0 0 0 0 0 0 0 0 0 0 0
15.4343 7.1482 0.3288 H 0 0 0 0 0 0 0 0 0 0 0 0
14.1864 7.7010 1.4533 H 0 0 0 0 0 0 0 0 0 0 0 0
13.9338 7.9187 -2.2552 H 0 0 0 0 0 0 0 0 0 0 0 0
13.0843 6.3832 -2.4748 H 0 0 0 0 0 0 0 0 0 0 0 0
14.7860 6.3909 -1.9932 H 0 0 0 0 0 0 0 0 0 0 0 0
4.3525 4.9158 -0.2596 H 0 0 0 0 0 0 0 0 0 0 0 0
2.6429 5.0049 0.1835 H 0 0 0 0 0 0 0 0 0 0 0 0
3.8770 5.6139 1.2940 H 0 0 0 0 0 0 0 0 0 0 0 0
1.4565 7.1855 0.2220 H 0 0 0 0 0 0 0 0 0 0 0 0
2.6550 7.8512 1.3393 H 0 0 0 0 0 0 0 0 0 0 0 0
2.3101 8.6806 -0.1842 H 0 0 0 0 0 0 0 0 0 0 0 0
3.9332 6.1647 -2.3893 H 0 0 0 0 0 0 0 0 0 0 0 0
2.2103 6.2177 -1.9935 H 0 0 0 0 0 0 0 0 0 0 0 0
3.0816 7.7143 -2.3542 H 0 0 0 0 0 0 0 0 0 0 0 0
1 2 1 0 0 0 0
1 3 4 0 0 0 0
1 4 4 0 0 0 0
2 5 1 0 0 0 0
2 6 1 0 0 0 0
2 7 1 0 0 0 0
3 8 1 0 0 0 0
3 9 4 0 0 0 0
4 10 4 0 0 0 0
5 11 4 0 0 0 0
5 12 4 0 0 0 0
8 11 1 0 0 0 0
9 13 4 0 0 0 0
10 13 4 0 0 0 0
11 14 4 0 0 0 0
12 15 4 0 0 0 0
14 16 4 0 0 0 0
15 16 4 0 0 0 0
15 17 1 0 0 0 0
17 18 1 0 0 0 0
17 19 1 0 0 0 0
17 20 1 0 0 0 0
10 21 1 0 0 0 0
21 22 1 0 0 0 0
21 23 1 0 0 0 0
21 24 1 0 0 0 0
4 25 1 0 0 0 0
6 26 1 0 0 0 0
6 27 1 0 0 0 0
6 28 1 0 0 0 0
7 29 1 0 0 0 0
7 30 1 0 0 0 0
7 31 1 0 0 0 0
9 32 1 0 0 0 0
12 33 1 0 0 0 0
13 34 1 0 0 0 0

```
 14 35  1  0  0  0  0
 16 36  1  0  0  0  0
 18 37  1  0  0  0  0
 18 38  1  0  0  0  0
 18 39  1  0  0  0  0
 19 40  1  0  0  0  0
 19 41  1  0  0  0  0
 19 42  1  0  0  0  0
 20 43  1  0  0  0  0
 20 44  1  0  0  0  0
 20 45  1  0  0  0  0
 22 46  1  0  0  0  0
 22 47  1  0  0  0  0
 22 48  1  0  0  0  0
 23 49  1  0  0  0  0
 23 50  1  0  0  0  0
 23 51  1  0  0  0  0
 24 52  1  0  0  0  0
 24 53  1  0  0  0  0
 24 54  1  0  0  0  0
M  END
>  <Alias name>
2,7-Di-t-butyl-9,9-dimethylxanthene

>  <deltaH°(c)>   kJ/mol
-12997.19

$$$$
2-Stearoylglycerol
  ChemBrain IXL

 67 66  0  0  0  0  0  0  0  0  1 V2000
   23.4826   12.6026   -0.8160 O   0  0  0  0  0  0  0  0  0  0  0  0
   24.8280   13.1877   -0.7245 C   0  0  0  0  0  0  0  0  0  0  0  0
   25.6354   12.4517    0.3623 C   0  0  0  0  0  0  0  0  0  0  0  0
   24.7153   14.6803   -0.3583 C   0  0  0  0  0  0  0  0  0  0  0  0
   23.4468   11.1838   -1.1988 C   0  0  0  0  0  0  0  0  0  0  0  0
   24.9480   12.5809    1.6552 O   0  0  0  0  0  0  0  0  0  0  0  0
   23.9499   15.3834   -1.3978 O   0  0  0  0  0  0  0  0  0  0  0  0
   22.1494   10.4816   -1.3168 C   0  0  0  0  0  0  0  0  0  0  0  0
   24.4867   10.5850   -1.4191 O   0  0  0  0  0  0  0  0  0  0  0  0
   21.0055   11.4546   -0.9711 C   0  0  0  0  0  0  0  0  0  0  0  0
   19.6545   10.7236   -1.0943 C   0  0  0  0  0  0  0  0  0  0  0  0
   18.5104   11.6973   -0.7513 C   0  0  0  0  0  0  0  0  0  0  0  0
   17.1591   10.9677   -0.8793 C   0  0  0  0  0  0  0  0  0  0  0  0
   16.0149   11.9422   -0.5390 C   0  0  0  0  0  0  0  0  0  0  0  0
   14.6634   11.2138   -0.6712 C   0  0  0  0  0  0  0  0  0  0  0  0
   13.5192   12.1884   -0.3311 C   0  0  0  0  0  0  0  0  0  0  0  0
   12.1676   11.4606   -0.4653 C   0  0  0  0  0  0  0  0  0  0  0  0
   11.0235   12.4346   -0.1228 C   0  0  0  0  0  0  0  0  0  0  0  0
    9.6719   11.7068   -0.2571 C   0  0  0  0  0  0  0  0  0  0  0  0
    8.5281   12.6797    0.0894 C   0  0  0  0  0  0  0  0  0  0  0  0
    7.1764   11.9517   -0.0439 C   0  0  0  0  0  0  0  0  0  0  0  0
    6.0329   12.9234    0.3063 C   0  0  0  0  0  0  0  0  0  0  0  0
    4.6813   12.1952    0.1739 C   0  0  0  0  0  0  0  0  0  0  0  0
    3.5379   13.1662    0.5265 C   0  0  0  0  0  0  0  0  0  0  0  0
    2.1863   12.4379    0.3943 C   0  0  0  0  0  0  0  0  0  0  0  0
   25.4009   12.1465    2.4118 H   0  0  0  0  0  0  0  0  0  0  0  0
   23.8302   16.3471   -1.2454 H   0  0  0  0  0  0  0  0  0  0  0  0
   25.3311   13.0870   -1.6783 H   0  0  0  0  0  0  0  0  0  0  0  0
   26.6244   12.8870    0.4342 H   0  0  0  0  0  0  0  0  0  0  0  0
   25.7202   11.4040    0.1017 H   0  0  0  0  0  0  0  0  0  0  0  0
   24.2086   14.7812    0.5935 H   0  0  0  0  0  0  0  0  0  0  0  0
```

```
 25.7067   15.1104   -0.2869 H   0  0  0  0  0  0  0  0  0  0  0  0
 22.0240   10.1228   -2.3309 H   0  0  0  0  0  0  0  0  0  0  0  0
 22.1292    9.6428   -0.6320 H   0  0  0  0  0  0  0  0  0  0  0  0
 21.1307   11.8132    0.0431 H   0  0  0  0  0  0  0  0  0  0  0  0
 21.0257   12.2935   -1.6558 H   0  0  0  0  0  0  0  0  0  0  0  0
 19.5301   10.3632   -2.1080 H   0  0  0  0  0  0  0  0  0  0  0  0
 19.6334    9.8859   -0.4082 H   0  0  0  0  0  0  0  0  0  0  0  0
 18.6329   12.0550    0.2636 H   0  0  0  0  0  0  0  0  0  0  0  0
 18.5336   12.5368   -1.4352 H   0  0  0  0  0  0  0  0  0  0  0  0
 17.0376   10.6083   -1.8936 H   0  0  0  0  0  0  0  0  0  0  0  0
 17.1352   10.1294   -0.1940 H   0  0  0  0  0  0  0  0  0  0  0  0
 16.1348   12.2992    0.4765 H   0  0  0  0  0  0  0  0  0  0  0  0
 16.0408   12.7820   -1.2223 H   0  0  0  0  0  0  0  0  0  0  0  0
 14.5436   10.8567   -1.6865 H   0  0  0  0  0  0  0  0  0  0  0  0
 14.6375   10.3741    0.0123 H   0  0  0  0  0  0  0  0  0  0  0  0
 13.6382   12.5444    0.6848 H   0  0  0  0  0  0  0  0  0  0  0  0
 13.5461   13.0289   -1.0136 H   0  0  0  0  0  0  0  0  0  0  0  0
 12.0478   11.1062   -1.4817 H   0  0  0  0  0  0  0  0  0  0  0  0
 12.1414   10.6191    0.2159 H   0  0  0  0  0  0  0  0  0  0  0  0
 11.1433   12.7890    0.8935 H   0  0  0  0  0  0  0  0  0  0  0  0
 11.0497   13.2761   -0.8040 H   0  0  0  0  0  0  0  0  0  0  0  0
  9.5508   11.3549   -1.2742 H   0  0  0  0  0  0  0  0  0  0  0  0
  9.6468   10.8636    0.4220 H   0  0  0  0  0  0  0  0  0  0  0  0
  8.6495   13.0321    1.1062 H   0  0  0  0  0  0  0  0  0  0  0  0
  8.5527   13.5225   -0.5902 H   0  0  0  0  0  0  0  0  0  0  0  0
  7.0538   11.6016   -1.0614 H   0  0  0  0  0  0  0  0  0  0  0  0
  7.1529   11.1072    0.6337 H   0  0  0  0  0  0  0  0  0  0  0  0
  6.1559   13.2740    1.3236 H   0  0  0  0  0  0  0  0  0  0  0  0
  6.0561   13.7676   -0.3717 H   0  0  0  0  0  0  0  0  0  0  0  0
  4.5575   11.8460   -0.8438 H   0  0  0  0  0  0  0  0  0  0  0  0
  4.6588   11.3500    0.8507 H   0  0  0  0  0  0  0  0  0  0  0  0
  3.6618   13.5155    1.5441 H   0  0  0  0  0  0  0  0  0  0  0  0
  3.5603   14.0113   -0.1505 H   0  0  0  0  0  0  0  0  0  0  0  0
  2.1639   11.5929    1.0713 H   0  0  0  0  0  0  0  0  0  0  0  0
  1.3827   13.1204    0.6421 H   0  0  0  0  0  0  0  0  0  0  0  0
  2.0624   12.0887   -0.6233 H   0  0  0  0  0  0  0  0  0  0  0  0
  1  2  1  0  0  0  0
  1  5  1  0  0  0  0
  2  3  1  0  0  0  0
  2  4  1  0  0  0  0
  3  6  1  0  0  0  0
  4  7  1  0  0  0  0
  5  8  1  0  0  0  0
  5  9  2  0  0  0  0
  8 10  1  0  0  0  0
 10 11  1  0  0  0  0
 11 12  1  0  0  0  0
 12 13  1  0  0  0  0
 13 14  1  0  0  0  0
 14 15  1  0  0  0  0
 15 16  1  0  0  0  0
 16 17  1  0  0  0  0
 17 18  1  0  0  0  0
 18 19  1  0  0  0  0
 19 20  1  0  0  0  0
 20 21  1  0  0  0  0
 21 22  1  0  0  0  0
 22 23  1  0  0  0  0
 23 24  1  0  0  0  0
 24 25  1  0  0  0  0
  6 26  1  0  0  0  0
  7 27  1  0  0  0  0
  2 28  1  0  0  0  0
```

```
  3 29  1  0  0  0  0
  3 30  1  0  0  0  0
  4 31  1  0  0  0  0
  4 32  1  0  0  0  0
  8 33  1  0  0  0  0
  8 34  1  0  0  0  0
 10 35  1  0  0  0  0
 10 36  1  0  0  0  0
 11 37  1  0  0  0  0
 11 38  1  0  0  0  0
 12 39  1  0  0  0  0
 12 40  1  0  0  0  0
 13 41  1  0  0  0  0
 13 42  1  0  0  0  0
 14 43  1  0  0  0  0
 14 44  1  0  0  0  0
 15 45  1  0  0  0  0
 15 46  1  0  0  0  0
 16 47  1  0  0  0  0
 16 48  1  0  0  0  0
 17 49  1  0  0  0  0
 17 50  1  0  0  0  0
 18 51  1  0  0  0  0
 18 52  1  0  0  0  0
 19 53  1  0  0  0  0
 19 54  1  0  0  0  0
 20 55  1  0  0  0  0
 20 56  1  0  0  0  0
 21 57  1  0  0  0  0
 21 58  1  0  0  0  0
 22 59  1  0  0  0  0
 22 60  1  0  0  0  0
 23 61  1  0  0  0  0
 23 62  1  0  0  0  0
 24 63  1  0  0  0  0
 24 64  1  0  0  0  0
 25 65  1  0  0  0  0
 25 66  1  0  0  0  0
 25 67  1  0  0  0  0
M  END
>  <Alias name>
2-Stearoylglycerol

>  <deltaH°(c)>   kJ/mol
-12944

$$$$
Tetraphenylmethane
  ChemBrain IXL

 45 48  0  0  0  0  0  0  0  0  1 V2000
    4.9248    7.8463    3.1719 C   0  0  0  0  0  0  0  0  0  0  0  0
    5.4626    9.1294    3.0706 C   0  0  0  0  0  0  0  0  0  0  0  0
    6.3019    9.4518    2.0040 C   0  0  0  0  0  0  0  0  0  0  0  0
    6.6034    8.4910    1.0385 C   0  0  0  0  0  0  0  0  0  0  0  0
    6.0651    7.2080    1.1395 C   0  0  0  0  0  0  0  0  0  0  0  0
    5.2263    6.8856    2.2065 C   0  0  0  0  0  0  0  0  0  0  0  0
    6.3852    6.1870    0.1171 C   0  0  0  0  0  0  0  0  0  0  0  0
    3.1825    5.9110   -2.6936 C   0  0  0  0  0  0  0  0  0  0  0  0
    4.4470    5.4247   -3.0262 C   0  0  0  0  0  0  0  0  0  0  0  0
    5.4928    5.5152   -2.1075 C   0  0  0  0  0  0  0  0  0  0  0  0
    5.2741    6.0913   -0.8559 C   0  0  0  0  0  0  0  0  0  0  0  0
    4.0099    6.5785   -0.5236 C   0  0  0  0  0  0  0  0  0  0  0  0
```

```
 2.9640 6.4879 -1.4424 C 0 0 0 0 0 0 0 0 0 0 0 0
 9.9563 7.2937 -1.9457 C 0 0 0 0 0 0 0 0 0 0 0 0
 10.0047 6.3345 -0.9339 C 0 0 0 0 0 0 0 0 0 0 0 0
 8.8383 5.9724 -0.2596 C 0 0 0 0 0 0 0 0 0 0 0 0
 7.6238 6.5701 -0.5966 C 0 0 0 0 0 0 0 0 0 0 0 0
 7.5754 7.5286 -1.6091 C 0 0 0 0 0 0 0 0 0 0 0 0
 8.7416 7.8907 -2.2834 C 0 0 0 0 0 0 0 0 0 0 0 0
 6.5778 4.8784 0.7812 C 0 0 0 0 0 0 0 0 0 0 0 0
 6.0234 3.7248 0.2263 C 0 0 0 0 0 0 0 0 0 0 0 0
 6.2028 2.4927 0.8555 C 0 0 0 0 0 0 0 0 0 0 0 0
 6.9369 2.4142 2.0392 C 0 0 0 0 0 0 0 0 0 0 0 0
 7.4916 3.5677 2.5937 C 0 0 0 0 0 0 0 0 0 0 0 0
 7.3122 4.7998 1.9647 C 0 0 0 0 0 0 0 0 0 0 0 0
 4.2732 7.5960 4.0000 H 0 0 0 0 0 0 0 0 0 0 0 0
 5.2285 9.8754 3.8201 H 0 0 0 0 0 0 0 0 0 0 0 0
 6.7195 10.4479 1.9255 H 0 0 0 0 0 0 0 0 0 0 0 0
 7.2553 8.7411 0.2107 H 0 0 0 0 0 0 0 0 0 0 0 0
 4.8090 5.8893 2.2853 H 0 0 0 0 0 0 0 0 0 0 0 0
 2.3705 5.8406 -3.4068 H 0 0 0 0 0 0 0 0 0 0 0 0
 4.6168 4.9769 -3.9976 H 0 0 0 0 0 0 0 0 0 0 0 0
 6.4746 5.1382 -2.3659 H 0 0 0 0 0 0 0 0 0 0 0 0
 3.8403 7.0269 0.4475 H 0 0 0 0 0 0 0 0 0 0 0 0
 1.9823 6.8656 -1.1843 H 0 0 0 0 0 0 0 0 0 0 0 0
 10.8618 7.5749 -2.4691 H 0 0 0 0 0 0 0 0 0 0 0 0
 10.9477 5.8709 -0.6720 H 0 0 0 0 0 0 0 0 0 0 0 0
 8.8758 5.2273 0.5255 H 0 0 0 0 0 0 0 0 0 0 0 0
 6.6322 7.9917 -1.8716 H 0 0 0 0 0 0 0 0 0 0 0 0
 8.7039 8.6352 -3.0690 H 0 0 0 0 0 0 0 0 0 0 0 0
 5.4539 3.7858 -0.6927 H 0 0 0 0 0 0 0 0 0 0 0 0
 5.7722 1.5971 0.4250 H 0 0 0 0 0 0 0 0 0 0 0 0
 7.0761 1.4576 2.5276 H 0 0 0 0 0 0 0 0 0 0 0 0
 8.0615 3.5068 3.5126 H 0 0 0 0 0 0 0 0 0 0 0 0
 7.7431 5.6953 2.3951 H 0 0 0 0 0 0 0 0 0 0 0 0
 1 2 4 0 0 0 0
 2 3 4 0 0 0 0
 3 4 4 0 0 0 0
 4 5 4 0 0 0 0
 5 6 4 0 0 0 0
 6 1 4 0 0 0 0
 5 7 1 0 0 0 0
 8 9 4 0 0 0 0
 9 10 4 0 0 0 0
10 11 4 0 0 0 0
11 12 4 0 0 0 0
12 13 4 0 0 0 0
13 8 4 0 0 0 0
11 7 1 0 0 0 0
14 15 4 0 0 0 0
15 16 4 0 0 0 0
16 17 4 0 0 0 0
17 18 4 0 0 0 0
18 19 4 0 0 0 0
19 14 4 0 0 0 0
20 21 4 0 0 0 0
21 22 4 0 0 0 0
22 23 4 0 0 0 0
23 24 4 0 0 0 0
24 25 4 0 0 0 0
25 20 4 0 0 0 0
17 7 1 0 0 0 0
20 7 1 0 0 0 0
 1 26 1 0 0 0 0
 2 27 1 0 0 0 0
```

```
  3 28  1  0  0  0  0
  4 29  1  0  0  0  0
  6 30  1  0  0  0  0
  8 31  1  0  0  0  0
  9 32  1  0  0  0  0
 10 33  1  0  0  0  0
 12 34  1  0  0  0  0
 13 35  1  0  0  0  0
 14 36  1  0  0  0  0
 15 37  1  0  0  0  0
 16 38  1  0  0  0  0
 18 39  1  0  0  0  0
 19 40  1  0  0  0  0
 21 41  1  0  0  0  0
 22 42  1  0  0  0  0
 23 43  1  0  0  0  0
 24 44  1  0  0  0  0
 25 45  1  0  0  0  0
M  END
>  <Alias name>
Tetraphenylmethane

>  <deltaH°(c)>   kJ/mol
-12943

$$$$
1-Stearoylglycerol
  ChemBrain IXL

 67 66  0  0  0  0  0  0  0  0  1 V2000
    5.4347   14.9891   -1.4025 C   0  0  0  0  0  0  0  0  0  0  0  0
    6.4830   14.0245   -0.8151 C   0  0  0  0  0  0  0  0  0  0  0  0
    7.8874   14.4365   -1.2974 C   0  0  0  0  0  0  0  0  0  0  0  0
    4.0853   14.5902   -0.9437 C   0  0  0  0  0  0  0  0  0  0  0  0
    8.9356   13.4705   -0.7119 C   0  0  0  0  0  0  0  0  0  0  0  0
    3.9223   13.4182   -0.0715 O   0  0  0  0  0  0  0  0  0  0  0  0
    3.1084   15.2340   -1.2893 O   0  0  0  0  0  0  0  0  0  0  0  0
   10.3389   13.8764   -1.2023 C   0  0  0  0  0  0  0  0  0  0  0  0
    2.5360   13.1206    0.3165 C   0  0  0  0  0  0  0  0  0  0  0  0
   11.3868   12.9089   -0.6187 C   0  0  0  0  0  0  0  0  0  0  0  0
    2.5127   11.8756    1.2244 C   0  0  0  0  0  0  0  0  0  0  0  0
   12.7889   13.3083   -1.1180 C   0  0  0  0  0  0  0  0  0  0  0  0
    3.1001   10.6725    0.4614 C   0  0  0  0  0  0  0  0  0  0  0  0
    3.3161   12.1320    2.4284 O   0  0  0  0  0  0  0  0  0  0  0  0
   13.8368   12.3406   -0.5347 C   0  0  0  0  0  0  0  0  0  0  0  0
    2.2953   10.4147   -0.7414 O   0  0  0  0  0  0  0  0  0  0  0  0
   15.2378   12.7343   -1.0415 C   0  0  0  0  0  0  0  0  0  0  0  0
   16.2860   11.7681   -0.4562 C   0  0  0  0  0  0  0  0  0  0  0  0
   17.6862   12.1576   -0.9685 C   0  0  0  0  0  0  0  0  0  0  0  0
   18.7349   11.1944   -0.3795 C   0  0  0  0  0  0  0  0  0  0  0  0
   20.1346   11.5813   -0.8951 C   0  0  0  0  0  0  0  0  0  0  0  0
   21.1840   10.6211   -0.3022 C   0  0  0  0  0  0  0  0  0  0  0  0
   22.5834   11.0066   -0.8197 C   0  0  0  0  0  0  0  0  0  0  0  0
   23.6332   10.0485   -0.2240 C   0  0  0  0  0  0  0  0  0  0  0  0
   25.0324   10.4333   -0.7424 C   0  0  0  0  0  0  0  0  0  0  0  0
    4.2616   12.3419    2.2600 H   0  0  0  0  0  0  0  0  0  0  0  0
    2.6011    9.6599   -1.2920 H   0  0  0  0  0  0  0  0  0  0  0  0
    5.6456   15.9979   -1.0695 H   0  0  0  0  0  0  0  0  0  0  0  0
    5.4743   14.9503   -2.4841 H   0  0  0  0  0  0  0  0  0  0  0  0
    6.2697   13.0152   -1.1448 H   0  0  0  0  0  0  0  0  0  0  0  0
    6.4461   14.0664    0.2665 H   0  0  0  0  0  0  0  0  0  0  0  0
    8.1014   15.4452   -0.9663 H   0  0  0  0  0  0  0  0  0  0  0  0
    7.9238   14.3962   -2.3791 H   0  0  0  0  0  0  0  0  0  0  0  0
```

```
  8.7175   12.4608   -1.0373 H   0  0  0  0  0  0  0  0  0  0  0  0
  8.9040   13.5159    0.3697 H   0  0  0  0  0  0  0  0  0  0  0  0
 10.5576   14.8855   -0.8755 H   0  0  0  0  0  0  0  0  0  0  0  0
 10.3699   13.8326   -2.2840 H   0  0  0  0  0  0  0  0  0  0  0  0
  1.9454   12.9311   -0.5713 H   0  0  0  0  0  0  0  0  0  0  0  0
  2.1217   13.9658    0.8522 H   0  0  0  0  0  0  0  0  0  0  0  0
 11.1636   11.8988   -0.9393 H   0  0  0  0  0  0  0  0  0  0  0  0
 11.3610   12.9584    0.4628 H   0  0  0  0  0  0  0  0  0  0  0  0
  1.4915   11.6573    1.5115 H   0  0  0  0  0  0  0  0  0  0  0  0
 13.0122   14.3183   -0.7972 H   0  0  0  0  0  0  0  0  0  0  0  0
 12.8146   13.2590   -2.1996 H   0  0  0  0  0  0  0  0  0  0  0  0
  3.0852    9.7979    1.1000 H   0  0  0  0  0  0  0  0  0  0  0  0
  4.1207   10.8914    0.1728 H   0  0  0  0  0  0  0  0  0  0  0  0
 13.6097   11.3297   -0.8500 H   0  0  0  0  0  0  0  0  0  0  0  0
 13.8157   12.3948    0.5468 H   0  0  0  0  0  0  0  0  0  0  0  0
 15.4642   13.7458   -0.7277 H   0  0  0  0  0  0  0  0  0  0  0  0
 15.2594   12.6784   -2.1228 H   0  0  0  0  0  0  0  0  0  0  0  0
 16.0568   10.7560   -0.7661 H   0  0  0  0  0  0  0  0  0  0  0  0
 16.2677   11.8275    0.6250 H   0  0  0  0  0  0  0  0  0  0  0  0
 17.9140   13.1709   -0.6614 H   0  0  0  0  0  0  0  0  0  0  0  0
 17.7054   12.0951   -2.0495 H   0  0  0  0  0  0  0  0  0  0  0  0
 18.5055   10.1807   -0.6841 H   0  0  0  0  0  0  0  0  0  0  0  0
 18.7178   11.2591    0.7014 H   0  0  0  0  0  0  0  0  0  0  0  0
 20.3626   12.5961   -0.5934 H   0  0  0  0  0  0  0  0  0  0  0  0
 20.1528   11.5132   -1.9758 H   0  0  0  0  0  0  0  0  0  0  0  0
 20.9550    9.6061   -0.6024 H   0  0  0  0  0  0  0  0  0  0  0  0
 21.1670   10.6904    0.7785 H   0  0  0  0  0  0  0  0  0  0  0  0
 22.8114   12.0224   -0.5215 H   0  0  0  0  0  0  0  0  0  0  0  0
 22.6011   10.9349   -1.9001 H   0  0  0  0  0  0  0  0  0  0  0  0
 23.4047    9.0326   -0.5215 H   0  0  0  0  0  0  0  0  0  0  0  0
 23.6160   10.1208    0.8564 H   0  0  0  0  0  0  0  0  0  0  0  0
 25.2606   11.4495   -0.4455 H   0  0  0  0  0  0  0  0  0  0  0  0
 25.0498   10.3603   -1.8228 H   0  0  0  0  0  0  0  0  0  0  0  0
 25.7703    9.7604   -0.3232 H   0  0  0  0  0  0  0  0  0  0  0  0
  1  2  1  0  0  0  0
  1  4  1  0  0  0  0
  2  3  1  0  0  0  0
  3  5  1  0  0  0  0
  4  6  1  0  0  0  0
  4  7  2  0  0  0  0
  5  8  1  0  0  0  0
  6  9  1  0  0  0  0
  8 10  1  0  0  0  0
  9 11  1  0  0  0  0
 10 12  1  0  0  0  0
 11 13  1  0  0  0  0
 11 14  1  0  0  0  0
 12 15  1  0  0  0  0
 13 16  1  0  0  0  0
 15 17  1  0  0  0  0
 17 18  1  0  0  0  0
 18 19  1  0  0  0  0
 19 20  1  0  0  0  0
 20 21  1  0  0  0  0
 21 22  1  0  0  0  0
 22 23  1  0  0  0  0
 23 24  1  0  0  0  0
 24 25  1  0  0  0  0
 14 26  1  0  0  0  0
 16 27  1  0  0  0  0
  1 28  1  0  0  0  0
  1 29  1  0  0  0  0
  2 30  1  0  0  0  0
```

```
  2 31  1  0  0  0  0
  3 32  1  0  0  0  0
  3 33  1  0  0  0  0
  5 34  1  0  0  0  0
  5 35  1  0  0  0  0
  8 36  1  0  0  0  0
  8 37  1  0  0  0  0
  9 38  1  0  0  0  0
  9 39  1  0  0  0  0
 10 40  1  0  0  0  0
 10 41  1  0  0  0  0
 11 42  1  0  0  0  0
 12 43  1  0  0  0  0
 12 44  1  0  0  0  0
 13 45  1  0  0  0  0
 13 46  1  0  0  0  0
 15 47  1  0  0  0  0
 15 48  1  0  0  0  0
 17 49  1  0  0  0  0
 17 50  1  0  0  0  0
 18 51  1  0  0  0  0
 18 52  1  0  0  0  0
 19 53  1  0  0  0  0
 19 54  1  0  0  0  0
 20 55  1  0  0  0  0
 20 56  1  0  0  0  0
 21 57  1  0  0  0  0
 21 58  1  0  0  0  0
 22 59  1  0  0  0  0
 22 60  1  0  0  0  0
 23 61  1  0  0  0  0
 23 62  1  0  0  0  0
 24 63  1  0  0  0  0
 24 64  1  0  0  0  0
 25 65  1  0  0  0  0
 25 66  1  0  0  0  0
 25 67  1  0  0  0  0
M  END
>  <Alias name>
1-Glyceryl stearate

>  <deltaH°(c)>   kJ/mol
-12928

$$$$
Diisobutylammonium diisobutyldithiocarbamate
  ChemBrain IXL

 59 57  0  0  0  0  0  0  0  0  1 V2000
    3.3102    4.8762   -2.2733 C   0  0  0  0  0  0  0  0  0  0  0  0
    3.2554    5.5052   -0.8643 C   0  0  0  0  0  0  0  0  0  0  0  0
    4.6171    5.8918   -0.4423 N   0  0  0  0  0  0  0  0  0  0  0  0
    5.6564    4.8636   -0.2996 C   0  0  0  0  0  0  0  0  0  0  0  0
    6.0928    4.7786    1.1748 C   0  0  0  0  0  0  0  0  0  0  0  0
    8.9213   10.0325    0.0596 C   0  0  0  0  0  0  0  0  0  0  0  0
   10.3577    9.4991   -0.1320 C   0  0  0  0  0  0  0  0  0  0  0  0
   10.3112    8.0820   -0.5273 N   0  3  0  0  0  0  0  0  0  0  0  0
   11.6825    7.5766   -0.7024 C   0  0  0  0  0  0  0  0  0  0  0  0
   11.6401    6.0858   -1.1034 C   0  0  0  0  0  0  0  0  0  0  0  0
    4.9250    7.2383   -0.1732 C   0  0  0  0  0  0  0  0  0  0  0  0
    6.6243    7.7034    0.3929 S   0  5  0  0  0  0  0  0  0  0  0  0
    3.7320    8.4526   -0.3674 S   0  0  0  0  0  0  0  0  0  0  0  0
    3.8651    5.9067   -3.2766 C   0  0  0  0  0  0  0  0  0  0  0  0
```

```
    1.8912    4.4532   -2.7040 C   0  0  0  0  0  0  0  0  0  0  0  0
    7.1967    3.7154    1.3224 C   0  0  0  0  0  0  0  0  0  0  0  0
    4.8827    4.3906    2.0475 C   0  0  0  0  0  0  0  0  0  0  0  0
   13.0792    5.5591   -1.2685 C   0  0  0  0  0  0  0  0  0  0  0  0
   10.8805    5.9280   -2.4369 C   0  0  0  0  0  0  0  0  0  0  0  0
    8.9778   11.5060    0.5074 C   0  0  0  0  0  0  0  0  0  0  0  0
    8.1479    9.9307   -1.2716 C   0  0  0  0  0  0  0  0  0  0  0  0
    9.8067    7.9915   -1.3944 H   0  0  0  0  0  0  0  0  0  0  0  0
    9.8450    7.5477    0.1907 H   0  0  0  0  0  0  0  0  0  0  0  0
    3.9551    4.0069   -2.2554 H   0  0  0  0  0  0  0  0  0  0  0  0
    2.6188    6.3792   -0.8852 H   0  0  0  0  0  0  0  0  0  0  0  0
    2.8532    4.7839   -0.1639 H   0  0  0  0  0  0  0  0  0  0  0  0
    6.5088    5.1250   -0.9141 H   0  0  0  0  0  0  0  0  0  0  0  0
    5.2635    3.9054   -0.6159 H   0  0  0  0  0  0  0  0  0  0  0  0
    6.4739    5.7408    1.4939 H   0  0  0  0  0  0  0  0  0  0  0  0
    8.4163    9.4452    0.8164 H   0  0  0  0  0  0  0  0  0  0  0  0
   10.9040    9.5951    0.7983 H   0  0  0  0  0  0  0  0  0  0  0  0
   10.8560   10.0732   -0.9035 H   0  0  0  0  0  0  0  0  0  0  0  0
   12.2267    7.6831    0.2280 H   0  0  0  0  0  0  0  0  0  0  0  0
   12.1805    8.1451   -1.4783 H   0  0  0  0  0  0  0  0  0  0  0  0
   11.1352    5.5186   -0.3313 H   0  0  0  0  0  0  0  0  0  0  0  0
    3.2180    6.7750   -3.2975 H   0  0  0  0  0  0  0  0  0  0  0  0
    3.9061    5.4632   -4.2640 H   0  0  0  0  0  0  0  0  0  0  0  0
    4.8607    6.2057   -2.9736 H   0  0  0  0  0  0  0  0  0  0  0  0
    1.9309    4.0096   -3.6914 H   0  0  0  0  0  0  0  0  0  0  0  0
    1.5004    3.7299   -1.9990 H   0  0  0  0  0  0  0  0  0  0  0  0
    1.2456    5.3225   -2.7246 H   0  0  0  0  0  0  0  0  0  0  0  0
    7.5047    3.6565    2.3587 H   0  0  0  0  0  0  0  0  0  0  0  0
    8.0458    3.9895    0.7083 H   0  0  0  0  0  0  0  0  0  0  0  0
    6.8162    2.7531    1.0034 H   0  0  0  0  0  0  0  0  0  0  0  0
    4.5017    3.4281    1.7291 H   0  0  0  0  0  0  0  0  0  0  0  0
    5.1892    4.3326    3.0844 H   0  0  0  0  0  0  0  0  0  0  0  0
    4.1067    5.1389    1.9417 H   0  0  0  0  0  0  0  0  0  0  0  0
   13.5847    6.1256   -2.0409 H   0  0  0  0  0  0  0  0  0  0  0  0
   13.0510    4.5132   -1.5481 H   0  0  0  0  0  0  0  0  0  0  0  0
   13.6132    5.6683   -0.3327 H   0  0  0  0  0  0  0  0  0  0  0  0
   10.8654    4.8834   -2.7219 H   0  0  0  0  0  0  0  0  0  0  0  0
    9.8651    6.2838   -2.3190 H   0  0  0  0  0  0  0  0  0  0  0  0
   11.3783    6.5049   -3.2065 H   0  0  0  0  0  0  0  0  0  0  0  0
    7.9708   11.8806    0.6438 H   0  0  0  0  0  0  0  0  0  0  0  0
    9.5197   11.5787    1.4422 H   0  0  0  0  0  0  0  0  0  0  0  0
    9.4823   12.0942   -0.2492 H   0  0  0  0  0  0  0  0  0  0  0  0
    8.0955    8.8949   -1.5811 H   0  0  0  0  0  0  0  0  0  0  0  0
    7.1454   10.3176   -1.1367 H   0  0  0  0  0  0  0  0  0  0  0  0
    8.6588   10.5093   -2.0313 H   0  0  0  0  0  0  0  0  0  0  0  0
  1  2  1  0  0  0  0
  2  3  1  0  0  0  0
  3  4  1  0  0  0  0
  4  5  1  0  0  0  0
  6  7  1  0  0  0  0
  7  8  1  0  0  0  0
  8  9  1  0  0  0  0
  9 10  1  0  0  0  0
  3 11  1  0  0  0  0
 11 12  1  0  0  0  0
 11 13  2  0  0  0  0
  1 14  1  0  0  0  0
  1 15  1  0  0  0  0
  5 16  1  0  0  0  0
  5 17  1  0  0  0  0
 10 18  1  0  0  0  0
 10 19  1  0  0  0  0
  6 20  1  0  0  0  0
```

```
  6 21  1  0  0  0  0
  8 22  1  0  0  0  0
  8 23  1  0  0  0  0
  1 24  1  0  0  0  0
  2 25  1  0  0  0  0
  2 26  1  0  0  0  0
  4 27  1  0  0  0  0
  4 28  1  0  0  0  0
  5 29  1  0  0  0  0
  6 30  1  0  0  0  0
  7 31  1  0  0  0  0
  7 32  1  0  0  0  0
  9 33  1  0  0  0  0
  9 34  1  0  0  0  0
 10 35  1  0  0  0  0
 14 36  1  0  0  0  0
 14 37  1  0  0  0  0
 14 38  1  0  0  0  0
 15 39  1  0  0  0  0
 15 40  1  0  0  0  0
 15 41  1  0  0  0  0
 16 42  1  0  0  0  0
 16 43  1  0  0  0  0
 16 44  1  0  0  0  0
 17 45  1  0  0  0  0
 17 46  1  0  0  0  0
 17 47  1  0  0  0  0
 18 48  1  0  0  0  0
 18 49  1  0  0  0  0
 18 50  1  0  0  0  0
 19 51  1  0  0  0  0
 19 52  1  0  0  0  0
 19 53  1  0  0  0  0
 20 54  1  0  0  0  0
 20 55  1  0  0  0  0
 20 56  1  0  0  0  0
 21 57  1  0  0  0  0
 21 58  1  0  0  0  0
 21 59  1  0  0  0  0
M  END
>  <Alias name>
Diisobutylammonium diisobutyldithiocarbamate

>  <deltaH°(c)>   kJ/mol
-12891

$$$$
3,7,11,15-Tetramethyl-1-hexadecyn-3-ol
  ChemBrain IXL

 59 58  0  0  0  0  0  0  0  0  1 V2000
    2.7141    7.9691   -0.0577 C   0  0  0  0  0  0  0  0  0  0  0  0
    2.0544    7.0598   -0.4903 C   0  0  0  0  0  0  0  0  0  0  0  0
    3.5590    9.1334    0.4961 C   0  0  0  0  0  0  0  0  0  0  0  0
    4.0449    9.9608   -0.6192 O   0  0  0  0  0  0  0  0  0  0  0  0
    4.7611    8.5692    1.2844 C   0  0  0  0  0  0  0  0  0  0  0  0
    2.6912    9.9959    1.4332 C   0  0  0  0  0  0  0  0  0  0  0  0
    6.8710    5.0651   -0.3031 C   0  0  0  0  0  0  0  0  0  0  0  0
    7.7033    6.2524    0.2236 C   0  0  0  0  0  0  0  0  0  0  0  0
    5.6290    7.6977    0.3534 C   0  0  0  0  0  0  0  0  0  0  0  0
    6.8258    7.1322    1.1481 C   0  0  0  0  0  0  0  0  0  0  0  0
    9.0493    8.2787   -0.4509 C   0  0  0  0  0  0  0  0  0  0  0  0
    8.2139    7.0911   -0.9703 C   0  0  0  0  0  0  0  0  0  0  0  0
```

```
   9.5708    9.1016   -1.6485 C   0  0  0  0  0  0  0  0  0  0  0  0
  12.4910    8.8694   -1.1659 C   0  0  0  0  0  0  0  0  0  0  0  0
  11.6029    9.7859   -0.2998 C   0  0  0  0  0  0  0  0  0  0  0  0
  13.6900    8.3774   -0.3297 C   0  0  0  0  0  0  0  0  0  0  0  0
  14.5939    7.4749   -1.1978 C   0  0  0  0  0  0  0  0  0  0  0  0
  10.4106   10.2992   -1.1391 C   0  0  0  0  0  0  0  0  0  0  0  0
   9.5278   11.2166   -0.2678 C   0  0  0  0  0  0  0  0  0  0  0  0
  15.8034    7.0084   -0.3640 C   0  0  0  0  0  0  0  0  0  0  0  0
  13.7948    6.2441   -1.6743 C   0  0  0  0  0  0  0  0  0  0  0  0
   4.6010    9.4878   -1.2770 H   0  0  0  0  0  0  0  0  0  0  0  0
   1.4609    6.2418   -0.8793 H   0  0  0  0  0  0  0  0  0  0  0  0
   4.4005    7.9666    2.1089 H   0  0  0  0  0  0  0  0  0  0  0  0
   5.3560    9.3881    1.6697 H   0  0  0  0  0  0  0  0  0  0  0  0
   2.3322    9.3877    2.2543 H   0  0  0  0  0  0  0  0  0  0  0  0
   3.2840   10.8144    1.8224 H   0  0  0  0  0  0  0  0  0  0  0  0
   1.8476   10.3917    0.8814 H   0  0  0  0  0  0  0  0  0  0  0  0
   7.4919    4.4414   -0.9343 H   0  0  0  0  0  0  0  0  0  0  0  0
   6.5054    4.4816    0.5327 H   0  0  0  0  0  0  0  0  0  0  0  0
   6.0328    5.4373   -0.8777 H   0  0  0  0  0  0  0  0  0  0  0  0
   8.5490    5.8756    0.7852 H   0  0  0  0  0  0  0  0  0  0  0  0
   5.9932    8.3004   -0.4677 H   0  0  0  0  0  0  0  0  0  0  0  0
   5.0339    6.8812   -0.0338 H   0  0  0  0  0  0  0  0  0  0  0  0
   6.4591    6.5327    1.9721 H   0  0  0  0  0  0  0  0  0  0  0  0
   7.4186    7.9504    1.5357 H   0  0  0  0  0  0  0  0  0  0  0  0
   8.4318    8.9067    0.1767 H   0  0  0  0  0  0  0  0  0  0  0  0
   9.8869    7.9055    0.1237 H   0  0  0  0  0  0  0  0  0  0  0  0
   8.8303    6.4698   -1.6082 H   0  0  0  0  0  0  0  0  0  0  0  0
   7.3710    7.4635   -1.5376 H   0  0  0  0  0  0  0  0  0  0  0  0
  10.1864    8.4713   -2.2769 H   0  0  0  0  0  0  0  0  0  0  0  0
   8.7308    9.4710   -2.2237 H   0  0  0  0  0  0  0  0  0  0  0  0
  12.8522    9.4228   -2.0238 H   0  0  0  0  0  0  0  0  0  0  0  0
  11.9127    8.0196   -1.5019 H   0  0  0  0  0  0  0  0  0  0  0  0
  11.2332    9.2300    0.5519 H   0  0  0  0  0  0  0  0  0  0  0  0
  12.1873   10.6290    0.0472 H   0  0  0  0  0  0  0  0  0  0  0  0
  13.3293    7.8144    0.5224 H   0  0  0  0  0  0  0  0  0  0  0  0
  14.2604    9.2301    0.0173 H   0  0  0  0  0  0  0  0  0  0  0  0
  14.9429    8.0335   -2.0573 H   0  0  0  0  0  0  0  0  0  0  0  0
  10.7837   10.8608   -1.9865 H   0  0  0  0  0  0  0  0  0  0  0  0
   9.1650   10.6629    0.5884 H   0  0  0  0  0  0  0  0  0  0  0  0
  10.1122   12.0628    0.0716 H   0  0  0  0  0  0  0  0  0  0  0  0
   8.6873   11.5699   -0.8522 H   0  0  0  0  0  0  0  0  0  0  0  0
  16.3660    7.8714   -0.0300 H   0  0  0  0  0  0  0  0  0  0  0  0
  16.4388    6.3765   -0.9721 H   0  0  0  0  0  0  0  0  0  0  0  0
  15.4552    6.4496    0.4959 H   0  0  0  0  0  0  0  0  0  0  0  0
  14.4362    5.6063   -2.2699 H   0  0  0  0  0  0  0  0  0  0  0  0
  13.4350    5.6922   -0.8147 H   0  0  0  0  0  0  0  0  0  0  0  0
  12.9536    6.5684   -2.2729 H   0  0  0  0  0  0  0  0  0  0  0  0
  1  2  3  0  0  0  0
  1  3  1  0  0  0  0
  3  4  1  0  0  0  0
  3  5  1  0  0  0  0
  3  6  1  0  0  0  0
  5  9  1  0  0  0  0
  7  8  1  0  0  0  0
  8 10  1  0  0  0  0
  8 12  1  0  0  0  0
  9 10  1  0  0  0  0
 11 12  1  0  0  0  0
 11 13  1  0  0  0  0
 13 18  1  0  0  0  0
 14 15  1  0  0  0  0
 14 16  1  0  0  0  0
 15 18  1  0  0  0  0
```

```
 16 17  1  0  0  0  0
 17 20  1  0  0  0  0
 17 21  1  0  0  0  0
 18 19  1  0  0  0  0
  4 22  1  0  0  0  0
  2 23  1  0  0  0  0
  5 24  1  0  0  0  0
  5 25  1  0  0  0  0
  6 26  1  0  0  0  0
  6 27  1  0  0  0  0
  6 28  1  0  0  0  0
  7 29  1  0  0  0  0
  7 30  1  0  0  0  0
  7 31  1  0  0  0  0
  8 32  1  0  0  0  0
  9 33  1  0  0  0  0
  9 34  1  0  0  0  0
 10 35  1  0  0  0  0
 10 36  1  0  0  0  0
 11 37  1  0  0  0  0
 11 38  1  0  0  0  0
 12 39  1  0  0  0  0
 12 40  1  0  0  0  0
 13 41  1  0  0  0  0
 13 42  1  0  0  0  0
 14 43  1  0  0  0  0
 14 44  1  0  0  0  0
 15 45  1  0  0  0  0
 15 46  1  0  0  0  0
 16 47  1  0  0  0  0
 16 48  1  0  0  0  0
 17 49  1  0  0  0  0
 18 50  1  0  0  0  0
 19 51  1  0  0  0  0
 19 52  1  0  0  0  0
 19 53  1  0  0  0  0
 20 54  1  0  0  0  0
 20 55  1  0  0  0  0
 20 56  1  0  0  0  0
 21 57  1  0  0  0  0
 21 58  1  0  0  0  0
 21 59  1  0  0  0  0
M  END
>  <Alias name>
3,7,11,15-Tetramethyl-1-hexadecyn-3-ol

>  <deltaH°(c)>   kJ/mol
-12860

$$$$
Tetraisobutylsucccinonitrile
  ChemBrain IXL

 58 57  0  0  0  0  0  0  0  0  1 V2000
    3.2600    6.7514   -1.5759 C   0  0  0  0  0  0  0  0  0  0  0  0
    3.4704    6.2017   -0.1488 C   0  0  0  0  0  0  0  0  0  0  0  0
    2.3630    6.7493    0.7721 C   0  0  0  0  0  0  0  0  0  0  0  0
    4.8573    6.6469    0.4021 C   0  0  0  0  0  0  0  0  0  0  0  0
    6.0148    6.0585   -0.4733 C   0  0  0  0  0  0  0  0  0  0  0  0
    6.0357    6.7703   -1.8408 C   0  0  0  0  0  0  0  0  0  0  0  0
    5.7914    4.5136   -0.7175 C   0  0  0  0  0  0  0  0  0  0  0  0
    7.4077    6.2840    0.2353 C   0  0  0  0  0  0  0  0  0  0  0  0
    6.0516    7.3049   -2.8677 N   0  0  0  0  0  0  0  0  0  0  0  0
```

```
5.8538 3.6900 0.6140 C 0 0 0 0 0 0 0 0 0 0 0 0
6.1136 2.2107 0.2688 C 0 0 0 0 0 0 0 0 0 0 0 0
4.5149 3.7868 1.3875 C 0 0 0 0 0 0 0 0 0 0 0 0
7.2574 6.0596 1.7536 C 0 0 0 0 0 0 0 0 0 0 0 0
8.4300 5.2576 -0.3382 C 0 0 0 0 0 0 0 0 0 0 0 0
7.9568 7.7349 -0.0447 C 0 0 0 0 0 0 0 0 0 0 0 0
7.1444 5.8910 2.8936 N 0 0 0 0 0 0 0 0 0 0 0 0
9.8703 5.5728 0.1626 C 0 0 0 0 0 0 0 0 0 0 0 0
9.9428 5.4654 1.7012 C 0 0 0 0 0 0 0 0 0 0 0 0
10.8519 4.5613 -0.4606 C 0 0 0 0 0 0 0 0 0 0 0 0
6.9322 8.8371 0.3397 C 0 0 0 0 0 0 0 0 0 0 0 0
6.5714 8.7432 1.8404 C 0 0 0 0 0 0 0 0 0 0 0 0
7.5572 10.2178 0.0607 C 0 0 0 0 0 0 0 0 0 0 0 0
2.2618 6.5016 -1.9134 H 0 0 0 0 0 0 0 0 0 0 0 0
3.9832 6.3105 -2.2443 H 0 0 0 0 0 0 0 0 0 0 0 0
3.3799 7.8277 -1.5702 H 0 0 0 0 0 0 0 0 0 0 0 0
3.4164 5.1242 -0.1684 H 0 0 0 0 0 0 0 0 0 0 0 0
1.3954 6.4399 0.3967 H 0 0 0 0 0 0 0 0 0 0 0 0
2.5022 6.3620 1.7738 H 0 0 0 0 0 0 0 0 0 0 0 0
2.4129 7.8310 0.7931 H 0 0 0 0 0 0 0 0 0 0 0 0
4.9074 7.7154 0.3964 H 0 0 0 0 0 0 0 0 0 0 0 0
4.9660 6.2988 1.4129 H 0 0 0 0 0 0 0 0 0 0 0 0
6.5700 4.1570 -1.3799 H 0 0 0 0 0 0 0 0 0 0 0 0
4.8311 4.3565 -1.1906 H 0 0 0 0 0 0 0 0 0 0 0 0
6.6589 4.0536 1.2301 H 0 0 0 0 0 0 0 0 0 0 0 0
7.0508 2.1242 -0.2669 H 0 0 0 0 0 0 0 0 0 0 0 0
5.3077 1.8376 -0.3511 H 0 0 0 0 0 0 0 0 0 0 0 0
6.1649 1.6306 1.1819 H 0 0 0 0 0 0 0 0 0 0 0 0
3.7090 3.4314 0.7576 H 0 0 0 0 0 0 0 0 0 0 0 0
4.5710 3.1732 2.2780 H 0 0 0 0 0 0 0 0 0 0 0 0
4.3266 4.7986 1.6687 H 0 0 0 0 0 0 0 0 0 0 0 0
8.1519 4.2595 -0.0277 H 0 0 0 0 0 0 0 0 0 0 0 0
8.4151 5.3113 -1.4198 H 0 0 0 0 0 0 0 0 0 0 0 0
8.8644 7.8836 0.5262 H 0 0 0 0 0 0 0 0 0 0 0 0
8.1861 7.8251 -1.0992 H 0 0 0 0 0 0 0 0 0 0 0 0
10.1484 6.5733 -0.1420 H 0 0 0 0 0 0 0 0 0 0 0 0
10.9689 5.5951 2.0221 H 0 0 0 0 0 0 0 0 0 0 0 0
9.3306 6.2330 2.1476 H 0 0 0 0 0 0 0 0 0 0 0 0
9.5882 4.4907 2.0131 H 0 0 0 0 0 0 0 0 0 0 0 0
11.8574 4.7782 -0.1217 H 0 0 0 0 0 0 0 0 0 0 0 0
10.8107 4.6365 -1.5401 H 0 0 0 0 0 0 0 0 0 0 0 0
10.5774 3.5583 -0.1578 H 0 0 0 0 0 0 0 0 0 0 0 0
6.0448 8.7282 -0.2575 H 0 0 0 0 0 0 0 0 0 0 0 0
7.4755 8.8036 2.4336 H 0 0 0 0 0 0 0 0 0 0 0 0
5.9131 9.5619 2.1031 H 0 0 0 0 0 0 0 0 0 0 0 0
6.0736 7.8095 2.0372 H 0 0 0 0 0 0 0 0 0 0 0 0
6.8477 10.9941 0.3191 H 0 0 0 0 0 0 0 0 0 0 0 0
8.4539 10.3353 0.6567 H 0 0 0 0 0 0 0 0 0 0 0 0
7.8093 10.2943 -0.9897 H 0 0 0 0 0 0 0 0 0 0 0 0
1 2 1 0 0 0 0
2 3 1 0 0 0 0
2 4 1 0 0 0 0
4 5 1 0 0 0 0
5 6 1 0 0 0 0
5 7 1 0 0 0 0
5 8 1 0 0 0 0
6 9 3 0 0 0 0
7 10 1 0 0 0 0
8 13 1 0 0 0 0
8 14 1 0 0 0 0
8 15 1 0 0 0 0
10 11 1 0 0 0 0
10 12 1 0 0 0 0
```

```
 13 16  3  0  0  0  0
 14 17  1  0  0  0  0
 15 20  1  0  0  0  0
 17 18  1  0  0  0  0
 17 19  1  0  0  0  0
 20 21  1  0  0  0  0
 20 22  1  0  0  0  0
  1 23  1  0  0  0  0
  1 24  1  0  0  0  0
  1 25  1  0  0  0  0
  2 26  1  0  0  0  0
  3 27  1  0  0  0  0
  3 28  1  0  0  0  0
  3 29  1  0  0  0  0
  4 30  1  0  0  0  0
  4 31  1  0  0  0  0
  7 32  1  0  0  0  0
  7 33  1  0  0  0  0
 10 34  1  0  0  0  0
 11 35  1  0  0  0  0
 11 36  1  0  0  0  0
 11 37  1  0  0  0  0
 12 38  1  0  0  0  0
 12 39  1  0  0  0  0
 12 40  1  0  0  0  0
 14 41  1  0  0  0  0
 14 42  1  0  0  0  0
 15 43  1  0  0  0  0
 15 44  1  0  0  0  0
 17 45  1  0  0  0  0
 18 46  1  0  0  0  0
 18 47  1  0  0  0  0
 18 48  1  0  0  0  0
 19 49  1  0  0  0  0
 19 50  1  0  0  0  0
 19 51  1  0  0  0  0
 20 52  1  0  0  0  0
 21 53  1  0  0  0  0
 21 54  1  0  0  0  0
 21 55  1  0  0  0  0
 22 56  1  0  0  0  0
 22 57  1  0  0  0  0
 22 58  1  0  0  0  0
M  END
>  <Alias name>
Tetraisobutylsucccinonitrile

>  <deltaH°(c)>   kJ/mol
-12750

$$$$
1,8-Di(3-formyl-4-methoxyphenyl)naphthalene
  ChemBrain IXL

 50 53  0  0  0  0  0  0  0  0  1 V2000
    3.7230    4.4109    2.5090 C   0  0  0  0  0  0  0  0  0  0  0  0
    2.6199    5.0840    3.0356 C   0  0  0  0  0  0  0  0  0  0  0  0
    2.3719    6.4104    2.6782 C   0  0  0  0  0  0  0  0  0  0  0  0
    3.2313    7.0740    1.8003 C   0  0  0  0  0  0  0  0  0  0  0  0
    4.3552    6.4164    1.2820 C   0  0  0  0  0  0  0  0  0  0  0  0
    4.5865    5.0606    1.6255 C   0  0  0  0  0  0  0  0  0  0  0  0
    5.2378    7.1205    0.4248 C   0  0  0  0  0  0  0  0  0  0  0  0
    4.9556    8.4405    0.0697 C   0  0  0  0  0  0  0  0  0  0  0  0
```

```
   3.8211    9.0766    0.5749 C   0  0  0  0  0  0  0  0  0  0  0  0  0
   2.9640    8.3968    1.4419 C   0  0  0  0  0  0  0  0  0  0  0  0  0
   7.9151    2.8813    0.0045 C   0  0  0  0  0  0  0  0  0  0  0  0  0
   6.8230    3.1876   -0.8044 C   0  0  0  0  0  0  0  0  0  0  0  0  0
   5.7387    3.8992   -0.2800 C   0  0  0  0  0  0  0  0  0  0  0  0  0
   5.7454    4.3034    1.0590 C   0  0  0  0  0  0  0  0  0  0  0  0  0
   6.8381    3.9859    1.8705 C   0  0  0  0  0  0  0  0  0  0  0  0  0
   7.9225    3.2781    1.3432 C   0  0  0  0  0  0  0  0  0  0  0  0  0
   6.8159    2.7568   -2.2199 C   0  0  0  0  0  0  0  0  0  0  0  0  0
   5.8794    3.0531   -2.9431 O   0  0  0  0  0  0  0  0  0  0  0  0  0
   9.0579    2.1390   -0.5564 O   0  0  0  0  0  0  0  0  0  0  0  0  0
  10.2463    2.0664    0.3081 C   0  0  0  0  0  0  0  0  0  0  0  0  0
   8.7920    5.2741   -1.1276 C   0  0  0  0  0  0  0  0  0  0  0  0  0
   8.7354    5.6609    0.2097 C   0  0  0  0  0  0  0  0  0  0  0  0  0
   7.5780    6.2592    0.7188 C   0  0  0  0  0  0  0  0  0  0  0  0  0
   6.4750    6.4757   -0.1135 C   0  0  0  0  0  0  0  0  0  0  0  0  0
   6.5386    6.0958   -1.4569 C   0  0  0  0  0  0  0  0  0  0  0  0  0
   7.6947    5.4935   -1.9625 C   0  0  0  0  0  0  0  0  0  0  0  0  0
   9.9047    5.4358    1.0881 C   0  0  0  0  0  0  0  0  0  0  0  0  0
   9.8478    5.7376    2.2685 O   0  0  0  0  0  0  0  0  0  0  0  0  0
  10.0105    4.6350   -1.6553 O   0  0  0  0  0  0  0  0  0  0  0  0  0
   9.9185    4.1556   -3.0434 C   0  0  0  0  0  0  0  0  0  0  0  0  0
   3.9101    3.3810    2.7863 H   0  0  0  0  0  0  0  0  0  0  0  0  0
   1.9550    4.5762    3.7228 H   0  0  0  0  0  0  0  0  0  0  0  0  0
   1.5104    6.9263    3.0829 H   0  0  0  0  0  0  0  0  0  0  0  0  0
   5.6198    8.9723   -0.6002 H   0  0  0  0  0  0  0  0  0  0  0  0  0
   3.6053   10.0997    0.2937 H   0  0  0  0  0  0  0  0  0  0  0  0  0
   2.0892    8.8967    1.8379 H   0  0  0  0  0  0  0  0  0  0  0  0  0
   4.8927    4.1373   -0.9124 H   0  0  0  0  0  0  0  0  0  0  0  0  0
   6.8450    4.2887    2.9100 H   0  0  0  0  0  0  0  0  0  0  0  0  0
   8.7702    3.0368    1.9736 H   0  0  0  0  0  0  0  0  0  0  0  0  0
   7.6427    2.1782   -2.6138 H   0  0  0  0  0  0  0  0  0  0  0  0  0
   9.9779    1.6123    1.2536 H   0  0  0  0  0  0  0  0  0  0  0  0  0
  10.6286    3.0648    0.4823 H   0  0  0  0  0  0  0  0  0  0  0  0  0
  11.0086    1.4674   -0.1764 H   0  0  0  0  0  0  0  0  0  0  0  0  0
   7.5365    6.5553    1.7595 H   0  0  0  0  0  0  0  0  0  0  0  0  0
   5.6908    6.2684   -2.1078 H   0  0  0  0  0  0  0  0  0  0  0  0  0
   7.7398    5.1964   -3.0035 H   0  0  0  0  0  0  0  0  0  0  0  0  0
  10.8111    5.0028    0.6825 H   0  0  0  0  0  0  0  0  0  0  0  0  0
  10.8667    3.7217   -3.3387 H   0  0  0  0  0  0  0  0  0  0  0  0  0
   9.6826    4.9857   -3.6974 H   0  0  0  0  0  0  0  0  0  0  0  0  0
   9.1405    3.4057   -3.1162 H   0  0  0  0  0  0  0  0  0  0  0  0  0
  1  2  4  0  0  0  0
  2  3  4  0  0  0  0
  3  4  4  0  0  0  0
  4  5  4  0  0  0  0
  5  6  4  0  0  0  0
  6  1  4  0  0  0  0
  5  7  4  0  0  0  0
  7  8  4  0  0  0  0
  8  9  4  0  0  0  0
  4 10  4  0  0  0  0
 10  9  4  0  0  0  0
 11 12  4  0  0  0  0
 12 13  4  0  0  0  0
 13 14  4  0  0  0  0
 14 15  4  0  0  0  0
 15 16  4  0  0  0  0
 16 11  4  0  0  0  0
 12 17  1  0  0  0  0
 17 18  2  0  0  0  0
 14  6  1  0  0  0  0
 11 19  1  0  0  0  0
```

19 20 1 0 0 0 0
21 22 4 0 0 0 0
22 23 4 0 0 0 0
23 24 4 0 0 0 0
24 25 4 0 0 0 0
25 26 4 0 0 0 0
26 21 4 0 0 0 0
22 27 1 0 0 0 0
27 28 2 0 0 0 0
21 29 1 0 0 0 0
29 30 1 0 0 0 0
24 7 1 0 0 0 0
1 31 1 0 0 0 0
2 32 1 0 0 0 0
3 33 1 0 0 0 0
8 34 1 0 0 0 0
9 35 1 0 0 0 0
10 36 1 0 0 0 0
13 37 1 0 0 0 0
15 38 1 0 0 0 0
16 39 1 0 0 0 0
17 40 1 0 0 0 0
20 41 1 0 0 0 0
20 42 1 0 0 0 0
20 43 1 0 0 0 0
23 44 1 0 0 0 0
25 45 1 0 0 0 0
26 46 1 0 0 0 0
27 47 1 0 0 0 0
30 48 1 0 0 0 0
30 49 1 0 0 0 0
30 50 1 0 0 0 0
M END
> <Alias name>
1,8-Di(3-formyl-4-methoxyphenyl)naphthalene

> <deltaH°(c)>   kJ/mol
-12731

$$$$
N,N-Bis(3-phenoxy-2-hydroxypropyl)aniline
ChemBrain IXL

56 58 0 0 0 0 0 0 0 0 1 V2000
16.1263   7.7875   -0.5513 C   0 0 0 0 0 0 0 0 0 0 0 0
17.0293   8.3786   -1.4361 C   0 0 0 0 0 0 0 0 0 0 0 0
16.5897   6.9186   0.4403 C   0 0 0 0 0 0 0 0 0 0 0 0
14.6874   8.0830   -0.6652 O   0 0 0 0 0 0 0 0 0 0 0 0
18.3931   8.1008   -1.3315 C   0 0 0 0 0 0 0 0 0 0 0 0
18.8550   7.2330   -0.3408 C   0 0 0 0 0 0 0 0 0 0 0 0
17.9532   6.6413   0.5445 C   0 0 0 0 0 0 0 0 0 0 0 0
13.8152   7.3680   0.2847 C   0 0 0 0 0 0 0 0 0 0 0 0
12.3529   7.7933   0.0520 C   0 0 0 0 0 0 0 0 0 0 0 0
11.9762   7.4996   -1.3392 O   0 0 0 0 0 0 0 0 0 0 0 0
12.2143   9.3090   0.3186 C   0 0 0 0 0 0 0 0 0 0 0 0
8.5819   9.5422   1.7623 O   0 0 0 0 0 0 0 0 0 0 0 0
8.4310   9.3144   0.3177 C   0 0 0 0 0 0 0 0 0 0 0 0
9.7654   8.7836   -0.2663 C   0 0 0 0 0 0 0 0 0 0 0 0
7.3121   8.2805   0.0750 C   0 0 0 0 0 0 0 0 0 0 0 0
10.8496   9.7661   -0.0188 N   0 0 0 0 0 0 0 0 0 0 0 0
10.5866   11.1407   -0.1094 C   0 0 0 0 0 0 0 0 0 0 0 0
6.0282   8.8267   0.5534 O   0 0 0 0 0 0 0 0 0 0 0 0
4.8602   7.9413   0.3838 C   0 0 0 0 0 0 0 0 0 0 0 0

5.0250	6.6480	-0.1207	C	0	0	0	0	0	0	0	0	0	0	0	0	0
3.5858	8.3962	0.7281	C	0	0	0	0	0	0	0	0	0	0	0	0	0
3.9178	5.8133	-0.2799	C	0	0	0	0	0	0	0	0	0	0	0	0	0
2.6448	6.2696	0.0647	C	0	0	0	0	0	0	0	0	0	0	0	0	0
2.4787	7.5608	0.5684	C	0	0	0	0	0	0	0	0	0	0	0	0	0
11.2430	12.0327	0.7403	C	0	0	0	0	0	0	0	0	0	0	0	0	0
9.6698	11.6188	-1.0481	C	0	0	0	0	0	0	0	0	0	0	0	0	0
10.9824	13.4007	0.6523	C	0	0	0	0	0	0	0	0	0	0	0	0	0
10.0658	13.8777	-0.2857	C	0	0	0	0	0	0	0	0	0	0	0	0	0
9.4096	12.9867	-1.1359	C	0	0	0	0	0	0	0	0	0	0	0	0	0
12.5173	7.9556	-2.0213	H	0	0	0	0	0	0	0	0	0	0	0	0	0
7.7784	9.8825	2.2153	H	0	0	0	0	0	0	0	0	0	0	0	0	0
16.6715	9.0520	-2.2048	H	0	0	0	0	0	0	0	0	0	0	0	0	0
15.8913	6.4587	1.1281	H	0	0	0	0	0	0	0	0	0	0	0	0	0
19.0931	8.5596	-2.0184	H	0	0	0	0	0	0	0	0	0	0	0	0	0
19.9132	7.0178	-0.2598	H	0	0	0	0	0	0	0	0	0	0	0	0	0
18.3116	5.9678	1.3133	H	0	0	0	0	0	0	0	0	0	0	0	0	0
14.1060	7.6127	1.2991	H	0	0	0	0	0	0	0	0	0	0	0	0	0
13.9101	6.3003	0.1288	H	0	0	0	0	0	0	0	0	0	0	0	0	0
11.7058	7.2461	0.7258	H	0	0	0	0	0	0	0	0	0	0	0	0	0
12.4140	9.5097	1.3641	H	0	0	0	0	0	0	0	0	0	0	0	0	0
12.9298	9.8454	-0.2922	H	0	0	0	0	0	0	0	0	0	0	0	0	0
8.1690	10.2425	-0.1665	H	0	0	0	0	0	0	0	0	0	0	0	0	0
10.0136	7.8448	0.2085	H	0	0	0	0	0	0	0	0	0	0	0	0	0
9.6557	8.6293	-1.3326	H	0	0	0	0	0	0	0	0	0	0	0	0	0
7.5409	7.3672	0.6109	H	0	0	0	0	0	0	0	0	0	0	0	0	0
7.2403	8.0692	-0.9851	H	0	0	0	0	0	0	0	0	0	0	0	0	0
6.0117	6.2920	-0.3887	H	0	0	0	0	0	0	0	0	0	0	0	0	0
3.4558	9.3977	1.1189	H	0	0	0	0	0	0	0	0	0	0	0	0	0
4.0468	4.8112	-0.6708	H	0	0	0	0	0	0	0	0	0	0	0	0	0
1.7857	5.6217	-0.0590	H	0	0	0	0	0	0	0	0	0	0	0	0	0
1.4905	7.9145	0.8356	H	0	0	0	0	0	0	0	0	0	0	0	0	0
11.9542	11.6632	1.4685	H	0	0	0	0	0	0	0	0	0	0	0	0	0
9.1603	10.9280	-1.7086	H	0	0	0	0	0	0	0	0	0	0	0	0	0
11.4915	14.0922	1.3121	H	0	0	0	0	0	0	0	0	0	0	0	0	0
9.8637	14.9392	-0.3538	H	0	0	0	0	0	0	0	0	0	0	0	0	0
8.6985	13.3567	-1.8640	H	0	0	0	0	0	0	0	0	0	0	0	0	0

2	1	4	0	0	0	0
1	3	4	0	0	0	0
1	4	1	0	0	0	0
5	2	4	0	0	0	0
3	7	4	0	0	0	0
4	8	1	0	0	0	0
6	5	4	0	0	0	0
7	6	4	0	0	0	0
8	9	1	0	0	0	0
9	10	1	0	0	0	0
9	11	1	0	0	0	0
11	16	1	0	0	0	0
12	13	1	0	0	0	0
13	14	1	0	0	0	0
13	15	1	0	0	0	0
14	16	1	0	0	0	0
15	18	1	0	0	0	0
16	17	1	0	0	0	0
25	17	4	0	0	0	0
17	26	4	0	0	0	0
18	19	1	0	0	0	0
20	19	4	0	0	0	0
19	21	4	0	0	0	0
22	20	4	0	0	0	0
21	24	4	0	0	0	0
23	22	4	0	0	0	0

24 23 4 0 0 0 0
27 25 4 0 0 0 0
26 29 4 0 0 0 0
28 27 4 0 0 0 0
29 28 4 0 0 0 0
10 30 1 0 0 0 0
12 31 1 0 0 0 0
2 32 1 0 0 0 0
3 33 1 0 0 0 0
5 34 1 0 0 0 0
6 35 1 0 0 0 0
7 36 1 0 0 0 0
8 37 1 0 0 0 0
8 38 1 0 0 0 0
9 39 1 0 0 0 0
11 40 1 0 0 0 0
11 41 1 0 0 0 0
13 42 1 0 0 0 0
14 43 1 0 0 0 0
14 44 1 0 0 0 0
15 45 1 0 0 0 0
15 46 1 0 0 0 0
20 47 1 0 0 0 0
21 48 1 0 0 0 0
22 49 1 0 0 0 0
23 50 1 0 0 0 0
24 51 1 0 0 0 0
25 52 1 0 0 0 0
26 53 1 0 0 0 0
27 54 1 0 0 0 0
28 55 1 0 0 0 0
29 56 1 0 0 0 0
M END
> <Alias name>
N,N-Bis(3-phenoxy-2-hydroxypropyl)aniline

> <deltaH°(c)>   kJ/mol
-12720

$$$$
Methyl 2-methylstearate
ChemBrain IXL

62 61 0 0 0 0 0 0 0 0 1 V2000
13.6112   13.7915   -0.2704 C   0 0 0 0 0 0 0 0 0 0 0 0
12.2905   13.1065   0.1318 C   0 0 0 0 0 0 0 0 0 0 0 0
11.0996   13.9079   -0.4284 C   0 0 0 0 0 0 0 0 0 0 0 0
9.7832   13.2206   -0.0157 C   0 0 0 0 0 0 0 0 0 0 0 0
8.5877   14.0136   -0.5777 C   0 0 0 0 0 0 0 0 0 0 0 0
7.2744   13.3244   -0.1579 C   0 0 0 0 0 0 0 0 0 0 0 0
6.0758   14.1113   -0.7220 C   0 0 0 0 0 0 0 0 0 0 0 0
4.7644   13.4213   -0.2979 C   0 0 0 0 0 0 0 0 0 0 0 0
3.5642   14.2051   -0.8636 C   0 0 0 0 0 0 0 0 0 0 0 0
14.7961   12.9805   0.2889 C   0 0 0 0 0 0 0 0 0 0 0 0
2.2536   13.5152   -0.4376 C   0 0 0 0 0 0 0 0 0 0 0 0
16.1216   13.6637   -0.0994 C   0 0 0 0 0 0 0 0 0 0 0 0
17.3000   12.8435   0.4606 C   0 0 0 0 0 0 0 0 0 0 0 0
18.6301   13.5264   0.0887 C   0 0 0 0 0 0 0 0 0 0 0 0
19.8024   12.6985   0.6506 C   0 0 0 0 0 0 0 0 0 0 0 0
21.1369   13.3846   0.2956 C   0 0 0 0 0 0 0 0 0 0 0 0
22.3109   12.5649   0.8765 C   0 0 0 0 0 0 0 0 0 0 0 0
23.5890   13.2495   0.5703 C   0 0 0 0 0 0 0 0 0 0 0 0
23.7941   14.3739   0.9985 O   0 0 0 0 0 0 0 0 0 0 0 0

```
 24.6113   12.5740   -0.2497 O   0  0  0  0  0  0  0  0  0  0  0  0
 25.8371   13.3524   -0.4898 C   0  0  0  0  0  0  0  0  0  0  0  0
 22.3184   11.1540    0.2501 C   0  0  0  0  0  0  0  0  0  0  0  0
 13.6361   14.7952    0.1356 H   0  0  0  0  0  0  0  0  0  0  0  0
 13.6813   13.8363   -1.3502 H   0  0  0  0  0  0  0  0  0  0  0  0
 12.2690   12.1010   -0.2700 H   0  0  0  0  0  0  0  0  0  0  0  0
 12.2207   13.0647    1.2117 H   0  0  0  0  0  0  0  0  0  0  0  0
 11.1220   14.9147   -0.0301 H   0  0  0  0  0  0  0  0  0  0  0  0
 11.1650   13.9449   -1.5088 H   0  0  0  0  0  0  0  0  0  0  0  0
  9.7640   12.2121   -0.4099 H   0  0  0  0  0  0  0  0  0  0  0  0
  9.7176   13.1867    1.0648 H   0  0  0  0  0  0  0  0  0  0  0  0
  8.6073   15.0230   -0.1859 H   0  0  0  0  0  0  0  0  0  0  0  0
  8.6504   14.0442   -1.6584 H   0  0  0  0  0  0  0  0  0  0  0  0
  7.2573   12.3137   -0.5465 H   0  0  0  0  0  0  0  0  0  0  0  0
  7.2112   13.2965    0.9229 H   0  0  0  0  0  0  0  0  0  0  0  0
  6.0933   15.1226   -0.3349 H   0  0  0  0  0  0  0  0  0  0  0  0
  6.1373   14.1371   -1.8029 H   0  0  0  0  0  0  0  0  0  0  0  0
  4.7483   12.4092   -0.6830 H   0  0  0  0  0  0  0  0  0  0  0  0
  4.7026   13.3972    0.7831 H   0  0  0  0  0  0  0  0  0  0  0  0
  3.5807   15.2174   -0.4794 H   0  0  0  0  0  0  0  0  0  0  0  0
  3.6252   14.2281   -1.9445 H   0  0  0  0  0  0  0  0  0  0  0  0
 14.7730   11.9788   -0.1219 H   0  0  0  0  0  0  0  0  0  0  0  0
 14.7201   12.9293    1.3680 H   0  0  0  0  0  0  0  0  0  0  0  0
  2.2374   12.5026   -0.8213 H   0  0  0  0  0  0  0  0  0  0  0  0
  2.1924   13.4926    0.6435 H   0  0  0  0  0  0  0  0  0  0  0  0
  1.4102   14.0656   -0.8355 H   0  0  0  0  0  0  0  0  0  0  0  0
 16.1478   14.6639    0.3151 H   0  0  0  0  0  0  0  0  0  0  0  0
 16.1986   13.7173   -1.1784 H   0  0  0  0  0  0  0  0  0  0  0  0
 17.2769   11.8459    0.0399 H   0  0  0  0  0  0  0  0  0  0  0  0
 17.2159   12.7821    1.5386 H   0  0  0  0  0  0  0  0  0  0  0  0
 18.6555   14.5228    0.5123 H   0  0  0  0  0  0  0  0  0  0  0  0
 18.7156   13.5894   -0.9891 H   0  0  0  0  0  0  0  0  0  0  0  0
 19.7805   11.7059    0.2201 H   0  0  0  0  0  0  0  0  0  0  0  0
 19.7094   12.6269    1.7271 H   0  0  0  0  0  0  0  0  0  0  0  0
 21.1513   14.3822    0.7170 H   0  0  0  0  0  0  0  0  0  0  0  0
 21.2367   13.4464   -0.7811 H   0  0  0  0  0  0  0  0  0  0  0  0
 22.1959   12.4821    1.9501 H   0  0  0  0  0  0  0  0  0  0  0  0
 25.5865   14.2698   -1.0083 H   0  0  0  0  0  0  0  0  0  0  0  0
 26.5211   12.7709   -1.0953 H   0  0  0  0  0  0  0  0  0  0  0  0
 26.3056   13.5896    0.4574 H   0  0  0  0  0  0  0  0  0  0  0  0
 23.1505   10.5870    0.6488 H   0  0  0  0  0  0  0  0  0  0  0  0
 21.3916   10.6481    0.4870 H   0  0  0  0  0  0  0  0  0  0  0  0
 22.4203   11.2368   -0.8250 H   0  0  0  0  0  0  0  0  0  0  0  0
  1  2  1  0  0  0  0
  2  3  1  0  0  0  0
  3  4  1  0  0  0  0
  4  5  1  0  0  0  0
  5  6  1  0  0  0  0
  6  7  1  0  0  0  0
  7  8  1  0  0  0  0
  8  9  1  0  0  0  0
  1 10  1  0  0  0  0
  9 11  1  0  0  0  0
 12 13  1  0  0  0  0
 13 14  1  0  0  0  0
 14 15  1  0  0  0  0
 15 16  1  0  0  0  0
 16 17  1  0  0  0  0
 17 18  1  0  0  0  0
 10 12  1  0  0  0  0
 18 19  2  0  0  0  0
 18 20  1  0  0  0  0
 20 21  1  0  0  0  0
```

```
 17 22  1  0  0  0  0
  1 23  1  0  0  0  0
  1 24  1  0  0  0  0
  2 25  1  0  0  0  0
  2 26  1  0  0  0  0
  3 27  1  0  0  0  0
  3 28  1  0  0  0  0
  4 29  1  0  0  0  0
  4 30  1  0  0  0  0
  5 31  1  0  0  0  0
  5 32  1  0  0  0  0
  6 33  1  0  0  0  0
  6 34  1  0  0  0  0
  7 35  1  0  0  0  0
  7 36  1  0  0  0  0
  8 37  1  0  0  0  0
  8 38  1  0  0  0  0
  9 39  1  0  0  0  0
  9 40  1  0  0  0  0
 10 41  1  0  0  0  0
 10 42  1  0  0  0  0
 11 43  1  0  0  0  0
 11 44  1  0  0  0  0
 11 45  1  0  0  0  0
 12 46  1  0  0  0  0
 12 47  1  0  0  0  0
 13 48  1  0  0  0  0
 13 49  1  0  0  0  0
 14 50  1  0  0  0  0
 14 51  1  0  0  0  0
 15 52  1  0  0  0  0
 15 53  1  0  0  0  0
 16 54  1  0  0  0  0
 16 55  1  0  0  0  0
 17 56  1  0  0  0  0
 21 57  1  0  0  0  0
 21 58  1  0  0  0  0
 21 59  1  0  0  0  0
 22 60  1  0  0  0  0
 22 61  1  0  0  0  0
 22 62  1  0  0  0  0
M  END
>  <Alias name>
Methyl 2-methylstearate

>  <deltaH°(c)>   kJ/mol
-12686

$$$$
Nonadecane
  ChemBrain IXL

 59 58  0  0  0  0  0  0  0  0  1 V2000
   11.2697    9.9525   -0.0732 C   0  0  0  0  0  0  0  0  0  0  0  0
   10.3757   10.9451    0.6951 C   0  0  0  0  0  0  0  0  0  0  0  0
    9.2440   10.1753    1.4033 C   0  0  0  0  0  0  0  0  0  0  0  0
    8.3523   11.1675    2.1749 C   0  0  0  0  0  0  0  0  0  0  0  0
    7.2228   10.3973    2.8861 C   0  0  0  0  0  0  0  0  0  0  0  0
    6.3330   11.3892    3.6603 C   0  0  0  0  0  0  0  0  0  0  0  0
    5.2050   10.6187    4.3736 C   0  0  0  0  0  0  0  0  0  0  0  0
    4.3163   11.6104    5.1494 C   0  0  0  0  0  0  0  0  0  0  0  0
    3.1890   10.8398    5.8636 C   0  0  0  0  0  0  0  0  0  0  0  0
   12.4038   10.7219   -0.7780 C   0  0  0  0  0  0  0  0  0  0  0  0
```

```
  2.3007   11.8314    6.6400 C   0  0  0  0  0  0  0  0  0  0  0  0
 13.3004    9.7288   -1.5428 C   0  0  0  0  0  0  0  0  0  0  0  0
 14.4369   10.4978   -2.2443 C   0  0  0  0  0  0  0  0  0  0  0  0
 15.3355    9.5043   -3.0061 C   0  0  0  0  0  0  0  0  0  0  0  0
 16.4738   10.2729   -3.7049 C   0  0  0  0  0  0  0  0  0  0  0  0
 17.3740    9.2792   -4.4647 C   0  0  0  0  0  0  0  0  0  0  0  0
 18.5134   10.0476   -5.1619 C   0  0  0  0  0  0  0  0  0  0  0  0
 19.4142    9.0538   -5.9207 C   0  0  0  0  0  0  0  0  0  0  0  0
 20.5540    9.8221   -6.6173 C   0  0  0  0  0  0  0  0  0  0  0  0
 10.6774    9.4297   -0.8095 H   0  0  0  0  0  0  0  0  0  0  0  0
 11.6941    9.2403    0.6191 H   0  0  0  0  0  0  0  0  0  0  0  0
  9.9492   11.6559    0.0027 H   0  0  0  0  0  0  0  0  0  0  0  0
 10.9688   11.4696    1.4296 H   0  0  0  0  0  0  0  0  0  0  0  0
  8.6493    9.6527    0.6687 H   0  0  0  0  0  0  0  0  0  0  0  0
  9.6708    9.4629    2.0939 H   0  0  0  0  0  0  0  0  0  0  0  0
  8.9477   11.6915    2.9079 H   0  0  0  0  0  0  0  0  0  0  0  0
  7.9237   11.8787    1.4842 H   0  0  0  0  0  0  0  0  0  0  0  0
  6.6262    9.8748    2.1531 H   0  0  0  0  0  0  0  0  0  0  0  0
  7.6516    9.6849    3.5754 H   0  0  0  0  0  0  0  0  0  0  0  0
  5.9030   12.1008    2.9710 H   0  0  0  0  0  0  0  0  0  0  0  0
  6.9301   11.9127    4.3923 H   0  0  0  0  0  0  0  0  0  0  0  0
  4.6071   10.0961    3.6416 H   0  0  0  0  0  0  0  0  0  0  0  0
  5.6351    9.9063    5.0620 H   0  0  0  0  0  0  0  0  0  0  0  0
  3.8856   12.3224    4.4609 H   0  0  0  0  0  0  0  0  0  0  0  0
  4.9144   12.1335    5.8809 H   0  0  0  0  0  0  0  0  0  0  0  0
  3.6197   10.1275    6.5518 H   0  0  0  0  0  0  0  0  0  0  0  0
  2.5906   10.3171    5.1321 H   0  0  0  0  0  0  0  0  0  0  0  0
 12.9945   11.2466   -0.0417 H   0  0  0  0  0  0  0  0  0  0  0  0
 11.9798   11.4324   -1.4721 H   0  0  0  0  0  0  0  0  0  0  0  0
 13.7225    9.0169   -0.8488 H   0  0  0  0  0  0  0  0  0  0  0  0
 12.7104    9.2057   -2.2809 H   0  0  0  0  0  0  0  0  0  0  0  0
 15.0254   11.0225   -1.5063 H   0  0  0  0  0  0  0  0  0  0  0  0
 14.0151   11.2082   -2.9399 H   0  0  0  0  0  0  0  0  0  0  0  0
 15.7557    8.7928   -2.3106 H   0  0  0  0  0  0  0  0  0  0  0  0
 14.7476    8.9808   -3.7455 H   0  0  0  0  0  0  0  0  0  0  0  0
 16.0539   10.9835   -4.4015 H   0  0  0  0  0  0  0  0  0  0  0  0
 17.0608   10.7976   -2.9656 H   0  0  0  0  0  0  0  0  0  0  0  0
 17.7930    8.5680   -3.7682 H   0  0  0  0  0  0  0  0  0  0  0  0
 16.7873    8.7554   -5.2049 H   0  0  0  0  0  0  0  0  0  0  0  0
 19.0996   10.5720   -4.4217 H   0  0  0  0  0  0  0  0  0  0  0  0
 18.0945   10.7584   -5.8589 H   0  0  0  0  0  0  0  0  0  0  0  0
 18.8282    8.5297   -6.6612 H   0  0  0  0  0  0  0  0  0  0  0  0
 19.8327    8.3428   -5.2237 H   0  0  0  0  0  0  0  0  0  0  0  0
  2.8991   12.3541    7.3715 H   0  0  0  0  0  0  0  0  0  0  0  0
  1.8699   12.5437    5.9519 H   0  0  0  0  0  0  0  0  0  0  0  0
  1.5107   11.2913    7.1406 H   0  0  0  0  0  0  0  0  0  0  0  0
 20.1356   10.5330   -7.3143 H   0  0  0  0  0  0  0  0  0  0  0  0
 21.1401   10.3462   -5.8768 H   0  0  0  0  0  0  0  0  0  0  0  0
 21.1853    9.1255   -7.1490 H   0  0  0  0  0  0  0  0  0  0  0  0
  1  2  1  0  0  0  0
  2  3  1  0  0  0  0
  3  4  1  0  0  0  0
  4  5  1  0  0  0  0
  5  6  1  0  0  0  0
  6  7  1  0  0  0  0
  7  8  1  0  0  0  0
  8  9  1  0  0  0  0
  1 20  1  0  0  0  0
  1 21  1  0  0  0  0
  1 10  1  0  0  0  0
  2 22  1  0  0  0  0
  2 23  1  0  0  0  0
  3 24  1  0  0  0  0
```

```
  3 25  1  0  0  0  0
  4 26  1  0  0  0  0
  4 27  1  0  0  0  0
  5 28  1  0  0  0  0
  5 29  1  0  0  0  0
  6 30  1  0  0  0  0
  6 31  1  0  0  0  0
  7 32  1  0  0  0  0
  7 33  1  0  0  0  0
  8 34  1  0  0  0  0
  8 35  1  0  0  0  0
  9 36  1  0  0  0  0
  9 37  1  0  0  0  0
  9 11  1  0  0  0  0
 10 38  1  0  0  0  0
 10 39  1  0  0  0  0
 12 13  1  0  0  0  0
 13 14  1  0  0  0  0
 14 15  1  0  0  0  0
 15 16  1  0  0  0  0
 16 17  1  0  0  0  0
 17 18  1  0  0  0  0
 12 40  1  0  0  0  0
 12 41  1  0  0  0  0
 13 42  1  0  0  0  0
 13 43  1  0  0  0  0
 14 44  1  0  0  0  0
 14 45  1  0  0  0  0
 15 46  1  0  0  0  0
 15 47  1  0  0  0  0
 16 48  1  0  0  0  0
 16 49  1  0  0  0  0
 17 50  1  0  0  0  0
 17 51  1  0  0  0  0
 18 52  1  0  0  0  0
 18 19  1  0  0  0  0
 18 53  1  0  0  0  0
 10 12  1  0  0  0  0
 11 54  1  0  0  0  0
 11 55  1  0  0  0  0
 11 56  1  0  0  0  0
 19 57  1  0  0  0  0
 19 58  1  0  0  0  0
 19 59  1  0  0  0  0
M  END
>  <Alias name>
Nonadecane

>  <deltaH°(c)>   kJ/mol
-12662

$$$$
1-Eicosanoic acid
  ChemBrain IXL

 62 61  0  0  0  0  0  0  0  0  1 V2000
    2.1562    4.4664    3.3883 C   0  0  0  0  0  0  0  0  0  0  0  0
    3.4778    4.5106    2.5970 C   0  0  0  0  0  0  0  0  0  0  0  0
    4.1173    5.9051    2.7425 C   0  0  0  0  0  0  0  0  0  0  0  0
    5.4389    5.9492    1.9513 C   0  0  0  0  0  0  0  0  0  0  0  0
    6.0784    7.3437    2.0968 C   0  0  0  0  0  0  0  0  0  0  0  0
    7.4000    7.3877    1.3056 C   0  0  0  0  0  0  0  0  0  0  0  0
    8.0397    8.7821    1.4511 C   0  0  0  0  0  0  0  0  0  0  0  0
```

9.3614	8.8260	0.6601	C	0	0	0	0	0	0	0	0	0	0	0	0
10.0012	10.2203	0.8057	C	0	0	0	0	0	0	0	0	0	0	0	0
11.3231	10.2639	0.0150	C	0	0	0	0	0	0	0	0	0	0	0	0
11.9631	11.6582	0.1607	C	0	0	0	0	0	0	0	0	0	0	0	0
13.2852	11.7015	-0.6298	C	0	0	0	0	0	0	0	0	0	0	0	0
13.9254	13.0957	-0.4841	C	0	0	0	0	0	0	0	0	0	0	0	0
15.2477	13.1388	-1.2742	C	0	0	0	0	0	0	0	0	0	0	0	0
15.8880	14.5329	-1.1284	C	0	0	0	0	0	0	0	0	0	0	0	0
17.2105	14.5758	-1.9183	C	0	0	0	0	0	0	0	0	0	0	0	0
17.8510	15.9698	-1.7724	C	0	0	0	0	0	0	0	0	0	0	0	0
19.1736	16.0125	-2.5620	C	0	0	0	0	0	0	0	0	0	0	0	0
19.8143	17.4063	-2.4158	C	0	0	0	0	0	0	0	0	0	0	0	0
21.0850	17.4471	-3.1735	C	0	0	0	0	0	0	0	0	0	0	0	0
21.7507	18.4693	-3.1937	O	0	0	0	0	0	0	0	0	0	0	0	0
21.5450	16.2560	-3.9019	O	0	0	0	0	0	0	0	0	0	0	0	0
22.3971	16.3646	-4.3799	H	0	0	0	0	0	0	0	0	0	0	0	0
1.7068	3.4864	3.2860	H	0	0	0	0	0	0	0	0	0	0	0	0
2.3549	4.6650	4.4342	H	0	0	0	0	0	0	0	0	0	0	0	0
1.4782	5.2168	3.0008	H	0	0	0	0	0	0	0	0	0	0	0	0
4.1558	3.7603	2.9845	H	0	0	0	0	0	0	0	0	0	0	0	0
3.2791	4.3119	1.5511	H	0	0	0	0	0	0	0	0	0	0	0	0
3.4392	6.6554	2.3549	H	0	0	0	0	0	0	0	0	0	0	0	0
4.3159	6.1038	3.7884	H	0	0	0	0	0	0	0	0	0	0	0	0
6.1169	5.1989	2.3388	H	0	0	0	0	0	0	0	0	0	0	0	0
5.2402	5.7505	0.9053	H	0	0	0	0	0	0	0	0	0	0	0	0
5.4004	8.0940	1.7091	H	0	0	0	0	0	0	0	0	0	0	0	0
6.2770	7.5424	3.1427	H	0	0	0	0	0	0	0	0	0	0	0	0
8.0780	6.6373	1.6932	H	0	0	0	0	0	0	0	0	0	0	0	0
7.2014	7.1890	0.2597	H	0	0	0	0	0	0	0	0	0	0	0	0
7.3618	9.5325	1.0634	H	0	0	0	0	0	0	0	0	0	0	0	0
8.2381	8.9808	2.4971	H	0	0	0	0	0	0	0	0	0	0	0	0
10.0392	8.0755	1.0478	H	0	0	0	0	0	0	0	0	0	0	0	0
9.1629	8.6273	-0.3858	H	0	0	0	0	0	0	0	0	0	0	0	0
9.3236	10.9707	0.4178	H	0	0	0	0	0	0	0	0	0	0	0	0
10.1995	10.4191	1.8517	H	0	0	0	0	0	0	0	0	0	0	0	0
12.0007	9.5134	0.4028	H	0	0	0	0	0	0	0	0	0	0	0	0
11.1248	10.0653	-1.0310	H	0	0	0	0	0	0	0	0	0	0	0	0
11.2857	12.4087	-0.2275	H	0	0	0	0	0	0	0	0	0	0	0	0
12.1612	11.8570	1.2067	H	0	0	0	0	0	0	0	0	0	0	0	0
13.9626	10.9509	-0.2418	H	0	0	0	0	0	0	0	0	0	0	0	0
13.0871	11.5028	-1.6758	H	0	0	0	0	0	0	0	0	0	0	0	0
13.2482	13.8463	-0.8724	H	0	0	0	0	0	0	0	0	0	0	0	0
14.1233	13.2945	0.5620	H	0	0	0	0	0	0	0	0	0	0	0	0
15.9249	12.3881	-0.8860	H	0	0	0	0	0	0	0	0	0	0	0	0
15.0498	12.9401	-2.3203	H	0	0	0	0	0	0	0	0	0	0	0	0
15.2110	15.2835	-1.5169	H	0	0	0	0	0	0	0	0	0	0	0	0
16.0857	14.7317	-0.0824	H	0	0	0	0	0	0	0	0	0	0	0	0
17.8875	13.8250	-1.5299	H	0	0	0	0	0	0	0	0	0	0	0	0
17.0128	14.3770	-2.9644	H	0	0	0	0	0	0	0	0	0	0	0	0
17.1741	16.7205	-2.1611	H	0	0	0	0	0	0	0	0	0	0	0	0
18.0484	16.1686	-0.7263	H	0	0	0	0	0	0	0	0	0	0	0	0
19.8504	15.2615	-2.1735	H	0	0	0	0	0	0	0	0	0	0	0	0
18.9761	15.8139	-3.6082	H	0	0	0	0	0	0	0	0	0	0	0	0
20.0114	17.6052	-1.3696	H	0	0	0	0	0	0	0	0	0	0	0	0
19.1378	18.1572	-2.8048	H	0	0	0	0	0	0	0	0	0	0	0	0

1	2	1	0	0	0	0
2	3	1	0	0	0	0
3	4	1	0	0	0	0
4	5	1	0	0	0	0
5	6	1	0	0	0	0
6	7	1	0	0	0	0
7	8	1	0	0	0	0
8	9	1	0	0	0	0

9 10 1 0 0 0 0
10 11 1 0 0 0 0
11 12 1 0 0 0 0
12 13 1 0 0 0 0
13 14 1 0 0 0 0
14 15 1 0 0 0 0
15 16 1 0 0 0 0
16 17 1 0 0 0 0
17 18 1 0 0 0 0
18 19 1 0 0 0 0
19 20 1 0 0 0 0
20 21 2 0 0 0 0
20 22 1 0 0 0 0
22 23 1 0 0 0 0
1 24 1 0 0 0 0
1 25 1 0 0 0 0
1 26 1 0 0 0 0
2 27 1 0 0 0 0
2 28 1 0 0 0 0
3 29 1 0 0 0 0
3 30 1 0 0 0 0
4 31 1 0 0 0 0
4 32 1 0 0 0 0
5 33 1 0 0 0 0
5 34 1 0 0 0 0
6 35 1 0 0 0 0
6 36 1 0 0 0 0
7 37 1 0 0 0 0
7 38 1 0 0 0 0
8 39 1 0 0 0 0
8 40 1 0 0 0 0
9 41 1 0 0 0 0
9 42 1 0 0 0 0
10 43 1 0 0 0 0
10 44 1 0 0 0 0
11 45 1 0 0 0 0
11 46 1 0 0 0 0
12 47 1 0 0 0 0
12 48 1 0 0 0 0
13 49 1 0 0 0 0
13 50 1 0 0 0 0
14 51 1 0 0 0 0
14 52 1 0 0 0 0
15 53 1 0 0 0 0
15 54 1 0 0 0 0
16 55 1 0 0 0 0
16 56 1 0 0 0 0
17 57 1 0 0 0 0
17 58 1 0 0 0 0
18 59 1 0 0 0 0
18 60 1 0 0 0 0
19 61 1 0 0 0 0
19 62 1 0 0 0 0
M END
> <Alias name>
Arachidic acid

> <deltaH°(c)> kJ/mol
-12655.9

$$$$
1,1-Diphenyl-3,3,5,5,7,7-hexamethylcyclotetrasiloxane
ChemBrain IXL

```
54 56  0  0  0  0  0  0  0  0  1 V2000
    3.5948    7.6070    0.6554 Si  0  0  0  0  0  0  0  0  0  0  0  0
    4.3399    6.1945    0.3146 O   0  0  0  0  0  0  0  0  0  0  0  0
    5.0349    6.1039   -1.1611 Si  0  0  0  0  0  0  0  0  0  0  0  0
    6.4302    6.9499   -1.1688 O   0  0  0  0  0  0  0  0  0  0  0  0
    7.2442    6.9669    0.2456 Si  0  0  0  0  0  0  0  0  0  0  0  0
    6.4942    7.9607    1.2968 O   0  0  0  0  0  0  0  0  0  0  0  0
    5.7930    9.2724    0.6278 Si  0  0  0  0  0  0  0  0  0  0  0  0
    4.3830    8.8401   -0.0706 O   0  0  0  0  0  0  0  0  0  0  0  0
    5.4692   10.5440    1.9496 C   0  0  0  0  0  0  0  0  0  0  0  0
    6.9256    9.9998   -0.6613 C   0  0  0  0  0  0  0  0  0  0  0  0
    7.4076    2.6677    2.0180 C   0  0  0  0  0  0  0  0  0  0  0  0
    6.4108    3.5546    2.4288 C   0  0  0  0  0  0  0  0  0  0  0  0
    6.3618    4.8442    1.8971 C   0  0  0  0  0  0  0  0  0  0  0  0
    7.3096    5.2470    0.9548 C   0  0  0  0  0  0  0  0  0  0  0  0
    8.3064    4.3605    0.5438 C   0  0  0  0  0  0  0  0  0  0  0  0
    8.3554    3.0707    1.0755 C   0  0  0  0  0  0  0  0  0  0  0  0
   11.5737    8.5022   -0.4900 C   0  0  0  0  0  0  0  0  0  0  0  0
   10.9757    8.6720    0.7601 C   0  0  0  0  0  0  0  0  0  0  0  0
    9.6767    8.2113    0.9806 C   0  0  0  0  0  0  0  0  0  0  0  0
    8.9758    7.5808   -0.0488 C   0  0  0  0  0  0  0  0  0  0  0  0
    9.5733    7.4109   -1.2988 C   0  0  0  0  0  0  0  0  0  0  0  0
   10.8724    7.8717   -1.5195 C   0  0  0  0  0  0  0  0  0  0  0  0
    3.5861    7.8670    2.5003 C   0  0  0  0  0  0  0  0  0  0  0  0
    1.8386    7.5470    0.0375 C   0  0  0  0  0  0  0  0  0  0  0  0
    5.3923    4.3207   -1.5633 C   0  0  0  0  0  0  0  0  0  0  0  0
    3.8819    6.8037   -2.4464 C   0  0  0  0  0  0  0  0  0  0  0  0
    4.8110   10.1255    2.7001 H   0  0  0  0  0  0  0  0  0  0  0  0
    6.4058   10.8315    2.4108 H   0  0  0  0  0  0  0  0  0  0  0  0
    5.0030   11.4150    1.5059 H   0  0  0  0  0  0  0  0  0  0  0  0
    6.4584   10.8720   -1.1013 H   0  0  0  0  0  0  0  0  0  0  0  0
    7.1146    9.2636   -1.4328 H   0  0  0  0  0  0  0  0  0  0  0  0
    7.8619   10.2864   -0.1987 H   0  0  0  0  0  0  0  0  0  0  0  0
    7.4457    1.6672    2.4305 H   0  0  0  0  0  0  0  0  0  0  0  0
    5.6756    3.2420    3.1599 H   0  0  0  0  0  0  0  0  0  0  0  0
    5.5883    5.5323    2.2156 H   0  0  0  0  0  0  0  0  0  0  0  0
    9.0415    4.6732   -0.1873 H   0  0  0  0  0  0  0  0  0  0  0  0
    9.1286    2.3828    0.7569 H   0  0  0  0  0  0  0  0  0  0  0  0
   12.5815    8.8596   -0.6611 H   0  0  0  0  0  0  0  0  0  0  0  0
   11.5197    9.1610    1.5585 H   0  0  0  0  0  0  0  0  0  0  0  0
    9.2127    8.3430    1.9502 H   0  0  0  0  0  0  0  0  0  0  0  0
    9.0292    6.9218   -2.0972 H   0  0  0  0  0  0  0  0  0  0  0  0
   11.3362    7.7401   -2.4891 H   0  0  0  0  0  0  0  0  0  0  0  0
    3.0588    7.0502    2.9773 H   0  0  0  0  0  0  0  0  0  0  0  0
    4.6052    7.9006    2.8651 H   0  0  0  0  0  0  0  0  0  0  0  0
    3.0890    8.8010    2.7309 H   0  0  0  0  0  0  0  0  0  0  0  0
    1.3143    6.7310    0.5190 H   0  0  0  0  0  0  0  0  0  0  0  0
    1.3425    8.4814    0.2691 H   0  0  0  0  0  0  0  0  0  0  0  0
    1.8376    7.3941   -1.0340 H   0  0  0  0  0  0  0  0  0  0  0  0
    6.0637    3.9103   -0.8198 H   0  0  0  0  0  0  0  0  0  0  0  0
    5.8536    4.2566   -2.5409 H   0  0  0  0  0  0  0  0  0  0  0  0
    4.4668    3.7583   -1.5633 H   0  0  0  0  0  0  0  0  0  0  0  0
    2.9571    6.2403   -2.4467 H   0  0  0  0  0  0  0  0  0  0  0  0
    3.6726    7.8413   -2.2173 H   0  0  0  0  0  0  0  0  0  0  0  0
    4.3459    6.7362   -3.4225 H   0  0  0  0  0  0  0  0  0  0  0  0
  1  2  1  0  0  0  0
  2  3  1  0  0  0  0
  3  4  1  0  0  0  0
  4  5  1  0  0  0  0
  5  6  1  0  0  0  0
  6  7  1  0  0  0  0
  7  8  1  0  0  0  0
```

```
  8  1  1  0  0  0  0
  7  9  1  0  0  0  0
  7 10  1  0  0  0  0
 11 12  4  0  0  0  0
 12 13  4  0  0  0  0
 13 14  4  0  0  0  0
 14 15  4  0  0  0  0
 15 16  4  0  0  0  0
 16 11  4  0  0  0  0
 17 18  4  0  0  0  0
 18 19  4  0  0  0  0
 19 20  4  0  0  0  0
 20 21  4  0  0  0  0
 21 22  4  0  0  0  0
 22 17  4  0  0  0  0
 14  5  1  0  0  0  0
 20  5  1  0  0  0  0
 24  1  1  0  0  0  0
 23  1  1  0  0  0  0
 26  3  1  0  0  0  0
 25  3  1  0  0  0  0
  9 27  1  0  0  0  0
  9 28  1  0  0  0  0
  9 29  1  0  0  0  0
 10 30  1  0  0  0  0
 10 31  1  0  0  0  0
 10 32  1  0  0  0  0
 11 33  1  0  0  0  0
 12 34  1  0  0  0  0
 13 35  1  0  0  0  0
 15 36  1  0  0  0  0
 16 37  1  0  0  0  0
 17 38  1  0  0  0  0
 18 39  1  0  0  0  0
 19 40  1  0  0  0  0
 21 41  1  0  0  0  0
 22 42  1  0  0  0  0
 23 43  1  0  0  0  0
 23 44  1  0  0  0  0
 23 45  1  0  0  0  0
 24 46  1  0  0  0  0
 24 47  1  0  0  0  0
 24 48  1  0  0  0  0
 25 49  1  0  0  0  0
 25 50  1  0  0  0  0
 25 51  1  0  0  0  0
 26 52  1  0  0  0  0
 26 53  1  0  0  0  0
 26 54  1  0  0  0  0
M  END
>  <Alias name>
1,1-Diphenyl-3,3,5,5,7,7-hexamethylcyclotetrasiloxane

>  <deltaH°(c)>   kJ/mol
-12593

$$$$
Cetylmethacrylate
  ChemBrain IXL

 60 59  0  0  0  0  0  0  0  0  1 V2000
   14.8412    4.8436   -6.3960 C   0  0  0  0  0  0  0  0  0  0  0  0
   15.2272    5.8845   -6.9036 O   0  0  0  0  0  0  0  0  0  0  0  0
```

13.9193 4.8810 -5.2469 O 0 0 0 0 0 0 0 0 0 0 0 0
13.5374 6.2293 -4.7965 C 0 0 0 0 0 0 0 0 0 0 0 0
12.5732 6.1189 -3.5996 C 0 0 0 0 0 0 0 0 0 0 0 0
12.1773 7.5322 -3.1281 C 0 0 0 0 0 0 0 0 0 0 0 0
11.2089 7.4246 -1.9339 C 0 0 0 0 0 0 0 0 0 0 0 0
10.8176 8.8387 -1.4615 C 0 0 0 0 0 0 0 0 0 0 0 0
9.8445 8.7329 -0.2710 C 0 0 0 0 0 0 0 0 0 0 0 0
9.4586 10.1476 0.2040 C 0 0 0 0 0 0 0 0 0 0 0 0
8.4809 10.0430 1.3909 C 0 0 0 0 0 0 0 0 0 0 0 0
8.1013 11.4579 1.8699 C 0 0 0 0 0 0 0 0 0 0 0 0
7.1201 11.3540 3.0539 C 0 0 0 0 0 0 0 0 0 0 0 0
6.7471 12.7691 3.5377 C 0 0 0 0 0 0 0 0 0 0 0 0
5.7633 12.6656 4.7195 C 0 0 0 0 0 0 0 0 0 0 0 0
5.3961 14.0806 5.2078 C 0 0 0 0 0 0 0 0 0 0 0 0
4.4103 13.9774 6.3880 C 0 0 0 0 0 0 0 0 0 0 0 0
4.0467 15.3924 6.8791 C 0 0 0 0 0 0 0 0 0 0 0 0
3.0598 15.2894 8.0582 C 0 0 0 0 0 0 0 0 0 0 0 0
15.3009 3.5426 -6.9369 C 0 0 0 0 0 0 0 0 0 0 0 0
16.2207 3.5052 -8.0975 C 0 0 0 0 0 0 0 0 0 0 0 0
14.8862 2.4030 -6.3780 C 0 0 0 0 0 0 0 0 0 0 0 0
14.4241 6.7741 -4.4964 H 0 0 0 0 0 0 0 0 0 0 0 0
13.0478 6.7553 -5.6068 H 0 0 0 0 0 0 0 0 0 0 0 0
11.6853 5.5765 -3.9001 H 0 0 0 0 0 0 0 0 0 0 0 0
13.0617 5.5916 -2.7897 H 0 0 0 0 0 0 0 0 0 0 0 0
13.0652 8.0733 -2.8250 H 0 0 0 0 0 0 0 0 0 0 0 0
11.6922 8.0606 -3.9394 H 0 0 0 0 0 0 0 0 0 0 0 0
10.3196 6.8864 -2.2377 H 0 0 0 0 0 0 0 0 0 0 0 0
11.6928 6.8941 -1.1232 H 0 0 0 0 0 0 0 0 0 0 0 0
11.7068 9.3754 -1.1542 H 0 0 0 0 0 0 0 0 0 0 0 0
10.3376 9.3706 -2.2736 H 0 0 0 0 0 0 0 0 0 0 0 0
8.9536 8.1999 -0.5792 H 0 0 0 0 0 0 0 0 0 0 0 0
10.3229 8.1979 0.5400 H 0 0 0 0 0 0 0 0 0 0 0 0
10.3492 10.6790 0.5158 H 0 0 0 0 0 0 0 0 0 0 0 0
8.9839 10.6841 -0.6082 H 0 0 0 0 0 0 0 0 0 0 0 0
7.5881 9.5160 1.0778 H 0 0 0 0 0 0 0 0 0 0 0 0
8.9537 9.5023 2.2015 H 0 0 0 0 0 0 0 0 0 0 0 0
8.9939 11.9834 2.1860 H 0 0 0 0 0 0 0 0 0 0 0 0
7.6316 11.9999 1.0584 H 0 0 0 0 0 0 0 0 0 0 0 0
6.2251 10.8334 2.7364 H 0 0 0 0 0 0 0 0 0 0 0 0
7.5877 10.8074 3.8634 H 0 0 0 0 0 0 0 0 0 0 0 0
7.6419 13.2886 3.8575 H 0 0 0 0 0 0 0 0 0 0 0 0
6.2817 13.3167 2.7275 H 0 0 0 0 0 0 0 0 0 0 0 0
4.8664 12.1504 4.3985 H 0 0 0 0 0 0 0 0 0 0 0 0
6.2269 12.1139 5.5279 H 0 0 0 0 0 0 0 0 0 0 0 0
6.2928 14.5950 5.5304 H 0 0 0 0 0 0 0 0 0 0 0 0
4.9342 14.6331 4.3990 H 0 0 0 0 0 0 0 0 0 0 0 0
3.5124 13.4656 6.0646 H 0 0 0 0 0 0 0 0 0 0 0 0
4.8712 13.4223 7.1958 H 0 0 0 0 0 0 0 0 0 0 0 0
4.9445 15.9036 7.2036 H 0 0 0 0 0 0 0 0 0 0 0 0
3.5870 15.9479 6.0712 H 0 0 0 0 0 0 0 0 0 0 0 0
3.5192 14.7333 8.8660 H 0 0 0 0 0 0 0 0 0 0 0 0
2.8051 16.2836 8.4035 H 0 0 0 0 0 0 0 0 0 0 0 0
2.1616 14.7788 7.7336 H 0 0 0 0 0 0 0 0 0 0 0 0
16.4370 4.5163 -8.4199 H 0 0 0 0 0 0 0 0 0 0 0 0
15.7557 2.9593 -8.9092 H 0 0 0 0 0 0 0 0 0 0 0 0
17.1420 3.0125 -7.8123 H 0 0 0 0 0 0 0 0 0 0 0 0
15.2221 1.4523 -6.7733 H 0 0 0 0 0 0 0 0 0 0 0 0
14.2137 2.4293 -5.5294 H 0 0 0 0 0 0 0 0 0 0 0 0
1 2 2 0 0 0 0
1 3 1 0 0 0 0
3 4 1 0 0 0 0
4 5 1 0 0 0 0
5 6 1 0 0 0 0

6 7 1 0 0 0 0
7 8 1 0 0 0 0
8 9 1 0 0 0 0
9 10 1 0 0 0 0
10 11 1 0 0 0 0
11 12 1 0 0 0 0
12 13 1 0 0 0 0
13 14 1 0 0 0 0
14 15 1 0 0 0 0
15 16 1 0 0 0 0
16 17 1 0 0 0 0
17 18 1 0 0 0 0
18 19 1 0 0 0 0
1 20 1 0 0 0 0
20 21 1 0 0 0 0
20 22 2 0 0 0 0
4 23 1 0 0 0 0
4 24 1 0 0 0 0
5 25 1 0 0 0 0
5 26 1 0 0 0 0
6 27 1 0 0 0 0
6 28 1 0 0 0 0
7 29 1 0 0 0 0
7 30 1 0 0 0 0
8 31 1 0 0 0 0
8 32 1 0 0 0 0
9 33 1 0 0 0 0
9 34 1 0 0 0 0
10 35 1 0 0 0 0
10 36 1 0 0 0 0
11 37 1 0 0 0 0
11 38 1 0 0 0 0
12 39 1 0 0 0 0
12 40 1 0 0 0 0
13 41 1 0 0 0 0
13 42 1 0 0 0 0
14 43 1 0 0 0 0
14 44 1 0 0 0 0
15 45 1 0 0 0 0
15 46 1 0 0 0 0
16 47 1 0 0 0 0
16 48 1 0 0 0 0
17 49 1 0 0 0 0
17 50 1 0 0 0 0
18 51 1 0 0 0 0
18 52 1 0 0 0 0
19 53 1 0 0 0 0
19 54 1 0 0 0 0
19 55 1 0 0 0 0
21 56 1 0 0 0 0
21 57 1 0 0 0 0
21 58 1 0 0 0 0
22 59 1 0 0 0 0
22 60 1 0 0 0 0
M END
> <Alias name>
Cetylmethacrylate

> <deltaH°(c)> kJ/mol
-12567.05

$$$$

Ethyl (E) 9-octadecenoate

ChemBrain IXL

```
60 59  0  0  0  0  0  0  0  0  1 V2000
  13.0607   12.2126    0.0848 C   0  0  0  0  0  0  0  0  0  0  0  0  0
  11.8356   12.6162    0.4293 C   0  0  0  0  0  0  0  0  0  0  0  0  0
  10.7840   11.6180    0.7271 C   0  0  0  0  0  0  0  0  0  0  0  0  0
   9.4722   12.3438    1.0835 C   0  0  0  0  0  0  0  0  0  0  0  0  0
   8.3779   11.3042    1.3933 C   0  0  0  0  0  0  0  0  0  0  0  0  0
   7.0661   12.0298    1.7496 C   0  0  0  0  0  0  0  0  0  0  0  0  0
   5.9718   10.9901    2.0593 C   0  0  0  0  0  0  0  0  0  0  0  0  0
   4.6598   11.7155    2.4155 C   0  0  0  0  0  0  0  0  0  0  0  0  0
   3.5654   10.6755    2.7251 C   0  0  0  0  0  0  0  0  0  0  0  0  0
  14.1122   13.2108   -0.2130 C   0  0  0  0  0  0  0  0  0  0  0  0  0
   2.2533   11.4008    3.0813 C   0  0  0  0  0  0  0  0  0  0  0  0  0
  15.4236   12.4851   -0.5715 C   0  0  0  0  0  0  0  0  0  0  0  0  0
  16.5174   13.5248   -0.8815 C   0  0  0  0  0  0  0  0  0  0  0  0  0
  17.8288   12.7998   -1.2401 C   0  0  0  0  0  0  0  0  0  0  0  0  0
  18.9218   13.8402   -1.5501 C   0  0  0  0  0  0  0  0  0  0  0  0  0
  20.2335   13.1164   -1.9089 C   0  0  0  0  0  0  0  0  0  0  0  0  0
  21.3260   14.1583   -2.2188 C   0  0  0  0  0  0  0  0  0  0  0  0  0
  22.5880   13.4655   -2.5642 C   0  0  0  0  0  0  0  0  0  0  0  0  0
  23.5806   14.1164   -2.8483 O   0  0  0  0  0  0  0  0  0  0  0  0  0
  22.6450   11.9932   -2.5647 O   0  0  0  0  0  0  0  0  0  0  0  0  0
  23.9507   11.4171   -2.9235 C   0  0  0  0  0  0  0  0  0  0  0  0  0
  23.8621    9.8797   -2.8827 C   0  0  0  0  0  0  0  0  0  0  0  0  0
  13.2850   11.1547    0.0233 H   0  0  0  0  0  0  0  0  0  0  0  0  0
  11.6113   13.6741    0.4908 H   0  0  0  0  0  0  0  0  0  0  0  0  0
  11.0955   11.0041    1.5632 H   0  0  0  0  0  0  0  0  0  0  0  0  0
  10.6251   10.9910   -0.1416 H   0  0  0  0  0  0  0  0  0  0  0  0  0
   9.1606   12.9577    0.2474 H   0  0  0  0  0  0  0  0  0  0  0  0  0
   9.6310   12.9707    1.9523 H   0  0  0  0  0  0  0  0  0  0  0  0  0
   8.6895   10.6904    2.2294 H   0  0  0  0  0  0  0  0  0  0  0  0  0
   8.2193   10.6773    0.5246 H   0  0  0  0  0  0  0  0  0  0  0  0  0
   6.7546   12.6437    0.9135 H   0  0  0  0  0  0  0  0  0  0  0  0  0
   7.2248   12.6567    2.6184 H   0  0  0  0  0  0  0  0  0  0  0  0  0
   6.2834   10.3763    2.8954 H   0  0  0  0  0  0  0  0  0  0  0  0  0
   5.8133   10.3632    1.1905 H   0  0  0  0  0  0  0  0  0  0  0  0  0
   4.3483   12.3294    1.5794 H   0  0  0  0  0  0  0  0  0  0  0  0  0
   4.8183   12.3424    3.2843 H   0  0  0  0  0  0  0  0  0  0  0  0  0
   3.8769   10.0617    3.5612 H   0  0  0  0  0  0  0  0  0  0  0  0  0
   3.4070   10.0487    1.8564 H   0  0  0  0  0  0  0  0  0  0  0  0  0
  13.8000   13.8256   -1.0482 H   0  0  0  0  0  0  0  0  0  0  0  0  0
  14.2721   13.8368    0.6561 H   0  0  0  0  0  0  0  0  0  0  0  0  0
   1.4845   10.6698    3.2988 H   0  0  0  0  0  0  0  0  0  0  0  0  0
   2.4116   12.0276    3.9501 H   0  0  0  0  0  0  0  0  0  0  0  0  0
   1.9416   12.0146    2.2452 H   0  0  0  0  0  0  0  0  0  0  0  0  0
  15.7360   11.8704    0.2637 H   0  0  0  0  0  0  0  0  0  0  0  0  0
  15.2638   11.8590   -1.4407 H   0  0  0  0  0  0  0  0  0  0  0  0  0
  16.2048   14.1396   -1.7166 H   0  0  0  0  0  0  0  0  0  0  0  0  0
  16.6771   14.1508   -0.0123 H   0  0  0  0  0  0  0  0  0  0  0  0  0
  18.1416   12.1853   -0.4050 H   0  0  0  0  0  0  0  0  0  0  0  0  0
  17.6692   12.1738   -2.1093 H   0  0  0  0  0  0  0  0  0  0  0  0  0
  18.6088   14.4549   -2.3850 H   0  0  0  0  0  0  0  0  0  0  0  0  0
  19.0812   14.4663   -0.6809 H   0  0  0  0  0  0  0  0  0  0  0  0  0
  20.5469   12.5019   -1.0739 H   0  0  0  0  0  0  0  0  0  0  0  0  0
  20.0743   12.4904   -2.7783 H   0  0  0  0  0  0  0  0  0  0  0  0  0
  21.0119   14.7729   -3.0534 H   0  0  0  0  0  0  0  0  0  0  0  0  0
  21.4844   14.7843   -1.3494 H   0  0  0  0  0  0  0  0  0  0  0  0  0
  24.7002   11.7543   -2.2182 H   0  0  0  0  0  0  0  0  0  0  0  0  0
  24.2244   11.7365   -3.9215 H   0  0  0  0  0  0  0  0  0  0  0  0  0
  24.8230    9.4557   -3.1468 H   0  0  0  0  0  0  0  0  0  0  0  0  0
  23.5884    9.5603   -1.8848 H   0  0  0  0  0  0  0  0  0  0  0  0  0
  23.1126    9.5426   -3.5880 H   0  0  0  0  0  0  0  0  0  0  0  0  0
```

1 2 2 0 0 0 0
2 3 1 0 0 0 0
3 4 1 0 0 0 0
4 5 1 0 0 0 0
5 6 1 0 0 0 0
6 7 1 0 0 0 0
7 8 1 0 0 0 0
8 9 1 0 0 0 0
1 10 1 0 0 0 0
9 11 1 0 0 0 0
12 13 1 0 0 0 0
13 14 1 0 0 0 0
14 15 1 0 0 0 0
15 16 1 0 0 0 0
16 17 1 0 0 0 0
17 18 1 0 0 0 0
10 12 1 0 0 0 0
18 19 2 0 0 0 0
18 20 1 0 0 0 0
20 21 1 0 0 0 0
21 22 1 0 0 0 0
1 23 1 0 0 0 0
2 24 1 0 0 0 0
3 25 1 0 0 0 0
3 26 1 0 0 0 0
4 27 1 0 0 0 0
4 28 1 0 0 0 0
5 29 1 0 0 0 0
5 30 1 0 0 0 0
6 31 1 0 0 0 0
6 32 1 0 0 0 0
7 33 1 0 0 0 0
7 34 1 0 0 0 0
8 35 1 0 0 0 0
8 36 1 0 0 0 0
9 37 1 0 0 0 0
9 38 1 0 0 0 0
10 39 1 0 0 0 0
10 40 1 0 0 0 0
11 41 1 0 0 0 0
11 42 1 0 0 0 0
11 43 1 0 0 0 0
12 44 1 0 0 0 0
12 45 1 0 0 0 0
13 46 1 0 0 0 0
13 47 1 0 0 0 0
14 48 1 0 0 0 0
14 49 1 0 0 0 0
15 50 1 0 0 0 0
15 51 1 0 0 0 0
16 52 1 0 0 0 0
16 53 1 0 0 0 0
17 54 1 0 0 0 0
17 55 1 0 0 0 0
21 56 1 0 0 0 0
21 57 1 0 0 0 0
22 58 1 0 0 0 0
22 59 1 0 0 0 0
22 60 1 0 0 0 0
M END
> <Alias name>
Ethyl elaidate

```
>  <deltaH°(c)>   kJ/mol
-12528

$$$$
Ethyl (Z) 9-octadecenoate
  ChemBrain IXL

 60 59  0  0  0  0  0  0  0  0  1 V2000
   13.0251    9.7473   -0.0863 C   0  0  0  0  0  0  0  0  0  0  0  0
   11.7021    9.5663   -0.0401 C   0  0  0  0  0  0  0  0  0  0  0  0
   10.7948   10.7357    0.0262 C   0  0  0  0  0  0  0  0  0  0  0  0
    9.3318   10.2570   -0.0415 C   0  0  0  0  0  0  0  0  0  0  0  0
    8.3900   11.4747    0.0277 C   0  0  0  0  0  0  0  0  0  0  0  0
    6.9266   10.9974   -0.0404 C   0  0  0  0  0  0  0  0  0  0  0  0
    5.9854   12.2157    0.0292 C   0  0  0  0  0  0  0  0  0  0  0  0
    4.5217   11.7388   -0.0389 C   0  0  0  0  0  0  0  0  0  0  0  0
    3.5806   12.9573    0.0310 C   0  0  0  0  0  0  0  0  0  0  0  0
   13.5877   11.1178   -0.0713 C   0  0  0  0  0  0  0  0  0  0  0  0
    2.1169   12.4807   -0.0371 C   0  0  0  0  0  0  0  0  0  0  0  0
   15.1266   11.0430   -0.0708 C   0  0  0  0  0  0  0  0  0  0  0  0
   15.7088   12.4694   -0.0560 C   0  0  0  0  0  0  0  0  0  0  0  0
   17.2476   12.3968   -0.0564 C   0  0  0  0  0  0  0  0  0  0  0  0
   17.8281   13.8237   -0.0426 C   0  0  0  0  0  0  0  0  0  0  0  0
   19.3669   13.7524   -0.0442 C   0  0  0  0  0  0  0  0  0  0  0  0
   19.9465   15.1802   -0.0311 C   0  0  0  0  0  0  0  0  0  0  0  0
   21.4256   15.1147   -0.0336 C   0  0  0  0  0  0  0  0  0  0  0  0
   22.0797   16.1450   -0.0398 O   0  0  0  0  0  0  0  0  0  0  0  0
   22.1062   13.8078   -0.0286 O   0  0  0  0  0  0  0  0  0  0  0  0
   23.5765   13.8674   -0.0318 C   0  0  0  0  0  0  0  0  0  0  0  0
   24.1485   12.4370   -0.0262 C   0  0  0  0  0  0  0  0  0  0  0  0
   13.6867    8.8912   -0.1347 H   0  0  0  0  0  0  0  0  0  0  0  0
   11.2928    8.5637   -0.0511 H   0  0  0  0  0  0  0  0  0  0  0  0
   10.9580   11.2668    0.9558 H   0  0  0  0  0  0  0  0  0  0  0  0
   10.9968   11.3970   -0.8074 H   0  0  0  0  0  0  0  0  0  0  0  0
    9.1681    9.7263   -0.9712 H   0  0  0  0  0  0  0  0  0  0  0  0
    9.1292    9.5960    0.7922 H   0  0  0  0  0  0  0  0  0  0  0  0
    8.5537   12.0053    0.9576 H   0  0  0  0  0  0  0  0  0  0  0  0
    8.5931   12.1359   -0.8057 H   0  0  0  0  0  0  0  0  0  0  0  0
    6.7627   10.4673   -0.9704 H   0  0  0  0  0  0  0  0  0  0  0  0
    6.7234   10.3361    0.7928 H   0  0  0  0  0  0  0  0  0  0  0  0
    6.1495   12.7458    0.9593 H   0  0  0  0  0  0  0  0  0  0  0  0
    6.1888   12.8770   -0.8039 H   0  0  0  0  0  0  0  0  0  0  0  0
    4.3576   11.2088   -0.9690 H   0  0  0  0  0  0  0  0  0  0  0  0
    4.3184   11.0774    0.7942 H   0  0  0  0  0  0  0  0  0  0  0  0
    3.7447   13.4873    0.9611 H   0  0  0  0  0  0  0  0  0  0  0  0
    3.7840   13.6187   -0.8021 H   0  0  0  0  0  0  0  0  0  0  0  0
   13.2530   11.6548   -0.9502 H   0  0  0  0  0  0  0  0  0  0  0  0
   13.2515   11.6362    0.8182 H   0  0  0  0  0  0  0  0  0  0  0  0
    1.9134   11.8193    0.7959 H   0  0  0  0  0  0  0  0  0  0  0  0
    1.4558   13.3370    0.0120 H   0  0  0  0  0  0  0  0  0  0  0  0
    1.9527   11.9508   -0.9673 H   0  0  0  0  0  0  0  0  0  0  0  0
   15.4619   10.5070    0.8085 H   0  0  0  0  0  0  0  0  0  0  0  0
   15.4634   10.5247   -0.9601 H   0  0  0  0  0  0  0  0  0  0  0  0
   15.3727   13.0055   -0.9349 H   0  0  0  0  0  0  0  0  0  0  0  0
   15.3722   12.9875    0.8335 H   0  0  0  0  0  0  0  0  0  0  0  0
   17.5841   11.8615    0.8229 H   0  0  0  0  0  0  0  0  0  0  0  0
   17.5844   11.8785   -0.9456 H   0  0  0  0  0  0  0  0  0  0  0  0
   17.4908   14.3591   -0.9215 H   0  0  0  0  0  0  0  0  0  0  0  0
   17.4917   14.3418    0.8469 H   0  0  0  0  0  0  0  0  0  0  0  0
   19.7045   13.2174    0.8349 H   0  0  0  0  0  0  0  0  0  0  0  0
   19.7034   13.2342   -0.9337 H   0  0  0  0  0  0  0  0  0  0  0  0
   19.6078   15.7151   -0.9098 H   0  0  0  0  0  0  0  0  0  0  0  0
   19.6099   15.6980    0.8585 H   0  0  0  0  0  0  0  0  0  0  0  0
```

23.9184	14.3963	0.8494	H	0	0	0	0	0	0	0	0	0	0	0	0
23.9149	14.3880	-0.9192	H	0	0	0	0	0	0	0	0	0	0	0	0
25.2306	12.4809	-0.0285	H	0	0	0	0	0	0	0	0	0	0	0	0
23.8068	11.9082	-0.9073	H	0	0	0	0	0	0	0	0	0	0	0	0
23.8102	11.9165	0.8612	H	0	0	0	0	0	0	0	0	0	0	0	0

1	2	2	0	0	0	0
2	3	1	0	0	0	0
3	4	1	0	0	0	0
4	5	1	0	0	0	0
5	6	1	0	0	0	0
6	7	1	0	0	0	0
7	8	1	0	0	0	0
8	9	1	0	0	0	0
1	10	1	0	0	0	0
9	11	1	0	0	0	0
12	13	1	0	0	0	0
13	14	1	0	0	0	0
14	15	1	0	0	0	0
15	16	1	0	0	0	0
16	17	1	0	0	0	0
17	18	1	0	0	0	0
10	12	1	0	0	0	0
18	19	2	0	0	0	0
18	20	1	0	0	0	0
20	21	1	0	0	0	0
21	22	1	0	0	0	0
1	23	1	0	0	0	0
2	24	1	0	0	0	0
3	25	1	0	0	0	0
3	26	1	0	0	0	0
4	27	1	0	0	0	0
4	28	1	0	0	0	0
5	29	1	0	0	0	0
5	30	1	0	0	0	0
6	31	1	0	0	0	0
6	32	1	0	0	0	0
7	33	1	0	0	0	0
7	34	1	0	0	0	0
8	35	1	0	0	0	0
8	36	1	0	0	0	0
9	37	1	0	0	0	0
9	38	1	0	0	0	0
10	39	1	0	0	0	0
10	40	1	0	0	0	0
11	41	1	0	0	0	0
11	42	1	0	0	0	0
11	43	1	0	0	0	0
12	44	1	0	0	0	0
12	45	1	0	0	0	0
13	46	1	0	0	0	0
13	47	1	0	0	0	0
14	48	1	0	0	0	0
14	49	1	0	0	0	0
15	50	1	0	0	0	0
15	51	1	0	0	0	0
16	52	1	0	0	0	0
16	53	1	0	0	0	0
17	54	1	0	0	0	0
17	55	1	0	0	0	0
21	56	1	0	0	0	0
21	57	1	0	0	0	0
22	58	1	0	0	0	0
22	59	1	0	0	0	0

```
 22 60  1  0  0  0  0
M  END
>  <Alias name>
Ethyl oleate

>  <deltaH°(c)>   kJ/mol
-12525

$$$$
1-Nonadecene
  ChemBrain IXL

 57 56  0  0  0  0  0  0  0  0  1 V2000
   13.4770   12.0768    0.0000 C   0  0  0  0  0  0  0  0  0  0  0  0
   12.2313   12.9828   -0.0466 C   0  0  0  0  0  0  0  0  0  0  0  0
   10.9603   12.1127    0.0009 C   0  0  0  0  0  0  0  0  0  0  0  0
    9.7153   13.0199   -0.0426 C   0  0  0  0  0  0  0  0  0  0  0  0
    8.4437   12.1506    0.0011 C   0  0  0  0  0  0  0  0  0  0  0  0
    7.1993   13.0587   -0.0412 C   0  0  0  0  0  0  0  0  0  0  0  0
    5.9273   12.1898   -0.0014 C   0  0  0  0  0  0  0  0  0  0  0  0
    4.6830   13.0984   -0.0435 C   0  0  0  0  0  0  0  0  0  0  0  0
    3.4605   12.2641   -0.0076 C   0  0  0  0  0  0  0  0  0  0  0  0
   14.7473   12.9478   -0.0504 C   0  0  0  0  0  0  0  0  0  0  0  0
    2.2570   12.8421   -0.0211 C   0  0  0  0  0  0  0  0  0  0  0  0
   15.9937   12.0431    0.0009 C   0  0  0  0  0  0  0  0  0  0  0  0
   17.2633   12.9151   -0.0510 C   0  0  0  0  0  0  0  0  0  0  0  0
   18.5105   12.0117    0.0056 C   0  0  0  0  0  0  0  0  0  0  0  0
   19.7795   12.8844   -0.0470 C   0  0  0  0  0  0  0  0  0  0  0  0
   21.0271   11.9819    0.0143 C   0  0  0  0  0  0  0  0  0  0  0  0
   22.2959   12.8552   -0.0387 C   0  0  0  0  0  0  0  0  0  0  0  0
   23.5439   11.9531    0.0254 C   0  0  0  0  0  0  0  0  0  0  0  0
   24.8124   12.8264   -0.0281 C   0  0  0  0  0  0  0  0  0  0  0  0
   13.4664   11.4043   -0.8489 H   0  0  0  0  0  0  0  0  0  0  0  0
   13.4703   11.5007    0.9171 H   0  0  0  0  0  0  0  0  0  0  0  0
   12.2412   13.6539    0.8033 H   0  0  0  0  0  0  0  0  0  0  0  0
   12.2391   13.5604   -0.9627 H   0  0  0  0  0  0  0  0  0  0  0  0
   10.9491   11.4432   -0.8502 H   0  0  0  0  0  0  0  0  0  0  0  0
   10.9531   11.5336    0.9160 H   0  0  0  0  0  0  0  0  0  0  0  0
    9.7254   13.6878    0.8098 H   0  0  0  0  0  0  0  0  0  0  0  0
    9.7240   13.6010   -0.9565 H   0  0  0  0  0  0  0  0  0  0  0  0
    8.4330   11.4835   -0.8520 H   0  0  0  0  0  0  0  0  0  0  0  0
    8.4352   11.5688    0.9145 H   0  0  0  0  0  0  0  0  0  0  0  0
    7.2089   13.7241    0.8132 H   0  0  0  0  0  0  0  0  0  0  0  0
    7.2092   13.6423   -0.9534 H   0  0  0  0  0  0  0  0  0  0  0  0
    5.9176   11.5246   -0.8560 H   0  0  0  0  0  0  0  0  0  0  0  0
    5.9173   11.6059    0.9108 H   0  0  0  0  0  0  0  0  0  0  0  0
    4.6940   13.6835   -0.9548 H   0  0  0  0  0  0  0  0  0  0  0  0
    4.6920   13.7626    0.8119 H   0  0  0  0  0  0  0  0  0  0  0  0
    3.5422   11.1848    0.0295 H   0  0  0  0  0  0  0  0  0  0  0  0
   14.7560   13.6225    0.7967 H   0  0  0  0  0  0  0  0  0  0  0  0
   14.7552   13.5216   -0.9689 H   0  0  0  0  0  0  0  0  0  0  0  0
    1.3629   12.2316    0.0051 H   0  0  0  0  0  0  0  0  0  0  0  0
    2.1748   13.9214   -0.0583 H   0  0  0  0  0  0  0  0  0  0  0  0
   15.9848   11.3676   -0.8456 H   0  0  0  0  0  0  0  0  0  0  0  0
   15.9865   11.4704    0.9200 H   0  0  0  0  0  0  0  0  0  0  0  0
   17.2703   13.5931    0.7935 H   0  0  0  0  0  0  0  0  0  0  0  0
   17.2721   13.4853   -0.9717 H   0  0  0  0  0  0  0  0  0  0  0  0
   18.5036   11.3332   -0.8385 H   0  0  0  0  0  0  0  0  0  0  0  0
   18.5020   11.4421    0.9267 H   0  0  0  0  0  0  0  0  0  0  0  0
   19.7847   13.5649    0.7955 H   0  0  0  0  0  0  0  0  0  0  0  0
   19.7894   13.4519   -0.9694 H   0  0  0  0  0  0  0  0  0  0  0  0
   21.0220   11.3011   -0.8279 H   0  0  0  0  0  0  0  0  0  0  0  0
   21.0175   11.4149    0.9370 H   0  0  0  0  0  0  0  0  0  0  0  0
```

22.3000	13.5373	0.8025	H	0	0	0	0	0	0	0	0	0	0	0	0
22.3064	13.4209	-0.9621	H	0	0	0	0	0	0	0	0	0	0	0	0
23.5397	11.2708	-0.8156	H	0	0	0	0	0	0	0	0	0	0	0	0
23.5336	11.3878	0.9490	H	0	0	0	0	0	0	0	0	0	0	0	0
24.8165	13.5090	0.8127	H	0	0	0	0	0	0	0	0	0	0	0	0
24.8230	13.3915	-0.9519	H	0	0	0	0	0	0	0	0	0	0	0	0
25.6892	12.1924	0.0174	H	0	0	0	0	0	0	0	0	0	0	0	0

```
  1  2  1  0  0  0  0
  2  3  1  0  0  0  0
  3  4  1  0  0  0  0
  4  5  1  0  0  0  0
  5  6  1  0  0  0  0
  6  7  1  0  0  0  0
  7  8  1  0  0  0  0
  8  9  1  0  0  0  0
  1 10  1  0  0  0  0
  9 11  2  0  0  0  0
 12 13  1  0  0  0  0
 13 14  1  0  0  0  0
 14 15  1  0  0  0  0
 15 16  1  0  0  0  0
 16 17  1  0  0  0  0
 17 18  1  0  0  0  0
 18 19  1  0  0  0  0
 10 12  1  0  0  0  0
  1 20  1  0  0  0  0
  1 21  1  0  0  0  0
  2 22  1  0  0  0  0
  2 23  1  0  0  0  0
  3 24  1  0  0  0  0
  3 25  1  0  0  0  0
  4 26  1  0  0  0  0
  4 27  1  0  0  0  0
  5 28  1  0  0  0  0
  5 29  1  0  0  0  0
  6 30  1  0  0  0  0
  6 31  1  0  0  0  0
  7 32  1  0  0  0  0
  7 33  1  0  0  0  0
  8 34  1  0  0  0  0
  8 35  1  0  0  0  0
  9 36  1  0  0  0  0
 10 37  1  0  0  0  0
 10 38  1  0  0  0  0
 11 39  1  0  0  0  0
 11 40  1  0  0  0  0
 12 41  1  0  0  0  0
 12 42  1  0  0  0  0
 13 43  1  0  0  0  0
 13 44  1  0  0  0  0
 14 45  1  0  0  0  0
 14 46  1  0  0  0  0
 15 47  1  0  0  0  0
 15 48  1  0  0  0  0
 16 49  1  0  0  0  0
 16 50  1  0  0  0  0
 17 51  1  0  0  0  0
 17 52  1  0  0  0  0
 18 53  1  0  0  0  0
 18 54  1  0  0  0  0
 19 55  1  0  0  0  0
 19 56  1  0  0  0  0
 19 57  1  0  0  0  0
```

```
M  END
>  <Alias name>
1-Nonadecene

>  <deltaH°(c)>   kJ/mol
-12516.49

$$$$
1,1,2-Tris(2-methylphenyl)ethane
  ChemBrain IXL

 47 49  0  0  0  0  0  0  0  0  1 V2000
    7.9774    9.4431   -0.3970 C   0  0  0  0  0  0  0  0  0  0  0  0
    9.1942    8.9573   -0.8758 C   0  0  0  0  0  0  0  0  0  0  0  0
    9.2252    7.8021   -1.6572 C   0  0  0  0  0  0  0  0  0  0  0  0
    8.0402    7.1281   -1.9531 C   0  0  0  0  0  0  0  0  0  0  0  0
    6.8230    7.6146   -1.4755 C   0  0  0  0  0  0  0  0  0  0  0  0
    6.7913    8.7745   -0.7009 C   0  0  0  0  0  0  0  0  0  0  0  0
    5.5653    6.9002   -1.7907 C   0  0  0  0  0  0  0  0  0  0  0  0
    5.4993    9.2960   -0.2016 C   0  0  0  0  0  0  0  0  0  0  0  0
    4.9753    3.1769   -1.8364 C   0  0  0  0  0  0  0  0  0  0  0  0
    6.1766    2.7040   -1.3081 C   0  0  0  0  0  0  0  0  0  0  0  0
    7.1501    3.6043   -0.8751 C   0  0  0  0  0  0  0  0  0  0  0  0
    6.9269    4.9770   -0.9823 C   0  0  0  0  0  0  0  0  0  0  0  0
    5.7532    5.4480   -1.5702 C   0  0  0  0  0  0  0  0  0  0  0  0
    4.7564    4.5497   -1.9504 C   0  0  0  0  0  0  0  0  0  0  0  0
    3.4671    5.0530   -2.4744 C   0  0  0  0  0  0  0  0  0  0  0  0
    4.4377    7.4175   -0.8761 C   0  0  0  0  0  0  0  0  0  0  0  0
    3.7723    6.3242    2.6714 C   0  0  0  0  0  0  0  0  0  0  0  0
    5.0591    6.0053    3.1056 C   0  0  0  0  0  0  0  0  0  0  0  0
    6.1419    6.1407    2.2366 C   0  0  0  0  0  0  0  0  0  0  0  0
    5.9379    6.5941    0.9332 C   0  0  0  0  0  0  0  0  0  0  0  0
    4.6521    6.9205    0.5015 C   0  0  0  0  0  0  0  0  0  0  0  0
    3.5684    6.7785    1.3683 C   0  0  0  0  0  0  0  0  0  0  0  0
    2.2023    7.1099    0.9055 C   0  0  0  0  0  0  0  0  0  0  0  0
    7.9535   10.3392    0.2106 H   0  0  0  0  0  0  0  0  0  0  0  0
   10.1149    9.4769   -0.6408 H   0  0  0  0  0  0  0  0  0  0  0  0
   10.1692    7.4285   -2.0342 H   0  0  0  0  0  0  0  0  0  0  0  0
    8.0651    6.2272   -2.5535 H   0  0  0  0  0  0  0  0  0  0  0  0
    5.2999    7.0766   -2.8257 H   0  0  0  0  0  0  0  0  0  0  0  0
    5.0331    8.5550    0.4359 H   0  0  0  0  0  0  0  0  0  0  0  0
    5.6708   10.2030    0.3648 H   0  0  0  0  0  0  0  0  0  0  0  0
    4.8487    9.5116   -1.0401 H   0  0  0  0  0  0  0  0  0  0  0  0
    4.2129    2.4783   -2.1580 H   0  0  0  0  0  0  0  0  0  0  0  0
    6.3534    1.6381   -1.2340 H   0  0  0  0  0  0  0  0  0  0  0  0
    8.0789    3.2377   -0.4559 H   0  0  0  0  0  0  0  0  0  0  0  0
    7.6647    5.6766   -0.6093 H   0  0  0  0  0  0  0  0  0  0  0  0
    3.1052    4.3844   -3.2457 H   0  0  0  0  0  0  0  0  0  0  0  0
    2.7441    5.1005   -1.6695 H   0  0  0  0  0  0  0  0  0  0  0  0
    3.6085    6.0424   -2.8915 H   0  0  0  0  0  0  0  0  0  0  0  0
    3.4831    7.0618   -1.2436 H   0  0  0  0  0  0  0  0  0  0  0  0
    4.4429    8.5005   -0.8740 H   0  0  0  0  0  0  0  0  0  0  0  0
    2.9316    6.2191    3.3461 H   0  0  0  0  0  0  0  0  0  0  0  0
    5.2174    5.6526    4.1172 H   0  0  0  0  0  0  0  0  0  0  0  0
    7.1410    5.8937    2.5739 H   0  0  0  0  0  0  0  0  0  0  0  0
    6.7777    6.6928    0.2565 H   0  0  0  0  0  0  0  0  0  0  0  0
    1.7931    6.2701    0.3576 H   0  0  0  0  0  0  0  0  0  0  0  0
    1.5733    7.3239    1.7608 H   0  0  0  0  0  0  0  0  0  0  0  0
    2.2407    7.9782    0.2594 H   0  0  0  0  0  0  0  0  0  0  0  0
  1  2  4  0  0  0  0
  2  3  4  0  0  0  0
  3  4  4  0  0  0  0
  4  5  4  0  0  0  0
```

```
  5  6  4  0  0  0  0
  6  1  4  0  0  0  0
  5  7  1  0  0  0  0
  6  8  1  0  0  0  0
  9 10  4  0  0  0  0
 10 11  4  0  0  0  0
 11 12  4  0  0  0  0
 12 13  4  0  0  0  0
 13 14  4  0  0  0  0
 14  9  4  0  0  0  0
 14 15  1  0  0  0  0
 13  7  1  0  0  0  0
  7 16  1  0  0  0  0
 17 18  4  0  0  0  0
 18 19  4  0  0  0  0
 19 20  4  0  0  0  0
 20 21  4  0  0  0  0
 21 22  4  0  0  0  0
 22 17  4  0  0  0  0
 22 23  1  0  0  0  0
 21 16  1  0  0  0  0
  1 24  1  0  0  0  0
  2 25  1  0  0  0  0
  3 26  1  0  0  0  0
  4 27  1  0  0  0  0
  7 28  1  0  0  0  0
  8 29  1  0  0  0  0
  8 30  1  0  0  0  0
  8 31  1  0  0  0  0
  9 32  1  0  0  0  0
 10 33  1  0  0  0  0
 11 34  1  0  0  0  0
 12 35  1  0  0  0  0
 15 36  1  0  0  0  0
 15 37  1  0  0  0  0
 15 38  1  0  0  0  0
 16 39  1  0  0  0  0
 16 40  1  0  0  0  0
 17 41  1  0  0  0  0
 18 42  1  0  0  0  0
 19 43  1  0  0  0  0
 20 44  1  0  0  0  0
 23 45  1  0  0  0  0
 23 46  1  0  0  0  0
 23 47  1  0  0  0  0
M  END
>  <Alias name>
1,1,2-Tris(2-methylphenyl)ethane

>  <deltaH°(c)>   kJ/mol
-12513.4

$$$$
1,1,2-Tritolylethane
  ChemBrain IXL

 47 49  0  0  0  0  0  0  0  0  1 V2000
    5.4931    9.5176    0.2890 C   0  0  0  0  0  0  0  0  0  0  0  0
    4.3920    9.6580   -0.5559 C   0  0  0  0  0  0  0  0  0  0  0  0
    5.3364    8.9392    1.5488 C   0  0  0  0  0  0  0  0  0  0  0  0
    6.8276    9.9822   -0.1512 C   0  0  0  0  0  0  0  0  0  0  0  0
    3.1372    9.2058   -0.1472 C   0  0  0  0  0  0  0  0  0  0  0  0
    2.9822    8.6192    1.1090 C   0  0  0  0  0  0  0  0  0  0  0  0
```

4.0816 8.4870 1.9574 C 0 0 0 0 0 0 0 0 0 0 0 0
1.6515 8.1372 1.5416 C 0 0 0 0 0 0 0 0 0 0 0 0
7.9187 9.2278 0.6331 C 0 0 0 0 0 0 0 0 0 0 0 0
9.2530 9.6995 0.1999 C 0 0 0 0 0 0 0 0 0 0 0 0
7.7998 7.7760 0.3713 C 0 0 0 0 0 0 0 0 0 0 0 0
10.3366 8.8210 0.1979 C 0 0 0 0 0 0 0 0 0 0 0 0
11.5915 9.2623 -0.2223 C 0 0 0 0 0 0 0 0 0 0 0 0
11.7639 10.5834 -0.6356 C 0 0 0 0 0 0 0 0 0 0 0 0
10.6819 11.4639 -0.6266 C 0 0 0 0 0 0 0 0 0 0 0 0
13.0947 11.0510 -1.0836 C 0 0 0 0 0 0 0 0 0 0 0 0
9.4270 11.0226 -0.2063 C 0 0 0 0 0 0 0 0 0 0 0 0
7.7561 6.8764 1.4366 C 0 0 0 0 0 0 0 0 0 0 0 0
7.6437 5.5080 1.1899 C 0 0 0 0 0 0 0 0 0 0 0 0
7.5752 5.0392 -0.1222 C 0 0 0 0 0 0 0 0 0 0 0 0
7.6191 5.9387 -1.1875 C 0 0 0 0 0 0 0 0 0 0 0 0
7.4558 3.5874 -0.3840 C 0 0 0 0 0 0 0 0 0 0 0 0
7.7315 7.3071 -0.9408 C 0 0 0 0 0 0 0 0 0 0 0 0
4.5113 10.1180 -1.5290 H 0 0 0 0 0 0 0 0 0 0 0 0
6.1889 8.8414 2.2095 H 0 0 0 0 0 0 0 0 0 0 0 0
6.9192 11.0452 0.0347 H 0 0 0 0 0 0 0 0 0 0 0 0
6.9457 9.7895 -1.2103 H 0 0 0 0 0 0 0 0 0 0 0 0
2.2833 9.3101 -0.8051 H 0 0 0 0 0 0 0 0 0 0 0 0
3.9610 8.0332 2.9333 H 0 0 0 0 0 0 0 0 0 0 0 0
1.6757 7.0600 1.6510 H 0 0 0 0 0 0 0 0 0 0 0 0
1.3942 8.5908 2.4908 H 0 0 0 0 0 0 0 0 0 0 0 0
0.9106 8.4081 0.7995 H 0 0 0 0 0 0 0 0 0 0 0 0
7.7962 9.4147 1.6928 H 0 0 0 0 0 0 0 0 0 0 0 0
10.2037 7.7964 0.5226 H 0 0 0 0 0 0 0 0 0 0 0 0
12.4318 8.5791 -0.2278 H 0 0 0 0 0 0 0 0 0 0 0 0
10.8161 12.4900 -0.9460 H 0 0 0 0 0 0 0 0 0 0 0 0
13.0825 12.1289 -1.1874 H 0 0 0 0 0 0 0 0 0 0 0 0
13.3336 10.5990 -2.0383 H 0 0 0 0 0 0 0 0 0 0 0 0
13.8419 10.7672 -0.3528 H 0 0 0 0 0 0 0 0 0 0 0 0
8.5880 11.7073 -0.1954 H 0 0 0 0 0 0 0 0 0 0 0 0
7.8094 7.2404 2.4552 H 0 0 0 0 0 0 0 0 0 0 0 0
7.6098 4.8097 2.0169 H 0 0 0 0 0 0 0 0 0 0 0 0
7.5660 5.5747 -2.2061 H 0 0 0 0 0 0 0 0 0 0 0 0
8.4215 3.1924 -0.6743 H 0 0 0 0 0 0 0 0 0 0 0 0
7.1154 3.0856 0.5133 H 0 0 0 0 0 0 0 0 0 0 0 0
6.7432 3.4220 -1.1825 H 0 0 0 0 0 0 0 0 0 0 0 0
7.7657 8.0055 -1.7678 H 0 0 0 0 0 0 0 0 0 0 0 0
2 1 4 0 0 0 0
1 3 4 0 0 0 0
1 4 1 0 0 0 0
5 2 4 0 0 0 0
3 7 4 0 0 0 0
4 9 1 0 0 0 0
6 5 4 0 0 0 0
7 6 4 0 0 0 0
6 8 1 0 0 0 0
9 10 1 0 0 0 0
9 11 1 0 0 0 0
12 10 4 0 0 0 0
10 17 4 0 0 0 0
18 11 4 0 0 0 0
11 23 4 0 0 0 0
13 12 4 0 0 0 0
14 13 4 0 0 0 0
15 14 4 0 0 0 0
14 16 1 0 0 0 0
17 15 4 0 0 0 0
19 18 4 0 0 0 0
20 19 4 0 0 0 0

```
 21 20  4  0  0  0  0
 20 22  1  0  0  0  0
 23 21  4  0  0  0  0
  2 24  1  0  0  0  0
  3 25  1  0  0  0  0
  4 26  1  0  0  0  0
  4 27  1  0  0  0  0
  5 28  1  0  0  0  0
  7 29  1  0  0  0  0
  8 30  1  0  0  0  0
  8 31  1  0  0  0  0
  8 32  1  0  0  0  0
  9 33  1  0  0  0  0
 12 34  1  0  0  0  0
 13 35  1  0  0  0  0
 15 36  1  0  0  0  0
 16 37  1  0  0  0  0
 16 38  1  0  0  0  0
 16 39  1  0  0  0  0
 17 40  1  0  0  0  0
 18 41  1  0  0  0  0
 19 42  1  0  0  0  0
 21 43  1  0  0  0  0
 22 44  1  0  0  0  0
 22 45  1  0  0  0  0
 22 46  1  0  0  0  0
 23 47  1  0  0  0  0
M  END
>  <Alias name>
1,1,2-Tris(4-methylphenyl)ethane

>  <deltaH°(c)>   kJ/mol
-12505.43

$$$$
Tetradecylcyclopentane
  ChemBrain IXL

 57 57  0  0  0  0  0  0  0  0  1 V2000
   19.9477   11.6362    0.3958 C   0  0  0  0  0  0  0  0  0  0  0  0
   21.2146   12.3115   -0.1082 C   0  0  0  0  0  0  0  0  0  0  0  0
   22.3056   11.2610    0.0831 C   0  0  0  0  0  0  0  0  0  0  0  0
   21.5923    9.8830   -0.1158 C   0  0  0  0  0  0  0  0  0  0  0  0
   20.0603   10.1977   -0.1044 C   0  0  0  0  0  0  0  0  0  0  0  0
   18.6820   12.3265   -0.1513 C   0  0  0  0  0  0  0  0  0  0  0  0
   17.4303   11.5820    0.3538 C   0  0  0  0  0  0  0  0  0  0  0  0
   16.1660   12.2739   -0.1916 C   0  0  0  0  0  0  0  0  0  0  0  0
   14.9146   11.5293    0.3128 C   0  0  0  0  0  0  0  0  0  0  0  0
   13.6502   12.2207   -0.2334 C   0  0  0  0  0  0  0  0  0  0  0  0
   12.3988   11.4755    0.2704 C   0  0  0  0  0  0  0  0  0  0  0  0
   11.1344   12.1663   -0.2767 C   0  0  0  0  0  0  0  0  0  0  0  0
    9.8831   11.4207    0.2265 C   0  0  0  0  0  0  0  0  0  0  0  0
    8.6187   12.1109   -0.3214 C   0  0  0  0  0  0  0  0  0  0  0  0
    7.3674   11.3648    0.1812 C   0  0  0  0  0  0  0  0  0  0  0  0
    6.1031   12.0546   -0.3671 C   0  0  0  0  0  0  0  0  0  0  0  0
    4.8517   11.3083    0.1351 C   0  0  0  0  0  0  0  0  0  0  0  0
    3.5873   11.9979   -0.4136 C   0  0  0  0  0  0  0  0  0  0  0  0
    2.3360   11.2517    0.0885 C   0  0  0  0  0  0  0  0  0  0  0  0
   19.9435   11.6431    1.4787 H   0  0  0  0  0  0  0  0  0  0  0  0
   21.4327   13.2137    0.4496 H   0  0  0  0  0  0  0  0  0  0  0  0
   21.1082   12.5351   -1.1626 H   0  0  0  0  0  0  0  0  0  0  0  0
   22.6683   11.3232    1.1016 H   0  0  0  0  0  0  0  0  0  0  0  0
   23.1243   11.3928   -0.6133 H   0  0  0  0  0  0  0  0  0  0  0  0
```

```
  21.8593    9.2190    0.6969 H   0  0  0  0  0  0  0  0  0  0  0  0
  21.8612    9.4390   -1.0662 H   0  0  0  0  0  0  0  0  0  0  0  0
  19.6873   10.1563   -1.1204 H   0  0  0  0  0  0  0  0  0  0  0  0
  19.5133    9.5047    0.5225 H   0  0  0  0  0  0  0  0  0  0  0  0
  18.7004   12.3086   -1.2340 H   0  0  0  0  0  0  0  0  0  0  0  0
  18.6542   13.3532    0.1922 H   0  0  0  0  0  0  0  0  0  0  0  0
  17.4125   11.5990    1.4365 H   0  0  0  0  0  0  0  0  0  0  0  0
  17.4570   10.5556    0.0093 H   0  0  0  0  0  0  0  0  0  0  0  0
  16.1840   12.2572   -1.2743 H   0  0  0  0  0  0  0  0  0  0  0  0
  16.1389   13.3002    0.1532 H   0  0  0  0  0  0  0  0  0  0  0  0
  14.8962   11.5464    1.3955 H   0  0  0  0  0  0  0  0  0  0  0  0
  14.9420   10.5029   -0.0316 H   0  0  0  0  0  0  0  0  0  0  0  0
  13.6688   12.2039   -1.3161 H   0  0  0  0  0  0  0  0  0  0  0  0
  13.6225   13.2470    0.1113 H   0  0  0  0  0  0  0  0  0  0  0  0
  12.3798   11.4928    1.3531 H   0  0  0  0  0  0  0  0  0  0  0  0
  12.4269   10.4491   -0.0738 H   0  0  0  0  0  0  0  0  0  0  0  0
  11.1537   12.1494   -1.3594 H   0  0  0  0  0  0  0  0  0  0  0  0
  11.1060   13.1926    0.0679 H   0  0  0  0  0  0  0  0  0  0  0  0
   9.8635   11.4380    1.3091 H   0  0  0  0  0  0  0  0  0  0  0  0
   9.9118   10.3942   -0.1177 H   0  0  0  0  0  0  0  0  0  0  0  0
   8.6386   12.0938   -1.4040 H   0  0  0  0  0  0  0  0  0  0  0  0
   8.5898   13.1372    0.0231 H   0  0  0  0  0  0  0  0  0  0  0  0
   7.3473   11.3822    1.2639 H   0  0  0  0  0  0  0  0  0  0  0  0
   7.3966   10.3384   -0.1629 H   0  0  0  0  0  0  0  0  0  0  0  0
   6.1233   12.0374   -1.4498 H   0  0  0  0  0  0  0  0  0  0  0  0
   6.0737   13.0810   -0.0228 H   0  0  0  0  0  0  0  0  0  0  0  0
   4.8314   11.3256    1.2178 H   0  0  0  0  0  0  0  0  0  0  0  0
   4.8812   10.2818   -0.2090 H   0  0  0  0  0  0  0  0  0  0  0  0
   3.6077   11.9807   -1.4962 H   0  0  0  0  0  0  0  0  0  0  0  0
   3.5577   13.0243   -0.0694 H   0  0  0  0  0  0  0  0  0  0  0  0
   2.3654   10.2252   -0.2557 H   0  0  0  0  0  0  0  0  0  0  0  0
   2.3155   11.2689    1.1711 H   0  0  0  0  0  0  0  0  0  0  0  0
   1.4477   11.7364   -0.2972 H   0  0  0  0  0  0  0  0  0  0  0  0
  1  2  1  0  0  0  0
  2  3  1  0  0  0  0
  3  4  1  0  0  0  0
  4  5  1  0  0  0  0
  1  5  1  0  0  0  0
  6  7  1  0  0  0  0
  7  8  1  0  0  0  0
  8  9  1  0  0  0  0
  9 10  1  0  0  0  0
 10 11  1  0  0  0  0
 11 12  1  0  0  0  0
 12 13  1  0  0  0  0
 13 14  1  0  0  0  0
 14 15  1  0  0  0  0
 15 16  1  0  0  0  0
  1  6  1  0  0  0  0
 16 17  1  0  0  0  0
 17 18  1  0  0  0  0
 18 19  1  0  0  0  0
  1 20  1  0  0  0  0
  2 21  1  0  0  0  0
  2 22  1  0  0  0  0
  3 23  1  0  0  0  0
  3 24  1  0  0  0  0
  4 25  1  0  0  0  0
  4 26  1  0  0  0  0
  5 27  1  0  0  0  0
  5 28  1  0  0  0  0
  6 29  1  0  0  0  0
  6 30  1  0  0  0  0
```

```
  7 31  1  0  0  0  0
  7 32  1  0  0  0  0
  8 33  1  0  0  0  0
  8 34  1  0  0  0  0
  9 35  1  0  0  0  0
  9 36  1  0  0  0  0
 10 37  1  0  0  0  0
 10 38  1  0  0  0  0
 11 39  1  0  0  0  0
 11 40  1  0  0  0  0
 12 41  1  0  0  0  0
 12 42  1  0  0  0  0
 13 43  1  0  0  0  0
 13 44  1  0  0  0  0
 14 45  1  0  0  0  0
 14 46  1  0  0  0  0
 15 47  1  0  0  0  0
 15 48  1  0  0  0  0
 16 49  1  0  0  0  0
 16 50  1  0  0  0  0
 17 51  1  0  0  0  0
 17 52  1  0  0  0  0
 18 53  1  0  0  0  0
 18 54  1  0  0  0  0
 19 55  1  0  0  0  0
 19 56  1  0  0  0  0
 19 57  1  0  0  0  0
M  END
>  <Alias name>
Tetradecylcyclopentane

>  <deltaH°(c)>   kJ/mol
-12446

$$$$
3',5'-Diisopropyl-4,4-dimethyl-3-phenyl-1,2-benzocyclobuten-3-ol
  ChemBrain IXL

 51 53  0  0  0  0  0  0  0  0  1 V2000
    5.2074    7.1081    0.7115 C   0  0  0  0  0  0  0  0  0  0  0  0
    4.7783    5.9941    0.0128 C   0  0  0  0  0  0  0  0  0  0  0  0
    5.7727    5.0752   -0.4536 C   0  0  0  0  0  0  0  0  0  0  0  0
    7.1432    5.3536   -0.1795 C   0  0  0  0  0  0  0  0  0  0  0  0
    7.4993    6.3064    0.4093 C   0  0  0  0  0  0  0  0  0  0  0  0
    6.5717    7.2929    0.9286 C   0  0  0  0  0  0  0  0  0  0  0  0
    8.8739    5.9725    0.2987 C   0  0  0  0  0  0  0  0  0  0  0  0
    8.4556    4.8557   -0.3730 C   0  0  0  0  0  0  0  0  0  0  0  0
    6.9712    8.5047    1.6902 C   0  0  0  0  0  0  0  0  0  0  0  0
    8.2601    8.2505    2.4541 C   0  0  0  0  0  0  0  0  0  0  0  0
    5.8307    8.8635    2.7004 C   0  0  0  0  0  0  0  0  0  0  0  0
    3.2627    5.7981   -0.2171 C   0  0  0  0  0  0  0  0  0  0  0  0
    2.5403    7.1833   -0.2302 C   0  0  0  0  0  0  0  0  0  0  0  0
    2.9927    5.0936   -1.5637 C   0  0  0  0  0  0  0  0  0  0  0  0
   10.8878    8.7577   -2.1485 C   0  0  0  0  0  0  0  0  0  0  0  0
   11.5751    7.6652   -1.6188 C   0  0  0  0  0  0  0  0  0  0  0  0
   10.9096    6.7507   -0.8120 C   0  0  0  0  0  0  0  0  0  0  0  0
    9.5684    6.9284   -0.5362 C   0  0  0  0  0  0  0  0  0  0  0  0
    8.8695    8.0168   -1.0612 C   0  0  0  0  0  0  0  0  0  0  0  0
    9.5332    8.9342   -1.8693 C   0  0  0  0  0  0  0  0  0  0  0  0
    8.8237    4.8238   -1.8545 C   0  0  0  0  0  0  0  0  0  0  0  0
    8.6541    3.5345    0.3819 C   0  0  0  0  0  0  0  0  0  0  0  0
    9.4979    5.6950    1.5923 O   0  0  0  0  0  0  0  0  0  0  0  0
   10.3498    5.2179    1.5453 H   0  0  0  0  0  0  0  0  0  0  0  0
```

```
   4.4844    7.8337    1.0865 H   0  0  0  0  0  0  0  0  0  0  0  0
   5.4815    4.1880   -1.0022 H   0  0  0  0  0  0  0  0  0  0  0  0
   7.1114    9.3304    1.0055 H   0  0  0  0  0  0  0  0  0  0  0  0
   8.4889    9.1149    3.0665 H   0  0  0  0  0  0  0  0  0  0  0  0
   9.0630    8.0805    1.7544 H   0  0  0  0  0  0  0  0  0  0  0  0
   8.1382    7.3816    3.0868 H   0  0  0  0  0  0  0  0  0  0  0  0
   4.9061    9.0403    2.1593 H   0  0  0  0  0  0  0  0  0  0  0  0
   5.6881    8.0408    3.3941 H   0  0  0  0  0  0  0  0  0  0  0  0
   6.0995    9.7560    3.2520 H   0  0  0  0  0  0  0  0  0  0  0  0
   2.8523    5.1974    0.5856 H   0  0  0  0  0  0  0  0  0  0  0  0
   1.4798    7.0368   -0.4295 H   0  0  0  0  0  0  0  0  0  0  0  0
   2.6600    7.6666    0.7306 H   0  0  0  0  0  0  0  0  0  0  0  0
   2.9664    7.8086   -1.0049 H   0  0  0  0  0  0  0  0  0  0  0  0
   3.6455    5.4979   -2.3229 H   0  0  0  0  0  0  0  0  0  0  0  0
   3.1690    4.0334   -1.4588 H   0  0  0  0  0  0  0  0  0  0  0  0
   1.9552    5.2595   -1.8538 H   0  0  0  0  0  0  0  0  0  0  0  0
  11.4059    9.4680   -2.7760 H   0  0  0  0  0  0  0  0  0  0  0  0
  12.6238    7.5278   -1.8343 H   0  0  0  0  0  0  0  0  0  0  0  0
  11.4357    5.9007   -0.3977 H   0  0  0  0  0  0  0  0  0  0  0  0
   7.8135    8.1481   -0.8398 H   0  0  0  0  0  0  0  0  0  0  0  0
   8.9980    9.7822   -2.2798 H   0  0  0  0  0  0  0  0  0  0  0  0
   9.8789    4.6522   -1.9590 H   0  0  0  0  0  0  0  0  0  0  0  0
   8.5598    5.7713   -2.3118 H   0  0  0  0  0  0  0  0  0  0  0  0
   8.2733    4.0242   -2.3424 H   0  0  0  0  0  0  0  0  0  0  0  0
   9.6977    3.2782    0.3947 H   0  0  0  0  0  0  0  0  0  0  0  0
   8.0906    2.7485   -0.1143 H   0  0  0  0  0  0  0  0  0  0  0  0
   8.2926    3.6444    1.4008 H   0  0  0  0  0  0  0  0  0  0  0  0
  1  2  4  0  0  0  0
  2  3  4  0  0  0  0
  3  4  4  0  0  0  0
  4  5  4  0  0  0  0
  5  6  4  0  0  0  0
  6  1  4  0  0  0  0
  5  7  1  0  0  0  0
  4  8  1  0  0  0  0
  7  8  1  0  0  0  0
  6  9  1  0  0  0  0
  9 10  1  0  0  0  0
  9 11  1  0  0  0  0
  2 12  1  0  0  0  0
 12 13  1  0  0  0  0
 12 14  1  0  0  0  0
 15 16  4  0  0  0  0
 16 17  4  0  0  0  0
 17 18  4  0  0  0  0
 18 19  4  0  0  0  0
 19 20  4  0  0  0  0
 20 15  4  0  0  0  0
 18  7  1  0  0  0  0
  8 21  1  0  0  0  0
  8 22  1  0  0  0  0
  7 23  1  0  0  0  0
 23 24  1  0  0  0  0
  1 25  1  0  0  0  0
  3 26  1  0  0  0  0
  9 27  1  0  0  0  0
 10 28  1  0  0  0  0
 10 29  1  0  0  0  0
 10 30  1  0  0  0  0
 11 31  1  0  0  0  0
 11 32  1  0  0  0  0
 11 33  1  0  0  0  0
 12 34  1  0  0  0  0
```

```
 13 35  1  0  0  0  0
 13 36  1  0  0  0  0
 13 37  1  0  0  0  0
 14 38  1  0  0  0  0
 14 39  1  0  0  0  0
 14 40  1  0  0  0  0
 15 41  1  0  0  0  0
 16 42  1  0  0  0  0
 17 43  1  0  0  0  0
 19 44  1  0  0  0  0
 20 45  1  0  0  0  0
 21 46  1  0  0  0  0
 21 47  1  0  0  0  0
 21 48  1  0  0  0  0
 22 49  1  0  0  0  0
 22 50  1  0  0  0  0
 22 51  1  0  0  0  0
M  END
>  <Alias name>
3',5'-Diisopropyl-4,4-dimethyl-3-phenyl-1,2-benzocyclobuten-3-ol

>  <deltaH°(c)>   kJ/mol
-12440

$$$$
Tetradecylbenzene
  ChemBrain IXL

 54 54  0  0  0  0  0  0  0  0  1 V2000
    3.4696   12.4136    2.4673 C   0  0  0  0  0  0  0  0  0  0  0  0
    2.3231   11.7423    2.8924 C   0  0  0  0  0  0  0  0  0  0  0  0
    2.3285   10.3507    2.9996 C   0  0  0  0  0  0  0  0  0  0  0  0
    3.4809    9.6293    2.6816 C   0  0  0  0  0  0  0  0  0  0  0  0
    4.6297   10.2986    2.2559 C   0  0  0  0  0  0  0  0  0  0  0  0
    4.6218   11.6940    2.1493 C   0  0  0  0  0  0  0  0  0  0  0  0
    5.8555    9.5240    1.9183 C   0  0  0  0  0  0  0  0  0  0  0  0
    7.0183   10.4902    1.5910 C   0  0  0  0  0  0  0  0  0  0  0  0
    8.2837    9.6750    1.2584 C   0  0  0  0  0  0  0  0  0  0  0  0
    9.4469   10.6337    0.9273 C   0  0  0  0  0  0  0  0  0  0  0  0
   10.7083    9.8097    0.5997 C   0  0  0  0  0  0  0  0  0  0  0  0
   11.8761   10.7575    0.2585 C   0  0  0  0  0  0  0  0  0  0  0  0
   13.1315    9.9216   -0.0621 C   0  0  0  0  0  0  0  0  0  0  0  0
   14.3045   10.8572   -0.4154 C   0  0  0  0  0  0  0  0  0  0  0  0
   15.5545   10.0099   -0.7270 C   0  0  0  0  0  0  0  0  0  0  0  0
   16.7318   10.9353   -1.0909 C   0  0  0  0  0  0  0  0  0  0  0  0
   17.9786   10.0799   -1.3932 C   0  0  0  0  0  0  0  0  0  0  0  0
   19.1584   10.9988   -1.7645 C   0  0  0  0  0  0  0  0  0  0  0  0
   20.4039   10.1394   -2.0601 C   0  0  0  0  0  0  0  0  0  0  0  0
   21.5844   11.0558   -2.4354 C   0  0  0  0  0  0  0  0  0  0  0  0
    3.4656   13.4936    2.3841 H   0  0  0  0  0  0  0  0  0  0  0  0
    1.4290   12.3012    3.1392 H   0  0  0  0  0  0  0  0  0  0  0  0
    1.4384    9.8304    3.3296 H   0  0  0  0  0  0  0  0  0  0  0  0
    3.4834    8.5500    2.7652 H   0  0  0  0  0  0  0  0  0  0  0  0
    5.5089   12.2186    1.8199 H   0  0  0  0  0  0  0  0  0  0  0  0
    5.6580    8.8956    1.0588 H   0  0  0  0  0  0  0  0  0  0  0  0
    6.1312    8.9031    2.7615 H   0  0  0  0  0  0  0  0  0  0  0  0
    6.7503   11.1034    0.7394 H   0  0  0  0  0  0  0  0  0  0  0  0
    7.2128   11.1250    2.4469 H   0  0  0  0  0  0  0  0  0  0  0  0
    8.5525    9.0632    2.1105 H   0  0  0  0  0  0  0  0  0  0  0  0
    8.0879    9.0381    0.4048 H   0  0  0  0  0  0  0  0  0  0  0  0
    9.1810   11.2433    0.0725 H   0  0  0  0  0  0  0  0  0  0  0  0
    9.6434   11.2722    1.7798 H   0  0  0  0  0  0  0  0  0  0  0  0
   10.9750    9.2038    1.4566 H   0  0  0  0  0  0  0  0  0  0  0  0
```

```
 10.5078    9.1663   -0.2480 H   0  0  0  0  0  0  0  0  0  0  0  0
 11.6133   11.3604   -0.6020 H   0  0  0  0  0  0  0  0  0  0  0  0
 12.0776   11.4031    1.1045 H   0  0  0  0  0  0  0  0  0  0  0  0
 13.3955    9.3233    0.8011 H   0  0  0  0  0  0  0  0  0  0  0  0
 12.9253    9.2702   -0.9023 H   0  0  0  0  0  0  0  0  0  0  0  0
 14.0446   11.4514   -1.2828 H   0  0  0  0  0  0  0  0  0  0  0  0
 14.5107   11.5115    0.4227 H   0  0  0  0  0  0  0  0  0  0  0  0
 15.8156    9.4198    0.1427 H   0  0  0  0  0  0  0  0  0  0  0  0
 15.3442    9.3504   -1.5599 H   0  0  0  0  0  0  0  0  0  0  0  0
 16.4745   11.5210   -1.9648 H   0  0  0  0  0  0  0  0  0  0  0  0
 16.9410   11.5981   -0.2603 H   0  0  0  0  0  0  0  0  0  0  0  0
 18.2370    9.4973   -0.5176 H   0  0  0  0  0  0  0  0  0  0  0  0
 17.7667    9.4133   -2.2201 H   0  0  0  0  0  0  0  0  0  0  0  0
 18.9024   11.5781   -2.6430 H   0  0  0  0  0  0  0  0  0  0  0  0
 19.3688   11.6678   -0.9394 H   0  0  0  0  0  0  0  0  0  0  0  0
 20.6608    9.5619   -1.1807 H   0  0  0  0  0  0  0  0  0  0  0  0
 20.1922    9.4684   -2.8834 H   0  0  0  0  0  0  0  0  0  0  0  0
 21.7957   11.7276   -1.6127 H   0  0  0  0  0  0  0  0  0  0  0  0
 21.3283   11.6323   -3.3156 H   0  0  0  0  0  0  0  0  0  0  0  0
 22.4590   10.4514   -2.6416 H   0  0  0  0  0  0  0  0  0  0  0  0
  1  2  4  0  0  0  0
  2  3  4  0  0  0  0
  3  4  4  0  0  0  0
  4  5  4  0  0  0  0
  5  6  4  0  0  0  0
  6  1  4  0  0  0  0
  5  7  1  0  0  0  0
  7  8  1  0  0  0  0
  8  9  1  0  0  0  0
  9 10  1  0  0  0  0
 10 11  1  0  0  0  0
 11 12  1  0  0  0  0
 12 13  1  0  0  0  0
 13 14  1  0  0  0  0
 14 15  1  0  0  0  0
 15 16  1  0  0  0  0
 16 17  1  0  0  0  0
 17 18  1  0  0  0  0
 18 19  1  0  0  0  0
 19 20  1  0  0  0  0
  1 21  1  0  0  0  0
  2 22  1  0  0  0  0
  3 23  1  0  0  0  0
  4 24  1  0  0  0  0
  6 25  1  0  0  0  0
  7 26  1  0  0  0  0
  7 27  1  0  0  0  0
  8 28  1  0  0  0  0
  8 29  1  0  0  0  0
  9 30  1  0  0  0  0
  9 31  1  0  0  0  0
 10 32  1  0  0  0  0
 10 33  1  0  0  0  0
 11 34  1  0  0  0  0
 11 35  1  0  0  0  0
 12 36  1  0  0  0  0
 12 37  1  0  0  0  0
 13 38  1  0  0  0  0
 13 39  1  0  0  0  0
 14 40  1  0  0  0  0
 14 41  1  0  0  0  0
 15 42  1  0  0  0  0
 15 43  1  0  0  0  0
```

```
 16 44  1  0  0  0  0
 16 45  1  0  0  0  0
 17 46  1  0  0  0  0
 17 47  1  0  0  0  0
 18 48  1  0  0  0  0
 18 49  1  0  0  0  0
 19 50  1  0  0  0  0
 19 51  1  0  0  0  0
 20 52  1  0  0  0  0
 20 53  1  0  0  0  0
 20 54  1  0  0  0  0
M  END
>  <Alias name>
Tetradecylbenzene

>  <deltaH°(c)>   kJ/mol
-12426.32

$$$$
Tridecylcyclohexane
  ChemBrain IXL

 57 57  0  0  0  0  0  0  0  0  1 V2000
    3.5010    9.3614    0.0761 C   0  0  0  0  0  0  0  0  0  0  0  0  0
    2.4327   10.3776   -0.4129 C   0  0  0  0  0  0  0  0  0  0  0  0  0
    2.4614   11.5981    0.4981 C   0  0  0  0  0  0  0  0  0  0  0  0  0
    3.7624   12.4007    0.2209 C   0  0  0  0  0  0  0  0  0  0  0  0  0
    4.9131   11.4200   -0.1417 C   0  0  0  0  0  0  0  0  0  0  0  0  0
    4.7251   10.1278    0.6499 C   0  0  0  0  0  0  0  0  0  0  0  0  0
    6.2719   12.0718    0.2003 C   0  0  0  0  0  0  0  0  0  0  0  0  0
    7.4212   11.1219   -0.1979 C   0  0  0  0  0  0  0  0  0  0  0  0  0
    8.7727   11.7913    0.1184 C   0  0  0  0  0  0  0  0  0  0  0  0  0
    9.9210   10.8435   -0.2790 C   0  0  0  0  0  0  0  0  0  0  0  0  0
   11.2726   11.5126    0.0379 C   0  0  0  0  0  0  0  0  0  0  0  0  0
   12.4209   10.5645   -0.3590 C   0  0  0  0  0  0  0  0  0  0  0  0  0
   13.7725   11.2333   -0.0414 C   0  0  0  0  0  0  0  0  0  0  0  0  0
   14.9208   10.2850   -0.4378 C   0  0  0  0  0  0  0  0  0  0  0  0  0
   16.2724   10.9536   -0.1196 C   0  0  0  0  0  0  0  0  0  0  0  0  0
   17.4207   10.0051   -0.5157 C   0  0  0  0  0  0  0  0  0  0  0  0  0
   18.7723   10.6735   -0.1971 C   0  0  0  0  0  0  0  0  0  0  0  0  0
   19.9207    9.7249   -0.5930 C   0  0  0  0  0  0  0  0  0  0  0  0  0
   21.2723   10.3931   -0.2743 C   0  0  0  0  0  0  0  0  0  0  0  0  0
    3.8109    8.7456   -0.7590 H   0  0  0  0  0  0  0  0  0  0  0  0  0
    3.0827    8.7325    0.8520 H   0  0  0  0  0  0  0  0  0  0  0  0  0
    2.6626   10.6876   -1.4248 H   0  0  0  0  0  0  0  0  0  0  0  0  0
    1.4538    9.9153   -0.3886 H   0  0  0  0  0  0  0  0  0  0  0  0  0
    1.6000   12.2265    0.3095 H   0  0  0  0  0  0  0  0  0  0  0  0  0
    2.4510   11.2683    1.5297 H   0  0  0  0  0  0  0  0  0  0  0  0  0
    4.0250   12.9646    1.1075 H   0  0  0  0  0  0  0  0  0  0  0  0  0
    3.6053   13.0819   -0.6060 H   0  0  0  0  0  0  0  0  0  0  0  0  0
    4.8759   11.1935   -1.2002 H   0  0  0  0  0  0  0  0  0  0  0  0  0
    4.5446   10.3743    1.6889 H   0  0  0  0  0  0  0  0  0  0  0  0  0
    5.6076    9.5082    0.5726 H   0  0  0  0  0  0  0  0  0  0  0  0  0
    6.3196   12.2708    1.2638 H   0  0  0  0  0  0  0  0  0  0  0  0  0
    6.3707   13.0024   -0.3448 H   0  0  0  0  0  0  0  0  0  0  0  0  0
    7.3632   10.9097   -1.2583 H   0  0  0  0  0  0  0  0  0  0  0  0  0
    7.3380   10.1990    0.3599 H   0  0  0  0  0  0  0  0  0  0  0  0  0
    8.8308   12.0040    1.1787 H   0  0  0  0  0  0  0  0  0  0  0  0  0
    8.8575   12.7152   -0.4403 H   0  0  0  0  0  0  0  0  0  0  0  0  0
    9.8632   10.6311   -1.3394 H   0  0  0  0  0  0  0  0  0  0  0  0  0
    9.8360    9.9194    0.2793 H   0  0  0  0  0  0  0  0  0  0  0  0  0
   11.3303   11.7252    1.0983 H   0  0  0  0  0  0  0  0  0  0  0  0  0
   11.3578   12.4365   -0.5207 H   0  0  0  0  0  0  0  0  0  0  0  0  0
```

12.3636 10.3522 -1.4195 H 0 0 0 0 0 0 0 0 0 0 0 0
12.3354 9.6405 0.1993 H 0 0 0 0 0 0 0 0 0 0 0 0
13.8297 11.4459 1.0190 H 0 0 0 0 0 0 0 0 0 0 0 0
13.8582 12.1572 -0.5999 H 0 0 0 0 0 0 0 0 0 0 0 0
14.8639 10.0727 -1.4983 H 0 0 0 0 0 0 0 0 0 0 0 0
14.8349 9.3610 0.1205 H 0 0 0 0 0 0 0 0 0 0 0 0
16.3292 11.1661 0.9408 H 0 0 0 0 0 0 0 0 0 0 0 0
16.3585 11.8775 -0.6781 H 0 0 0 0 0 0 0 0 0 0 0 0
17.3641 9.7928 -1.5761 H 0 0 0 0 0 0 0 0 0 0 0 0
17.3345 9.0811 0.0426 H 0 0 0 0 0 0 0 0 0 0 0 0
18.8289 10.8859 0.8634 H 0 0 0 0 0 0 0 0 0 0 0 0
18.8586 11.5975 -0.7554 H 0 0 0 0 0 0 0 0 0 0 0 0
19.8642 9.5126 -1.6534 H 0 0 0 0 0 0 0 0 0 0 0 0
19.8344 8.8010 -0.0347 H 0 0 0 0 0 0 0 0 0 0 0 0
21.3587 11.3171 -0.8326 H 0 0 0 0 0 0 0 0 0 0 0 0
22.0790 9.7264 -0.5525 H 0 0 0 0 0 0 0 0 0 0 0 0
21.3289 10.6054 0.7862 H 0 0 0 0 0 0 0 0 0 0 0 0
1 2 1 0 0 0 0
2 3 1 0 0 0 0
3 4 1 0 0 0 0
4 5 1 0 0 0 0
5 6 1 0 0 0 0
6 1 1 0 0 0 0
5 7 1 0 0 0 0
7 8 1 0 0 0 0
8 9 1 0 0 0 0
9 10 1 0 0 0 0
10 11 1 0 0 0 0
11 12 1 0 0 0 0
12 13 1 0 0 0 0
13 14 1 0 0 0 0
14 15 1 0 0 0 0
15 16 1 0 0 0 0
16 17 1 0 0 0 0
17 18 1 0 0 0 0
18 19 1 0 0 0 0
1 20 1 0 0 0 0
1 21 1 0 0 0 0
2 22 1 0 0 0 0
2 23 1 0 0 0 0
3 24 1 0 0 0 0
3 25 1 0 0 0 0
4 26 1 0 0 0 0
4 27 1 0 0 0 0
5 28 1 0 0 0 0
6 29 1 0 0 0 0
6 30 1 0 0 0 0
7 31 1 0 0 0 0
7 32 1 0 0 0 0
8 33 1 0 0 0 0
8 34 1 0 0 0 0
9 35 1 0 0 0 0
9 36 1 0 0 0 0
10 37 1 0 0 0 0
10 38 1 0 0 0 0
11 39 1 0 0 0 0
11 40 1 0 0 0 0
12 41 1 0 0 0 0
12 42 1 0 0 0 0
13 43 1 0 0 0 0
13 44 1 0 0 0 0
14 45 1 0 0 0 0
14 46 1 0 0 0 0

```
 15 47  1  0  0  0  0
 15 48  1  0  0  0  0
 16 49  1  0  0  0  0
 16 50  1  0  0  0  0
 17 51  1  0  0  0  0
 17 52  1  0  0  0  0
 18 53  1  0  0  0  0
 18 54  1  0  0  0  0
 19 55  1  0  0  0  0
 19 56  1  0  0  0  0
 19 57  1  0  0  0  0
M  END
>  <Alias name>
Tridecylcyclohexane

>  <deltaH°(c)>   kJ/mol
-12422

$$$$
1,1'-Diphenyl-1,1'-bicyclopentyl
  ChemBrain IXL

 48 51  0  0  0  0  0  0  0  0  1 V2000
    7.1049    5.4901    1.6147 C   0  0  0  0  0  0  0  0  0  0  0  0
    7.2102    3.9523    1.5564 C   0  0  0  0  0  0  0  0  0  0  0  0
    6.7545    5.9766    0.2009 C   0  0  0  0  0  0  0  0  0  0  0  0
    7.2159    3.5439    0.0654 C   0  0  0  0  0  0  0  0  0  0  0  0
    7.0997    4.8367   -0.7693 C   0  0  0  0  0  0  0  0  0  0  0  0
    5.2996    6.2439    0.1313 C   0  0  0  0  0  0  0  0  0  0  0  0
    7.5722    7.2718   -0.1559 C   0  0  0  0  0  0  0  0  0  0  0  0
    3.2385    6.9003    1.2090 C   0  0  0  0  0  0  0  0  0  0  0  0
    2.5540    6.7276    0.0057 C   0  0  0  0  0  0  0  0  0  0  0  0
    4.6109    6.6578    1.2719 C   0  0  0  0  0  0  0  0  0  0  0  0
    3.2421    6.3123   -1.1345 C   0  0  0  0  0  0  0  0  0  0  0  0
    4.6145    6.0698   -1.0717 C   0  0  0  0  0  0  0  0  0  0  0  0
    7.1211    9.7048   -0.0171 C   0  0  0  0  0  0  0  0  0  0  0  0
    7.2292    8.4107    0.8153 C   0  0  0  0  0  0  0  0  0  0  0  0
    7.1062    9.2973   -1.5083 C   0  0  0  0  0  0  0  0  0  0  0  0
    7.2196    7.7595   -1.5693 C   0  0  0  0  0  0  0  0  0  0  0  0
    9.0271    7.0040   -0.0882 C   0  0  0  0  0  0  0  0  0  0  0  0
    9.7233    7.2284    1.0999 C   0  0  0  0  0  0  0  0  0  0  0  0
   11.0956    6.9855    1.1612 C   0  0  0  0  0  0  0  0  0  0  0  0
    9.7048    6.5393   -1.2160 C   0  0  0  0  0  0  0  0  0  0  0  0
   11.0772    6.2964   -1.1545 C   0  0  0  0  0  0  0  0  0  0  0  0
   11.7727    6.5194    0.0340 C   0  0  0  0  0  0  0  0  0  0  0  0
    8.0530    5.9121    1.9254 H   0  0  0  0  0  0  0  0  0  0  0  0
    6.3252    5.7814    2.3092 H   0  0  0  0  0  0  0  0  0  0  0  0
    6.3566    3.5072    2.0557 H   0  0  0  0  0  0  0  0  0  0  0  0
    8.1262    3.6262    2.0319 H   0  0  0  0  0  0  0  0  0  0  0  0
    6.3703    2.8962   -0.1415 H   0  0  0  0  0  0  0  0  0  0  0  0
    8.1397    3.0315   -0.1735 H   0  0  0  0  0  0  0  0  0  0  0  0
    8.0426    5.0435   -1.2602 H   0  0  0  0  0  0  0  0  0  0  0  0
    6.3144    4.7304   -1.5077 H   0  0  0  0  0  0  0  0  0  0  0  0
    2.7043    7.2227    2.0943 H   0  0  0  0  0  0  0  0  0  0  0  0
    1.4886    6.9160   -0.0432 H   0  0  0  0  0  0  0  0  0  0  0  0
    5.1419    6.7909    2.2064 H   0  0  0  0  0  0  0  0  0  0  0  0
    2.7105    6.1781   -2.0687 H   0  0  0  0  0  0  0  0  0  0  0  0
    5.1482    5.7465   -1.9568 H   0  0  0  0  0  0  0  0  0  0  0  0
    7.9770   10.3408    0.1811 H   0  0  0  0  0  0  0  0  0  0  0  0
    6.2074   10.2290    0.2315 H   0  0  0  0  0  0  0  0  0  0  0  0
    6.2836    8.2071    1.3030 H   0  0  0  0  0  0  0  0  0  0  0  0
    8.0133    8.5123    1.5568 H   0  0  0  0  0  0  0  0  0  0  0  0
    7.9496    9.7483   -2.0210 H   0  0  0  0  0  0  0  0  0  0  0  0
```

```
  6.1803    9.6183   -1.9694 H   0  0  0  0  0  0  0  0  0  0  0  0
  6.2742    7.3331   -1.8810 H   0  0  0  0  0  0  0  0  0  0  0  0
  8.0005    7.4739   -2.2635 H   0  0  0  0  0  0  0  0  0  0  0  0
  9.1981    7.5913    1.9747 H   0  0  0  0  0  0  0  0  0  0  0  0
 11.6357    7.1587    2.0840 H   0  0  0  0  0  0  0  0  0  0  0  0
  9.1653    6.3671   -2.1390 H   0  0  0  0  0  0  0  0  0  0  0  0
 11.6029    5.9346   -2.0297 H   0  0  0  0  0  0  0  0  0  0  0  0
 12.8381    6.3306    0.0817 H   0  0  0  0  0  0  0  0  0  0  0  0
  2  1  1  0  0  0  0
  1  3  1  0  0  0  0
  4  2  1  0  0  0  0
  3  5  1  0  0  0  0
  3  6  1  0  0  0  0
  3  7  1  0  0  0  0
  5  4  1  0  0  0  0
 12  6  4  0  0  0  0
  6 10  4  0  0  0  0
  7 14  1  0  0  0  0
 16  7  1  0  0  0  0
  7 17  1  0  0  0  0
  8  9  4  0  0  0  0
 10  8  4  0  0  0  0
  9 11  4  0  0  0  0
 11 12  4  0  0  0  0
 14 13  1  0  0  0  0
 13 15  1  0  0  0  0
 15 16  1  0  0  0  0
 18 17  4  0  0  0  0
 17 20  4  0  0  0  0
 19 18  4  0  0  0  0
 22 19  4  0  0  0  0
 20 21  4  0  0  0  0
 21 22  4  0  0  0  0
  1 23  1  0  0  0  0
  1 24  1  0  0  0  0
  2 25  1  0  0  0  0
  2 26  1  0  0  0  0
  4 27  1  0  0  0  0
  4 28  1  0  0  0  0
  5 29  1  0  0  0  0
  5 30  1  0  0  0  0
  8 31  1  0  0  0  0
  9 32  1  0  0  0  0
 10 33  1  0  0  0  0
 11 34  1  0  0  0  0
 12 35  1  0  0  0  0
 13 36  1  0  0  0  0
 13 37  1  0  0  0  0
 14 38  1  0  0  0  0
 14 39  1  0  0  0  0
 15 40  1  0  0  0  0
 15 41  1  0  0  0  0
 16 42  1  0  0  0  0
 16 43  1  0  0  0  0
 18 44  1  0  0  0  0
 19 45  1  0  0  0  0
 20 46  1  0  0  0  0
 21 47  1  0  0  0  0
 22 48  1  0  0  0  0
M  END
>  <Alias name>
1,1'-Diphenyl-1,1'-bicyclopentyl
```

```
>  <deltaH°(c)>   kJ/mol
-12343

$$$$
Tris(2-methylphenyl)ethylene
  ChemBrain IXL

 45 47  0  0  0  0  0  0  0  0  1 V2000
    8.3742    7.6868   -0.6022 C   0  0  0  0  0  0  0  0  0  0  0  0
    9.1279    8.8518   -0.7470 C   0  0  0  0  0  0  0  0  0  0  0  0
    9.2904    9.7165    0.3356 C   0  0  0  0  0  0  0  0  0  0  0  0
    8.7029    9.4140    1.5641 C   0  0  0  0  0  0  0  0  0  0  0  0
    7.9533    8.2466    1.7104 C   0  0  0  0  0  0  0  0  0  0  0  0
    7.7806    7.3878    0.6246 C   0  0  0  0  0  0  0  0  0  0  0  0
    8.2045    6.7682   -1.7501 C   0  0  0  0  0  0  0  0  0  0  0  0
    9.3736    3.8544   -0.7062 C   0  0  0  0  0  0  0  0  0  0  0  0
   10.7240    3.7637   -1.0445 C   0  0  0  0  0  0  0  0  0  0  0  0
   11.6393    4.6709   -0.5106 C   0  0  0  0  0  0  0  0  0  0  0  0
   11.2050    5.6661    0.3653 C   0  0  0  0  0  0  0  0  0  0  0  0
    9.8551    5.7552    0.7058 C   0  0  0  0  0  0  0  0  0  0  0  0
    8.9374    4.8565    0.1611 C   0  0  0  0  0  0  0  0  0  0  0  0
    8.4039    2.8876   -1.2674 C   0  0  0  0  0  0  0  0  0  0  0  0
    4.8851    5.1113    1.6476 C   0  0  0  0  0  0  0  0  0  0  0  0
    3.5578    5.2006    2.0672 C   0  0  0  0  0  0  0  0  0  0  0  0
    2.9090    6.4356    2.0710 C   0  0  0  0  0  0  0  0  0  0  0  0
    3.5866    7.5807    1.6521 C   0  0  0  0  0  0  0  0  0  0  0  0
    4.9115    7.4901    1.2250 C   0  0  0  0  0  0  0  0  0  0  0  0
    5.5609    6.2556    1.2242 C   0  0  0  0  0  0  0  0  0  0  0  0
    5.5765    3.8027    1.6515 C   0  0  0  0  0  0  0  0  0  0  0  0
    6.9669    6.1598    0.7743 C   0  0  0  0  0  0  0  0  0  0  0  0
    7.5009    4.9658    0.5036 C   0  0  0  0  0  0  0  0  0  0  0  0
    9.5868    9.0850   -1.6999 H   0  0  0  0  0  0  0  0  0  0  0  0
    9.8726   10.6227    0.2222 H   0  0  0  0  0  0  0  0  0  0  0  0
    8.8287   10.0856    2.4044 H   0  0  0  0  0  0  0  0  0  0  0  0
    7.5056    8.0069    2.6668 H   0  0  0  0  0  0  0  0  0  0  0  0
    9.1775    6.4729   -2.1230 H   0  0  0  0  0  0  0  0  0  0  0  0
    7.6540    7.2704   -2.5360 H   0  0  0  0  0  0  0  0  0  0  0  0
    7.6580    5.8893   -1.4314 H   0  0  0  0  0  0  0  0  0  0  0  0
   11.0617    2.9889   -1.7217 H   0  0  0  0  0  0  0  0  0  0  0  0
   12.6871    4.6026   -0.7760 H   0  0  0  0  0  0  0  0  0  0  0  0
   11.9159    6.3695    0.7809 H   0  0  0  0  0  0  0  0  0  0  0  0
    9.5195    6.5220    1.3929 H   0  0  0  0  0  0  0  0  0  0  0  0
    8.9369    2.1097   -1.8001 H   0  0  0  0  0  0  0  0  0  0  0  0
    7.8285    2.4455   -0.4635 H   0  0  0  0  0  0  0  0  0  0  0  0
    7.7368    3.4001   -1.9494 H   0  0  0  0  0  0  0  0  0  0  0  0
    3.0310    4.3112    2.3900 H   0  0  0  0  0  0  0  0  0  0  0  0
    1.8794    6.5053    2.3991 H   0  0  0  0  0  0  0  0  0  0  0  0
    3.0842    8.5401    1.6585 H   0  0  0  0  0  0  0  0  0  0  0  0
    5.4358    8.3781    0.8940 H   0  0  0  0  0  0  0  0  0  0  0  0
    6.5802    3.9259    2.0393 H   0  0  0  0  0  0  0  0  0  0  0  0
    5.6248    3.4169    0.6407 H   0  0  0  0  0  0  0  0  0  0  0  0
    5.0304    3.1078    2.2773 H   0  0  0  0  0  0  0  0  0  0  0  0
    6.8829    4.0771    0.5346 H   0  0  0  0  0  0  0  0  0  0  0  0
  1  2  4  0  0  0  0
  2  3  4  0  0  0  0
  3  4  4  0  0  0  0
  4  5  4  0  0  0  0
  5  6  4  0  0  0  0
  6  1  4  0  0  0  0
  1  7  1  0  0  0  0
  8  9  4  0  0  0  0
  9 10  4  0  0  0  0
 10 11  4  0  0  0  0
```

```
 11 12  4  0  0  0  0
 12 13  4  0  0  0  0
 13  8  4  0  0  0  0
  8 14  1  0  0  0  0
 15 16  4  0  0  0  0
 16 17  4  0  0  0  0
 17 18  4  0  0  0  0
 18 19  4  0  0  0  0
 19 20  4  0  0  0  0
 20 15  4  0  0  0  0
 15 21  1  0  0  0  0
  6 22  1  0  0  0  0
 22 23  2  0  0  0  0
 13 23  1  0  0  0  0
 20 22  1  0  0  0  0
  2 24  1  0  0  0  0
  3 25  1  0  0  0  0
  4 26  1  0  0  0  0
  5 27  1  0  0  0  0
  7 28  1  0  0  0  0
  7 29  1  0  0  0  0
  7 30  1  0  0  0  0
  9 31  1  0  0  0  0
 10 32  1  0  0  0  0
 11 33  1  0  0  0  0
 12 34  1  0  0  0  0
 14 35  1  0  0  0  0
 14 36  1  0  0  0  0
 14 37  1  0  0  0  0
 16 38  1  0  0  0  0
 17 39  1  0  0  0  0
 18 40  1  0  0  0  0
 19 41  1  0  0  0  0
 21 42  1  0  0  0  0
 21 43  1  0  0  0  0
 21 44  1  0  0  0  0
 23 45  1  0  0  0  0
M  END
>  <Alias name>
Tris(2-methylphenyl)ethylene

>  <deltaH°(c)>   kJ/mol
-12328.2

$$$$
Tris(4-methylphenyl)ethylene
  ChemBrain IXL

 45 47  0  0  0  0  0  0  0  0  1 V2000
    9.2914    7.2267   -0.8259 C   0  0  0  0  0  0  0  0  0  0  0  0
    9.8718    8.4870   -0.9705 C   0  0  0  0  0  0  0  0  0  0  0  0
    9.4078    9.5600   -0.2093 C   0  0  0  0  0  0  0  0  0  0  0  0
    8.3643    9.3725    0.6973 C   0  0  0  0  0  0  0  0  0  0  0  0
    7.7848    8.1119    0.8428 C   0  0  0  0  0  0  0  0  0  0  0  0
    8.2475    7.0393    0.0802 C   0  0  0  0  0  0  0  0  0  0  0  0
   10.6690    3.8725   -0.3750 C   0  0  0  0  0  0  0  0  0  0  0  0
   12.0575    4.0065   -0.3636 C   0  0  0  0  0  0  0  0  0  0  0  0
   12.6536    5.0279    0.3764 C   0  0  0  0  0  0  0  0  0  0  0  0
   11.8614    5.9136    1.1070 C   0  0  0  0  0  0  0  0  0  0  0  0
   10.4729    5.7788    1.0967 C   0  0  0  0  0  0  0  0  0  0  0  0
    9.8766    4.7595    0.3541 C   0  0  0  0  0  0  0  0  0  0  0  0
    5.5769    4.3316    0.5544 C   0  0  0  0  0  0  0  0  0  0  0  0
    4.1882    4.2039    0.5864 C   0  0  0  0  0  0  0  0  0  0  0  0
```

3.3797	5.3119	0.3320	C	0	0	0	0	0	0	0	0	0	0	0	0
3.9601	6.5472	0.0436	C	0	0	0	0	0	0	0	0	0	0	0	0
5.3488	6.6744	0.0089	C	0	0	0	0	0	0	0	0	0	0	0	0
6.1573	5.5669	0.2659	C	0	0	0	0	0	0	0	0	0	0	0	0
7.6307	5.7026	0.2324	C	0	0	0	0	0	0	0	0	0	0	0	0
8.4034	4.6192	0.3396	C	0	0	0	0	0	0	0	0	0	0	0	0
1.9064	5.1769	0.3681	C	0	0	0	0	0	0	0	0	0	0	0	0
10.0226	10.8974	-0.3637	C	0	0	0	0	0	0	0	0	0	0	0	0
14.1266	5.1717	0.3863	C	0	0	0	0	0	0	0	0	0	0	0	0
9.6514	6.3937	-1.4171	H	0	0	0	0	0	0	0	0	0	0	0	0
10.6826	8.6323	-1.6736	H	0	0	0	0	0	0	0	0	0	0	0	0
8.0041	10.2055	1.2882	H	0	0	0	0	0	0	0	0	0	0	0	0
6.9758	7.9659	1.5479	H	0	0	0	0	0	0	0	0	0	0	0	0
10.2061	3.0799	-0.9498	H	0	0	0	0	0	0	0	0	0	0	0	0
12.6726	3.3177	-0.9292	H	0	0	0	0	0	0	0	0	0	0	0	0
12.3242	6.7059	1.6822	H	0	0	0	0	0	0	0	0	0	0	0	0
9.8580	6.4654	1.6653	H	0	0	0	0	0	0	0	0	0	0	0	0
6.2046	3.4718	0.7535	H	0	0	0	0	0	0	0	0	0	0	0	0
3.7376	3.2445	0.8088	H	0	0	0	0	0	0	0	0	0	0	0	0
3.3325	7.4076	-0.1533	H	0	0	0	0	0	0	0	0	0	0	0	0
5.7994	7.6328	-0.2176	H	0	0	0	0	0	0	0	0	0	0	0	0
7.9521	3.6377	0.4159	H	0	0	0	0	0	0	0	0	0	0	0	0
1.6401	4.1271	0.3745	H	0	0	0	0	0	0	0	0	0	0	0	0
1.5221	5.6522	1.2621	H	0	0	0	0	0	0	0	0	0	0	0	0
1.4787	5.6524	-0.5059	H	0	0	0	0	0	0	0	0	0	0	0	0
9.2448	11.6501	-0.4010	H	0	0	0	0	0	0	0	0	0	0	0	0
10.5976	10.9267	-1.2810	H	0	0	0	0	0	0	0	0	0	0	0	0
10.6752	11.0940	0.4779	H	0	0	0	0	0	0	0	0	0	0	0	0
14.3866	6.2230	0.3899	H	0	0	0	0	0	0	0	0	0	0	0	0
14.5412	4.7002	-0.4961	H	0	0	0	0	0	0	0	0	0	0	0	0
14.5297	4.6971	1.2724	H	0	0	0	0	0	0	0	0	0	0	0	0

1	2	4	0	0	0	0
2	3	4	0	0	0	0
3	4	4	0	0	0	0
4	5	4	0	0	0	0
5	6	4	0	0	0	0
6	1	4	0	0	0	0
7	8	4	0	0	0	0
8	9	4	0	0	0	0
9	10	4	0	0	0	0
10	11	4	0	0	0	0
11	12	4	0	0	0	0
12	7	4	0	0	0	0
13	14	4	0	0	0	0
14	15	4	0	0	0	0
15	16	4	0	0	0	0
16	17	4	0	0	0	0
17	18	4	0	0	0	0
18	13	4	0	0	0	0
6	19	1	0	0	0	0
19	20	2	0	0	0	0
12	20	1	0	0	0	0
18	19	1	0	0	0	0
15	21	1	0	0	0	0
3	22	1	0	0	0	0
9	23	1	0	0	0	0
1	24	1	0	0	0	0
2	25	1	0	0	0	0
4	26	1	0	0	0	0
5	27	1	0	0	0	0
7	28	1	0	0	0	0
8	29	1	0	0	0	0
10	30	1	0	0	0	0

```
 11 31  1  0  0  0  0
 13 32  1  0  0  0  0
 14 33  1  0  0  0  0
 16 34  1  0  0  0  0
 17 35  1  0  0  0  0
 20 36  1  0  0  0  0
 21 37  1  0  0  0  0
 21 38  1  0  0  0  0
 21 39  1  0  0  0  0
 22 40  1  0  0  0  0
 22 41  1  0  0  0  0
 22 42  1  0  0  0  0
 23 43  1  0  0  0  0
 23 44  1  0  0  0  0
 23 45  1  0  0  0  0
M  END
>  <Alias name>
Tris(4-methylphenyl)ethylene

>  <deltaH°(c)>   kJ/mol
-12324.2

$$$$
1,3,5-Triphenylbenzene
  ChemBrain IXL

 42 45  0  0  0  0  0  0  0  0  1 V2000
    7.0122    8.7136    0.0000 C   0  0  0  0  0  0  0  0  0  0  0  0
    8.1102    9.5741   -0.0001 C   0  0  0  0  0  0  0  0  0  0  0  0
    9.4044    9.0535   -0.0048 C   0  0  0  0  0  0  0  0  0  0  0  0
    9.6005    7.6723   -0.0094 C   0  0  0  0  0  0  0  0  0  0  0  0
    8.5025    6.8119   -0.0093 C   0  0  0  0  0  0  0  0  0  0  0  0
    7.2084    7.3325   -0.0046 C   0  0  0  0  0  0  0  0  0  0  0  0
    7.6127    4.4861   -0.0130 C   0  0  0  0  0  0  0  0  0  0  0  0
    8.7107    5.3466   -0.0141 C   0  0  0  0  0  0  0  0  0  0  0  0
   10.0049    4.8259   -0.0198 C   0  0  0  0  0  0  0  0  0  0  0  0
   10.2011    3.4448   -0.0243 C   0  0  0  0  0  0  0  0  0  0  0  0
    9.1031    2.5843   -0.0232 C   0  0  0  0  0  0  0  0  0  0  0  0
    7.8089    3.1050   -0.0176 C   0  0  0  0  0  0  0  0  0  0  0  0
   12.7652   11.6873   -0.0052 C   0  0  0  0  0  0  0  0  0  0  0  0
   12.9613   10.3064    0.0172 C   0  0  0  0  0  0  0  0  0  0  0  0
   11.8633    9.4459    0.0173 C   0  0  0  0  0  0  0  0  0  0  0  0
   10.5692    9.9664   -0.0049 C   0  0  0  0  0  0  0  0  0  0  0  0
   10.3732   11.3474   -0.0273 C   0  0  0  0  0  0  0  0  0  0  0  0
   11.4712   12.2078   -0.0274 C   0  0  0  0  0  0  0  0  0  0  0  0
    3.0507   10.3073    0.0144 C   0  0  0  0  0  0  0  0  0  0  0  0
    4.1483   11.1672   -0.0293 C   0  0  0  0  0  0  0  0  0  0  0  0
    5.4425   10.6466   -0.0340 C   0  0  0  0  0  0  0  0  0  0  0  0
    5.6391    9.2660    0.0050 C   0  0  0  0  0  0  0  0  0  0  0  0
    4.5415    8.4061    0.0487 C   0  0  0  0  0  0  0  0  0  0  0  0
    3.2473    8.9268    0.0533 C   0  0  0  0  0  0  0  0  0  0  0  0
    7.9579   10.6464    0.0036 H   0  0  0  0  0  0  0  0  0  0  0  0
   10.6053    7.2681   -0.0131 H   0  0  0  0  0  0  0  0  0  0  0  0
    6.3559    6.6645   -0.0046 H   0  0  0  0  0  0  0  0  0  0  0  0
    6.6080    4.8903   -0.0086 H   0  0  0  0  0  0  0  0  0  0  0  0
   10.8573    5.4940   -0.0206 H   0  0  0  0  0  0  0  0  0  0  0  0
   11.2058    3.0406   -0.0287 H   0  0  0  0  0  0  0  0  0  0  0  0
    9.2554    1.5121   -0.0268 H   0  0  0  0  0  0  0  0  0  0  0  0
    6.9565    2.4369   -0.0167 H   0  0  0  0  0  0  0  0  0  0  0  0
   13.6177   12.3554   -0.0053 H   0  0  0  0  0  0  0  0  0  0  0  0
   13.9660    9.9023    0.0345 H   0  0  0  0  0  0  0  0  0  0  0  0
   12.0155    8.3738    0.0347 H   0  0  0  0  0  0  0  0  0  0  0  0
    9.3685   11.7514   -0.0446 H   0  0  0  0  0  0  0  0  0  0  0  0
```

11.3190   13.2799   -0.0448 H   0 0 0 0 0 0 0 0 0 0 0 0
2.0460   10.7115   0.0180 H   0 0 0 0 0 0 0 0 0 0 0 0
3.9957   12.2390   -0.0596 H   0 0 0 0 0 0 0 0 0 0 0 0
6.2947   11.3141   -0.0679 H   0 0 0 0 0 0 0 0 0 0 0 0
4.6941   7.3344   0.0790 H   0 0 0 0 0 0 0 0 0 0 0 0
2.3952   8.2592   0.0872 H   0 0 0 0 0 0 0 0 0 0 0 0
1 2 4 0 0 0 0
2 3 4 0 0 0 0
3 4 4 0 0 0 0
4 5 4 0 0 0 0
5 6 4 0 0 0 0
6 1 4 0 0 0 0
7 8 4 0 0 0 0
8 9 4 0 0 0 0
9 10 4 0 0 0 0
10 11 4 0 0 0 0
11 12 4 0 0 0 0
12 7 4 0 0 0 0
13 14 4 0 0 0 0
14 15 4 0 0 0 0
15 16 4 0 0 0 0
16 17 4 0 0 0 0
17 18 4 0 0 0 0
18 13 4 0 0 0 0
19 20 4 0 0 0 0
20 21 4 0 0 0 0
21 22 4 0 0 0 0
22 23 4 0 0 0 0
23 24 4 0 0 0 0
24 19 4 0 0 0 0
22 1 1 0 0 0 0
8 5 1 0 0 0 0
16 3 1 0 0 0 0
2 25 1 0 0 0 0
4 26 1 0 0 0 0
6 27 1 0 0 0 0
7 28 1 0 0 0 0
9 29 1 0 0 0 0
10 30 1 0 0 0 0
11 31 1 0 0 0 0
12 32 1 0 0 0 0
13 33 1 0 0 0 0
14 34 1 0 0 0 0
15 35 1 0 0 0 0
17 36 1 0 0 0 0
18 37 1 0 0 0 0
19 38 1 0 0 0 0
20 39 1 0 0 0 0
21 40 1 0 0 0 0
23 41 1 0 0 0 0
24 42 1 0 0 0 0
M END
> <Alias name>
1,3,5-Triphenylbenzene

> <deltaH°(c)>   kJ/mol
-12279.34

$$$$
Brucine
ChemBrain IXL

55 61 0 0 0 0 0 0 0 0 1 V2000

```
 11.2782   -3.2303   -5.3082 C   0  0  0  0  0  0  0  0  0  0  0  0  0
 11.5978   -4.7189   -4.8292 C   0  0  0  0  0  0  0  0  0  0  0  0  0
  9.9180   -3.1056   -5.0256 C   0  0  0  0  0  0  0  0  0  0  0  0  0
 12.1444   -2.0803   -4.6413 C   0  0  0  0  0  0  0  0  0  0  0  0  0
 11.4626   -3.0804   -6.8002 C   0  0  0  0  0  0  0  0  0  0  0  0  0
 12.4082   -4.7870   -3.6795 C   0  0  0  0  0  0  0  0  0  0  0  0  0
 10.4015   -5.1719   -4.4551 N   0  0  0  0  0  0  0  0  0  0  0  0  0
  9.4241   -4.2196   -4.5786 C   0  0  0  0  0  0  0  0  0  0  0  0  0
  9.1401   -1.9624   -5.2027 C   0  0  0  0  0  0  0  0  0  0  0  0  0
 13.0064   -1.5788   -5.7415 N   0  0  0  0  0  0  0  0  0  0  0  0  0
 13.0277   -2.6086   -3.4717 C   0  0  0  0  0  0  0  0  0  0  0  0  0
 12.2660   -1.7891   -6.9925 C   0  0  0  0  0  0  0  0  0  0  0  0  0
 13.5019   -3.8955   -3.8842 C   0  0  0  0  0  0  0  0  0  0  0  0  0
 12.8282   -6.2267   -3.4526 C   0  0  0  0  0  0  0  0  0  0  0  0  0
 10.2905   -6.4542   -3.8876 C   0  0  0  0  0  0  0  0  0  0  0  0  0
  8.0445   -4.3286   -4.3027 C   0  0  0  0  0  0  0  0  0  0  0  0  0
  7.7694   -2.0196   -4.9481 C   0  0  0  0  0  0  0  0  0  0  0  0  0
 14.1930   -2.4180   -5.7979 C   0  0  0  0  0  0  0  0  0  0  0  0  0
 13.8147   -3.7537   -5.3372 C   0  0  0  0  0  0  0  0  0  0  0  0  0
 11.5299   -7.1919   -3.4433 C   0  0  0  0  0  0  0  0  0  0  0  0  0
 13.7237   -6.6006   -4.5213 O   0  0  0  0  0  0  0  0  0  0  0  0  0
  9.1894   -6.9733   -3.7637 O   0  0  0  0  0  0  0  0  0  0  0  0  0
  7.2184   -3.1938   -4.4462 C   0  0  0  0  0  0  0  0  0  0  0  0  0
  6.9065   -0.8474   -5.2086 O   0  0  0  0  0  0  0  0  0  0  0  0  0
 13.7396   -4.8021   -6.2395 C   0  0  0  0  0  0  0  0  0  0  0  0  0
 13.2502   -6.2102   -5.8576 C   0  0  0  0  0  0  0  0  0  0  0  0  0
  5.7702   -3.2220   -4.0643 O   0  0  0  0  0  0  0  0  0  0  0  0  0
  7.5179    0.4613   -4.9184 C   0  0  0  0  0  0  0  0  0  0  0  0  0
  5.0584   -4.5103   -4.2491 C   0  0  0  0  0  0  0  0  0  0  0  0  0
 12.0016   -5.3164   -5.6353 H   0  0  0  0  0  0  0  0  0  0  0  0  0
 11.4955   -1.2897   -4.2854 H   0  0  0  0  0  0  0  0  0  0  0  0  0
 10.4978   -2.9992   -7.2882 H   0  0  0  0  0  0  0  0  0  0  0  0  0
 12.0110   -3.9265   -7.1940 H   0  0  0  0  0  0  0  0  0  0  0  0  0
 11.8308   -4.4508   -2.8229 H   0  0  0  0  0  0  0  0  0  0  0  0  0
  9.5937   -1.0385   -5.5327 H   0  0  0  0  0  0  0  0  0  0  0  0  0
 12.4314   -2.7061   -2.5676 H   0  0  0  0  0  0  0  0  0  0  0  0  0
 13.8594   -1.9447   -3.2991 H   0  0  0  0  0  0  0  0  0  0  0  0  0
 11.5938   -0.9586   -7.1683 H   0  0  0  0  0  0  0  0  0  0  0  0  0
 12.9515   -1.8968   -7.8183 H   0  0  0  0  0  0  0  0  0  0  0  0  0
 14.3700   -4.2014   -3.3147 H   0  0  0  0  0  0  0  0  0  0  0  0  0
 13.3381   -6.3093   -2.5015 H   0  0  0  0  0  0  0  0  0  0  0  0  0
  7.6177   -5.2745   -3.9851 H   0  0  0  0  0  0  0  0  0  0  0  0  0
 14.9600   -2.0149   -5.1511 H   0  0  0  0  0  0  0  0  0  0  0  0  0
 14.5570   -2.4723   -6.8126 H   0  0  0  0  0  0  0  0  0  0  0  0  0
 11.6989   -8.0288   -4.1092 H   0  0  0  0  0  0  0  0  0  0  0  0  0
 11.3702   -7.5685   -2.4359 H   0  0  0  0  0  0  0  0  0  0  0  0  0
 14.0349   -4.6226   -7.2616 H   0  0  0  0  0  0  0  0  0  0  0  0  0
 13.6278   -6.9257   -6.5840 H   0  0  0  0  0  0  0  0  0  0  0  0  0
 12.1644   -6.2324   -5.8722 H   0  0  0  0  0  0  0  0  0  0  0  0  0
  6.7931    1.2477   -5.0984 H   0  0  0  0  0  0  0  0  0  0  0  0  0
  8.3763    0.6084   -5.5613 H   0  0  0  0  0  0  0  0  0  0  0  0  0
  7.8329    0.4917   -3.8827 H   0  0  0  0  0  0  0  0  0  0  0  0  0
  5.3000   -4.9264   -5.2192 H   0  0  0  0  0  0  0  0  0  0  0  0  0
  5.3591   -5.2019   -3.4733 H   0  0  0  0  0  0  0  0  0  0  0  0  0
  3.9848   -4.3435   -4.1861 H   0  0  0  0  0  0  0  0  0  0  0  0  0
  1  4  1  0  0  0  0
  1  5  1  0  0  0  0
  2  6  1  0  0  0  0
  2  7  1  0  0  0  0
  3  8  4  0  0  0  0
  3  9  4  0  0  0  0
  4 10  1  0  0  0  0
  4 11  1  0  0  0  0
```

```
  5 12  1  0  0  0  0
  6 13  1  0  0  0  0
  6 14  1  0  0  0  0
  7 15  1  0  0  0  0
  7  8  1  0  0  0  0
  8 16  4  0  0  0  0
  9 17  4  0  0  0  0
 10 18  1  0  0  0  0
 10 12  1  0  0  0  0
 11 13  1  0  0  0  0
 13 19  1  0  0  0  0
 14 20  1  0  0  0  0
 14 21  1  0  0  0  0
 15 22  2  0  0  0  0
 15 20  1  0  0  0  0
 16 23  4  0  0  0  0
 17 24  1  0  0  0  0
 17 23  4  0  0  0  0
 18 19  1  0  0  0  0
 19 25  2  0  0  0  0
 21 26  1  0  0  0  0
 23 27  1  0  0  0  0
 24 28  1  0  0  0  0
 25 26  1  0  0  0  0
 27 29  1  0  0  0  0
  1  2  1  0  0  0  0
  1  3  1  0  0  0  0
  2 30  1  0  0  0  0
  4 31  1  0  0  0  0
  5 32  1  0  0  0  0
  5 33  1  0  0  0  0
  6 34  1  0  0  0  0
  9 35  1  0  0  0  0
 11 36  1  0  0  0  0
 11 37  1  0  0  0  0
 12 38  1  0  0  0  0
 12 39  1  0  0  0  0
 13 40  1  0  0  0  0
 14 41  1  0  0  0  0
 16 42  1  0  0  0  0
 18 43  1  0  0  0  0
 18 44  1  0  0  0  0
 20 45  1  0  0  0  0
 20 46  1  0  0  0  0
 25 47  1  0  0  0  0
 26 48  1  0  0  0  0
 26 49  1  0  0  0  0
 28 50  1  0  0  0  0
 28 51  1  0  0  0  0
 28 52  1  0  0  0  0
 29 53  1  0  0  0  0
 29 54  1  0  0  0  0
 29 55  1  0  0  0  0
M  END
>  <Alias name>
Brucine

>  <deltaH°(c)>   kJ/mol
-12279

$$$$
Tetraphenoxysilane
  ChemBrain IXL
```

```
49 52  0  0  0  0  0  0  0  0  1 V2000
   7.3619    6.6233    0.3258 Si  0  0  0  0  0  0  0  0  0  0  0  0
   5.7982    7.1096    0.3538 O   0  0  0  0  0  0  0  0  0  0  0  0
   3.3097    5.3809    3.2334 C   0  0  0  0  0  0  0  0  0  0  0  0
   2.8091    6.3381    2.3506 C   0  0  0  0  0  0  0  0  0  0  0  0
   3.6466    6.9106    1.3918 C   0  0  0  0  0  0  0  0  0  0  0  0
   4.9877    6.5285    1.3117 C   0  0  0  0  0  0  0  0  0  0  0  0
   5.4879    5.5665    2.2000 C   0  0  0  0  0  0  0  0  0  0  0  0
   4.6484    4.9954    3.1580 C   0  0  0  0  0  0  0  0  0  0  0  0
   8.0993    7.0806    1.7146 O   0  0  0  0  0  0  0  0  0  0  0  0
  12.1859    6.2749    2.0638 C   0  0  0  0  0  0  0  0  0  0  0  0
  11.5078    5.9083    0.9010 C   0  0  0  0  0  0  0  0  0  0  0  0
  10.1430    6.1728    0.7743 C   0  0  0  0  0  0  0  0  0  0  0  0
   9.4510    6.8073    1.8150 C   0  0  0  0  0  0  0  0  0  0  0  0
  10.1350    7.1721    2.9769 C   0  0  0  0  0  0  0  0  0  0  0  0
  11.4998    6.9066    3.1013 C   0  0  0  0  0  0  0  0  0  0  0  0
   7.4262    4.9929    0.1867 O   0  0  0  0  0  0  0  0  0  0  0  0
   5.7028    3.2951   -3.2220 C   0  0  0  0  0  0  0  0  0  0  0  0
   6.3000    2.4859   -2.2552 C   0  0  0  0  0  0  0  0  0  0  0  0
   6.8733    3.0607   -1.1197 C   0  0  0  0  0  0  0  0  0  0  0  0
   6.8519    4.4464   -0.9462 C   0  0  0  0  0  0  0  0  0  0  0  0
   6.2511    5.2570   -1.9192 C   0  0  0  0  0  0  0  0  0  0  0  0
   5.6784    4.6799   -3.0539 C   0  0  0  0  0  0  0  0  0  0  0  0
   8.1243    7.3095   -0.9512 O   0  0  0  0  0  0  0  0  0  0  0  0
   8.2944   11.4847   -1.0609 C   0  0  0  0  0  0  0  0  0  0  0  0
   7.6588   10.8484    0.0056 C   0  0  0  0  0  0  0  0  0  0  0  0
   7.5965    9.4547    0.0519 C   0  0  0  0  0  0  0  0  0  0  0  0
   8.1722    8.6914   -0.9730 C   0  0  0  0  0  0  0  0  0  0  0  0
   8.8073    9.3338   -2.0383 C   0  0  0  0  0  0  0  0  0  0  0  0
   8.8684   10.7277   -2.0824 C   0  0  0  0  0  0  0  0  0  0  0  0
   2.6594    4.9372    3.9770 H   0  0  0  0  0  0  0  0  0  0  0  0
   1.7700    6.6372    2.4093 H   0  0  0  0  0  0  0  0  0  0  0  0
   3.2553    7.6534    0.7078 H   0  0  0  0  0  0  0  0  0  0  0  0
   6.5248    5.2632    2.1466 H   0  0  0  0  0  0  0  0  0  0  0  0
   5.0367    4.2523    3.8433 H   0  0  0  0  0  0  0  0  0  0  0  0
  13.2446    6.0692    2.1611 H   0  0  0  0  0  0  0  0  0  0  0  0
  12.0407    5.4180    0.0959 H   0  0  0  0  0  0  0  0  0  0  0  0
   9.6231    5.8861   -0.1298 H   0  0  0  0  0  0  0  0  0  0  0  0
   9.6049    7.6626    3.7837 H   0  0  0  0  0  0  0  0  0  0  0  0
  12.0262    7.1911    4.0038 H   0  0  0  0  0  0  0  0  0  0  0  0
   5.2582    2.8486   -4.1027 H   0  0  0  0  0  0  0  0  0  0  0  0
   6.3189    1.4111   -2.3858 H   0  0  0  0  0  0  0  0  0  0  0  0
   7.3362    2.4301   -0.3708 H   0  0  0  0  0  0  0  0  0  0  0  0
   6.2289    6.3312   -1.7948 H   0  0  0  0  0  0  0  0  0  0  0  0
   5.2147    5.3077   -3.8045 H   0  0  0  0  0  0  0  0  0  0  0  0
   8.3422   12.5660   -1.0958 H   0  0  0  0  0  0  0  0  0  0  0  0
   7.2134   11.4364    0.7984 H   0  0  0  0  0  0  0  0  0  0  0  0
   7.1023    8.9674    0.8815 H   0  0  0  0  0  0  0  0  0  0  0  0
   9.2538    8.7486   -2.8326 H   0  0  0  0  0  0  0  0  0  0  0  0
   9.3618   11.2216   -2.9102 H   0  0  0  0  0  0  0  0  0  0  0  0
  2  1  1  0  0  0  0
  3  4  4  0  0  0  0
  4  5  4  0  0  0  0
  5  6  4  0  0  0  0
  6  7  4  0  0  0  0
  7  8  4  0  0  0  0
  8  3  4  0  0  0  0
  6  2  1  0  0  0  0
 10 11  4  0  0  0  0
 11 12  4  0  0  0  0
 12 13  4  0  0  0  0
 13 14  4  0  0  0  0
```

```
 14 15  4  0  0  0  0
 15 10  4  0  0  0  0
 13  9  1  0  0  0  0
  9  1  1  0  0  0  0
 17 18  4  0  0  0  0
 18 19  4  0  0  0  0
 19 20  4  0  0  0  0
 20 21  4  0  0  0  0
 21 22  4  0  0  0  0
 22 17  4  0  0  0  0
 20 16  1  0  0  0  0
 24 25  4  0  0  0  0
 25 26  4  0  0  0  0
 26 27  4  0  0  0  0
 27 28  4  0  0  0  0
 28 29  4  0  0  0  0
 29 24  4  0  0  0  0
 27 23  1  0  0  0  0
 23  1  1  0  0  0  0
 16  1  1  0  0  0  0
  3 30  1  0  0  0  0
  4 31  1  0  0  0  0
  5 32  1  0  0  0  0
  7 33  1  0  0  0  0
  8 34  1  0  0  0  0
 10 35  1  0  0  0  0
 11 36  1  0  0  0  0
 12 37  1  0  0  0  0
 14 38  1  0  0  0  0
 15 39  1  0  0  0  0
 17 40  1  0  0  0  0
 18 41  1  0  0  0  0
 19 42  1  0  0  0  0
 21 43  1  0  0  0  0
 22 44  1  0  0  0  0
 24 45  1  0  0  0  0
 25 46  1  0  0  0  0
 26 47  1  0  0  0  0
 28 48  1  0  0  0  0
 29 49  1  0  0  0  0
M  END
>  <Alias name>
Tetraphenoxysilane

>  <deltaH°(c)>   kJ/mol
-12270

$$$$
1,2,4-Triphenylbenzene
  ChemBrain IXL

 42 45  0  0  0  0  0  0  0  0  1 V2000
    8.9793    4.5694   -0.0531 C   0  0  0  0  0  0  0  0  0  0  0  0
    7.5849    4.5395   -0.0576 C   0  0  0  0  0  0  0  0  0  0  0  0
    6.8620    5.7321   -0.0462 C   0  0  0  0  0  0  0  0  0  0  0  0
    7.5360    6.9571   -0.0305 C   0  0  0  0  0  0  0  0  0  0  0  0
    8.9298    6.9851   -0.0252 C   0  0  0  0  0  0  0  0  0  0  0  0
    9.6509    5.7920   -0.0371 C   0  0  0  0  0  0  0  0  0  0  0  0
   11.8507    4.6308   -0.0270 C   0  0  0  0  0  0  0  0  0  0  0  0
   11.1283    5.8236   -0.0328 C   0  0  0  0  0  0  0  0  0  0  0  0
   11.7988    7.0463   -0.0345 C   0  0  0  0  0  0  0  0  0  0  0  0
   13.1931    7.0764   -0.0306 C   0  0  0  0  0  0  0  0  0  0  0  0
   13.9163    5.8836   -0.0249 C   0  0  0  0  0  0  0  0  0  0  0  0
```

```
 13.2451    4.6607   -0.0231 C   0  0  0  0  0  0  0  0  0  0  0  0
  5.3540   10.6298    0.0142 C   0  0  0  0  0  0  0  0  0  0  0  0
  6.4818   10.4977   -0.7961 C   0  0  0  0  0  0  0  0  0  0  0  0
  7.1904    9.2961   -0.8152 C   0  0  0  0  0  0  0  0  0  0  0  0
  6.7745    8.2278   -0.0199 C   0  0  0  0  0  0  0  0  0  0  0  0
  5.6427    8.3580    0.7858 C   0  0  0  0  0  0  0  0  0  0  0  0
  4.9341    9.5598    0.8047 C   0  0  0  0  0  0  0  0  0  0  0  0
  2.5900    5.6192   -0.0653 C   0  0  0  0  0  0  0  0  0  0  0  0
  3.3091    4.6999    0.6989 C   0  0  0  0  0  0  0  0  0  0  0  0
  4.7038    4.7389    0.7073 C   0  0  0  0  0  0  0  0  0  0  0  0
  5.3802    5.6957   -0.0505 C   0  0  0  0  0  0  0  0  0  0  0  0
  4.6604    6.6167   -0.8128 C   0  0  0  0  0  0  0  0  0  0  0  0
  3.2656    6.5777   -0.8209 C   0  0  0  0  0  0  0  0  0  0  0  0
  9.5406    3.6433   -0.0621 H   0  0  0  0  0  0  0  0  0  0  0  0
  7.0635    3.5902   -0.0699 H   0  0  0  0  0  0  0  0  0  0  0  0
  9.4522    7.9335   -0.0118 H   0  0  0  0  0  0  0  0  0  0  0  0
 11.3291    3.6816   -0.0256 H   0  0  0  0  0  0  0  0  0  0  0  0
 11.2368    7.9722   -0.0389 H   0  0  0  0  0  0  0  0  0  0  0  0
 13.7141    8.0257   -0.0320 H   0  0  0  0  0  0  0  0  0  0  0  0
 14.9988    5.9068   -0.0219 H   0  0  0  0  0  0  0  0  0  0  0  0
 13.8064    3.7347   -0.0187 H   0  0  0  0  0  0  0  0  0  0  0  0
  4.8044   11.5630    0.0296 H   0  0  0  0  0  0  0  0  0  0  0  0
  6.8073   11.3281   -1.4104 H   0  0  0  0  0  0  0  0  0  0  0  0
  8.0635    9.1925   -1.4474 H   0  0  0  0  0  0  0  0  0  0  0  0
  5.3142    7.5262    1.3965 H   0  0  0  0  0  0  0  0  0  0  0  0
  4.0579    9.6620    1.4332 H   0  0  0  0  0  0  0  0  0  0  0  0
  1.5073    5.5887   -0.0719 H   0  0  0  0  0  0  0  0  0  0  0  0
  2.7845    3.9558    1.2857 H   0  0  0  0  0  0  0  0  0  0  0  0
  5.2615    4.0263    1.3021 H   0  0  0  0  0  0  0  0  0  0  0  0
  5.1844    7.3620   -1.3982 H   0  0  0  0  0  0  0  0  0  0  0  0
  2.7073    7.2916   -1.4139 H   0  0  0  0  0  0  0  0  0  0  0  0
  1  2  4  0  0  0  0
  2  3  4  0  0  0  0
  3  4  4  0  0  0  0
  4  5  4  0  0  0  0
  5  6  4  0  0  0  0
  6  1  4  0  0  0  0
  7  8  4  0  0  0  0
  8  9  4  0  0  0  0
  9 10  4  0  0  0  0
 10 11  4  0  0  0  0
 11 12  4  0  0  0  0
 12  7  4  0  0  0  0
 13 14  4  0  0  0  0
 14 15  4  0  0  0  0
 15 16  4  0  0  0  0
 16 17  4  0  0  0  0
 17 18  4  0  0  0  0
 18 13  4  0  0  0  0
 19 20  4  0  0  0  0
 20 21  4  0  0  0  0
 21 22  4  0  0  0  0
 22 23  4  0  0  0  0
 23 24  4  0  0  0  0
 24 19  4  0  0  0  0
 22  3  1  0  0  0  0
 16  4  1  0  0  0  0
  8  6  1  0  0  0  0
  1 25  1  0  0  0  0
  2 26  1  0  0  0  0
  5 27  1  0  0  0  0
  7 28  1  0  0  0  0
  9 29  1  0  0  0  0
```

```
 10 30  1  0  0  0  0
 11 31  1  0  0  0  0
 12 32  1  0  0  0  0
 13 33  1  0  0  0  0
 14 34  1  0  0  0  0
 15 35  1  0  0  0  0
 17 36  1  0  0  0  0
 18 37  1  0  0  0  0
 19 38  1  0  0  0  0
 20 39  1  0  0  0  0
 21 40  1  0  0  0  0
 23 41  1  0  0  0  0
 24 42  1  0  0  0  0
M  END
>  <Alias name>
1,2,4-Triphenylbenzene

>  <deltaH°(c)>   kJ/mol
-12260

$$$$
1,2,3-Triphenylbenzene
  ChemBrain IXL

 42 45  0  0  0  0  0  0  0  0  1 V2000
    7.0026    3.2212    0.8870 C   0  0  0  0  0  0  0  0  0  0  0  0
    5.9071    3.9508    0.4251 C   0  0  0  0  0  0  0  0  0  0  0  0
    6.0258    5.3232    0.2049 C   0  0  0  0  0  0  0  0  0  0  0  0
    7.2406    5.9657    0.4448 C   0  0  0  0  0  0  0  0  0  0  0  0
    8.3364    5.2359    0.9058 C   0  0  0  0  0  0  0  0  0  0  0  0
    8.2172    3.8638    1.1278 C   0  0  0  0  0  0  0  0  0  0  0  0
   10.8253    5.2819    0.8387 C   0  0  0  0  0  0  0  0  0  0  0  0
    9.6255    5.9173    1.1595 C   0  0  0  0  0  0  0  0  0  0  0  0
    9.6408    7.1949    1.7193 C   0  0  0  0  0  0  0  0  0  0  0  0
   10.8561    7.8340    1.9655 C   0  0  0  0  0  0  0  0  0  0  0  0
   12.0560    7.1969    1.6488 C   0  0  0  0  0  0  0  0  0  0  0  0
   12.0406    5.9209    1.0851 C   0  0  0  0  0  0  0  0  0  0  0  0
    7.6041   10.1659   -0.2326 C   0  0  0  0  0  0  0  0  0  0  0  0
    8.5699    9.2925   -0.7330 C   0  0  0  0  0  0  0  0  0  0  0  0
    8.4513    7.9203   -0.5115 C   0  0  0  0  0  0  0  0  0  0  0  0
    7.3667    7.4215    0.2103 C   0  0  0  0  0  0  0  0  0  0  0  0
    6.4011    8.2949    0.7111 C   0  0  0  0  0  0  0  0  0  0  0  0
    6.5198    9.6671    0.4895 C   0  0  0  0  0  0  0  0  0  0  0  0
    2.6692    7.5493   -1.2131 C   0  0  0  0  0  0  0  0  0  0  0  0
    3.9496    7.8434   -1.6821 C   0  0  0  0  0  0  0  0  0  0  0  0
    5.0467    7.1187   -1.2159 C   0  0  0  0  0  0  0  0  0  0  0  0
    4.8629    6.0977   -0.2834 C   0  0  0  0  0  0  0  0  0  0  0  0
    3.5830    5.8066    0.1890 C   0  0  0  0  0  0  0  0  0  0  0  0
    2.4859    6.5311   -0.2774 C   0  0  0  0  0  0  0  0  0  0  0  0
    6.9103    2.1559    1.0587 H   0  0  0  0  0  0  0  0  0  0  0  0
    4.9644    3.4518    0.2374 H   0  0  0  0  0  0  0  0  0  0  0  0
    9.0675    3.2975    1.4871 H   0  0  0  0  0  0  0  0  0  0  0  0
   10.8132    4.2926    0.3983 H   0  0  0  0  0  0  0  0  0  0  0  0
    8.7092    7.6906    1.9629 H   0  0  0  0  0  0  0  0  0  0  0  0
   10.8680    8.8249    2.4026 H   0  0  0  0  0  0  0  0  0  0  0  0
   12.9995    7.6928    1.8405 H   0  0  0  0  0  0  0  0  0  0  0  0
   12.9721    5.4265    0.8388 H   0  0  0  0  0  0  0  0  0  0  0  0
    7.6962   11.2311   -0.4046 H   0  0  0  0  0  0  0  0  0  0  0  0
    9.4118    9.6797   -1.2935 H   0  0  0  0  0  0  0  0  0  0  0  0
    9.2012    7.2422   -0.8999 H   0  0  0  0  0  0  0  0  0  0  0  0
    5.5594    7.9077    1.2718 H   0  0  0  0  0  0  0  0  0  0  0  0
    5.7700   10.3452    0.8781 H   0  0  0  0  0  0  0  0  0  0  0  0
    1.8174    8.1114   -1.5755 H   0  0  0  0  0  0  0  0  0  0  0  0
```

```
    4.0920    8.6343   -2.4081 H   0  0  0  0  0  0  0  0  0  0  0  0
    6.0411    7.3489   -1.5780 H   0  0  0  0  0  0  0  0  0  0  0  0
    3.4411    5.0181    0.9178 H   0  0  0  0  0  0  0  0  0  0  0  0
    1.4919    6.3030    0.0870 H   0  0  0  0  0  0  0  0  0  0  0  0
  1  2  4  0  0  0  0
  2  3  4  0  0  0  0
  3  4  4  0  0  0  0
  4  5  4  0  0  0  0
  5  6  4  0  0  0  0
  6  1  4  0  0  0  0
  7  8  4  0  0  0  0
  8  9  4  0  0  0  0
  9 10  4  0  0  0  0
 10 11  4  0  0  0  0
 11 12  4  0  0  0  0
 12  7  4  0  0  0  0
 13 14  4  0  0  0  0
 14 15  4  0  0  0  0
 15 16  4  0  0  0  0
 16 17  4  0  0  0  0
 17 18  4  0  0  0  0
 18 13  4  0  0  0  0
 19 20  4  0  0  0  0
 20 21  4  0  0  0  0
 21 22  4  0  0  0  0
 22 23  4  0  0  0  0
 23 24  4  0  0  0  0
 24 19  4  0  0  0  0
 22  3  1  0  0  0  0
 16  4  1  0  0  0  0
  8  5  1  0  0  0  0
  1 25  1  0  0  0  0
  2 26  1  0  0  0  0
  6 27  1  0  0  0  0
  7 28  1  0  0  0  0
  9 29  1  0  0  0  0
 10 30  1  0  0  0  0
 11 31  1  0  0  0  0
 12 32  1  0  0  0  0
 13 33  1  0  0  0  0
 14 34  1  0  0  0  0
 15 35  1  0  0  0  0
 17 36  1  0  0  0  0
 18 37  1  0  0  0  0
 19 38  1  0  0  0  0
 20 39  1  0  0  0  0
 21 40  1  0  0  0  0
 23 41  1  0  0  0  0
 24 42  1  0  0  0  0
M  END
>  <Alias name>
1,2,3-Triphenylbenzene

>  <deltaH°(c)>   kJ/mol
-12259

$$$$
p-Quaterphenyl
  ChemBrain IXL

 42 45  0  0  0  0  0  0  0  0  1 V2000
   11.7640    8.5792   -0.3900 C   0  0  0  0  0  0  0  0  0  0  0  0
   10.8048    9.5322   -0.0467 C   0  0  0  0  0  0  0  0  0  0  0  0
```

11.1993 10.8221 0.3093 C 0 0 0 0 0 0 0 0 0 0 0 0 0
12.5529 11.1589 0.3222 C 0 0 0 0 0 0 0 0 0 0 0 0 0
13.5121 10.2059 -0.0210 C 0 0 0 0 0 0 0 0 0 0 0 0 0
13.1176 8.9161 -0.3771 C 0 0 0 0 0 0 0 0 0 0 0 0 0
17.6556 11.2369 0.0183 C 0 0 0 0 0 0 0 0 0 0 0 0 0
16.6983 12.1810 0.3902 C 0 0 0 0 0 0 0 0 0 0 0 0 0
15.3447 11.8442 0.3774 C 0 0 0 0 0 0 0 0 0 0 0 0 0
14.9483 10.5632 -0.0074 C 0 0 0 0 0 0 0 0 0 0 0 0 0
15.9055 9.6191 -0.3793 C 0 0 0 0 0 0 0 0 0 0 0 0 0
17.2592 9.9559 -0.3665 C 0 0 0 0 0 0 0 0 0 0 0 0 0
2.9070 8.7853 0.1302 C 0 0 0 0 0 0 0 0 0 0 0 0 0
2.5178 7.4703 -0.1254 C 0 0 0 0 0 0 0 0 0 0 0 0 0
3.4823 6.4921 -0.3681 C 0 0 0 0 0 0 0 0 0 0 0 0 0
4.8360 6.8289 -0.3553 C 0 0 0 0 0 0 0 0 0 0 0 0 0
5.2252 8.1439 -0.0997 C 0 0 0 0 0 0 0 0 0 0 0 0 0
4.2607 9.1221 0.1430 C 0 0 0 0 0 0 0 0 0 0 0 0 0
9.3686 9.1749 -0.0604 C 0 0 0 0 0 0 0 0 0 0 0 0 0
8.4088 10.1308 0.2729 C 0 0 0 0 0 0 0 0 0 0 0 0 0
7.0552 9.7939 0.2601 C 0 0 0 0 0 0 0 0 0 0 0 0 0
6.6613 8.5012 -0.0861 C 0 0 0 0 0 0 0 0 0 0 0 0 0
7.6211 7.5454 -0.4194 C 0 0 0 0 0 0 0 0 0 0 0 0 0
8.9748 7.8822 -0.4066 C 0 0 0 0 0 0 0 0 0 0 0 0 0
11.4577 7.5779 -0.6664 H 0 0 0 0 0 0 0 0 0 0 0 0 0
10.4546 11.5619 0.5758 H 0 0 0 0 0 0 0 0 0 0 0 0 0
12.8592 12.1602 0.5986 H 0 0 0 0 0 0 0 0 0 0 0 0 0
13.8623 8.1762 -0.6435 H 0 0 0 0 0 0 0 0 0 0 0 0 0
18.7065 11.4984 0.0283 H 0 0 0 0 0 0 0 0 0 0 0 0 0
17.0061 13.1755 0.6889 H 0 0 0 0 0 0 0 0 0 0 0 0 0
14.6015 12.5771 0.6661 H 0 0 0 0 0 0 0 0 0 0 0 0 0
15.5978 8.6247 -0.6781 H 0 0 0 0 0 0 0 0 0 0 0 0 0
18.0023 9.2230 -0.6552 H 0 0 0 0 0 0 0 0 0 0 0 0 0
2.1582 9.5447 0.3187 H 0 0 0 0 0 0 0 0 0 0 0 0 0
1.4669 7.2088 -0.1354 H 0 0 0 0 0 0 0 0 0 0 0 0 0
3.1802 5.4712 -0.5666 H 0 0 0 0 0 0 0 0 0 0 0 0 0
5.5848 6.0695 -0.5437 H 0 0 0 0 0 0 0 0 0 0 0 0 0
4.5628 10.1430 0.3414 H 0 0 0 0 0 0 0 0 0 0 0 0 0
8.7146 11.1344 0.5417 H 0 0 0 0 0 0 0 0 0 0 0 0 0
6.3100 10.5360 0.5189 H 0 0 0 0 0 0 0 0 0 0 0 0 0
7.3153 6.5418 -0.6882 H 0 0 0 0 0 0 0 0 0 0 0 0 0
9.7199 7.1401 -0.6653 H 0 0 0 0 0 0 0 0 0 0 0 0 0
1 2 4 0 0 0 0
2 3 4 0 0 0 0
3 4 4 0 0 0 0
4 5 4 0 0 0 0
5 6 4 0 0 0 0
6 1 4 0 0 0 0
7 8 4 0 0 0 0
8 9 4 0 0 0 0
9 10 4 0 0 0 0
10 11 4 0 0 0 0
11 12 4 0 0 0 0
12 7 4 0 0 0 0
10 5 1 0 0 0 0
13 14 4 0 0 0 0
14 15 4 0 0 0 0
15 16 4 0 0 0 0
16 17 4 0 0 0 0
17 18 4 0 0 0 0
18 13 4 0 0 0 0
19 20 4 0 0 0 0
20 21 4 0 0 0 0
21 22 4 0 0 0 0
22 23 4 0 0 0 0

```
 23 24  4  0  0  0  0
 24 19  4  0  0  0  0
 22 17  1  0  0  0  0
 19  2  1  0  0  0  0
  1 25  1  0  0  0  0
  3 26  1  0  0  0  0
  4 27  1  0  0  0  0
  6 28  1  0  0  0  0
  7 29  1  0  0  0  0
  8 30  1  0  0  0  0
  9 31  1  0  0  0  0
 11 32  1  0  0  0  0
 12 33  1  0  0  0  0
 13 34  1  0  0  0  0
 14 35  1  0  0  0  0
 15 36  1  0  0  0  0
 16 37  1  0  0  0  0
 18 38  1  0  0  0  0
 20 39  1  0  0  0  0
 21 40  1  0  0  0  0
 23 41  1  0  0  0  0
 24 42  1  0  0  0  0
M  END
>  <Alias name>
p-Quaterphenyl

>  <deltaH°(c)>   kJ/mol
-12244

$$$$
Trihexylamine
  ChemBrain IXL

 58 57  0  0  0  0  0  0  0  0  1 V2000
   11.8854   13.3395    0.0632 C   0  0  0  0  0  0  0  0  0  0  0  0
   11.0039   12.2602   -0.5945 C   0  0  0  0  0  0  0  0  0  0  0  0
   11.2191   10.9133    0.1231 C   0  0  0  0  0  0  0  0  0  0  0  0
   10.3372    9.8341   -0.5345 C   0  0  0  0  0  0  0  0  0  0  0  0
   10.5528    8.4874    0.1827 C   0  0  0  0  0  0  0  0  0  0  0  0
    9.6502    7.4042   -0.4784 C   0  0  0  0  0  0  0  0  0  0  0  0
    9.7585    6.0972    0.2446 N   0  0  0  0  0  0  0  0  0  0  0  0
   17.2816    6.0468    0.6802 C   0  0  0  0  0  0  0  0  0  0  0  0
   16.0196    5.2163    0.3765 C   0  0  0  0  0  0  0  0  0  0  0  0
   14.7782    6.1278    0.4346 C   0  0  0  0  0  0  0  0  0  0  0  0
   13.5163    5.2976    0.1290 C   0  0  0  0  0  0  0  0  0  0  0  0
   12.2755    6.2097    0.1851 C   0  0  0  0  0  0  0  0  0  0  0  0
   11.0215    5.3787   -0.1300 C   0  0  0  0  0  0  0  0  0  0  0  0
    2.2882    5.3568    0.5771 C   0  0  0  0  0  0  0  0  0  0  0  0
    3.6074    4.6912    0.1394 C   0  0  0  0  0  0  0  0  0  0  0  0
    4.7870    5.6316    0.4549 C   0  0  0  0  0  0  0  0  0  0  0  0
    6.1060    4.9667    0.0156 C   0  0  0  0  0  0  0  0  0  0  0  0
    7.2852    5.9077    0.3295 C   0  0  0  0  0  0  0  0  0  0  0  0
    8.6076    5.2393   -0.1141 C   0  0  0  0  0  0  0  0  0  0  0  0
   11.6151   13.4396    1.1072 H   0  0  0  0  0  0  0  0  0  0  0  0
   11.7341   14.2858   -0.4412 H   0  0  0  0  0  0  0  0  0  0  0  0
   12.9266   13.0517   -0.0141 H   0  0  0  0  0  0  0  0  0  0  0  0
   11.2741   12.1603   -1.6384 H   0  0  0  0  0  0  0  0  0  0  0  0
    9.9628   12.5481   -0.5171 H   0  0  0  0  0  0  0  0  0  0  0  0
   10.9491   11.0133    1.1671 H   0  0  0  0  0  0  0  0  0  0  0  0
   12.2602   10.6252    0.0454 H   0  0  0  0  0  0  0  0  0  0  0  0
   10.6070    9.7342   -1.5785 H   0  0  0  0  0  0  0  0  0  0  0  0
    9.2960   10.1219   -0.4566 H   0  0  0  0  0  0  0  0  0  0  0  0
   10.2820    8.5908    1.2261 H   0  0  0  0  0  0  0  0  0  0  0  0
```

```
 11.5730    8.2150    0.1107 H   0  0  0  0  0  0  0  0  0  0  0  0
  9.9263    7.2709   -1.5172 H   0  0  0  0  0  0  0  0  0  0  0  0
  8.6294    7.7449   -0.4265 H   0  0  0  0  0  0  0  0  0  0  0  0
 17.3792    6.8369   -0.0541 H   0  0  0  0  0  0  0  0  0  0  0  0
 18.1538    5.4062    0.6392 H   0  0  0  0  0  0  0  0  0  0  0  0
 17.1988    6.4811    1.6688 H   0  0  0  0  0  0  0  0  0  0  0  0
 15.9221    4.4263    1.1108 H   0  0  0  0  0  0  0  0  0  0  0  0
 16.1026    4.7821   -0.6121 H   0  0  0  0  0  0  0  0  0  0  0  0
 14.8763    6.9185   -0.2989 H   0  0  0  0  0  0  0  0  0  0  0  0
 14.6944    6.5612    1.4236 H   0  0  0  0  0  0  0  0  0  0  0  0
 13.4172    4.5076    0.8631 H   0  0  0  0  0  0  0  0  0  0  0  0
 13.6006    4.8634   -0.8596 H   0  0  0  0  0  0  0  0  0  0  0  0
 12.3820    6.9911   -0.5485 H   0  0  0  0  0  0  0  0  0  0  0  0
 12.1892    6.6404    1.1752 H   0  0  0  0  0  0  0  0  0  0  0  0
 11.0045    5.1523   -1.1891 H   0  0  0  0  0  0  0  0  0  0  0  0
 11.0686    4.4513    0.4276 H   0  0  0  0  0  0  0  0  0  0  0  0
  1.4596    4.6958    0.3552 H   0  0  0  0  0  0  0  0  0  0  0  0
  2.3186    5.5529    1.6417 H   0  0  0  0  0  0  0  0  0  0  0  0
  2.1594    6.2894    0.0417 H   0  0  0  0  0  0  0  0  0  0  0  0
  3.5768    4.4951   -0.9252 H   0  0  0  0  0  0  0  0  0  0  0  0
  3.7360    3.7586    0.6747 H   0  0  0  0  0  0  0  0  0  0  0  0
  4.8182    5.8269    1.5197 H   0  0  0  0  0  0  0  0  0  0  0  0
  4.6577    6.5646   -0.0796 H   0  0  0  0  0  0  0  0  0  0  0  0
  6.0743    4.7706   -1.0490 H   0  0  0  0  0  0  0  0  0  0  0  0
  6.2360    4.0341    0.5507 H   0  0  0  0  0  0  0  0  0  0  0  0
  7.3181    6.1012    1.3945 H   0  0  0  0  0  0  0  0  0  0  0  0
  7.1510    6.8379   -0.2019 H   0  0  0  0  0  0  0  0  0  0  0  0
  8.5927    5.0836   -1.1859 H   0  0  0  0  0  0  0  0  0  0  0  0
  8.7072    4.2835    0.3857 H   0  0  0  0  0  0  0  0  0  0  0  0
  1  2  1  0  0  0  0
  2  3  1  0  0  0  0
  3  4  1  0  0  0  0
  4  5  1  0  0  0  0
  5  6  1  0  0  0  0
  6  7  1  0  0  0  0
  8  9  1  0  0  0  0
  9 10  1  0  0  0  0
 10 11  1  0  0  0  0
 11 12  1  0  0  0  0
 12 13  1  0  0  0  0
 14 15  1  0  0  0  0
 15 16  1  0  0  0  0
 16 17  1  0  0  0  0
 17 18  1  0  0  0  0
 18 19  1  0  0  0  0
 13  7  1  0  0  0  0
 19  7  1  0  0  0  0
  1 20  1  0  0  0  0
  1 21  1  0  0  0  0
  1 22  1  0  0  0  0
  2 23  1  0  0  0  0
  2 24  1  0  0  0  0
  3 25  1  0  0  0  0
  3 26  1  0  0  0  0
  4 27  1  0  0  0  0
  4 28  1  0  0  0  0
  5 29  1  0  0  0  0
  5 30  1  0  0  0  0
  6 31  1  0  0  0  0
  6 32  1  0  0  0  0
  8 33  1  0  0  0  0
  8 34  1  0  0  0  0
  8 35  1  0  0  0  0
```

```
 9 36  1  0  0  0  0
 9 37  1  0  0  0  0
10 38  1  0  0  0  0
10 39  1  0  0  0  0
11 40  1  0  0  0  0
11 41  1  0  0  0  0
12 42  1  0  0  0  0
12 43  1  0  0  0  0
13 44  1  0  0  0  0
13 45  1  0  0  0  0
14 46  1  0  0  0  0
14 47  1  0  0  0  0
14 48  1  0  0  0  0
15 49  1  0  0  0  0
15 50  1  0  0  0  0
16 51  1  0  0  0  0
16 52  1  0  0  0  0
17 53  1  0  0  0  0
17 54  1  0  0  0  0
18 55  1  0  0  0  0
18 56  1  0  0  0  0
19 57  1  0  0  0  0
19 58  1  0  0  0  0
M  END
>  <Alias name>
Trihexylamine

>  <deltaH°(c)>   kJ/mol
-12232.32

$$$$
1,8-Di(4-methoxyphenyl)naphthalene
  ChemBrain IXL

 46 49  0  0  0  0  0  0  0  0  1 V2000
    5.0888    4.0711    1.0178 C   0  0  0  0  0  0  0  0  0  0  0  0
    3.8011    3.7843    1.4712 C   0  0  0  0  0  0  0  0  0  0  0  0
    2.9268    4.8222    1.7981 C   0  0  0  0  0  0  0  0  0  0  0  0
    3.3394    6.1508    1.6803 C   0  0  0  0  0  0  0  0  0  0  0  0
    4.6344    6.4474    1.2421 C   0  0  0  0  0  0  0  0  0  0  0  0
    5.5081    5.3962    0.8922 C   0  0  0  0  0  0  0  0  0  0  0  0
    5.0479    7.7933    1.1564 C   0  0  0  0  0  0  0  0  0  0  0  0
    4.1555    8.8184    1.4751 C   0  0  0  0  0  0  0  0  0  0  0  0
    2.8606    8.5136    1.8956 C   0  0  0  0  0  0  0  0  0  0  0  0
    2.4546    7.1823    2.0016 C   0  0  0  0  0  0  0  0  0  0  0  0
    8.3457    6.4914   -1.3599 C   0  0  0  0  0  0  0  0  0  0  0  0
    9.4569    6.2093   -0.5645 C   0  0  0  0  0  0  0  0  0  0  0  0
    9.2808    5.6580    0.7063 C   0  0  0  0  0  0  0  0  0  0  0  0
    7.9937    5.3913    1.1809 C   0  0  0  0  0  0  0  0  0  0  0  0
    6.8803    5.6794    0.3868 C   0  0  0  0  0  0  0  0  0  0  0  0
    7.0592    6.2261   -0.8855 C   0  0  0  0  0  0  0  0  0  0  0  0
   10.8135    6.4935   -1.0681 O   0  0  0  0  0  0  0  0  0  0  0  0
   11.9120    6.1438   -0.1512 C   0  0  0  0  0  0  0  0  0  0  0  0
    7.9534    9.0416   -0.9253 C   0  0  0  0  0  0  0  0  0  0  0  0
    9.0316    8.7855   -0.0756 C   0  0  0  0  0  0  0  0  0  0  0  0
    8.8084    8.2156    1.1783 C   0  0  0  0  0  0  0  0  0  0  0  0
    7.5102    7.8938    1.5804 C   0  0  0  0  0  0  0  0  0  0  0  0
    6.4315    8.1405    0.7289 C   0  0  0  0  0  0  0  0  0  0  0  0
    6.6541    8.7215   -0.5226 C   0  0  0  0  0  0  0  0  0  0  0  0
   10.4046    9.1165   -0.4997 O   0  0  0  0  0  0  0  0  0  0  0  0
   10.5344    9.6182   -1.8784 C   0  0  0  0  0  0  0  0  0  0  0  0
    5.7649    3.2637    0.7623 H   0  0  0  0  0  0  0  0  0  0  0  0
    3.4796    2.7550    1.5695 H   0  0  0  0  0  0  0  0  0  0  0  0
```

```
    1.9265    4.5964    2.1438 H   0  0  0  0  0  0  0  0  0  0  0  0
    4.4693    9.8527    1.3961 H   0  0  0  0  0  0  0  0  0  0  0  0
    2.1697    9.3112    2.1400 H   0  0  0  0  0  0  0  0  0  0  0  0
    1.4513    6.9490    2.3337 H   0  0  0  0  0  0  0  0  0  0  0  0
    8.4818    6.9160   -2.3465 H   0  0  0  0  0  0  0  0  0  0  0  0
   10.1431    5.4364    1.3247 H   0  0  0  0  0  0  0  0  0  0  0  0
    7.8592    4.9603    2.1657 H   0  0  0  0  0  0  0  0  0  0  0  0
    6.1995    6.4444   -1.5055 H   0  0  0  0  0  0  0  0  0  0  0  0
   12.8596    6.4104   -0.6051 H   0  0  0  0  0  0  0  0  0  0  0  0
   11.8935    5.0791    0.0458 H   0  0  0  0  0  0  0  0  0  0  0  0
   11.7929    6.6852    0.7789 H   0  0  0  0  0  0  0  0  0  0  0  0
    8.1247    9.4893   -1.8975 H   0  0  0  0  0  0  0  0  0  0  0  0
    9.6432    8.0234    1.8404 H   0  0  0  0  0  0  0  0  0  0  0  0
    7.3402    7.4529    2.5539 H   0  0  0  0  0  0  0  0  0  0  0  0
    5.8185    8.9245   -1.1814 H   0  0  0  0  0  0  0  0  0  0  0  0
    9.9465   10.5204   -1.9916 H   0  0  0  0  0  0  0  0  0  0  0  0
   10.1790    8.8662   -2.5718 H   0  0  0  0  0  0  0  0  0  0  0  0
   11.5755    9.8375   -2.0862 H   0  0  0  0  0  0  0  0  0  0  0  0
  1  2  4  0  0  0  0
  2  3  4  0  0  0  0
  3  4  4  0  0  0  0
  4  5  4  0  0  0  0
  5  6  4  0  0  0  0
  6  1  4  0  0  0  0
  5  7  4  0  0  0  0
  7  8  4  0  0  0  0
  8  9  4  0  0  0  0
  4 10  4  0  0  0  0
 10  9  4  0  0  0  0
 11 12  4  0  0  0  0
 12 13  4  0  0  0  0
 13 14  4  0  0  0  0
 14 15  4  0  0  0  0
 15 16  4  0  0  0  0
 16 11  4  0  0  0  0
 12 17  1  0  0  0  0
 17 18  1  0  0  0  0
 15  6  1  0  0  0  0
 19 20  4  0  0  0  0
 20 21  4  0  0  0  0
 21 22  4  0  0  0  0
 22 23  4  0  0  0  0
 23 24  4  0  0  0  0
 24 19  4  0  0  0  0
 20 25  1  0  0  0  0
 25 26  1  0  0  0  0
 23  7  1  0  0  0  0
  1 27  1  0  0  0  0
  2 28  1  0  0  0  0
  3 29  1  0  0  0  0
  8 30  1  0  0  0  0
  9 31  1  0  0  0  0
 10 32  1  0  0  0  0
 11 33  1  0  0  0  0
 13 34  1  0  0  0  0
 14 35  1  0  0  0  0
 16 36  1  0  0  0  0
 18 37  1  0  0  0  0
 18 38  1  0  0  0  0
 18 39  1  0  0  0  0
 19 40  1  0  0  0  0
 21 41  1  0  0  0  0
 22 42  1  0  0  0  0
```

```
 24 43  1  0  0  0  0
 26 44  1  0  0  0  0
 26 45  1  0  0  0  0
 26 46  1  0  0  0  0
M  END
>  <Alias name>
1,8-Di(4-methoxyphenyl)naphthalene

>  <deltaH°(c)>   kJ/mol
-12218

$$$$
Methyltris(4-methylphenoxy)silane
  ChemBrain IXL

 50 52  0  0  0  0  0  0  0  0  1 V2000
    8.3418   11.8511    0.4364 C   0  0  0  0  0  0  0  0  0  0  0  0  0
    8.3453   10.0109    0.7270 Si  0  0  0  0  0  0  0  0  0  0  0  0  0
    8.2767    5.0874   -0.4562 C   0  0  0  0  0  0  0  0  0  0  0  0  0
    8.2888    5.8562    0.7078 C   0  0  0  0  0  0  0  0  0  0  0  0  0
    8.3090    7.2486    0.6238 C   0  0  0  0  0  0  0  0  0  0  0  0  0
    8.3167    7.8720   -0.6241 C   0  0  0  0  0  0  0  0  0  0  0  0  0
    8.3059    7.1032   -1.7880 C   0  0  0  0  0  0  0  0  0  0  0  0  0
    8.2857    5.7109   -1.7040 C   0  0  0  0  0  0  0  0  0  0  0  0  0
    8.3348    9.2344   -0.7062 O   0  0  0  0  0  0  0  0  0  0  0  0  0
    3.4029   10.1159   -0.3904 C   0  0  0  0  0  0  0  0  0  0  0  0  0
    4.6036   10.3046   -1.0753 C   0  0  0  0  0  0  0  0  0  0  0  0  0
    5.8185   10.1657   -0.4039 C   0  0  0  0  0  0  0  0  0  0  0  0  0
    5.8330    9.8050    0.9435 C   0  0  0  0  0  0  0  0  0  0  0  0  0
    4.6325    9.6610    1.6391 C   0  0  0  0  0  0  0  0  0  0  0  0  0
    3.4173    9.7988    0.9681 C   0  0  0  0  0  0  0  0  0  0  0  0  0
    7.0212    9.5931    1.5809 O   0  0  0  0  0  0  0  0  0  0  0  0  0
   13.2723   10.1526   -0.4535 C   0  0  0  0  0  0  0  0  0  0  0  0  0
   12.0619   10.3322   -1.1234 C   0  0  0  0  0  0  0  0  0  0  0  0  0
   10.8564   10.1825   -0.4375 C   0  0  0  0  0  0  0  0  0  0  0  0  0
   10.8614    9.8193    0.9093 C   0  0  0  0  0  0  0  0  0  0  0  0  0
   12.0714    9.6853    1.5903 C   0  0  0  0  0  0  0  0  0  0  0  0  0
   13.2772    9.8340    0.9047 C   0  0  0  0  0  0  0  0  0  0  0  0  0
    9.6829    9.5950    1.5605 O   0  0  0  0  0  0  0  0  0  0  0  0  0
    8.2541    3.6003   -0.3665 C   0  0  0  0  0  0  0  0  0  0  0  0  0
    2.1053   10.2529   -1.1098 C   0  0  0  0  0  0  0  0  0  0  0  0  0
   14.5599   10.3011   -1.1886 C   0  0  0  0  0  0  0  0  0  0  0  0  0
    7.4536   12.1272   -0.1184 H   0  0  0  0  0  0  0  0  0  0  0  0  0
    9.2221   12.1288   -0.1300 H   0  0  0  0  0  0  0  0  0  0  0  0  0
    8.3476   12.3669    1.3886 H   0  0  0  0  0  0  0  0  0  0  0  0  0
    8.2824    5.3722    1.6766 H   0  0  0  0  0  0  0  0  0  0  0  0  0
    8.3188    7.8454    1.5275 H   0  0  0  0  0  0  0  0  0  0  0  0  0
    8.3133    7.5872   -2.7568 H   0  0  0  0  0  0  0  0  0  0  0  0  0
    8.2769    5.1140   -2.6077 H   0  0  0  0  0  0  0  0  0  0  0  0  0
    4.5925   10.5589   -2.1279 H   0  0  0  0  0  0  0  0  0  0  0  0  0
    6.7502   10.3379   -0.9286 H   0  0  0  0  0  0  0  0  0  0  0  0  0
    4.6439    9.4423    2.6997 H   0  0  0  0  0  0  0  0  0  0  0  0  0
    2.4852    9.6597    1.5016 H   0  0  0  0  0  0  0  0  0  0  0  0  0
   12.0580   10.5878   -2.1758 H   0  0  0  0  0  0  0  0  0  0  0  0  0
    9.9171   10.3481   -0.9504 H   0  0  0  0  0  0  0  0  0  0  0  0  0
   12.0748    9.4660    2.6509 H   0  0  0  0  0  0  0  0  0  0  0  0  0
   14.2169    9.7021    1.4267 H   0  0  0  0  0  0  0  0  0  0  0  0  0
    9.2696    3.2245   -0.3446 H   0  0  0  0  0  0  0  0  0  0  0  0  0
    7.7389    3.3009    0.5378 H   0  0  0  0  0  0  0  0  0  0  0  0  0
    7.7375    3.1945   -1.2275 H   0  0  0  0  0  0  0  0  0  0  0  0  0
    1.6853    9.2706   -1.2871 H   0  0  0  0  0  0  0  0  0  0  0  0  0
    1.4189   10.8359   -0.5083 H   0  0  0  0  0  0  0  0  0  0  0  0  0
    2.2687   10.7520   -2.0570 H   0  0  0  0  0  0  0  0  0  0  0  0  0
```

14.7502 11.3508 -1.3750 H 0 0 0 0 0 0 0 0 0 0 0 0
14.5002 9.7720 -2.1316 H 0 0 0 0 0 0 0 0 0 0 0 0
15.3650 9.8883 -0.5934 H 0 0 0 0 0 0 0 0 0 0 0 0
1 2 1 0 0 0 0
3 4 4 0 0 0 0
4 5 4 0 0 0 0
5 6 4 0 0 0 0
6 7 4 0 0 0 0
7 8 4 0 0 0 0
8 3 4 0 0 0 0
6 9 1 0 0 0 0
10 11 4 0 0 0 0
11 12 4 0 0 0 0
12 13 4 0 0 0 0
13 14 4 0 0 0 0
14 15 4 0 0 0 0
15 10 4 0 0 0 0
13 16 1 0 0 0 0
9 2 1 0 0 0 0
17 18 4 0 0 0 0
18 19 4 0 0 0 0
19 20 4 0 0 0 0
20 21 4 0 0 0 0
21 22 4 0 0 0 0
22 17 4 0 0 0 0
20 23 1 0 0 0 0
23 2 1 0 0 0 0
16 2 1 0 0 0 0
3 24 1 0 0 0 0
10 25 1 0 0 0 0
17 26 1 0 0 0 0
1 27 1 0 0 0 0
1 28 1 0 0 0 0
1 29 1 0 0 0 0
4 30 1 0 0 0 0
5 31 1 0 0 0 0
7 32 1 0 0 0 0
8 33 1 0 0 0 0
11 34 1 0 0 0 0
12 35 1 0 0 0 0
14 36 1 0 0 0 0
15 37 1 0 0 0 0
18 38 1 0 0 0 0
19 39 1 0 0 0 0
21 40 1 0 0 0 0
22 41 1 0 0 0 0
24 42 1 0 0 0 0
24 43 1 0 0 0 0
24 44 1 0 0 0 0
25 45 1 0 0 0 0
25 46 1 0 0 0 0
25 47 1 0 0 0 0
26 48 1 0 0 0 0
26 49 1 0 0 0 0
26 50 1 0 0 0 0
M END
> <Alias name>
Methyltris(4-methylphenoxy)silane

> <deltaH°(c)> kJ/mol
-12166

$$$$

```
Methyltris(2-methylphenoxy)silane
  ChemBrain IXL

 50 52  0  0  0  0  0  0  0  0  1 V2000
    7.3990   10.8032    0.0246 C   0  0  0  0  0  0  0  0  0  0  0  0  0
    7.5370    9.0023    0.4821 Si  0  0  0  0  0  0  0  0  0  0  0  0  0
    7.6692    3.9390   -0.3998 C   0  0  0  0  0  0  0  0  0  0  0  0  0
    7.4574    4.4947   -1.6617 C   0  0  0  0  0  0  0  0  0  0  0  0  0
    7.3657    5.8797   -1.8092 C   0  0  0  0  0  0  0  0  0  0  0  0  0
    7.4852    6.7145   -0.6954 C   0  0  0  0  0  0  0  0  0  0  0  0  0
    7.6979    6.1534    0.5712 C   0  0  0  0  0  0  0  0  0  0  0  0  0
    7.7893    4.7680    0.7160 C   0  0  0  0  0  0  0  0  0  0  0  0  0
    7.1394    6.4694   -3.1590 C   0  0  0  0  0  0  0  0  0  0  0  0  0
    7.3908    8.0832   -0.8661 O   0  0  0  0  0  0  0  0  0  0  0  0  0
    2.4350    8.4785    0.0710 C   0  0  0  0  0  0  0  0  0  0  0  0  0
    2.6893    8.2478    1.4231 C   0  0  0  0  0  0  0  0  0  0  0  0  0
    3.9982    8.2957    1.9065 C   0  0  0  0  0  0  0  0  0  0  0  0  0
    5.0580    8.5752    1.0398 C   0  0  0  0  0  0  0  0  0  0  0  0  0
    4.7987    8.8055   -0.3183 C   0  0  0  0  0  0  0  0  0  0  0  0  0
    3.4892    8.7569   -0.7990 C   0  0  0  0  0  0  0  0  0  0  0  0  0
    4.2648    8.0482    3.3518 C   0  0  0  0  0  0  0  0  0  0  0  0  0
    6.3476    8.6191    1.5422 O   0  0  0  0  0  0  0  0  0  0  0  0  0
   12.3677    8.8983   -1.2865 C   0  0  0  0  0  0  0  0  0  0  0  0  0
   12.5046    8.6607    0.0813 C   0  0  0  0  0  0  0  0  0  0  0  0  0
   11.3737    8.6052    0.8981 C   0  0  0  0  0  0  0  0  0  0  0  0  0
   10.1013    8.7876    0.3503 C   0  0  0  0  0  0  0  0  0  0  0  0  0
    9.9675    9.0252   -1.0245 C   0  0  0  0  0  0  0  0  0  0  0  0  0
   11.0997    9.0802   -1.8389 C   0  0  0  0  0  0  0  0  0  0  0  0  0
   11.5248    8.3507    2.3588 C   0  0  0  0  0  0  0  0  0  0  0  0  0
    8.9944    8.7299    1.1802 O   0  0  0  0  0  0  0  0  0  0  0  0  0
    6.4354   10.9864   -0.4342 H   0  0  0  0  0  0  0  0  0  0  0  0  0
    7.4956   11.4104    0.9160 H   0  0  0  0  0  0  0  0  0  0  0  0  0
    8.1859   11.0597   -0.6737 H   0  0  0  0  0  0  0  0  0  0  0  0  0
    7.7402    2.8645   -0.2862 H   0  0  0  0  0  0  0  0  0  0  0  0  0
    7.3642    3.8510   -2.5276 H   0  0  0  0  0  0  0  0  0  0  0  0  0
    7.7919    6.7907    1.4400 H   0  0  0  0  0  0  0  0  0  0  0  0  0
    7.9537    4.3363    1.6953 H   0  0  0  0  0  0  0  0  0  0  0  0  0
    6.1398    6.2252   -3.4966 H   0  0  0  0  0  0  0  0  0  0  0  0  0
    7.2497    7.5457   -3.1063 H   0  0  0  0  0  0  0  0  0  0  0  0  0
    7.8642    6.0660   -3.8553 H   0  0  0  0  0  0  0  0  0  0  0  0  0
    1.4194    8.4413   -0.3030 H   0  0  0  0  0  0  0  0  0  0  0  0  0
    1.8707    8.0314    2.0982 H   0  0  0  0  0  0  0  0  0  0  0  0  0
    5.6116    9.0210   -0.9984 H   0  0  0  0  0  0  0  0  0  0  0  0  0
    3.2915    8.9356   -1.8486 H   0  0  0  0  0  0  0  0  0  0  0  0  0
    5.2527    8.4131    3.6046 H   0  0  0  0  0  0  0  0  0  0  0  0  0
    4.2105    6.9853    3.5518 H   0  0  0  0  0  0  0  0  0  0  0  0  0
    3.5248    8.5662    3.9493 H   0  0  0  0  0  0  0  0  0  0  0  0  0
   13.2454    8.9415   -1.9192 H   0  0  0  0  0  0  0  0  0  0  0  0  0
   13.4891    8.5193    0.5097 H   0  0  0  0  0  0  0  0  0  0  0  0  0
    8.9873    9.1661   -1.4593 H   0  0  0  0  0  0  0  0  0  0  0  0  0
   10.9937    9.2643   -2.9007 H   0  0  0  0  0  0  0  0  0  0  0  0  0
   12.5042    8.6799    2.6834 H   0  0  0  0  0  0  0  0  0  0  0  0  0
   11.4174    7.2908    2.5534 H   0  0  0  0  0  0  0  0  0  0  0  0  0
   10.7625    8.8963    2.9011 H   0  0  0  0  0  0  0  0  0  0  0  0  0
  1  2  1  0  0  0  0
  3  4  4  0  0  0  0
  4  5  4  0  0  0  0
  5  6  4  0  0  0  0
  6  7  4  0  0  0  0
  7  8  4  0  0  0  0
  8  3  4  0  0  0  0
  5  9  1  0  0  0  0
  6 10  1  0  0  0  0
```

```
 11 12  4  0  0  0  0
 12 13  4  0  0  0  0
 13 14  4  0  0  0  0
 14 15  4  0  0  0  0
 15 16  4  0  0  0  0
 16 11  4  0  0  0  0
 13 17  1  0  0  0  0
 14 18  1  0  0  0  0
 10  2  1  0  0  0  0
 19 20  4  0  0  0  0
 20 21  4  0  0  0  0
 21 22  4  0  0  0  0
 22 23  4  0  0  0  0
 23 24  4  0  0  0  0
 24 19  4  0  0  0  0
 21 25  1  0  0  0  0
 22 26  1  0  0  0  0
 26  2  1  0  0  0  0
 18  2  1  0  0  0  0
  1 27  1  0  0  0  0
  1 28  1  0  0  0  0
  1 29  1  0  0  0  0
  3 30  1  0  0  0  0
  4 31  1  0  0  0  0
  7 32  1  0  0  0  0
  8 33  1  0  0  0  0
  9 34  1  0  0  0  0
  9 35  1  0  0  0  0
  9 36  1  0  0  0  0
 11 37  1  0  0  0  0
 12 38  1  0  0  0  0
 15 39  1  0  0  0  0
 16 40  1  0  0  0  0
 17 41  1  0  0  0  0
 17 42  1  0  0  0  0
 17 43  1  0  0  0  0
 19 44  1  0  0  0  0
 20 45  1  0  0  0  0
 23 46  1  0  0  0  0
 24 47  1  0  0  0  0
 25 48  1  0  0  0  0
 25 49  1  0  0  0  0
 25 50  1  0  0  0  0
M  END
>  <Alias name>
Methyltris(2-methylphenoxy)silane

>  <deltaH°(c)>   kJ/mol
-12159

$$$$
Methyltris(3-methylphenoxy)silane
  ChemBrain IXL

 50 52  0  0  0  0  0  0  0  0  1 V2000
    7.5916    8.9628    1.0885 C   0  0  0  0  0  0  0  0  0  0  0  0
    6.9574    7.2147    0.9712 Si  0  0  0  0  0  0  0  0  0  0  0  0
    4.4677    3.2733   -1.2049 C   0  0  0  0  0  0  0  0  0  0  0  0
    5.2246    3.4676   -0.0489 C   0  0  0  0  0  0  0  0  0  0  0  0
    5.7675    4.7237    0.2289 C   0  0  0  0  0  0  0  0  0  0  0  0
    5.5531    5.7916   -0.6529 C   0  0  0  0  0  0  0  0  0  0  0  0
    4.7946    5.5910   -1.8084 C   0  0  0  0  0  0  0  0  0  0  0  0
    4.2529    4.3346   -2.0841 C   0  0  0  0  0  0  0  0  0  0  0  0
```

```
   6.0787    7.0454   -0.4025 O   0  0  0  0  0  0  0  0  0  0  0  0
   2.3582    8.8951    2.5500 C   0  0  0  0  0  0  0  0  0  0  0  0
   3.1580    9.0725    1.4205 C   0  0  0  0  0  0  0  0  0  0  0  0
   4.3812    8.4077    1.3184 C   0  0  0  0  0  0  0  0  0  0  0  0
   4.8093    7.5615    2.3502 C   0  0  0  0  0  0  0  0  0  0  0  0
   4.0041    7.3872    3.4782 C   0  0  0  0  0  0  0  0  0  0  0  0
   2.7812    8.0526    3.5782 C   0  0  0  0  0  0  0  0  0  0  0  0
   6.0182    6.8923    2.2750 O   0  0  0  0  0  0  0  0  0  0  0  0
  10.6761    6.2101   -2.4393 C   0  0  0  0  0  0  0  0  0  0  0  0
   9.5361    7.0138   -2.4051 C   0  0  0  0  0  0  0  0  0  0  0  0
   8.7115    7.0103   -1.2787 C   0  0  0  0  0  0  0  0  0  0  0  0
   9.0271    6.2004   -0.1794 C   0  0  0  0  0  0  0  0  0  0  0  0
  10.1693    5.3974   -0.2196 C   0  0  0  0  0  0  0  0  0  0  0  0
  10.9923    5.4022   -1.3470 C   0  0  0  0  0  0  0  0  0  0  0  0
   8.2280    6.1796    0.9500 O   0  0  0  0  0  0  0  0  0  0  0  0
   2.7067    9.9724    0.3218 C   0  0  0  0  0  0  0  0  0  0  0  0
   5.4535    2.3331    0.8897 C   0  0  0  0  0  0  0  0  0  0  0  0
   9.1989    7.8768   -3.5723 C   0  0  0  0  0  0  0  0  0  0  0  0
   6.7549    9.6500    1.1075 H   0  0  0  0  0  0  0  0  0  0  0  0
   8.2165    9.1786    0.2310 H   0  0  0  0  0  0  0  0  0  0  0  0
   8.1721    9.0759    1.9957 H   0  0  0  0  0  0  0  0  0  0  0  0
   4.0470    2.2987   -1.4195 H   0  0  0  0  0  0  0  0  0  0  0  0
   6.3534    4.8684    1.1266 H   0  0  0  0  0  0  0  0  0  0  0  0
   4.6262    6.4132   -2.4928 H   0  0  0  0  0  0  0  0  0  0  0  0
   3.6654    4.1837   -2.9813 H   0  0  0  0  0  0  0  0  0  0  0  0
   1.4093    9.4111    2.6284 H   0  0  0  0  0  0  0  0  0  0  0  0
   4.9960    8.5482    0.4397 H   0  0  0  0  0  0  0  0  0  0  0  0
   4.3301    6.7341    4.2781 H   0  0  0  0  0  0  0  0  0  0  0  0
   2.1603    7.9148    4.4547 H   0  0  0  0  0  0  0  0  0  0  0  0
  11.3152    6.2135   -3.3135 H   0  0  0  0  0  0  0  0  0  0  0  0
   7.8283    7.6344   -1.2587 H   0  0  0  0  0  0  0  0  0  0  0  0
  10.4174    4.7694    0.6270 H   0  0  0  0  0  0  0  0  0  0  0  0
  11.8770    4.7783   -1.3737 H   0  0  0  0  0  0  0  0  0  0  0  0
   1.8100   10.4964    0.6290 H   0  0  0  0  0  0  0  0  0  0  0  0
   2.4959    9.3843   -0.5628 H   0  0  0  0  0  0  0  0  0  0  0  0
   3.4865   10.6906    0.1003 H   0  0  0  0  0  0  0  0  0  0  0  0
   6.3193    1.7678    0.5681 H   0  0  0  0  0  0  0  0  0  0  0  0
   5.6241    2.7185    1.8874 H   0  0  0  0  0  0  0  0  0  0  0  0
   4.5832    1.6883    0.8959 H   0  0  0  0  0  0  0  0  0  0  0  0
   8.3137    8.4590   -3.3475 H   0  0  0  0  0  0  0  0  0  0  0  0
   9.0114    7.2548   -4.4387 H   0  0  0  0  0  0  0  0  0  0  0  0
  10.0267    8.5438   -3.7790 H   0  0  0  0  0  0  0  0  0  0  0  0
  1  2  1  0  0  0  0
  3  4  4  0  0  0  0
  4  5  4  0  0  0  0
  5  6  4  0  0  0  0
  6  7  4  0  0  0  0
  7  8  4  0  0  0  0
  8  3  4  0  0  0  0
  6  9  1  0  0  0  0
 10 11  4  0  0  0  0
 11 12  4  0  0  0  0
 12 13  4  0  0  0  0
 13 14  4  0  0  0  0
 14 15  4  0  0  0  0
 15 10  4  0  0  0  0
 13 16  1  0  0  0  0
  9  2  1  0  0  0  0
 17 18  4  0  0  0  0
 18 19  4  0  0  0  0
 19 20  4  0  0  0  0
 20 21  4  0  0  0  0
 21 22  4  0  0  0  0
```

```
 22 17  4  0  0  0  0
 20 23  1  0  0  0  0
 23  2  1  0  0  0  0
 16  2  1  0  0  0  0
 11 24  1  0  0  0  0
  4 25  1  0  0  0  0
 18 26  1  0  0  0  0
  1 27  1  0  0  0  0
  1 28  1  0  0  0  0
  1 29  1  0  0  0  0
  3 30  1  0  0  0  0
  5 31  1  0  0  0  0
  7 32  1  0  0  0  0
  8 33  1  0  0  0  0
 10 34  1  0  0  0  0
 12 35  1  0  0  0  0
 14 36  1  0  0  0  0
 15 37  1  0  0  0  0
 17 38  1  0  0  0  0
 19 39  1  0  0  0  0
 21 40  1  0  0  0  0
 22 41  1  0  0  0  0
 24 42  1  0  0  0  0
 24 43  1  0  0  0  0
 24 44  1  0  0  0  0
 25 45  1  0  0  0  0
 25 46  1  0  0  0  0
 25 47  1  0  0  0  0
 26 48  1  0  0  0  0
 26 49  1  0  0  0  0
 26 50  1  0  0  0  0
M  END
>  <Alias name>
Methyltris(3-methylphenoxy)silane

>  <deltaH°(c)>   kJ/mol
-12150

$$$$
Octadecyl isocyanate
  ChemBrain IXL

 58 57  0  0  0  0  0  0  0  0  1 V2000
   14.6746   13.4064    0.0000 C   0  0  0  0  0  0  0  0  0  0  0  0
   13.4133   12.5208    0.0000 C   0  0  0  0  0  0  0  0  0  0  0  0
   12.1579   13.4147    0.0000 C   0  0  0  0  0  0  0  0  0  0  0  0
   15.9300   12.5126    0.0000 C   0  0  0  0  0  0  0  0  0  0  0  0
   10.8966   12.5292    0.0000 C   0  0  0  0  0  0  0  0  0  0  0  0
   17.1912   13.3983    0.0000 C   0  0  0  0  0  0  0  0  0  0  0  0
    9.6413   13.4231    0.0000 C   0  0  0  0  0  0  0  0  0  0  0  0
   18.4467   12.5046    0.0000 C   0  0  0  0  0  0  0  0  0  0  0  0
    8.3799   12.5377    0.0000 C   0  0  0  0  0  0  0  0  0  0  0  0
   19.7079   13.3903    0.0000 C   0  0  0  0  0  0  0  0  0  0  0  0
    7.1246   13.4316    0.0000 C   0  0  0  0  0  0  0  0  0  0  0  0
   20.9634   12.4966    0.0001 C   0  0  0  0  0  0  0  0  0  0  0  0
    5.8632   12.5462    0.0000 C   0  0  0  0  0  0  0  0  0  0  0  0
   22.2246   13.3823    0.0001 C   0  0  0  0  0  0  0  0  0  0  0  0
    4.6080   13.4400    0.0000 C   0  0  0  0  0  0  0  0  0  0  0  0
   23.4801   12.4887    0.0001 C   0  0  0  0  0  0  0  0  0  0  0  0
    3.4621   12.6350   -0.0001 N   0  0  0  0  0  0  0  0  0  0  0  0
   24.7414   13.3745    0.0002 C   0  0  0  0  0  0  0  0  0  0  0  0
    2.3100   13.1697    0.0003 C   0  0  0  0  0  0  0  0  0  0  0  0
   25.9969   12.4810    0.0002 C   0  0  0  0  0  0  0  0  0  0  0  0
```

```
  1.2035   13.6834    0.0006 O   0  0  0  0  0  0  0  0  0  0  0  0
 14.6766   14.0316    0.8843 H   0  0  0  0  0  0  0  0  0  0  0  0
 14.6766   14.0317   -0.8842 H   0  0  0  0  0  0  0  0  0  0  0  0
 13.4113   11.8956   -0.8843 H   0  0  0  0  0  0  0  0  0  0  0  0
 13.4113   11.8955    0.8842 H   0  0  0  0  0  0  0  0  0  0  0  0
 12.1600   14.0399    0.8843 H   0  0  0  0  0  0  0  0  0  0  0  0
 12.1600   14.0400   -0.8842 H   0  0  0  0  0  0  0  0  0  0  0  0
 15.9280   11.8874   -0.8843 H   0  0  0  0  0  0  0  0  0  0  0  0
 15.9280   11.8874    0.8842 H   0  0  0  0  0  0  0  0  0  0  0  0
 10.8945   11.9040   -0.8843 H   0  0  0  0  0  0  0  0  0  0  0  0
 10.8945   11.9039    0.8842 H   0  0  0  0  0  0  0  0  0  0  0  0
 17.1932   14.0235    0.8843 H   0  0  0  0  0  0  0  0  0  0  0  0
 17.1932   14.0236   -0.8842 H   0  0  0  0  0  0  0  0  0  0  0  0
  9.6434   14.0483    0.8843 H   0  0  0  0  0  0  0  0  0  0  0  0
  9.6434   14.0484   -0.8842 H   0  0  0  0  0  0  0  0  0  0  0  0
 18.4447   11.8793   -0.8843 H   0  0  0  0  0  0  0  0  0  0  0  0
 18.4447   11.8793    0.8843 H   0  0  0  0  0  0  0  0  0  0  0  0
  8.3778   11.9124   -0.8843 H   0  0  0  0  0  0  0  0  0  0  0  0
  8.3778   11.9123    0.8842 H   0  0  0  0  0  0  0  0  0  0  0  0
 19.7099   14.0155    0.8843 H   0  0  0  0  0  0  0  0  0  0  0  0
 19.7099   14.0155   -0.8842 H   0  0  0  0  0  0  0  0  0  0  0  0
  7.1267   14.0568    0.8843 H   0  0  0  0  0  0  0  0  0  0  0  0
  7.1268   14.0569   -0.8842 H   0  0  0  0  0  0  0  0  0  0  0  0
 20.9615   11.8713   -0.8842 H   0  0  0  0  0  0  0  0  0  0  0  0
 20.9614   11.8713    0.8843 H   0  0  0  0  0  0  0  0  0  0  0  0
  5.8610   11.9210   -0.8843 H   0  0  0  0  0  0  0  0  0  0  0  0
  5.8610   11.9209    0.8841 H   0  0  0  0  0  0  0  0  0  0  0  0
 22.2265   14.0076    0.8844 H   0  0  0  0  0  0  0  0  0  0  0  0
 22.2265   14.0076   -0.8842 H   0  0  0  0  0  0  0  0  0  0  0  0
  4.6102   14.0654   -0.8844 H   0  0  0  0  0  0  0  0  0  0  0  0
  4.6102   14.0653    0.8845 H   0  0  0  0  0  0  0  0  0  0  0  0
 23.4782   11.8634   -0.8841 H   0  0  0  0  0  0  0  0  0  0  0  0
 23.4781   11.8634    0.8844 H   0  0  0  0  0  0  0  0  0  0  0  0
 24.7433   13.9997    0.8844 H   0  0  0  0  0  0  0  0  0  0  0  0
 24.7434   13.9997   -0.8841 H   0  0  0  0  0  0  0  0  0  0  0  0
 26.8829   13.1036    0.0002 H   0  0  0  0  0  0  0  0  0  0  0  0
 25.9951   11.8557   -0.8841 H   0  0  0  0  0  0  0  0  0  0  0  0
 25.9950   11.8557    0.8845 H   0  0  0  0  0  0  0  0  0  0  0  0
  1  2  1  0  0  0  0
  1  4  1  0  0  0  0
  2  3  1  0  0  0  0
  3  5  1  0  0  0  0
  4  6  1  0  0  0  0
  5  7  1  0  0  0  0
  6  8  1  0  0  0  0
  7  9  1  0  0  0  0
  8 10  1  0  0  0  0
  9 11  1  0  0  0  0
 10 12  1  0  0  0  0
 11 13  1  0  0  0  0
 12 14  1  0  0  0  0
 13 15  1  0  0  0  0
 14 16  1  0  0  0  0
 15 17  1  0  0  0  0
 16 18  1  0  0  0  0
 17 19  2  0  0  0  0
 18 20  1  0  0  0  0
 19 21  2  0  0  0  0
  1 22  1  0  0  0  0
  1 23  1  0  0  0  0
  2 24  1  0  0  0  0
  2 25  1  0  0  0  0
  3 26  1  0  0  0  0
```

```
  3 27  1  0  0  0  0
  4 28  1  0  0  0  0
  4 29  1  0  0  0  0
  5 30  1  0  0  0  0
  5 31  1  0  0  0  0
  6 32  1  0  0  0  0
  6 33  1  0  0  0  0
  7 34  1  0  0  0  0
  7 35  1  0  0  0  0
  8 36  1  0  0  0  0
  8 37  1  0  0  0  0
  9 38  1  0  0  0  0
  9 39  1  0  0  0  0
 10 40  1  0  0  0  0
 10 41  1  0  0  0  0
 11 42  1  0  0  0  0
 11 43  1  0  0  0  0
 12 44  1  0  0  0  0
 12 45  1  0  0  0  0
 13 46  1  0  0  0  0
 13 47  1  0  0  0  0
 14 48  1  0  0  0  0
 14 49  1  0  0  0  0
 15 50  1  0  0  0  0
 15 51  1  0  0  0  0
 16 52  1  0  0  0  0
 16 53  1  0  0  0  0
 18 54  1  0  0  0  0
 18 55  1  0  0  0  0
 20 56  1  0  0  0  0
 20 57  1  0  0  0  0
 20 58  1  0  0  0  0
M  END
>  <Alias name>
Octadecyl isocyanate

>  <deltaH°(c)>   kJ/mol
-12146

$$$$
Isopropyl palmitate
  ChemBrain IXL

 59 58  0  0  0  0  0  0  0  0  1 V2000
   20.9758   13.3942   -0.3370 C   0  0  0  0  0  0  0  0  0  0  0  0
   22.3610   12.9526   -0.1198 O   0  0  0  0  0  0  0  0  0  0  0  0
   19.8517   12.4800   -0.0350 C   0  0  0  0  0  0  0  0  0  0  0  0
   20.7527   14.5142   -0.7660 O   0  0  0  0  0  0  0  0  0  0  0  0
   22.5047   11.5696    0.3572 C   0  0  0  0  0  0  0  0  0  0  0  0
   18.5184   13.2369   -0.1895 C   0  0  0  0  0  0  0  0  0  0  0  0
   23.9398   11.3511    0.8744 C   0  0  0  0  0  0  0  0  0  0  0  0
   17.3472   12.2804    0.1074 C   0  0  0  0  0  0  0  0  0  0  0  0
   16.0129   13.0255   -0.0900 C   0  0  0  0  0  0  0  0  0  0  0  0
   14.8418   12.0735    0.2214 C   0  0  0  0  0  0  0  0  0  0  0  0
   13.5072   12.8084   -0.0095 C   0  0  0  0  0  0  0  0  0  0  0  0
   12.3360   11.8598    0.3117 C   0  0  0  0  0  0  0  0  0  0  0  0
   11.0015   12.5848    0.0507 C   0  0  0  0  0  0  0  0  0  0  0  0
    9.8301   11.6398    0.3818 C   0  0  0  0  0  0  0  0  0  0  0  0
    8.4959   12.3574    0.0995 C   0  0  0  0  0  0  0  0  0  0  0  0
    7.3242   11.4172    0.4429 C   0  0  0  0  0  0  0  0  0  0  0  0
    5.9902   12.1307    0.1495 C   0  0  0  0  0  0  0  0  0  0  0  0
    4.8183   11.1953    0.5050 C   0  0  0  0  0  0  0  0  0  0  0  0
    3.4844   11.9071    0.2071 C   0  0  0  0  0  0  0  0  0  0  0  0
```

2.3124 10.9746 0.5698 C 0 0 0 0 0 0 0 0 0 0 0 0 0
22.2224 10.5944 -0.8021 C 0 0 0 0 0 0 0 0 0 0 0 0 0
19.9449 12.1165 0.9809 H 0 0 0 0 0 0 0 0 0 0 0 0 0
19.8731 11.6426 -0.7215 H 0 0 0 0 0 0 0 0 0 0 0 0 0
21.8003 11.3896 1.1599 H 0 0 0 0 0 0 0 0 0 0 0 0 0
18.4310 13.6113 -1.2019 H 0 0 0 0 0 0 0 0 0 0 0 0 0
18.4918 14.0668 0.5058 H 0 0 0 0 0 0 0 0 0 0 0 0 0
24.1374 12.0351 1.6905 H 0 0 0 0 0 0 0 0 0 0 0 0 0
24.6442 11.5331 0.0722 H 0 0 0 0 0 0 0 0 0 0 0 0 0
24.0462 10.3316 1.2239 H 0 0 0 0 0 0 0 0 0 0 0 0 0
17.4173 11.9303 1.1298 H 0 0 0 0 0 0 0 0 0 0 0 0 0
17.3918 11.4347 -0.5677 H 0 0 0 0 0 0 0 0 0 0 0 0 0
15.9381 13.3665 -1.1152 H 0 0 0 0 0 0 0 0 0 0 0 0 0
15.9729 13.8773 0.5776 H 0 0 0 0 0 0 0 0 0 0 0 0 0
14.9029 11.7516 1.2537 H 0 0 0 0 0 0 0 0 0 0 0 0 0
14.8957 11.2098 -0.4298 H 0 0 0 0 0 0 0 0 0 0 0 0 0
13.4430 13.1240 -1.0435 H 0 0 0 0 0 0 0 0 0 0 0 0 0
13.4565 13.6761 0.6366 H 0 0 0 0 0 0 0 0 0 0 0 0 0
12.3876 11.5617 1.3516 H 0 0 0 0 0 0 0 0 0 0 0 0 0
12.3992 10.9817 -0.3191 H 0 0 0 0 0 0 0 0 0 0 0 0 0
10.9468 12.8764 -0.9909 H 0 0 0 0 0 0 0 0 0 0 0 0 0
10.9416 13.4669 0.6761 H 0 0 0 0 0 0 0 0 0 0 0 0 0
9.8758 11.3607 1.4272 H 0 0 0 0 0 0 0 0 0 0 0 0 0
9.8989 10.7506 -0.2327 H 0 0 0 0 0 0 0 0 0 0 0 0 0
8.4463 12.6283 -0.9479 H 0 0 0 0 0 0 0 0 0 0 0 0 0
8.4313 13.2515 0.7071 H 0 0 0 0 0 0 0 0 0 0 0 0 0
7.3691 11.1529 1.4922 H 0 0 0 0 0 0 0 0 0 0 0 0 0
7.3934 10.5194 -0.1588 H 0 0 0 0 0 0 0 0 0 0 0 0 0
5.9415 12.3871 -0.9015 H 0 0 0 0 0 0 0 0 0 0 0 0 0
5.9250 13.0332 0.7446 H 0 0 0 0 0 0 0 0 0 0 0 0 0
4.8651 10.9416 1.5568 H 0 0 0 0 0 0 0 0 0 0 0 0 0
4.8854 10.2914 -0.0878 H 0 0 0 0 0 0 0 0 0 0 0 0 0
3.4353 12.1560 -0.8457 H 0 0 0 0 0 0 0 0 0 0 0 0 0
3.4198 12.8138 0.7958 H 0 0 0 0 0 0 0 0 0 0 0 0 0
2.3777 10.0673 -0.0180 H 0 0 0 0 0 0 0 0 0 0 0 0 0
2.3609 10.7267 1.6229 H 0 0 0 0 0 0 0 0 0 0 0 0 0
1.3750 11.4744 0.3593 H 0 0 0 0 0 0 0 0 0 0 0 0 0
22.3454 9.5759 -0.4552 H 0 0 0 0 0 0 0 0 0 0 0 0 0
21.2080 10.7359 -1.1540 H 0 0 0 0 0 0 0 0 0 0 0 0 0
22.9155 10.7861 -1.6120 H 0 0 0 0 0 0 0 0 0 0 0 0 0
1 2 1 0 0 0 0
1 3 1 0 0 0 0
1 4 2 0 0 0 0
2 5 1 0 0 0 0
3 6 1 0 0 0 0
5 7 1 0 0 0 0
6 8 1 0 0 0 0
8 9 1 0 0 0 0
9 10 1 0 0 0 0
10 11 1 0 0 0 0
11 12 1 0 0 0 0
12 13 1 0 0 0 0
13 14 1 0 0 0 0
14 15 1 0 0 0 0
15 16 1 0 0 0 0
16 17 1 0 0 0 0
17 18 1 0 0 0 0
18 19 1 0 0 0 0
19 20 1 0 0 0 0
5 21 1 0 0 0 0
3 22 1 0 0 0 0
3 23 1 0 0 0 0
5 24 1 0 0 0 0

```
  6 25  1  0  0  0  0
  6 26  1  0  0  0  0
  7 27  1  0  0  0  0
  7 28  1  0  0  0  0
  7 29  1  0  0  0  0
  8 30  1  0  0  0  0
  8 31  1  0  0  0  0
  9 32  1  0  0  0  0
  9 33  1  0  0  0  0
 10 34  1  0  0  0  0
 10 35  1  0  0  0  0
 11 36  1  0  0  0  0
 11 37  1  0  0  0  0
 12 38  1  0  0  0  0
 12 39  1  0  0  0  0
 13 40  1  0  0  0  0
 13 41  1  0  0  0  0
 14 42  1  0  0  0  0
 14 43  1  0  0  0  0
 15 44  1  0  0  0  0
 15 45  1  0  0  0  0
 16 46  1  0  0  0  0
 16 47  1  0  0  0  0
 17 48  1  0  0  0  0
 17 49  1  0  0  0  0
 18 50  1  0  0  0  0
 18 51  1  0  0  0  0
 19 52  1  0  0  0  0
 19 53  1  0  0  0  0
 20 54  1  0  0  0  0
 20 55  1  0  0  0  0
 20 56  1  0  0  0  0
 21 57  1  0  0  0  0
 21 58  1  0  0  0  0
 21 59  1  0  0  0  0
M  END
>  <Alias name>
Isopropyl palmitate

>  <deltaH°(c)>   kJ/mol
-12082.33

$$$$
Tetra-n-butylammonium thiocyanate
  ChemBrain IXL

 56 54  0  0  0  0  0  0  0  0  1 V2000
    8.2666   10.6424   -1.6844 C   0  0  0  0  0  0  0  0  0  0  0  0
    7.0804    9.7844   -1.2037 C   0  0  0  0  0  0  0  0  0  0  0  0
    7.5823    8.7333   -0.1951 C   0  0  0  0  0  0  0  0  0  0  0  0
    6.3906    7.8734    0.2863 C   0  0  0  0  0  0  0  0  0  0  0  0
    6.8570    6.8502    1.2557 N   0  3  0  0  0  0  0  0  0  0  0  0
    9.7841    2.8377    1.8447 C   0  0  0  0  0  0  0  0  0  0  0  0
    9.3139    3.9313    0.8663 C   0  0  0  0  0  0  0  0  0  0  0  0
    8.2993    4.8509    1.5727 C   0  0  0  0  0  0  0  0  0  0  0  0
    7.8311    5.9490    0.5899 C   0  0  0  0  0  0  0  0  0  0  0  0
    6.2395   10.1431    4.9695 C   0  0  0  0  0  0  0  0  0  0  0  0
    7.2209    9.1703    4.2876 C   0  0  0  0  0  0  0  0  0  0  0  0
    6.5246    8.4918    3.0922 C   0  0  0  0  0  0  0  0  0  0  0  0
    7.5116    7.5170    2.4094 C   0  0  0  0  0  0  0  0  0  0  0  0
    3.1474    3.7829   -0.1042 C   0  0  0  0  0  0  0  0  0  0  0  0
    3.8179    4.5178    1.0727 C   0  0  0  0  0  0  0  0  0  0  0  0
    5.0235    5.3253    0.5544 C   0  0  0  0  0  0  0  0  0  0  0  0
```

```
   5.6948    6.0615    1.7370 C   0  0  0  0  0  0  0  0  0  0  0  0
  10.5532    9.7539    0.6974 N   0  5  0  0  0  0  0  0  0  0  0  0
  10.7369    8.5846    1.1576 C   0  0  0  0  0  0  0  0  0  0  0  0
  10.9842    7.0102    1.7773 S   0  0  0  0  0  0  0  0  0  0  0  0
   7.9138   11.3812   -2.3933 H   0  0  0  0  0  0  0  0  0  0  0  0
   9.0021   10.0066   -2.1616 H   0  0  0  0  0  0  0  0  0  0  0  0
   8.7176   11.1425   -0.8362 H   0  0  0  0  0  0  0  0  0  0  0  0
   6.6295    9.2843   -2.0518 H   0  0  0  0  0  0  0  0  0  0  0  0
   6.3449   10.4202   -0.7266 H   0  0  0  0  0  0  0  0  0  0  0  0
   8.0326    9.2344    0.6511 H   0  0  0  0  0  0  0  0  0  0  0  0
   8.3170    8.0995   -0.6728 H   0  0  0  0  0  0  0  0  0  0  0  0
   5.9389    7.3820   -0.5649 H   0  0  0  0  0  0  0  0  0  0  0  0
   5.6574    8.5114    0.7609 H   0  0  0  0  0  0  0  0  0  0  0  0
  10.2535    3.3000    2.7043 H   0  0  0  0  0  0  0  0  0  0  0  0
   8.9322    2.2532    2.1694 H   0  0  0  0  0  0  0  0  0  0  0  0
  10.4971    2.1915    1.3481 H   0  0  0  0  0  0  0  0  0  0  0  0
  10.1658    4.5157    0.5417 H   0  0  0  0  0  0  0  0  0  0  0  0
   8.8446    3.4690    0.0067 H   0  0  0  0  0  0  0  0  0  0  0  0
   7.4483    4.2660    1.8948 H   0  0  0  0  0  0  0  0  0  0  0  0
   8.7685    5.3099    2.4322 H   0  0  0  0  0  0  0  0  0  0  0  0
   8.6871    6.5239    0.2639 H   0  0  0  0  0  0  0  0  0  0  0  0
   7.3643    5.4856   -0.2687 H   0  0  0  0  0  0  0  0  0  0  0  0
   5.3732    9.5955    5.3197 H   0  0  0  0  0  0  0  0  0  0  0  0
   5.9265   10.8976    4.2583 H   0  0  0  0  0  0  0  0  0  0  0  0
   6.7291   10.6199    5.8095 H   0  0  0  0  0  0  0  0  0  0  0  0
   7.5339    8.4159    4.9988 H   0  0  0  0  0  0  0  0  0  0  0  0
   8.0872    9.7179    3.9375 H   0  0  0  0  0  0  0  0  0  0  0  0
   6.2120    9.2466    2.3833 H   0  0  0  0  0  0  0  0  0  0  0  0
   5.6598    7.9452    3.4432 H   0  0  0  0  0  0  0  0  0  0  0  0
   7.8264    6.7694    3.1249 H   0  0  0  0  0  0  0  0  0  0  0  0
   8.3758    8.0670    2.0641 H   0  0  0  0  0  0  0  0  0  0  0  0
   3.8614    3.1107   -0.5639 H   0  0  0  0  0  0  0  0  0  0  0  0
   2.3002    3.2154    0.2602 H   0  0  0  0  0  0  0  0  0  0  0  0
   2.8096    4.5062   -0.8361 H   0  0  0  0  0  0  0  0  0  0  0  0
   3.1039    5.1899    1.5323 H   0  0  0  0  0  0  0  0  0  0  0  0
   4.1556    3.7945    1.8045 H   0  0  0  0  0  0  0  0  0  0  0  0
   5.7353    4.6527    0.0953 H   0  0  0  0  0  0  0  0  0  0  0  0
   4.6848    6.0470   -0.1766 H   0  0  0  0  0  0  0  0  0  0  0  0
   4.9759    6.7259    2.1973 H   0  0  0  0  0  0  0  0  0  0  0  0
   6.0291    5.3364    2.4666 H   0  0  0  0  0  0  0  0  0  0  0  0
  1  2  1  0  0  0  0
  2  3  1  0  0  0  0
  3  4  1  0  0  0  0
  4  5  1  0  0  0  0
  6  7  1  0  0  0  0
  7  8  1  0  0  0  0
  8  9  1  0  0  0  0
  9  5  1  0  0  0  0
 10 11  1  0  0  0  0
 11 12  1  0  0  0  0
 12 13  1  0  0  0  0
 14 15  1  0  0  0  0
 15 16  1  0  0  0  0
 16 17  1  0  0  0  0
 13  5  1  0  0  0  0
 17  5  1  0  0  0  0
 18 19  2  0  0  0  0
 19 20  2  0  0  0  0
  1 21  1  0  0  0  0
  1 22  1  0  0  0  0
  1 23  1  0  0  0  0
  2 24  1  0  0  0  0
  2 25  1  0  0  0  0
```

```
  3 26  1  0  0  0  0
  3 27  1  0  0  0  0
  4 28  1  0  0  0  0
  4 29  1  0  0  0  0
  6 30  1  0  0  0  0
  6 31  1  0  0  0  0
  6 32  1  0  0  0  0
  7 33  1  0  0  0  0
  7 34  1  0  0  0  0
  8 35  1  0  0  0  0
  8 36  1  0  0  0  0
  9 37  1  0  0  0  0
  9 38  1  0  0  0  0
 10 39  1  0  0  0  0
 10 40  1  0  0  0  0
 10 41  1  0  0  0  0
 11 42  1  0  0  0  0
 11 43  1  0  0  0  0
 12 44  1  0  0  0  0
 12 45  1  0  0  0  0
 13 46  1  0  0  0  0
 13 47  1  0  0  0  0
 14 48  1  0  0  0  0
 14 49  1  0  0  0  0
 14 50  1  0  0  0  0
 15 51  1  0  0  0  0
 15 52  1  0  0  0  0
 16 53  1  0  0  0  0
 16 54  1  0  0  0  0
 17 55  1  0  0  0  0
 17 56  1  0  0  0  0
M  END
>  <Alias name>
Tetra-n-butylammonium thiocyanate

>  <deltaH°(c)>   kJ/mol
-12078

$$$$
trans-Heptacyclene
  ChemBrain IXL

 40 46  0  0  0  0  0  0  0  0  1 V2000
    4.6093    6.4070    0.0000 C   0  0  0  0  0  0  0  0  0  0  0  0
    5.3525    5.3578    0.0857 C   0  0  0  0  0  0  0  0  0  0  0  0
    5.2520    7.4805    0.3033 C   0  0  0  0  0  0  0  0  0  0  0  0
    3.3041    6.3844   -0.3650 C   0  0  0  0  0  0  0  0  0  0  0  0
    6.6787    5.6932    0.4803 C   0  0  0  0  0  0  0  0  0  0  0  0
    4.8297    4.1007   -0.1979 C   0  0  0  0  0  0  0  0  0  0  0  0
    6.5980    7.1989    0.6404 C   0  0  0  0  0  0  0  0  0  0  0  0
    4.6216    8.7170    0.2598 C   0  0  0  0  0  0  0  0  0  0  0  0
    2.7157    5.1593   -0.6630 C   0  0  0  0  0  0  0  0  0  0  0  0
    2.6084    7.5879   -0.4272 C   0  0  0  0  0  0  0  0  0  0  0  0
    3.4888    4.0013   -0.5772 C   0  0  0  0  0  0  0  0  0  0  0  0
    3.2771    8.7706   -0.1113 C   0  0  0  0  0  0  0  0  0  0  0  0
    9.6145    6.7102   -0.0313 C   0  0  0  0  0  0  0  0  0  0  0  0
    8.9995    5.6216   -0.3427 C   0  0  0  0  0  0  0  0  0  0  0  0
    8.8458    7.7398   -0.1125 C   0  0  0  0  0  0  0  0  0  0  0  0
   10.9177    6.7665    0.3373 C   0  0  0  0  0  0  0  0  0  0  0  0
    7.6381    5.8727   -0.6754 C   0  0  0  0  0  0  0  0  0  0  0  0
    9.6706    4.4037   -0.3204 C   0  0  0  0  0  0  0  0  0  0  0  0
    7.5384    7.3775   -0.5176 C   0  0  0  0  0  0  0  0  0  0  0  0
    9.3263    9.0058    0.1952 C   0  0  0  0  0  0  0  0  0  0  0  0
```

```
  11.6503    5.5844    0.3811 C   0  0  0  0  0  0  0  0  0  0  0  0
  11.4671    8.0042    0.6572 C   0  0  0  0  0  0  0  0  0  0  0  0
  11.0184    4.3867    0.0466 C   0  0  0  0  0  0  0  0  0  0  0  0
  10.6595    9.1394    0.5861 C   0  0  0  0  0  0  0  0  0  0  0  0
   7.0663    5.1776    1.3527 H   0  0  0  0  0  0  0  0  0  0  0  0
   5.4488    3.2139   -0.1272 H   0  0  0  0  0  0  0  0  0  0  0  0
   6.9616    7.5681    1.5954 H   0  0  0  0  0  0  0  0  0  0  0  0
   5.1613    9.6229    0.5098 H   0  0  0  0  0  0  0  0  0  0  0  0
   1.6711    5.1046   -0.9581 H   0  0  0  0  0  0  0  0  0  0  0  0
   1.5616    7.6070   -0.7182 H   0  0  0  0  0  0  0  0  0  0  0  0
   3.0516    3.0333   -0.8044 H   0  0  0  0  0  0  0  0  0  0  0  0
   2.7566    9.7224   -0.1542 H   0  0  0  0  0  0  0  0  0  0  0  0
   7.3033    5.5079   -1.6407 H   0  0  0  0  0  0  0  0  0  0  0  0
   9.1597    3.4843   -0.5819 H   0  0  0  0  0  0  0  0  0  0  0  0
   7.1219    7.8918   -1.3793 H   0  0  0  0  0  0  0  0  0  0  0  0
   8.6802    9.8737    0.1325 H   0  0  0  0  0  0  0  0  0  0  0  0
  12.6978    5.5933    0.6714 H   0  0  0  0  0  0  0  0  0  0  0  0
  12.5079    8.0859    0.9589 H   0  0  0  0  0  0  0  0  0  0  0  0
  11.5697    3.4511    0.0728 H   0  0  0  0  0  0  0  0  0  0  0  0
  11.0645   10.1160    0.8326 H   0  0  0  0  0  0  0  0  0  0  0  0
  1  2  4  0  0  0  0
  1  3  4  0  0  0  0
  1  4  4  0  0  0  0
  2  5  1  0  0  0  0
  2  6  4  0  0  0  0
  3  7  1  0  0  0  0
  3  8  4  0  0  0  0
  4  9  4  0  0  0  0
  4 10  4  0  0  0  0
  5  7  1  0  0  0  0
  6 11  4  0  0  0  0
  8 12  4  0  0  0  0
  9 11  4  0  0  0  0
 10 12  4  0  0  0  0
 13 14  4  0  0  0  0
 13 15  4  0  0  0  0
 13 16  4  0  0  0  0
 14 17  1  0  0  0  0
 14 18  4  0  0  0  0
 15 19  1  0  0  0  0
 15 20  4  0  0  0  0
 16 21  4  0  0  0  0
 16 22  4  0  0  0  0
 17 19  1  0  0  0  0
 18 23  4  0  0  0  0
 20 24  4  0  0  0  0
 21 23  4  0  0  0  0
 22 24  4  0  0  0  0
 17  5  1  0  0  0  0
 19  7  1  0  0  0  0
  5 25  1  0  0  0  0
  6 26  1  0  0  0  0
  7 27  1  0  0  0  0
  8 28  1  0  0  0  0
  9 29  1  0  0  0  0
 10 30  1  0  0  0  0
 11 31  1  0  0  0  0
 12 32  1  0  0  0  0
 17 33  1  0  0  0  0
 18 34  1  0  0  0  0
 19 35  1  0  0  0  0
 20 36  1  0  0  0  0
 21 37  1  0  0  0  0
```

```
 22 38  1  0  0  0  0
 23 39  1  0  0  0  0
 24 40  1  0  0  0  0
M  END
>  <Alias name>
trans-Heptacyclene

>  <deltaH°(c)>   kJ/mol
-12056.4

$$$$
Octadecane
  ChemBrain IXL

 56 55  0  0  0  0  0  0  0  0  1 V2000
    9.4370    7.9130   -0.4689 C   0  0  0  0  0  0  0  0  0  0  0  0  0
    8.7065    8.8986    0.4638 C   0  0  0  0  0  0  0  0  0  0  0  0  0
    7.7837    8.1153    1.4176 C   0  0  0  0  0  0  0  0  0  0  0  0  0
    7.0529    9.1010    2.3500 C   0  0  0  0  0  0  0  0  0  0  0  0  0
    6.1298    8.3177    3.3036 C   0  0  0  0  0  0  0  0  0  0  0  0  0
    5.3988    9.3034    4.2358 C   0  0  0  0  0  0  0  0  0  0  0  0  0
    4.4754    8.5202    5.1892 C   0  0  0  0  0  0  0  0  0  0  0  0  0
    3.7443    9.5060    6.1212 C   0  0  0  0  0  0  0  0  0  0  0  0  0
    2.8208    8.7228    7.0745 C   0  0  0  0  0  0  0  0  0  0  0  0  0
   10.3596    8.6963   -1.4230 C   0  0  0  0  0  0  0  0  0  0  0  0  0
    2.0896    9.7084    8.0066 C   0  0  0  0  0  0  0  0  0  0  0  0  0
   11.0897    7.7108   -2.3560 C   0  0  0  0  0  0  0  0  0  0  0  0  0
   12.0120    8.4941   -3.3103 C   0  0  0  0  0  0  0  0  0  0  0  0  0
   12.7419    7.5086   -4.2435 C   0  0  0  0  0  0  0  0  0  0  0  0  0
   13.6640    8.2919   -5.1980 C   0  0  0  0  0  0  0  0  0  0  0  0  0
   14.3937    7.3064   -6.1314 C   0  0  0  0  0  0  0  0  0  0  0  0  0
   15.3157    8.0898   -7.0859 C   0  0  0  0  0  0  0  0  0  0  0  0  0
   16.0454    7.1043   -8.0193 C   0  0  0  0  0  0  0  0  0  0  0  0  0
    8.7116    7.3576   -1.0449 H   0  0  0  0  0  0  0  0  0  0  0  0  0
   10.0277    7.2287    0.1220 H   0  0  0  0  0  0  0  0  0  0  0  0  0
    8.1160    9.5831   -0.1271 H   0  0  0  0  0  0  0  0  0  0  0  0  0
    9.4319    9.4538    1.0400 H   0  0  0  0  0  0  0  0  0  0  0  0  0
    7.0584    7.5599    0.8414 H   0  0  0  0  0  0  0  0  0  0  0  0  0
    8.3742    7.4310    2.0087 H   0  0  0  0  0  0  0  0  0  0  0  0  0
    7.7781    9.6562    2.9264 H   0  0  0  0  0  0  0  0  0  0  0  0  0
    6.4626    9.7854    1.7589 H   0  0  0  0  0  0  0  0  0  0  0  0  0
    5.4047    7.7623    2.7272 H   0  0  0  0  0  0  0  0  0  0  0  0  0
    6.7201    7.6335    3.8949 H   0  0  0  0  0  0  0  0  0  0  0  0  0
    4.8085    9.9878    3.6444 H   0  0  0  0  0  0  0  0  0  0  0  0  0
    6.1238    9.8588    4.8123 H   0  0  0  0  0  0  0  0  0  0  0  0  0
    3.7504    7.9648    4.6127 H   0  0  0  0  0  0  0  0  0  0  0  0  0
    5.0657    7.8360    5.7806 H   0  0  0  0  0  0  0  0  0  0  0  0  0
    3.1541   10.1903    5.5298 H   0  0  0  0  0  0  0  0  0  0  0  0  0
    4.4692   10.0613    6.6978 H   0  0  0  0  0  0  0  0  0  0  0  0  0
    3.4110    8.0385    7.6660 H   0  0  0  0  0  0  0  0  0  0  0  0  0
    2.0959    8.1673    6.4980 H   0  0  0  0  0  0  0  0  0  0  0  0  0
   11.0851    9.2515   -0.8470 H   0  0  0  0  0  0  0  0  0  0  0  0  0
    9.7688    9.3808   -2.0137 H   0  0  0  0  0  0  0  0  0  0  0  0  0
   11.6806    7.0264   -1.7653 H   0  0  0  0  0  0  0  0  0  0  0  0  0
   10.3641    7.1554   -2.9318 H   0  0  0  0  0  0  0  0  0  0  0  0  0
   12.7377    9.0493   -2.7345 H   0  0  0  0  0  0  0  0  0  0  0  0  0
   11.4211    9.1786   -3.9008 H   0  0  0  0  0  0  0  0  0  0  0  0  0
   13.3329    6.8241   -3.6530 H   0  0  0  0  0  0  0  0  0  0  0  0  0
   12.0161    6.9533   -4.8192 H   0  0  0  0  0  0  0  0  0  0  0  0  0
   13.0730    8.9765   -5.7884 H   0  0  0  0  0  0  0  0  0  0  0  0  0
   14.3898    8.8471   -4.6223 H   0  0  0  0  0  0  0  0  0  0  0  0  0
   14.9848    6.6220   -5.5410 H   0  0  0  0  0  0  0  0  0  0  0  0  0
   13.6679    6.7512   -6.7070 H   0  0  0  0  0  0  0  0  0  0  0  0  0
```

```
 16.0416    8.6450   -6.5103 H   0  0  0  0  0  0  0  0  0  0  0  0
 14.7247    8.7743   -7.6763 H   0  0  0  0  0  0  0  0  0  0  0  0
 16.6915    7.6534   -8.6882 H   0  0  0  0  0  0  0  0  0  0  0  0
 16.6365    6.4198   -7.4289 H   0  0  0  0  0  0  0  0  0  0  0  0
 15.3195    6.5491   -8.5949 H   0  0  0  0  0  0  0  0  0  0  0  0
  1.4994   10.3927    7.4151 H   0  0  0  0  0  0  0  0  0  0  0  0
  2.8145   10.2638    8.5831 H   0  0  0  0  0  0  0  0  0  0  0  0
  1.4425    9.1595    8.6746 H   0  0  0  0  0  0  0  0  0  0  0  0
  1  2  1  0  0  0  0
  2  3  1  0  0  0  0
  3  4  1  0  0  0  0
  4  5  1  0  0  0  0
  5  6  1  0  0  0  0
  6  7  1  0  0  0  0
  7  8  1  0  0  0  0
  8  9  1  0  0  0  0
  1 19  1  0  0  0  0
  1 20  1  0  0  0  0
  1 10  1  0  0  0  0
  2 21  1  0  0  0  0
  2 22  1  0  0  0  0
  3 23  1  0  0  0  0
  3 24  1  0  0  0  0
  4 25  1  0  0  0  0
  4 26  1  0  0  0  0
  5 27  1  0  0  0  0
  5 28  1  0  0  0  0
  6 29  1  0  0  0  0
  6 30  1  0  0  0  0
  7 31  1  0  0  0  0
  7 32  1  0  0  0  0
  8 33  1  0  0  0  0
  8 34  1  0  0  0  0
  9 35  1  0  0  0  0
  9 36  1  0  0  0  0
  9 11  1  0  0  0  0
 10 37  1  0  0  0  0
 10 38  1  0  0  0  0
 12 13  1  0  0  0  0
 13 14  1  0  0  0  0
 14 15  1  0  0  0  0
 15 16  1  0  0  0  0
 16 17  1  0  0  0  0
 17 18  1  0  0  0  0
 12 39  1  0  0  0  0
 12 40  1  0  0  0  0
 13 41  1  0  0  0  0
 13 42  1  0  0  0  0
 14 43  1  0  0  0  0
 14 44  1  0  0  0  0
 15 45  1  0  0  0  0
 15 46  1  0  0  0  0
 16 47  1  0  0  0  0
 16 48  1  0  0  0  0
 17 49  1  0  0  0  0
 17 50  1  0  0  0  0
 18 51  1  0  0  0  0
 18 52  1  0  0  0  0
 18 53  1  0  0  0  0
 10 12  1  0  0  0  0
 11 54  1  0  0  0  0
 11 55  1  0  0  0  0
 11 56  1  0  0  0  0
```

```
M  END
>  <Alias name>
Octadecane

>  <deltaH°(c)>   kJ/mol
-12009

$$$$
cis-Heptacyclene
  ChemBrain IXL

 40 46  0  0  0  0  0  0  0  0  1 V2000
    4.2099    5.4229   -0.2854 C   0  0  0  0  0  0  0  0  0  0  0  0
    4.5553    4.5707   -1.1915 C   0  0  0  0  0  0  0  0  0  0  0  0
    4.6249    6.6146   -0.5606 C   0  0  0  0  0  0  0  0  0  0  0  0
    3.4900    5.1035    0.8310 C   0  0  0  0  0  0  0  0  0  0  0  0
    5.3199    5.2023   -2.2554 C   0  0  0  0  0  0  0  0  0  0  0  0
    4.1894    3.2294   -1.0534 C   0  0  0  0  0  0  0  0  0  0  0  0
    5.3617    6.6495   -1.8145 C   0  0  0  0  0  0  0  0  0  0  0  0
    4.3416    7.6647    0.3154 C   0  0  0  0  0  0  0  0  0  0  0  0
    3.0952    3.7867    1.0229 C   0  0  0  0  0  0  0  0  0  0  0  0
    3.1756    6.1057    1.7389 C   0  0  0  0  0  0  0  0  0  0  0  0
    3.4489    2.8362    0.0693 C   0  0  0  0  0  0  0  0  0  0  0  0
    3.6068    7.4035    1.4792 C   0  0  0  0  0  0  0  0  0  0  0  0
    7.5274    5.2463    0.0763 C   0  0  0  0  0  0  0  0  0  0  0  0
    7.2666    4.4166   -0.8784 C   0  0  0  0  0  0  0  0  0  0  0  0
    7.3393    6.4714   -0.2846 C   0  0  0  0  0  0  0  0  0  0  0  0
    7.9530    4.8716    1.3193 C   0  0  0  0  0  0  0  0  0  0  0  0
    6.8360    5.1059   -2.0851 C   0  0  0  0  0  0  0  0  0  0  0  0
    7.4151    3.0449   -0.6618 C   0  0  0  0  0  0  0  0  0  0  0  0
    6.8799    6.5582   -1.6617 C   0  0  0  0  0  0  0  0  0  0  0  0
    7.5728    7.5038    0.6272 C   0  0  0  0  0  0  0  0  0  0  0  0
    8.1211    3.5212    1.5946 C   0  0  0  0  0  0  0  0  0  0  0  0
    8.2042    5.8527    2.2685 C   0  0  0  0  0  0  0  0  0  0  0  0
    7.8485    2.5947    0.5920 C   0  0  0  0  0  0  0  0  0  0  0  0
    8.0118    7.1865    1.9194 C   0  0  0  0  0  0  0  0  0  0  0  0
    4.9602    5.0705   -3.2650 H   0  0  0  0  0  0  0  0  0  0  0  0
    4.4712    2.5023   -1.7996 H   0  0  0  0  0  0  0  0  0  0  0  0
    5.0080    7.3481   -2.5570 H   0  0  0  0  0  0  0  0  0  0  0  0
    4.6817    8.6666    0.1015 H   0  0  0  0  0  0  0  0  0  0  0  0
    2.5195    3.5027    1.9036 H   0  0  0  0  0  0  0  0  0  0  0  0
    2.6023    5.8805    2.6379 H   0  0  0  0  0  0  0  0  0  0  0  0
    3.1513    1.7997    0.1988 H   0  0  0  0  0  0  0  0  0  0  0  0
    3.3729    8.2038    2.1754 H   0  0  0  0  0  0  0  0  0  0  0  0
    7.3944    4.8904   -2.9830 H   0  0  0  0  0  0  0  0  0  0  0  0
    7.2005    2.3373   -1.4482 H   0  0  0  0  0  0  0  0  0  0  0  0
    7.4492    7.1848   -2.3320 H   0  0  0  0  0  0  0  0  0  0  0  0
    7.4186    8.5338    0.3439 H   0  0  0  0  0  0  0  0  0  0  0  0
    8.4599    3.1929    2.5769 H   0  0  0  0  0  0  0  0  0  0  0  0
    8.5453    5.5836    3.2679 H   0  0  0  0  0  0  0  0  0  0  0  0
    7.9726    1.5328    0.7845 H   0  0  0  0  0  0  0  0  0  0  0  0
    8.2015    7.9712    2.6460 H   0  0  0  0  0  0  0  0  0  0  0  0
  1  2  4  0  0  0  0
  1  3  4  0  0  0  0
  1  4  4  0  0  0  0
  2  5  1  0  0  0  0
  2  6  4  0  0  0  0
  3  7  1  0  0  0  0
  3  8  4  0  0  0  0
  4  9  4  0  0  0  0
  4 10  4  0  0  0  0
  5  7  1  0  0  0  0
  6 11  4  0  0  0  0
```

```
  8 12  4  0  0  0  0
  9 11  4  0  0  0  0
 10 12  4  0  0  0  0
 13 14  4  0  0  0  0
 13 15  4  0  0  0  0
 13 16  4  0  0  0  0
 14 17  1  0  0  0  0
 14 18  4  0  0  0  0
 15 19  1  0  0  0  0
 15 20  4  0  0  0  0
 16 21  4  0  0  0  0
 16 22  4  0  0  0  0
 17 19  1  0  0  0  0
 18 23  4  0  0  0  0
 20 24  4  0  0  0  0
 21 23  4  0  0  0  0
 22 24  4  0  0  0  0
 17  5  1  0  0  0  0
 19  7  1  0  0  0  0
  5 25  1  0  0  0  0
  6 26  1  0  0  0  0
  7 27  1  0  0  0  0
  8 28  1  0  0  0  0
  9 29  1  0  0  0  0
 10 30  1  0  0  0  0
 11 31  1  0  0  0  0
 12 32  1  0  0  0  0
 17 33  1  0  0  0  0
 18 34  1  0  0  0  0
 19 35  1  0  0  0  0
 20 36  1  0  0  0  0
 21 37  1  0  0  0  0
 22 38  1  0  0  0  0
 23 39  1  0  0  0  0
 24 40  1  0  0  0  0
M  END
>  <Alias name>
cis-Heptacyclene

>  <deltaH°(c)>   kJ/mol
-12008

$$$$
Progesterone
  ChemBrain IXL

 53 56  0  0  0  0  0  0  0  0  1 V2000
    5.4251    6.7689    0.0995 C   0  0  0  0  0  0  0  0  0  0  0  0
    4.3657    6.4808   -0.9809 C   0  0  0  0  0  0  0  0  0  0  0  0
    3.0113    6.2524   -0.3163 C   0  0  0  0  0  0  0  0  0  0  0  0
    2.6368    7.4947    0.4964 C   0  0  0  0  0  0  0  0  0  0  0  0
    3.6810    8.3106    1.1199 C   0  0  0  0  0  0  0  0  0  0  0  0
    4.9823    8.0165    0.9596 C   0  0  0  0  0  0  0  0  0  0  0  0
    5.5631    5.5397    1.0157 C   0  0  0  0  0  0  0  0  0  0  0  0
    6.7820    7.0352   -0.5976 C   0  0  0  0  0  0  0  0  0  0  0  0
    6.0361    8.8918    1.6895 C   0  0  0  0  0  0  0  0  0  0  0  0
    7.7891    7.6584    0.4174 C   0  0  0  0  0  0  0  0  0  0  0  0
    7.2870    9.0436    0.8196 C   0  0  0  0  0  0  0  0  0  0  0  0
    7.3439    5.6755   -1.1795 C   0  0  0  0  0  0  0  0  0  0  0  0
    9.1287    7.7869   -0.2728 C   0  0  0  0  0  0  0  0  0  0  0  0
    8.7886    5.8735   -1.7462 C   0  0  0  0  0  0  0  0  0  0  0  0
    9.6369    6.4535   -0.6445 C   0  0  0  0  0  0  0  0  0  0  0  0
   10.2376    8.3958    0.5755 C   0  0  0  0  0  0  0  0  0  0  0  0
```

9.5766	5.5421	0.5991	C	0	0	0	0	0	0	0	0	0	0	0	0
11.1014	6.6881	-1.0174	C	0	0	0	0	0	0	0	0	0	0	0	0
11.5638	7.8409	-0.0583	C	0	0	0	0	0	0	0	0	0	0	0	0
11.9446	5.4148	-0.8078	C	0	0	0	0	0	0	0	0	0	0	0	0
13.4156	5.5290	-0.3629	C	0	0	0	0	0	0	0	0	0	0	0	0
1.4644	7.8096	0.6186	O	0	0	0	0	0	0	0	0	0	0	0	0
11.4662	4.3199	-1.0562	O	0	0	0	0	0	0	0	0	0	0	0	0
4.6482	5.5992	-1.5374	H	0	0	0	0	0	0	0	0	0	0	0	0
4.2991	7.3246	-1.6539	H	0	0	0	0	0	0	0	0	0	0	0	0
2.2616	6.0759	-1.0741	H	0	0	0	0	0	0	0	0	0	0	0	0
3.0709	5.3964	0.3389	H	0	0	0	0	0	0	0	0	0	0	0	0
3.3995	9.1611	1.7228	H	0	0	0	0	0	0	0	0	0	0	0	0
4.6168	5.3388	1.4936	H	0	0	0	0	0	0	0	0	0	0	0	0
6.3131	5.7344	1.7676	H	0	0	0	0	0	0	0	0	0	0	0	0
5.8582	4.6838	0.4258	H	0	0	0	0	0	0	0	0	0	0	0	0
6.6289	7.7295	-1.4132	H	0	0	0	0	0	0	0	0	0	0	0	0
5.6158	9.8683	1.8845	H	0	0	0	0	0	0	0	0	0	0	0	0
6.3049	8.4251	2.6234	H	0	0	0	0	0	0	0	0	0	0	0	0
7.8770	7.0279	1.2882	H	0	0	0	0	0	0	0	0	0	0	0	0
8.0546	9.5617	1.3741	H	0	0	0	0	0	0	0	0	0	0	0	0
7.0408	9.6103	-0.0707	H	0	0	0	0	0	0	0	0	0	0	0	0
6.6953	5.3285	-1.9689	H	0	0	0	0	0	0	0	0	0	0	0	0
7.3703	4.9372	-0.3900	H	0	0	0	0	0	0	0	0	0	0	0	0
9.0075	8.3802	-1.1665	H	0	0	0	0	0	0	0	0	0	0	0	0
9.1925	4.9192	-2.0524	H	0	0	0	0	0	0	0	0	0	0	0	0
8.7637	6.5480	-2.5865	H	0	0	0	0	0	0	0	0	0	0	0	0
10.2102	9.4698	0.5303	H	0	0	0	0	0	0	0	0	0	0	0	0
10.1474	8.0566	1.6024	H	0	0	0	0	0	0	0	0	0	0	0	0
8.5500	5.4319	0.9165	H	0	0	0	0	0	0	0	0	0	0	0	0
10.1527	5.9907	1.3993	H	0	0	0	0	0	0	0	0	0	0	0	0
9.9867	4.5743	0.3610	H	0	0	0	0	0	0	0	0	0	0	0	0
11.1726	7.0135	-2.0448	H	0	0	0	0	0	0	0	0	0	0	0	0
12.2165	7.4534	0.7086	H	0	0	0	0	0	0	0	0	0	0	0	0
12.0605	8.6172	-0.6198	H	0	0	0	0	0	0	0	0	0	0	0	0
13.4748	6.1303	0.5321	H	0	0	0	0	0	0	0	0	0	0	0	0
13.9946	5.9940	-1.1476	H	0	0	0	0	0	0	0	0	0	0	0	0
13.8086	4.5435	-0.1611	H	0	0	0	0	0	0	0	0	0	0	0	0

1	2	1	0	0	0	0
2	3	1	0	0	0	0
3	4	1	0	0	0	0
4	5	1	0	0	0	0
5	6	2	0	0	0	0
6	1	1	0	0	0	0
1	7	1	0	0	0	0
1	8	1	0	0	0	0
6	9	1	0	0	0	0
10	8	1	0	0	0	0
9	11	1	0	0	0	0
11	10	1	0	0	0	0
8	12	1	0	0	0	0
10	13	1	0	0	0	0
12	14	1	0	0	0	0
14	15	1	0	0	0	0
15	13	1	0	0	0	0
13	16	1	0	0	0	0
15	17	1	0	0	0	0
15	18	1	0	0	0	0
16	19	1	0	0	0	0
19	18	1	0	0	0	0
18	20	1	0	0	0	0
20	21	1	0	0	0	0
4	22	2	0	0	0	0
20	23	2	0	0	0	0

```
  2 24  1  0  0  0  0
  2 25  1  0  0  0  0
  3 26  1  0  0  0  0
  3 27  1  0  0  0  0
  5 28  1  0  0  0  0
  7 29  1  0  0  0  0
  7 30  1  0  0  0  0
  7 31  1  0  0  0  0
  8 32  1  0  0  0  0
  9 33  1  0  0  0  0
  9 34  1  0  0  0  0
 10 35  1  0  0  0  0
 11 36  1  0  0  0  0
 11 37  1  0  0  0  0
 12 38  1  0  0  0  0
 12 39  1  0  0  0  0
 13 40  1  0  0  0  0
 14 41  1  0  0  0  0
 14 42  1  0  0  0  0
 16 43  1  0  0  0  0
 16 44  1  0  0  0  0
 17 45  1  0  0  0  0
 17 46  1  0  0  0  0
 17 47  1  0  0  0  0
 18 48  1  0  0  0  0
 19 49  1  0  0  0  0
 19 50  1  0  0  0  0
 21 51  1  0  0  0  0
 21 52  1  0  0  0  0
 21 53  1  0  0  0  0
M  END
>  <Alias name>
Progesterone

>  <deltaH°(c)>   kJ/mol
-12008

$$$$
Propyl palmitate
  ChemBrain IXL

 59 58  0  0  0  0  0  0  0  0  1 V2000
   20.6370    9.9856   -0.0832 C   0  0  0  0  0  0  0  0  0  0  0  0
   20.8687   11.4372   -0.0696 O   0  0  0  0  0  0  0  0  0  0  0  0
   19.2548    9.4571   -0.1116 C   0  0  0  0  0  0  0  0  0  0  0  0
   21.5839    9.2166   -0.0711 O   0  0  0  0  0  0  0  0  0  0  0  0
   22.3774   13.3784   -0.0309 C   0  0  0  0  0  0  0  0  0  0  0  0
   18.2555   10.6291   -0.0606 C   0  0  0  0  0  0  0  0  0  0  0  0
   23.8591   13.8011   -0.0044 C   0  0  0  0  0  0  0  0  0  0  0  0
   16.8164   10.0788   -0.0919 C   0  0  0  0  0  0  0  0  0  0  0  0
   15.8170   11.2508   -0.0428 C   0  0  0  0  0  0  0  0  0  0  0  0
   14.3780   10.7004   -0.0780 C   0  0  0  0  0  0  0  0  0  0  0  0
   13.3784   11.8723   -0.0297 C   0  0  0  0  0  0  0  0  0  0  0  0
   11.9395   11.3220   -0.0677 C   0  0  0  0  0  0  0  0  0  0  0  0
   10.9399   12.4937   -0.0182 C   0  0  0  0  0  0  0  0  0  0  0  0
    9.5010   11.9433   -0.0574 C   0  0  0  0  0  0  0  0  0  0  0  0
    8.5012   13.1148   -0.0054 C   0  0  0  0  0  0  0  0  0  0  0  0
    7.0624   12.5644   -0.0447 C   0  0  0  0  0  0  0  0  0  0  0  0
    6.0626   13.7357    0.0099 C   0  0  0  0  0  0  0  0  0  0  0  0
    4.6238   13.1852   -0.0291 C   0  0  0  0  0  0  0  0  0  0  0  0
    3.6240   14.3565    0.0271 C   0  0  0  0  0  0  0  0  0  0  0  0
    2.1852   13.8061   -0.0119 C   0  0  0  0  0  0  0  0  0  0  0  0
   22.2821   11.8404   -0.0437 C   0  0  0  0  0  0  0  0  0  0  0  0
```

```
 19.1028    8.8926   -1.0233 H   0  0  0  0  0  0  0  0  0  0  0  0
 19.0976    8.8113    0.7434 H   0  0  0  0  0  0  0  0  0  0  0  0
 21.9042   13.7768   -0.9198 H   0  0  0  0  0  0  0  0  0  0  0  0
 21.8763   13.7638    0.8484 H   0  0  0  0  0  0  0  0  0  0  0  0
 18.4066   11.1929    0.8516 H   0  0  0  0  0  0  0  0  0  0  0  0
 18.4134   11.2757   -0.9149 H   0  0  0  0  0  0  0  0  0  0  0  0
 23.9261   14.8820    0.0046 H   0  0  0  0  0  0  0  0  0  0  0  0
 24.3602   13.4157   -0.8838 H   0  0  0  0  0  0  0  0  0  0  0  0
 24.3323   13.4026    0.8845 H   0  0  0  0  0  0  0  0  0  0  0  0
 16.6658    9.5139   -1.0036 H   0  0  0  0  0  0  0  0  0  0  0  0
 16.6581    9.4333    0.7631 H   0  0  0  0  0  0  0  0  0  0  0  0
 15.9658   11.8142    0.8700 H   0  0  0  0  0  0  0  0  0  0  0  0
 15.9770   11.8977   -0.8965 H   0  0  0  0  0  0  0  0  0  0  0  0
 14.2294   10.1366   -0.9906 H   0  0  0  0  0  0  0  0  0  0  0  0
 14.2178   10.0539    0.7760 H   0  0  0  0  0  0  0  0  0  0  0  0
 13.5257   12.4350    0.8839 H   0  0  0  0  0  0  0  0  0  0  0  0
 13.5399   12.5199   -0.8826 H   0  0  0  0  0  0  0  0  0  0  0  0
 11.7920   10.7599   -0.9816 H   0  0  0  0  0  0  0  0  0  0  0  0
 11.7784   10.6737    0.7847 H   0  0  0  0  0  0  0  0  0  0  0  0
 11.0868   13.0552    0.8961 H   0  0  0  0  0  0  0  0  0  0  0  0
 11.1016   13.1424   -0.8702 H   0  0  0  0  0  0  0  0  0  0  0  0
  9.3536   11.3832   -0.9726 H   0  0  0  0  0  0  0  0  0  0  0  0
  9.3399   11.2931    0.7936 H   0  0  0  0  0  0  0  0  0  0  0  0
  8.6487   13.6748    0.9098 H   0  0  0  0  0  0  0  0  0  0  0  0
  8.6624   13.7650   -0.8564 H   0  0  0  0  0  0  0  0  0  0  0  0
  6.9145   12.0059   -0.9607 H   0  0  0  0  0  0  0  0  0  0  0  0
  6.9019   11.9127    0.8053 H   0  0  0  0  0  0  0  0  0  0  0  0
  6.2107   14.2943    0.9259 H   0  0  0  0  0  0  0  0  0  0  0  0
  6.2231   14.3872   -0.8402 H   0  0  0  0  0  0  0  0  0  0  0  0
  4.4754   12.6276   -0.9456 H   0  0  0  0  0  0  0  0  0  0  0  0
  4.4637   12.5328    0.8204 H   0  0  0  0  0  0  0  0  0  0  0  0
  3.7724   14.9142    0.9436 H   0  0  0  0  0  0  0  0  0  0  0  0
  3.7841   15.0089   -0.8224 H   0  0  0  0  0  0  0  0  0  0  0  0
  2.0252   13.1536    0.8375 H   0  0  0  0  0  0  0  0  0  0  0  0
  1.4826   14.6292    0.0277 H   0  0  0  0  0  0  0  0  0  0  0  0
  2.0367   13.2485   -0.9284 H   0  0  0  0  0  0  0  0  0  0  0  0
 22.7549   11.4420    0.8455 H   0  0  0  0  0  0  0  0  0  0  0  0
 22.7837   11.4549   -0.9227 H   0  0  0  0  0  0  0  0  0  0  0  0
  1  2  1  0  0  0  0
  1  3  1  0  0  0  0
  1  4  2  0  0  0  0
  3  6  1  0  0  0  0
  5  7  1  0  0  0  0
  6  8  1  0  0  0  0
  8  9  1  0  0  0  0
  9 10  1  0  0  0  0
 10 11  1  0  0  0  0
 11 12  1  0  0  0  0
 12 13  1  0  0  0  0
 13 14  1  0  0  0  0
 14 15  1  0  0  0  0
 15 16  1  0  0  0  0
 16 17  1  0  0  0  0
 17 18  1  0  0  0  0
 18 19  1  0  0  0  0
 19 20  1  0  0  0  0
  5 21  1  0  0  0  0
 21  2  1  0  0  0  0
  3 22  1  0  0  0  0
  3 23  1  0  0  0  0
  5 24  1  0  0  0  0
  5 25  1  0  0  0  0
  6 26  1  0  0  0  0
```

```
  6 27  1  0  0  0  0
  7 28  1  0  0  0  0
  7 29  1  0  0  0  0
  7 30  1  0  0  0  0
  8 31  1  0  0  0  0
  8 32  1  0  0  0  0
  9 33  1  0  0  0  0
  9 34  1  0  0  0  0
 10 35  1  0  0  0  0
 10 36  1  0  0  0  0
 11 37  1  0  0  0  0
 11 38  1  0  0  0  0
 12 39  1  0  0  0  0
 12 40  1  0  0  0  0
 13 41  1  0  0  0  0
 13 42  1  0  0  0  0
 14 43  1  0  0  0  0
 14 44  1  0  0  0  0
 15 45  1  0  0  0  0
 15 46  1  0  0  0  0
 16 47  1  0  0  0  0
 16 48  1  0  0  0  0
 17 49  1  0  0  0  0
 17 50  1  0  0  0  0
 18 51  1  0  0  0  0
 18 52  1  0  0  0  0
 19 53  1  0  0  0  0
 19 54  1  0  0  0  0
 20 55  1  0  0  0  0
 20 56  1  0  0  0  0
 20 57  1  0  0  0  0
 21 58  1  0  0  0  0
 21 59  1  0  0  0  0
M  END
>  <Alias name>
Propyl palmitate

>  <deltaH°(c)>   kJ/mol
-12003

$$$$
1-Nonadecanoic acid
  ChemBrain IXL

 59 58  0  0  0  0  0  0  0  0  1 V2000
   12.0645   11.4295    0.2381 C   0  0  0  0  0  0  0  0  0  0  0  0
   11.3367   10.1252   -0.1413 C   0  0  0  0  0  0  0  0  0  0  0  0
    9.8548   10.2258    0.2692 C   0  0  0  0  0  0  0  0  0  0  0  0
    9.1247    8.9256   -0.1194 C   0  0  0  0  0  0  0  0  0  0  0  0
    7.6448    9.0228    0.2988 C   0  0  0  0  0  0  0  0  0  0  0  0
    6.9132    7.7252   -0.0955 C   0  0  0  0  0  0  0  0  0  0  0  0
    5.4353    7.8188    0.3311 C   0  0  0  0  0  0  0  0  0  0  0  0
    4.7028    6.5226   -0.0664 C   0  0  0  0  0  0  0  0  0  0  0  0
    3.2265    6.6138    0.3656 C   0  0  0  0  0  0  0  0  0  0  0  0
   13.5474   11.3272   -0.1681 C   0  0  0  0  0  0  0  0  0  0  0  0
    2.4935    5.3183   -0.0335 C   0  0  0  0  0  0  0  0  0  0  0  0
   14.2719   12.6370    0.1982 C   0  0  0  0  0  0  0  0  0  0  0  0
   15.7550   12.5345   -0.2074 C   0  0  0  0  0  0  0  0  0  0  0  0
   16.4759   13.8502    0.1445 C   0  0  0  0  0  0  0  0  0  0  0  0
   17.9592   13.7475   -0.2607 C   0  0  0  0  0  0  0  0  0  0  0  0
   18.6776   15.0673    0.0809 C   0  0  0  0  0  0  0  0  0  0  0  0
   20.1616   14.9633   -0.3209 C   0  0  0  0  0  0  0  0  0  0  0  0
   20.8800   16.2832    0.0205 C   0  0  0  0  0  0  0  0  0  0  0  0
```

```
22.3066 16.1811 -0.3598 C 0 0 0 0 0 0 0 0 0 0 0 0
23.1567 17.3804 -0.3531 O 0 0 0 0 0 0 0 0 0 0 0 0
22.7771 15.1017 -0.6789 O 0 0 0 0 0 0 0 0 0 0 0 0
22.7031 18.2096 -0.0831 H 0 0 0 0 0 0 0 0 0 0 0 0
11.9930 11.5848 1.3075 H 0 0 0 0 0 0 0 0 0 0 0 0
11.6051 12.2628 -0.2792 H 0 0 0 0 0 0 0 0 0 0 0 0
11.4054 9.9716 -1.2111 H 0 0 0 0 0 0 0 0 0 0 0 0
11.7981 9.2915 0.3735 H 0 0 0 0 0 0 0 0 0 0 0 0
9.7860 10.3723 1.3400 H 0 0 0 0 0 0 0 0 0 0 0 0
9.3953 11.0639 -0.2401 H 0 0 0 0 0 0 0 0 0 0 0 0
9.1884 8.7823 -1.1910 H 0 0 0 0 0 0 0 0 0 0 0 0
9.5881 8.0867 0.3851 H 0 0 0 0 0 0 0 0 0 0 0 0
7.5810 9.1617 1.3710 H 0 0 0 0 0 0 0 0 0 0 0 0
7.1826 9.8644 -0.2023 H 0 0 0 0 0 0 0 0 0 0 0 0
6.9713 7.5897 -1.1684 H 0 0 0 0 0 0 0 0 0 0 0 0
7.3794 6.8829 0.4005 H 0 0 0 0 0 0 0 0 0 0 0 0
5.3771 7.9517 1.4044 H 0 0 0 0 0 0 0 0 0 0 0 0
4.9697 8.6626 -0.1629 H 0 0 0 0 0 0 0 0 0 0 0 0
4.7574 6.3919 -1.1401 H 0 0 0 0 0 0 0 0 0 0 0 0
5.1711 5.6783 0.4243 H 0 0 0 0 0 0 0 0 0 0 0 0
3.1719 6.7432 1.4395 H 0 0 0 0 0 0 0 0 0 0 0 0
2.7585 7.4588 -0.1241 H 0 0 0 0 0 0 0 0 0 0 0 0
13.6186 11.1618 -1.2361 H 0 0 0 0 0 0 0 0 0 0 0 0
14.0093 10.5002 0.3568 H 0 0 0 0 0 0 0 0 0 0 0 0
2.9619 4.4732 0.4555 H 0 0 0 0 0 0 0 0 0 0 0 0
1.4561 5.3821 0.2708 H 0 0 0 0 0 0 0 0 0 0 0 0
2.5474 5.1893 -1.1075 H 0 0 0 0 0 0 0 0 0 0 0 0
14.2003 12.8026 1.2661 H 0 0 0 0 0 0 0 0 0 0 0 0
13.8103 13.4640 -0.3271 H 0 0 0 0 0 0 0 0 0 0 0 0
15.8263 12.3577 -1.2735 H 0 0 0 0 0 0 0 0 0 0 0 0
16.2194 11.7145 0.3263 H 0 0 0 0 0 0 0 0 0 0 0 0
16.4044 14.0271 1.2105 H 0 0 0 0 0 0 0 0 0 0 0 0
16.0118 14.6702 -0.3895 H 0 0 0 0 0 0 0 0 0 0 0 0
18.0306 13.5628 -1.3255 H 0 0 0 0 0 0 0 0 0 0 0 0
18.4253 12.9325 0.2791 H 0 0 0 0 0 0 0 0 0 0 0 0
18.6039 15.2534 1.1453 H 0 0 0 0 0 0 0 0 0 0 0 0
18.2131 15.8819 -0.4609 H 0 0 0 0 0 0 0 0 0 0 0 0
20.2353 14.7770 -1.3852 H 0 0 0 0 0 0 0 0 0 0 0 0
20.6261 14.1488 0.2211 H 0 0 0 0 0 0 0 0 0 0 0 0
20.8025 16.4717 1.0842 H 0 0 0 0 0 0 0 0 0 0 0 0
20.4183 17.0970 -0.5249 H 0 0 0 0 0 0 0 0 0 0 0 0
1 2 1 0 0 0 0
2 3 1 0 0 0 0
3 4 1 0 0 0 0
4 5 1 0 0 0 0
5 6 1 0 0 0 0
6 7 1 0 0 0 0
7 8 1 0 0 0 0
8 9 1 0 0 0 0
1 10 1 0 0 0 0
9 11 1 0 0 0 0
12 13 1 0 0 0 0
13 14 1 0 0 0 0
14 15 1 0 0 0 0
15 16 1 0 0 0 0
16 17 1 0 0 0 0
17 18 1 0 0 0 0
18 19 1 0 0 0 0
10 12 1 0 0 0 0
19 20 1 0 0 0 0
19 21 2 0 0 0 0
20 22 1 0 0 0 0
1 23 1 0 0 0 0
```

```
  1 24  1  0  0  0  0
  2 25  1  0  0  0  0
  2 26  1  0  0  0  0
  3 27  1  0  0  0  0
  3 28  1  0  0  0  0
  4 29  1  0  0  0  0
  4 30  1  0  0  0  0
  5 31  1  0  0  0  0
  5 32  1  0  0  0  0
  6 33  1  0  0  0  0
  6 34  1  0  0  0  0
  7 35  1  0  0  0  0
  7 36  1  0  0  0  0
  8 37  1  0  0  0  0
  8 38  1  0  0  0  0
  9 39  1  0  0  0  0
  9 40  1  0  0  0  0
 10 41  1  0  0  0  0
 10 42  1  0  0  0  0
 11 43  1  0  0  0  0
 11 44  1  0  0  0  0
 11 45  1  0  0  0  0
 12 46  1  0  0  0  0
 12 47  1  0  0  0  0
 13 48  1  0  0  0  0
 13 49  1  0  0  0  0
 14 50  1  0  0  0  0
 14 51  1  0  0  0  0
 15 52  1  0  0  0  0
 15 53  1  0  0  0  0
 16 54  1  0  0  0  0
 16 55  1  0  0  0  0
 17 56  1  0  0  0  0
 17 57  1  0  0  0  0
 18 58  1  0  0  0  0
 18 59  1  0  0  0  0
M  END
>  <Alias name>
1-Nonadecanoic acid

>  <deltaH°(c)>   kJ/mol
-11999.68

$$$$
Methylprednisolone
  ChemBrain IXL

 57 60  0  0  0  0  0  0  0  0  1 V2000
    4.9598    5.3341    0.0936 C   0  0  0  0  0  0  0  0  0  0  0  0
    4.8276    4.1457   -0.8265 C   0  0  0  0  0  0  0  0  0  0  0  0
    3.6991    3.9008   -1.5079 C   0  0  0  0  0  0  0  0  0  0  0  0
    2.5361    4.7959   -1.3627 C   0  0  0  0  0  0  0  0  0  0  0  0
    2.6161    5.9548   -0.4573 C   0  0  0  0  0  0  0  0  0  0  0  0
    3.7434    6.1995    0.2203 C   0  0  0  0  0  0  0  0  0  0  0  0
    5.3398    4.8213    1.4878 C   0  0  0  0  0  0  0  0  0  0  0  0
    6.0103    6.1817   -0.4292 C   0  0  0  0  0  0  0  0  0  0  0  0
    3.7974    7.3580    1.1026 C   0  0  0  0  0  0  0  0  0  0  0  0
    6.2723    7.3165    0.5921 C   0  0  0  0  0  0  0  0  0  0  0  0
    5.0056    8.2218    0.7037 C   0  0  0  0  0  0  0  0  0  0  0  0
    7.2825    5.3194   -0.7122 C   0  0  0  0  0  0  0  0  0  0  0  0
    7.4031    8.0980    0.0819 C   0  0  0  0  0  0  0  0  0  0  0  0
    8.4647    6.2416   -1.0622 C   0  0  0  0  0  0  0  0  0  0  0  0
    8.5941    7.2285    0.0502 C   0  0  0  0  0  0  0  0  0  0  0  0
```

7.7955 9.2915 0.9389 C 0 0 0 0 0 0 0 0 0 0 0 0
8.6873 6.4650 1.3905 C 0 0 0 0 0 0 0 0 0 0 0 0
9.7578 8.1603 -0.0753 C 0 0 0 0 0 0 0 0 0 0 0 0
9.3375 9.4299 0.7388 C 0 0 0 0 0 0 0 0 0 0 0 0
10.9734 7.5355 0.4616 C 0 0 0 0 0 0 0 0 0 0 0 0
11.0413 7.2403 1.6433 O 0 0 0 0 0 0 0 0 0 0 0 0
1.5103 4.5781 -1.9901 O 0 0 0 0 0 0 0 0 0 0 0 0
7.6085 4.5295 0.4847 O 0 0 0 0 0 0 0 0 0 0 0 0
12.1066 7.2636 -0.4397 C 0 0 0 0 0 0 0 0 0 0 0 0
12.1302 8.2735 -1.5098 O 0 0 0 0 0 0 0 0 0 0 0 0
9.9518 8.5170 -1.4862 O 0 0 0 0 0 0 0 0 0 0 0 0
2.4874 8.1730 0.9668 C 0 0 0 0 0 0 0 0 0 0 0 0
8.2881 3.8330 0.3511 H 0 0 0 0 0 0 0 0 0 0 0 0
11.3181 8.3113 -2.0641 H 0 0 0 0 0 0 0 0 0 0 0 0
9.1421 8.8331 -1.9487 H 0 0 0 0 0 0 0 0 0 0 0 0
5.6656 3.4733 -0.9432 H 0 0 0 0 0 0 0 0 0 0 0 0
3.6388 3.0424 -2.1685 H 0 0 0 0 0 0 0 0 0 0 0 0
1.7592 6.6101 -0.3407 H 0 0 0 0 0 0 0 0 0 0 0 0
5.4904 5.6584 2.1524 H 0 0 0 0 0 0 0 0 0 0 0 0
6.2484 4.2397 1.4229 H 0 0 0 0 0 0 0 0 0 0 0 0
4.5372 4.1956 1.8699 H 0 0 0 0 0 0 0 0 0 0 0 0
5.6721 6.6208 -1.3608 H 0 0 0 0 0 0 0 0 0 0 0 0
3.9112 7.0260 2.1262 H 0 0 0 0 0 0 0 0 0 0 0 0
6.5138 6.8956 1.5597 H 0 0 0 0 0 0 0 0 0 0 0 0
5.1676 8.9844 1.4517 H 0 0 0 0 0 0 0 0 0 0 0 0
4.8107 8.6895 -0.2550 H 0 0 0 0 0 0 0 0 0 0 0 0
7.0863 4.6505 -1.5431 H 0 0 0 0 0 0 0 0 0 0 0 0
7.1787 8.4360 -0.9218 H 0 0 0 0 0 0 0 0 0 0 0 0
9.3724 5.6587 -1.1465 H 0 0 0 0 0 0 0 0 0 0 0 0
8.2672 6.7563 -1.9934 H 0 0 0 0 0 0 0 0 0 0 0 0
7.2847 10.1838 0.6008 H 0 0 0 0 0 0 0 0 0 0 0 0
7.5658 9.0956 1.9814 H 0 0 0 0 0 0 0 0 0 0 0 0
9.6034 5.8904 1.4168 H 0 0 0 0 0 0 0 0 0 0 0 0
8.6802 7.1723 2.2105 H 0 0 0 0 0 0 0 0 0 0 0 0
7.8382 5.7955 1.4848 H 0 0 0 0 0 0 0 0 0 0 0 0
9.8400 9.4478 1.6962 H 0 0 0 0 0 0 0 0 0 0 0 0
9.5611 10.3272 0.1776 H 0 0 0 0 0 0 0 0 0 0 0 0
13.0311 7.3072 0.1194 H 0 0 0 0 0 0 0 0 0 0 0 0
11.9925 6.2791 -0.8755 H 0 0 0 0 0 0 0 0 0 0 0 0
1.6440 7.5511 1.2507 H 0 0 0 0 0 0 0 0 0 0 0 0
2.5306 9.0380 1.6160 H 0 0 0 0 0 0 0 0 0 0 0 0
2.3660 8.4968 -0.0594 H 0 0 0 0 0 0 0 0 0 0 0 0
1 2 1 0 0 0 0
2 3 2 0 0 0 0
3 4 1 0 0 0 0
4 5 1 0 0 0 0
5 6 2 0 0 0 0
6 1 1 0 0 0 0
1 7 1 0 0 0 0
1 8 1 0 0 0 0
6 9 1 0 0 0 0
10 8 1 0 0 0 0
9 11 1 0 0 0 0
11 10 1 0 0 0 0
8 12 1 0 0 0 0
10 13 1 0 0 0 0
12 14 1 0 0 0 0
14 15 1 0 0 0 0
15 13 1 0 0 0 0
13 16 1 0 0 0 0
15 17 1 0 0 0 0
15 18 1 0 0 0 0
16 19 1 0 0 0 0

```
 19 18  1  0  0  0  0
 18 20  1  0  0  0  0
 20 21  2  0  0  0  0
  4 22  2  0  0  0  0
 23 12  1  0  0  0  0
 20 24  1  0  0  0  0
 24 25  1  0  0  0  0
 18 26  1  0  0  0  0
  9 27  1  0  0  0  0
 23 28  1  0  0  0  0
 25 29  1  0  0  0  0
 26 30  1  0  0  0  0
  2 31  1  0  0  0  0
  3 32  1  0  0  0  0
  5 33  1  0  0  0  0
  7 34  1  0  0  0  0
  7 35  1  0  0  0  0
  7 36  1  0  0  0  0
  8 37  1  0  0  0  0
  9 38  1  0  0  0  0
 10 39  1  0  0  0  0
 11 40  1  0  0  0  0
 11 41  1  0  0  0  0
 12 42  1  0  0  0  0
 13 43  1  0  0  0  0
 14 44  1  0  0  0  0
 14 45  1  0  0  0  0
 16 46  1  0  0  0  0
 16 47  1  0  0  0  0
 17 48  1  0  0  0  0
 17 49  1  0  0  0  0
 17 50  1  0  0  0  0
 19 51  1  0  0  0  0
 19 52  1  0  0  0  0
 24 53  1  0  0  0  0
 24 54  1  0  0  0  0
 27 55  1  0  0  0  0
 27 56  1  0  0  0  0
 27 57  1  0  0  0  0
M  END
>  <Alias name>
Methylprednisolone

>  <deltaH°(c)>   kJ/mol
-11899

$$$$
(1,8)Paracyclophane
  ChemBrain IXL

 47 49  0  0  0  0  0  0  0  0  1 V2000
    8.1528    9.4191    1.6929 C   0  0  0  0  0  0  0  0  0  0  0  0
    8.2341    8.3673    2.6042 C   0  0  0  0  0  0  0  0  0  0  0  0
    8.3190    7.0594    2.1294 C   0  0  0  0  0  0  0  0  0  0  0  0
    8.3396    6.8318    0.7533 C   0  0  0  0  0  0  0  0  0  0  0  0
    8.5788    7.8946   -0.1171 C   0  0  0  0  0  0  0  0  0  0  0  0
    8.5109    9.2003    0.3639 C   0  0  0  0  0  0  0  0  0  0  0  0
    5.6074    5.4855    0.3046 C   0  0  0  0  0  0  0  0  0  0  0  0
    6.7754    5.4296   -0.4549 C   0  0  0  0  0  0  0  0  0  0  0  0
    6.6903    5.3446   -1.8446 C   0  0  0  0  0  0  0  0  0  0  0  0
    5.4760    5.6171   -2.4727 C   0  0  0  0  0  0  0  0  0  0  0  0
    4.3768    5.9872   -1.6994 C   0  0  0  0  0  0  0  0  0  0  0  0
    4.3925    5.7406   -0.3278 C   0  0  0  0  0  0  0  0  0  0  0  0
```

8.1025 5.4661 0.2149 C 0 0 0 0 0 0 0 0 0 0 0 0
7.6837 10.7618 2.1321 C 0 0 0 0 0 0 0 0 0 0 0 0
3.1991 6.6438 -2.3300 C 0 0 0 0 0 0 0 0 0 0 0 0
6.3191 11.0506 1.5074 C 0 0 0 0 0 0 0 0 0 0 0 0
3.1219 8.0981 -1.8665 C 0 0 0 0 0 0 0 0 0 0 0 0
6.1808 10.2654 0.2004 C 0 0 0 0 0 0 0 0 0 0 0 0
3.7943 8.2327 -0.4977 C 0 0 0 0 0 0 0 0 0 0 0 0
6.0147 8.7763 0.5137 C 0 0 0 0 0 0 0 0 0 0 0 0
5.3096 8.0844 -0.6557 C 0 0 0 0 0 0 0 0 0 0 0 0
8.2329 8.5640 3.6691 H 0 0 0 0 0 0 0 0 0 0 0 0
8.3692 6.2282 2.8220 H 0 0 0 0 0 0 0 0 0 0 0 0
8.8152 7.7066 -1.1572 H 0 0 0 0 0 0 0 0 0 0 0 0
8.7355 10.0357 -0.2874 H 0 0 0 0 0 0 0 0 0 0 0 0
5.6443 5.3318 1.3761 H 0 0 0 0 0 0 0 0 0 0 0 0
7.5591 5.0690 -2.4299 H 0 0 0 0 0 0 0 0 0 0 0 0
5.3874 5.5406 -3.5492 H 0 0 0 0 0 0 0 0 0 0 0 0
3.4705 5.7457 0.2401 H 0 0 0 0 0 0 0 0 0 0 0 0
8.1186 4.7489 1.0264 H 0 0 0 0 0 0 0 0 0 0 0 0
8.8767 5.2198 -0.5015 H 0 0 0 0 0 0 0 0 0 0 0 0
7.5987 10.7796 3.2114 H 0 0 0 0 0 0 0 0 0 0 0 0
8.3940 11.5146 1.8129 H 0 0 0 0 0 0 0 0 0 0 0 0
3.3017 6.6124 -3.4075 H 0 0 0 0 0 0 0 0 0 0 0 0
2.2957 6.1228 -2.0375 H 0 0 0 0 0 0 0 0 0 0 0 0
6.2327 12.1104 1.3029 H 0 0 0 0 0 0 0 0 0 0 0 0
5.5372 10.7502 2.1937 H 0 0 0 0 0 0 0 0 0 0 0 0
3.6297 8.7324 -2.5823 H 0 0 0 0 0 0 0 0 0 0 0 0
2.0843 8.3980 -1.7902 H 0 0 0 0 0 0 0 0 0 0 0 0
5.3133 10.6188 -0.3428 H 0 0 0 0 0 0 0 0 0 0 0 0
7.0683 10.4102 -0.4033 H 0 0 0 0 0 0 0 0 0 0 0 0
3.5687 9.2054 -0.0789 H 0 0 0 0 0 0 0 0 0 0 0 0
3.4233 7.4599 0.1643 H 0 0 0 0 0 0 0 0 0 0 0 0
5.4217 8.6603 1.4124 H 0 0 0 0 0 0 0 0 0 0 0 0
6.9893 8.3280 0.6639 H 0 0 0 0 0 0 0 0 0 0 0 0
5.5707 7.0332 -0.6637 H 0 0 0 0 0 0 0 0 0 0 0 0
5.6220 8.5410 -1.5867 H 0 0 0 0 0 0 0 0 0 0 0 0
1 2 4 0 0 0 0
2 3 4 0 0 0 0
3 4 4 0 0 0 0
4 5 4 0 0 0 0
5 6 4 0 0 0 0
6 1 4 0 0 0 0
7 8 4 0 0 0 0
8 9 4 0 0 0 0
9 10 4 0 0 0 0
10 11 4 0 0 0 0
11 12 4 0 0 0 0
12 7 4 0 0 0 0
4 13 1 0 0 0 0
13 8 1 0 0 0 0
1 14 1 0 0 0 0
11 15 1 0 0 0 0
14 16 1 0 0 0 0
15 17 1 0 0 0 0
16 18 1 0 0 0 0
17 19 1 0 0 0 0
18 20 1 0 0 0 0
19 21 1 0 0 0 0
20 21 1 0 0 0 0
2 22 1 0 0 0 0
3 23 1 0 0 0 0
5 24 1 0 0 0 0
6 25 1 0 0 0 0
7 26 1 0 0 0 0

```
 9 27  1  0  0  0  0
10 28  1  0  0  0  0
12 29  1  0  0  0  0
13 30  1  0  0  0  0
13 31  1  0  0  0  0
14 32  1  0  0  0  0
14 33  1  0  0  0  0
15 34  1  0  0  0  0
15 35  1  0  0  0  0
16 36  1  0  0  0  0
16 37  1  0  0  0  0
17 38  1  0  0  0  0
17 39  1  0  0  0  0
18 40  1  0  0  0  0
18 41  1  0  0  0  0
19 42  1  0  0  0  0
19 43  1  0  0  0  0
20 44  1  0  0  0  0
20 45  1  0  0  0  0
21 46  1  0  0  0  0
21 47  1  0  0  0  0
M  END
>  <Alias name>
[1,8]Paracyclophane

>  <deltaH°(c)>   kJ/mol
-11898

$$$$
Elaidic acid methylester
  ChemBrain IXL

 57 56  0  0  0  0  0  0  0  0  1 V2000
    2.2767   11.6463    0.0876 C   0  0  0  0  0  0  0  0  0  0  0  0
    3.6293   10.9090    0.0512 C   0  0  0  0  0  0  0  0  0  0  0  0
    4.7765   11.9374    0.0902 C   0  0  0  0  0  0  0  0  0  0  0  0
    6.1288   11.1997    0.0533 C   0  0  0  0  0  0  0  0  0  0  0  0
    7.2761   12.2275    0.0907 C   0  0  0  0  0  0  0  0  0  0  0  0
    8.6278   11.4891    0.0524 C   0  0  0  0  0  0  0  0  0  0  0  0
    9.7758   12.5161    0.0872 C   0  0  0  0  0  0  0  0  0  0  0  0
   11.1270   11.7765    0.0478 C   0  0  0  0  0  0  0  0  0  0  0  0
   12.2307   12.7618    0.0792 C   0  0  0  0  0  0  0  0  0  0  0  0
   13.4982   12.3433    0.0927 C   0  0  0  0  0  0  0  0  0  0  0  0
   14.6022   13.3283    0.1241 C   0  0  0  0  0  0  0  0  0  0  0  0
   15.9506   12.5894    0.0365 C   0  0  0  0  0  0  0  0  0  0  0  0
   17.1012   13.6127    0.0679 C   0  0  0  0  0  0  0  0  0  0  0  0
   18.4474   12.8705   -0.0232 C   0  0  0  0  0  0  0  0  0  0  0  0
   19.6007   13.8902    0.0076 C   0  0  0  0  0  0  0  0  0  0  0  0
   20.9446   13.1444   -0.0891 C   0  0  0  0  0  0  0  0  0  0  0  0
   22.1006   14.1610   -0.0563 C   0  0  0  0  0  0  0  0  0  0  0  0
   23.3907   13.4433   -0.1561 C   0  0  0  0  0  0  0  0  0  0  0  0
   24.3271   13.9550   -0.7484 O   0  0  0  0  0  0  0  0  0  0  0  0
   23.5432   12.1135    0.4588 O   0  0  0  0  0  0  0  0  0  0  0  0
   24.8685   11.4956    0.2938 C   0  0  0  0  0  0  0  0  0  0  0  0
    1.4708   10.9235    0.0599 H   0  0  0  0  0  0  0  0  0  0  0  0
    2.2077   12.2293    0.9976 H   0  0  0  0  0  0  0  0  0  0  0  0
    2.2010   12.3042   -0.7693 H   0  0  0  0  0  0  0  0  0  0  0  0
    3.7049   10.2511    0.9081 H   0  0  0  0  0  0  0  0  0  0  0  0
    3.6981   10.3259   -0.8588 H   0  0  0  0  0  0  0  0  0  0  0  0
    4.7007   12.5954   -0.7666 H   0  0  0  0  0  0  0  0  0  0  0  0
    4.7080   12.5204    1.0004 H   0  0  0  0  0  0  0  0  0  0  0  0
    6.2049   10.5424    0.9106 H   0  0  0  0  0  0  0  0  0  0  0  0
    6.1967   10.6160   -0.8564 H   0  0  0  0  0  0  0  0  0  0  0  0
```

```
    7.1997   12.8853   -0.7664 H   0  0  0  0  0  0  0  0  0  0  0  0
    7.2089   12.8109    1.0007 H   0  0  0  0  0  0  0  0  0  0  0  0
    8.7048   10.8325    0.9102 H   0  0  0  0  0  0  0  0  0  0  0  0
    8.6941   10.9045   -0.8568 H   0  0  0  0  0  0  0  0  0  0  0  0
    9.6986   13.1731   -0.7703 H   0  0  0  0  0  0  0  0  0  0  0  0
    9.7103   13.1003    0.9968 H   0  0  0  0  0  0  0  0  0  0  0  0
   11.2045   11.1203    0.9059 H   0  0  0  0  0  0  0  0  0  0  0  0
   11.1916   11.1912   -0.8611 H   0  0  0  0  0  0  0  0  0  0  0  0
   12.0102   13.8222    0.0911 H   0  0  0  0  0  0  0  0  0  0  0  0
   13.7185   11.2829    0.0808 H   0  0  0  0  0  0  0  0  0  0  0  0
   14.5062   14.0053   -0.7158 H   0  0  0  0  0  0  0  0  0  0  0  0
   14.5581   13.8910    1.0484 H   0  0  0  0  0  0  0  0  0  0  0  0
   16.0465   11.9127    0.8767 H   0  0  0  0  0  0  0  0  0  0  0  0
   15.9939   12.0260   -0.8874 H   0  0  0  0  0  0  0  0  0  0  0  0
   17.0049   14.2905   -0.7713 H   0  0  0  0  0  0  0  0  0  0  0  0
   17.0601   14.1746    0.9928 H   0  0  0  0  0  0  0  0  0  0  0  0
   18.5430   12.1923    0.8157 H   0  0  0  0  0  0  0  0  0  0  0  0
   18.4875   12.3085   -0.9480 H   0  0  0  0  0  0  0  0  0  0  0  0
   19.5041   14.5706   -0.8295 H   0  0  0  0  0  0  0  0  0  0  0  0
   19.5637   14.4498    0.9342 H   0  0  0  0  0  0  0  0  0  0  0  0
   21.0399   12.4625    0.7469 H   0  0  0  0  0  0  0  0  0  0  0  0
   20.9812   12.5860   -1.0163 H   0  0  0  0  0  0  0  0  0  0  0  0
   22.0031   14.8456   -0.8899 H   0  0  0  0  0  0  0  0  0  0  0  0
   22.0671   14.7164    0.8728 H   0  0  0  0  0  0  0  0  0  0  0  0
   25.6214   12.1305    0.7446 H   0  0  0  0  0  0  0  0  0  0  0  0
   25.0820   11.3755   -0.7612 H   0  0  0  0  0  0  0  0  0  0  0  0
   24.8775   10.5264    0.7766 H   0  0  0  0  0  0  0  0  0  0  0  0
  1  2  1  0  0  0  0
  2  3  1  0  0  0  0
  3  4  1  0  0  0  0
  4  5  1  0  0  0  0
  5  6  1  0  0  0  0
  6  7  1  0  0  0  0
  7  8  1  0  0  0  0
  8  9  1  0  0  0  0
  9 10  2  0  0  0  0
 10 11  1  0  0  0  0
 11 12  1  0  0  0  0
 12 13  1  0  0  0  0
 13 14  1  0  0  0  0
 14 15  1  0  0  0  0
 15 16  1  0  0  0  0
 16 17  1  0  0  0  0
 17 18  1  0  0  0  0
 18 19  2  0  0  0  0
 18 20  1  0  0  0  0
 20 21  1  0  0  0  0
  1 22  1  0  0  0  0
  1 23  1  0  0  0  0
  1 24  1  0  0  0  0
  2 25  1  0  0  0  0
  2 26  1  0  0  0  0
  3 27  1  0  0  0  0
  3 28  1  0  0  0  0
  4 29  1  0  0  0  0
  4 30  1  0  0  0  0
  5 31  1  0  0  0  0
  5 32  1  0  0  0  0
  6 33  1  0  0  0  0
  6 34  1  0  0  0  0
  7 35  1  0  0  0  0
  7 36  1  0  0  0  0
  8 37  1  0  0  0  0
```

8 38 1 0 0 0 0
9 39 1 0 0 0 0
10 40 1 0 0 0 0
11 41 1 0 0 0 0
11 42 1 0 0 0 0
12 43 1 0 0 0 0
12 44 1 0 0 0 0
13 45 1 0 0 0 0
13 46 1 0 0 0 0
14 47 1 0 0 0 0
14 48 1 0 0 0 0
15 49 1 0 0 0 0
15 50 1 0 0 0 0
16 51 1 0 0 0 0
16 52 1 0 0 0 0
17 53 1 0 0 0 0
17 54 1 0 0 0 0
21 55 1 0 0 0 0
21 56 1 0 0 0 0
21 57 1 0 0 0 0
M END
> <Alias name>
Methylelaidate

> <deltaH°(c)> kJ/mol
-11894

$$$$
1-Octadecene
ChemBrain IXL

54 53 0 0 0 0 0 0 0 0 1 V2000
13.4291 11.6054 0.0000 C 0 0 0 0 0 0 0 0 0 0 0 0
12.1468 12.4602 -0.0038 C 0 0 0 0 0 0 0 0 0 0 0 0
10.9134 11.5363 -0.0015 C 0 0 0 0 0 0 0 0 0 0 0 0
9.6312 12.3912 -0.0065 C 0 0 0 0 0 0 0 0 0 0 0 0
8.3978 11.4674 -0.0056 C 0 0 0 0 0 0 0 0 0 0 0 0
7.1157 12.3223 -0.0116 C 0 0 0 0 0 0 0 0 0 0 0 0
5.8823 11.3985 -0.0116 C 0 0 0 0 0 0 0 0 0 0 0 0
4.5999 12.2535 -0.0182 C 0 0 0 0 0 0 0 0 0 0 0 0
3.4147 11.3663 -0.0187 C 0 0 0 0 0 0 0 0 0 0 0 0
14.6625 12.5292 -0.0036 C 0 0 0 0 0 0 0 0 0 0 0 0
2.1873 11.8916 -0.0228 C 0 0 0 0 0 0 0 0 0 0 0 0
15.9447 11.6744 -0.0011 C 0 0 0 0 0 0 0 0 0 0 0 0
17.1781 12.5983 -0.0058 C 0 0 0 0 0 0 0 0 0 0 0 0
18.4604 11.7435 -0.0042 C 0 0 0 0 0 0 0 0 0 0 0 0
19.6938 12.6674 -0.0099 C 0 0 0 0 0 0 0 0 0 0 0 0
20.9761 11.8125 -0.0088 C 0 0 0 0 0 0 0 0 0 0 0 0
22.2097 12.7364 -0.0148 C 0 0 0 0 0 0 0 0 0 0 0 0
23.4919 11.8817 -0.0140 C 0 0 0 0 0 0 0 0 0 0 0 0
13.4461 10.9839 0.8868 H 0 0 0 0 0 0 0 0 0 0 0 0
13.4464 10.9767 -0.8817 H 0 0 0 0 0 0 0 0 0 0 0 0
12.1303 13.0821 -0.8902 H 0 0 0 0 0 0 0 0 0 0 0 0
12.1291 13.0882 0.8783 H 0 0 0 0 0 0 0 0 0 0 0 0
10.9295 10.9148 0.8853 H 0 0 0 0 0 0 0 0 0 0 0 0
10.9316 10.9078 -0.8833 H 0 0 0 0 0 0 0 0 0 0 0 0
9.6156 13.0132 -0.8929 H 0 0 0 0 0 0 0 0 0 0 0 0
9.6126 13.0192 0.8756 H 0 0 0 0 0 0 0 0 0 0 0 0
8.4131 10.8458 0.8811 H 0 0 0 0 0 0 0 0 0 0 0 0
8.4168 10.8390 -0.8874 H 0 0 0 0 0 0 0 0 0 0 0 0
7.1008 12.9444 -0.8980 H 0 0 0 0 0 0 0 0 0 0 0 0
7.0964 12.9503 0.8706 H 0 0 0 0 0 0 0 0 0 0 0 0
5.8970 10.7766 0.8750 H 0 0 0 0 0 0 0 0 0 0 0 0

5.9018	10.7703	-0.8936	H	0	0	0	0	0	0	0	0	0	0	0	0
4.5855	12.8756	-0.9046	H	0	0	0	0	0	0	0	0	0	0	0	0
4.5803	12.8815	0.8640	H	0	0	0	0	0	0	0	0	0	0	0	0
3.5431	10.2908	-0.0159	H	0	0	0	0	0	0	0	0	0	0	0	0
14.6450	13.1511	-0.8901	H	0	0	0	0	0	0	0	0	0	0	0	0
14.6456	13.1574	0.8784	H	0	0	0	0	0	0	0	0	0	0	0	0
1.3205	11.2424	-0.0232	H	0	0	0	0	0	0	0	0	0	0	0	0
2.0584	12.9670	-0.0256	H	0	0	0	0	0	0	0	0	0	0	0	0
15.9626	11.0530	0.8858	H	0	0	0	0	0	0	0	0	0	0	0	0
15.9612	11.0458	-0.8828	H	0	0	0	0	0	0	0	0	0	0	0	0
17.1599	13.2201	-0.8924	H	0	0	0	0	0	0	0	0	0	0	0	0
17.1620	13.2266	0.8761	H	0	0	0	0	0	0	0	0	0	0	0	0
18.4789	11.1221	0.8825	H	0	0	0	0	0	0	0	0	0	0	0	0
18.4762	11.1149	-0.8860	H	0	0	0	0	0	0	0	0	0	0	0	0
19.6751	13.2891	-0.8965	H	0	0	0	0	0	0	0	0	0	0	0	0
19.6782	13.2958	0.8721	H	0	0	0	0	0	0	0	0	0	0	0	0
20.9949	11.1910	0.8779	H	0	0	0	0	0	0	0	0	0	0	0	0
20.9916	11.1840	-0.8907	H	0	0	0	0	0	0	0	0	0	0	0	0
22.1909	13.3580	-0.9014	H	0	0	0	0	0	0	0	0	0	0	0	0
22.1943	13.3649	0.8670	H	0	0	0	0	0	0	0	0	0	0	0	0
24.3585	12.5311	-0.0182	H	0	0	0	0	0	0	0	0	0	0	0	0
23.5074	11.2533	-0.8959	H	0	0	0	0	0	0	0	0	0	0	0	0
23.5109	11.2601	0.8726	H	0	0	0	0	0	0	0	0	0	0	0	0

1	2	1	0	0	0	0
2	3	1	0	0	0	0
3	4	1	0	0	0	0
4	5	1	0	0	0	0
5	6	1	0	0	0	0
6	7	1	0	0	0	0
7	8	1	0	0	0	0
8	9	1	0	0	0	0
1	10	1	0	0	0	0
9	11	2	0	0	0	0
12	13	1	0	0	0	0
13	14	1	0	0	0	0
14	15	1	0	0	0	0
15	16	1	0	0	0	0
16	17	1	0	0	0	0
17	18	1	0	0	0	0
10	12	1	0	0	0	0
1	19	1	0	0	0	0
1	20	1	0	0	0	0
2	21	1	0	0	0	0
2	22	1	0	0	0	0
3	23	1	0	0	0	0
3	24	1	0	0	0	0
4	25	1	0	0	0	0
4	26	1	0	0	0	0
5	27	1	0	0	0	0
5	28	1	0	0	0	0
6	29	1	0	0	0	0
6	30	1	0	0	0	0
7	31	1	0	0	0	0
7	32	1	0	0	0	0
8	33	1	0	0	0	0
8	34	1	0	0	0	0
9	35	1	0	0	0	0
10	36	1	0	0	0	0
10	37	1	0	0	0	0
11	38	1	0	0	0	0
11	39	1	0	0	0	0
12	40	1	0	0	0	0
12	41	1	0	0	0	0

```
 13 42  1  0  0  0  0
 13 43  1  0  0  0  0
 14 44  1  0  0  0  0
 14 45  1  0  0  0  0
 15 46  1  0  0  0  0
 15 47  1  0  0  0  0
 16 48  1  0  0  0  0
 16 49  1  0  0  0  0
 17 50  1  0  0  0  0
 17 51  1  0  0  0  0
 18 52  1  0  0  0  0
 18 53  1  0  0  0  0
 18 54  1  0  0  0  0
M  END
>  <Alias name>
1-Octadecene

>  <deltaH°(c)>   kJ/mol
-11861.52

$$$$
1-Chlorooctadecane
  ChemBrain IXL

 56 55  0  0  0  0  0  0  0  0  1 V2000
   13.3631   11.0286   -0.1610 C   0  0  0  0  0  0  0  0  0  0  0  0
   12.1723   11.9388    0.1976 C   0  0  0  0  0  0  0  0  0  0  0  0
   11.0162   11.0803    0.7464 C   0  0  0  0  0  0  0  0  0  0  0  0
    9.8256   11.9907    1.1051 C   0  0  0  0  0  0  0  0  0  0  0  0
    8.6694   11.1324    1.6543 C   0  0  0  0  0  0  0  0  0  0  0  0
    7.4790   12.0429    2.0131 C   0  0  0  0  0  0  0  0  0  0  0  0
    6.3228   11.1847    2.5624 C   0  0  0  0  0  0  0  0  0  0  0  0
    5.1325   12.0954    2.9213 C   0  0  0  0  0  0  0  0  0  0  0  0
    3.9763   11.2372    3.4707 C   0  0  0  0  0  0  0  0  0  0  0  0
   14.5192   11.8873   -0.7096 C   0  0  0  0  0  0  0  0  0  0  0  0
    2.7860   12.1479    3.8296 C   0  0  0  0  0  0  0  0  0  0  0  0
   15.7101   10.9772   -1.0681 C   0  0  0  0  0  0  0  0  0  0  0  0
   16.8663   11.8360   -1.6164 C   0  0  0  0  0  0  0  0  0  0  0  0
   18.0573   10.9261   -1.9748 C   0  0  0  0  0  0  0  0  0  0  0  0
   19.2134   11.7851   -2.5230 C   0  0  0  0  0  0  0  0  0  0  0  0
   20.4046   10.8752   -2.8814 C   0  0  0  0  0  0  0  0  0  0  0  0
   21.5608   11.7343   -3.4295 C   0  0  0  0  0  0  0  0  0  0  0  0
   22.7519   10.8246   -3.7879 C   0  0  0  0  0  0  0  0  0  0  0  0
    1.4478   11.1543    4.4656 Cl  0  0  0  0  0  0  0  0  0  0  0  0
   13.0564   10.3127   -0.9136 H   0  0  0  0  0  0  0  0  0  0  0  0
   13.6940   10.5013    0.7252 H   0  0  0  0  0  0  0  0  0  0  0  0
   12.4791   12.6547    0.9501 H   0  0  0  0  0  0  0  0  0  0  0  0
   11.8413   12.4660   -0.6886 H   0  0  0  0  0  0  0  0  0  0  0  0
   10.7093   10.3644   -0.0061 H   0  0  0  0  0  0  0  0  0  0  0  0
   11.3472   10.5530    1.6326 H   0  0  0  0  0  0  0  0  0  0  0  0
   10.1325   12.7067    1.8575 H   0  0  0  0  0  0  0  0  0  0  0  0
    9.4945   12.5179    0.2189 H   0  0  0  0  0  0  0  0  0  0  0  0
    8.3624   10.4165    0.9019 H   0  0  0  0  0  0  0  0  0  0  0  0
    9.0006   10.6052    2.5404 H   0  0  0  0  0  0  0  0  0  0  0  0
    7.7861   12.7589    2.7654 H   0  0  0  0  0  0  0  0  0  0  0  0
    7.1478   12.5701    1.1269 H   0  0  0  0  0  0  0  0  0  0  0  0
    6.0156   10.4688    1.8101 H   0  0  0  0  0  0  0  0  0  0  0  0
    6.6540   10.6575    3.4485 H   0  0  0  0  0  0  0  0  0  0  0  0
    5.4397   12.8114    3.6735 H   0  0  0  0  0  0  0  0  0  0  0  0
    4.8012   12.6225    2.0351 H   0  0  0  0  0  0  0  0  0  0  0  0
    3.6690   10.5213    2.7184 H   0  0  0  0  0  0  0  0  0  0  0  0
    4.3075   10.7101    4.3568 H   0  0  0  0  0  0  0  0  0  0  0  0
   14.8258   12.6032    0.0430 H   0  0  0  0  0  0  0  0  0  0  0  0
```

14.1883 12.4145 -1.5958 H 0 0 0 0 0 0 0 0 0 0 0 0
3.0932 12.8638 4.5819 H 0 0 0 0 0 0 0 0 0 0 0 0
2.4547 12.6750 2.9435 H 0 0 0 0 0 0 0 0 0 0 0 0
15.4037 10.2614 -1.8208 H 0 0 0 0 0 0 0 0 0 0 0 0
16.0410 10.4498 -0.1819 H 0 0 0 0 0 0 0 0 0 0 0 0
17.1726 12.5519 -0.8637 H 0 0 0 0 0 0 0 0 0 0 0 0
16.5354 12.3633 -2.5027 H 0 0 0 0 0 0 0 0 0 0 0 0
17.7510 10.2103 -2.7276 H 0 0 0 0 0 0 0 0 0 0 0 0
18.3881 10.3987 -1.0886 H 0 0 0 0 0 0 0 0 0 0 0 0
19.5197 12.5009 -1.7702 H 0 0 0 0 0 0 0 0 0 0 0 0
18.8826 12.3124 -3.4092 H 0 0 0 0 0 0 0 0 0 0 0 0
20.0983 10.1594 -3.6342 H 0 0 0 0 0 0 0 0 0 0 0 0
20.7354 10.3478 -1.9952 H 0 0 0 0 0 0 0 0 0 0 0 0
21.8670 12.4501 -2.6767 H 0 0 0 0 0 0 0 0 0 0 0 0
21.2300 12.2616 -4.3157 H 0 0 0 0 0 0 0 0 0 0 0 0
23.5642 11.4285 -4.1730 H 0 0 0 0 0 0 0 0 0 0 0 0
22.4458 10.1088 -4.5407 H 0 0 0 0 0 0 0 0 0 0 0 0
23.0828 10.2973 -2.9017 H 0 0 0 0 0 0 0 0 0 0 0 0
1 2 1 0 0 0 0
2 3 1 0 0 0 0
3 4 1 0 0 0 0
4 5 1 0 0 0 0
5 6 1 0 0 0 0
6 7 1 0 0 0 0
7 8 1 0 0 0 0
8 9 1 0 0 0 0
1 10 1 0 0 0 0
9 11 1 0 0 0 0
12 13 1 0 0 0 0
13 14 1 0 0 0 0
14 15 1 0 0 0 0
15 16 1 0 0 0 0
16 17 1 0 0 0 0
17 18 1 0 0 0 0
10 12 1 0 0 0 0
11 19 1 0 0 0 0
1 20 1 0 0 0 0
1 21 1 0 0 0 0
2 22 1 0 0 0 0
2 23 1 0 0 0 0
3 24 1 0 0 0 0
3 25 1 0 0 0 0
4 26 1 0 0 0 0
4 27 1 0 0 0 0
5 28 1 0 0 0 0
5 29 1 0 0 0 0
6 30 1 0 0 0 0
6 31 1 0 0 0 0
7 32 1 0 0 0 0
7 33 1 0 0 0 0
8 34 1 0 0 0 0
8 35 1 0 0 0 0
9 36 1 0 0 0 0
9 37 1 0 0 0 0
10 38 1 0 0 0 0
10 39 1 0 0 0 0
11 40 1 0 0 0 0
11 41 1 0 0 0 0
12 42 1 0 0 0 0
12 43 1 0 0 0 0
13 44 1 0 0 0 0
13 45 1 0 0 0 0
14 46 1 0 0 0 0

```
 14 47  1  0  0  0  0
 15 48  1  0  0  0  0
 15 49  1  0  0  0  0
 16 50  1  0  0  0  0
 16 51  1  0  0  0  0
 17 52  1  0  0  0  0
 17 53  1  0  0  0  0
 18 54  1  0  0  0  0
 18 55  1  0  0  0  0
 18 56  1  0  0  0  0
M  END
>  <Alias name>
1-Chlorooctadecane

>  <deltaH°(c)>   kJ/mol
-11850

$$$$
2,7-Di-t-butylfluorene
  ChemBrain IXL

 47 49  0  0  0  0  0  0  0  0  1 V2000
   13.6711    6.5195    1.2868 C   0  0  0  0  0  0  0  0  0  0  0  0
   13.0800    7.6287    0.3879 C   0  0  0  0  0  0  0  0  0  0  0  0
   13.3667    9.0043    1.0208 C   0  0  0  0  0  0  0  0  0  0  0  0
   13.7348    7.5576   -1.0066 C   0  0  0  0  0  0  0  0  0  0  0  0
   11.6120    7.4292    0.2545 C   0  0  0  0  0  0  0  0  0  0  0  0
   10.7161    8.4996    0.4151 C   0  0  0  0  0  0  0  0  0  0  0  0
    9.3516    8.2605    0.2802 C   0  0  0  0  0  0  0  0  0  0  0  0
    8.9198    7.0827    0.0136 C   0  0  0  0  0  0  0  0  0  0  0  0
    9.7466    5.9870   -0.1569 C   0  0  0  0  0  0  0  0  0  0  0  0
   11.1153    6.1630   -0.0346 C   0  0  0  0  0  0  0  0  0  0  0  0
    7.5862    7.1005   -0.0672 C   0  0  0  0  0  0  0  0  0  0  0  0
    7.1567    8.2897    0.1474 C   0  0  0  0  0  0  0  0  0  0  0  0
    8.2550    9.1750    0.3966 C   0  0  0  0  0  0  0  0  0  0  0  0
    5.7924    8.5653    0.1171 C   0  0  0  0  0  0  0  0  0  0  0  0
    4.8940    7.5188   -0.1520 C   0  0  0  0  0  0  0  0  0  0  0  0
    5.3894    6.2392   -0.3813 C   0  0  0  0  0  0  0  0  0  0  0  0
    6.7575    6.0269   -0.3378 C   0  0  0  0  0  0  0  0  0  0  0  0
    3.4247    7.7583   -0.1959 C   0  0  0  0  0  0  0  0  0  0  0  0
    3.0791    9.0390    0.5891 C   0  0  0  0  0  0  0  0  0  0  0  0
    2.9742    7.9159   -1.6620 C   0  0  0  0  0  0  0  0  0  0  0  0
    2.6867    6.5572    0.4387 C   0  0  0  0  0  0  0  0  0  0  0  0
   14.7406    6.6621    1.3802 H   0  0  0  0  0  0  0  0  0  0  0  0
   13.4746    5.5511    0.8425 H   0  0  0  0  0  0  0  0  0  0  0  0
   13.2134    6.5661    2.2667 H   0  0  0  0  0  0  0  0  0  0  0  0
   12.9793    9.7848    0.3784 H   0  0  0  0  0  0  0  0  0  0  0  0
   14.4352    9.1310    1.1387 H   0  0  0  0  0  0  0  0  0  0  0  0
   12.8864    9.0624    1.9898 H   0  0  0  0  0  0  0  0  0  0  0  0
   13.3003    8.3134   -1.6485 H   0  0  0  0  0  0  0  0  0  0  0  0
   13.5641    6.5775   -1.4360 H   0  0  0  0  0  0  0  0  0  0  0  0
   14.7999    7.7306   -0.9148 H   0  0  0  0  0  0  0  0  0  0  0  0
   11.0784    9.4927    0.6402 H   0  0  0  0  0  0  0  0  0  0  0  0
    9.3331    5.0116   -0.3813 H   0  0  0  0  0  0  0  0  0  0  0  0
   11.7884    5.3250   -0.1623 H   0  0  0  0  0  0  0  0  0  0  0  0
    8.2003    9.5950    1.3925 H   0  0  0  0  0  0  0  0  0  0  0  0
    8.3110    9.9499   -0.3572 H   0  0  0  0  0  0  0  0  0  0  0  0
    5.4324    9.5677    0.2984 H   0  0  0  0  0  0  0  0  0  0  0  0
    4.7160    5.4187   -0.5905 H   0  0  0  0  0  0  0  0  0  0  0  0
    7.1693    5.0406   -0.5124 H   0  0  0  0  0  0  0  0  0  0  0  0
    3.4088    8.9335    1.6149 H   0  0  0  0  0  0  0  0  0  0  0  0
    3.5765    9.8862    0.1349 H   0  0  0  0  0  0  0  0  0  0  0  0
    2.0080    9.1964    0.5678 H   0  0  0  0  0  0  0  0  0  0  0  0
```

```
    1.9035    8.0747   -1.6959 H   0  0  0  0  0  0  0  0  0  0  0  0
    3.4795    8.7640   -2.1060 H   0  0  0  0  0  0  0  0  0  0  0  0
    3.2233    7.0175   -2.2146 H   0  0  0  0  0  0  0  0  0  0  0  0
    3.0332    6.4158    1.4546 H   0  0  0  0  0  0  0  0  0  0  0  0
    1.6210    6.7504    0.4441 H   0  0  0  0  0  0  0  0  0  0  0  0
    2.8871    5.6632   -0.1388 H   0  0  0  0  0  0  0  0  0  0  0  0
  1  2  1  0  0  0  0
  2  3  1  0  0  0  0
  2  4  1  0  0  0  0
  2  5  1  0  0  0  0
  5  6  4  0  0  0  0
  6  7  4  0  0  0  0
  7  8  4  0  0  0  0
  8  9  4  0  0  0  0
  9 10  4  0  0  0  0
  5 10  4  0  0  0  0
  8 11  1  0  0  0  0
 11 12  4  0  0  0  0
 12 13  1  0  0  0  0
  7 13  1  0  0  0  0
 12 14  4  0  0  0  0
 14 15  4  0  0  0  0
 15 16  4  0  0  0  0
 16 17  4  0  0  0  0
 11 17  4  0  0  0  0
 15 18  1  0  0  0  0
 18 19  1  0  0  0  0
 18 20  1  0  0  0  0
 18 21  1  0  0  0  0
  1 22  1  0  0  0  0
  1 23  1  0  0  0  0
  1 24  1  0  0  0  0
  3 25  1  0  0  0  0
  3 26  1  0  0  0  0
  3 27  1  0  0  0  0
  4 28  1  0  0  0  0
  4 29  1  0  0  0  0
  4 30  1  0  0  0  0
  6 31  1  0  0  0  0
  9 32  1  0  0  0  0
 10 33  1  0  0  0  0
 13 34  1  0  0  0  0
 13 35  1  0  0  0  0
 14 36  1  0  0  0  0
 16 37  1  0  0  0  0
 17 38  1  0  0  0  0
 19 39  1  0  0  0  0
 19 40  1  0  0  0  0
 19 41  1  0  0  0  0
 20 42  1  0  0  0  0
 20 43  1  0  0  0  0
 20 44  1  0  0  0  0
 21 45  1  0  0  0  0
 21 46  1  0  0  0  0
 21 47  1  0  0  0  0
M  END
>  <Alias name>
2,7-Di-t-butylfluorene

>  <deltaH°(c)>   kJ/mol
-11838

$$$$
```

```
Methyloleate
  ChemBrain IXL

 57 56  0  0  0  0  0  0  0  0  1 V2000
    2.0646   13.1307    0.0000 C   0  0  0  0  0  0  0  0  0  0  0  0
    3.5596   13.4865    0.1130 C   0  0  0  0  0  0  0  0  0  0  0  0
    4.4046   12.2046   -0.0209 C   0  0  0  0  0  0  0  0  0  0  0  0
    5.8996   12.5605    0.0920 C   0  0  0  0  0  0  0  0  0  0  0  0
    6.7444   11.2786   -0.0406 C   0  0  0  0  0  0  0  0  0  0  0  0
    8.2392   11.6348    0.0718 C   0  0  0  0  0  0  0  0  0  0  0  0
    9.0844   10.3531   -0.0601 C   0  0  0  0  0  0  0  0  0  0  0  0
   10.5789   10.7106    0.0507 C   0  0  0  0  0  0  0  0  0  0  0  0
   11.3934    9.4794   -0.0772 C   0  0  0  0  0  0  0  0  0  0  0  0
   12.7234    9.5634   -0.1728 C   0  0  0  0  0  0  0  0  0  0  0  0
   13.3872   10.8878   -0.1510 C   0  0  0  0  0  0  0  0  0  0  0  0
   14.9154   10.6981   -0.1930 C   0  0  0  0  0  0  0  0  0  0  0  0
   15.6045   12.0758   -0.1661 C   0  0  0  0  0  0  0  0  0  0  0  0
   17.1323   11.8858   -0.2090 C   0  0  0  0  0  0  0  0  0  0  0  0
   17.8224   13.2625   -0.1749 C   0  0  0  0  0  0  0  0  0  0  0  0
   19.3499   13.0709   -0.2198 C   0  0  0  0  0  0  0  0  0  0  0  0
   20.0418   14.4466   -0.1782 C   0  0  0  0  0  0  0  0  0  0  0  0
   21.5092   14.2624   -0.2260 C   0  0  0  0  0  0  0  0  0  0  0  0
   22.2072   15.0854   -0.7962 O   0  0  0  0  0  0  0  0  0  0  0  0
   22.1254   13.0873    0.4138 O   0  0  0  0  0  0  0  0  0  0  0  0
   23.5905   13.0088    0.3004 C   0  0  0  0  0  0  0  0  0  0  0  0
    1.7977   12.4383    0.7888 H   0  0  0  0  0  0  0  0  0  0  0  0
    1.4710   14.0315    0.0943 H   0  0  0  0  0  0  0  0  0  0  0  0
    1.8743   12.6722   -0.9625 H   0  0  0  0  0  0  0  0  0  0  0  0
    3.7497   13.9453    1.0754 H   0  0  0  0  0  0  0  0  0  0  0  0
    3.8265   14.1788   -0.6759 H   0  0  0  0  0  0  0  0  0  0  0  0
    4.2144   11.7458   -0.9833 H   0  0  0  0  0  0  0  0  0  0  0  0
    4.1377   11.5124    0.7681 H   0  0  0  0  0  0  0  0  0  0  0  0
    6.0896   13.0201    1.0541 H   0  0  0  0  0  0  0  0  0  0  0  0
    6.1666   13.2521   -0.6975 H   0  0  0  0  0  0  0  0  0  0  0  0
    6.5542   10.8188   -1.0026 H   0  0  0  0  0  0  0  0  0  0  0  0
    6.4776   10.5871    0.7491 H   0  0  0  0  0  0  0  0  0  0  0  0
    8.4292   12.0951    1.0335 H   0  0  0  0  0  0  0  0  0  0  0  0
    8.5059   12.3259   -0.7182 H   0  0  0  0  0  0  0  0  0  0  0  0
    8.8937    9.8922   -1.0214 H   0  0  0  0  0  0  0  0  0  0  0  0
    8.8187    9.6624    0.7306 H   0  0  0  0  0  0  0  0  0  0  0  0
   10.7695   11.1711    1.0122 H   0  0  0  0  0  0  0  0  0  0  0  0
   10.8437   11.4023   -0.7395 H   0  0  0  0  0  0  0  0  0  0  0  0
   10.9102    8.5104   -0.0933 H   0  0  0  0  0  0  0  0  0  0  0  0
   13.3168    8.6623   -0.2662 H   0  0  0  0  0  0  0  0  0  0  0  0
   13.0714   11.4641   -1.0120 H   0  0  0  0  0  0  0  0  0  0  0  0
   13.1137   11.4138    0.7554 H   0  0  0  0  0  0  0  0  0  0  0  0
   15.2311   10.1198    0.6665 H   0  0  0  0  0  0  0  0  0  0  0  0
   15.1895   10.1749   -1.1008 H   0  0  0  0  0  0  0  0  0  0  0  0
   15.2883   12.6547   -1.0251 H   0  0  0  0  0  0  0  0  0  0  0  0
   15.3310   12.5985    0.7421 H   0  0  0  0  0  0  0  0  0  0  0  0
   17.4477   11.3029    0.6475 H   0  0  0  0  0  0  0  0  0  0  0  0
   17.4061   11.3671   -1.1194 H   0  0  0  0  0  0  0  0  0  0  0  0
   17.5063   13.8464   -1.0306 H   0  0  0  0  0  0  0  0  0  0  0  0
   17.5500   13.7805    0.7364 H   0  0  0  0  0  0  0  0  0  0  0  0
   19.6651   12.4825    0.6331 H   0  0  0  0  0  0  0  0  0  0  0  0
   19.6225   12.5571   -1.1335 H   0  0  0  0  0  0  0  0  0  0  0  0
   19.7242   15.0363   -1.0294 H   0  0  0  0  0  0  0  0  0  0  0  0
   19.7718   14.9591    0.7370 H   0  0  0  0  0  0  0  0  0  0  0  0
   24.0358   13.8799    0.7649 H   0  0  0  0  0  0  0  0  0  0  0  0
   23.8704   12.9746   -0.7454 H   0  0  0  0  0  0  0  0  0  0  0  0
   23.9436   12.1141    0.7980 H   0  0  0  0  0  0  0  0  0  0  0  0
  1  2  1  0  0  0  0
  2  3  1  0  0  0  0
```

```
  3  4  1  0  0  0  0
  4  5  1  0  0  0  0
  5  6  1  0  0  0  0
  6  7  1  0  0  0  0
  7  8  1  0  0  0  0
  8  9  1  0  0  0  0
  9 10  2  0  0  0  0
 10 11  1  0  0  0  0
 11 12  1  0  0  0  0
 12 13  1  0  0  0  0
 13 14  1  0  0  0  0
 14 15  1  0  0  0  0
 15 16  1  0  0  0  0
 16 17  1  0  0  0  0
 17 18  1  0  0  0  0
 18 19  2  0  0  0  0
 18 20  1  0  0  0  0
 20 21  1  0  0  0  0
  1 22  1  0  0  0  0
  1 23  1  0  0  0  0
  1 24  1  0  0  0  0
  2 25  1  0  0  0  0
  2 26  1  0  0  0  0
  3 27  1  0  0  0  0
  3 28  1  0  0  0  0
  4 29  1  0  0  0  0
  4 30  1  0  0  0  0
  5 31  1  0  0  0  0
  5 32  1  0  0  0  0
  6 33  1  0  0  0  0
  6 34  1  0  0  0  0
  7 35  1  0  0  0  0
  7 36  1  0  0  0  0
  8 37  1  0  0  0  0
  8 38  1  0  0  0  0
  9 39  1  0  0  0  0
 10 40  1  0  0  0  0
 11 41  1  0  0  0  0
 11 42  1  0  0  0  0
 12 43  1  0  0  0  0
 12 44  1  0  0  0  0
 13 45  1  0  0  0  0
 13 46  1  0  0  0  0
 14 47  1  0  0  0  0
 14 48  1  0  0  0  0
 15 49  1  0  0  0  0
 15 50  1  0  0  0  0
 16 51  1  0  0  0  0
 16 52  1  0  0  0  0
 17 53  1  0  0  0  0
 17 54  1  0  0  0  0
 21 55  1  0  0  0  0
 21 56  1  0  0  0  0
 21 57  1  0  0  0  0
M  END
>  <Alias name>
Methyl (Z) 9-octadecenoate

>  <deltaH°(c)>   kJ/mol
-11832.4

$$$$
Stearic alcohol
```

ChemBrain IXL

```
57 56  0  0  0  0  0  0  0  0  1 V2000
   11.6802   11.4526    0.3034 C   0  0  0  0  0  0  0  0  0  0  0  0
   11.0221   10.1773   -0.2582 C   0  0  0  0  0  0  0  0  0  0  0  0
   13.1432   11.5292   -0.1747 C   0  0  0  0  0  0  0  0  0  0  0  0
    9.5586   10.1014    0.2188 C   0  0  0  0  0  0  0  0  0  0  0  0
   13.8009   12.8052    0.3857 C   0  0  0  0  0  0  0  0  0  0  0  0
    8.9001    8.8268   -0.3438 C   0  0  0  0  0  0  0  0  0  0  0  0
   15.2636   12.8824   -0.0934 C   0  0  0  0  0  0  0  0  0  0  0  0
    7.4364    8.7516    0.1323 C   0  0  0  0  0  0  0  0  0  0  0  0
   15.9210   14.1590    0.4662 C   0  0  0  0  0  0  0  0  0  0  0  0
    6.7775    7.4774   -0.4313 C   0  0  0  0  0  0  0  0  0  0  0  0
   17.3834   14.2366   -0.0136 C   0  0  0  0  0  0  0  0  0  0  0  0
    5.3135    7.4026    0.0441 C   0  0  0  0  0  0  0  0  0  0  0  0
   18.0405   15.5137    0.5453 C   0  0  0  0  0  0  0  0  0  0  0  0
    4.6545    6.1289   -0.5200 C   0  0  0  0  0  0  0  0  0  0  0  0
   19.5030   15.5915    0.0651 C   0  0  0  0  0  0  0  0  0  0  0  0
    3.1902    6.0542   -0.0448 C   0  0  0  0  0  0  0  0  0  0  0  0
   20.1600   16.8686    0.6237 C   0  0  0  0  0  0  0  0  0  0  0  0
    2.5311    4.7807   -0.6091 C   0  0  0  0  0  0  0  0  0  0  0  0
   21.5546   16.9425    0.1655 O   0  0  0  0  0  0  0  0  0  0  0  0
   22.0528   17.7307    0.4767 H   0  0  0  0  0  0  0  0  0  0  0  0
   11.6551   11.4252    1.3857 H   0  0  0  0  0  0  0  0  0  0  0  0
   11.1395   12.3226   -0.0484 H   0  0  0  0  0  0  0  0  0  0  0  0
   11.0479   10.2044   -1.3406 H   0  0  0  0  0  0  0  0  0  0  0  0
   11.5622    9.3072    0.0941 H   0  0  0  0  0  0  0  0  0  0  0  0
   13.1683   11.5558   -1.2571 H   0  0  0  0  0  0  0  0  0  0  0  0
   13.6841   10.6596    0.1776 H   0  0  0  0  0  0  0  0  0  0  0  0
    9.5327   10.0735    1.3012 H   0  0  0  0  0  0  0  0  0  0  0  0
    9.0188   10.9720   -0.1328 H   0  0  0  0  0  0  0  0  0  0  0  0
   13.7765   12.7784    1.4681 H   0  0  0  0  0  0  0  0  0  0  0  0
   13.2595   13.6747    0.0339 H   0  0  0  0  0  0  0  0  0  0  0  0
    8.9267    8.8545   -1.4262 H   0  0  0  0  0  0  0  0  0  0  0  0
    9.4395    7.9562    0.0084 H   0  0  0  0  0  0  0  0  0  0  0  0
   15.2880   12.9085   -1.1758 H   0  0  0  0  0  0  0  0  0  0  0  0
   15.8052   12.0133    0.2590 H   0  0  0  0  0  0  0  0  0  0  0  0
    7.4098    8.7232    1.2146 H   0  0  0  0  0  0  0  0  0  0  0  0
    6.8972    9.6225   -0.2194 H   0  0  0  0  0  0  0  0  0  0  0  0
   15.8971   14.1327    1.5486 H   0  0  0  0  0  0  0  0  0  0  0  0
   15.3790   15.0281    0.1142 H   0  0  0  0  0  0  0  0  0  0  0  0
    6.8046    7.5056   -1.5136 H   0  0  0  0  0  0  0  0  0  0  0  0
    7.3164    6.6065   -0.0793 H   0  0  0  0  0  0  0  0  0  0  0  0
   17.4073   14.2625   -1.0961 H   0  0  0  0  0  0  0  0  0  0  0  0
   17.9255   13.3678    0.3387 H   0  0  0  0  0  0  0  0  0  0  0  0
    5.2864    7.3741    1.1264 H   0  0  0  0  0  0  0  0  0  0  0  0
    4.7748    8.2738   -0.3076 H   0  0  0  0  0  0  0  0  0  0  0  0
   18.0169   15.4876    1.6278 H   0  0  0  0  0  0  0  0  0  0  0  0
   17.4982   16.3824    0.1932 H   0  0  0  0  0  0  0  0  0  0  0  0
    4.6818    6.1573   -1.6023 H   0  0  0  0  0  0  0  0  0  0  0  0
    5.1930    5.2576   -0.1681 H   0  0  0  0  0  0  0  0  0  0  0  0
   19.5266   15.6174   -1.0173 H   0  0  0  0  0  0  0  0  0  0  0  0
   20.0453   14.7229    0.4174 H   0  0  0  0  0  0  0  0  0  0  0  0
    3.1629    6.0256    1.0374 H   0  0  0  0  0  0  0  0  0  0  0  0
    2.6517    6.9255   -0.3966 H   0  0  0  0  0  0  0  0  0  0  0  0
   20.1366   16.8428    1.7062 H   0  0  0  0  0  0  0  0  0  0  0  0
   19.6177   17.7373    0.2715 H   0  0  0  0  0  0  0  0  0  0  0  0
    2.5583    4.8093   -1.6913 H   0  0  0  0  0  0  0  0  0  0  0  0
    3.0694    3.9093   -0.2573 H   0  0  0  0  0  0  0  0  0  0  0  0
    1.5023    4.7285   -0.2751 H   0  0  0  0  0  0  0  0  0  0  0  0
  1  2  1  0  0  0  0
  1  3  1  0  0  0  0
  2  4  1  0  0  0  0
```

 3 5 1 0 0 0 0
 4 6 1 0 0 0 0
 5 7 1 0 0 0 0
 6 8 1 0 0 0 0
 7 9 1 0 0 0 0
 8 10 1 0 0 0 0
 9 11 1 0 0 0 0
10 12 1 0 0 0 0
11 13 1 0 0 0 0
12 14 1 0 0 0 0
13 15 1 0 0 0 0
14 16 1 0 0 0 0
15 17 1 0 0 0 0
16 18 1 0 0 0 0
17 19 1 0 0 0 0
19 20 1 0 0 0 0
 1 21 1 0 0 0 0
 1 22 1 0 0 0 0
 2 23 1 0 0 0 0
 2 24 1 0 0 0 0
 3 25 1 0 0 0 0
 3 26 1 0 0 0 0
 4 27 1 0 0 0 0
 4 28 1 0 0 0 0
 5 29 1 0 0 0 0
 5 30 1 0 0 0 0
 6 31 1 0 0 0 0
 6 32 1 0 0 0 0
 7 33 1 0 0 0 0
 7 34 1 0 0 0 0
 8 35 1 0 0 0 0
 8 36 1 0 0 0 0
 9 37 1 0 0 0 0
 9 38 1 0 0 0 0
10 39 1 0 0 0 0
10 40 1 0 0 0 0
11 41 1 0 0 0 0
11 42 1 0 0 0 0
12 43 1 0 0 0 0
12 44 1 0 0 0 0
13 45 1 0 0 0 0
13 46 1 0 0 0 0
14 47 1 0 0 0 0
14 48 1 0 0 0 0
15 49 1 0 0 0 0
15 50 1 0 0 0 0
16 51 1 0 0 0 0
16 52 1 0 0 0 0
17 53 1 0 0 0 0
17 54 1 0 0 0 0
18 55 1 0 0 0 0
18 56 1 0 0 0 0
18 57 1 0 0 0 0
M END
> <Alias name>
1-Octadecanol

> <deltaH°(c)>   kJ/mol
-11820

$$$$
Tridecylcyclopentane
  ChemBrain IXL

```
54 54  0  0  0  0  0  0  0  0  1 V2000
   18.6754   10.4252   -0.1500 C   0  0  0  0  0  0  0  0  0  0  0  0  0
   19.9008    9.7129    0.4031 C   0  0  0  0  0  0  0  0  0  0  0  0  0
   21.0372   10.7154    0.2184 C   0  0  0  0  0  0  0  0  0  0  0  0  0
   20.3740   12.1244    0.3673 C   0  0  0  0  0  0  0  0  0  0  0  0  0
   18.8316   11.8694    0.3211 C   0  0  0  0  0  0  0  0  0  0  0  0  0
   17.3695    9.7976    0.3776 C   0  0  0  0  0  0  0  0  0  0  0  0  0
   16.1618   10.5780   -0.1783 C   0  0  0  0  0  0  0  0  0  0  0  0  0
   14.8572    9.9487    0.3479 C   0  0  0  0  0  0  0  0  0  0  0  0  0
   13.6499   10.7286   -0.2080 C   0  0  0  0  0  0  0  0  0  0  0  0  0
   12.3453   10.0999    0.3191 C   0  0  0  0  0  0  0  0  0  0  0  0  0
   11.1378   10.8795   -0.2370 C   0  0  0  0  0  0  0  0  0  0  0  0  0
    9.8333   10.2513    0.2910 C   0  0  0  0  0  0  0  0  0  0  0  0  0
    8.6258   11.0307   -0.2653 C   0  0  0  0  0  0  0  0  0  0  0  0  0
    7.3214   10.4029    0.2634 C   0  0  0  0  0  0  0  0  0  0  0  0  0
    6.1138   11.1822   -0.2929 C   0  0  0  0  0  0  0  0  0  0  0  0  0
    4.8094   10.5548    0.2363 C   0  0  0  0  0  0  0  0  0  0  0  0  0
    3.6017   11.3339   -0.3202 C   0  0  0  0  0  0  0  0  0  0  0  0  0
    2.2973   10.7069    0.2091 C   0  0  0  0  0  0  0  0  0  0  0  0  0
   18.6995   10.3932   -1.2322 H   0  0  0  0  0  0  0  0  0  0  0  0  0
   19.7579    9.5182    1.4589 H   0  0  0  0  0  0  0  0  0  0  0  0  0
   20.0978    8.7902   -0.1284 H   0  0  0  0  0  0  0  0  0  0  0  0  0
   21.8314   10.5680    0.9396 H   0  0  0  0  0  0  0  0  0  0  0  0  0
   21.4239   10.6155   -0.7882 H   0  0  0  0  0  0  0  0  0  0  0  0  0
   20.6351   12.5796    1.3145 H   0  0  0  0  0  0  0  0  0  0  0  0  0
   20.6884   12.7584   -0.4524 H   0  0  0  0  0  0  0  0  0  0  0  0  0
   18.3292   12.5685   -0.3358 H   0  0  0  0  0  0  0  0  0  0  0  0  0
   18.4338   11.9489    1.3253 H   0  0  0  0  0  0  0  0  0  0  0  0  0
   17.3103    8.7650    0.0567 H   0  0  0  0  0  0  0  0  0  0  0  0  0
   17.3598    9.8403    1.4597 H   0  0  0  0  0  0  0  0  0  0  0  0  0
   16.2198   11.6104    0.1434 H   0  0  0  0  0  0  0  0  0  0  0  0  0
   16.1720   10.5359   -1.2604 H   0  0  0  0  0  0  0  0  0  0  0  0  0
   14.7991    8.9162    0.0263 H   0  0  0  0  0  0  0  0  0  0  0  0  0
   14.8470    9.9909    1.4300 H   0  0  0  0  0  0  0  0  0  0  0  0  0
   13.7083   11.7612    0.1131 H   0  0  0  0  0  0  0  0  0  0  0  0  0
   13.6597   10.6858   -1.2901 H   0  0  0  0  0  0  0  0  0  0  0  0  0
   12.2869    9.0671   -0.0017 H   0  0  0  0  0  0  0  0  0  0  0  0  0
   12.3354   10.1428    1.4012 H   0  0  0  0  0  0  0  0  0  0  0  0  0
   11.1965   11.9123    0.0833 H   0  0  0  0  0  0  0  0  0  0  0  0  0
   11.1473   10.8359   -1.3190 H   0  0  0  0  0  0  0  0  0  0  0  0  0
    9.7747    9.2183   -0.0291 H   0  0  0  0  0  0  0  0  0  0  0  0  0
    9.8238   10.2950    1.3731 H   0  0  0  0  0  0  0  0  0  0  0  0  0
    8.6847   12.0637    0.0544 H   0  0  0  0  0  0  0  0  0  0  0  0  0
    8.6350   10.9865   -1.3473 H   0  0  0  0  0  0  0  0  0  0  0  0  0
    7.2626    9.3698   -0.0561 H   0  0  0  0  0  0  0  0  0  0  0  0  0
    7.3121   10.4473    1.3455 H   0  0  0  0  0  0  0  0  0  0  0  0  0
    6.1728   12.2154    0.0262 H   0  0  0  0  0  0  0  0  0  0  0  0  0
    6.1228   11.1375   -1.3750 H   0  0  0  0  0  0  0  0  0  0  0  0  0
    4.7504    9.5215   -0.0828 H   0  0  0  0  0  0  0  0  0  0  0  0  0
    4.8004   10.5996    1.3183 H   0  0  0  0  0  0  0  0  0  0  0  0  0
    3.6607   12.3672   -0.0014 H   0  0  0  0  0  0  0  0  0  0  0  0  0
    3.6105   11.2890   -1.4022 H   0  0  0  0  0  0  0  0  0  0  0  0  0
    2.2382    9.6736   -0.1097 H   0  0  0  0  0  0  0  0  0  0  0  0  0
    2.2883   10.7519    1.2912 H   0  0  0  0  0  0  0  0  0  0  0  0  0
    1.4489   11.2545   -0.1820 H   0  0  0  0  0  0  0  0  0  0  0  0  0
  1  2  1  0  0  0  0
  2  3  1  0  0  0  0
  3  4  1  0  0  0  0
  4  5  1  0  0  0  0
  1  5  1  0  0  0  0
  6  7  1  0  0  0  0
  7  8  1  0  0  0  0
```

```
  8  9  1  0  0  0  0
  9 10  1  0  0  0  0
 10 11  1  0  0  0  0
 11 12  1  0  0  0  0
 12 13  1  0  0  0  0
 13 14  1  0  0  0  0
 14 15  1  0  0  0  0
 15 16  1  0  0  0  0
  1  6  1  0  0  0  0
 16 17  1  0  0  0  0
 17 18  1  0  0  0  0
  1 19  1  0  0  0  0
  2 20  1  0  0  0  0
  2 21  1  0  0  0  0
  3 22  1  0  0  0  0
  3 23  1  0  0  0  0
  4 24  1  0  0  0  0
  4 25  1  0  0  0  0
  5 26  1  0  0  0  0
  5 27  1  0  0  0  0
  6 28  1  0  0  0  0
  6 29  1  0  0  0  0
  7 30  1  0  0  0  0
  7 31  1  0  0  0  0
  8 32  1  0  0  0  0
  8 33  1  0  0  0  0
  9 34  1  0  0  0  0
  9 35  1  0  0  0  0
 10 36  1  0  0  0  0
 10 37  1  0  0  0  0
 11 38  1  0  0  0  0
 11 39  1  0  0  0  0
 12 40  1  0  0  0  0
 12 41  1  0  0  0  0
 13 42  1  0  0  0  0
 13 43  1  0  0  0  0
 14 44  1  0  0  0  0
 14 45  1  0  0  0  0
 15 46  1  0  0  0  0
 15 47  1  0  0  0  0
 16 48  1  0  0  0  0
 16 49  1  0  0  0  0
 17 50  1  0  0  0  0
 17 51  1  0  0  0  0
 18 52  1  0  0  0  0
 18 53  1  0  0  0  0
 18 54  1  0  0  0  0
M  END
>  <Alias name>
Tridecylcyclopentane

>  <deltaH°(c)>   kJ/mol
-11791

$$$$
Tridecylbenzene
  ChemBrain IXL

 51 51  0  0  0  0  0  0  0  0  1 V2000
    3.5402    9.4154    0.0000 C   0  0  0  0  0  0  0  0  0  0  0  0
    2.3939   10.2103   -0.0067 C   0  0  0  0  0  0  0  0  0  0  0  0
    2.5092   11.6013    0.0014 C   0  0  0  0  0  0  0  0  0  0  0  0
    3.7713   12.1984    0.0163 C   0  0  0  0  0  0  0  0  0  0  0  0
```

```
    4.9203   11.4053    0.0230 C   0  0  0  0  0  0  0  0  0  0  0  0
    4.8020   10.0107    0.0148 C   0  0  0  0  0  0  0  0  0  0  0  0
    6.2637   12.0480    0.0390 C   0  0  0  0  0  0  0  0  0  0  0  0
    7.3688   10.9653    0.0169 C   0  0  0  0  0  0  0  0  0  0  0  0
    8.7534   11.6443    0.0426 C   0  0  0  0  0  0  0  0  0  0  0  0
    9.8600   10.5683    0.0140 C   0  0  0  0  0  0  0  0  0  0  0  0
   11.2412   11.2544    0.0515 C   0  0  0  0  0  0  0  0  0  0  0  0
   12.3540   10.1864    0.0107 C   0  0  0  0  0  0  0  0  0  0  0  0
   13.7305   10.8816    0.0548 C   0  0  0  0  0  0  0  0  0  0  0  0
   14.8494    9.8218   -0.0010 C   0  0  0  0  0  0  0  0  0  0  0  0
   16.2218   10.5244    0.0460 C   0  0  0  0  0  0  0  0  0  0  0  0
   17.3450    9.4708   -0.0236 C   0  0  0  0  0  0  0  0  0  0  0  0
   18.7147   10.1778    0.0248 C   0  0  0  0  0  0  0  0  0  0  0  0
   19.8400    9.1272   -0.0520 C   0  0  0  0  0  0  0  0  0  0  0  0
   21.2082    9.8353   -0.0024 C   0  0  0  0  0  0  0  0  0  0  0  0
    3.4509    8.3359   -0.0063 H   0  0  0  0  0  0  0  0  0  0  0  0
    1.4146    9.7479   -0.0182 H   0  0  0  0  0  0  0  0  0  0  0  0
    1.6191   12.2175   -0.0037 H   0  0  0  0  0  0  0  0  0  0  0  0
    3.8589   13.2773    0.0226 H   0  0  0  0  0  0  0  0  0  0  0  0
    5.6884    9.3903    0.0200 H   0  0  0  0  0  0  0  0  0  0  0  0
    6.3648   12.6458    0.9360 H   0  0  0  0  0  0  0  0  0  0  0  0
    6.3675   12.6847   -0.8307 H   0  0  0  0  0  0  0  0  0  0  0  0
    7.2749   10.3711   -0.8840 H   0  0  0  0  0  0  0  0  0  0  0  0
    7.2653   10.3252    0.8846 H   0  0  0  0  0  0  0  0  0  0  0  0
    8.8482   12.2348    0.9454 H   0  0  0  0  0  0  0  0  0  0  0  0
    8.8550   12.2886   -0.8219 H   0  0  0  0  0  0  0  0  0  0  0  0
    9.7715    9.9829   -0.8929 H   0  0  0  0  0  0  0  0  0  0  0  0
    9.7556    9.9192    0.8747 H   0  0  0  0  0  0  0  0  0  0  0  0
   11.3310   11.8336    0.9621 H   0  0  0  0  0  0  0  0  0  0  0  0
   11.3407   11.9106   -0.8042 H   0  0  0  0  0  0  0  0  0  0  0  0
   12.2688    9.6106   -0.9026 H   0  0  0  0  0  0  0  0  0  0  0  0
   12.2543    9.5272    0.8642 H   0  0  0  0  0  0  0  0  0  0  0  0
   13.8179   11.4500    0.9724 H   0  0  0  0  0  0  0  0  0  0  0  0
   13.8243   11.5489   -0.7929 H   0  0  0  0  0  0  0  0  0  0  0  0
   14.7648    9.2553   -0.9201 H   0  0  0  0  0  0  0  0  0  0  0  0
   14.7566    9.1529    0.8457 H   0  0  0  0  0  0  0  0  0  0  0  0
   16.3089   11.0842    0.9690 H   0  0  0  0  0  0  0  0  0  0  0  0
   16.3096   11.2006   -0.7954 H   0  0  0  0  0  0  0  0  0  0  0  0
   17.2594    8.9123   -0.9475 H   0  0  0  0  0  0  0  0  0  0  0  0
   17.2580    8.7938    0.8172 H   0  0  0  0  0  0  0  0  0  0  0  0
   18.8018   10.7328    0.9507 H   0  0  0  0  0  0  0  0  0  0  0  0
   18.7991   10.8586   -0.8132 H   0  0  0  0  0  0  0  0  0  0  0  0
   19.7538    8.5730   -0.9784 H   0  0  0  0  0  0  0  0  0  0  0  0
   19.7557    8.4459    0.7855 H   0  0  0  0  0  0  0  0  0  0  0  0
   21.2923   10.5175   -0.8394 H   0  0  0  0  0  0  0  0  0  0  0  0
   21.9988    9.0973   -0.0574 H   0  0  0  0  0  0  0  0  0  0  0  0
   21.2950   10.3888    0.9245 H   0  0  0  0  0  0  0  0  0  0  0  0
  1  2  4  0  0  0  0
  2  3  4  0  0  0  0
  3  4  4  0  0  0  0
  4  5  4  0  0  0  0
  5  6  4  0  0  0  0
  6  1  4  0  0  0  0
  5  7  1  0  0  0  0
  7  8  1  0  0  0  0
  8  9  1  0  0  0  0
  9 10  1  0  0  0  0
 10 11  1  0  0  0  0
 11 12  1  0  0  0  0
 12 13  1  0  0  0  0
 13 14  1  0  0  0  0
 14 15  1  0  0  0  0
 15 16  1  0  0  0  0
```

16 17 1 0 0 0 0
17 18 1 0 0 0 0
18 19 1 0 0 0 0
1 20 1 0 0 0 0
2 21 1 0 0 0 0
3 22 1 0 0 0 0
4 23 1 0 0 0 0
6 24 1 0 0 0 0
7 25 1 0 0 0 0
7 26 1 0 0 0 0
8 27 1 0 0 0 0
8 28 1 0 0 0 0
9 29 1 0 0 0 0
9 30 1 0 0 0 0
10 31 1 0 0 0 0
10 32 1 0 0 0 0
11 33 1 0 0 0 0
11 34 1 0 0 0 0
12 35 1 0 0 0 0
12 36 1 0 0 0 0
13 37 1 0 0 0 0
13 38 1 0 0 0 0
14 39 1 0 0 0 0
14 40 1 0 0 0 0
15 41 1 0 0 0 0
15 42 1 0 0 0 0
16 43 1 0 0 0 0
16 44 1 0 0 0 0
17 45 1 0 0 0 0
17 46 1 0 0 0 0
18 47 1 0 0 0 0
18 48 1 0 0 0 0
19 49 1 0 0 0 0
19 50 1 0 0 0 0
19 51 1 0 0 0 0
M END
> <Alias name>
Tridecylbenzene

> <deltaH°(c)>   kJ/mol
-11769.29

$$$$
Dodecylcyclohexane
ChemBrain IXL

54 54 0 0 0 0 0 0 0 0 1 V2000
17.6131   11.1622   -0.3624 C   0 0 0 0 0 0 0 0 0 0 0 0
18.9170   11.8598   0.0748 C   0 0 0 0 0 0 0 0 0 0 0 0
20.1180   10.9411   -0.2246 C   0 0 0 0 0 0 0 0 0 0 0 0
19.9592   9.6185   0.5510 C   0 0 0 0 0 0 0 0 0 0 0 0
18.6574   8.9186   0.1130 C   0 0 0 0 0 0 0 0 0 0 0 0
17.4535   9.8356   0.4118 C   0 0 0 0 0 0 0 0 0 0 0 0
16.1517   9.1307   -0.0279 C   0 0 0 0 0 0 0 0 0 0 0 0
14.9401   10.0300   0.2929 C   0 0 0 0 0 0 0 0 0 0 0 0
13.6428   9.3127   -0.1279 C   0 0 0 0 0 0 0 0 0 0 0 0
12.4321   10.2109   0.1917 C   0 0 0 0 0 0 0 0 0 0 0 0
11.1347   9.4938   -0.2297 C   0 0 0 0 0 0 0 0 0 0 0 0
9.9241   10.3926   0.0890 C   0 0 0 0 0 0 0 0 0 0 0 0
8.6267   9.6758   -0.3329 C   0 0 0 0 0 0 0 0 0 0 0 0
7.4162   10.5750   -0.0152 C   0 0 0 0 0 0 0 0 0 0 0 0
6.1188   9.8585   -0.4375 C   0 0 0 0 0 0 0 0 0 0 0 0
4.9083   10.7580   -0.1203 C   0 0 0 0 0 0 0 0 0 0 0 0

```
   3.6108   10.0415   -0.5429 C   0  0  0  0  0  0  0  0  0  0  0  0
   2.4004   10.9410   -0.2260 C   0  0  0  0  0  0  0  0  0  0  0  0
  17.6520   10.9575   -1.4252 H   0  0  0  0  0  0  0  0  0  0  0  0
  16.7713   11.8081   -0.1501 H   0  0  0  0  0  0  0  0  0  0  0  0
  18.8768   12.0667    1.1371 H   0  0  0  0  0  0  0  0  0  0  0  0
  19.0290   12.7890   -0.4698 H   0  0  0  0  0  0  0  0  0  0  0  0
  21.0331   11.4319    0.0825 H   0  0  0  0  0  0  0  0  0  0  0  0
  20.1583   10.7341   -1.2868 H   0  0  0  0  0  0  0  0  0  0  0  0
  19.9181    9.8255    1.6133 H   0  0  0  0  0  0  0  0  0  0  0  0
  20.8036    8.9737    0.3415 H   0  0  0  0  0  0  0  0  0  0  0  0
  18.6987    8.7119   -0.9493 H   0  0  0  0  0  0  0  0  0  0  0  0
  18.5468    7.9891    0.6575 H   0  0  0  0  0  0  0  0  0  0  0  0
  17.4115   10.0405    1.4744 H   0  0  0  0  0  0  0  0  0  0  0  0
  16.1871    8.9390   -1.0932 H   0  0  0  0  0  0  0  0  0  0  0  0
  16.0537    8.1925    0.5040 H   0  0  0  0  0  0  0  0  0  0  0  0
  14.9120   10.2320    1.3565 H   0  0  0  0  0  0  0  0  0  0  0  0
  15.0280   10.9624   -0.2493 H   0  0  0  0  0  0  0  0  0  0  0  0
  13.6711    9.1100   -1.1914 H   0  0  0  0  0  0  0  0  0  0  0  0
  13.5536    8.3799    0.4151 H   0  0  0  0  0  0  0  0  0  0  0  0
  12.4036   10.4134    1.2552 H   0  0  0  0  0  0  0  0  0  0  0  0
  12.5215   11.1438   -0.3511 H   0  0  0  0  0  0  0  0  0  0  0  0
  11.1636    9.2908   -1.2931 H   0  0  0  0  0  0  0  0  0  0  0  0
  11.0449    8.5613    0.3136 H   0  0  0  0  0  0  0  0  0  0  0  0
   9.8950   10.5954    1.1524 H   0  0  0  0  0  0  0  0  0  0  0  0
  10.0141   11.3253   -0.4540 H   0  0  0  0  0  0  0  0  0  0  0  0
   8.6561    9.4725   -1.3963 H   0  0  0  0  0  0  0  0  0  0  0  0
   8.5363    8.7434    0.2105 H   0  0  0  0  0  0  0  0  0  0  0  0
   7.3866   10.7782    1.0482 H   0  0  0  0  0  0  0  0  0  0  0  0
   7.5067   11.5075   -0.5584 H   0  0  0  0  0  0  0  0  0  0  0  0
   6.1486    9.6550   -1.5008 H   0  0  0  0  0  0  0  0  0  0  0  0
   6.0280    8.9262    0.1061 H   0  0  0  0  0  0  0  0  0  0  0  0
   4.8785   10.9614    0.9430 H   0  0  0  0  0  0  0  0  0  0  0  0
   4.9992   11.6903   -0.6638 H   0  0  0  0  0  0  0  0  0  0  0  0
   3.6407    9.8379   -1.6061 H   0  0  0  0  0  0  0  0  0  0  0  0
   3.5198    9.1093    0.0007 H   0  0  0  0  0  0  0  0  0  0  0  0
   1.4889   10.4373   -0.5230 H   0  0  0  0  0  0  0  0  0  0  0  0
   2.3704   11.1446    0.8373 H   0  0  0  0  0  0  0  0  0  0  0  0
   2.4913   11.8733   -0.7697 H   0  0  0  0  0  0  0  0  0  0  0  0
  1  2  1  0  0  0  0
  2  3  1  0  0  0  0
  3  4  1  0  0  0  0
  4  5  1  0  0  0  0
  5  6  1  0  0  0  0
  6  1  1  0  0  0  0
  6  7  1  0  0  0  0
  7  8  1  0  0  0  0
  8  9  1  0  0  0  0
  9 10  1  0  0  0  0
 10 11  1  0  0  0  0
 11 12  1  0  0  0  0
 12 13  1  0  0  0  0
 13 14  1  0  0  0  0
 14 15  1  0  0  0  0
 15 16  1  0  0  0  0
 16 17  1  0  0  0  0
 17 18  1  0  0  0  0
  1 19  1  0  0  0  0
  1 20  1  0  0  0  0
  2 21  1  0  0  0  0
  2 22  1  0  0  0  0
  3 23  1  0  0  0  0
  3 24  1  0  0  0  0
  4 25  1  0  0  0  0
```

```
  4 26  1  0  0  0  0
  5 27  1  0  0  0  0
  5 28  1  0  0  0  0
  6 29  1  0  0  0  0
  7 30  1  0  0  0  0
  7 31  1  0  0  0  0
  8 32  1  0  0  0  0
  8 33  1  0  0  0  0
  9 34  1  0  0  0  0
  9 35  1  0  0  0  0
 10 36  1  0  0  0  0
 10 37  1  0  0  0  0
 11 38  1  0  0  0  0
 11 39  1  0  0  0  0
 12 40  1  0  0  0  0
 12 41  1  0  0  0  0
 13 42  1  0  0  0  0
 13 43  1  0  0  0  0
 14 44  1  0  0  0  0
 14 45  1  0  0  0  0
 15 46  1  0  0  0  0
 15 47  1  0  0  0  0
 16 48  1  0  0  0  0
 16 49  1  0  0  0  0
 17 50  1  0  0  0  0
 17 51  1  0  0  0  0
 18 52  1  0  0  0  0
 18 53  1  0  0  0  0
 18 54  1  0  0  0  0
M  END
>  <Alias name>
Dodecylcyclohexane

>  <deltaH°(c)>   kJ/mol
-11760.6

$$$$
2,4,6-Triphenyl-pyridine
  ChemBrain IXL

 41 44  0  0  0  0  0  0  0  0  1 V2000
    8.9368   11.0337    0.0000 C   0  0  0  0  0  0  0  0  0  0  0  0
    8.9371    9.6375    0.0000 C   0  0  0  0  0  0  0  0  0  0  0  0
    7.7288   11.7316    0.0000 C   0  0  0  0  0  0  0  0  0  0  0  0
    7.7288    8.9374    0.0000 C   0  0  0  0  0  0  0  0  0  0  0  0
    6.5208   11.0337    0.0000 C   0  0  0  0  0  0  0  0  0  0  0  0
    6.5204    9.6376    0.0000 C   0  0  0  0  0  0  0  0  0  0  0  0
    7.7288    7.4501    0.0000 C   0  0  0  0  0  0  0  0  0  0  0  0
    7.7288    4.6860    0.0000 N   0  0  0  0  0  0  0  0  0  0  0  0
    6.5498    5.3548    0.0000 C   0  0  0  0  0  0  0  0  0  0  0  0
    8.9077    5.3548    0.0000 C   0  0  0  0  0  0  0  0  0  0  0  0
    6.5313    6.7471    0.0000 C   0  0  0  0  0  0  0  0  0  0  0  0
    5.2708    4.6119    0.0000 C   0  0  0  0  0  0  0  0  0  0  0  0
    8.9263    6.7471    0.0000 C   0  0  0  0  0  0  0  0  0  0  0  0
   10.1867    4.6119    0.0000 C   0  0  0  0  0  0  0  0  0  0  0  0
    2.8567    3.2142    0.0000 C   0  0  0  0  0  0  0  0  0  0  0  0
    2.8546    4.6091    0.0000 C   0  0  0  0  0  0  0  0  0  0  0  0
    4.0659    2.5185    0.0000 C   0  0  0  0  0  0  0  0  0  0  0  0
    4.0617    5.3081    0.0000 C   0  0  0  0  0  0  0  0  0  0  0  0
    5.2730    3.2175    0.0000 C   0  0  0  0  0  0  0  0  0  0  0  0
   12.6008    3.2142    0.0000 C   0  0  0  0  0  0  0  0  0  0  0  0
   11.3916    2.5185    0.0000 C   0  0  0  0  0  0  0  0  0  0  0  0
   12.6029    4.6092    0.0000 C   0  0  0  0  0  0  0  0  0  0  0  0
```

10.1845	3.2175	0.0000	C	0	0	0	0	0	0	0	0	0	0	0	0
11.3958	5.3081	0.0000	C	0	0	0	0	0	0	0	0	0	0	0	0
9.8748	11.5758	0.0000	H	0	0	0	0	0	0	0	0	0	0	0	0
9.8755	9.0975	0.0000	H	0	0	0	0	0	0	0	0	0	0	0	0
7.7288	12.8153	0.0000	H	0	0	0	0	0	0	0	0	0	0	0	0
5.5828	11.5758	0.0000	H	0	0	0	0	0	0	0	0	0	0	0	0
5.5821	9.0975	0.0000	H	0	0	0	0	0	0	0	0	0	0	0	0
5.5878	7.2793	0.0000	H	0	0	0	0	0	0	0	0	0	0	0	0
9.8698	7.2793	0.0000	H	0	0	0	0	0	0	0	0	0	0	0	0
1.9197	2.6714	0.0000	H	0	0	0	0	0	0	0	0	0	0	0	0
1.9158	5.1494	0.0000	H	0	0	0	0	0	0	0	0	0	0	0	0
4.0675	1.4357	0.0000	H	0	0	0	0	0	0	0	0	0	0	0	0
4.0602	6.3913	0.0000	H	0	0	0	0	0	0	0	0	0	0	0	0
6.2117	2.6776	0.0000	H	0	0	0	0	0	0	0	0	0	0	0	0
13.5379	2.6714	0.0000	H	0	0	0	0	0	0	0	0	0	0	0	0
11.3900	1.4357	0.0000	H	0	0	0	0	0	0	0	0	0	0	0	0
13.5417	5.1495	0.0000	H	0	0	0	0	0	0	0	0	0	0	0	0
9.2458	2.6776	0.0000	H	0	0	0	0	0	0	0	0	0	0	0	0
11.3973	6.3913	0.0000	H	0	0	0	0	0	0	0	0	0	0	0	0

1	2	4	0	0	0	0
3	1	4	0	0	0	0
2	4	4	0	0	0	0
5	3	4	0	0	0	0
4	6	4	0	0	0	0
4	7	1	0	0	0	0
6	5	4	0	0	0	0
11	7	4	0	0	0	0
7	13	4	0	0	0	0
8	9	4	0	0	0	0
10	8	4	0	0	0	0
9	11	4	0	0	0	0
9	12	1	0	0	0	0
13	10	4	0	0	0	0
10	14	1	0	0	0	0
18	12	4	0	0	0	0
12	19	4	0	0	0	0
23	14	4	0	0	0	0
14	24	4	0	0	0	0
15	16	4	0	0	0	0
17	15	4	0	0	0	0
16	18	4	0	0	0	0
19	17	4	0	0	0	0
20	21	4	0	0	0	0
22	20	4	0	0	0	0
21	23	4	0	0	0	0
24	22	4	0	0	0	0
1	25	1	0	0	0	0
2	26	1	0	0	0	0
3	27	1	0	0	0	0
5	28	1	0	0	0	0
6	29	1	0	0	0	0
11	30	1	0	0	0	0
13	31	1	0	0	0	0
15	32	1	0	0	0	0
16	33	1	0	0	0	0
17	34	1	0	0	0	0
18	35	1	0	0	0	0
19	36	1	0	0	0	0
20	37	1	0	0	0	0
21	38	1	0	0	0	0
22	39	1	0	0	0	0
23	40	1	0	0	0	0
24	41	1	0	0	0	0

```
M  END
>  <Alias name>
2,4,6-Triphenylpyridine

>  <deltaH°(c)>   kJ/mol
-11757

$$$$
Androstan
  ChemBrain IXL

 51 54  0  0  0  0  0  0  0  0  1 V2000
    4.9190    6.2644    0.3784 C   0  0  0  0  0  0  0  0  0  0  0  0  0
    3.5821    5.7619   -0.1955 C   0  0  0  0  0  0  0  0  0  0  0  0  0
    2.4249    6.2653    0.6889 C   0  0  0  0  0  0  0  0  0  0  0  0  0
    2.4260    7.8078    0.7106 C   0  0  0  0  0  0  0  0  0  0  0  0  0
    3.7655    8.3140    1.2795 C   0  0  0  0  0  0  0  0  0  0  0  0  0
    4.9202    7.8108    0.3968 C   0  0  0  0  0  0  0  0  0  0  0  0  0
    5.1002    5.7309    1.8101 C   0  0  0  0  0  0  0  0  0  0  0  0  0
    6.0741    5.7746   -0.5021 C   0  0  0  0  0  0  0  0  0  0  0  0  0
    6.2637    8.3275    0.9654 C   0  0  0  0  0  0  0  0  0  0  0  0  0
    7.4156    6.2697    0.1007 C   0  0  0  0  0  0  0  0  0  0  0  0  0
    7.4288    7.8066    0.1009 C   0  0  0  0  0  0  0  0  0  0  0  0  0
    6.0667    4.1999   -0.5980 C   0  0  0  0  0  0  0  0  0  0  0  0  0
    8.5168    5.7545   -0.7740 C   0  0  0  0  0  0  0  0  0  0  0  0  0
    7.3270    3.7055   -1.4010 C   0  0  0  0  0  0  0  0  0  0  0  0  0
    8.5558    4.2729   -0.7218 C   0  0  0  0  0  0  0  0  0  0  0  0  0
    9.9157    6.1945   -0.3815 C   0  0  0  0  0  0  0  0  0  0  0  0  0
    8.5872    3.8164    0.7524 C   0  0  0  0  0  0  0  0  0  0  0  0  0
    9.8833    3.9046   -1.3641 C   0  0  0  0  0  0  0  0  0  0  0  0  0
   10.8511    5.0724   -0.9555 C   0  0  0  0  0  0  0  0  0  0  0  0  0
    4.7905    8.1816   -0.6100 H   0  0  0  0  0  0  0  0  0  0  0  0  0
    5.9600    6.1866   -1.4936 H   0  0  0  0  0  0  0  0  0  0  0  0  0
    7.5324    5.8951    1.1066 H   0  0  0  0  0  0  0  0  0  0  0  0  0
    8.3272    6.0634   -1.7897 H   0  0  0  0  0  0  0  0  0  0  0  0  0
    3.4576    6.1384   -1.2005 H   0  0  0  0  0  0  0  0  0  0  0  0  0
    3.5798    4.6823   -0.2124 H   0  0  0  0  0  0  0  0  0  0  0  0  0
    2.5516    5.8913    1.6939 H   0  0  0  0  0  0  0  0  0  0  0  0  0
    1.4864    5.9117    0.2890 H   0  0  0  0  0  0  0  0  0  0  0  0  0
    2.2974    8.1822   -0.2943 H   0  0  0  0  0  0  0  0  0  0  0  0  0
    1.6159    8.1594    1.3320 H   0  0  0  0  0  0  0  0  0  0  0  0  0
    3.8934    7.9427    2.2852 H   0  0  0  0  0  0  0  0  0  0  0  0  0
    3.7665    9.3942    1.2917 H   0  0  0  0  0  0  0  0  0  0  0  0  0
    5.1012    4.6513    1.7945 H   0  0  0  0  0  0  0  0  0  0  0  0  0
    6.0384    6.0862    2.2101 H   0  0  0  0  0  0  0  0  0  0  0  0  0
    4.2886    6.0812    2.4299 H   0  0  0  0  0  0  0  0  0  0  0  0  0
    6.2657    9.4076    0.9565 H   0  0  0  0  0  0  0  0  0  0  0  0  0
    6.3841    7.9765    1.9784 H   0  0  0  0  0  0  0  0  0  0  0  0  0
    7.3142    8.1669   -0.9125 H   0  0  0  0  0  0  0  0  0  0  0  0  0
    8.3653    8.1601    0.5050 H   0  0  0  0  0  0  0  0  0  0  0  0  0
    5.1688    3.8743   -1.1001 H   0  0  0  0  0  0  0  0  0  0  0  0  0
    6.0916    3.7819    0.3989 H   0  0  0  0  0  0  0  0  0  0  0  0  0
    7.2738    4.0570   -2.4195 H   0  0  0  0  0  0  0  0  0  0  0  0  0
    7.3681    2.6262   -1.3871 H   0  0  0  0  0  0  0  0  0  0  0  0  0
   10.1524    7.1490   -0.8155 H   0  0  0  0  0  0  0  0  0  0  0  0  0
   10.0034    6.2350    0.6986 H   0  0  0  0  0  0  0  0  0  0  0  0  0
    8.6624    2.7384    0.7933 H   0  0  0  0  0  0  0  0  0  0  0  0  0
    7.6853    4.1336    1.2496 H   0  0  0  0  0  0  0  0  0  0  0  0  0
    9.4444    4.2549    1.2459 H   0  0  0  0  0  0  0  0  0  0  0  0  0
   10.2410    2.9612   -0.9883 H   0  0  0  0  0  0  0  0  0  0  0  0  0
    9.7779    3.8678   -2.4422 H   0  0  0  0  0  0  0  0  0  0  0  0  0
   11.3858    5.4372   -1.8186 H   0  0  0  0  0  0  0  0  0  0  0  0  0
   11.5433    4.7382   -0.1970 H   0  0  0  0  0  0  0  0  0  0  0  0  0
```

```
  1  2  1  0  0  0  0
  2  3  1  0  0  0  0
  3  4  1  0  0  0  0
  4  5  1  0  0  0  0
  5  6  1  0  0  0  0
  6  1  1  0  0  0  0
  1  7  1  0  0  0  0
  1  8  1  0  0  0  0
  6 20  1  0  0  0  0
  6  9  1  0  0  0  0
 10  8  1  0  0  0  0
  9 11  1  0  0  0  0
 11 10  1  0  0  0  0
  8 21  1  0  0  0  0
  8 12  1  0  0  0  0
 10 22  1  0  0  0  0
 10 13  1  0  0  0  0
 12 14  1  0  0  0  0
 14 15  1  0  0  0  0
 15 13  1  0  0  0  0
 13 23  1  0  0  0  0
 13 16  1  0  0  0  0
 15 17  1  0  0  0  0
 15 18  1  0  0  0  0
 16 19  1  0  0  0  0
 19 18  1  0  0  0  0
  2 24  1  0  0  0  0
  2 25  1  0  0  0  0
  3 26  1  0  0  0  0
  3 27  1  0  0  0  0
  4 28  1  0  0  0  0
  4 29  1  0  0  0  0
  5 30  1  0  0  0  0
  5 31  1  0  0  0  0
  7 32  1  0  0  0  0
  7 33  1  0  0  0  0
  7 34  1  0  0  0  0
  9 35  1  0  0  0  0
  9 36  1  0  0  0  0
 11 37  1  0  0  0  0
 11 38  1  0  0  0  0
 12 39  1  0  0  0  0
 12 40  1  0  0  0  0
 14 41  1  0  0  0  0
 14 42  1  0  0  0  0
 16 43  1  0  0  0  0
 16 44  1  0  0  0  0
 17 45  1  0  0  0  0
 17 46  1  0  0  0  0
 17 47  1  0  0  0  0
 18 48  1  0  0  0  0
 18 49  1  0  0  0  0
 19 50  1  0  0  0  0
 19 51  1  0  0  0  0
M  END
>  <Alias name>
Androstane

>  <deltaH°(c)>   kJ/mol
-11740

$$$$
1-Decylnaphthalene
```

ChemBrain IXL

```
48 49  0  0  0  0  0  0  0  0  1 V2000
    2.9811    6.5585    4.7156 C   0  0  0  0  0  0  0  0  0  0  0  0
    2.2722    7.6385    5.2433 C   0  0  0  0  0  0  0  0  0  0  0  0
    2.6147    8.9420    4.8817 C   0  0  0  0  0  0  0  0  0  0  0  0
    3.6663    9.1663    3.9922 C   0  0  0  0  0  0  0  0  0  0  0  0
    4.3769    8.0857    3.4632 C   0  0  0  0  0  0  0  0  0  0  0  0
    4.0328    6.7815    3.8261 C   0  0  0  0  0  0  0  0  0  0  0  0
    5.4305    8.3104    2.5719 C   0  0  0  0  0  0  0  0  0  0  0  0
    5.7691    9.6201    2.2132 C   0  0  0  0  0  0  0  0  0  0  0  0
    5.0582   10.6973    2.7428 C   0  0  0  0  0  0  0  0  0  0  0  0
    4.0072   10.4708    3.6319 C   0  0  0  0  0  0  0  0  0  0  0  0
    6.1881    7.1572    2.0079 C   0  0  0  0  0  0  0  0  0  0  0  0
    7.2711    7.6678    1.0318 C   0  0  0  0  0  0  0  0  0  0  0  0
    8.0482    6.4710    0.4502 C   0  0  0  0  0  0  0  0  0  0  0  0
    9.1251    6.9854   -0.5260 C   0  0  0  0  0  0  0  0  0  0  0  0
    9.9060    5.7911   -1.1072 C   0  0  0  0  0  0  0  0  0  0  0  0
   10.9803    6.3064   -2.0851 C   0  0  0  0  0  0  0  0  0  0  0  0
   11.7652    5.1121   -2.6615 C   0  0  0  0  0  0  0  0  0  0  0  0
   12.8390    5.6262   -3.6401 C   0  0  0  0  0  0  0  0  0  0  0  0
   13.6271    4.4313   -4.2114 C   0  0  0  0  0  0  0  0  0  0  0  0
   14.7009    4.9441   -5.1904 C   0  0  0  0  0  0  0  0  0  0  0  0
    2.7151    5.5471    4.9964 H   0  0  0  0  0  0  0  0  0  0  0  0
    1.4562    7.4651    5.9335 H   0  0  0  0  0  0  0  0  0  0  0  0
    2.0643    9.7797    5.2915 H   0  0  0  0  0  0  0  0  0  0  0  0
    4.5819    5.9423    3.4175 H   0  0  0  0  0  0  0  0  0  0  0  0
    6.5834    9.8010    1.5240 H   0  0  0  0  0  0  0  0  0  0  0  0
    5.3225   11.7097    2.4634 H   0  0  0  0  0  0  0  0  0  0  0  0
    3.4558   11.3076    4.0425 H   0  0  0  0  0  0  0  0  0  0  0  0
    5.5063    6.5035    1.4782 H   0  0  0  0  0  0  0  0  0  0  0  0
    6.6600    6.6083    2.8133 H   0  0  0  0  0  0  0  0  0  0  0  0
    7.9561    8.3188    1.5610 H   0  0  0  0  0  0  0  0  0  0  0  0
    6.8003    8.2173    0.2258 H   0  0  0  0  0  0  0  0  0  0  0  0
    7.3643    5.8183   -0.0779 H   0  0  0  0  0  0  0  0  0  0  0  0
    8.5223    5.9226    1.2547 H   0  0  0  0  0  0  0  0  0  0  0  0
    9.8075    7.6401    0.0018 H   0  0  0  0  0  0  0  0  0  0  0  0
    8.6504    7.5324   -1.3313 H   0  0  0  0  0  0  0  0  0  0  0  0
    9.2237    5.1352   -1.6335 H   0  0  0  0  0  0  0  0  0  0  0  0
   10.3826    5.2455   -0.3022 H   0  0  0  0  0  0  0  0  0  0  0  0
   11.6604    6.9656   -1.5597 H   0  0  0  0  0  0  0  0  0  0  0  0
   10.5035    6.8486   -2.8924 H   0  0  0  0  0  0  0  0  0  0  0  0
   11.0850    4.4525   -3.1860 H   0  0  0  0  0  0  0  0  0  0  0  0
   12.2424    4.5707   -1.8541 H   0  0  0  0  0  0  0  0  0  0  0  0
   13.5171    6.2888   -3.1165 H   0  0  0  0  0  0  0  0  0  0  0  0
   12.3617    6.1644   -4.4497 H   0  0  0  0  0  0  0  0  0  0  0  0
   12.9489    3.7685   -4.7344 H   0  0  0  0  0  0  0  0  0  0  0  0
   14.1044    3.8935   -3.4016 H   0  0  0  0  0  0  0  0  0  0  0  0
   15.3787    5.6076   -4.6676 H   0  0  0  0  0  0  0  0  0  0  0  0
   14.2236    5.4810   -6.0009 H   0  0  0  0  0  0  0  0  0  0  0  0
   15.2550    4.1042   -5.5907 H   0  0  0  0  0  0  0  0  0  0  0  0
  1  2  4  0  0  0  0
  2  3  4  0  0  0  0
  3  4  4  0  0  0  0
  4  5  4  0  0  0  0
  5  6  4  0  0  0  0
  6  1  4  0  0  0  0
  5  7  4  0  0  0  0
  7  8  4  0  0  0  0
  8  9  4  0  0  0  0
  9 10  4  0  0  0  0
 10  4  4  0  0  0  0
  7 11  1  0  0  0  0
```

```
 11 12  1  0  0  0  0
 12 13  1  0  0  0  0
 13 14  1  0  0  0  0
 14 15  1  0  0  0  0
 15 16  1  0  0  0  0
 16 17  1  0  0  0  0
 17 18  1  0  0  0  0
 18 19  1  0  0  0  0
 19 20  1  0  0  0  0
  1 21  1  0  0  0  0
  2 22  1  0  0  0  0
  3 23  1  0  0  0  0
  6 24  1  0  0  0  0
  8 25  1  0  0  0  0
  9 26  1  0  0  0  0
 10 27  1  0  0  0  0
 11 28  1  0  0  0  0
 11 29  1  0  0  0  0
 12 30  1  0  0  0  0
 12 31  1  0  0  0  0
 13 32  1  0  0  0  0
 13 33  1  0  0  0  0
 14 34  1  0  0  0  0
 14 35  1  0  0  0  0
 15 36  1  0  0  0  0
 15 37  1  0  0  0  0
 16 38  1  0  0  0  0
 16 39  1  0  0  0  0
 17 40  1  0  0  0  0
 17 41  1  0  0  0  0
 18 42  1  0  0  0  0
 18 43  1  0  0  0  0
 19 44  1  0  0  0  0
 19 45  1  0  0  0  0
 20 46  1  0  0  0  0
 20 47  1  0  0  0  0
 20 48  1  0  0  0  0
M  END
>  <Alias name>
1-Decylnaphthalene

>  <deltaH°(c)>   kJ/mol
-11723.49

$$$$
1-Tetradecyl-3-methylimidazolium bromide
  ChemBrain IXL

 56 55  0  0  0  0  0  0  0  0  1 V2000
   20.0188    8.4872   -0.0009 C   0  0  0  0  0  0  0  0  0  0  0  0
   18.7490    8.7470    0.0044 C   0  0  0  0  0  0  0  0  0  0  0  0
   20.1513    7.1053   -0.0135 N   0  0  0  0  0  0  0  0  0  0  0  0
   18.9636    6.5621   -0.0157 C   0  3  0  0  0  0  0  0  0  0  0  0
   18.0842    7.5275   -0.0050 N   0  0  0  0  0  0  0  0  0  0  0  0
   16.6230    7.3485   -0.0033 C   0  0  0  0  0  0  0  0  0  0  0  0
   21.4246    6.3708   -0.0231 C   0  0  0  0  0  0  0  0  0  0  0  0
   15.9322    8.7270   -0.0068 C   0  0  0  0  0  0  0  0  0  0  0  0
   14.4032    8.5356   -0.0057 C   0  0  0  0  0  0  0  0  0  0  0  0
   13.7129    9.9134   -0.0093 C   0  0  0  0  0  0  0  0  0  0  0  0
   12.1837    9.7221   -0.0085 C   0  0  0  0  0  0  0  0  0  0  0  0
   11.4935   11.1000   -0.0124 C   0  0  0  0  0  0  0  0  0  0  0  0
    9.9643   10.9088   -0.0121 C   0  0  0  0  0  0  0  0  0  0  0  0
    9.2743   12.2868   -0.0162 C   0  0  0  0  0  0  0  0  0  0  0  0
```

7.7451	12.0958	-0.0164	C	0	0	0	0	0	0	0	0	0	0	0	0
7.0551	13.4738	-0.0208	C	0	0	0	0	0	0	0	0	0	0	0	0
5.5259	13.2828	-0.0212	C	0	0	0	0	0	0	0	0	0	0	0	0
4.8360	14.6609	-0.0258	C	0	0	0	0	0	0	0	0	0	0	0	0
3.3066	14.4700	-0.0264	C	0	0	0	0	0	0	0	0	0	0	0	0
2.6167	15.8480	-0.0310	C	0	0	0	0	0	0	0	0	0	0	0	0
-2.1364	6.3838	-0.0202	Br	0	0	0	0	0	0	0	0	0	0	0	0
18.7474	5.5041	-0.0247	H	0	0	0	0	0	0	0	0	0	0	0	0
20.8241	9.2112	0.0038	H	0	0	0	0	0	0	0	0	0	0	0	0
18.2930	9.7291	0.0142	H	0	0	0	0	0	0	0	0	0	0	0	0
16.3268	6.7946	-0.8855	H	0	0	0	0	0	0	0	0	0	0	0	0
16.3282	6.7999	0.8827	H	0	0	0	0	0	0	0	0	0	0	0	0
22.2476	7.0749	-0.0256	H	0	0	0	0	0	0	0	0	0	0	0	0
21.4772	5.7509	-0.9095	H	0	0	0	0	0	0	0	0	0	0	0	0
21.4872	5.7455	0.8589	H	0	0	0	0	0	0	0	0	0	0	0	0
16.2272	9.2809	0.8758	H	0	0	0	0	0	0	0	0	0	0	0	0
16.2265	9.2760	-0.8928	H	0	0	0	0	0	0	0	0	0	0	0	0
14.1080	7.9816	-0.8883	H	0	0	0	0	0	0	0	0	0	0	0	0
14.1087	7.9866	0.8803	H	0	0	0	0	0	0	0	0	0	0	0	0
14.0078	10.4672	0.8734	H	0	0	0	0	0	0	0	0	0	0	0	0
14.0075	10.4624	-0.8951	H	0	0	0	0	0	0	0	0	0	0	0	0
11.8888	9.1682	-0.8912	H	0	0	0	0	0	0	0	0	0	0	0	0
11.8890	9.1732	0.8773	H	0	0	0	0	0	0	0	0	0	0	0	0
11.7882	11.6538	0.8704	H	0	0	0	0	0	0	0	0	0	0	0	0
11.7885	11.6490	-0.8981	H	0	0	0	0	0	0	0	0	0	0	0	0
9.6696	10.3549	-0.8948	H	0	0	0	0	0	0	0	0	0	0	0	0
9.6693	10.3600	0.8737	H	0	0	0	0	0	0	0	0	0	0	0	0
9.5688	12.8406	0.8666	H	0	0	0	0	0	0	0	0	0	0	0	0
9.5695	12.8357	-0.9019	H	0	0	0	0	0	0	0	0	0	0	0	0
7.4506	11.5418	-0.8992	H	0	0	0	0	0	0	0	0	0	0	0	0
7.4497	11.5470	0.8694	H	0	0	0	0	0	0	0	0	0	0	0	0
7.3495	14.0277	0.8621	H	0	0	0	0	0	0	0	0	0	0	0	0
7.3506	14.0226	-0.9064	H	0	0	0	0	0	0	0	0	0	0	0	0
5.2315	12.7289	-0.9041	H	0	0	0	0	0	0	0	0	0	0	0	0
5.2303	12.7341	0.8645	H	0	0	0	0	0	0	0	0	0	0	0	0
5.1303	15.2148	0.8571	H	0	0	0	0	0	0	0	0	0	0	0	0
5.1316	15.2097	-0.9115	H	0	0	0	0	0	0	0	0	0	0	0	0
3.0123	13.9161	-0.9092	H	0	0	0	0	0	0	0	0	0	0	0	0
3.0110	13.9214	0.8592	H	0	0	0	0	0	0	0	0	0	0	0	0
1.5422	15.7136	-0.0315	H	0	0	0	0	0	0	0	0	0	0	0	0
2.9109	16.4020	0.8518	H	0	0	0	0	0	0	0	0	0	0	0	0
2.9123	16.3967	-0.9167	H	0	0	0	0	0	0	0	0	0	0	0	0

1	2	2	0	0	0	0
1	3	1	0	0	0	0
3	4	4	0	0	0	0
4	5	4	0	0	0	0
5	2	1	0	0	0	0
5	6	1	0	0	0	0
3	7	1	0	0	0	0
6	8	1	0	0	0	0
8	9	1	0	0	0	0
9	10	1	0	0	0	0
11	12	1	0	0	0	0
12	13	1	0	0	0	0
13	14	1	0	0	0	0
11	10	1	0	0	0	0
14	15	1	0	0	0	0
15	16	1	0	0	0	0
16	17	1	0	0	0	0
17	18	1	0	0	0	0
18	19	1	0	0	0	0
19	20	1	0	0	0	0
4	22	1	0	0	0	0

```
  1 23  1  0  0  0  0
  2 24  1  0  0  0  0
  6 25  1  0  0  0  0
  6 26  1  0  0  0  0
  7 27  1  0  0  0  0
  7 28  1  0  0  0  0
  7 29  1  0  0  0  0
  8 30  1  0  0  0  0
  8 31  1  0  0  0  0
  9 32  1  0  0  0  0
  9 33  1  0  0  0  0
 10 34  1  0  0  0  0
 10 35  1  0  0  0  0
 11 36  1  0  0  0  0
 11 37  1  0  0  0  0
 12 38  1  0  0  0  0
 12 39  1  0  0  0  0
 13 40  1  0  0  0  0
 13 41  1  0  0  0  0
 14 42  1  0  0  0  0
 14 43  1  0  0  0  0
 15 44  1  0  0  0  0
 15 45  1  0  0  0  0
 16 46  1  0  0  0  0
 16 47  1  0  0  0  0
 17 48  1  0  0  0  0
 17 49  1  0  0  0  0
 18 50  1  0  0  0  0
 18 51  1  0  0  0  0
 19 52  1  0  0  0  0
 19 53  1  0  0  0  0
 20 54  1  0  0  0  0
 20 55  1  0  0  0  0
 20 56  1  0  0  0  0
M  END
>  <Alias name>
1-Tetradecyl-3-methylimidazolium bromide

>  <deltaH°(c)>   kJ/mol
-11718

$$$$
Methyl linoleate
  ChemBrain IXL

 55 54  0  0  0  0  0  0  0  0  1 V2000
    3.7427   13.7233    0.0000 C   0  0  0  0  0  0  0  0  0  0  0  0
    2.7854   12.5161    0.0044 C   0  0  0  0  0  0  0  0  0  0  0  0
    3.6047   11.2116    0.0181 C   0  0  0  0  0  0  0  0  0  0  0  0
    2.6486   10.0037    0.0207 C   0  0  0  0  0  0  0  0  0  0  0  0
    3.4700    8.7011    0.0336 C   0  0  0  0  0  0  0  0  0  0  0  0
    2.5540    7.5376    0.0326 C   0  0  0  0  0  0  0  0  0  0  0  0
    3.0451    6.3010   -0.0795 C   0  0  0  0  0  0  0  0  0  0  0  0
    4.5060    6.0929   -0.2028 C   0  0  0  0  0  0  0  0  0  0  0  0
    4.8083    4.6437   -0.1525 C   0  0  0  0  0  0  0  0  0  0  0  0
    6.0718    4.2212   -0.2467 C   0  0  0  0  0  0  0  0  0  0  0  0
    7.1707    5.2016   -0.4014 C   0  0  0  0  0  0  0  0  0  0  0  0
    8.5131    4.5382   -0.0369 C   0  0  0  0  0  0  0  0  0  0  0  0
    9.6451    5.5737   -0.1776 C   0  0  0  0  0  0  0  0  0  0  0  0
   10.9932    4.9250    0.1918 C   0  0  0  0  0  0  0  0  0  0  0  0
   12.1137    5.9763    0.0656 C   0  0  0  0  0  0  0  0  0  0  0  0
   13.4680    5.3399    0.4333 C   0  0  0  0  0  0  0  0  0  0  0  0
   14.5811    6.4031    0.3120 C   0  0  0  0  0  0  0  0  0  0  0  0
```

```
   15.8912    5.7999    0.6610 C   0  0  0  0  0  0  0  0  0  0  0  0
   17.1108    6.6233    0.6401 O   0  0  0  0  0  0  0  0  0  0  0  0
   15.9594    4.6202    0.9659 O   0  0  0  0  0  0  0  0  0  0  0  0
   16.9091    8.0297    0.2603 C   0  0  0  0  0  0  0  0  0  0  0  0
    3.1671   14.6404   -0.0096 H   0  0  0  0  0  0  0  0  0  0  0  0
    4.3713   13.6824   -0.8810 H   0  0  0  0  0  0  0  0  0  0  0  0
    4.3628   13.6956    0.8875 H   0  0  0  0  0  0  0  0  0  0  0  0
    2.1655   12.5437   -0.8832 H   0  0  0  0  0  0  0  0  0  0  0  0
    2.1568   12.5569    0.8853 H   0  0  0  0  0  0  0  0  0  0  0  0
    4.2240   11.1836    0.9062 H   0  0  0  0  0  0  0  0  0  0  0  0
    4.2343   11.1715   -0.8623 H   0  0  0  0  0  0  0  0  0  0  0  0
    2.0292   10.0314   -0.8673 H   0  0  0  0  0  0  0  0  0  0  0  0
    2.0194   10.0427    0.9014 H   0  0  0  0  0  0  0  0  0  0  0  0
    4.0880    8.6730    0.9225 H   0  0  0  0  0  0  0  0  0  0  0  0
    4.1011    8.6641   -0.8459 H   0  0  0  0  0  0  0  0  0  0  0  0
    1.4854    7.6895    0.1228 H   0  0  0  0  0  0  0  0  0  0  0  0
    2.3745    5.4503   -0.0801 H   0  0  0  0  0  0  0  0  0  0  0  0
    5.0114    6.5949    0.6128 H   0  0  0  0  0  0  0  0  0  0  0  0
    4.8498    6.5004   -1.1455 H   0  0  0  0  0  0  0  0  0  0  0  0
    4.0047    3.9261   -0.0394 H   0  0  0  0  0  0  0  0  0  0  0  0
    6.2924    3.1614   -0.2098 H   0  0  0  0  0  0  0  0  0  0  0  0
    7.2053    5.5455   -1.4276 H   0  0  0  0  0  0  0  0  0  0  0  0
    6.9966    6.0445    0.2555 H   0  0  0  0  0  0  0  0  0  0  0  0
    8.4745    4.1816    0.9850 H   0  0  0  0  0  0  0  0  0  0  0  0
    8.6987    3.7053   -0.7041 H   0  0  0  0  0  0  0  0  0  0  0  0
    9.6840    5.9284   -1.2001 H   0  0  0  0  0  0  0  0  0  0  0  0
    9.4536    6.4079    0.4859 H   0  0  0  0  0  0  0  0  0  0  0  0
   10.9521    4.5608    1.2110 H   0  0  0  0  0  0  0  0  0  0  0  0
   11.1937    4.0992   -0.4797 H   0  0  0  0  0  0  0  0  0  0  0  0
   12.1520    6.3413   -0.9532 H   0  0  0  0  0  0  0  0  0  0  0  0
   11.9098    6.8016    0.7365 H   0  0  0  0  0  0  0  0  0  0  0  0
   13.4297    4.9709    1.4510 H   0  0  0  0  0  0  0  0  0  0  0  0
   13.6767    4.5187   -0.2414 H   0  0  0  0  0  0  0  0  0  0  0  0
   14.6151    6.7741   -0.7047 H   0  0  0  0  0  0  0  0  0  0  0  0
   14.3708    7.2229    0.9876 H   0  0  0  0  0  0  0  0  0  0  0  0
   16.2327    8.5001    0.9634 H   0  0  0  0  0  0  0  0  0  0  0  0
   17.8602    8.5469    0.2733 H   0  0  0  0  0  0  0  0  0  0  0  0
   16.4856    8.0777   -0.7353 H   0  0  0  0  0  0  0  0  0  0  0  0
 10 11  1  0  0  0  0
  5  6  1  0  0  0  0
 11 12  1  0  0  0  0
  1  2  1  0  0  0  0
 12 13  1  0  0  0  0
  6  7  2  0  0  0  0
 13 14  1  0  0  0  0
  3  4  1  0  0  0  0
 14 15  1  0  0  0  0
  7  8  1  0  0  0  0
 15 16  1  0  0  0  0
 16 17  1  0  0  0  0
  8  9  1  0  0  0  0
 17 18  1  0  0  0  0
  4  5  1  0  0  0  0
 18 19  1  0  0  0  0
  9 10  2  0  0  0  0
 18 20  2  0  0  0  0
  2  3  1  0  0  0  0
 19 21  1  0  0  0  0
  1 22  1  0  0  0  0
  1 23  1  0  0  0  0
  1 24  1  0  0  0  0
  2 25  1  0  0  0  0
  2 26  1  0  0  0  0
```

```
  3 27  1  0  0  0  0
  3 28  1  0  0  0  0
  4 29  1  0  0  0  0
  4 30  1  0  0  0  0
  5 31  1  0  0  0  0
  5 32  1  0  0  0  0
  6 33  1  0  0  0  0
  7 34  1  0  0  0  0
  8 35  1  0  0  0  0
  8 36  1  0  0  0  0
  9 37  1  0  0  0  0
 10 38  1  0  0  0  0
 11 39  1  0  0  0  0
 11 40  1  0  0  0  0
 12 41  1  0  0  0  0
 12 42  1  0  0  0  0
 13 43  1  0  0  0  0
 13 44  1  0  0  0  0
 14 45  1  0  0  0  0
 14 46  1  0  0  0  0
 15 47  1  0  0  0  0
 15 48  1  0  0  0  0
 16 49  1  0  0  0  0
 16 50  1  0  0  0  0
 17 51  1  0  0  0  0
 17 52  1  0  0  0  0
 21 53  1  0  0  0  0
 21 54  1  0  0  0  0
 21 55  1  0  0  0  0
M  END
>  <Alias name>
Methyl Z,Z-9,12-octadecadieneoate

>  <deltaH°(c)>   kJ/mol
-11690

$$$$
2-Palmitoylglycerol
  ChemBrain IXL

 61 60  0  0  0  0  0  0  0  0  1 V2000
    4.6570   12.3079    0.8316 O   0  0  0  0  0  0  0  0  0  0  0  0
    3.3016   11.7375    0.7546 C   0  0  0  0  0  0  0  0  0  0  0  0
    4.7206   13.7276    1.2225 C   0  0  0  0  0  0  0  0  0  0  0  0
    3.3932   10.2470    0.3733 C   0  0  0  0  0  0  0  0  0  0  0  0
    2.4840   12.4918   -0.3141 C   0  0  0  0  0  0  0  0  0  0  0  0
    6.0301   14.4134    1.3197 C   0  0  0  0  0  0  0  0  0  0  0  0
    3.6941   14.3404    1.4669 O   0  0  0  0  0  0  0  0  0  0  0  0
    4.1708    9.5275    1.3922 O   0  0  0  0  0  0  0  0  0  0  0  0
    3.1476   12.3646   -1.6198 O   0  0  0  0  0  0  0  0  0  0  0  0
    7.1562   13.4358    0.9291 C   0  0  0  0  0  0  0  0  0  0  0  0
    8.5165   14.1504    1.0373 C   0  0  0  0  0  0  0  0  0  0  0  0
    9.6427   13.1717    0.6537 C   0  0  0  0  0  0  0  0  0  0  0  0
   11.0038   13.8834    0.7730 C   0  0  0  0  0  0  0  0  0  0  0  0
   12.1309   12.9027    0.3968 C   0  0  0  0  0  0  0  0  0  0  0  0
   13.4927   13.6112    0.5277 C   0  0  0  0  0  0  0  0  0  0  0  0
   14.6205   12.6292    0.1566 C   0  0  0  0  0  0  0  0  0  0  0  0
   15.9828   13.3353    0.2966 C   0  0  0  0  0  0  0  0  0  0  0  0
   17.1110   12.3529   -0.0724 C   0  0  0  0  0  0  0  0  0  0  0  0
   18.4735   13.0575    0.0734 C   0  0  0  0  0  0  0  0  0  0  0  0
   19.6020   12.0753   -0.2958 C   0  0  0  0  0  0  0  0  0  0  0  0
   20.9647   12.7791   -0.1469 C   0  0  0  0  0  0  0  0  0  0  0  0
   22.0934   11.7971   -0.5169 C   0  0  0  0  0  0  0  0  0  0  0  0
```

23.4562	12.5005	-0.3667	C	0	0	0	0	0	0	0	0	0	0	0	0	0
4.2800	8.5646	1.2282	H	0	0	0	0	0	0	0	0	0	0	0	0	0
2.6916	12.8177	-2.3634	H	0	0	0	0	0	0	0	0	0	0	0	0	0
2.8142	11.8341	1.7169	H	0	0	0	0	0	0	0	0	0	0	0	0	0
3.8813	10.1498	-0.5886	H	0	0	0	0	0	0	0	0	0	0	0	0	0
2.3966	9.8270	0.3165	H	0	0	0	0	0	0	0	0	0	0	0	0	0
1.4887	12.0688	-0.3723	H	0	0	0	0	0	0	0	0	0	0	0	0	0
2.4159	13.5384	-0.0444	H	0	0	0	0	0	0	0	0	0	0	0	0	0
6.1844	14.7533	2.3363	H	0	0	0	0	0	0	0	0	0	0	0	0	0
6.0424	15.2639	0.6493	H	0	0	0	0	0	0	0	0	0	0	0	0	0
7.0051	13.0989	-0.0891	H	0	0	0	0	0	0	0	0	0	0	0	0	0
7.1421	12.5836	1.5972	H	0	0	0	0	0	0	0	0	0	0	0	0	0
8.6653	14.4907	2.0546	H	0	0	0	0	0	0	0	0	0	0	0	0	0
8.5325	15.0004	0.3663	H	0	0	0	0	0	0	0	0	0	0	0	0	0
9.4983	12.8366	-0.3660	H	0	0	0	0	0	0	0	0	0	0	0	0	0
9.6219	12.3186	1.3205	H	0	0	0	0	0	0	0	0	0	0	0	0	0
11.1456	14.2221	1.7919	H	0	0	0	0	0	0	0	0	0	0	0	0	0
11.0267	14.7341	0.1033	H	0	0	0	0	0	0	0	0	0	0	0	0	0
11.9937	12.5692	-0.6244	H	0	0	0	0	0	0	0	0	0	0	0	0	0
12.1031	12.0487	1.0623	H	0	0	0	0	0	0	0	0	0	0	0	0	0
13.6280	13.9470	1.5484	H	0	0	0	0	0	0	0	0	0	0	0	0	0
13.5220	14.4637	-0.1397	H	0	0	0	0	0	0	0	0	0	0	0	0	0
14.4888	12.2977	-0.8659	H	0	0	0	0	0	0	0	0	0	0	0	0	0
14.5874	11.7742	0.8205	H	0	0	0	0	0	0	0	0	0	0	0	0	0
16.1136	13.6676	1.3190	H	0	0	0	0	0	0	0	0	0	0	0	0	0
16.0165	14.1899	-0.3678	H	0	0	0	0	0	0	0	0	0	0	0	0	0
16.9824	12.0235	-1.0961	H	0	0	0	0	0	0	0	0	0	0	0	0	0
17.0748	11.4966	0.5897	H	0	0	0	0	0	0	0	0	0	0	0	0	0
18.6020	13.3866	1.0971	H	0	0	0	0	0	0	0	0	0	0	0	0	0
18.5097	13.9141	-0.5884	H	0	0	0	0	0	0	0	0	0	0	0	0	0
19.4747	11.7478	-1.3202	H	0	0	0	0	0	0	0	0	0	0	0	0	0
19.5645	11.2178	0.3646	H	0	0	0	0	0	0	0	0	0	0	0	0	0
21.0921	13.1059	0.8778	H	0	0	0	0	0	0	0	0	0	0	0	0	0
21.0019	13.6371	-0.8067	H	0	0	0	0	0	0	0	0	0	0	0	0	0
21.9665	11.4710	-1.5418	H	0	0	0	0	0	0	0	0	0	0	0	0	0
22.0556	10.9388	0.1423	H	0	0	0	0	0	0	0	0	0	0	0	0	0
23.4939	13.3590	-1.0257	H	0	0	0	0	0	0	0	0	0	0	0	0	0
24.2491	11.8104	-0.6270	H	0	0	0	0	0	0	0	0	0	0	0	0	0
23.5833	12.8263	0.6583	H	0	0	0	0	0	0	0	0	0	0	0	0	0

1	2	1	0	0	0	0
1	3	1	0	0	0	0
2	4	1	0	0	0	0
2	5	1	0	0	0	0
3	6	1	0	0	0	0
3	7	2	0	0	0	0
4	8	1	0	0	0	0
5	9	1	0	0	0	0
6	10	1	0	0	0	0
10	11	1	0	0	0	0
11	12	1	0	0	0	0
12	13	1	0	0	0	0
13	14	1	0	0	0	0
14	15	1	0	0	0	0
15	16	1	0	0	0	0
16	17	1	0	0	0	0
17	18	1	0	0	0	0
18	19	1	0	0	0	0
19	20	1	0	0	0	0
20	21	1	0	0	0	0
21	22	1	0	0	0	0
22	23	1	0	0	0	0
8	24	1	0	0	0	0
9	25	1	0	0	0	0

```
  2 26  1  0  0  0  0
  4 27  1  0  0  0  0
  4 28  1  0  0  0  0
  5 29  1  0  0  0  0
  5 30  1  0  0  0  0
  6 31  1  0  0  0  0
  6 32  1  0  0  0  0
 10 33  1  0  0  0  0
 10 34  1  0  0  0  0
 11 35  1  0  0  0  0
 11 36  1  0  0  0  0
 12 37  1  0  0  0  0
 12 38  1  0  0  0  0
 13 39  1  0  0  0  0
 13 40  1  0  0  0  0
 14 41  1  0  0  0  0
 14 42  1  0  0  0  0
 15 43  1  0  0  0  0
 15 44  1  0  0  0  0
 16 45  1  0  0  0  0
 16 46  1  0  0  0  0
 17 47  1  0  0  0  0
 17 48  1  0  0  0  0
 18 49  1  0  0  0  0
 18 50  1  0  0  0  0
 19 51  1  0  0  0  0
 19 52  1  0  0  0  0
 20 53  1  0  0  0  0
 20 54  1  0  0  0  0
 21 55  1  0  0  0  0
 21 56  1  0  0  0  0
 22 57  1  0  0  0  0
 22 58  1  0  0  0  0
 23 59  1  0  0  0  0
 23 60  1  0  0  0  0
 23 61  1  0  0  0  0
M  END
>  <Alias name>
2-Monopalmitin

>  <deltaH°(c)>   kJ/mol
-11685

$$$$
Adamantylideneadamantane
  ChemBrain IXL

 48 53  0  0  0  0  0  0  0  0  1 V2000
    8.4737    3.0273   -1.4555 C   0  0  0  0  0  0  0  0  0  0  0  0
    9.3981    4.2775   -1.4208 C   0  0  0  0  0  0  0  0  0  0  0  0
   10.3541    4.1699   -0.2101 C   0  0  0  0  0  0  0  0  0  0  0  0
    9.5238    4.0989    1.0925 C   0  0  0  0  0  0  0  0  0  0  0  0
    8.5985    2.8498    1.0422 C   0  0  0  0  0  0  0  0  0  0  0  0
    7.6414    2.9837   -0.1587 C   0  0  0  0  0  0  0  0  0  0  0  0
    6.9166    4.2403   -0.0332 C   0  0  0  0  0  0  0  0  0  0  0  0
    8.5346    5.5632   -1.2784 C   0  0  0  0  0  0  0  0  0  0  0  0
    8.6596    5.3857    1.2193 C   0  0  0  0  0  0  0  0  0  0  0  0
    7.7011    5.4660    0.0147 C   0  0  0  0  0  0  0  0  0  0  0  0
    3.9424    2.9343    1.2691 C   0  0  0  0  0  0  0  0  0  0  0  0
    4.7763    3.0431   -0.0229 C   0  0  0  0  0  0  0  0  0  0  0  0
    5.5614    4.2680    0.0366 C   0  0  0  0  0  0  0  0  0  0  0  0
    4.8372    5.5237    0.1737 C   0  0  0  0  0  0  0  0  0  0  0  0
    4.0047    5.4684    1.4699 C   0  0  0  0  0  0  0  0  0  0  0  0
```

```
   3.0796    4.2190    1.4234 C   0  0  0  0  0  0  0  0  0  0  0  0
   2.1239    4.3383    0.2135 C   0  0  0  0  0  0  0  0  0  0  0  0
   3.8181    3.1351   -1.2269 C   0  0  0  0  0  0  0  0  0  0  0  0
   3.8803    5.6692   -1.0261 C   0  0  0  0  0  0  0  0  0  0  0  0
   2.9544    4.4211   -1.0883 C   0  0  0  0  0  0  0  0  0  0  0  0
   7.8120    3.0892   -2.3106 H   0  0  0  0  0  0  0  0  0  0  0  0
   9.0783    2.1315   -1.5288 H   0  0  0  0  0  0  0  0  0  0  0  0
   9.9762    4.3276   -2.3359 H   0  0  0  0  0  0  0  0  0  0  0  0
  10.9999    5.0395   -0.1806 H   0  0  0  0  0  0  0  0  0  0  0  0
  10.9574    3.2749   -0.3039 H   0  0  0  0  0  0  0  0  0  0  0  0
  10.1902    4.0234    1.9436 H   0  0  0  0  0  0  0  0  0  0  0  0
   8.0254    2.7859    1.9588 H   0  0  0  0  0  0  0  0  0  0  0  0
   9.2011    1.9569    0.9280 H   0  0  0  0  0  0  0  0  0  0  0  0
   6.9495    2.1507   -0.1833 H   0  0  0  0  0  0  0  0  0  0  0  0
   7.8735    5.6524   -2.1315 H   0  0  0  0  0  0  0  0  0  0  0  0
   9.1816    6.4306   -1.2285 H   0  0  0  0  0  0  0  0  0  0  0  0
   8.0872    5.3490    2.1379 H   0  0  0  0  0  0  0  0  0  0  0  0
   9.3046    6.2559    1.2283 H   0  0  0  0  0  0  0  0  0  0  0  0
   7.0499    6.3264    0.1084 H   0  0  0  0  0  0  0  0  0  0  0  0
   4.6033    2.8368    2.1215 H   0  0  0  0  0  0  0  0  0  0  0  0
   3.2950    2.0678    1.2110 H   0  0  0  0  0  0  0  0  0  0  0  0
   5.4271    2.1833   -0.1245 H   0  0  0  0  0  0  0  0  0  0  0  0
   5.5294    6.3560    0.2062 H   0  0  0  0  0  0  0  0  0  0  0  0
   4.6663    5.3981    2.3244 H   0  0  0  0  0  0  0  0  0  0  0  0
   3.4005    6.3638    1.5515 H   0  0  0  0  0  0  0  0  0  0  0  0
   2.5013    4.1607    2.3378 H   0  0  0  0  0  0  0  0  0  0  0  0
   1.4777    3.4694    0.1758 H   0  0  0  0  0  0  0  0  0  0  0  0
   1.5210    5.2328    0.3155 H   0  0  0  0  0  0  0  0  0  0  0  0
   4.3908    3.1801   -2.1450 H   0  0  0  0  0  0  0  0  0  0  0  0
   3.1727    2.2653   -1.2441 H   0  0  0  0  0  0  0  0  0  0  0  0
   4.4536    5.7415   -1.9420 H   0  0  0  0  0  0  0  0  0  0  0  0
   3.2781    6.5613   -0.9036 H   0  0  0  0  0  0  0  0  0  0  0  0
   2.2882    4.5048   -1.9387 H   0  0  0  0  0  0  0  0  0  0  0  0
  1  2  1  0  0  0  0
  2  3  1  0  0  0  0
  3  4  1  0  0  0  0
  4  5  1  0  0  0  0
  5  6  1  0  0  0  0
  6  1  1  0  0  0  0
  6  7  1  0  0  0  0
  2  8  1  0  0  0  0
  4  9  1  0  0  0  0
  7 10  1  0  0  0  0
 10  9  1  0  0  0  0
 10  8  1  0  0  0  0
 11 12  1  0  0  0  0
 12 13  1  0  0  0  0
 13 14  1  0  0  0  0
 14 15  1  0  0  0  0
 15 16  1  0  0  0  0
 16 11  1  0  0  0  0
 16 17  1  0  0  0  0
 12 18  1  0  0  0  0
 14 19  1  0  0  0  0
 17 20  1  0  0  0  0
 20 19  1  0  0  0  0
 20 18  1  0  0  0  0
 13  7  2  0  0  0  0
  1 21  1  0  0  0  0
  1 22  1  0  0  0  0
  2 23  1  0  0  0  0
  3 24  1  0  0  0  0
  3 25  1  0  0  0  0
```

```
  4 26  1  0  0  0  0
  5 27  1  0  0  0  0
  5 28  1  0  0  0  0
  6 29  1  0  0  0  0
  8 30  1  0  0  0  0
  8 31  1  0  0  0  0
  9 32  1  0  0  0  0
  9 33  1  0  0  0  0
 10 34  1  0  0  0  0
 11 35  1  0  0  0  0
 11 36  1  0  0  0  0
 12 37  1  0  0  0  0
 14 38  1  0  0  0  0
 15 39  1  0  0  0  0
 15 40  1  0  0  0  0
 16 41  1  0  0  0  0
 17 42  1  0  0  0  0
 17 43  1  0  0  0  0
 18 44  1  0  0  0  0
 18 45  1  0  0  0  0
 19 46  1  0  0  0  0
 19 47  1  0  0  0  0
 20 48  1  0  0  0  0
M  END
>  <Alias name>
2-(2-Adamantylidene)adamantane

>  <deltaH°(c)>   kJ/mol
-11632

$$$$
1-Palmitoylglycerol
  ChemBrain IXL

 61 60  0  0  0  0  0  0  0  0  1 V2000
    7.4736    9.0513    0.6643 C   0  0  0  0  0  0  0  0  0  0  0  0
    6.5736    7.9522    0.2493 C   0  0  0  0  0  0  0  0  0  0  0  0
    8.8114    8.9328   -0.0912 C   0  0  0  0  0  0  0  0  0  0  0  0
    5.2309    7.8400    0.8371 O   0  0  0  0  0  0  0  0  0  0  0  0
    6.9460    7.1392   -0.5805 O   0  0  0  0  0  0  0  0  0  0  0  0
    9.7474   10.0789    0.3389 C   0  0  0  0  0  0  0  0  0  0  0  0
    4.8963    8.8766    1.8242 C   0  0  0  0  0  0  0  0  0  0  0  0
   11.0855    9.9602   -0.4162 C   0  0  0  0  0  0  0  0  0  0  0  0
    3.4689    8.6395    2.3544 C   0  0  0  0  0  0  0  0  0  0  0  0
   12.0205   11.1078    0.0121 C   0  0  0  0  0  0  0  0  0  0  0  0
    2.4687    8.7047    1.1839 C   0  0  0  0  0  0  0  0  0  0  0  0
    3.3955    7.3153    2.9885 O   0  0  0  0  0  0  0  0  0  0  0  0
   13.3597   10.9875   -0.7409 C   0  0  0  0  0  0  0  0  0  0  0  0
    2.5413   10.0294    0.5507 O   0  0  0  0  0  0  0  0  0  0  0  0
   14.2939   12.1363   -0.3140 C   0  0  0  0  0  0  0  0  0  0  0  0
   15.6347   12.0136   -1.0636 C   0  0  0  0  0  0  0  0  0  0  0  0
   16.5684   13.1631   -0.6375 C   0  0  0  0  0  0  0  0  0  0  0  0
   17.9111   13.0376   -1.3832 C   0  0  0  0  0  0  0  0  0  0  0  0
   18.8443   14.1877   -0.9578 C   0  0  0  0  0  0  0  0  0  0  0  0
   20.1887   14.0597   -1.7000 C   0  0  0  0  0  0  0  0  0  0  0  0
   21.1216   15.2103   -1.2753 C   0  0  0  0  0  0  0  0  0  0  0  0
   22.4669   15.0808   -2.0155 C   0  0  0  0  0  0  0  0  0  0  0  0
   23.3995   16.2319   -1.5913 C   0  0  0  0  0  0  0  0  0  0  0  0
    3.6025    6.5588    2.3958 H   0  0  0  0  0  0  0  0  0  0  0  0
    1.9313   10.1607   -0.2088 H   0  0  0  0  0  0  0  0  0  0  0  0
    7.0115   10.0031    0.4330 H   0  0  0  0  0  0  0  0  0  0  0  0
    7.6540    8.9872    1.7303 H   0  0  0  0  0  0  0  0  0  0  0  0
    9.2745    7.9819    0.1417 H   0  0  0  0  0  0  0  0  0  0  0  0
```

8.6308	8.9949	-1.1572	H	0	0	0	0	0	0	0	0	0	0	0	0	0	0
9.2845	11.0298	0.1057	H	0	0	0	0	0	0	0	0	0	0	0	0	0	0
9.9278	10.0170	1.4049	H	0	0	0	0	0	0	0	0	0	0	0	0	0	0
5.5991	8.8319	2.6470	H	0	0	0	0	0	0	0	0	0	0	0	0	0	0
4.9501	9.8517	1.3561	H	0	0	0	0	0	0	0	0	0	0	0	0	0	0
11.5493	9.0100	-0.1818	H	0	0	0	0	0	0	0	0	0	0	0	0	0	0
10.9050	10.0204	-1.4824	H	0	0	0	0	0	0	0	0	0	0	0	0	0	0
3.2229	9.4025	3.0825	H	0	0	0	0	0	0	0	0	0	0	0	0	0	0
11.5576	12.0579	-0.2242	H	0	0	0	0	0	0	0	0	0	0	0	0	0	0
12.1995	11.0486	1.0785	H	0	0	0	0	0	0	0	0	0	0	0	0	0	0
2.7153	7.9423	0.4554	H	0	0	0	0	0	0	0	0	0	0	0	0	0	0
1.4657	8.5372	1.5564	H	0	0	0	0	0	0	0	0	0	0	0	0	0	0
13.8233	10.0380	-0.5036	H	0	0	0	0	0	0	0	0	0	0	0	0	0	0
13.1806	11.0453	-1.8075	H	0	0	0	0	0	0	0	0	0	0	0	0	0	0
13.8316	13.0858	-0.5542	H	0	0	0	0	0	0	0	0	0	0	0	0	0	0
14.4704	12.0804	0.7530	H	0	0	0	0	0	0	0	0	0	0	0	0	0	0
16.0974	11.0645	-0.8227	H	0	0	0	0	0	0	0	0	0	0	0	0	0	0
15.4581	12.0686	-2.1307	H	0	0	0	0	0	0	0	0	0	0	0	0	0	0
16.1072	14.1121	-0.8817	H	0	0	0	0	0	0	0	0	0	0	0	0	0	0
16.7420	13.1103	0.4301	H	0	0	0	0	0	0	0	0	0	0	0	0	0	0
18.3726	12.0889	-1.1385	H	0	0	0	0	0	0	0	0	0	0	0	0	0	0
17.7374	13.0898	-2.4509	H	0	0	0	0	0	0	0	0	0	0	0	0	0	0
18.3842	15.1363	-1.2053	H	0	0	0	0	0	0	0	0	0	0	0	0	0	0
19.0155	14.1374	0.1104	H	0	0	0	0	0	0	0	0	0	0	0	0	0	0
20.6491	13.1113	-1.4520	H	0	0	0	0	0	0	0	0	0	0	0	0	0	0
20.0175	14.1094	-2.7683	H	0	0	0	0	0	0	0	0	0	0	0	0	0	0
20.6620	16.1586	-1.5250	H	0	0	0	0	0	0	0	0	0	0	0	0	0	0
21.2913	15.1617	-0.2067	H	0	0	0	0	0	0	0	0	0	0	0	0	0	0
22.9268	14.1327	-1.7653	H	0	0	0	0	0	0	0	0	0	0	0	0	0	0
22.2972	15.1288	-3.0840	H	0	0	0	0	0	0	0	0	0	0	0	0	0	0
22.9399	17.1799	-1.8420	H	0	0	0	0	0	0	0	0	0	0	0	0	0	0
24.3452	16.1405	-2.1111	H	0	0	0	0	0	0	0	0	0	0	0	0	0	0
23.5688	16.1842	-0.5227	H	0	0	0	0	0	0	0	0	0	0	0	0	0	0

1	2	1	0	0	0	0
1	3	1	0	0	0	0
2	4	1	0	0	0	0
2	5	2	0	0	0	0
3	6	1	0	0	0	0
4	7	1	0	0	0	0
6	8	1	0	0	0	0
7	9	1	0	0	0	0
8	10	1	0	0	0	0
9	11	1	0	0	0	0
9	12	1	0	0	0	0
10	13	1	0	0	0	0
11	14	1	0	0	0	0
13	15	1	0	0	0	0
15	16	1	0	0	0	0
16	17	1	0	0	0	0
17	18	1	0	0	0	0
18	19	1	0	0	0	0
19	20	1	0	0	0	0
20	21	1	0	0	0	0
21	22	1	0	0	0	0
22	23	1	0	0	0	0
12	24	1	0	0	0	0
14	25	1	0	0	0	0
1	26	1	0	0	0	0
1	27	1	0	0	0	0
3	28	1	0	0	0	0
3	29	1	0	0	0	0
6	30	1	0	0	0	0
6	31	1	0	0	0	0

```
  7 32  1  0  0  0  0
  7 33  1  0  0  0  0
  8 34  1  0  0  0  0
  8 35  1  0  0  0  0
  9 36  1  0  0  0  0
 10 37  1  0  0  0  0
 10 38  1  0  0  0  0
 11 39  1  0  0  0  0
 11 40  1  0  0  0  0
 13 41  1  0  0  0  0
 13 42  1  0  0  0  0
 15 43  1  0  0  0  0
 15 44  1  0  0  0  0
 16 45  1  0  0  0  0
 16 46  1  0  0  0  0
 17 47  1  0  0  0  0
 17 48  1  0  0  0  0
 18 49  1  0  0  0  0
 18 50  1  0  0  0  0
 19 51  1  0  0  0  0
 19 52  1  0  0  0  0
 20 53  1  0  0  0  0
 20 54  1  0  0  0  0
 21 55  1  0  0  0  0
 21 56  1  0  0  0  0
 22 57  1  0  0  0  0
 22 58  1  0  0  0  0
 23 59  1  0  0  0  0
 23 60  1  0  0  0  0
 23 61  1  0  0  0  0
M  END
>  <Alias name>
1-Palmitoylglycerol

>  <deltaH°(c)>   kJ/mol
-11626

$$$$
Fitone
  ChemBrain IXL

 55 54  0  0  0  0  0  0  0  0  1 V2000
    4.0733    6.0871   -1.4646 C   0  0  0  0  0  0  0  0  0  0  0  0
    5.2836    5.2687   -1.9769 C   0  0  0  0  0  0  0  0  0  0  0  0
    4.5347    7.0734   -0.3669 C   0  0  0  0  0  0  0  0  0  0  0  0
    3.0119    5.1315   -0.8816 C   0  0  0  0  0  0  0  0  0  0  0  0
    5.9353    4.5076   -0.8034 C   0  0  0  0  0  0  0  0  0  0  0  0
    5.6173    8.0133   -0.9354 C   0  0  0  0  0  0  0  0  0  0  0  0
    7.1520    3.7142   -1.3196 C   0  0  0  0  0  0  0  0  0  0  0  0
    6.0682    8.9997    0.1610 C   0  0  0  0  0  0  0  0  0  0  0  0
    7.8070    2.9476   -0.1484 C   0  0  0  0  0  0  0  0  0  0  0  0
    7.1488    9.9468   -0.4049 C   0  0  0  0  0  0  0  0  0  0  0  0
    8.2489    3.9453    0.9513 C   0  0  0  0  0  0  0  0  0  0  0  0
    9.0351    2.1714   -0.6671 C   0  0  0  0  0  0  0  0  0  0  0  0
    7.5753   10.9474    0.6869 C   0  0  0  0  0  0  0  0  0  0  0  0
    8.3754    9.1253   -0.8541 C   0  0  0  0  0  0  0  0  0  0  0  0
    9.2390    4.9728    0.3615 C   0  0  0  0  0  0  0  0  0  0  0  0
    9.6672    5.9637    1.4635 C   0  0  0  0  0  0  0  0  0  0  0  0
   10.6162    6.9536    0.9009 C   0  0  0  0  0  0  0  0  0  0  0  0
   11.1663    8.0232    1.7624 C   0  0  0  0  0  0  0  0  0  0  0  0
   10.9449    6.8882   -0.2727 O   0  0  0  0  0  0  0  0  0  0  0  0
    3.6421    6.6423   -2.2883 H   0  0  0  0  0  0  0  0  0  0  0  0
    4.9486    4.5602   -2.7242 H   0  0  0  0  0  0  0  0  0  0  0  0
```

```
   6.0101    5.9387   -2.4178 H   0  0  0  0  0  0  0  0  0  0  0  0
   3.6883    7.6600   -0.0316 H   0  0  0  0  0  0  0  0  0  0  0  0
   4.9415    6.5193    0.4690 H   0  0  0  0  0  0  0  0  0  0  0  0
   3.4359    4.5841   -0.0497 H   0  0  0  0  0  0  0  0  0  0  0  0
   2.1597    5.7051   -0.5388 H   0  0  0  0  0  0  0  0  0  0  0  0
   2.6936    4.4352   -1.6475 H   0  0  0  0  0  0  0  0  0  0  0  0
   6.2595    5.2141   -0.0519 H   0  0  0  0  0  0  0  0  0  0  0  0
   5.2162    3.8242   -0.3709 H   0  0  0  0  0  0  0  0  0  0  0  0
   6.4652    7.4284   -1.2644 H   0  0  0  0  0  0  0  0  0  0  0  0
   5.2120    8.5657   -1.7740 H   0  0  0  0  0  0  0  0  0  0  0  0
   6.8281    3.0090   -2.0749 H   0  0  0  0  0  0  0  0  0  0  0  0
   7.8714    4.3980   -1.7512 H   0  0  0  0  0  0  0  0  0  0  0  0
   5.2175    9.5819    0.4930 H   0  0  0  0  0  0  0  0  0  0  0  0
   6.4756    8.4470    0.9986 H   0  0  0  0  0  0  0  0  0  0  0  0
   7.0918    2.2494    0.2683 H   0  0  0  0  0  0  0  0  0  0  0  0
   6.7466   10.4880   -1.2523 H   0  0  0  0  0  0  0  0  0  0  0  0
   7.3807    4.4637    1.3367 H   0  0  0  0  0  0  0  0  0  0  0  0
   8.7297    3.4032    1.7560 H   0  0  0  0  0  0  0  0  0  0  0  0
   9.7465    2.8649   -1.0965 H   0  0  0  0  0  0  0  0  0  0  0  0
   8.7207    1.4631   -1.4236 H   0  0  0  0  0  0  0  0  0  0  0  0
   9.4994    1.6398    0.1542 H   0  0  0  0  0  0  0  0  0  0  0  0
   6.7155   11.5251    1.0029 H   0  0  0  0  0  0  0  0  0  0  0  0
   8.3318   11.6133    0.2906 H   0  0  0  0  0  0  0  0  0  0  0  0
   7.9783   10.4069    1.5345 H   0  0  0  0  0  0  0  0  0  0  0  0
   9.1369    9.7939   -1.2360 H   0  0  0  0  0  0  0  0  0  0  0  0
   8.0818    8.4325   -1.6319 H   0  0  0  0  0  0  0  0  0  0  0  0
   8.7697    8.5743   -0.0095 H   0  0  0  0  0  0  0  0  0  0  0  0
  10.1116    4.4576   -0.0186 H   0  0  0  0  0  0  0  0  0  0  0  0
   8.7605    5.5153   -0.4435 H   0  0  0  0  0  0  0  0  0  0  0  0
  10.1470    5.4217    2.2691 H   0  0  0  0  0  0  0  0  0  0  0  0
   8.7939    6.4781    1.8449 H   0  0  0  0  0  0  0  0  0  0  0  0
  10.4373    8.8179    1.8609 H   0  0  0  0  0  0  0  0  0  0  0  0
  12.0712    8.4165    1.3159 H   0  0  0  0  0  0  0  0  0  0  0  0
  11.3931    7.6179    2.7408 H   0  0  0  0  0  0  0  0  0  0  0  0
  1  2  1  0  0  0  0
  1  3  1  0  0  0  0
  1  4  1  0  0  0  0
  2  5  1  0  0  0  0
  3  6  1  0  0  0  0
  5  7  1  0  0  0  0
  6  8  1  0  0  0  0
  7  9  1  0  0  0  0
  8 10  1  0  0  0  0
  9 11  1  0  0  0  0
  9 12  1  0  0  0  0
 10 13  1  0  0  0  0
 10 14  1  0  0  0  0
 11 15  1  0  0  0  0
 15 16  1  0  0  0  0
 16 17  1  0  0  0  0
 17 18  1  0  0  0  0
 17 19  2  0  0  0  0
  1 20  1  0  0  0  0
  2 21  1  0  0  0  0
  2 22  1  0  0  0  0
  3 23  1  0  0  0  0
  3 24  1  0  0  0  0
  4 25  1  0  0  0  0
  4 26  1  0  0  0  0
  4 27  1  0  0  0  0
  5 28  1  0  0  0  0
  5 29  1  0  0  0  0
  6 30  1  0  0  0  0
```

```
  6 31  1  0  0  0  0
  7 32  1  0  0  0  0
  7 33  1  0  0  0  0
  8 34  1  0  0  0  0
  8 35  1  0  0  0  0
  9 36  1  0  0  0  0
 10 37  1  0  0  0  0
 11 38  1  0  0  0  0
 11 39  1  0  0  0  0
 12 40  1  0  0  0  0
 12 41  1  0  0  0  0
 12 42  1  0  0  0  0
 13 43  1  0  0  0  0
 13 44  1  0  0  0  0
 13 45  1  0  0  0  0
 14 46  1  0  0  0  0
 14 47  1  0  0  0  0
 14 48  1  0  0  0  0
 15 49  1  0  0  0  0
 15 50  1  0  0  0  0
 16 51  1  0  0  0  0
 16 52  1  0  0  0  0
 18 53  1  0  0  0  0
 18 54  1  0  0  0  0
 18 55  1  0  0  0  0
M  END
>  <Alias name>
Hexahydrofarnesyl acetone

>  <deltaH°(c)>   kJ/mol
-11602

$$$$
2,2',6,6'-Tetraethylazobenzene N,N'-dioxide
  ChemBrain IXL

 50 51  0  0  0  0  0  0  0  0  1 V2000
    7.1020    5.8286   -0.3518 N   0  3  0  0  0  0  0  0  0  0  0  0  0
    5.6523    5.5509   -0.5836 C   0  0  0  0  0  0  0  0  0  0  0  0  0
    7.8671    4.9007   -0.0497 O   0  5  0  0  0  0  0  0  0  0  0  0  0
    4.9242    6.2931   -1.5271 C   0  0  0  0  0  0  0  0  0  0  0  0  0
    5.0072    4.5395    0.1406 C   0  0  0  0  0  0  0  0  0  0  0  0  0
    3.5685    6.0299   -1.7317 C   0  0  0  0  0  0  0  0  0  0  0  0  0
    5.5840    7.3595   -2.3129 C   0  0  0  0  0  0  0  0  0  0  0  0  0
    3.6523    4.2781   -0.0694 C   0  0  0  0  0  0  0  0  0  0  0  0  0
    5.7572    3.7420    1.1323 C   0  0  0  0  0  0  0  0  0  0  0  0  0
    2.9329    5.0234   -1.0038 C   0  0  0  0  0  0  0  0  0  0  0  0  0
    7.6119    7.1544   -0.4733 N   0  3  0  0  0  0  0  0  0  0  0  0  0
    9.0657    7.4285   -0.2656 C   0  0  0  0  0  0  0  0  0  0  0  0  0
    6.8422    8.0864   -0.7505 O   0  5  0  0  0  0  0  0  0  0  0  0  0
   10.0366    6.4970   -0.6703 C   0  0  0  0  0  0  0  0  0  0  0  0  0
    9.4718    8.6267    0.3344 C   0  0  0  0  0  0  0  0  0  0  0  0  0
   11.3915    6.7629   -0.4649 C   0  0  0  0  0  0  0  0  0  0  0  0  0
    9.6361    5.2267   -1.3172 C   0  0  0  0  0  0  0  0  0  0  0  0  0
   10.8279    8.8893    0.5353 C   0  0  0  0  0  0  0  0  0  0  0  0  0
    8.4665    9.6211    0.7589 C   0  0  0  0  0  0  0  0  0  0  0  0  0
   11.7871    7.9577    0.1372 C   0  0  0  0  0  0  0  0  0  0  0  0  0
    9.1615   10.7401    1.5636 C   0  0  0  0  0  0  0  0  0  0  0  0  0
   10.9041    4.4651   -1.7745 C   0  0  0  0  0  0  0  0  0  0  0  0  0
    4.5528    7.9969   -3.2744 C   0  0  0  0  0  0  0  0  0  0  0  0  0
    4.7949    2.7559    1.8301 C   0  0  0  0  0  0  0  0  0  0  0  0  0
    3.0092    6.6085   -2.4572 H   0  0  0  0  0  0  0  0  0  0  0  0  0
    5.9697    8.1174   -1.6418 H   0  0  0  0  0  0  0  0  0  0  0  0  0
```

6.3995	6.9349	-2.8863	H	0	0	0	0	0	0	0	0	0	0	0	0
3.1587	3.4950	0.4944	H	0	0	0	0	0	0	0	0	0	0	0	0
6.1954	4.4024	1.8696	H	0	0	0	0	0	0	0	0	0	0	0	0
6.5411	3.1884	0.6315	H	0	0	0	0	0	0	0	0	0	0	0	0
1.8802	4.8206	-1.1643	H	0	0	0	0	0	0	0	0	0	0	0	0
12.1369	6.0398	-0.7738	H	0	0	0	0	0	0	0	0	0	0	0	0
9.0113	5.4389	-2.1767	H	0	0	0	0	0	0	0	0	0	0	0	0
9.0845	4.6189	-0.6101	H	0	0	0	0	0	0	0	0	0	0	0	0
11.1358	9.8181	1.0011	H	0	0	0	0	0	0	0	0	0	0	0	0
7.9894	10.0470	-0.1145	H	0	0	0	0	0	0	0	0	0	0	0	0
7.7215	9.1391	1.3790	H	0	0	0	0	0	0	0	0	0	0	0	0
12.8397	8.1622	0.2957	H	0	0	0	0	0	0	0	0	0	0	0	0
8.4234	11.4613	1.8910	H	0	0	0	0	0	0	0	0	0	0	0	0
9.8952	11.2334	0.9371	H	0	0	0	0	0	0	0	0	0	0	0	0
9.6551	10.3110	2.4274	H	0	0	0	0	0	0	0	0	0	0	0	0
11.4717	5.0857	-2.4583	H	0	0	0	0	0	0	0	0	0	0	0	0
10.6162	3.5481	-2.2742	H	0	0	0	0	0	0	0	0	0	0	0	0
11.5150	4.2289	-0.9109	H	0	0	0	0	0	0	0	0	0	0	0	0
4.1603	7.2364	-3.9394	H	0	0	0	0	0	0	0	0	0	0	0	0
3.7406	8.4269	-2.6998	H	0	0	0	0	0	0	0	0	0	0	0	0
5.0324	8.7734	-3.8577	H	0	0	0	0	0	0	0	0	0	0	0	0
4.3632	2.0892	1.0930	H	0	0	0	0	0	0	0	0	0	0	0	0
5.3408	2.1768	2.5644	H	0	0	0	0	0	0	0	0	0	0	0	0
4.0042	3.3096	2.3225	H	0	0	0	0	0	0	0	0	0	0	0	0

1	2	1	0	0	0	0
1	3	1	0	0	0	0
2	4	4	0	0	0	0
2	5	4	0	0	0	0
4	6	4	0	0	0	0
4	7	1	0	0	0	0
5	8	4	0	0	0	0
5	9	1	0	0	0	0
6	10	4	0	0	0	0
8	10	4	0	0	0	0
11	12	1	0	0	0	0
11	13	1	0	0	0	0
12	14	4	0	0	0	0
12	15	4	0	0	0	0
14	16	4	0	0	0	0
14	17	1	0	0	0	0
15	18	4	0	0	0	0
15	19	1	0	0	0	0
16	20	4	0	0	0	0
18	20	4	0	0	0	0
11	1	2	0	0	0	0
19	21	1	0	0	0	0
17	22	1	0	0	0	0
7	23	1	0	0	0	0
9	24	1	0	0	0	0
6	25	1	0	0	0	0
7	26	1	0	0	0	0
7	27	1	0	0	0	0
8	28	1	0	0	0	0
9	29	1	0	0	0	0
9	30	1	0	0	0	0
10	31	1	0	0	0	0
16	32	1	0	0	0	0
17	33	1	0	0	0	0
17	34	1	0	0	0	0
18	35	1	0	0	0	0
19	36	1	0	0	0	0
19	37	1	0	0	0	0
20	38	1	0	0	0	0

21 39 1 0 0 0 0
21 40 1 0 0 0 0
21 41 1 0 0 0 0
22 42 1 0 0 0 0
22 43 1 0 0 0 0
22 44 1 0 0 0 0
23 45 1 0 0 0 0
23 46 1 0 0 0 0
23 47 1 0 0 0 0
24 48 1 0 0 0 0
24 49 1 0 0 0 0
24 50 1 0 0 0 0
M END
> <Alias name>
2,2',6,6'-Tetraethylazobenzene N,N'-dioxide

> <deltaH°(c)>   kJ/mol
-11593.23

$$$$
1,1-Dicyclohexylhexane
ChemBrain IXL

52 53 0 0 0 0 0 0 0 0 1 V2000
7.3168   5.1988   -0.0481 C   0 0 0 0 0 0 0 0 0 0 0 0
8.5047   5.9980   0.5475 C   0 0 0 0 0 0 0 0 0 0 0 0
9.8302   5.5149   -0.1500 C   0 0 0 0 0 0 0 0 0 0 0 0
8.2585   7.5278   0.3165 C   0 0 0 0 0 0 0 0 0 0 0 0
6.0072   5.5857   0.6743 C   0 0 0 0 0 0 0 0 0 0 0 0
10.0459   4.0153   0.1807 C   0 0 0 0 0 0 0 0 0 0 0 0
11.0501   6.3330   0.3466 C   0 0 0 0 0 0 0 0 0 0 0 0
8.6571   7.9155   -1.1255 C   0 0 0 0 0 0 0 0 0 0 0 0
9.0709   8.3688   1.3398 C   0 0 0 0 0 0 0 0 0 0 0 0
4.8437   4.7486   0.1084 C   0 0 0 0 0 0 0 0 0 0 0 0
11.3378   3.5117   -0.4843 C   0 0 0 0 0 0 0 0 0 0 0 0
12.3438   5.7962   -0.3061 C   0 0 0 0 0 0 0 0 0 0 0 0
8.3880   9.4173   -1.3461 C   0 0 0 0 0 0 0 0 0 0 0 0
8.7978   9.8668   1.0998 C   0 0 0 0 0 0 0 0 0 0 0 0
3.5365   5.1324   0.8289 C   0 0 0 0 0 0 0 0 0 0 0 0
12.5308   4.3144   0.0496 C   0 0 0 0 0 0 0 0 0 0 0 0
9.2135   10.2431   -0.3374 C   0 0 0 0 0 0 0 0 0 0 0 0
2.3729   4.2955   0.2630 C   0 0 0 0 0 0 0 0 0 0 0 0
7.4953   4.1405   0.0832 H   0 0 0 0 0 0 0 0 0 0 0 0
7.2259   5.4198   -1.1044 H   0 0 0 0 0 0 0 0 0 0 0 0
8.5630   5.8026   1.6112 H   0 0 0 0 0 0 0 0 0 0 0 0
9.7348   5.6337   -1.2220 H   0 0 0 0 0 0 0 0 0 0 0 0
7.2102   7.7385   0.4597 H   0 0 0 0 0 0 0 0 0 0 0 0
5.8025   6.6349   0.5186 H   0 0 0 0 0 0 0 0 0 0 0 0
6.1090   5.3908   1.7347 H   0 0 0 0 0 0 0 0 0 0 0 0
9.2072   3.4411   -0.1883 H   0 0 0 0 0 0 0 0 0 0 0 0
10.1224   3.8911   1.2538 H   0 0 0 0 0 0 0 0 0 0 0 0
10.9259   7.3624   0.0690 H   0 0 0 0 0 0 0 0 0 0 0 0
11.1270   6.2524   1.4239 H   0 0 0 0 0 0 0 0 0 0 0 0
9.7071   7.7127   -1.2790 H   0 0 0 0 0 0 0 0 0 0 0 0
8.0717   7.3373   -1.8293 H   0 0 0 0 0 0 0 0 0 0 0 0
8.7638   8.1051   2.3442 H   0 0 0 0 0 0 0 0 0 0 0 0
10.1143   8.1780   1.2289 H   0 0 0 0 0 0 0 0 0 0 0 0
5.0466   3.6963   0.2648 H   0 0 0 0 0 0 0 0 0 0 0 0
4.7418   4.9428   -0.9522 H   0 0 0 0 0 0 0 0 0 0 0 0
11.4766   2.4628   -0.2537 H   0 0 0 0 0 0 0 0 0 0 0 0
11.2664   3.6395   -1.5574 H   0 0 0 0 0 0 0 0 0 0 0 0
13.1917   6.3634   0.0570 H   0 0 0 0 0 0 0 0 0 0 0 0
12.2745   5.9015   -1.3817 H   0 0 0 0 0 0 0 0 0 0 0 0

8.6730 9.6902 -2.3545 H 0 0 0 0 0 0 0 0 0 0 0 0
7.3341 9.6203 -1.2016 H 0 0 0 0 0 0 0 0 0 0 0 0
7.7419 10.0672 1.2338 H 0 0 0 0 0 0 0 0 0 0 0 0
9.3692 10.4552 1.8067 H 0 0 0 0 0 0 0 0 0 0 0 0
3.3337 6.1847 0.6725 H 0 0 0 0 0 0 0 0 0 0 0 0
3.6384 4.9381 1.8894 H 0 0 0 0 0 0 0 0 0 0 0 0
12.5898 4.2042 1.1254 H 0 0 0 0 0 0 0 0 0 0 0 0
13.4440 3.9474 -0.4021 H 0 0 0 0 0 0 0 0 0 0 0 0
9.0310 11.2979 -0.5006 H 0 0 0 0 0 0 0 0 0 0 0 0
10.2671 10.0336 -0.4753 H 0 0 0 0 0 0 0 0 0 0 0 0
2.5756 3.2432 0.4194 H 0 0 0 0 0 0 0 0 0 0 0 0
1.4545 4.5654 0.7694 H 0 0 0 0 0 0 0 0 0 0 0 0
2.2709 4.4898 -0.7976 H 0 0 0 0 0 0 0 0 0 0 0 0
1 2 1 0 0 0 0
1 5 1 0 0 0 0
2 3 1 0 0 0 0
2 4 1 0 0 0 0
6 3 1 0 0 0 0
3 7 1 0 0 0 0
8 4 1 0 0 0 0
4 9 1 0 0 0 0
5 10 1 0 0 0 0
11 6 1 0 0 0 0
7 12 1 0 0 0 0
13 8 1 0 0 0 0
9 14 1 0 0 0 0
10 15 1 0 0 0 0
16 11 1 0 0 0 0
12 16 1 0 0 0 0
17 13 1 0 0 0 0
14 17 1 0 0 0 0
15 18 1 0 0 0 0
1 19 1 0 0 0 0
1 20 1 0 0 0 0
2 21 1 0 0 0 0
3 22 1 0 0 0 0
4 23 1 0 0 0 0
5 24 1 0 0 0 0
5 25 1 0 0 0 0
6 26 1 0 0 0 0
6 27 1 0 0 0 0
7 28 1 0 0 0 0
7 29 1 0 0 0 0
8 30 1 0 0 0 0
8 31 1 0 0 0 0
9 32 1 0 0 0 0
9 33 1 0 0 0 0
10 34 1 0 0 0 0
10 35 1 0 0 0 0
11 36 1 0 0 0 0
11 37 1 0 0 0 0
12 38 1 0 0 0 0
12 39 1 0 0 0 0
13 40 1 0 0 0 0
13 41 1 0 0 0 0
14 42 1 0 0 0 0
14 43 1 0 0 0 0
15 44 1 0 0 0 0
15 45 1 0 0 0 0
16 46 1 0 0 0 0
16 47 1 0 0 0 0
17 48 1 0 0 0 0
17 49 1 0 0 0 0

```
 18 50  1  0  0  0  0
 18 51  1  0  0  0  0
 18 52  1  0  0  0  0
M  END
>  <Alias name>
1,1-Dicyclohexylhexane

>  <deltaH°(c)>   kJ/mol
-11560

$$$$
1,6-Dicyclohexylhexane
  ChemBrain IXL

 52 53  0  0  0  0  0  0  0  0  1 V2000
    7.6301    8.8608   -0.6153 C   0  0  0  0  0  0  0  0  0  0  0  0
    6.5571    7.7526   -0.6153 C   0  0  0  0  0  0  0  0  0  0  0  0
    5.1508    8.3903   -0.6369 C   0  0  0  0  0  0  0  0  0  0  0  0
    9.0310    8.2187   -0.6156 C   0  0  0  0  0  0  0  0  0  0  0  0
    4.0845    7.2761   -0.6652 C   0  0  0  0  0  0  0  0  0  0  0  0
    4.9544    9.2579    0.6255 C   0  0  0  0  0  0  0  0  0  0  0  0
   10.1035    9.3253   -0.6157 C   0  0  0  0  0  0  0  0  0  0  0  0
    2.6788    7.9086   -0.6872 C   0  0  0  0  0  0  0  0  0  0  0  0
    3.5470    9.8878    0.6035 C   0  0  0  0  0  0  0  0  0  0  0  0
   11.5043    8.6832   -0.6153 C   0  0  0  0  0  0  0  0  0  0  0  0
    2.4837    8.7720    0.5747 C   0  0  0  0  0  0  0  0  0  0  0  0
   12.5774    9.7914   -0.6154 C   0  0  0  0  0  0  0  0  0  0  0  0
   13.9837    9.1537   -0.6369 C   0  0  0  0  0  0  0  0  0  0  0  0
   14.1801    8.2863    0.6255 C   0  0  0  0  0  0  0  0  0  0  0  0
   15.0500   10.2680   -0.6653 C   0  0  0  0  0  0  0  0  0  0  0  0
   15.5875    7.6564    0.6035 C   0  0  0  0  0  0  0  0  0  0  0  0
   16.4556    9.6355   -0.6874 C   0  0  0  0  0  0  0  0  0  0  0  0
   16.6507    8.7722    0.5746 C   0  0  0  0  0  0  0  0  0  0  0  0
    7.5147    9.4752   -1.4997 H   0  0  0  0  0  0  0  0  0  0  0  0
    7.5159    9.4750    0.2683 H   0  0  0  0  0  0  0  0  0  0  0  0
    6.6647    7.1457    0.2752 H   0  0  0  0  0  0  0  0  0  0  0  0
    6.6830    7.1304   -1.4927 H   0  0  0  0  0  0  0  0  0  0  0  0
    5.0488    9.0093   -1.5197 H   0  0  0  0  0  0  0  0  0  0  0  0
    9.1465    7.6041    0.2686 H   0  0  0  0  0  0  0  0  0  0  0  0
    9.1463    7.6042   -1.5000 H   0  0  0  0  0  0  0  0  0  0  0  0
    4.1872    6.6557    0.2166 H   0  0  0  0  0  0  0  0  0  0  0  0
    4.2207    6.6688   -1.5514 H   0  0  0  0  0  0  0  0  0  0  0  0
    5.0593    8.6395    1.5084 H   0  0  0  0  0  0  0  0  0  0  0  0
    5.6990   10.0427    0.6442 H   0  0  0  0  0  0  0  0  0  0  0  0
    9.9882    9.9397   -1.5001 H   0  0  0  0  0  0  0  0  0  0  0  0
    9.9879    9.9400    0.2685 H   0  0  0  0  0  0  0  0  0  0  0  0
    2.5759    8.5292   -1.5688 H   0  0  0  0  0  0  0  0  0  0  0  0
    1.9311    7.1256   -0.7078 H   0  0  0  0  0  0  0  0  0  0  0  0
    3.4430   10.5081   -0.2781 H   0  0  0  0  0  0  0  0  0  0  0  0
    3.4094   10.4945    1.4898 H   0  0  0  0  0  0  0  0  0  0  0  0
   11.6186    8.0691    0.2683 H   0  0  0  0  0  0  0  0  0  0  0  0
   11.6198    8.0688   -1.4996 H   0  0  0  0  0  0  0  0  0  0  0  0
    1.4959    9.2152    0.5594 H   0  0  0  0  0  0  0  0  0  0  0  0
    2.5874    8.1516    1.4563 H   0  0  0  0  0  0  0  0  0  0  0  0
   12.4514   10.4136   -1.4929 H   0  0  0  0  0  0  0  0  0  0  0  0
   12.4697   10.3984    0.2751 H   0  0  0  0  0  0  0  0  0  0  0  0
   14.0856    8.5347   -1.5197 H   0  0  0  0  0  0  0  0  0  0  0  0
   13.4355    7.5014    0.6443 H   0  0  0  0  0  0  0  0  0  0  0  0
   14.0752    8.9047    1.5084 H   0  0  0  0  0  0  0  0  0  0  0  0
   14.9137   10.8751   -1.5516 H   0  0  0  0  0  0  0  0  0  0  0  0
   14.9472   10.8884    0.2164 H   0  0  0  0  0  0  0  0  0  0  0  0
   15.7251    7.0498    1.4899 H   0  0  0  0  0  0  0  0  0  0  0  0
   15.6915    7.0360   -0.2780 H   0  0  0  0  0  0  0  0  0  0  0  0
```

```
   17.2033   10.4185   -0.7080 H   0  0  0  0  0  0  0  0  0  0  0  0
   16.5585    9.0148   -1.5689 H   0  0  0  0  0  0  0  0  0  0  0  0
   16.5470    9.3927    1.4562 H   0  0  0  0  0  0  0  0  0  0  0  0
   17.6386    8.3289    0.5594 H   0  0  0  0  0  0  0  0  0  0  0  0
  1  2  1  0  0  0  0
  1  4  1  0  0  0  0
  2  3  1  0  0  0  0
  5  3  1  0  0  0  0
  3  6  1  0  0  0  0
  4  7  1  0  0  0  0
  8  5  1  0  0  0  0
  6  9  1  0  0  0  0
  7 10  1  0  0  0  0
 11  8  1  0  0  0  0
  9 11  1  0  0  0  0
 10 12  1  0  0  0  0
 12 13  1  0  0  0  0
 13 14  1  0  0  0  0
 15 13  1  0  0  0  0
 14 16  1  0  0  0  0
 17 15  1  0  0  0  0
 16 18  1  0  0  0  0
 18 17  1  0  0  0  0
  1 19  1  0  0  0  0
  1 20  1  0  0  0  0
  2 21  1  0  0  0  0
  2 22  1  0  0  0  0
  3 23  1  0  0  0  0
  4 24  1  0  0  0  0
  4 25  1  0  0  0  0
  5 26  1  0  0  0  0
  5 27  1  0  0  0  0
  6 28  1  0  0  0  0
  6 29  1  0  0  0  0
  7 30  1  0  0  0  0
  7 31  1  0  0  0  0
  8 32  1  0  0  0  0
  8 33  1  0  0  0  0
  9 34  1  0  0  0  0
  9 35  1  0  0  0  0
 10 36  1  0  0  0  0
 10 37  1  0  0  0  0
 11 38  1  0  0  0  0
 11 39  1  0  0  0  0
 12 40  1  0  0  0  0
 12 41  1  0  0  0  0
 13 42  1  0  0  0  0
 14 43  1  0  0  0  0
 14 44  1  0  0  0  0
 15 45  1  0  0  0  0
 15 46  1  0  0  0  0
 16 47  1  0  0  0  0
 16 48  1  0  0  0  0
 17 49  1  0  0  0  0
 17 50  1  0  0  0  0
 18 51  1  0  0  0  0
 18 52  1  0  0  0  0
M  END
>  <Alias name>
1,6-Dicyclohexylhexane

>  <deltaH°(c)>   kJ/mol
-11560
```

```
$$$$
Methyl linolenate
  ChemBrain IXL

 53 52  0  0  0  0  0  0  0  0  1 V2000
    4.2392   13.1540    0.1870 C   0  0  0  0  0  0  0  0  0  0  0  0
    4.1521   11.6252    0.3623 C   0  0  0  0  0  0  0  0  0  0  0  0
    2.7612   11.1793    0.1215 C   0  0  0  0  0  0  0  0  0  0  0  0
    2.4878    9.8775    0.0035 C   0  0  0  0  0  0  0  0  0  0  0  0
    3.5756    8.8791    0.1132 C   0  0  0  0  0  0  0  0  0  0  0  0
    2.9957    7.5168    0.1211 C   0  0  0  0  0  0  0  0  0  0  0  0
    3.7977    6.4489    0.1247 C   0  0  0  0  0  0  0  0  0  0  0  0
    5.2678    6.6261    0.1208 C   0  0  0  0  0  0  0  0  0  0  0  0
    5.9247    5.3007    0.1827 C   0  0  0  0  0  0  0  0  0  0  0  0
    7.2554    5.2173    0.2612 C   0  0  0  0  0  0  0  0  0  0  0  0
    8.0743    6.4505    0.2866 C   0  0  0  0  0  0  0  0  0  0  0  0
    9.5582    6.0898    0.0669 C   0  0  0  0  0  0  0  0  0  0  0  0
   10.4053    7.3782    0.0795 C   0  0  0  0  0  0  0  0  0  0  0  0
   11.8877    7.0263   -0.1602 C   0  0  0  0  0  0  0  0  0  0  0  0
   12.7259    8.3210   -0.1676 C   0  0  0  0  0  0  0  0  0  0  0  0
   14.2054    7.9786   -0.4345 C   0  0  0  0  0  0  0  0  0  0  0  0
   15.0360    9.2782   -0.4588 C   0  0  0  0  0  0  0  0  0  0  0  0
   16.4537    8.9548   -0.7369 C   0  0  0  0  0  0  0  0  0  0  0  0
   17.4868    9.9910   -0.5914 O   0  0  0  0  0  0  0  0  0  0  0  0
   16.7649    7.8283   -1.0870 O   0  0  0  0  0  0  0  0  0  0  0  0
   16.9914   11.3075   -0.1635 C   0  0  0  0  0  0  0  0  0  0  0  0
    3.5773   13.6370    0.8953 H   0  0  0  0  0  0  0  0  0  0  0  0
    3.9448   13.4192   -0.8209 H   0  0  0  0  0  0  0  0  0  0  0  0
    5.2568   13.4803    0.3635 H   0  0  0  0  0  0  0  0  0  0  0  0
    4.4468   11.3601    1.3702 H   0  0  0  0  0  0  0  0  0  0  0  0
    4.8138   11.1424   -0.3461 H   0  0  0  0  0  0  0  0  0  0  0  0
    1.9650   11.9091    0.0410 H   0  0  0  0  0  0  0  0  0  0  0  0
    1.4701    9.5520   -0.1726 H   0  0  0  0  0  0  0  0  0  0  0  0
    4.1248    9.0431    1.0321 H   0  0  0  0  0  0  0  0  0  0  0  0
    4.2460    8.9813   -0.7312 H   0  0  0  0  0  0  0  0  0  0  0  0
    1.9206    7.3863    0.1239 H   0  0  0  0  0  0  0  0  0  0  0  0
    3.3726    5.4529    0.1305 H   0  0  0  0  0  0  0  0  0  0  0  0
    5.5626    7.2157    0.9800 H   0  0  0  0  0  0  0  0  0  0  0  0
    5.5676    7.1356   -0.7867 H   0  0  0  0  0  0  0  0  0  0  0  0
    5.3260    4.3984    0.1643 H   0  0  0  0  0  0  0  0  0  0  0  0
    7.7353    4.2475    0.3065 H   0  0  0  0  0  0  0  0  0  0  0  0
    7.7453    7.1182   -0.5000 H   0  0  0  0  0  0  0  0  0  0  0  0
    7.9603    6.9395    1.2461 H   0  0  0  0  0  0  0  0  0  0  0  0
    9.8909    5.4308    0.8594 H   0  0  0  0  0  0  0  0  0  0  0  0
    9.6715    5.5921   -0.8883 H   0  0  0  0  0  0  0  0  0  0  0  0
   10.0617    8.0431   -0.7031 H   0  0  0  0  0  0  0  0  0  0  0  0
   10.3020    7.8681    1.0398 H   0  0  0  0  0  0  0  0  0  0  0  0
   12.2369    6.3742    0.6309 H   0  0  0  0  0  0  0  0  0  0  0  0
   11.9902    6.5239   -1.1143 H   0  0  0  0  0  0  0  0  0  0  0  0
   12.3627    8.9811   -0.9456 H   0  0  0  0  0  0  0  0  0  0  0  0
   12.6375    8.8131    0.7930 H   0  0  0  0  0  0  0  0  0  0  0  0
   14.5733    7.3294    0.3505 H   0  0  0  0  0  0  0  0  0  0  0  0
   14.2932    7.4758   -1.3898 H   0  0  0  0  0  0  0  0  0  0  0  0
   14.6570    9.9341   -1.2328 H   0  0  0  0  0  0  0  0  0  0  0  0
   14.9605    9.7726    0.5018 H   0  0  0  0  0  0  0  0  0  0  0  0
   16.2760   11.6769   -0.8878 H   0  0  0  0  0  0  0  0  0  0  0  0
   16.5120   11.2161    0.8033 H   0  0  0  0  0  0  0  0  0  0  0  0
   17.8215   11.9994   -0.0908 H   0  0  0  0  0  0  0  0  0  0  0  0
 10 11  1  0  0  0  0
  5  6  1  0  0  0  0
 11 12  1  0  0  0  0
  1  2  1  0  0  0  0
```

```
 12 13  1  0  0  0  0
  6  7  2  0  0  0  0
 13 14  1  0  0  0  0
  3  4  2  0  0  0  0
 14 15  1  0  0  0  0
  7  8  1  0  0  0  0
 15 16  1  0  0  0  0
 16 17  1  0  0  0  0
  8  9  1  0  0  0  0
 17 18  1  0  0  0  0
  4  5  1  0  0  0  0
 18 19  1  0  0  0  0
  9 10  2  0  0  0  0
 18 20  2  0  0  0  0
  2  3  1  0  0  0  0
 19 21  1  0  0  0  0
  1 22  1  0  0  0  0
  1 23  1  0  0  0  0
  1 24  1  0  0  0  0
  2 25  1  0  0  0  0
  2 26  1  0  0  0  0
  3 27  1  0  0  0  0
  4 28  1  0  0  0  0
  5 29  1  0  0  0  0
  5 30  1  0  0  0  0
  6 31  1  0  0  0  0
  7 32  1  0  0  0  0
  8 33  1  0  0  0  0
  8 34  1  0  0  0  0
  9 35  1  0  0  0  0
 10 36  1  0  0  0  0
 11 37  1  0  0  0  0
 11 38  1  0  0  0  0
 12 39  1  0  0  0  0
 12 40  1  0  0  0  0
 13 41  1  0  0  0  0
 13 42  1  0  0  0  0
 14 43  1  0  0  0  0
 14 44  1  0  0  0  0
 15 45  1  0  0  0  0
 15 46  1  0  0  0  0
 16 47  1  0  0  0  0
 16 48  1  0  0  0  0
 17 49  1  0  0  0  0
 17 50  1  0  0  0  0
 21 51  1  0  0  0  0
 21 52  1  0  0  0  0
 21 53  1  0  0  0  0
M  END
>  <Alias name>
Methyl linolenate

>  <deltaH°(c)>   kJ/mol
-11506

$$$$
4,4'-Di-t-butylbiphenyl
  ChemBrain IXL

 46 47  0  0  0  0  0  0  0  0  1 V2000
    8.9075    7.4229   -0.1060 C   0  0  0  0  0  0  0  0  0  0  0  0
    9.8568    8.4451   -0.1212 C   0  0  0  0  0  0  0  0  0  0  0  0
   11.2047    8.1423   -0.3144 C   0  0  0  0  0  0  0  0  0  0  0  0
```

```
 11.6034    6.8174   -0.4928 C   0  0  0  0  0  0  0  0  0  0  0  0
 10.6542    5.7953   -0.4774 C   0  0  0  0  0  0  0  0  0  0  0  0
  9.3063    6.0981   -0.2842 C   0  0  0  0  0  0  0  0  0  0  0  0
  7.4774    7.7442    0.0990 C   0  0  0  0  0  0  0  0  0  0  0  0
  6.5293    6.7212    0.1229 C   0  0  0  0  0  0  0  0  0  0  0  0
  5.1813    7.0240    0.3161 C   0  0  0  0  0  0  0  0  0  0  0  0
  4.7815    8.3497    0.4856 C   0  0  0  0  0  0  0  0  0  0  0  0
  5.7297    9.3726    0.4616 C   0  0  0  0  0  0  0  0  0  0  0  0
  7.0777    9.0699    0.2684 C   0  0  0  0  0  0  0  0  0  0  0  0
  3.3515    8.6709    0.6910 C   0  0  0  0  0  0  0  0  0  0  0  0
  2.4810    7.6145   -0.0169 C   0  0  0  0  0  0  0  0  0  0  0  0
  3.0483   10.0632    0.1042 C   0  0  0  0  0  0  0  0  0  0  0  0
  3.0361    8.6693    2.1993 C   0  0  0  0  0  0  0  0  0  0  0  0
 13.0335    6.4963   -0.6984 C   0  0  0  0  0  0  0  0  0  0  0  0
 13.9040    7.5497    0.0138 C   0  0  0  0  0  0  0  0  0  0  0  0
 13.3498    6.5031   -2.2066 C   0  0  0  0  0  0  0  0  0  0  0  0
 13.3356    5.1017   -0.1166 C   0  0  0  0  0  0  0  0  0  0  0  0
  9.5472    9.4736    0.0170 H   0  0  0  0  0  0  0  0  0  0  0  0
 11.9417    8.9358   -0.3259 H   0  0  0  0  0  0  0  0  0  0  0  0
 10.9638    4.7667   -0.6154 H   0  0  0  0  0  0  0  0  0  0  0  0
  8.5693    5.3045   -0.2725 H   0  0  0  0  0  0  0  0  0  0  0  0
  6.8396    5.6920   -0.0085 H   0  0  0  0  0  0  0  0  0  0  0  0
  4.4452    6.2299    0.3344 H   0  0  0  0  0  0  0  0  0  0  0  0
  5.4193   10.4019    0.5930 H   0  0  0  0  0  0  0  0  0  0  0  0
  7.8138    9.8640    0.2500 H   0  0  0  0  0  0  0  0  0  0  0  0
  2.6714    6.6405    0.4165 H   0  0  0  0  0  0  0  0  0  0  0  0
  1.4351    7.8666    0.1072 H   0  0  0  0  0  0  0  0  0  0  0  0
  2.7246    7.5941   -1.0719 H   0  0  0  0  0  0  0  0  0  0  0  0
  1.9957   10.2858    0.2281 H   0  0  0  0  0  0  0  0  0  0  0  0
  3.6384   10.8094    0.6219 H   0  0  0  0  0  0  0  0  0  0  0  0
  3.2978   10.0730   -0.9496 H   0  0  0  0  0  0  0  0  0  0  0  0
  3.2490    7.6908    2.6117 H   0  0  0  0  0  0  0  0  0  0  0  0
  3.6480    9.4116    2.6968 H   0  0  0  0  0  0  0  0  0  0  0  0
  1.9897    8.9045    2.3496 H   0  0  0  0  0  0  0  0  0  0  0  0
 13.7146    8.5253   -0.4165 H   0  0  0  0  0  0  0  0  0  0  0  0
 14.9499    7.2973   -0.1104 H   0  0  0  0  0  0  0  0  0  0  0  0
 13.6595    7.5666    1.0687 H   0  0  0  0  0  0  0  0  0  0  0  0
 13.1376    7.4833   -2.6155 H   0  0  0  0  0  0  0  0  0  0  0  0
 12.7378    5.7630   -2.7072 H   0  0  0  0  0  0  0  0  0  0  0  0
 14.3962    6.2679   -2.3571 H   0  0  0  0  0  0  0  0  0  0  0  0
 14.3882    4.8791   -0.2406 H   0  0  0  0  0  0  0  0  0  0  0  0
 12.7454    4.3577   -0.6372 H   0  0  0  0  0  0  0  0  0  0  0  0
 13.0855    5.0883    0.9371 H   0  0  0  0  0  0  0  0  0  0  0  0
  1  2  4  0  0  0  0
  2  3  4  0  0  0  0
  3  4  4  0  0  0  0
  4  5  4  0  0  0  0
  5  6  4  0  0  0  0
  1  6  4  0  0  0  0
  7  8  4  0  0  0  0
  8  9  4  0  0  0  0
  9 10  4  0  0  0  0
 10 11  4  0  0  0  0
 11 12  4  0  0  0  0
  7 12  4  0  0  0  0
  1  7  1  0  0  0  0
 13 14  1  0  0  0  0
 13 16  1  0  0  0  0
 13 15  1  0  0  0  0
 13 10  1  0  0  0  0
 17 18  1  0  0  0  0
 17 20  1  0  0  0  0
 17 19  1  0  0  0  0
```

```
 17  4  1  0  0  0  0
  2 21  1  0  0  0  0
  3 22  1  0  0  0  0
  5 23  1  0  0  0  0
  6 24  1  0  0  0  0
  8 25  1  0  0  0  0
  9 26  1  0  0  0  0
 11 27  1  0  0  0  0
 12 28  1  0  0  0  0
 14 29  1  0  0  0  0
 14 30  1  0  0  0  0
 14 31  1  0  0  0  0
 15 32  1  0  0  0  0
 15 33  1  0  0  0  0
 15 34  1  0  0  0  0
 16 35  1  0  0  0  0
 16 36  1  0  0  0  0
 16 37  1  0  0  0  0
 18 38  1  0  0  0  0
 18 39  1  0  0  0  0
 18 40  1  0  0  0  0
 19 41  1  0  0  0  0
 19 42  1  0  0  0  0
 19 43  1  0  0  0  0
 20 44  1  0  0  0  0
 20 45  1  0  0  0  0
 20 46  1  0  0  0  0
M  END
>  <Alias name>
4,4'-Di-t-butylbiphenyl

>  <deltaH°(c)>   kJ/mol
-11471.63

$$$$
3-Methoxy-1,3,3-triphenylpropyne
  ChemBrain IXL

 41 43  0  0  0  0  0  0  0  0  1 V2000
    9.9229    8.7302    0.2862 C   0  0  0  0  0  0  0  0  0  0  0  0  0
   10.1149    9.9734   -0.3167 C   0  0  0  0  0  0  0  0  0  0  0  0  0
    9.3611    7.6812   -0.4418 C   0  0  0  0  0  0  0  0  0  0  0  0  0
    9.7461   10.1674   -1.6481 C   0  0  0  0  0  0  0  0  0  0  0  0  0
    9.1855    9.1180   -2.3765 C   0  0  0  0  0  0  0  0  0  0  0  0  0
    8.9934    7.8748   -1.7735 C   0  0  0  0  0  0  0  0  0  0  0  0  0
    9.1552    6.3630    0.1986 C   0  0  0  0  0  0  0  0  0  0  0  0  0
   10.0587    5.3674   -0.4202 C   0  0  0  0  0  0  0  0  0  0  0  0  0
    7.6921    5.9201    0.0037 C   0  0  0  0  0  0  0  0  0  0  0  0  0
    9.4490    6.4649    1.6354 O   0  0  0  0  0  0  0  0  0  0  0  0  0
    9.5682    4.4822   -1.3804 C   0  0  0  0  0  0  0  0  0  0  0  0  0
   10.4213    3.5482   -1.9684 C   0  0  0  0  0  0  0  0  0  0  0  0  0
   11.7642    3.4974   -1.5942 C   0  0  0  0  0  0  0  0  0  0  0  0  0
   12.2539    4.3801   -0.6314 C   0  0  0  0  0  0  0  0  0  0  0  0  0
   11.4007    5.3141   -0.0433 C   0  0  0  0  0  0  0  0  0  0  0  0  0
    6.5490    5.5740   -0.1487 C   0  0  0  0  0  0  0  0  0  0  0  0  0
    9.2835    5.2164    2.3935 C   0  0  0  0  0  0  0  0  0  0  0  0  0
    5.1439    5.1487   -0.3359 C   0  0  0  0  0  0  0  0  0  0  0  0  0
    4.1023    6.0006    0.0320 C   0  0  0  0  0  0  0  0  0  0  0  0  0
    2.7779    5.6005   -0.1465 C   0  0  0  0  0  0  0  0  0  0  0  0  0
    2.4950    4.3481   -0.6919 C   0  0  0  0  0  0  0  0  0  0  0  0  0
    3.5366    3.4957   -1.0587 C   0  0  0  0  0  0  0  0  0  0  0  0  0
    4.8610    3.8958   -0.8802 C   0  0  0  0  0  0  0  0  0  0  0  0  0
   10.2099    8.5795    1.3196 H   0  0  0  0  0  0  0  0  0  0  0  0  0
```

10.5504 10.7880 0.2487 H 0 0 0 0 0 0 0 0 0 0 0 0
9.8950 11.1326 -2.1161 H 0 0 0 0 0 0 0 0 0 0 0 0
8.8993 9.2685 -3.4101 H 0 0 0 0 0 0 0 0 0 0 0 0
8.5589 7.0599 -2.3392 H 0 0 0 0 0 0 0 0 0 0 0 0
8.5252 4.5202 -1.6693 H 0 0 0 0 0 0 0 0 0 0 0 0
10.0411 2.8625 -2.7155 H 0 0 0 0 0 0 0 0 0 0 0 0
12.4267 2.7726 -2.0511 H 0 0 0 0 0 0 0 0 0 0 0 0
13.2963 4.3404 -0.3404 H 0 0 0 0 0 0 0 0 0 0 0 0
11.7803 5.9979 0.7059 H 0 0 0 0 0 0 0 0 0 0 0 0
9.9480 4.4605 1.9935 H 0 0 0 0 0 0 0 0 0 0 0 0
9.5221 5.3922 3.4351 H 0 0 0 0 0 0 0 0 0 0 0 0
8.2586 4.8767 2.3102 H 0 0 0 0 0 0 0 0 0 0 0 0
4.3219 6.9726 0.4561 H 0 0 0 0 0 0 0 0 0 0 0 0
1.9693 6.2622 0.1385 H 0 0 0 0 0 0 0 0 0 0 0 0
1.4668 4.0376 -0.8305 H 0 0 0 0 0 0 0 0 0 0 0 0
3.3170 2.5233 -1.4819 H 0 0 0 0 0 0 0 0 0 0 0 0
5.6696 3.2337 -1.1643 H 0 0 0 0 0 0 0 0 0 0 0 0
2 1 4 0 0 0 0
1 3 4 0 0 0 0
4 2 4 0 0 0 0
3 6 4 0 0 0 0
3 7 1 0 0 0 0
5 4 4 0 0 0 0
6 5 4 0 0 0 0
7 8 1 0 0 0 0
7 9 1 0 0 0 0
7 10 1 0 0 0 0
11 8 4 0 0 0 0
8 15 4 0 0 0 0
9 16 3 0 0 0 0
10 17 1 0 0 0 0
12 11 4 0 0 0 0
13 12 4 0 0 0 0
14 13 4 0 0 0 0
15 14 4 0 0 0 0
16 18 1 0 0 0 0
19 18 4 0 0 0 0
18 23 4 0 0 0 0
20 19 4 0 0 0 0
21 20 4 0 0 0 0
22 21 4 0 0 0 0
23 22 4 0 0 0 0
1 24 1 0 0 0 0
2 25 1 0 0 0 0
4 26 1 0 0 0 0
5 27 1 0 0 0 0
6 28 1 0 0 0 0
11 29 1 0 0 0 0
12 30 1 0 0 0 0
13 31 1 0 0 0 0
14 32 1 0 0 0 0
15 33 1 0 0 0 0
17 34 1 0 0 0 0
17 35 1 0 0 0 0
17 36 1 0 0 0 0
19 37 1 0 0 0 0
20 38 1 0 0 0 0
21 39 1 0 0 0 0
22 40 1 0 0 0 0
23 41 1 0 0 0 0
M END
> <Alias name>

3-Methoxy-1,3,3-triphenylpropyne

> <deltaH°(c)>   kJ/mol
-11450

$$$$
Ethoxytriphenylsilane
  ChemBrain IXL

 42 44 0 0 0 0 0 0 0 0 1 V2000
    7.1146   6.6799   0.7077 Si 0 0 0 0 0 0 0 0 0 0 0 0
    6.7404   6.4011   -0.8541 O   0 0 0 0 0 0 0 0 0 0 0 0
    7.6823   2.6227   2.9166 C   0 0 0 0 0 0 0 0 0 0 0 0
    6.3985   3.0776   2.6103 C   0 0 0 0 0 0 0 0 0 0 0 0
    6.2288   4.2946   1.9479 C   0 0 0 0 0 0 0 0 0 0 0 0
    7.3428   5.0568   1.5917 C   0 0 0 0 0 0 0 0 0 0 0 0
    8.6268   4.6020   1.8983 C   0 0 0 0 0 0 0 0 0 0 0 0
    8.7964   3.3850   2.5607 C   0 0 0 0 0 0 0 0 0 0 0 0
    5.5019   5.7175   -1.0682 C   0 0 0 0 0 0 0 0 0 0 0 0
    5.2762   5.5307   -2.5692 C   0 0 0 0 0 0 0 0 0 0 0 0
    8.7314   8.8466   1.5479 C   0 0 0 0 0 0 0 0 0 0 0 0
    9.9126   9.5874   1.6175 C   0 0 0 0 0 0 0 0 0 0 0 0
   11.0521   9.1496   0.9400 C   0 0 0 0 0 0 0 0 0 0 0 0
   11.0104   7.9710   0.1930 C   0 0 0 0 0 0 0 0 0 0 0 0
    9.8293   7.2302   0.1233 C   0 0 0 0 0 0 0 0 0 0 0 0
    8.6899   7.6680   0.8008 C   0 0 0 0 0 0 0 0 0 0 0 0
    5.7354   7.6349   1.5150 C   0 0 0 0 0 0 0 0 0 0 0 0
    5.4080   7.3933   2.8503 C   0 0 0 0 0 0 0 0 0 0 0 0
    4.3738   8.1093   3.4557 C   0 0 0 0 0 0 0 0 0 0 0 0
    3.6673   9.0669   2.7257 C   0 0 0 0 0 0 0 0 0 0 0 0
    3.9950   9.3084   1.3903 C   0 0 0 0 0 0 0 0 0 0 0 0
    5.0291   8.5924   0.7849 C   0 0 0 0 0 0 0 0 0 0 0 0
    7.8139   1.6786   3.4304 H   0 0 0 0 0 0 0 0 0 0 0 0
    5.5343   2.4864   2.8864 H   0 0 0 0 0 0 0 0 0 0 0 0
    5.2329   4.6475   1.7104 H   0 0 0 0 0 0 0 0 0 0 0 0
    9.4910   5.1928   1.6226 H   0 0 0 0 0 0 0 0 0 0 0 0
    9.7921   3.0321   2.7984 H   0 0 0 0 0 0 0 0 0 0 0 0
    4.6902   6.3009   -0.6512 H   0 0 0 0 0 0 0 0 0 0 0 0
    5.5356   4.7488   -0.5851 H   0 0 0 0 0 0 0 0 0 0 0 0
    5.2424   6.4993   -3.0524 H   0 0 0 0 0 0 0 0 0 0 0 0
    4.3388   5.0133   -2.7317 H   0 0 0 0 0 0 0 0 0 0 0 0
    6.0875   4.9472   -2.9864 H   0 0 0 0 0 0 0 0 0 0 0 0
    7.8476   9.1862   2.0734 H   0 0 0 0 0 0 0 0 0 0 0 0
    9.9449   10.5016   2.1969 H   0 0 0 0 0 0 0 0 0 0 0 0
   11.9683   9.7242   0.9940 H   0 0 0 0 0 0 0 0 0 0 0 0
   11.8942   7.6314   -0.3325 H   0 0 0 0 0 0 0 0 0 0 0 0
    9.7970   6.3159   -0.4561 H   0 0 0 0 0 0 0 0 0 0 0 0
    5.9560   6.6505   3.4166 H   0 0 0 0 0 0 0 0 0 0 0 0
    4.1196   7.9219   4.4915 H   0 0 0 0 0 0 0 0 0 0 0 0
    2.8651   9.6223   3.1954 H   0 0 0 0 0 0 0 0 0 0 0 0
    3.4471   10.0512   0.8242 H   0 0 0 0 0 0 0 0 0 0 0 0
    5.2833   8.7796   -0.2509 H   0 0 0 0 0 0 0 0 0 0 0 0
  2 1 1 0 0 0 0
  3 4 4 0 0 0 0
  4 5 4 0 0 0 0
  5 6 4 0 0 0 0
  6 7 4 0 0 0 0
  7 8 4 0 0 0 0
  8 3 4 0 0 0 0
  2 9 1 0 0 0 0
  9 10 1 0 0 0 0
 11 12 4 0 0 0 0
 12 13 4 0 0 0 0
 13 14 4 0 0 0 0

```
 14 15  4  0  0  0  0
 15 16  4  0  0  0  0
 16 11  4  0  0  0  0
 17 18  4  0  0  0  0
 18 19  4  0  0  0  0
 19 20  4  0  0  0  0
 20 21  4  0  0  0  0
 21 22  4  0  0  0  0
 22 17  4  0  0  0  0
  6  1  1  0  0  0  0
 17  1  1  0  0  0  0
 16  1  1  0  0  0  0
  3 23  1  0  0  0  0
  4 24  1  0  0  0  0
  5 25  1  0  0  0  0
  7 26  1  0  0  0  0
  8 27  1  0  0  0  0
  9 28  1  0  0  0  0
  9 29  1  0  0  0  0
 10 30  1  0  0  0  0
 10 31  1  0  0  0  0
 10 32  1  0  0  0  0
 11 33  1  0  0  0  0
 12 34  1  0  0  0  0
 13 35  1  0  0  0  0
 14 36  1  0  0  0  0
 15 37  1  0  0  0  0
 18 38  1  0  0  0  0
 19 39  1  0  0  0  0
 20 40  1  0  0  0  0
 21 41  1  0  0  0  0
 22 42  1  0  0  0  0
M  END
>  <Alias name>
Ethoxytriphenylsilane

>  <deltaH°(c)>   kJ/mol
-11446

$$$$
3-Phenyl-5-phenoxymethyl-2-N-phenyliminooxazolidine
  ChemBrain IXL

 46 49  0  0  0  0  0  0  0  0  1 V2000
    7.3893    2.9652   -0.6385 C   0  0  0  0  0  0  0  0  0  0  0  0
    6.0505    3.6841   -1.0897 C   0  0  0  0  0  0  0  0  0  0  0  0
    8.2534    4.0667   -0.3340 N   0  0  0  0  0  0  0  0  0  0  0  0
    7.6838    5.2264   -0.5578 C   0  0  0  0  0  0  0  0  0  0  0  0
    6.4114    5.1018   -1.1444 O   0  0  0  0  0  0  0  0  0  0  0  0
    5.0053    3.5236   -0.0193 C   0  0  0  0  0  0  0  0  0  0  0  0
    5.6185    3.8617    1.2871 O   0  0  0  0  0  0  0  0  0  0  0  0
    2.6515    5.2287    3.0674 C   0  0  0  0  0  0  0  0  0  0  0  0
    3.4277    4.6654    2.0618 C   0  0  0  0  0  0  0  0  0  0  0  0
    4.8062    4.7269    2.1471 C   0  0  0  0  0  0  0  0  0  0  0  0
    5.4047    5.6074    3.0517 C   0  0  0  0  0  0  0  0  0  0  0  0
    4.6342    6.1842    4.0590 C   0  0  0  0  0  0  0  0  0  0  0  0
    3.2560    5.9779    4.0761 C   0  0  0  0  0  0  0  0  0  0  0  0
    8.2428    6.3518   -0.2426 N   0  0  0  0  0  0  0  0  0  0  0  0
    8.8360    9.7235   -1.6945 C   0  0  0  0  0  0  0  0  0  0  0  0
    9.1209    8.4516   -1.1908 C   0  0  0  0  0  0  0  0  0  0  0  0
    8.0916    7.5057   -1.0679 C   0  0  0  0  0  0  0  0  0  0  0  0
    6.9074    7.7151   -1.7593 C   0  0  0  0  0  0  0  0  0  0  0  0
    6.6092    8.9839   -2.2542 C   0  0  0  0  0  0  0  0  0  0  0  0
```

```
    7.5690    9.9946   -2.2075 C   0  0  0  0  0  0  0  0  0  0  0  0
   11.3510    2.6720    1.2175 C   0  0  0  0  0  0  0  0  0  0  0  0
   10.0904    2.7366    0.6128 C   0  0  0  0  0  0  0  0  0  0  0  0
    9.5946    3.9586    0.1480 C   0  0  0  0  0  0  0  0  0  0  0  0
   10.4302    5.0858    0.1644 C   0  0  0  0  0  0  0  0  0  0  0  0
   11.6910    5.0203    0.7685 C   0  0  0  0  0  0  0  0  0  0  0  0
   12.1474    3.8149    1.3014 C   0  0  0  0  0  0  0  0  0  0  0  0
    7.2214    2.3598    0.2401 H   0  0  0  0  0  0  0  0  0  0  0  0
    7.7989    2.3701   -1.4463 H   0  0  0  0  0  0  0  0  0  0  0  0
    5.7068    3.3239   -2.0511 H   0  0  0  0  0  0  0  0  0  0  0  0
    4.6488    2.5113   -0.0055 H   0  0  0  0  0  0  0  0  0  0  0  0
    4.1831    4.2024   -0.2187 H   0  0  0  0  0  0  0  0  0  0  0  0
    1.5821    5.0843    3.0659 H   0  0  0  0  0  0  0  0  0  0  0  0
    2.9609    4.1797    1.2162 H   0  0  0  0  0  0  0  0  0  0  0  0
    6.4628    5.8438    2.9724 H   0  0  0  0  0  0  0  0  0  0  0  0
    5.1043    6.7904    4.8267 H   0  0  0  0  0  0  0  0  0  0  0  0
    2.6557    6.3985    4.8712 H   0  0  0  0  0  0  0  0  0  0  0  0
    9.5974   10.4995   -1.6871 H   0  0  0  0  0  0  0  0  0  0  0  0
   10.1367    8.2005   -0.9005 H   0  0  0  0  0  0  0  0  0  0  0  0
    6.2216    6.8960   -1.9132 H   0  0  0  0  0  0  0  0  0  0  0  0
    5.6338    9.1839   -2.6736 H   0  0  0  0  0  0  0  0  0  0  0  0
    7.3300   10.9887   -2.5691 H   0  0  0  0  0  0  0  0  0  0  0  0
   11.7109    1.7333    1.6217 H   0  0  0  0  0  0  0  0  0  0  0  0
    9.4995    1.8386    0.5047 H   0  0  0  0  0  0  0  0  0  0  0  0
   10.1015    6.0108   -0.2916 H   0  0  0  0  0  0  0  0  0  0  0  0
   12.3153    5.9067    0.8234 H   0  0  0  0  0  0  0  0  0  0  0  0
   13.1208    3.7661    1.7807 H   0  0  0  0  0  0  0  0  0  0  0  0
  1  2  1  0  0  0  0
  1  3  1  0  0  0  0
  3  4  1  0  0  0  0
  4  5  1  0  0  0  0
  5  2  1  0  0  0  0
  2  6  1  0  0  0  0
  6  7  1  0  0  0  0
  8  9  4  0  0  0  0
  9 10  4  0  0  0  0
 10 11  4  0  0  0  0
 11 12  4  0  0  0  0
 12 13  4  0  0  0  0
 13  8  4  0  0  0  0
 10  7  1  0  0  0  0
  4 14  2  0  0  0  0
 15 16  4  0  0  0  0
 16 17  4  0  0  0  0
 17 18  4  0  0  0  0
 18 19  4  0  0  0  0
 19 20  4  0  0  0  0
 20 15  4  0  0  0  0
 21 22  4  0  0  0  0
 22 23  4  0  0  0  0
 23 24  4  0  0  0  0
 24 25  4  0  0  0  0
 25 26  4  0  0  0  0
 26 21  4  0  0  0  0
 23  3  1  0  0  0  0
 17 14  1  0  0  0  0
  1 27  1  0  0  0  0
  1 28  1  0  0  0  0
  2 29  1  0  0  0  0
  6 30  1  0  0  0  0
  6 31  1  0  0  0  0
  8 32  1  0  0  0  0
  9 33  1  0  0  0  0
```

11 34 1 0 0 0 0
12 35 1 0 0 0 0
13 36 1 0 0 0 0
15 37 1 0 0 0 0
16 38 1 0 0 0 0
18 39 1 0 0 0 0
19 40 1 0 0 0 0
20 41 1 0 0 0 0
21 42 1 0 0 0 0
22 43 1 0 0 0 0
24 44 1 0 0 0 0
25 45 1 0 0 0 0
26 46 1 0 0 0 0
M END
> <Alias name>
3-Phenyl-5-phenoxymethyl-2-N-phenyliminooxazolidine

> <deltaH°(c)> kJ/mol
-11441

$$$$
Myristic acid t-butylperoxyester
ChemBrain IXL

57 56 0 0 0 0 0 0 0 0 1 V2000
8.0575 9.8224 0.6906 C 0 0 0 0 0 0 0 0 0 0 0 0
7.0667 8.6654 0.4572 C 0 0 0 0 0 0 0 0 0 0 0 0
5.6839 9.1503 0.6651 C 0 0 0 0 0 0 0 0 0 0 0 0
5.4447 10.5525 1.0361 O 0 0 0 0 0 0 0 0 0 0 0 0
4.7426 8.3864 0.5284 O 0 0 0 0 0 0 0 0 0 0 0 0
9.4972 9.3172 0.4747 C 0 0 0 0 0 0 0 0 0 0 0 0
10.4882 10.4741 0.7080 C 0 0 0 0 0 0 0 0 0 0 0 0
11.9278 9.9681 0.4932 C 0 0 0 0 0 0 0 0 0 0 0 0
12.9191 11.1247 0.7264 C 0 0 0 0 0 0 0 0 0 0 0 0
14.3587 10.6181 0.5126 C 0 0 0 0 0 0 0 0 0 0 0 0
15.3503 11.7744 0.7456 C 0 0 0 0 0 0 0 0 0 0 0 0
16.7898 11.2672 0.5327 C 0 0 0 0 0 0 0 0 0 0 0 0
4.0225 10.9152 1.2265 O 0 0 0 0 0 0 0 0 0 0 0 0
3.5271 11.9750 0.3364 C 0 0 0 0 0 0 0 0 0 0 0 0
4.3456 13.2607 0.5635 C 0 0 0 0 0 0 0 0 0 0 0 0
3.6742 11.5247 -1.1300 C 0 0 0 0 0 0 0 0 0 0 0 0
2.0422 12.2506 0.6426 C 0 0 0 0 0 0 0 0 0 0 0 0
17.7817 12.4234 0.7656 C 0 0 0 0 0 0 0 0 0 0 0 0
19.2212 11.9158 0.5533 C 0 0 0 0 0 0 0 0 0 0 0 0
20.2131 13.0718 0.7862 C 0 0 0 0 0 0 0 0 0 0 0 0
21.6526 12.5640 0.5742 C 0 0 0 0 0 0 0 0 0 0 0 0
7.8476 10.6237 -0.0071 H 0 0 0 0 0 0 0 0 0 0 0 0
7.9518 10.1893 1.7041 H 0 0 0 0 0 0 0 0 0 0 0 0
7.2769 7.8640 1.1547 H 0 0 0 0 0 0 0 0 0 0 0 0
7.1722 8.2988 -0.5564 H 0 0 0 0 0 0 0 0 0 0 0 0
9.7070 8.5159 1.1724 H 0 0 0 0 0 0 0 0 0 0 0 0
9.6028 8.9503 -0.5388 H 0 0 0 0 0 0 0 0 0 0 0 0
10.2790 11.2750 0.0096 H 0 0 0 0 0 0 0 0 0 0 0 0
10.3820 10.8416 1.7212 H 0 0 0 0 0 0 0 0 0 0 0 0
12.1369 9.1672 1.1916 H 0 0 0 0 0 0 0 0 0 0 0 0
12.0339 9.6005 -0.5199 H 0 0 0 0 0 0 0 0 0 0 0 0
12.7106 11.9253 0.0274 H 0 0 0 0 0 0 0 0 0 0 0 0
12.8126 11.4929 1.7393 H 0 0 0 0 0 0 0 0 0 0 0 0
14.5672 9.8175 1.2115 H 0 0 0 0 0 0 0 0 0 0 0 0
14.4652 10.2499 -0.5003 H 0 0 0 0 0 0 0 0 0 0 0 0
15.1424 12.5748 0.0463 H 0 0 0 0 0 0 0 0 0 0 0 0
15.2435 12.1431 1.7583 H 0 0 0 0 0 0 0 0 0 0 0 0
16.9977 10.4669 1.2321 H 0 0 0 0 0 0 0 0 0 0 0 0

16.8966	10.8986	-0.4800	H	0	0	0	0	0	0	0	0	0	0	0	0	0
3.9815	14.0412	-0.0932	H	0	0	0	0	0	0	0	0	0	0	0	0	0
5.3894	13.0667	0.3494	H	0	0	0	0	0	0	0	0	0	0	0	0	0
4.2413	13.5779	1.5937	H	0	0	0	0	0	0	0	0	0	0	0	0	0
4.7176	11.3296	-1.3448	H	0	0	0	0	0	0	0	0	0	0	0	0	0
3.3106	12.3061	-1.7858	H	0	0	0	0	0	0	0	0	0	0	0	0	0
3.0978	10.6219	-1.2899	H	0	0	0	0	0	0	0	0	0	0	0	0	0
1.4670	11.3469	0.4831	H	0	0	0	0	0	0	0	0	0	0	0	0	0
1.9389	12.5671	1.6732	H	0	0	0	0	0	0	0	0	0	0	0	0	0
1.6772	13.0313	-0.0132	H	0	0	0	0	0	0	0	0	0	0	0	0	0
17.5742	13.2236	0.0660	H	0	0	0	0	0	0	0	0	0	0	0	0	0
17.6747	12.7924	1.7782	H	0	0	0	0	0	0	0	0	0	0	0	0	0
19.4286	11.1156	1.2530	H	0	0	0	0	0	0	0	0	0	0	0	0	0
19.3281	11.5467	-0.4592	H	0	0	0	0	0	0	0	0	0	0	0	0	0
20.0058	13.8719	0.0864	H	0	0	0	0	0	0	0	0	0	0	0	0	0
20.1060	13.4410	1.7987	H	0	0	0	0	0	0	0	0	0	0	0	0	0
22.3497	13.3765	0.7378	H	0	0	0	0	0	0	0	0	0	0	0	0	0
21.7597	12.1948	-0.4382	H	0	0	0	0	0	0	0	0	0	0	0	0	0
21.8599	11.7640	1.2741	H	0	0	0	0	0	0	0	0	0	0	0	0	0

1	2	1	0	0	0	0
2	3	1	0	0	0	0
3	4	1	0	0	0	0
3	5	2	0	0	0	0
1	6	1	0	0	0	0
6	7	1	0	0	0	0
7	8	1	0	0	0	0
8	9	1	0	0	0	0
9	10	1	0	0	0	0
10	11	1	0	0	0	0
11	12	1	0	0	0	0
4	13	1	0	0	0	0
13	14	1	0	0	0	0
14	15	1	0	0	0	0
14	16	1	0	0	0	0
14	17	1	0	0	0	0
12	18	1	0	0	0	0
18	19	1	0	0	0	0
19	20	1	0	0	0	0
20	21	1	0	0	0	0
1	22	1	0	0	0	0
1	23	1	0	0	0	0
2	24	1	0	0	0	0
2	25	1	0	0	0	0
6	26	1	0	0	0	0
6	27	1	0	0	0	0
7	28	1	0	0	0	0
7	29	1	0	0	0	0
8	30	1	0	0	0	0
8	31	1	0	0	0	0
9	32	1	0	0	0	0
9	33	1	0	0	0	0
10	34	1	0	0	0	0
10	35	1	0	0	0	0
11	36	1	0	0	0	0
11	37	1	0	0	0	0
12	38	1	0	0	0	0
12	39	1	0	0	0	0
15	40	1	0	0	0	0
15	41	1	0	0	0	0
15	42	1	0	0	0	0
16	43	1	0	0	0	0
16	44	1	0	0	0	0
16	45	1	0	0	0	0

```
 17 46  1  0  0  0  0
 17 47  1  0  0  0  0
 17 48  1  0  0  0  0
 18 49  1  0  0  0  0
 18 50  1  0  0  0  0
 19 51  1  0  0  0  0
 19 52  1  0  0  0  0
 20 53  1  0  0  0  0
 20 54  1  0  0  0  0
 21 55  1  0  0  0  0
 21 56  1  0  0  0  0
 21 57  1  0  0  0  0
M  END
>  <Alias name>
Myristic acid t-butylperoxyester

>  <deltaH°(c)>   kJ/mol
-11440

$$$$
Tetra-cyclopentadiene
  ChemBrain IXL

 44 50  0  0  0  0  0  0  0  0  1 V2000
    2.0786    5.7926   -1.1079 C   0  0  0  0  0  0  0  0  0  0  0  0
    1.9868    6.3149    0.1053 C   0  0  0  0  0  0  0  0  0  0  0  0
    3.2979    4.8570   -1.1224 C   0  0  0  0  0  0  0  0  0  0  0  0
    3.1510    5.7691    0.9344 C   0  0  0  0  0  0  0  0  0  0  0  0
    4.3948    6.5003    0.3549 C   0  0  0  0  0  0  0  0  0  0  0  0
    3.3011    4.4149    0.3095 C   0  0  0  0  0  0  0  0  0  0  0  0
    4.5064    5.8201   -1.1343 C   0  0  0  0  0  0  0  0  0  0  0  0
    5.7202    6.0688    1.0002 C   0  0  0  0  0  0  0  0  0  0  0  0
    5.8725    5.0945   -1.0530 C   0  0  0  0  0  0  0  0  0  0  0  0
    6.9263    6.2145   -1.1478 C   0  0  0  0  0  0  0  0  0  0  0  0
    5.9431    4.7229    0.3891 C   0  0  0  0  0  0  0  0  0  0  0  0
    6.8045    6.9282    0.2962 C   0  0  0  0  0  0  0  0  0  0  0  0
    8.3863    5.6903   -1.1284 C   0  0  0  0  0  0  0  0  0  0  0  0
    8.2150    6.7319    0.8720 C   0  0  0  0  0  0  0  0  0  0  0  0
    9.1194    7.7047    0.0569 C   0  0  0  0  0  0  0  0  0  0  0  0
    8.5970    5.4109    0.3128 C   0  0  0  0  0  0  0  0  0  0  0  0
    9.2624    6.9389   -1.3447 C   0  0  0  0  0  0  0  0  0  0  0  0
   10.5803    7.8032    0.6224 C   0  0  0  0  0  0  0  0  0  0  0  0
   10.7385    6.6079   -1.4399 C   0  0  0  0  0  0  0  0  0  0  0  0
   11.4461    7.1262   -0.4533 C   0  0  0  0  0  0  0  0  0  0  0  0
    1.4168    5.9929   -1.9411 H   0  0  0  0  0  0  0  0  0  0  0  0
    1.2249    7.0033    0.4494 H   0  0  0  0  0  0  0  0  0  0  0  0
    3.2957    4.0698   -1.8664 H   0  0  0  0  0  0  0  0  0  0  0  0
    3.0319    5.7895    2.0110 H   0  0  0  0  0  0  0  0  0  0  0  0
    4.2735    7.5749    0.3115 H   0  0  0  0  0  0  0  0  0  0  0  0
    4.2411    3.9510    0.5819 H   0  0  0  0  0  0  0  0  0  0  0  0
    2.4543    3.7765    0.5319 H   0  0  0  0  0  0  0  0  0  0  0  0
    4.4593    6.5526   -1.9300 H   0  0  0  0  0  0  0  0  0  0  0  0
    5.7395    6.0912    2.0829 H   0  0  0  0  0  0  0  0  0  0  0  0
    6.0040    4.2729   -1.7468 H   0  0  0  0  0  0  0  0  0  0  0  0
    6.7372    6.9014   -1.9630 H   0  0  0  0  0  0  0  0  0  0  0  0
    5.1521    4.0325    0.6567 H   0  0  0  0  0  0  0  0  0  0  0  0
    6.9189    4.3296    0.6467 H   0  0  0  0  0  0  0  0  0  0  0  0
    6.5257    7.9709    0.2161 H   0  0  0  0  0  0  0  0  0  0  0  0
    8.5901    4.8593   -1.7927 H   0  0  0  0  0  0  0  0  0  0  0  0
    8.2933    6.8159    1.9489 H   0  0  0  0  0  0  0  0  0  0  0  0
    8.6621    8.6789   -0.0632 H   0  0  0  0  0  0  0  0  0  0  0  0
    7.9354    4.6279    0.6631 H   0  0  0  0  0  0  0  0  0  0  0  0
    9.6343    5.1795    0.5215 H   0  0  0  0  0  0  0  0  0  0  0  0
```

```
  8.9198    7.5440   -2.1750 H   0  0  0  0  0  0  0  0  0  0  0  0
 10.6593    7.2718    1.5629 H   0  0  0  0  0  0  0  0  0  0  0  0
 10.8696    8.8394    0.7454 H   0  0  0  0  0  0  0  0  0  0  0  0
 11.1663    6.0032   -2.2298 H   0  0  0  0  0  0  0  0  0  0  0  0
 12.5272    7.0863   -0.4099 H   0  0  0  0  0  0  0  0  0  0  0  0
  1  2  2  0  0  0  0
  3  1  1  0  0  0  0
  2  4  1  0  0  0  0
  7  3  1  0  0  0  0
  3  6  1  0  0  0  0
  4  5  1  0  0  0  0
  6  4  1  0  0  0  0
  7  5  1  0  0  0  0
  5  8  1  0  0  0  0
  9  7  1  0  0  0  0
  8 12  1  0  0  0  0
 11  8  1  0  0  0  0
 10  9  1  0  0  0  0
  9 11  1  0  0  0  0
 10 12  1  0  0  0  0
 13 10  1  0  0  0  0
 12 14  1  0  0  0  0
 17 13  1  0  0  0  0
 13 16  1  0  0  0  0
 14 15  1  0  0  0  0
 16 14  1  0  0  0  0
 15 17  1  0  0  0  0
 15 18  1  0  0  0  0
 19 17  1  0  0  0  0
 18 20  1  0  0  0  0
 20 19  2  0  0  0  0
  1 21  1  0  0  0  0
  2 22  1  0  0  0  0
  3 23  1  0  0  0  0
  4 24  1  0  0  0  0
  5 25  1  0  0  0  0
  6 26  1  0  0  0  0
  6 27  1  0  0  0  0
  7 28  1  0  0  0  0
  8 29  1  0  0  0  0
  9 30  1  0  0  0  0
 10 31  1  0  0  0  0
 11 32  1  0  0  0  0
 11 33  1  0  0  0  0
 12 34  1  0  0  0  0
 13 35  1  0  0  0  0
 14 36  1  0  0  0  0
 15 37  1  0  0  0  0
 16 38  1  0  0  0  0
 16 39  1  0  0  0  0
 17 40  1  0  0  0  0
 18 41  1  0  0  0  0
 18 42  1  0  0  0  0
 19 43  1  0  0  0  0
 20 44  1  0  0  0  0
M  END
>  <Alias name>
Tetra-cyclopentadiene

>  <deltaH°(c)>   kJ/mol
-11426

$$$$
```

```
Glyceryltribenzoate
  ChemBrain IXL

 50 52  0  0  0  0  0  0  0  0  1 V2000
    4.3564   10.6630   -0.0987 C   0  0  0  0  0  0  0  0  0  0  0  0  0
    4.7644   10.2354   -1.3622 C   0  0  0  0  0  0  0  0  0  0  0  0  0
    4.9326    8.8744   -1.6172 C   0  0  0  0  0  0  0  0  0  0  0  0  0
    4.7018    7.9420   -0.6058 C   0  0  0  0  0  0  0  0  0  0  0  0  0
    4.3435    8.3736    0.6711 C   0  0  0  0  0  0  0  0  0  0  0  0  0
    4.1270    9.7300    0.9125 C   0  0  0  0  0  0  0  0  0  0  0  0  0
    4.1930    7.3926    1.7695 C   0  0  0  0  0  0  0  0  0  0  0  0  0
    3.1369    7.3122    2.3750 O   0  0  0  0  0  0  0  0  0  0  0  0  0
    4.4894    2.0733   -1.8940 C   0  0  0  0  0  0  0  0  0  0  0  0  0
    5.2180    1.3508   -2.8390 C   0  0  0  0  0  0  0  0  0  0  0  0  0
    6.4436    1.8366   -3.2952 C   0  0  0  0  0  0  0  0  0  0  0  0  0
    6.9394    3.0460   -2.8079 C   0  0  0  0  0  0  0  0  0  0  0  0  0
    6.2062    3.7733   -1.8701 C   0  0  0  0  0  0  0  0  0  0  0  0  0
    4.9855    3.2825   -1.4064 C   0  0  0  0  0  0  0  0  0  0  0  0  0
    6.7233    5.0655   -1.3671 C   0  0  0  0  0  0  0  0  0  0  0  0  0
    7.2805    5.8379   -2.1296 O   0  0  0  0  0  0  0  0  0  0  0  0  0
   13.7074    7.0256    0.4118 C   0  0  0  0  0  0  0  0  0  0  0  0  0
   13.3085    6.8288    1.7339 C   0  0  0  0  0  0  0  0  0  0  0  0  0
   11.9573    6.6607    2.0372 C   0  0  0  0  0  0  0  0  0  0  0  0  0
   11.0057    6.6840    1.0176 C   0  0  0  0  0  0  0  0  0  0  0  0  0
   11.4076    6.8512   -0.3076 C   0  0  0  0  0  0  0  0  0  0  0  0  0
   12.7555    7.0489   -0.6076 C   0  0  0  0  0  0  0  0  0  0  0  0  0
   10.4042    6.8190   -1.3953 C   0  0  0  0  0  0  0  0  0  0  0  0  0
   10.7460    7.0317   -2.5469 O   0  0  0  0  0  0  0  0  0  0  0  0  0
    6.6504    7.0730    1.8678 C   0  0  0  0  0  0  0  0  0  0  0  0  0
    7.0653    6.7510    0.4191 C   0  0  0  0  0  0  0  0  0  0  0  0  0
    8.6022    6.7720    0.3078 C   0  0  0  0  0  0  0  0  0  0  0  0  0
    8.9958    6.5307   -1.0879 O   0  0  0  0  0  0  0  0  0  0  0  0  0
    6.5656    5.4184    0.0511 O   0  0  0  0  0  0  0  0  0  0  0  0  0
    5.3160    6.5151    2.1308 O   0  0  0  0  0  0  0  0  0  0  0  0  0
    4.2177   11.7190    0.0972 H   0  0  0  0  0  0  0  0  0  0  0  0  0
    4.9506   10.9601   -2.1451 H   0  0  0  0  0  0  0  0  0  0  0  0  0
    5.2422    8.5416   -2.6002 H   0  0  0  0  0  0  0  0  0  0  0  0  0
    4.8009    6.8834   -0.8119 H   0  0  0  0  0  0  0  0  0  0  0  0  0
    3.7808   10.0588    1.8846 H   0  0  0  0  0  0  0  0  0  0  0  0  0
    3.5385    1.6955   -1.5390 H   0  0  0  0  0  0  0  0  0  0  0  0  0
    4.8323    0.4126   -3.2185 H   0  0  0  0  0  0  0  0  0  0  0  0  0
    7.0101    1.2749   -4.0276 H   0  0  0  0  0  0  0  0  0  0  0  0  0
    7.8936    3.4204   -3.1577 H   0  0  0  0  0  0  0  0  0  0  0  0  0
    4.4229    3.8402   -0.6679 H   0  0  0  0  0  0  0  0  0  0  0  0  0
   14.7559    7.1603    0.1767 H   0  0  0  0  0  0  0  0  0  0  0  0  0
   14.0479    6.8064    2.5250 H   0  0  0  0  0  0  0  0  0  0  0  0  0
   11.6472    6.5122    3.0641 H   0  0  0  0  0  0  0  0  0  0  0  0  0
    9.9549    6.5722    1.2548 H   0  0  0  0  0  0  0  0  0  0  0  0  0
   13.0628    7.2204   -1.6318 H   0  0  0  0  0  0  0  0  0  0  0  0  0
    7.3659    6.6363    2.5537 H   0  0  0  0  0  0  0  0  0  0  0  0  0
    6.6265    8.1468    2.0063 H   0  0  0  0  0  0  0  0  0  0  0  0  0
    6.6448    7.4916   -0.2499 H   0  0  0  0  0  0  0  0  0  0  0  0  0
    9.0205    5.9983    0.9398 H   0  0  0  0  0  0  0  0  0  0  0  0  0
    8.9740    7.7382    0.6259 H   0  0  0  0  0  0  0  0  0  0  0  0  0
  1  2  4  0  0  0  0
  2  3  4  0  0  0  0
  3  4  4  0  0  0  0
  4  5  4  0  0  0  0
  5  6  4  0  0  0  0
  6  1  4  0  0  0  0
  5  7  1  0  0  0  0
  7  8  2  0  0  0  0
  9 10  4  0  0  0  0
```

```
 10 11  4  0  0  0  0
 11 12  4  0  0  0  0
 12 13  4  0  0  0  0
 13 14  4  0  0  0  0
 14  9  4  0  0  0  0
 13 15  1  0  0  0  0
 15 16  2  0  0  0  0
 17 18  4  0  0  0  0
 18 19  4  0  0  0  0
 19 20  4  0  0  0  0
 20 21  4  0  0  0  0
 21 22  4  0  0  0  0
 22 17  4  0  0  0  0
 21 23  1  0  0  0  0
 23 24  2  0  0  0  0
 25 26  1  0  0  0  0
 26 27  1  0  0  0  0
 27 28  1  0  0  0  0
 26 29  1  0  0  0  0
 25 30  1  0  0  0  0
 15 29  1  0  0  0  0
 23 28  1  0  0  0  0
  7 30  1  0  0  0  0
  1 31  1  0  0  0  0
  2 32  1  0  0  0  0
  3 33  1  0  0  0  0
  4 34  1  0  0  0  0
  6 35  1  0  0  0  0
  9 36  1  0  0  0  0
 10 37  1  0  0  0  0
 11 38  1  0  0  0  0
 12 39  1  0  0  0  0
 14 40  1  0  0  0  0
 17 41  1  0  0  0  0
 18 42  1  0  0  0  0
 19 43  1  0  0  0  0
 20 44  1  0  0  0  0
 22 45  1  0  0  0  0
 25 46  1  0  0  0  0
 25 47  1  0  0  0  0
 26 48  1  0  0  0  0
 27 49  1  0  0  0  0
 27 50  1  0  0  0  0
M  END
>  <Alias name>
Glyceryltribenzoate

>  <deltaH°(c)>   kJ/mol
-11413

$$$$
Tribenzylamine
  ChemBrain IXL

 43 45  0  0  0  0  0  0  0  0  1 V2000
    7.1336    6.0751   -1.7029 N   0  0  0  0  0  0  0  0  0  0  0  0
    8.3598    6.6205   -2.3032 C   0  0  0  0  0  0  0  0  0  0  0  0
    6.0128    6.9913   -1.9592 C   0  0  0  0  0  0  0  0  0  0  0  0
    7.3226    5.9255   -0.2527 C   0  0  0  0  0  0  0  0  0  0  0  0
    9.4531    5.6303   -2.1781 C   0  0  0  0  0  0  0  0  0  0  0  0
    4.7524    6.3719   -1.4911 C   0  0  0  0  0  0  0  0  0  0  0  0
    7.6221    7.2460    0.3449 C   0  0  0  0  0  0  0  0  0  0  0  0
   10.7507    5.9714   -2.5594 C   0  0  0  0  0  0  0  0  0  0  0  0
```

```
  9.1860    4.3555   -1.6789 C   0  0  0  0  0  0  0  0  0  0  0  0
  4.7508    5.0550   -1.0315 C   0  0  0  0  0  0  0  0  0  0  0  0
  3.5658    7.1049   -1.5093 C   0  0  0  0  0  0  0  0  0  0  0  0
  7.8441    7.3561    1.7177 C   0  0  0  0  0  0  0  0  0  0  0  0
  7.6823    8.3807   -0.4645 C   0  0  0  0  0  0  0  0  0  0  0  0
 11.7932    5.0635   -2.3739 C   0  0  0  0  0  0  0  0  0  0  0  0
 10.2282    3.4472   -1.4932 C   0  0  0  0  0  0  0  0  0  0  0  0
  3.5784    4.4955   -0.5232 C   0  0  0  0  0  0  0  0  0  0  0  0
  2.3932    6.5459   -1.0010 C   0  0  0  0  0  0  0  0  0  0  0  0
  8.1258    8.6007    2.2812 C   0  0  0  0  0  0  0  0  0  0  0  0
  7.9641    9.6253    0.0990 C   0  0  0  0  0  0  0  0  0  0  0  0
 11.5325    3.8026   -1.8372 C   0  0  0  0  0  0  0  0  0  0  0  0
  2.4004    5.2424   -0.5044 C   0  0  0  0  0  0  0  0  0  0  0  0
  8.1858    9.7353    1.4718 C   0  0  0  0  0  0  0  0  0  0  0  0
  8.1848    6.8347   -3.3503 H   0  0  0  0  0  0  0  0  0  0  0  0
  8.6384    7.5325   -1.7898 H   0  0  0  0  0  0  0  0  0  0  0  0
  6.1774    7.9206   -1.4280 H   0  0  0  0  0  0  0  0  0  0  0  0
  5.9446    7.1900   -3.0216 H   0  0  0  0  0  0  0  0  0  0  0  0
  6.4185    5.5257    0.1897 H   0  0  0  0  0  0  0  0  0  0  0  0
  8.1466    5.2486   -0.0640 H   0  0  0  0  0  0  0  0  0  0  0  0
 10.9487    6.9411   -2.9994 H   0  0  0  0  0  0  0  0  0  0  0  0
  8.1698    4.0711   -1.4355 H   0  0  0  0  0  0  0  0  0  0  0  0
  5.6595    4.4670   -1.0693 H   0  0  0  0  0  0  0  0  0  0  0  0
  3.5549    8.1078   -1.9181 H   0  0  0  0  0  0  0  0  0  0  0  0
  7.7977    6.4752    2.3460 H   0  0  0  0  0  0  0  0  0  0  0  0
  7.5104    8.2952   -1.5304 H   0  0  0  0  0  0  0  0  0  0  0  0
 12.8049    5.3374   -2.6465 H   0  0  0  0  0  0  0  0  0  0  0  0
 10.0247    2.4661   -1.0821 H   0  0  0  0  0  0  0  0  0  0  0  0
  3.5828    3.4814   -0.1431 H   0  0  0  0  0  0  0  0  0  0  0  0
  1.4774    7.1239   -0.9920 H   0  0  0  0  0  0  0  0  0  0  0  0
  8.2980    8.6861    3.3470 H   0  0  0  0  0  0  0  0  0  0  0  0
  8.0107   10.5062   -0.5294 H   0  0  0  0  0  0  0  0  0  0  0  0
 12.3427    3.0996   -1.6876 H   0  0  0  0  0  0  0  0  0  0  0  0
  1.4916    4.8103   -0.1043 H   0  0  0  0  0  0  0  0  0  0  0  0
  8.4045   10.7016    1.9093 H   0  0  0  0  0  0  0  0  0  0  0  0
  1  2  1  0  0  0  0
  1  3  1  0  0  0  0
  1  4  1  0  0  0  0
  2  5  1  0  0  0  0
  3  6  1  0  0  0  0
  4  7  1  0  0  0  0
  5  8  4  0  0  0  0
  5  9  4  0  0  0  0
  6 10  4  0  0  0  0
  6 11  4  0  0  0  0
  7 12  4  0  0  0  0
  7 13  4  0  0  0  0
  8 14  4  0  0  0  0
  9 15  4  0  0  0  0
 10 16  4  0  0  0  0
 11 17  4  0  0  0  0
 12 18  4  0  0  0  0
 13 19  4  0  0  0  0
 14 20  4  0  0  0  0
 15 20  4  0  0  0  0
 16 21  4  0  0  0  0
 17 21  4  0  0  0  0
 18 22  4  0  0  0  0
 19 22  4  0  0  0  0
  2 23  1  0  0  0  0
  2 24  1  0  0  0  0
  3 25  1  0  0  0  0
  3 26  1  0  0  0  0
```

```
  4 27  1  0  0  0  0
  4 28  1  0  0  0  0
  8 29  1  0  0  0  0
  9 30  1  0  0  0  0
 10 31  1  0  0  0  0
 11 32  1  0  0  0  0
 12 33  1  0  0  0  0
 13 34  1  0  0  0  0
 14 35  1  0  0  0  0
 15 36  1  0  0  0  0
 16 37  1  0  0  0  0
 17 38  1  0  0  0  0
 18 39  1  0  0  0  0
 19 40  1  0  0  0  0
 20 41  1  0  0  0  0
 21 42  1  0  0  0  0
 22 43  1  0  0  0  0
M  END
>  <Alias name>
Tribenzylamine

>  <deltaH°(c)>   kJ/mol
-11406

$$$$
Stearic peroxyacid
  ChemBrain IXL

 57 56  0  0  0  0  0  0  0  0  1 V2000
   13.5764   11.9438   -0.0859 C   0  0  0  0  0  0  0  0  0  0  0  0
   12.1918   12.6023    0.0695 C   0  0  0  0  0  0  0  0  0  0  0  0
   11.0931   11.5319   -0.0773 C   0  0  0  0  0  0  0  0  0  0  0  0
    9.7095   12.1930    0.0765 C   0  0  0  0  0  0  0  0  0  0  0  0
    8.6092   11.1237   -0.0670 C   0  0  0  0  0  0  0  0  0  0  0  0
    7.2264   11.7866    0.0861 C   0  0  0  0  0  0  0  0  0  0  0  0
    6.1252   10.7179   -0.0543 C   0  0  0  0  0  0  0  0  0  0  0  0
    4.7428   11.3819    0.0983 C   0  0  0  0  0  0  0  0  0  0  0  0
    3.6411   10.3132   -0.0402 C   0  0  0  0  0  0  0  0  0  0  0  0
   14.6732   13.0158    0.0635 C   0  0  0  0  0  0  0  0  0  0  0  0
    2.2589   10.9775    0.1120 C   0  0  0  0  0  0  0  0  0  0  0  0
   16.0590   12.3606   -0.0944 C   0  0  0  0  0  0  0  0  0  0  0  0
   17.1537   13.4346    0.0565 C   0  0  0  0  0  0  0  0  0  0  0  0
   18.5406   12.7827   -0.1045 C   0  0  0  0  0  0  0  0  0  0  0  0
   19.6334   13.8587    0.0467 C   0  0  0  0  0  0  0  0  0  0  0  0
   21.0212   13.2095   -0.1171 C   0  0  0  0  0  0  0  0  0  0  0  0
   22.1132   14.2875    0.0338 C   0  0  0  0  0  0  0  0  0  0  0  0
   23.4485   13.6666   -0.1249 C   0  0  0  0  0  0  0  0  0  0  0  0
   24.4537   14.3517   -0.0314 O   0  0  0  0  0  0  0  0  0  0  0  0
   23.5633   12.2247   -0.3950 O   0  0  0  0  0  0  0  0  0  0  0  0
   24.9483   11.7212   -0.5330 O   0  0  0  0  0  0  0  0  0  0  0  0
   25.2051   11.0287    0.1156 H   0  0  0  0  0  0  0  0  0  0  0  0
   13.7044   11.1872    0.6783 H   0  0  0  0  0  0  0  0  0  0  0  0
   13.6505   11.4852   -1.0643 H   0  0  0  0  0  0  0  0  0  0  0  0
   12.0638   13.3580   -0.6956 H   0  0  0  0  0  0  0  0  0  0  0  0
   12.1188   13.0625    1.0471 H   0  0  0  0  0  0  0  0  0  0  0  0
   11.2198   10.7769    0.6887 H   0  0  0  0  0  0  0  0  0  0  0  0
   11.1663   11.0707   -1.0545 H   0  0  0  0  0  0  0  0  0  0  0  0
    9.5824   12.9467   -0.6908 H   0  0  0  0  0  0  0  0  0  0  0  0
    9.6374   12.6561    1.0528 H   0  0  0  0  0  0  0  0  0  0  0  0
    8.7355   10.3705    0.7009 H   0  0  0  0  0  0  0  0  0  0  0  0
    8.6814   10.6599   -1.0430 H   0  0  0  0  0  0  0  0  0  0  0  0
    7.0997   12.5386   -0.6829 H   0  0  0  0  0  0  0  0  0  0  0  0
    7.1553   12.2521    1.0613 H   0  0  0  0  0  0  0  0  0  0  0  0
```

```
   6.2516    9.9662    0.7151 H   0  0  0  0  0  0  0  0  0  0  0  0
   6.1964   10.2520   -1.0294 H   0  0  0  0  0  0  0  0  0  0  0  0
   4.6162   12.1328   -0.6718 H   0  0  0  0  0  0  0  0  0  0  0  0
   4.6723   11.8488    1.0729 H   0  0  0  0  0  0  0  0  0  0  0  0
   3.7675    9.5625    0.7300 H   0  0  0  0  0  0  0  0  0  0  0  0
   3.7117    9.8461   -1.0147 H   0  0  0  0  0  0  0  0  0  0  0  0
  14.5435   13.7735   -0.6993 H   0  0  0  0  0  0  0  0  0  0  0  0
  14.5998   13.4731    1.0425 H   0  0  0  0  0  0  0  0  0  0  0  0
   1.4849   10.2264    0.0149 H   0  0  0  0  0  0  0  0  0  0  0  0
   2.1883   11.4448    1.0864 H   0  0  0  0  0  0  0  0  0  0  0  0
   2.1323   11.7280   -0.6584 H   0  0  0  0  0  0  0  0  0  0  0  0
  16.1892   11.6025    0.6681 H   0  0  0  0  0  0  0  0  0  0  0  0
  16.1332   11.9043   -1.0738 H   0  0  0  0  0  0  0  0  0  0  0  0
  17.0216   14.1939   -0.7044 H   0  0  0  0  0  0  0  0  0  0  0  0
  17.0804   13.8894    1.0366 H   0  0  0  0  0  0  0  0  0  0  0  0
  18.6735   12.0236    0.6565 H   0  0  0  0  0  0  0  0  0  0  0  0
  18.6144   12.3282   -1.0847 H   0  0  0  0  0  0  0  0  0  0  0  0
  19.4988   14.6188   -0.7129 H   0  0  0  0  0  0  0  0  0  0  0  0
  19.5605   14.3119    1.0276 H   0  0  0  0  0  0  0  0  0  0  0  0
  21.1563   12.4496    0.6428 H   0  0  0  0  0  0  0  0  0  0  0  0
  21.0942   12.7563   -1.0980 H   0  0  0  0  0  0  0  0  0  0  0  0
  21.9767   15.0479   -0.7251 H   0  0  0  0  0  0  0  0  0  0  0  0
  22.0402   14.7400    1.0150 H   0  0  0  0  0  0  0  0  0  0  0  0
  1  2  1  0  0  0  0
  2  3  1  0  0  0  0
  3  4  1  0  0  0  0
  4  5  1  0  0  0  0
  5  6  1  0  0  0  0
  6  7  1  0  0  0  0
  7  8  1  0  0  0  0
  8  9  1  0  0  0  0
  1 10  1  0  0  0  0
  9 11  1  0  0  0  0
 12 13  1  0  0  0  0
 13 14  1  0  0  0  0
 14 15  1  0  0  0  0
 15 16  1  0  0  0  0
 16 17  1  0  0  0  0
 17 18  1  0  0  0  0
 10 12  1  0  0  0  0
 18 19  2  0  0  0  0
 18 20  1  0  0  0  0
 20 21  1  0  0  0  0
 21 22  1  0  0  0  0
  1 23  1  0  0  0  0
  1 24  1  0  0  0  0
  2 25  1  0  0  0  0
  2 26  1  0  0  0  0
  3 27  1  0  0  0  0
  3 28  1  0  0  0  0
  4 29  1  0  0  0  0
  4 30  1  0  0  0  0
  5 31  1  0  0  0  0
  5 32  1  0  0  0  0
  6 33  1  0  0  0  0
  6 34  1  0  0  0  0
  7 35  1  0  0  0  0
  7 36  1  0  0  0  0
  8 37  1  0  0  0  0
  8 38  1  0  0  0  0
  9 39  1  0  0  0  0
  9 40  1  0  0  0  0
 10 41  1  0  0  0  0
```

```
 10 42  1  0  0  0  0
 11 43  1  0  0  0  0
 11 44  1  0  0  0  0
 11 45  1  0  0  0  0
 12 46  1  0  0  0  0
 12 47  1  0  0  0  0
 13 48  1  0  0  0  0
 13 49  1  0  0  0  0
 14 50  1  0  0  0  0
 14 51  1  0  0  0  0
 15 52  1  0  0  0  0
 15 53  1  0  0  0  0
 16 54  1  0  0  0  0
 16 55  1  0  0  0  0
 17 56  1  0  0  0  0
 17 57  1  0  0  0  0
M  END
>  <Alias name>
Peroxyoctadecanoic acid

>  <deltaH°(c)>   kJ/mol
-11378

$$$$
t-Butyl tetradecanoate
  ChemBrain IXL

 56 55  0  0  0  0  0  0  0  0  1 V2000
   22.3507   11.2947    0.4772 C   0  0  0  0  0  0  0  0  0  0  0  0
   21.0616   11.0684   -0.3361 C   0  0  0  0  0  0  0  0  0  0  0  0
   19.8366   11.2593    0.5791 C   0  0  0  0  0  0  0  0  0  0  0  0
   18.5473   11.0414   -0.2362 C   0  0  0  0  0  0  0  0  0  0  0  0
   17.3222   11.2380    0.6777 C   0  0  0  0  0  0  0  0  0  0  0  0
   16.0326   11.0400   -0.1423 C   0  0  0  0  0  0  0  0  0  0  0  0
   14.8074   11.2502    0.7685 C   0  0  0  0  0  0  0  0  0  0  0  0
   13.5179   11.0821   -0.0582 C   0  0  0  0  0  0  0  0  0  0  0  0
   12.2930   11.3126    0.8481 C   0  0  0  0  0  0  0  0  0  0  0  0
   11.0043   11.1790    0.0137 C   0  0  0  0  0  0  0  0  0  0  0  0
    9.7803   11.4324    0.9151 C   0  0  0  0  0  0  0  0  0  0  0  0
    8.4932   11.3312    0.0738 C   0  0  0  0  0  0  0  0  0  0  0  0
    7.2703   11.6026    0.9714 C   0  0  0  0  0  0  0  0  0  0  0  0
    6.0356   11.5283    0.1586 C   0  0  0  0  0  0  0  0  0  0  0  0
    6.1074   11.5044   -1.0590 O   0  0  0  0  0  0  0  0  0  0  0  0
    4.7229   11.4835    0.8185 O   0  0  0  0  0  0  0  0  0  0  0  0
    3.5720   11.4020   -0.0924 C   0  0  0  0  0  0  0  0  0  0  0  0
    3.6762   10.1180   -0.9381 C   0  0  0  0  0  0  0  0  0  0  0  0
    3.5653   12.6305   -1.0227 C   0  0  0  0  0  0  0  0  0  0  0  0
    2.2682   11.3721    0.7284 C   0  0  0  0  0  0  0  0  0  0  0  0
   22.3530   12.3009    0.8777 H   0  0  0  0  0  0  0  0  0  0  0  0
   23.2117   11.1602   -0.1659 H   0  0  0  0  0  0  0  0  0  0  0  0
   22.3936   10.5820    1.2915 H   0  0  0  0  0  0  0  0  0  0  0  0
   21.0186   11.7813   -1.1502 H   0  0  0  0  0  0  0  0  0  0  0  0
   21.0595   10.0622   -0.7368 H   0  0  0  0  0  0  0  0  0  0  0  0
   19.8765   10.5424    1.3899 H   0  0  0  0  0  0  0  0  0  0  0  0
   19.8420   12.2635    0.9846 H   0  0  0  0  0  0  0  0  0  0  0  0
   18.5093   11.7556   -1.0494 H   0  0  0  0  0  0  0  0  0  0  0  0
   18.5400   10.0358   -0.6383 H   0  0  0  0  0  0  0  0  0  0  0  0
   17.3527   10.5144    1.4829 H   0  0  0  0  0  0  0  0  0  0  0  0
   17.3371   12.2389    1.0911 H   0  0  0  0  0  0  0  0  0  0  0  0
   16.0068   11.7573   -0.9533 H   0  0  0  0  0  0  0  0  0  0  0  0
   16.0131   10.0360   -0.5476 H   0  0  0  0  0  0  0  0  0  0  0  0
   14.8219   10.5192    1.5675 H   0  0  0  0  0  0  0  0  0  0  0  0
   14.8382   12.2470    1.1907 H   0  0  0  0  0  0  0  0  0  0  0  0
```

13.5102	11.8035	-0.8659	H	0	0	0	0	0	0	0	0	0	0	0	0	0
13.4801	10.0805	-0.4684	H	0	0	0	0	0	0	0	0	0	0	0	0	0
12.2873	10.5756	1.6416	H	0	0	0	0	0	0	0	0	0	0	0	0	0
12.3436	12.3054	1.2778	H	0	0	0	0	0	0	0	0	0	0	0	0	0
11.0175	11.9051	-0.7897	H	0	0	0	0	0	0	0	0	0	0	0	0	0
10.9456	10.1810	-0.4026	H	0	0	0	0	0	0	0	0	0	0	0	0	0
9.7544	10.6921	1.7051	H	0	0	0	0	0	0	0	0	0	0	0	0	0
9.8507	12.4219	1.3497	H	0	0	0	0	0	0	0	0	0	0	0	0	0
8.5249	12.0628	-0.7240	H	0	0	0	0	0	0	0	0	0	0	0	0	0
8.4163	10.3376	-0.3503	H	0	0	0	0	0	0	0	0	0	0	0	0	0
7.3556	12.5895	1.4090	H	0	0	0	0	0	0	0	0	0	0	0	0	0
7.2290	10.8606	1.7592	H	0	0	0	0	0	0	0	0	0	0	0	0	0
4.5892	10.1421	-1.5201	H	0	0	0	0	0	0	0	0	0	0	0	0	0
3.6878	9.2549	-0.2840	H	0	0	0	0	0	0	0	0	0	0	0	0	0
2.8249	10.0545	-1.6045	H	0	0	0	0	0	0	0	0	0	0	0	0	0
2.7133	12.5742	-1.6889	H	0	0	0	0	0	0	0	0	0	0	0	0	0
4.4783	12.6475	-1.6049	H	0	0	0	0	0	0	0	0	0	0	0	0	0
3.4995	13.5333	-0.4280	H	0	0	0	0	0	0	0	0	0	0	0	0	0
2.2730	10.5087	1.3822	H	0	0	0	0	0	0	0	0	0	0	0	0	0
2.1950	12.2745	1.3227	H	0	0	0	0	0	0	0	0	0	0	0	0	0
1.4204	11.3120	0.0573	H	0	0	0	0	0	0	0	0	0	0	0	0	0

1	2	1	0	0	0	0
2	3	1	0	0	0	0
3	4	1	0	0	0	0
4	5	1	0	0	0	0
5	6	1	0	0	0	0
6	7	1	0	0	0	0
7	8	1	0	0	0	0
8	9	1	0	0	0	0
9	10	1	0	0	0	0
10	11	1	0	0	0	0
11	12	1	0	0	0	0
12	13	1	0	0	0	0
14	15	2	0	0	0	0
14	16	1	0	0	0	0
14	13	1	0	0	0	0
16	17	1	0	0	0	0
17	18	1	0	0	0	0
17	19	1	0	0	0	0
17	20	1	0	0	0	0
1	21	1	0	0	0	0
1	22	1	0	0	0	0
1	23	1	0	0	0	0
2	24	1	0	0	0	0
2	25	1	0	0	0	0
3	26	1	0	0	0	0
3	27	1	0	0	0	0
4	28	1	0	0	0	0
4	29	1	0	0	0	0
5	30	1	0	0	0	0
5	31	1	0	0	0	0
6	32	1	0	0	0	0
6	33	1	0	0	0	0
7	34	1	0	0	0	0
7	35	1	0	0	0	0
8	36	1	0	0	0	0
8	37	1	0	0	0	0
9	38	1	0	0	0	0
9	39	1	0	0	0	0
10	40	1	0	0	0	0
10	41	1	0	0	0	0
11	42	1	0	0	0	0
11	43	1	0	0	0	0

```
 12 44  1  0  0  0  0
 12 45  1  0  0  0  0
 13 46  1  0  0  0  0
 13 47  1  0  0  0  0
 18 48  1  0  0  0  0
 18 49  1  0  0  0  0
 18 50  1  0  0  0  0
 19 51  1  0  0  0  0
 19 52  1  0  0  0  0
 19 53  1  0  0  0  0
 20 54  1  0  0  0  0
 20 55  1  0  0  0  0
 20 56  1  0  0  0  0
M  END
>  <Alias name>
t-Butyl tetradecanoate

>  <deltaH°(c)>   kJ/mol
-11373.6

$$$$
Ethyl palmitate
  ChemBrain IXL

 56 55  0  0  0  0  0  0  0  0  1 V2000
   19.7510   12.4036   -2.6071 C   0  0  0  0  0  0  0  0  0  0  0  0
   20.9537   11.8704   -3.2630 O   0  0  0  0  0  0  0  0  0  0  0  0
   18.6089   11.5088   -2.3152 C   0  0  0  0  0  0  0  0  0  0  0  0
   19.6943   13.5839   -2.3034 O   0  0  0  0  0  0  0  0  0  0  0  0
   20.9026   10.4366   -3.5834 C   0  0  0  0  0  0  0  0  0  0  0  0
   17.3851   12.3532   -1.9105 C   0  0  0  0  0  0  0  0  0  0  0  0
   22.2277   10.0105   -4.2445 C   0  0  0  0  0  0  0  0  0  0  0  0
   16.2103   11.4197   -1.5594 C   0  0  0  0  0  0  0  0  0  0  0  0
   15.0016   12.2624   -1.1083 C   0  0  0  0  0  0  0  0  0  0  0  0
   13.8492   11.3270   -0.6941 C   0  0  0  0  0  0  0  0  0  0  0  0
   12.6561   12.1687   -0.2015 C   0  0  0  0  0  0  0  0  0  0  0  0
   11.5248   11.2323    0.2654 C   0  0  0  0  0  0  0  0  0  0  0  0
   10.3418   12.0737    0.7822 C   0  0  0  0  0  0  0  0  0  0  0  0
    9.2241   11.1371    1.2804 C   0  0  0  0  0  0  0  0  0  0  0  0
    8.0459   11.9785    1.8081 C   0  0  0  0  0  0  0  0  0  0  0  0
    6.9337   11.0418    2.3184 C   0  0  0  0  0  0  0  0  0  0  0  0
    5.7577   11.8833    2.8511 C   0  0  0  0  0  0  0  0  0  0  0  0
    4.6463   10.9466    3.3628 C   0  0  0  0  0  0  0  0  0  0  0  0
    3.4712   11.7880    3.8976 C   0  0  0  0  0  0  0  0  0  0  0  0
    2.3592   10.8513    4.4081 C   0  0  0  0  0  0  0  0  0  0  0  0
   18.8714   10.8414   -1.5037 H   0  0  0  0  0  0  0  0  0  0  0  0
   18.3708   10.9278   -3.1977 H   0  0  0  0  0  0  0  0  0  0  0  0
   20.0822   10.2480   -4.2648 H   0  0  0  0  0  0  0  0  0  0  0  0
   20.7540    9.8685   -2.6735 H   0  0  0  0  0  0  0  0  0  0  0  0
   17.1015   12.9960   -2.7346 H   0  0  0  0  0  0  0  0  0  0  0  0
   17.6341   12.9598   -1.0485 H   0  0  0  0  0  0  0  0  0  0  0  0
   22.3751   10.5768   -5.1557 H   0  0  0  0  0  0  0  0  0  0  0  0
   22.1911    8.9536   -4.4782 H   0  0  0  0  0  0  0  0  0  0  0  0
   23.0484   10.2015   -3.5640 H   0  0  0  0  0  0  0  0  0  0  0  0
   16.5054   10.7538   -0.7579 H   0  0  0  0  0  0  0  0  0  0  0  0
   15.9391   10.8372   -2.4312 H   0  0  0  0  0  0  0  0  0  0  0  0
   14.6773   12.8948   -1.9255 H   0  0  0  0  0  0  0  0  0  0  0  0
   15.2863   12.8796   -0.2652 H   0  0  0  0  0  0  0  0  0  0  0  0
   14.1832   10.6734    0.1022 H   0  0  0  0  0  0  0  0  0  0  0  0
   13.5437   10.7315   -1.5456 H   0  0  0  0  0  0  0  0  0  0  0  0
   12.2970   12.7937   -1.0098 H   0  0  0  0  0  0  0  0  0  0  0  0
   12.9718   12.7934    0.6250 H   0  0  0  0  0  0  0  0  0  0  0  0
   11.8893   10.5947    1.0614 H   0  0  0  0  0  0  0  0  0  0  0  0
```

11.1967 10.6203 -0.5657 H 0 0 0 0 0 0 0 0 0 0 0 0
9.9621 12.6939 -0.0203 H 0 0 0 0 0 0 0 0 0 0 0 0
10.6756 12.7032 1.5978 H 0 0 0 0 0 0 0 0 0 0 0 0
9.6062 10.5111 2.0773 H 0 0 0 0 0 0 0 0 0 0 0 0
8.8846 10.5134 0.4627 H 0 0 0 0 0 0 0 0 0 0 0 0
7.6579 12.5977 1.0088 H 0 0 0 0 0 0 0 0 0 0 0 0
8.3876 12.6089 2.6198 H 0 0 0 0 0 0 0 0 0 0 0 0
7.3228 10.4200 3.1152 H 0 0 0 0 0 0 0 0 0 0 0 0
6.5895 10.4140 1.5059 H 0 0 0 0 0 0 0 0 0 0 0 0
5.3679 12.5042 2.0539 H 0 0 0 0 0 0 0 0 0 0 0 0
6.1022 12.5120 3.6628 H 0 0 0 0 0 0 0 0 0 0 0 0
5.0365 10.3245 4.1588 H 0 0 0 0 0 0 0 0 0 0 0 0
4.3007 10.3190 2.5507 H 0 0 0 0 0 0 0 0 0 0 0 0
3.0816 12.4108 3.1019 H 0 0 0 0 0 0 0 0 0 0 0 0
3.8166 12.4148 4.7105 H 0 0 0 0 0 0 0 0 0 0 0 0
2.7489 10.2284 5.2038 H 0 0 0 0 0 0 0 0 0 0 0 0
1.5334 11.4426 4.7841 H 0 0 0 0 0 0 0 0 0 0 0 0
2.0137 10.2245 3.5953 H 0 0 0 0 0 0 0 0 0 0 0 0
1 2 1 0 0 0 0
1 3 1 0 0 0 0
1 4 2 0 0 0 0
2 5 1 0 0 0 0
3 6 1 0 0 0 0
5 7 1 0 0 0 0
6 8 1 0 0 0 0
8 9 1 0 0 0 0
9 10 1 0 0 0 0
10 11 1 0 0 0 0
11 12 1 0 0 0 0
12 13 1 0 0 0 0
13 14 1 0 0 0 0
14 15 1 0 0 0 0
15 16 1 0 0 0 0
16 17 1 0 0 0 0
17 18 1 0 0 0 0
18 19 1 0 0 0 0
19 20 1 0 0 0 0
3 21 1 0 0 0 0
3 22 1 0 0 0 0
5 23 1 0 0 0 0
5 24 1 0 0 0 0
6 25 1 0 0 0 0
6 26 1 0 0 0 0
7 27 1 0 0 0 0
7 28 1 0 0 0 0
7 29 1 0 0 0 0
8 30 1 0 0 0 0
8 31 1 0 0 0 0
9 32 1 0 0 0 0
9 33 1 0 0 0 0
10 34 1 0 0 0 0
10 35 1 0 0 0 0
11 36 1 0 0 0 0
11 37 1 0 0 0 0
12 38 1 0 0 0 0
12 39 1 0 0 0 0
13 40 1 0 0 0 0
13 41 1 0 0 0 0
14 42 1 0 0 0 0
14 43 1 0 0 0 0
15 44 1 0 0 0 0
15 45 1 0 0 0 0
16 46 1 0 0 0 0

```
 16 47  1  0  0  0  0
 17 48  1  0  0  0  0
 17 49  1  0  0  0  0
 18 50  1  0  0  0  0
 18 51  1  0  0  0  0
 19 52  1  0  0  0  0
 19 53  1  0  0  0  0
 20 54  1  0  0  0  0
 20 55  1  0  0  0  0
 20 56  1  0  0  0  0
M  END
>  <Alias name>
Ethyl hexadecanoate

>  <deltaH°(c)>   kJ/mol
-11368

$$$$
Heptadecane
  ChemBrain IXL

 53 52  0  0  0  0  0  0  0  0  1 V2000
   10.5849    9.4780    0.0692 C   0  0  0  0  0  0  0  0  0  0  0  0
    9.6258   10.3905    0.8581 C   0  0  0  0  0  0  0  0  0  0  0  0
    8.4608    9.5505    1.4168 C   0  0  0  0  0  0  0  0  0  0  0  0
    7.5040   10.4622    2.2094 C   0  0  0  0  0  0  0  0  0  0  0  0
    6.3411    9.6215    2.7714 C   0  0  0  0  0  0  0  0  0  0  0  0
    5.3859   10.5327    3.5667 C   0  0  0  0  0  0  0  0  0  0  0  0
    4.2244    9.6916    4.1308 C   0  0  0  0  0  0  0  0  0  0  0  0
    3.2701   10.6024    4.9274 C   0  0  0  0  0  0  0  0  0  0  0  0
    2.1091    9.7612    5.4924 C   0  0  0  0  0  0  0  0  0  0  0  0
   11.7524   10.3172   -0.4856 C   0  0  0  0  0  0  0  0  0  0  0  0
   12.7140    9.4040   -1.2706 C   0  0  0  0  0  0  0  0  0  0  0  0
   13.8838   10.2424   -1.8216 C   0  0  0  0  0  0  0  0  0  0  0  0
   14.8475    9.3284   -2.6032 C   0  0  0  0  0  0  0  0  0  0  0  0
   16.0189   10.1663   -3.1515 C   0  0  0  0  0  0  0  0  0  0  0  0
   16.9839    9.2519   -3.9310 C   0  0  0  0  0  0  0  0  0  0  0  0
   18.1562   10.0896   -4.4780 C   0  0  0  0  0  0  0  0  0  0  0  0
   19.1217    9.1751   -5.2567 C   0  0  0  0  0  0  0  0  0  0  0  0
   10.0519    9.0172   -0.7493 H   0  0  0  0  0  0  0  0  0  0  0  0
   10.9721    8.7112    0.7238 H   0  0  0  0  0  0  0  0  0  0  0  0
    9.2363   11.1557    0.2031 H   0  0  0  0  0  0  0  0  0  0  0  0
   10.1598   10.8533    1.6749 H   0  0  0  0  0  0  0  0  0  0  0  0
    7.9251    9.0896    0.6000 H   0  0  0  0  0  0  0  0  0  0  0  0
    8.8507    8.7837    2.0697 H   0  0  0  0  0  0  0  0  0  0  0  0
    8.0405   10.9247    3.0247 H   0  0  0  0  0  0  0  0  0  0  0  0
    7.1121   11.2277    1.5560 H   0  0  0  0  0  0  0  0  0  0  0  0
    5.8033    9.1604    1.9562 H   0  0  0  0  0  0  0  0  0  0  0  0
    6.7333    8.8549    3.4232 H   0  0  0  0  0  0  0  0  0  0  0  0
    4.9925   11.2984    2.9146 H   0  0  0  0  0  0  0  0  0  0  0  0
    5.9242   10.9949    4.3810 H   0  0  0  0  0  0  0  0  0  0  0  0
    3.6855    9.2300    3.3166 H   0  0  0  0  0  0  0  0  0  0  0  0
    4.6180    8.9253    4.7822 H   0  0  0  0  0  0  0  0  0  0  0  0
    2.8760   11.3684    4.2760 H   0  0  0  0  0  0  0  0  0  0  0  0
    3.8092   11.0644    5.7413 H   0  0  0  0  0  0  0  0  0  0  0  0
    2.5032    8.9952    6.1438 H   0  0  0  0  0  0  0  0  0  0  0  0
    1.5700    9.2992    4.6785 H   0  0  0  0  0  0  0  0  0  0  0  0
    1.4404   10.3996    6.0507 H   0  0  0  0  0  0  0  0  0  0  0  0
   12.2836   10.7801    0.3330 H   0  0  0  0  0  0  0  0  0  0  0  0
   11.3657   11.0824   -1.1423 H   0  0  0  0  0  0  0  0  0  0  0  0
   13.0986    8.6373   -0.6142 H   0  0  0  0  0  0  0  0  0  0  0  0
   12.1837    8.9429   -2.0907 H   0  0  0  0  0  0  0  0  0  0  0  0
   14.4125   10.7052   -1.0014 H   0  0  0  0  0  0  0  0  0  0  0  0
```

```
  13.4997   11.0076   -2.4798 H   0  0  0  0  0  0  0  0  0  0  0  0
  15.2300    8.5622   -1.9453 H   0  0  0  0  0  0  0  0  0  0  0  0
  14.3194    8.8670   -3.4246 H   0  0  0  0  0  0  0  0  0  0  0  0
  15.6367   10.9317   -3.8107 H   0  0  0  0  0  0  0  0  0  0  0  0
  16.5460   10.6289   -2.3302 H   0  0  0  0  0  0  0  0  0  0  0  0
  17.3655    8.4861   -3.2720 H   0  0  0  0  0  0  0  0  0  0  0  0
  16.4572    8.7900   -4.7530 H   0  0  0  0  0  0  0  0  0  0  0  0
  18.6825   10.5520   -3.6561 H   0  0  0  0  0  0  0  0  0  0  0  0
  17.7748   10.8551   -5.1375 H   0  0  0  0  0  0  0  0  0  0  0  0
  19.9432    9.7621   -5.6399 H   0  0  0  0  0  0  0  0  0  0  0  0
  19.5031    8.4095   -4.5972 H   0  0  0  0  0  0  0  0  0  0  0  0
  18.5954    8.7126   -6.0786 H   0  0  0  0  0  0  0  0  0  0  0  0
  1  2  1  0  0  0  0
  2  3  1  0  0  0  0
  3  4  1  0  0  0  0
  4  5  1  0  0  0  0
  5  6  1  0  0  0  0
  6  7  1  0  0  0  0
  7  8  1  0  0  0  0
  8  9  1  0  0  0  0
  1 18  1  0  0  0  0
  1 19  1  0  0  0  0
  1 10  1  0  0  0  0
  2 20  1  0  0  0  0
  2 21  1  0  0  0  0
  3 22  1  0  0  0  0
  3 23  1  0  0  0  0
  4 24  1  0  0  0  0
  4 25  1  0  0  0  0
  5 26  1  0  0  0  0
  5 27  1  0  0  0  0
  6 28  1  0  0  0  0
  6 29  1  0  0  0  0
  7 30  1  0  0  0  0
  7 31  1  0  0  0  0
  8 32  1  0  0  0  0
  8 33  1  0  0  0  0
  9 34  1  0  0  0  0
  9 35  1  0  0  0  0
  9 36  1  0  0  0  0
 10 37  1  0  0  0  0
 10 38  1  0  0  0  0
 11 12  1  0  0  0  0
 12 13  1  0  0  0  0
 13 14  1  0  0  0  0
 14 15  1  0  0  0  0
 15 16  1  0  0  0  0
 16 17  1  0  0  0  0
 11 39  1  0  0  0  0
 11 40  1  0  0  0  0
 12 41  1  0  0  0  0
 12 42  1  0  0  0  0
 13 43  1  0  0  0  0
 13 44  1  0  0  0  0
 14 45  1  0  0  0  0
 14 46  1  0  0  0  0
 15 47  1  0  0  0  0
 15 48  1  0  0  0  0
 16 49  1  0  0  0  0
 16 50  1  0  0  0  0
 17 51  1  0  0  0  0
 17 52  1  0  0  0  0
 17 53  1  0  0  0  0
```

```
 10 11  1  0  0  0  0
M  END
>  <Alias name>
Heptadecane

>  <deltaH°(c)>   kJ/mol
-11351

$$$$
n-Octadecanoic acid
  ChemBrain IXL

 56 55  0  0  0  0  0  0  0  0  1 V2000
   11.9079   10.5766   -0.1479 C   0  0  0  0  0  0  0  0  0  0  0  0  0
   10.8359   11.6304    0.1913 C   0  0  0  0  0  0  0  0  0  0  0  0  0
    9.7507   10.9929    1.0805 C   0  0  0  0  0  0  0  0  0  0  0  0  0
    8.6795   12.0480    1.4184 C   0  0  0  0  0  0  0  0  0  0  0  0  0
    7.5932   11.4120    2.3074 C   0  0  0  0  0  0  0  0  0  0  0  0  0
    6.5227   12.4682    2.6443 C   0  0  0  0  0  0  0  0  0  0  0  0  0
    5.4358   11.8333    3.5332 C   0  0  0  0  0  0  0  0  0  0  0  0  0
    4.3657   12.8901    3.8695 C   0  0  0  0  0  0  0  0  0  0  0  0  0
    3.2783   12.2557    4.7585 C   0  0  0  0  0  0  0  0  0  0  0  0  0
   12.9917   11.2158   -1.0376 C   0  0  0  0  0  0  0  0  0  0  0  0  0
    2.2084   13.3126    5.0946 C   0  0  0  0  0  0  0  0  0  0  0  0  0
   14.0646   10.1636   -1.3785 C   0  0  0  0  0  0  0  0  0  0  0  0  0
   15.1468   10.8046   -2.2689 C   0  0  0  0  0  0  0  0  0  0  0  0  0
   16.2204    9.7536   -2.6114 C   0  0  0  0  0  0  0  0  0  0  0  0  0
   17.3012   10.3961   -3.5025 C   0  0  0  0  0  0  0  0  0  0  0  0  0
   18.3753    9.3461   -3.8462 C   0  0  0  0  0  0  0  0  0  0  0  0  0
   19.4560    9.9896   -4.7381 C   0  0  0  0  0  0  0  0  0  0  0  0  0
   20.4897    8.9819   -5.0698 C   0  0  0  0  0  0  0  0  0  0  0  0  0
   21.4354    9.2888   -5.7767 O   0  0  0  0  0  0  0  0  0  0  0  0  0
   20.3771    7.6115   -4.5475 O   0  0  0  0  0  0  0  0  0  0  0  0  0
   21.1029    7.0045   -4.8135 H   0  0  0  0  0  0  0  0  0  0  0  0  0
   12.3596   10.2135    0.7670 H   0  0  0  0  0  0  0  0  0  0  0  0  0
   11.4480    9.7506   -0.6762 H   0  0  0  0  0  0  0  0  0  0  0  0  0
   10.3849   11.9938   -0.7238 H   0  0  0  0  0  0  0  0  0  0  0  0  0
   11.2960   12.4562    0.7196 H   0  0  0  0  0  0  0  0  0  0  0  0  0
   10.2013   10.6298    1.9959 H   0  0  0  0  0  0  0  0  0  0  0  0  0
    9.2902   10.1671    0.5523 H   0  0  0  0  0  0  0  0  0  0  0  0  0
    8.2294   12.4113    0.5028 H   0  0  0  0  0  0  0  0  0  0  0  0  0
    9.1402   12.8737    1.9465 H   0  0  0  0  0  0  0  0  0  0  0  0  0
    8.0431   11.0489    3.2232 H   0  0  0  0  0  0  0  0  0  0  0  0  0
    7.1323   10.5864    1.7794 H   0  0  0  0  0  0  0  0  0  0  0  0  0
    6.0732   12.8313    1.7283 H   0  0  0  0  0  0  0  0  0  0  0  0  0
    6.9838   13.2938    3.1721 H   0  0  0  0  0  0  0  0  0  0  0  0  0
    5.8851   11.4702    4.4492 H   0  0  0  0  0  0  0  0  0  0  0  0  0
    4.9745   11.0077    3.0054 H   0  0  0  0  0  0  0  0  0  0  0  0  0
    3.9165   13.2532    2.9534 H   0  0  0  0  0  0  0  0  0  0  0  0  0
    4.8271   13.7157    4.3972 H   0  0  0  0  0  0  0  0  0  0  0  0  0
    3.7274   11.8927    5.6746 H   0  0  0  0  0  0  0  0  0  0  0  0  0
    2.8169   11.4302    4.2308 H   0  0  0  0  0  0  0  0  0  0  0  0  0
   12.5396   11.5794   -1.9521 H   0  0  0  0  0  0  0  0  0  0  0  0  0
   13.4514   12.0418   -0.5091 H   0  0  0  0  0  0  0  0  0  0  0  0  0
    1.4444   12.8668    5.7193 H   0  0  0  0  0  0  0  0  0  0  0  0  0
    2.6698   14.1382    5.6224 H   0  0  0  0  0  0  0  0  0  0  0  0  0
    1.7592   13.6757    4.1785 H   0  0  0  0  0  0  0  0  0  0  0  0  0
   14.5176    9.8004   -0.4643 H   0  0  0  0  0  0  0  0  0  0  0  0  0
   13.6051    9.3373   -1.9068 H   0  0  0  0  0  0  0  0  0  0  0  0  0
   14.6933   11.1682   -3.1827 H   0  0  0  0  0  0  0  0  0  0  0  0  0
   15.6062   11.6307   -1.7404 H   0  0  0  0  0  0  0  0  0  0  0  0  0
   16.6747    9.3905   -1.6978 H   0  0  0  0  0  0  0  0  0  0  0  0  0
   15.7612    8.9272   -3.1397 H   0  0  0  0  0  0  0  0  0  0  0  0  0
```

```
   16.8467   10.7596   -4.4159 H   0  0  0  0  0  0  0  0  0  0  0  0
   17.7603   11.2224   -2.9741 H   0  0  0  0  0  0  0  0  0  0  0  0
   18.8301    8.9828   -2.9329 H   0  0  0  0  0  0  0  0  0  0  0  0
   17.9162    8.5196   -4.3745 H   0  0  0  0  0  0  0  0  0  0  0  0
   19.9146   10.8162   -4.2097 H   0  0  0  0  0  0  0  0  0  0  0  0
   19.0008   10.3531   -5.6510 H   0  0  0  0  0  0  0  0  0  0  0  0
  1  2  1  0  0  0  0
  2  3  1  0  0  0  0
  3  4  1  0  0  0  0
  4  5  1  0  0  0  0
  5  6  1  0  0  0  0
  6  7  1  0  0  0  0
  7  8  1  0  0  0  0
  8  9  1  0  0  0  0
  1 10  1  0  0  0  0
  9 11  1  0  0  0  0
 12 13  1  0  0  0  0
 13 14  1  0  0  0  0
 14 15  1  0  0  0  0
 15 16  1  0  0  0  0
 16 17  1  0  0  0  0
 17 18  1  0  0  0  0
 10 12  1  0  0  0  0
 18 19  2  0  0  0  0
 18 20  1  0  0  0  0
 20 21  1  0  0  0  0
  1 22  1  0  0  0  0
  1 23  1  0  0  0  0
  2 24  1  0  0  0  0
  2 25  1  0  0  0  0
  3 26  1  0  0  0  0
  3 27  1  0  0  0  0
  4 28  1  0  0  0  0
  4 29  1  0  0  0  0
  5 30  1  0  0  0  0
  5 31  1  0  0  0  0
  6 32  1  0  0  0  0
  6 33  1  0  0  0  0
  7 34  1  0  0  0  0
  7 35  1  0  0  0  0
  8 36  1  0  0  0  0
  8 37  1  0  0  0  0
  9 38  1  0  0  0  0
  9 39  1  0  0  0  0
 10 40  1  0  0  0  0
 10 41  1  0  0  0  0
 11 42  1  0  0  0  0
 11 43  1  0  0  0  0
 11 44  1  0  0  0  0
 12 45  1  0  0  0  0
 12 46  1  0  0  0  0
 13 47  1  0  0  0  0
 13 48  1  0  0  0  0
 14 49  1  0  0  0  0
 14 50  1  0  0  0  0
 15 51  1  0  0  0  0
 15 52  1  0  0  0  0
 16 53  1  0  0  0  0
 16 54  1  0  0  0  0
 17 55  1  0  0  0  0
 17 56  1  0  0  0  0
M  END
>  <Alias name>
```

Stearic acid

> <deltaH°(c)> kJ/mol
-11336.8

$$$$
Dioctyl sulfide
  ChemBrain IXL

```
 51 50  0  0  0  0  0  0  0  0  1 V2000
    2.1797   10.5977    3.9084 C   0  0  0  0  0  0  0  0  0  0  0  0
    3.4242   11.3705    3.4303 C   0  0  0  0  0  0  0  0  0  0  0  0
    4.4922   10.3728    2.9411 C   0  0  0  0  0  0  0  0  0  0  0  0
    5.7369   11.1457    2.4631 C   0  0  0  0  0  0  0  0  0  0  0  0
    6.8049   10.1481    1.9740 C   0  0  0  0  0  0  0  0  0  0  0  0
    8.0496   10.9210    1.4962 C   0  0  0  0  0  0  0  0  0  0  0  0
    9.1177    9.9233    1.0074 C   0  0  0  0  0  0  0  0  0  0  0  0
   10.3624   10.6962    0.5300 C   0  0  0  0  0  0  0  0  0  0  0  0
   11.6214    9.5201   -0.0457 S   0  0  0  0  0  0  0  0  0  0  0  0
   21.0824   10.8152   -3.8858 C   0  0  0  0  0  0  0  0  0  0  0  0
   19.8476   11.5594   -3.3418 C   0  0  0  0  0  0  0  0  0  0  0  0
   18.7651   10.5370   -2.9441 C   0  0  0  0  0  0  0  0  0  0  0  0
   17.5303   11.2814   -2.3999 C   0  0  0  0  0  0  0  0  0  0  0  0
   16.4479   10.2590   -2.0021 C   0  0  0  0  0  0  0  0  0  0  0  0
   15.2132   11.0034   -1.4577 C   0  0  0  0  0  0  0  0  0  0  0  0
   14.1310    9.9810   -1.0597 C   0  0  0  0  0  0  0  0  0  0  0  0
   12.8967   10.7253   -0.5150 C   0  0  0  0  0  0  0  0  0  0  0  0
    2.4552    9.9368    4.7209 H   0  0  0  0  0  0  0  0  0  0  0  0
    1.4294   11.2989    4.2521 H   0  0  0  0  0  0  0  0  0  0  0  0
    1.7798   10.0144    3.0882 H   0  0  0  0  0  0  0  0  0  0  0  0
    3.8240   11.9538    4.2505 H   0  0  0  0  0  0  0  0  0  0  0  0
    3.1486   12.0314    2.6179 H   0  0  0  0  0  0  0  0  0  0  0  0
    4.0925    9.7895    2.1208 H   0  0  0  0  0  0  0  0  0  0  0  0
    4.7678    9.7118    3.7535 H   0  0  0  0  0  0  0  0  0  0  0  0
    6.1366   11.7291    3.2833 H   0  0  0  0  0  0  0  0  0  0  0  0
    5.4614   11.8067    1.6506 H   0  0  0  0  0  0  0  0  0  0  0  0
    6.4052    9.5648    1.1537 H   0  0  0  0  0  0  0  0  0  0  0  0
    7.0803    9.4870    2.7865 H   0  0  0  0  0  0  0  0  0  0  0  0
    8.4491   11.5044    2.3165 H   0  0  0  0  0  0  0  0  0  0  0  0
    7.7742   11.5819    0.6837 H   0  0  0  0  0  0  0  0  0  0  0  0
    8.7182    9.3401    0.1870 H   0  0  0  0  0  0  0  0  0  0  0  0
    9.3929    9.2623    1.8200 H   0  0  0  0  0  0  0  0  0  0  0  0
   10.0873   11.3570   -0.2827 H   0  0  0  0  0  0  0  0  0  0  0  0
   10.7618   11.2796    1.3504 H   0  0  0  0  0  0  0  0  0  0  0  0
   21.8429   11.5338   -4.1652 H   0  0  0  0  0  0  0  0  0  0  0  0
   20.7995   10.2330   -4.7541 H   0  0  0  0  0  0  0  0  0  0  0  0
   21.4727   10.1557   -3.1205 H   0  0  0  0  0  0  0  0  0  0  0  0
   19.4575   12.2189   -4.1071 H   0  0  0  0  0  0  0  0  0  0  0  0
   20.1306   12.1416   -2.4736 H   0  0  0  0  0  0  0  0  0  0  0  0
   19.1553    9.8774   -2.1788 H   0  0  0  0  0  0  0  0  0  0  0  0
   18.4820    9.9548   -3.8123 H   0  0  0  0  0  0  0  0  0  0  0  0
   17.1401   11.9409   -3.1652 H   0  0  0  0  0  0  0  0  0  0  0  0
   17.8134   11.8636   -1.5317 H   0  0  0  0  0  0  0  0  0  0  0  0
   16.8382    9.5994   -1.2370 H   0  0  0  0  0  0  0  0  0  0  0  0
   16.1647    9.6769   -2.8704 H   0  0  0  0  0  0  0  0  0  0  0  0
   14.8229   11.6629   -2.2229 H   0  0  0  0  0  0  0  0  0  0  0  0
   15.4965   11.5855   -0.5895 H   0  0  0  0  0  0  0  0  0  0  0  0
   14.5215    9.3214   -0.2947 H   0  0  0  0  0  0  0  0  0  0  0  0
   13.8476    9.3990   -1.9280 H   0  0  0  0  0  0  0  0  0  0  0  0
   12.5060   11.3848   -1.2800 H   0  0  0  0  0  0  0  0  0  0  0  0
   13.1801   11.3074    0.3532 H   0  0  0  0  0  0  0  0  0  0  0  0
  1  2  1  0  0  0  0
  2  3  1  0  0  0  0
```

```
  3  4  1  0  0  0  0
  4  5  1  0  0  0  0
  5  6  1  0  0  0  0
  6  7  1  0  0  0  0
  7  8  1  0  0  0  0
  8  9  1  0  0  0  0
 10 11  1  0  0  0  0
 11 12  1  0  0  0  0
 12 13  1  0  0  0  0
 13 14  1  0  0  0  0
 14 15  1  0  0  0  0
 15 16  1  0  0  0  0
 16 17  1  0  0  0  0
 17  9  1  0  0  0  0
  1 18  1  0  0  0  0
  1 19  1  0  0  0  0
  1 20  1  0  0  0  0
  2 21  1  0  0  0  0
  2 22  1  0  0  0  0
  3 23  1  0  0  0  0
  3 24  1  0  0  0  0
  4 25  1  0  0  0  0
  4 26  1  0  0  0  0
  5 27  1  0  0  0  0
  5 28  1  0  0  0  0
  6 29  1  0  0  0  0
  6 30  1  0  0  0  0
  7 31  1  0  0  0  0
  7 32  1  0  0  0  0
  8 33  1  0  0  0  0
  8 34  1  0  0  0  0
 10 35  1  0  0  0  0
 10 36  1  0  0  0  0
 10 37  1  0  0  0  0
 11 38  1  0  0  0  0
 11 39  1  0  0  0  0
 12 40  1  0  0  0  0
 12 41  1  0  0  0  0
 13 42  1  0  0  0  0
 13 43  1  0  0  0  0
 14 44  1  0  0  0  0
 14 45  1  0  0  0  0
 15 46  1  0  0  0  0
 15 47  1  0  0  0  0
 16 48  1  0  0  0  0
 16 49  1  0  0  0  0
 17 50  1  0  0  0  0
 17 51  1  0  0  0  0
M  END
>  <Alias name>
Dioctyl sulfide

>  <deltaH°(c)>   kJ/mol
-11329

$$$$
Bis(O-ethyl-N-benzoylthiocarbamato)nickel(II)
  ChemBrain IXL

 49 52  0  0  0  0  0  0  0  0  1 V2000
    9.1599   -8.7311    3.0047 C   0  0  0  0  0  0  0  0  0  0  0  0
   10.4768   -8.4053    3.3274 C   0  0  0  0  0  0  0  0  0  0  0  0
   11.0317   -7.2153    2.8620 C   0  0  0  0  0  0  0  0  0  0  0  0
```

```
  10.3033   -6.4055    1.9963 C   0  0  0  0  0  0  0  0  0  0  0  0  0
   8.9509   -6.6606    1.7848 C   0  0  0  0  0  0  0  0  0  0  0  0  0
   8.3945   -7.8557    2.2354 C   0  0  0  0  0  0  0  0  0  0  0  0  0
  10.9647   -5.2785    1.3038 C   0  0  0  0  0  0  0  0  0  0  0  0  0
  10.2924   -4.5087    0.6183 O   0  0  0  0  0  0  0  0  0  0  0  0  0
  12.3654   -5.1077    1.4319 N   0  0  0  0  0  0  0  0  0  0  0  0  0
  12.9215   -3.9775    1.1958 C   0  0  0  0  0  0  0  0  0  0  0  0  0
  14.3590   -3.9515    0.8289 O   0  0  0  0  0  0  0  0  0  0  0  0  0
  11.9293   -2.3633    1.3205 S   0  5  0  0  0  0  0  0  0  0  0  0  0
  14.8224   -2.7735    0.0750 C   0  0  0  0  0  0  0  0  0  0  0  0  0
  16.3517   -2.8766   -0.1409 C   0  0  0  0  0  0  0  0  0  0  0  0  0
   9.7534   -2.8229    1.2514 Ni  0  0  0  0  0  0  0  0  0  0  0  0  0
   8.3919    3.4931    2.8568 C   0  0  0  0  0  0  0  0  0  0  0  0  0
   7.2747    2.8150    3.3286 C   0  0  0  0  0  0  0  0  0  0  0  0  0
   7.3113    1.4372    3.4042 C   0  0  0  0  0  0  0  0  0  0  0  0  0
   8.4183    0.7933    2.9128 C   0  0  0  0  0  0  0  0  0  0  0  0  0
   9.6109    1.4582    2.9517 C   0  0  0  0  0  0  0  0  0  0  0  0  0
   9.5927    2.8128    2.7197 C   0  0  0  0  0  0  0  0  0  0  0  0  0
   8.3166   -0.5659    2.3452 C   0  0  0  0  0  0  0  0  0  0  0  0  0
   9.1907   -0.9712    1.5290 O   0  0  0  0  0  0  0  0  0  0  0  0  0
   7.2141   -1.3661    2.7433 N   0  0  0  0  0  0  0  0  0  0  0  0  0
   6.7341   -2.2311    1.9652 C   0  0  0  0  0  0  0  0  0  0  0  0  0
   5.3470   -2.1593    1.4982 O   0  0  0  0  0  0  0  0  0  0  0  0  0
   7.7775   -3.5735    1.4244 S   0  5  0  0  0  0  0  0  0  0  0  0  0
   4.9837   -3.2059    0.5218 C   0  0  0  0  0  0  0  0  0  0  0  0  0
   3.6149   -2.8827   -0.1017 C   0  0  0  0  0  0  0  0  0  0  0  0  0
   8.7322   -9.6629    3.3512 H   0  0  0  0  0  0  0  0  0  0  0  0  0
  11.0674   -9.0756    3.9385 H   0  0  0  0  0  0  0  0  0  0  0  0  0
  12.0265   -6.9206    3.1727 H   0  0  0  0  0  0  0  0  0  0  0  0  0
   8.3337   -5.9322    1.2725 H   0  0  0  0  0  0  0  0  0  0  0  0  0
   7.3699   -8.1039    1.9883 H   0  0  0  0  0  0  0  0  0  0  0  0  0
  14.3238   -2.7363   -0.8867 H   0  0  0  0  0  0  0  0  0  0  0  0  0
  14.5970   -1.8758    0.6344 H   0  0  0  0  0  0  0  0  0  0  0  0  0
  16.7784   -1.8825   -0.1906 H   0  0  0  0  0  0  0  0  0  0  0  0  0
  16.5525   -3.4023   -1.0673 H   0  0  0  0  0  0  0  0  0  0  0  0  0
  16.7969   -3.4192    0.6849 H   0  0  0  0  0  0  0  0  0  0  0  0  0
   8.3263    4.5365    2.5973 H   0  0  0  0  0  0  0  0  0  0  0  0  0
   6.3899    3.3544    3.6318 H   0  0  0  0  0  0  0  0  0  0  0  0  0
   6.4940    0.8770    3.8474 H   0  0  0  0  0  0  0  0  0  0  0  0  0
  10.5362    0.9341    3.1736 H   0  0  0  0  0  0  0  0  0  0  0  0  0
  10.4957    3.3314    2.4303 H   0  0  0  0  0  0  0  0  0  0  0  0  0
   4.9342   -4.1659    1.0224 H   0  0  0  0  0  0  0  0  0  0  0  0  0
   5.7344   -3.2460   -0.2590 H   0  0  0  0  0  0  0  0  0  0  0  0  0
   3.0615   -3.8018   -0.2530 H   0  0  0  0  0  0  0  0  0  0  0  0  0
   3.0607   -2.2326    0.5621 H   0  0  0  0  0  0  0  0  0  0  0  0  0
   3.7603   -2.3882   -1.0542 H   0  0  0  0  0  0  0  0  0  0  0  0  0
  1  2  4  0  0  0  0
  2  3  4  0  0  0  0
  3  4  4  0  0  0  0
  4  5  4  0  0  0  0
  5  6  4  0  0  0  0
  6  1  4  0  0  0  0
  4  7  1  0  0  0  0
  7  8  2  0  0  0  0
  7  9  1  0  0  0  0
  9 10  2  0  0  0  0
 10 11  1  0  0  0  0
 10 12  1  0  0  0  0
 11 13  1  0  0  0  0
 13 14  1  0  0  0  0
 15 12  1  0  0  0  0
 15  8  1  0  0  0  0
 16 17  4  0  0  0  0
```

17 18 4 0 0 0 0
18 19 4 0 0 0 0
19 20 4 0 0 0 0
20 21 4 0 0 0 0
21 16 4 0 0 0 0
19 22 1 0 0 0 0
22 23 2 0 0 0 0
22 24 1 0 0 0 0
24 25 2 0 0 0 0
25 26 1 0 0 0 0
25 27 1 0 0 0 0
26 28 1 0 0 0 0
28 29 1 0 0 0 0
27 15 1 0 0 0 0
23 15 1 0 0 0 0
1 30 1 0 0 0 0
2 31 1 0 0 0 0
3 32 1 0 0 0 0
5 33 1 0 0 0 0
6 34 1 0 0 0 0
13 35 1 0 0 0 0
13 36 1 0 0 0 0
14 37 1 0 0 0 0
14 38 1 0 0 0 0
14 39 1 0 0 0 0
16 40 1 0 0 0 0
17 41 1 0 0 0 0
18 42 1 0 0 0 0
20 43 1 0 0 0 0
21 44 1 0 0 0 0
28 45 1 0 0 0 0
28 46 1 0 0 0 0
29 47 1 0 0 0 0
29 48 1 0 0 0 0
29 49 1 0 0 0 0
M END
> <Alias name>
Bis(O-ethyl-N-benzoylthiocarbamato)nickel(II)

> <deltaH°(c)> kJ/mol
-11321.2

$$$$
2,6-Di-t-butyl-4-phenylphenol
ChemBrain IXL

47 48 0 0 0 0 0 0 0 0 1 V2000
5.6751 7.1151 0.0000 C 0 0 0 0 0 0 0 0 0 0 0 0
6.2537 5.8457 -0.0057 C 0 0 0 0 0 0 0 0 0 0 0 0
4.2865 7.2487 -0.0067 C 0 0 0 0 0 0 0 0 0 0 0 0
5.4438 4.7100 -0.0177 C 0 0 0 0 0 0 0 0 0 0 0 0
7.7269 5.7040 0.0011 C 0 0 0 0 0 0 0 0 0 0 0 0
4.0552 4.8436 -0.0244 C 0 0 0 0 0 0 0 0 0 0 0 0
3.4766 6.1129 -0.0194 C 0 0 0 0 0 0 0 0 0 0 0 0
3.1959 3.6387 -0.0369 C 0 0 0 0 0 0 0 0 0 0 0 0
2.0134 6.2537 -0.0275 O 0 0 0 0 0 0 0 0 0 0 0 0
3.6727 8.5953 -0.0004 C 0 0 0 0 0 0 0 0 0 0 0 0
8.5369 6.8398 0.0013 C 0 0 0 0 0 0 0 0 0 0 0 0
9.9255 6.7062 0.0074 C 0 0 0 0 0 0 0 0 0 0 0 0
10.5041 5.4368 0.0134 C 0 0 0 0 0 0 0 0 0 0 0 0
9.6941 4.3011 0.0134 C 0 0 0 0 0 0 0 0 0 0 0 0
8.3055 4.4347 0.0073 C 0 0 0 0 0 0 0 0 0 0 0 0
2.2976 3.6613 -1.2888 C 0 0 0 0 0 0 0 0 0 0 0 0

4.0806	2.3771	-0.0621	C	0	0	0	0	0	0	0	0	0	0	0	0
2.3149	3.6230	1.2273	C	0	0	0	0	0	0	0	0	0	0	0	0
2.7939	8.7652	-1.2548	C	0	0	0	0	0	0	0	0	0	0	0	0
2.8033	8.7576	1.2616	C	0	0	0	0	0	0	0	0	0	0	0	0
4.7816	9.6654	-0.0013	C	0	0	0	0	0	0	0	0	0	0	0	0
1.5144	5.4072	-0.0013	H	0	0	0	0	0	0	0	0	0	0	0	0
6.3039	7.9968	0.0096	H	0	0	0	0	0	0	0	0	0	0	0	0
5.8930	3.7246	-0.0219	H	0	0	0	0	0	0	0	0	0	0	0	0
8.0877	7.8252	-0.0033	H	0	0	0	0	0	0	0	0	0	0	0	0
10.5543	7.5879	0.0074	H	0	0	0	0	0	0	0	0	0	0	0	0
11.5821	5.3331	0.0181	H	0	0	0	0	0	0	0	0	0	0	0	0
10.1432	3.3156	0.0181	H	0	0	0	0	0	0	0	0	0	0	0	0
7.6767	3.5529	0.0074	H	0	0	0	0	0	0	0	0	0	0	0	0
1.6592	2.7865	-1.2908	H	0	0	0	0	0	0	0	0	0	0	0	0
1.6858	4.5548	-1.2780	H	0	0	0	0	0	0	0	0	0	0	0	0
2.9167	3.6584	-2.1774	H	0	0	0	0	0	0	0	0	0	0	0	0
4.7010	2.3890	-0.9497	H	0	0	0	0	0	0	0	0	0	0	0	0
3.4518	1.4955	-0.0730	H	0	0	0	0	0	0	0	0	0	0	0	0
4.7106	2.3604	0.8186	H	0	0	0	0	0	0	0	0	0	0	0	0
1.6768	2.7481	1.2118	H	0	0	0	0	0	0	0	0	0	0	0	0
2.9461	3.5940	2.1069	H	0	0	0	0	0	0	0	0	0	0	0	0
1.7026	4.5160	1.2517	H	0	0	0	0	0	0	0	0	0	0	0	0
2.0163	8.0114	-1.2556	H	0	0	0	0	0	0	0	0	0	0	0	0
2.3424	9.7496	-1.2488	H	0	0	0	0	0	0	0	0	0	0	0	0
3.4054	8.6539	-2.1417	H	0	0	0	0	0	0	0	0	0	0	0	0
2.0262	8.0033	1.2641	H	0	0	0	0	0	0	0	0	0	0	0	0
3.4216	8.6420	2.1432	H	0	0	0	0	0	0	0	0	0	0	0	0
2.3511	9.7417	1.2643	H	0	0	0	0	0	0	0	0	0	0	0	0
4.3324	10.6508	0.0016	H	0	0	0	0	0	0	0	0	0	0	0	0
5.3936	9.5502	-0.8874	H	0	0	0	0	0	0	0	0	0	0	0	0
5.3981	9.5471	0.8811	H	0	0	0	0	0	0	0	0	0	0	0	0

2	1	4	0	0	0	0
1	3	4	0	0	0	0
4	2	4	0	0	0	0
2	5	1	0	0	0	0
3	7	4	0	0	0	0
3	10	1	0	0	0	0
6	4	4	0	0	0	0
11	5	4	0	0	0	0
5	15	4	0	0	0	0
7	6	4	0	0	0	0
6	8	1	0	0	0	0
7	9	1	0	0	0	0
8	16	1	0	0	0	0
8	17	1	0	0	0	0
8	18	1	0	0	0	0
10	19	1	0	0	0	0
10	20	1	0	0	0	0
10	21	1	0	0	0	0
12	11	4	0	0	0	0
13	12	4	0	0	0	0
14	13	4	0	0	0	0
15	14	4	0	0	0	0
9	22	1	0	0	0	0
1	23	1	0	0	0	0
4	24	1	0	0	0	0
11	25	1	0	0	0	0
12	26	1	0	0	0	0
13	27	1	0	0	0	0
14	28	1	0	0	0	0
15	29	1	0	0	0	0
16	30	1	0	0	0	0
16	31	1	0	0	0	0

16 32 1 0 0 0 0
17 33 1 0 0 0 0
17 34 1 0 0 0 0
17 35 1 0 0 0 0
18 36 1 0 0 0 0
18 37 1 0 0 0 0
18 38 1 0 0 0 0
19 39 1 0 0 0 0
19 40 1 0 0 0 0
19 41 1 0 0 0 0
20 42 1 0 0 0 0
20 43 1 0 0 0 0
20 44 1 0 0 0 0
21 45 1 0 0 0 0
21 46 1 0 0 0 0
21 47 1 0 0 0 0
M END
> <Alias name>
2,6-Di-t-butyl-4-phenylphenol

> <deltaH°(c)>   kJ/mol
-11280

$$$$
Tripentylsilane
  ChemBrain IXL

50 49 0 0 0 0 0 0 0 0 1 V2000
6.6034   3.0016   1.6723 Si 0 0 0 0 0 0 0 0 0 0 0 0
6.6212   4.5288   2.7801 C   0 0 0 0 0 0 0 0 0 0 0 0
6.4963   5.8210   1.9462 C   0 0 0 0 0 0 0 0 0 0 0 0
3.6548   5.1347   -2.2462 C   0 0 0 0 0 0 0 0 0 0 0 0
3.6764   4.0142   -1.1883 C   0 0 0 0 0 0 0 0 0 0 0 0
5.0189   4.0468   -0.4331 C   0 0 0 0 0 0 0 0 0 0 0 0
5.0379   2.9212   0.6221 C   0 0 0 0 0 0 0 0 0 0 0 0
8.3279   4.1556   -0.2591 C   0 0 0 0 0 0 0 0 0 0 0 0
8.1599   2.8927   0.6114 C   0 0 0 0 0 0 0 0 0 0 0 0
9.5427   3.9749   -1.1894 C   0 0 0 0 0 0 0 0 0 0 0 0
6.6306   7.0421   2.8761 C   0 0 0 0 0 0 0 0 0 0 0 0
9.7165   5.2360   -2.0581 C   0 0 0 0 0 0 0 0 0 0 0 0
6.5026   8.3353   2.0476 C   0 0 0 0 0 0 0 0 0 0 0 0
10.9313   5.0557   -2.9887 C   0 0 0 0 0 0 0 0 0 0 0 0
2.3121   5.1024   -3.0017 C   0 0 0 0 0 0 0 0 0 0 0 0
6.6369   9.5568   2.9773 C   0 0 0 0 0 0 0 0 0 0 0 0
6.5957   1.8175   2.5600 H   0 0 0 0 0 0 0 0 0 0 0 0
5.7892   4.4754   3.4712 H   0 0 0 0 0 0 0 0 0 0 0 0
7.5479   4.5506   3.3400 H   0 0 0 0 0 0 0 0 0 0 0 0
7.2704   5.8520   1.2051 H   0 0 0 0 0 0 0 0 0 0 0 0
5.5343   5.8466   1.4651 H   0 0 0 0 0 0 0 0 0 0 0 0
3.7713   6.0943   -1.7580 H   0 0 0 0 0 0 0 0 0 0 0 0
4.4669   4.9852   -2.9469 H   0 0 0 0 0 0 0 0 0 0 0 0
3.5600   3.0545   -1.6765 H   0 0 0 0 0 0 0 0 0 0 0 0
2.8643   4.1636   -0.4876 H   0 0 0 0 0 0 0 0 0 0 0 0
5.1331   4.9982   0.0476 H   0 0 0 0 0 0 0 0 0 0 0 0
5.8224   3.8969   -1.1328 H   0 0 0 0 0 0 0 0 0 0 0 0
4.9991   1.9648   0.1157 H   0 0 0 0 0 0 0 0 0 0 0 0
4.1728   3.0180   1.2663 H   0 0 0 0 0 0 0 0 0 0 0 0
7.4472   4.3096   -0.8507 H   0 0 0 0 0 0 0 0 0 0 0 0
8.4913   5.0096   0.3747 H   0 0 0 0 0 0 0 0 0 0 0 0
9.0235   2.7945   1.2574 H   0 0 0 0 0 0 0 0 0 0 0 0
8.0934   2.0215   -0.0284 H   0 0 0 0 0 0 0 0 0 0 0 0
10.4340   3.8214   -0.5936 H   0 0 0 0 0 0 0 0 0 0 0 0
9.3832   3.1154   -1.8286 H   0 0 0 0 0 0 0 0 0 0 0 0

```
    5.8482    7.0138    3.6244 H   0  0  0  0  0  0  0  0  0  0  0  0
    7.5974    7.0200    3.3637 H   0  0  0  0  0  0  0  0  0  0  0  0
    8.8252    5.3896   -2.6537 H   0  0  0  0  0  0  0  0  0  0  0  0
    9.8760    6.0955   -1.4188 H   0  0  0  0  0  0  0  0  0  0  0  0
    7.2849    8.3635    1.2993 H   0  0  0  0  0  0  0  0  0  0  0  0
    5.5358    8.3574    1.5601 H   0  0  0  0  0  0  0  0  0  0  0  0
   11.0532    5.9419   -3.5990 H   0  0  0  0  0  0  0  0  0  0  0  0
   10.7719    4.1962   -3.6281 H   0  0  0  0  0  0  0  0  0  0  0  0
   11.8227    4.9021   -2.3931 H   0  0  0  0  0  0  0  0  0  0  0  0
    2.1956    4.1428   -3.4900 H   0  0  0  0  0  0  0  0  0  0  0  0
    1.5000    5.2520   -2.3011 H   0  0  0  0  0  0  0  0  0  0  0  0
    2.2972    5.8898   -3.7450 H   0  0  0  0  0  0  0  0  0  0  0  0
    5.8546    9.5287    3.7256 H   0  0  0  0  0  0  0  0  0  0  0  0
    6.5469   10.4653    2.3949 H   0  0  0  0  0  0  0  0  0  0  0  0
    7.6038    9.5348    3.4648 H   0  0  0  0  0  0  0  0  0  0  0  0
  1  2  1  0  0  0  0
  2  3  1  0  0  0  0
  4  5  1  0  0  0  0
  5  6  1  0  0  0  0
  6  7  1  0  0  0  0
  7  1  1  0  0  0  0
  8  9  1  0  0  0  0
  9  1  1  0  0  0  0
  8 10  1  0  0  0  0
  3 11  1  0  0  0  0
 10 12  1  0  0  0  0
 11 13  1  0  0  0  0
 12 14  1  0  0  0  0
  4 15  1  0  0  0  0
 13 16  1  0  0  0  0
  1 17  1  0  0  0  0
  2 18  1  0  0  0  0
  2 19  1  0  0  0  0
  3 20  1  0  0  0  0
  3 21  1  0  0  0  0
  4 22  1  0  0  0  0
  4 23  1  0  0  0  0
  5 24  1  0  0  0  0
  5 25  1  0  0  0  0
  6 26  1  0  0  0  0
  6 27  1  0  0  0  0
  7 28  1  0  0  0  0
  7 29  1  0  0  0  0
  8 30  1  0  0  0  0
  8 31  1  0  0  0  0
  9 32  1  0  0  0  0
  9 33  1  0  0  0  0
 10 34  1  0  0  0  0
 10 35  1  0  0  0  0
 11 36  1  0  0  0  0
 11 37  1  0  0  0  0
 12 38  1  0  0  0  0
 12 39  1  0  0  0  0
 13 40  1  0  0  0  0
 13 41  1  0  0  0  0
 14 42  1  0  0  0  0
 14 43  1  0  0  0  0
 14 44  1  0  0  0  0
 15 45  1  0  0  0  0
 15 46  1  0  0  0  0
 15 47  1  0  0  0  0
 16 48  1  0  0  0  0
 16 49  1  0  0  0  0
```

```
 16 50  1  0  0  0  0
M  END
>  <Alias name>
Tripentylsilane

>  <deltaH°(c)>   kJ/mol
-11251

$$$$
Strychnine
  ChemBrain IXL

 47 53  0  0  0  0  0  0  0  0  1 V2000
    2.5152    5.4947    0.4256 C   0  0  0  0  0  0  0  0  0  0  0  0
    2.7684    6.8572    0.2974 C   0  0  0  0  0  0  0  0  0  0  0  0
    4.0862    7.3091    0.3209 C   0  0  0  0  0  0  0  0  0  0  0  0
    5.1183    6.3853    0.4708 C   0  0  0  0  0  0  0  0  0  0  0  0
    4.8946    5.1181    0.5830 C   0  0  0  0  0  0  0  0  0  0  0  0
    3.5884    4.6103    0.5729 C   0  0  0  0  0  0  0  0  0  0  0  0
    6.0520    4.4388    0.7034 N   0  0  0  0  0  0  0  0  0  0  0  0
    7.1167    5.2235    0.6271 C   0  0  0  0  0  0  0  0  0  0  0  0
    6.4850    6.6615    0.5421 C   0  0  0  0  0  0  0  0  0  0  0  0
    6.2283    3.0826    0.7712 C   0  0  0  0  0  0  0  0  0  0  0  0
    7.6049    2.5599    0.7217 C   0  0  0  0  0  0  0  0  0  0  0  0
    8.5162    3.4912   -0.2430 C   0  0  0  0  0  0  0  0  0  0  0  0
    7.7970    4.8138   -0.5343 C   0  0  0  0  0  0  0  0  0  0  0  0
    5.2676    2.3313    0.8760 O   0  0  0  0  0  0  0  0  0  0  0  0
    8.6771    5.8900   -0.9246 C   0  0  0  0  0  0  0  0  0  0  0  0
    7.8505    6.8073   -1.6772 C   0  0  0  0  0  0  0  0  0  0  0  0
    6.9866    7.5781   -0.6413 C   0  0  0  0  0  0  0  0  0  0  0  0
    9.7562    3.8041    0.4035 O   0  0  0  0  0  0  0  0  0  0  0  0
    9.5946    4.5297    1.6627 C   0  0  0  0  0  0  0  0  0  0  0  0
    9.5892    6.0577    1.4476 C   0  0  0  0  0  0  0  0  0  0  0  0
    9.1682    6.6644    0.2718 C   0  0  0  0  0  0  0  0  0  0  0  0
    9.1816    8.1404    0.1628 C   0  0  0  0  0  0  0  0  0  0  0  0
    7.7924    8.6300    0.0175 N   0  0  0  0  0  0  0  0  0  0  0  0
    7.2385    8.8076    1.3702 C   0  0  0  0  0  0  0  0  0  0  0  0
    6.7528    7.4305    1.8133 C   0  0  0  0  0  0  0  0  0  0  0  0
    1.4907    5.1252    0.4109 H   0  0  0  0  0  0  0  0  0  0  0  0
    1.9463    7.5604    0.1803 H   0  0  0  0  0  0  0  0  0  0  0  0
    4.3025    8.3647    0.2231 H   0  0  0  0  0  0  0  0  0  0  0  0
    3.4049    3.5472    0.6776 H   0  0  0  0  0  0  0  0  0  0  0  0
    7.7344    5.1163    1.5055 H   0  0  0  0  0  0  0  0  0  0  0  0
    8.0169    2.5627    1.7235 H   0  0  0  0  0  0  0  0  0  0  0  0
    7.5856    1.5436    0.3445 H   0  0  0  0  0  0  0  0  0  0  0  0
    8.7027    2.9654   -1.1714 H   0  0  0  0  0  0  0  0  0  0  0  0
    7.0675    4.6482   -1.3253 H   0  0  0  0  0  0  0  0  0  0  0  0
    9.4977    5.5209   -1.5233 H   0  0  0  0  0  0  0  0  0  0  0  0
    7.2057    6.2524   -2.3562 H   0  0  0  0  0  0  0  0  0  0  0  0
    8.4681    7.4937   -2.2320 H   0  0  0  0  0  0  0  0  0  0  0  0
    6.1336    8.0261   -1.1396 H   0  0  0  0  0  0  0  0  0  0  0  0
   10.4146    4.2676    2.3208 H   0  0  0  0  0  0  0  0  0  0  0  0
    8.6602    4.2288    2.1277 H   0  0  0  0  0  0  0  0  0  0  0  0
    9.9276    6.6864    2.2545 H   0  0  0  0  0  0  0  0  0  0  0  0
    9.6203    8.5608    1.0552 H   0  0  0  0  0  0  0  0  0  0  0  0
    9.7610    8.4314   -0.6999 H   0  0  0  0  0  0  0  0  0  0  0  0
    7.9979    9.1701    2.0428 H   0  0  0  0  0  0  0  0  0  0  0  0
    6.4028    9.4980    1.3393 H   0  0  0  0  0  0  0  0  0  0  0  0
    7.5213    6.9317    2.3912 H   0  0  0  0  0  0  0  0  0  0  0  0
    5.8436    7.5185    2.3970 H   0  0  0  0  0  0  0  0  0  0  0  0
  1  2  4  0  0  0  0
  2  3  4  0  0  0  0
  3  4  4  0  0  0  0
```

```
  4  5  4  0  0  0  0
  5  6  4  0  0  0  0
  6  1  4  0  0  0  0
  5  7  1  0  0  0  0
  7  8  1  0  0  0  0
  8  9  1  0  0  0  0
  9  4  1  0  0  0  0
  7 10  1  0  0  0  0
 10 11  1  0  0  0  0
 11 12  1  0  0  0  0
  8 13  1  0  0  0  0
 12 13  1  0  0  0  0
 10 14  2  0  0  0  0
 13 15  1  0  0  0  0
 15 16  1  0  0  0  0
 16 17  1  0  0  0  0
 17  9  1  0  0  0  0
 12 18  1  0  0  0  0
 18 19  1  0  0  0  0
 19 20  1  0  0  0  0
 20 21  2  0  0  0  0
 21 15  1  0  0  0  0
 21 22  1  0  0  0  0
 22 23  1  0  0  0  0
 23 17  1  0  0  0  0
 23 24  1  0  0  0  0
 24 25  1  0  0  0  0
 25  9  1  0  0  0  0
  1 26  1  0  0  0  0
  2 27  1  0  0  0  0
  3 28  1  0  0  0  0
  6 29  1  0  0  0  0
  8 30  1  0  0  0  0
 11 31  1  0  0  0  0
 11 32  1  0  0  0  0
 12 33  1  0  0  0  0
 13 34  1  0  0  0  0
 15 35  1  0  0  0  0
 16 36  1  0  0  0  0
 16 37  1  0  0  0  0
 17 38  1  0  0  0  0
 19 39  1  0  0  0  0
 19 40  1  0  0  0  0
 20 41  1  0  0  0  0
 22 42  1  0  0  0  0
 22 43  1  0  0  0  0
 24 44  1  0  0  0  0
 24 45  1  0  0  0  0
 25 46  1  0  0  0  0
 25 47  1  0  0  0  0
M  END
>  <Alias name>
Strychnine

>  <deltaH°(c)>   kJ/mol
-11244

$$$$
Triisopentylsilane
  ChemBrain IXL

 50 49  0  0  0  0  0  0  0  0  1 V2000
    5.1742    5.2984    1.8213 Si  0  0  0  0  0  0  0  0  0  0  0  0
```

```
   4.2564    3.8756    0.9903 C   0  0  0  0  0  0  0  0  0  0  0  0
   4.6523    3.7546   -0.4973 C   0  0  0  0  0  0  0  0  0  0  0  0
   9.7963    4.2434   -0.6506 C   0  0  0  0  0  0  0  0  0  0  0  0
   9.1732    4.5074    0.7341 C   0  0  0  0  0  0  0  0  0  0  0  0
   7.6440    4.6581    0.5878 C   0  0  0  0  0  0  0  0  0  0  0  0
   7.0198    4.9406    1.9717 C   0  0  0  0  0  0  0  0  0  0  0  0
   5.5012    6.9520   -0.4568 C   0  0  0  0  0  0  0  0  0  0  0  0
   4.8538    6.9374    0.9450 C   0  0  0  0  0  0  0  0  0  0  0  0
   5.1029    8.2483   -1.1941 C   0  0  0  0  0  0  0  0  0  0  0  0
   3.9894    2.5005   -1.1059 C   0  0  0  0  0  0  0  0  0  0  0  0
   5.6071    9.4718   -0.4012 C   0  0  0  0  0  0  0  0  0  0  0  0
   4.4003    2.3714   -2.5857 C   0  0  0  0  0  0  0  0  0  0  0  0
   5.7353    8.2528   -2.5996 C   0  0  0  0  0  0  0  0  0  0  0  0
   9.4904    3.3251    1.6729 C   0  0  0  0  0  0  0  0  0  0  0  0
   2.4550    2.6262   -1.0090 C   0  0  0  0  0  0  0  0  0  0  0  0
   4.6382    5.4233    3.1950 H   0  0  0  0  0  0  0  0  0  0  0  0
   3.1910    4.0542    1.0570 H   0  0  0  0  0  0  0  0  0  0  0  0
   4.4953    2.9517    1.5023 H   0  0  0  0  0  0  0  0  0  0  0  0
   5.7177    3.6693   -0.5832 H   0  0  0  0  0  0  0  0  0  0  0  0
   4.3162    4.6272   -1.0307 H   0  0  0  0  0  0  0  0  0  0  0  0
  10.8695    4.1387   -0.5495 H   0  0  0  0  0  0  0  0  0  0  0  0
   9.3822    3.3326   -1.0652 H   0  0  0  0  0  0  0  0  0  0  0  0
   9.5751    5.0734   -1.3101 H   0  0  0  0  0  0  0  0  0  0  0  0
   9.5877    5.4182    1.1480 H   0  0  0  0  0  0  0  0  0  0  0  0
   7.4301    5.4701   -0.0790 H   0  0  0  0  0  0  0  0  0  0  0  0
   7.2347    3.7461    0.1884 H   0  0  0  0  0  0  0  0  0  0  0  0
   7.1624    4.0744    2.6049 H   0  0  0  0  0  0  0  0  0  0  0  0
   7.5117    5.7945    2.4205 H   0  0  0  0  0  0  0  0  0  0  0  0
   5.1633    6.1053   -1.0212 H   0  0  0  0  0  0  0  0  0  0  0  0
   6.5726    6.9155   -0.3581 H   0  0  0  0  0  0  0  0  0  0  0  0
   5.2736    7.7423    1.5344 H   0  0  0  0  0  0  0  0  0  0  0  0
   3.7854    7.0860    0.8492 H   0  0  0  0  0  0  0  0  0  0  0  0
   4.0248    8.2957   -1.2839 H   0  0  0  0  0  0  0  0  0  0  0  0
   4.3145    1.6213   -0.5637 H   0  0  0  0  0  0  0  0  0  0  0  0
   5.3371   10.3789   -0.9276 H   0  0  0  0  0  0  0  0  0  0  0  0
   6.6843    9.4190   -0.3022 H   0  0  0  0  0  0  0  0  0  0  0  0
   5.1541    9.4774    0.5818 H   0  0  0  0  0  0  0  0  0  0  0  0
   5.4774    2.2819   -2.6547 H   0  0  0  0  0  0  0  0  0  0  0  0
   4.0756    3.2504   -3.1286 H   0  0  0  0  0  0  0  0  0  0  0  0
   3.9368    1.4911   -3.0135 H   0  0  0  0  0  0  0  0  0  0  0  0
   6.8136    8.2053   -2.5105 H   0  0  0  0  0  0  0  0  0  0  0  0
   5.3810    7.3948   -3.1575 H   0  0  0  0  0  0  0  0  0  0  0  0
   5.4555    9.1613   -3.1184 H   0  0  0  0  0  0  0  0  0  0  0  0
  10.5639    3.2143    1.7632 H   0  0  0  0  0  0  0  0  0  0  0  0
   9.0669    2.4159    1.2643 H   0  0  0  0  0  0  0  0  0  0  0  0
   9.0645    3.5146    2.6497 H   0  0  0  0  0  0  0  0  0  0  0  0
   1.9930    1.7507   -1.4482 H   0  0  0  0  0  0  0  0  0  0  0  0
   2.1634    2.7041    0.0305 H   0  0  0  0  0  0  0  0  0  0  0  0
   2.1308    3.5113   -1.5424 H   0  0  0  0  0  0  0  0  0  0  0  0
  1  2  1  0  0  0  0
  2  3  1  0  0  0  0
  4  5  1  0  0  0  0
  5  6  1  0  0  0  0
  6  7  1  0  0  0  0
  7  1  1  0  0  0  0
  8  9  1  0  0  0  0
  9  1  1  0  0  0  0
  8 10  1  0  0  0  0
  3 11  1  0  0  0  0
 10 12  1  0  0  0  0
 11 13  1  0  0  0  0
 10 14  1  0  0  0  0
  5 15  1  0  0  0  0
```

```
 11 16  1  0  0  0  0
  1 17  1  0  0  0  0
  2 18  1  0  0  0  0
  2 19  1  0  0  0  0
  3 20  1  0  0  0  0
  3 21  1  0  0  0  0
  4 22  1  0  0  0  0
  4 23  1  0  0  0  0
  4 24  1  0  0  0  0
  5 25  1  0  0  0  0
  6 26  1  0  0  0  0
  6 27  1  0  0  0  0
  7 28  1  0  0  0  0
  7 29  1  0  0  0  0
  8 30  1  0  0  0  0
  8 31  1  0  0  0  0
  9 32  1  0  0  0  0
  9 33  1  0  0  0  0
 10 34  1  0  0  0  0
 11 35  1  0  0  0  0
 12 36  1  0  0  0  0
 12 37  1  0  0  0  0
 12 38  1  0  0  0  0
 13 39  1  0  0  0  0
 13 40  1  0  0  0  0
 13 41  1  0  0  0  0
 14 42  1  0  0  0  0
 14 43  1  0  0  0  0
 14 44  1  0  0  0  0
 15 45  1  0  0  0  0
 15 46  1  0  0  0  0
 15 47  1  0  0  0  0
 16 48  1  0  0  0  0
 16 49  1  0  0  0  0
 16 50  1  0  0  0  0
M  END
>  <Alias name>
Triisopentylsilane

>  <deltaH°(c)>   kJ/mol
-11244

$$$$
Propyltributylsilane
  ChemBrain IXL

 50 49  0  0  0  0  0  0  0  0  1 V2000
    8.6876    8.8357   -1.2340 C   0  0  0  0  0  0  0  0  0  0  0  0
    7.5026    8.9489   -0.2542 C   0  0  0  0  0  0  0  0  0  0  0  0
    7.6193    7.6231    1.0689 Si  0  0  0  0  0  0  0  0  0  0  0  0
    9.1397    7.8366    2.1486 C   0  0  0  0  0  0  0  0  0  0  0  0
   10.4223    7.6616    1.3112 C   0  0  0  0  0  0  0  0  0  0  0  0
    5.4864    4.1903   -2.3989 C   0  0  0  0  0  0  0  0  0  0  0  0
    6.6854    4.3999   -1.4541 C   0  0  0  0  0  0  0  0  0  0  0  0
    6.5169    5.7319   -0.6979 C   0  0  0  0  0  0  0  0  0  0  0  0
    7.7181    5.9394    0.2459 C   0  0  0  0  0  0  0  0  0  0  0  0
    4.8208    7.6728    1.3561 C   0  0  0  0  0  0  0  0  0  0  0  0
    6.1173    7.7755    2.1840 C   0  0  0  0  0  0  0  0  0  0  0  0
    3.6079    7.9243    2.2728 C   0  0  0  0  0  0  0  0  0  0  0  0
    8.5031    9.8508   -2.3783 C   0  0  0  0  0  0  0  0  0  0  0  0
   11.6506    7.7180    2.2400 C   0  0  0  0  0  0  0  0  0  0  0  0
    2.3105    7.8227    1.4477 C   0  0  0  0  0  0  0  0  0  0  0  0
   12.9342    7.5429    1.4057 C   0  0  0  0  0  0  0  0  0  0  0  0
```

9.6039 9.0463 -0.7182 H 0 0 0 0 0 0 0 0 0 0 0 0
8.7237 7.8338 -1.6437 H 0 0 0 0 0 0 0 0 0 0 0 0
6.5777 8.8200 -0.8026 H 0 0 0 0 0 0 0 0 0 0 0 0
7.5093 9.9266 0.2114 H 0 0 0 0 0 0 0 0 0 0 0 0
9.1246 7.0889 2.9318 H 0 0 0 0 0 0 0 0 0 0 0 0
9.1306 8.8230 2.5953 H 0 0 0 0 0 0 0 0 0 0 0 0
10.3965 6.7041 0.8058 H 0 0 0 0 0 0 0 0 0 0 0 0
10.4903 8.4491 0.5862 H 0 0 0 0 0 0 0 0 0 0 0 0
5.6049 3.2542 -2.9303 H 0 0 0 0 0 0 0 0 0 0 0 0
4.5712 4.1638 -1.8205 H 0 0 0 0 0 0 0 0 0 0 0 0
5.4403 5.0058 -3.1102 H 0 0 0 0 0 0 0 0 0 0 0 0
6.7314 3.5845 -0.7429 H 0 0 0 0 0 0 0 0 0 0 0 0
7.6005 4.4265 -2.0326 H 0 0 0 0 0 0 0 0 0 0 0 0
6.4713 6.5470 -1.4097 H 0 0 0 0 0 0 0 0 0 0 0 0
5.6098 5.7024 -0.1266 H 0 0 0 0 0 0 0 0 0 0 0 0
7.7217 5.1657 1.0036 H 0 0 0 0 0 0 0 0 0 0 0 0
8.6334 5.8808 -0.3298 H 0 0 0 0 0 0 0 0 0 0 0 0
4.8358 8.4143 0.5670 H 0 0 0 0 0 0 0 0 0 0 0 0
4.7431 6.6929 0.9272 H 0 0 0 0 0 0 0 0 0 0 0 0
6.1431 8.7368 2.6819 H 0 0 0 0 0 0 0 0 0 0 0 0
6.1364 6.9874 2.9265 H 0 0 0 0 0 0 0 0 0 0 0 0
3.5925 7.1834 3.0624 H 0 0 0 0 0 0 0 0 0 0 0 0
3.6828 8.9135 2.7072 H 0 0 0 0 0 0 0 0 0 0 0 0
7.5786 9.6406 -2.9018 H 0 0 0 0 0 0 0 0 0 0 0 0
8.4666 10.8530 -1.9694 H 0 0 0 0 0 0 0 0 0 0 0 0
9.3343 9.7723 -3.0679 H 0 0 0 0 0 0 0 0 0 0 0 0
11.6768 8.6751 2.7461 H 0 0 0 0 0 0 0 0 0 0 0 0
11.5855 6.9236 2.9732 H 0 0 0 0 0 0 0 0 0 0 0 0
2.2356 6.8335 1.0131 H 0 0 0 0 0 0 0 0 0 0 0 0
1.4582 7.9994 2.0920 H 0 0 0 0 0 0 0 0 0 0 0 0
2.3258 8.5637 0.6580 H 0 0 0 0 0 0 0 0 0 0 0 0
12.9994 8.3373 0.6725 H 0 0 0 0 0 0 0 0 0 0 0 0
12.9081 6.5858 0.8996 H 0 0 0 0 0 0 0 0 0 0 0 0
13.7973 7.5826 2.0586 H 0 0 0 0 0 0 0 0 0 0 0 0
1 2 1 0 0 0 0
2 3 1 0 0 0 0
3 4 1 0 0 0 0
4 5 1 0 0 0 0
6 7 1 0 0 0 0
7 8 1 0 0 0 0
8 9 1 0 0 0 0
9 3 1 0 0 0 0
10 11 1 0 0 0 0
11 3 1 0 0 0 0
10 12 1 0 0 0 0
1 13 1 0 0 0 0
5 14 1 0 0 0 0
12 15 1 0 0 0 0
14 16 1 0 0 0 0
1 17 1 0 0 0 0
1 18 1 0 0 0 0
2 19 1 0 0 0 0
2 20 1 0 0 0 0
4 21 1 0 0 0 0
4 22 1 0 0 0 0
5 23 1 0 0 0 0
5 24 1 0 0 0 0
6 25 1 0 0 0 0
6 26 1 0 0 0 0
6 27 1 0 0 0 0
7 28 1 0 0 0 0
7 29 1 0 0 0 0
8 30 1 0 0 0 0

 8 31 1 0 0 0 0
 9 32 1 0 0 0 0
 9 33 1 0 0 0 0
10 34 1 0 0 0 0
10 35 1 0 0 0 0
11 36 1 0 0 0 0
11 37 1 0 0 0 0
12 38 1 0 0 0 0
12 39 1 0 0 0 0
13 40 1 0 0 0 0
13 41 1 0 0 0 0
13 42 1 0 0 0 0
14 43 1 0 0 0 0
14 44 1 0 0 0 0
15 45 1 0 0 0 0
15 46 1 0 0 0 0
15 47 1 0 0 0 0
16 48 1 0 0 0 0
16 49 1 0 0 0 0
16 50 1 0 0 0 0
M END
> <Alias name>
Propyltributylsilane

> <deltaH°(c)>   kJ/mol
-11211

$$$$
6,10,14-Trimethylpentadeca-3,5-diene-2-one
  ChemBrain IXL

 51 50 0 0 0 0 0 0 0 0 1 V2000
    3.8491   10.2893   0.7482 C   0 0 0 0 0 0 0 0 0 0 0 0
    2.8113   9.1617   0.9106 C   0 0 0 0 0 0 0 0 0 0 0 0
    3.5251   7.7967   0.8680 C   0 0 0 0 0 0 0 0 0 0 0 0
    2.0872   9.3208   2.2615 C   0 0 0 0 0 0 0 0 0 0 0 0
    4.2306   7.6281   -0.4916 C   0 0 0 0 0 0 0 0 0 0 0 0
    4.9503   6.2661   -0.5314 C   0 0 0 0 0 0 0 0 0 0 0 0
    5.5969   6.0671   -1.9160 C   0 0 0 0 0 0 0 0 0 0 0 0
    6.6253   7.1867   -2.1684 C   0 0 0 0 0 0 0 0 0 0 0 0
    6.3053   4.6993   -1.9603 C   0 0 0 0 0 0 0 0 0 0 0 0
    7.7002   7.1552   -1.0645 C   0 0 0 0 0 0 0 0 0 0 0 0
   13.7028   10.0794   1.9516 C   0 0 0 0 0 0 0 0 0 0 0 0
   12.6727   10.1066   0.8892 C   0 0 0 0 0 0 0 0 0 0 0 0
   13.7017   8.9921   2.9557 C   0 0 0 0 0 0 0 0 0 0 0 0
   14.5528   10.9532   1.9997 O   0 0 0 0 0 0 0 0 0 0 0 0
   11.7500   9.1436   0.8293 C   0 0 0 0 0 0 0 0 0 0 0 0
   10.7204   9.1705   -0.2335 C   0 0 0 0 0 0 0 0 0 0 0 0
    9.7730   8.2305   -0.2690 C   0 0 0 0 0 0 0 0 0 0 0 0
    8.7438   8.2570   -1.3323 C   0 0 0 0 0 0 0 0 0 0 0 0
    9.7519   7.1621   0.7549 C   0 0 0 0 0 0 0 0 0 0 0 0
    3.3503   11.2492   0.7997 H   0 0 0 0 0 0 0 0 0 0 0 0
    4.3429   10.1901   -0.2106 H   0 0 0 0 0 0 0 0 0 0 0 0
    4.5834   10.2210   1.5412 H   0 0 0 0 0 0 0 0 0 0 0 0
    2.0893   9.2169   0.1052 H   0 0 0 0 0 0 0 0 0 0 0 0
    2.7978   7.0048   0.9980 H   0 0 0 0 0 0 0 0 0 0 0 0
    4.2582   7.7477   1.6636 H   0 0 0 0 0 0 0 0 0 0 0 0
    1.3549   8.5308   2.3738 H   0 0 0 0 0 0 0 0 0 0 0 0
    2.8086   9.2614   3.0670 H   0 0 0 0 0 0 0 0 0 0 0 0
    1.5891   10.2819   2.2931 H   0 0 0 0 0 0 0 0 0 0 0 0
    4.9543   8.4229   -0.6238 H   0 0 0 0 0 0 0 0 0 0 0 0
    3.4968   7.6720   -1.2869 H   0 0 0 0 0 0 0 0 0 0 0 0
    4.2344   5.4740   -0.3501 H   0 0 0 0 0 0 0 0 0 0 0 0

```
5.7177 6.2409 0.2323 H 0 0 0 0 0 0 0 0 0 0 0 0
4.8307 6.1017 -2.6806 H 0 0 0 0 0 0 0 0 0 0 0 0
7.0940 7.0356 -3.1330 H 0 0 0 0 0 0 0 0 0 0 0 0
6.1241 8.1466 -2.1569 H 0 0 0 0 0 0 0 0 0 0 0 0
7.0494 4.6525 -1.1748 H 0 0 0 0 0 0 0 0 0 0 0 0
6.7872 4.5733 -2.9220 H 0 0 0 0 0 0 0 0 0 0 0 0
5.5772 3.9108 -1.8153 H 0 0 0 0 0 0 0 0 0 0 0 0
7.2342 7.3256 -0.1019 H 0 0 0 0 0 0 0 0 0 0 0 0
8.1882 6.1884 -1.0633 H 0 0 0 0 0 0 0 0 0 0 0 0
12.6675 10.9077 0.1605 H 0 0 0 0 0 0 0 0 0 0 0 0
13.9048 8.0479 2.4657 H 0 0 0 0 0 0 0 0 0 0 0 0
14.4662 9.1854 3.6980 H 0 0 0 0 0 0 0 0 0 0 0 0
12.7331 8.9473 3.4383 H 0 0 0 0 0 0 0 0 0 0 0 0
11.7549 8.3428 1.5584 H 0 0 0 0 0 0 0 0 0 0 0 0
10.7355 9.9526 -0.9824 H 0 0 0 0 0 0 0 0 0 0 0 0
9.2128 8.0842 -2.2930 H 0 0 0 0 0 0 0 0 0 0 0 0
8.2551 9.2234 -1.3377 H 0 0 0 0 0 0 0 0 0 0 0 0
10.4873 6.4075 0.5044 H 0 0 0 0 0 0 0 0 0 0 0 0
9.9852 7.5859 1.7238 H 0 0 0 0 0 0 0 0 0 0 0 0
8.7679 6.7109 0.7857 H 0 0 0 0 0 0 0 0 0 0 0 0
1 2 1 0 0 0 0
2 3 1 0 0 0 0
2 4 1 0 0 0 0
3 5 1 0 0 0 0
5 6 1 0 0 0 0
6 7 1 0 0 0 0
7 8 1 0 0 0 0
7 9 1 0 0 0 0
8 10 1 0 0 0 0
10 18 1 0 0 0 0
11 12 1 0 0 0 0
11 13 1 0 0 0 0
11 14 2 0 0 0 0
12 15 2 0 0 0 0
15 16 1 0 0 0 0
16 17 2 0 0 0 0
17 18 1 0 0 0 0
17 19 1 0 0 0 0
1 20 1 0 0 0 0
1 21 1 0 0 0 0
1 22 1 0 0 0 0
2 23 1 0 0 0 0
3 24 1 0 0 0 0
3 25 1 0 0 0 0
4 26 1 0 0 0 0
4 27 1 0 0 0 0
4 28 1 0 0 0 0
5 29 1 0 0 0 0
5 30 1 0 0 0 0
6 31 1 0 0 0 0
6 32 1 0 0 0 0
7 33 1 0 0 0 0
8 34 1 0 0 0 0
8 35 1 0 0 0 0
9 36 1 0 0 0 0
9 37 1 0 0 0 0
9 38 1 0 0 0 0
10 39 1 0 0 0 0
10 40 1 0 0 0 0
12 41 1 0 0 0 0
13 42 1 0 0 0 0
13 43 1 0 0 0 0
13 44 1 0 0 0 0
```

15 45 1 0 0 0 0
16 46 1 0 0 0 0
18 47 1 0 0 0 0
18 48 1 0 0 0 0
19 49 1 0 0 0 0
19 50 1 0 0 0 0
19 51 1 0 0 0 0
M END
> <Alias name>
6,10,14-Trimethylpentadeca-3,5-diene-2-one

> <deltaH°(c)>   kJ/mol
-11209

$$$$
1-Heptadecene
  ChemBrain IXL

 51 50 0 0 0 0 0 0 0 0 1 V2000
   12.0222   10.6261   0.3103 C   0 0 0 0 0 0 0 0 0 0 0 0
   10.7805   11.4960   0.5861 C   0 0 0 0 0 0 0 0 0 0 0 0
    9.5665   10.5870   0.8593 C   0 0 0 0 0 0 0 0 0 0 0 0
    8.3250   11.4573   1.1350 C   0 0 0 0 0 0 0 0 0 0 0 0
    7.1108   10.5486   1.4084 C   0 0 0 0 0 0 0 0 0 0 0 0
    5.8695   11.4192   1.6840 C   0 0 0 0 0 0 0 0 0 0 0 0
    4.6550   10.5107   1.9575 C   0 0 0 0 0 0 0 0 0 0 0 0
    3.4623   11.3470   2.2223 C   0 0 0 0 0 0 0 0 0 0 0 0
    2.2885   10.7702   2.4904 C   0 0 0 0 0 0 0 0 0 0 0 0
   13.2360   11.5354   0.0372 C   0 0 0 0 0 0 0 0 0 0 0 0
   14.4779   10.6659   -0.2389 C   0 0 0 0 0 0 0 0 0 0 0 0
   15.6915   11.5756   -0.5117 C   0 0 0 0 0 0 0 0 0 0 0 0
   16.9337   10.7063   -0.7880 C   0 0 0 0 0 0 0 0 0 0 0 0
   18.1471   11.6163   -1.0606 C   0 0 0 0 0 0 0 0 0 0 0 0
   19.3894   10.7472   -1.3371 C   0 0 0 0 0 0 0 0 0 0 0 0
   20.6030   11.6574   -1.6096 C   0 0 0 0 0 0 0 0 0 0 0 0
   21.8452   10.7886   -1.8861 C   0 0 0 0 0 0 0 0 0 0 0 0
   12.2249   10.0026   1.1723 H   0 0 0 0 0 0 0 0 0 0 0 0
   11.8391   9.9992   -0.5537 H   0 0 0 0 0 0 0 0 0 0 0 0
   10.5778   12.1195   -0.2759 H   0 0 0 0 0 0 0 0 0 0 0 0
   10.9637   12.1229   1.4500 H   0 0 0 0 0 0 0 0 0 0 0 0
    9.7691   9.9636   1.7215 H   0 0 0 0 0 0 0 0 0 0 0 0
    9.3833   9.9600   -0.0045 H   0 0 0 0 0 0 0 0 0 0 0 0
    8.1225   12.0806   0.2729 H   0 0 0 0 0 0 0 0 0 0 0 0
    8.5083   12.0843   1.9988 H   0 0 0 0 0 0 0 0 0 0 0 0
    7.3133   9.9253   2.2706 H   0 0 0 0 0 0 0 0 0 0 0 0
    6.9274   9.9216   0.5447 H   0 0 0 0 0 0 0 0 0 0 0 0
    5.6672   12.0425   0.8218 H   0 0 0 0 0 0 0 0 0 0 0 0
    6.0530   12.0463   2.5478 H   0 0 0 0 0 0 0 0 0 0 0 0
    4.4716   9.8835   1.0938 H   0 0 0 0 0 0 0 0 0 0 0 0
    4.8574   9.8874   2.8198 H   0 0 0 0 0 0 0 0 0 0 0 0
    3.5418   12.4268   2.1985 H   0 0 0 0 0 0 0 0 0 0 0 0
    2.2084   9.6903   2.5144 H   0 0 0 0 0 0 0 0 0 0 0 0
    1.4162   11.3821   2.6840 H   0 0 0 0 0 0 0 0 0 0 0 0
   13.0332   12.1590   -0.8247 H   0 0 0 0 0 0 0 0 0 0 0 0
   13.4190   12.1622   0.9012 H   0 0 0 0 0 0 0 0 0 0 0 0
   14.6808   10.0422   0.6230 H   0 0 0 0 0 0 0 0 0 0 0 0
   14.2950   10.0392   -1.1030 H   0 0 0 0 0 0 0 0 0 0 0 0
   15.4886   12.1993   -1.3735 H   0 0 0 0 0 0 0 0 0 0 0 0
   15.8744   12.2022   0.3524 H   0 0 0 0 0 0 0 0 0 0 0 0
   17.1366   10.0825   0.0737 H   0 0 0 0 0 0 0 0 0 0 0 0
   16.7508   10.0798   -1.6522 H   0 0 0 0 0 0 0 0 0 0 0 0
   17.9441   12.2402   -1.9223 H   0 0 0 0 0 0 0 0 0 0 0 0
   18.3299   12.2428   -0.1963 H   0 0 0 0 0 0 0 0 0 0 0 0

19.5924 10.1233 -0.4754 H 0 0 0 0 0 0 0 0 0 0 0 0
19.2067 10.1208 -2.2014 H 0 0 0 0 0 0 0 0 0 0 0 0
20.3999 12.2814 -2.4711 H 0 0 0 0 0 0 0 0 0 0 0 0
20.7857 12.2838 -0.7453 H 0 0 0 0 0 0 0 0 0 0 0 0
22.6978 11.4283 -2.0775 H 0 0 0 0 0 0 0 0 0 0 0 0
21.6626 10.1622 -2.7505 H 0 0 0 0 0 0 0 0 0 0 0 0
22.0484 10.1646 -1.0246 H 0 0 0 0 0 0 0 0 0 0 0 0
1 2 1 0 0 0 0
2 3 1 0 0 0 0
3 4 1 0 0 0 0
4 5 1 0 0 0 0
5 6 1 0 0 0 0
6 7 1 0 0 0 0
7 8 1 0 0 0 0
8 9 2 0 0 0 0
1 10 1 0 0 0 0
11 12 1 0 0 0 0
12 13 1 0 0 0 0
13 14 1 0 0 0 0
14 15 1 0 0 0 0
15 16 1 0 0 0 0
16 17 1 0 0 0 0
10 11 1 0 0 0 0
1 18 1 0 0 0 0
1 19 1 0 0 0 0
2 20 1 0 0 0 0
2 21 1 0 0 0 0
3 22 1 0 0 0 0
3 23 1 0 0 0 0
4 24 1 0 0 0 0
4 25 1 0 0 0 0
5 26 1 0 0 0 0
5 27 1 0 0 0 0
6 28 1 0 0 0 0
6 29 1 0 0 0 0
7 30 1 0 0 0 0
7 31 1 0 0 0 0
8 32 1 0 0 0 0
9 33 1 0 0 0 0
9 34 1 0 0 0 0
10 35 1 0 0 0 0
10 36 1 0 0 0 0
11 37 1 0 0 0 0
11 38 1 0 0 0 0
12 39 1 0 0 0 0
12 40 1 0 0 0 0
13 41 1 0 0 0 0
13 42 1 0 0 0 0
14 43 1 0 0 0 0
14 44 1 0 0 0 0
15 45 1 0 0 0 0
15 46 1 0 0 0 0
16 47 1 0 0 0 0
16 48 1 0 0 0 0
17 49 1 0 0 0 0
17 50 1 0 0 0 0
17 51 1 0 0 0 0
M END
> <Alias name>
1-Heptadecene

> <deltaH°(c)> kJ/mol
-11206.67

```
$$$$
Cortisone
  ChemBrain IXL

 54 57  0  0  0  0  0  0  0  0  1 V2000
    8.9126    5.9127   -0.0435 C   0  0  0  0  0  0  0  0  0  0  0  0
    4.8980    5.7010    1.3979 C   0  0  0  0  0  0  0  0  0  0  0  0
   10.2473    6.3401   -0.6160 C   0  0  0  0  0  0  0  0  0  0  0  0
    6.0294    6.1779    0.5281 C   0  0  0  0  0  0  0  0  0  0  0  0
    7.1286    6.8170    1.4075 C   0  0  0  0  0  0  0  0  0  0  0  0
    8.2806    7.1763    0.4701 C   0  0  0  0  0  0  0  0  0  0  0  0
    6.6507    5.0119   -0.2806 C   0  0  0  0  0  0  0  0  0  0  0  0
    4.2978    6.9045    2.0435 C   0  0  0  0  0  0  0  0  0  0  0  0
    7.9866    5.2531   -1.0231 C   0  0  0  0  0  0  0  0  0  0  0  0
    3.1236    6.7979    2.6625 C   0  0  0  0  0  0  0  0  0  0  0  0
   11.2478    5.1726   -0.5931 C   0  0  0  0  0  0  0  0  0  0  0  0
   10.7156    7.5203    0.3041 C   0  0  0  0  0  0  0  0  0  0  0  0
    9.4273    7.9710    1.0987 C   0  0  0  0  0  0  0  0  0  0  0  0
    6.5785    8.0962    2.1376 C   0  0  0  0  0  0  0  0  0  0  0  0
    3.8270    5.0034    0.5485 C   0  0  0  0  0  0  0  0  0  0  0  0
    5.0231    8.2683    1.9087 C   0  0  0  0  0  0  0  0  0  0  0  0
    2.3627    5.5441    2.5723 C   0  0  0  0  0  0  0  0  0  0  0  0
    6.1361    3.9057   -0.2708 O   0  0  0  0  0  0  0  0  0  0  0  0
   12.0232    5.0461    0.3398 O   0  0  0  0  0  0  0  0  0  0  0  0
    1.4691    5.3224    3.3729 O   0  0  0  0  0  0  0  0  0  0  0  0
   10.0690    6.8210   -1.9945 O   0  0  0  0  0  0  0  0  0  0  0  0
    9.1559    4.9522    1.1360 C   0  0  0  0  0  0  0  0  0  0  0  0
    2.6855    4.4924    1.4676 C   0  0  0  0  0  0  0  0  0  0  0  0
    5.4286    4.7331    2.4735 C   0  0  0  0  0  0  0  0  0  0  0  0
   11.2681    4.1639   -1.7622 C   0  0  0  0  0  0  0  0  0  0  0  0
   11.6421    4.8598   -3.0036 O   0  0  0  0  0  0  0  0  0  0  0  0
    5.6538    6.9211   -0.1595 H   0  0  0  0  0  0  0  0  0  0  0  0
    7.4683    6.0995    2.1403 H   0  0  0  0  0  0  0  0  0  0  0  0
    7.8870    7.7348   -0.3660 H   0  0  0  0  0  0  0  0  0  0  0  0
    8.4029    4.3119   -1.3499 H   0  0  0  0  0  0  0  0  0  0  0  0
    7.8249    5.9009   -1.8725 H   0  0  0  0  0  0  0  0  0  0  0  0
    2.7386    7.6249    3.2408 H   0  0  0  0  0  0  0  0  0  0  0  0
   11.4775    7.1796    0.9895 H   0  0  0  0  0  0  0  0  0  0  0  0
   11.0893    8.3364   -0.2959 H   0  0  0  0  0  0  0  0  0  0  0  0
    9.2617    9.0306    0.9766 H   0  0  0  0  0  0  0  0  0  0  0  0
    9.5262    7.7230    2.1455 H   0  0  0  0  0  0  0  0  0  0  0  0
    6.7713    8.0074    3.1971 H   0  0  0  0  0  0  0  0  0  0  0  0
    7.0903    8.9673    1.7575 H   0  0  0  0  0  0  0  0  0  0  0  0
    4.2694    4.1683    0.0262 H   0  0  0  0  0  0  0  0  0  0  0  0
    3.4246    5.7038   -0.1687 H   0  0  0  0  0  0  0  0  0  0  0  0
    4.8507    8.6675    0.9208 H   0  0  0  0  0  0  0  0  0  0  0  0
    4.6294    8.9547    2.6440 H   0  0  0  0  0  0  0  0  0  0  0  0
    9.5650    6.2461   -2.5587 H   0  0  0  0  0  0  0  0  0  0  0  0
    9.7939    5.4295    1.8639 H   0  0  0  0  0  0  0  0  0  0  0  0
    9.6324    4.0523    0.7743 H   0  0  0  0  0  0  0  0  0  0  0  0
    8.2115    4.6993    1.5950 H   0  0  0  0  0  0  0  0  0  0  0  0
    1.8006    4.3173    0.8731 H   0  0  0  0  0  0  0  0  0  0  0  0
    2.9901    3.5674    1.9340 H   0  0  0  0  0  0  0  0  0  0  0  0
    5.9353    3.9073    1.9960 H   0  0  0  0  0  0  0  0  0  0  0  0
    6.1195    5.2550    3.1185 H   0  0  0  0  0  0  0  0  0  0  0  0
    4.6022    4.3579    3.0593 H   0  0  0  0  0  0  0  0  0  0  0  0
   11.9864    3.3855   -1.5553 H   0  0  0  0  0  0  0  0  0  0  0  0
   10.2863    3.7268   -1.8775 H   0  0  0  0  0  0  0  0  0  0  0  0
   10.9781    5.4434   -3.3531 H   0  0  0  0  0  0  0  0  0  0  0  0
  2  4  1  0  0  0  0
  3  1  1  0  0  0  0
  4  7  1  0  0  0  0
```

```
  5  6  1  0  0  0  0
  6  1  1  0  0  0  0
  7  9  1  0  0  0  0
  8  2  1  0  0  0  0
  9  1  1  0  0  0  0
 10  8  2  0  0  0  0
  3 11  1  0  0  0  0
 12  3  1  0  0  0  0
 13  6  1  0  0  0  0
 14  5  1  0  0  0  0
 15  2  1  0  0  0  0
 16 14  1  0  0  0  0
 17 23  1  0  0  0  0
 18  7  2  0  0  0  0
 19 11  2  0  0  0  0
 20 17  2  0  0  0  0
 21  3  1  0  0  0  0
  1 22  1  0  0  0  0
 23 15  1  0  0  0  0
  2 24  1  0  0  0  0
 25 11  1  0  0  0  0
 26 25  1  0  0  0  0
 12 13  1  0  0  0  0
  4  5  1  0  0  0  0
  8 16  1  0  0  0  0
 17 10  1  0  0  0  0
  4 27  1  0  0  0  0
  5 28  1  0  0  0  0
  6 29  1  0  0  0  0
  9 30  1  0  0  0  0
  9 31  1  0  0  0  0
 10 32  1  0  0  0  0
 12 33  1  0  0  0  0
 12 34  1  0  0  0  0
 13 35  1  0  0  0  0
 13 36  1  0  0  0  0
 14 37  1  0  0  0  0
 14 38  1  0  0  0  0
 15 39  1  0  0  0  0
 15 40  1  0  0  0  0
 16 41  1  0  0  0  0
 16 42  1  0  0  0  0
 21 43  1  0  0  0  0
 22 44  1  0  0  0  0
 22 45  1  0  0  0  0
 22 46  1  0  0  0  0
 23 47  1  0  0  0  0
 23 48  1  0  0  0  0
 24 49  1  0  0  0  0
 24 50  1  0  0  0  0
 24 51  1  0  0  0  0
 25 52  1  0  0  0  0
 25 53  1  0  0  0  0
 26 54  1  0  0  0  0
M  END
>  <Alias name>
Cortisone

>  <deltaH°(c)>   kJ/mol
-11204

$$$$
1,8-Diphenylnaphthalene
```

ChemBrain IXL

```
38 41  0  0  0  0  0  0  0  0  1 V2000
    4.3090    8.0087   -0.1741 C   0  0  0  0  0  0  0  0  0  0  0  0
    4.4761    9.3873   -0.0461 C   0  0  0  0  0  0  0  0  0  0  0  0
    5.7310    9.9178    0.2628 C   0  0  0  0  0  0  0  0  0  0  0  0
    6.8296    9.0735    0.4492 C   0  0  0  0  0  0  0  0  0  0  0  0
    6.6815    7.6824    0.3283 C   0  0  0  0  0  0  0  0  0  0  0  0
    5.3930    7.1490    0.0066 C   0  0  0  0  0  0  0  0  0  0  0  0
    7.8241    6.8447    0.5308 C   0  0  0  0  0  0  0  0  0  0  0  0
    9.0603    7.4153    0.8358 C   0  0  0  0  0  0  0  0  0  0  0  0
    9.1872    8.7995    0.9480 C   0  0  0  0  0  0  0  0  0  0  0  0
    8.0768    9.6252    0.7569 C   0  0  0  0  0  0  0  0  0  0  0  0
    4.7235    2.9274   -0.4535 C   0  0  0  0  0  0  0  0  0  0  0  0
    4.1347    3.6154    0.6088 C   0  0  0  0  0  0  0  0  0  0  0  0
    4.3515    4.9887    0.7602 C   0  0  0  0  0  0  0  0  0  0  0  0
    5.1608    5.6777   -0.1508 C   0  0  0  0  0  0  0  0  0  0  0  0
    5.7482    4.9838   -1.2144 C   0  0  0  0  0  0  0  0  0  0  0  0
    5.5294    3.6114   -1.3650 C   0  0  0  0  0  0  0  0  0  0  0  0
    7.5884    2.5612    0.2516 C   0  0  0  0  0  0  0  0  0  0  0  0
    8.3272    3.2819   -0.6882 C   0  0  0  0  0  0  0  0  0  0  0  0
    8.4048    4.6754   -0.5991 C   0  0  0  0  0  0  0  0  0  0  0  0
    7.7409    5.3525    0.4306 C   0  0  0  0  0  0  0  0  0  0  0  0
    7.0023    4.6257    1.3707 C   0  0  0  0  0  0  0  0  0  0  0  0
    6.9268    3.2327    1.2808 C   0  0  0  0  0  0  0  0  0  0  0  0
    3.3340    7.6024   -0.4166 H   0  0  0  0  0  0  0  0  0  0  0  0
    3.6307   10.0472   -0.1883 H   0  0  0  0  0  0  0  0  0  0  0  0
    5.8530   10.9877    0.3588 H   0  0  0  0  0  0  0  0  0  0  0  0
    9.9244    6.7790    0.9877 H   0  0  0  0  0  0  0  0  0  0  0  0
   10.1492    9.2341    1.1852 H   0  0  0  0  0  0  0  0  0  0  0  0
    8.1827   10.6973    0.8472 H   0  0  0  0  0  0  0  0  0  0  0  0
    4.5551    1.8625   -0.5706 H   0  0  0  0  0  0  0  0  0  0  0  0
    3.5091    3.0837    1.3162 H   0  0  0  0  0  0  0  0  0  0  0  0
    3.8930    5.5185    1.5848 H   0  0  0  0  0  0  0  0  0  0  0  0
    6.3732    5.5105   -1.9233 H   0  0  0  0  0  0  0  0  0  0  0  0
    5.9859    3.0770   -2.1897 H   0  0  0  0  0  0  0  0  0  0  0  0
    7.5285    1.4806    0.1821 H   0  0  0  0  0  0  0  0  0  0  0  0
    8.8407    2.7599   -1.4873 H   0  0  0  0  0  0  0  0  0  0  0  0
    8.9788    5.2305   -1.3294 H   0  0  0  0  0  0  0  0  0  0  0  0
    6.4883    5.1424    2.1703 H   0  0  0  0  0  0  0  0  0  0  0  0
    6.3538    2.6728    2.0104 H   0  0  0  0  0  0  0  0  0  0  0  0
  1  2  4  0  0  0  0
  2  3  4  0  0  0  0
  3  4  4  0  0  0  0
  4  5  4  0  0  0  0
  5  6  4  0  0  0  0
  6  1  4  0  0  0  0
  5  7  4  0  0  0  0
  7  8  4  0  0  0  0
  8  9  4  0  0  0  0
  4 10  4  0  0  0  0
 10  9  4  0  0  0  0
 11 12  4  0  0  0  0
 12 13  4  0  0  0  0
 13 14  4  0  0  0  0
 14 15  4  0  0  0  0
 15 16  4  0  0  0  0
 16 11  4  0  0  0  0
 17 18  4  0  0  0  0
 18 19  4  0  0  0  0
 19 20  4  0  0  0  0
 20 21  4  0  0  0  0
 21 22  4  0  0  0  0
```

```
 22 17  4  0  0  0  0
 20  7  1  0  0  0  0
 14  6  1  0  0  0  0
  1 23  1  0  0  0  0
  2 24  1  0  0  0  0
  3 25  1  0  0  0  0
  8 26  1  0  0  0  0
  9 27  1  0  0  0  0
 10 28  1  0  0  0  0
 11 29  1  0  0  0  0
 12 30  1  0  0  0  0
 13 31  1  0  0  0  0
 15 32  1  0  0  0  0
 16 33  1  0  0  0  0
 17 34  1  0  0  0  0
 18 35  1  0  0  0  0
 19 36  1  0  0  0  0
 21 37  1  0  0  0  0
 22 38  1  0  0  0  0
M  END
>  <Alias name>
1,8-Diphenylnaphthalene

>  <deltaH°(c)>   kJ/mol
-11187

$$$$
Di-n-hexylphthalate
  ChemBrain IXL

 54 54  0  0  0  0  0  0  0  0  1 V2000
    2.4850    9.4884    1.4684 C   0  0  0  0  0  0  0  0  0  0  0  0
    2.7534   10.7830    1.0204 C   0  0  0  0  0  0  0  0  0  0  0  0
    4.0534   11.1482    0.6688 C   0  0  0  0  0  0  0  0  0  0  0  0
    5.0926   10.2223    0.7635 C   0  0  0  0  0  0  0  0  0  0  0  0
    4.8188    8.9041    1.2151 C   0  0  0  0  0  0  0  0  0  0  0  0
    3.5151    8.5523    1.5657 C   0  0  0  0  0  0  0  0  0  0  0  0
    6.4792   10.6512    0.3777 C   0  0  0  0  0  0  0  0  0  0  0  0
    6.6260   11.5340   -0.4519 O   0  0  0  0  0  0  0  0  0  0  0  0
    7.6774   10.0198    0.9935 O   0  0  0  0  0  0  0  0  0  0  0  0
    5.9015    7.8702    1.3313 C   0  0  0  0  0  0  0  0  0  0  0  0
    5.8007    6.9858    2.1660 O   0  0  0  0  0  0  0  0  0  0  0  0
    7.0936    7.9045    0.4422 O   0  0  0  0  0  0  0  0  0  0  0  0
    8.9804   10.4872    0.4429 C   0  0  0  0  0  0  0  0  0  0  0  0
   10.1576    9.8307    1.2161 C   0  0  0  0  0  0  0  0  0  0  0  0
   11.5049   10.3126    0.6113 C   0  0  0  0  0  0  0  0  0  0  0  0
   12.6851    9.6015    1.3185 C   0  0  0  0  0  0  0  0  0  0  0  0
   14.0221   10.2001    0.8187 C   0  0  0  0  0  0  0  0  0  0  0  0
   15.2032    9.4610    1.4839 C   0  0  0  0  0  0  0  0  0  0  0  0
    8.1280    6.8769    0.7467 C   0  0  0  0  0  0  0  0  0  0  0  0
    9.2702    6.9363   -0.3049 C   0  0  0  0  0  0  0  0  0  0  0  0
   10.3636    5.8953    0.0607 C   0  0  0  0  0  0  0  0  0  0  0  0
   11.5311    5.9787   -0.9534 C   0  0  0  0  0  0  0  0  0  0  0  0
   12.6282    4.9596   -0.5621 C   0  0  0  0  0  0  0  0  0  0  0  0
   13.7869    5.0274   -1.5803 C   0  0  0  0  0  0  0  0  0  0  0  0
    1.4757    9.2098    1.7411 H   0  0  0  0  0  0  0  0  0  0  0  0
    1.9514   11.5055    0.9455 H   0  0  0  0  0  0  0  0  0  0  0  0
    4.2571   12.1535    0.3211 H   0  0  0  0  0  0  0  0  0  0  0  0
    3.3027    7.5491    1.9143 H   0  0  0  0  0  0  0  0  0  0  0  0
    9.0453   11.5642    0.5396 H   0  0  0  0  0  0  0  0  0  0  0  0
    9.0452   10.2171   -0.6039 H   0  0  0  0  0  0  0  0  0  0  0  0
   10.0894    8.7533    1.1323 H   0  0  0  0  0  0  0  0  0  0  0  0
   10.1085   10.1158    2.2598 H   0  0  0  0  0  0  0  0  0  0  0  0
```

```
 11.5979   11.3831    0.7466 H   0  0  0  0  0  0  0  0  0  0  0  0
 11.5281   10.0807   -0.4464 H   0  0  0  0  0  0  0  0  0  0  0  0
 12.6573    8.5428    1.0921 H   0  0  0  0  0  0  0  0  0  0  0  0
 12.6041    9.7441    2.3888 H   0  0  0  0  0  0  0  0  0  0  0  0
 14.0650   11.2514    1.0754 H   0  0  0  0  0  0  0  0  0  0  0  0
 14.0877   10.0889   -0.2567 H   0  0  0  0  0  0  0  0  0  0  0  0
 16.1337    9.9412    1.2036 H   0  0  0  0  0  0  0  0  0  0  0  0
 15.2163    8.4296    1.1538 H   0  0  0  0  0  0  0  0  0  0  0  0
 15.0905    9.4942    2.5603 H   0  0  0  0  0  0  0  0  0  0  0  0
  7.6741    5.8935    0.7267 H   0  0  0  0  0  0  0  0  0  0  0  0
  8.5386    7.0618    1.7317 H   0  0  0  0  0  0  0  0  0  0  0  0
  9.7045    7.9282   -0.3116 H   0  0  0  0  0  0  0  0  0  0  0  0
  8.8708    6.7102   -1.2859 H   0  0  0  0  0  0  0  0  0  0  0  0
  9.9363    4.9003    0.0352 H   0  0  0  0  0  0  0  0  0  0  0  0
 10.7375    6.1004    1.0562 H   0  0  0  0  0  0  0  0  0  0  0  0
 11.9476    6.9782   -0.9436 H   0  0  0  0  0  0  0  0  0  0  0  0
 11.1644    5.7500   -1.9464 H   0  0  0  0  0  0  0  0  0  0  0  0
 12.2089    3.9609   -0.5600 H   0  0  0  0  0  0  0  0  0  0  0  0
 13.0033    5.1949    0.4262 H   0  0  0  0  0  0  0  0  0  0  0  0
 14.1820    6.0351   -1.6118 H   0  0  0  0  0  0  0  0  0  0  0  0
 13.4222    4.7516   -2.5620 H   0  0  0  0  0  0  0  0  0  0  0  0
 14.5712    4.3416   -1.2810 H   0  0  0  0  0  0  0  0  0  0  0  0
  1  2  4  0  0  0  0
  2  3  4  0  0  0  0
  3  4  4  0  0  0  0
  4  5  4  0  0  0  0
  5  6  4  0  0  0  0
  6  1  4  0  0  0  0
  7  8  2  0  0  0  0
  7  9  1  0  0  0  0
  7  4  1  0  0  0  0
 10 11  2  0  0  0  0
 10 12  1  0  0  0  0
 10  5  1  0  0  0  0
  9 13  1  0  0  0  0
 13 14  1  0  0  0  0
 14 15  1  0  0  0  0
 15 16  1  0  0  0  0
 16 17  1  0  0  0  0
 17 18  1  0  0  0  0
 19 20  1  0  0  0  0
 20 21  1  0  0  0  0
 21 22  1  0  0  0  0
 22 23  1  0  0  0  0
 23 24  1  0  0  0  0
 19 12  1  0  0  0  0
  1 25  1  0  0  0  0
  2 26  1  0  0  0  0
  3 27  1  0  0  0  0
  6 28  1  0  0  0  0
 13 29  1  0  0  0  0
 13 30  1  0  0  0  0
 14 31  1  0  0  0  0
 14 32  1  0  0  0  0
 15 33  1  0  0  0  0
 15 34  1  0  0  0  0
 16 35  1  0  0  0  0
 16 36  1  0  0  0  0
 17 37  1  0  0  0  0
 17 38  1  0  0  0  0
 18 39  1  0  0  0  0
 18 40  1  0  0  0  0
 18 41  1  0  0  0  0
```

```
 19 42  1  0  0  0  0
 19 43  1  0  0  0  0
 20 44  1  0  0  0  0
 20 45  1  0  0  0  0
 21 46  1  0  0  0  0
 21 47  1  0  0  0  0
 22 48  1  0  0  0  0
 22 49  1  0  0  0  0
 23 50  1  0  0  0  0
 23 51  1  0  0  0  0
 24 52  1  0  0  0  0
 24 53  1  0  0  0  0
 24 54  1  0  0  0  0
M  END
>  <Alias name>
Dihexyl phthalate

>  <deltaH°(c)>   kJ/mol
-11177.97

$$$$
1',2',3',4'-Tetrahydro-1',2-binaphthylmethane
  ChemBrain IXL

 41 44  0  0  0  0  0  0  0  0  1 V2000
    9.1037    7.6747    1.0668 C   0  0  0  0  0  0  0  0  0  0  0  0
    9.0228    6.3104    0.3319 C   0  0  0  0  0  0  0  0  0  0  0  0
    7.7067    6.1768   -0.3624 C   0  0  0  0  0  0  0  0  0  0  0  0
   10.4385    7.7859    1.7769 C   0  0  0  0  0  0  0  0  0  0  0  0
    8.9443    8.8086    0.0468 C   0  0  0  0  0  0  0  0  0  0  0  0
    6.6351    7.0083   -0.0179 C   0  0  0  0  0  0  0  0  0  0  0  0
    7.5414    5.2147   -1.3616 C   0  0  0  0  0  0  0  0  0  0  0  0
   11.2179    8.9790    1.7395 C   0  0  0  0  0  0  0  0  0  0  0  0
   10.9124    6.6831    2.4905 C   0  0  0  0  0  0  0  0  0  0  0  0
    9.2644   10.1292    0.7223 C   0  0  0  0  0  0  0  0  0  0  0  0
    5.4053    6.8755   -0.6706 C   0  0  0  0  0  0  0  0  0  0  0  0
    6.3115    5.0803   -2.0100 C   0  0  0  0  0  0  0  0  0  0  0  0
   10.7673   10.1955    0.9868 C   0  0  0  0  0  0  0  0  0  0  0  0
   12.4374    9.0310    2.4150 C   0  0  0  0  0  0  0  0  0  0  0  0
   12.1373    6.7490    3.1587 C   0  0  0  0  0  0  0  0  0  0  0  0
    5.2434    5.9091   -1.6640 C   0  0  0  0  0  0  0  0  0  0  0  0
    4.3371    7.7092   -0.3301 C   0  0  0  0  0  0  0  0  0  0  0  0
   12.8970    7.9200    3.1214 C   0  0  0  0  0  0  0  0  0  0  0  0
    4.0136    5.7715   -2.3113 C   0  0  0  0  0  0  0  0  0  0  0  0
    3.1083    7.5719   -0.9781 C   0  0  0  0  0  0  0  0  0  0  0  0
    2.9462    6.6023   -1.9679 C   0  0  0  0  0  0  0  0  0  0  0  0
    8.3051    7.7361    1.7957 H   0  0  0  0  0  0  0  0  0  0  0  0
    9.1315    5.5091    1.0509 H   0  0  0  0  0  0  0  0  0  0  0  0
    9.8202    6.2487   -0.3979 H   0  0  0  0  0  0  0  0  0  0  0  0
    9.6245    8.6513   -0.7811 H   0  0  0  0  0  0  0  0  0  0  0  0
    7.9248    8.8270   -0.3201 H   0  0  0  0  0  0  0  0  0  0  0  0
    6.7574    7.7563    0.7555 H   0  0  0  0  0  0  0  0  0  0  0  0
    8.3683    4.5719   -1.6341 H   0  0  0  0  0  0  0  0  0  0  0  0
   10.3274    5.7731    2.5257 H   0  0  0  0  0  0  0  0  0  0  0  0
    8.9726   10.9486    0.0747 H   0  0  0  0  0  0  0  0  0  0  0  0
    8.7261   10.1982    1.6596 H   0  0  0  0  0  0  0  0  0  0  0  0
    6.1860    4.3317   -2.7826 H   0  0  0  0  0  0  0  0  0  0  0  0
   10.9887   11.0809    1.5705 H   0  0  0  0  0  0  0  0  0  0  0  0
   11.2945   10.2487    0.0422 H   0  0  0  0  0  0  0  0  0  0  0  0
   13.0291    9.9385    2.3902 H   0  0  0  0  0  0  0  0  0  0  0  0
   12.4990    5.8896    3.7074 H   0  0  0  0  0  0  0  0  0  0  0  0
    4.4618    8.4629    0.4374 H   0  0  0  0  0  0  0  0  0  0  0  0
   13.8450    7.9662    3.6418 H   0  0  0  0  0  0  0  0  0  0  0  0
```

3.8874 5.0194 -3.0805 H 0 0 0 0 0 0 0 0 0 0 0 0
2.2799 8.2184 -0.7127 H 0 0 0 0 0 0 0 0 0 0 0 0
1.9917 6.4945 -2.4699 H 0 0 0 0 0 0 0 0 0 0 0 0
1 2 1 0 0 0 0
4 1 1 0 0 0 0
1 5 1 0 0 0 0
2 3 1 0 0 0 0
3 6 4 0 0 0 0
7 3 4 0 0 0 0
8 4 4 0 0 0 0
9 4 4 0 0 0 0
5 10 1 0 0 0 0
6 11 4 0 0 0 0
12 7 4 0 0 0 0
13 8 1 0 0 0 0
8 14 4 0 0 0 0
15 9 4 0 0 0 0
10 13 1 0 0 0 0
16 11 4 0 0 0 0
11 17 4 0 0 0 0
16 12 4 0 0 0 0
14 18 4 0 0 0 0
18 15 4 0 0 0 0
19 16 4 0 0 0 0
17 20 4 0 0 0 0
21 19 4 0 0 0 0
20 21 4 0 0 0 0
1 22 1 0 0 0 0
2 23 1 0 0 0 0
2 24 1 0 0 0 0
5 25 1 0 0 0 0
5 26 1 0 0 0 0
6 27 1 0 0 0 0
7 28 1 0 0 0 0
9 29 1 0 0 0 0
10 30 1 0 0 0 0
10 31 1 0 0 0 0
12 32 1 0 0 0 0
13 33 1 0 0 0 0
13 34 1 0 0 0 0
14 35 1 0 0 0 0
15 36 1 0 0 0 0
17 37 1 0 0 0 0
18 38 1 0 0 0 0
19 39 1 0 0 0 0
20 40 1 0 0 0 0
21 41 1 0 0 0 0
M END
> <Alias name>
1',2',3',4'-Tetrahydro-1',2-binaphthylmethane

> <deltaH°(c)> kJ/mol
-11173.5

$$$$
1,4-Diphenylnaphthalene
ChemBrain IXL

38 41 0 0 0 0 0 0 0 0 1 V2000
8.8911 5.2394 0.8997 C 0 0 0 0 0 0 0 0 0 0 0 0
7.4961 5.2432 0.8990 C 0 0 0 0 0 0 0 0 0 0 0 0
6.8019 6.4029 0.5537 C 0 0 0 0 0 0 0 0 0 0 0 0
7.5028 7.5588 0.2091 C 0 0 0 0 0 0 0 0 0 0 0 0

```
  8.8978    7.5550    0.2098 C   0  0  0  0  0  0  0  0  0  0  0  0
  9.5919    6.3953    0.5551 C   0  0  0  0  0  0  0  0  0  0  0  0
  9.5986    8.7109   -0.1348 C   0  0  0  0  0  0  0  0  0  0  0  0
  8.9044    9.8704   -0.4807 C   0  0  0  0  0  0  0  0  0  0  0  0
  7.5094    9.8742   -0.4814 C   0  0  0  0  0  0  0  0  0  0  0  0
  6.8086    8.7185   -0.1362 C   0  0  0  0  0  0  0  0  0  0  0  0
  2.5387    8.7383   -0.1501 C   0  0  0  0  0  0  0  0  0  0  0  0
  3.2392    9.7932    0.4351 C   0  0  0  0  0  0  0  0  0  0  0  0
  4.6341    9.7843    0.4442 C   0  0  0  0  0  0  0  0  0  0  0  0
  5.3286    8.7228   -0.1363 C   0  0  0  0  0  0  0  0  0  0  0  0
  4.6281    7.6653   -0.7168 C   0  0  0  0  0  0  0  0  0  0  0  0
  3.2331    7.6741   -0.7257 C   0  0  0  0  0  0  0  0  0  0  0  0
 13.8686    8.7074   -0.1442 C   0  0  0  0  0  0  0  0  0  0  0  0
 13.1689    7.6494   -0.7248 C   0  0  0  0  0  0  0  0  0  0  0  0
 11.7739    7.6481   -0.7174 C   0  0  0  0  0  0  0  0  0  0  0  0
 11.0786    8.7071   -0.1333 C   0  0  0  0  0  0  0  0  0  0  0  0
 11.7783    9.7624    0.4521 C   0  0  0  0  0  0  0  0  0  0  0  0
 13.1732    9.7637    0.4446 C   0  0  0  0  0  0  0  0  0  0  0  0
  9.4300    4.3391    1.1678 H   0  0  0  0  0  0  0  0  0  0  0  0
  6.9520    4.3458    1.1666 H   0  0  0  0  0  0  0  0  0  0  0  0
  5.7189    6.4059    0.5531 H   0  0  0  0  0  0  0  0  0  0  0  0
 10.6749    6.3923    0.5555 H   0  0  0  0  0  0  0  0  0  0  0  0
  9.4485   10.7677   -0.7487 H   0  0  0  0  0  0  0  0  0  0  0  0
  6.9705   10.7744   -0.7500 H   0  0  0  0  0  0  0  0  0  0  0  0
  1.4557    8.7455   -0.1577 H   0  0  0  0  0  0  0  0  0  0  0  0
  2.7000   10.6191    0.8825 H   0  0  0  0  0  0  0  0  0  0  0  0
  5.1779   10.6014    0.9018 H   0  0  0  0  0  0  0  0  0  0  0  0
  5.1672    6.8375   -1.1606 H   0  0  0  0  0  0  0  0  0  0  0  0
  2.6893    6.8548   -1.1794 H   0  0  0  0  0  0  0  0  0  0  0  0
 14.9515    8.7087   -0.1506 H   0  0  0  0  0  0  0  0  0  0  0  0
 13.7087    6.8289   -1.1812 H   0  0  0  0  0  0  0  0  0  0  0  0
 11.2308    6.8250   -1.1651 H   0  0  0  0  0  0  0  0  0  0  0  0
 11.2385   10.5807    0.9125 H   0  0  0  0  0  0  0  0  0  0  0  0
 13.7164   10.5849    0.8959 H   0  0  0  0  0  0  0  0  0  0  0  0
  1  2  4  0  0  0  0
  2  3  4  0  0  0  0
  3  4  4  0  0  0  0
  4  5  4  0  0  0  0
  5  6  4  0  0  0  0
  6  1  4  0  0  0  0
  5  7  4  0  0  0  0
  7  8  4  0  0  0  0
  8  9  4  0  0  0  0
  4 10  4  0  0  0  0
 10  9  4  0  0  0  0
 11 12  4  0  0  0  0
 12 13  4  0  0  0  0
 13 14  4  0  0  0  0
 14 15  4  0  0  0  0
 15 16  4  0  0  0  0
 16 11  4  0  0  0  0
 17 18  4  0  0  0  0
 18 19  4  0  0  0  0
 19 20  4  0  0  0  0
 20 21  4  0  0  0  0
 21 22  4  0  0  0  0
 22 17  4  0  0  0  0
 20  7  1  0  0  0  0
 14 10  1  0  0  0  0
  1 23  1  0  0  0  0
  2 24  1  0  0  0  0
  3 25  1  0  0  0  0
  6 26  1  0  0  0  0
```

```
  8 27  1  0  0  0  0
  9 28  1  0  0  0  0
 11 29  1  0  0  0  0
 12 30  1  0  0  0  0
 13 31  1  0  0  0  0
 15 32  1  0  0  0  0
 16 33  1  0  0  0  0
 17 34  1  0  0  0  0
 18 35  1  0  0  0  0
 19 36  1  0  0  0  0
 21 37  1  0  0  0  0
 22 38  1  0  0  0  0
M  END
>  <Alias name>
1,4-Diphenylnaphthalene

>  <deltaH°(c)>   kJ/mol
-11167

$$$$
1',2',3',4'-Tetrahydro-1,2-dinaphthylmethane
  ChemBrain IXL

 41 44  0  0  0  0  0  0  0  0  1 V2000
   10.0020    7.6897    0.5915 C   0  0  0  0  0  0  0  0  0  0  0  0
    9.0555    8.8501    0.4985 C   0  0  0  0  0  0  0  0  0  0  0  0
    7.5701    8.3588    0.5815 C   0  0  0  0  0  0  0  0  0  0  0  0
   11.3801    7.8898    0.4271 C   0  0  0  0  0  0  0  0  0  0  0  0
    9.5181    6.4023    0.8440 C   0  0  0  0  0  0  0  0  0  0  0  0
    6.6252    9.5781    0.6057 C   0  0  0  0  0  0  0  0  0  0  0  0
    7.2332    7.5094   -0.6313 C   0  0  0  0  0  0  0  0  0  0  0  0
   12.2623    6.8082    0.5121 C   0  0  0  0  0  0  0  0  0  0  0  0
   11.8766    9.1704    0.1775 C   0  0  0  0  0  0  0  0  0  0  0  0
   10.4032    5.3229    0.9281 C   0  0  0  0  0  0  0  0  0  0  0  0
    5.1848    9.1457    0.4480 C   0  0  0  0  0  0  0  0  0  0  0  0
    5.8751    6.8309   -0.3994 C   0  0  0  0  0  0  0  0  0  0  0  0
   11.7738    5.5264    0.7619 C   0  0  0  0  0  0  0  0  0  0  0  0
   13.6341    7.0083    0.3473 C   0  0  0  0  0  0  0  0  0  0  0  0
   13.2490    9.3697    0.0130 C   0  0  0  0  0  0  0  0  0  0  0  0
    4.8332    7.8505   -0.0296 C   0  0  0  0  0  0  0  0  0  0  0  0
    4.1743   10.0512    0.7802 C   0  0  0  0  0  0  0  0  0  0  0  0
   14.1274    8.2888    0.0978 C   0  0  0  0  0  0  0  0  0  0  0  0
    3.4866    7.5050   -0.1602 C   0  0  0  0  0  0  0  0  0  0  0  0
    2.8320    9.6908    0.6450 C   0  0  0  0  0  0  0  0  0  0  0  0
    2.4892    8.4211    0.1760 C   0  0  0  0  0  0  0  0  0  0  0  0
    9.2502    9.5341    1.3154 H   0  0  0  0  0  0  0  0  0  0  0  0
    9.2108    9.3631   -0.4414 H   0  0  0  0  0  0  0  0  0  0  0  0
    7.4309    7.7744    1.4825 H   0  0  0  0  0  0  0  0  0  0  0  0
    8.4559    6.2402    0.9748 H   0  0  0  0  0  0  0  0  0  0  0  0
    6.7399   10.0985    1.5476 H   0  0  0  0  0  0  0  0  0  0  0  0
    6.8872   10.2472   -0.2055 H   0  0  0  0  0  0  0  0  0  0  0  0
    7.9982    6.7548   -0.7710 H   0  0  0  0  0  0  0  0  0  0  0  0
    7.1788    8.1386   -1.5116 H   0  0  0  0  0  0  0  0  0  0  0  0
   11.1970   10.0101    0.1115 H   0  0  0  0  0  0  0  0  0  0  0  0
   10.0256    4.3261    1.1224 H   0  0  0  0  0  0  0  0  0  0  0  0
    5.5710    6.3218   -1.3058 H   0  0  0  0  0  0  0  0  0  0  0  0
    5.9689    6.1082    0.4018 H   0  0  0  0  0  0  0  0  0  0  0  0
   12.4591    4.6886    0.8269 H   0  0  0  0  0  0  0  0  0  0  0  0
   14.3164    6.1688    0.4131 H   0  0  0  0  0  0  0  0  0  0  0  0
   13.6326   10.3638   -0.1807 H   0  0  0  0  0  0  0  0  0  0  0  0
    4.4336   11.0374    1.1443 H   0  0  0  0  0  0  0  0  0  0  0  0
   15.1923    8.4441   -0.0301 H   0  0  0  0  0  0  0  0  0  0  0  0
    3.2155    6.5214   -0.5241 H   0  0  0  0  0  0  0  0  0  0  0  0
```

```
    2.0546   10.3977    0.9047 H   0  0  0  0  0  0  0  0  0  0  0  0
    1.4470    8.1464    0.0728 H   0  0  0  0  0  0  0  0  0  0  0  0
  1  2  1  0  0  0  0
  1  4  4  0  0  0  0
  5  1  4  0  0  0  0
  2  3  1  0  0  0  0
  6  3  1  0  0  0  0
  3  7  1  0  0  0  0
  4  8  4  0  0  0  0
  4  9  4  0  0  0  0
 10  5  4  0  0  0  0
 11  6  1  0  0  0  0
  7 12  1  0  0  0  0
  8 13  4  0  0  0  0
 14  8  4  0  0  0  0
  9 15  4  0  0  0  0
 13 10  4  0  0  0  0
 11 16  4  0  0  0  0
 17 11  4  0  0  0  0
 12 16  1  0  0  0  0
 18 14  4  0  0  0  0
 15 18  4  0  0  0  0
 16 19  4  0  0  0  0
 20 17  4  0  0  0  0
 19 21  4  0  0  0  0
 21 20  4  0  0  0  0
  2 22  1  0  0  0  0
  2 23  1  0  0  0  0
  3 24  1  0  0  0  0
  5 25  1  0  0  0  0
  6 26  1  0  0  0  0
  6 27  1  0  0  0  0
  7 28  1  0  0  0  0
  7 29  1  0  0  0  0
  9 30  1  0  0  0  0
 10 31  1  0  0  0  0
 12 32  1  0  0  0  0
 12 33  1  0  0  0  0
 13 34  1  0  0  0  0
 14 35  1  0  0  0  0
 15 36  1  0  0  0  0
 17 37  1  0  0  0  0
 18 38  1  0  0  0  0
 19 39  1  0  0  0  0
 20 40  1  0  0  0  0
 21 41  1  0  0  0  0
M  END
>  <Alias name>
1',2',3',4'-Tetrahydro-1,2-dinaphthylmethane

>  <deltaH°(c)>   kJ/mol
-11166

$$$$
Oleic acid
  ChemBrain IXL

 54 53  0  0  0  0  0  0  0  0  1 V2000
    2.0982   13.4575    3.2558 C   0  0  0  0  0  0  0  0  0  0  0  0
    3.5417   13.4223    2.7178 C   0  0  0  0  0  0  0  0  0  0  0  0
    4.0271   11.9616    2.6442 C   0  0  0  0  0  0  0  0  0  0  0  0
    5.4710   11.9269    2.1073 C   0  0  0  0  0  0  0  0  0  0  0  0
    5.9582   10.4667    2.0367 C   0  0  0  0  0  0  0  0  0  0  0  0
```

```
7.4029 10.4334 1.5019 C 0 0 0 0 0 0 0 0 0 0 0 0
7.8930 8.9741 1.4353 C 0 0 0 0 0 0 0 0 0 0 0 0
9.3382 8.9424 0.9019 C 0 0 0 0 0 0 0 0 0 0 0 0
9.8110 7.5417 0.8396 C 0 0 0 0 0 0 0 0 0 0 0 0
11.0126 7.2654 0.3286 C 0 0 0 0 0 0 0 0 0 0 0 0
11.8708 8.3602 -0.1757 C 0 0 0 0 0 0 0 0 0 0 0 0
13.2074 7.7780 -0.6753 C 0 0 0 0 0 0 0 0 0 0 0 0
14.0983 8.9195 -1.2023 C 0 0 0 0 0 0 0 0 0 0 0 0
15.4350 8.3400 -1.7045 C 0 0 0 0 0 0 0 0 0 0 0 0
16.3223 9.4836 -2.2336 C 0 0 0 0 0 0 0 0 0 0 0 0
17.6593 8.9069 -2.7383 C 0 0 0 0 0 0 0 0 0 0 0 0
18.5441 10.0521 -3.2686 C 0 0 0 0 0 0 0 0 0 0 0 0
19.8283 9.4998 -3.7546 C 0 0 0 0 0 0 0 0 0 0 0 0
20.6714 10.2431 -4.2289 O 0 0 0 0 0 0 0 0 0 0 0 0
20.0879 8.0556 -3.6658 O 0 0 0 0 0 0 0 0 0 0 0 0
20.9630 7.7690 -4.0097 H 0 0 0 0 0 0 0 0 0 0 0 0
2.0708 13.0174 4.2450 H 0 0 0 0 0 0 0 0 0 0 0 0
1.7573 14.4842 3.3079 H 0 0 0 0 0 0 0 0 0 0 0 0
1.4520 12.8958 2.5927 H 0 0 0 0 0 0 0 0 0 0 0 0
4.1879 13.9844 3.3807 H 0 0 0 0 0 0 0 0 0 0 0 0
3.5691 13.8620 1.7285 H 0 0 0 0 0 0 0 0 0 0 0 0
3.3816 11.3996 1.9807 H 0 0 0 0 0 0 0 0 0 0 0 0
3.9991 11.5215 3.6334 H 0 0 0 0 0 0 0 0 0 0 0 0
6.1160 12.4910 2.7696 H 0 0 0 0 0 0 0 0 0 0 0 0
5.4985 12.3648 1.1172 H 0 0 0 0 0 0 0 0 0 0 0 0
5.3144 9.9029 1.3731 H 0 0 0 0 0 0 0 0 0 0 0 0
5.9294 10.0282 3.0266 H 0 0 0 0 0 0 0 0 0 0 0 0
8.0459 11.0002 2.1638 H 0 0 0 0 0 0 0 0 0 0 0 0
7.4310 10.8691 0.5109 H 0 0 0 0 0 0 0 0 0 0 0 0
7.2509 8.4073 0.7725 H 0 0 0 0 0 0 0 0 0 0 0 0
7.8642 8.5379 2.4262 H 0 0 0 0 0 0 0 0 0 0 0 0
9.9799 9.5108 1.5637 H 0 0 0 0 0 0 0 0 0 0 0 0
9.3666 9.3772 -0.0896 H 0 0 0 0 0 0 0 0 0 0 0 0
9.1821 6.7408 1.2086 H 0 0 0 0 0 0 0 0 0 0 0 0
11.3594 6.2404 0.2831 H 0 0 0 0 0 0 0 0 0 0 0 0
11.3694 8.8648 -0.9923 H 0 0 0 0 0 0 0 0 0 0 0 0
12.0610 9.0667 0.6227 H 0 0 0 0 0 0 0 0 0 0 0 0
13.7102 7.2751 0.1415 H 0 0 0 0 0 0 0 0 0 0 0 0
13.0175 7.0704 -1.4730 H 0 0 0 0 0 0 0 0 0 0 0 0
13.5943 9.4232 -2.0178 H 0 0 0 0 0 0 0 0 0 0 0 0
14.2886 9.6263 -0.4042 H 0 0 0 0 0 0 0 0 0 0 0 0
15.9405 7.8385 -0.8885 H 0 0 0 0 0 0 0 0 0 0 0 0
15.2450 7.6318 -2.5015 H 0 0 0 0 0 0 0 0 0 0 0 0
15.8156 9.9860 -3.0483 H 0 0 0 0 0 0 0 0 0 0 0 0
16.5127 10.1911 -1.4361 H 0 0 0 0 0 0 0 0 0 0 0 0
18.1670 8.4059 -1.9234 H 0 0 0 0 0 0 0 0 0 0 0 0
17.4692 8.1987 -3.5352 H 0 0 0 0 0 0 0 0 0 0 0 0
18.0357 10.5536 -4.0828 H 0 0 0 0 0 0 0 0 0 0 0 0
18.7343 10.7600 -2.4714 H 0 0 0 0 0 0 0 0 0 0 0 0
1 2 1 0 0 0 0
2 3 1 0 0 0 0
3 4 1 0 0 0 0
4 5 1 0 0 0 0
5 6 1 0 0 0 0
6 7 1 0 0 0 0
7 8 1 0 0 0 0
8 9 1 0 0 0 0
9 10 2 0 0 0 0
10 11 1 0 0 0 0
11 12 1 0 0 0 0
12 13 1 0 0 0 0
13 14 1 0 0 0 0
14 15 1 0 0 0 0
```

```
 15 16  1  0  0  0  0
 16 17  1  0  0  0  0
 17 18  1  0  0  0  0
 18 19  2  0  0  0  0
 18 20  1  0  0  0  0
 20 21  1  0  0  0  0
  1 22  1  0  0  0  0
  1 23  1  0  0  0  0
  1 24  1  0  0  0  0
  2 25  1  0  0  0  0
  2 26  1  0  0  0  0
  3 27  1  0  0  0  0
  3 28  1  0  0  0  0
  4 29  1  0  0  0  0
  4 30  1  0  0  0  0
  5 31  1  0  0  0  0
  5 32  1  0  0  0  0
  6 33  1  0  0  0  0
  6 34  1  0  0  0  0
  7 35  1  0  0  0  0
  7 36  1  0  0  0  0
  8 37  1  0  0  0  0
  8 38  1  0  0  0  0
  9 39  1  0  0  0  0
 10 40  1  0  0  0  0
 11 41  1  0  0  0  0
 11 42  1  0  0  0  0
 12 43  1  0  0  0  0
 12 44  1  0  0  0  0
 13 45  1  0  0  0  0
 13 46  1  0  0  0  0
 14 47  1  0  0  0  0
 14 48  1  0  0  0  0
 15 49  1  0  0  0  0
 15 50  1  0  0  0  0
 16 51  1  0  0  0  0
 16 52  1  0  0  0  0
 17 53  1  0  0  0  0
 17 54  1  0  0  0  0
M  END
>  <Alias name>
(9Z)-Octadecenoic acid

>  <deltaH°(c)>   kJ/mol
-11160.7

$$$$
1-(Biphenyl-4-yl)naphthalene
  ChemBrain IXL

 38 41  0  0  0  0  0  0  0  0  1 V2000
    4.0926    5.5276   -0.7973 C   0  0  0  0  0  0  0  0  0  0  0  0
    2.7778    5.9903   -0.7406 C   0  0  0  0  0  0  0  0  0  0  0  0
    2.5183    7.3110   -0.3738 C   0  0  0  0  0  0  0  0  0  0  0  0
    3.5737    8.1690   -0.0639 C   0  0  0  0  0  0  0  0  0  0  0  0
    4.8885    7.7064   -0.1208 C   0  0  0  0  0  0  0  0  0  0  0  0
    5.1479    6.3856   -0.4871 C   0  0  0  0  0  0  0  0  0  0  0  0
    5.9439    8.5646    0.1885 C   0  0  0  0  0  0  0  0  0  0  0  0
    5.6843    9.8846    0.5577 C   0  0  0  0  0  0  0  0  0  0  0  0
    4.3695   10.3475    0.6136 C   0  0  0  0  0  0  0  0  0  0  0  0
    3.3142    9.4897    0.3030 C   0  0  0  0  0  0  0  0  0  0  0  0
    8.9833    6.3725    0.6118 C   0  0  0  0  0  0  0  0  0  0  0  0
    7.6663    6.8297    0.6625 C   0  0  0  0  0  0  0  0  0  0  0  0
```

```
   7.3390    8.0747    0.1249 C   0  0  0  0  0  0  0  0  0  0  0  0
   8.3268    8.8579   -0.4726 C   0  0  0  0  0  0  0  0  0  0  0  0
   9.6438    8.4008   -0.5230 C   0  0  0  0  0  0  0  0  0  0  0  0
   9.9722    7.1585    0.0200 C   0  0  0  0  0  0  0  0  0  0  0  0
  12.9636    4.8637    0.1069 C   0  0  0  0  0  0  0  0  0  0  0  0
  11.6464    5.3203    0.1577 C   0  0  0  0  0  0  0  0  0  0  0  0
  11.3698    6.6744   -0.0318 C   0  0  0  0  0  0  0  0  0  0  0  0
  12.4106    7.5722   -0.2701 C   0  0  0  0  0  0  0  0  0  0  0  0
  13.7278    7.1156   -0.3209 C   0  0  0  0  0  0  0  0  0  0  0  0
  14.0043    5.7613   -0.1325 C   0  0  0  0  0  0  0  0  0  0  0  0
   4.2940    4.5024   -1.0821 H   0  0  0  0  0  0  0  0  0  0  0  0
   1.9585    5.3243   -0.9813 H   0  0  0  0  0  0  0  0  0  0  0  0
   1.4976    7.6702   -0.3296 H   0  0  0  0  0  0  0  0  0  0  0  0
   6.1686    6.0262   -0.5306 H   0  0  0  0  0  0  0  0  0  0  0  0
   6.5034   10.5499    0.8010 H   0  0  0  0  0  0  0  0  0  0  0  0
   4.1681   11.3729    0.8981 H   0  0  0  0  0  0  0  0  0  0  0  0
   2.2935    9.8490    0.3467 H   0  0  0  0  0  0  0  0  0  0  0  0
   9.2380    5.4074    1.0320 H   0  0  0  0  0  0  0  0  0  0  0  0
   6.8980    6.2182    1.1191 H   0  0  0  0  0  0  0  0  0  0  0  0
   8.0711    9.8208   -0.8973 H   0  0  0  0  0  0  0  0  0  0  0  0
  10.4113    9.0105   -0.9836 H   0  0  0  0  0  0  0  0  0  0  0  0
  13.1783    3.8123    0.2533 H   0  0  0  0  0  0  0  0  0  0  0  0
  10.8386    4.6235    0.3441 H   0  0  0  0  0  0  0  0  0  0  0  0
  12.1960    8.6237   -0.4157 H   0  0  0  0  0  0  0  0  0  0  0  0
  14.5357    7.8125   -0.5066 H   0  0  0  0  0  0  0  0  0  0  0  0
  15.0268    5.4068   -0.1720 H   0  0  0  0  0  0  0  0  0  0  0  0
  1  2  4  0  0  0  0
  2  3  4  0  0  0  0
  3  4  4  0  0  0  0
  4  5  4  0  0  0  0
  5  6  4  0  0  0  0
  6  1  4  0  0  0  0
  5  7  4  0  0  0  0
  7  8  4  0  0  0  0
  8  9  4  0  0  0  0
  9 10  4  0  0  0  0
 10  4  4  0  0  0  0
 11 12  4  0  0  0  0
 12 13  4  0  0  0  0
 13 14  4  0  0  0  0
 14 15  4  0  0  0  0
 15 16  4  0  0  0  0
 16 11  4  0  0  0  0
 13  7  1  0  0  0  0
 17 18  4  0  0  0  0
 18 19  4  0  0  0  0
 19 20  4  0  0  0  0
 20 21  4  0  0  0  0
 21 22  4  0  0  0  0
 22 17  4  0  0  0  0
 19 16  1  0  0  0  0
  1 23  1  0  0  0  0
  2 24  1  0  0  0  0
  3 25  1  0  0  0  0
  6 26  1  0  0  0  0
  8 27  1  0  0  0  0
  9 28  1  0  0  0  0
 10 29  1  0  0  0  0
 11 30  1  0  0  0  0
 12 31  1  0  0  0  0
 14 32  1  0  0  0  0
 15 33  1  0  0  0  0
 17 34  1  0  0  0  0
```

```
 18 35  1  0  0  0  0
 20 36  1  0  0  0  0
 21 37  1  0  0  0  0
 22 38  1  0  0  0  0
M  END
>  <Alias name>
1-(Biphenyl-4-yl)naphthalene

>  <deltaH°(c)>   kJ/mol
-11156

$$$$
Elaidic acid
  ChemBrain IXL

 54 53  0  0  0  0  0  0  0  0  1 V2000
    2.1454    8.0454    3.2948 C   0  0  0  0  0  0  0  0  0  0  0  0
    3.4655    7.5987    2.6371 C   0  0  0  0  0  0  0  0  0  0  0  0
    4.4183    8.8048    2.5268 C   0  0  0  0  0  0  0  0  0  0  0  0
    5.7383    8.3582    1.8688 C   0  0  0  0  0  0  0  0  0  0  0  0
    6.6907    9.5645    1.7577 C   0  0  0  0  0  0  0  0  0  0  0  0
    8.0104    9.1180    1.0991 C   0  0  0  0  0  0  0  0  0  0  0  0
    8.9623   10.3247    0.9867 C   0  0  0  0  0  0  0  0  0  0  0  0
   10.2819    9.8783    0.3278 C   0  0  0  0  0  0  0  0  0  0  0  0
   11.1959   11.0373    0.2194 C   0  0  0  0  0  0  0  0  0  0  0  0
   12.4081   10.8834   -0.3182 C   0  0  0  0  0  0  0  0  0  0  0  0
   13.3214   12.0428   -0.4285 C   0  0  0  0  0  0  0  0  0  0  0  0
   14.6325   11.6012   -1.1073 C   0  0  0  0  0  0  0  0  0  0  0  0
   15.5829   12.8087   -1.2230 C   0  0  0  0  0  0  0  0  0  0  0  0
   16.8937   12.3673   -1.9025 C   0  0  0  0  0  0  0  0  0  0  0  0
   17.8435   13.5751   -2.0197 C   0  0  0  0  0  0  0  0  0  0  0  0
   19.1538   13.1341   -2.7004 C   0  0  0  0  0  0  0  0  0  0  0  0
   20.1033   14.3421   -2.8181 C   0  0  0  0  0  0  0  0  0  0  0  0
   21.3618   13.9185   -3.4717 C   0  0  0  0  0  0  0  0  0  0  0  0
   22.2427   14.7349   -3.6861 O   0  0  0  0  0  0  0  0  0  0  0  0
   21.5505   12.5140   -3.8624 O   0  0  0  0  0  0  0  0  0  0  0  0
   22.4123   12.3142   -4.2911 H   0  0  0  0  0  0  0  0  0  0  0  0
    1.4757    7.1978    3.3721 H   0  0  0  0  0  0  0  0  0  0  0  0
    2.3491    8.4359    4.2841 H   0  0  0  0  0  0  0  0  0  0  0  0
    1.6837    8.8163    2.6903 H   0  0  0  0  0  0  0  0  0  0  0  0
    3.2618    7.2079    1.6478 H   0  0  0  0  0  0  0  0  0  0  0  0
    3.9273    6.8279    3.2417 H   0  0  0  0  0  0  0  0  0  0  0  0
    4.6222    9.1954    3.5161 H   0  0  0  0  0  0  0  0  0  0  0  0
    3.9564    9.5756    1.9224 H   0  0  0  0  0  0  0  0  0  0  0  0
    5.5343    7.9669    0.8798 H   0  0  0  0  0  0  0  0  0  0  0  0
    6.2005    7.5878    2.4736 H   0  0  0  0  0  0  0  0  0  0  0  0
    6.8951    9.9554    2.7468 H   0  0  0  0  0  0  0  0  0  0  0  0
    6.2283   10.3351    1.1534 H   0  0  0  0  0  0  0  0  0  0  0  0
    8.4734    8.3481    1.7039 H   0  0  0  0  0  0  0  0  0  0  0  0
    7.8059    8.7262    0.1104 H   0  0  0  0  0  0  0  0  0  0  0  0
    8.4993   11.0946    0.3820 H   0  0  0  0  0  0  0  0  0  0  0  0
    9.1669   10.7164    1.9754 H   0  0  0  0  0  0  0  0  0  0  0  0
   10.7451    9.1086    0.9327 H   0  0  0  0  0  0  0  0  0  0  0  0
   10.0772    9.4862   -0.6608 H   0  0  0  0  0  0  0  0  0  0  0  0
   10.8813   12.0102    0.5762 H   0  0  0  0  0  0  0  0  0  0  0  0
   12.7233    9.9101   -0.6736 H   0  0  0  0  0  0  0  0  0  0  0  0
   12.8511   12.8176   -1.0213 H   0  0  0  0  0  0  0  0  0  0  0  0
   13.5384   12.4267    0.5606 H   0  0  0  0  0  0  0  0  0  0  0  0
   15.1031   10.8269   -0.5141 H   0  0  0  0  0  0  0  0  0  0  0  0
   14.4153   11.2167   -2.0961 H   0  0  0  0  0  0  0  0  0  0  0  0
   15.1121   13.5832   -1.8156 H   0  0  0  0  0  0  0  0  0  0  0  0
   15.8006   13.1928   -0.2341 H   0  0  0  0  0  0  0  0  0  0  0  0
   17.3652   11.5936   -1.3093 H   0  0  0  0  0  0  0  0  0  0  0  0
```

```
   16.6760   11.9820   -2.8910 H   0  0  0  0  0  0  0  0  0  0  0  0
   17.3717   14.3492   -2.6122 H   0  0  0  0  0  0  0  0  0  0  0  0
   18.0619   13.9599   -1.0312 H   0  0  0  0  0  0  0  0  0  0  0  0
   19.6258   12.3603   -2.1076 H   0  0  0  0  0  0  0  0  0  0  0  0
   18.9353   12.7490   -3.6887 H   0  0  0  0  0  0  0  0  0  0  0  0
   20.3217   14.7273   -1.8298 H   0  0  0  0  0  0  0  0  0  0  0  0
   19.6314   15.1158   -3.4110 H   0  0  0  0  0  0  0  0  0  0  0  0
  1  2  1  0  0  0  0
  2  3  1  0  0  0  0
  3  4  1  0  0  0  0
  4  5  1  0  0  0  0
  5  6  1  0  0  0  0
  6  7  1  0  0  0  0
  7  8  1  0  0  0  0
  8  9  1  0  0  0  0
  9 10  2  0  0  0  0
 10 11  1  0  0  0  0
 11 12  1  0  0  0  0
 12 13  1  0  0  0  0
 13 14  1  0  0  0  0
 14 15  1  0  0  0  0
 15 16  1  0  0  0  0
 16 17  1  0  0  0  0
 17 18  1  0  0  0  0
 18 19  2  0  0  0  0
 18 20  1  0  0  0  0
 20 21  1  0  0  0  0
  1 22  1  0  0  0  0
  1 23  1  0  0  0  0
  1 24  1  0  0  0  0
  2 25  1  0  0  0  0
  2 26  1  0  0  0  0
  3 27  1  0  0  0  0
  3 28  1  0  0  0  0
  4 29  1  0  0  0  0
  4 30  1  0  0  0  0
  5 31  1  0  0  0  0
  5 32  1  0  0  0  0
  6 33  1  0  0  0  0
  6 34  1  0  0  0  0
  7 35  1  0  0  0  0
  7 36  1  0  0  0  0
  8 37  1  0  0  0  0
  8 38  1  0  0  0  0
  9 39  1  0  0  0  0
 10 40  1  0  0  0  0
 11 41  1  0  0  0  0
 11 42  1  0  0  0  0
 12 43  1  0  0  0  0
 12 44  1  0  0  0  0
 13 45  1  0  0  0  0
 13 46  1  0  0  0  0
 14 47  1  0  0  0  0
 14 48  1  0  0  0  0
 15 49  1  0  0  0  0
 15 50  1  0  0  0  0
 16 51  1  0  0  0  0
 16 52  1  0  0  0  0
 17 53  1  0  0  0  0
 17 54  1  0  0  0  0
M  END
>  <Alias name>
Elaidic acid
```

```
>  <deltaH°(c)>   kJ/mol
-11153.2

$$$$
Adipoyl-bis(N,N-diethylthiourea)
  ChemBrain IXL

 54 53  0  0  0  0  0  0  0  0  1 V2000
    8.4424   10.2197    0.5924 C   0  0  0  0  0  0  0  0  0  0  0  0  0
    8.5508    9.0672    0.9776 O   0  0  0  0  0  0  0  0  0  0  0  0  0
    7.1750   10.7542    0.3317 N   0  0  0  0  0  0  0  0  0  0  0  0  0
    6.0784    9.9019    0.1561 C   0  0  0  0  0  0  0  0  0  0  0  0  0
    6.3279    8.2625   -0.2615 S   0  0  0  0  0  0  0  0  0  0  0  0  0
    4.7774   10.3917    0.3223 N   0  0  0  0  0  0  0  0  0  0  0  0  0
    3.6245    9.5131    0.0781 C   0  0  0  0  0  0  0  0  0  0  0  0  0
    4.5644   11.7845    0.7411 C   0  0  0  0  0  0  0  0  0  0  0  0  0
    5.0551   11.9659    2.1906 C   0  0  0  0  0  0  0  0  0  0  0  0  0
    2.3229   10.3305    0.1901 C   0  0  0  0  0  0  0  0  0  0  0  0  0
   14.5481   10.9323   -0.5106 C   0  0  0  0  0  0  0  0  0  0  0  0  0
   14.4393   12.0869   -0.8892 O   0  0  0  0  0  0  0  0  0  0  0  0  0
   15.8159   10.3953   -0.2568 N   0  0  0  0  0  0  0  0  0  0  0  0  0
   16.9139   11.2459   -0.0809 C   0  0  0  0  0  0  0  0  0  0  0  0  0
   16.6673   12.8835    0.3451 S   0  0  0  0  0  0  0  0  0  0  0  0  0
   18.2138   10.7556   -0.2538 N   0  0  0  0  0  0  0  0  0  0  0  0  0
   18.4242    9.3641   -0.6782 C   0  0  0  0  0  0  0  0  0  0  0  0  0
   19.3684   11.6325   -0.0108 C   0  0  0  0  0  0  0  0  0  0  0  0  0
   20.6688   10.8143   -0.1298 C   0  0  0  0  0  0  0  0  0  0  0  0  0
   17.9307    9.1889   -2.1275 C   0  0  0  0  0  0  0  0  0  0  0  0  0
    9.6505   11.0527    0.4009 C   0  0  0  0  0  0  0  0  0  0  0  0  0
   10.8662   10.1423    0.1404 C   0  0  0  0  0  0  0  0  0  0  0  0  0
   12.1242   11.0097   -0.0591 C   0  0  0  0  0  0  0  0  0  0  0  0  0
   13.3399   10.0992   -0.3194 C   0  0  0  0  0  0  0  0  0  0  0  0  0
    7.0519   11.7538    0.2704 H   0  0  0  0  0  0  0  0  0  0  0  0  0
   15.9383    9.3954   -0.2008 H   0  0  0  0  0  0  0  0  0  0  0  0  0
    3.6152    8.7171    0.8125 H   0  0  0  0  0  0  0  0  0  0  0  0  0
    3.6990    9.0872   -0.9149 H   0  0  0  0  0  0  0  0  0  0  0  0  0
    5.1180   12.4468    0.0870 H   0  0  0  0  0  0  0  0  0  0  0  0  0
    3.5090   12.0210    0.6850 H   0  0  0  0  0  0  0  0  0  0  0  0  0
    4.9127   12.9958    2.4937 H   0  0  0  0  0  0  0  0  0  0  0  0  0
    6.1068   11.7142    2.2498 H   0  0  0  0  0  0  0  0  0  0  0  0  0
    4.4906   11.3150    2.8469 H   0  0  0  0  0  0  0  0  0  0  0  0  0
    1.4739    9.6848    0.0026 H   0  0  0  0  0  0  0  0  0  0  0  0  0
    2.2446   10.7500    1.1855 H   0  0  0  0  0  0  0  0  0  0  0  0  0
    2.3356   11.1311   -0.5391 H   0  0  0  0  0  0  0  0  0  0  0  0  0
   17.8708    8.7001   -0.0257 H   0  0  0  0  0  0  0  0  0  0  0  0  0
   19.4793    9.1260   -0.6249 H   0  0  0  0  0  0  0  0  0  0  0  0  0
   19.3758   12.4307   -0.7427 H   0  0  0  0  0  0  0  0  0  0  0  0  0
   19.2976   12.0553    0.9837 H   0  0  0  0  0  0  0  0  0  0  0  0  0
   21.5191   11.4586    0.0567 H   0  0  0  0  0  0  0  0  0  0  0  0  0
   20.7433   10.3977   -1.1267 H   0  0  0  0  0  0  0  0  0  0  0  0  0
   20.6580   10.0115    0.5970 H   0  0  0  0  0  0  0  0  0  0  0  0  0
   18.0712    8.1600   -2.4348 H   0  0  0  0  0  0  0  0  0  0  0  0  0
   16.8792    9.4422   -2.1839 H   0  0  0  0  0  0  0  0  0  0  0  0  0
   18.4948    9.8415   -2.7823 H   0  0  0  0  0  0  0  0  0  0  0  0  0
    9.5044   11.7111   -0.4466 H   0  0  0  0  0  0  0  0  0  0  0  0  0
    9.8264   11.6438    1.2911 H   0  0  0  0  0  0  0  0  0  0  0  0  0
   11.0123    9.4840    0.9879 H   0  0  0  0  0  0  0  0  0  0  0  0  0
   10.6904    9.5511   -0.7498 H   0  0  0  0  0  0  0  0  0  0  0  0  0
   11.9781   11.6679   -0.9066 H   0  0  0  0  0  0  0  0  0  0  0  0  0
   12.2999   11.6009    0.8311 H   0  0  0  0  0  0  0  0  0  0  0  0  0
   13.4858    9.4407    0.5279 H   0  0  0  0  0  0  0  0  0  0  0  0  0
   13.1643    9.5083   -1.2099 H   0  0  0  0  0  0  0  0  0  0  0  0  0
```

```
  1  2  2  0  0  0  0
  1  3  1  0  0  0  0
  4  5  2  0  0  0  0
  4  6  1  0  0  0  0
  4  3  1  0  0  0  0
  6  7  1  0  0  0  0
  6  8  1  0  0  0  0
  8  9  1  0  0  0  0
  7 10  1  0  0  0  0
 11 12  2  0  0  0  0
 11 13  1  0  0  0  0
 14 15  2  0  0  0  0
 14 16  1  0  0  0  0
 14 13  1  0  0  0  0
 16 17  1  0  0  0  0
 16 18  1  0  0  0  0
 18 19  1  0  0  0  0
 17 20  1  0  0  0  0
 21 22  1  0  0  0  0
 22 23  1  0  0  0  0
 23 24  1  0  0  0  0
  1 21  1  0  0  0  0
 11 24  1  0  0  0  0
  3 25  1  0  0  0  0
 13 26  1  0  0  0  0
  7 27  1  0  0  0  0
  7 28  1  0  0  0  0
  8 29  1  0  0  0  0
  8 30  1  0  0  0  0
  9 31  1  0  0  0  0
  9 32  1  0  0  0  0
  9 33  1  0  0  0  0
 10 34  1  0  0  0  0
 10 35  1  0  0  0  0
 10 36  1  0  0  0  0
 17 37  1  0  0  0  0
 17 38  1  0  0  0  0
 18 39  1  0  0  0  0
 18 40  1  0  0  0  0
 19 41  1  0  0  0  0
 19 42  1  0  0  0  0
 19 43  1  0  0  0  0
 20 44  1  0  0  0  0
 20 45  1  0  0  0  0
 20 46  1  0  0  0  0
 21 47  1  0  0  0  0
 21 48  1  0  0  0  0
 22 49  1  0  0  0  0
 22 50  1  0  0  0  0
 23 51  1  0  0  0  0
 23 52  1  0  0  0  0
 24 53  1  0  0  0  0
 24 54  1  0  0  0  0
M  END
>  <Alias name>
Adipoyl-bis(N,N-diethylthiourea)

>  <deltaH°(c)>   kJ/mol
-11143

$$$$
2-(Biphenyl-4-yl)naphthalene
  ChemBrain IXL
```

```
38 41  0  0  0  0  0  0  0  0  1 V2000
    3.1162    6.8916    0.0000 C   0  0  0  0  0  0  0  0  0  0  0  0
    2.5297    8.1573    0.0024 C   0  0  0  0  0  0  0  0  0  0  0  0
    3.3325    9.2982    0.0036 C   0  0  0  0  0  0  0  0  0  0  0  0
    4.7219    9.1733    0.0023 C   0  0  0  0  0  0  0  0  0  0  0  0
    5.3085    7.9076   -0.0001 C   0  0  0  0  0  0  0  0  0  0  0  0
    4.5056    6.7668   -0.0013 C   0  0  0  0  0  0  0  0  0  0  0  0
    6.6979    7.7827   -0.0014 C   0  0  0  0  0  0  0  0  0  0  0  0
    7.5007    8.9235   -0.0003 C   0  0  0  0  0  0  0  0  0  0  0  0
    6.9142   10.1892    0.0021 C   0  0  0  0  0  0  0  0  0  0  0  0
    5.5248   10.3141    0.0034 C   0  0  0  0  0  0  0  0  0  0  0  0
   10.9507    7.4005   -0.0145 C   0  0  0  0  0  0  0  0  0  0  0  0
    9.5613    7.5254   -0.0132 C   0  0  0  0  0  0  0  0  0  0  0  0
    8.9748    8.7910   -0.0016 C   0  0  0  0  0  0  0  0  0  0  0  0
    9.7776    9.9318    0.0086 C   0  0  0  0  0  0  0  0  0  0  0  0
   11.1670    9.8069    0.0073 C   0  0  0  0  0  0  0  0  0  0  0  0
   11.7536    8.5413   -0.0042 C   0  0  0  0  0  0  0  0  0  0  0  0
   15.2036    7.0183   -0.0176 C   0  0  0  0  0  0  0  0  0  0  0  0
   13.8142    7.1431   -0.0163 C   0  0  0  0  0  0  0  0  0  0  0  0
   13.2276    8.4088   -0.0056 C   0  0  0  0  0  0  0  0  0  0  0  0
   14.0305    9.5496    0.0039 C   0  0  0  0  0  0  0  0  0  0  0  0
   15.4199    9.4247    0.0026 C   0  0  0  0  0  0  0  0  0  0  0  0
   16.0064    8.1590   -0.0081 C   0  0  0  0  0  0  0  0  0  0  0  0
    2.4929    6.0060   -0.0009 H   0  0  0  0  0  0  0  0  0  0  0  0
    1.4510    8.2543    0.0034 H   0  0  0  0  0  0  0  0  0  0  0  0
    2.8772   10.2808    0.0055 H   0  0  0  0  0  0  0  0  0  0  0  0
    4.9610    5.7842   -0.0032 H   0  0  0  0  0  0  0  0  0  0  0  0
    7.1532    6.8001   -0.0033 H   0  0  0  0  0  0  0  0  0  0  0  0
    7.5375   11.0749    0.0030 H   0  0  0  0  0  0  0  0  0  0  0  0
    5.0694   11.2967    0.0053 H   0  0  0  0  0  0  0  0  0  0  0  0
   11.4061    6.4179   -0.0235 H   0  0  0  0  0  0  0  0  0  0  0  0
    8.9380    6.6398   -0.0212 H   0  0  0  0  0  0  0  0  0  0  0  0
    9.3223   10.9144    0.0176 H   0  0  0  0  0  0  0  0  0  0  0  0
   11.7903   10.6925    0.0153 H   0  0  0  0  0  0  0  0  0  0  0  0
   15.6590    6.0357   -0.0260 H   0  0  0  0  0  0  0  0  0  0  0  0
   13.1909    6.2575   -0.0237 H   0  0  0  0  0  0  0  0  0  0  0  0
   13.5751   10.5321    0.0122 H   0  0  0  0  0  0  0  0  0  0  0  0
   16.0432   10.3103    0.0100 H   0  0  0  0  0  0  0  0  0  0  0  0
   17.0851    8.0621   -0.0091 H   0  0  0  0  0  0  0  0  0  0  0  0
  1  2  4  0  0  0  0
  2  3  4  0  0  0  0
  3  4  4  0  0  0  0
  4  5  4  0  0  0  0
  5  6  4  0  0  0  0
  6  1  4  0  0  0  0
  5  7  4  0  0  0  0
  7  8  4  0  0  0  0
  8  9  4  0  0  0  0
  9 10  4  0  0  0  0
 10  4  4  0  0  0  0
 11 12  4  0  0  0  0
 12 13  4  0  0  0  0
 13 14  4  0  0  0  0
 14 15  4  0  0  0  0
 15 16  4  0  0  0  0
 16 11  4  0  0  0  0
 17 18  4  0  0  0  0
 18 19  4  0  0  0  0
 19 20  4  0  0  0  0
 20 21  4  0  0  0  0
 21 22  4  0  0  0  0
 22 17  4  0  0  0  0
```

```
 19 16  1  0  0  0  0
  8 13  1  0  0  0  0
  1 23  1  0  0  0  0
  2 24  1  0  0  0  0
  3 25  1  0  0  0  0
  6 26  1  0  0  0  0
  7 27  1  0  0  0  0
  9 28  1  0  0  0  0
 10 29  1  0  0  0  0
 11 30  1  0  0  0  0
 12 31  1  0  0  0  0
 14 32  1  0  0  0  0
 15 33  1  0  0  0  0
 17 34  1  0  0  0  0
 18 35  1  0  0  0  0
 20 36  1  0  0  0  0
 21 37  1  0  0  0  0
 22 38  1  0  0  0  0
M  END
>  <Alias name>
2-(Biphenyl-4-yl)naphthalene

>  <deltaH°(c)>   kJ/mol
-11142

$$$$
Epiandrosterone
  ChemBrain IXL

 51 54  0  0  0  0  0  0  0  0  1 V2000
    6.2748    6.1364   -0.2768 C   0  0  0  0  0  0  0  0  0  0  0  0  0
   10.5078    6.0022    0.0112 C   0  0  0  0  0  0  0  0  0  0  0  0  0
    7.7264    6.4799   -0.6349 C   0  0  0  0  0  0  0  0  0  0  0  0  0
    9.8306    7.2968    0.2415 C   0  0  0  0  0  0  0  0  0  0  0  0  0
    8.4479    7.0174    0.6377 C   0  0  0  0  0  0  0  0  0  0  0  0  0
    5.5713    7.4304    0.2015 C   0  0  0  0  0  0  0  0  0  0  0  0  0
   11.9430    6.3195    0.1717 C   0  0  0  0  0  0  0  0  0  0  0  0  0
   10.0691    5.4764   -1.3275 C   0  0  0  0  0  0  0  0  0  0  0  0  0
    8.4984    5.1962   -1.2316 C   0  0  0  0  0  0  0  0  0  0  0  0  0
    5.5482    5.5830   -1.5250 C   0  0  0  0  0  0  0  0  0  0  0  0  0
   10.7308    8.0737    1.2819 C   0  0  0  0  0  0  0  0  0  0  0  0  0
    7.7626    8.2976    1.1163 C   0  0  0  0  0  0  0  0  0  0  0  0  0
    6.2904    7.9873    1.4554 C   0  0  0  0  0  0  0  0  0  0  0  0  0
   12.1742    7.5287    1.0282 C   0  0  0  0  0  0  0  0  0  0  0  0  0
    4.1102    7.1253    0.5451 C   0  0  0  0  0  0  0  0  0  0  0  0  0
   12.8406    5.6765   -0.3502 O   0  0  0  0  0  0  0  0  0  0  0  0  0
    4.0633    5.3103   -1.1866 C   0  0  0  0  0  0  0  0  0  0  0  0  0
    3.3931    6.6146   -0.7115 C   0  0  0  0  0  0  0  0  0  0  0  0  0
    6.2464    5.0835    0.8487 C   0  0  0  0  0  0  0  0  0  0  0  0  0
   10.0964    4.9942    1.1030 C   0  0  0  0  0  0  0  0  0  0  0  0  0
    1.9807    6.3580   -0.3984 O   0  0  0  0  0  0  0  0  0  0  0  0  0
    7.7225    7.2573   -1.3836 H   0  0  0  0  0  0  0  0  0  0  0  0  0
    9.8187    7.8502   -0.6851 H   0  0  0  0  0  0  0  0  0  0  0  0  0
    8.4291    6.2707    1.4186 H   0  0  0  0  0  0  0  0  0  0  0  0  0
    5.6066    8.1677   -0.5873 H   0  0  0  0  0  0  0  0  0  0  0  0  0
   10.2652    6.2107   -2.0943 H   0  0  0  0  0  0  0  0  0  0  0  0  0
   10.5937    4.5585   -1.5537 H   0  0  0  0  0  0  0  0  0  0  0  0  0
    8.3285    4.3469   -0.5845 H   0  0  0  0  0  0  0  0  0  0  0  0  0
    8.1147    4.9747   -2.2164 H   0  0  0  0  0  0  0  0  0  0  0  0  0
    5.6075    6.3059   -2.3251 H   0  0  0  0  0  0  0  0  0  0  0  0  0
    6.0200    4.6627   -1.8368 H   0  0  0  0  0  0  0  0  0  0  0  0  0
   10.6906    9.1352    1.0988 H   0  0  0  0  0  0  0  0  0  0  0  0  0
   10.4179    7.8503    2.2912 H   0  0  0  0  0  0  0  0  0  0  0  0  0
```

7.8047	9.0419	0.3349	H	0	0	0	0	0	0	0	0	0	0	0	0	0
8.2663	8.6689	1.9962	H	0	0	0	0	0	0	0	0	0	0	0	0	0
6.2512	7.2548	2.2474	H	0	0	0	0	0	0	0	0	0	0	0	0	0
5.7991	8.8926	1.7785	H	0	0	0	0	0	0	0	0	0	0	0	0	0
12.7691	8.2591	0.4994	H	0	0	0	0	0	0	0	0	0	0	0	0	0
12.6474	7.2509	1.9580	H	0	0	0	0	0	0	0	0	0	0	0	0	0
4.0709	6.3703	1.3158	H	0	0	0	0	0	0	0	0	0	0	0	0	0
3.6272	8.0253	0.8960	H	0	0	0	0	0	0	0	0	0	0	0	0	0
4.0029	4.5693	-0.4036	H	0	0	0	0	0	0	0	0	0	0	0	0	0
3.5551	4.9456	-2.0670	H	0	0	0	0	0	0	0	0	0	0	0	0	0
3.4597	7.3589	-1.4916	H	0	0	0	0	0	0	0	0	0	0	0	0	0
5.2215	4.8761	1.1210	H	0	0	0	0	0	0	0	0	0	0	0	0	0
6.7180	4.1750	0.5045	H	0	0	0	0	0	0	0	0	0	0	0	0	0
6.7777	5.4615	1.7092	H	0	0	0	0	0	0	0	0	0	0	0	0	0
9.0545	4.7396	0.9849	H	0	0	0	0	0	0	0	0	0	0	0	0	0
10.6981	4.0991	1.0098	H	0	0	0	0	0	0	0	0	0	0	0	0	0
10.2532	5.4322	2.0761	H	0	0	0	0	0	0	0	0	0	0	0	0	0
1.4990	7.1100	-0.0730	H	0	0	0	0	0	0	0	0	0	0	0	0	0

2	8	1	0	0	0	0
3	1	1	0	0	0	0
4	5	1	0	0	0	0
5	3	1	0	0	0	0
6	1	1	0	0	0	0
7	2	1	0	0	0	0
8	9	1	0	0	0	0
9	3	1	0	0	0	0
10	1	1	0	0	0	0
11	4	1	0	0	0	0
12	13	1	0	0	0	0
13	6	1	0	0	0	0
14	11	1	0	0	0	0
15	6	1	0	0	0	0
16	7	2	0	0	0	0
17	10	1	0	0	0	0
18	17	1	0	0	0	0
1	19	1	0	0	0	0
2	20	1	0	0	0	0
18	21	1	0	0	0	0
18	15	1	0	0	0	0
12	5	1	0	0	0	0
2	4	1	0	0	0	0
14	7	1	0	0	0	0
3	22	1	0	0	0	0
4	23	1	0	0	0	0
5	24	1	0	0	0	0
6	25	1	0	0	0	0
8	26	1	0	0	0	0
8	27	1	0	0	0	0
9	28	1	0	0	0	0
9	29	1	0	0	0	0
10	30	1	0	0	0	0
10	31	1	0	0	0	0
11	32	1	0	0	0	0
11	33	1	0	0	0	0
12	34	1	0	0	0	0
12	35	1	0	0	0	0
13	36	1	0	0	0	0
13	37	1	0	0	0	0
14	38	1	0	0	0	0
14	39	1	0	0	0	0
15	40	1	0	0	0	0
15	41	1	0	0	0	0
17	42	1	0	0	0	0

```
 17 43  1  0  0  0  0
 18 44  1  0  0  0  0
 19 45  1  0  0  0  0
 19 46  1  0  0  0  0
 19 47  1  0  0  0  0
 20 48  1  0  0  0  0
 20 49  1  0  0  0  0
 20 50  1  0  0  0  0
 21 51  1  0  0  0  0
M  END
>  <Alias name>
Epiandrosterone

>  <deltaH°(c)>   kJ/mol
-11137

$$$$
Dodecylcyclopentane
  ChemBrain IXL

 51 51  0  0  0  0  0  0  0  0  1 V2000
   17.4772   10.5576    0.4280 C   0  0  0  0  0  0  0  0  0  0  0  0
   18.7431   11.2369   -0.0731 C   0  0  0  0  0  0  0  0  0  0  0  0
   19.8366   10.1892    0.1192 C   0  0  0  0  0  0  0  0  0  0  0  0
   19.1274    8.8094   -0.0822 C   0  0  0  0  0  0  0  0  0  0  0  0
   17.5946    9.1199   -0.0732 C   0  0  0  0  0  0  0  0  0  0  0  0
   16.2106   11.2449   -0.1208 C   0  0  0  0  0  0  0  0  0  0  0  0
   14.9600   10.4965    0.3813 C   0  0  0  0  0  0  0  0  0  0  0  0
   13.6948   11.1849   -0.1665 C   0  0  0  0  0  0  0  0  0  0  0  0
   12.4446   10.4360    0.3345 C   0  0  0  0  0  0  0  0  0  0  0  0
   11.1793   11.1233   -0.2149 C   0  0  0  0  0  0  0  0  0  0  0  0
    9.9291   10.3735    0.2850 C   0  0  0  0  0  0  0  0  0  0  0  0
    8.6639   11.0596   -0.2661 C   0  0  0  0  0  0  0  0  0  0  0  0
    7.4138   10.3091    0.2328 C   0  0  0  0  0  0  0  0  0  0  0  0
    6.1487   10.9942   -0.3196 C   0  0  0  0  0  0  0  0  0  0  0  0
    4.8985   10.2431    0.1786 C   0  0  0  0  0  0  0  0  0  0  0  0
    3.6333   10.9278   -0.3745 C   0  0  0  0  0  0  0  0  0  0  0  0
    2.3832   10.1767    0.1233 C   0  0  0  0  0  0  0  0  0  0  0  0
   17.4710   10.5637    1.5110 H   0  0  0  0  0  0  0  0  0  0  0  0
   18.9578   12.1392    0.4859 H   0  0  0  0  0  0  0  0  0  0  0  0
   18.6379   11.4611   -1.1275 H   0  0  0  0  0  0  0  0  0  0  0  0
   20.1974   10.2515    1.1384 H   0  0  0  0  0  0  0  0  0  0  0  0
   20.6562   10.3238   -0.5756 H   0  0  0  0  0  0  0  0  0  0  0  0
   19.3949    8.1455    0.7305 H   0  0  0  0  0  0  0  0  0  0  0  0
   19.3993    8.3669   -1.0324 H   0  0  0  0  0  0  0  0  0  0  0  0
   17.2235    9.0783   -1.0899 H   0  0  0  0  0  0  0  0  0  0  0  0
   17.0484    8.4248    0.5522 H   0  0  0  0  0  0  0  0  0  0  0  0
   16.2310   11.2280   -1.2035 H   0  0  0  0  0  0  0  0  0  0  0  0
   16.1792   12.2712    0.2236 H   0  0  0  0  0  0  0  0  0  0  0  0
   14.9398   10.5129    1.4640 H   0  0  0  0  0  0  0  0  0  0  0  0
   14.9905    9.4704    0.0364 H   0  0  0  0  0  0  0  0  0  0  0  0
   13.7153   11.1690   -1.2492 H   0  0  0  0  0  0  0  0  0  0  0  0
   13.6637   12.2108    0.1790 H   0  0  0  0  0  0  0  0  0  0  0  0
   12.4233   10.4529    1.4172 H   0  0  0  0  0  0  0  0  0  0  0  0
   12.4763    9.4098   -0.0100 H   0  0  0  0  0  0  0  0  0  0  0  0
   11.2011   11.1070   -1.2976 H   0  0  0  0  0  0  0  0  0  0  0  0
   11.1470   12.1493    0.1301 H   0  0  0  0  0  0  0  0  0  0  0  0
    9.9067   10.3908    1.3676 H   0  0  0  0  0  0  0  0  0  0  0  0
    9.9621    9.3472   -0.0591 H   0  0  0  0  0  0  0  0  0  0  0  0
    8.6869   11.0429   -1.3487 H   0  0  0  0  0  0  0  0  0  0  0  0
    8.6305   12.0857    0.0786 H   0  0  0  0  0  0  0  0  0  0  0  0
    7.3903   10.3265    1.3154 H   0  0  0  0  0  0  0  0  0  0  0  0
    7.4478    9.2827   -0.1111 H   0  0  0  0  0  0  0  0  0  0  0  0
```

```
    6.1724   10.9771   -1.4022 H   0  0  0  0  0  0  0  0  0  0  0  0
    6.1143   12.0204    0.0247 H   0  0  0  0  0  0  0  0  0  0  0  0
    4.8745   10.2605    1.2612 H   0  0  0  0  0  0  0  0  0  0  0  0
    4.9331    9.2167   -0.1653 H   0  0  0  0  0  0  0  0  0  0  0  0
    3.6574   10.9105   -1.4570 H   0  0  0  0  0  0  0  0  0  0  0  0
    3.5985   11.9540   -0.0305 H   0  0  0  0  0  0  0  0  0  0  0  0
    2.4178    9.1503   -0.2206 H   0  0  0  0  0  0  0  0  0  0  0  0
    2.3589   10.1940    1.2059 H   0  0  0  0  0  0  0  0  0  0  0  0
    1.4944   10.6579   -0.2654 H   0  0  0  0  0  0  0  0  0  0  0  0
  1  2  1  0  0  0  0
  2  3  1  0  0  0  0
  3  4  1  0  0  0  0
  4  5  1  0  0  0  0
  1  5  1  0  0  0  0
  6  7  1  0  0  0  0
  7  8  1  0  0  0  0
  8  9  1  0  0  0  0
  9 10  1  0  0  0  0
 10 11  1  0  0  0  0
 11 12  1  0  0  0  0
 12 13  1  0  0  0  0
 13 14  1  0  0  0  0
 14 15  1  0  0  0  0
 15 16  1  0  0  0  0
  1  6  1  0  0  0  0
 16 17  1  0  0  0  0
  1 18  1  0  0  0  0
  2 19  1  0  0  0  0
  2 20  1  0  0  0  0
  3 21  1  0  0  0  0
  3 22  1  0  0  0  0
  4 23  1  0  0  0  0
  4 24  1  0  0  0  0
  5 25  1  0  0  0  0
  5 26  1  0  0  0  0
  6 27  1  0  0  0  0
  6 28  1  0  0  0  0
  7 29  1  0  0  0  0
  7 30  1  0  0  0  0
  8 31  1  0  0  0  0
  8 32  1  0  0  0  0
  9 33  1  0  0  0  0
  9 34  1  0  0  0  0
 10 35  1  0  0  0  0
 10 36  1  0  0  0  0
 11 37  1  0  0  0  0
 11 38  1  0  0  0  0
 12 39  1  0  0  0  0
 12 40  1  0  0  0  0
 13 41  1  0  0  0  0
 13 42  1  0  0  0  0
 14 43  1  0  0  0  0
 14 44  1  0  0  0  0
 15 45  1  0  0  0  0
 15 46  1  0  0  0  0
 16 47  1  0  0  0  0
 16 48  1  0  0  0  0
 17 49  1  0  0  0  0
 17 50  1  0  0  0  0
 17 51  1  0  0  0  0
M  END
>  <Alias name>
Dodecylcyclopentane
```

```
>  <deltaH°(c)>   kJ/mol
-11135

$$$$
1,2,4-Tri-t-butylbenzene
  ChemBrain IXL

 48 48  0  0  0  0  0  0  0  0  1 V2000
    7.4348    6.9207   -0.3164 C   0  0  0  0  0  0  0  0  0  0  0  0
    6.3511    7.7822   -0.4880 C   0  0  0  0  0  0  0  0  0  0  0  0
    5.0489    7.3002   -0.3546 C   0  0  0  0  0  0  0  0  0  0  0  0
    4.8302    5.9560   -0.0526 C   0  0  0  0  0  0  0  0  0  0  0  0
    5.9139    5.0948    0.1206 C   0  0  0  0  0  0  0  0  0  0  0  0
    7.2161    5.5765   -0.0141 C   0  0  0  0  0  0  0  0  0  0  0  0
    5.6820    3.6700    0.4472 C   0  0  0  0  0  0  0  0  0  0  0  0
    5.3336    3.5358    1.9423 C   0  0  0  0  0  0  0  0  0  0  0  0
    6.9563    2.8590    0.1419 C   0  0  0  0  0  0  0  0  0  0  0  0
    4.5146    3.1316   -0.4026 C   0  0  0  0  0  0  0  0  0  0  0  0
    3.4485    5.4435    0.0842 C   0  0  0  0  0  0  0  0  0  0  0  0
    3.3701    4.4950    1.2961 C   0  0  0  0  0  0  0  0  0  0  0  0
    3.0559    4.6775   -1.1940 C   0  0  0  0  0  0  0  0  0  0  0  0
    2.4809    6.6246    0.2928 C   0  0  0  0  0  0  0  0  0  0  0  0
    8.8165    7.4327   -0.4550 C   0  0  0  0  0  0  0  0  0  0  0  0
    9.2367    7.3824   -1.9367 C   0  0  0  0  0  0  0  0  0  0  0  0
    9.7737    6.5606    0.3803 C   0  0  0  0  0  0  0  0  0  0  0  0
    8.8776    8.8883    0.0471 C   0  0  0  0  0  0  0  0  0  0  0  0
    6.5209    8.8253   -0.7248 H   0  0  0  0  0  0  0  0  0  0  0  0
    4.2076    7.9696   -0.4855 H   0  0  0  0  0  0  0  0  0  0  0  0
    8.0573    4.9069    0.1160 H   0  0  0  0  0  0  0  0  0  0  0  0
    4.9825    2.5311    2.1424 H   0  0  0  0  0  0  0  0  0  0  0  0
    6.2160    3.7352    2.5378 H   0  0  0  0  0  0  0  0  0  0  0  0
    4.5577    4.2469    2.1974 H   0  0  0  0  0  0  0  0  0  0  0  0
    7.7711    3.2223    0.7558 H   0  0  0  0  0  0  0  0  0  0  0  0
    7.2141    2.9719   -0.9039 H   0  0  0  0  0  0  0  0  0  0  0  0
    6.7792    1.8130    0.3591 H   0  0  0  0  0  0  0  0  0  0  0  0
    4.3749    2.0775   -0.1974 H   0  0  0  0  0  0  0  0  0  0  0  0
    3.6088    3.6709   -0.1545 H   0  0  0  0  0  0  0  0  0  0  0  0
    4.7398    3.2682   -1.4531 H   0  0  0  0  0  0  0  0  0  0  0  0
    4.0190    3.6438    1.1309 H   0  0  0  0  0  0  0  0  0  0  0  0
    3.6858    5.0219    2.1880 H   0  0  0  0  0  0  0  0  0  0  0  0
    2.3502    4.1526    1.4211 H   0  0  0  0  0  0  0  0  0  0  0  0
    3.8483    3.9888   -1.4598 H   0  0  0  0  0  0  0  0  0  0  0  0
    2.1415    4.1248   -1.0171 H   0  0  0  0  0  0  0  0  0  0  0  0
    2.9020    5.3805   -2.0033 H   0  0  0  0  0  0  0  0  0  0  0  0
    1.4730    6.2481    0.4163 H   0  0  0  0  0  0  0  0  0  0  0  0
    2.7710    7.1769    1.1782 H   0  0  0  0  0  0  0  0  0  0  0  0
    2.5188    7.2789   -0.5693 H   0  0  0  0  0  0  0  0  0  0  0  0
   10.2452    7.7638   -2.0391 H   0  0  0  0  0  0  0  0  0  0  0  0
    8.5597    7.9896   -2.5248 H   0  0  0  0  0  0  0  0  0  0  0  0
    9.2006    6.3585   -2.2876 H   0  0  0  0  0  0  0  0  0  0  0  0
   10.7766    6.9646    0.3189 H   0  0  0  0  0  0  0  0  0  0  0  0
    9.7681    5.5482   -0.0043 H   0  0  0  0  0  0  0  0  0  0  0  0
    9.4490    6.5559    1.4135 H   0  0  0  0  0  0  0  0  0  0  0  0
    8.2265    9.5067   -0.5583 H   0  0  0  0  0  0  0  0  0  0  0  0
    9.8944    9.2536   -0.0276 H   0  0  0  0  0  0  0  0  0  0  0  0
    8.5549    8.9277    1.0802 H   0  0  0  0  0  0  0  0  0  0  0  0
  1  2  4  0  0  0  0
  2  3  4  0  0  0  0
  3  4  4  0  0  0  0
  4  5  4  0  0  0  0
  5  6  4  0  0  0  0
  6  1  4  0  0  0  0
```

```
  7  8  1  0  0  0  0
  7  9  1  0  0  0  0
  7 10  1  0  0  0  0
  4 11  1  0  0  0  0
 11 12  1  0  0  0  0
 11 13  1  0  0  0  0
 11 14  1  0  0  0  0
 15 16  1  0  0  0  0
 15 17  1  0  0  0  0
 15 18  1  0  0  0  0
  7  5  1  0  0  0  0
 15  1  1  0  0  0  0
  2 19  1  0  0  0  0
  3 20  1  0  0  0  0
  6 21  1  0  0  0  0
  8 22  1  0  0  0  0
  8 23  1  0  0  0  0
  8 24  1  0  0  0  0
  9 25  1  0  0  0  0
  9 26  1  0  0  0  0
  9 27  1  0  0  0  0
 10 28  1  0  0  0  0
 10 29  1  0  0  0  0
 10 30  1  0  0  0  0
 12 31  1  0  0  0  0
 12 32  1  0  0  0  0
 12 33  1  0  0  0  0
 13 34  1  0  0  0  0
 13 35  1  0  0  0  0
 13 36  1  0  0  0  0
 14 37  1  0  0  0  0
 14 38  1  0  0  0  0
 14 39  1  0  0  0  0
 16 40  1  0  0  0  0
 16 41  1  0  0  0  0
 16 42  1  0  0  0  0
 17 43  1  0  0  0  0
 17 44  1  0  0  0  0
 17 45  1  0  0  0  0
 18 46  1  0  0  0  0
 18 47  1  0  0  0  0
 18 48  1  0  0  0  0
M  END
>  <Alias name>
1,2,4-Tri-t-butylbenzene

>  <deltaH°(c)>   kJ/mol
-11126.6

$$$$
Cycloheptadecane
  ChemBrain IXL

 51 51  0  0  0  0  0  0  0  0  1 V2000
    4.3838    5.2877   -0.7231 C   0  0  0  0  0  0  0  0  0  0  0  0
    5.3744    4.5143    0.1679 C   0  0  0  0  0  0  0  0  0  0  0  0
    3.1343    5.6439    0.0962 C   0  0  0  0  0  0  0  0  0  0  0  0
    5.4667    7.8928   -0.1250 C   0  0  0  0  0  0  0  0  0  0  0  0
    6.8796    8.4706    0.1457 C   0  0  0  0  0  0  0  0  0  0  0  0
    4.3980    8.8756    0.3517 C   0  0  0  0  0  0  0  0  0  0  0  0
    7.9178    7.7438   -0.7491 C   0  0  0  0  0  0  0  0  0  0  0  0
    9.2946    7.7265   -0.0509 C   0  0  0  0  0  0  0  0  0  0  0  0
    6.7119    4.4016   -0.5671 C   0  0  0  0  0  0  0  0  0  0  0  0
```

7.8644 4.3448 0.4600 C 0 0 0 0 0 0 0 0 0 0 0 0
2.5685 7.0531 -0.3083 C 0 0 0 0 0 0 0 0 0 0 0 0
3.0443 8.1501 0.7378 C 0 0 0 0 0 0 0 0 0 0 0 0
10.4084 7.3100 -1.0633 C 0 0 0 0 0 0 0 0 0 0 0 0
11.6751 6.8520 -0.2890 C 0 0 0 0 0 0 0 0 0 0 0 0
9.1793 4.6932 -0.2556 C 0 0 0 0 0 0 0 0 0 0 0 0
10.3218 4.8799 0.7779 C 0 0 0 0 0 0 0 0 0 0 0 0
11.6361 5.3001 0.0469 C 0 0 0 0 0 0 0 0 0 0 0 0
4.8463 6.1701 -1.0575 H 0 0 0 0 0 0 0 0 0 0 0 0
4.1035 4.6894 -1.5811 H 0 0 0 0 0 0 0 0 0 0 0 0
4.9893 3.5294 0.4010 H 0 0 0 0 0 0 0 0 0 0 0 0
5.5233 5.0712 1.0846 H 0 0 0 0 0 0 0 0 0 0 0 0
2.3806 4.8865 -0.0793 H 0 0 0 0 0 0 0 0 0 0 0 0
3.3814 5.6600 1.1506 H 0 0 0 0 0 0 0 0 0 0 0 0
5.3425 7.7278 -1.1861 H 0 0 0 0 0 0 0 0 0 0 0 0
5.3650 6.9603 0.4081 H 0 0 0 0 0 0 0 0 0 0 0 0
7.1269 8.3166 1.1888 H 0 0 0 0 0 0 0 0 0 0 0 0
6.8966 9.5305 -0.0762 H 0 0 0 0 0 0 0 0 0 0 0 0
4.7663 9.3989 1.2255 H 0 0 0 0 0 0 0 0 0 0 0 0
4.2092 9.5953 -0.4351 H 0 0 0 0 0 0 0 0 0 0 0 0
7.9906 8.2541 -1.7015 H 0 0 0 0 0 0 0 0 0 0 0 0
7.6026 6.7382 -0.9104 H 0 0 0 0 0 0 0 0 0 0 0 0
9.2635 7.0243 0.7635 H 0 0 0 0 0 0 0 0 0 0 0 0
9.5185 8.7127 0.3364 H 0 0 0 0 0 0 0 0 0 0 0 0
6.8398 5.2723 -1.1812 H 0 0 0 0 0 0 0 0 0 0 0 0
6.7212 3.5190 -1.1943 H 0 0 0 0 0 0 0 0 0 0 0 0
7.9255 3.3564 0.8981 H 0 0 0 0 0 0 0 0 0 0 0 0
7.6825 5.0763 1.2377 H 0 0 0 0 0 0 0 0 0 0 0 0
2.8751 7.3174 -1.3129 H 0 0 0 0 0 0 0 0 0 0 0 0
1.4876 7.0028 -0.2683 H 0 0 0 0 0 0 0 0 0 0 0 0
2.2723 8.9053 0.8166 H 0 0 0 0 0 0 0 0 0 0 0 0
3.1615 7.6752 1.7040 H 0 0 0 0 0 0 0 0 0 0 0 0
10.6556 8.1680 -1.6759 H 0 0 0 0 0 0 0 0 0 0 0 0
10.0671 6.5103 -1.6992 H 0 0 0 0 0 0 0 0 0 0 0 0
11.7559 7.4217 0.6285 H 0 0 0 0 0 0 0 0 0 0 0 0
12.5467 7.0457 -0.9017 H 0 0 0 0 0 0 0 0 0 0 0 0
9.0429 5.5950 -0.7962 H 0 0 0 0 0 0 0 0 0 0 0 0
9.4383 3.8987 -0.9442 H 0 0 0 0 0 0 0 0 0 0 0 0
10.4866 3.9384 1.2870 H 0 0 0 0 0 0 0 0 0 0 0 0
10.0507 5.6335 1.5027 H 0 0 0 0 0 0 0 0 0 0 0 0
12.4793 5.0672 0.6851 H 0 0 0 0 0 0 0 0 0 0 0 0
11.7241 4.7327 -0.8713 H 0 0 0 0 0 0 0 0 0 0 0 0
2 1 1 0 0 0 0
3 1 1 0 0 0 0
9 2 1 0 0 0 0
3 11 1 0 0 0 0
4 5 1 0 0 0 0
4 6 1 0 0 0 0
5 7 1 0 0 0 0
12 6 1 0 0 0 0
7 8 1 0 0 0 0
8 13 1 0 0 0 0
10 9 1 0 0 0 0
15 10 1 0 0 0 0
11 12 1 0 0 0 0
14 13 1 0 0 0 0
14 17 1 0 0 0 0
15 16 1 0 0 0 0
17 16 1 0 0 0 0
1 18 1 0 0 0 0
1 19 1 0 0 0 0
2 20 1 0 0 0 0
2 21 1 0 0 0 0

```
  3 22  1  0  0  0  0
  3 23  1  0  0  0  0
  4 24  1  0  0  0  0
  4 25  1  0  0  0  0
  5 26  1  0  0  0  0
  5 27  1  0  0  0  0
  6 28  1  0  0  0  0
  6 29  1  0  0  0  0
  7 30  1  0  0  0  0
  7 31  1  0  0  0  0
  8 32  1  0  0  0  0
  8 33  1  0  0  0  0
  9 34  1  0  0  0  0
  9 35  1  0  0  0  0
 10 36  1  0  0  0  0
 10 37  1  0  0  0  0
 11 38  1  0  0  0  0
 11 39  1  0  0  0  0
 12 40  1  0  0  0  0
 12 41  1  0  0  0  0
 13 42  1  0  0  0  0
 13 43  1  0  0  0  0
 14 44  1  0  0  0  0
 14 45  1  0  0  0  0
 15 46  1  0  0  0  0
 15 47  1  0  0  0  0
 16 48  1  0  0  0  0
 16 49  1  0  0  0  0
 17 50  1  0  0  0  0
 17 51  1  0  0  0  0
M  END
>  <Alias name>
Cycloheptadecane

>  <deltaH°(c)>   kJ/mol
-11126.1

$$$$
1-Heptadecanol
  ChemBrain IXL

 54 53  0  0  0  0  0  0  0  0  1 V2000
   19.4603   12.3467   -1.3900 C   0  0  0  0  0  0  0  0  0  0  0  0
   20.7225   11.4958   -1.6315 C   0  0  0  0  0  0  0  0  0  0  0  0
   21.9308   12.4234   -1.8638 C   0  0  0  0  0  0  0  0  0  0  0  0
   23.1344   11.6116   -2.0940 O   0  0  0  0  0  0  0  0  0  0  0  0
   18.2519   11.4189   -1.1577 C   0  0  0  0  0  0  0  0  0  0  0  0
   16.9898   12.2696   -0.9162 C   0  0  0  0  0  0  0  0  0  0  0  0
   15.7814   11.3418   -0.6838 C   0  0  0  0  0  0  0  0  0  0  0  0
   14.5193   12.1925   -0.4422 C   0  0  0  0  0  0  0  0  0  0  0  0
   13.3110   11.2647   -0.2097 C   0  0  0  0  0  0  0  0  0  0  0  0
   12.0488   12.1153    0.0320 C   0  0  0  0  0  0  0  0  0  0  0  0
   10.8406   11.1874    0.2645 C   0  0  0  0  0  0  0  0  0  0  0  0
    9.5784   12.0380    0.5063 C   0  0  0  0  0  0  0  0  0  0  0  0
    8.3702   11.1100    0.7388 C   0  0  0  0  0  0  0  0  0  0  0  0
    7.1080   11.9605    0.9806 C   0  0  0  0  0  0  0  0  0  0  0  0
    5.8998   11.0325    1.2132 C   0  0  0  0  0  0  0  0  0  0  0  0
    4.6376   11.8830    1.4551 C   0  0  0  0  0  0  0  0  0  0  0  0
    3.4293   10.9548    1.6877 C   0  0  0  0  0  0  0  0  0  0  0  0
    2.1672   11.8052    1.9296 C   0  0  0  0  0  0  0  0  0  0  0  0
   23.9576   12.1249   -2.2521 H   0  0  0  0  0  0  0  0  0  0  0  0
   19.2749   12.9711   -2.2553 H   0  0  0  0  0  0  0  0  0  0  0  0
   19.6079   12.9722   -0.5184 H   0  0  0  0  0  0  0  0  0  0  0  0
```

20.9078 10.8714 -0.7663 H 0 0 0 0 0 0 0 0 0 0 0 0
20.5748 10.8703 -2.5031 H 0 0 0 0 0 0 0 0 0 0 0 0
22.0786 13.0489 -0.9921 H 0 0 0 0 0 0 0 0 0 0 0 0
21.7456 13.0478 -2.7290 H 0 0 0 0 0 0 0 0 0 0 0 0
18.4373 10.7945 -0.2925 H 0 0 0 0 0 0 0 0 0 0 0 0
18.1042 10.7933 -2.0293 H 0 0 0 0 0 0 0 0 0 0 0 0
16.8043 12.8940 -1.7814 H 0 0 0 0 0 0 0 0 0 0 0 0
17.1375 12.8952 -0.0445 H 0 0 0 0 0 0 0 0 0 0 0 0
15.9669 10.7174 0.1814 H 0 0 0 0 0 0 0 0 0 0 0 0
15.6337 10.7163 -1.5554 H 0 0 0 0 0 0 0 0 0 0 0 0
14.3338 12.8169 -1.3074 H 0 0 0 0 0 0 0 0 0 0 0 0
14.6670 12.8181 0.4295 H 0 0 0 0 0 0 0 0 0 0 0 0
13.4965 10.6402 0.6555 H 0 0 0 0 0 0 0 0 0 0 0 0
13.1632 10.6391 -1.0813 H 0 0 0 0 0 0 0 0 0 0 0 0
11.8632 12.7397 -0.8332 H 0 0 0 0 0 0 0 0 0 0 0 0
12.1966 12.7409 0.9036 H 0 0 0 0 0 0 0 0 0 0 0 0
11.0262 10.5630 1.1297 H 0 0 0 0 0 0 0 0 0 0 0 0
10.6928 10.5619 -0.6071 H 0 0 0 0 0 0 0 0 0 0 0 0
9.3927 12.6624 -0.3589 H 0 0 0 0 0 0 0 0 0 0 0 0
9.7262 12.6635 1.3779 H 0 0 0 0 0 0 0 0 0 0 0 0
8.5559 10.4855 1.6040 H 0 0 0 0 0 0 0 0 0 0 0 0
8.2224 10.4845 -0.1328 H 0 0 0 0 0 0 0 0 0 0 0 0
6.9223 12.5849 0.1155 H 0 0 0 0 0 0 0 0 0 0 0 0
7.2558 12.5860 1.8523 H 0 0 0 0 0 0 0 0 0 0 0 0
6.0856 10.4080 2.0784 H 0 0 0 0 0 0 0 0 0 0 0 0
5.7521 10.4069 0.3416 H 0 0 0 0 0 0 0 0 0 0 0 0
4.4518 12.5074 0.5899 H 0 0 0 0 0 0 0 0 0 0 0 0
4.7854 12.5085 2.3267 H 0 0 0 0 0 0 0 0 0 0 0 0
3.6151 10.3304 2.5528 H 0 0 0 0 0 0 0 0 0 0 0 0
3.2815 10.3293 0.8161 H 0 0 0 0 0 0 0 0 0 0 0 0
1.3183 11.1528 2.0930 H 0 0 0 0 0 0 0 0 0 0 0 0
2.3148 12.4307 2.8012 H 0 0 0 0 0 0 0 0 0 0 0 0
1.9813 12.4296 1.0644 H 0 0 0 0 0 0 0 0 0 0 0 0
1 2 1 0 0 0 0
2 3 1 0 0 0 0
3 4 1 0 0 0 0
1 5 1 0 0 0 0
5 6 1 0 0 0 0
6 7 1 0 0 0 0
7 8 1 0 0 0 0
8 9 1 0 0 0 0
9 10 1 0 0 0 0
10 11 1 0 0 0 0
11 12 1 0 0 0 0
12 13 1 0 0 0 0
13 14 1 0 0 0 0
14 15 1 0 0 0 0
15 16 1 0 0 0 0
16 17 1 0 0 0 0
17 18 1 0 0 0 0
4 19 1 0 0 0 0
1 20 1 0 0 0 0
1 21 1 0 0 0 0
2 22 1 0 0 0 0
2 23 1 0 0 0 0
3 24 1 0 0 0 0
3 25 1 0 0 0 0
5 26 1 0 0 0 0
5 27 1 0 0 0 0
6 28 1 0 0 0 0
6 29 1 0 0 0 0
7 30 1 0 0 0 0
7 31 1 0 0 0 0

```
  8 32  1  0  0  0  0
  8 33  1  0  0  0  0
  9 34  1  0  0  0  0
  9 35  1  0  0  0  0
 10 36  1  0  0  0  0
 10 37  1  0  0  0  0
 11 38  1  0  0  0  0
 11 39  1  0  0  0  0
 12 40  1  0  0  0  0
 12 41  1  0  0  0  0
 13 42  1  0  0  0  0
 13 43  1  0  0  0  0
 14 44  1  0  0  0  0
 14 45  1  0  0  0  0
 15 46  1  0  0  0  0
 15 47  1  0  0  0  0
 16 48  1  0  0  0  0
 16 49  1  0  0  0  0
 17 50  1  0  0  0  0
 17 51  1  0  0  0  0
 18 52  1  0  0  0  0
 18 53  1  0  0  0  0
 18 54  1  0  0  0  0
M  END
>  <Alias name>
1-Heptadecanol

>  <deltaH°(c)>   kJ/mol
-11119.67

$$$$
Undecylcyclohexane
  ChemBrain IXL

 51 51  0  0  0  0  0  0  0  0  1 V2000
   16.2713   10.5969   -0.4102 C   0  0  0  0  0  0  0  0  0  0  0  0
   17.6186   11.2087    0.0242 C   0  0  0  0  0  0  0  0  0  0  0  0
   18.7629   10.2388   -0.3307 C   0  0  0  0  0  0  0  0  0  0  0  0
   18.5478    8.8999    0.4021 C   0  0  0  0  0  0  0  0  0  0  0  0
   17.2025    8.2858   -0.0333 C   0  0  0  0  0  0  0  0  0  0  0  0
   16.0552    9.2540    0.3211 C   0  0  0  0  0  0  0  0  0  0  0  0
   14.7096    8.6350   -0.1161 C   0  0  0  0  0  0  0  0  0  0  0  0
   13.5540    9.5850    0.2602 C   0  0  0  0  0  0  0  0  0  0  0  0
   12.2127    8.9528   -0.1591 C   0  0  0  0  0  0  0  0  0  0  0  0
   11.0578    9.9015    0.2165 C   0  0  0  0  0  0  0  0  0  0  0  0
    9.7164    9.2697   -0.2037 C   0  0  0  0  0  0  0  0  0  0  0  0
    8.5616   10.2186    0.1714 C   0  0  0  0  0  0  0  0  0  0  0  0
    7.2203    9.5872   -0.2496 C   0  0  0  0  0  0  0  0  0  0  0  0
    6.0654   10.5363    0.1252 C   0  0  0  0  0  0  0  0  0  0  0  0
    4.7241    9.9052   -0.2964 C   0  0  0  0  0  0  0  0  0  0  0  0
    3.5692   10.8545    0.0782 C   0  0  0  0  0  0  0  0  0  0  0  0
    2.2280   10.2236   -0.3435 C   0  0  0  0  0  0  0  0  0  0  0  0
   16.2797   10.4281   -1.4800 H   0  0  0  0  0  0  0  0  0  0  0  0
   15.4695   11.2785   -0.1588 H   0  0  0  0  0  0  0  0  0  0  0  0
   17.6091   11.3797    1.0935 H   0  0  0  0  0  0  0  0  0  0  0  0
   17.7702   12.1493   -0.4904 H   0  0  0  0  0  0  0  0  0  0  0  0
   19.7085   10.6693   -0.0256 H   0  0  0  0  0  0  0  0  0  0  0  0
   18.7726   10.0676   -1.4001 H   0  0  0  0  0  0  0  0  0  0  0  0
   18.5373    9.0712    1.4714 H   0  0  0  0  0  0  0  0  0  0  0  0
   19.3524    8.2192    0.1535 H   0  0  0  0  0  0  0  0  0  0  0  0
   17.2132    8.1148   -1.1027 H   0  0  0  0  0  0  0  0  0  0  0  0
   17.0523    7.3448    0.4811 H   0  0  0  0  0  0  0  0  0  0  0  0
   16.0438    9.4232    1.3908 H   0  0  0  0  0  0  0  0  0  0  0  0
```

```
  14.7150    8.4798   -1.1880 H   0  0  0  0  0  0  0  0  0  0  0  0
  14.5714    7.6849    0.3848 H   0  0  0  0  0  0  0  0  0  0  0  0
  13.5562    9.7499    1.3306 H   0  0  0  0  0  0  0  0  0  0  0  0
  13.6817   10.5303   -0.2509 H   0  0  0  0  0  0  0  0  0  0  0  0
  12.2105    8.7875   -1.2294 H   0  0  0  0  0  0  0  0  0  0  0  0
  12.0838    8.0070    0.3525 H   0  0  0  0  0  0  0  0  0  0  0  0
  11.0596   10.0663    1.2869 H   0  0  0  0  0  0  0  0  0  0  0  0
  11.1870   10.8475   -0.2947 H   0  0  0  0  0  0  0  0  0  0  0  0
   9.7148    9.1047   -1.2741 H   0  0  0  0  0  0  0  0  0  0  0  0
   9.5870    8.3238    0.3076 H   0  0  0  0  0  0  0  0  0  0  0  0
   8.5628   10.3833    1.2418 H   0  0  0  0  0  0  0  0  0  0  0  0
   8.6913   11.1647   -0.3395 H   0  0  0  0  0  0  0  0  0  0  0  0
   7.2191    9.4224   -1.3200 H   0  0  0  0  0  0  0  0  0  0  0  0
   7.0904    8.6413    0.2615 H   0  0  0  0  0  0  0  0  0  0  0  0
   6.0663   10.7009    1.1956 H   0  0  0  0  0  0  0  0  0  0  0  0
   6.1955   11.4824   -0.3856 H   0  0  0  0  0  0  0  0  0  0  0  0
   4.7233    9.7406   -1.3668 H   0  0  0  0  0  0  0  0  0  0  0  0
   4.5940    8.9592    0.2144 H   0  0  0  0  0  0  0  0  0  0  0  0
   3.5699   11.0190    1.1486 H   0  0  0  0  0  0  0  0  0  0  0  0
   3.6994   11.8006   -0.4324 H   0  0  0  0  0  0  0  0  0  0  0  0
   2.0976    9.2775    0.1672 H   0  0  0  0  0  0  0  0  0  0  0  0
   1.4167   10.8908   -0.0802 H   0  0  0  0  0  0  0  0  0  0  0  0
   2.2272   10.0592   -1.4140 H   0  0  0  0  0  0  0  0  0  0  0  0
  1  2  1  0  0  0  0
  2  3  1  0  0  0  0
  3  4  1  0  0  0  0
  4  5  1  0  0  0  0
  5  6  1  0  0  0  0
  6  1  1  0  0  0  0
  6  7  1  0  0  0  0
  7  8  1  0  0  0  0
  8  9  1  0  0  0  0
  9 10  1  0  0  0  0
 10 11  1  0  0  0  0
 11 12  1  0  0  0  0
 12 13  1  0  0  0  0
 13 14  1  0  0  0  0
 14 15  1  0  0  0  0
 15 16  1  0  0  0  0
 16 17  1  0  0  0  0
  1 18  1  0  0  0  0
  1 19  1  0  0  0  0
  2 20  1  0  0  0  0
  2 21  1  0  0  0  0
  3 22  1  0  0  0  0
  3 23  1  0  0  0  0
  4 24  1  0  0  0  0
  4 25  1  0  0  0  0
  5 26  1  0  0  0  0
  5 27  1  0  0  0  0
  6 28  1  0  0  0  0
  7 29  1  0  0  0  0
  7 30  1  0  0  0  0
  8 31  1  0  0  0  0
  8 32  1  0  0  0  0
  9 33  1  0  0  0  0
  9 34  1  0  0  0  0
 10 35  1  0  0  0  0
 10 36  1  0  0  0  0
 11 37  1  0  0  0  0
 11 38  1  0  0  0  0
 12 39  1  0  0  0  0
 12 40  1  0  0  0  0
```

```
 13 41  1  0  0  0  0
 13 42  1  0  0  0  0
 14 43  1  0  0  0  0
 14 44  1  0  0  0  0
 15 45  1  0  0  0  0
 15 46  1  0  0  0  0
 16 47  1  0  0  0  0
 16 48  1  0  0  0  0
 17 49  1  0  0  0  0
 17 50  1  0  0  0  0
 17 51  1  0  0  0  0
M  END
>  <Alias name>
Undecylcyclohexane

>  <deltaH°(c)>   kJ/mol
-11114

$$$$
Dodecylbenzene
  ChemBrain IXL

 48 48  0  0  0  0  0  0  0  0  1 V2000
    3.4601   10.5725    3.7164 C   0  0  0  0  0  0  0  0  0  0  0  0
    2.3705    9.9185    4.2919 C   0  0  0  0  0  0  0  0  0  0  0  0
    2.3045    8.5252    4.2742 C   0  0  0  0  0  0  0  0  0  0  0  0
    3.3279    7.7858    3.6810 C   0  0  0  0  0  0  0  0  0  0  0  0
    4.4175    8.4396    3.1051 C   0  0  0  0  0  0  0  0  0  0  0  0
    4.4836    9.8331    3.1231 C   0  0  0  0  0  0  0  0  0  0  0  0
    5.5030    7.6540    2.4753 C   0  0  0  0  0  0  0  0  0  0  0  0
    6.5964    8.6085    1.9528 C   0  0  0  0  0  0  0  0  0  0  0  0
    7.7211    7.7851    1.2936 C   0  0  0  0  0  0  0  0  0  0  0  0
    8.8181    8.7329    0.7677 C   0  0  0  0  0  0  0  0  0  0  0  0
    9.9316    7.9027    0.0975 C   0  0  0  0  0  0  0  0  0  0  0  0
   11.0329    8.8433   -0.4314 C   0  0  0  0  0  0  0  0  0  0  0  0
   12.1354    8.0070   -1.1122 C   0  0  0  0  0  0  0  0  0  0  0  0
   13.2408    8.9415   -1.6423 C   0  0  0  0  0  0  0  0  0  0  0  0
   14.3349    8.1012   -2.3313 C   0  0  0  0  0  0  0  0  0  0  0  0
   15.4433    9.0321   -2.8609 C   0  0  0  0  0  0  0  0  0  0  0  0
   16.5326    8.1900   -3.5545 C   0  0  0  0  0  0  0  0  0  0  0  0
   17.6427    9.1194   -4.0827 C   0  0  0  0  0  0  0  0  0  0  0  0
    3.5113   11.6542    3.7302 H   0  0  0  0  0  0  0  0  0  0  0  0
    1.5759   10.4925    4.7525 H   0  0  0  0  0  0  0  0  0  0  0  0
    1.4586    8.0175    4.7211 H   0  0  0  0  0  0  0  0  0  0  0  0
    3.2766    6.7041    3.6674 H   0  0  0  0  0  0  0  0  0  0  0  0
    5.3295   10.3409    2.6764 H   0  0  0  0  0  0  0  0  0  0  0  0
    5.1005    7.0804    1.6496 H   0  0  0  0  0  0  0  0  0  0  0  0
    5.9319    6.9806    3.2070 H   0  0  0  0  0  0  0  0  0  0  0  0
    6.1677    9.2841    1.2229 H   0  0  0  0  0  0  0  0  0  0  0  0
    7.0027    9.1792    2.7787 H   0  0  0  0  0  0  0  0  0  0  0  0
    8.1485    7.1094    2.0241 H   0  0  0  0  0  0  0  0  0  0  0  0
    7.3133    7.2133    0.4692 H   0  0  0  0  0  0  0  0  0  0  0  0
    8.3899    9.4149    0.0434 H   0  0  0  0  0  0  0  0  0  0  0  0
    9.2347    9.2970    1.5931 H   0  0  0  0  0  0  0  0  0  0  0  0
   10.3580    7.2203    0.8223 H   0  0  0  0  0  0  0  0  0  0  0  0
    9.5135    7.3381   -0.7266 H   0  0  0  0  0  0  0  0  0  0  0  0
   10.6058    9.5318   -1.1501 H   0  0  0  0  0  0  0  0  0  0  0  0
   11.4594    9.4004    0.3936 H   0  0  0  0  0  0  0  0  0  0  0  0
   12.5605    7.3174   -0.3933 H   0  0  0  0  0  0  0  0  0  0  0  0
   11.7080    7.4500   -1.9368 H   0  0  0  0  0  0  0  0  0  0  0  0
   12.8152    9.6358   -2.3563 H   0  0  0  0  0  0  0  0  0  0  0  0
   13.6746    9.4926   -0.8171 H   0  0  0  0  0  0  0  0  0  0  0  0
   14.7585    7.4055   -1.6175 H   0  0  0  0  0  0  0  0  0  0  0  0
```

```
   13.9008    7.5511   -3.1571 H   0  0  0  0  0  0  0  0  0  0  0  0
   15.0194    9.7304   -3.5718 H   0  0  0  0  0  0  0  0  0  0  0  0
   15.8810    9.5788   -2.0348 H   0  0  0  0  0  0  0  0  0  0  0  0
   16.9553    7.4906   -2.8438 H   0  0  0  0  0  0  0  0  0  0  0  0
   16.0950    7.6443   -4.3813 H   0  0  0  0  0  0  0  0  0  0  0  0
   17.2200    9.8194   -4.7927 H   0  0  0  0  0  0  0  0  0  0  0  0
   18.4072    8.5274   -4.5708 H   0  0  0  0  0  0  0  0  0  0  0  0
   18.0811    9.6643   -3.2559 H   0  0  0  0  0  0  0  0  0  0  0  0
  1  2  4  0  0  0  0
  2  3  4  0  0  0  0
  3  4  4  0  0  0  0
  4  5  4  0  0  0  0
  5  6  4  0  0  0  0
  6  1  4  0  0  0  0
  5  7  1  0  0  0  0
  7  8  1  0  0  0  0
  8  9  1  0  0  0  0
  9 10  1  0  0  0  0
 10 11  1  0  0  0  0
 11 12  1  0  0  0  0
 12 13  1  0  0  0  0
 13 14  1  0  0  0  0
 14 15  1  0  0  0  0
 15 16  1  0  0  0  0
 16 17  1  0  0  0  0
 17 18  1  0  0  0  0
  1 19  1  0  0  0  0
  2 20  1  0  0  0  0
  3 21  1  0  0  0  0
  4 22  1  0  0  0  0
  6 23  1  0  0  0  0
  7 24  1  0  0  0  0
  7 25  1  0  0  0  0
  8 26  1  0  0  0  0
  8 27  1  0  0  0  0
  9 28  1  0  0  0  0
  9 29  1  0  0  0  0
 10 30  1  0  0  0  0
 10 31  1  0  0  0  0
 11 32  1  0  0  0  0
 11 33  1  0  0  0  0
 12 34  1  0  0  0  0
 12 35  1  0  0  0  0
 13 36  1  0  0  0  0
 13 37  1  0  0  0  0
 14 38  1  0  0  0  0
 14 39  1  0  0  0  0
 15 40  1  0  0  0  0
 15 41  1  0  0  0  0
 16 42  1  0  0  0  0
 16 43  1  0  0  0  0
 17 44  1  0  0  0  0
 17 45  1  0  0  0  0
 18 46  1  0  0  0  0
 18 47  1  0  0  0  0
 18 48  1  0  0  0  0
M  END
>  <Alias name>
1-Phenyldodecane

>  <deltaH°(c)>   kJ/mol
-11113.44
```

```
$$$$
Androsterone
  ChemBrain IXL

 51 54  0  0  0  0  0  0  0  0  1 V2000
    5.2574    4.8056    0.1232 C   0  0  0  0  0  0  0  0  0  0  0  0
    9.0460    6.6045   -0.1251 C   0  0  0  0  0  0  0  0  0  0  0  0
    6.4108    5.5569   -0.5359 C   0  0  0  0  0  0  0  0  0  0  0  0
    7.8619    7.4099   -0.2585 C   0  0  0  0  0  0  0  0  0  0  0  0
    6.8010    6.7481    0.3787 C   0  0  0  0  0  0  0  0  0  0  0  0
    4.0761    5.8044    0.3067 C   0  0  0  0  0  0  0  0  0  0  0  0
   10.0952    7.5388   -0.2672 C   0  0  0  0  0  0  0  0  0  0  0  0
    8.9741    5.4805   -1.1476 C   0  0  0  0  0  0  0  0  0  0  0  0
    7.6846    4.6001   -0.7882 C   0  0  0  0  0  0  0  0  0  0  0  0
    4.8031    3.6320   -0.7754 C   0  0  0  0  0  0  0  0  0  0  0  0
    8.2454    8.7916    0.3072 C   0  0  0  0  0  0  0  0  0  0  0  0
    5.6500    7.7009    0.5182 C   0  0  0  0  0  0  0  0  0  0  0  0
    4.5104    6.9810    1.1922 C   0  0  0  0  0  0  0  0  0  0  0  0
    9.7295    8.8877    0.0311 C   0  0  0  0  0  0  0  0  0  0  0  0
    2.9011    5.1011    0.9475 C   0  0  0  0  0  0  0  0  0  0  0  0
   11.2224    7.2154   -0.6169 O   0  0  0  0  0  0  0  0  0  0  0  0
    3.5543    2.9549   -0.1510 C   0  0  0  0  0  0  0  0  0  0  0  0
    2.4268    3.9942    0.0124 C   0  0  0  0  0  0  0  0  0  0  0  0
    5.7122    4.2639    1.4925 C   0  0  0  0  0  0  0  0  0  0  0  0
    9.1328    5.9753    1.2817 C   0  0  0  0  0  0  0  0  0  0  0  0
    2.0969    4.5719   -1.3010 O   0  0  0  0  0  0  0  0  0  0  0  0
    1.4990    5.3525   -1.2745 H   0  0  0  0  0  0  0  0  0  0  0  0
    6.0757    5.9441   -1.4891 H   0  0  0  0  0  0  0  0  0  0  0  0
    7.6292    7.5144   -1.3099 H   0  0  0  0  0  0  0  0  0  0  0  0
    7.1143    6.3878    1.3518 H   0  0  0  0  0  0  0  0  0  0  0  0
    3.7791    6.1827   -0.6643 H   0  0  0  0  0  0  0  0  0  0  0  0
    9.8684    4.8648   -1.0883 H   0  0  0  0  0  0  0  0  0  0  0  0
    8.8749    5.8896   -2.1447 H   0  0  0  0  0  0  0  0  0  0  0  0
    7.4710    3.9221   -1.6072 H   0  0  0  0  0  0  0  0  0  0  0  0
    7.8842    4.0240    0.1098 H   0  0  0  0  0  0  0  0  0  0  0  0
    5.6041    2.9068   -0.8554 H   0  0  0  0  0  0  0  0  0  0  0  0
    4.5559    4.0067   -1.7611 H   0  0  0  0  0  0  0  0  0  0  0  0
    7.7123    9.5763   -0.2000 H   0  0  0  0  0  0  0  0  0  0  0  0
    8.0631    8.8268    1.3733 H   0  0  0  0  0  0  0  0  0  0  0  0
    5.9539    8.5512    1.1149 H   0  0  0  0  0  0  0  0  0  0  0  0
    5.3407    8.0373   -0.4646 H   0  0  0  0  0  0  0  0  0  0  0  0
    3.6828    7.6625    1.3248 H   0  0  0  0  0  0  0  0  0  0  0  0
    4.8367    6.6113    2.1553 H   0  0  0  0  0  0  0  0  0  0  0  0
   10.2671    9.2441    0.8982 H   0  0  0  0  0  0  0  0  0  0  0  0
    9.9182    9.5238   -0.8271 H   0  0  0  0  0  0  0  0  0  0  0  0
    2.0999    5.8118    1.1089 H   0  0  0  0  0  0  0  0  0  0  0  0
    3.2062    4.6750    1.8941 H   0  0  0  0  0  0  0  0  0  0  0  0
    3.2160    2.1557   -0.7965 H   0  0  0  0  0  0  0  0  0  0  0  0
    3.8125    2.5501    0.8197 H   0  0  0  0  0  0  0  0  0  0  0  0
    1.5503    3.5192    0.4302 H   0  0  0  0  0  0  0  0  0  0  0  0
    6.1129    5.0753    2.0870 H   0  0  0  0  0  0  0  0  0  0  0  0
    6.4792    3.5112    1.3459 H   0  0  0  0  0  0  0  0  0  0  0  0
    4.8678    3.8230    2.0049 H   0  0  0  0  0  0  0  0  0  0  0  0
   10.0655    5.4179    1.3681 H   0  0  0  0  0  0  0  0  0  0  0  0
    9.1101    6.7526    2.0289 H   0  0  0  0  0  0  0  0  0  0  0  0
    8.2996    5.3024    1.4301 H   0  0  0  0  0  0  0  0  0  0  0  0
  2  8  1  0  0  0  0
  3  1  1  0  0  0  0
  4  5  1  0  0  0  0
  5  3  1  0  0  0  0
  6  1  1  0  0  0  0
  7  2  1  0  0  0  0
  8  9  1  0  0  0  0
```

9 3 1 0 0 0 0
10 1 1 0 0 0 0
11 4 1 0 0 0 0
12 13 1 0 0 0 0
13 6 1 0 0 0 0
14 11 1 0 0 0 0
15 6 1 0 0 0 0
16 7 2 0 0 0 0
17 10 1 0 0 0 0
18 17 1 0 0 0 0
1 19 1 0 0 0 0
2 20 1 0 0 0 0
18 21 1 0 0 0 0
18 15 1 0 0 0 0
12 5 1 0 0 0 0
2 4 1 0 0 0 0
14 7 1 0 0 0 0
21 22 1 0 0 0 0
3 23 1 0 0 0 0
4 24 1 0 0 0 0
5 25 1 0 0 0 0
6 26 1 0 0 0 0
8 27 1 0 0 0 0
8 28 1 0 0 0 0
9 29 1 0 0 0 0
9 30 1 0 0 0 0
10 31 1 0 0 0 0
10 32 1 0 0 0 0
11 33 1 0 0 0 0
11 34 1 0 0 0 0
12 35 1 0 0 0 0
12 36 1 0 0 0 0
13 37 1 0 0 0 0
13 38 1 0 0 0 0
14 39 1 0 0 0 0
14 40 1 0 0 0 0
15 41 1 0 0 0 0
15 42 1 0 0 0 0
17 43 1 0 0 0 0
17 44 1 0 0 0 0
18 45 1 0 0 0 0
19 46 1 0 0 0 0
19 47 1 0 0 0 0
19 48 1 0 0 0 0
20 49 1 0 0 0 0
20 50 1 0 0 0 0
20 51 1 0 0 0 0
M END
> <Alias name>
Androsterone

> <deltaH°(c)> kJ/mol
-11095

$$$$
1-Nonylnaphthalene
ChemBrain IXL

45 46 0 0 0 0 0 0 0 0 1 V2000
3.5419 6.7676 1.7710 C 0 0 0 0 0 0 0 0 0 0 0 0
2.4631 7.6511 1.8121 C 0 0 0 0 0 0 0 0 0 0 0 0
2.6514 8.9969 1.4970 C 0 0 0 0 0 0 0 0 0 0 0 0
3.9184 9.4592 1.1406 C 0 0 0 0 0 0 0 0 0 0 0 0

4.9971	8.5757	1.0994	C	0	0	0	0	0	0	0	0	0	0	0	0
4.8089	7.2299	1.4146	C	0	0	0	0	0	0	0	0	0	0	0	0
6.2641	9.0381	0.7430	C	0	0	0	0	0	0	0	0	0	0	0	0
6.4524	10.3839	0.4280	C	0	0	0	0	0	0	0	0	0	0	0	0
5.3736	11.2674	0.4692	C	0	0	0	0	0	0	0	0	0	0	0	0
4.1066	10.8051	0.8255	C	0	0	0	0	0	0	0	0	0	0	0	0
7.4086	8.1007	0.6990	C	0	0	0	0	0	0	0	0	0	0	0	0
8.6796	8.8614	0.2742	C	0	0	0	0	0	0	0	0	0	0	0	0
9.8718	7.8860	0.2311	C	0	0	0	0	0	0	0	0	0	0	0	0
11.1430	8.6469	-0.1929	C	0	0	0	0	0	0	0	0	0	0	0	0
12.3360	7.6723	-0.2322	C	0	0	0	0	0	0	0	0	0	0	0	0
13.6074	8.4335	-0.6550	C	0	0	0	0	0	0	0	0	0	0	0	0
14.8009	7.4594	-0.6918	C	0	0	0	0	0	0	0	0	0	0	0	0
16.0725	8.2208	-1.1139	C	0	0	0	0	0	0	0	0	0	0	0	0
17.2662	7.2469	-1.1497	C	0	0	0	0	0	0	0	0	0	0	0	0
3.3958	5.7227	2.0156	H	0	0	0	0	0	0	0	0	0	0	0	0
1.4795	7.2921	2.0888	H	0	0	0	0	0	0	0	0	0	0	0	0
1.8139	9.6828	1.5290	H	0	0	0	0	0	0	0	0	0	0	0	0
5.6464	6.5440	1.3827	H	0	0	0	0	0	0	0	0	0	0	0	0
7.4360	10.7429	0.1515	H	0	0	0	0	0	0	0	0	0	0	0	0
5.5197	12.3123	0.2246	H	0	0	0	0	0	0	0	0	0	0	0	0
3.2691	11.4910	0.8575	H	0	0	0	0	0	0	0	0	0	0	0	0
7.2031	7.3134	-0.0157	H	0	0	0	0	0	0	0	0	0	0	0	0
7.5582	7.6674	1.6802	H	0	0	0	0	0	0	0	0	0	0	0	0
8.8839	9.6499	0.9879	H	0	0	0	0	0	0	0	0	0	0	0	0
8.5307	9.2930	-0.7079	H	0	0	0	0	0	0	0	0	0	0	0	0
9.6678	7.0978	-0.4831	H	0	0	0	0	0	0	0	0	0	0	0	0
10.0203	7.4538	1.2129	H	0	0	0	0	0	0	0	0	0	0	0	0
11.3455	9.4368	0.5198	H	0	0	0	0	0	0	0	0	0	0	0	0
10.9954	9.0767	-1.1759	H	0	0	0	0	0	0	0	0	0	0	0	0
12.1339	6.8828	-0.9456	H	0	0	0	0	0	0	0	0	0	0	0	0
12.4830	7.2418	0.7506	H	0	0	0	0	0	0	0	0	0	0	0	0
13.8085	9.2241	0.0573	H	0	0	0	0	0	0	0	0	0	0	0	0
13.4611	8.8624	-1.6386	H	0	0	0	0	0	0	0	0	0	0	0	0
14.6001	6.6691	-1.4045	H	0	0	0	0	0	0	0	0	0	0	0	0
14.9470	7.0301	0.2916	H	0	0	0	0	0	0	0	0	0	0	0	0
16.2729	9.0115	-0.4016	H	0	0	0	0	0	0	0	0	0	0	0	0
15.9267	8.6495	-2.0977	H	0	0	0	0	0	0	0	0	0	0	0	0
18.1599	7.7820	-1.4464	H	0	0	0	0	0	0	0	0	0	0	0	0
17.0658	6.4561	-1.8620	H	0	0	0	0	0	0	0	0	0	0	0	0
17.4120	6.8182	-0.1659	H	0	0	0	0	0	0	0	0	0	0	0	0

1	2	4	0	0	0	0
2	3	4	0	0	0	0
3	4	4	0	0	0	0
4	5	4	0	0	0	0
5	6	4	0	0	0	0
6	1	4	0	0	0	0
5	7	4	0	0	0	0
7	8	4	0	0	0	0
8	9	4	0	0	0	0
9	10	4	0	0	0	0
10	4	4	0	0	0	0
7	11	1	0	0	0	0
11	12	1	0	0	0	0
12	13	1	0	0	0	0
13	14	1	0	0	0	0
14	15	1	0	0	0	0
15	16	1	0	0	0	0
16	17	1	0	0	0	0
17	18	1	0	0	0	0
18	19	1	0	0	0	0
1	20	1	0	0	0	0
2	21	1	0	0	0	0

```
  3 22  1  0  0  0  0
  6 23  1  0  0  0  0
  8 24  1  0  0  0  0
  9 25  1  0  0  0  0
 10 26  1  0  0  0  0
 11 27  1  0  0  0  0
 11 28  1  0  0  0  0
 12 29  1  0  0  0  0
 12 30  1  0  0  0  0
 13 31  1  0  0  0  0
 13 32  1  0  0  0  0
 14 33  1  0  0  0  0
 14 34  1  0  0  0  0
 15 35  1  0  0  0  0
 15 36  1  0  0  0  0
 16 37  1  0  0  0  0
 16 38  1  0  0  0  0
 17 39  1  0  0  0  0
 17 40  1  0  0  0  0
 18 41  1  0  0  0  0
 18 42  1  0  0  0  0
 19 43  1  0  0  0  0
 19 44  1  0  0  0  0
 19 45  1  0  0  0  0
M  END
>  <Alias name>
1-Nonylnaphthalene

>  <deltaH°(c)>   kJ/mol
-11067.39

$$$$
1,3,5-Tri-t-butylbenzene
  ChemBrain IXL

 48 48  0  0  0  0  0  0  0  0  1 V2000
    6.0151    7.4803    0.1157 C   0  0  0  0  0  0  0  0  0  0  0  0
    4.8322    6.7452    0.0348 C   0  0  0  0  0  0  0  0  0  0  0  0
    4.8701    5.3526    0.1072 C   0  0  0  0  0  0  0  0  0  0  0  0
    6.0909    4.6950    0.2597 C   0  0  0  0  0  0  0  0  0  0  0  0
    7.2737    5.4301    0.3409 C   0  0  0  0  0  0  0  0  0  0  0  0
    7.2359    6.8227    0.2683 C   0  0  0  0  0  0  0  0  0  0  0  0
    8.4909    7.6025    0.3532 C   0  0  0  0  0  0  0  0  0  0  0  0
    9.5771    6.7541    1.0424 C   0  0  0  0  0  0  0  0  0  0  0  0
    8.2445    8.8848    1.1716 C   0  0  0  0  0  0  0  0  0  0  0  0
    8.9578    7.9805   -1.0658 C   0  0  0  0  0  0  0  0  0  0  0  0
    6.1312    3.2175    0.3353 C   0  0  0  0  0  0  0  0  0  0  0  0
    7.3878    2.7769    1.1109 C   0  0  0  0  0  0  0  0  0  0  0  0
    6.1749    2.6313   -1.0892 C   0  0  0  0  0  0  0  0  0  0  0  0
    4.8728    2.7059    1.0629 C   0  0  0  0  0  0  0  0  0  0  0  0
    3.5371    7.4428   -0.1279 C   0  0  0  0  0  0  0  0  0  0  0  0
    3.2565    7.6622   -1.6271 C   0  0  0  0  0  0  0  0  0  0  0  0
    3.5943    8.8058    0.5888 C   0  0  0  0  0  0  0  0  0  0  0  0
    2.4120    6.5868    0.4854 C   0  0  0  0  0  0  0  0  0  0  0  0
    5.9856    8.5614    0.0600 H   0  0  0  0  0  0  0  0  0  0  0  0
    3.9517    4.7820    0.0450 H   0  0  0  0  0  0  0  0  0  0  0  0
    8.2214    4.9197    0.4604 H   0  0  0  0  0  0  0  0  0  0  0  0
   10.4852    7.3369    1.1357 H   0  0  0  0  0  0  0  0  0  0  0  0
    9.7750    5.8699    0.4493 H   0  0  0  0  0  0  0  0  0  0  0  0
    9.2346    6.4594    2.0267 H   0  0  0  0  0  0  0  0  0  0  0  0
    7.4945    9.4900    0.6773 H   0  0  0  0  0  0  0  0  0  0  0  0
    7.8984    8.6195    2.1629 H   0  0  0  0  0  0  0  0  0  0  0  0
    9.1673    9.4462    1.2496 H   0  0  0  0  0  0  0  0  0  0  0  0
```

```
    9.8747    8.5535   -1.0035 H   0  0  0  0  0  0  0  0  0  0  0  0
    8.1932    8.5744   -1.5512 H   0  0  0  0  0  0  0  0  0  0  0  0
    9.1336    7.0793   -1.6401 H   0  0  0  0  0  0  0  0  0  0  0  0
    7.3740    3.2168    2.1004 H   0  0  0  0  0  0  0  0  0  0  0  0
    8.2730    3.1070    0.5814 H   0  0  0  0  0  0  0  0  0  0  0  0
    7.3995    1.6973    1.1959 H   0  0  0  0  0  0  0  0  0  0  0  0
    7.0602    2.9894   -1.5999 H   0  0  0  0  0  0  0  0  0  0  0  0
    6.2025    1.5500   -1.0339 H   0  0  0  0  0  0  0  0  0  0  0  0
    5.2926    2.9424   -1.6348 H   0  0  0  0  0  0  0  0  0  0  0  0
    4.9142    1.6265    1.1401 H   0  0  0  0  0  0  0  0  0  0  0  0
    4.8291    3.1374    2.0553 H   0  0  0  0  0  0  0  0  0  0  0  0
    3.9907    2.9944    0.5048 H   0  0  0  0  0  0  0  0  0  0  0  0
    3.2155    6.7043   -2.1307 H   0  0  0  0  0  0  0  0  0  0  0  0
    2.3093    8.1735   -1.7461 H   0  0  0  0  0  0  0  0  0  0  0  0
    4.0477    8.2630   -2.0583 H   0  0  0  0  0  0  0  0  0  0  0  0
    2.6373    9.3039    0.4942 H   0  0  0  0  0  0  0  0  0  0  0  0
    4.3660    9.4182    0.1389 H   0  0  0  0  0  0  0  0  0  0  0  0
    3.8198    8.6533    1.6371 H   0  0  0  0  0  0  0  0  0  0  0  0
    2.3498    5.6421   -0.0404 H   0  0  0  0  0  0  0  0  0  0  0  0
    2.6263    6.4045    1.5312 H   0  0  0  0  0  0  0  0  0  0  0  0
    1.4692    7.1123    0.3964 H   0  0  0  0  0  0  0  0  0  0  0  0
  1  2  4  0  0  0  0
  2  3  4  0  0  0  0
  3  4  4  0  0  0  0
  4  5  4  0  0  0  0
  5  6  4  0  0  0  0
  6  1  4  0  0  0  0
  6  7  1  0  0  0  0
  7  8  1  0  0  0  0
  7  9  1  0  0  0  0
  7 10  1  0  0  0  0
  4 11  1  0  0  0  0
 11 12  1  0  0  0  0
 11 13  1  0  0  0  0
 11 14  1  0  0  0  0
  2 15  1  0  0  0  0
 15 16  1  0  0  0  0
 15 17  1  0  0  0  0
 15 18  1  0  0  0  0
  1 19  1  0  0  0  0
  3 20  1  0  0  0  0
  5 21  1  0  0  0  0
  8 22  1  0  0  0  0
  8 23  1  0  0  0  0
  8 24  1  0  0  0  0
  9 25  1  0  0  0  0
  9 26  1  0  0  0  0
  9 27  1  0  0  0  0
 10 28  1  0  0  0  0
 10 29  1  0  0  0  0
 10 30  1  0  0  0  0
 12 31  1  0  0  0  0
 12 32  1  0  0  0  0
 12 33  1  0  0  0  0
 13 34  1  0  0  0  0
 13 35  1  0  0  0  0
 13 36  1  0  0  0  0
 14 37  1  0  0  0  0
 14 38  1  0  0  0  0
 14 39  1  0  0  0  0
 16 40  1  0  0  0  0
 16 41  1  0  0  0  0
 16 42  1  0  0  0  0
```

```
 17 43  1  0  0  0  0
 17 44  1  0  0  0  0
 17 45  1  0  0  0  0
 18 46  1  0  0  0  0
 18 47  1  0  0  0  0
 18 48  1  0  0  0  0
M  END
>  <Alias name>
1,3,5-Tri-t-butylbenzene

>  <deltaH°(c)>   kJ/mol
-11058.23

$$$$
Pyridine-2,6-dicarbonyl-bis(N,N-diethylthiourea)
  ChemBrain IXL

 51 51  0  0  0  0  0  0  0  0  1 V2000
   12.0968    9.1429    1.1956 C   0  0  0  0  0  0  0  0  0  0  0  0  0
   12.0813   10.5021    0.9035 C   0  0  0  0  0  0  0  0  0  0  0  0  0
   10.9021   11.1596    0.7667 N   0  0  0  0  0  0  0  0  0  0  0  0  0
    9.7212   10.5064    0.9088 C   0  0  0  0  0  0  0  0  0  0  0  0  0
    9.7022    9.1473    1.2012 C   0  0  0  0  0  0  0  0  0  0  0  0  0
   10.8986    8.4602    1.3452 C   0  0  0  0  0  0  0  0  0  0  0  0  0
    8.4404   11.2298    0.7546 C   0  0  0  0  0  0  0  0  0  0  0  0  0
    8.4374   12.4385    0.5890 O   0  0  0  0  0  0  0  0  0  0  0  0  0
    7.2310   10.5251    0.7994 N   0  0  0  0  0  0  0  0  0  0  0  0  0
    6.0277   11.2103    1.0061 C   0  0  0  0  0  0  0  0  0  0  0  0  0
    6.0334   12.7311    1.7877 S   0  0  0  0  0  0  0  0  0  0  0  0  0
    4.8199   10.6503    0.5729 N   0  0  0  0  0  0  0  0  0  0  0  0  0
    3.5417   11.2312    1.0107 C   0  0  0  0  0  0  0  0  0  0  0  0  0
    4.8300    9.4820   -0.3196 C   0  0  0  0  0  0  0  0  0  0  0  0  0
    5.7798    9.7486   -1.5037 C   0  0  0  0  0  0  0  0  0  0  0  0  0
    2.3828   10.3125    0.5763 C   0  0  0  0  0  0  0  0  0  0  0  0  0
   13.3639   11.2208    0.7439 C   0  0  0  0  0  0  0  0  0  0  0  0  0
   13.3725   12.4337    0.6127 O   0  0  0  0  0  0  0  0  0  0  0  0  0
   14.5684   10.5068    0.7441 N   0  0  0  0  0  0  0  0  0  0  0  0  0
   15.7747   11.1675    1.0048 C   0  0  0  0  0  0  0  0  0  0  0  0  0
   15.7674   12.6711    1.8188 S   0  0  0  0  0  0  0  0  0  0  0  0  0
   16.9869   10.5970    0.5989 N   0  0  0  0  0  0  0  0  0  0  0  0  0
   16.9863    9.3779   -0.2229 C   0  0  0  0  0  0  0  0  0  0  0  0  0
   18.2601   11.2174    0.9947 C   0  0  0  0  0  0  0  0  0  0  0  0  0
   19.4320   10.3359    0.5202 C   0  0  0  0  0  0  0  0  0  0  0  0  0
   16.2319    9.6466   -1.5397 C   0  0  0  0  0  0  0  0  0  0  0  0  0
    7.2268    9.5227    0.6830 H   0  0  0  0  0  0  0  0  0  0  0  0  0
   14.5669    9.5152    0.5567 H   0  0  0  0  0  0  0  0  0  0  0  0  0
   13.0389    8.6202    1.3058 H   0  0  0  0  0  0  0  0  0  0  0  0  0
    8.7588    8.6279    1.3160 H   0  0  0  0  0  0  0  0  0  0  0  0  0
   10.8973    7.4012    1.5721 H   0  0  0  0  0  0  0  0  0  0  0  0  0
    3.4161   12.2080    0.5602 H   0  0  0  0  0  0  0  0  0  0  0  0  0
    3.5401   11.3285    2.0893 H   0  0  0  0  0  0  0  0  0  0  0  0  0
    3.8291    9.3037   -0.6926 H   0  0  0  0  0  0  0  0  0  0  0  0  0
    5.1715    8.6122    0.2282 H   0  0  0  0  0  0  0  0  0  0  0  0  0
    5.4329   10.6123   -2.0574 H   0  0  0  0  0  0  0  0  0  0  0  0  0
    5.7954    8.8841   -2.1559 H   0  0  0  0  0  0  0  0  0  0  0  0  0
    6.7788    9.9368   -1.1300 H   0  0  0  0  0  0  0  0  0  0  0  0  0
    2.5308    9.3234    0.9917 H   0  0  0  0  0  0  0  0  0  0  0  0  0
    1.4456   10.7188    0.9367 H   0  0  0  0  0  0  0  0  0  0  0  0  0
    2.3571   10.2501   -0.5046 H   0  0  0  0  0  0  0  0  0  0  0  0  0
   16.4950    8.5783    0.3178 H   0  0  0  0  0  0  0  0  0  0  0  0  0
   18.0068    9.0906   -0.4436 H   0  0  0  0  0  0  0  0  0  0  0  0  0
   18.2947   11.3138    2.0729 H   0  0  0  0  0  0  0  0  0  0  0  0  0
   18.3398   12.1980    0.5420 H   0  0  0  0  0  0  0  0  0  0  0  0  0
```

```
   20.3687   10.7848    0.8272 H   0  0  0  0  0  0  0  0  0  0  0  0
   19.4079   10.2536   -0.5594 H   0  0  0  0  0  0  0  0  0  0  0  0
   19.3432    9.3500    0.9594 H   0  0  0  0  0  0  0  0  0  0  0  0
   16.2179    8.7442   -2.1384 H   0  0  0  0  0  0  0  0  0  0  0  0
   15.2156    9.9487   -1.3184 H   0  0  0  0  0  0  0  0  0  0  0  0
   16.7318   10.4357   -2.0876 H   0  0  0  0  0  0  0  0  0  0  0  0
  1  2  4  0  0  0  0
  2  3  4  0  0  0  0
  3  4  4  0  0  0  0
  4  5  4  0  0  0  0
  5  6  4  0  0  0  0
  6  1  4  0  0  0  0
  7  8  2  0  0  0  0
  7  9  1  0  0  0  0
  7  4  1  0  0  0  0
 10 11  2  0  0  0  0
 10 12  1  0  0  0  0
 10  9  1  0  0  0  0
 12 13  1  0  0  0  0
 12 14  1  0  0  0  0
 14 15  1  0  0  0  0
 13 16  1  0  0  0  0
 17 18  2  0  0  0  0
 17 19  1  0  0  0  0
 20 21  2  0  0  0  0
 20 22  1  0  0  0  0
 20 19  1  0  0  0  0
 22 23  1  0  0  0  0
 22 24  1  0  0  0  0
 24 25  1  0  0  0  0
 23 26  1  0  0  0  0
 17  2  1  0  0  0  0
  9 27  1  0  0  0  0
 19 28  1  0  0  0  0
  1 29  1  0  0  0  0
  5 30  1  0  0  0  0
  6 31  1  0  0  0  0
 13 32  1  0  0  0  0
 13 33  1  0  0  0  0
 14 34  1  0  0  0  0
 14 35  1  0  0  0  0
 15 36  1  0  0  0  0
 15 37  1  0  0  0  0
 15 38  1  0  0  0  0
 16 39  1  0  0  0  0
 16 40  1  0  0  0  0
 16 41  1  0  0  0  0
 23 42  1  0  0  0  0
 23 43  1  0  0  0  0
 24 44  1  0  0  0  0
 24 45  1  0  0  0  0
 25 46  1  0  0  0  0
 25 47  1  0  0  0  0
 25 48  1  0  0  0  0
 26 49  1  0  0  0  0
 26 50  1  0  0  0  0
 26 51  1  0  0  0  0
M  END
>  <Alias name>
Pyridine-2,6-dicarbonyl-bis(N,N-diethylthiourea)

>  <deltaH°(c)>   kJ/mol
-11041
```

```
$$$$
Hexaethylbenzene
  ChemBrain IXL

 48 48  0  0  0  0  0  0  0  0  1 V2000
    6.1123    4.2589   -0.2366 C   0  0  0  0  0  0  0  0  0  0  0  0
    4.9385    4.9882   -0.4271 C   0  0  0  0  0  0  0  0  0  0  0  0
    4.9221    6.3609   -0.1792 C   0  0  0  0  0  0  0  0  0  0  0  0
    6.0801    7.0046    0.2576 C   0  0  0  0  0  0  0  0  0  0  0  0
    7.2539    6.2753    0.4481 C   0  0  0  0  0  0  0  0  0  0  0  0
    7.2703    4.9027    0.2001 C   0  0  0  0  0  0  0  0  0  0  0  0
    8.5162    4.1293    0.4003 C   0  0  0  0  0  0  0  0  0  0  0  0
    8.5639    3.5973    1.8458 C   0  0  0  0  0  0  0  0  0  0  0  0
    8.4819    6.9580    0.9134 C   0  0  0  0  0  0  0  0  0  0  0  0
    9.2946    7.4396   -0.3041 C   0  0  0  0  0  0  0  0  0  0  0  0
    6.0632    8.4613    0.5188 C   0  0  0  0  0  0  0  0  0  0  0  0
    5.6871    8.7148    1.9915 C   0  0  0  0  0  0  0  0  0  0  0  0
    3.6762    7.1342   -0.3794 C   0  0  0  0  0  0  0  0  0  0  0  0
    3.6286    7.6661   -1.8249 C   0  0  0  0  0  0  0  0  0  0  0  0
    3.7105    4.3055   -0.8924 C   0  0  0  0  0  0  0  0  0  0  0  0
    2.8965    3.8264    0.3252 C   0  0  0  0  0  0  0  0  0  0  0  0
    6.1292    2.8022   -0.4978 C   0  0  0  0  0  0  0  0  0  0  0  0
    6.5025    2.5488   -1.9712 C   0  0  0  0  0  0  0  0  0  0  0  0
    8.5394    3.2966   -0.2918 H   0  0  0  0  0  0  0  0  0  0  0  0
    9.3712    4.7700    0.2230 H   0  0  0  0  0  0  0  0  0  0  0  0
    8.5406    4.4300    2.5379 H   0  0  0  0  0  0  0  0  0  0  0  0
    7.7090    2.9566    2.0230 H   0  0  0  0  0  0  0  0  0  0  0  0
    9.4756    3.0315    1.9923 H   0  0  0  0  0  0  0  0  0  0  0  0
    8.2117    7.8088    1.5265 H   0  0  0  0  0  0  0  0  0  0  0  0
    9.0796    6.2682    1.4963 H   0  0  0  0  0  0  0  0  0  0  0  0
    9.5649    6.5887   -0.9172 H   0  0  0  0  0  0  0  0  0  0  0  0
    8.6969    8.1293   -0.8872 H   0  0  0  0  0  0  0  0  0  0  0  0
   10.1931    7.9392    0.0363 H   0  0  0  0  0  0  0  0  0  0  0  0
    7.0443    8.8750    0.3204 H   0  0  0  0  0  0  0  0  0  0  0  0
    5.3343    8.9354   -0.1268 H   0  0  0  0  0  0  0  0  0  0  0  0
    6.4161    8.2407    2.6370 H   0  0  0  0  0  0  0  0  0  0  0  0
    4.7061    8.3010    2.1899 H   0  0  0  0  0  0  0  0  0  0  0  0
    5.6747    9.7807    2.1826 H   0  0  0  0  0  0  0  0  0  0  0  0
    3.6531    7.9669    0.3126 H   0  0  0  0  0  0  0  0  0  0  0  0
    2.8212    6.4936   -0.2021 H   0  0  0  0  0  0  0  0  0  0  0  0
    3.6518    6.8334   -2.5169 H   0  0  0  0  0  0  0  0  0  0  0  0
    4.4835    8.3068   -2.0022 H   0  0  0  0  0  0  0  0  0  0  0  0
    2.7168    8.2320   -1.9714 H   0  0  0  0  0  0  0  0  0  0  0  0
    3.9807    3.4535   -1.5039 H   0  0  0  0  0  0  0  0  0  0  0  0
    3.1138    4.9948   -1.4770 H   0  0  0  0  0  0  0  0  0  0  0  0
    3.4932    3.1373    0.9099 H   0  0  0  0  0  0  0  0  0  0  0  0
    1.9979    3.3268   -0.0152 H   0  0  0  0  0  0  0  0  0  0  0  0
    2.6262    4.6784    0.9367 H   0  0  0  0  0  0  0  0  0  0  0  0
    5.1487    2.3882   -0.2975 H   0  0  0  0  0  0  0  0  0  0  0  0
    6.8596    2.3284    0.1463 H   0  0  0  0  0  0  0  0  0  0  0  0
    5.7721    3.0226   -2.6154 H   0  0  0  0  0  0  0  0  0  0  0  0
    7.4830    2.9629   -2.1716 H   0  0  0  0  0  0  0  0  0  0  0  0
    6.5149    1.4829   -2.1623 H   0  0  0  0  0  0  0  0  0  0  0  0
  1  2  4  0  0  0  0
  2  3  4  0  0  0  0
  3  4  4  0  0  0  0
  4  5  4  0  0  0  0
  5  6  4  0  0  0  0
  6  1  4  0  0  0  0
  6  7  1  0  0  0  0
  7  8  1  0  0  0  0
  5  9  1  0  0  0  0
```

```
  9 10  1  0  0  0  0
  4 11  1  0  0  0  0
 11 12  1  0  0  0  0
  3 13  1  0  0  0  0
 13 14  1  0  0  0  0
  2 15  1  0  0  0  0
 15 16  1  0  0  0  0
  1 17  1  0  0  0  0
 17 18  1  0  0  0  0
  7 19  1  0  0  0  0
  7 20  1  0  0  0  0
  8 21  1  0  0  0  0
  8 22  1  0  0  0  0
  8 23  1  0  0  0  0
  9 24  1  0  0  0  0
  9 25  1  0  0  0  0
 10 26  1  0  0  0  0
 10 27  1  0  0  0  0
 10 28  1  0  0  0  0
 11 29  1  0  0  0  0
 11 30  1  0  0  0  0
 12 31  1  0  0  0  0
 12 32  1  0  0  0  0
 12 33  1  0  0  0  0
 13 34  1  0  0  0  0
 13 35  1  0  0  0  0
 14 36  1  0  0  0  0
 14 37  1  0  0  0  0
 14 38  1  0  0  0  0
 15 39  1  0  0  0  0
 15 40  1  0  0  0  0
 16 41  1  0  0  0  0
 16 42  1  0  0  0  0
 16 43  1  0  0  0  0
 17 44  1  0  0  0  0
 17 45  1  0  0  0  0
 18 46  1  0  0  0  0
 18 47  1  0  0  0  0
 18 48  1  0  0  0  0
M  END
>  <Alias name>
Hexaethylbenzene

>  <deltaH°(c)>   kJ/mol
-11027

$$$$
2,4,6-Tri-t-butylnitrosobenzene
  ChemBrain IXL

 49 49  0  0  0  0  0  0  0  0  1 V2000
    7.2621    4.4510   -0.1562 C   0  0  0  0  0  0  0  0  0  0  0  0
    7.1997    5.8431   -0.0936 C   0  0  0  0  0  0  0  0  0  0  0  0
    5.9720    6.4786    0.0932 C   0  0  0  0  0  0  0  0  0  0  0  0
    4.8076    5.7216    0.2245 C   0  0  0  0  0  0  0  0  0  0  0  0
    4.8701    4.3294    0.1619 C   0  0  0  0  0  0  0  0  0  0  0  0
    6.0957    3.6946   -0.0401 C   0  0  0  0  0  0  0  0  0  0  0  0
    6.1552    2.2985   -0.1262 N   0  0  0  0  0  0  0  0  0  0  0  0
    5.4083    1.6016    0.5767 O   0  0  0  0  0  0  0  0  0  0  0  0
    8.5657    3.7764   -0.3457 C   0  0  0  0  0  0  0  0  0  0  0  0
    9.6813    4.8344   -0.4492 C   0  0  0  0  0  0  0  0  0  0  0  0
    8.5284    2.9409   -1.6401 C   0  0  0  0  0  0  0  0  0  0  0  0
    8.8448    2.8514    0.8548 C   0  0  0  0  0  0  0  0  0  0  0  0
```

```
   3.6360    3.5259    0.3096 C   0  0  0  0  0  0  0  0  0  0  0  0
   3.5730    2.4703   -0.8113 C   0  0  0  0  0  0  0  0  0  0  0  0
   3.6433    2.8205    1.6797 C   0  0  0  0  0  0  0  0  0  0  0  0
   2.4067    4.4502    0.2144 C   0  0  0  0  0  0  0  0  0  0  0  0
   5.9047    7.9559    0.1522 C   0  0  0  0  0  0  0  0  0  0  0  0
   7.2831    8.5173    0.5516 C   0  0  0  0  0  0  0  0  0  0  0  0
   4.8546    8.3810    1.1968 C   0  0  0  0  0  0  0  0  0  0  0  0
   5.5065    8.5075   -1.2305 C   0  0  0  0  0  0  0  0  0  0  0  0
   8.1044    6.4306   -0.1901 H   0  0  0  0  0  0  0  0  0  0  0  0
   3.8552    6.2147    0.3751 H   0  0  0  0  0  0  0  0  0  0  0  0
   9.4827    5.4873   -1.2901 H   0  0  0  0  0  0  0  0  0  0  0  0
  10.6346    4.3409   -0.5926 H   0  0  0  0  0  0  0  0  0  0  0  0
   9.7106    5.4185    0.4623 H   0  0  0  0  0  0  0  0  0  0  0  0
   9.4824    2.4476   -1.7789 H   0  0  0  0  0  0  0  0  0  0  0  0
   7.7446    2.1971   -1.5672 H   0  0  0  0  0  0  0  0  0  0  0  0
   8.3319    3.5909   -2.4837 H   0  0  0  0  0  0  0  0  0  0  0  0
   9.7964    2.3540    0.7137 H   0  0  0  0  0  0  0  0  0  0  0  0
   8.0581    2.1109    0.9305 H   0  0  0  0  0  0  0  0  0  0  0  0
   8.8759    3.4392    1.7639 H   0  0  0  0  0  0  0  0  0  0  0  0
   4.4294    1.8116   -0.7354 H   0  0  0  0  0  0  0  0  0  0  0  0
   3.5827    2.9655   -1.7744 H   0  0  0  0  0  0  0  0  0  0  0  0
   2.6628    1.8920   -0.7119 H   0  0  0  0  0  0  0  0  0  0  0  0
   4.5015    2.1629    1.7425 H   0  0  0  0  0  0  0  0  0  0  0  0
   3.6982    3.5619    2.4672 H   0  0  0  0  0  0  0  0  0  0  0  0
   2.7354    2.2409    1.7921 H   0  0  0  0  0  0  0  0  0  0  0  0
   1.5031    3.8585    0.2938 H   0  0  0  0  0  0  0  0  0  0  0  0
   2.4361    5.1742    1.0193 H   0  0  0  0  0  0  0  0  0  0  0  0
   2.4169    4.9676   -0.7370 H   0  0  0  0  0  0  0  0  0  0  0  0
   7.2249    9.5959    0.6293 H   0  0  0  0  0  0  0  0  0  0  0  0
   8.0142    8.2485   -0.2008 H   0  0  0  0  0  0  0  0  0  0  0  0
   7.5790    8.1019    1.5071 H   0  0  0  0  0  0  0  0  0  0  0  0
   5.1252    7.9786    2.1651 H   0  0  0  0  0  0  0  0  0  0  0  0
   4.8179    9.4620    1.2524 H   0  0  0  0  0  0  0  0  0  0  0  0
   3.8827    8.0012    0.9068 H   0  0  0  0  0  0  0  0  0  0  0  0
   4.5412    8.1070   -1.5146 H   0  0  0  0  0  0  0  0  0  0  0  0
   5.4498    9.5881   -1.1854 H   0  0  0  0  0  0  0  0  0  0  0  0
   6.2485    8.2152   -1.9632 H   0  0  0  0  0  0  0  0  0  0  0  0
  1  2  4  0  0  0  0
  2  3  4  0  0  0  0
  3  4  4  0  0  0  0
  4  5  4  0  0  0  0
  5  6  4  0  0  0  0
  6  1  4  0  0  0  0
  6  7  1  0  0  0  0
  7  8  2  0  0  0  0
  1  9  1  0  0  0  0
  9 10  1  0  0  0  0
  9 11  1  0  0  0  0
  9 12  1  0  0  0  0
  5 13  1  0  0  0  0
 13 14  1  0  0  0  0
 13 15  1  0  0  0  0
 13 16  1  0  0  0  0
  3 17  1  0  0  0  0
 17 18  1  0  0  0  0
 17 19  1  0  0  0  0
 17 20  1  0  0  0  0
  2 21  1  0  0  0  0
  4 22  1  0  0  0  0
 10 23  1  0  0  0  0
 10 24  1  0  0  0  0
 10 25  1  0  0  0  0
 11 26  1  0  0  0  0
```

```
 11 27  1  0  0  0  0
 11 28  1  0  0  0  0
 12 29  1  0  0  0  0
 12 30  1  0  0  0  0
 12 31  1  0  0  0  0
 14 32  1  0  0  0  0
 14 33  1  0  0  0  0
 14 34  1  0  0  0  0
 15 35  1  0  0  0  0
 15 36  1  0  0  0  0
 15 37  1  0  0  0  0
 16 38  1  0  0  0  0
 16 39  1  0  0  0  0
 16 40  1  0  0  0  0
 18 41  1  0  0  0  0
 18 42  1  0  0  0  0
 18 43  1  0  0  0  0
 19 44  1  0  0  0  0
 19 45  1  0  0  0  0
 19 46  1  0  0  0  0
 20 47  1  0  0  0  0
 20 48  1  0  0  0  0
 20 49  1  0  0  0  0
M  END
>  <Alias name>
2,4,6-Tri-t-butylnitrosobenzene

>  <deltaH°(c)>   kJ/mol
-11019

$$$$
N,N'-Bis(o-methoxyphenyl)terephthalamide
  ChemBrain IXL

 48 50  0  0  0  0  0  0  0  0  1 V2000
   15.5081   10.2716    0.0000 C   0  0  0  0  0  0  0  0  0  0  0  0
   16.3323   11.3971   -0.0001 C   0  0  0  0  0  0  0  0  0  0  0  0
   16.0707    8.9951   -0.0009 C   0  0  0  0  0  0  0  0  0  0  0  0
   14.1163   10.4231    0.0010 N   0  0  0  0  0  0  0  0  0  0  0  0
   17.7191   11.2461   -0.0008 C   0  0  0  0  0  0  0  0  0  0  0  0
   15.7394   12.7423    0.0007 O   0  0  0  0  0  0  0  0  0  0  0  0
   18.2817    9.9696   -0.0018 C   0  0  0  0  0  0  0  0  0  0  0  0
   17.4575    8.8441   -0.0018 C   0  0  0  0  0  0  0  0  0  0  0  0
   16.7080   13.8481    0.0007 C   0  0  0  0  0  0  0  0  0  0  0  0
   13.2892    9.2936    0.0013 C   0  0  0  0  0  0  0  0  0  0  0  0
   11.8179    9.4538    0.0014 C   0  0  0  0  0  0  0  0  0  0  0  0
   13.7813    8.1772    0.0015 O   0  0  0  0  0  0  0  0  0  0  0  0
    9.8685   10.8813    0.0016 C   0  0  0  0  0  0  0  0  0  0  0  0
   11.2553   10.7303    0.0015 C   0  0  0  0  0  0  0  0  0  0  0  0
    9.0443    9.7558    0.0017 C   0  0  0  0  0  0  0  0  0  0  0  0
   10.9937    8.3283    0.0014 C   0  0  0  0  0  0  0  0  0  0  0  0
    9.6069    8.4793    0.0016 C   0  0  0  0  0  0  0  0  0  0  0  0
    7.5730    9.9160    0.0018 C   0  0  0  0  0  0  0  0  0  0  0  0
    7.0809   11.0324    0.0024 O   0  0  0  0  0  0  0  0  0  0  0  0
    6.7459    8.7864    0.0011 N   0  0  0  0  0  0  0  0  0  0  0  0
    5.3541    8.9380    0.0001 C   0  0  0  0  0  0  0  0  0  0  0  0
    3.4047   10.3655   -0.0018 C   0  0  0  0  0  0  0  0  0  0  0  0
    4.7915   10.2145   -0.0008 C   0  0  0  0  0  0  0  0  0  0  0  0
    2.5805    9.2400   -0.0018 C   0  0  0  0  0  0  0  0  0  0  0  0
    4.5299    7.8125    0.0000 C   0  0  0  0  0  0  0  0  0  0  0  0
    3.1431    7.9635   -0.0008 C   0  0  0  0  0  0  0  0  0  0  0  0
    5.1228    6.4674    0.0008 O   0  0  0  0  0  0  0  0  0  0  0  0
    4.1543    5.3615    0.0008 C   0  0  0  0  0  0  0  0  0  0  0  0
```

```
 13.7094   11.3464    0.0014 H   0  0  0  0  0  0  0  0  0  0  0  0
  7.1528    7.8632    0.0014 H   0  0  0  0  0  0  0  0  0  0  0  0
 15.4309    8.1213   -0.0009 H   0  0  0  0  0  0  0  0  0  0  0  0
 18.3589   12.1199   -0.0007 H   0  0  0  0  0  0  0  0  0  0  0  0
 19.3584    9.8524   -0.0024 H   0  0  0  0  0  0  0  0  0  0  0  0
 17.8943    7.8531   -0.0025 H   0  0  0  0  0  0  0  0  0  0  0  0
 16.1777   14.7924    0.0014 H   0  0  0  0  0  0  0  0  0  0  0  0
 17.3294   13.7836   -0.8839 H   0  0  0  0  0  0  0  0  0  0  0  0
 17.3303   13.7829    0.8846 H   0  0  0  0  0  0  0  0  0  0  0  0
  9.4317   11.8723    0.0017 H   0  0  0  0  0  0  0  0  0  0  0  0
 11.8951   11.6041    0.0015 H   0  0  0  0  0  0  0  0  0  0  0  0
 11.4305    7.3373    0.0014 H   0  0  0  0  0  0  0  0  0  0  0  0
  8.9671    7.6055    0.0016 H   0  0  0  0  0  0  0  0  0  0  0  0
  2.9679   11.3565   -0.0026 H   0  0  0  0  0  0  0  0  0  0  0  0
  5.4313   11.0883   -0.0008 H   0  0  0  0  0  0  0  0  0  0  0  0
  1.5039    9.3572   -0.0025 H   0  0  0  0  0  0  0  0  0  0  0  0
  2.5033    7.0897   -0.0007 H   0  0  0  0  0  0  0  0  0  0  0  0
  4.6845    4.4172    0.0015 H   0  0  0  0  0  0  0  0  0  0  0  0
  3.5319    5.4267    0.8847 H   0  0  0  0  0  0  0  0  0  0  0  0
  3.5329    5.4259   -0.8839 H   0  0  0  0  0  0  0  0  0  0  0  0
  2  1  4  0  0  0  0
  1  3  4  0  0  0  0
  1  4  1  0  0  0  0
  5  2  4  0  0  0  0
  2  6  1  0  0  0  0
  3  8  4  0  0  0  0
  4 10  1  0  0  0  0
  7  5  4  0  0  0  0
  6  9  1  0  0  0  0
  8  7  4  0  0  0  0
 10 11  1  0  0  0  0
 10 12  2  0  0  0  0
 11 14  4  0  0  0  0
 16 11  4  0  0  0  0
 14 13  4  0  0  0  0
 13 15  4  0  0  0  0
 15 17  4  0  0  0  0
 15 18  1  0  0  0  0
 17 16  4  0  0  0  0
 18 19  2  0  0  0  0
 18 20  1  0  0  0  0
 20 21  1  0  0  0  0
 21 23  4  0  0  0  0
 25 21  4  0  0  0  0
 23 22  4  0  0  0  0
 22 24  4  0  0  0  0
 24 26  4  0  0  0  0
 26 25  4  0  0  0  0
 25 27  1  0  0  0  0
 27 28  1  0  0  0  0
  4 29  1  0  0  0  0
 20 30  1  0  0  0  0
  3 31  1  0  0  0  0
  5 32  1  0  0  0  0
  7 33  1  0  0  0  0
  8 34  1  0  0  0  0
  9 35  1  0  0  0  0
  9 36  1  0  0  0  0
  9 37  1  0  0  0  0
 13 38  1  0  0  0  0
 14 39  1  0  0  0  0
 16 40  1  0  0  0  0
 17 41  1  0  0  0  0
```

```
 22 42  1  0  0  0  0
 23 43  1  0  0  0  0
 24 44  1  0  0  0  0
 26 45  1  0  0  0  0
 28 46  1  0  0  0  0
 28 47  1  0  0  0  0
 28 48  1  0  0  0  0
M  END
>  <Alias name>
N,N'-Bis(o-methoxyphenyl)terephthalamide

>  <deltaH°(c)>   kJ/mol
-11007

$$$$
Linoleic acid
  ChemBrain IXL

 52 51  0  0  0  0  0  0  0  0  1 V2000
    2.7277   11.7459   -0.7793 C   0  0  0  0  0  0  0  0  0  0  0  0  0
    2.2784   10.3321   -1.2124 C   0  0  0  0  0  0  0  0  0  0  0  0  0
    3.4707    9.3614   -1.1187 C   0  0  0  0  0  0  0  0  0  0  0  0  0
    3.0295    7.9434   -1.5503 C   0  0  0  0  0  0  0  0  0  0  0  0  0
    4.2357    7.0031   -1.4470 C   0  0  0  0  0  0  0  0  0  0  0  0  0
    3.8691    5.6422   -1.8464 C   0  0  0  0  0  0  0  0  0  0  0  0  0
    4.7971    4.7098   -1.8095 C   0  0  0  0  0  0  0  0  0  0  0  0  0
    6.1271    5.0984   -1.3727 C   0  0  0  0  0  0  0  0  0  0  0  0  0
    7.0619    3.9864   -1.3766 C   0  0  0  0  0  0  0  0  0  0  0  0  0
    8.2939    4.2356   -1.0072 C   0  0  0  0  0  0  0  0  0  0  0  0  0
    8.6162    5.6001   -0.6269 C   0  0  0  0  0  0  0  0  0  0  0  0  0
   10.0777    5.7512   -0.2111 C   0  0  0  0  0  0  0  0  0  0  0  0  0
   10.2989    7.2307    0.1641 C   0  0  0  0  0  0  0  0  0  0  0  0  0
   11.7463    7.4757    0.5950 C   0  0  0  0  0  0  0  0  0  0  0  0  0
   11.9108    8.9700    0.9567 C   0  0  0  0  0  0  0  0  0  0  0  0  0
   13.3590    9.2389    1.3921 C   0  0  0  0  0  0  0  0  0  0  0  0  0
   13.5229   10.7299    1.7524 C   0  0  0  0  0  0  0  0  0  0  0  0  0
   14.9205   10.9824    2.1704 C   0  0  0  0  0  0  0  0  0  0  0  0  0
   15.3377   12.3360    2.5622 O   0  0  0  0  0  0  0  0  0  0  0  0  0
   15.7266   10.0666    2.1896 O   0  0  0  0  0  0  0  0  0  0  0  0  0
   14.6310   13.0179    2.5183 H   0  0  0  0  0  0  0  0  0  0  0  0  0
    1.8877   12.4269   -0.8435 H   0  0  0  0  0  0  0  0  0  0  0  0  0
    3.5211   12.0894   -1.4322 H   0  0  0  0  0  0  0  0  0  0  0  0  0
    3.0889   11.7140    0.2415 H   0  0  0  0  0  0  0  0  0  0  0  0  0
    1.9198   10.3648   -2.2329 H   0  0  0  0  0  0  0  0  0  0  0  0  0
    1.4843    9.9912   -0.5611 H   0  0  0  0  0  0  0  0  0  0  0  0  0
    3.8309    9.3300   -0.0969 H   0  0  0  0  0  0  0  0  0  0  0  0  0
    4.2667    9.7037   -1.7698 H   0  0  0  0  0  0  0  0  0  0  0  0  0
    2.6735    7.9677   -2.5734 H   0  0  0  0  0  0  0  0  0  0  0  0  0
    2.2380    7.5933   -0.8983 H   0  0  0  0  0  0  0  0  0  0  0  0  0
    4.5912    6.9872   -0.4255 H   0  0  0  0  0  0  0  0  0  0  0  0  0
    5.0251    7.3602   -2.0945 H   0  0  0  0  0  0  0  0  0  0  0  0  0
    2.8571    5.4055   -2.1657 H   0  0  0  0  0  0  0  0  0  0  0  0  0
    4.5740    3.6800   -2.0975 H   0  0  0  0  0  0  0  0  0  0  0  0  0
    6.0629    5.4927   -0.3722 H   0  0  0  0  0  0  0  0  0  0  0  0  0
    6.4990    5.8652   -2.0318 H   0  0  0  0  0  0  0  0  0  0  0  0  0
    6.7532    2.9814   -1.6751 H   0  0  0  0  0  0  0  0  0  0  0  0  0
    9.0472    3.4472   -0.9869 H   0  0  0  0  0  0  0  0  0  0  0  0  0
    8.4199    6.2501   -1.4668 H   0  0  0  0  0  0  0  0  0  0  0  0  0
    7.9832    5.8889    0.1993 H   0  0  0  0  0  0  0  0  0  0  0  0  0
   10.2857    5.1178    0.6444 H   0  0  0  0  0  0  0  0  0  0  0  0  0
   10.7265    5.4782   -1.0363 H   0  0  0  0  0  0  0  0  0  0  0  0  0
   10.0721    7.8517   -0.6941 H   0  0  0  0  0  0  0  0  0  0  0  0  0
    9.6347    7.4930    0.9787 H   0  0  0  0  0  0  0  0  0  0  0  0  0
```

```
   11.9765    6.8655    1.4587 H   0  0  0  0  0  0  0  0  0  0  0  0
   12.4150    7.2231   -0.2177 H   0  0  0  0  0  0  0  0  0  0  0  0
   11.6749    9.5788    0.0915 H   0  0  0  0  0  0  0  0  0  0  0  0
   11.2372    9.2212    1.7679 H   0  0  0  0  0  0  0  0  0  0  0  0
   13.5937    8.6316    2.2566 H   0  0  0  0  0  0  0  0  0  0  0  0
   14.0310    8.9883    0.5816 H   0  0  0  0  0  0  0  0  0  0  0  0
   13.2886   11.3395    0.8876 H   0  0  0  0  0  0  0  0  0  0  0  0
   12.8515   10.9823    2.5647 H   0  0  0  0  0  0  0  0  0  0  0  0
 10 11  1  0  0  0  0
  5  6  1  0  0  0  0
 11 12  1  0  0  0  0
  1  2  1  0  0  0  0
 12 13  1  0  0  0  0
  6  7  2  0  0  0  0
 13 14  1  0  0  0  0
  3  4  1  0  0  0  0
 14 15  1  0  0  0  0
  7  8  1  0  0  0  0
 15 16  1  0  0  0  0
 16 17  1  0  0  0  0
  8  9  1  0  0  0  0
 17 18  1  0  0  0  0
  4  5  1  0  0  0  0
 18 19  1  0  0  0  0
  9 10  2  0  0  0  0
 18 20  2  0  0  0  0
  2  3  1  0  0  0  0
 19 21  1  0  0  0  0
  1 22  1  0  0  0  0
  1 23  1  0  0  0  0
  1 24  1  0  0  0  0
  2 25  1  0  0  0  0
  2 26  1  0  0  0  0
  3 27  1  0  0  0  0
  3 28  1  0  0  0  0
  4 29  1  0  0  0  0
  4 30  1  0  0  0  0
  5 31  1  0  0  0  0
  5 32  1  0  0  0  0
  6 33  1  0  0  0  0
  7 34  1  0  0  0  0
  8 35  1  0  0  0  0
  8 36  1  0  0  0  0
  9 37  1  0  0  0  0
 10 38  1  0  0  0  0
 11 39  1  0  0  0  0
 11 40  1  0  0  0  0
 12 41  1  0  0  0  0
 12 42  1  0  0  0  0
 13 43  1  0  0  0  0
 13 44  1  0  0  0  0
 14 45  1  0  0  0  0
 14 46  1  0  0  0  0
 15 47  1  0  0  0  0
 15 48  1  0  0  0  0
 16 49  1  0  0  0  0
 16 50  1  0  0  0  0
 17 51  1  0  0  0  0
 17 52  1  0  0  0  0
M  END
>  <Alias name>
Linoleic acid
```

```
> <deltaH°(c)>  kJ/mol
-10990.21

$$$$
N,N'-Bis-(m-methoxyphenyl)terephtalamide
  ChemBrain IXL

 48 50  0  0  0  0  0  0  0  0  1 V2000
    2.4646    8.2153    0.0000 C   0  0  0  0  0  0  0  0  0  0  0  0
    1.9189    9.5802    0.0001 O   0  0  0  0  0  0  0  0  0  0  0  0
    2.9248   10.6514    0.0379 C   0  0  0  0  0  0  0  0  0  0  0  0
    4.2826   10.3325    0.0675 C   0  0  0  0  0  0  0  0  0  0  0  0
    5.2372   11.3491    0.1029 C   0  0  0  0  0  0  0  0  0  0  0  0
    2.5217   11.9869    0.0443 C   0  0  0  0  0  0  0  0  0  0  0  0
    3.4764   13.0035    0.0797 C   0  0  0  0  0  0  0  0  0  0  0  0
    4.8341   12.6846    0.1091 C   0  0  0  0  0  0  0  0  0  0  0  0
    6.5998   11.0291    0.1322 N   0  0  0  0  0  0  0  0  0  0  0  0
    6.1689    8.7996    0.1261 O   0  0  0  0  0  0  0  0  0  0  0  0
    7.0042    9.6887    0.1330 C   0  0  0  0  0  0  0  0  0  0  0  0
    8.4450    9.3505    0.1424 C   0  0  0  0  0  0  0  0  0  0  0  0
   10.2061    7.6962    0.1474 C   0  0  0  0  0  0  0  0  0  0  0  0
    8.8480    8.0150    0.1387 C   0  0  0  0  0  0  0  0  0  0  0  0
   11.1612    8.7129    0.1598 C   0  0  0  0  0  0  0  0  0  0  0  0
    9.4001   10.3672    0.1548 C   0  0  0  0  0  0  0  0  0  0  0  0
   10.7582   10.0484    0.1636 C   0  0  0  0  0  0  0  0  0  0  0  0
   13.4371    9.2636    0.1947 O   0  0  0  0  0  0  0  0  0  0  0  0
   12.6020    8.3746    0.1689 C   0  0  0  0  0  0  0  0  0  0  0  0
   13.0066    7.0345    0.1478 N   0  0  0  0  0  0  0  0  0  0  0  0
   14.3692    6.7151    0.1140 C   0  0  0  0  0  0  0  0  0  0  0  0
   15.3233    7.7322    0.0760 C   0  0  0  0  0  0  0  0  0  0  0  0
   16.6811    7.4140    0.0414 C   0  0  0  0  0  0  0  0  0  0  0  0
   14.7730    5.3799    0.1183 C   0  0  0  0  0  0  0  0  0  0  0  0
   16.1308    5.0616    0.0841 C   0  0  0  0  0  0  0  0  0  0  0  0
   17.0848    6.0787    0.0457 C   0  0  0  0  0  0  0  0  0  0  0  0
   17.6864    8.4857    0.0005 O   0  0  0  0  0  0  0  0  0  0  0  0
   17.1401    9.8504    0.0034 C   0  0  0  0  0  0  0  0  0  0  0  0
    7.2904   11.7644    0.1528 H   0  0  0  0  0  0  0  0  0  0  0  0
   12.3161    6.2989    0.1570 H   0  0  0  0  0  0  0  0  0  0  0  0
    3.0963    8.0791   -0.8691 H   0  0  0  0  0  0  0  0  0  0  0  0
    1.6509    7.5012   -0.0297 H   0  0  0  0  0  0  0  0  0  0  0  0
    3.0486    8.0599    0.8987 H   0  0  0  0  0  0  0  0  0  0  0  0
    4.5955    9.2957    0.0630 H   0  0  0  0  0  0  0  0  0  0  0  0
    1.4676   12.2345    0.0216 H   0  0  0  0  0  0  0  0  0  0  0  0
    3.1634   14.0403    0.0845 H   0  0  0  0  0  0  0  0  0  0  0  0
    5.5752   13.4738    0.1366 H   0  0  0  0  0  0  0  0  0  0  0  0
   10.5189    6.6593    0.1446 H   0  0  0  0  0  0  0  0  0  0  0  0
    8.1065    7.2257    0.1291 H   0  0  0  0  0  0  0  0  0  0  0  0
    9.0873   11.4040    0.1577 H   0  0  0  0  0  0  0  0  0  0  0  0
   11.4996   10.8377    0.1733 H   0  0  0  0  0  0  0  0  0  0  0  0
   15.0099    8.7689    0.0733 H   0  0  0  0  0  0  0  0  0  0  0  0
   14.0324    4.5903    0.1482 H   0  0  0  0  0  0  0  0  0  0  0  0
   16.4442    4.0250    0.0872 H   0  0  0  0  0  0  0  0  0  0  0  0
   18.1389    5.8316    0.0193 H   0  0  0  0  0  0  0  0  0  0  0  0
   16.5052    9.9870   -0.8633 H   0  0  0  0  0  0  0  0  0  0  0  0
   16.5592   10.0048    0.9043 H   0  0  0  0  0  0  0  0  0  0  0  0
   17.9534   10.5649   -0.0287 H   0  0  0  0  0  0  0  0  0  0  0  0
  1  2  1  0  0  0  0
  2  3  1  0  0  0  0
  4  3  4  0  0  0  0
  3  6  4  0  0  0  0
  5  4  4  0  0  0  0
  8  5  4  0  0  0  0
  5  9  1  0  0  0  0
```

```
  6  7  4  0  0  0  0
  7  8  4  0  0  0  0
  9 11  1  0  0  0  0
 10 11  2  0  0  0  0
 11 12  1  0  0  0  0
 14 12  4  0  0  0  0
 12 16  4  0  0  0  0
 13 14  4  0  0  0  0
 15 13  4  0  0  0  0
 17 15  4  0  0  0  0
 15 19  1  0  0  0  0
 16 17  4  0  0  0  0
 18 19  2  0  0  0  0
 19 20  1  0  0  0  0
 20 21  1  0  0  0  0
 22 21  4  0  0  0  0
 21 24  4  0  0  0  0
 23 22  4  0  0  0  0
 26 23  4  0  0  0  0
 23 27  1  0  0  0  0
 24 25  4  0  0  0  0
 25 26  4  0  0  0  0
 27 28  1  0  0  0  0
  9 29  1  0  0  0  0
 20 30  1  0  0  0  0
  1 31  1  0  0  0  0
  1 32  1  0  0  0  0
  1 33  1  0  0  0  0
  4 34  1  0  0  0  0
  6 35  1  0  0  0  0
  7 36  1  0  0  0  0
  8 37  1  0  0  0  0
 13 38  1  0  0  0  0
 14 39  1  0  0  0  0
 16 40  1  0  0  0  0
 17 41  1  0  0  0  0
 22 42  1  0  0  0  0
 24 43  1  0  0  0  0
 25 44  1  0  0  0  0
 26 45  1  0  0  0  0
 28 46  1  0  0  0  0
 28 47  1  0  0  0  0
 28 48  1  0  0  0  0
M  END
>  <Alias name>
N,N'-Bis-(m-methoxyphenyl)terephtalamide

>  <deltaH°(c)>   kJ/mol
-10966

$$$$
2,4,6-Tri-t-butylnitrobenzene
  ChemBrain IXL

 50 50  0  0  0  0  0  0  0  0  1 V2000
    7.1941    4.8354    0.3931 C   0  0  0  0  0  0  0  0  0  0  0  0
    6.8111    6.1677    0.5673 C   0  0  0  0  0  0  0  0  0  0  0  0
    5.4790    6.5511    0.3917 C   0  0  0  0  0  0  0  0  0  0  0  0
    4.5156    5.6025    0.0383 C   0  0  0  0  0  0  0  0  0  0  0  0
    4.8718    4.2635   -0.1420 C   0  0  0  0  0  0  0  0  0  0  0  0
    6.2206    3.8714    0.0358 C   0  0  0  0  0  0  0  0  0  0  0  0
    8.6409    4.4501    0.5898 C   0  0  0  0  0  0  0  0  0  0  0  0
    9.4642    5.6964    0.9700 C   0  0  0  0  0  0  0  0  0  0  0  0
```

```
   9.2086    3.8572   -0.7226 C   0  0  0  0  0  0  0  0  0  0  0  0
   8.7562    3.4080    1.7284 C   0  0  0  0  0  0  0  0  0  0  0  0
   3.8091    3.2604   -0.5238 C   0  0  0  0  0  0  0  0  0  0  0  0
   4.1630    2.6141   -1.8851 C   0  0  0  0  0  0  0  0  0  0  0  0
   3.7061    2.1651    0.5650 C   0  0  0  0  0  0  0  0  0  0  0  0
   2.4456    3.9686   -0.6484 C   0  0  0  0  0  0  0  0  0  0  0  0
   5.0869    7.9692    0.5803 C   0  0  0  0  0  0  0  0  0  0  0  0
   5.9957    8.6208    1.6444 C   0  0  0  0  0  0  0  0  0  0  0  0
   3.6185    8.0435    1.0508 C   0  0  0  0  0  0  0  0  0  0  0  0
   5.2377    8.7211   -0.7552 C   0  0  0  0  0  0  0  0  0  0  0  0
   7.7905    2.0991    0.0013 O   0  5  0  0  0  0  0  0  0  0  0  0
   6.6151    2.4462   -0.1527 N   0  3  0  0  0  0  0  0  0  0  0  0
   5.7696    1.6015   -0.4636 O   0  0  0  0  0  0  0  0  0  0  0  0
   7.5492    6.9058    0.8394 H   0  0  0  0  0  0  0  0  0  0  0  0
   3.4893    5.9060   -0.0961 H   0  0  0  0  0  0  0  0  0  0  0  0
   9.3942    6.4311    0.1780 H   0  0  0  0  0  0  0  0  0  0  0  0
  10.5009    5.4142    1.1095 H   0  0  0  0  0  0  0  0  0  0  0  0
   9.0786    6.1174    1.8898 H   0  0  0  0  0  0  0  0  0  0  0  0
  10.2487    3.5906   -0.5781 H   0  0  0  0  0  0  0  0  0  0  0  0
   9.1311    4.5915   -1.5142 H   0  0  0  0  0  0  0  0  0  0  0  0
   8.6451    2.9735   -0.9941 H   0  0  0  0  0  0  0  0  0  0  0  0
   9.7968    3.1417    1.8700 H   0  0  0  0  0  0  0  0  0  0  0  0
   8.1907    2.5224    1.4672 H   0  0  0  0  0  0  0  0  0  0  0  0
   8.3637    3.8292    2.6450 H   0  0  0  0  0  0  0  0  0  0  0  0
   5.1143    2.1031   -1.8069 H   0  0  0  0  0  0  0  0  0  0  0  0
   3.3933    1.9020   -2.1576 H   0  0  0  0  0  0  0  0  0  0  0  0
   4.2280    3.3830   -2.6443 H   0  0  0  0  0  0  0  0  0  0  0  0
   4.6556    1.6526    0.6538 H   0  0  0  0  0  0  0  0  0  0  0  0
   3.4523    2.6210    1.5134 H   0  0  0  0  0  0  0  0  0  0  0  0
   2.9372    1.4533    0.2894 H   0  0  0  0  0  0  0  0  0  0  0  0
   1.6877    3.2445   -0.9227 H   0  0  0  0  0  0  0  0  0  0  0  0
   2.1853    4.4209    0.3000 H   0  0  0  0  0  0  0  0  0  0  0  0
   2.5048    4.7347   -1.4110 H   0  0  0  0  0  0  0  0  0  0  0  0
   5.6854    9.6450    1.8047 H   0  0  0  0  0  0  0  0  0  0  0  0
   7.0221    8.6055    1.3028 H   0  0  0  0  0  0  0  0  0  0  0  0
   5.9158    8.0697    2.5738 H   0  0  0  0  0  0  0  0  0  0  0  0
   3.5052    7.4816    1.9702 H   0  0  0  0  0  0  0  0  0  0  0  0
   3.3476    9.0767    1.2239 H   0  0  0  0  0  0  0  0  0  0  0  0
   2.9728    7.6252    0.2901 H   0  0  0  0  0  0  0  0  0  0  0  0
   4.5982    8.2640   -1.5007 H   0  0  0  0  0  0  0  0  0  0  0  0
   6.2687    8.6697   -1.0837 H   0  0  0  0  0  0  0  0  0  0  0  0
   4.9527    9.7564   -0.6208 H   0  0  0  0  0  0  0  0  0  0  0  0
  1  2  4  0  0  0  0
  2  3  4  0  0  0  0
  3  4  4  0  0  0  0
  4  5  4  0  0  0  0
  5  6  4  0  0  0  0
  6  1  4  0  0  0  0
  1  7  1  0  0  0  0
  7  8  1  0  0  0  0
  7  9  1  0  0  0  0
  7 10  1  0  0  0  0
  5 11  1  0  0  0  0
 11 12  1  0  0  0  0
 11 13  1  0  0  0  0
 11 14  1  0  0  0  0
  3 15  1  0  0  0  0
 15 16  1  0  0  0  0
 15 17  1  0  0  0  0
 15 18  1  0  0  0  0
 19 20  1  0  0  0  0
 20 21  2  0  0  0  0
 20  6  1  0  0  0  0
```

```
  2 22  1  0  0  0  0
  4 23  1  0  0  0  0
  8 24  1  0  0  0  0
  8 25  1  0  0  0  0
  8 26  1  0  0  0  0
  9 27  1  0  0  0  0
  9 28  1  0  0  0  0
  9 29  1  0  0  0  0
 10 30  1  0  0  0  0
 10 31  1  0  0  0  0
 10 32  1  0  0  0  0
 12 33  1  0  0  0  0
 12 34  1  0  0  0  0
 12 35  1  0  0  0  0
 13 36  1  0  0  0  0
 13 37  1  0  0  0  0
 13 38  1  0  0  0  0
 14 39  1  0  0  0  0
 14 40  1  0  0  0  0
 14 41  1  0  0  0  0
 16 42  1  0  0  0  0
 16 43  1  0  0  0  0
 16 44  1  0  0  0  0
 17 45  1  0  0  0  0
 17 46  1  0  0  0  0
 17 47  1  0  0  0  0
 18 48  1  0  0  0  0
 18 49  1  0  0  0  0
 18 50  1  0  0  0  0
M  END
>  <Alias name>
2,4,6-Tri-t-butylnitrobenzene

>  <deltaH°(c)>   kJ/mol
-10926.4

$$$$
Di-n-octylamine
  ChemBrain IXL

 52 51  0  0  0  0  0  0  0  0  1 V2000
    2.1222    9.2185    6.0351 C   0  0  0  0  0  0  0  0  0  0  0  0
    3.3965    9.9795    5.6207 C   0  0  0  0  0  0  0  0  0  0  0  0
    4.1365    9.1854    4.5265 C   0  0  0  0  0  0  0  0  0  0  0  0
    5.4109    9.9465    4.1122 C   0  0  0  0  0  0  0  0  0  0  0  0
    6.1511    9.1522    3.0184 C   0  0  0  0  0  0  0  0  0  0  0  0
    7.4255    9.9132    2.6042 C   0  0  0  0  0  0  0  0  0  0  0  0
    8.1661    9.1186    1.5109 C   0  0  0  0  0  0  0  0  0  0  0  0
    9.4407    9.8793    1.0969 C   0  0  0  0  0  0  0  0  0  0  0  0
   10.1476    9.1203    0.0544 N   0  0  0  0  0  0  0  0  0  0  0  0
   11.3638    9.8461   -0.3417 C   0  0  0  0  0  0  0  0  0  0  0  0
   12.1037    9.0504   -1.4344 C   0  0  0  0  0  0  0  0  0  0  0  0
   13.3785    9.8104   -1.8493 C   0  0  0  0  0  0  0  0  0  0  0  0
   14.1189    9.0142   -2.9415 C   0  0  0  0  0  0  0  0  0  0  0  0
   15.3939    9.7741   -3.3562 C   0  0  0  0  0  0  0  0  0  0  0  0
   16.1347    8.9775   -4.4479 C   0  0  0  0  0  0  0  0  0  0  0  0
   17.4098    9.7374   -4.8626 C   0  0  0  0  0  0  0  0  0  0  0  0
   18.1508    8.9408   -5.9539 C   0  0  0  0  0  0  0  0  0  0  0  0
   10.4000    8.2114    0.4127 H   0  0  0  0  0  0  0  0  0  0  0  0
    2.3932    8.2425    6.4184 H   0  0  0  0  0  0  0  0  0  0  0  0
    1.6025    9.7766    6.8039 H   0  0  0  0  0  0  0  0  0  0  0  0
    1.4755    9.1016    5.1743 H   0  0  0  0  0  0  0  0  0  0  0  0
    3.1255   10.9555    5.2375 H   0  0  0  0  0  0  0  0  0  0  0  0
```

```
 4.0431   10.0965    6.4816 H   0  0  0  0  0  0  0  0  0  0  0  0
 4.4075    8.2093    4.9098 H   0  0  0  0  0  0  0  0  0  0  0  0
 3.4899    9.0684    3.6657 H   0  0  0  0  0  0  0  0  0  0  0  0
 5.1399   10.9224    3.7287 H   0  0  0  0  0  0  0  0  0  0  0  0
 6.0574   10.0637    4.9731 H   0  0  0  0  0  0  0  0  0  0  0  0
 6.4220    8.1763    3.4018 H   0  0  0  0  0  0  0  0  0  0  0  0
 5.5046    9.0351    2.1574 H   0  0  0  0  0  0  0  0  0  0  0  0
 7.1546   10.8890    2.2203 H   0  0  0  0  0  0  0  0  0  0  0  0
 8.0717   10.0308    3.4653 H   0  0  0  0  0  0  0  0  0  0  0  0
 8.4369    8.1428    1.8947 H   0  0  0  0  0  0  0  0  0  0  0  0
 7.5201    9.0012    0.6497 H   0  0  0  0  0  0  0  0  0  0  0  0
10.0865    9.9972    1.9584 H   0  0  0  0  0  0  0  0  0  0  0  0
 9.1698   10.8550    0.7125 H   0  0  0  0  0  0  0  0  0  0  0  0
11.0930   10.8216   -0.7264 H   0  0  0  0  0  0  0  0  0  0  0  0
12.0104    9.9642    0.5192 H   0  0  0  0  0  0  0  0  0  0  0  0
12.3741    8.0748   -1.0497 H   0  0  0  0  0  0  0  0  0  0  0  0
11.4573    8.9325   -2.2952 H   0  0  0  0  0  0  0  0  0  0  0  0
13.1080   10.7858   -2.2345 H   0  0  0  0  0  0  0  0  0  0  0  0
14.0246    9.9288   -0.9883 H   0  0  0  0  0  0  0  0  0  0  0  0
14.3893    8.0388   -2.5563 H   0  0  0  0  0  0  0  0  0  0  0  0
13.4729    8.8959   -3.8026 H   0  0  0  0  0  0  0  0  0  0  0  0
15.1235   10.7494   -3.7417 H   0  0  0  0  0  0  0  0  0  0  0  0
16.0397    9.8927   -2.4949 H   0  0  0  0  0  0  0  0  0  0  0  0
16.4050    8.0022   -4.0624 H   0  0  0  0  0  0  0  0  0  0  0  0
15.4889    8.8589   -5.3092 H   0  0  0  0  0  0  0  0  0  0  0  0
17.1395   10.7126   -5.2483 H   0  0  0  0  0  0  0  0  0  0  0  0
18.0555    9.8561   -4.0013 H   0  0  0  0  0  0  0  0  0  0  0  0
19.0467    9.4749   -6.2452 H   0  0  0  0  0  0  0  0  0  0  0  0
17.5052    8.8221   -6.8154 H   0  0  0  0  0  0  0  0  0  0  0  0
18.4212    7.9656   -5.5683 H   0  0  0  0  0  0  0  0  0  0  0  0
 1  2  1  0  0  0  0
 2  3  1  0  0  0  0
 3  4  1  0  0  0  0
 4  5  1  0  0  0  0
 5  6  1  0  0  0  0
 6  7  1  0  0  0  0
 7  8  1  0  0  0  0
 8  9  1  0  0  0  0
10 11  1  0  0  0  0
11 12  1  0  0  0  0
12 13  1  0  0  0  0
13 14  1  0  0  0  0
14 15  1  0  0  0  0
15 16  1  0  0  0  0
16 17  1  0  0  0  0
10  9  1  0  0  0  0
 9 18  1  0  0  0  0
 1 19  1  0  0  0  0
 1 20  1  0  0  0  0
 1 21  1  0  0  0  0
 2 22  1  0  0  0  0
 2 23  1  0  0  0  0
 3 24  1  0  0  0  0
 3 25  1  0  0  0  0
 4 26  1  0  0  0  0
 4 27  1  0  0  0  0
 5 28  1  0  0  0  0
 5 29  1  0  0  0  0
 6 30  1  0  0  0  0
 6 31  1  0  0  0  0
 7 32  1  0  0  0  0
 7 33  1  0  0  0  0
 8 34  1  0  0  0  0
```

```
  8 35  1  0  0  0  0
 10 36  1  0  0  0  0
 10 37  1  0  0  0  0
 11 38  1  0  0  0  0
 11 39  1  0  0  0  0
 12 40  1  0  0  0  0
 12 41  1  0  0  0  0
 13 42  1  0  0  0  0
 13 43  1  0  0  0  0
 14 44  1  0  0  0  0
 14 45  1  0  0  0  0
 15 46  1  0  0  0  0
 15 47  1  0  0  0  0
 16 48  1  0  0  0  0
 16 49  1  0  0  0  0
 17 50  1  0  0  0  0
 17 51  1  0  0  0  0
 17 52  1  0  0  0  0
M  END
>  <Alias name>
Dioctylamine

>  <deltaH°(c)>   kJ/mol
-10891

$$$$
Di(2-ethylhexyl)amine
  ChemBrain IXL

 52 51  0  0  0  0  0  0  0  0  1 V2000
   13.7833    9.9548    0.8114 C   0  0  0  0  0  0  0  0  0  0  0  0
   13.0643    9.2926   -0.3800 C   0  0  0  0  0  0  0  0  0  0  0  0
   12.3963    7.9843    0.0857 C   0  0  0  0  0  0  0  0  0  0  0  0
   11.6768    7.3220   -1.1074 C   0  0  0  0  0  0  0  0  0  0  0  0
   11.0246    5.9966   -0.6521 C   0  0  0  0  0  0  0  0  0  0  0  0
    9.9934    6.2780    0.4667 C   0  0  0  0  0  0  0  0  0  0  0  0
    8.9404    7.1702   -0.0437 N   0  0  0  0  0  0  0  0  0  0  0  0
   10.3163    5.3363   -1.8558 C   0  0  0  0  0  0  0  0  0  0  0  0
    9.6856    3.9985   -1.4178 C   0  0  0  0  0  0  0  0  0  0  0  0
    2.5782   10.7593    1.6645 C   0  0  0  0  0  0  0  0  0  0  0  0
    3.6826    9.8221    2.1904 C   0  0  0  0  0  0  0  0  0  0  0  0
    4.7047    9.5524    1.0690 C   0  0  0  0  0  0  0  0  0  0  0  0
    5.8099    8.6143    1.5961 C   0  0  0  0  0  0  0  0  0  0  0  0
    6.8503    8.3581    0.4819 C   0  0  0  0  0  0  0  0  0  0  0  0
    7.9557    7.4248    1.0182 C   0  0  0  0  0  0  0  0  0  0  0  0
    6.1624    7.7001   -0.7407 C   0  0  0  0  0  0  0  0  0  0  0  0
    5.5128    6.3635   -0.3220 C   0  0  0  0  0  0  0  0  0  0  0  0
    9.3513    8.0429   -0.3368 H   0  0  0  0  0  0  0  0  0  0  0  0
   13.0638   10.1743    1.5905 H   0  0  0  0  0  0  0  0  0  0  0  0
   14.2526   10.8742    0.4840 H   0  0  0  0  0  0  0  0  0  0  0  0
   14.5387    9.2812    1.1969 H   0  0  0  0  0  0  0  0  0  0  0  0
   13.7838    9.0732   -1.1590 H   0  0  0  0  0  0  0  0  0  0  0  0
   12.3091    9.9663   -0.7654 H   0  0  0  0  0  0  0  0  0  0  0  0
   11.6779    8.2043    0.8645 H   0  0  0  0  0  0  0  0  0  0  0  0
   13.1517    7.3106    0.4708 H   0  0  0  0  0  0  0  0  0  0  0  0
   12.3951    7.1178   -1.8917 H   0  0  0  0  0  0  0  0  0  0  0  0
   10.9132    7.9901   -1.4844 H   0  0  0  0  0  0  0  0  0  0  0  0
   11.7908    5.3302   -0.2758 H   0  0  0  0  0  0  0  0  0  0  0  0
   10.4896    6.7482    1.3056 H   0  0  0  0  0  0  0  0  0  0  0  0
    9.5493    5.3454    0.7895 H   0  0  0  0  0  0  0  0  0  0  0  0
   11.0385    5.1529   -2.6416 H   0  0  0  0  0  0  0  0  0  0  0  0
    9.5415    5.9966   -2.2255 H   0  0  0  0  0  0  0  0  0  0  0  0
   10.4584    3.3431   -1.0356 H   0  0  0  0  0  0  0  0  0  0  0  0
```

8.9514 4.1807 -0.6439 H 0 0 0 0 0 0 0 0 0 0 0 0
9.2044 3.5314 -2.2681 H 0 0 0 0 0 0 0 0 0 0 0 0
2.0780 10.2925 0.8250 H 0 0 0 0 0 0 0 0 0 0 0 0
1.8601 10.9488 2.4527 H 0 0 0 0 0 0 0 0 0 0 0 0
3.0203 11.6954 1.3464 H 0 0 0 0 0 0 0 0 0 0 0 0
3.2404 8.8862 2.5085 H 0 0 0 0 0 0 0 0 0 0 0 0
4.1826 10.2890 3.0299 H 0 0 0 0 0 0 0 0 0 0 0 0
5.1469 10.4884 0.7510 H 0 0 0 0 0 0 0 0 0 0 0 0
4.2042 9.0862 0.2303 H 0 0 0 0 0 0 0 0 0 0 0 0
5.3696 7.6746 1.9032 H 0 0 0 0 0 0 0 0 0 0 0 0
6.2989 9.0765 2.4446 H 0 0 0 0 0 0 0 0 0 0 0 0
7.2920 9.2997 0.1802 H 0 0 0 0 0 0 0 0 0 0 0 0
8.4472 7.8951 1.8610 H 0 0 0 0 0 0 0 0 0 0 0 0
7.5158 6.4879 1.3348 H 0 0 0 0 0 0 0 0 0 0 0 0
6.9005 7.5149 -1.5112 H 0 0 0 0 0 0 0 0 0 0 0 0
5.4005 8.3648 -1.1263 H 0 0 0 0 0 0 0 0 0 0 0 0
6.2733 5.6962 0.0620 H 0 0 0 0 0 0 0 0 0 0 0 0
4.7716 6.5460 0.4451 H 0 0 0 0 0 0 0 0 0 0 0 0
5.0365 5.9097 -1.1821 H 0 0 0 0 0 0 0 0 0 0 0 0
1 2 1 0 0 0 0
2 3 1 0 0 0 0
3 4 1 0 0 0 0
4 5 1 0 0 0 0
5 6 1 0 0 0 0
6 7 1 0 0 0 0
5 8 1 0 0 0 0
8 9 1 0 0 0 0
10 11 1 0 0 0 0
11 12 1 0 0 0 0
12 13 1 0 0 0 0
13 14 1 0 0 0 0
14 15 1 0 0 0 0
14 16 1 0 0 0 0
16 17 1 0 0 0 0
15 7 1 0 0 0 0
7 18 1 0 0 0 0
1 19 1 0 0 0 0
1 20 1 0 0 0 0
1 21 1 0 0 0 0
2 22 1 0 0 0 0
2 23 1 0 0 0 0
3 24 1 0 0 0 0
3 25 1 0 0 0 0
4 26 1 0 0 0 0
4 27 1 0 0 0 0
5 28 1 0 0 0 0
6 29 1 0 0 0 0
6 30 1 0 0 0 0
8 31 1 0 0 0 0
8 32 1 0 0 0 0
9 33 1 0 0 0 0
9 34 1 0 0 0 0
9 35 1 0 0 0 0
10 36 1 0 0 0 0
10 37 1 0 0 0 0
10 38 1 0 0 0 0
11 39 1 0 0 0 0
11 40 1 0 0 0 0
12 41 1 0 0 0 0
12 42 1 0 0 0 0
13 43 1 0 0 0 0
13 44 1 0 0 0 0
14 45 1 0 0 0 0

```
 15 46  1  0  0  0  0
 15 47  1  0  0  0  0
 16 48  1  0  0  0  0
 16 49  1  0  0  0  0
 17 50  1  0  0  0  0
 17 51  1  0  0  0  0
 17 52  1  0  0  0  0
M  END
>  <Alias name>
Di(2-ethylhexyl)amine

>  <deltaH°(c)>   kJ/mol
-10889.83

$$$$
2,4,6-Tri-t-butylphenol
  ChemBrain IXL

 49 49  0  0  0  0  0  0  0  0  1 V2000
    6.0875    7.6978    0.2749 C   0  0  0  0  0  0  0  0  0  0  0  0
    7.2970    7.0300    0.1058 C   0  0  0  0  0  0  0  0  0  0  0  0
    7.3049    5.6669   -0.2112 C   0  0  0  0  0  0  0  0  0  0  0  0
    6.1016    4.9687   -0.3600 C   0  0  0  0  0  0  0  0  0  0  0  0
    4.8868    5.6361   -0.1905 C   0  0  0  0  0  0  0  0  0  0  0  0
    4.8661    6.9980    0.1264 C   0  0  0  0  0  0  0  0  0  0  0  0
    3.5415    7.6934    0.3034 C   0  0  0  0  0  0  0  0  0  0  0  0
    2.3948    6.6829    0.0834 C   0  0  0  0  0  0  0  0  0  0  0  0
    3.4028    8.8362   -0.7352 C   0  0  0  0  0  0  0  0  0  0  0  0
    3.4327    8.2631    1.7412 C   0  0  0  0  0  0  0  0  0  0  0  0
    6.1128    3.5158   -0.6980 C   0  0  0  0  0  0  0  0  0  0  0  0
    4.8610    3.1656   -1.5332 C   0  0  0  0  0  0  0  0  0  0  0  0
    6.1085    2.6875    0.6015 C   0  0  0  0  0  0  0  0  0  0  0  0
    7.3782    3.1808   -1.5163 C   0  0  0  0  0  0  0  0  0  0  0  0
    8.5733    7.7765    0.2648 C   0  0  0  0  0  0  0  0  0  0  0  0
    8.6375    8.3832    1.6813 C   0  0  0  0  0  0  0  0  0  0  0  0
    8.6369    8.9083   -0.7808 C   0  0  0  0  0  0  0  0  0  0  0  0
    9.7690    6.8231    0.0612 C   0  0  0  0  0  0  0  0  0  0  0  0
    6.1137    9.1377    0.6093 O   0  0  0  0  0  0  0  0  0  0  0  0
    5.2444    9.5642    0.7164 H   0  0  0  0  0  0  0  0  0  0  0  0
    8.2443    5.1511   -0.3418 H   0  0  0  0  0  0  0  0  0  0  0  0
    3.9589    5.0967   -0.3050 H   0  0  0  0  0  0  0  0  0  0  0  0
    2.4781    5.8789    0.8042 H   0  0  0  0  0  0  0  0  0  0  0  0
    1.4432    7.1843    0.2110 H   0  0  0  0  0  0  0  0  0  0  0  0
    2.4571    6.2779   -0.9191 H   0  0  0  0  0  0  0  0  0  0  0  0
    2.4562    9.3419   -0.5895 H   0  0  0  0  0  0  0  0  0  0  0  0
    4.2067    9.5406   -0.6151 H   0  0  0  0  0  0  0  0  0  0  0  0
    3.4376    8.4196   -1.7342 H   0  0  0  0  0  0  0  0  0  0  0  0
    2.4857    8.7764    1.8542 H   0  0  0  0  0  0  0  0  0  0  0  0
    3.4881    7.4502    2.4546 H   0  0  0  0  0  0  0  0  0  0  0  0
    4.2373    8.9529    1.9244 H   0  0  0  0  0  0  0  0  0  0  0  0
    4.8896    2.1173   -1.8030 H   0  0  0  0  0  0  0  0  0  0  0  0
    3.9704    3.3625   -0.9513 H   0  0  0  0  0  0  0  0  0  0  0  0
    4.8461    3.7692   -2.4324 H   0  0  0  0  0  0  0  0  0  0  0  0
    6.9870    2.9296    1.1868 H   0  0  0  0  0  0  0  0  0  0  0  0
    6.1167    1.6326    0.3574 H   0  0  0  0  0  0  0  0  0  0  0  0
    5.2184    2.9187    1.1740 H   0  0  0  0  0  0  0  0  0  0  0  0
    7.3971    3.7845   -2.4154 H   0  0  0  0  0  0  0  0  0  0  0  0
    7.3655    2.1323   -1.7861 H   0  0  0  0  0  0  0  0  0  0  0  0
    8.2587    3.3885   -0.9231 H   0  0  0  0  0  0  0  0  0  0  0  0
    7.8015    9.0573    1.8236 H   0  0  0  0  0  0  0  0  0  0  0  0
    9.5652    8.9291    1.7986 H   0  0  0  0  0  0  0  0  0  0  0  0
    8.5902    7.5894    2.4165 H   0  0  0  0  0  0  0  0  0  0  0  0
    7.8005    9.5814   -0.6356 H   0  0  0  0  0  0  0  0  0  0  0  0
```

```
    8.5895    8.4835   -1.7760 H   0  0  0  0  0  0  0  0  0  0  0  0
    9.5643    9.4549   -0.6654 H   0  0  0  0  0  0  0  0  0  0  0  0
   10.6936    7.3736    0.1810 H   0  0  0  0  0  0  0  0  0  0  0  0
    9.7280    6.3995   -0.9348 H   0  0  0  0  0  0  0  0  0  0  0  0
    9.7255    6.0272    0.7947 H   0  0  0  0  0  0  0  0  0  0  0  0
  1  2  4  0  0  0  0
  2  3  4  0  0  0  0
  3  4  4  0  0  0  0
  4  5  4  0  0  0  0
  5  6  4  0  0  0  0
  6  1  4  0  0  0  0
  6  7  1  0  0  0  0
  7  8  1  0  0  0  0
  7  9  1  0  0  0  0
  7 10  1  0  0  0  0
  4 11  1  0  0  0  0
 11 12  1  0  0  0  0
 11 13  1  0  0  0  0
 11 14  1  0  0  0  0
  2 15  1  0  0  0  0
 15 16  1  0  0  0  0
 15 17  1  0  0  0  0
 15 18  1  0  0  0  0
  1 19  1  0  0  0  0
 19 20  1  0  0  0  0
  3 21  1  0  0  0  0
  5 22  1  0  0  0  0
  8 23  1  0  0  0  0
  8 24  1  0  0  0  0
  8 25  1  0  0  0  0
  9 26  1  0  0  0  0
  9 27  1  0  0  0  0
  9 28  1  0  0  0  0
 10 29  1  0  0  0  0
 10 30  1  0  0  0  0
 10 31  1  0  0  0  0
 12 32  1  0  0  0  0
 12 33  1  0  0  0  0
 12 34  1  0  0  0  0
 13 35  1  0  0  0  0
 13 36  1  0  0  0  0
 13 37  1  0  0  0  0
 14 38  1  0  0  0  0
 14 39  1  0  0  0  0
 14 40  1  0  0  0  0
 16 41  1  0  0  0  0
 16 42  1  0  0  0  0
 16 43  1  0  0  0  0
 17 44  1  0  0  0  0
 17 45  1  0  0  0  0
 17 46  1  0  0  0  0
 18 47  1  0  0  0  0
 18 48  1  0  0  0  0
 18 49  1  0  0  0  0
M  END
>  <Alias name>
2,4,6-Tri-t-butylphenol

>  <deltaH°(c)>   kJ/mol
-10870

$$$$
Tetrabutyl silicate
```

ChemBrain IXL


```
57 56  0  0  0  0  0  0  0  0  1 V2000
    8.0234    8.6021   -0.8637 O   0  0  0  0  0  0  0  0  0  0  0  0
    9.2868    8.6252    0.1659 Si  0  0  0  0  0  0  0  0  0  0  0  0
   10.6786    8.6372   -0.6825 O   0  0  0  0  0  0  0  0  0  0  0  0
    9.2057    9.9615    1.0957 O   0  0  0  0  0  0  0  0  0  0  0  0
    9.2397    7.3002    1.1139 O   0  0  0  0  0  0  0  0  0  0  0  0
   15.5898    8.6795   -0.8838 C   0  0  0  0  0  0  0  0  0  0  0  0
   14.3670    8.6685    0.0358 C   0  0  0  0  0  0  0  0  0  0  0  0
   13.0920    8.6580   -0.8104 C   0  0  0  0  0  0  0  0  0  0  0  0
   11.8697    8.6471    0.1090 C   0  0  0  0  0  0  0  0  0  0  0  0
    9.2525   11.1996    0.3814 C   0  0  0  0  0  0  0  0  0  0  0  0
    9.1676   12.3622    1.3716 C   0  0  0  0  0  0  0  0  0  0  0  0
    9.2177   13.6875    0.6080 C   0  0  0  0  0  0  0  0  0  0  0  0
    9.1327   14.8506    1.5984 C   0  0  0  0  0  0  0  0  0  0  0  0
    9.2834    6.0536    0.4142 C   0  0  0  0  0  0  0  0  0  0  0  0
    9.2325    4.9029    1.4205 C   0  0  0  0  0  0  0  0  0  0  0  0
    9.2791    3.5686    0.6726 C   0  0  0  0  0  0  0  0  0  0  0  0
    9.2282    2.4173    1.6791 C   0  0  0  0  0  0  0  0  0  0  0  0
    6.7357    8.6011   -0.2415 C   0  0  0  0  0  0  0  0  0  0  0  0
    5.6499    8.5783   -1.3184 C   0  0  0  0  0  0  0  0  0  0  0  0
    4.2716    8.5773   -0.6535 C   0  0  0  0  0  0  0  0  0  0  0  0
    3.1852    8.5543   -1.7307 C   0  0  0  0  0  0  0  0  0  0  0  0
   15.5639    9.5640   -1.5084 H   0  0  0  0  0  0  0  0  0  0  0  0
   15.5791    7.7955   -1.5093 H   0  0  0  0  0  0  0  0  0  0  0  0
   16.4919    8.6869   -0.2848 H   0  0  0  0  0  0  0  0  0  0  0  0
   14.3931    7.7841    0.6603 H   0  0  0  0  0  0  0  0  0  0  0  0
   14.3779    9.5525    0.6613 H   0  0  0  0  0  0  0  0  0  0  0  0
   13.0658    9.5424   -1.4350 H   0  0  0  0  0  0  0  0  0  0  0  0
   13.0810    7.7739   -1.4358 H   0  0  0  0  0  0  0  0  0  0  0  0
   11.8958    7.7625    0.7337 H   0  0  0  0  0  0  0  0  0  0  0  0
   11.8806    9.5315    0.7345 H   0  0  0  0  0  0  0  0  0  0  0  0
   10.1821   11.2631   -0.1711 H   0  0  0  0  0  0  0  0  0  0  0  0
    8.4184   11.2508   -0.3078 H   0  0  0  0  0  0  0  0  0  0  0  0
    8.2383   12.2987    1.9239 H   0  0  0  0  0  0  0  0  0  0  0  0
   10.0014   12.3109    2.0607 H   0  0  0  0  0  0  0  0  0  0  0  0
   10.1470   13.7510    0.0557 H   0  0  0  0  0  0  0  0  0  0  0  0
    8.3839   13.7387   -0.0812 H   0  0  0  0  0  0  0  0  0  0  0  0
    8.2033   14.7874    2.1508 H   0  0  0  0  0  0  0  0  0  0  0  0
    9.1682   15.7883    1.0578 H   0  0  0  0  0  0  0  0  0  0  0  0
    9.9665   14.7996    2.2877 H   0  0  0  0  0  0  0  0  0  0  0  0
   10.2012    5.9924   -0.1580 H   0  0  0  0  0  0  0  0  0  0  0  0
    8.4348    5.9852   -0.2556 H   0  0  0  0  0  0  0  0  0  0  0  0
    8.3151    4.9641    1.9927 H   0  0  0  0  0  0  0  0  0  0  0  0
   10.0809    4.9712    2.0901 H   0  0  0  0  0  0  0  0  0  0  0  0
   10.1965    3.5073    0.1003 H   0  0  0  0  0  0  0  0  0  0  0  0
    8.4307    3.5002    0.0030 H   0  0  0  0  0  0  0  0  0  0  0  0
   10.0767    2.4854    2.3487 H   0  0  0  0  0  0  0  0  0  0  0  0
    9.2612    1.4732    1.1496 H   0  0  0  0  0  0  0  0  0  0  0  0
    8.3108    2.4783    2.2514 H   0  0  0  0  0  0  0  0  0  0  0  0
    6.6389    7.7244    0.3873 H   0  0  0  0  0  0  0  0  0  0  0  0
    6.6258    9.4932    0.3630 H   0  0  0  0  0  0  0  0  0  0  0  0
    5.7467    9.4547   -1.9472 H   0  0  0  0  0  0  0  0  0  0  0  0
    5.7597    7.6864   -1.9229 H   0  0  0  0  0  0  0  0  0  0  0  0
    4.1748    7.7009   -0.0246 H   0  0  0  0  0  0  0  0  0  0  0  0
    4.1617    9.4692   -0.0491 H   0  0  0  0  0  0  0  0  0  0  0  0
    3.2949    7.6624   -2.3351 H   0  0  0  0  0  0  0  0  0  0  0  0
    2.2100    8.5537   -1.2598 H   0  0  0  0  0  0  0  0  0  0  0  0
    3.2818    9.4307   -2.3596 H   0  0  0  0  0  0  0  0  0  0  0  0
  1  2  1  0  0  0  0
  2  3  1  0  0  0  0
  2  4  1  0  0  0  0
```

```
  2  5  1  0  0  0  0
  6  7  1  0  0  0  0
  7  8  1  0  0  0  0
  8  9  1  0  0  0  0
 10 11  1  0  0  0  0
 11 12  1  0  0  0  0
 12 13  1  0  0  0  0
 14 15  1  0  0  0  0
 15 16  1  0  0  0  0
 16 17  1  0  0  0  0
 18 19  1  0  0  0  0
 19 20  1  0  0  0  0
 20 21  1  0  0  0  0
  9  3  1  0  0  0  0
 10  4  1  0  0  0  0
 14  5  1  0  0  0  0
 18  1  1  0  0  0  0
  6 22  1  0  0  0  0
  6 23  1  0  0  0  0
  6 24  1  0  0  0  0
  7 25  1  0  0  0  0
  7 26  1  0  0  0  0
  8 27  1  0  0  0  0
  8 28  1  0  0  0  0
  9 29  1  0  0  0  0
  9 30  1  0  0  0  0
 10 31  1  0  0  0  0
 10 32  1  0  0  0  0
 11 33  1  0  0  0  0
 11 34  1  0  0  0  0
 12 35  1  0  0  0  0
 12 36  1  0  0  0  0
 13 37  1  0  0  0  0
 13 38  1  0  0  0  0
 13 39  1  0  0  0  0
 14 40  1  0  0  0  0
 14 41  1  0  0  0  0
 15 42  1  0  0  0  0
 15 43  1  0  0  0  0
 16 44  1  0  0  0  0
 16 45  1  0  0  0  0
 17 46  1  0  0  0  0
 17 47  1  0  0  0  0
 17 48  1  0  0  0  0
 18 49  1  0  0  0  0
 18 50  1  0  0  0  0
 19 51  1  0  0  0  0
 19 52  1  0  0  0  0
 20 53  1  0  0  0  0
 20 54  1  0  0  0  0
 21 55  1  0  0  0  0
 21 56  1  0  0  0  0
 21 57  1  0  0  0  0
M  END
>  <Alias name>
Tetrabutoxysilane

>  <deltaH°(c)>   kJ/mol
-10847

$$$$
Dibenzo(ac)anthracene
  ChemBrain IXL
```

```
36 40  0  0  0  0  0  0  0  0  1 V2000
    2.4486    5.4127   -0.0859 C   0  0  0  0  0  0  0  0  0  0  0  0
    2.4398    6.8074   -0.1163 C   0  0  0  0  0  0  0  0  0  0  0  0
    3.6433    7.5128   -0.1062 C   0  0  0  0  0  0  0  0  0  0  0  0
    4.8555    6.8237   -0.0657 C   0  0  0  0  0  0  0  0  0  0  0  0
    4.8642    5.4290   -0.0353 C   0  0  0  0  0  0  0  0  0  0  0  0
    3.6608    4.7236   -0.0454 C   0  0  0  0  0  0  0  0  0  0  0  0
    7.2799    5.4453    0.0154 C   0  0  0  0  0  0  0  0  0  0  0  0
    7.2711    6.8400   -0.0150 C   0  0  0  0  0  0  0  0  0  0  0  0
    8.4745    7.5455   -0.0049 C   0  0  0  0  0  0  0  0  0  0  0  0
    9.6867    6.8563    0.0357 C   0  0  0  0  0  0  0  0  0  0  0  0
    9.6955    5.4617    0.0661 C   0  0  0  0  0  0  0  0  0  0  0  0
    8.4921    4.7562    0.0560 C   0  0  0  0  0  0  0  0  0  0  0  0
    6.0764    4.7399    0.0053 C   0  0  0  0  0  0  0  0  0  0  0  0
    6.0589    7.5292   -0.0555 C   0  0  0  0  0  0  0  0  0  0  0  0
    8.5009    3.3615    0.0864 C   0  0  0  0  0  0  0  0  0  0  0  0
   10.9077    4.7725    0.1066 C   0  0  0  0  0  0  0  0  0  0  0  0
    9.7131    2.6724    0.1269 C   0  0  0  0  0  0  0  0  0  0  0  0
   10.9165    3.3779    0.1370 C   0  0  0  0  0  0  0  0  0  0  0  0
   10.8901    7.5618    0.0458 C   0  0  0  0  0  0  0  0  0  0  0  0
    8.4657    8.9401   -0.0353 C   0  0  0  0  0  0  0  0  0  0  0  0
   10.8814    8.9564    0.0154 C   0  0  0  0  0  0  0  0  0  0  0  0
    9.6692    9.6456   -0.0251 C   0  0  0  0  0  0  0  0  0  0  0  0
    1.5143    4.8650   -0.0938 H   0  0  0  0  0  0  0  0  0  0  0  0
    1.4988    7.3424   -0.1478 H   0  0  0  0  0  0  0  0  0  0  0  0
    3.6365    8.5956   -0.1298 H   0  0  0  0  0  0  0  0  0  0  0  0
    3.6676    3.6408   -0.0218 H   0  0  0  0  0  0  0  0  0  0  0  0
    6.0833    3.6572    0.0289 H   0  0  0  0  0  0  0  0  0  0  0  0
    6.0521    8.6119   -0.0791 H   0  0  0  0  0  0  0  0  0  0  0  0
    7.5666    2.8138    0.0785 H   0  0  0  0  0  0  0  0  0  0  0  0
   11.8420    5.3202    0.1145 H   0  0  0  0  0  0  0  0  0  0  0  0
    9.7199    1.5897    0.1505 H   0  0  0  0  0  0  0  0  0  0  0  0
   11.8576    2.8428    0.1685 H   0  0  0  0  0  0  0  0  0  0  0  0
   11.8312    7.0267    0.0773 H   0  0  0  0  0  0  0  0  0  0  0  0
    7.5246    9.4751   -0.0667 H   0  0  0  0  0  0  0  0  0  0  0  0
   11.8156    9.5041    0.0233 H   0  0  0  0  0  0  0  0  0  0  0  0
    9.6623   10.7283   -0.0487 H   0  0  0  0  0  0  0  0  0  0  0  0
  1  2  4  0  0  0  0
  2  3  4  0  0  0  0
  3  4  4  0  0  0  0
  4  5  4  0  0  0  0
  5  6  4  0  0  0  0
  6  1  4  0  0  0  0
  7  8  4  0  0  0  0
  8  9  4  0  0  0  0
  9 10  4  0  0  0  0
 10 11  4  0  0  0  0
 11 12  4  0  0  0  0
 12  7  4  0  0  0  0
  5 13  4  0  0  0  0
 13  7  4  0  0  0  0
  4 14  4  0  0  0  0
 14  8  4  0  0  0  0
 12 15  4  0  0  0  0
 11 16  4  0  0  0  0
 15 17  4  0  0  0  0
 16 18  4  0  0  0  0
 10 19  4  0  0  0  0
  9 20  4  0  0  0  0
 19 21  4  0  0  0  0
 20 22  4  0  0  0  0
 22 21  4  0  0  0  0
```

```
 17 18  4  0  0  0  0
  1 23  1  0  0  0  0
  2 24  1  0  0  0  0
  3 25  1  0  0  0  0
  6 26  1  0  0  0  0
 13 27  1  0  0  0  0
 14 28  1  0  0  0  0
 15 29  1  0  0  0  0
 16 30  1  0  0  0  0
 17 31  1  0  0  0  0
 18 32  1  0  0  0  0
 19 33  1  0  0  0  0
 20 34  1  0  0  0  0
 21 35  1  0  0  0  0
 22 36  1  0  0  0  0
M  END
>  <Alias name>
Benzo[b]triphenylene

>  <deltaH°(c)>   kJ/mol
-10842

$$$$
Tetra-n-butylammonium nitrate
  ChemBrain IXL

 57 55  0  0  0  0  0  0  0  0  1 V2000
    9.4306    8.4013   -3.0362 C   0  0  0  0  0  0  0  0  0  0  0  0
    8.1006    8.2838   -2.2669 C   0  0  0  0  0  0  0  0  0  0  0  0
    8.3448    7.5658   -0.9257 C   0  0  0  0  0  0  0  0  0  0  0  0
    7.0093    7.4496   -0.1550 C   0  0  0  0  0  0  0  0  0  0  0  0
    7.2246    6.7558    1.1398 N   0  3  0  0  0  0  0  0  0  0  0  0
    8.6352    2.4546    3.2668 C   0  0  0  0  0  0  0  0  0  0  0  0
    8.4440    3.2082    1.9365 C   0  0  0  0  0  0  0  0  0  0  0  0
    7.9168    4.6277    2.2212 C   0  0  0  0  0  0  0  0  0  0  0  0
    7.7260    5.3818    0.8849 C   0  0  0  0  0  0  0  0  0  0  0  0
    8.3013   11.1506    3.2712 C   0  0  0  0  0  0  0  0  0  0  0  0
    8.7827    9.7068    3.0299 C   0  0  0  0  0  0  0  0  0  0  0  0
    7.7372    8.9507    2.1877 C   0  0  0  0  0  0  0  0  0  0  0  0
    8.2224    7.5024    1.9469 C   0  0  0  0  0  0  0  0  0  0  0  0
    2.5348    5.0184    1.0583 C   0  0  0  0  0  0  0  0  0  0  0  0
    3.5740    5.8258    1.8600 C   0  0  0  0  0  0  0  0  0  0  0  0
    4.8995    5.8790    1.0761 C   0  0  0  0  0  0  0  0  0  0  0  0
    5.9406    6.6894    1.8826 C   0  0  0  0  0  0  0  0  0  0  0  0
   10.6932    5.7140    1.6478 O   0  5  0  0  0  0  0  0  0  0  0  0
   11.4338    6.3006    0.8452 N   0  3  0  0  0  0  0  0  0  0  0  0
   12.5567    5.8420    0.5880 O   0  0  0  0  0  0  0  0  0  0  0  0
   11.0516    7.3466    0.2993 O   0  5  0  0  0  0  0  0  0  0  0  0
    9.2589    8.9060   -3.9788 H   0  0  0  0  0  0  0  0  0  0  0  0
    9.8278    7.4114   -3.2238 H   0  0  0  0  0  0  0  0  0  0  0  0
   10.1397    8.9691   -2.4466 H   0  0  0  0  0  0  0  0  0  0  0  0
    7.3916    7.7160   -2.8565 H   0  0  0  0  0  0  0  0  0  0  0  0
    7.7035    9.2737   -2.0794 H   0  0  0  0  0  0  0  0  0  0  0  0
    9.0538    8.1337   -0.3385 H   0  0  0  0  0  0  0  0  0  0  0  0
    8.7414    6.5774   -1.1145 H   0  0  0  0  0  0  0  0  0  0  0  0
    6.3021    6.8879   -0.7501 H   0  0  0  0  0  0  0  0  0  0  0  0
    6.6146    8.4398    0.0284 H   0  0  0  0  0  0  0  0  0  0  0  0
    9.3480    2.9872    3.8842 H   0  0  0  0  0  0  0  0  0  0  0  0
    9.0057    1.4569    3.0665 H   0  0  0  0  0  0  0  0  0  0  0  0
    7.6863    2.3901    3.7848 H   0  0  0  0  0  0  0  0  0  0  0  0
    9.3929    3.2726    1.4185 H   0  0  0  0  0  0  0  0  0  0  0  0
    7.7312    2.6755    1.3191 H   0  0  0  0  0  0  0  0  0  0  0  0
    6.9693    4.5616    2.7386 H   0  0  0  0  0  0  0  0  0  0  0  0
```

```
8.6294 5.1582 2.8382 H 0 0 0 0 0 0 0 0 0 0 0 0
8.6744 5.4361 0.3678 H 0 0 0 0 0 0 0 0 0 0 0 0
7.0143 4.8470 0.2706 H 0 0 0 0 0 0 0 0 0 0 0 0
7.3564 11.1325 3.8000 H 0 0 0 0 0 0 0 0 0 0 0 0
9.0362 11.6819 3.8631 H 0 0 0 0 0 0 0 0 0 0 0 0
8.1731 11.6521 2.3199 H 0 0 0 0 0 0 0 0 0 0 0 0
8.9110 9.2054 3.9812 H 0 0 0 0 0 0 0 0 0 0 0 0
9.7276 9.7250 2.5011 H 0 0 0 0 0 0 0 0 0 0 0 0
7.6101 9.4528 1.2382 H 0 0 0 0 0 0 0 0 0 0 0 0
6.7940 8.9337 2.7167 H 0 0 0 0 0 0 0 0 0 0 0 0
8.3557 7.0082 2.8997 H 0 0 0 0 0 0 0 0 0 0 0 0
9.1671 7.5244 1.4207 H 0 0 0 0 0 0 0 0 0 0 0 0
2.9023 4.0117 0.9019 H 0 0 0 0 0 0 0 0 0 0 0 0
1.6034 4.9811 1.6094 H 0 0 0 0 0 0 0 0 0 0 0 0
2.3685 5.4949 0.1001 H 0 0 0 0 0 0 0 0 0 0 0 0
3.2064 6.8324 2.0164 H 0 0 0 0 0 0 0 0 0 0 0 0
3.7402 5.3493 2.8182 H 0 0 0 0 0 0 0 0 0 0 0 0
5.2649 4.8729 0.9204 H 0 0 0 0 0 0 0 0 0 0 0 0
4.7319 6.3548 0.1193 H 0 0 0 0 0 0 0 0 0 0 0 0
5.5661 7.6915 2.0423 H 0 0 0 0 0 0 0 0 0 0 0 0
6.1026 6.2119 2.8395 H 0 0 0 0 0 0 0 0 0 0 0 0
1 2 1 0 0 0 0
2 3 1 0 0 0 0
3 4 1 0 0 0 0
4 5 1 0 0 0 0
6 7 1 0 0 0 0
7 8 1 0 0 0 0
8 9 1 0 0 0 0
9 5 1 0 0 0 0
10 11 1 0 0 0 0
11 12 1 0 0 0 0
12 13 1 0 0 0 0
14 15 1 0 0 0 0
15 16 1 0 0 0 0
16 17 1 0 0 0 0
13 5 1 0 0 0 0
17 5 1 0 0 0 0
18 19 1 0 0 0 0
19 20 2 0 0 0 0
19 21 1 0 0 0 0
1 22 1 0 0 0 0
1 23 1 0 0 0 0
1 24 1 0 0 0 0
2 25 1 0 0 0 0
2 26 1 0 0 0 0
3 27 1 0 0 0 0
3 28 1 0 0 0 0
4 29 1 0 0 0 0
4 30 1 0 0 0 0
6 31 1 0 0 0 0
6 32 1 0 0 0 0
6 33 1 0 0 0 0
7 34 1 0 0 0 0
7 35 1 0 0 0 0
8 36 1 0 0 0 0
8 37 1 0 0 0 0
9 38 1 0 0 0 0
9 39 1 0 0 0 0
10 40 1 0 0 0 0
10 41 1 0 0 0 0
10 42 1 0 0 0 0
11 43 1 0 0 0 0
11 44 1 0 0 0 0
```

```
 12 45  1  0  0  0  0
 12 46  1  0  0  0  0
 13 47  1  0  0  0  0
 13 48  1  0  0  0  0
 14 49  1  0  0  0  0
 14 50  1  0  0  0  0
 14 51  1  0  0  0  0
 15 52  1  0  0  0  0
 15 53  1  0  0  0  0
 16 54  1  0  0  0  0
 16 55  1  0  0  0  0
 17 56  1  0  0  0  0
 17 57  1  0  0  0  0
M  END
>  <Alias name>
Tetra-n-butylammonium nitrate

>  <deltaH°(c)>   kJ/mol
-10841

$$$$
Dibenzo(ah)anthracene
  ChemBrain IXL

 36 40  0  0  0  0  0  0  0  0  1 V2000
    6.2241    7.4786   -0.1447 C   0  0  0  0  0  0  0  0  0  0  0  0
    4.9624    8.0607   -0.2682 C   0  0  0  0  0  0  0  0  0  0  0  0
    6.3414    6.1016    0.0453 C   0  0  0  0  0  0  0  0  0  0  0  0
    7.3685    8.2735   -0.2114 C   0  0  0  0  0  0  0  0  0  0  0  0
    3.8180    7.2658   -0.2015 C   0  0  0  0  0  0  0  0  0  0  0  0
    4.8451    9.4377   -0.4582 C   0  0  0  0  0  0  0  0  0  0  0  0
    7.6032    5.5195    0.1687 C   0  0  0  0  0  0  0  0  0  0  0  0
    5.1970    5.3067    0.1120 C   0  0  0  0  0  0  0  0  0  0  0  0
    8.6303    7.6914   -0.0880 C   0  0  0  0  0  0  0  0  0  0  0  0
    3.9353    5.8888   -0.0114 C   0  0  0  0  0  0  0  0  0  0  0  0
    2.5562    7.8479   -0.3249 C   0  0  0  0  0  0  0  0  0  0  0  0
    3.5834   10.0198   -0.5817 C   0  0  0  0  0  0  0  0  0  0  0  0
    8.7476    6.3144    0.1021 C   0  0  0  0  0  0  0  0  0  0  0  0
    9.7747    8.4863   -0.1546 C   0  0  0  0  0  0  0  0  0  0  0  0
    2.4389    9.2249   -0.5150 C   0  0  0  0  0  0  0  0  0  0  0  0
   10.0093    5.7323    0.2255 C   0  0  0  0  0  0  0  0  0  0  0  0
   11.0364    7.9043   -0.0312 C   0  0  0  0  0  0  0  0  0  0  0  0
   11.1537    6.5272    0.1589 C   0  0  0  0  0  0  0  0  0  0  0  0
   10.1266    4.3553    0.4156 C   0  0  0  0  0  0  0  0  0  0  0  0
   12.4155    5.9452    0.2823 C   0  0  0  0  0  0  0  0  0  0  0  0
   11.3884    3.7732    0.5390 C   0  0  0  0  0  0  0  0  0  0  0  0
   12.5328    4.5682    0.4723 C   0  0  0  0  0  0  0  0  0  0  0  0
    7.2775    9.3425   -0.3590 H   0  0  0  0  0  0  0  0  0  0  0  0
    5.7336   10.0548   -0.5100 H   0  0  0  0  0  0  0  0  0  0  0  0
    7.6942    4.4505    0.3163 H   0  0  0  0  0  0  0  0  0  0  0  0
    5.2881    4.2377    0.2595 H   0  0  0  0  0  0  0  0  0  0  0  0
    3.0468    5.2716    0.0403 H   0  0  0  0  0  0  0  0  0  0  0  0
    1.6678    7.2307   -0.2732 H   0  0  0  0  0  0  0  0  0  0  0  0
    3.4923   11.0888   -0.7292 H   0  0  0  0  0  0  0  0  0  0  0  0
    9.6836    9.5554   -0.3022 H   0  0  0  0  0  0  0  0  0  0  0  0
    1.4594    9.6768   -0.6108 H   0  0  0  0  0  0  0  0  0  0  0  0
   11.9249    8.5214   -0.0830 H   0  0  0  0  0  0  0  0  0  0  0  0
    9.2382    3.7382    0.4673 H   0  0  0  0  0  0  0  0  0  0  0  0
   13.3039    6.5623    0.2305 H   0  0  0  0  0  0  0  0  0  0  0  0
   11.4794    2.7042    0.6866 H   0  0  0  0  0  0  0  0  0  0  0  0
   13.5123    4.1163    0.5682 H   0  0  0  0  0  0  0  0  0  0  0  0
  1  2  4  0  0  0  0
  1  3  4  0  0  0  0
```

```
  1  4  4  0  0  0  0
  2  5  4  0  0  0  0
  2  6  4  0  0  0  0
  3  7  4  0  0  0  0
  3  8  4  0  0  0  0
  4  9  4  0  0  0  0
  5 10  4  0  0  0  0
  5 11  4  0  0  0  0
  6 12  4  0  0  0  0
  7 13  4  0  0  0  0
  8 10  4  0  0  0  0
  9 14  4  0  0  0  0
  9 13  4  0  0  0  0
 11 15  4  0  0  0  0
 12 15  4  0  0  0  0
 13 16  4  0  0  0  0
 14 17  4  0  0  0  0
 16 18  4  0  0  0  0
 16 19  4  0  0  0  0
 17 18  4  0  0  0  0
 18 20  4  0  0  0  0
 19 21  4  0  0  0  0
 20 22  4  0  0  0  0
 21 22  4  0  0  0  0
  4 23  1  0  0  0  0
  6 24  1  0  0  0  0
  7 25  1  0  0  0  0
  8 26  1  0  0  0  0
 10 27  1  0  0  0  0
 11 28  1  0  0  0  0
 12 29  1  0  0  0  0
 14 30  1  0  0  0  0
 15 31  1  0  0  0  0
 17 32  1  0  0  0  0
 19 33  1  0  0  0  0
 20 34  1  0  0  0  0
 21 35  1  0  0  0  0
 22 36  1  0  0  0  0
M  END
>  <Alias name>
Dibenz[a,h]anthracene

>  <deltaH°(c)>   kJ/mol
-10836

$$$$
2,4,5-Triphenylimidazole
  ChemBrain IXL

 39 42  0  0  0  0  0  0  0  0  1 V2000
    6.8162    7.0022   -0.2281 C   0  0  0  0  0  0  0  0  0  0  0  0
    6.4294    8.2760   -0.1052 C   0  0  0  0  0  0  0  0  0  0  0  0
    8.1559    6.9401    0.1847 N   0  0  0  0  0  0  0  0  0  0  0  0
    8.5880    8.0878    0.5296 C   0  0  0  0  0  0  0  0  0  0  0  0
    7.5346    8.9917    0.3573 N   0  0  0  0  0  0  0  0  0  0  0  0
   12.5043    9.0277    1.9473 C   0  0  0  0  0  0  0  0  0  0  0  0
   11.5456   10.0333    1.8237 C   0  0  0  0  0  0  0  0  0  0  0  0
   10.2667    9.7240    1.3616 C   0  0  0  0  0  0  0  0  0  0  0  0
    9.9459    8.4096    1.0222 C   0  0  0  0  0  0  0  0  0  0  0  0
   10.9039    7.4032    1.1467 C   0  0  0  0  0  0  0  0  0  0  0  0
   12.1837    7.7127    1.6088 C   0  0  0  0  0  0  0  0  0  0  0  0
    5.9951    5.8673   -0.7125 C   0  0  0  0  0  0  0  0  0  0  0  0
    6.2368    4.5762   -0.2374 C   0  0  0  0  0  0  0  0  0  0  0  0
```

```
    5.4545    3.5099   -0.6836 C   0  0  0  0  0  0  0  0  0  0  0  0
    4.4323    3.7331   -1.6059 C   0  0  0  0  0  0  0  0  0  0  0  0
    4.1962    5.0211   -2.0868 C   0  0  0  0  0  0  0  0  0  0  0  0
    4.9814    6.0868   -1.6448 C   0  0  0  0  0  0  0  0  0  0  0  0
    4.9623   10.1690   -0.7834 C   0  0  0  0  0  0  0  0  0  0  0  0
    3.7044   10.6975   -1.0737 C   0  0  0  0  0  0  0  0  0  0  0  0
    2.5703    9.8920   -0.9746 C   0  0  0  0  0  0  0  0  0  0  0  0
    2.6935    8.5600   -0.5780 C   0  0  0  0  0  0  0  0  0  0  0  0
    3.9513    8.0329   -0.2802 C   0  0  0  0  0  0  0  0  0  0  0  0
    5.0878    8.8340   -0.3952 C   0  0  0  0  0  0  0  0  0  0  0  0
    7.5744    9.9875    0.5149 H   0  0  0  0  0  0  0  0  0  0  0  0
   13.4976    9.2679    2.3060 H   0  0  0  0  0  0  0  0  0  0  0  0
   11.7942   11.0542    2.0864 H   0  0  0  0  0  0  0  0  0  0  0  0
    9.5221   10.5047    1.2659 H   0  0  0  0  0  0  0  0  0  0  0  0
   10.6547    6.3823    0.8847 H   0  0  0  0  0  0  0  0  0  0  0  0
   12.9283    6.9321    1.7047 H   0  0  0  0  0  0  0  0  0  0  0  0
    7.0318    4.4014    0.4775 H   0  0  0  0  0  0  0  0  0  0  0  0
    5.6410    2.5085   -0.3139 H   0  0  0  0  0  0  0  0  0  0  0  0
    3.8217    2.9063   -1.9488 H   0  0  0  0  0  0  0  0  0  0  0  0
    3.4032    5.1940   -2.8035 H   0  0  0  0  0  0  0  0  0  0  0  0
    4.8039    7.0840   -2.0257 H   0  0  0  0  0  0  0  0  0  0  0  0
    5.8429   10.7956   -0.8595 H   0  0  0  0  0  0  0  0  0  0  0  0
    3.6084   11.7334   -1.3762 H   0  0  0  0  0  0  0  0  0  0  0  0
    1.5940   10.3005   -1.2056 H   0  0  0  0  0  0  0  0  0  0  0  0
    1.8126    7.9350   -0.5013 H   0  0  0  0  0  0  0  0  0  0  0  0
    4.0446    7.0032    0.0396 H   0  0  0  0  0  0  0  0  0  0  0  0
  1  2  2  0  0  0  0
  1  3  1  0  0  0  0
  3  4  2  0  0  0  0
  4  5  1  0  0  0  0
  5  2  1  0  0  0  0
  6  7  4  0  0  0  0
  7  8  4  0  0  0  0
  8  9  4  0  0  0  0
  9 10  4  0  0  0  0
 10 11  4  0  0  0  0
 11  6  4  0  0  0  0
  9  4  1  0  0  0  0
 12 13  4  0  0  0  0
 13 14  4  0  0  0  0
 14 15  4  0  0  0  0
 15 16  4  0  0  0  0
 16 17  4  0  0  0  0
 17 12  4  0  0  0  0
 12  1  1  0  0  0  0
 18 19  4  0  0  0  0
 19 20  4  0  0  0  0
 20 21  4  0  0  0  0
 21 22  4  0  0  0  0
 22 23  4  0  0  0  0
 23 18  4  0  0  0  0
 23  2  1  0  0  0  0
  5 24  1  0  0  0  0
  6 25  1  0  0  0  0
  7 26  1  0  0  0  0
  8 27  1  0  0  0  0
 10 28  1  0  0  0  0
 11 29  1  0  0  0  0
 13 30  1  0  0  0  0
 14 31  1  0  0  0  0
 15 32  1  0  0  0  0
 16 33  1  0  0  0  0
 17 34  1  0  0  0  0
```

```
 18 35  1  0  0  0  0
 19 36  1  0  0  0  0
 20 37  1  0  0  0  0
 21 38  1  0  0  0  0
 22 39  1  0  0  0  0
M  END
>  <Alias name>
Lophine

>  <deltaH°(c)>   kJ/mol
-10831

$$$$
5,6,6a,7,8,12b,12c-Octahydrodibenzo(a,i)biphenylene
  ChemBrain IXL

 40 44  0  0  0  0  0  0  0  0  1 V2000
    6.0125    6.0551   -0.5117 C   0  0  0  0  0  0  0  0  0  0  0  0
    5.9142    4.4984   -0.6700 C   0  0  0  0  0  0  0  0  0  0  0  0
    7.1354    5.8786    0.5232 C   0  0  0  0  0  0  0  0  0  0  0  0
    4.8073    6.7072    0.0708 C   0  0  0  0  0  0  0  0  0  0  0  0
    7.0442    4.3223    0.3559 C   0  0  0  0  0  0  0  0  0  0  0  0
    4.5865    3.8892   -0.1759 C   0  0  0  0  0  0  0  0  0  0  0  0
    8.2952    3.6732   -0.2697 C   0  0  0  0  0  0  0  0  0  0  0  0
    8.4174    6.4872    0.0724 C   0  0  0  0  0  0  0  0  0  0  0  0
    3.4279    4.7669   -0.6200 C   0  0  0  0  0  0  0  0  0  0  0  0
    3.5603    6.0815    0.0169 C   0  0  0  0  0  0  0  0  0  0  0  0
    2.4400    6.6968    0.5696 C   0  0  0  0  0  0  0  0  0  0  0  0
    4.9254    7.9598    0.6701 C   0  0  0  0  0  0  0  0  0  0  0  0
    9.5795    5.7187   -0.0194 C   0  0  0  0  0  0  0  0  0  0  0  0
    9.5451    4.2949    0.3313 C   0  0  0  0  0  0  0  0  0  0  0  0
   10.7724    6.2956   -0.4474 C   0  0  0  0  0  0  0  0  0  0  0  0
    8.4582    7.8413   -0.2544 C   0  0  0  0  0  0  0  0  0  0  0  0
    3.7999    8.5871    1.2048 C   0  0  0  0  0  0  0  0  0  0  0  0
    2.5574    7.9551    1.1552 C   0  0  0  0  0  0  0  0  0  0  0  0
   10.8142    7.6524   -0.7596 C   0  0  0  0  0  0  0  0  0  0  0  0
    9.6574    8.4256   -0.6624 C   0  0  0  0  0  0  0  0  0  0  0  0
    6.3617    6.5385   -1.4153 H   0  0  0  0  0  0  0  0  0  0  0  0
    6.1990    4.1615   -1.6596 H   0  0  0  0  0  0  0  0  0  0  0  0
    6.8354    6.2037    1.5112 H   0  0  0  0  0  0  0  0  0  0  0  0
    6.7086    3.8254    1.2584 H   0  0  0  0  0  0  0  0  0  0  0  0
    4.4676    2.8956   -0.5933 H   0  0  0  0  0  0  0  0  0  0  0  0
    4.5979    3.8264    0.9052 H   0  0  0  0  0  0  0  0  0  0  0  0
    8.2888    2.6079   -0.0675 H   0  0  0  0  0  0  0  0  0  0  0  0
    8.2900    3.8363   -1.3403 H   0  0  0  0  0  0  0  0  0  0  0  0
    3.4494    4.8793   -1.6973 H   0  0  0  0  0  0  0  0  0  0  0  0
    2.4914    4.3101   -0.3212 H   0  0  0  0  0  0  0  0  0  0  0  0
    1.4788    6.1967    0.5438 H   0  0  0  0  0  0  0  0  0  0  0  0
    5.8912    8.4457    0.7202 H   0  0  0  0  0  0  0  0  0  0  0  0
    9.5237    4.1846    1.4087 H   0  0  0  0  0  0  0  0  0  0  0  0
   10.4237    3.8004   -0.0671 H   0  0  0  0  0  0  0  0  0  0  0  0
   11.6663    5.6893   -0.5372 H   0  0  0  0  0  0  0  0  0  0  0  0
    7.5583    8.4394   -0.1912 H   0  0  0  0  0  0  0  0  0  0  0  0
    3.8908    9.5647    1.6587 H   0  0  0  0  0  0  0  0  0  0  0  0
    1.6846    8.4416    1.5710 H   0  0  0  0  0  0  0  0  0  0  0  0
   11.7444    8.1058   -1.0772 H   0  0  0  0  0  0  0  0  0  0  0  0
    9.6902    9.4793   -0.9040 H   0  0  0  0  0  0  0  0  0  0  0  0
  2  1  1  0  0  0  0
  1  3  1  0  0  0  0
  4  1  1  0  0  0  0
  5  2  1  0  0  0  0
  2  6  1  0  0  0  0
  3  5  1  0  0  0  0
```

```
  3  8  1  0  0  0  0
 10  4  4  0  0  0  0
 12  4  4  0  0  0  0
  7  5  1  0  0  0  0
  6  9  1  0  0  0  0
 14  7  1  0  0  0  0
  8 13  4  0  0  0  0
  8 16  4  0  0  0  0
  9 10  1  0  0  0  0
 10 11  4  0  0  0  0
 11 18  4  0  0  0  0
 17 12  4  0  0  0  0
 13 14  1  0  0  0  0
 15 13  4  0  0  0  0
 19 15  4  0  0  0  0
 16 20  4  0  0  0  0
 18 17  4  0  0  0  0
 20 19  4  0  0  0  0
  1 21  1  0  0  0  0
  2 22  1  0  0  0  0
  3 23  1  0  0  0  0
  5 24  1  0  0  0  0
  6 25  1  0  0  0  0
  6 26  1  0  0  0  0
  7 27  1  0  0  0  0
  7 28  1  0  0  0  0
  9 29  1  0  0  0  0
  9 30  1  0  0  0  0
 11 31  1  0  0  0  0
 12 32  1  0  0  0  0
 14 33  1  0  0  0  0
 14 34  1  0  0  0  0
 15 35  1  0  0  0  0
 16 36  1  0  0  0  0
 17 37  1  0  0  0  0
 18 38  1  0  0  0  0
 19 39  1  0  0  0  0
 20 40  1  0  0  0  0
M  END
>  <Alias name>
5,6,6a,7,8,12b,12c-Octahydrodibenzo[a,i]biphenylene

>  <deltaH°(c)>   kJ/mol
-10808

$$$$
Triisopentylborine
  ChemBrain IXL

 49 48  0  0  0  0  0  0  0  0  1 V2000
    6.9463    7.3218   -0.6112 B   0  0  0  0  0  0  0  0  0  0  0  0
    8.3480    7.6274   -1.2314 C   0  0  0  0  0  0  0  0  0  0  0  0
    6.5632    5.8552   -0.2305 C   0  0  0  0  0  0  0  0  0  0  0  0
    5.9278    8.4827   -0.3716 C   0  0  0  0  0  0  0  0  0  0  0  0
    8.5051    9.1500   -1.4306 C   0  0  0  0  0  0  0  0  0  0  0  0
    7.7340    4.9091   -0.5721 C   0  0  0  0  0  0  0  0  0  0  0  0
    4.5942    7.9027    0.1457 C   0  0  0  0  0  0  0  0  0  0  0  0
    7.3489    3.4566   -0.2169 C   0  0  0  0  0  0  0  0  0  0  0  0
    3.5796    9.0463    0.3626 C   0  0  0  0  0  0  0  0  0  0  0  0
    9.8820    9.4522   -2.0608 C   0  0  0  0  0  0  0  0  0  0  0  0
    4.1241   10.0304    1.4187 C   0  0  0  0  0  0  0  0  0  0  0  0
    2.2422    8.4577    0.8530 C   0  0  0  0  0  0  0  0  0  0  0  0
   10.0217   10.9713   -2.2809 C   0  0  0  0  0  0  0  0  0  0  0  0
```

11.0027	8.9659	-1.1186	C	0	0	0	0	0	0	0	0	0	0	0	0
8.5116	2.5143	-0.5850	C	0	0	0	0	0	0	0	0	0	0	0	0
7.0623	3.3501	1.2954	C	0	0	0	0	0	0	0	0	0	0	0	0
9.1217	7.2686	-0.5654	H	0	0	0	0	0	0	0	0	0	0	0	0
8.4353	7.1277	-2.1882	H	0	0	0	0	0	0	0	0	0	0	0	0
5.6840	5.5564	-0.7876	H	0	0	0	0	0	0	0	0	0	0	0	0
6.3534	5.8022	0.8298	H	0	0	0	0	0	0	0	0	0	0	0	0
6.3314	9.1695	0.3609	H	0	0	0	0	0	0	0	0	0	0	0	0
5.7552	9.0084	-1.3024	H	0	0	0	0	0	0	0	0	0	0	0	0
7.7227	9.5095	-2.0875	H	0	0	0	0	0	0	0	0	0	0	0	0
8.4291	9.6495	-0.4725	H	0	0	0	0	0	0	0	0	0	0	0	0
8.6101	5.1991	-0.0051	H	0	0	0	0	0	0	0	0	0	0	0	0
7.9520	4.9743	-1.6310	H	0	0	0	0	0	0	0	0	0	0	0	0
4.1989	7.2050	-0.5824	H	0	0	0	0	0	0	0	0	0	0	0	0
4.7636	7.3877	1.0834	H	0	0	0	0	0	0	0	0	0	0	0	0
6.4644	3.1714	-0.7728	H	0	0	0	0	0	0	0	0	0	0	0	0
3.4214	9.5705	-0.5716	H	0	0	0	0	0	0	0	0	0	0	0	0
9.9634	8.9415	-3.0123	H	0	0	0	0	0	0	0	0	0	0	0	0
3.4032	10.8225	1.5790	H	0	0	0	0	0	0	0	0	0	0	0	0
5.0556	10.4567	1.0697	H	0	0	0	0	0	0	0	0	0	0	0	0
4.2942	9.5035	2.3495	H	0	0	0	0	0	0	0	0	0	0	0	0
2.3995	7.9331	1.7873	H	0	0	0	0	0	0	0	0	0	0	0	0
1.5291	9.2586	1.0042	H	0	0	0	0	0	0	0	0	0	0	0	0
1.8581	7.7677	0.1118	H	0	0	0	0	0	0	0	0	0	0	0	0
9.2364	11.3138	-2.9433	H	0	0	0	0	0	0	0	0	0	0	0	0
10.9865	11.1846	-2.7243	H	0	0	0	0	0	0	0	0	0	0	0	0
9.9404	11.4829	-1.3298	H	0	0	0	0	0	0	0	0	0	0	0	0
11.9664	9.1934	-1.5570	H	0	0	0	0	0	0	0	0	0	0	0	0
10.9159	7.8968	-0.9749	H	0	0	0	0	0	0	0	0	0	0	0	0
10.9137	9.4672	-0.1627	H	0	0	0	0	0	0	0	0	0	0	0	0
9.3967	2.7988	-0.0295	H	0	0	0	0	0	0	0	0	0	0	0	0
8.7129	2.5870	-1.6467	H	0	0	0	0	0	0	0	0	0	0	0	0
8.2423	1.4949	-0.3377	H	0	0	0	0	0	0	0	0	0	0	0	0
6.8075	2.3271	1.5429	H	0	0	0	0	0	0	0	0	0	0	0	0
7.9428	3.6474	1.8515	H	0	0	0	0	0	0	0	0	0	0	0	0
6.2360	3.9996	1.5536	H	0	0	0	0	0	0	0	0	0	0	0	0

1	2	1	0	0	0	0
1	3	1	0	0	0	0
1	4	1	0	0	0	0
2	5	1	0	0	0	0
3	6	1	0	0	0	0
4	7	1	0	0	0	0
6	8	1	0	0	0	0
7	9	1	0	0	0	0
5	10	1	0	0	0	0
9	11	1	0	0	0	0
9	12	1	0	0	0	0
10	13	1	0	0	0	0
10	14	1	0	0	0	0
8	15	1	0	0	0	0
8	16	1	0	0	0	0
2	17	1	0	0	0	0
2	18	1	0	0	0	0
3	19	1	0	0	0	0
3	20	1	0	0	0	0
4	21	1	0	0	0	0
4	22	1	0	0	0	0
5	23	1	0	0	0	0
5	24	1	0	0	0	0
6	25	1	0	0	0	0
6	26	1	0	0	0	0
7	27	1	0	0	0	0
7	28	1	0	0	0	0

8 29 1 0 0 0 0
 9 30 1 0 0 0 0
10 31 1 0 0 0 0
11 32 1 0 0 0 0
11 33 1 0 0 0 0
11 34 1 0 0 0 0
12 35 1 0 0 0 0
12 36 1 0 0 0 0
12 37 1 0 0 0 0
13 38 1 0 0 0 0
13 39 1 0 0 0 0
13 40 1 0 0 0 0
14 41 1 0 0 0 0
14 42 1 0 0 0 0
14 43 1 0 0 0 0
15 44 1 0 0 0 0
15 45 1 0 0 0 0
15 46 1 0 0 0 0
16 47 1 0 0 0 0
16 48 1 0 0 0 0
16 49 1 0 0 0 0
M END
> <Alias name>
Triisopentylborane

> <deltaH°(c)>   kJ/mol
-10802

$$$$
N-(Diethylaminothiocarbonyl)-N'-phenylbenzamidine
  ChemBrain IXL

 43 44 0 0 0 0 0 0 0 0 1 V2000
   10.9044   7.8199   -0.7820 C   0 0 0 0 0 0 0 0 0 0 0 0
   10.2152   6.8755   -1.5434 C   0 0 0 0 0 0 0 0 0 0 0 0
    8.8210   6.9078   -1.6003 C   0 0 0 0 0 0 0 0 0 0 0 0
    8.1127   7.8857   -0.8945 C   0 0 0 0 0 0 0 0 0 0 0 0
    8.8047   8.8300   -0.1338 C   0 0 0 0 0 0 0 0 0 0 0 0
   10.1994   8.7969   -0.0775 C   0 0 0 0 0 0 0 0 0 0 0 0
    6.6259   7.9236   -0.9516 C   0 0 0 0 0 0 0 0 0 0 0 0
    5.8966   6.7704   -1.3204 N   0 0 0 0 0 0 0 0 0 0 0 0
    6.0120   9.0039   -0.6674 N   0 0 0 0 0 0 0 0 0 0 0 0
    4.6071   9.0565   -0.7151 C   0 0 0 0 0 0 0 0 0 0 0 0
    3.8299   10.4703   -1.2817 S   0 0 0 0 0 0 0 0 0 0 0 0
    3.8384   7.9520   -0.2981 N   0 0 0 0 0 0 0 0 0 0 0 0
    4.2832   7.1236   0.8417 C   0 0 0 0 0 0 0 0 0 0 0 0
    2.5829   7.6247   -0.9967 C   0 0 0 0 0 0 0 0 0 0 0 0
    2.3627   6.0960   -0.9777 C   0 0 0 0 0 0 0 0 0 0 0 0
    5.1235   7.9791   1.8120 C   0 0 0 0 0 0 0 0 0 0 0 0
    6.3958   5.4836   -1.0363 C   0 0 0 0 0 0 0 0 0 0 0 0
    7.2967   5.2935   0.0162 C   0 0 0 0 0 0 0 0 0 0 0 0
    7.7887   4.0173   0.2950 C   0 0 0 0 0 0 0 0 0 0 0 0
    7.3819   2.9283   -0.4766 C   0 0 0 0 0 0 0 0 0 0 0 0
    6.4824   3.1153   -1.5273 C   0 0 0 0 0 0 0 0 0 0 0 0
    5.9895   4.3918   -1.8070 C   0 0 0 0 0 0 0 0 0 0 0 0
    5.0083   6.8689   -1.7876 H   0 0 0 0 0 0 0 0 0 0 0 0
   11.9863   7.7946   -0.7378 H   0 0 0 0 0 0 0 0 0 0 0 0
   10.7623   6.1171   -2.0904 H   0 0 0 0 0 0 0 0 0 0 0 0
    8.2891   6.1741   -2.1920 H   0 0 0 0 0 0 0 0 0 0 0 0
    8.2593   9.5883   0.4134 H   0 0 0 0 0 0 0 0 0 0 0 0
   10.7347   9.5296   0.5132 H   0 0 0 0 0 0 0 0 0 0 0 0
    4.8810   6.3006   0.4776 H   0 0 0 0 0 0 0 0 0 0 0 0
    3.4179   6.7358   1.3635 H   0 0 0 0 0 0 0 0 0 0 0 0

```
    2.6424    7.9663   -2.0230 H   0  0  0  0  0  0  0  0  0  0  0  0
    1.7551    8.1149   -0.5010 H   0  0  0  0  0  0  0  0  0  0  0  0
    3.1986    5.6044   -1.4606 H   0  0  0  0  0  0  0  0  0  0  0  0
    2.2862    5.7543    0.0466 H   0  0  0  0  0  0  0  0  0  0  0  0
    1.4487    5.8563   -1.5067 H   0  0  0  0  0  0  0  0  0  0  0  0
    4.5261    8.8063    2.1734 H   0  0  0  0  0  0  0  0  0  0  0  0
    5.9943    8.3616    1.2957 H   0  0  0  0  0  0  0  0  0  0  0  0
    5.4397    7.3692    2.6489 H   0  0  0  0  0  0  0  0  0  0  0  0
    7.6136    6.1361    0.6167 H   0  0  0  0  0  0  0  0  0  0  0  0
    8.4865    3.8722    1.1107 H   0  0  0  0  0  0  0  0  0  0  0  0
    7.7640    1.9380   -0.2600 H   0  0  0  0  0  0  0  0  0  0  0  0
    6.1668    2.2696   -2.1259 H   0  0  0  0  0  0  0  0  0  0  0  0
    5.2919    4.5349   -2.6224 H   0  0  0  0  0  0  0  0  0  0  0  0
  1  2  4  0  0  0  0
  2  3  4  0  0  0  0
  3  4  4  0  0  0  0
  4  5  4  0  0  0  0
  5  6  4  0  0  0  0
  6  1  4  0  0  0  0
  7  8  1  0  0  0  0
  7  9  2  0  0  0  0
  7  4  1  0  0  0  0
 10 11  2  0  0  0  0
 10 12  1  0  0  0  0
 10  9  1  0  0  0  0
 12 13  1  0  0  0  0
 12 14  1  0  0  0  0
 14 15  1  0  0  0  0
 13 16  1  0  0  0  0
 17 18  4  0  0  0  0
 18 19  4  0  0  0  0
 19 20  4  0  0  0  0
 20 21  4  0  0  0  0
 21 22  4  0  0  0  0
 22 17  4  0  0  0  0
 17  8  1  0  0  0  0
  8 23  1  0  0  0  0
  1 24  1  0  0  0  0
  2 25  1  0  0  0  0
  3 26  1  0  0  0  0
  5 27  1  0  0  0  0
  6 28  1  0  0  0  0
 13 29  1  0  0  0  0
 13 30  1  0  0  0  0
 14 31  1  0  0  0  0
 14 32  1  0  0  0  0
 15 33  1  0  0  0  0
 15 34  1  0  0  0  0
 15 35  1  0  0  0  0
 16 36  1  0  0  0  0
 16 37  1  0  0  0  0
 16 38  1  0  0  0  0
 18 39  1  0  0  0  0
 19 40  1  0  0  0  0
 20 41  1  0  0  0  0
 21 42  1  0  0  0  0
 22 43  1  0  0  0  0
M  END
>  <Alias name>
N-(Diethylaminothiocarbonyl)-N'-phenylbenzamidine

>  <deltaH°(c)>   kJ/mol
-10801
```

```
$$$$
Di-n-butylsebacate
  ChemBrain IXL

 56 55  0  0  0  0  0  0  0  0  1 V2000
    9.3042   14.5979   -0.2406 C   0  0  0  0  0  0  0  0  0  0  0  0  0
    9.7063   13.1105   -0.2302 C   0  0  0  0  0  0  0  0  0  0  0  0  0
   11.2420   12.9947   -0.2075 C   0  0  0  0  0  0  0  0  0  0  0  0  0
   11.6445   11.5081   -0.2043 C   0  0  0  0  0  0  0  0  0  0  0  0  0
   13.1804   11.3924   -0.2029 C   0  0  0  0  0  0  0  0  0  0  0  0  0
   13.5828    9.9058   -0.2100 C   0  0  0  0  0  0  0  0  0  0  0  0  0
   15.1186    9.7899   -0.2298 C   0  0  0  0  0  0  0  0  0  0  0  0  0
   15.5207    8.3026   -0.2442 C   0  0  0  0  0  0  0  0  0  0  0  0  0
    7.8286   14.7119   -0.2767 C   0  0  0  0  0  0  0  0  0  0  0  0  0
    7.3069   15.8040   -0.4341 O   0  0  0  0  0  0  0  0  0  0  0  0  0
    6.9918   13.5090   -0.1229 O   0  0  0  0  0  0  0  0  0  0  0  0  0
   16.9963    8.1886   -0.2776 C   0  0  0  0  0  0  0  0  0  0  0  0  0
   17.5184    7.0962   -0.4316 O   0  0  0  0  0  0  0  0  0  0  0  0  0
   17.8328    9.3920   -0.1253 O   0  0  0  0  0  0  0  0  0  0  0  0  0
    5.5405   13.7451   -0.1769 C   0  0  0  0  0  0  0  0  0  0  0  0  0
    4.7957   12.4073   -0.0066 C   0  0  0  0  0  0  0  0  0  0  0  0  0
    3.2755   12.6549   -0.0644 C   0  0  0  0  0  0  0  0  0  0  0  0  0
    2.5301   11.3169    0.1047 C   0  0  0  0  0  0  0  0  0  0  0  0  0
   19.2842    9.1558   -0.1763 C   0  0  0  0  0  0  0  0  0  0  0  0  0
   20.0286   10.4940   -0.0080 C   0  0  0  0  0  0  0  0  0  0  0  0  0
   21.5489   10.2465   -0.0628 C   0  0  0  0  0  0  0  0  0  0  0  0  0
   22.2940   11.5849    0.1043 C   0  0  0  0  0  0  0  0  0  0  0  0  0
    9.6818   15.0801    0.6526 H   0  0  0  0  0  0  0  0  0  0  0  0  0
    9.7242   15.0789   -1.1153 H   0  0  0  0  0  0  0  0  0  0  0  0  0
    9.3211   12.6264   -1.1191 H   0  0  0  0  0  0  0  0  0  0  0  0  0
    9.2946   12.6310    0.6494 H   0  0  0  0  0  0  0  0  0  0  0  0  0
   11.6278   13.4754    0.6830 H   0  0  0  0  0  0  0  0  0  0  0  0  0
   11.6530   13.4777   -1.0854 H   0  0  0  0  0  0  0  0  0  0  0  0  0
   11.2474   11.0250   -1.0885 H   0  0  0  0  0  0  0  0  0  0  0  0  0
   11.2448   11.0273    0.6801 H   0  0  0  0  0  0  0  0  0  0  0  0  0
   13.5782   11.8703    0.6839 H   0  0  0  0  0  0  0  0  0  0  0  0  0
   13.5793   11.8782   -1.0847 H   0  0  0  0  0  0  0  0  0  0  0  0  0
   13.1737    9.4255   -1.0902 H   0  0  0  0  0  0  0  0  0  0  0  0  0
   13.1952    9.4222    0.6782 H   0  0  0  0  0  0  0  0  0  0  0  0  0
   15.5284   10.2666    0.6521 H   0  0  0  0  0  0  0  0  0  0  0  0  0
   15.5056   10.2769   -1.1163 H   0  0  0  0  0  0  0  0  0  0  0  0  0
   15.1024    7.8244   -1.1213 H   0  0  0  0  0  0  0  0  0  0  0  0  0
   15.1412    7.8175    0.6467 H   0  0  0  0  0  0  0  0  0  0  0  0  0
    5.2814   14.1834   -1.1328 H   0  0  0  0  0  0  0  0  0  0  0  0  0
    5.2549   14.4212    0.6196 H   0  0  0  0  0  0  0  0  0  0  0  0  0
    5.0541   11.9694    0.9495 H   0  0  0  0  0  0  0  0  0  0  0  0  0
    5.0819   11.7311   -0.8026 H   0  0  0  0  0  0  0  0  0  0  0  0  0
    3.0173   13.0935   -1.0203 H   0  0  0  0  0  0  0  0  0  0  0  0  0
    2.9893   13.3306    0.7320 H   0  0  0  0  0  0  0  0  0  0  0  0  0
    2.8162   10.6410   -0.6917 H   0  0  0  0  0  0  0  0  0  0  0  0  0
    2.7880   10.8782    1.0606 H   0  0  0  0  0  0  0  0  0  0  0  0  0
    1.4620   11.4912    0.0640 H   0  0  0  0  0  0  0  0  0  0  0  0  0
   19.5685    8.4816    0.6221 H   0  0  0  0  0  0  0  0  0  0  0  0  0
   19.5449    8.7153   -1.1308 H   0  0  0  0  0  0  0  0  0  0  0  0  0
   19.7436   11.1684   -0.8060 H   0  0  0  0  0  0  0  0  0  0  0  0  0
   19.7686   10.9341    0.9467 H   0  0  0  0  0  0  0  0  0  0  0  0  0
   21.8339    9.5726    0.7355 H   0  0  0  0  0  0  0  0  0  0  0  0  0
   21.8086    9.8057   -1.0173 H   0  0  0  0  0  0  0  0  0  0  0  0  0
   22.0090   12.2589   -0.6940 H   0  0  0  0  0  0  0  0  0  0  0  0  0
   23.3621   11.4107    0.0657 H   0  0  0  0  0  0  0  0  0  0  0  0  0
   22.0345   12.0258    1.0589 H   0  0  0  0  0  0  0  0  0  0  0  0  0
  1  2  1  0  0  0  0
```

```
  2  3  1  0  0  0  0
  3  4  1  0  0  0  0
  4  5  1  0  0  0  0
  5  6  1  0  0  0  0
  6  7  1  0  0  0  0
  7  8  1  0  0  0  0
  9 10  2  0  0  0  0
  9 11  1  0  0  0  0
  9  1  1  0  0  0  0
 12 13  2  0  0  0  0
 12 14  1  0  0  0  0
 12  8  1  0  0  0  0
 11 15  1  0  0  0  0
 15 16  1  0  0  0  0
 16 17  1  0  0  0  0
 17 18  1  0  0  0  0
 14 19  1  0  0  0  0
 19 20  1  0  0  0  0
 20 21  1  0  0  0  0
 21 22  1  0  0  0  0
  1 23  1  0  0  0  0
  1 24  1  0  0  0  0
  2 25  1  0  0  0  0
  2 26  1  0  0  0  0
  3 27  1  0  0  0  0
  3 28  1  0  0  0  0
  4 29  1  0  0  0  0
  4 30  1  0  0  0  0
  5 31  1  0  0  0  0
  5 32  1  0  0  0  0
  6 33  1  0  0  0  0
  6 34  1  0  0  0  0
  7 35  1  0  0  0  0
  7 36  1  0  0  0  0
  8 37  1  0  0  0  0
  8 38  1  0  0  0  0
 15 39  1  0  0  0  0
 15 40  1  0  0  0  0
 16 41  1  0  0  0  0
 16 42  1  0  0  0  0
 17 43  1  0  0  0  0
 17 44  1  0  0  0  0
 18 45  1  0  0  0  0
 18 46  1  0  0  0  0
 18 47  1  0  0  0  0
 19 48  1  0  0  0  0
 19 49  1  0  0  0  0
 20 50  1  0  0  0  0
 20 51  1  0  0  0  0
 21 52  1  0  0  0  0
 21 53  1  0  0  0  0
 22 54  1  0  0  0  0
 22 55  1  0  0  0  0
 22 56  1  0  0  0  0
M  END
>  <Alias name>
Dibutyl decanedioate

>  <deltaH°(c)>   kJ/mol
-10793.57

$$$$
Di-n-hexyladipate
```

ChemBrain IXL

```
56 55  0  0  0  0  0  0  0  0  1 V2000
   11.0726   10.9900    0.3305 C   0  0  0  0  0  0  0  0  0  0  0  0  0
   12.5933   11.0557    0.0896 C   0  0  0  0  0  0  0  0  0  0  0  0  0
   13.0516   12.5269    0.0982 C   0  0  0  0  0  0  0  0  0  0  0  0  0
   14.5762   12.5912   -0.1168 C   0  0  0  0  0  0  0  0  0  0  0  0  0
   10.6419    9.5750    0.3820 C   0  0  0  0  0  0  0  0  0  0  0  0  0
   11.4640    8.6822    0.2570 O   0  0  0  0  0  0  0  0  0  0  0  0  0
    9.2237    9.2452    0.5836 O   0  0  0  0  0  0  0  0  0  0  0  0  0
   15.0251   13.9998   -0.0481 C   0  0  0  0  0  0  0  0  0  0  0  0  0
   14.2200   14.8859    0.1865 O   0  0  0  0  0  0  0  0  0  0  0  0  0
   16.4412   14.3312   -0.2625 O   0  0  0  0  0  0  0  0  0  0  0  0  0
    8.3253   10.4040    0.6887 C   0  0  0  0  0  0  0  0  0  0  0  0  0
    6.8833    9.9160    0.9277 C   0  0  0  0  0  0  0  0  0  0  0  0  0
    5.9394   11.1303    1.0245 C   0  0  0  0  0  0  0  0  0  0  0  0  0
    4.5004   10.6431    1.2823 C   0  0  0  0  0  0  0  0  0  0  0  0  0
    3.5537   11.8564    1.3616 C   0  0  0  0  0  0  0  0  0  0  0  0  0
    2.1165   11.3699    1.6304 C   0  0  0  0  0  0  0  0  0  0  0  0  0
   17.3083   13.1842   -0.5680 C   0  0  0  0  0  0  0  0  0  0  0  0  0
   18.7618   13.6688   -0.7334 C   0  0  0  0  0  0  0  0  0  0  0  0  0
   19.6691   12.4679   -1.0642 C   0  0  0  0  0  0  0  0  0  0  0  0  0
   21.1260   12.9490   -1.2075 C   0  0  0  0  0  0  0  0  0  0  0  0  0
   22.0310   11.7507   -1.5534 C   0  0  0  0  0  0  0  0  0  0  0  0  0
   23.4899   12.2296   -1.6838 C   0  0  0  0  0  0  0  0  0  0  0  0  0
   10.5571   11.4952   -0.4769 H   0  0  0  0  0  0  0  0  0  0  0  0  0
   10.8347   11.4741    1.2696 H   0  0  0  0  0  0  0  0  0  0  0  0  0
   13.1068   10.5130    0.8737 H   0  0  0  0  0  0  0  0  0  0  0  0  0
   12.8263   10.6105   -0.8698 H   0  0  0  0  0  0  0  0  0  0  0  0  0
   12.5518   13.0654   -0.6975 H   0  0  0  0  0  0  0  0  0  0  0  0  0
   12.8021   12.9772    1.0510 H   0  0  0  0  0  0  0  0  0  0  0  0  0
   15.0740   12.0156    0.6537 H   0  0  0  0  0  0  0  0  0  0  0  0  0
   14.8213   12.1812   -1.0888 H   0  0  0  0  0  0  0  0  0  0  0  0  0
    8.6370   11.0285    1.5169 H   0  0  0  0  0  0  0  0  0  0  0  0  0
    8.3652   10.9762   -0.2299 H   0  0  0  0  0  0  0  0  0  0  0  0  0
    6.5749    9.2839    0.1041 H   0  0  0  0  0  0  0  0  0  0  0  0  0
    6.8415    9.3517    1.8511 H   0  0  0  0  0  0  0  0  0  0  0  0  0
    6.2557   11.7692    1.8398 H   0  0  0  0  0  0  0  0  0  0  0  0  0
    5.9712   11.6871    0.0962 H   0  0  0  0  0  0  0  0  0  0  0  0  0
    4.1886    9.9942    0.4733 H   0  0  0  0  0  0  0  0  0  0  0  0  0
    4.4662   10.0969    2.2169 H   0  0  0  0  0  0  0  0  0  0  0  0  0
    3.8702   12.5093    2.1656 H   0  0  0  0  0  0  0  0  0  0  0  0  0
    3.5820   12.3982    0.4242 H   0  0  0  0  0  0  0  0  0  0  0  0  0
    1.8016   10.7135    0.8287 H   0  0  0  0  0  0  0  0  0  0  0  0  0
    2.0873   10.8320    2.5700 H   0  0  0  0  0  0  0  0  0  0  0  0  0
    1.4505   12.2224    1.6817 H   0  0  0  0  0  0  0  0  0  0  0  0  0
   16.9771   12.7158   -1.4866 H   0  0  0  0  0  0  0  0  0  0  0  0  0
   17.2569   12.4670    0.2418 H   0  0  0  0  0  0  0  0  0  0  0  0  0
   19.0954   14.1304    0.1876 H   0  0  0  0  0  0  0  0  0  0  0  0  0
   18.8119   14.3916   -1.5384 H   0  0  0  0  0  0  0  0  0  0  0  0  0
   19.3451   12.0158   -1.9934 H   0  0  0  0  0  0  0  0  0  0  0  0  0
   19.6068   11.7379   -0.2666 H   0  0  0  0  0  0  0  0  0  0  0  0  0
   21.4535   13.3914   -0.2748 H   0  0  0  0  0  0  0  0  0  0  0  0  0
   21.1864   13.6870   -1.9979 H   0  0  0  0  0  0  0  0  0  0  0  0  0
   21.7092   11.3140   -2.4908 H   0  0  0  0  0  0  0  0  0  0  0  0  0
   21.9635   11.0086   -0.7675 H   0  0  0  0  0  0  0  0  0  0  0  0  0
   23.5566   12.9748   -2.4668 H   0  0  0  0  0  0  0  0  0  0  0  0  0
   24.1253   11.3882   -1.9311 H   0  0  0  0  0  0  0  0  0  0  0  0  0
   23.8131   12.6623   -0.7451 H   0  0  0  0  0  0  0  0  0  0  0  0  0
  1  2  1  0  0  0  0
  2  3  1  0  0  0  0
  3  4  1  0  0  0  0
  5  6  2  0  0  0  0
```

```
  5  7  1  0  0  0  0
  8  9  2  0  0  0  0
  8 10  1  0  0  0  0
  5  1  1  0  0  0  0
  8  4  1  0  0  0  0
  7 11  1  0  0  0  0
 11 12  1  0  0  0  0
 12 13  1  0  0  0  0
 13 14  1  0  0  0  0
 14 15  1  0  0  0  0
 15 16  1  0  0  0  0
 10 17  1  0  0  0  0
 17 18  1  0  0  0  0
 18 19  1  0  0  0  0
 19 20  1  0  0  0  0
 20 21  1  0  0  0  0
 21 22  1  0  0  0  0
  1 23  1  0  0  0  0
  1 24  1  0  0  0  0
  2 25  1  0  0  0  0
  2 26  1  0  0  0  0
  3 27  1  0  0  0  0
  3 28  1  0  0  0  0
  4 29  1  0  0  0  0
  4 30  1  0  0  0  0
 11 31  1  0  0  0  0
 11 32  1  0  0  0  0
 12 33  1  0  0  0  0
 12 34  1  0  0  0  0
 13 35  1  0  0  0  0
 13 36  1  0  0  0  0
 14 37  1  0  0  0  0
 14 38  1  0  0  0  0
 15 39  1  0  0  0  0
 15 40  1  0  0  0  0
 16 41  1  0  0  0  0
 16 42  1  0  0  0  0
 16 43  1  0  0  0  0
 17 44  1  0  0  0  0
 17 45  1  0  0  0  0
 18 46  1  0  0  0  0
 18 47  1  0  0  0  0
 19 48  1  0  0  0  0
 19 49  1  0  0  0  0
 20 50  1  0  0  0  0
 20 51  1  0  0  0  0
 21 52  1  0  0  0  0
 21 53  1  0  0  0  0
 22 54  1  0  0  0  0
 22 55  1  0  0  0  0
 22 56  1  0  0  0  0
M  END
>  <Alias name>
Dihexyladipate

>  <deltaH°(c)>   kJ/mol
-10788.67

$$$$
Bis(2,4,5-trimethylphenyl)-methane
  ChemBrain IXL

 43 44  0  0  0  0  0  0  0  0  1 V2000
```

```
   3.4048    5.7793    0.4928 C   0  0  0  0  0  0  0  0  0  0  0  0
   3.4180    7.1639    0.3176 C   0  0  0  0  0  0  0  0  0  0  0  0
   4.5563    7.7923   -0.1936 C   0  0  0  0  0  0  0  0  0  0  0  0
   5.6801    7.0353   -0.5289 C   0  0  0  0  0  0  0  0  0  0  0  0
   5.6697    5.6494   -0.3510 C   0  0  0  0  0  0  0  0  0  0  0  0
   4.5288    5.0215    0.1590 C   0  0  0  0  0  0  0  0  0  0  0  0
   6.8699    4.8450   -0.7040 C   0  0  0  0  0  0  0  0  0  0  0  0
   4.5095    3.5520    0.3462 C   0  0  0  0  0  0  0  0  0  0  0  0
   2.2243    7.9658    0.6738 C   0  0  0  0  0  0  0  0  0  0  0  0
   4.5724    9.2619   -0.3800 C   0  0  0  0  0  0  0  0  0  0  0  0
  10.4302    6.1213   -0.7280 C   0  0  0  0  0  0  0  0  0  0  0  0
  10.4153    6.9985    0.3577 C   0  0  0  0  0  0  0  0  0  0  0  0
   9.2446    7.1715    1.0995 C   0  0  0  0  0  0  0  0  0  0  0  0
   8.0902    6.4669    0.7555 C   0  0  0  0  0  0  0  0  0  0  0  0
   8.1021    5.5910   -0.3337 C   0  0  0  0  0  0  0  0  0  0  0  0
   9.2753    5.4175   -1.0742 C   0  0  0  0  0  0  0  0  0  0  0  0
   9.2955    4.4870   -2.2268 C   0  0  0  0  0  0  0  0  0  0  0  0
  11.6417    7.7452    0.7225 C   0  0  0  0  0  0  0  0  0  0  0  0
   9.2265    8.1028    2.2515 C   0  0  0  0  0  0  0  0  0  0  0  0
   2.5220    5.2923    0.8882 H   0  0  0  0  0  0  0  0  0  0  0  0
   6.5613    7.5236   -0.9262 H   0  0  0  0  0  0  0  0  0  0  0  0
   6.8450    3.9050   -0.1680 H   0  0  0  0  0  0  0  0  0  0  0  0
   6.8719    4.6517   -1.7693 H   0  0  0  0  0  0  0  0  0  0  0  0
   5.2006    3.2794    1.1340 H   0  0  0  0  0  0  0  0  0  0  0  0
   3.5099    3.2354    0.6174 H   0  0  0  0  0  0  0  0  0  0  0  0
   4.8038    3.0667   -0.5762 H   0  0  0  0  0  0  0  0  0  0  0  0
   1.4799    7.3236    1.1283 H   0  0  0  0  0  0  0  0  0  0  0  0
   2.5068    8.7423    1.3739 H   0  0  0  0  0  0  0  0  0  0  0  0
   1.8133    8.4181   -0.2203 H   0  0  0  0  0  0  0  0  0  0  0  0
   3.6594    9.5736   -0.8720 H   0  0  0  0  0  0  0  0  0  0  0  0
   5.4236    9.5396   -0.9894 H   0  0  0  0  0  0  0  0  0  0  0  0
   4.6460    9.7473    0.5853 H   0  0  0  0  0  0  0  0  0  0  0  0
  11.3384    5.9866   -1.3024 H   0  0  0  0  0  0  0  0  0  0  0  0
   7.1843    6.6004    1.3335 H   0  0  0  0  0  0  0  0  0  0  0  0
  10.3175    4.3383   -2.5531 H   0  0  0  0  0  0  0  0  0  0  0  0
   8.7146    4.9059   -3.0390 H   0  0  0  0  0  0  0  0  0  0  0  0
   8.8693    3.5363   -1.9309 H   0  0  0  0  0  0  0  0  0  0  0  0
  11.4537    8.8088    0.6432 H   0  0  0  0  0  0  0  0  0  0  0  0
  12.4457    7.4698    0.0511 H   0  0  0  0  0  0  0  0  0  0  0  0
  11.9224    7.5031    1.7401 H   0  0  0  0  0  0  0  0  0  0  0  0
   8.2276    8.1429    2.6682 H   0  0  0  0  0  0  0  0  0  0  0  0
   9.9194    7.7552    3.0076 H   0  0  0  0  0  0  0  0  0  0  0  0
   9.5195    9.0917    1.9208 H   0  0  0  0  0  0  0  0  0  0  0  0
  1  2  4  0  0  0  0
  2  3  4  0  0  0  0
  3  4  4  0  0  0  0
  4  5  4  0  0  0  0
  5  6  4  0  0  0  0
  6  1  4  0  0  0  0
  5  7  1  0  0  0  0
  6  8  1  0  0  0  0
  2  9  1  0  0  0  0
  3 10  1  0  0  0  0
 11 12  4  0  0  0  0
 12 13  4  0  0  0  0
 13 14  4  0  0  0  0
 14 15  4  0  0  0  0
 15 16  4  0  0  0  0
 16 11  4  0  0  0  0
 16 17  1  0  0  0  0
 12 18  1  0  0  0  0
 13 19  1  0  0  0  0
 15  7  1  0  0  0  0
```

1 20 1 0 0 0 0
4 21 1 0 0 0 0
7 22 1 0 0 0 0
7 23 1 0 0 0 0
8 24 1 0 0 0 0
8 25 1 0 0 0 0
8 26 1 0 0 0 0
9 27 1 0 0 0 0
9 28 1 0 0 0 0
9 29 1 0 0 0 0
10 30 1 0 0 0 0
10 31 1 0 0 0 0
10 32 1 0 0 0 0
11 33 1 0 0 0 0
14 34 1 0 0 0 0
17 35 1 0 0 0 0
17 36 1 0 0 0 0
17 37 1 0 0 0 0
18 38 1 0 0 0 0
18 39 1 0 0 0 0
18 40 1 0 0 0 0
19 41 1 0 0 0 0
19 42 1 0 0 0 0
19 43 1 0 0 0 0
M END
> <Alias name>
Bis(2,4,5-trimethylphenyl)-methane

> <deltaH°(c)>   kJ/mol
-10759.5

$$$$
Triphenylphosphine sulfide
ChemBrain IXL

35 37 0 0 0 0 0 0 0 0 1 V2000
6.0727 5.9496 1.0258 P 0 0 0 0 0 0 0 0 0 0 0 0
4.8644 7.0557 0.1238 C 0 0 0 0 0 0 0 0 0 0 0 0
6.5113 4.4906 -0.0586 C 0 0 0 0 0 0 0 0 0 0 0 0
7.6224 6.9160 1.4272 C 0 0 0 0 0 0 0 0 0 0 0 0
5.2968 5.3393 2.6022 S 0 0 0 0 0 0 0 0 0 0 0 0
5.2007 8.3824 -0.1457 C 0 0 0 0 0 0 0 0 0 0 0 0
3.6268 6.5540 -0.2795 C 0 0 0 0 0 0 0 0 0 0 0 0
5.9472 4.3711 -1.3288 C 0 0 0 0 0 0 0 0 0 0 0 0
7.4025 3.5216 0.4028 C 0 0 0 0 0 0 0 0 0 0 0 0
8.8567 6.4906 0.9356 C 0 0 0 0 0 0 0 0 0 0 0 0
7.5443 8.0624 2.2183 C 0 0 0 0 0 0 0 0 0 0 0 0
4.2990 9.2078 -0.8179 C 0 0 0 0 0 0 0 0 0 0 0 0
2.7251 7.3794 -0.9517 C 0 0 0 0 0 0 0 0 0 0 0 0
6.2737 3.2823 -2.1374 C 0 0 0 0 0 0 0 0 0 0 0 0
7.7291 2.4328 -0.4058 C 0 0 0 0 0 0 0 0 0 0 0 0
10.0129 7.2110 1.2358 C 0 0 0 0 0 0 0 0 0 0 0 0
8.7005 8.7829 2.5184 C 0 0 0 0 0 0 0 0 0 0 0 0
3.0612 8.7062 -1.2209 C 0 0 0 0 0 0 0 0 0 0 0 0
7.1646 2.3131 -1.6759 C 0 0 0 0 0 0 0 0 0 0 0 0
9.9348 8.3572 2.0272 C 0 0 0 0 0 0 0 0 0 0 0 0
6.1617 8.7717 0.1668 H 0 0 0 0 0 0 0 0 0 0 0 0
3.3659 5.5238 -0.0708 H 0 0 0 0 0 0 0 0 0 0 0 0
5.2558 5.1237 -1.6873 H 0 0 0 0 0 0 0 0 0 0 0 0
7.8409 3.6147 1.3887 H 0 0 0 0 0 0 0 0 0 0 0 0
8.9173 5.6010 0.3210 H 0 0 0 0 0 0 0 0 0 0 0 0
6.5860 8.3930 2.5994 H 0 0 0 0 0 0 0 0 0 0 0 0
4.5599 10.2379 -1.0269 H 0 0 0 0 0 0 0 0 0 0 0 0

```
    1.7642    6.9900   -1.2646 H   0  0  0  0  0  0  0  0  0  0  0  0  0
    5.8355    3.1893   -3.1234 H   0  0  0  0  0  0  0  0  0  0  0  0  0
    8.4208    1.6804   -0.0475 H   0  0  0  0  0  0  0  0  0  0  0  0  0
   10.9711    6.8806    0.8544 H   0  0  0  0  0  0  0  0  0  0  0  0  0
    8.6399    9.6727    3.1327 H   0  0  0  0  0  0  0  0  0  0  0  0  0
    2.3612    9.3470   -1.7427 H   0  0  0  0  0  0  0  0  0  0  0  0  0
    7.4181    1.4677   -2.3036 H   0  0  0  0  0  0  0  0  0  0  0  0  0
   10.8324    8.9165    2.2602 H   0  0  0  0  0  0  0  0  0  0  0  0  0
  1  2  1  0  0  0  0
  1  3  1  0  0  0  0
  1  4  1  0  0  0  0
  1  5  2  0  0  0  0
  2  6  4  0  0  0  0
  2  7  4  0  0  0  0
  3  8  4  0  0  0  0
  3  9  4  0  0  0  0
  4 10  4  0  0  0  0
  4 11  4  0  0  0  0
  6 12  4  0  0  0  0
  7 13  4  0  0  0  0
  8 14  4  0  0  0  0
  9 15  4  0  0  0  0
 10 16  4  0  0  0  0
 11 17  4  0  0  0  0
 12 18  4  0  0  0  0
 13 18  4  0  0  0  0
 14 19  4  0  0  0  0
 15 19  4  0  0  0  0
 16 20  4  0  0  0  0
 17 20  4  0  0  0  0
  6 21  1  0  0  0  0
  7 22  1  0  0  0  0
  8 23  1  0  0  0  0
  9 24  1  0  0  0  0
 10 25  1  0  0  0  0
 11 26  1  0  0  0  0
 12 27  1  0  0  0  0
 13 28  1  0  0  0  0
 14 29  1  0  0  0  0
 15 30  1  0  0  0  0
 16 31  1  0  0  0  0
 17 32  1  0  0  0  0
 18 33  1  0  0  0  0
 19 34  1  0  0  0  0
 20 35  1  0  0  0  0
M  END
>  <Alias name>
Triphenylphosphine sulfide

>  <deltaH°(c)>   kJ/mol
-10753

$$$$
Methyl 2-hexyldecanoate
  ChemBrain IXL

 53 52  0  0  0  0  0  0  0  0  1 V2000
   12.2670    4.8484    0.8178 C   0  0  0  0  0  0  0  0  0  0  0  0  0
   11.1393    4.6488   -0.2133 C   0  0  0  0  0  0  0  0  0  0  0  0  0
   12.2333    6.2972    1.3419 C   0  0  0  0  0  0  0  0  0  0  0  0  0
   13.5746    4.5863    0.1758 C   0  0  0  0  0  0  0  0  0  0  0  0  0
    9.7768    4.9033    0.4600 C   0  0  0  0  0  0  0  0  0  0  0  0  0
   12.4118    7.2740    0.1634 C   0  0  0  0  0  0  0  0  0  0  0  0  0
```

14.6717 5.5539 0.3208 O 0 0 0 0 0 0 0 0 0 0 0 0
13.7418 3.5672 -0.4737 O 0 0 0 0 0 0 0 0 0 0 0 0
8.6473 4.6697 -0.5620 C 0 0 0 0 0 0 0 0 0 0 0 0
12.4083 8.7217 0.6913 C 0 0 0 0 0 0 0 0 0 0 0 0
15.9157 5.1912 -0.3733 C 0 0 0 0 0 0 0 0 0 0 0 0
7.2847 4.9253 0.1110 C 0 0 0 0 0 0 0 0 0 0 0 0
6.1558 4.7026 -0.9140 C 0 0 0 0 0 0 0 0 0 0 0 0
4.7932 4.9562 -0.2406 C 0 0 0 0 0 0 0 0 0 0 0 0
12.5830 9.6987 -0.4876 C 0 0 0 0 0 0 0 0 0 0 0 0
12.5810 11.1464 0.0404 C 0 0 0 0 0 0 0 0 0 0 0 0
12.7534 12.1235 -1.1387 C 0 0 0 0 0 0 0 0 0 0 0 0
3.6645 4.7379 -1.2668 C 0 0 0 0 0 0 0 0 0 0 0 0
2.3017 4.9898 -0.5929 C 0 0 0 0 0 0 0 0 0 0 0 0
12.1265 4.1623 1.6439 H 0 0 0 0 0 0 0 0 0 0 0 0
11.1713 3.6344 -0.5913 H 0 0 0 0 0 0 0 0 0 0 0 0
11.2725 5.3439 -1.0330 H 0 0 0 0 0 0 0 0 0 0 0 0
11.2823 6.4843 1.8250 H 0 0 0 0 0 0 0 0 0 0 0 0
13.0353 6.4418 2.0552 H 0 0 0 0 0 0 0 0 0 0 0 0
9.7355 5.9252 0.8164 H 0 0 0 0 0 0 0 0 0 0 0 0
9.6542 4.2246 1.2949 H 0 0 0 0 0 0 0 0 0 0 0 0
13.3532 7.0729 -0.3328 H 0 0 0 0 0 0 0 0 0 0 0 0
11.5982 7.1441 -0.5395 H 0 0 0 0 0 0 0 0 0 0 0 0
8.6881 3.6477 -0.9178 H 0 0 0 0 0 0 0 0 0 0 0 0
8.7703 5.3480 -1.3973 H 0 0 0 0 0 0 0 0 0 0 0 0
11.4679 8.9220 1.1898 H 0 0 0 0 0 0 0 0 0 0 0 0
13.2235 8.8524 1.3922 H 0 0 0 0 0 0 0 0 0 0 0 0
16.6590 5.9612 -0.2079 H 0 0 0 0 0 0 0 0 0 0 0 0
15.7227 5.0977 -1.4348 H 0 0 0 0 0 0 0 0 0 0 0 0
16.2818 4.2474 0.0116 H 0 0 0 0 0 0 0 0 0 0 0 0
7.2469 5.9450 0.4738 H 0 0 0 0 0 0 0 0 0 0 0 0
7.1584 4.2418 0.9414 H 0 0 0 0 0 0 0 0 0 0 0 0
6.1945 3.6833 -1.2779 H 0 0 0 0 0 0 0 0 0 0 0 0
6.2814 5.3871 -1.7438 H 0 0 0 0 0 0 0 0 0 0 0 0
4.7557 5.9745 0.1261 H 0 0 0 0 0 0 0 0 0 0 0 0
4.6662 4.2695 0.5872 H 0 0 0 0 0 0 0 0 0 0 0 0
13.5229 9.4978 -0.9867 H 0 0 0 0 0 0 0 0 0 0 0 0
11.7673 9.5688 -1.1881 H 0 0 0 0 0 0 0 0 0 0 0 0
11.6418 11.3468 0.5409 H 0 0 0 0 0 0 0 0 0 0 0 0
13.3977 11.2768 0.7396 H 0 0 0 0 0 0 0 0 0 0 0 0
11.9366 11.9932 -1.8378 H 0 0 0 0 0 0 0 0 0 0 0 0
13.6925 11.9229 -1.6394 H 0 0 0 0 0 0 0 0 0 0 0 0
12.7522 13.1409 -0.7676 H 0 0 0 0 0 0 0 0 0 0 0 0
3.7027 3.7199 -1.6344 H 0 0 0 0 0 0 0 0 0 0 0 0
3.7907 5.4254 -2.0940 H 0 0 0 0 0 0 0 0 0 0 0 0
2.1751 4.3018 0.2338 H 0 0 0 0 0 0 0 0 0 0 0 0
2.2638 6.0076 -0.2247 H 0 0 0 0 0 0 0 0 0 0 0 0
1.5085 4.8371 -1.3143 H 0 0 0 0 0 0 0 0 0 0 0 0
1 2 1 0 0 0 0
1 3 1 0 0 0 0
1 4 1 0 0 0 0
2 5 1 0 0 0 0
3 6 1 0 0 0 0
4 7 1 0 0 0 0
4 8 2 0 0 0 0
5 9 1 0 0 0 0
6 10 1 0 0 0 0
7 11 1 0 0 0 0
9 12 1 0 0 0 0
12 13 1 0 0 0 0
13 14 1 0 0 0 0
10 15 1 0 0 0 0
15 16 1 0 0 0 0
16 17 1 0 0 0 0

```
 14 18  1  0  0  0  0
 18 19  1  0  0  0  0
  1 20  1  0  0  0  0
  2 21  1  0  0  0  0
  2 22  1  0  0  0  0
  3 23  1  0  0  0  0
  3 24  1  0  0  0  0
  5 25  1  0  0  0  0
  5 26  1  0  0  0  0
  6 27  1  0  0  0  0
  6 28  1  0  0  0  0
  9 29  1  0  0  0  0
  9 30  1  0  0  0  0
 10 31  1  0  0  0  0
 10 32  1  0  0  0  0
 11 33  1  0  0  0  0
 11 34  1  0  0  0  0
 11 35  1  0  0  0  0
 12 36  1  0  0  0  0
 12 37  1  0  0  0  0
 13 38  1  0  0  0  0
 13 39  1  0  0  0  0
 14 40  1  0  0  0  0
 14 41  1  0  0  0  0
 15 42  1  0  0  0  0
 15 43  1  0  0  0  0
 16 44  1  0  0  0  0
 16 45  1  0  0  0  0
 17 46  1  0  0  0  0
 17 47  1  0  0  0  0
 17 48  1  0  0  0  0
 18 49  1  0  0  0  0
 18 50  1  0  0  0  0
 19 51  1  0  0  0  0
 19 52  1  0  0  0  0
 19 53  1  0  0  0  0
M  END
>  <Alias name>
Methyl 2-hexyldecanoate

>  <deltaH°(c)>   kJ/mol
-10743

$$$$
Isopropyl myristate
  ChemBrain IXL

 53 52  0  0  0  0  0  0  0  0  1 V2000
    2.4928    7.1851   -0.6599 C   0  0  0  0  0  0  0  0  0  0  0  0
    3.3612    8.3022   -0.0494 C   0  0  0  0  0  0  0  0  0  0  0  0
    4.8128    8.1530   -0.5448 C   0  0  0  0  0  0  0  0  0  0  0  0
    5.6805    9.2721    0.0629 C   0  0  0  0  0  0  0  0  0  0  0  0
    7.1349    9.1147   -0.4214 C   0  0  0  0  0  0  0  0  0  0  0  0
    8.0023   10.2351    0.1845 C   0  0  0  0  0  0  0  0  0  0  0  0
    9.4619   10.0619   -0.2783 C   0  0  0  0  0  0  0  0  0  0  0  0
   10.3289   11.1832    0.3263 C   0  0  0  0  0  0  0  0  0  0  0  0
   11.7951   10.9887   -0.1064 C   0  0  0  0  0  0  0  0  0  0  0  0
   12.6610   12.1141    0.4922 C   0  0  0  0  0  0  0  0  0  0  0  0
   14.1334   11.8971    0.0926 C   0  0  0  0  0  0  0  0  0  0  0  0
   14.9968   13.0322    0.6765 C   0  0  0  0  0  0  0  0  0  0  0  0
   16.4753   12.7905    0.3158 C   0  0  0  0  0  0  0  0  0  0  0  0
   17.3050   13.8786    0.8797 C   0  0  0  0  0  0  0  0  0  0  0  0
   16.8107   14.6868    1.6483 O   0  0  0  0  0  0  0  0  0  0  0  0
```

```
 18.7247   13.9855    0.5137 O   0  0  0  0  0  0  0  0  0  0  0  0
 19.2154   12.9313   -0.3857 C   0  0  0  0  0  0  0  0  0  0  0  0
 20.5905   13.3372   -0.9506 C   0  0  0  0  0  0  0  0  0  0  0  0
 19.3510   11.6107    0.3967 C   0  0  0  0  0  0  0  0  0  0  0  0
  2.8814    6.2202   -0.3583 H   0  0  0  0  0  0  0  0  0  0  0  0
  1.4730    7.2889   -0.3103 H   0  0  0  0  0  0  0  0  0  0  0  0
  2.5136    7.2612   -1.7400 H   0  0  0  0  0  0  0  0  0  0  0  0
  2.9737    9.2670   -0.3524 H   0  0  0  0  0  0  0  0  0  0  0  0
  3.3390    8.2272    1.0308 H   0  0  0  0  0  0  0  0  0  0  0  0
  5.2011    7.1891   -0.2398 H   0  0  0  0  0  0  0  0  0  0  0  0
  4.8348    8.2256   -1.6252 H   0  0  0  0  0  0  0  0  0  0  0  0
  5.2981   10.2358   -0.2500 H   0  0  0  0  0  0  0  0  0  0  0  0
  5.6507    9.2055    1.1435 H   0  0  0  0  0  0  0  0  0  0  0  0
  7.5179    8.1517   -0.1071 H   0  0  0  0  0  0  0  0  0  0  0  0
  7.1645    9.1797   -1.5021 H   0  0  0  0  0  0  0  0  0  0  0  0
  7.6308   11.1975   -0.1452 H   0  0  0  0  0  0  0  0  0  0  0  0
  7.9575   10.1818    1.2653 H   0  0  0  0  0  0  0  0  0  0  0  0
  9.8337    9.1000    0.0524 H   0  0  0  0  0  0  0  0  0  0  0  0
  9.5065   10.1141   -1.3591 H   0  0  0  0  0  0  0  0  0  0  0  0
  9.9732   12.1439   -0.0250 H   0  0  0  0  0  0  0  0  0  0  0  0
 10.2636   11.1472    1.4068 H   0  0  0  0  0  0  0  0  0  0  0  0
 12.1524   10.0303    0.2494 H   0  0  0  0  0  0  0  0  0  0  0  0
 11.8596   11.0196   -1.1870 H   0  0  0  0  0  0  0  0  0  0  0  0
 12.3216   13.0706    0.1143 H   0  0  0  0  0  0  0  0  0  0  0  0
 12.5742   12.1011    1.5717 H   0  0  0  0  0  0  0  0  0  0  0  0
 14.4765   10.9462    0.4811 H   0  0  0  0  0  0  0  0  0  0  0  0
 14.2183   11.8978   -0.9870 H   0  0  0  0  0  0  0  0  0  0  0  0
 14.6746   13.9800    0.2631 H   0  0  0  0  0  0  0  0  0  0  0  0
 14.8866   13.0520    1.7537 H   0  0  0  0  0  0  0  0  0  0  0  0
 16.7967   11.8414    0.7268 H   0  0  0  0  0  0  0  0  0  0  0  0
 16.5859   12.7733   -0.7614 H   0  0  0  0  0  0  0  0  0  0  0  0
 18.5154   12.7977   -1.2011 H   0  0  0  0  0  0  0  0  0  0  0  0
 20.4951   14.2648   -1.5013 H   0  0  0  0  0  0  0  0  0  0  0  0
 20.9526   12.5601   -1.6123 H   0  0  0  0  0  0  0  0  0  0  0  0
 21.2903   13.4719   -0.1351 H   0  0  0  0  0  0  0  0  0  0  0  0
 19.7272   10.8378   -0.2621 H   0  0  0  0  0  0  0  0  0  0  0  0
 20.0393   11.7486    1.2214 H   0  0  0  0  0  0  0  0  0  0  0  0
 18.3817   11.3175    0.7807 H   0  0  0  0  0  0  0  0  0  0  0  0
  1  2  1  0  0  0  0
  2  3  1  0  0  0  0
  3  4  1  0  0  0  0
  4  5  1  0  0  0  0
  5  6  1  0  0  0  0
  6  7  1  0  0  0  0
  7  8  1  0  0  0  0
  8  9  1  0  0  0  0
  9 10  1  0  0  0  0
 10 11  1  0  0  0  0
 11 12  1  0  0  0  0
 12 13  1  0  0  0  0
 14 15  2  0  0  0  0
 14 16  1  0  0  0  0
 14 13  1  0  0  0  0
 16 17  1  0  0  0  0
 17 18  1  0  0  0  0
 17 19  1  0  0  0  0
  1 20  1  0  0  0  0
  1 21  1  0  0  0  0
  1 22  1  0  0  0  0
  2 23  1  0  0  0  0
  2 24  1  0  0  0  0
  3 25  1  0  0  0  0
  3 26  1  0  0  0  0
```

```
  4 27  1  0  0  0  0
  4 28  1  0  0  0  0
  5 29  1  0  0  0  0
  5 30  1  0  0  0  0
  6 31  1  0  0  0  0
  6 32  1  0  0  0  0
  7 33  1  0  0  0  0
  7 34  1  0  0  0  0
  8 35  1  0  0  0  0
  8 36  1  0  0  0  0
  9 37  1  0  0  0  0
  9 38  1  0  0  0  0
 10 39  1  0  0  0  0
 10 40  1  0  0  0  0
 11 41  1  0  0  0  0
 11 42  1  0  0  0  0
 12 43  1  0  0  0  0
 12 44  1  0  0  0  0
 13 45  1  0  0  0  0
 13 46  1  0  0  0  0
 17 47  1  0  0  0  0
 18 48  1  0  0  0  0
 18 49  1  0  0  0  0
 18 50  1  0  0  0  0
 19 51  1  0  0  0  0
 19 52  1  0  0  0  0
 19 53  1  0  0  0  0
M  END
>  <Alias name>
Isopropyl myristate

>  <deltaH°(c)>   kJ/mol
-10736.17

$$$$
N,N'-Bis(salicylaldehydo)cyclohexanediimine
  ChemBrain IXL

 46 48  0  0  0  0  0  0  0  0  1 V2000
    2.3229    9.2367   -0.7654 C   0  0  0  0  0  0  0  0  0  0  0  0
    2.9213   10.4336   -1.1599 C   0  0  0  0  0  0  0  0  0  0  0  0
    4.3110   10.5545   -1.1515 C   0  0  0  0  0  0  0  0  0  0  0  0
    5.1025    9.4777   -0.7511 C   0  0  0  0  0  0  0  0  0  0  0  0
    4.5040    8.2815   -0.3549 C   0  0  0  0  0  0  0  0  0  0  0  0
    3.1142    8.1617   -0.3602 C   0  0  0  0  0  0  0  0  0  0  0  0
    5.3436    7.1396    0.0710 C   0  0  0  0  0  0  0  0  0  0  0  0
    6.5670    9.6036   -0.7467 O   0  0  0  0  0  0  0  0  0  0  0  0
    6.6034    7.1852   -0.0835 N   0  0  0  0  0  0  0  0  0  0  0  0
   13.0407   10.5129    0.9667 C   0  0  0  0  0  0  0  0  0  0  0  0
   12.1791   11.5275    1.3840 C   0  0  0  0  0  0  0  0  0  0  0  0
   10.8004   11.3146    1.3870 C   0  0  0  0  0  0  0  0  0  0  0  0
   10.2835   10.0861    0.9751 C   0  0  0  0  0  0  0  0  0  0  0  0
   11.1450    9.0721    0.5559 C   0  0  0  0  0  0  0  0  0  0  0  0
   12.5235    9.2862    0.5498 C   0  0  0  0  0  0  0  0  0  0  0  0
   10.5966    7.7692    0.1177 C   0  0  0  0  0  0  0  0  0  0  0  0
    8.8311    9.8600    0.9828 O   0  0  0  0  0  0  0  0  0  0  0  0
    9.3644    7.5105    0.2843 N   0  0  0  0  0  0  0  0  0  0  0  0
    8.8348    6.2994   -0.1768 C   0  0  0  0  0  0  0  0  0  0  0  0
    9.7142    5.1336    0.3156 C   0  0  0  0  0  0  0  0  0  0  0  0
    9.1313    3.8005   -0.1920 C   0  0  0  0  0  0  0  0  0  0  0  0
    7.6987    3.6273    0.3488 C   0  0  0  0  0  0  0  0  0  0  0  0
    6.8194    4.7930   -0.1437 C   0  0  0  0  0  0  0  0  0  0  0  0
    7.4023    6.1261    0.3639 C   0  0  0  0  0  0  0  0  0  0  0  0
```

```
   7.0526    8.7964   -0.4656 H   0  0  0  0  0  0  0  0  0  0  0  0
   8.5485    8.9643    0.6928 H   0  0  0  0  0  0  0  0  0  0  0  0
   1.2441    9.1422   -0.7738 H   0  0  0  0  0  0  0  0  0  0  0  0
   2.3070   11.2688   -1.4726 H   0  0  0  0  0  0  0  0  0  0  0  0
   4.7755   11.4843   -1.4558 H   0  0  0  0  0  0  0  0  0  0  0  0
   2.6495    7.2340   -0.0497 H   0  0  0  0  0  0  0  0  0  0  0  0
   4.8838    6.2651    0.5145 H   0  0  0  0  0  0  0  0  0  0  0  0
  14.1111   10.6775    0.9663 H   0  0  0  0  0  0  0  0  0  0  0  0
  12.5805   12.4806    1.7057 H   0  0  0  0  0  0  0  0  0  0  0  0
  10.1314   12.1029    1.7091 H   0  0  0  0  0  0  0  0  0  0  0  0
  13.1921    8.5001    0.2215 H   0  0  0  0  0  0  0  0  0  0  0  0
  11.2460    7.0364   -0.3450 H   0  0  0  0  0  0  0  0  0  0  0  0
   8.8140    6.3034   -1.2596 H   0  0  0  0  0  0  0  0  0  0  0  0
   9.7351    5.1296    1.3984 H   0  0  0  0  0  0  0  0  0  0  0  0
  10.7210    5.2554   -0.0644 H   0  0  0  0  0  0  0  0  0  0  0  0
   9.7493    2.9812    0.1541 H   0  0  0  0  0  0  0  0  0  0  0  0
   9.1104    3.8046   -1.2747 H   0  0  0  0  0  0  0  0  0  0  0  0
   7.2891    2.6903   -0.0079 H   0  0  0  0  0  0  0  0  0  0  0  0
   7.7196    3.6232    1.4316 H   0  0  0  0  0  0  0  0  0  0  0  0
   6.7985    4.7971   -1.2265 H   0  0  0  0  0  0  0  0  0  0  0  0
   5.8126    4.6712    0.2363 H   0  0  0  0  0  0  0  0  0  0  0  0
   7.4231    6.1221    1.4467 H   0  0  0  0  0  0  0  0  0  0  0  0
  1  2  4  0  0  0  0
  2  3  4  0  0  0  0
  3  4  4  0  0  0  0
  4  5  4  0  0  0  0
  5  6  4  0  0  0  0
  6  1  4  0  0  0  0
  5  7  1  0  0  0  0
  4  8  1  0  0  0  0
  7  9  2  0  0  0  0
 10 11  4  0  0  0  0
 11 12  4  0  0  0  0
 12 13  4  0  0  0  0
 13 14  4  0  0  0  0
 14 15  4  0  0  0  0
 15 10  4  0  0  0  0
 14 16  1  0  0  0  0
 13 17  1  0  0  0  0
 16 18  2  0  0  0  0
 19 20  1  0  0  0  0
 20 21  1  0  0  0  0
 21 22  1  0  0  0  0
 22 23  1  0  0  0  0
 23 24  1  0  0  0  0
 24 19  1  0  0  0  0
  9 24  1  0  0  0  0
 18 19  1  0  0  0  0
  8 25  1  0  0  0  0
 17 26  1  0  0  0  0
  1 27  1  0  0  0  0
  2 28  1  0  0  0  0
  3 29  1  0  0  0  0
  6 30  1  0  0  0  0
  7 31  1  0  0  0  0
 10 32  1  0  0  0  0
 11 33  1  0  0  0  0
 12 34  1  0  0  0  0
 15 35  1  0  0  0  0
 16 36  1  0  0  0  0
 19 37  1  0  0  0  0
 20 38  1  0  0  0  0
 20 39  1  0  0  0  0
```

```
 21 40  1  0  0  0  0
 21 41  1  0  0  0  0
 22 42  1  0  0  0  0
 22 43  1  0  0  0  0
 23 44  1  0  0  0  0
 23 45  1  0  0  0  0
 24 46  1  0  0  0  0
M  END
>  <Alias name>
N,N'-Bis(salicylaldehydo)cyclohexanediimine

>  <deltaH°(c)>   kJ/mol
-10733

$$$$
Cycloheptadecanone
  ChemBrain IXL

 50 50  0  0  0  0  0  0  0  0  1 V2000
    4.2755    5.2998   -0.6235 C   0  0  0  0  0  0  0  0  0  0  0  0
    5.2907    4.4691    0.1852 C   0  0  0  0  0  0  0  0  0  0  0  0
    3.0896    5.6851    0.2820 C   0  0  0  0  0  0  0  0  0  0  0  0
    5.3174    7.9191   -0.1749 C   0  0  0  0  0  0  0  0  0  0  0  0
    6.6903    8.5993   -0.0096 C   0  0  0  0  0  0  0  0  0  0  0  0
    4.2031    8.9103    0.2131 C   0  0  0  0  0  0  0  0  0  0  0  0
    7.7543    7.8059   -0.7926 C   0  0  0  0  0  0  0  0  0  0  0  0
    9.1108    7.9136   -0.0694 C   0  0  0  0  0  0  0  0  0  0  0  0
    6.6022    4.3414   -0.6136 C   0  0  0  0  0  0  0  0  0  0  0  0
    7.7822    4.1632    0.3613 C   0  0  0  0  0  0  0  0  0  0  0  0
    2.5387    7.0580   -0.1494 C   0  0  0  0  0  0  0  0  0  0  0  0
    3.0235    8.1388    0.8362 C   0  0  0  0  0  0  0  0  0  0  0  0
   10.2043    7.2476   -0.9269 C   0  0  0  0  0  0  0  0  0  0  0  0
   11.2786    6.7365   -0.0464 C   0  0  0  0  0  0  0  0  0  0  0  0
    9.0801    4.6561   -0.3074 C   0  0  0  0  0  0  0  0  0  0  0  0
   10.2261    4.6421    0.7227 C   0  0  0  0  0  0  0  0  0  0  0  0
   11.4841    5.2782    0.1001 C   0  0  0  0  0  0  0  0  0  0  0  0
   11.9946    7.5173    0.5585 O   0  0  0  0  0  0  0  0  0  0  0  0
    4.7556    6.1982   -0.9913 H   0  0  0  0  0  0  0  0  0  0  0  0
    3.9153    4.7145   -1.4605 H   0  0  0  0  0  0  0  0  0  0  0  0
    4.8836    3.4828    0.3709 H   0  0  0  0  0  0  0  0  0  0  0  0
    5.4894    4.9613    1.1292 H   0  0  0  0  0  0  0  0  0  0  0  0
    2.3098    4.9389    0.1927 H   0  0  0  0  0  0  0  0  0  0  0  0
    3.4232    5.7371    1.3111 H   0  0  0  0  0  0  0  0  0  0  0  0
    5.1871    7.6150   -1.2061 H   0  0  0  0  0  0  0  0  0  0  0  0
    5.2661    7.0487    0.4675 H   0  0  0  0  0  0  0  0  0  0  0  0
    6.9574    8.6231    1.0397 H   0  0  0  0  0  0  0  0  0  0  0  0
    6.6403    9.6112   -0.3924 H   0  0  0  0  0  0  0  0  0  0  0  0
    4.5881    9.6231    0.9318 H   0  0  0  0  0  0  0  0  0  0  0  0
    3.8639    9.4362   -0.6708 H   0  0  0  0  0  0  0  0  0  0  0  0
    7.8448    8.2126   -1.7922 H   0  0  0  0  0  0  0  0  0  0  0  0
    7.4582    6.7658   -0.8515 H   0  0  0  0  0  0  0  0  0  0  0  0
    9.0494    7.4138    0.8894 H   0  0  0  0  0  0  0  0  0  0  0  0
    9.3569    8.9573    0.0826 H   0  0  0  0  0  0  0  0  0  0  0  0
    6.7539    5.2366   -1.2041 H   0  0  0  0  0  0  0  0  0  0  0  0
    6.5431    3.4819   -1.2698 H   0  0  0  0  0  0  0  0  0  0  0  0
    7.8835    3.1159    0.6179 H   0  0  0  0  0  0  0  0  0  0  0  0
    7.5981    4.7388    1.2600 H   0  0  0  0  0  0  0  0  0  0  0  0
    2.8934    7.2925   -1.1454 H   0  0  0  0  0  0  0  0  0  0  0  0
    1.4561    7.0288   -0.1493 H   0  0  0  0  0  0  0  0  0  0  0  0
    2.2131    8.8260    1.0457 H   0  0  0  0  0  0  0  0  0  0  0  0
    3.3457    7.6691    1.7573 H   0  0  0  0  0  0  0  0  0  0  0  0
   10.6169    7.9753   -1.6147 H   0  0  0  0  0  0  0  0  0  0  0  0
    9.7742    6.4258   -1.4859 H   0  0  0  0  0  0  0  0  0  0  0  0
```

```
    8.9366    5.6650   -0.6741 H   0  0  0  0  0  0  0  0  0  0  0  0
    9.3303    4.0035   -1.1347 H   0  0  0  0  0  0  0  0  0  0  0  0
    9.9314    5.2073    1.5982 H   0  0  0  0  0  0  0  0  0  0  0  0
   10.4421    3.6201    1.0085 H   0  0  0  0  0  0  0  0  0  0  0  0
   12.3364    5.0966    0.7432 H   0  0  0  0  0  0  0  0  0  0  0  0
   11.6664    4.8397   -0.8732 H   0  0  0  0  0  0  0  0  0  0  0  0
  2  1  1  0  0  0  0
  3  1  1  0  0  0  0
  9  2  1  0  0  0  0
  3 11  1  0  0  0  0
  4  5  1  0  0  0  0
  4  6  1  0  0  0  0
  5  7  1  0  0  0  0
 12  6  1  0  0  0  0
  7  8  1  0  0  0  0
  8 13  1  0  0  0  0
 10  9  1  0  0  0  0
 15 10  1  0  0  0  0
 11 12  1  0  0  0  0
 14 13  1  0  0  0  0
 14 17  1  0  0  0  0
 14 18  2  0  0  0  0
 15 16  1  0  0  0  0
 17 16  1  0  0  0  0
  1 19  1  0  0  0  0
  1 20  1  0  0  0  0
  2 21  1  0  0  0  0
  2 22  1  0  0  0  0
  3 23  1  0  0  0  0
  3 24  1  0  0  0  0
  4 25  1  0  0  0  0
  4 26  1  0  0  0  0
  5 27  1  0  0  0  0
  5 28  1  0  0  0  0
  6 29  1  0  0  0  0
  6 30  1  0  0  0  0
  7 31  1  0  0  0  0
  7 32  1  0  0  0  0
  8 33  1  0  0  0  0
  8 34  1  0  0  0  0
  9 35  1  0  0  0  0
  9 36  1  0  0  0  0
 10 37  1  0  0  0  0
 10 38  1  0  0  0  0
 11 39  1  0  0  0  0
 11 40  1  0  0  0  0
 12 41  1  0  0  0  0
 12 42  1  0  0  0  0
 13 43  1  0  0  0  0
 13 44  1  0  0  0  0
 15 45  1  0  0  0  0
 15 46  1  0  0  0  0
 16 47  1  0  0  0  0
 16 48  1  0  0  0  0
 17 49  1  0  0  0  0
 17 50  1  0  0  0  0
M  END
>  <Alias name>
Cycloheptadecanone

>  <deltaH°(c)>   kJ/mol
-10727
```

```
$$$$
Diphenyl(phenylethynyl)carbinol
  ChemBrain IXL

 38 40  0  0  0  0  0  0  0  0  1 V2000
    9.6552    9.7958    0.1406 C   0  0  0  0  0  0  0  0  0  0  0  0
   10.9729   10.2111    0.3339 C   0  0  0  0  0  0  0  0  0  0  0  0
   12.0280    9.3645   -0.0067 C   0  0  0  0  0  0  0  0  0  0  0  0
   11.7655    8.1027   -0.5406 C   0  0  0  0  0  0  0  0  0  0  0  0
   10.4479    7.6875   -0.7339 C   0  0  0  0  0  0  0  0  0  0  0  0
    9.3927    8.5340   -0.3932 C   0  0  0  0  0  0  0  0  0  0  0  0
    7.9948    8.0934   -0.5981 C   0  0  0  0  0  0  0  0  0  0  0  0
    6.8575    7.7349   -0.7649 C   0  0  0  0  0  0  0  0  0  0  0  0
    5.4020    7.2762   -0.9782 C   0  0  0  0  0  0  0  0  0  0  0  0
    5.2552    5.8746   -0.5261 C   0  0  0  0  0  0  0  0  0  0  0  0
    5.8128    5.4712    0.6873 C   0  0  0  0  0  0  0  0  0  0  0  0
    5.6732    4.1504    1.1142 C   0  0  0  0  0  0  0  0  0  0  0  0
    4.9766    3.2330    0.3274 C   0  0  0  0  0  0  0  0  0  0  0  0
    4.4197    3.6363   -0.8864 C   0  0  0  0  0  0  0  0  0  0  0  0
    4.5592    4.9570   -1.3132 C   0  0  0  0  0  0  0  0  0  0  0  0
    4.4917    8.1441   -0.1982 C   0  0  0  0  0  0  0  0  0  0  0  0
    3.2759    8.5519   -0.7472 C   0  0  0  0  0  0  0  0  0  0  0  0
    2.4173    9.3689   -0.0114 C   0  0  0  0  0  0  0  0  0  0  0  0
    2.7749    9.7786    1.2733 C   0  0  0  0  0  0  0  0  0  0  0  0
    3.9910    9.3714    1.8220 C   0  0  0  0  0  0  0  0  0  0  0  0
    4.8496    8.5545    1.0862 C   0  0  0  0  0  0  0  0  0  0  0  0
    5.0634    7.3685   -2.4057 O   0  0  0  0  0  0  0  0  0  0  0  0
    5.1409    8.2684   -2.7937 H   0  0  0  0  0  0  0  0  0  0  0  0
    8.8360   10.4530    0.4050 H   0  0  0  0  0  0  0  0  0  0  0  0
   11.1766   11.1906    0.7483 H   0  0  0  0  0  0  0  0  0  0  0  0
   13.0510    9.6869    0.1434 H   0  0  0  0  0  0  0  0  0  0  0  0
   12.5847    7.4455   -0.8050 H   0  0  0  0  0  0  0  0  0  0  0  0
   10.2441    6.7079   -1.1484 H   0  0  0  0  0  0  0  0  0  0  0  0
    6.3540    6.1833    1.2979 H   0  0  0  0  0  0  0  0  0  0  0  0
    6.1057    3.8373    2.0565 H   0  0  0  0  0  0  0  0  0  0  0  0
    4.8682    2.2077    0.6589 H   0  0  0  0  0  0  0  0  0  0  0  0
    3.8790    2.9240   -1.4973 H   0  0  0  0  0  0  0  0  0  0  0  0
    4.1273    5.2699   -2.2558 H   0  0  0  0  0  0  0  0  0  0  0  0
    2.9985    8.2343   -1.7447 H   0  0  0  0  0  0  0  0  0  0  0  0
    1.4732    9.6851   -0.4375 H   0  0  0  0  0  0  0  0  0  0  0  0
    2.1083   10.4128    1.8446 H   0  0  0  0  0  0  0  0  0  0  0  0
    4.2686    9.6896    2.8193 H   0  0  0  0  0  0  0  0  0  0  0  0
    5.7940    8.2388    1.5121 H   0  0  0  0  0  0  0  0  0  0  0  0
  2  1  4  0  0  0  0
  3  2  4  0  0  0  0
  4  3  4  0  0  0  0
  5  4  4  0  0  0  0
  6  5  4  0  0  0  0
  1  6  4  0  0  0  0
  6  7  1  0  0  0  0
  7  8  3  0  0  0  0
  8  9  1  0  0  0  0
  9 10  1  0  0  0  0
 11 10  4  0  0  0  0
 12 11  4  0  0  0  0
 13 12  4  0  0  0  0
 14 13  4  0  0  0  0
 15 14  4  0  0  0  0
 10 15  4  0  0  0  0
  9 16  1  0  0  0  0
 17 16  4  0  0  0  0
 18 17  4  0  0  0  0
 19 18  4  0  0  0  0
```

```
 20 19  4  0  0  0  0
 21 20  4  0  0  0  0
 16 21  4  0  0  0  0
  9 22  1  0  0  0  0
 22 23  1  0  0  0  0
  1 24  1  0  0  0  0
  2 25  1  0  0  0  0
  3 26  1  0  0  0  0
  4 27  1  0  0  0  0
  5 28  1  0  0  0  0
 11 29  1  0  0  0  0
 12 30  1  0  0  0  0
 13 31  1  0  0  0  0
 14 32  1  0  0  0  0
 15 33  1  0  0  0  0
 17 34  1  0  0  0  0
 18 35  1  0  0  0  0
 19 36  1  0  0  0  0
 20 37  1  0  0  0  0
 21 38  1  0  0  0  0
M  END
>  <Alias name>
Diphenyl(phenylethynyl)carbinol

>  <deltaH°(c)>   kJ/mol
-10720

$$$$
Methyl palmitate
  ChemBrain IXL

 53 52  0  0  0  0  0  0  0  0  1 V2000
   19.3516   11.3132   -3.7959 C   0  0  0  0  0  0  0  0  0  0  0  0
   20.6034   10.7306   -4.3002 O   0  0  0  0  0  0  0  0  0  0  0  0
   18.2885   10.4332   -3.2612 C   0  0  0  0  0  0  0  0  0  0  0  0
   19.1889   12.5221   -3.8181 O   0  0  0  0  0  0  0  0  0  0  0  0
   20.6833    9.2650   -4.2197 C   0  0  0  0  0  0  0  0  0  0  0  0
   17.0968   11.2919   -2.7952 C   0  0  0  0  0  0  0  0  0  0  0  0
   15.9887   10.3753   -2.2416 C   0  0  0  0  0  0  0  0  0  0  0  0
   14.7982   11.2341   -1.7726 C   0  0  0  0  0  0  0  0  0  0  0  0
   13.6872   10.3168   -1.2258 C   0  0  0  0  0  0  0  0  0  0  0  0
   12.5002   11.1758   -0.7484 C   0  0  0  0  0  0  0  0  0  0  0  0
   11.3861   10.2578   -0.2092 C   0  0  0  0  0  0  0  0  0  0  0  0
   10.2057   11.1172    0.2835 C   0  0  0  0  0  0  0  0  0  0  0  0
    9.0892   10.1987    0.8170 C   0  0  0  0  0  0  0  0  0  0  0  0
    7.9177   11.0586    1.3298 C   0  0  0  0  0  0  0  0  0  0  0  0
    6.8004   10.1398    1.8610 C   0  0  0  0  0  0  0  0  0  0  0  0
    5.6376   10.9998    2.3930 C   0  0  0  0  0  0  0  0  0  0  0  0
    4.5198   10.0809    2.9230 C   0  0  0  0  0  0  0  0  0  0  0  0
    3.3628   10.9408    3.4674 C   0  0  0  0  0  0  0  0  0  0  0  0
    2.2440   10.0219    3.9952 C   0  0  0  0  0  0  0  0  0  0  0  0
   18.6758    9.8678   -2.4226 H   0  0  0  0  0  0  0  0  0  0  0  0
   17.9609    9.7511   -4.0360 H   0  0  0  0  0  0  0  0  0  0  0  0
   21.6363    8.9333   -4.6130 H   0  0  0  0  0  0  0  0  0  0  0  0
   19.8809    8.8273   -4.8006 H   0  0  0  0  0  0  0  0  0  0  0  0
   20.5916    8.9547   -3.1862 H   0  0  0  0  0  0  0  0  0  0  0  0
   16.7110   11.8591   -3.6333 H   0  0  0  0  0  0  0  0  0  0  0  0
   17.4239   11.9724   -2.0188 H   0  0  0  0  0  0  0  0  0  0  0  0
   16.3752    9.8065   -1.4050 H   0  0  0  0  0  0  0  0  0  0  0  0
   15.6601    9.6963   -3.0187 H   0  0  0  0  0  0  0  0  0  0  0  0
   14.4148   11.8067   -2.6081 H   0  0  0  0  0  0  0  0  0  0  0  0
   15.1256   11.9095   -0.9919 H   0  0  0  0  0  0  0  0  0  0  0  0
   14.0724    9.7395   -0.3944 H   0  0  0  0  0  0  0  0  0  0  0  0
```

```
 13.3554    9.6456   -2.0083 H   0  0  0  0  0  0  0  0  0  0  0  0
 12.1187   11.7575   -1.5784 H   0  0  0  0  0  0  0  0  0  0  0  0
 12.8306   11.8428    0.0383 H   0  0  0  0  0  0  0  0  0  0  0  0
 11.7710    9.6677    0.6132 H   0  0  0  0  0  0  0  0  0  0  0  0
 11.0476    9.5987   -0.9991 H   0  0  0  0  0  0  0  0  0  0  0  0
  9.8234   11.7106   -0.5379 H   0  0  0  0  0  0  0  0  0  0  0  0
 10.5431   11.7733    1.0764 H   0  0  0  0  0  0  0  0  0  0  0  0
  9.4757    9.5945    1.6283 H   0  0  0  0  0  0  0  0  0  0  0  0
  8.7413    9.5532    0.0200 H   0  0  0  0  0  0  0  0  0  0  0  0
  7.5323   11.6641    0.5189 H   0  0  0  0  0  0  0  0  0  0  0  0
  8.2652   11.7029    2.1280 H   0  0  0  0  0  0  0  0  0  0  0  0
  7.1898    9.5238    2.6621 H   0  0  0  0  0  0  0  0  0  0  0  0
  6.4429    9.5056    1.0591 H   0  0  0  0  0  0  0  0  0  0  0  0
  5.2487   11.6163    1.5921 H   0  0  0  0  0  0  0  0  0  0  0  0
  5.9949   11.6334    3.1954 H   0  0  0  0  0  0  0  0  0  0  0  0
  4.9112    9.4577    3.7176 H   0  0  0  0  0  0  0  0  0  0  0  0
  4.1561    9.4539    2.1184 H   0  0  0  0  0  0  0  0  0  0  0  0
  2.9724   11.5652    2.6732 H   0  0  0  0  0  0  0  0  0  0  0  0
  3.7262   11.5666    4.2731 H   0  0  0  0  0  0  0  0  0  0  0  0
  2.6348    9.3963    4.7882 H   0  0  0  0  0  0  0  0  0  0  0  0
  1.4316   10.6262    4.3794 H   0  0  0  0  0  0  0  0  0  0  0  0
  1.8795    9.3973    3.1891 H   0  0  0  0  0  0  0  0  0  0  0  0
  1  2  1  0  0  0  0
  1  3  1  0  0  0  0
  1  4  2  0  0  0  0
  2  5  1  0  0  0  0
  3  6  1  0  0  0  0
  6  7  1  0  0  0  0
  7  8  1  0  0  0  0
  8  9  1  0  0  0  0
  9 10  1  0  0  0  0
 10 11  1  0  0  0  0
 11 12  1  0  0  0  0
 12 13  1  0  0  0  0
 13 14  1  0  0  0  0
 14 15  1  0  0  0  0
 15 16  1  0  0  0  0
 16 17  1  0  0  0  0
 17 18  1  0  0  0  0
 18 19  1  0  0  0  0
  3 20  1  0  0  0  0
  3 21  1  0  0  0  0
  5 22  1  0  0  0  0
  5 23  1  0  0  0  0
  5 24  1  0  0  0  0
  6 25  1  0  0  0  0
  6 26  1  0  0  0  0
  7 27  1  0  0  0  0
  7 28  1  0  0  0  0
  8 29  1  0  0  0  0
  8 30  1  0  0  0  0
  9 31  1  0  0  0  0
  9 32  1  0  0  0  0
 10 33  1  0  0  0  0
 10 34  1  0  0  0  0
 11 35  1  0  0  0  0
 11 36  1  0  0  0  0
 12 37  1  0  0  0  0
 12 38  1  0  0  0  0
 13 39  1  0  0  0  0
 13 40  1  0  0  0  0
 14 41  1  0  0  0  0
 14 42  1  0  0  0  0
```

 15 43 1 0 0 0 0
 15 44 1 0 0 0 0
 16 45 1 0 0 0 0
 16 46 1 0 0 0 0
 17 47 1 0 0 0 0
 17 48 1 0 0 0 0
 18 49 1 0 0 0 0
 18 50 1 0 0 0 0
 19 51 1 0 0 0 0
 19 52 1 0 0 0 0
 19 53 1 0 0 0 0
M END
> <Alias name>
Methyl hexadecanoate

> <deltaH°(c)>   kJ/mol
-10718.33

$$$$
2,4,6-Triphenyltriazine
  ChemBrain IXL

 39 42 0 0 0 0 0 0 0 0 1 V2000
    6.9604   8.5982   0.0000 C   0 0 0 0 0 0 0 0 0 0 0 0 0
    5.5866   9.1487   -0.0006 C   0 0 0 0 0 0 0 0 0 0 0 0 0
    8.0188   9.4298   0.0000 N   0 0 0 0 0 0 0 0 0 0 0 0 0
    7.1515   7.2658   0.0005 N   0 0 0 0 0 0 0 0 0 0 0 0 0
    5.3886   10.5296   -0.0031 C   0 0 0 0 0 0 0 0 0 0 0 0 0
    4.4898   8.2868   0.0013 C   0 0 0 0 0 0 0 0 0 0 0 0 0
    9.2681   8.9291   0.0005 C   0 0 0 0 0 0 0 0 0 0 0 0 0
    8.4009   6.7651   0.0010 C   0 0 0 0 0 0 0 0 0 0 0 0 0
    4.0938   11.0486   -0.0037 C   0 0 0 0 0 0 0 0 0 0 0 0 0
    3.1949   8.8058   0.0008 C   0 0 0 0 0 0 0 0 0 0 0 0 0
   10.4318   9.8435   0.0005 C   0 0 0 0 0 0 0 0 0 0 0 0 0
    9.4592   7.5967   0.0010 N   0 0 0 0 0 0 0 0 0 0 0 0 0
    8.6109   5.3000   0.0016 C   0 0 0 0 0 0 0 0 0 0 0 0 0
    2.9969   10.1867   -0.0017 C   0 0 0 0 0 0 0 0 0 0 0 0 0
   10.2338   11.2244   0.0031 C   0 0 0 0 0 0 0 0 0 0 0 0 0
   11.7267   9.3246   -0.0022 C   0 0 0 0 0 0 0 0 0 0 0 0 0
    9.9058   4.7811   -0.0048 C   0 0 0 0 0 0 0 0 0 0 0 0 0
    7.5141   4.4381   0.0085 C   0 0 0 0 0 0 0 0 0 0 0 0 0
   11.3307   12.0863   0.0031 C   0 0 0 0 0 0 0 0 0 0 0 0 0
   12.8236   10.1865   -0.0023 C   0 0 0 0 0 0 0 0 0 0 0 0 0
   10.1038   3.4002   -0.0043 C   0 0 0 0 0 0 0 0 0 0 0 0 0
    7.7121   3.0572   0.0091 C   0 0 0 0 0 0 0 0 0 0 0 0 0
   12.6256   11.5673   0.0004 C   0 0 0 0 0 0 0 0 0 0 0 0 0
    9.0070   2.5383   0.0026 C   0 0 0 0 0 0 0 0 0 0 0 0 0
    6.2402   11.1988   -0.0045 H   0 0 0 0 0 0 0 0 0 0 0 0 0
    4.6435   7.2148   0.0033 H   0 0 0 0 0 0 0 0 0 0 0 0 0
    3.9400   12.1206   -0.0056 H   0 0 0 0 0 0 0 0 0 0 0 0 0
    2.3433   8.1367   0.0023 H   0 0 0 0 0 0 0 0 0 0 0 0 0
    1.9916   10.5896   -0.0022 H   0 0 0 0 0 0 0 0 0 0 0 0 0
    9.2286   11.6273   0.0052 H   0 0 0 0 0 0 0 0 0 0 0 0 0
   11.8804   8.2525   -0.0043 H   0 0 0 0 0 0 0 0 0 0 0 0 0
   10.7573   5.4502   -0.0102 H   0 0 0 0 0 0 0 0 0 0 0 0 0
    6.5088   4.8410   0.0135 H   0 0 0 0 0 0 0 0 0 0 0 0 0
   11.1770   13.1583   0.0052 H   0 0 0 0 0 0 0 0 0 0 0 0 0
   13.8289   9.7836   -0.0044 H   0 0 0 0 0 0 0 0 0 0 0 0 0
   11.1091   2.9973   -0.0093 H   0 0 0 0 0 0 0 0 0 0 0 0 0
    6.8606   2.3881   0.0145 H   0 0 0 0 0 0 0 0 0 0 0 0 0
   13.4771   12.2365   0.0003 H   0 0 0 0 0 0 0 0 0 0 0 0 0
    9.1607   1.4663   0.0030 H   0 0 0 0 0 0 0 0 0 0 0 0 0
  1 2 1 0 0 0 0

```
  1  3  4  0  0  0  0
  1  4  4  0  0  0  0
  2  5  4  0  0  0  0
  2  6  4  0  0  0  0
  3  7  4  0  0  0  0
  4  8  4  0  0  0  0
  5  9  4  0  0  0  0
  6 10  4  0  0  0  0
  7 11  1  0  0  0  0
  7 12  4  0  0  0  0
  8 13  1  0  0  0  0
  8 12  4  0  0  0  0
  9 14  4  0  0  0  0
 10 14  4  0  0  0  0
 11 15  4  0  0  0  0
 11 16  4  0  0  0  0
 13 17  4  0  0  0  0
 13 18  4  0  0  0  0
 15 19  4  0  0  0  0
 16 20  4  0  0  0  0
 17 21  4  0  0  0  0
 18 22  4  0  0  0  0
 19 23  4  0  0  0  0
 20 23  4  0  0  0  0
 21 24  4  0  0  0  0
 22 24  4  0  0  0  0
  5 25  1  0  0  0  0
  6 26  1  0  0  0  0
  9 27  1  0  0  0  0
 10 28  1  0  0  0  0
 14 29  1  0  0  0  0
 15 30  1  0  0  0  0
 16 31  1  0  0  0  0
 17 32  1  0  0  0  0
 18 33  1  0  0  0  0
 19 34  1  0  0  0  0
 20 35  1  0  0  0  0
 21 36  1  0  0  0  0
 22 37  1  0  0  0  0
 23 38  1  0  0  0  0
 24 39  1  0  0  0  0
M  END
>  <Alias name>
2,4,6-Triphenyltriazine

>  <deltaH°(c)>   kJ/mol
-10717.5

$$$$
1,2'-Dinaphthylmethane
  ChemBrain IXL

 37 40  0  0  0  0  0  0  0  0  1 V2000
    9.1246    7.1133   -0.5396 C   0  0  0  0  0  0  0  0  0  0  0  0
    8.8736    5.7475   -0.0276 C   0  0  0  0  0  0  0  0  0  0  0  0
    7.4151    5.5247    0.0896 C   0  0  0  0  0  0  0  0  0  0  0  0
   10.3431    7.7418   -0.2819 C   0  0  0  0  0  0  0  0  0  0  0  0
    8.1428    7.7721   -1.2799 C   0  0  0  0  0  0  0  0  0  0  0  0
    6.6301    6.4154    0.8221 C   0  0  0  0  0  0  0  0  0  0  0  0
    6.8254    4.4241   -0.5323 C   0  0  0  0  0  0  0  0  0  0  0  0
   10.5794    9.0294   -0.7639 C   0  0  0  0  0  0  0  0  0  0  0  0
   11.3252    7.0827    0.4576 C   0  0  0  0  0  0  0  0  0  0  0  0
    8.3791    9.0597   -1.7619 C   0  0  0  0  0  0  0  0  0  0  0  0
```

```
    5.2556    6.2048    0.9335 C   0  0  0  0  0  0  0  0  0  0  0  0
    5.4509    4.2134   -0.4209 C   0  0  0  0  0  0  0  0  0  0  0  0
    9.5974    9.6884   -1.5038 C   0  0  0  0  0  0  0  0  0  0  0  0
   11.7977    9.6581   -0.5058 C   0  0  0  0  0  0  0  0  0  0  0  0
   12.5435    7.7114    0.7156 C   0  0  0  0  0  0  0  0  0  0  0  0
    4.6660    5.1038    0.3121 C   0  0  0  0  0  0  0  0  0  0  0  0
    4.4706    7.0952    1.6664 C   0  0  0  0  0  0  0  0  0  0  0  0
   12.7798    8.9991    0.2339 C   0  0  0  0  0  0  0  0  0  0  0  0
    3.2915    4.8932    0.4236 C   0  0  0  0  0  0  0  0  0  0  0  0
    3.0961    6.8846    1.7779 C   0  0  0  0  0  0  0  0  0  0  0  0
    2.5066    5.7835    1.1565 C   0  0  0  0  0  0  0  0  0  0  0  0
    9.3352    5.6364    0.9458 H   0  0  0  0  0  0  0  0  0  0  0  0
    9.2957    5.0221   -0.7122 H   0  0  0  0  0  0  0  0  0  0  0  0
    7.1971    7.2840   -1.4804 H   0  0  0  0  0  0  0  0  0  0  0  0
    7.0878    7.2703    1.3043 H   0  0  0  0  0  0  0  0  0  0  0  0
    7.4347    3.7331   -1.1017 H   0  0  0  0  0  0  0  0  0  0  0  0
   11.1419    6.0830    0.8315 H   0  0  0  0  0  0  0  0  0  0  0  0
    7.6168    9.5713   -2.3364 H   0  0  0  0  0  0  0  0  0  0  0  0
    4.9932    3.3587   -0.9033 H   0  0  0  0  0  0  0  0  0  0  0  0
    9.7808   10.6881   -1.8778 H   0  0  0  0  0  0  0  0  0  0  0  0
   11.9811   10.6578   -0.8798 H   0  0  0  0  0  0  0  0  0  0  0  0
   13.3060    7.1997    1.2899 H   0  0  0  0  0  0  0  0  0  0  0  0
    4.9283    7.9501    2.1487 H   0  0  0  0  0  0  0  0  0  0  0  0
   13.7256    9.4871    0.4343 H   0  0  0  0  0  0  0  0  0  0  0  0
    2.8338    4.0384   -0.0588 H   0  0  0  0  0  0  0  0  0  0  0  0
    2.4868    7.5759    2.3468 H   0  0  0  0  0  0  0  0  0  0  0  0
    1.4395    5.6200    1.2431 H   0  0  0  0  0  0  0  0  0  0  0  0
  1  2  1  0  0  0  0
  4  1  4  0  0  0  0
  1  5  4  0  0  0  0
  2  3  1  0  0  0  0
  3  6  4  0  0  0  0
  7  3  4  0  0  0  0
  8  4  4  0  0  0  0
  9  4  4  0  0  0  0
  5 10  4  0  0  0  0
  6 11  4  0  0  0  0
 12  7  4  0  0  0  0
 13  8  4  0  0  0  0
  8 14  4  0  0  0  0
 15  9  4  0  0  0  0
 10 13  4  0  0  0  0
 16 11  4  0  0  0  0
 11 17  4  0  0  0  0
 16 12  4  0  0  0  0
 14 18  4  0  0  0  0
 18 15  4  0  0  0  0
 19 16  4  0  0  0  0
 17 20  4  0  0  0  0
 21 19  4  0  0  0  0
 20 21  4  0  0  0  0
  2 22  1  0  0  0  0
  2 23  1  0  0  0  0
  5 24  1  0  0  0  0
  6 25  1  0  0  0  0
  7 26  1  0  0  0  0
  9 27  1  0  0  0  0
 10 28  1  0  0  0  0
 12 29  1  0  0  0  0
 13 30  1  0  0  0  0
 14 31  1  0  0  0  0
 15 32  1  0  0  0  0
 17 33  1  0  0  0  0
```

```
 18 34  1  0  0  0  0
 19 35  1  0  0  0  0
 20 36  1  0  0  0  0
 21 37  1  0  0  0  0
M  END
>  <Alias name>
1,2'-Dinaphthylmethane

>  <deltaH°(c)>   kJ/mol
-10712

$$$$
Hexadecane
  ChemBrain IXL

 50 49  0  0  0  0  0  0  0  0  1 V2000
   11.0854    7.3547   -3.4109 C   0  0  0  0  0  0  0  0  0  0  0  0
   10.2173    8.1955   -2.4547 C   0  0  0  0  0  0  0  0  0  0  0  0
    9.4779    7.2617   -1.4768 C   0  0  0  0  0  0  0  0  0  0  0  0
    8.6110    8.1026   -0.5197 C   0  0  0  0  0  0  0  0  0  0  0  0
    7.8728    7.1688    0.4592 C   0  0  0  0  0  0  0  0  0  0  0  0
    7.0071    8.0098    1.4175 C   0  0  0  0  0  0  0  0  0  0  0  0
    6.2702    7.0762    2.3973 C   0  0  0  0  0  0  0  0  0  0  0  0
    5.4057    7.9173    3.3565 C   0  0  0  0  0  0  0  0  0  0  0  0
    4.6696    6.9837    4.3371 C   0  0  0  0  0  0  0  0  0  0  0  0
   11.8256    8.2887   -4.3881 C   0  0  0  0  0  0  0  0  0  0  0  0
    3.8058    7.8249    5.2970 C   0  0  0  0  0  0  0  0  0  0  0  0
   12.6946    7.4480   -5.3436 C   0  0  0  0  0  0  0  0  0  0  0  0
    3.0701    6.8914    6.2780 C   0  0  0  0  0  0  0  0  0  0  0  0
   13.4352    8.3820   -6.3204 C   0  0  0  0  0  0  0  0  0  0  0  0
    2.2068    7.7325    7.2381 C   0  0  0  0  0  0  0  0  0  0  0  0
   14.3045    7.5413   -7.2757 C   0  0  0  0  0  0  0  0  0  0  0  0
   10.4550    6.6774   -3.9679 H   0  0  0  0  0  0  0  0  0  0  0  0
   11.8056    6.7883   -2.8391 H   0  0  0  0  0  0  0  0  0  0  0  0
    9.4966    8.7615   -3.0264 H   0  0  0  0  0  0  0  0  0  0  0  0
   10.8476    8.8733   -1.8982 H   0  0  0  0  0  0  0  0  0  0  0  0
    8.8469    6.5845   -2.0332 H   0  0  0  0  0  0  0  0  0  0  0  0
   10.1986    6.6951   -0.9058 H   0  0  0  0  0  0  0  0  0  0  0  0
    9.2420    8.7804    0.0360 H   0  0  0  0  0  0  0  0  0  0  0  0
    7.8897    8.6685   -1.0905 H   0  0  0  0  0  0  0  0  0  0  0  0
    7.2410    6.4917   -0.0963 H   0  0  0  0  0  0  0  0  0  0  0  0
    8.5941    6.6022    1.0293 H   0  0  0  0  0  0  0  0  0  0  0  0
    6.2852    8.5759    0.8475 H   0  0  0  0  0  0  0  0  0  0  0  0
    7.6390    8.6876    1.9723 H   0  0  0  0  0  0  0  0  0  0  0  0
    5.6376    6.3990    1.8426 H   0  0  0  0  0  0  0  0  0  0  0  0
    6.9922    6.5096    2.9666 H   0  0  0  0  0  0  0  0  0  0  0  0
    4.6832    8.4835    2.7874 H   0  0  0  0  0  0  0  0  0  0  0  0
    6.0382    8.5949    3.9107 H   0  0  0  0  0  0  0  0  0  0  0  0
    5.3921    6.4172    4.9058 H   0  0  0  0  0  0  0  0  0  0  0  0
    4.0366    6.3065    3.7830 H   0  0  0  0  0  0  0  0  0  0  0  0
   12.4555    8.9664   -3.8309 H   0  0  0  0  0  0  0  0  0  0  0  0
   11.1055    8.8547   -4.9603 H   0  0  0  0  0  0  0  0  0  0  0  0
    3.0831    8.3912    4.7284 H   0  0  0  0  0  0  0  0  0  0  0  0
    4.4388    8.5023    5.8509 H   0  0  0  0  0  0  0  0  0  0  0  0
   12.0647    6.7705   -5.9010 H   0  0  0  0  0  0  0  0  0  0  0  0
   13.4145    6.8817   -4.7713 H   0  0  0  0  0  0  0  0  0  0  0  0
    3.7929    6.3249    6.8464 H   0  0  0  0  0  0  0  0  0  0  0  0
    2.4370    6.2141    5.7241 H   0  0  0  0  0  0  0  0  0  0  0  0
   14.0649    9.0596   -5.7630 H   0  0  0  0  0  0  0  0  0  0  0  0
   12.7154    8.9481   -6.8929 H   0  0  0  0  0  0  0  0  0  0  0  0
    2.8399    8.4098    7.7919 H   0  0  0  0  0  0  0  0  0  0  0  0
    1.4840    8.2990    6.6697 H   0  0  0  0  0  0  0  0  0  0  0  0
    1.6912    7.0783    7.9255 H   0  0  0  0  0  0  0  0  0  0  0  0
```

```
   15.0243    6.9752   -6.7032 H   0  0  0  0  0  0  0  0  0  0  0  0
   13.6748    6.8637   -7.8331 H   0  0  0  0  0  0  0  0  0  0  0  0
   14.8235    8.1959   -7.9602 H   0  0  0  0  0  0  0  0  0  0  0  0
  1  2  1  0  0  0  0
  2  3  1  0  0  0  0
  3  4  1  0  0  0  0
  4  5  1  0  0  0  0
  5  6  1  0  0  0  0
  6  7  1  0  0  0  0
  7  8  1  0  0  0  0
  8  9  1  0  0  0  0
  1 17  1  0  0  0  0
  1 18  1  0  0  0  0
  1 10  1  0  0  0  0
  2 19  1  0  0  0  0
  2 20  1  0  0  0  0
  3 21  1  0  0  0  0
  3 22  1  0  0  0  0
  4 23  1  0  0  0  0
  4 24  1  0  0  0  0
  5 25  1  0  0  0  0
  5 26  1  0  0  0  0
  6 27  1  0  0  0  0
  6 28  1  0  0  0  0
  7 29  1  0  0  0  0
  7 30  1  0  0  0  0
  8 31  1  0  0  0  0
  8 32  1  0  0  0  0
  9 33  1  0  0  0  0
  9 34  1  0  0  0  0
  9 11  1  0  0  0  0
 10 35  1  0  0  0  0
 10 12  1  0  0  0  0
 10 36  1  0  0  0  0
 11 13  1  0  0  0  0
 12 14  1  0  0  0  0
 11 37  1  0  0  0  0
 11 38  1  0  0  0  0
 12 39  1  0  0  0  0
 12 40  1  0  0  0  0
 13 15  1  0  0  0  0
 13 41  1  0  0  0  0
 13 42  1  0  0  0  0
 14 16  1  0  0  0  0
 14 43  1  0  0  0  0
 14 44  1  0  0  0  0
 15 45  1  0  0  0  0
 15 46  1  0  0  0  0
 15 47  1  0  0  0  0
 16 48  1  0  0  0  0
 16 49  1  0  0  0  0
 16 50  1  0  0  0  0
M  END
>  <Alias name>
Cetane

>  <deltaH°(c)>   kJ/mol
-10699

$$$$
2,2,4,4,6,8,8-Heptamethylnonane
  ChemBrain IXL
```

```
50 49  0  0  0  0  0  0  0  0  1 V2000
  11.4777    5.9367   -2.0669 C   0  0  0  0  0  0  0  0  0  0  0  0
  10.0904    6.5917   -1.9149 C   0  0  0  0  0  0  0  0  0  0  0  0
   9.1114    5.5597   -1.2634 C   0  0  0  0  0  0  0  0  0  0  0  0
   7.6642    6.1284   -1.1785 C   0  0  0  0  0  0  0  0  0  0  0  0
   6.8911    5.3503   -0.0743 C   0  0  0  0  0  0  0  0  0  0  0  0
   5.7067    6.2005    0.4970 C   0  0  0  0  0  0  0  0  0  0  0  0
   4.8748    5.2750    1.4796 C   0  0  0  0  0  0  0  0  0  0  0  0
   3.4547    5.8623    1.8599 C   0  0  0  0  0  0  0  0  0  0  0  0
   2.9269    5.0931    3.0873 C   0  0  0  0  0  0  0  0  0  0  0  0
   2.4523    5.6567    0.6897 C   0  0  0  0  0  0  0  0  0  0  0  0
   3.5433    7.3604    2.2205 C   0  0  0  0  0  0  0  0  0  0  0  0
   4.8286    6.7233   -0.6545 C   0  0  0  0  0  0  0  0  0  0  0  0
   6.3017    7.4051    1.2770 C   0  0  0  0  0  0  0  0  0  0  0  0
   6.9452    5.9286   -2.5538 C   0  0  0  0  0  0  0  0  0  0  0  0
   9.5857    7.0302   -3.3010 C   0  0  0  0  0  0  0  0  0  0  0  0
  10.2141    7.8314   -1.0041 C   0  0  0  0  0  0  0  0  0  0  0  0
  12.1660    6.6471   -2.5078 H   0  0  0  0  0  0  0  0  0  0  0  0
  11.3979    5.0666   -2.7067 H   0  0  0  0  0  0  0  0  0  0  0  0
  11.8437    5.6363   -1.0929 H   0  0  0  0  0  0  0  0  0  0  0  0
   9.4609    5.3382   -0.2625 H   0  0  0  0  0  0  0  0  0  0  0  0
   9.1031    4.6467   -1.8459 H   0  0  0  0  0  0  0  0  0  0  0  0
   7.7032    7.1764   -0.9205 H   0  0  0  0  0  0  0  0  0  0  0  0
   7.5715    5.1201    0.7363 H   0  0  0  0  0  0  0  0  0  0  0  0
   6.5099    4.4258   -0.4902 H   0  0  0  0  0  0  0  0  0  0  0  0
   5.4493    5.1373    2.3873 H   0  0  0  0  0  0  0  0  0  0  0  0
   4.7308    4.3094    1.0108 H   0  0  0  0  0  0  0  0  0  0  0  0
   2.8493    4.0399    2.8473 H   0  0  0  0  0  0  0  0  0  0  0  0
   3.6102    5.2249    3.9172 H   0  0  0  0  0  0  0  0  0  0  0  0
   1.9504    5.4742    3.3598 H   0  0  0  0  0  0  0  0  0  0  0  0
   2.3846    4.6012    0.4570 H   0  0  0  0  0  0  0  0  0  0  0  0
   1.4766    6.0217    0.9857 H   0  0  0  0  0  0  0  0  0  0  0  0
   2.7806    6.1895   -0.1762 H   0  0  0  0  0  0  0  0  0  0  0  0
   2.5770    7.7019    2.5705 H   0  0  0  0  0  0  0  0  0  0  0  0
   3.8260    7.9283    1.3518 H   0  0  0  0  0  0  0  0  0  0  0  0
   4.2787    7.5010    3.0031 H   0  0  0  0  0  0  0  0  0  0  0  0
   4.4323    5.8882   -1.2191 H   0  0  0  0  0  0  0  0  0  0  0  0
   4.0207    7.3061   -0.2554 H   0  0  0  0  0  0  0  0  0  0  0  0
   5.4246    7.3493   -1.3009 H   0  0  0  0  0  0  0  0  0  0  0  0
   5.5141    8.0169    1.6644 H   0  0  0  0  0  0  0  0  0  0  0  0
   6.9169    7.9956    0.6112 H   0  0  0  0  0  0  0  0  0  0  0  0
   6.9084    7.0387    2.0957 H   0  0  0  0  0  0  0  0  0  0  0  0
   6.9446    4.8754   -2.8056 H   0  0  0  0  0  0  0  0  0  0  0  0
   5.9341    6.2762   -2.4931 H   0  0  0  0  0  0  0  0  0  0  0  0
   7.4594    6.4731   -3.3178 H   0  0  0  0  0  0  0  0  0  0  0  0
   9.3771    6.1559   -3.9051 H   0  0  0  0  0  0  0  0  0  0  0  0
   8.6875    7.6184   -3.1874 H   0  0  0  0  0  0  0  0  0  0  0  0
  10.3456    7.6306   -3.7857 H   0  0  0  0  0  0  0  0  0  0  0  0
   9.2460    8.3039   -0.9022 H   0  0  0  0  0  0  0  0  0  0  0  0
  10.9129    8.5329   -1.4428 H   0  0  0  0  0  0  0  0  0  0  0  0
  10.5723    7.5280   -0.0281 H   0  0  0  0  0  0  0  0  0  0  0  0
  1  2  1  0  0  0  0
  2  3  1  0  0  0  0
  3  4  1  0  0  0  0
  4  5  1  0  0  0  0
  5  6  1  0  0  0  0
  6  7  1  0  0  0  0
  7  8  1  0  0  0  0
  8  9  1  0  0  0  0
  8 10  1  0  0  0  0
  8 11  1  0  0  0  0
  6 12  1  0  0  0  0
  6 13  1  0  0  0  0
```

```
  4 14  1  0  0  0  0
  2 15  1  0  0  0  0
  2 16  1  0  0  0  0
  1 17  1  0  0  0  0
  1 18  1  0  0  0  0
  1 19  1  0  0  0  0
  3 20  1  0  0  0  0
  3 21  1  0  0  0  0
  4 22  1  0  0  0  0
  5 23  1  0  0  0  0
  5 24  1  0  0  0  0
  7 25  1  0  0  0  0
  7 26  1  0  0  0  0
  9 27  1  0  0  0  0
  9 28  1  0  0  0  0
  9 29  1  0  0  0  0
 10 30  1  0  0  0  0
 10 31  1  0  0  0  0
 10 32  1  0  0  0  0
 11 33  1  0  0  0  0
 11 34  1  0  0  0  0
 11 35  1  0  0  0  0
 12 36  1  0  0  0  0
 12 37  1  0  0  0  0
 12 38  1  0  0  0  0
 13 39  1  0  0  0  0
 13 40  1  0  0  0  0
 13 41  1  0  0  0  0
 14 42  1  0  0  0  0
 14 43  1  0  0  0  0
 14 44  1  0  0  0  0
 15 45  1  0  0  0  0
 15 46  1  0  0  0  0
 15 47  1  0  0  0  0
 16 48  1  0  0  0  0
 16 49  1  0  0  0  0
 16 50  1  0  0  0  0
M  END
>  <Alias name>
2,2,4,4,6,8,8-Heptamethylnonane

>  <deltaH°(c)>   kJ/mol
-10685.85

$$$$
N,N-Dibutylaminotriethylsilane
  ChemBrain IXL

 49 48  0  0  0  0  0  0  0  0  1 V2000
    4.7377    4.6580   -0.3185 Si  0  0  0  0  0  0  0  0  0  0  0  0
    5.7757    5.8014    0.0940 N   0  0  0  0  0  0  0  0  0  0  0  0
    3.4144    5.3573   -1.4443 C   0  0  0  0  0  0  0  0  0  0  0  0
    3.9056    3.9811    1.2287 C   0  0  0  0  0  0  0  0  0  0  0  0
    5.6267    3.2942   -1.2637 C   0  0  0  0  0  0  0  0  0  0  0  0
    5.0857    7.1168    0.1820 C   0  0  0  0  0  0  0  0  0  0  0  0
    6.8586    5.8774   -0.9134 C   0  0  0  0  0  0  0  0  0  0  0  0
    3.9705    7.0682    1.2492 C   0  0  0  0  0  0  0  0  0  0  0  0
    7.8801    6.9593   -0.5026 C   0  0  0  0  0  0  0  0  0  0  0  0
    6.7081    2.6354   -0.3805 C   0  0  0  0  0  0  0  0  0  0  0  0
    4.0586    5.8830   -2.7445 C   0  0  0  0  0  0  0  0  0  0  0  0
    4.9669    3.5008    2.2404 C   0  0  0  0  0  0  0  0  0  0  0  0
    3.3407    8.4667    1.3950 C   0  0  0  0  0  0  0  0  0  0  0  0
    2.2267    8.4201    2.4585 C   0  0  0  0  0  0  0  0  0  0  0  0
```

9.0267	6.9982	-1.5314	C	0	0	0	0	0	0	0	0	0	0	0	0	
10.0479	8.0772	-1.1222	C	0	0	0	0	0	0	0	0	0	0	0	0	
2.7036	4.5776	-1.6878	H	0	0	0	0	0	0	0	0	0	0	0	0	
2.9015	6.1667	-0.9422	H	0	0	0	0	0	0	0	0	0	0	0	0	
3.2598	3.1546	0.9595	H	0	0	0	0	0	0	0	0	0	0	0	0	
3.3149	4.7597	1.6855	H	0	0	0	0	0	0	0	0	0	0	0	0	
4.9098	2.5428	-1.5701	H	0	0	0	0	0	0	0	0	0	0	0	0	
6.0949	3.7200	-2.1424	H	0	0	0	0	0	0	0	0	0	0	0	0	
5.8035	7.8765	0.4578	H	0	0	0	0	0	0	0	0	0	0	0	0	
4.6545	7.3653	-0.7768	H	0	0	0	0	0	0	0	0	0	0	0	0	
7.3588	4.9236	-0.9771	H	0	0	0	0	0	0	0	0	0	0	0	0	
6.4393	6.1271	-1.8779	H	0	0	0	0	0	0	0	0	0	0	0	0	
3.2112	6.3651	0.9465	H	0	0	0	0	0	0	0	0	0	0	0	0	
4.3915	6.7614	2.1986	H	0	0	0	0	0	0	0	0	0	0	0	0	
7.3932	7.9243	-0.4711	H	0	0	0	0	0	0	0	0	0	0	0	0	
8.2810	6.7244	0.4755	H	0	0	0	0	0	0	0	0	0	0	0	0	
7.3590	3.3962	0.0278	H	0	0	0	0	0	0	0	0	0	0	0	0	
7.2902	1.9470	-0.9803	H	0	0	0	0	0	0	0	0	0	0	0	0	
6.2381	2.0960	0.4204	H	0	0	0	0	0	0	0	0	0	0	0	0	
3.2859	6.2641	-3.4005	H	0	0	0	0	0	0	0	0	0	0	0	0	
4.5849	5.0756	-3.2385	H	0	0	0	0	0	0	0	0	0	0	0	0	
4.7537	6.6759	-2.5101	H	0	0	0	0	0	0	0	0	0	0	0	0	
5.6425	4.3158	2.4691	H	0	0	0	0	0	0	0	0	0	0	0	0	
5.5200	2.6834	1.8242	H	0	0	0	0	0	0	0	0	0	0	0	0	
4.4755	3.1750	3.1486	H	0	0	0	0	0	0	0	0	0	0	0	0	
4.1011	9.1752	1.6995	H	0	0	0	0	0	0	0	0	0	0	0	0	
2.9205	8.7741	0.4454	H	0	0	0	0	0	0	0	0	0	0	0	0	
1.4663	7.7116	2.1540	H	0	0	0	0	0	0	0	0	0	0	0	0	
2.6469	8.1128	3.4082	H	0	0	0	0	0	0	0	0	0	0	0	0	
1.7840	9.4031	2.5608	H	0	0	0	0	0	0	0	0	0	0	0	0	
9.5156	6.0324	-1.5638	H	0	0	0	0	0	0	0	0	0	0	0	0	
8.6261	7.2329	-2.5098	H	0	0	0	0	0	0	0	0	0	0	0	0	
9.5591	9.0431	-1.0899	H	0	0	0	0	0	0	0	0	0	0	0	0	
10.8536	8.1044	-1.8453	H	0	0	0	0	0	0	0	0	0	0	0	0	
10.4487	7.8425	-0.1439	H	0	0	0	0	0	0	0	0	0	0	0	0	
1	2	1	0	0	0	0										
1	3	1	0	0	0	0										
1	4	1	0	0	0	0										
1	5	1	0	0	0	0										
2	6	1	0	0	0	0										
2	7	1	0	0	0	0										
6	8	1	0	0	0	0										
7	9	1	0	0	0	0										
5	10	1	0	0	0	0										
3	11	1	0	0	0	0										
4	12	1	0	0	0	0										
8	13	1	0	0	0	0										
13	14	1	0	0	0	0										
9	15	1	0	0	0	0										
15	16	1	0	0	0	0										
3	17	1	0	0	0	0										
3	18	1	0	0	0	0										
4	19	1	0	0	0	0										
4	20	1	0	0	0	0										
5	21	1	0	0	0	0										
5	22	1	0	0	0	0										
6	23	1	0	0	0	0										
6	24	1	0	0	0	0										
7	25	1	0	0	0	0										
7	26	1	0	0	0	0										
8	27	1	0	0	0	0										
8	28	1	0	0	0	0										
9	29	1	0	0	0	0										

 9 30 1 0 0 0 0
10 31 1 0 0 0 0
10 32 1 0 0 0 0
10 33 1 0 0 0 0
11 34 1 0 0 0 0
11 35 1 0 0 0 0
11 36 1 0 0 0 0
12 37 1 0 0 0 0
12 38 1 0 0 0 0
12 39 1 0 0 0 0
13 40 1 0 0 0 0
13 41 1 0 0 0 0
14 42 1 0 0 0 0
14 43 1 0 0 0 0
14 44 1 0 0 0 0
15 45 1 0 0 0 0
15 46 1 0 0 0 0
16 47 1 0 0 0 0
16 48 1 0 0 0 0
16 49 1 0 0 0 0
M END
> <Alias name>
N,N-Dibutylaminotriethylsilane

> <deltaH°(c)>   kJ/mol
-10684

$$$$
N-Octylaminotriethylsilane
  ChemBrain IXL

 49 48 0 0 0 0 0 0 0 0 1 V2000
   13.3984   8.0185   -1.1614 Si 0 0 0 0 0 0 0 0 0 0 0 0
   12.3701   7.8962   0.0254 N   0 0 0 0 0 0 0 0 0 0 0 0
   13.0755   9.6042   -2.0885 C   0 0 0 0 0 0 0 0 0 0 0 0
   13.1608   6.5847   -2.3495 C   0 0 0 0 0 0 0 0 0 0 0 0
   15.1403   8.0911   -0.4667 C   0 0 0 0 0 0 0 0 0 0 0 0
   10.9988   7.8696   -0.5092 C   0 0 0 0 0 0 0 0 0 0 0 0
    9.9972   7.7520   0.6554 C   0 0 0 0 0 0 0 0 0 0 0 0
   15.4321   6.8375   0.3820 C   0 0 0 0 0 0 0 0 0 0 0 0
   13.2340   10.8006   -1.1307 C   0 0 0 0 0 0 0 0 0 0 0 0
   13.3273   5.2423   -1.6099 C   0 0 0 0 0 0 0 0 0 0 0 0
    8.5611   7.7252   0.0971 C   0 0 0 0 0 0 0 0 0 0 0 0
    7.5591   7.6079   1.2621 C   0 0 0 0 0 0 0 0 0 0 0 0
    6.1229   7.5818   0.7039 C   0 0 0 0 0 0 0 0 0 0 0 0
    5.1208   7.4646   1.8689 C   0 0 0 0 0 0 0 0 0 0 0 0
    3.6845   7.4389   1.3107 C   0 0 0 0 0 0 0 0 0 0 0 0
    2.6824   7.3217   2.4756 C   0 0 0 0 0 0 0 0 0 0 0 0
   12.5478   7.0455   0.5379 H   0 0 0 0 0 0 0 0 0 0 0 0
   13.7838   9.6970   -2.9024 H   0 0 0 0 0 0 0 0 0 0 0 0
   12.0686   9.5913   -2.4863 H   0 0 0 0 0 0 0 0 0 0 0 0
   13.8898   6.6515   -3.1475 H   0 0 0 0 0 0 0 0 0 0 0 0
   12.1644   6.6350   -2.7701 H   0 0 0 0 0 0 0 0 0 0 0 0
   15.2357   8.9706   0.1578 H   0 0 0 0 0 0 0 0 0 0 0 0
   15.8521   8.1511   -1.2805 H   0 0 0 0 0 0 0 0 0 0 0 0
   10.8067   8.7827   -1.0587 H   0 0 0 0 0 0 0 0 0 0 0 0
   10.8843   7.0193   -1.1701 H   0 0 0 0 0 0 0 0 0 0 0 0
   10.1882   6.8385   1.2048 H   0 0 0 0 0 0 0 0 0 0 0 0
   10.1116   8.6018   1.3169 H   0 0 0 0 0 0 0 0 0 0 0 0
   16.4079   6.9343   0.8414 H   0 0 0 0 0 0 0 0 0 0 0 0
   15.4203   5.9665   -0.2449 H   0 0 0 0 0 0 0 0 0 0 0 0
   14.6787   6.7396   1.1536 H   0 0 0 0 0 0 0 0 0 0 0 0
   12.5261   10.7073   -0.3163 H   0 0 0 0 0 0 0 0 0 0 0 0

```
 14.2415   10.8143   -0.7335 H   0  0  0  0  0  0  0  0  0  0  0  0
 13.0457   11.7212   -1.6689 H   0  0  0  0  0  0  0  0  0  0  0  0
 14.3362    5.1472   -1.2569 H   0  0  0  0  0  0  0  0  0  0  0  0
 13.1081    4.4284   -2.2897 H   0  0  0  0  0  0  0  0  0  0  0  0
 12.6441    5.2047   -0.7705 H   0  0  0  0  0  0  0  0  0  0  0  0
  8.3704    8.6387   -0.4524 H   0  0  0  0  0  0  0  0  0  0  0  0
  8.4468    6.8754   -0.5644 H   0  0  0  0  0  0  0  0  0  0  0  0
  7.7495    6.6941    1.8114 H   0  0  0  0  0  0  0  0  0  0  0  0
  7.6738    8.4574    1.9239 H   0  0  0  0  0  0  0  0  0  0  0  0
  5.9325    8.4956    0.1546 H   0  0  0  0  0  0  0  0  0  0  0  0
  6.0081    6.7322    0.0422 H   0  0  0  0  0  0  0  0  0  0  0  0
  5.3111    6.5507    2.4181 H   0  0  0  0  0  0  0  0  0  0  0  0
  5.2358    8.3140    2.5308 H   0  0  0  0  0  0  0  0  0  0  0  0
  3.4942    8.3528    0.7616 H   0  0  0  0  0  0  0  0  0  0  0  0
  3.5695    6.5895    0.6489 H   0  0  0  0  0  0  0  0  0  0  0  0
  2.8725    6.4078    3.0247 H   0  0  0  0  0  0  0  0  0  0  0  0
  2.7973    8.1711    3.1375 H   0  0  0  0  0  0  0  0  0  0  0  0
  1.6733    7.3037    2.0831 H   0  0  0  0  0  0  0  0  0  0  0  0
  1  2  1  0  0  0  0
  1  3  1  0  0  0  0
  1  4  1  0  0  0  0
  1  5  1  0  0  0  0
  2  6  1  0  0  0  0
  6  7  1  0  0  0  0
  5  8  1  0  0  0  0
  3  9  1  0  0  0  0
  4 10  1  0  0  0  0
  7 11  1  0  0  0  0
 11 12  1  0  0  0  0
 12 13  1  0  0  0  0
 13 14  1  0  0  0  0
 14 15  1  0  0  0  0
 15 16  1  0  0  0  0
  2 17  1  0  0  0  0
  3 18  1  0  0  0  0
  3 19  1  0  0  0  0
  4 20  1  0  0  0  0
  4 21  1  0  0  0  0
  5 22  1  0  0  0  0
  5 23  1  0  0  0  0
  6 24  1  0  0  0  0
  6 25  1  0  0  0  0
  7 26  1  0  0  0  0
  7 27  1  0  0  0  0
  8 28  1  0  0  0  0
  8 29  1  0  0  0  0
  8 30  1  0  0  0  0
  9 31  1  0  0  0  0
  9 32  1  0  0  0  0
  9 33  1  0  0  0  0
 10 34  1  0  0  0  0
 10 35  1  0  0  0  0
 10 36  1  0  0  0  0
 11 37  1  0  0  0  0
 11 38  1  0  0  0  0
 12 39  1  0  0  0  0
 12 40  1  0  0  0  0
 13 41  1  0  0  0  0
 13 42  1  0  0  0  0
 14 43  1  0  0  0  0
 14 44  1  0  0  0  0
 15 45  1  0  0  0  0
 15 46  1  0  0  0  0
```

16 47 1 0 0 0 0
16 48 1 0 0 0 0
16 49 1 0 0 0 0
M END
> <Alias name>
N-Octylaminotriethylsilane

> <deltaH°(c)>   kJ/mol
-10669

$$$$
N,N-Diisobutylaminotriethylsilane
  ChemBrain IXL

49 48 0 0 0 0 0 0 0 0 1 V2000
5.4784   4.4218   0.5870 Si 0 0 0 0 0 0 0 0 0 0 0 0
6.0066   5.4548   -0.5183 N   0 0 0 0 0 0 0 0 0 0 0 0
3.6834   4.7968   1.0201 C   0 0 0 0 0 0 0 0 0 0 0 0
6.4823   4.5984   2.1579 C   0 0 0 0 0 0 0 0 0 0 0 0
5.6713   2.6571   -0.0454 C   0 0 0 0 0 0 0 0 0 0 0 0
7.4650   5.7068   -0.3325 C   0 0 0 0 0 0 0 0 0 0 0 0
5.2682   6.7331   -0.3948 C   0 0 0 0 0 0 0 0 0 0 0 0
8.2709   4.3992   -0.5104 C   0 0 0 0 0 0 0 0 0 0 0 0
5.7497   7.7229   -1.4780 C   0 0 0 0 0 0 0 0 0 0 0 0
4.8961   2.4701   -1.3678 C   0 0 0 0 0 0 0 0 0 0 0 0
2.7740   4.6258   -0.2176 C   0 0 0 0 0 0 0 0 0 0 0 0
6.2916   6.0148   2.7434 C   0 0 0 0 0 0 0 0 0 0 0 0
9.7683   4.6896   -0.2920 C   0 0 0 0 0 0 0 0 0 0 0 0
4.9413   9.0307   -1.3726 C   0 0 0 0 0 0 0 0 0 0 0 0
5.5403   7.1006   -2.8727 C   0 0 0 0 0 0 0 0 0 0 0 0
8.0604   3.8523   -1.9357 C   0 0 0 0 0 0 0 0 0 0 0 0
3.6116   5.8168   1.3765 H   0 0 0 0 0 0 0 0 0 0 0 0
3.3535   4.1225   1.8005 H   0 0 0 0 0 0 0 0 0 0 0 0
6.1462   3.8658   2.8811 H   0 0 0 0 0 0 0 0 0 0 0 0
7.5292   4.4340   1.9404 H   0 0 0 0 0 0 0 0 0 0 0 0
5.2919   1.9648   0.6958 H   0 0 0 0 0 0 0 0 0 0 0 0
6.7166   2.4537   -0.2194 H   0 0 0 0 0 0 0 0 0 0 0 0
7.6392   6.1022   0.6561 H   0 0 0 0 0 0 0 0 0 0 0 0
7.7984   6.4264   -1.0668 H   0 0 0 0 0 0 0 0 0 0 0 0
4.2135   6.5538   -0.5233 H   0 0 0 0 0 0 0 0 0 0 0 0
5.4425   7.1588   0.5841 H   0 0 0 0 0 0 0 0 0 0 0 0
7.9407   3.6694   0.2107 H   0 0 0 0 0 0 0 0 0 0 0 0
6.7993   7.9373   -1.3325 H   0 0 0 0 0 0 0 0 0 0 0 0
5.1046   1.4864   -1.7695 H   0 0 0 0 0 0 0 0 0 0 0 0
3.8451   2.5616   -1.1860 H   0 0 0 0 0 0 0 0 0 0 0 0
5.2064   3.2228   -2.0798 H   0 0 0 0 0 0 0 0 0 0 0 0
2.7260   3.5890   -0.4874 H   0 0 0 0 0 0 0 0 0 0 0 0
1.7779   4.9786   0.0185 H   0 0 0 0 0 0 0 0 0 0 0 0
3.1711   5.1970   -1.0447 H   0 0 0 0 0 0 0 0 0 0 0 0
5.2425   6.1788   2.9561 H   0 0 0 0 0 0 0 0 0 0 0 0
6.8632   6.1060   3.6586 H   0 0 0 0 0 0 0 0 0 0 0 0
6.6338   6.7514   2.0316 H   0 0 0 0 0 0 0 0 0 0 0 0
10.1031   5.4231   -1.0150 H   0 0 0 0 0 0 0 0 0 0 0 0
10.3352   3.7752   -0.4162 H   0 0 0 0 0 0 0 0 0 0 0 0
9.9190   5.0745   0.7090 H   0 0 0 0 0 0 0 0 0 0 0 0
3.8895   8.8179   -1.5187 H   0 0 0 0 0 0 0 0 0 0 0 0
5.0884   9.4680   -0.3928 H   0 0 0 0 0 0 0 0 0 0 0 0
5.2779   9.7254   -2.1322 H   0 0 0 0 0 0 0 0 0 0 0 0
6.1086   6.1815   -2.9469 H   0 0 0 0 0 0 0 0 0 0 0 0
4.4885   6.8874   -3.0188 H   0 0 0 0 0 0 0 0 0 0 0 0
5.8767   7.7954   -3.6323 H   0 0 0 0 0 0 0 0 0 0 0 0
8.6206   2.9334   -2.0561 H   0 0 0 0 0 0 0 0 0 0 0 0
7.0113   3.6576   -2.0955 H   0 0 0 0 0 0 0 0 0 0 0 0

```
   8.4055    4.5826   -2.6571 H   0  0  0  0  0  0  0  0  0  0  0  0
  1  2  1  0  0  0  0
  1  3  1  0  0  0  0
  1  4  1  0  0  0  0
  1  5  1  0  0  0  0
  2  6  1  0  0  0  0
  2  7  1  0  0  0  0
  6  8  1  0  0  0  0
  7  9  1  0  0  0  0
  5 10  1  0  0  0  0
  3 11  1  0  0  0  0
  4 12  1  0  0  0  0
  8 13  1  0  0  0  0
  9 14  1  0  0  0  0
  9 15  1  0  0  0  0
  8 16  1  0  0  0  0
  3 17  1  0  0  0  0
  3 18  1  0  0  0  0
  4 19  1  0  0  0  0
  4 20  1  0  0  0  0
  5 21  1  0  0  0  0
  5 22  1  0  0  0  0
  6 23  1  0  0  0  0
  6 24  1  0  0  0  0
  7 25  1  0  0  0  0
  7 26  1  0  0  0  0
  8 27  1  0  0  0  0
  9 28  1  0  0  0  0
 10 29  1  0  0  0  0
 10 30  1  0  0  0  0
 10 31  1  0  0  0  0
 11 32  1  0  0  0  0
 11 33  1  0  0  0  0
 11 34  1  0  0  0  0
 12 35  1  0  0  0  0
 12 36  1  0  0  0  0
 12 37  1  0  0  0  0
 13 38  1  0  0  0  0
 13 39  1  0  0  0  0
 13 40  1  0  0  0  0
 14 41  1  0  0  0  0
 14 42  1  0  0  0  0
 14 43  1  0  0  0  0
 15 44  1  0  0  0  0
 15 45  1  0  0  0  0
 15 46  1  0  0  0  0
 16 47  1  0  0  0  0
 16 48  1  0  0  0  0
 16 49  1  0  0  0  0
M  END
>  <Alias name>
N,N-Diisobutylaminotriethylsilane

>  <deltaH°(c)>   kJ/mol
-10667

$$$$
Dicyclohexyl phthalate
  ChemBrain IXL

 50 52  0  0  0  0  0  0  0  0  1 V2000
   11.6158    6.4432    0.9529 C   0  0  0  0  0  0  0  0  0  0  0  0
   10.5293    7.1622    0.4548 C   0  0  0  0  0  0  0  0  0  0  0  0
```

9.2370	6.6552	0.5840	C	0	0	0	0	0	0	0	0	0	0	0	0	0
9.0341	5.4033	1.1989	C	0	0	0	0	0	0	0	0	0	0	0	0	0
10.1240	4.6937	1.7013	C	0	0	0	0	0	0	0	0	0	0	0	0	0
11.4134	5.2109	1.5760	C	0	0	0	0	0	0	0	0	0	0	0	0	0
7.6669	4.8200	1.3206	C	0	0	0	0	0	0	0	0	0	0	0	0	0
7.5323	3.6146	1.4524	O	0	0	0	0	0	0	0	0	0	0	0	0	0
6.4727	5.6903	1.2850	O	0	0	0	0	0	0	0	0	0	0	0	0	0
8.0874	7.4525	0.0678	C	0	0	0	0	0	0	0	0	0	0	0	0	0
8.2093	8.6555	-0.0950	O	0	0	0	0	0	0	0	0	0	0	0	0	0
6.8014	6.7975	-0.2503	O	0	0	0	0	0	0	0	0	0	0	0	0	0
3.5255	3.4665	0.3026	C	0	0	0	0	0	0	0	0	0	0	0	0	0
4.8674	4.2028	0.1433	C	0	0	0	0	0	0	0	0	0	0	0	0	0
5.1818	4.9822	1.4374	C	0	0	0	0	0	0	0	0	0	0	0	0	0
4.0525	6.0065	1.7118	C	0	0	0	0	0	0	0	0	0	0	0	0	0
2.6982	5.2680	1.8385	C	0	0	0	0	0	0	0	0	0	0	0	0	0
2.4093	4.4949	0.5387	C	0	0	0	0	0	0	0	0	0	0	0	0	0
4.1255	9.5220	-0.1656	C	0	0	0	0	0	0	0	0	0	0	0	0	0
5.2127	8.5785	0.3780	C	0	0	0	0	0	0	0	0	0	0	0	0	0
5.7540	7.7037	-0.7717	C	0	0	0	0	0	0	0	0	0	0	0	0	0
4.5965	6.8717	-1.3777	C	0	0	0	0	0	0	0	0	0	0	0	0	0
3.4876	7.8200	-1.8941	C	0	0	0	0	0	0	0	0	0	0	0	0	0
2.9676	8.6865	-0.7320	C	0	0	0	0	0	0	0	0	0	0	0	0	0
12.6172	6.8417	0.8557	H	0	0	0	0	0	0	0	0	0	0	0	0	0
10.6890	8.1159	-0.0330	H	0	0	0	0	0	0	0	0	0	0	0	0	0
9.9685	3.7394	2.1894	H	0	0	0	0	0	0	0	0	0	0	0	0	0
12.2578	4.6556	1.9628	H	0	0	0	0	0	0	0	0	0	0	0	0	0
3.3131	2.9010	-0.5961	H	0	0	0	0	0	0	0	0	0	0	0	0	0
3.5810	2.7931	1.1494	H	0	0	0	0	0	0	0	0	0	0	0	0	0
5.6539	3.4833	-0.0454	H	0	0	0	0	0	0	0	0	0	0	0	0	0
4.8029	4.8941	-0.6880	H	0	0	0	0	0	0	0	0	0	0	0	0	0
5.2476	4.2894	2.2669	H	0	0	0	0	0	0	0	0	0	0	0	0	0
4.2613	6.5346	2.6341	H	0	0	0	0	0	0	0	0	0	0	0	0	0
4.0011	6.7151	0.8947	H	0	0	0	0	0	0	0	0	0	0	0	0	0
1.9083	5.9887	2.0123	H	0	0	0	0	0	0	0	0	0	0	0	0	0
2.7417	4.5736	2.6686	H	0	0	0	0	0	0	0	0	0	0	0	0	0
2.3739	5.1872	-0.2936	H	0	0	0	0	0	0	0	0	0	0	0	0	0
1.4581	3.9837	0.6246	H	0	0	0	0	0	0	0	0	0	0	0	0	0
3.7595	10.1529	0.6348	H	0	0	0	0	0	0	0	0	0	0	0	0	0
4.5430	10.1400	-0.9513	H	0	0	0	0	0	0	0	0	0	0	0	0	0
4.7881	7.9436	1.1459	H	0	0	0	0	0	0	0	0	0	0	0	0	0
6.0209	9.1631	0.7987	H	0	0	0	0	0	0	0	0	0	0	0	0	0
6.1784	8.3401	-1.5381	H	0	0	0	0	0	0	0	0	0	0	0	0	0
4.9729	6.2771	-2.2010	H	0	0	0	0	0	0	0	0	0	0	0	0	0
4.1862	6.2173	-0.6188	H	0	0	0	0	0	0	0	0	0	0	0	0	0
3.8918	8.4606	-2.6683	H	0	0	0	0	0	0	0	0	0	0	0	0	0
2.6716	7.2345	-2.3002	H	0	0	0	0	0	0	0	0	0	0	0	0	0
2.1875	9.3461	-1.0924	H	0	0	0	0	0	0	0	0	0	0	0	0	0
2.5693	8.0468	0.0460	H	0	0	0	0	0	0	0	0	0	0	0	0	0

1	2	4	0	0	0	0
2	3	4	0	0	0	0
3	4	4	0	0	0	0
4	5	4	0	0	0	0
5	6	4	0	0	0	0
6	1	4	0	0	0	0
7	8	2	0	0	0	0
7	9	1	0	0	0	0
7	4	1	0	0	0	0
10	11	2	0	0	0	0
10	12	1	0	0	0	0
10	3	1	0	0	0	0
13	14	1	0	0	0	0
14	15	1	0	0	0	0
15	16	1	0	0	0	0

```
 16 17  1  0  0  0  0
 17 18  1  0  0  0  0
 18 13  1  0  0  0  0
 19 20  1  0  0  0  0
 20 21  1  0  0  0  0
 21 22  1  0  0  0  0
 22 23  1  0  0  0  0
 23 24  1  0  0  0  0
 24 19  1  0  0  0  0
 15  9  1  0  0  0  0
 21 12  1  0  0  0  0
  1 25  1  0  0  0  0
  2 26  1  0  0  0  0
  5 27  1  0  0  0  0
  6 28  1  0  0  0  0
 13 29  1  0  0  0  0
 13 30  1  0  0  0  0
 14 31  1  0  0  0  0
 14 32  1  0  0  0  0
 15 33  1  0  0  0  0
 16 34  1  0  0  0  0
 16 35  1  0  0  0  0
 17 36  1  0  0  0  0
 17 37  1  0  0  0  0
 18 38  1  0  0  0  0
 18 39  1  0  0  0  0
 19 40  1  0  0  0  0
 19 41  1  0  0  0  0
 20 42  1  0  0  0  0
 20 43  1  0  0  0  0
 21 44  1  0  0  0  0
 22 45  1  0  0  0  0
 22 46  1  0  0  0  0
 23 47  1  0  0  0  0
 23 48  1  0  0  0  0
 24 49  1  0  0  0  0
 24 50  1  0  0  0  0
M  END
>  <Alias name>
Dicyclohexyl phthalate

>  <deltaH°(c)>   kJ/mol
-10655

$$$$
1,3,3-Triphenyl-2-propen-1-one
  ChemBrain IXL

 38 40  0  0  0  0  0  0  0  0  1 V2000
    7.6131    3.2080   -0.2873 C   0  0  0  0  0  0  0  0  0  0  0  0
    6.7682    4.1865   -0.8116 C   0  0  0  0  0  0  0  0  0  0  0  0
    7.0205    5.5348   -0.5578 C   0  0  0  0  0  0  0  0  0  0  0  0
    8.1187    5.9047    0.2188 C   0  0  0  0  0  0  0  0  0  0  0  0
    8.9623    4.9262    0.7450 C   0  0  0  0  0  0  0  0  0  0  0  0
    8.7100    3.5779    0.4911 C   0  0  0  0  0  0  0  0  0  0  0  0
   10.7661    7.0785   -0.2109 C   0  0  0  0  0  0  0  0  0  0  0  0
    9.7663    7.8632    0.3640 C   0  0  0  0  0  0  0  0  0  0  0  0
   10.0647    9.1455    0.8253 C   0  0  0  0  0  0  0  0  0  0  0  0
   11.3635    9.6421    0.7136 C   0  0  0  0  0  0  0  0  0  0  0  0
   12.3636    8.8569    0.1399 C   0  0  0  0  0  0  0  0  0  0  0  0
   12.0649    7.5751   -0.3224 C   0  0  0  0  0  0  0  0  0  0  0  0
    8.3888    7.3354    0.4846 C   0  0  0  0  0  0  0  0  0  0  0  0
    7.3901    8.1499    0.8330 C   0  0  0  0  0  0  0  0  0  0  0  0
```

```
  6.0145    7.6197    0.9647 C   0  0  0  0  0  0  0  0  0  0  0  0
  5.8345    6.4876    1.3822 O   0  0  0  0  0  0  0  0  0  0  0  0
  2.6761   10.0522   -0.1172 C   0  0  0  0  0  0  0  0  0  0  0  0
  3.9722   10.5544   -0.2347 C   0  0  0  0  0  0  0  0  0  0  0  0
  5.0629    9.7599    0.1191 C   0  0  0  0  0  0  0  0  0  0  0  0
  4.8574    8.4629    0.5900 C   0  0  0  0  0  0  0  0  0  0  0  0
  3.5612    7.9608    0.7078 C   0  0  0  0  0  0  0  0  0  0  0  0
  2.4706    8.7554    0.3540 C   0  0  0  0  0  0  0  0  0  0  0  0
  7.4174    2.1613   -0.4846 H   0  0  0  0  0  0  0  0  0  0  0  0
  5.9165    3.8993   -1.4157 H   0  0  0  0  0  0  0  0  0  0  0  0
  6.3638    6.2943   -0.9637 H   0  0  0  0  0  0  0  0  0  0  0  0
  9.8130    5.2133    1.3506 H   0  0  0  0  0  0  0  0  0  0  0  0
  9.3658    2.8183    0.8984 H   0  0  0  0  0  0  0  0  0  0  0  0
 10.5340    6.0837   -0.5706 H   0  0  0  0  0  0  0  0  0  0  0  0
  9.2882    9.7553    1.2702 H   0  0  0  0  0  0  0  0  0  0  0  0
 11.5954   10.6374    1.0723 H   0  0  0  0  0  0  0  0  0  0  0  0
 13.3720    9.2424    0.0534 H   0  0  0  0  0  0  0  0  0  0  0  0
 12.8413    6.9655   -0.7679 H   0  0  0  0  0  0  0  0  0  0  0  0
  7.5863    9.1985    1.0192 H   0  0  0  0  0  0  0  0  0  0  0  0
  1.8294   10.6691   -0.3919 H   0  0  0  0  0  0  0  0  0  0  0  0
  4.1318   11.5612   -0.6005 H   0  0  0  0  0  0  0  0  0  0  0  0
  6.0691   10.1498    0.0281 H   0  0  0  0  0  0  0  0  0  0  0  0
  3.4017    6.9542    1.0739 H   0  0  0  0  0  0  0  0  0  0  0  0
  1.4643    8.3655    0.4453 H   0  0  0  0  0  0  0  0  0  0  0  0
  1  2  4  0  0  0  0
  2  3  4  0  0  0  0
  3  4  4  0  0  0  0
  4  5  4  0  0  0  0
  5  6  4  0  0  0  0
  6  1  4  0  0  0  0
  7  8  4  0  0  0  0
  8  9  4  0  0  0  0
  9 10  4  0  0  0  0
 10 11  4  0  0  0  0
 11 12  4  0  0  0  0
 12  7  4  0  0  0  0
  4 13  1  0  0  0  0
 13  8  1  0  0  0  0
 13 14  2  0  0  0  0
 14 15  1  0  0  0  0
 15 16  2  0  0  0  0
 17 18  4  0  0  0  0
 18 19  4  0  0  0  0
 19 20  4  0  0  0  0
 20 21  4  0  0  0  0
 21 22  4  0  0  0  0
 22 17  4  0  0  0  0
 20 15  1  0  0  0  0
  1 23  1  0  0  0  0
  2 24  1  0  0  0  0
  3 25  1  0  0  0  0
  5 26  1  0  0  0  0
  6 27  1  0  0  0  0
  7 28  1  0  0  0  0
  9 29  1  0  0  0  0
 10 30  1  0  0  0  0
 11 31  1  0  0  0  0
 12 32  1  0  0  0  0
 14 33  1  0  0  0  0
 17 34  1  0  0  0  0
 18 35  1  0  0  0  0
 19 36  1  0  0  0  0
 21 37  1  0  0  0  0
```

```
 22 38  1  0  0  0  0
M  END
>  <Alias name>
1,3,3-Triphenyl-2-propen-1-one

>  <deltaH°(c)>   kJ/mol
-10640

$$$$
3-Hydroxy-1,3,3-triphenyl-1-propanone
  ChemBrain IXL

 41 43  0  0  0  0  0  0  0  0  1 V2000
    7.1889    5.3677   -1.1982 C   0  0  0  0  0  0  0  0  0  0  0  0
    5.9214    6.2409   -1.1235 C   0  0  0  0  0  0  0  0  0  0  0  0
    5.6100    6.7677   -2.4600 O   0  0  0  0  0  0  0  0  0  0  0  0
    4.7868    5.4234   -0.6387 C   0  0  0  0  0  0  0  0  0  0  0  0
    6.1523    7.3667   -0.1910 C   0  0  0  0  0  0  0  0  0  0  0  0
    8.2786    6.1343   -1.8428 C   0  0  0  0  0  0  0  0  0  0  0  0
    4.9854    4.4858    0.3750 C   0  0  0  0  0  0  0  0  0  0  0  0
    3.5187    5.5905   -1.1956 C   0  0  0  0  0  0  0  0  0  0  0  0
    5.7867    8.6621   -0.5575 C   0  0  0  0  0  0  0  0  0  0  0  0
    6.7357    7.1325    1.0543 C   0  0  0  0  0  0  0  0  0  0  0  0
    8.0135    7.0111   -2.6487 O   0  0  0  0  0  0  0  0  0  0  0  0
    9.6898    5.8374   -1.5097 C   0  0  0  0  0  0  0  0  0  0  0  0
    3.9147    3.7197    0.8361 C   0  0  0  0  0  0  0  0  0  0  0  0
    2.4479    4.8244   -0.7344 C   0  0  0  0  0  0  0  0  0  0  0  0
    6.0091    9.7240    0.3194 C   0  0  0  0  0  0  0  0  0  0  0  0
    6.9582    8.1944    1.9311 C   0  0  0  0  0  0  0  0  0  0  0  0
   10.7134    6.6099   -2.0588 C   0  0  0  0  0  0  0  0  0  0  0  0
    9.9963    4.7851   -0.6467 C   0  0  0  0  0  0  0  0  0  0  0  0
    2.6459    3.8893    0.2816 C   0  0  0  0  0  0  0  0  0  0  0  0
    6.5951    9.4902    1.5635 C   0  0  0  0  0  0  0  0  0  0  0  0
   12.0436    6.3294   -1.7458 C   0  0  0  0  0  0  0  0  0  0  0  0
   11.3264    4.5046   -0.3336 C   0  0  0  0  0  0  0  0  0  0  0  0
   12.3501    5.2767   -0.8832 C   0  0  0  0  0  0  0  0  0  0  0  0
    6.3684    7.1781   -2.9319 H   0  0  0  0  0  0  0  0  0  0  0  0
    7.4907    5.0831   -0.1977 H   0  0  0  0  0  0  0  0  0  0  0  0
    6.9807    4.4780   -1.7794 H   0  0  0  0  0  0  0  0  0  0  0  0
    5.9709    4.3527    0.8039 H   0  0  0  0  0  0  0  0  0  0  0  0
    3.3655    6.3149   -1.9859 H   0  0  0  0  0  0  0  0  0  0  0  0
    5.3302    8.8433   -1.5227 H   0  0  0  0  0  0  0  0  0  0  0  0
    7.0158    6.1262    1.3404 H   0  0  0  0  0  0  0  0  0  0  0  0
    4.0684    2.9934    1.6245 H   0  0  0  0  0  0  0  0  0  0  0  0
    1.4630    4.9557   -1.1652 H   0  0  0  0  0  0  0  0  0  0  0  0
    5.7268   10.7299    0.0342 H   0  0  0  0  0  0  0  0  0  0  0  0
    7.4128    8.0129    2.8972 H   0  0  0  0  0  0  0  0  0  0  0  0
   10.4754    7.4273   -2.7282 H   0  0  0  0  0  0  0  0  0  0  0  0
    9.2016    4.1859   -0.2198 H   0  0  0  0  0  0  0  0  0  0  0  0
    1.8145    3.2949    0.6400 H   0  0  0  0  0  0  0  0  0  0  0  0
    6.7682   10.3147    2.2441 H   0  0  0  0  0  0  0  0  0  0  0  0
   12.8383    6.9288   -2.1724 H   0  0  0  0  0  0  0  0  0  0  0  0
   11.5644    3.6875    0.3361 H   0  0  0  0  0  0  0  0  0  0  0  0
   13.3828    5.0589   -0.6402 H   0  0  0  0  0  0  0  0  0  0  0  0
  1  2  1  0  0  0  0
  3  2  1  0  0  0  0
  4  2  1  0  0  0  0
  5  2  1  0  0  0  0
  6  1  1  0  0  0  0
  4  7  4  0  0  0  0
  8  4  4  0  0  0  0
  9  5  4  0  0  0  0
  5 10  4  0  0  0  0
```

```
 11  6  2  0  0  0  0
 12  6  1  0  0  0  0
  7 13  4  0  0  0  0
 14  8  4  0  0  0  0
 15  9  4  0  0  0  0
 10 16  4  0  0  0  0
 17 12  4  0  0  0  0
 12 18  4  0  0  0  0
 13 19  4  0  0  0  0
 20 15  4  0  0  0  0
 21 17  4  0  0  0  0
 18 22  4  0  0  0  0
 23 21  4  0  0  0  0
 19 14  4  0  0  0  0
 16 20  4  0  0  0  0
 22 23  4  0  0  0  0
  3 24  1  0  0  0  0
  1 25  1  0  0  0  0
  1 26  1  0  0  0  0
  7 27  1  0  0  0  0
  8 28  1  0  0  0  0
  9 29  1  0  0  0  0
 10 30  1  0  0  0  0
 13 31  1  0  0  0  0
 14 32  1  0  0  0  0
 15 33  1  0  0  0  0
 16 34  1  0  0  0  0
 17 35  1  0  0  0  0
 18 36  1  0  0  0  0
 19 37  1  0  0  0  0
 20 38  1  0  0  0  0
 21 39  1  0  0  0  0
 22 40  1  0  0  0  0
 23 41  1  0  0  0  0
M  END
>  <Alias name>
3-Hydroxy-1,3,3-triphenyl-1-propanone

>  <deltaH°(c)>   kJ/mol
-10600

$$$$
1,1,1-Triphenylethane
  ChemBrain IXL

 38 40  0  0  0  0  0  0  0  0  1 V2000
    7.5791    2.3259    3.1279 C   0  0  0  0  0  0  0  0  0  0  0  0
    8.4800    3.3909    3.1196 C   0  0  0  0  0  0  0  0  0  0  0  0
    8.3269    4.4268    2.1979 C   0  0  0  0  0  0  0  0  0  0  0  0
    7.2730    4.3976    1.2844 C   0  0  0  0  0  0  0  0  0  0  0  0
    6.3719    3.3326    1.2929 C   0  0  0  0  0  0  0  0  0  0  0  0
    6.5251    2.2968    2.2146 C   0  0  0  0  0  0  0  0  0  0  0  0
    5.6116    6.4100    2.0721 C   0  0  0  0  0  0  0  0  0  0  0  0
    4.9514    7.4787    2.6788 C   0  0  0  0  0  0  0  0  0  0  0  0
    5.0894    8.7674    2.1628 C   0  0  0  0  0  0  0  0  0  0  0  0
    5.8875    8.9873    1.0400 C   0  0  0  0  0  0  0  0  0  0  0  0
    6.5476    7.9186    0.4333 C   0  0  0  0  0  0  0  0  0  0  0  0
    6.4099    6.6300    0.9495 C   0  0  0  0  0  0  0  0  0  0  0  0
    6.9394    4.3052   -1.8739 C   0  0  0  0  0  0  0  0  0  0  0  0
    6.3176    5.0166   -0.8475 C   0  0  0  0  0  0  0  0  0  0  0  0
    4.9483    5.2758   -0.9087 C   0  0  0  0  0  0  0  0  0  0  0  0
    4.2008    4.8239   -1.9963 C   0  0  0  0  0  0  0  0  0  0  0  0
    4.8225    4.1128   -3.0229 C   0  0  0  0  0  0  0  0  0  0  0  0
```

6.1919 3.8535 -2.9616 C 0 0 0 0 0 0 0 0 0 0 0 0
7.1108 5.4963 0.3062 C 0 0 0 0 0 0 0 0 0 0 0 0
8.4976 5.9594 -0.1807 C 0 0 0 0 0 0 0 0 0 0 0 0
7.6981 1.5216 3.8434 H 0 0 0 0 0 0 0 0 0 0 0 0
9.2983 3.4136 3.8286 H 0 0 0 0 0 0 0 0 0 0 0 0
9.0261 5.2538 2.1916 H 0 0 0 0 0 0 0 0 0 0 0 0
5.5536 3.3101 0.5840 H 0 0 0 0 0 0 0 0 0 0 0 0
5.8256 1.4700 2.2212 H 0 0 0 0 0 0 0 0 0 0 0 0
5.5044 5.4095 2.4727 H 0 0 0 0 0 0 0 0 0 0 0 0
4.3317 7.3079 3.5505 H 0 0 0 0 0 0 0 0 0 0 0 0
4.5770 9.5971 2.6339 H 0 0 0 0 0 0 0 0 0 0 0 0
5.9946 9.9878 0.6394 H 0 0 0 0 0 0 0 0 0 0 0 0
7.1670 8.0894 -0.4386 H 0 0 0 0 0 0 0 0 0 0 0 0
8.0024 4.1037 -1.8263 H 0 0 0 0 0 0 0 0 0 0 0 0
4.4656 5.8278 -0.1117 H 0 0 0 0 0 0 0 0 0 0 0 0
3.1377 5.0252 -2.0438 H 0 0 0 0 0 0 0 0 0 0 0 0
4.2422 3.7621 -3.8673 H 0 0 0 0 0 0 0 0 0 0 0 0
6.6746 3.3014 -3.7585 H 0 0 0 0 0 0 0 0 0 0 0 0
8.3774 6.7249 -0.9374 H 0 0 0 0 0 0 0 0 0 0 0 0
9.0324 5.1168 -0.6014 H 0 0 0 0 0 0 0 0 0 0 0 0
9.0575 6.3619 0.6543 H 0 0 0 0 0 0 0 0 0 0 0 0
1 2 4 0 0 0 0
2 3 4 0 0 0 0
3 4 4 0 0 0 0
4 5 4 0 0 0 0
5 6 4 0 0 0 0
6 1 4 0 0 0 0
7 8 4 0 0 0 0
8 9 4 0 0 0 0
9 10 4 0 0 0 0
10 11 4 0 0 0 0
11 12 4 0 0 0 0
12 7 4 0 0 0 0
13 14 4 0 0 0 0
14 15 4 0 0 0 0
15 16 4 0 0 0 0
16 17 4 0 0 0 0
17 18 4 0 0 0 0
18 13 4 0 0 0 0
4 19 1 0 0 0 0
19 14 1 0 0 0 0
19 12 1 0 0 0 0
19 20 1 0 0 0 0
1 21 1 0 0 0 0
2 22 1 0 0 0 0
3 23 1 0 0 0 0
5 24 1 0 0 0 0
6 25 1 0 0 0 0
7 26 1 0 0 0 0
8 27 1 0 0 0 0
9 28 1 0 0 0 0
10 29 1 0 0 0 0
11 30 1 0 0 0 0
13 31 1 0 0 0 0
15 32 1 0 0 0 0
16 33 1 0 0 0 0
17 34 1 0 0 0 0
18 35 1 0 0 0 0
20 36 1 0 0 0 0
20 37 1 0 0 0 0
20 38 1 0 0 0 0
M END
> <Alias name>

1,1,1-Triphenylethane

```
>  <deltaH°(c)>   kJ/mol
-10599

$$$$
Ethyldihexylsilane
  ChemBrain IXL

 47 46  0  0  0  0  0  0  0  0  1 V2000
    9.7281    8.4653   -1.8240 C   0  0  0  0  0  0  0  0  0  0  0  0  0
   10.9320    9.2514   -1.2642 C   0  0  0  0  0  0  0  0  0  0  0  0  0
   10.8963    9.2971    0.6221 Si  0  0  0  0  0  0  0  0  0  0  0  0  0
   10.8867    7.5610    1.3611 C   0  0  0  0  0  0  0  0  0  0  0  0  0
    9.5568    6.8378    1.0627 C   0  0  0  0  0  0  0  0  0  0  0  0  0
    9.6366    5.3898    1.5832 C   0  0  0  0  0  0  0  0  0  0  0  0  0
    8.3081    4.6654    1.2916 C   0  0  0  0  0  0  0  0  0  0  0  0  0
    8.3874    3.2170    1.8124 C   0  0  0  0  0  0  0  0  0  0  0  0  0
    3.1127   10.3555    0.9795 C   0  0  0  0  0  0  0  0  0  0  0  0  0
    4.4629   10.8827    1.5025 C   0  0  0  0  0  0  0  0  0  0  0  0  0
    5.6115   10.0797    0.8610 C   0  0  0  0  0  0  0  0  0  0  0  0  0
    6.9618   10.6069    1.3841 C   0  0  0  0  0  0  0  0  0  0  0  0  0
    8.1097    9.8035    0.7433 C   0  0  0  0  0  0  0  0  0  0  0  0  0
    9.4596   10.3365    1.2670 C   0  0  0  0  0  0  0  0  0  0  0  0  0
    7.0588    2.4927    1.5213 C   0  0  0  0  0  0  0  0  0  0  0  0  0
   12.1454    9.9544    1.0668 H   0  0  0  0  0  0  0  0  0  0  0  0  0
    9.8017    7.4344   -1.5251 H   0  0  0  0  0  0  0  0  0  0  0  0  0
    8.8145    8.8841   -1.4506 H   0  0  0  0  0  0  0  0  0  0  0  0  0
    9.7342    8.5240   -2.9053 H   0  0  0  0  0  0  0  0  0  0  0  0  0
   11.8464    8.7715   -1.5901 H   0  0  0  0  0  0  0  0  0  0  0  0  0
   10.9072   10.2650   -1.6446 H   0  0  0  0  0  0  0  0  0  0  0  0  0
   11.0147    7.6315    2.4341 H   0  0  0  0  0  0  0  0  0  0  0  0  0
   11.7071    6.9919    0.9417 H   0  0  0  0  0  0  0  0  0  0  0  0  0
    9.3782    6.8261    0.0056 H   0  0  0  0  0  0  0  0  0  0  0  0  0
    8.7508    7.3485    1.5596 H   0  0  0  0  0  0  0  0  0  0  0  0  0
    9.8157    5.3995    2.6512 H   0  0  0  0  0  0  0  0  0  0  0  0  0
   10.4472    4.8716    1.0858 H   0  0  0  0  0  0  0  0  0  0  0  0  0
    8.1291    4.6556    0.2235 H   0  0  0  0  0  0  0  0  0  0  0  0  0
    7.4976    5.1839    1.7888 H   0  0  0  0  0  0  0  0  0  0  0  0  0
    8.5667    3.2267    2.8804 H   0  0  0  0  0  0  0  0  0  0  0  0  0
    9.1977    2.6984    1.3151 H   0  0  0  0  0  0  0  0  0  0  0  0  0
    3.0094    9.3088    1.2377 H   0  0  0  0  0  0  0  0  0  0  0  0  0
    2.3058   10.9198    1.4304 H   0  0  0  0  0  0  0  0  0  0  0  0  0
    3.0739   10.4674   -0.0970 H   0  0  0  0  0  0  0  0  0  0  0  0  0
    4.5016   10.7709    2.5790 H   0  0  0  0  0  0  0  0  0  0  0  0  0
    4.5661   11.9294    1.2444 H   0  0  0  0  0  0  0  0  0  0  0  0  0
    5.5729   10.1916   -0.2155 H   0  0  0  0  0  0  0  0  0  0  0  0  0
    5.5082    9.0330    1.1192 H   0  0  0  0  0  0  0  0  0  0  0  0  0
    7.0002   10.4954    2.4607 H   0  0  0  0  0  0  0  0  0  0  0  0  0
    7.0654   11.6535    1.1256 H   0  0  0  0  0  0  0  0  0  0  0  0  0
    8.0684    9.9183   -0.3256 H   0  0  0  0  0  0  0  0  0  0  0  0  0
    8.0038    8.7677    0.9983 H   0  0  0  0  0  0  0  0  0  0  0  0  0
    9.5852   11.3598    0.9358 H   0  0  0  0  0  0  0  0  0  0  0  0  0
    9.4604   10.3104    2.3497 H   0  0  0  0  0  0  0  0  0  0  0  0  0
    7.1148    1.4750    1.8873 H   0  0  0  0  0  0  0  0  0  0  0  0  0
    6.2484    3.0111    2.0187 H   0  0  0  0  0  0  0  0  0  0  0  0  0
    6.8794    2.4828    0.4533 H   0  0  0  0  0  0  0  0  0  0  0  0  0
  1  2  1  0  0  0  0
  2  3  1  0  0  0  0
  3  4  1  0  0  0  0
  4  5  1  0  0  0  0
  5  6  1  0  0  0  0
  6  7  1  0  0  0  0
```

7 8 1 0 0 0 0
9 10 1 0 0 0 0
10 11 1 0 0 0 0
11 12 1 0 0 0 0
12 13 1 0 0 0 0
13 14 1 0 0 0 0
14 3 1 0 0 0 0
8 15 1 0 0 0 0
3 16 1 0 0 0 0
1 17 1 0 0 0 0
1 18 1 0 0 0 0
1 19 1 0 0 0 0
2 20 1 0 0 0 0
2 21 1 0 0 0 0
4 22 1 0 0 0 0
4 23 1 0 0 0 0
5 24 1 0 0 0 0
5 25 1 0 0 0 0
6 26 1 0 0 0 0
6 27 1 0 0 0 0
7 28 1 0 0 0 0
7 29 1 0 0 0 0
8 30 1 0 0 0 0
8 31 1 0 0 0 0
9 32 1 0 0 0 0
9 33 1 0 0 0 0
9 34 1 0 0 0 0
10 35 1 0 0 0 0
10 36 1 0 0 0 0
11 37 1 0 0 0 0
11 38 1 0 0 0 0
12 39 1 0 0 0 0
12 40 1 0 0 0 0
13 41 1 0 0 0 0
13 42 1 0 0 0 0
14 43 1 0 0 0 0
14 44 1 0 0 0 0
15 45 1 0 0 0 0
15 46 1 0 0 0 0
15 47 1 0 0 0 0
M END
> <Alias name>
Ethyldihexylsilane

> <deltaH°(c)>   kJ/mol
-10596

$$$$
2,3-Dimethyl-2,3-bis(phenylazo)butane
ChemBrain IXL

44 45 0 0 0 0 0 0 0 0 1 V2000
6.3594   7.9382   0.9210 C   0 0 0 0 0 0 0 0 0 0 0 0
7.5898   7.5615   0.3825 C   0 0 0 0 0 0 0 0 0 0 0 0
5.8347   9.2011   0.6455 C   0 0 0 0 0 0 0 0 0 0 0 0
8.2957   8.4476   -0.4314 C   0 0 0 0 0 0 0 0 0 0 0 0
8.1163   6.2940   0.6589 N   0 0 0 0 0 0 0 0 0 0 0 0
7.7710   9.7105   -0.7070 C   0 0 0 0 0 0 0 0 0 0 0 0
6.5405   10.0872   -0.1686 C   0 0 0 0 0 0 0 0 0 0 0 0
9.2912   5.9362   0.1485 N   0 0 0 0 0 0 0 0 0 0 0 0
9.8152   4.6668   0.4205 C   0 0 0 0 0 0 0 0 0 0 0 0
9.0914   3.6163   -0.4440 C   0 0 0 0 0 0 0 0 0 0 0 0
9.6098   4.3349   1.9113 C   0 0 0 0 0 0 0 0 0 0 0 0

```
 11.3210    4.6518    0.0937 C   0  0  0  0  0  0  0  0  0  0  0  0
  7.7200    3.6465   -0.1644 N   0  0  0  0  0  0  0  0  0  0  0  0
  9.6500    2.2159   -0.1256 C   0  0  0  0  0  0  0  0  0  0  0  0
  9.3188    3.9332   -1.9349 C   0  0  0  0  0  0  0  0  0  0  0  0
  6.9338    4.5213   -0.7852 N   0  0  0  0  0  0  0  0  0  0  0  0
  5.5634    4.5559   -0.5006 C   0  0  0  0  0  0  0  0  0  0  0  0
  2.8325    4.6249    0.0664 C   0  0  0  0  0  0  0  0  0  0  0  0
  3.6589    3.7142    0.7250 C   0  0  0  0  0  0  0  0  0  0  0  0
  3.3716    5.5012   -0.8757 C   0  0  0  0  0  0  0  0  0  0  0  0
  5.0243    3.6797    0.4415 C   0  0  0  0  0  0  0  0  0  0  0  0
  4.7371    5.4667   -1.1592 C   0  0  0  0  0  0  0  0  0  0  0  0
  5.8114    7.2503    1.5530 H   0  0  0  0  0  0  0  0  0  0  0  0
  4.8794    9.4936    1.0635 H   0  0  0  0  0  0  0  0  0  0  0  0
  9.2510    8.1552   -0.8494 H   0  0  0  0  0  0  0  0  0  0  0  0
  8.3190   10.3984   -1.3390 H   0  0  0  0  0  0  0  0  0  0  0  0
  6.1332   11.0676   -0.3825 H   0  0  0  0  0  0  0  0  0  0  0  0
 10.0319    3.3609    2.1258 H   0  0  0  0  0  0  0  0  0  0  0  0
 10.1025    5.0837    2.5190 H   0  0  0  0  0  0  0  0  0  0  0  0
  8.5505    4.3269    2.1367 H   0  0  0  0  0  0  0  0  0  0  0  0
 11.8295    5.3908    0.7006 H   0  0  0  0  0  0  0  0  0  0  0  0
 11.4653    4.8840   -0.9542 H   0  0  0  0  0  0  0  0  0  0  0  0
 11.7266    3.6701    0.3051 H   0  0  0  0  0  0  0  0  0  0  0  0
 10.7106    2.1922   -0.3431 H   0  0  0  0  0  0  0  0  0  0  0  0
  9.1404    1.4775   -0.7322 H   0  0  0  0  0  0  0  0  0  0  0  0
  9.4914    1.9937    0.9225 H   0  0  0  0  0  0  0  0  0  0  0  0
  8.9433    4.9250   -2.1545 H   0  0  0  0  0  0  0  0  0  0  0  0
  8.7945    3.2060   -2.5425 H   0  0  0  0  0  0  0  0  0  0  0  0
 10.3782    3.8912   -2.1554 H   0  0  0  0  0  0  0  0  0  0  0  0
  1.7725    4.6517    0.2866 H   0  0  0  0  0  0  0  0  0  0  0  0
  3.2404    3.0339    1.4564 H   0  0  0  0  0  0  0  0  0  0  0  0
  2.7301    6.2082   -1.3869 H   0  0  0  0  0  0  0  0  0  0  0  0
  5.6659    2.9726    0.9528 H   0  0  0  0  0  0  0  0  0  0  0  0
  5.1556    6.1470   -1.8906 H   0  0  0  0  0  0  0  0  0  0  0  0
  2  1  4  0  0  0  0
  1  3  4  0  0  0  0
  4  2  4  0  0  0  0
  2  5  1  0  0  0  0
  3  7  4  0  0  0  0
  6  4  4  0  0  0  0
  5  8  2  0  0  0  0
  7  6  4  0  0  0  0
  8  9  1  0  0  0  0
  9 10  1  0  0  0  0
  9 11  1  0  0  0  0
  9 12  1  0  0  0  0
 10 13  1  0  0  0  0
 10 14  1  0  0  0  0
 10 15  1  0  0  0  0
 13 16  2  0  0  0  0
 16 17  1  0  0  0  0
 21 17  4  0  0  0  0
 17 22  4  0  0  0  0
 18 19  4  0  0  0  0
 20 18  4  0  0  0  0
 19 21  4  0  0  0  0
 22 20  4  0  0  0  0
  1 23  1  0  0  0  0
  3 24  1  0  0  0  0
  4 25  1  0  0  0  0
  6 26  1  0  0  0  0
  7 27  1  0  0  0  0
 11 28  1  0  0  0  0
 11 29  1  0  0  0  0
```

```
 11 30  1  0  0  0  0
 12 31  1  0  0  0  0
 12 32  1  0  0  0  0
 12 33  1  0  0  0  0
 14 34  1  0  0  0  0
 14 35  1  0  0  0  0
 14 36  1  0  0  0  0
 15 37  1  0  0  0  0
 15 38  1  0  0  0  0
 15 39  1  0  0  0  0
 18 40  1  0  0  0  0
 19 41  1  0  0  0  0
 20 42  1  0  0  0  0
 21 43  1  0  0  0  0
 22 44  1  0  0  0  0
M  END
>  <Alias name>
2,3-Dimethyl-2,3-bis(phenylazo)butane

>  <deltaH°(c)>   kJ/mol
-10589

$$$$
Di-n-octylether
  ChemBrain IXL

 51 50  0  0  0  0  0  0  0  0  1 V2000
    6.6224    8.7834    2.4398 C   0  0  0  0  0  0  0  0  0  0  0  0
    7.4918    9.9704    1.9811 C   0  0  0  0  0  0  0  0  0  0  0  0
    8.7556    9.4396    1.2765 C   0  0  0  0  0  0  0  0  0  0  0  0
    9.6245   10.6263    0.8171 C   0  0  0  0  0  0  0  0  0  0  0  0
   10.8292   10.1196    0.1442 O   0  0  0  0  0  0  0  0  0  0  0  0
   11.7488   11.1557   -0.3471 C   0  0  0  0  0  0  0  0  0  0  0  0
   12.9590   10.4872   -1.0273 C   0  0  0  0  0  0  0  0  0  0  0  0
   13.9231   11.5731   -1.5435 C   0  0  0  0  0  0  0  0  0  0  0  0
   15.1331   10.9045   -2.2244 C   0  0  0  0  0  0  0  0  0  0  0  0
   16.0967   11.9904   -2.7413 C   0  0  0  0  0  0  0  0  0  0  0  0
   17.3065   11.3218   -3.4227 C   0  0  0  0  0  0  0  0  0  0  0  0
   18.2701   12.4078   -3.9399 C   0  0  0  0  0  0  0  0  0  0  0  0
   19.4797   11.7393   -4.6215 C   0  0  0  0  0  0  0  0  0  0  0  0
    5.3584    9.3142    3.1438 C   0  0  0  0  0  0  0  0  0  0  0  0
    4.4888    8.1273    3.6021 C   0  0  0  0  0  0  0  0  0  0  0  0
    3.2245    8.6581    4.3058 C   0  0  0  0  0  0  0  0  0  0  0  0
    2.3547    7.4713    4.7639 C   0  0  0  0  0  0  0  0  0  0  0  0
    7.1872    8.1671    3.1283 H   0  0  0  0  0  0  0  0  0  0  0  0
    6.3350    8.1926    1.5789 H   0  0  0  0  0  0  0  0  0  0  0  0
    6.9269   10.5869    1.2930 H   0  0  0  0  0  0  0  0  0  0  0  0
    7.7797   10.5609    2.8421 H   0  0  0  0  0  0  0  0  0  0  0  0
    9.3208    8.8234    1.9647 H   0  0  0  0  0  0  0  0  0  0  0  0
    8.4677    8.8487    0.4158 H   0  0  0  0  0  0  0  0  0  0  0  0
    9.0590   11.2428    0.1293 H   0  0  0  0  0  0  0  0  0  0  0  0
    9.9131   11.2169    1.6778 H   0  0  0  0  0  0  0  0  0  0  0  0
   12.0901   11.7595    0.4847 H   0  0  0  0  0  0  0  0  0  0  0  0
   11.2345   11.7850   -1.0629 H   0  0  0  0  0  0  0  0  0  0  0  0
   12.6175    9.8830   -1.8586 H   0  0  0  0  0  0  0  0  0  0  0  0
   13.4736    9.8584   -0.3113 H   0  0  0  0  0  0  0  0  0  0  0  0
   14.2650   12.1771   -0.7121 H   0  0  0  0  0  0  0  0  0  0  0  0
   13.4083   12.2023   -2.2591 H   0  0  0  0  0  0  0  0  0  0  0  0
   14.7911   10.3002   -3.0555 H   0  0  0  0  0  0  0  0  0  0  0  0
   15.6482   10.2757   -1.5088 H   0  0  0  0  0  0  0  0  0  0  0  0
   16.4390   12.5946   -1.9102 H   0  0  0  0  0  0  0  0  0  0  0  0
   15.5815   12.6194   -3.4567 H   0  0  0  0  0  0  0  0  0  0  0  0
   16.9643   10.7174   -4.2537 H   0  0  0  0  0  0  0  0  0  0  0  0
```

```
 17.8219   10.6930   -2.7072 H   0  0  0  0  0  0  0  0  0  0  0  0
 18.6125   13.0120   -3.1090 H   0  0  0  0  0  0  0  0  0  0  0  0
 17.7548   13.0366   -4.6554 H   0  0  0  0  0  0  0  0  0  0  0  0
 20.1566   12.5026   -4.9849 H   0  0  0  0  0  0  0  0  0  0  0  0
 19.1375   11.1350   -5.4526 H   0  0  0  0  0  0  0  0  0  0  0  0
 19.9953   11.1105   -3.9062 H   0  0  0  0  0  0  0  0  0  0  0  0
  4.7938    9.9308    2.4554 H   0  0  0  0  0  0  0  0  0  0  0  0
  5.6459    9.9048    4.0049 H   0  0  0  0  0  0  0  0  0  0  0  0
  5.0533    7.5108    4.2907 H   0  0  0  0  0  0  0  0  0  0  0  0
  4.2015    7.5366    2.7410 H   0  0  0  0  0  0  0  0  0  0  0  0
  2.6600    9.2746    3.6173 H   0  0  0  0  0  0  0  0  0  0  0  0
  3.5117    9.2487    5.1669 H   0  0  0  0  0  0  0  0  0  0  0  0
  2.0674    6.8807    3.9028 H   0  0  0  0  0  0  0  0  0  0  0  0
  2.9191    6.8548    5.4526 H   0  0  0  0  0  0  0  0  0  0  0  0
  1.4665    7.8445    5.2583 H   0  0  0  0  0  0  0  0  0  0  0  0
  1  2  1  0  0  0  0
  2  3  1  0  0  0  0
  3  4  1  0  0  0  0
  4  5  1  0  0  0  0
  5  6  1  0  0  0  0
  6  7  1  0  0  0  0
  7  8  1  0  0  0  0
  8  9  1  0  0  0  0
  9 10  1  0  0  0  0
 10 11  1  0  0  0  0
 11 12  1  0  0  0  0
 12 13  1  0  0  0  0
  1 14  1  0  0  0  0
 14 15  1  0  0  0  0
 15 16  1  0  0  0  0
 16 17  1  0  0  0  0
  1 18  1  0  0  0  0
  1 19  1  0  0  0  0
  2 20  1  0  0  0  0
  2 21  1  0  0  0  0
  3 22  1  0  0  0  0
  3 23  1  0  0  0  0
  4 24  1  0  0  0  0
  4 25  1  0  0  0  0
  6 26  1  0  0  0  0
  6 27  1  0  0  0  0
  7 28  1  0  0  0  0
  7 29  1  0  0  0  0
  8 30  1  0  0  0  0
  8 31  1  0  0  0  0
  9 32  1  0  0  0  0
  9 33  1  0  0  0  0
 10 34  1  0  0  0  0
 10 35  1  0  0  0  0
 11 36  1  0  0  0  0
 11 37  1  0  0  0  0
 12 38  1  0  0  0  0
 12 39  1  0  0  0  0
 13 40  1  0  0  0  0
 13 41  1  0  0  0  0
 13 42  1  0  0  0  0
 14 43  1  0  0  0  0
 14 44  1  0  0  0  0
 15 45  1  0  0  0  0
 15 46  1  0  0  0  0
 16 47  1  0  0  0  0
 16 48  1  0  0  0  0
 17 49  1  0  0  0  0
```

```
 17 50  1  0  0  0  0
 17 51  1  0  0  0  0
M  END
>  <Alias name>
Di-n-octylether

>  <deltaH°(c)>   kJ/mol
-10579.97

$$$$
1-(4'-Methoxybenzylidene)-2-phenazin-1-oylhydrazine
  ChemBrain IXL

 43 46  0  0  0  0  0  0  0  0  1 V2000
    5.9409   12.1267   -0.0612 C   0  0  0  0  0  0  0  0  0  0  0  0
    4.5824   12.4435   -0.0795 C   0  0  0  0  0  0  0  0  0  0  0  0
    3.6289   11.4253   -0.0918 C   0  0  0  0  0  0  0  0  0  0  0  0
    4.0338   10.0904   -0.0858 C   0  0  0  0  0  0  0  0  0  0  0  0
    5.3923    9.7736   -0.0676 C   0  0  0  0  0  0  0  0  0  0  0  0
    6.3458   10.7918   -0.0557 C   0  0  0  0  0  0  0  0  0  0  0  0
    4.8629    7.5032   -0.0735 C   0  0  0  0  0  0  0  0  0  0  0  0
    3.5045    7.8199   -0.0919 C   0  0  0  0  0  0  0  0  0  0  0  0
    2.5510    6.8018   -0.1043 C   0  0  0  0  0  0  0  0  0  0  0  0
    2.9559    5.4668   -0.0983 C   0  0  0  0  0  0  0  0  0  0  0  0
    4.3143    5.1501   -0.0800 C   0  0  0  0  0  0  0  0  0  0  0  0
    5.2679    6.1683   -0.0675 C   0  0  0  0  0  0  0  0  0  0  0  0
    5.7830    8.4856   -0.0615 N   0  0  0  0  0  0  0  0  0  0  0  0
    3.1138    9.1080   -0.0977 N   0  0  0  0  0  0  0  0  0  0  0  0
    7.7870   10.4558   -0.0372 C   0  0  0  0  0  0  0  0  0  0  0  0
    8.6210   11.3462   -0.0310 O   0  0  0  0  0  0  0  0  0  0  0  0
    8.1934    9.1161   -0.0269 N   0  0  0  0  0  0  0  0  0  0  0  0
    9.6055    8.7868   -0.0205 N   0  0  0  0  0  0  0  0  0  0  0  0
    9.9741    7.5728    0.0367 C   0  0  0  0  0  0  0  0  0  0  0  0
   11.4155    7.2372    0.0551 C   0  0  0  0  0  0  0  0  0  0  0  0
   11.8205    5.9047    0.1340 C   0  0  0  0  0  0  0  0  0  0  0  0
   13.1790    5.5883    0.1494 C   0  0  0  0  0  0  0  0  0  0  0  0
   14.1326    6.6045    0.0865 C   0  0  0  0  0  0  0  0  0  0  0  0
   13.7276    7.9372    0.0088 C   0  0  0  0  0  0  0  0  0  0  0  0
   12.3691    8.2536   -0.0065 C   0  0  0  0  0  0  0  0  0  0  0  0
   15.5642    6.2711    0.1021 O   0  0  0  0  0  0  0  0  0  0  0  0
   16.4717    7.4245    0.0189 C   0  0  0  0  0  0  0  0  0  0  0  0
    7.5036    8.3797   -0.0239 H   0  0  0  0  0  0  0  0  0  0  0  0
    6.6811   12.9172   -0.0514 H   0  0  0  0  0  0  0  0  0  0  0  0
    4.2681   13.4799   -0.0841 H   0  0  0  0  0  0  0  0  0  0  0  0
    2.5743   11.6712   -0.1060 H   0  0  0  0  0  0  0  0  0  0  0  0
    1.4964    7.0477   -0.1186 H   0  0  0  0  0  0  0  0  0  0  0  0
    2.2156    4.6764   -0.1080 H   0  0  0  0  0  0  0  0  0  0  0  0
    4.6287    4.1137   -0.0753 H   0  0  0  0  0  0  0  0  0  0  0  0
    6.3225    5.9224   -0.0533 H   0  0  0  0  0  0  0  0  0  0  0  0
    9.2338    6.7831    0.0720 H   0  0  0  0  0  0  0  0  0  0  0  0
   11.0802    5.1157    0.1834 H   0  0  0  0  0  0  0  0  0  0  0  0
   13.4934    4.5537    0.2102 H   0  0  0  0  0  0  0  0  0  0  0  0
   14.4680    8.7261   -0.0396 H   0  0  0  0  0  0  0  0  0  0  0  0
   12.0547    9.2882   -0.0662 H   0  0  0  0  0  0  0  0  0  0  0  0
   16.2943    8.0847    0.8589 H   0  0  0  0  0  0  0  0  0  0  0  0
   16.2915    7.9597   -0.9052 H   0  0  0  0  0  0  0  0  0  0  0  0
   17.4978    7.0790    0.0418 H   0  0  0  0  0  0  0  0  0  0  0  0
  1  2  4  0  0  0  0
  2  3  4  0  0  0  0
  3  4  4  0  0  0  0
  4  5  4  0  0  0  0
  5  6  4  0  0  0  0
  6  1  4  0  0  0  0
```

```
  7  8  4  0  0  0  0
  8  9  4  0  0  0  0
  9 10  4  0  0  0  0
 10 11  4  0  0  0  0
 11 12  4  0  0  0  0
 12  7  4  0  0  0  0
  5 13  4  0  0  0  0
 13  7  4  0  0  0  0
  4 14  4  0  0  0  0
 14  8  4  0  0  0  0
 15 16  2  0  0  0  0
 15 17  1  0  0  0  0
 15  6  1  0  0  0  0
 17 18  1  0  0  0  0
 18 19  2  0  0  0  0
 20 21  4  0  0  0  0
 21 22  4  0  0  0  0
 22 23  4  0  0  0  0
 23 24  4  0  0  0  0
 24 25  4  0  0  0  0
 25 20  4  0  0  0  0
 20 19  1  0  0  0  0
 23 26  1  0  0  0  0
 26 27  1  0  0  0  0
 17 28  1  0  0  0  0
  1 29  1  0  0  0  0
  2 30  1  0  0  0  0
  3 31  1  0  0  0  0
  9 32  1  0  0  0  0
 10 33  1  0  0  0  0
 11 34  1  0  0  0  0
 12 35  1  0  0  0  0
 19 36  1  0  0  0  0
 21 37  1  0  0  0  0
 22 38  1  0  0  0  0
 24 39  1  0  0  0  0
 25 40  1  0  0  0  0
 27 41  1  0  0  0  0
 27 42  1  0  0  0  0
 27 43  1  0  0  0  0
M  END
>  <Alias name>
1-(4'-Methoxybenzylidene)-2-phenazin-1-oylhydrazine

>  <deltaH°(c)>   kJ/mol
-10578

$$$$
2,5-Dimethyl-2-t-butylperoxy-5-trimethylsilylperoxyhexyne
  ChemBrain IXL

 50 49  0  0  0  0  0  0  0  0  1 V2000
    4.5858    7.7543    1.1159 C   0  0  0  0  0  0  0  0  0  0  0  0
    4.1299    7.3359   -0.1758 O   0  0  0  0  0  0  0  0  0  0  0  0
    5.3615    6.6153    1.7809 C   0  0  0  0  0  0  0  0  0  0  0  0
    3.3824    8.1276    1.9850 C   0  0  0  0  0  0  0  0  0  0  0  0
    5.4998    8.9716    0.9624 C   0  0  0  0  0  0  0  0  0  0  0  0
    3.1984    6.1987   -0.1726 O   0  0  0  0  0  0  0  0  0  0  0  0
    3.6286    5.0499   -0.9139 C   0  0  0  0  0  0  0  0  0  0  0  0
    4.9236    4.4974   -0.3078 C   0  0  0  0  0  0  0  0  0  0  0  0
    3.8699    5.4444   -2.3736 C   0  0  0  0  0  0  0  0  0  0  0  0
    2.5433    3.9730   -0.8530 C   0  0  0  0  0  0  0  0  0  0  0  0
    5.9226    4.0653    0.1631 C   0  0  0  0  0  0  0  0  0  0  0  0
```

```
    7.2092    3.5003    0.7754 C   0  0  0  0  0  0  0  0  0  0  0  0
    8.3506    4.1698    0.2248 O   0  0  0  0  0  0  0  0  0  0  0  0
    7.1828    3.7044    2.2921 C   0  0  0  0  0  0  0  0  0  0  0  0
    7.3018    2.0045    0.4661 C   0  0  0  0  0  0  0  0  0  0  0  0
    8.4256    5.6124    0.4934 O   0  0  0  0  0  0  0  0  0  0  0  0
    8.4944    6.5589   -0.8313 Si  0  0  0  0  0  0  0  0  0  0  0  0
    9.7237    5.8486   -2.0366 C   0  0  0  0  0  0  0  0  0  0  0  0
    6.8196    6.6354   -1.6346 C   0  0  0  0  0  0  0  0  0  0  0  0
    9.0321    8.2734   -0.3405 C   0  0  0  0  0  0  0  0  0  0  0  0
    6.2141    6.3530    1.1675 H   0  0  0  0  0  0  0  0  0  0  0  0
    4.7152    5.7532    1.8891 H   0  0  0  0  0  0  0  0  0  0  0  0
    5.7042    6.9340    2.7576 H   0  0  0  0  0  0  0  0  0  0  0  0
    2.7346    7.2665    2.0937 H   0  0  0  0  0  0  0  0  0  0  0  0
    3.7268    8.4457    2.9614 H   0  0  0  0  0  0  0  0  0  0  0  0
    2.8341    8.9347    1.5150 H   0  0  0  0  0  0  0  0  0  0  0  0
    4.9491    9.7802    0.4981 H   0  0  0  0  0  0  0  0  0  0  0  0
    6.3471    8.7102    0.3427 H   0  0  0  0  0  0  0  0  0  0  0  0
    5.8493    9.2863    1.9380 H   0  0  0  0  0  0  0  0  0  0  0  0
    4.6226    6.2192   -2.4186 H   0  0  0  0  0  0  0  0  0  0  0  0
    2.9474    5.8131   -2.8047 H   0  0  0  0  0  0  0  0  0  0  0  0
    4.2081    4.5793   -2.9305 H   0  0  0  0  0  0  0  0  0  0  0  0
    2.8649    3.1040   -1.4137 H   0  0  0  0  0  0  0  0  0  0  0  0
    2.3701    3.6931    0.1788 H   0  0  0  0  0  0  0  0  0  0  0  0
    1.6266    4.3597   -1.2808 H   0  0  0  0  0  0  0  0  0  0  0  0
    6.3225    3.1962    2.7100 H   0  0  0  0  0  0  0  0  0  0  0  0
    8.0880    3.2988    2.7267 H   0  0  0  0  0  0  0  0  0  0  0  0
    7.1193    4.7626    2.5129 H   0  0  0  0  0  0  0  0  0  0  0  0
    8.2079    1.6013    0.9012 H   0  0  0  0  0  0  0  0  0  0  0  0
    6.4425    1.4952    0.8848 H   0  0  0  0  0  0  0  0  0  0  0  0
    7.3206    1.8583   -0.6069 H   0  0  0  0  0  0  0  0  0  0  0  0
    9.7714    6.4781   -2.9165 H   0  0  0  0  0  0  0  0  0  0  0  0
    9.4137    4.8511   -2.3226 H   0  0  0  0  0  0  0  0  0  0  0  0
   10.7007    5.8036   -1.5715 H   0  0  0  0  0  0  0  0  0  0  0  0
    6.5140    5.6385   -1.9255 H   0  0  0  0  0  0  0  0  0  0  0  0
    6.1016    7.0435   -0.9350 H   0  0  0  0  0  0  0  0  0  0  0  0
    6.8687    7.2682   -2.5119 H   0  0  0  0  0  0  0  0  0  0  0  0
    8.3216    8.6872    0.3632 H   0  0  0  0  0  0  0  0  0  0  0  0
    9.0774    8.9032   -1.2204 H   0  0  0  0  0  0  0  0  0  0  0  0
   10.0108    8.2265    0.1206 H   0  0  0  0  0  0  0  0  0  0  0  0
  1  2  1  0  0  0  0
  1  3  1  0  0  0  0
  1  4  1  0  0  0  0
  1  5  1  0  0  0  0
  2  6  1  0  0  0  0
  6  7  1  0  0  0  0
  7  8  1  0  0  0  0
  7  9  1  0  0  0  0
  7 10  1  0  0  0  0
  8 11  3  0  0  0  0
 11 12  1  0  0  0  0
 12 13  1  0  0  0  0
 12 14  1  0  0  0  0
 12 15  1  0  0  0  0
 13 16  1  0  0  0  0
 16 17  1  0  0  0  0
 17 18  1  0  0  0  0
 17 19  1  0  0  0  0
 17 20  1  0  0  0  0
  3 21  1  0  0  0  0
  3 22  1  0  0  0  0
  3 23  1  0  0  0  0
  4 24  1  0  0  0  0
  4 25  1  0  0  0  0
```

```
  4 26  1  0  0  0  0
  5 27  1  0  0  0  0
  5 28  1  0  0  0  0
  5 29  1  0  0  0  0
  9 30  1  0  0  0  0
  9 31  1  0  0  0  0
  9 32  1  0  0  0  0
 10 33  1  0  0  0  0
 10 34  1  0  0  0  0
 10 35  1  0  0  0  0
 14 36  1  0  0  0  0
 14 37  1  0  0  0  0
 14 38  1  0  0  0  0
 15 39  1  0  0  0  0
 15 40  1  0  0  0  0
 15 41  1  0  0  0  0
 18 42  1  0  0  0  0
 18 43  1  0  0  0  0
 18 44  1  0  0  0  0
 19 45  1  0  0  0  0
 19 46  1  0  0  0  0
 19 47  1  0  0  0  0
 20 48  1  0  0  0  0
 20 49  1  0  0  0  0
 20 50  1  0  0  0  0
M  END
>  <Alias name>
2,5-Dimethyl-2-t-butylperoxy-5-trimethylsilylperoxyhexyne

>  <deltaH°(c)>   kJ/mol
-10576

$$$$
1,1,2-Triphenylethane
  ChemBrain IXL

 38 40  0  0  0  0  0  0  0  0  1 V2000
    6.6964    4.1347    0.0000 C   0  0  0  0  0  0  0  0  0  0  0  0
    6.1569    3.6673   -1.1985 C   0  0  0  0  0  0  0  0  0  0  0  0
    5.9489    4.5479   -2.2602 C   0  0  0  0  0  0  0  0  0  0  0  0
    6.2801    5.8965   -2.1229 C   0  0  0  0  0  0  0  0  0  0  0  0
    6.8176    6.3645   -0.9230 C   0  0  0  0  0  0  0  0  0  0  0  0
    7.0277    5.4830    0.1374 C   0  0  0  0  0  0  0  0  0  0  0  0
    7.1681    7.7962   -0.7764 C   0  0  0  0  0  0  0  0  0  0  0  0
    6.7551    8.2951    0.6269 C   0  0  0  0  0  0  0  0  0  0  0  0
    2.5277    7.9159    1.1241 C   0  0  0  0  0  0  0  0  0  0  0  0
    3.3409    8.1773    2.2271 C   0  0  0  0  0  0  0  0  0  0  0  0
    4.7204    8.3042    2.0627 C   0  0  0  0  0  0  0  0  0  0  0  0
    5.2883    8.1666    0.7961 C   0  0  0  0  0  0  0  0  0  0  0  0
    4.4741    7.9093   -0.3084 C   0  0  0  0  0  0  0  0  0  0  0  0
    3.0941    7.7822   -0.1437 C   0  0  0  0  0  0  0  0  0  0  0  0
   11.3830    8.2670   -1.2451 C   0  0  0  0  0  0  0  0  0  0  0  0
   10.7679    7.0747   -1.6270 C   0  0  0  0  0  0  0  0  0  0  0  0
    9.3897    6.9220   -1.4767 C   0  0  0  0  0  0  0  0  0  0  0  0
    8.6276    7.9607   -0.9425 C   0  0  0  0  0  0  0  0  0  0  0  0
    9.2417    9.1538   -0.5634 C   0  0  0  0  0  0  0  0  0  0  0  0
   10.6199    9.3067   -0.7135 C   0  0  0  0  0  0  0  0  0  0  0  0
    6.8581    3.4509    0.8240 H   0  0  0  0  0  0  0  0  0  0  0  0
    5.8995    2.6205   -1.3048 H   0  0  0  0  0  0  0  0  0  0  0  0
    5.5304    4.1849   -3.1910 H   0  0  0  0  0  0  0  0  0  0  0  0
    6.1198    6.5798   -2.9478 H   0  0  0  0  0  0  0  0  0  0  0  0
    7.4478    5.8453    1.0672 H   0  0  0  0  0  0  0  0  0  0  0  0
    6.6509    8.3742   -1.5326 H   0  0  0  0  0  0  0  0  0  0  0  0
```

```
    7.2575    7.7035    1.3814 H   0  0  0  0  0  0  0  0  0  0  0  0
    7.0394    9.3346    0.7365 H   0  0  0  0  0  0  0  0  0  0  0  0
    1.4567    7.8167    1.2522 H   0  0  0  0  0  0  0  0  0  0  0  0
    2.9012    8.2817    3.2110 H   0  0  0  0  0  0  0  0  0  0  0  0
    5.3508    8.5096    2.9186 H   0  0  0  0  0  0  0  0  0  0  0  0
    4.9131    7.8083   -1.2934 H   0  0  0  0  0  0  0  0  0  0  0  0
    2.4624    7.5798   -1.0003 H   0  0  0  0  0  0  0  0  0  0  0  0
   12.4530    8.3856   -1.3613 H   0  0  0  0  0  0  0  0  0  0  0  0
   11.3603    6.2677   -2.0401 H   0  0  0  0  0  0  0  0  0  0  0  0
    8.9114    5.9970   -1.7748 H   0  0  0  0  0  0  0  0  0  0  0  0
    8.6486    9.9614   -0.1524 H   0  0  0  0  0  0  0  0  0  0  0  0
   11.0972   10.2324   -0.4174 H   0  0  0  0  0  0  0  0  0  0  0  0
  1  2  4  0  0  0  0
  2  3  4  0  0  0  0
  3  4  4  0  0  0  0
  4  5  4  0  0  0  0
  5  6  4  0  0  0  0
  6  1  4  0  0  0  0
  5  7  1  0  0  0  0
  7  8  1  0  0  0  0
  9 10  4  0  0  0  0
 10 11  4  0  0  0  0
 11 12  4  0  0  0  0
 12 13  4  0  0  0  0
 13 14  4  0  0  0  0
 14  9  4  0  0  0  0
 15 16  4  0  0  0  0
 16 17  4  0  0  0  0
 17 18  4  0  0  0  0
 18 19  4  0  0  0  0
 19 20  4  0  0  0  0
 20 15  4  0  0  0  0
 18  7  1  0  0  0  0
 12  8  1  0  0  0  0
  1 21  1  0  0  0  0
  2 22  1  0  0  0  0
  3 23  1  0  0  0  0
  4 24  1  0  0  0  0
  6 25  1  0  0  0  0
  7 26  1  0  0  0  0
  8 27  1  0  0  0  0
  8 28  1  0  0  0  0
  9 29  1  0  0  0  0
 10 30  1  0  0  0  0
 11 31  1  0  0  0  0
 13 32  1  0  0  0  0
 14 33  1  0  0  0  0
 15 34  1  0  0  0  0
 16 35  1  0  0  0  0
 17 36  1  0  0  0  0
 19 37  1  0  0  0  0
 20 38  1  0  0  0  0
M  END
>  <Alias name>
1,1,2-Triphenylethane

>  <deltaH°(c)>   kJ/mol
-10572

$$$$
1-Bromohexadecane
  ChemBrain IXL
```

```
50 49  0  0  0  0  0  0  0  0  1 V2000
  13.1038   11.0736   -1.8456 C   0  0  0  0  0  0  0  0  0  0  0  0
  11.7312   11.1921   -1.1549 C   0  0  0  0  0  0  0  0  0  0  0  0
  11.1871    9.7833   -0.8479 C   0  0  0  0  0  0  0  0  0  0  0  0
   9.8146    9.9017   -0.1571 C   0  0  0  0  0  0  0  0  0  0  0  0
   9.2706    8.4929    0.1501 C   0  0  0  0  0  0  0  0  0  0  0  0
   7.8981    8.6114    0.8410 C   0  0  0  0  0  0  0  0  0  0  0  0
   7.3542    7.2026    1.1483 C   0  0  0  0  0  0  0  0  0  0  0  0
   5.9818    7.3211    1.8393 C   0  0  0  0  0  0  0  0  0  0  0  0
   5.4379    5.9123    2.1467 C   0  0  0  0  0  0  0  0  0  0  0  0
  13.6479   12.4824   -2.1525 C   0  0  0  0  0  0  0  0  0  0  0  0
   4.0656    6.0309    2.8379 C   0  0  0  0  0  0  0  0  0  0  0  0
  15.0205   12.3640   -2.8432 C   0  0  0  0  0  0  0  0  0  0  0  0
   3.5217    4.6221    3.1453 C   0  0  0  0  0  0  0  0  0  0  0  0
  15.5647   13.7729   -3.1501 C   0  0  0  0  0  0  0  0  0  0  0  0
   2.1495    4.7405    3.8365 C   0  0  0  0  0  0  0  0  0  0  0  0
  16.9372   13.6546   -3.8406 C   0  0  0  0  0  0  0  0  0  0  0  0
   1.4690    2.9772    4.2212 Br  0  0  0  0  0  0  0  0  0  0  0  0
  12.9963   10.5190   -2.7696 H   0  0  0  0  0  0  0  0  0  0  0  0
  13.7935   10.5549   -1.1913 H   0  0  0  0  0  0  0  0  0  0  0  0
  11.8387   11.7468   -0.2310 H   0  0  0  0  0  0  0  0  0  0  0  0
  11.0414   11.7107   -1.8092 H   0  0  0  0  0  0  0  0  0  0  0  0
  11.0796    9.2286   -1.7718 H   0  0  0  0  0  0  0  0  0  0  0  0
  11.8769    9.2646   -0.1937 H   0  0  0  0  0  0  0  0  0  0  0  0
   9.9222   10.4565    0.7668 H   0  0  0  0  0  0  0  0  0  0  0  0
   9.1248   10.4203   -0.8114 H   0  0  0  0  0  0  0  0  0  0  0  0
   9.1629    7.9382   -0.7738 H   0  0  0  0  0  0  0  0  0  0  0  0
   9.9605    7.9743    0.8043 H   0  0  0  0  0  0  0  0  0  0  0  0
   8.0058    9.1662    1.7648 H   0  0  0  0  0  0  0  0  0  0  0  0
   7.2082    9.1300    0.1868 H   0  0  0  0  0  0  0  0  0  0  0  0
   7.2465    6.6478    0.2244 H   0  0  0  0  0  0  0  0  0  0  0  0
   8.0442    6.6840    1.8024 H   0  0  0  0  0  0  0  0  0  0  0  0
   6.0895    7.8760    2.7631 H   0  0  0  0  0  0  0  0  0  0  0  0
   5.2918    7.8397    1.1851 H   0  0  0  0  0  0  0  0  0  0  0  0
   5.3301    5.3575    1.2229 H   0  0  0  0  0  0  0  0  0  0  0  0
   6.1279    5.3938    2.8009 H   0  0  0  0  0  0  0  0  0  0  0  0
  13.7553   13.0371   -1.2286 H   0  0  0  0  0  0  0  0  0  0  0  0
  12.9581   13.0011   -2.8069 H   0  0  0  0  0  0  0  0  0  0  0  0
   4.1734    6.5857    3.7616 H   0  0  0  0  0  0  0  0  0  0  0  0
   3.3755    6.5494    2.1837 H   0  0  0  0  0  0  0  0  0  0  0  0
  14.9130   11.8093   -3.7671 H   0  0  0  0  0  0  0  0  0  0  0  0
  15.7102   11.8453   -2.1888 H   0  0  0  0  0  0  0  0  0  0  0  0
   3.4139    4.0672    2.2215 H   0  0  0  0  0  0  0  0  0  0  0  0
   4.2118    4.1036    3.7994 H   0  0  0  0  0  0  0  0  0  0  0  0
  15.6721   14.3276   -2.2262 H   0  0  0  0  0  0  0  0  0  0  0  0
  14.8750   14.2916   -3.8044 H   0  0  0  0  0  0  0  0  0  0  0  0
   2.2572    5.2954    4.7603 H   0  0  0  0  0  0  0  0  0  0  0  0
   1.4594    5.2590    3.1824 H   0  0  0  0  0  0  0  0  0  0  0  0
  17.3193   14.6447   -4.0562 H   0  0  0  0  0  0  0  0  0  0  0  0
  16.8299   13.1000   -4.7646 H   0  0  0  0  0  0  0  0  0  0  0  0
  17.6270   13.1360   -3.1863 H   0  0  0  0  0  0  0  0  0  0  0  0
  1  2  1  0  0  0  0
  2  3  1  0  0  0  0
  3  4  1  0  0  0  0
  4  5  1  0  0  0  0
  5  6  1  0  0  0  0
  6  7  1  0  0  0  0
  7  8  1  0  0  0  0
  8  9  1  0  0  0  0
  1 10  1  0  0  0  0
  9 11  1  0  0  0  0
 10 12  1  0  0  0  0
 11 13  1  0  0  0  0
```

```
 12 14  1  0  0  0  0
 13 15  1  0  0  0  0
 14 16  1  0  0  0  0
 15 17  1  0  0  0  0
  1 18  1  0  0  0  0
  1 19  1  0  0  0  0
  2 20  1  0  0  0  0
  2 21  1  0  0  0  0
  3 22  1  0  0  0  0
  3 23  1  0  0  0  0
  4 24  1  0  0  0  0
  4 25  1  0  0  0  0
  5 26  1  0  0  0  0
  5 27  1  0  0  0  0
  6 28  1  0  0  0  0
  6 29  1  0  0  0  0
  7 30  1  0  0  0  0
  7 31  1  0  0  0  0
  8 32  1  0  0  0  0
  8 33  1  0  0  0  0
  9 34  1  0  0  0  0
  9 35  1  0  0  0  0
 10 36  1  0  0  0  0
 10 37  1  0  0  0  0
 11 38  1  0  0  0  0
 11 39  1  0  0  0  0
 12 40  1  0  0  0  0
 12 41  1  0  0  0  0
 13 42  1  0  0  0  0
 13 43  1  0  0  0  0
 14 44  1  0  0  0  0
 14 45  1  0  0  0  0
 15 46  1  0  0  0  0
 15 47  1  0  0  0  0
 16 48  1  0  0  0  0
 16 49  1  0  0  0  0
 16 50  1  0  0  0  0
M  END
>  <Alias name>
1-Bromohexadecane

>  <deltaH°(c)>   kJ/mol
-10568

$$$$
Dipropyldibutylsilane
  ChemBrain IXL

 47 46  0  0  0  0  0  0  0  0  1 V2000
    9.1691    4.1801    1.3484 C   0  0  0  0  0  0  0  0  0  0  0  0
    7.8090    3.5464    1.6995 C   0  0  0  0  0  0  0  0  0  0  0  0
   10.3057    3.2727    1.8582 C   0  0  0  0  0  0  0  0  0  0  0  0
    6.4345    4.6446    1.0840 Si  0  0  0  0  0  0  0  0  0  0  0  0
    6.6350    4.7920   -0.7863 C   0  0  0  0  0  0  0  0  0  0  0  0
    5.6172    5.7900   -1.3774 C   0  0  0  0  0  0  0  0  0  0  0  0
    5.7415    5.7943   -2.9131 C   0  0  0  0  0  0  0  0  0  0  0  0
    4.5482    9.5848    1.8818 C   0  0  0  0  0  0  0  0  0  0  0  0
    5.7181    8.6410    2.2208 C   0  0  0  0  0  0  0  0  0  0  0  0
    5.4596    7.2570    1.5946 C   0  0  0  0  0  0  0  0  0  0  0  0
    6.6313    6.3144    1.9399 C   0  0  0  0  0  0  0  0  0  0  0  0
    3.6026    4.5810    0.9655 C   0  0  0  0  0  0  0  0  0  0  0  0
    4.8066    3.8110    1.5474 C   0  0  0  0  0  0  0  0  0  0  0  0
    2.2958    3.9322    1.4607 C   0  0  0  0  0  0  0  0  0  0  0  0
```

```
   11.6658    3.9062    1.5071 C   0  0  0  0  0  0  0  0  0  0  0  0
    9.2460    5.1530    1.8177 H   0  0  0  0  0  0  0  0  0  0  0  0
    9.2496    4.2901    0.2741 H   0  0  0  0  0  0  0  0  0  0  0  0
    7.7289    3.4357    2.7738 H   0  0  0  0  0  0  0  0  0  0  0  0
    7.7317    2.5738    1.2296 H   0  0  0  0  0  0  0  0  0  0  0  0
   10.2288    2.2997    1.3889 H   0  0  0  0  0  0  0  0  0  0  0  0
   10.2254    3.1626    2.9326 H   0  0  0  0  0  0  0  0  0  0  0  0
    7.6363    5.1386   -1.0095 H   0  0  0  0  0  0  0  0  0  0  0  0
    6.4869    3.8186   -1.2371 H   0  0  0  0  0  0  0  0  0  0  0  0
    4.6223    5.5007   -1.1023 H   0  0  0  0  0  0  0  0  0  0  0  0
    5.8230    6.7772   -1.0028 H   0  0  0  0  0  0  0  0  0  0  0  0
    5.0304    6.4961   -3.3307 H   0  0  0  0  0  0  0  0  0  0  0  0
    5.5360    4.8019   -3.2947 H   0  0  0  0  0  0  0  0  0  0  0  0
    6.7456    6.0880   -3.1932 H   0  0  0  0  0  0  0  0  0  0  0  0
    3.6274    9.1754    2.2786 H   0  0  0  0  0  0  0  0  0  0  0  0
    4.7299   10.5574    2.3218 H   0  0  0  0  0  0  0  0  0  0  0  0
    4.4649    9.6848    0.8066 H   0  0  0  0  0  0  0  0  0  0  0  0
    5.8013    8.5410    3.2959 H   0  0  0  0  0  0  0  0  0  0  0  0
    6.6388    9.0505    1.8240 H   0  0  0  0  0  0  0  0  0  0  0  0
    5.3757    7.3585    0.5307 H   0  0  0  0  0  0  0  0  0  0  0  0
    4.5450    6.8538    1.9924 H   0  0  0  0  0  0  0  0  0  0  0  0
    6.6556    6.1628    3.0119 H   0  0  0  0  0  0  0  0  0  0  0  0
    7.5624    6.7679    1.6236 H   0  0  0  0  0  0  0  0  0  0  0  0
    3.6352    5.6035    1.2860 H   0  0  0  0  0  0  0  0  0  0  0  0
    3.6339    4.5381   -0.1090 H   0  0  0  0  0  0  0  0  0  0  0  0
    4.8006    2.8018    1.1549 H   0  0  0  0  0  0  0  0  0  0  0  0
    4.7214    3.7735    2.6264 H   0  0  0  0  0  0  0  0  0  0  0  0
    2.2599    2.8995    1.1364 H   0  0  0  0  0  0  0  0  0  0  0  0
    2.2601    3.9723    2.5424 H   0  0  0  0  0  0  0  0  0  0  0  0
    1.4488    4.4687    1.0515 H   0  0  0  0  0  0  0  0  0  0  0  0
   11.7427    4.8792    1.9764 H   0  0  0  0  0  0  0  0  0  0  0  0
   11.7462    4.0163    0.4327 H   0  0  0  0  0  0  0  0  0  0  0  0
   12.4643    3.2685    1.8654 H   0  0  0  0  0  0  0  0  0  0  0  0
  1  2  1  0  0  0  0
  1  3  1  0  0  0  0
  2  4  1  0  0  0  0
  4  5  1  0  0  0  0
  5  6  1  0  0  0  0
  6  7  1  0  0  0  0
  8  9  1  0  0  0  0
  9 10  1  0  0  0  0
 10 11  1  0  0  0  0
 11  4  1  0  0  0  0
 12 13  1  0  0  0  0
 12 14  1  0  0  0  0
 13  4  1  0  0  0  0
  3 15  1  0  0  0  0
  1 16  1  0  0  0  0
  1 17  1  0  0  0  0
  2 18  1  0  0  0  0
  2 19  1  0  0  0  0
  3 20  1  0  0  0  0
  3 21  1  0  0  0  0
  5 22  1  0  0  0  0
  5 23  1  0  0  0  0
  6 24  1  0  0  0  0
  6 25  1  0  0  0  0
  7 26  1  0  0  0  0
  7 27  1  0  0  0  0
  7 28  1  0  0  0  0
  8 29  1  0  0  0  0
  8 30  1  0  0  0  0
  8 31  1  0  0  0  0
```

```
  9 32  1  0  0  0  0
  9 33  1  0  0  0  0
 10 34  1  0  0  0  0
 10 35  1  0  0  0  0
 11 36  1  0  0  0  0
 11 37  1  0  0  0  0
 12 38  1  0  0  0  0
 12 39  1  0  0  0  0
 13 40  1  0  0  0  0
 13 41  1  0  0  0  0
 14 42  1  0  0  0  0
 14 43  1  0  0  0  0
 14 44  1  0  0  0  0
 15 45  1  0  0  0  0
 15 46  1  0  0  0  0
 15 47  1  0  0  0  0
M  END
>  <Alias name>
Dipropyldibutylsilane

>  <deltaH°(c)>   kJ/mol
-10555

$$$$
Methyl (Z) 9-hexadecenoate
  ChemBrain IXL

 51 50  0  0  0  0  0  0  0  0  1 V2000
   10.1490    8.5819    0.0746 C   0  0  0  0  0  0  0  0  0  0  0  0
    8.8159    8.5687   -0.0092 C   0  0  0  0  0  0  0  0  0  0  0  0
    8.0648    9.8432   -0.0860 C   0  0  0  0  0  0  0  0  0  0  0  0
    6.5538    9.5556   -0.0027 C   0  0  0  0  0  0  0  0  0  0  0  0
    5.7738   10.8819   -0.0835 C   0  0  0  0  0  0  0  0  0  0  0  0
    4.2623   10.5947    0.0004 C   0  0  0  0  0  0  0  0  0  0  0  0
    3.4818   11.9211   -0.0809 C   0  0  0  0  0  0  0  0  0  0  0  0
    1.9703   11.6338    0.0033 C   0  0  0  0  0  0  0  0  0  0  0  0
   10.8787    9.8709    0.0910 C   0  0  0  0  0  0  0  0  0  0  0  0
   12.3935    9.6054    0.0049 C   0  0  0  0  0  0  0  0  0  0  0  0
   13.1519   10.9465    0.0261 C   0  0  0  0  0  0  0  0  0  0  0  0
   14.6670   10.6805   -0.0592 C   0  0  0  0  0  0  0  0  0  0  0  0
   15.4266   12.0207   -0.0301 C   0  0  0  0  0  0  0  0  0  0  0  0
   16.9417   11.7538   -0.1136 C   0  0  0  0  0  0  0  0  0  0  0  0
   17.7020   13.0936   -0.0759 C   0  0  0  0  0  0  0  0  0  0  0  0
   19.1570   12.8383   -0.1578 C   0  0  0  0  0  0  0  0  0  0  0  0
   19.8826   13.6327   -0.7339 O   0  0  0  0  0  0  0  0  0  0  0  0
   19.7276   11.6270    0.4553 O   0  0  0  0  0  0  0  0  0  0  0  0
   21.1838   11.4789    0.3089 C   0  0  0  0  0  0  0  0  0  0  0  0
   10.6966    7.6493    0.1307 H   0  0  0  0  0  0  0  0  0  0  0  0
    8.2840    7.6254   -0.0211 H   0  0  0  0  0  0  0  0  0  0  0  0
    8.3559   10.4840    0.7372 H   0  0  0  0  0  0  0  0  0  0  0  0
    8.2870   10.3371   -1.0239 H   0  0  0  0  0  0  0  0  0  0  0  0
    6.2624    8.9146   -0.8256 H   0  0  0  0  0  0  0  0  0  0  0  0
    6.3313    9.0624    0.9354 H   0  0  0  0  0  0  0  0  0  0  0  0
    6.0657   11.5231    0.7391 H   0  0  0  0  0  0  0  0  0  0  0  0
    5.9960   11.3748   -1.0219 H   0  0  0  0  0  0  0  0  0  0  0  0
    3.9706    9.9532   -0.8220 H   0  0  0  0  0  0  0  0  0  0  0  0
    4.0401   10.1020    0.9389 H   0  0  0  0  0  0  0  0  0  0  0  0
    3.7737   12.5626    0.7413 H   0  0  0  0  0  0  0  0  0  0  0  0
    3.7039   12.4136   -1.0195 H   0  0  0  0  0  0  0  0  0  0  0  0
    1.6784   10.9922   -0.8189 H   0  0  0  0  0  0  0  0  0  0  0  0
    1.7481   11.1414    0.9419 H   0  0  0  0  0  0  0  0  0  0  0  0
    1.4220   12.5659   -0.0540 H   0  0  0  0  0  0  0  0  0  0  0  0
   10.5691   10.4720   -0.7551 H   0  0  0  0  0  0  0  0  0  0  0  0
```

```
  10.6567   10.3996    1.0098 H   0  0  0  0  0  0  0  0  0  0  0  0
  12.7028    9.0025    0.8496 H   0  0  0  0  0  0  0  0  0  0  0  0
  12.6160    9.0792   -0.9151 H   0  0  0  0  0  0  0  0  0  0  0  0
  12.8429   11.5496   -0.8188 H   0  0  0  0  0  0  0  0  0  0  0  0
  12.9289   11.4726    0.9461 H   0  0  0  0  0  0  0  0  0  0  0  0
  14.9747   10.0731    0.7830 H   0  0  0  0  0  0  0  0  0  0  0  0
  14.8906   10.1586   -0.9814 H   0  0  0  0  0  0  0  0  0  0  0  0
  15.1199   12.6281   -0.8727 H   0  0  0  0  0  0  0  0  0  0  0  0
  15.2023   12.5427    0.8919 H   0  0  0  0  0  0  0  0  0  0  0  0
  17.2472   11.1417    0.7260 H   0  0  0  0  0  0  0  0  0  0  0  0
  17.1668   11.2367   -1.0383 H   0  0  0  0  0  0  0  0  0  0  0  0
  17.3955   13.7063   -0.9147 H   0  0  0  0  0  0  0  0  0  0  0  0
  17.4777   13.6100    0.8492 H   0  0  0  0  0  0  0  0  0  0  0  0
  21.6808   12.3217    0.7732 H   0  0  0  0  0  0  0  0  0  0  0  0
  21.4390   11.4440   -0.7431 H   0  0  0  0  0  0  0  0  0  0  0  0
  21.5034   10.5623    0.7885 H   0  0  0  0  0  0  0  0  0  0  0  0
  1  2  2  0  0  0  0
  2  3  1  0  0  0  0
  3  4  1  0  0  0  0
  4  5  1  0  0  0  0
  5  6  1  0  0  0  0
  6  7  1  0  0  0  0
  7  8  1  0  0  0  0
  1  9  1  0  0  0  0
 10 11  1  0  0  0  0
 11 12  1  0  0  0  0
 12 13  1  0  0  0  0
 13 14  1  0  0  0  0
 14 15  1  0  0  0  0
 15 16  1  0  0  0  0
  9 10  1  0  0  0  0
 16 17  2  0  0  0  0
 16 18  1  0  0  0  0
 18 19  1  0  0  0  0
  1 20  1  0  0  0  0
  2 21  1  0  0  0  0
  3 22  1  0  0  0  0
  3 23  1  0  0  0  0
  4 24  1  0  0  0  0
  4 25  1  0  0  0  0
  5 26  1  0  0  0  0
  5 27  1  0  0  0  0
  6 28  1  0  0  0  0
  6 29  1  0  0  0  0
  7 30  1  0  0  0  0
  7 31  1  0  0  0  0
  8 32  1  0  0  0  0
  8 33  1  0  0  0  0
  8 34  1  0  0  0  0
  9 35  1  0  0  0  0
  9 36  1  0  0  0  0
 10 37  1  0  0  0  0
 10 38  1  0  0  0  0
 11 39  1  0  0  0  0
 11 40  1  0  0  0  0
 12 41  1  0  0  0  0
 12 42  1  0  0  0  0
 13 43  1  0  0  0  0
 13 44  1  0  0  0  0
 14 45  1  0  0  0  0
 14 46  1  0  0  0  0
 15 47  1  0  0  0  0
 15 48  1  0  0  0  0
```

```
 19 49  1  0  0  0  0
 19 50  1  0  0  0  0
 19 51  1  0  0  0  0
M  END
>  <Alias name>
Methyl palmitoleate

>  <deltaH°(c)>   kJ/mol
-10548

$$$$
1-Hexadecene
  ChemBrain IXL

 48 47  0  0  0  0  0  0  0  0  1 V2000
    9.5650   10.0099    0.6673 C   0  0  0  0  0  0  0  0  0  0  0  0
    8.3498   10.9363    0.8666 C   0  0  0  0  0  0  0  0  0  0  0  0
    7.1057   10.0884    1.1952 C   0  0  0  0  0  0  0  0  0  0  0  0
    5.8911   11.0150    1.3973 C   0  0  0  0  0  0  0  0  0  0  0  0
    4.6471   10.1671    1.7270 C   0  0  0  0  0  0  0  0  0  0  0  0
    3.4803   11.0572    1.9226 C   0  0  0  0  0  0  0  0  0  0  0  0
    2.2786   10.5360    2.1812 C   0  0  0  0  0  0  0  0  0  0  0  0
   10.8095   10.8579    0.3404 C   0  0  0  0  0  0  0  0  0  0  0  0
   12.0254    9.9317    0.1445 C   0  0  0  0  0  0  0  0  0  0  0  0
   13.2703   10.7798   -0.1806 C   0  0  0  0  0  0  0  0  0  0  0  0
   14.4868    9.8536   -0.3735 C   0  0  0  0  0  0  0  0  0  0  0  0
   15.7321   10.7017   -0.6973 C   0  0  0  0  0  0  0  0  0  0  0  0
   16.9490    9.7756   -0.8880 C   0  0  0  0  0  0  0  0  0  0  0  0
   18.1945   10.6237   -1.2112 C   0  0  0  0  0  0  0  0  0  0  0  0
   19.4117    9.6975   -1.4009 C   0  0  0  0  0  0  0  0  0  0  0  0
   20.6572   10.5454   -1.7240 C   0  0  0  0  0  0  0  0  0  0  0  0
    9.3684    9.3272   -0.1501 H   0  0  0  0  0  0  0  0  0  0  0  0
    9.7411    9.4454    1.5746 H   0  0  0  0  0  0  0  0  0  0  0  0
    8.5469   11.6195    1.6834 H   0  0  0  0  0  0  0  0  0  0  0  0
    8.1729   11.5001   -0.0410 H   0  0  0  0  0  0  0  0  0  0  0  0
    6.9074    9.4063    0.3777 H   0  0  0  0  0  0  0  0  0  0  0  0
    7.2833    9.5233    2.1019 H   0  0  0  0  0  0  0  0  0  0  0  0
    6.0898   11.6974    2.2144 H   0  0  0  0  0  0  0  0  0  0  0  0
    5.7132   11.5798    0.4904 H   0  0  0  0  0  0  0  0  0  0  0  0
    4.4479    9.4852    0.9095 H   0  0  0  0  0  0  0  0  0  0  0  0
    4.8255    9.6016    2.6333 H   0  0  0  0  0  0  0  0  0  0  0  0
    3.6013   12.1314    1.8559 H   0  0  0  0  0  0  0  0  0  0  0  0
    2.1571    9.4618    2.2480 H   0  0  0  0  0  0  0  0  0  0  0  0
    1.4254   11.1873    2.3242 H   0  0  0  0  0  0  0  0  0  0  0  0
   11.0047   11.5419    1.1571 H   0  0  0  0  0  0  0  0  0  0  0  0
   10.6343   11.4209   -0.5680 H   0  0  0  0  0  0  0  0  0  0  0  0
   11.8306    9.2482   -0.6728 H   0  0  0  0  0  0  0  0  0  0  0  0
   12.1997    9.3680    1.0527 H   0  0  0  0  0  0  0  0  0  0  0  0
   13.4638   11.4645    0.6359 H   0  0  0  0  0  0  0  0  0  0  0  0
   13.0968   11.3420   -1.0898 H   0  0  0  0  0  0  0  0  0  0  0  0
   14.2936    9.1693   -1.1904 H   0  0  0  0  0  0  0  0  0  0  0  0
   14.6597    9.2909    0.5355 H   0  0  0  0  0  0  0  0  0  0  0  0
   15.9244   11.3869    0.1191 H   0  0  0  0  0  0  0  0  0  0  0  0
   15.5598   11.2634   -1.6071 H   0  0  0  0  0  0  0  0  0  0  0  0
   16.7569    9.0906   -1.7046 H   0  0  0  0  0  0  0  0  0  0  0  0
   17.1210    9.2137    0.0217 H   0  0  0  0  0  0  0  0  0  0  0  0
   18.3862   11.3091   -0.3949 H   0  0  0  0  0  0  0  0  0  0  0  0
   18.0227   11.1851   -2.1213 H   0  0  0  0  0  0  0  0  0  0  0  0
   19.2201    9.0121   -2.2172 H   0  0  0  0  0  0  0  0  0  0  0  0
   19.5834    9.1361   -0.4909 H   0  0  0  0  0  0  0  0  0  0  0  0
   20.8490   11.2309   -0.9078 H   0  0  0  0  0  0  0  0  0  0  0  0
   20.4857   11.1067   -2.6341 H   0  0  0  0  0  0  0  0  0  0  0  0
   21.5124    9.8944   -1.8571 H   0  0  0  0  0  0  0  0  0  0  0  0
```

1 2 1 0 0 0 0
2 3 1 0 0 0 0
3 4 1 0 0 0 0
4 5 1 0 0 0 0
5 6 1 0 0 0 0
6 7 2 0 0 0 0
1 8 1 0 0 0 0
8 9 1 0 0 0 0
9 10 1 0 0 0 0
10 11 1 0 0 0 0
11 12 1 0 0 0 0
12 13 1 0 0 0 0
13 14 1 0 0 0 0
14 15 1 0 0 0 0
15 16 1 0 0 0 0
1 17 1 0 0 0 0
1 18 1 0 0 0 0
2 19 1 0 0 0 0
2 20 1 0 0 0 0
3 21 1 0 0 0 0
3 22 1 0 0 0 0
4 23 1 0 0 0 0
4 24 1 0 0 0 0
5 25 1 0 0 0 0
5 26 1 0 0 0 0
6 27 1 0 0 0 0
7 28 1 0 0 0 0
7 29 1 0 0 0 0
8 30 1 0 0 0 0
8 31 1 0 0 0 0
9 32 1 0 0 0 0
9 33 1 0 0 0 0
10 34 1 0 0 0 0
10 35 1 0 0 0 0
11 36 1 0 0 0 0
11 37 1 0 0 0 0
12 38 1 0 0 0 0
12 39 1 0 0 0 0
13 40 1 0 0 0 0
13 41 1 0 0 0 0
14 42 1 0 0 0 0
14 43 1 0 0 0 0
15 44 1 0 0 0 0
15 45 1 0 0 0 0
16 46 1 0 0 0 0
16 47 1 0 0 0 0
16 48 1 0 0 0 0
M END
> <Alias name>
Cetene

> <deltaH°(c)> kJ/mol
-10539

$$$$
Benzo(ghi)perylene
ChemBrain IXL

34 39 0 0 0 0 0 0 0 0 1 V2000
7.7735 5.3654 0.0000 C 0 0 0 0 0 0 0 0 0 0 0 0
7.7641 6.7601 -0.0258 C 0 0 0 0 0 0 0 0 0 0 0 0
6.5703 4.6603 0.0361 C 0 0 0 0 0 0 0 0 0 0 0 0
8.9860 4.6757 -0.0103 C 0 0 0 0 0 0 0 0 0 0 0 0

```
    6.5516    7.4498   -0.0154 C   0  0  0  0  0  0  0  0  0  0  0  0
    8.9673    7.4652   -0.0619 C   0  0  0  0  0  0  0  0  0  0  0  0
    5.3578    5.3500    0.0465 C   0  0  0  0  0  0  0  0  0  0  0  0
    6.5797    3.2656    0.0619 C   0  0  0  0  0  0  0  0  0  0  0  0
   10.1892    5.3808   -0.0464 C   0  0  0  0  0  0  0  0  0  0  0  0
    4.1640    3.2502    0.1083 C   0  0  0  0  0  0  0  0  0  0  0  0
    5.3484    6.7448    0.0207 C   0  0  0  0  0  0  0  0  0  0  0  0
    6.5422    8.8445   -0.0412 C   0  0  0  0  0  0  0  0  0  0  0  0
   10.1798    6.7755   -0.0722 C   0  0  0  0  0  0  0  0  0  0  0  0
    8.9579    8.8599   -0.0876 C   0  0  0  0  0  0  0  0  0  0  0  0
    4.1546    4.6450    0.0826 C   0  0  0  0  0  0  0  0  0  0  0  0
    5.3765    2.5606    0.0980 C   0  0  0  0  0  0  0  0  0  0  0  0
    4.1359    7.4344    0.0311 C   0  0  0  0  0  0  0  0  0  0  0  0
    7.7454    9.5496   -0.0773 C   0  0  0  0  0  0  0  0  0  0  0  0
    2.9420    5.3347    0.0929 C   0  0  0  0  0  0  0  0  0  0  0  0
    2.9327    6.7294    0.0672 C   0  0  0  0  0  0  0  0  0  0  0  0
    7.7922    2.5759    0.0515 C   0  0  0  0  0  0  0  0  0  0  0  0
    8.9954    3.2810    0.0154 C   0  0  0  0  0  0  0  0  0  0  0  0
   11.1305    4.8453   -0.0545 H   0  0  0  0  0  0  0  0  0  0  0  0
    3.2299    2.7029    0.1363 H   0  0  0  0  0  0  0  0  0  0  0  0
    5.6009    9.3800   -0.0331 H   0  0  0  0  0  0  0  0  0  0  0  0
   11.1139    7.3229   -0.1002 H   0  0  0  0  0  0  0  0  0  0  0  0
    9.8920    9.4073   -0.1156 H   0  0  0  0  0  0  0  0  0  0  0  0
    5.3838    1.4778    0.1180 H   0  0  0  0  0  0  0  0  0  0  0  0
    4.1286    8.5172    0.0111 H   0  0  0  0  0  0  0  0  0  0  0  0
    7.7381   10.6324   -0.0972 H   0  0  0  0  0  0  0  0  0  0  0  0
    2.0080    4.7873    0.1209 H   0  0  0  0  0  0  0  0  0  0  0  0
    1.9913    7.2648    0.0752 H   0  0  0  0  0  0  0  0  0  0  0  0
    7.7995    1.4931    0.0715 H   0  0  0  0  0  0  0  0  0  0  0  0
    9.9367    2.7456    0.0074 H   0  0  0  0  0  0  0  0  0  0  0  0
  1  2  4  0  0  0  0
  1  3  4  0  0  0  0
  1  4  4  0  0  0  0
  2  5  4  0  0  0  0
  2  6  4  0  0  0  0
  3  7  4  0  0  0  0
  3  8  4  0  0  0  0
  4  9  4  0  0  0  0
  5 11  4  0  0  0  0
  5 12  4  0  0  0  0
  6 13  4  0  0  0  0
  6 14  4  0  0  0  0
  7 15  4  0  0  0  0
  7 11  4  0  0  0  0
  8 16  4  0  0  0  0
  9 13  4  0  0  0  0
 10 16  4  0  0  0  0
 11 17  4  0  0  0  0
 12 18  4  0  0  0  0
 14 18  4  0  0  0  0
 15 19  4  0  0  0  0
 17 20  4  0  0  0  0
 19 20  4  0  0  0  0
 10 15  4  0  0  0  0
  8 21  4  0  0  0  0
 21 22  4  0  0  0  0
 22  4  4  0  0  0  0
  9 23  1  0  0  0  0
 10 24  1  0  0  0  0
 12 25  1  0  0  0  0
 13 26  1  0  0  0  0
 14 27  1  0  0  0  0
 16 28  1  0  0  0  0
```

```
 17 29  1  0  0  0  0
 18 30  1  0  0  0  0
 19 31  1  0  0  0  0
 20 32  1  0  0  0  0
 21 33  1  0  0  0  0
 22 34  1  0  0  0  0
M  END
>  <Alias name>
Benzo[g,h,i]perylene

>  <deltaH°(c)>   kJ/mol
-10529

$$$$
Cetyl alcohol
  ChemBrain IXL

 51 50  0  0  0  0  0  0  0  0  1 V2000
   11.5132   10.2099    0.0000 C   0  0  0  0  0  0  0  0  0  0  0  0  0  0
    9.9887   10.4296    0.0498 C   0  0  0  0  0  0  0  0  0  0  0  0  0  0
   12.2337   11.5520    0.2338 C   0  0  0  0  0  0  0  0  0  0  0  0  0  0
    9.2682    9.0873   -0.1832 C   0  0  0  0  0  0  0  0  0  0  0  0  0  0
   13.7583   11.3322    0.1847 C   0  0  0  0  0  0  0  0  0  0  0  0  0  0
    7.7437    9.3069   -0.1326 C   0  0  0  0  0  0  0  0  0  0  0  0  0  0
   14.4788   12.6741    0.4192 C   0  0  0  0  0  0  0  0  0  0  0  0  0  0
    7.0232    7.9645   -0.3649 C   0  0  0  0  0  0  0  0  0  0  0  0  0  0
   16.0033   12.4542    0.3707 C   0  0  0  0  0  0  0  0  0  0  0  0  0  0
    5.4986    8.1840   -0.3139 C   0  0  0  0  0  0  0  0  0  0  0  0  0  0
   16.7238   13.7961    0.6058 C   0  0  0  0  0  0  0  0  0  0  0  0  0  0
    4.7781    6.8416   -0.5457 C   0  0  0  0  0  0  0  0  0  0  0  0  0  0
   18.2485   13.5762    0.5577 C   0  0  0  0  0  0  0  0  0  0  0  0  0  0
    3.2535    7.0610   -0.4944 C   0  0  0  0  0  0  0  0  0  0  0  0  0  0
   18.9689   14.9179    0.7930 C   0  0  0  0  0  0  0  0  0  0  0  0  0  0
    2.5329    5.7187   -0.7261 C   0  0  0  0  0  0  0  0  0  0  0  0  0  0
   20.4230   14.7077    0.7472 O   0  0  0  0  0  0  0  0  0  0  0  0  0  0
   20.9641   15.5160    0.8883 H   0  0  0  0  0  0  0  0  0  0  0  0  0  0
   11.7897    9.8159   -0.9702 H   0  0  0  0  0  0  0  0  0  0  0  0  0  0
   11.8019    9.5062    0.7710 H   0  0  0  0  0  0  0  0  0  0  0  0  0  0
    9.7124   10.8239    1.0199 H   0  0  0  0  0  0  0  0  0  0  0  0  0  0
    9.6999   11.1329   -0.7214 H   0  0  0  0  0  0  0  0  0  0  0  0  0  0
   11.9569   11.9461    1.2038 H   0  0  0  0  0  0  0  0  0  0  0  0  0  0
   11.9454   12.2556   -0.5375 H   0  0  0  0  0  0  0  0  0  0  0  0  0  0
    9.5442    8.6931   -1.1534 H   0  0  0  0  0  0  0  0  0  0  0  0  0  0
    9.5573    8.3839    0.5879 H   0  0  0  0  0  0  0  0  0  0  0  0  0  0
   14.0352   10.9384   -0.7854 H   0  0  0  0  0  0  0  0  0  0  0  0  0  0
   14.0465   10.6282    0.9556 H   0  0  0  0  0  0  0  0  0  0  0  0  0  0
    7.4678    9.7015    0.8375 H   0  0  0  0  0  0  0  0  0  0  0  0  0  0
    7.4545   10.0100   -0.9039 H   0  0  0  0  0  0  0  0  0  0  0  0  0  0
   14.2015   13.0680    1.3892 H   0  0  0  0  0  0  0  0  0  0  0  0  0  0
   14.1909   13.3780   -0.3520 H   0  0  0  0  0  0  0  0  0  0  0  0  0  0
    7.2988    7.5701   -1.3351 H   0  0  0  0  0  0  0  0  0  0  0  0  0  0
    7.3126    7.2613    0.4062 H   0  0  0  0  0  0  0  0  0  0  0  0  0  0
   16.2806   12.0607   -0.5993 H   0  0  0  0  0  0  0  0  0  0  0  0  0  0
   16.2911   11.7502    1.1417 H   0  0  0  0  0  0  0  0  0  0  0  0  0  0
    5.2231    8.5787    0.6563 H   0  0  0  0  0  0  0  0  0  0  0  0  0  0
    5.2091    8.8870   -1.0851 H   0  0  0  0  0  0  0  0  0  0  0  0  0  0
   16.4463   14.1898    1.5758 H   0  0  0  0  0  0  0  0  0  0  0  0  0  0
   16.4362   14.5002   -0.1653 H   0  0  0  0  0  0  0  0  0  0  0  0  0  0
    5.0536    6.4469   -1.5159 H   0  0  0  0  0  0  0  0  0  0  0  0  0  0
    5.0678    6.1385    0.2254 H   0  0  0  0  0  0  0  0  0  0  0  0  0  0
   18.5260   13.1827   -0.4123 H   0  0  0  0  0  0  0  0  0  0  0  0  0  0
   18.5361   12.8721    1.3286 H   0  0  0  0  0  0  0  0  0  0  0  0  0  0
    2.9780    7.4557    0.4757 H   0  0  0  0  0  0  0  0  0  0  0  0  0  0
```

2.9638 7.7639 -1.2656 H 0 0 0 0 0 0 0 0 0 0 0 0
18.6915 15.3114 1.7630 H 0 0 0 0 0 0 0 0 0 0 0 0
18.6815 15.6220 0.0220 H 0 0 0 0 0 0 0 0 0 0 0 0
2.8225 5.0157 0.0451 H 0 0 0 0 0 0 0 0 0 0 0 0
1.4617 5.8731 -0.6900 H 0 0 0 0 0 0 0 0 0 0 0 0
2.8082 5.3239 -1.6963 H 0 0 0 0 0 0 0 0 0 0 0 0
1 2 1 0 0 0 0
1 3 1 0 0 0 0
2 4 1 0 0 0 0
3 5 1 0 0 0 0
4 6 1 0 0 0 0
5 7 1 0 0 0 0
6 8 1 0 0 0 0
7 9 1 0 0 0 0
8 10 1 0 0 0 0
9 11 1 0 0 0 0
10 12 1 0 0 0 0
11 13 1 0 0 0 0
12 14 1 0 0 0 0
13 15 1 0 0 0 0
14 16 1 0 0 0 0
15 17 1 0 0 0 0
17 18 1 0 0 0 0
1 19 1 0 0 0 0
1 20 1 0 0 0 0
2 21 1 0 0 0 0
2 22 1 0 0 0 0
3 23 1 0 0 0 0
3 24 1 0 0 0 0
4 25 1 0 0 0 0
4 26 1 0 0 0 0
5 27 1 0 0 0 0
5 28 1 0 0 0 0
6 29 1 0 0 0 0
6 30 1 0 0 0 0
7 31 1 0 0 0 0
7 32 1 0 0 0 0
8 33 1 0 0 0 0
8 34 1 0 0 0 0
9 35 1 0 0 0 0
9 36 1 0 0 0 0
10 37 1 0 0 0 0
10 38 1 0 0 0 0
11 39 1 0 0 0 0
11 40 1 0 0 0 0
12 41 1 0 0 0 0
12 42 1 0 0 0 0
13 43 1 0 0 0 0
13 44 1 0 0 0 0
14 45 1 0 0 0 0
14 46 1 0 0 0 0
15 47 1 0 0 0 0
15 48 1 0 0 0 0
16 49 1 0 0 0 0
16 50 1 0 0 0 0
16 51 1 0 0 0 0
M END
> <Alias name>
1-Hexadecanol

> <deltaH°(c)> kJ/mol
-10510

```
$$$$
Cyclohexadecane
  ChemBrain IXL

 48 48  0  0  0  0  0  0  0  0  1 V2000
    6.7876    8.6284   -0.2684 C   0  0  0  0  0  0  0  0  0  0  0  0
    5.4858    7.9381   -0.7784 C   0  0  0  0  0  0  0  0  0  0  0  0
    5.8041    6.8870   -1.8564 C   0  0  0  0  0  0  0  0  0  0  0  0
    4.5563    6.0131   -2.1070 C   0  0  0  0  0  0  0  0  0  0  0  0
    4.3846    4.9985   -0.9381 C   0  0  0  0  0  0  0  0  0  0  0  0
    3.1739    4.0941   -1.2328 C   0  0  0  0  0  0  0  0  0  0  0  0
    3.1418    2.8361   -0.2916 C   0  0  0  0  0  0  0  0  0  0  0  0
    2.8505    3.2268    1.1934 C   0  0  0  0  0  0  0  0  0  0  0  0
    4.1587    3.5748    1.9926 C   0  0  0  0  0  0  0  0  0  0  0  0
    7.5828    7.6327    0.6050 C   0  0  0  0  0  0  0  0  0  0  0  0
    5.1615    2.3738    1.9450 C   0  0  0  0  0  0  0  0  0  0  0  0
    8.5826    6.7984   -0.2439 C   0  0  0  0  0  0  0  0  0  0  0  0
    6.1531    2.5959    0.7968 C   0  0  0  0  0  0  0  0  0  0  0  0
    8.7717    5.4384    0.4451 C   0  0  0  0  0  0  0  0  0  0  0  0
    7.2150    3.6274    1.2512 C   0  0  0  0  0  0  0  0  0  0  0  0
    7.5709    4.5410    0.0817 C   0  0  0  0  0  0  0  0  0  0  0  0
    7.3926    8.9640   -1.1017 H   0  0  0  0  0  0  0  0  0  0  0  0
    6.5090    9.4793    0.3405 H   0  0  0  0  0  0  0  0  0  0  0  0
    4.8246    8.6854   -1.1990 H   0  0  0  0  0  0  0  0  0  0  0  0
    4.9928    7.4569    0.0569 H   0  0  0  0  0  0  0  0  0  0  0  0
    6.6057    6.2615   -1.5318 H   0  0  0  0  0  0  0  0  0  0  0  0
    6.0865    7.3904   -2.7725 H   0  0  0  0  0  0  0  0  0  0  0  0
    3.6782    6.6425   -2.1821 H   0  0  0  0  0  0  0  0  0  0  0  0
    4.6867    5.4650   -3.0318 H   0  0  0  0  0  0  0  0  0  0  0  0
    4.2317    5.5271   -0.0065 H   0  0  0  0  0  0  0  0  0  0  0  0
    5.2673    4.3971   -0.8710 H   0  0  0  0  0  0  0  0  0  0  0  0
    3.2381    3.7543   -2.2591 H   0  0  0  0  0  0  0  0  0  0  0  0
    2.2613    4.6631   -1.1062 H   0  0  0  0  0  0  0  0  0  0  0  0
    2.3469    2.1844   -0.6319 H   0  0  0  0  0  0  0  0  0  0  0  0
    4.0745    2.3043   -0.3528 H   0  0  0  0  0  0  0  0  0  0  0  0
    2.1747    4.0728    1.2151 H   0  0  0  0  0  0  0  0  0  0  0  0
    2.3732    2.3853    1.6799 H   0  0  0  0  0  0  0  0  0  0  0  0
    3.8878    3.7631    3.0240 H   0  0  0  0  0  0  0  0  0  0  0  0
    4.6275    4.4584    1.5805 H   0  0  0  0  0  0  0  0  0  0  0  0
    6.8829    6.9675    1.0958 H   0  0  0  0  0  0  0  0  0  0  0  0
    8.1390    8.1778    1.3574 H   0  0  0  0  0  0  0  0  0  0  0  0
    5.7115    2.3219    2.8764 H   0  0  0  0  0  0  0  0  0  0  0  0
    4.6137    1.4515    1.7962 H   0  0  0  0  0  0  0  0  0  0  0  0
    9.5285    7.3226   -0.2997 H   0  0  0  0  0  0  0  0  0  0  0  0
    8.1989    6.6460   -1.2352 H   0  0  0  0  0  0  0  0  0  0  0  0
    6.6420    1.6660    0.5348 H   0  0  0  0  0  0  0  0  0  0  0  0
    5.6258    2.9718   -0.0505 H   0  0  0  0  0  0  0  0  0  0  0  0
    8.8078    5.5886    1.5172 H   0  0  0  0  0  0  0  0  0  0  0  0
    9.6888    4.9693    0.1114 H   0  0  0  0  0  0  0  0  0  0  0  0
    6.8041    4.2343    2.0486 H   0  0  0  0  0  0  0  0  0  0  0  0
    8.0963    3.1102    1.6099 H   0  0  0  0  0  0  0  0  0  0  0  0
    7.8101    3.9523   -0.7952 H   0  0  0  0  0  0  0  0  0  0  0  0
    6.7203    5.1656   -0.1293 H   0  0  0  0  0  0  0  0  0  0  0  0
  1  2  1  0  0  0  0
  2  3  1  0  0  0  0
  3  4  1  0  0  0  0
  4  5  1  0  0  0  0
  5  6  1  0  0  0  0
  6  7  1  0  0  0  0
  7  8  1  0  0  0  0
  8  9  1  0  0  0  0
  1 10  1  0  0  0  0
  9 11  1  0  0  0  0
```

10 12 1 0 0 0 0
11 13 1 0 0 0 0
12 14 1 0 0 0 0
13 15 1 0 0 0 0
14 16 1 0 0 0 0
15 16 1 0 0 0 0
 1 17 1 0 0 0 0
 1 18 1 0 0 0 0
 2 19 1 0 0 0 0
 2 20 1 0 0 0 0
 3 21 1 0 0 0 0
 3 22 1 0 0 0 0
 4 23 1 0 0 0 0
 4 24 1 0 0 0 0
 5 25 1 0 0 0 0
 5 26 1 0 0 0 0
 6 27 1 0 0 0 0
 6 28 1 0 0 0 0
 7 29 1 0 0 0 0
 7 30 1 0 0 0 0
 8 31 1 0 0 0 0
 8 32 1 0 0 0 0
 9 33 1 0 0 0 0
 9 34 1 0 0 0 0
10 35 1 0 0 0 0
10 36 1 0 0 0 0
11 37 1 0 0 0 0
11 38 1 0 0 0 0
12 39 1 0 0 0 0
12 40 1 0 0 0 0
13 41 1 0 0 0 0
13 42 1 0 0 0 0
14 43 1 0 0 0 0
14 44 1 0 0 0 0
15 45 1 0 0 0 0
15 46 1 0 0 0 0
16 47 1 0 0 0 0
16 48 1 0 0 0 0
M END
> <Alias name>
Cyclohexadecane

> <deltaH°(c)>   kJ/mol
-10466

$$$$
p-Diacetylbenzene diethyl ketal
  ChemBrain IXL

 52 52 0 0 0 0 0 0 0 0 1 V2000
    6.4038   6.0711   -1.7465 C   0 0 0 0 0 0 0 0 0 0 0 0
    5.6026   6.9234   -0.9849 C   0 0 0 0 0 0 0 0 0 0 0 0
    6.4109   4.6997   -1.4851 C   0 0 0 0 0 0 0 0 0 0 0 0
    4.8075   6.4052   0.0390 C   0 0 0 0 0 0 0 0 0 0 0 0
    4.8145   5.0337   0.3003 C   0 0 0 0 0 0 0 0 0 0 0 0
    3.9565   7.3107   0.8479 C   0 0 0 0 0 0 0 0 0 0 0 0
    5.6158   4.1815   -0.4613 C   0 0 0 0 0 0 0 0 0 0 0 0
    7.2619   3.7942   -2.2940 C   0 0 0 0 0 0 0 0 0 0 0 0
    2.6697   7.6390   0.0641 C   0 0 0 0 0 0 0 0 0 0 0 0
    4.6907   8.5560   1.1279 O   0 0 0 0 0 0 0 0 0 0 0 0
    3.6061   6.6507   2.1182 O   0 0 0 0 0 0 0 0 0 0 0 0
    5.9382   8.3881   1.8896 C   0 0 0 0 0 0 0 0 0 0 0 0
    6.5927   9.7647   2.1122 C   0 0 0 0 0 0 0 0 0 0 0 0

```
   2.7780    7.4511    3.0338 C   0  0  0  0  0  0  0  0  0  0  0  0
   2.5128    6.6470    4.3209 C   0  0  0  0  0  0  0  0  0  0  0  0
   6.3968    3.1016   -3.3664 C   0  0  0  0  0  0  0  0  0  0  0  0
   7.8664    2.7756   -1.4193 O   0  0  0  0  0  0  0  0  0  0  0  0
   8.3271    4.5753   -2.9478 O   0  0  0  0  0  0  0  0  0  0  0  0
   8.7356    3.3018   -0.3553 C   0  0  0  0  0  0  0  0  0  0  0  0
   9.2992    2.1314    0.4726 C   0  0  0  0  0  0  0  0  0  0  0  0
   9.2648    3.7934   -3.7689 C   0  0  0  0  0  0  0  0  0  0  0  0
  10.3297    4.7329   -4.3661 C   0  0  0  0  0  0  0  0  0  0  0  0
   7.0203    6.4740   -2.5405 H   0  0  0  0  0  0  0  0  0  0  0  0
   5.5977    7.9871   -1.1883 H   0  0  0  0  0  0  0  0  0  0  0  0
   4.1981    4.6309    1.0943 H   0  0  0  0  0  0  0  0  0  0  0  0
   5.6206    3.1178   -0.2579 H   0  0  0  0  0  0  0  0  0  0  0  0
   2.1250    6.7244   -0.1350 H   0  0  0  0  0  0  0  0  0  0  0  0
   2.0510    8.3076    0.6489 H   0  0  0  0  0  0  0  0  0  0  0  0
   2.9297    8.1156   -0.8730 H   0  0  0  0  0  0  0  0  0  0  0  0
   5.7178    7.9340    2.8478 H   0  0  0  0  0  0  0  0  0  0  0  0
   6.6168    7.7512    1.3359 H   0  0  0  0  0  0  0  0  0  0  0  0
   5.9141   10.4016    2.6661 H   0  0  0  0  0  0  0  0  0  0  0  0
   6.8130   10.2188    1.1540 H   0  0  0  0  0  0  0  0  0  0  0  0
   7.5109    9.6414    2.6729 H   0  0  0  0  0  0  0  0  0  0  0  0
   1.8358    7.6877    2.5561 H   0  0  0  0  0  0  0  0  0  0  0  0
   3.2975    8.3684    3.2820 H   0  0  0  0  0  0  0  0  0  0  0  0
   1.9935    5.7297    4.0728 H   0  0  0  0  0  0  0  0  0  0  0  0
   3.4552    6.4104    4.7992 H   0  0  0  0  0  0  0  0  0  0  0  0
   1.9035    7.2358    4.9952 H   0  0  0  0  0  0  0  0  0  0  0  0
   5.9585    3.8498   -4.0151 H   0  0  0  0  0  0  0  0  0  0  0  0
   5.6092    2.5357   -2.8843 H   0  0  0  0  0  0  0  0  0  0  0  0
   7.0143    2.4328   -3.9523 H   0  0  0  0  0  0  0  0  0  0  0  0
   8.1622    3.9565    0.2891 H   0  0  0  0  0  0  0  0  0  0  0  0
   9.5531    3.8570   -0.7986 H   0  0  0  0  0  0  0  0  0  0  0  0
   9.8728    1.4768   -0.1718 H   0  0  0  0  0  0  0  0  0  0  0  0
   9.9392    2.5185    1.2558 H   0  0  0  0  0  0  0  0  0  0  0  0
   8.4818    1.5762    0.9157 H   0  0  0  0  0  0  0  0  0  0  0  0
   9.7482    3.0459   -3.1520 H   0  0  0  0  0  0  0  0  0  0  0  0
   8.7242    3.3058   -4.5701 H   0  0  0  0  0  0  0  0  0  0  0  0
   9.8465    5.4805   -4.9828 H   0  0  0  0  0  0  0  0  0  0  0  0
  11.0203    4.1579   -4.9704 H   0  0  0  0  0  0  0  0  0  0  0  0
  10.8709    5.2207   -3.5648 H   0  0  0  0  0  0  0  0  0  0  0  0
  1  2  4  0  0  0  0
  3  1  4  0  0  0  0
  2  4  4  0  0  0  0
  7  3  4  0  0  0  0
  3  8  1  0  0  0  0
  4  5  4  0  0  0  0
  4  6  1  0  0  0  0
  5  7  4  0  0  0  0
  6  9  1  0  0  0  0
  6 10  1  0  0  0  0
  6 11  1  0  0  0  0
  8 16  1  0  0  0  0
  8 17  1  0  0  0  0
  8 18  1  0  0  0  0
 10 12  1  0  0  0  0
 11 14  1  0  0  0  0
 12 13  1  0  0  0  0
 14 15  1  0  0  0  0
 17 19  1  0  0  0  0
 18 21  1  0  0  0  0
 19 20  1  0  0  0  0
 21 22  1  0  0  0  0
  1 23  1  0  0  0  0
  2 24  1  0  0  0  0
```

```
  5 25  1  0  0  0  0
  7 26  1  0  0  0  0
  9 27  1  0  0  0  0
  9 28  1  0  0  0  0
  9 29  1  0  0  0  0
 12 30  1  0  0  0  0
 12 31  1  0  0  0  0
 13 32  1  0  0  0  0
 13 33  1  0  0  0  0
 13 34  1  0  0  0  0
 14 35  1  0  0  0  0
 14 36  1  0  0  0  0
 15 37  1  0  0  0  0
 15 38  1  0  0  0  0
 15 39  1  0  0  0  0
 16 40  1  0  0  0  0
 16 41  1  0  0  0  0
 16 42  1  0  0  0  0
 19 43  1  0  0  0  0
 19 44  1  0  0  0  0
 20 45  1  0  0  0  0
 20 46  1  0  0  0  0
 20 47  1  0  0  0  0
 21 48  1  0  0  0  0
 21 49  1  0  0  0  0
 22 50  1  0  0  0  0
 22 51  1  0  0  0  0
 22 52  1  0  0  0  0
M  END
>  <Alias name>
p-Diacetylbenzene diethyl ketal

>  <deltaH°(c)>   kJ/mol
-10465

$$$$
Undecylbenzene
  ChemBrain IXL

 45 45  0  0  0  0  0  0  0  0  1 V2000
    3.4874   10.0673    1.6450 C   0  0  0  0  0  0  0  0  0  0  0  0
    2.4760    9.1253    1.8339 C   0  0  0  0  0  0  0  0  0  0  0  0
    2.7733    7.7625    1.7838 C   0  0  0  0  0  0  0  0  0  0  0  0
    4.0827    7.3407    1.5447 C   0  0  0  0  0  0  0  0  0  0  0  0
    5.0968    8.2813    1.3553 C   0  0  0  0  0  0  0  0  0  0  0  0
    4.7961    9.6472    1.4060 C   0  0  0  0  0  0  0  0  0  0  0  0
    6.4914    7.8262    1.1007 C   0  0  0  0  0  0  0  0  0  0  0  0
    7.4208    9.0494    0.9293 C   0  0  0  0  0  0  0  0  0  0  0  0
    8.8618    8.5693    0.6667 C   0  0  0  0  0  0  0  0  0  0  0  0
    9.7878    9.7910    0.4961 C   0  0  0  0  0  0  0  0  0  0  0  0
   11.2288    9.3106    0.2337 C   0  0  0  0  0  0  0  0  0  0  0  0
   12.1559   10.5307    0.0633 C   0  0  0  0  0  0  0  0  0  0  0  0
   13.5961   10.0477   -0.1986 C   0  0  0  0  0  0  0  0  0  0  0  0
   14.5252   11.2655   -0.3690 C   0  0  0  0  0  0  0  0  0  0  0  0
   15.9645   10.7798   -0.6305 C   0  0  0  0  0  0  0  0  0  0  0  0
   16.8954   11.9962   -0.8010 C   0  0  0  0  0  0  0  0  0  0  0  0
   18.3341   11.5094   -1.0623 C   0  0  0  0  0  0  0  0  0  0  0  0
    3.2568   11.1249    1.6838 H   0  0  0  0  0  0  0  0  0  0  0  0
    1.4602    9.4517    2.0193 H   0  0  0  0  0  0  0  0  0  0  0  0
    1.9879    7.0321    1.9305 H   0  0  0  0  0  0  0  0  0  0  0  0
    4.3117    6.2833    1.5062 H   0  0  0  0  0  0  0  0  0  0  0  0
    5.5775   10.3813    1.2601 H   0  0  0  0  0  0  0  0  0  0  0  0
    6.8331    7.2296    1.9373 H   0  0  0  0  0  0  0  0  0  0  0  0
```

6.5151 7.2276 0.1987 H 0 0 0 0 0 0 0 0 0 0 0 0
7.0817 9.6461 0.0914 H 0 0 0 0 0 0 0 0 0 0 0 0
7.3996 9.6484 1.8315 H 0 0 0 0 0 0 0 0 0 0 0 0
9.2018 7.9729 1.5041 H 0 0 0 0 0 0 0 0 0 0 0 0
8.8840 7.9708 -0.2355 H 0 0 0 0 0 0 0 0 0 0 0 0
9.4480 10.3875 -0.3415 H 0 0 0 0 0 0 0 0 0 0 0 0
9.7656 10.3897 1.3983 H 0 0 0 0 0 0 0 0 0 0 0 0
11.5683 8.7138 1.0712 H 0 0 0 0 0 0 0 0 0 0 0 0
11.2509 8.7120 -0.6685 H 0 0 0 0 0 0 0 0 0 0 0 0
11.8172 11.1275 -0.7745 H 0 0 0 0 0 0 0 0 0 0 0 0
12.1343 11.1295 0.9655 H 0 0 0 0 0 0 0 0 0 0 0 0
13.9342 9.4504 0.6391 H 0 0 0 0 0 0 0 0 0 0 0 0
13.6173 9.4488 -1.1006 H 0 0 0 0 0 0 0 0 0 0 0 0
14.1878 11.8628 -1.2070 H 0 0 0 0 0 0 0 0 0 0 0 0
14.5046 11.8646 0.5330 H 0 0 0 0 0 0 0 0 0 0 0 0
16.3016 10.1822 0.2074 H 0 0 0 0 0 0 0 0 0 0 0 0
15.9849 10.1807 -1.5324 H 0 0 0 0 0 0 0 0 0 0 0 0
16.5588 12.5938 -1.6391 H 0 0 0 0 0 0 0 0 0 0 0 0
16.8753 12.5954 0.1009 H 0 0 0 0 0 0 0 0 0 0 0 0
18.9882 12.3640 -1.1821 H 0 0 0 0 0 0 0 0 0 0 0 0
18.3542 10.9101 -1.9642 H 0 0 0 0 0 0 0 0 0 0 0 0
18.6708 10.9117 -0.2242 H 0 0 0 0 0 0 0 0 0 0 0 0
1 2 4 0 0 0 0
2 3 4 0 0 0 0
3 4 4 0 0 0 0
4 5 4 0 0 0 0
5 6 4 0 0 0 0
6 1 4 0 0 0 0
5 7 1 0 0 0 0
7 8 1 0 0 0 0
8 9 1 0 0 0 0
9 10 1 0 0 0 0
10 11 1 0 0 0 0
11 12 1 0 0 0 0
12 13 1 0 0 0 0
13 14 1 0 0 0 0
14 15 1 0 0 0 0
15 16 1 0 0 0 0
16 17 1 0 0 0 0
1 18 1 0 0 0 0
2 19 1 0 0 0 0
3 20 1 0 0 0 0
4 21 1 0 0 0 0
6 22 1 0 0 0 0
7 23 1 0 0 0 0
7 24 1 0 0 0 0
8 25 1 0 0 0 0
8 26 1 0 0 0 0
9 27 1 0 0 0 0
9 28 1 0 0 0 0
10 29 1 0 0 0 0
10 30 1 0 0 0 0
11 31 1 0 0 0 0
11 32 1 0 0 0 0
12 33 1 0 0 0 0
12 34 1 0 0 0 0
13 35 1 0 0 0 0
13 36 1 0 0 0 0
14 37 1 0 0 0 0
14 38 1 0 0 0 0
15 39 1 0 0 0 0
15 40 1 0 0 0 0
16 41 1 0 0 0 0

```
 16 42  1  0  0  0  0
 17 43  1  0  0  0  0
 17 44  1  0  0  0  0
 17 45  1  0  0  0  0
M  END
>  <Alias name>
Undecylbenzene

>  <deltaH°(c)>   kJ/mol
-10457.25

$$$$
Decylcyclohexane
  ChemBrain IXL

 48 48  0  0  0  0  0  0  0  0  1 V2000
   14.7977    8.6218   -1.4064 C   0  0  0  0  0  0  0  0  0  0  0  0
   16.2017    7.9903   -1.4913 C   0  0  0  0  0  0  0  0  0  0  0  0
   16.5210    7.2659   -0.1672 C   0  0  0  0  0  0  0  0  0  0  0  0
   16.4808    8.2777    0.9966 C   0  0  0  0  0  0  0  0  0  0  0  0
   15.0767    8.9091    1.0808 C   0  0  0  0  0  0  0  0  0  0  0  0
   14.7606    9.6353   -0.2435 C   0  0  0  0  0  0  0  0  0  0  0  0
   13.3584   10.2843   -0.1611 C   0  0  0  0  0  0  0  0  0  0  0  0
   12.2895    9.1969    0.0842 C   0  0  0  0  0  0  0  0  0  0  0  0
   10.8985    9.8549    0.1658 C   0  0  0  0  0  0  0  0  0  0  0  0
    9.8310    8.7708    0.4111 C   0  0  0  0  0  0  0  0  0  0  0  0
    8.4400    9.4289    0.4944 C   0  0  0  0  0  0  0  0  0  0  0  0
    7.3725    8.3449    0.7403 C   0  0  0  0  0  0  0  0  0  0  0  0
    5.9817    9.0032    0.8252 C   0  0  0  0  0  0  0  0  0  0  0  0
    4.9143    7.9192    1.0716 C   0  0  0  0  0  0  0  0  0  0  0  0
    3.5234    8.5776    1.1573 C   0  0  0  0  0  0  0  0  0  0  0  0
    2.4561    7.4938    1.4038 C   0  0  0  0  0  0  0  0  0  0  0  0
   14.5737    9.1302   -2.3359 H   0  0  0  0  0  0  0  0  0  0  0  0
   14.0638    7.8455   -1.2334 H   0  0  0  0  0  0  0  0  0  0  0  0
   16.9367    8.7666   -1.6645 H   0  0  0  0  0  0  0  0  0  0  0  0
   16.2287    7.2791   -2.3074 H   0  0  0  0  0  0  0  0  0  0  0  0
   17.5076    6.8236   -0.2268 H   0  0  0  0  0  0  0  0  0  0  0  0
   15.7869    6.4886    0.0049 H   0  0  0  0  0  0  0  0  0  0  0  0
   16.7036    7.7681    1.9256 H   0  0  0  0  0  0  0  0  0  0  0  0
   17.2161    9.0542    0.8255 H   0  0  0  0  0  0  0  0  0  0  0  0
   14.3427    8.1325    1.2519 H   0  0  0  0  0  0  0  0  0  0  0  0
   15.0486    9.6192    1.8978 H   0  0  0  0  0  0  0  0  0  0  0  0
   15.5020   10.4057   -0.4156 H   0  0  0  0  0  0  0  0  0  0  0  0
   13.1438   10.7959   -1.0911 H   0  0  0  0  0  0  0  0  0  0  0  0
   13.3396   10.9971    0.6540 H   0  0  0  0  0  0  0  0  0  0  0  0
   12.5006    8.6854    1.0142 H   0  0  0  0  0  0  0  0  0  0  0  0
   12.3037    8.4853   -0.7308 H   0  0  0  0  0  0  0  0  0  0  0  0
   10.6861   10.3667   -0.7647 H   0  0  0  0  0  0  0  0  0  0  0  0
   10.8834   10.5673    0.9813 H   0  0  0  0  0  0  0  0  0  0  0  0
   10.0441    8.2582    1.3410 H   0  0  0  0  0  0  0  0  0  0  0  0
    9.8453    8.0589   -0.4050 H   0  0  0  0  0  0  0  0  0  0  0  0
    8.2267    9.9412   -0.4356 H   0  0  0  0  0  0  0  0  0  0  0  0
    8.4259   10.1410    1.3103 H   0  0  0  0  0  0  0  0  0  0  0  0
    7.5864    7.8320    1.6699 H   0  0  0  0  0  0  0  0  0  0  0  0
    7.3860    7.6333   -0.0760 H   0  0  0  0  0  0  0  0  0  0  0  0
    5.7677    9.5159   -0.1045 H   0  0  0  0  0  0  0  0  0  0  0  0
    5.9684    9.7149    1.6413 H   0  0  0  0  0  0  0  0  0  0  0  0
    5.1287    7.4062    2.0010 H   0  0  0  0  0  0  0  0  0  0  0  0
    4.9272    7.2077    0.2552 H   0  0  0  0  0  0  0  0  0  0  0  0
    3.3090    9.0905    0.2279 H   0  0  0  0  0  0  0  0  0  0  0  0
    3.5105    9.2891    1.9736 H   0  0  0  0  0  0  0  0  0  0  0  0
    2.6704    6.9808    2.3332 H   0  0  0  0  0  0  0  0  0  0  0  0
    1.4789    7.9567    1.4641 H   0  0  0  0  0  0  0  0  0  0  0  0
```

```
   2.4687    6.7823    0.5874 H   0  0  0  0  0  0  0  0  0  0  0  0
  1  2  1  0  0  0  0
  2  3  1  0  0  0  0
  3  4  1  0  0  0  0
  4  5  1  0  0  0  0
  5  6  1  0  0  0  0
  6  1  1  0  0  0  0
  6  7  1  0  0  0  0
  7  8  1  0  0  0  0
  8  9  1  0  0  0  0
  9 10  1  0  0  0  0
 10 11  1  0  0  0  0
 11 12  1  0  0  0  0
 12 13  1  0  0  0  0
 13 14  1  0  0  0  0
 14 15  1  0  0  0  0
 15 16  1  0  0  0  0
  1 17  1  0  0  0  0
  1 18  1  0  0  0  0
  2 19  1  0  0  0  0
  2 20  1  0  0  0  0
  3 21  1  0  0  0  0
  3 22  1  0  0  0  0
  4 23  1  0  0  0  0
  4 24  1  0  0  0  0
  5 25  1  0  0  0  0
  5 26  1  0  0  0  0
  6 27  1  0  0  0  0
  7 28  1  0  0  0  0
  7 29  1  0  0  0  0
  8 30  1  0  0  0  0
  8 31  1  0  0  0  0
  9 32  1  0  0  0  0
  9 33  1  0  0  0  0
 10 34  1  0  0  0  0
 10 35  1  0  0  0  0
 11 36  1  0  0  0  0
 11 37  1  0  0  0  0
 12 38  1  0  0  0  0
 12 39  1  0  0  0  0
 13 40  1  0  0  0  0
 13 41  1  0  0  0  0
 14 42  1  0  0  0  0
 14 43  1  0  0  0  0
 15 44  1  0  0  0  0
 15 45  1  0  0  0  0
 16 46  1  0  0  0  0
 16 47  1  0  0  0  0
 16 48  1  0  0  0  0
M  END
>  <Alias name>
Decylcyclohexane

>  <deltaH°(c)>   kJ/mol
-10451

$$$$
N-(Diethylaminothiocarbonyl)-N',N'-diethylbenzamidine
  ChemBrain IXL

 45 45  0  0  0  0  0  0  0  0  1 V2000
    4.2893    5.9697   -0.1327 N   0  0  0  0  0  0  0  0  0  0  0  0
    5.4083    6.4999    0.5206 C   0  0  0  0  0  0  0  0  0  0  0  0
```

5.3277	8.0540	1.2294	S	0	0	0	0	0	0	0	0	0	0	0	0
6.5932	5.7579	0.5935	N	0	0	0	0	0	0	0	0	0	0	0	0
3.1143	6.8168	-0.3828	C	0	0	0	0	0	0	0	0	0	0	0	0
4.2894	4.5657	-0.5684	C	0	0	0	0	0	0	0	0	0	0	0	0
2.3233	6.2571	-1.5810	C	0	0	0	0	0	0	0	0	0	0	0	0
4.9806	3.6964	0.4999	C	0	0	0	0	0	0	0	0	0	0	0	0
11.3319	4.0870	0.6854	C	0	0	0	0	0	0	0	0	0	0	0	0
11.3320	5.3152	0.0240	C	0	0	0	0	0	0	0	0	0	0	0	0
10.1478	6.0425	-0.0967	C	0	0	0	0	0	0	0	0	0	0	0	0
8.9617	5.5376	0.4365	C	0	0	0	0	0	0	0	0	0	0	0	0
8.9638	4.3146	1.1076	C	0	0	0	0	0	0	0	0	0	0	0	0
10.1480	3.5871	1.2281	C	0	0	0	0	0	0	0	0	0	0	0	0
7.7013	6.2994	0.2904	C	0	0	0	0	0	0	0	0	0	0	0	0
7.7304	7.6170	-0.1821	N	0	0	0	0	0	0	0	0	0	0	0	0
6.9260	7.9960	-1.3526	C	0	0	0	0	0	0	0	0	0	0	0	0
8.5654	8.6213	0.4924	C	0	0	0	0	0	0	0	0	0	0	0	0
6.6503	9.5118	-1.3174	C	0	0	0	0	0	0	0	0	0	0	0	0
8.6591	8.2870	1.9938	C	0	0	0	0	0	0	0	0	0	0	0	0
3.4377	7.8265	-0.6037	H	0	0	0	0	0	0	0	0	0	0	0	0
2.4812	6.8246	0.4958	H	0	0	0	0	0	0	0	0	0	0	0	0
4.8244	4.4788	-1.5060	H	0	0	0	0	0	0	0	0	0	0	0	0
3.2688	4.2293	-0.7026	H	0	0	0	0	0	0	0	0	0	0	0	0
1.9696	5.2607	-1.3464	H	0	0	0	0	0	0	0	0	0	0	0	0
1.4774	6.9010	-1.7877	H	0	0	0	0	0	0	0	0	0	0	0	0
2.9668	6.2162	-2.4511	H	0	0	0	0	0	0	0	0	0	0	0	0
5.9973	4.0410	0.6433	H	0	0	0	0	0	0	0	0	0	0	0	0
4.9921	2.6640	0.1730	H	0	0	0	0	0	0	0	0	0	0	0	0
4.4382	3.7733	1.4341	H	0	0	0	0	0	0	0	0	0	0	0	0
12.2509	3.5216	0.7777	H	0	0	0	0	0	0	0	0	0	0	0	0
12.2515	5.7041	-0.3958	H	0	0	0	0	0	0	0	0	0	0	0	0
10.1492	6.9992	-0.6044	H	0	0	0	0	0	0	0	0	0	0	0	0
8.0460	3.9299	1.5350	H	0	0	0	0	0	0	0	0	0	0	0	0
10.1481	2.6343	1.7429	H	0	0	0	0	0	0	0	0	0	0	0	0
7.4667	7.7484	-2.2577	H	0	0	0	0	0	0	0	0	0	0	0	0
5.9865	7.4577	-1.3347	H	0	0	0	0	0	0	0	0	0	0	0	0
9.5577	8.6159	0.0587	H	0	0	0	0	0	0	0	0	0	0	0	0
8.1223	9.6017	0.3678	H	0	0	0	0	0	0	0	0	0	0	0	0
6.0554	9.7906	-2.1783	H	0	0	0	0	0	0	0	0	0	0	0	0
6.1121	9.7598	-0.4110	H	0	0	0	0	0	0	0	0	0	0	0	0
7.5898	10.0501	-1.3382	H	0	0	0	0	0	0	0	0	0	0	0	0
9.2665	9.0322	2.4923	H	0	0	0	0	0	0	0	0	0	0	0	0
9.1107	7.3106	2.1187	H	0	0	0	0	0	0	0	0	0	0	0	0
7.6658	8.2832	2.4254	H	0	0	0	0	0	0	0	0	0	0	0	0

1	2	1	0	0	0	0
2	3	2	0	0	0	0
2	4	1	0	0	0	0
1	5	1	0	0	0	0
1	6	1	0	0	0	0
5	7	1	0	0	0	0
6	8	1	0	0	0	0
9	10	4	0	0	0	0
10	11	4	0	0	0	0
11	12	4	0	0	0	0
12	13	4	0	0	0	0
13	14	4	0	0	0	0
14	9	4	0	0	0	0
15	16	1	0	0	0	0
15	12	1	0	0	0	0
15	4	2	0	0	0	0
16	17	1	0	0	0	0
16	18	1	0	0	0	0
17	19	1	0	0	0	0
18	20	1	0	0	0	0

5 21 1 0 0 0 0
5 22 1 0 0 0 0
6 23 1 0 0 0 0
6 24 1 0 0 0 0
7 25 1 0 0 0 0
7 26 1 0 0 0 0
7 27 1 0 0 0 0
8 28 1 0 0 0 0
8 29 1 0 0 0 0
8 30 1 0 0 0 0
9 31 1 0 0 0 0
10 32 1 0 0 0 0
11 33 1 0 0 0 0
13 34 1 0 0 0 0
14 35 1 0 0 0 0
17 36 1 0 0 0 0
17 37 1 0 0 0 0
18 38 1 0 0 0 0
18 39 1 0 0 0 0
19 40 1 0 0 0 0
19 41 1 0 0 0 0
19 42 1 0 0 0 0
20 43 1 0 0 0 0
20 44 1 0 0 0 0
20 45 1 0 0 0 0
M END
> <Alias name>
N-(Diethylaminothiocarbonyl)-N',N'-diethylbenzamidine

> <deltaH°(c)>   kJ/mol
-10435

$$$$
1-Dodecyl-3-methylimidazolium bromide
ChemBrain IXL

50 49 0 0 0 0 0 0 0 0 1 V2000
18.8033   11.6909   -0.0015 C   0 0 0 0 0 0 0 0 0 0 0 0
17.5510   11.3568   -0.0013 C   0 0 0 0 0 0 0 0 0 0 0 0
19.5385   10.5132   0.0088 N   0 0 0 0 0 0 0 0 0 0 0 0
18.7180   9.4972   0.0150 C   0 3 0 0 0 0 0 0 0 0 0 0
17.5003   9.9688   0.0092 N   0 0 0 0 0 0 0 0 0 0 0 0
16.2724   9.1566   0.0132 C   0 0 0 0 0 0 0 0 0 0 0 0
21.0058   10.4241   0.0121 C   0 0 0 0 0 0 0 0 0 0 0 0
15.0391   10.0821   0.0110 C   0 0 0 0 0 0 0 0 0 0 0 0
13.7561   9.2284   0.0151 C   0 0 0 0 0 0 0 0 0 0 0 0
12.5235   10.1534   0.0127 C   0 0 0 0 0 0 0 0 0 0 0 0
11.2405   9.2997   0.0164 C   0 0 0 0 0 0 0 0 0 0 0 0
10.0078   10.2246   0.0136 C   0 0 0 0 0 0 0 0 0 0 0 0
8.7248   9.3709   0.0168 C   0 0 0 0 0 0 0 0 0 0 0 0
7.4921   10.2958   0.0136 C   0 0 0 0 0 0 0 0 0 0 0 0
6.2091   9.4421   0.0164 C   0 0 0 0 0 0 0 0 0 0 0 0
4.9764   10.3670   0.0129 C   0 0 0 0 0 0 0 0 0 0 0 0
3.6933   9.5132   0.0155 C   0 0 0 0 0 0 0 0 0 0 0 0
2.4606   10.4379   0.0119 C   0 0 0 0 0 0 0 0 0 0 0 0
2.4519   1.0339   0.0282 Br 0 0 0 0 0 0 0 0 0 0 0 0
18.9966   8.4539   0.0235 H   0 0 0 0 0 0 0 0 0 0 0 0
19.2010   12.6981   -0.0085 H   0 0 0 0 0 0 0 0 0 0 0 0
16.7047   12.0323   -0.0079 H   0 0 0 0 0 0 0 0 0 0 0 0
16.2547   8.5345   0.8994 H   0 0 0 0 0 0 0 0 0 0 0 0
16.2531   8.5283   -0.8687 H   0 0 0 0 0 0 0 0 0 0 0 0
21.4283   11.4213   0.0127 H   0 0 0 0 0 0 0 0 0 0 0 0
21.3371   9.8923   -0.8713 H   0 0 0 0 0 0 0 0 0 0 0 0

21.3331 9.8928 0.8972 H 0 0 0 0 0 0 0 0 0 0 0 0
15.0575 10.7102 0.8931 H 0 0 0 0 0 0 0 0 0 0 0 0
15.0559 10.7040 -0.8754 H 0 0 0 0 0 0 0 0 0 0 0 0
13.7378 8.6002 -0.8669 H 0 0 0 0 0 0 0 0 0 0 0 0
13.7391 8.6066 0.9016 H 0 0 0 0 0 0 0 0 0 0 0 0
12.5417 10.7814 0.8948 H 0 0 0 0 0 0 0 0 0 0 0 0
12.5407 10.7754 -0.8737 H 0 0 0 0 0 0 0 0 0 0 0 0
11.2224 8.6715 -0.8656 H 0 0 0 0 0 0 0 0 0 0 0 0
11.2232 8.6778 0.9029 H 0 0 0 0 0 0 0 0 0 0 0 0
10.0257 10.8526 0.8957 H 0 0 0 0 0 0 0 0 0 0 0 0
10.0253 10.8466 -0.8728 H 0 0 0 0 0 0 0 0 0 0 0 0
8.7070 8.7427 -0.8652 H 0 0 0 0 0 0 0 0 0 0 0 0
8.7072 8.7490 0.9033 H 0 0 0 0 0 0 0 0 0 0 0 0
7.5097 10.9238 0.8957 H 0 0 0 0 0 0 0 0 0 0 0 0
7.5098 10.9178 -0.8728 H 0 0 0 0 0 0 0 0 0 0 0 0
6.1916 8.8139 -0.8657 H 0 0 0 0 0 0 0 0 0 0 0 0
6.1913 8.8202 0.9029 H 0 0 0 0 0 0 0 0 0 0 0 0
4.9939 10.9951 0.8950 H 0 0 0 0 0 0 0 0 0 0 0 0
4.9943 10.9889 -0.8736 H 0 0 0 0 0 0 0 0 0 0 0 0
3.6758 8.8851 -0.8665 H 0 0 0 0 0 0 0 0 0 0 0 0
3.6753 8.8913 0.9019 H 0 0 0 0 0 0 0 0 0 0 0 0
1.5592 9.8378 0.0137 H 0 0 0 0 0 0 0 0 0 0 0 0
2.4779 11.0660 0.8940 H 0 0 0 0 0 0 0 0 0 0 0 0
2.4784 11.0599 -0.8746 H 0 0 0 0 0 0 0 0 0 0 0 0
1 2 2 0 0 0 0
1 3 1 0 0 0 0
3 4 4 0 0 0 0
4 5 4 0 0 0 0
5 2 1 0 0 0 0
5 6 1 0 0 0 0
3 7 1 0 0 0 0
6 8 1 0 0 0 0
8 9 1 0 0 0 0
9 10 1 0 0 0 0
11 12 1 0 0 0 0
12 13 1 0 0 0 0
13 14 1 0 0 0 0
11 10 1 0 0 0 0
14 15 1 0 0 0 0
15 16 1 0 0 0 0
16 17 1 0 0 0 0
17 18 1 0 0 0 0
4 20 1 0 0 0 0
1 21 1 0 0 0 0
2 22 1 0 0 0 0
6 23 1 0 0 0 0
6 24 1 0 0 0 0
7 25 1 0 0 0 0
7 26 1 0 0 0 0
7 27 1 0 0 0 0
8 28 1 0 0 0 0
8 29 1 0 0 0 0
9 30 1 0 0 0 0
9 31 1 0 0 0 0
10 32 1 0 0 0 0
10 33 1 0 0 0 0
11 34 1 0 0 0 0
11 35 1 0 0 0 0
12 36 1 0 0 0 0
12 37 1 0 0 0 0
13 38 1 0 0 0 0
13 39 1 0 0 0 0
14 40 1 0 0 0 0

```
 14 41  1  0  0  0  0
 15 42  1  0  0  0  0
 15 43  1  0  0  0  0
 16 44  1  0  0  0  0
 16 45  1  0  0  0  0
 17 46  1  0  0  0  0
 17 47  1  0  0  0  0
 18 48  1  0  0  0  0
 18 49  1  0  0  0  0
 18 50  1  0  0  0  0
M  END
>  <Alias name>
1-Dodecyl-3-methylimidazolium bromide

>  <deltaH°(c)>   kJ/mol
-10406

$$$$
Triphenylethylene
  ChemBrain IXL

 36 38  0  0  0  0  0  0  0  0  1 V2000
    8.8087    8.5305    0.9272 C   0  0  0  0  0  0  0  0  0  0  0  0
    8.4552    9.3955   -0.1085 C   0  0  0  0  0  0  0  0  0  0  0  0
    7.5540    8.9787   -1.0885 C   0  0  0  0  0  0  0  0  0  0  0  0
    7.0063    7.6970   -1.0325 C   0  0  0  0  0  0  0  0  0  0  0  0
    7.3591    6.8323    0.0038 C   0  0  0  0  0  0  0  0  0  0  0  0
    8.2608    7.2489    0.9833 C   0  0  0  0  0  0  0  0  0  0  0  0
    6.7767    5.4731    0.0641 C   0  0  0  0  0  0  0  0  0  0  0  0
    7.5768    4.4057    0.1149 C   0  0  0  0  0  0  0  0  0  0  0  0
    2.5370    4.9653    0.0774 C   0  0  0  0  0  0  0  0  0  0  0  0
    3.0884    6.2315    0.2739 C   0  0  0  0  0  0  0  0  0  0  0  0
    4.4735    6.3974    0.2698 C   0  0  0  0  0  0  0  0  0  0  0  0
    5.3072    5.2972    0.0681 C   0  0  0  0  0  0  0  0  0  0  0  0
    4.7557    4.0312   -0.1297 C   0  0  0  0  0  0  0  0  0  0  0  0
    3.3706    3.8651   -0.1242 C   0  0  0  0  0  0  0  0  0  0  0  0
   11.8163    4.9147    0.0810 C   0  0  0  0  0  0  0  0  0  0  0  0
   11.2154    4.1334    1.0682 C   0  0  0  0  0  0  0  0  0  0  0  0
    9.8305    3.9665    1.0788 C   0  0  0  0  0  0  0  0  0  0  0  0
    9.0463    4.5812    0.1025 C   0  0  0  0  0  0  0  0  0  0  0  0
    9.6473    5.3613   -0.8856 C   0  0  0  0  0  0  0  0  0  0  0  0
   11.0322    5.5286   -0.8959 C   0  0  0  0  0  0  0  0  0  0  0  0
    9.5085    8.8540    1.6878 H   0  0  0  0  0  0  0  0  0  0  0  0
    8.8803   10.3907   -0.1519 H   0  0  0  0  0  0  0  0  0  0  0  0
    7.2797    9.6502   -1.8927 H   0  0  0  0  0  0  0  0  0  0  0  0
    6.3071    7.3732   -1.7935 H   0  0  0  0  0  0  0  0  0  0  0  0
    8.5356    6.5772    1.7872 H   0  0  0  0  0  0  0  0  0  0  0  0
    7.1507    3.4113    0.1651 H   0  0  0  0  0  0  0  0  0  0  0  0
    1.4617    4.8364    0.0813 H   0  0  0  0  0  0  0  0  0  0  0  0
    2.4412    7.0858    0.4296 H   0  0  0  0  0  0  0  0  0  0  0  0
    4.9017    7.3803    0.4232 H   0  0  0  0  0  0  0  0  0  0  0  0
    5.4028    3.1773   -0.2877 H   0  0  0  0  0  0  0  0  0  0  0  0
    2.9425    2.8820   -0.2763 H   0  0  0  0  0  0  0  0  0  0  0  0
   12.8914    5.0446    0.0730 H   0  0  0  0  0  0  0  0  0  0  0  0
   11.8241    3.6564    1.8264 H   0  0  0  0  0  0  0  0  0  0  0  0
    9.3640    3.3597    1.8451 H   0  0  0  0  0  0  0  0  0  0  0  0
    9.0387    5.8371   -1.6446 H   0  0  0  0  0  0  0  0  0  0  0  0
   11.4987    6.1351   -1.6624 H   0  0  0  0  0  0  0  0  0  0  0  0
  1  2  4  0  0  0  0
  2  3  4  0  0  0  0
  3  4  4  0  0  0  0
  4  5  4  0  0  0  0
  5  6  4  0  0  0  0
```

```
  6  1  4  0  0  0  0
  5  7  1  0  0  0  0
  7  8  2  0  0  0  0
  9 10  4  0  0  0  0
 10 11  4  0  0  0  0
 11 12  4  0  0  0  0
 12 13  4  0  0  0  0
 13 14  4  0  0  0  0
 14  9  4  0  0  0  0
 12  7  1  0  0  0  0
 15 16  4  0  0  0  0
 16 17  4  0  0  0  0
 17 18  4  0  0  0  0
 18 19  4  0  0  0  0
 19 20  4  0  0  0  0
 20 15  4  0  0  0  0
 18  8  1  0  0  0  0
  1 21  1  0  0  0  0
  2 22  1  0  0  0  0
  3 23  1  0  0  0  0
  4 24  1  0  0  0  0
  6 25  1  0  0  0  0
  8 26  1  0  0  0  0
  9 27  1  0  0  0  0
 10 28  1  0  0  0  0
 11 29  1  0  0  0  0
 13 30  1  0  0  0  0
 14 31  1  0  0  0  0
 15 32  1  0  0  0  0
 16 33  1  0  0  0  0
 17 34  1  0  0  0  0
 19 35  1  0  0  0  0
 20 36  1  0  0  0  0
M  END
>  <Alias name>
Triphenylethylene

>  <deltaH°(c)>   kJ/mol
-10397

$$$$
Papaverine
  ChemBrain IXL

 46 48  0  0  0  0  0  0  0  0  1 V2000
   10.2571    9.4068    0.0531 C   0  0  0  0  0  0  0  0  0  0  0  0  0
   10.3136   10.7063    0.5295 C   0  0  0  0  0  0  0  0  0  0  0  0  0
    9.0148    8.8099   -0.1700 C   0  0  0  0  0  0  0  0  0  0  0  0  0
   11.4398    8.7050   -0.1997 C   0  0  0  0  0  0  0  0  0  0  0  0  0
   11.5519   11.3187    0.7574 C   0  0  0  0  0  0  0  0  0  0  0  0  0
    9.1271   11.3894    0.7763 C   0  0  0  0  0  0  0  0  0  0  0  0  0
    8.9496    7.4286   -0.6805 C   0  0  0  0  0  0  0  0  0  0  0  0  0
    7.8722    9.4891    0.0786 N   0  0  0  0  0  0  0  0  0  0  0  0  0
   12.6785    9.3130    0.0261 C   0  0  0  0  0  0  0  0  0  0  0  0  0
   12.7362   10.6185    0.5059 C   0  0  0  0  0  0  0  0  0  0  0  0  0
    7.9052   10.7576    0.5421 C   0  0  0  0  0  0  0  0  0  0  0  0  0
    7.5364    7.0101   -0.7840 C   0  0  0  0  0  0  0  0  0  0  0  0  0
   13.9245    8.5790   -0.2412 O   0  0  0  0  0  0  0  0  0  0  0  0  0
   14.0484   11.2531    0.7455 O   0  0  0  0  0  0  0  0  0  0  0  0  0
    7.2216    5.6637   -0.9566 C   0  0  0  0  0  0  0  0  0  0  0  0  0
    6.5187    7.9616   -0.7087 C   0  0  0  0  0  0  0  0  0  0  0  0  0
   13.7473    7.2021   -0.7164 C   0  0  0  0  0  0  0  0  0  0  0  0  0
   14.0030   12.6432    1.2271 C   0  0  0  0  0  0  0  0  0  0  0  0  0
```

```
5.8891 5.2678 -1.0621 C 0 0 0 0 0 0 0 0 0 0 0 0 0
5.1852 7.5656 -0.8143 C 0 0 0 0 0 0 0 0 0 0 0 0 0
4.8704 6.2181 -0.9928 C 0 0 0 0 0 0 0 0 0 0 0 0 0
5.5592 3.8495 -1.2463 O 0 0 0 0 0 0 0 0 0 0 0 0 0
3.4652 5.7986 -1.1081 O 0 0 0 0 0 0 0 0 0 0 0 0 0
6.7187 2.9540 -1.3519 C 0 0 0 0 0 0 0 0 0 0 0 0 0
2.4780 6.8794 -0.9669 C 0 0 0 0 0 0 0 0 0 0 0 0 0
11.3967 7.6893 -0.5709 H 0 0 0 0 0 0 0 0 0 0 0 0 0
11.5935 12.3359 1.1290 H 0 0 0 0 0 0 0 0 0 0 0 0 0
9.1525 12.4071 1.1485 H 0 0 0 0 0 0 0 0 0 0 0 0 0
9.4736 6.7678 -0.0012 H 0 0 0 0 0 0 0 0 0 0 0 0 0
9.4129 7.3829 -1.6583 H 0 0 0 0 0 0 0 0 0 0 0 0 0
6.9801 11.2871 0.7331 H 0 0 0 0 0 0 0 0 0 0 0 0 0
8.0119 4.9255 -1.0087 H 0 0 0 0 0 0 0 0 0 0 0 0 0
6.7638 9.0076 -0.5683 H 0 0 0 0 0 0 0 0 0 0 0 0 0
14.7176 6.7483 -0.8772 H 0 0 0 0 0 0 0 0 0 0 0 0 0
13.2006 6.6324 0.0243 H 0 0 0 0 0 0 0 0 0 0 0 0 0
13.1938 7.2101 -1.6468 H 0 0 0 0 0 0 0 0 0 0 0 0 0
13.4746 12.6817 2.1711 H 0 0 0 0 0 0 0 0 0 0 0 0 0
13.4906 13.2611 0.5006 H 0 0 0 0 0 0 0 0 0 0 0 0 0
15.0140 13.0106 1.3642 H 0 0 0 0 0 0 0 0 0 0 0 0 0
4.3947 8.3041 -0.7577 H 0 0 0 0 0 0 0 0 0 0 0 0 0
7.3058 3.0153 -0.4438 H 0 0 0 0 0 0 0 0 0 0 0 0 0
7.3282 3.2508 -2.1965 H 0 0 0 0 0 0 0 0 0 0 0 0 0
6.3771 1.9364 -1.4930 H 0 0 0 0 0 0 0 0 0 0 0 0 0
1.4794 6.4702 -1.0592 H 0 0 0 0 0 0 0 0 0 0 0 0 0
2.5888 7.3453 0.0045 H 0 0 0 0 0 0 0 0 0 0 0 0 0
2.6382 7.6185 -1.7421 H 0 0 0 0 0 0 0 0 0 0 0 0 0
1 2 4 0 0 0 0
1 3 4 0 0 0 0
1 4 4 0 0 0 0
2 5 4 0 0 0 0
2 6 4 0 0 0 0
3 7 1 0 0 0 0
3 8 4 0 0 0 0
4 9 4 0 0 0 0
5 10 4 0 0 0 0
6 11 4 0 0 0 0
7 12 1 0 0 0 0
8 11 4 0 0 0 0
9 13 1 0 0 0 0
9 10 4 0 0 0 0
10 14 1 0 0 0 0
12 15 4 0 0 0 0
12 16 4 0 0 0 0
13 17 1 0 0 0 0
14 18 1 0 0 0 0
15 19 4 0 0 0 0
16 20 4 0 0 0 0
19 21 4 0 0 0 0
19 22 1 0 0 0 0
20 21 4 0 0 0 0
21 23 1 0 0 0 0
22 24 1 0 0 0 0
23 25 1 0 0 0 0
4 26 1 0 0 0 0
5 27 1 0 0 0 0
6 28 1 0 0 0 0
7 29 1 0 0 0 0
7 30 1 0 0 0 0
11 31 1 0 0 0 0
15 32 1 0 0 0 0
16 33 1 0 0 0 0
```

```
 17 34  1  0  0  0  0
 17 35  1  0  0  0  0
 17 36  1  0  0  0  0
 18 37  1  0  0  0  0
 18 38  1  0  0  0  0
 18 39  1  0  0  0  0
 20 40  1  0  0  0  0
 24 41  1  0  0  0  0
 24 42  1  0  0  0  0
 24 43  1  0  0  0  0
 25 44  1  0  0  0  0
 25 45  1  0  0  0  0
 25 46  1  0  0  0  0
M  END
>  <Alias name>
Papaverine

>  <deltaH°(c)>   kJ/mol
-10375

$$$$
2,6-Di-t-butylnaphthalene
  ChemBrain IXL

 42 43  0  0  0  0  0  0  0  0  1 V2000
    4.9677    7.0577   -0.9282 C   0  0  0  0  0  0  0  0  0  0  0  0
    5.2501    5.7006   -1.0855 C   0  0  0  0  0  0  0  0  0  0  0  0
    6.4815    5.1919   -0.6723 C   0  0  0  0  0  0  0  0  0  0  0  0
    7.4305    6.0402   -0.1014 C   0  0  0  0  0  0  0  0  0  0  0  0
    7.1480    7.3972    0.0561 C   0  0  0  0  0  0  0  0  0  0  0  0
    5.9167    7.9059   -0.3573 C   0  0  0  0  0  0  0  0  0  0  0  0
    8.0970    8.2455    0.6270 C   0  0  0  0  0  0  0  0  0  0  0  0
    9.3285    7.7368    1.0403 C   0  0  0  0  0  0  0  0  0  0  0  0
    9.6108    6.3798    0.8830 C   0  0  0  0  0  0  0  0  0  0  0  0
    8.6619    5.5315    0.3121 C   0  0  0  0  0  0  0  0  0  0  0  0
   10.3354    8.6369    1.6456 C   0  0  0  0  0  0  0  0  0  0  0  0
    9.6208    9.7979    2.3640 C   0  0  0  0  0  0  0  0  0  0  0  0
    4.2431    4.8005   -1.6907 C   0  0  0  0  0  0  0  0  0  0  0  0
    4.9577    3.6400   -2.4099 C   0  0  0  0  0  0  0  0  0  0  0  0
    3.3937    5.5890   -2.7063 C   0  0  0  0  0  0  0  0  0  0  0  0
   11.1841    7.8486    2.6619 C   0  0  0  0  0  0  0  0  0  0  0  0
    3.3294    4.2353   -0.5860 C   0  0  0  0  0  0  0  0  0  0  0  0
   11.2498    9.2013    0.5410 C   0  0  0  0  0  0  0  0  0  0  0  0
    4.0118    7.4526   -1.2494 H   0  0  0  0  0  0  0  0  0  0  0  0
    6.7009    4.1385   -0.7947 H   0  0  0  0  0  0  0  0  0  0  0  0
    5.6974    8.9594   -0.2350 H   0  0  0  0  0  0  0  0  0  0  0  0
    7.8777    9.2990    0.7494 H   0  0  0  0  0  0  0  0  0  0  0  0
   10.5667    5.9848    1.2042 H   0  0  0  0  0  0  0  0  0  0  0  0
    8.8812    4.4780    0.1897 H   0  0  0  0  0  0  0  0  0  0  0  0
    9.0490   10.3705    1.6442 H   0  0  0  0  0  0  0  0  0  0  0  0
   10.3564   10.4395    2.8331 H   0  0  0  0  0  0  0  0  0  0  0  0
    8.9548    9.3997    3.1194 H   0  0  0  0  0  0  0  0  0  0  0  0
    5.5300    3.0673   -1.6906 H   0  0  0  0  0  0  0  0  0  0  0  0
    4.2220    2.9984   -2.8790 H   0  0  0  0  0  0  0  0  0  0  0  0
    5.6232    4.0388   -3.1655 H   0  0  0  0  0  0  0  0  0  0  0  0
    2.6761    4.9238   -3.1704 H   0  0  0  0  0  0  0  0  0  0  0  0
    2.8687    6.3868   -2.1956 H   0  0  0  0  0  0  0  0  0  0  0  0
    4.0394    6.0107   -3.4667 H   0  0  0  0  0  0  0  0  0  0  0  0
   11.9017    8.5138    3.1260 H   0  0  0  0  0  0  0  0  0  0  0  0
   11.7090    7.0504    2.1518 H   0  0  0  0  0  0  0  0  0  0  0  0
   10.5379    7.4276    3.4223 H   0  0  0  0  0  0  0  0  0  0  0  0
    3.9263    3.6811    0.1277 H   0  0  0  0  0  0  0  0  0  0  0  0
    2.8273    5.0509   -0.0804 H   0  0  0  0  0  0  0  0  0  0  0  0
```

2.5924   3.5768   -1.0288 H   0 0 0 0 0 0 0 0 0 0 0 0
10.6534   9.7555   -0.1732 H   0 0 0 0 0 0 0 0 0 0 0 0
11.7519   8.3854   0.0360 H   0 0 0 0 0 0 0 0 0 0 0 0
11.9867   9.8598   0.9840 H   0 0 0 0 0 0 0 0 0 0 0 0
1 2 4 0 0 0 0
2 3 4 0 0 0 0
3 4 4 0 0 0 0
4 5 4 0 0 0 0
5 6 4 0 0 0 0
6 1 4 0 0 0 0
5 7 4 0 0 0 0
7 8 4 0 0 0 0
8 9 4 0 0 0 0
9 10 4 0 0 0 0
10 4 4 0 0 0 0
8 11 1 0 0 0 0
11 12 1 0 0 0 0
2 13 1 0 0 0 0
13 14 1 0 0 0 0
13 15 1 0 0 0 0
11 16 1 0 0 0 0
13 17 1 0 0 0 0
11 18 1 0 0 0 0
1 19 1 0 0 0 0
3 20 1 0 0 0 0
6 21 1 0 0 0 0
7 22 1 0 0 0 0
9 23 1 0 0 0 0
10 24 1 0 0 0 0
12 25 1 0 0 0 0
12 26 1 0 0 0 0
12 27 1 0 0 0 0
14 28 1 0 0 0 0
14 29 1 0 0 0 0
14 30 1 0 0 0 0
15 31 1 0 0 0 0
15 32 1 0 0 0 0
15 33 1 0 0 0 0
16 34 1 0 0 0 0
16 35 1 0 0 0 0
16 36 1 0 0 0 0
17 37 1 0 0 0 0
17 38 1 0 0 0 0
17 39 1 0 0 0 0
18 40 1 0 0 0 0
18 41 1 0 0 0 0
18 42 1 0 0 0 0
M END
> <Alias name>
2,6-Di-t-butylnaphthalene

> <deltaH°(c)>   kJ/mol
-10351

$$$$
Dodecahydrotriphenyl
ChemBrain IXL

42 45 0 0 0 0 0 0 0 0 1 V2000
5.8708   5.0740   -0.2097 C   0 0 0 0 0 0 0 0 0 0 0 0
5.7198   6.4852   -0.1013 C   0 0 0 0 0 0 0 0 0 0 0 0
7.1405   4.5047   -0.1706 C   0 0 0 0 0 0 0 0 0 0 0 0
4.6908   4.1632   -0.3718 C   0 0 0 0 0 0 0 0 0 0 0 0

```
6.8403    7.2951    0.0424 C   0  0  0  0  0  0  0  0  0  0  0  0
4.3735    7.1429   -0.1316 C   0  0  0  0  0  0  0  0  0  0  0  0
8.2883    5.3336   -0.0222 C   0  0  0  0  0  0  0  0  0  0  0  0
7.2528    3.0153   -0.2929 C   0  0  0  0  0  0  0  0  0  0  0  0
3.4041    4.8770    0.0352 C   0  0  0  0  0  0  0  0  0  0  0  0
8.1391    6.7134    0.0827 C   0  0  0  0  0  0  0  0  0  0  0  0
6.6312    8.7754    0.1459 C   0  0  0  0  0  0  0  0  0  0  0  0
3.3244    6.1929   -0.7055 C   0  0  0  0  0  0  0  0  0  0  0  0
9.6768    4.7638    0.0312 C   0  0  0  0  0  0  0  0  0  0  0  0
8.6126    2.5397    0.2095 C   0  0  0  0  0  0  0  0  0  0  0  0
9.3735    7.5479    0.2411 C   0  0  0  0  0  0  0  0  0  0  0  0
7.8779    9.4559    0.7070 C   0  0  0  0  0  0  0  0  0  0  0  0
9.6920    3.3288   -0.4961 C   0  0  0  0  0  0  0  0  0  0  0  0
9.0785    9.0071   -0.0951 C   0  0  0  0  0  0  0  0  0  0  0  0
4.8261    3.2879    0.2513 H   0  0  0  0  0  0  0  0  0  0  0  0
4.6164    3.8573   -1.4063 H   0  0  0  0  0  0  0  0  0  0  0  0
4.4246    8.0329   -0.7470 H   0  0  0  0  0  0  0  0  0  0  0  0
4.0901    7.4202    0.8744 H   0  0  0  0  0  0  0  0  0  0  0  0
7.1380    2.7331   -1.3306 H   0  0  0  0  0  0  0  0  0  0  0  0
6.4718    2.5480    0.2937 H   0  0  0  0  0  0  0  0  0  0  0  0
2.5501    4.2621   -0.2229 H   0  0  0  0  0  0  0  0  0  0  0  0
3.4097    5.0610    1.1032 H   0  0  0  0  0  0  0  0  0  0  0  0
6.4194    9.1745   -0.8364 H   0  0  0  0  0  0  0  0  0  0  0  0
5.7910    8.9723    0.8009 H   0  0  0  0  0  0  0  0  0  0  0  0
3.5167    6.0289   -1.7594 H   0  0  0  0  0  0  0  0  0  0  0  0
2.3377    6.6222   -0.5789 H   0  0  0  0  0  0  0  0  0  0  0  0
10.0248    4.7701    1.0548 H   0  0  0  0  0  0  0  0  0  0  0  0
10.3364    5.3730   -0.5744 H   0  0  0  0  0  0  0  0  0  0  0  0
8.7321    1.4849   -0.0081 H   0  0  0  0  0  0  0  0  0  0  0  0
8.6848    2.7006    1.2788 H   0  0  0  0  0  0  0  0  0  0  0  0
9.7198    7.4810    1.2634 H   0  0  0  0  0  0  0  0  0  0  0  0
10.1441    7.1769   -0.4230 H   0  0  0  0  0  0  0  0  0  0  0  0
7.7705   10.5312    0.6300 H   0  0  0  0  0  0  0  0  0  0  0  0
8.0105    9.1775    1.7460 H   0  0  0  0  0  0  0  0  0  0  0  0
10.6581    2.8795   -0.2990 H   0  0  0  0  0  0  0  0  0  0  0  0
9.5026    3.3290   -1.5630 H   0  0  0  0  0  0  0  0  0  0  0  0
9.9354    9.6192    0.1594 H   0  0  0  0  0  0  0  0  0  0  0  0
8.8649    9.1028   -1.1533 H   0  0  0  0  0  0  0  0  0  0  0  0
1  2  4  0  0  0  0
1  3  4  0  0  0  0
1  4  1  0  0  0  0
2  5  4  0  0  0  0
2  6  1  0  0  0  0
3  7  4  0  0  0  0
3  8  1  0  0  0  0
4  9  1  0  0  0  0
5 10  4  0  0  0  0
5 11  1  0  0  0  0
6 12  1  0  0  0  0
7 13  1  0  0  0  0
7 10  4  0  0  0  0
8 14  1  0  0  0  0
9 12  1  0  0  0  0
10 15  1  0  0  0  0
11 16  1  0  0  0  0
13 17  1  0  0  0  0
14 17  1  0  0  0  0
15 18  1  0  0  0  0
16 18  1  0  0  0  0
4 19  1  0  0  0  0
4 20  1  0  0  0  0
6 21  1  0  0  0  0
6 22  1  0  0  0  0
```

```
  8 23  1  0  0  0  0
  8 24  1  0  0  0  0
  9 25  1  0  0  0  0
  9 26  1  0  0  0  0
 11 27  1  0  0  0  0
 11 28  1  0  0  0  0
 12 29  1  0  0  0  0
 12 30  1  0  0  0  0
 13 31  1  0  0  0  0
 13 32  1  0  0  0  0
 14 33  1  0  0  0  0
 14 34  1  0  0  0  0
 15 35  1  0  0  0  0
 15 36  1  0  0  0  0
 16 37  1  0  0  0  0
 16 38  1  0  0  0  0
 17 39  1  0  0  0  0
 17 40  1  0  0  0  0
 18 41  1  0  0  0  0
 18 42  1  0  0  0  0
M  END
>  <Alias name>
Tritetralin

>  <deltaH°(c)>   kJ/mol
-10319

$$$$
1,12-Dimethyl(4)helicene
  ChemBrain IXL

 36 39  0  0  0  0  0  0  0  0  1 V2000
    5.0026    7.2872   -0.5909 C   0  0  0  0  0  0  0  0  0  0  0  0
    6.3357    7.8939   -0.8040 C   0  0  0  0  0  0  0  0  0  0  0  0
    4.8307    5.9106   -0.7223 C   0  0  0  0  0  0  0  0  0  0  0  0
    3.9141    8.0914   -0.2591 C   0  0  0  0  0  0  0  0  0  0  0  0
    5.9298    5.0631   -0.8207 C   0  0  0  0  0  0  0  0  0  0  0  0
    3.5538    5.3756   -0.7584 C   0  0  0  0  0  0  0  0  0  0  0  0
    2.6406    7.5315   -0.1685 C   0  0  0  0  0  0  0  0  0  0  0  0
    7.1907    5.4683   -0.3942 C   0  0  0  0  0  0  0  0  0  0  0  0
    5.7665    3.8000   -1.3500 C   0  0  0  0  0  0  0  0  0  0  0  0
    3.3776    4.0428   -1.1249 C   0  0  0  0  0  0  0  0  0  0  0  0
    2.4582    6.1731   -0.4282 C   0  0  0  0  0  0  0  0  0  0  0  0
    8.3127    4.8023   -0.8588 C   0  0  0  0  0  0  0  0  0  0  0  0
    7.3338    6.5323    0.4945 C   0  0  0  0  0  0  0  0  0  0  0  0
    6.8861    3.0749   -1.7531 C   0  0  0  0  0  0  0  0  0  0  0  0
    4.4844    3.2683   -1.4736 C   0  0  0  0  0  0  0  0  0  0  0  0
    8.1617    3.6038   -1.5536 C   0  0  0  0  0  0  0  0  0  0  0  0
    9.5809    5.3352   -0.6281 C   0  0  0  0  0  0  0  0  0  0  0  0
    6.1492    7.0884    1.1853 C   0  0  0  0  0  0  0  0  0  0  0  0
    8.5971    7.0705    0.7317 C   0  0  0  0  0  0  0  0  0  0  0  0
    9.7219    6.4770    0.1608 C   0  0  0  0  0  0  0  0  0  0  0  0
    6.2205    8.8597   -1.2809 H   0  0  0  0  0  0  0  0  0  0  0  0
    6.9294    7.2472   -1.4376 H   0  0  0  0  0  0  0  0  0  0  0  0
    6.8318    8.0195    0.1503 H   0  0  0  0  0  0  0  0  0  0  0  0
    4.0573    9.1490   -0.0718 H   0  0  0  0  0  0  0  0  0  0  0  0
    1.7944    8.1508    0.1033 H   0  0  0  0  0  0  0  0  0  0  0  0
    2.3851    3.6102   -1.1387 H   0  0  0  0  0  0  0  0  0  0  0  0
    1.4679    5.7391   -0.3732 H   0  0  0  0  0  0  0  0  0  0  0  0
    6.7668    2.1053   -2.2194 H   0  0  0  0  0  0  0  0  0  0  0  0
    4.3476    2.2583   -1.8383 H   0  0  0  0  0  0  0  0  0  0  0  0
    9.0313    3.0854   -1.9374 H   0  0  0  0  0  0  0  0  0  0  0  0
   10.4545    4.8641   -1.0606 H   0  0  0  0  0  0  0  0  0  0  0  0
```

5.3154 6.4061 1.0779 H 0 0 0 0 0 0 0 0 0 0 0 0
6.3739 7.2212 2.2366 H 0 0 0 0 0 0 0 0 0 0 0 0
5.8922 8.0450 0.7475 H 0 0 0 0 0 0 0 0 0 0 0 0
8.7046 7.9480 1.3581 H 0 0 0 0 0 0 0 0 0 0 0 0
10.7038 6.9024 0.3298 H 0 0 0 0 0 0 0 0 0 0 0 0
1 2 1 0 0 0 0
1 3 4 0 0 0 0
4 1 4 0 0 0 0
3 5 4 0 0 0 0
6 3 4 0 0 0 0
7 4 4 0 0 0 0
5 8 4 0 0 0 0
9 5 4 0 0 0 0
10 6 4 0 0 0 0
6 11 4 0 0 0 0
11 7 4 0 0 0 0
12 8 4 0 0 0 0
8 13 4 0 0 0 0
14 9 4 0 0 0 0
9 15 4 0 0 0 0
15 10 4 0 0 0 0
12 16 4 0 0 0 0
17 12 4 0 0 0 0
13 18 1 0 0 0 0
13 19 4 0 0 0 0
16 14 4 0 0 0 0
20 17 4 0 0 0 0
19 20 4 0 0 0 0
2 21 1 0 0 0 0
2 22 1 0 0 0 0
2 23 1 0 0 0 0
4 24 1 0 0 0 0
7 25 1 0 0 0 0
10 26 1 0 0 0 0
11 27 1 0 0 0 0
14 28 1 0 0 0 0
15 29 1 0 0 0 0
16 30 1 0 0 0 0
17 31 1 0 0 0 0
18 32 1 0 0 0 0
18 33 1 0 0 0 0
18 34 1 0 0 0 0
19 35 1 0 0 0 0
20 36 1 0 0 0 0
M END
> <Alias name>
1,12-Dimethylbenzo[c]phenanthrene

> <deltaH°(c)> kJ/mol
-10311

$$$$
7,12-Dimethylbenz(a)anthracene
ChemBrain IXL

36 39 0 0 0 0 0 0 0 0 1 V2000
3.1253 4.2576 0.0000 C 0 0 0 0 0 0 0 0 0 0 0 0
2.5400 5.4779 -0.3382 C 0 0 0 0 0 0 0 0 0 0 0 0
3.3258 6.6269 -0.4294 C 0 0 0 0 0 0 0 0 0 0 0 0
4.6968 6.5557 -0.1821 C 0 0 0 0 0 0 0 0 0 0 0 0
5.2822 5.3354 0.1557 C 0 0 0 0 0 0 0 0 0 0 0 0
4.4964 4.1864 0.2469 C 0 0 0 0 0 0 0 0 0 0 0 0
7.4392 6.4130 0.3107 C 0 0 0 0 0 0 0 0 0 0 0 0

```
   6.8539    7.6333   -0.0271 C   0  0  0  0  0  0  0  0  0  0  0  0
   7.6398    8.7821   -0.1191 C   0  0  0  0  0  0  0  0  0  0  0  0
   9.0111    8.7107    0.1270 C   0  0  0  0  0  0  0  0  0  0  0  0
   9.5964    7.4904    0.4651 C   0  0  0  0  0  0  0  0  0  0  0  0
   8.8105    6.3416    0.5570 C   0  0  0  0  0  0  0  0  0  0  0  0
   6.6534    5.2640    0.4022 C   0  0  0  0  0  0  0  0  0  0  0  0
   5.4825    7.7048   -0.2727 C   0  0  0  0  0  0  0  0  0  0  0  0
   9.3958    5.1213    0.8951 C   0  0  0  0  0  0  0  0  0  0  0  0
  10.7670    5.0499    1.1414 C   0  0  0  0  0  0  0  0  0  0  0  0
  11.5530    6.1988    1.0495 C   0  0  0  0  0  0  0  0  0  0  0  0
  10.9676    7.4190    0.7114 C   0  0  0  0  0  0  0  0  0  0  0  0
   7.2744    3.9694    0.7609 C   0  0  0  0  0  0  0  0  0  0  0  0
   4.8612    8.9999   -0.6295 C   0  0  0  0  0  0  0  0  0  0  0  0
   2.5152    3.3656    0.0709 H   0  0  0  0  0  0  0  0  0  0  0  0
   1.4755    5.5332   -0.5300 H   0  0  0  0  0  0  0  0  0  0  0  0
   2.8714    7.5742   -0.6920 H   0  0  0  0  0  0  0  0  0  0  0  0
   4.9508    3.2390    0.5095 H   0  0  0  0  0  0  0  0  0  0  0  0
   7.1854    9.7295   -0.3817 H   0  0  0  0  0  0  0  0  0  0  0  0
   9.6212    9.6026    0.0556 H   0  0  0  0  0  0  0  0  0  0  0  0
   8.7857    4.2294    0.9665 H   0  0  0  0  0  0  0  0  0  0  0  0
  11.2215    4.1025    1.4038 H   0  0  0  0  0  0  0  0  0  0  0  0
  12.6175    6.1433    1.2406 H   0  0  0  0  0  0  0  0  0  0  0  0
  11.5778    8.3110    0.6401 H   0  0  0  0  0  0  0  0  0  0  0  0
   8.0266    3.7136    0.0250 H   0  0  0  0  0  0  0  0  0  0  0  0
   6.5143    3.1983    0.7832 H   0  0  0  0  0  0  0  0  0  0  0  0
   7.7367    4.0489    1.7371 H   0  0  0  0  0  0  0  0  0  0  0  0
   3.8176    8.9914   -0.3404 H   0  0  0  0  0  0  0  0  0  0  0  0
   5.3731    9.8009   -0.1107 H   0  0  0  0  0  0  0  0  0  0  0  0
   4.9384    9.1549   -1.6986 H   0  0  0  0  0  0  0  0  0  0  0  0
  1  2  4  0  0  0  0
  2  3  4  0  0  0  0
  3  4  4  0  0  0  0
  4  5  4  0  0  0  0
  5  6  4  0  0  0  0
  6  1  4  0  0  0  0
  7  8  4  0  0  0  0
  8  9  4  0  0  0  0
  9 10  4  0  0  0  0
 10 11  4  0  0  0  0
 11 12  4  0  0  0  0
 12  7  4  0  0  0  0
  5 13  4  0  0  0  0
 13  7  4  0  0  0  0
  4 14  4  0  0  0  0
 14  8  4  0  0  0  0
 12 15  4  0  0  0  0
 15 16  4  0  0  0  0
 16 17  4  0  0  0  0
 17 18  4  0  0  0  0
 18 11  4  0  0  0  0
 13 19  1  0  0  0  0
 14 20  1  0  0  0  0
  1 21  1  0  0  0  0
  2 22  1  0  0  0  0
  3 23  1  0  0  0  0
  6 24  1  0  0  0  0
  9 25  1  0  0  0  0
 10 26  1  0  0  0  0
 15 27  1  0  0  0  0
 16 28  1  0  0  0  0
 17 29  1  0  0  0  0
 18 30  1  0  0  0  0
 19 31  1  0  0  0  0
```

```
 19 32  1  0  0  0  0
 19 33  1  0  0  0  0
 20 34  1  0  0  0  0
 20 35  1  0  0  0  0
 20 36  1  0  0  0  0
M  END
>  <Alias name>
7,12-Dimethylbenz[a]anthracene

>  <deltaH°(c)>   kJ/mol
-10307.4

$$$$
6,13-Pentacenequinone
  ChemBrain IXL

 36 40  0  0  0  0  0  0  0  0  1 V2000
    7.2658    7.1558    0.0000 C   0  0  0  0  0  0  0  0  0  0  0  0
    7.2658    8.5508    0.0000 C   0  0  0  0  0  0  0  0  0  0  0  0
    8.5476    6.4158    0.0000 C   0  0  0  0  0  0  0  0  0  0  0  0
    6.0577    6.4583    0.0000 C   0  0  0  0  0  0  0  0  0  0  0  0
    8.5476    9.2908    0.0000 C   0  0  0  0  0  0  0  0  0  0  0  0
    6.0577    9.2483    0.0000 C   0  0  0  0  0  0  0  0  0  0  0  0
    9.8293    7.1558    0.0000 C   0  0  0  0  0  0  0  0  0  0  0  0
    8.5476    5.1958    0.0001 O   0  0  0  0  0  0  0  0  0  0  0  0
    4.8496    7.1558   -0.0001 C   0  0  0  0  0  0  0  0  0  0  0  0
    9.8293    8.5508    0.0000 C   0  0  0  0  0  0  0  0  0  0  0  0
    8.5476   10.5108    0.0001 O   0  0  0  0  0  0  0  0  0  0  0  0
    4.8496    8.5508   -0.0001 C   0  0  0  0  0  0  0  0  0  0  0  0
   11.0374    6.4583    0.0000 C   0  0  0  0  0  0  0  0  0  0  0  0
    3.6415    6.4583   -0.0001 C   0  0  0  0  0  0  0  0  0  0  0  0
   11.0374    9.2483    0.0000 C   0  0  0  0  0  0  0  0  0  0  0  0
    3.6415    9.2483   -0.0001 C   0  0  0  0  0  0  0  0  0  0  0  0
   12.2455    7.1558   -0.0001 C   0  0  0  0  0  0  0  0  0  0  0  0
    2.4334    7.1558   -0.0002 C   0  0  0  0  0  0  0  0  0  0  0  0
   12.2455    8.5508   -0.0001 C   0  0  0  0  0  0  0  0  0  0  0  0
    2.4334    8.5508   -0.0002 C   0  0  0  0  0  0  0  0  0  0  0  0
   13.4536    6.4583   -0.0001 C   0  0  0  0  0  0  0  0  0  0  0  0
   13.4536    9.2483   -0.0001 C   0  0  0  0  0  0  0  0  0  0  0  0
   14.6617    7.1558   -0.0002 C   0  0  0  0  0  0  0  0  0  0  0  0
   14.6617    8.5508   -0.0002 C   0  0  0  0  0  0  0  0  0  0  0  0
    6.0577    5.3753    0.0000 H   0  0  0  0  0  0  0  0  0  0  0  0
    6.0577   10.3313    0.0000 H   0  0  0  0  0  0  0  0  0  0  0  0
   11.0374    5.3753    0.0000 H   0  0  0  0  0  0  0  0  0  0  0  0
    3.6415    5.3753   -0.0001 H   0  0  0  0  0  0  0  0  0  0  0  0
   11.0374   10.3313    0.0000 H   0  0  0  0  0  0  0  0  0  0  0  0
    3.6415   10.3313   -0.0001 H   0  0  0  0  0  0  0  0  0  0  0  0
    1.4955    6.6143   -0.0003 H   0  0  0  0  0  0  0  0  0  0  0  0
    1.4955    9.0923   -0.0003 H   0  0  0  0  0  0  0  0  0  0  0  0
   13.4536    5.3753   -0.0001 H   0  0  0  0  0  0  0  0  0  0  0  0
   13.4536   10.3313   -0.0001 H   0  0  0  0  0  0  0  0  0  0  0  0
   15.5996    6.6143   -0.0003 H   0  0  0  0  0  0  0  0  0  0  0  0
   15.5996    9.0923   -0.0003 H   0  0  0  0  0  0  0  0  0  0  0  0
  1  2  4  0  0  0  0
  1  3  1  0  0  0  0
  1  4  4  0  0  0  0
  2  5  1  0  0  0  0
  2  6  4  0  0  0  0
  3  7  1  0  0  0  0
  3  8  2  0  0  0  0
  4  9  4  0  0  0  0
  5 10  1  0  0  0  0
  5 11  2  0  0  0  0
```

```
  6 12  4  0  0  0  0
  7 13  4  0  0  0  0
  7 10  4  0  0  0  0
  9 14  4  0  0  0  0
  9 12  4  0  0  0  0
 10 15  4  0  0  0  0
 12 16  4  0  0  0  0
 13 17  4  0  0  0  0
 14 18  4  0  0  0  0
 15 19  4  0  0  0  0
 16 20  4  0  0  0  0
 17 21  4  0  0  0  0
 17 19  4  0  0  0  0
 18 20  4  0  0  0  0
 19 22  4  0  0  0  0
 21 23  4  0  0  0  0
 22 24  4  0  0  0  0
 23 24  4  0  0  0  0
  4 25  1  0  0  0  0
  6 26  1  0  0  0  0
 13 27  1  0  0  0  0
 14 28  1  0  0  0  0
 15 29  1  0  0  0  0
 16 30  1  0  0  0  0
 18 31  1  0  0  0  0
 20 32  1  0  0  0  0
 21 33  1  0  0  0  0
 22 34  1  0  0  0  0
 23 35  1  0  0  0  0
 24 36  1  0  0  0  0
M  END
>  <Alias name>
6,13-Pentacenedione

>  <deltaH°(c)>   kJ/mol
-10299

$$$$
Triphenylphosphine
  ChemBrain IXL

 34 36  0  0  0  0  0  0  0  0  1 V2000
    6.9131    6.4991    1.7386 P   0  0  0  0  0  0  0  0  0  0  0  0
    6.7774    4.8820    0.8090 C   0  0  0  0  0  0  0  0  0  0  0  0
    5.2541    7.2547    1.3203 C   0  0  0  0  0  0  0  0  0  0  0  0
    8.0114    7.4567    0.5662 C   0  0  0  0  0  0  0  0  0  0  0  0
    7.7400    4.5407   -0.1412 C   0  0  0  0  0  0  0  0  0  0  0  0
    5.7136    4.0169    1.0656 C   0  0  0  0  0  0  0  0  0  0  0  0
    5.0705    8.6339    1.4205 C   0  0  0  0  0  0  0  0  0  0  0  0
    4.2001    6.4392    0.9081 C   0  0  0  0  0  0  0  0  0  0  0  0
    7.5820    8.6793    0.0496 C   0  0  0  0  0  0  0  0  0  0  0  0
    9.2601    6.9484    0.2081 C   0  0  0  0  0  0  0  0  0  0  0  0
    7.6628    3.3151   -0.8029 C   0  0  0  0  0  0  0  0  0  0  0  0
    5.6360    2.7915    0.4035 C   0  0  0  0  0  0  0  0  0  0  0  0
    3.8220    9.1930    1.1472 C   0  0  0  0  0  0  0  0  0  0  0  0
    2.9514    6.9981    0.6353 C   0  0  0  0  0  0  0  0  0  0  0  0
    8.4184    9.4132   -0.7917 C   0  0  0  0  0  0  0  0  0  0  0  0
   10.0964    7.6820   -0.6335 C   0  0  0  0  0  0  0  0  0  0  0  0
    6.6118    2.4396   -0.5291 C   0  0  0  0  0  0  0  0  0  0  0  0
    2.7619    8.3748    0.7566 C   0  0  0  0  0  0  0  0  0  0  0  0
    9.6764    8.9155   -1.1317 C   0  0  0  0  0  0  0  0  0  0  0  0
    8.5472    5.2272   -0.3651 H   0  0  0  0  0  0  0  0  0  0  0  0
    4.9481    4.2966    1.7788 H   0  0  0  0  0  0  0  0  0  0  0  0
```

5.8972 9.2706 1.7105 H 0 0 0 0 0 0 0 0 0 0 0 0
4.3512 5.3723 0.7999 H 0 0 0 0 0 0 0 0 0 0 0 0
6.5995 9.0586 0.3017 H 0 0 0 0 0 0 0 0 0 0 0 0
9.5803 5.9843 0.5834 H 0 0 0 0 0 0 0 0 0 0 0 0
8.4188 3.0433 -1.5293 H 0 0 0 0 0 0 0 0 0 0 0 0
4.8181 2.1134 0.6136 H 0 0 0 0 0 0 0 0 0 0 0 0
3.6758 10.2622 1.2383 H 0 0 0 0 0 0 0 0 0 0 0 0
2.1291 6.3632 0.3292 H 0 0 0 0 0 0 0 0 0 0 0 0
8.0911 10.3694 -1.1808 H 0 0 0 0 0 0 0 0 0 0 0 0
11.0718 7.2940 -0.9001 H 0 0 0 0 0 0 0 0 0 0 0 0
6.5535 1.4868 -1.0406 H 0 0 0 0 0 0 0 0 0 0 0 0
1.7917 8.8084 0.5475 H 0 0 0 0 0 0 0 0 0 0 0 0
10.3270 9.4867 -1.7823 H 0 0 0 0 0 0 0 0 0 0 0 0
1 2 1 0 0 0 0
1 3 1 0 0 0 0
1 4 1 0 0 0 0
2 5 4 0 0 0 0
2 6 4 0 0 0 0
3 7 4 0 0 0 0
3 8 4 0 0 0 0
4 9 4 0 0 0 0
4 10 4 0 0 0 0
5 11 4 0 0 0 0
6 12 4 0 0 0 0
7 13 4 0 0 0 0
8 14 4 0 0 0 0
9 15 4 0 0 0 0
10 16 4 0 0 0 0
11 17 4 0 0 0 0
12 17 4 0 0 0 0
13 18 4 0 0 0 0
14 18 4 0 0 0 0
15 19 4 0 0 0 0
16 19 4 0 0 0 0
5 20 1 0 0 0 0
6 21 1 0 0 0 0
7 22 1 0 0 0 0
8 23 1 0 0 0 0
9 24 1 0 0 0 0
10 25 1 0 0 0 0
11 26 1 0 0 0 0
12 27 1 0 0 0 0
13 28 1 0 0 0 0
14 29 1 0 0 0 0
15 30 1 0 0 0 0
16 31 1 0 0 0 0
17 32 1 0 0 0 0
18 33 1 0 0 0 0
19 34 1 0 0 0 0
M END
> <Alias name>
Triphenylphosphane

> <deltaH°(c)> kJ/mol
-10299

$$$$
5,6-Dimethylchrysene
ChemBrain IXL

36 39 0 0 0 0 0 0 0 0 1 V2000
8.3301 8.8281 -0.0905 C 0 0 0 0 0 0 0 0 0 0 0 0
7.2422 7.8259 -0.1415 C 0 0 0 0 0 0 0 0 0 0 0 0

```
    7.5468    6.4726   -0.2890 C   0  0  0  0  0  0  0  0  0  0  0  0
    6.5214    5.5280   -0.3369 C   0  0  0  0  0  0  0  0  0  0  0  0
    5.1914    5.9367   -0.2374 C   0  0  0  0  0  0  0  0  0  0  0  0
    4.8867    7.2900   -0.0900 C   0  0  0  0  0  0  0  0  0  0  0  0
    5.9121    8.2346   -0.0421 C   0  0  0  0  0  0  0  0  0  0  0  0
    5.5889    9.6704    0.1142 C   0  0  0  0  0  0  0  0  0  0  0  0
    3.5566    7.6987    0.0096 C   0  0  0  0  0  0  0  0  0  0  0  0
    2.5312    6.7541   -0.0383 C   0  0  0  0  0  0  0  0  0  0  0  0
    2.8359    5.4008   -0.1859 C   0  0  0  0  0  0  0  0  0  0  0  0
    4.1660    4.9921   -0.2854 C   0  0  0  0  0  0  0  0  0  0  0  0
    6.8261    4.1747   -0.4844 C   0  0  0  0  0  0  0  0  0  0  0  0
    8.1562    3.7660   -0.5839 C   0  0  0  0  0  0  0  0  0  0  0  0
    9.1816    4.7106   -0.5359 C   0  0  0  0  0  0  0  0  0  0  0  0
    8.8769    6.0639   -0.3885 C   0  0  0  0  0  0  0  0  0  0  0  0
    9.9023    7.0085   -0.3405 C   0  0  0  0  0  0  0  0  0  0  0  0
   11.2324    6.5998   -0.4400 C   0  0  0  0  0  0  0  0  0  0  0  0
   11.5370    5.2465   -0.5874 C   0  0  0  0  0  0  0  0  0  0  0  0
   10.5116    4.3019   -0.6354 C   0  0  0  0  0  0  0  0  0  0  0  0
    7.9046    9.8171    0.0268 H   0  0  0  0  0  0  0  0  0  0  0  0
    8.9015    8.7876   -1.0096 H   0  0  0  0  0  0  0  0  0  0  0  0
    8.9802    8.6128    0.7485 H   0  0  0  0  0  0  0  0  0  0  0  0
    4.5140    9.7959    0.1570 H   0  0  0  0  0  0  0  0  0  0  0  0
    6.0325   10.0428    1.0293 H   0  0  0  0  0  0  0  0  0  0  0  0
    5.9836   10.2232   -0.7293 H   0  0  0  0  0  0  0  0  0  0  0  0
    3.3201    8.7494    0.1242 H   0  0  0  0  0  0  0  0  0  0  0  0
    1.4987    7.0714    0.0390 H   0  0  0  0  0  0  0  0  0  0  0  0
    2.0398    4.6675   -0.2231 H   0  0  0  0  0  0  0  0  0  0  0  0
    4.4025    3.9415   -0.3999 H   0  0  0  0  0  0  0  0  0  0  0  0
    6.0300    3.4413   -0.5217 H   0  0  0  0  0  0  0  0  0  0  0  0
    8.3927    2.7153   -0.6984 H   0  0  0  0  0  0  0  0  0  0  0  0
    9.6658    8.0591   -0.2260 H   0  0  0  0  0  0  0  0  0  0  0  0
   12.0285    7.3331   -0.4027 H   0  0  0  0  0  0  0  0  0  0  0  0
   12.5696    4.9292   -0.6647 H   0  0  0  0  0  0  0  0  0  0  0  0
   10.7482    3.2512   -0.7499 H   0  0  0  0  0  0  0  0  0  0  0  0
  1  2  1  0  0  0  0
  2  7  4  0  0  0  0
  2  3  4  0  0  0  0
  3 16  4  0  0  0  0
  3  4  4  0  0  0  0
  4 13  4  0  0  0  0
  4  5  4  0  0  0  0
  5 12  4  0  0  0  0
  5  6  4  0  0  0  0
  6  9  4  0  0  0  0
  6  7  4  0  0  0  0
  7  8  1  0  0  0  0
  9 10  4  0  0  0  0
 10 11  4  0  0  0  0
 11 12  4  0  0  0  0
 13 14  4  0  0  0  0
 14 15  4  0  0  0  0
 15 20  4  0  0  0  0
 15 16  4  0  0  0  0
 16 17  4  0  0  0  0
 17 18  4  0  0  0  0
 18 19  4  0  0  0  0
 19 20  4  0  0  0  0
  1 21  1  0  0  0  0
  1 22  1  0  0  0  0
  1 23  1  0  0  0  0
  8 24  1  0  0  0  0
  8 25  1  0  0  0  0
  8 26  1  0  0  0  0
```

```
  9 27  1  0  0  0  0
 10 28  1  0  0  0  0
 11 29  1  0  0  0  0
 12 30  1  0  0  0  0
 13 31  1  0  0  0  0
 14 32  1  0  0  0  0
 17 33  1  0  0  0  0
 18 34  1  0  0  0  0
 19 35  1  0  0  0  0
 20 36  1  0  0  0  0
M  END
>  <Alias name>
5,6-Dimethylchrysene

>  <deltaH°(c)>   kJ/mol
-10296.1

$$$$
1,12-Dimethylbenzo(a)anthracene
  ChemBrain IXL

 36 39  0  0  0  0  0  0  0  0  1 V2000
    7.5190    5.3920    0.9562 C   0  0  0  0  0  0  0  0  0  0  0  0
    7.8550    6.7681    0.5203 C   0  0  0  0  0  0  0  0  0  0  0  0
    8.4862    4.3933    0.9451 C   0  0  0  0  0  0  0  0  0  0  0  0
    6.2305    5.0856    1.3769 C   0  0  0  0  0  0  0  0  0  0  0  0
    9.8432    4.7058    0.9471 C   0  0  0  0  0  0  0  0  0  0  0  0
    8.0933    3.0718    0.9313 C   0  0  0  0  0  0  0  0  0  0  0  0
    5.8601    3.7549    1.5389 C   0  0  0  0  0  0  0  0  0  0  0  0
    5.3148    6.1054    1.6361 C   0  0  0  0  0  0  0  0  0  0  0  0
   10.7600    3.7525    0.5343 C   0  0  0  0  0  0  0  0  0  0  0  0
   10.2870    5.9625    1.3611 C   0  0  0  0  0  0  0  0  0  0  0  0
    6.7804    2.7419    1.2658 C   0  0  0  0  0  0  0  0  0  0  0  0
    9.0149    2.0863    0.5830 C   0  0  0  0  0  0  0  0  0  0  0  0
    4.5726    3.4387    1.9732 C   0  0  0  0  0  0  0  0  0  0  0  0
    4.0280    5.7910    2.0716 C   0  0  0  0  0  0  0  0  0  0  0  0
   10.3455    2.4348    0.3483 C   0  0  0  0  0  0  0  0  0  0  0  0
   12.0868    4.1184    0.3085 C   0  0  0  0  0  0  0  0  0  0  0  0
    9.3550    6.9010    2.0294 C   0  0  0  0  0  0  0  0  0  0  0  0
   11.6131    6.3332    1.1449 C   0  0  0  0  0  0  0  0  0  0  0  0
    3.6570    4.4572    2.2415 C   0  0  0  0  0  0  0  0  0  0  0  0
   12.5100    5.4153    0.6003 C   0  0  0  0  0  0  0  0  0  0  0  0
    7.1679    7.0785   -0.2574 H   0  0  0  0  0  0  0  0  0  0  0  0
    8.8672    6.7885    0.1364 H   0  0  0  0  0  0  0  0  0  0  0  0
    7.7748    7.4437    1.3633 H   0  0  0  0  0  0  0  0  0  0  0  0
    5.6029    7.1408    1.4992 H   0  0  0  0  0  0  0  0  0  0  0  0
    6.4761    1.7042    1.3136 H   0  0  0  0  0  0  0  0  0  0  0  0
    8.6996    1.0547    0.4944 H   0  0  0  0  0  0  0  0  0  0  0  0
    4.2836    2.4034    2.1021 H   0  0  0  0  0  0  0  0  0  0  0  0
    3.3170    6.5821    2.2777 H   0  0  0  0  0  0  0  0  0  0  0  0
   11.0542    1.6840    0.0225 H   0  0  0  0  0  0  0  0  0  0  0  0
   12.7874    3.3973   -0.0930 H   0  0  0  0  0  0  0  0  0  0  0  0
    8.4512    6.3749    2.3091 H   0  0  0  0  0  0  0  0  0  0  0  0
    9.8257    7.3068    2.9168 H   0  0  0  0  0  0  0  0  0  0  0  0
    9.1076    7.7086    1.3511 H   0  0  0  0  0  0  0  0  0  0  0  0
   11.9460    7.3325    1.3994 H   0  0  0  0  0  0  0  0  0  0  0  0
    2.6585    4.2122    2.5817 H   0  0  0  0  0  0  0  0  0  0  0  0
   13.5339    5.7091    0.4043 H   0  0  0  0  0  0  0  0  0  0  0  0
  1  2  1  0  0  0  0
  1  3  4  0  0  0  0
  4  1  4  0  0  0  0
  3  5  4  0  0  0  0
  6  3  4  0  0  0  0
```

```
  7  4  4  0  0  0  0
  8  4  4  0  0  0  0
  9  5  4  0  0  0  0
  5 10  4  0  0  0  0
  6 11  4  0  0  0  0
 12  6  4  0  0  0  0
 11  7  4  0  0  0  0
  7 13  4  0  0  0  0
 14  8  4  0  0  0  0
  9 15  4  0  0  0  0
 16  9  4  0  0  0  0
 10 17  1  0  0  0  0
 10 18  4  0  0  0  0
 15 12  4  0  0  0  0
 13 19  4  0  0  0  0
 19 14  4  0  0  0  0
 20 16  4  0  0  0  0
 18 20  4  0  0  0  0
  2 21  1  0  0  0  0
  2 22  1  0  0  0  0
  2 23  1  0  0  0  0
  8 24  1  0  0  0  0
 11 25  1  0  0  0  0
 12 26  1  0  0  0  0
 13 27  1  0  0  0  0
 14 28  1  0  0  0  0
 15 29  1  0  0  0  0
 16 30  1  0  0  0  0
 17 31  1  0  0  0  0
 17 32  1  0  0  0  0
 17 33  1  0  0  0  0
 18 34  1  0  0  0  0
 19 35  1  0  0  0  0
 20 36  1  0  0  0  0
M  END
>  <Alias name>
1,12-Dimethylbenz[a]anthracene

>  <deltaH°(c)>   kJ/mol
-10293.62

$$$$
2,4,6-Triphenoxy-s-triazine
  ChemBrain IXL

 42 45  0  0  0  0  0  0  0  0  1 V2000
    6.9290    8.3356   -0.2084 C   0  0  0  0  0  0  0  0  0  0  0  0
    8.7931    9.7356   -0.1974 C   0  0  0  0  0  0  0  0  0  0  0  0
    9.0735    7.4214   -0.2240 C   0  0  0  0  0  0  0  0  0  0  0  0
    7.4569    9.5737   -0.1956 N   0  0  0  0  0  0  0  0  0  0  0  0
    7.7373    7.2594   -0.2217 N   0  0  0  0  0  0  0  0  0  0  0  0
    9.6014    8.6595   -0.2108 N   0  0  0  0  0  0  0  0  0  0  0  0
    9.3696   11.0878   -0.1847 O   0  0  0  0  0  0  0  0  0  0  0  0
    5.4697    8.1588   -0.2079 O   0  0  0  0  0  0  0  0  0  0  0  0
    9.9563    6.2461   -0.2411 O   0  0  0  0  0  0  0  0  0  0  0  0
    4.6932    9.4028   -0.1063 C   0  0  0  0  0  0  0  0  0  0  0  0
   10.8386   11.1366   -0.1609 C   0  0  0  0  0  0  0  0  0  0  0  0
    9.2647    4.9492   -0.2172 C   0  0  0  0  0  0  0  0  0  0  0  0
    5.3494   10.6240    0.0487 C   0  0  0  0  0  0  0  0  0  0  0  0
    3.3000    9.3622   -0.1650 C   0  0  0  0  0  0  0  0  0  0  0  0
   11.4958   12.3671   -0.1682 C   0  0  0  0  0  0  0  0  0  0  0  0
   11.5755    9.9525   -0.1310 C   0  0  0  0  0  0  0  0  0  0  0  0
   10.0017    3.7651   -0.2435 C   0  0  0  0  0  0  0  0  0  0  0  0
```

```
   7.8713    4.9026   -0.1682 C   0  0  0  0  0  0  0  0  0  0  0  0
   2.5631   10.5427   -0.0675 C   0  0  0  0  0  0  0  0  0  0  0  0
   4.6125   11.8044    0.1462 C   0  0  0  0  0  0  0  0  0  0  0  0
  12.8899   12.4134   -0.1455 C   0  0  0  0  0  0  0  0  0  0  0  0
  12.9695    9.9988   -0.1083 C   0  0  0  0  0  0  0  0  0  0  0  0
   9.3453    2.5343   -0.2217 C   0  0  0  0  0  0  0  0  0  0  0  0
   7.2149    3.6719   -0.1464 C   0  0  0  0  0  0  0  0  0  0  0  0
   3.2193   11.7638    0.0881 C   0  0  0  0  0  0  0  0  0  0  0  0
  13.6267   11.2293   -0.1156 C   0  0  0  0  0  0  0  0  0  0  0  0
   7.9519    2.4877   -0.1732 C   0  0  0  0  0  0  0  0  0  0  0  0
   6.4311   10.6556    0.0934 H   0  0  0  0  0  0  0  0  0  0  0  0
   2.7905    8.4142   -0.2862 H   0  0  0  0  0  0  0  0  0  0  0  0
  10.9238   13.2864   -0.1915 H   0  0  0  0  0  0  0  0  0  0  0  0
  11.0653    8.9972   -0.1254 H   0  0  0  0  0  0  0  0  0  0  0  0
  11.0834    3.8012   -0.2808 H   0  0  0  0  0  0  0  0  0  0  0  0
   7.2992    5.8219   -0.1471 H   0  0  0  0  0  0  0  0  0  0  0  0
   1.4815   10.5111   -0.1127 H   0  0  0  0  0  0  0  0  0  0  0  0
   5.1220   12.7525    0.2670 H   0  0  0  0  0  0  0  0  0  0  0  0
  13.4001   13.3687   -0.1512 H   0  0  0  0  0  0  0  0  0  0  0  0
  13.5416    9.0795   -0.0851 H   0  0  0  0  0  0  0  0  0  0  0  0
   9.9174    1.6150   -0.2424 H   0  0  0  0  0  0  0  0  0  0  0  0
   6.1332    3.6357   -0.1086 H   0  0  0  0  0  0  0  0  0  0  0  0
   2.6472   12.6802    0.1639 H   0  0  0  0  0  0  0  0  0  0  0  0
  14.7090   11.2653   -0.0980 H   0  0  0  0  0  0  0  0  0  0  0  0
   7.4423    1.5323   -0.1563 H   0  0  0  0  0  0  0  0  0  0  0  0
  2  4  4  0  0  0  0
  3  5  4  0  0  0  0
  4  1  4  0  0  0  0
  5  1  4  0  0  0  0
  6  3  4  0  0  0  0
  7  2  1  0  0  0  0
  8  1  1  0  0  0  0
  9  3  1  0  0  0  0
 10  8  1  0  0  0  0
 11  7  1  0  0  0  0
 12  9  1  0  0  0  0
 13 10  4  0  0  0  0
 14 10  4  0  0  0  0
 15 11  4  0  0  0  0
 16 11  4  0  0  0  0
 17 12  4  0  0  0  0
 18 12  4  0  0  0  0
 19 14  4  0  0  0  0
 20 13  4  0  0  0  0
 21 15  4  0  0  0  0
 22 16  4  0  0  0  0
 23 17  4  0  0  0  0
 24 18  4  0  0  0  0
 25 20  4  0  0  0  0
 26 22  4  0  0  0  0
 27 24  4  0  0  0  0
  6  2  4  0  0  0  0
 25 19  4  0  0  0  0
 27 23  4  0  0  0  0
 26 21  4  0  0  0  0
 13 28  1  0  0  0  0
 14 29  1  0  0  0  0
 15 30  1  0  0  0  0
 16 31  1  0  0  0  0
 17 32  1  0  0  0  0
 18 33  1  0  0  0  0
 19 34  1  0  0  0  0
 20 35  1  0  0  0  0
```

21 36 1 0 0 0 0
22 37 1 0 0 0 0
23 38 1 0 0 0 0
24 39 1 0 0 0 0
25 40 1 0 0 0 0
26 41 1 0 0 0 0
27 42 1 0 0 0 0
M END
> <Alias name>
2,4,6-Triphenoxy-s-triazine

> <deltaH°(c)>   kJ/mol
-10289

$$$$
Triisopentylamine
  ChemBrain IXL

49 48 0 0 0 0 0 0 0 0 1 V2000
    3.6016   4.2177   -0.5213 C   0 0 0 0 0 0 0 0 0 0 0 0
    4.8641   4.4121   0.3459 C   0 0 0 0 0 0 0 0 0 0 0 0
    2.3476   4.3325   0.3673 C   0 0 0 0 0 0 0 0 0 0 0 0
    3.5535   5.3019   -1.6179 C   0 0 0 0 0 0 0 0 0 0 0 0
    6.1254   4.2717   -0.5353 C   0 0 0 0 0 0 0 0 0 0 0 0
    7.3370   4.4066   0.2994 N   0 0 0 0 0 0 0 0 0 0 0 0
    8.5375   4.2587   -0.5491 C   0 0 0 0 0 0 0 0 0 0 0 0
    9.8103   4.3842   0.3175 C   0 0 0 0 0 0 0 0 0 0 0 0
   11.0603   4.1738   -0.5638 C   0 0 0 0 0 0 0 0 0 0 0 0
   12.3257   4.2743   0.3103 C   0 0 0 0 0 0 0 0 0 0 0 0
   11.1089   5.2560   -1.6623 C   0 0 0 0 0 0 0 0 0 0 0 0
    7.3682   8.2341   0.6113 C   0 0 0 0 0 0 0 0 0 0 0 0
    7.3455   6.8613   -0.0945 C   0 0 0 0 0 0 0 0 0 0 0 0
    7.3954   9.3535   -0.4478 C   0 0 0 0 0 0 0 0 0 0 0 0
    6.1076   8.3885   1.4875 C   0 0 0 0 0 0 0 0 0 0 0 0
    7.3475   5.7329   0.9650 C   0 0 0 0 0 0 0 0 0 0 0 0
    3.6278   3.2384   -0.9827 H   0 0 0 0 0 0 0 0 0 0 0 0
    4.8434   5.3961   0.7949 H   0 0 0 0 0 0 0 0 0 0 0 0
    4.8843   3.6603   1.1250 H   0 0 0 0 0 0 0 0 0 0 0 0
    2.3796   3.5711   1.1369 H   0 0 0 0 0 0 0 0 0 0 0 0
    2.3207   5.3117   0.8292 H   0 0 0 0 0 0 0 0 0 0 0 0
    1.4613   4.1952   -0.2396 H   0 0 0 0 0 0 0 0 0 0 0 0
    2.6595   5.1715   -2.2149 H   0 0 0 0 0 0 0 0 0 0 0 0
    3.5397   6.2817   -1.1567 H   0 0 0 0 0 0 0 0 0 0 0 0
    4.4260   5.2138   -2.2522 H   0 0 0 0 0 0 0 0 0 0 0 0
    6.1243   3.2982   -1.0096 H   0 0 0 0 0 0 0 0 0 0 0 0
    6.1226   5.0404   -1.2953 H   0 0 0 0 0 0 0 0 0 0 0 0
    8.5403   5.0274   -1.3090 H   0 0 0 0 0 0 0 0 0 0 0 0
    8.5222   3.2853   -1.0237 H   0 0 0 0 0 0 0 0 0 0 0 0
    9.7898   3.6333   1.0975 H   0 0 0 0 0 0 0 0 0 0 0 0
    9.8481   5.3682   0.7654 H   0 0 0 0 0 0 0 0 0 0 0 0
   11.0170   3.1943   -1.0237 H   0 0 0 0 0 0 0 0 0 0 0 0
   12.3697   5.2536   0.7706 H   0 0 0 0 0 0 0 0 0 0 0 0
   13.2033   4.1256   -0.3066 H   0 0 0 0 0 0 0 0 0 0 0 0
   12.2934   3.5142   1.0810 H   0 0 0 0 0 0 0 0 0 0 0 0
   10.2282   5.1779   -2.2863 H   0 0 0 0 0 0 0 0 0 0 0 0
   11.9944   5.1141   -2.2694 H   0 0 0 0 0 0 0 0 0 0 0 0
   11.1400   6.2361   -1.2024 H   0 0 0 0 0 0 0 0 0 0 0 0
    8.2515   8.3055   1.2337 H   0 0 0 0 0 0 0 0 0 0 0 0
    8.2207   6.7678   -0.7230 H   0 0 0 0 0 0 0 0 0 0 0 0
    6.4544   6.7853   -0.7032 H   0 0 0 0 0 0 0 0 0 0 0 0
    6.5123   9.2827   -1.0707 H   0 0 0 0 0 0 0 0 0 0 0 0
    8.2805   9.2470   -1.0628 H   0 0 0 0 0 0 0 0 0 0 0 0
    7.4127   10.3172   0.0459 H   0 0 0 0 0 0 0 0 0 0 0 0

```
    6.0945    7.6131    2.2423 H   0  0  0  0  0  0  0  0  0  0  0  0
    6.1192    9.3589    1.9681 H   0  0  0  0  0  0  0  0  0  0  0  0
    5.2240    8.3036    0.8670 H   0  0  0  0  0  0  0  0  0  0  0  0
    6.4713    5.8265    1.5902 H   0  0  0  0  0  0  0  0  0  0  0  0
    8.2334    5.8196    1.5789 H   0  0  0  0  0  0  0  0  0  0  0  0
  1  2  1  0  0  0  0
  1  3  1  0  0  0  0
  1  4  1  0  0  0  0
  2  5  1  0  0  0  0
  5  6  1  0  0  0  0
  6  7  1  0  0  0  0
  7  8  1  0  0  0  0
  8  9  1  0  0  0  0
  9 10  1  0  0  0  0
  9 11  1  0  0  0  0
 12 13  1  0  0  0  0
 12 14  1  0  0  0  0
 12 15  1  0  0  0  0
 13 16  1  0  0  0  0
 16  6  1  0  0  0  0
  1 17  1  0  0  0  0
  2 18  1  0  0  0  0
  2 19  1  0  0  0  0
  3 20  1  0  0  0  0
  3 21  1  0  0  0  0
  3 22  1  0  0  0  0
  4 23  1  0  0  0  0
  4 24  1  0  0  0  0
  4 25  1  0  0  0  0
  5 26  1  0  0  0  0
  5 27  1  0  0  0  0
  7 28  1  0  0  0  0
  7 29  1  0  0  0  0
  8 30  1  0  0  0  0
  8 31  1  0  0  0  0
  9 32  1  0  0  0  0
 10 33  1  0  0  0  0
 10 34  1  0  0  0  0
 10 35  1  0  0  0  0
 11 36  1  0  0  0  0
 11 37  1  0  0  0  0
 11 38  1  0  0  0  0
 12 39  1  0  0  0  0
 13 40  1  0  0  0  0
 13 41  1  0  0  0  0
 14 42  1  0  0  0  0
 14 43  1  0  0  0  0
 14 44  1  0  0  0  0
 15 45  1  0  0  0  0
 15 46  1  0  0  0  0
 15 47  1  0  0  0  0
 16 48  1  0  0  0  0
 16 49  1  0  0  0  0
M  END
>  <Alias name>
Triisoamylamine

>  <deltaH°(c)>   kJ/mol
-10277

$$$$
2,2',4,4',6,6'-Hexamethylazobenzene N,N'-dioxide
  ChemBrain IXL
```

```
44 45  0  0  0  0  0  0  0  0  1 V2000
    8.2321    7.9272   -0.1016 N   0  3  0  0  0  0  0  0  0  0  0  0
    9.6412    7.9468   -0.6672 C   0  0  0  0  0  0  0  0  0  0  0  0
    7.6919    8.9933    0.2308 O   0  5  0  0  0  0  0  0  0  0  0  0
   10.0901    6.9565   -1.5731 C   0  0  0  0  0  0  0  0  0  0  0  0
   10.5256    8.9708   -0.2956 C   0  0  0  0  0  0  0  0  0  0  0  0
   11.3937    6.9964   -2.0767 C   0  0  0  0  0  0  0  0  0  0  0  0
    9.1937    5.8635   -2.0042 C   0  0  0  0  0  0  0  0  0  0  0  0
   11.8286    9.0041   -0.8069 C   0  0  0  0  0  0  0  0  0  0  0  0
   10.0841   10.0239    0.6416 C   0  0  0  0  0  0  0  0  0  0  0  0
   12.2618    8.0173   -1.6952 C   0  0  0  0  0  0  0  0  0  0  0  0
   13.6439    8.0525   -2.2341 C   0  0  0  0  0  0  0  0  0  0  0  0
    7.5064    6.6738    0.0630 N   0  3  0  0  0  0  0  0  0  0  0  0
    6.0902    6.6556    0.6109 C   0  0  0  0  0  0  0  0  0  0  0  0
    8.0571    5.6052   -0.2433 O   0  5  0  0  0  0  0  0  0  0  0  0
    5.1917    7.7261    0.3831 C   0  0  0  0  0  0  0  0  0  0  0  0
    5.6483    5.5520    1.3553 C   0  0  0  0  0  0  0  0  0  0  0  0
    3.8948    7.6842    0.9031 C   0  0  0  0  0  0  0  0  0  0  0  0
    5.6037    8.9064   -0.4062 C   0  0  0  0  0  0  0  0  0  0  0  0
    4.3467    5.5177    1.8699 C   0  0  0  0  0  0  0  0  0  0  0  0
    6.5591    4.4153    1.6008 C   0  0  0  0  0  0  0  0  0  0  0  0
    3.4722    6.5834    1.6451 C   0  0  0  0  0  0  0  0  0  0  0  0
    2.0942    6.5471    2.1940 C   0  0  0  0  0  0  0  0  0  0  0  0
   11.7316    6.2311   -2.7666 H   0  0  0  0  0  0  0  0  0  0  0  0
    9.7476    5.1727   -2.6333 H   0  0  0  0  0  0  0  0  0  0  0  0
    8.8198    5.3385   -1.1365 H   0  0  0  0  0  0  0  0  0  0  0  0
    8.3652    6.2746   -2.5647 H   0  0  0  0  0  0  0  0  0  0  0  0
   12.5046    9.7982   -0.5130 H   0  0  0  0  0  0  0  0  0  0  0  0
    9.1389    9.7396    1.0844 H   0  0  0  0  0  0  0  0  0  0  0  0
   10.8263   10.1468    1.4211 H   0  0  0  0  0  0  0  0  0  0  0  0
    9.9661   10.9574    0.1052 H   0  0  0  0  0  0  0  0  0  0  0  0
   13.9842    9.0790   -2.2906 H   0  0  0  0  0  0  0  0  0  0  0  0
   13.6568    7.6131   -3.2247 H   0  0  0  0  0  0  0  0  0  0  0  0
   14.3007    7.4899   -1.5817 H   0  0  0  0  0  0  0  0  0  0  0  0
    3.2144    8.5105    0.7291 H   0  0  0  0  0  0  0  0  0  0  0  0
    4.8056    9.6435   -0.3917 H   0  0  0  0  0  0  0  0  0  0  0  0
    5.7992    8.6088   -1.4273 H   0  0  0  0  0  0  0  0  0  0  0  0
    6.4972    9.3359    0.0242 H   0  0  0  0  0  0  0  0  0  0  0  0
    4.0152    4.6615    2.4450 H   0  0  0  0  0  0  0  0  0  0  0  0
    7.5649    4.6899    1.3120 H   0  0  0  0  0  0  0  0  0  0  0  0
    6.2356    3.5617    1.0179 H   0  0  0  0  0  0  0  0  0  0  0  0
    6.5420    4.1607    2.6537 H   0  0  0  0  0  0  0  0  0  0  0  0
    1.7526    5.5209    2.2465 H   0  0  0  0  0  0  0  0  0  0  0  0
    2.0892    6.9804    3.1868 H   0  0  0  0  0  0  0  0  0  0  0  0
    1.4335    7.1148    1.5494 H   0  0  0  0  0  0  0  0  0  0  0  0
  1  2  1  0  0  0  0
  1  3  1  0  0  0  0
  2  4  4  0  0  0  0
  2  5  4  0  0  0  0
  4  6  4  0  0  0  0
  4  7  1  0  0  0  0
  5  8  4  0  0  0  0
  5  9  1  0  0  0  0
  6 10  4  0  0  0  0
  8 10  4  0  0  0  0
 10 11  1  0  0  0  0
 12 13  1  0  0  0  0
 12 14  1  0  0  0  0
 13 15  4  0  0  0  0
 13 16  4  0  0  0  0
 15 17  4  0  0  0  0
 15 18  1  0  0  0  0
```

```
 16 19  4  0  0  0  0
 16 20  1  0  0  0  0
 17 21  4  0  0  0  0
 19 21  4  0  0  0  0
 21 22  1  0  0  0  0
 12  1  2  0  0  0  0
  6 23  1  0  0  0  0
  7 24  1  0  0  0  0
  7 25  1  0  0  0  0
  7 26  1  0  0  0  0
  8 27  1  0  0  0  0
  9 28  1  0  0  0  0
  9 29  1  0  0  0  0
  9 30  1  0  0  0  0
 11 31  1  0  0  0  0
 11 32  1  0  0  0  0
 11 33  1  0  0  0  0
 17 34  1  0  0  0  0
 18 35  1  0  0  0  0
 18 36  1  0  0  0  0
 18 37  1  0  0  0  0
 19 38  1  0  0  0  0
 20 39  1  0  0  0  0
 20 40  1  0  0  0  0
 20 41  1  0  0  0  0
 22 42  1  0  0  0  0
 22 43  1  0  0  0  0
 22 44  1  0  0  0  0
M  END
>  <Alias name>
2,2',4,4',6,6'-Hexamethylazobenzene N,N'-dioxide

>  <deltaH°(c)>   kJ/mol
-10267.64

$$$$
5,8-Dimethylbenzo(c)phenanthrene
  ChemBrain IXL

 36 39  0  0  0  0  0  0  0  0  1 V2000
    7.7788    3.6095   -0.1824 C   0  0  0  0  0  0  0  0  0  0  0  0
    8.9517    2.7945   -0.5761 C   0  0  0  0  0  0  0  0  0  0  0  0
    7.9615    4.8845    0.3493 C   0  0  0  0  0  0  0  0  0  0  0  0
    6.4883    3.1045   -0.3428 C   0  0  0  0  0  0  0  0  0  0  0  0
    6.8562    5.6876    0.6081 C   0  0  0  0  0  0  0  0  0  0  0  0
    9.2462    5.3559    0.6218 C   0  0  0  0  0  0  0  0  0  0  0  0
    5.3888    3.9189   -0.0764 C   0  0  0  0  0  0  0  0  0  0  0  0
    5.5861    5.2503    0.2366 C   0  0  0  0  0  0  0  0  0  0  0  0
    7.0228    6.9215    1.2380 C   0  0  0  0  0  0  0  0  0  0  0  0
    9.4181    6.6082    1.2122 C   0  0  0  0  0  0  0  0  0  0  0  0
    4.0929    3.4075   -0.1215 C   0  0  0  0  0  0  0  0  0  0  0  0
    4.5122    6.1363    0.1760 C   0  0  0  0  0  0  0  0  0  0  0  0
    8.3052    7.3849    1.5341 C   0  0  0  0  0  0  0  0  0  0  0  0
    3.0042    4.2650    0.0387 C   0  0  0  0  0  0  0  0  0  0  0  0
    3.2142    5.6394    0.1308 C   0  0  0  0  0  0  0  0  0  0  0  0
    4.7324    7.5140    0.1584 C   0  0  0  0  0  0  0  0  0  0  0  0
    1.6293    3.7180    0.1114 C   0  0  0  0  0  0  0  0  0  0  0  0
    2.1293    6.5154    0.1775 C   0  0  0  0  0  0  0  0  0  0  0  0
    3.6490    8.3929    0.1787 C   0  0  0  0  0  0  0  0  0  0  0  0
    2.3470    7.8931    0.2040 C   0  0  0  0  0  0  0  0  0  0  0  0
    9.7852    3.4478   -0.8044 H   0  0  0  0  0  0  0  0  0  0  0  0
    8.7051    2.2054   -1.4507 H   0  0  0  0  0  0  0  0  0  0  0  0
    9.2231    2.1348    0.2389 H   0  0  0  0  0  0  0  0  0  0  0  0
```

```
    6.3414    2.0840   -0.6733 H   0  0  0  0  0  0  0  0  0  0  0  0
   10.1097    4.7503    0.3752 H   0  0  0  0  0  0  0  0  0  0  0  0
    6.1578    7.5193    1.4972 H   0  0  0  0  0  0  0  0  0  0  0  0
   10.4153    6.9772    1.4206 H   0  0  0  0  0  0  0  0  0  0  0  0
    3.9317    2.3485   -0.2801 H   0  0  0  0  0  0  0  0  0  0  0  0
    8.4366    8.3475    2.0137 H   0  0  0  0  0  0  0  0  0  0  0  0
    5.7434    7.9012    0.1288 H   0  0  0  0  0  0  0  0  0  0  0  0
    1.6629    2.7060    0.4954 H   0  0  0  0  0  0  0  0  0  0  0  0
    1.0297    4.3343    0.7705 H   0  0  0  0  0  0  0  0  0  0  0  0
    1.1892    3.7141   -0.8783 H   0  0  0  0  0  0  0  0  0  0  0  0
    1.1185    6.1264    0.1934 H   0  0  0  0  0  0  0  0  0  0  0  0
    3.8186    9.4629    0.1749 H   0  0  0  0  0  0  0  0  0  0  0  0
    1.5056    8.5744    0.2441 H   0  0  0  0  0  0  0  0  0  0  0  0
  1  2  1  0  0  0  0
  1  3  4  0  0  0  0
  4  1  4  0  0  0  0
  3  5  4  0  0  0  0
  3  6  4  0  0  0  0
  7  4  4  0  0  0  0
  5  8  4  0  0  0  0
  9  5  4  0  0  0  0
  6 10  4  0  0  0  0
  7  8  4  0  0  0  0
 11  7  4  0  0  0  0
  8 12  4  0  0  0  0
 13  9  4  0  0  0  0
 10 13  4  0  0  0  0
 14 11  4  0  0  0  0
 15 12  4  0  0  0  0
 12 16  4  0  0  0  0
 15 14  4  0  0  0  0
 14 17  1  0  0  0  0
 18 15  4  0  0  0  0
 16 19  4  0  0  0  0
 20 18  4  0  0  0  0
 19 20  4  0  0  0  0
  2 21  1  0  0  0  0
  2 22  1  0  0  0  0
  2 23  1  0  0  0  0
  4 24  1  0  0  0  0
  6 25  1  0  0  0  0
  9 26  1  0  0  0  0
 10 27  1  0  0  0  0
 11 28  1  0  0  0  0
 13 29  1  0  0  0  0
 16 30  1  0  0  0  0
 17 31  1  0  0  0  0
 17 32  1  0  0  0  0
 17 33  1  0  0  0  0
 18 34  1  0  0  0  0
 19 35  1  0  0  0  0
 20 36  1  0  0  0  0
M  END
>  <Alias name>
5,8-Dimethylbenzo[c]phenanthrene

>  <deltaH°(c)>   kJ/mol
-10265

$$$$
1,2-Dicyclohexylbutane
  ChemBrain IXL
```

```
46 47  0  0  0  0  0  0  0  0  1 V2000
    6.6714    5.8432   -0.7362 C   0  0  0  0  0  0  0  0  0  0  0  0
    7.6221    6.3854    0.3603 C   0  0  0  0  0  0  0  0  0  0  0  0
    7.3438    7.8925    0.5951 C   0  0  0  0  0  0  0  0  0  0  0  0
    9.0922    6.1886   -0.0877 C   0  0  0  0  0  0  0  0  0  0  0  0
    5.2013    6.0670   -0.3150 C   0  0  0  0  0  0  0  0  0  0  0  0
    7.5796    8.6795   -0.7118 C   0  0  0  0  0  0  0  0  0  0  0  0
   10.0424    6.7410    0.9979 C   0  0  0  0  0  0  0  0  0  0  0  0
    9.3709    4.6836   -0.2957 C   0  0  0  0  0  0  0  0  0  0  0  0
    4.9232    5.3287    1.0122 C   0  0  0  0  0  0  0  0  0  0  0  0
    4.2666    5.5193   -1.4128 C   0  0  0  0  0  0  0  0  0  0  0  0
   11.5051    6.5390    0.5543 C   0  0  0  0  0  0  0  0  0  0  0  0
   10.8349    4.4861   -0.7367 C   0  0  0  0  0  0  0  0  0  0  0  0
    3.4541    5.5489    1.4257 C   0  0  0  0  0  0  0  0  0  0  0  0
    2.7988    5.7408   -0.9972 C   0  0  0  0  0  0  0  0  0  0  0  0
   11.7783    5.0364    0.3502 C   0  0  0  0  0  0  0  0  0  0  0  0
    2.5222    5.0013    0.3267 C   0  0  0  0  0  0  0  0  0  0  0  0
    6.8475    4.7844   -0.8735 H   0  0  0  0  0  0  0  0  0  0  0  0
    6.8615    6.3623   -1.6666 H   0  0  0  0  0  0  0  0  0  0  0  0
    7.4536    5.8431    1.2816 H   0  0  0  0  0  0  0  0  0  0  0  0
    6.3181    8.0237    0.9134 H   0  0  0  0  0  0  0  0  0  0  0  0
    8.0074    8.2656    1.3639 H   0  0  0  0  0  0  0  0  0  0  0  0
    9.2606    6.7172   -1.0170 H   0  0  0  0  0  0  0  0  0  0  0  0
    5.0199    7.1257   -0.1839 H   0  0  0  0  0  0  0  0  0  0  0  0
    7.3827    9.7303   -0.5398 H   0  0  0  0  0  0  0  0  0  0  0  0
    6.9146    8.3100   -1.4816 H   0  0  0  0  0  0  0  0  0  0  0  0
    8.6062    8.5531   -1.0301 H   0  0  0  0  0  0  0  0  0  0  0  0
    9.8532    7.7966    1.1405 H   0  0  0  0  0  0  0  0  0  0  0  0
    9.8717    6.2146    1.9288 H   0  0  0  0  0  0  0  0  0  0  0  0
    8.7103    4.2961   -1.0601 H   0  0  0  0  0  0  0  0  0  0  0  0
    9.1990    4.1530    0.6327 H   0  0  0  0  0  0  0  0  0  0  0  0
    5.5756    5.7151    1.7843 H   0  0  0  0  0  0  0  0  0  0  0  0
    5.1066    4.2694    0.8814 H   0  0  0  0  0  0  0  0  0  0  0  0
    4.4481    4.4599   -1.5459 H   0  0  0  0  0  0  0  0  0  0  0  0
    4.4603    6.0383   -2.3433 H   0  0  0  0  0  0  0  0  0  0  0  0
   12.1698    6.9262    1.3164 H   0  0  0  0  0  0  0  0  0  0  0  0
   11.6764    7.0666   -0.3758 H   0  0  0  0  0  0  0  0  0  0  0  0
   11.0286    3.4307   -0.8819 H   0  0  0  0  0  0  0  0  0  0  0  0
   11.0066    5.0157   -1.6656 H   0  0  0  0  0  0  0  0  0  0  0  0
    3.2716    6.6081    1.5589 H   0  0  0  0  0  0  0  0  0  0  0  0
    3.2594    5.0295    2.3557 H   0  0  0  0  0  0  0  0  0  0  0  0
    2.1434    5.3565   -1.7688 H   0  0  0  0  0  0  0  0  0  0  0  0
    2.6171    6.8001   -0.8639 H   0  0  0  0  0  0  0  0  0  0  0  0
   11.6054    4.5083    1.2797 H   0  0  0  0  0  0  0  0  0  0  0  0
   12.8064    4.8954    0.0407 H   0  0  0  0  0  0  0  0  0  0  0  0
    2.7044    3.9421    0.1935 H   0  0  0  0  0  0  0  0  0  0  0  0
    1.4908    5.1563    0.6179 H   0  0  0  0  0  0  0  0  0  0  0  0
  1  2  1  0  0  0  0
  1  5  1  0  0  0  0
  2  3  1  0  0  0  0
  2  4  1  0  0  0  0
  3  6  1  0  0  0  0
  7  4  1  0  0  0  0
  4  8  1  0  0  0  0
  5  9  1  0  0  0  0
 10  5  1  0  0  0  0
 11  7  1  0  0  0  0
  8 12  1  0  0  0  0
  9 13  1  0  0  0  0
 14 10  1  0  0  0  0
 15 11  1  0  0  0  0
 12 15  1  0  0  0  0
 13 16  1  0  0  0  0
```

```
 16 14  1  0  0  0  0
  1 17  1  0  0  0  0
  1 18  1  0  0  0  0
  2 19  1  0  0  0  0
  3 20  1  0  0  0  0
  3 21  1  0  0  0  0
  4 22  1  0  0  0  0
  5 23  1  0  0  0  0
  6 24  1  0  0  0  0
  6 25  1  0  0  0  0
  6 26  1  0  0  0  0
  7 27  1  0  0  0  0
  7 28  1  0  0  0  0
  8 29  1  0  0  0  0
  8 30  1  0  0  0  0
  9 31  1  0  0  0  0
  9 32  1  0  0  0  0
 10 33  1  0  0  0  0
 10 34  1  0  0  0  0
 11 35  1  0  0  0  0
 11 36  1  0  0  0  0
 12 37  1  0  0  0  0
 12 38  1  0  0  0  0
 13 39  1  0  0  0  0
 13 40  1  0  0  0  0
 14 41  1  0  0  0  0
 14 42  1  0  0  0  0
 15 43  1  0  0  0  0
 15 44  1  0  0  0  0
 16 45  1  0  0  0  0
 16 46  1  0  0  0  0
M  END
>  <Alias name>
1,2-Dicyclohexylbutane

>  <deltaH°(c)>   kJ/mol
-10260

$$$$
Diisopropylammonium diisopropyldithiocarbamate
  ChemBrain IXL

 47 45  0  0  0  0  0  0  0  0  1 V2000
    2.2817    6.6272    0.0998 C   0  0  0  0  0  0  0  0  0  0  0  0
    3.4722    5.6879   -0.1744 C   0  0  0  0  0  0  0  0  0  0  0  0
    4.6665    6.1964    0.5155 N   0  0  0  0  0  0  0  0  0  0  0  0
    5.6411    5.2561    1.0875 C   0  0  0  0  0  0  0  0  0  0  0  0
    6.6685    6.0327    1.9337 C   0  0  0  0  0  0  0  0  0  0  0  0
    8.0923    8.4415   -1.7786 C   0  0  0  0  0  0  0  0  0  0  0  0
    9.4047    7.6638   -1.5609 C   0  0  0  0  0  0  0  0  0  0  0  0
    9.3708    7.0033   -0.2481 N   0  3  0  0  0  0  0  0  0  0  0  0
   10.6063    6.2301   -0.0566 C   0  0  0  0  0  0  0  0  0  0  0  0
   10.3719    5.1431    1.0102 C   0  0  0  0  0  0  0  0  0  0  0  0
    4.8758    7.5761    0.6278 C   0  0  0  0  0  0  0  0  0  0  0  0
    5.4199    8.3009    2.2406 S   0  5  0  0  0  0  0  0  0  0  0  0
    4.6286    8.5913   -0.7258 S   0  0  0  0  0  0  0  0  0  0  0  0
    3.1418    4.2750    0.3444 C   0  0  0  0  0  0  0  0  0  0  0  0
    6.3686    4.5147   -0.0508 C   0  0  0  0  0  0  0  0  0  0  0  0
   11.7344    7.1714    0.4085 C   0  0  0  0  0  0  0  0  0  0  0  0
   10.5954    8.6406   -1.6139 C   0  0  0  0  0  0  0  0  0  0  0  0
    8.5759    6.3834   -0.2039 H   0  0  0  0  0  0  0  0  0  0  0  0
    9.2945    7.7004    0.4773 H   0  0  0  0  0  0  0  0  0  0  0  0
    1.3992    6.2468   -0.3996 H   0  0  0  0  0  0  0  0  0  0  0  0
```

```
   2.0996    6.6773    1.1662 H   0  0  0  0  0  0  0  0  0  0  0  0
   2.5096    7.6176   -0.2744 H   0  0  0  0  0  0  0  0  0  0  0  0
   3.6611    5.6462   -1.2399 H   0  0  0  0  0  0  0  0  0  0  0  0
   5.1257    4.5388    1.7141 H   0  0  0  0  0  0  0  0  0  0  0  0
   6.1568    6.5563    2.7318 H   0  0  0  0  0  0  0  0  0  0  0  0
   7.1860    6.7479    1.3063 H   0  0  0  0  0  0  0  0  0  0  0  0
   7.3846    5.3397    2.3576 H   0  0  0  0  0  0  0  0  0  0  0  0
   7.2566    7.7531   -1.7553 H   0  0  0  0  0  0  0  0  0  0  0  0
   7.9749    9.1785   -0.9937 H   0  0  0  0  0  0  0  0  0  0  0  0
   8.1230    8.9396   -2.7398 H   0  0  0  0  0  0  0  0  0  0  0  0
   9.5152    6.9175   -2.3378 H   0  0  0  0  0  0  0  0  0  0  0  0
  10.8886    5.7630   -0.9920 H   0  0  0  0  0  0  0  0  0  0  0  0
  11.2849    4.5798    1.1583 H   0  0  0  0  0  0  0  0  0  0  0  0
  10.0802    5.6096    1.9430 H   0  0  0  0  0  0  0  0  0  0  0  0
   9.5858    4.4758    0.6790 H   0  0  0  0  0  0  0  0  0  0  0  0
   3.9749    3.6128    0.1438 H   0  0  0  0  0  0  0  0  0  0  0  0
   2.9616    4.3152    1.4116 H   0  0  0  0  0  0  0  0  0  0  0  0
   2.2567    3.9040   -0.1574 H   0  0  0  0  0  0  0  0  0  0  0  0
   7.0721    3.8075    0.3708 H   0  0  0  0  0  0  0  0  0  0  0  0
   6.9005    5.2302   -0.6656 H   0  0  0  0  0  0  0  0  0  0  0  0
   5.6443    3.9853   -0.6575 H   0  0  0  0  0  0  0  0  0  0  0  0
  11.4245    7.6896    1.3075 H   0  0  0  0  0  0  0  0  0  0  0  0
  11.9456    7.8939   -0.3701 H   0  0  0  0  0  0  0  0  0  0  0  0
  12.6258    6.5920    0.6149 H   0  0  0  0  0  0  0  0  0  0  0  0
  10.6451    9.1998   -0.6877 H   0  0  0  0  0  0  0  0  0  0  0  0
  10.4640    9.3254   -2.4425 H   0  0  0  0  0  0  0  0  0  0  0  0
  11.5140    8.0830   -1.7486 H   0  0  0  0  0  0  0  0  0  0  0  0
  1  2  1  0  0  0  0
  2  3  1  0  0  0  0
  3  4  1  0  0  0  0
  4  5  1  0  0  0  0
  6  7  1  0  0  0  0
  7  8  1  0  0  0  0
  8  9  1  0  0  0  0
  9 10  1  0  0  0  0
  3 11  1  0  0  0  0
 11 12  1  0  0  0  0
 11 13  2  0  0  0  0
  2 14  1  0  0  0  0
  4 15  1  0  0  0  0
  9 16  1  0  0  0  0
  7 17  1  0  0  0  0
  8 18  1  0  0  0  0
  8 19  1  0  0  0  0
  1 20  1  0  0  0  0
  1 21  1  0  0  0  0
  1 22  1  0  0  0  0
  2 23  1  0  0  0  0
  4 24  1  0  0  0  0
  5 25  1  0  0  0  0
  5 26  1  0  0  0  0
  5 27  1  0  0  0  0
  6 28  1  0  0  0  0
  6 29  1  0  0  0  0
  6 30  1  0  0  0  0
  7 31  1  0  0  0  0
  9 32  1  0  0  0  0
 10 33  1  0  0  0  0
 10 34  1  0  0  0  0
 10 35  1  0  0  0  0
 14 36  1  0  0  0  0
 14 37  1  0  0  0  0
 14 38  1  0  0  0  0
```

```
 15 39  1  0  0  0  0
 15 40  1  0  0  0  0
 15 41  1  0  0  0  0
 16 42  1  0  0  0  0
 16 43  1  0  0  0  0
 16 44  1  0  0  0  0
 17 45  1  0  0  0  0
 17 46  1  0  0  0  0
 17 47  1  0  0  0  0
M  END
>  <Alias name>
Diisopropylammonium diisopropyldithiocarbamate

>  <deltaH°(c)>   kJ/mol
-10260

$$$$
Tri-n-pentylamine
  ChemBrain IXL

 49 48  0  0  0  0  0  0  0  0  1 V2000
   12.9401    2.4885    0.1991 C   0  0  0  0  0  0  0  0  0  0  0  0  0
   12.0792    3.6732   -0.2806 C   0  0  0  0  0  0  0  0  0  0  0  0  0
   10.6365    3.5018    0.2339 C   0  0  0  0  0  0  0  0  0  0  0  0  0
    9.7758    4.6867   -0.2457 C   0  0  0  0  0  0  0  0  0  0  0  0  0
    8.3309    4.5136    0.2701 C   0  0  0  0  0  0  0  0  0  0  0  0  0
    7.4866    5.6240   -0.2208 N   0  0  0  0  0  0  0  0  0  0  0  0  0
    2.0445    2.4692    0.1991 C   0  0  0  0  0  0  0  0  0  0  0  0  0
    3.5009    2.6223   -0.2806 C   0  0  0  0  0  0  0  0  0  0  0  0  0
    4.0739    3.9573    0.2340 C   0  0  0  0  0  0  0  0  0  0  0  0  0
    5.5304    4.1102   -0.2456 C   0  0  0  0  0  0  0  0  0  0  0  0  0
    6.1030    5.4481    0.2701 C   0  0  0  0  0  0  0  0  0  0  0  0  0
    7.4756   11.9145    0.1991 C   0  0  0  0  0  0  0  0  0  0  0  0  0
    6.8800   10.5766   -0.2806 C   0  0  0  0  0  0  0  0  0  0  0  0  0
    7.7497    9.4129    0.2339 C   0  0  0  0  0  0  0  0  0  0  0  0  0
    7.1538    8.0751   -0.2456 C   0  0  0  0  0  0  0  0  0  0  0  0  0
    8.0261    6.9103    0.2701 C   0  0  0  0  0  0  0  0  0  0  0  0  0
   13.9537    2.6091   -0.1626 H   0  0  0  0  0  0  0  0  0  0  0  0  0
   12.5275    1.5636   -0.1848 H   0  0  0  0  0  0  0  0  0  0  0  0  0
   12.9440    2.4601    1.2817 H   0  0  0  0  0  0  0  0  0  0  0  0  0
   12.4919    4.5979    0.1032 H   0  0  0  0  0  0  0  0  0  0  0  0  0
   12.0754    3.7015   -1.3632 H   0  0  0  0  0  0  0  0  0  0  0  0  0
   10.2238    2.5771   -0.1500 H   0  0  0  0  0  0  0  0  0  0  0  0  0
   10.6404    3.4733    1.3165 H   0  0  0  0  0  0  0  0  0  0  0  0  0
   10.1896    5.6094    0.1378 H   0  0  0  0  0  0  0  0  0  0  0  0  0
    9.7718    4.7147   -1.3282 H   0  0  0  0  0  0  0  0  0  0  0  0  0
    7.9347    3.5743   -0.0902 H   0  0  0  0  0  0  0  0  0  0  0  0  0
    8.3322    4.5122    1.3532 H   0  0  0  0  0  0  0  0  0  0  0  0  0
    1.6421    1.5311   -0.1625 H   0  0  0  0  0  0  0  0  0  0  0  0  0
    2.0180    2.4799    1.2817 H   0  0  0  0  0  0  0  0  0  0  0  0  0
    1.4499    3.2889   -0.1847 H   0  0  0  0  0  0  0  0  0  0  0  0  0
    4.0954    1.8025    0.1032 H   0  0  0  0  0  0  0  0  0  0  0  0  0
    3.5273    2.6115   -1.3632 H   0  0  0  0  0  0  0  0  0  0  0  0  0
    3.4794    4.7772   -0.1500 H   0  0  0  0  0  0  0  0  0  0  0  0  0
    4.0473    3.9682    1.3166 H   0  0  0  0  0  0  0  0  0  0  0  0  0
    6.1225    3.2906    0.1379 H   0  0  0  0  0  0  0  0  0  0  0  0  0
    5.5566    4.0998   -1.3281 H   0  0  0  0  0  0  0  0  0  0  0  0  0
    5.4876    6.2608   -0.0901 H   0  0  0  0  0  0  0  0  0  0  0  0  0
    6.1010    5.4476    1.3533 H   0  0  0  0  0  0  0  0  0  0  0  0  0
    6.8643   12.7320   -0.1626 H   0  0  0  0  0  0  0  0  0  0  0  0  0
    8.4828   12.0195   -0.1848 H   0  0  0  0  0  0  0  0  0  0  0  0  0
    7.4982   11.9321    1.2817 H   0  0  0  0  0  0  0  0  0  0  0  0  0
    6.8573   10.5591   -1.3632 H   0  0  0  0  0  0  0  0  0  0  0  0  0
```

```
   5.8728   10.4717    0.1033 H   0  0  0  0  0  0  0  0  0  0  0  0
   7.7724    9.4305    1.3166 H   0  0  0  0  0  0  0  0  0  0  0  0
   8.7569    9.5178   -0.1501 H   0  0  0  0  0  0  0  0  0  0  0  0
   7.1316    8.0576   -1.3281 H   0  0  0  0  0  0  0  0  0  0  0  0
   6.1479    7.9721    0.1379 H   0  0  0  0  0  0  0  0  0  0  0  0
   8.0267    6.9122    1.3532 H   0  0  0  0  0  0  0  0  0  0  0  0
   9.0376    7.0369   -0.0902 H   0  0  0  0  0  0  0  0  0  0  0  0
  1  2  1  0  0  0  0
  2  3  1  0  0  0  0
  3  4  1  0  0  0  0
  4  5  1  0  0  0  0
  5  6  1  0  0  0  0
  7  8  1  0  0  0  0
  8  9  1  0  0  0  0
  9 10  1  0  0  0  0
 10 11  1  0  0  0  0
 11  6  1  0  0  0  0
 12 13  1  0  0  0  0
 13 14  1  0  0  0  0
 14 15  1  0  0  0  0
 15 16  1  0  0  0  0
 16  6  1  0  0  0  0
  1 17  1  0  0  0  0
  1 18  1  0  0  0  0
  1 19  1  0  0  0  0
  2 20  1  0  0  0  0
  2 21  1  0  0  0  0
  3 22  1  0  0  0  0
  3 23  1  0  0  0  0
  4 24  1  0  0  0  0
  4 25  1  0  0  0  0
  5 26  1  0  0  0  0
  5 27  1  0  0  0  0
  7 28  1  0  0  0  0
  7 29  1  0  0  0  0
  7 30  1  0  0  0  0
  8 31  1  0  0  0  0
  8 32  1  0  0  0  0
  9 33  1  0  0  0  0
  9 34  1  0  0  0  0
 10 35  1  0  0  0  0
 10 36  1  0  0  0  0
 11 37  1  0  0  0  0
 11 38  1  0  0  0  0
 12 39  1  0  0  0  0
 12 40  1  0  0  0  0
 12 41  1  0  0  0  0
 13 42  1  0  0  0  0
 13 43  1  0  0  0  0
 14 44  1  0  0  0  0
 14 45  1  0  0  0  0
 15 46  1  0  0  0  0
 15 47  1  0  0  0  0
 16 48  1  0  0  0  0
 16 49  1  0  0  0  0
M  END
>  <Alias name>
Tri-n-pentylamine

>  <deltaH°(c)>   kJ/mol
-10259.09

$$$$
```

Dipropylammonium dipropyldithiocarbamate
 ChemBrain IXL

```
 47 45  0  0  0  0  0  0  0  0  1 V2000
    4.9037    4.8402   -1.9231 C   0  0  0  0  0  0  0  0  0  0  0  0
    3.4825    4.8937   -1.3297 C   0  0  0  0  0  0  0  0  0  0  0  0
    3.4949    5.6993   -0.1001 N   0  0  0  0  0  0  0  0  0  0  0  0
    4.3902    6.8600    0.0091 C   0  0  0  0  0  0  0  0  0  0  0  0
    3.5696    8.1567   -0.1316 C   0  0  0  0  0  0  0  0  0  0  0  0
   10.1261    8.8152   -1.3519 C   0  0  0  0  0  0  0  0  0  0  0  0
   10.5550    7.5734   -0.5466 C   0  0  0  0  0  0  0  0  0  0  0  0
    9.3920    7.0261    0.1668 N   0  3  0  0  0  0  0  0  0  0  0  0
    9.7973    5.8308    0.9204 C   0  0  0  0  0  0  0  0  0  0  0  0
    8.5824    5.2683    1.6834 C   0  0  0  0  0  0  0  0  0  0  0  0
    2.6541    5.3609    0.9669 C   0  0  0  0  0  0  0  0  0  0  0  0
    2.7123    6.3385    2.5364 S   0  5  0  0  0  0  0  0  0  0  0  0
    1.5733    4.0440    0.8194 S   0  0  0  0  0  0  0  0  0  0  0  0
    4.5049    9.3739    0.0042 C   0  0  0  0  0  0  0  0  0  0  0  0
    4.9063    3.9334   -3.1690 C   0  0  0  0  0  0  0  0  0  0  0  0
    9.5942    9.8938   -0.3884 C   0  0  0  0  0  0  0  0  0  0  0  0
    7.4868    4.8636    0.6781 C   0  0  0  0  0  0  0  0  0  0  0  0
    8.6758    6.7772   -0.4989 H   0  0  0  0  0  0  0  0  0  0  0  0
    9.0318    7.7199    0.8048 H   0  0  0  0  0  0  0  0  0  0  0  0
    5.5883    4.4412   -1.1848 H   0  0  0  0  0  0  0  0  0  0  0  0
    5.2160    5.8387   -2.2027 H   0  0  0  0  0  0  0  0  0  0  0  0
    2.8079    5.3424   -2.0484 H   0  0  0  0  0  0  0  0  0  0  0  0
    3.1491    3.8893   -1.0999 H   0  0  0  0  0  0  0  0  0  0  0  0
    4.8821    6.8470    0.9739 H   0  0  0  0  0  0  0  0  0  0  0  0
    5.1347    6.8169   -0.7762 H   0  0  0  0  0  0  0  0  0  0  0  0
    3.0898    8.1767   -1.1024 H   0  0  0  0  0  0  0  0  0  0  0  0
    2.8153    8.1927    0.6447 H   0  0  0  0  0  0  0  0  0  0  0  0
   10.9779    9.2049   -1.8954 H   0  0  0  0  0  0  0  0  0  0  0  0
    9.3466    8.5401   -2.0516 H   0  0  0  0  0  0  0  0  0  0  0  0
   11.3185    7.8531    0.1689 H   0  0  0  0  0  0  0  0  0  0  0  0
   10.9499    6.8241   -1.2215 H   0  0  0  0  0  0  0  0  0  0  0  0
   10.5756    6.0953    1.6255 H   0  0  0  0  0  0  0  0  0  0  0  0
   10.1714    5.0811    0.2342 H   0  0  0  0  0  0  0  0  0  0  0  0
    8.8858    4.4002    2.2555 H   0  0  0  0  0  0  0  0  0  0  0  0
    8.1951    6.0253    2.3540 H   0  0  0  0  0  0  0  0  0  0  0  0
    3.9282   10.2852   -0.0951 H   0  0  0  0  0  0  0  0  0  0  0  0
    5.2594    9.3377   -0.7719 H   0  0  0  0  0  0  0  0  0  0  0  0
    4.9844    9.3541    0.9750 H   0  0  0  0  0  0  0  0  0  0  0  0
    5.9040    3.8996   -3.5889 H   0  0  0  0  0  0  0  0  0  0  0  0
    4.5986    2.9336   -2.8885 H   0  0  0  0  0  0  0  0  0  0  0  0
    4.2180    4.3297   -3.9053 H   0  0  0  0  0  0  0  0  0  0  0  0
    8.7307    9.5107    0.1412 H   0  0  0  0  0  0  0  0  0  0  0  0
   10.3675   10.1550    0.3234 H   0  0  0  0  0  0  0  0  0  0  0  0
    9.3107   10.7737   -0.9525 H   0  0  0  0  0  0  0  0  0  0  0  0
    7.8802    4.1210   -0.0051 H   0  0  0  0  0  0  0  0  0  0  0  0
    6.6410    4.4498    1.2130 H   0  0  0  0  0  0  0  0  0  0  0  0
    7.1693    5.7356    0.1197 H   0  0  0  0  0  0  0  0  0  0  0  0
  1  2  1  0  0  0  0
  2  3  1  0  0  0  0
  3  4  1  0  0  0  0
  4  5  1  0  0  0  0
  6  7  1  0  0  0  0
  7  8  1  0  0  0  0
  8  9  1  0  0  0  0
  9 10  1  0  0  0  0
  3 11  1  0  0  0  0
 11 12  1  0  0  0  0
 11 13  2  0  0  0  0
  5 14  1  0  0  0  0
```

1 15 1 0 0 0 0
6 16 1 0 0 0 0
10 17 1 0 0 0 0
8 18 1 0 0 0 0
8 19 1 0 0 0 0
1 20 1 0 0 0 0
1 21 1 0 0 0 0
2 22 1 0 0 0 0
2 23 1 0 0 0 0
4 24 1 0 0 0 0
4 25 1 0 0 0 0
5 26 1 0 0 0 0
5 27 1 0 0 0 0
6 28 1 0 0 0 0
6 29 1 0 0 0 0
7 30 1 0 0 0 0
7 31 1 0 0 0 0
9 32 1 0 0 0 0
9 33 1 0 0 0 0
10 34 1 0 0 0 0
10 35 1 0 0 0 0
14 36 1 0 0 0 0
14 37 1 0 0 0 0
14 38 1 0 0 0 0
15 39 1 0 0 0 0
15 40 1 0 0 0 0
15 41 1 0 0 0 0
16 42 1 0 0 0 0
16 43 1 0 0 0 0
16 44 1 0 0 0 0
17 45 1 0 0 0 0
17 46 1 0 0 0 0
17 47 1 0 0 0 0
M END
> <Alias name>
Dipropylammonium dipropyldithiocarbamate

> <deltaH°(c)> kJ/mol
-10252

$$$$
2,2-Dicyclohexylbutane
ChemBrain IXL

46 47 0 0 0 0 0 0 0 0 1 V2000
6.2494 5.1132 0.4058 C 0 0 0 0 0 0 0 0 0 0 0 0
5.8635 3.8005 -0.3529 C 0 0 0 0 0 0 0 0 0 0 0 0
6.6295 4.7514 1.8612 C 0 0 0 0 0 0 0 0 0 0 0 0
5.0416 6.1236 0.4398 C 0 0 0 0 0 0 0 0 0 0 0 0
7.4730 5.7763 -0.2930 C 0 0 0 0 0 0 0 0 0 0 0 0
6.0082 3.9879 -1.8916 C 0 0 0 0 0 0 0 0 0 0 0 0
4.5438 6.4406 -0.9797 C 0 0 0 0 0 0 0 0 0 0 0 0
3.8749 5.5242 1.2540 C 0 0 0 0 0 0 0 0 0 0 0 0
7.8631 7.0740 0.4501 C 0 0 0 0 0 0 0 0 0 0 0 0
8.6728 4.8010 -0.2795 C 0 0 0 0 0 0 0 0 0 0 0 0
3.4260 7.4937 -0.8975 C 0 0 0 0 0 0 0 0 0 0 0 0
2.7299 6.5585 1.3407 C 0 0 0 0 0 0 0 0 0 0 0 0
9.0685 7.7274 -0.2563 C 0 0 0 0 0 0 0 0 0 0 0 0
9.8739 5.4560 -0.9881 C 0 0 0 0 0 0 0 0 0 0 0 0
2.2475 6.9206 -0.0814 C 0 0 0 0 0 0 0 0 0 0 0 0
10.2626 6.7516 -0.2481 C 0 0 0 0 0 0 0 0 0 0 0 0
6.5278 3.0052 -0.0383 H 0 0 0 0 0 0 0 0 0 0 0 0
4.8449 3.5289 -0.1106 H 0 0 0 0 0 0 0 0 0 0 0 0

```
    6.8867    5.6524    2.4027 H   0  0  0  0  0  0  0  0  0  0  0  0
    7.4767    4.0788    1.8569 H   0  0  0  0  0  0  0  0  0  0  0  0
    5.7908    4.2681    2.3440 H   0  0  0  0  0  0  0  0  0  0  0  0
    5.3643    7.0404    0.9141 H   0  0  0  0  0  0  0  0  0  0  0  0
    7.2161    6.0149   -1.3168 H   0  0  0  0  0  0  0  0  0  0  0  0
    5.7130    3.0745   -2.3927 H   0  0  0  0  0  0  0  0  0  0  0  0
    5.3873    4.7909   -2.2254 H   0  0  0  0  0  0  0  0  0  0  0  0
    7.0390    4.2098   -2.1319 H   0  0  0  0  0  0  0  0  0  0  0  0
    5.3614    6.8233   -1.5771 H   0  0  0  0  0  0  0  0  0  0  0  0
    4.1492    5.5453   -1.4304 H   0  0  0  0  0  0  0  0  0  0  0  0
    3.5159    4.6268    0.7673 H   0  0  0  0  0  0  0  0  0  0  0  0
    4.2156    5.2829    2.2516 H   0  0  0  0  0  0  0  0  0  0  0  0
    7.0276    7.7606    0.4422 H   0  0  0  0  0  0  0  0  0  0  0  0
    8.1286    6.8397    1.4728 H   0  0  0  0  0  0  0  0  0  0  0  0
    8.9407    4.5715    0.7433 H   0  0  0  0  0  0  0  0  0  0  0  0
    8.4039    3.8885   -0.7949 H   0  0  0  0  0  0  0  0  0  0  0  0
    3.8034    8.3856   -0.4128 H   0  0  0  0  0  0  0  0  0  0  0  0
    3.0874    7.7402   -1.8960 H   0  0  0  0  0  0  0  0  0  0  0  0
    1.9065    6.1391    1.9052 H   0  0  0  0  0  0  0  0  0  0  0  0
    3.0891    7.4516    1.8370 H   0  0  0  0  0  0  0  0  0  0  0  0
    8.8026    7.9635   -1.2793 H   0  0  0  0  0  0  0  0  0  0  0  0
    9.3422    8.6366    0.2642 H   0  0  0  0  0  0  0  0  0  0  0  0
   10.7131    4.7718   -0.9826 H   0  0  0  0  0  0  0  0  0  0  0  0
    9.6060    5.6905   -2.0108 H   0  0  0  0  0  0  0  0  0  0  0  0
    1.8695    6.0315   -0.5708 H   0  0  0  0  0  0  0  0  0  0  0  0
    1.4589    7.6599   -0.0177 H   0  0  0  0  0  0  0  0  0  0  0  0
   10.5297    6.5167    0.7749 H   0  0  0  0  0  0  0  0  0  0  0  0
   11.1076    7.2113   -0.7452 H   0  0  0  0  0  0  0  0  0  0  0  0
  1  2  1  0  0  0  0
  1  3  1  0  0  0  0
  1  4  1  0  0  0  0
  1  5  1  0  0  0  0
  2  6  1  0  0  0  0
  7  4  1  0  0  0  0
  4  8  1  0  0  0  0
  9  5  1  0  0  0  0
  5 10  1  0  0  0  0
 11  7  1  0  0  0  0
  8 12  1  0  0  0  0
 13  9  1  0  0  0  0
 10 14  1  0  0  0  0
 15 11  1  0  0  0  0
 12 15  1  0  0  0  0
 16 13  1  0  0  0  0
 14 16  1  0  0  0  0
  2 17  1  0  0  0  0
  2 18  1  0  0  0  0
  3 19  1  0  0  0  0
  3 20  1  0  0  0  0
  3 21  1  0  0  0  0
  4 22  1  0  0  0  0
  5 23  1  0  0  0  0
  6 24  1  0  0  0  0
  6 25  1  0  0  0  0
  6 26  1  0  0  0  0
  7 27  1  0  0  0  0
  7 28  1  0  0  0  0
  8 29  1  0  0  0  0
  8 30  1  0  0  0  0
  9 31  1  0  0  0  0
  9 32  1  0  0  0  0
 10 33  1  0  0  0  0
 10 34  1  0  0  0  0
```

```
 11 35  1  0  0  0  0
 11 36  1  0  0  0  0
 12 37  1  0  0  0  0
 12 38  1  0  0  0  0
 13 39  1  0  0  0  0
 13 40  1  0  0  0  0
 14 41  1  0  0  0  0
 14 42  1  0  0  0  0
 15 43  1  0  0  0  0
 15 44  1  0  0  0  0
 16 45  1  0  0  0  0
 16 46  1  0  0  0  0
M  END
>  <Alias name>
2,2-Dicyclohexylbutane

>  <deltaH°(c)>   kJ/mol
-10240

$$$$
3,9-Dimethylbenz(a)anthracene
  ChemBrain IXL

 36 39  0  0  0  0  0  0  0  0  1 V2000
    5.7330    5.3410    0.0000 C   0  0  0  0  0  0  0  0  0  0  0  0
    5.7578    6.7358    0.0000 C   0  0  0  0  0  0  0  0  0  0  0  0
    4.5127    4.6651    0.0000 C   0  0  0  0  0  0  0  0  0  0  0  0
    6.9285    4.6222    0.0000 C   0  0  0  0  0  0  0  0  0  0  0  0
    4.5623    7.4546    0.0001 C   0  0  0  0  0  0  0  0  0  0  0  0
    6.9781    7.4117    0.0000 C   0  0  0  0  0  0  0  0  0  0  0  0
    3.3420    6.7787    0.0001 C   0  0  0  0  0  0  0  0  0  0  0  0
    3.3172    5.3839    0.0000 C   0  0  0  0  0  0  0  0  0  0  0  0
    2.0225    4.6668   -0.0002 C   0  0  0  0  0  0  0  0  0  0  0  0
    8.1488    5.2981    0.0000 C   0  0  0  0  0  0  0  0  0  0  0  0
    8.1736    6.6929    0.0000 C   0  0  0  0  0  0  0  0  0  0  0  0
    9.3444    4.5793   -0.0001 C   0  0  0  0  0  0  0  0  0  0  0  0
    9.3939    7.3688    0.0000 C   0  0  0  0  0  0  0  0  0  0  0  0
   10.5647    5.2552    0.0000 C   0  0  0  0  0  0  0  0  0  0  0  0
   10.5894    6.6500    0.0000 C   0  0  0  0  0  0  0  0  0  0  0  0
   11.8097    7.3259    0.0000 C   0  0  0  0  0  0  0  0  0  0  0  0
    9.4187    8.7636    0.0001 C   0  0  0  0  0  0  0  0  0  0  0  0
   11.8345    8.7207    0.0000 C   0  0  0  0  0  0  0  0  0  0  0  0
   10.6390    9.4396    0.0000 C   0  0  0  0  0  0  0  0  0  0  0  0
   13.1292    9.4378   -0.0002 C   0  0  0  0  0  0  0  0  0  0  0  0
    4.4935    3.5823    0.0000 H   0  0  0  0  0  0  0  0  0  0  0  0
    6.9093    3.5393    0.0000 H   0  0  0  0  0  0  0  0  0  0  0  0
    4.5815    8.5375    0.0001 H   0  0  0  0  0  0  0  0  0  0  0  0
    6.9973    8.4946    0.0001 H   0  0  0  0  0  0  0  0  0  0  0  0
    2.4138    7.3368    0.0001 H   0  0  0  0  0  0  0  0  0  0  0  0
    2.2015    3.5987   -0.0003 H   0  0  0  0  0  0  0  0  0  0  0  0
    1.4593    4.9384    0.8841 H   0  0  0  0  0  0  0  0  0  0  0  0
    1.4594    4.9386   -0.8844 H   0  0  0  0  0  0  0  0  0  0  0  0
    9.3252    3.4964   -0.0001 H   0  0  0  0  0  0  0  0  0  0  0  0
   11.4928    4.6971   -0.0001 H   0  0  0  0  0  0  0  0  0  0  0  0
   12.7379    6.7679    0.0000 H   0  0  0  0  0  0  0  0  0  0  0  0
    8.4905    9.3217    0.0001 H   0  0  0  0  0  0  0  0  0  0  0  0
   10.6582   10.5224    0.0001 H   0  0  0  0  0  0  0  0  0  0  0  0
   12.9502   10.5060   -0.0003 H   0  0  0  0  0  0  0  0  0  0  0  0
   13.6923    9.1661   -0.8844 H   0  0  0  0  0  0  0  0  0  0  0  0
   13.6924    9.1662    0.8841 H   0  0  0  0  0  0  0  0  0  0  0  0
  1  2  4  0  0  0  0
  1  3  4  0  0  0  0
  4  1  4  0  0  0  0
```

```
  5  2  4  0  0  0  0
  2  6  4  0  0  0  0
  3  8  4  0  0  0  0
 10  4  4  0  0  0  0
  7  5  4  0  0  0  0
  6 11  4  0  0  0  0
  8  7  4  0  0  0  0
  8  9  1  0  0  0  0
 10 11  4  0  0  0  0
 12 10  4  0  0  0  0
 11 13  4  0  0  0  0
 14 12  4  0  0  0  0
 15 13  4  0  0  0  0
 13 17  4  0  0  0  0
 15 14  4  0  0  0  0
 16 15  4  0  0  0  0
 18 16  4  0  0  0  0
 17 19  4  0  0  0  0
 19 18  4  0  0  0  0
 18 20  1  0  0  0  0
  3 21  1  0  0  0  0
  4 22  1  0  0  0  0
  5 23  1  0  0  0  0
  6 24  1  0  0  0  0
  7 25  1  0  0  0  0
  9 26  1  0  0  0  0
  9 27  1  0  0  0  0
  9 28  1  0  0  0  0
 12 29  1  0  0  0  0
 14 30  1  0  0  0  0
 16 31  1  0  0  0  0
 17 32  1  0  0  0  0
 19 33  1  0  0  0  0
 20 34  1  0  0  0  0
 20 35  1  0  0  0  0
 20 36  1  0  0  0  0
M  END
>  <Alias name>
3,9-Dimethylbenzo[a]anthracene

>  <deltaH°(c)>   kJ/mol
-10239.82

$$$$
1,1-Diphenylhexane
  ChemBrain IXL

 40 41  0  0  0  0  0  0  0  0  1 V2000
    5.9909    4.8015    1.7865 C   0  0  0  0  0  0  0  0  0  0  0  0
    4.6440    5.3811    1.3123 C   0  0  0  0  0  0  0  0  0  0  0  0
    3.9644    4.3986    0.4387 C   0  0  0  0  0  0  0  0  0  0  0  0
    6.8840    4.5116    0.5647 C   0  0  0  0  0  0  0  0  0  0  0  0
    2.9451    3.5964    0.9522 C   0  0  0  0  0  0  0  0  0  0  0  0
    4.3430    4.2747   -0.8982 C   0  0  0  0  0  0  0  0  0  0  0  0
    8.2371    3.9475    1.0398 C   0  0  0  0  0  0  0  0  0  0  0  0
    2.2992    2.6758    0.1269 C   0  0  0  0  0  0  0  0  0  0  0  0
    3.6971    3.3539   -1.7235 C   0  0  0  0  0  0  0  0  0  0  0  0
    9.1331    3.6647   -0.1816 C   0  0  0  0  0  0  0  0  0  0  0  0
    2.6750    2.5548   -1.2111 C   0  0  0  0  0  0  0  0  0  0  0  0
   10.4867    3.1021    0.2936 C   0  0  0  0  0  0  0  0  0  0  0  0
    4.8835    6.6308    0.5563 C   0  0  0  0  0  0  0  0  0  0  0  0
    3.9789    7.0365   -0.4251 C   0  0  0  0  0  0  0  0  0  0  0  0
    6.0138    7.4029    0.8252 C   0  0  0  0  0  0  0  0  0  0  0  0
```

```
4.1998    8.2192   -1.1311 C   0  0  0  0  0  0  0  0  0  0  0  0
6.2346    8.5856    0.1192 C   0  0  0  0  0  0  0  0  0  0  0  0
5.3274    8.9940   -0.8587 C   0  0  0  0  0  0  0  0  0  0  0  0
6.4842    5.5170    2.4327 H   0  0  0  0  0  0  0  0  0  0  0  0
5.8163    3.8825    2.3322 H   0  0  0  0  0  0  0  0  0  0  0  0
4.0197    5.5930    2.1714 H   0  0  0  0  0  0  0  0  0  0  0  0
6.3958    3.7875   -0.0757 H   0  0  0  0  0  0  0  0  0  0  0  0
7.0491    5.4286    0.0125 H   0  0  0  0  0  0  0  0  0  0  0  0
2.6552    3.6885    1.9916 H   0  0  0  0  0  0  0  0  0  0  0  0
5.1383    4.8932   -1.2953 H   0  0  0  0  0  0  0  0  0  0  0  0
8.7229    4.6695    1.6844 H   0  0  0  0  0  0  0  0  0  0  0  0
8.0725    3.0278    1.5874 H   0  0  0  0  0  0  0  0  0  0  0  0
1.5061    2.0549    0.5249 H   0  0  0  0  0  0  0  0  0  0  0  0
3.9893    3.2596   -2.7621 H   0  0  0  0  0  0  0  0  0  0  0  0
8.6478    2.9418   -0.8257 H   0  0  0  0  0  0  0  0  0  0  0  0
9.2968    4.5842   -0.7299 H   0  0  0  0  0  0  0  0  0  0  0  0
2.1731    1.8405   -1.8520 H   0  0  0  0  0  0  0  0  0  0  0  0
10.9712    3.8242    0.9391 H   0  0  0  0  0  0  0  0  0  0  0  0
11.1172    2.9051   -0.5647 H   0  0  0  0  0  0  0  0  0  0  0  0
10.3232    2.1817    0.8403 H   0  0  0  0  0  0  0  0  0  0  0  0
3.1052    6.4333   -0.6389 H   0  0  0  0  0  0  0  0  0  0  0  0
6.7198    7.0842    1.5820 H   0  0  0  0  0  0  0  0  0  0  0  0
3.4958    8.5359   -1.8908 H   0  0  0  0  0  0  0  0  0  0  0  0
7.1104    9.1868    0.3302 H   0  0  0  0  0  0  0  0  0  0  0  0
5.4985    9.9125   -1.4063 H   0  0  0  0  0  0  0  0  0  0  0  0
1  2  1  0  0  0  0
1  4  1  0  0  0  0
2  3  1  0  0  0  0
5  3  4  0  0  0  0
3  6  4  0  0  0  0
4  7  1  0  0  0  0
8  5  4  0  0  0  0
6  9  4  0  0  0  0
7 10  1  0  0  0  0
11  8  4  0  0  0  0
9 11  4  0  0  0  0
10 12  1  0  0  0  0
13 14  4  0  0  0  0
15 13  4  0  0  0  0
14 16  4  0  0  0  0
17 15  4  0  0  0  0
16 18  4  0  0  0  0
18 17  4  0  0  0  0
13  2  1  0  0  0  0
1 19  1  0  0  0  0
1 20  1  0  0  0  0
2 21  1  0  0  0  0
4 22  1  0  0  0  0
4 23  1  0  0  0  0
5 24  1  0  0  0  0
6 25  1  0  0  0  0
7 26  1  0  0  0  0
7 27  1  0  0  0  0
8 28  1  0  0  0  0
9 29  1  0  0  0  0
10 30  1  0  0  0  0
10 31  1  0  0  0  0
11 32  1  0  0  0  0
12 33  1  0  0  0  0
12 34  1  0  0  0  0
12 35  1  0  0  0  0
14 36  1  0  0  0  0
15 37  1  0  0  0  0
```

 16 38 1 0 0 0 0
 17 39 1 0 0 0 0
 18 40 1 0 0 0 0
M END
> <Alias name>
1,1-Diphenylhexane

> <deltaH°(c)>   kJ/mol
-10230

$$$$
1,6-Diphenylhexane
  ChemBrain IXL

 40 41 0 0 0 0 0 0 0 0 1 V2000
    7.2786   7.9887   0.0000 C   0 0 0 0 0 0 0 0 0 0 0 0 0
    6.0036   8.8542   0.0001 C   0 0 0 0 0 0 0 0 0 0 0 0 0
    4.8118   7.9769   0.0003 C   0 0 0 0 0 0 0 0 0 0 0 0 0
    8.5196   8.9023   0.0000 C   0 0 0 0 0 0 0 0 0 0 0 0 0
    3.5338   8.5363   0.0003 C   0 0 0 0 0 0 0 0 0 0 0 0 0
    4.9662   6.5904   0.0003 C   0 0 0 0 0 0 0 0 0 0 0 0 0
    9.7946   8.0367   0.0000 C   0 0 0 0 0 0 0 0 0 0 0 0 0
    2.4104   7.7093   0.0003 C   0 0 0 0 0 0 0 0 0 0 0 0 0
    3.8428   5.7634   0.0003 C   0 0 0 0 0 0 0 0 0 0 0 0 0
   11.0356   8.9503   0.0000 C   0 0 0 0 0 0 0 0 0 0 0 0 0
    2.5649   6.3229   0.0002 C   0 0 0 0 0 0 0 0 0 0 0 0 0
   12.3106   8.0847   0.0001 C   0 0 0 0 0 0 0 0 0 0 0 0 0
   13.5025   8.9621   0.0003 C   0 0 0 0 0 0 0 0 0 0 0 0 0
   13.3480   10.3485   0.0003 C   0 0 0 0 0 0 0 0 0 0 0 0 0
   14.7804   8.4027   0.0003 C   0 0 0 0 0 0 0 0 0 0 0 0 0
   14.4715   11.1755   0.0003 C   0 0 0 0 0 0 0 0 0 0 0 0 0
   15.9038   9.2297   0.0003 C   0 0 0 0 0 0 0 0 0 0 0 0 0
   15.7494   10.6161   0.0002 C   0 0 0 0 0 0 0 0 0 0 0 0 0
    7.2906   7.3635   0.8842 H   0 0 0 0 0 0 0 0 0 0 0 0 0
    7.2905   7.3636   -0.8843 H   0 0 0 0 0 0 0 0 0 0 0 0 0
    5.9916   9.4793   -0.8842 H   0 0 0 0 0 0 0 0 0 0 0 0 0
    5.9918   9.4795   0.8843 H   0 0 0 0 0 0 0 0 0 0 0 0 0
    8.5077   9.5274   -0.8842 H   0 0 0 0 0 0 0 0 0 0 0 0 0
    8.5077   9.5274   0.8843 H   0 0 0 0 0 0 0 0 0 0 0 0 0
    3.4139   9.6126   0.0005 H   0 0 0 0 0 0 0 0 0 0 0 0 0
    5.9583   6.1561   0.0005 H   0 0 0 0 0 0 0 0 0 0 0 0 0
    9.8065   7.4116   0.8843 H   0 0 0 0 0 0 0 0 0 0 0 0 0
    9.8065   7.4116   -0.8842 H   0 0 0 0 0 0 0 0 0 0 0 0 0
    1.4183   8.1436   0.0002 H   0 0 0 0 0 0 0 0 0 0 0 0 0
    3.9627   4.6871   0.0002 H   0 0 0 0 0 0 0 0 0 0 0 0 0
   11.0237   9.5754   -0.8843 H   0 0 0 0 0 0 0 0 0 0 0 0 0
   11.0237   9.5755   0.8842 H   0 0 0 0 0 0 0 0 0 0 0 0 0
    1.6927   5.6808   0.0001 H   0 0 0 0 0 0 0 0 0 0 0 0 0
   12.3224   7.4595   0.8843 H   0 0 0 0 0 0 0 0 0 0 0 0 0
   12.3226   7.4596   -0.8842 H   0 0 0 0 0 0 0 0 0 0 0 0 0
   12.3559   10.7829   0.0005 H   0 0 0 0 0 0 0 0 0 0 0 0 0
   14.9003   7.3263   0.0005 H   0 0 0 0 0 0 0 0 0 0 0 0 0
   14.3515   12.2519   0.0002 H   0 0 0 0 0 0 0 0 0 0 0 0 0
   16.8959   8.7953   0.0002 H   0 0 0 0 0 0 0 0 0 0 0 0 0
   16.6215   11.2581   0.0001 H   0 0 0 0 0 0 0 0 0 0 0 0 0
  1 2 1 0 0 0 0
  1 4 1 0 0 0 0
  2 3 1 0 0 0 0
  5 3 4 0 0 0 0
  3 6 4 0 0 0 0
  4 7 1 0 0 0 0
  8 5 4 0 0 0 0
  6 9 4 0 0 0 0

7 10 1 0 0 0 0
11 8 4 0 0 0 0
9 11 4 0 0 0 0
10 12 1 0 0 0 0
12 13 1 0 0 0 0
13 14 4 0 0 0 0
15 13 4 0 0 0 0
14 16 4 0 0 0 0
17 15 4 0 0 0 0
16 18 4 0 0 0 0
18 17 4 0 0 0 0
1 19 1 0 0 0 0
1 20 1 0 0 0 0
2 21 1 0 0 0 0
2 22 1 0 0 0 0
4 23 1 0 0 0 0
4 24 1 0 0 0 0
5 25 1 0 0 0 0
6 26 1 0 0 0 0
7 27 1 0 0 0 0
7 28 1 0 0 0 0
8 29 1 0 0 0 0
9 30 1 0 0 0 0
10 31 1 0 0 0 0
10 32 1 0 0 0 0
11 33 1 0 0 0 0
12 34 1 0 0 0 0
12 35 1 0 0 0 0
14 36 1 0 0 0 0
15 37 1 0 0 0 0
16 38 1 0 0 0 0
17 39 1 0 0 0 0
18 40 1 0 0 0 0
M END
> <Alias name>
1,6-Diphenylhexane

> <deltaH°(c)> kJ/mol
-10230

$$$$
1,1-Dicyclohexylbutane
ChemBrain IXL

46 47 0 0 0 0 0 0 0 0 1 V2000
7.7245 3.9842 0.0407 C 0 0 0 0 0 0 0 0 0 0 0 0
6.5688 4.8552 0.5979 C 0 0 0 0 0 0 0 0 0 0 0 0
5.2336 4.4294 -0.1183 C 0 0 0 0 0 0 0 0 0 0 0 0
9.0382 4.3149 0.7835 C 0 0 0 0 0 0 0 0 0 0 0 0
4.9330 2.9494 0.2332 C 0 0 0 0 0 0 0 0 0 0 0 0
4.0490 5.3190 0.3393 C 0 0 0 0 0 0 0 0 0 0 0 0
10.1667 3.4078 0.2564 C 0 0 0 0 0 0 0 0 0 0 0 0
3.6297 2.5020 -0.4494 C 0 0 0 0 0 0 0 0 0 0 0 0
2.7418 4.8388 -0.3306 C 0 0 0 0 0 0 0 0 0 0 0 0
6.8997 6.3656 0.3457 C 0 0 0 0 0 0 0 0 0 0 0 0
2.4702 3.3755 0.0463 C 0 0 0 0 0 0 0 0 0 0 0 0
6.1130 7.2664 1.3377 C 0 0 0 0 0 0 0 0 0 0 0 0
6.5502 6.7473 -1.1105 C 0 0 0 0 0 0 0 0 0 0 0 0
6.4692 8.7434 1.0776 C 0 0 0 0 0 0 0 0 0 0 0 0
6.9023 8.2287 -1.3514 C 0 0 0 0 0 0 0 0 0 0 0 0
6.1020 9.1148 -0.3740 C 0 0 0 0 0 0 0 0 0 0 0 0
7.8478 4.1810 -1.0171 H 0 0 0 0 0 0 0 0 0 0 0 0
7.4878 2.9392 0.1861 H 0 0 0 0 0 0 0 0 0 0 0 0

```
    6.4794    4.6826    1.6633 H   0  0  0  0  0  0  0  0  0  0  0  0
    5.3561    4.5238   -1.1900 H   0  0  0  0  0  0  0  0  0  0  0  0
    8.9053    4.1449    1.8447 H   0  0  0  0  0  0  0  0  0  0  0  0
    9.3011    5.3488    0.6142 H   0  0  0  0  0  0  0  0  0  0  0  0
    4.8291    2.8487    1.3065 H   0  0  0  0  0  0  0  0  0  0  0  0
    5.7474    2.3256   -0.1087 H   0  0  0  0  0  0  0  0  0  0  0  0
    3.9468    5.2619    1.4159 H   0  0  0  0  0  0  0  0  0  0  0  0
    4.2325    6.3353    0.0469 H   0  0  0  0  0  0  0  0  0  0  0  0
   11.0878    3.6380    0.7774 H   0  0  0  0  0  0  0  0  0  0  0  0
   10.3002    3.5773   -0.8049 H   0  0  0  0  0  0  0  0  0  0  0  0
    9.9054    2.3706    0.4264 H   0  0  0  0  0  0  0  0  0  0  0  0
    3.7286    2.6066   -1.5228 H   0  0  0  0  0  0  0  0  0  0  0  0
    3.4314    1.4662   -0.2040 H   0  0  0  0  0  0  0  0  0  0  0  0
    2.8376    4.9209   -1.4062 H   0  0  0  0  0  0  0  0  0  0  0  0
    1.9180    5.4560    0.0054 H   0  0  0  0  0  0  0  0  0  0  0  0
    7.9547    6.5237    0.5067 H   0  0  0  0  0  0  0  0  0  0  0  0
    1.5481    3.0486   -0.4178 H   0  0  0  0  0  0  0  0  0  0  0  0
    2.3846    3.2879    1.1223 H   0  0  0  0  0  0  0  0  0  0  0  0
    5.0632    7.1285    1.2088 H   0  0  0  0  0  0  0  0  0  0  0  0
    6.3860    7.0053    2.3525 H   0  0  0  0  0  0  0  0  0  0  0  0
    7.1179    6.1265   -1.7923 H   0  0  0  0  0  0  0  0  0  0  0  0
    5.4942    6.5969   -1.2819 H   0  0  0  0  0  0  0  0  0  0  0  0
    5.9159    9.3738    1.7624 H   0  0  0  0  0  0  0  0  0  0  0  0
    7.5313    8.8907    1.2296 H   0  0  0  0  0  0  0  0  0  0  0  0
    7.9624    8.3788   -1.1889 H   0  0  0  0  0  0  0  0  0  0  0  0
    6.6518    8.4977   -2.3699 H   0  0  0  0  0  0  0  0  0  0  0  0
    5.0417    8.9582   -0.5298 H   0  0  0  0  0  0  0  0  0  0  0  0
    6.3429   10.1554   -0.5516 H   0  0  0  0  0  0  0  0  0  0  0  0
  1  2  1  0  0  0  0
  1  4  1  0  0  0  0
  2  3  1  0  0  0  0
  5  3  1  0  0  0  0
  3  6  1  0  0  0  0
  4  7  1  0  0  0  0
  8  5  1  0  0  0  0
  6  9  1  0  0  0  0
 11  8  1  0  0  0  0
  9 11  1  0  0  0  0
 10 12  1  0  0  0  0
 13 10  1  0  0  0  0
 12 14  1  0  0  0  0
 15 13  1  0  0  0  0
 14 16  1  0  0  0  0
 16 15  1  0  0  0  0
 10  2  1  0  0  0  0
  1 17  1  0  0  0  0
  1 18  1  0  0  0  0
  2 19  1  0  0  0  0
  3 20  1  0  0  0  0
  4 21  1  0  0  0  0
  4 22  1  0  0  0  0
  5 23  1  0  0  0  0
  5 24  1  0  0  0  0
  6 25  1  0  0  0  0
  6 26  1  0  0  0  0
  7 27  1  0  0  0  0
  7 28  1  0  0  0  0
  7 29  1  0  0  0  0
  8 30  1  0  0  0  0
  8 31  1  0  0  0  0
  9 32  1  0  0  0  0
  9 33  1  0  0  0  0
 10 34  1  0  0  0  0
```

```
 11 35  1  0  0  0  0
 11 36  1  0  0  0  0
 12 37  1  0  0  0  0
 12 38  1  0  0  0  0
 13 39  1  0  0  0  0
 13 40  1  0  0  0  0
 14 41  1  0  0  0  0
 14 42  1  0  0  0  0
 15 43  1  0  0  0  0
 15 44  1  0  0  0  0
 16 45  1  0  0  0  0
 16 46  1  0  0  0  0
M  END
>  <Alias name>
1,1-Dicyclohexylbutane

>  <deltaH°(c)>   kJ/mol
-10220

$$$$
1,3-Dicyclohexylbutane
  ChemBrain IXL

 46 47  0  0  0  0  0  0  0  0  1 V2000
    7.1257    7.6218   -0.5863 C   0  0  0  0  0  0  0  0  0  0  0  0
    6.4975    6.8246    0.5770 C   0  0  0  0  0  0  0  0  0  0  0  0
    4.9715    6.7180    0.3686 C   0  0  0  0  0  0  0  0  0  0  0  0
    8.6483    7.7595   -0.3597 C   0  0  0  0  0  0  0  0  0  0  0  0
    4.6788    5.9692   -0.9498 C   0  0  0  0  0  0  0  0  0  0  0  0
    4.3472    5.9445    1.5481 C   0  0  0  0  0  0  0  0  0  0  0  0
    9.2671    8.5628   -1.5245 C   0  0  0  0  0  0  0  0  0  0  0  0
    3.1541    5.8588   -1.1517 C   0  0  0  0  0  0  0  0  0  0  0  0
    2.8231    5.8356    1.3437 C   0  0  0  0  0  0  0  0  0  0  0  0
    9.2978    6.3516   -0.2878 C   0  0  0  0  0  0  0  0  0  0  0  0
    2.5330    5.0857    0.0286 C   0  0  0  0  0  0  0  0  0  0  0  0
   10.8174    6.4939   -0.0591 C   0  0  0  0  0  0  0  0  0  0  0  0
    9.0468    5.5891   -1.6078 C   0  0  0  0  0  0  0  0  0  0  0  0
   11.4567    5.0939    0.0275 C   0  0  0  0  0  0  0  0  0  0  0  0
    9.6882    4.1895   -1.5215 C   0  0  0  0  0  0  0  0  0  0  0  0
   11.2062    4.3319   -1.2896 C   0  0  0  0  0  0  0  0  0  0  0  0
    6.9452    7.1041   -1.5184 H   0  0  0  0  0  0  0  0  0  0  0  0
    6.6785    8.6071   -0.6296 H   0  0  0  0  0  0  0  0  0  0  0  0
    6.6981    7.3338    1.5115 H   0  0  0  0  0  0  0  0  0  0  0  0
    6.9267    5.8316    0.6084 H   0  0  0  0  0  0  0  0  0  0  0  0
    4.5434    7.7117    0.3223 H   0  0  0  0  0  0  0  0  0  0  0  0
    8.8259    8.2838    0.5711 H   0  0  0  0  0  0  0  0  0  0  0  0
    5.1098    4.9767   -0.9046 H   0  0  0  0  0  0  0  0  0  0  0  0
    5.1125    6.5134   -1.7785 H   0  0  0  0  0  0  0  0  0  0  0  0
    4.5505    6.4705    2.4725 H   0  0  0  0  0  0  0  0  0  0  0  0
    4.7766    4.9514    1.5961 H   0  0  0  0  0  0  0  0  0  0  0  0
    8.8109    9.5439   -1.5676 H   0  0  0  0  0  0  0  0  0  0  0  0
    9.0905    8.0425   -2.4568 H   0  0  0  0  0  0  0  0  0  0  0  0
   10.3323    8.6680   -1.3657 H   0  0  0  0  0  0  0  0  0  0  0  0
    2.9497    5.3320   -2.0754 H   0  0  0  0  0  0  0  0  0  0  0  0
    2.7235    6.8513   -1.1994 H   0  0  0  0  0  0  0  0  0  0  0  0
    2.3935    6.8286    1.2955 H   0  0  0  0  0  0  0  0  0  0  0  0
    2.3858    5.2930    2.1725 H   0  0  0  0  0  0  0  0  0  0  0  0
    8.8634    5.7979    0.5341 H   0  0  0  0  0  0  0  0  0  0  0  0
    1.4624    5.0085   -0.1142 H   0  0  0  0  0  0  0  0  0  0  0  0
    2.9634    4.0931    0.0765 H   0  0  0  0  0  0  0  0  0  0  0  0
   10.9949    7.0295    0.8651 H   0  0  0  0  0  0  0  0  0  0  0  0
   11.2578    7.0400   -0.8830 H   0  0  0  0  0  0  0  0  0  0  0  0
    9.4865    6.1365   -2.4313 H   0  0  0  0  0  0  0  0  0  0  0  0
```

```
    7.9817    5.4870   -1.7696 H   0  0  0  0  0  0  0  0  0  0  0  0
   11.0157    4.5460    0.8511 H   0  0  0  0  0  0  0  0  0  0  0  0
   12.5226    5.1934    0.1897 H   0  0  0  0  0  0  0  0  0  0  0  0
    9.5132    3.6548   -2.4467 H   0  0  0  0  0  0  0  0  0  0  0  0
    9.2473    3.6407   -0.6985 H   0  0  0  0  0  0  0  0  0  0  0  0
   11.6556    3.3487   -1.2286 H   0  0  0  0  0  0  0  0  0  0  0  0
   11.6472    4.8799   -2.1130 H   0  0  0  0  0  0  0  0  0  0  0  0
  1  2  1  0  0  0  0
  1  4  1  0  0  0  0
  2  3  1  0  0  0  0
  5  3  1  0  0  0  0
  3  6  1  0  0  0  0
  4  7  1  0  0  0  0
  8  5  1  0  0  0  0
  6  9  1  0  0  0  0
 11  8  1  0  0  0  0
  9 11  1  0  0  0  0
 10 12  1  0  0  0  0
 13 10  1  0  0  0  0
 12 14  1  0  0  0  0
 15 13  1  0  0  0  0
 14 16  1  0  0  0  0
 16 15  1  0  0  0  0
 10  4  1  0  0  0  0
  1 17  1  0  0  0  0
  1 18  1  0  0  0  0
  2 19  1  0  0  0  0
  2 20  1  0  0  0  0
  3 21  1  0  0  0  0
  4 22  1  0  0  0  0
  5 23  1  0  0  0  0
  5 24  1  0  0  0  0
  6 25  1  0  0  0  0
  6 26  1  0  0  0  0
  7 27  1  0  0  0  0
  7 28  1  0  0  0  0
  7 29  1  0  0  0  0
  8 30  1  0  0  0  0
  8 31  1  0  0  0  0
  9 32  1  0  0  0  0
  9 33  1  0  0  0  0
 10 34  1  0  0  0  0
 11 35  1  0  0  0  0
 11 36  1  0  0  0  0
 12 37  1  0  0  0  0
 12 38  1  0  0  0  0
 13 39  1  0  0  0  0
 13 40  1  0  0  0  0
 14 41  1  0  0  0  0
 14 42  1  0  0  0  0
 15 43  1  0  0  0  0
 15 44  1  0  0  0  0
 16 45  1  0  0  0  0
 16 46  1  0  0  0  0
M  END
>  <Alias name>
1,3-Dicyclohexylbutane

>  <deltaH°(c)>   kJ/mol
-10220

$$$$
1,4-Dicyclohexylbutane
```

ChemBrain IXL

```
46 47  0  0  0  0  0  0  0  0  1 V2000
    7.6960    7.5876   -0.2710 C   0  0  0  0  0  0  0  0  0  0  0  0  0
    6.6887    8.6729    0.1615 C   0  0  0  0  0  0  0  0  0  0  0  0  0
    5.2916    8.3422   -0.4072 C   0  0  0  0  0  0  0  0  0  0  0  0  0
    9.0923    7.9343    0.2806 C   0  0  0  0  0  0  0  0  0  0  0  0  0
    4.8122    6.9839    0.1495 C   0  0  0  0  0  0  0  0  0  0  0  0  0
    4.2969    9.4464    0.0060 C   0  0  0  0  0  0  0  0  0  0  0  0  0
   10.0996    6.8490   -0.1518 C   0  0  0  0  0  0  0  0  0  0  0  0  0
    3.4145    6.6602   -0.4157 C   0  0  0  0  0  0  0  0  0  0  0  0  0
    2.9007    9.1203   -0.5604 C   0  0  0  0  0  0  0  0  0  0  0  0  0
   11.4966    7.1797    0.4169 C   0  0  0  0  0  0  0  0  0  0  0  0  0
    2.4229    7.7655   -0.0017 C   0  0  0  0  0  0  0  0  0  0  0  0  0
   11.9761    8.5380   -0.1399 C   0  0  0  0  0  0  0  0  0  0  0  0  0
   12.4913    6.0755    0.0037 C   0  0  0  0  0  0  0  0  0  0  0  0  0
   13.3738    8.8617    0.4253 C   0  0  0  0  0  0  0  0  0  0  0  0  0
   13.8875    6.4016    0.5701 C   0  0  0  0  0  0  0  0  0  0  0  0  0
   14.3654    7.7564    0.0114 C   0  0  0  0  0  0  0  0  0  0  0  0  0
    7.7361    7.5427   -1.3523 H   0  0  0  0  0  0  0  0  0  0  0  0  0
    7.3831    6.6277    0.1188 H   0  0  0  0  0  0  0  0  0  0  0  0  0
    6.6391    8.7092    1.2428 H   0  0  0  0  0  0  0  0  0  0  0  0  0
    7.0123    9.6347   -0.2168 H   0  0  0  0  0  0  0  0  0  0  0  0  0
    5.3448    8.2910   -1.4877 H   0  0  0  0  0  0  0  0  0  0  0  0  0
    9.0521    7.9792    1.3619 H   0  0  0  0  0  0  0  0  0  0  0  0  0
    9.4052    8.8942   -0.1091 H   0  0  0  0  0  0  0  0  0  0  0  0  0
    4.7618    7.0342    1.2302 H   0  0  0  0  0  0  0  0  0  0  0  0  0
    5.5069    6.2077   -0.1435 H   0  0  0  0  0  0  0  0  0  0  0  0  0
    4.2447    9.4992    1.0865 H   0  0  0  0  0  0  0  0  0  0  0  0  0
    4.6318   10.3987   -0.3860 H   0  0  0  0  0  0  0  0  0  0  0  0  0
   10.1492    6.8127   -1.2331 H   0  0  0  0  0  0  0  0  0  0  0  0  0
    9.7760    5.8872    0.2264 H   0  0  0  0  0  0  0  0  0  0  0  0  0
    3.4655    6.6075   -1.4962 H   0  0  0  0  0  0  0  0  0  0  0  0  0
    3.0783    5.7086   -0.0235 H   0  0  0  0  0  0  0  0  0  0  0  0  0
    2.9528    9.0672   -1.6408 H   0  0  0  0  0  0  0  0  0  0  0  0  0
    2.2035    9.8964   -0.2703 H   0  0  0  0  0  0  0  0  0  0  0  0  0
   11.4434    7.2309    1.4974 H   0  0  0  0  0  0  0  0  0  0  0  0  0
    1.4417    7.5373   -0.3988 H   0  0  0  0  0  0  0  0  0  0  0  0  0
    2.3716    7.8184    1.0788 H   0  0  0  0  0  0  0  0  0  0  0  0  0
   12.0265    8.4877   -1.2205 H   0  0  0  0  0  0  0  0  0  0  0  0  0
   11.2814    9.3142    0.1531 H   0  0  0  0  0  0  0  0  0  0  0  0  0
   12.5436    6.0227   -1.0768 H   0  0  0  0  0  0  0  0  0  0  0  0  0
   12.1565    5.1232    0.3957 H   0  0  0  0  0  0  0  0  0  0  0  0  0
   13.3228    8.9144    1.5059 H   0  0  0  0  0  0  0  0  0  0  0  0  0
   13.7100    9.8134    0.0331 H   0  0  0  0  0  0  0  0  0  0  0  0  0
   13.8355    6.4547    1.6505 H   0  0  0  0  0  0  0  0  0  0  0  0  0
   14.5848    5.6255    0.2800 H   0  0  0  0  0  0  0  0  0  0  0  0  0
   15.3466    7.9846    0.4085 H   0  0  0  0  0  0  0  0  0  0  0  0  0
   14.4167    7.7035   -1.0691 H   0  0  0  0  0  0  0  0  0  0  0  0  0
  1  2  1  0  0  0  0
  1  4  1  0  0  0  0
  2  3  1  0  0  0  0
  5  3  1  0  0  0  0
  3  6  1  0  0  0  0
  4  7  1  0  0  0  0
  8  5  1  0  0  0  0
  6  9  1  0  0  0  0
  7 10  1  0  0  0  0
 11  8  1  0  0  0  0
  9 11  1  0  0  0  0
 10 12  1  0  0  0  0
 13 10  1  0  0  0  0
 12 14  1  0  0  0  0
```

```
 15 13  1  0  0  0  0
 14 16  1  0  0  0  0
 16 15  1  0  0  0  0
  1 17  1  0  0  0  0
  1 18  1  0  0  0  0
  2 19  1  0  0  0  0
  2 20  1  0  0  0  0
  3 21  1  0  0  0  0
  4 22  1  0  0  0  0
  4 23  1  0  0  0  0
  5 24  1  0  0  0  0
  5 25  1  0  0  0  0
  6 26  1  0  0  0  0
  6 27  1  0  0  0  0
  7 28  1  0  0  0  0
  7 29  1  0  0  0  0
  8 30  1  0  0  0  0
  8 31  1  0  0  0  0
  9 32  1  0  0  0  0
  9 33  1  0  0  0  0
 10 34  1  0  0  0  0
 11 35  1  0  0  0  0
 11 36  1  0  0  0  0
 12 37  1  0  0  0  0
 12 38  1  0  0  0  0
 13 39  1  0  0  0  0
 13 40  1  0  0  0  0
 14 41  1  0  0  0  0
 14 42  1  0  0  0  0
 15 43  1  0  0  0  0
 15 44  1  0  0  0  0
 16 45  1  0  0  0  0
 16 46  1  0  0  0  0
M  END
>  <Alias name>
1,4-Dicyclohexylbutane

>  <deltaH°(c)>   kJ/mol
-10220

$$$$
Methyltriphenoxysilane
  ChemBrain IXL

 41 43  0  0  0  0  0  0  0  0  1 V2000
    7.4625    2.8419    0.1111 C   0  0  0  0  0  0  0  0  0  0  0  0
    7.4872    4.6795    0.4172 Si  0  0  0  0  0  0  0  0  0  0  0  0
    6.4893    5.0329    1.6686 O   0  0  0  0  0  0  0  0  0  0  0  0
    9.0134    5.1447    0.7934 O   0  0  0  0  0  0  0  0  0  0  0  0
    6.9844    5.4634   -0.9322 O   0  0  0  0  0  0  0  0  0  0  0  0
    6.9091    9.6402   -0.8149 C   0  0  0  0  0  0  0  0  0  0  0  0
    6.5179    8.9589   -1.9676 C   0  0  0  0  0  0  0  0  0  0  0  0
    6.5461    7.5638   -1.9982 C   0  0  0  0  0  0  0  0  0  0  0  0
    6.9654    6.8443   -0.8769 C   0  0  0  0  0  0  0  0  0  0  0  0
    7.3578    7.5312    0.2798 C   0  0  0  0  0  0  0  0  0  0  0  0
    7.3288    8.9266    0.3082 C   0  0  0  0  0  0  0  0  0  0  0  0
    3.2643    4.4490   -0.0416 C   0  0  0  0  0  0  0  0  0  0  0  0
    2.3754    4.6802    1.0085 C   0  0  0  0  0  0  0  0  0  0  0  0
    2.8612    5.0274    2.2693 C   0  0  0  0  0  0  0  0  0  0  0  0
    4.2360    5.1435    2.4810 C   0  0  0  0  0  0  0  0  0  0  0  0
    5.1308    4.9126    1.4335 C   0  0  0  0  0  0  0  0  0  0  0  0
    4.6395    4.5645    0.1679 C   0  0  0  0  0  0  0  0  0  0  0  0
    9.5693    4.7851   -1.5421 C   0  0  0  0  0  0  0  0  0  0  0  0
```

```
 10.5240    4.7761   -2.5604 C   0  0  0  0  0  0  0  0  0  0  0  0
 11.8490    5.1098   -2.2793 C   0  0  0  0  0  0  0  0  0  0  0  0
 12.2206    5.4530   -0.9792 C   0  0  0  0  0  0  0  0  0  0  0  0
 11.2676    5.4626    0.0407 C   0  0  0  0  0  0  0  0  0  0  0  0
  9.9395    5.1288   -0.2348 C   0  0  0  0  0  0  0  0  0  0  0  0
  8.1241    2.6041   -0.7125 H   0  0  0  0  0  0  0  0  0  0  0  0
  6.4548    2.5302   -0.1336 H   0  0  0  0  0  0  0  0  0  0  0  0
  7.7944    2.3233    1.0020 H   0  0  0  0  0  0  0  0  0  0  0  0
  6.8869   10.7226   -0.7918 H   0  0  0  0  0  0  0  0  0  0  0  0
  6.1921    9.5127   -2.8393 H   0  0  0  0  0  0  0  0  0  0  0  0
  6.2420    7.0373   -2.8943 H   0  0  0  0  0  0  0  0  0  0  0  0
  7.6842    6.9837    1.1536 H   0  0  0  0  0  0  0  0  0  0  0  0
  7.6324    9.4558    1.2028 H   0  0  0  0  0  0  0  0  0  0  0  0
  2.8870    4.1796   -1.0202 H   0  0  0  0  0  0  0  0  0  0  0  0
  1.3087    4.5901    0.8450 H   0  0  0  0  0  0  0  0  0  0  0  0
  2.1711    5.2070    3.0843 H   0  0  0  0  0  0  0  0  0  0  0  0
  4.6102    5.4131    3.4607 H   0  0  0  0  0  0  0  0  0  0  0  0
  5.3234    4.3848   -0.6506 H   0  0  0  0  0  0  0  0  0  0  0  0
  8.5435    4.5263   -1.7668 H   0  0  0  0  0  0  0  0  0  0  0  0
 10.2357    4.5100   -3.5697 H   0  0  0  0  0  0  0  0  0  0  0  0
 12.5889    5.1023   -3.0700 H   0  0  0  0  0  0  0  0  0  0  0  0
 13.2492    5.7121   -0.7611 H   0  0  0  0  0  0  0  0  0  0  0  0
 11.5589    5.7293    1.0489 H   0  0  0  0  0  0  0  0  0  0  0  0
  1  2  1  0  0  0  0
  3  2  1  0  0  0  0
  4  2  1  0  0  0  0
  5  2  1  0  0  0  0
  6  7  4  0  0  0  0
  7  8  4  0  0  0  0
  8  9  4  0  0  0  0
  9 10  4  0  0  0  0
 10 11  4  0  0  0  0
 11  6  4  0  0  0  0
  9  5  1  0  0  0  0
 12 13  4  0  0  0  0
 13 14  4  0  0  0  0
 14 15  4  0  0  0  0
 15 16  4  0  0  0  0
 16 17  4  0  0  0  0
 17 12  4  0  0  0  0
 16  3  1  0  0  0  0
 18 19  4  0  0  0  0
 19 20  4  0  0  0  0
 20 21  4  0  0  0  0
 21 22  4  0  0  0  0
 22 23  4  0  0  0  0
 23 18  4  0  0  0  0
 23  4  1  0  0  0  0
  1 24  1  0  0  0  0
  1 25  1  0  0  0  0
  1 26  1  0  0  0  0
  6 27  1  0  0  0  0
  7 28  1  0  0  0  0
  8 29  1  0  0  0  0
 10 30  1  0  0  0  0
 11 31  1  0  0  0  0
 12 32  1  0  0  0  0
 13 33  1  0  0  0  0
 14 34  1  0  0  0  0
 15 35  1  0  0  0  0
 17 36  1  0  0  0  0
 18 37  1  0  0  0  0
 19 38  1  0  0  0  0
```

20 39 1 0 0 0 0
21 40 1 0 0 0 0
22 41 1 0 0 0 0
M END
> <Alias name>
Methyltriphenoxysilane

> <deltaH°(c)>   kJ/mol
-10220

$$$$
4-Isopropyldicyclohexylmethane
  ChemBrain IXL

46 47 0 0 0 0 0 0 0 0 1 V2000
    8.8103   5.4517   0.6328 C   0 0 0 0 0 0 0 0 0 0 0 0 0
    7.5751   6.0771   -0.0550 C   0 0 0 0 0 0 0 0 0 0 0 0 0
   10.0338   6.3813   0.4638 C   0 0 0 0 0 0 0 0 0 0 0 0 0
    7.2619   7.4459   0.5849 C   0 0 0 0 0 0 0 0 0 0 0 0 0
    6.3641   5.1401   0.1212 C   0 0 0 0 0 0 0 0 0 0 0 0 0
   11.2576   5.7452   1.1545 C   0 0 0 0 0 0 0 0 0 0 0 0 0
   10.3351   6.5760   -1.0382 C   0 0 0 0 0 0 0 0 0 0 0 0 0
    6.0281   8.0654   -0.1016 C   0 0 0 0 0 0 0 0 0 0 0 0 0
    5.1305   5.7567   -0.5671 C   0 0 0 0 0 0 0 0 0 0 0 0 0
   12.4805   6.6689   0.9891 C   0 0 0 0 0 0 0 0 0 0 0 0 0
   11.5601   7.4981   -1.2022 C   0 0 0 0 0 0 0 0 0 0 0 0 0
    4.8139   7.1268   0.0711 C   0 0 0 0 0 0 0 0 0 0 0 0 0
   12.7811   6.8602   -0.5105 C   0 0 0 0 0 0 0 0 0 0 0 0 0
    3.5764   7.7477   -0.6218 C   0 0 0 0 0 0 0 0 0 0 0 0 0
    2.3622   6.8114   -0.4494 C   0 0 0 0 0 0 0 0 0 0 0 0 0
    3.2587   9.1167   0.0151 C   0 0 0 0 0 0 0 0 0 0 0 0 0
    8.6045   5.3166   1.6874 H   0 0 0 0 0 0 0 0 0 0 0 0 0
    9.0242   4.4904   0.1822 H   0 0 0 0 0 0 0 0 0 0 0 0 0
    7.7769   6.2094   -1.1099 H   0 0 0 0 0 0 0 0 0 0 0 0 0
    9.8240   7.3411   0.9172 H   0 0 0 0 0 0 0 0 0 0 0 0 0
    7.0590   7.3130   1.6404 H   0 0 0 0 0 0 0 0 0 0 0 0 0
    8.1107   8.1054   0.4609 H   0 0 0 0 0 0 0 0 0 0 0 0 0
    6.1602   5.0083   1.1766 H   0 0 0 0 0 0 0 0 0 0 0 0 0
    6.5838   4.1788   -0.3265 H   0 0 0 0 0 0 0 0 0 0 0 0 0
   11.4679   4.7837   0.7025 H   0 0 0 0 0 0 0 0 0 0 0 0 0
   11.0473   5.6102   2.2082 H   0 0 0 0 0 0 0 0 0 0 0 0 0
    9.4790   7.0264   -1.5231 H   0 0 0 0 0 0 0 0 0 0 0 0 0
   10.5431   5.6151   -1.4923 H   0 0 0 0 0 0 0 0 0 0 0 0 0
    6.2323   8.2008   -1.1566 H   0 0 0 0 0 0 0 0 0 0 0 0 0
    5.8104   9.0245   0.3493 H   0 0 0 0 0 0 0 0 0 0 0 0 0
    5.3343   5.8885   -1.6226 H   0 0 0 0 0 0 0 0 0 0 0 0 0
    4.2827   5.0960   -0.4433 H   0 0 0 0 0 0 0 0 0 0 0 0 0
   13.3387   6.2218   1.4750 H   0 0 0 0 0 0 0 0 0 0 0 0 0
   12.2703   7.6306   1.4408 H   0 0 0 0 0 0 0 0 0 0 0 0 0
   11.3510   8.4598   -0.7503 H   0 0 0 0 0 0 0 0 0 0 0 0 0
   11.7718   7.6328   -2.2556 H   0 0 0 0 0 0 0 0 0 0 0 0 0
    4.6080   6.9942   1.1261 H   0 0 0 0 0 0 0 0 0 0 0 0 0
   13.6409   7.5083   -0.6264 H   0 0 0 0 0 0 0 0 0 0 0 0 0
   12.9905   5.8985   -0.9623 H   0 0 0 0 0 0 0 0 0 0 0 0 0
    3.7819   7.8803   -1.6768 H   0 0 0 0 0 0 0 0 0 0 0 0 0
    2.5734   5.8561   -0.9117 H   0 0 0 0 0 0 0 0 0 0 0 0 0
    2.1650   6.6664   0.6056 H   0 0 0 0 0 0 0 0 0 0 0 0 0
    1.4945   7.2557   -0.9209 H   0 0 0 0 0 0 0 0 0 0 0 0 0
    2.3832   9.5411   -0.4604 H   0 0 0 0 0 0 0 0 0 0 0 0 0
    3.0674   8.9869   1.0732 H   0 0 0 0 0 0 0 0 0 0 0 0 0
    4.1004   9.7830   -0.1205 H   0 0 0 0 0 0 0 0 0 0 0 0 0
  1 2 1 0 0 0 0
  1 3 1 0 0 0 0

```
  4  2  1  0  0  0  0
  2  5  1  0  0  0  0
  6  3  1  0  0  0  0
  3  7  1  0  0  0  0
  8  4  1  0  0  0  0
  5  9  1  0  0  0  0
 10  6  1  0  0  0  0
  7 11  1  0  0  0  0
 12  8  1  0  0  0  0
  9 12  1  0  0  0  0
 13 10  1  0  0  0  0
 11 13  1  0  0  0  0
 12 14  1  0  0  0  0
 14 15  1  0  0  0  0
 14 16  1  0  0  0  0
  1 17  1  0  0  0  0
  1 18  1  0  0  0  0
  2 19  1  0  0  0  0
  3 20  1  0  0  0  0
  4 21  1  0  0  0  0
  4 22  1  0  0  0  0
  5 23  1  0  0  0  0
  5 24  1  0  0  0  0
  6 25  1  0  0  0  0
  6 26  1  0  0  0  0
  7 27  1  0  0  0  0
  7 28  1  0  0  0  0
  8 29  1  0  0  0  0
  8 30  1  0  0  0  0
  9 31  1  0  0  0  0
  9 32  1  0  0  0  0
 10 33  1  0  0  0  0
 10 34  1  0  0  0  0
 11 35  1  0  0  0  0
 11 36  1  0  0  0  0
 12 37  1  0  0  0  0
 13 38  1  0  0  0  0
 13 39  1  0  0  0  0
 14 40  1  0  0  0  0
 15 41  1  0  0  0  0
 15 42  1  0  0  0  0
 15 43  1  0  0  0  0
 16 44  1  0  0  0  0
 16 45  1  0  0  0  0
 16 46  1  0  0  0  0
M  END
>  <Alias name>
4-Isopropyldicyclohexylmethane

>  <deltaH°(c)>   kJ/mol
-10200

$$$$
4,4'-(1-Phenylethylidene) biphenol
  ChemBrain IXL

 40 42  0  0  0  0  0  0  0  0  1 V2000
    5.8307    6.3499   -0.9185 C   0  0  0  0  0  0  0  0  0  0  0  0
    4.8184    7.0796   -0.1052 C   0  0  0  0  0  0  0  0  0  0  0  0
    7.1162    7.1025   -0.9078 C   0  0  0  0  0  0  0  0  0  0  0  0
    6.0442    4.9928   -0.3439 C   0  0  0  0  0  0  0  0  0  0  0  0
    4.1650    8.1950   -0.6361 C   0  0  0  0  0  0  0  0  0  0  0  0
    4.5190    6.6519    1.1919 C   0  0  0  0  0  0  0  0  0  0  0  0
```

```
   8.0905    6.8376   -1.8740 C   0  0  0  0  0  0  0  0  0  0  0  0
   7.3529    8.0751    0.0683 C   0  0  0  0  0  0  0  0  0  0  0  0
   7.1607    4.7377    0.4577 C   0  0  0  0  0  0  0  0  0  0  0  0
   5.1281    3.9701   -0.6038 C   0  0  0  0  0  0  0  0  0  0  0  0
   3.2165    8.8787    0.1261 C   0  0  0  0  0  0  0  0  0  0  0  0
   3.5706    7.3364    1.9529 C   0  0  0  0  0  0  0  0  0  0  0  0
   9.2953    7.5422   -1.8644 C   0  0  0  0  0  0  0  0  0  0  0  0
   8.5580    8.7789    0.0768 C   0  0  0  0  0  0  0  0  0  0  0  0
   7.3593    3.4656    0.9959 C   0  0  0  0  0  0  0  0  0  0  0  0
   5.3279    2.6983   -0.0650 C   0  0  0  0  0  0  0  0  0  0  0  0
   2.9193    8.4495    1.4203 C   0  0  0  0  0  0  0  0  0  0  0  0
   9.5291    8.5127   -0.8893 C   0  0  0  0  0  0  0  0  0  0  0  0
   6.4432    2.4461    0.7346 C   0  0  0  0  0  0  0  0  0  0  0  0
   1.9201    9.1699    2.2229 O   0  0  0  0  0  0  0  0  0  0  0  0
  10.7984    9.2544   -0.8800 O   0  0  0  0  0  0  0  0  0  0  0  0
   5.3238    6.2191   -2.3748 C   0  0  0  0  0  0  0  0  0  0  0  0
   1.5059    9.9414    1.7763 H   0  0  0  0  0  0  0  0  0  0  0  0
  10.8855    9.9204   -0.1621 H   0  0  0  0  0  0  0  0  0  0  0  0
   4.3936    8.5301   -1.6393 H   0  0  0  0  0  0  0  0  0  0  0  0
   5.0226    5.7887    1.6079 H   0  0  0  0  0  0  0  0  0  0  0  0
   7.9119    6.0854   -2.6312 H   0  0  0  0  0  0  0  0  0  0  0  0
   6.6017    8.2839    0.8193 H   0  0  0  0  0  0  0  0  0  0  0  0
   7.8731    5.5267    0.6624 H   0  0  0  0  0  0  0  0  0  0  0  0
   4.2622    4.1630   -1.2234 H   0  0  0  0  0  0  0  0  0  0  0  0
   2.7110    9.7427   -0.2870 H   0  0  0  0  0  0  0  0  0  0  0  0
   3.3399    7.0036    2.9572 H   0  0  0  0  0  0  0  0  0  0  0  0
  10.0490    7.3357   -2.6139 H   0  0  0  0  0  0  0  0  0  0  0  0
   8.7396    9.5320    0.8336 H   0  0  0  0  0  0  0  0  0  0  0  0
   8.2247    3.2696    1.6166 H   0  0  0  0  0  0  0  0  0  0  0  0
   4.6171    1.9070   -0.2676 H   0  0  0  0  0  0  0  0  0  0  0  0
   6.5978    1.4592    1.1524 H   0  0  0  0  0  0  0  0  0  0  0  0
   6.0384    5.6517   -2.9567 H   0  0  0  0  0  0  0  0  0  0  0  0
   4.3690    5.7098   -2.3810 H   0  0  0  0  0  0  0  0  0  0  0  0
   5.2089    7.2046   -2.8073 H   0  0  0  0  0  0  0  0  0  0  0  0
  1  2  1  0  0  0  0
  1  3  1  0  0  0  0
  1  4  1  0  0  0  0
  1 22  1  0  0  0  0
  2  5  4  0  0  0  0
  2  6  4  0  0  0  0
  3  7  4  0  0  0  0
  3  8  4  0  0  0  0
  4  9  4  0  0  0  0
  4 10  4  0  0  0  0
  5 11  4  0  0  0  0
  6 12  4  0  0  0  0
  7 13  4  0  0  0  0
  8 14  4  0  0  0  0
  9 15  4  0  0  0  0
 10 16  4  0  0  0  0
 11 17  4  0  0  0  0
 12 17  4  0  0  0  0
 13 18  4  0  0  0  0
 14 18  4  0  0  0  0
 15 19  4  0  0  0  0
 16 19  4  0  0  0  0
 17 20  1  0  0  0  0
 18 21  1  0  0  0  0
 20 23  1  0  0  0  0
 21 24  1  0  0  0  0
  5 25  1  0  0  0  0
  6 26  1  0  0  0  0
  7 27  1  0  0  0  0
```

```
  8 28  1  0  0  0  0
  9 29  1  0  0  0  0
 10 30  1  0  0  0  0
 11 31  1  0  0  0  0
 12 32  1  0  0  0  0
 13 33  1  0  0  0  0
 14 34  1  0  0  0  0
 15 35  1  0  0  0  0
 16 36  1  0  0  0  0
 19 37  1  0  0  0  0
 22 38  1  0  0  0  0
 22 39  1  0  0  0  0
 22 40  1  0  0  0  0
M  END
>  <Alias name>
Bisphenol AP

>  <deltaH°(c)>   kJ/mol
-10194

$$$$
2,3-Dimethyl-2,3-diphenylbutane
  ChemBrain IXL

 40 41  0  0  0  0  0  0  0  0  1 V2000
    6.2273    7.1065    1.5385 C   0  0  0  0  0  0  0  0  0  0  0  0
    6.0680    6.9540    0.0134 C   0  0  0  0  0  0  0  0  0  0  0  0
    7.3521    6.3414   -0.5786 C   0  0  0  0  0  0  0  0  0  0  0  0
    8.5537    7.2473   -0.2466 C   0  0  0  0  0  0  0  0  0  0  0  0
    5.8206    8.3378   -0.6181 C   0  0  0  0  0  0  0  0  0  0  0  0
    7.2063    6.2214   -2.1080 C   0  0  0  0  0  0  0  0  0  0  0  0
    4.9192    6.0670   -0.2763 C   0  0  0  0  0  0  0  0  0  0  0  0
    4.5792    5.0515    0.6176 C   0  0  0  0  0  0  0  0  0  0  0  0
    3.4991    4.2127    0.3424 C   0  0  0  0  0  0  0  0  0  0  0  0
    2.7577    4.3906   -0.8258 C   0  0  0  0  0  0  0  0  0  0  0  0
    3.0962    5.4077   -1.7186 C   0  0  0  0  0  0  0  0  0  0  0  0
    4.1763    6.2465   -1.4434 C   0  0  0  0  0  0  0  0  0  0  0  0
    7.0534    3.8698   -0.6330 C   0  0  0  0  0  0  0  0  0  0  0  0
    7.5714    4.9982    0.0029 C   0  0  0  0  0  0  0  0  0  0  0  0
    8.2960    4.8606    1.1870 C   0  0  0  0  0  0  0  0  0  0  0  0
    8.4994    3.5949    1.7372 C   0  0  0  0  0  0  0  0  0  0  0  0
    7.9795    2.4667    1.1025 C   0  0  0  0  0  0  0  0  0  0  0  0
    7.2567    2.6041   -0.0827 C   0  0  0  0  0  0  0  0  0  0  0  0
    5.3120    7.5041    1.9592 H   0  0  0  0  0  0  0  0  0  0  0  0
    7.0458    7.7835    1.7501 H   0  0  0  0  0  0  0  0  0  0  0  0
    6.4363    6.1390    1.9782 H   0  0  0  0  0  0  0  0  0  0  0  0
    8.4049    8.2225   -0.6935 H   0  0  0  0  0  0  0  0  0  0  0  0
    9.4598    6.8039   -0.6408 H   0  0  0  0  0  0  0  0  0  0  0  0
    8.6409    7.3520    0.8278 H   0  0  0  0  0  0  0  0  0  0  0  0
    5.7362    8.2342   -1.6928 H   0  0  0  0  0  0  0  0  0  0  0  0
    4.9034    8.7563   -0.2226 H   0  0  0  0  0  0  0  0  0  0  0  0
    6.6484    8.9952   -0.3827 H   0  0  0  0  0  0  0  0  0  0  0  0
    8.0926    5.7548   -2.5196 H   0  0  0  0  0  0  0  0  0  0  0  0
    6.3385    5.6171   -2.3416 H   0  0  0  0  0  0  0  0  0  0  0  0
    7.0852    7.2082   -2.5376 H   0  0  0  0  0  0  0  0  0  0  0  0
    5.1538    4.9143    1.5253 H   0  0  0  0  0  0  0  0  0  0  0  0
    3.2360    3.4234    1.0357 H   0  0  0  0  0  0  0  0  0  0  0  0
    1.9194    3.7392   -1.0397 H   0  0  0  0  0  0  0  0  0  0  0  0
    2.5205    5.5460   -2.6254 H   0  0  0  0  0  0  0  0  0  0  0  0
    4.4382    7.0371   -2.1357 H   0  0  0  0  0  0  0  0  0  0  0  0
    6.4934    3.9763   -1.5538 H   0  0  0  0  0  0  0  0  0  0  0  0
    8.7008    5.7364    1.6790 H   0  0  0  0  0  0  0  0  0  0  0  0
    9.0608    3.4882    2.6572 H   0  0  0  0  0  0  0  0  0  0  0  0
```

```
    8.1371    1.4841    1.5298 H   0  0  0  0  0  0  0  0  0  0  0  0
    6.8534    1.7282   -0.5757 H   0  0  0  0  0  0  0  0  0  0  0  0
  1  2  1  0  0  0  0
  2  3  1  0  0  0  0
  3  4  1  0  0  0  0
  2  5  1  0  0  0  0
  3  6  1  0  0  0  0
  7  8  4  0  0  0  0
  8  9  4  0  0  0  0
  9 10  4  0  0  0  0
 10 11  4  0  0  0  0
 11 12  4  0  0  0  0
 12  7  4  0  0  0  0
 13 14  4  0  0  0  0
 14 15  4  0  0  0  0
 15 16  4  0  0  0  0
 16 17  4  0  0  0  0
 17 18  4  0  0  0  0
 18 13  4  0  0  0  0
  7  2  1  0  0  0  0
 14  3  1  0  0  0  0
  1 19  1  0  0  0  0
  1 20  1  0  0  0  0
  1 21  1  0  0  0  0
  4 22  1  0  0  0  0
  4 23  1  0  0  0  0
  4 24  1  0  0  0  0
  5 25  1  0  0  0  0
  5 26  1  0  0  0  0
  5 27  1  0  0  0  0
  6 28  1  0  0  0  0
  6 29  1  0  0  0  0
  6 30  1  0  0  0  0
  8 31  1  0  0  0  0
  9 32  1  0  0  0  0
 10 33  1  0  0  0  0
 11 34  1  0  0  0  0
 12 35  1  0  0  0  0
 13 36  1  0  0  0  0
 15 37  1  0  0  0  0
 16 38  1  0  0  0  0
 17 39  1  0  0  0  0
 18 40  1  0  0  0  0
M  END
>  <Alias name>
Dicumyl

>  <deltaH°(c)>   kJ/mol
-10188

$$$$
Lauric acid t-butylperoxyester
  ChemBrain IXL

 51 50  0  0  0  0  0  0  0  0  1 V2000
    8.1030    8.7897    0.6867 C   0  0  0  0  0  0  0  0  0  0  0  0
    7.1568    7.5952    0.4582 C   0  0  0  0  0  0  0  0  0  0  0  0
    5.7567    8.0283    0.6645 C   0  0  0  0  0  0  0  0  0  0  0  0
    5.4648    9.4220    1.0299 O   0  0  0  0  0  0  0  0  0  0  0  0
    4.8449    7.2288    0.5314 O   0  0  0  0  0  0  0  0  0  0  0  0
    9.5608    8.3388    0.4715 C   0  0  0  0  0  0  0  0  0  0  0  0
   10.5070    9.5336    0.6993 C   0  0  0  0  0  0  0  0  0  0  0  0
   11.9648    9.0827    0.4843 C   0  0  0  0  0  0  0  0  0  0  0  0
```

```
 12.9108   10.2777    0.7113 C   0  0  0  0  0  0  0  0  0  0  0  0  0
 14.3686    9.8270    0.4962 C   0  0  0  0  0  0  0  0  0  0  0  0  0
 15.3146   11.0223    0.7223 C   0  0  0  0  0  0  0  0  0  0  0  0  0
 16.7724   10.5716    0.5070 C   0  0  0  0  0  0  0  0  0  0  0  0  0
  4.0299    9.7316    1.2191 O   0  0  0  0  0  0  0  0  0  0  0  0  0
  3.4949   10.7685    0.3250 C   0  0  0  0  0  0  0  0  0  0  0  0  0
  4.2648   12.0849    0.5464 C   0  0  0  0  0  0  0  0  0  0  0  0  0
  3.6583   10.3180   -1.1396 C   0  0  0  0  0  0  0  0  0  0  0  0  0
  2.0008   10.9895    0.6308 C   0  0  0  0  0  0  0  0  0  0  0  0  0
 17.7183   11.7671    0.7326 C   0  0  0  0  0  0  0  0  0  0  0  0  0
 19.1761   11.3164    0.5173 C   0  0  0  0  0  0  0  0  0  0  0  0  0
  7.8625    9.5800   -0.0137 H   0  0  0  0  0  0  0  0  0  0  0  0  0
  7.9842    9.1560    1.6989 H   0  0  0  0  0  0  0  0  0  0  0  0  0
  7.3972    6.8050    1.1588 H   0  0  0  0  0  0  0  0  0  0  0  0  0
  7.2759    7.2287   -0.5539 H   0  0  0  0  0  0  0  0  0  0  0  0  0
  9.8015    7.5489    1.1722 H   0  0  0  0  0  0  0  0  0  0  0  0  0
  9.6796    7.9722   -0.5406 H   0  0  0  0  0  0  0  0  0  0  0  0  0
 10.2663   10.3234   -0.0015 H   0  0  0  0  0  0  0  0  0  0  0  0  0
 10.3881    9.9004    1.7114 H   0  0  0  0  0  0  0  0  0  0  0  0  0
 12.2057    8.2933    1.1856 H   0  0  0  0  0  0  0  0  0  0  0  0  0
 12.0835    8.7153   -0.5275 H   0  0  0  0  0  0  0  0  0  0  0  0  0
 12.6699   11.0672    0.0101 H   0  0  0  0  0  0  0  0  0  0  0  0  0
 12.7921   10.6450    1.7232 H   0  0  0  0  0  0  0  0  0  0  0  0  0
 14.6098    9.0380    1.1978 H   0  0  0  0  0  0  0  0  0  0  0  0  0
 14.4872    9.4591   -0.5155 H   0  0  0  0  0  0  0  0  0  0  0  0  0
 15.0733   11.8113    0.0208 H   0  0  0  0  0  0  0  0  0  0  0  0  0
 15.1961   11.3900    1.7340 H   0  0  0  0  0  0  0  0  0  0  0  0  0
 17.0138    9.7828    1.2087 H   0  0  0  0  0  0  0  0  0  0  0  0  0
 16.8908   10.2035   -0.5046 H   0  0  0  0  0  0  0  0  0  0  0  0  0
  3.8717   12.8484   -0.1135 H   0  0  0  0  0  0  0  0  0  0  0  0  0
  5.3151   11.9292    0.3330 H   0  0  0  0  0  0  0  0  0  0  0  0  0
  4.1486   12.4023    1.5753 H   0  0  0  0  0  0  0  0  0  0  0  0  0
  4.7082   10.1611   -1.3541 H   0  0  0  0  0  0  0  0  0  0  0  0  0
  3.2656   11.0826   -1.7985 H   0  0  0  0  0  0  0  0  0  0  0  0  0
  3.1159    9.3937   -1.2957 H   0  0  0  0  0  0  0  0  0  0  0  0  0
  1.4598   10.0642    0.4754 H   0  0  0  0  0  0  0  0  0  0  0  0  0
  1.8860   11.3063    1.6600 H   0  0  0  0  0  0  0  0  0  0  0  0  0
  1.6066   11.7532   -0.0281 H   0  0  0  0  0  0  0  0  0  0  0  0  0
 17.4768   12.5559    0.0309 H   0  0  0  0  0  0  0  0  0  0  0  0  0
 17.5999   12.1351    1.7443 H   0  0  0  0  0  0  0  0  0  0  0  0  0
 19.8408   12.1566    0.6757 H   0  0  0  0  0  0  0  0  0  0  0  0  0
 19.2944   10.9482   -0.4943 H   0  0  0  0  0  0  0  0  0  0  0  0  0
 19.4175   10.5277    1.2191 H   0  0  0  0  0  0  0  0  0  0  0  0  0
  1  2  1  0  0  0  0
  2  3  1  0  0  0  0
  3  4  1  0  0  0  0
  3  5  2  0  0  0  0
  1  6  1  0  0  0  0
  6  7  1  0  0  0  0
  7  8  1  0  0  0  0
  8  9  1  0  0  0  0
  9 10  1  0  0  0  0
 10 11  1  0  0  0  0
 11 12  1  0  0  0  0
  4 13  1  0  0  0  0
 13 14  1  0  0  0  0
 14 15  1  0  0  0  0
 14 16  1  0  0  0  0
 14 17  1  0  0  0  0
 12 18  1  0  0  0  0
 18 19  1  0  0  0  0
  1 20  1  0  0  0  0
  1 21  1  0  0  0  0
```

```
  2 22  1  0  0  0  0
  2 23  1  0  0  0  0
  6 24  1  0  0  0  0
  6 25  1  0  0  0  0
  7 26  1  0  0  0  0
  7 27  1  0  0  0  0
  8 28  1  0  0  0  0
  8 29  1  0  0  0  0
  9 30  1  0  0  0  0
  9 31  1  0  0  0  0
 10 32  1  0  0  0  0
 10 33  1  0  0  0  0
 11 34  1  0  0  0  0
 11 35  1  0  0  0  0
 12 36  1  0  0  0  0
 12 37  1  0  0  0  0
 15 38  1  0  0  0  0
 15 39  1  0  0  0  0
 15 40  1  0  0  0  0
 16 41  1  0  0  0  0
 16 42  1  0  0  0  0
 16 43  1  0  0  0  0
 17 44  1  0  0  0  0
 17 45  1  0  0  0  0
 17 46  1  0  0  0  0
 18 47  1  0  0  0  0
 18 48  1  0  0  0  0
 19 49  1  0  0  0  0
 19 50  1  0  0  0  0
 19 51  1  0  0  0  0
M  END
>  <Alias name>
t-Butyl peroxydodecanoate

>  <deltaH°(c)>   kJ/mol
-10138

$$$$
N-Benzyl-pivalophenone imine
  ChemBrain IXL

 40 41  0  0  0  0  0  0  0  0  1 V2000
    4.6885    7.0327    0.4137 C   0  0  0  0  0  0  0  0  0  0  0  0
    5.9149    6.3079    0.0126 C   0  0  0  0  0  0  0  0  0  0  0  0
    4.4784    8.3417   -0.0204 C   0  0  0  0  0  0  0  0  0  0  0  0
    3.7425    6.4069    1.2258 C   0  0  0  0  0  0  0  0  0  0  0  0
    7.0252    6.9219   -0.0449 N   0  0  0  0  0  0  0  0  0  0  0  0
    3.3165    9.0205    0.3473 C   0  0  0  0  0  0  0  0  0  0  0  0
    2.5804    7.0855    1.5933 C   0  0  0  0  0  0  0  0  0  0  0  0
    9.3648    7.0528    0.1566 C   0  0  0  0  0  0  0  0  0  0  0  0
    2.3671    8.3921    1.1534 C   0  0  0  0  0  0  0  0  0  0  0  0
   10.6623    6.5472    0.0724 C   0  0  0  0  0  0  0  0  0  0  0  0
    9.1524    8.3586    0.5991 C   0  0  0  0  0  0  0  0  0  0  0  0
   11.7471    7.3467    0.4329 C   0  0  0  0  0  0  0  0  0  0  0  0
   10.2373    9.1580    0.9597 C   0  0  0  0  0  0  0  0  0  0  0  0
   11.5346    8.6520    0.8766 C   0  0  0  0  0  0  0  0  0  0  0  0
    8.2137    6.2038   -0.2236 C   0  0  0  0  0  0  0  0  0  0  0  0
    5.8476    4.8677   -0.3214 C   0  0  0  0  0  0  0  0  0  0  0  0
    4.4220    4.5147   -0.7882 C   0  0  0  0  0  0  0  0  0  0  0  0
    6.2009    4.0341    0.9255 C   0  0  0  0  0  0  0  0  0  0  0  0
    6.8497    4.5546   -1.4495 C   0  0  0  0  0  0  0  0  0  0  0  0
    5.2173    8.8309   -0.6429 H   0  0  0  0  0  0  0  0  0  0  0  0
    3.9103    5.3942    1.5711 H   0  0  0  0  0  0  0  0  0  0  0  0
```

```
    3.1515   10.0353    0.0070 H   0  0  0  0  0  0  0  0  0  0  0  0
    1.8439    6.5981    2.2199 H   0  0  0  0  0  0  0  0  0  0  0  0
    1.4644    8.9186    1.4378 H   0  0  0  0  0  0  0  0  0  0  0  0
   10.8273    5.5341   -0.2728 H   0  0  0  0  0  0  0  0  0  0  0  0
    8.1453    8.7517    0.6628 H   0  0  0  0  0  0  0  0  0  0  0  0
   12.7543    6.9539    0.3682 H   0  0  0  0  0  0  0  0  0  0  0  0
   10.0723   10.1715    1.3041 H   0  0  0  0  0  0  0  0  0  0  0  0
   12.3769    9.2726    1.1567 H   0  0  0  0  0  0  0  0  0  0  0  0
    8.1973    5.3179    0.3991 H   0  0  0  0  0  0  0  0  0  0  0  0
    8.3071    5.9131   -1.2626 H   0  0  0  0  0  0  0  0  0  0  0  0
    4.1449    5.1542   -1.6171 H   0  0  0  0  0  0  0  0  0  0  0  0
    3.7275    4.6624    0.0296 H   0  0  0  0  0  0  0  0  0  0  0  0
    4.3918    3.4795   -1.1050 H   0  0  0  0  0  0  0  0  0  0  0  0
    6.1415    2.9800    0.6839 H   0  0  0  0  0  0  0  0  0  0  0  0
    7.2067    4.2744    1.2473 H   0  0  0  0  0  0  0  0  0  0  0  0
    5.5030    4.2619    1.7217 H   0  0  0  0  0  0  0  0  0  0  0  0
    6.6296    3.5801   -1.8677 H   0  0  0  0  0  0  0  0  0  0  0  0
    6.7677    5.3069   -2.2242 H   0  0  0  0  0  0  0  0  0  0  0  0
    7.8560    4.5567   -1.0493 H   0  0  0  0  0  0  0  0  0  0  0  0
  1  2  1  0  0  0  0
  1  3  4  0  0  0  0
  1  4  4  0  0  0  0
  2  5  2  0  0  0  0
  3  6  4  0  0  0  0
  4  7  4  0  0  0  0
  6  9  4  0  0  0  0
  7  9  4  0  0  0  0
  8 10  4  0  0  0  0
  8 11  4  0  0  0  0
 10 12  4  0  0  0  0
 11 13  4  0  0  0  0
 12 14  4  0  0  0  0
 13 14  4  0  0  0  0
  5 15  1  0  0  0  0
  8 15  1  0  0  0  0
  2 16  1  0  0  0  0
 16 17  1  0  0  0  0
 16 18  1  0  0  0  0
 16 19  1  0  0  0  0
  3 20  1  0  0  0  0
  4 21  1  0  0  0  0
  6 22  1  0  0  0  0
  7 23  1  0  0  0  0
  9 24  1  0  0  0  0
 10 25  1  0  0  0  0
 11 26  1  0  0  0  0
 12 27  1  0  0  0  0
 13 28  1  0  0  0  0
 14 29  1  0  0  0  0
 15 30  1  0  0  0  0
 15 31  1  0  0  0  0
 17 32  1  0  0  0  0
 17 33  1  0  0  0  0
 17 34  1  0  0  0  0
 18 35  1  0  0  0  0
 18 36  1  0  0  0  0
 18 37  1  0  0  0  0
 19 38  1  0  0  0  0
 19 39  1  0  0  0  0
 19 40  1  0  0  0  0
M  END
>  <Alias name>
N-Benzyl-pivalophenone imine
```

```
>  <deltaH°(c)>   kJ/mol
-10131

$$$$
Tritylazide
  ChemBrain IXL

 37 39  0  0  0  0  0  0  0  0  1 V2000
    7.5604    5.8519   -0.6834 C   0  0  0  0  0  0  0  0  0  0  0  0
    8.3317    5.7292   -1.8453 N   0  0  0  0  0  0  0  0  0  0  0  0
    6.1317    5.6573   -1.0169 C   0  0  0  0  0  0  0  0  0  0  0  0
    7.7526    7.2000   -0.1038 C   0  0  0  0  0  0  0  0  0  0  0  0
    7.9817    4.8280    0.2987 C   0  0  0  0  0  0  0  0  0  0  0  0
    9.5853    5.3578   -1.7632 N   0  3  0  0  0  0  0  0  0  0  0  0
   10.8580    4.9807   -1.6798 N   0  5  0  0  0  0  0  0  0  0  0  0
    5.2696    5.0819   -0.0832 C   0  0  0  0  0  0  0  0  0  0  0  0
    3.9235    4.8959   -0.3986 C   0  0  0  0  0  0  0  0  0  0  0  0
    5.6471    6.0492   -2.2649 C   0  0  0  0  0  0  0  0  0  0  0  0
    4.3010    5.8632   -2.5803 C   0  0  0  0  0  0  0  0  0  0  0  0
    3.4392    5.2864   -1.6472 C   0  0  0  0  0  0  0  0  0  0  0  0
    6.6768    7.8668    0.4828 C   0  0  0  0  0  0  0  0  0  0  0  0
    6.8569    9.1393    1.0253 C   0  0  0  0  0  0  0  0  0  0  0  0
    9.0094    7.8039   -0.1441 C   0  0  0  0  0  0  0  0  0  0  0  0
    9.1895    9.0765    0.3983 C   0  0  0  0  0  0  0  0  0  0  0  0
    8.1132    9.7443    0.9828 C   0  0  0  0  0  0  0  0  0  0  0  0
    8.3015    5.2032    1.6037 C   0  0  0  0  0  0  0  0  0  0  0  0
    8.6971    4.2379    2.5299 C   0  0  0  0  0  0  0  0  0  0  0  0
    8.0589    3.4877   -0.0805 C   0  0  0  0  0  0  0  0  0  0  0  0
    8.4545    2.5225    0.8457 C   0  0  0  0  0  0  0  0  0  0  0  0
    8.7735    2.8976    2.1510 C   0  0  0  0  0  0  0  0  0  0  0  0
    5.6453    4.7797    0.8865 H   0  0  0  0  0  0  0  0  0  0  0  0
    3.2544    4.4483    0.3259 H   0  0  0  0  0  0  0  0  0  0  0  0
    6.3159    6.4979   -2.9890 H   0  0  0  0  0  0  0  0  0  0  0  0
    3.9250    6.1666   -3.5496 H   0  0  0  0  0  0  0  0  0  0  0  0
    2.3942    5.1417   -1.8922 H   0  0  0  0  0  0  0  0  0  0  0  0
    5.7018    7.3965    0.5172 H   0  0  0  0  0  0  0  0  0  0  0  0
    6.0214    9.6576    1.4794 H   0  0  0  0  0  0  0  0  0  0  0  0
    9.8453    7.2848   -0.5965 H   0  0  0  0  0  0  0  0  0  0  0  0
   10.1649    9.5459    0.3657 H   0  0  0  0  0  0  0  0  0  0  0  0
    8.2529   10.7323    1.4036 H   0  0  0  0  0  0  0  0  0  0  0  0
    8.2428    6.2439    1.8978 H   0  0  0  0  0  0  0  0  0  0  0  0
    8.9449    4.5292    3.5432 H   0  0  0  0  0  0  0  0  0  0  0  0
    7.8118    3.1966   -1.0939 H   0  0  0  0  0  0  0  0  0  0  0  0
    8.5139    1.4819    0.5515 H   0  0  0  0  0  0  0  0  0  0  0  0
    9.0804    2.1482    2.8700 H   0  0  0  0  0  0  0  0  0  0  0  0
  1  2  1  0  0  0  0
  1  3  1  0  0  0  0
  1  4  1  0  0  0  0
  1  5  1  0  0  0  0
  2  6  2  0  0  0  0
  3  8  4  0  0  0  0
 10  3  4  0  0  0  0
  4 13  4  0  0  0  0
 15  4  4  0  0  0  0
  5 18  4  0  0  0  0
 20  5  4  0  0  0  0
  6  7  2  0  0  0  0
  8  9  4  0  0  0  0
  9 12  4  0  0  0  0
 11 10  4  0  0  0  0
 12 11  4  0  0  0  0
 13 14  4  0  0  0  0
```

```
 14 17  4  0  0  0  0
 16 15  4  0  0  0  0
 17 16  4  0  0  0  0
 18 19  4  0  0  0  0
 19 22  4  0  0  0  0
 21 20  4  0  0  0  0
 22 21  4  0  0  0  0
  8 23  1  0  0  0  0
  9 24  1  0  0  0  0
 10 25  1  0  0  0  0
 11 26  1  0  0  0  0
 12 27  1  0  0  0  0
 13 28  1  0  0  0  0
 14 29  1  0  0  0  0
 15 30  1  0  0  0  0
 16 31  1  0  0  0  0
 17 32  1  0  0  0  0
 18 33  1  0  0  0  0
 19 34  1  0  0  0  0
 20 35  1  0  0  0  0
 21 36  1  0  0  0  0
 22 37  1  0  0  0  0
M  END
>  <Alias name>
Tritylazide

>  <deltaH°(c)>   kJ/mol
-10113.04

$$$$
1-Pentadecanoic acid methylester
  ChemBrain IXL

 50 49  0  0  0  0  0  0  0  0  1 V2000
   16.9902   10.9850   -1.2450 C   0  0  0  0  0  0  0  0  0  0  0  0
   18.1324   11.9858   -1.5004 C   0  0  0  0  0  0  0  0  0  0  0  0
   19.3835   11.2457   -1.7777 C   0  0  0  0  0  0  0  0  0  0  0  0
   19.3284    9.9149   -2.4069 O   0  0  0  0  0  0  0  0  0  0  0  0
   15.6908   11.7541   -0.9411 C   0  0  0  0  0  0  0  0  0  0  0  0
   14.5515   10.7506   -0.6837 C   0  0  0  0  0  0  0  0  0  0  0  0
   13.2545   11.5154   -0.3596 C   0  0  0  0  0  0  0  0  0  0  0  0
   12.1199   10.5077   -0.0955 C   0  0  0  0  0  0  0  0  0  0  0  0
   10.8256   11.2677    0.2503 C   0  0  0  0  0  0  0  0  0  0  0  0
    9.6962   10.2561    0.5229 C   0  0  0  0  0  0  0  0  0  0  0  0
    8.4045   11.0118    0.8877 C   0  0  0  0  0  0  0  0  0  0  0  0
    7.2794    9.9969    1.1674 C   0  0  0  0  0  0  0  0  0  0  0  0
    5.9896   10.7497    1.5457 C   0  0  0  0  0  0  0  0  0  0  0  0
    4.8670    9.7329    1.8294 C   0  0  0  0  0  0  0  0  0  0  0  0
    3.5779   10.4842    2.2143 C   0  0  0  0  0  0  0  0  0  0  0  0
    2.4561    9.4668    2.4988 C   0  0  0  0  0  0  0  0  0  0  0  0
   20.4602   11.7398   -1.4840 O   0  0  0  0  0  0  0  0  0  0  0  0
   20.6339    9.2739   -2.6317 C   0  0  0  0  0  0  0  0  0  0  0  0
   17.2433   10.3568   -0.3999 H   0  0  0  0  0  0  0  0  0  0  0  0
   16.8476   10.3679   -2.1235 H   0  0  0  0  0  0  0  0  0  0  0  0
   17.8842   12.6077   -2.3517 H   0  0  0  0  0  0  0  0  0  0  0  0
   18.2685   12.6092   -0.6253 H   0  0  0  0  0  0  0  0  0  0  0  0
   15.4358   12.3817   -1.7861 H   0  0  0  0  0  0  0  0  0  0  0  0
   15.8337   12.3712   -0.0626 H   0  0  0  0  0  0  0  0  0  0  0  0
   14.8135   10.1148    0.1530 H   0  0  0  0  0  0  0  0  0  0  0  0
   14.4007   10.1414   -1.5663 H   0  0  0  0  0  0  0  0  0  0  0  0
   12.9886   12.1483   -1.1972 H   0  0  0  0  0  0  0  0  0  0  0  0
   13.4067   12.1268    0.5213 H   0  0  0  0  0  0  0  0  0  0  0  0
   12.3933    9.8664    0.7332 H   0  0  0  0  0  0  0  0  0  0  0  0
```

```
 11.9593    9.9047   -0.9806 H   0  0  0  0  0  0  0  0  0  0  0  0
 10.5476   11.9053   -0.5798 H   0  0  0  0  0  0  0  0  0  0  0  0
 10.9880   11.8739    1.1329 H   0  0  0  0  0  0  0  0  0  0  0  0
  9.9808    9.6112    1.3451 H   0  0  0  0  0  0  0  0  0  0  0  0
  9.5263    9.6572   -0.3633 H   0  0  0  0  0  0  0  0  0  0  0  0
  8.1162   11.6535    0.0643 H   0  0  0  0  0  0  0  0  0  0  0  0
  8.5759   11.6134    1.7718 H   0  0  0  0  0  0  0  0  0  0  0  0
  7.5724    9.3501    1.9852 H   0  0  0  0  0  0  0  0  0  0  0  0
  7.1027    9.4004    0.2810 H   0  0  0  0  0  0  0  0  0  0  0  0
  5.6945   11.3947    0.7273 H   0  0  0  0  0  0  0  0  0  0  0  0
  6.1672   11.3477    2.4310 H   0  0  0  0  0  0  0  0  0  0  0  0
  5.1644    9.0853    2.6449 H   0  0  0  0  0  0  0  0  0  0  0  0
  4.6869    9.1374    0.9429 H   0  0  0  0  0  0  0  0  0  0  0  0
  3.2800   11.1314    1.3987 H   0  0  0  0  0  0  0  0  0  0  0  0
  3.7583   11.0799    3.1005 H   0  0  0  0  0  0  0  0  0  0  0  0
  2.7543    8.8192    3.3141 H   0  0  0  0  0  0  0  0  0  0  0  0
  1.5504    9.9947    2.7700 H   0  0  0  0  0  0  0  0  0  0  0  0
  2.2754    8.8714    1.6125 H   0  0  0  0  0  0  0  0  0  0  0  0
 21.2345    9.8968   -3.2831 H   0  0  0  0  0  0  0  0  0  0  0  0
 20.4859    8.3058   -3.0937 H   0  0  0  0  0  0  0  0  0  0  0  0
 21.1422    9.1476   -1.6836 H   0  0  0  0  0  0  0  0  0  0  0  0
  1  2  1  0  0  0  0
  2  3  1  0  0  0  0
  3  4  1  0  0  0  0
  1  5  1  0  0  0  0
  5  6  1  0  0  0  0
  6  7  1  0  0  0  0
  7  8  1  0  0  0  0
  8  9  1  0  0  0  0
  9 10  1  0  0  0  0
 10 11  1  0  0  0  0
 11 12  1  0  0  0  0
 12 13  1  0  0  0  0
 13 14  1  0  0  0  0
 14 15  1  0  0  0  0
 15 16  1  0  0  0  0
  3 17  2  0  0  0  0
  4 18  1  0  0  0  0
  1 19  1  0  0  0  0
  1 20  1  0  0  0  0
  2 21  1  0  0  0  0
  2 22  1  0  0  0  0
  5 23  1  0  0  0  0
  5 24  1  0  0  0  0
  6 25  1  0  0  0  0
  6 26  1  0  0  0  0
  7 27  1  0  0  0  0
  7 28  1  0  0  0  0
  8 29  1  0  0  0  0
  8 30  1  0  0  0  0
  9 31  1  0  0  0  0
  9 32  1  0  0  0  0
 10 33  1  0  0  0  0
 10 34  1  0  0  0  0
 11 35  1  0  0  0  0
 11 36  1  0  0  0  0
 12 37  1  0  0  0  0
 12 38  1  0  0  0  0
 13 39  1  0  0  0  0
 13 40  1  0  0  0  0
 14 41  1  0  0  0  0
 14 42  1  0  0  0  0
 15 43  1  0  0  0  0
```

```
 15 44  1  0  0  0  0
 16 45  1  0  0  0  0
 16 46  1  0  0  0  0
 16 47  1  0  0  0  0
 18 48  1  0  0  0  0
 18 49  1  0  0  0  0
 18 50  1  0  0  0  0
M  END
>  <Alias name>
Methyl pentadecanoate

>  <deltaH°(c)>   kJ/mol
-10105

$$$$
3,5,3',5'-Tetramethyl-4,4'-diethyldipyrrylmethene
  ChemBrain IXL

 43 44  0  0  0  0  0  0  0  0  1 V2000
    8.8421    9.0445    0.7798 C   0  0  0  0  0  0  0  0  0  0  0  0  0
   10.0345    9.7474    0.6507 N   0  0  0  0  0  0  0  0  0  0  0  0  0
    7.6095    9.5488    0.6398 C   0  0  0  0  0  0  0  0  0  0  0  0  0
    9.2042    7.6606    1.0941 C   0  0  0  0  0  0  0  0  0  0  0  0  0
    6.4374    8.6550    0.4847 C   0  0  0  0  0  0  0  0  0  0  0  0  0
    6.4187    7.4120   -0.1702 N   0  0  0  0  0  0  0  0  0  0  0  0  0
    5.2084    8.8926    0.9476 C   0  0  0  0  0  0  0  0  0  0  0  0  0
   12.7524    6.4842    0.6676 C   0  0  0  0  0  0  0  0  0  0  0  0  0
   11.3974    6.3521    1.3933 C   0  0  0  0  0  0  0  0  0  0  0  0  0
    2.4883    6.0888    0.9481 C   0  0  0  0  0  0  0  0  0  0  0  0  0
    2.8597    7.5845    0.9142 C   0  0  0  0  0  0  0  0  0  0  0  0  0
   10.5389    7.6073    1.1214 C   0  0  0  0  0  0  0  0  0  0  0  0  0
    8.2376    6.4881    1.3491 C   0  0  0  0  0  0  0  0  0  0  0  0  0
    4.3612    7.7427    0.5841 C   0  0  0  0  0  0  0  0  0  0  0  0  0
    4.7532   10.1447    1.7224 C   0  0  0  0  0  0  0  0  0  0  0  0  0
    5.1235    6.8710   -0.0790 C   0  0  0  0  0  0  0  0  0  0  0  0  0
    4.6683    5.5105   -0.6423 C   0  0  0  0  0  0  0  0  0  0  0  0  0
   11.0494    8.9865    0.8369 C   0  0  0  0  0  0  0  0  0  0  0  0  0
   12.5288    9.4171    0.7797 C   0  0  0  0  0  0  0  0  0  0  0  0  0
    7.2008    6.9862   -0.6444 H   0  0  0  0  0  0  0  0  0  0  0  0  0
    7.4674   10.6222    0.6398 H   0  0  0  0  0  0  0  0  0  0  0  0  0
   13.3002    7.3247    1.0752 H   0  0  0  0  0  0  0  0  0  0  0  0  0
   12.5820    6.6429   -0.3899 H   0  0  0  0  0  0  0  0  0  0  0  0  0
   13.3272    5.5764    0.8083 H   0  0  0  0  0  0  0  0  0  0  0  0  0
   10.8781    5.4742    1.0295 H   0  0  0  0  0  0  0  0  0  0  0  0  0
   11.5672    6.2549    2.4585 H   0  0  0  0  0  0  0  0  0  0  0  0  0
    3.1337    5.5718    1.6471 H   0  0  0  0  0  0  0  0  0  0  0  0  0
    1.4565    5.9806    1.2615 H   0  0  0  0  0  0  0  0  0  0  0  0  0
    2.6115    5.6636   -0.0398 H   0  0  0  0  0  0  0  0  0  0  0  0  0
    2.2695    8.0852    0.1570 H   0  0  0  0  0  0  0  0  0  0  0  0  0
    2.6552    8.0278    1.8815 H   0  0  0  0  0  0  0  0  0  0  0  0  0
    8.5858    5.9119    2.1981 H   0  0  0  0  0  0  0  0  0  0  0  0  0
    7.2479    6.8739    1.5562 H   0  0  0  0  0  0  0  0  0  0  0  0  0
    8.2018    5.8528    0.4725 H   0  0  0  0  0  0  0  0  0  0  0  0  0
    3.6774   10.1203    1.8474 H   0  0  0  0  0  0  0  0  0  0  0  0  0
    5.2292   10.1606    2.6950 H   0  0  0  0  0  0  0  0  0  0  0  0  0
    5.0321   11.0328    1.1693 H   0  0  0  0  0  0  0  0  0  0  0  0  0
    5.5010    5.0297   -1.1410 H   0  0  0  0  0  0  0  0  0  0  0  0  0
    4.3212    4.8816    0.1682 H   0  0  0  0  0  0  0  0  0  0  0  0  0
    3.8636    5.6640   -1.3504 H   0  0  0  0  0  0  0  0  0  0  0  0  0
   12.5874   10.4826    0.5928 H   0  0  0  0  0  0  0  0  0  0  0  0  0
   13.0299    8.8829   -0.0179 H   0  0  0  0  0  0  0  0  0  0  0  0  0
   13.0081    9.1895    1.7237 H   0  0  0  0  0  0  0  0  0  0  0  0  0
  2  1  1  0  0  0  0
```

```
  1  3  2  0  0  0  0
  1  4  1  0  0  0  0
 18  2  2  0  0  0  0
  3  5  1  0  0  0  0
  4 12  2  0  0  0  0
  4 13  1  0  0  0  0
  5  6  1  0  0  0  0
  7  5  2  0  0  0  0
  6 16  1  0  0  0  0
 14  7  1  0  0  0  0
  7 15  1  0  0  0  0
  8  9  1  0  0  0  0
  9 12  1  0  0  0  0
 10 11  1  0  0  0  0
 11 14  1  0  0  0  0
 12 18  1  0  0  0  0
 16 14  2  0  0  0  0
 16 17  1  0  0  0  0
 18 19  1  0  0  0  0
  6 20  1  0  0  0  0
  3 21  1  0  0  0  0
  8 22  1  0  0  0  0
  8 23  1  0  0  0  0
  8 24  1  0  0  0  0
  9 25  1  0  0  0  0
  9 26  1  0  0  0  0
 10 27  1  0  0  0  0
 10 28  1  0  0  0  0
 10 29  1  0  0  0  0
 11 30  1  0  0  0  0
 11 31  1  0  0  0  0
 13 32  1  0  0  0  0
 13 33  1  0  0  0  0
 13 34  1  0  0  0  0
 15 35  1  0  0  0  0
 15 36  1  0  0  0  0
 15 37  1  0  0  0  0
 17 38  1  0  0  0  0
 17 39  1  0  0  0  0
 17 40  1  0  0  0  0
 19 41  1  0  0  0  0
 19 42  1  0  0  0  0
 19 43  1  0  0  0  0
M  END
>  <Alias name>
3,5,3',5'-Tetramethyl-4,4'-diethyldipyrrylmethene

>  <deltaH°(c)>   kJ/mol
-10092

$$$$
Diisobutyl azelate
  ChemBrain IXL

 53 52  0  0  0  0  0  0  0  0  1 V2000
   13.8207    7.1787    0.4025 C   0  0  0  0  0  0  0  0  0  0  0  0
   15.2842    7.0628    0.3235 O   0  0  0  0  0  0  0  0  0  0  0  0
   13.1758    8.5059    0.2859 C   0  0  0  0  0  0  0  0  0  0  0  0
   13.1377    6.1808    0.5643 O   0  0  0  0  0  0  0  0  0  0  0  0
   15.9989    8.3335    0.1347 C   0  0  0  0  0  0  0  0  0  0  0  0
   11.6441    8.3332    0.2614 C   0  0  0  0  0  0  0  0  0  0  0  0
   17.5155    8.0662    0.0761 C   0  0  0  0  0  0  0  0  0  0  0  0
   10.9732    9.7166    0.1522 C   0  0  0  0  0  0  0  0  0  0  0  0
```

```
   18.2653    9.3994   -0.1112 C   0  0  0  0  0  0  0  0  0  0  0  0
   17.8270    7.1310   -1.1086 C   0  0  0  0  0  0  0  0  0  0  0  0
    9.4410    9.5466    0.1386 C   0  0  0  0  0  0  0  0  0  0  0  0
    8.7722   10.9322    0.0430 C   0  0  0  0  0  0  0  0  0  0  0  0
    7.2395   10.7660    0.0416 C   0  0  0  0  0  0  0  0  0  0  0  0
    6.5734   12.1536   -0.0453 C   0  0  0  0  0  0  0  0  0  0  0  0
    5.1011   11.9975   -0.0381 C   0  0  0  0  0  0  0  0  0  0  0  0
    4.5034   10.6540   -0.0196 O   0  0  0  0  0  0  0  0  0  0  0  0
    4.3833   12.9840   -0.0476 O   0  0  0  0  0  0  0  0  0  0  0  0
    3.0331   10.6283   -0.0150 C   0  0  0  0  0  0  0  0  0  0  0  0
    2.5443    9.1666    0.0051 C   0  0  0  0  0  0  0  0  0  0  0  0
    3.0734    8.4653    1.2711 C   0  0  0  0  0  0  0  0  0  0  0  0
    3.0669    8.4328   -1.2451 C   0  0  0  0  0  0  0  0  0  0  0  0
   13.4563    9.1192    1.1332 H   0  0  0  0  0  0  0  0  0  0  0  0
   13.4996    8.9853   -0.6296 H   0  0  0  0  0  0  0  0  0  0  0  0
   15.6776    8.7943   -0.7912 H   0  0  0  0  0  0  0  0  0  0  0  0
   15.7811    8.9967    0.9627 H   0  0  0  0  0  0  0  0  0  0  0  0
   11.3626    7.7271   -0.5908 H   0  0  0  0  0  0  0  0  0  0  0  0
   11.3210    7.8463    1.1733 H   0  0  0  0  0  0  0  0  0  0  0  0
   17.8356    7.5985    0.9990 H   0  0  0  0  0  0  0  0  0  0  0  0
   11.2612   10.3253    1.0004 H   0  0  0  0  0  0  0  0  0  0  0  0
   11.2907   10.2002   -0.7634 H   0  0  0  0  0  0  0  0  0  0  0  0
   18.0473   10.0562    0.7219 H   0  0  0  0  0  0  0  0  0  0  0  0
   17.9443    9.8676   -1.0335 H   0  0  0  0  0  0  0  0  0  0  0  0
   19.3310    9.2115   -0.1535 H   0  0  0  0  0  0  0  0  0  0  0  0
   18.8928    6.9432   -1.1497 H   0  0  0  0  0  0  0  0  0  0  0  0
   17.5069    7.5986   -2.0315 H   0  0  0  0  0  0  0  0  0  0  0  0
   17.3001    6.1940   -0.9770 H   0  0  0  0  0  0  0  0  0  0  0  0
    9.1518    8.9462   -0.7151 H   0  0  0  0  0  0  0  0  0  0  0  0
    9.1242    9.0548    1.0500 H   0  0  0  0  0  0  0  0  0  0  0  0
    9.0689   11.5353    0.8921 H   0  0  0  0  0  0  0  0  0  0  0  0
    9.0829   11.4203   -0.8725 H   0  0  0  0  0  0  0  0  0  0  0  0
    6.9418   10.1683   -0.8111 H   0  0  0  0  0  0  0  0  0  0  0  0
    6.9289   10.2728    0.9544 H   0  0  0  0  0  0  0  0  0  0  0  0
    6.8768   12.7532    0.8040 H   0  0  0  0  0  0  0  0  0  0  0  0
    6.8797   12.6441   -0.9610 H   0  0  0  0  0  0  0  0  0  0  0  0
    2.6607   11.1211   -0.9046 H   0  0  0  0  0  0  0  0  0  0  0  0
    2.6658   11.1438    0.8637 H   0  0  0  0  0  0  0  0  0  0  0  0
    1.4614    9.1482    0.0082 H   0  0  0  0  0  0  0  0  0  0  0  0
    4.1562    8.4832    1.2680 H   0  0  0  0  0  0  0  0  0  0  0  0
    2.7294    7.4385    1.2855 H   0  0  0  0  0  0  0  0  0  0  0  0
    2.7064    8.9812    2.1498 H   0  0  0  0  0  0  0  0  0  0  0  0
    4.1497    8.4507   -1.2480 H   0  0  0  0  0  0  0  0  0  0  0  0
    2.6953    8.9259   -2.1348 H   0  0  0  0  0  0  0  0  0  0  0  0
    2.7228    7.4060   -1.2311 H   0  0  0  0  0  0  0  0  0  0  0  0
  1  2  1  0  0  0  0
  1  3  1  0  0  0  0
  1  4  2  0  0  0  0
  2  5  1  0  0  0  0
  3  6  1  0  0  0  0
  5  7  1  0  0  0  0
  6  8  1  0  0  0  0
  7  9  1  0  0  0  0
  7 10  1  0  0  0  0
  8 11  1  0  0  0  0
 11 12  1  0  0  0  0
 12 13  1  0  0  0  0
 13 14  1  0  0  0  0
 14 15  1  0  0  0  0
 15 16  1  0  0  0  0
 15 17  2  0  0  0  0
 16 18  1  0  0  0  0
 18 19  1  0  0  0  0
```

```
 19 20  1  0  0  0  0
 19 21  1  0  0  0  0
  3 22  1  0  0  0  0
  3 23  1  0  0  0  0
  5 24  1  0  0  0  0
  5 25  1  0  0  0  0
  6 26  1  0  0  0  0
  6 27  1  0  0  0  0
  7 28  1  0  0  0  0
  8 29  1  0  0  0  0
  8 30  1  0  0  0  0
  9 31  1  0  0  0  0
  9 32  1  0  0  0  0
  9 33  1  0  0  0  0
 10 34  1  0  0  0  0
 10 35  1  0  0  0  0
 10 36  1  0  0  0  0
 11 37  1  0  0  0  0
 11 38  1  0  0  0  0
 12 39  1  0  0  0  0
 12 40  1  0  0  0  0
 13 41  1  0  0  0  0
 13 42  1  0  0  0  0
 14 43  1  0  0  0  0
 14 44  1  0  0  0  0
 18 45  1  0  0  0  0
 18 46  1  0  0  0  0
 19 47  1  0  0  0  0
 20 48  1  0  0  0  0
 20 49  1  0  0  0  0
 20 50  1  0  0  0  0
 21 51  1  0  0  0  0
 21 52  1  0  0  0  0
 21 53  1  0  0  0  0
M  END
>  <Alias name>
Diisobutyl azelate

>  <deltaH°(c)>   kJ/mol
-10092

$$$$
Benzyldiphenylamine
  ChemBrain IXL

 37 39  0  0  0  0  0  0  0  0  1 V2000
    2.7925    9.0980   -0.8558 C   0  0  0  0  0  0  0  0  0  0  0  0
    2.5000    8.5422    0.3905 C   0  0  0  0  0  0  0  0  0  0  0  0
    3.5346    8.1186    1.2257 C   0  0  0  0  0  0  0  0  0  0  0  0
    4.8619    8.2506    0.8146 C   0  0  0  0  0  0  0  0  0  0  0  0
    5.1553    8.8065   -0.4324 C   0  0  0  0  0  0  0  0  0  0  0  0
    4.1197    9.2301   -1.2672 C   0  0  0  0  0  0  0  0  0  0  0  0
    6.5659    8.9459   -0.8703 C   0  0  0  0  0  0  0  0  0  0  0  0
    7.2926    7.6842   -0.6115 N   0  0  0  0  0  0  0  0  0  0  0  0
    8.6819    7.7023   -0.3667 C   0  0  0  0  0  0  0  0  0  0  0  0
    6.5957    6.4636   -0.6121 C   0  0  0  0  0  0  0  0  0  0  0  0
    5.5036    6.2814   -1.4636 C   0  0  0  0  0  0  0  0  0  0  0  0
    4.8114    5.0700   -1.4630 C   0  0  0  0  0  0  0  0  0  0  0  0
    5.2097    4.0391   -0.6112 C   0  0  0  0  0  0  0  0  0  0  0  0
    6.3004    4.2195    0.2402 C   0  0  0  0  0  0  0  0  0  0  0  0
    6.9931    5.4307    0.2400 C   0  0  0  0  0  0  0  0  0  0  0  0
    9.4563    6.5749   -0.6534 C   0  0  0  0  0  0  0  0  0  0  0  0
   10.8311    6.5900   -0.4125 C   0  0  0  0  0  0  0  0  0  0  0  0
```

```
  11.4355    7.7316    0.1154 C   0  0  0  0  0  0  0  0  0  0  0  0
  10.6656    8.8588    0.4028 C   0  0  0  0  0  0  0  0  0  0  0  0
   9.2913    8.8452    0.1624 C   0  0  0  0  0  0  0  0  0  0  0  0
   1.9895    9.4267   -1.5037 H   0  0  0  0  0  0  0  0  0  0  0  0
   1.4701    8.4397    0.7095 H   0  0  0  0  0  0  0  0  0  0  0  0
   3.3075    7.6873    2.1928 H   0  0  0  0  0  0  0  0  0  0  0  0
   5.6643    7.9214    1.4631 H   0  0  0  0  0  0  0  0  0  0  0  0
   4.3459    9.6612   -2.2343 H   0  0  0  0  0  0  0  0  0  0  0  0
   7.0318    9.7518   -0.3226 H   0  0  0  0  0  0  0  0  0  0  0  0
   6.5943    9.1671   -1.9299 H   0  0  0  0  0  0  0  0  0  0  0  0
   5.1934    7.0801   -2.1251 H   0  0  0  0  0  0  0  0  0  0  0  0
   3.9650    4.9299   -2.1237 H   0  0  0  0  0  0  0  0  0  0  0  0
   4.6723    3.0991   -0.6109 H   0  0  0  0  0  0  0  0  0  0  0  0
   6.6092    3.4195    0.9013 H   0  0  0  0  0  0  0  0  0  0  0  0
   7.8389    5.5696    0.9016 H   0  0  0  0  0  0  0  0  0  0  0  0
   8.9904    5.6882   -1.0633 H   0  0  0  0  0  0  0  0  0  0  0  0
  11.4290    5.7155   -0.6355 H   0  0  0  0  0  0  0  0  0  0  0  0
  12.5021    7.7426    0.3021 H   0  0  0  0  0  0  0  0  0  0  0  0
  11.1347    9.7448    0.8126 H   0  0  0  0  0  0  0  0  0  0  0  0
   8.6991    9.7211    0.3870 H   0  0  0  0  0  0  0  0  0  0  0  0
  2  1  4  0  0  0  0
  3  2  4  0  0  0  0
  4  3  4  0  0  0  0
  5  4  4  0  0  0  0
  6  5  4  0  0  0  0
  1  6  4  0  0  0  0
  5  7  1  0  0  0  0
  7  8  1  0  0  0  0
  8  9  1  0  0  0  0
  8 10  1  0  0  0  0
 11 10  4  0  0  0  0
 12 11  4  0  0  0  0
 13 12  4  0  0  0  0
 14 13  4  0  0  0  0
 15 14  4  0  0  0  0
 10 15  4  0  0  0  0
 16  9  4  0  0  0  0
 17 16  4  0  0  0  0
 18 17  4  0  0  0  0
 19 18  4  0  0  0  0
 20 19  4  0  0  0  0
  9 20  4  0  0  0  0
  1 21  1  0  0  0  0
  2 22  1  0  0  0  0
  3 23  1  0  0  0  0
  4 24  1  0  0  0  0
  6 25  1  0  0  0  0
  7 26  1  0  0  0  0
  7 27  1  0  0  0  0
 11 28  1  0  0  0  0
 12 29  1  0  0  0  0
 13 30  1  0  0  0  0
 14 31  1  0  0  0  0
 15 32  1  0  0  0  0
 16 33  1  0  0  0  0
 17 34  1  0  0  0  0
 18 35  1  0  0  0  0
 19 36  1  0  0  0  0
 20 37  1  0  0  0  0
M  END
>  <Alias name>
Benzyldiphenylamine
```

```
>  <deltaH°(c)>   kJ/mol
-10091

$$$$
Triptycene
  ChemBrain IXL

 34 38  0  0  0  0  0  0  0  0  1 V2000
    6.3213    4.3547    0.4565 C   0  0  0  0  0  0  0  0  0  0  0  0
    5.1442    4.9463   -0.0654 C   0  0  0  0  0  0  0  0  0  0  0  0
    7.0973    4.0102   -0.6883 C   0  0  0  0  0  0  0  0  0  0  0  0
    7.0308    5.4228    1.0695 C   0  0  0  0  0  0  0  0  0  0  0  0
    5.3778    5.9683   -0.8828 C   0  0  0  0  0  0  0  0  0  0  0  0
    3.8505    4.5307    0.2223 C   0  0  0  0  0  0  0  0  0  0  0  0
    7.3325    5.0339   -1.5033 C   0  0  0  0  0  0  0  0  0  0  0  0
    7.5844    2.7405   -0.9713 C   0  0  0  0  0  0  0  0  0  0  0  0
    7.2714    6.4432    0.2516 C   0  0  0  0  0  0  0  0  0  0  0  0
    7.4535    5.4436    2.3933 C   0  0  0  0  0  0  0  0  0  0  0  0
    6.7635    6.2538   -1.0629 C   0  0  0  0  0  0  0  0  0  0  0  0
    4.3331    6.6617   -1.4823 C   0  0  0  0  0  0  0  0  0  0  0  0
    2.7777    5.2013   -0.3602 C   0  0  0  0  0  0  0  0  0  0  0  0
    8.0726    4.8768   -2.6675 C   0  0  0  0  0  0  0  0  0  0  0  0
    8.3320    2.5432   -2.1310 C   0  0  0  0  0  0  0  0  0  0  0  0
    7.9600    7.5694    0.6865 C   0  0  0  0  0  0  0  0  0  0  0  0
    8.1458    6.5567    2.8665 C   0  0  0  0  0  0  0  0  0  0  0  0
    3.0202    6.2739   -1.2178 C   0  0  0  0  0  0  0  0  0  0  0  0
    8.5771    3.6182   -2.9847 C   0  0  0  0  0  0  0  0  0  0  0  0
    8.4013    7.6262    2.0074 C   0  0  0  0  0  0  0  0  0  0  0  0
    6.1264    3.5233    1.1226 H   0  0  0  0  0  0  0  0  0  0  0  0
    3.6808    3.6958    0.8910 H   0  0  0  0  0  0  0  0  0  0  0  0
    7.3841    1.9165   -0.2976 H   0  0  0  0  0  0  0  0  0  0  0  0
    7.2465    4.6049    3.0464 H   0  0  0  0  0  0  0  0  0  0  0  0
    6.9587    7.0851   -1.7290 H   0  0  0  0  0  0  0  0  0  0  0  0
    4.5390    7.4923   -2.1462 H   0  0  0  0  0  0  0  0  0  0  0  0
    1.7621    4.8909   -0.1479 H   0  0  0  0  0  0  0  0  0  0  0  0
    8.2536    5.7233   -3.3184 H   0  0  0  0  0  0  0  0  0  0  0  0
    8.7203    1.5602   -2.3674 H   0  0  0  0  0  0  0  0  0  0  0  0
    8.1498    8.3904    0.0062 H   0  0  0  0  0  0  0  0  0  0  0  0
    8.4831    6.5902    3.8951 H   0  0  0  0  0  0  0  0  0  0  0  0
    2.1936    6.8029   -1.6757 H   0  0  0  0  0  0  0  0  0  0  0  0
    9.1561    3.4764   -3.8889 H   0  0  0  0  0  0  0  0  0  0  0  0
    8.9392    8.4959    2.3639 H   0  0  0  0  0  0  0  0  0  0  0  0
  1  2  1  0  0  0  0
  1  3  1  0  0  0  0
  1  4  1  0  0  0  0
  2  5  4  0  0  0  0
  2  6  4  0  0  0  0
  3  7  4  0  0  0  0
  3  8  4  0  0  0  0
  4  9  4  0  0  0  0
  4 10  4  0  0  0  0
  5 11  1  0  0  0  0
  5 12  4  0  0  0  0
  6 13  4  0  0  0  0
  7 14  4  0  0  0  0
  7 11  1  0  0  0  0
  8 15  4  0  0  0  0
  9 16  4  0  0  0  0
 11  9  1  0  0  0  0
 10 17  4  0  0  0  0
 12 18  4  0  0  0  0
 13 18  4  0  0  0  0
 14 19  4  0  0  0  0
```

```
 15 19  4  0  0  0  0
 16 20  4  0  0  0  0
 17 20  4  0  0  0  0
  1 21  1  0  0  0  0
  6 22  1  0  0  0  0
  8 23  1  0  0  0  0
 10 24  1  0  0  0  0
 11 25  1  0  0  0  0
 12 26  1  0  0  0  0
 13 27  1  0  0  0  0
 14 28  1  0  0  0  0
 15 29  1  0  0  0  0
 16 30  1  0  0  0  0
 17 31  1  0  0  0  0
 18 32  1  0  0  0  0
 19 33  1  0  0  0  0
 20 34  1  0  0  0  0
M  END
>  <Alias name>
Triptycene

>  <deltaH°(c)>   kJ/mol
-10088

$$$$
Palmitic peroxyacid
  ChemBrain IXL

 51 50  0  0  0  0  0  0  0  0  1 V2000
   18.5023   10.3051    0.0781 C   0  0  0  0  0  0  0  0  0  0  0  0
   19.5753    9.2157   -0.1132 C   0  0  0  0  0  0  0  0  0  0  0  0
   20.9191    9.8109    0.0611 C   0  0  0  0  0  0  0  0  0  0  0  0
   21.0563   11.2486    0.3355 O   0  0  0  0  0  0  0  0  0  0  0  0
   21.9130    9.1084   -0.0229 O   0  0  0  0  0  0  0  0  0  0  0  0
   17.1032    9.6858   -0.1054 C   0  0  0  0  0  0  0  0  0  0  0  0
   16.0302   10.7755    0.0840 C   0  0  0  0  0  0  0  0  0  0  0  0
   14.6313   10.1569   -0.1027 C   0  0  0  0  0  0  0  0  0  0  0  0
   13.5584   11.2471    0.0843 C   0  0  0  0  0  0  0  0  0  0  0  0
   12.1595   10.6291   -0.1057 C   0  0  0  0  0  0  0  0  0  0  0  0
   11.0867   11.7197    0.0797 C   0  0  0  0  0  0  0  0  0  0  0  0
    9.6880   11.1022   -0.1126 C   0  0  0  0  0  0  0  0  0  0  0  0
    8.6152   12.1930    0.0720 C   0  0  0  0  0  0  0  0  0  0  0  0
    7.2165   11.5758   -0.1215 C   0  0  0  0  0  0  0  0  0  0  0  0
    6.1437   12.6665    0.0628 C   0  0  0  0  0  0  0  0  0  0  0  0
    4.7451   12.0494   -0.1312 C   0  0  0  0  0  0  0  0  0  0  0  0
    3.6723   13.1402    0.0531 C   0  0  0  0  0  0  0  0  0  0  0  0
    2.2736   12.5231   -0.1408 C   0  0  0  0  0  0  0  0  0  0  0  0
   22.4483   11.7244    0.4975 O   0  0  0  0  0  0  0  0  0  0  0  0
   22.7327   12.4055   -0.1517 H   0  0  0  0  0  0  0  0  0  0  0  0
   18.5847   10.7221    1.0742 H   0  0  0  0  0  0  0  0  0  0  0  0
   18.6490   11.0889   -0.6547 H   0  0  0  0  0  0  0  0  0  0  0  0
   19.4920    8.7978   -1.1088 H   0  0  0  0  0  0  0  0  0  0  0  0
   19.4295    8.4326    0.6205 H   0  0  0  0  0  0  0  0  0  0  0  0
   17.0213    9.2676   -1.1010 H   0  0  0  0  0  0  0  0  0  0  0  0
   16.9560    8.9028    0.6282 H   0  0  0  0  0  0  0  0  0  0  0  0
   16.1107   11.1921    1.0804 H   0  0  0  0  0  0  0  0  0  0  0  0
   16.1789   11.5596   -0.6482 H   0  0  0  0  0  0  0  0  0  0  0  0
   14.5514    9.7389   -1.0986 H   0  0  0  0  0  0  0  0  0  0  0  0
   14.4820    9.3739    0.6304 H   0  0  0  0  0  0  0  0  0  0  0  0
   13.6368   11.6635    1.0810 H   0  0  0  0  0  0  0  0  0  0  0  0
   13.7090   12.0312   -0.6474 H   0  0  0  0  0  0  0  0  0  0  0  0
   12.0816   10.2117   -1.1019 H   0  0  0  0  0  0  0  0  0  0  0  0
   12.0084    9.8458    0.6268 H   0  0  0  0  0  0  0  0  0  0  0  0
```

```
 11.1636   12.1360    1.0766 H   0  0  0  0  0  0  0  0  0  0  0  0
 11.2388   12.5038   -0.6517 H   0  0  0  0  0  0  0  0  0  0  0  0
  9.6113   10.6854   -1.1092 H   0  0  0  0  0  0  0  0  0  0  0  0
  9.5356   10.3185    0.6192 H   0  0  0  0  0  0  0  0  0  0  0  0
  8.6913   12.6092    1.0689 H   0  0  0  0  0  0  0  0  0  0  0  0
  8.7681   12.9771   -0.6592 H   0  0  0  0  0  0  0  0  0  0  0  0
  7.1404   11.1594   -1.1184 H   0  0  0  0  0  0  0  0  0  0  0  0
  7.0635   10.7918    0.6098 H   0  0  0  0  0  0  0  0  0  0  0  0
  6.2196   13.0827    1.0597 H   0  0  0  0  0  0  0  0  0  0  0  0
  6.2968   13.4507   -0.6684 H   0  0  0  0  0  0  0  0  0  0  0  0
  4.6692   11.6332   -1.1281 H   0  0  0  0  0  0  0  0  0  0  0  0
  4.5919   11.2653    0.6000 H   0  0  0  0  0  0  0  0  0  0  0  0
  3.7481   13.5564    1.0501 H   0  0  0  0  0  0  0  0  0  0  0  0
  3.8254   13.9243   -0.6780 H   0  0  0  0  0  0  0  0  0  0  0  0
  2.1204   11.7391    0.5905 H   0  0  0  0  0  0  0  0  0  0  0  0
  1.5197   13.2897   -0.0116 H   0  0  0  0  0  0  0  0  0  0  0  0
  2.1978   12.1066   -1.1377 H   0  0  0  0  0  0  0  0  0  0  0  0
  1  2  1  0  0  0  0
  2  3  1  0  0  0  0
  3  4  1  0  0  0  0
  3  5  2  0  0  0  0
  1  6  1  0  0  0  0
  6  7  1  0  0  0  0
  7  8  1  0  0  0  0
  8  9  1  0  0  0  0
  9 10  1  0  0  0  0
 10 11  1  0  0  0  0
 11 12  1  0  0  0  0
 12 13  1  0  0  0  0
 13 14  1  0  0  0  0
 14 15  1  0  0  0  0
 15 16  1  0  0  0  0
 16 17  1  0  0  0  0
 17 18  1  0  0  0  0
  4 19  1  0  0  0  0
 19 20  1  0  0  0  0
  1 21  1  0  0  0  0
  1 22  1  0  0  0  0
  2 23  1  0  0  0  0
  2 24  1  0  0  0  0
  6 25  1  0  0  0  0
  6 26  1  0  0  0  0
  7 27  1  0  0  0  0
  7 28  1  0  0  0  0
  8 29  1  0  0  0  0
  8 30  1  0  0  0  0
  9 31  1  0  0  0  0
  9 32  1  0  0  0  0
 10 33  1  0  0  0  0
 10 34  1  0  0  0  0
 11 35  1  0  0  0  0
 11 36  1  0  0  0  0
 12 37  1  0  0  0  0
 12 38  1  0  0  0  0
 13 39  1  0  0  0  0
 13 40  1  0  0  0  0
 14 41  1  0  0  0  0
 14 42  1  0  0  0  0
 15 43  1  0  0  0  0
 15 44  1  0  0  0  0
 16 45  1  0  0  0  0
 16 46  1  0  0  0  0
 17 47  1  0  0  0  0
```

```
 17 48  1  0  0  0  0
 18 49  1  0  0  0  0
 18 50  1  0  0  0  0
 18 51  1  0  0  0  0
M  END
>  <Alias name>
Peroxyhexadecanoic acid

>  <deltaH°(c)>   kJ/mol
-10074

$$$$
4,6-Diethyl-4,6-dimethylnonane
  ChemBrain IXL

 47 46  0  0  0  0  0  0  0  0  1 V2000
    2.4302    6.4951   -0.4860 C   0  0  0  0  0  0  0  0  0  0  0  0
    3.5332    5.6489    0.1782 C   0  0  0  0  0  0  0  0  0  0  0  0
    4.8877    6.3797    0.0505 C   0  0  0  0  0  0  0  0  0  0  0  0
    6.0064    5.5267    0.6952 C   0  0  0  0  0  0  0  0  0  0  0  0
    7.3756    6.3256    0.7397 C   0  0  0  0  0  0  0  0  0  0  0  0
    7.7366    7.0446   -0.6302 C   0  0  0  0  0  0  0  0  0  0  0  0
    9.2277    7.4704   -0.5877 C   0  0  0  0  0  0  0  0  0  0  0  0
   10.1318    6.2214   -0.4779 C   0  0  0  0  0  0  0  0  0  0  0  0
   11.6089    6.6591   -0.4551 C   0  0  0  0  0  0  0  0  0  0  0  0
    6.1709    4.1915   -0.0989 C   0  0  0  0  0  0  0  0  0  0  0  0
    7.3048    3.3449    0.5212 C   0  0  0  0  0  0  0  0  0  0  0  0
    6.8667    8.3263   -0.8263 C   0  0  0  0  0  0  0  0  0  0  0  0
    7.0681    9.2882    0.3657 C   0  0  0  0  0  0  0  0  0  0  0  0
    5.5996    5.1862    2.1465 C   0  0  0  0  0  0  0  0  0  0  0  0
    7.5276    6.0963   -1.8280 C   0  0  0  0  0  0  0  0  0  0  0  0
    1.4799    5.9834   -0.3971 H   0  0  0  0  0  0  0  0  0  0  0  0
    2.3689    7.4583    0.0054 H   0  0  0  0  0  0  0  0  0  0  0  0
    2.6664    6.6385   -1.5332 H   0  0  0  0  0  0  0  0  0  0  0  0
    3.5937    4.6866   -0.3133 H   0  0  0  0  0  0  0  0  0  0  0  0
    3.2959    5.5063    1.2240 H   0  0  0  0  0  0  0  0  0  0  0  0
    4.8274    7.3366    0.5543 H   0  0  0  0  0  0  0  0  0  0  0  0
    5.1079    6.5329   -0.9925 H   0  0  0  0  0  0  0  0  0  0  0  0
    7.3156    7.0754    1.5151 H   0  0  0  0  0  0  0  0  0  0  0  0
    8.1644    5.6358    0.9899 H   0  0  0  0  0  0  0  0  0  0  0  0
    9.3954    8.1115    0.2679 H   0  0  0  0  0  0  0  0  0  0  0  0
    9.4720    8.0100   -1.4944 H   0  0  0  0  0  0  0  0  0  0  0  0
    9.9613    5.5755   -1.3294 H   0  0  0  0  0  0  0  0  0  0  0  0
    9.9022    5.6862    0.4326 H   0  0  0  0  0  0  0  0  0  0  0  0
   11.7809    7.3053    0.3968 H   0  0  0  0  0  0  0  0  0  0  0  0
   11.8408    7.1948   -1.3673 H   0  0  0  0  0  0  0  0  0  0  0  0
   12.2433    5.7848   -0.3787 H   0  0  0  0  0  0  0  0  0  0  0  0
    6.4039    4.4000   -1.1215 H   0  0  0  0  0  0  0  0  0  0  0  0
    5.2447    3.6336   -0.0523 H   0  0  0  0  0  0  0  0  0  0  0  0
    8.2382    3.8867    0.4574 H   0  0  0  0  0  0  0  0  0  0  0  0
    7.3936    2.4114   -0.0202 H   0  0  0  0  0  0  0  0  0  0  0  0
    7.0766    3.1387    1.5586 H   0  0  0  0  0  0  0  0  0  0  0  0
    7.1680    8.8237   -1.7398 H   0  0  0  0  0  0  0  0  0  0  0  0
    5.8339    8.0614   -0.8958 H   0  0  0  0  0  0  0  0  0  0  0  0
    6.4786   10.1829    0.2093 H   0  0  0  0  0  0  0  0  0  0  0  0
    8.1138    9.5554    0.4425 H   0  0  0  0  0  0  0  0  0  0  0  0
    6.7508    8.8048    1.2792 H   0  0  0  0  0  0  0  0  0  0  0  0
    6.3891    4.6161    2.6185 H   0  0  0  0  0  0  0  0  0  0  0  0
    5.4371    6.1027    2.7000 H   0  0  0  0  0  0  0  0  0  0  0  0
    4.6888    4.6022    2.1398 H   0  0  0  0  0  0  0  0  0  0  0  0
    8.0634    5.1714   -1.6564 H   0  0  0  0  0  0  0  0  0  0  0  0
    6.4771    5.8902   -1.9477 H   0  0  0  0  0  0  0  0  0  0  0  0
    7.9027    6.5679   -2.7277 H   0  0  0  0  0  0  0  0  0  0  0  0
```

```
  1  2  1  0  0  0  0
  2  3  1  0  0  0  0
  3  4  1  0  0  0  0
  4  5  1  0  0  0  0
  5  6  1  0  0  0  0
  6  7  1  0  0  0  0
  7  8  1  0  0  0  0
  8  9  1  0  0  0  0
  4 10  1  0  0  0  0
 10 11  1  0  0  0  0
  6 12  1  0  0  0  0
 12 13  1  0  0  0  0
  4 14  1  0  0  0  0
  6 15  1  0  0  0  0
  1 16  1  0  0  0  0
  1 17  1  0  0  0  0
  1 18  1  0  0  0  0
  2 19  1  0  0  0  0
  2 20  1  0  0  0  0
  3 21  1  0  0  0  0
  3 22  1  0  0  0  0
  5 23  1  0  0  0  0
  5 24  1  0  0  0  0
  7 25  1  0  0  0  0
  7 26  1  0  0  0  0
  8 27  1  0  0  0  0
  8 28  1  0  0  0  0
  9 29  1  0  0  0  0
  9 30  1  0  0  0  0
  9 31  1  0  0  0  0
 10 32  1  0  0  0  0
 10 33  1  0  0  0  0
 11 34  1  0  0  0  0
 11 35  1  0  0  0  0
 11 36  1  0  0  0  0
 12 37  1  0  0  0  0
 12 38  1  0  0  0  0
 13 39  1  0  0  0  0
 13 40  1  0  0  0  0
 13 41  1  0  0  0  0
 14 42  1  0  0  0  0
 14 43  1  0  0  0  0
 14 44  1  0  0  0  0
 15 45  1  0  0  0  0
 15 46  1  0  0  0  0
 15 47  1  0  0  0  0
M  END
>  <Alias name>
4,6-Diethyl-4,6-dimethylnonane

>  <deltaH°(c)>   kJ/mol
-10068.2

$$$$
Ethyl myristate
  ChemBrain IXL

 50 49  0  0  0  0  0  0  0  0  1 V2000
    2.3296    9.8282    3.2033 C   0  0  0  0  0  0  0  0  0  0  0  0
    3.4690   10.7822    2.7952 C   0  0  0  0  0  0  0  0  0  0  0  0
    4.7122    9.9599    2.4042 C   0  0  0  0  0  0  0  0  0  0  0  0
    5.8474   10.9135    1.9837 C   0  0  0  0  0  0  0  0  0  0  0  0
    7.0899   10.0911    1.5906 C   0  0  0  0  0  0  0  0  0  0  0  0
```

8.2142	11.0432	1.1390	C	0	0	0	0	0	0	0	0	0	0	0	0	0
9.4524	10.2201	0.7337	C	0	0	0	0	0	0	0	0	0	0	0	0	0
10.5579	11.1688	0.2312	C	0	0	0	0	0	0	0	0	0	0	0	0	0
11.7855	10.3436	-0.2009	C	0	0	0	0	0	0	0	0	0	0	0	0	0
12.8650	11.2865	-0.7669	C	0	0	0	0	0	0	0	0	0	0	0	0	0
14.0757	10.4574	-1.2376	C	0	0	0	0	0	0	0	0	0	0	0	0	0
15.1264	11.3934	-1.8656	C	0	0	0	0	0	0	0	0	0	0	0	0	0
16.3199	10.5600	-2.3713	C	0	0	0	0	0	0	0	0	0	0	0	0	0
17.3100	11.4544	-3.0118	C	0	0	0	0	0	0	0	0	0	0	0	0	0
16.9777	12.5698	-3.3776	O	0	0	0	0	0	0	0	0	0	0	0	0	0
18.6938	10.9986	-3.2074	O	0	0	0	0	0	0	0	0	0	0	0	0	0
18.9752	9.6404	-2.7206	C	0	0	0	0	0	0	0	0	0	0	0	0	0
20.4482	9.2877	-3.0049	C	0	0	0	0	0	0	0	0	0	0	0	0	0
2.6464	9.2277	4.0471	H	0	0	0	0	0	0	0	0	0	0	0	0	0
1.4562	10.4061	3.4790	H	0	0	0	0	0	0	0	0	0	0	0	0	0
2.0857	9.1802	2.3705	H	0	0	0	0	0	0	0	0	0	0	0	0	0
3.1526	11.3819	1.9508	H	0	0	0	0	0	0	0	0	0	0	0	0	0
3.7123	11.4308	3.6277	H	0	0	0	0	0	0	0	0	0	0	0	0	0
5.0340	9.3671	3.2515	H	0	0	0	0	0	0	0	0	0	0	0	0	0
4.4664	9.3047	1.5777	H	0	0	0	0	0	0	0	0	0	0	0	0	0
5.5250	11.5074	1.1374	H	0	0	0	0	0	0	0	0	0	0	0	0	0
6.0943	11.5677	2.8107	H	0	0	0	0	0	0	0	0	0	0	0	0	0
7.4259	9.5146	2.4436	H	0	0	0	0	0	0	0	0	0	0	0	0	0
6.8369	9.4205	0.7787	H	0	0	0	0	0	0	0	0	0	0	0	0	0
7.8751	11.6261	0.2916	H	0	0	0	0	0	0	0	0	0	0	0	0	0
8.4735	11.7078	1.9538	H	0	0	0	0	0	0	0	0	0	0	0	0	0
9.8142	9.6663	1.5912	H	0	0	0	0	0	0	0	0	0	0	0	0	0
9.1838	9.5287	-0.0554	H	0	0	0	0	0	0	0	0	0	0	0	0	0
10.1894	11.7371	-0.6138	H	0	0	0	0	0	0	0	0	0	0	0	0	0
10.8407	11.8471	1.0267	H	0	0	0	0	0	0	0	0	0	0	0	0	0
12.1830	9.8125	0.6552	H	0	0	0	0	0	0	0	0	0	0	0	0	0
11.4922	9.6320	-0.9628	H	0	0	0	0	0	0	0	0	0	0	0	0	0
12.4582	11.8392	-1.6048	H	0	0	0	0	0	0	0	0	0	0	0	0	0
13.1795	11.9791	0.0039	H	0	0	0	0	0	0	0	0	0	0	0	0	0
14.5115	9.9421	-0.3907	H	0	0	0	0	0	0	0	0	0	0	0	0	0
13.7522	9.7322	-1.9740	H	0	0	0	0	0	0	0	0	0	0	0	0	0
14.6826	11.9290	-2.6957	H	0	0	0	0	0	0	0	0	0	0	0	0	0
15.4699	12.1014	-1.1215	H	0	0	0	0	0	0	0	0	0	0	0	0	0
16.7825	10.0484	-1.5364	H	0	0	0	0	0	0	0	0	0	0	0	0	0
15.9715	9.8315	-3.0929	H	0	0	0	0	0	0	0	0	0	0	0	0	0
18.3304	8.9340	-3.2287	H	0	0	0	0	0	0	0	0	0	0	0	0	0
18.7922	9.5939	-1.6542	H	0	0	0	0	0	0	0	0	0	0	0	0	0
21.0930	9.9928	-2.4950	H	0	0	0	0	0	0	0	0	0	0	0	0	0
20.6317	9.3363	-4.0711	H	0	0	0	0	0	0	0	0	0	0	0	0	0
20.6549	8.2862	-2.6483	H	0	0	0	0	0	0	0	0	0	0	0	0	0

1	2	1	0	0	0	0
2	3	1	0	0	0	0
3	4	1	0	0	0	0
4	5	1	0	0	0	0
5	6	1	0	0	0	0
6	7	1	0	0	0	0
7	8	1	0	0	0	0
8	9	1	0	0	0	0
9	10	1	0	0	0	0
10	11	1	0	0	0	0
11	12	1	0	0	0	0
12	13	1	0	0	0	0
14	15	2	0	0	0	0
14	16	1	0	0	0	0
14	13	1	0	0	0	0
16	17	1	0	0	0	0
17	18	1	0	0	0	0
1	19	1	0	0	0	0

```
  1 20  1  0  0  0  0
  1 21  1  0  0  0  0
  2 22  1  0  0  0  0
  2 23  1  0  0  0  0
  3 24  1  0  0  0  0
  3 25  1  0  0  0  0
  4 26  1  0  0  0  0
  4 27  1  0  0  0  0
  5 28  1  0  0  0  0
  5 29  1  0  0  0  0
  6 30  1  0  0  0  0
  6 31  1  0  0  0  0
  7 32  1  0  0  0  0
  7 33  1  0  0  0  0
  8 34  1  0  0  0  0
  8 35  1  0  0  0  0
  9 36  1  0  0  0  0
  9 37  1  0  0  0  0
 10 38  1  0  0  0  0
 10 39  1  0  0  0  0
 11 40  1  0  0  0  0
 11 41  1  0  0  0  0
 12 42  1  0  0  0  0
 12 43  1  0  0  0  0
 13 44  1  0  0  0  0
 13 45  1  0  0  0  0
 17 46  1  0  0  0  0
 17 47  1  0  0  0  0
 18 48  1  0  0  0  0
 18 49  1  0  0  0  0
 18 50  1  0  0  0  0
M  END
>  <Alias name>
Ethyl myristate

>  <deltaH°(c)>   kJ/mol
-10067

$$$$
2-Hexyldecanoic acid
  ChemBrain IXL

 50 49  0  0  0  0  0  0  0  0  1 V2000
   11.1122   11.3989   -0.4301 C   0  0  0  0  0  0  0  0  0  0  0  0
    9.8544   10.5779   -0.7746 C   0  0  0  0  0  0  0  0  0  0  0  0
    9.8582    9.3194    0.0042 C   0  0  0  0  0  0  0  0  0  0  0  0
    9.8700    9.3644    1.4735 O   0  0  0  0  0  0  0  0  0  0  0  0
    9.8515    8.2447   -0.5731 O   0  0  0  0  0  0  0  0  0  0  0  0
   12.3709   10.5764   -0.7674 C   0  0  0  0  0  0  0  0  0  0  0  0
   13.6286   11.4004   -0.4299 C   0  0  0  0  0  0  0  0  0  0  0  0
   14.8873   10.5737   -0.7565 C   0  0  0  0  0  0  0  0  0  0  0  0
   16.1450   11.3999   -0.4242 C   0  0  0  0  0  0  0  0  0  0  0  0
   17.4038   10.5711   -0.7457 C   0  0  0  0  0  0  0  0  0  0  0  0
    8.5958   11.3951   -0.4244 C   0  0  0  0  0  0  0  0  0  0  0  0
    7.3380   10.5724   -0.7649 C   0  0  0  0  0  0  0  0  0  0  0  0
    6.0794   11.3904   -0.4164 C   0  0  0  0  0  0  0  0  0  0  0  0
    4.8215   10.5655   -0.7512 C   0  0  0  0  0  0  0  0  0  0  0  0
   18.6614   11.3991   -0.4179 C   0  0  0  0  0  0  0  0  0  0  0  0
   19.9202   10.5696   -0.7371 C   0  0  0  0  0  0  0  0  0  0  0  0
    3.5630   11.3830   -0.4014 C   0  0  0  0  0  0  0  0  0  0  0  0
    2.3052   10.5575   -0.7347 C   0  0  0  0  0  0  0  0  0  0  0  0
    9.8747   10.2676    1.8614 H   0  0  0  0  0  0  0  0  0  0  0  0
   11.1144   12.3153   -1.0073 H   0  0  0  0  0  0  0  0  0  0  0  0
```

11.1093	11.6377	0.6262	H	0	0	0	0	0	0	0	0	0	0	0	0
9.8524	10.3475	-1.8329	H	0	0	0	0	0	0	0	0	0	0	0	0
12.3714	9.6625	-0.1863	H	0	0	0	0	0	0	0	0	0	0	0	0
12.3711	10.3331	-1.8227	H	0	0	0	0	0	0	0	0	0	0	0	0
13.6321	12.3105	-1.0170	H	0	0	0	0	0	0	0	0	0	0	0	0
13.6243	11.6505	0.6238	H	0	0	0	0	0	0	0	0	0	0	0	0
14.8858	9.6654	-0.1666	H	0	0	0	0	0	0	0	0	0	0	0	0
14.8896	10.3202	-1.8094	H	0	0	0	0	0	0	0	0	0	0	0	0
16.1484	12.3063	-1.0169	H	0	0	0	0	0	0	0	0	0	0	0	0
16.1407	11.6565	0.6279	H	0	0	0	0	0	0	0	0	0	0	0	0
17.4020	9.6663	-0.1506	H	0	0	0	0	0	0	0	0	0	0	0	0
17.4063	10.3117	-1.7972	H	0	0	0	0	0	0	0	0	0	0	0	0
8.5994	11.6280	0.6333	H	0	0	0	0	0	0	0	0	0	0	0	0
8.5915	12.3146	-0.9966	H	0	0	0	0	0	0	0	0	0	0	0	0
7.3350	10.3383	-1.8223	H	0	0	0	0	0	0	0	0	0	0	0	0
7.3415	9.6536	-0.1917	H	0	0	0	0	0	0	0	0	0	0	0	0
6.0844	11.6282	0.6402	H	0	0	0	0	0	0	0	0	0	0	0	0
6.0737	12.3073	-0.9927	H	0	0	0	0	0	0	0	0	0	0	0	0
4.8159	10.3286	-1.8079	H	0	0	0	0	0	0	0	0	0	0	0	0
4.8277	9.6482	-0.1756	H	0	0	0	0	0	0	0	0	0	0	0	0
18.6640	12.3032	-1.0142	H	0	0	0	0	0	0	0	0	0	0	0	0
18.6580	11.6600	0.6332	H	0	0	0	0	0	0	0	0	0	0	0	0
20.8040	11.1518	-0.5077	H	0	0	0	0	0	0	0	0	0	0	0	0
19.9231	10.3078	-1.7880	H	0	0	0	0	0	0	0	0	0	0	0	0
19.9181	9.6660	-0.1400	H	0	0	0	0	0	0	0	0	0	0	0	0
3.5691	11.6208	0.6552	H	0	0	0	0	0	0	0	0	0	0	0	0
3.5563	12.2999	-0.9777	H	0	0	0	0	0	0	0	0	0	0	0	0
2.3117	9.6408	-0.1582	H	0	0	0	0	0	0	0	0	0	0	0	0
1.4207	11.1322	-0.4891	H	0	0	0	0	0	0	0	0	0	0	0	0
2.2991	10.3195	-1.7912	H	0	0	0	0	0	0	0	0	0	0	0	0

1	2	1	0	0	0	0
2	3	1	0	0	0	0
3	4	1	0	0	0	0
3	5	2	0	0	0	0
1	6	1	0	0	0	0
6	7	1	0	0	0	0
7	8	1	0	0	0	0
8	9	1	0	0	0	0
9	10	1	0	0	0	0
2	11	1	0	0	0	0
11	12	1	0	0	0	0
12	13	1	0	0	0	0
13	14	1	0	0	0	0
10	15	1	0	0	0	0
15	16	1	0	0	0	0
14	17	1	0	0	0	0
17	18	1	0	0	0	0
4	19	1	0	0	0	0
1	20	1	0	0	0	0
1	21	1	0	0	0	0
2	22	1	0	0	0	0
6	23	1	0	0	0	0
6	24	1	0	0	0	0
7	25	1	0	0	0	0
7	26	1	0	0	0	0
8	27	1	0	0	0	0
8	28	1	0	0	0	0
9	29	1	0	0	0	0
9	30	1	0	0	0	0
10	31	1	0	0	0	0
10	32	1	0	0	0	0
11	33	1	0	0	0	0
11	34	1	0	0	0	0

```
 12 35  1  0  0  0  0
 12 36  1  0  0  0  0
 13 37  1  0  0  0  0
 13 38  1  0  0  0  0
 14 39  1  0  0  0  0
 14 40  1  0  0  0  0
 15 41  1  0  0  0  0
 15 42  1  0  0  0  0
 16 43  1  0  0  0  0
 16 44  1  0  0  0  0
 16 45  1  0  0  0  0
 17 46  1  0  0  0  0
 17 47  1  0  0  0  0
 18 48  1  0  0  0  0
 18 49  1  0  0  0  0
 18 50  1  0  0  0  0
M  END
>  <Alias name>
2-Hexyldecanoic acid

>  <deltaH°(c)>   kJ/mol
-10065

$$$$
1,3,5-Triphenyltriazine-2,4,6-trione
  ChemBrain IXL

 42 45  0  0  0  0  0  0  0  0  1 V2000
    8.0003    9.4180    0.0000 C   0  0  0  0  0  0  0  0  0  0  0  0
    6.9023    8.5546   -0.0646 N   0  0  0  0  0  0  0  0  0  0  0  0
    9.2887    8.8965    0.1524 N   0  0  0  0  0  0  0  0  0  0  0  0
    7.8344   10.6243   -0.0765 O   0  0  0  0  0  0  0  0  0  0  0  0
    7.1038    7.1705   -0.0632 C   0  0  0  0  0  0  0  0  0  0  0  0
    5.6038    9.0747   -0.1300 C   0  0  0  0  0  0  0  0  0  0  0  0
    8.3809    6.6525    0.1752 N   0  0  0  0  0  0  0  0  0  0  0  0
    6.1673    6.4164   -0.2697 O   0  0  0  0  0  0  0  0  0  0  0  0
    9.4666    7.5205    0.3279 C   0  0  0  0  0  0  0  0  0  0  0  0
    8.5720    5.2673    0.2608 C   0  0  0  0  0  0  0  0  0  0  0  0
   10.5671    7.0782    0.6139 O   0  0  0  0  0  0  0  0  0  0  0  0
   10.3983    9.7502    0.1288 C   0  0  0  0  0  0  0  0  0  0  0  0
   11.6536    9.2533   -0.2229 C   0  0  0  0  0  0  0  0  0  0  0  0
   12.7590   10.1040   -0.2466 C   0  0  0  0  0  0  0  0  0  0  0  0
   12.6093   11.4516    0.0814 C   0  0  0  0  0  0  0  0  0  0  0  0
   11.3541   11.9485    0.4332 C   0  0  0  0  0  0  0  0  0  0  0  0
   10.2487   11.0978    0.4569 C   0  0  0  0  0  0  0  0  0  0  0  0
    5.3899   10.3539   -0.6439 C   0  0  0  0  0  0  0  0  0  0  0  0
    4.0963   10.8723   -0.7089 C   0  0  0  0  0  0  0  0  0  0  0  0
    3.0166   10.1115   -0.2601 C   0  0  0  0  0  0  0  0  0  0  0  0
    3.2304    8.8323    0.2536 C   0  0  0  0  0  0  0  0  0  0  0  0
    4.5239    8.3139    0.3186 C   0  0  0  0  0  0  0  0  0  0  0  0
    7.5268    4.4431    0.6790 C   0  0  0  0  0  0  0  0  0  0  0  0
    7.7173    3.0637    0.7645 C   0  0  0  0  0  0  0  0  0  0  0  0
    8.9529    2.5082    0.4316 C   0  0  0  0  0  0  0  0  0  0  0  0
    9.9980    3.3320    0.0133 C   0  0  0  0  0  0  0  0  0  0  0  0
    9.8076    4.7115   -0.0722 C   0  0  0  0  0  0  0  0  0  0  0  0
   11.7699    8.2071   -0.4776 H   0  0  0  0  0  0  0  0  0  0  0  0
   13.7334    9.7182   -0.5198 H   0  0  0  0  0  0  0  0  0  0  0  0
   13.4674   12.1120    0.0631 H   0  0  0  0  0  0  0  0  0  0  0  0
   11.2379   12.9947    0.6880 H   0  0  0  0  0  0  0  0  0  0  0  0
    9.2743   11.4837    0.7301 H   0  0  0  0  0  0  0  0  0  0  0  0
    6.2280   10.9446   -0.9924 H   0  0  0  0  0  0  0  0  0  0  0  0
    3.9304   11.8654   -1.1078 H   0  0  0  0  0  0  0  0  0  0  0  0
    2.0124   10.5140   -0.3106 H   0  0  0  0  0  0  0  0  0  0  0  0
```

```
    2.3921    8.2417    0.6020 H   0  0  0  0  0  0  0  0  0  0  0  0
    4.6897    7.3207    0.7174 H   0  0  0  0  0  0  0  0  0  0  0  0
    6.5675    4.8742    0.9373 H   0  0  0  0  0  0  0  0  0  0  0  0
    6.9059    2.4241    1.0892 H   0  0  0  0  0  0  0  0  0  0  0  0
    9.1008    1.4373    0.4980 H   0  0  0  0  0  0  0  0  0  0  0  0
   10.9573    2.9007   -0.2452 H   0  0  0  0  0  0  0  0  0  0  0  0
   10.6191    5.3509   -0.3970 H   0  0  0  0  0  0  0  0  0  0  0  0
  2  1  1  0  0  0  0
  1  3  1  0  0  0  0
  1  4  2  0  0  0  0
  5  2  1  0  0  0  0
  2  6  1  0  0  0  0
  3  9  1  0  0  0  0
  3 12  1  0  0  0  0
  7  5  1  0  0  0  0
  5  8  2  0  0  0  0
 18  6  4  0  0  0  0
  6 22  4  0  0  0  0
  9  7  1  0  0  0  0
  7 10  1  0  0  0  0
  9 11  2  0  0  0  0
 23 10  4  0  0  0  0
 10 27  4  0  0  0  0
 13 12  4  0  0  0  0
 12 17  4  0  0  0  0
 14 13  4  0  0  0  0
 15 14  4  0  0  0  0
 16 15  4  0  0  0  0
 17 16  4  0  0  0  0
 19 18  4  0  0  0  0
 20 19  4  0  0  0  0
 21 20  4  0  0  0  0
 22 21  4  0  0  0  0
 24 23  4  0  0  0  0
 25 24  4  0  0  0  0
 26 25  4  0  0  0  0
 27 26  4  0  0  0  0
 13 28  1  0  0  0  0
 14 29  1  0  0  0  0
 15 30  1  0  0  0  0
 16 31  1  0  0  0  0
 17 32  1  0  0  0  0
 18 33  1  0  0  0  0
 19 34  1  0  0  0  0
 20 35  1  0  0  0  0
 21 36  1  0  0  0  0
 22 37  1  0  0  0  0
 23 38  1  0  0  0  0
 24 39  1  0  0  0  0
 25 40  1  0  0  0  0
 26 41  1  0  0  0  0
 27 42  1  0  0  0  0
M  END
>  <Alias name>
1,3,5-Triphenyltriazine-2,4,6-trione

>  <deltaH°(c)>   kJ/mol
-10063

$$$$
Dicumylperoxide
  ChemBrain IXL
```

```
42 43  0  0  0  0  0  0  0  0  1 V2000
    3.8995    7.0330   -1.7755 C   0  0  0  0  0  0  0  0  0  0  0  0
    2.6327    6.9768   -1.1939 C   0  0  0  0  0  0  0  0  0  0  0  0
    2.4824    7.2056    0.1739 C   0  0  0  0  0  0  0  0  0  0  0  0
    3.5989    7.4907    0.9602 C   0  0  0  0  0  0  0  0  0  0  0  0
    4.8656    7.5473    0.3786 C   0  0  0  0  0  0  0  0  0  0  0  0
    5.0159    7.3180   -0.9892 C   0  0  0  0  0  0  0  0  0  0  0  0
    6.0500    7.8505    1.2127 C   0  0  0  0  0  0  0  0  0  0  0  0
    7.2686    7.4371    0.5021 O   0  0  0  0  0  0  0  0  0  0  0  0
    6.1072    9.3648    1.4924 C   0  0  0  0  0  0  0  0  0  0  0  0
    5.9485    7.0853    2.5464 C   0  0  0  0  0  0  0  0  0  0  0  0
    7.3452    5.9948    0.1788 O   0  0  0  0  0  0  0  0  0  0  0  0
   10.9037    7.0705   -1.6145 C   0  0  0  0  0  0  0  0  0  0  0  0
   12.0690    7.0958   -0.8481 C   0  0  0  0  0  0  0  0  0  0  0  0
   12.0762    6.5350    0.4292 C   0  0  0  0  0  0  0  0  0  0  0  0
   10.9180    5.9487    0.9400 C   0  0  0  0  0  0  0  0  0  0  0  0
    9.7528    5.9228    0.1734 C   0  0  0  0  0  0  0  0  0  0  0  0
    9.7455    6.4842   -1.1037 C   0  0  0  0  0  0  0  0  0  0  0  0
    8.5244    5.2998    0.7149 C   0  0  0  0  0  0  0  0  0  0  0  0
    8.4770    3.8155    0.3034 C   0  0  0  0  0  0  0  0  0  0  0  0
    8.5289    5.4068    2.2522 C   0  0  0  0  0  0  0  0  0  0  0  0
    4.0161    6.8554   -2.8374 H   0  0  0  0  0  0  0  0  0  0  0  0
    1.7660    6.7556   -1.8044 H   0  0  0  0  0  0  0  0  0  0  0  0
    1.4990    7.1619    0.6254 H   0  0  0  0  0  0  0  0  0  0  0  0
    3.4822    7.6682    2.0222 H   0  0  0  0  0  0  0  0  0  0  0  0
    5.9994    7.3613   -1.4406 H   0  0  0  0  0  0  0  0  0  0  0  0
    5.2108    9.6689    2.0187 H   0  0  0  0  0  0  0  0  0  0  0  0
    6.1754    9.9027    0.5549 H   0  0  0  0  0  0  0  0  0  0  0  0
    6.9756    9.5871    2.1002 H   0  0  0  0  0  0  0  0  0  0  0  0
    6.8240    7.2927    3.1492 H   0  0  0  0  0  0  0  0  0  0  0  0
    5.0604    7.4033    3.0784 H   0  0  0  0  0  0  0  0  0  0  0  0
    5.8898    6.0220    2.3490 H   0  0  0  0  0  0  0  0  0  0  0  0
   10.8982    7.5059   -2.6061 H   0  0  0  0  0  0  0  0  0  0  0  0
   12.9682    7.5509   -1.2447 H   0  0  0  0  0  0  0  0  0  0  0  0
   12.9808    6.5548    1.0243 H   0  0  0  0  0  0  0  0  0  0  0  0
   10.9234    5.5136    1.9318 H   0  0  0  0  0  0  0  0  0  0  0  0
    8.8407    6.4649   -1.6985 H   0  0  0  0  0  0  0  0  0  0  0  0
    9.3426    3.3022    0.7034 H   0  0  0  0  0  0  0  0  0  0  0  0
    8.4798    3.7401   -0.7769 H   0  0  0  0  0  0  0  0  0  0  0  0
    7.5751    3.3611    0.6947 H   0  0  0  0  0  0  0  0  0  0  0  0
    7.6141    4.9817    2.6463 H   0  0  0  0  0  0  0  0  0  0  0  0
    9.3788    4.8657    2.6492 H   0  0  0  0  0  0  0  0  0  0  0  0
    8.5969    6.4483    2.5413 H   0  0  0  0  0  0  0  0  0  0  0  0
  1  2  4  0  0  0  0
  2  3  4  0  0  0  0
  3  4  4  0  0  0  0
  4  5  4  0  0  0  0
  5  6  4  0  0  0  0
  6  1  4  0  0  0  0
  5  7  1  0  0  0  0
  7  8  1  0  0  0  0
  7  9  1  0  0  0  0
  7 10  1  0  0  0  0
  8 11  1  0  0  0  0
 12 13  4  0  0  0  0
 13 14  4  0  0  0  0
 14 15  4  0  0  0  0
 15 16  4  0  0  0  0
 16 17  4  0  0  0  0
 17 12  4  0  0  0  0
 16 18  1  0  0  0  0
 18 19  1  0  0  0  0
 18 20  1  0  0  0  0
```

```
 18 11  1  0  0  0  0
  1 21  1  0  0  0  0
  2 22  1  0  0  0  0
  3 23  1  0  0  0  0
  4 24  1  0  0  0  0
  6 25  1  0  0  0  0
  9 26  1  0  0  0  0
  9 27  1  0  0  0  0
  9 28  1  0  0  0  0
 10 29  1  0  0  0  0
 10 30  1  0  0  0  0
 10 31  1  0  0  0  0
 12 32  1  0  0  0  0
 13 33  1  0  0  0  0
 14 34  1  0  0  0  0
 15 35  1  0  0  0  0
 17 36  1  0  0  0  0
 19 37  1  0  0  0  0
 19 38  1  0  0  0  0
 19 39  1  0  0  0  0
 20 40  1  0  0  0  0
 20 41  1  0  0  0  0
 20 42  1  0  0  0  0
M  END
>  <Alias name>
DCP

>  <deltaH°(c)>   kJ/mol
-10062

$$$$
t-Butyl laurate
  ChemBrain IXL

 50 49  0  0  0  0  0  0  0  0  1 V2000
   16.0871    9.7193    0.1341 O   0  0  0  0  0  0  0  0  0  0  0  0
   15.9788    8.2550    0.0622 C   0  0  0  0  0  0  0  0  0  0  0  0
   17.4543   10.2474    0.0210 C   0  0  0  0  0  0  0  0  0  0  0  0
   16.9821    7.5754   -0.0790 O   0  0  0  0  0  0  0  0  0  0  0  0
   14.6527    7.6053    0.1612 C   0  0  0  0  0  0  0  0  0  0  0  0
   18.0580    9.8246   -1.3323 C   0  0  0  0  0  0  0  0  0  0  0  0
   17.4197   11.7855    0.1099 C   0  0  0  0  0  0  0  0  0  0  0  0
   18.3182    9.6859    1.1668 C   0  0  0  0  0  0  0  0  0  0  0  0
    8.6194    9.4881    0.8118 C   0  0  0  0  0  0  0  0  0  0  0  0
    9.7093    8.4100    0.6556 C   0  0  0  0  0  0  0  0  0  0  0  0
    7.2354    8.8137    0.8777 C   0  0  0  0  0  0  0  0  0  0  0  0
   11.0923    9.0851    0.5771 C   0  0  0  0  0  0  0  0  0  0  0  0
   12.1818    8.0071    0.4174 C   0  0  0  0  0  0  0  0  0  0  0  0
   13.5636    8.6831    0.3248 C   0  0  0  0  0  0  0  0  0  0  0  0
    2.2859    9.6228    1.2993 C   0  0  0  0  0  0  0  0  0  0  0  0
    3.6708   10.2964    1.2456 C   0  0  0  0  0  0  0  0  0  0  0  0
    4.7608    9.2182    1.0902 C   0  0  0  0  0  0  0  0  0  0  0  0
    6.1454    9.8920    1.0327 C   0  0  0  0  0  0  0  0  0  0  0  0
   14.4627    7.0337   -0.7388 H   0  0  0  0  0  0  0  0  0  0  0  0
   14.6379    6.9440    1.0188 H   0  0  0  0  0  0  0  0  0  0  0  0
   19.0632   10.2184   -1.4180 H   0  0  0  0  0  0  0  0  0  0  0  0
   17.4477   10.2145   -2.1375 H   0  0  0  0  0  0  0  0  0  0  0  0
   18.0875    8.7437   -1.3925 H   0  0  0  0  0  0  0  0  0  0  0  0
   16.8123   12.1801   -0.6951 H   0  0  0  0  0  0  0  0  0  0  0  0
   16.9957   12.0826    1.0612 H   0  0  0  0  0  0  0  0  0  0  0  0
   18.4270   12.1746    0.0263 H   0  0  0  0  0  0  0  0  0  0  0  0
   19.3241   10.0791    1.0866 H   0  0  0  0  0  0  0  0  0  0  0  0
   18.3468    8.6053    1.1012 H   0  0  0  0  0  0  0  0  0  0  0  0
```

```
 17.8910    9.9787    2.1180 H   0  0  0  0  0  0  0  0  0  0  0  0
  8.7925   10.0469    1.7233 H   0  0  0  0  0  0  0  0  0  0  0  0
  8.6531   10.1611   -0.0361 H   0  0  0  0  0  0  0  0  0  0  0  0
  9.5312    7.8454   -0.2513 H   0  0  0  0  0  0  0  0  0  0  0  0
  9.6814    7.7422    1.5078 H   0  0  0  0  0  0  0  0  0  0  0  0
  7.0627    8.2542   -0.0333 H   0  0  0  0  0  0  0  0  0  0  0  0
  7.2014    8.1415    1.7262 H   0  0  0  0  0  0  0  0  0  0  0  0
 11.2714    9.6477    1.4850 H   0  0  0  0  0  0  0  0  0  0  0  0
 11.1194    9.7547   -0.2737 H   0  0  0  0  0  0  0  0  0  0  0  0
 11.9971    7.4381   -0.4854 H   0  0  0  0  0  0  0  0  0  0  0  0
 12.1612    7.3435    1.2729 H   0  0  0  0  0  0  0  0  0  0  0  0
 13.7494    9.2500    1.2286 H   0  0  0  0  0  0  0  0  0  0  0  0
 13.5833    9.3487   -0.5293 H   0  0  0  0  0  0  0  0  0  0  0  0
  2.2466    8.9450    2.1431 H   0  0  0  0  0  0  0  0  0  0  0  0
  1.5199   10.3806    1.4089 H   0  0  0  0  0  0  0  0  0  0  0  0
  2.1178    9.0695    0.3836 H   0  0  0  0  0  0  0  0  0  0  0  0
  3.8387   10.8501    2.1611 H   0  0  0  0  0  0  0  0  0  0  0  0
  3.7103   10.9739    0.4015 H   0  0  0  0  0  0  0  0  0  0  0  0
  4.5914    8.6627    0.1761 H   0  0  0  0  0  0  0  0  0  0  0  0
  4.7230    8.5423    1.9356 H   0  0  0  0  0  0  0  0  0  0  0  0
  6.3147   10.4477    1.9467 H   0  0  0  0  0  0  0  0  0  0  0  0
  6.1833   10.5677    0.1872 H   0  0  0  0  0  0  0  0  0  0  0  0
  1  2  1  0  0  0  0
  1  3  1  0  0  0  0
  2  4  2  0  0  0  0
  2  5  1  0  0  0  0
  3  6  1  0  0  0  0
  3  7  1  0  0  0  0
  3  8  1  0  0  0  0
  5 14  1  0  0  0  0
  9 10  1  0  0  0  0
  9 11  1  0  0  0  0
 10 12  1  0  0  0  0
 11 18  1  0  0  0  0
 12 13  1  0  0  0  0
 13 14  1  0  0  0  0
 15 16  1  0  0  0  0
 16 17  1  0  0  0  0
 17 18  1  0  0  0  0
  5 19  1  0  0  0  0
  5 20  1  0  0  0  0
  6 21  1  0  0  0  0
  6 22  1  0  0  0  0
  6 23  1  0  0  0  0
  7 24  1  0  0  0  0
  7 25  1  0  0  0  0
  7 26  1  0  0  0  0
  8 27  1  0  0  0  0
  8 28  1  0  0  0  0
  8 29  1  0  0  0  0
  9 30  1  0  0  0  0
  9 31  1  0  0  0  0
 10 32  1  0  0  0  0
 10 33  1  0  0  0  0
 11 34  1  0  0  0  0
 11 35  1  0  0  0  0
 12 36  1  0  0  0  0
 12 37  1  0  0  0  0
 13 38  1  0  0  0  0
 13 39  1  0  0  0  0
 14 40  1  0  0  0  0
 14 41  1  0  0  0  0
 15 42  1  0  0  0  0
```

```
 15 43  1  0  0  0  0
 15 44  1  0  0  0  0
 16 45  1  0  0  0  0
 16 46  1  0  0  0  0
 17 47  1  0  0  0  0
 17 48  1  0  0  0  0
 18 49  1  0  0  0  0
 18 50  1  0  0  0  0
M  END
>  <Alias name>
t-Butyl dodecanoate

>  <deltaH°(c)>   kJ/mol
-10058.9

$$$$
5,5,7,7-Tetramethylundecane
  ChemBrain IXL

 47 46  0  0  0  0  0  0  0  0  1 V2000
    9.6936    7.1788   -0.2743 C   0  0  0  0  0  0  0  0  0  0  0  0
    8.3260    7.6014   -0.8647 C   0  0  0  0  0  0  0  0  0  0  0  0
    7.3141    7.8457    0.2765 C   0  0  0  0  0  0  0  0  0  0  0  0
    7.0626    6.5241    1.1416 C   0  0  0  0  0  0  0  0  0  0  0  0
    6.8110    5.2026    0.2764 C   0  0  0  0  0  0  0  0  0  0  0  0
    5.7991    5.4470   -0.8647 C   0  0  0  0  0  0  0  0  0  0  0  0
    4.4314    5.8696   -0.2743 C   0  0  0  0  0  0  0  0  0  0  0  0
    3.4491    6.1753   -1.4219 C   0  0  0  0  0  0  0  0  0  0  0  0
    2.0880    6.5956   -0.8347 C   0  0  0  0  0  0  0  0  0  0  0  0
   10.6759    6.8733   -1.4221 C   0  0  0  0  0  0  0  0  0  0  0  0
   12.0371    6.4530   -0.8350 C   0  0  0  0  0  0  0  0  0  0  0  0
    6.2432    4.1153    1.2131 C   0  0  0  0  0  0  0  0  0  0  0  0
    8.1430    4.6732   -0.3181 C   0  0  0  0  0  0  0  0  0  0  0  0
    5.9821    8.3751   -0.3178 C   0  0  0  0  0  0  0  0  0  0  0  0
    7.8819    8.9328    1.2133 C   0  0  0  0  0  0  0  0  0  0  0  0
   10.0887    7.9837    0.3315 H   0  0  0  0  0  0  0  0  0  0  0  0
    9.5693    6.2986    0.3372 H   0  0  0  0  0  0  0  0  0  0  0  0
    7.9532    6.8341   -1.5190 H   0  0  0  0  0  0  0  0  0  0  0  0
    8.4523    8.5169   -1.4291 H   0  0  0  0  0  0  0  0  0  0  0  0
    6.1998    6.6920    1.7742 H   0  0  0  0  0  0  0  0  0  0  0  0
    7.9254    6.3560    1.7741 H   0  0  0  0  0  0  0  0  0  0  0  0
    6.1718    6.2144   -1.5190 H   0  0  0  0  0  0  0  0  0  0  0  0
    5.6726    4.5316   -1.4293 H   0  0  0  0  0  0  0  0  0  0  0  0
    4.0364    5.0646    0.3315 H   0  0  0  0  0  0  0  0  0  0  0  0
    4.5559    6.7497    0.3374 H   0  0  0  0  0  0  0  0  0  0  0  0
    3.8435    6.9797   -2.0302 H   0  0  0  0  0  0  0  0  0  0  0  0
    3.3209    5.2903   -2.0327 H   0  0  0  0  0  0  0  0  0  0  0  0
    1.6933    5.7912   -0.2263 H   0  0  0  0  0  0  0  0  0  0  0  0
    2.2162    7.4807   -0.2238 H   0  0  0  0  0  0  0  0  0  0  0  0
    1.3978    6.8104   -1.6410 H   0  0  0  0  0  0  0  0  0  0  0  0
   10.2815    6.0689   -2.0304 H   0  0  0  0  0  0  0  0  0  0  0  0
   10.8040    7.7583   -2.0328 H   0  0  0  0  0  0  0  0  0  0  0  0
   12.4317    7.2573   -0.2266 H   0  0  0  0  0  0  0  0  0  0  0  0
   12.7272    6.2383   -1.6414 H   0  0  0  0  0  0  0  0  0  0  0  0
   11.9090    5.5678   -0.2242 H   0  0  0  0  0  0  0  0  0  0  0  0
    6.9584    3.9076    1.9994 H   0  0  0  0  0  0  0  0  0  0  0  0
    5.3170    4.4631    1.6517 H   0  0  0  0  0  0  0  0  0  0  0  0
    6.0576    3.2113    0.6463 H   0  0  0  0  0  0  0  0  0  0  0  0
    8.5391    5.3743   -1.0164 H   0  0  0  0  0  0  0  0  0  0  0  0
    7.9596    3.7329   -0.8230 H   0  0  0  0  0  0  0  0  0  0  0  0
    8.8565    4.5168    0.4813 H   0  0  0  0  0  0  0  0  0  0  0  0
    5.5859    7.6741   -1.0162 H   0  0  0  0  0  0  0  0  0  0  0  0
    5.2686    8.5314    0.4816 H   0  0  0  0  0  0  0  0  0  0  0  0
```

```
   6.1654    9.3155   -0.8226 H   0  0  0  0  0  0  0  0  0  0  0  0
   7.1668    9.1404    1.9998 H   0  0  0  0  0  0  0  0  0  0  0  0
   8.0675    9.8369    0.6467 H   0  0  0  0  0  0  0  0  0  0  0  0
   8.8081    8.5850    1.6518 H   0  0  0  0  0  0  0  0  0  0  0  0
  1  2  1  0  0  0  0
  2  3  1  0  0  0  0
  3  4  1  0  0  0  0
  4  5  1  0  0  0  0
  5  6  1  0  0  0  0
  6  7  1  0  0  0  0
  7  8  1  0  0  0  0
  8  9  1  0  0  0  0
  1 10  1  0  0  0  0
 10 11  1  0  0  0  0
  5 12  1  0  0  0  0
  5 13  1  0  0  0  0
  3 14  1  0  0  0  0
  3 15  1  0  0  0  0
  1 16  1  0  0  0  0
  1 17  1  0  0  0  0
  2 18  1  0  0  0  0
  2 19  1  0  0  0  0
  4 20  1  0  0  0  0
  4 21  1  0  0  0  0
  6 22  1  0  0  0  0
  6 23  1  0  0  0  0
  7 24  1  0  0  0  0
  7 25  1  0  0  0  0
  8 26  1  0  0  0  0
  8 27  1  0  0  0  0
  9 28  1  0  0  0  0
  9 29  1  0  0  0  0
  9 30  1  0  0  0  0
 10 31  1  0  0  0  0
 10 32  1  0  0  0  0
 11 33  1  0  0  0  0
 11 34  1  0  0  0  0
 11 35  1  0  0  0  0
 12 36  1  0  0  0  0
 12 37  1  0  0  0  0
 12 38  1  0  0  0  0
 13 39  1  0  0  0  0
 13 40  1  0  0  0  0
 13 41  1  0  0  0  0
 14 42  1  0  0  0  0
 14 43  1  0  0  0  0
 14 44  1  0  0  0  0
 15 45  1  0  0  0  0
 15 46  1  0  0  0  0
 15 47  1  0  0  0  0
M  END
>  <Alias name>
5,5,7,7-Tetramethylundecane

>  <deltaH°(c)>   kJ/mol
-10049.4

$$$$
Pentadecane
  ChemBrain IXL

 47 46  0  0  0  0  0  0  0  0  1 V2000
   14.5659    9.2987   -2.3409 C   0  0  0  0  0  0  0  0  0  0  0  0
```

```
13.3838 10.0663 -1.7176 C 0 0 0 0 0 0 0 0 0 0 0 0
12.3328 9.0626 -1.2048 C 0 0 0 0 0 0 0 0 0 0 0 0
11.1508 9.8302 -0.5812 C 0 0 0 0 0 0 0 0 0 0 0 0
10.1000 8.8266 -0.0681 C 0 0 0 0 0 0 0 0 0 0 0 0
8.9181 9.5941 0.5557 C 0 0 0 0 0 0 0 0 0 0 0 0
7.8674 8.5905 1.0691 C 0 0 0 0 0 0 0 0 0 0 0 0
6.6857 9.3580 1.6932 C 0 0 0 0 0 0 0 0 0 0 0 0
5.6351 8.3543 2.2069 C 0 0 0 0 0 0 0 0 0 0 0 0
15.6170 10.3024 -2.8536 C 0 0 0 0 0 0 0 0 0 0 0 0
4.4535 9.1219 2.8311 C 0 0 0 0 0 0 0 0 0 0 0 0
16.7992 9.5349 -3.4768 C 0 0 0 0 0 0 0 0 0 0 0 0
3.4029 8.1182 3.3449 C 0 0 0 0 0 0 0 0 0 0 0 0
17.8503 10.5385 -3.9894 C 0 0 0 0 0 0 0 0 0 0 0 0
2.2214 8.8857 3.9693 C 0 0 0 0 0 0 0 0 0 0 0 0
14.2104 8.6974 -3.1647 H 0 0 0 0 0 0 0 0 0 0 0 0
15.0131 8.6588 -1.5947 H 0 0 0 0 0 0 0 0 0 0 0 0
12.9365 10.7062 -2.4639 H 0 0 0 0 0 0 0 0 0 0 0 0
13.7393 10.6677 -0.8940 H 0 0 0 0 0 0 0 0 0 0 0 0
11.9772 8.4613 -2.0284 H 0 0 0 0 0 0 0 0 0 0 0 0
12.7802 8.4227 -0.4587 H 0 0 0 0 0 0 0 0 0 0 0 0
11.5065 10.4316 0.2423 H 0 0 0 0 0 0 0 0 0 0 0 0
10.7033 10.4700 -1.3274 H 0 0 0 0 0 0 0 0 0 0 0 0
9.7442 8.2252 -0.8917 H 0 0 0 0 0 0 0 0 0 0 0 0
10.5475 8.1866 0.6779 H 0 0 0 0 0 0 0 0 0 0 0 0
8.4704 10.2339 -0.1904 H 0 0 0 0 0 0 0 0 0 0 0 0
9.2740 10.1955 1.3791 H 0 0 0 0 0 0 0 0 0 0 0 0
7.5114 7.9891 0.2457 H 0 0 0 0 0 0 0 0 0 0 0 0
8.3152 7.9505 1.8151 H 0 0 0 0 0 0 0 0 0 0 0 0
6.2379 9.9978 0.9472 H 0 0 0 0 0 0 0 0 0 0 0 0
7.0418 9.9595 2.5166 H 0 0 0 0 0 0 0 0 0 0 0 0
6.0830 7.7144 2.9527 H 0 0 0 0 0 0 0 0 0 0 0 0
5.2790 7.7530 1.3835 H 0 0 0 0 0 0 0 0 0 0 0 0
15.9723 10.9038 -2.0299 H 0 0 0 0 0 0 0 0 0 0 0 0
15.1698 10.9423 -3.5999 H 0 0 0 0 0 0 0 0 0 0 0 0
4.0056 9.7617 2.0853 H 0 0 0 0 0 0 0 0 0 0 0 0
4.8097 9.7233 3.6545 H 0 0 0 0 0 0 0 0 0 0 0 0
16.4438 8.9336 -4.3006 H 0 0 0 0 0 0 0 0 0 0 0 0
17.2463 8.8949 -2.7306 H 0 0 0 0 0 0 0 0 0 0 0 0
3.8509 7.4783 4.0908 H 0 0 0 0 0 0 0 0 0 0 0 0
3.0467 7.5168 2.5216 H 0 0 0 0 0 0 0 0 0 0 0 0
18.6787 10.0006 -4.4261 H 0 0 0 0 0 0 0 0 0 0 0 0
18.2057 11.1398 -3.1657 H 0 0 0 0 0 0 0 0 0 0 0 0
17.4031 11.1785 -4.7357 H 0 0 0 0 0 0 0 0 0 0 0 0
1.7734 9.5256 3.2234 H 0 0 0 0 0 0 0 0 0 0 0 0
1.4852 8.1823 4.3293 H 0 0 0 0 0 0 0 0 0 0 0 0
2.5776 9.4871 4.7926 H 0 0 0 0 0 0 0 0 0 0 0 0
1 2 1 0 0 0 0
2 3 1 0 0 0 0
3 4 1 0 0 0 0
4 5 1 0 0 0 0
5 6 1 0 0 0 0
6 7 1 0 0 0 0
7 8 1 0 0 0 0
8 9 1 0 0 0 0
1 16 1 0 0 0 0
1 17 1 0 0 0 0
1 10 1 0 0 0 0
2 18 1 0 0 0 0
2 19 1 0 0 0 0
3 20 1 0 0 0 0
3 21 1 0 0 0 0
4 22 1 0 0 0 0
4 23 1 0 0 0 0
```

```
  5 24  1  0  0  0  0
  5 25  1  0  0  0  0
  6 26  1  0  0  0  0
  6 27  1  0  0  0  0
  7 28  1  0  0  0  0
  7 29  1  0  0  0  0
  8 30  1  0  0  0  0
  8 31  1  0  0  0  0
  9 32  1  0  0  0  0
  9 33  1  0  0  0  0
  9 11  1  0  0  0  0
 10 34  1  0  0  0  0
 10 12  1  0  0  0  0
 10 35  1  0  0  0  0
 11 13  1  0  0  0  0
 12 14  1  0  0  0  0
 11 36  1  0  0  0  0
 11 37  1  0  0  0  0
 12 38  1  0  0  0  0
 12 39  1  0  0  0  0
 13 15  1  0  0  0  0
 13 40  1  0  0  0  0
 13 41  1  0  0  0  0
 14 42  1  0  0  0  0
 14 43  1  0  0  0  0
 14 44  1  0  0  0  0
 15 45  1  0  0  0  0
 15 46  1  0  0  0  0
 15 47  1  0  0  0  0
M  END
>  <Alias name>
Pentadecane

>  <deltaH°(c)>   kJ/mol
-10046

$$$$
1,1'-Binaphthyl
  ChemBrain IXL

 34 37  0  0  0  0  0  0  0  0  1 V2000
    5.0742    6.1859    0.3258 C   0  0  0  0  0  0  0  0  0  0  0  0
    4.0425    5.2849    0.5902 C   0  0  0  0  0  0  0  0  0  0  0  0
    4.1604    3.9532    0.1918 C   0  0  0  0  0  0  0  0  0  0  0  0
    5.3080    3.5235   -0.4749 C   0  0  0  0  0  0  0  0  0  0  0  0
    6.3363    4.4263   -0.7462 C   0  0  0  0  0  0  0  0  0  0  0  0
    6.2175    5.7585   -0.3497 C   0  0  0  0  0  0  0  0  0  0  0  0
    9.6503    7.2963   -0.6201 C   0  0  0  0  0  0  0  0  0  0  0  0
    8.6239    6.3985   -0.3259 C   0  0  0  0  0  0  0  0  0  0  0  0
    7.3043    6.7185   -0.6458 C   0  0  0  0  0  0  0  0  0  0  0  0
    7.0092    7.9432   -1.2449 C   0  0  0  0  0  0  0  0  0  0  0  0
    8.0347    8.8443   -1.5320 C   0  0  0  0  0  0  0  0  0  0  0  0
    9.3554    8.5201   -1.2212 C   0  0  0  0  0  0  0  0  0  0  0  0
    4.9626    7.5142    0.7370 C   0  0  0  0  0  0  0  0  0  0  0  0
    3.8128    7.9451    1.3990 C   0  0  0  0  0  0  0  0  0  0  0  0
    2.7775    7.0461    1.6558 C   0  0  0  0  0  0  0  0  0  0  0  0
    2.8931    5.7156    1.2529 C   0  0  0  0  0  0  0  0  0  0  0  0
    8.9171    5.1809    0.2884 C   0  0  0  0  0  0  0  0  0  0  0  0
   10.2384    4.8544    0.5944 C   0  0  0  0  0  0  0  0  0  0  0  0
   11.2657    5.7485    0.2924 C   0  0  0  0  0  0  0  0  0  0  0  0
   10.9714    6.9702   -0.3133 C   0  0  0  0  0  0  0  0  0  0  0  0
    3.3609    3.2529    0.4000 H   0  0  0  0  0  0  0  0  0  0  0  0
    5.4006    2.4890   -0.7818 H   0  0  0  0  0  0  0  0  0  0  0  0
```

```
   7.2268    4.0930   -1.2646 H   0  0  0  0  0  0  0  0  0  0  0  0
   5.9840    8.1945   -1.4871 H   0  0  0  0  0  0  0  0  0  0  0  0
   7.8055    9.7956   -1.9962 H   0  0  0  0  0  0  0  0  0  0  0  0
  10.1518    9.2186   -1.4465 H   0  0  0  0  0  0  0  0  0  0  0  0
   5.7686    8.2109    0.5423 H   0  0  0  0  0  0  0  0  0  0  0  0
   3.7239    8.9776    1.7135 H   0  0  0  0  0  0  0  0  0  0  0  0
   1.8840    7.3812    2.1679 H   0  0  0  0  0  0  0  0  0  0  0  0
   2.0904    5.0170    1.4546 H   0  0  0  0  0  0  0  0  0  0  0  0
   8.1190    4.4890    0.5277 H   0  0  0  0  0  0  0  0  0  0  0  0
  10.4666    3.9068    1.0665 H   0  0  0  0  0  0  0  0  0  0  0  0
  12.2917    5.4942    0.5282 H   0  0  0  0  0  0  0  0  0  0  0  0
  11.7687    7.6655   -0.5453 H   0  0  0  0  0  0  0  0  0  0  0  0
  1  2  4  0  0  0  0
  2  3  4  0  0  0  0
  3  4  4  0  0  0  0
  4  5  4  0  0  0  0
  5  6  4  0  0  0  0
  6  1  4  0  0  0  0
  7  8  4  0  0  0  0
  8  9  4  0  0  0  0
  9 10  4  0  0  0  0
 10 11  4  0  0  0  0
 11 12  4  0  0  0  0
 12  7  4  0  0  0  0
  9  6  1  0  0  0  0
  1 13  4  0  0  0  0
 13 14  4  0  0  0  0
 14 15  4  0  0  0  0
 15 16  4  0  0  0  0
 16  2  4  0  0  0  0
  8 17  4  0  0  0  0
 17 18  4  0  0  0  0
 18 19  4  0  0  0  0
 19 20  4  0  0  0  0
 20  7  4  0  0  0  0
  3 21  1  0  0  0  0
  4 22  1  0  0  0  0
  5 23  1  0  0  0  0
 10 24  1  0  0  0  0
 11 25  1  0  0  0  0
 12 26  1  0  0  0  0
 13 27  1  0  0  0  0
 14 28  1  0  0  0  0
 15 29  1  0  0  0  0
 16 30  1  0  0  0  0
 17 31  1  0  0  0  0
 18 32  1  0  0  0  0
 19 33  1  0  0  0  0
 20 34  1  0  0  0  0
M  END
>  <Alias name>
1,1'-Binaphthyl

>  <deltaH°(c)>   kJ/mol
-10040

$$$$
n-Hexadecanoic acid
  ChemBrain IXL

 50 49  0  0  0  0  0  0  0  0  1 V2000
  16.2702   12.7983   -0.6808 C   0  0  0  0  0  0  0  0  0  0  0  0
  17.7825   12.6398   -0.9366 C   0  0  0  0  0  0  0  0  0  0  0  0
```

18.4234 13.9749 -0.9634 C 0 0 0 0 0 0 0 0 0 0 0 0
17.6143 15.1849 -0.7523 O 0 0 0 0 0 0 0 0 0 0 0 0
19.6235 14.0710 -1.1607 O 0 0 0 0 0 0 0 0 0 0 0 0
15.6051 11.4086 -0.6559 C 0 0 0 0 0 0 0 0 0 0 0 0
14.0931 11.5666 -0.4035 C 0 0 0 0 0 0 0 0 0 0 0 0
13.4283 10.1767 -0.3826 C 0 0 0 0 0 0 0 0 0 0 0 0
11.9154 10.3338 -0.1347 C 0 0 0 0 0 0 0 0 0 0 0 0
11.2512 8.9434 -0.1172 C 0 0 0 0 0 0 0 0 0 0 0 0
9.7377 9.0994 0.1268 C 0 0 0 0 0 0 0 0 0 0 0 0
9.0745 7.7085 0.1431 C 0 0 0 0 0 0 0 0 0 0 0 0
7.5605 7.8635 0.3852 C 0 0 0 0 0 0 0 0 0 0 0 0
6.8982 6.4721 0.4025 C 0 0 0 0 0 0 0 0 0 0 0 0
5.3841 6.6264 0.6443 C 0 0 0 0 0 0 0 0 0 0 0 0
4.7226 5.2347 0.6637 C 0 0 0 0 0 0 0 0 0 0 0 0
3.2084 5.3886 0.9059 C 0 0 0 0 0 0 0 0 0 0 0 0
2.5471 3.9969 0.9265 C 0 0 0 0 0 0 0 0 0 0 0 0
18.1147 16.0303 -0.7853 H 0 0 0 0 0 0 0 0 0 0 0 0
16.1146 13.2901 0.2716 H 0 0 0 0 0 0 0 0 0 0 0 0
15.8309 13.3945 -1.4710 H 0 0 0 0 0 0 0 0 0 0 0 0
18.2217 12.0446 -0.1456 H 0 0 0 0 0 0 0 0 0 0 0 0
17.9375 12.1462 -1.8880 H 0 0 0 0 0 0 0 0 0 0 0 0
15.7625 10.9162 -1.6076 H 0 0 0 0 0 0 0 0 0 0 0 0
16.0430 10.8129 0.1355 H 0 0 0 0 0 0 0 0 0 0 0 0
13.9353 12.0568 0.5493 H 0 0 0 0 0 0 0 0 0 0 0 0
13.6558 12.1641 -1.1938 H 0 0 0 0 0 0 0 0 0 0 0 0
13.5888 9.6856 -1.3344 H 0 0 0 0 0 0 0 0 0 0 0 0
13.8638 9.5800 0.4094 H 0 0 0 0 0 0 0 0 0 0 0 0
11.7544 10.8231 0.8180 H 0 0 0 0 0 0 0 0 0 0 0 0
11.4803 10.9318 -0.9258 H 0 0 0 0 0 0 0 0 0 0 0 0
11.4146 8.4535 -1.0691 H 0 0 0 0 0 0 0 0 0 0 0 0
11.6850 8.3462 0.6753 H 0 0 0 0 0 0 0 0 0 0 0 0
9.5740 9.5888 1.0790 H 0 0 0 0 0 0 0 0 0 0 0 0
9.3040 9.6970 -0.6655 H 0 0 0 0 0 0 0 0 0 0 0 0
9.2395 7.2188 -0.8086 H 0 0 0 0 0 0 0 0 0 0 0 0
9.5075 7.1115 0.9362 H 0 0 0 0 0 0 0 0 0 0 0 0
7.3953 8.3538 1.3366 H 0 0 0 0 0 0 0 0 0 0 0 0
7.1272 8.4598 -0.4083 H 0 0 0 0 0 0 0 0 0 0 0 0
7.0637 5.9817 -0.5488 H 0 0 0 0 0 0 0 0 0 0 0 0
7.3316 5.8760 1.1961 H 0 0 0 0 0 0 0 0 0 0 0 0
5.2187 7.1180 1.5951 H 0 0 0 0 0 0 0 0 0 0 0 0
4.9505 7.2213 -0.1500 H 0 0 0 0 0 0 0 0 0 0 0 0
4.8879 4.7431 -0.2871 H 0 0 0 0 0 0 0 0 0 0 0 0
5.1565 4.6398 1.4578 H 0 0 0 0 0 0 0 0 0 0 0 0
3.0430 5.8809 1.8562 H 0 0 0 0 0 0 0 0 0 0 0 0
2.7743 5.9828 0.1113 H 0 0 0 0 0 0 0 0 0 0 0 0
1.4831 4.1053 1.0967 H 0 0 0 0 0 0 0 0 0 0 0 0
2.9811 3.4027 1.7212 H 0 0 0 0 0 0 0 0 0 0 0 0
2.7123 3.5045 -0.0239 H 0 0 0 0 0 0 0 0 0 0 0 0
1 2 1 0 0 0 0
2 3 1 0 0 0 0
3 4 1 0 0 0 0
3 5 2 0 0 0 0
1 6 1 0 0 0 0
6 7 1 0 0 0 0
7 8 1 0 0 0 0
8 9 1 0 0 0 0
9 10 1 0 0 0 0
10 11 1 0 0 0 0
11 12 1 0 0 0 0
12 13 1 0 0 0 0
13 14 1 0 0 0 0
14 15 1 0 0 0 0
15 16 1 0 0 0 0

```
 16 17  1  0  0  0  0
 17 18  1  0  0  0  0
  4 19  1  0  0  0  0
  1 20  1  0  0  0  0
  1 21  1  0  0  0  0
  2 22  1  0  0  0  0
  2 23  1  0  0  0  0
  6 24  1  0  0  0  0
  6 25  1  0  0  0  0
  7 26  1  0  0  0  0
  7 27  1  0  0  0  0
  8 28  1  0  0  0  0
  8 29  1  0  0  0  0
  9 30  1  0  0  0  0
  9 31  1  0  0  0  0
 10 32  1  0  0  0  0
 10 33  1  0  0  0  0
 11 34  1  0  0  0  0
 11 35  1  0  0  0  0
 12 36  1  0  0  0  0
 12 37  1  0  0  0  0
 13 38  1  0  0  0  0
 13 39  1  0  0  0  0
 14 40  1  0  0  0  0
 14 41  1  0  0  0  0
 15 42  1  0  0  0  0
 15 43  1  0  0  0  0
 16 44  1  0  0  0  0
 16 45  1  0  0  0  0
 17 46  1  0  0  0  0
 17 47  1  0  0  0  0
 18 48  1  0  0  0  0
 18 49  1  0  0  0  0
 18 50  1  0  0  0  0
M  END
>  <Alias name>
Palmitic acid

>  <deltaH°(c)>   kJ/mol
-10028.6

$$$$
m-Chlorobenzyliden-5,6,7,8-tetrahydronaphthyl-2-acetonitrile
  ChemBrain IXL

 37 39  0  0  0  0  0  0  0  0  1 V2000
    2.4835    6.9220    0.3699 C   0  0  0  0  0  0  0  0  0  0  0  0  0
    2.6937    8.2329   -0.3619 C   0  0  0  0  0  0  0  0  0  0  0  0  0
    3.5145    5.9069   -0.1267 C   0  0  0  0  0  0  0  0  0  0  0  0  0
    3.9896    8.8731    0.1344 C   0  0  0  0  0  0  0  0  0  0  0  0  0
    5.1229    7.8897    0.0582 C   0  0  0  0  0  0  0  0  0  0  0  0  0
    4.8992    6.4885   -0.0596 C   0  0  0  0  0  0  0  0  0  0  0  0  0
    6.4270    8.3758    0.1077 C   0  0  0  0  0  0  0  0  0  0  0  0  0
    5.9884    5.6202   -0.1177 C   0  0  0  0  0  0  0  0  0  0  0  0  0
    7.2879    6.1218   -0.0654 C   0  0  0  0  0  0  0  0  0  0  0  0  0
    7.5048    7.4951    0.0459 C   0  0  0  0  0  0  0  0  0  0  0  0  0
    8.8792    8.0174    0.0982 C   0  0  0  0  0  0  0  0  0  0  0  0  0
   11.2920    7.7180   -0.0795 C   0  0  0  0  0  0  0  0  0  0  0  0  0
    9.9075    7.1994   -0.1308 C   0  0  0  0  0  0  0  0  0  0  0  0  0
   11.5692    9.0160   -0.5110 C   0  0  0  0  0  0  0  0  0  0  0  0  0
   12.3203    6.9080    0.4004 C   0  0  0  0  0  0  0  0  0  0  0  0  0
    9.1139    9.4467    0.4045 C   0  0  0  0  0  0  0  0  0  0  0  0  0
    9.2963   10.5650    0.6442 N   0  0  0  0  0  0  0  0  0  0  0  0  0
```

```
  12.8761    9.5022   -0.4639 C   0  0  0  0  0  0  0  0  0  0  0  0
  13.6269    7.3939    0.4471 C   0  0  0  0  0  0  0  0  0  0  0  0
  13.9049    8.6910    0.0151 C   0  0  0  0  0  0  0  0  0  0  0  0
  14.9422    6.3563    1.0593 Cl  0  0  0  0  0  0  0  0  0  0  0  0
   1.4839    6.5520    0.1717 H   0  0  0  0  0  0  0  0  0  0  0  0
   2.6084    7.0764    1.4345 H   0  0  0  0  0  0  0  0  0  0  0  0
   2.7638    8.0474   -1.4265 H   0  0  0  0  0  0  0  0  0  0  0  0
   1.8604    8.8974   -0.1633 H   0  0  0  0  0  0  0  0  0  0  0  0
   3.2872    5.6395   -1.1513 H   0  0  0  0  0  0  0  0  0  0  0  0
   3.4683    5.0198    0.4929 H   0  0  0  0  0  0  0  0  0  0  0  0
   3.8599    9.1921    1.1612 H   0  0  0  0  0  0  0  0  0  0  0  0
   4.2205    9.7340   -0.4811 H   0  0  0  0  0  0  0  0  0  0  0  0
   6.6025    9.4412    0.1946 H   0  0  0  0  0  0  0  0  0  0  0  0
   5.8236    4.5535   -0.2038 H   0  0  0  0  0  0  0  0  0  0  0  0
   8.1298    5.4443   -0.1113 H   0  0  0  0  0  0  0  0  0  0  0  0
   9.7281    6.1552   -0.3541 H   0  0  0  0  0  0  0  0  0  0  0  0
  10.7700    9.6462   -0.8826 H   0  0  0  0  0  0  0  0  0  0  0  0
  12.1042    5.9018    0.7368 H   0  0  0  0  0  0  0  0  0  0  0  0
  13.0919   10.5095   -0.7992 H   0  0  0  0  0  0  0  0  0  0  0  0
  14.9196    9.0681    0.0516 H   0  0  0  0  0  0  0  0  0  0  0  0
  1  2  1  0  0  0  0
  3  1  1  0  0  0  0
  2  4  1  0  0  0  0
  6  3  1  0  0  0  0
  4  5  1  0  0  0  0
  6  5  4  0  0  0  0
  5  7  4  0  0  0  0
  8  6  4  0  0  0  0
  7 10  4  0  0  0  0
  9  8  4  0  0  0  0
 10  9  4  0  0  0  0
 10 11  1  0  0  0  0
 11 13  2  0  0  0  0
 11 16  1  0  0  0  0
 12 13  1  0  0  0  0
 12 14  4  0  0  0  0
 15 12  4  0  0  0  0
 14 18  4  0  0  0  0
 19 15  4  0  0  0  0
 16 17  3  0  0  0  0
 18 20  4  0  0  0  0
 20 19  4  0  0  0  0
 19 21  1  0  0  0  0
  1 22  1  0  0  0  0
  1 23  1  0  0  0  0
  2 24  1  0  0  0  0
  2 25  1  0  0  0  0
  3 26  1  0  0  0  0
  3 27  1  0  0  0  0
  4 28  1  0  0  0  0
  4 29  1  0  0  0  0
  7 30  1  0  0  0  0
  8 31  1  0  0  0  0
  9 32  1  0  0  0  0
 13 33  1  0  0  0  0
 14 34  1  0  0  0  0
 15 35  1  0  0  0  0
 18 36  1  0  0  0  0
 20 37  1  0  0  0  0
M  END
>  <Alias name>
m-Chlorobenzyliden-5,6,7,8-tetrahydronaphthyl-2-acetonitrile
```

```
>  <deltaH°(c)>   kJ/mol
-9986.8

$$$$
9-Phenyl-9-phosphafluorene
  ChemBrain IXL

 32 35  0  0  0  0  0  0  0  0  1 V2000
    6.5338    6.4097   -1.4887 P   0  0  0  0  0  0  0  0  0  0  0  0
    5.0758    6.8849   -0.6429 C   0  0  0  0  0  0  0  0  0  0  0  0
    6.4555    4.8470   -0.7076 C   0  0  0  0  0  0  0  0  0  0  0  0
    7.9040    7.2931   -0.5021 C   0  0  0  0  0  0  0  0  0  0  0  0
    4.4603    8.1262   -0.7062 C   0  0  0  0  0  0  0  0  0  0  0  0
    4.5870    5.9074    0.0878 C   0  0  0  0  0  0  0  0  0  0  0  0
    7.3768    3.8203   -0.8419 C   0  0  0  0  0  0  0  0  0  0  0  0
    5.3880    4.7248    0.0506 C   0  0  0  0  0  0  0  0  0  0  0  0
    7.7443    8.6273   -0.1080 C   0  0  0  0  0  0  0  0  0  0  0  0
    9.0886    6.6192   -0.1831 C   0  0  0  0  0  0  0  0  0  0  0  0
    3.2799    8.3287    0.0032 C   0  0  0  0  0  0  0  0  0  0  0  0
    3.4059    6.0615    0.8021 C   0  0  0  0  0  0  0  0  0  0  0  0
    7.1474    2.6202   -0.1760 C   0  0  0  0  0  0  0  0  0  0  0  0
    5.1135    3.5418    0.7235 C   0  0  0  0  0  0  0  0  0  0  0  0
    8.7600    9.2796    0.5986 C   0  0  0  0  0  0  0  0  0  0  0  0
   10.1031    7.2731    0.5235 C   0  0  0  0  0  0  0  0  0  0  0  0
    2.7457    7.2872    0.7589 C   0  0  0  0  0  0  0  0  0  0  0  0
    6.0047    2.4777    0.6084 C   0  0  0  0  0  0  0  0  0  0  0  0
    9.9387    8.6026    0.9136 C   0  0  0  0  0  0  0  0  0  0  0  0
    4.8907    8.9235   -1.2980 H   0  0  0  0  0  0  0  0  0  0  0  0
    8.2590    3.9506   -1.4550 H   0  0  0  0  0  0  0  0  0  0  0  0
    6.8331    9.1572   -0.3519 H   0  0  0  0  0  0  0  0  0  0  0  0
    9.2217    5.5892   -0.4853 H   0  0  0  0  0  0  0  0  0  0  0  0
    2.7807    9.2898   -0.0324 H   0  0  0  0  0  0  0  0  0  0  0  0
    3.0044    5.2424    1.3855 H   0  0  0  0  0  0  0  0  0  0  0  0
    7.8527    1.8033   -0.2674 H   0  0  0  0  0  0  0  0  0  0  0  0
    4.2214    3.4475    1.3300 H   0  0  0  0  0  0  0  0  0  0  0  0
    8.6324   10.3123    0.9030 H   0  0  0  0  0  0  0  0  0  0  0  0
   11.0185    6.7472    0.7694 H   0  0  0  0  0  0  0  0  0  0  0  0
    1.8239    7.4308    1.3096 H   0  0  0  0  0  0  0  0  0  0  0  0
    5.8120    1.5459    1.1257 H   0  0  0  0  0  0  0  0  0  0  0  0
   10.7262    9.1091    1.4619 H   0  0  0  0  0  0  0  0  0  0  0  0
  1  2  1  0  0  0  0
  1  3  1  0  0  0  0
  1  4  1  0  0  0  0
  2  5  4  0  0  0  0
  2  6  4  0  0  0  0
  3  7  4  0  0  0  0
  3  8  4  0  0  0  0
  4  9  4  0  0  0  0
  4 10  4  0  0  0  0
  5 11  4  0  0  0  0
  6 12  4  0  0  0  0
  7 13  4  0  0  0  0
  8 14  4  0  0  0  0
  9 15  4  0  0  0  0
 10 16  4  0  0  0  0
 11 17  4  0  0  0  0
 12 17  4  0  0  0  0
 13 18  4  0  0  0  0
 14 18  4  0  0  0  0
 15 19  4  0  0  0  0
 16 19  4  0  0  0  0
  8  6  1  0  0  0  0
  5 20  1  0  0  0  0
```

```
  7 21  1  0  0  0  0
  9 22  1  0  0  0  0
 10 23  1  0  0  0  0
 11 24  1  0  0  0  0
 12 25  1  0  0  0  0
 13 26  1  0  0  0  0
 14 27  1  0  0  0  0
 15 28  1  0  0  0  0
 16 29  1  0  0  0  0
 17 30  1  0  0  0  0
 18 31  1  0  0  0  0
 19 32  1  0  0  0  0
M  END
>  <Alias name>
9-Phenyl-9-phosphafluorene

>  <deltaH°(c)>   kJ/mol
-9982.2

$$$$
p-Chlorobenzyliden-5,6,7,8-tetrahydronaphthyl-2-acetonitrile
  ChemBrain IXL

 37 39  0  0  0  0  0  0  0  0  1 V2000
    2.5106    8.2571    0.3347 C   0  0  0  0  0  0  0  0  0  0  0  0  0
    2.9982    9.5079   -0.3685 C   0  0  0  0  0  0  0  0  0  0  0  0  0
    3.3285    7.0616   -0.1578 C   0  0  0  0  0  0  0  0  0  0  0  0  0
    4.3842    9.8668    0.1669 C   0  0  0  0  0  0  0  0  0  0  0  0  0
    5.2976    8.6764    0.0974 C   0  0  0  0  0  0  0  0  0  0  0  0  0
    4.7994    7.3509   -0.0502 C   0  0  0  0  0  0  0  0  0  0  0  0  0
    6.6711    8.8889    0.1836 C   0  0  0  0  0  0  0  0  0  0  0  0  0
    5.6923    6.2816   -0.1002 C   0  0  0  0  0  0  0  0  0  0  0  0  0
    7.0645    6.5100   -0.0109 C   0  0  0  0  0  0  0  0  0  0  0  0  0
    7.5507    7.8101    0.1297 C   0  0  0  0  0  0  0  0  0  0  0  0  0
    9.0003    8.0448    0.2219 C   0  0  0  0  0  0  0  0  0  0  0  0  0
   11.3057    7.2520    0.1771 C   0  0  0  0  0  0  0  0  0  0  0  0  0
    9.8470    7.0228    0.0861 C   0  0  0  0  0  0  0  0  0  0  0  0  0
   11.8602    8.4341   -0.3159 C   0  0  0  0  0  0  0  0  0  0  0  0  0
   12.1264    6.2850    0.7560 C   0  0  0  0  0  0  0  0  0  0  0  0  0
    9.5117    9.4129    0.4646 C   0  0  0  0  0  0  0  0  0  0  0  0  0
    9.9105   10.4835    0.6545 N   0  0  0  0  0  0  0  0  0  0  0  0  0
   13.2363    8.6471   -0.2310 C   0  0  0  0  0  0  0  0  0  0  0  0  0
   13.5022    6.4978    0.8405 C   0  0  0  0  0  0  0  0  0  0  0  0  0
   14.0573    7.6786    0.3471 C   0  0  0  0  0  0  0  0  0  0  0  0  0
   15.8171    7.9502    0.4557 Cl  0  0  0  0  0  0  0  0  0  0  0  0  0
    1.4623    8.0987    0.1084 H   0  0  0  0  0  0  0  0  0  0  0  0  0
    2.6376    8.3692    1.4045 H   0  0  0  0  0  0  0  0  0  0  0  0  0
    3.0556    9.3263   -1.4346 H   0  0  0  0  0  0  0  0  0  0  0  0  0
    2.3112   10.3242   -0.1758 H   0  0  0  0  0  0  0  0  0  0  0  0  0
    3.0777    6.8593   -1.1917 H   0  0  0  0  0  0  0  0  0  0  0  0  0
    3.0894    6.1938    0.4444 H   0  0  0  0  0  0  0  0  0  0  0  0  0
    4.2962   10.1916    1.1964 H   0  0  0  0  0  0  0  0  0  0  0  0  0
    4.7989   10.6716   -0.4277 H   0  0  0  0  0  0  0  0  0  0  0  0  0
    7.0554    9.8956    0.2927 H   0  0  0  0  0  0  0  0  0  0  0  0  0
    5.3179    5.2713   -0.2088 H   0  0  0  0  0  0  0  0  0  0  0  0  0
    7.7535    5.6770   -0.0508 H   0  0  0  0  0  0  0  0  0  0  0  0  0
    9.4658    6.0250   -0.0905 H   0  0  0  0  0  0  0  0  0  0  0  0  0
   11.2224    9.1863   -0.7644 H   0  0  0  0  0  0  0  0  0  0  0  0  0
   11.6953    5.3690    1.1399 H   0  0  0  0  0  0  0  0  0  0  0  0  0
   13.6674    9.5642   -0.6140 H   0  0  0  0  0  0  0  0  0  0  0  0  0
   14.1393    5.7462    1.2890 H   0  0  0  0  0  0  0  0  0  0  0  0  0
  1  2  1  0  0  0  0
  3  1  1  0  0  0  0
```

```
  2  4  1  0  0  0  0
  6  3  1  0  0  0  0
  4  5  1  0  0  0  0
  6  5  4  0  0  0  0
  5  7  4  0  0  0  0
  8  6  4  0  0  0  0
  7 10  4  0  0  0  0
  9  8  4  0  0  0  0
 10  9  4  0  0  0  0
 10 11  1  0  0  0  0
 11 13  2  0  0  0  0
 11 16  1  0  0  0  0
 12 13  1  0  0  0  0
 12 14  4  0  0  0  0
 15 12  4  0  0  0  0
 14 18  4  0  0  0  0
 19 15  4  0  0  0  0
 16 17  3  0  0  0  0
 18 20  4  0  0  0  0
 20 19  4  0  0  0  0
 20 21  1  0  0  0  0
  1 22  1  0  0  0  0
  1 23  1  0  0  0  0
  2 24  1  0  0  0  0
  2 25  1  0  0  0  0
  3 26  1  0  0  0  0
  3 27  1  0  0  0  0
  4 28  1  0  0  0  0
  4 29  1  0  0  0  0
  7 30  1  0  0  0  0
  8 31  1  0  0  0  0
  9 32  1  0  0  0  0
 13 33  1  0  0  0  0
 14 34  1  0  0  0  0
 15 35  1  0  0  0  0
 18 36  1  0  0  0  0
 19 37  1  0  0  0  0
M  END
>  <Alias name>
p-Chlorobenzyliden-5,6,7,8-tetrahydronaphthyl-2-acetonitrile

>  <deltaH°(c)>   kJ/mol
-9982.2

$$$$
(3,3)Paracyclophane
  ChemBrain IXL

 38 40  0  0  0  0  0  0  0  0  1 V2000
    7.4024    3.7003    0.1118 C   0  0  0  0  0  0  0  0  0  0  0  0
    8.8750    3.8957   -0.0038 C   0  0  0  0  0  0  0  0  0  0  0  0
    6.7743    3.8477    1.3486 C   0  0  0  0  0  0  0  0  0  0  0  0
    6.6519    3.3701   -1.0167 C   0  0  0  0  0  0  0  0  0  0  0  0
    8.5227    6.3259   -0.4649 C   0  0  0  0  0  0  0  0  0  0  0  0
    5.3941    3.6750    1.4549 C   0  0  0  0  0  0  0  0  0  0  0  0
    5.2717    3.1974   -0.9105 C   0  0  0  0  0  0  0  0  0  0  0  0
    7.0486    6.1564   -0.3293 C   0  0  0  0  0  0  0  0  0  0  0  0
    4.6421    3.3550    0.3243 C   0  0  0  0  0  0  0  0  0  0  0  0
    6.2783    5.8309   -1.4459 C   0  0  0  0  0  0  0  0  0  0  0  0
    6.4388    6.3234    0.9142 C   0  0  0  0  0  0  0  0  0  0  0  0
    3.1664    3.1815    0.4357 C   0  0  0  0  0  0  0  0  0  0  0  0
    4.8995    5.6623   -1.3169 C   0  0  0  0  0  0  0  0  0  0  0  0
    5.0600    6.1548    1.0431 C   0  0  0  0  0  0  0  0  0  0  0  0
```

```
  2.8200    5.6285    0.0684 C   0  0  0  0  0  0  0  0  0  0  0  0
  4.2911    5.8192   -0.0714 C   0  0  0  0  0  0  0  0  0  0  0  0
  2.5452    4.4556    1.0117 C   0  0  0  0  0  0  0  0  0  0  0  0
  9.2371    5.3159    0.4354 C   0  0  0  0  0  0  0  0  0  0  0  0
  9.3849    3.1808    0.6301 H   0  0  0  0  0  0  0  0  0  0  0  0
  9.1792    3.7474   -1.0326 H   0  0  0  0  0  0  0  0  0  0  0  0
  7.3581    4.0962    2.2263 H   0  0  0  0  0  0  0  0  0  0  0  0
  7.1407    3.2477   -1.9754 H   0  0  0  0  0  0  0  0  0  0  0  0
  8.7992    7.3307   -0.1705 H   0  0  0  0  0  0  0  0  0  0  0  0
  8.8120    6.1590   -1.4951 H   0  0  0  0  0  0  0  0  0  0  0  0
  4.9064    3.7893    2.4151 H   0  0  0  0  0  0  0  0  0  0  0  0
  4.6890    2.9411   -1.7866 H   0  0  0  0  0  0  0  0  0  0  0  0
  6.7506    5.7090   -2.4128 H   0  0  0  0  0  0  0  0  0  0  0  0
  7.0357    6.5839    1.7794 H   0  0  0  0  0  0  0  0  0  0  0  0
  2.7505    2.9888   -0.5455 H   0  0  0  0  0  0  0  0  0  0  0  0
  2.9495    2.3464    1.0902 H   0  0  0  0  0  0  0  0  0  0  0  0
  4.3015    5.4096   -2.1837 H   0  0  0  0  0  0  0  0  0  0  0  0
  4.5866    6.2843    2.0085 H   0  0  0  0  0  0  0  0  0  0  0  0
  2.3892    5.4192   -0.9029 H   0  0  0  0  0  0  0  0  0  0  0  0
  2.3761    6.5293    0.4737 H   0  0  0  0  0  0  0  0  0  0  0  0
  1.4759    4.3200    1.1172 H   0  0  0  0  0  0  0  0  0  0  0  0
  2.9802    4.6628    1.9816 H   0  0  0  0  0  0  0  0  0  0  0  0
  8.9281    5.4660    1.4624 H   0  0  0  0  0  0  0  0  0  0  0  0
 10.3081    5.4562    0.3564 H   0  0  0  0  0  0  0  0  0  0  0  0
  1  2  1  0  0  0  0
  1  3  4  0  0  0  0
  1  4  4  0  0  0  0
  3  6  4  0  0  0  0
  4  7  4  0  0  0  0
  5  8  1  0  0  0  0
  6  9  4  0  0  0  0
  7  9  4  0  0  0  0
  8 10  4  0  0  0  0
  8 11  4  0  0  0  0
  9 12  1  0  0  0  0
 10 13  4  0  0  0  0
 11 14  4  0  0  0  0
 13 16  4  0  0  0  0
 14 16  4  0  0  0  0
 15 16  1  0  0  0  0
 12 17  1  0  0  0  0
 17 15  1  0  0  0  0
  2 18  1  0  0  0  0
 18  5  1  0  0  0  0
  2 19  1  0  0  0  0
  2 20  1  0  0  0  0
  3 21  1  0  0  0  0
  4 22  1  0  0  0  0
  5 23  1  0  0  0  0
  5 24  1  0  0  0  0
  6 25  1  0  0  0  0
  7 26  1  0  0  0  0
 10 27  1  0  0  0  0
 11 28  1  0  0  0  0
 12 29  1  0  0  0  0
 12 30  1  0  0  0  0
 13 31  1  0  0  0  0
 14 32  1  0  0  0  0
 15 33  1  0  0  0  0
 15 34  1  0  0  0  0
 17 35  1  0  0  0  0
 17 36  1  0  0  0  0
 18 37  1  0  0  0  0
```

```
 18 38  1  0  0  0  0
M  END
>  <Alias name>
[3,3]Paracyclophane

>  <deltaH°(c)>   kJ/mol
-9967.8

$$$$
Diacylperoxide IV
  ChemBrain IXL

 56 55  0  0  0  0  0  0  0  0  1 V2000
   14.9290   13.0195   -1.1284 C   0  0  0  0  0  0  0  0  0  0  0  0  0
   16.3932   12.8217   -1.0413 C   0  0  0  0  0  0  0  0  0  0  0  0  0
   17.1244   13.2938   -1.8962 O   0  0  0  0  0  0  0  0  0  0  0  0  0
   16.9664   12.0562    0.0751 O   0  0  0  0  0  0  0  0  0  0  0  0  0
   12.0413   11.8853    1.1984 C   0  0  0  0  0  0  0  0  0  0  0  0  0
   10.5772   12.0837    1.1118 C   0  0  0  0  0  0  0  0  0  0  0  0  0
    9.8462   11.6142    1.9684 O   0  0  0  0  0  0  0  0  0  0  0  0  0
   10.0037   12.8464   -0.0064 O   0  0  0  0  0  0  0  0  0  0  0  0  0
    6.5636   14.6025    0.0448 C   0  0  0  0  0  0  0  0  0  0  0  0  0
    8.0220   14.3501    0.0356 C   0  0  0  0  0  0  0  0  0  0  0  0  0
    8.8032   15.2871    0.0474 O   0  0  0  0  0  0  0  0  0  0  0  0  0
    8.5290   12.9705    0.0123 O   0  0  0  0  0  0  0  0  0  0  0  0  0
   20.4063   10.2999    0.0200 C   0  0  0  0  0  0  0  0  0  0  0  0  0
   18.9480   10.5524    0.0306 C   0  0  0  0  0  0  0  0  0  0  0  0  0
   18.1666    9.6155    0.0179 O   0  0  0  0  0  0  0  0  0  0  0  0  0
   18.4411   11.9320    0.0564 O   0  0  0  0  0  0  0  0  0  0  0  0  0
   12.7227   12.5534   -0.0115 C   0  0  0  0  0  0  0  0  0  0  0  0  0
   14.2476   12.3512    0.0815 C   0  0  0  0  0  0  0  0  0  0  0  0  0
   21.1605   11.6434    0.0500 C   0  0  0  0  0  0  0  0  0  0  0  0  0
   22.6788   11.3807    0.0326 C   0  0  0  0  0  0  0  0  0  0  0  0  0
   23.4330   12.7242    0.0611 C   0  0  0  0  0  0  0  0  0  0  0  0  0
    5.8096   13.2587    0.0255 C   0  0  0  0  0  0  0  0  0  0  0  0  0
    4.2913   13.5214    0.0420 C   0  0  0  0  0  0  0  0  0  0  0  0  0
    3.5372   12.1776    0.0244 C   0  0  0  0  0  0  0  0  0  0  0  0  0
   24.9513   12.4614    0.0433 C   0  0  0  0  0  0  0  0  0  0  0  0  0
    2.0189   12.4403    0.0414 C   0  0  0  0  0  0  0  0  0  0  0  0  0
   14.5583   12.5739   -2.0432 H   0  0  0  0  0  0  0  0  0  0  0  0  0
   14.7073   14.0796   -1.1275 H   0  0  0  0  0  0  0  0  0  0  0  0  0
   12.4124   12.3307    2.1132 H   0  0  0  0  0  0  0  0  0  0  0  0  0
   12.2626   10.8251    1.1975 H   0  0  0  0  0  0  0  0  0  0  0  0  0
    6.2920   15.1815   -0.8292 H   0  0  0  0  0  0  0  0  0  0  0  0  0
    6.2981   15.1526    0.9391 H   0  0  0  0  0  0  0  0  0  0  0  0  0
   20.6776    9.7142    0.8896 H   0  0  0  0  0  0  0  0  0  0  0  0  0
   20.6721    9.7567   -0.8784 H   0  0  0  0  0  0  0  0  0  0  0  0  0
   12.3542   12.1055   -0.9261 H   0  0  0  0  0  0  0  0  0  0  0  0  0
   12.4984   13.6129   -0.0125 H   0  0  0  0  0  0  0  0  0  0  0  0  0
   14.6160   12.7989    0.9962 H   0  0  0  0  0  0  0  0  0  0  0  0  0
   14.4719   11.2917    0.0823 H   0  0  0  0  0  0  0  0  0  0  0  0  0
   20.8858   12.2310   -0.8173 H   0  0  0  0  0  0  0  0  0  0  0  0  0
   20.8980   12.1847    0.9506 H   0  0  0  0  0  0  0  0  0  0  0  0  0
   22.9536   10.7940    0.9005 H   0  0  0  0  0  0  0  0  0  0  0  0  0
   22.9411   10.8385   -0.8674 H   0  0  0  0  0  0  0  0  0  0  0  0  0
   23.1580   13.3110   -0.8066 H   0  0  0  0  0  0  0  0  0  0  0  0  0
   23.1710   13.2662    0.9613 H   0  0  0  0  0  0  0  0  0  0  0  0  0
    6.0850   12.6777    0.8970 H   0  0  0  0  0  0  0  0  0  0  0  0  0
    6.0715   12.7107   -0.8711 H   0  0  0  0  0  0  0  0  0  0  0  0  0
    4.0157   14.1014   -0.8301 H   0  0  0  0  0  0  0  0  0  0  0  0  0
    4.0296   14.0704    0.9381 H   0  0  0  0  0  0  0  0  0  0  0  0  0
    3.8130   11.5974    0.8963 H   0  0  0  0  0  0  0  0  0  0  0  0  0
    3.7986   11.6287   -0.8719 H   0  0  0  0  0  0  0  0  0  0  0  0  0
```

```
   25.4814   13.4056    0.0631 H   0  0  0  0  0  0  0  0  0  0  0  0
   25.2133   11.9192   -0.8569 H   0  0  0  0  0  0  0  0  0  0  0  0
   25.2264   11.8747    0.9110 H   0  0  0  0  0  0  0  0  0  0  0  0
    1.4890   11.4959    0.0290 H   0  0  0  0  0  0  0  0  0  0  0  0
    1.7575   12.9892    0.9376 H   0  0  0  0  0  0  0  0  0  0  0  0
    1.7431   13.0204   -0.8306 H   0  0  0  0  0  0  0  0  0  0  0  0
  1  2  1  0  0  0  0
  2  3  2  0  0  0  0
  2  4  1  0  0  0  0
  5  6  1  0  0  0  0
  6  7  2  0  0  0  0
  6  8  1  0  0  0  0
  9 10  1  0  0  0  0
 10 11  2  0  0  0  0
 10 12  1  0  0  0  0
 13 14  1  0  0  0  0
 14 15  2  0  0  0  0
 14 16  1  0  0  0  0
 16  4  1  0  0  0  0
 12  8  1  0  0  0  0
  5 17  1  0  0  0  0
  1 18  1  0  0  0  0
 17 18  1  0  0  0  0
 13 19  1  0  0  0  0
 19 20  1  0  0  0  0
 20 21  1  0  0  0  0
  9 22  1  0  0  0  0
 22 23  1  0  0  0  0
 23 24  1  0  0  0  0
 21 25  1  0  0  0  0
 24 26  1  0  0  0  0
  1 27  1  0  0  0  0
  1 28  1  0  0  0  0
  5 29  1  0  0  0  0
  5 30  1  0  0  0  0
  9 31  1  0  0  0  0
  9 32  1  0  0  0  0
 13 33  1  0  0  0  0
 13 34  1  0  0  0  0
 17 35  1  0  0  0  0
 17 36  1  0  0  0  0
 18 37  1  0  0  0  0
 18 38  1  0  0  0  0
 19 39  1  0  0  0  0
 19 40  1  0  0  0  0
 20 41  1  0  0  0  0
 20 42  1  0  0  0  0
 21 43  1  0  0  0  0
 21 44  1  0  0  0  0
 22 45  1  0  0  0  0
 22 46  1  0  0  0  0
 23 47  1  0  0  0  0
 23 48  1  0  0  0  0
 24 49  1  0  0  0  0
 24 50  1  0  0  0  0
 25 51  1  0  0  0  0
 25 52  1  0  0  0  0
 25 53  1  0  0  0  0
 26 54  1  0  0  0  0
 26 55  1  0  0  0  0
 26 56  1  0  0  0  0
M  END
>  <Alias name>
```

Diacylperoxide IV

> <deltaH°(c)>   kJ/mol
-9966.8

$$$$
Di-t-amylperoxypyromellitate
  ChemBrain IXL

```
 56 56  0  0  0  0  0  0  0  0  1 V2000
   10.6607    7.9642   -0.3135 C   0  0  0  0  0  0  0  0  0  0  0  0  0
   11.0432    9.3035   -0.4372 C   0  0  0  0  0  0  0  0  0  0  0  0  0
    9.3153    7.6227   -0.3765 C   0  0  0  0  0  0  0  0  0  0  0  0  0
   11.6763    6.9062   -0.1140 C   0  0  0  0  0  0  0  0  0  0  0  0  0
   10.0814   10.3045   -0.3724 C   0  0  0  0  0  0  0  0  0  0  0  0  0
   12.4638    9.6652   -0.6383 C   0  0  0  0  0  0  0  0  0  0  0  0  0
    8.7359    9.9629   -0.3125 C   0  0  0  0  0  0  0  0  0  0  0  0  0
    8.3537    8.6238   -0.4403 C   0  0  0  0  0  0  0  0  0  0  0  0  0
    7.7201   11.0204   -0.1116 C   0  0  0  0  0  0  0  0  0  0  0  0  0
    6.9333    8.2627   -0.6449 C   0  0  0  0  0  0  0  0  0  0  0  0  0
   13.3994    8.6857   -1.2079 O   0  0  0  0  0  0  0  0  0  0  0  0  0
   14.7799    9.1817   -1.4038 O   0  0  0  0  0  0  0  0  0  0  0  0  0
   12.8581   10.7780   -0.3317 O   0  0  0  0  0  0  0  0  0  0  0  0  0
   15.8275    8.3778   -0.7565 C   0  0  0  0  0  0  0  0  0  0  0  0  0
   15.5959    8.3615    0.7675 C   0  0  0  0  0  0  0  0  0  0  0  0  0
   15.5249    9.8087    1.2921 C   0  0  0  0  0  0  0  0  0  0  0  0  0
   17.2072    8.9929   -1.0595 C   0  0  0  0  0  0  0  0  0  0  0  0  0
   15.7779    6.9358   -1.2985 C   0  0  0  0  0  0  0  0  0  0  0  0  0
    6.6220   10.7352    0.3380 O   0  0  0  0  0  0  0  0  0  0  0  0  0
    8.0358   12.4158   -0.4533 O   0  0  0  0  0  0  0  0  0  0  0  0  0
   12.7757    7.1912    0.3325 O   0  0  0  0  0  0  0  0  0  0  0  0  0
   11.3587    5.5105   -0.4531 O   0  0  0  0  0  0  0  0  0  0  0  0  0
    6.5412    7.1451   -0.3533 O   0  0  0  0  0  0  0  0  0  0  0  0  0
    5.9954    9.2484   -1.2000 O   0  0  0  0  0  0  0  0  0  0  0  0  0
    4.6140    8.7548   -1.3949 O   0  0  0  0  0  0  0  0  0  0  0  0  0
    3.5685    9.5579   -0.7428 C   0  0  0  0  0  0  0  0  0  0  0  0  0
    2.1863    8.9552   -1.0592 C   0  0  0  0  0  0  0  0  0  0  0  0  0
    3.6298   11.0057   -1.2677 C   0  0  0  0  0  0  0  0  0  0  0  0  0
    3.7933    9.5548    0.7822 C   0  0  0  0  0  0  0  0  0  0  0  0  0
    3.8363    8.1014    1.2925 C   0  0  0  0  0  0  0  0  0  0  0  0  0
    8.9359   12.5599   -0.8226 H   0  0  0  0  0  0  0  0  0  0  0  0  0
   10.4579    5.3667   -0.8206 H   0  0  0  0  0  0  0  0  0  0  0  0  0
    9.0176    6.5813   -0.3756 H   0  0  0  0  0  0  0  0  0  0  0  0  0
   10.3791   11.3459   -0.3685 H   0  0  0  0  0  0  0  0  0  0  0  0  0
   14.6653    7.8520    0.9857 H   0  0  0  0  0  0  0  0  0  0  0  0  0
   16.4137    7.8421    1.2515 H   0  0  0  0  0  0  0  0  0  0  0  0  0
   14.5556   10.2307    1.0572 H   0  0  0  0  0  0  0  0  0  0  0  0  0
   16.3002   10.4013    0.8222 H   0  0  0  0  0  0  0  0  0  0  0  0  0
   15.6692    9.8110    2.3655 H   0  0  0  0  0  0  0  0  0  0  0  0  0
   17.1710   10.0617   -0.8888 H   0  0  0  0  0  0  0  0  0  0  0  0  0
   17.4692    8.8008   -2.0927 H   0  0  0  0  0  0  0  0  0  0  0  0  0
   17.9512    8.5484   -0.4100 H   0  0  0  0  0  0  0  0  0  0  0  0  0
   16.7143    6.4365   -1.0822 H   0  0  0  0  0  0  0  0  0  0  0  0  0
   14.9649    6.3997   -0.8244 H   0  0  0  0  0  0  0  0  0  0  0  0  0
   15.6197    6.9577   -2.3696 H   0  0  0  0  0  0  0  0  0  0  0  0  0
    2.2138    7.8848   -0.8971 H   0  0  0  0  0  0  0  0  0  0  0  0  0
    1.4416    9.3997   -0.4106 H   0  0  0  0  0  0  0  0  0  0  0  0  0
    1.9320    9.1576   -2.0924 H   0  0  0  0  0  0  0  0  0  0  0  0  0
    2.6963   11.5091   -1.0479 H   0  0  0  0  0  0  0  0  0  0  0  0  0
    3.7906   10.9953   -2.3385 H   0  0  0  0  0  0  0  0  0  0  0  0  0
    4.4453   11.5305   -0.7852 H   0  0  0  0  0  0  0  0  0  0  0  0  0
    2.9825   10.0838    1.2678 H   0  0  0  0  0  0  0  0  0  0  0  0  0
    4.7317   10.0455    1.0098 H   0  0  0  0  0  0  0  0  0  0  0  0  0
```

```
    4.7983    7.6640    1.0560 H   0  0  0  0  0  0  0  0  0  0  0  0
    3.6889    8.0911    2.3654 H   0  0  0  0  0  0  0  0  0  0  0  0
    3.0516    7.5278    0.8148 H   0  0  0  0  0  0  0  0  0  0  0  0
  2  1  4  0  0  0  0
  1  3  4  0  0  0  0
  1  4  1  0  0  0  0
  5  2  4  0  0  0  0
  2  6  1  0  0  0  0
  3  8  4  0  0  0  0
  4 21  2  0  0  0  0
  4 22  1  0  0  0  0
  7  5  4  0  0  0  0
  6 11  1  0  0  0  0
  6 13  2  0  0  0  0
  8  7  4  0  0  0  0
  7  9  1  0  0  0  0
  8 10  1  0  0  0  0
  9 19  2  0  0  0  0
  9 20  1  0  0  0  0
 10 23  2  0  0  0  0
 10 24  1  0  0  0  0
 11 12  1  0  0  0  0
 12 14  1  0  0  0  0
 14 15  1  0  0  0  0
 14 17  1  0  0  0  0
 14 18  1  0  0  0  0
 15 16  1  0  0  0  0
 24 25  1  0  0  0  0
 25 26  1  0  0  0  0
 26 27  1  0  0  0  0
 26 28  1  0  0  0  0
 26 29  1  0  0  0  0
 29 30  1  0  0  0  0
 20 31  1  0  0  0  0
 22 32  1  0  0  0  0
  3 33  1  0  0  0  0
  5 34  1  0  0  0  0
 15 35  1  0  0  0  0
 15 36  1  0  0  0  0
 16 37  1  0  0  0  0
 16 38  1  0  0  0  0
 16 39  1  0  0  0  0
 17 40  1  0  0  0  0
 17 41  1  0  0  0  0
 17 42  1  0  0  0  0
 18 43  1  0  0  0  0
 18 44  1  0  0  0  0
 18 45  1  0  0  0  0
 27 46  1  0  0  0  0
 27 47  1  0  0  0  0
 27 48  1  0  0  0  0
 28 49  1  0  0  0  0
 28 50  1  0  0  0  0
 28 51  1  0  0  0  0
 29 52  1  0  0  0  0
 29 53  1  0  0  0  0
 30 54  1  0  0  0  0
 30 55  1  0  0  0  0
 30 56  1  0  0  0  0
M  END
>  <Alias name>
Di-t-amylperoxypyromellitate
```

> <deltaH°(c)>   kJ/mol
-9945.4

$$$$
(Z)-1,2-Bis(benzylthio)ethylene
  ChemBrain IXL

 34 35 0 0 0 0 0 0 0 0 0 1 V2000
    4.8039   8.1432   0.0000 C   0 0 0 0 0 0 0 0 0 0 0 0 0
    5.2488   9.4654   0.0029 C   0 0 0 0 0 0 0 0 0 0 0 0 0
    3.4364   7.8674   0.0003 C   0 0 0 0 0 0 0 0 0 0 0 0 0
    5.7827   7.0331   -0.0034 C   0 0 0 0 0 0 0 0 0 0 0 0 0
    4.3262   10.5117   0.0056 C   0 0 0 0 0 0 0 0 0 0 0 0 0
    2.9587   10.2359   0.0056 C   0 0 0 0 0 0 0 0 0 0 0 0 0
    2.5139   8.9138   0.0030 C   0 0 0 0 0 0 0 0 0 0 0 0 0
   12.3041   7.0253   -0.0079 C   0 0 0 0 0 0 0 0 0 0 0 0 0
   10.6202   7.7080   -0.0043 S   0 0 0 0 0 0 0 0 0 0 0 0 0
   13.2855   8.1331   -0.0042 C   0 0 0 0 0 0 0 0 0 0 0 0 0
    9.7098   6.1355   -0.0074 C   0 0 0 0 0 0 0 0 0 0 0 0 0
    8.3748   6.1371   -0.0067 C   0 0 0 0 0 0 0 0 0 0 0 0 0
    7.4682   7.7117   -0.0027 S   0 0 0 0 0 0 0 0 0 0 0 0 0
   13.7689   10.5004   -0.0013 C   0 0 0 0 0 0 0 0 0 0 0 0 0
   12.8438   9.4563   -0.0041 C   0 0 0 0 0 0 0 0 0 0 0 0 0
   15.1357   10.2213   0.0017 C   0 0 0 0 0 0 0 0 0 0 0 0 0
   14.6523   7.8540   -0.0007 C   0 0 0 0 0 0 0 0 0 0 0 0 0
   15.5774   8.8981   0.0021 C   0 0 0 0 0 0 0 0 0 0 0 0 0
    6.3104   9.6795   0.0032 H   0 0 0 0 0 0 0 0 0 0 0 0 0
    3.0911   6.8410   -0.0016 H   0 0 0 0 0 0 0 0 0 0 0 0 0
    5.6380   6.4270   -0.8892 H   0 0 0 0 0 0 0 0 0 0 0 0 0
    5.6390   6.4224   0.8794 H   0 0 0 0 0 0 0 0 0 0 0 0 0
    4.6716   11.5382   0.0077 H   0 0 0 0 0 0 0 0 0 0 0 0 0
    2.2425   11.0483   0.0077 H   0 0 0 0 0 0 0 0 0 0 0 0 0
    1.4522   8.6997   0.0030 H   0 0 0 0 0 0 0 0 0 0 0 0 0
   12.4473   6.4131   0.8739 H   0 0 0 0 0 0 0 0 0 0 0 0 0
   12.4463   6.4200   -0.8946 H   0 0 0 0 0 0 0 0 0 0 0 0 0
   10.2502   5.1969   -0.0098 H   0 0 0 0 0 0 0 0 0 0 0 0 0
    7.8322   5.1998   -0.0085 H   0 0 0 0 0 0 0 0 0 0 0 0 0
   13.4260   11.5277   -0.0016 H   0 0 0 0 0 0 0 0 0 0 0 0 0
   11.7827   9.6729   -0.0063 H   0 0 0 0 0 0 0 0 0 0 0 0 0
   15.8539   11.0320   0.0039 H   0 0 0 0 0 0 0 0 0 0 0 0 0
   14.9952   6.8267   -0.0001 H   0 0 0 0 0 0 0 0 0 0 0 0 0
   16.6385   8.6815   0.0046 H   0 0 0 0 0 0 0 0 0 0 0 0 0
  2 1 4 0 0 0 0
  1 3 4 0 0 0 0
  1 4 1 0 0 0 0
  5 2 4 0 0 0 0
  3 7 4 0 0 0 0
  4 13 1 0 0 0 0
  6 5 4 0 0 0 0
  7 6 4 0 0 0 0
  8 9 1 0 0 0 0
  8 10 1 0 0 0 0
  9 11 1 0 0 0 0
 10 15 4 0 0 0 0
 17 10 4 0 0 0 0
 11 12 2 0 0 0 0
 12 13 1 0 0 0 0
 15 14 4 0 0 0 0
 14 16 4 0 0 0 0
 16 18 4 0 0 0 0
 18 17 4 0 0 0 0
  2 19 1 0 0 0 0
  3 20 1 0 0 0 0

```
  4 21  1  0  0  0  0
  4 22  1  0  0  0  0
  5 23  1  0  0  0  0
  6 24  1  0  0  0  0
  7 25  1  0  0  0  0
  8 26  1  0  0  0  0
  8 27  1  0  0  0  0
 11 28  1  0  0  0  0
 12 29  1  0  0  0  0
 14 30  1  0  0  0  0
 15 31  1  0  0  0  0
 16 32  1  0  0  0  0
 17 33  1  0  0  0  0
 18 34  1  0  0  0  0
M  END
>  <Alias name>
(Z)-1,2-Bis(benzylthio)ethylene

>  <deltaH°(c)>   kJ/mol
-9937

$$$$
Triphenylmethane
  ChemBrain IXL

 35 37  0  0  0  0  0  0  0  0  1 V2000
    9.5903    2.3065   -0.6070 C   0  0  0  0  0  0  0  0  0  0  0  0
    9.4981    3.1770   -1.6933 C   0  0  0  0  0  0  0  0  0  0  0  0
    8.5951    4.2400   -1.6608 C   0  0  0  0  0  0  0  0  0  0  0  0
    7.7842    4.4326   -0.5420 C   0  0  0  0  0  0  0  0  0  0  0  0
    7.8765    3.5620    0.5444 C   0  0  0  0  0  0  0  0  0  0  0  0
    8.7795    2.4990    0.5119 C   0  0  0  0  0  0  0  0  0  0  0  0
    8.4352    6.5588    1.1833 C   0  0  0  0  0  0  0  0  0  0  0  0
    8.9980    7.6274    1.8817 C   0  0  0  0  0  0  0  0  0  0  0  0
    8.5349    8.9250    1.6631 C   0  0  0  0  0  0  0  0  0  0  0  0
    7.5089    9.1542    0.7460 C   0  0  0  0  0  0  0  0  0  0  0  0
    6.9460    8.0857    0.0474 C   0  0  0  0  0  0  0  0  0  0  0  0
    7.4090    6.7878    0.2660 C   0  0  0  0  0  0  0  0  0  0  0  0
    5.0212    3.8550    0.0310 C   0  0  0  0  0  0  0  0  0  0  0  0
    5.4892    5.1590    0.1964 C   0  0  0  0  0  0  0  0  0  0  0  0
    4.7830    6.0568    0.9977 C   0  0  0  0  0  0  0  0  0  0  0  0
    3.6093    5.6506    1.6333 C   0  0  0  0  0  0  0  0  0  0  0  0
    3.1416    4.3468    1.4677 C   0  0  0  0  0  0  0  0  0  0  0  0
    3.8476    3.4490    0.6666 C   0  0  0  0  0  0  0  0  0  0  0  0
    6.7865    5.6073   -0.5064 C   0  0  0  0  0  0  0  0  0  0  0  0
   10.2893    1.4837   -0.6322 H   0  0  0  0  0  0  0  0  0  0  0  0
   10.1258    3.0279   -2.5594 H   0  0  0  0  0  0  0  0  0  0  0  0
    8.5238    4.9138   -2.5019 H   0  0  0  0  0  0  0  0  0  0  0  0
    7.2489    3.7109    1.4106 H   0  0  0  0  0  0  0  0  0  0  0  0
    8.8509    1.8252    1.3528 H   0  0  0  0  0  0  0  0  0  0  0  0
    8.7940    5.5545    1.3528 H   0  0  0  0  0  0  0  0  0  0  0  0
    9.7923    7.4500    2.5917 H   0  0  0  0  0  0  0  0  0  0  0  0
    8.9706    9.7522    2.2038 H   0  0  0  0  0  0  0  0  0  0  0  0
    7.1504   10.1588    0.5767 H   0  0  0  0  0  0  0  0  0  0  0  0
    6.1518    8.2633   -0.6625 H   0  0  0  0  0  0  0  0  0  0  0  0
    5.5676    3.1598   -0.5892 H   0  0  0  0  0  0  0  0  0  0  0  0
    5.1446    7.0661    1.1261 H   0  0  0  0  0  0  0  0  0  0  0  0
    3.0628    6.3457    2.2535 H   0  0  0  0  0  0  0  0  0  0  0  0
    2.2331    4.0324    1.9598 H   0  0  0  0  0  0  0  0  0  0  0  0
    3.4855    2.4396    0.5384 H   0  0  0  0  0  0  0  0  0  0  0  0
    6.5599    5.9175   -1.5158 H   0  0  0  0  0  0  0  0  0  0  0  0
  1  2  4  0  0  0  0
  2  3  4  0  0  0  0
```

```
  3  4  4  0  0  0  0
  4  5  4  0  0  0  0
  5  6  4  0  0  0  0
  6  1  4  0  0  0  0
  7  8  4  0  0  0  0
  8  9  4  0  0  0  0
  9 10  4  0  0  0  0
 10 11  4  0  0  0  0
 11 12  4  0  0  0  0
 12  7  4  0  0  0  0
 13 14  4  0  0  0  0
 14 15  4  0  0  0  0
 15 16  4  0  0  0  0
 16 17  4  0  0  0  0
 17 18  4  0  0  0  0
 18 13  4  0  0  0  0
  4 19  1  0  0  0  0
 19 14  1  0  0  0  0
 19 12  1  0  0  0  0
  1 20  1  0  0  0  0
  2 21  1  0  0  0  0
  3 22  1  0  0  0  0
  5 23  1  0  0  0  0
  6 24  1  0  0  0  0
  7 25  1  0  0  0  0
  8 26  1  0  0  0  0
  9 27  1  0  0  0  0
 10 28  1  0  0  0  0
 11 29  1  0  0  0  0
 13 30  1  0  0  0  0
 15 31  1  0  0  0  0
 16 32  1  0  0  0  0
 17 33  1  0  0  0  0
 18 34  1  0  0  0  0
 19 35  1  0  0  0  0
M  END
>  <Alias name>
Tritane

>  <deltaH°(c)>   kJ/mol
-9934.1

$$$$
Methyldihexylsilane
  ChemBrain IXL

 44 43  0  0  0  0  0  0  0  0  1 V2000
    3.6919    4.0232   -0.0473 Si  0  0  0  0  0  0  0  0  0  0  0  0
    3.8418    4.1862    1.8038 C   0  0  0  0  0  0  0  0  0  0  0  0
    5.1195    2.9924   -0.6960 C   0  0  0  0  0  0  0  0  0  0  0  0
    6.4655    3.6349   -0.3055 C   0  0  0  0  0  0  0  0  0  0  0  0
    7.6188    2.7123   -0.7447 C   0  0  0  0  0  0  0  0  0  0  0  0
    8.9654    3.3522   -0.3547 C   0  0  0  0  0  0  0  0  0  0  0  0
   10.1192    2.4298   -0.7942 C   0  0  0  0  0  0  0  0  0  0  0  0
   11.4658    3.0695   -0.4040 C   0  0  0  0  0  0  0  0  0  0  0  0
    3.6341    5.7336   -0.8173 C   0  0  0  0  0  0  0  0  0  0  0  0
    4.8941    6.5361   -0.4359 C   0  0  0  0  0  0  0  0  0  0  0  0
    4.7687    7.9727   -0.9788 C   0  0  0  0  0  0  0  0  0  0  0  0
    6.0276    8.7767   -0.5998 C   0  0  0  0  0  0  0  0  0  0  0  0
    5.9026   10.2136   -1.1429 C   0  0  0  0  0  0  0  0  0  0  0  0
    7.1617   11.0174   -0.7645 C   0  0  0  0  0  0  0  0  0  0  0  0
    2.4280    3.3241   -0.3697 H   0  0  0  0  0  0  0  0  0  0  0  0
    3.0028    4.7549    2.1852 H   0  0  0  0  0  0  0  0  0  0  0  0
```

4.7650 4.6972 2.0474 H 0 0 0 0 0 0 0 0 0 0 0 0
3.8447 3.2012 2.2538 H 0 0 0 0 0 0 0 0 0 0 0 0
5.0635 2.0002 -0.2656 H 0 0 0 0 0 0 0 0 0 0 0 0
5.0524 2.9191 -1.7743 H 0 0 0 0 0 0 0 0 0 0 0 0
6.5028 3.7722 0.7681 H 0 0 0 0 0 0 0 0 0 0 0 0
6.5668 4.5852 -0.7932 H 0 0 0 0 0 0 0 0 0 0 0 0
7.5821 2.5746 -1.8182 H 0 0 0 0 0 0 0 0 0 0 0 0
7.5198 1.7521 -0.2538 H 0 0 0 0 0 0 0 0 0 0 0 0
9.0022 3.4898 0.7189 H 0 0 0 0 0 0 0 0 0 0 0 0
9.0642 4.3127 -0.8453 H 0 0 0 0 0 0 0 0 0 0 0 0
10.0826 2.2924 -1.8678 H 0 0 0 0 0 0 0 0 0 0 0 0
10.0204 1.4693 -0.3038 H 0 0 0 0 0 0 0 0 0 0 0 0
11.5648 4.0300 -0.8944 H 0 0 0 0 0 0 0 0 0 0 0 0
12.2763 2.4212 -0.7130 H 0 0 0 0 0 0 0 0 0 0 0 0
11.5026 3.2068 0.6696 H 0 0 0 0 0 0 0 0 0 0 0 0
3.5744 5.6435 -1.8948 H 0 0 0 0 0 0 0 0 0 0 0 0
2.7585 6.2561 -0.4527 H 0 0 0 0 0 0 0 0 0 0 0 0
4.9916 6.5655 0.6422 H 0 0 0 0 0 0 0 0 0 0 0 0
5.7614 6.0705 -0.8629 H 0 0 0 0 0 0 0 0 0 0 0 0
4.6698 7.9438 -2.0568 H 0 0 0 0 0 0 0 0 0 0 0 0
3.8949 8.4462 -0.5486 H 0 0 0 0 0 0 0 0 0 0 0 0
6.1265 8.8056 0.4783 H 0 0 0 0 0 0 0 0 0 0 0 0
6.9015 8.3030 -1.0298 H 0 0 0 0 0 0 0 0 0 0 0 0
5.8034 10.1846 -2.2209 H 0 0 0 0 0 0 0 0 0 0 0 0
5.0291 10.6874 -0.7126 H 0 0 0 0 0 0 0 0 0 0 0 0
7.0736 12.0270 -1.1462 H 0 0 0 0 0 0 0 0 0 0 0 0
8.0353 10.5437 -1.1950 H 0 0 0 0 0 0 0 0 0 0 0 0
7.2611 11.0464 0.3135 H 0 0 0 0 0 0 0 0 0 0 0 0
1 2 1 0 0 0 0
1 3 1 0 0 0 0
3 4 1 0 0 0 0
4 5 1 0 0 0 0
5 6 1 0 0 0 0
6 7 1 0 0 0 0
7 8 1 0 0 0 0
9 10 1 0 0 0 0
10 11 1 0 0 0 0
11 12 1 0 0 0 0
12 13 1 0 0 0 0
13 14 1 0 0 0 0
9 1 1 0 0 0 0
1 15 1 0 0 0 0
2 16 1 0 0 0 0
2 17 1 0 0 0 0
2 18 1 0 0 0 0
3 19 1 0 0 0 0
3 20 1 0 0 0 0
4 21 1 0 0 0 0
4 22 1 0 0 0 0
5 23 1 0 0 0 0
5 24 1 0 0 0 0
6 25 1 0 0 0 0
6 26 1 0 0 0 0
7 27 1 0 0 0 0
7 28 1 0 0 0 0
8 29 1 0 0 0 0
8 30 1 0 0 0 0
8 31 1 0 0 0 0
9 32 1 0 0 0 0
9 33 1 0 0 0 0
10 34 1 0 0 0 0
10 35 1 0 0 0 0
11 36 1 0 0 0 0

```
 11 37  1  0  0  0  0
 12 38  1  0  0  0  0
 12 39  1  0  0  0  0
 13 40  1  0  0  0  0
 13 41  1  0  0  0  0
 14 42  1  0  0  0  0
 14 43  1  0  0  0  0
 14 44  1  0  0  0  0
M  END
>  <Alias name>
Methyldihexylsilane

>  <deltaH°(c)>   kJ/mol
-9934

$$$$
1,2-Dimethyl-1,2-diphenlethylene glycol dimethyl ether
  ChemBrain IXL

 42 43  0  0  0  0  0  0  0  0  1 V2000
    6.4062    3.7005   -0.2387 C   0  0  0  0  0  0  0  0  0  0  0  0
    6.5999    3.9742    1.1925 O   0  0  0  0  0  0  0  0  0  0  0  0
    6.5919    2.1938   -0.5033 C   0  0  0  0  0  0  0  0  0  0  0  0
    7.4396    4.4971   -1.0585 C   0  0  0  0  0  0  0  0  0  0  0  0
    5.0403    4.1068   -0.6383 C   0  0  0  0  0  0  0  0  0  0  0  0
    5.6855    3.2558    2.0917 C   0  0  0  0  0  0  0  0  0  0  0  0
    8.7962    4.1197   -0.6364 O   0  0  0  0  0  0  0  0  0  0  0  0
    7.2612    4.1812   -2.5562 C   0  0  0  0  0  0  0  0  0  0  0  0
    7.2378    5.9458   -0.8325 C   0  0  0  0  0  0  0  0  0  0  0  0
    9.8827    4.7772   -1.3766 C   0  0  0  0  0  0  0  0  0  0  0  0
    4.3709    3.4086   -1.6434 C   0  0  0  0  0  0  0  0  0  0  0  0
    3.0834    3.7916   -2.0201 C   0  0  0  0  0  0  0  0  0  0  0  0
    4.4224    5.1881   -0.0099 C   0  0  0  0  0  0  0  0  0  0  0  0
    3.1350    5.5712   -0.3866 C   0  0  0  0  0  0  0  0  0  0  0  0
    2.4655    4.8729   -1.3917 C   0  0  0  0  0  0  0  0  0  0  0  0
    7.8155    6.5660    0.2755 C   0  0  0  0  0  0  0  0  0  0  0  0
    7.6207    7.9303    0.4915 C   0  0  0  0  0  0  0  0  0  0  0  0
    6.4698    6.6910   -1.7274 C   0  0  0  0  0  0  0  0  0  0  0  0
    6.2750    8.0554   -1.5114 C   0  0  0  0  0  0  0  0  0  0  0  0
    6.8502    8.6750   -0.4017 C   0  0  0  0  0  0  0  0  0  0  0  0
    7.5847    1.8924   -0.1926 H   0  0  0  0  0  0  0  0  0  0  0  0
    6.4687    1.9950   -1.5607 H   0  0  0  0  0  0  0  0  0  0  0  0
    5.8530    1.6350    0.0576 H   0  0  0  0  0  0  0  0  0  0  0  0
    5.8288    2.1887    1.9751 H   0  0  0  0  0  0  0  0  0  0  0  0
    5.8916    3.5378    3.1168 H   0  0  0  0  0  0  0  0  0  0  0  0
    4.6623    3.5116    1.8457 H   0  0  0  0  0  0  0  0  0  0  0  0
    7.4582    3.1304   -2.7294 H   0  0  0  0  0  0  0  0  0  0  0  0
    6.2463    4.4123   -2.8554 H   0  0  0  0  0  0  0  0  0  0  0  0
    7.9535    4.7787   -3.1362 H   0  0  0  0  0  0  0  0  0  0  0  0
    9.7996    5.8509   -1.2618 H   0  0  0  0  0  0  0  0  0  0  0  0
    9.8118    4.5198   -2.4262 H   0  0  0  0  0  0  0  0  0  0  0  0
   10.8372    4.4453   -0.9872 H   0  0  0  0  0  0  0  0  0  0  0  0
    4.8506    2.5691   -2.1313 H   0  0  0  0  0  0  0  0  0  0  0  0
    2.5637    3.2495   -2.8004 H   0  0  0  0  0  0  0  0  0  0  0  0
    4.9421    5.7302    0.7705 H   0  0  0  0  0  0  0  0  0  0  0  0
    2.6553    6.4107    0.1013 H   0  0  0  0  0  0  0  0  0  0  0  0
    1.4660    5.1703   -1.6841 H   0  0  0  0  0  0  0  0  0  0  0  0
    8.4153    5.9883    0.9679 H   0  0  0  0  0  0  0  0  0  0  0  0
    8.0676    8.4114    1.3528 H   0  0  0  0  0  0  0  0  0  0  0  0
    6.0249    6.2104   -2.5899 H   0  0  0  0  0  0  0  0  0  0  0  0
    5.6773    8.6335   -2.2051 H   0  0  0  0  0  0  0  0  0  0  0  0
    6.6986    9.7341   -0.2338 H   0  0  0  0  0  0  0  0  0  0  0  0
  1  2  1  0  0  0  0
```

```
  1  3  1  0  0  0  0
  1  4  1  0  0  0  0
  1  5  1  0  0  0  0
  2  6  1  0  0  0  0
  4  7  1  0  0  0  0
  4  8  1  0  0  0  0
  4  9  1  0  0  0  0
  5 11  4  0  0  0  0
 13  5  4  0  0  0  0
  7 10  1  0  0  0  0
  9 16  4  0  0  0  0
 18  9  4  0  0  0  0
 11 12  4  0  0  0  0
 12 15  4  0  0  0  0
 14 13  4  0  0  0  0
 15 14  4  0  0  0  0
 16 17  4  0  0  0  0
 17 20  4  0  0  0  0
 19 18  4  0  0  0  0
 20 19  4  0  0  0  0
  3 21  1  0  0  0  0
  3 22  1  0  0  0  0
  3 23  1  0  0  0  0
  6 24  1  0  0  0  0
  6 25  1  0  0  0  0
  6 26  1  0  0  0  0
  8 27  1  0  0  0  0
  8 28  1  0  0  0  0
  8 29  1  0  0  0  0
 10 30  1  0  0  0  0
 10 31  1  0  0  0  0
 10 32  1  0  0  0  0
 11 33  1  0  0  0  0
 12 34  1  0  0  0  0
 13 35  1  0  0  0  0
 14 36  1  0  0  0  0
 15 37  1  0  0  0  0
 16 38  1  0  0  0  0
 17 39  1  0  0  0  0
 18 40  1  0  0  0  0
 19 41  1  0  0  0  0
 20 42  1  0  0  0  0
M  END
>  <Alias name>
1,2-Dimethyl-1,2-diphenlethylene glycol dimethyl ether

>  <deltaH°(c)>   kJ/mol
-9929.9

$$$$
N,N'-(1,3-Phenylene)bis(phthalimide)
  ChemBrain IXL

 40 44  0  0  0  0  0  0  0  0  1 V2000
    2.5968   10.3871    0.0000 C   0  0  0  0  0  0  0  0  0  0  0  0
    3.3049   11.6087    0.0076 C   0  0  0  0  0  0  0  0  0  0  0  0
    4.7137   11.5767    0.0438 C   0  0  0  0  0  0  0  0  0  0  0  0
    5.3653   10.3203    0.0131 C   0  0  0  0  0  0  0  0  0  0  0  0
    4.7382    9.2137   -0.0142 C   0  0  0  0  0  0  0  0  0  0  0  0
    3.3233    9.1650   -0.0133 C   0  0  0  0  0  0  0  0  0  0  0  0
    6.7213   10.0298    0.0187 C   0  0  0  0  0  0  0  0  0  0  0  0
    7.6257   10.8828    0.0332 O   0  0  0  0  0  0  0  0  0  0  0  0
    6.8853    8.7151    0.0041 N   0  0  0  0  0  0  0  0  0  0  0  0
```

```
   5.7092    8.1746   -0.0304 C   0  0  0  0  0  0  0  0  0  0  0  0
   5.4891    6.9340   -0.0771 O   0  0  0  0  0  0  0  0  0  0  0  0
  15.4109   11.6037   -0.0134 C   0  0  0  0  0  0  0  0  0  0  0  0
  16.1239   10.3876    0.0003 C   0  0  0  0  0  0  0  0  0  0  0  0
  15.4040    9.1833    0.0131 C   0  0  0  0  0  0  0  0  0  0  0  0
  14.0014    9.2390    0.0119 C   0  0  0  0  0  0  0  0  0  0  0  0
  13.3498   10.3239    0.0037 C   0  0  0  0  0  0  0  0  0  0  0  0
  13.9913   11.5706   -0.0082 C   0  0  0  0  0  0  0  0  0  0  0  0
  13.0959    8.2150    0.0234 C   0  0  0  0  0  0  0  0  0  0  0  0
  13.3985    7.0129    0.0294 O   0  0  0  0  0  0  0  0  0  0  0  0
  11.8819    8.7186    0.0264 N   0  0  0  0  0  0  0  0  0  0  0  0
  11.9891    9.9979    0.0117 C   0  0  0  0  0  0  0  0  0  0  0  0
  11.0308   10.8016    0.0058 O   0  0  0  0  0  0  0  0  0  0  0  0
   8.1755    7.9796    0.0191 C   0  0  0  0  0  0  0  0  0  0  0  0
   9.4154    8.6778    0.0432 C   0  0  0  0  0  0  0  0  0  0  0  0
  10.6386    7.9786    0.0376 C   0  0  0  0  0  0  0  0  0  0  0  0
  10.6336    6.5607    0.0424 C   0  0  0  0  0  0  0  0  0  0  0  0
   9.4179    5.8558    0.0078 C   0  0  0  0  0  0  0  0  0  0  0  0
   8.1989    6.5551    0.0090 C   0  0  0  0  0  0  0  0  0  0  0  0
   1.5056   10.3863    0.0049 H   0  0  0  0  0  0  0  0  0  0  0  0
   2.7721   12.5618   -0.0139 H   0  0  0  0  0  0  0  0  0  0  0  0
   5.2877   12.5027    0.0964 H   0  0  0  0  0  0  0  0  0  0  0  0
   2.8031    8.2074   -0.0228 H   0  0  0  0  0  0  0  0  0  0  0  0
  15.9477   12.5533   -0.0277 H   0  0  0  0  0  0  0  0  0  0  0  0
  17.2145   10.3776    0.0010 H   0  0  0  0  0  0  0  0  0  0  0  0
  15.9220    8.2262    0.0242 H   0  0  0  0  0  0  0  0  0  0  0  0
  13.4041   12.4873   -0.0129 H   0  0  0  0  0  0  0  0  0  0  0  0
   9.4308    9.7615    0.0674 H   0  0  0  0  0  0  0  0  0  0  0  0
  11.5707    6.0117    0.0733 H   0  0  0  0  0  0  0  0  0  0  0  0
   9.4180    4.7683   -0.0205 H   0  0  0  0  0  0  0  0  0  0  0  0
   7.2714    5.9941    0.0035 H   0  0  0  0  0  0  0  0  0  0  0  0
  1  2  4  0  0  0  0
  2  3  4  0  0  0  0
  3  4  4  0  0  0  0
  4  5  4  0  0  0  0
  5  6  4  0  0  0  0
  6  1  4  0  0  0  0
  7  8  2  0  0  0  0
  7  9  1  0  0  0  0
  7  4  1  0  0  0  0
  5 10  1  0  0  0  0
 10  9  1  0  0  0  0
 10 11  2  0  0  0  0
 12 13  4  0  0  0  0
 13 14  4  0  0  0  0
 14 15  4  0  0  0  0
 15 16  4  0  0  0  0
 16 17  4  0  0  0  0
 17 12  4  0  0  0  0
 18 19  2  0  0  0  0
 18 20  1  0  0  0  0
 18 15  1  0  0  0  0
 16 21  1  0  0  0  0
 21 20  1  0  0  0  0
 21 22  2  0  0  0  0
 23 24  4  0  0  0  0
 24 25  4  0  0  0  0
 25 26  4  0  0  0  0
 26 27  4  0  0  0  0
 27 28  4  0  0  0  0
 28 23  4  0  0  0  0
 23  9  1  0  0  0  0
 20 25  1  0  0  0  0
```

```
  1 29  1  0  0  0  0
  2 30  1  0  0  0  0
  3 31  1  0  0  0  0
  6 32  1  0  0  0  0
 12 33  1  0  0  0  0
 13 34  1  0  0  0  0
 14 35  1  0  0  0  0
 17 36  1  0  0  0  0
 24 37  1  0  0  0  0
 26 38  1  0  0  0  0
 27 39  1  0  0  0  0
 28 40  1  0  0  0  0
M  END
>  <Alias name>
N,N'-(1,3-Phenylene)bis(phthalimide)

>  <deltaH°(c)>   kJ/mol
-9910.9

$$$$
1-Pentadecene
  ChemBrain IXL

 45 44  0  0  0  0  0  0  0  0  1 V2000
   15.1189    8.9093   -1.4849 C   0  0  0  0  0  0  0  0  0  0  0  0
   13.9874    9.8516   -1.0302 C   0  0  0  0  0  0  0  0  0  0  0  0
   12.7797    9.0163   -0.5627 C   0  0  0  0  0  0  0  0  0  0  0  0
   11.6471    9.9587   -0.1111 C   0  0  0  0  0  0  0  0  0  0  0  0
   10.4384    9.1235    0.3539 C   0  0  0  0  0  0  0  0  0  0  0  0
    9.3045   10.0659    0.8020 C   0  0  0  0  0  0  0  0  0  0  0  0
    8.0949    9.2307    1.2644 C   0  0  0  0  0  0  0  0  0  0  0  0
    6.9598   10.1732    1.7096 C   0  0  0  0  0  0  0  0  0  0  0  0
    5.7495    9.3379    2.1702 C   0  0  0  0  0  0  0  0  0  0  0  0
   16.3258    9.7448   -1.9542 C   0  0  0  0  0  0  0  0  0  0  0  0
    4.6136   10.2804    2.6139 C   0  0  0  0  0  0  0  0  0  0  0  0
   17.4568    8.8024   -2.4106 C   0  0  0  0  0  0  0  0  0  0  0  0
    3.4503    9.4782    3.0557 C   0  0  0  0  0  0  0  0  0  0  0  0
   18.6635    9.6377   -2.8805 C   0  0  0  0  0  0  0  0  0  0  0  0
    2.3208   10.0869    3.4247 C   0  0  0  0  0  0  0  0  0  0  0  0
   15.4170    8.2775   -0.6573 H   0  0  0  0  0  0  0  0  0  0  0  0
   14.7677    8.2918   -2.3023 H   0  0  0  0  0  0  0  0  0  0  0  0
   14.3381   10.4685   -0.2121 H   0  0  0  0  0  0  0  0  0  0  0  0
   13.6902   10.4841   -1.8576 H   0  0  0  0  0  0  0  0  0  0  0  0
   12.4303    8.3981   -1.3804 H   0  0  0  0  0  0  0  0  0  0  0  0
   13.0763    8.3852    0.2659 H   0  0  0  0  0  0  0  0  0  0  0  0
   11.9959   10.5760    0.7076 H   0  0  0  0  0  0  0  0  0  0  0  0
   11.3518   10.5908   -0.9394 H   0  0  0  0  0  0  0  0  0  0  0  0
   10.0912    8.5047   -0.4643 H   0  0  0  0  0  0  0  0  0  0  0  0
   10.7330    8.4929    1.1837 H   0  0  0  0  0  0  0  0  0  0  0  0
    9.6511   10.6837    1.6212 H   0  0  0  0  0  0  0  0  0  0  0  0
    9.0112   10.6975   -0.0274 H   0  0  0  0  0  0  0  0  0  0  0  0
    7.7496    8.6116    0.4457 H   0  0  0  0  0  0  0  0  0  0  0  0
    8.3876    8.6005    2.0951 H   0  0  0  0  0  0  0  0  0  0  0  0
    7.3046   10.7916    2.5291 H   0  0  0  0  0  0  0  0  0  0  0  0
    6.6679   10.8041    0.8792 H   0  0  0  0  0  0  0  0  0  0  0  0
    5.4055    8.7188    1.3509 H   0  0  0  0  0  0  0  0  0  0  0  0
    6.0411    8.7076    3.0012 H   0  0  0  0  0  0  0  0  0  0  0  0
   16.0274   10.3773   -2.7811 H   0  0  0  0  0  0  0  0  0  0  0  0
   16.6778   10.3616   -1.1365 H   0  0  0  0  0  0  0  0  0  0  0  0
    4.9575   10.8992    3.4335 H   0  0  0  0  0  0  0  0  0  0  0  0
    4.3225   10.9110    1.7829 H   0  0  0  0  0  0  0  0  0  0  0  0
   17.7556    8.1702   -1.5837 H   0  0  0  0  0  0  0  0  0  0  0  0
   17.1048    8.1854   -3.2279 H   0  0  0  0  0  0  0  0  0  0  0  0
```

```
    3.5154    8.3973    3.0796 H   0  0  0  0  0  0  0  0  0  0  0  0
   18.3647   10.2701   -3.7074 H   0  0  0  0  0  0  0  0  0  0  0  0
   19.4580    8.9754   -3.2012 H   0  0  0  0  0  0  0  0  0  0  0  0
   19.0158   10.2547   -2.0631 H   0  0  0  0  0  0  0  0  0  0  0  0
    1.4701    9.4998    3.7478 H   0  0  0  0  0  0  0  0  0  0  0  0
    2.2552   11.1677    3.4009 H   0  0  0  0  0  0  0  0  0  0  0  0
  1  2  1  0  0  0  0
  2  3  1  0  0  0  0
  3  4  1  0  0  0  0
  4  5  1  0  0  0  0
  5  6  1  0  0  0  0
  6  7  1  0  0  0  0
  7  8  1  0  0  0  0
  8  9  1  0  0  0  0
  1 10  1  0  0  0  0
  9 11  1  0  0  0  0
 10 12  1  0  0  0  0
 11 13  1  0  0  0  0
 12 14  1  0  0  0  0
 13 15  2  0  0  0  0
  1 16  1  0  0  0  0
  1 17  1  0  0  0  0
  2 18  1  0  0  0  0
  2 19  1  0  0  0  0
  3 20  1  0  0  0  0
  3 21  1  0  0  0  0
  4 22  1  0  0  0  0
  4 23  1  0  0  0  0
  5 24  1  0  0  0  0
  5 25  1  0  0  0  0
  6 26  1  0  0  0  0
  6 27  1  0  0  0  0
  7 28  1  0  0  0  0
  7 29  1  0  0  0  0
  8 30  1  0  0  0  0
  8 31  1  0  0  0  0
  9 32  1  0  0  0  0
  9 33  1  0  0  0  0
 10 34  1  0  0  0  0
 10 35  1  0  0  0  0
 11 36  1  0  0  0  0
 11 37  1  0  0  0  0
 12 38  1  0  0  0  0
 12 39  1  0  0  0  0
 13 40  1  0  0  0  0
 14 41  1  0  0  0  0
 14 42  1  0  0  0  0
 14 43  1  0  0  0  0
 15 44  1  0  0  0  0
 15 45  1  0  0  0  0
M  END
>  <Alias name>
1-Pentadecene

>  <deltaH°(c)>   kJ/mol
-9895.79

$$$$
trans-2-Pentadecene
  ChemBrain IXL

 45 44  0  0  0  0  0  0  0  0  1 V2000
   15.9550    9.8294    0.0000 C   0  0  0  0  0  0  0  0  0  0  0  0
```

```
 14.6793   10.6898    0.0850 C   0  0  0  0  0  0  0  0  0  0  0  0
 13.4389    9.7783    0.0103 C   0  0  0  0  0  0  0  0  0  0  0  0
 12.1630   10.6391    0.0883 C   0  0  0  0  0  0  0  0  0  0  0  0
 10.9227    9.7280    0.0079 C   0  0  0  0  0  0  0  0  0  0  0  0
  9.6468   10.5894    0.0785 C   0  0  0  0  0  0  0  0  0  0  0  0
  8.4066    9.6785   -0.0069 C   0  0  0  0  0  0  0  0  0  0  0  0
  7.1306   10.5403    0.0579 C   0  0  0  0  0  0  0  0  0  0  0  0
  5.8903    9.6294   -0.0302 C   0  0  0  0  0  0  0  0  0  0  0  0
 17.1955   10.7413    0.0704 C   0  0  0  0  0  0  0  0  0  0  0  0
  4.6643   10.4572    0.0293 C   0  0  0  0  0  0  0  0  0  0  0  0
 18.4713    9.8812   -0.0187 C   0  0  0  0  0  0  0  0  0  0  0  0
  3.4631    9.8754   -0.0112 C   0  0  0  0  0  0  0  0  0  0  0  0
 19.7116   10.7930    0.0500 C   0  0  0  0  0  0  0  0  0  0  0  0
  2.2373   10.7029    0.0483 C   0  0  0  0  0  0  0  0  0  0  0  0
 15.9741    9.1293    0.8261 H   0  0  0  0  0  0  0  0  0  0  0  0
 15.9607    9.2841   -0.9356 H   0  0  0  0  0  0  0  0  0  0  0  0
 14.6616   11.3914   -0.7397 H   0  0  0  0  0  0  0  0  0  0  0  0
 14.6720   11.2334    1.0217 H   0  0  0  0  0  0  0  0  0  0  0  0
 13.4541    9.0793    0.8374 H   0  0  0  0  0  0  0  0  0  0  0  0
 13.4487    9.2317   -0.9246 H   0  0  0  0  0  0  0  0  0  0  0  0
 12.1498   11.3402   -0.7370 H   0  0  0  0  0  0  0  0  0  0  0  0
 12.1513   11.1834    1.0246 H   0  0  0  0  0  0  0  0  0  0  0  0
 10.9333    9.0297    0.8357 H   0  0  0  0  0  0  0  0  0  0  0  0
 10.9372    9.1805   -0.9264 H   0  0  0  0  0  0  0  0  0  0  0  0
  9.6379   11.2894   -0.7478 H   0  0  0  0  0  0  0  0  0  0  0  0
  9.6306   11.1348    1.0140 H   0  0  0  0  0  0  0  0  0  0  0  0
  8.4135    8.9806    0.8212 H   0  0  0  0  0  0  0  0  0  0  0  0
  8.4249    9.1306   -0.9409 H   0  0  0  0  0  0  0  0  0  0  0  0
  7.1248   11.2392   -0.7694 H   0  0  0  0  0  0  0  0  0  0  0  0
  7.1114   11.0871    0.9926 H   0  0  0  0  0  0  0  0  0  0  0  0
  5.9107    9.0813   -0.9641 H   0  0  0  0  0  0  0  0  0  0  0  0
  5.8953    8.9317    0.7981 H   0  0  0  0  0  0  0  0  0  0  0  0
 17.1749   11.4429   -0.7543 H   0  0  0  0  0  0  0  0  0  0  0  0
 17.1912   11.2849    1.0071 H   0  0  0  0  0  0  0  0  0  0  0  0
  4.7416   11.5349    0.1056 H   0  0  0  0  0  0  0  0  0  0  0  0
 18.4924    9.1801    0.8064 H   0  0  0  0  0  0  0  0  0  0  0  0
 18.4749    9.3369   -0.9550 H   0  0  0  0  0  0  0  0  0  0  0  0
  3.3856    8.7978   -0.0875 H   0  0  0  0  0  0  0  0  0  0  0  0
 19.6904   11.4942   -0.7751 H   0  0  0  0  0  0  0  0  0  0  0  0
 20.6079   10.1884   -0.0131 H   0  0  0  0  0  0  0  0  0  0  0  0
 19.7083   11.3370    0.9864 H   0  0  0  0  0  0  0  0  0  0  0  0
  1.3669   10.0586    0.0552 H   0  0  0  0  0  0  0  0  0  0  0  0
  2.2498   11.3024    0.9501 H   0  0  0  0  0  0  0  0  0  0  0  0
  2.1984   11.3531   -0.8170 H   0  0  0  0  0  0  0  0  0  0  0  0
  1  2  1  0  0  0  0
  2  3  1  0  0  0  0
  3  4  1  0  0  0  0
  4  5  1  0  0  0  0
  5  6  1  0  0  0  0
  6  7  1  0  0  0  0
  7  8  1  0  0  0  0
  8  9  1  0  0  0  0
  1 10  1  0  0  0  0
  9 11  1  0  0  0  0
 10 12  1  0  0  0  0
 11 13  2  0  0  0  0
 12 14  1  0  0  0  0
 13 15  1  0  0  0  0
  1 16  1  0  0  0  0
  1 17  1  0  0  0  0
  2 18  1  0  0  0  0
  2 19  1  0  0  0  0
  3 20  1  0  0  0  0
```

```
  3 21  1  0  0  0  0
  4 22  1  0  0  0  0
  4 23  1  0  0  0  0
  5 24  1  0  0  0  0
  5 25  1  0  0  0  0
  6 26  1  0  0  0  0
  6 27  1  0  0  0  0
  7 28  1  0  0  0  0
  7 29  1  0  0  0  0
  8 30  1  0  0  0  0
  8 31  1  0  0  0  0
  9 32  1  0  0  0  0
  9 33  1  0  0  0  0
 10 34  1  0  0  0  0
 10 35  1  0  0  0  0
 11 36  1  0  0  0  0
 12 37  1  0  0  0  0
 12 38  1  0  0  0  0
 13 39  1  0  0  0  0
 14 40  1  0  0  0  0
 14 41  1  0  0  0  0
 14 42  1  0  0  0  0
 15 43  1  0  0  0  0
 15 44  1  0  0  0  0
 15 45  1  0  0  0  0
M  END
>  <Alias name>
trans-2-Pentadecene

>  <deltaH°(c)>   kJ/mol
-9877.41

$$$$
Palmitoleic acid
  ChemBrain IXL

 48 47  0  0  0  0  0  0  0  0  1 V2000
   19.1071   12.4766   -0.0713 C   0  0  0  0  0  0  0  0  0  0  0  0
   17.6431   12.6935   -0.0659 C   0  0  0  0  0  0  0  0  0  0  0  0
   16.9218   11.3317   -0.0796 C   0  0  0  0  0  0  0  0  0  0  0  0
   15.3976   11.5581   -0.0747 C   0  0  0  0  0  0  0  0  0  0  0  0
   14.6757   10.1967   -0.0904 C   0  0  0  0  0  0  0  0  0  0  0  0
   13.1517   10.4241   -0.0873 C   0  0  0  0  0  0  0  0  0  0  0  0
   12.4289    9.0634   -0.1063 C   0  0  0  0  0  0  0  0  0  0  0  0
   10.9050    9.2918   -0.1051 C   0  0  0  0  0  0  0  0  0  0  0  0
   10.2100    7.9855   -0.1244 C   0  0  0  0  0  0  0  0  0  0  0  0
    8.8771    7.9381   -0.0697 C   0  0  0  0  0  0  0  0  0  0  0  0
    8.0951    9.1918    0.0118 C   0  0  0  0  0  0  0  0  0  0  0  0
    6.5916    8.8661   -0.0756 C   0  0  0  0  0  0  0  0  0  0  0  0
    5.7778   10.1718    0.0082 C   0  0  0  0  0  0  0  0  0  0  0  0
    4.2742    9.8466   -0.0789 C   0  0  0  0  0  0  0  0  0  0  0  0
    3.4606   11.1525    0.0060 C   0  0  0  0  0  0  0  0  0  0  0  0
    1.9569   10.8274   -0.0808 C   0  0  0  0  0  0  0  0  0  0  0  0
   19.8653   13.4324   -0.0784 O   0  0  0  0  0  0  0  0  0  0  0  0
   19.6476   11.1097   -0.0681 O   0  0  0  0  0  0  0  0  0  0  0  0
   20.6289   11.0509   -0.0723 H   0  0  0  0  0  0  0  0  0  0  0  0
   17.3590   13.2613   -0.9433 H   0  0  0  0  0  0  0  0  0  0  0  0
   17.3627   13.2416    0.8251 H   0  0  0  0  0  0  0  0  0  0  0  0
   17.2054   10.7642    0.7981 H   0  0  0  0  0  0  0  0  0  0  0  0
   17.2026   10.7834   -0.9703 H   0  0  0  0  0  0  0  0  0  0  0  0
   15.1144   12.1270   -0.9516 H   0  0  0  0  0  0  0  0  0  0  0  0
   15.1168   12.1052    0.8167 H   0  0  0  0  0  0  0  0  0  0  0  0
   14.9578    9.6283    0.7872 H   0  0  0  0  0  0  0  0  0  0  0  0
```

```
14.9573 9.6490 -0.9813 H 0 0 0 0 0 0 0 0 0 0 0 0
12.8701 10.9946 -0.9637 H 0 0 0 0 0 0 0 0 0 0 0 0
12.8700 10.9699 0.8046 H 0 0 0 0 0 0 0 0 0 0 0 0
12.7092 8.4933 0.7707 H 0 0 0 0 0 0 0 0 0 0 0 0
12.7113 8.5169 -0.9977 H 0 0 0 0 0 0 0 0 0 0 0 0
10.6249 9.8627 -0.9818 H 0 0 0 0 0 0 0 0 0 0 0 0
10.6226 9.8378 0.7866 H 0 0 0 0 0 0 0 0 0 0 0 0
10.7827 7.0682 -0.1830 H 0 0 0 0 0 0 0 0 0 0 0 0
8.3681 6.9823 -0.0848 H 0 0 0 0 0 0 0 0 0 0 0 0
8.3033 9.6866 0.9524 H 0 0 0 0 0 0 0 0 0 0 0 0
8.3715 9.8435 -0.8079 H 0 0 0 0 0 0 0 0 0 0 0 0
6.3836 8.3707 -1.0159 H 0 0 0 0 0 0 0 0 0 0 0 0
6.3150 8.2150 0.7445 H 0 0 0 0 0 0 0 0 0 0 0 0
5.9861 10.6673 0.9485 H 0 0 0 0 0 0 0 0 0 0 0 0
6.0545 10.8228 -0.8119 H 0 0 0 0 0 0 0 0 0 0 0 0
4.0658 9.3519 -1.0195 H 0 0 0 0 0 0 0 0 0 0 0 0
3.9977 9.1950 0.7407 H 0 0 0 0 0 0 0 0 0 0 0 0
3.6693 11.6474 0.9464 H 0 0 0 0 0 0 0 0 0 0 0 0
3.7370 11.8040 -0.8139 H 0 0 0 0 0 0 0 0 0 0 0 0
1.6805 10.1759 0.7389 H 0 0 0 0 0 0 0 0 0 0 0 0
1.3851 11.7453 -0.0210 H 0 0 0 0 0 0 0 0 0 0 0 0
1.7482 10.3327 -1.0213 H 0 0 0 0 0 0 0 0 0 0 0 0
1 2 1 0 0 0 0
2 3 1 0 0 0 0
3 4 1 0 0 0 0
4 5 1 0 0 0 0
5 6 1 0 0 0 0
6 7 1 0 0 0 0
7 8 1 0 0 0 0
8 9 1 0 0 0 0
9 10 2 0 0 0 0
10 11 1 0 0 0 0
11 12 1 0 0 0 0
12 13 1 0 0 0 0
13 14 1 0 0 0 0
14 15 1 0 0 0 0
15 16 1 0 0 0 0
1 17 2 0 0 0 0
1 18 1 0 0 0 0
18 19 1 0 0 0 0
2 20 1 0 0 0 0
2 21 1 0 0 0 0
3 22 1 0 0 0 0
3 23 1 0 0 0 0
4 24 1 0 0 0 0
4 25 1 0 0 0 0
5 26 1 0 0 0 0
5 27 1 0 0 0 0
6 28 1 0 0 0 0
6 29 1 0 0 0 0
7 30 1 0 0 0 0
7 31 1 0 0 0 0
8 32 1 0 0 0 0
8 33 1 0 0 0 0
9 34 1 0 0 0 0
10 35 1 0 0 0 0
11 36 1 0 0 0 0
11 37 1 0 0 0 0
12 38 1 0 0 0 0
12 39 1 0 0 0 0
13 40 1 0 0 0 0
13 41 1 0 0 0 0
14 42 1 0 0 0 0
```

14 43 1 0 0 0 0
15 44 1 0 0 0 0
15 45 1 0 0 0 0
16 46 1 0 0 0 0
16 47 1 0 0 0 0
16 48 1 0 0 0 0
M END
> <Alias name>
(9Z)-hexadecenoic acid

> <deltaH°(c)>   kJ/mol
-9877.3

$$$$
Dipentyl phthalate
  ChemBrain IXL

48 48 0 0 0 0 0 0 0 0 1 V2000
    2.5047   5.5834   -0.5218 C   0 0 0 0 0 0 0 0 0 0 0 0
    2.8627   4.3854   0.0981 C   0 0 0 0 0 0 0 0 0 0 0 0
    4.1946   4.1437   0.4357 C   0 0 0 0 0 0 0 0 0 0 0 0
    5.1717   5.0993   0.1558 C   0 0 0 0 0 0 0 0 0 0 0 0
    4.8114   6.3053   -0.4708 C   0 0 0 0 0 0 0 0 0 0 0 0
    3.4778   6.5421   -0.8052 C   0 0 0 0 0 0 0 0 0 0 0 0
    6.5884   4.8281   0.5282 C   0 0 0 0 0 0 0 0 0 0 0 0
    6.8373   4.1455   1.5083 O   0 0 0 0 0 0 0 0 0 0 0 0
    7.6947   5.3787   -0.2821 O   0 0 0 0 0 0 0 0 0 0 0 0
    5.8389   7.3370   -0.7854 C   0 0 0 0 0 0 0 0 0 0 0 0
    5.6704   8.0920   -1.7289 O   0 0 0 0 0 0 0 0 0 0 0 0
    7.0605   7.4537   0.0380 O   0 0 0 0 0 0 0 0 0 0 0 0
    9.0447   5.1623   0.2805 C   0 0 0 0 0 0 0 0 0 0 0 0
   10.1160   5.6197   -0.7364 C   0 0 0 0 0 0 0 0 0 0 0 0
   11.5212   5.4613   -0.1104 C   0 0 0 0 0 0 0 0 0 0 0 0
    8.0472   8.4405   -0.4506 C   0 0 0 0 0 0 0 0 0 0 0 0
    9.2138   8.5590   0.5575 C   0 0 0 0 0 0 0 0 0 0 0 0
   10.2573   9.5666   0.0213 C   0 0 0 0 0 0 0 0 0 0 0 0
   11.4241   9.6881   1.0248 C   0 0 0 0 0 0 0 0 0 0 0 0
   12.4542   10.7086   0.4963 C   0 0 0 0 0 0 0 0 0 0 0 0
   12.5991   5.7885   -1.1660 C   0 0 0 0 0 0 0 0 0 0 0 0
   13.9993   5.6597   -0.5299 C   0 0 0 0 0 0 0 0 0 0 0 0
    1.4706   5.7691   -0.7831 H   0 0 0 0 0 0 0 0 0 0 0 0
    2.1061   3.6427   0.3172 H   0 0 0 0 0 0 0 0 0 0 0 0
    4.4707   3.2130   0.9159 H   0 0 0 0 0 0 0 0 0 0 0 0
    3.1979   7.4716   -1.2855 H   0 0 0 0 0 0 0 0 0 0 0 0
    9.1495   5.7335   1.1946 H   0 0 0 0 0 0 0 0 0 0 0 0
    9.1803   4.1097   0.4965 H   0 0 0 0 0 0 0 0 0 0 0 0
   10.0487   5.0119   -1.6303 H   0 0 0 0 0 0 0 0 0 0 0 0
    9.9504   6.6587   -0.9930 H   0 0 0 0 0 0 0 0 0 0 0 0
   11.6209   6.1393   0.7283 H   0 0 0 0 0 0 0 0 0 0 0 0
   11.6505   4.4422   0.2326 H   0 0 0 0 0 0 0 0 0 0 0 0
    8.4326   8.1194   -1.4103 H   0 0 0 0 0 0 0 0 0 0 0 0
    7.5674   9.4054   -0.5602 H   0 0 0 0 0 0 0 0 0 0 0 0
    8.8340   8.9058   1.5106 H   0 0 0 0 0 0 0 0 0 0 0 0
    9.6804   7.5901   0.6856 H   0 0 0 0 0 0 0 0 0 0 0 0
   10.6367   9.2199   -0.9320 H   0 0 0 0 0 0 0 0 0 0 0 0
    9.7904   10.5353   -0.1073 H   0 0 0 0 0 0 0 0 0 0 0 0
   11.0439   10.0226   1.9820 H   0 0 0 0 0 0 0 0 0 0 0 0
   11.9008   8.7230   1.1430 H   0 0 0 0 0 0 0 0 0 0 0 0
   11.9779   11.6739   0.3765 H   0 0 0 0 0 0 0 0 0 0 0 0
   12.8366   10.3737   -0.4602 H   0 0 0 0 0 0 0 0 0 0 0 0
   13.2720   10.7948   1.2016 H   0 0 0 0 0 0 0 0 0 0 0 0
   12.5146   5.0966   -1.9948 H   0 0 0 0 0 0 0 0 0 0 0 0
   12.4578   6.8004   -1.5248 H   0 0 0 0 0 0 0 0 0 0 0 0

```
   14.1101    6.4002    0.2527 H   0  0  0  0  0  0  0  0  0  0  0  0
   14.7566    5.8197   -1.2879 H   0  0  0  0  0  0  0  0  0  0  0  0
   14.1148    4.6688   -0.1081 H   0  0  0  0  0  0  0  0  0  0  0  0
  1  2  4  0  0  0  0
  2  3  4  0  0  0  0
  3  4  4  0  0  0  0
  4  5  4  0  0  0  0
  5  6  4  0  0  0  0
  6  1  4  0  0  0  0
  7  8  2  0  0  0  0
  7  9  1  0  0  0  0
  7  4  1  0  0  0  0
 10 11  2  0  0  0  0
 10 12  1  0  0  0  0
 10  5  1  0  0  0  0
  9 13  1  0  0  0  0
 13 14  1  0  0  0  0
 14 15  1  0  0  0  0
 16 17  1  0  0  0  0
 17 18  1  0  0  0  0
 16 12  1  0  0  0  0
 18 19  1  0  0  0  0
 19 20  1  0  0  0  0
 15 21  1  0  0  0  0
 21 22  1  0  0  0  0
  1 23  1  0  0  0  0
  2 24  1  0  0  0  0
  3 25  1  0  0  0  0
  6 26  1  0  0  0  0
 13 27  1  0  0  0  0
 13 28  1  0  0  0  0
 14 29  1  0  0  0  0
 14 30  1  0  0  0  0
 15 31  1  0  0  0  0
 15 32  1  0  0  0  0
 16 33  1  0  0  0  0
 16 34  1  0  0  0  0
 17 35  1  0  0  0  0
 17 36  1  0  0  0  0
 18 37  1  0  0  0  0
 18 38  1  0  0  0  0
 19 39  1  0  0  0  0
 19 40  1  0  0  0  0
 20 41  1  0  0  0  0
 20 42  1  0  0  0  0
 20 43  1  0  0  0  0
 21 44  1  0  0  0  0
 21 45  1  0  0  0  0
 22 46  1  0  0  0  0
 22 47  1  0  0  0  0
 22 48  1  0  0  0  0
M  END
>  <Alias name>
Dipentyl phthalate

>  <deltaH°(c)>   kJ/mol
-9875

$$$$
N-Phenylbenzophenone imine
  ChemBrain IXL

 35 37  0  0  0  0  0  0  0  0  1 V2000
```

```
  4.8448    7.5648    0.4250 C   0  0  0  0  0  0  0  0  0  0  0  0
  6.0634    6.7247    0.4226 C   0  0  0  0  0  0  0  0  0  0  0  0
  4.9074    8.8833    0.8762 C   0  0  0  0  0  0  0  0  0  0  0  0
  3.6336    7.0381   -0.0239 C   0  0  0  0  0  0  0  0  0  0  0  0
  7.1945    7.2457    0.6725 N   0  0  0  0  0  0  0  0  0  0  0  0
  3.7532    9.6662    0.9046 C   0  0  0  0  0  0  0  0  0  0  0  0
  2.4794    7.8209    0.0045 C   0  0  0  0  0  0  0  0  0  0  0  0
  8.3565    6.4687    0.5918 C   0  0  0  0  0  0  0  0  0  0  0  0
  2.5389    9.1346    0.4702 C   0  0  0  0  0  0  0  0  0  0  0  0
  8.4008    5.2146    1.2012 C   0  0  0  0  0  0  0  0  0  0  0  0
  9.4700    6.9485   -0.0979 C   0  0  0  0  0  0  0  0  0  0  0  0
  9.5726    4.4591    1.1574 C   0  0  0  0  0  0  0  0  0  0  0  0
 10.6419    6.1930   -0.1419 C   0  0  0  0  0  0  0  0  0  0  0  0
 10.6939    4.9492    0.4876 C   0  0  0  0  0  0  0  0  0  0  0  0
  6.8459    4.6873   -0.7765 C   0  0  0  0  0  0  0  0  0  0  0  0
  5.9639    5.2775    0.1289 C   0  0  0  0  0  0  0  0  0  0  0  0
  6.7571    3.3217   -1.0474 C   0  0  0  0  0  0  0  0  0  0  0  0
  4.9881    4.5038    0.7575 C   0  0  0  0  0  0  0  0  0  0  0  0
  5.7841    2.5471   -0.4156 C   0  0  0  0  0  0  0  0  0  0  0  0
  4.8993    3.1382    0.4865 C   0  0  0  0  0  0  0  0  0  0  0  0
  5.8520    9.2990    1.2044 H   0  0  0  0  0  0  0  0  0  0  0  0
  3.5894    6.0214   -0.3945 H   0  0  0  0  0  0  0  0  0  0  0  0
  3.7999   10.6868    1.2640 H   0  0  0  0  0  0  0  0  0  0  0  0
  1.5371    7.4089   -0.3350 H   0  0  0  0  0  0  0  0  0  0  0  0
  1.6424    9.7416    0.4944 H   0  0  0  0  0  0  0  0  0  0  0  0
  7.5256    4.8277    1.7082 H   0  0  0  0  0  0  0  0  0  0  0  0
  9.4248    7.9074   -0.5992 H   0  0  0  0  0  0  0  0  0  0  0  0
  9.6119    3.4920    1.6434 H   0  0  0  0  0  0  0  0  0  0  0  0
 11.5113    6.5720   -0.6648 H   0  0  0  0  0  0  0  0  0  0  0  0
 11.6047    4.3641    0.4563 H   0  0  0  0  0  0  0  0  0  0  0  0
  7.5996    5.2891   -1.2690 H   0  0  0  0  0  0  0  0  0  0  0  0
  7.4435    2.8629   -1.7482 H   0  0  0  0  0  0  0  0  0  0  0  0
  4.2995    4.9632    1.4558 H   0  0  0  0  0  0  0  0  0  0  0  0
  5.7155    1.4868   -0.6254 H   0  0  0  0  0  0  0  0  0  0  0  0
  4.1434    2.5370    0.9765 H   0  0  0  0  0  0  0  0  0  0  0  0
  1  2  1  0  0  0  0
  1  3  4  0  0  0  0
  1  4  4  0  0  0  0
  2  5  2  0  0  0  0
  3  6  4  0  0  0  0
  4  7  4  0  0  0  0
  5  8  1  0  0  0  0
  6  9  4  0  0  0  0
  7  9  4  0  0  0  0
  8 10  4  0  0  0  0
  8 11  4  0  0  0  0
 10 12  4  0  0  0  0
 11 13  4  0  0  0  0
 12 14  4  0  0  0  0
 13 14  4  0  0  0  0
 15 16  4  0  0  0  0
 15 17  4  0  0  0  0
 16 18  4  0  0  0  0
 17 19  4  0  0  0  0
 18 20  4  0  0  0  0
 19 20  4  0  0  0  0
 16  2  1  0  0  0  0
  3 21  1  0  0  0  0
  4 22  1  0  0  0  0
  6 23  1  0  0  0  0
  7 24  1  0  0  0  0
  9 25  1  0  0  0  0
 10 26  1  0  0  0  0
```

```
 11 27  1  0  0  0  0
 12 28  1  0  0  0  0
 13 29  1  0  0  0  0
 14 30  1  0  0  0  0
 15 31  1  0  0  0  0
 17 32  1  0  0  0  0
 18 33  1  0  0  0  0
 19 34  1  0  0  0  0
 20 35  1  0  0  0  0
M  END
>  <Alias name>
Benzophenone anilide

>  <deltaH°(c)>   kJ/mol
-9872.9

$$$$
Diisopentyl phthalate
  ChemBrain IXL

 48 48  0  0  0  0  0  0  0  0  1 V2000
    2.7549    4.4465    0.1933 C   0  0  0  0  0  0  0  0  0  0  0  0
    4.0564    4.2250    0.6448 C   0  0  0  0  0  0  0  0  0  0  0  0
    5.0870    5.0819    0.2568 C   0  0  0  0  0  0  0  0  0  0  0  0
    4.8117    6.1656   -0.5958 C   0  0  0  0  0  0  0  0  0  0  0  0
    3.5078    6.3838   -1.0419 C   0  0  0  0  0  0  0  0  0  0  0  0
    2.4809    5.5247   -0.6492 C   0  0  0  0  0  0  0  0  0  0  0  0
    5.8984    7.0862   -1.0327 C   0  0  0  0  0  0  0  0  0  0  0  0
    5.8310    7.6286   -2.1233 O   0  0  0  0  0  0  0  0  0  0  0  0
    7.0571    7.3487   -0.1537 O   0  0  0  0  0  0  0  0  0  0  0  0
    6.4696    4.8367    0.7552 C   0  0  0  0  0  0  0  0  0  0  0  0
    6.6373    4.2603    1.8173 O   0  0  0  0  0  0  0  0  0  0  0  0
    7.6399    5.2852   -0.0285 O   0  0  0  0  0  0  0  0  0  0  0  0
    8.9474    5.0609    0.6250 C   0  0  0  0  0  0  0  0  0  0  0  0
    8.1138    8.1903   -0.7542 C   0  0  0  0  0  0  0  0  0  0  0  0
   10.0912    5.4268   -0.3495 C   0  0  0  0  0  0  0  0  0  0  0  0
    9.1825    8.5305    0.3105 C   0  0  0  0  0  0  0  0  0  0  0  0
   10.2974    9.3866   -0.3347 C   0  0  0  0  0  0  0  0  0  0  0  0
   11.3329    9.7831    0.7380 C   0  0  0  0  0  0  0  0  0  0  0  0
   10.9948    8.5734   -1.4428 C   0  0  0  0  0  0  0  0  0  0  0  0
   11.4538    5.1460    0.3270 C   0  0  0  0  0  0  0  0  0  0  0  0
   11.6314    6.0842    1.5371 C   0  0  0  0  0  0  0  0  0  0  0  0
   12.5942    5.3916   -0.6826 C   0  0  0  0  0  0  0  0  0  0  0  0
    1.9567    3.7811    0.4971 H   0  0  0  0  0  0  0  0  0  0  0  0
    4.2673    3.3868    1.2973 H   0  0  0  0  0  0  0  0  0  0  0  0
    3.2927    7.2213   -1.6940 H   0  0  0  0  0  0  0  0  0  0  0  0
    1.4704    5.6949   -0.9986 H   0  0  0  0  0  0  0  0  0  0  0  0
    9.0181    5.6800    1.5107 H   0  0  0  0  0  0  0  0  0  0  0  0
    9.0349    4.0189    0.9066 H   0  0  0  0  0  0  0  0  0  0  0  0
    8.5793    7.6544   -1.5721 H   0  0  0  0  0  0  0  0  0  0  0  0
    7.6747    9.1068   -1.1283 H   0  0  0  0  0  0  0  0  0  0  0  0
   10.0050    4.8292   -1.2486 H   0  0  0  0  0  0  0  0  0  0  0  0
   10.0255    6.4769   -0.6062 H   0  0  0  0  0  0  0  0  0  0  0  0
    9.6101    7.6146    0.6993 H   0  0  0  0  0  0  0  0  0  0  0  0
    8.7232    9.0861    1.1188 H   0  0  0  0  0  0  0  0  0  0  0  0
    9.8628   10.2810   -0.7636 H   0  0  0  0  0  0  0  0  0  0  0  0
   10.8592   10.4092    1.4837 H   0  0  0  0  0  0  0  0  0  0  0  0
   12.1448   10.3288    0.2716 H   0  0  0  0  0  0  0  0  0  0  0  0
   11.7233    8.8906    1.2109 H   0  0  0  0  0  0  0  0  0  0  0  0
   11.8026    9.1584   -1.8659 H   0  0  0  0  0  0  0  0  0  0  0  0
   10.2791    8.3325   -2.2186 H   0  0  0  0  0  0  0  0  0  0  0  0
   11.3937    7.6585   -1.0225 H   0  0  0  0  0  0  0  0  0  0  0  0
   11.4848    4.1165    0.6618 H   0  0  0  0  0  0  0  0  0  0  0  0
```

```
   10.8283    5.9178    2.2439 H   0  0  0  0  0  0  0  0  0  0  0  0
   11.6101    7.1138    1.2019 H   0  0  0  0  0  0  0  0  0  0  0  0
   12.5817    5.8801    2.0157 H   0  0  0  0  0  0  0  0  0  0  0  0
   12.5684    6.4225   -1.0133 H   0  0  0  0  0  0  0  0  0  0  0  0
   12.4698    4.7357   -1.5351 H   0  0  0  0  0  0  0  0  0  0  0  0
   13.5469    5.1889   -0.2073 H   0  0  0  0  0  0  0  0  0  0  0  0
  1  2  4  0  0  0  0
  2  3  4  0  0  0  0
  3  4  4  0  0  0  0
  4  5  4  0  0  0  0
  5  6  4  0  0  0  0
  6  1  4  0  0  0  0
  7  8  2  0  0  0  0
  7  9  1  0  0  0  0
  7  4  1  0  0  0  0
 10 11  2  0  0  0  0
 10 12  1  0  0  0  0
 10  3  1  0  0  0  0
 12 13  1  0  0  0  0
  9 14  1  0  0  0  0
 13 15  1  0  0  0  0
 14 16  1  0  0  0  0
 16 17  1  0  0  0  0
 17 18  1  0  0  0  0
 17 19  1  0  0  0  0
 15 20  1  0  0  0  0
 20 21  1  0  0  0  0
 20 22  1  0  0  0  0
  1 23  1  0  0  0  0
  2 24  1  0  0  0  0
  5 25  1  0  0  0  0
  6 26  1  0  0  0  0
 13 27  1  0  0  0  0
 13 28  1  0  0  0  0
 14 29  1  0  0  0  0
 14 30  1  0  0  0  0
 15 31  1  0  0  0  0
 15 32  1  0  0  0  0
 16 33  1  0  0  0  0
 16 34  1  0  0  0  0
 17 35  1  0  0  0  0
 18 36  1  0  0  0  0
 18 37  1  0  0  0  0
 18 38  1  0  0  0  0
 19 39  1  0  0  0  0
 19 40  1  0  0  0  0
 19 41  1  0  0  0  0
 20 42  1  0  0  0  0
 21 43  1  0  0  0  0
 21 44  1  0  0  0  0
 21 45  1  0  0  0  0
 22 46  1  0  0  0  0
 22 47  1  0  0  0  0
 22 48  1  0  0  0  0
M  END
>  <Alias name>
Diisoamyl phthalate

>  <deltaH°(c)>   kJ/mol
-9857.9

$$$$
Decylcyclopentane
```

ChemBrain IXL

```
45 45  0  0  0  0  0  0  0  0  1 V2000
   14.7350    7.9066   -1.6772 C   0  0  0  0  0  0  0  0  0  0  0  0
   15.9861    7.2703   -1.0763 C   0  0  0  0  0  0  0  0  0  0  0  0
   16.1152    7.8846    0.3583 C   0  0  0  0  0  0  0  0  0  0  0  0
   15.1063    9.0798    0.3963 C   0  0  0  0  0  0  0  0  0  0  0  0
   14.6846    9.2971   -1.0571 C   0  0  0  0  0  0  0  0  0  0  0  0
   13.2699    9.9283   -1.1616 C   0  0  0  0  0  0  0  0  0  0  0  0
   12.2234    9.0141   -0.4815 C   0  0  0  0  0  0  0  0  0  0  0  0
   10.8285    9.6581   -0.5995 C   0  0  0  0  0  0  0  0  0  0  0  0
    9.7839    8.7490    0.0767 C   0  0  0  0  0  0  0  0  0  0  0  0
    8.3888    9.3928   -0.0417 C   0  0  0  0  0  0  0  0  0  0  0  0
    7.3441    8.4835    0.6342 C   0  0  0  0  0  0  0  0  0  0  0  0
    5.9489    9.1271    0.5154 C   0  0  0  0  0  0  0  0  0  0  0  0
    4.9043    8.2176    1.1912 C   0  0  0  0  0  0  0  0  0  0  0  0
    3.5089    8.8611    1.0721 C   0  0  0  0  0  0  0  0  0  0  0  0
    2.4642    7.9518    1.7477 C   0  0  0  0  0  0  0  0  0  0  0  0
   14.7967    7.9552   -2.7571 H   0  0  0  0  0  0  0  0  0  0  0  0
   13.8641    7.3436   -1.3738 H   0  0  0  0  0  0  0  0  0  0  0  0
   15.9185    6.1900   -1.0451 H   0  0  0  0  0  0  0  0  0  0  0  0
   16.8430    7.5738   -1.6649 H   0  0  0  0  0  0  0  0  0  0  0  0
   15.8446    7.1612    1.1174 H   0  0  0  0  0  0  0  0  0  0  0  0
   17.1307    8.2275    0.5127 H   0  0  0  0  0  0  0  0  0  0  0  0
   15.5528    9.9672    0.8274 H   0  0  0  0  0  0  0  0  0  0  0  0
   14.2391    8.7847    0.9677 H   0  0  0  0  0  0  0  0  0  0  0  0
   15.4119    9.9314   -1.5485 H   0  0  0  0  0  0  0  0  0  0  0  0
   13.0104   10.0539   -2.2055 H   0  0  0  0  0  0  0  0  0  0  0  0
   13.2721   10.8954   -0.6742 H   0  0  0  0  0  0  0  0  0  0  0  0
   12.4735    8.8900    0.5626 H   0  0  0  0  0  0  0  0  0  0  0  0
   12.2148    8.0486   -0.9680 H   0  0  0  0  0  0  0  0  0  0  0  0
   10.5757    9.7826   -1.6452 H   0  0  0  0  0  0  0  0  0  0  0  0
   10.8349   10.6252   -0.1121 H   0  0  0  0  0  0  0  0  0  0  0  0
   10.0365    8.6247    1.1225 H   0  0  0  0  0  0  0  0  0  0  0  0
    9.7777    7.7818   -0.4106 H   0  0  0  0  0  0  0  0  0  0  0  0
    8.1362    9.5172   -1.0875 H   0  0  0  0  0  0  0  0  0  0  0  0
    8.3949   10.3599    0.4457 H   0  0  0  0  0  0  0  0  0  0  0  0
    7.5965    8.3593    1.6801 H   0  0  0  0  0  0  0  0  0  0  0  0
    7.3382    7.5163    0.1470 H   0  0  0  0  0  0  0  0  0  0  0  0
    5.6966    9.2514   -0.5305 H   0  0  0  0  0  0  0  0  0  0  0  0
    5.9548   10.0942    1.0028 H   0  0  0  0  0  0  0  0  0  0  0  0
    5.1564    8.0934    2.2371 H   0  0  0  0  0  0  0  0  0  0  0  0
    4.8985    7.2505    0.7039 H   0  0  0  0  0  0  0  0  0  0  0  0
    3.2567    8.9854    0.0263 H   0  0  0  0  0  0  0  0  0  0  0  0
    3.5146    9.8282    1.5594 H   0  0  0  0  0  0  0  0  0  0  0  0
    2.4584    6.9846    1.2604 H   0  0  0  0  0  0  0  0  0  0  0  0
    2.7162    7.8275    2.7936 H   0  0  0  0  0  0  0  0  0  0  0  0
    1.4839    8.4041    1.6639 H   0  0  0  0  0  0  0  0  0  0  0  0
  1  2  1  0  0  0  0
  3  4  1  0  0  0  0
  4  5  1  0  0  0  0
  5  1  1  0  0  0  0
  5  6  1  0  0  0  0
  6  7  1  0  0  0  0
  7  8  1  0  0  0  0
  8  9  1  0  0  0  0
  9 10  1  0  0  0  0
 10 11  1  0  0  0  0
 11 12  1  0  0  0  0
 12 13  1  0  0  0  0
 13 14  1  0  0  0  0
 14 15  1  0  0  0  0
  2  3  1  0  0  0  0
```

```
  1 16  1  0  0  0  0
  1 17  1  0  0  0  0
  2 18  1  0  0  0  0
  2 19  1  0  0  0  0
  3 20  1  0  0  0  0
  3 21  1  0  0  0  0
  4 22  1  0  0  0  0
  4 23  1  0  0  0  0
  5 24  1  0  0  0  0
  6 25  1  0  0  0  0
  6 26  1  0  0  0  0
  7 27  1  0  0  0  0
  7 28  1  0  0  0  0
  8 29  1  0  0  0  0
  8 30  1  0  0  0  0
  9 31  1  0  0  0  0
  9 32  1  0  0  0  0
 10 33  1  0  0  0  0
 10 34  1  0  0  0  0
 11 35  1  0  0  0  0
 11 36  1  0  0  0  0
 12 37  1  0  0  0  0
 12 38  1  0  0  0  0
 13 39  1  0  0  0  0
 13 40  1  0  0  0  0
 14 41  1  0  0  0  0
 14 42  1  0  0  0  0
 15 43  1  0  0  0  0
 15 44  1  0  0  0  0
 15 45  1  0  0  0  0
M  END
>  <Alias name>
Decylcyclopentane

>  <deltaH°(c)>   kJ/mol
-9829.61

$$$$
1-Pentadecanol
  ChemBrain IXL

 48 47  0  0  0  0  0  0  0  0  1 V2000
   12.7582    7.0506   -3.9170 C   0  0  0  0  0  0  0  0  0  0  0  0
   13.4350    5.9355   -4.7379 C   0  0  0  0  0  0  0  0  0  0  0  0
   14.5190    6.5504   -5.6442 C   0  0  0  0  0  0  0  0  0  0  0  0
   15.1643    5.4867   -6.4269 O   0  0  0  0  0  0  0  0  0  0  0  0
   11.6741    6.4355   -3.0106 C   0  0  0  0  0  0  0  0  0  0  0  0
   10.9973    7.5504   -2.1897 C   0  0  0  0  0  0  0  0  0  0  0  0
    9.9133    6.9353   -1.2833 C   0  0  0  0  0  0  0  0  0  0  0  0
    9.2366    8.0503   -0.4623 C   0  0  0  0  0  0  0  0  0  0  0  0
    8.1526    7.4351    0.4441 C   0  0  0  0  0  0  0  0  0  0  0  0
    7.4759    8.5500    1.2652 C   0  0  0  0  0  0  0  0  0  0  0  0
    6.3920    7.9348    2.1716 C   0  0  0  0  0  0  0  0  0  0  0  0
    5.7153    9.0496    2.9927 C   0  0  0  0  0  0  0  0  0  0  0  0
    4.6314    8.4344    3.8992 C   0  0  0  0  0  0  0  0  0  0  0  0
    3.9547    9.5492    4.7204 C   0  0  0  0  0  0  0  0  0  0  0  0
    2.8707    8.9340    5.6269 C   0  0  0  0  0  0  0  0  0  0  0  0
    2.1940   10.0486    6.4480 C   0  0  0  0  0  0  0  0  0  0  0  0
   15.8731    5.7911   -7.0361 H   0  0  0  0  0  0  0  0  0  0  0  0
   12.3028    7.7677   -4.5887 H   0  0  0  0  0  0  0  0  0  0  0  0
   13.4998    7.5493   -3.3053 H   0  0  0  0  0  0  0  0  0  0  0  0
   13.8903    5.2184   -4.0662 H   0  0  0  0  0  0  0  0  0  0  0  0
   12.6934    5.4368   -5.3496 H   0  0  0  0  0  0  0  0  0  0  0  0
```

15.2607 7.0491 -5.0325 H 0 0 0 0 0 0 0 0 0 0 0 0
14.0638 7.2675 -6.3160 H 0 0 0 0 0 0 0 0 0 0 0 0
12.1294 5.7183 -2.3389 H 0 0 0 0 0 0 0 0 0 0 0 0
10.9324 5.9368 -3.6223 H 0 0 0 0 0 0 0 0 0 0 0 0
10.5419 8.2676 -2.8614 H 0 0 0 0 0 0 0 0 0 0 0 0
11.7390 8.0492 -1.5780 H 0 0 0 0 0 0 0 0 0 0 0 0
10.3687 6.2182 -0.6116 H 0 0 0 0 0 0 0 0 0 0 0 0
9.1716 6.4367 -1.8950 H 0 0 0 0 0 0 0 0 0 0 0 0
8.7812 8.7674 -1.1340 H 0 0 0 0 0 0 0 0 0 0 0 0
9.9783 8.5490 0.1494 H 0 0 0 0 0 0 0 0 0 0 0 0
8.6080 6.7179 1.1158 H 0 0 0 0 0 0 0 0 0 0 0 0
7.4109 6.9364 -0.1676 H 0 0 0 0 0 0 0 0 0 0 0 0
7.0205 9.2672 0.5935 H 0 0 0 0 0 0 0 0 0 0 0 0
8.2176 9.0487 1.8768 H 0 0 0 0 0 0 0 0 0 0 0 0
6.8474 7.2176 2.8432 H 0 0 0 0 0 0 0 0 0 0 0 0
5.6503 7.4361 1.5600 H 0 0 0 0 0 0 0 0 0 0 0 0
5.2598 9.7668 2.3211 H 0 0 0 0 0 0 0 0 0 0 0 0
6.4570 9.5483 3.6044 H 0 0 0 0 0 0 0 0 0 0 0 0
5.0869 7.7172 4.5708 H 0 0 0 0 0 0 0 0 0 0 0 0
3.8897 7.9357 3.2875 H 0 0 0 0 0 0 0 0 0 0 0 0
3.4992 10.2664 4.0488 H 0 0 0 0 0 0 0 0 0 0 0 0
4.6964 10.0479 5.3320 H 0 0 0 0 0 0 0 0 0 0 0 0
3.3261 8.2168 6.2984 H 0 0 0 0 0 0 0 0 0 0 0 0
2.1290 8.4353 5.0153 H 0 0 0 0 0 0 0 0 0 0 0 0
1.4324 9.6161 7.0849 H 0 0 0 0 0 0 0 0 0 0 0 0
2.9356 10.5473 7.0598 H 0 0 0 0 0 0 0 0 0 0 0 0
1.7384 10.7658 5.7766 H 0 0 0 0 0 0 0 0 0 0 0 0
1 2 1 0 0 0 0
2 3 1 0 0 0 0
3 4 1 0 0 0 0
1 5 1 0 0 0 0
5 6 1 0 0 0 0
6 7 1 0 0 0 0
7 8 1 0 0 0 0
8 9 1 0 0 0 0
9 10 1 0 0 0 0
10 11 1 0 0 0 0
11 12 1 0 0 0 0
12 13 1 0 0 0 0
13 14 1 0 0 0 0
14 15 1 0 0 0 0
15 16 1 0 0 0 0
4 17 1 0 0 0 0
1 18 1 0 0 0 0
1 19 1 0 0 0 0
2 20 1 0 0 0 0
2 21 1 0 0 0 0
3 22 1 0 0 0 0
3 23 1 0 0 0 0
5 24 1 0 0 0 0
5 25 1 0 0 0 0
6 26 1 0 0 0 0
6 27 1 0 0 0 0
7 28 1 0 0 0 0
7 29 1 0 0 0 0
8 30 1 0 0 0 0
8 31 1 0 0 0 0
9 32 1 0 0 0 0
9 33 1 0 0 0 0
10 34 1 0 0 0 0
10 35 1 0 0 0 0
11 36 1 0 0 0 0
11 37 1 0 0 0 0

```
 12 38  1  0  0  0  0
 12 39  1  0  0  0  0
 13 40  1  0  0  0  0
 13 41  1  0  0  0  0
 14 42  1  0  0  0  0
 14 43  1  0  0  0  0
 15 44  1  0  0  0  0
 15 45  1  0  0  0  0
 16 46  1  0  0  0  0
 16 47  1  0  0  0  0
 16 48  1  0  0  0  0
M  END
>  <Alias name>
1-Pentadecanol

>  <deltaH°(c)>   kJ/mol
-9817.7

$$$$
Cyclopentadecane
  ChemBrain IXL

 45 45  0  0  0  0  0  0  0  0  1 V2000
    7.8261    3.6300   -0.3996 C   0  0  0  0  0  0  0  0  0  0  0  0
    6.3638    3.1868    0.0358 C   0  0  0  0  0  0  0  0  0  0  0  0
    5.6541    2.2961   -1.0284 C   0  0  0  0  0  0  0  0  0  0  0  0
    4.9916    3.1705   -2.1194 C   0  0  0  0  0  0  0  0  0  0  0  0
    4.0307    4.2579   -1.4789 C   0  0  0  0  0  0  0  0  0  0  0  0
    2.6309    3.6687   -1.0805 C   0  0  0  0  0  0  0  0  0  0  0  0
    2.6978    2.7740    0.1962 C   0  0  0  0  0  0  0  0  0  0  0  0
    3.3050    3.5364    1.4403 C   0  0  0  0  0  0  0  0  0  0  0  0
    2.5174    4.8698    1.7690 C   0  0  0  0  0  0  0  0  0  0  0  0
    7.8656    4.5423   -1.6906 C   0  0  0  0  0  0  0  0  0  0  0  0
    3.0300    6.0315    0.8761 C   0  0  0  0  0  0  0  0  0  0  0  0
    6.7963    5.6688   -1.6433 C   0  0  0  0  0  0  0  0  0  0  0  0
    4.5398    6.2601    1.1516 C   0  0  0  0  0  0  0  0  0  0  0  0
    6.6184    6.2137   -0.2195 C   0  0  0  0  0  0  0  0  0  0  0  0
    5.1963    6.7915   -0.1209 C   0  0  0  0  0  0  0  0  0  0  0  0
    8.2693    4.1819    0.4199 H   0  0  0  0  0  0  0  0  0  0  0  0
    8.4218    2.7441   -0.5811 H   0  0  0  0  0  0  0  0  0  0  0  0
    5.7717    4.0454    0.2237 H   0  0  0  0  0  0  0  0  0  0  0  0
    6.4466    2.6169    0.9527 H   0  0  0  0  0  0  0  0  0  0  0  0
    6.3752    1.6270   -1.4810 H   0  0  0  0  0  0  0  0  0  0  0  0
    4.8939    1.7152   -0.5427 H   0  0  0  0  0  0  0  0  0  0  0  0
    5.7671    3.6720   -2.6763 H   0  0  0  0  0  0  0  0  0  0  0  0
    4.4243    2.5418   -2.7942 H   0  0  0  0  0  0  0  0  0  0  0  0
    3.8697    5.0389   -2.2115 H   0  0  0  0  0  0  0  0  0  0  0  0
    4.4926    4.6839   -0.6182 H   0  0  0  0  0  0  0  0  0  0  0  0
    2.2483    3.0810   -1.9056 H   0  0  0  0  0  0  0  0  0  0  0  0
    1.9502    4.4862   -0.8914 H   0  0  0  0  0  0  0  0  0  0  0  0
    3.2981    1.9080   -0.0109 H   0  0  0  0  0  0  0  0  0  0  0  0
    1.6945    2.4502    0.4435 H   0  0  0  0  0  0  0  0  0  0  0  0
    4.3320    3.7654    1.2513 H   0  0  0  0  0  0  0  0  0  0  0  0
    3.2466    2.8812    2.3003 H   0  0  0  0  0  0  0  0  0  0  0  0
    1.4581    4.7161    1.6045 H   0  0  0  0  0  0  0  0  0  0  0  0
    2.6841    5.1309    2.8066 H   0  0  0  0  0  0  0  0  0  0  0  0
    8.8478    4.9922   -1.7632 H   0  0  0  0  0  0  0  0  0  0  0  0
    7.6930    3.9318   -2.5675 H   0  0  0  0  0  0  0  0  0  0  0  0
    2.8958    5.7650   -0.1551 H   0  0  0  0  0  0  0  0  0  0  0  0
    2.4777    6.9386    1.0872 H   0  0  0  0  0  0  0  0  0  0  0  0
    5.8640    5.2610   -1.9636 H   0  0  0  0  0  0  0  0  0  0  0  0
    7.0758    6.4718   -2.3137 H   0  0  0  0  0  0  0  0  0  0  0  0
    4.9896    5.3174    1.3968 H   0  0  0  0  0  0  0  0  0  0  0  0
```

```
    4.6819    6.9494    1.9745 H   0  0  0  0  0  0  0  0  0  0  0  0
    7.3601    6.9688    0.0088 H   0  0  0  0  0  0  0  0  0  0  0  0
    6.7105    5.4026    0.4739 H   0  0  0  0  0  0  0  0  0  0  0  0
    5.2132    7.8741   -0.1328 H   0  0  0  0  0  0  0  0  0  0  0  0
    4.6274    6.4353   -0.9656 H   0  0  0  0  0  0  0  0  0  0  0  0
  1  2  1  0  0  0  0
  2  3  1  0  0  0  0
  3  4  1  0  0  0  0
  4  5  1  0  0  0  0
  5  6  1  0  0  0  0
  6  7  1  0  0  0  0
  7  8  1  0  0  0  0
  8  9  1  0  0  0  0
  1 10  1  0  0  0  0
  9 11  1  0  0  0  0
 10 12  1  0  0  0  0
 11 13  1  0  0  0  0
 12 14  1  0  0  0  0
 13 15  1  0  0  0  0
 15 14  1  0  0  0  0
  1 16  1  0  0  0  0
  1 17  1  0  0  0  0
  2 18  1  0  0  0  0
  2 19  1  0  0  0  0
  3 20  1  0  0  0  0
  3 21  1  0  0  0  0
  4 22  1  0  0  0  0
  4 23  1  0  0  0  0
  5 24  1  0  0  0  0
  5 25  1  0  0  0  0
  6 26  1  0  0  0  0
  6 27  1  0  0  0  0
  7 28  1  0  0  0  0
  7 29  1  0  0  0  0
  8 30  1  0  0  0  0
  8 31  1  0  0  0  0
  9 32  1  0  0  0  0
  9 33  1  0  0  0  0
 10 34  1  0  0  0  0
 10 35  1  0  0  0  0
 11 36  1  0  0  0  0
 11 37  1  0  0  0  0
 12 38  1  0  0  0  0
 12 39  1  0  0  0  0
 13 40  1  0  0  0  0
 13 41  1  0  0  0  0
 14 42  1  0  0  0  0
 14 43  1  0  0  0  0
 15 44  1  0  0  0  0
 15 45  1  0  0  0  0
M  END
>  <Alias name>
Cyclopentadecane

>  <deltaH°(c)>   kJ/mol
-9814

$$$$
Nonylcyclohexane
  ChemBrain IXL

 45 45  0  0  0  0  0  0  0  0  1 V2000
    2.2771    9.0486   -0.2397 C   0  0  0  0  0  0  0  0  0  0  0  0
```

```
    3.6553    9.6611   -0.5563 C   0  0  0  0  0  0  0  0  0  0  0  0
    4.7633    8.6654   -0.1611 C   0  0  0  0  0  0  0  0  0  0  0  0
    6.1416    9.2781   -0.4775 C   0  0  0  0  0  0  0  0  0  0  0  0
    7.2495    8.2826   -0.0822 C   0  0  0  0  0  0  0  0  0  0  0  0
    8.6277    8.8955   -0.3983 C   0  0  0  0  0  0  0  0  0  0  0  0
    9.7357    7.9001   -0.0026 C   0  0  0  0  0  0  0  0  0  0  0  0
   11.1138    8.5132   -0.3182 C   0  0  0  0  0  0  0  0  0  0  0  0
   12.2227    7.5156    0.0781 C   0  0  0  0  0  0  0  0  0  0  0  0
   16.1290    9.0497   -0.5335 C   0  0  0  0  0  0  0  0  0  0  0  0
   15.1039   10.1086   -0.0425 C   0  0  0  0  0  0  0  0  0  0  0  0
   13.8491    9.3929    0.5304 C   0  0  0  0  0  0  0  0  0  0  0  0
   13.6074    8.1110   -0.2632 C   0  0  0  0  0  0  0  0  0  0  0  0
   14.7163    7.0826    0.0975 C   0  0  0  0  0  0  0  0  0  0  0  0
   16.0496    7.8300    0.3755 C   0  0  0  0  0  0  0  0  0  0  0  0
    2.1532    8.1298   -0.7996 H   0  0  0  0  0  0  0  0  0  0  0  0
    2.2109    8.8371    0.8204 H   0  0  0  0  0  0  0  0  0  0  0  0
    1.4987    9.7484   -0.5175 H   0  0  0  0  0  0  0  0  0  0  0  0
    3.7791   10.5798    0.0035 H   0  0  0  0  0  0  0  0  0  0  0  0
    3.7213    9.8726   -1.6163 H   0  0  0  0  0  0  0  0  0  0  0  0
    4.6396    7.7467   -0.7210 H   0  0  0  0  0  0  0  0  0  0  0  0
    4.6972    8.4538    0.8990 H   0  0  0  0  0  0  0  0  0  0  0  0
    6.2653   10.1969    0.0823 H   0  0  0  0  0  0  0  0  0  0  0  0
    6.2078    9.4896   -1.5376 H   0  0  0  0  0  0  0  0  0  0  0  0
    7.1260    7.3639   -0.6422 H   0  0  0  0  0  0  0  0  0  0  0  0
    7.1832    8.0709    0.9779 H   0  0  0  0  0  0  0  0  0  0  0  0
    8.7512    9.8143    0.1616 H   0  0  0  0  0  0  0  0  0  0  0  0
    8.6942    9.1070   -1.4583 H   0  0  0  0  0  0  0  0  0  0  0  0
    9.6125    6.9814   -0.5627 H   0  0  0  0  0  0  0  0  0  0  0  0
    9.6690    7.6883    1.0574 H   0  0  0  0  0  0  0  0  0  0  0  0
   11.2355    9.4310    0.2411 H   0  0  0  0  0  0  0  0  0  0  0  0
   11.1804    8.7245   -1.3782 H   0  0  0  0  0  0  0  0  0  0  0  0
   12.0851    6.5909   -0.4685 H   0  0  0  0  0  0  0  0  0  0  0  0
   12.1670    7.3171    1.1413 H   0  0  0  0  0  0  0  0  0  0  0  0
   17.1262    9.4708   -0.5087 H   0  0  0  0  0  0  0  0  0  0  0  0
   15.8861    8.7511   -1.5459 H   0  0  0  0  0  0  0  0  0  0  0  0
   15.5482   10.7184    0.7343 H   0  0  0  0  0  0  0  0  0  0  0  0
   14.8197   10.7381   -0.8765 H   0  0  0  0  0  0  0  0  0  0  0  0
   12.9931   10.0488    0.4544 H   0  0  0  0  0  0  0  0  0  0  0  0
   14.0194    9.1374    1.5690 H   0  0  0  0  0  0  0  0  0  0  0  0
   13.6537    8.3374   -1.3213 H   0  0  0  0  0  0  0  0  0  0  0  0
   14.4308    6.5287    0.9832 H   0  0  0  0  0  0  0  0  0  0  0  0
   14.8448    6.3968   -0.7307 H   0  0  0  0  0  0  0  0  0  0  0  0
   16.0740    8.1574    1.4076 H   0  0  0  0  0  0  0  0  0  0  0  0
   16.8842    7.1666    0.1856 H   0  0  0  0  0  0  0  0  0  0  0  0
  1  2  1  0  0  0  0
  2  3  1  0  0  0  0
  3  4  1  0  0  0  0
  4  5  1  0  0  0  0
  5  6  1  0  0  0  0
  6  7  1  0  0  0  0
  7  8  1  0  0  0  0
  8  9  1  0  0  0  0
 10 11  1  0  0  0  0
 11 12  1  0  0  0  0
 12 13  1  0  0  0  0
 13 14  1  0  0  0  0
 14 15  1  0  0  0  0
 15 10  1  0  0  0  0
 13  9  1  0  0  0  0
  1 16  1  0  0  0  0
  1 17  1  0  0  0  0
  1 18  1  0  0  0  0
  2 19  1  0  0  0  0
```

```
  2 20  1  0  0  0  0
  3 21  1  0  0  0  0
  3 22  1  0  0  0  0
  4 23  1  0  0  0  0
  4 24  1  0  0  0  0
  5 25  1  0  0  0  0
  5 26  1  0  0  0  0
  6 27  1  0  0  0  0
  6 28  1  0  0  0  0
  7 29  1  0  0  0  0
  7 30  1  0  0  0  0
  8 31  1  0  0  0  0
  8 32  1  0  0  0  0
  9 33  1  0  0  0  0
  9 34  1  0  0  0  0
 10 35  1  0  0  0  0
 10 36  1  0  0  0  0
 11 37  1  0  0  0  0
 11 38  1  0  0  0  0
 12 39  1  0  0  0  0
 12 40  1  0  0  0  0
 13 41  1  0  0  0  0
 14 42  1  0  0  0  0
 14 43  1  0  0  0  0
 15 44  1  0  0  0  0
 15 45  1  0  0  0  0
M  END
>  <Alias name>
Nonylcyclohexane

>  <deltaH°(c)>   kJ/mol
-9806

$$$$
Decylbenzene
  ChemBrain IXL

 42 42  0  0  0  0  0  0  0  0  1 V2000
    3.6313   10.3305    1.3774 C   0  0  0  0  0  0  0  0  0  0  0  0
    2.4292    9.6946    1.6882 C   0  0  0  0  0  0  0  0  0  0  0  0
    2.3923    8.3083    1.8397 C   0  0  0  0  0  0  0  0  0  0  0  0
    3.5574    7.5580    1.6805 C   0  0  0  0  0  0  0  0  0  0  0  0
    4.7596    8.1938    1.3699 C   0  0  0  0  0  0  0  0  0  0  0  0
    4.7965    9.5801    1.2182 C   0  0  0  0  0  0  0  0  0  0  0  0
    5.9958    7.3974    1.2014 C   0  0  0  0  0  0  0  0  0  0  0  0
    7.1661    8.3332    0.8397 C   0  0  0  0  0  0  0  0  0  0  0  0
    8.4538    7.5028    0.6736 C   0  0  0  0  0  0  0  0  0  0  0  0
    9.6266    8.4366    0.3157 C   0  0  0  0  0  0  0  0  0  0  0  0
   10.9162    7.6056    0.1679 C   0  0  0  0  0  0  0  0  0  0  0  0
   12.0917    8.5370   -0.1866 C   0  0  0  0  0  0  0  0  0  0  0  0
   13.3827    7.7055   -0.3183 C   0  0  0  0  0  0  0  0  0  0  0  0
   14.5599    8.6350   -0.6714 C   0  0  0  0  0  0  0  0  0  0  0  0
   15.8516    7.8032   -0.7937 C   0  0  0  0  0  0  0  0  0  0  0  0
   17.0295    8.7318   -1.1472 C   0  0  0  0  0  0  0  0  0  0  0  0
    3.6600   11.4067    1.2597 H   0  0  0  0  0  0  0  0  0  0  0  0
    1.5246   10.2771    1.8118 H   0  0  0  0  0  0  0  0  0  0  0  0
    1.4590    7.8147    2.0809 H   0  0  0  0  0  0  0  0  0  0  0  0
    3.5287    6.4818    1.7980 H   0  0  0  0  0  0  0  0  0  0  0  0
    5.7298   10.0738    0.9768 H   0  0  0  0  0  0  0  0  0  0  0  0
    5.8536    6.6741    0.4080 H   0  0  0  0  0  0  0  0  0  0  0  0
    6.2202    6.8797    2.1258 H   0  0  0  0  0  0  0  0  0  0  0  0
    6.9449    8.8461   -0.0882 H   0  0  0  0  0  0  0  0  0  0  0  0
    7.3046    9.0604    1.6302 H   0  0  0  0  0  0  0  0  0  0  0  0
```

```
    8.6728    6.9876    1.6006 H   0  0  0  0  0  0  0  0  0  0  0  0
    8.3156    6.7773   -0.1185 H   0  0  0  0  0  0  0  0  0  0  0  0
    9.4138    8.9423   -0.6181 H   0  0  0  0  0  0  0  0  0  0  0  0
    9.7575    9.1698    1.1019 H   0  0  0  0  0  0  0  0  0  0  0  0
   11.1270    7.0977    1.1009 H   0  0  0  0  0  0  0  0  0  0  0  0
   10.7856    6.8738   -0.6197 H   0  0  0  0  0  0  0  0  0  0  0  0
   11.8864    9.0364   -1.1254 H   0  0  0  0  0  0  0  0  0  0  0  0
   12.2160    9.2755    0.5958 H   0  0  0  0  0  0  0  0  0  0  0  0
   13.5870    7.2051    0.6202 H   0  0  0  0  0  0  0  0  0  0  0  0
   13.2583    6.9675   -1.1011 H   0  0  0  0  0  0  0  0  0  0  0  0
   14.3590    9.1304   -1.6133 H   0  0  0  0  0  0  0  0  0  0  0  0
   14.6807    9.3769    0.1083 H   0  0  0  0  0  0  0  0  0  0  0  0
   16.0526    7.3079    0.1482 H   0  0  0  0  0  0  0  0  0  0  0  0
   15.7306    7.0610   -1.5731 H   0  0  0  0  0  0  0  0  0  0  0  0
   17.1498    9.4747   -0.3684 H   0  0  0  0  0  0  0  0  0  0  0  0
   16.8292    9.2260   -2.0898 H   0  0  0  0  0  0  0  0  0  0  0  0
   17.9372    8.1472   -1.2318 H   0  0  0  0  0  0  0  0  0  0  0  0
  1  2  4  0  0  0  0
  2  3  4  0  0  0  0
  3  4  4  0  0  0  0
  4  5  4  0  0  0  0
  5  6  4  0  0  0  0
  6  1  4  0  0  0  0
  5  7  1  0  0  0  0
  7  8  1  0  0  0  0
  8  9  1  0  0  0  0
  9 10  1  0  0  0  0
 10 11  1  0  0  0  0
 11 12  1  0  0  0  0
 12 13  1  0  0  0  0
 13 14  1  0  0  0  0
 14 15  1  0  0  0  0
 15 16  1  0  0  0  0
  1 17  1  0  0  0  0
  2 18  1  0  0  0  0
  3 19  1  0  0  0  0
  4 20  1  0  0  0  0
  6 21  1  0  0  0  0
  7 22  1  0  0  0  0
  7 23  1  0  0  0  0
  8 24  1  0  0  0  0
  8 25  1  0  0  0  0
  9 26  1  0  0  0  0
  9 27  1  0  0  0  0
 10 28  1  0  0  0  0
 10 29  1  0  0  0  0
 11 30  1  0  0  0  0
 11 31  1  0  0  0  0
 12 32  1  0  0  0  0
 12 33  1  0  0  0  0
 13 34  1  0  0  0  0
 13 35  1  0  0  0  0
 14 36  1  0  0  0  0
 14 37  1  0  0  0  0
 15 38  1  0  0  0  0
 15 39  1  0  0  0  0
 16 40  1  0  0  0  0
 16 41  1  0  0  0  0
 16 42  1  0  0  0  0
M  END
>  <Alias name>
Decylbenzene
```

```
>  <deltaH°(c)>   kJ/mol
-9793

$$$$
1,2-Diphenylbenzimidazole
  ChemBrain IXL

 35 38  0  0  0  0  0  0  0  0  1 V2000
    5.9781    5.6332    0.0611 N   0  0  0  0  0  0  0  0  0  0  0  0
    6.8724    4.5838    0.0342 C   0  0  0  0  0  0  0  0  0  0  0  0
    4.7104    5.1122    0.1116 C   0  0  0  0  0  0  0  0  0  0  0  0
    6.2921    6.9705    0.0633 C   0  0  0  0  0  0  0  0  0  0  0  0
    6.1920    3.5001    0.0890 N   0  0  0  0  0  0  0  0  0  0  0  0
    8.3593    4.6726   -0.0460 C   0  0  0  0  0  0  0  0  0  0  0  0
    4.8504    3.7530    0.1441 C   0  0  0  0  0  0  0  0  0  0  0  0
    3.4695    5.7221    0.1206 C   0  0  0  0  0  0  0  0  0  0  0  0
    7.4333    7.4260    0.7254 C   0  0  0  0  0  0  0  0  0  0  0  0
    5.4632    7.8790   -0.5986 C   0  0  0  0  0  0  0  0  0  0  0  0
    9.1410    3.6446    0.4844 C   0  0  0  0  0  0  0  0  0  0  0  0
    8.9711    5.7715   -0.6495 C   0  0  0  0  0  0  0  0  0  0  0  0
    3.7656    2.9016    0.2243 C   0  0  0  0  0  0  0  0  0  0  0  0
    2.3346    4.8905    0.1821 C   0  0  0  0  0  0  0  0  0  0  0  0
    7.7447    8.7874    0.7241 C   0  0  0  0  0  0  0  0  0  0  0  0
    5.7741    9.2394   -0.5979 C   0  0  0  0  0  0  0  0  0  0  0  0
   10.5320    3.7158    0.4146 C   0  0  0  0  0  0  0  0  0  0  0  0
   10.3640    5.8435   -0.7173 C   0  0  0  0  0  0  0  0  0  0  0  0
    2.4832    3.4797    0.2357 C   0  0  0  0  0  0  0  0  0  0  0  0
    6.9150    9.6935    0.0629 C   0  0  0  0  0  0  0  0  0  0  0  0
   11.1439    4.8158   -0.1852 C   0  0  0  0  0  0  0  0  0  0  0  0
    3.3733    6.8001    0.0818 H   0  0  0  0  0  0  0  0  0  0  0  0
    8.0772    6.7244    1.2399 H   0  0  0  0  0  0  0  0  0  0  0  0
    4.5776    7.5276   -1.1134 H   0  0  0  0  0  0  0  0  0  0  0  0
    8.6663    2.7900    0.9513 H   0  0  0  0  0  0  0  0  0  0  0  0
    8.3669    6.5678   -1.0649 H   0  0  0  0  0  0  0  0  0  0  0  0
    3.8979    1.8281    0.2774 H   0  0  0  0  0  0  0  0  0  0  0  0
    1.3442    5.3287    0.1886 H   0  0  0  0  0  0  0  0  0  0  0  0
    8.6309    9.1407    1.2367 H   0  0  0  0  0  0  0  0  0  0  0  0
    5.1291    9.9432   -1.1107 H   0  0  0  0  0  0  0  0  0  0  0  0
   11.1371    2.9165    0.8261 H   0  0  0  0  0  0  0  0  0  0  0  0
   10.8399    6.6972   -1.1830 H   0  0  0  0  0  0  0  0  0  0  0  0
    1.6063    2.8459    0.2854 H   0  0  0  0  0  0  0  0  0  0  0  0
    7.1569   10.7499    0.0626 H   0  0  0  0  0  0  0  0  0  0  0  0
   12.2245    4.8723   -0.2378 H   0  0  0  0  0  0  0  0  0  0  0  0
  1  2  1  0  0  0  0
  3  1  1  0  0  0  0
  1  4  1  0  0  0  0
  2  5  2  0  0  0  0
  2  6  1  0  0  0  0
  7  3  4  0  0  0  0
  8  3  4  0  0  0  0
  9  4  4  0  0  0  0
  4 10  4  0  0  0  0
  5  7  1  0  0  0  0
 11  6  4  0  0  0  0
  6 12  4  0  0  0  0
  7 13  4  0  0  0  0
 14  8  4  0  0  0  0
 15  9  4  0  0  0  0
 10 16  4  0  0  0  0
 17 11  4  0  0  0  0
 12 18  4  0  0  0  0
 13 19  4  0  0  0  0
 19 14  4  0  0  0  0
```

```
 20 15  4  0  0  0  0
 16 20  4  0  0  0  0
 21 17  4  0  0  0  0
 18 21  4  0  0  0  0
  8 22  1  0  0  0  0
  9 23  1  0  0  0  0
 10 24  1  0  0  0  0
 11 25  1  0  0  0  0
 12 26  1  0  0  0  0
 13 27  1  0  0  0  0
 14 28  1  0  0  0  0
 15 29  1  0  0  0  0
 16 30  1  0  0  0  0
 17 31  1  0  0  0  0
 18 32  1  0  0  0  0
 19 33  1  0  0  0  0
 20 34  1  0  0  0  0
 21 35  1  0  0  0  0
M  END
>  <Alias name>
1,2-Diphenylbenzimidazole

>  <deltaH°(c)>   kJ/mol
-9774

$$$$
Perylene
  ChemBrain IXL

 32 36  0  0  0  0  0  0  0  0  1 V2000
    7.8100    5.3906   -0.0424 C   0  0  0  0  0  0  0  0  0  0  0  0
    7.7970    6.7855   -0.0560 C   0  0  0  0  0  0  0  0  0  0  0  0
    6.6089    4.6823   -0.0032 C   0  0  0  0  0  0  0  0  0  0  0  0
    9.0242    4.7041   -0.0682 C   0  0  0  0  0  0  0  0  0  0  0  0
    6.5829    7.4720   -0.0302 C   0  0  0  0  0  0  0  0  0  0  0  0
    8.9982    7.4939   -0.0952 C   0  0  0  0  0  0  0  0  0  0  0  0
    5.3948    5.3688    0.0225 C   0  0  0  0  0  0  0  0  0  0  0  0
    6.6219    3.2874    0.0103 C   0  0  0  0  0  0  0  0  0  0  0  0
   10.2253    5.4125   -0.1074 C   0  0  0  0  0  0  0  0  0  0  0  0
    4.2067    3.2655    0.0753 C   0  0  0  0  0  0  0  0  0  0  0  0
    5.3818    6.7636    0.0090 C   0  0  0  0  0  0  0  0  0  0  0  0
    6.5699    8.8669   -0.0438 C   0  0  0  0  0  0  0  0  0  0  0  0
   10.2123    6.8074   -0.1209 C   0  0  0  0  0  0  0  0  0  0  0  0
    8.9852    8.8887   -0.1087 C   0  0  0  0  0  0  0  0  0  0  0  0
    4.1937    4.6604    0.0618 C   0  0  0  0  0  0  0  0  0  0  0  0
    5.4208    2.5790    0.0496 C   0  0  0  0  0  0  0  0  0  0  0  0
    4.1677    7.4501    0.0347 C   0  0  0  0  0  0  0  0  0  0  0  0
    7.7711    9.5752   -0.0830 C   0  0  0  0  0  0  0  0  0  0  0  0
    2.9796    5.3469    0.0875 C   0  0  0  0  0  0  0  0  0  0  0  0
    2.9666    6.7418    0.0740 C   0  0  0  0  0  0  0  0  0  0  0  0
    9.0342    3.6212   -0.0577 H   0  0  0  0  0  0  0  0  0  0  0  0
    7.5645    2.7544   -0.0097 H   0  0  0  0  0  0  0  0  0  0  0  0
   11.1679    4.8795   -0.1274 H   0  0  0  0  0  0  0  0  0  0  0  0
    3.2742    2.7156    0.1057 H   0  0  0  0  0  0  0  0  0  0  0  0
    5.6274    9.3998   -0.0238 H   0  0  0  0  0  0  0  0  0  0  0  0
   11.1448    7.3573   -0.1514 H   0  0  0  0  0  0  0  0  0  0  0  0
    9.9177    9.4387   -0.1392 H   0  0  0  0  0  0  0  0  0  0  0  0
    5.4309    1.4961    0.0600 H   0  0  0  0  0  0  0  0  0  0  0  0
    4.1576    8.5330    0.0242 H   0  0  0  0  0  0  0  0  0  0  0  0
    7.7610   10.6582   -0.0935 H   0  0  0  0  0  0  0  0  0  0  0  0
    2.0471    4.7970    0.1179 H   0  0  0  0  0  0  0  0  0  0  0  0
    2.0240    7.2747    0.0939 H   0  0  0  0  0  0  0  0  0  0  0  0
  1  2  4  0  0  0  0
```

```
  1  3  4  0  0  0  0
  1  4  4  0  0  0  0
  2  5  4  0  0  0  0
  2  6  4  0  0  0  0
  3  7  4  0  0  0  0
  3  8  4  0  0  0  0
  4  9  4  0  0  0  0
  5 11  4  0  0  0  0
  5 12  4  0  0  0  0
  6 13  4  0  0  0  0
  6 14  4  0  0  0  0
  7 15  4  0  0  0  0
  7 11  4  0  0  0  0
  8 16  4  0  0  0  0
  9 13  4  0  0  0  0
 10 16  4  0  0  0  0
 11 17  4  0  0  0  0
 12 18  4  0  0  0  0
 14 18  4  0  0  0  0
 15 19  4  0  0  0  0
 17 20  4  0  0  0  0
 19 20  4  0  0  0  0
 10 15  4  0  0  0  0
  4 21  1  0  0  0  0
  8 22  1  0  0  0  0
  9 23  1  0  0  0  0
 10 24  1  0  0  0  0
 12 25  1  0  0  0  0
 13 26  1  0  0  0  0
 14 27  1  0  0  0  0
 16 28  1  0  0  0  0
 17 29  1  0  0  0  0
 18 30  1  0  0  0  0
 19 31  1  0  0  0  0
 20 32  1  0  0  0  0
M  END
>  <Alias name>
Perylene

>  <deltaH°(c)>   kJ/mol
-9772.8

$$$$
Triphenylcarbinol
  ChemBrain IXL

 36 38  0  0  0  0  0  0  0  0  1 V2000
    6.1775    5.9072    0.5019 C   0  0  0  0  0  0  0  0  0  0  0  0
    5.5375    4.7140   -0.0956 C   0  0  0  0  0  0  0  0  0  0  0  0
    6.8935    6.6621   -0.5506 C   0  0  0  0  0  0  0  0  0  0  0  0
    5.1406    6.7709    1.1098 C   0  0  0  0  0  0  0  0  0  0  0  0
    7.1318    5.4847    1.5371 O   0  0  0  0  0  0  0  0  0  0  0  0
    4.2099    4.7734   -0.5199 C   0  0  0  0  0  0  0  0  0  0  0  0
    6.2618    3.5299   -0.2345 C   0  0  0  0  0  0  0  0  0  0  0  0
    7.9983    7.4460   -0.2176 C   0  0  0  0  0  0  0  0  0  0  0  0
    6.4635    6.5898   -1.8757 C   0  0  0  0  0  0  0  0  0  0  0  0
    4.6815    7.8965    0.4255 C   0  0  0  0  0  0  0  0  0  0  0  0
    4.6225    6.4593    2.3669 C   0  0  0  0  0  0  0  0  0  0  0  0
    3.6056    3.6477   -1.0799 C   0  0  0  0  0  0  0  0  0  0  0  0
    5.6575    2.4042   -0.7945 C   0  0  0  0  0  0  0  0  0  0  0  0
    8.6757    8.1542   -1.2103 C   0  0  0  0  0  0  0  0  0  0  0  0
    7.1410    7.2979   -2.8685 C   0  0  0  0  0  0  0  0  0  0  0  0
    3.7058    8.7118    0.9994 C   0  0  0  0  0  0  0  0  0  0  0  0
```

3.6469 7.2747 2.9409 C 0 0 0 0 0 0 0 0 0 0 0 0
4.3293 2.4630 -1.2170 C 0 0 0 0 0 0 0 0 0 0 0 0
8.2472 8.0800 -2.5358 C 0 0 0 0 0 0 0 0 0 0 0 0
3.1887 8.4010 2.2572 C 0 0 0 0 0 0 0 0 0 0 0 0
6.7414 4.9663 2.2754 H 0 0 0 0 0 0 0 0 0 0 0 0
3.6484 5.6935 -0.4147 H 0 0 0 0 0 0 0 0 0 0 0 0
7.2933 3.4846 0.0924 H 0 0 0 0 0 0 0 0 0 0 0 0
8.3300 7.5048 0.8117 H 0 0 0 0 0 0 0 0 0 0 0 0
5.6038 5.9838 -2.1338 H 0 0 0 0 0 0 0 0 0 0 0 0
5.0824 8.1373 -0.5513 H 0 0 0 0 0 0 0 0 0 0 0 0
4.9776 5.5844 2.8974 H 0 0 0 0 0 0 0 0 0 0 0 0
2.5746 3.6935 -1.4082 H 0 0 0 0 0 0 0 0 0 0 0 0
6.2194 1.4846 -0.9012 H 0 0 0 0 0 0 0 0 0 0 0 0
9.5343 8.7617 -0.9520 H 0 0 0 0 0 0 0 0 0 0 0 0
6.8081 7.2406 -3.8975 H 0 0 0 0 0 0 0 0 0 0 0 0
3.3499 9.5861 0.4686 H 0 0 0 0 0 0 0 0 0 0 0 0
3.2453 7.0333 3.9173 H 0 0 0 0 0 0 0 0 0 0 0 0
3.8600 1.5890 -1.6514 H 0 0 0 0 0 0 0 0 0 0 0 0
8.7734 8.6295 -3.3066 H 0 0 0 0 0 0 0 0 0 0 0 0
2.4315 9.0342 2.7029 H 0 0 0 0 0 0 0 0 0 0 0 0
1 2 1 0 0 0 0
1 3 1 0 0 0 0
1 4 1 0 0 0 0
1 5 1 0 0 0 0
2 6 4 0 0 0 0
2 7 4 0 0 0 0
3 8 4 0 0 0 0
3 9 4 0 0 0 0
4 10 4 0 0 0 0
4 11 4 0 0 0 0
6 12 4 0 0 0 0
7 13 4 0 0 0 0
8 14 4 0 0 0 0
9 15 4 0 0 0 0
10 16 4 0 0 0 0
11 17 4 0 0 0 0
12 18 4 0 0 0 0
13 18 4 0 0 0 0
14 19 4 0 0 0 0
15 19 4 0 0 0 0
16 20 4 0 0 0 0
17 20 4 0 0 0 0
5 21 1 0 0 0 0
6 22 1 0 0 0 0
7 23 1 0 0 0 0
8 24 1 0 0 0 0
9 25 1 0 0 0 0
10 26 1 0 0 0 0
11 27 1 0 0 0 0
12 28 1 0 0 0 0
13 29 1 0 0 0 0
14 30 1 0 0 0 0
15 31 1 0 0 0 0
16 32 1 0 0 0 0
17 33 1 0 0 0 0
18 34 1 0 0 0 0
19 35 1 0 0 0 0
20 36 1 0 0 0 0
M END
> <Alias name>
Triphenylmethanol

> <deltaH°(c)> kJ/mol

-9760.9

$$$$
Pentaethylbenzene
  ChemBrain IXL

```
 42 42  0  0  0  0  0  0  0  0  1 V2000
    6.9467    4.7237   -0.4763 C   0  0  0  0  0  0  0  0  0  0  0  0
    7.5200    5.9001    0.0069 C   0  0  0  0  0  0  0  0  0  0  0  0
    6.7179    6.8728    0.6040 C   0  0  0  0  0  0  0  0  0  0  0  0
    5.3420    6.6711    0.7140 C   0  0  0  0  0  0  0  0  0  0  0  0
    4.7685    5.4954    0.2294 C   0  0  0  0  0  0  0  0  0  0  0  0
    5.5708    4.5219   -0.3661 C   0  0  0  0  0  0  0  0  0  0  0  0
    4.9622    3.2748   -0.8809 C   0  0  0  0  0  0  0  0  0  0  0  0
    6.0574    2.3883   -1.5049 C   0  0  0  0  0  0  0  0  0  0  0  0
    3.3088    5.2808    0.3471 C   0  0  0  0  0  0  0  0  0  0  0  0
    2.5819    6.0816   -0.7507 C   0  0  0  0  0  0  0  0  0  0  0  0
    4.4906    7.7044    1.3448 C   0  0  0  0  0  0  0  0  0  0  0  0
    4.3933    8.9284    0.4137 C   0  0  0  0  0  0  0  0  0  0  0  0
    7.3268    8.1189    1.1208 C   0  0  0  0  0  0  0  0  0  0  0  0
    7.8405    8.9672   -0.0586 C   0  0  0  0  0  0  0  0  0  0  0  0
    8.9791    6.1162   -0.1140 C   0  0  0  0  0  0  0  0  0  0  0  0
    9.7102    4.7650    0.0059 C   0  0  0  0  0  0  0  0  0  0  0  0
    7.5698    3.9672   -0.9371 H   0  0  0  0  0  0  0  0  0  0  0  0
    4.2224    3.5181   -1.6334 H   0  0  0  0  0  0  0  0  0  0  0  0
    4.4871    2.7421   -0.0665 H   0  0  0  0  0  0  0  0  0  0  0  0
    6.5336    2.9217   -2.3183 H   0  0  0  0  0  0  0  0  0  0  0  0
    5.6119    1.4765   -1.8829 H   0  0  0  0  0  0  0  0  0  0  0  0
    6.7965    2.1439   -0.7519 H   0  0  0  0  0  0  0  0  0  0  0  0
    3.0883    4.2269    0.2310 H   0  0  0  0  0  0  0  0  0  0  0  0
    2.9722    5.6151    1.3206 H   0  0  0  0  0  0  0  0  0  0  0  0
    1.5154    5.9076   -0.6781 H   0  0  0  0  0  0  0  0  0  0  0  0
    2.7850    7.1375   -0.6217 H   0  0  0  0  0  0  0  0  0  0  0  0
    2.9343    5.7624   -1.7237 H   0  0  0  0  0  0  0  0  0  0  0  0
    3.5002    7.3011    1.5157 H   0  0  0  0  0  0  0  0  0  0  0  0
    4.9266    8.0035    2.2900 H   0  0  0  0  0  0  0  0  0  0  0  0
    3.7536    9.6761    0.8661 H   0  0  0  0  0  0  0  0  0  0  0  0
    3.9764    8.6251   -0.5387 H   0  0  0  0  0  0  0  0  0  0  0  0
    5.3814    9.3441    0.2594 H   0  0  0  0  0  0  0  0  0  0  0  0
    6.5843    8.6812    1.6735 H   0  0  0  0  0  0  0  0  0  0  0  0
    8.1537    7.8722    1.7752 H   0  0  0  0  0  0  0  0  0  0  0  0
    8.2758    9.8842    0.3188 H   0  0  0  0  0  0  0  0  0  0  0  0
    7.0156    9.2043   -0.7191 H   0  0  0  0  0  0  0  0  0  0  0  0
    8.5912    8.4092   -0.6046 H   0  0  0  0  0  0  0  0  0  0  0  0
    9.3126    6.7784    0.6754 H   0  0  0  0  0  0  0  0  0  0  0  0
    9.1996    6.5618   -1.0762 H   0  0  0  0  0  0  0  0  0  0  0  0
   10.7796    4.9271   -0.0477 H   0  0  0  0  0  0  0  0  0  0  0  0
    9.4025    4.1150   -0.8039 H   0  0  0  0  0  0  0  0  0  0  0  0
    9.4622    4.3032    0.9536 H   0  0  0  0  0  0  0  0  0  0  0  0
  1  2  4  0  0  0  0
  2  3  4  0  0  0  0
  3  4  4  0  0  0  0
  4  5  4  0  0  0  0
  5  6  4  0  0  0  0
  6  1  4  0  0  0  0
  6  7  1  0  0  0  0
  7  8  1  0  0  0  0
  5  9  1  0  0  0  0
  9 10  1  0  0  0  0
  4 11  1  0  0  0  0
 11 12  1  0  0  0  0
  3 13  1  0  0  0  0
 13 14  1  0  0  0  0
```

2 15 1 0 0 0 0
15 16 1 0 0 0 0
1 17 1 0 0 0 0
7 18 1 0 0 0 0
7 19 1 0 0 0 0
8 20 1 0 0 0 0
8 21 1 0 0 0 0
8 22 1 0 0 0 0
9 23 1 0 0 0 0
9 24 1 0 0 0 0
10 25 1 0 0 0 0
10 26 1 0 0 0 0
10 27 1 0 0 0 0
11 28 1 0 0 0 0
11 29 1 0 0 0 0
12 30 1 0 0 0 0
12 31 1 0 0 0 0
12 32 1 0 0 0 0
13 33 1 0 0 0 0
13 34 1 0 0 0 0
14 35 1 0 0 0 0
14 36 1 0 0 0 0
14 37 1 0 0 0 0
15 38 1 0 0 0 0
15 39 1 0 0 0 0
16 40 1 0 0 0 0
16 41 1 0 0 0 0
16 42 1 0 0 0 0
M END
> <Alias name>
Pentaethylbenzene

> <deltaH°(c)> kJ/mol
-9760.54

$$$$
3,6-Diphenyl-4-cyclohexene-1,2-dicarboxylic anhydride
ChemBrain IXL

39 42 0 0 0 0 0 0 0 0 1 V2000
13.8272 8.1586 0.1060 C 0 0 0 0 0 0 0 0 0 0 0 0
13.2283 7.8087 -1.1053 C 0 0 0 0 0 0 0 0 0 0 0 0
13.0327 8.4683 1.2115 C 0 0 0 0 0 0 0 0 0 0 0 0
11.6364 8.4277 1.1054 C 0 0 0 0 0 0 0 0 0 0 0 0
11.8346 7.7677 -1.2134 C 0 0 0 0 0 0 0 0 0 0 0 0
11.0304 8.0808 -0.1124 C 0 0 0 0 0 0 0 0 0 0 0 0
9.5299 8.0100 -0.2415 C 0 0 0 0 0 0 0 0 0 0 0 0
10.4885 5.3448 0.1721 O 0 0 0 0 0 0 0 0 0 0 0 0
9.3357 5.7272 0.3088 C 0 0 0 0 0 0 0 0 0 0 0 0
8.8630 9.2736 0.0148 C 0 0 0 0 0 0 0 0 0 0 0 0
8.9944 7.0177 0.7401 C 0 0 0 0 0 0 0 0 0 0 0 0
8.2476 4.9322 0.0419 O 0 0 0 0 0 0 0 0 0 0 0 0
7.5538 9.2696 0.0100 C 0 0 0 0 0 0 0 0 0 0 0 0
7.4427 6.9988 0.7357 C 0 0 0 0 0 0 0 0 0 0 0 0
7.1371 5.6979 0.3046 C 0 0 0 0 0 0 0 0 0 0 0 0
6.8940 8.0000 -0.2520 C 0 0 0 0 0 0 0 0 0 0 0 0
5.3972 8.0729 -0.1200 C 0 0 0 0 0 0 0 0 0 0 0 0
5.9960 5.2793 0.1749 O 0 0 0 0 0 0 0 0 0 0 0 0
4.5891 7.7513 -1.2165 C 0 0 0 0 0 0 0 0 0 0 0 0
4.7982 8.4353 1.0949 C 0 0 0 0 0 0 0 0 0 0 0 0
3.4027 8.4802 1.2057 C 0 0 0 0 0 0 0 0 0 0 0 0
3.1957 7.7967 -1.1038 C 0 0 0 0 0 0 0 0 0 0 0 0
2.6028 8.1606 0.1065 C 0 0 0 0 0 0 0 0 0 0 0 0

```
   14.9099    8.1890    0.1881 H   0  0  0  0  0  0  0  0  0  0  0  0
   13.8455    7.5671   -1.9631 H   0  0  0  0  0  0  0  0  0  0  0  0
   13.4997    8.7393    2.1540 H   0  0  0  0  0  0  0  0  0  0  0  0
   11.0252    8.6670    1.9671 H   0  0  0  0  0  0  0  0  0  0  0  0
   11.3764    7.4941   -2.1535 H   0  0  0  0  0  0  0  0  0  0  0  0
    9.2826    7.6866   -1.2451 H   0  0  0  0  0  0  0  0  0  0  0  0
    9.4231   10.1877    0.2104 H   0  0  0  0  0  0  0  0  0  0  0  0
    9.3785    7.2067    1.7386 H   0  0  0  0  0  0  0  0  0  0  0  0
    6.9875   10.1805    0.2013 H   0  0  0  0  0  0  0  0  0  0  0  0
    7.0473    7.1734    1.7292 H   0  0  0  0  0  0  0  0  0  0  0  0
    7.1404    7.6832   -1.2578 H   0  0  0  0  0  0  0  0  0  0  0  0
    5.0440    7.4671   -2.1564 H   0  0  0  0  0  0  0  0  0  0  0  0
    5.4146    8.6827    1.9513 H   0  0  0  0  0  0  0  0  0  0  0  0
    2.9403    8.7624    2.1468 H   0  0  0  0  0  0  0  0  0  0  0  0
    2.5738    7.5479   -1.9571 H   0  0  0  0  0  0  0  0  0  0  0  0
    1.5208    8.1944    0.1929 H   0  0  0  0  0  0  0  0  0  0  0  0
  2  1  4  0  0  0  0
  1  3  4  0  0  0  0
  5  2  4  0  0  0  0
  3  4  4  0  0  0  0
  4  6  4  0  0  0  0
  6  5  4  0  0  0  0
  6  7  1  0  0  0  0
  7 10  1  0  0  0  0
 11  7  1  0  0  0  0
  8  9  2  0  0  0  0
 12  9  1  0  0  0  0
  9 11  1  0  0  0  0
 10 13  2  0  0  0  0
 11 14  1  0  0  0  0
 15 12  1  0  0  0  0
 13 16  1  0  0  0  0
 16 14  1  0  0  0  0
 14 15  1  0  0  0  0
 15 18  2  0  0  0  0
 16 17  1  0  0  0  0
 19 17  4  0  0  0  0
 17 20  4  0  0  0  0
 22 19  4  0  0  0  0
 20 21  4  0  0  0  0
 21 23  4  0  0  0  0
 23 22  4  0  0  0  0
  1 24  1  0  0  0  0
  2 25  1  0  0  0  0
  3 26  1  0  0  0  0
  4 27  1  0  0  0  0
  5 28  1  0  0  0  0
  7 29  1  0  0  0  0
 10 30  1  0  0  0  0
 11 31  1  0  0  0  0
 13 32  1  0  0  0  0
 14 33  1  0  0  0  0
 16 34  1  0  0  0  0
 19 35  1  0  0  0  0
 20 36  1  0  0  0  0
 21 37  1  0  0  0  0
 22 38  1  0  0  0  0
 23 39  1  0  0  0  0
M  END
>  <Alias name>
3,6-Diphenyl-4-cyclohexene-1,2-dicarboxylic anhydride

>  <deltaH°(c)>   kJ/mol
```

```
-9759.6

$$$$
Benzo(a)pyrene
  ChemBrain IXL

 32 36  0  0  0  0  0  0  0  0  1 V2000
    2.4562    7.5879   -0.1316 C   0  0  0  0  0  0  0  0  0  0  0  0
    3.6071    8.3758   -0.1049 C   0  0  0  0  0  0  0  0  0  0  0  0
    4.8646    7.7730   -0.0623 C   0  0  0  0  0  0  0  0  0  0  0  0
    4.9717    6.3817   -0.0463 C   0  0  0  0  0  0  0  0  0  0  0  0
    3.8198    5.5940   -0.0730 C   0  0  0  0  0  0  0  0  0  0  0  0
    2.5626    6.1971   -0.1156 C   0  0  0  0  0  0  0  0  0  0  0  0
    6.2305    5.7794   -0.0036 C   0  0  0  0  0  0  0  0  0  0  0  0
    7.3808    6.5692    0.0230 C   0  0  0  0  0  0  0  0  0  0  0  0
    8.6393    5.9680    0.0657 C   0  0  0  0  0  0  0  0  0  0  0  0
    8.7480    4.5772    0.0817 C   0  0  0  0  0  0  0  0  0  0  0  0
    7.5985    3.7874    0.0551 C   0  0  0  0  0  0  0  0  0  0  0  0
    6.3401    4.3881    0.0125 C   0  0  0  0  0  0  0  0  0  0  0  0
    6.0149    8.5618   -0.0356 C   0  0  0  0  0  0  0  0  0  0  0  0
    7.2727    7.9601    0.0070 C   0  0  0  0  0  0  0  0  0  0  0  0
    8.4227    8.7496    0.0337 C   0  0  0  0  0  0  0  0  0  0  0  0
    9.6808    8.1484    0.0763 C   0  0  0  0  0  0  0  0  0  0  0  0
    9.7892    6.7577    0.0923 C   0  0  0  0  0  0  0  0  0  0  0  0
   11.0475    6.1566    0.1350 C   0  0  0  0  0  0  0  0  0  0  0  0
   11.1560    4.7658    0.1510 C   0  0  0  0  0  0  0  0  0  0  0  0
   10.0063    3.9761    0.1244 C   0  0  0  0  0  0  0  0  0  0  0  0
    1.4829    8.0547   -0.1646 H   0  0  0  0  0  0  0  0  0  0  0  0
    3.5246    9.4526   -0.1173 H   0  0  0  0  0  0  0  0  0  0  0  0
    3.9010    4.5176   -0.0607 H   0  0  0  0  0  0  0  0  0  0  0  0
    1.6715    5.5872   -0.1363 H   0  0  0  0  0  0  0  0  0  0  0  0
    7.6827    2.7107    0.0675 H   0  0  0  0  0  0  0  0  0  0  0  0
    5.4512    3.7759   -0.0081 H   0  0  0  0  0  0  0  0  0  0  0  0
    5.9316    9.6385   -0.0480 H   0  0  0  0  0  0  0  0  0  0  0  0
    8.3389    9.8263    0.0213 H   0  0  0  0  0  0  0  0  0  0  0  0
   10.5710    8.7597    0.0969 H   0  0  0  0  0  0  0  0  0  0  0  0
   11.9376    6.7679    0.1556 H   0  0  0  0  0  0  0  0  0  0  0  0
   12.1301    4.3005    0.1841 H   0  0  0  0  0  0  0  0  0  0  0  0
   10.0903    2.8994    0.1368 H   0  0  0  0  0  0  0  0  0  0  0  0
  1  2  4  0  0  0  0
  2  3  4  0  0  0  0
  3  4  4  0  0  0  0
  4  5  4  0  0  0  0
  5  6  4  0  0  0  0
  6  1  4  0  0  0  0
  7  8  4  0  0  0  0
  8  9  4  0  0  0  0
  9 10  4  0  0  0  0
 10 11  4  0  0  0  0
 11 12  4  0  0  0  0
 12  7  4  0  0  0  0
  4  7  4  0  0  0  0
  3 13  4  0  0  0  0
 13 14  4  0  0  0  0
 14  8  4  0  0  0  0
 14 15  4  0  0  0  0
 15 16  4  0  0  0  0
 16 17  4  0  0  0  0
 17  9  4  0  0  0  0
 17 18  4  0  0  0  0
 18 19  4  0  0  0  0
 19 20  4  0  0  0  0
 20 10  4  0  0  0  0
```

```
  1 21  1  0  0  0  0
  2 22  1  0  0  0  0
  5 23  1  0  0  0  0
  6 24  1  0  0  0  0
 11 25  1  0  0  0  0
 12 26  1  0  0  0  0
 13 27  1  0  0  0  0
 15 28  1  0  0  0  0
 16 29  1  0  0  0  0
 18 30  1  0  0  0  0
 19 31  1  0  0  0  0
 20 32  1  0  0  0  0
M  END
>  <Alias name>
Benzo[a]pyrene

>  <deltaH°(c)>   kJ/mol
-9759.1

$$$$
Benzo(k)fluoranthene
  ChemBrain IXL

 32 36  0  0  0  0  0  0  0  0  1 V2000
    5.2734    9.2285    0.0488 C   0  0  0  0  0  0  0  0  0  0  0  0
    5.1575   10.6064    0.2142 C   0  0  0  0  0  0  0  0  0  0  0  0
    6.3177   11.3680    0.4019 C   0  0  0  0  0  0  0  0  0  0  0  0
    7.5460   10.7209    0.4090 C   0  0  0  0  0  0  0  0  0  0  0  0
    7.5533    9.4020    0.2358 C   0  0  0  0  0  0  0  0  0  0  0  0
    6.5317    8.6860    0.0747 C   0  0  0  0  0  0  0  0  0  0  0  0
    8.7663   11.3647    0.5863 C   0  0  0  0  0  0  0  0  0  0  0  0
    9.9366   10.6001    0.5682 C   0  0  0  0  0  0  0  0  0  0  0  0
    9.8392    9.2217    0.3708 C   0  0  0  0  0  0  0  0  0  0  0  0
    8.5774    8.6788    0.2075 C   0  0  0  0  0  0  0  0  0  0  0  0
    8.2241    4.9889   -0.2966 C   0  0  0  0  0  0  0  0  0  0  0  0
    6.8372    5.0080   -0.3422 C   0  0  0  0  0  0  0  0  0  0  0  0
    6.1742    6.2329   -0.2272 C   0  0  0  0  0  0  0  0  0  0  0  0
    6.9309    7.3883   -0.0564 C   0  0  0  0  0  0  0  0  0  0  0  0
    8.1724    7.3771    0.0053 C   0  0  0  0  0  0  0  0  0  0  0  0
    8.9121    6.1982   -0.1208 C   0  0  0  0  0  0  0  0  0  0  0  0
    6.1231    3.8110   -0.5032 C   0  0  0  0  0  0  0  0  0  0  0  0
    8.9106    3.7694   -0.4230 C   0  0  0  0  0  0  0  0  0  0  0  0
    6.8053    2.5962   -0.6131 C   0  0  0  0  0  0  0  0  0  0  0  0
    8.1983    2.5753   -0.5758 C   0  0  0  0  0  0  0  0  0  0  0  0
    4.4058    8.5963   -0.0972 H   0  0  0  0  0  0  0  0  0  0  0  0
    4.1825   11.0779    0.1987 H   0  0  0  0  0  0  0  0  0  0  0  0
    6.2643   12.4414    0.5380 H   0  0  0  0  0  0  0  0  0  0  0  0
    8.8055   12.4371    0.7357 H   0  0  0  0  0  0  0  0  0  0  0  0
   10.9039   11.0691    0.7041 H   0  0  0  0  0  0  0  0  0  0  0  0
   10.7206    8.5918    0.3469 H   0  0  0  0  0  0  0  0  0  0  0  0
    5.0929    6.2834   -0.2661 H   0  0  0  0  0  0  0  0  0  0  0  0
    9.9946    6.2190   -0.0816 H   0  0  0  0  0  0  0  0  0  0  0  0
    5.0407    3.8245   -0.5400 H   0  0  0  0  0  0  0  0  0  0  0  0
    9.9933    3.7493   -0.4010 H   0  0  0  0  0  0  0  0  0  0  0  0
    6.2540    1.6708   -0.7292 H   0  0  0  0  0  0  0  0  0  0  0  0
    8.7263    1.6334   -0.6649 H   0  0  0  0  0  0  0  0  0  0  0  0
  1  2  4  0  0  0  0
  2  3  4  0  0  0  0
  3  4  4  0  0  0  0
  4  5  4  0  0  0  0
  5  6  4  0  0  0  0
  6  1  4  0  0  0  0
  7  8  4  0  0  0  0
```

```
  8  9  4  0  0  0  0
  9 10  4  0  0  0  0
  7  4  4  0  0  0  0
 10  5  4  0  0  0  0
 11 12  4  0  0  0  0
 12 13  4  0  0  0  0
 13 14  4  0  0  0  0
 14 15  4  0  0  0  0
 15 16  4  0  0  0  0
 16 11  4  0  0  0  0
 14  6  1  0  0  0  0
 15 10  1  0  0  0  0
 12 17  4  0  0  0  0
 11 18  4  0  0  0  0
 17 19  4  0  0  0  0
 18 20  4  0  0  0  0
 19 20  4  0  0  0  0
  1 21  1  0  0  0  0
  2 22  1  0  0  0  0
  3 23  1  0  0  0  0
  7 24  1  0  0  0  0
  8 25  1  0  0  0  0
  9 26  1  0  0  0  0
 13 27  1  0  0  0  0
 16 28  1  0  0  0  0
 17 29  1  0  0  0  0
 18 30  1  0  0  0  0
 19 31  1  0  0  0  0
 20 32  1  0  0  0  0
M  END
>  <Alias name>
Benzo[k]fluoranthene

>  <deltaH°(c)>   kJ/mol
-9757.6

$$$$
N-Benzoyl-o-aminodiphenylamine
  ChemBrain IXL

 38 40  0  0  0  0  0  0  0  0  1 V2000
    2.4636    5.5561   -0.3145 C   0  0  0  0  0  0  0  0  0  0  0  0
    3.2028    4.8163    0.6086 C   0  0  0  0  0  0  0  0  0  0  0  0
    3.0236    6.6855   -0.9119 C   0  0  0  0  0  0  0  0  0  0  0  0
    4.5021    5.2059    0.9344 C   0  0  0  0  0  0  0  0  0  0  0  0
    5.0625    6.3346    0.3361 C   0  0  0  0  0  0  0  0  0  0  0  0
    4.3229    7.0750   -0.5863 C   0  0  0  0  0  0  0  0  0  0  0  0
    6.3673    6.7244    0.6614 N   0  0  0  0  0  0  0  0  0  0  0  0
    6.7576    8.0601    0.5083 C   0  0  0  0  0  0  0  0  0  0  0  0
    5.8079    9.0784    0.5936 C   0  0  0  0  0  0  0  0  0  0  0  0
    8.0961    8.3728    0.2703 C   0  0  0  0  0  0  0  0  0  0  0  0
    6.1975   10.4097    0.4453 C   0  0  0  0  0  0  0  0  0  0  0  0
    8.4856    9.7040    0.1217 C   0  0  0  0  0  0  0  0  0  0  0  0
    7.5364   10.7225    0.2095 C   0  0  0  0  0  0  0  0  0  0  0  0
    9.0485    7.3505    0.1806 N   0  0  0  0  0  0  0  0  0  0  0  0
   10.4055    7.6679    0.0473 C   0  0  0  0  0  0  0  0  0  0  0  0
   11.4086    6.5876   -0.0845 C   0  0  0  0  0  0  0  0  0  0  0  0
   10.7612    8.8348    0.0399 O   0  0  0  0  0  0  0  0  0  0  0  0
   10.9994    5.2541   -0.1018 C   0  0  0  0  0  0  0  0  0  0  0  0
   12.7633    6.9030   -0.1914 C   0  0  0  0  0  0  0  0  0  0  0  0
   11.9449    4.2359   -0.2254 C   0  0  0  0  0  0  0  0  0  0  0  0
   13.7088    5.8848   -0.3150 C   0  0  0  0  0  0  0  0  0  0  0  0
   13.2996    4.5512   -0.3320 C   0  0  0  0  0  0  0  0  0  0  0  0
```

```
    7.0263    6.0426    1.0063 H   0  0  0  0  0  0  0  0  0  0  0  0
    8.7568    6.3851    0.2119 H   0  0  0  0  0  0  0  0  0  0  0  0
    1.4549    5.2537   -0.5674 H   0  0  0  0  0  0  0  0  0  0  0  0
    2.7681    3.9395    1.0724 H   0  0  0  0  0  0  0  0  0  0  0  0
    2.4496    7.2599   -1.6285 H   0  0  0  0  0  0  0  0  0  0  0  0
    5.0757    4.6321    1.6518 H   0  0  0  0  0  0  0  0  0  0  0  0
    4.7575    7.9521   -1.0496 H   0  0  0  0  0  0  0  0  0  0  0  0
    4.7683    8.8355    0.7750 H   0  0  0  0  0  0  0  0  0  0  0  0
    5.4606   11.2004    0.5132 H   0  0  0  0  0  0  0  0  0  0  0  0
    9.5250    9.9468   -0.0618 H   0  0  0  0  0  0  0  0  0  0  0  0
    7.8389   11.7560    0.0946 H   0  0  0  0  0  0  0  0  0  0  0  0
    9.9476    5.0093   -0.0193 H   0  0  0  0  0  0  0  0  0  0  0  0
   13.0809    7.9383   -0.1785 H   0  0  0  0  0  0  0  0  0  0  0  0
   11.6272    3.2006   -0.2386 H   0  0  0  0  0  0  0  0  0  0  0  0
   14.7605    6.1296   -0.3978 H   0  0  0  0  0  0  0  0  0  0  0  0
   14.0337    3.7608   -0.4278 H   0  0  0  0  0  0  0  0  0  0  0  0
  1  2  4  0  0  0  0
  3  1  4  0  0  0  0
  2  4  4  0  0  0  0
  6  3  4  0  0  0  0
  4  5  4  0  0  0  0
  5  6  4  0  0  0  0
  5  7  1  0  0  0  0
  7  8  1  0  0  0  0
  9  8  4  0  0  0  0
  8 10  4  0  0  0  0
 11  9  4  0  0  0  0
 10 12  4  0  0  0  0
 10 14  1  0  0  0  0
 13 11  4  0  0  0  0
 12 13  4  0  0  0  0
 14 15  1  0  0  0  0
 15 16  1  0  0  0  0
 15 17  2  0  0  0  0
 18 16  4  0  0  0  0
 16 19  4  0  0  0  0
 20 18  4  0  0  0  0
 19 21  4  0  0  0  0
 22 20  4  0  0  0  0
 21 22  4  0  0  0  0
  7 23  1  0  0  0  0
 14 24  1  0  0  0  0
  1 25  1  0  0  0  0
  2 26  1  0  0  0  0
  3 27  1  0  0  0  0
  4 28  1  0  0  0  0
  6 29  1  0  0  0  0
  9 30  1  0  0  0  0
 11 31  1  0  0  0  0
 12 32  1  0  0  0  0
 13 33  1  0  0  0  0
 18 34  1  0  0  0  0
 19 35  1  0  0  0  0
 20 36  1  0  0  0  0
 21 37  1  0  0  0  0
 22 38  1  0  0  0  0
M  END
>  <Alias name>
N-Benzoyl-o-aminodiphenylamine

>  <deltaH°(c)>   kJ/mol
-9753
```

```
$$$$
Benzylidene-p-isopropylphenylacetonitrile
  ChemBrain IXL

 36 37  0  0  0  0  0  0  0  0  1 V2000
    4.7966    7.5147    0.2665 C   0  0  0  0  0  0  0  0  0  0  0  0
    5.2311    8.7524   -0.2081 C   0  0  0  0  0  0  0  0  0  0  0  0
    5.7273    6.5492    0.6508 C   0  0  0  0  0  0  0  0  0  0  0  0
    3.3482    7.2258    0.3622 C   0  0  0  0  0  0  0  0  0  0  0  0
    6.5963    9.0254   -0.2966 C   0  0  0  0  0  0  0  0  0  0  0  0
    7.5270    8.0604    0.0890 C   0  0  0  0  0  0  0  0  0  0  0  0
    7.0925    6.8222    0.5625 C   0  0  0  0  0  0  0  0  0  0  0  0
    8.9753    8.3502   -0.0042 C   0  0  0  0  0  0  0  0  0  0  0  0
    3.0403    5.8992   -0.3587 C   0  0  0  0  0  0  0  0  0  0  0  0
    2.5520    8.3671   -0.2998 C   0  0  0  0  0  0  0  0  0  0  0  0
    9.8308    7.3788   -0.3310 C   0  0  0  0  0  0  0  0  0  0  0  0
   11.7339    8.9847   -0.3454 C   0  0  0  0  0  0  0  0  0  0  0  0
   11.2794    7.6680   -0.4214 C   0  0  0  0  0  0  0  0  0  0  0  0
   13.0994    9.2572   -0.4298 C   0  0  0  0  0  0  0  0  0  0  0  0
   12.1904    6.6239   -0.5825 C   0  0  0  0  0  0  0  0  0  0  0  0
    9.3305    6.0128   -0.6030 C   0  0  0  0  0  0  0  0  0  0  0  0
   13.5559    6.8964   -0.6669 C   0  0  0  0  0  0  0  0  0  0  0  0
   14.0104    8.2130   -0.5905 C   0  0  0  0  0  0  0  0  0  0  0  0
    8.9390    4.9439   -0.8158 N   0  0  0  0  0  0  0  0  0  0  0  0
    4.5086    9.5014   -0.5079 H   0  0  0  0  0  0  0  0  0  0  0  0
    5.3900    5.5877    1.0178 H   0  0  0  0  0  0  0  0  0  0  0  0
    3.0644    7.1448    1.4042 H   0  0  0  0  0  0  0  0  0  0  0  0
    6.9336    9.9864   -0.6648 H   0  0  0  0  0  0  0  0  0  0  0  0
    7.8150    6.0730    0.8616 H   0  0  0  0  0  0  0  0  0  0  0  0
    9.3412    9.3503    0.1928 H   0  0  0  0  0  0  0  0  0  0  0  0
    3.5956    5.0962    0.1101 H   0  0  0  0  0  0  0  0  0  0  0  0
    1.9795    5.6906   -0.2935 H   0  0  0  0  0  0  0  0  0  0  0  0
    3.3293    5.9783   -1.3995 H   0  0  0  0  0  0  0  0  0  0  0  0
    1.4930    8.1467   -0.2464 H   0  0  0  0  0  0  0  0  0  0  0  0
    2.7540    9.2962    0.2187 H   0  0  0  0  0  0  0  0  0  0  0  0
    2.8494    8.4604   -1.3370 H   0  0  0  0  0  0  0  0  0  0  0  0
   11.0267    9.7954   -0.2210 H   0  0  0  0  0  0  0  0  0  0  0  0
   13.4523   10.2794   -0.3706 H   0  0  0  0  0  0  0  0  0  0  0  0
```
[truncated: 371,863 more chars]
